# Propagation of [D1,2]-type spliceosomal twin introns (stwintrons) in *Hypoxylaceae* and *Xylariaceae* fungi

**DOI:** 10.1128/spectrum.02926-24

**Published:** 2025-08-08

**Authors:** Erzsébet Fekete, Norbert Ág, Viktória Ág-Rácz, Alexandra Márton, Erzsébet Sándor, Claudio Scazzocchio, Michel Flipphi, Levente Karaffa

**Affiliations:** 1 Department of Biochemical Engineering, Faculty of Science and Technology, University of Debrecen, Debrecen, Hungary; 2 Juhász-Nagy Pál Doctoral School of Biology and Environmental Sciences, University of Debrecen 37599, Debrecen, Hungary; 3 Institute of Food Science, Faculty of Agricultural and Food Science and Environmental Management, University of Debrecen 37599, Debrecen, Hungary; 4 Department of Life Sciences, Imperial College London, London, United Kingdom; 5 Institute for Integrative Biology of the Cell (I2BC), Université Paris-Saclay, CEA and CNRS (Unité mixte de Recherche UMR 9198), Gif-sur-Yvette, Île-de-France, France; University of Natural Resources and Life Sciences Vienna, Vienna, Austria

**Keywords:** spliceosomal introns, splicing, [D1,2] stwintron propagation, *Xylariales*, near-identical stwintrons, near-terminal double-stranded RNA stem structure, [D1,2] missplicing using distal splice sites

## Abstract

[D1,2] stwintrons consist of nested U2 introns, where an internal intron splits the 5′-donor of an external intron between its first and second nucleotide. Almost all stwintrons described to date are of the [D1,2] type, suggesting unique means for their duplication. Sequence-similar [D1,2] stwintrons are typically integrated at new intron positions, specific for one species. Two hundred eighty-eight sequence-similar [D1,2] stwintrons were identified in the genomes of 14 *Xylariales*. Occasional missplicing was apparent, where almost the entire stwintron was excised as one canonical intron. Near-identical sister stwintrons were identified in *Xylaria* sp. MSU SB201401 and *Xylaria longipes* that share near-terminal inverted repeat elements of 10-nt length named 5′-NTIRE-10 and 3′-NTIRE-10, which are complementary as RNA. Complementary NTIRE-10 partners were also present in three *Hypoxylaceae* species. These NTIRE-10s can form a near-terminal double-stranded RNA stem structure that brings in close proximity the terminal G’s of the [D1,2] stwintron and of its alternative misspliced intron. The exact folding of the interior stwintron RNA appears irrelevant. Ten of the stwintrons with complementary NTIRE-10s are present in *Xylaria* sp. MSU SB201401, implying that [D1,2] stwintron duplication occurs frequently in this species.

**IMPORTANCE:**

Spliceosomal introns are excised from pre-mRNAs by a ribonucleoprotein complex, the U2 spliceosome. Excision does not necessarily occur by one splicing reaction. We had identified and validated intronic sequences in fungi that consist of two nested U2 introns and called them spliceosomal twin introns (stwintrons). In this bioinformatics-based study, we identified and validated almost 300 [D1,2] stwintrons with sequence similarity in the genomes of 14 species in the *Xylariales* order of *Ascomycota*. Thirty-seven of them feature small, fully complementary RNA inverted repeat elements. These elements form a near-terminal RNA stem structure, bringing the terminal G’s of the stwintron in close proximity. We further demonstrated the existence of an alternative splicing pattern involving one excision event that removes the two constituent introns from the transcript together. This work contributes to the understanding of mechanisms of stwintron gain in fungi.

## INTRODUCTION

**Fig 1:**
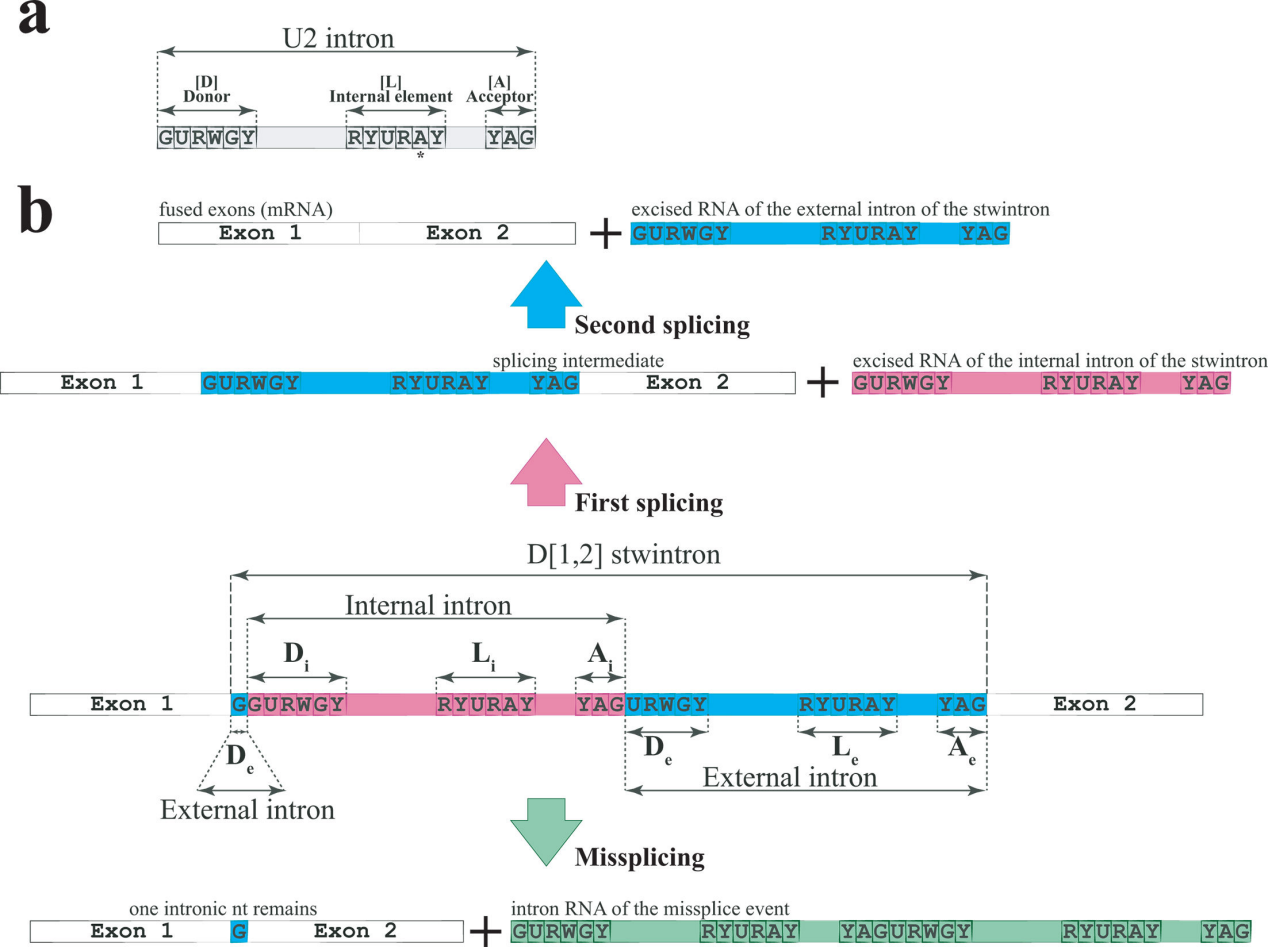
Schematic structure of fungal spliceosomal introns, types of spliceosomal twin introns, and sequential excision of nested introns. (**a**) Schematic structure of spliceosomal U2 fungal introns considering the location of the three splicing consensus sequences at or near the splice sites. The intron consensus sequences in the model fungus *Aspergillus nidulans*—[GURWGY], [RYURAY], and [YAG], respectively—were described by Kupfer et al. (40). (**b**) Consecutive excision of the constituent canonical introns of a [D1,2] stwintron versus the missplicing of the same by one canonical splicing reaction. In a [D1,2] stwintron, the internal intron is located in the 5′-donor of the discontinuous external intron between G_1_ and U_2_. Two lariat intron RNAs (magenta and turquoise, respectively) are released by two consecutive canonical splicing reactions, and the adjacent exons are eventually ligated together, resulting in the mature mRNA. Missplicing of a [D1,2] stwintron occurs when a canonical U2 intron nearly the size of the whole stwintron (i.e., one G smaller) is defined from the 5′-donor of the internal intron to the sequence element including the branch point adenosine and associated 3′-acceptor of the external intron, and excised in one canonical splicing reaction. One lariat intron RNA (green) is released, and one intronic G remains present between the exons bounding the stwintron.

Spliceosomal intronic sequences are intrinsic to eukaryotic nuclear transcriptomes. They are excised from pre-mRNAs by a ribonucleoprotein complex, the U2 spliceosome, which includes five short nuclear RNAs (snRNAs) essential for its assembly and function. Proper exon splicing requires initially the U1 and U2 snRNAs to interact by base pairing with conserved sequence elements at the boundaries of the intron sequence, the 5′-donor ([D]) and the 3′-acceptor ([A]), respectively, as well as with an internal sequence ([L]) carrying a key adenosine—the branch point A—at a short distance from the 3′-splice site (Fig. 1a). These three short intronic sequence elements are conserved in all U2 introns.

Continuous intronic sequences are not necessarily excised by one canonical splicing reaction. Splice site pairing in Ascomycota is determined by intron definition rather than by exon definition (1–3). We have identified and validated intronic sequences in ascomycete fungi that consist of two nested U2 introns from which the internal intron has to be removed prior to proper excision of the external intron by two consecutive canonical splicing reactions (1, 4–10). It is possible to detect a transient RNA species—the splicing intermediate—from which only the internal U2 intron was removed. We have called them spliceosomal twin introns or stwintrons (1) (Fig. 1b). Stwintrons are spliceosomal analogs of the group-II twin introns originally defined in the *Euglena gracilis* plastid genome (11). Stwintrons can be assigned to three classes dependent on which of the three consensus sequences of the external intron is disrupted by the internal intron. Figure 1b shows the consecutive excision of the constituent U2 introns according to intron definition, to remove a [D1,2] complex intronic sequence—in which the internal intron interrupts the donor of the external intron between its nt G_1_ and Y_2_—and join the bordering exons correctly.

In reference 5, we presented a path for the formation of a fungal stwintron, regardless of type. Stwintron structures could form following the integration or appearance of a functional U2 intron within one of the three consensus splice sequences of a host intron. Such events can explain the gain and loss of any type of stwintron in orthologous fungal genes. We illustrated this principle in two genes; one where a [D1,2] stwintron was generated from an extant canonical intron, and a second in which a [D2,3] stwintron evolved at a very ancient intron position. Nevertheless, almost all stwintrons we found to date are [D1,2]: we rarely encountered other stwintron types. This suggests a means for [D1,2] duplication not available to stwintrons of other types.

Recently, we searched for sequence-similar [D1,2] stwintrons with Blastn screens of whole genome sequences of an unnamed species of *Hypoxylon*, *Hypoxylon* sp. strain CO27-5 (9) and by concerted model-based sequence motif searches in the same genome sequences (10). The Blastn approach yielded 23 sequence-similar stwintrons at gene positions unique to strain CO27-5. We have called these 23 stwintrons sister stwintrons. With the motif search method, we found more than 100 genuine [D1,2]’s in the CO27-5 genome, which included the 23 sister stwintrons, together with many additional [D1,2] stwintrons. At variance with sister stwintrons, many of these additional [D1,2] stwintrons are found at gene positions that are also occupied by seemingly sequence-unrelated [D1,2] stwintrons in the orthologous genes in related species. Despite the loss of primary sequence similarity, this position conservation suggests common ancestry. However, we cannot exclude the possibility that some of these seemingly sequence-unrelated [D1,2] stwintrons could be gained in parallel at the same location as pre-existing in other *Xylariales* species.

In this bioinformatics-based study, we cross-identify and validate almost 300 [D1,2] stwintrons with sequence similarity in the genome sequences of 14 species in the *Xylariales* order of *Ascomycota*. Among them, near-identical stwintrons (>97% identity) were found in different genes and in different species, that feature small but fully complementary RNA inverted repeat elements. These elements arguably form a near-terminal RNA stem structure bringing the terminal G’s of the stwintron (i.e., the G_1_ of the internal donor and the G_3_ of the external acceptor) in close proximity. We further demonstrated the existence of an alternative splicing pattern involving one seemingly canonical U2 excision event that removes the two constituent introns from the transcript together as one big intron.

## MATERIALS AND METHODS

### Mining putative sequence-similar [D1,2] stwintrons from NCBI DNA databases

Blastn screens were mostly performed in individual whole genome sequences from the Whole Genome Shotgun Contigs Database at the servers of the National Center for Biotechnology Information (NCBI) (12). Initially, query sequences were selected amongst the 14 most sequence-similar [D1,2]’s, earlier found in the genomes of the strongly related taxa *Hypoxylon* sp. CO27-5 and EC38 (9, 10). We focused on the presence of an intron splice consensus core sequence, i.e., six nt for a 5′-donor or six nt for an internal sequence element including the branch point A near the 3′ end of U2 introns, in the Blast hits. Putative full-length [D1,2] stwintrons—their distal 5′- and 3′ splice sites—were defined by “walking” the sequence contig from the Blastn hit in divergent directions until satisfactory terminally consensus splice sequences could be included.

By this procedure, we found sequence-similar stwintrons in some other *Hypoxylariacae* genomes (*Hypoxylon*, *Daldinia*) as well as in a number of species of the sister family, the *Xylariaceae*. Subsequently, newly found [D1,2] stwintrons from the primary Blastn screens were used to cross-identify more sequence-similar stwintrons in the whole genome sequences, in particular, from those species where the secondary queries were originally found. In some genomes, we encountered only a sole [D1,2], sequence-similar to confirmed *Hypoxylon* sp. CO27-5/EC38 sister sequences. We ignored these “solo” sister stwintrons in the subsequent investigations for the absence of evidence that they can duplicate in their present form. In yet other sequenced species of the two families, we could not identify any sequence-similar intronic sequences reminiscent of *Hypoxylon* sp. CO27-5 sister stwintrons by Blastn.

### Comparative analysis of the intron-exon structure of orthologs of the gene harboring [D1,2] stwintron(s)

We manually predicted the intron-exon structure of each gene guided by comparative genomics of coding sequences, taking the intron phase into account. The coding sequences (ATG-stop) of the ortholog genes carrying identified [D1,2] stwintrons were mined in the *Hypoxylaceae* or *Xylariaceae* genomes by Tblastn screening of the NCBI’s Whole Genome Shotgun contigs (WGS) database to track intron position conservation patterns as well as to appreciate the level of sequence conservation amongst position-conserved “ortholog” stwintrons. The sequence-derived information thus collected for each of the 288 *Xylariales* [D1,2] stwintrons is listed in Table S1. We found *Xylariaceae* sister stwintrons, of which the internal intron’s donor starts with 5′-GC. One *Hypoxylon* sp. CO27-5 stwintron is split over two sequence contigs (GenBank MW477887). Occasionally, a sister stwintron is located in an open reading frame that appears unique to one species. We ignored computer-estimated model mRNAs and automatically deduced amino acid sequences, as contemporary annotation software is not trained to recognize stwintrons and often disregards available expression information.

We also recorded in Table S1 details about the gene models of the genes carrying the listed [D1,2] stwintrons, the stwintron phase, its/their position in the deduced intron-exon structure as well as of the gene’s peptide product and function. The coordinates of the coding region of the stwintron-carrying genes (start to stop) were predicted. After translation of the predicted mRNAs, we functionally annotated most of the genes carrying [D1,2] stwintrons with one or more protein domains categorized in the Protein Families (Pfam) database (13), essentially as described in references 9, 10.

### Verification of stwintron excision and the typical two-step splicing mode

A lot of the gene model predictions could be corroborated by RNA sequence reads in expression resources for seven of the investigated species (genome sequences): relevant sequence read archive (SRA) accessions are listed in Table 1. Perfectly or near-perfectly matching SRA reads were identified upon Blastn screening of the NCBI’s Sequence Read Archive for individual species using 60 nt long query sequences containing the manually predicted exon/exon fusion site at its center. To detect the stwintron splicing intermediate—the RNA species that lacks the predicted internal intron of the [D1,2] stwintron but still contains its external intron and abbreviated as “splinter” from here on—we used query sequences (60 nt) containing in their center the predicted fusion site of the upstream exon and the external intron of the stwintron. SRAs that confirm putative stwintrons and their predicted splinter are listed in Table S1 for all species with accessible SRA data. For conciseness, we recorded only one SRA read in Table S1 but in most cases, there are (many) more of them, especially for the excision of the complete stwintron. We did not record the number of reads covering the stwintron, nor its splinter, nor the underlying pre-mRNA, as such data may leave the impression that one can compare between stwintrons. For *Hypoxylon rubiginosum* and *Xylaria longipes*, the utilized SRA databases were generated in a different but intimately related strain from the strain in which genome we originally identified the sister stwintrons.

**TABLE 1:** Whole genome sequences of 14 taxa of *Xylariales* and SRA expression data used in this study

Genome (species) (14)	Strain/isolate [backup strain]	Master accession no. (NCBI)	Reference [to WGS]	No. of contigs	No. of sister stwintrons selected [code to unique stwintron names]	Accessible RNA resources (NCBI SRA/DOE-JGI)
*Hypoxylaceae* (7)					[73]	
*Daldinia childiae*	JS-1345	VYXO	(14)	133	19 [Dchc000X]	None
*Daldinia concentrica*	CBS 113277	CADCSW	(15)	69	9 [Dcoc00X]	None
*Daldinia eschscholzii*	IFB-TL01 [EC12]	AKGB [MDGZ]	(16) (17)	875 847	6 [Desc000X]	NCBI SRX872671–SRX872676
*Hypoxylon rubiginosum*	MUCL52887 [CBS 119005]	CADCXA [JAJKKX]	(15) (18)	70 [673]	5 [Hruc00X]	None [CBS 119005] NCBI SRX7582399
*Hypoxylon* sp. E7406B [*H. pulicicidum* clade]	E7406B	JYCQ	(19)	745	9 [HE7c000X]	None
*Hypoxylon* sp. CO27-5	CO27-5	MDCL	(17)	580	23 [HCOc000X]	NCBI SRX875229–SRX875234 (7, 8)
*Hypoxylon* sp. EC38	EC38	MDCK	(17)	599	2 [HECc000X]	NCBI SRX872662–SRX872667
*Xylariaceae* (7)					[215]	
*Nemania abortiva*	FL1152	JAJKKU	(18)	929	25 [Naboc000X]	https://mycocosm.jgi.doe.gov/Nemabo1/Nemabo1.home.html NCBI SRX7514343–SRX7514344
*Xylaria arbuscula*	FL1030 [VT107]	JAJLLY [JANPWZ]	(18) [JANPWZ: released 10 Jan 2023]	1,391 [3,895]	61 [Xarbc0000X]	https://mycocosm.jgi.doe.gov/XylarbFL1030/XylarbFL1030.home.html NCBI SRX7514331–SRX7514332
*Xylaria bambusicola*	CBS 139988	JAJJWZ	(18)	377	48 [Xbamc000X]	https://mycocosm.jgi.doe.gov/Xylbam139988_1/Xylbam139988_1.home.html NCBI SRX7582577
*Xylaria longipes*	IHI A66 [aka DSM 107183]	NQIL	(20)	1,006	4 [Xlonc0000X]	None [CBS 148.73] NCBI SRX7582590
*Xylaria* sp. BCC 1067	BCC 1067	SSCS	(21)	43	8 [Xbccc00X]	None
*Xylaria* sp. MSU SB201401	MSUSB201401	NPFG	(22)	5,995	60 [Xmsuc0000X]	None
*Xylaria striata* [backup MSU SB201401]	RK1-1	LOBO	[LOBO: released 3 Nov 2017]	79,106	[53]^*a*^ [Xstr00000X]	NCBI SRX1453632–SRX1453641
*Xylariaceae* sp. FL1651	FL1651	JAJJJS	(18)	252	9 [X1651c000X]	https://mycocosm.jgi.doe.gov/XylFL1651/XylFL1651.home.html NCBI SRX7514358–SRX7514359

^*a*^ [D1,2] stwintrons identified in *X. striata* RK1-1 are almost all orthologs of those found in MSU SB201401 (Fig. S2). One may consider the two specimen strains of the same species, or cryptic species. The 53 *X. striata* stwintrons thus do not add to the sum of the total number of sister stwintrons in *Xylariaceae* because they are already counted as MSU SB201401 stwintrons.

### Other informatics methods

The sequence logos of the opposite repeat elements near the 5′- and 3′-end of the stwintron RNA (5′-NTIRE-10 and 3′-NTIRE-10, respectively) were created by Skylign (23) using default settings. Note that the 3′ element, normally located between the external branch point sequence element and the associated acceptor, could not be defined in all stwintrons while apparently truncated NTIREs were discarded. Multiple sequence alignment with Fast Fourier Transform (MAFFT, version 7) (24) was used for the alignments of each single-stranded stwintron RNA sequence with its own reverse complement sequence, using the E-INS-i module while comparing results obtained with one of three scoring matrices, 200PAM (default), 20PAM, or 1PAM. RNAfold (25, 26) was used online (ViennaRNA web suite) with the default settings, except that isolated base pairs were not avoided, to predict the optimal secondary structure of single-stranded debranched stwintron RNAs, i.e., the structure with the lowest calculated minimum free energy (ΔG). For each stwintron, the single “optimal” RNAfold structure was compared with the secondary structures proposed by another online predictor, Mfold (UNAfold web suite) (27, 28).

### Phylogenetic analyses of groups of sequence-similar stwintrons

Selections of sister stwintrons (listed in Table S1) were aligned by MAFFT (24) using the E-INS-i iterative refinement module and the 200PAM scoring matrix; in almost all experiments, the conserved sequences at the 5′ and 3′ termini (i.e., 5′-GGT or 5′-GGC and 5′-AG, respectively) aligned perfectly. Subsequently, consecutive positions in the multiple sequence alignment (MSA) that were only occupied in one or two of the aligned stwintrons were eliminated, removing most of the “gappy regions” introduced by alignment while trimming down the number of informative nucleotides (nt). For each analysis, the number of informative nt is given in the legends of the respective supplemental figures. The resultant modified MSA was then used to infer maximum likelihood trees with PhyML version 3 (29, 30). The best suitable substitution model was identified automatically by the Smart Model Selection (SMS) module according to the Akaike Information Criterion. In most cases, SMS selected GTR + I + G (31). Ten thousand iterations of transfer bootstraps (32) were run to estimate branch support.

## RESULTS

### Identification of new [D1,2] sister stwintrons in the genome sequences of multiple *Xylariales*

Sister stwintrons are sequence-similar stwintrons that appear to be recent as they occur at positions in genes which are not occupied by (stw)introns in closely related species or strains (9, 10). In *Hypoxylon* sp. CO27-5, they cross-identify in Blastn screens of genomic DNA contigs. Here, we searched for sequence-similar sister stwintrons in other species of the *Hypoxylaceae* and species of its sister family, the *Xylariaceae*, using the six most sequence-similar of the 23 CO27-5 sister stwintrons as initial Blastn queries. The corresponding new [D1,2] stwintrons initially found in individual genomes were subsequently used as secondary queries to cross-identify more sister stwintrons in the individual species, while any stwintrons then identified in the secondary Blastn screens were again back-tested as genome-specific Blastn queries until no new sequence-similar [D1,2] stwintrons were encountered. Table 1 lists the genome resources (species) from which we selected 263 new [D1,2] stwintrons.

We have investigated the interrelations between groups of these *Xylariales* sister stwintrons with maximum likelihood phylogenies. This is by no means compatible with proteome-based whole-genome phylogenetics as intronic sequences are non-coding and small in fungi, with a much lower information content. The resulting stwintron phylogenies should not be used to estimate taxonomic relations amongst the species in our study. Nevertheless, phylogenetic analyses of sister stwintrons suggest the existence of species-specific clades amongst the majority of species investigated, implying occasional duplication of a stwintron (see Fig. S1 for a phylogeny of sister stwintrons in five species of *Xylariaceae*). Essentially, the [D1,2] stwintrons in the crown clades reside in unrelated genes at positions that are not occupied in the orthologous genes in the other species. All investigated species thus must have inherited the ability to duplicate stwintrons vertically and maintained this ability after speciation. Duplication-competent stwintrons in different species are thus not identical or extremely similar in sequence to one another, but they should somehow have retained common features important to stwintron duplication. However, where the compared taxa are very closely related, such as in the case of *Xylaria* sp. MSU SB201401 and *X. striata* RK1-1 (this work, maximum likelihood phylogeny in Fig. S2), many clades with short terminal branches consistent of the two orthologous stwintrons can be observed, i.e., sequence-similar stwintrons residing at the very same intron position in their orthologous genes in the compared taxa.

Some of the selected fungi harbor more than 40 sister stwintrons, while others carry less than 10 sequence-similar [D1,2] stwintrons. This does not necessarily mean that more duplication events have taken place in species with higher numbers of sister stwintrons; it could equally be that extant stwintrons are eliminated faster in species showing lower numbers. In 25 instances, the sister stwintron is the sole intronic sequence in the gene carrying it (Table S1), supporting the conjecture that sister stwintrons are recent, interrupting previously continuous coding sequences. Moreover, a few genes harbored more than one [D1,2] stwintron (Table S1).

### Verification of newly identified [D1,2] sister stwintrons by comparative analysis

The newly identified sister stwintrons were confirmed by comparative analysis of the gene harboring the stwintron(s) of interest, assessing the intron-exon structures of the orthologous genes in related *Xylariaceae* or *Hypoxylaceae* species from which the sister stwintron is absent. Intron position conservation in related species is typical for gene orthology (cf. [33]), but we looked specifically for “species-specific” intronic sequences in orthologous genes in a group of species of the same genus or family. The comparative approach was crucial to confirm stwintrons in genes apparently silent under the conditions used to generate the freely accessible RNA sequence resources. In Table S1, all 288 [D1,2] stwintrons from 14 species of *Hypoxylaceae* and *Xylariaceae* are detailed (see Materials and Methods for details).

From the sequence logo in Fig. S3, one can appreciate that the newly identified [D1,2] stwintrons have integrated seamlessly into seemingly random sites. We found no signs of target site duplication (TSD) in the exons bounding the stwintrons (Fig. S3). There is no evidence for splice site co-option (34, 35) in the 288 fungal [D1,2] stwintrons tested (Table S1). Some 5′ exons end at 5′-AG and some 3′ exons start with 5′-GU, but their frequencies are quite close to what could be expected if integration sites are random (i.e., one in every 16 cases).

### Confirmation of newly identified [D1,2] sister stwintrons with expression data and of instances of their missplicing

For six species*—Nemania abortiva*, *Xylariaceae* sp. FL1651, *Xylaria arbuscula* FL1030, *Xylaria bambusicola*, *X. longipes,* and *H. rubiginosum*—we verified the existence of the proposed [D1,2] stwintrons by analyzing covering RNA sequence reads (SRAs), publicly accessible at the NCBI or alternatively, in associated JGI-based EST resources (assembled EST contigs), in which the bounding exons are correctly joined. For most of them, we also found reads of the legitimate splicing intermediate (splinter), the transient RNA species in which the bounding exons are separated by the external intron of the predicted [D1,2] (see Table S1). However, consecutive excision of the internal before the external intron does not always take place. We identified SRA reads (Table S2) which provide direct evidence for missplicing of some [D1,2] stwintrons, in which almost the whole [D1,2] sequence is excised by one seemingly canonical splicing reaction using the internal donor at its 5′-splice site, and the distal external branch point sequence element and associated acceptor at its 3′-splice site (Fig. 1b: [D1,2] missplicing). Such an event is not in agreement with the mechanism of splice site pairing implied by intron definition theory (2), which ensures correct stwintron excision by consecutive canonical splicing reactions removing the internal and external introns, respectively. Splice site pairing by intron definition is essential to the very existence of stwintrons. However, the internal splice sites within the stwintron may be temporarily obscured by secondary structure or by RNA-binding proteins. The missplice event is characterized by the retention of one intronic G between the neighboring exons, causing a frameshift in the product mRNA.

### A short near-terminal double-stranded RNA structure potentially brings and keeps in close proximity the termini of the misspliced intron RNA

In our previous work, we have shown that all the [D1.2] stwintrons in *Hypoxylon* sp. CO27-5 consistently exhibits symmetry in alignments of the stwintron sequence with its reverse complement sequence, but that in the large majority of the 23 sister stwintrons, this symmetry is more striking than in uniquely occurring stwintrons, in particular, near the stwintrons' termini (10). The sequence-similar sister stwintrons at unique gene positions are arguably more recent (“younger”) than uniquely occurring (i.e., not sequence-similar) stwintrons present at gene positions also occupied by seemingly unrelated [D1,2] stwintrons in less directly related species of *Hypoxylaceae*. In the current work, we identified two small clusters of near-identical sister stwintrons with >97% identity (i.e., not more than five single-nucleotide polymorphisms [SNPs]) in two not closely related *Xylariaceae* species, which presumably are the products of the most recent stwintron duplications included in our set of 215 *Xylariaceae* sequence-similar stwintrons. The first cluster consists of four near-identical stwintrons present in *Xylaria* sp. MSU SB201401 but absent from the four orthologous genes in the very closely related *Xylaria striata* RK1-1 strain (see Fig. S2). The second cluster consists of three of the four sister stwintrons we identified in *Xylaria longipes*, those that share a rare 5′-G_1_C_2_ donor for their internal intron and for their misspliced intron.

**Fig 2:**
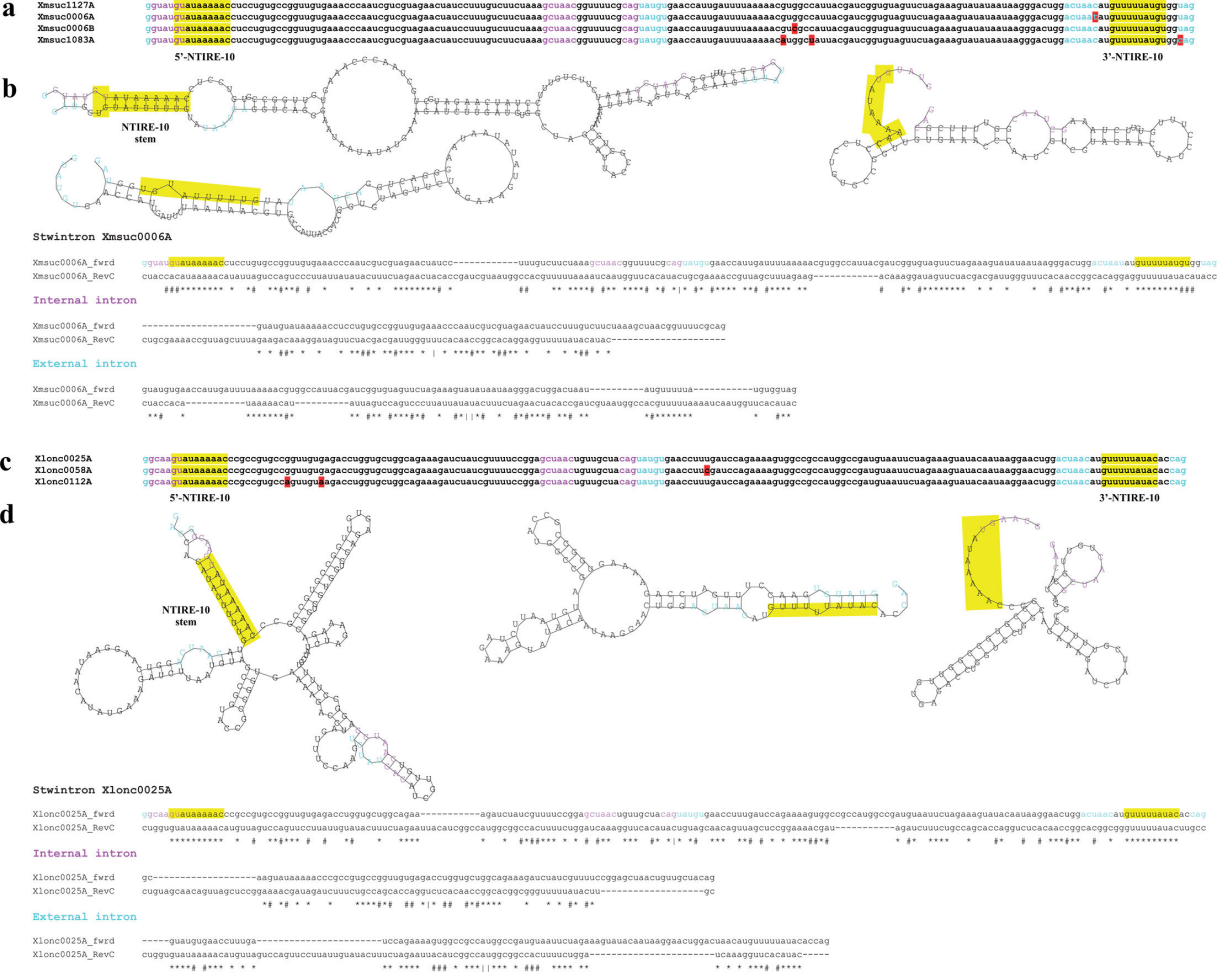
Near-identical stwintrons in *Xylaria* sp. MSU SB201401 and in *Xylaria longipes*, and their NTIRE-10 elements. The 5′-NTIRE-10 is identical in the seven stwintrons, while the 3′-NTIRE-10s are species specific but both fully complementary to the 5′-NTIRE-10 due to GU wobble base pairing. (**a**) MAFFT alignment of the four near-identical sister stwintrons unique to *Xylaria* sp. MSU SB201401. All four are 185 nt long but are located in four completely different genes (see Table S1). SNP positions are highlighted in red background. Consensus splicing sequences for the external intron are highlighted in turquoise letters and those of the internal intron in magenta letters. The NTIRE-10 inverted repeat elements 5′-GUAUAAAAAC and 5′-GUUUUUAUGU are 100% complementary and highlighted in yellow. The first two nt of the 5′-NTIRE-10 (i.e., 5′-GU) are the same as nt-5 and nt-6 of the core 5′-donor of the internal intron, 5′-GUAUGU, but are here presented as part of the 5′-NTIRE-10. (**b**) Secondary structures of the least minimal free energy (ΔG) of stwintron Xmsuc0006A and of its internal and external introns predicted by RNAfold, and the alignment of Xmsuc0006A and its constituent introns with their own reverse complement sequences, revealing terminal symmetry. The 5′-donors, internal sequence elements including the branch point A, and 3′-acceptors are highlighted in colored letters, as above. The repeat elements NTIRE-10 near either of the stwintron extremities can base pair, and the resulting 10-nt double-stranded RNA stem structure is highlighted in yellow background. In the diagnostic two-way alignment, the consensus line underneath the aligned sequences indicates nucleotide identity with the star symbol and the GU interactions with the number symbol. The center of the symmetry is the G shared by the overlapping constituent introns of the stwintron and indicated by the vertical line symbol | in the consensus line. (**c**) MAFFT alignment of the three near-identical sister stwintrons present in *Xylaria longipes* (in both sequenced strains). They are located in three completely different genes (see Table S1). They are 184 nt long and differ by 1, 2, or 3 nt; the SNP positions are highlighted in red. The two NTIRE-10 elements 5′-GUAUAAAAAC and 5′-GUUUUUAUAC are 100% complementary and highlighted in yellow. All other annotations are as described for panel a. (**d**) Secondary structures of the least minimal free energy (ΔG) of stwintron Xlonc0025A and its internal and external introns predicted by RNAfold, and the alignment of Xlonc0025A and of its constituent introns with their own reverse complement sequences, revealing terminal symmetry. RNAfold does not predict a hairpin structure for Xlonc0025A, but the 5′-NTIRE-10 close to the 5′ end of the [D1,2] (5′-GUAUAAAAAC) and its counterpart 3′-NTIRE-10 located between the sequence element around the branch point A and the acceptor of the external intron (5′-GUUUUUAUAC) are present and can form the double-stranded RNA stem. Like in Xmsuc0006A (panel b), the center of the symmetry in Xlonc0025A is the G shared by the overlapping constituent introns of the stwintron and indicated by the vertical line symbol | in the consensus line underneath its diagnostic alignment.

**Fig 3:**
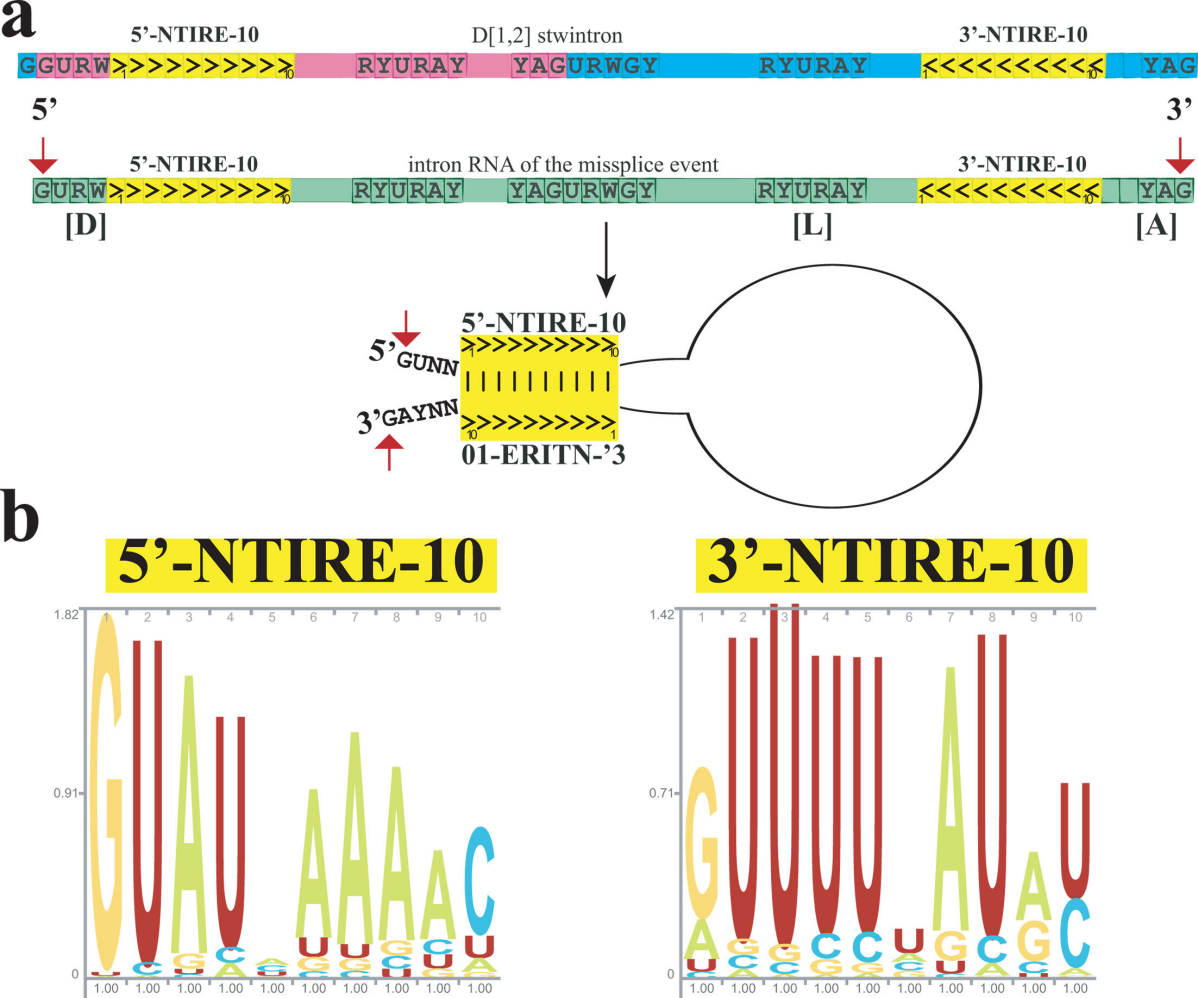
Missplicing retains the symmetry of the product intronic RNA. (**a**) Conventional [D1,2] stwintron splicing uses the internal and external (distal) splice sites according to intron definition and physically separates the stwintron’s inverted repeat elements in two product intron RNAs (magenta and turquoise, respectively), most of which themselves do not appear terminally symmetrical (see Data S1–S4). Missplicing retains the stwintron’s symmetry in a single product intronic RNA (green) after excision using only the distal splice sites, in contradiction to intron definition. A pair of a 5′ near-terminal inverted repeat element of 10 nt (5′-NTIRE-10) and its 3′-NTIRE-10 partner are indicated in the yellow boxes near each end of the RNA species that results from the missplicing event, while the > and < symbols clarify that these elements are inverted in sequence. The 6-nt core 5′-donor thus overlaps with the first 2 nt of the 5′-NTIRE-10. When the intron RNA folds back on itself after debranching, these two elements can base pair and form a double-stranded RNA stem. This double-stranded structure thus brings in close proximity the donor G_1_ of the internal intron of the original [D1,2] and the acceptor G_3_ of its external intron and may protect the intact intron RNA from rapid exonuclease degradation. Both G’s are highlighted with the red arrows for convenience. (**b**) Sequence logos of the 5′-NTIRE-10 and 3′-NTIRE-10 elements as they occur in the single-stranded (stw)intron RNA in *Xylariales* sister stwintrons. See Wheeler et al. (23) for technical information. The heights of the individual nt correspond to their frequency at each position.

**Fig 4:**
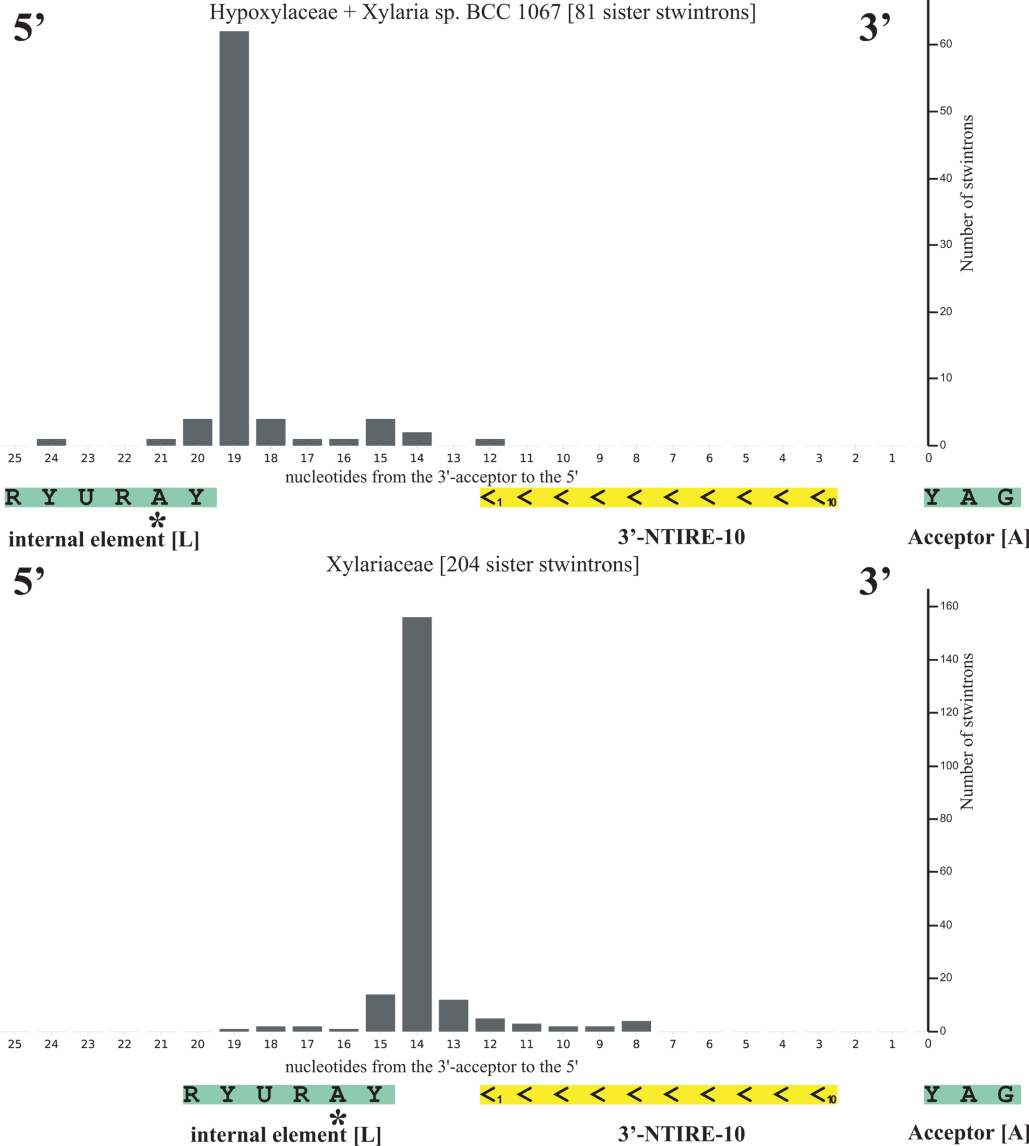
The distance between the sequence element around the branch point A [L] and the 3′-acceptor [A] of the terminally symmetrical misspliced intron of sister stwintrons is large enough to contain the entire 3′-NTIRE-10 element. The very same sequences shown are those of the original external intron of the underlying sister stwintron, too. The distance between the conserved internal sequence element (5′-NYUNAN: A is the branch point adenosine) and the associated 3′ acceptor (5′-HAG) in nucleotides is plotted against the number of sister stwintrons with a given distance. Individual distances per stwintron are found in Table S1. The plots are anchored at the 3′ splice site. The three relevant sequence elements are represented by the colored boxes underneath the horizontal axis. For the [D1,2] sister stwintrons in the *Xylariaceae* investigated, the distance is almost invariably 14 nt. The intermittent spacers are very short, 2 nt both 5′ and 3′ of the 3′-NTIRE-10 element. For the [D1,2] sister stwintrons in the *Hypoxylaceae* and those in *Xylaria* sp. BCC_1067, the distance is almost always 19 nt. The spacer with the 3′-acceptor is 2 nt, keeping the same short distance to the acceptor G_3_, while the spacer behind the consensus sequence around the branch point A is 7 nt in this second group of species. The conserved sequence element around the branch point A thus does not interfere directly with the formation of the NTIRE-10 stem structure (and vice versa). Both these distances between the internal sequence element around the branch point A and the associated 3′-acceptor appear to be considerably larger in the external introns of *Xylariales* sister stwintrons than in some other *Xylariales* U2 introns; for instance, in virtually all the internal introns of these 288 sister stwintrons, where this distance is too small to contain the entire NTIRE-10 element (Table S1).

Unlike in *Hypoxylon* sp. CO27-5 and EC38, the terminal symmetry is interrupted at several positions in both clusters of near-identical *Xylaria* sister stwintrons (Fig. 2). Nevertheless, an inverted repeat element of 10 nt is found very close to the termini of the misspliced introns and those of the underlying stwintrons in the pre-mRNAs, not only in all seven near-identical sister stwintrons (Fig. 2) but in at least 14 other sister stwintrons investigated (schematically summarized in Fig. 3). We have named this small element, the near-terminal inverted repeat element of 9 or 10 nt, NTIRE-9 or NTIRE-10. There appears to be a correlation between the presence of the 3′-repeat (3′-NTIRE) at its subterminal location and the distance between the two conserved sequence elements near the (stw)introns 3′ involved in excision, the sequence element around the branch point A and the associated 3′-acceptor (Fig. 4). Many U2 introns in the investigated 14 species cannot contain an intact 10-nt-long 3′-NTIRE between their conserved sequence element including the branch point A and its associated 3′-acceptor, because the distance between these conserved intron splicing elements is shorter than 10 nt. This is the case for virtually all internal introns of the investigated sister stwintrons (see Table S1).

### Fully complementary sequence variants of NTIRE-10 and near-identical stwintron copies

The two clusters of near-identical stwintrons are widely divergent in maximum likelihood phylogenies of sister stwintrons in *Xylariaceae* species (Fig. S1). Over their complete width, the three *X. longipes* stwintrons are considerably less similar (~75% identity) to the four MSU SB201401 stwintrons. However, the sequence of the 5′-NTIRE-10 is identical in the seven near-identical stwintrons from *X. longipes* and *Xylaria* sp. MSU SB201401 (Fig. 2). In the four MSU SB201401 stwintrons, the sequence of the 3′-NTIRE-10 is 100% complementary to the 5′-NTIRE-10, i.e., all 10 ribonucleotides base pair due to the occurrence of non-Watson-and-Crick GU interactions. The two NTIRE-10 sequences are fully complementary as DNA in the three stwintrons in *X. longipes* (Fig. 2). In *Hypoxylon* sp. CO27-5 and EC38, there is a third homogeneous group of near-identical intronic sequences, the type-2 cropped sister introns of regular intron size (cf. [9]). Of these, HCOc105A, HCOc153A, and HCOc171A contain the exact 5′- and 3′-NTIRE-10 present in the *X. longipes* near-identical stwintrons. Both elements are also identical in sequence in the type-2 cropped sister intron HECc321B, which is unique to EC38. We have argued (9, 10) that such EC38-specific intronic sequences show ongoing formation of new (stw)introns in the taxon, generated after the recent separation from CO27-5. These observations in three different clades of near-identical (stw)introns seem to suggest that 5′-GUAUAAAAAC and either of its fully complementary 3′-NTIRE-10 partners are at least facilitating duplication of competent [D1,2] sister stwintrons in the *Xylariales* order.

Further analysis of the secondary RNAfold structures and the diagnostic alignments of the stwintron’s sequence with their own reverse complement (Data S1 and S4) implies that there are 25 intronic sequences that feature 100% complementary, fully base pairing NTIRE-10 partner elements. Their ribonucleic sequences are listed in Table S3. Four of them are *Hypoxylon* sp. strain CO27-5/EC38 type-2 cropped sister introns, which directly derive from a sister stwintron (9, 10). Remarkably, 10 of the 21 sister stwintrons occur in MSU SB201401, albeit with sequence variations in one of the NTIRE-10. Two of the 10 stwintrons have a third alternative fully complementary 3′ NTIRE-10 (5′-GUUUUUAUAU, Table S3). Three more MSU SB201401 stwintrons have a fully complementary pair, with a sequence variant in the 5′-NTIRE-10 instead (Table S3). On the other hand, *Xylariaceae* sp. FL1651 (X1651c156A) has one fully complementary couple of NTIRE-10 (5′-GUAUAAAAGC; 5′-GCUUUUAUGU), with a compensating point mutation in one of the elements (Table S3). *Xylaria bambusicola* also features one stwintron (Xbamc159A) with a fully complementary couple of NTIRE-10 with two compensatory point mutations (5′-GUAUUAAAAU; 5′-AUUUUGAUAU; see Table S3). In *Hypoxylaceae* other than CO27-5 and EC38, there are fully complementary NTIRE-10 partners in two *Hypoxylon rubiginosum* stwintrons and in four *Daldinia childiae* stwintrons (Table S3). There are a further 17 fully complementary 9-nt long elements (NTIRE-9), eight of them in *X. bambusicola* and four of them in MSU SB201401. We analyzed the constituent introns of the sister stwintrons (see Table S1 for sequences). Most constituent introns do not appear terminally symmetrical, i.e., not involving the local NTIRE element near one of their termini (Data S1–S4). Where terminal symmetry was found by both methods, it concerns mostly the external intron (Data S3 and S4). The external intron has sufficient space between [L] and [A] for the complete 3′-NTIRE element.

Remarkable about the complementary NTIRE-10 elements in the mentioned near-identical sister (stw)introns is that the subterminal stem structure consists mostly of weaker AU and GU pairs. However, our observations and comparisons do not rule out that the secondary stem structure, rather than its primary structure, is a crucial element to enhance [D1,2] stwintron duplication.

## DISCUSSION

Almost 48 years after their first description (36, 37), the mechanisms driving the continuous generation of new introns at previously unoccupied gene positions remain to be elucidated. A number of non-mutually exclusive mechanisms have been proposed, including mechanisms involving transposition or double-stranded DNA break (DSB) repair (for a review, see reference 38). Our current work contributes to the understanding of mechanisms of stwintron gain in fungi by investigating the occurrence and apparent duplication of sequence-similar spliceosomal twin introns. Contemporary work by Gozashti et al. (35) describes a formidable bioinformatics search for sequence-similar introns across the eukaryotic domain using publicly available gene-annotated whole genome sequences (NCBI). In a broadening of the concept of the introner—originally coined by Worden et al. (39)—all introns with sequence similarity across their complete width are now termed introners, also when they exhibit characteristics of transposable elements. Analysis of thousands of introners present in 175 carefully vetted gene-annotated genome sequences allowed a classification in 548 species-specific families of at least five introners, “most” of which support a mechanism of intron propagation and proliferation involving a specialized non-autonomous DNA transposon. These transposons and the corresponding introners exhibit typical terminal inverted repeats (TIR). This mechanism—first described by Huff et al. (34)—proceeds without RNA intermediates. The transposon carries at one end a sequence that resembles an intron splice site, i.e., either the 5′-donor or the 3′-acceptor. The other splice site is co-opted from the TSD sequence, a small direct repeat in the bordering exons which typically results from transposon integration at its new location and consequently is different for each new integration. However, the necessity to co-opt the opposite splice site restricts the exact gene locations where the transposon can successfully maintain itself as an intron, i.e., the local exonic sequence is biased at at least one end of the new intron.

Here, we identified 288 sequence-similar stwintrons in 14 different taxa of *Hypoxylaceae* and *Xylariaceae*, using proven *Hypoxylon* sp. CO27-5 sister stwintrons as initial queries in a simple reciprocal blast-driven strategy, not unlike the mining strategy from reference 35. We looked for stwintrons that were not reported by Gozashti and co-workers (35) despite the sometimes considerable sequence similarity between sister stwintrons across the complex intronic sequence. Gozashti et al. (35) mention that fungal or ascomycete introner families are outliers, with no TIRs or TSDs. The integration sites of our 288 stwintrons and the cropped sister introns that directly derive from a sister stwintron seem to be seamless and random; despite the clear presence of TIRs, there is no evidence for TSDs crucial for splice site co-option. Moreover, there is no nucleotide bias in the exons immediately bordering at both 5′ and 3′ (Fig. S3). It seems unlikely that the mechanism to duplicate stwintrons and stwintron-derived cropped sister introns (in *Hypoxylon* sp. CO27-5 and EC38) involves the specialized DNA transposon presented by Huff et al. (34), although our data do not explicitly rule it out. In the mechanism we proposed for the propagation of symmetrical stwintrons in *Hypoxylon* sp. CO27-5 and EC38 (9), the actual integration site is “determined” by DSB, during which repair intact intron RNA is ligated into the break to temporarily tether the broken DNA ends and is accidentally not removed afterward and converted into DNA. A disadvantage of our hypothetical mechanism is that for stable integration, the stwintron requires an additional G to be inserted in the break (either at 5′ or 3′) to allow consecutive removal of the constituent introns of the stwintron without affecting the resulting mRNA. However, our model can account for the occasional formation of new canonical introns at random sites in genes without the need for such additional nt. Indeed, we found that type-2 cropped sister introns that derive directly from a parent [D1,2] sister stwintron by internal deletion propagate as canonical introns integrated at random exonic locations (9). In principle, any intact intron RNA can be inserted into a DSB and result in a stable new intron at a completely random gene position, including inside a pre-extant intron.

We observed symmetric elements at less than 5 nt from the terminal G’s on both sides of the intronic RNA excised by a missplicing of [D1,2] sister stwintrons from pre-mRNA in which the spliceosome is assembled at the distal splice sites, ignorant of the internal splice sites of the stwintron. These small TIRs are shared by the two clusters of near-identical sister stwintrons (>97% identity) in *Xylaria longipes* and in *Xylaria* sp. MSU SB201401, two not closely related taxa. These seven [D1,2] stwintrons occur in completely different genes. The two near-terminal inverted repeat elements of 10 nt length (NTIRE-10) next to each extremity in these near-identical sister stwintrons, 5′-NTIRE-10 and 3′-NTIRE-10, are 100% complementary in their RNA sequence. They can base pair to form a near-terminal stem structure and bring and keep in close proximity the two terminal Gs. The near-terminal stem may protect the excised and debranched intron RNA against rapid exonuclease degradation. The very same NTIRE elements are found in four type-2 cropped sister introns in *Hypoxylon* sp. CO27-5 and EC38. It would appear that the complementarity at the RNA level is associated with recent [D1,2] stwintron duplication. This provides circumstantial evidence for the involvement of an RNA intermediate in [D1,2] stwintron propagation.

## Data Availability

Data are contained within the article and the associated supplemental material.

## SUPPLEMENTAL MATERIAL

Data S1 spectrum.02926-24-s0001.pdf Collection of the RNAFold predicted secondary structures of 288 [D1,2] sister stwintrons in 14 taxa of Xylariales.

Data S2 spectrum.02926-24-s0002.pdf Collection of the RNAFold predicted secondary structures of the internal introns of the 288 [D1,2] sister stwintrons.

Data S3 spectrum.02926-24-s0003.pdf Collection of the RNAFold predicted secondary structures of the external introns of the 288 [D1,2] sister stwintrons.

Data S4 spectrum.02926-24-s0004.pdf Collection of the one-on-one sequence alignments of the 288 stwintrons and of their constituent U2 introns, with their respective reverse complement sequences.

Supplemental material spectrum.02926-24-s0005.pdf Fig. S1 to S3; Tables S2 and S3.

Table S1 spectrum.02926-24-s0006.xlsx Identifiers, localization, statistics, and other information concerning 288 sequence-similar sister stwintrons.

ASM does not own the copyrights to Supplemental Material that may be linked to, or accessed through, an article. The authors have granted ASM a non-exclusive, world-wide license to publish the Supplemental Material files. Please contact the corresponding author directly for reuse.
