# Supplementary material for: Propagation of [D1,2]-type spliceosomal twin introns (stwintrons) in Hypoxylaceae and Xylariaceae fungi
Source: Microbiol Spectr. 2025 Aug 8;13(9):e02926-24. doi: 10.1128/spectrum.02926-24 (PMC12403724; doi:10.1128/spectrum.02926-24)

Supplementary datafile S1. Collection of the RNAFold predicted secondary structures of 288 [D1,2] sister stwintrons in 14 taxa of Xylariales. The taxa are listed in Table 1 of the main text. The sequence of each stwintron is given in Table S1. The internal intron interrupts the 5'-donor of the external intron between its first and second nt ([D1,2]). The 5'-donor (6-nt core) [D] and 3'-acceptor (3-nt core) [A] elements at the splice sites of the constituent U2 introns are marked with circles around the nt. The conserved sequence element including the branch point adenosine [L] (6-nt core) near the 3'-splice site is likewise highlighted. These three conserved intronic sequence elements are indicated magenta for the internal intron- and turquoise for the external intron, as in Figure 2 of the main text. In the structures, the location of the NTIRE-9 or NTIRE-10 repeats fully complementary as RNA, is highlighted by the green line.

Dchc001A - Stwintron

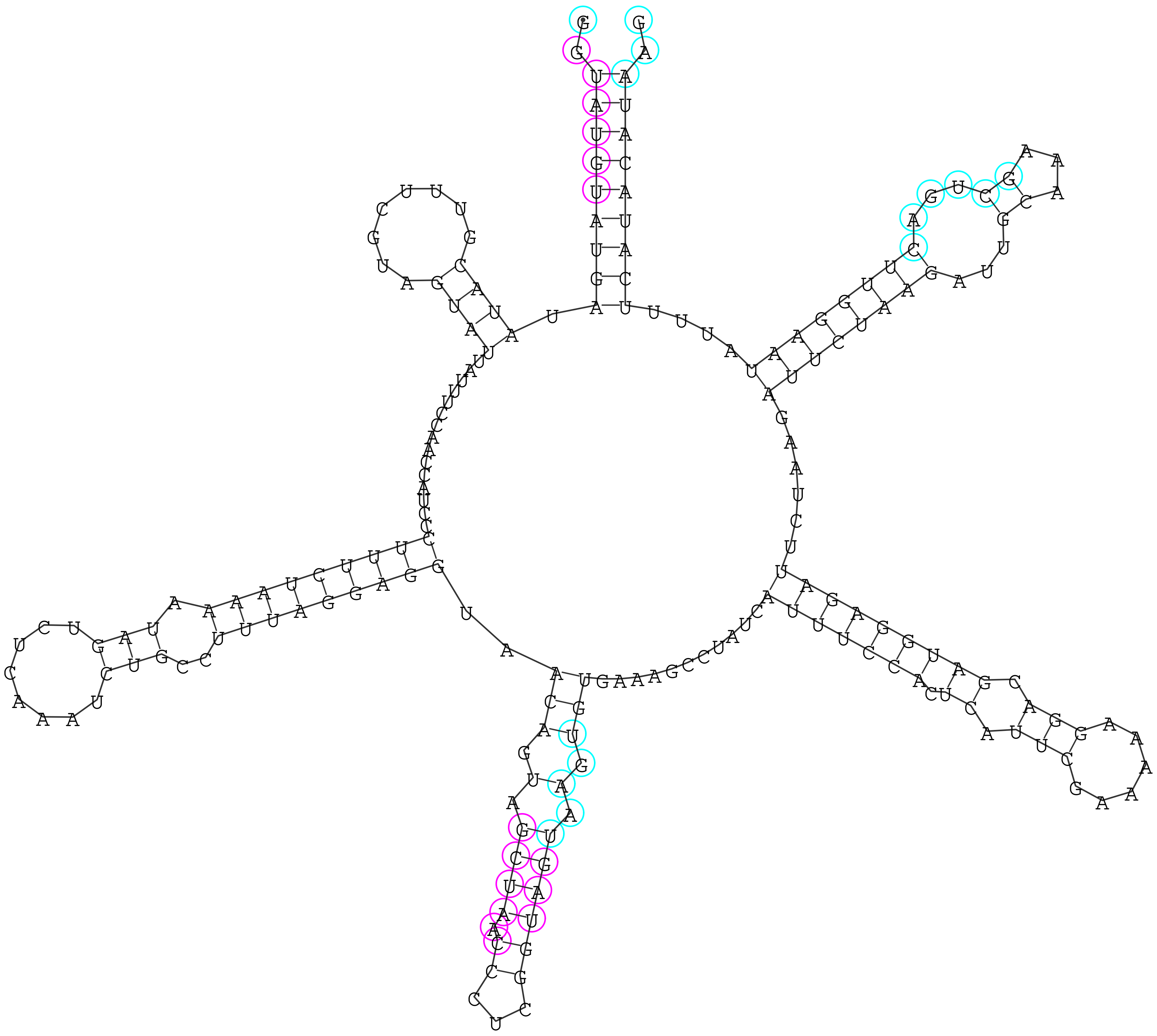

Dchc001B - Stwintron

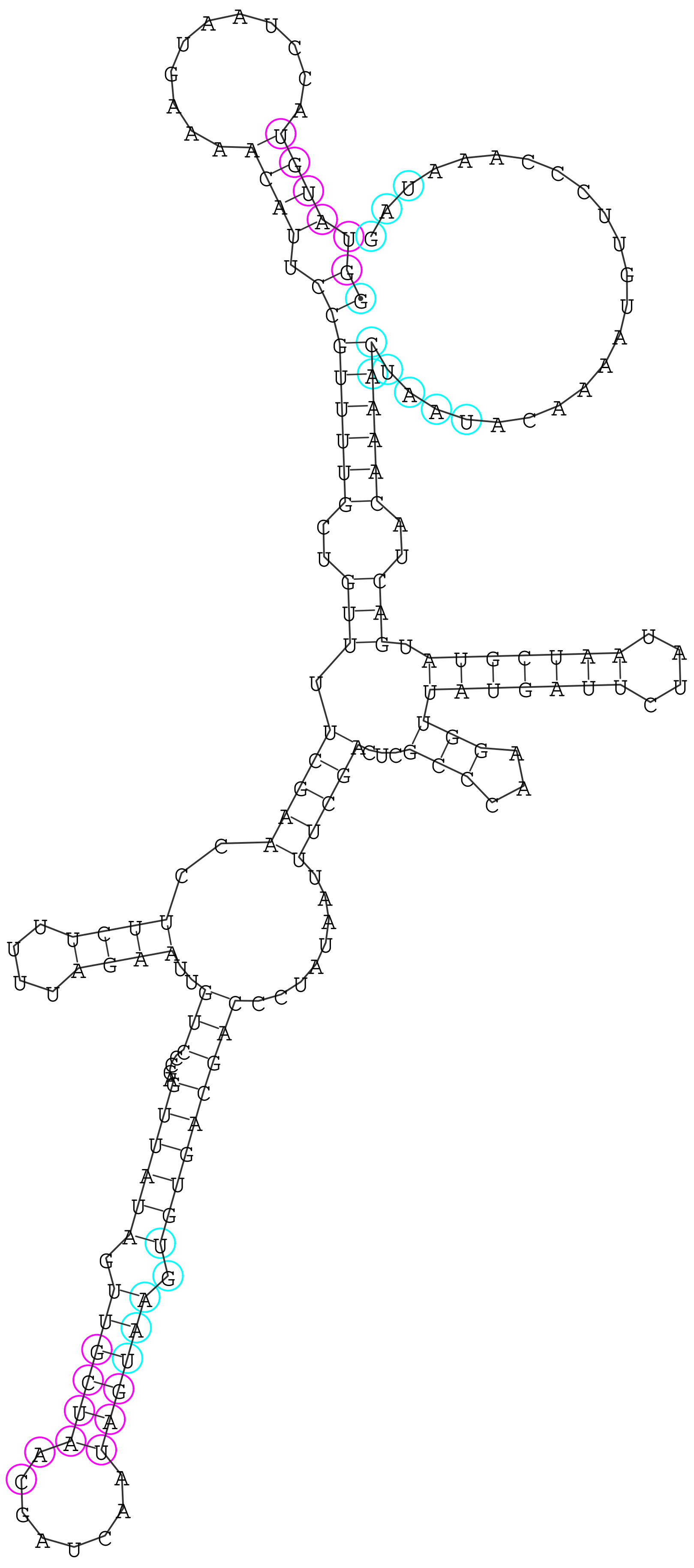

Dchc001C - Stwintron

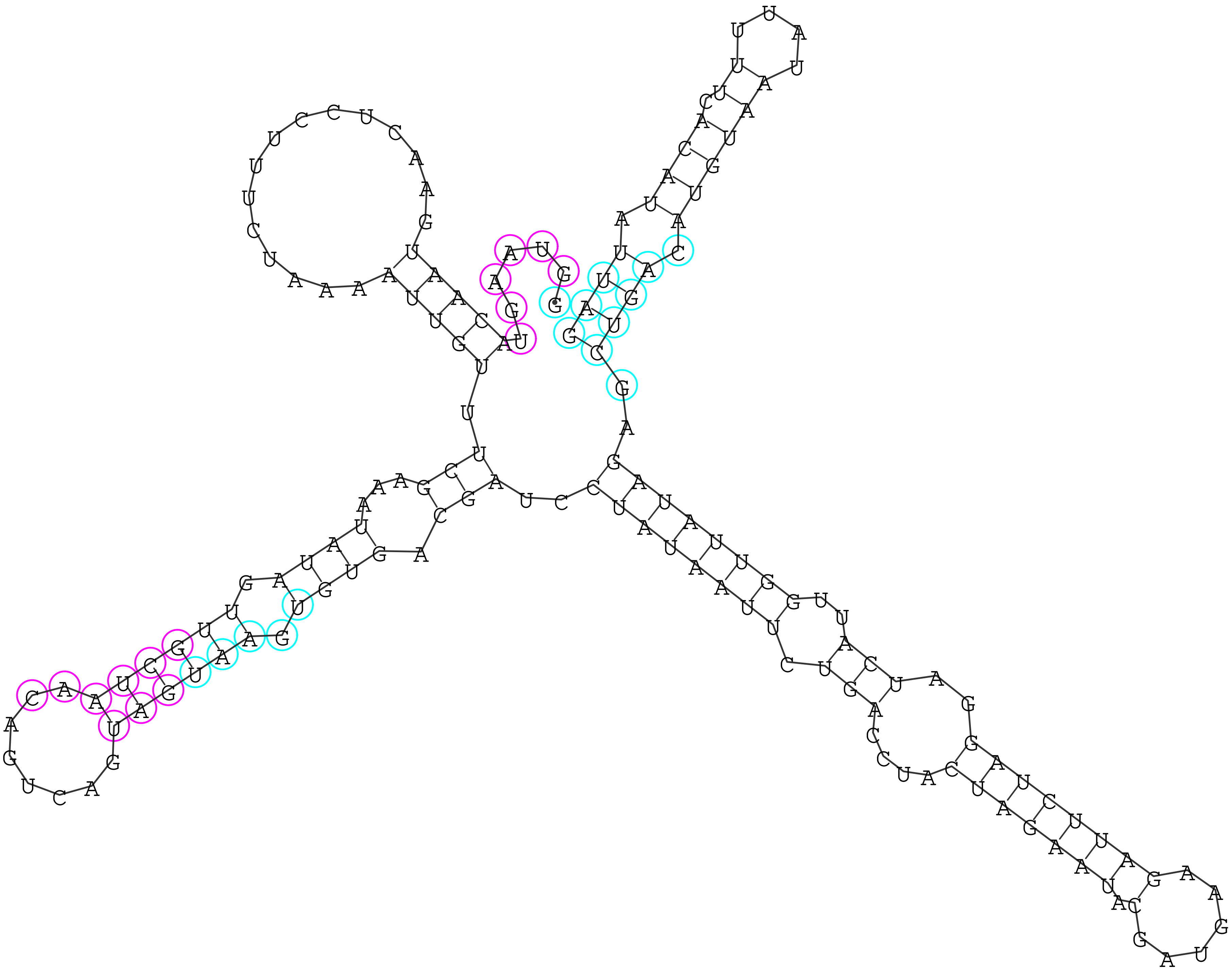

Dchc001D - Stwintron

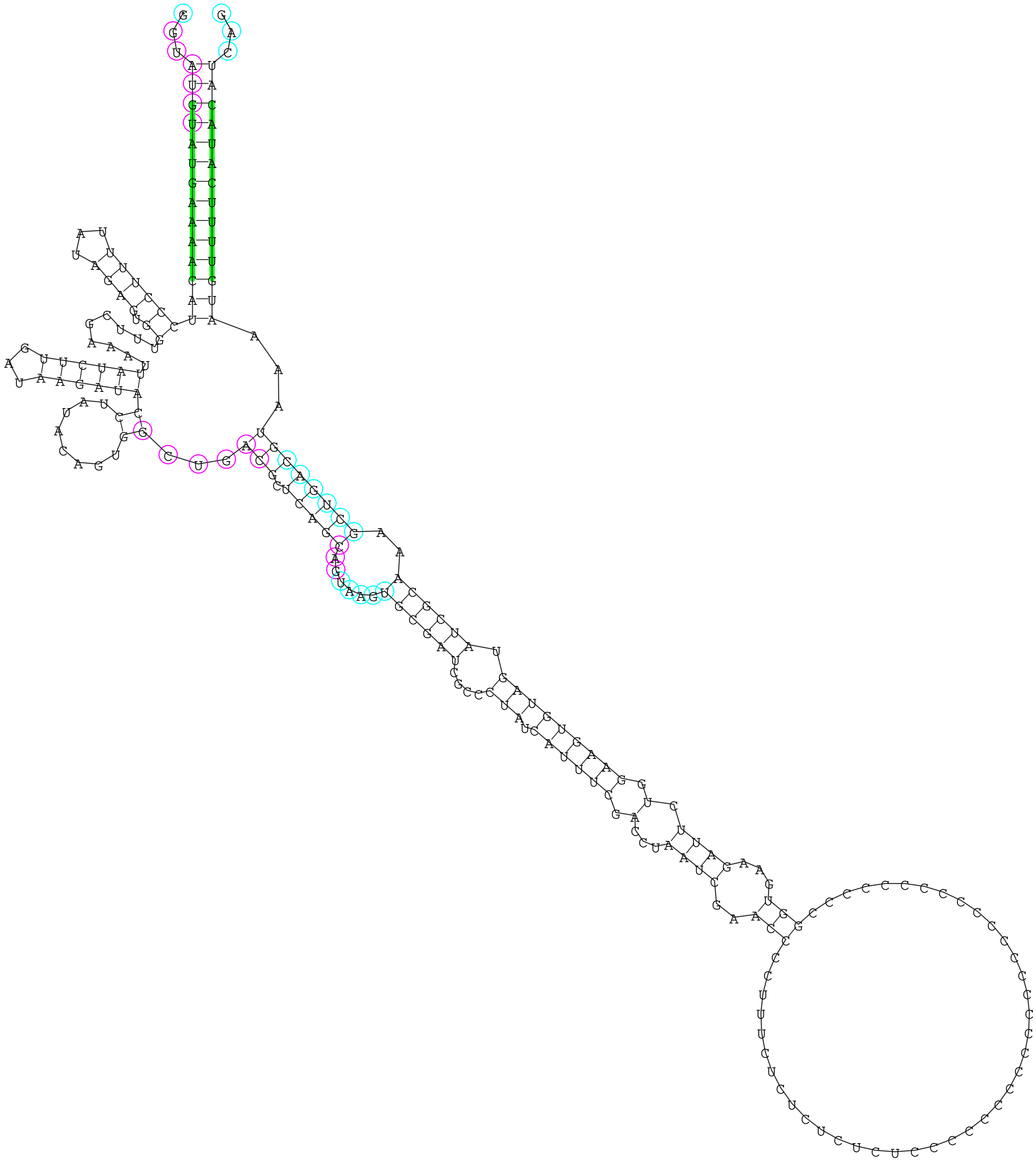

Dchc002A - Stwintron

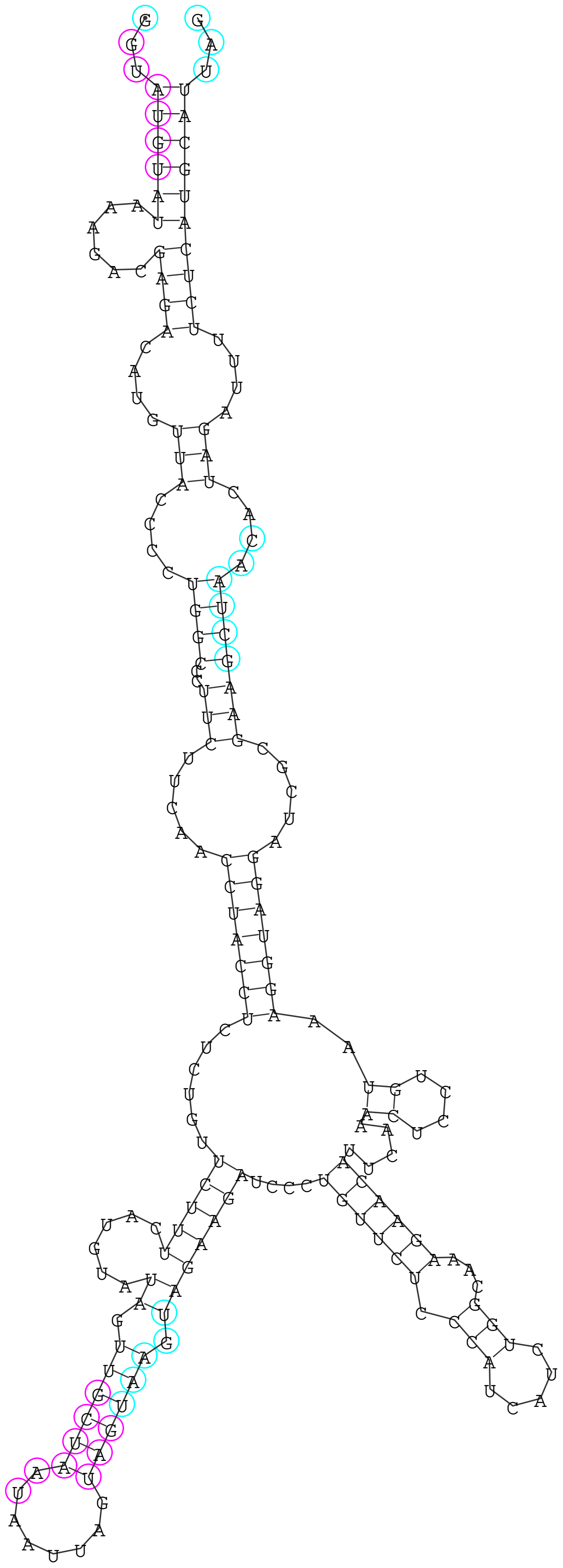

Dchc003A - Stwintron

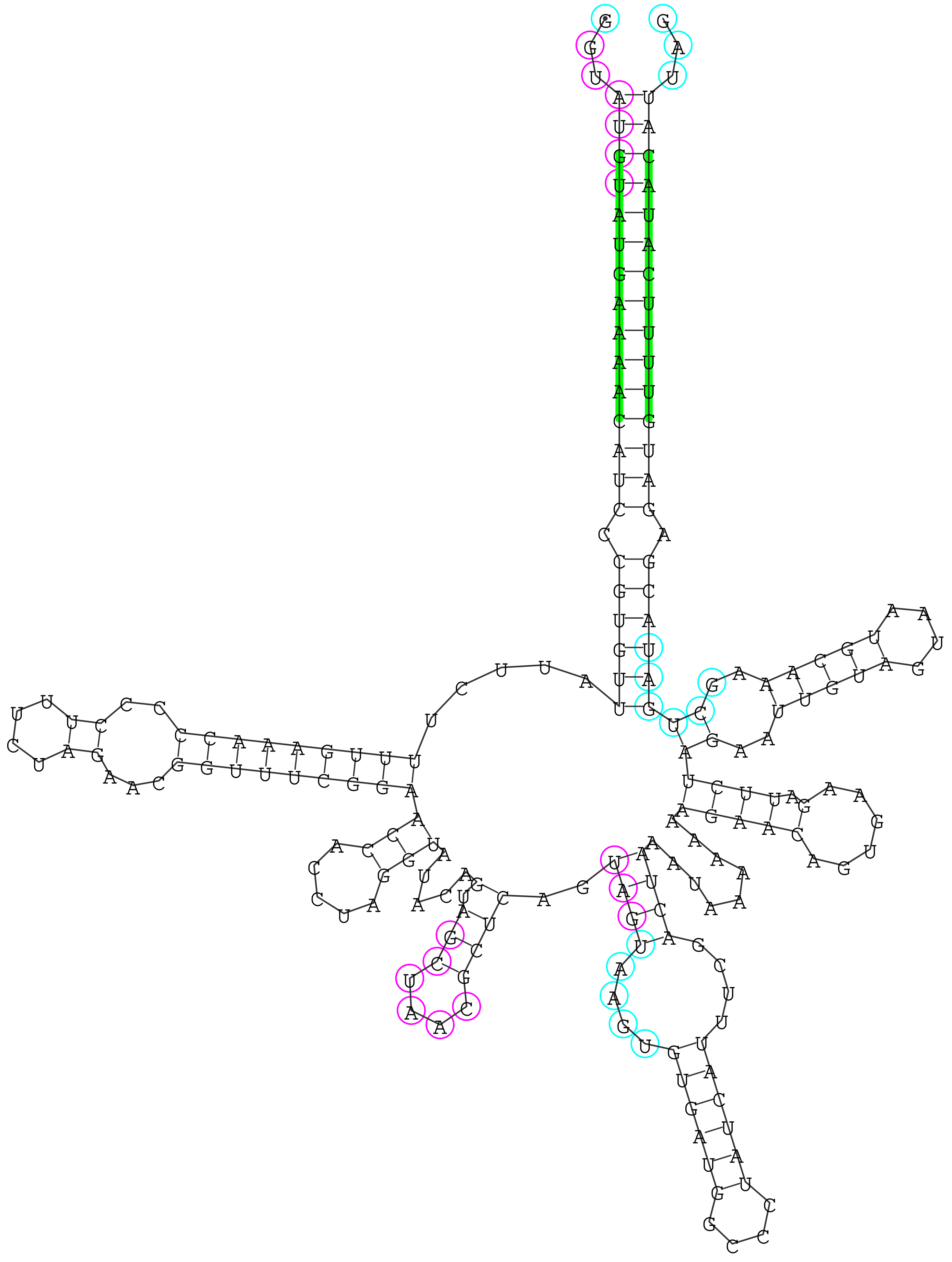

Dchc003B - Stwintron

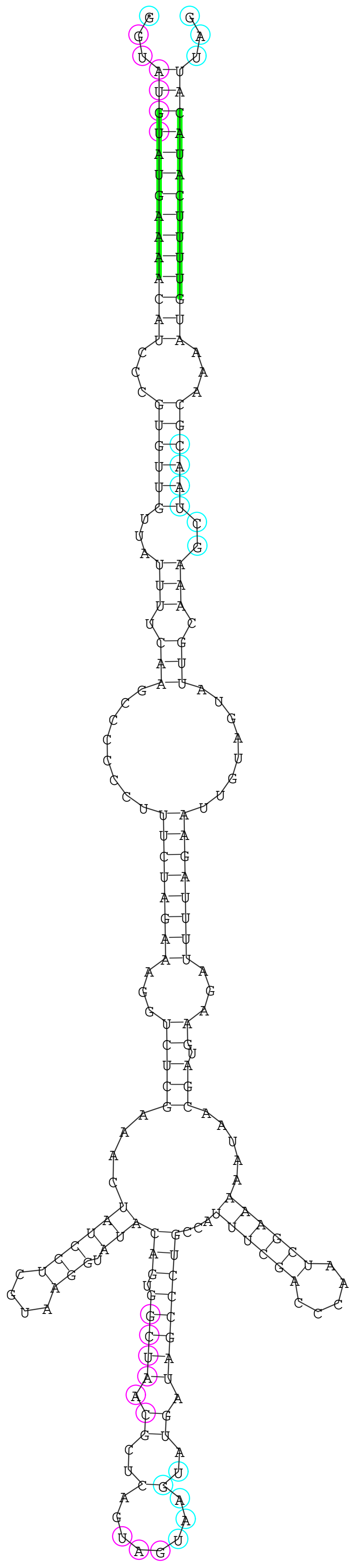

Dchc003C - Stwintron

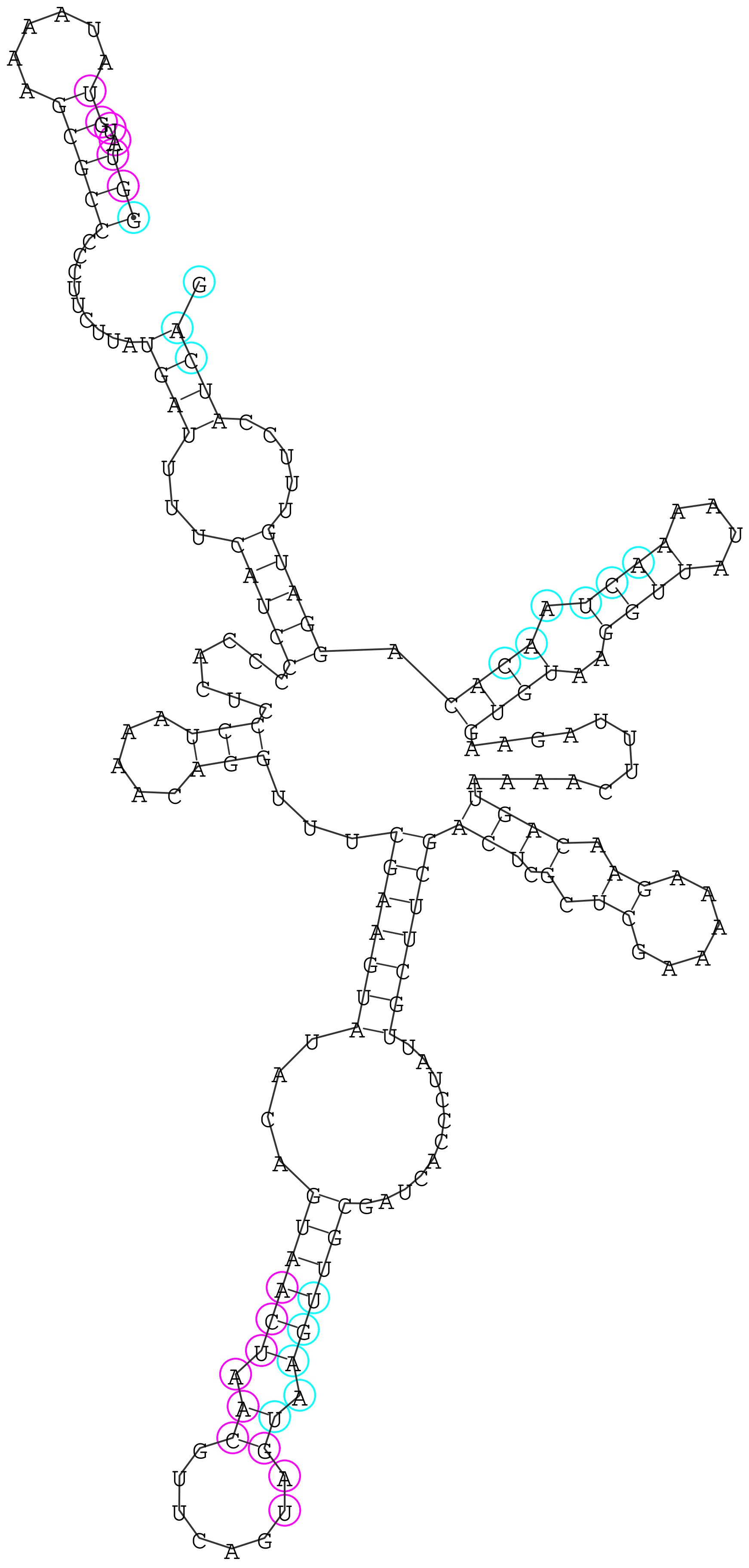

Dchc004A - Stwintron

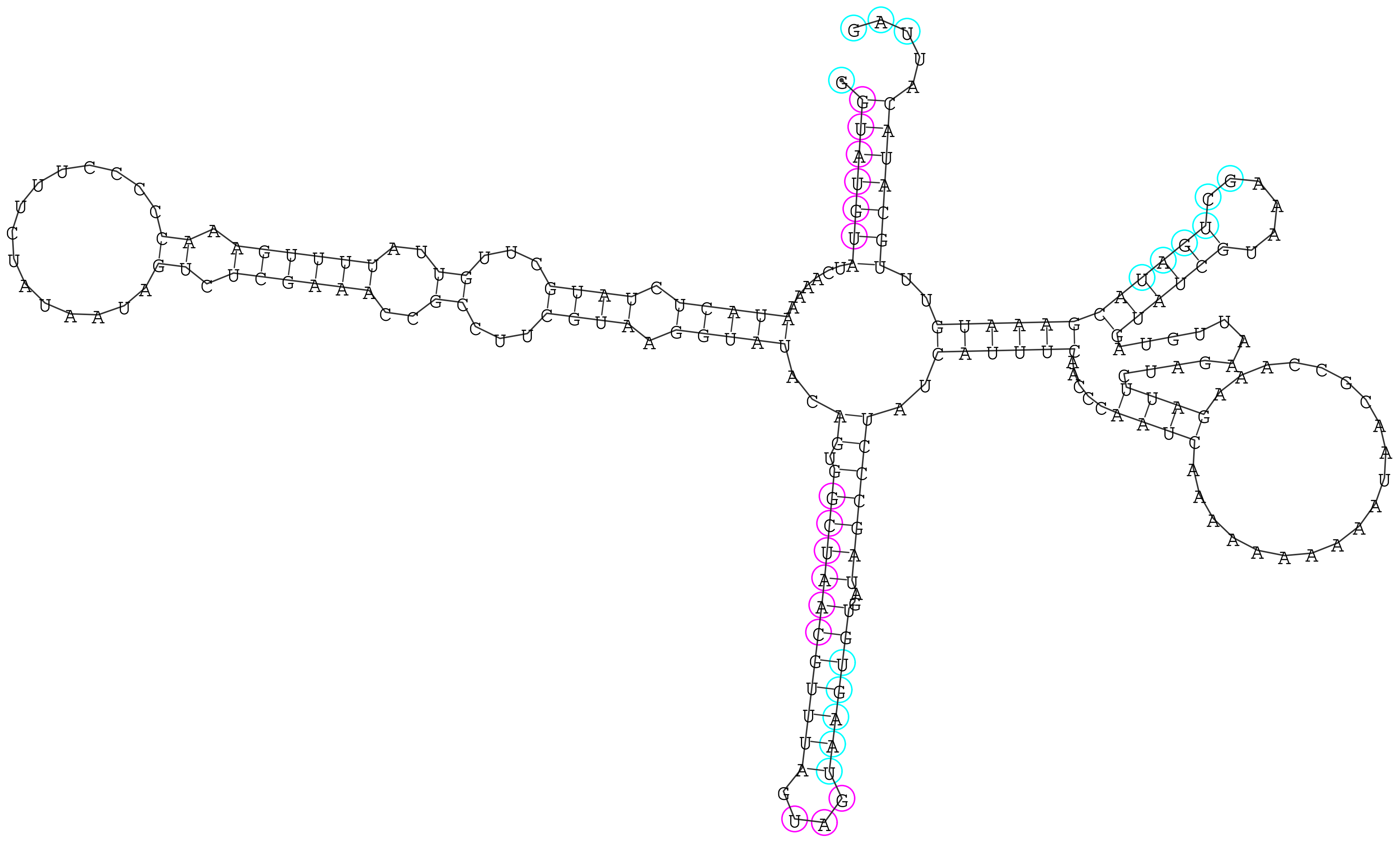

Dchc004B - Stwintron

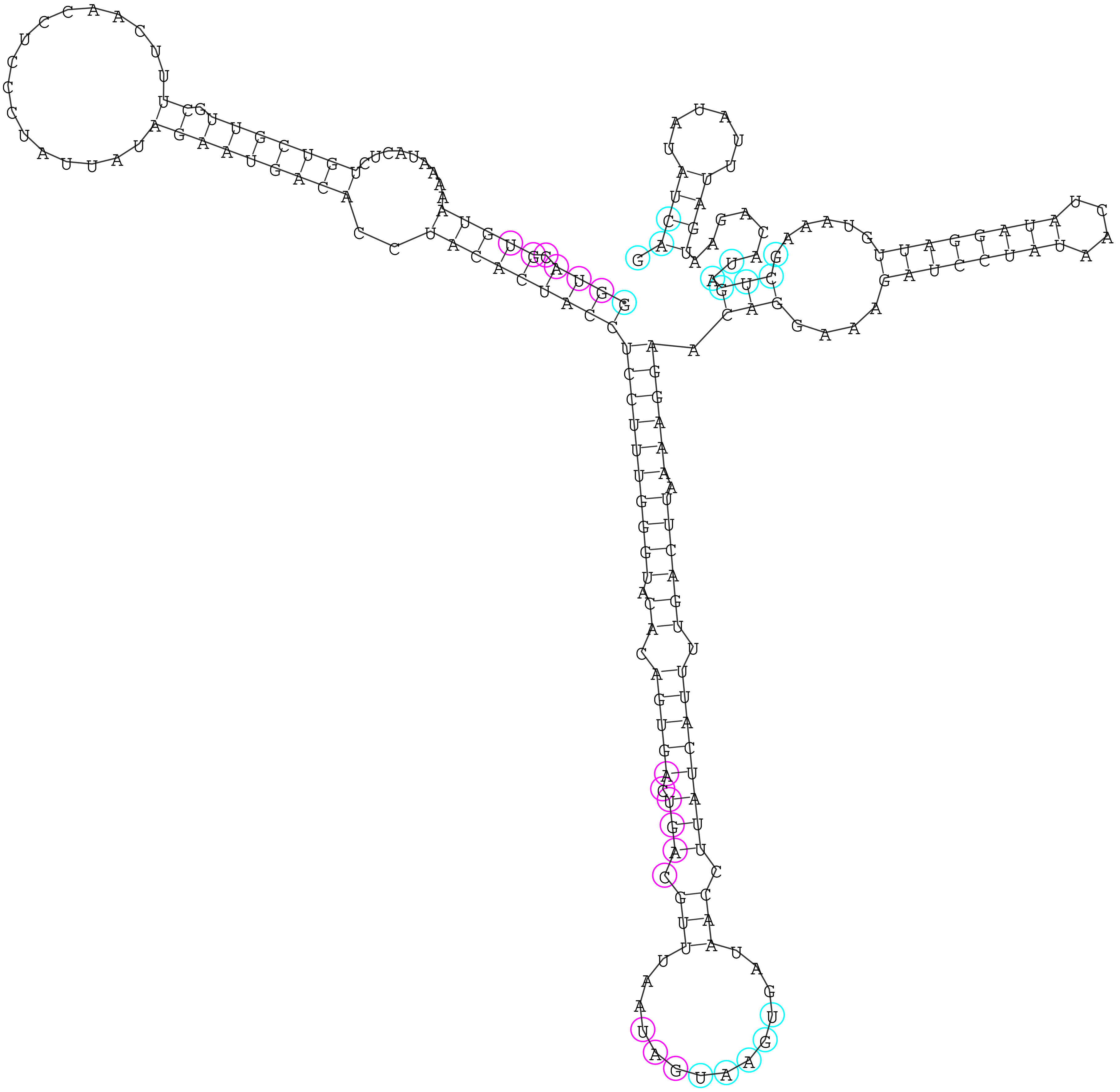

Dchc004C - Stwintron

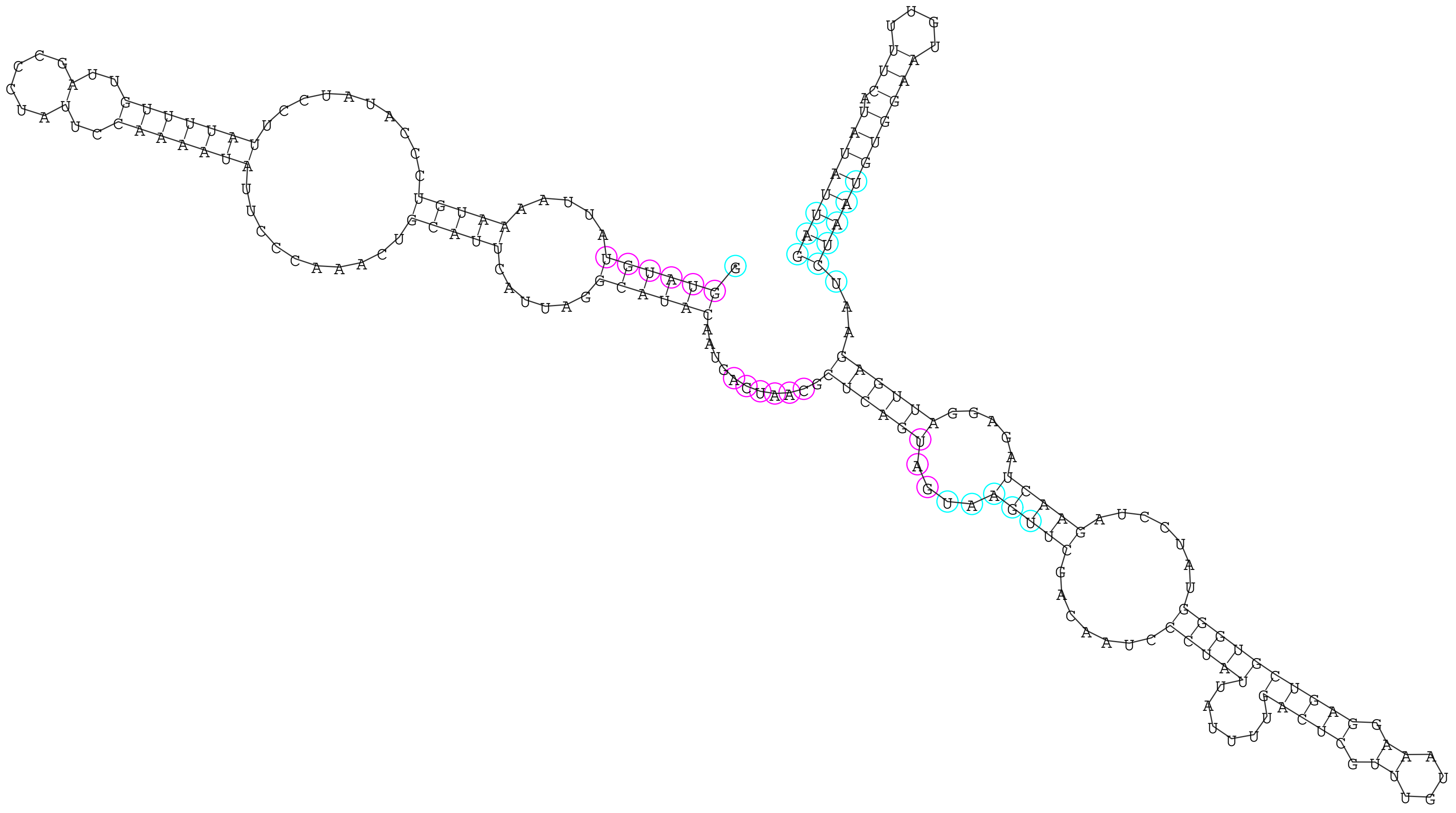

Dchc005A - Stwintron

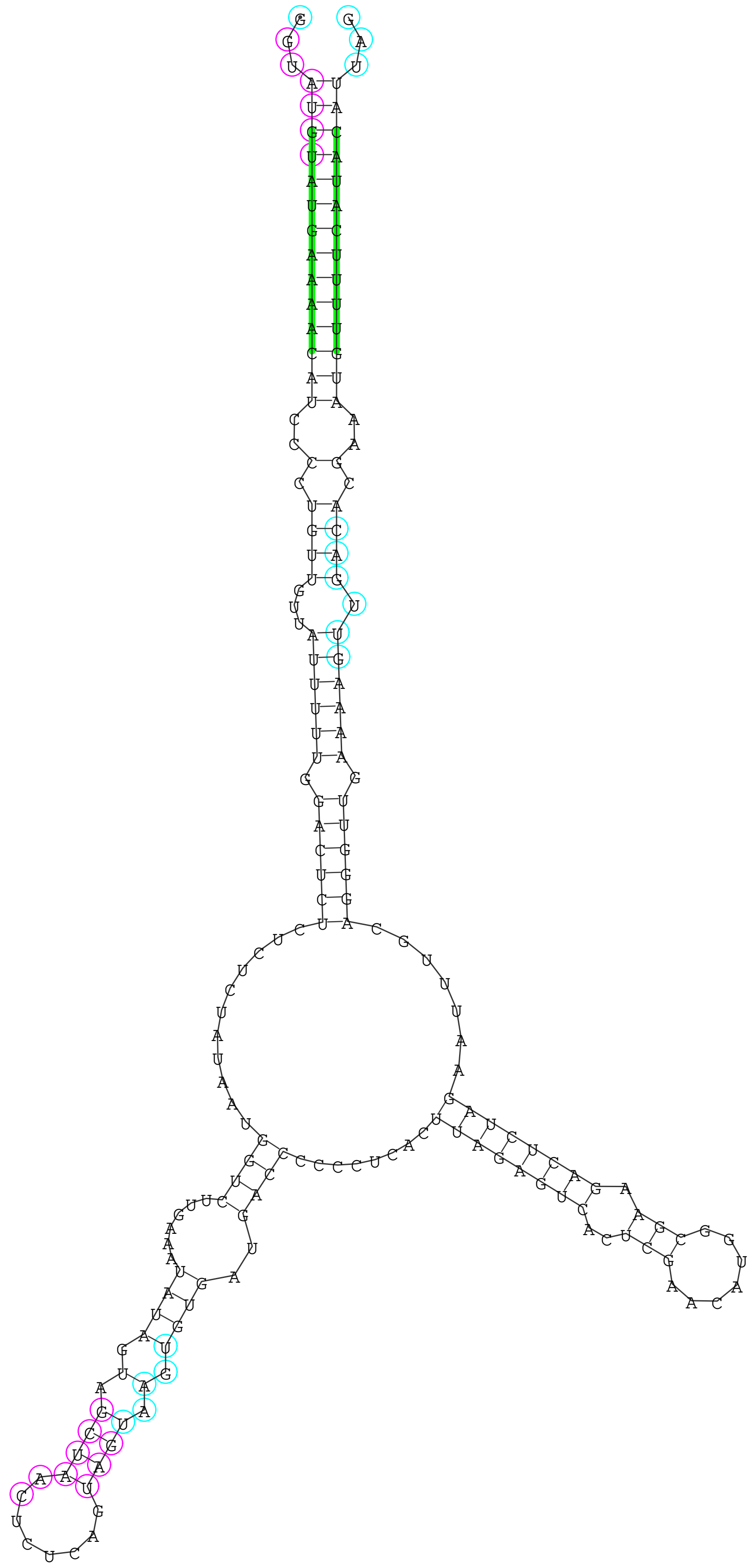

Dchc005B - Stwintron

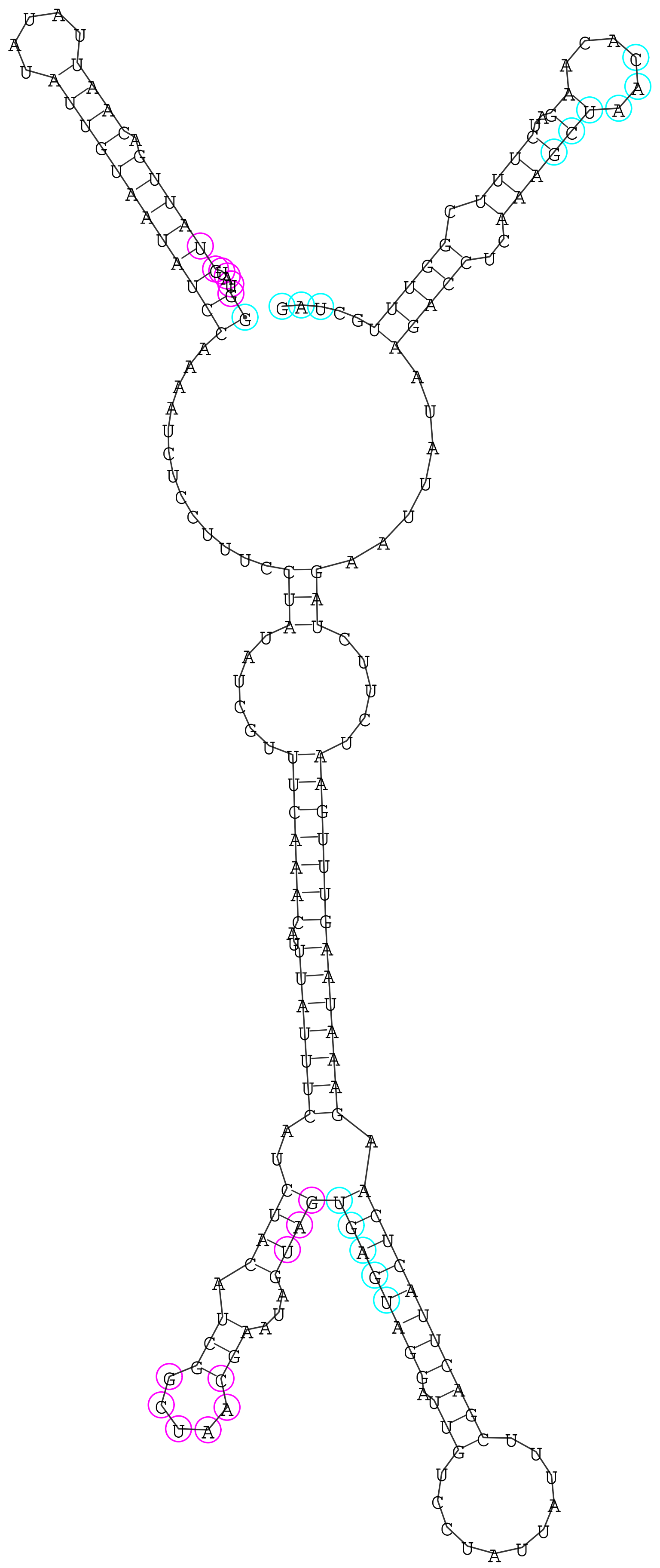

Dchc007A - Stwintron

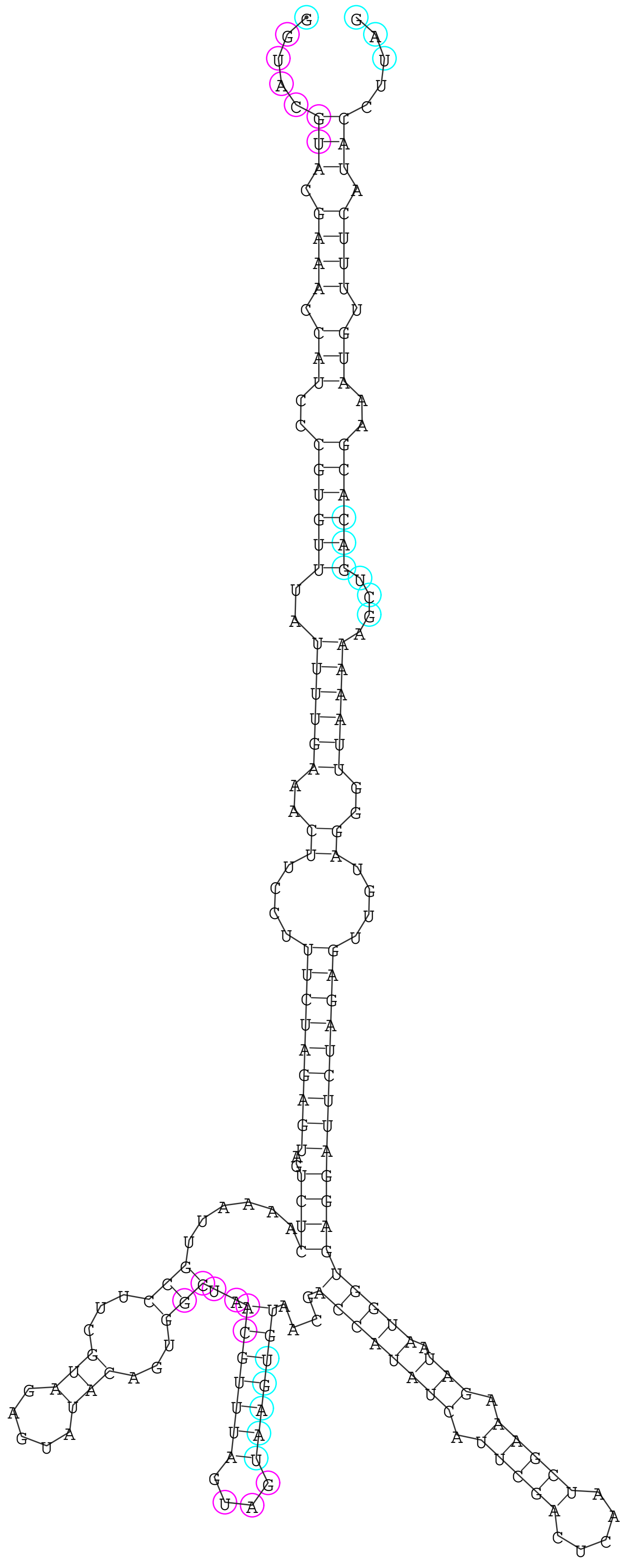

Dchc007B - Stwintron

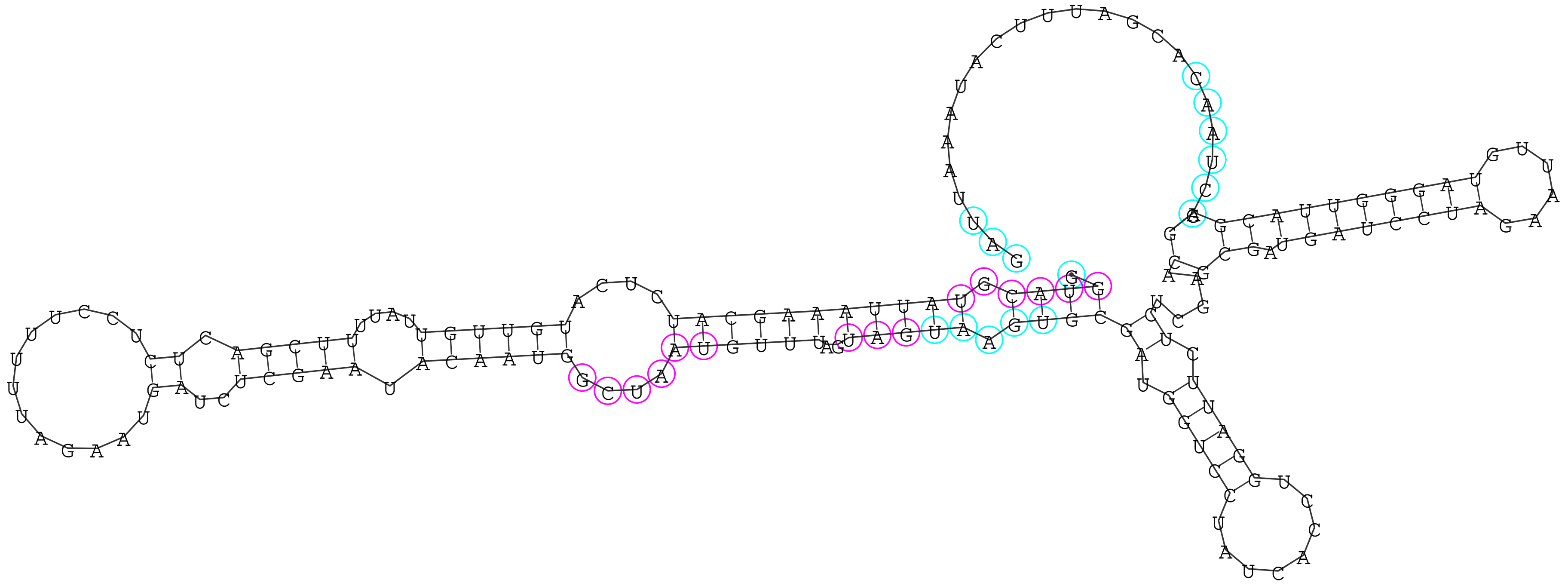

Dchc008A - Stwintron

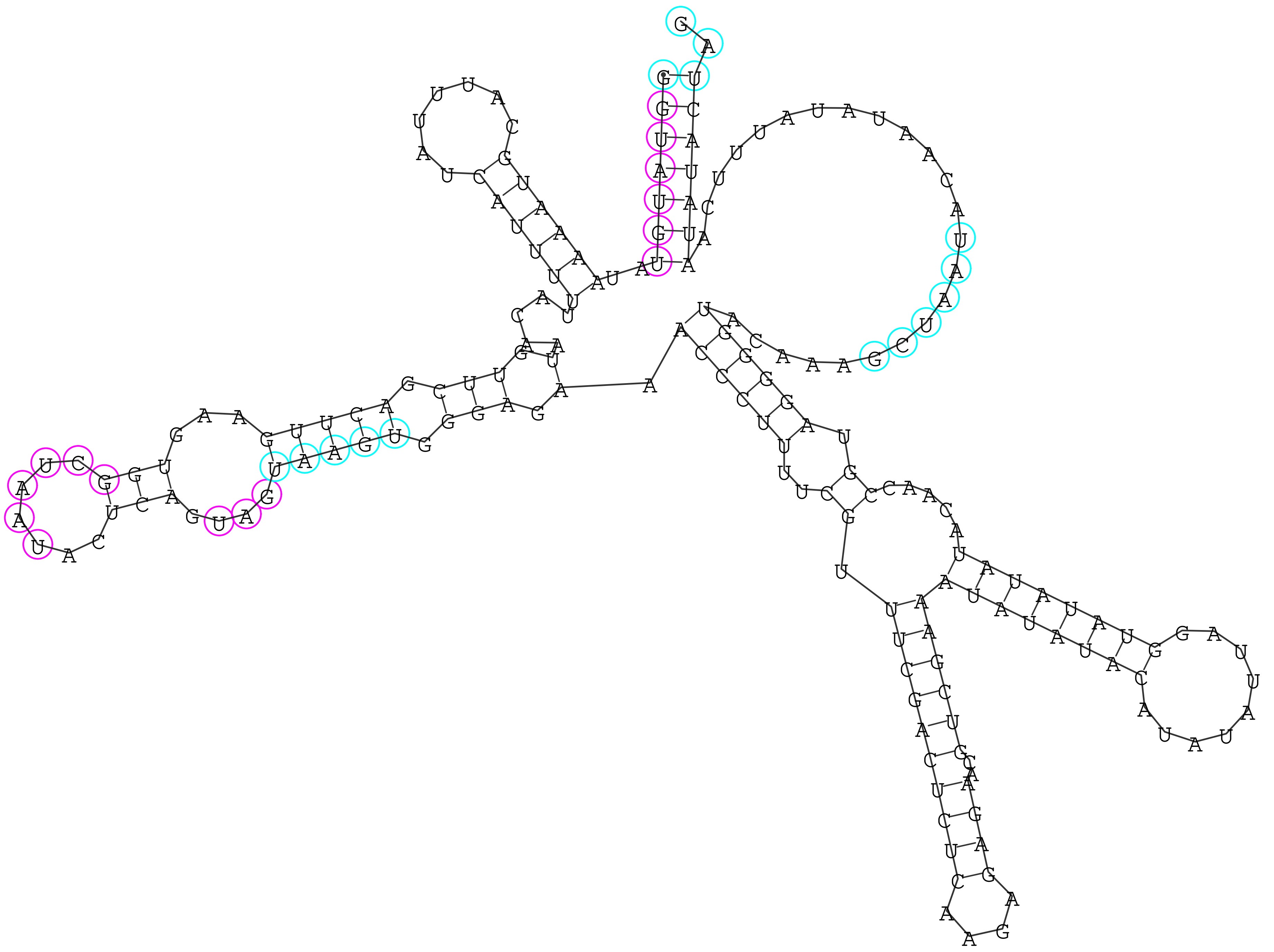

Dchc011A - Stwintron

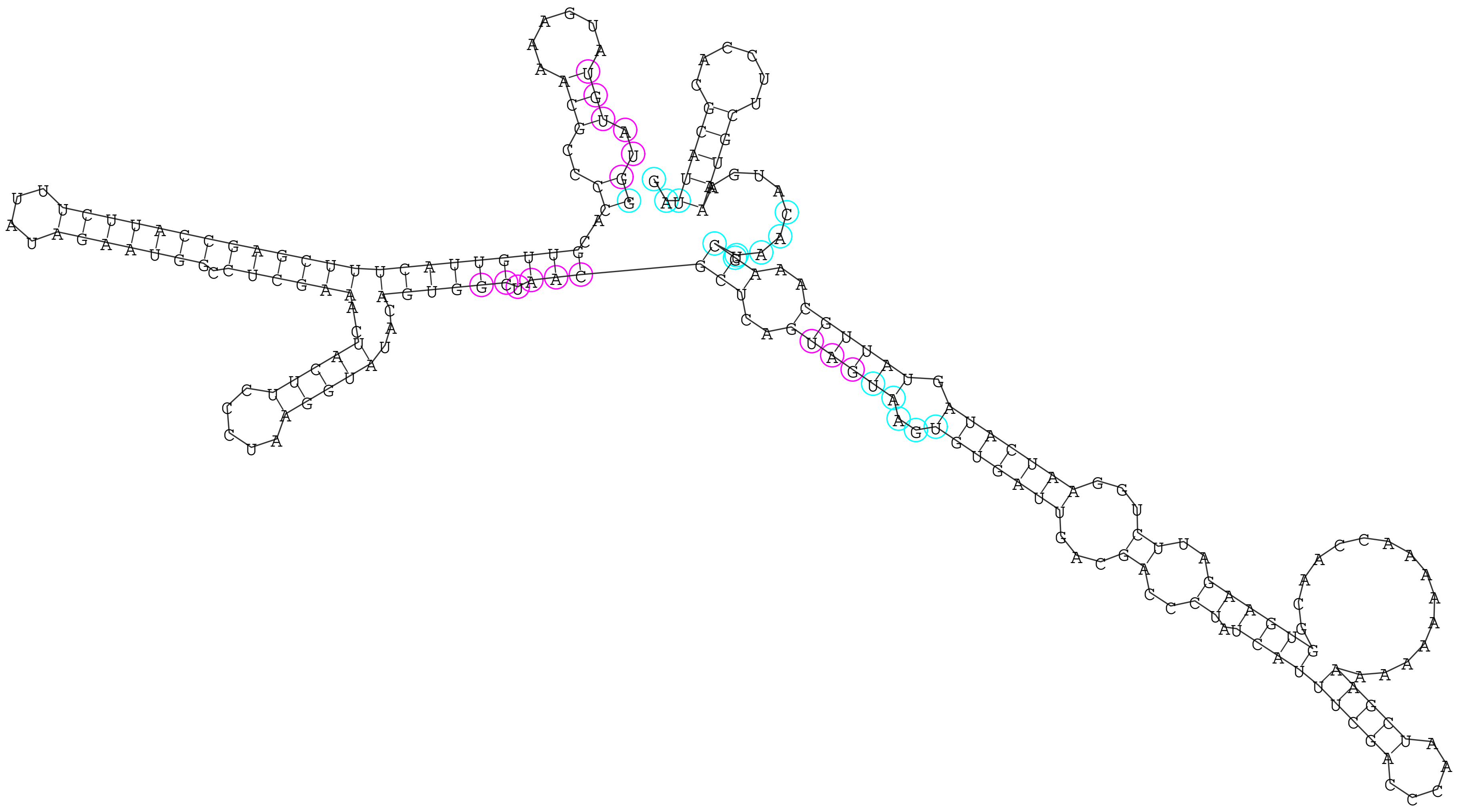

Dchc013A - Stwintron

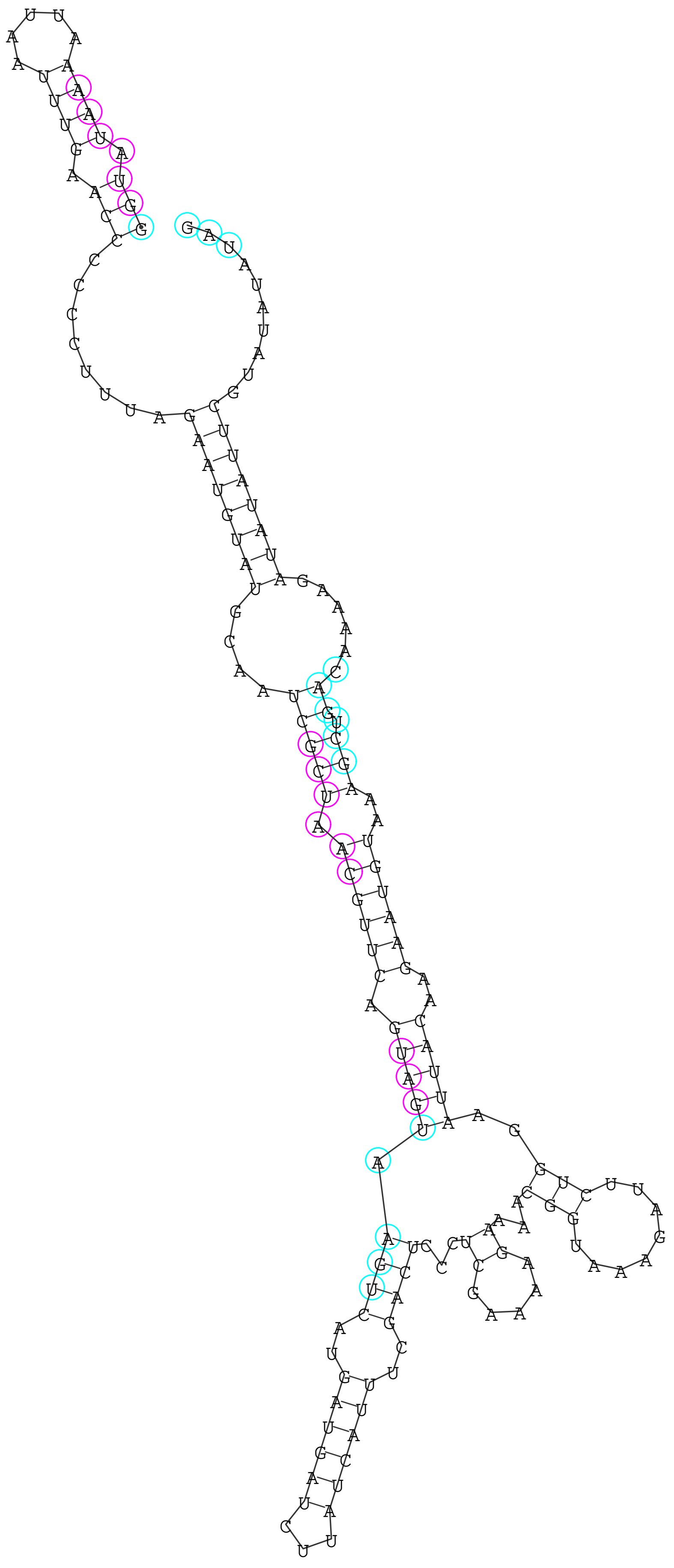

Dchc014A - Stwintron

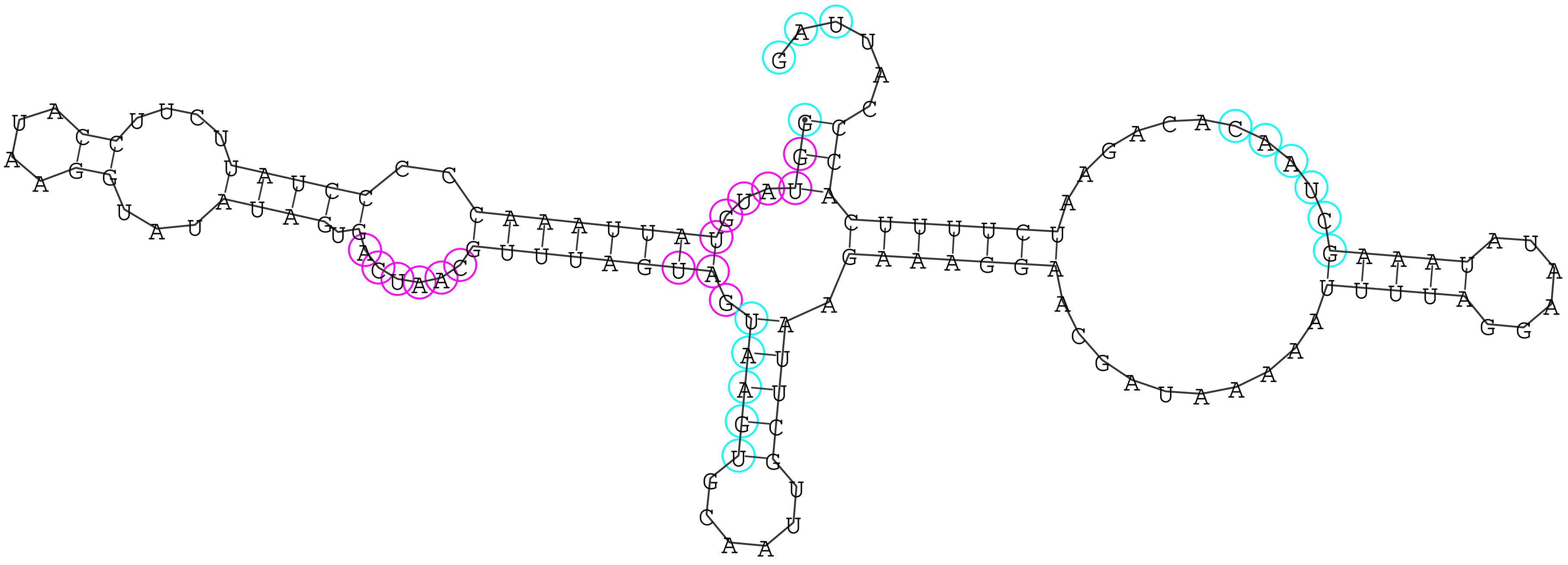

Dcoc02A - Stwintron

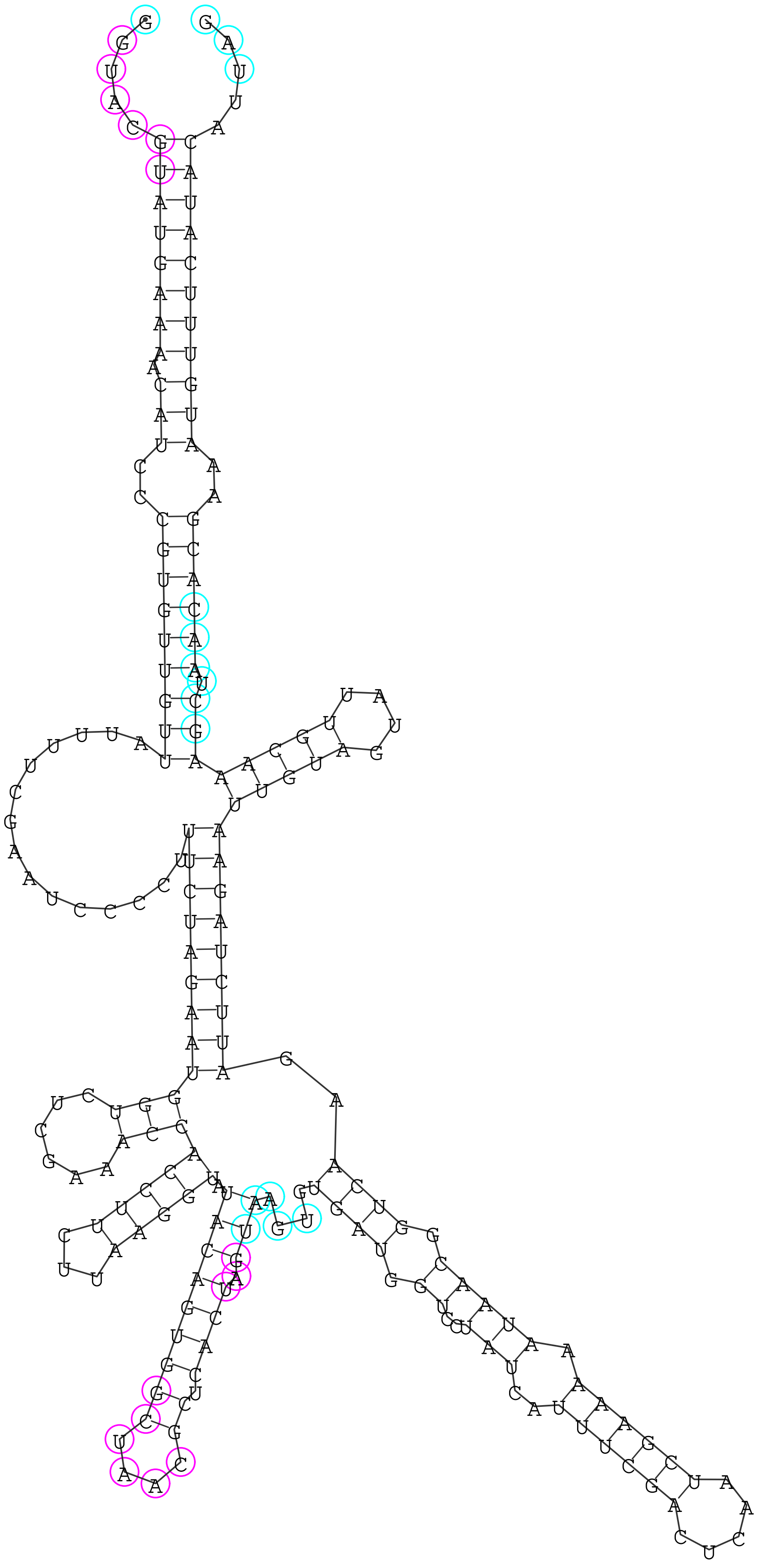

Dcoc02B - Stwintron

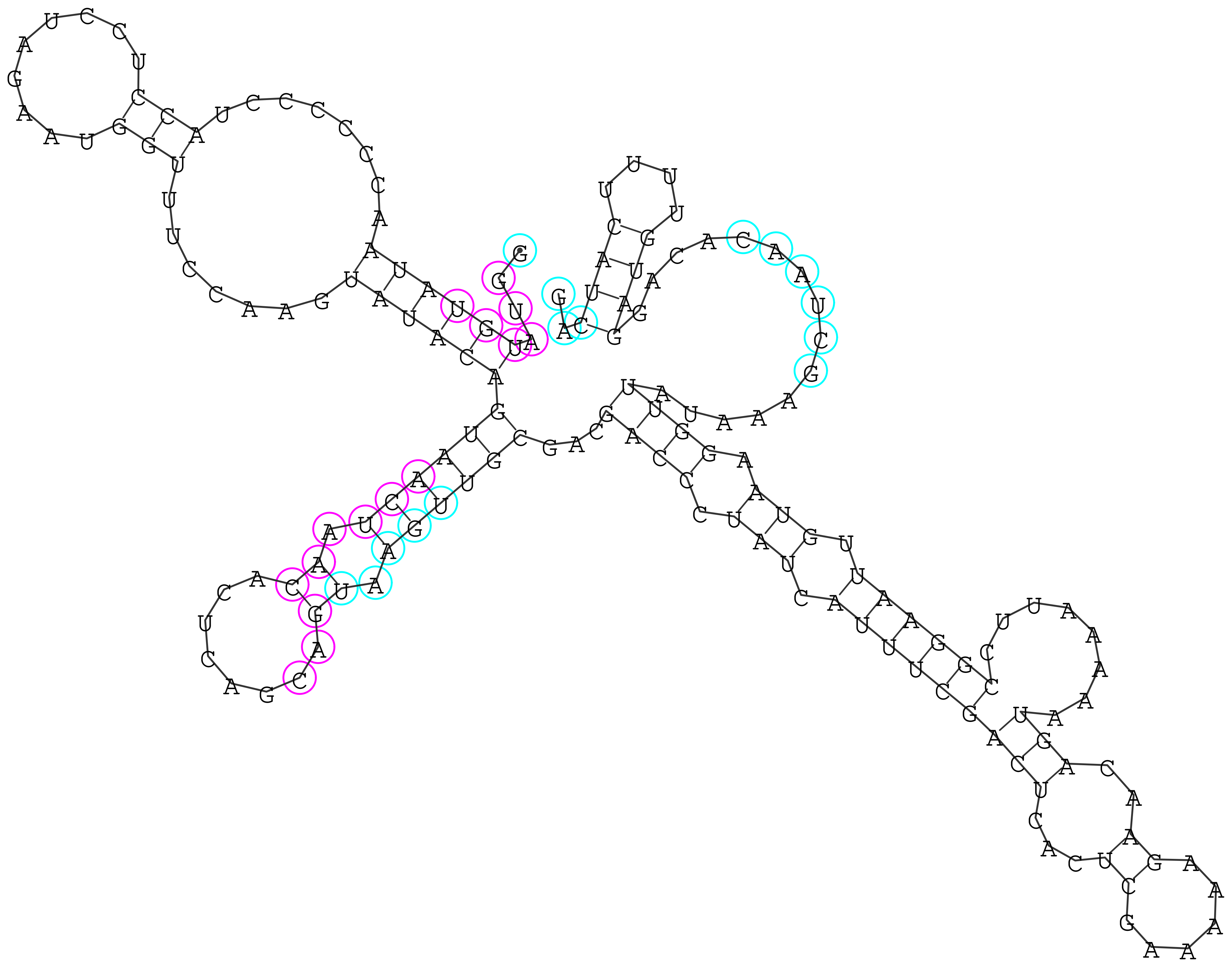

Dcoc03A - Stwintron

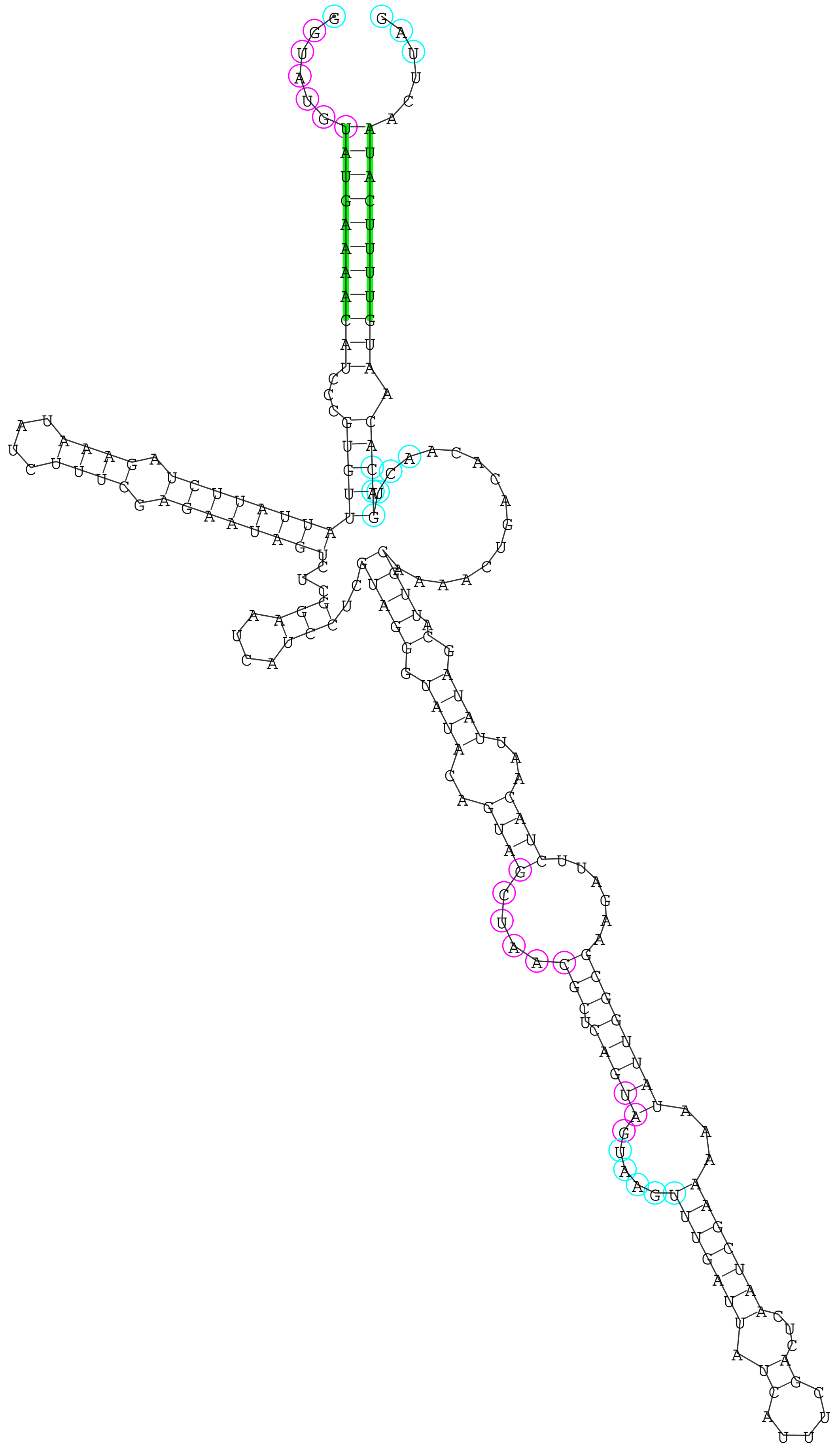

Dcoc05A - Stwintron

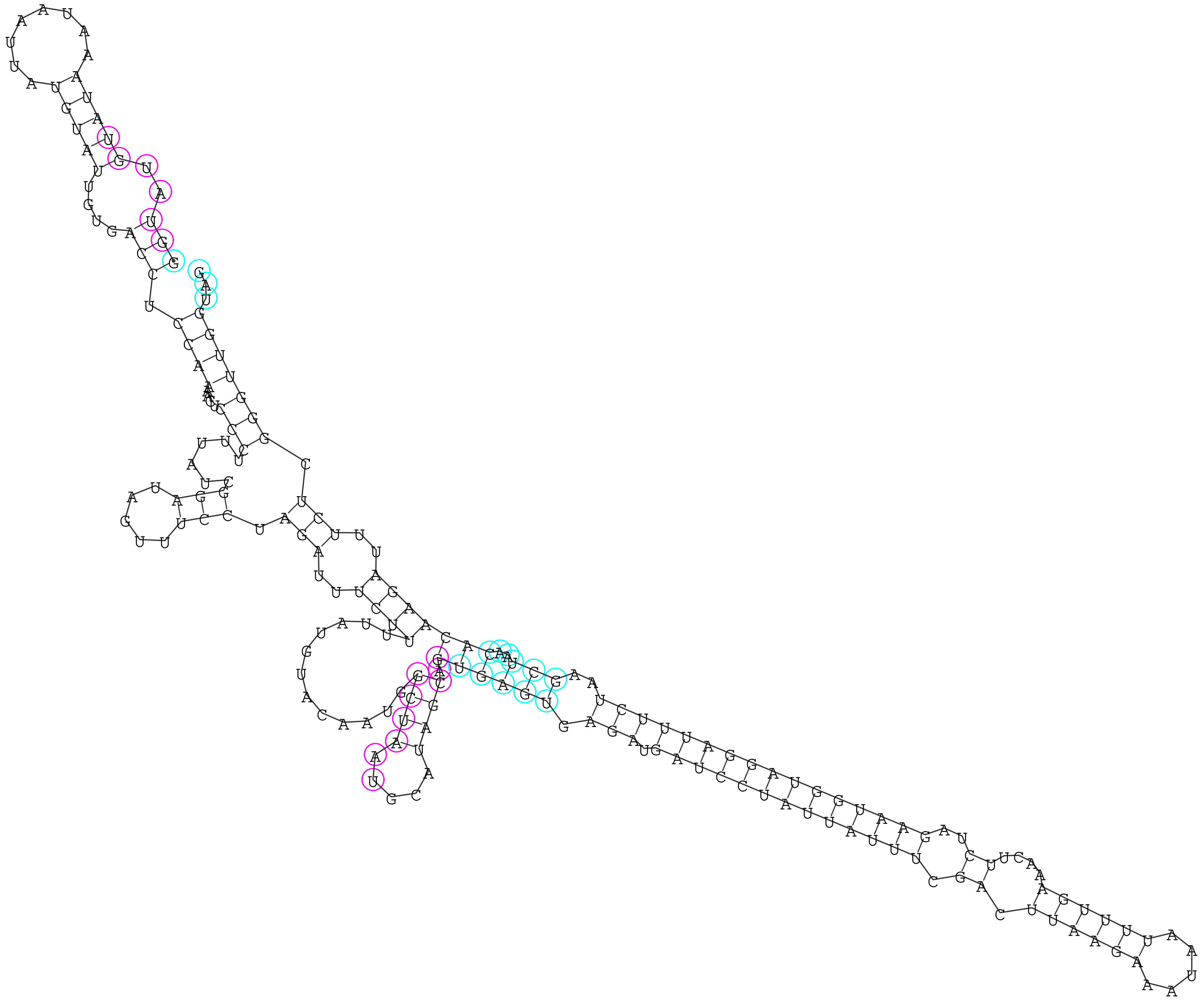

Dcoc06A - Stwintron

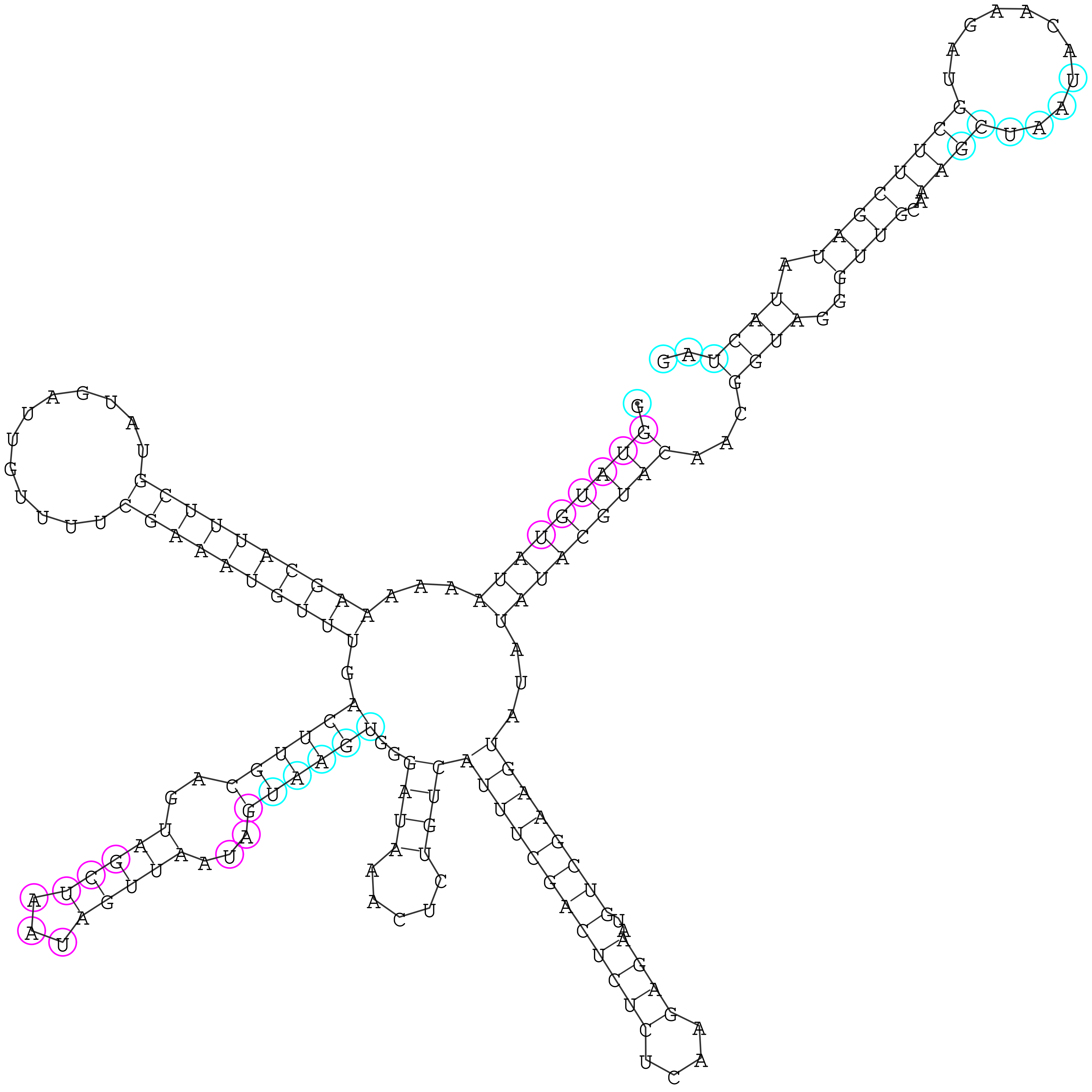

Dcoc11A - Stwintron

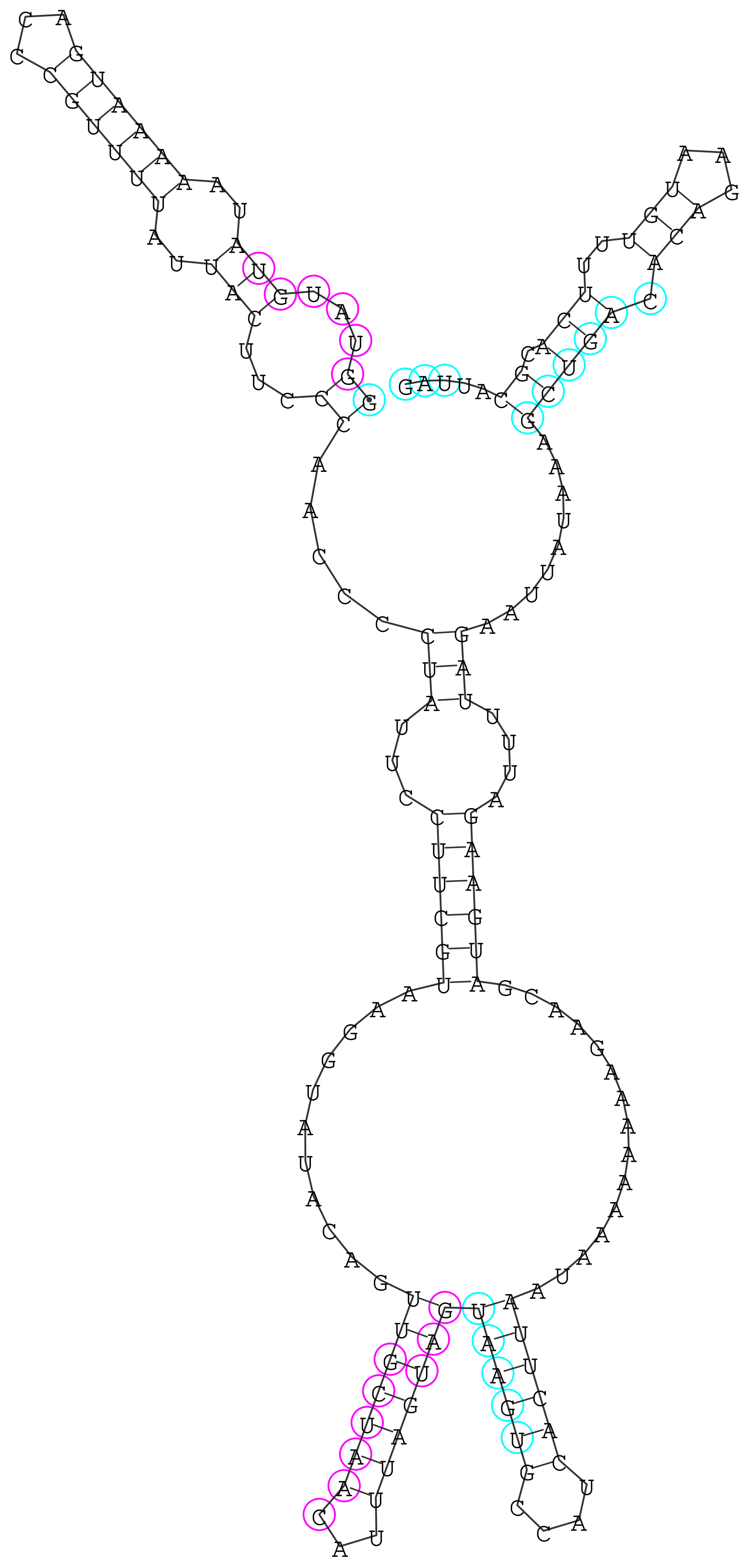

Dcoc20A - Stwintron

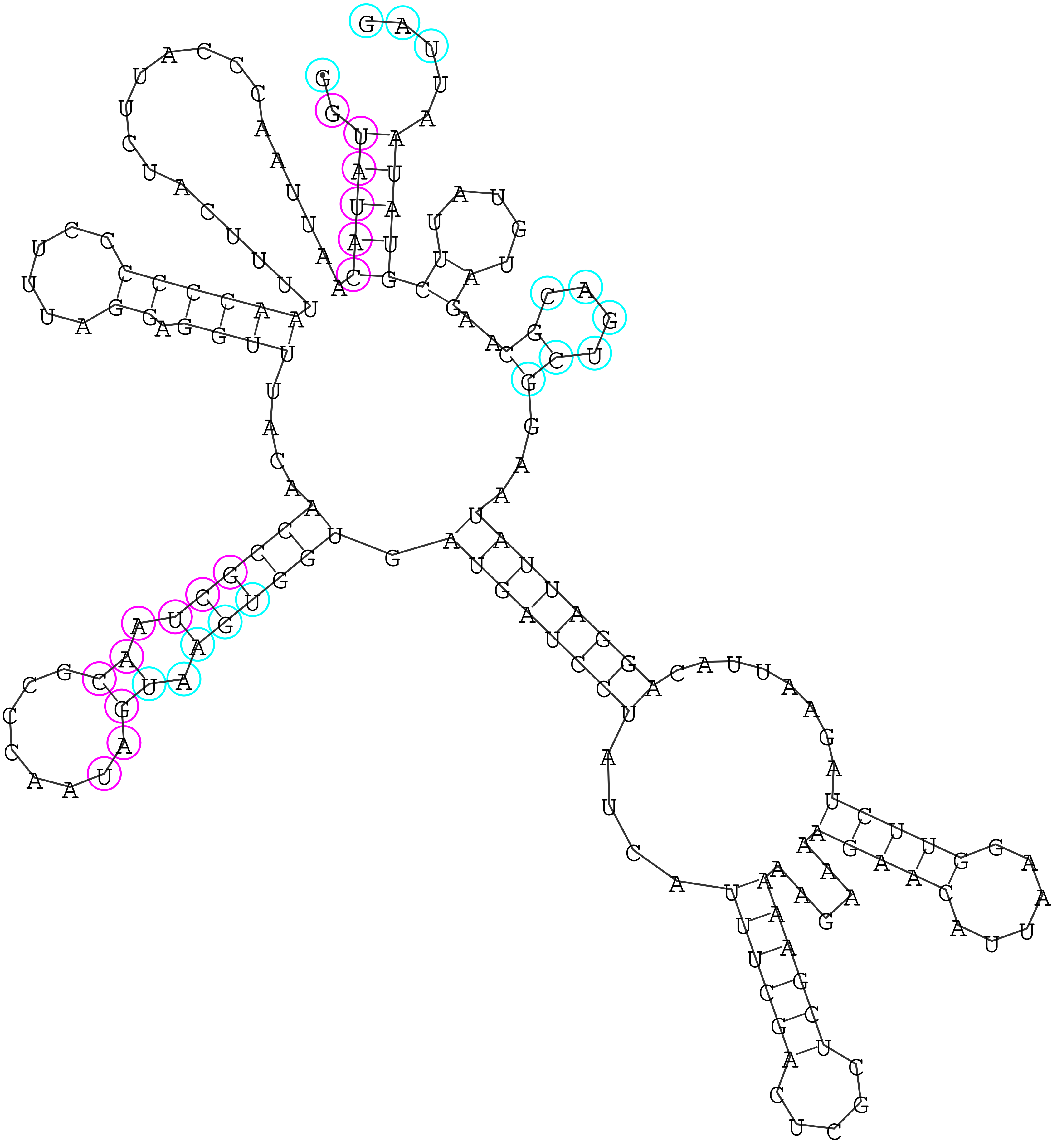

Dcoc38A - Stwintron

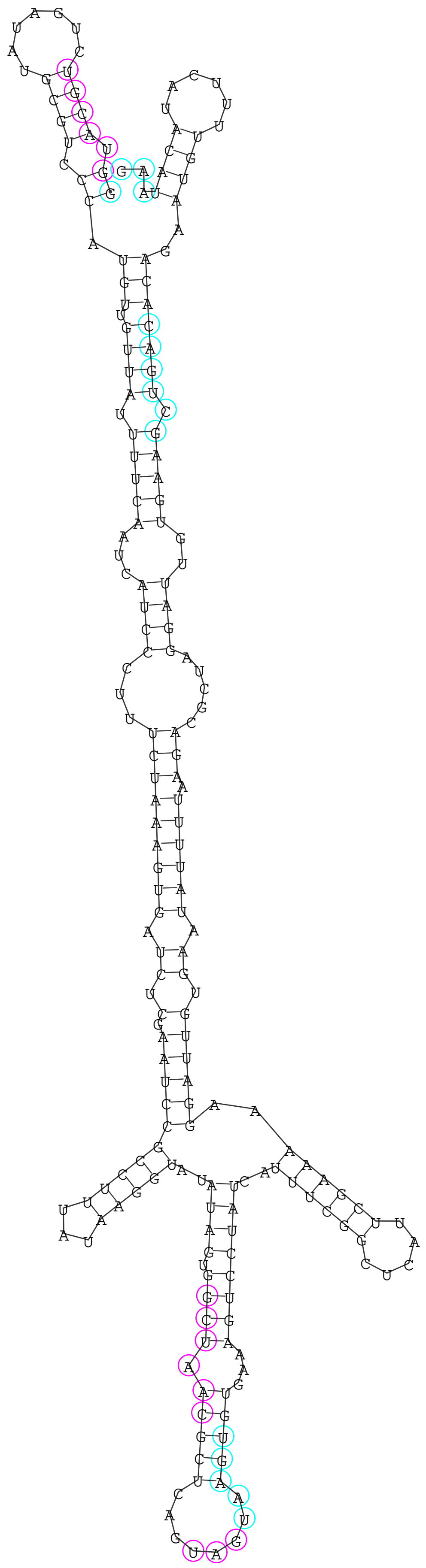

Dcoc41A - Stwintron

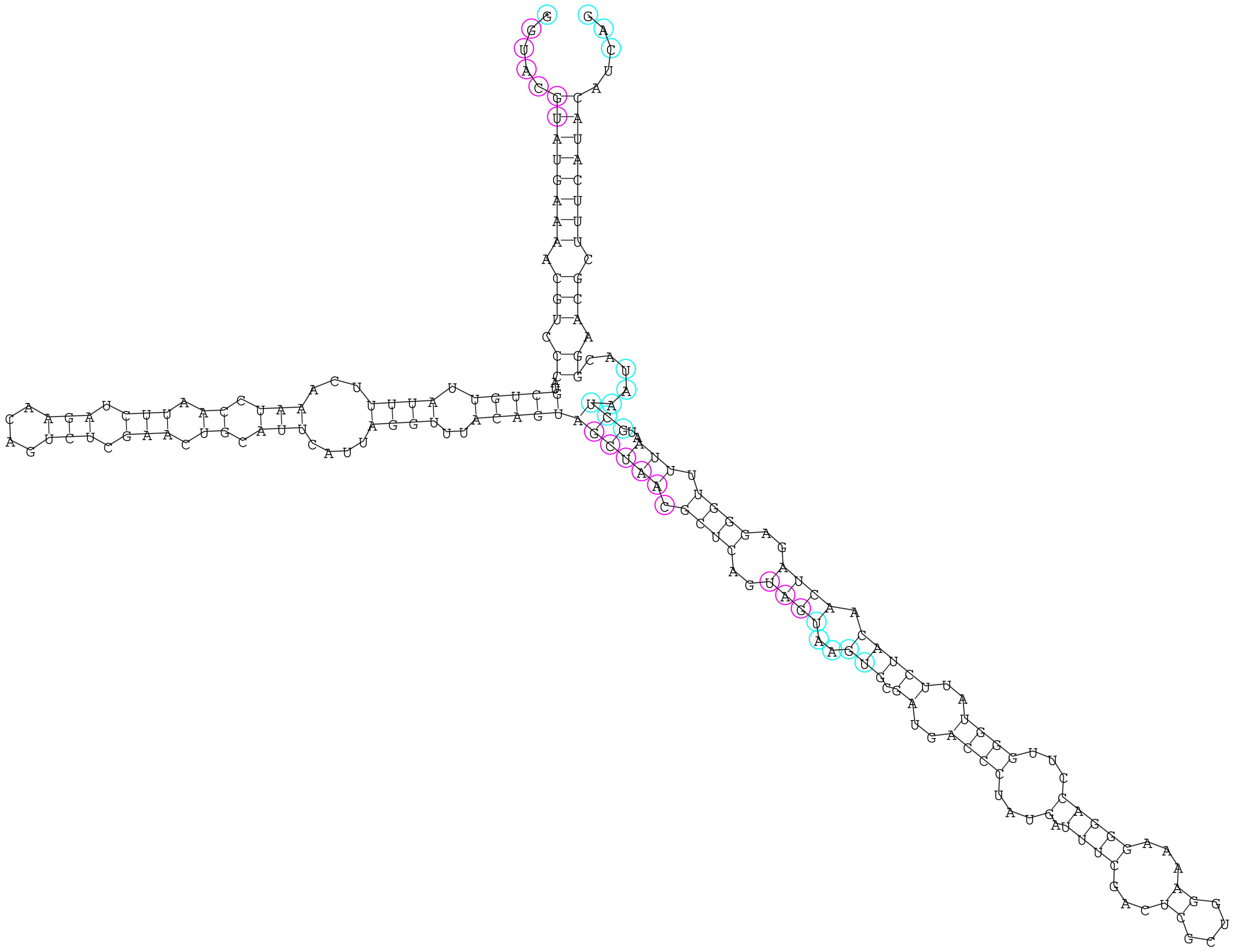

Desc187A - Stwintron

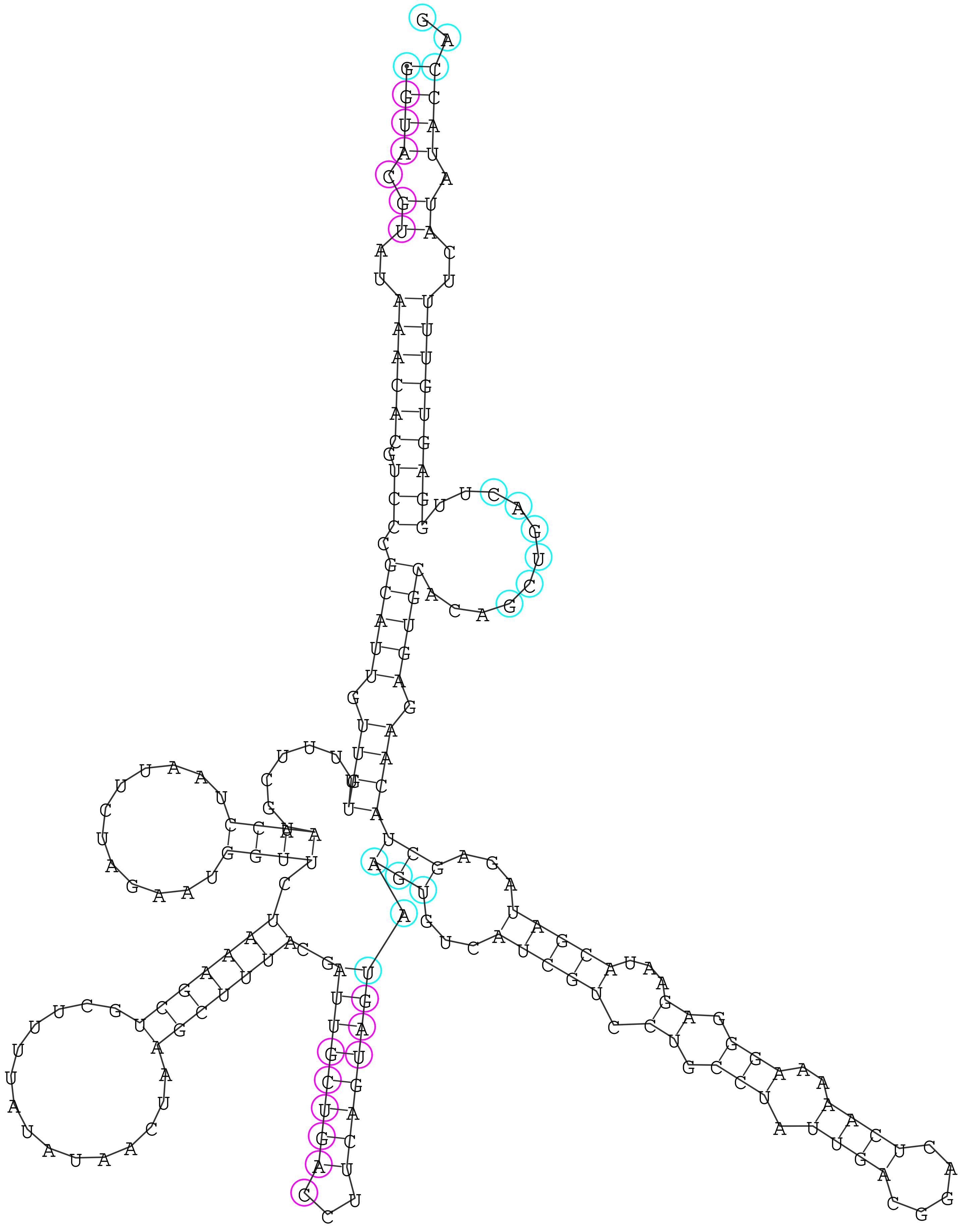

Desc198A - Stwintron

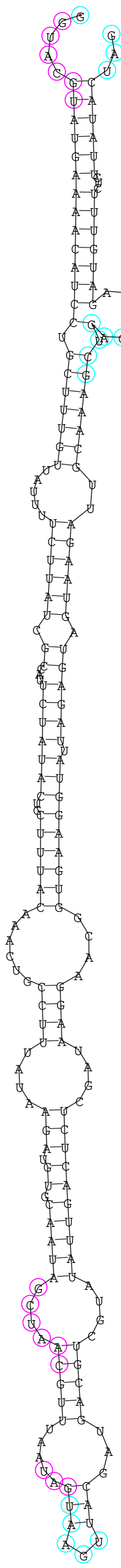

Desc274A - Stwintron

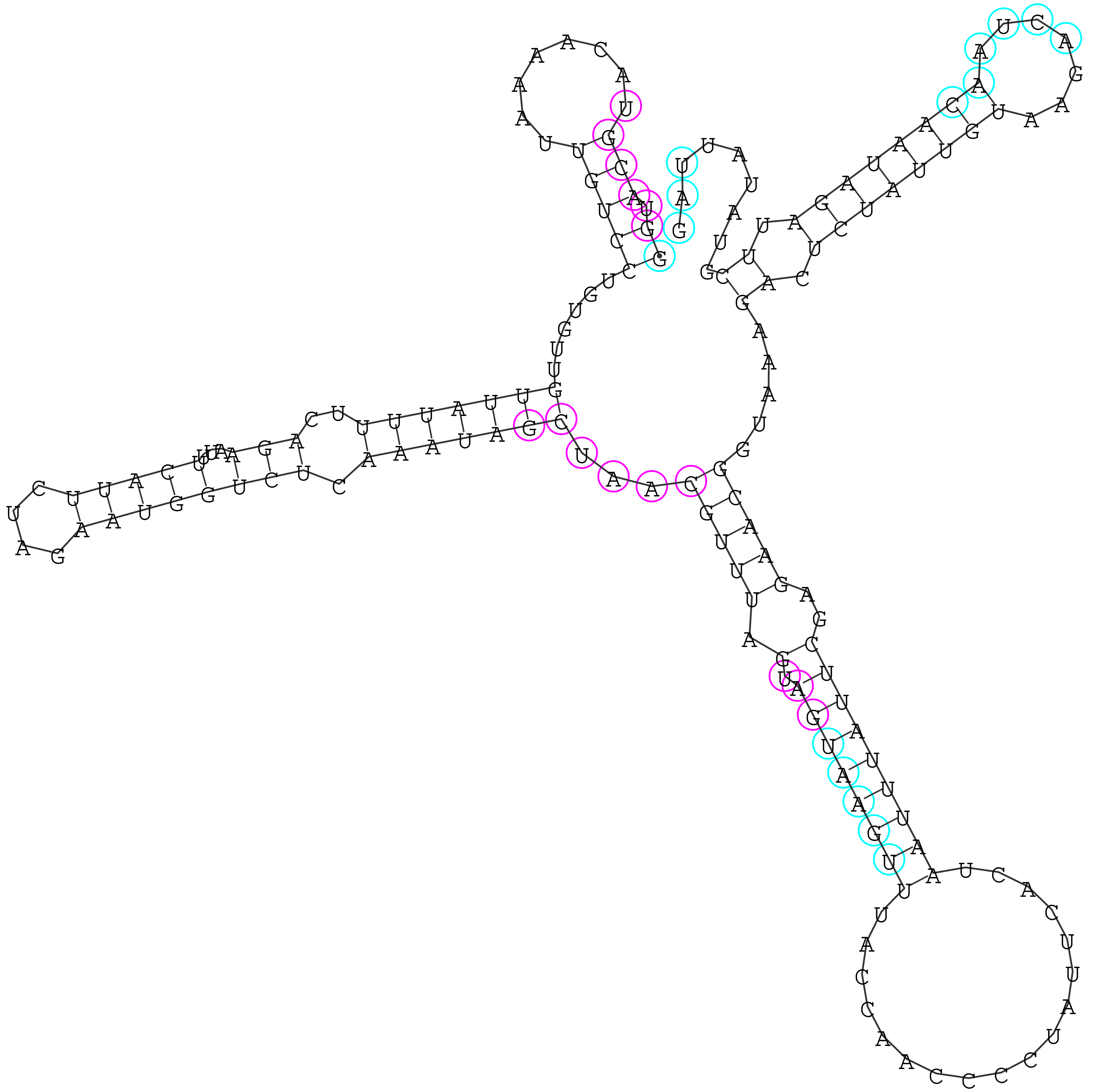

Desc420A - Stwintron

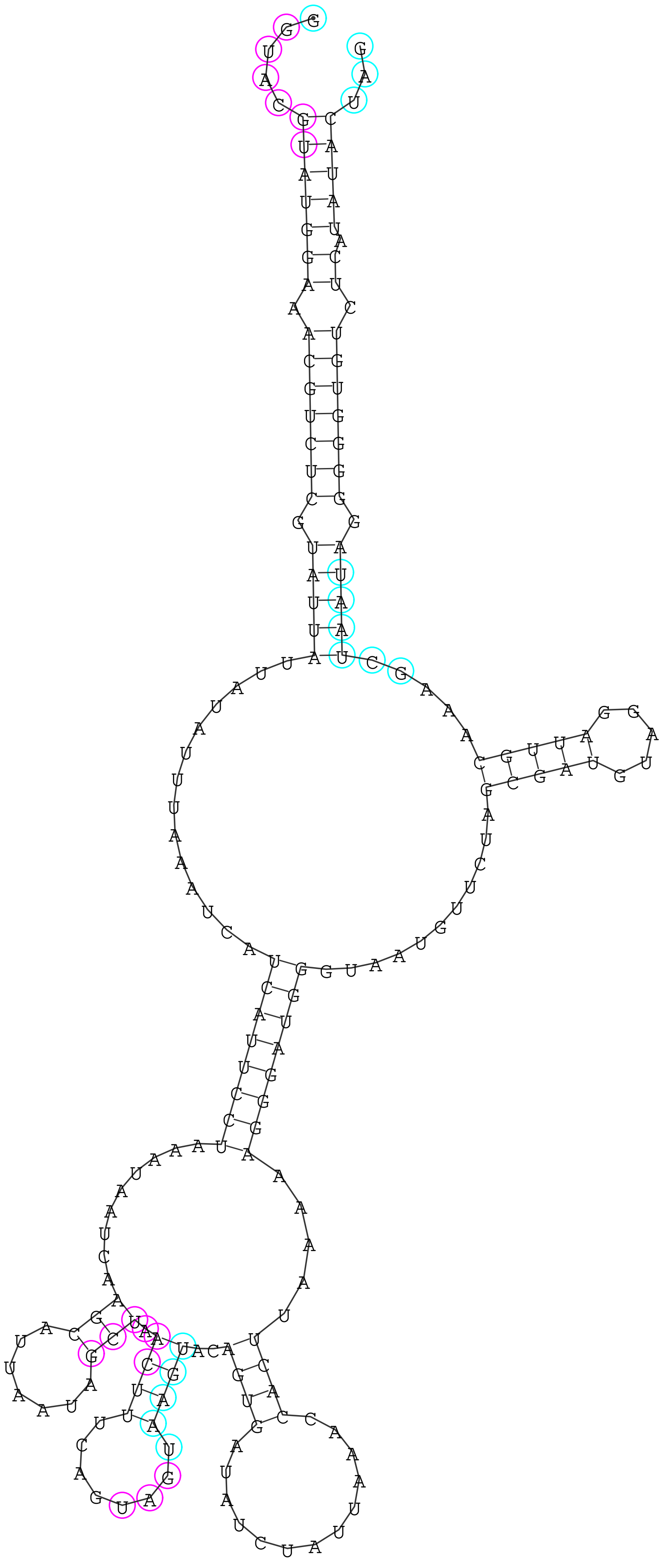

Desc618A - Stwintron

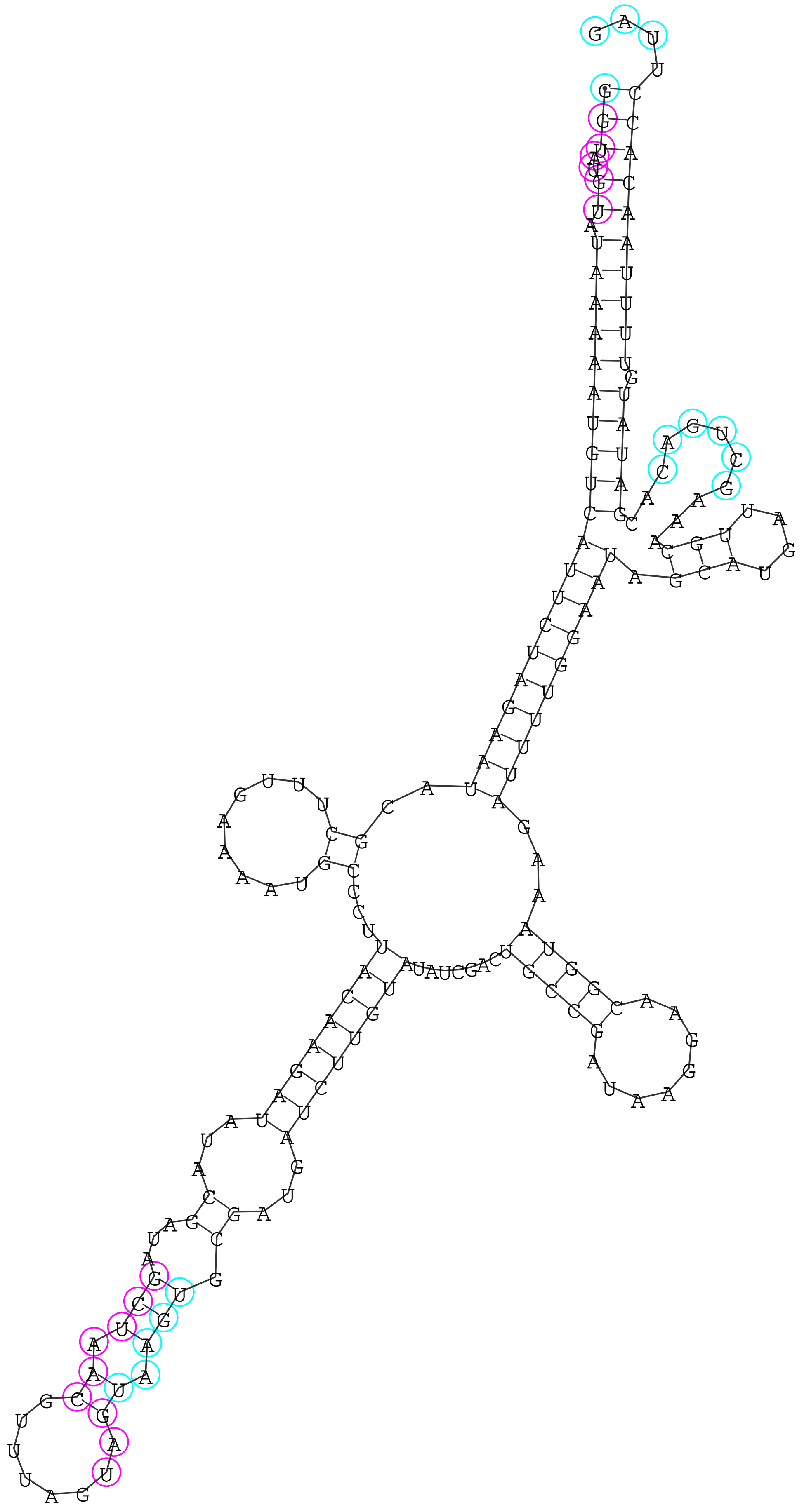

Desc640A - Stwintron

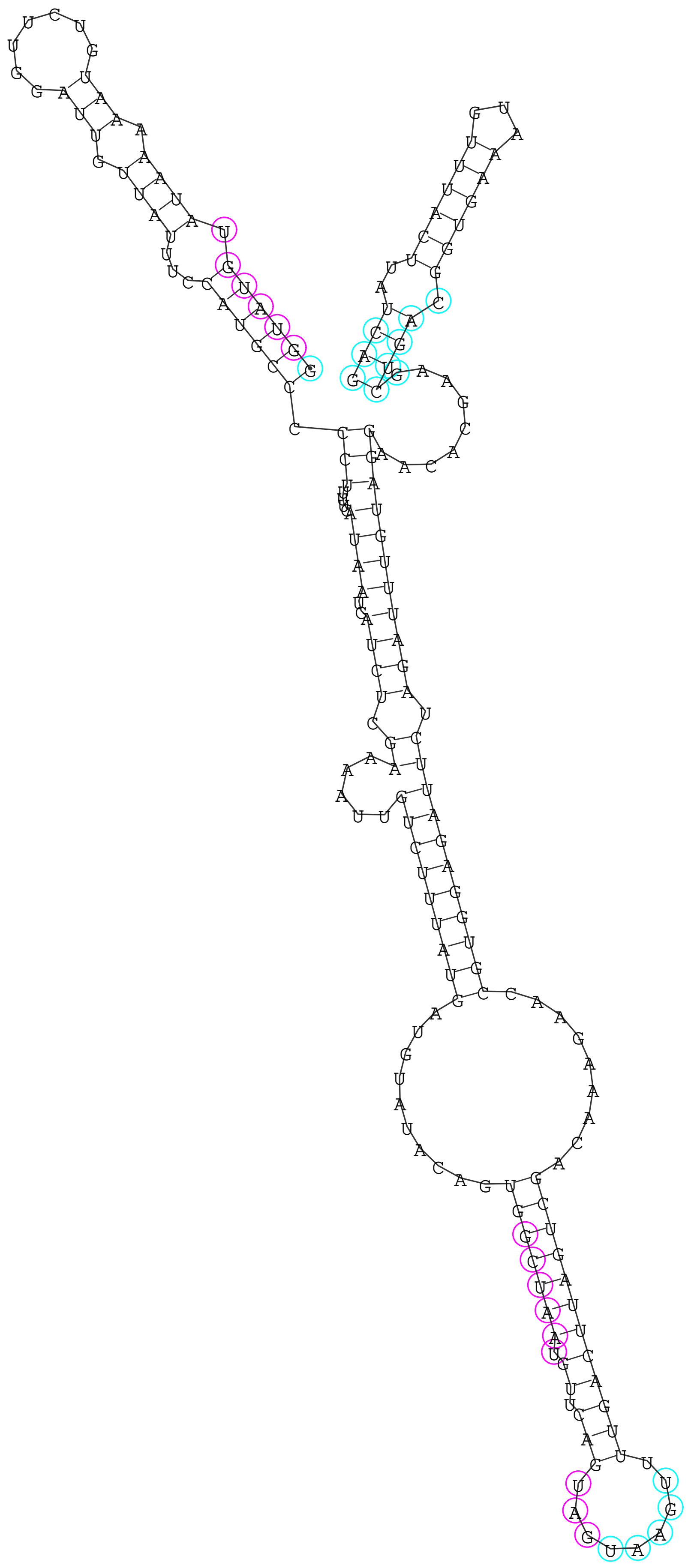

HCOc002A - Stwintron

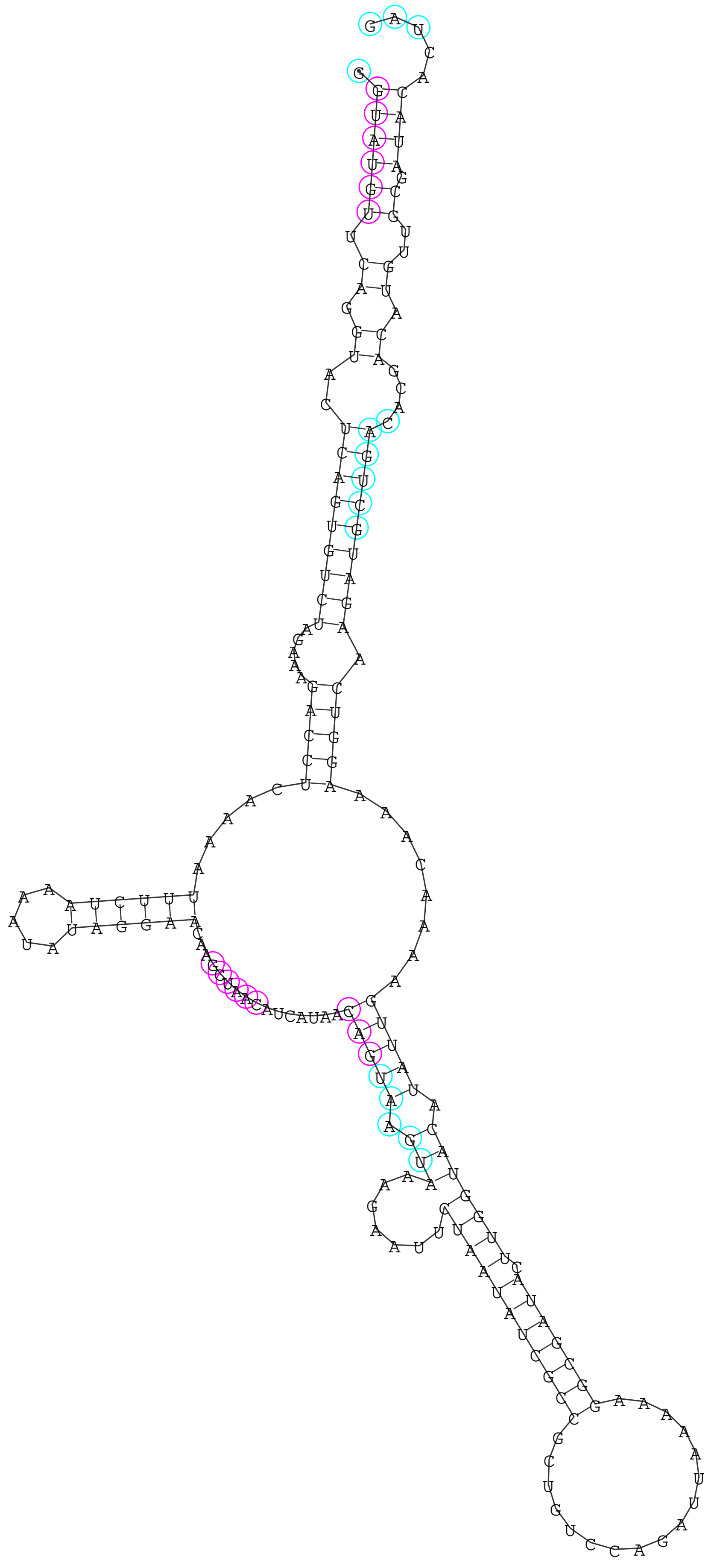

HCOc004A - Stwintron

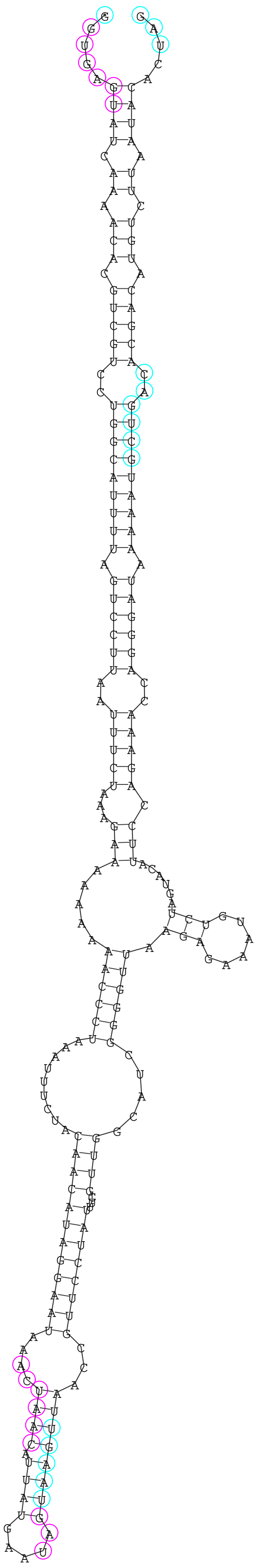

HCOc017A - Stwintron

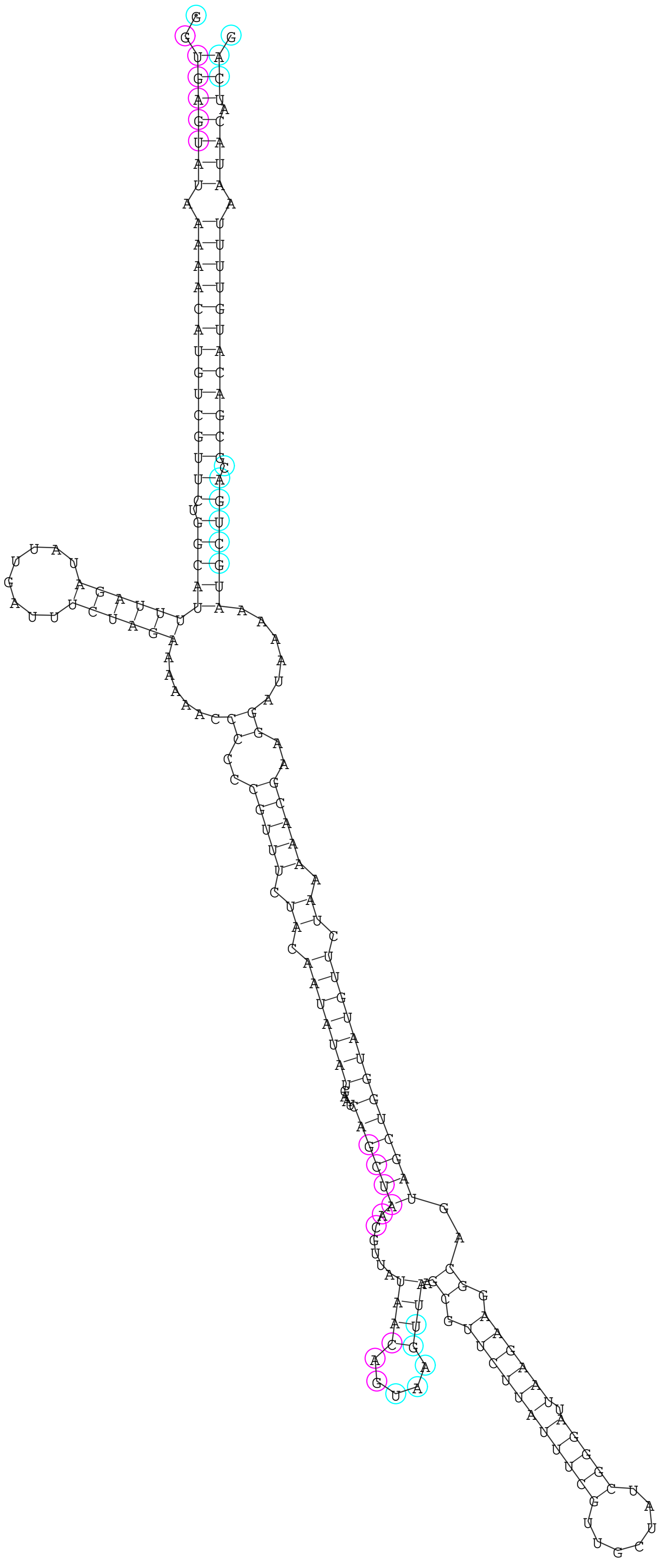

HCOc017B - Stwintron

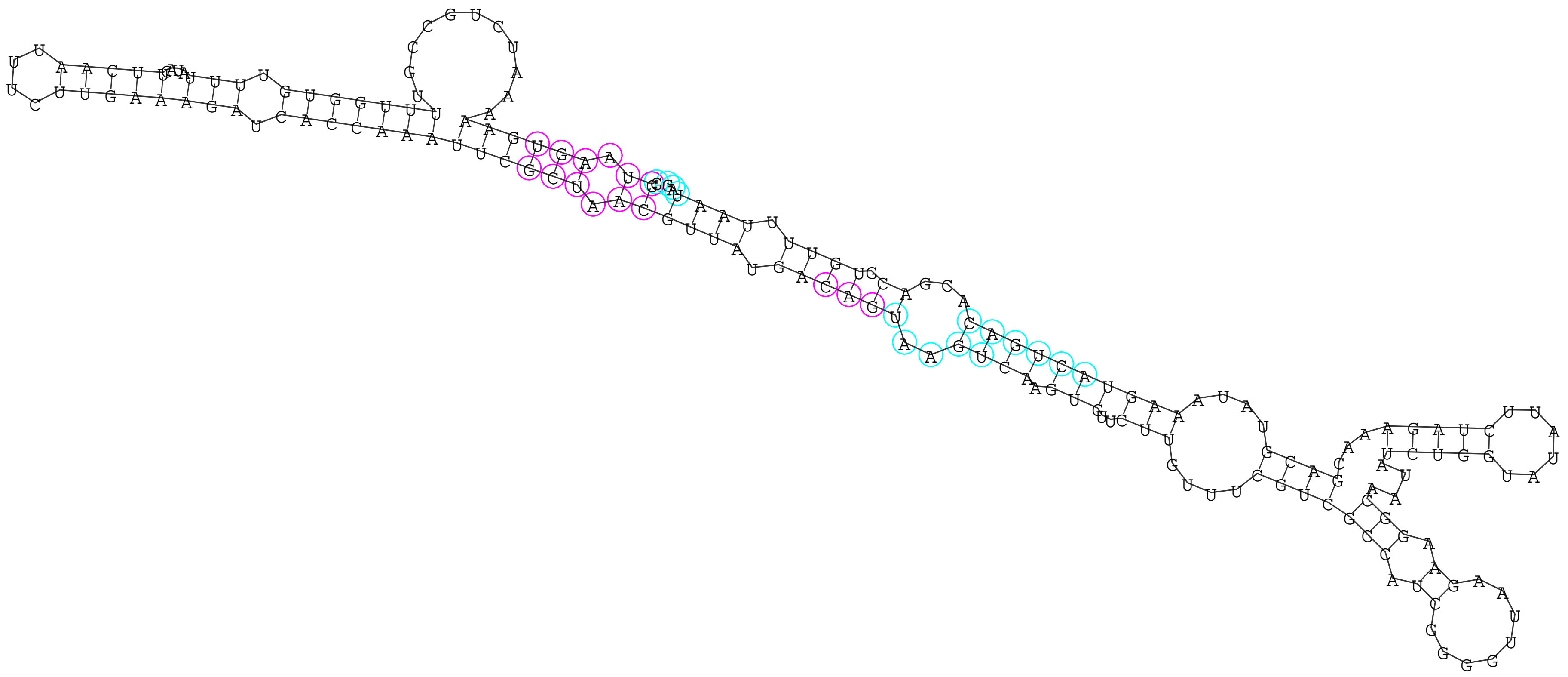

HCOc021A - Stwintron

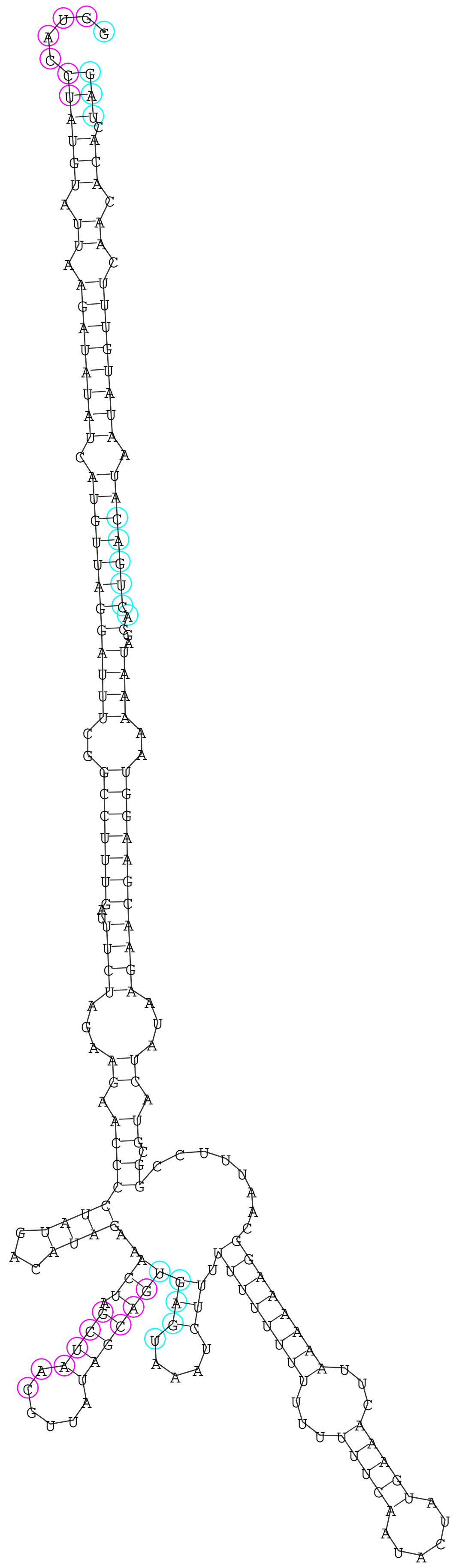

HCOc047A - Stwintron

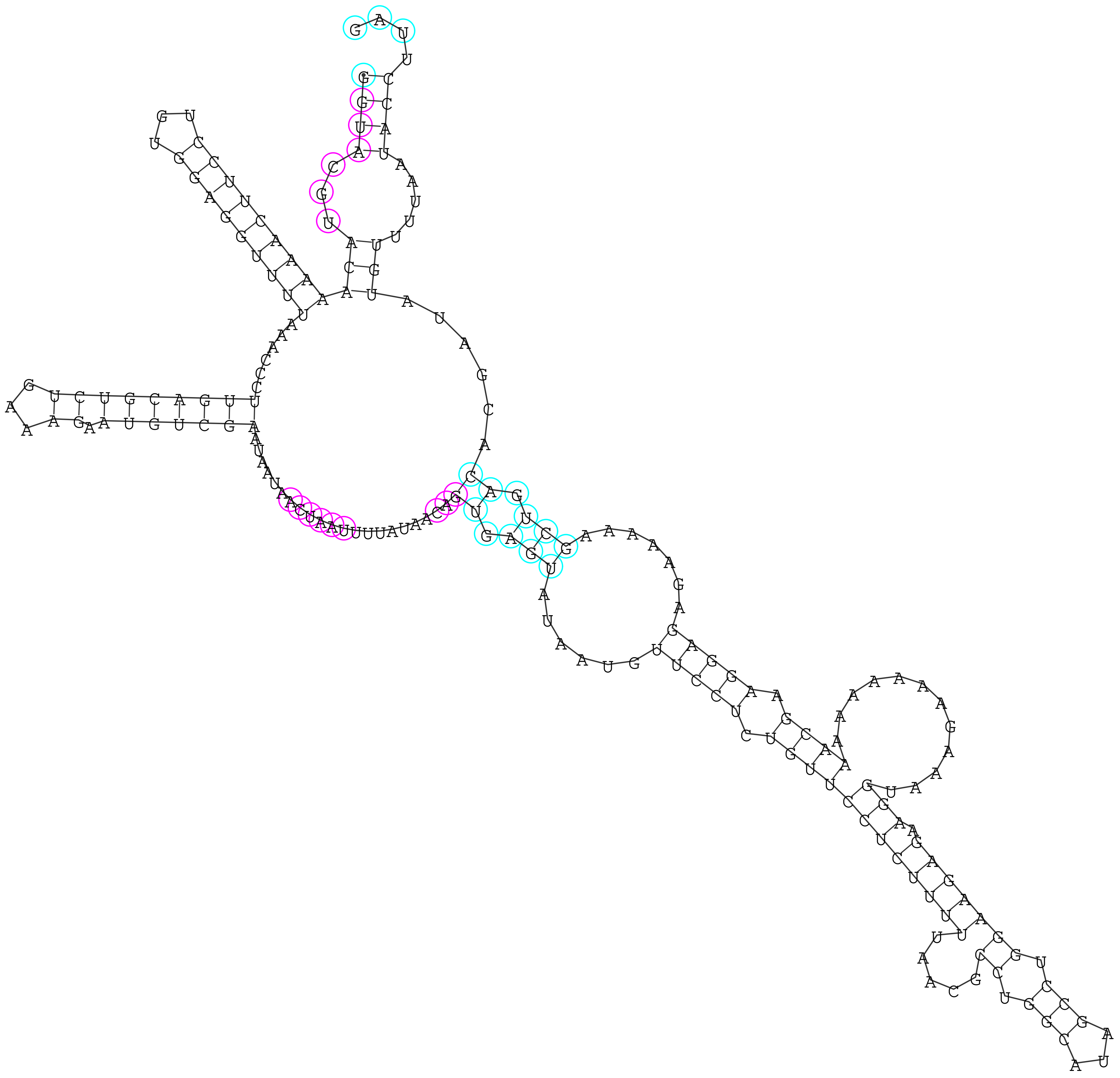

HCOc052A - Stwintron

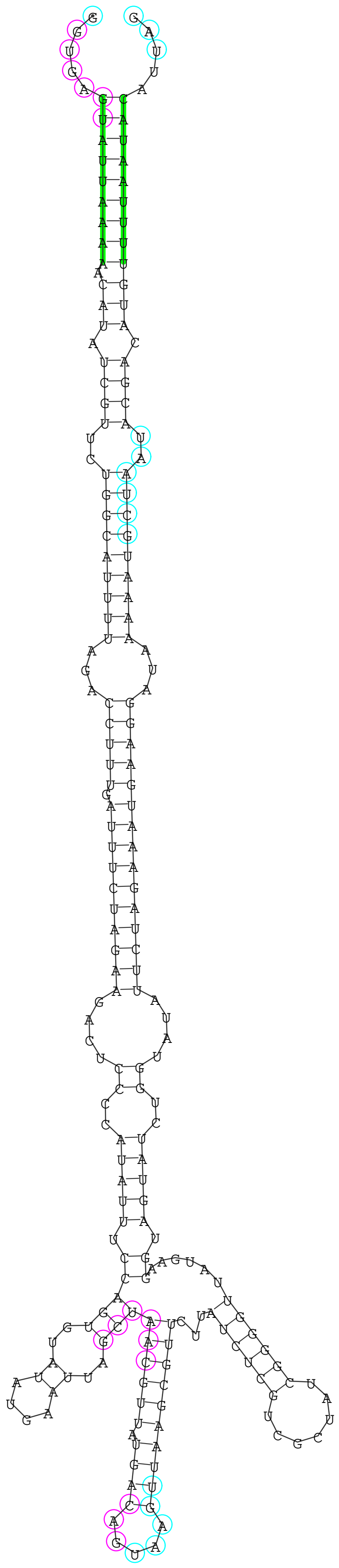

HCOc058A - Stwintron

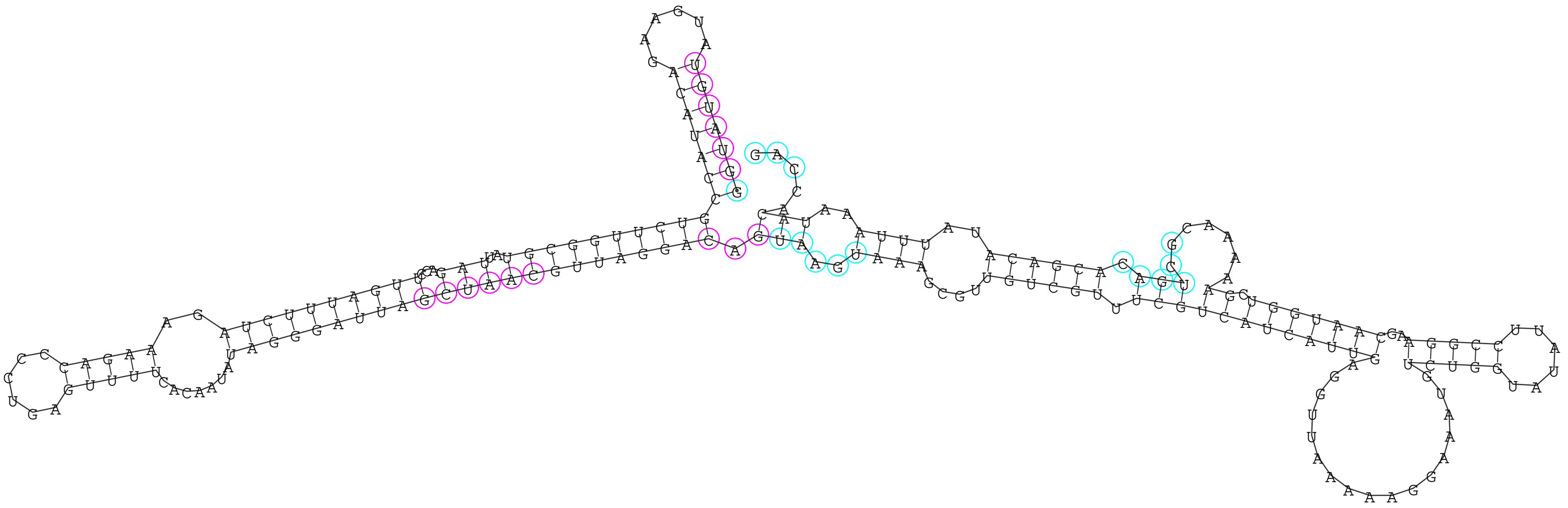

HCOc061A - Stwintron

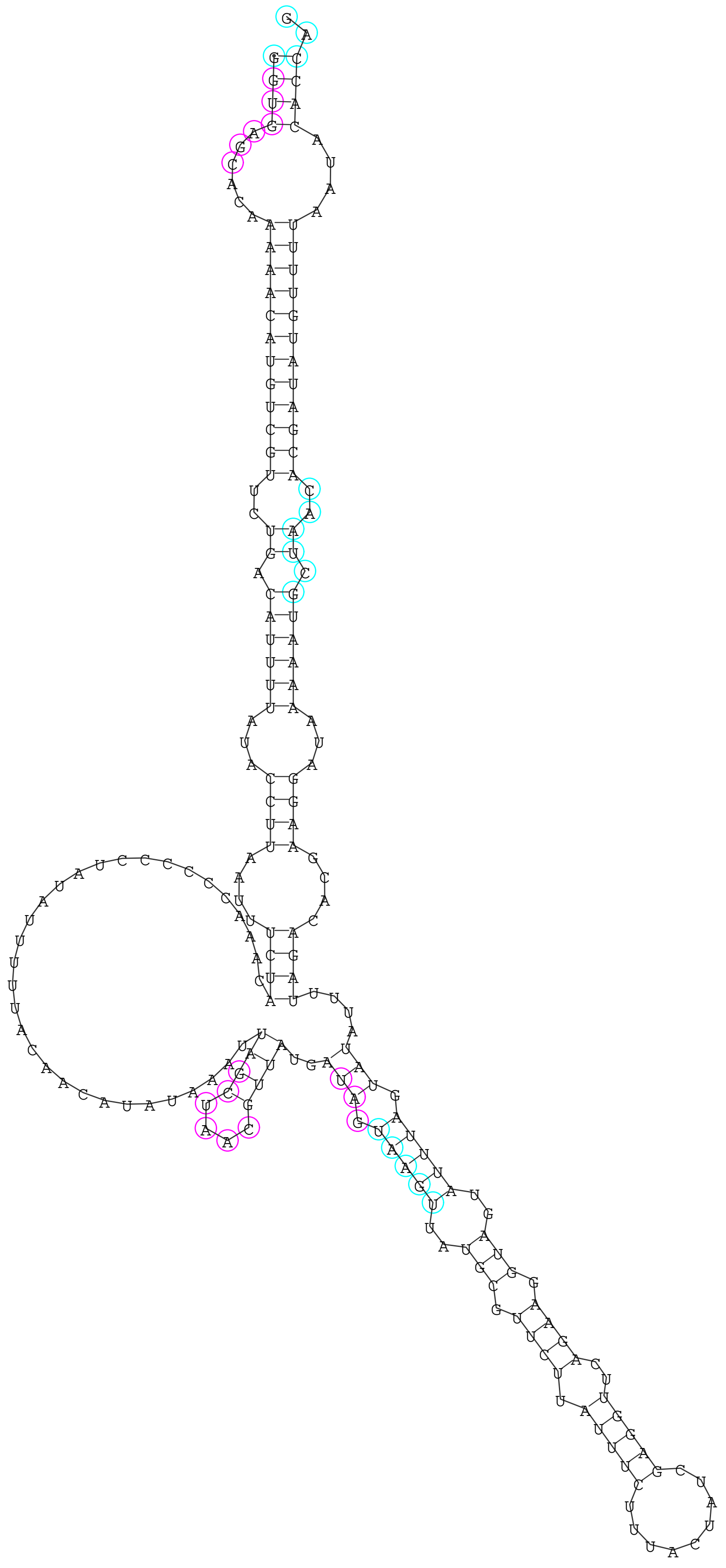

HCOc066A - Stwintron

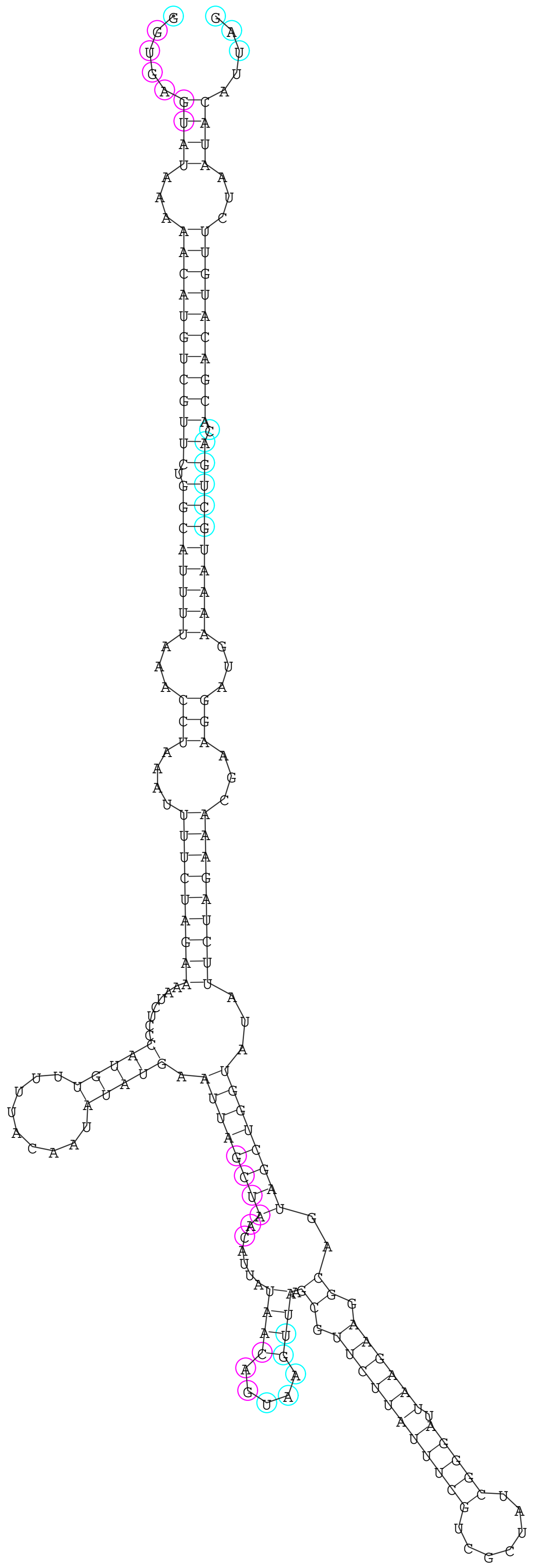

HCOc070A - Stwintron

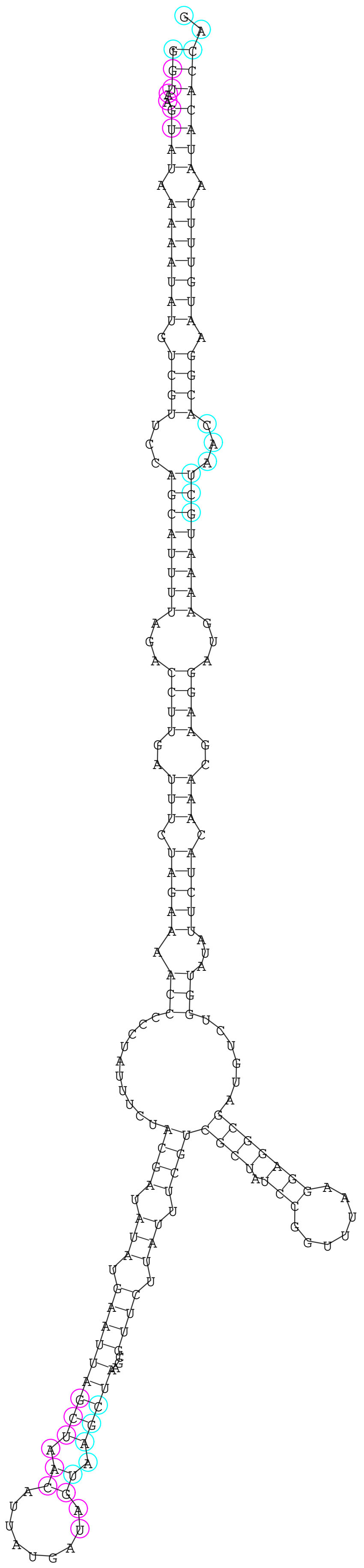

HCOc076A - Stwintron

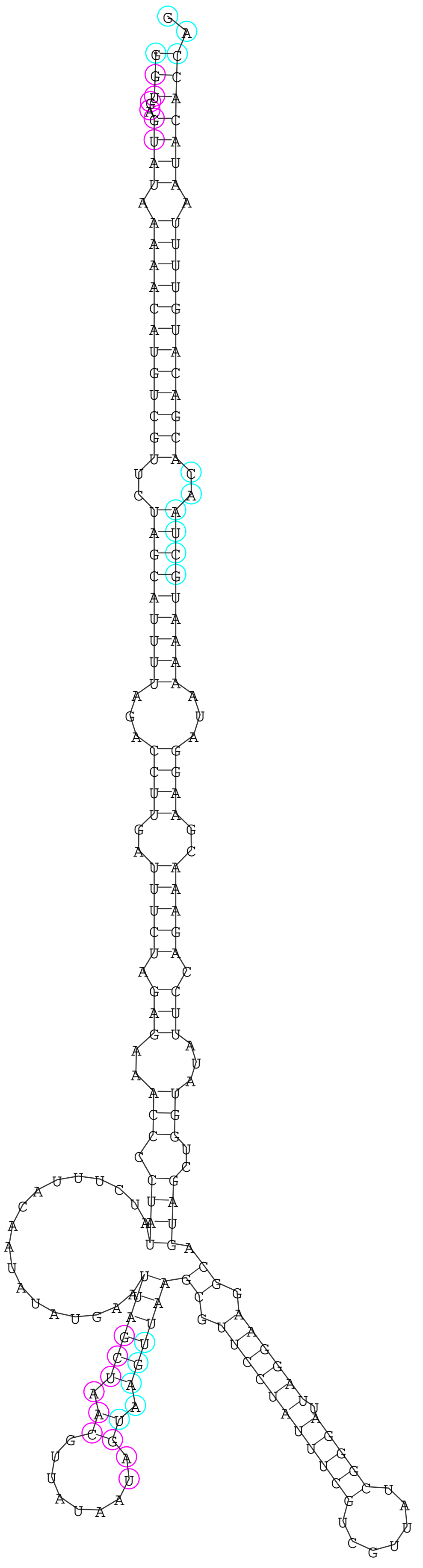

HCOc102A - Stwintron

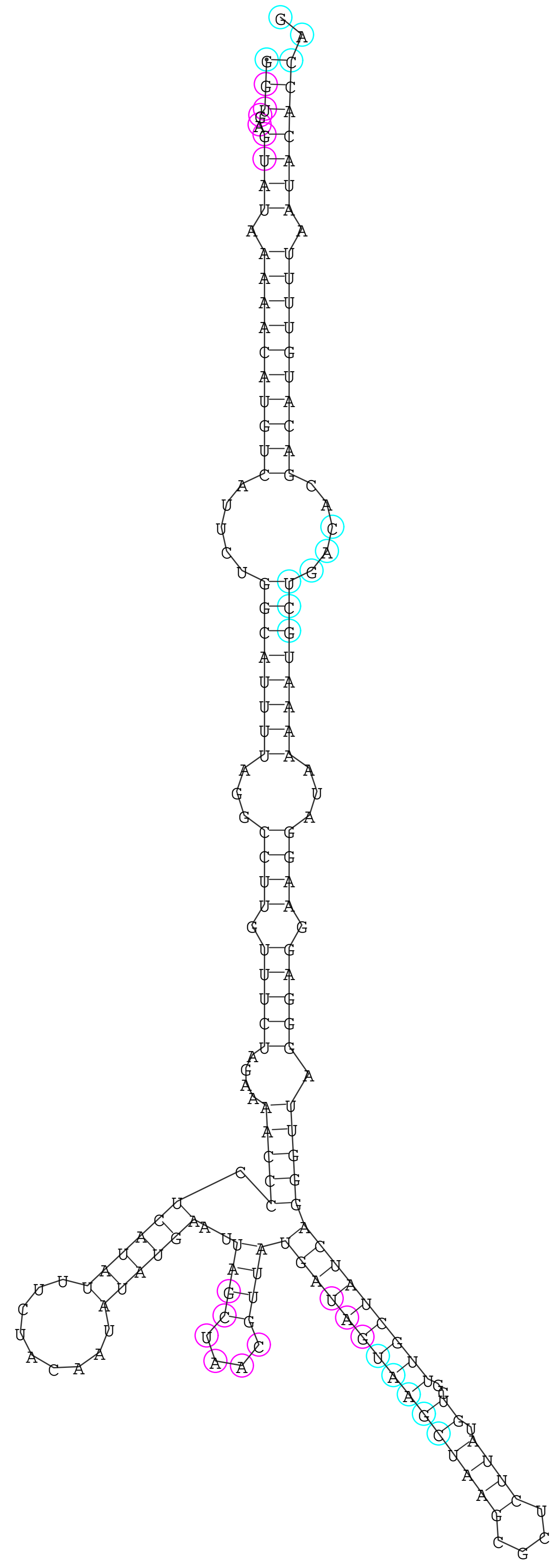

HCOc164A - Stwintron

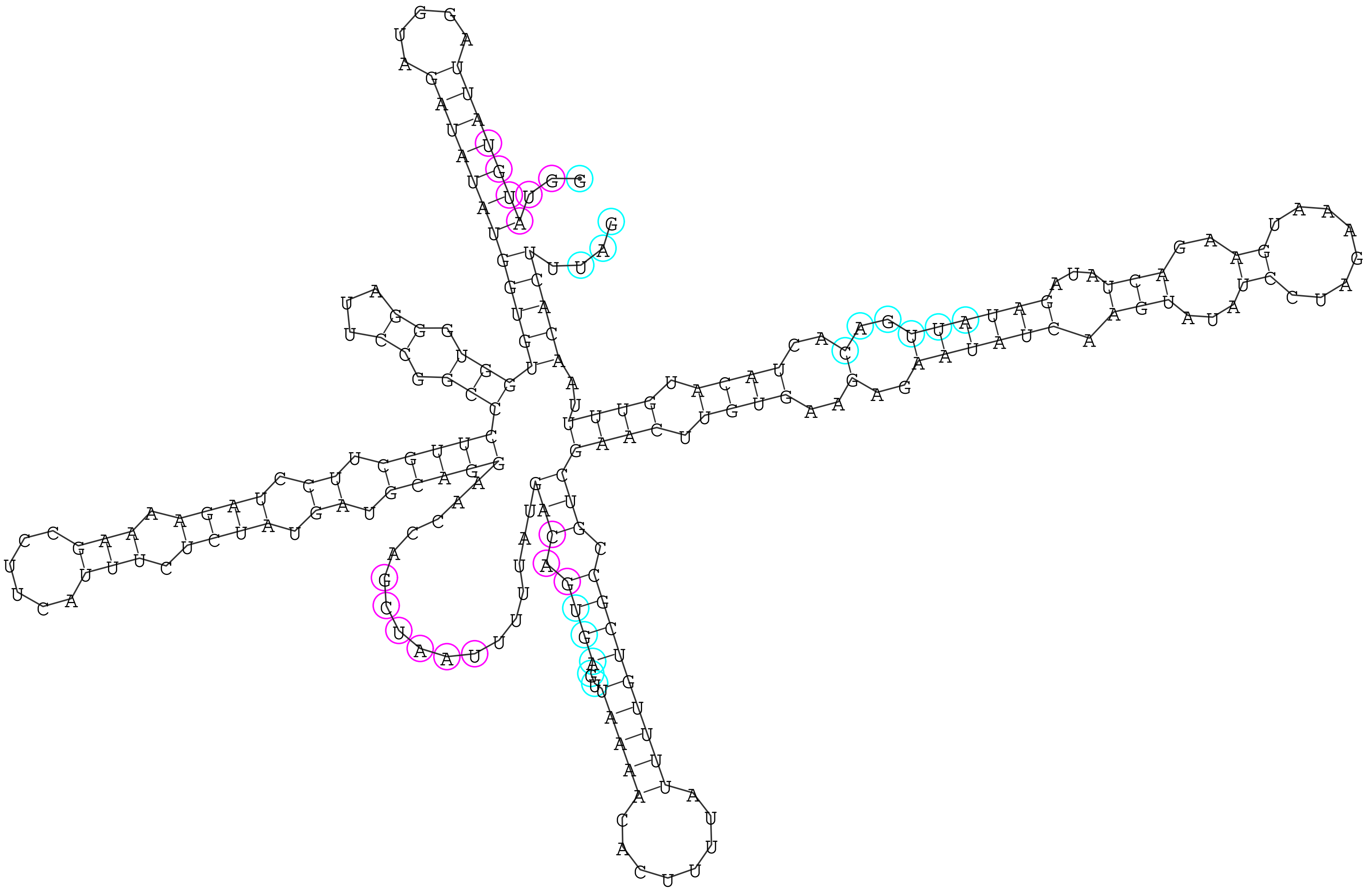

HCOc178A - Stwintron

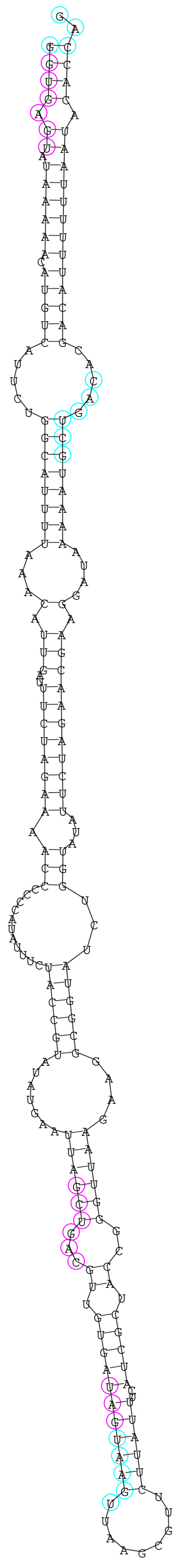

HCOc224-179 - Stwintron

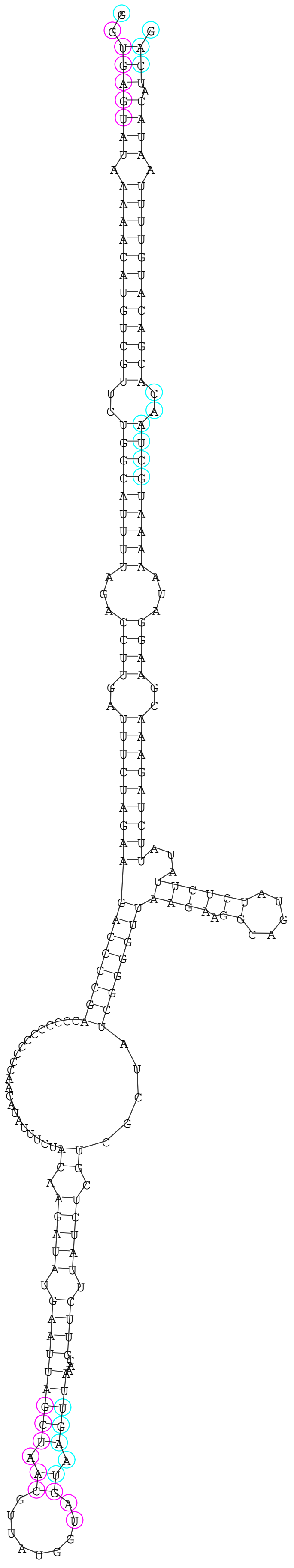

HCOc236A - Stwintron

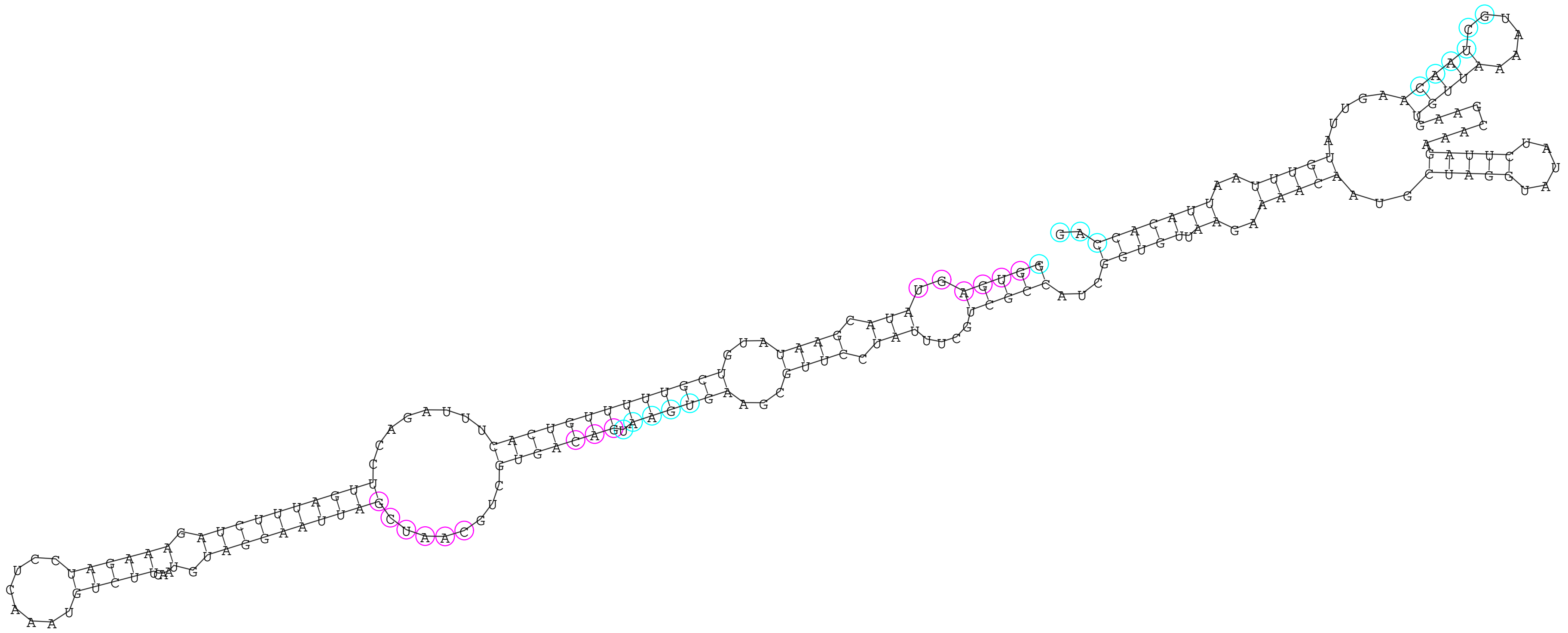

HCOc252A - Stwintron

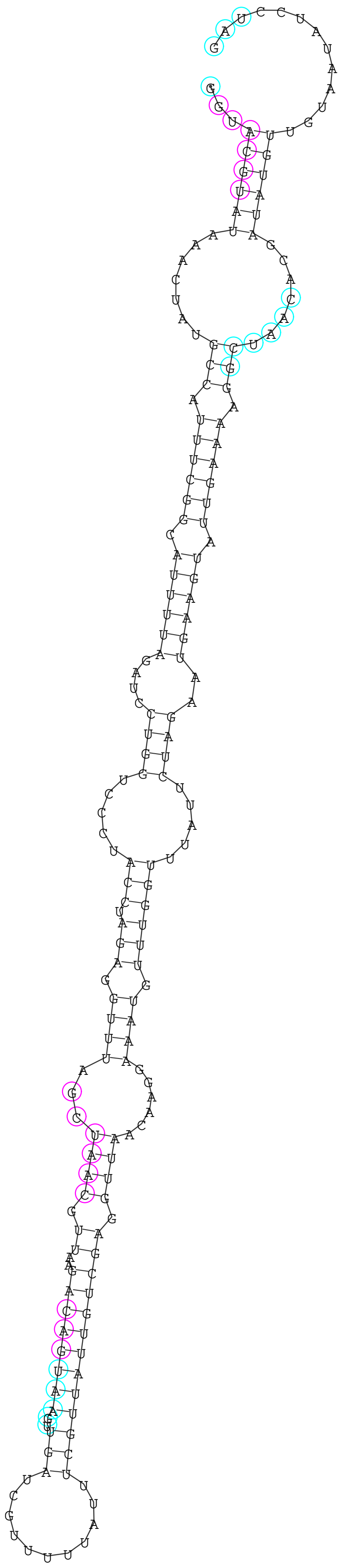

HCOc271A - Stwintron

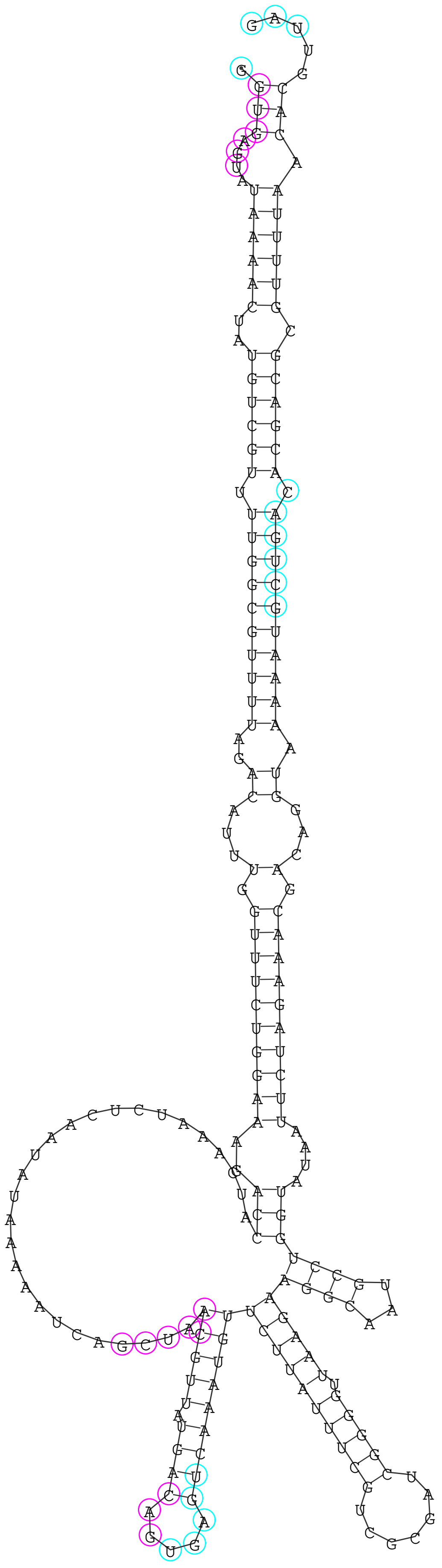

HCOc332A - Stwintron

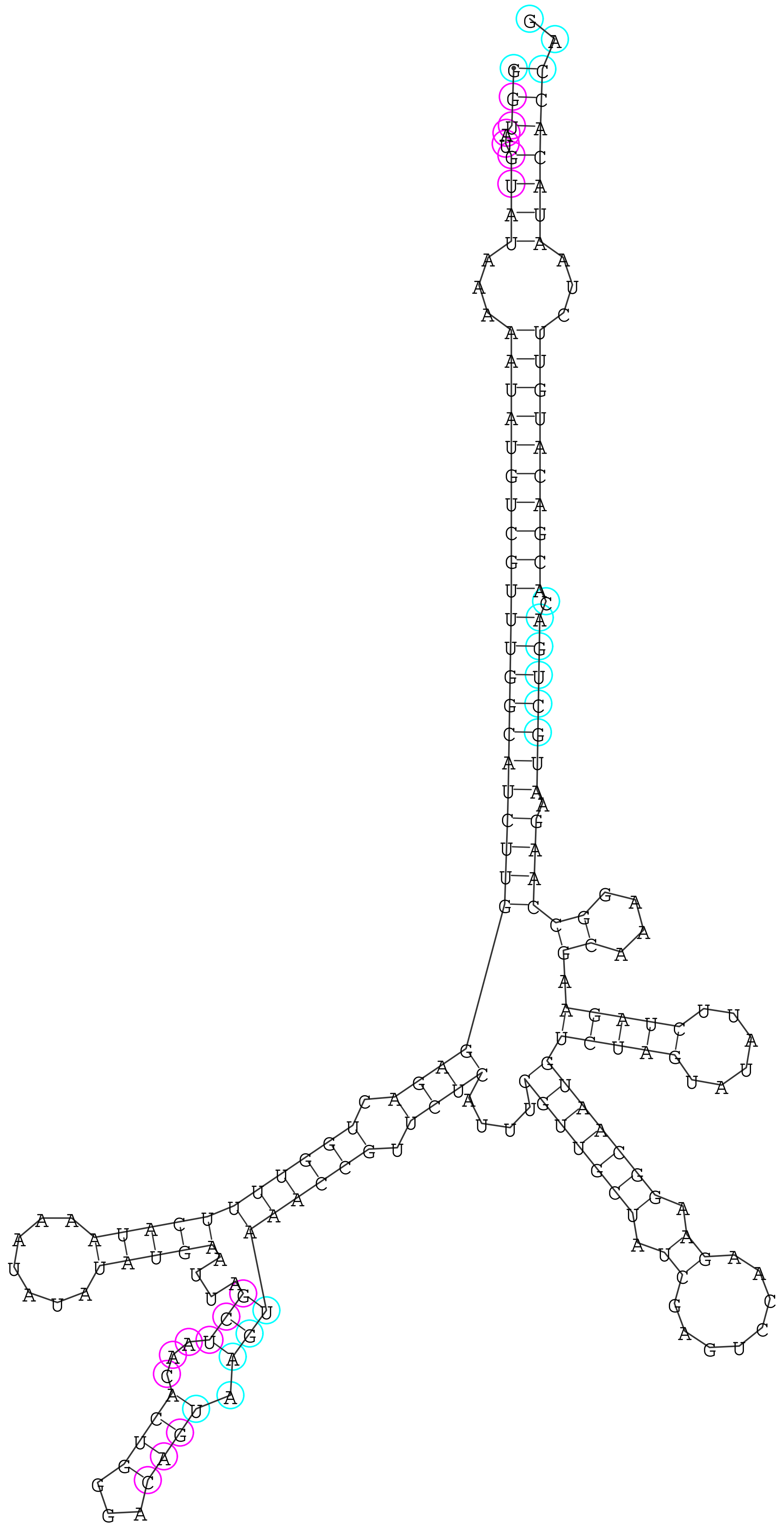

HCOc378A - Stwintron

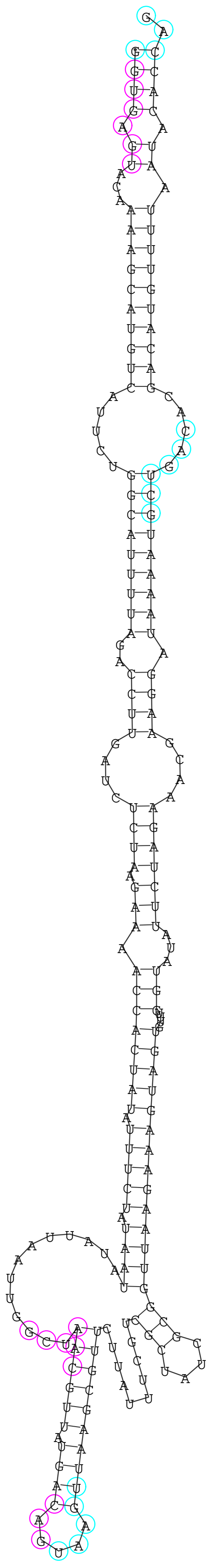

HCOc406A - Stwintron

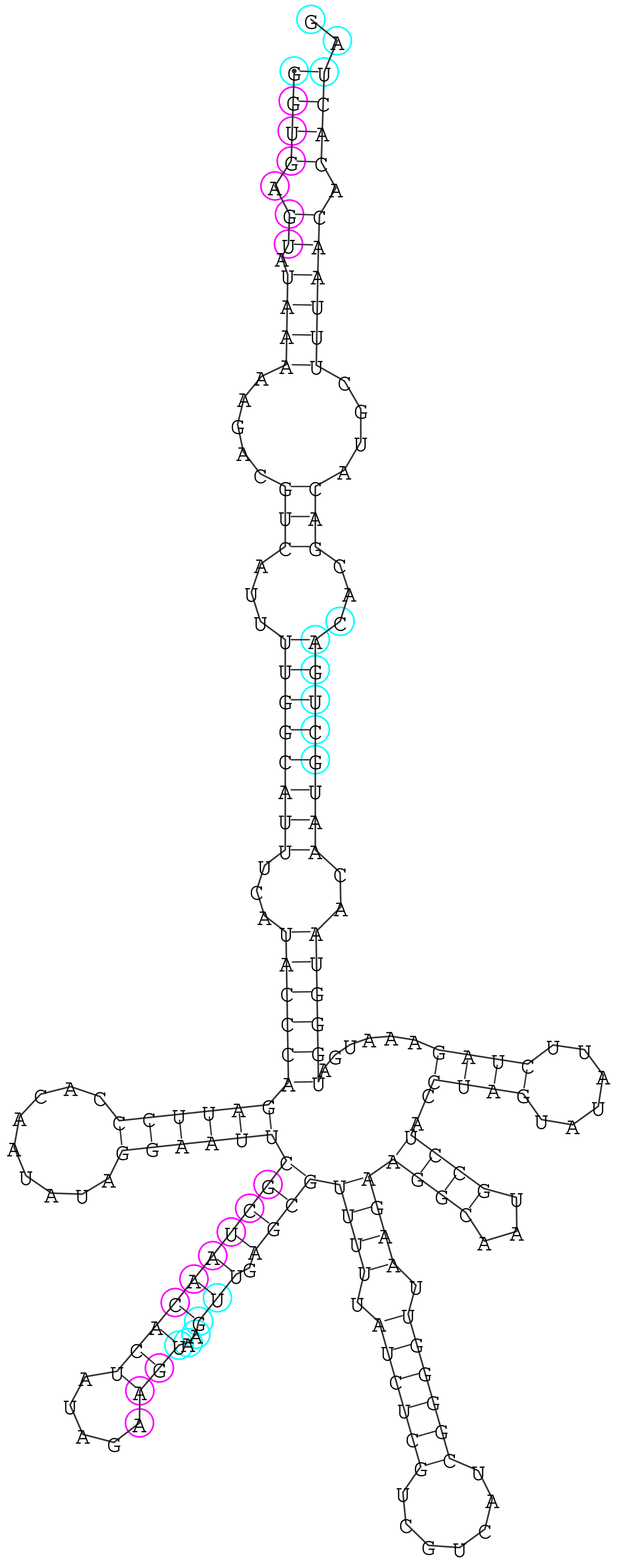

HCOc522A - Stwintron

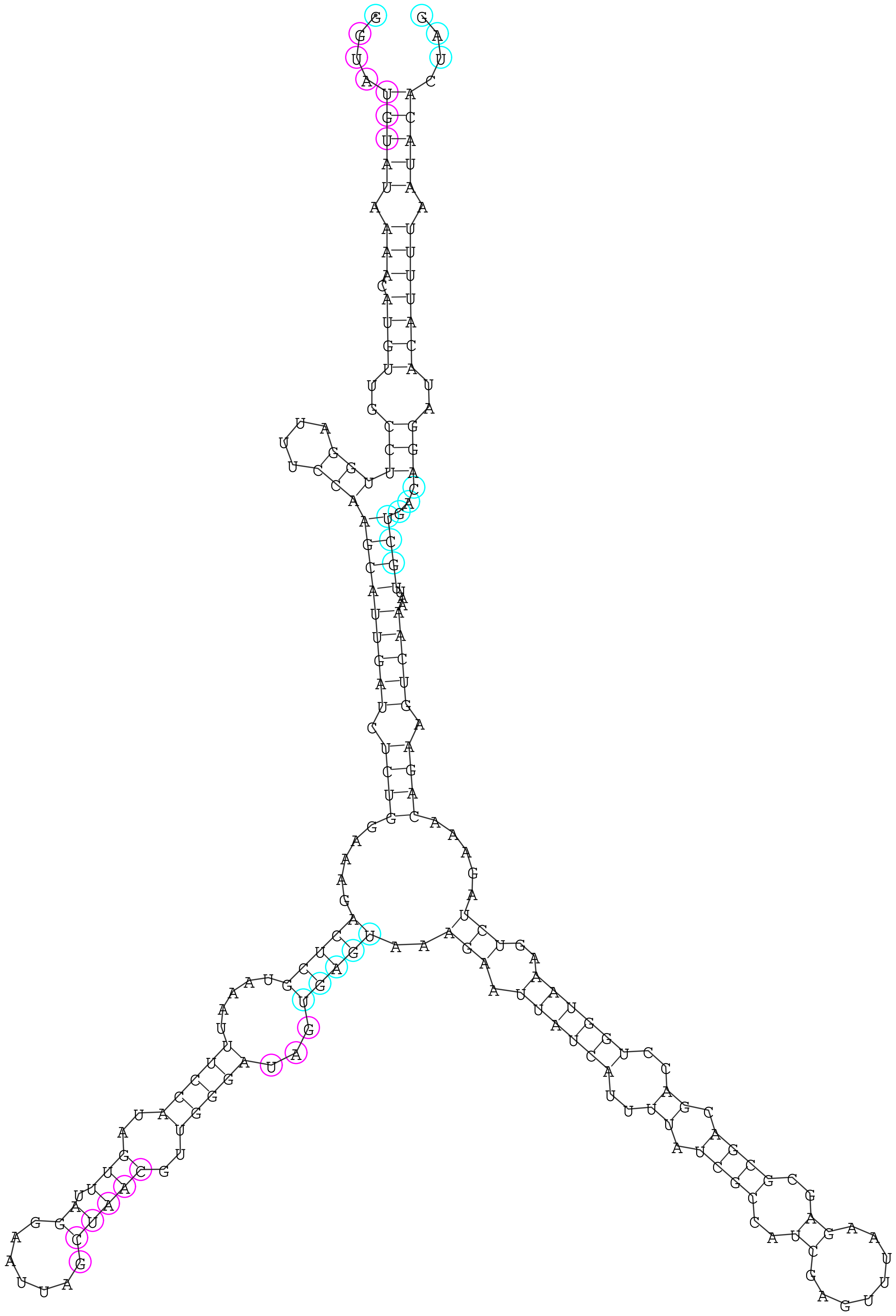

HE7c016A - Stwintron

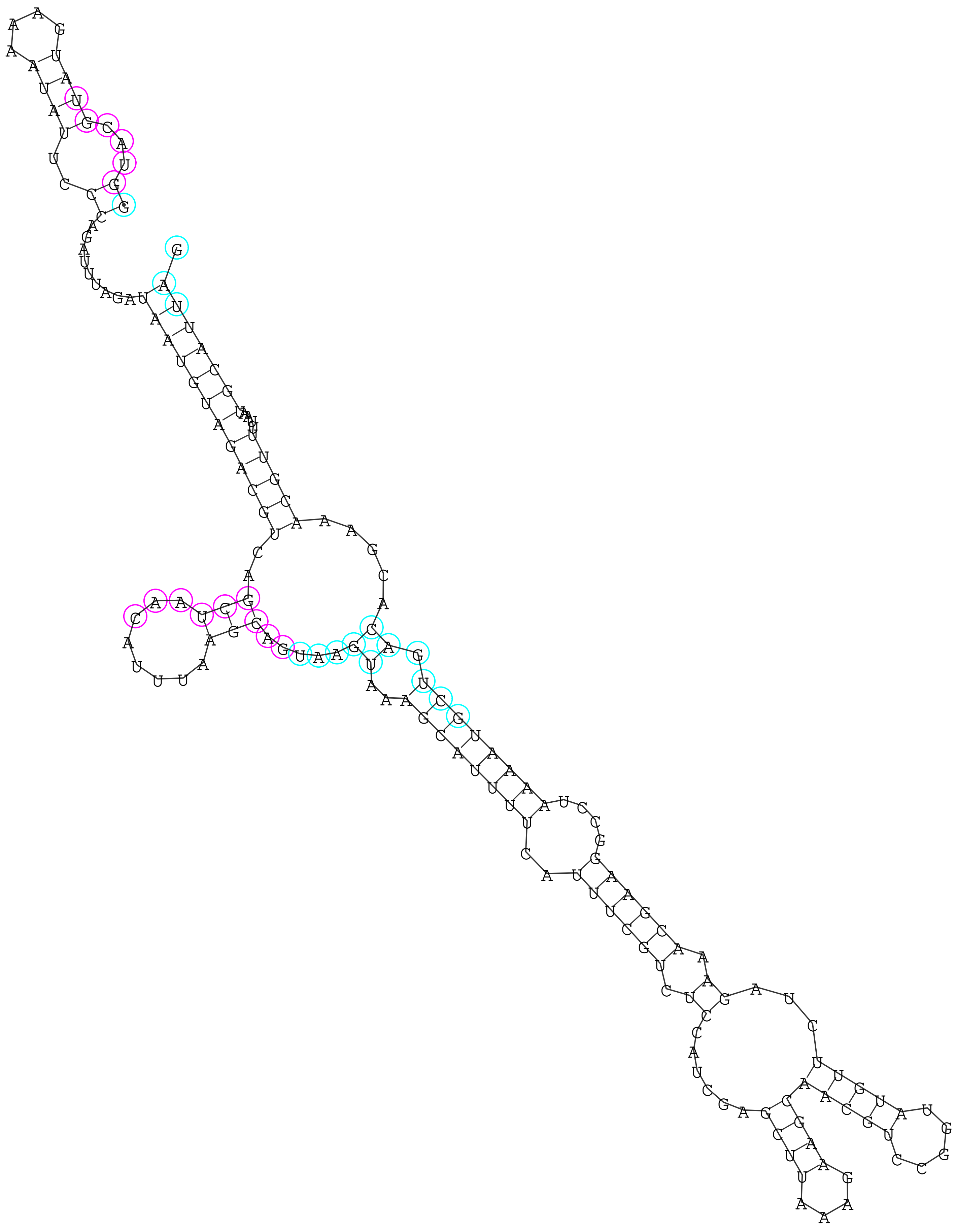

HE7c026A - Stwintron

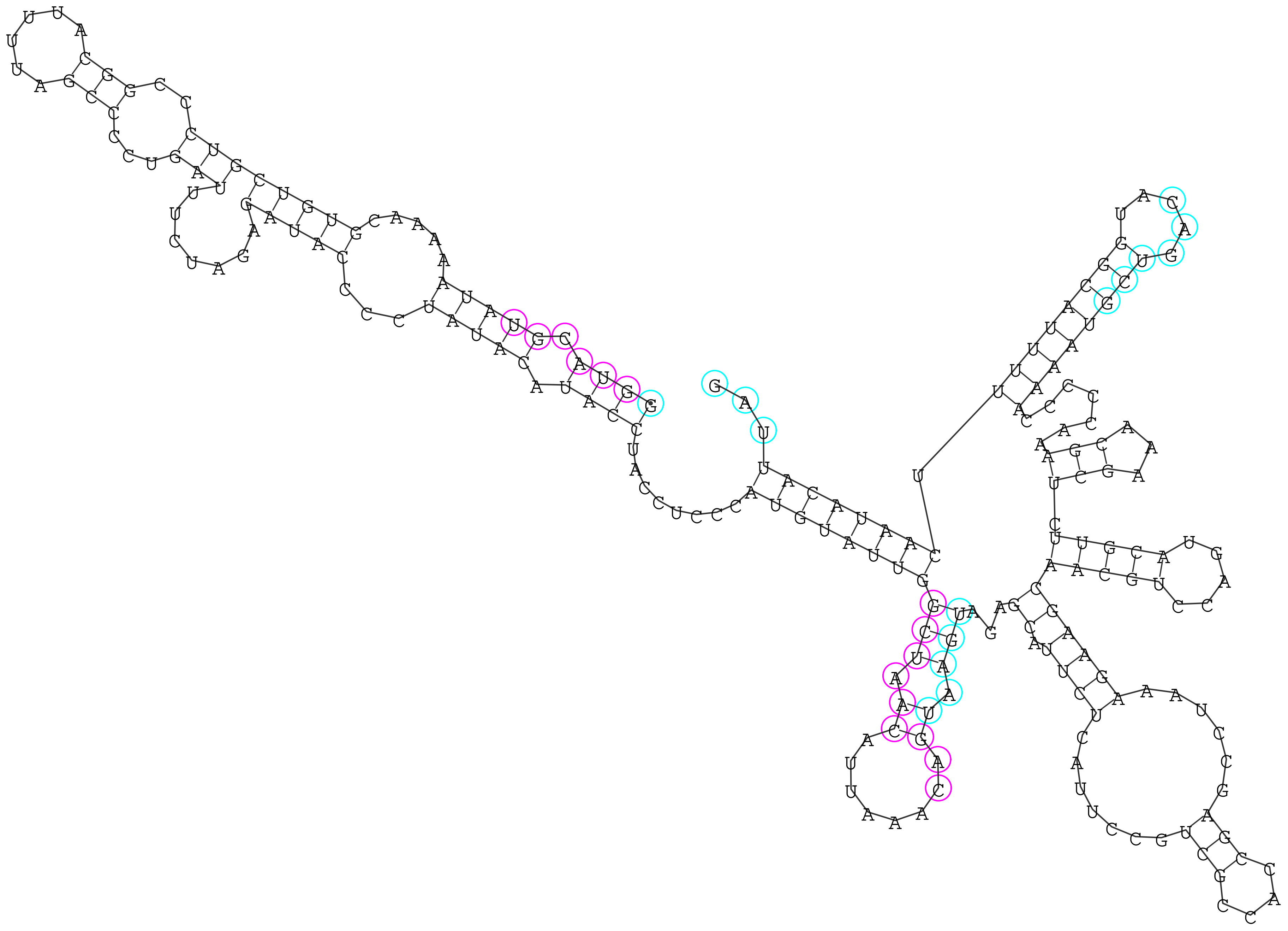

HE7c026B - Stwintron

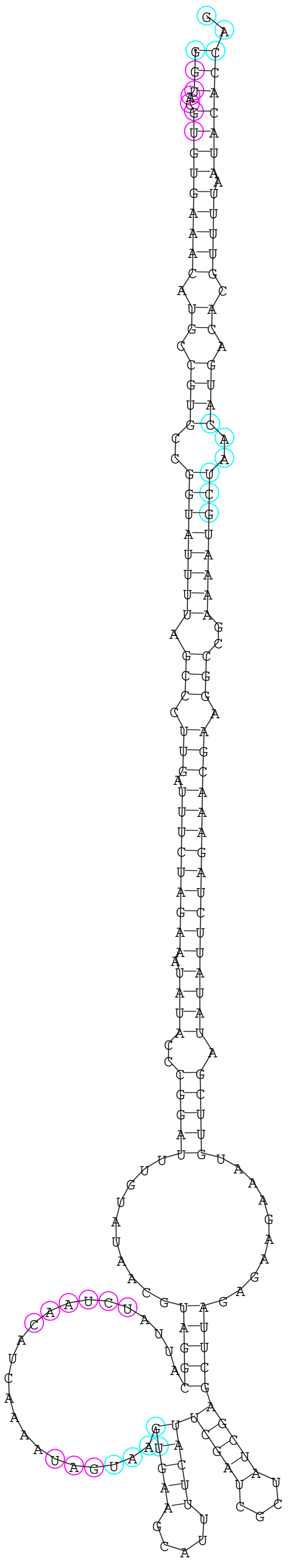

HE7c035A - Stwintron

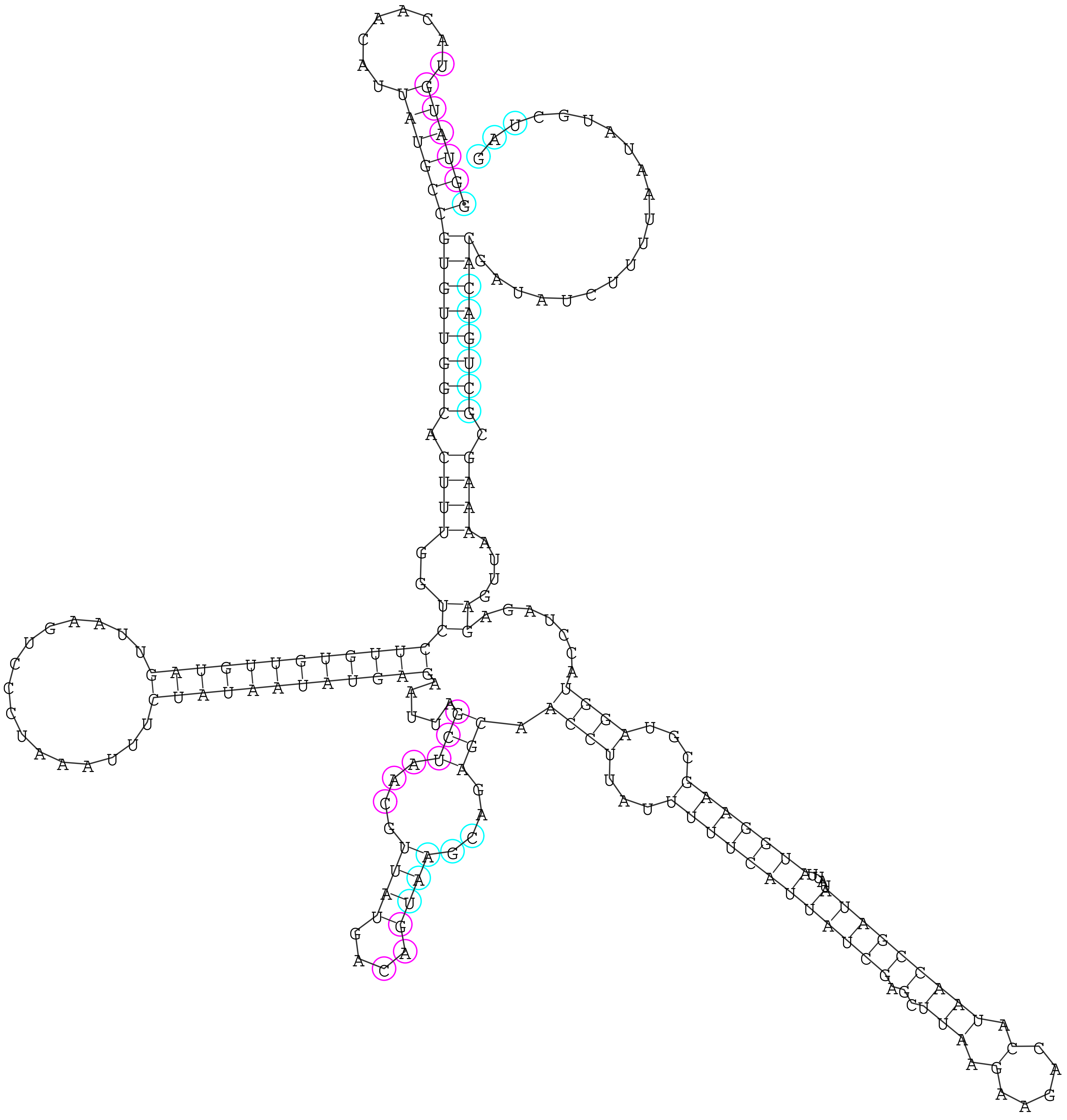

HE7c050A - Stwintron

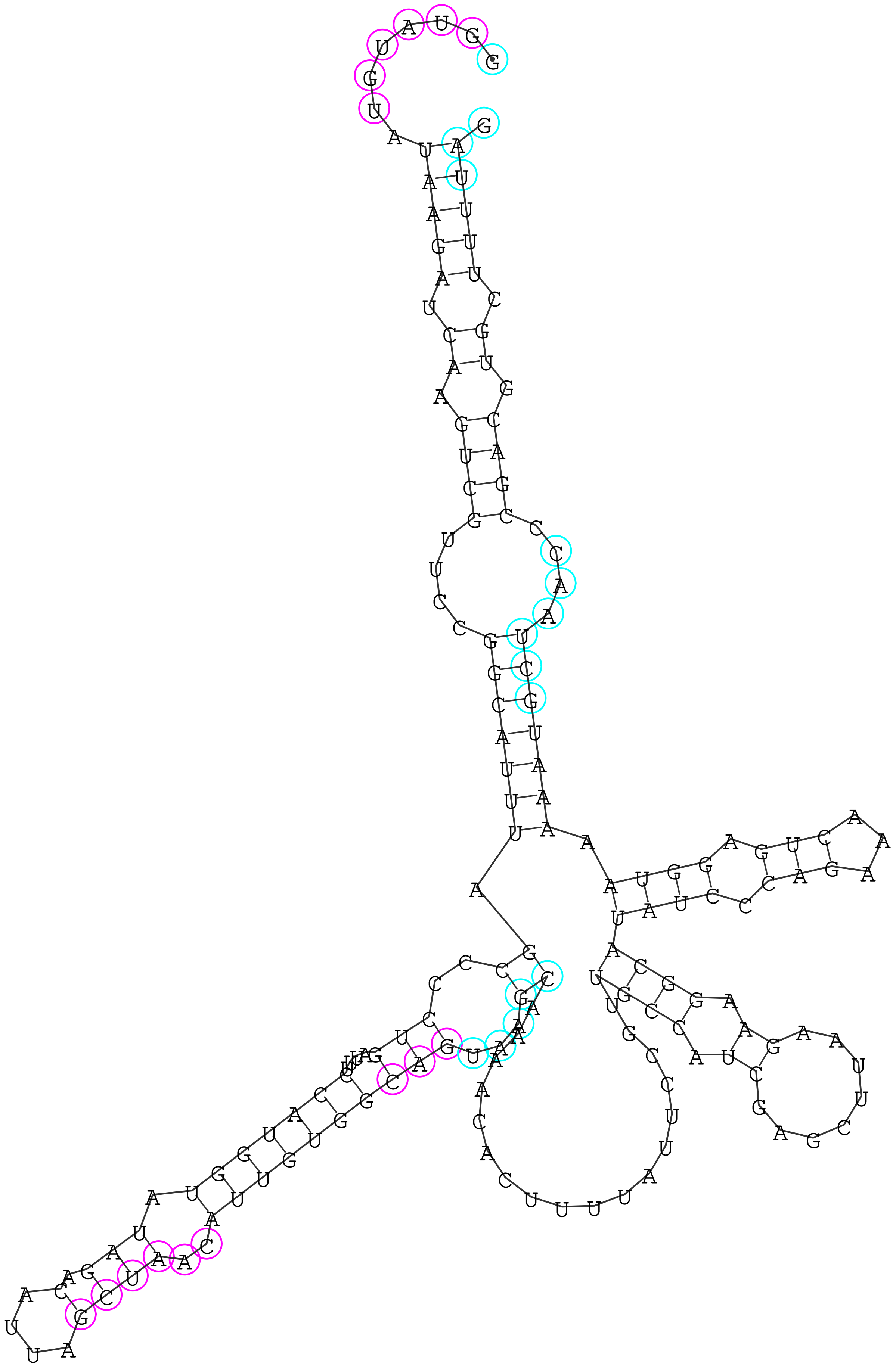

HE7c129A - Stwintron

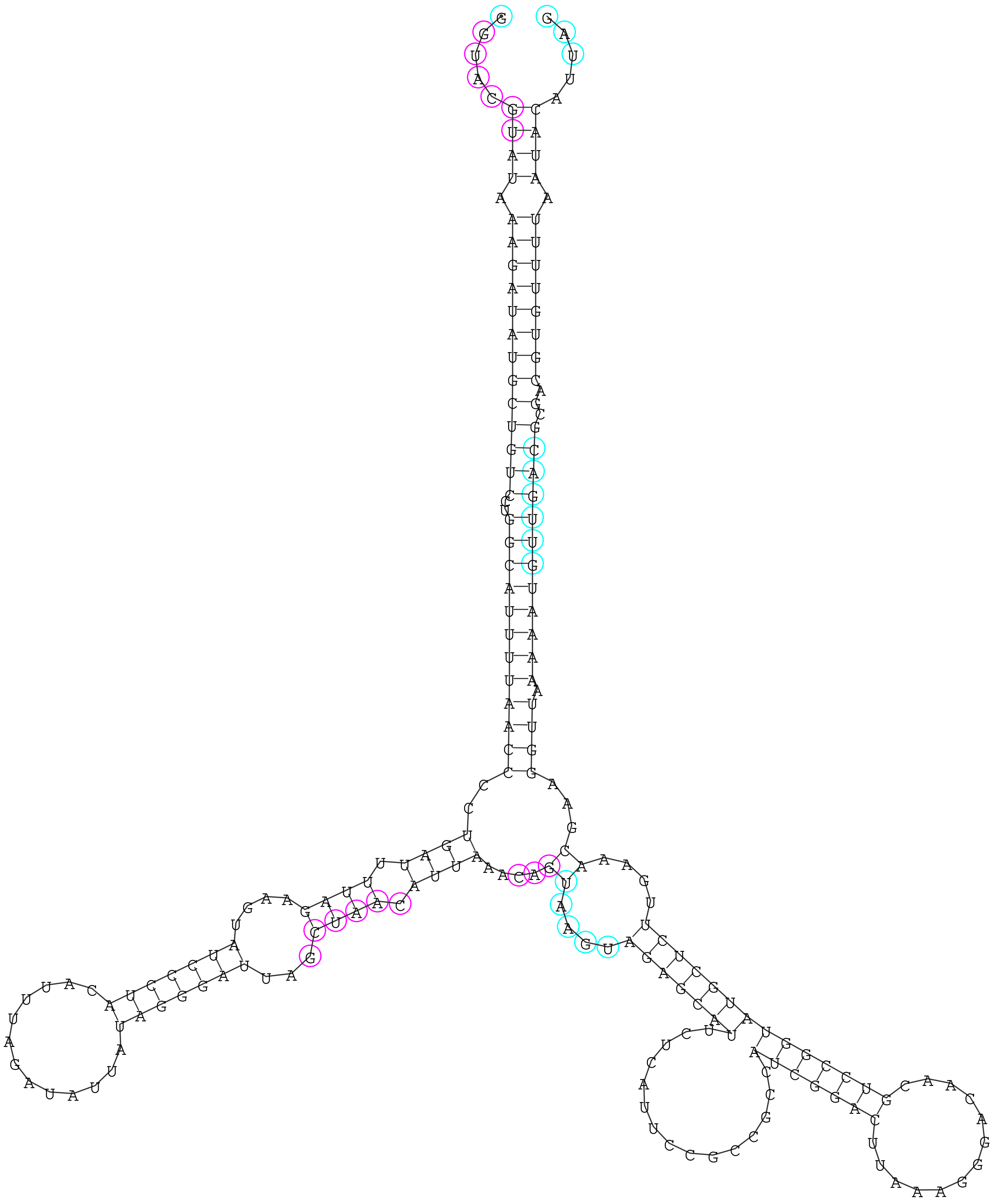

HE7c137A - Stwintron

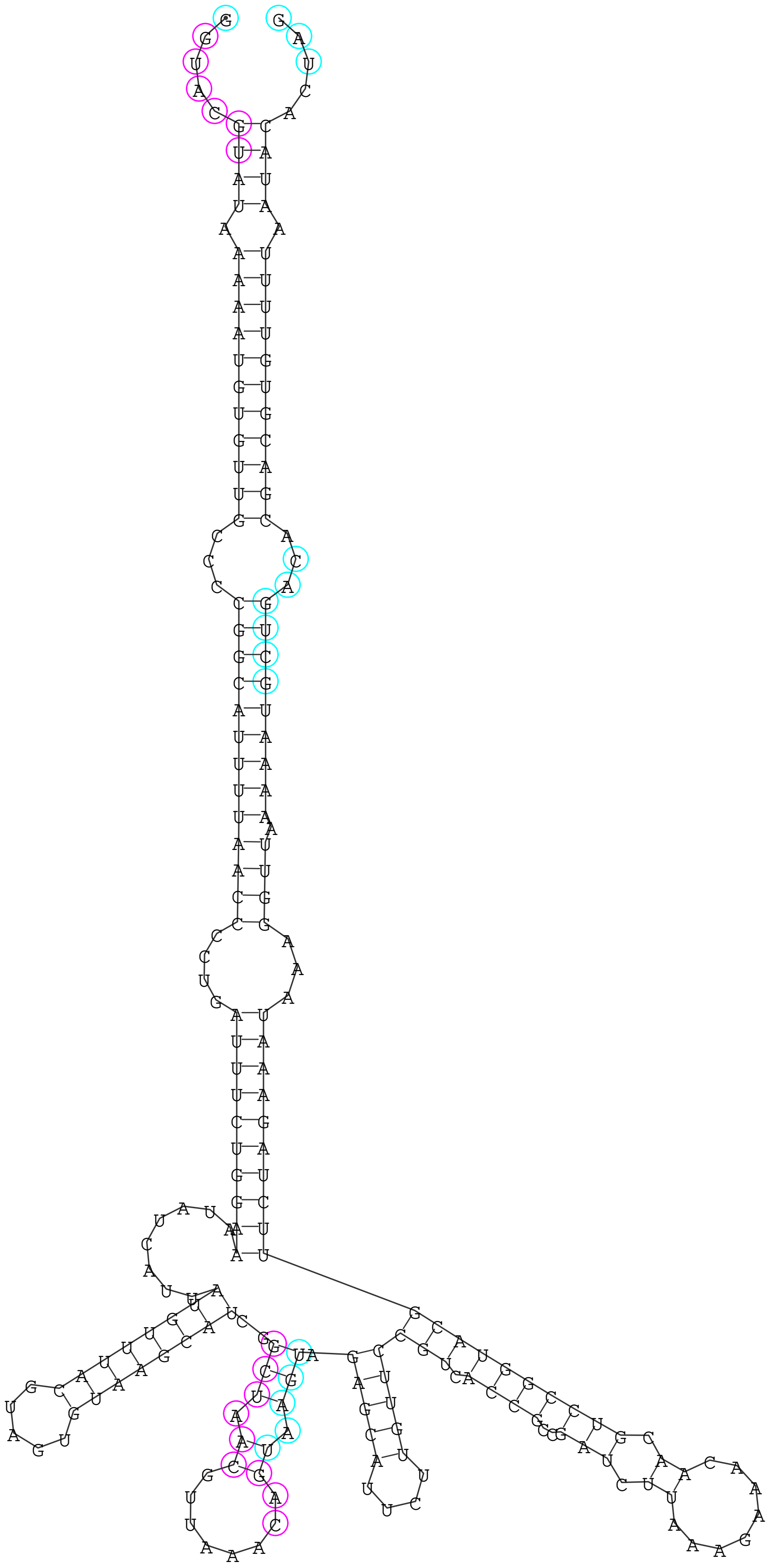

HE7c276A - Stwintron

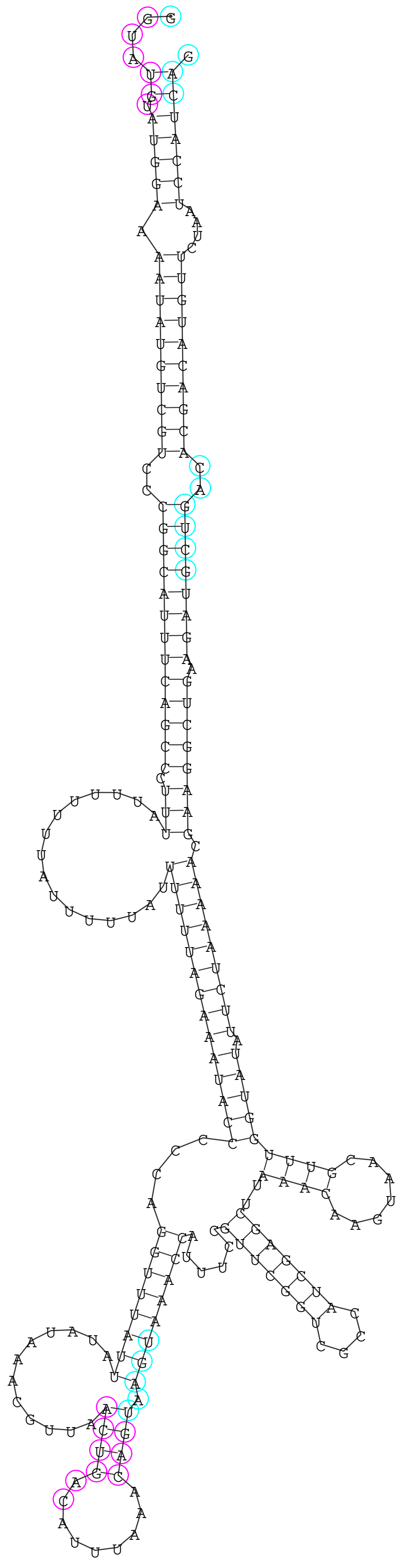

HE7c301A - Stwintron

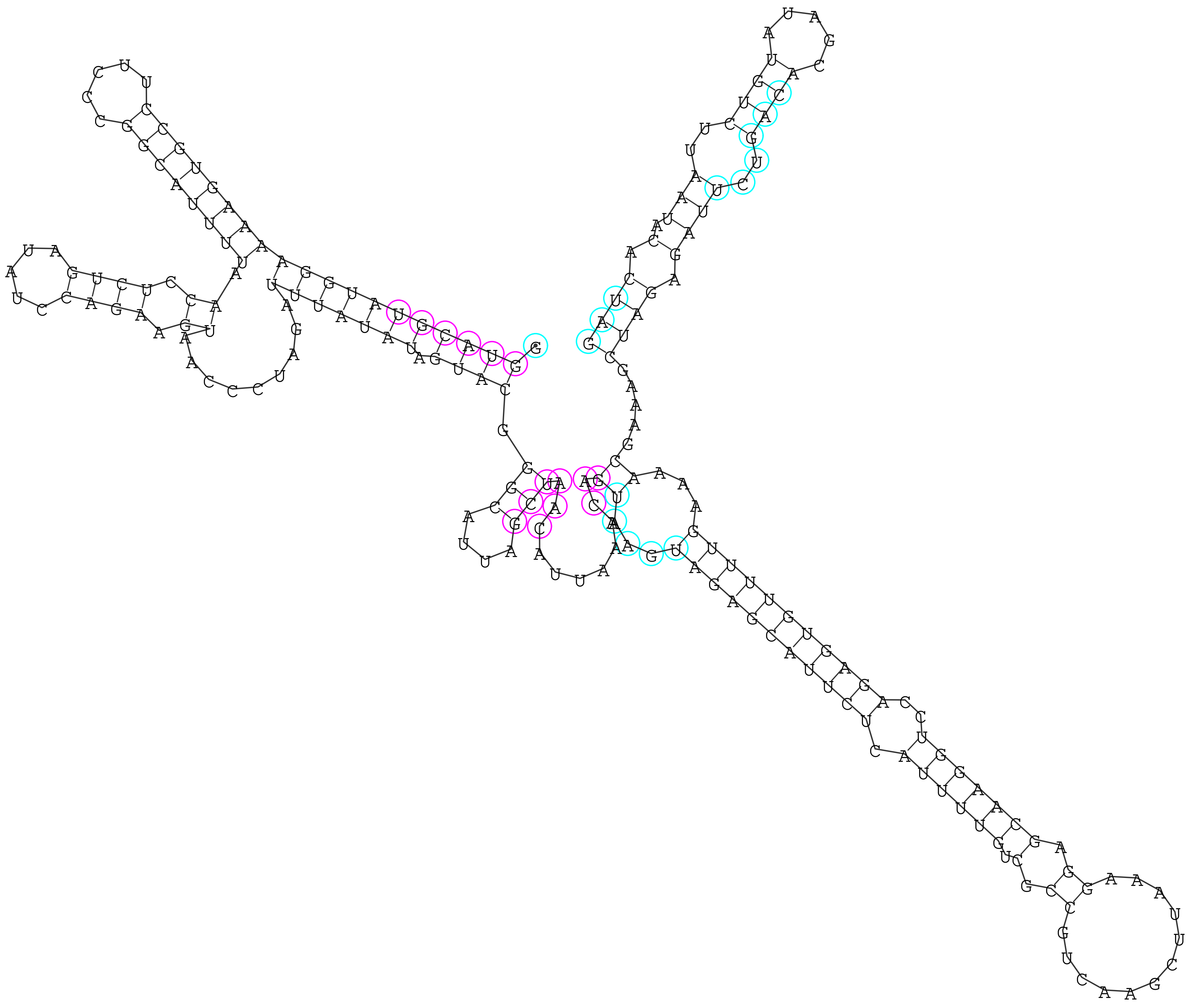

HECc114A - Stwintron

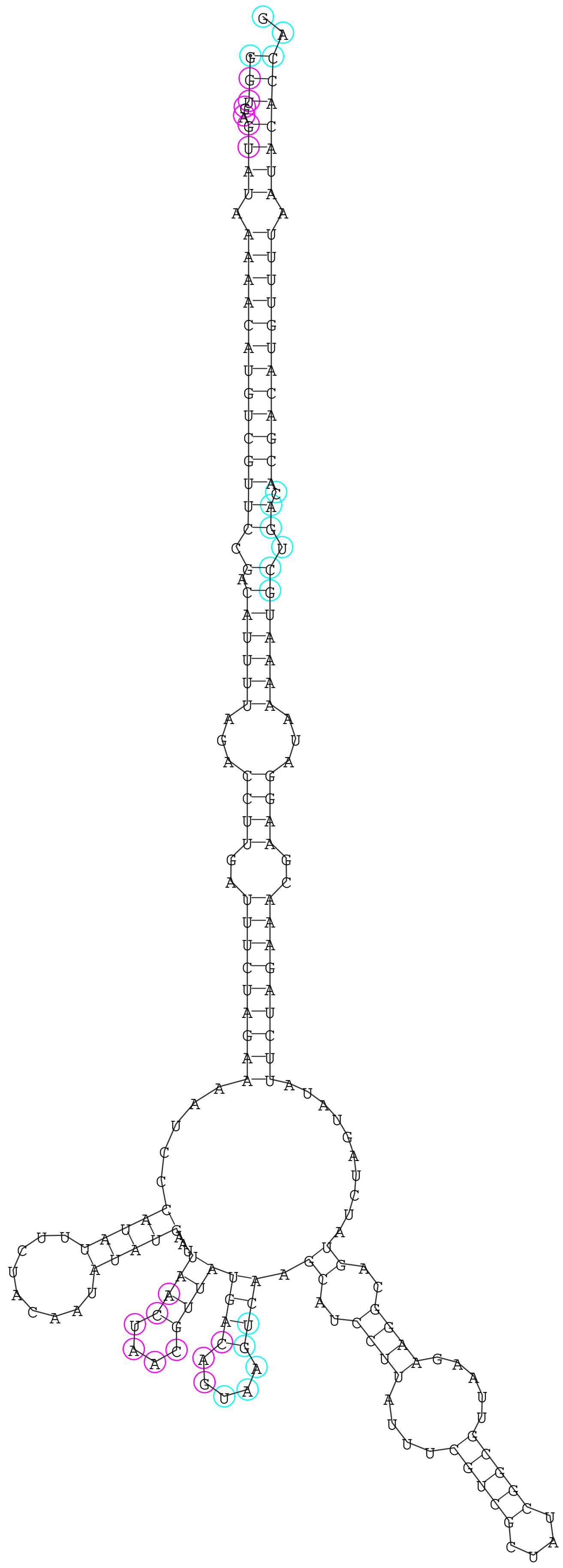

HECc217A - Stwintron

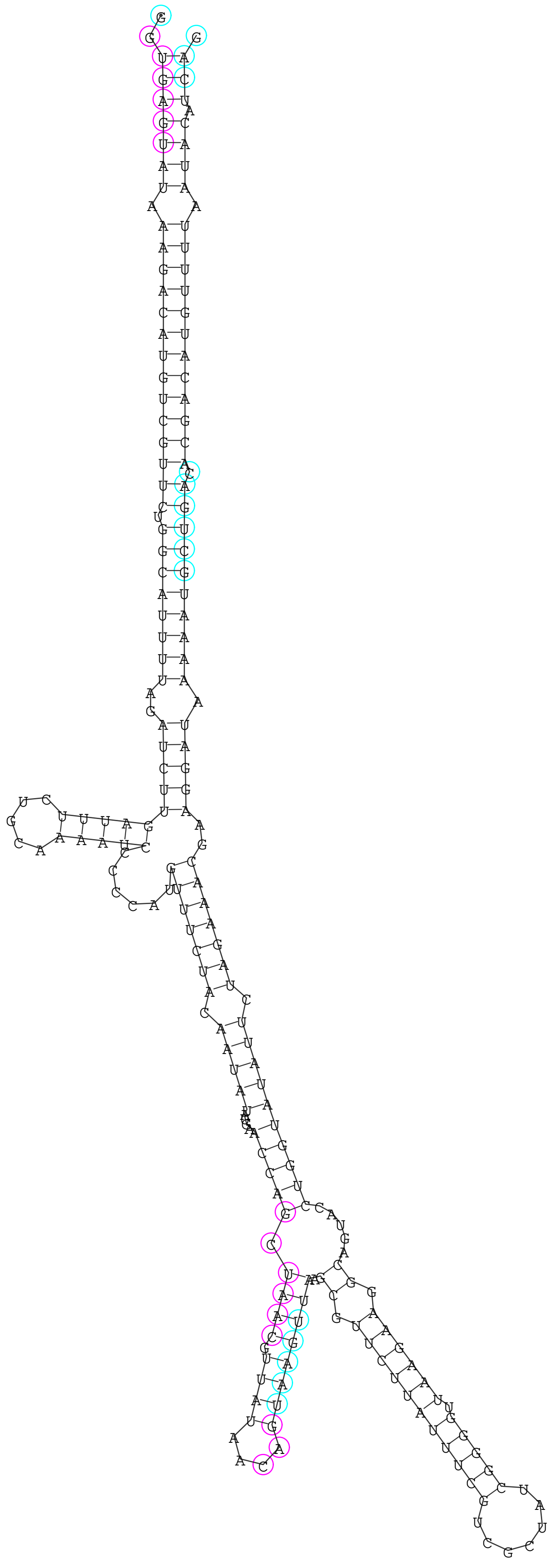

Hruc29A - Stwintron

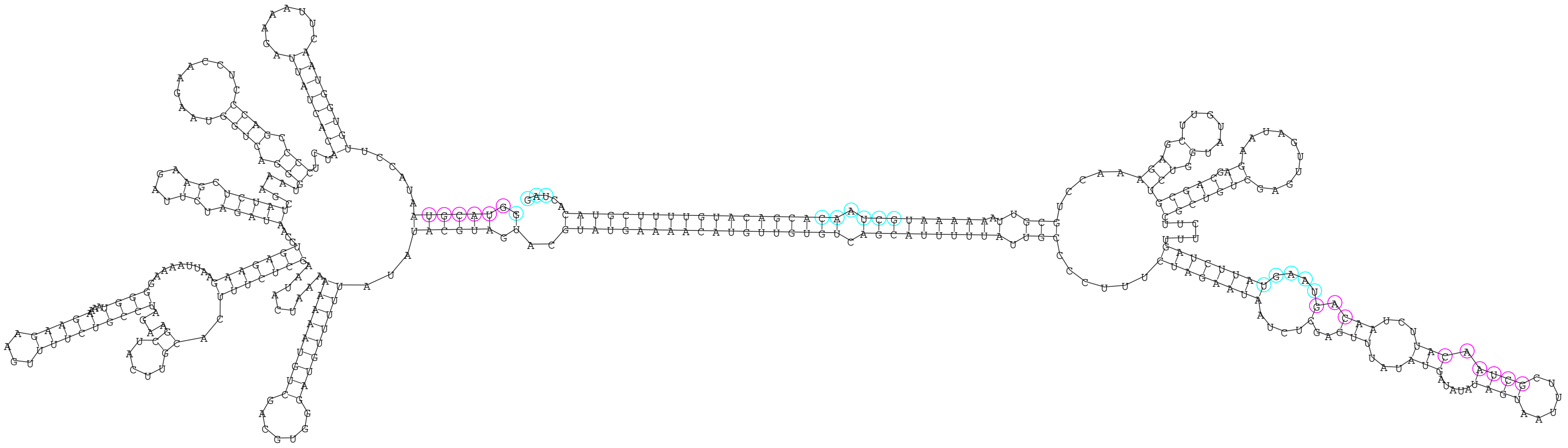

Hruc31A - Stwintron

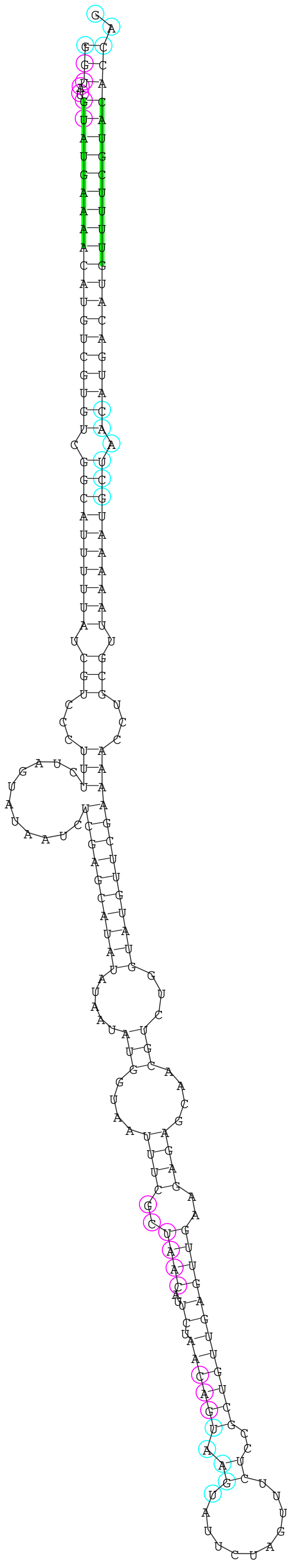

Hruc55A - Stwintron

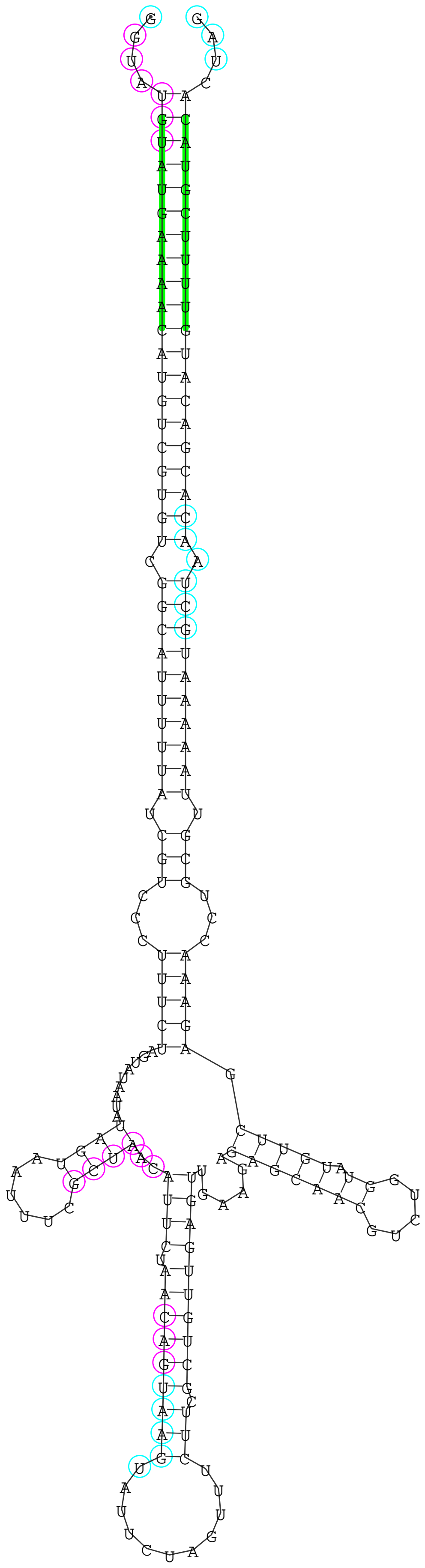

Hruc56A - Stwintron

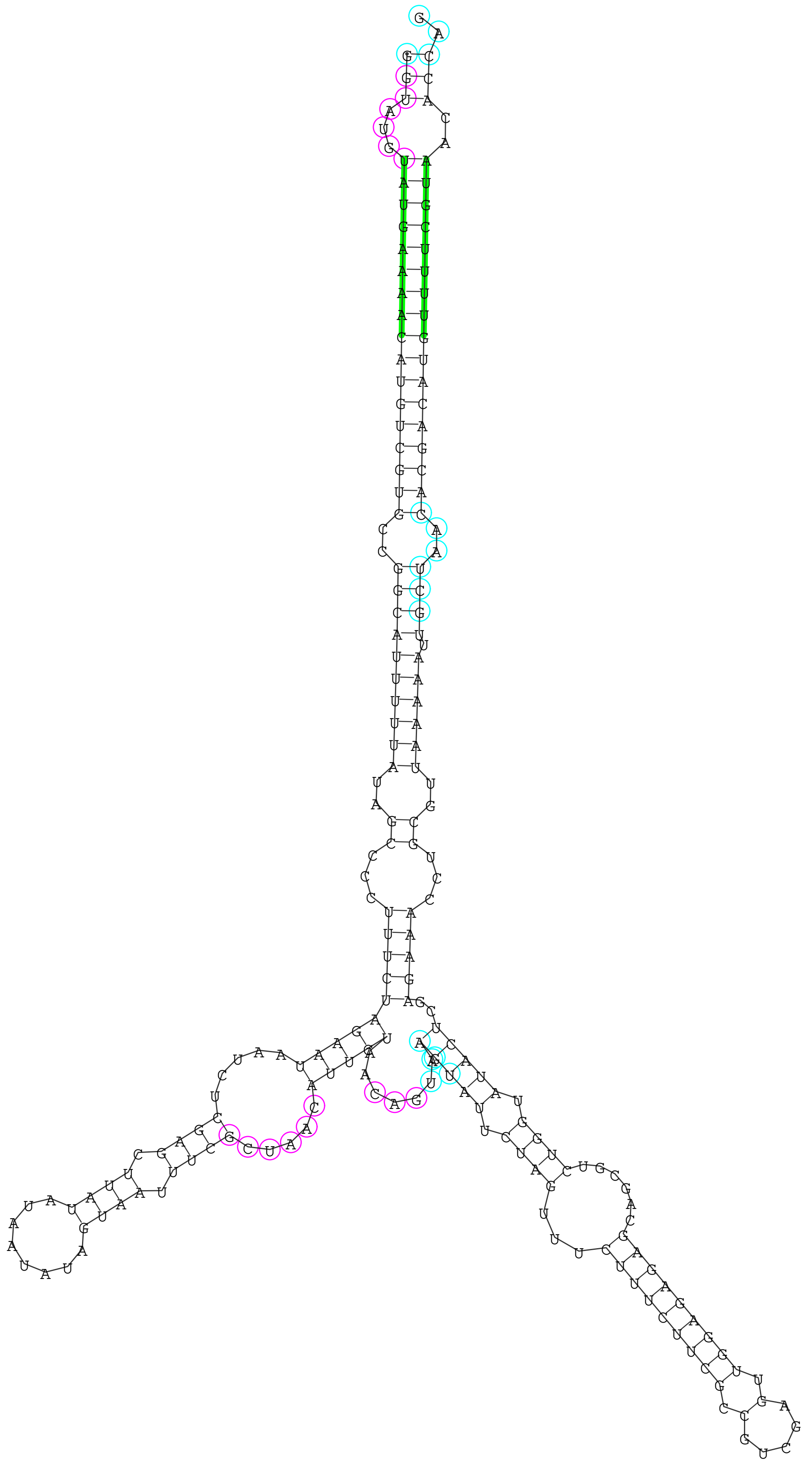

Hruc59A - Stwintron

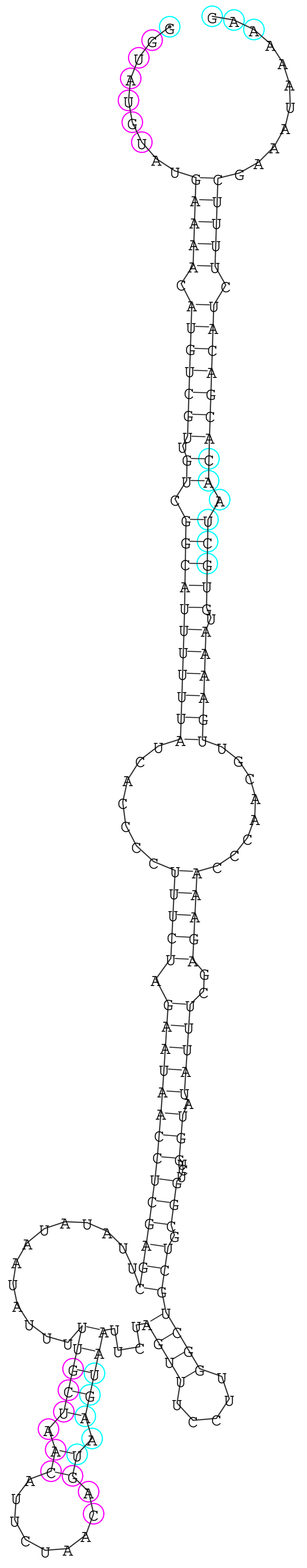

Naboc005A - Stwintron

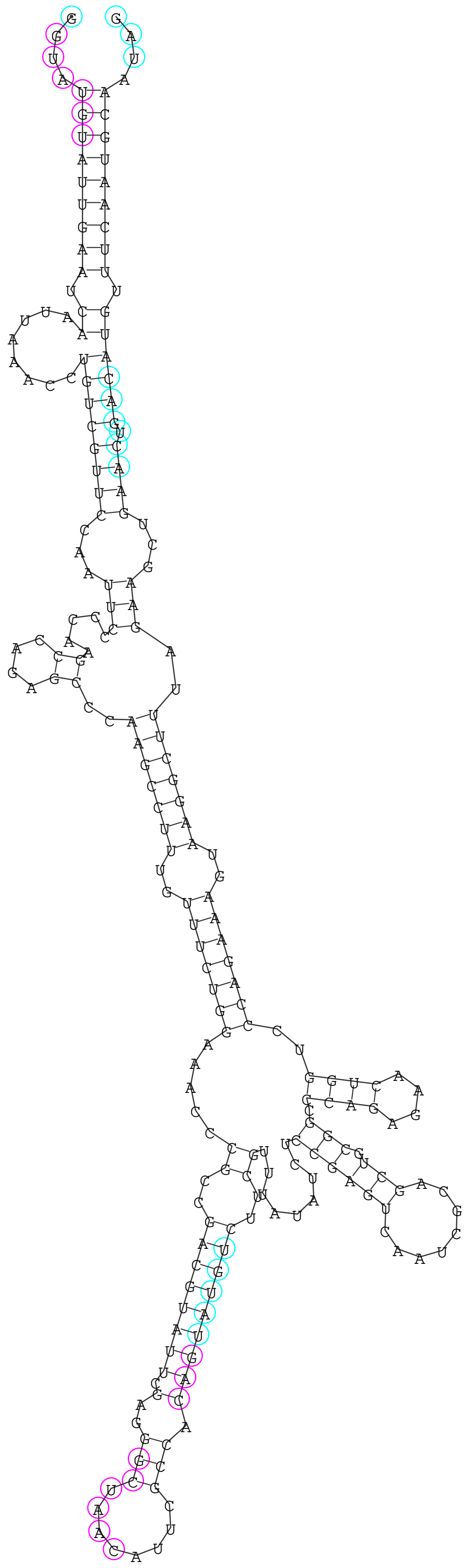

Naboc011A - Stwintron

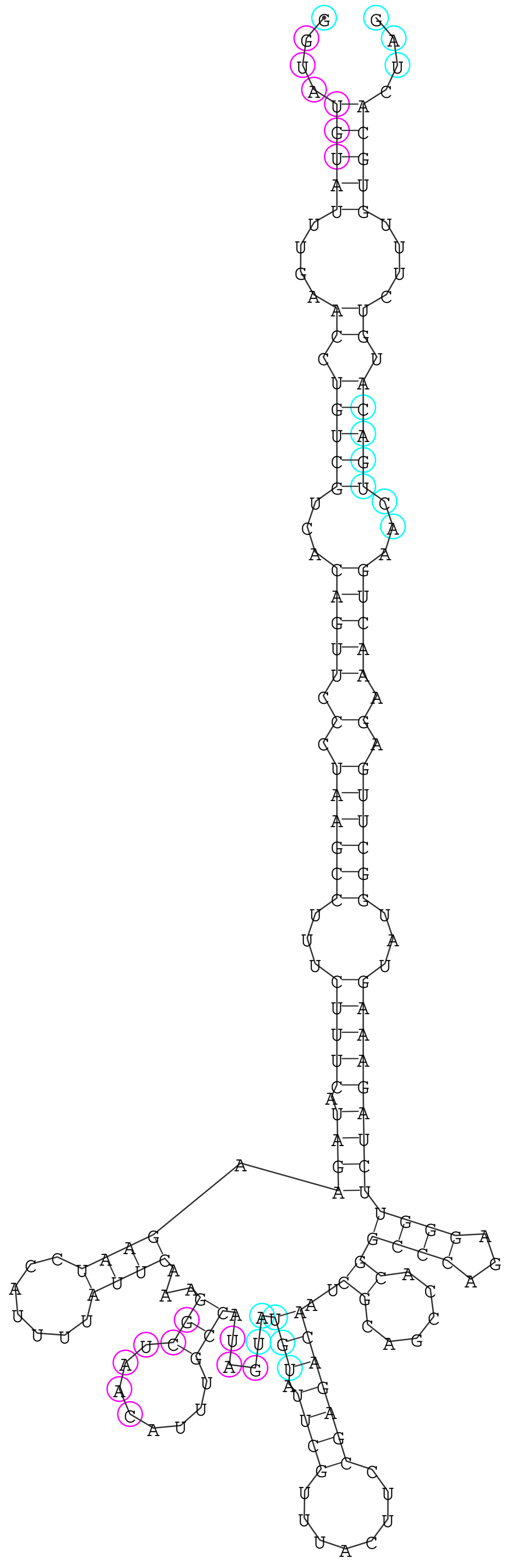

Naboc037A - Stwintron

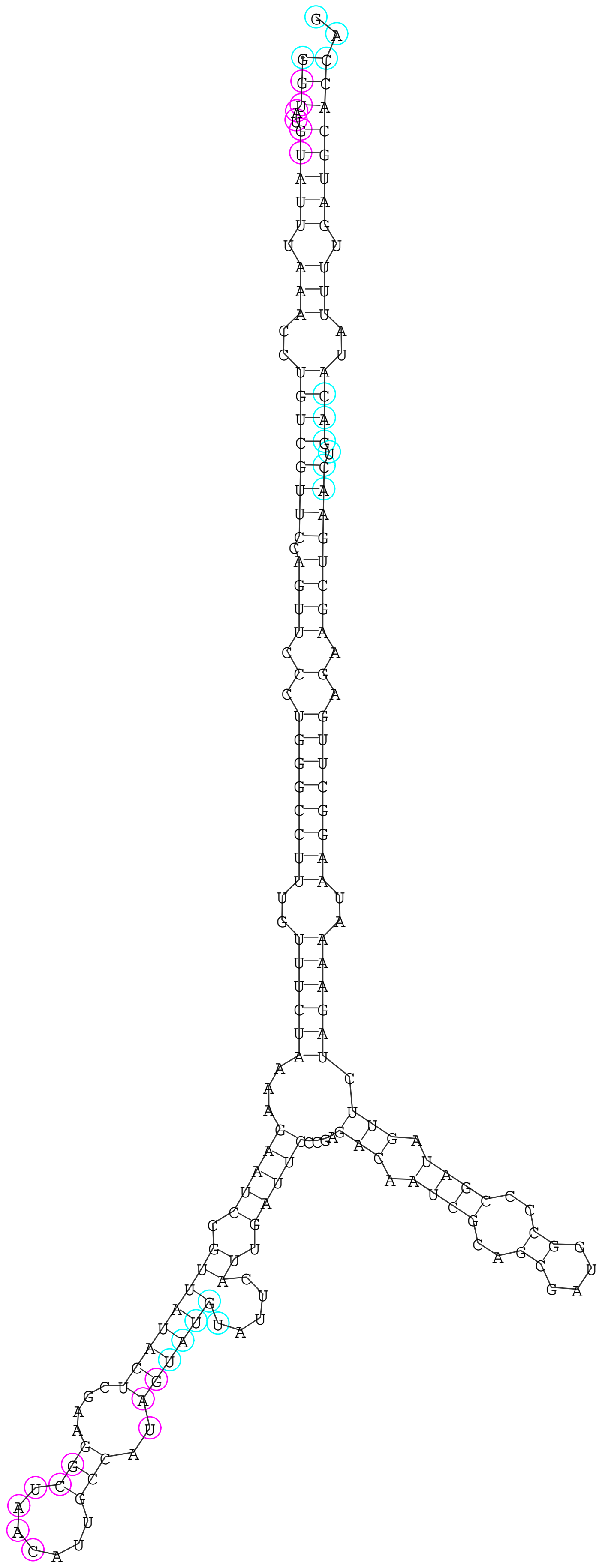

Naboc037B - Stwintron

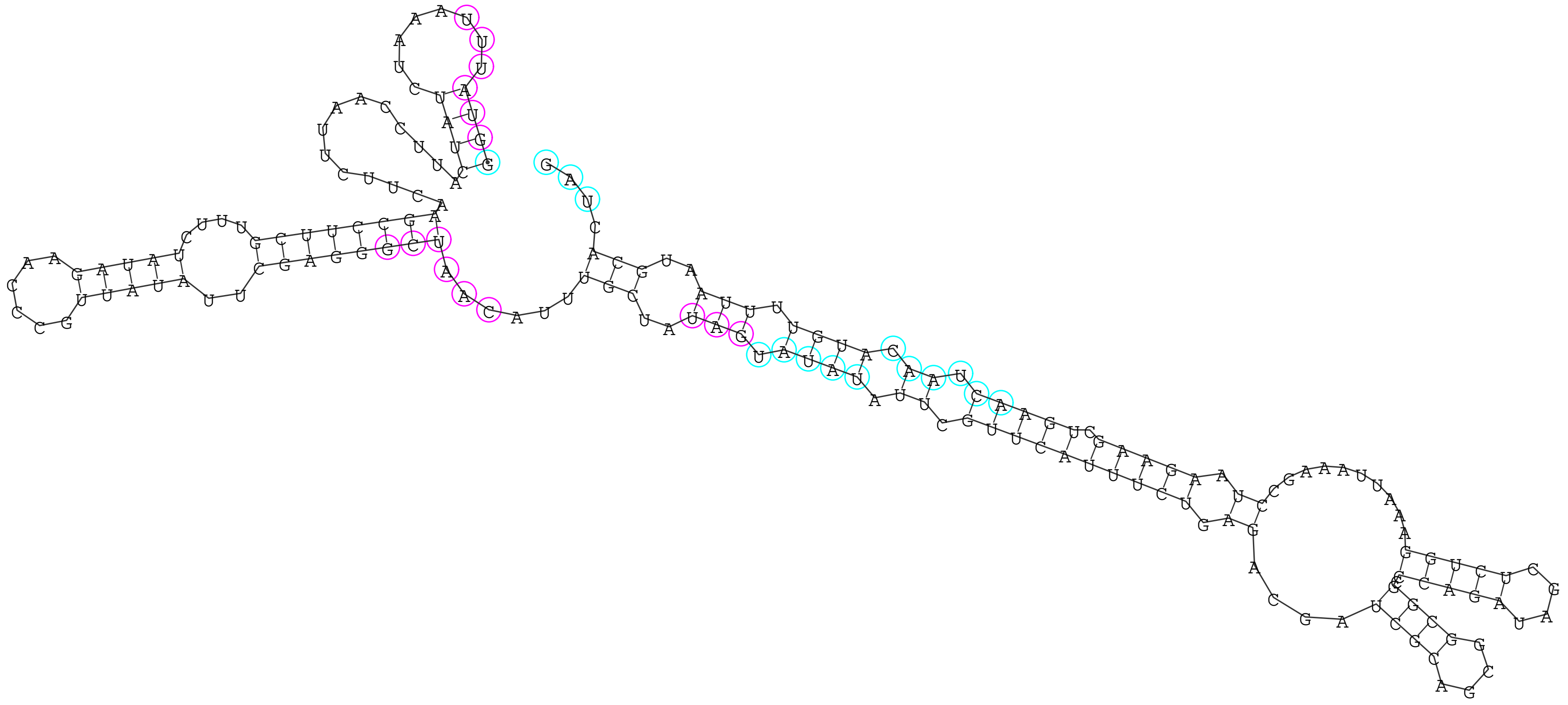

Naboc056A - Stwintron

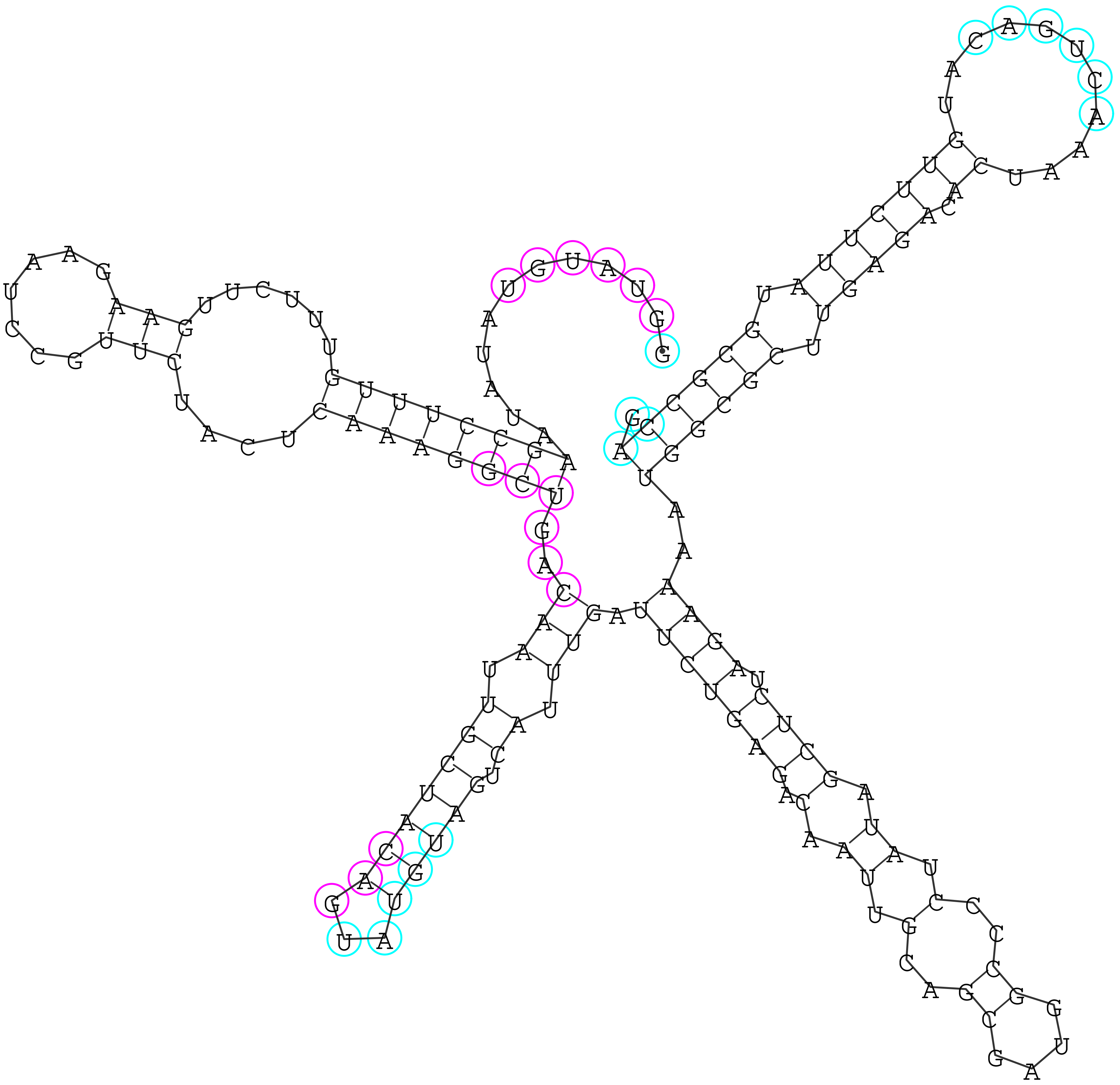

Naboc066A - Stwintron

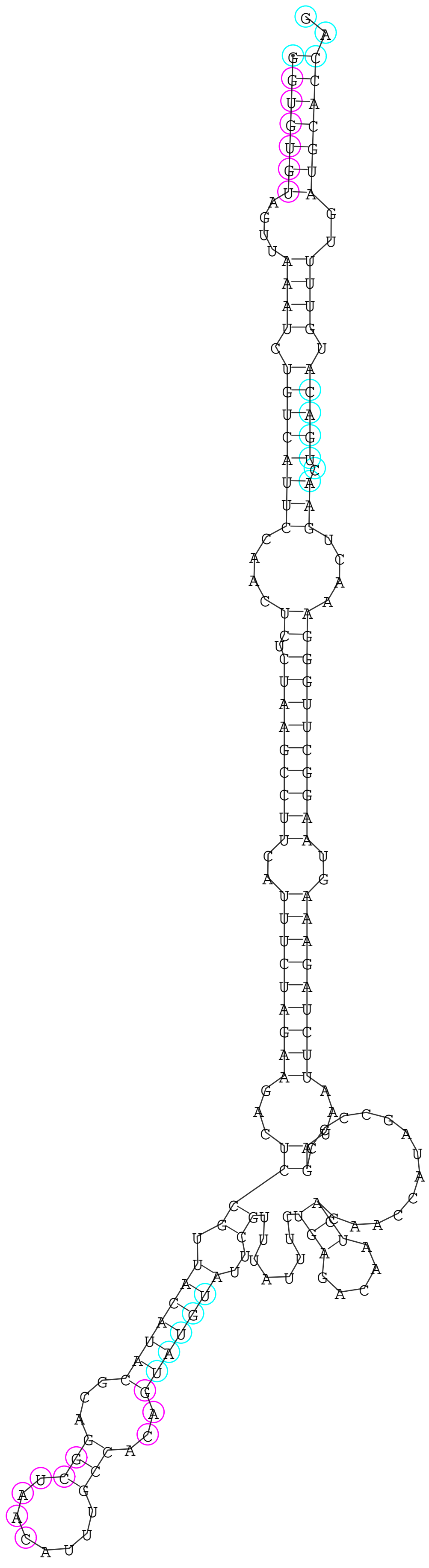

Naboc073A - Stwintron

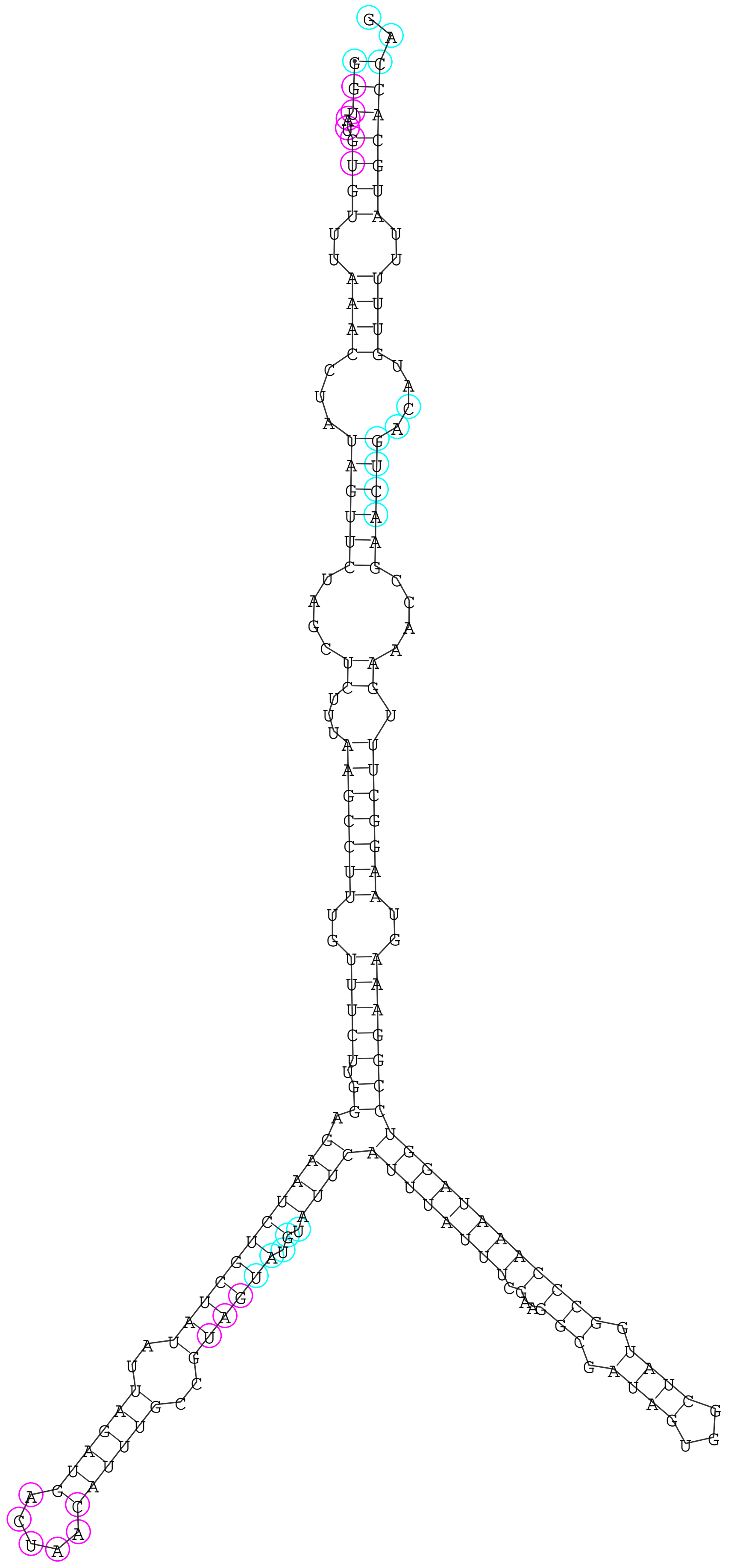

Naboc079A - Stwintron

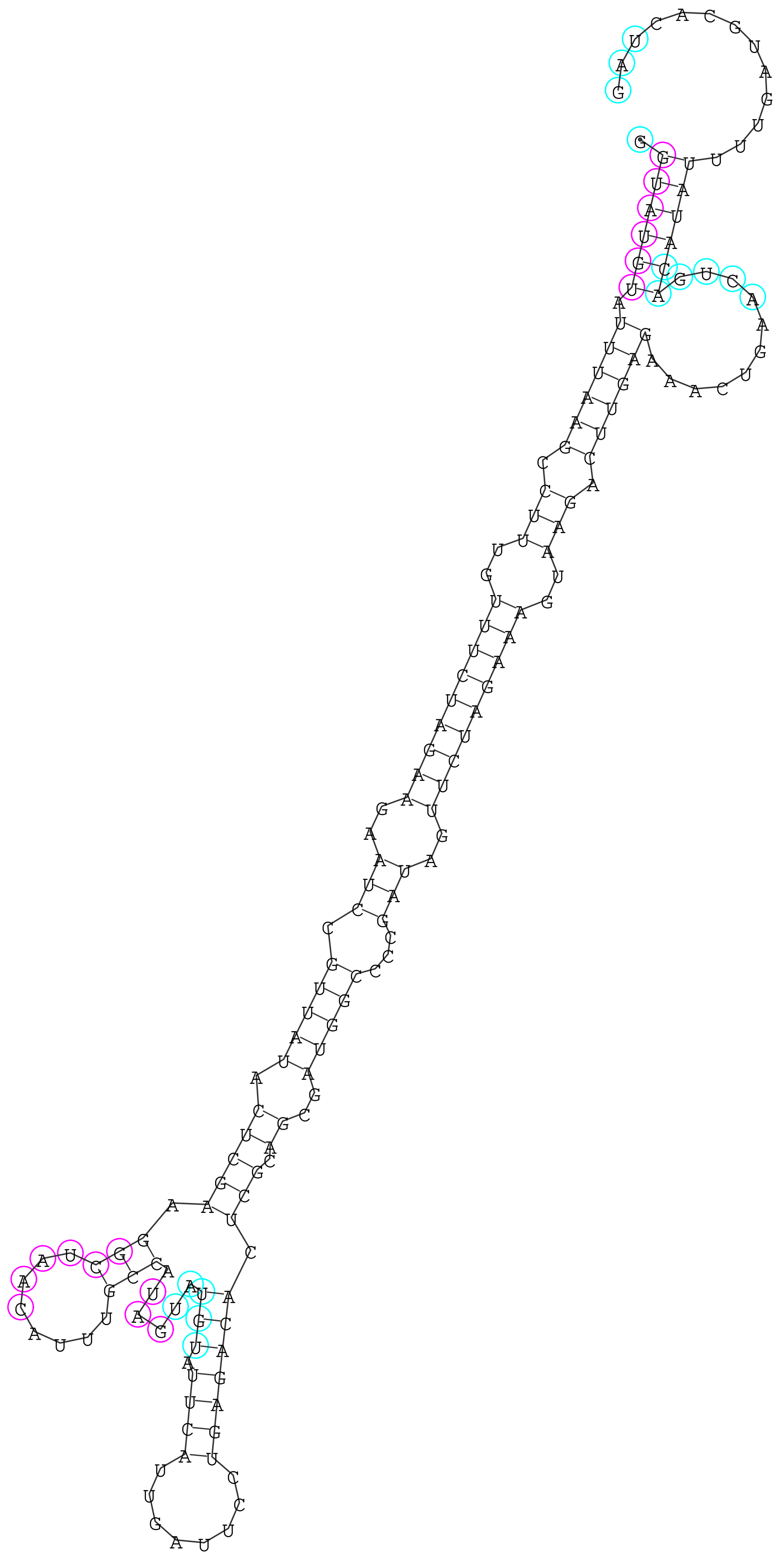

Naboc124A - Stwintron

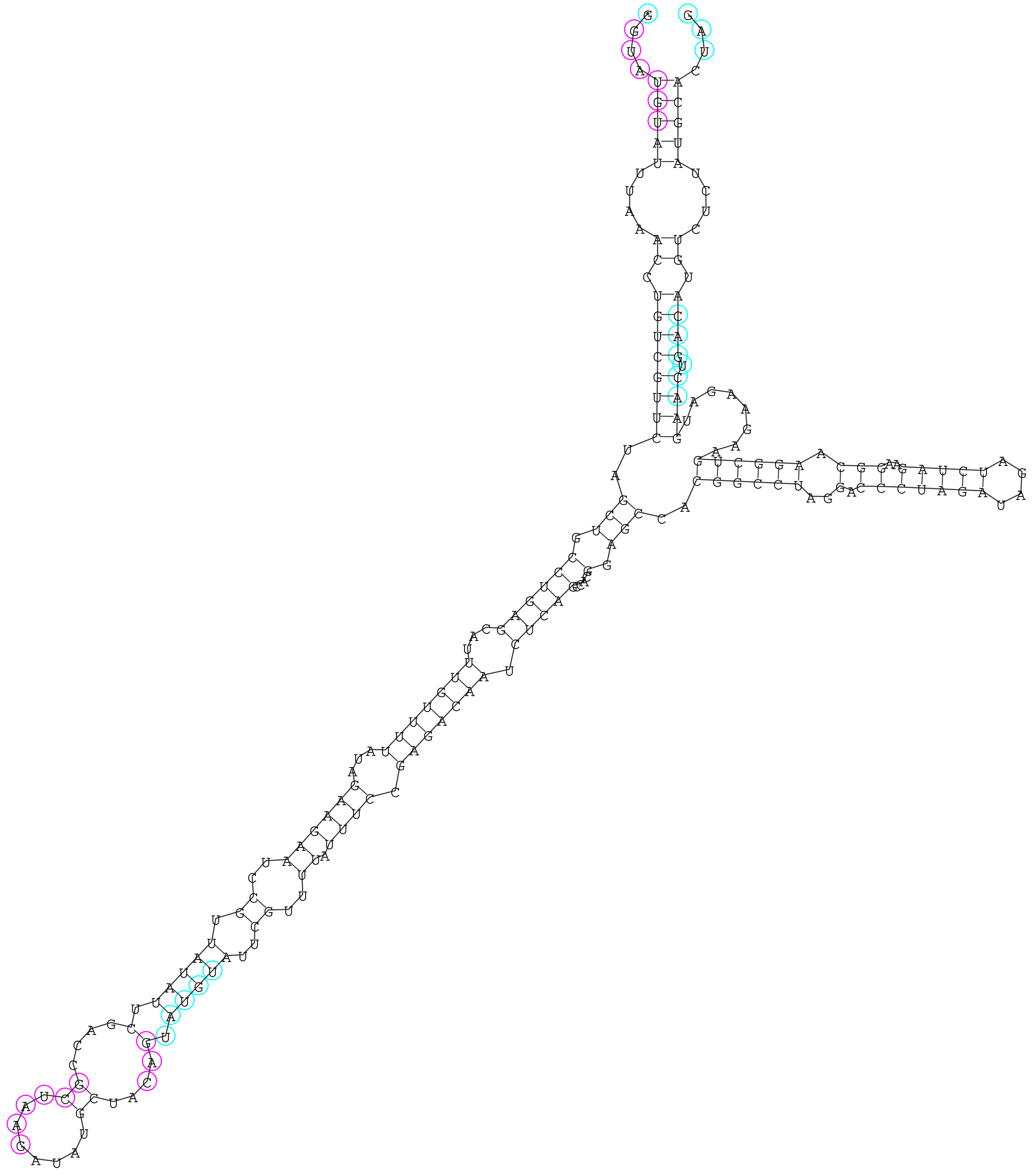

Naboc173A - Stwintron

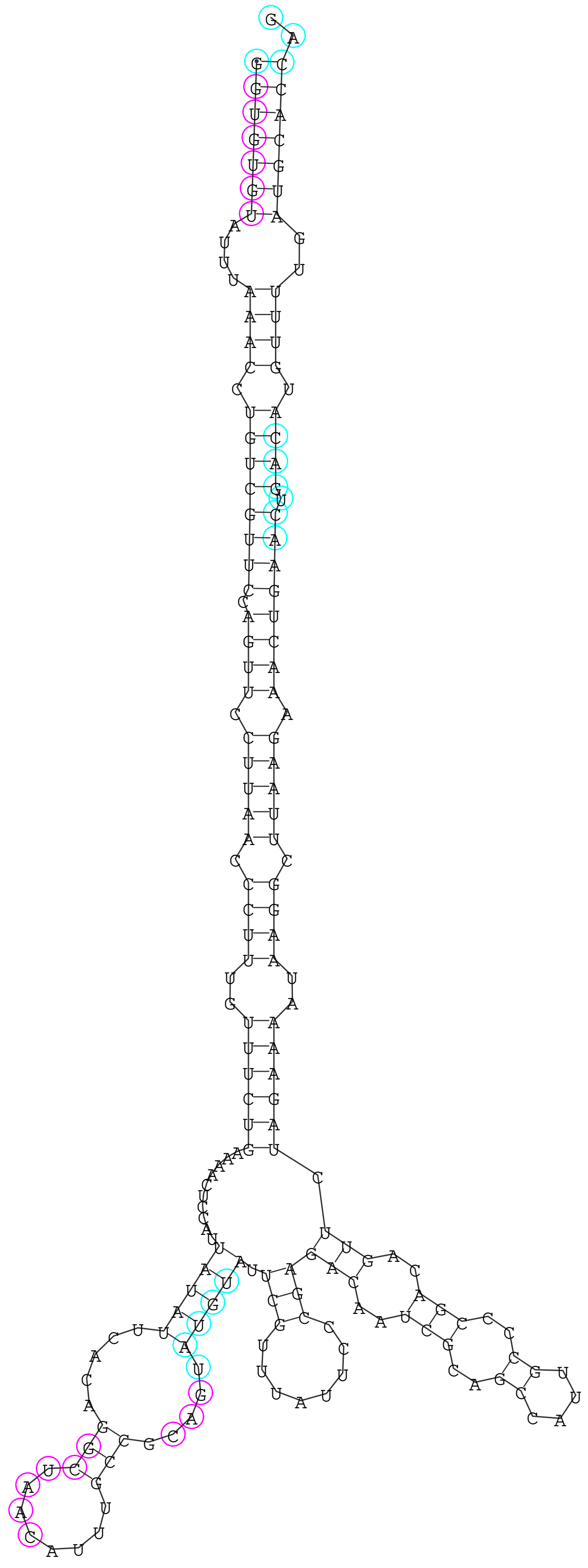

Naboc184A - Stwintron

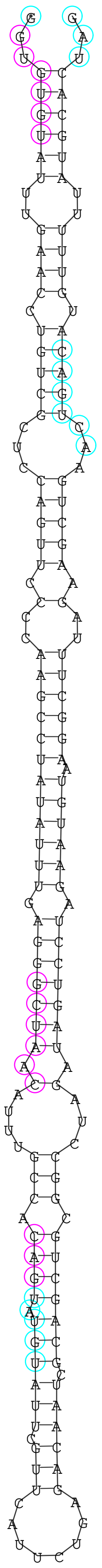

Naboc196A - Stwintron

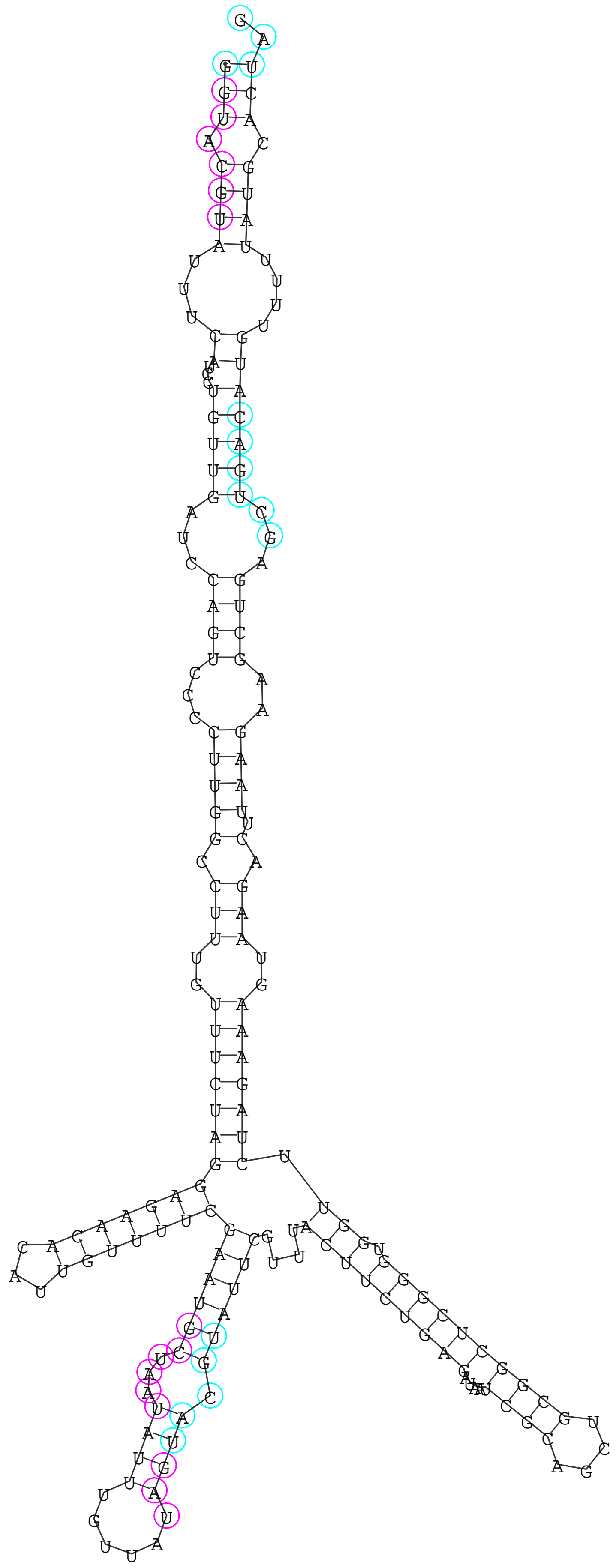

Naboc196B - Stwintron

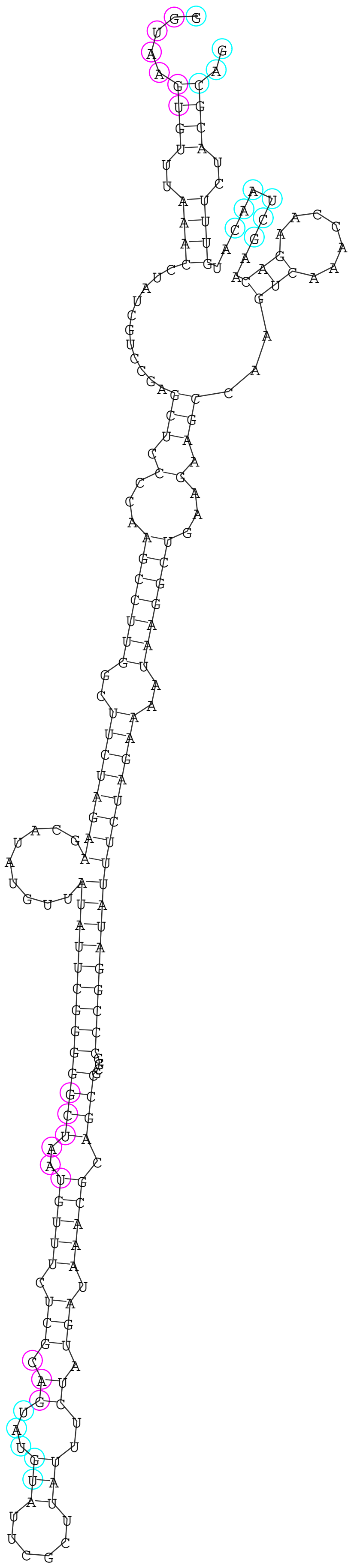

Naboc199A - Stwintron

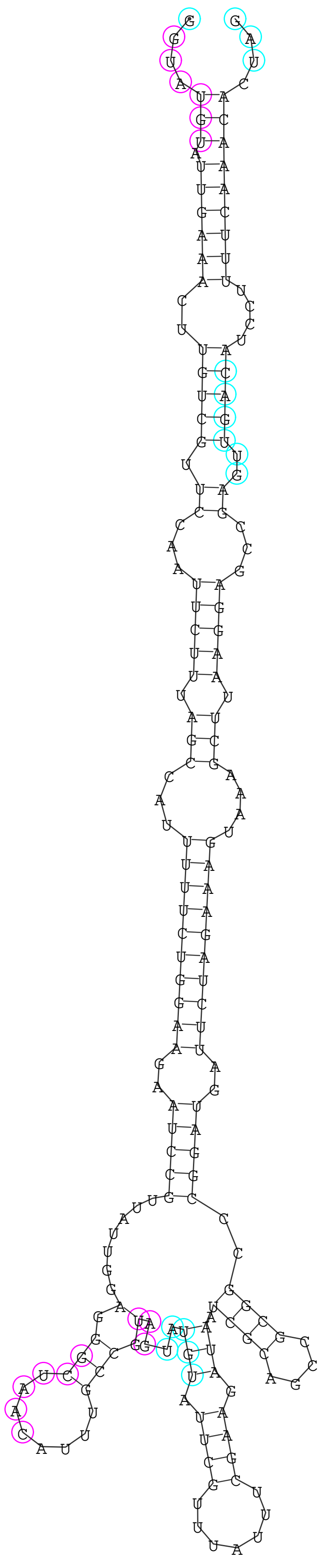

Naboc199B - Stwintron

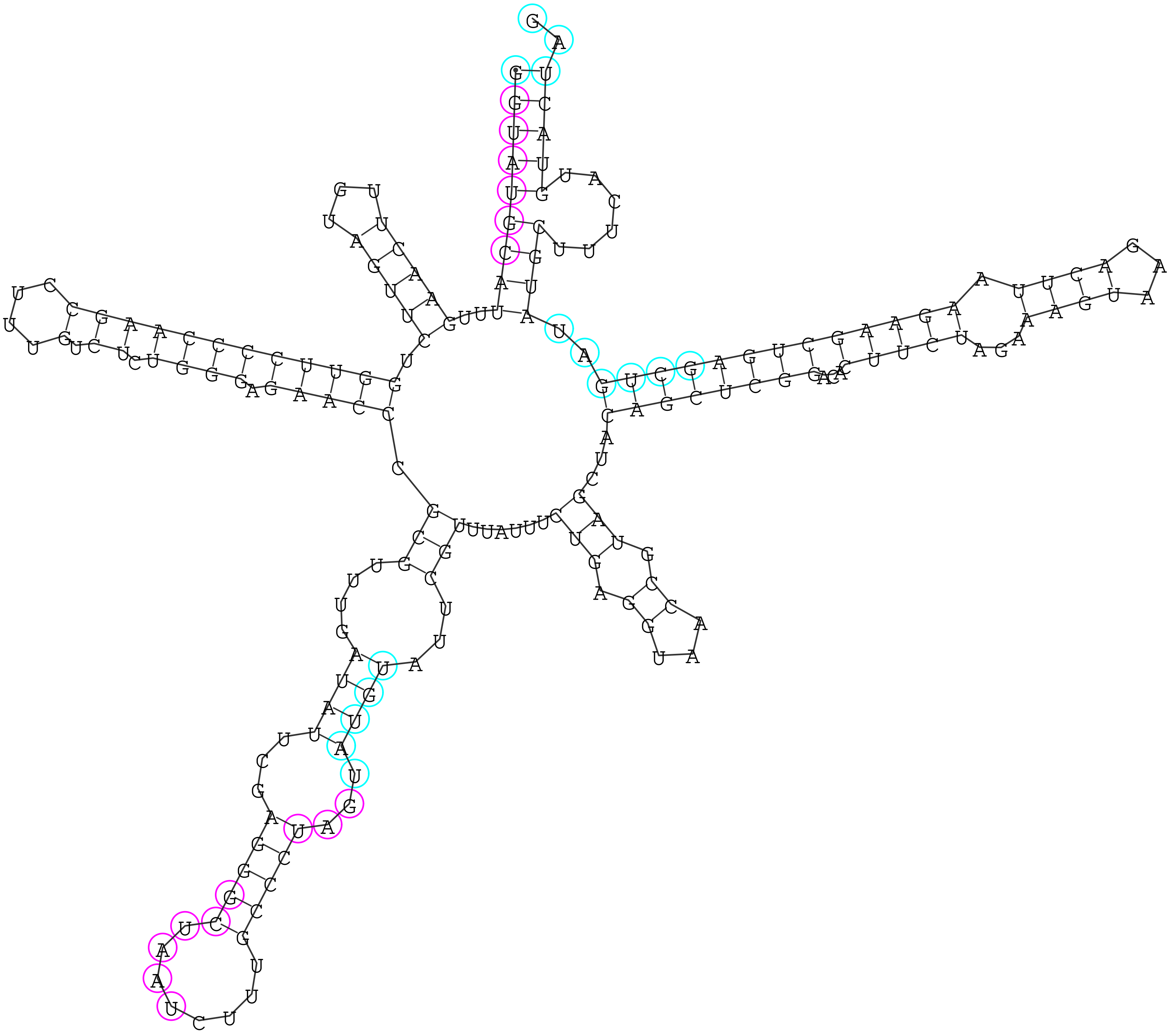

Naboc202A - Stwintron

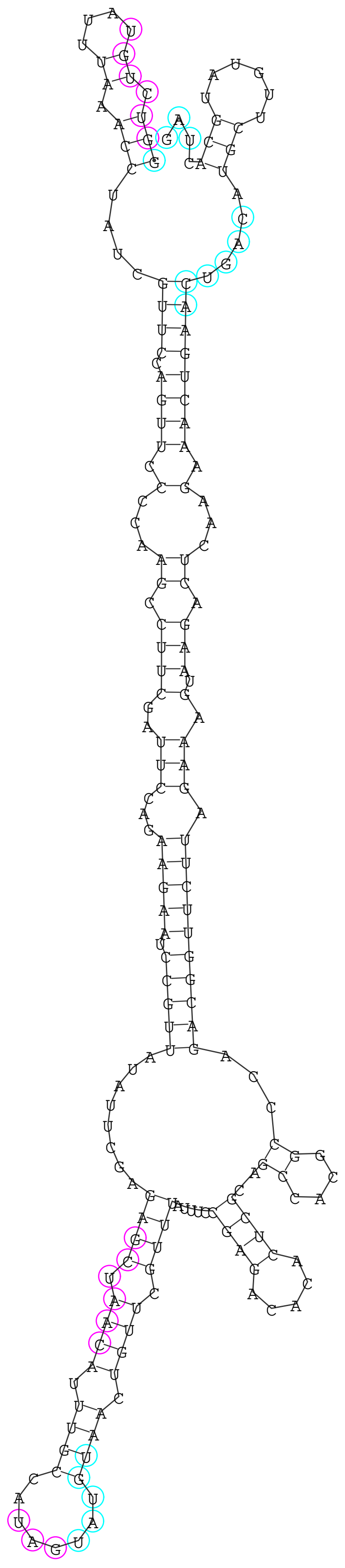

Naboc249A - Stwintron

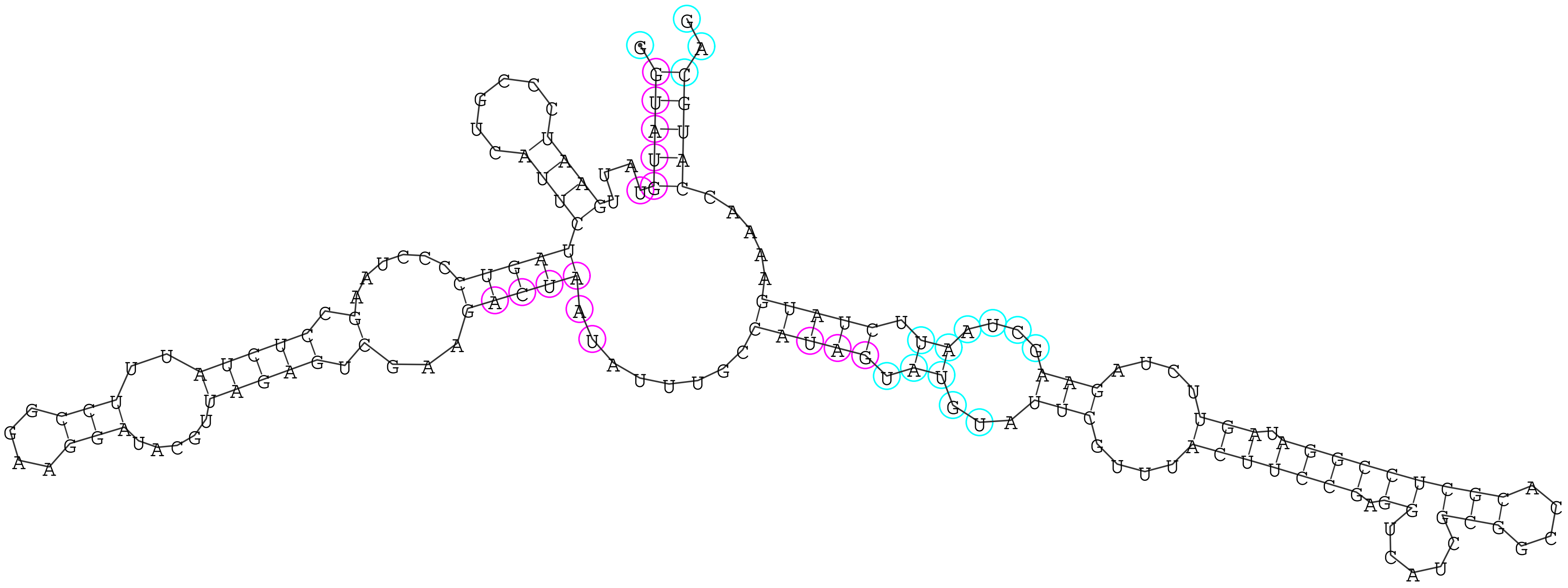

Naboc268A - Stwintron

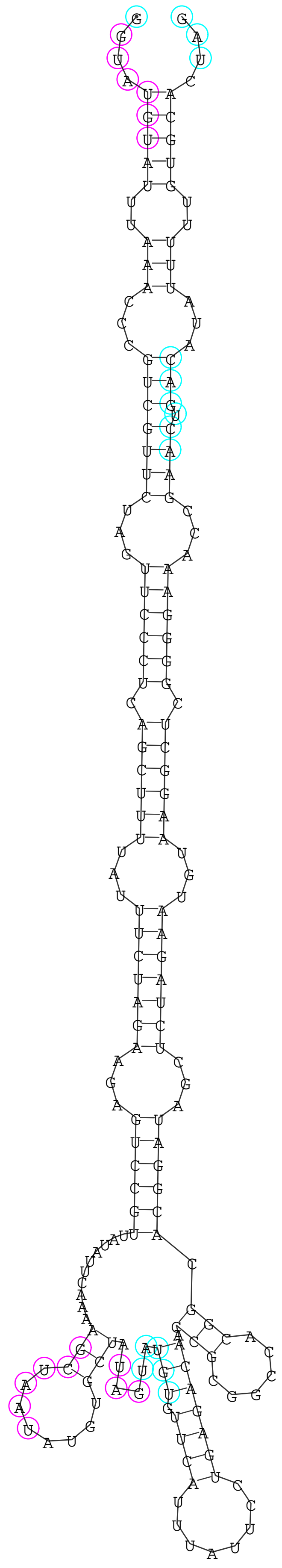

Naboc268B - Stwintron

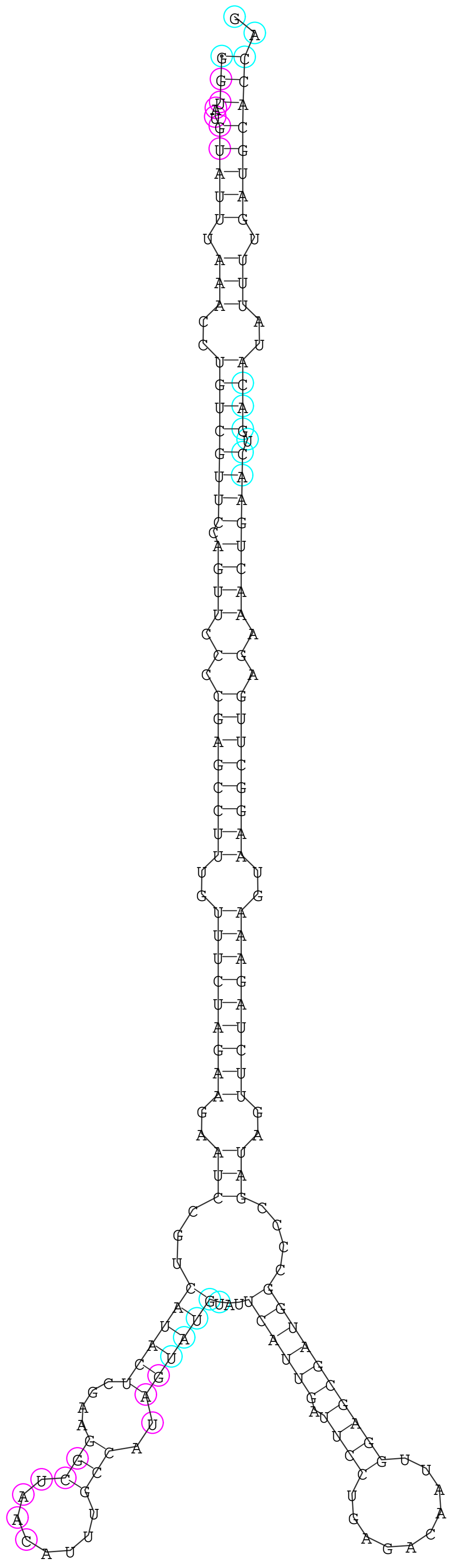

Naboc285A - Stwintron

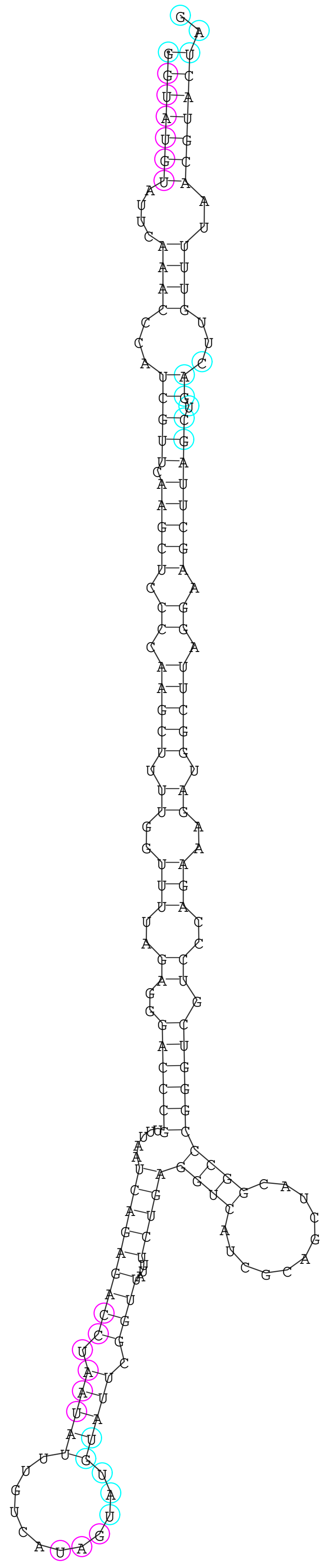

Naboc289A - Stwintron

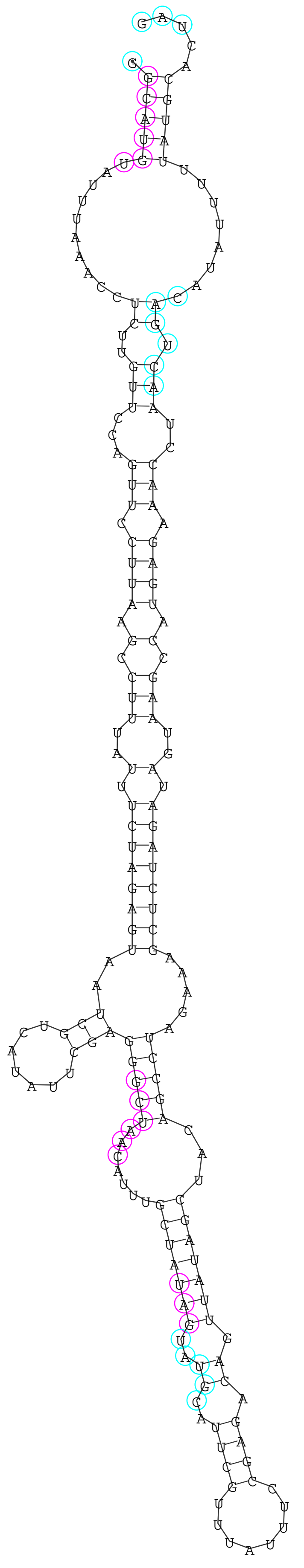

Naboc294A - Stwintron

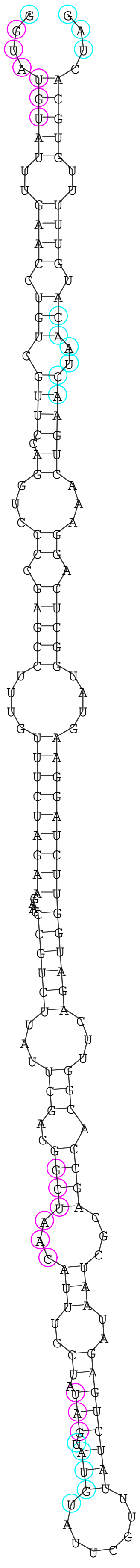

Naboc300A - Stwintron

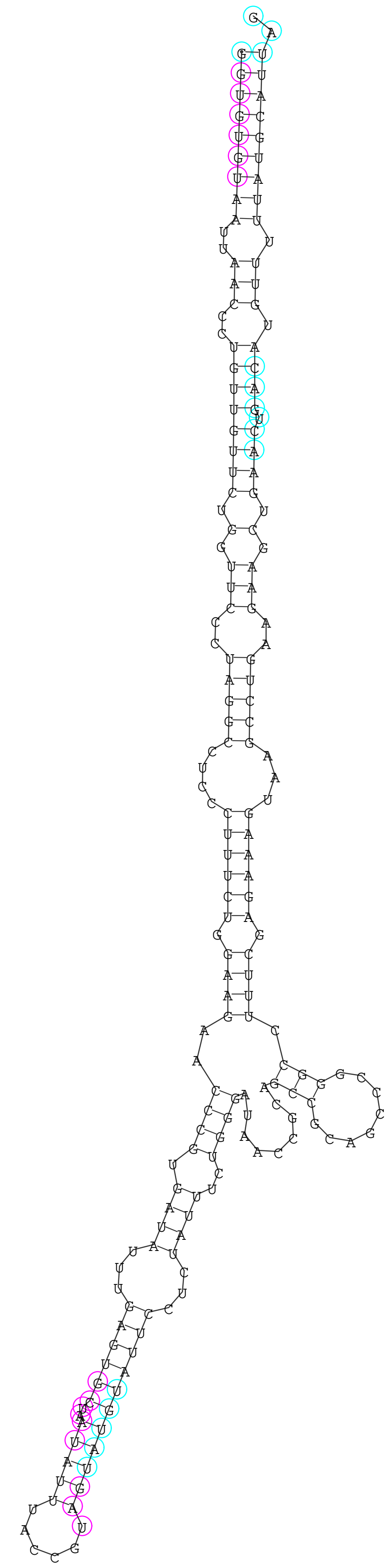

Naboc349A - Stwintron

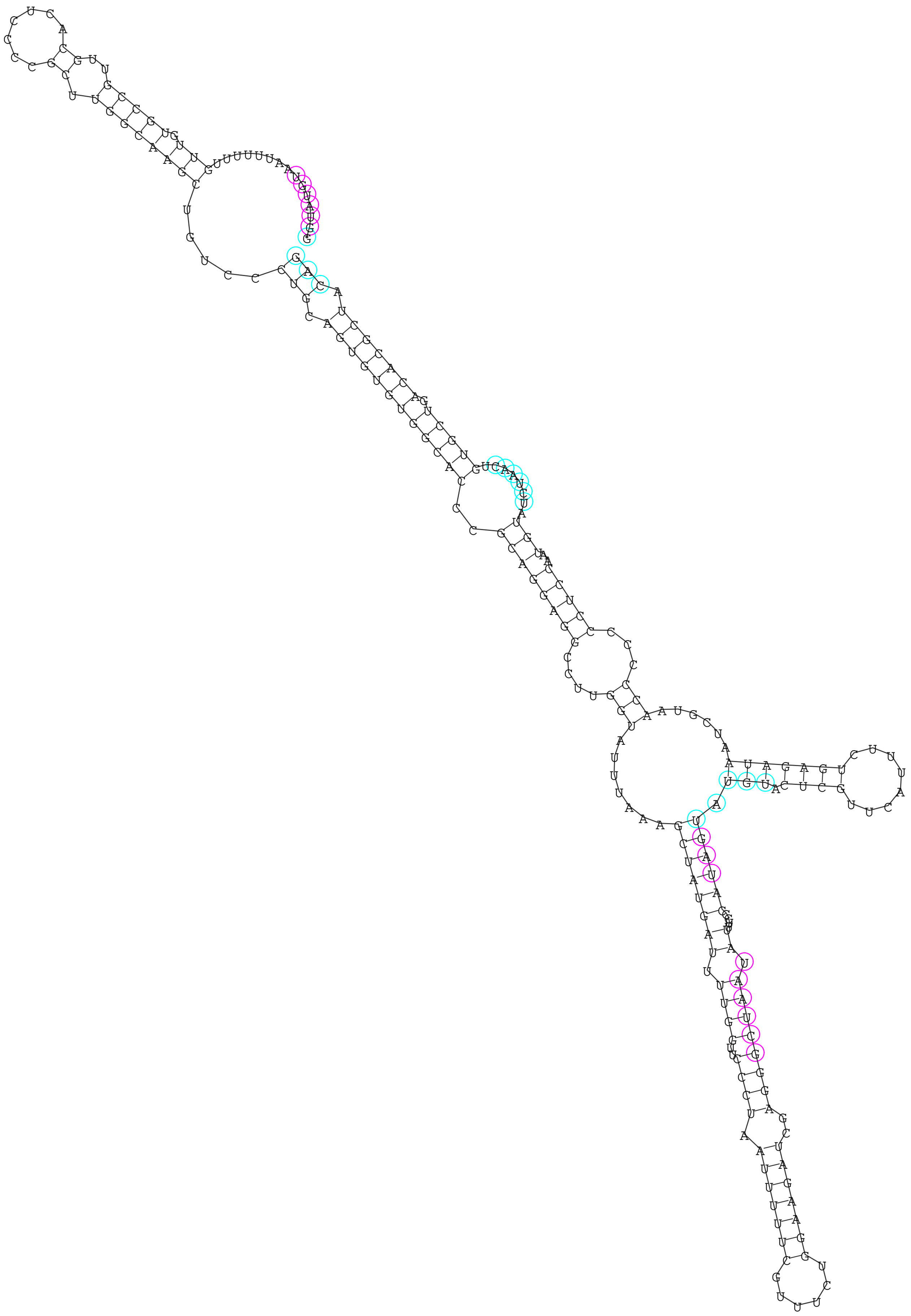

Naboc414A - Stwintron

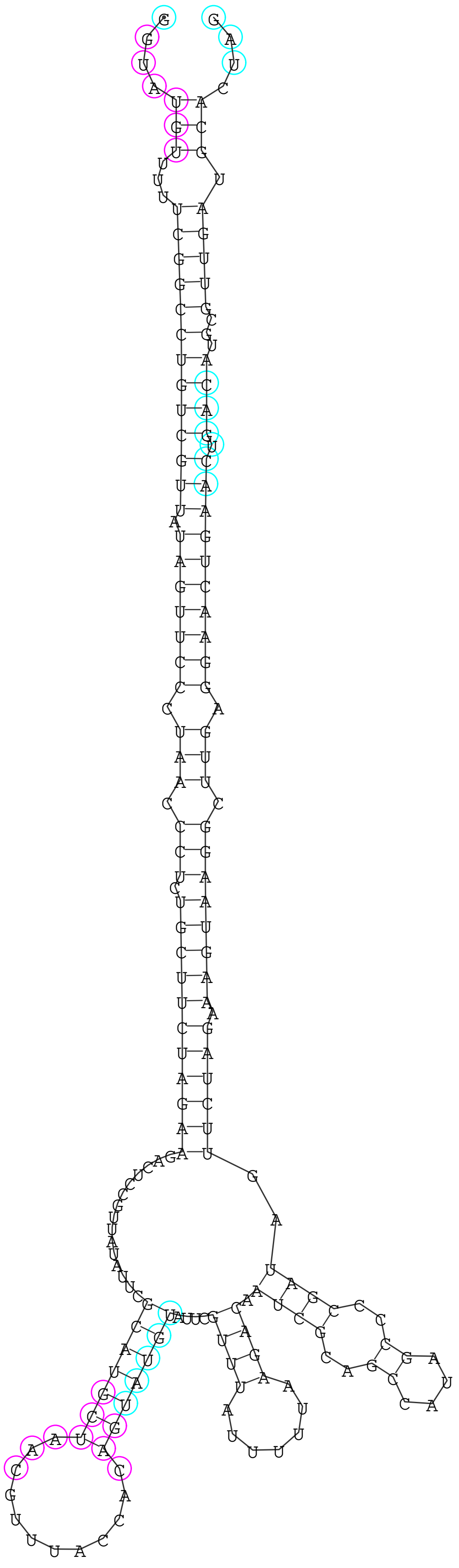

X1651c009A - Stwintron

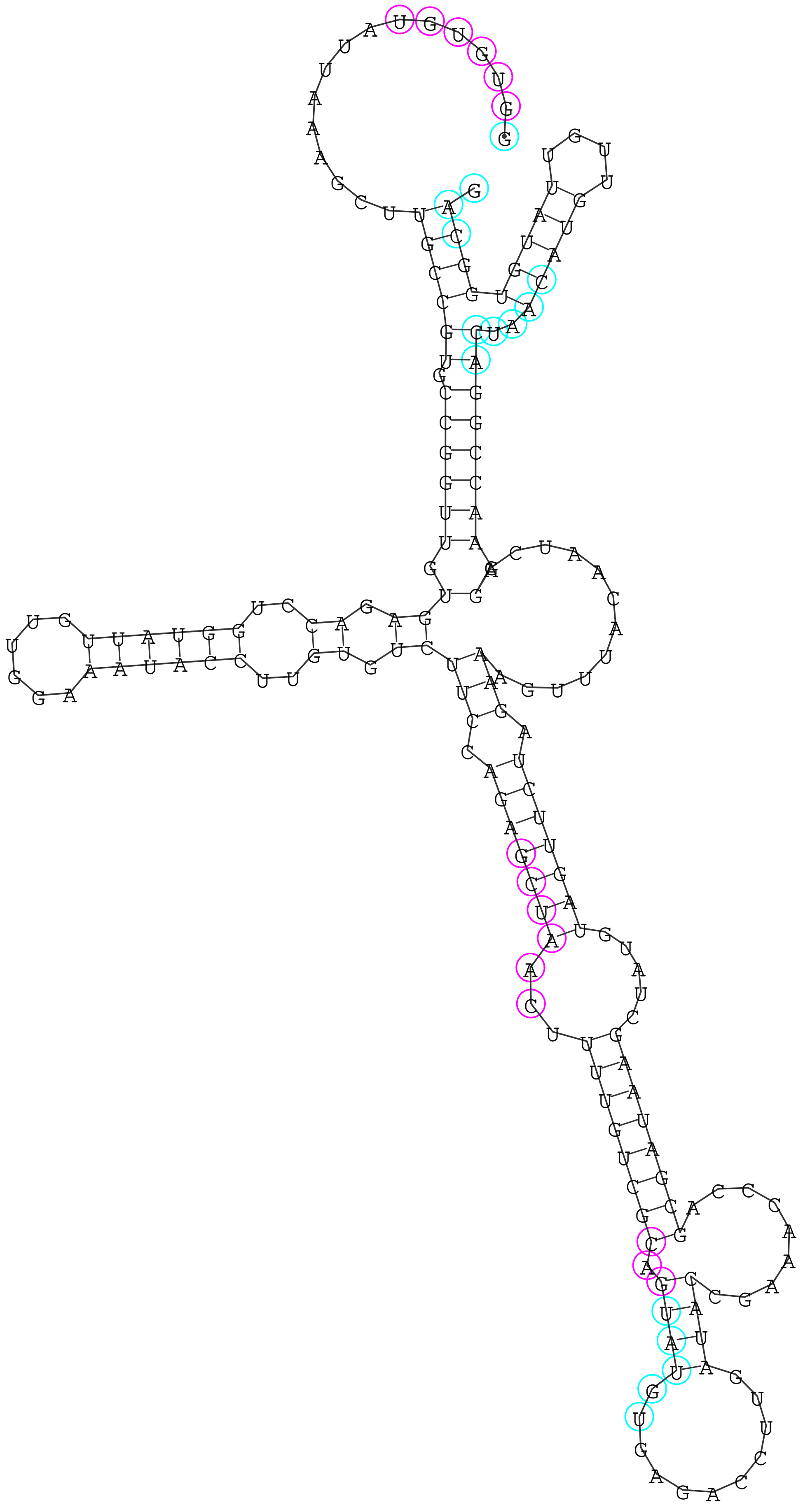

X1651c011A - Stwintron

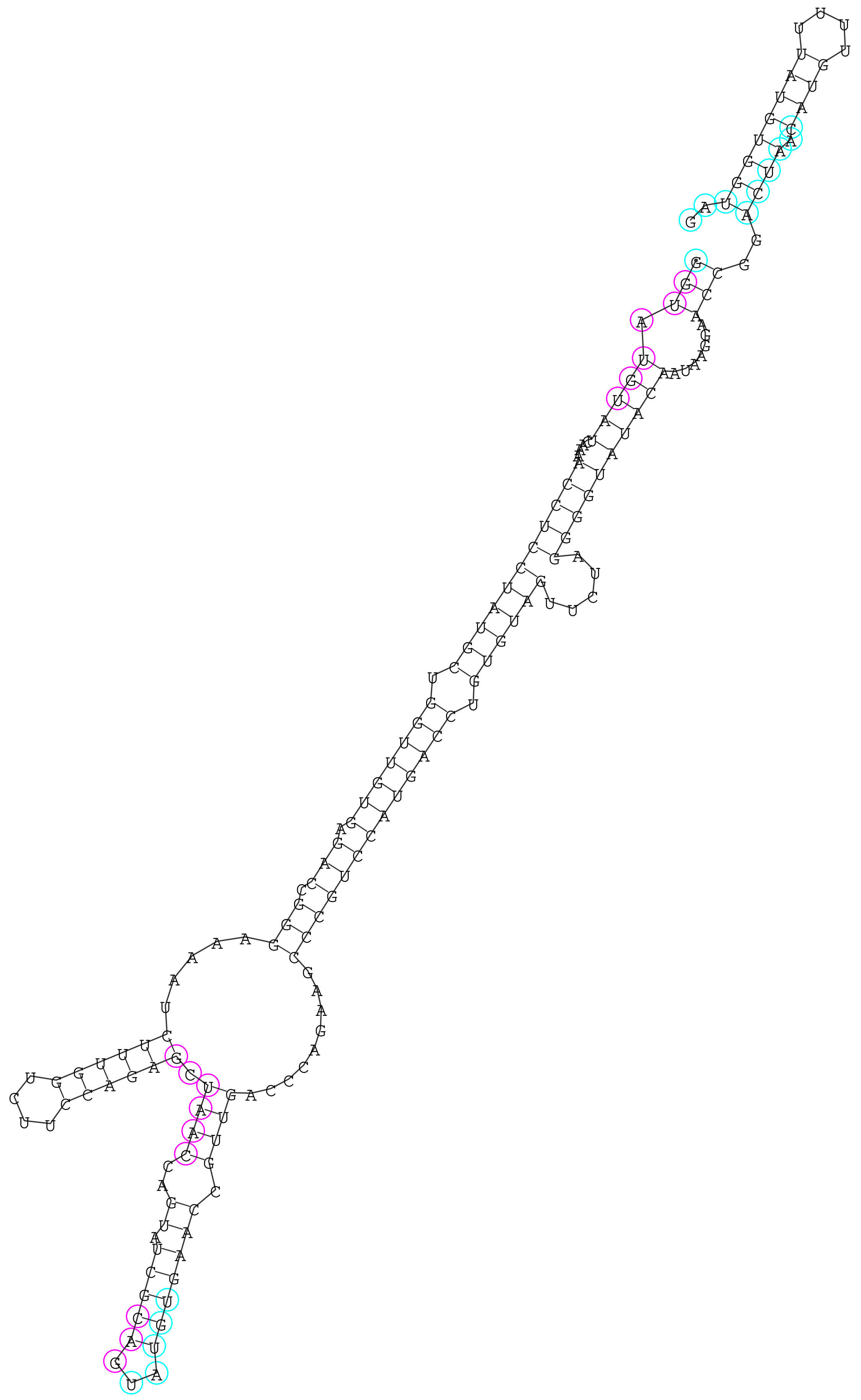

X1651c016A - Stwintron

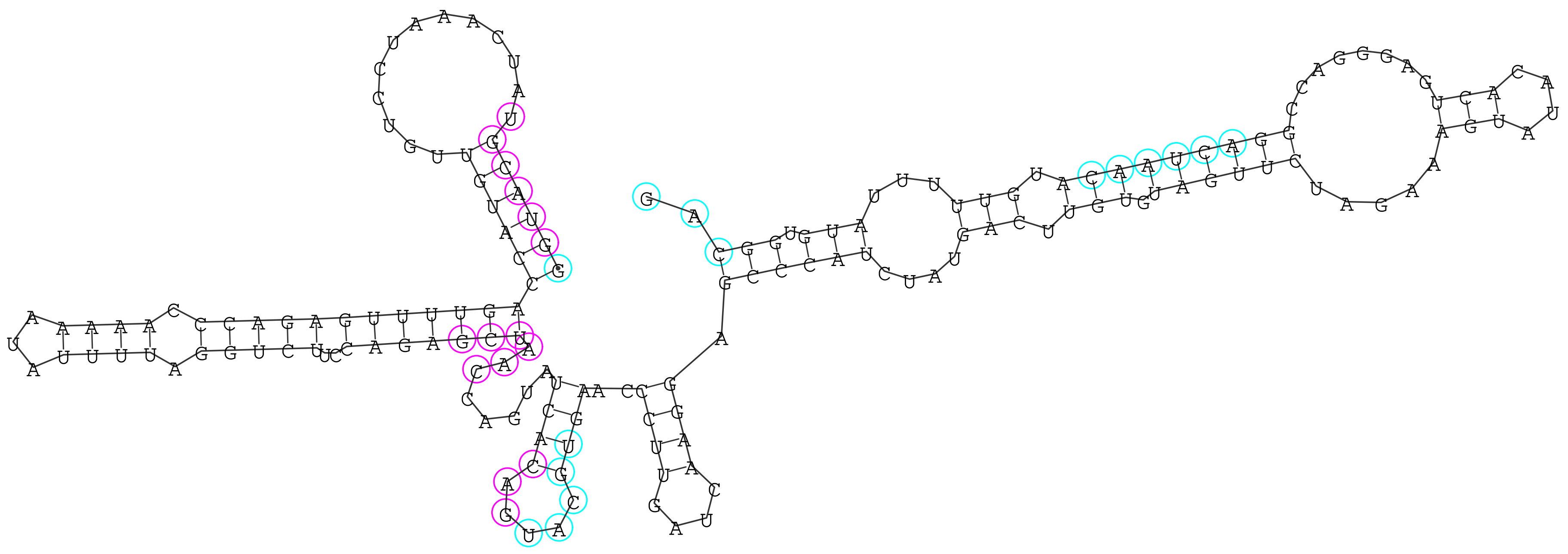

X1651c025A - Stwintron

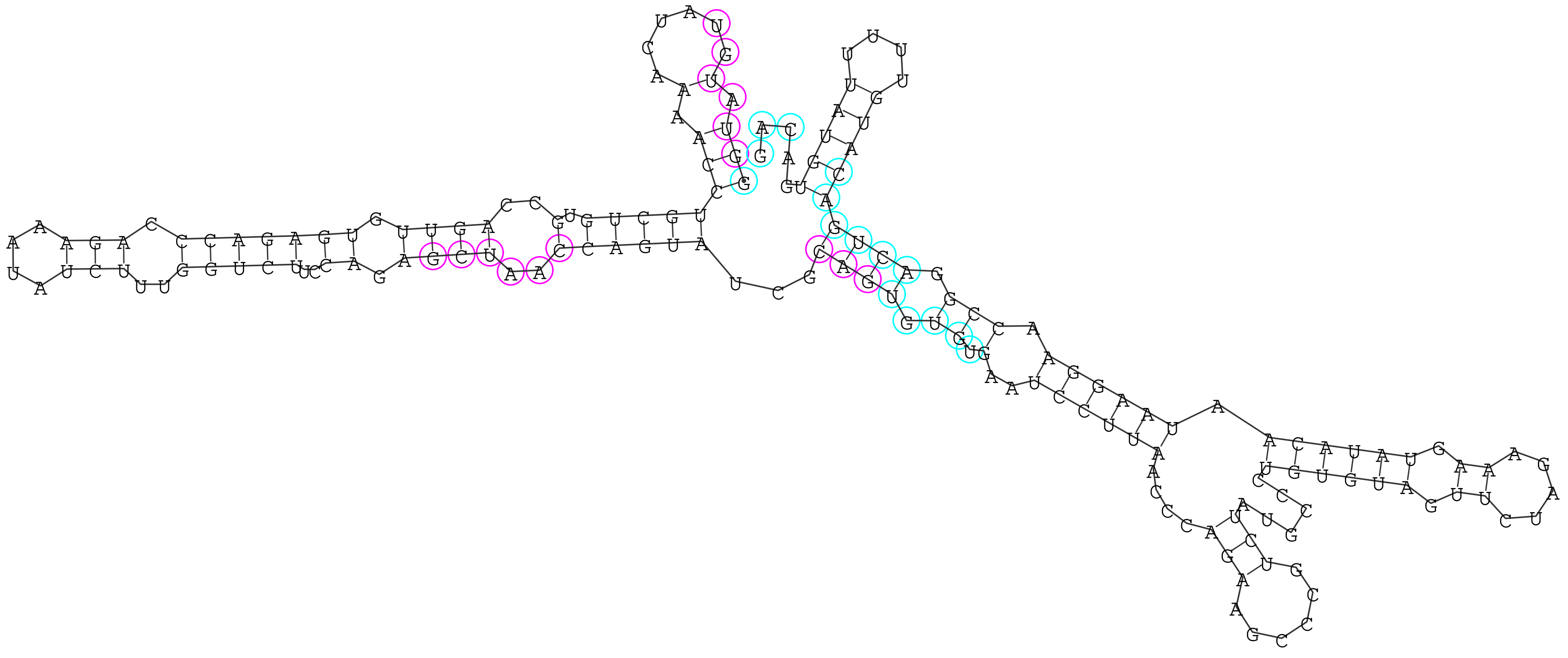

X1651c036A - Stwintron

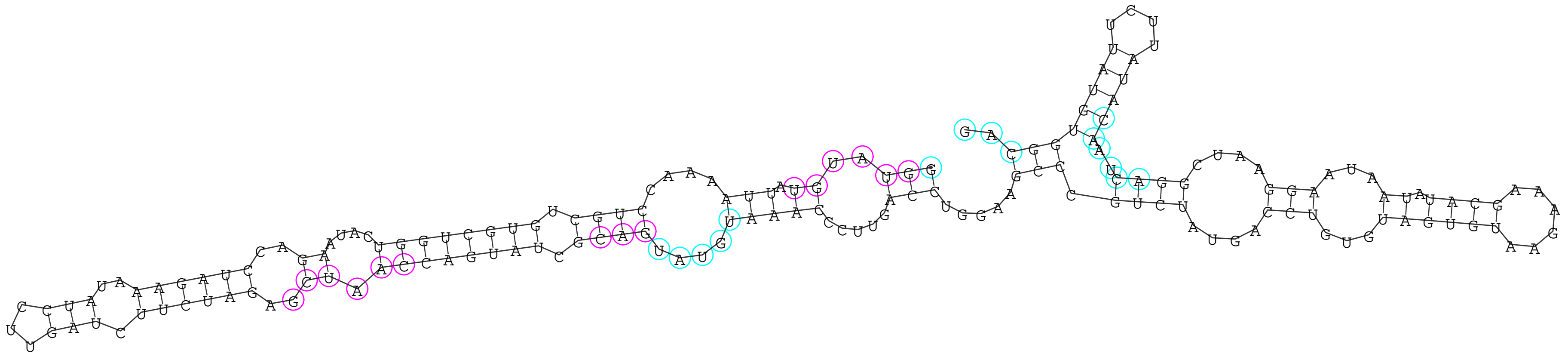

X1651c075A - Stwintron

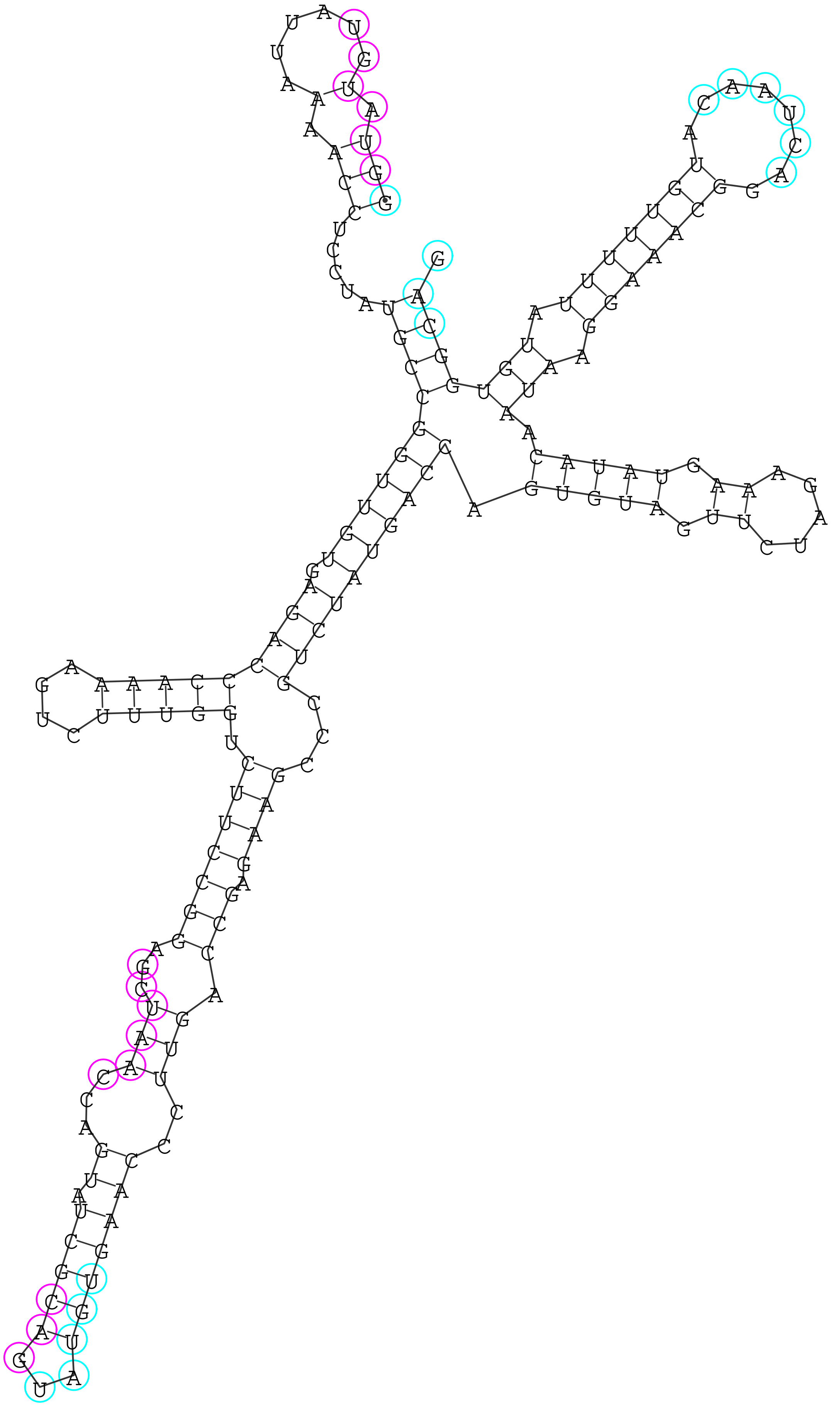

X1651c093A - Stwintron

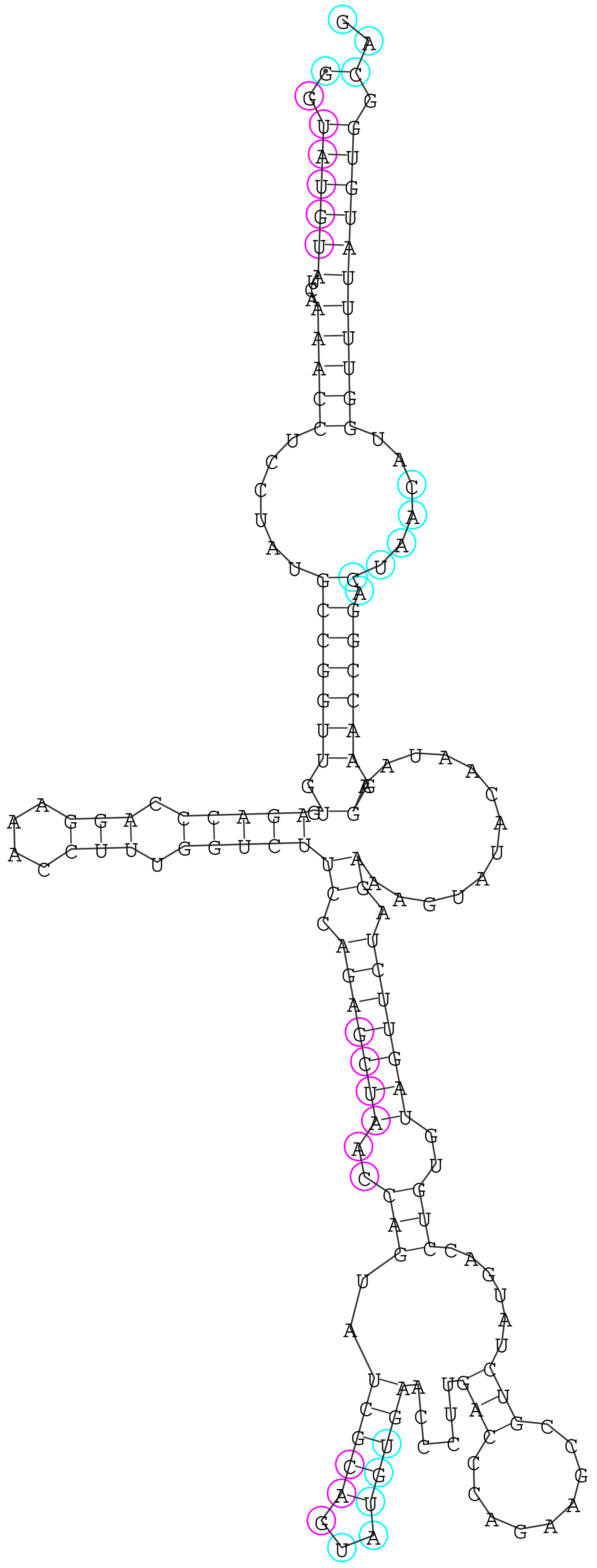

X1651c156A - Stwintron

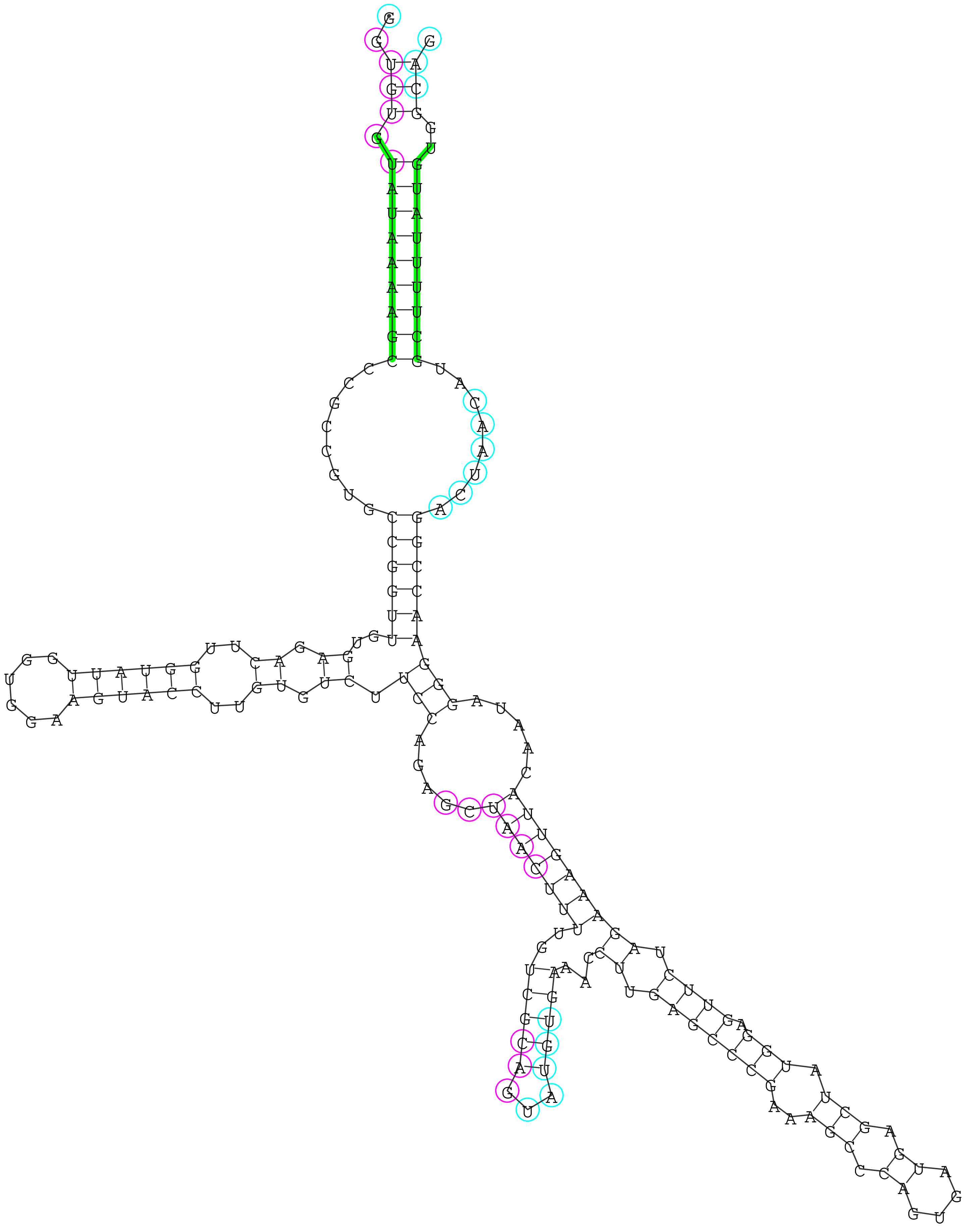

X1651c189A - Stwintron

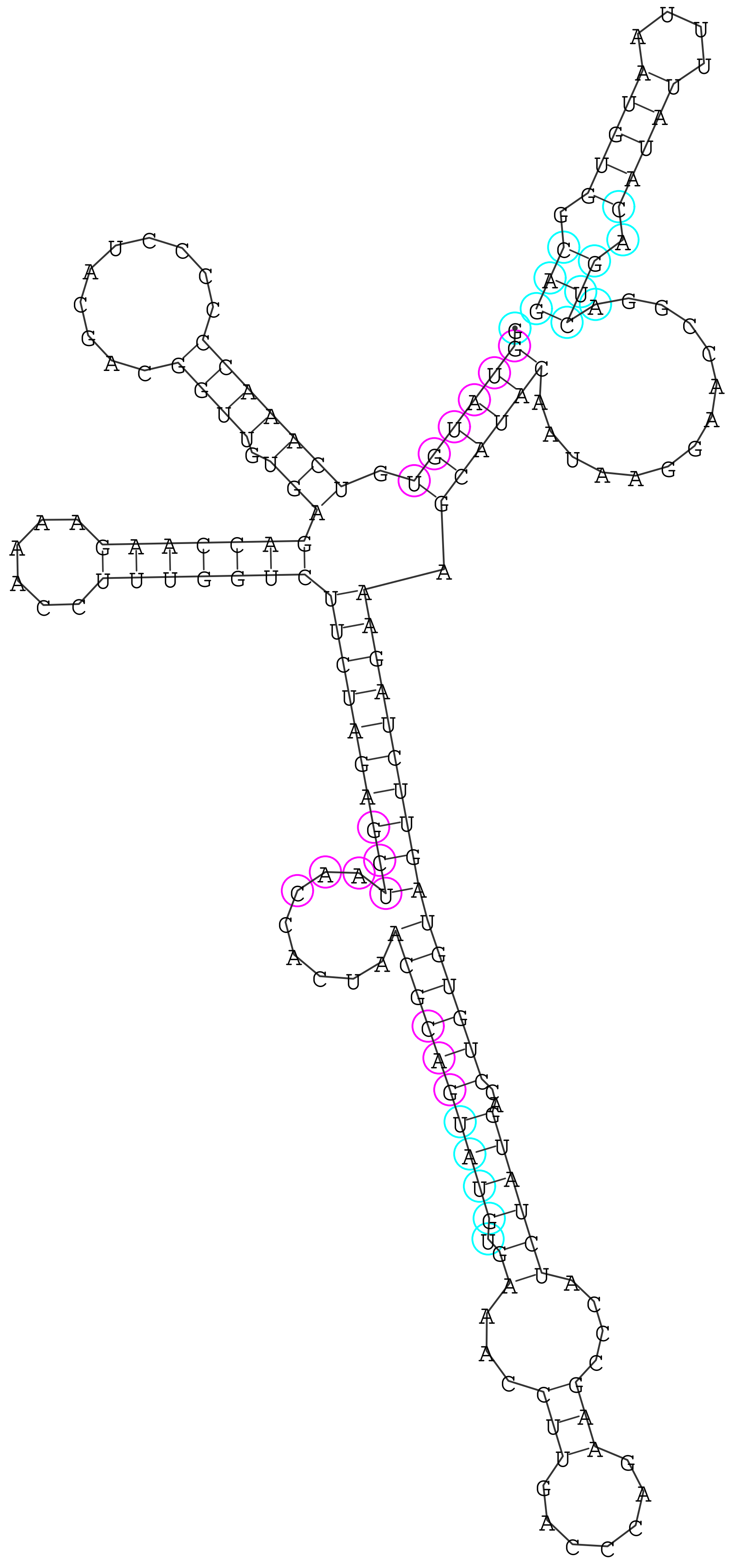

Xarbc0002A - Stwintron

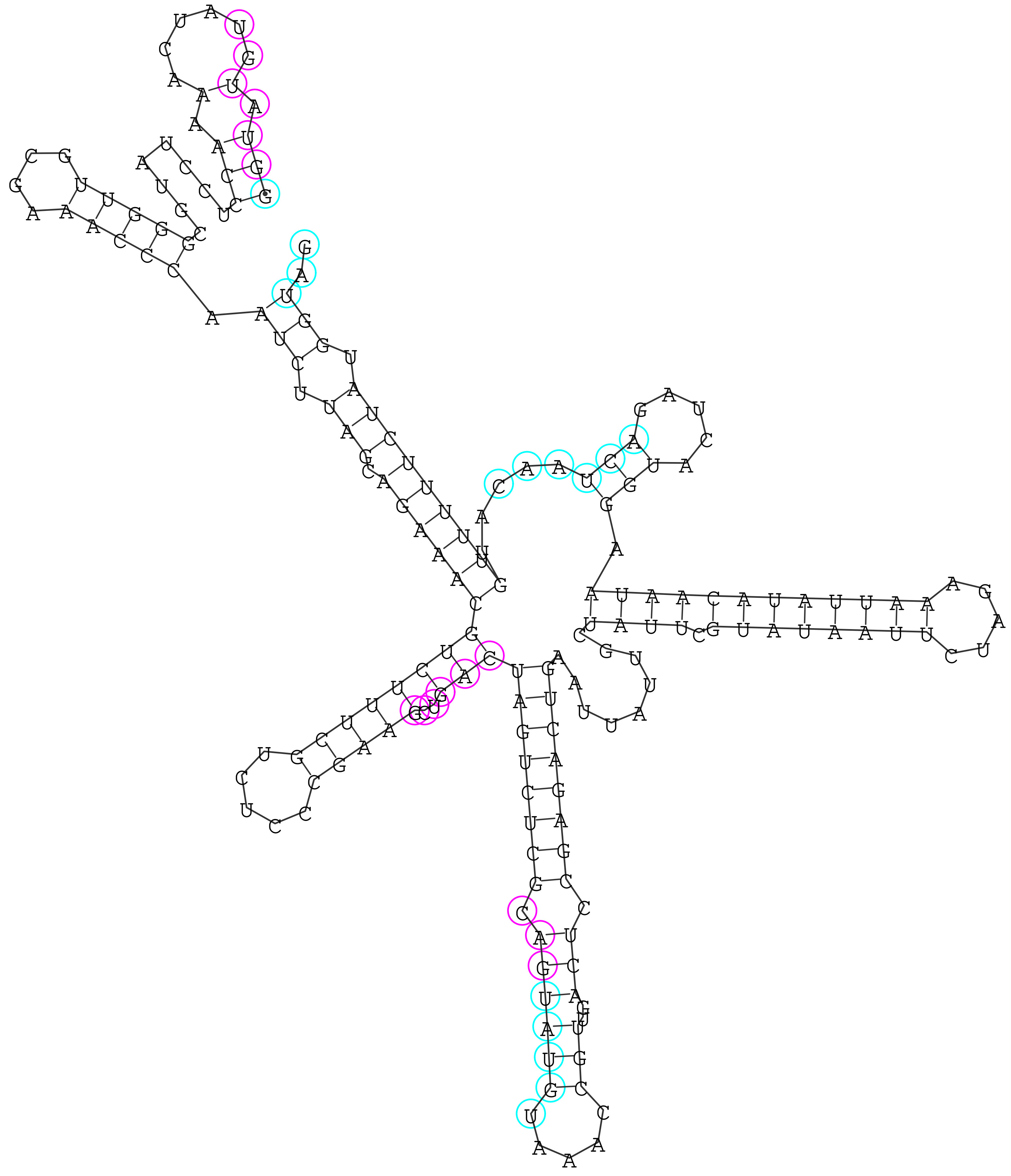

Xarbc0002B - Stwintron

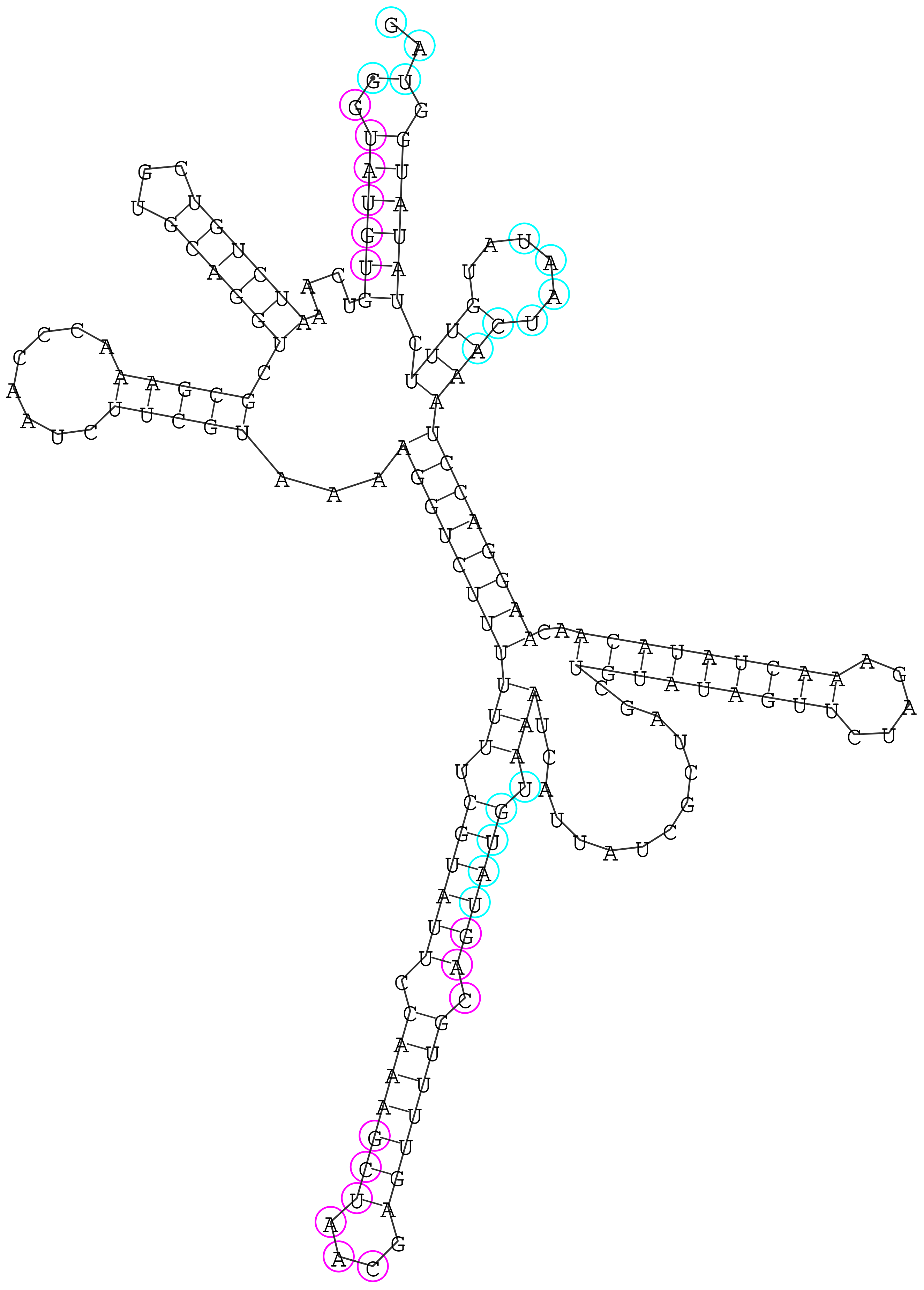

Xarbc0003A - Stwintron

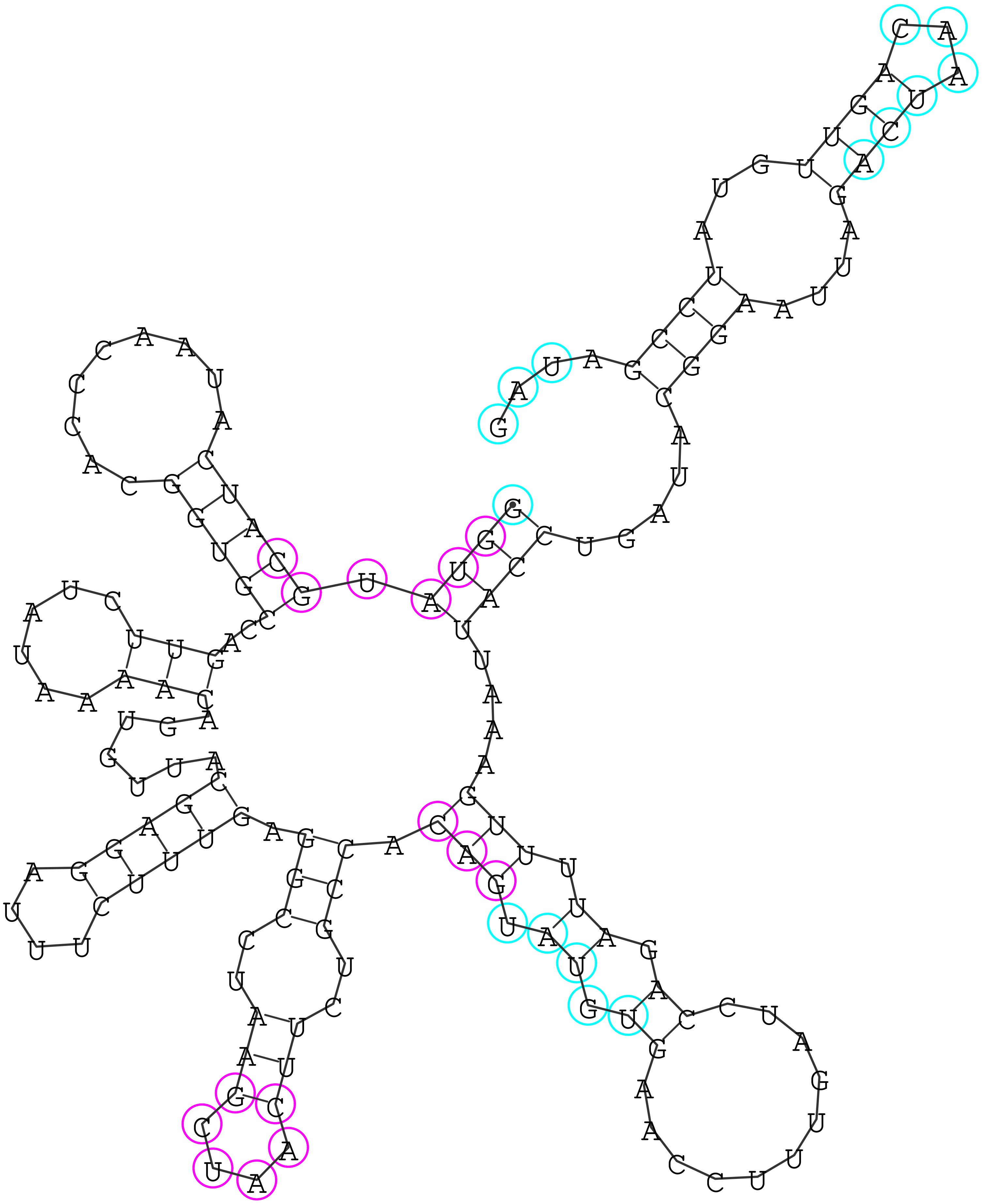

Xarbc0003B - Stwintron

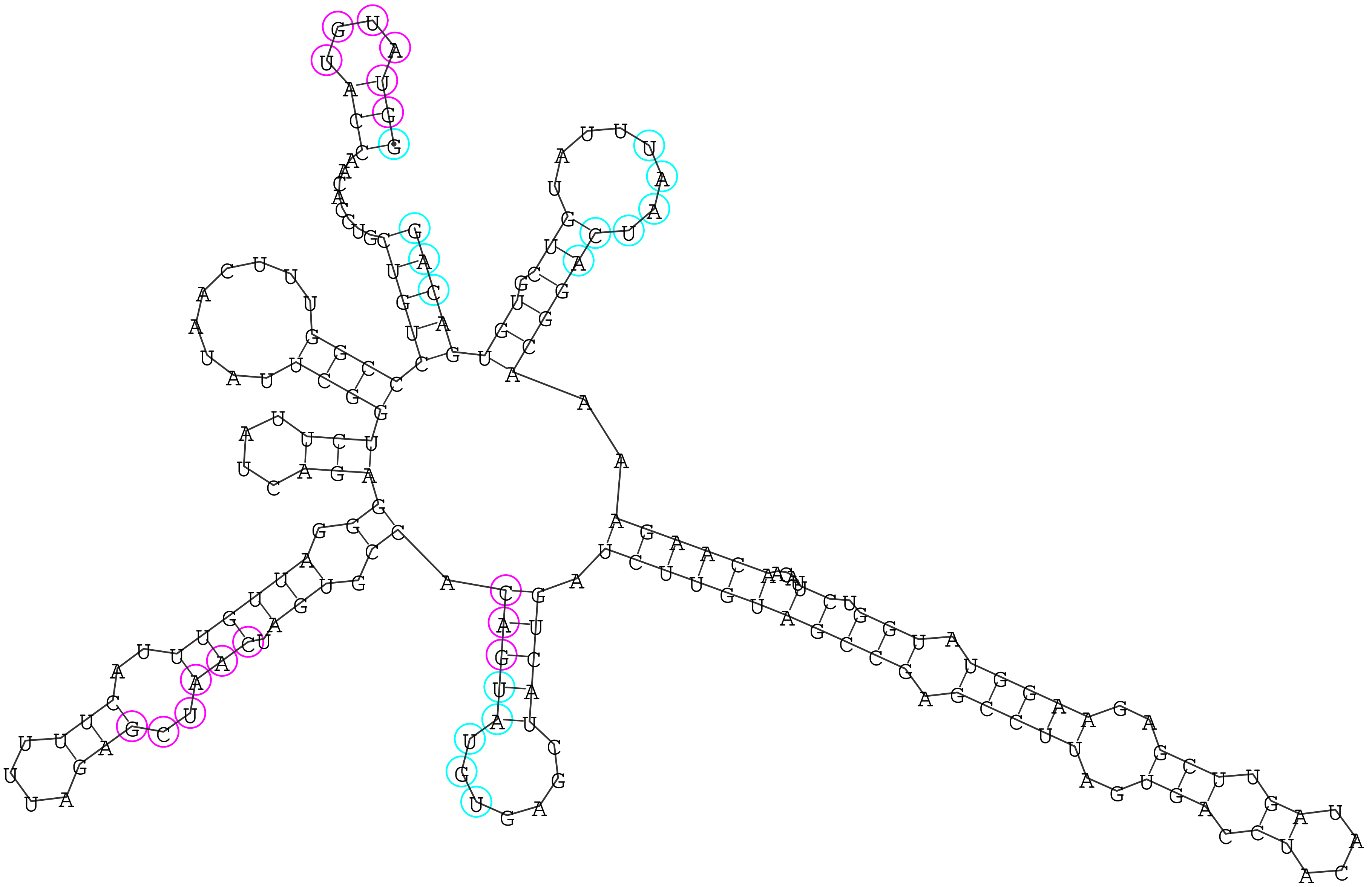

Xarbc0003C - Stwintron

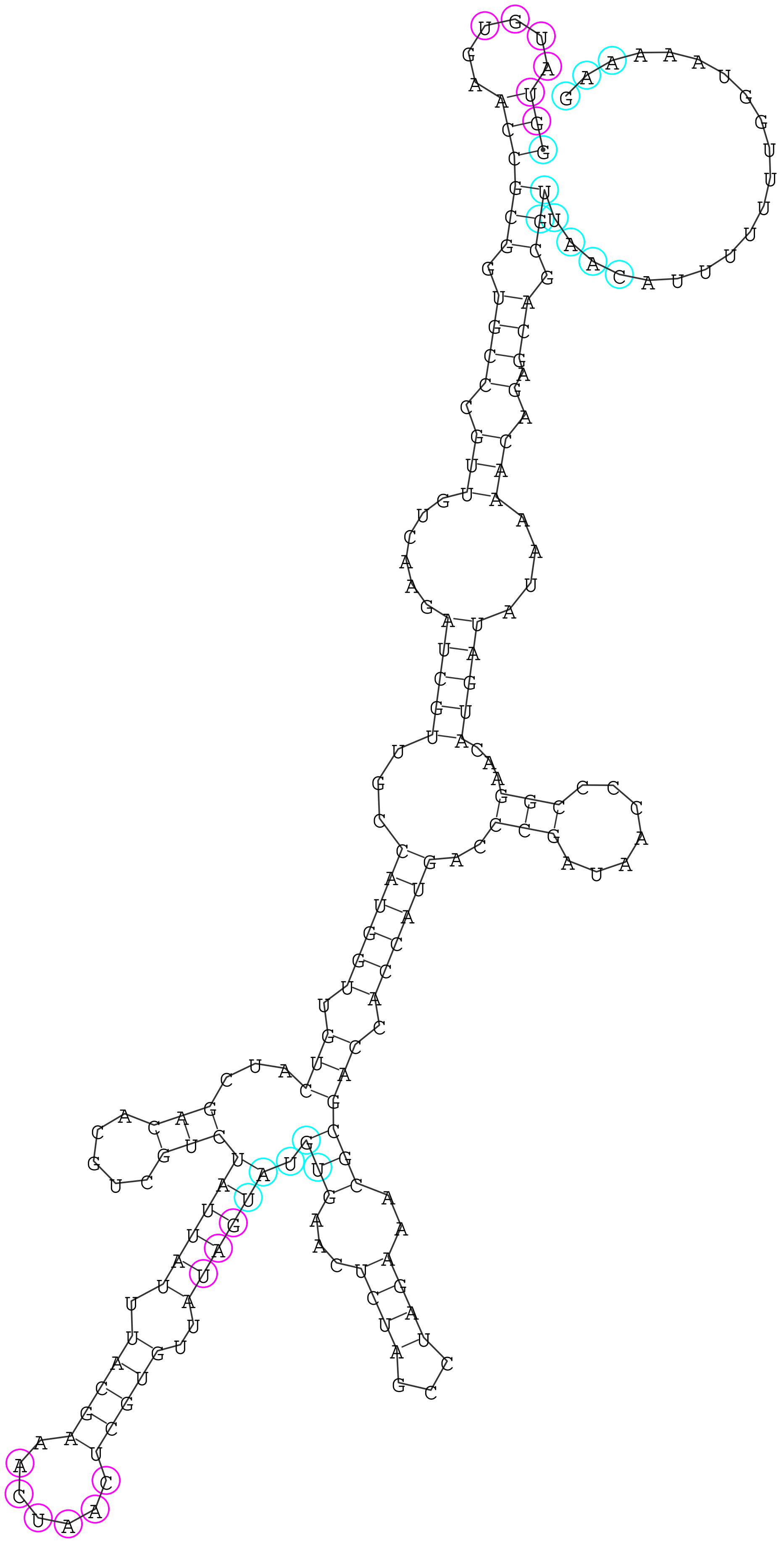

Xarbc0006A - Stwintron

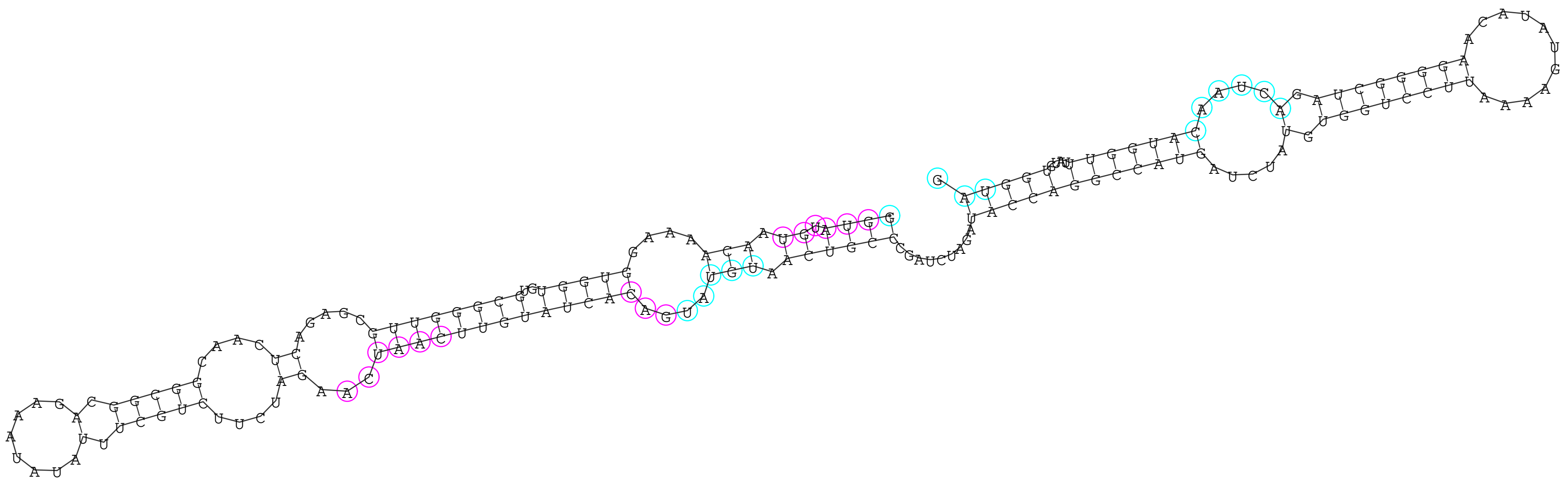

Xarbc0006B - Stwintron

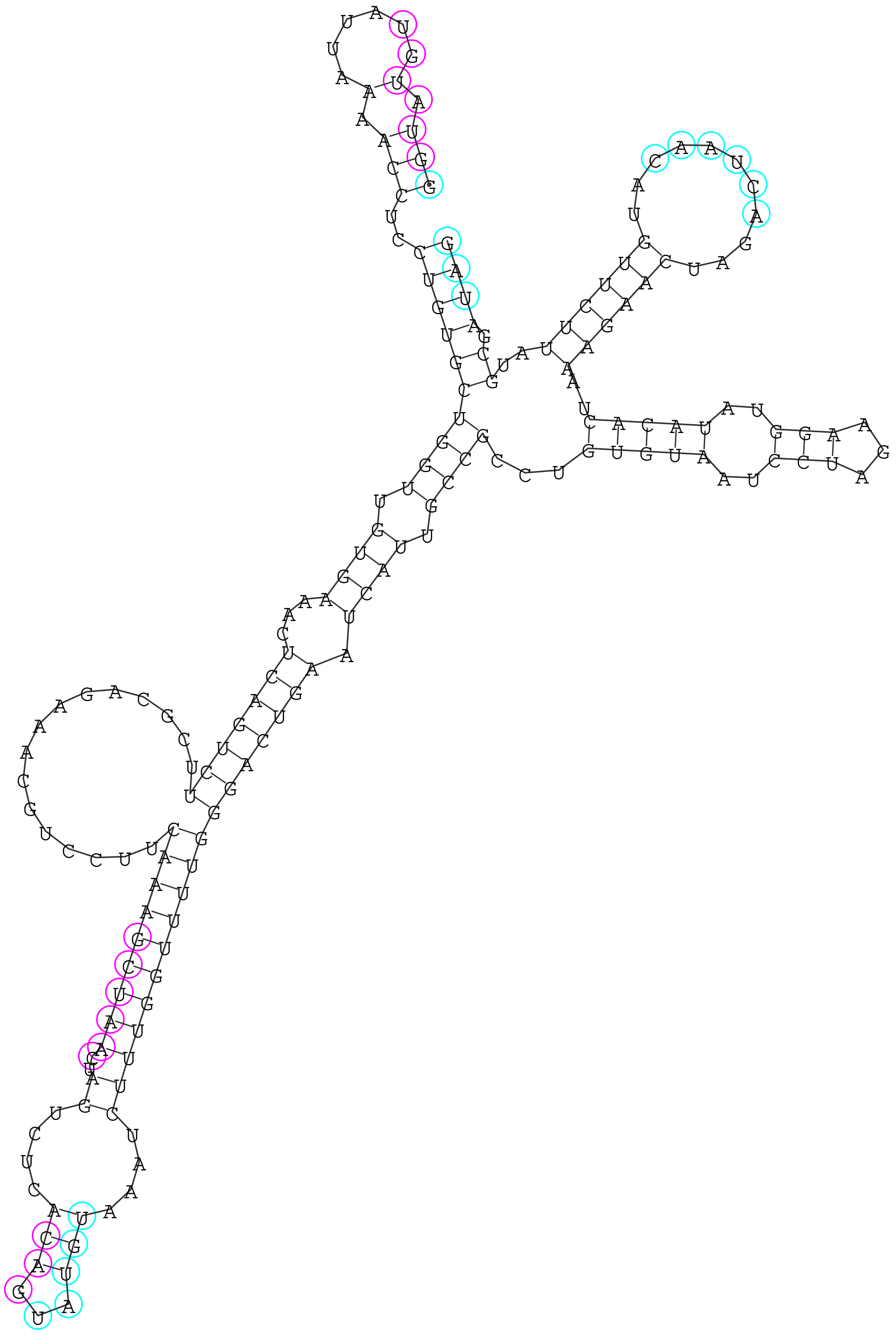

Xarbc0009A - Stwintron

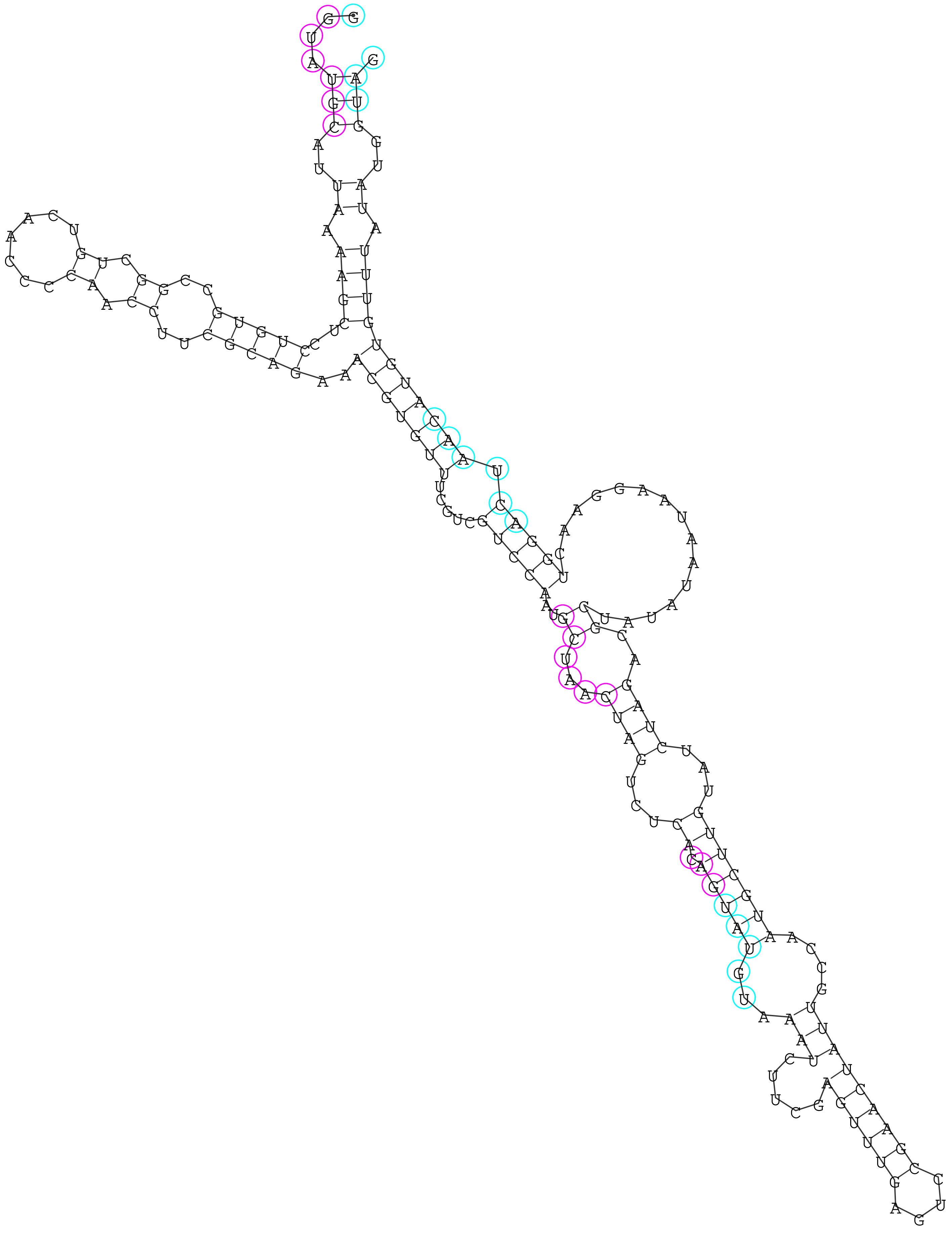

Xarbc0010A - Stwintron

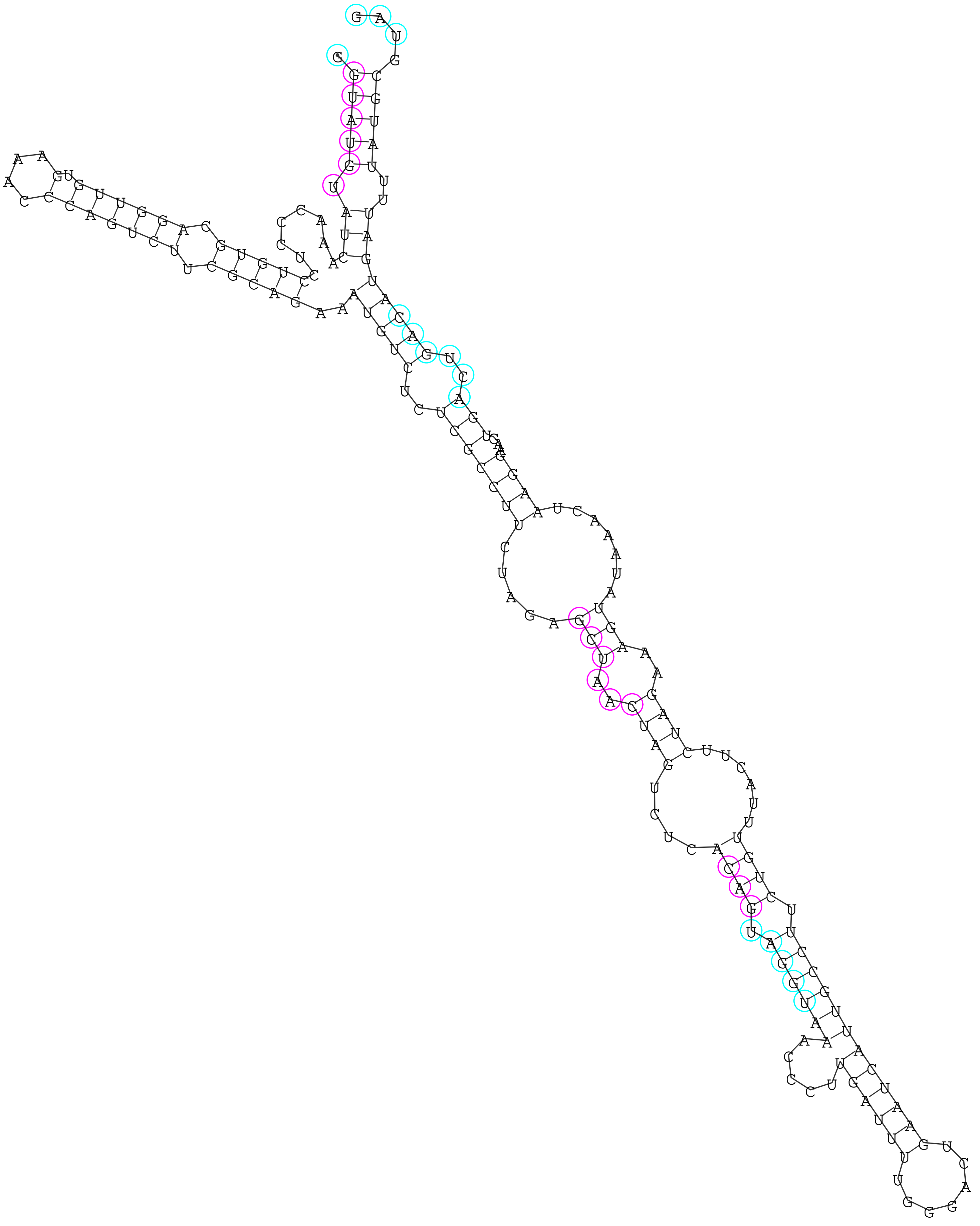

Xarbc0010B - Stwintron

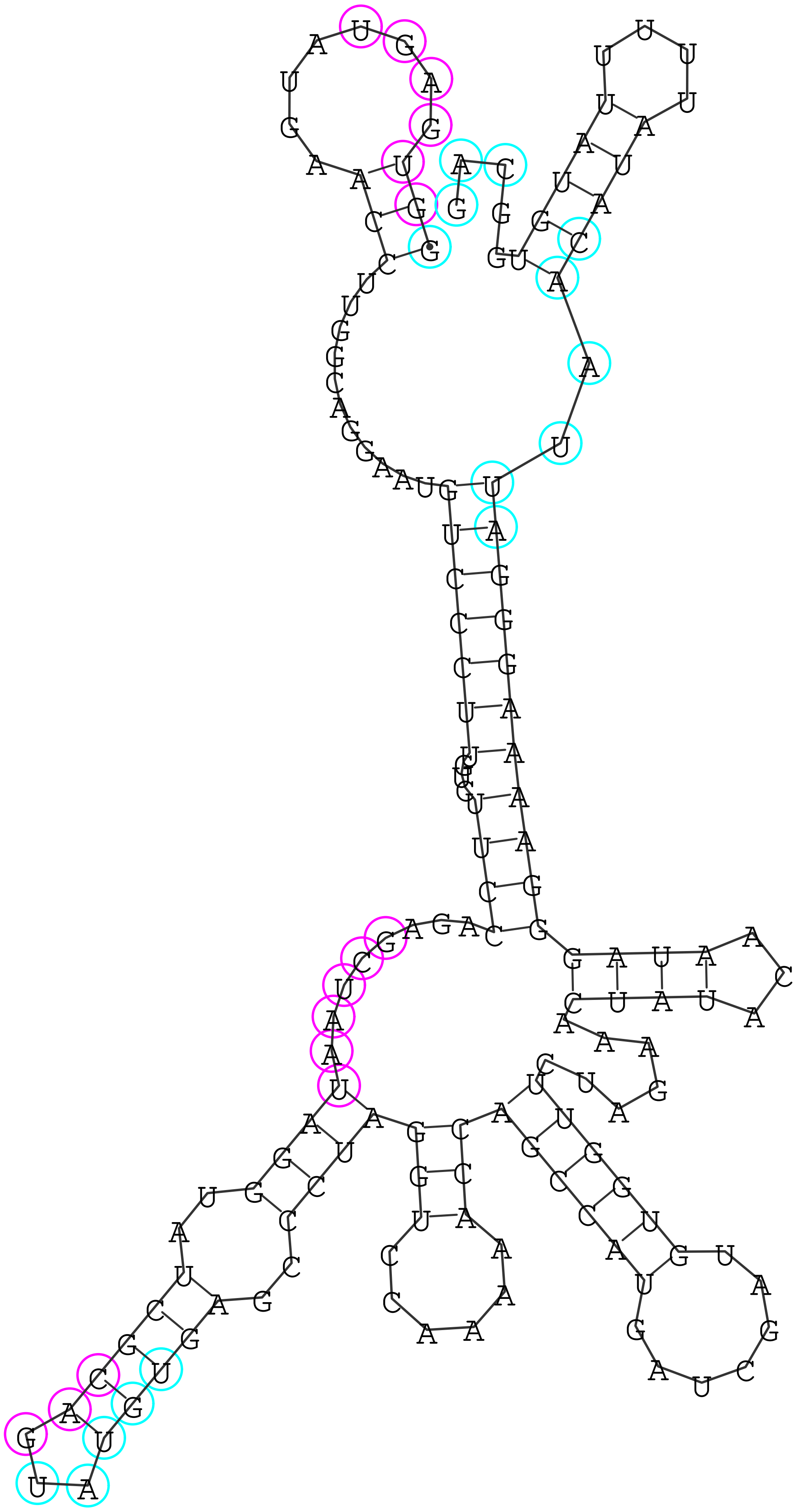

Xarbc0011A - Stwintron

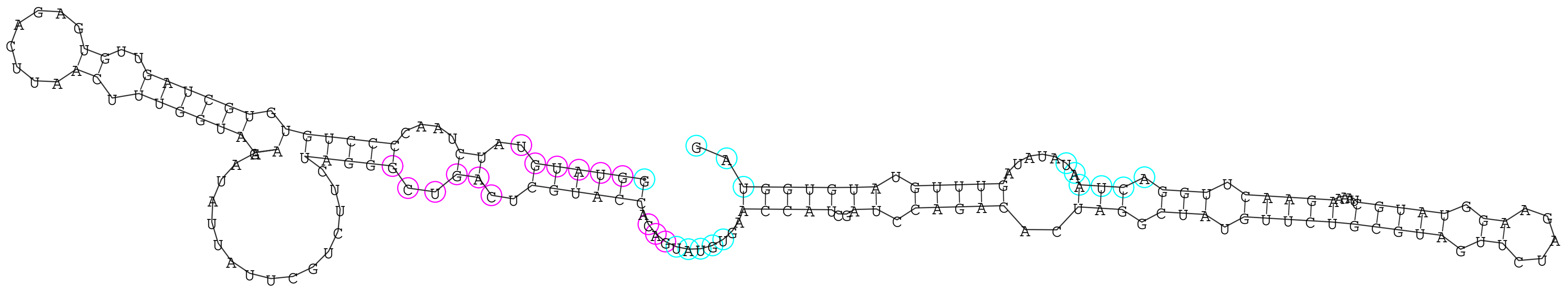

Xarbc0012A - Stwintron

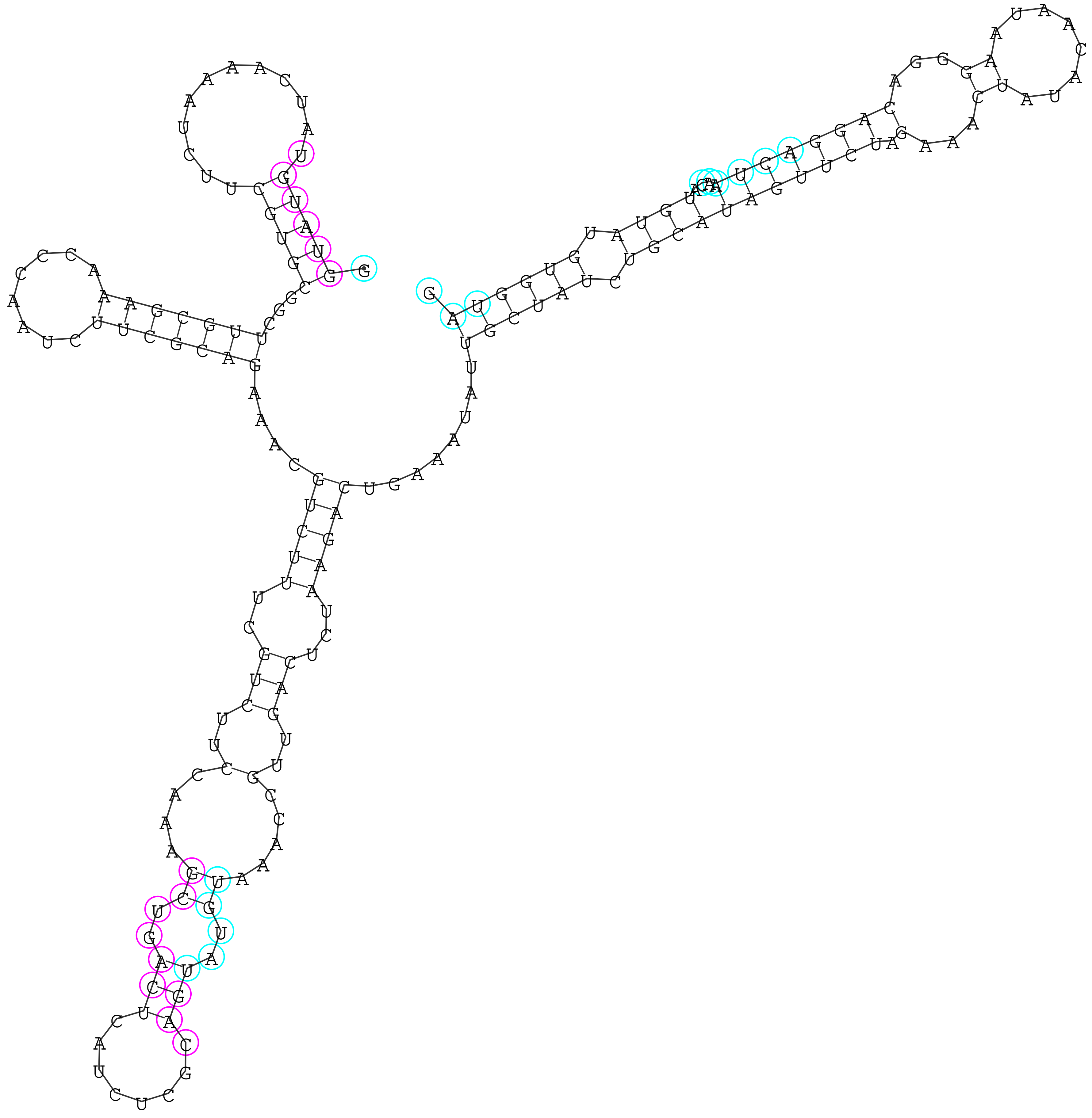

Xarbc0012B - Stwintron

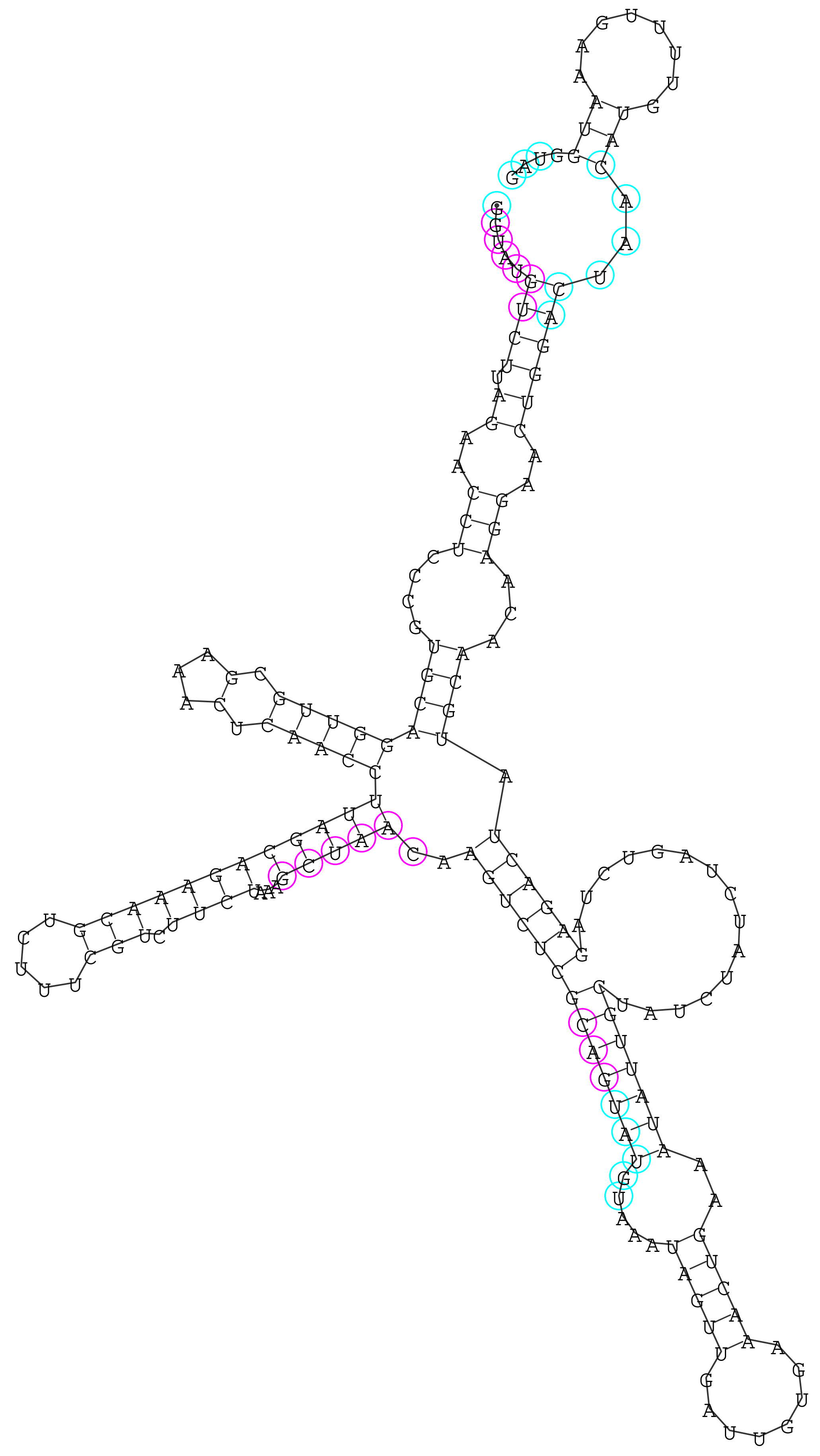

Xarbc0012D - Stwintron

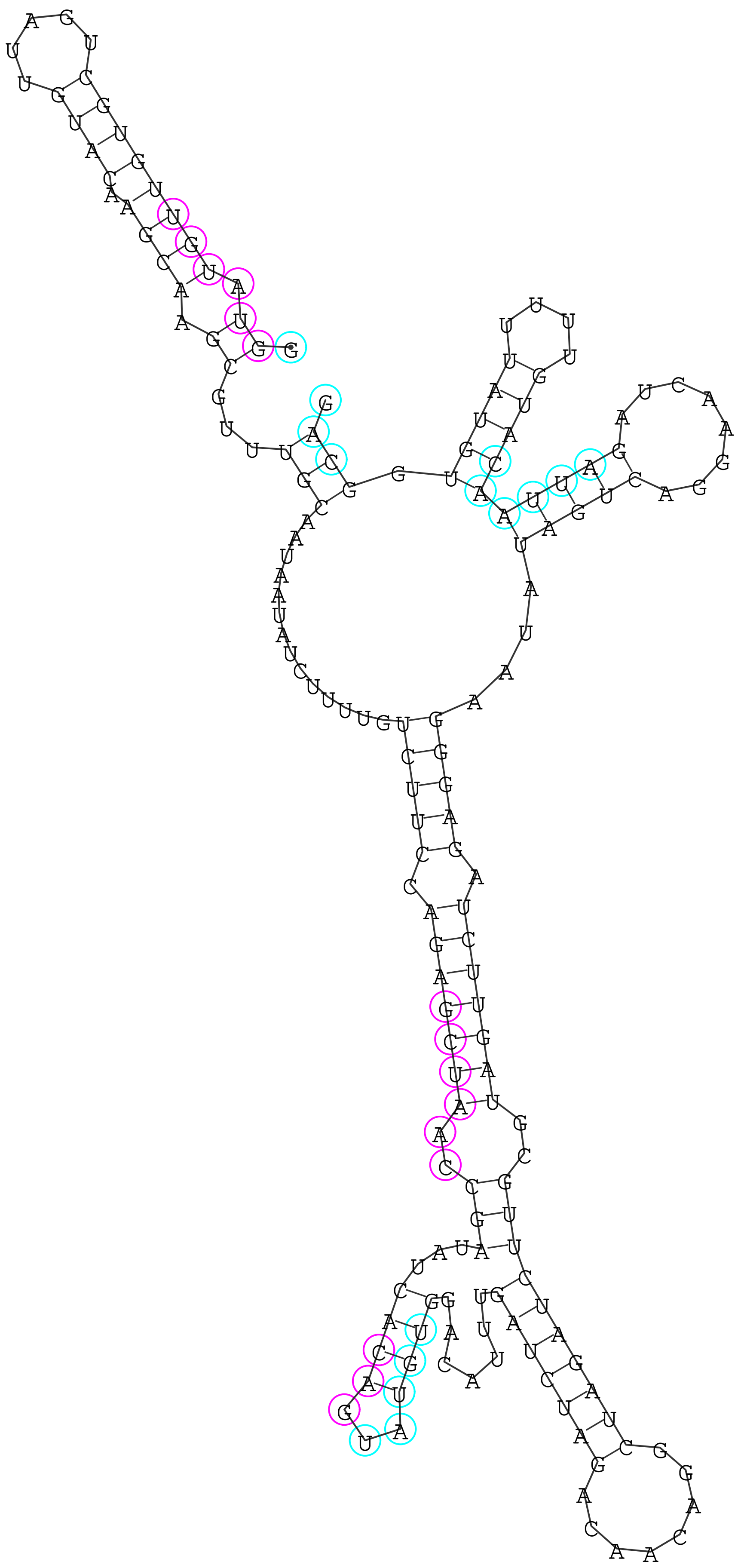

Xarbc0013A - Stwintron

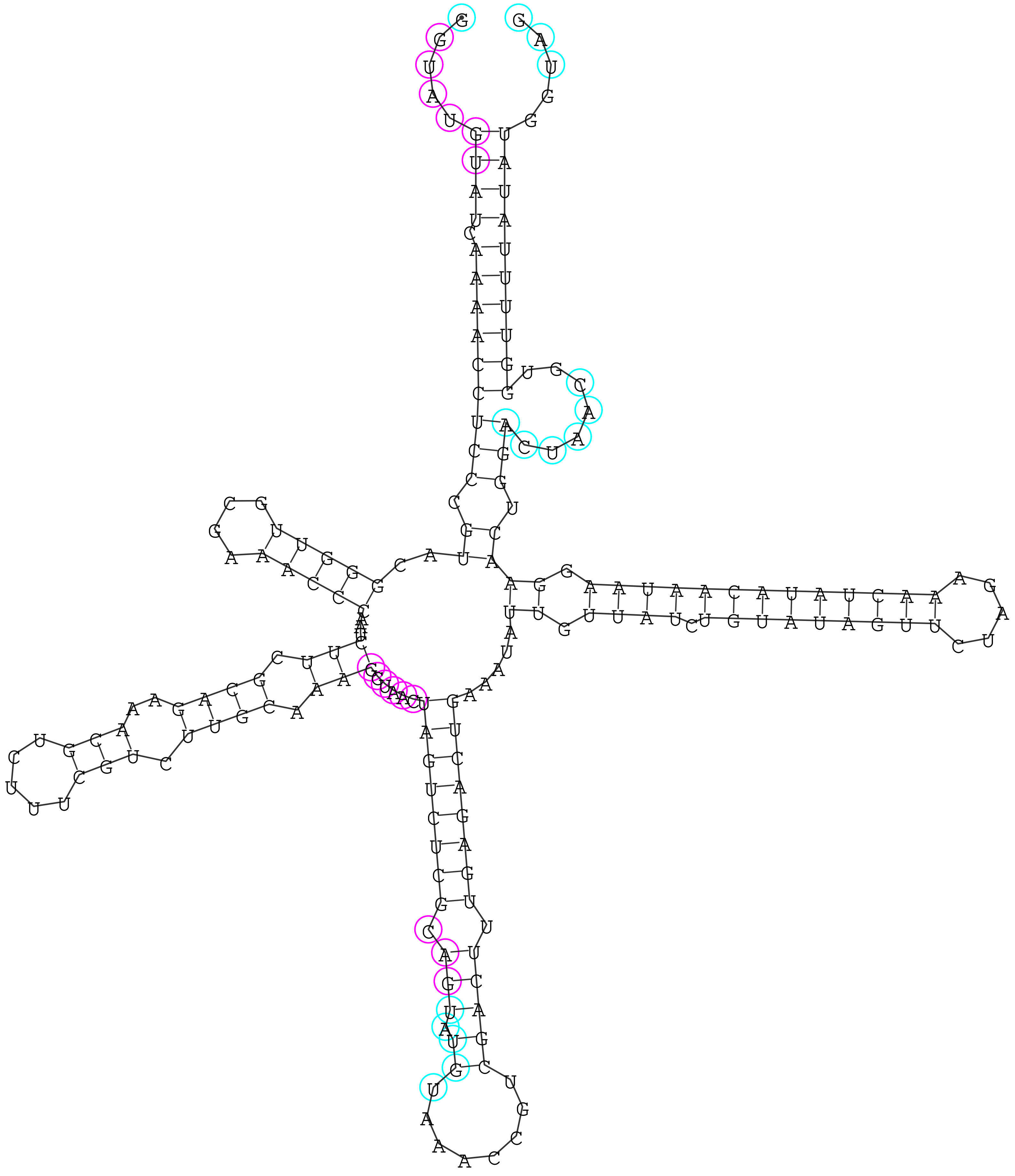

Xarbc0014A - Stwintron

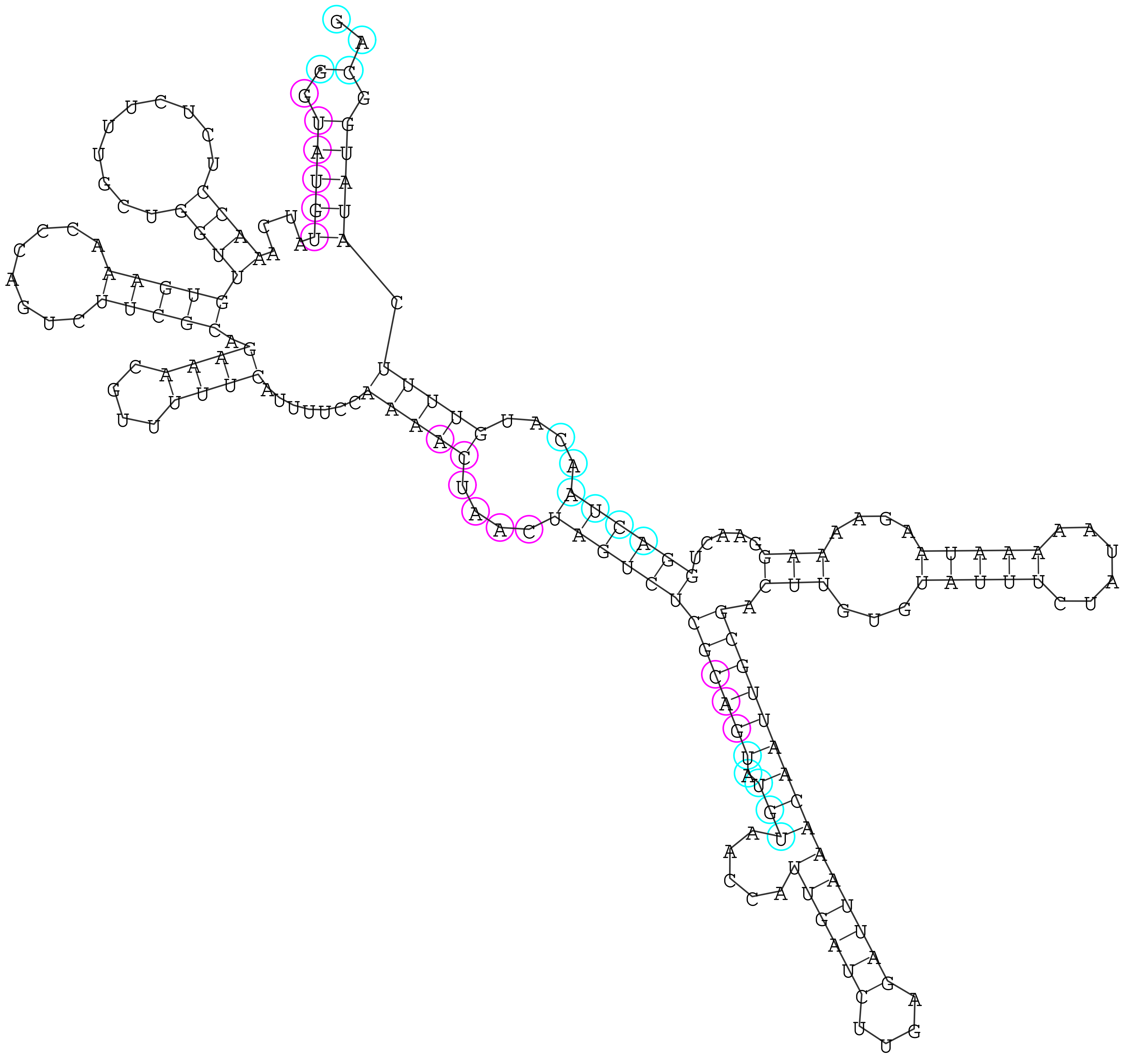

Xarbc0014B - Stwintron

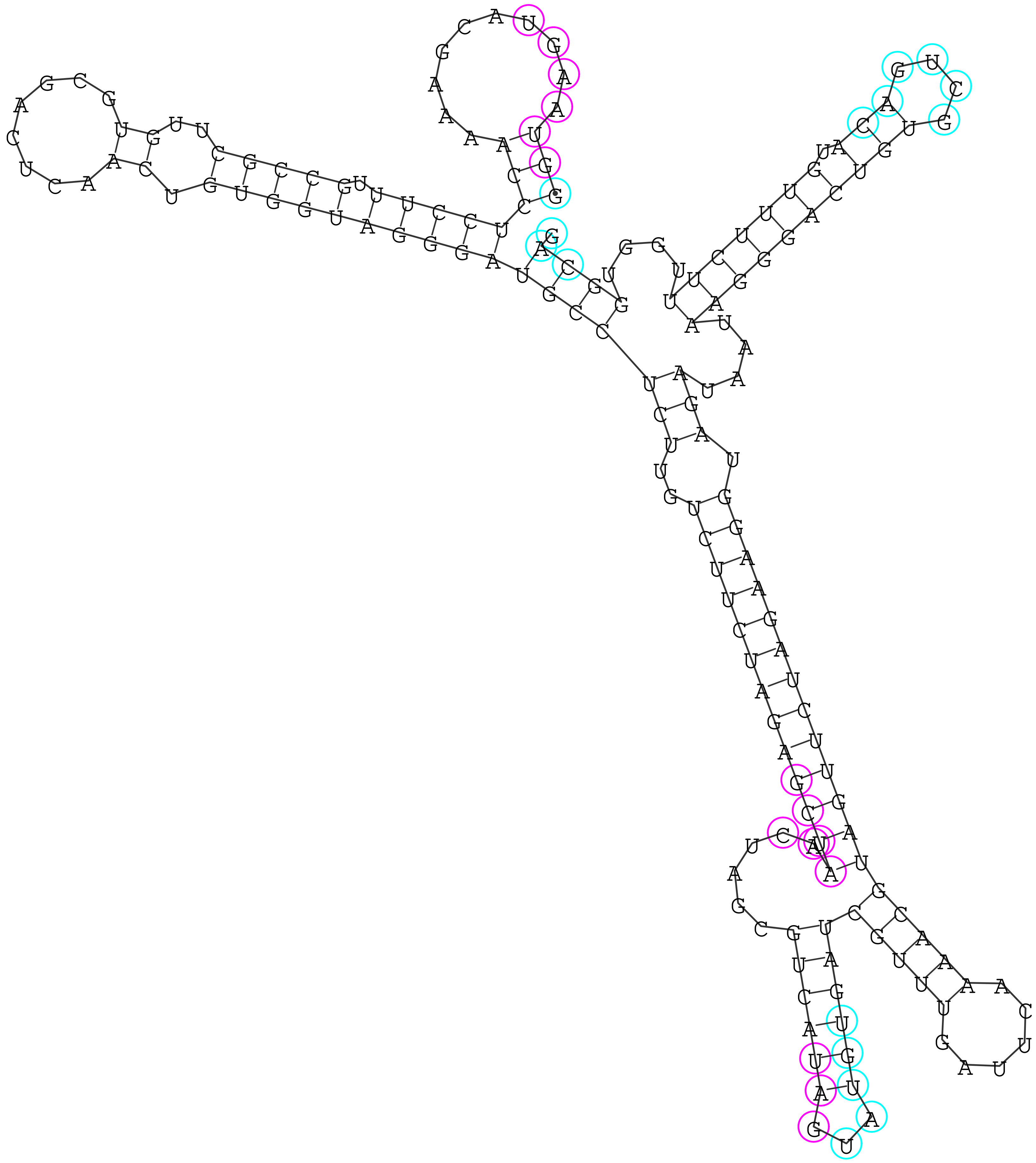

Xarbc0016A - Stwintron

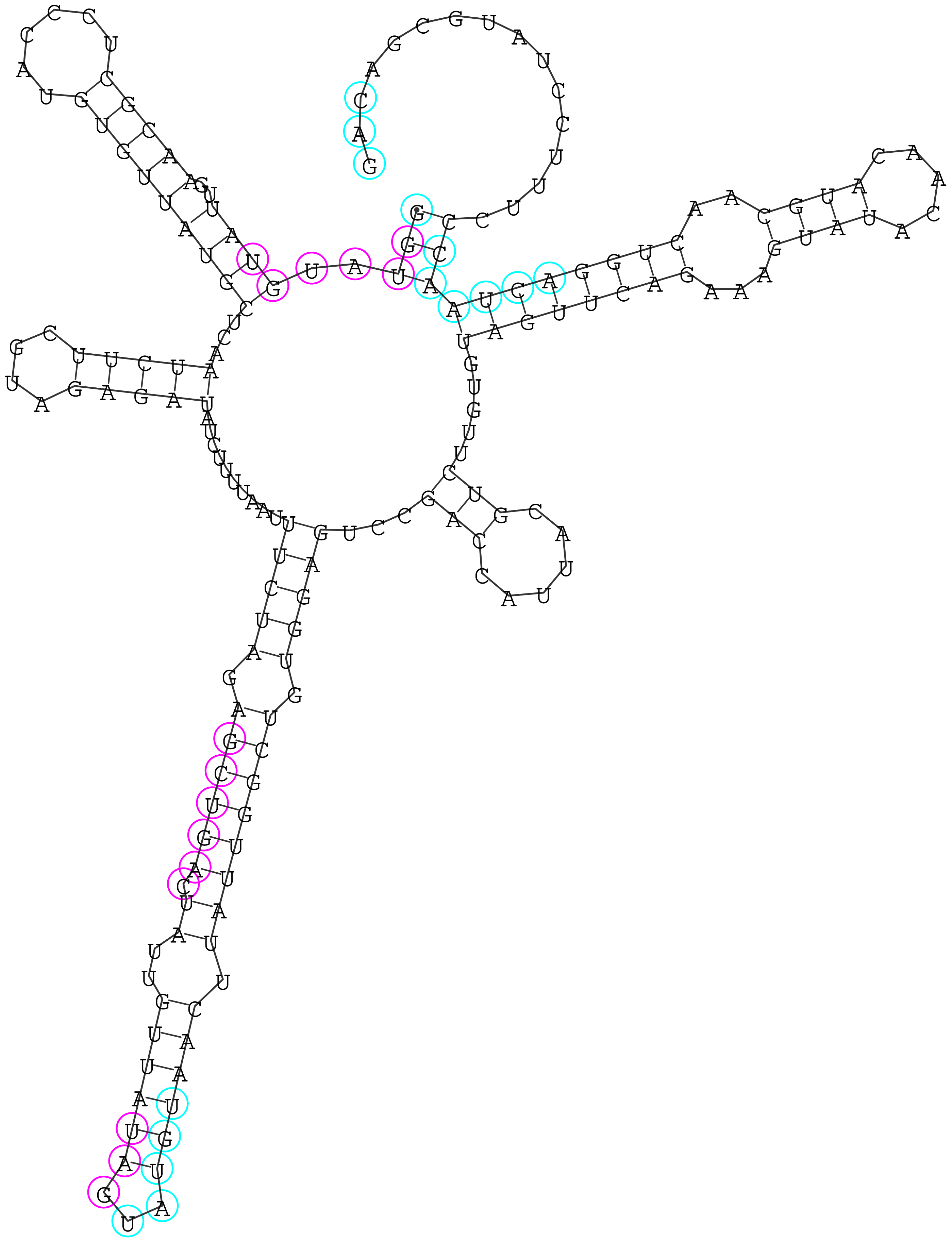

Xarbc0021A - Stwintron

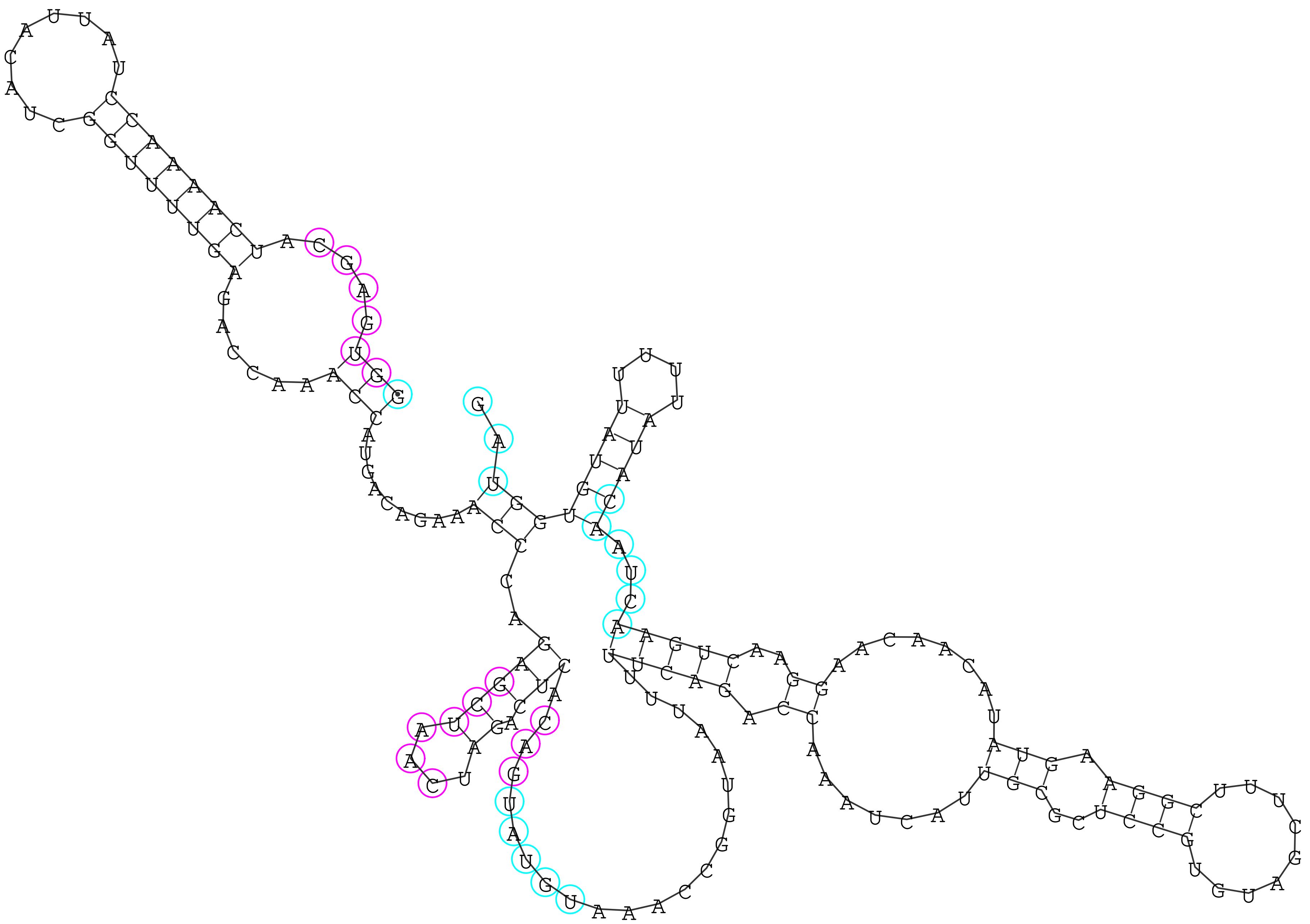

Xarbc0023A - Stwintron

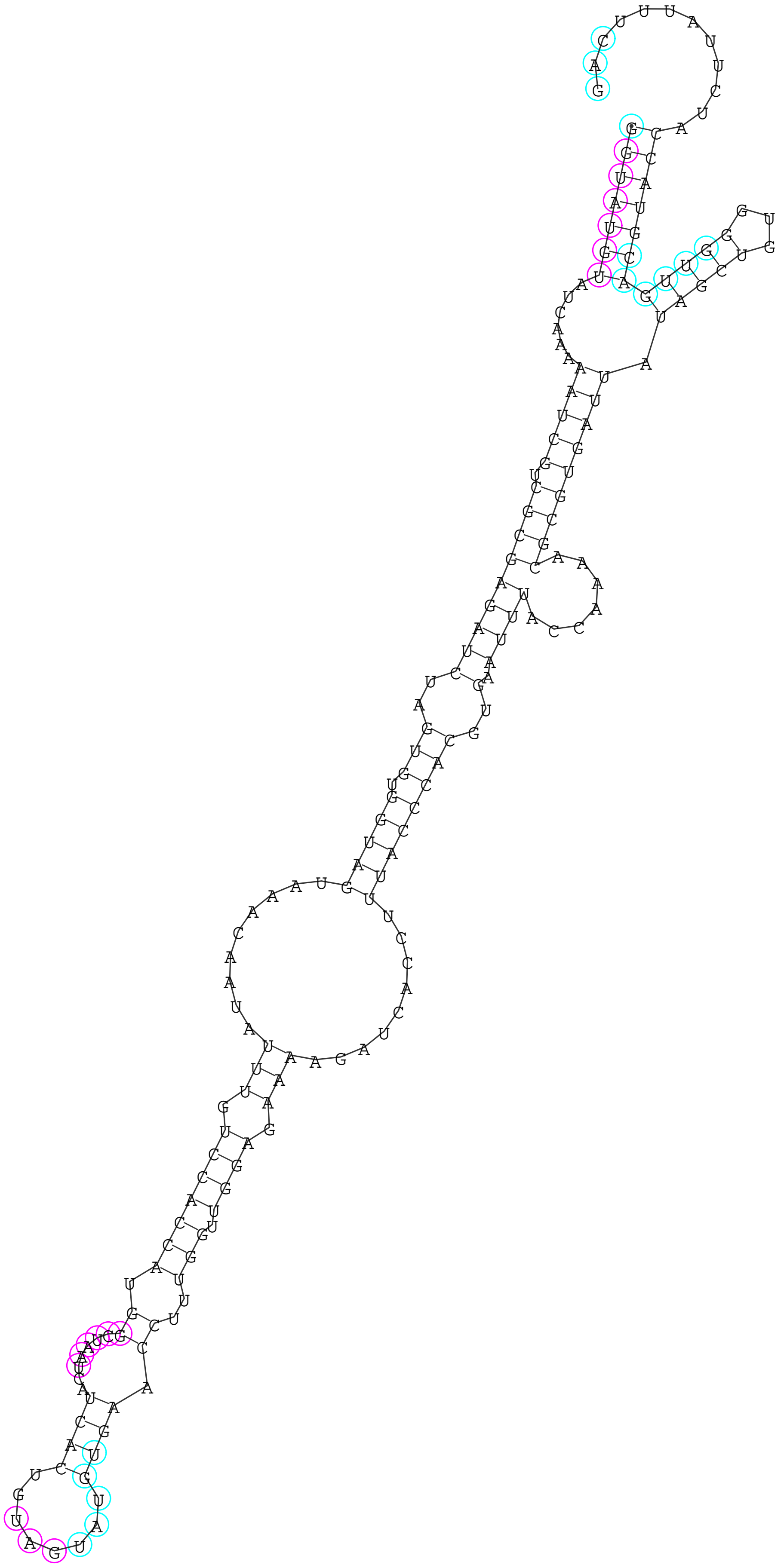

Xarbc0024A - Stwintron

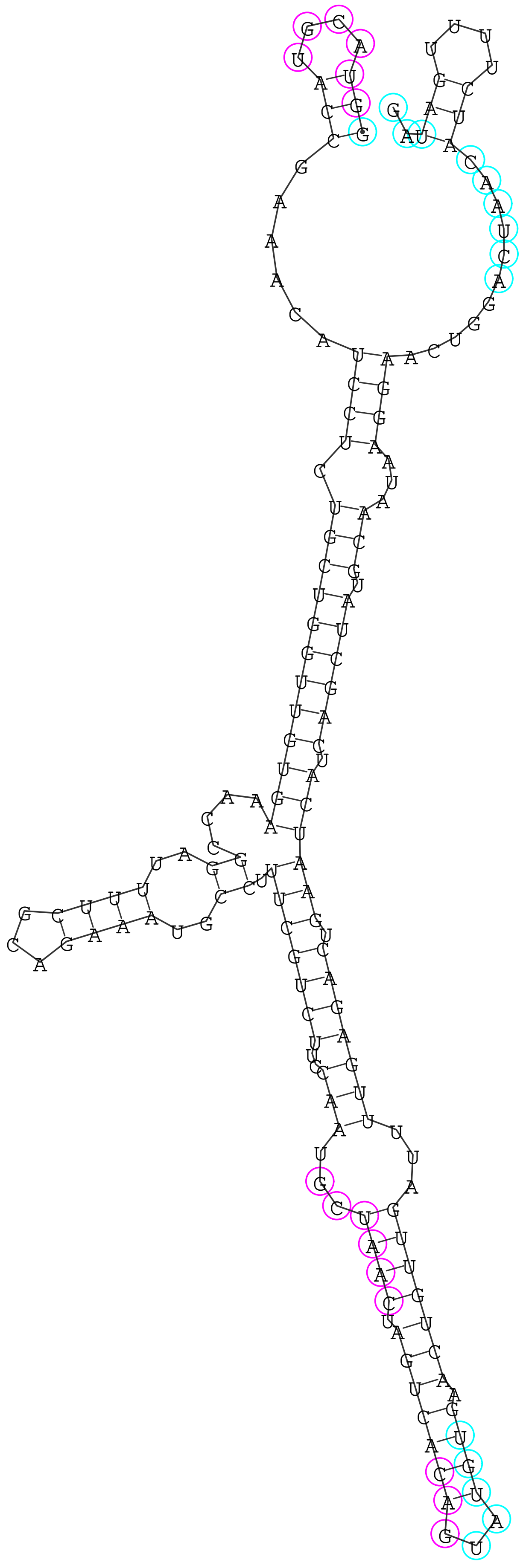

Xarbc0024B - Stwintron

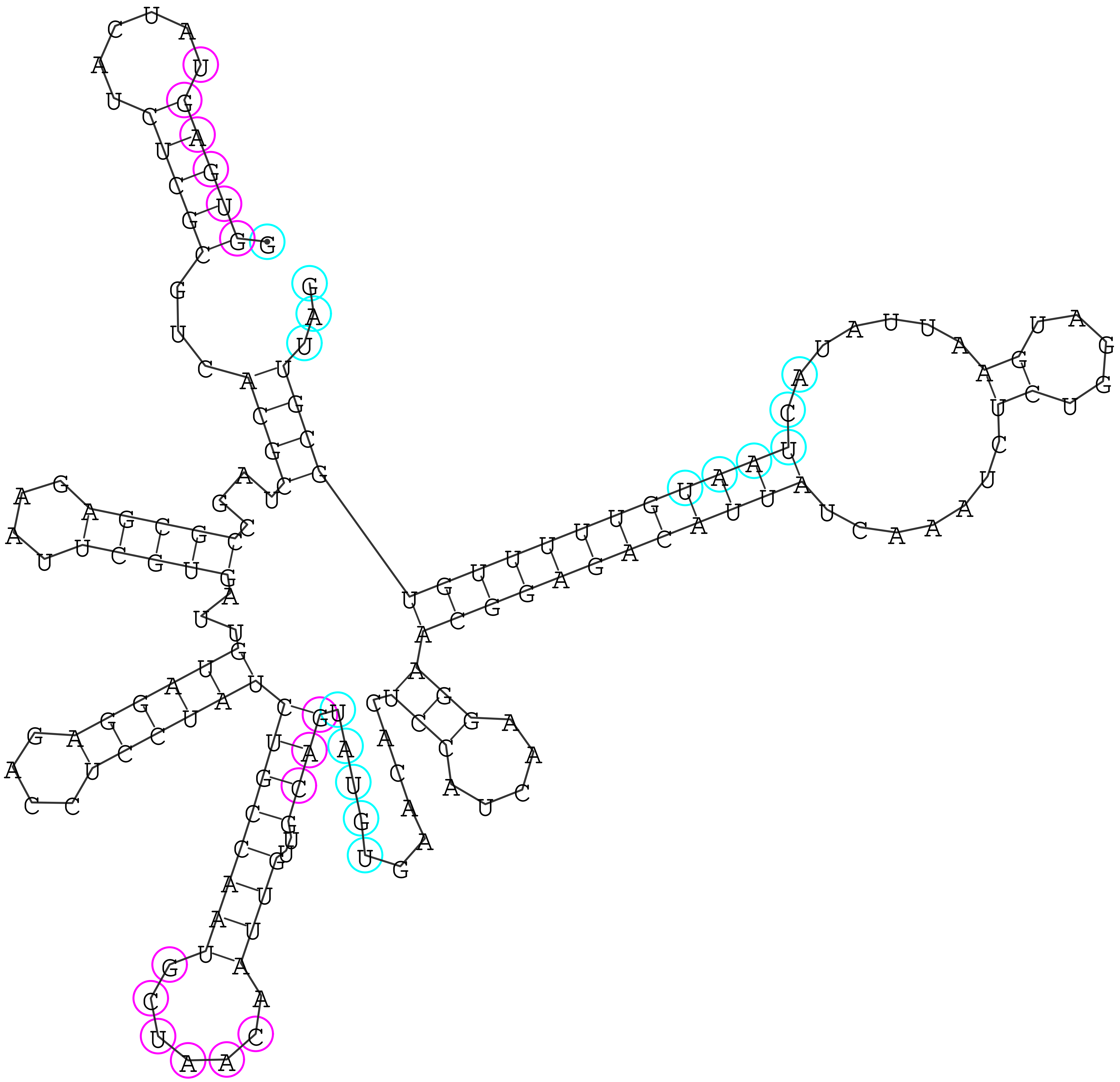

Xarbc0024C - Stwintron

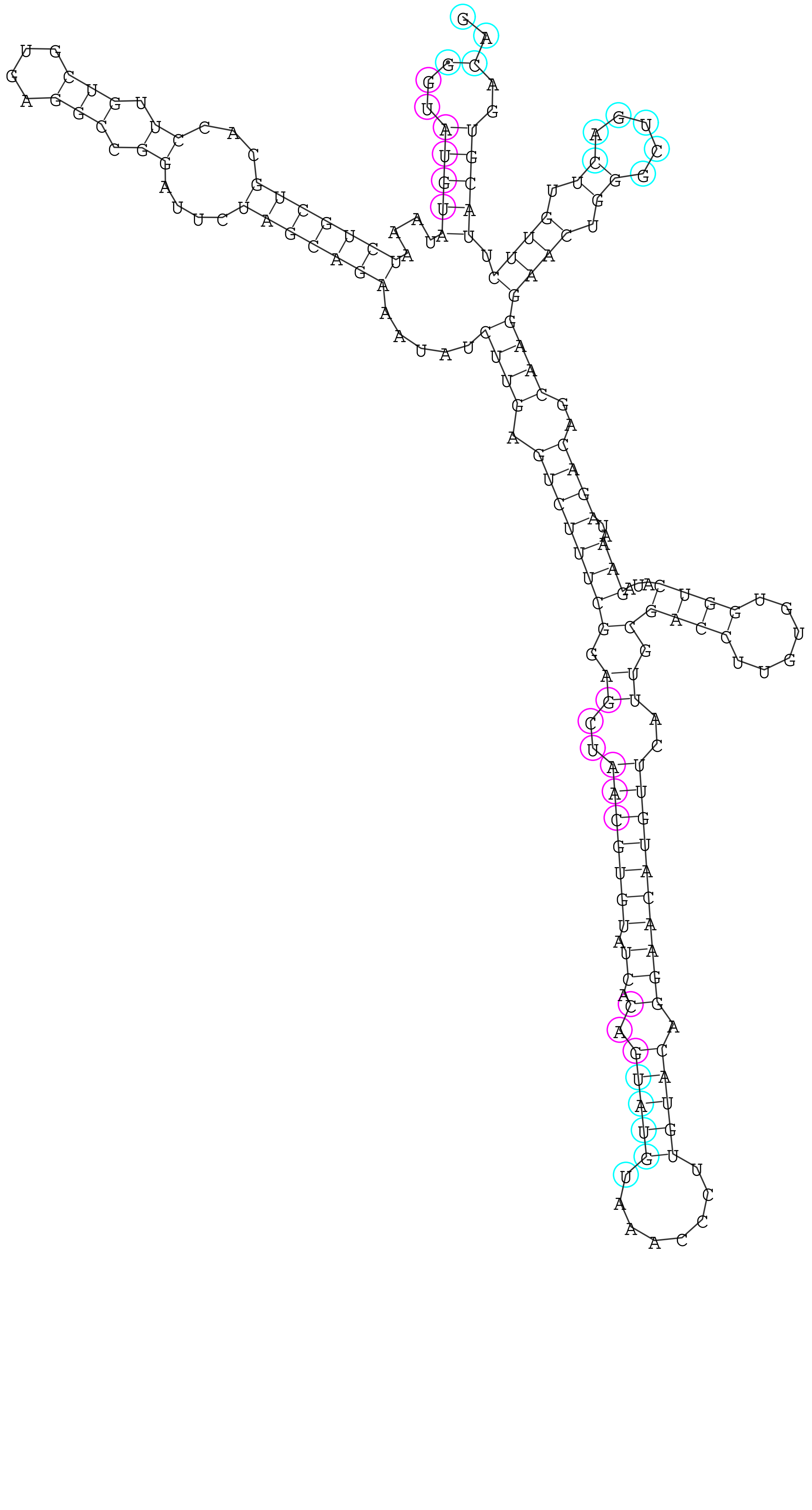

Xarbc0026A - Stwintron

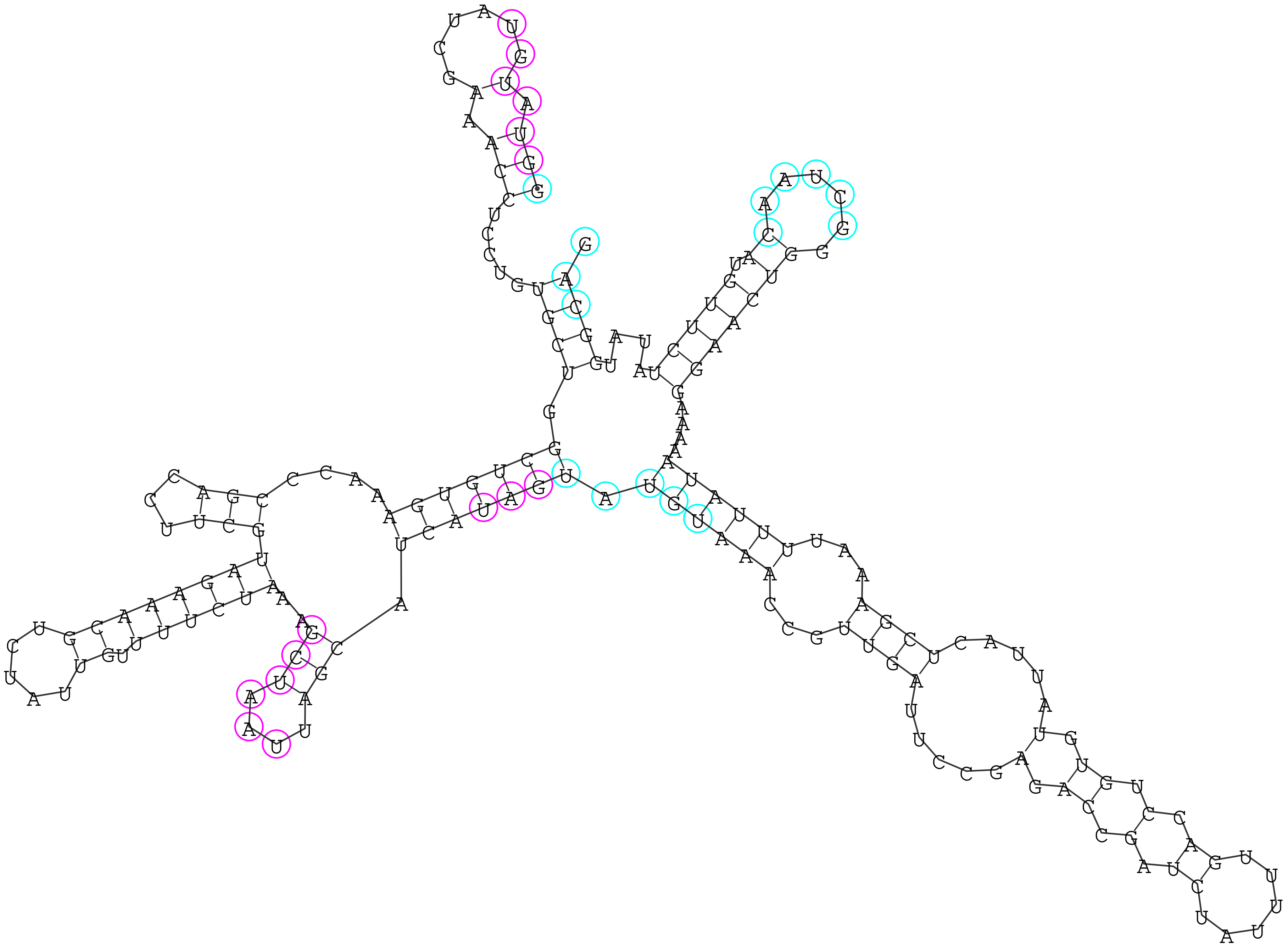

Xarbc0028A - Stwintron

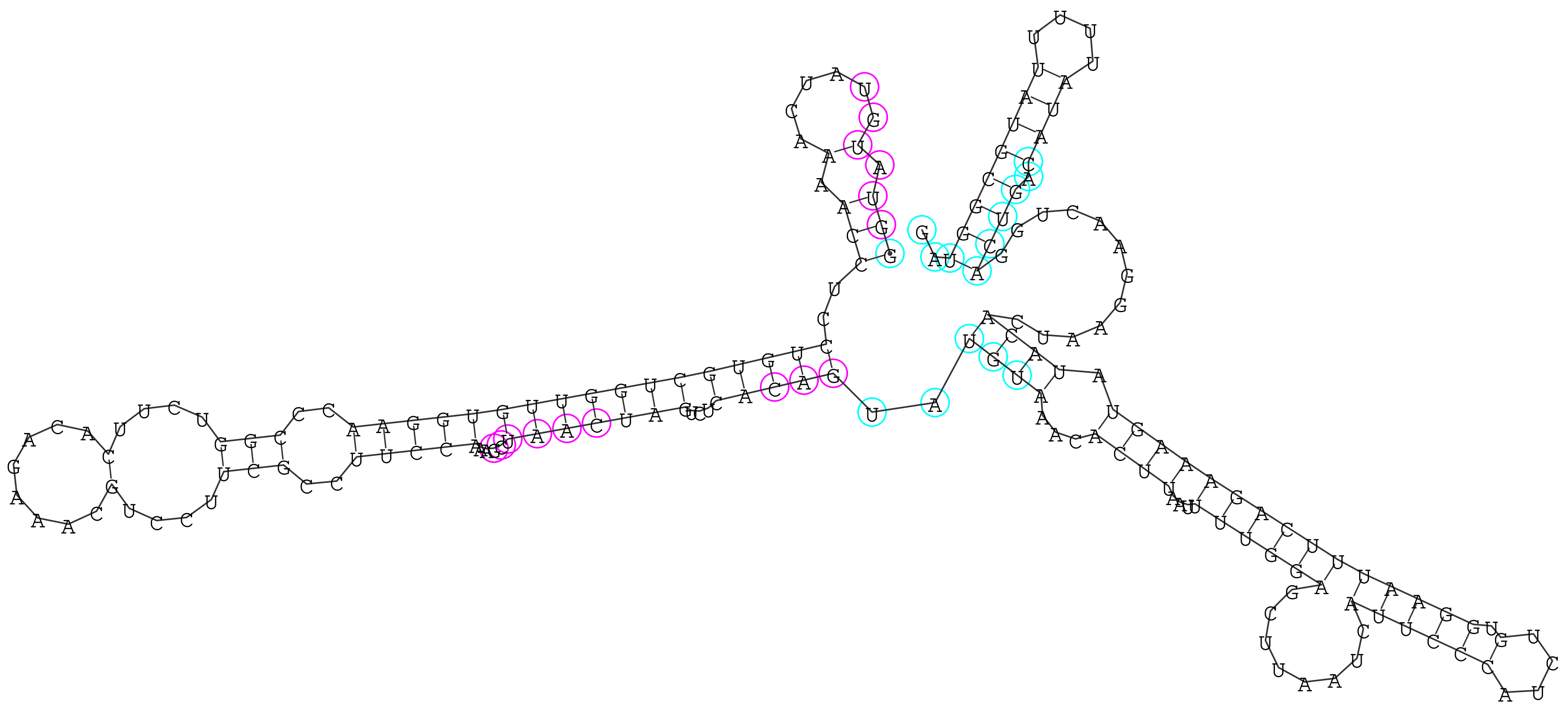

Xarbc0034A - Stwintron

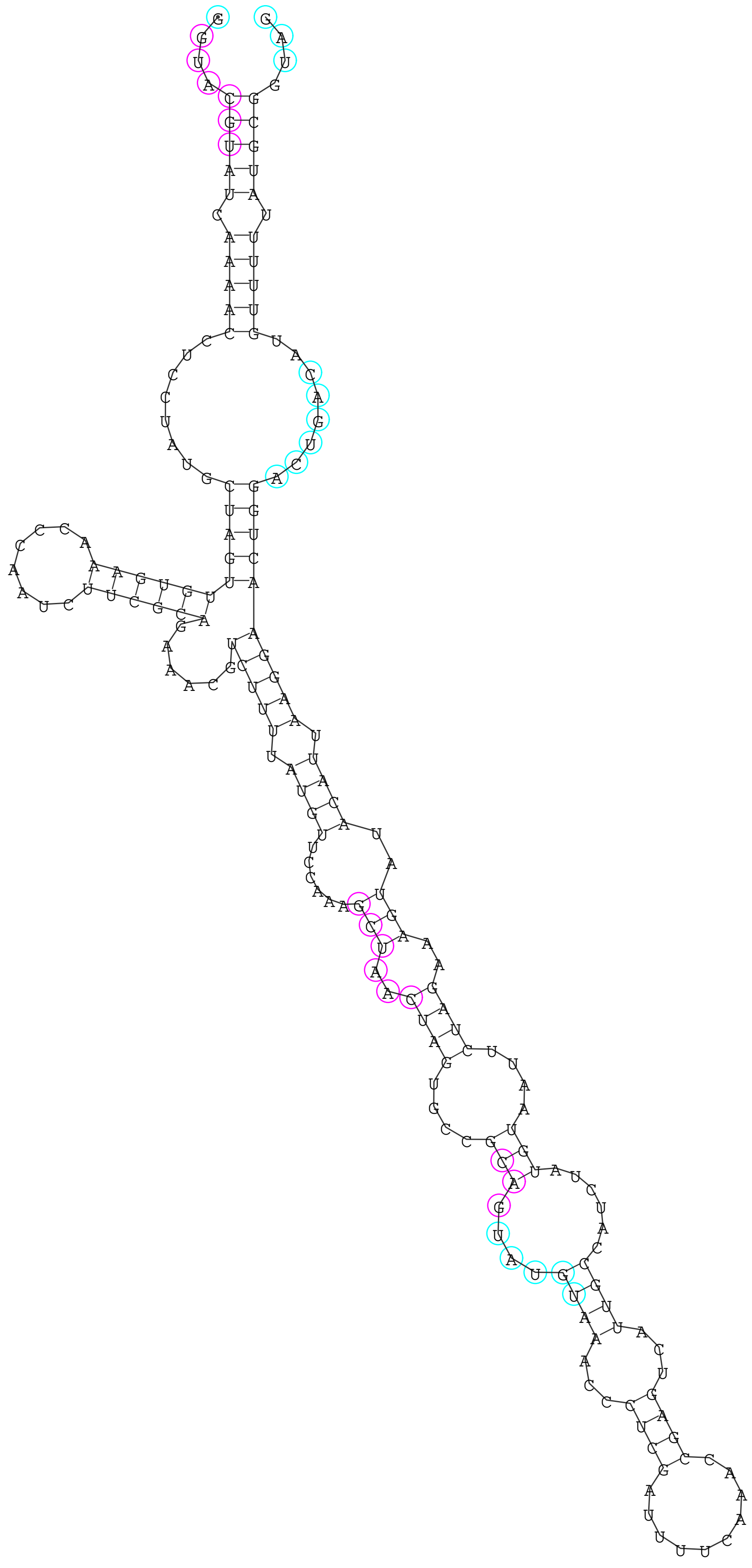

Xarbc0044A - Stwintron

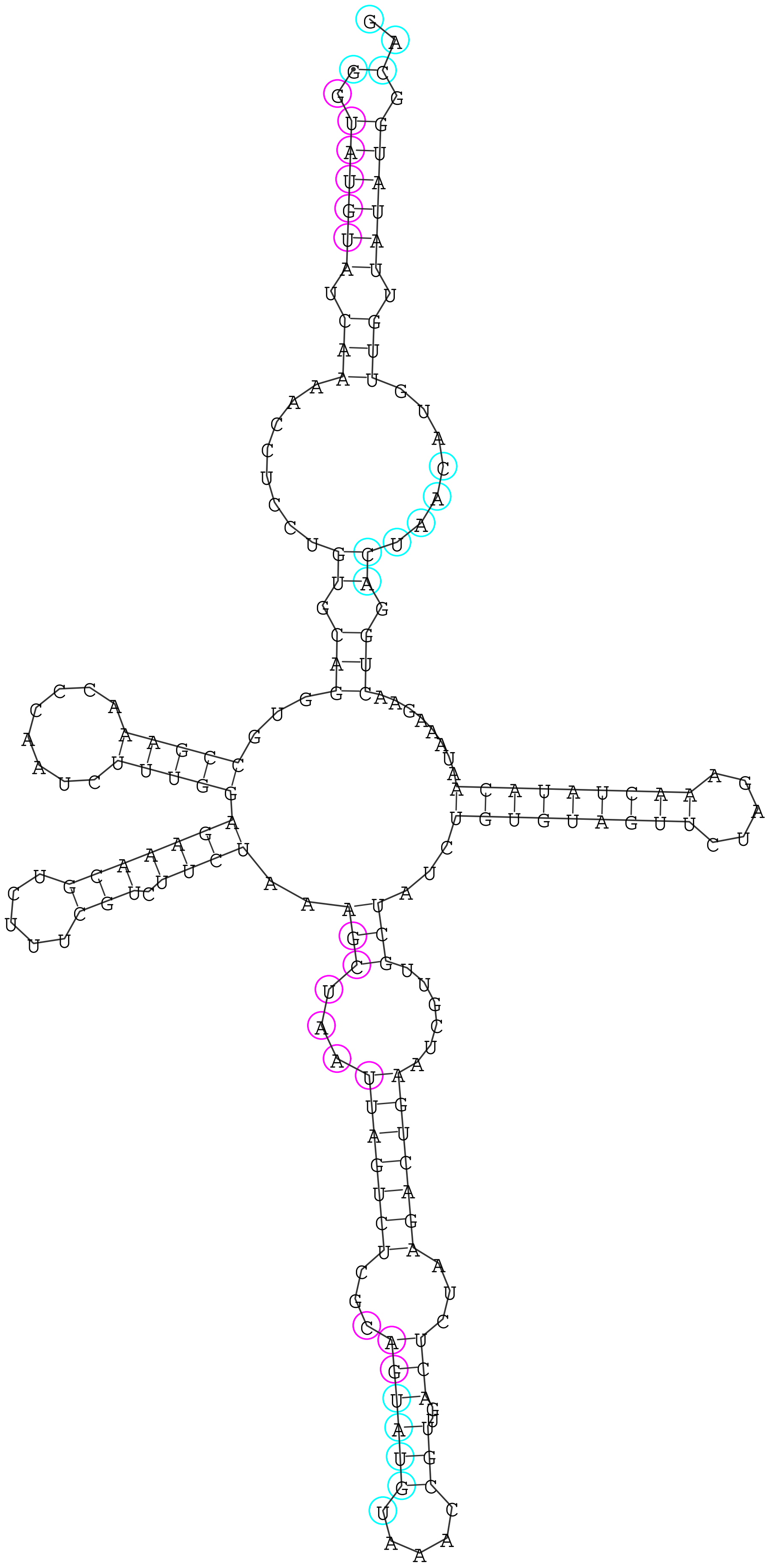

Xarbc0059A - Stwintron

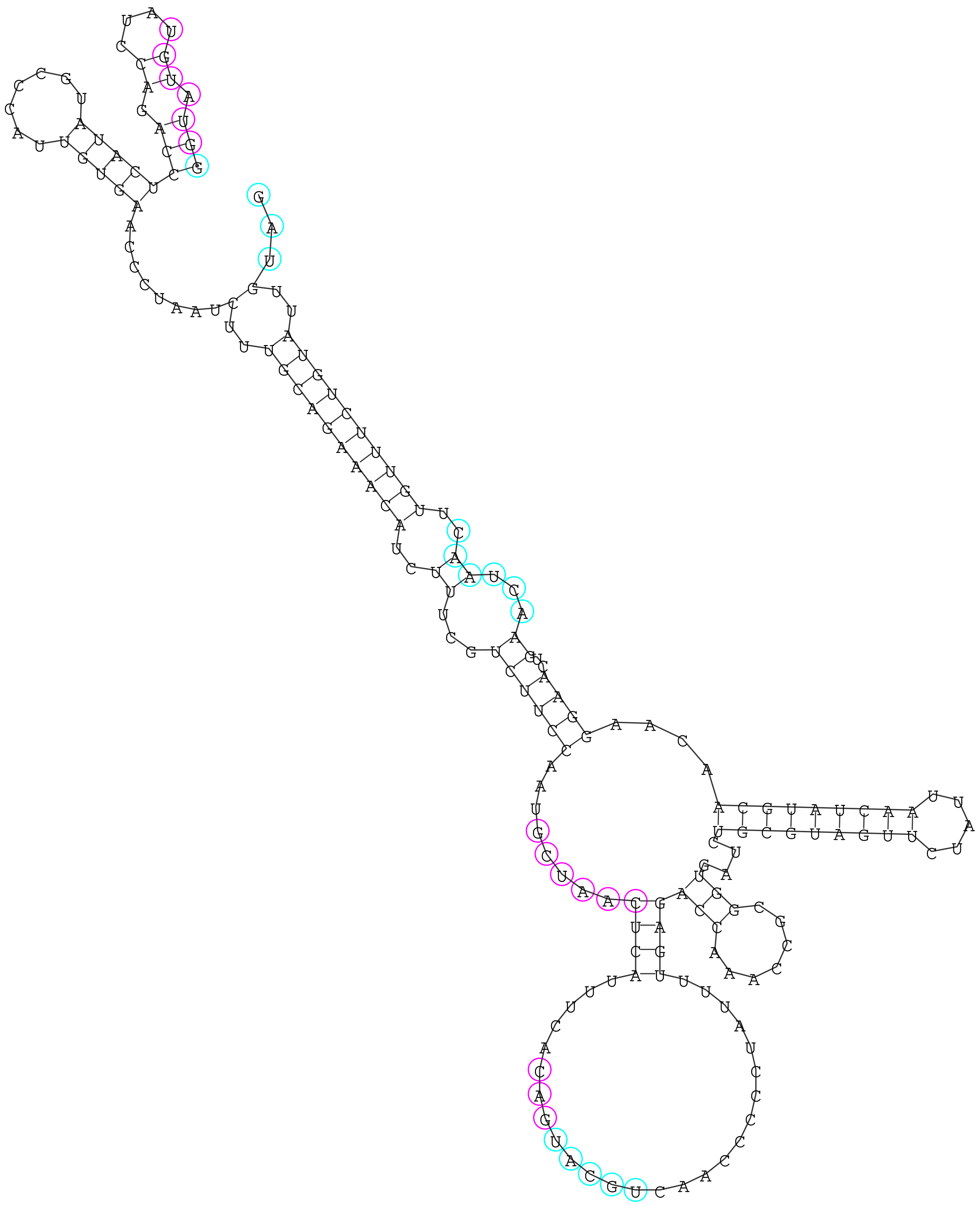

Xarbc0060A - Stwintron

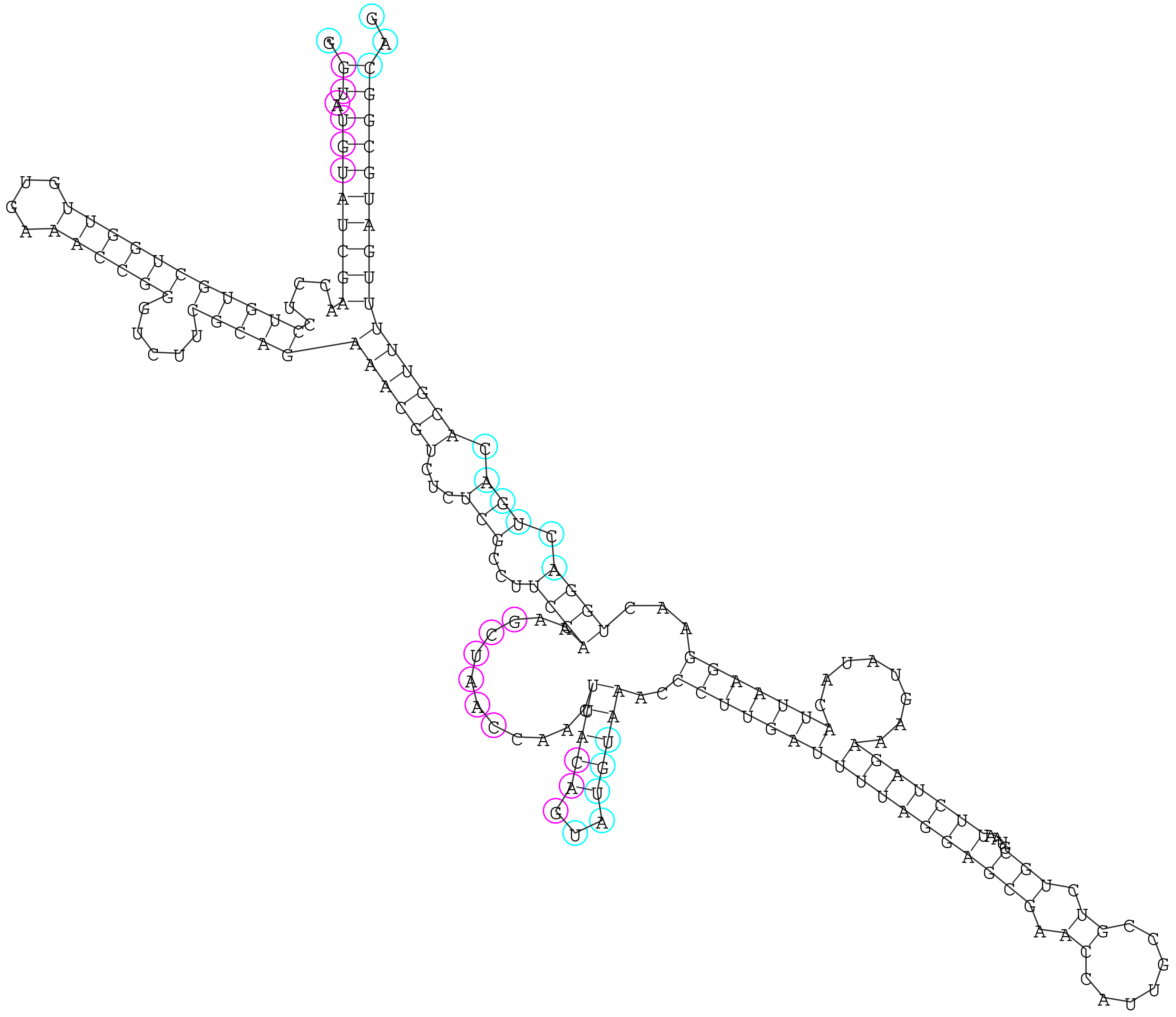

Xarbc0061A - Stwintron

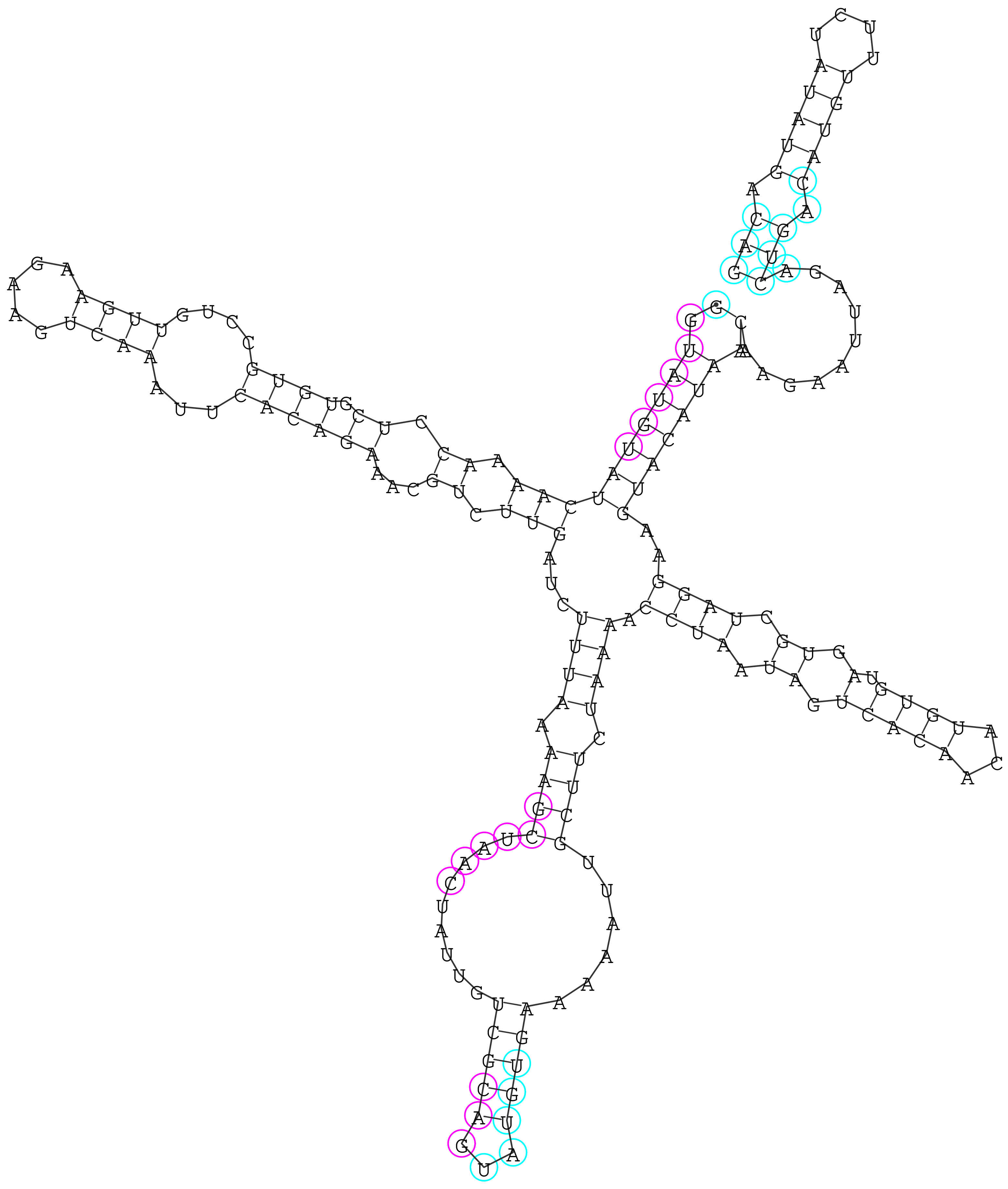

Xarbc0064A - Stwintron

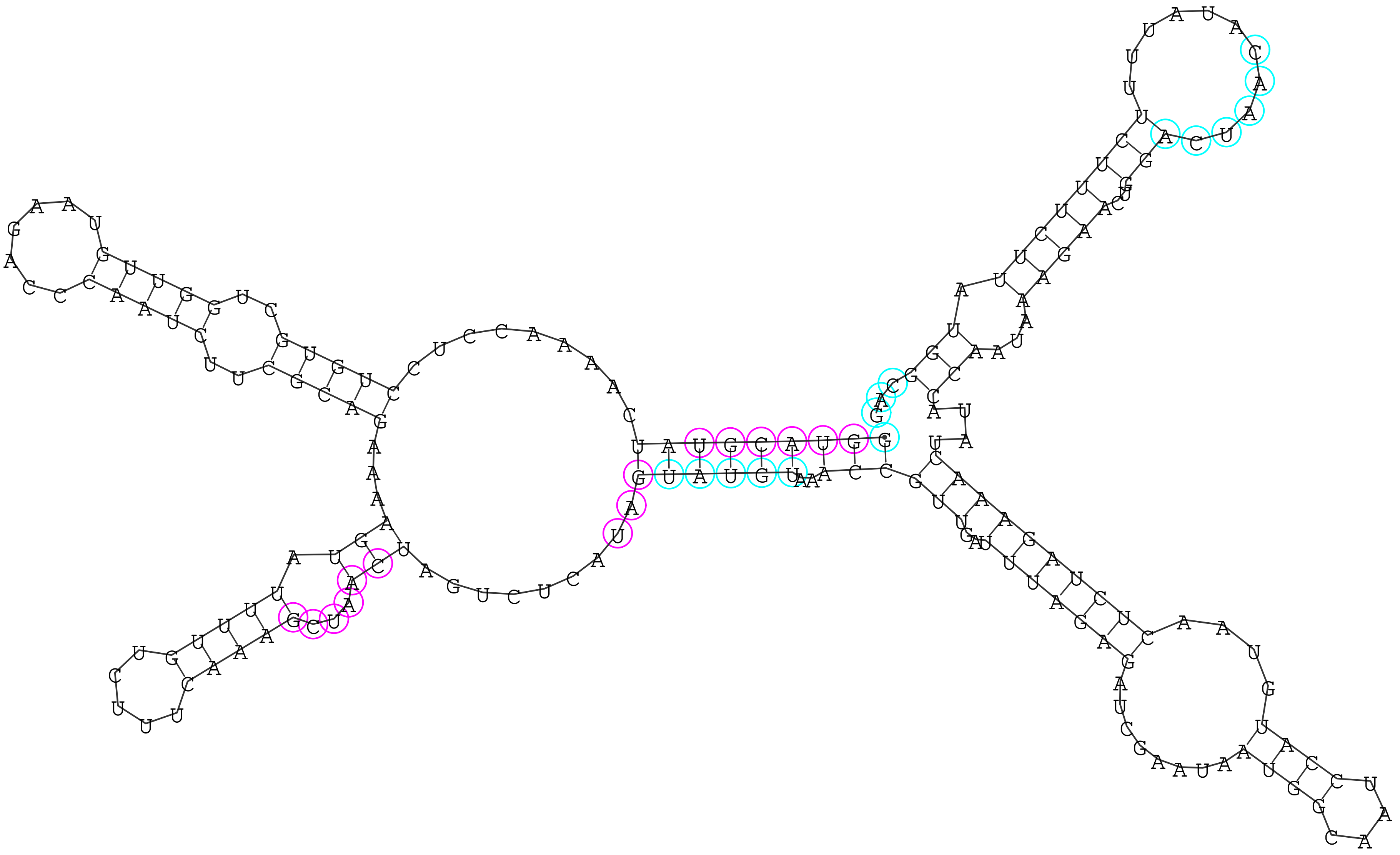

Xarbc0064B - Stwintron

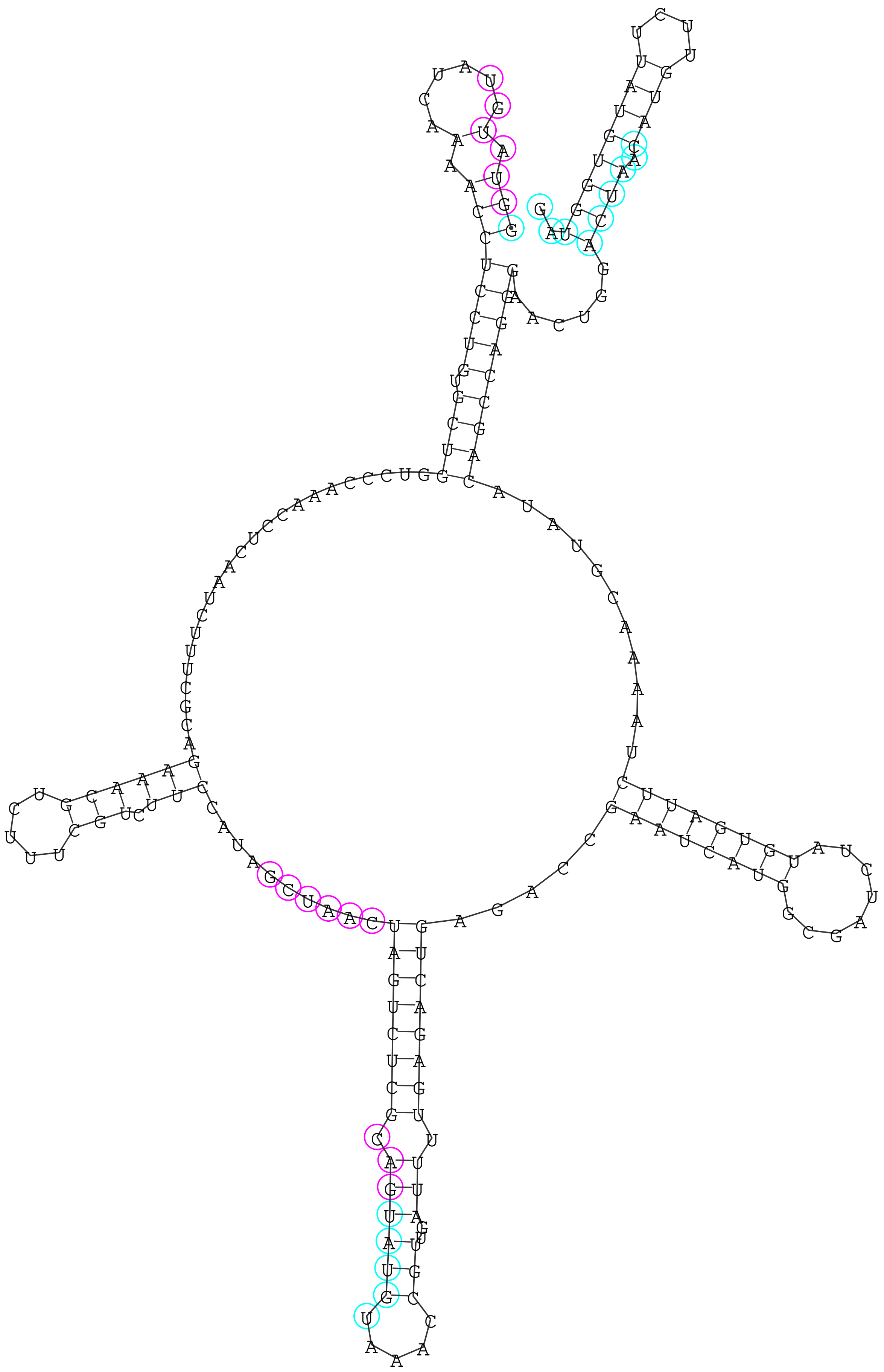

Xarbc0072A - Stwintron

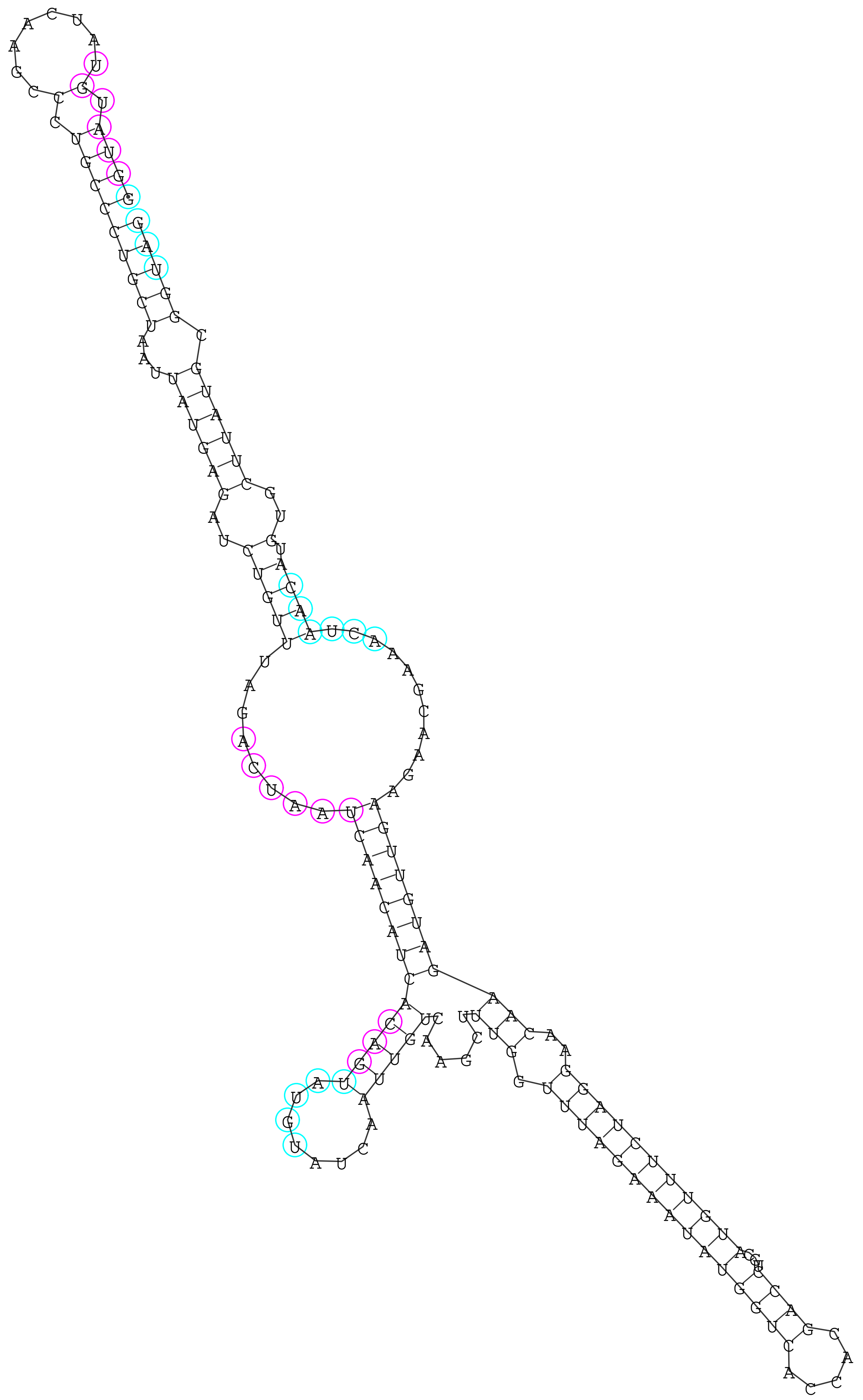

Xarbc0074A - Stwintron

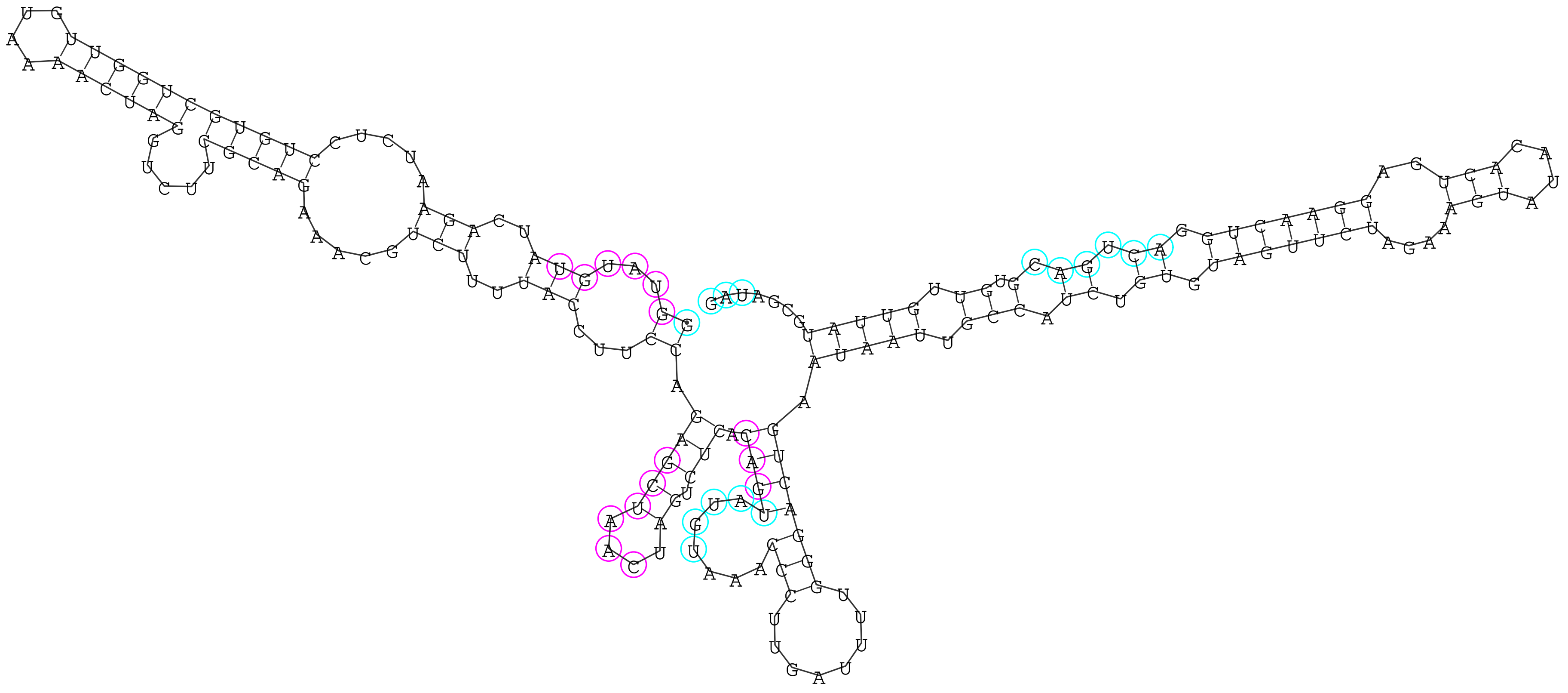

Xarbc0080A - Stwintron

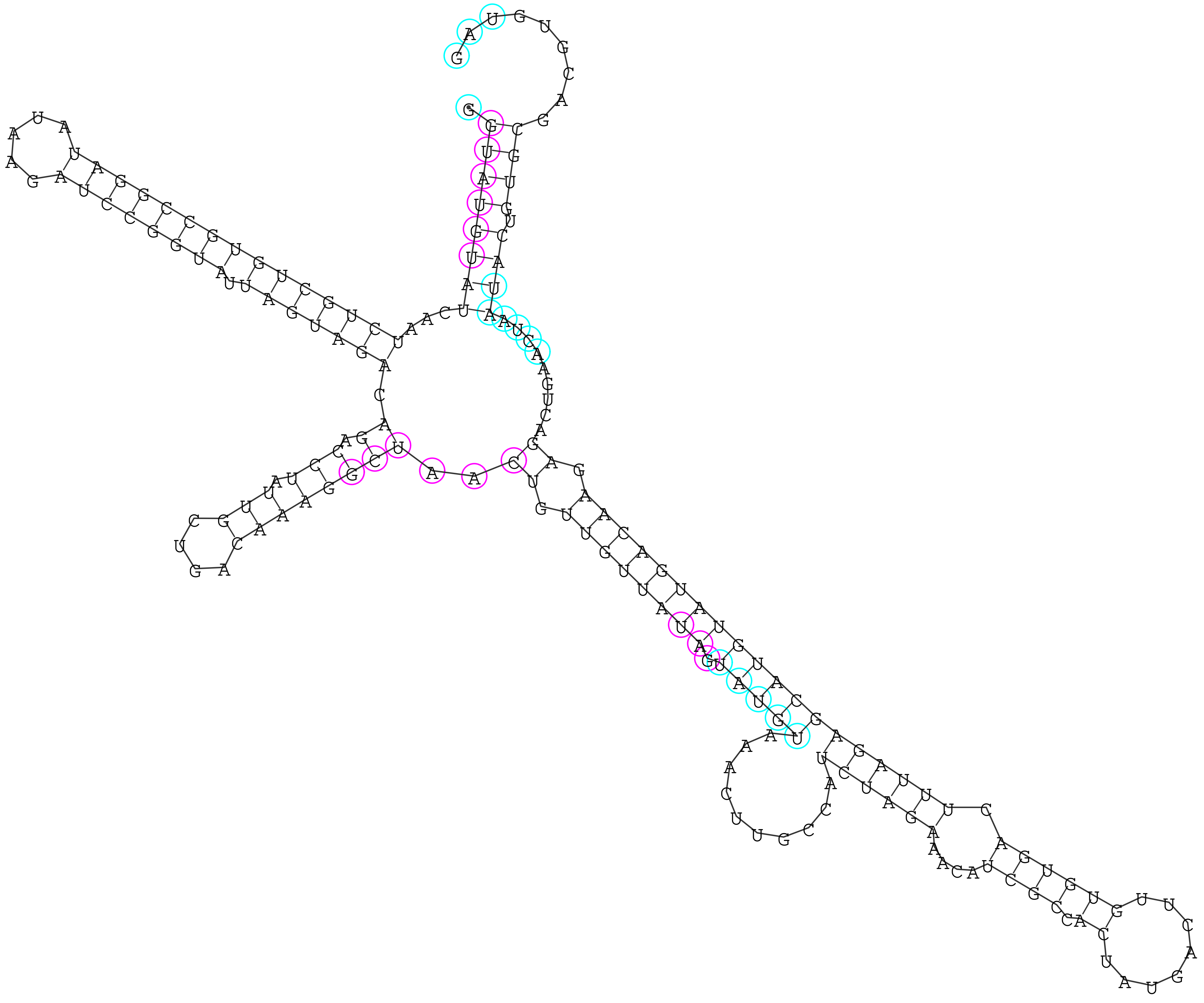

Xarbc0093A - Stwintron

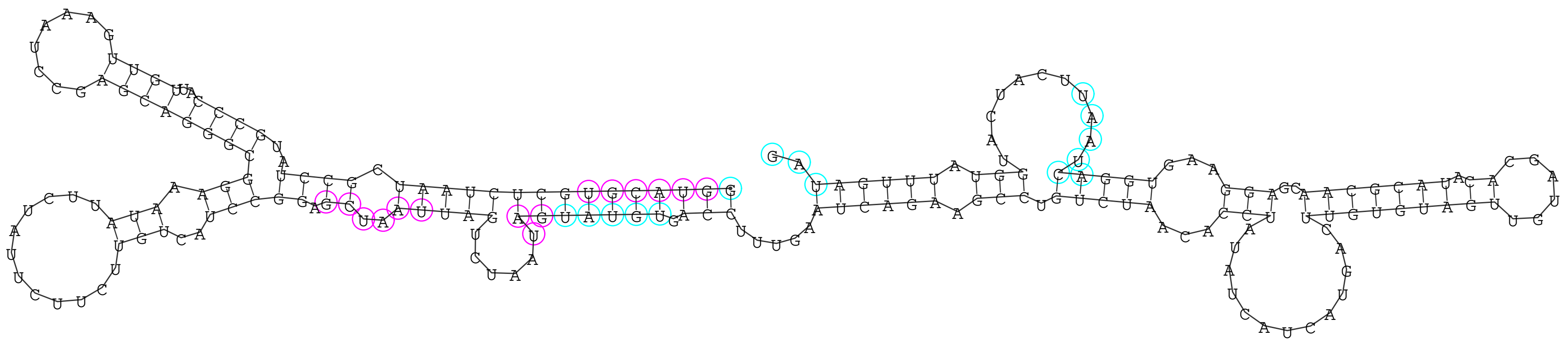

Xarbc0096A - Stwintron

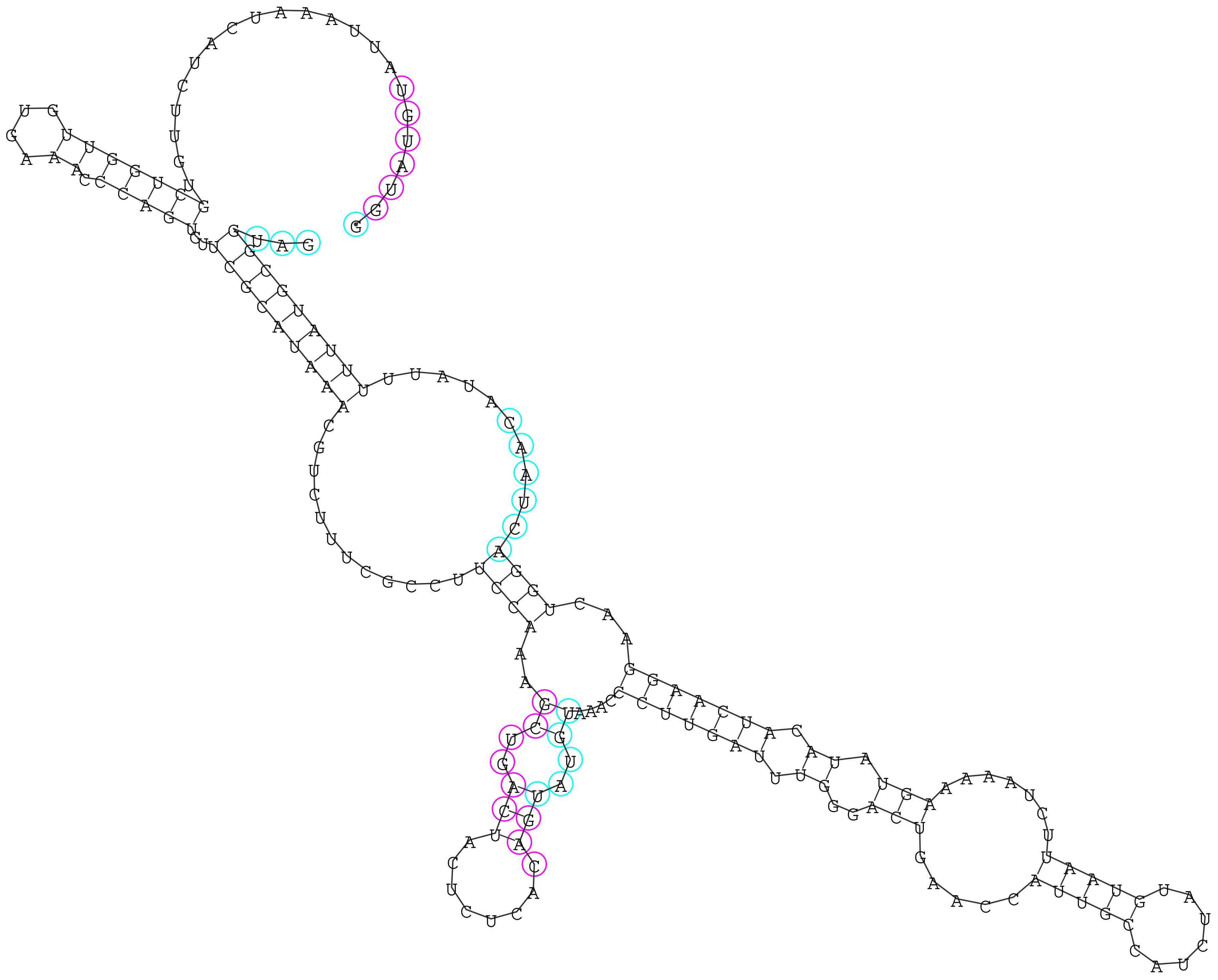

Xarbc0099A - Stwintron

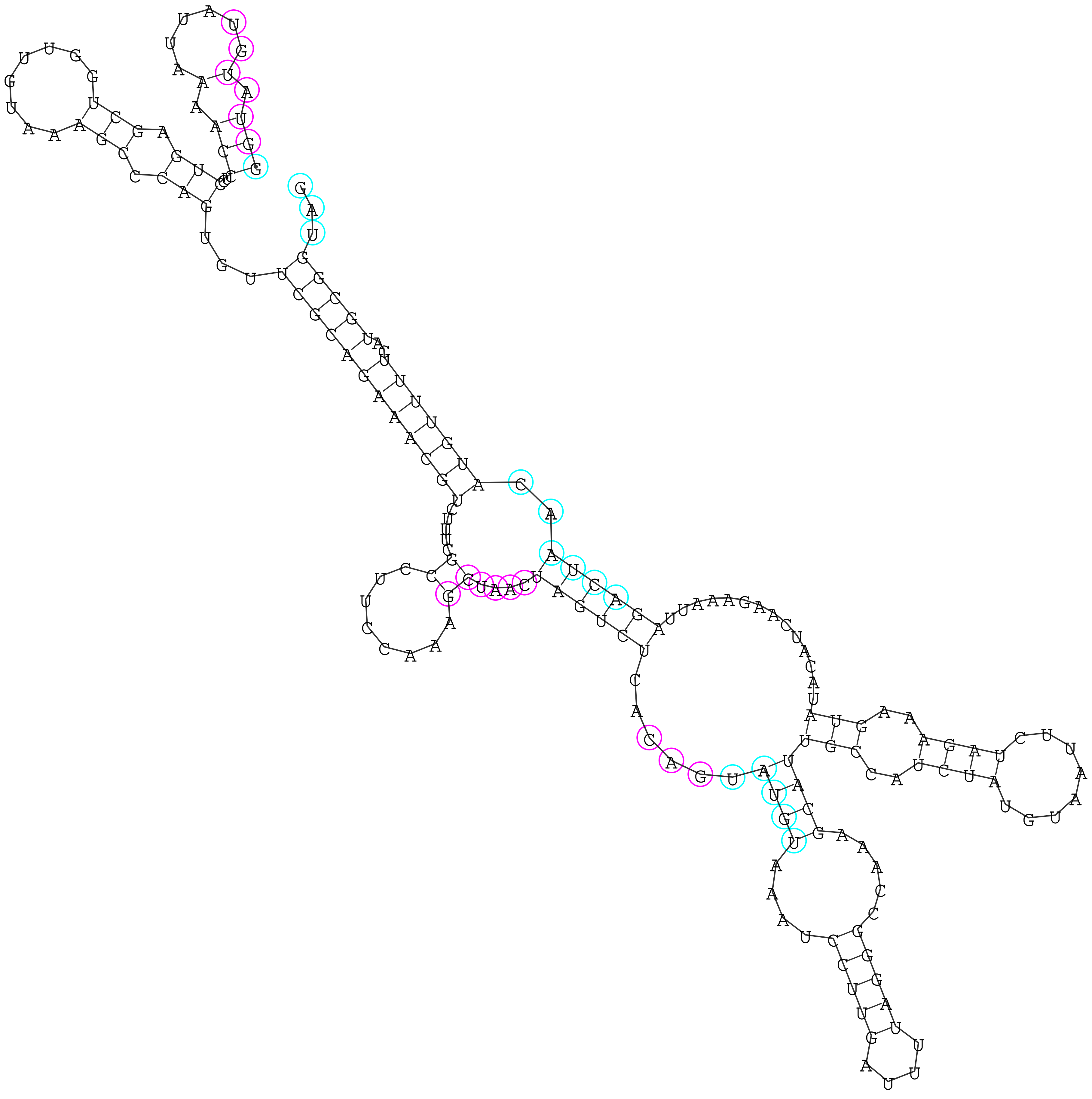

Xarbc0101A - Stwintron

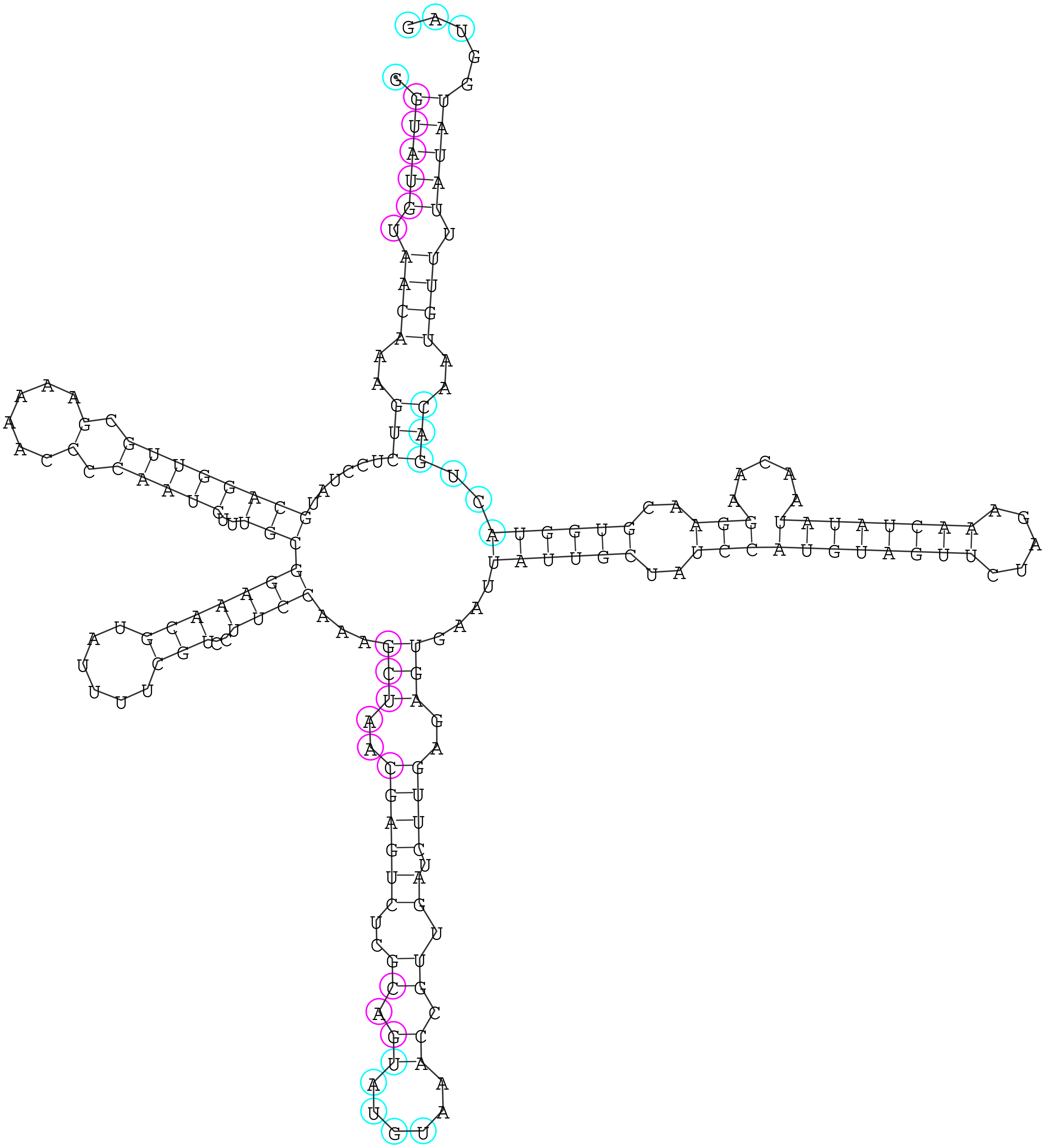

Xarbc0108A - Stwintron

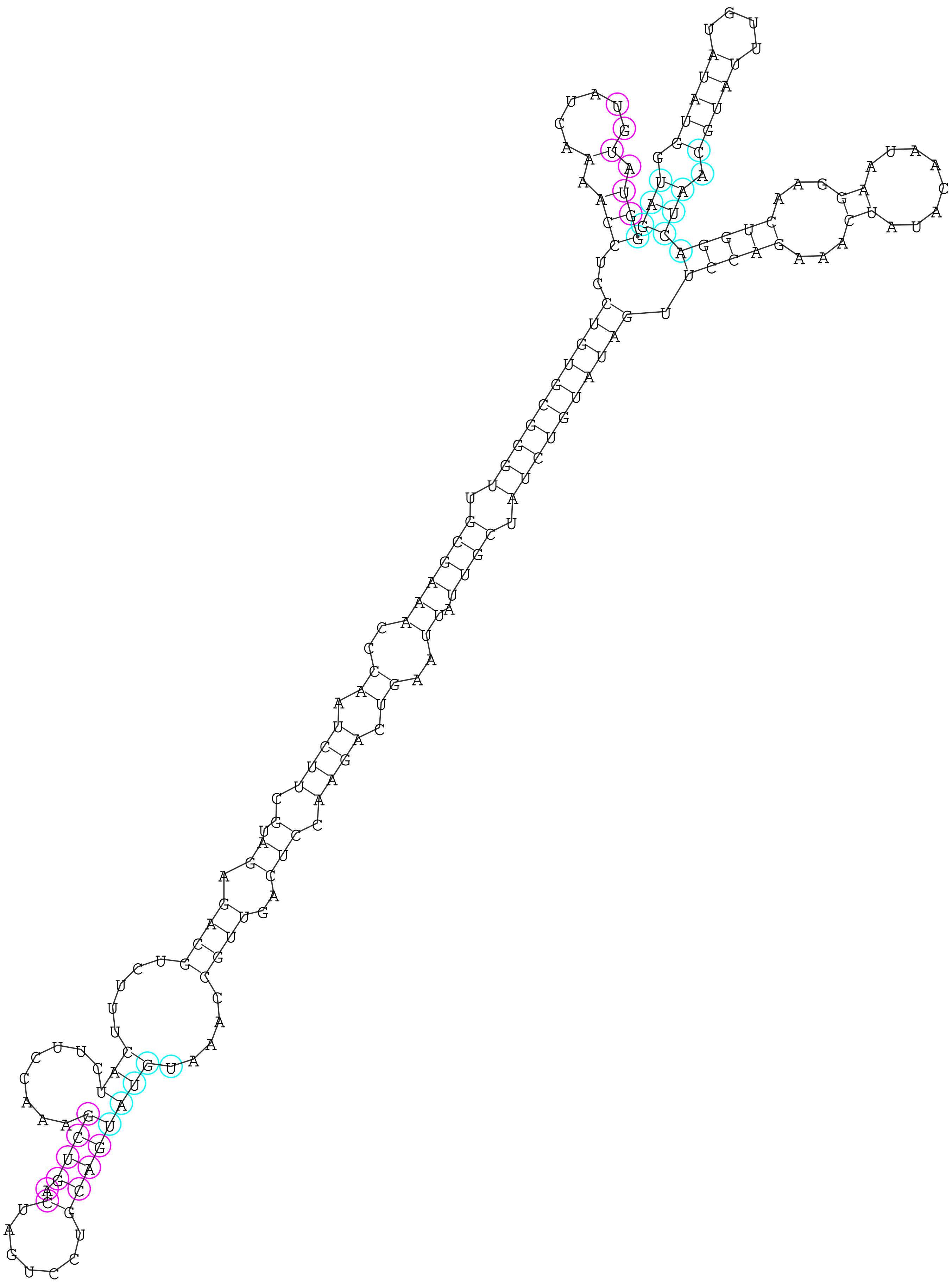

Xarbc0134A - Stwintron

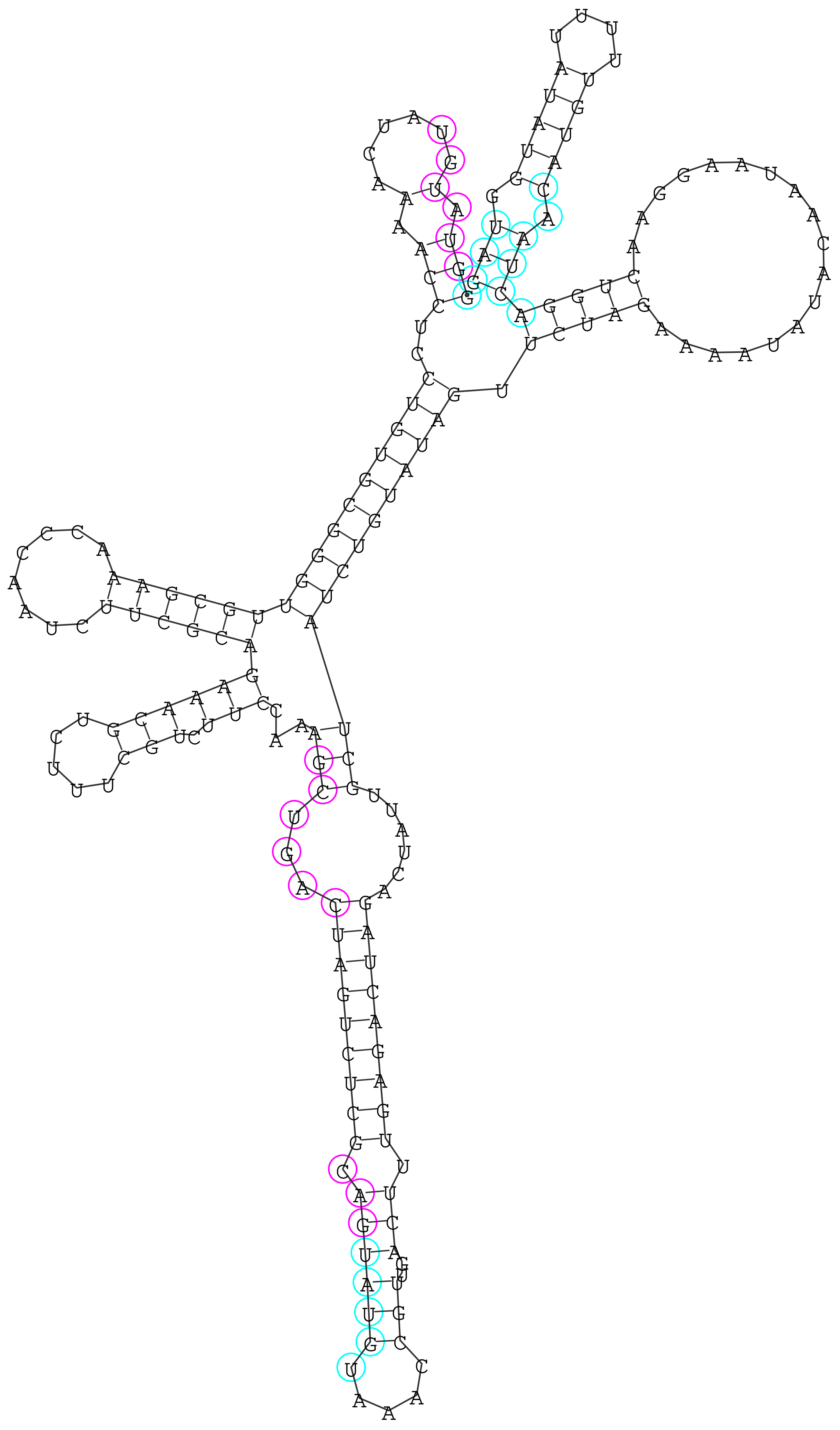

Xarbc0143A - Stwintron

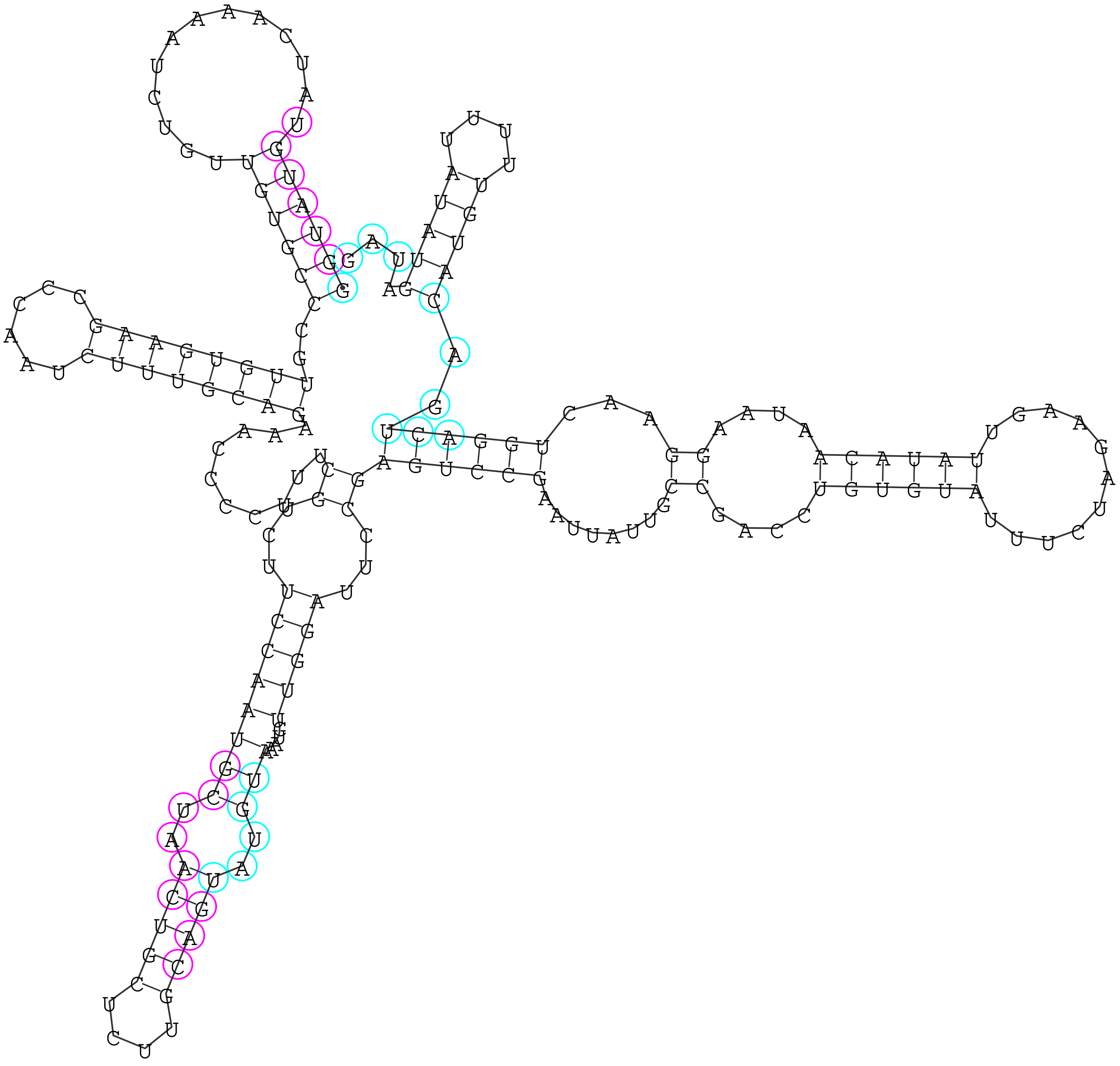

Xarbc0164A - Stwintron

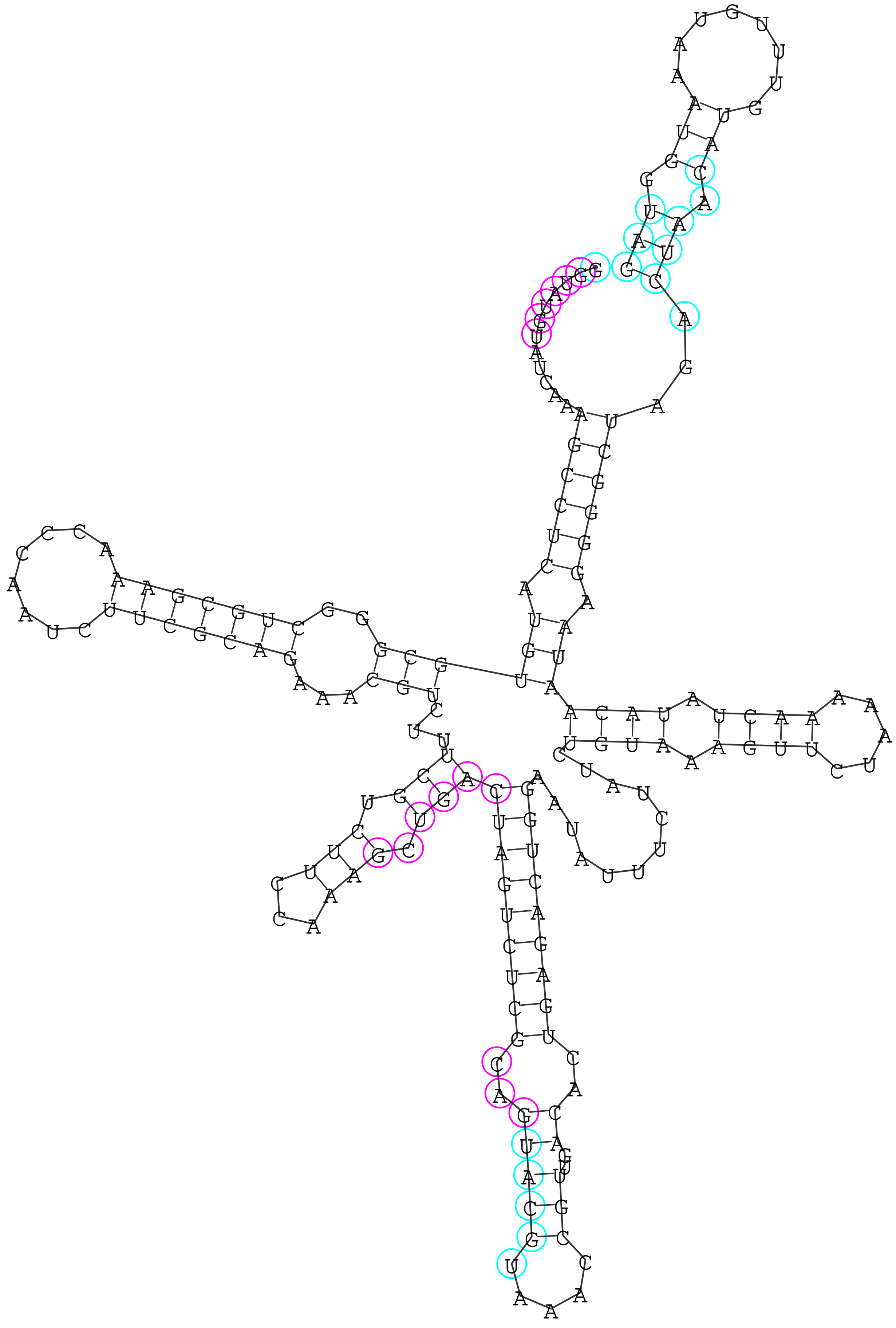

Xarbc0169A - Stwintron

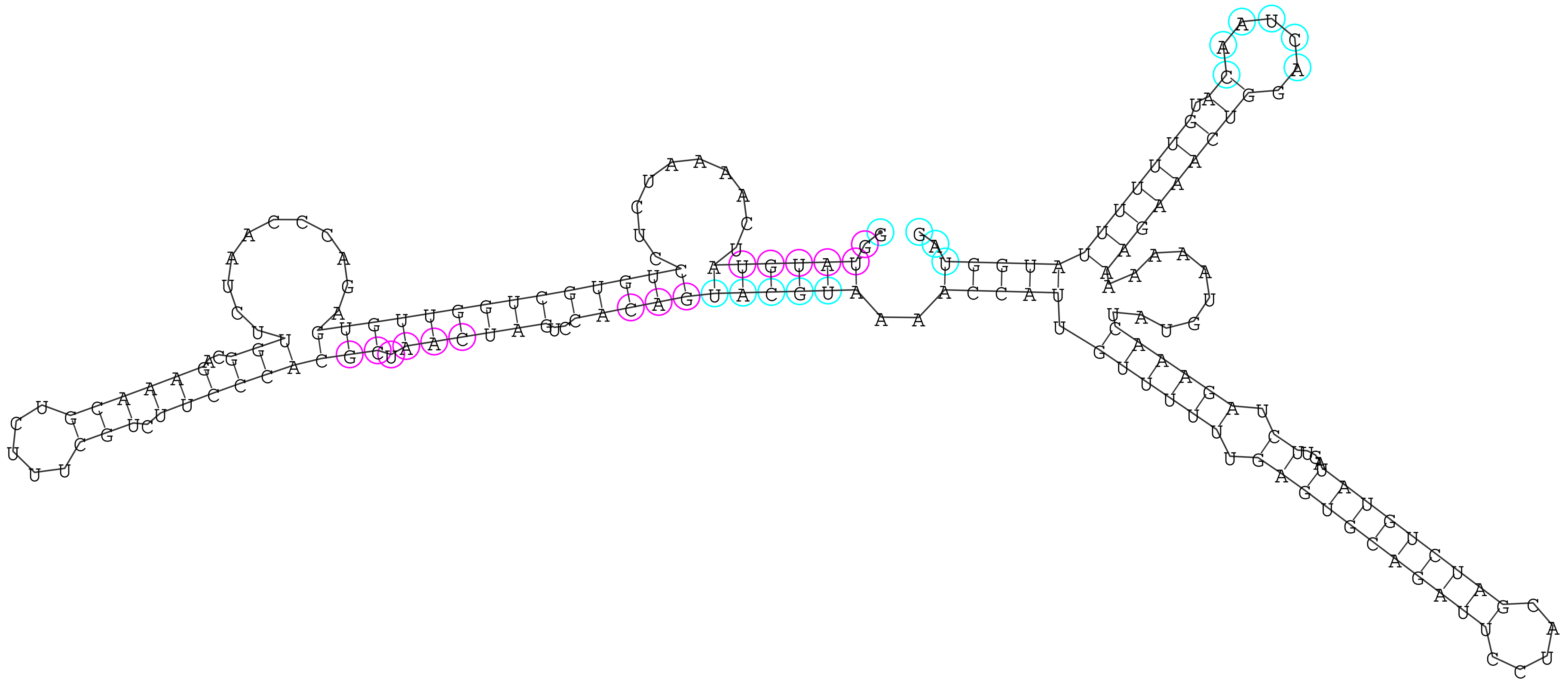

Xarbc0172A - Stwintron

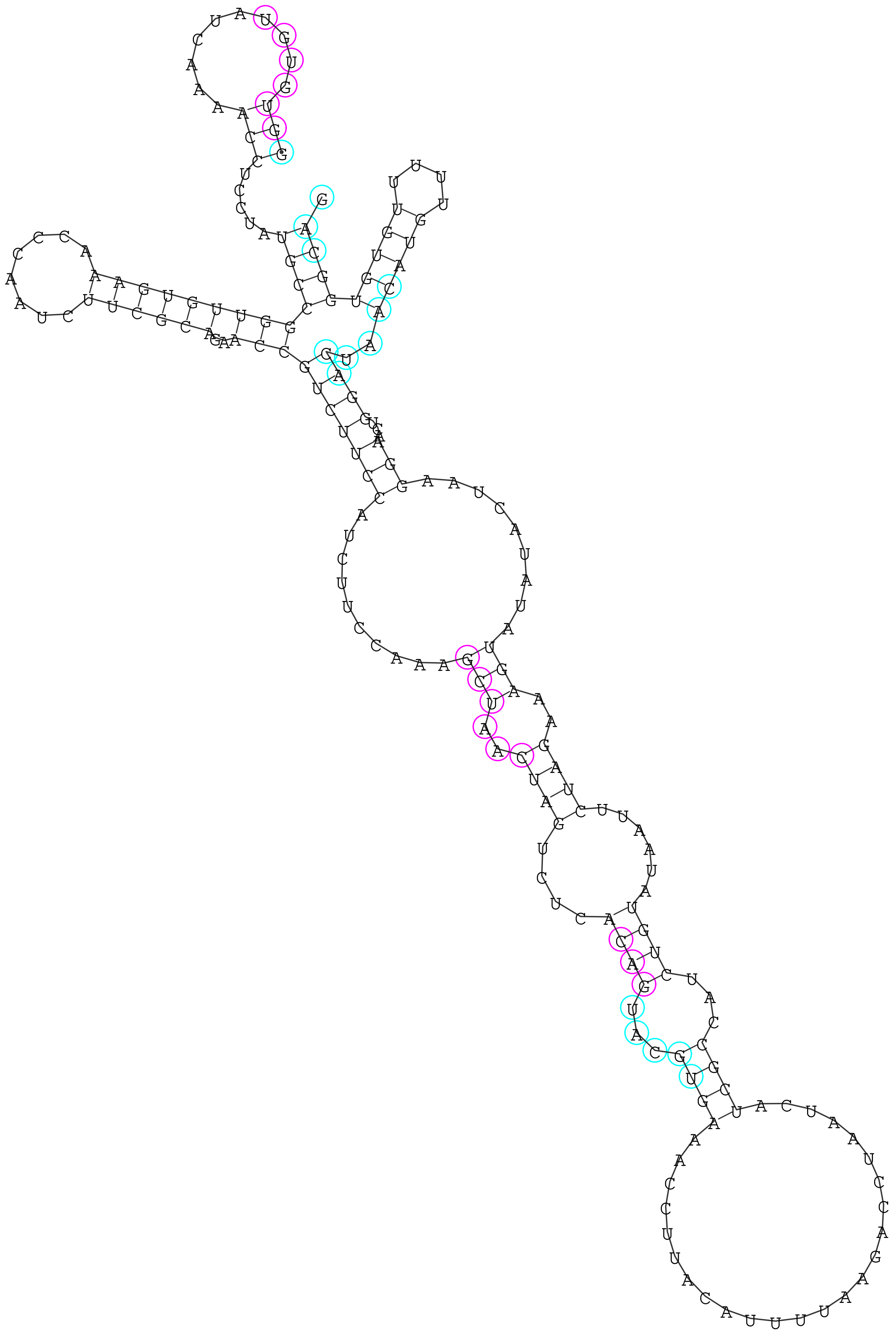

Xarbc0174A - Stwintron

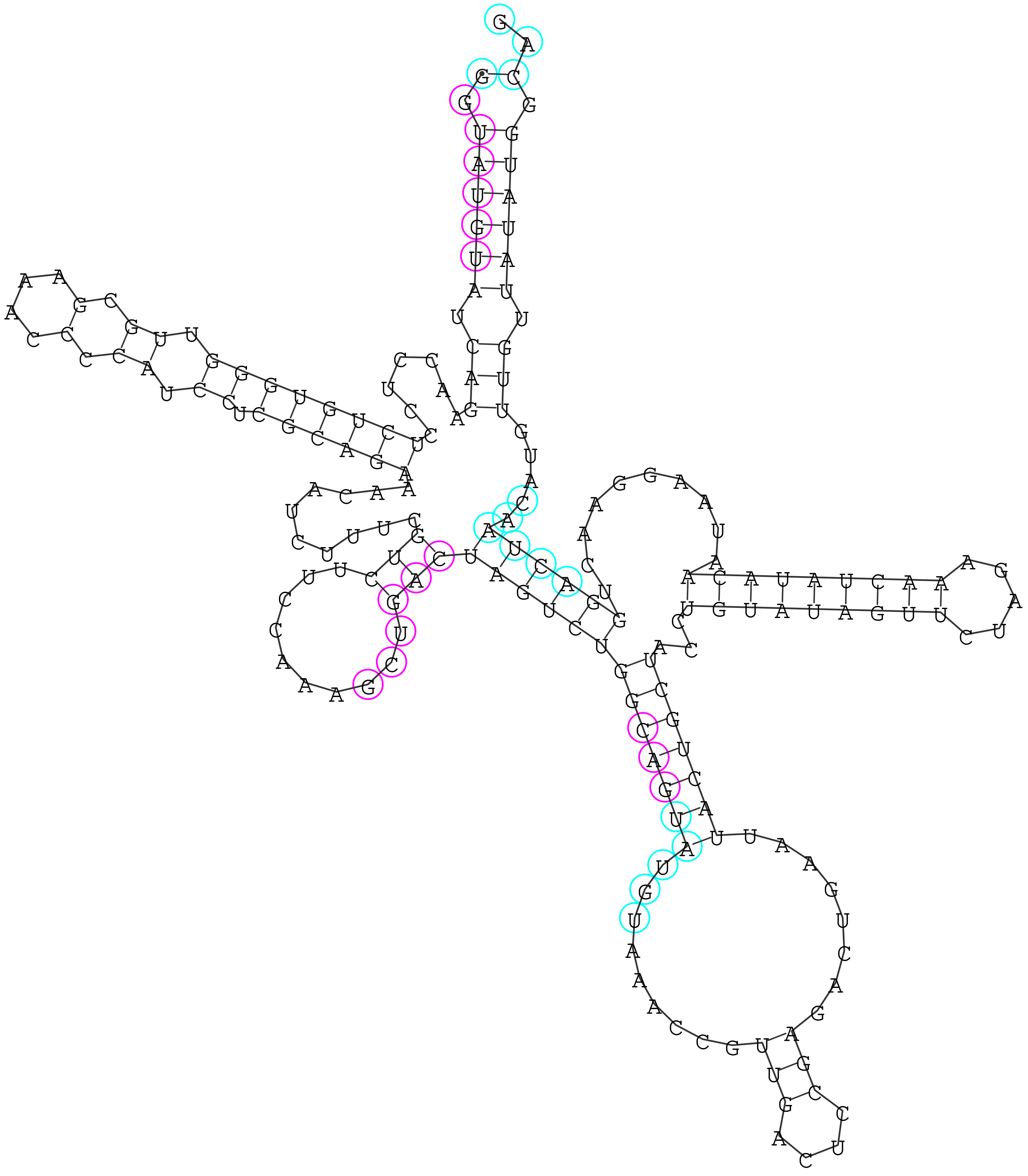

Xarbc0175A - Stwintron

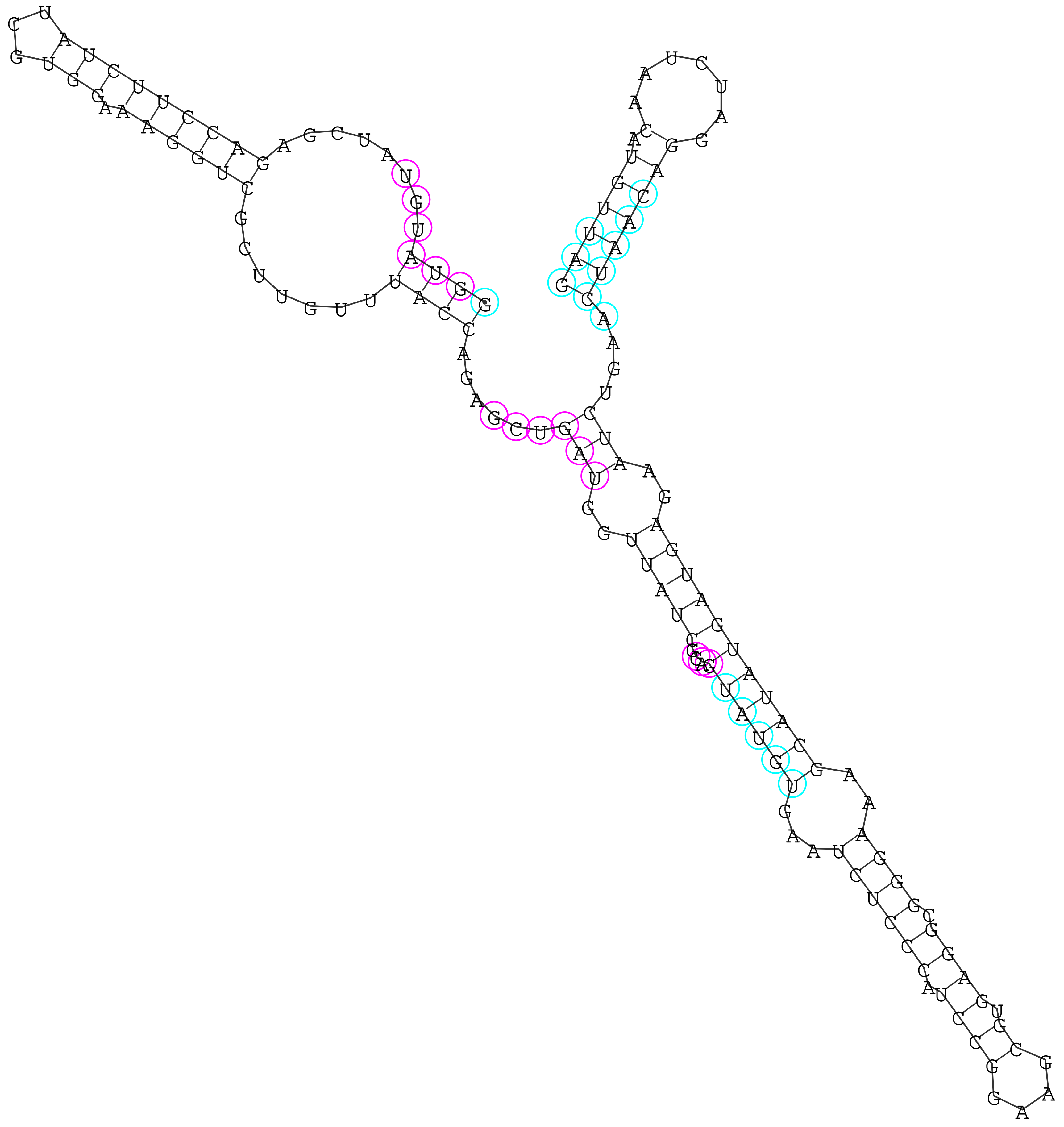

Xarbc0192A - Stwintron

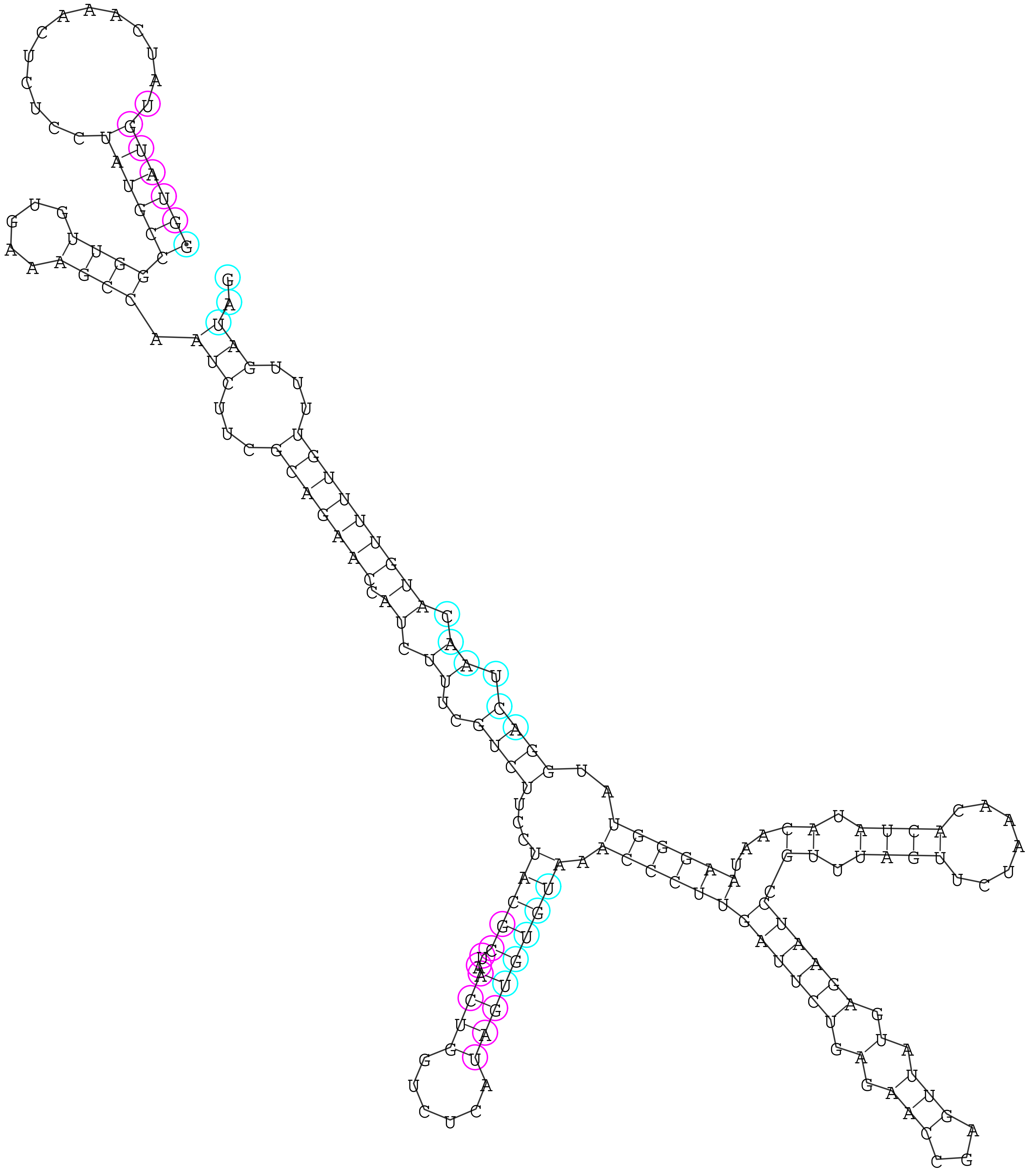

Xarbc0195A - Stwintron

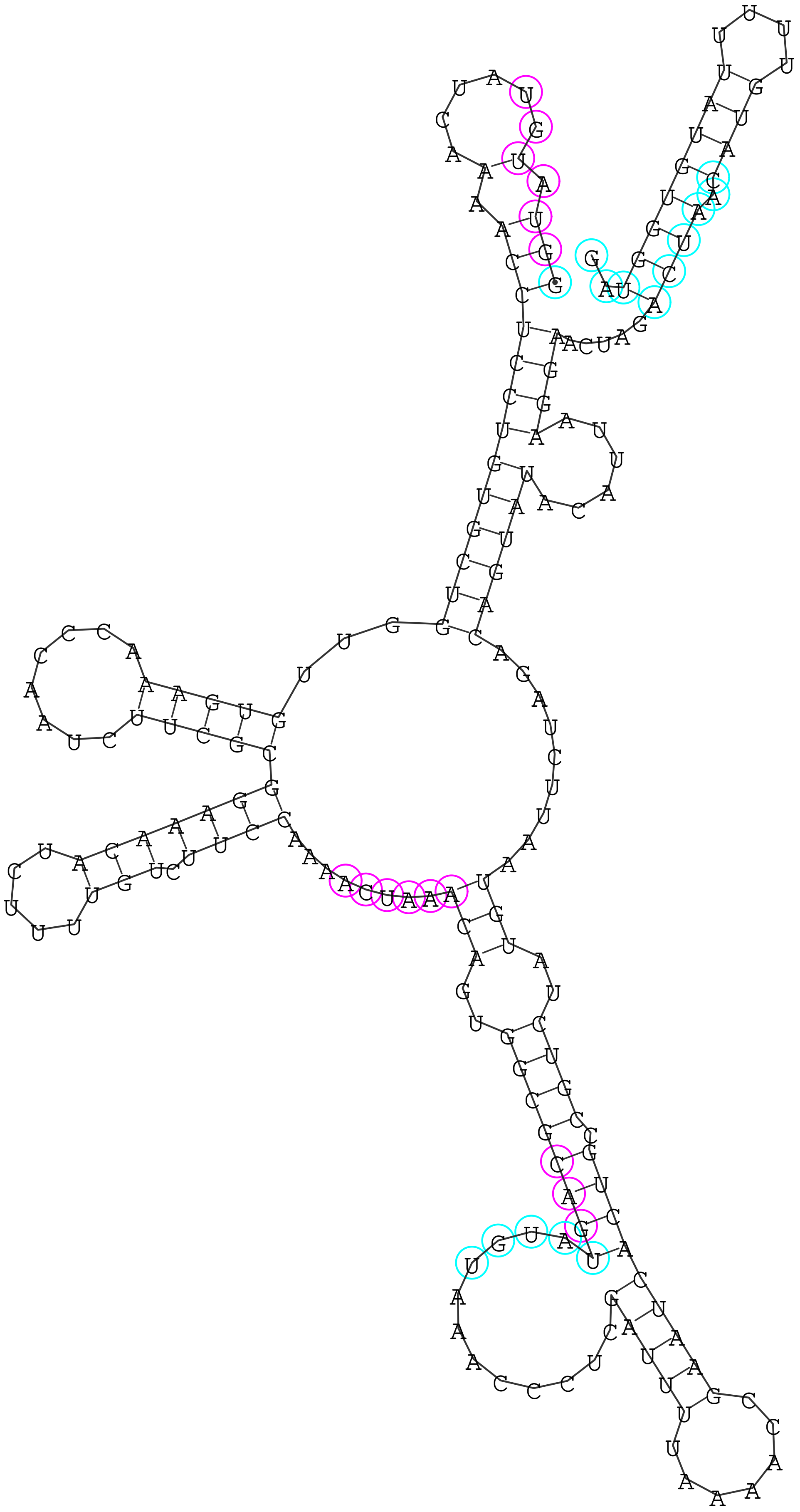

Xarbc0220A - Stwintron

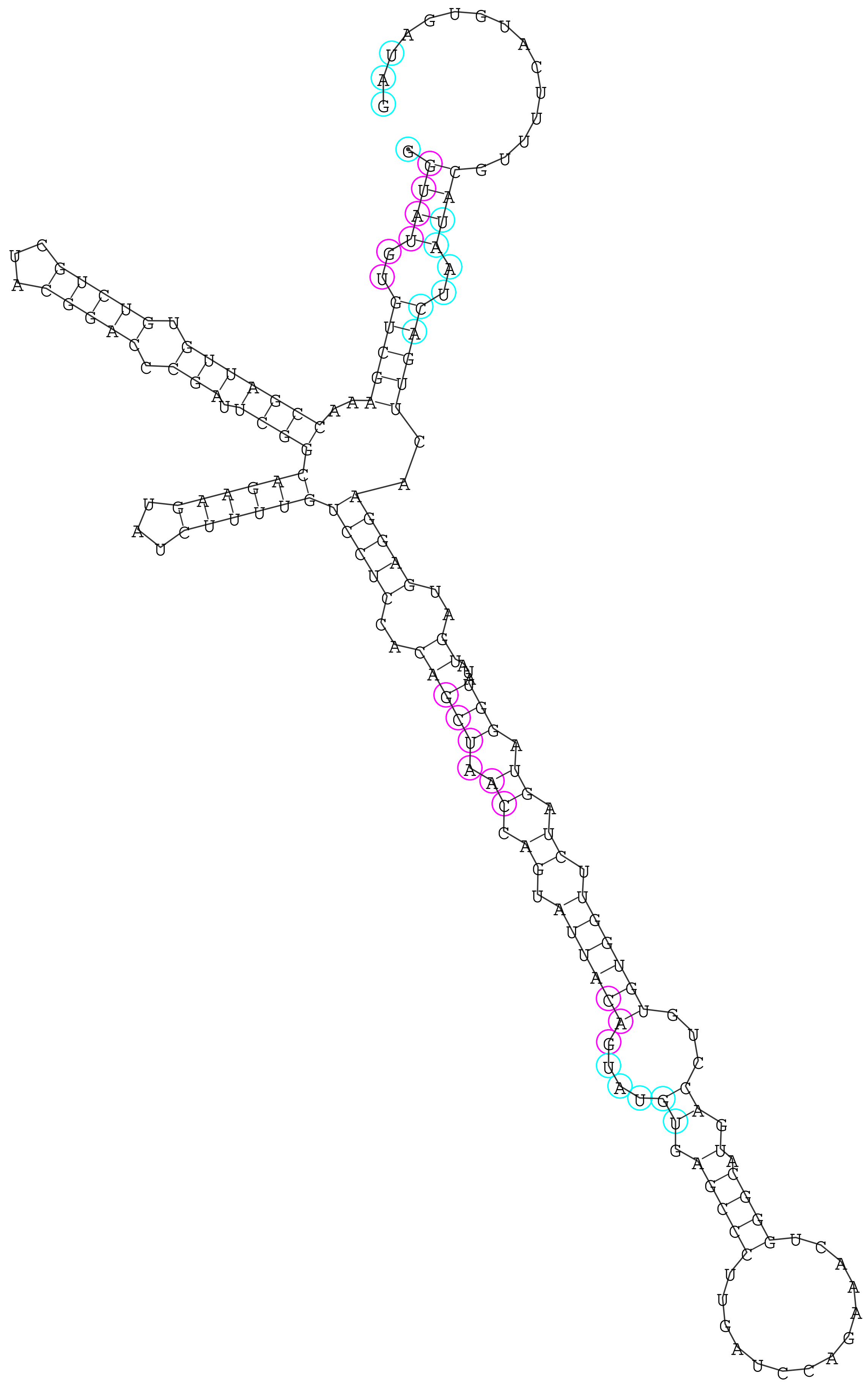

Xarbc0240A - Stwintron

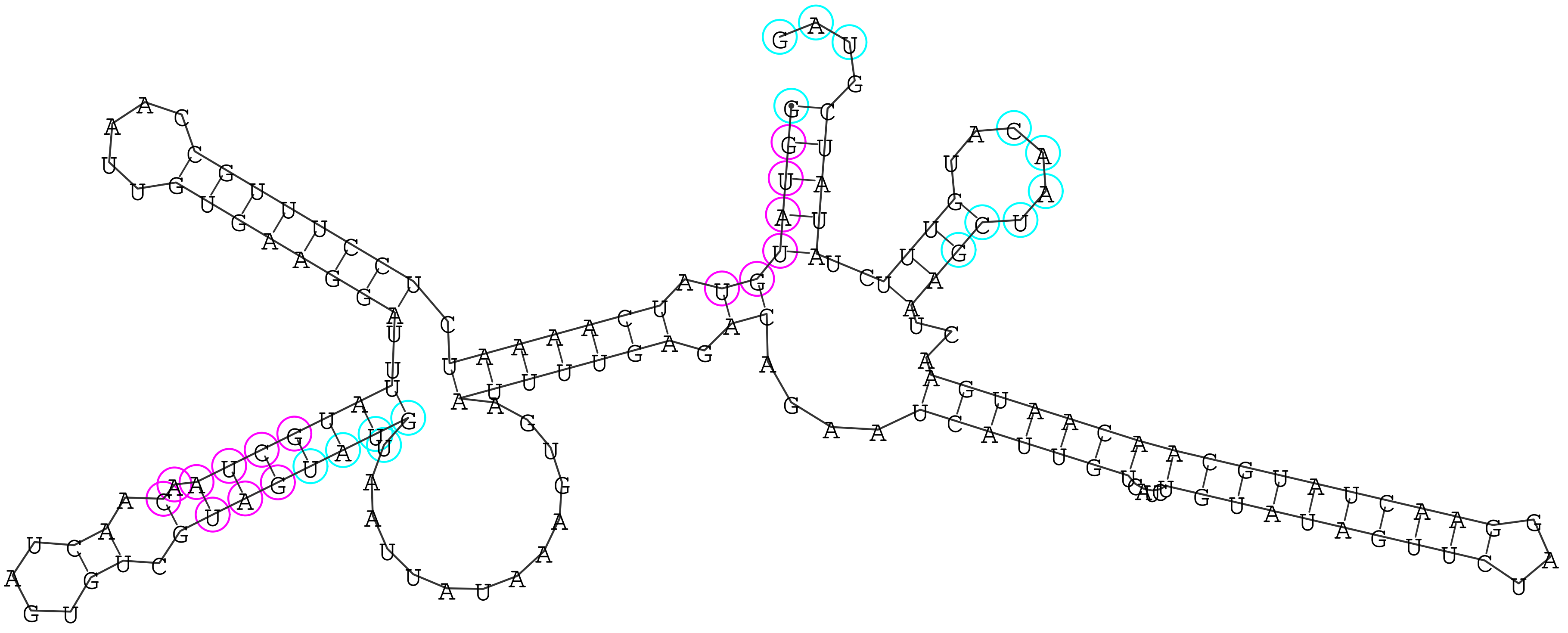

Xarbc0240B - Stwintron

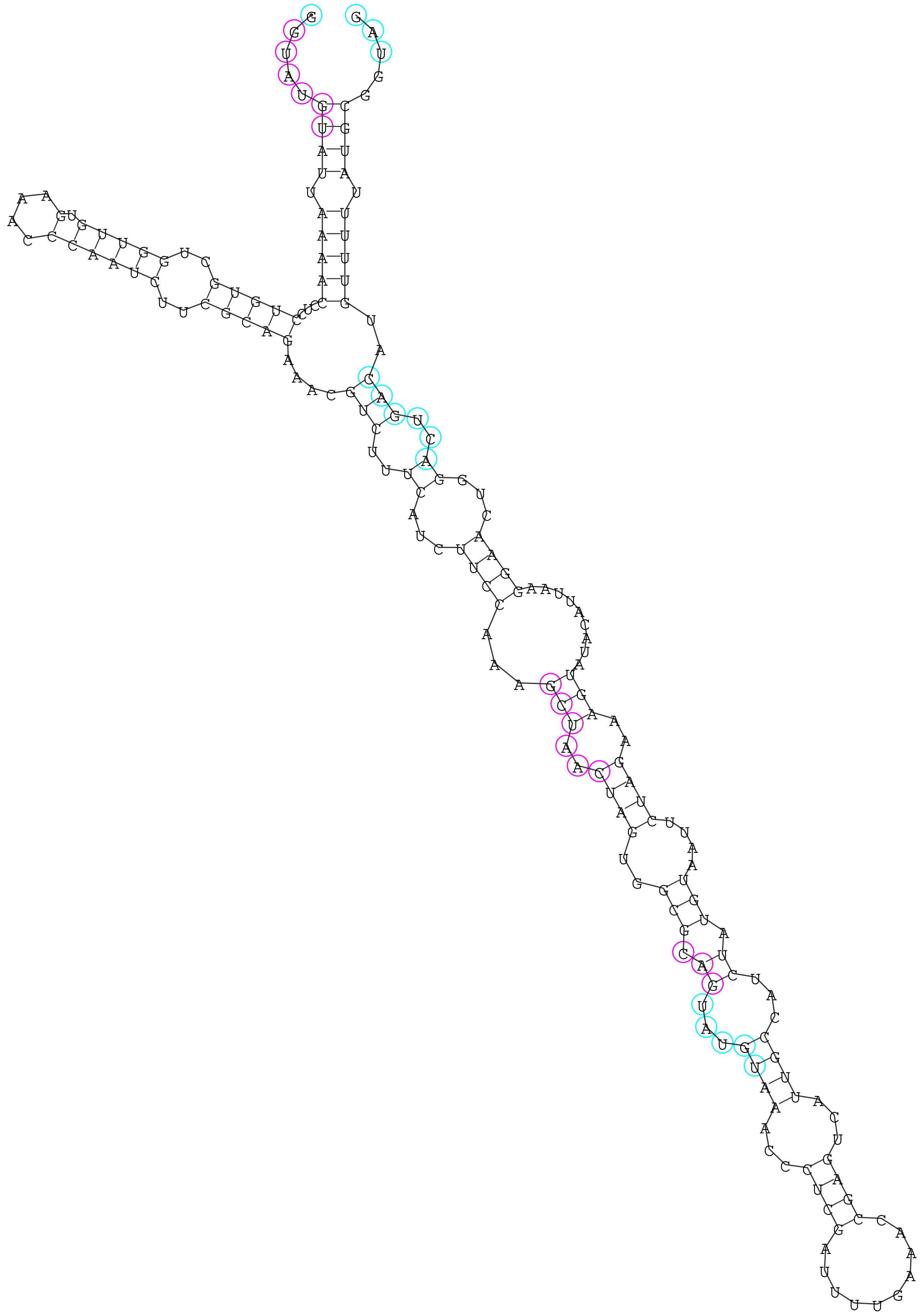

Xarbc0253A - Stwintron

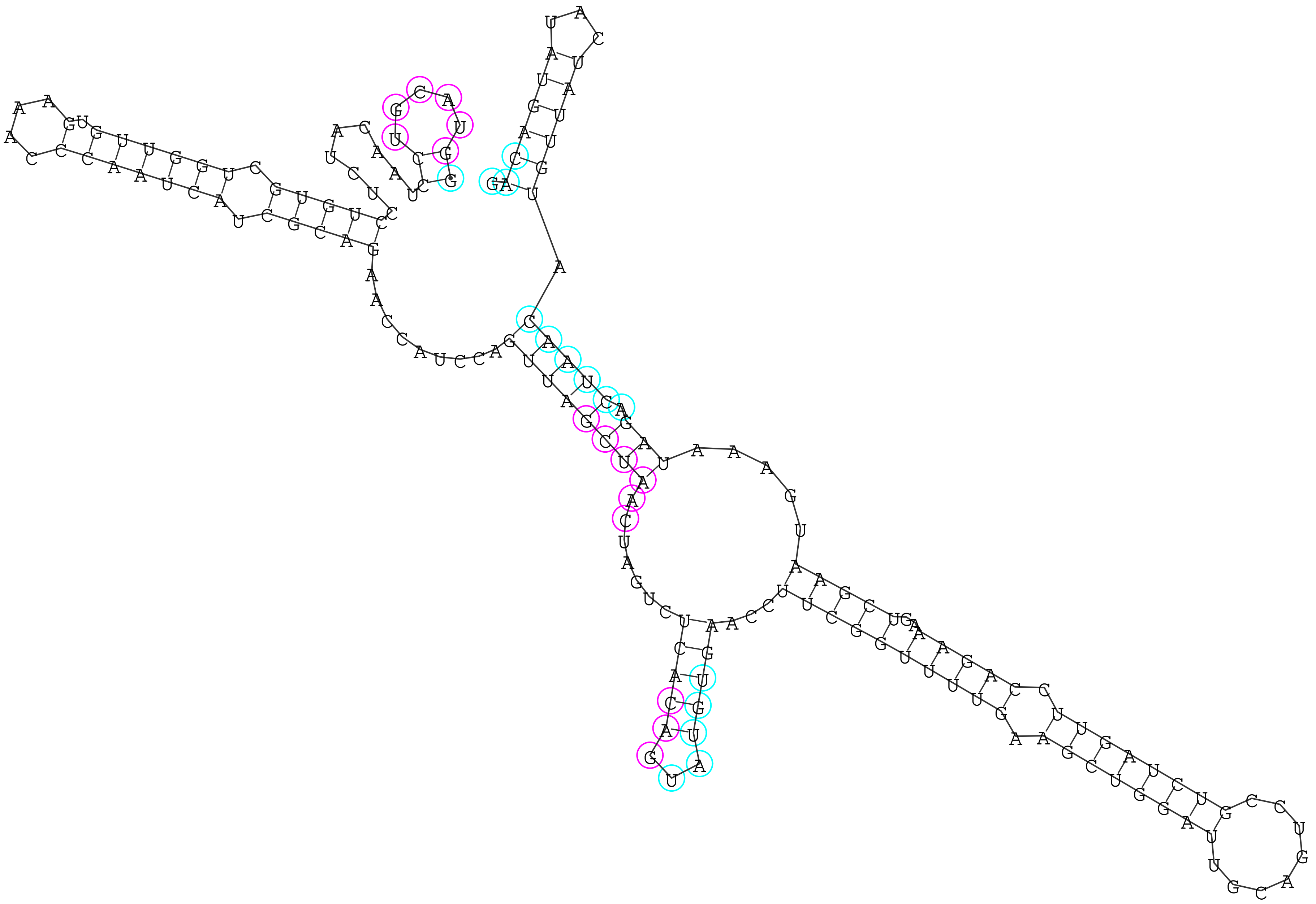

Xarbc0274A - Stwintron

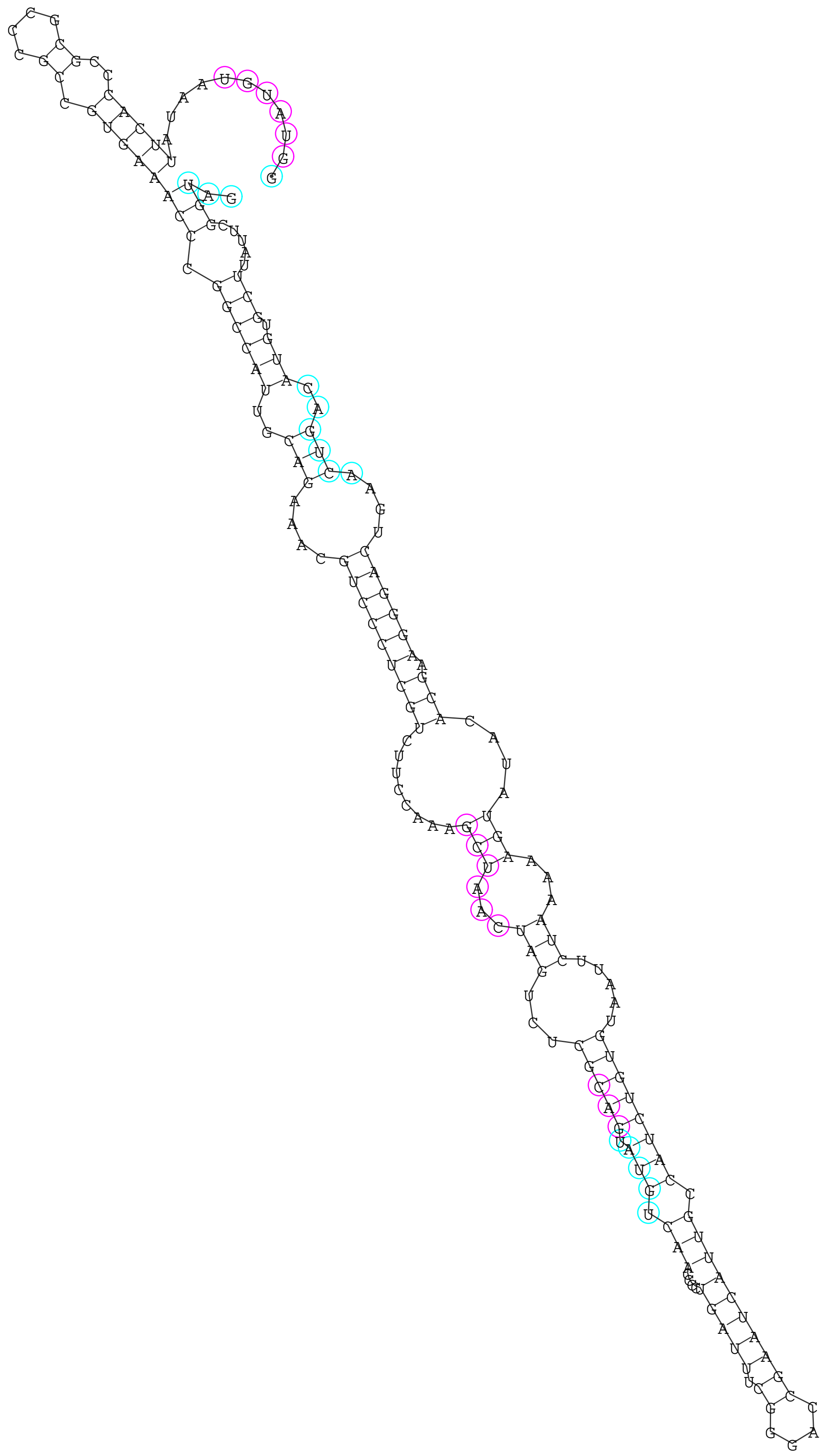

Xarbc0299A - Stwintron

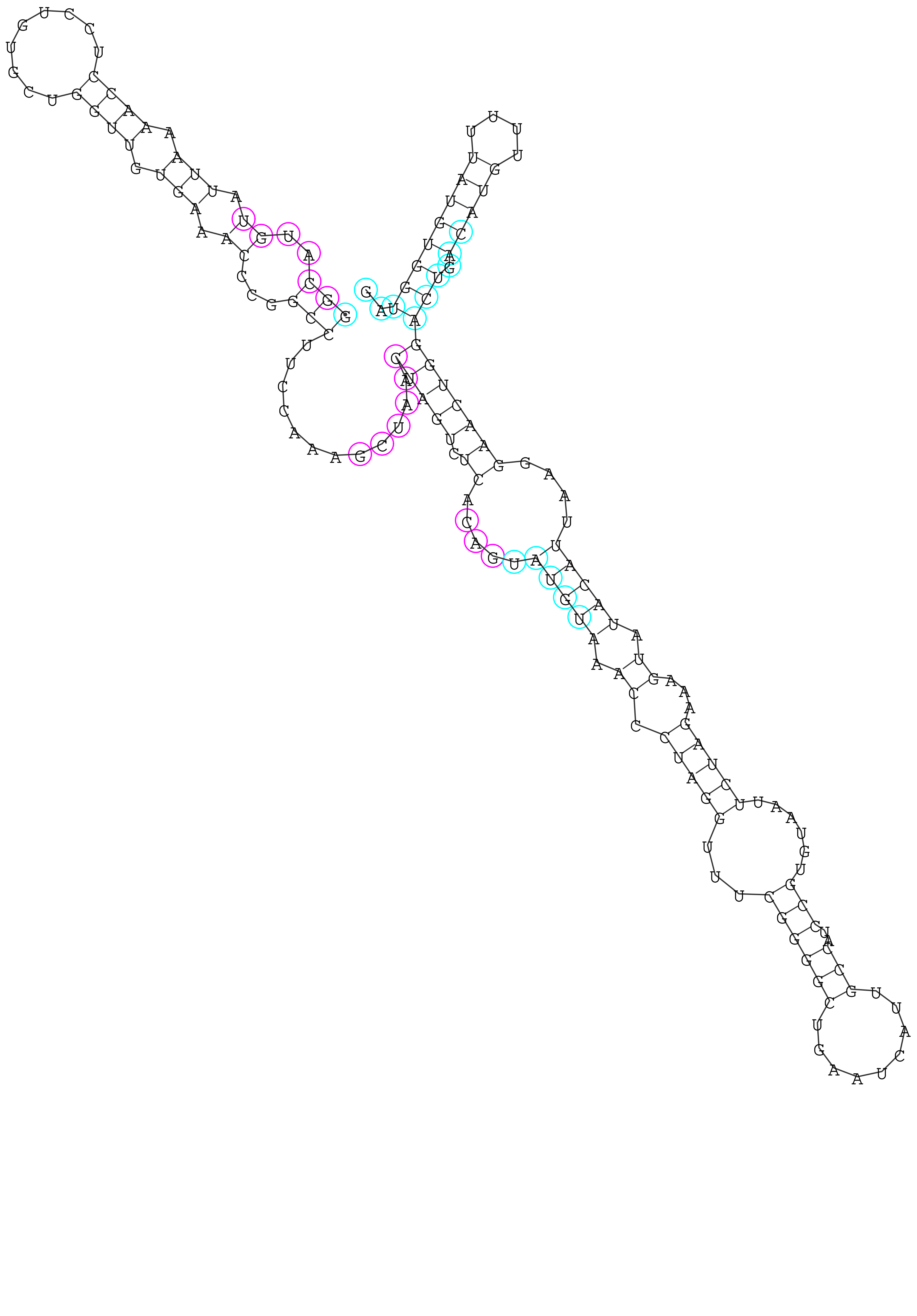

Xarbc0299B - Stwintron

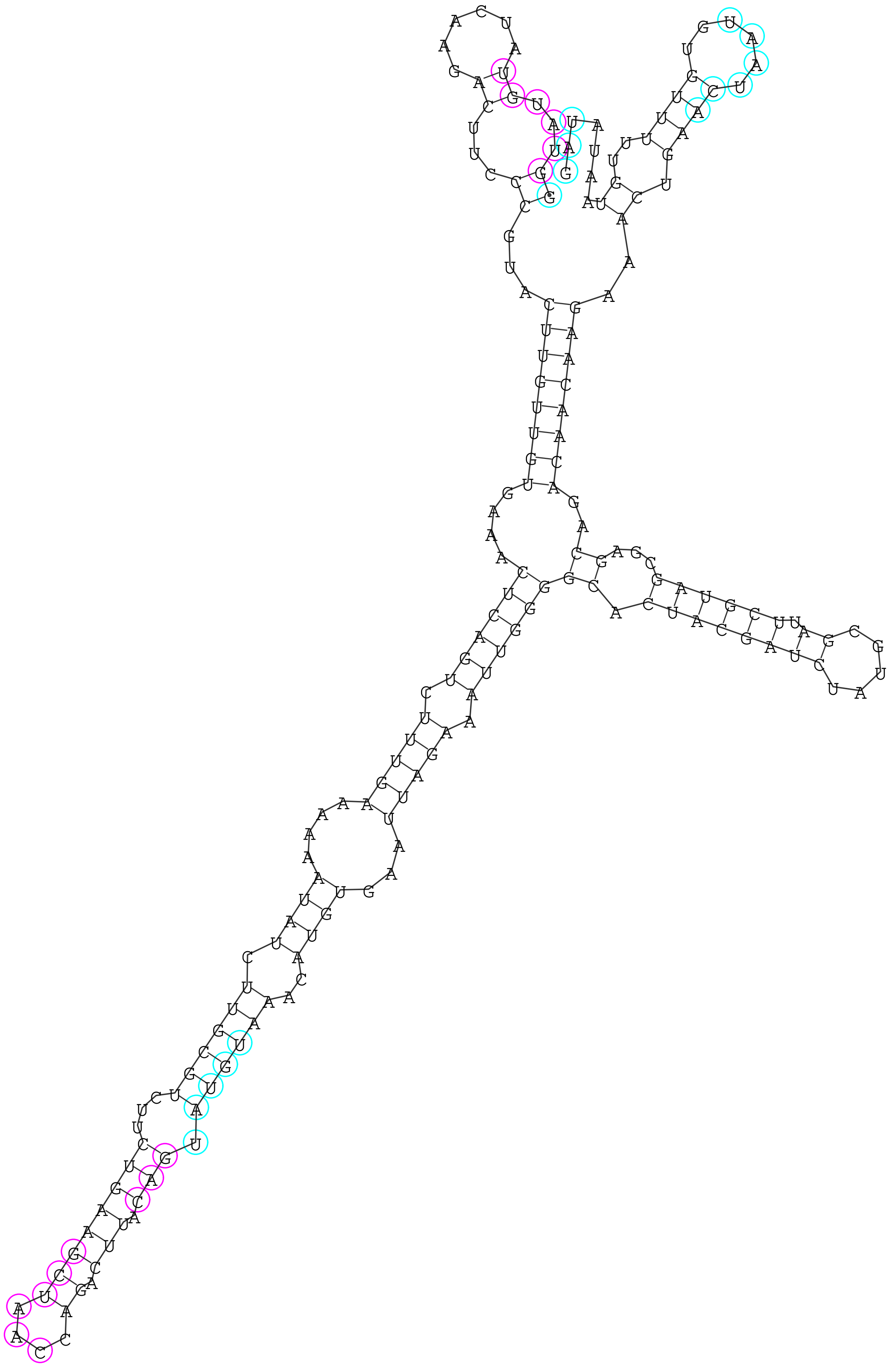

Xarbc0301A - Stwintron

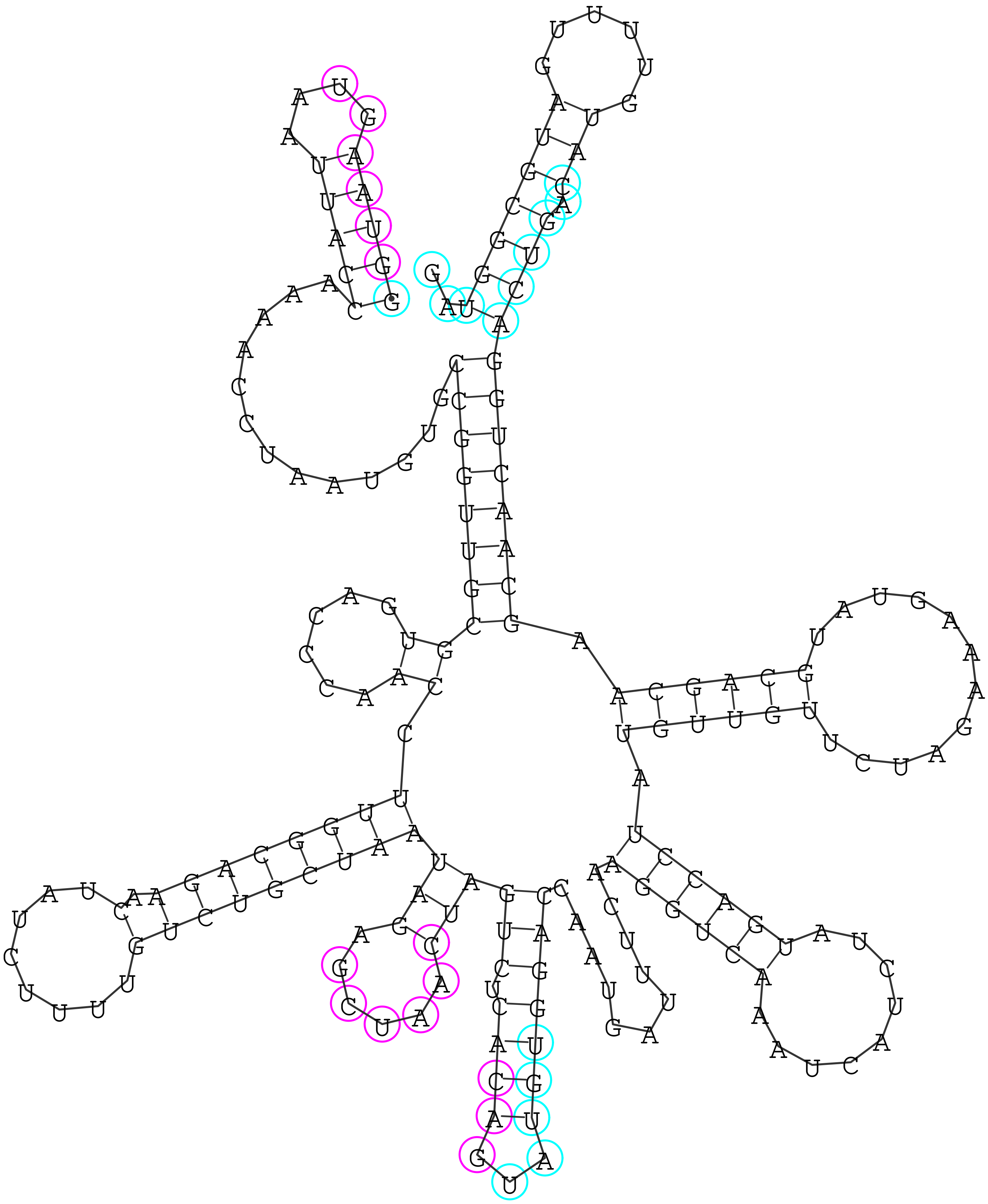

Xarbc0309A - Stwintron

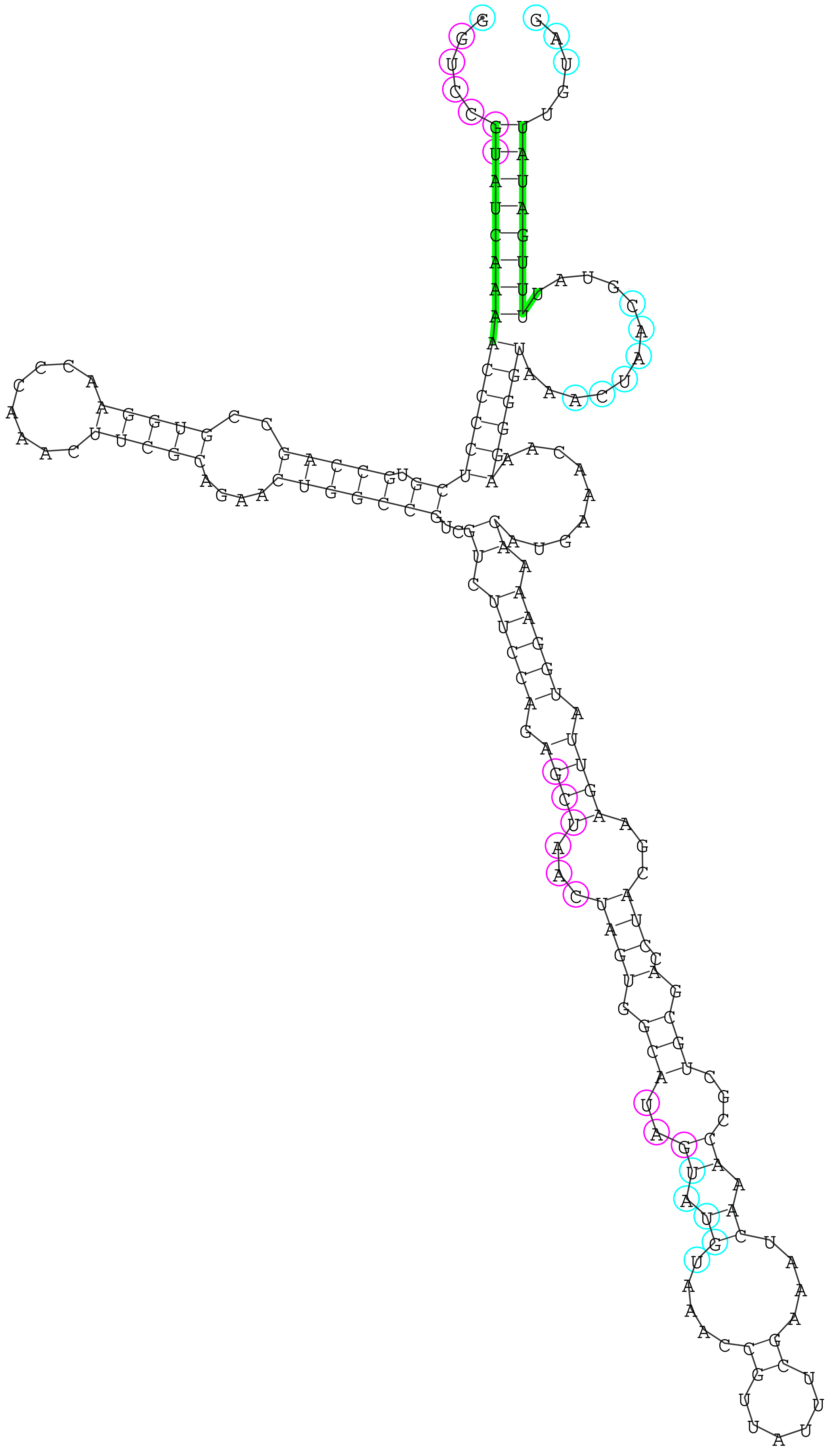

Xarbc0311A - Stwintron

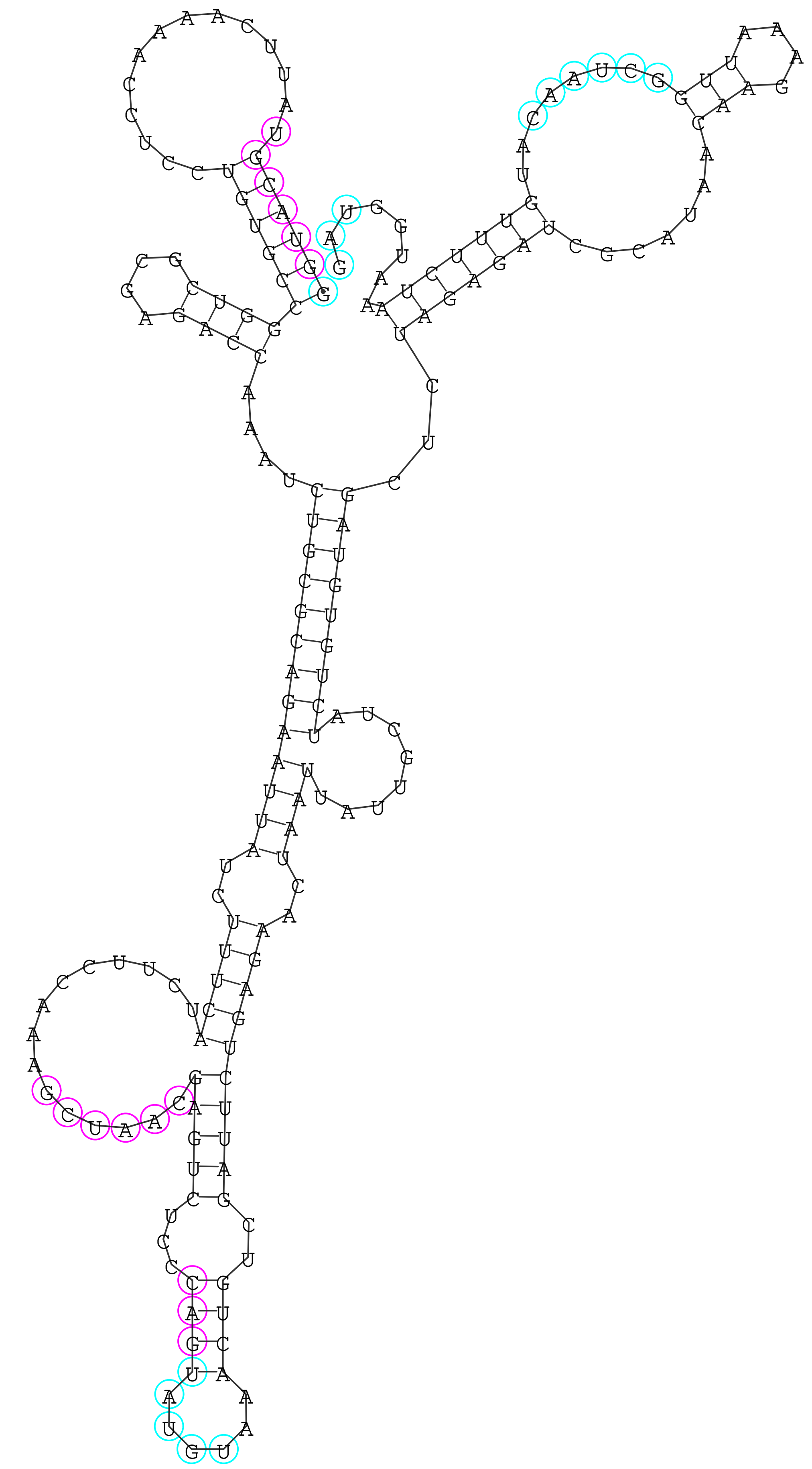

Xarbc0324A - Stwintron

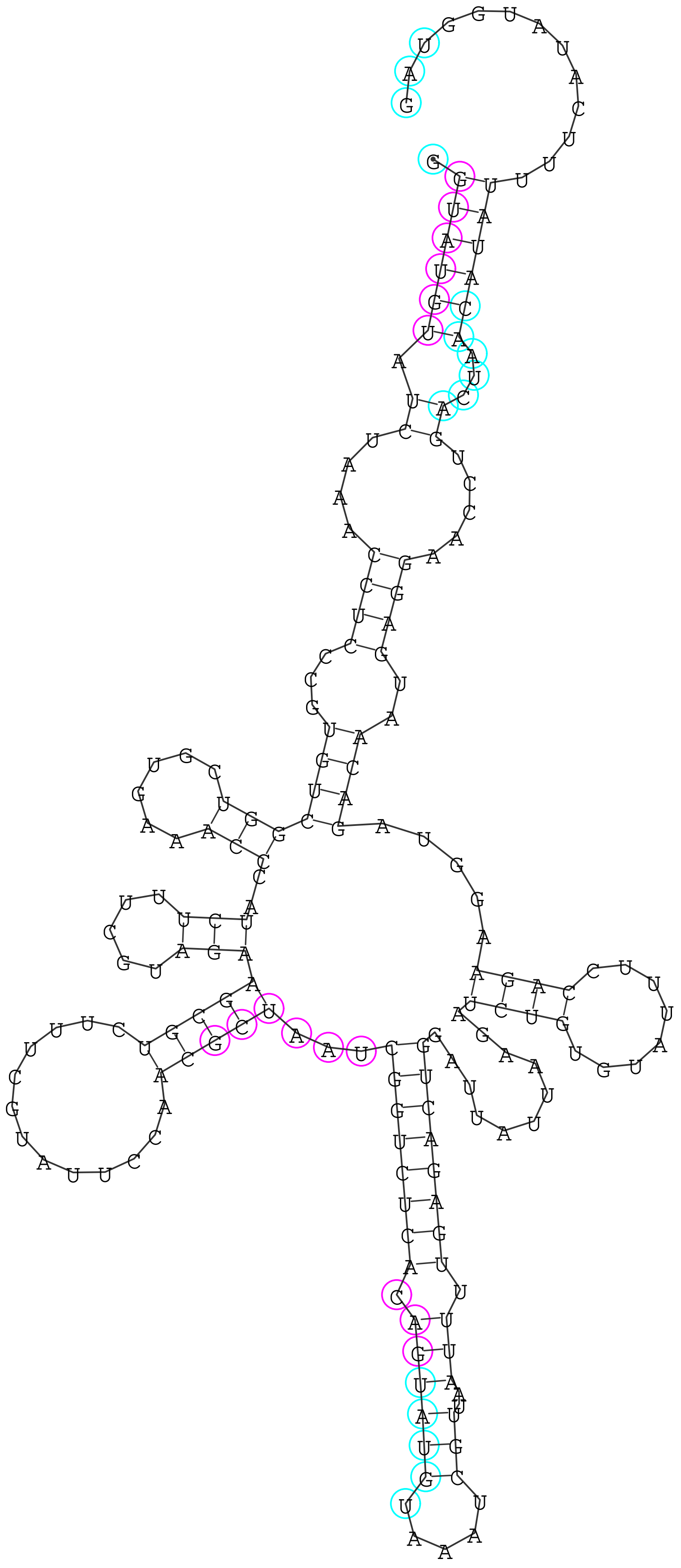

Xarbc0447A - Stwintron

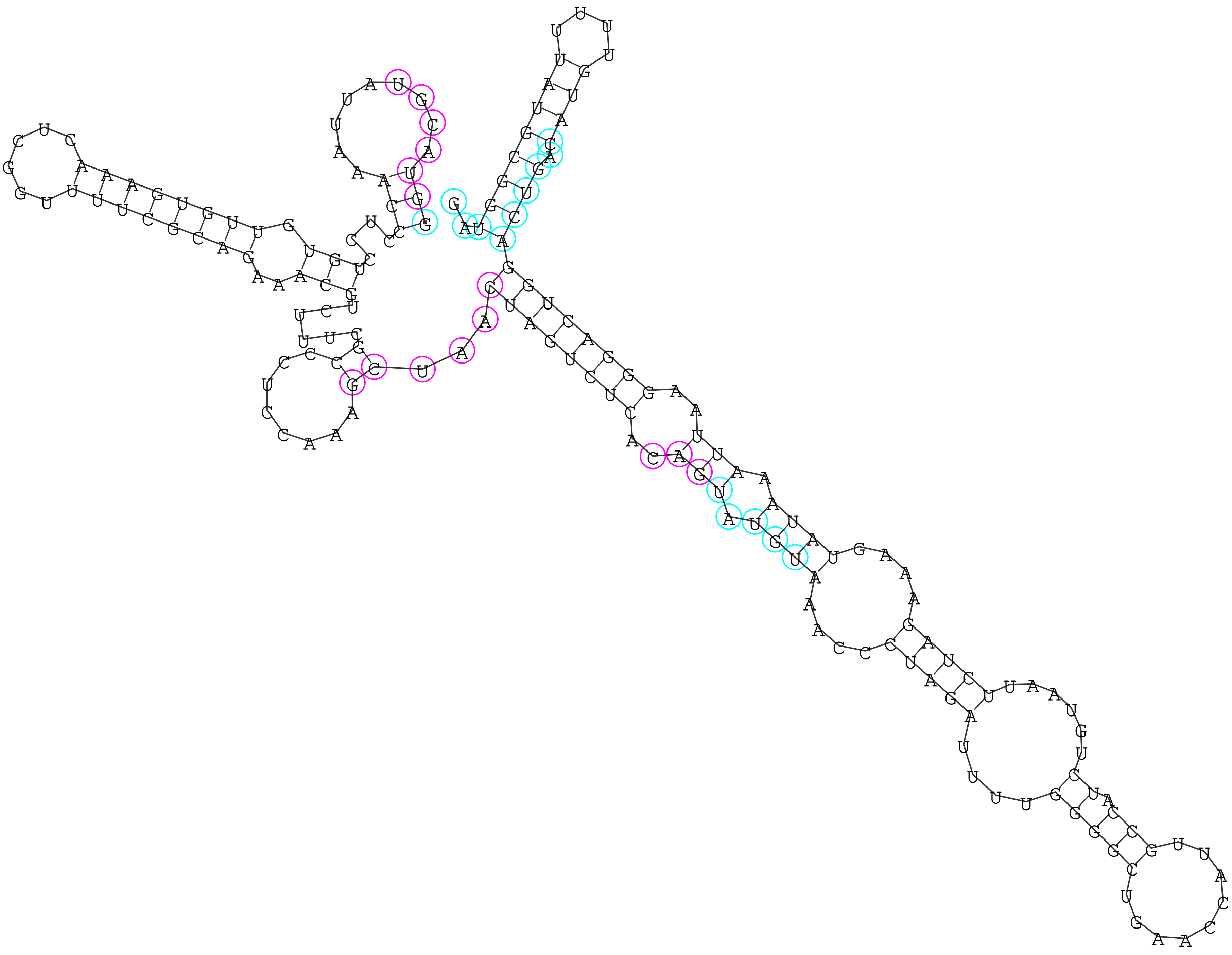

Xbamc009A - Stwintron

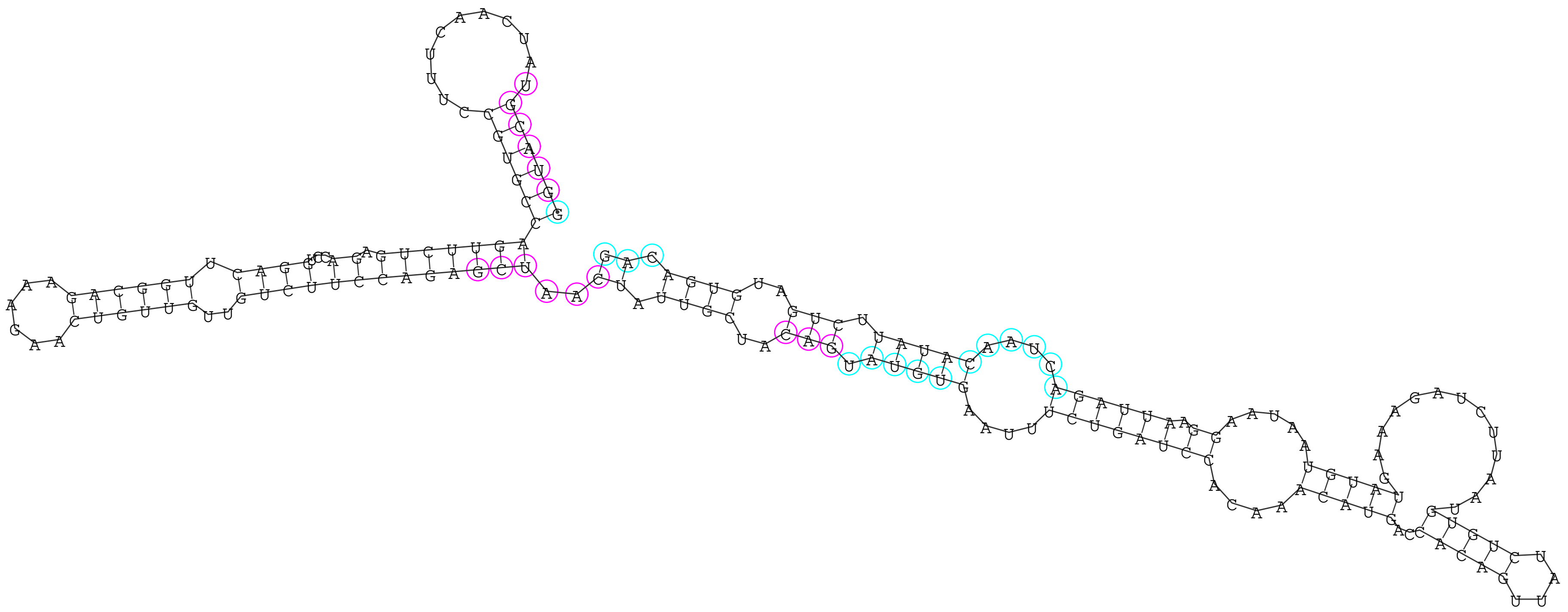

Xbamc015A - Stwintron

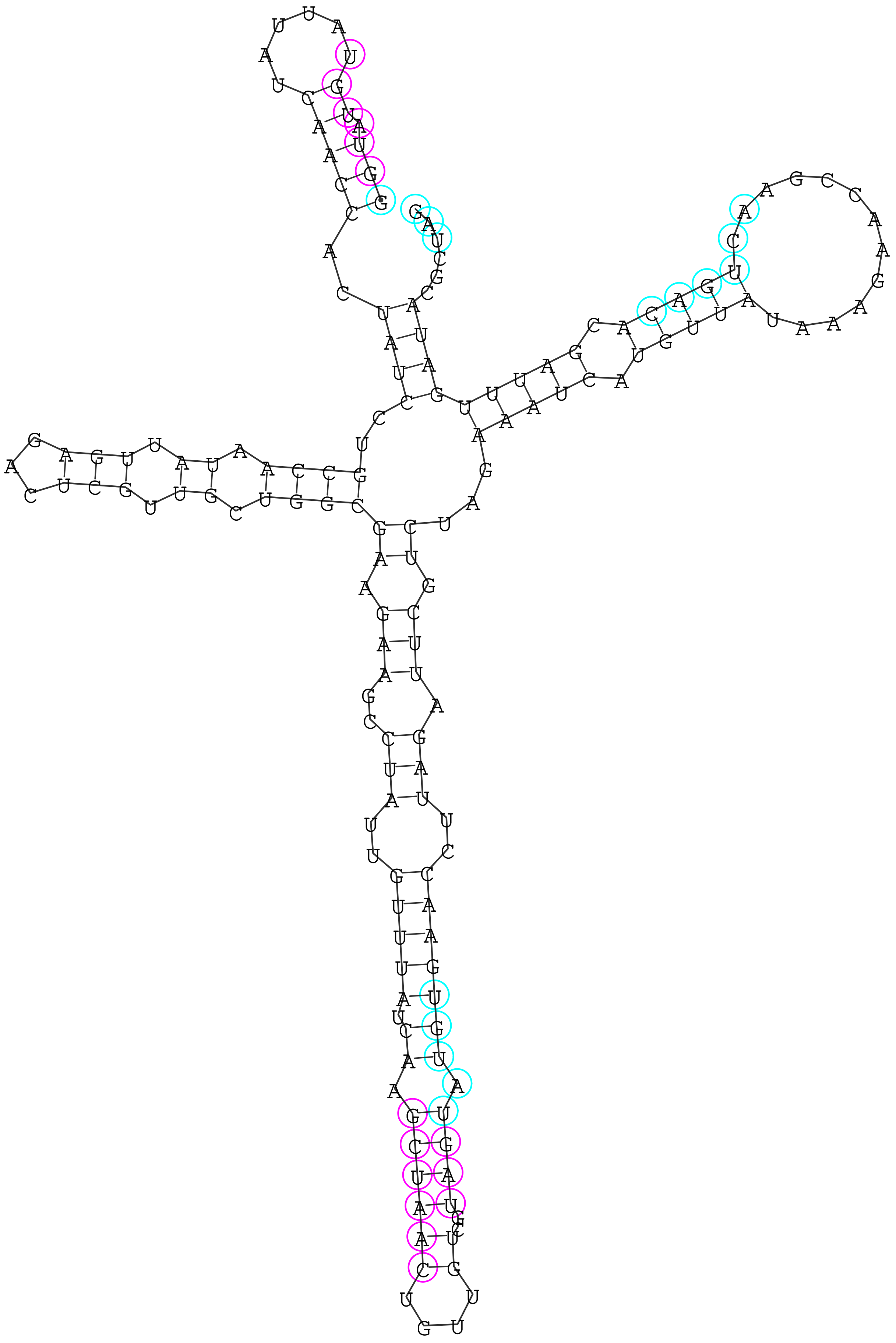

Xbamc019A - Stwintron

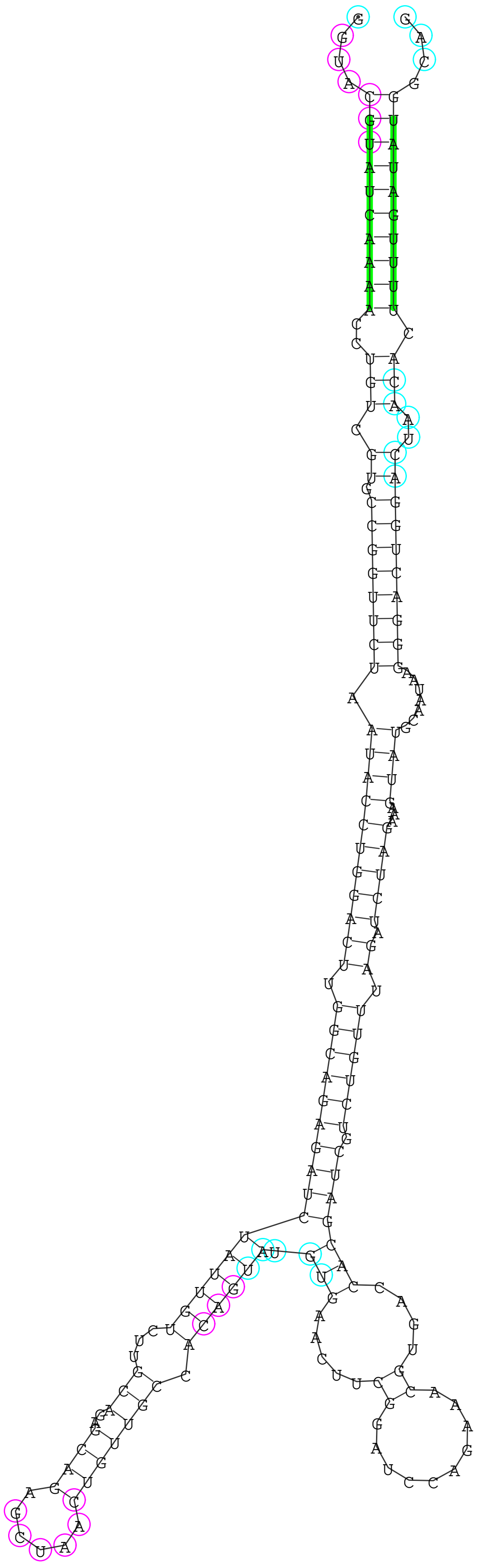

Xbamc020A - Stwintron

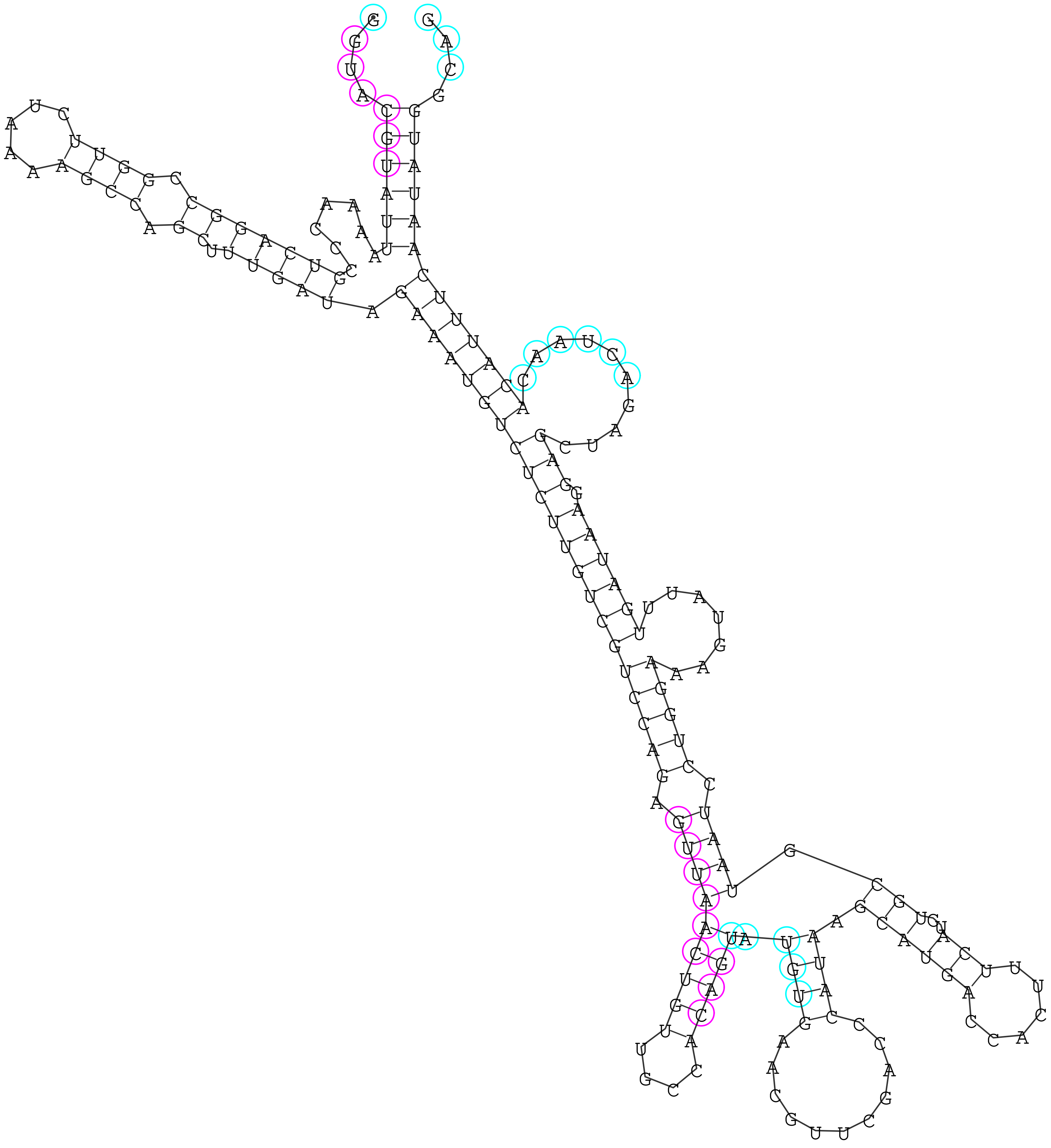

Xbamc022A - Stwintron

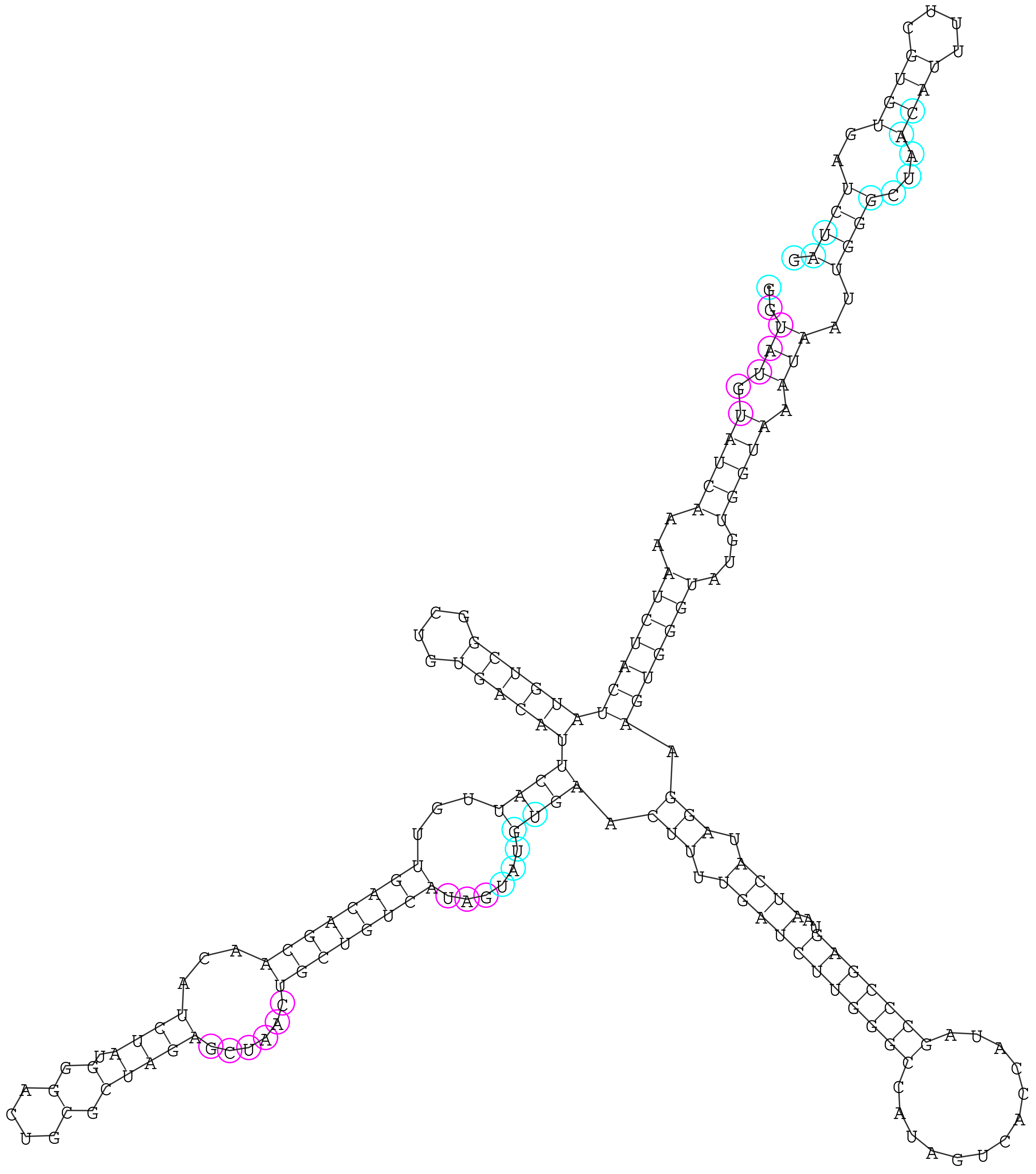

Xbamc024A - Stwintron

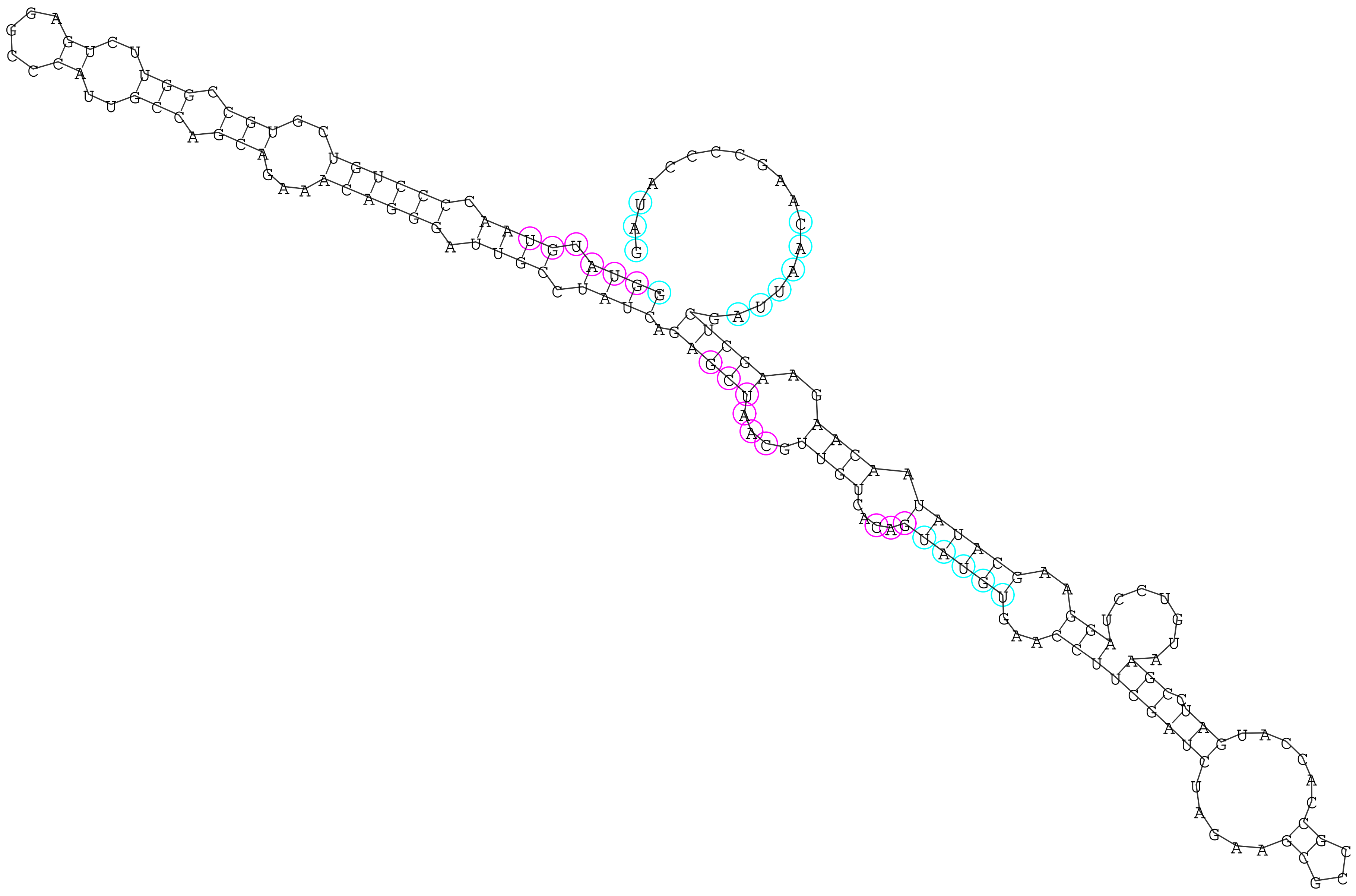

Xbamc025A - Stwintron

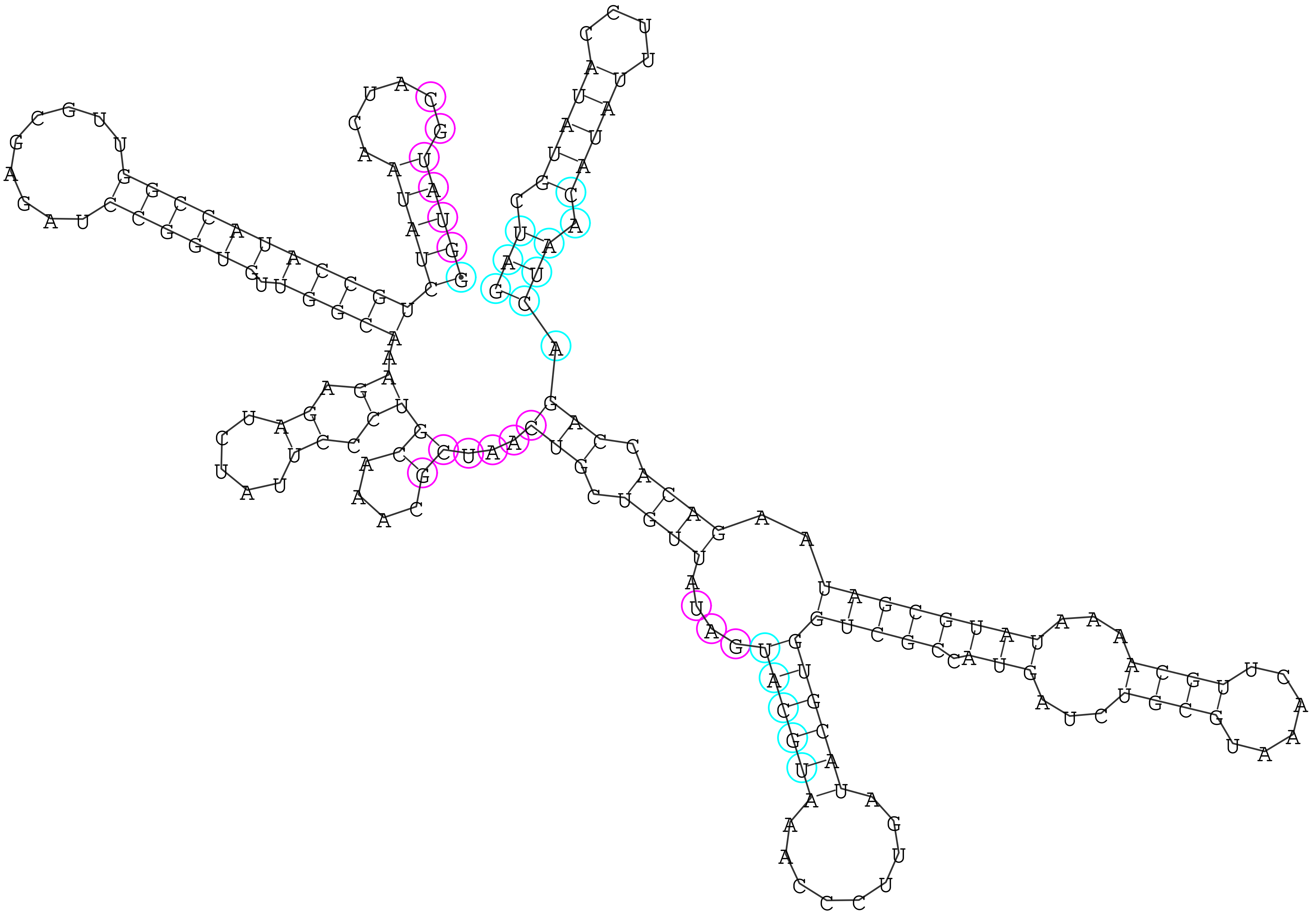

Xbamc027A - Stwintron

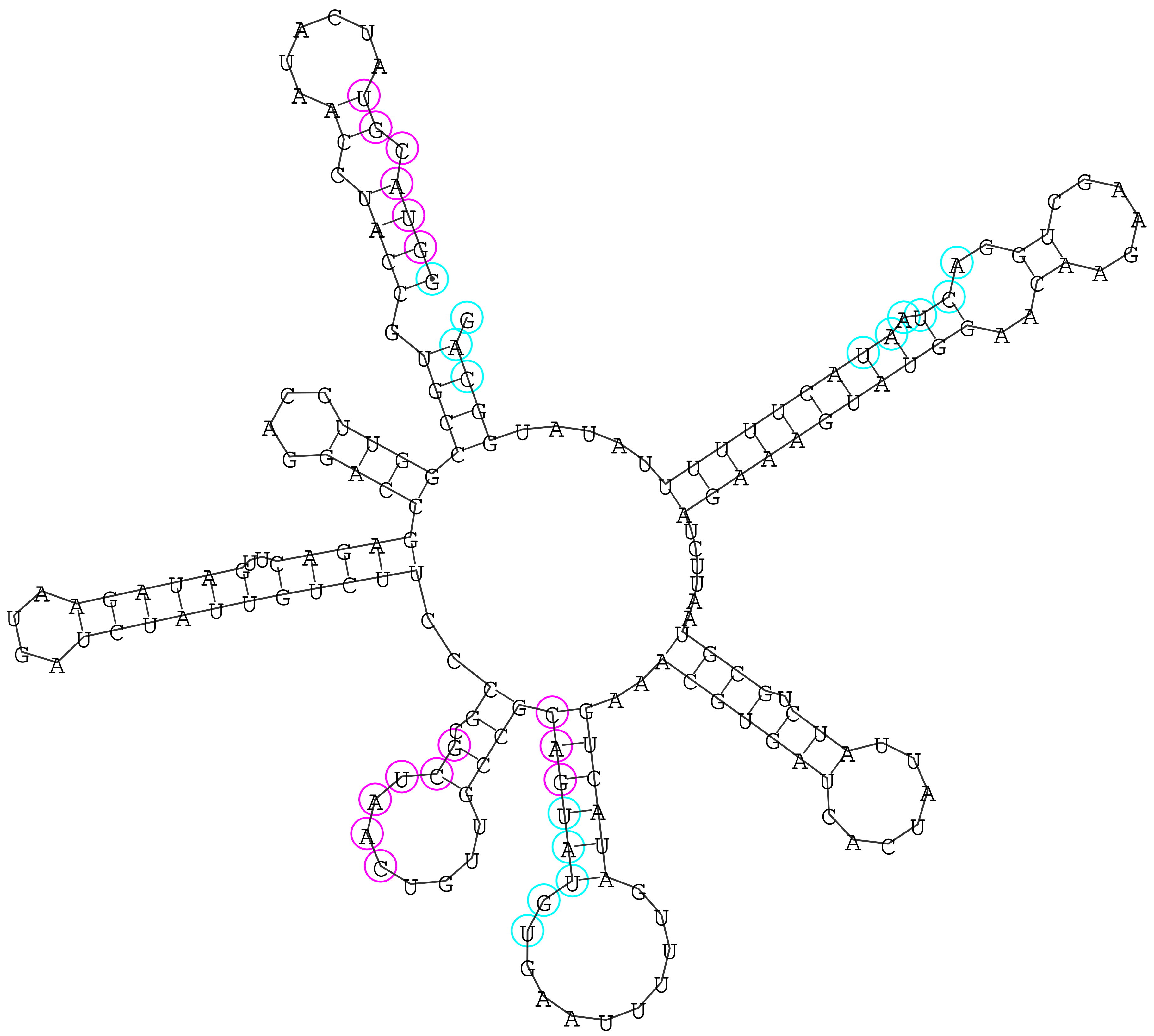

Xbamc027B - Stwintron

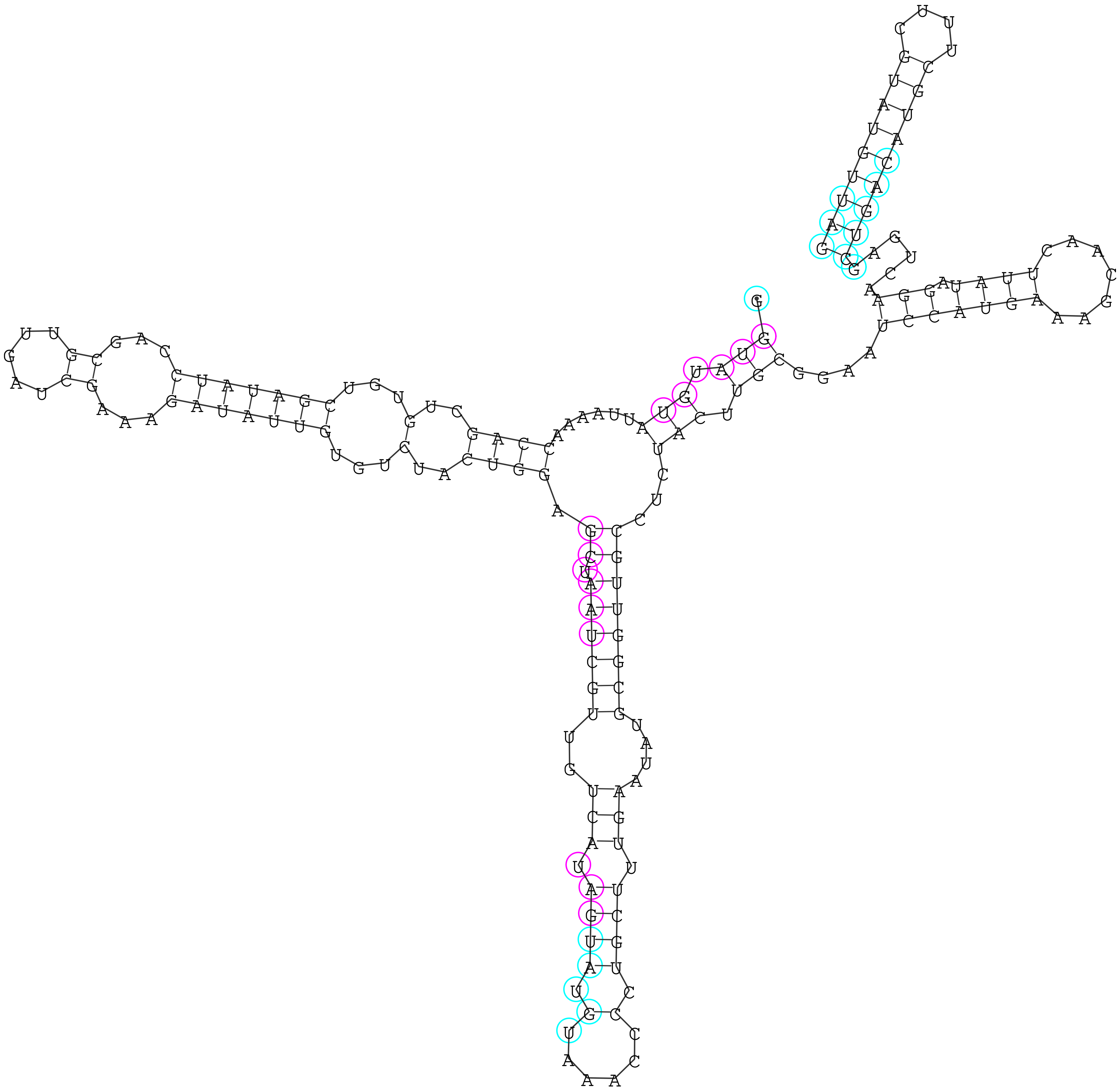

Xbamc027C - Stwintron

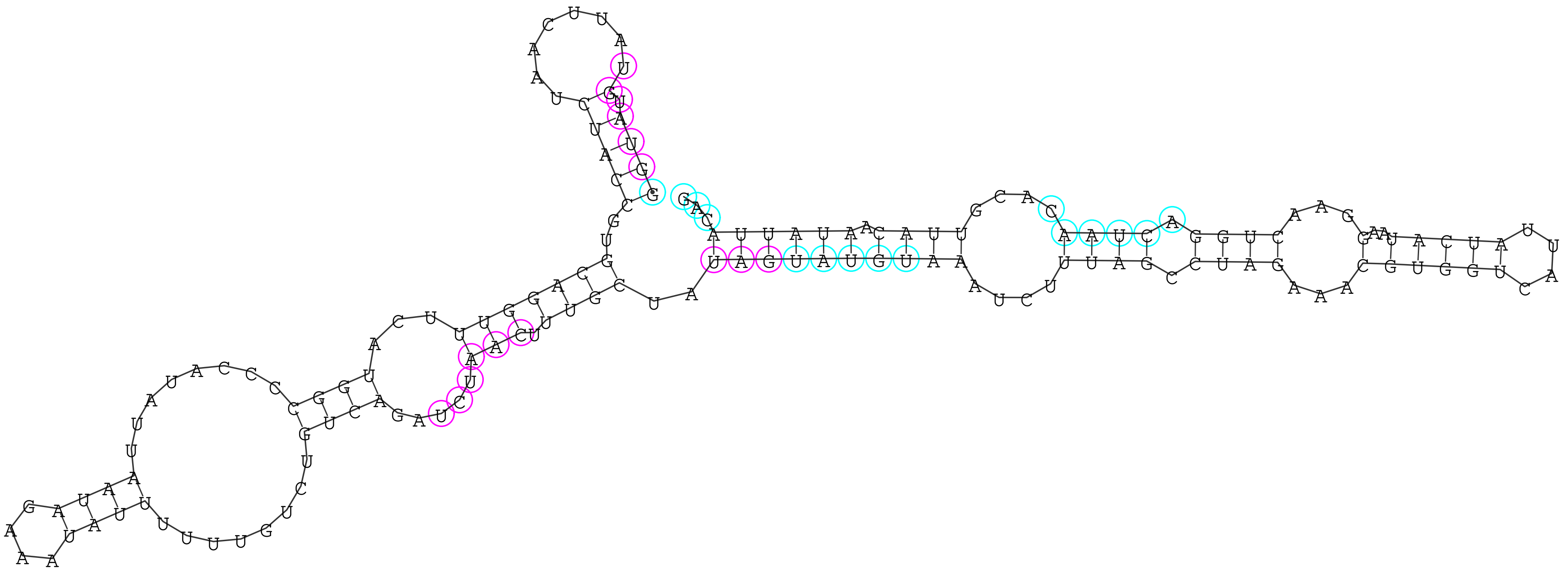

Xbamc040A - Stwintron

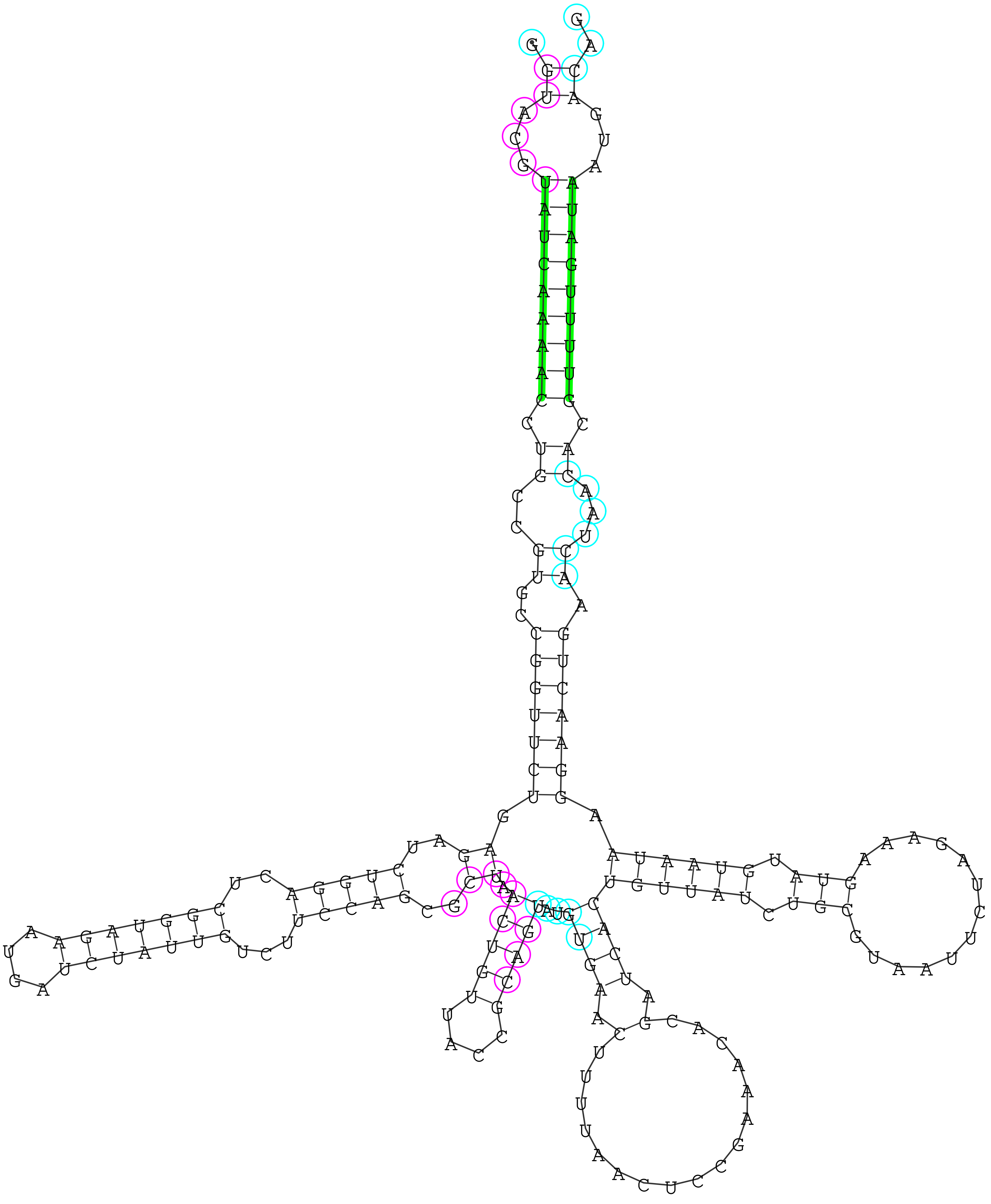

Xbamc041A - Stwintron

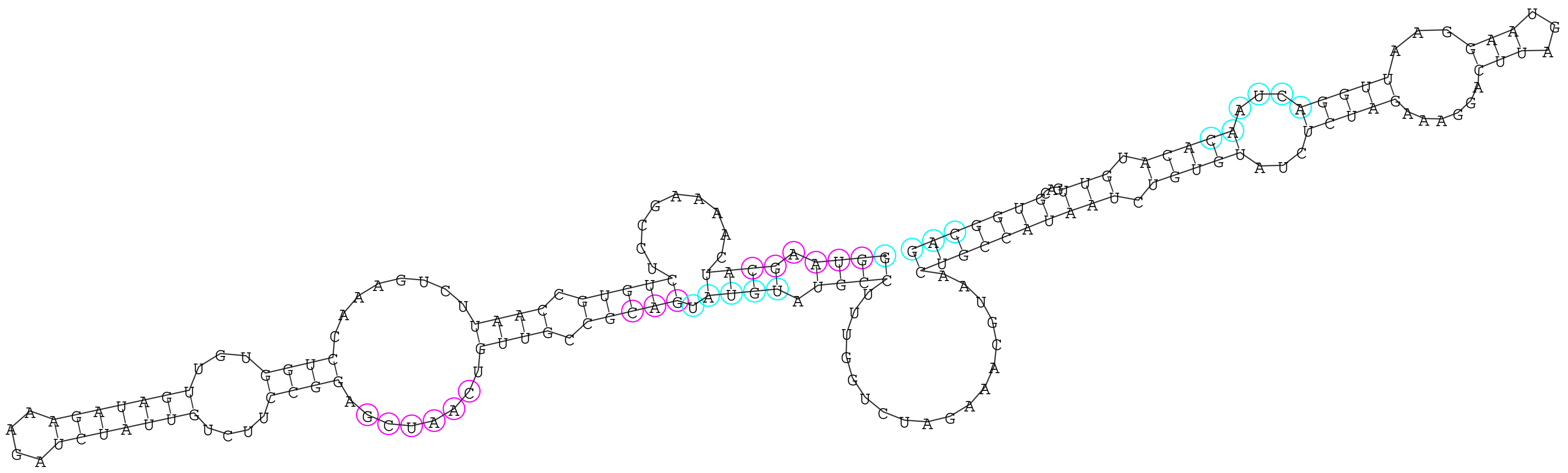

Xbamc051A - Stwintron

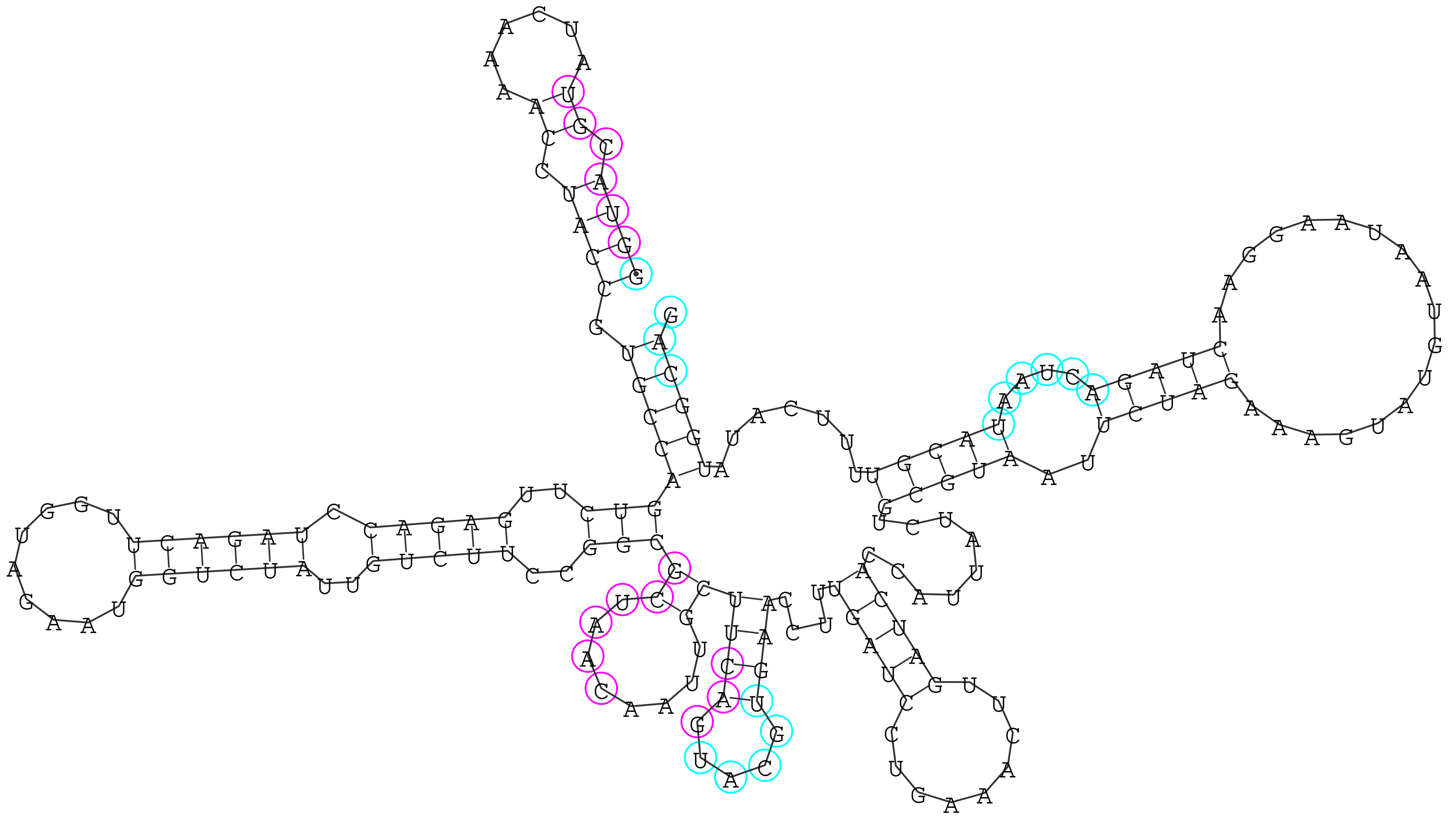

Xbamc053A - Stwintron

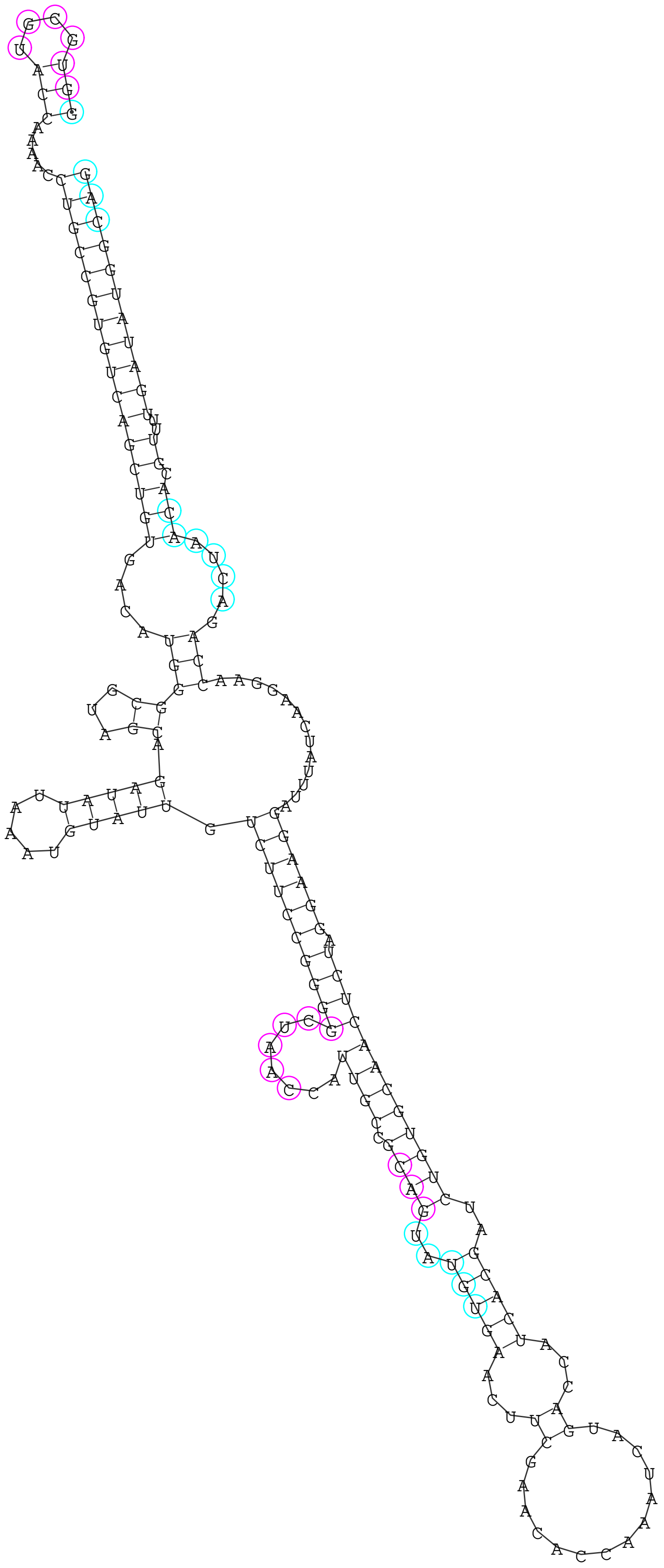

Xbamc053B - Stwintron

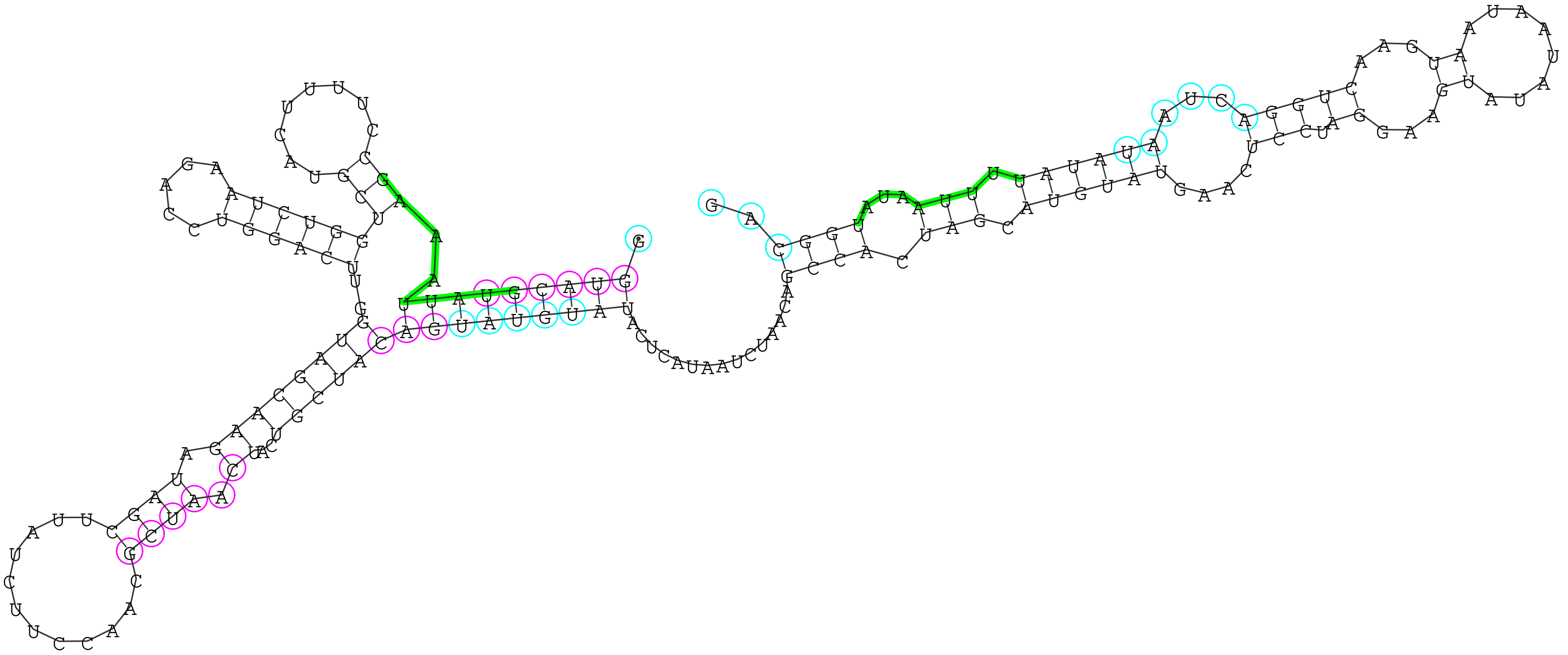

Xbamc053C - Stwintron

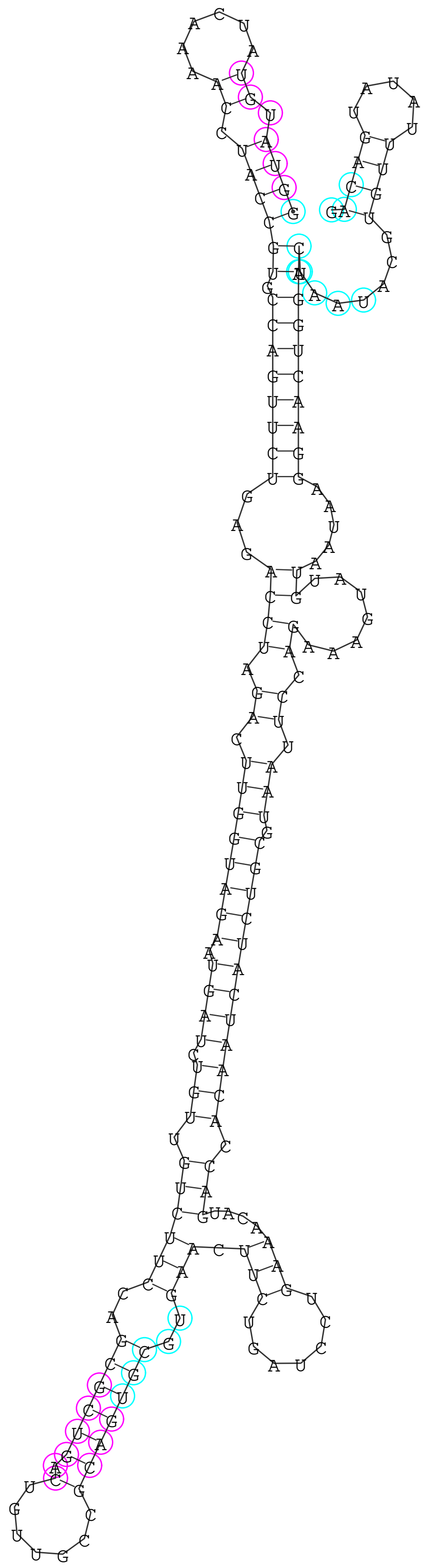

Xbamc067A - Stwintron

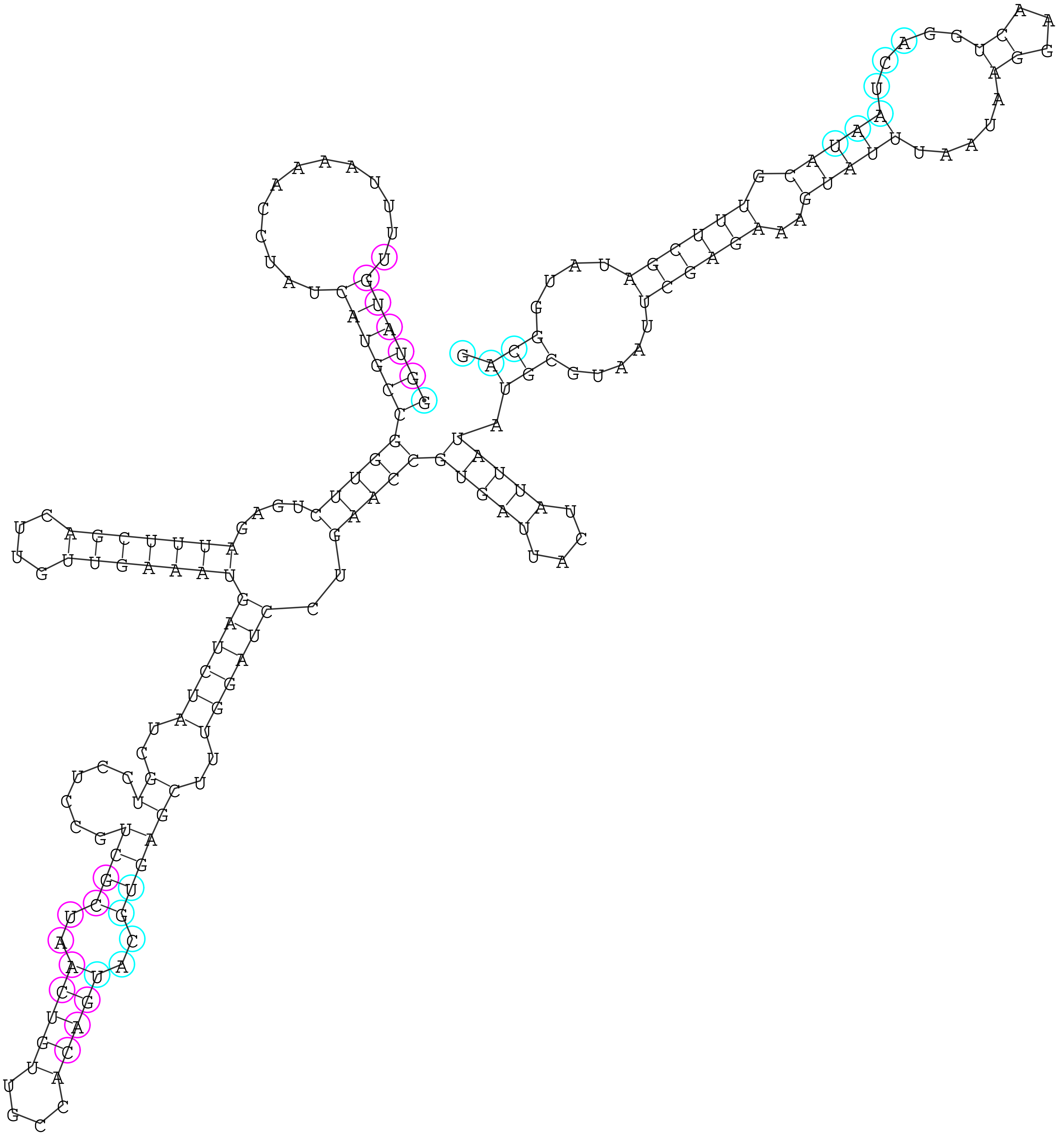

Xbamc067B - Stwintron

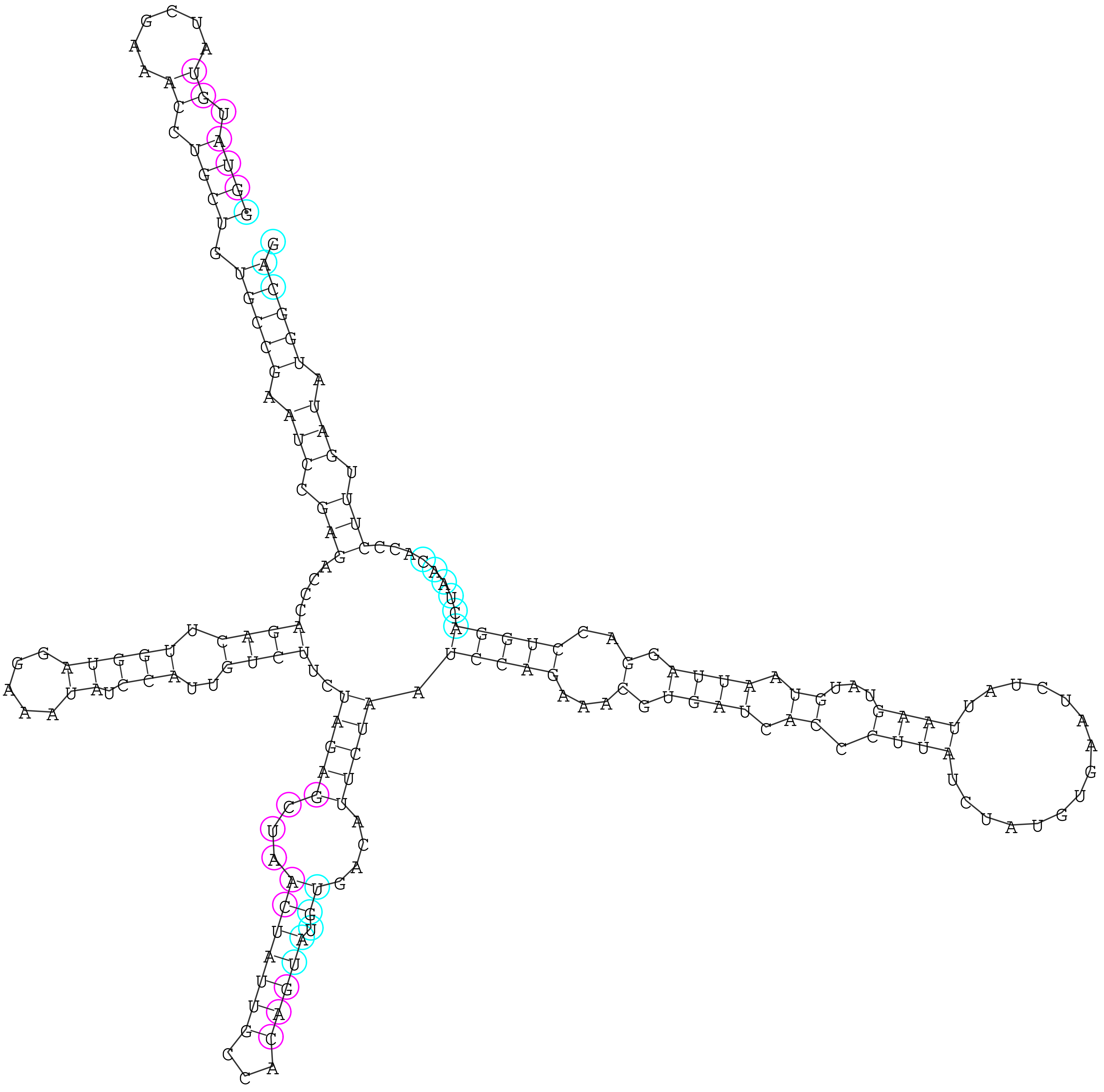

Xbamc080A - Stwintron

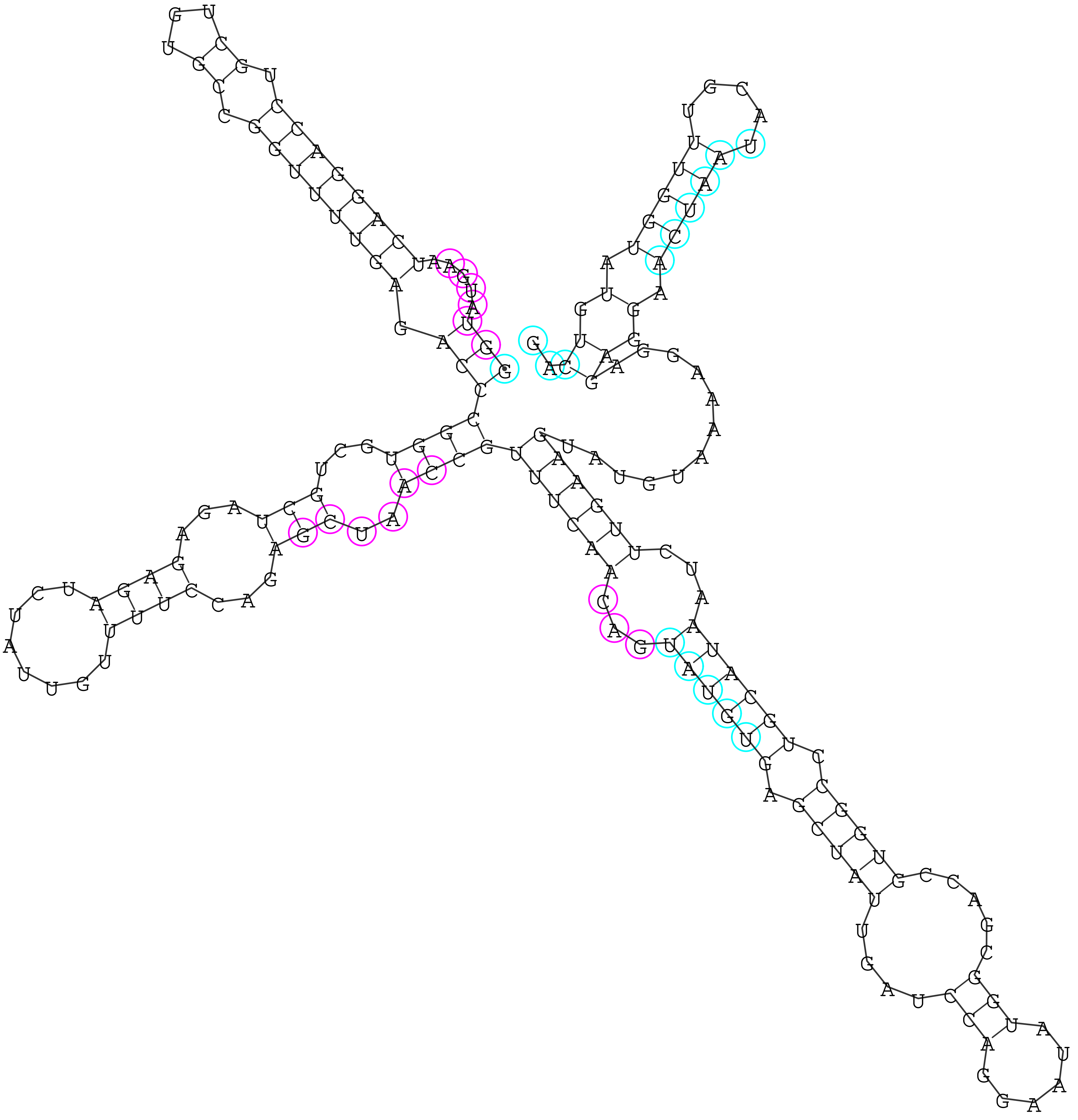

Xbamc083A - Stwintron

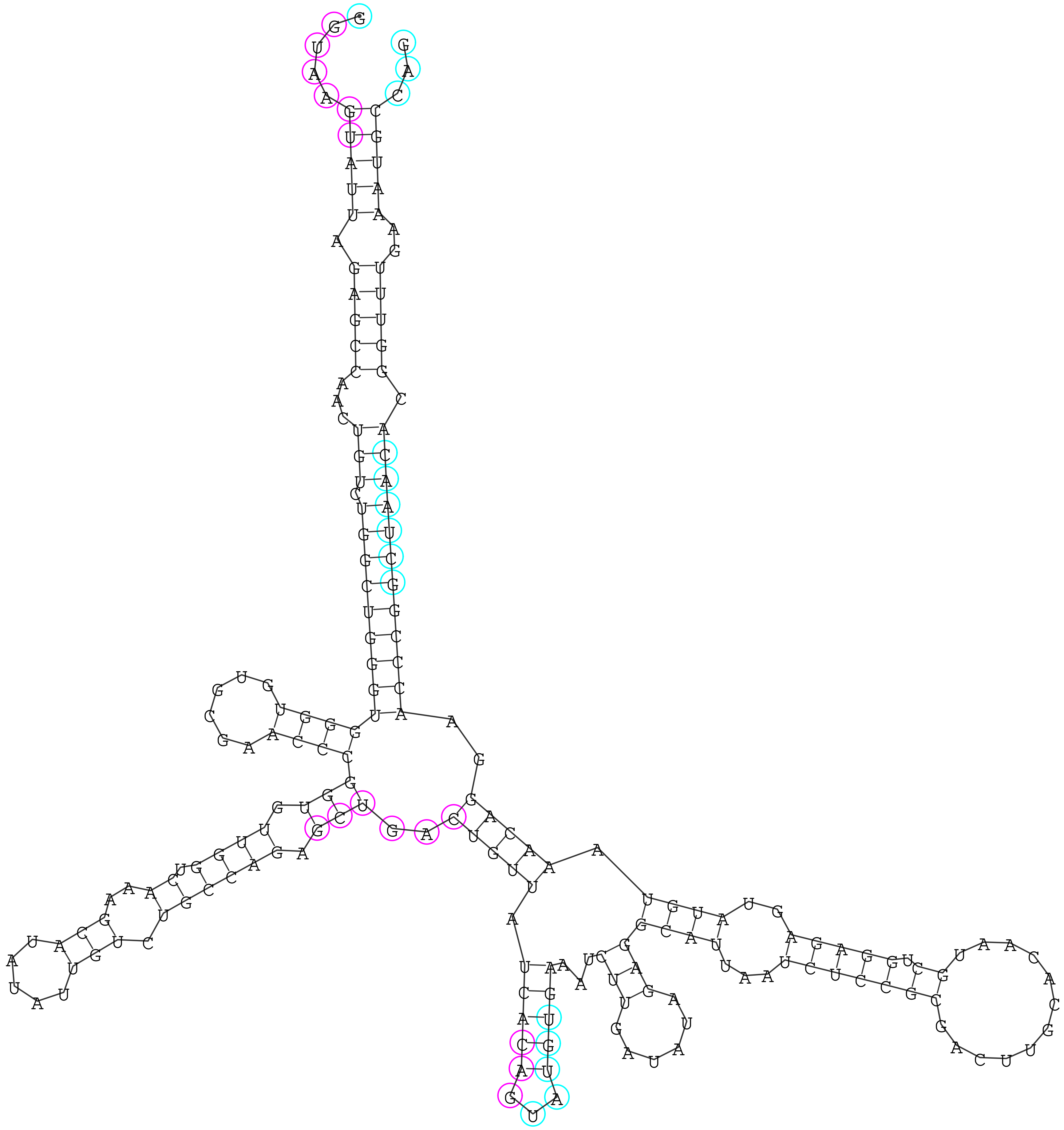

Xbamc083B - Stwintron

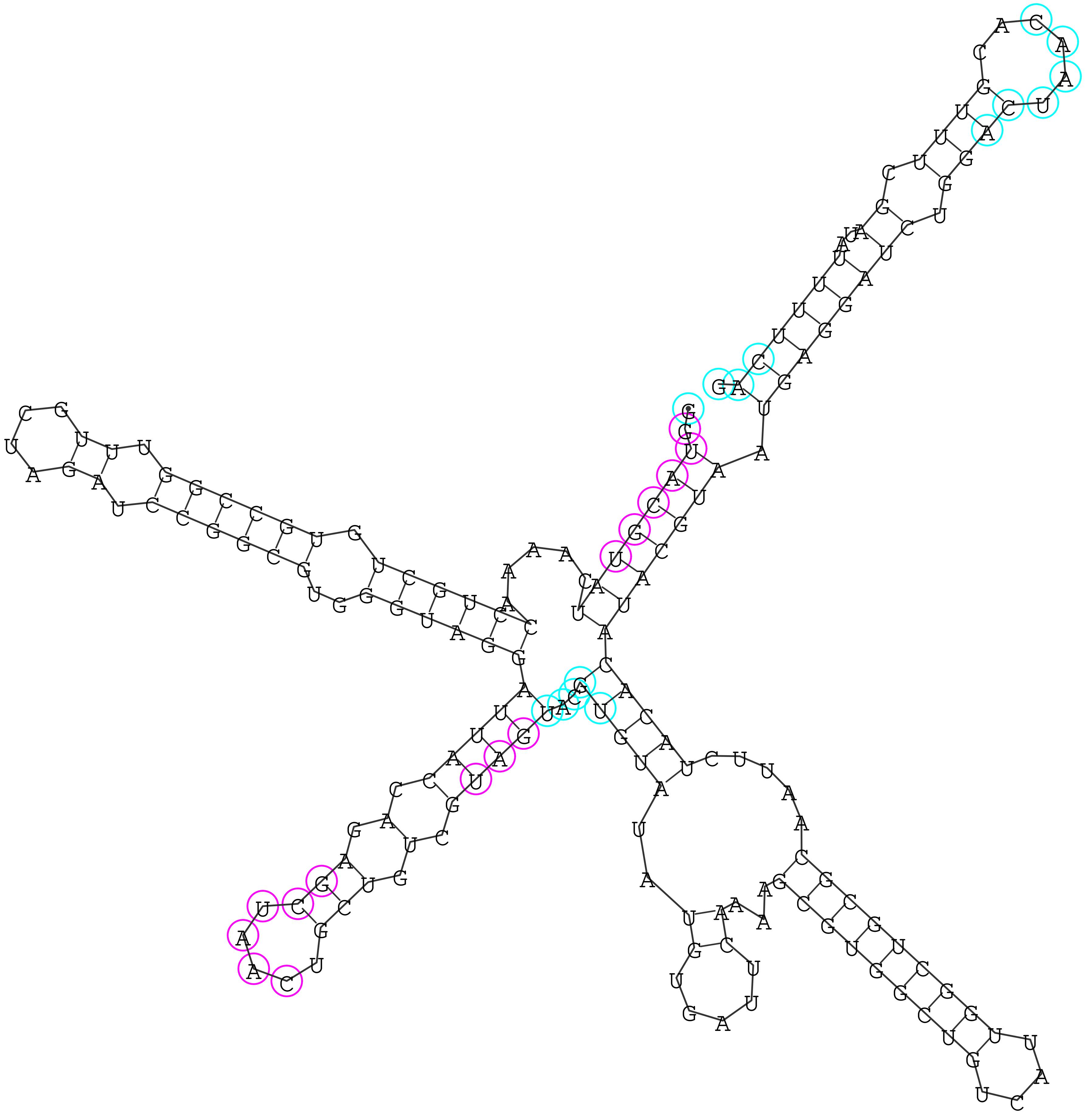

Xbamc084A - Stwintron

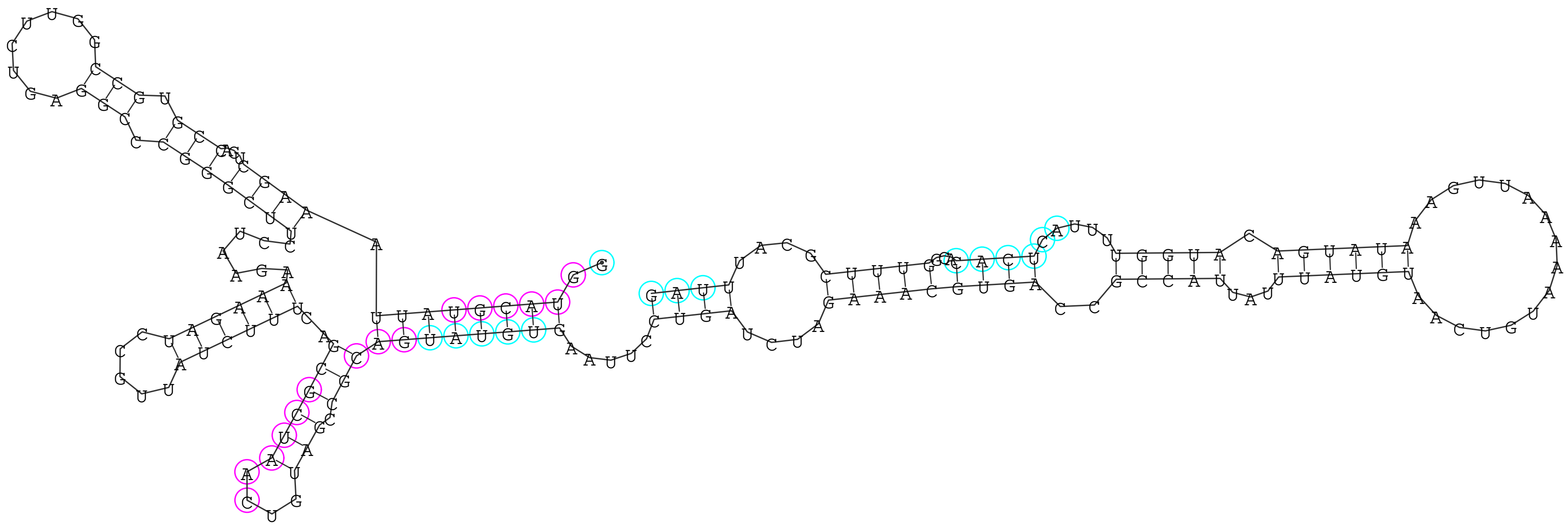

Xbamc086A - Stwintron

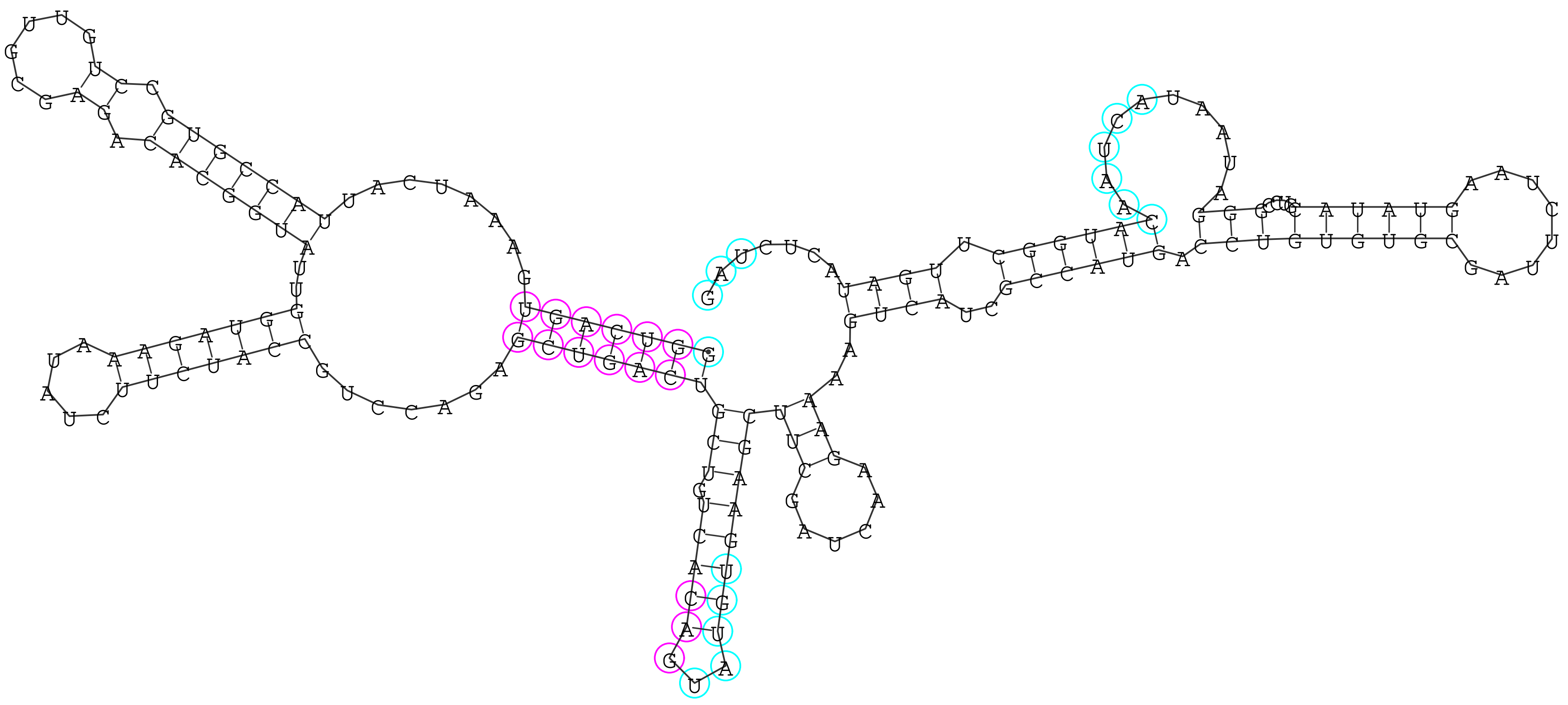

Xbamc089A - Stwintron

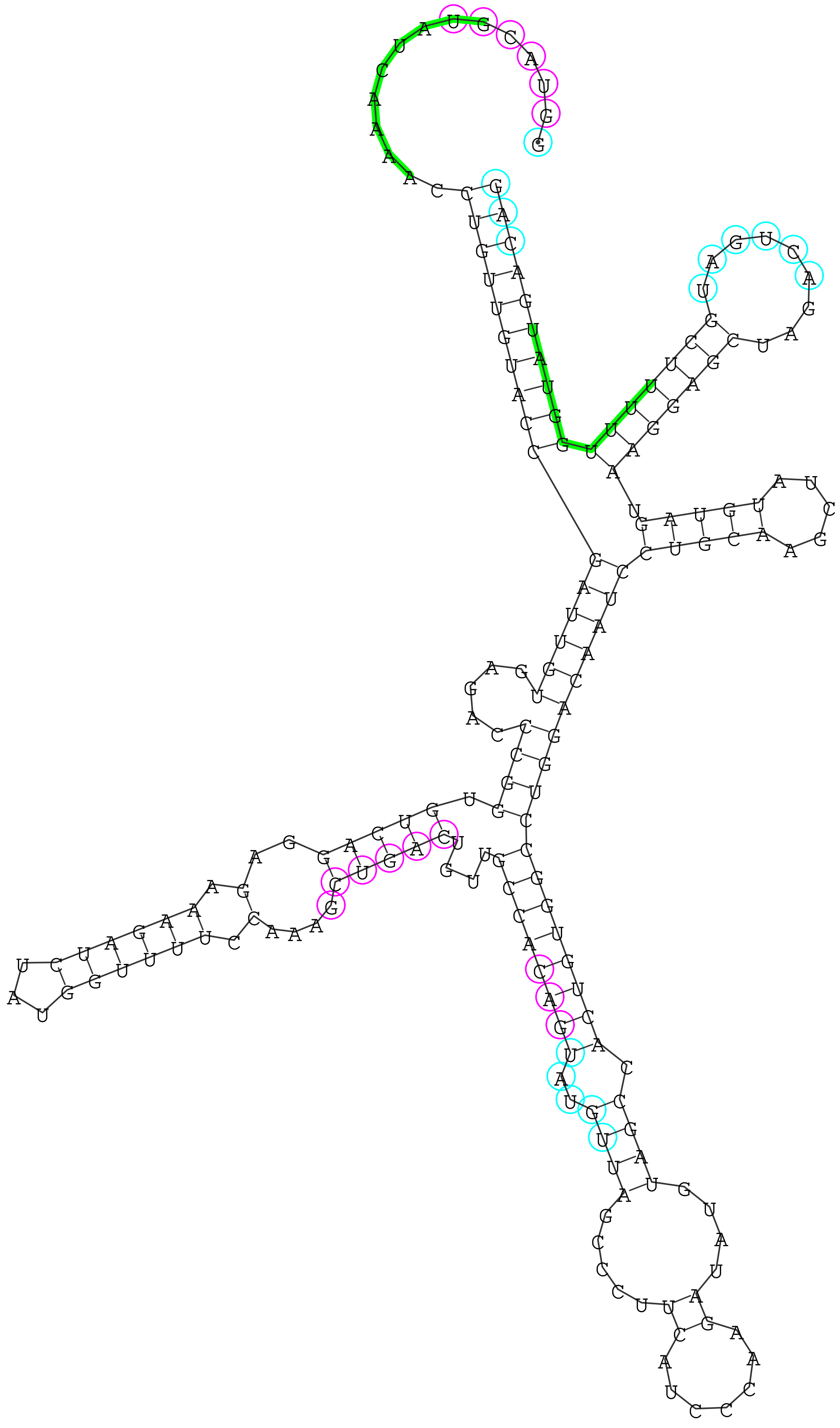

Xbamc096A - Stwintron

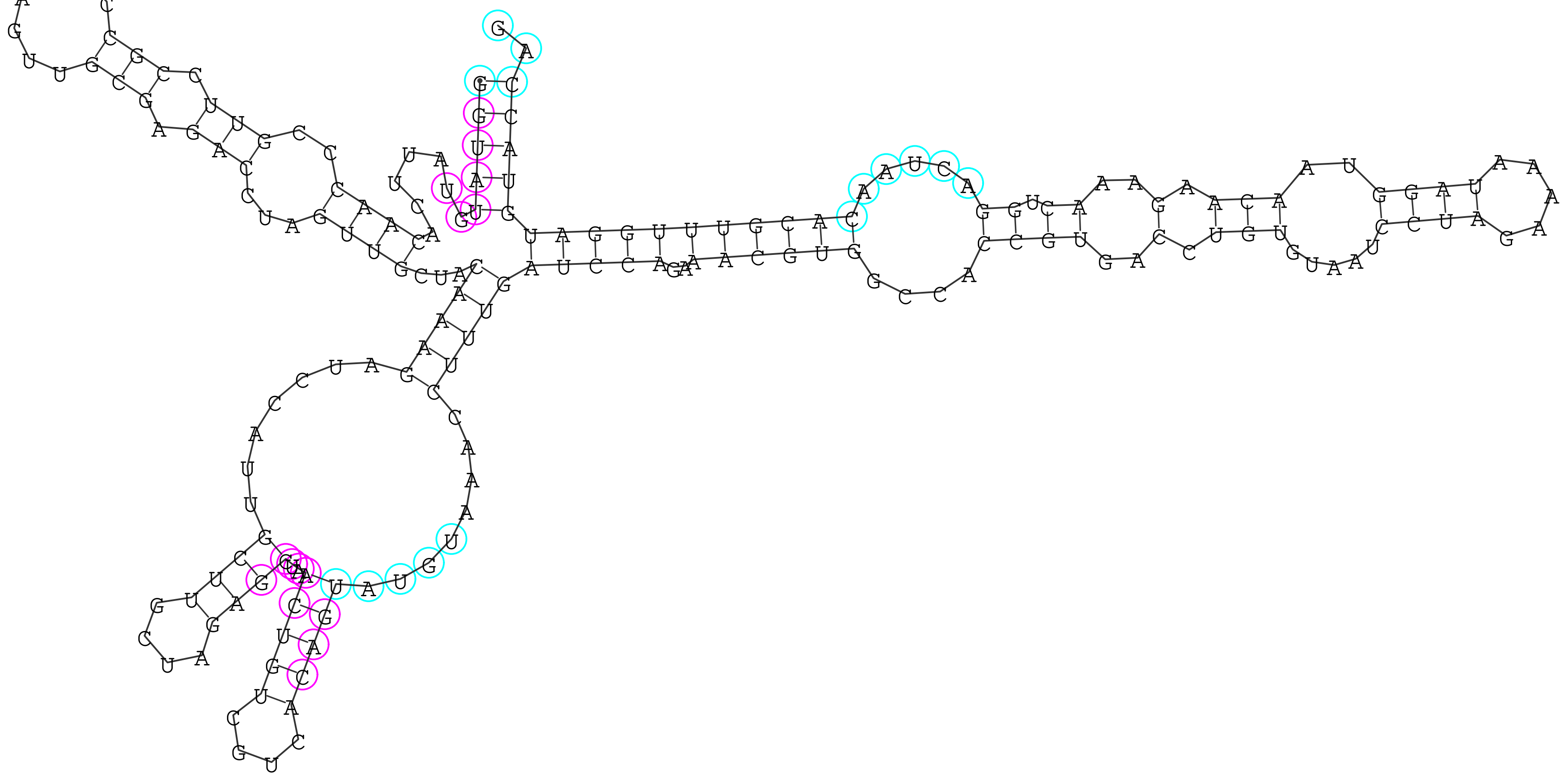

Xbamc102A - Stwintron

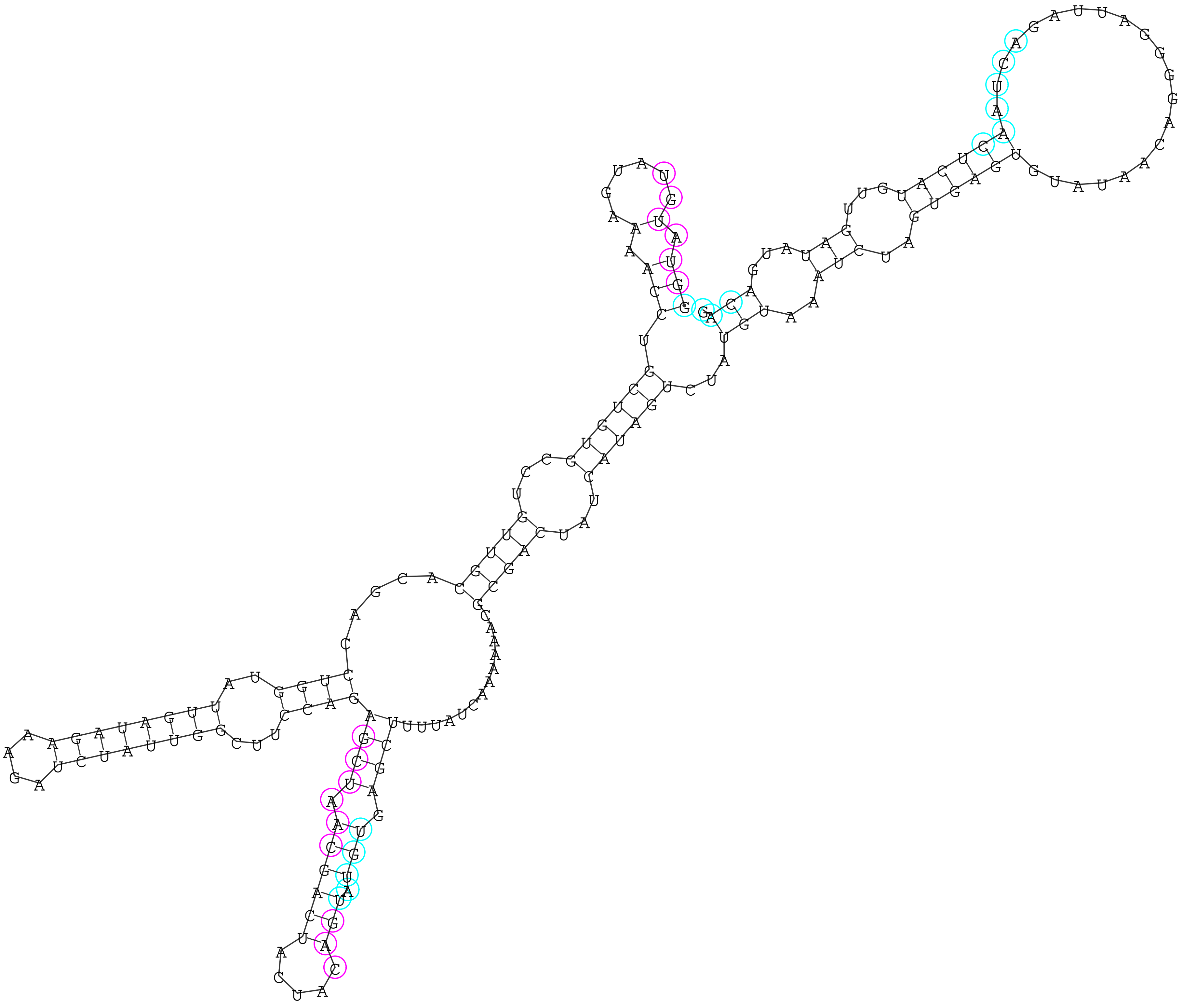

Xbamc106A - Stwintron

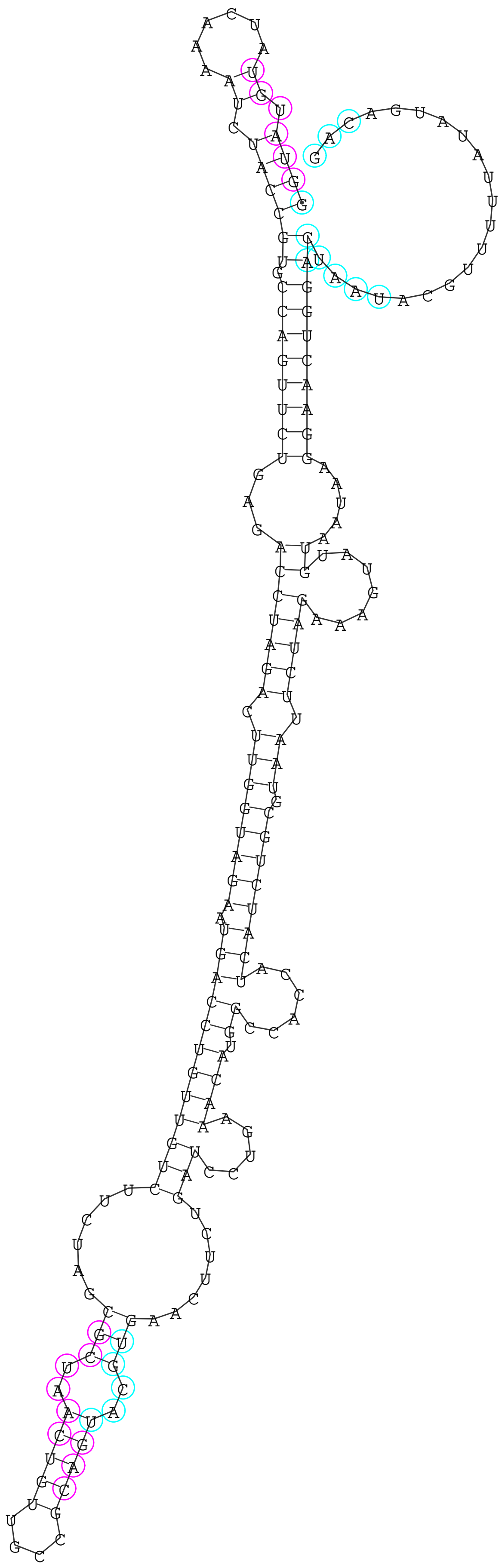

Xbamc106B - Stwintron

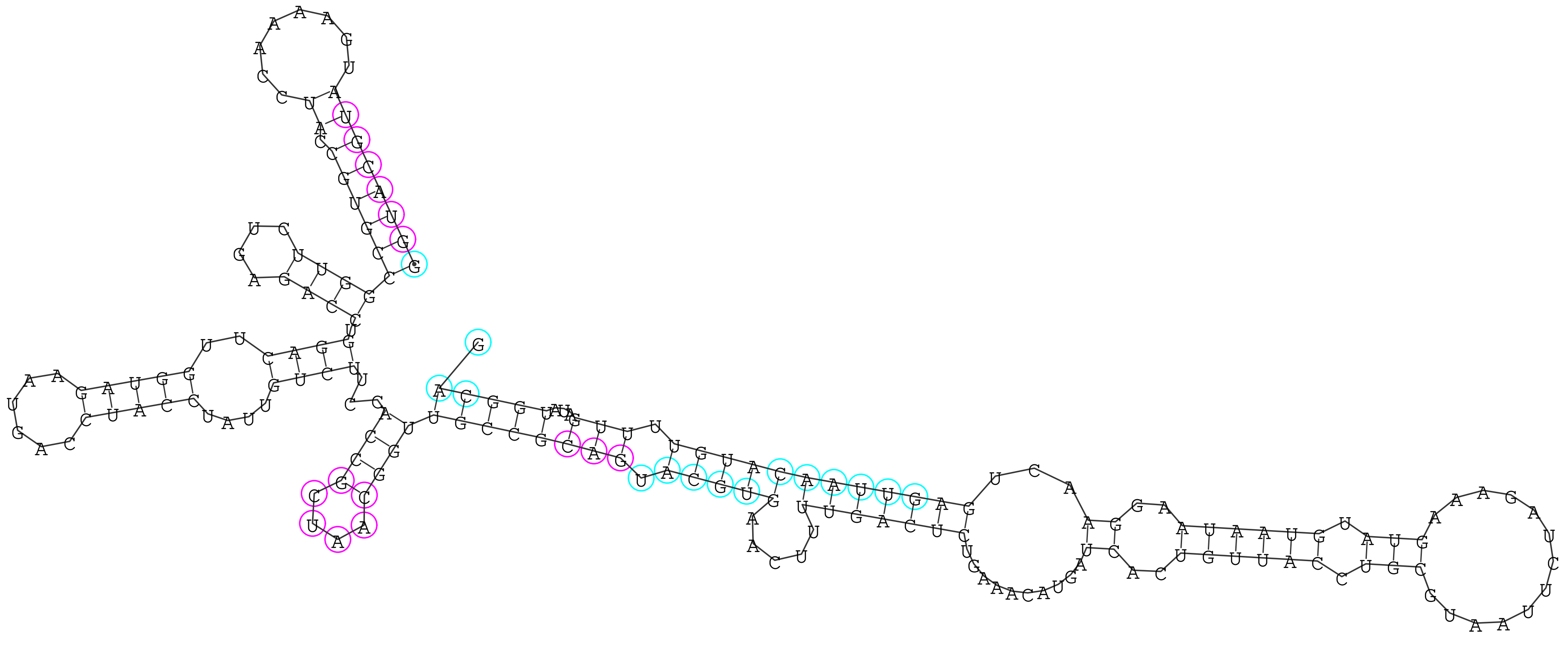

Xbamc107A - Stwintron

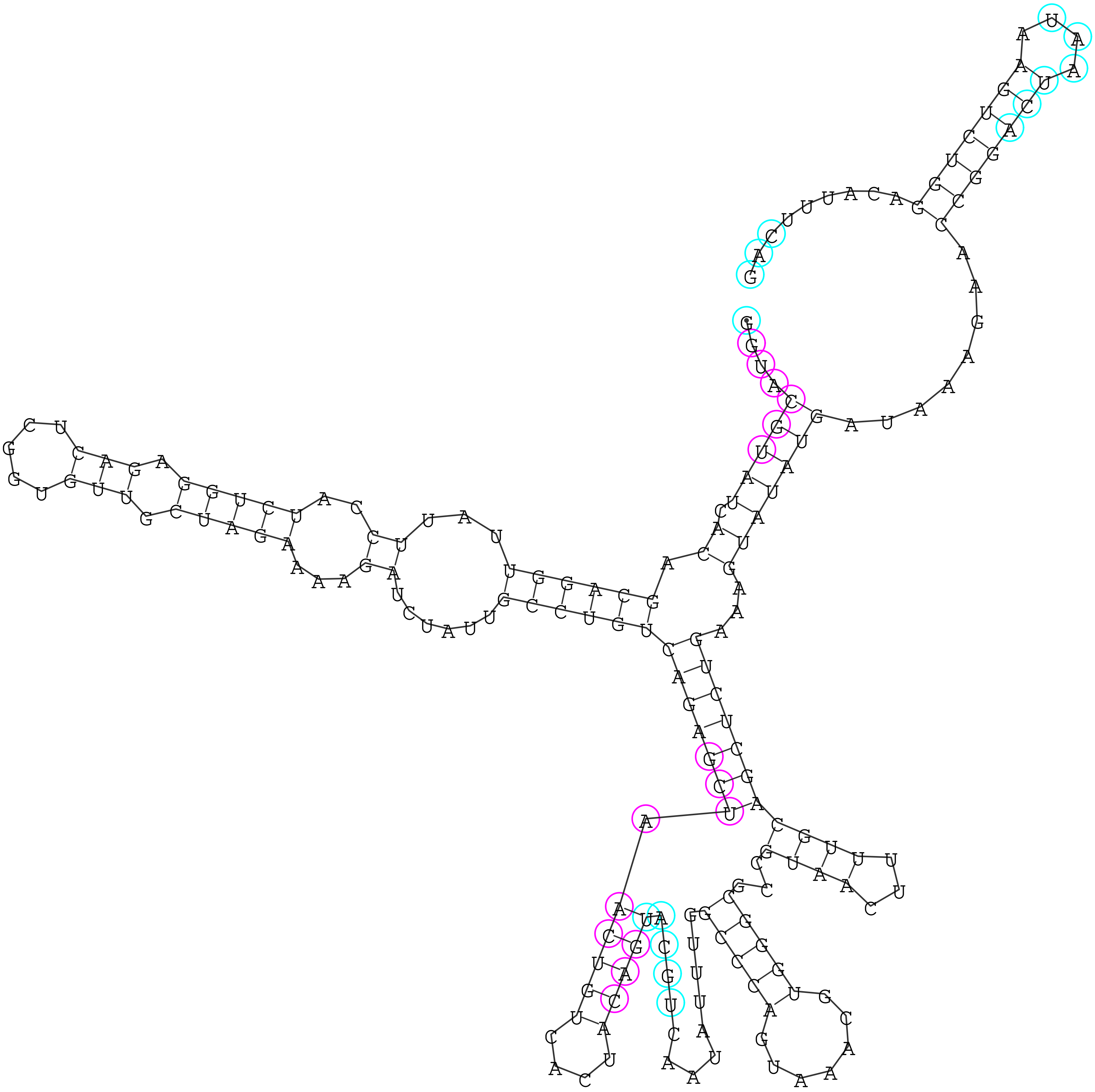

Xbamc109A - Stwintron

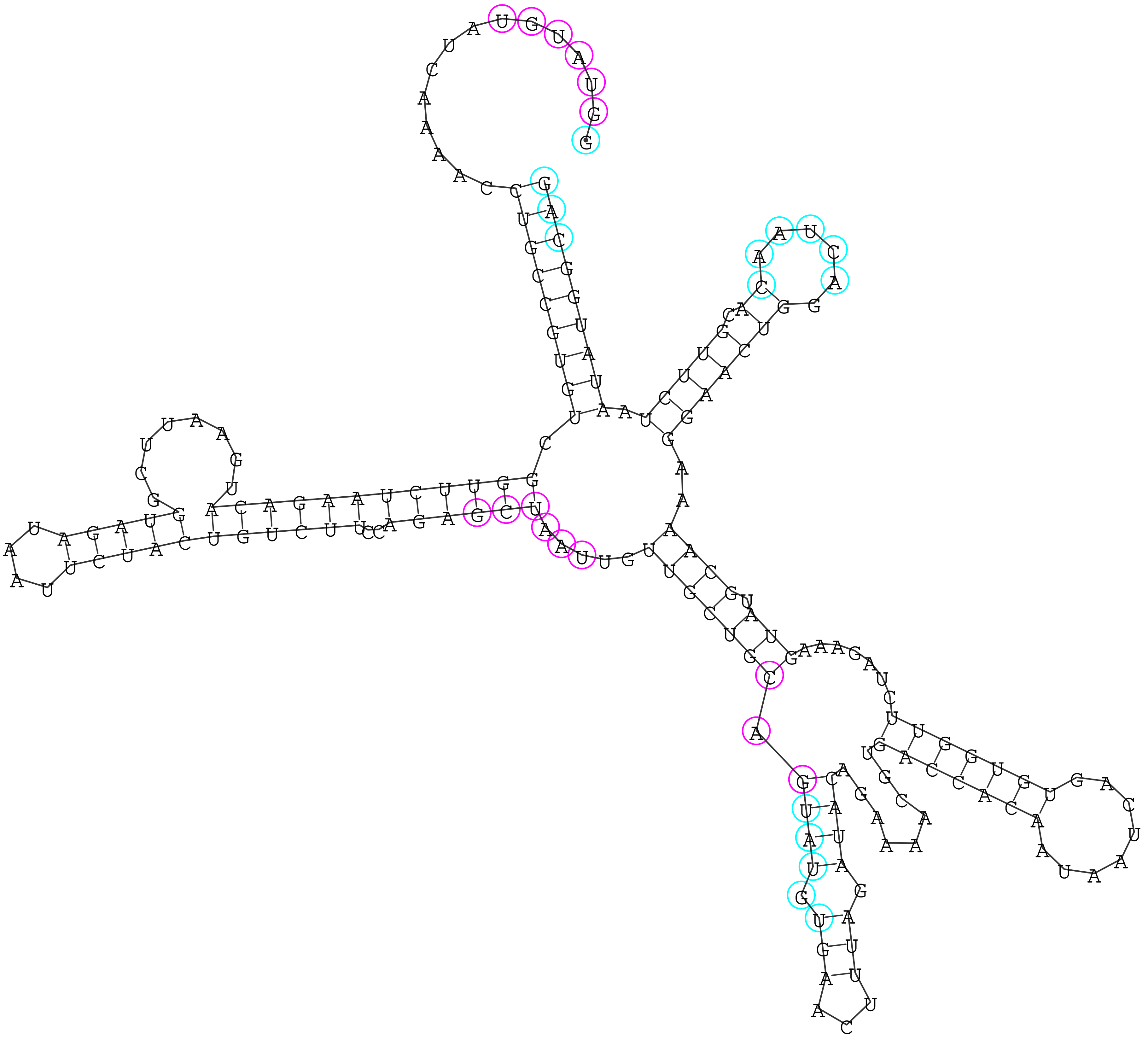

Xbamc110A - Stwintron

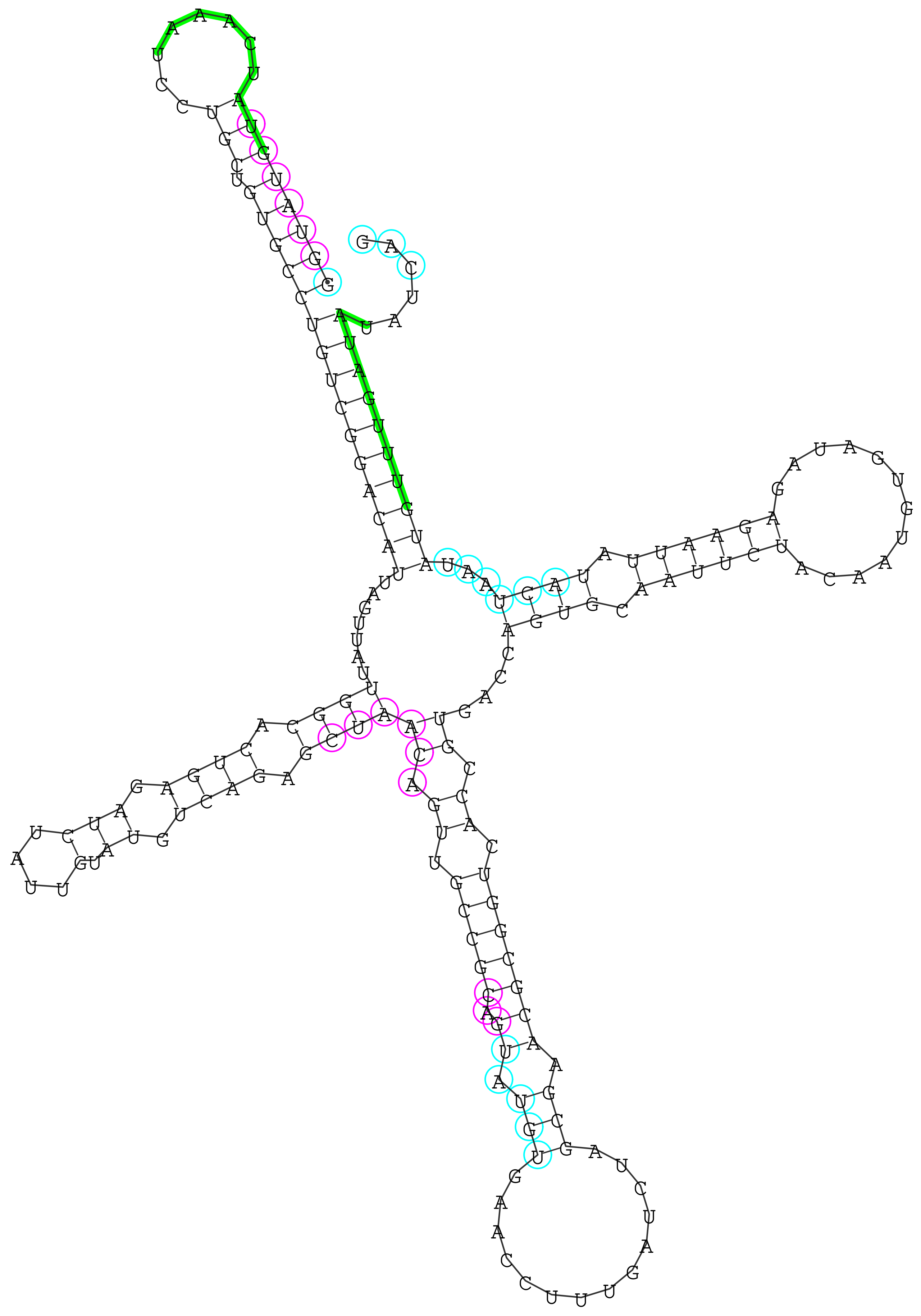

Xbamc124A - Stwintron

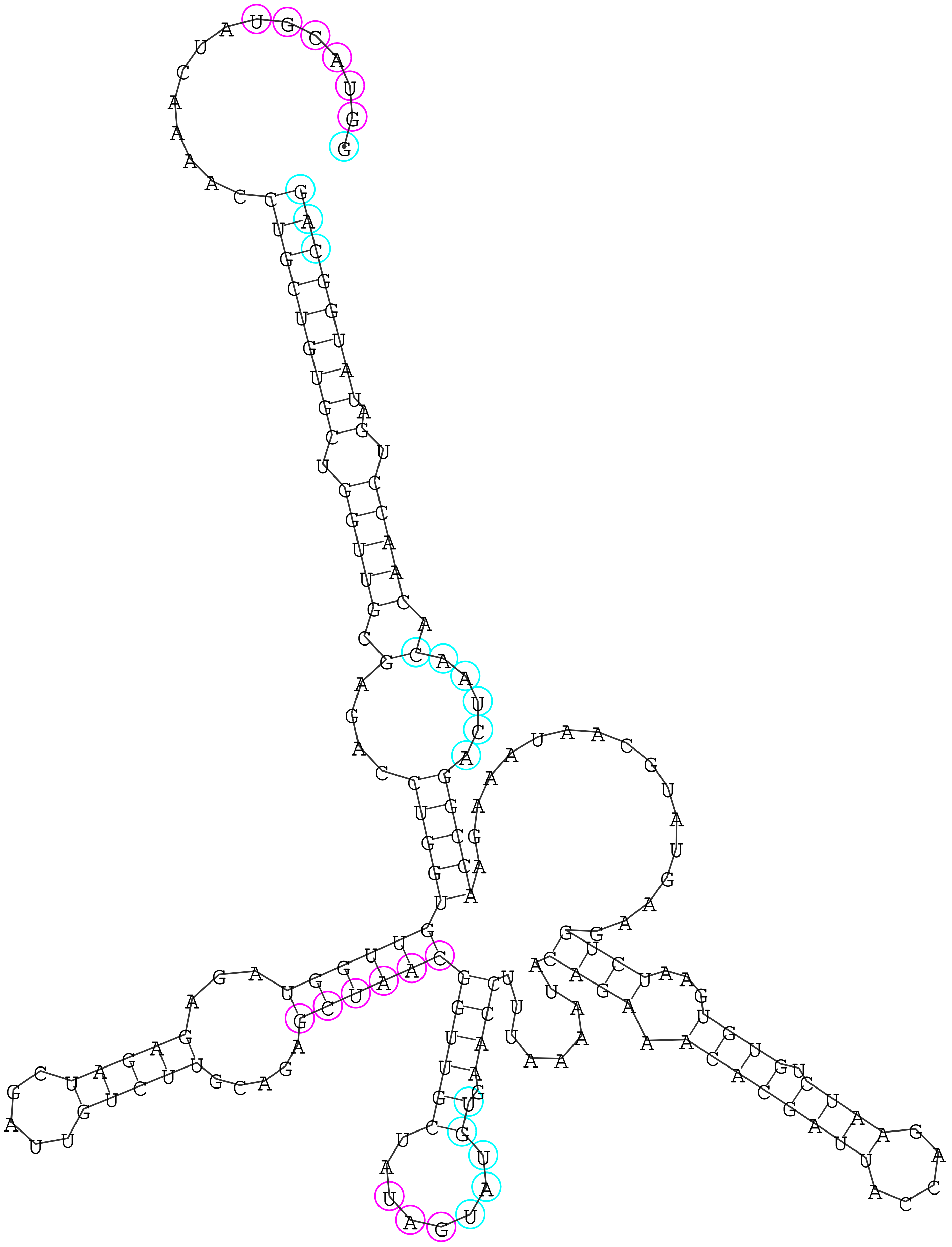

Xbamc132A - Stwintron

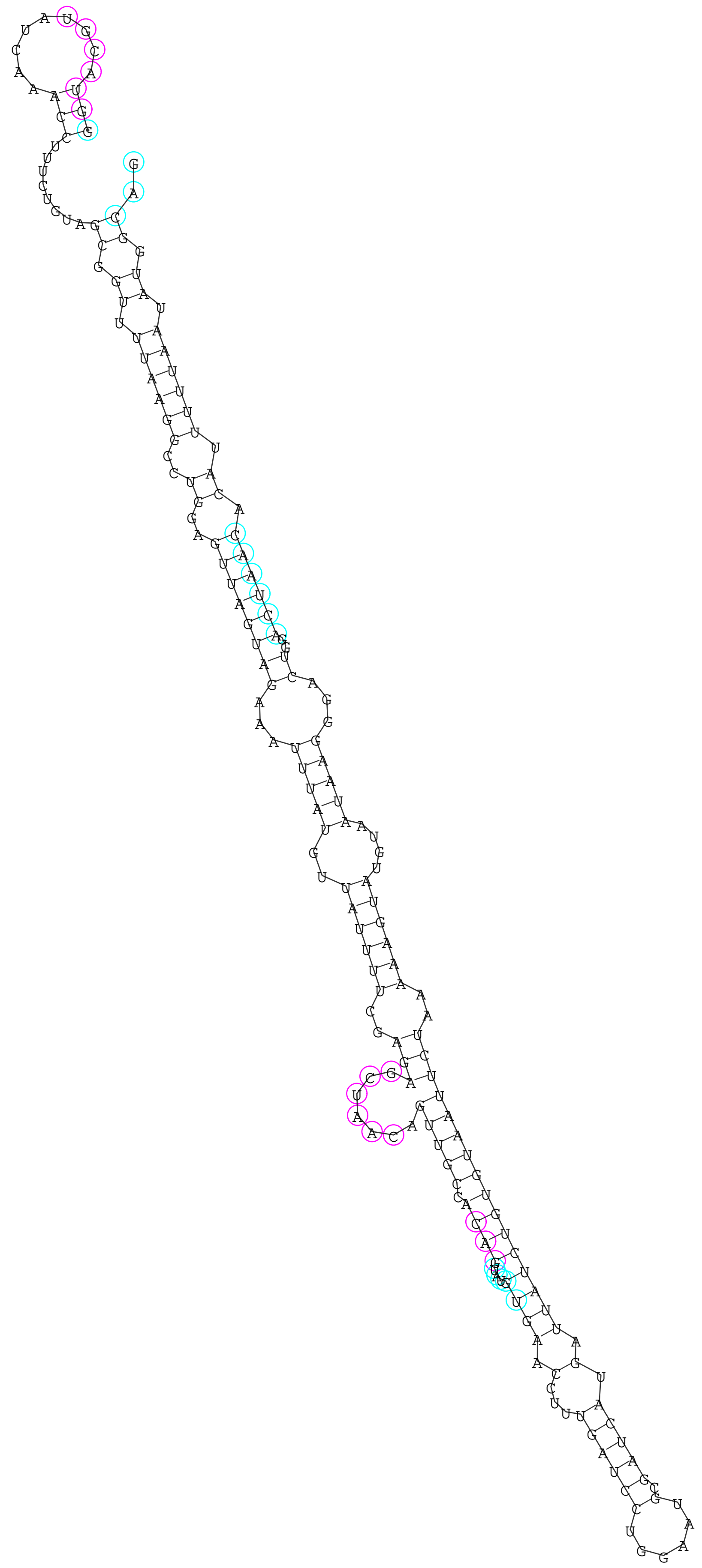

Xbamc132B - Stwintron

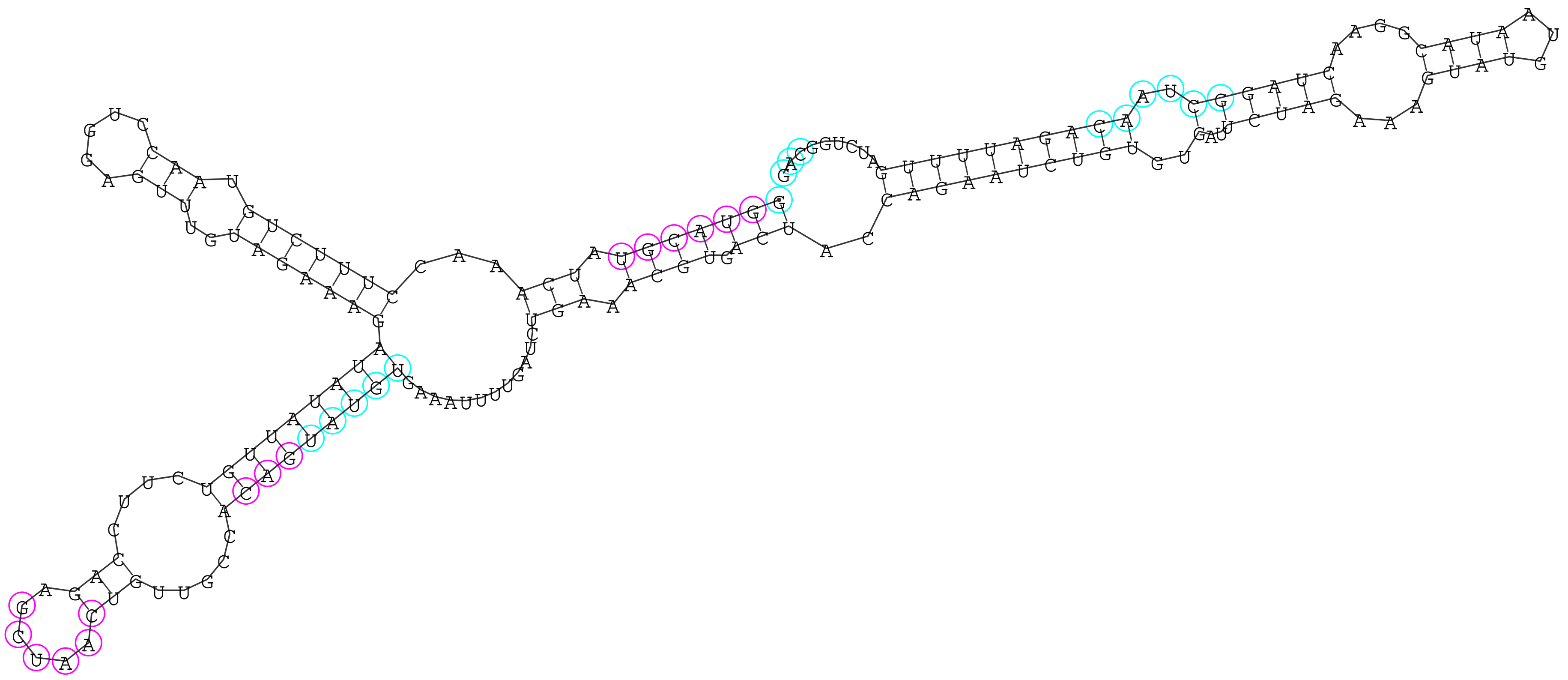

Xbamc152A - Stwintron

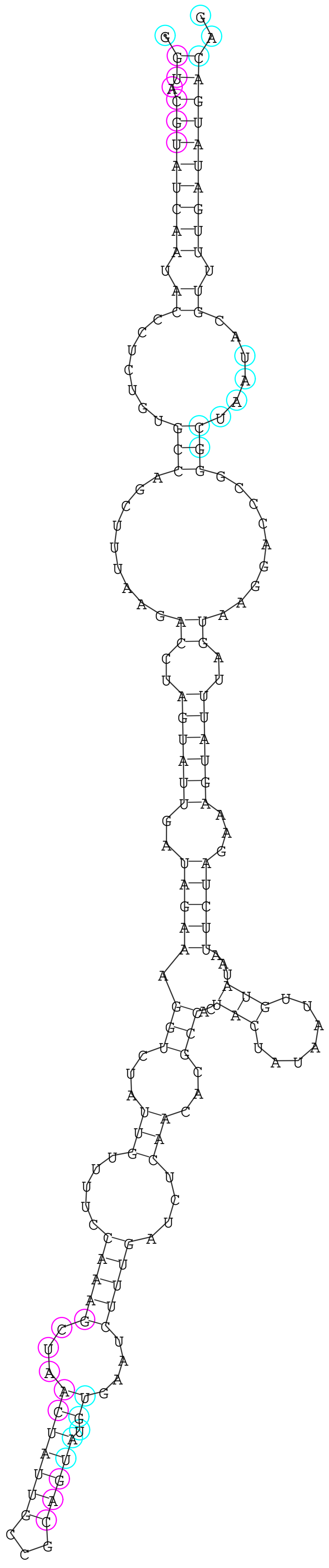

Xbamc152B - Stwintron

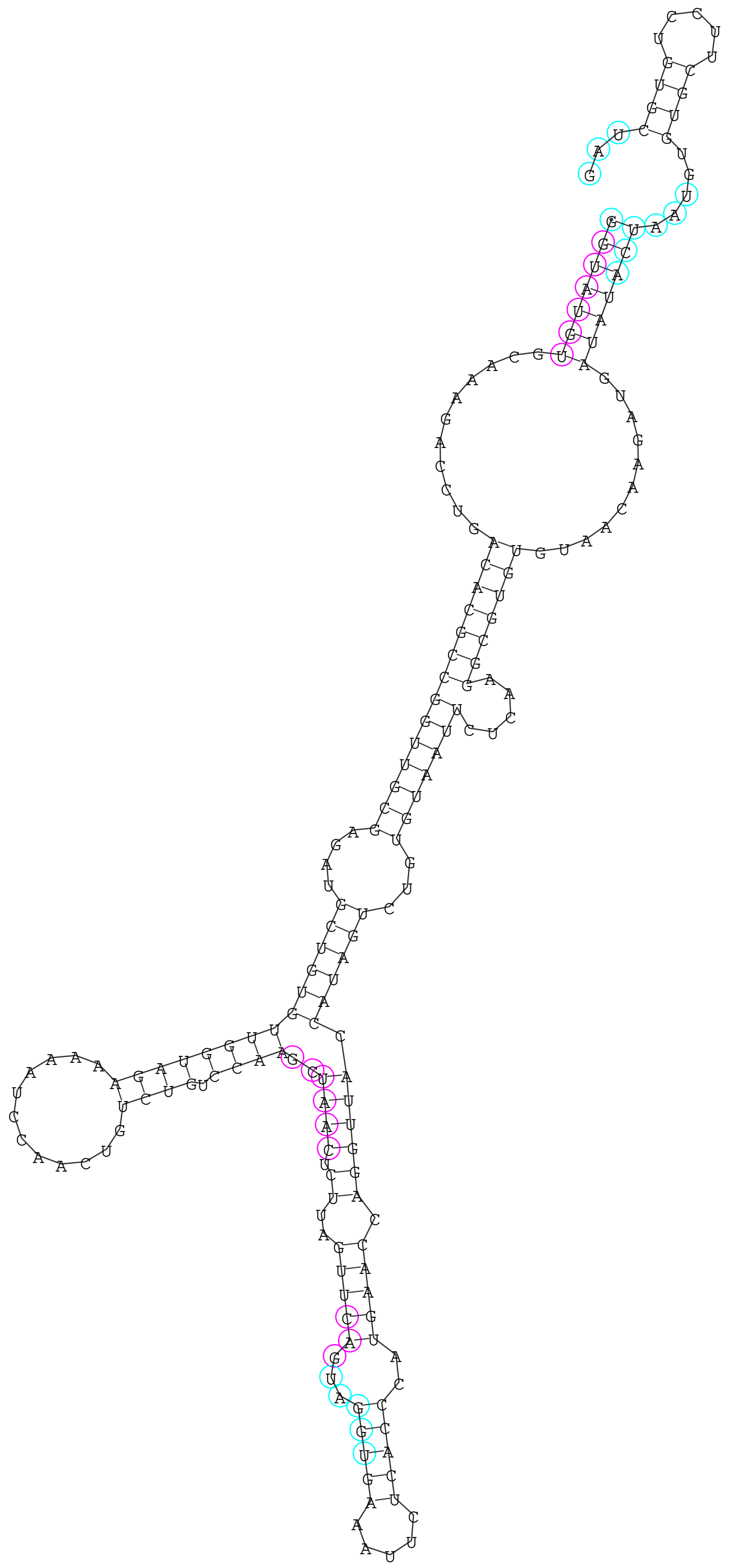

Xbamc152C - Stwintron

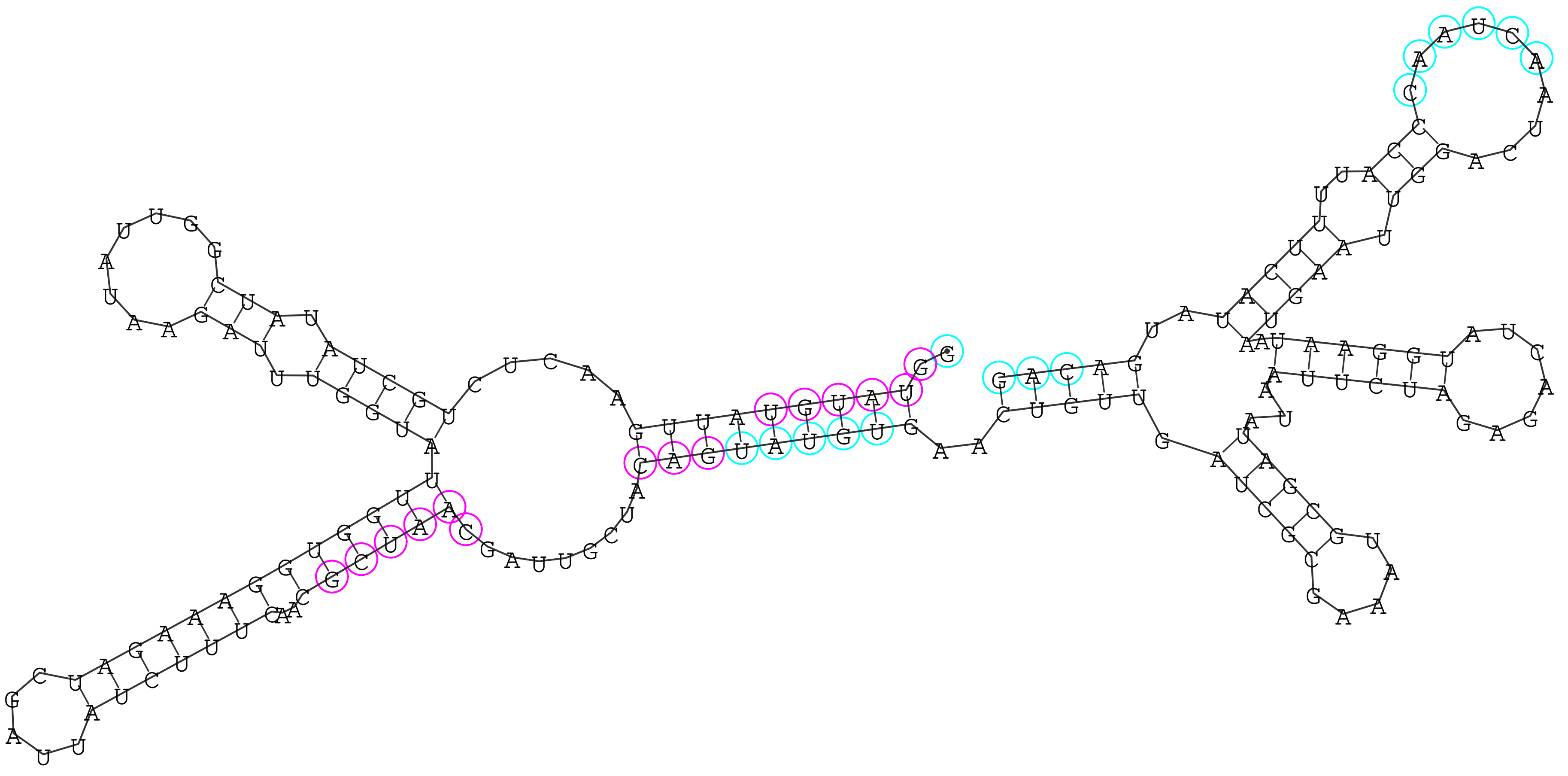

Xbamc153A - Stwintron

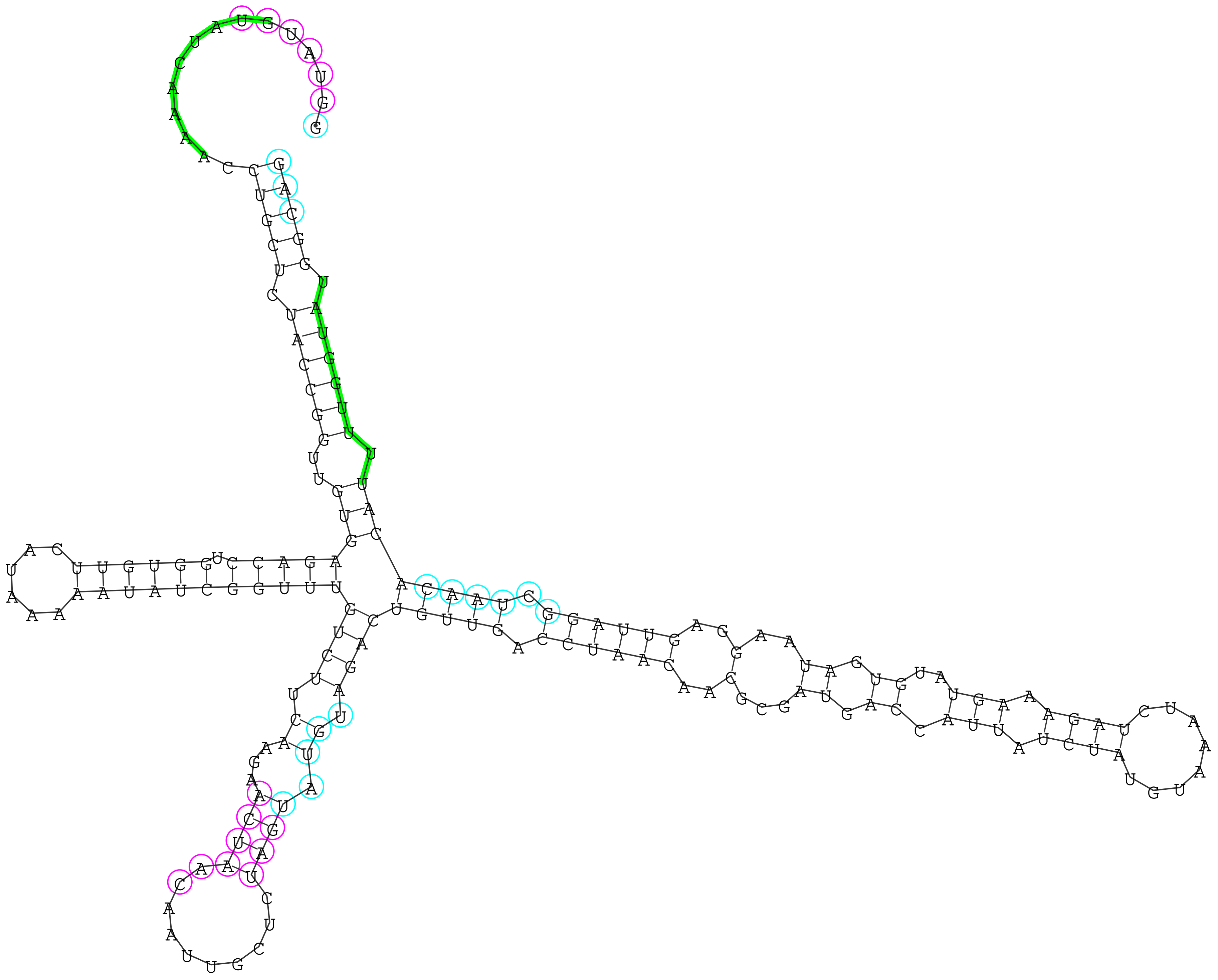

Xbamc155A - Stwintron

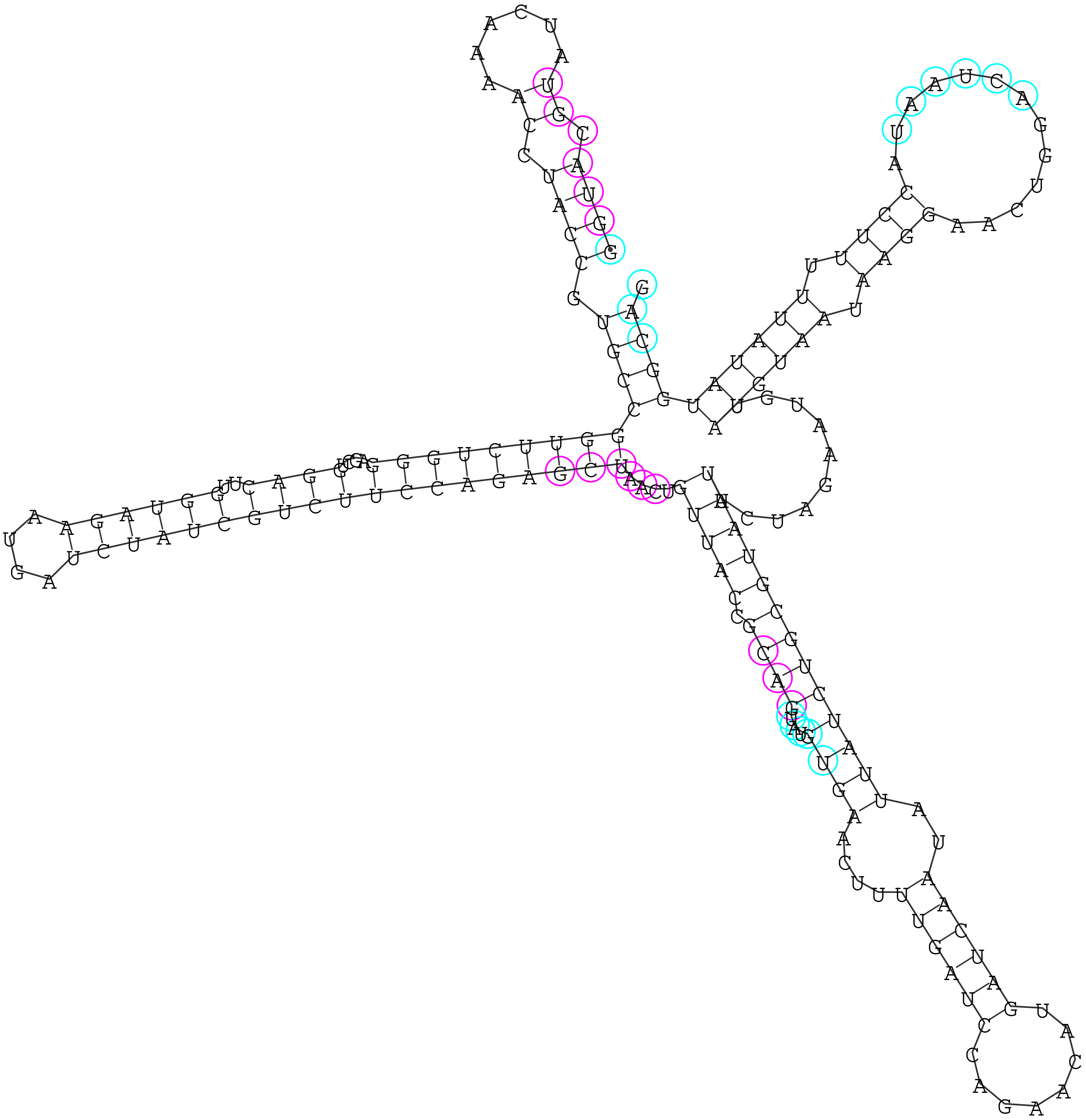

Xbamc156A - Stwintron

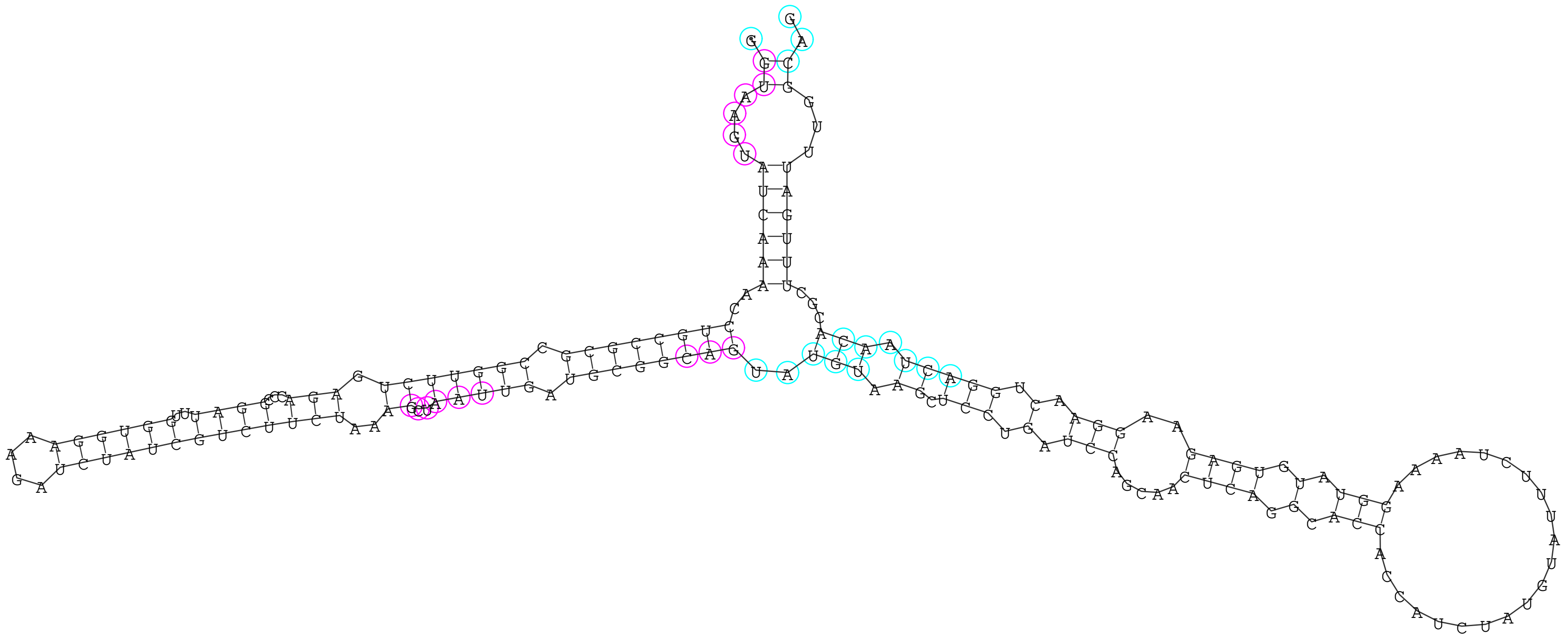

Xbamc159A - Stwintron

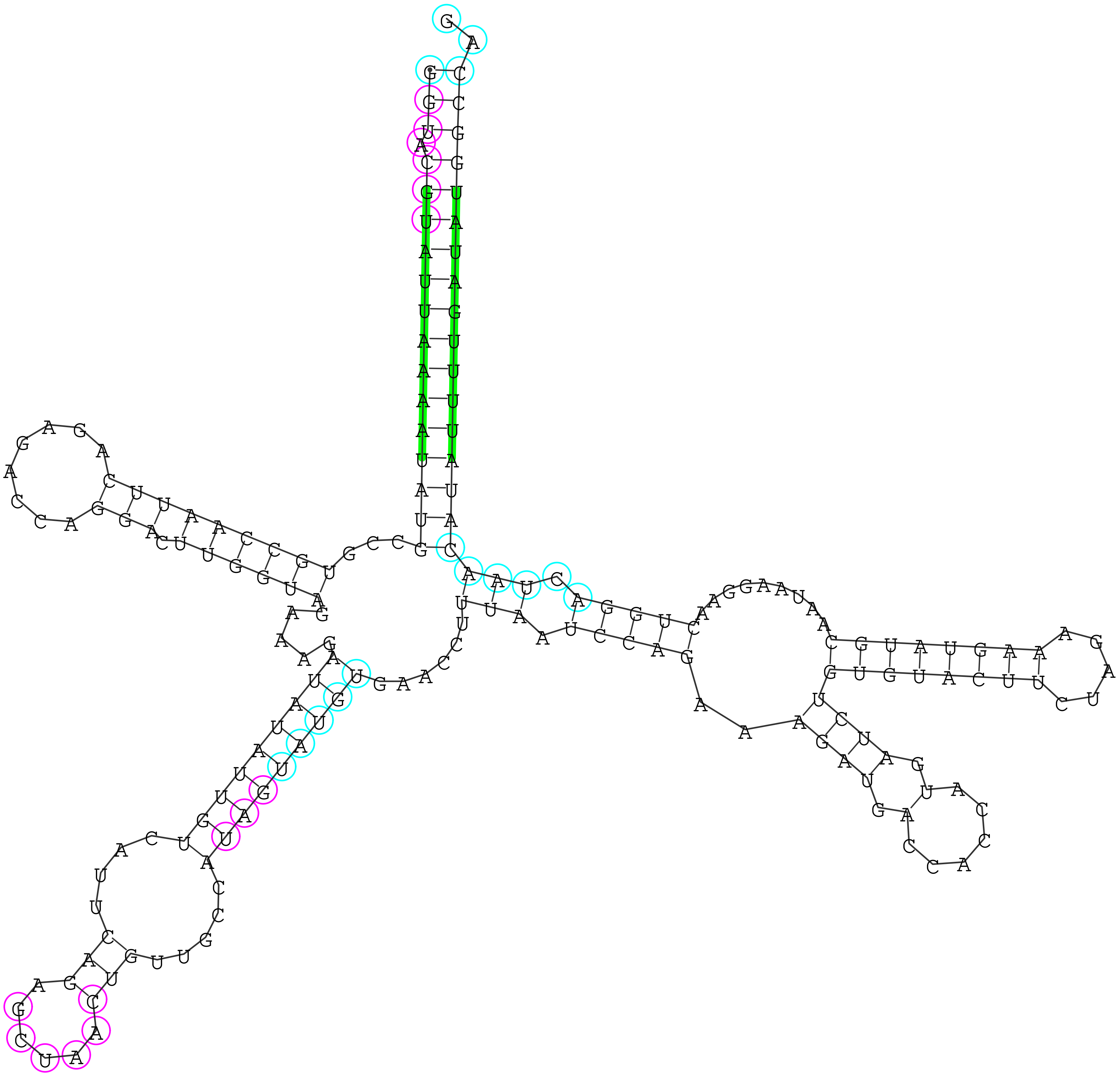

Xbamc164A - Stwintron

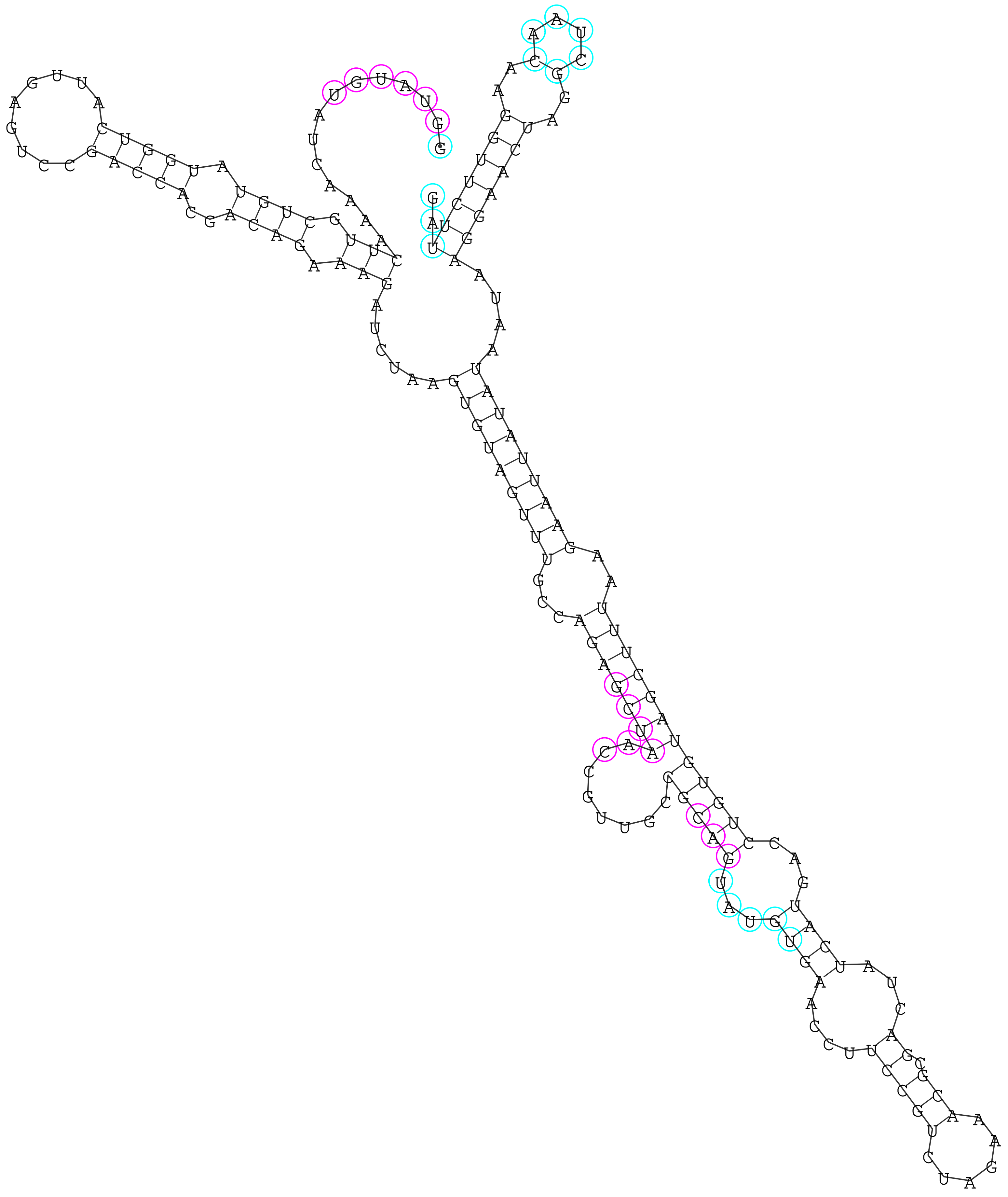

Xbamc176A - Stwintron

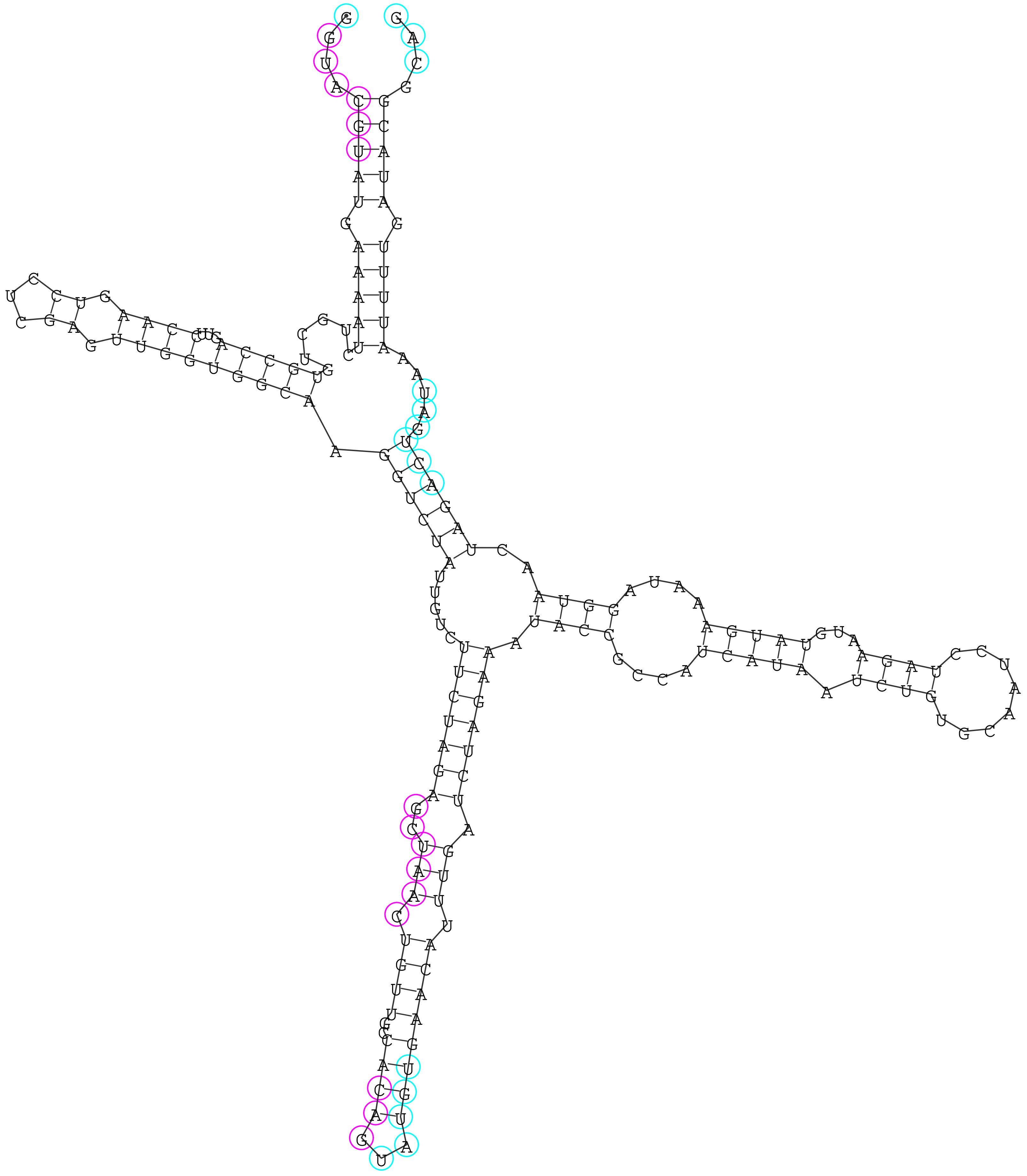

Xbamc177A - Stwintron

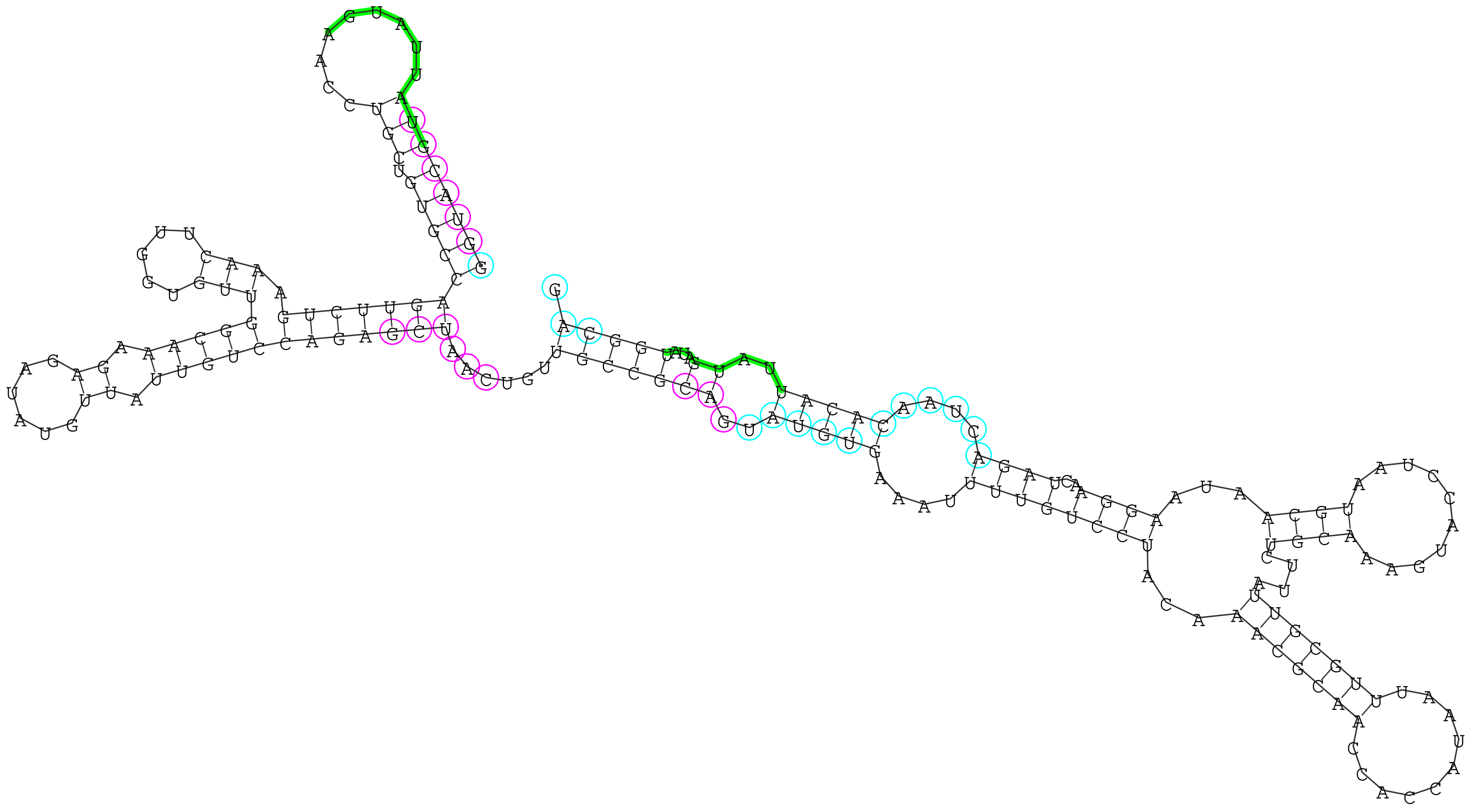

Xbamc191A - Stwintron

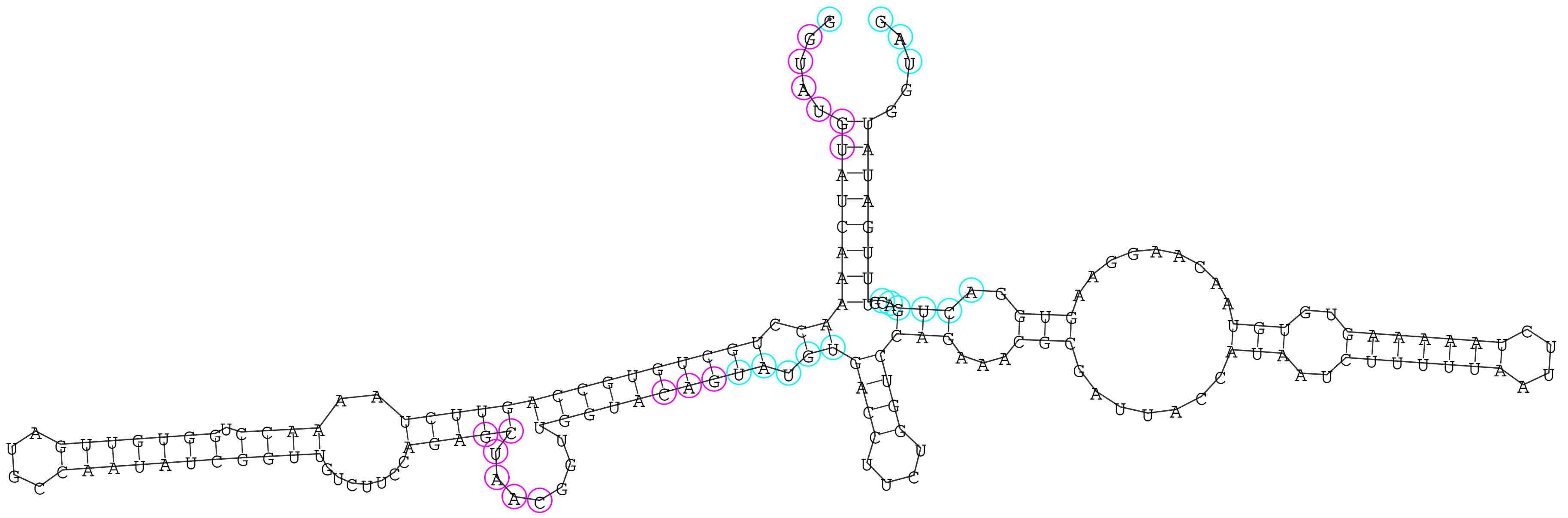

Xbamc198A - Stwintron

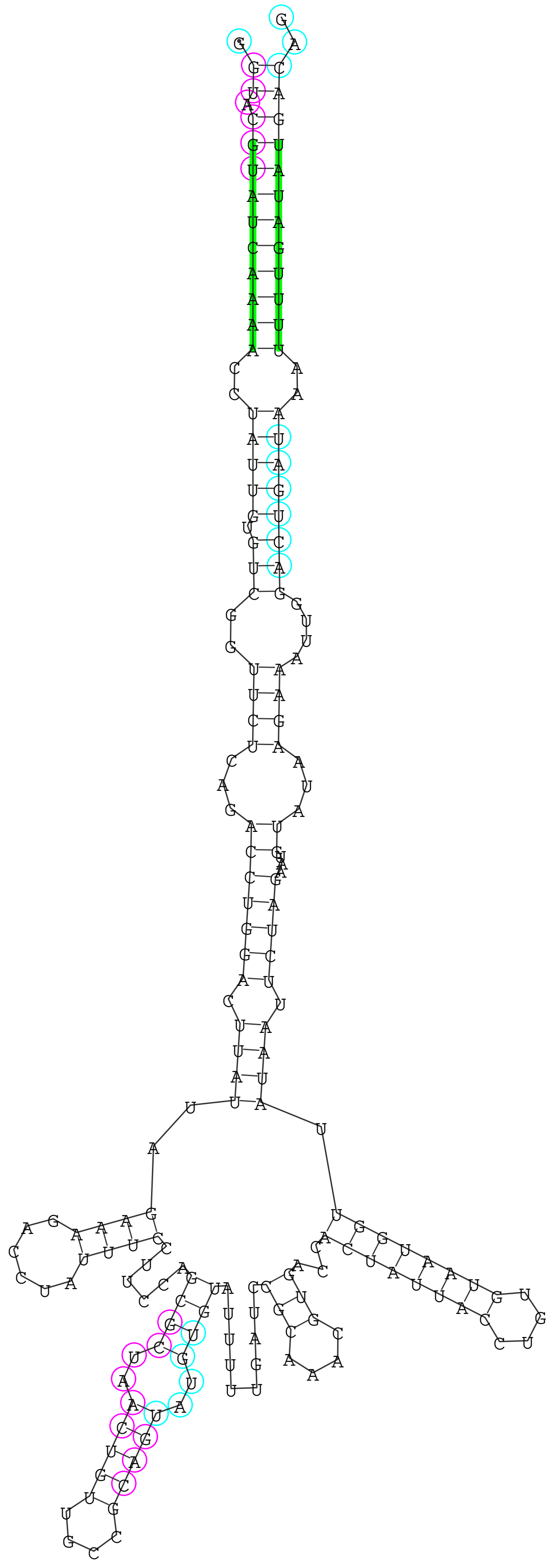

Xbamc199A - Stwintron

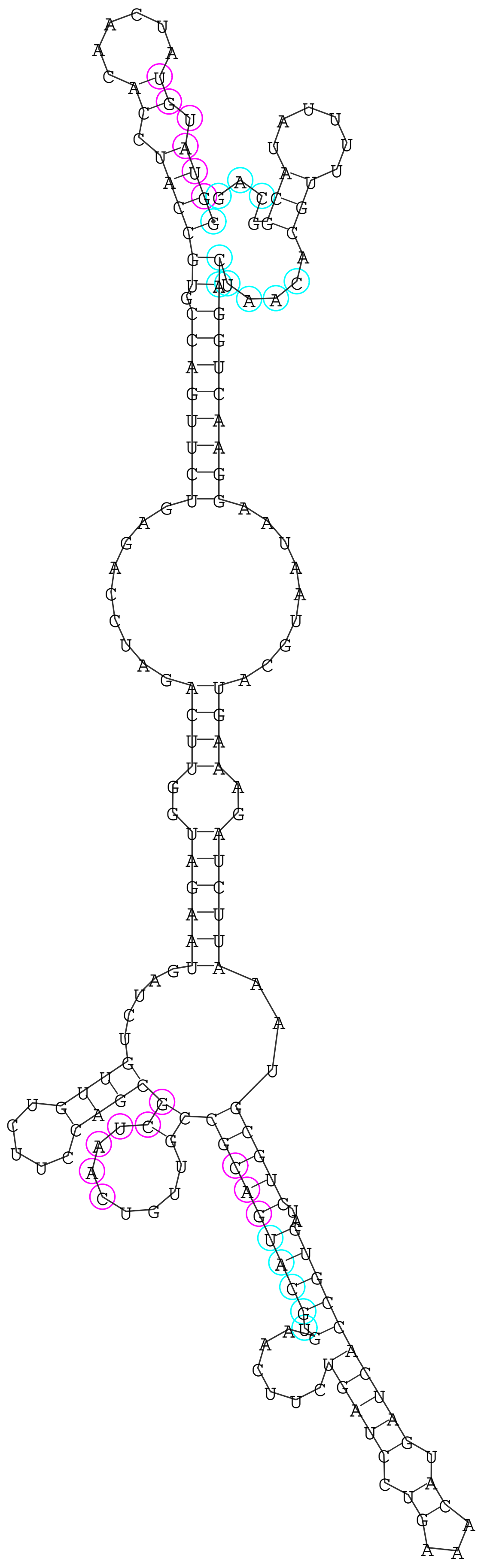

Xbamc236C - Stwintron

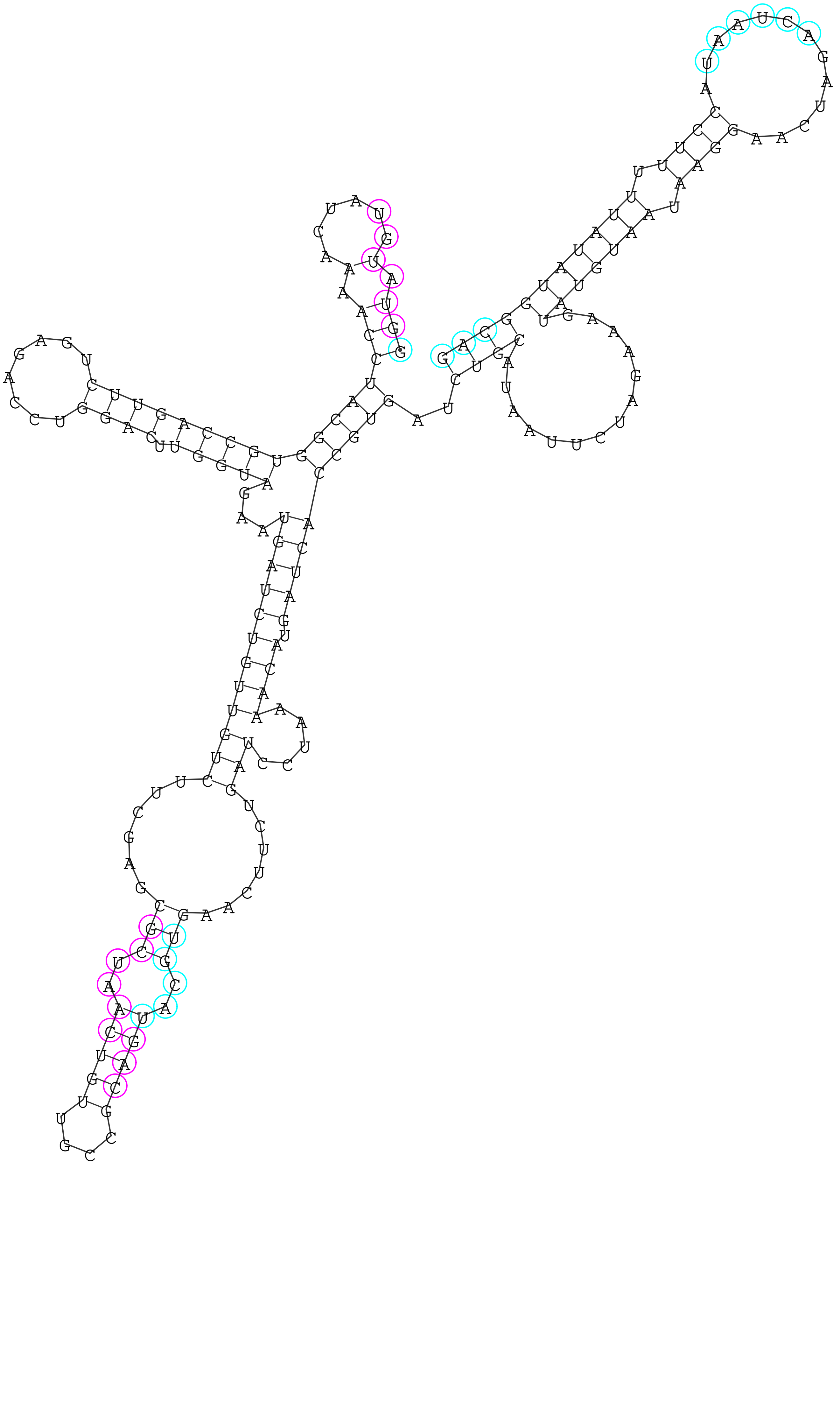

Xbccc01A - Stwintron

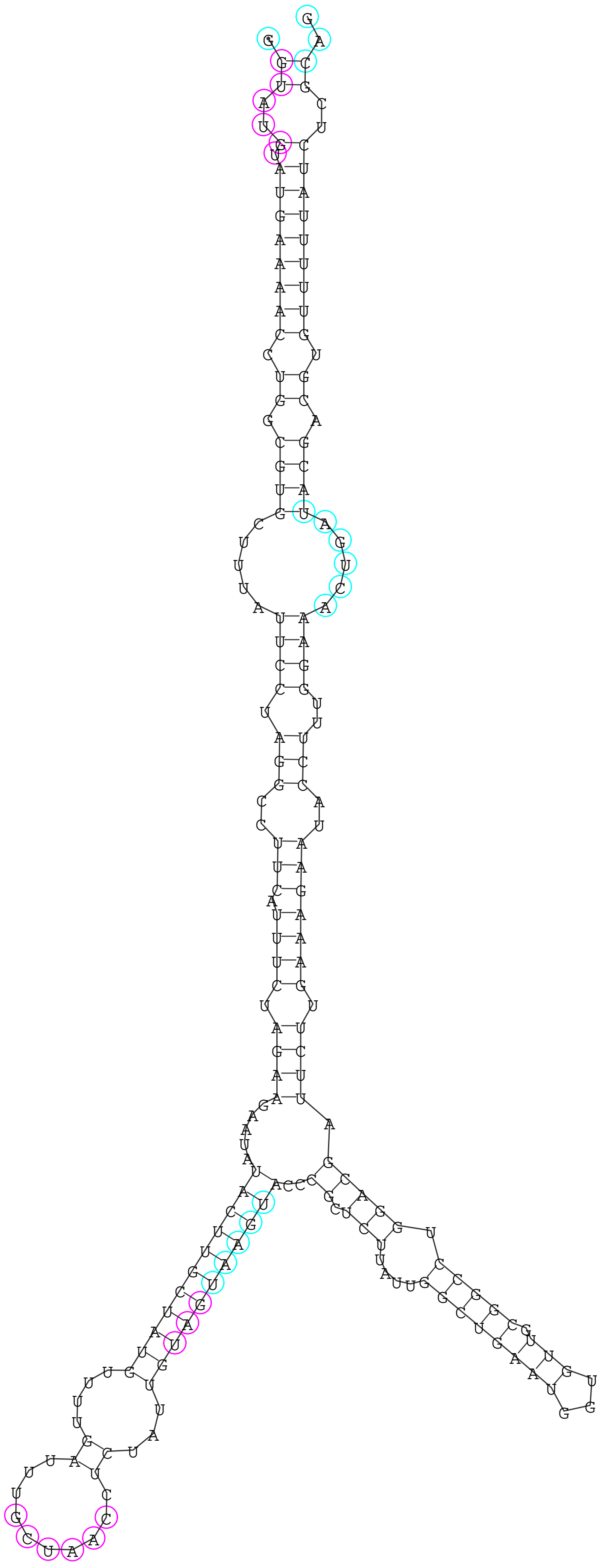

Xbccc01B - Stwintron

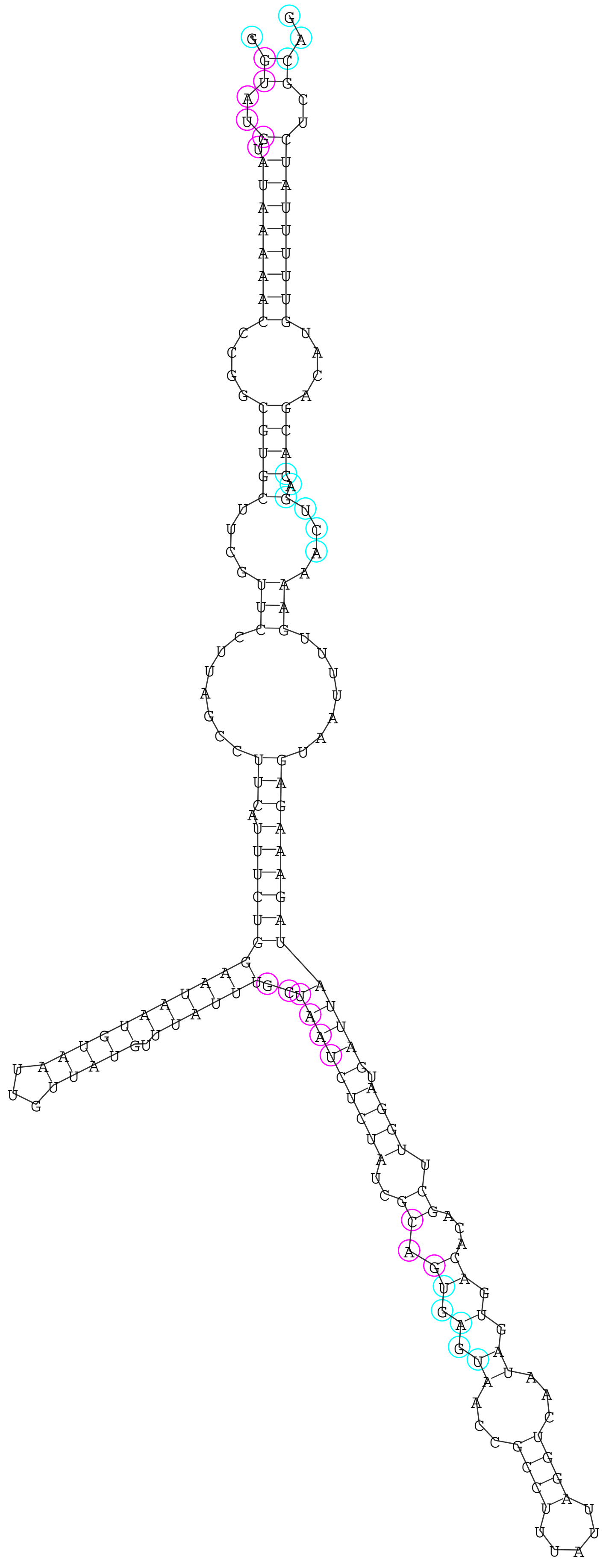

Xbccc05A - Stwintron

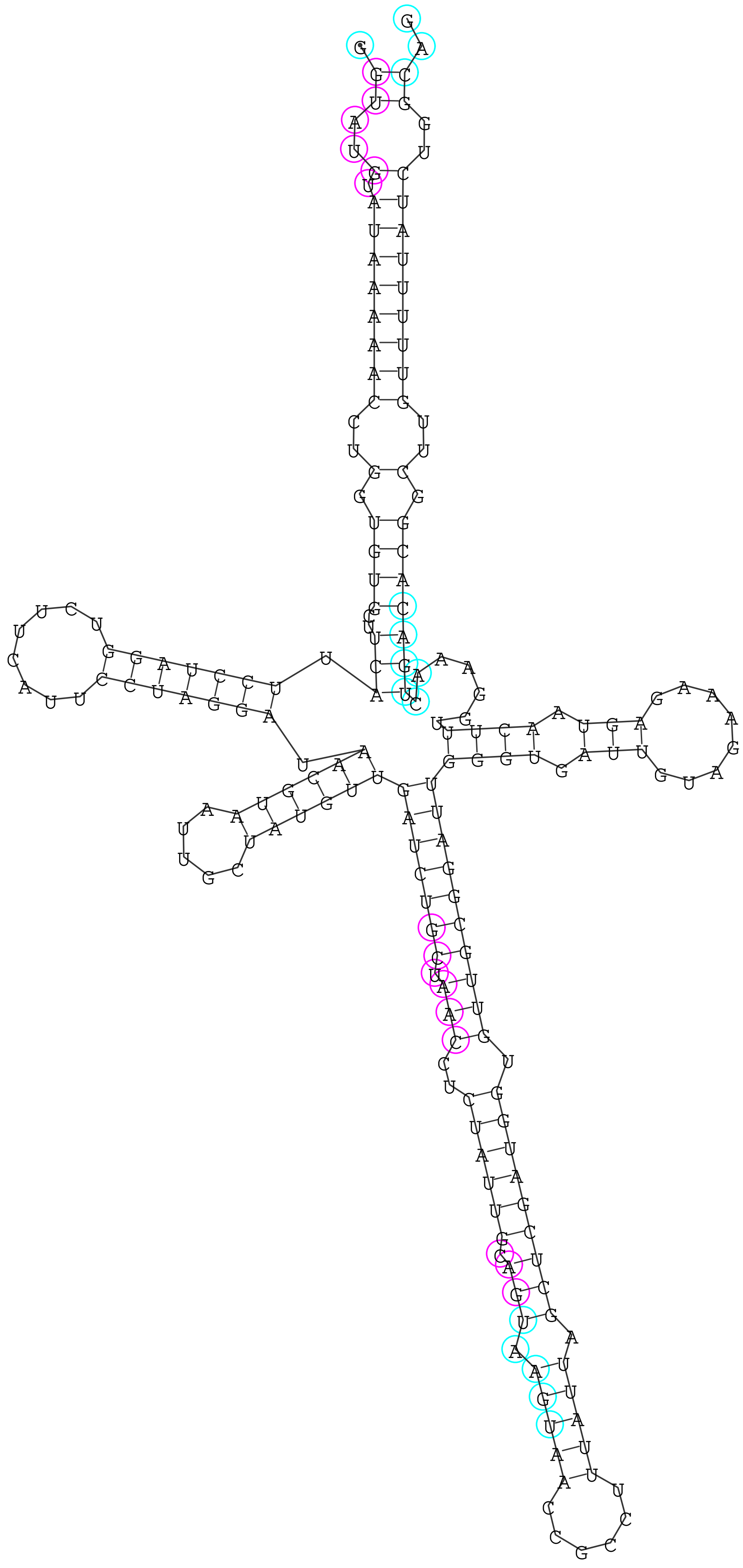

Xbccc05B - Stwintron

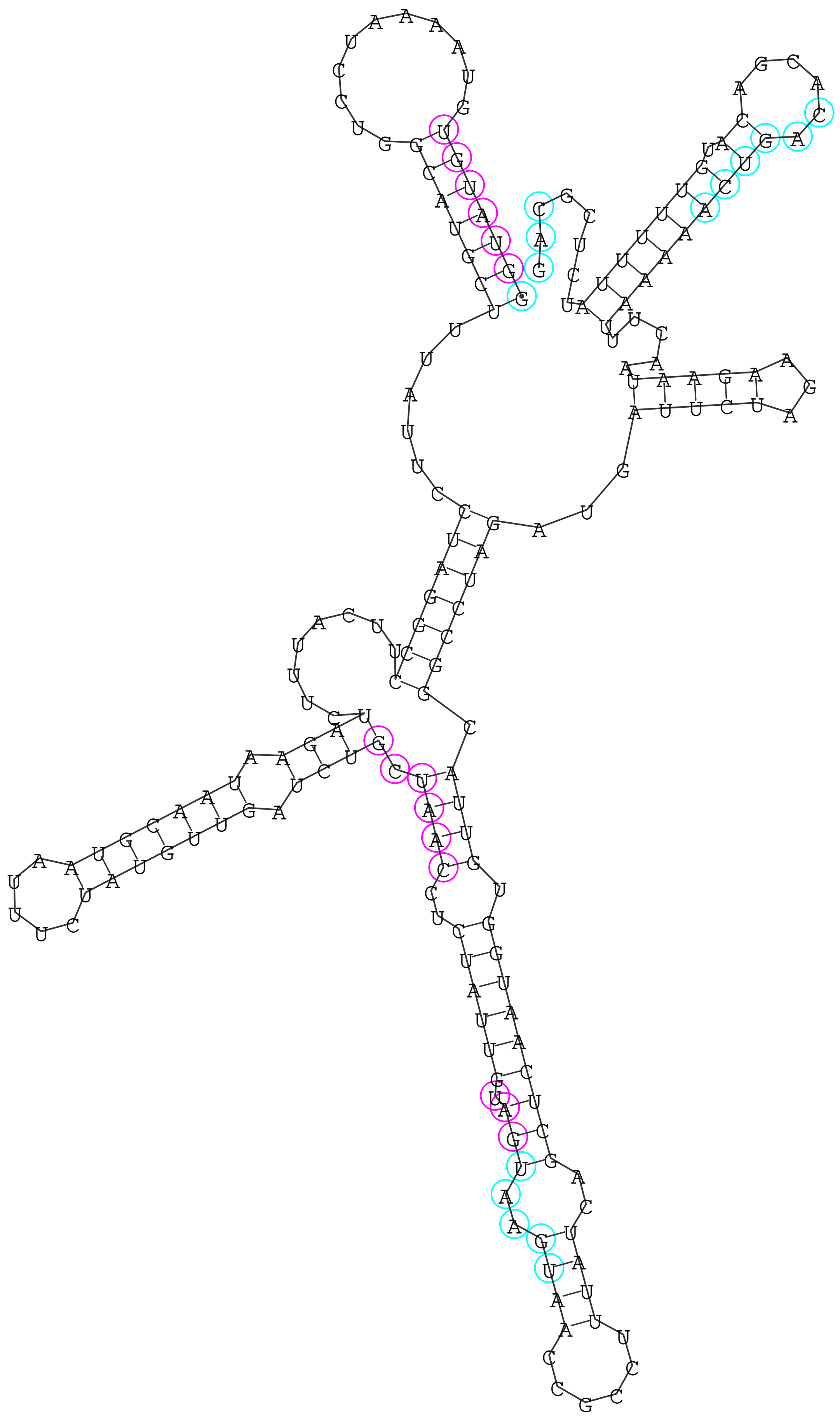

Xbccc05C - Stwintron

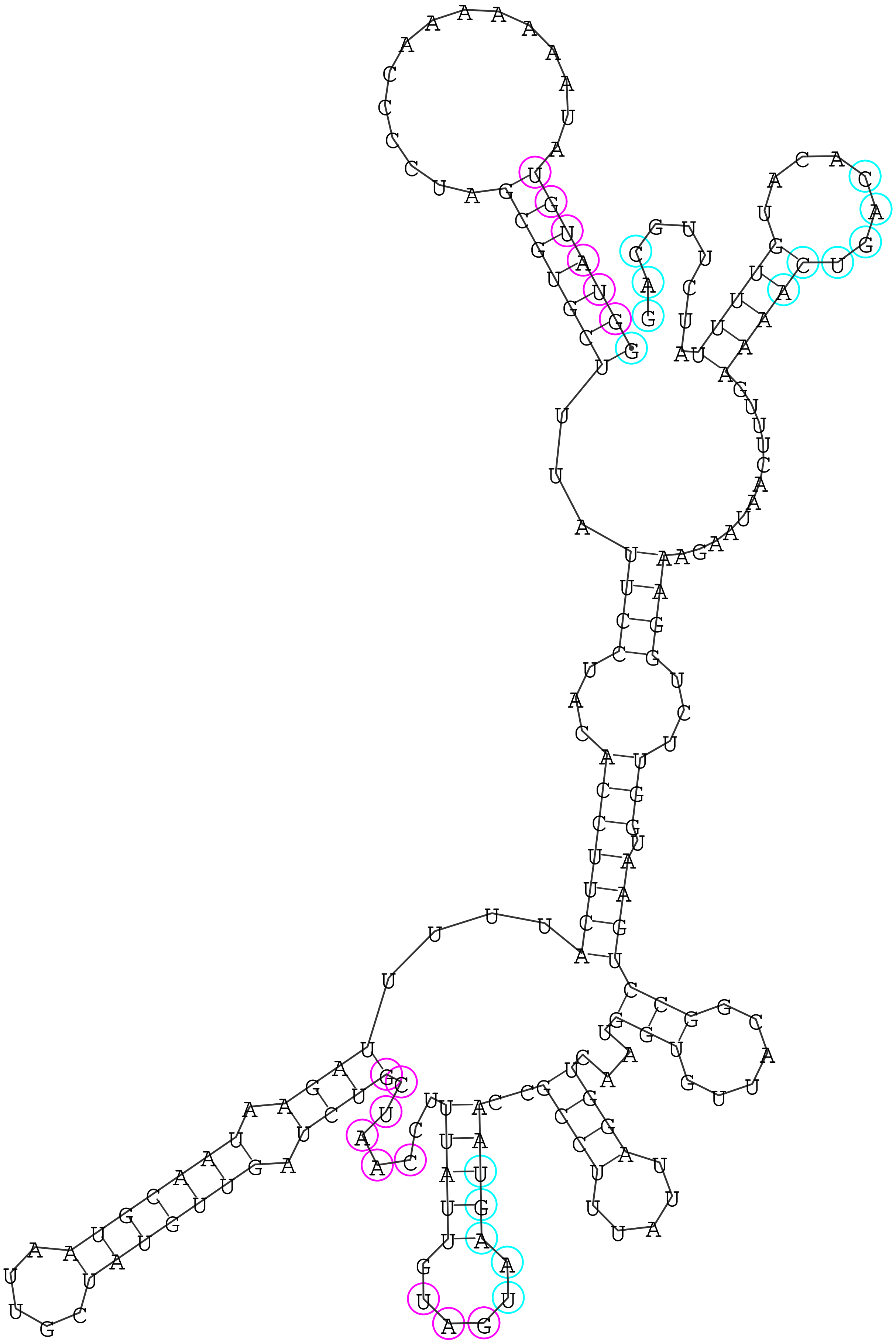

Xbccc06A - Stwintron

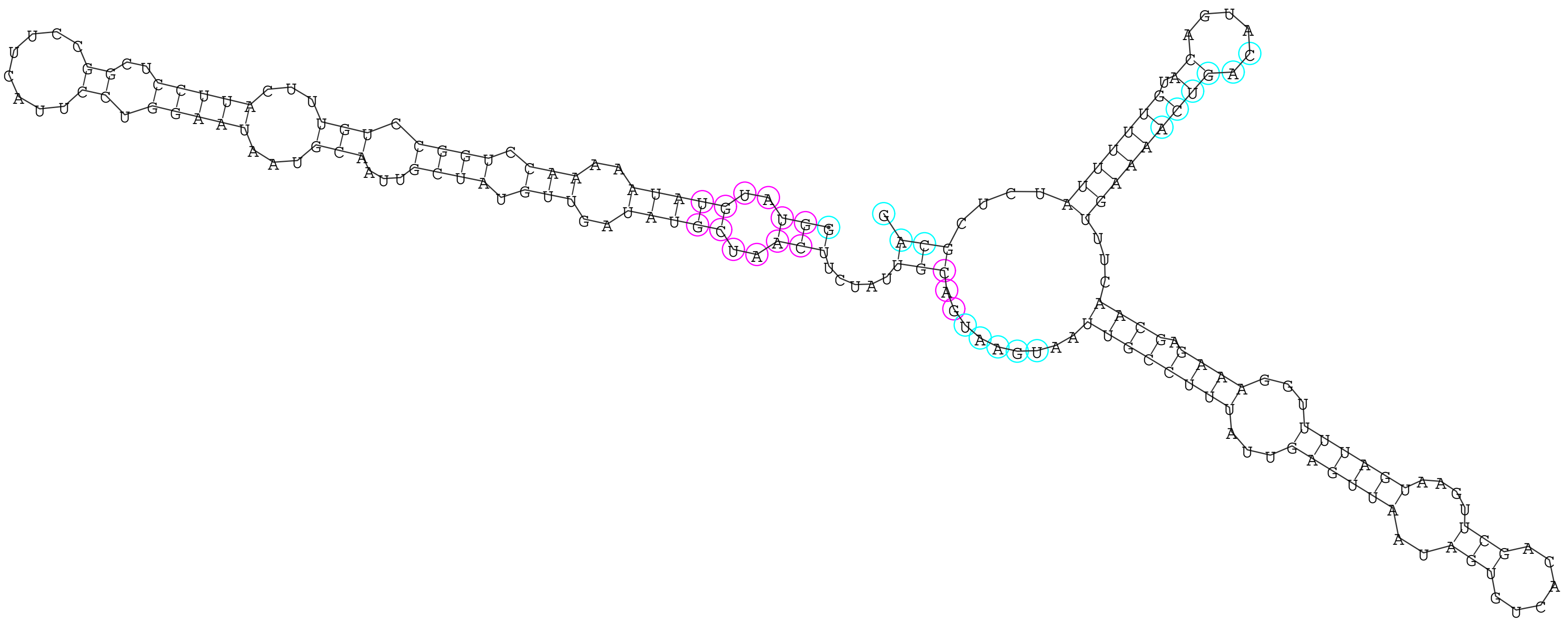

Xbccc07A - Stwintron

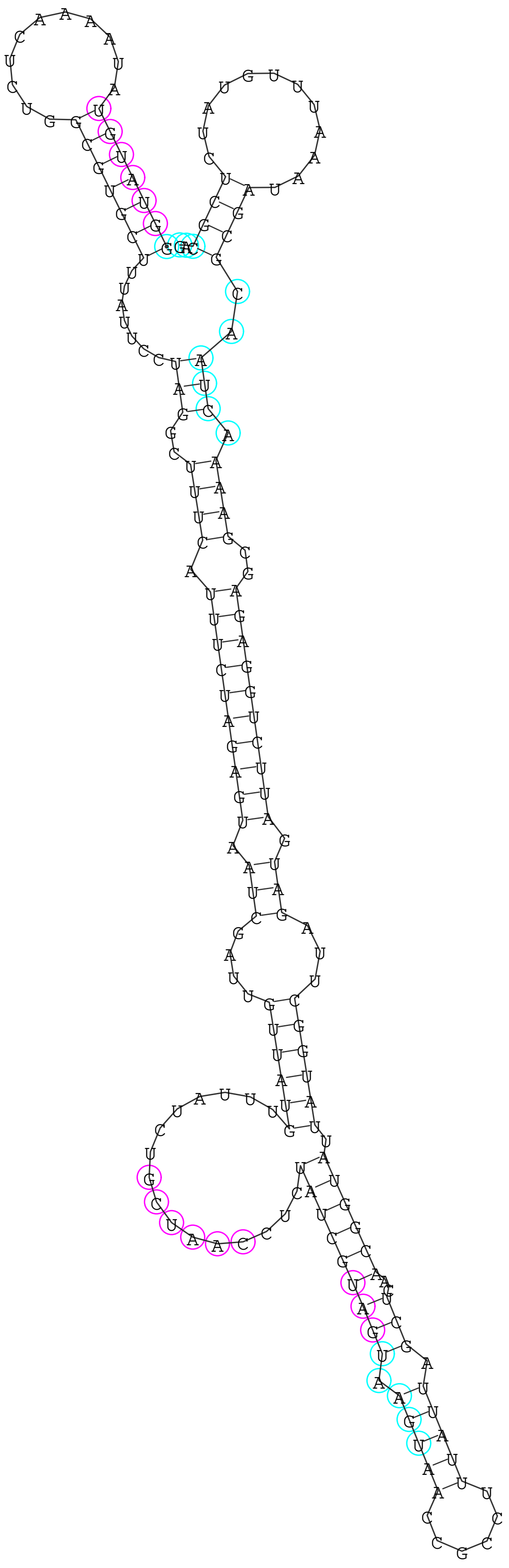

Xbccc09A - Stwintron

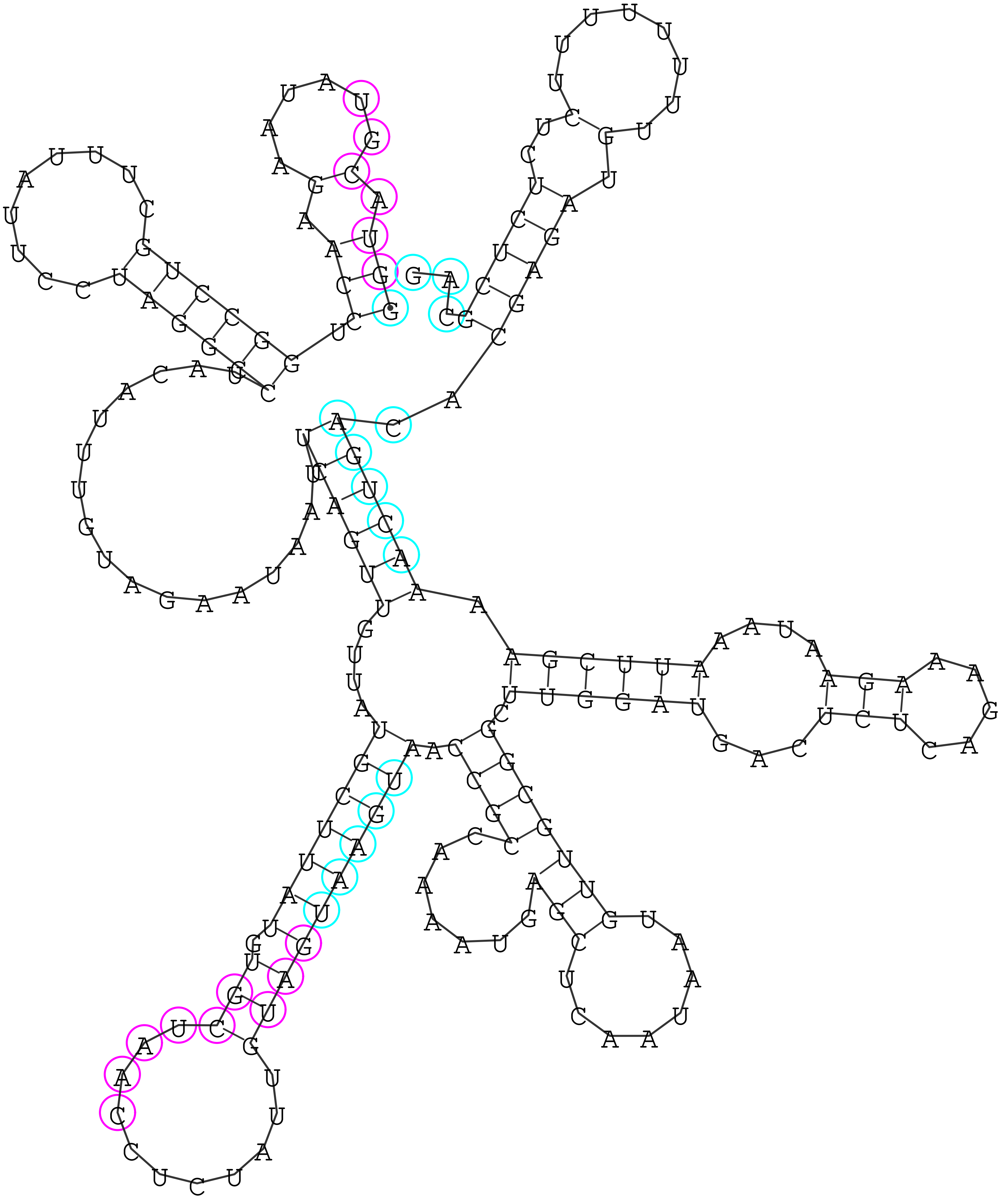

Xlonc0002A - Stwintron

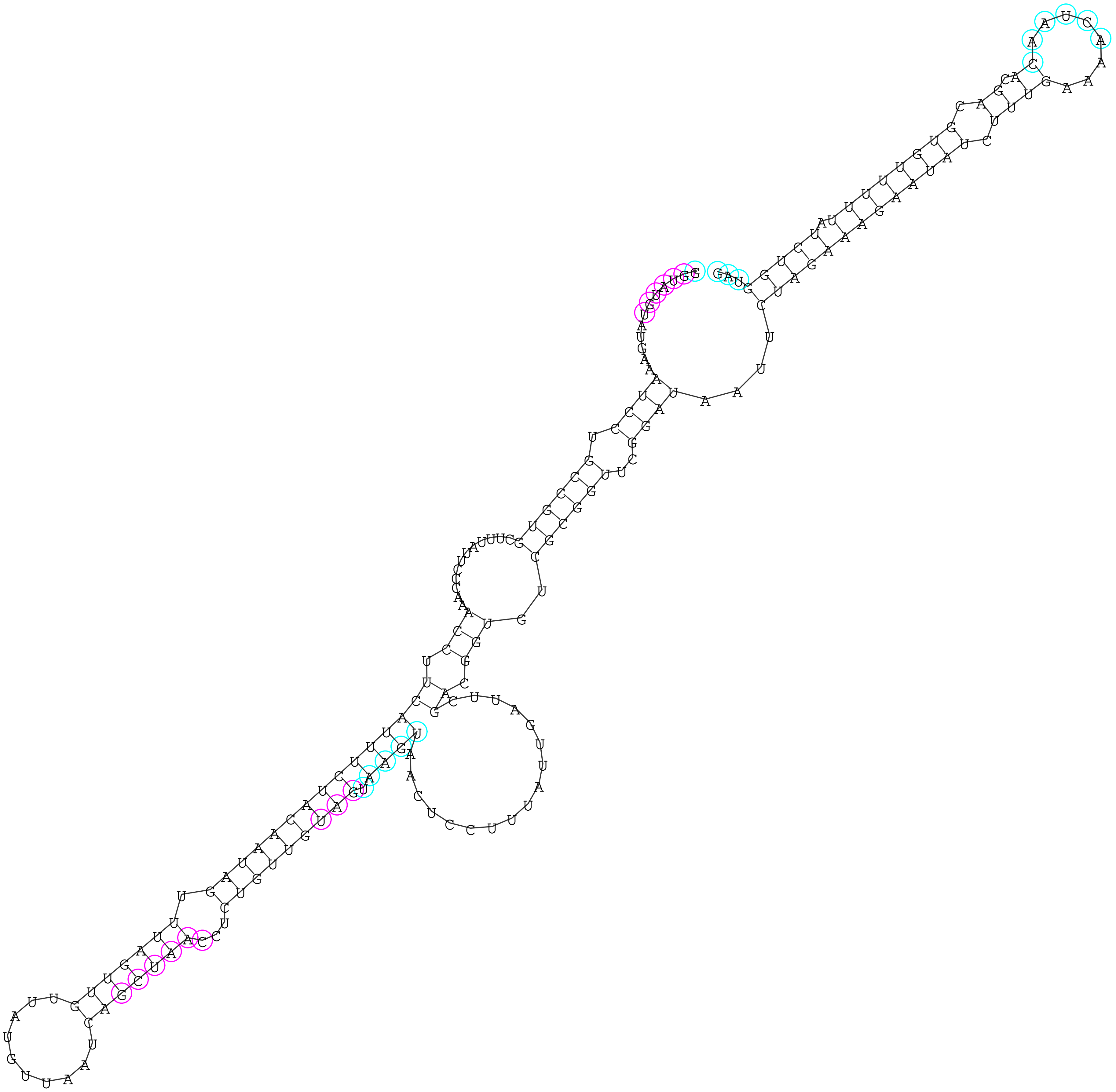

Xlonc0025A - Stwintron

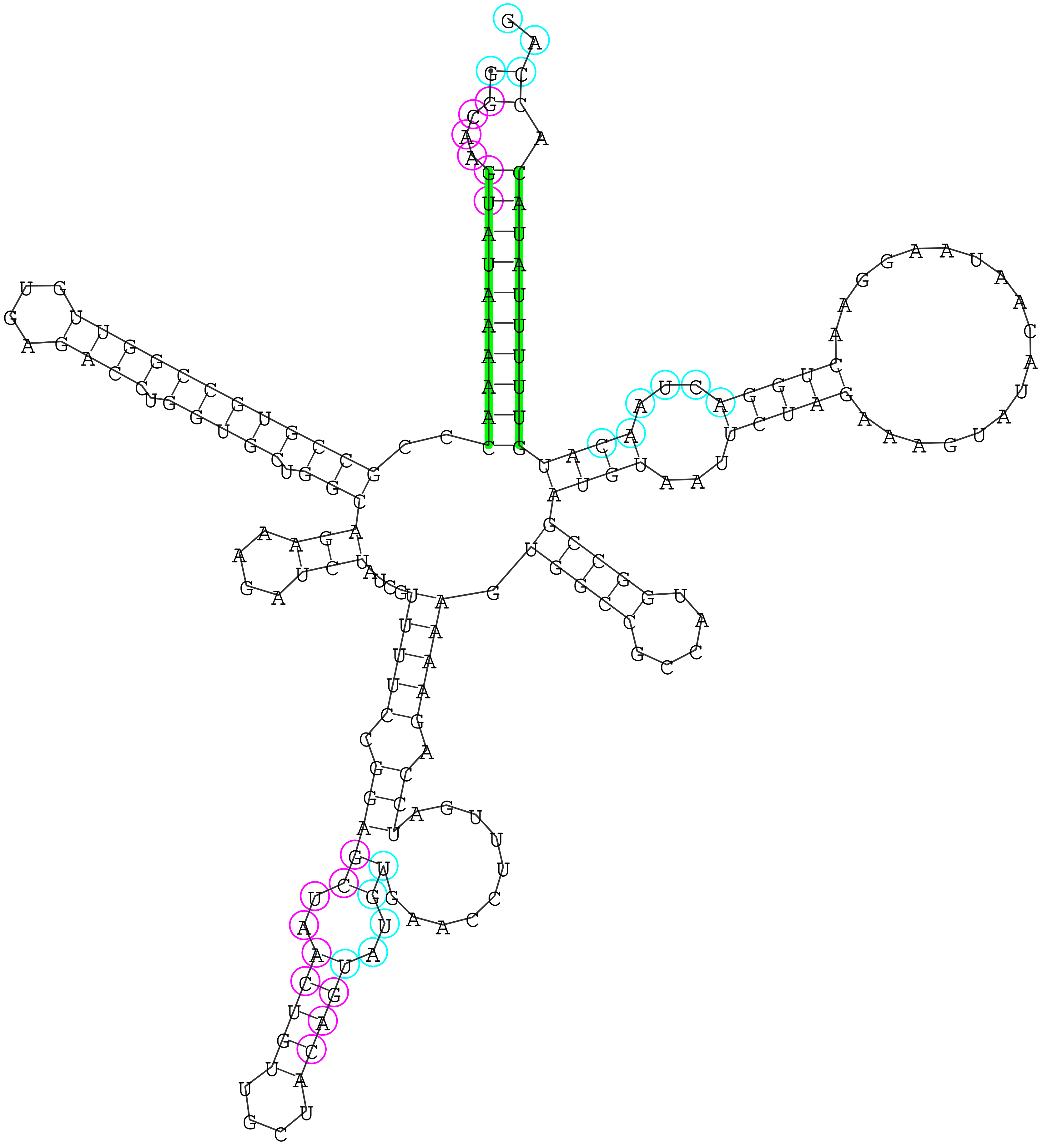

Xlonc0058A - Stwintron

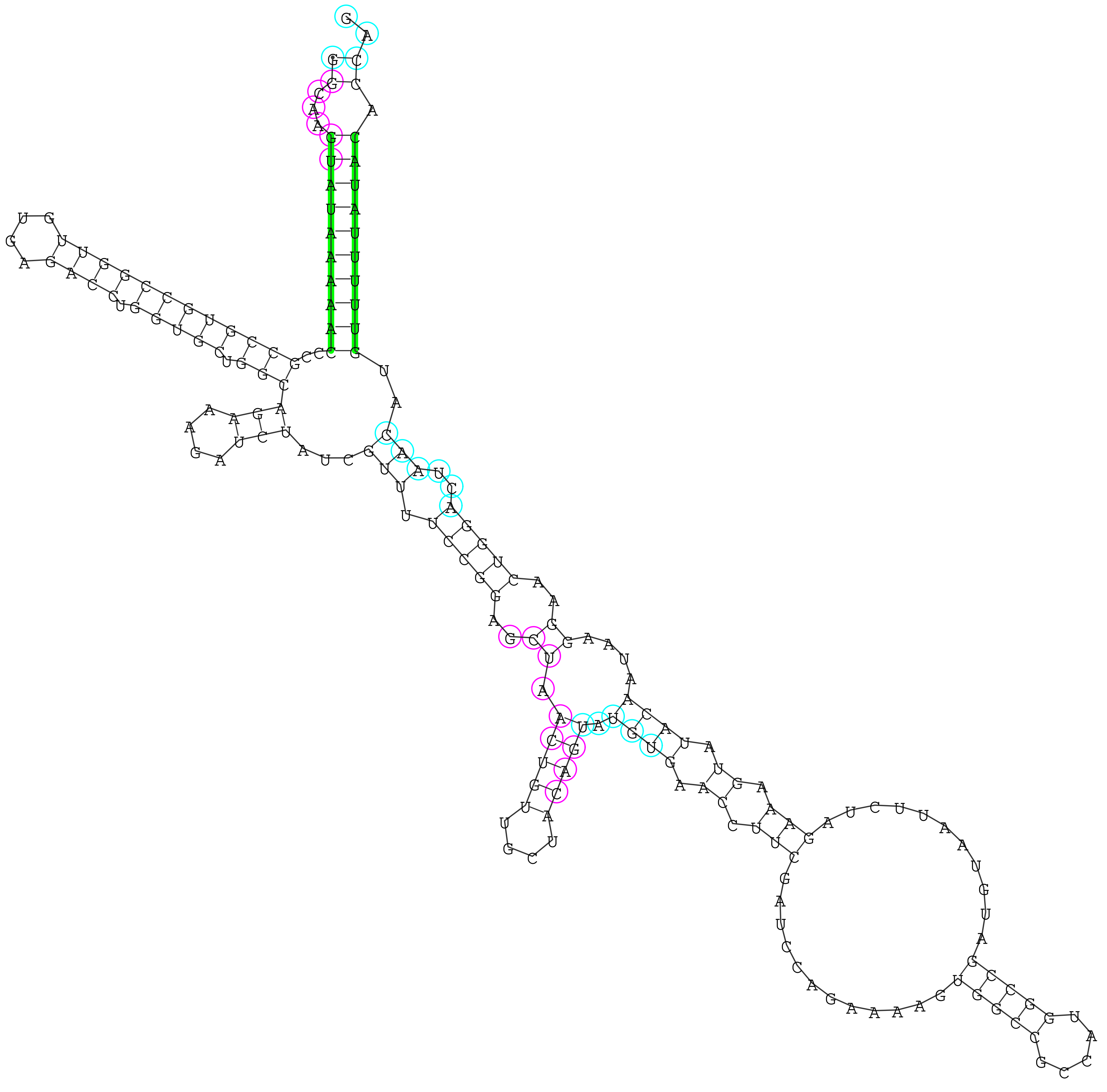

Xlonc0112A - Stwintron

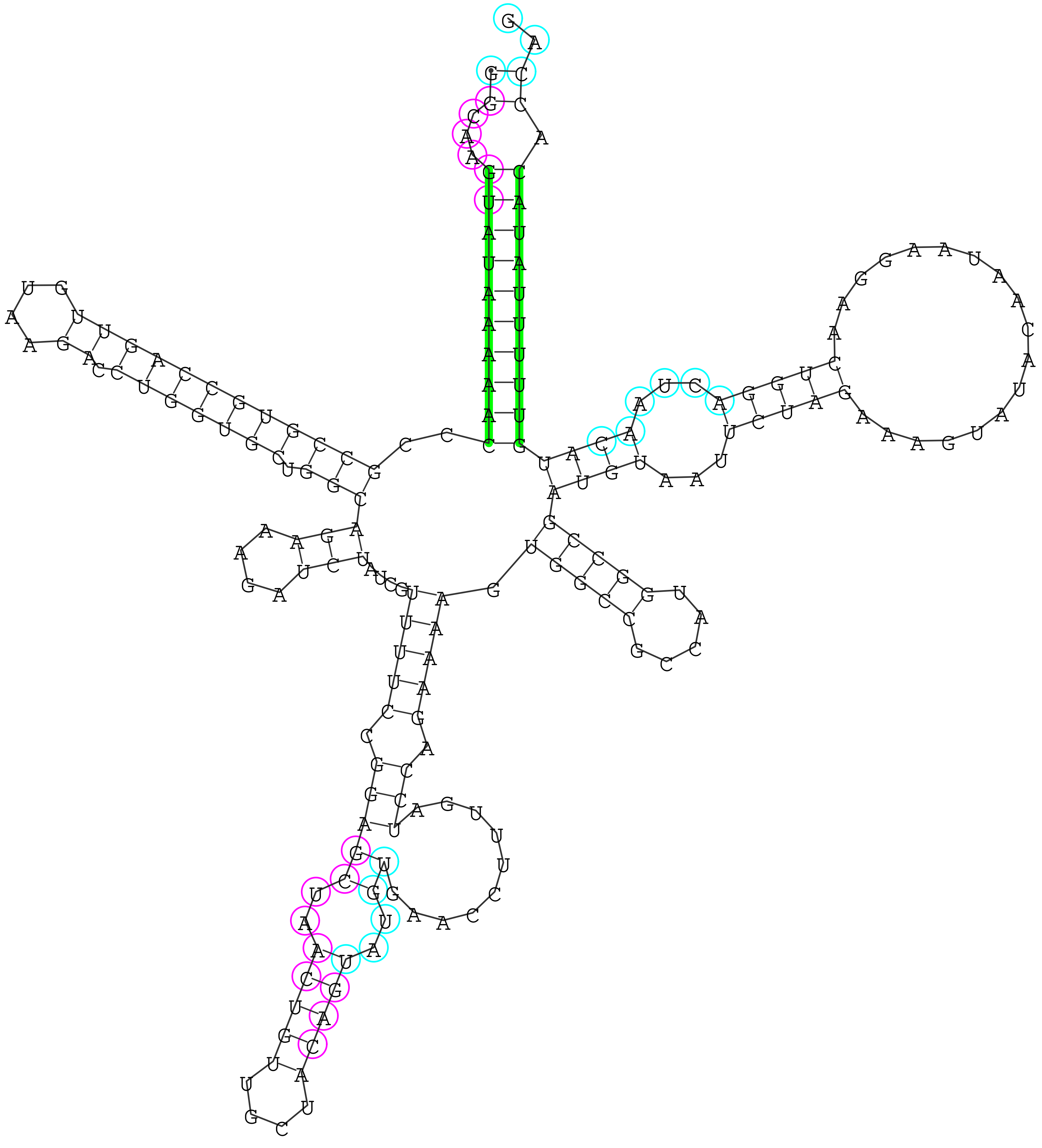

Xmsuc0005A - Stwintron

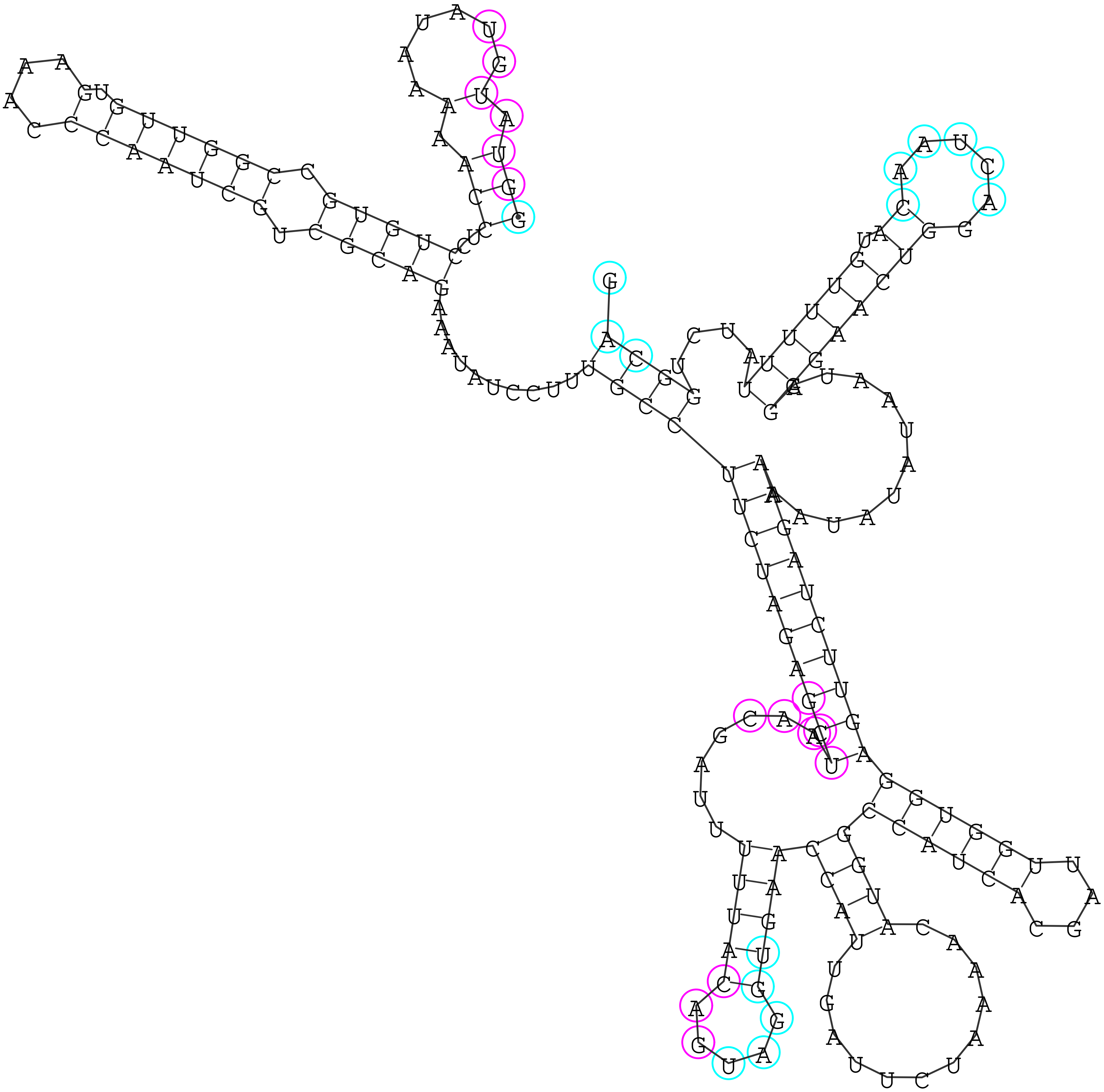

Xmsuc0006A - Stwintron

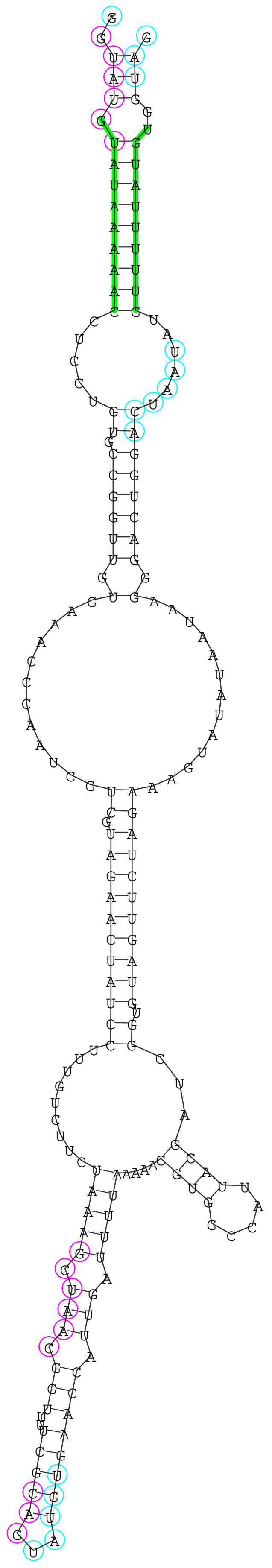

Xmsuc0006B - Stwintron

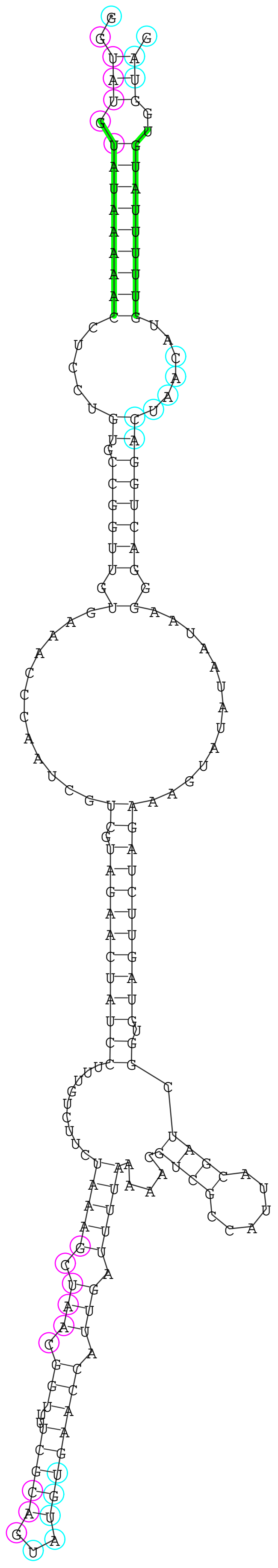

Xmsuc0009A - Stwintron

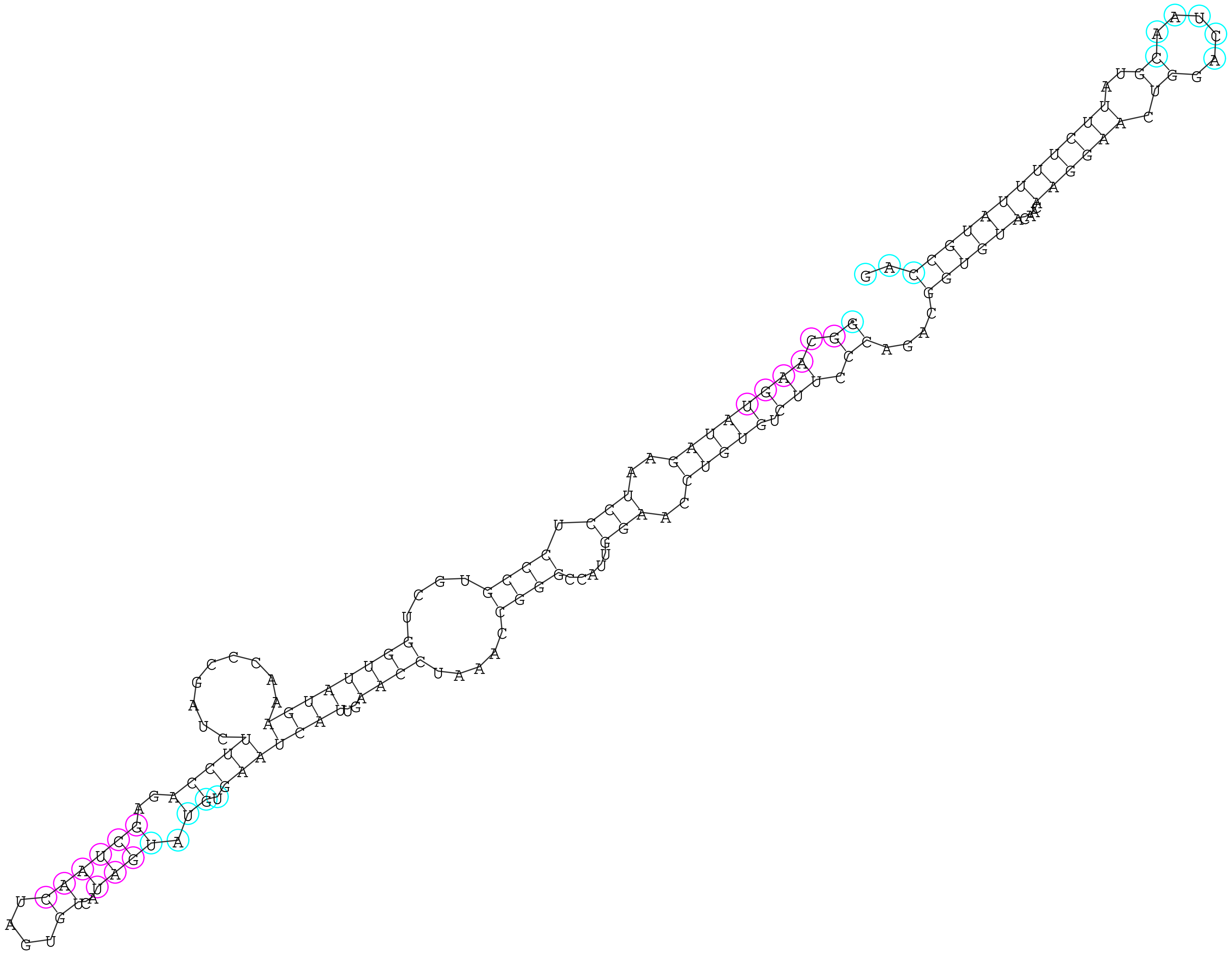

Xmsuc0018A - Stwintron

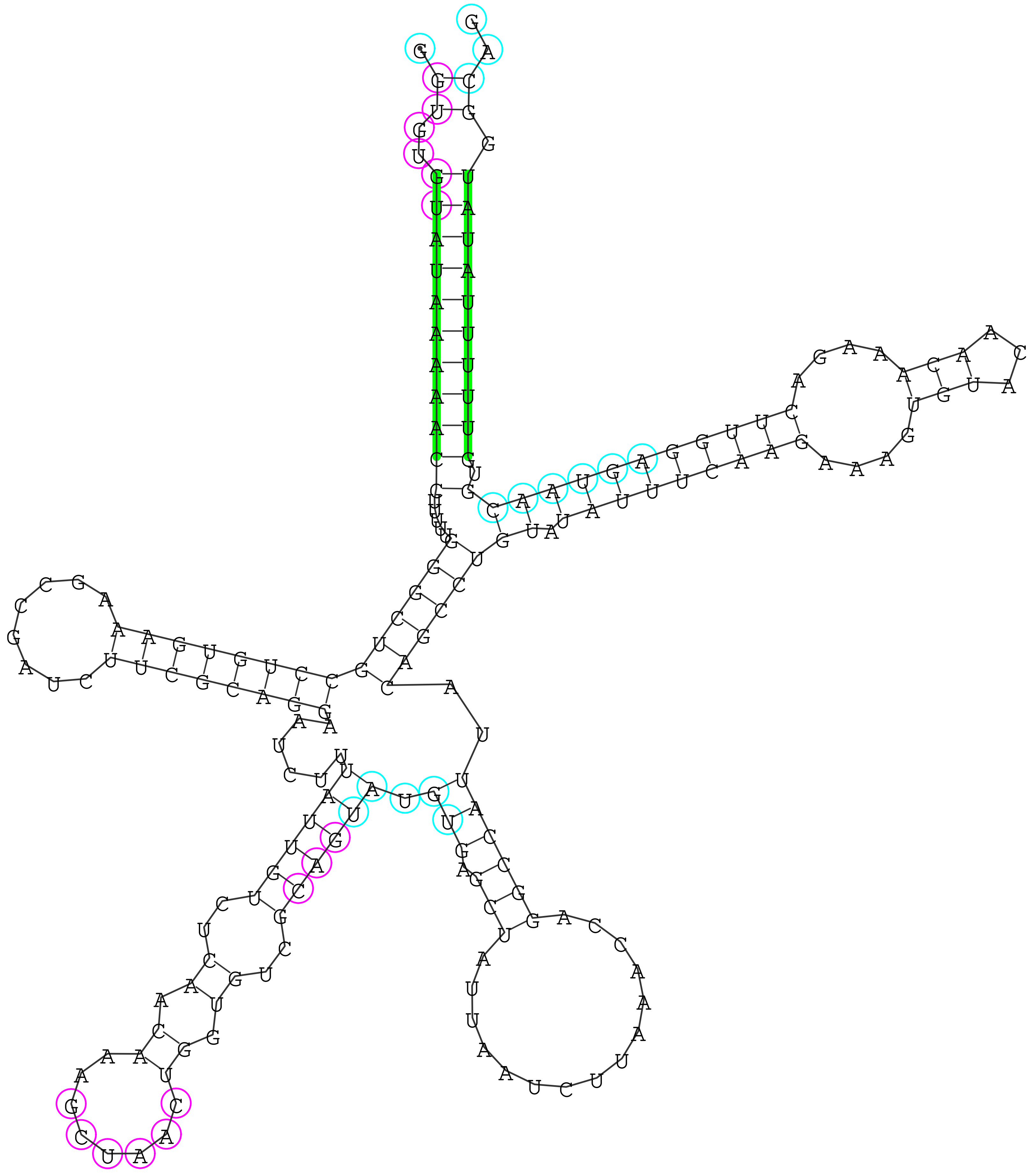

Xmsuc0018B - Stwintron

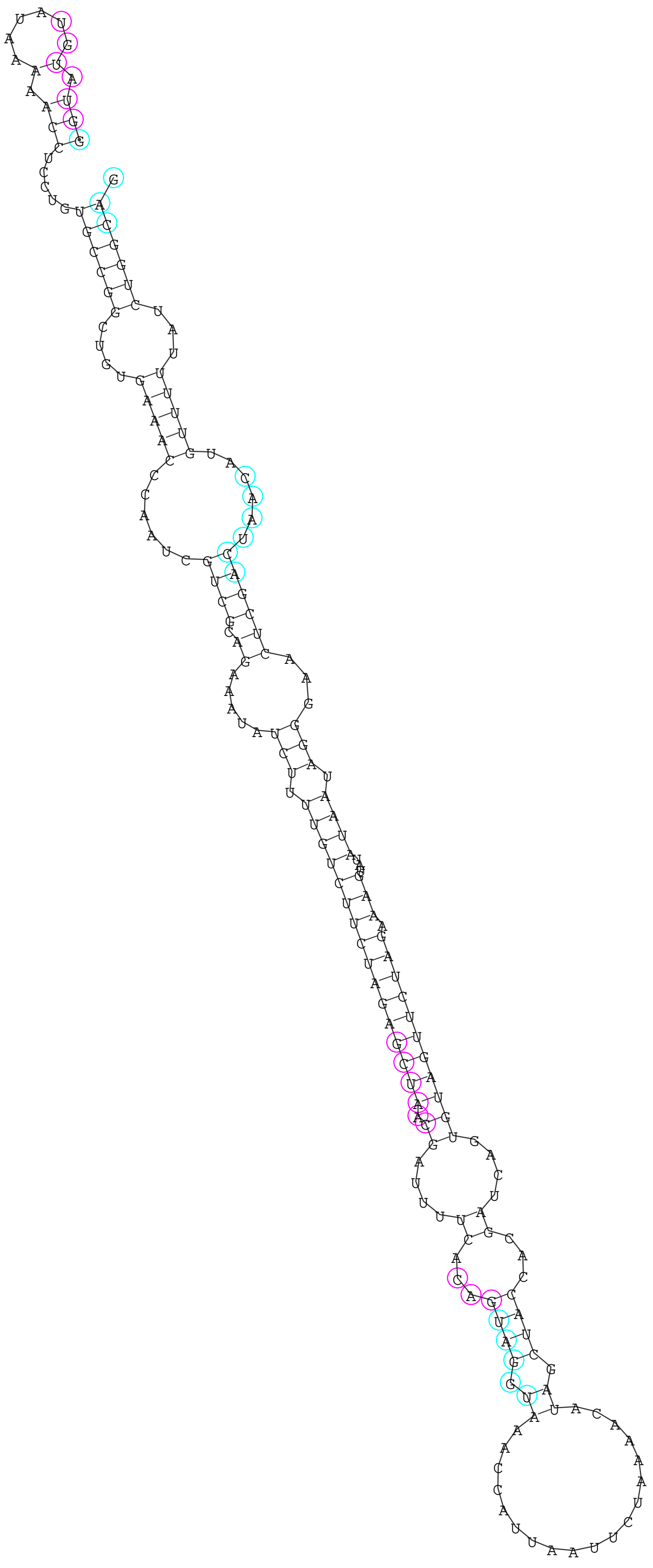

Xmsuc0019A - Stwintron

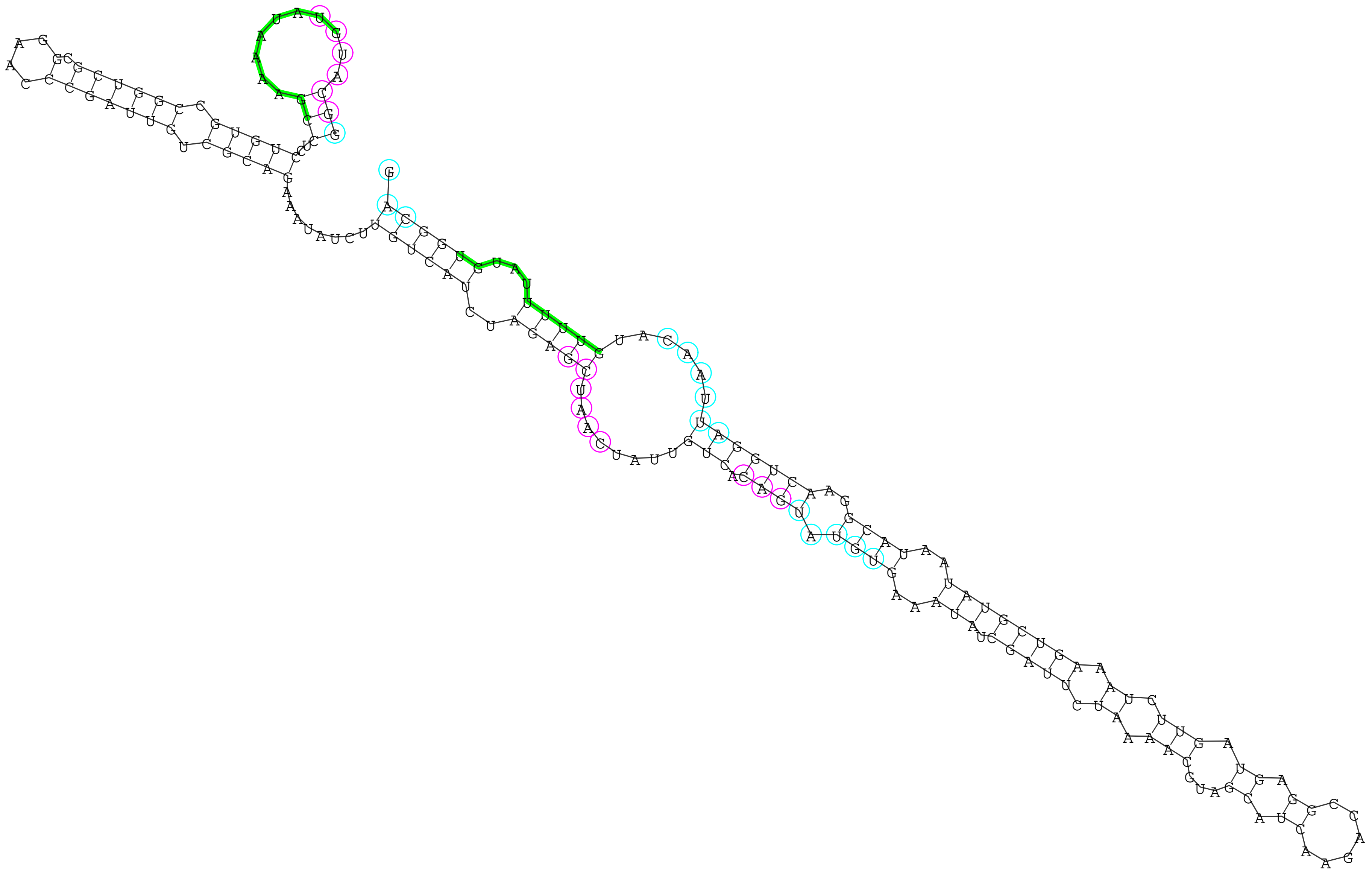

Xmsuc0028A - Stwintron

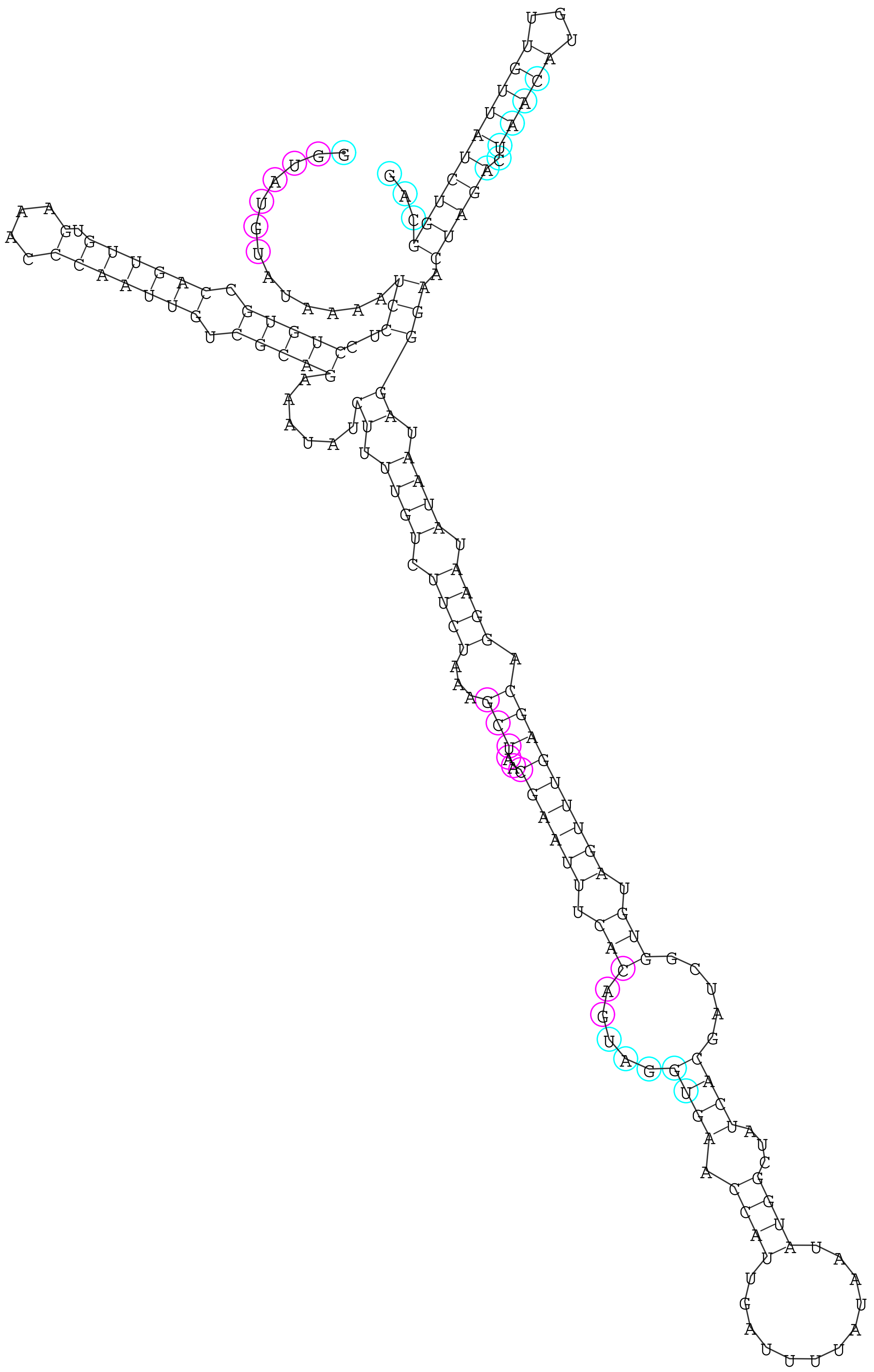

Xmsuc0031A - Stwintron

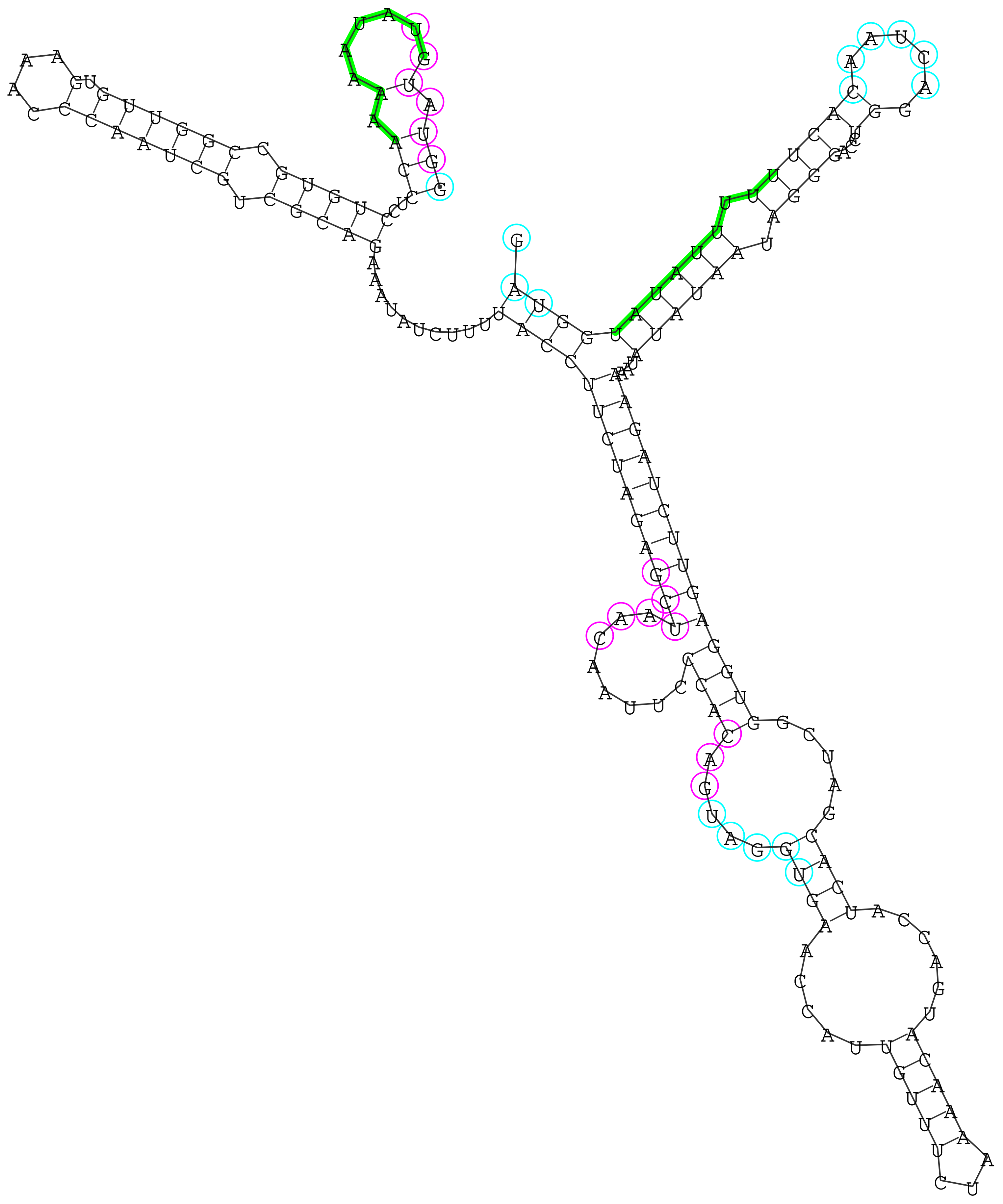

Xmsuc0045A - Stwintron

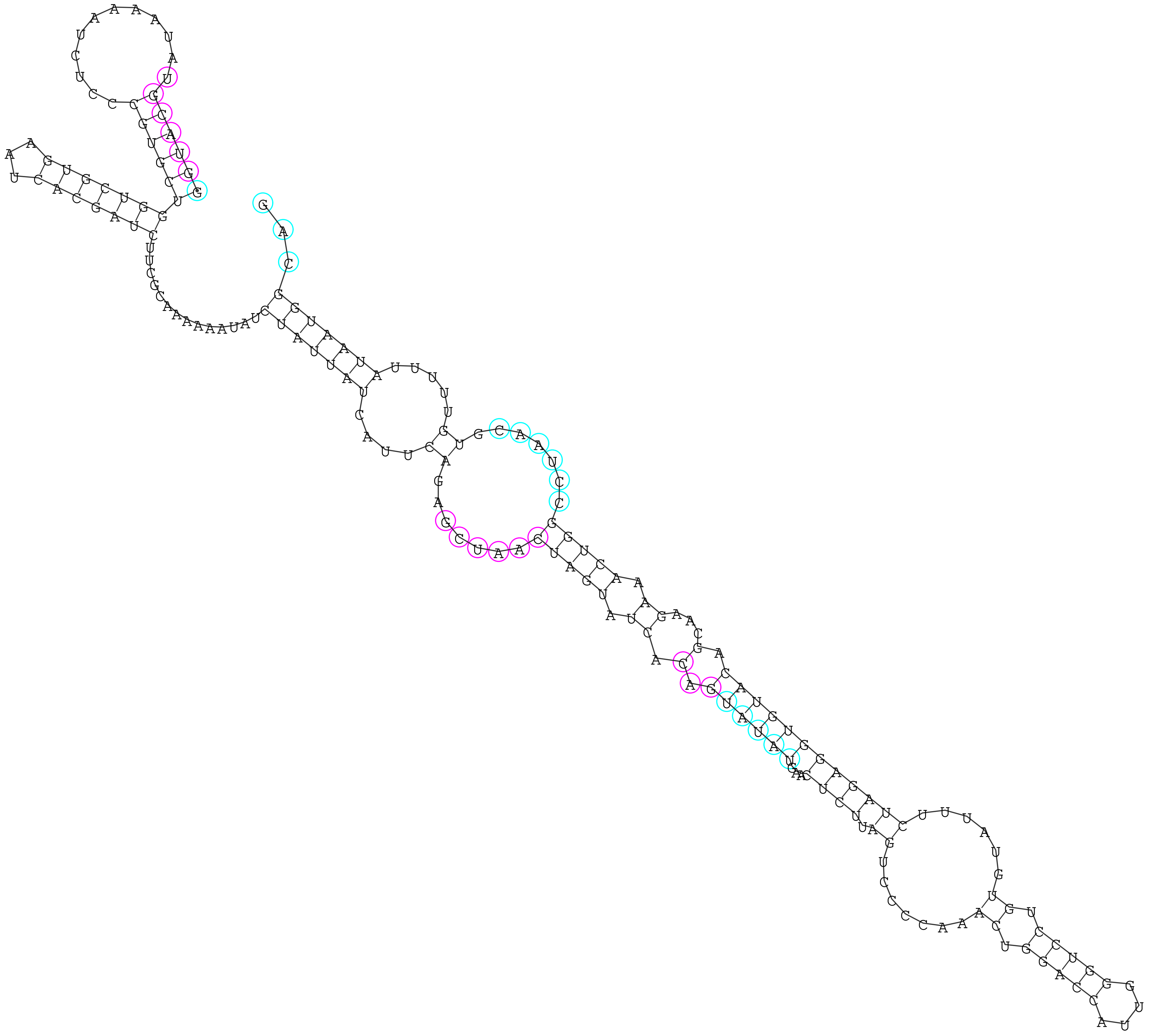

Xmsuc0067A - Stwintron

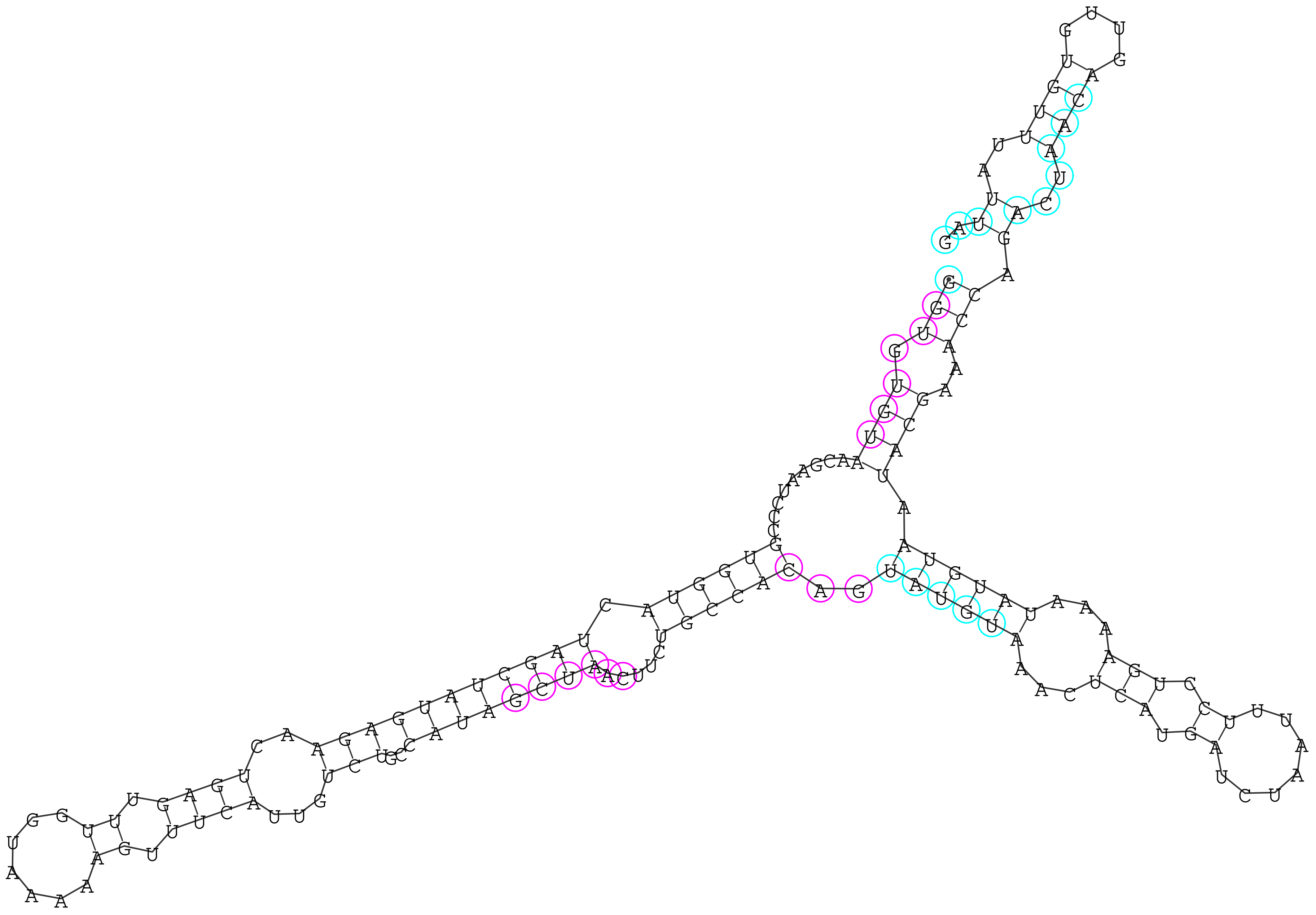

Xmsuc0070A - Stwintron

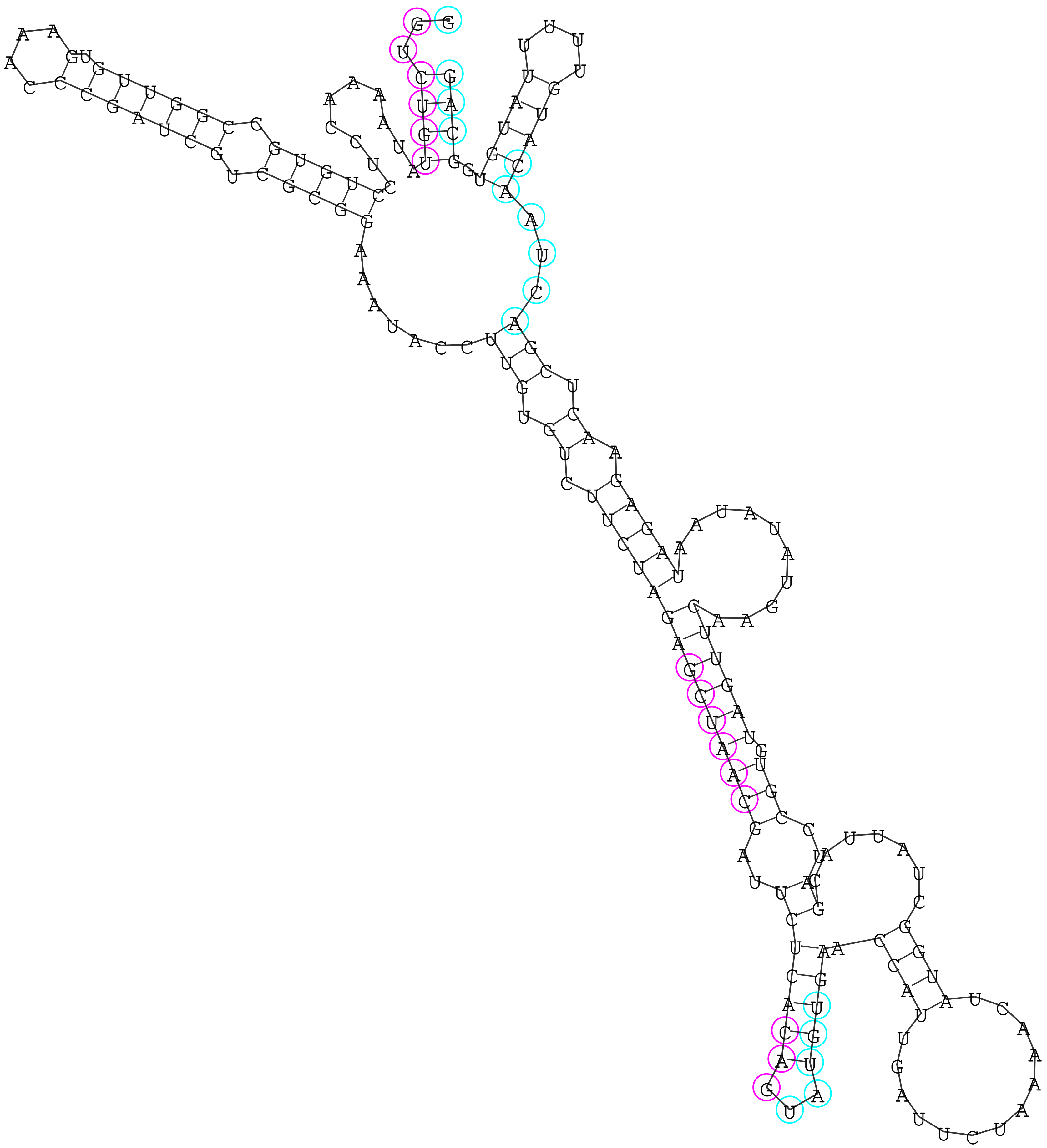

Xmsuc0075A - Stwintron

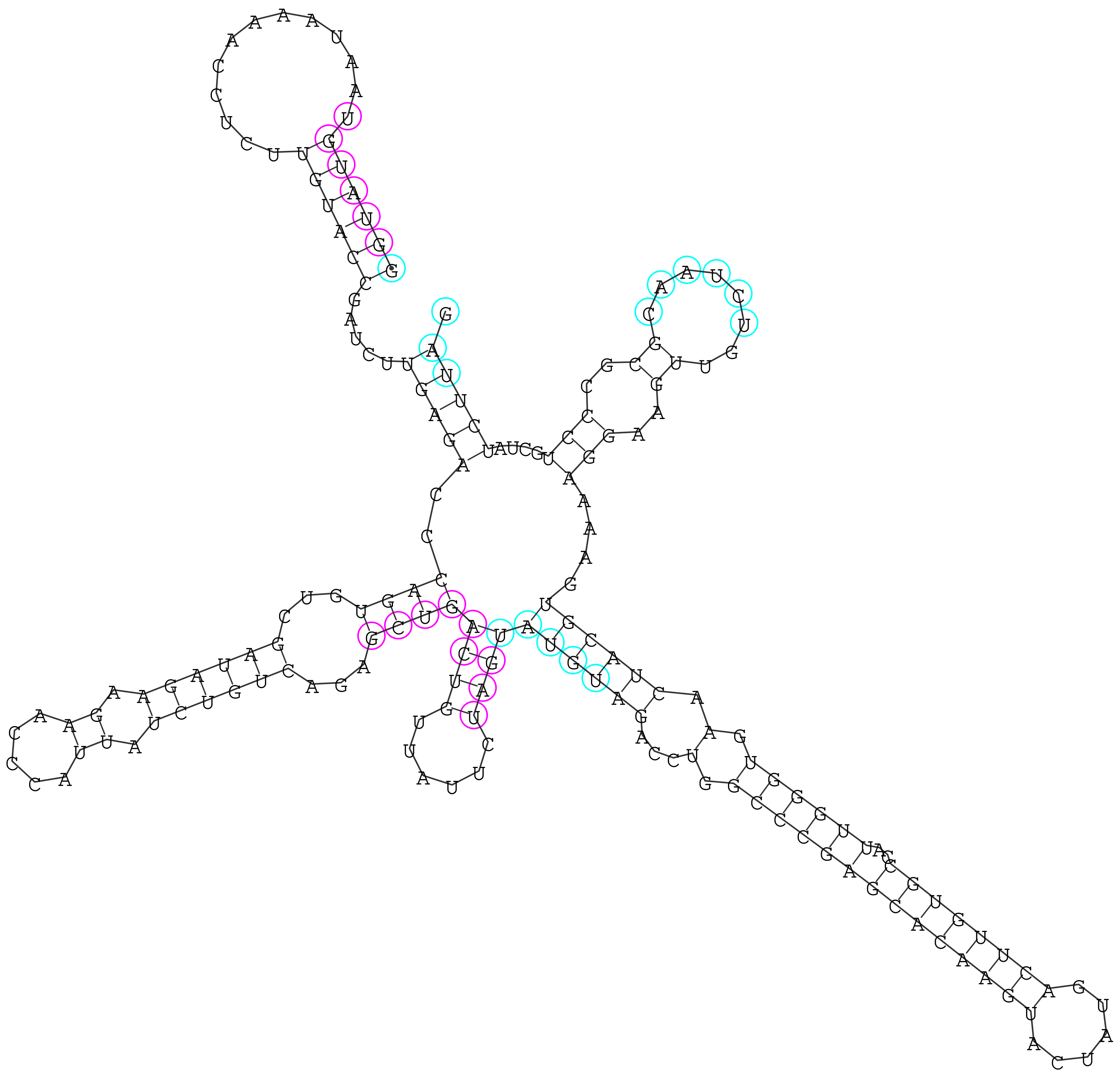

Xmsuc0077A - Stwintron

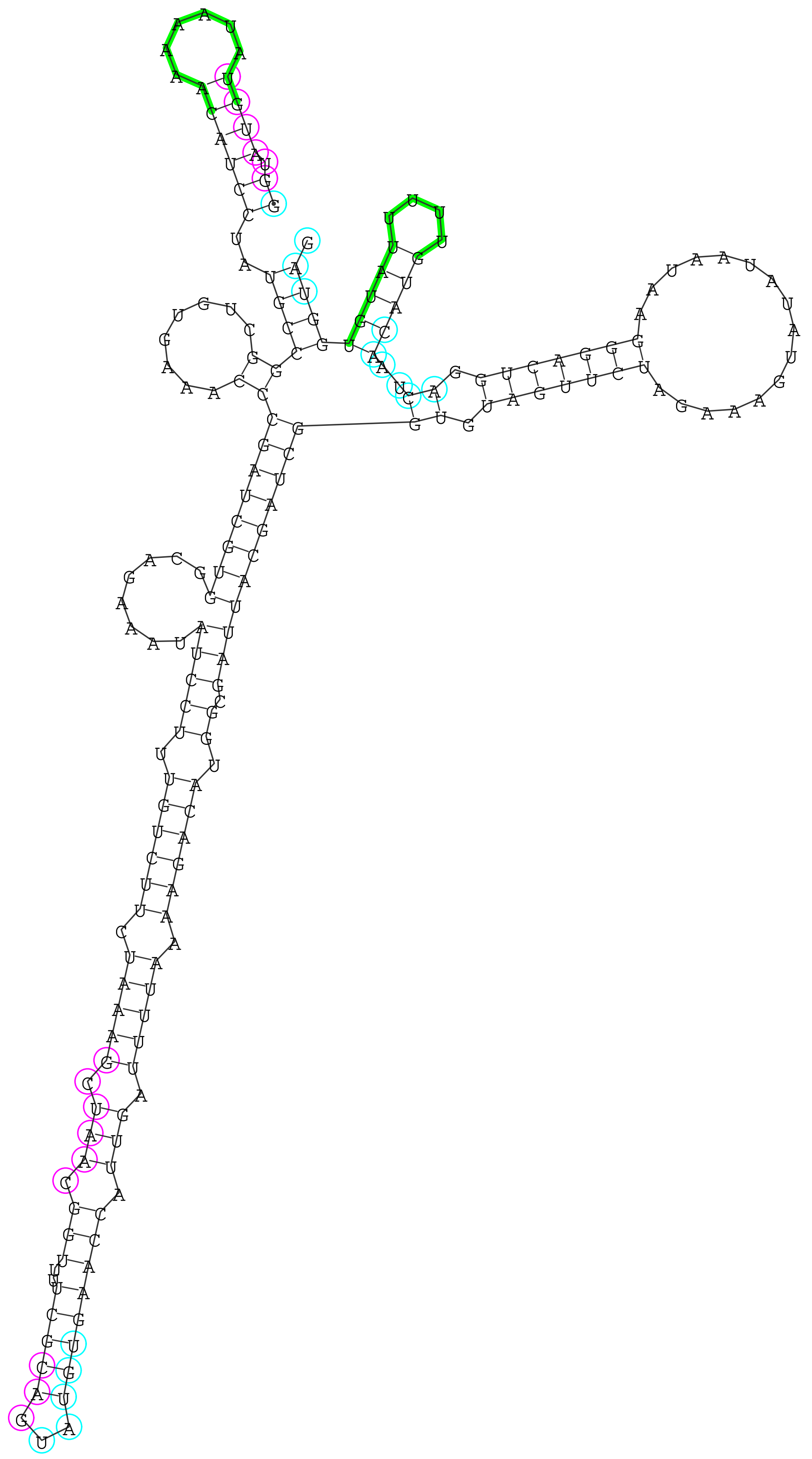

Xmsuc0082A - Stwintron

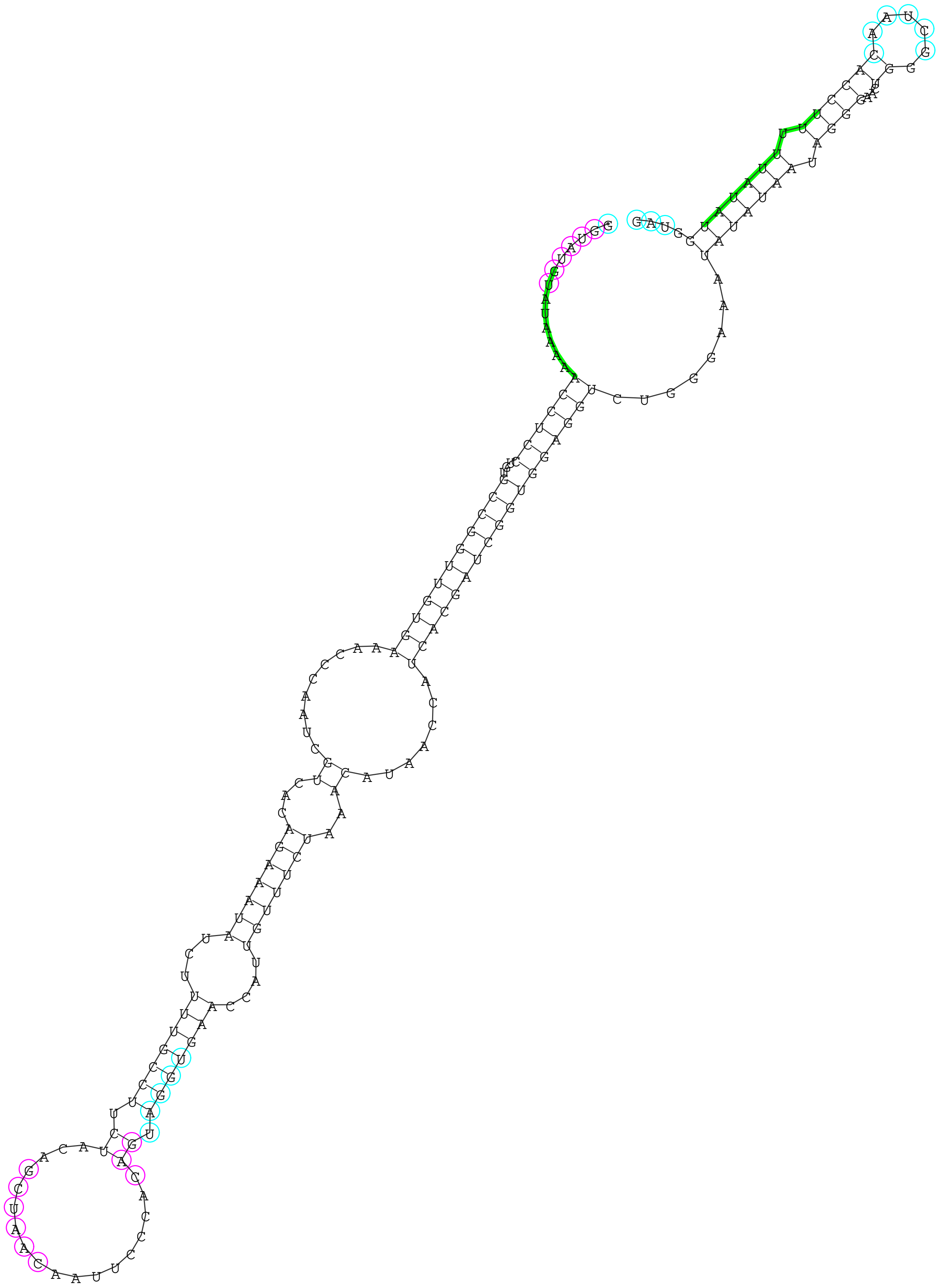

Xmsuc0086A - Stwintron

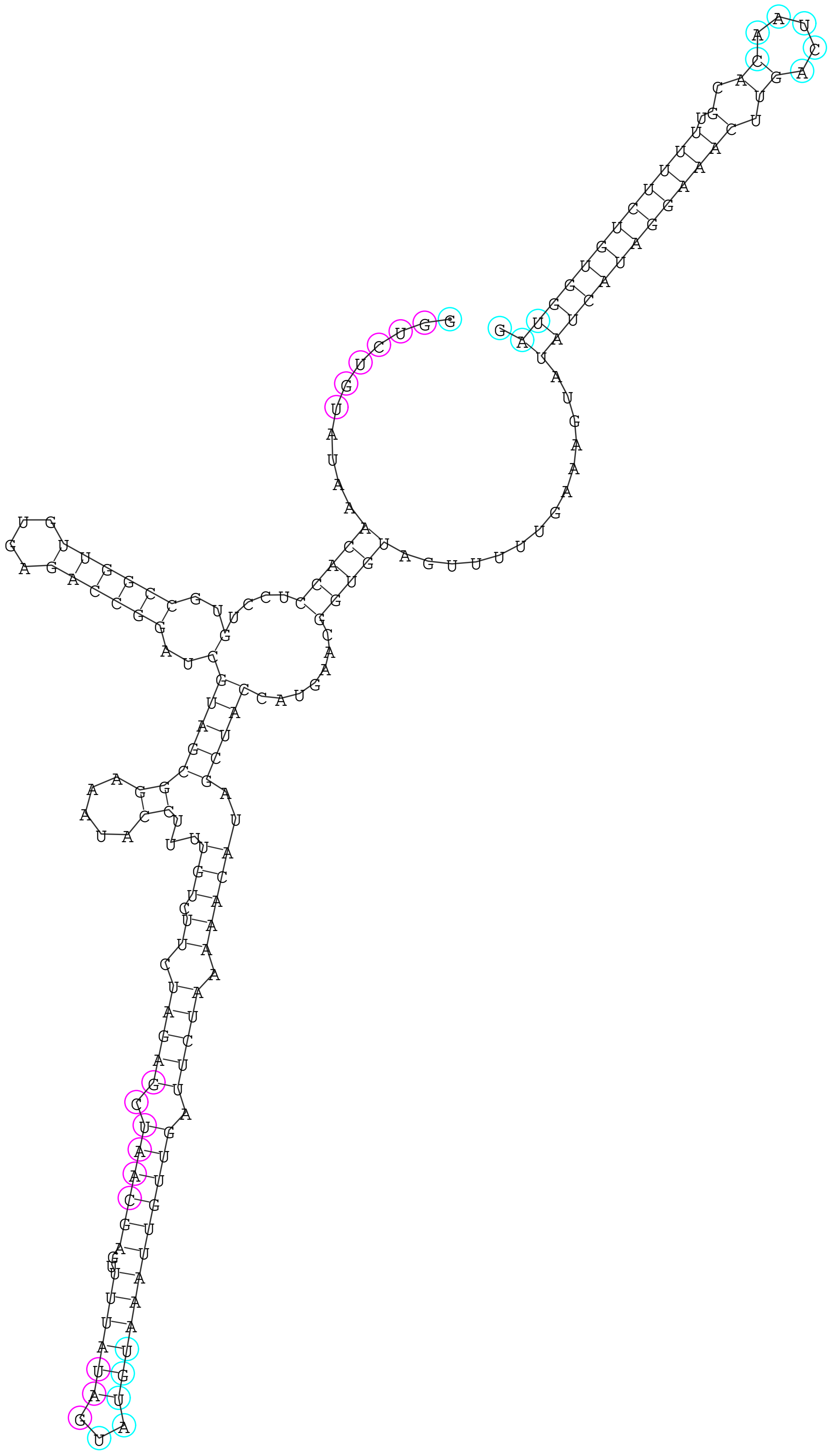

Xmsuc0086B - Stwintron

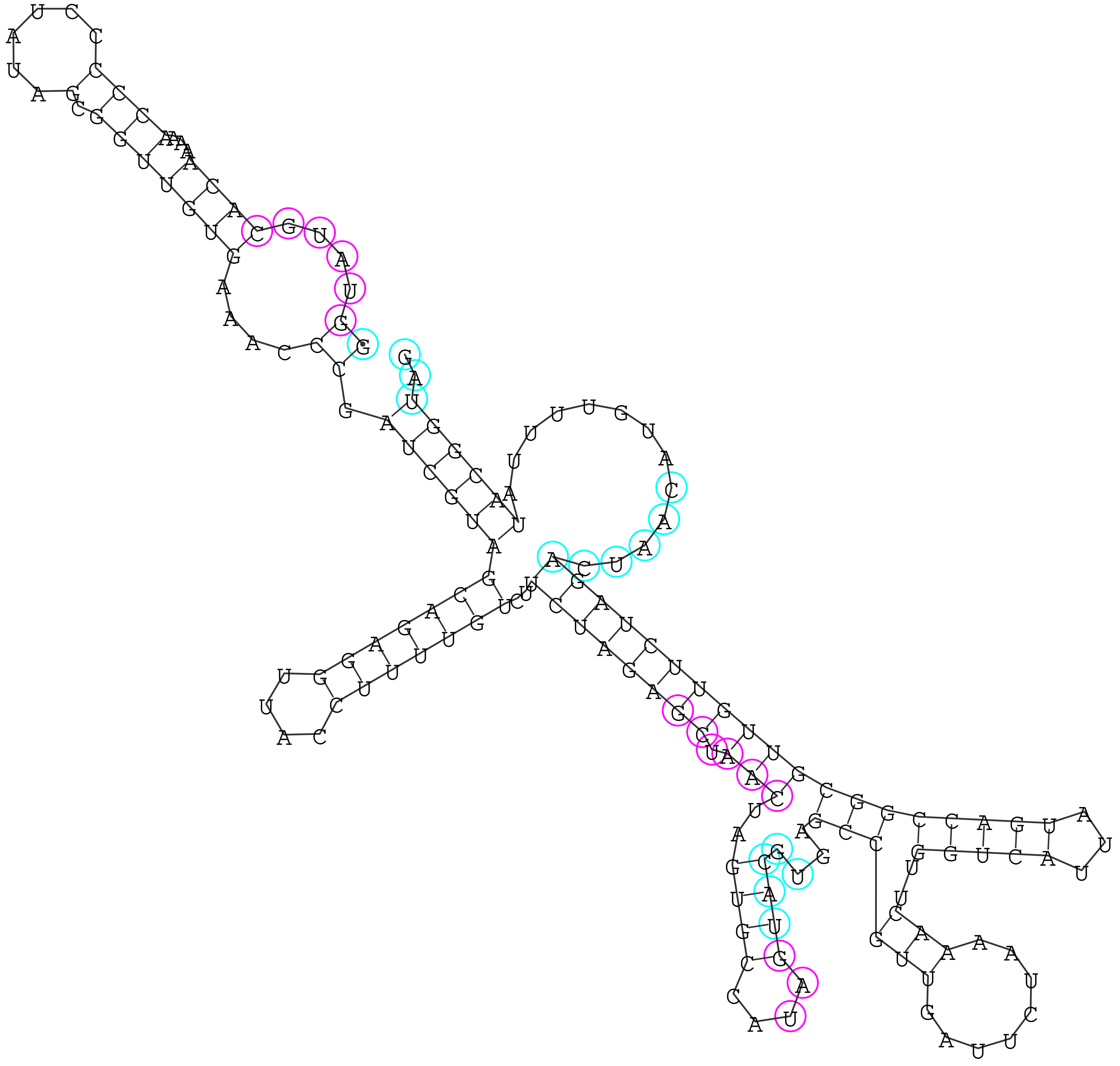

Xmsuc0098A - Stwintron

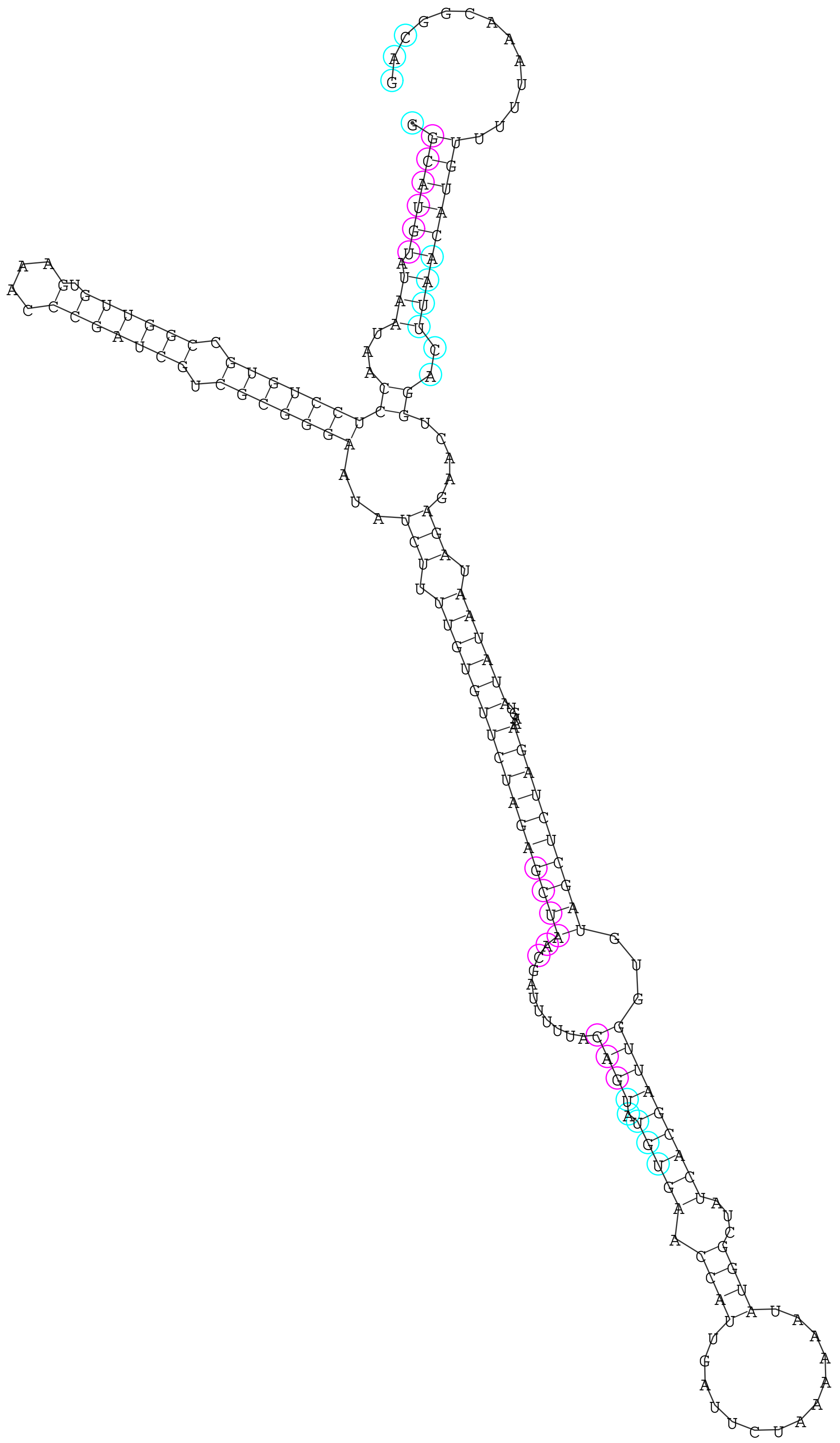

Xmsuc0110A - Stwintron

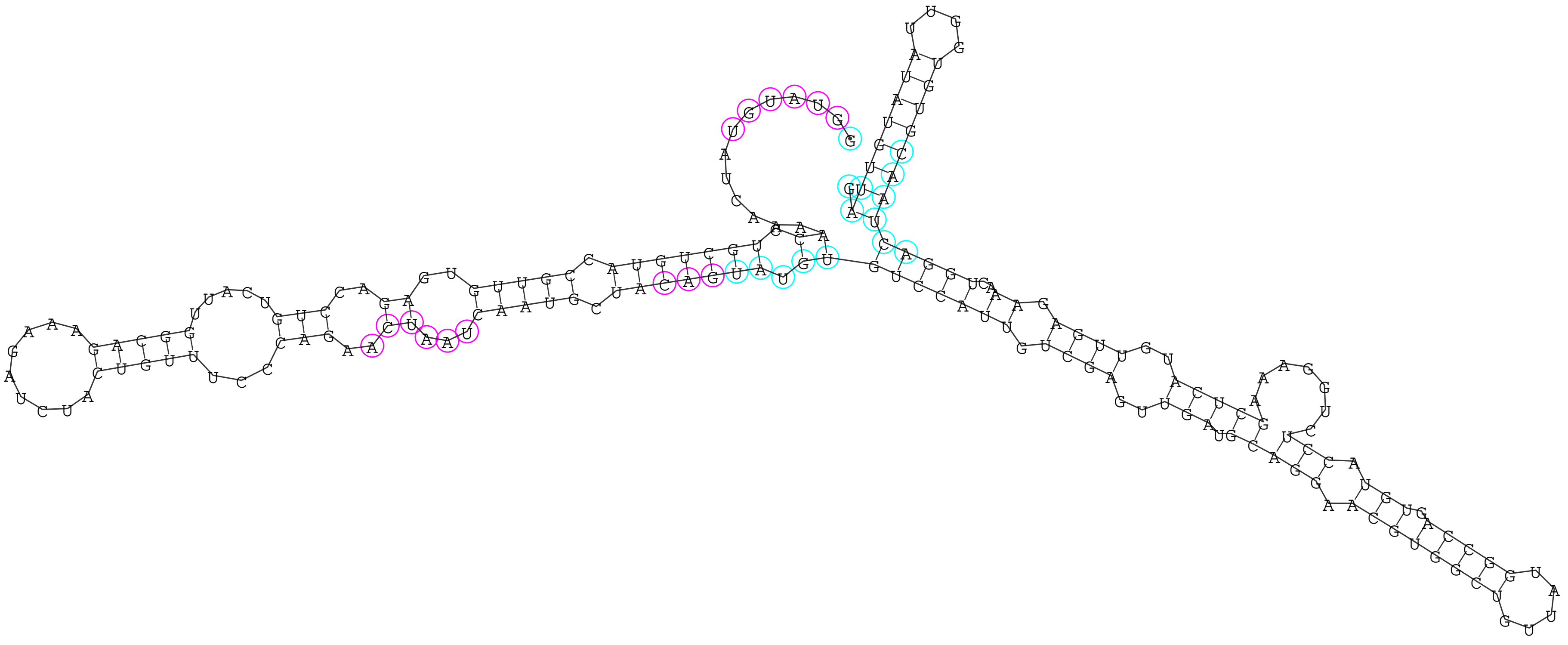

Xmsuc0111A - Stwintron

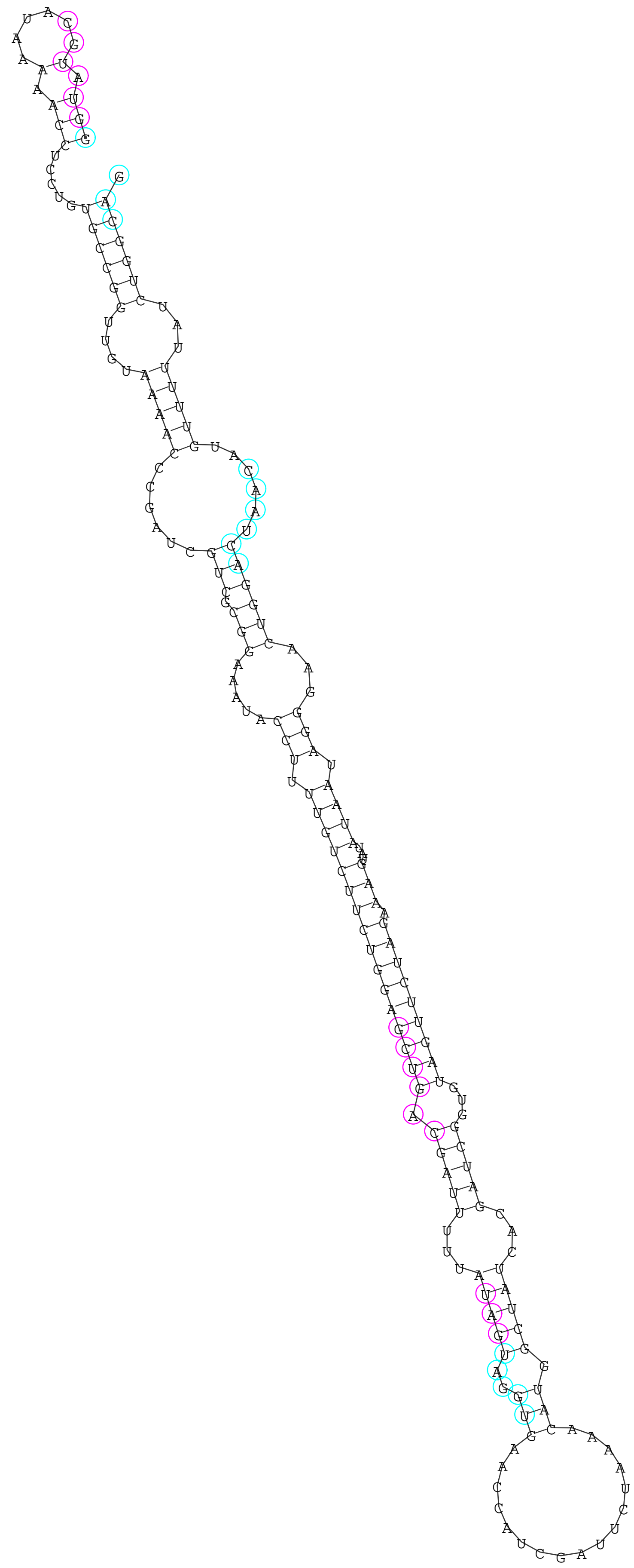

Xmsuc0114A - Stwintron

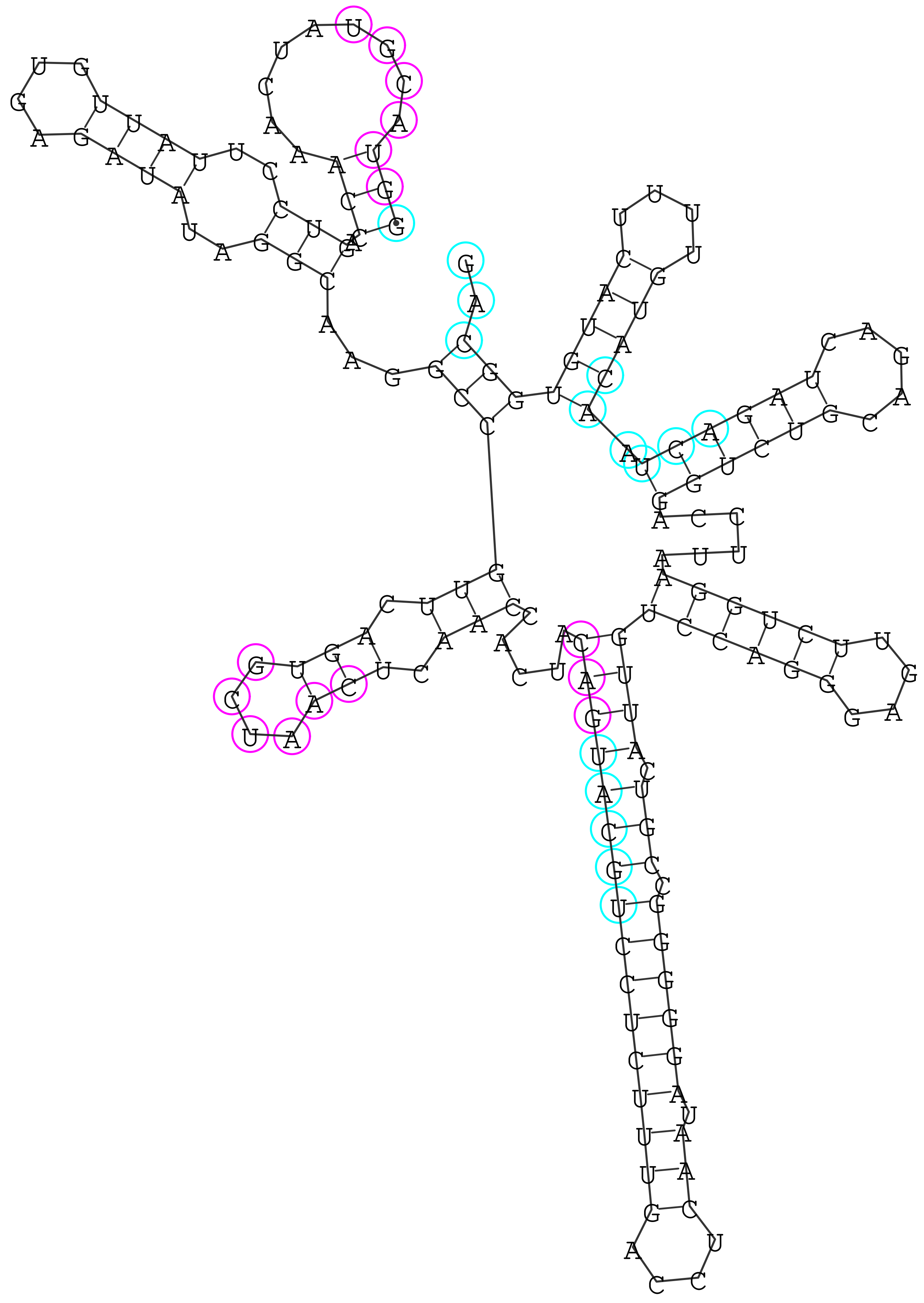

Xmsuc0121A - Stwintron

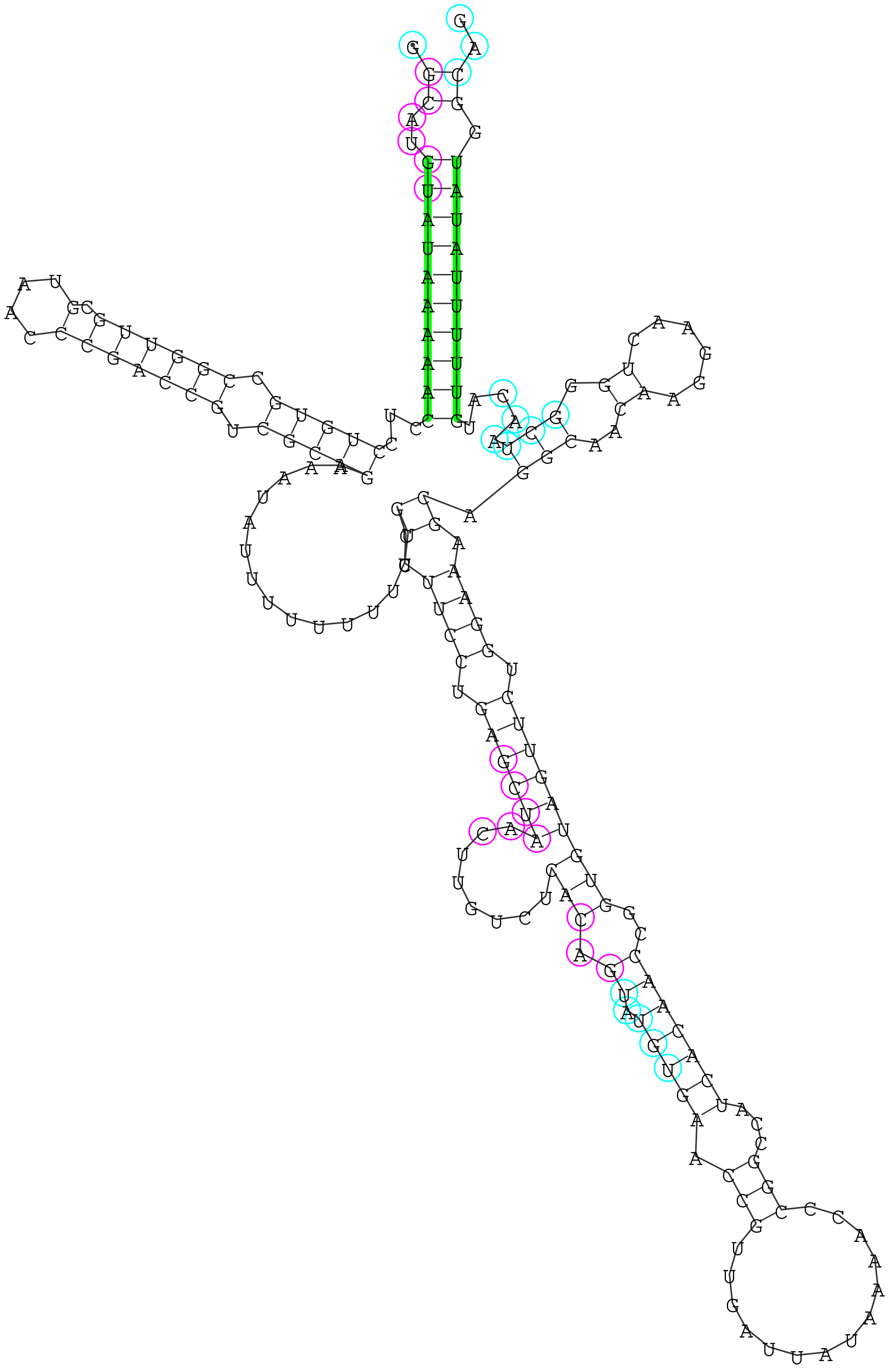

Xmsuc0131A - Stwintron

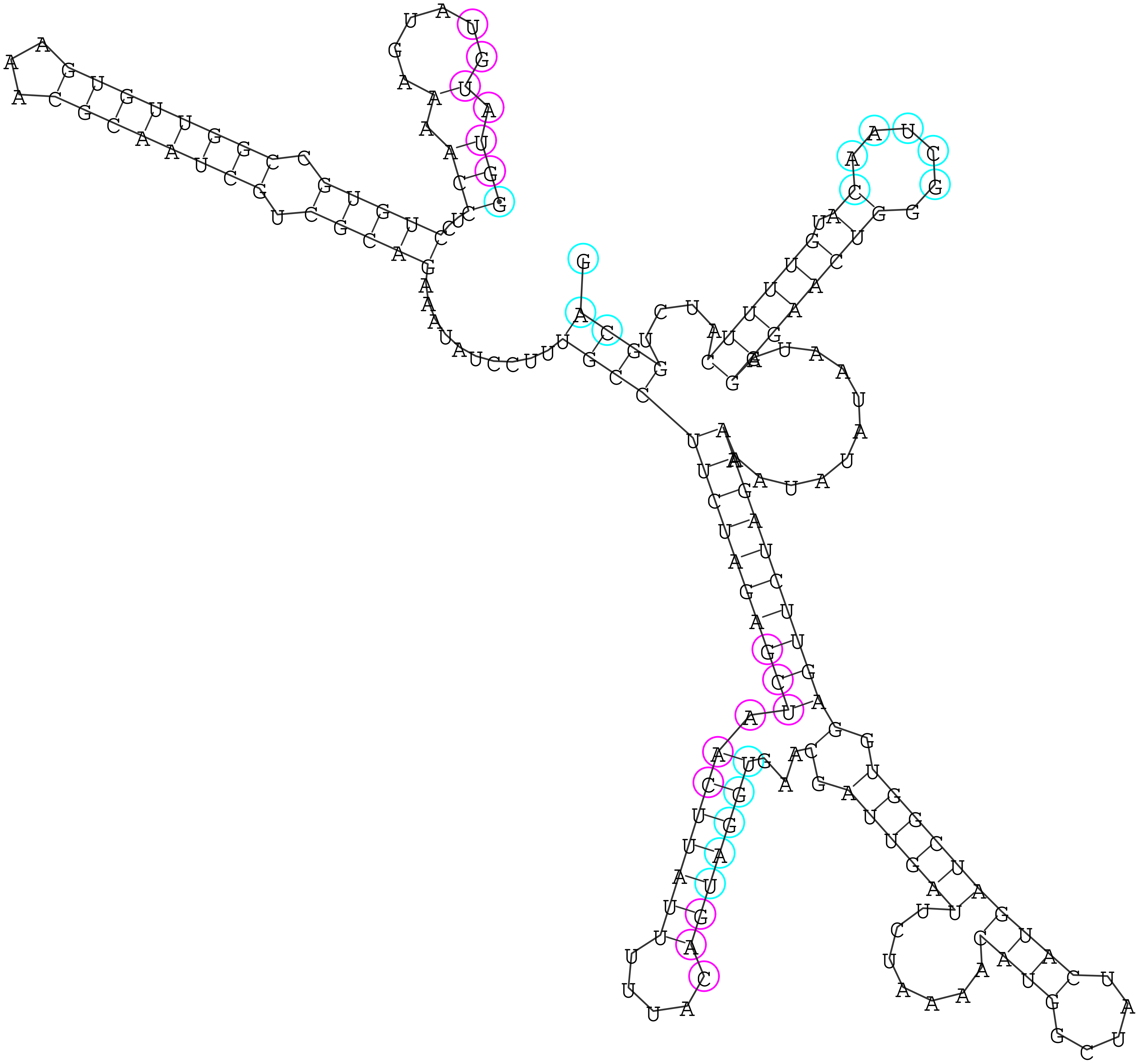

Xmsuc0137A - Stwintron

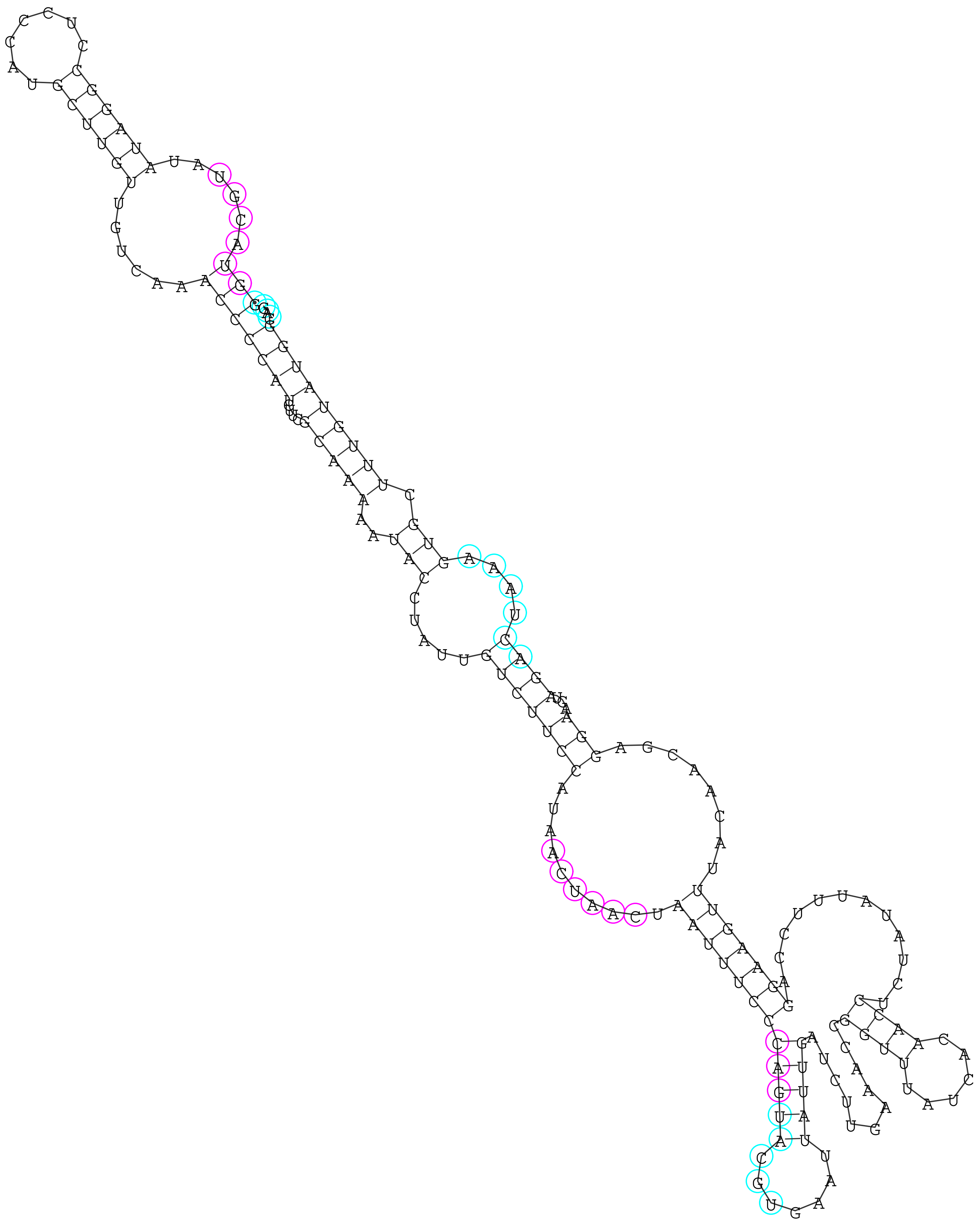

Xmsuc0141A - Stwintron

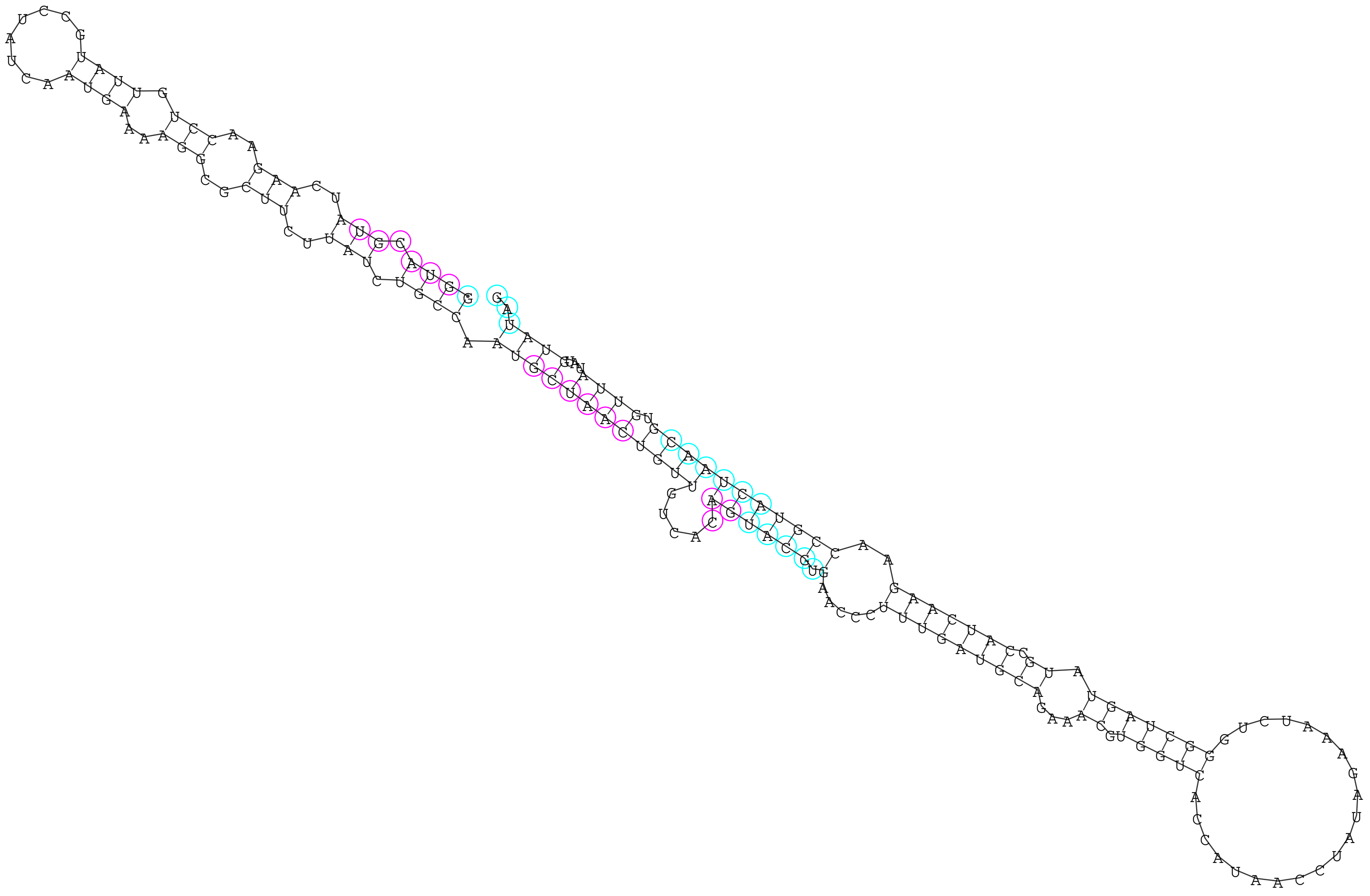

Xmsuc0143A - Stwintron

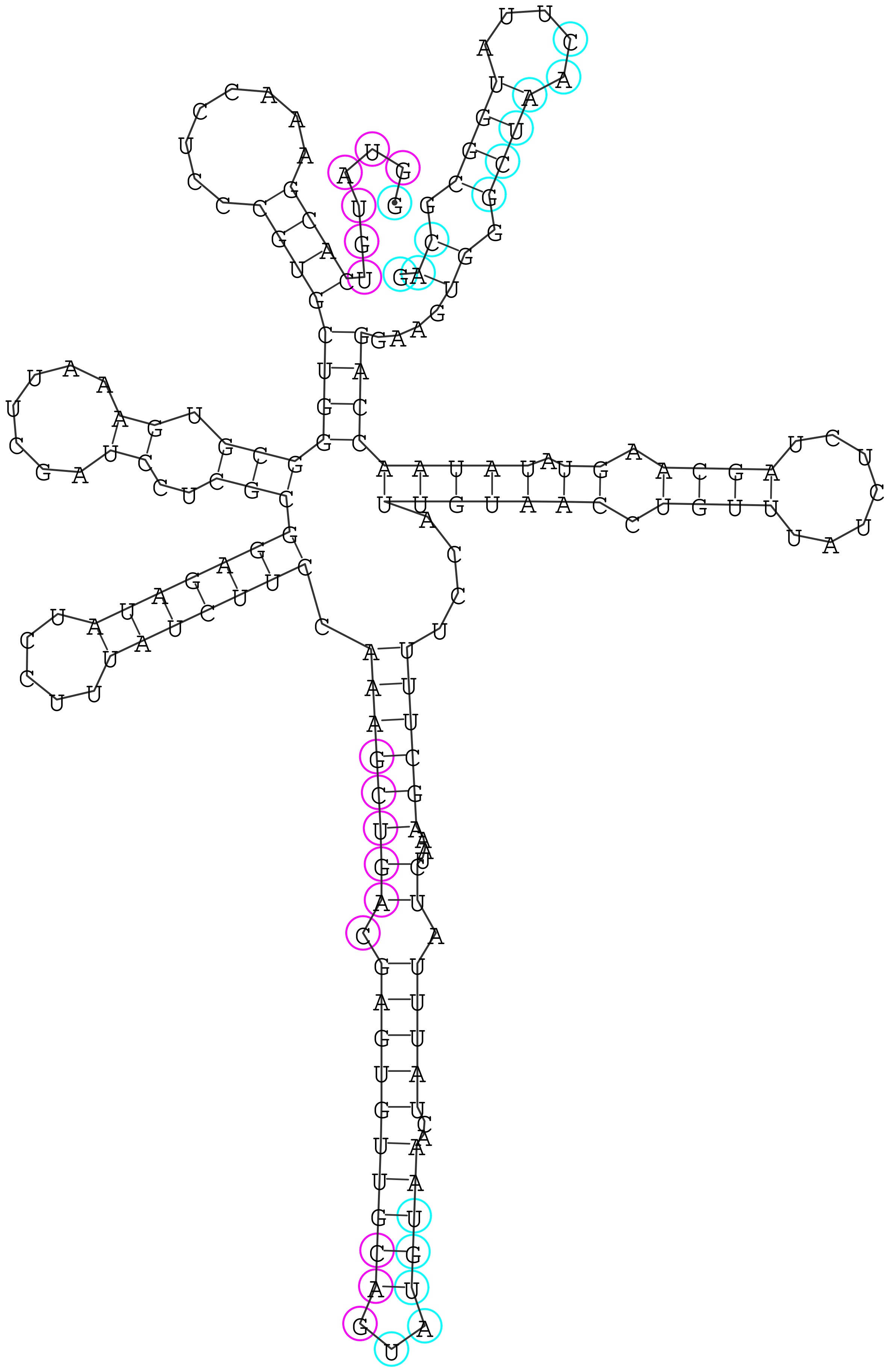

Xmsuc0146A - Stwintron

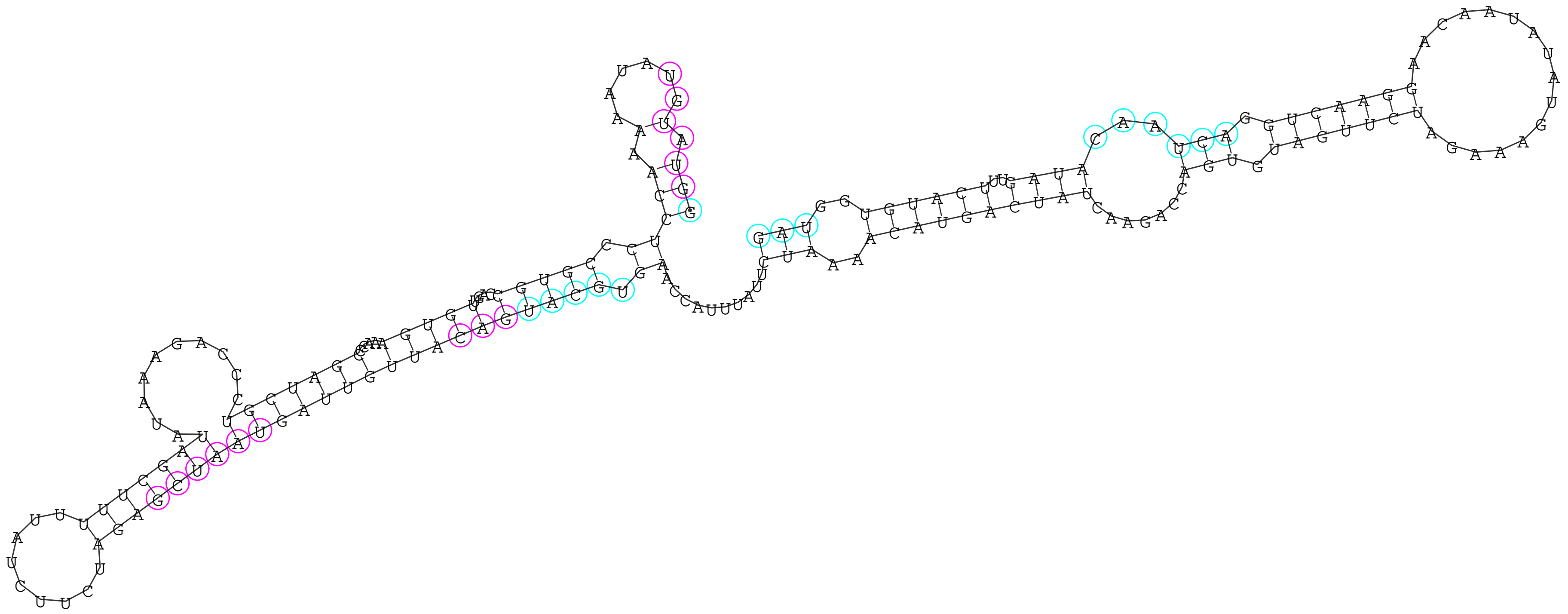

Xmsuc0153A - Stwintron

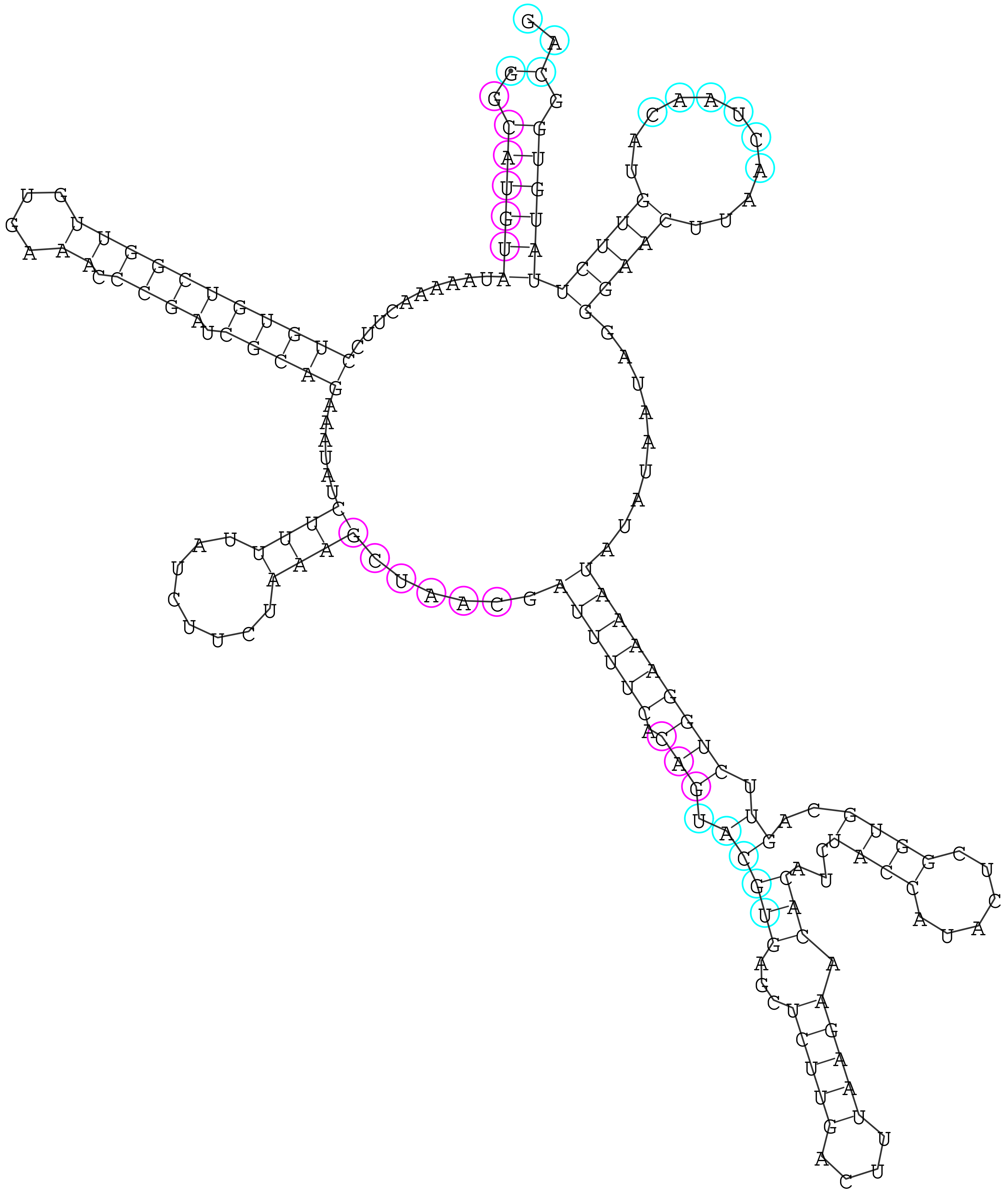

Xmsuc0159A - Stwintron

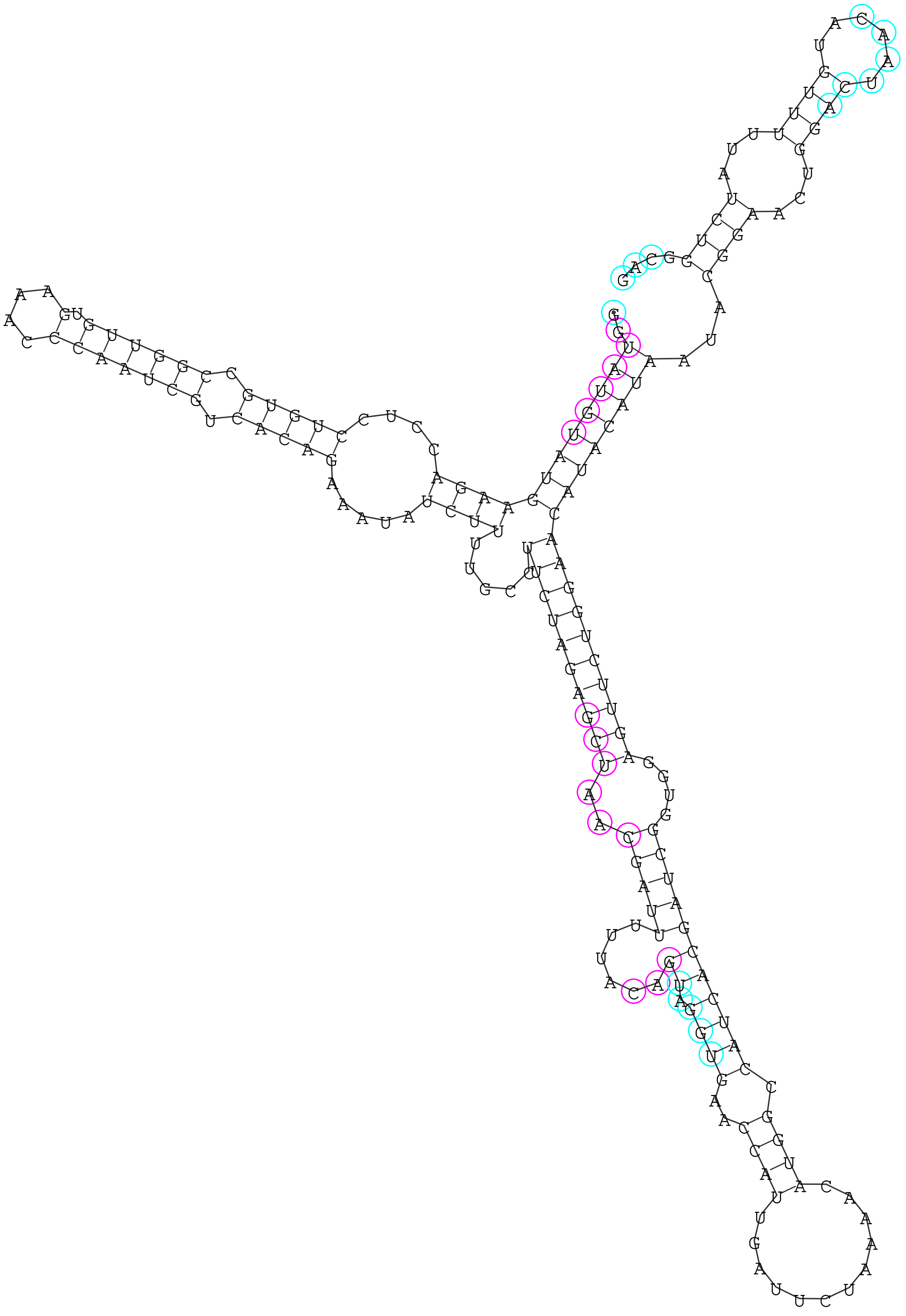

Xmsuc0162A - Stwintron

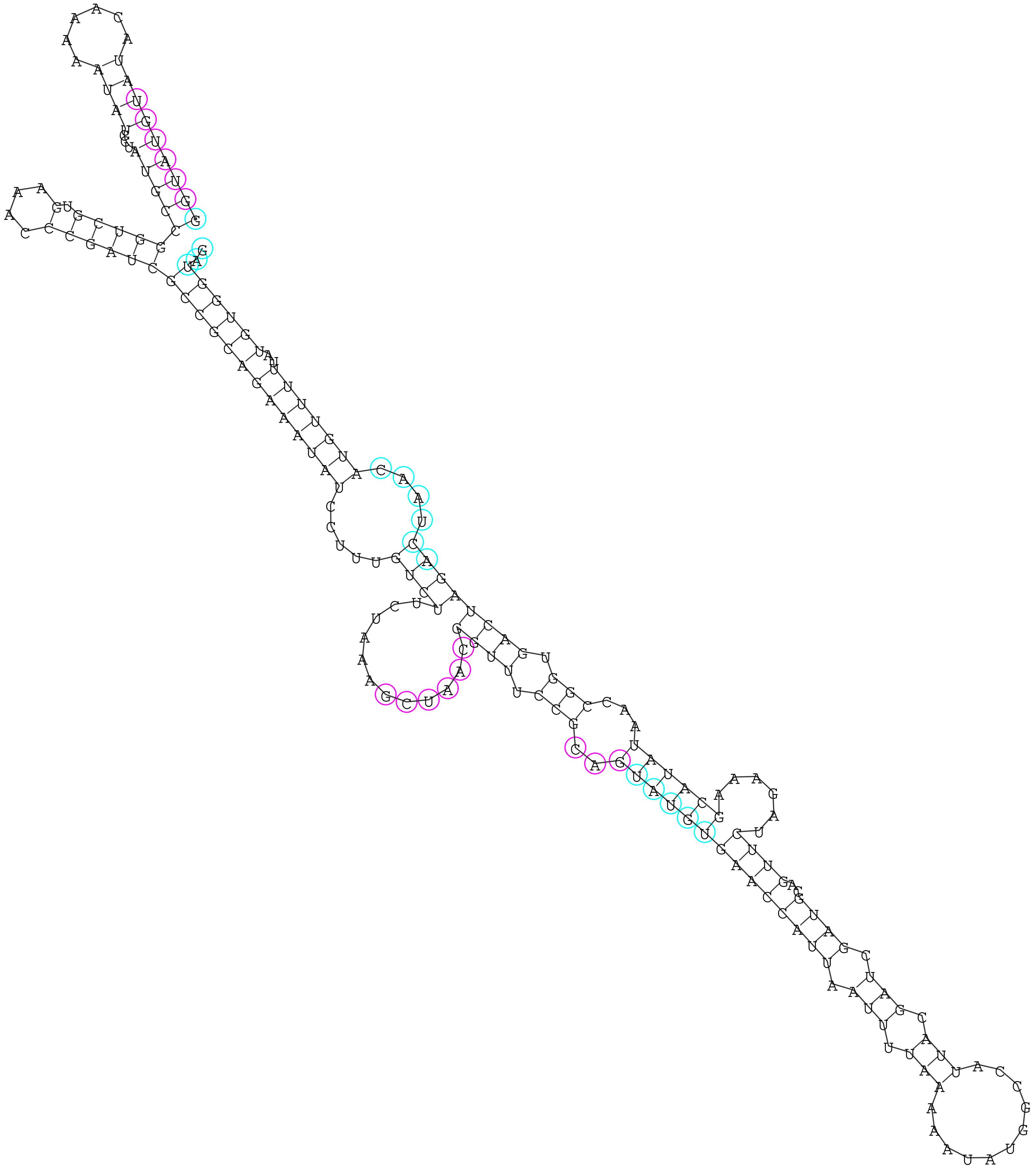

Xmsuc0168A - Stwintron

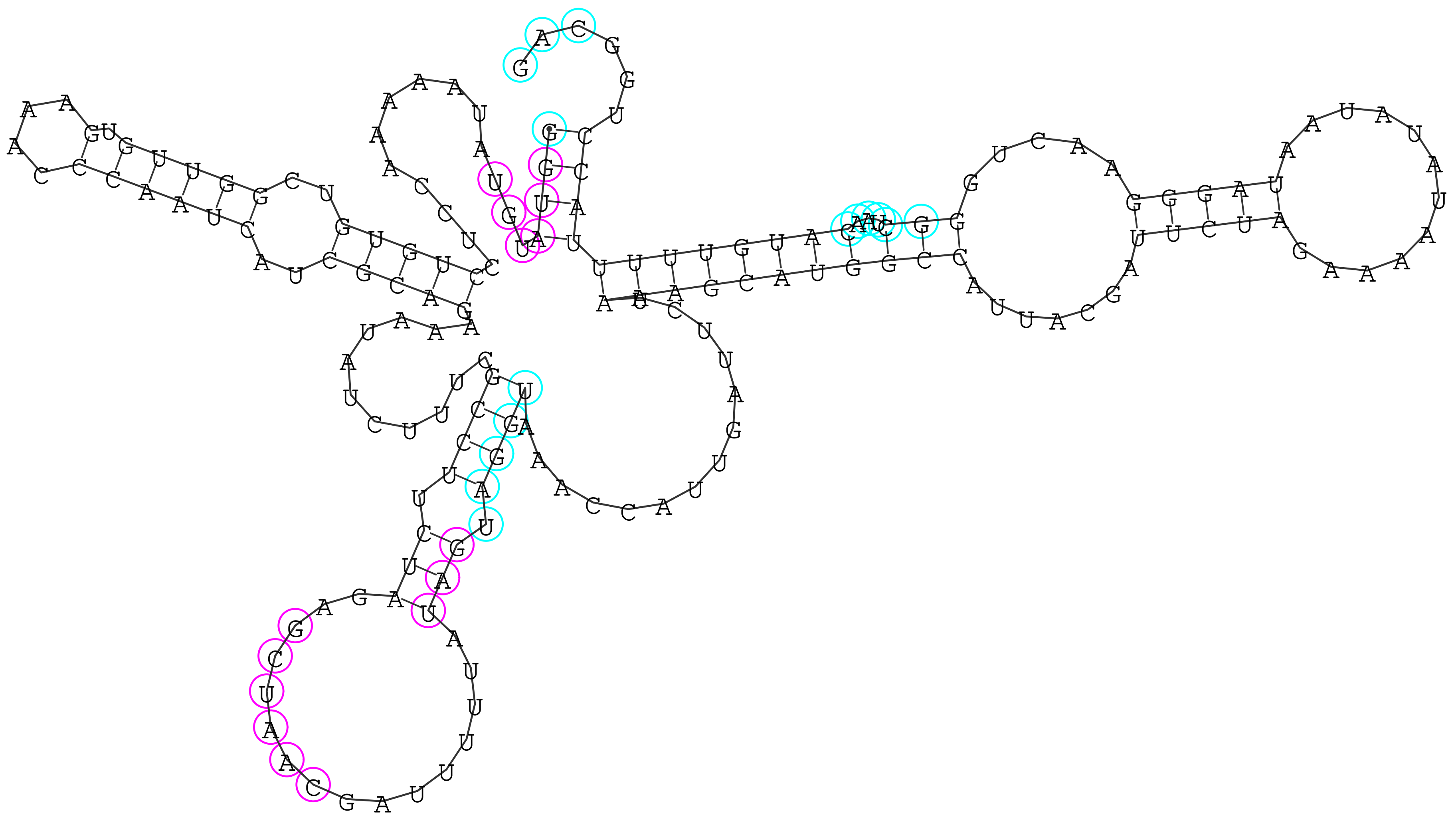

Xmsuc0170A - Stwintron

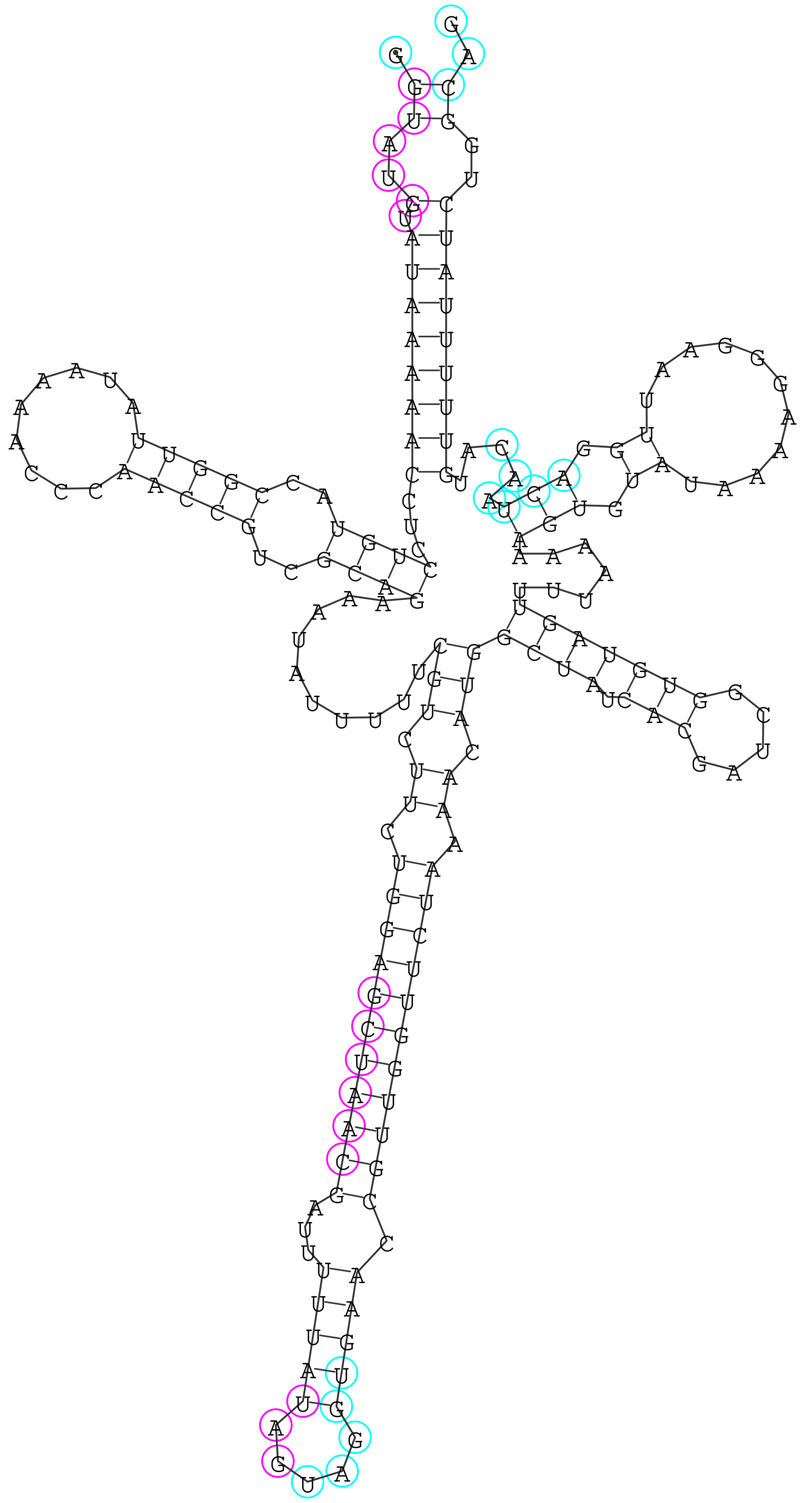

Xmsuc0171A - Stwintron

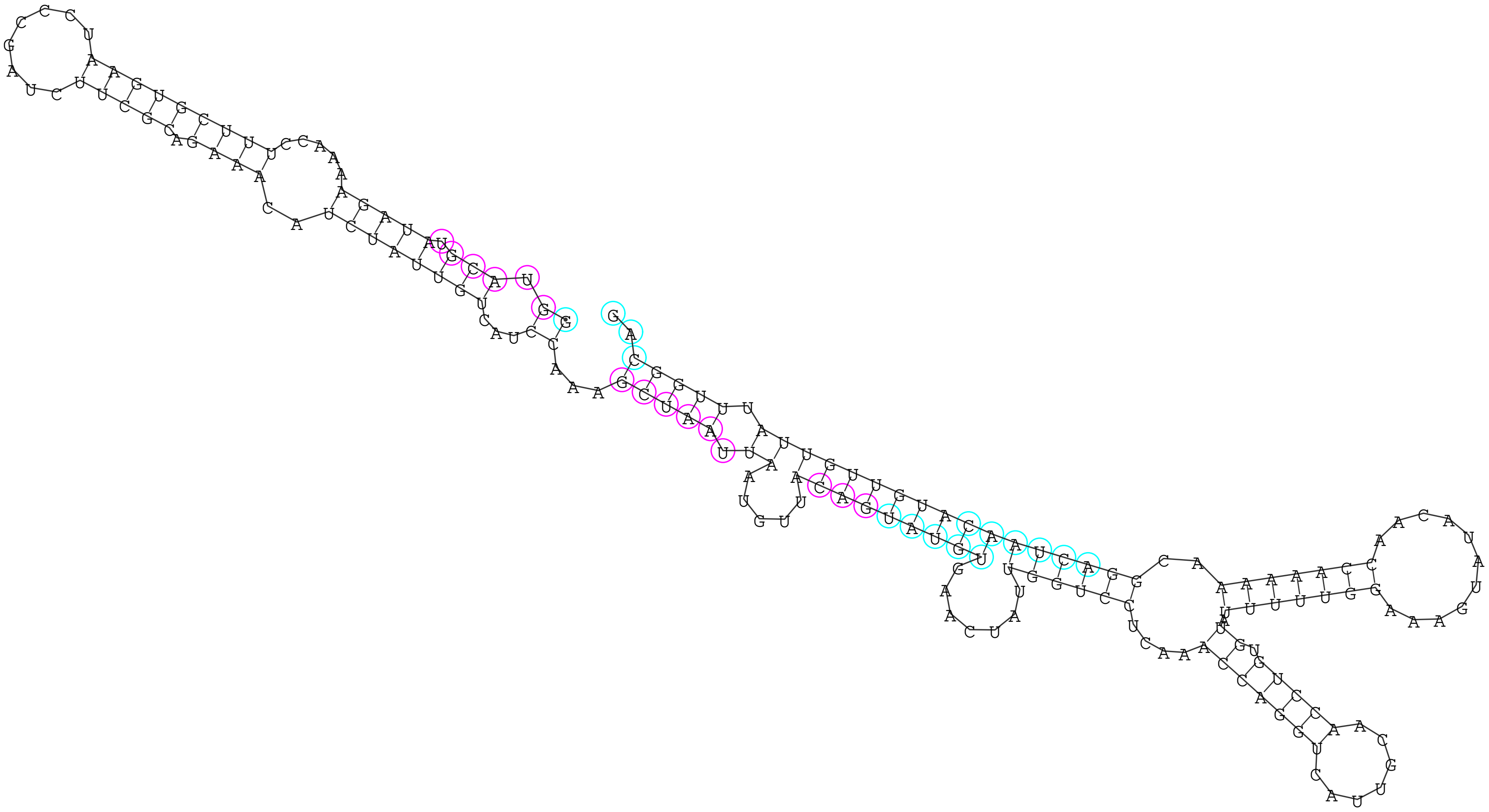

Xmsuc0178A - Stwintron

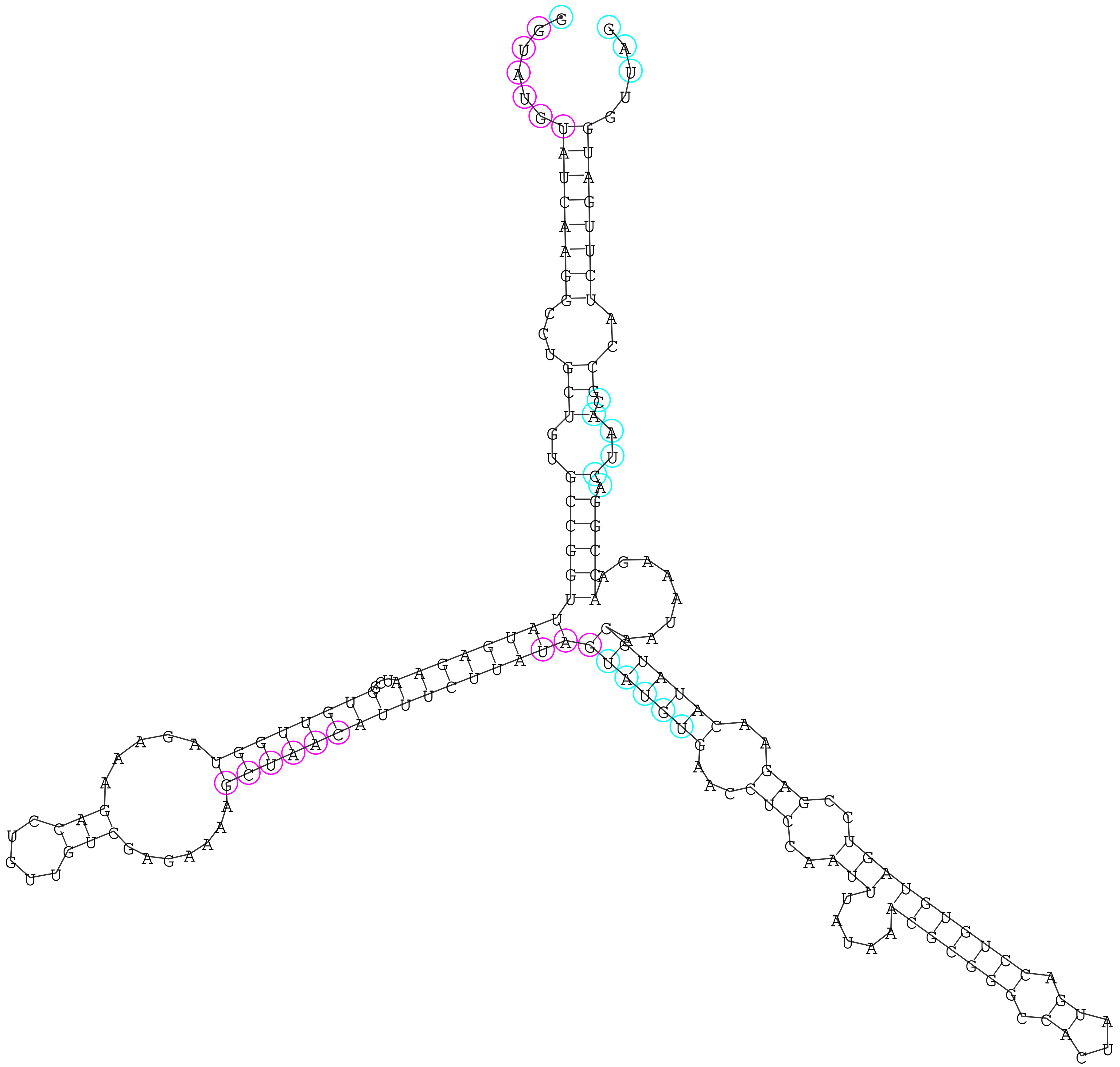

Xmsuc0185A - Stwintron

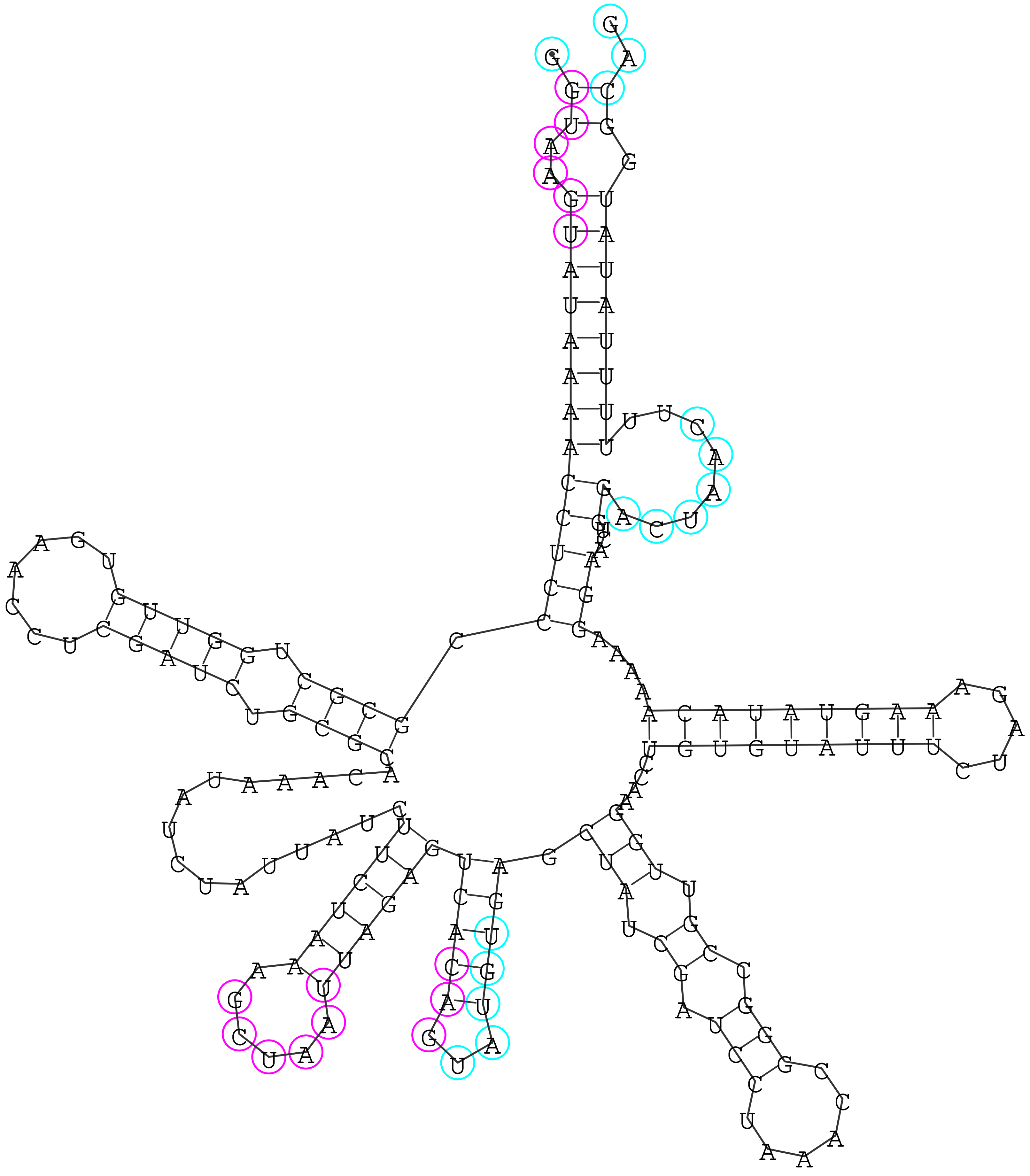

Xmsuc0187A - Stwintron

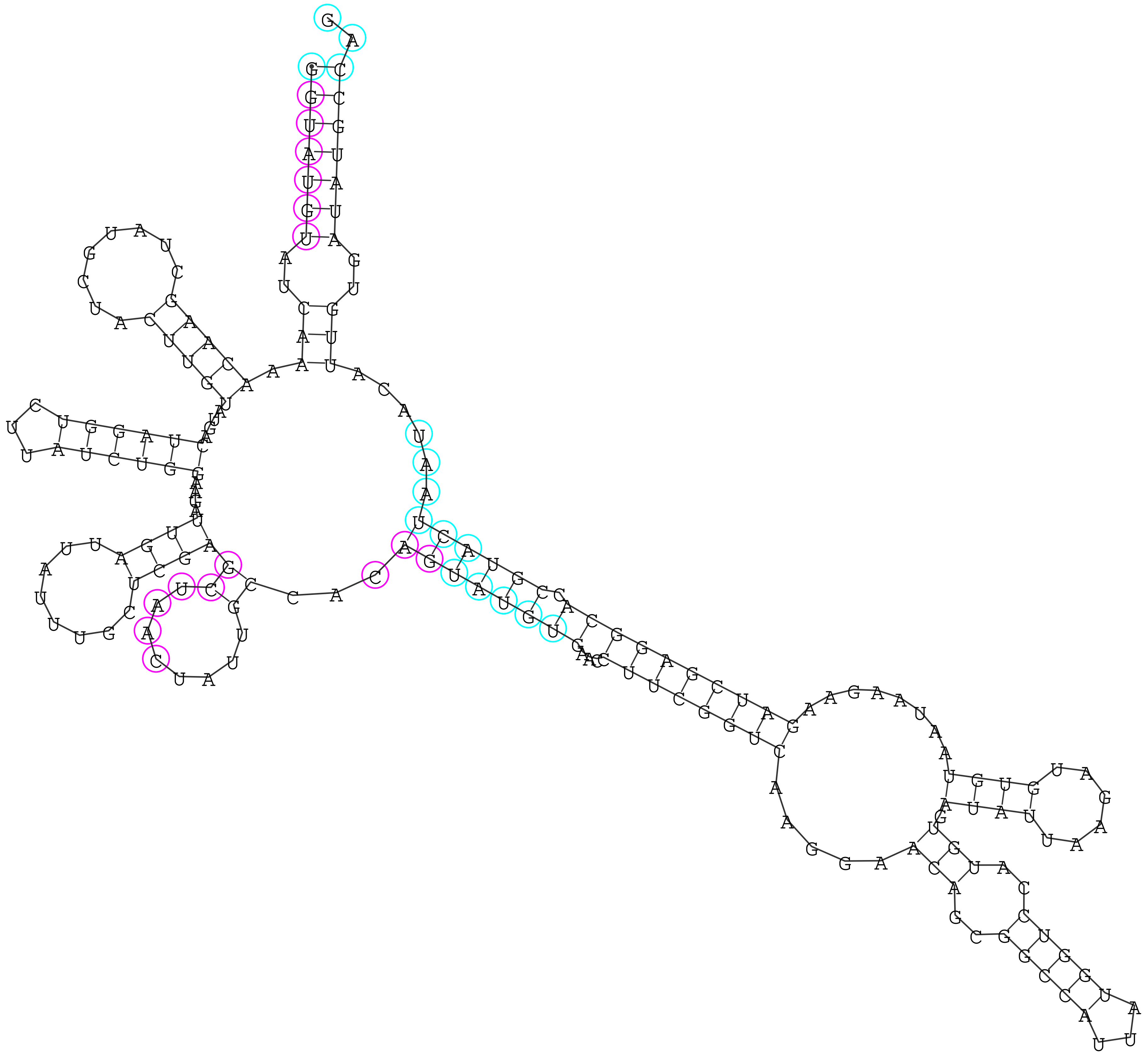

Xmsuc0237A - Stwintron

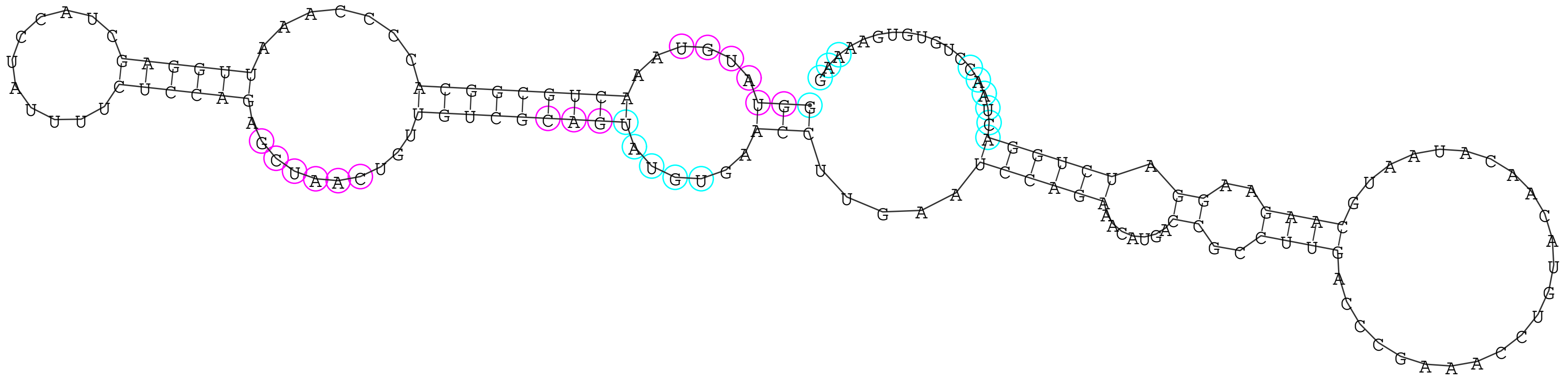

Xmsuc0285A - Stwintron

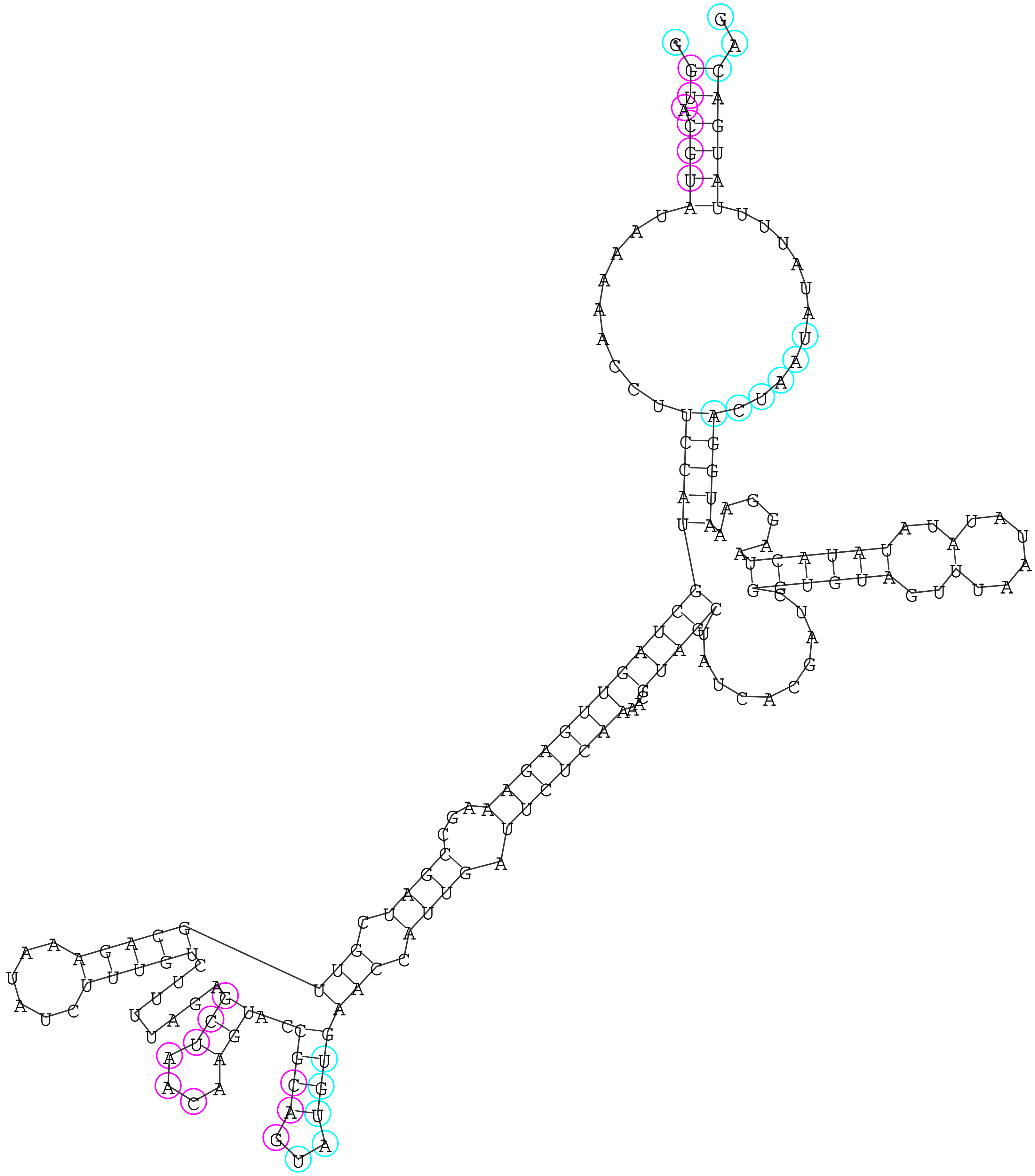

Xmsuc0293A - Stwintron

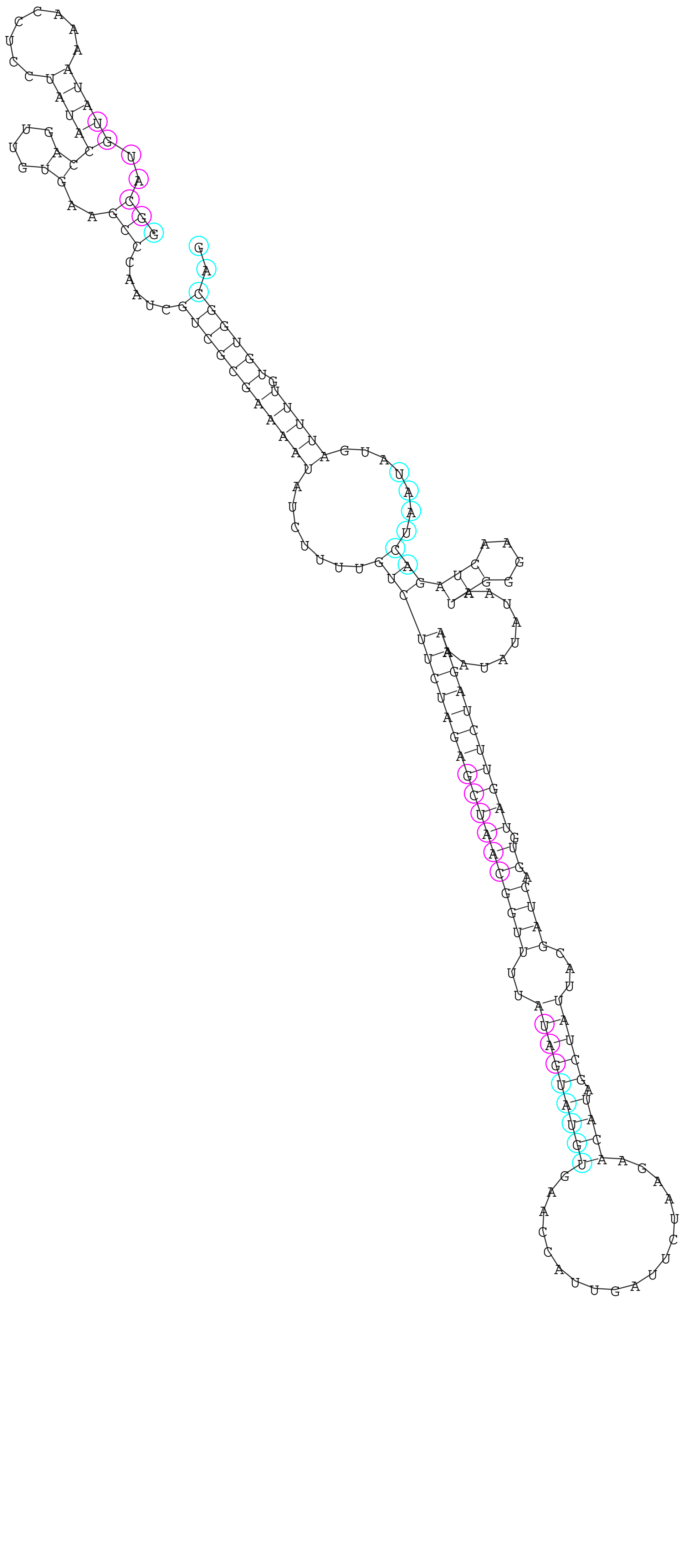

Xmsuc0301A - Stwintron

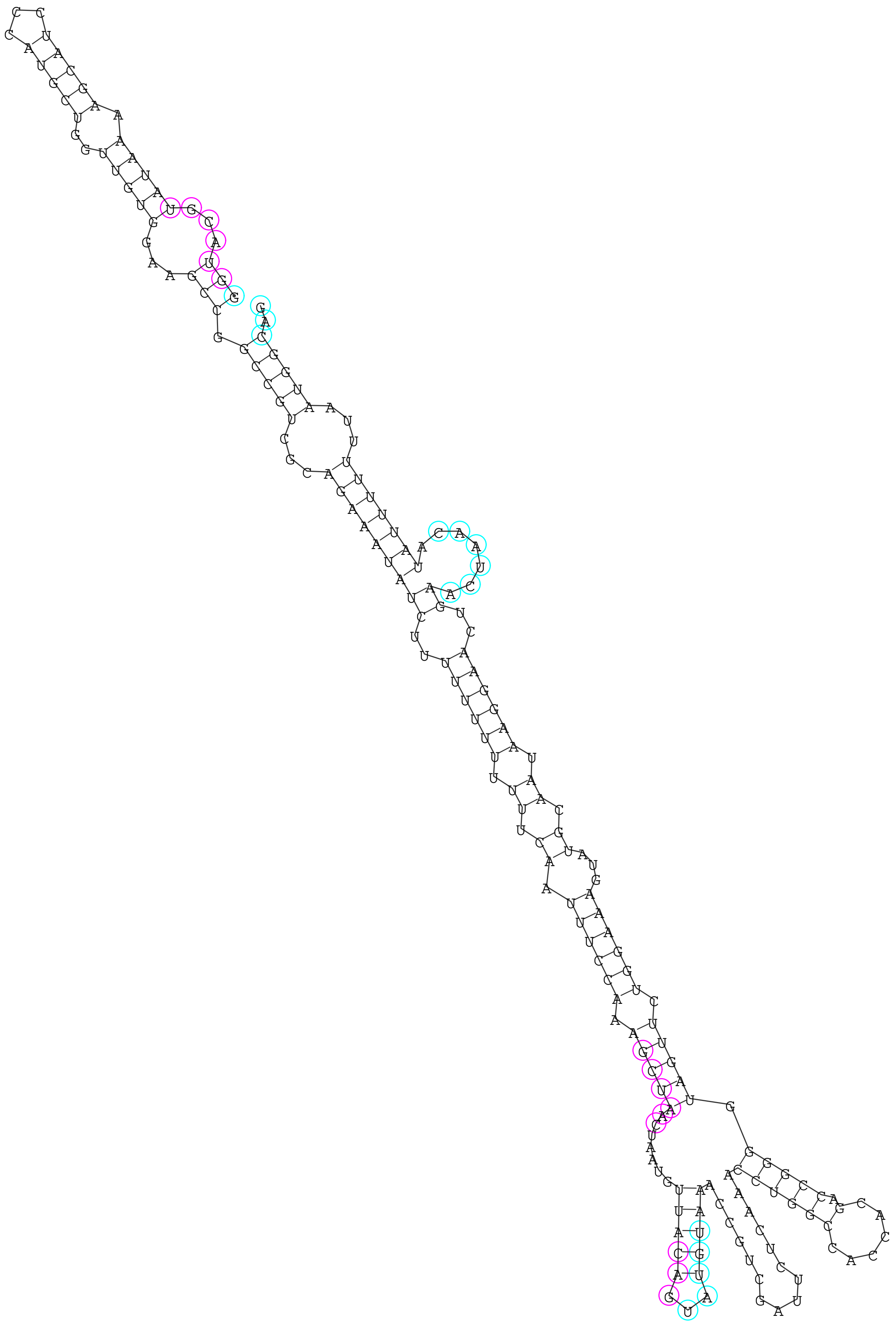

Xmsuc0306A - Stwintron

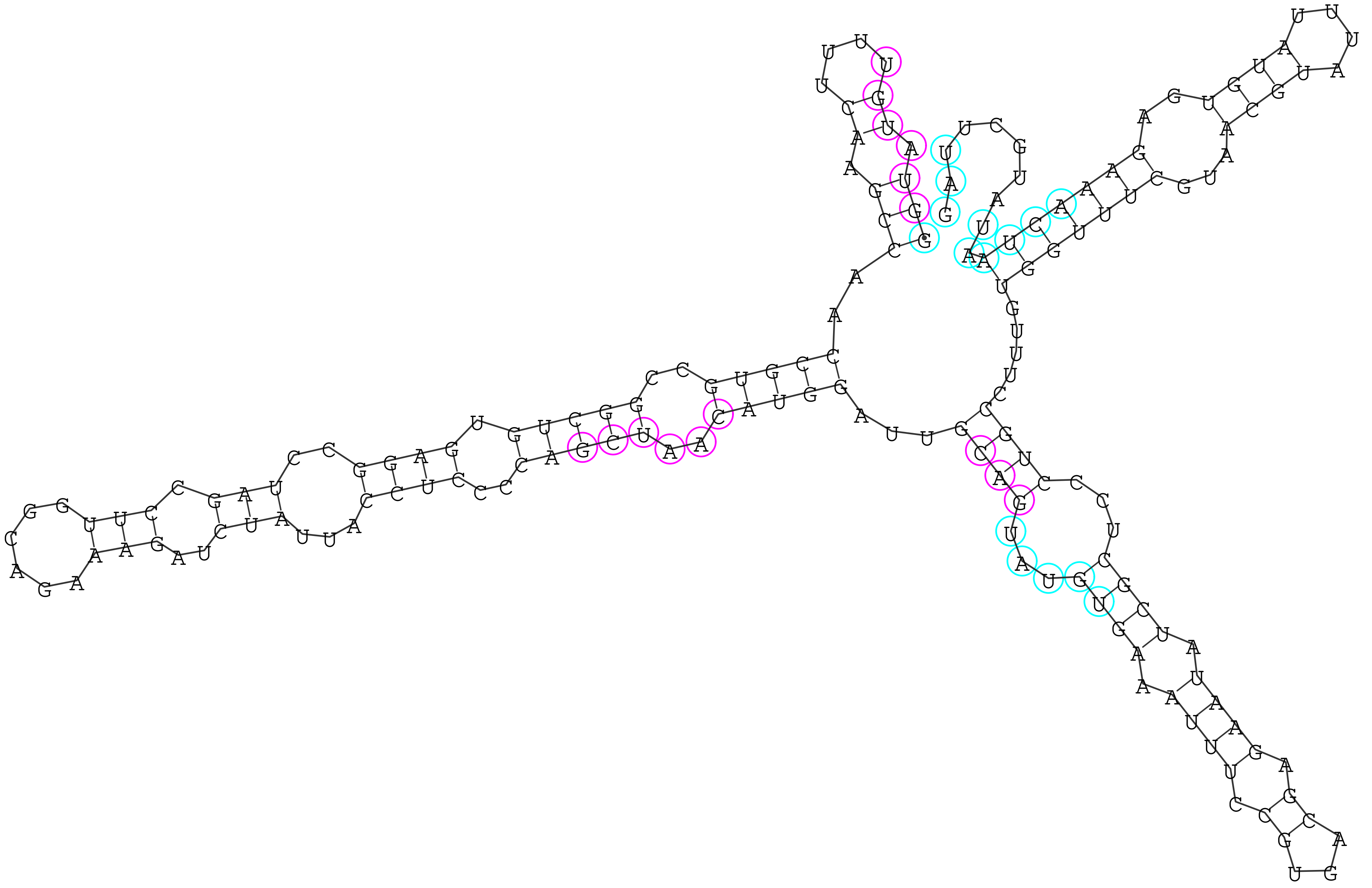

Xmsuc0348A - Stwintron

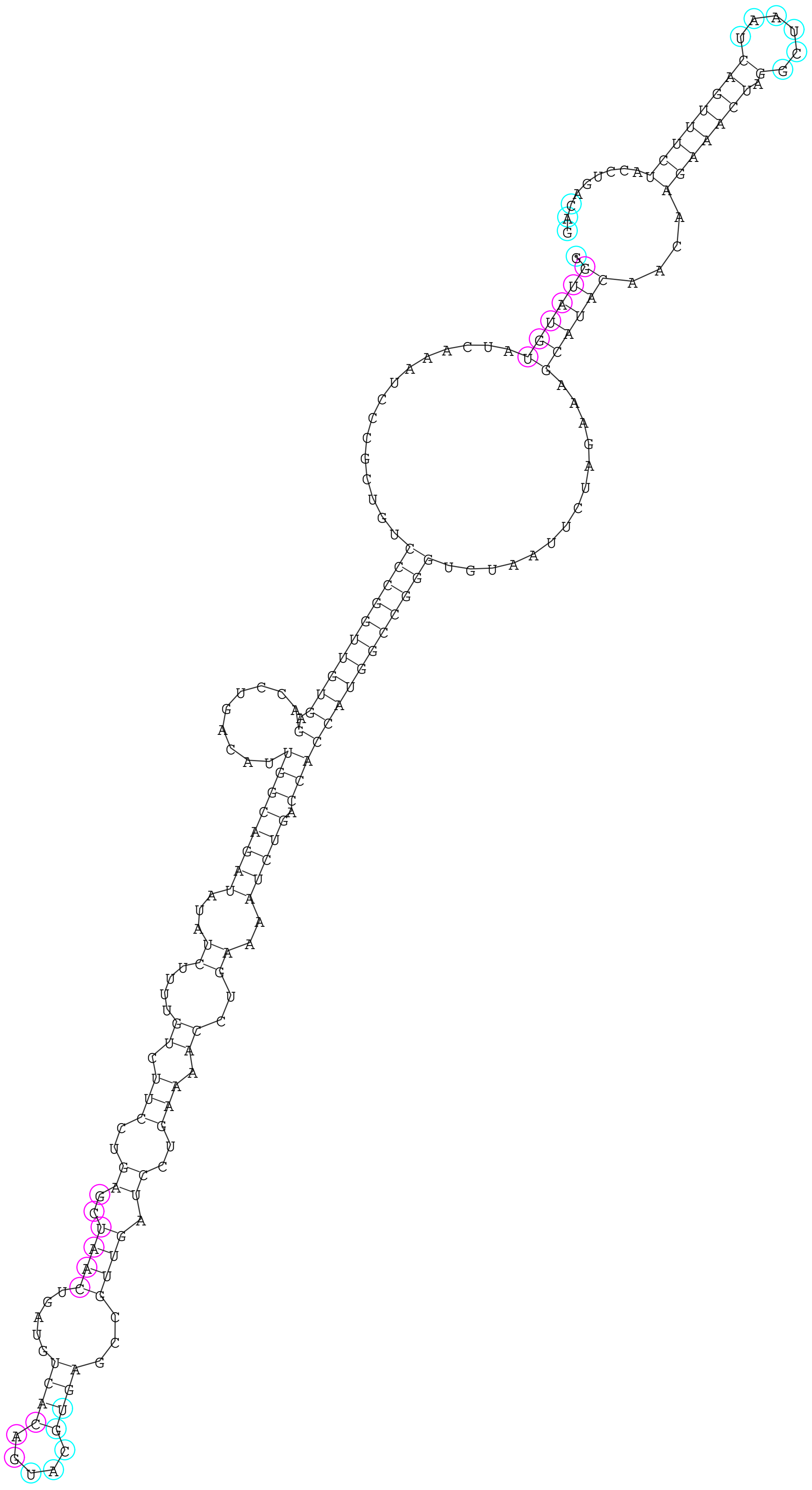

Xmsuc0374A - Stwintron

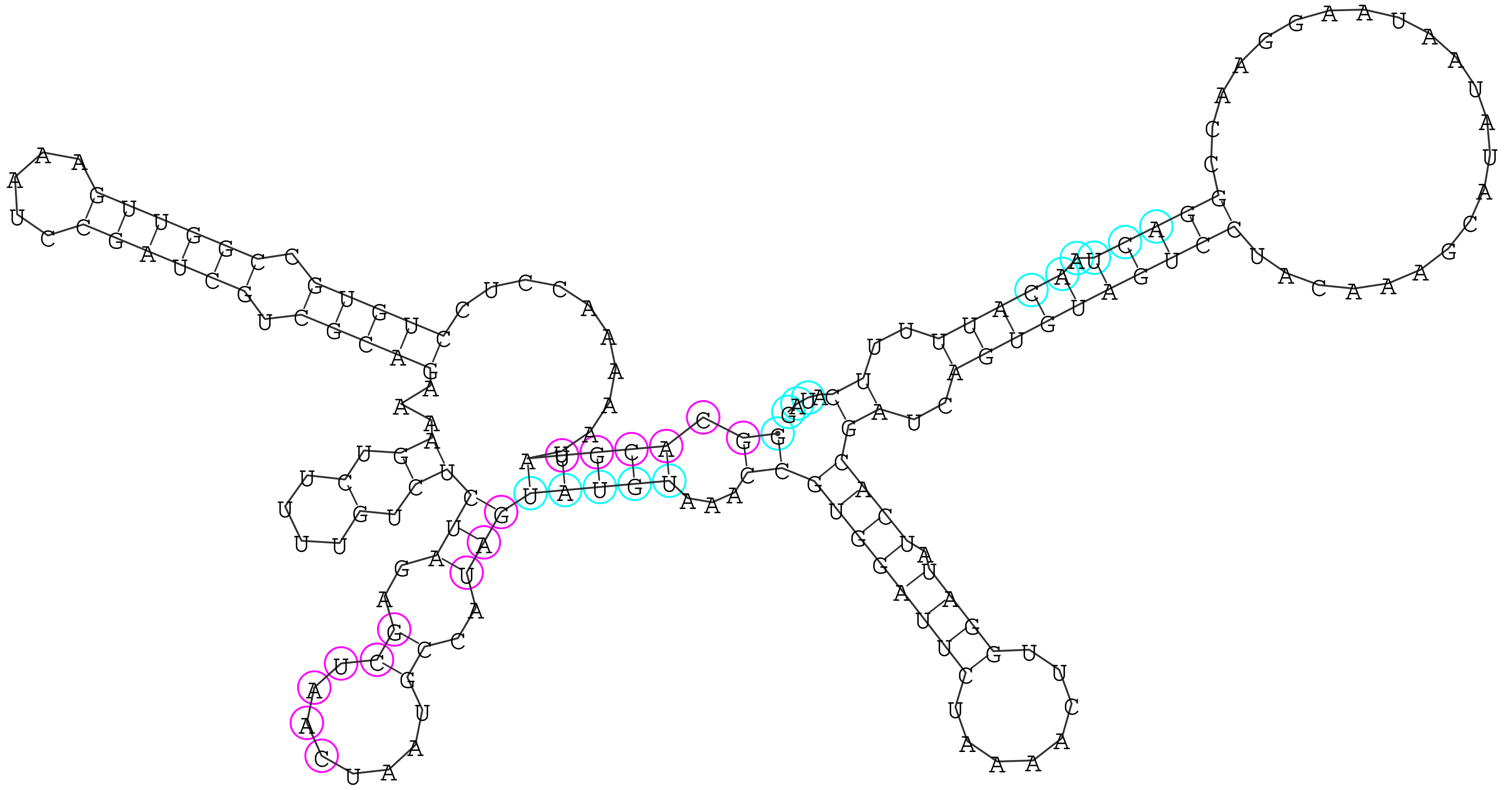

Xmsuc0374B - Stwintron

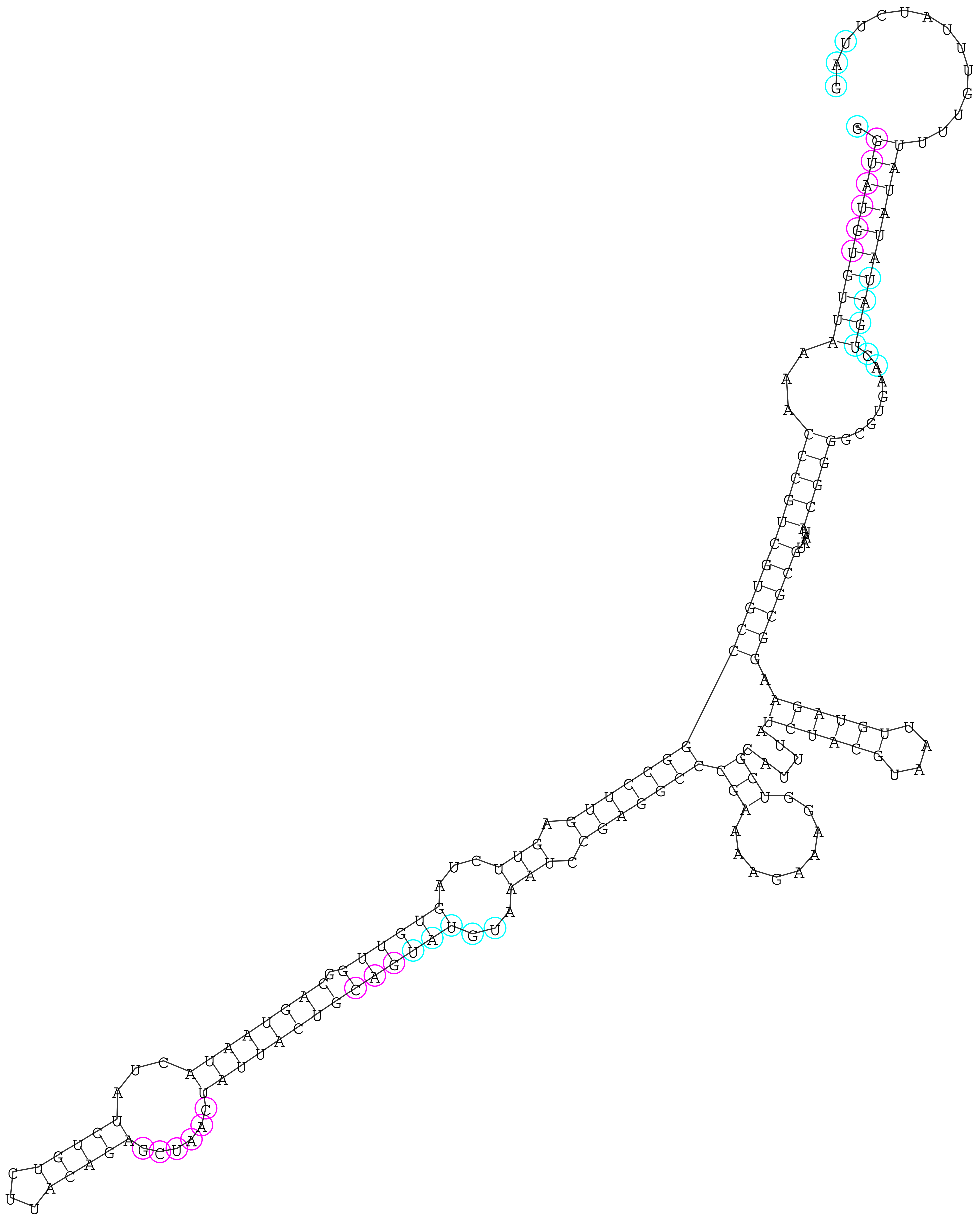

Xmsuc0378A - Stwintron

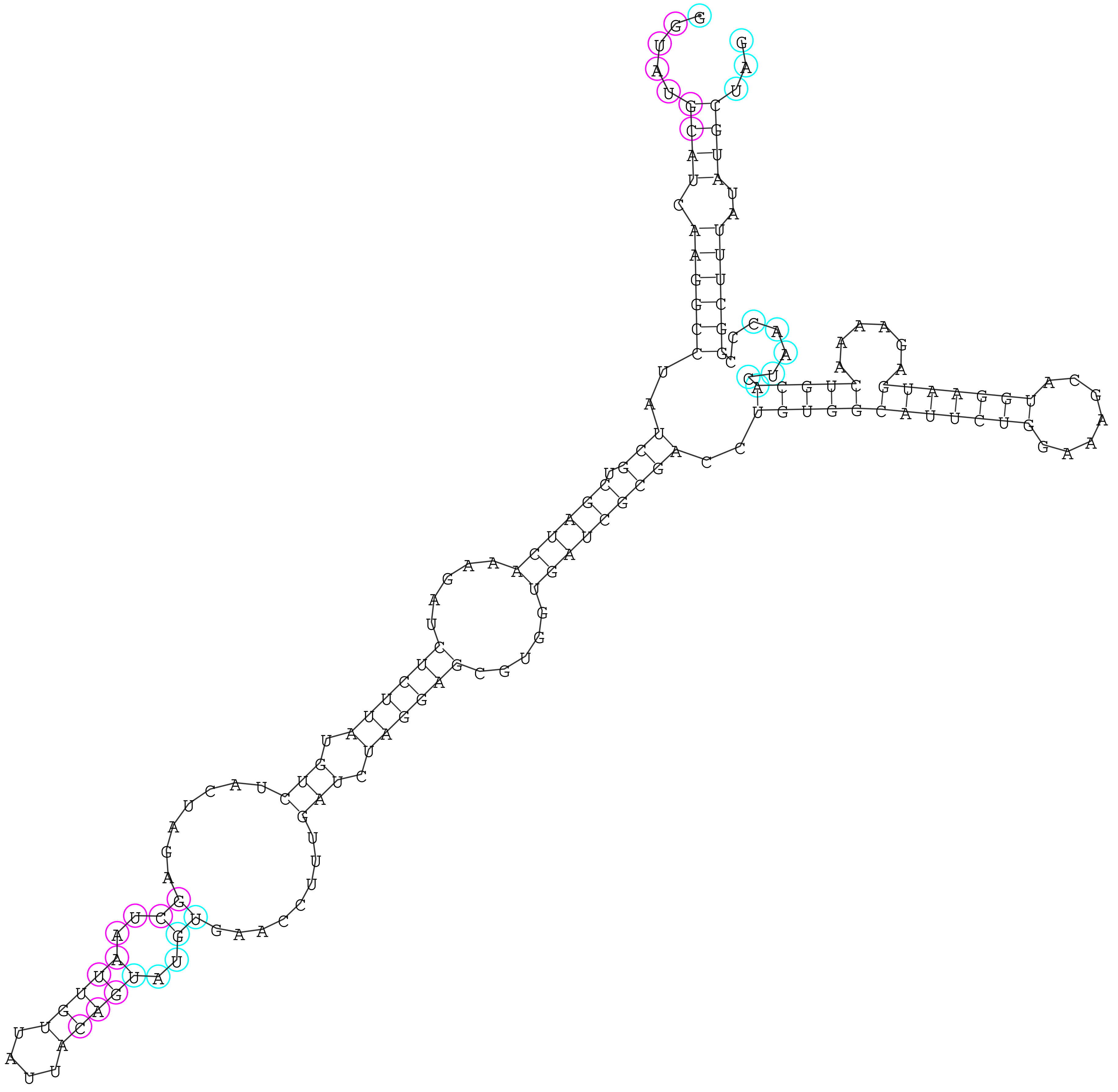

Xmsuc0385A - Stwintron

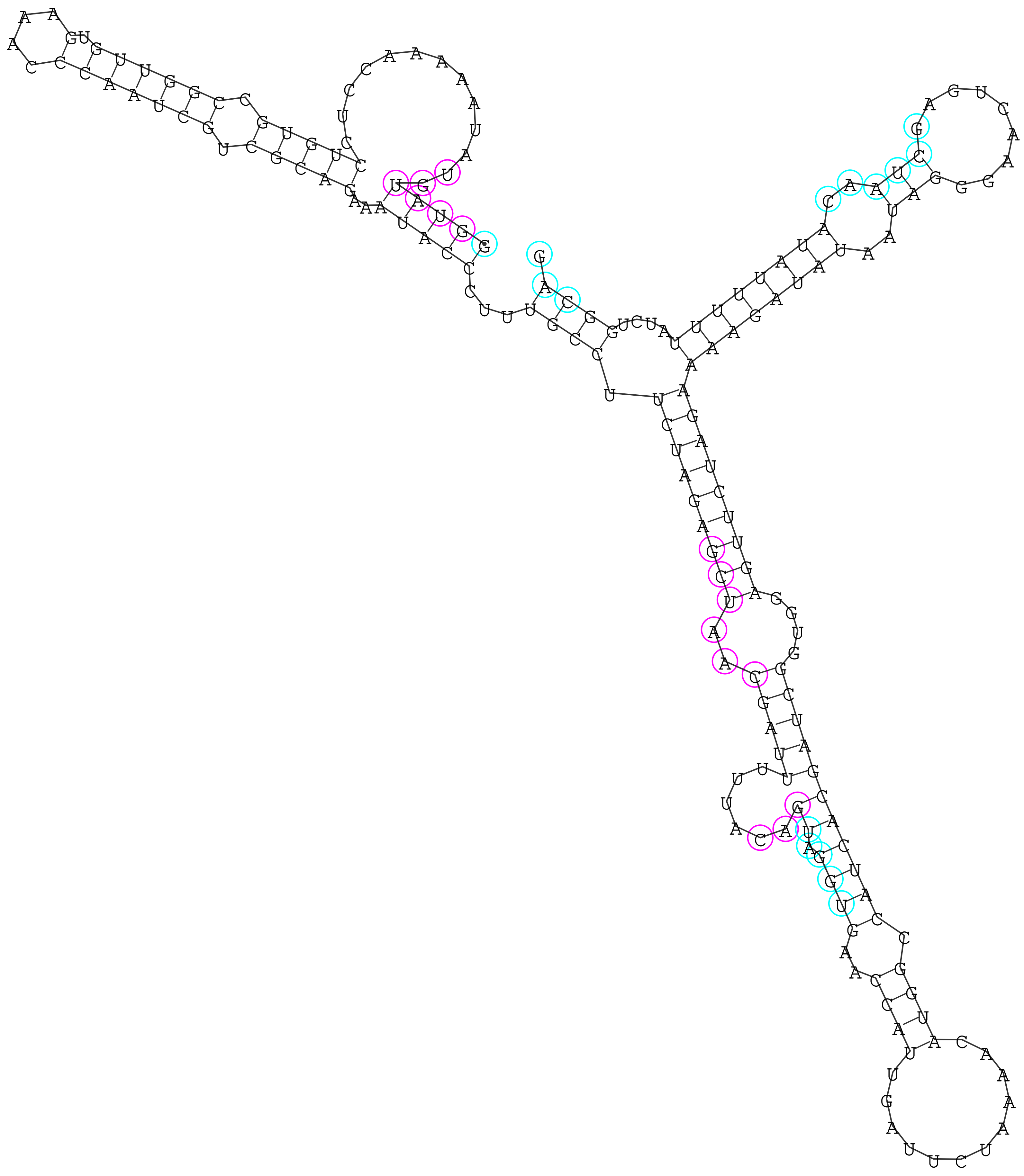

Xmsuc0412A - Stwintron

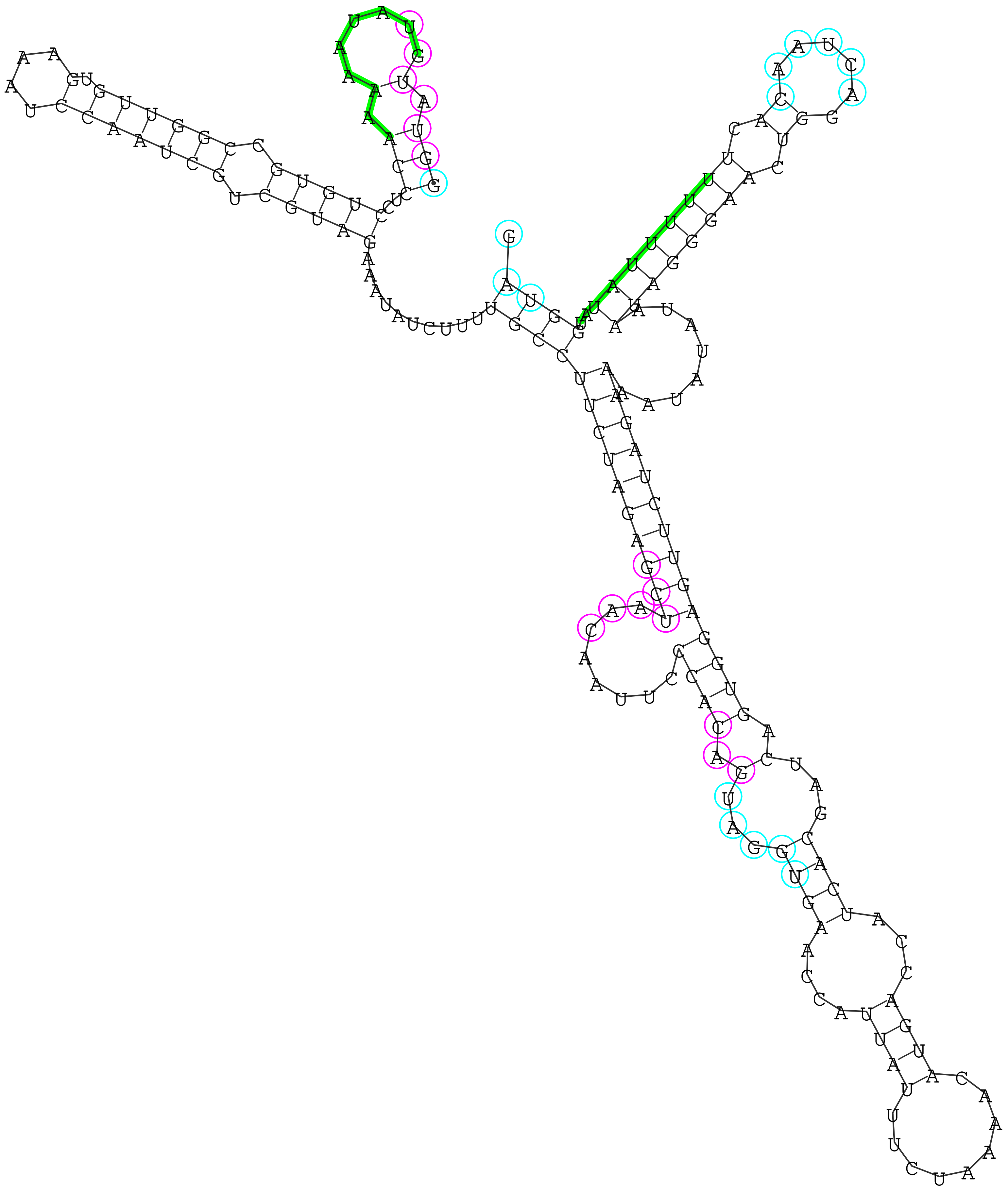

Xmsuc0520A - Stwintron

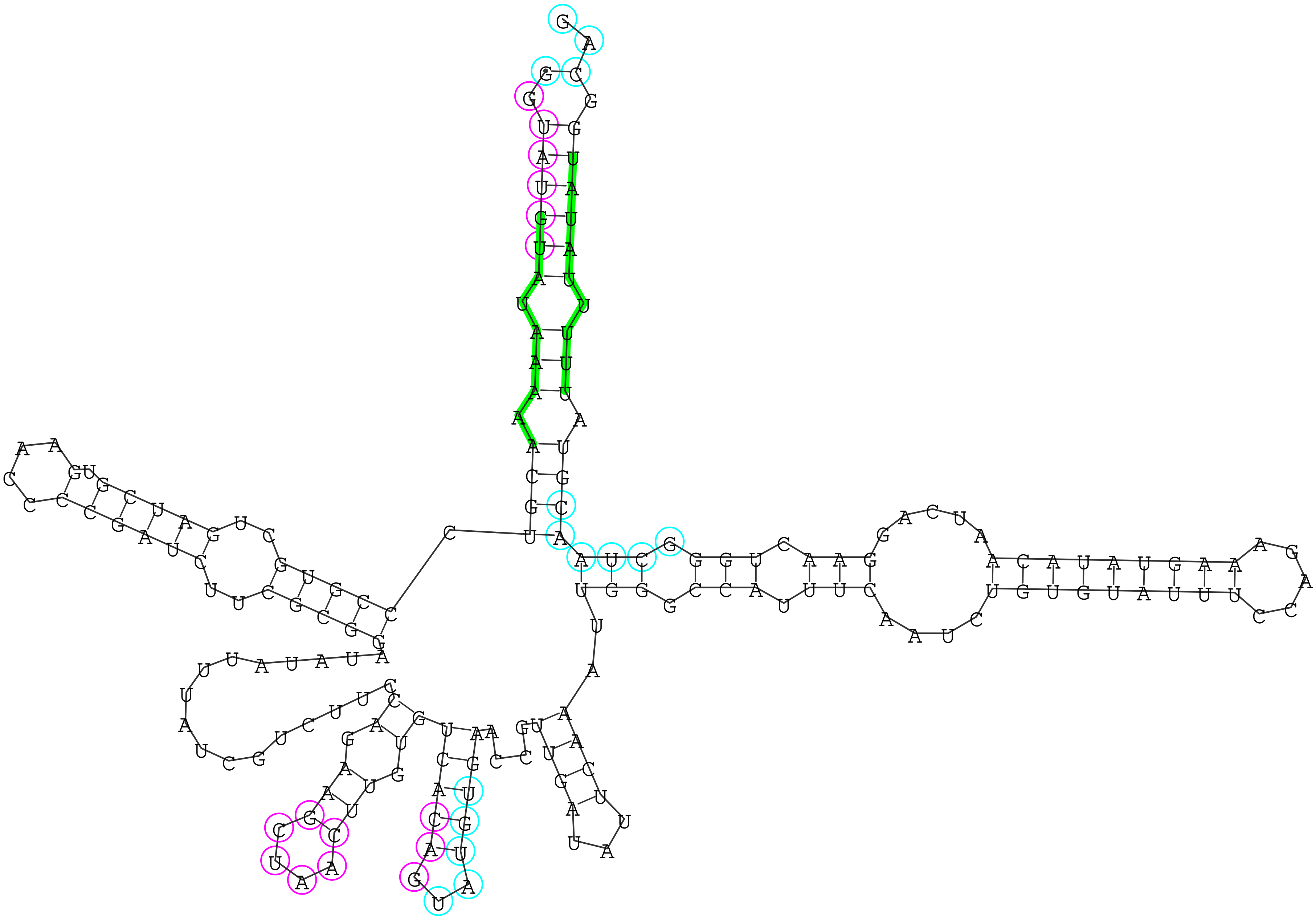

Xmsuc0671A - Stwintron

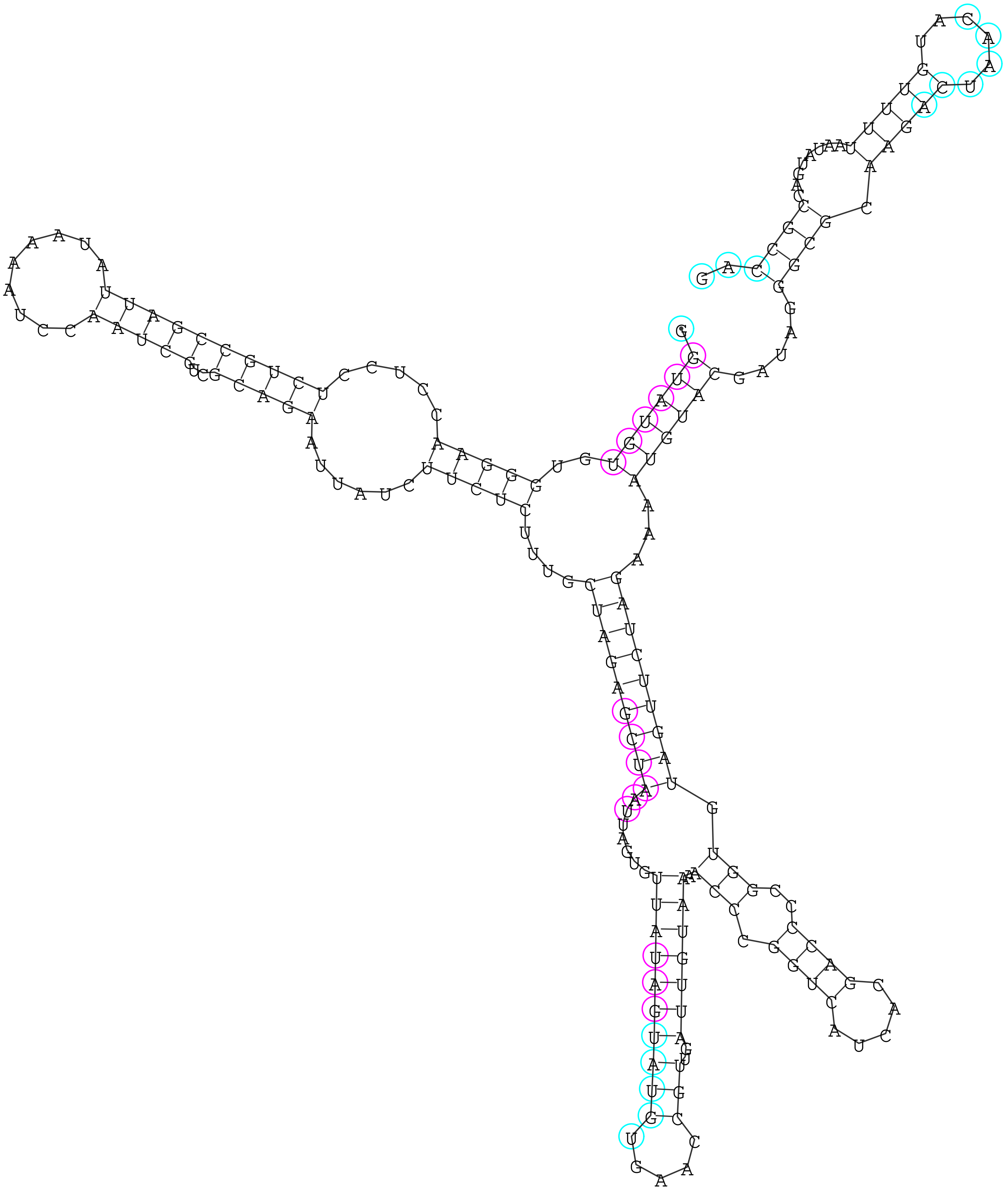

Xmsuc0710A - Stwintron

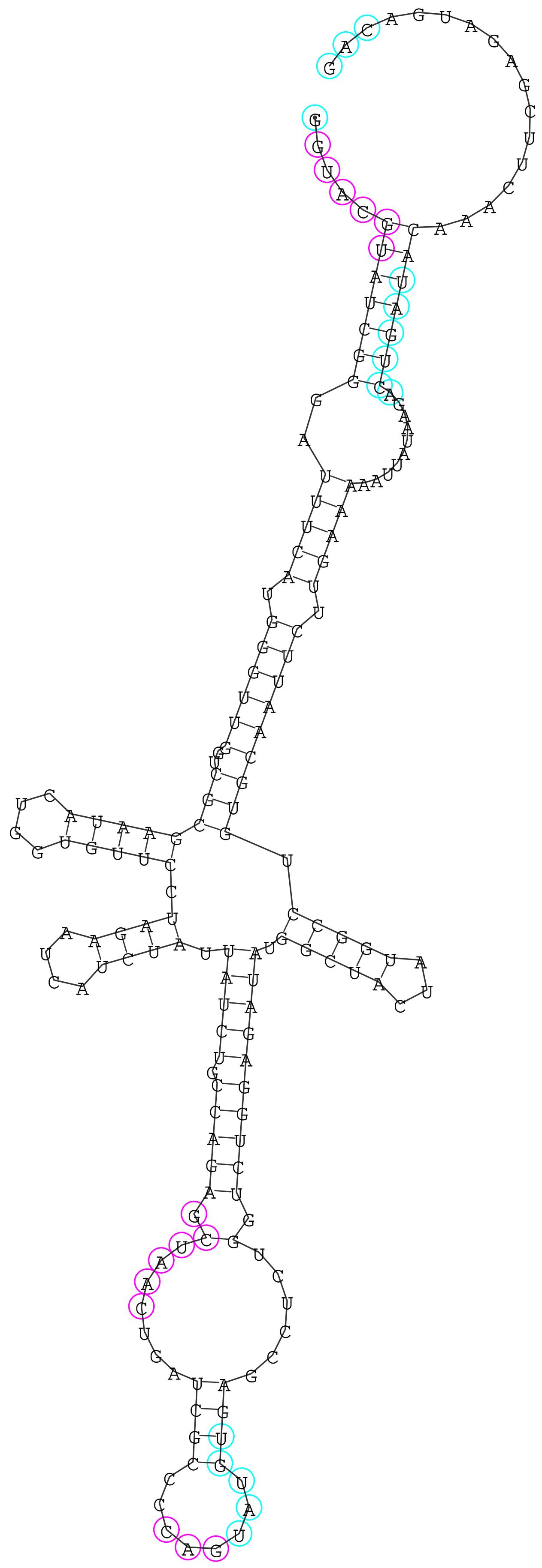

Xmsuc0775A - Stwintron

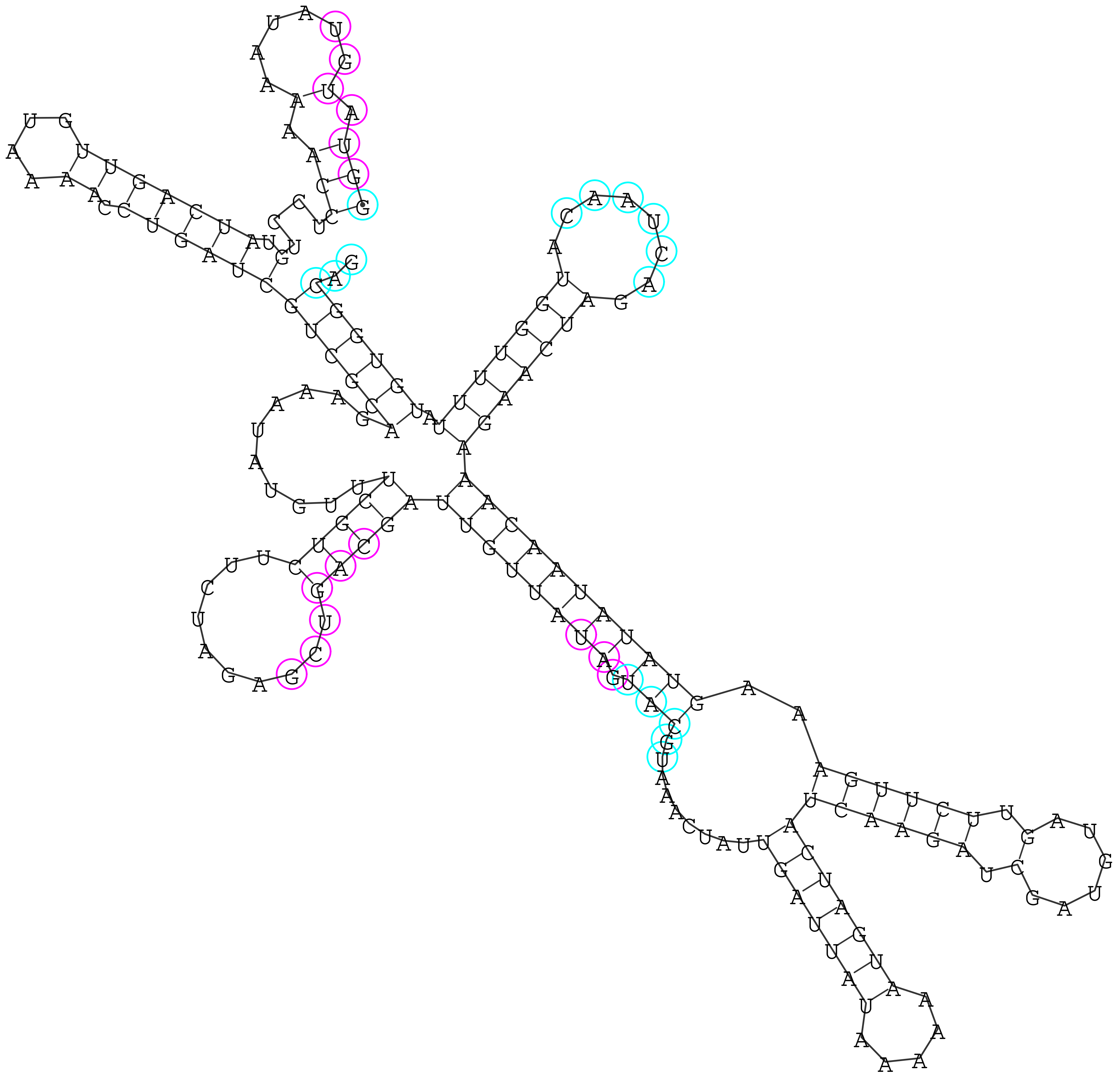

Xmsuc0776A - Stwintron

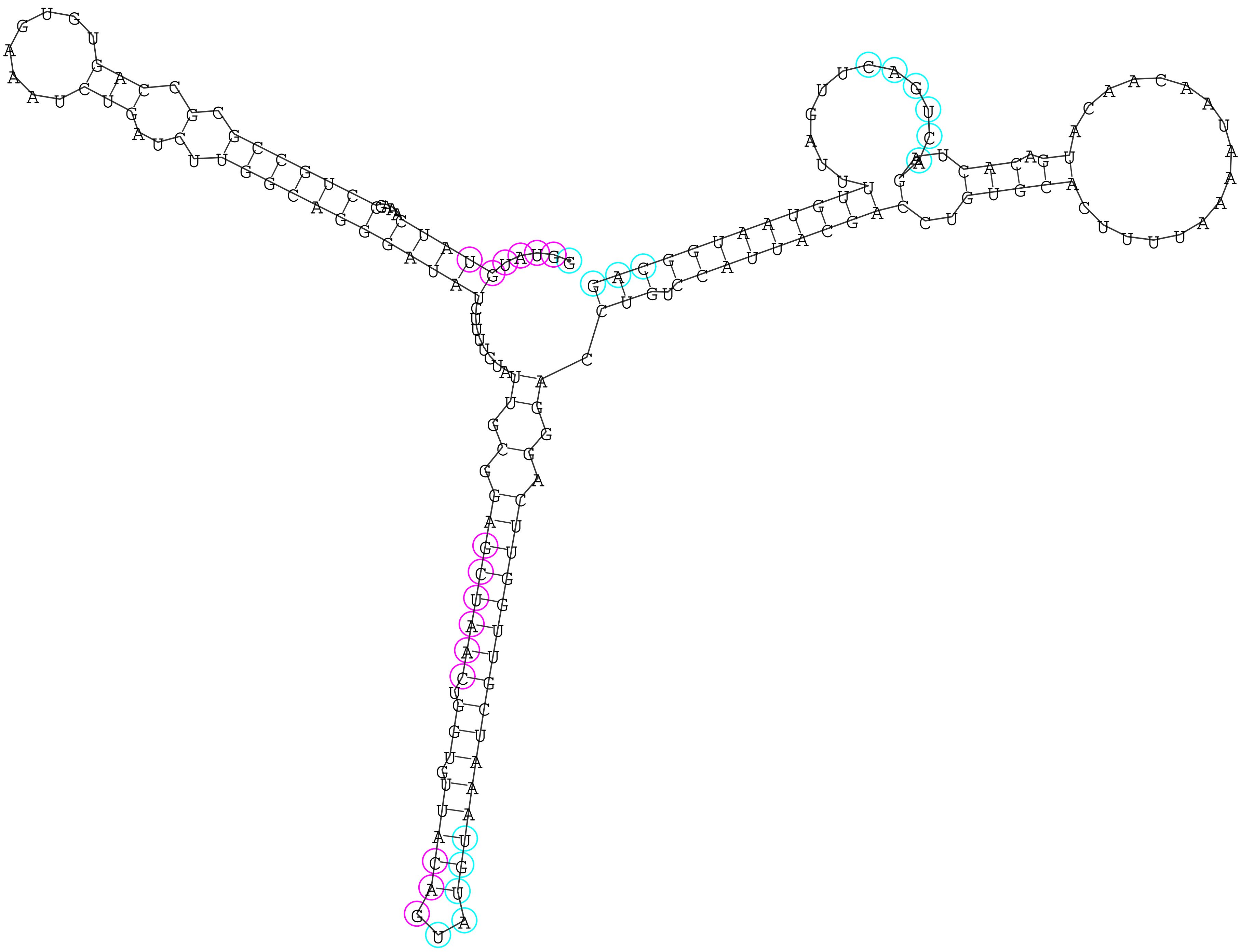

Xmsuc0776B - Stwintron

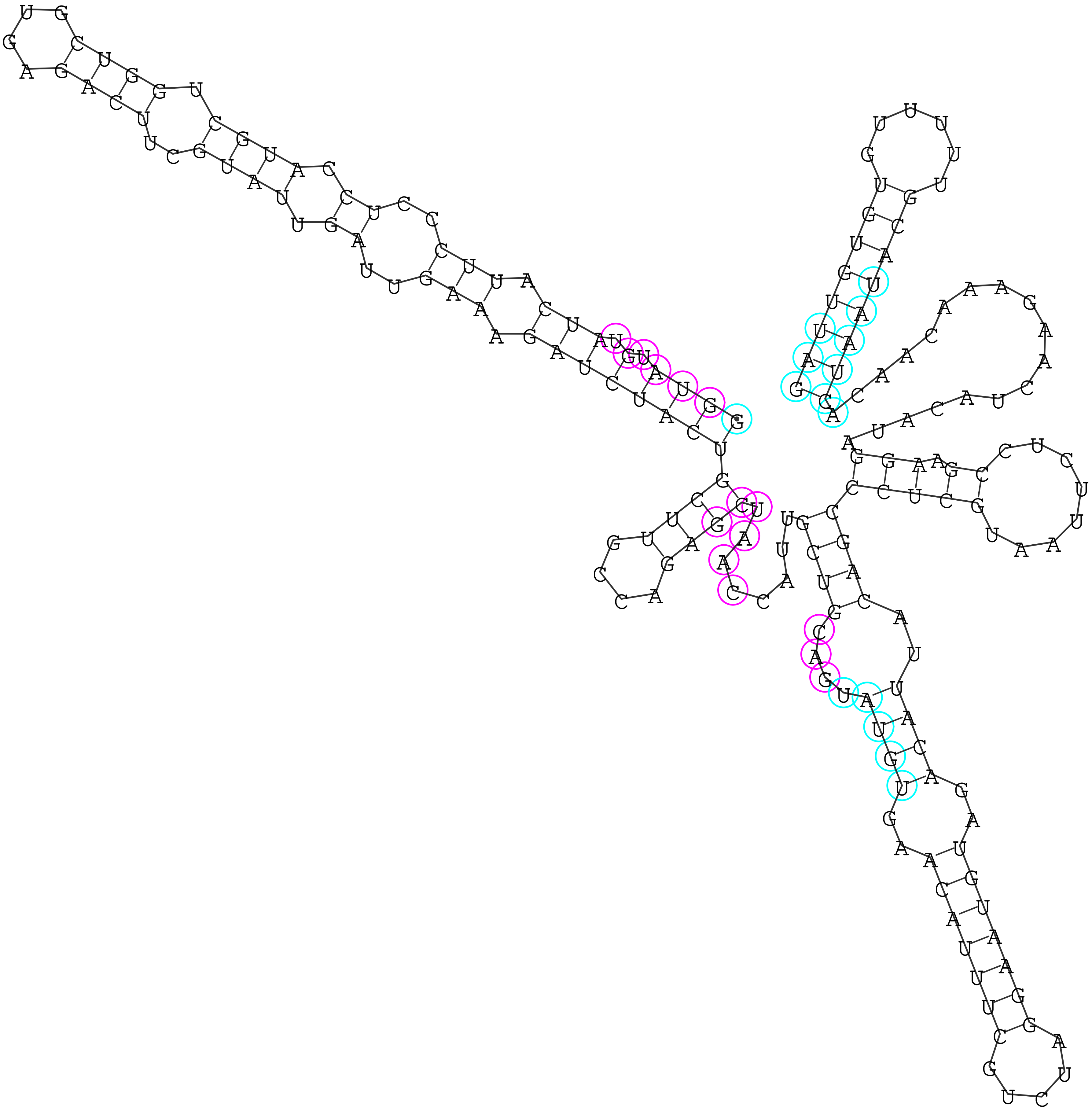

Xmsuc0776C - Stwintron

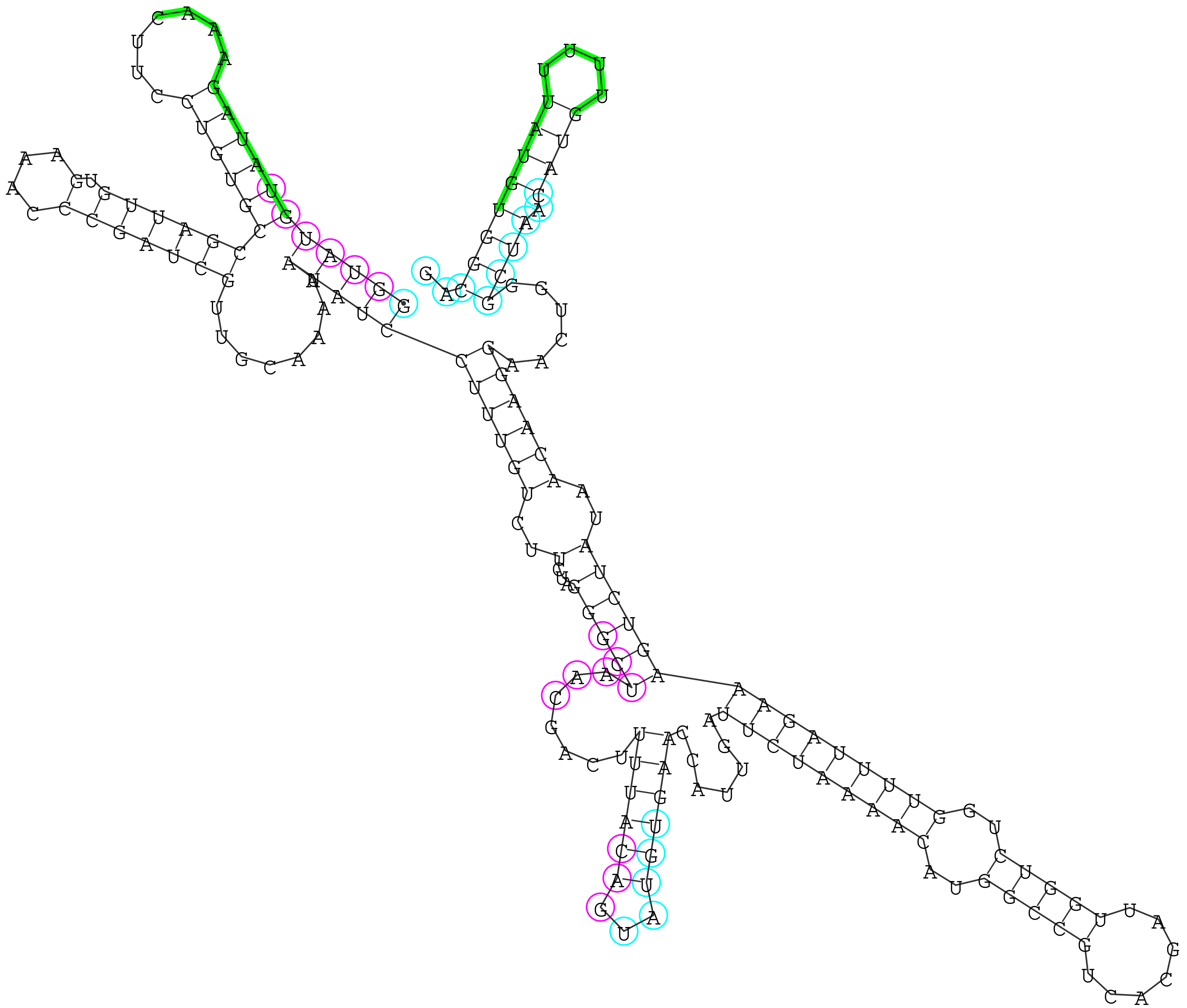

Xmsuc0808A - Stwintron

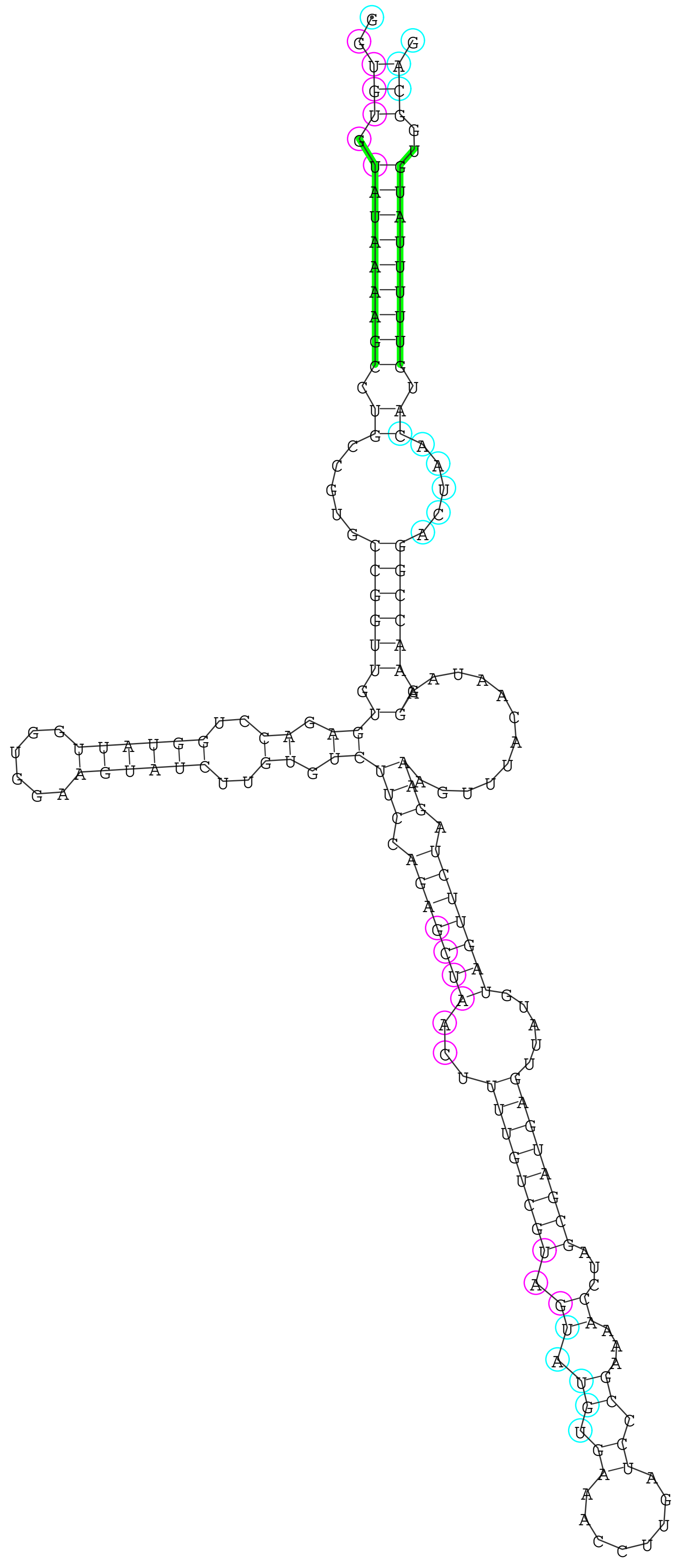

Xmsuc0819A - Stwintron

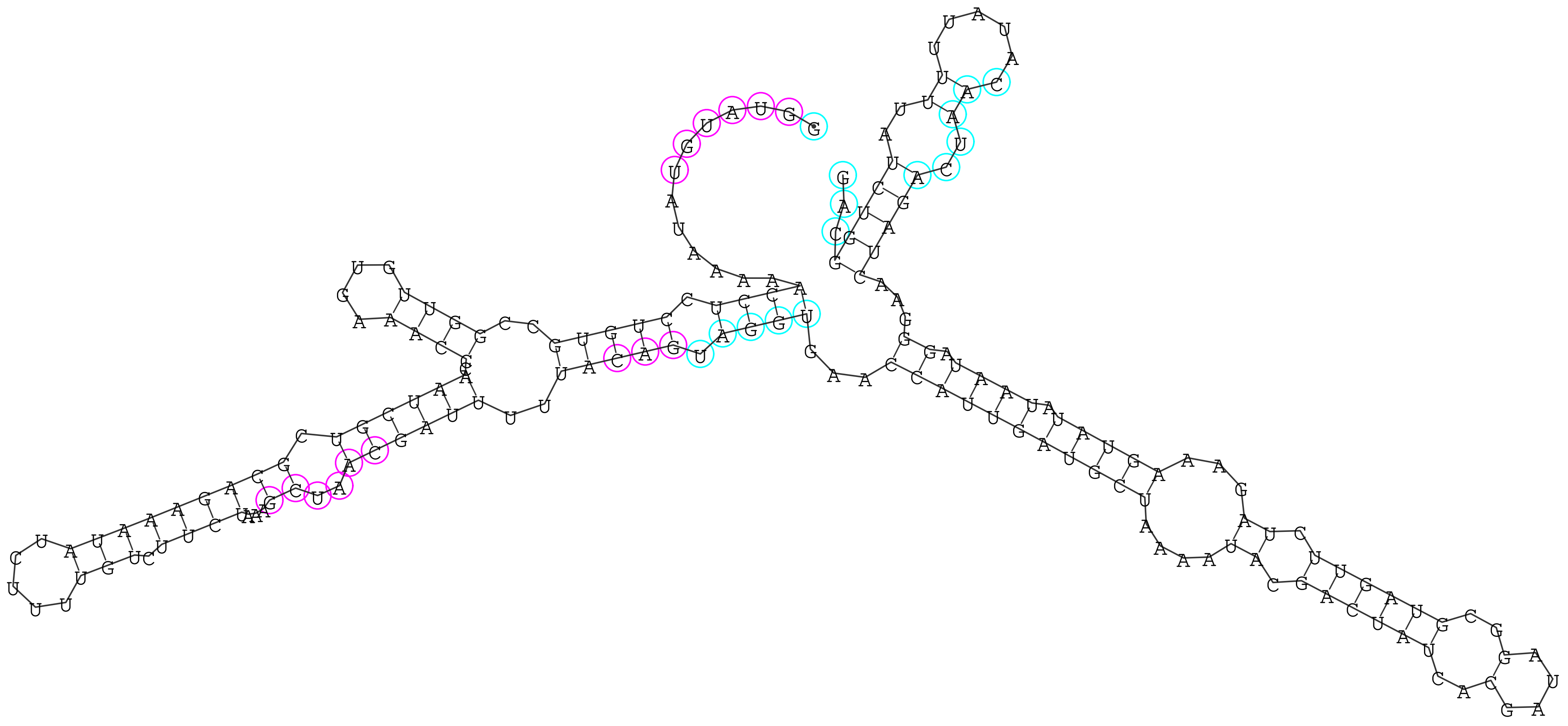

Xmsuc0904A - Stwintron

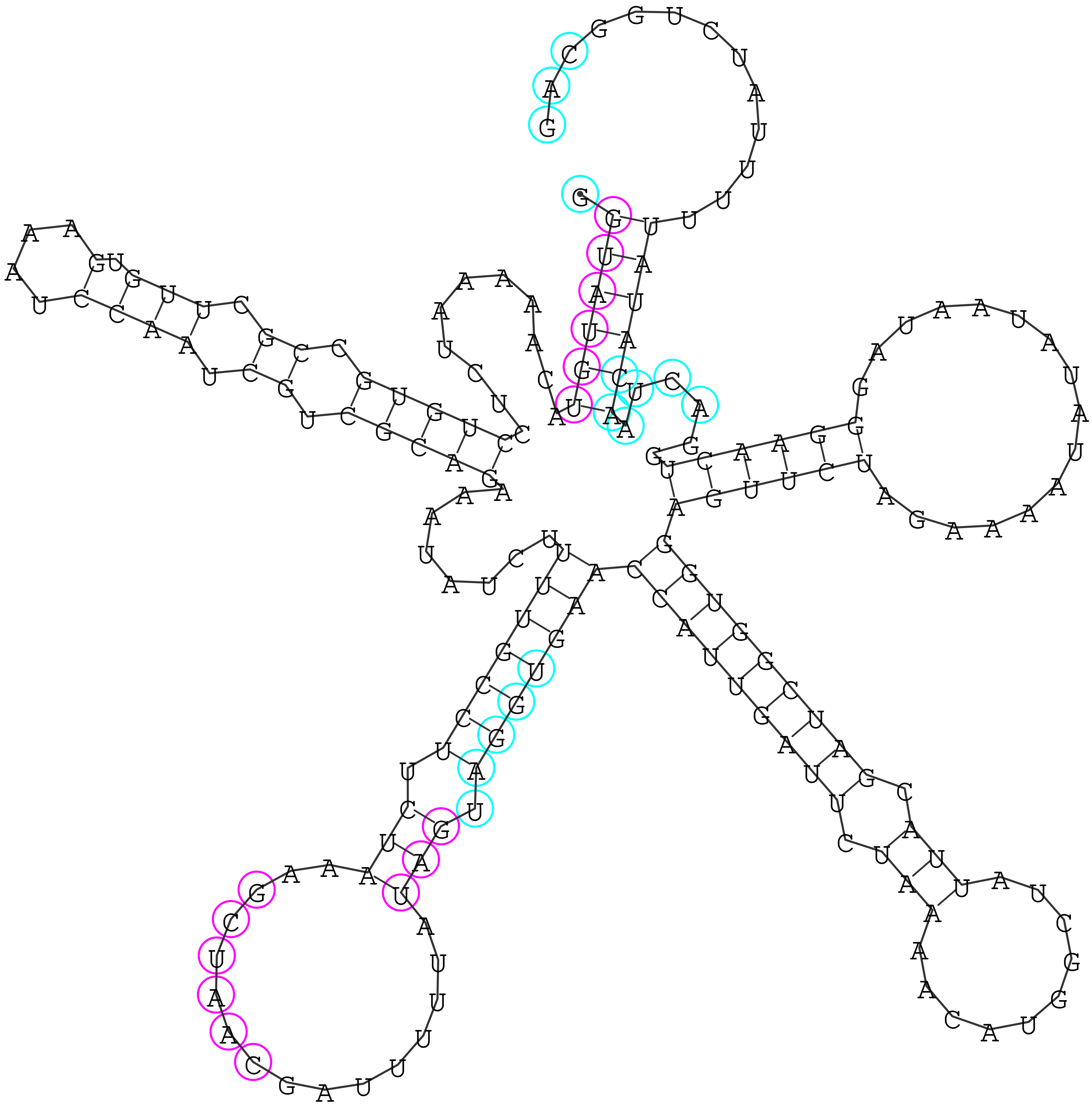

Xmsuc1083A - Stwintron

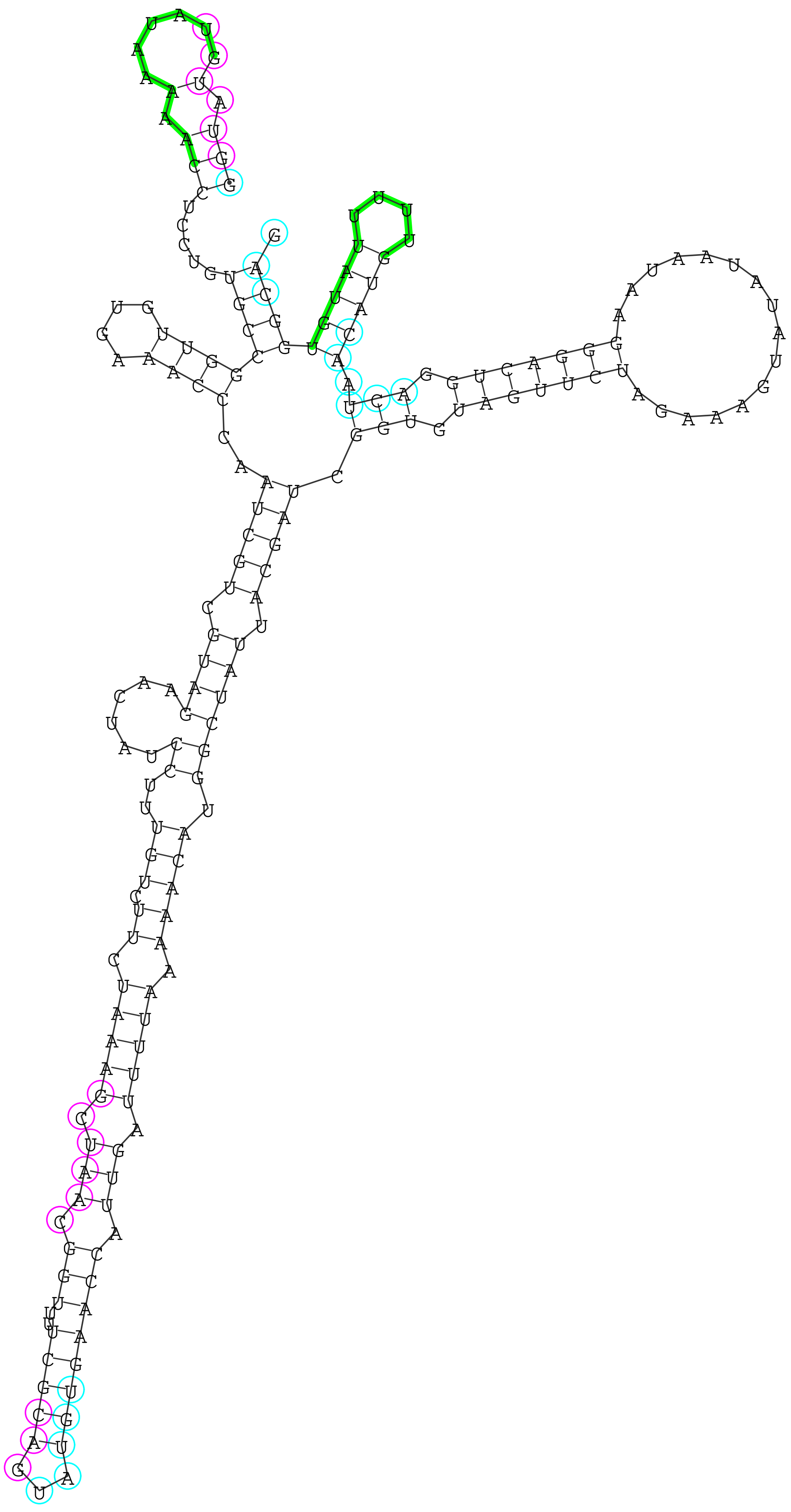

Xmsuc1127A - Stwintron

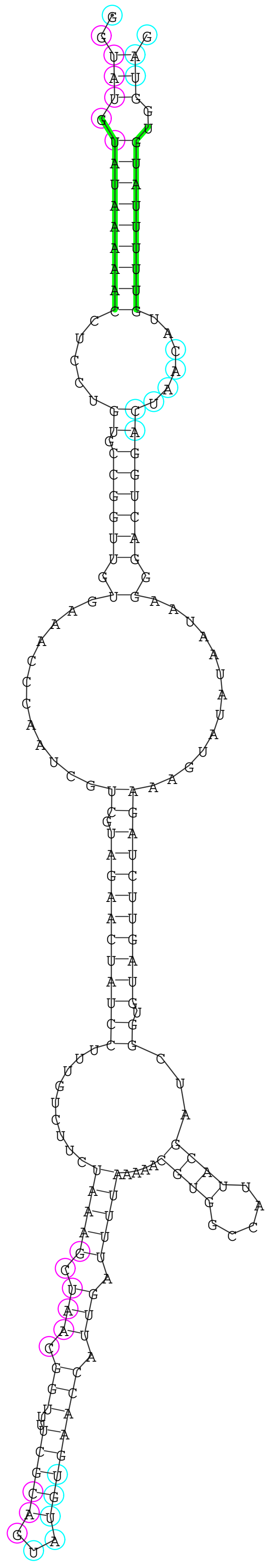

Xmsuc1145A - Stwintron

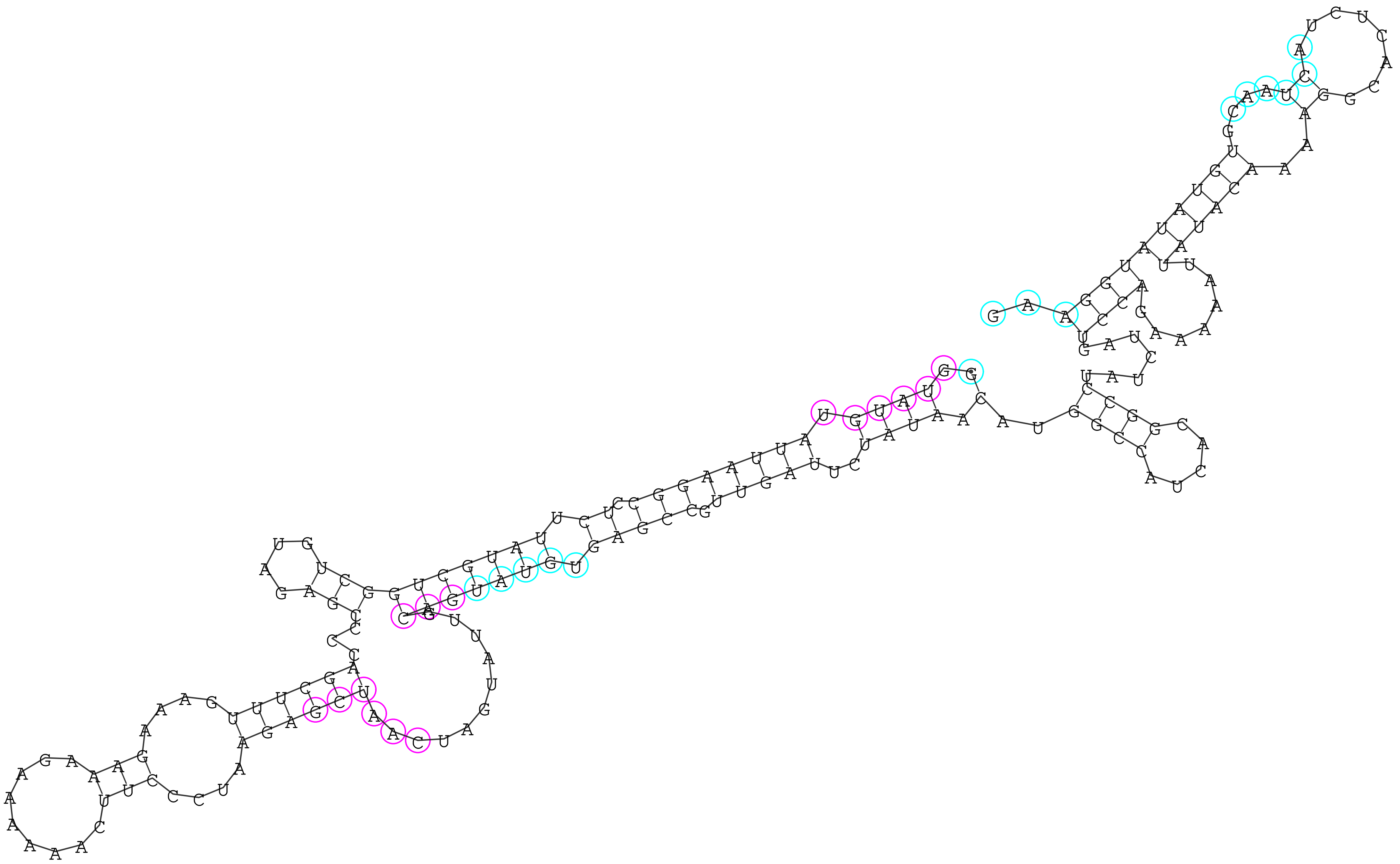

Supplement: Data S1 — Collection of the RNAFold predicted secondary structures of 288 [D1,2] sister stwintrons in 14 taxa of Xylariales. [file spectrum.02926-24-s0001.pdf]
