# Supplementary material for: Propagation of [D1,2]-type spliceosomal twin introns (stwintrons) in Hypoxylaceae and Xylariaceae fungi
Source: Microbiol Spectr. 2025 Aug 8;13(9):e02926-24. doi: 10.1128/spectrum.02926-24 (PMC12403724; doi:10.1128/spectrum.02926-24)

Supplementary datafile S2. Collection of the RNAFold predicted secondary structures of the internal introns of the 288 [D1,2] sister stwintrons. The intron sequences are given in Table S1. The internal intron interrupts the 5'-donor of the external intron between its first and second nt ([D1,2]). The 5'-donor (6-nt core) [D] and 3'-acceptor (3-nt core) [A] elements at the splice sites are marked with magenta circles around the nt. The conserved sequence element including the branch point adenosine [L] (6-nt core) near the 3'-splice site is likewise highlighted.

Dchc001A - Internal intron

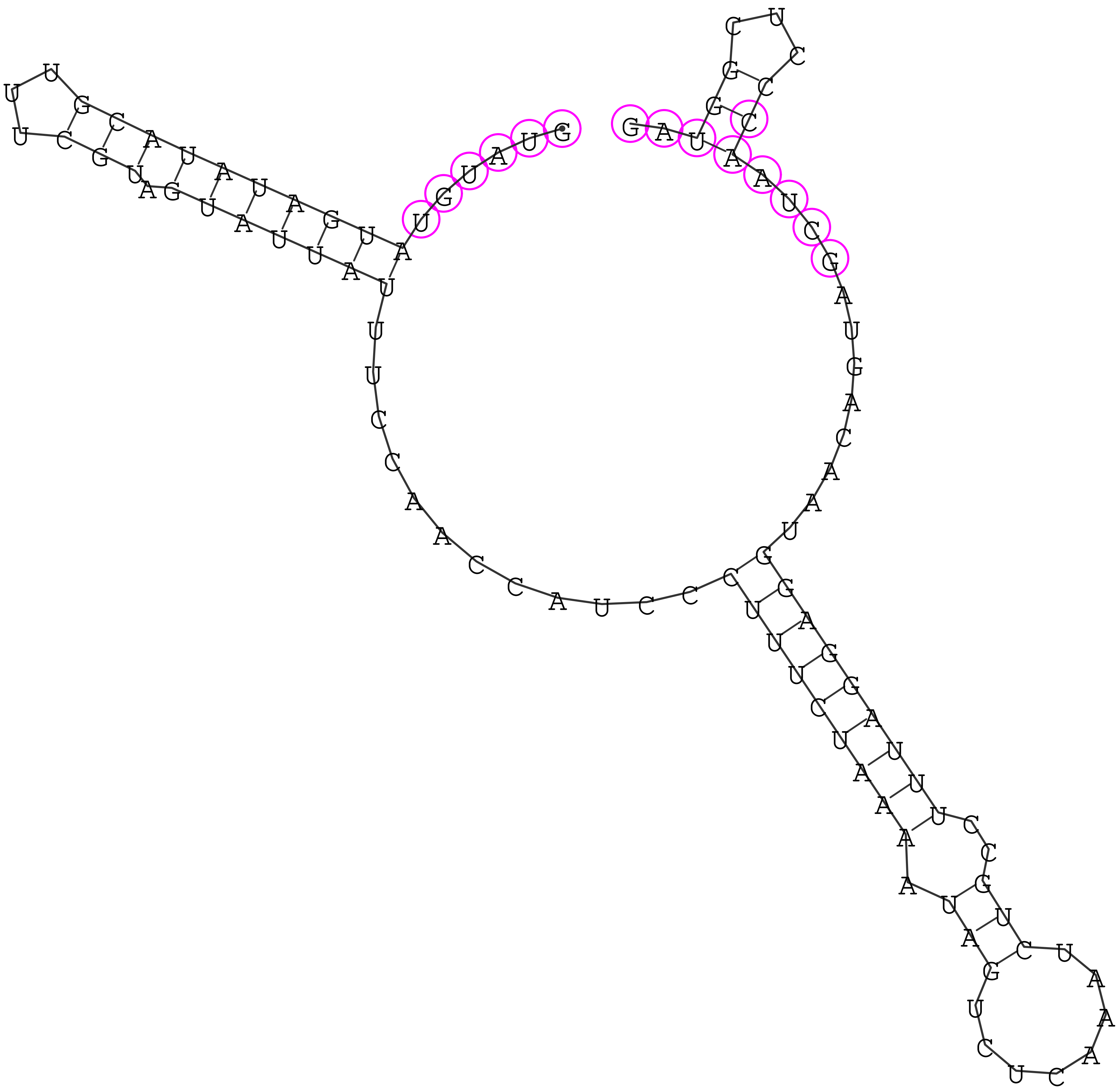

Dchc001B - Internal intron

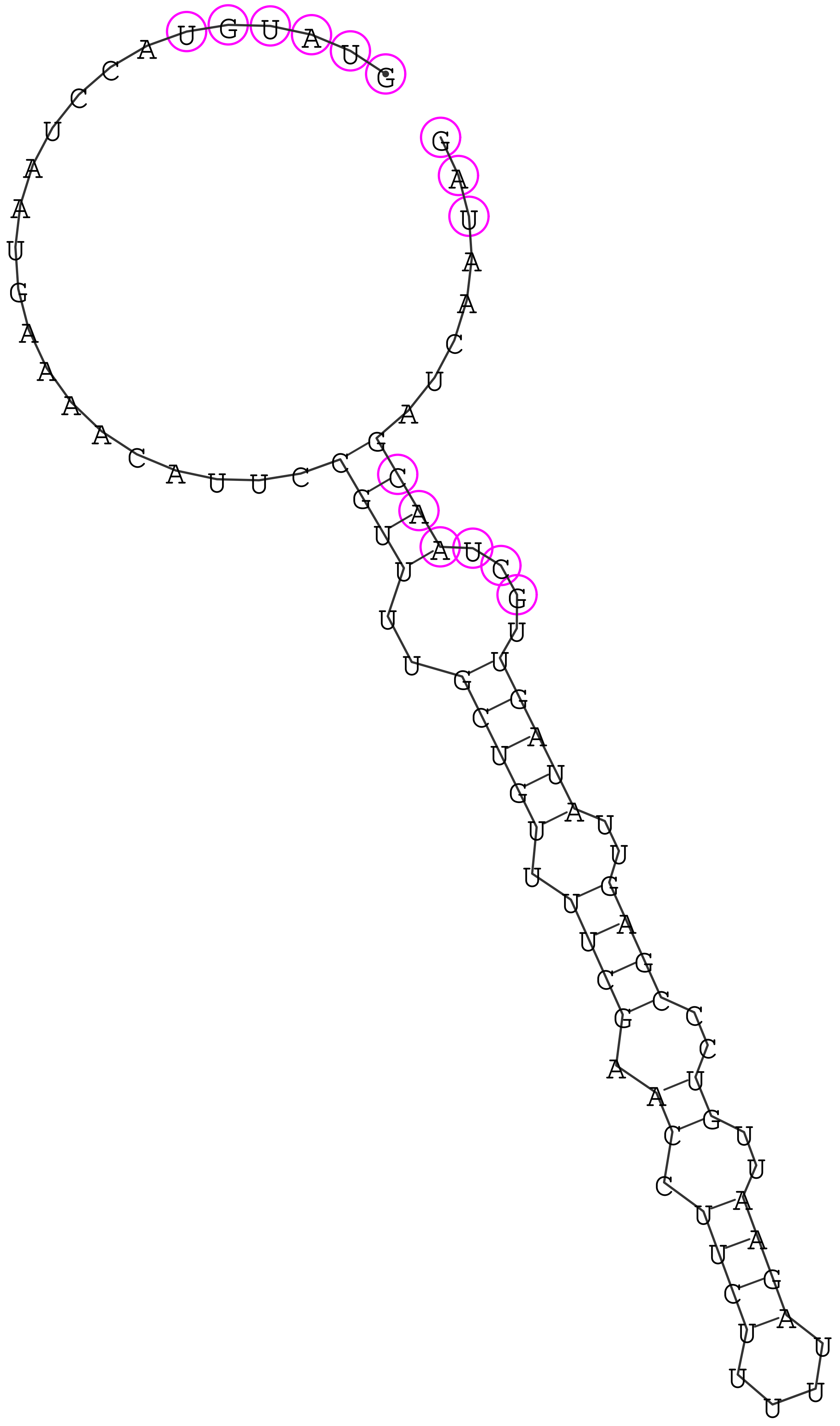

Dchc001C - Internal intron

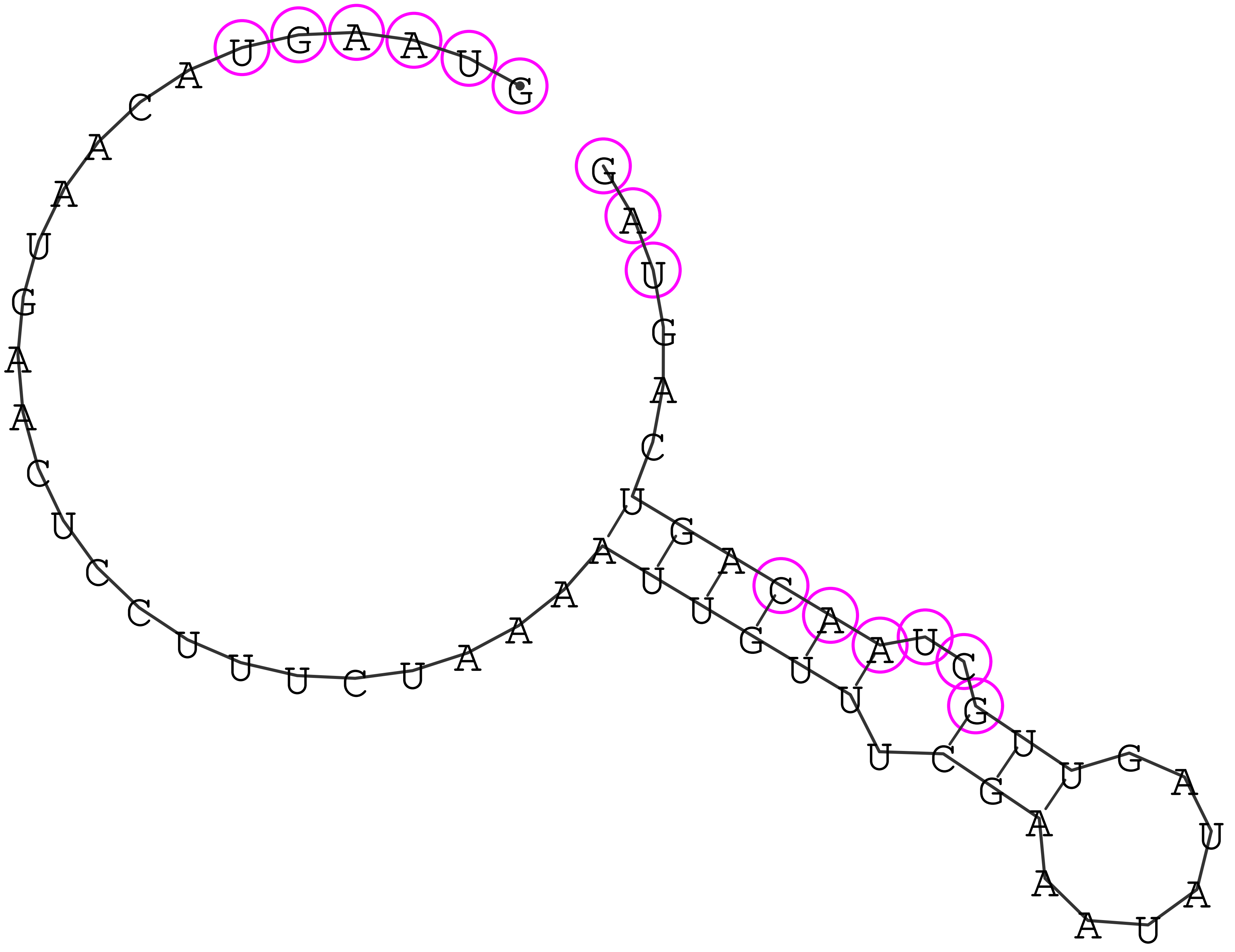

Dchc001D - Internal intron

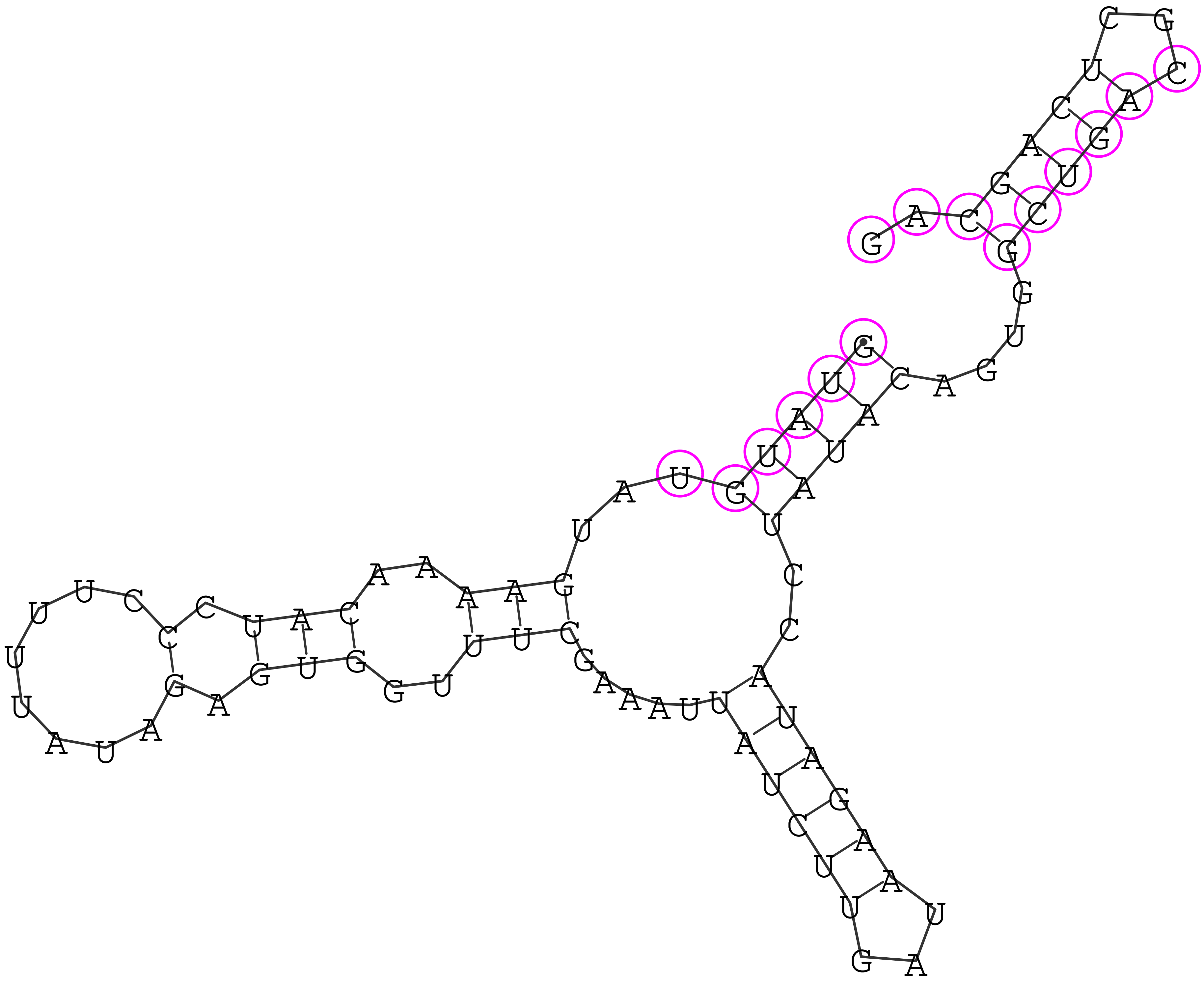

Dchc002A - Internal intron

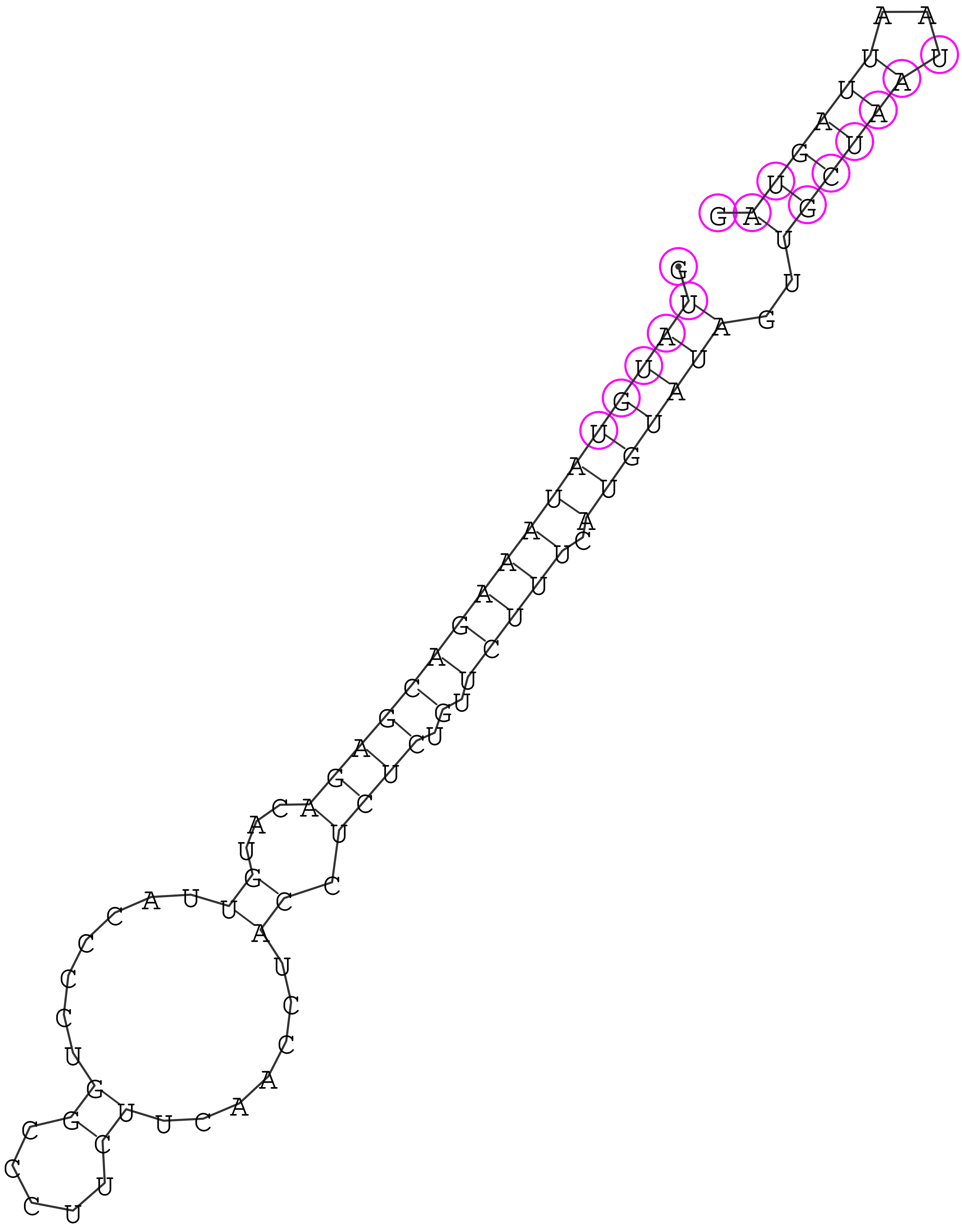

Dchc003A - Internal intron

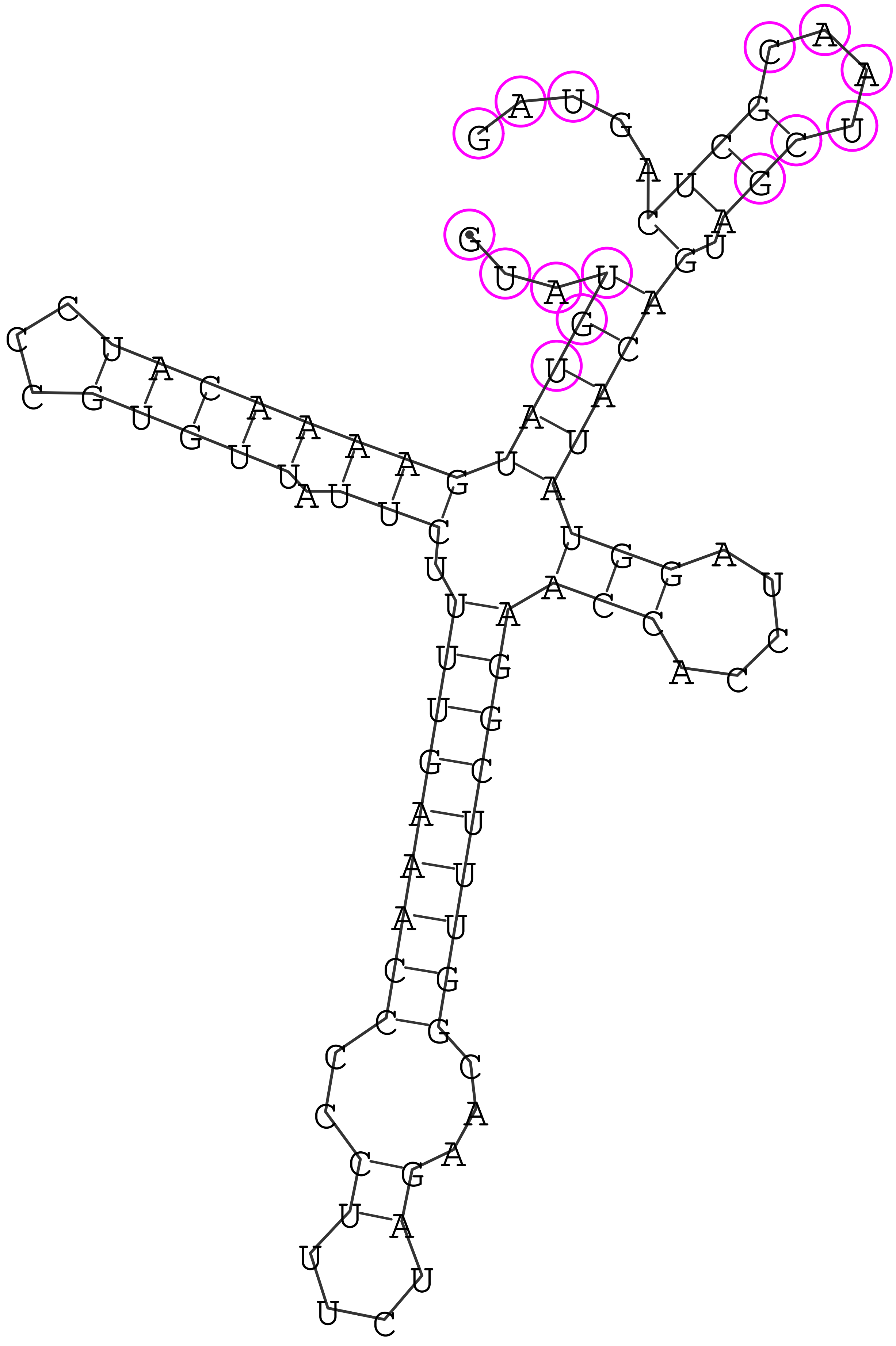

Dchc003B - Internal intron

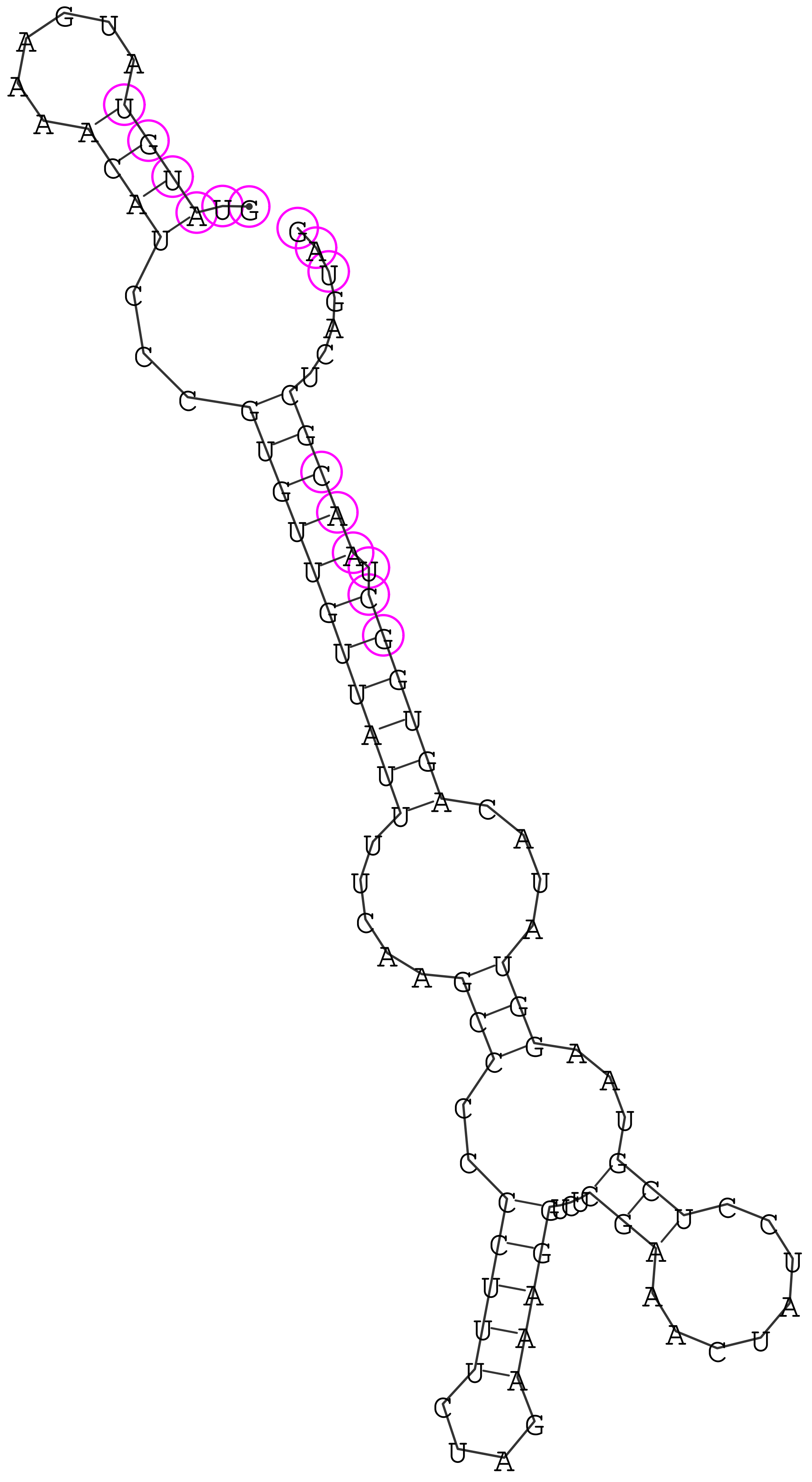

Dchc003C - Internal intron

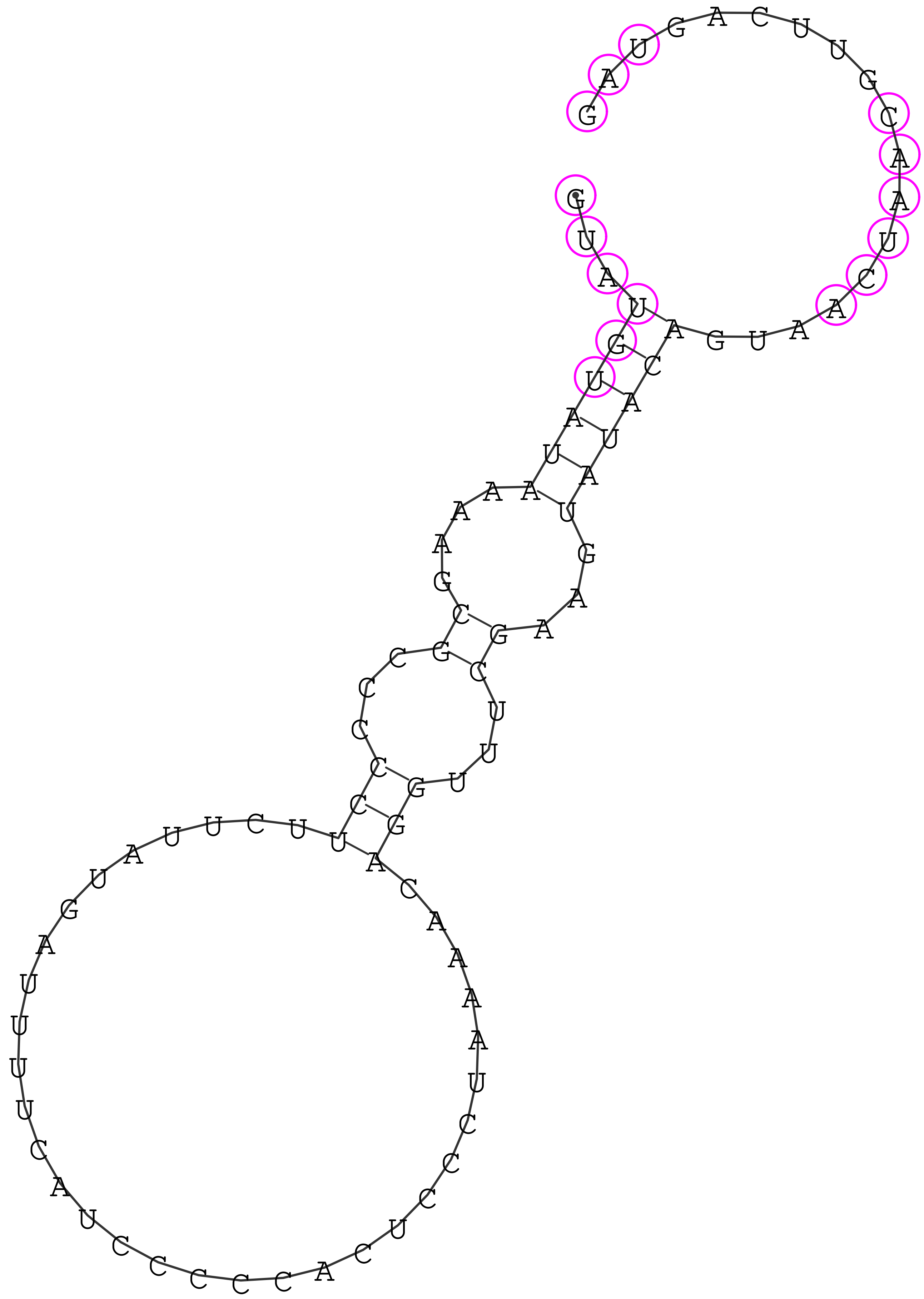

Dchc004A - Internal intron

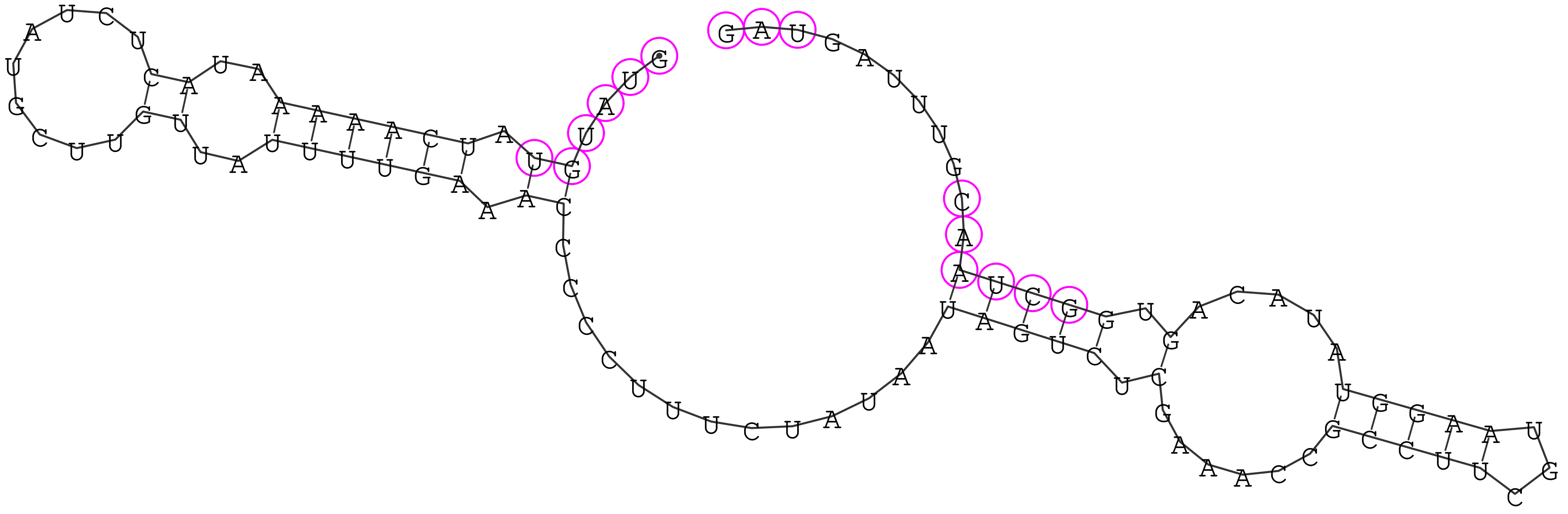

Dchc004B - Internal intron

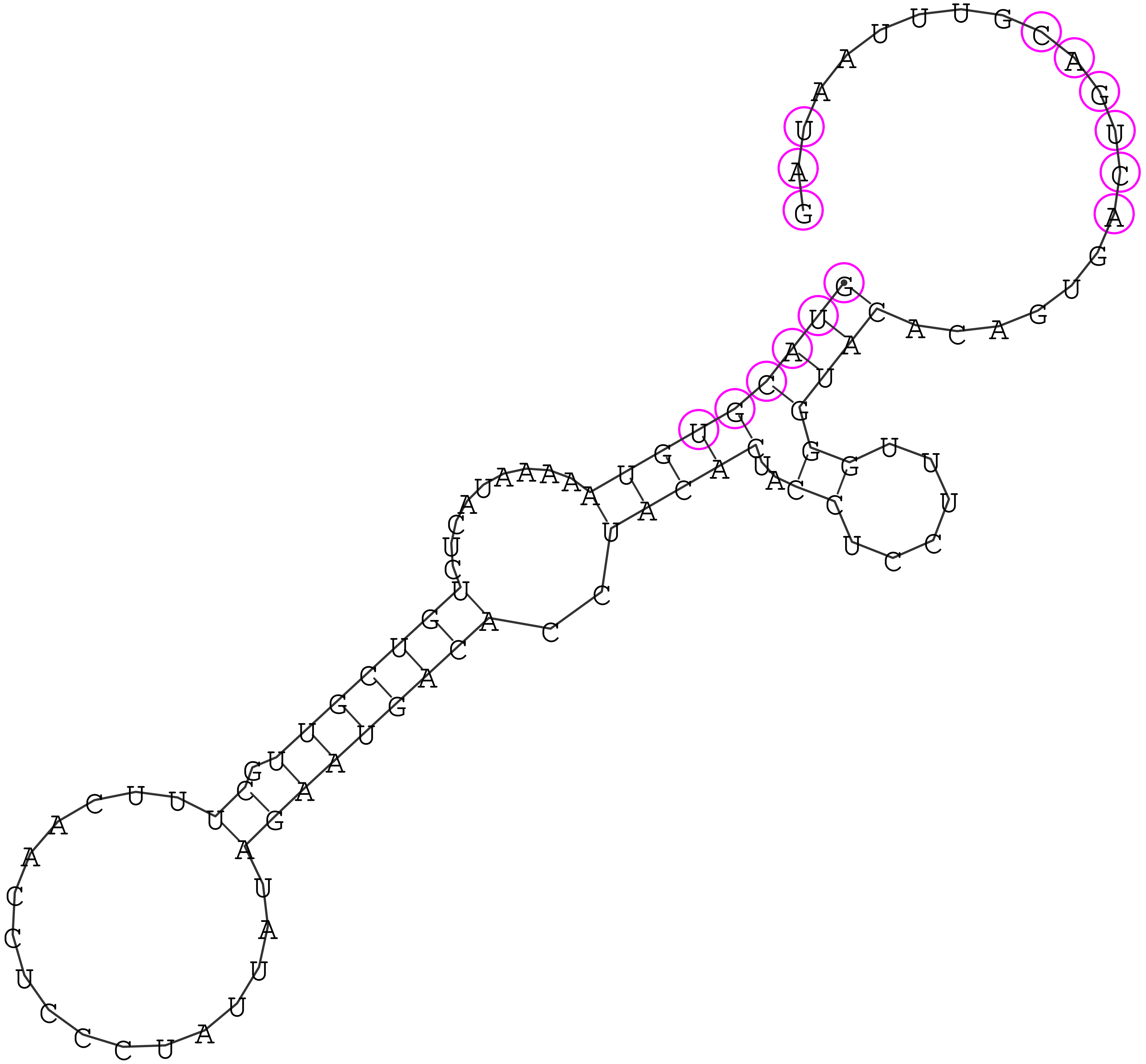

Dchc004C - Internal intron

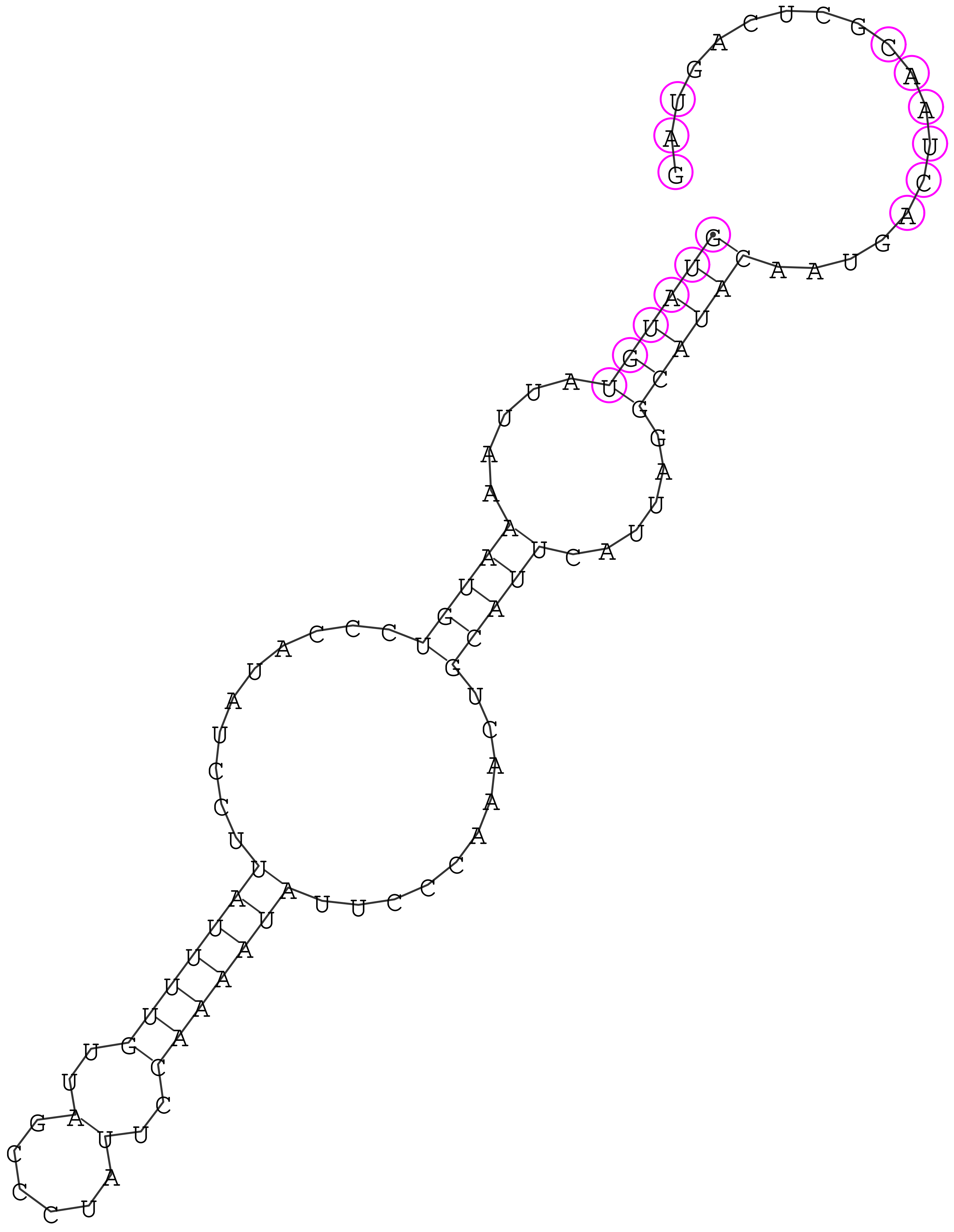

Dchc005A - Internal intron

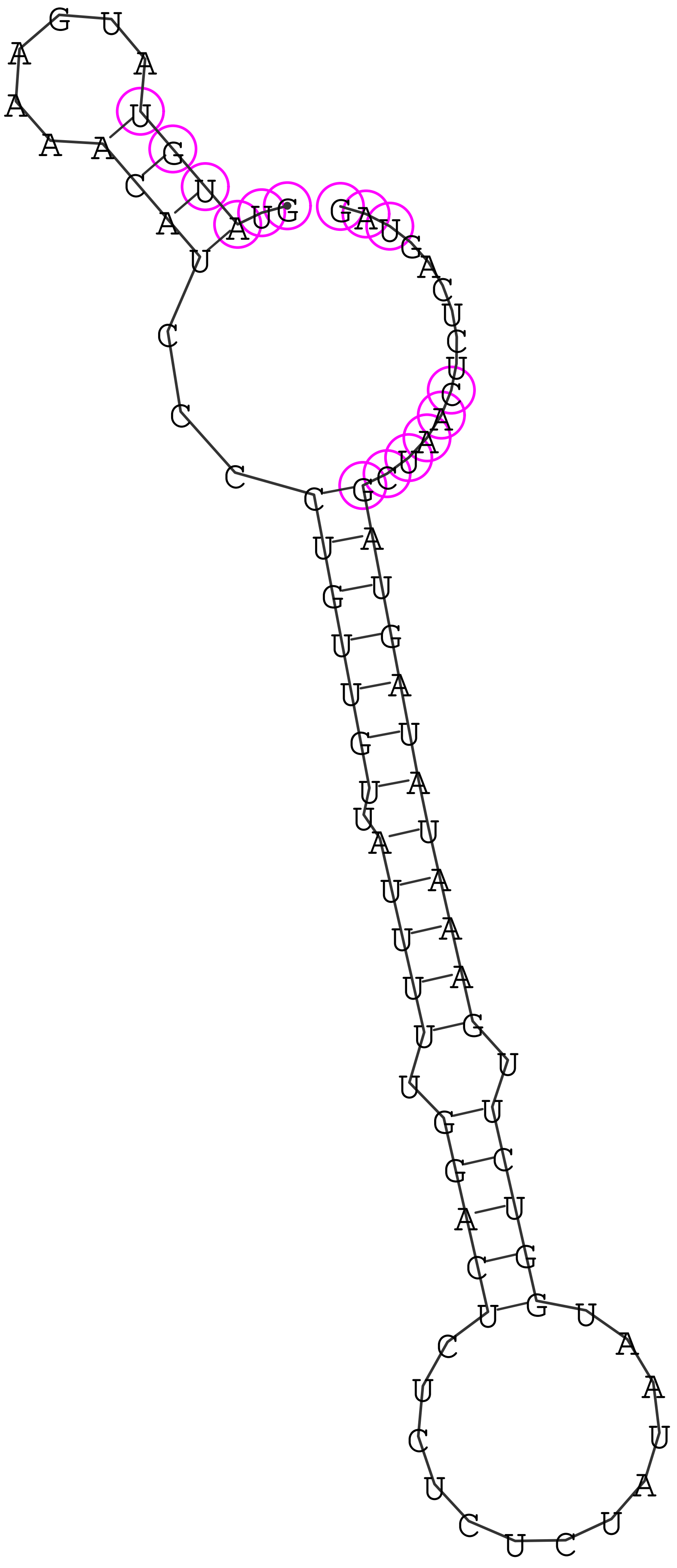

Dchc005B - Internal intron

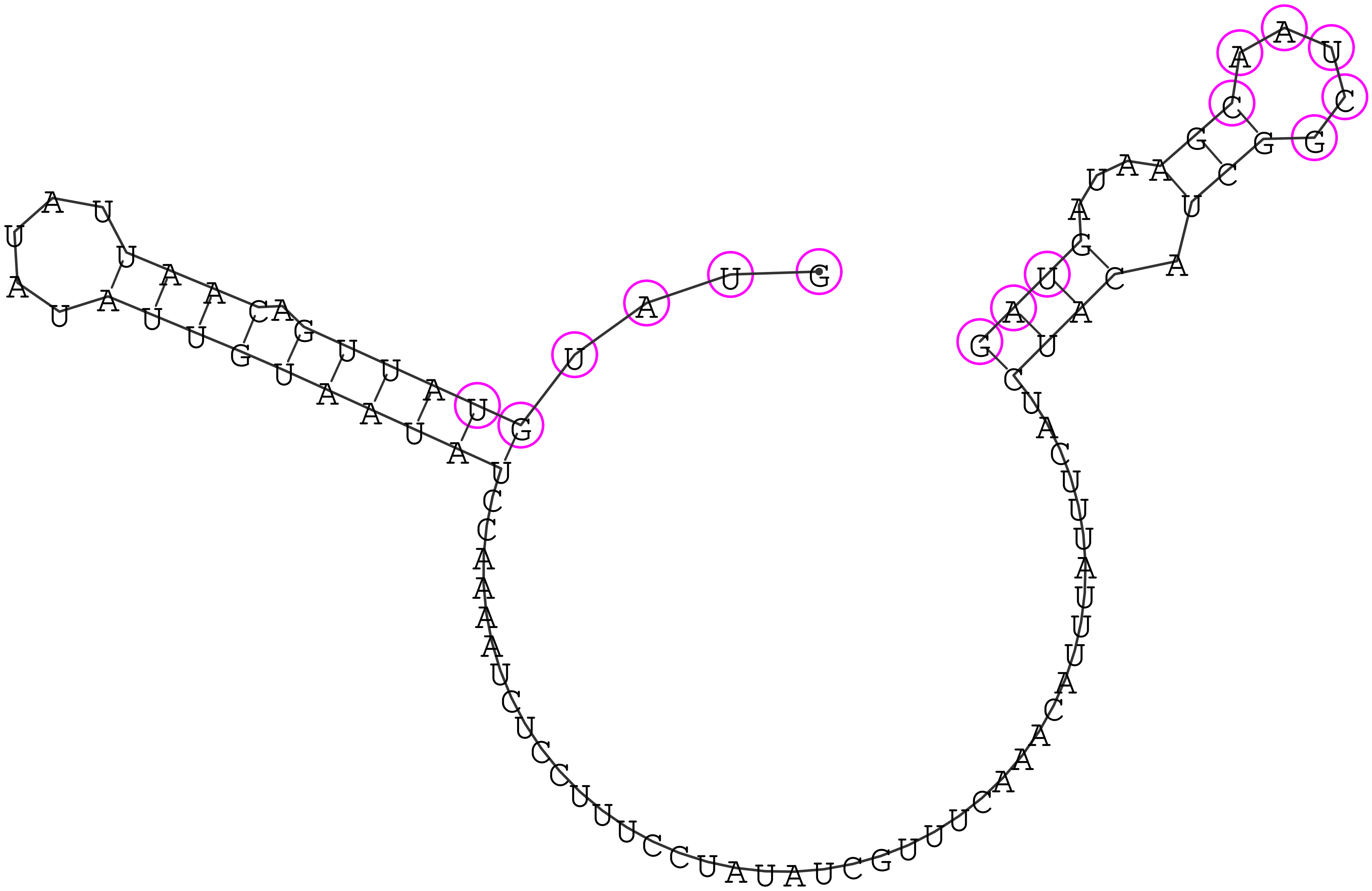

Dchc007A - Internal intron

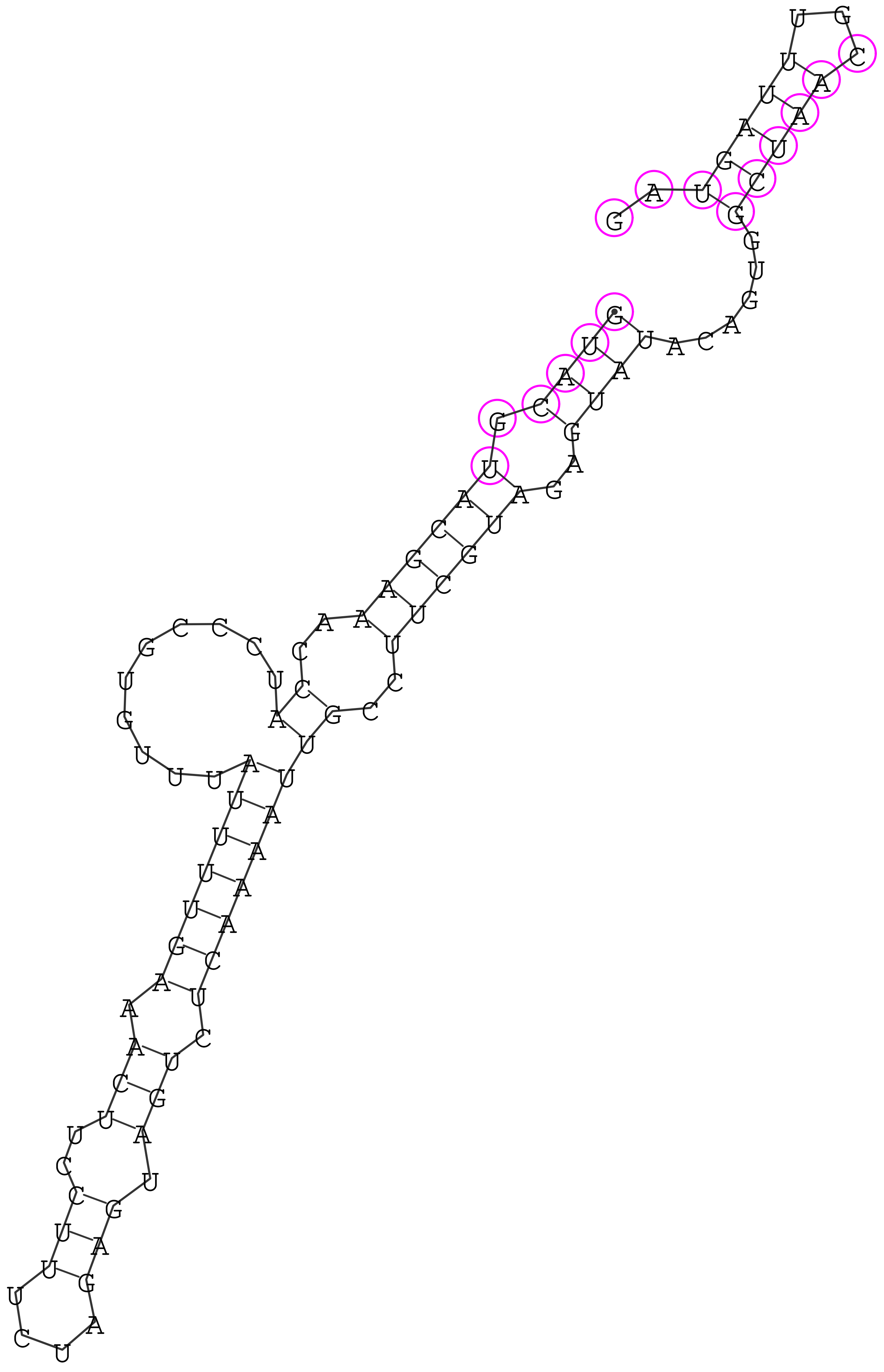

Dchc007B - Internal intron

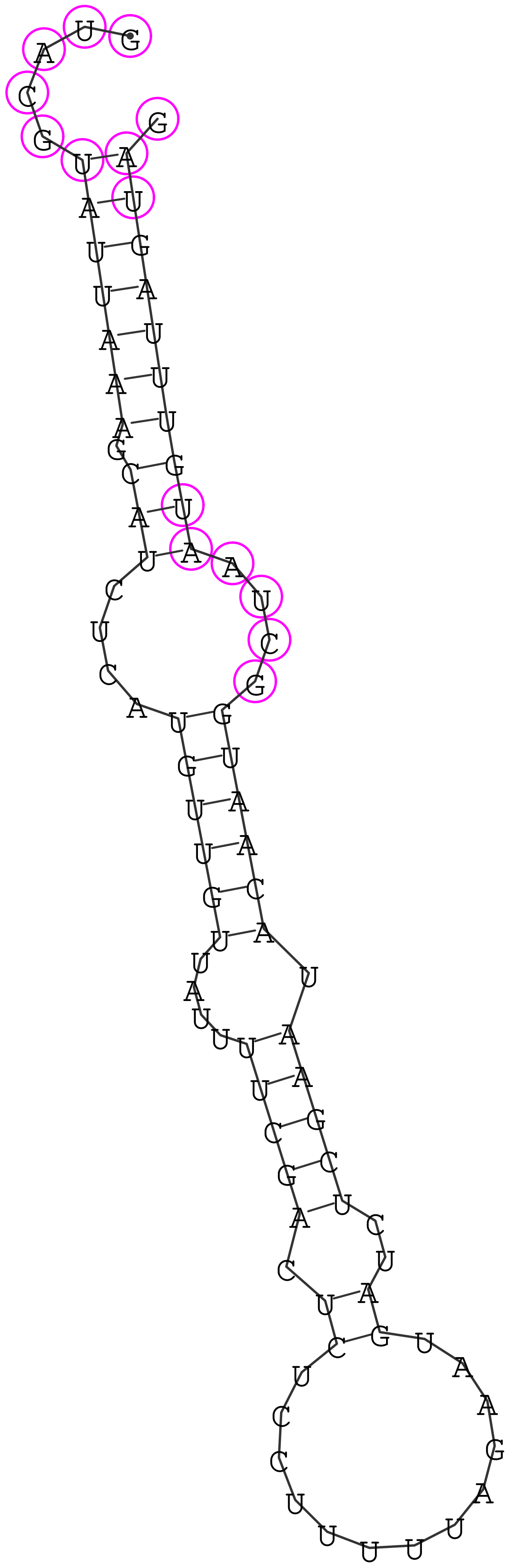

Dchc008A - Internal intron

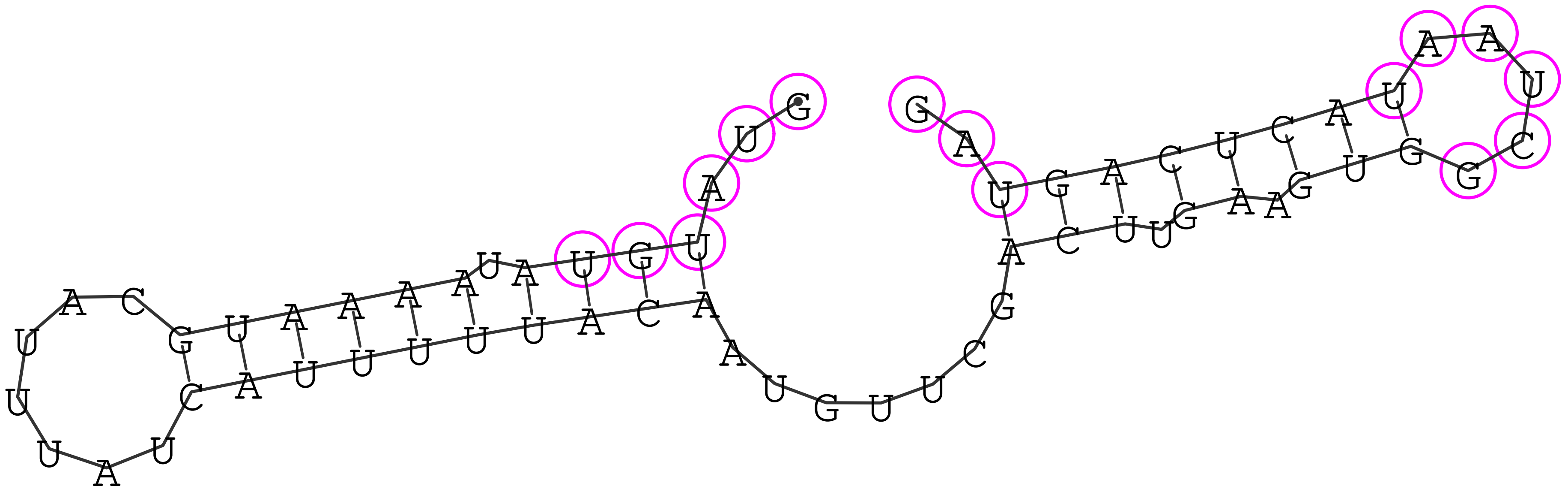

Dchc011A - Internal intron

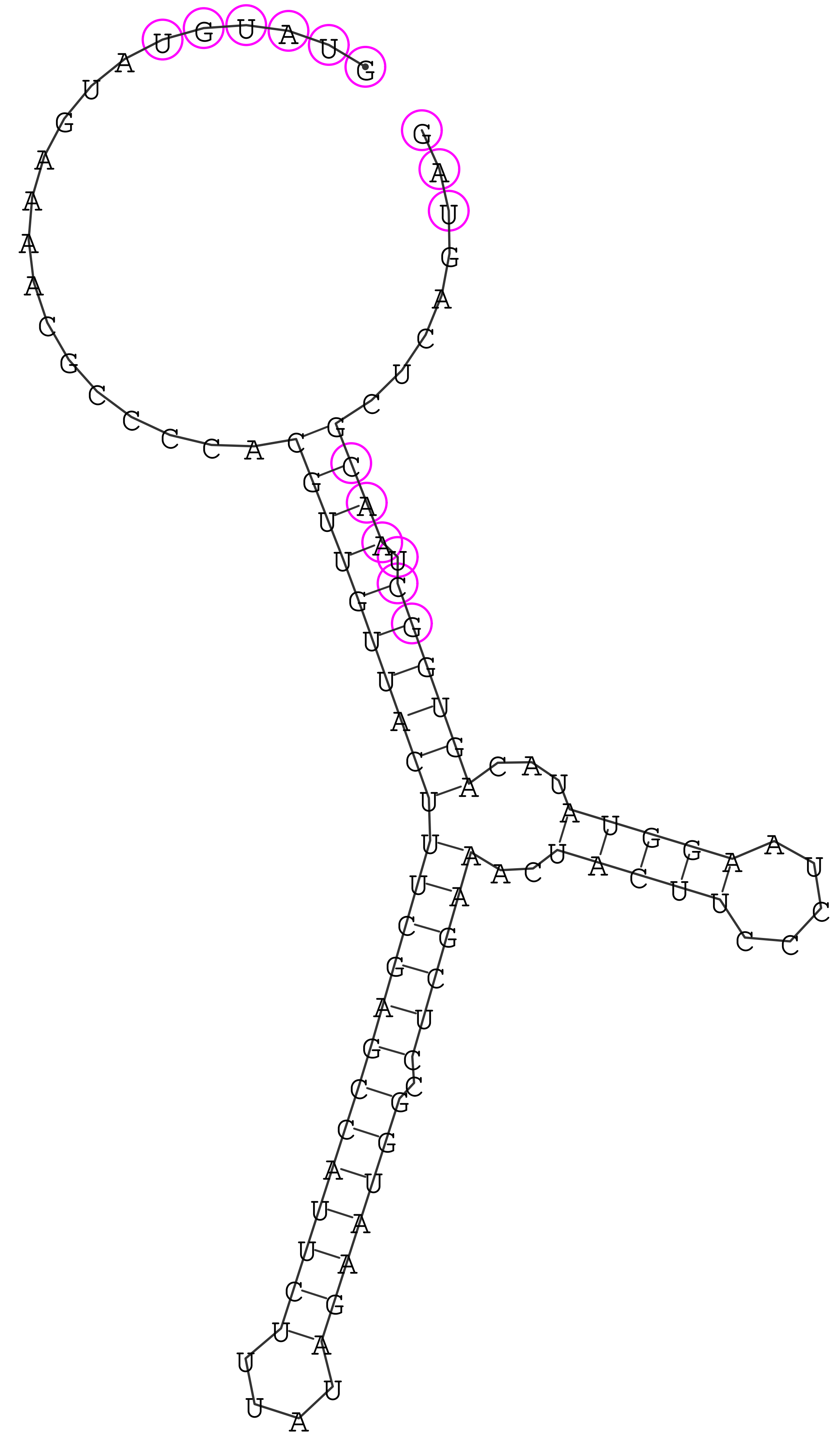

Dchc013A - Internal intron

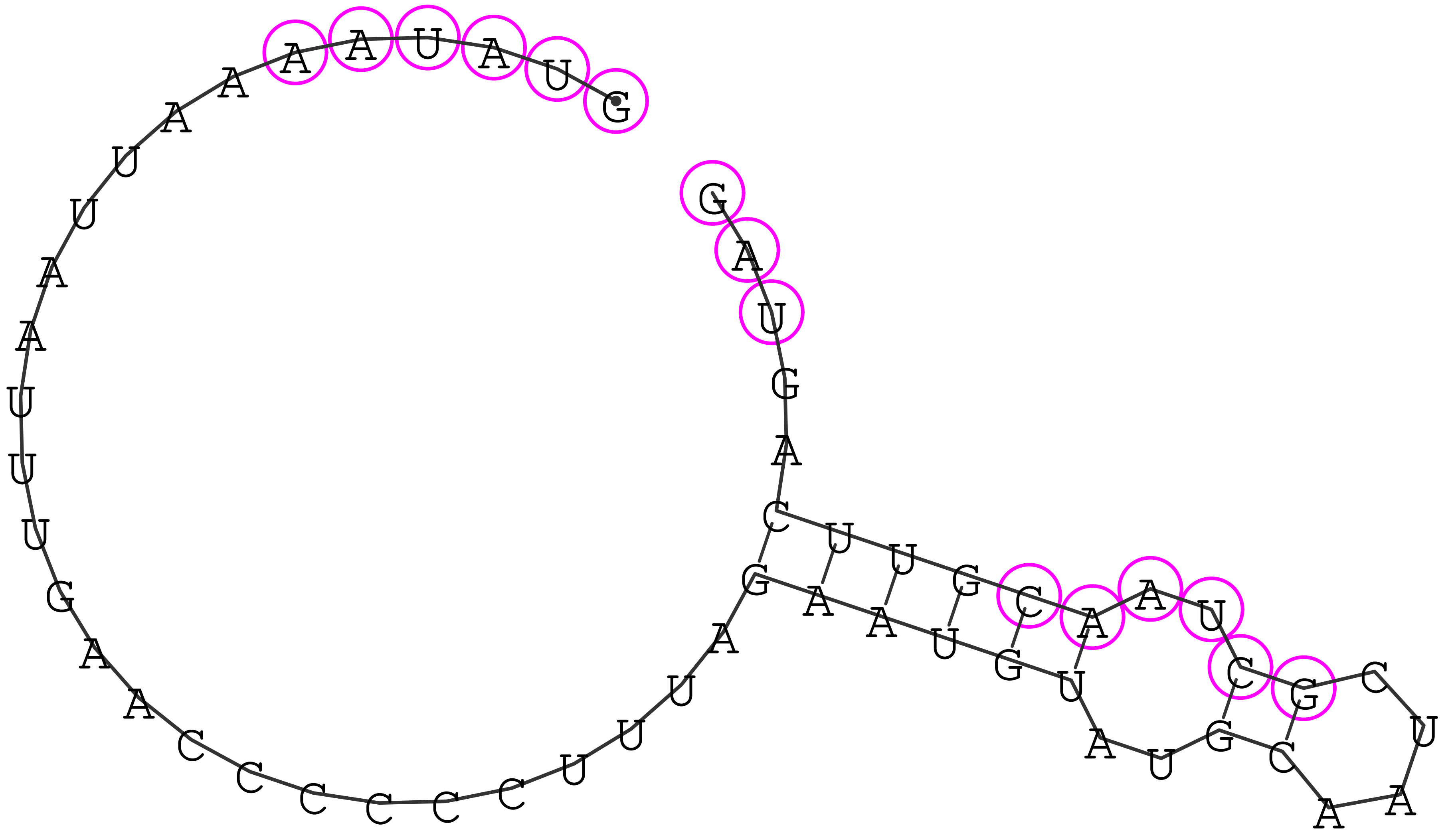

Dchc014A - Internal intron

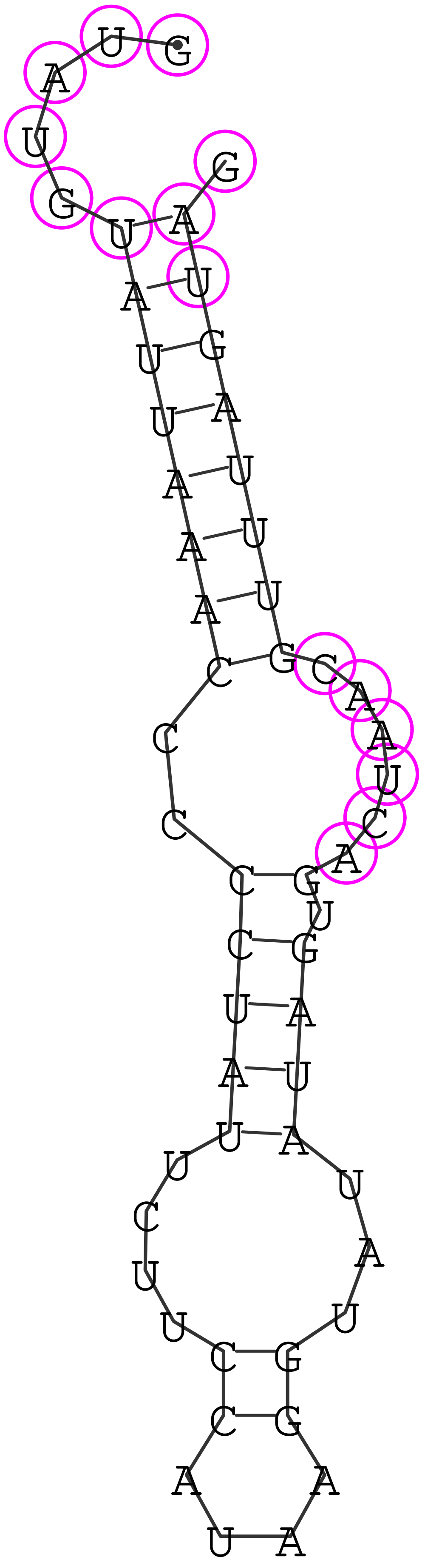

Dcoc02A - Internal intron

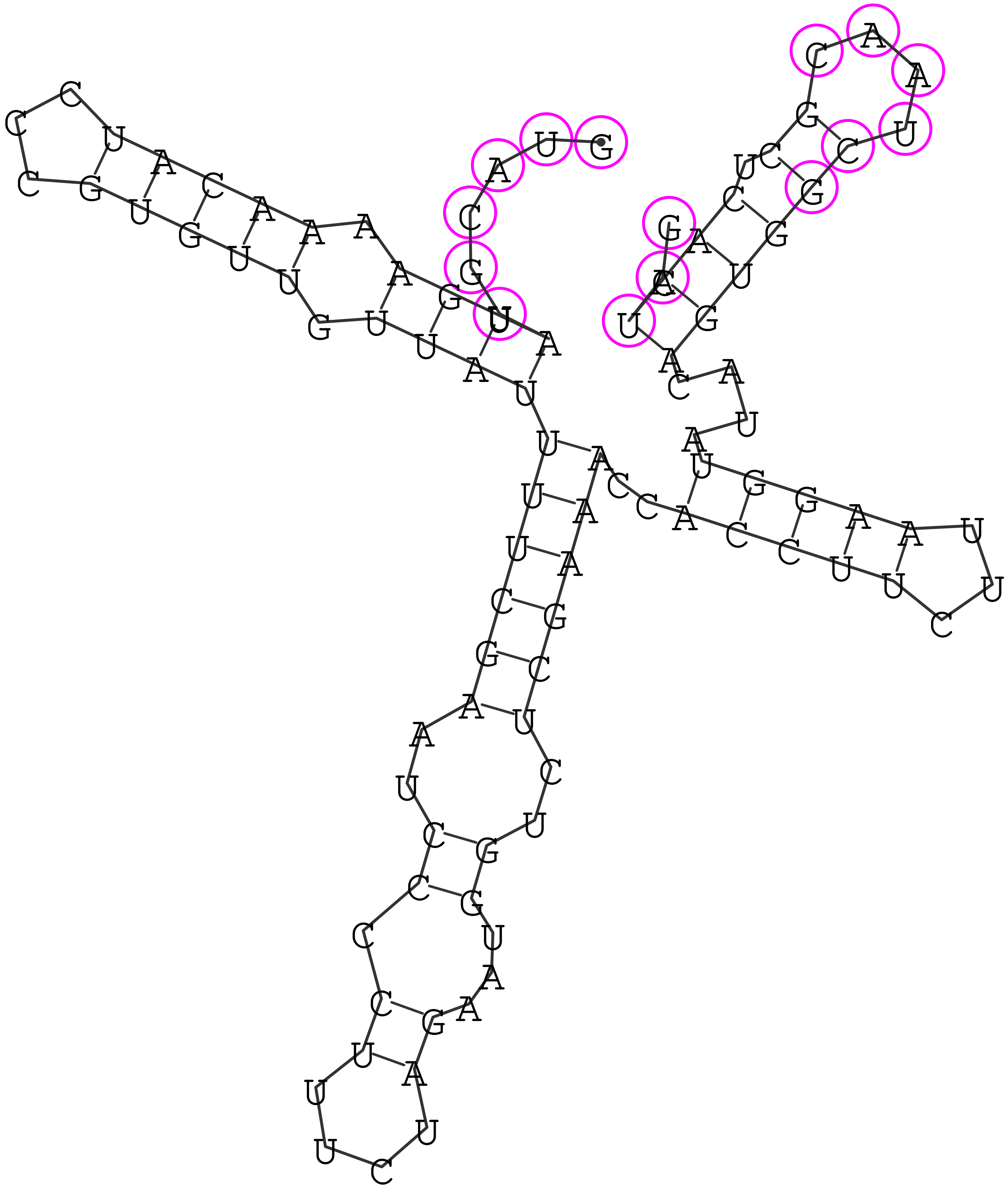

Dcoc02B - Internal intron

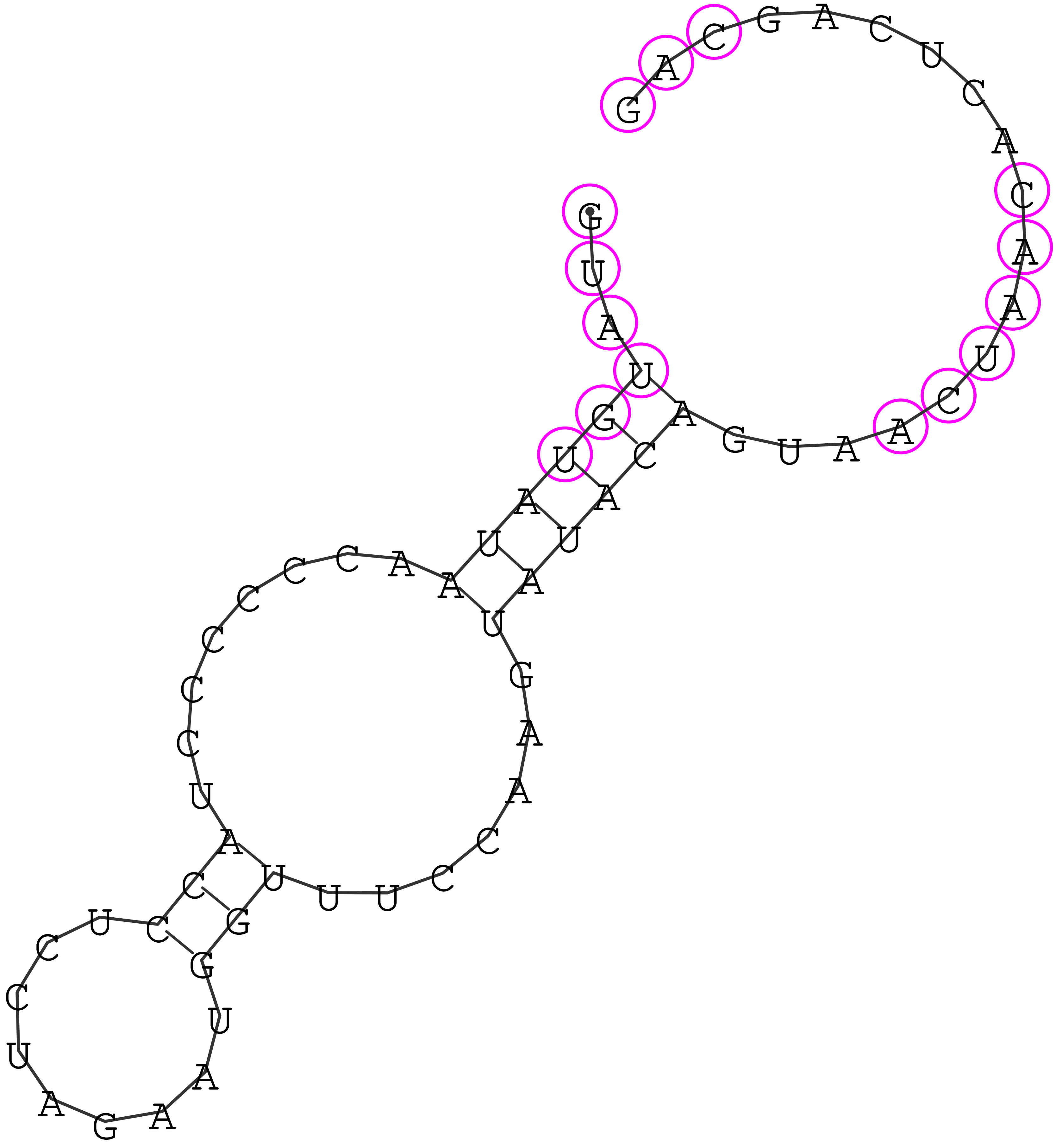

Dcoc03A - Internal intron

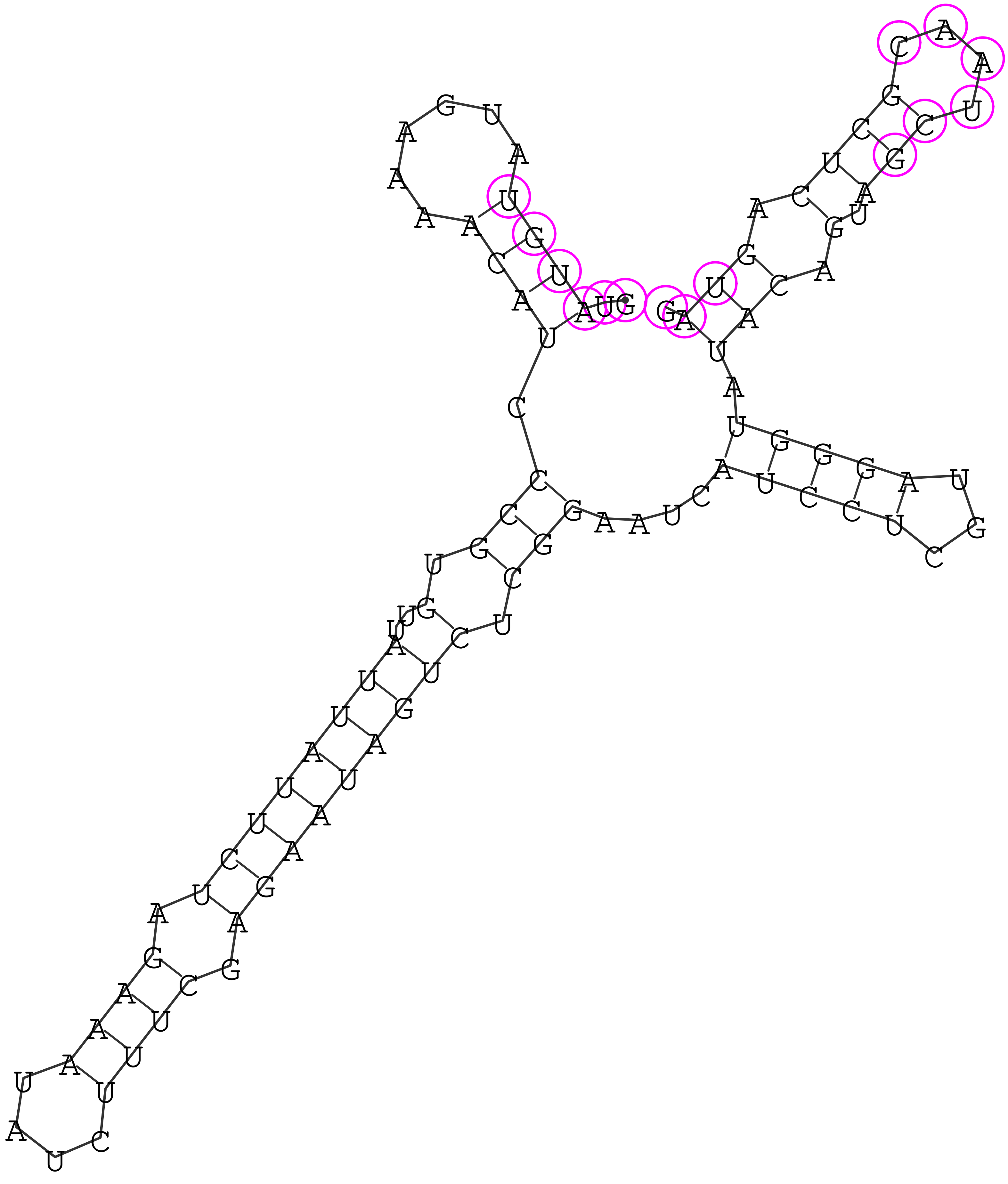

Dcoc05A - Internal intron

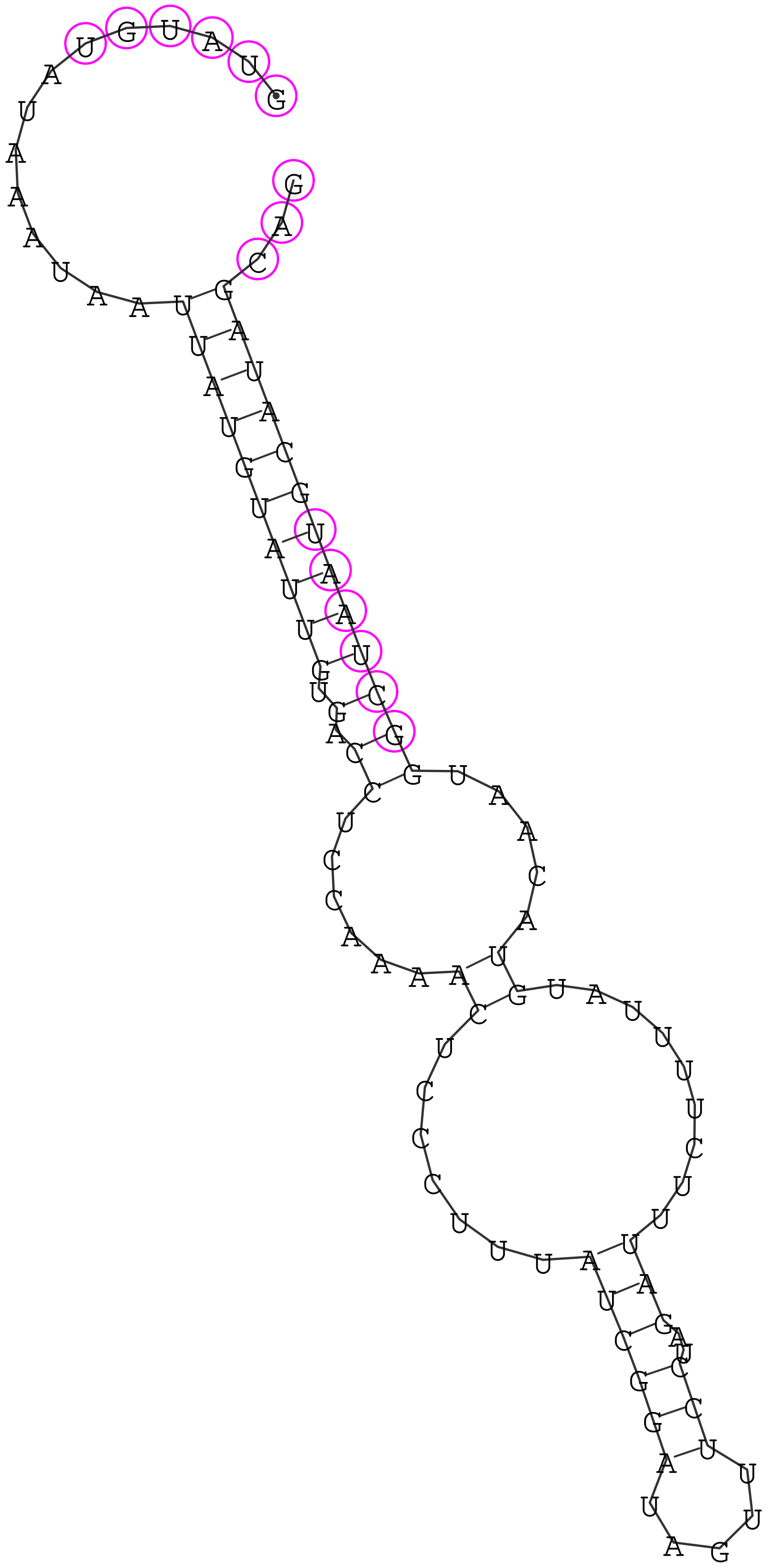

Dcoc06A - Internal intron

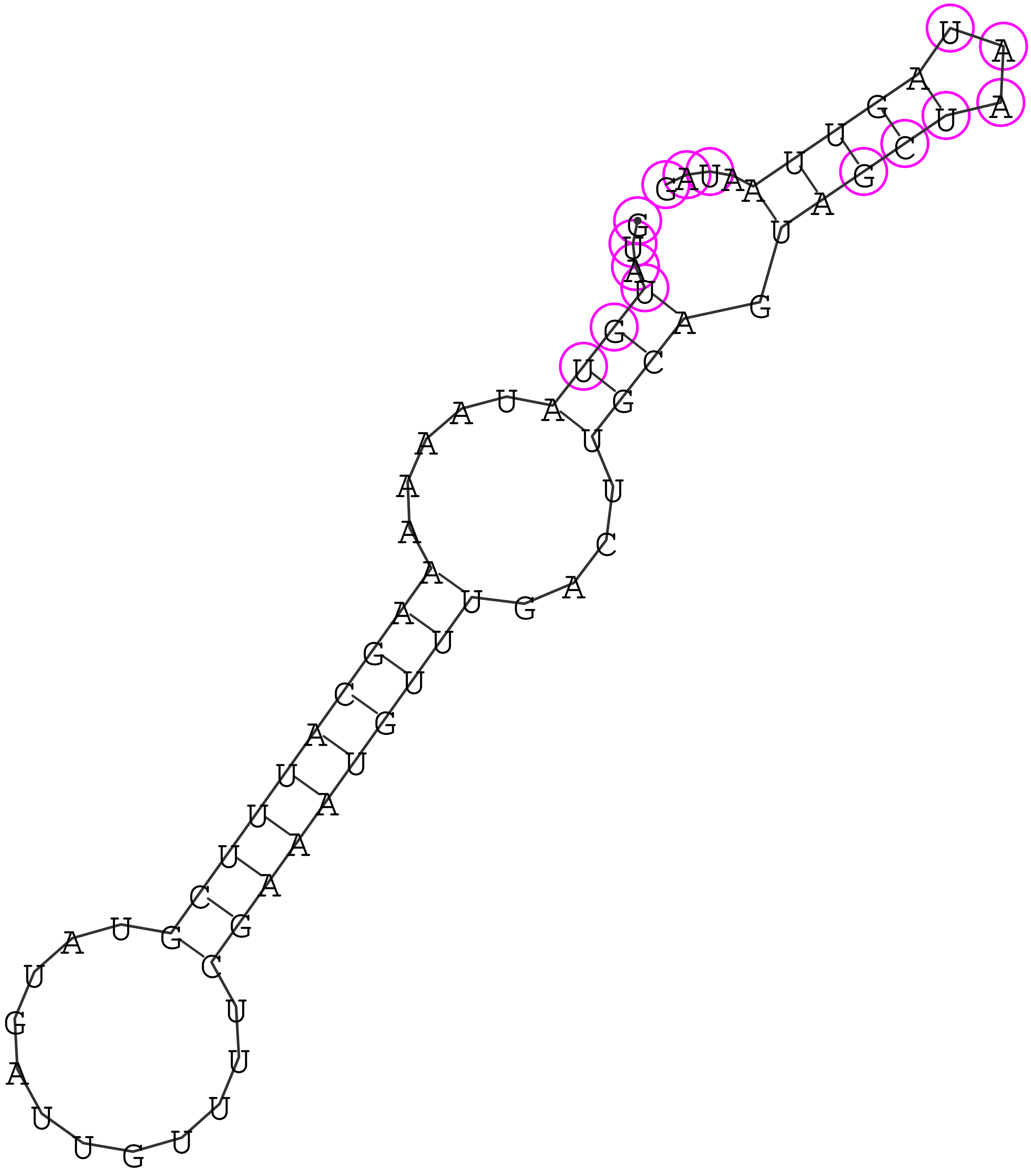

Dcoc11A - Internal intron

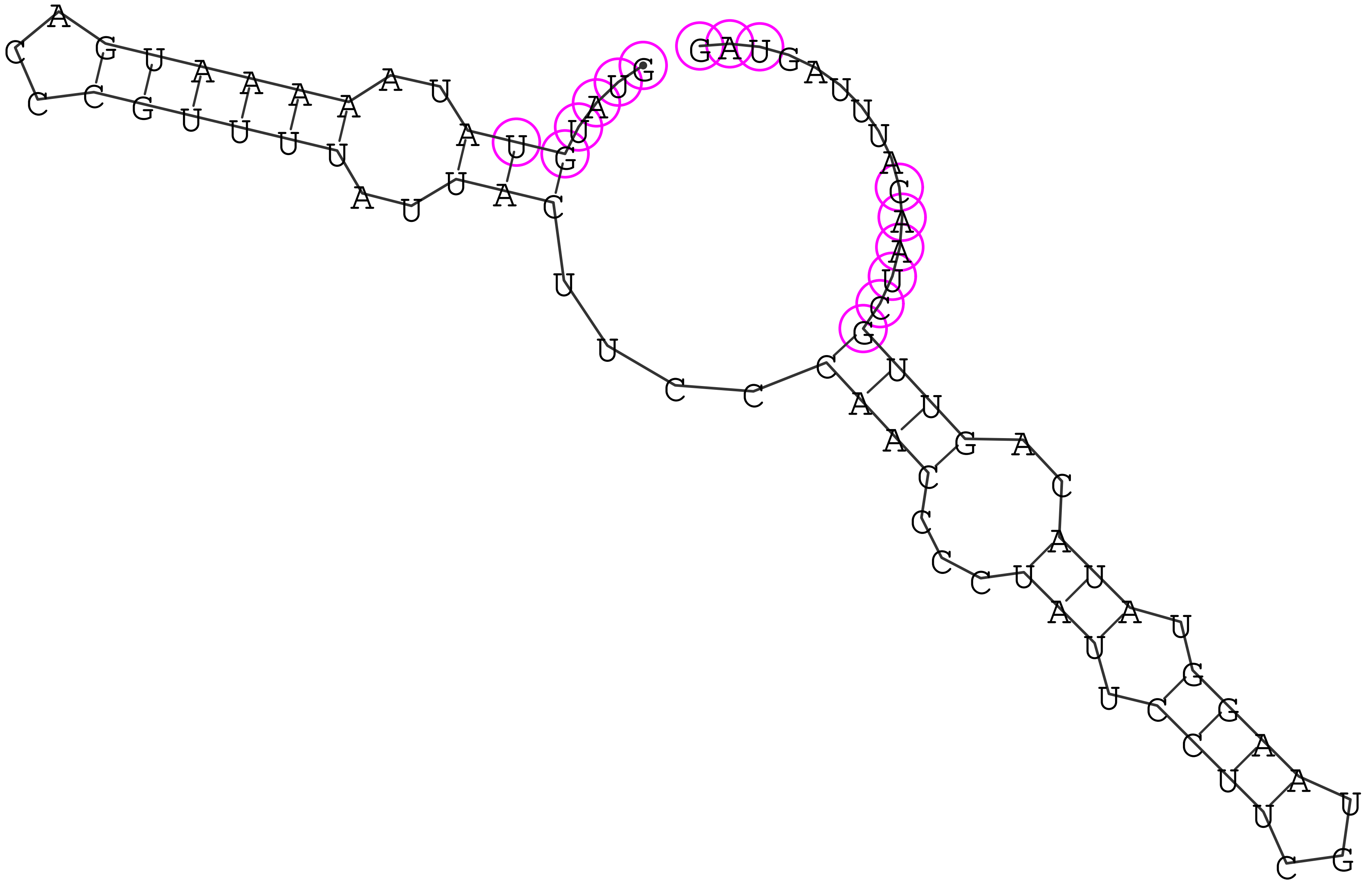

Dcoc20A - Internal intron

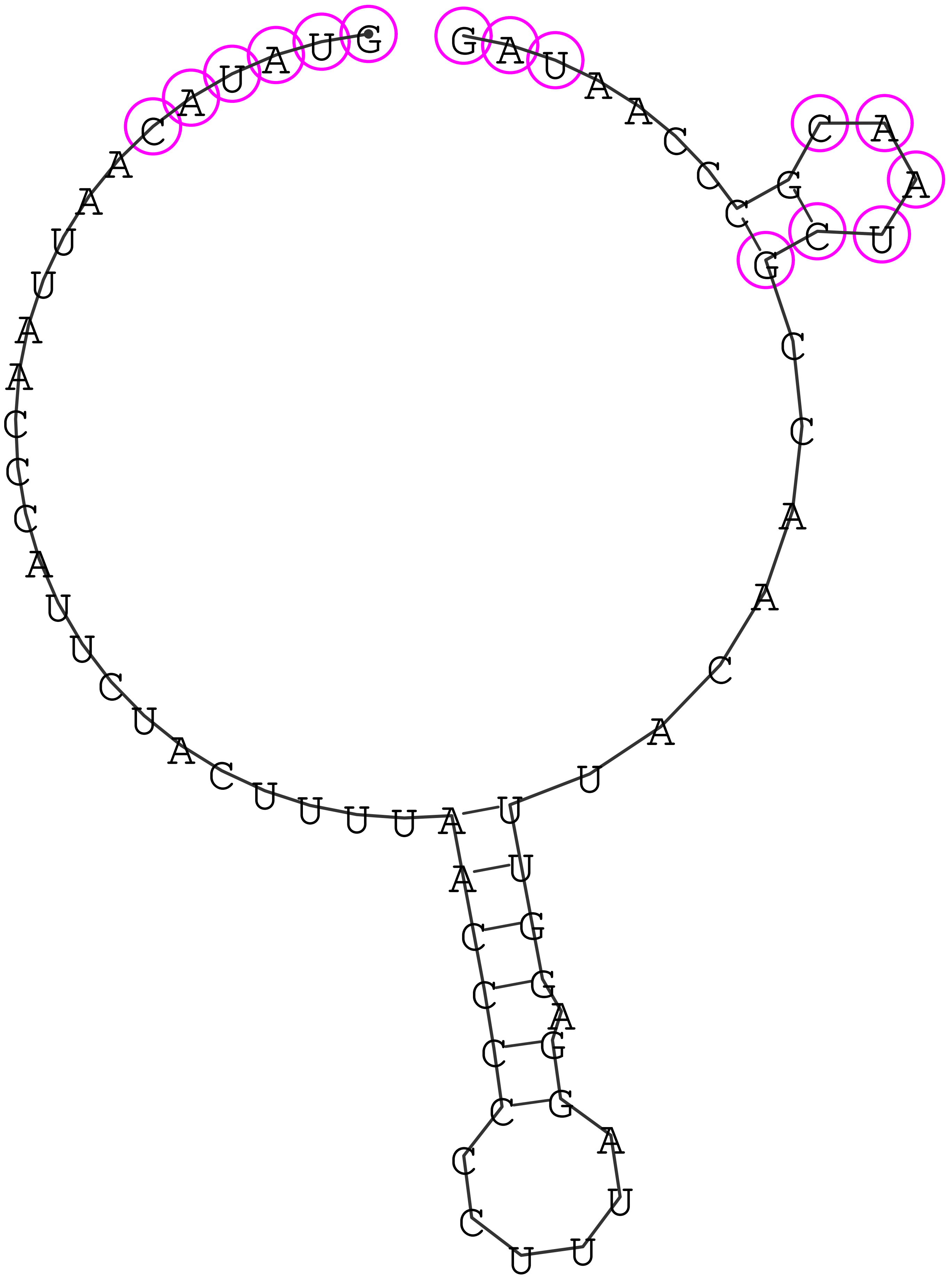

Dcoc38A - Internal intron

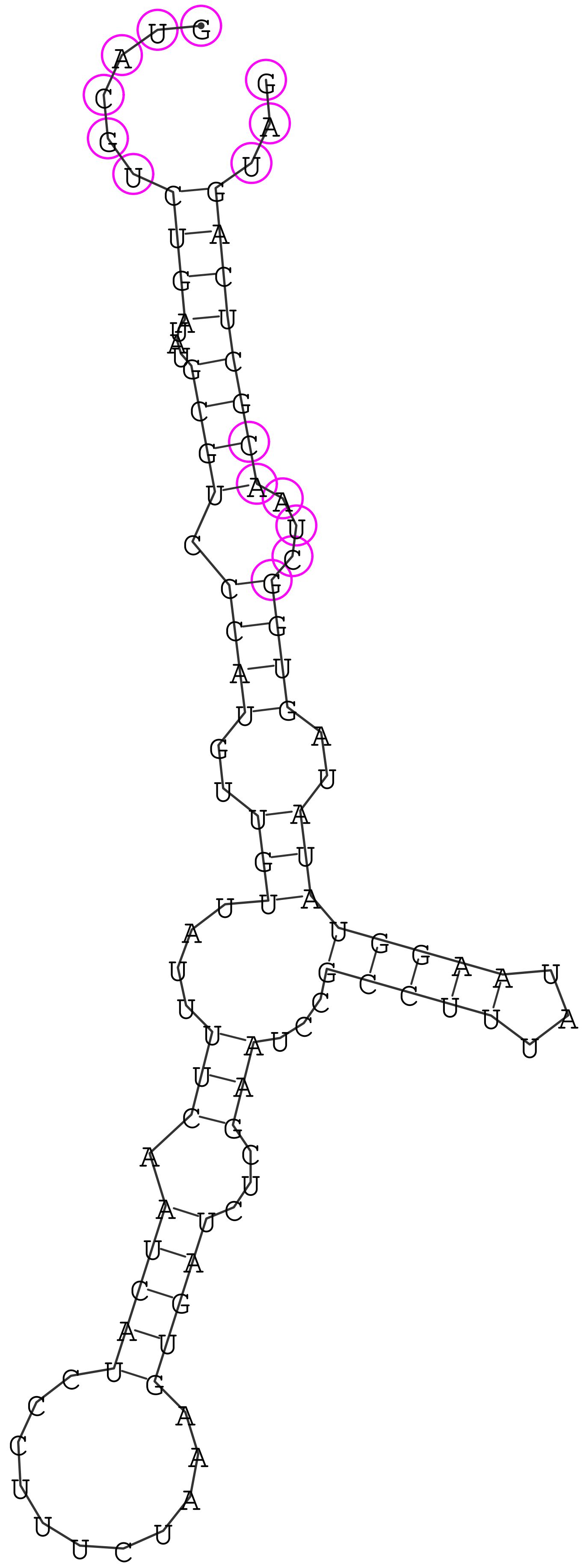

Dcoc41A - Internal intron

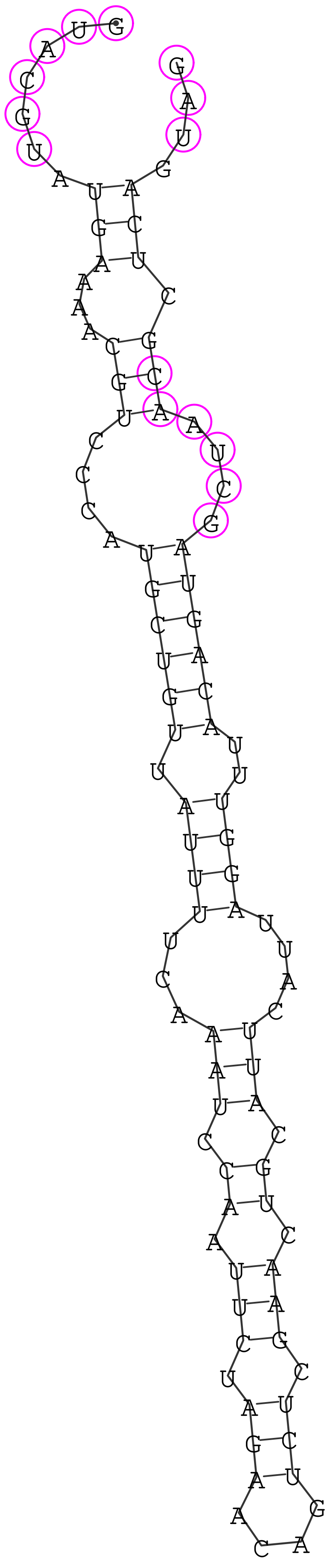

Desc187A - Internal intron

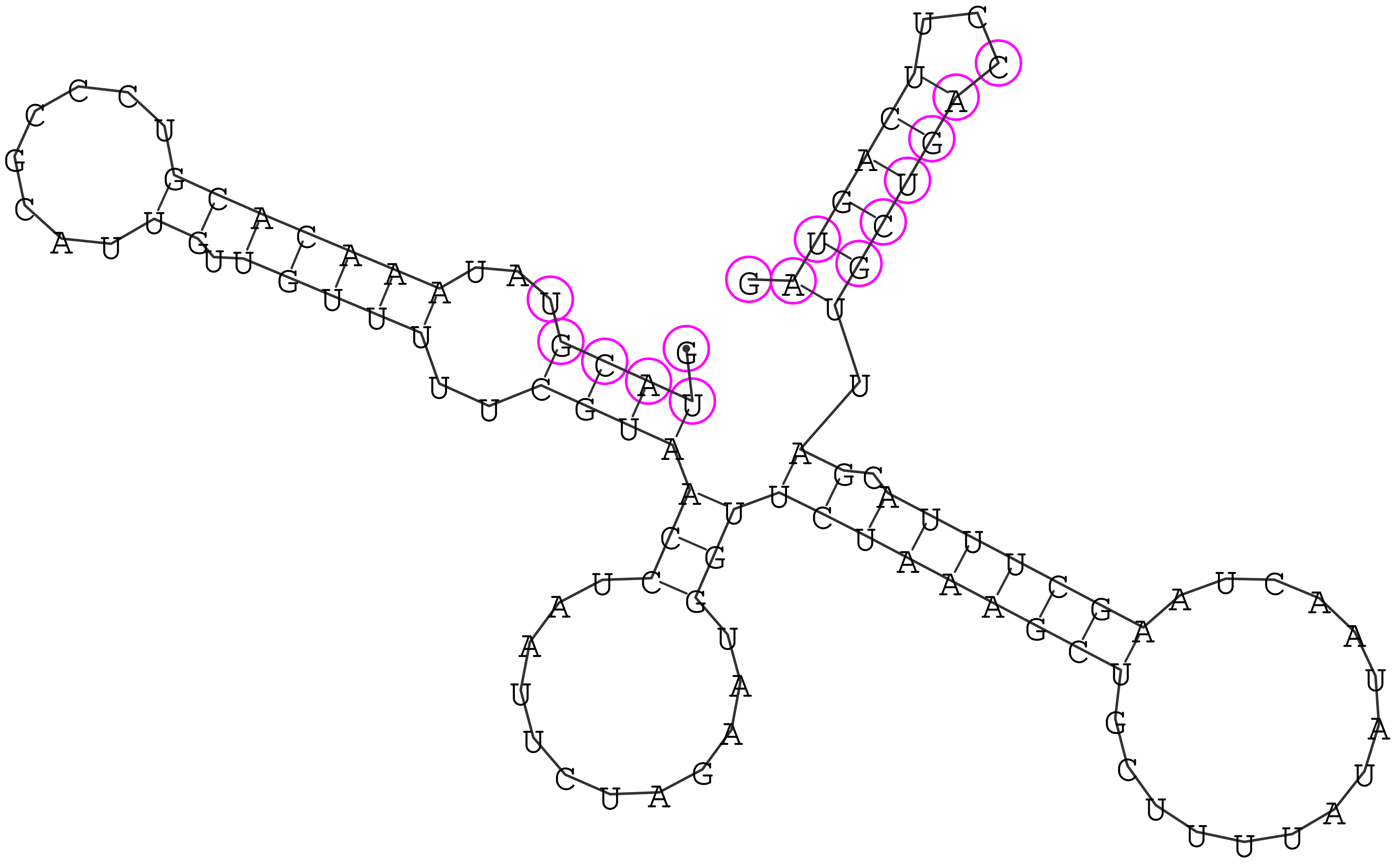

Desc198A - Internal intron

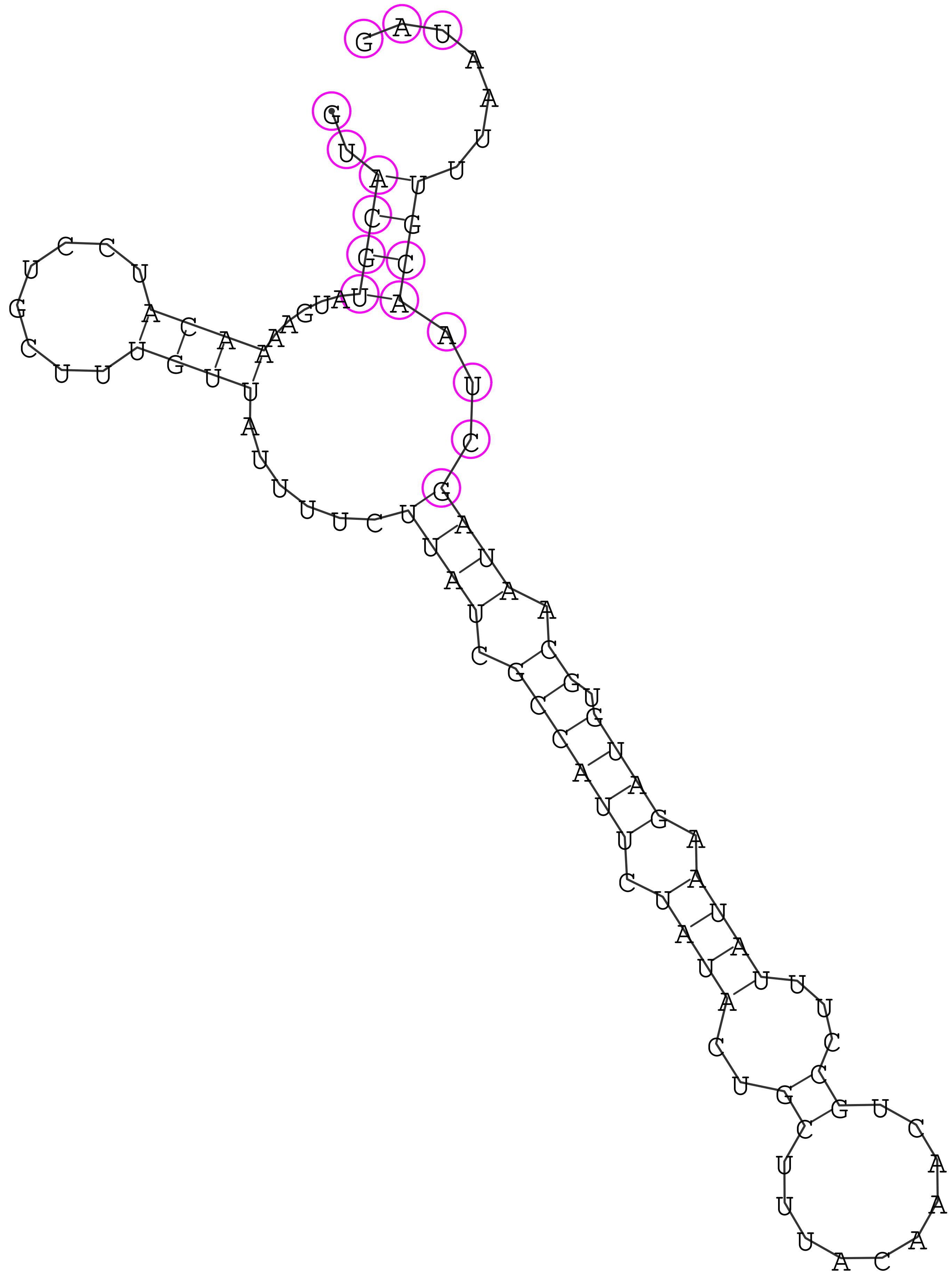

Desc274A - Internal intron

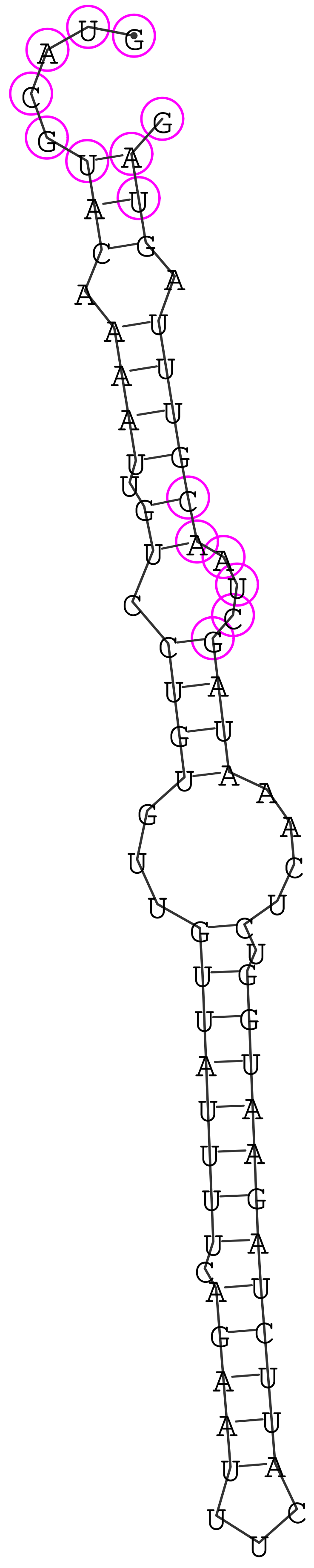

Desc420A - Internal intron

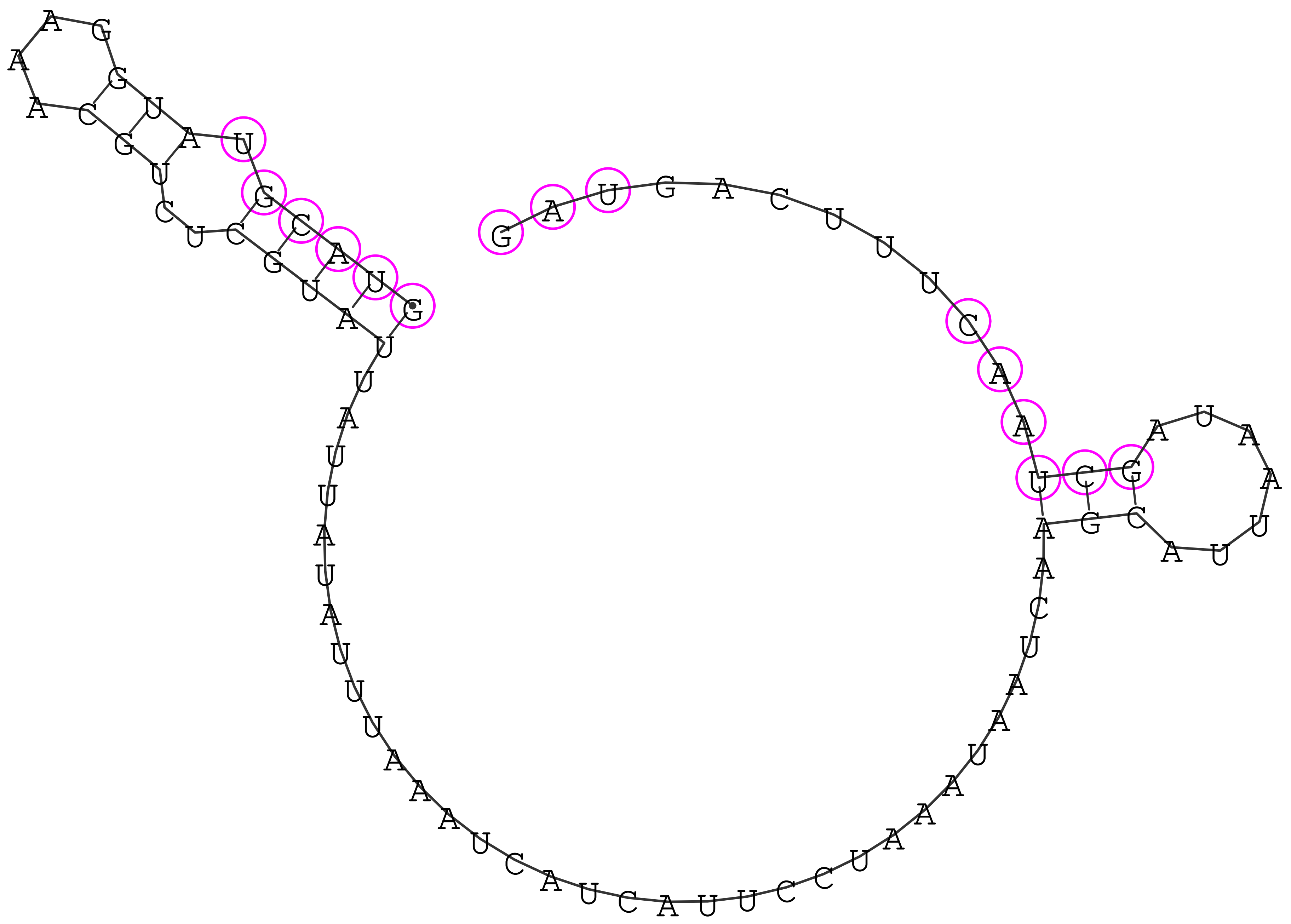

Desc618A - Internal intron

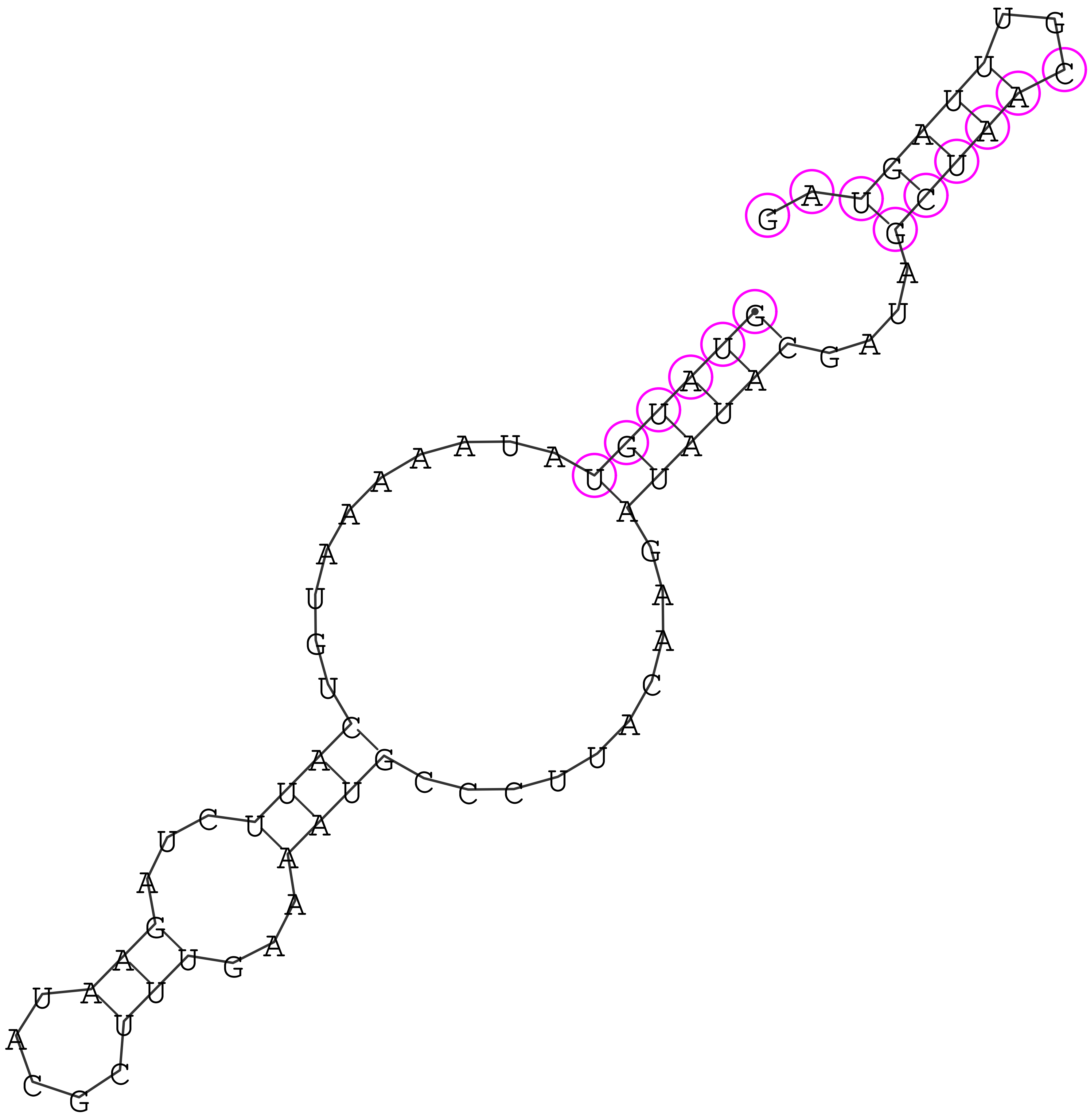

Desc640A - Internal intron

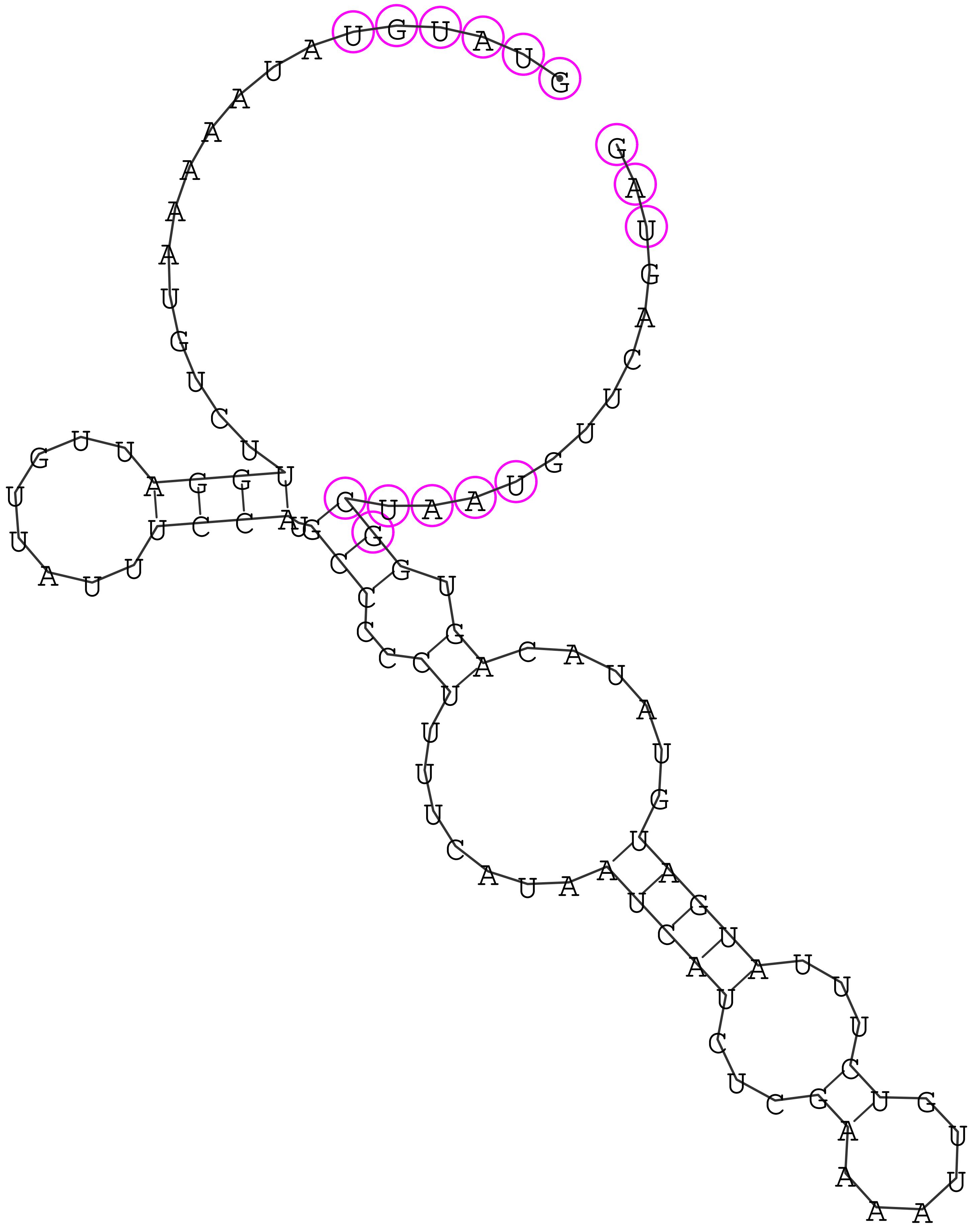

HCOc002A - Internal intron

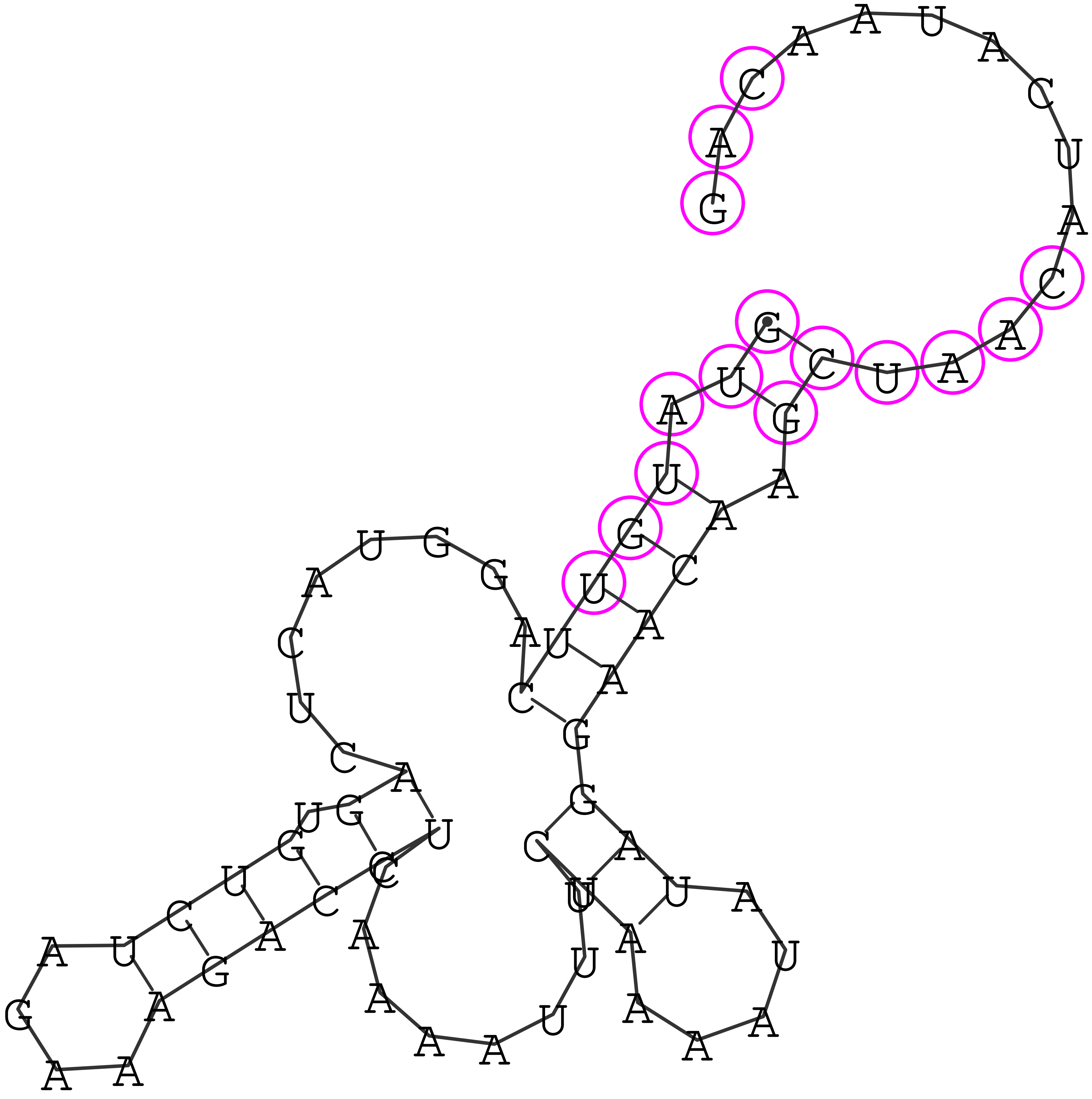

HCOc004A - Internal intron

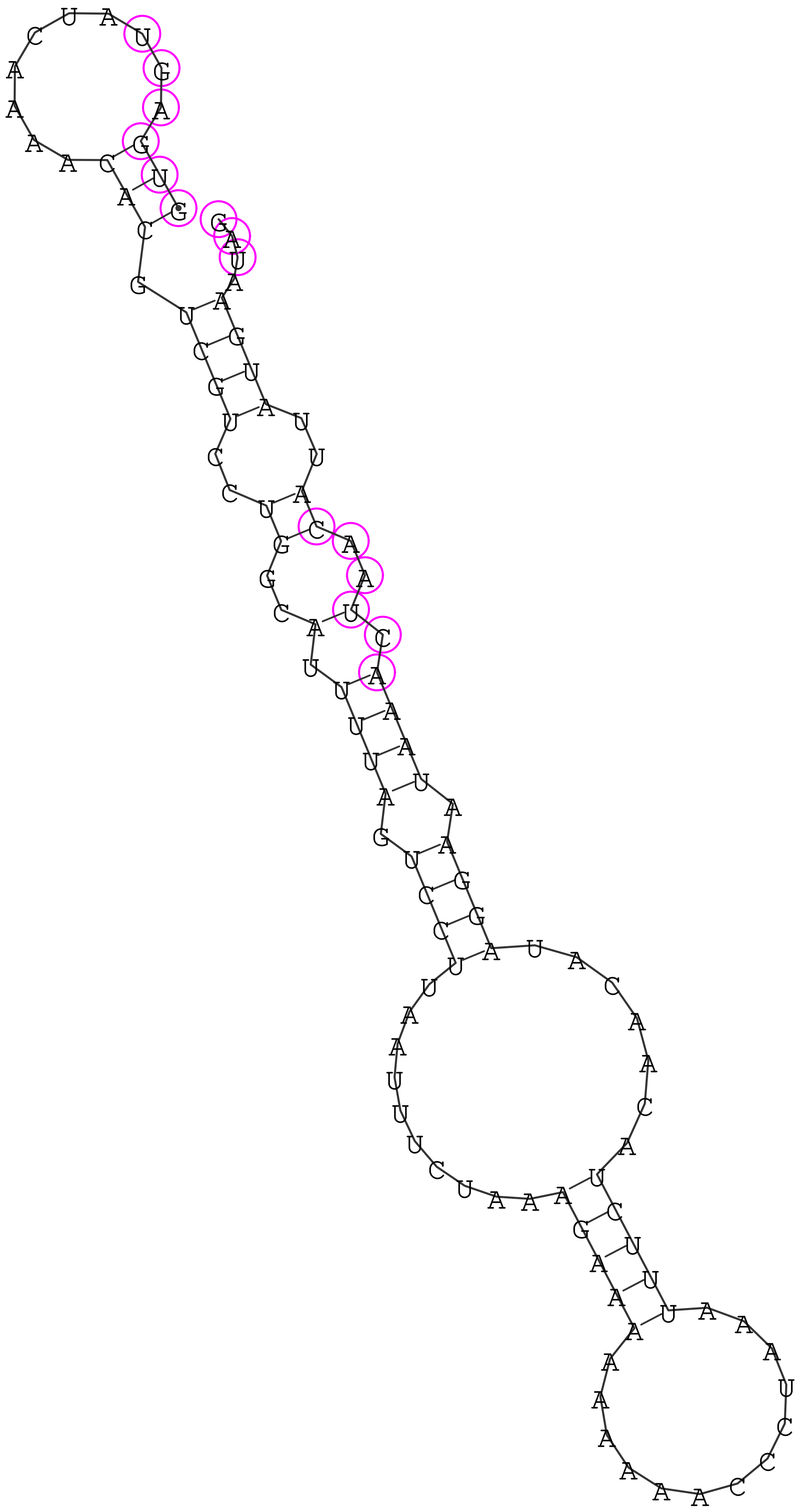

HCOc017A - Internal intron

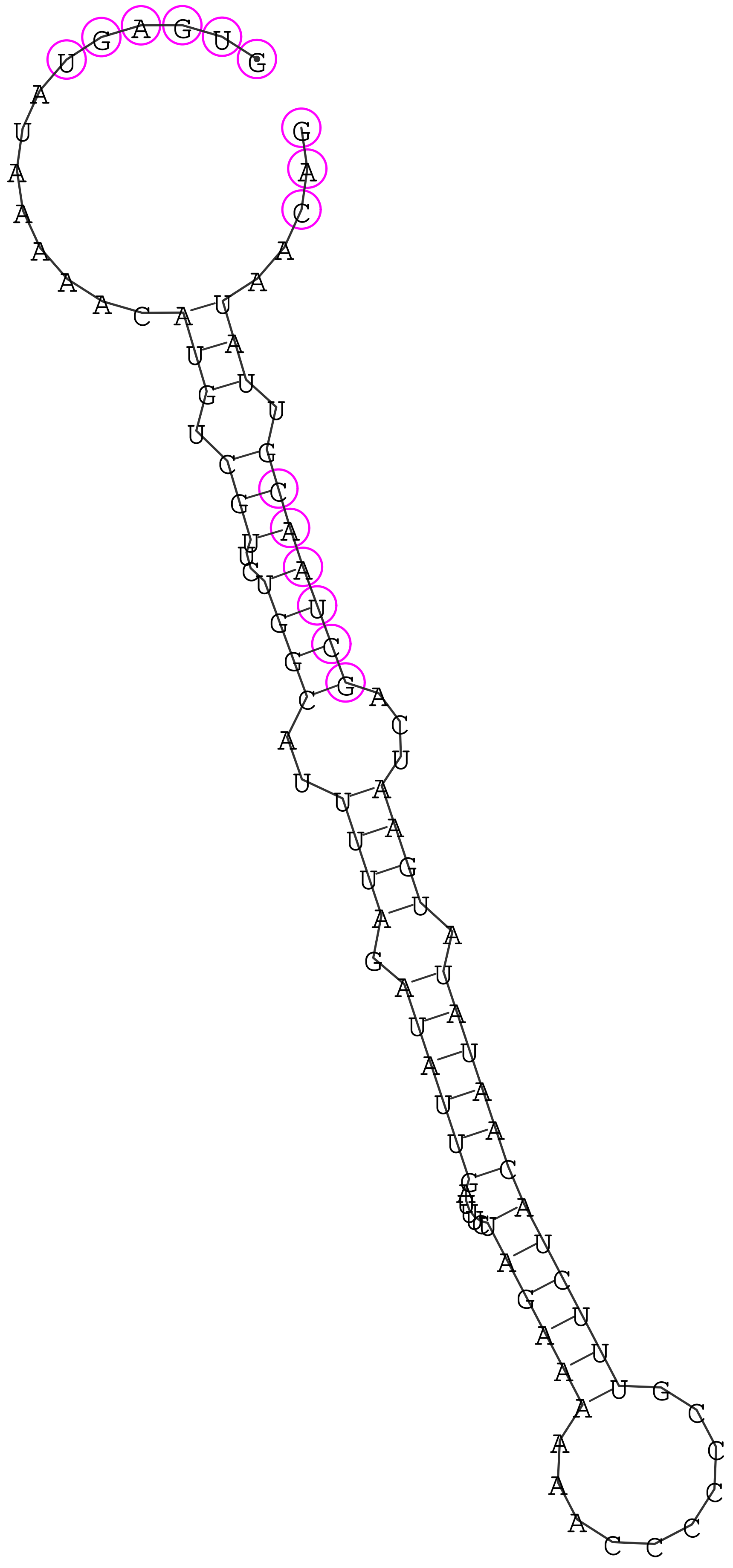

HCOc017B - Internal intron

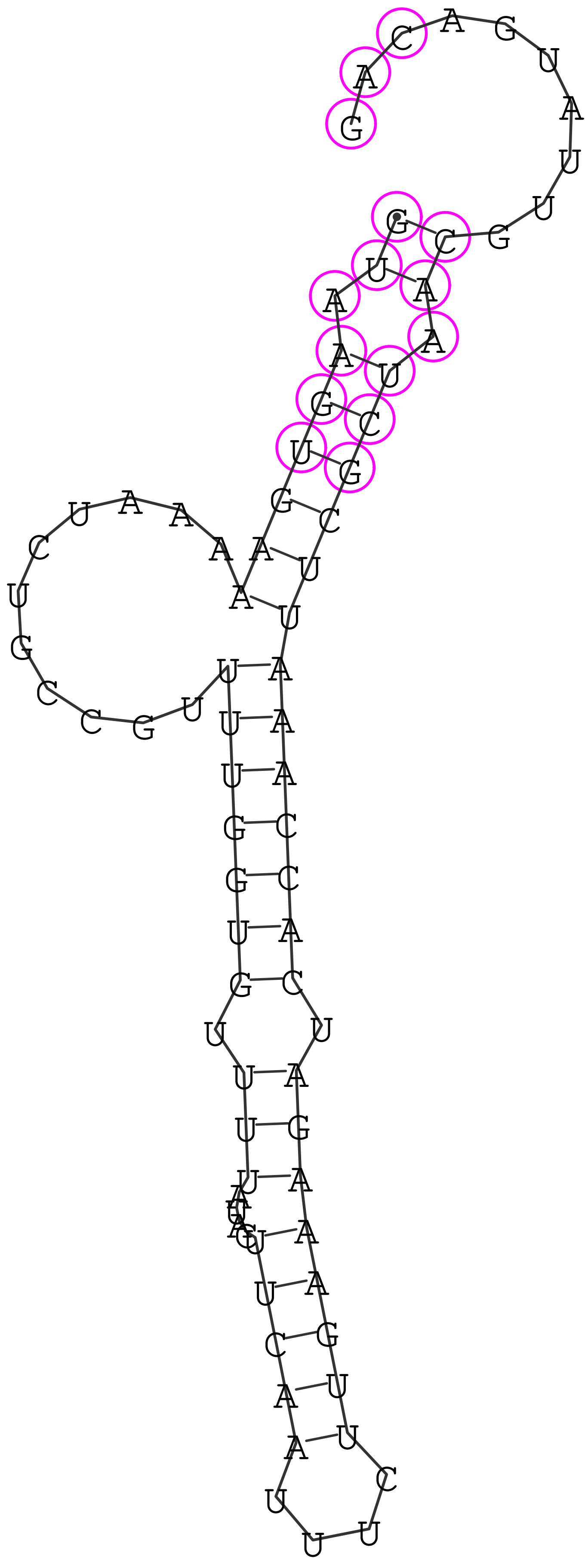

HCOc021A - Internal intron

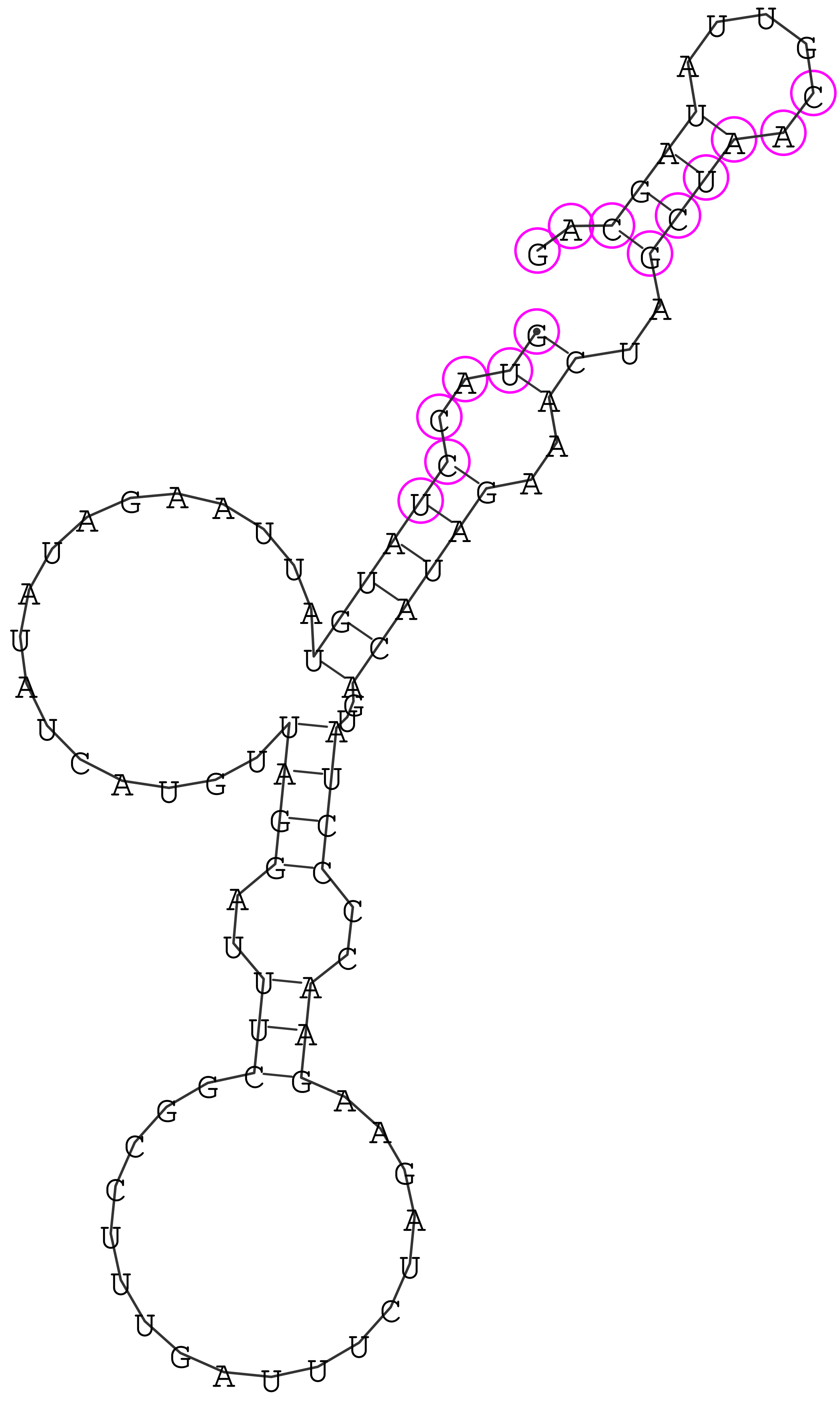

HCOc047A - Internal intron

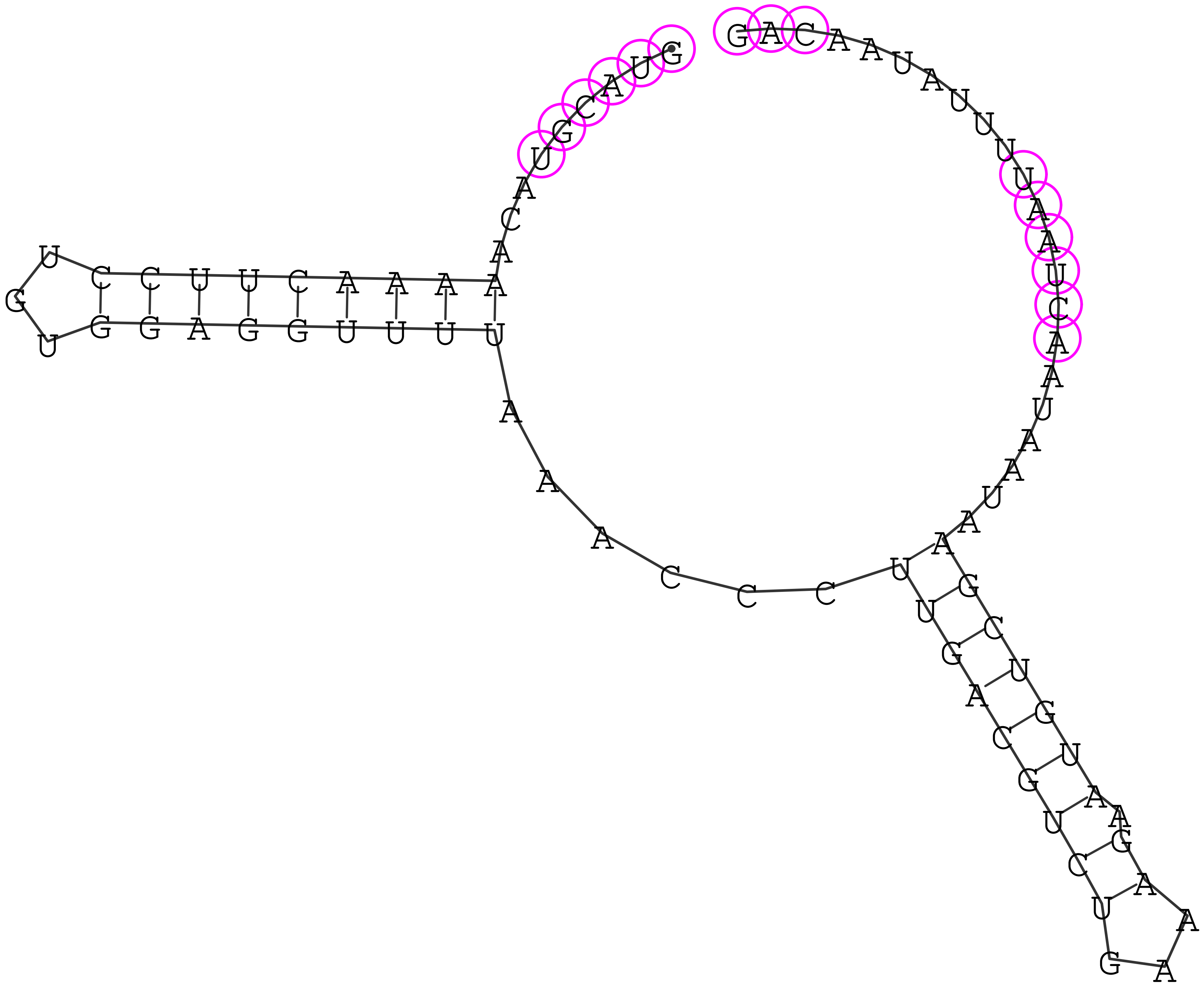

HCOc052A - Internal intron

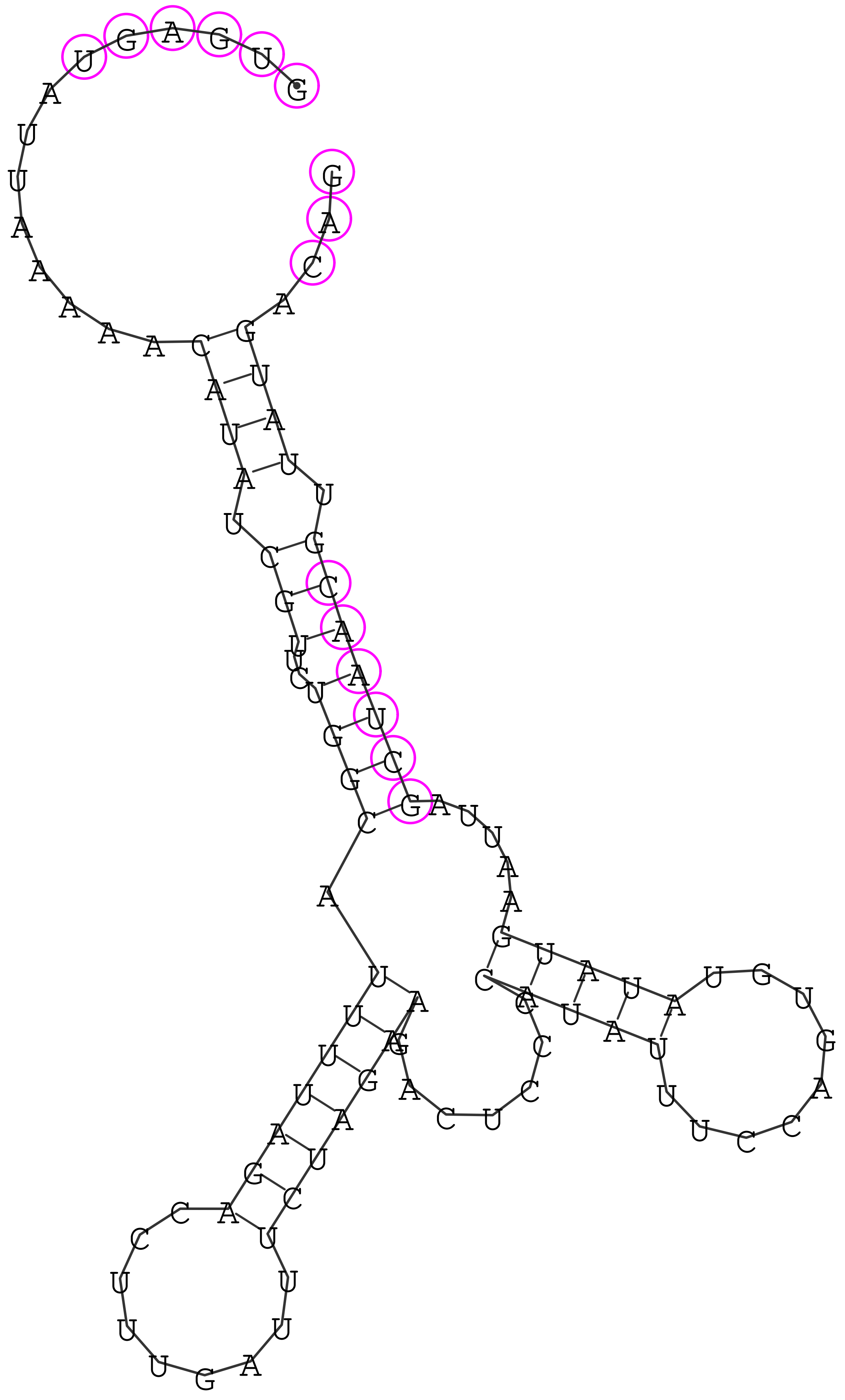

HCOc058A - Internal intron

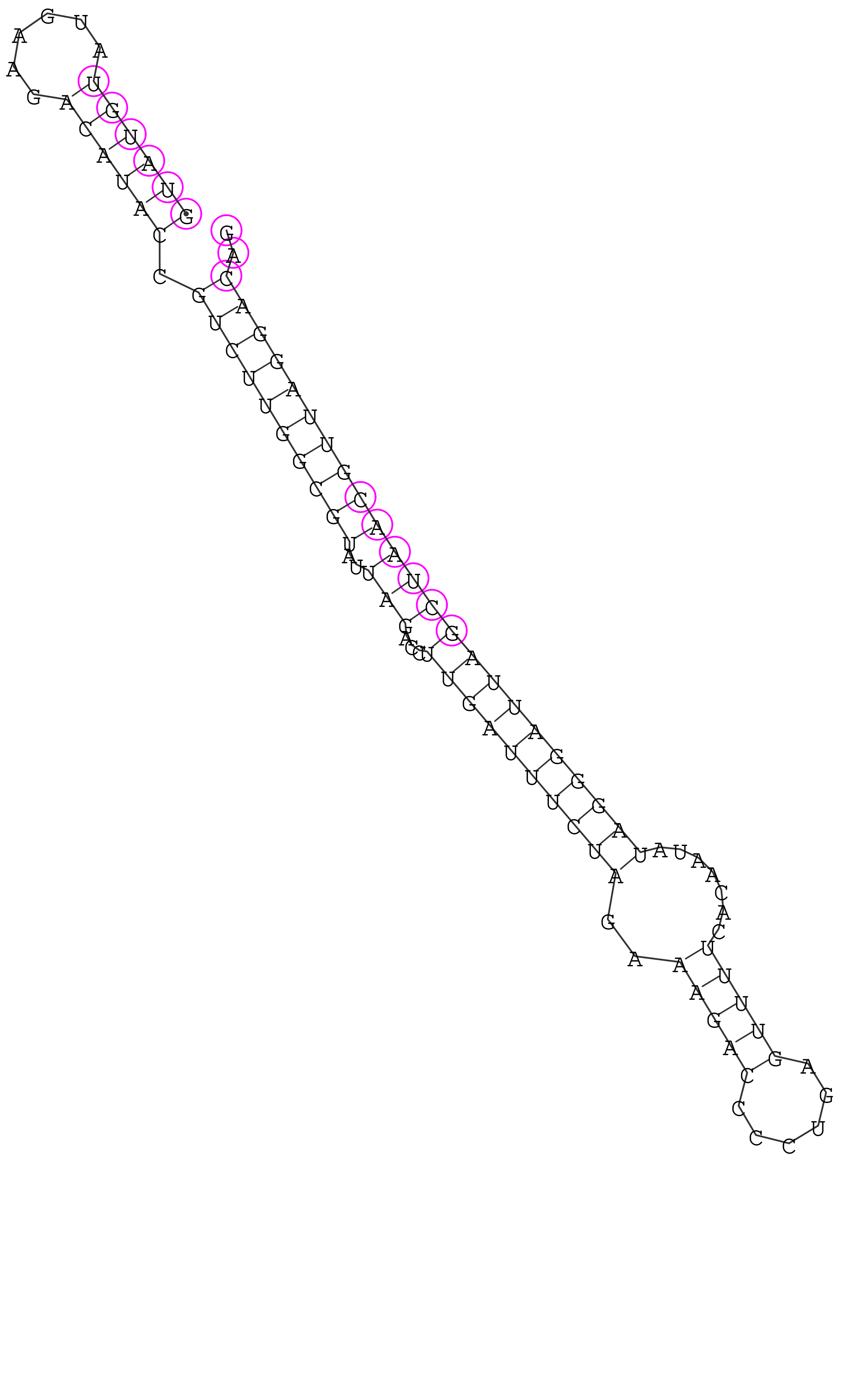

HCOc061A - Internal intron

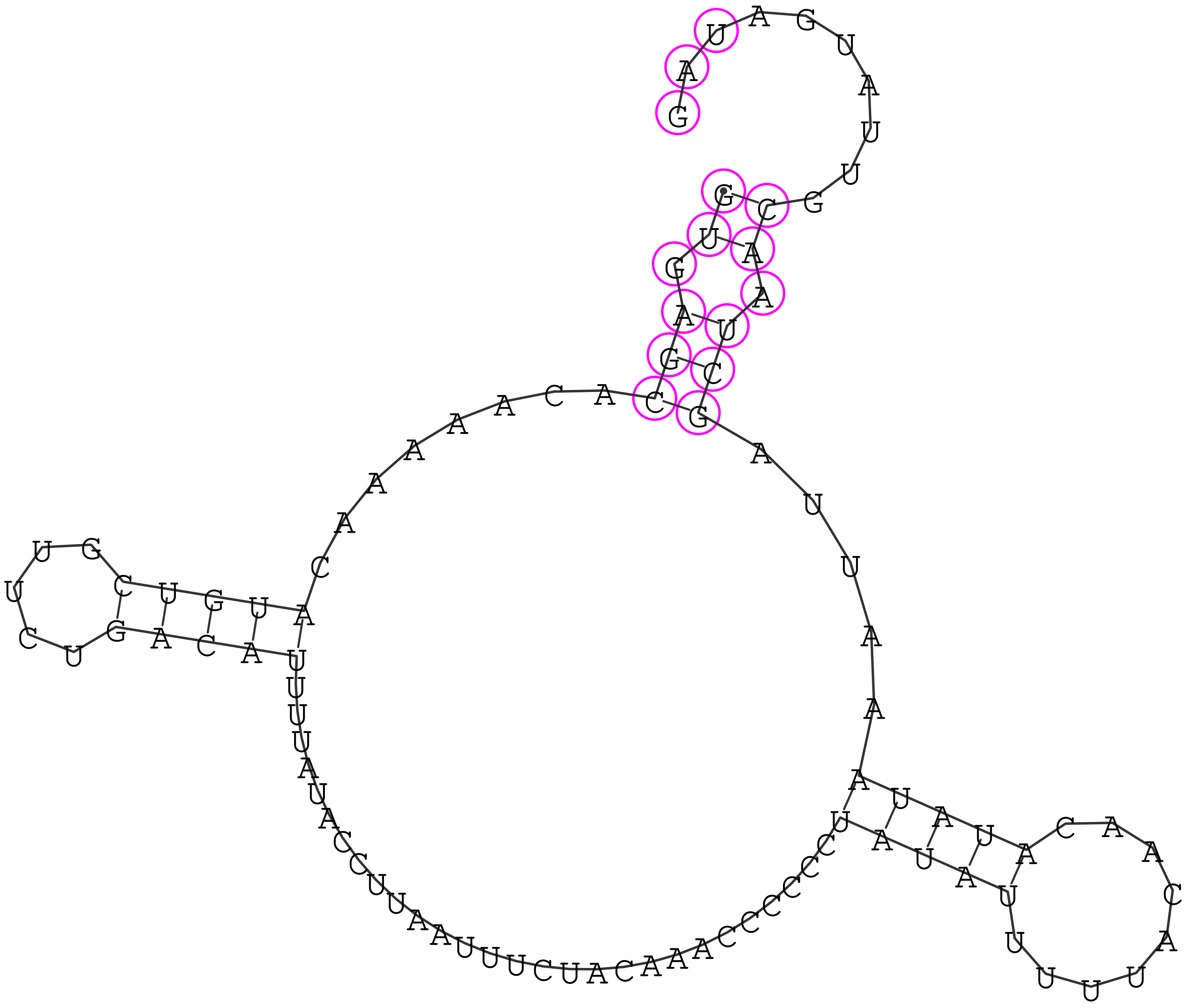

HCOc066A - Internal intron

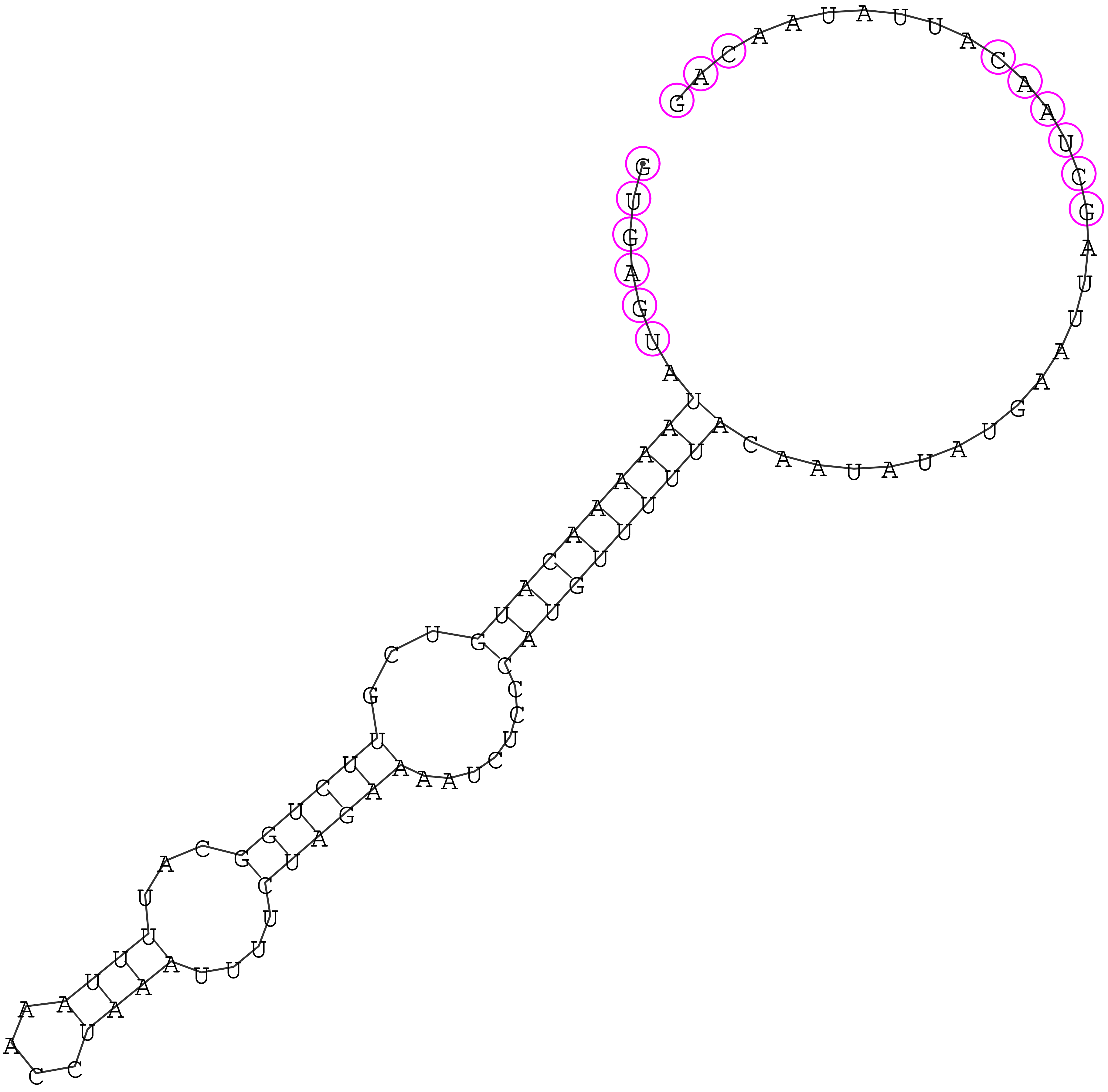

HCOc070A - Internal intron

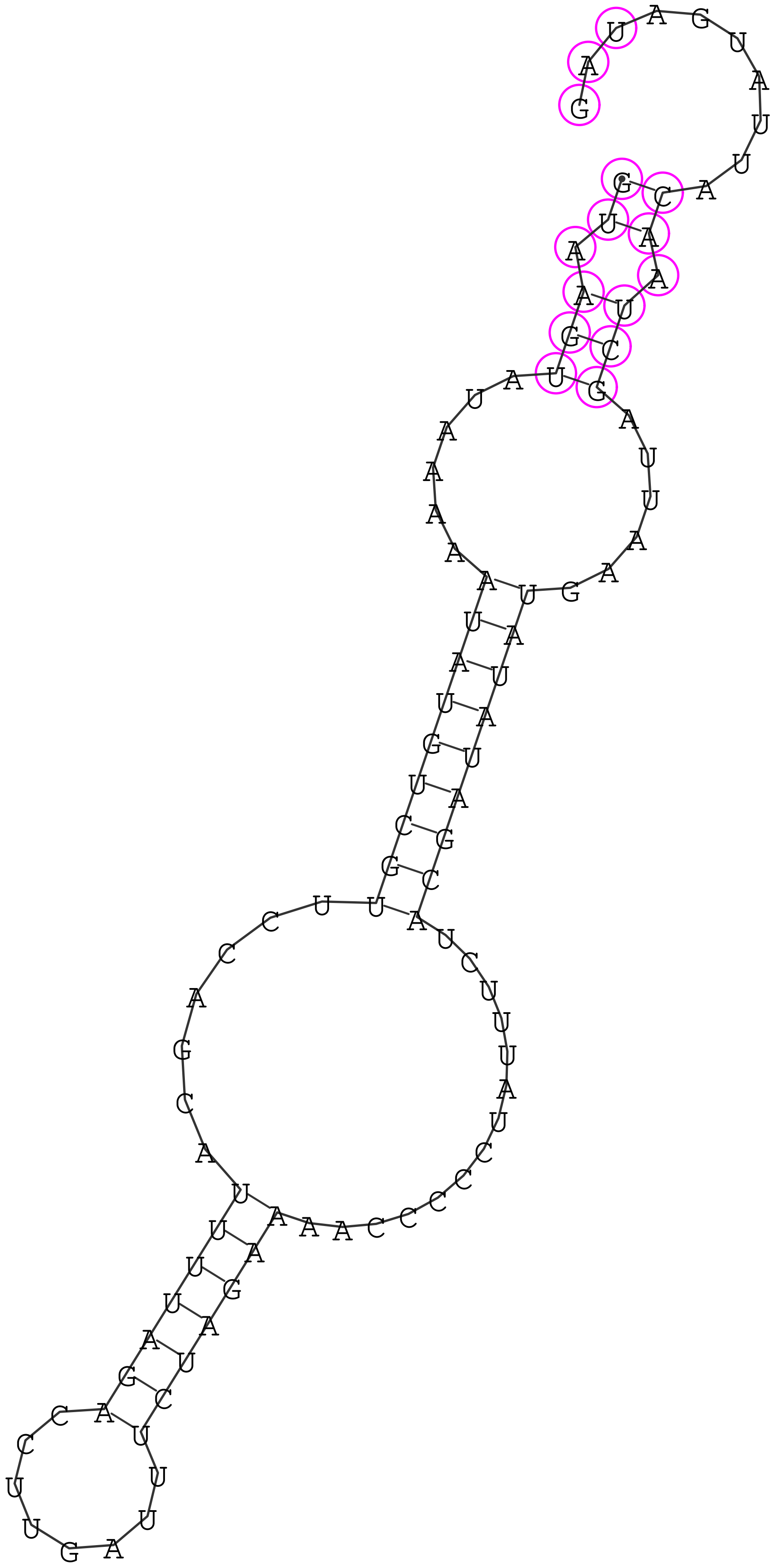

HCOc076A - Internal intron

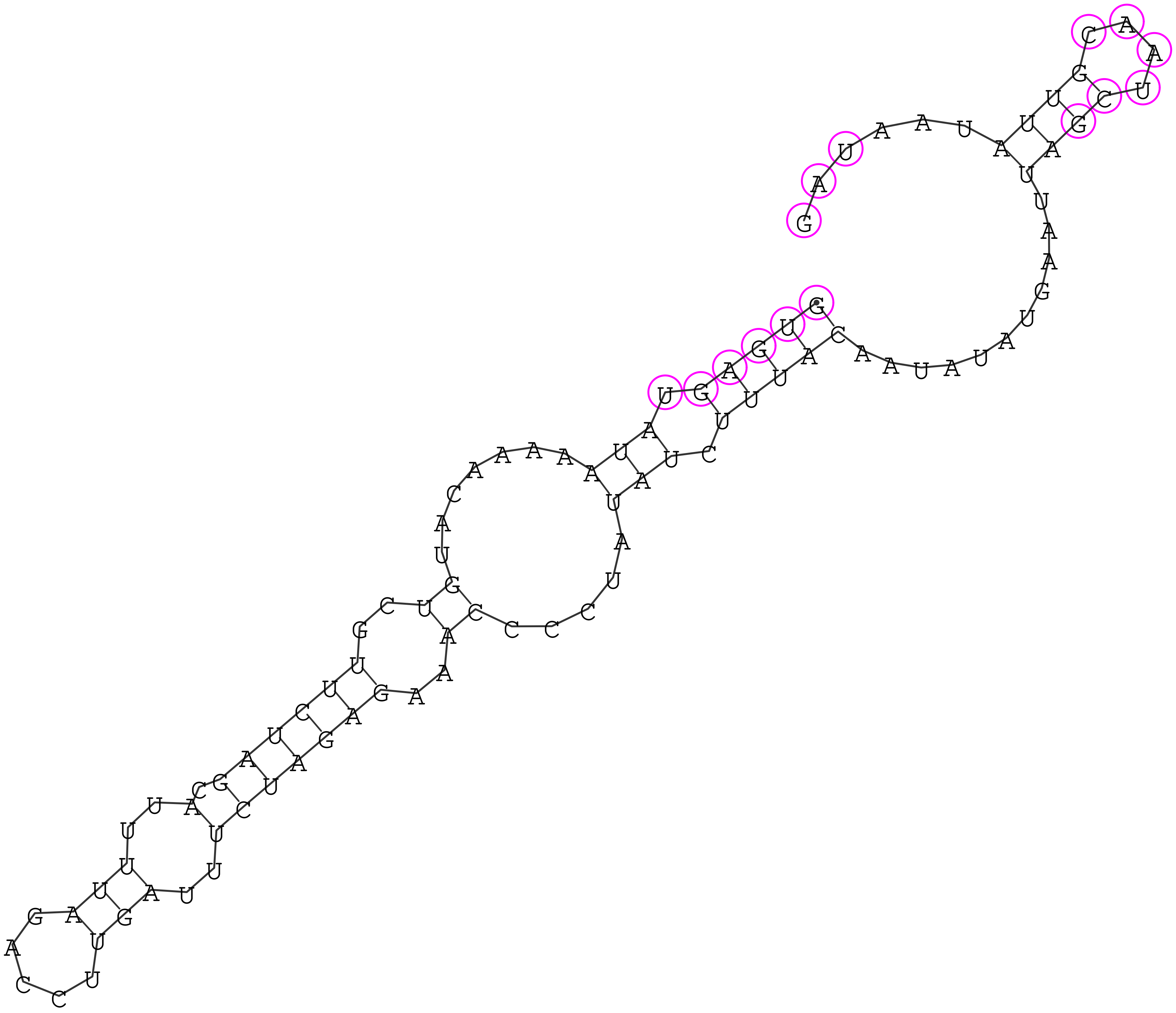

HCOc102A - Internal intron

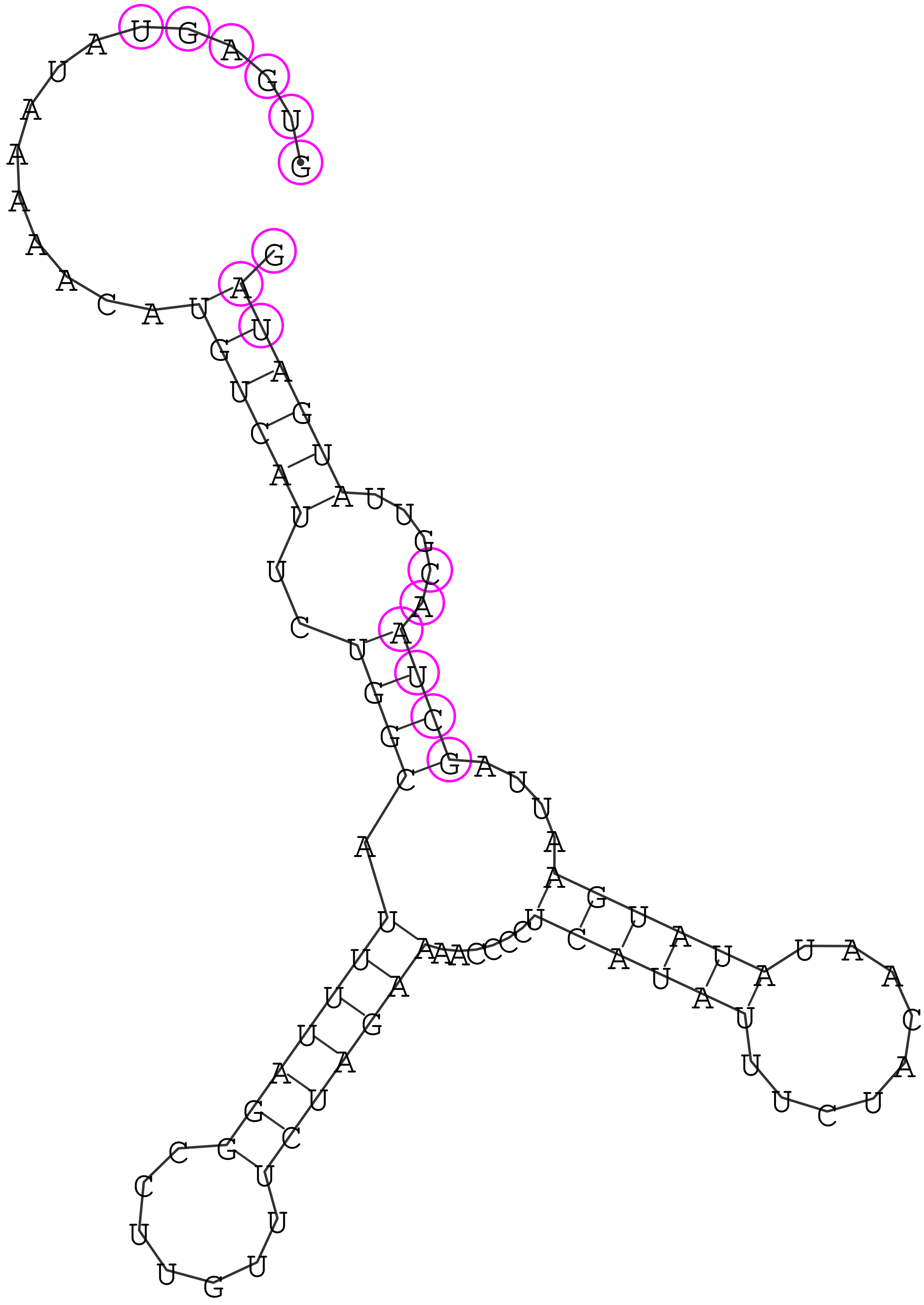

HCOc164A - Internal intron

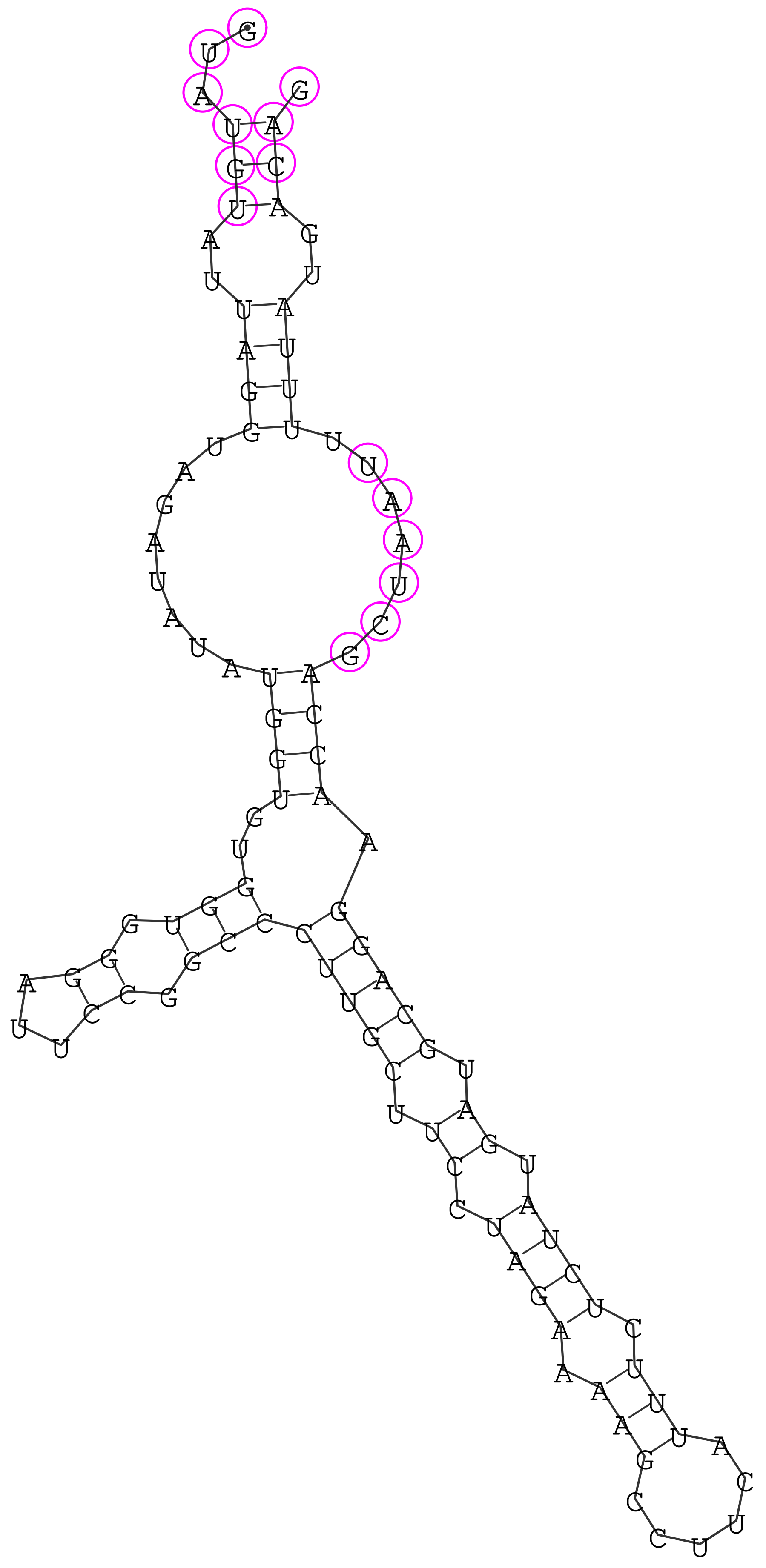

HCOc178A - Internal intron

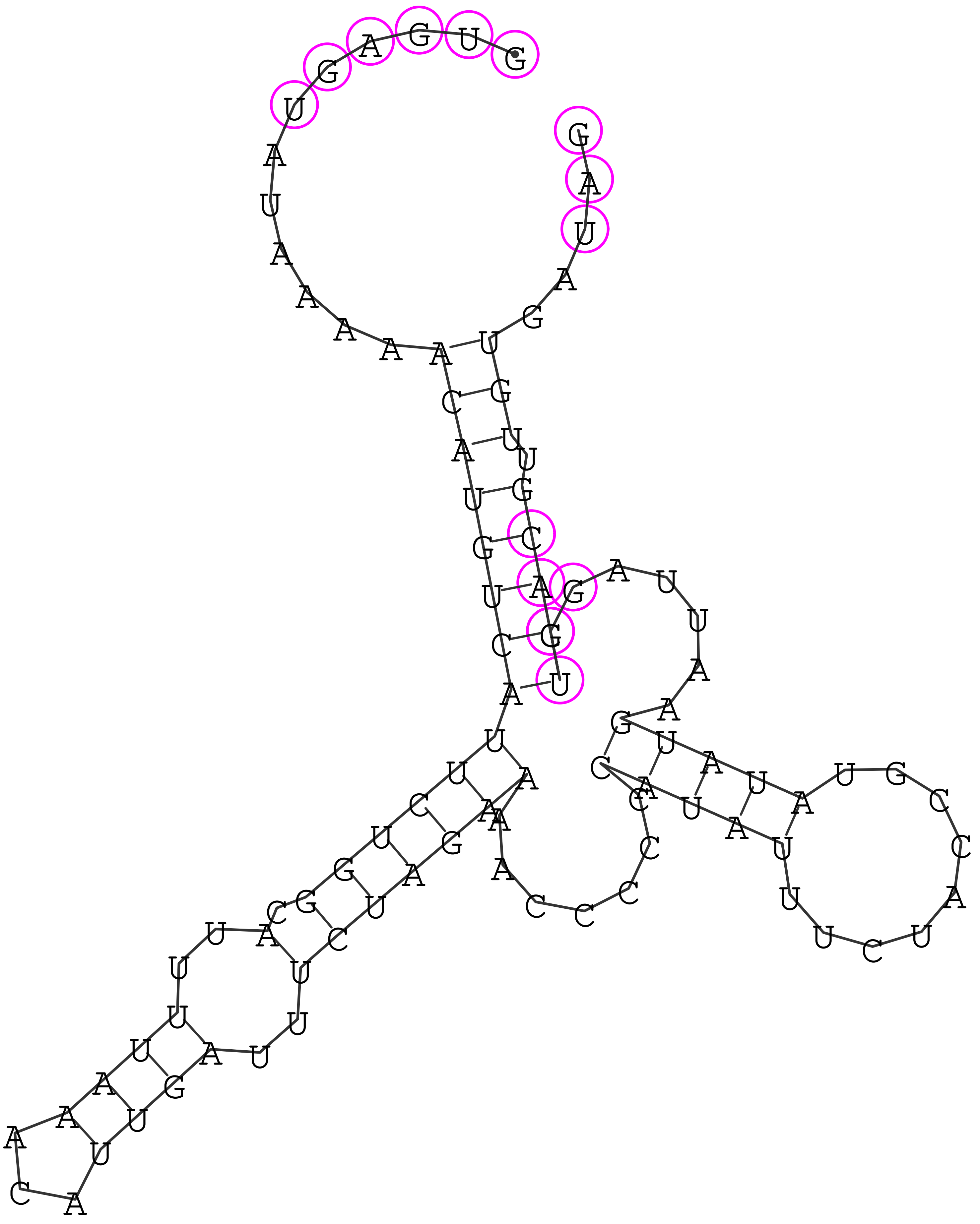

HCOc224-179 - Internal intron

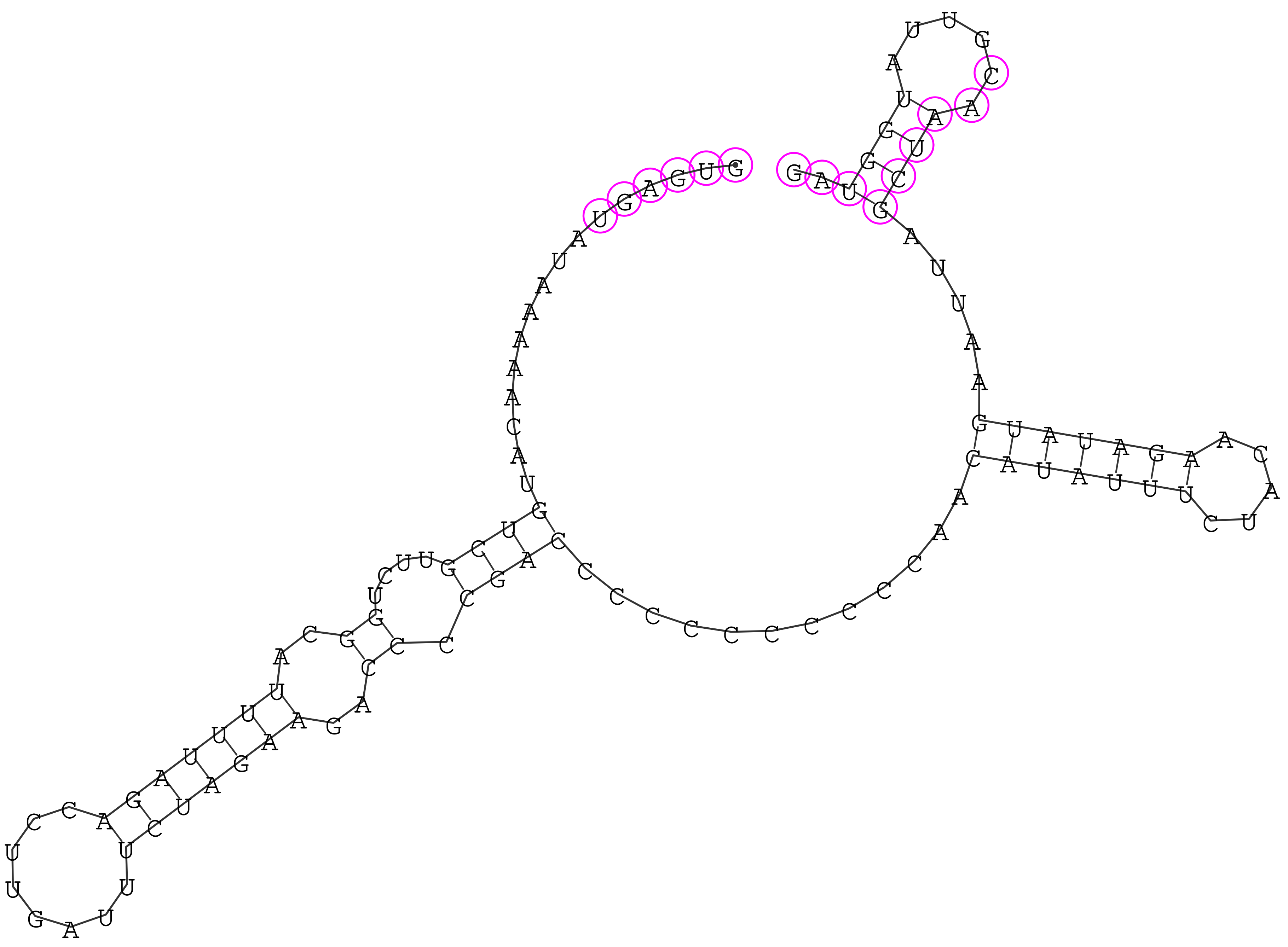

HCOc236A - Internal intron

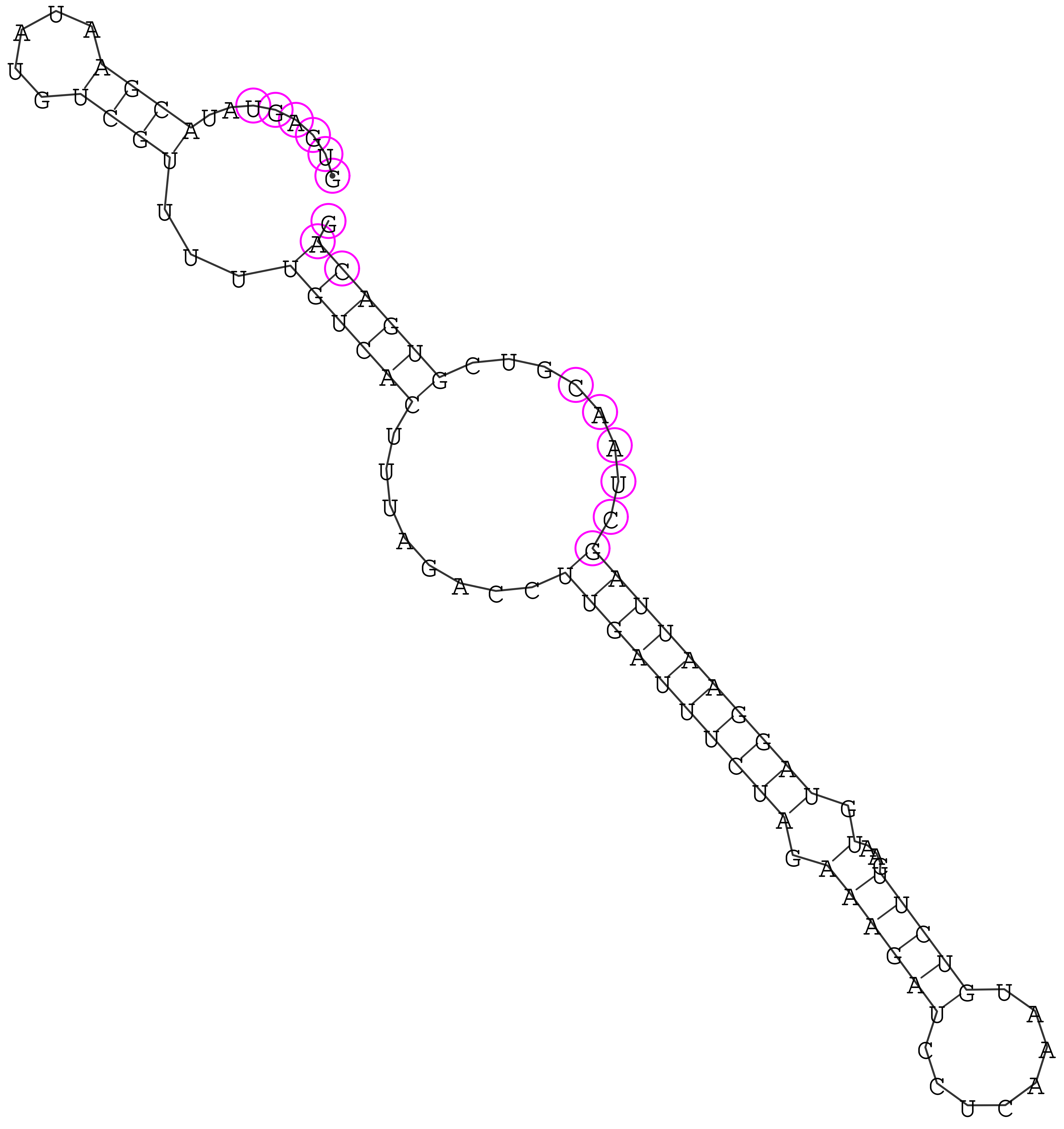

HCOc252A - Internal intron

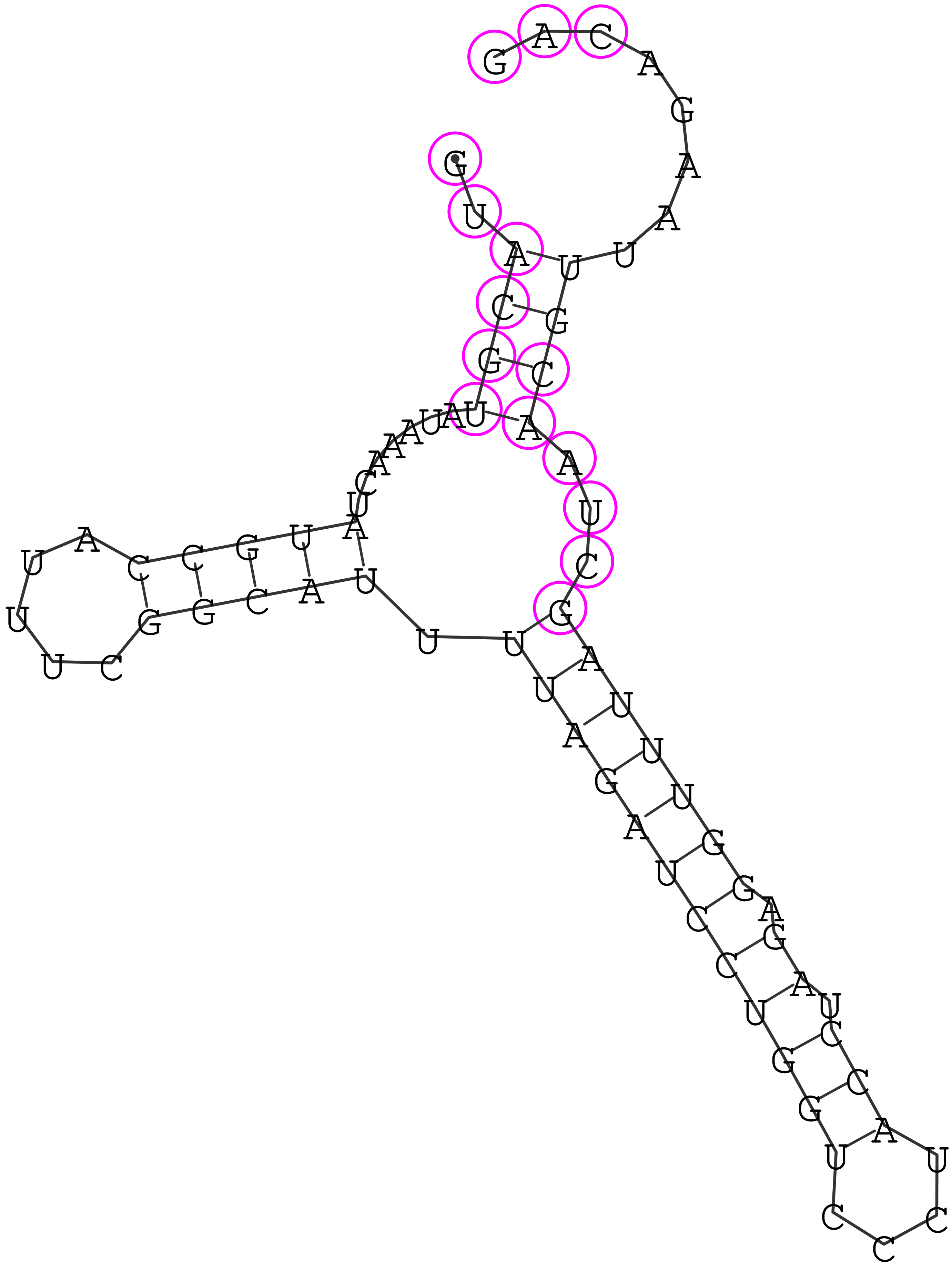

HCOc271A - Internal intron

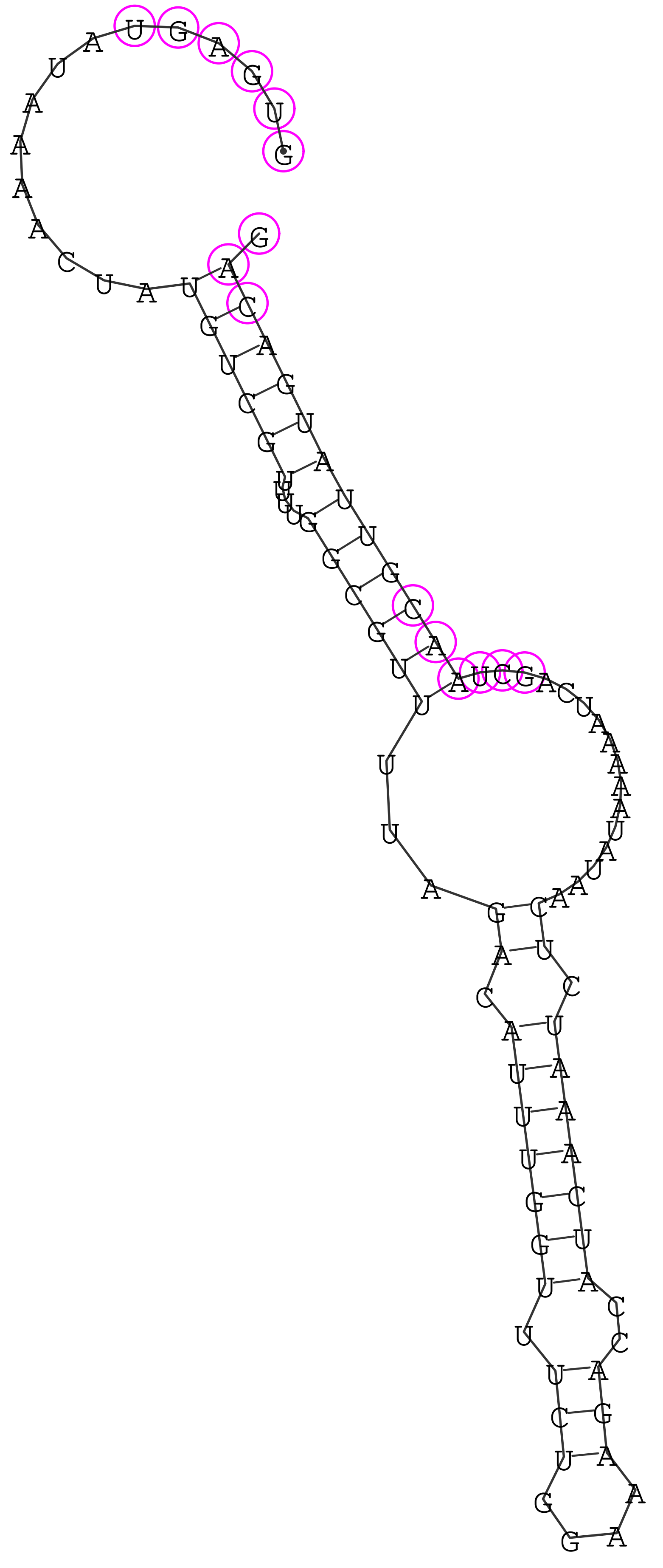

HCOc332A - Internal intron

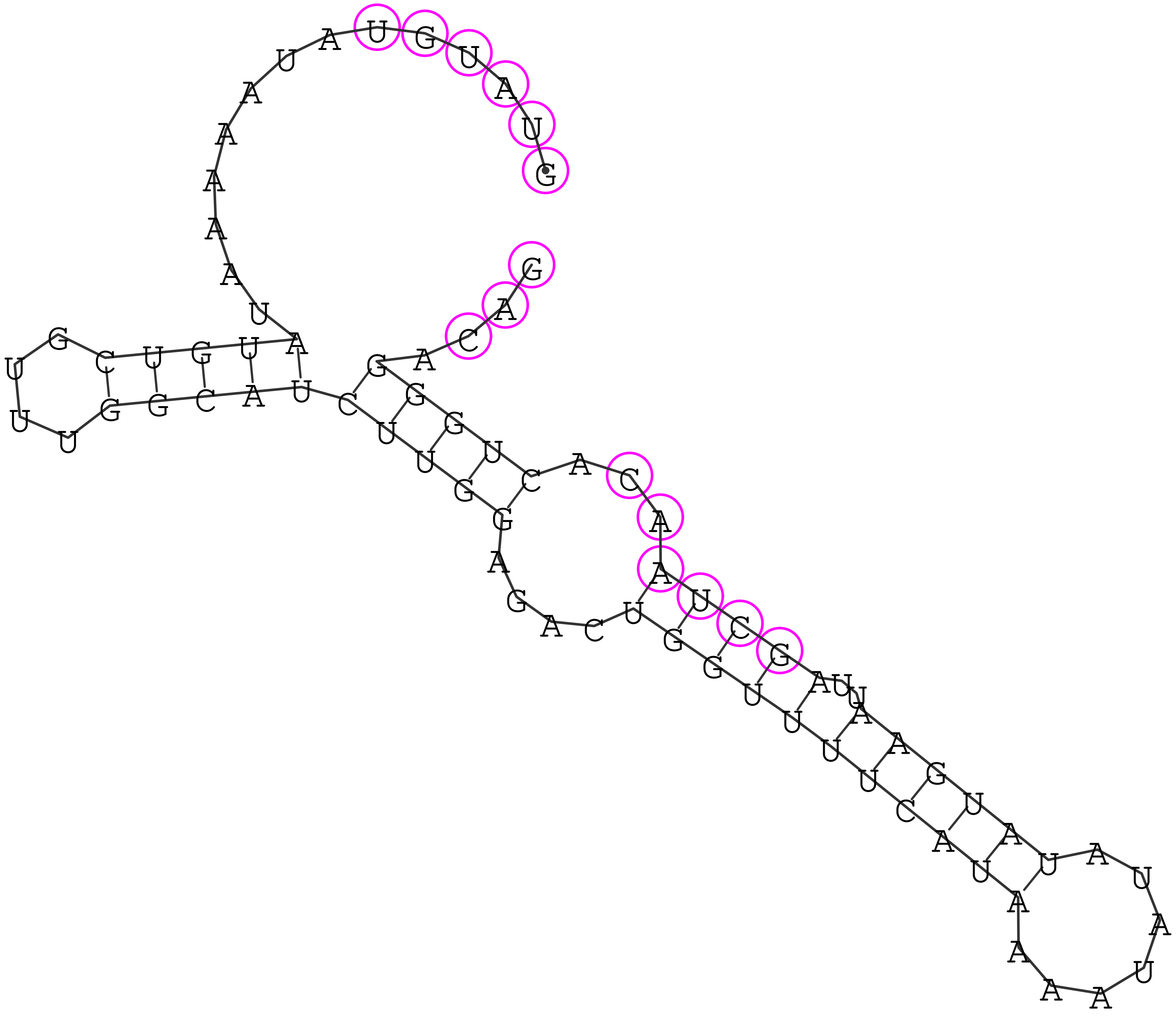

HCOc378A - Internal intron

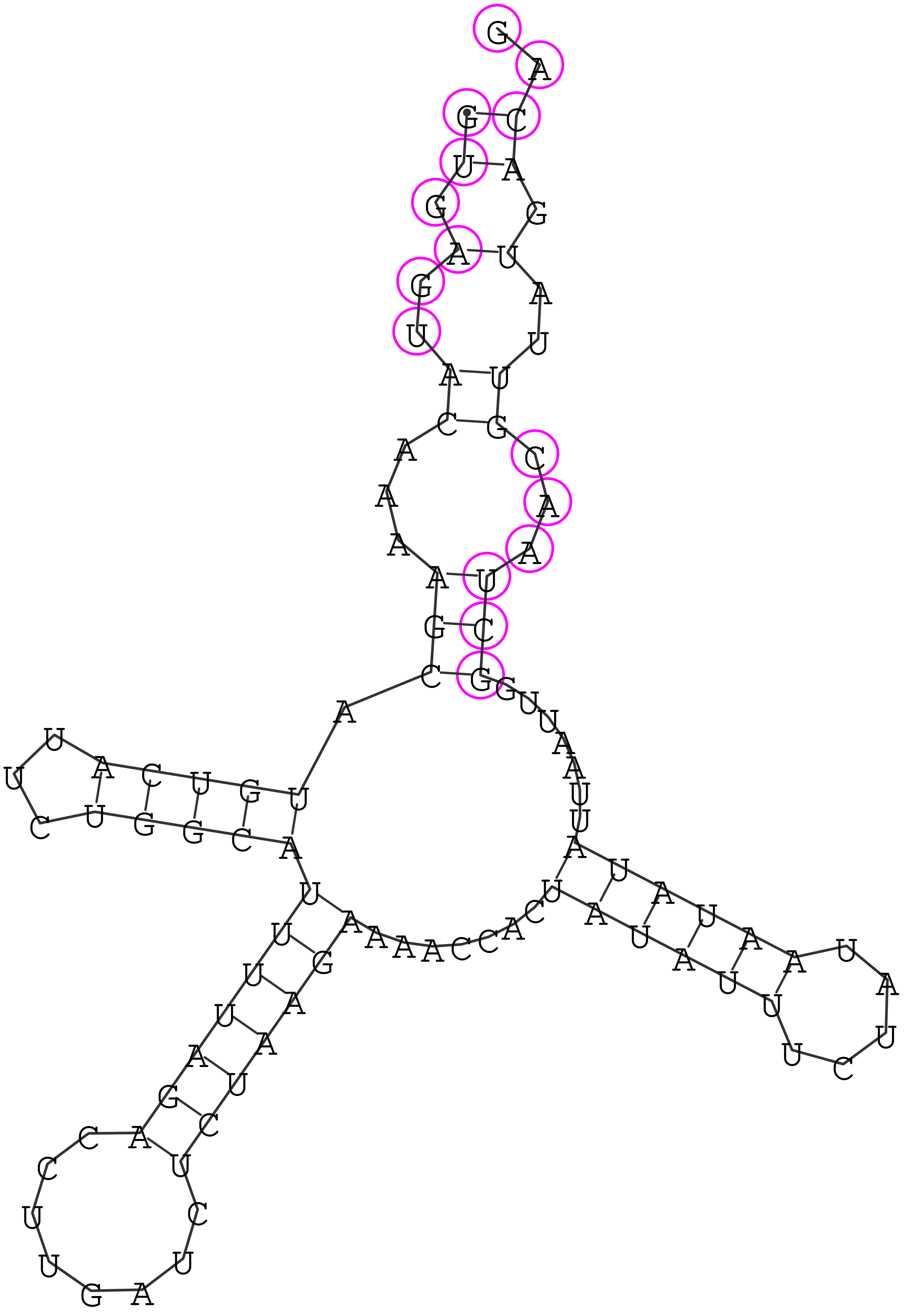

HCOc406A - Internal intron

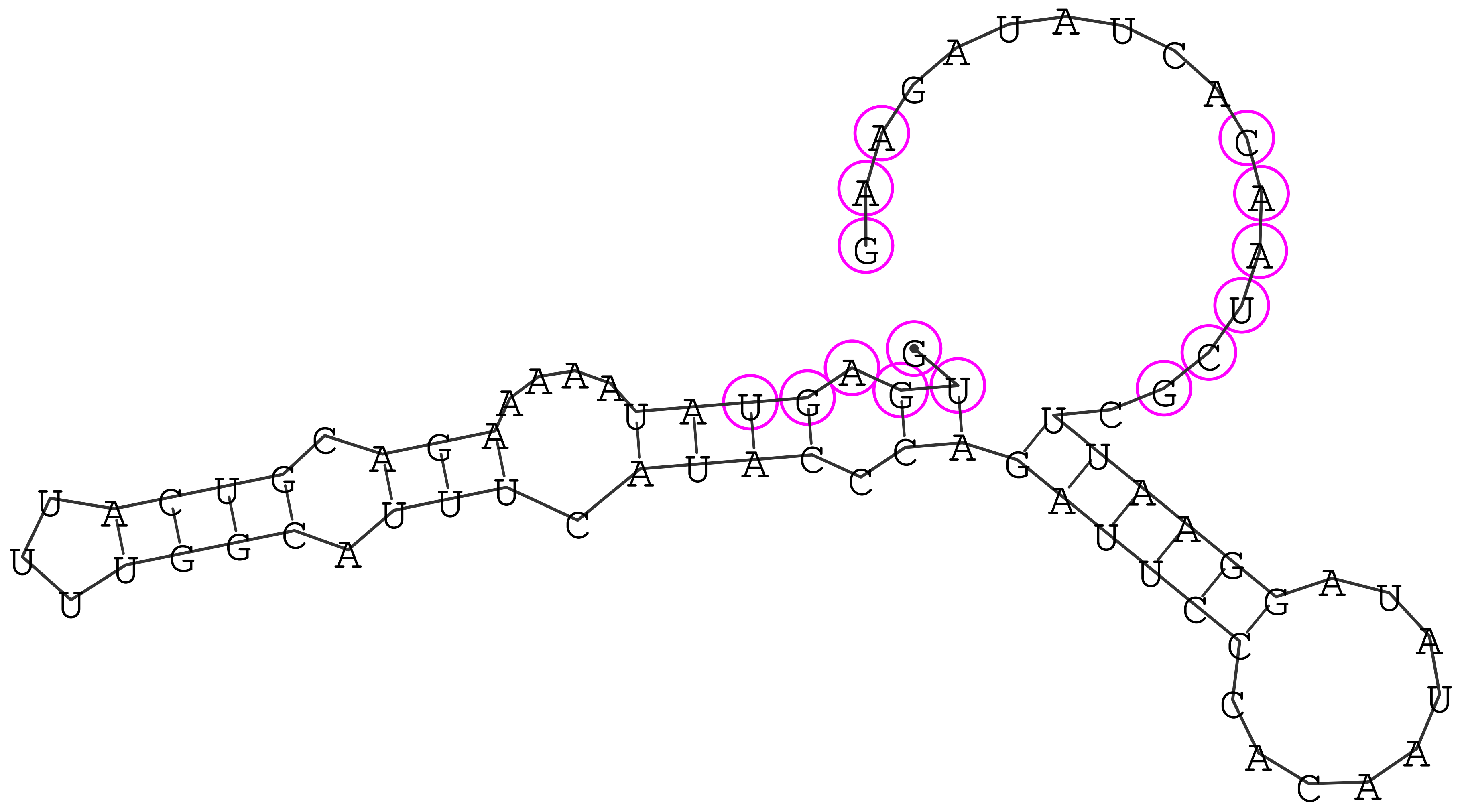

HCOc522A - Internal intron

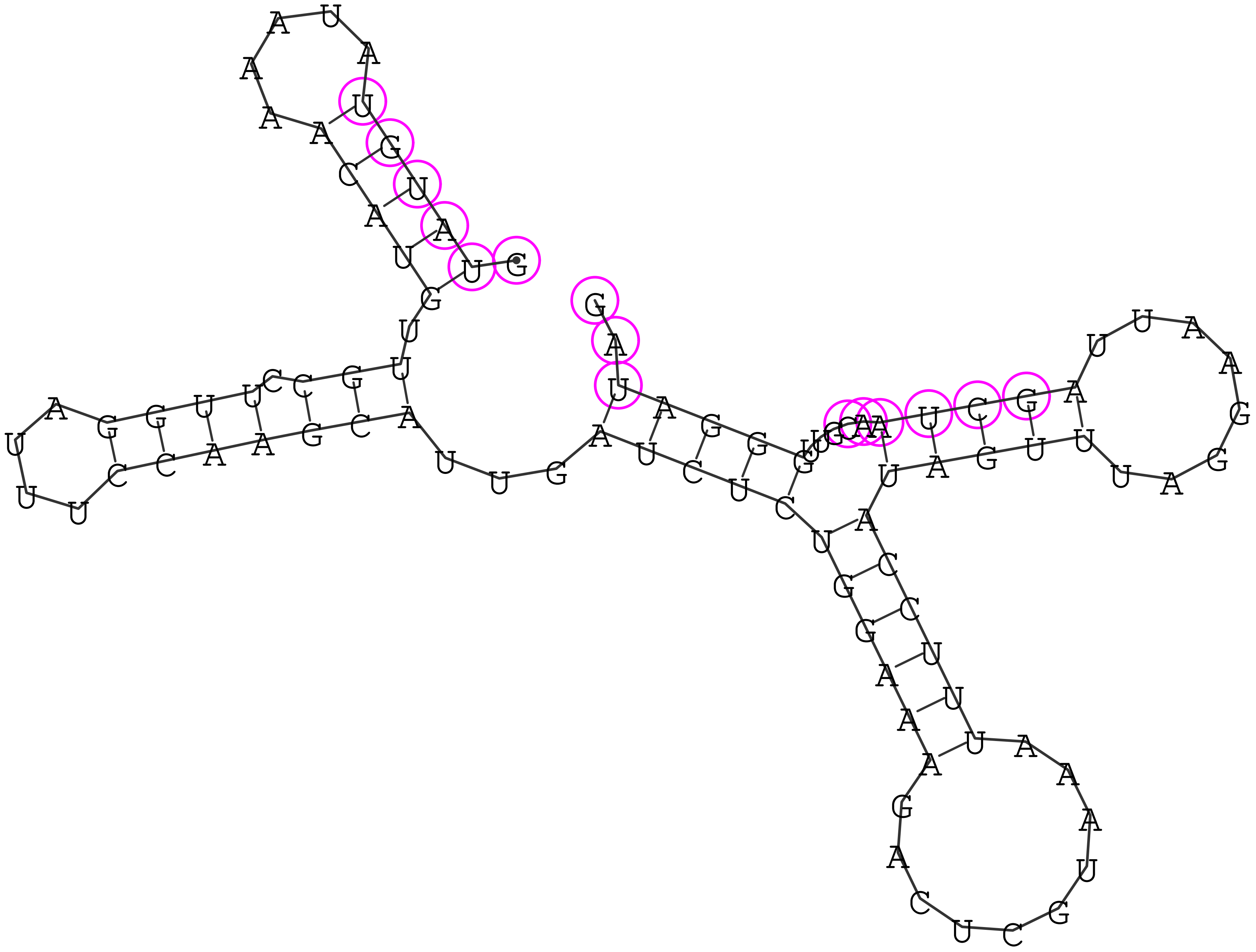

HE7c016A - Internal intron

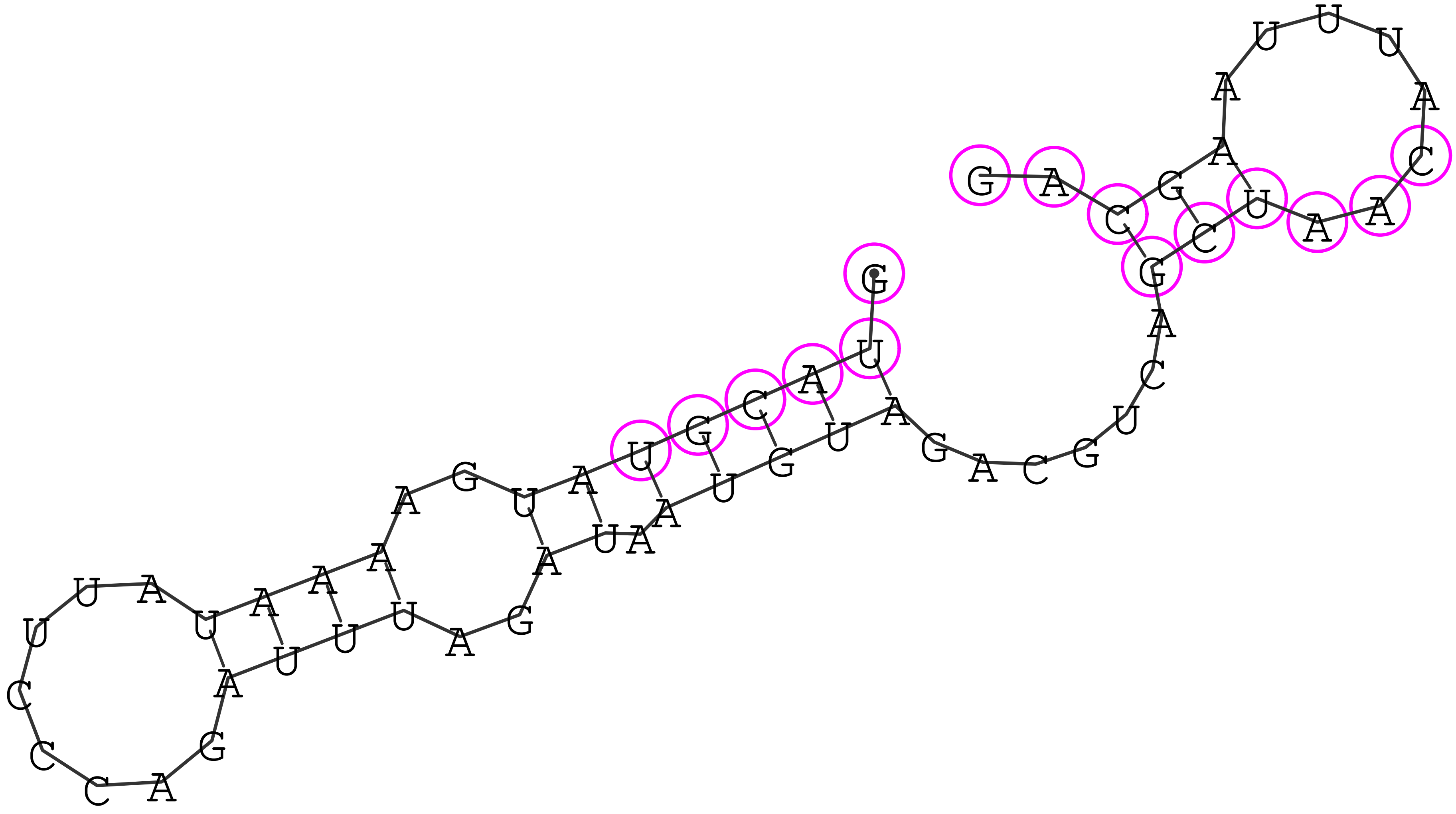

HE7c026A - Internal intron

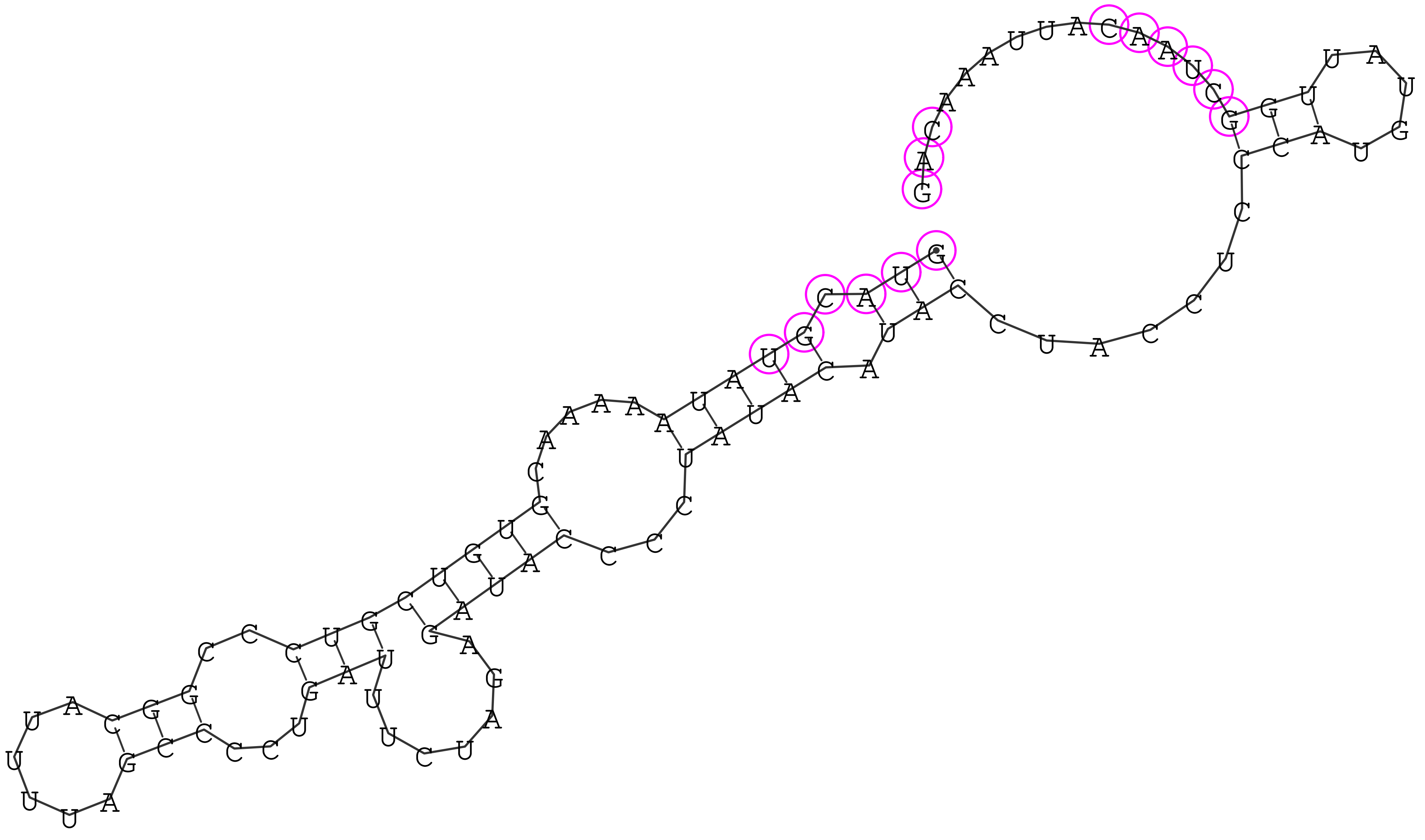

HE7c026B - Internal intron

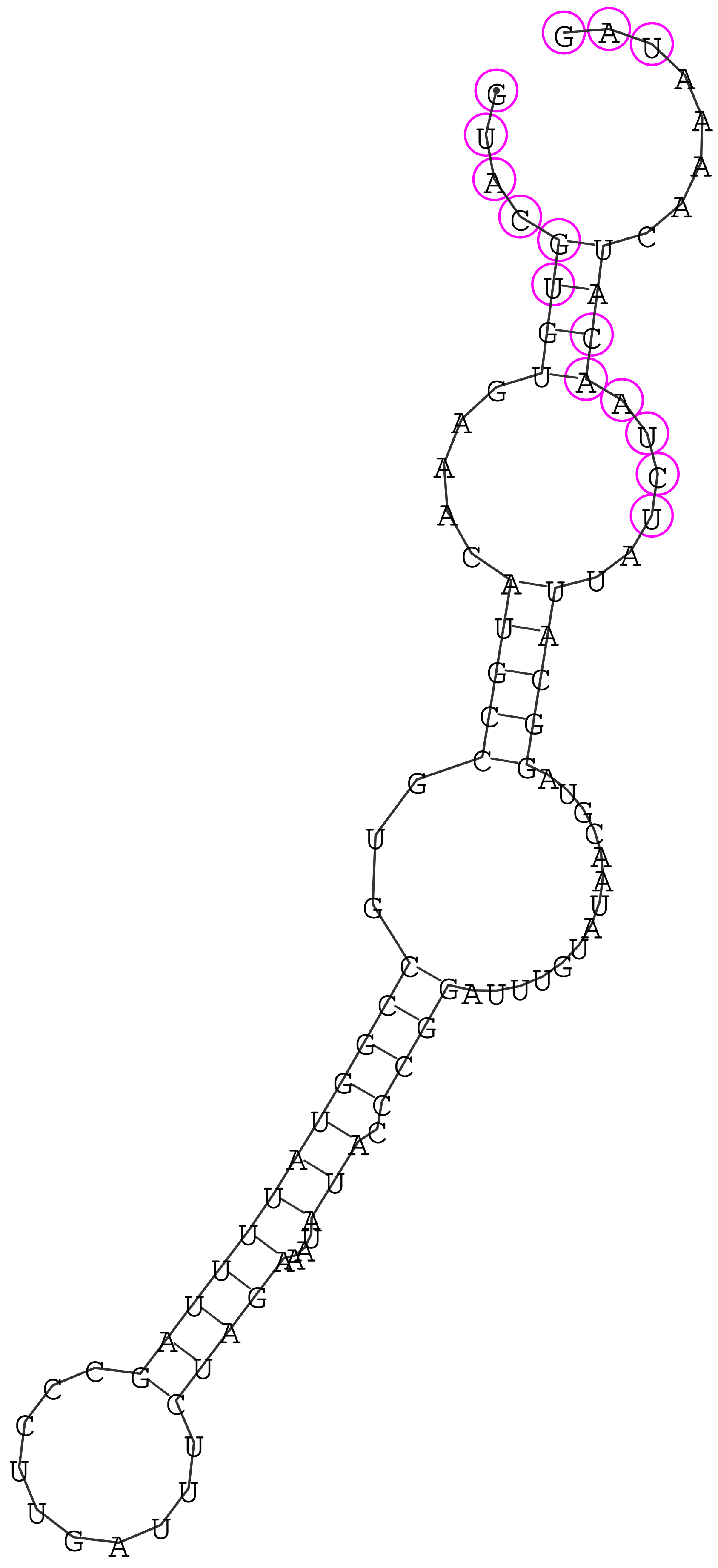

HE7c035A - Internal intron

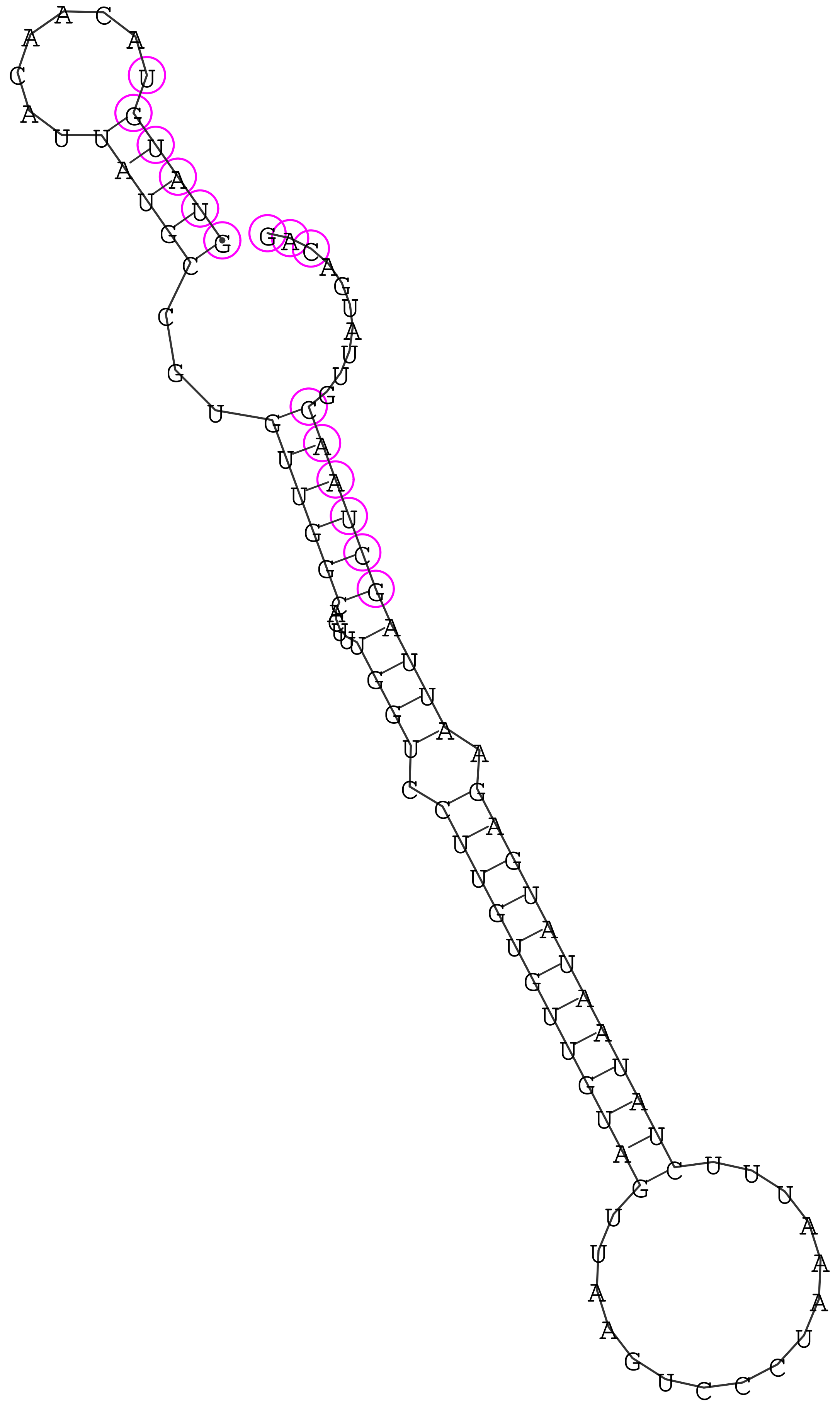

HE7c050A - Internal intron

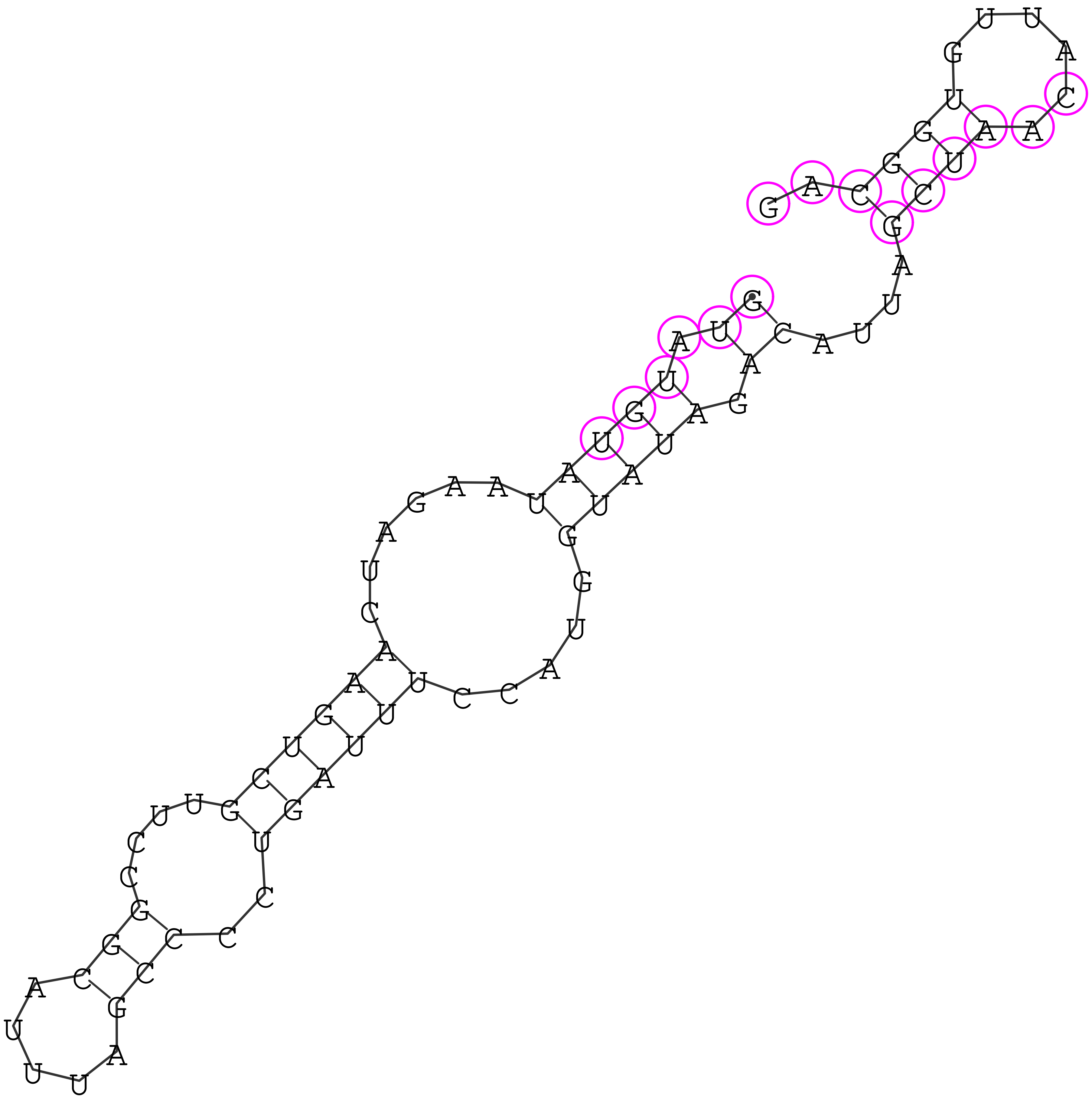

HE7c129A - Internal intron

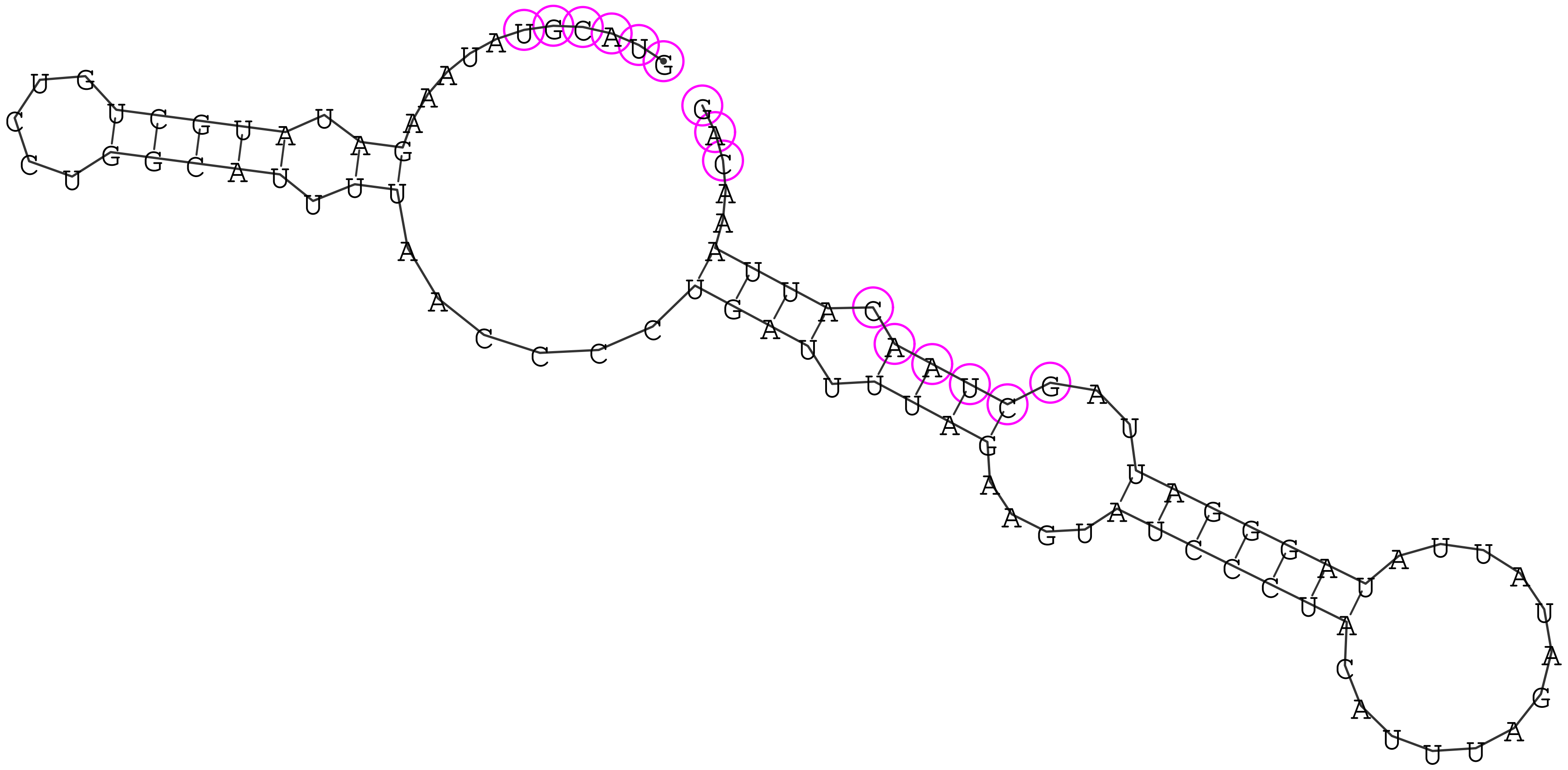

HE7c137A - Internal intron

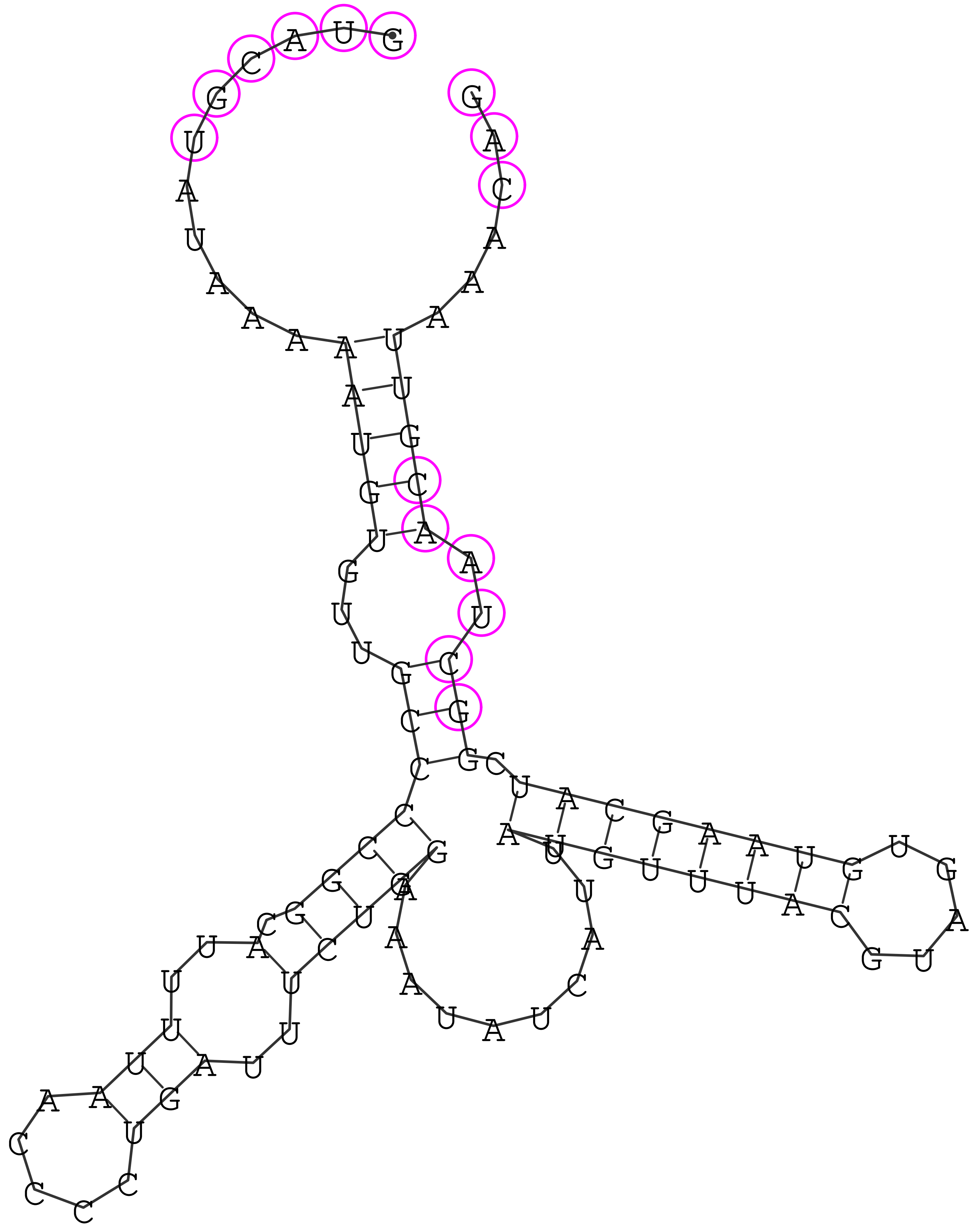

HE7c276A - Internal intron

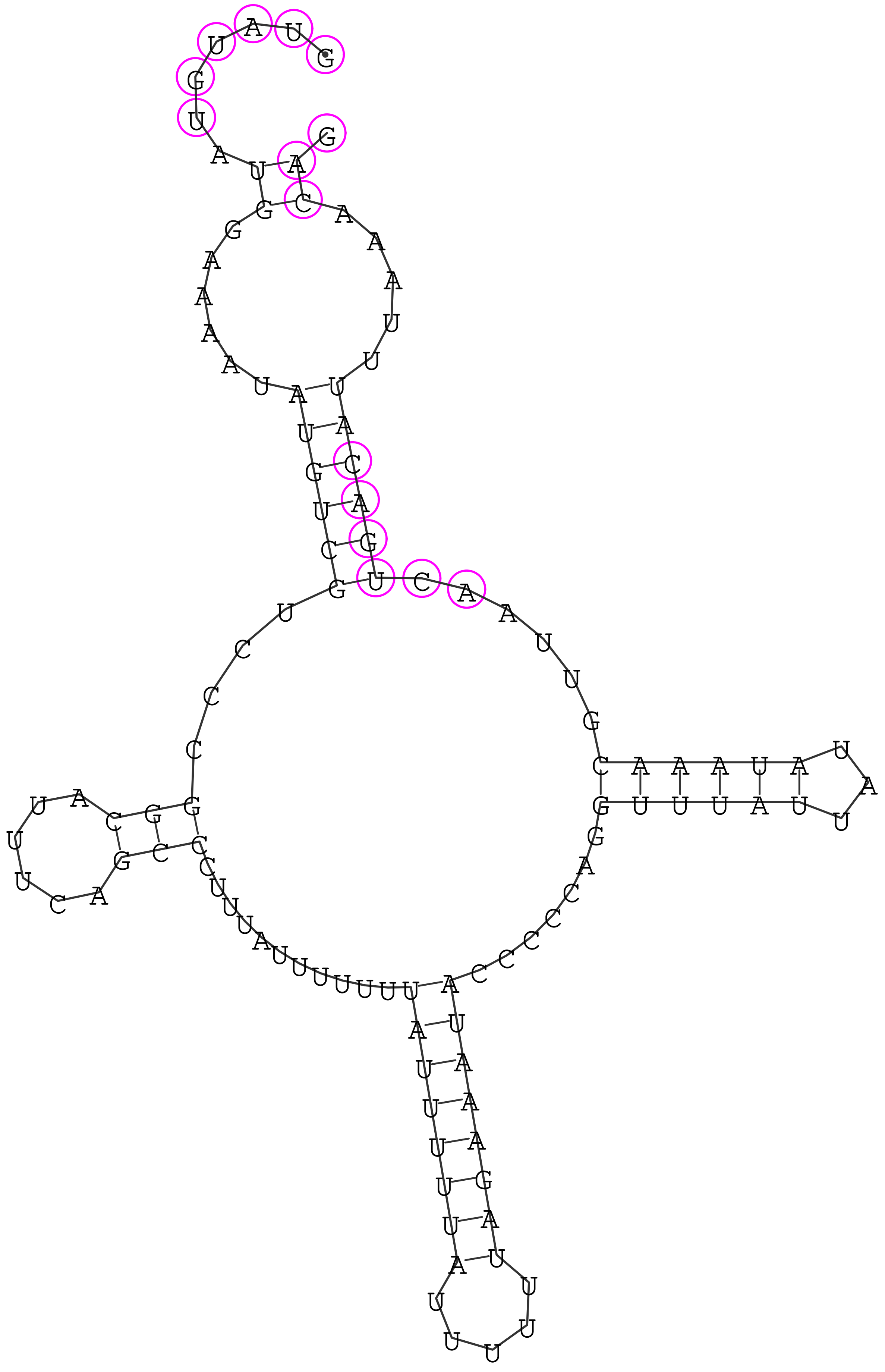

HE7c301A - Internal intron

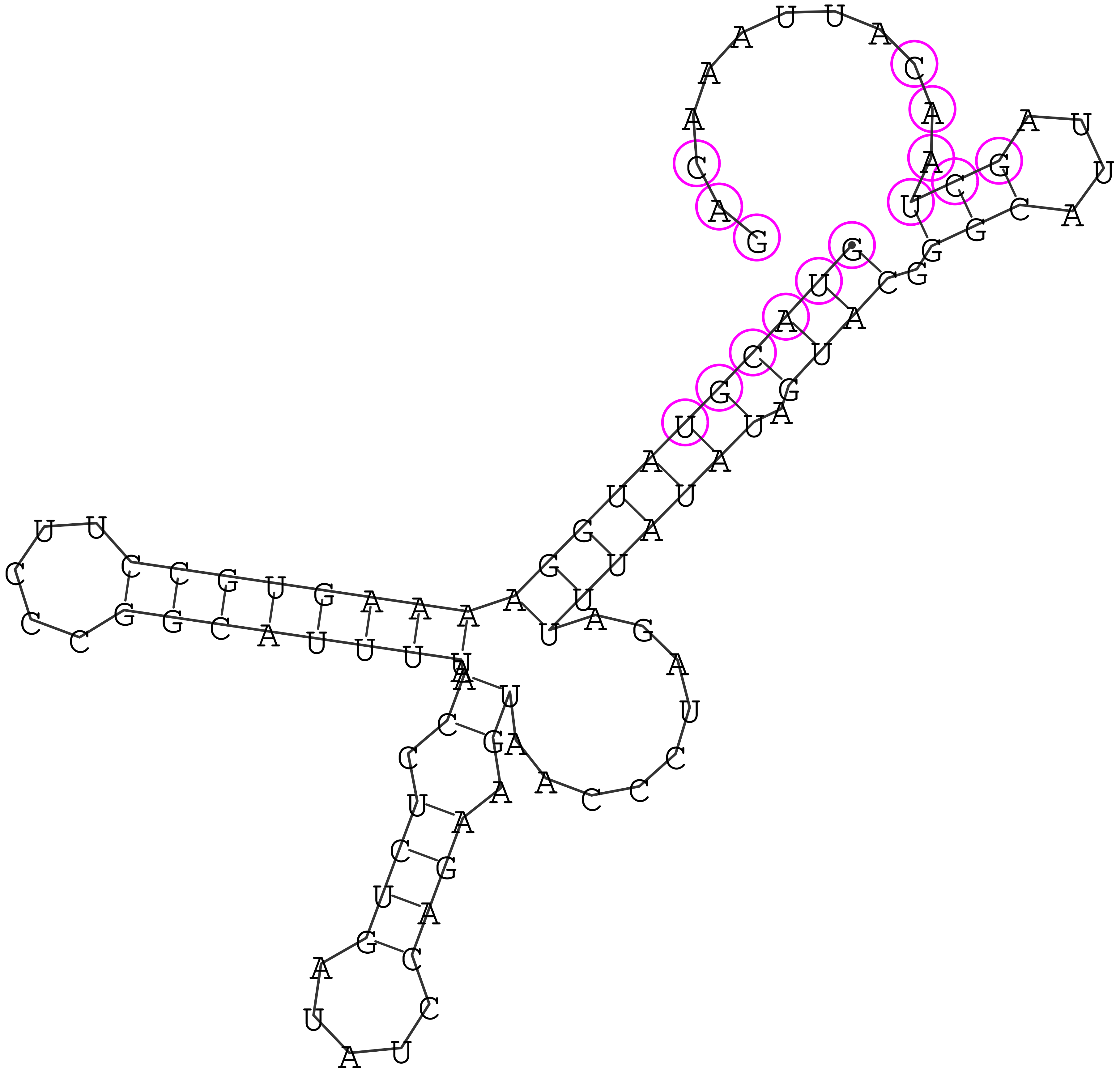

HECc114A - Internal intron

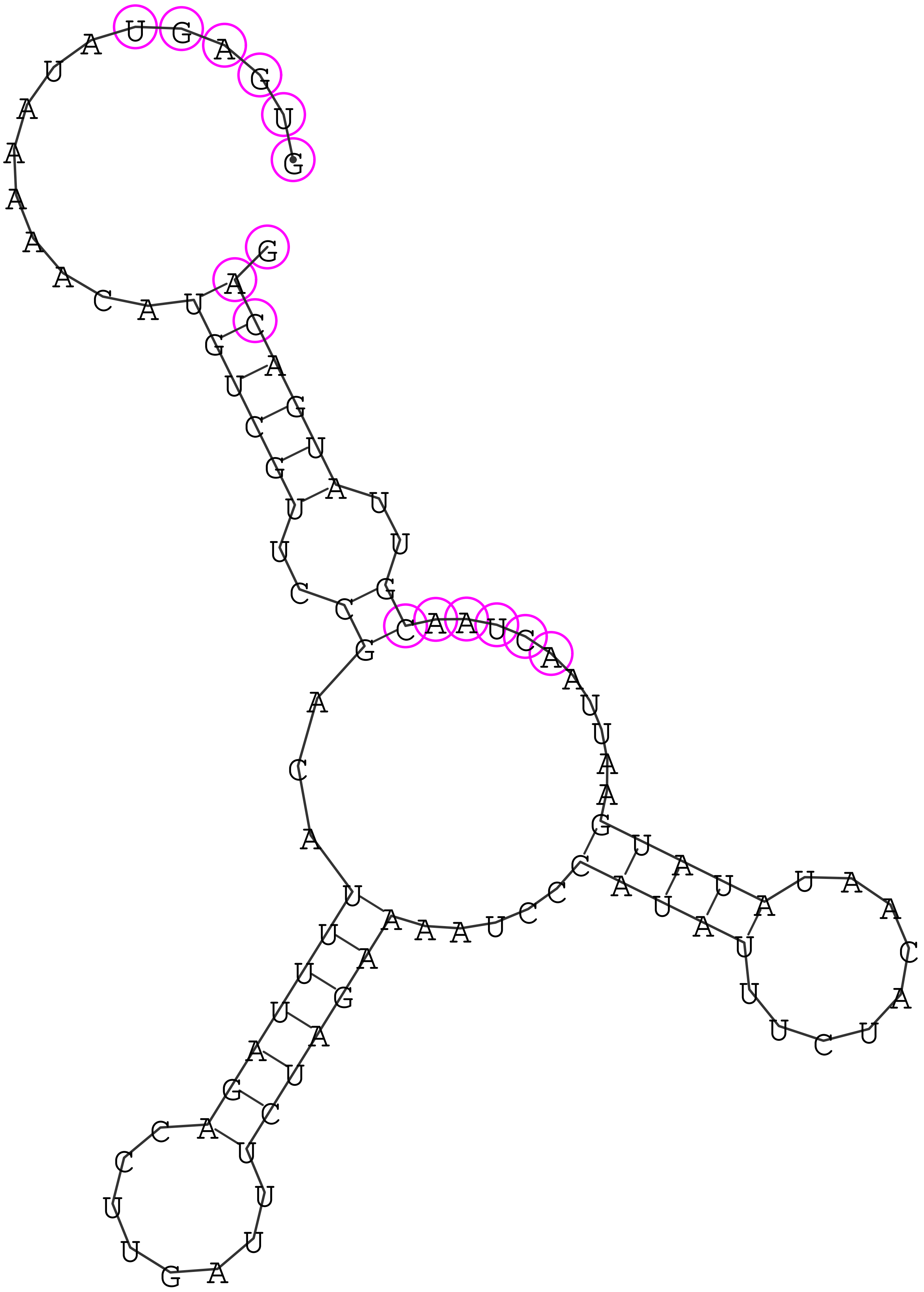

HECc217A - Internal intron

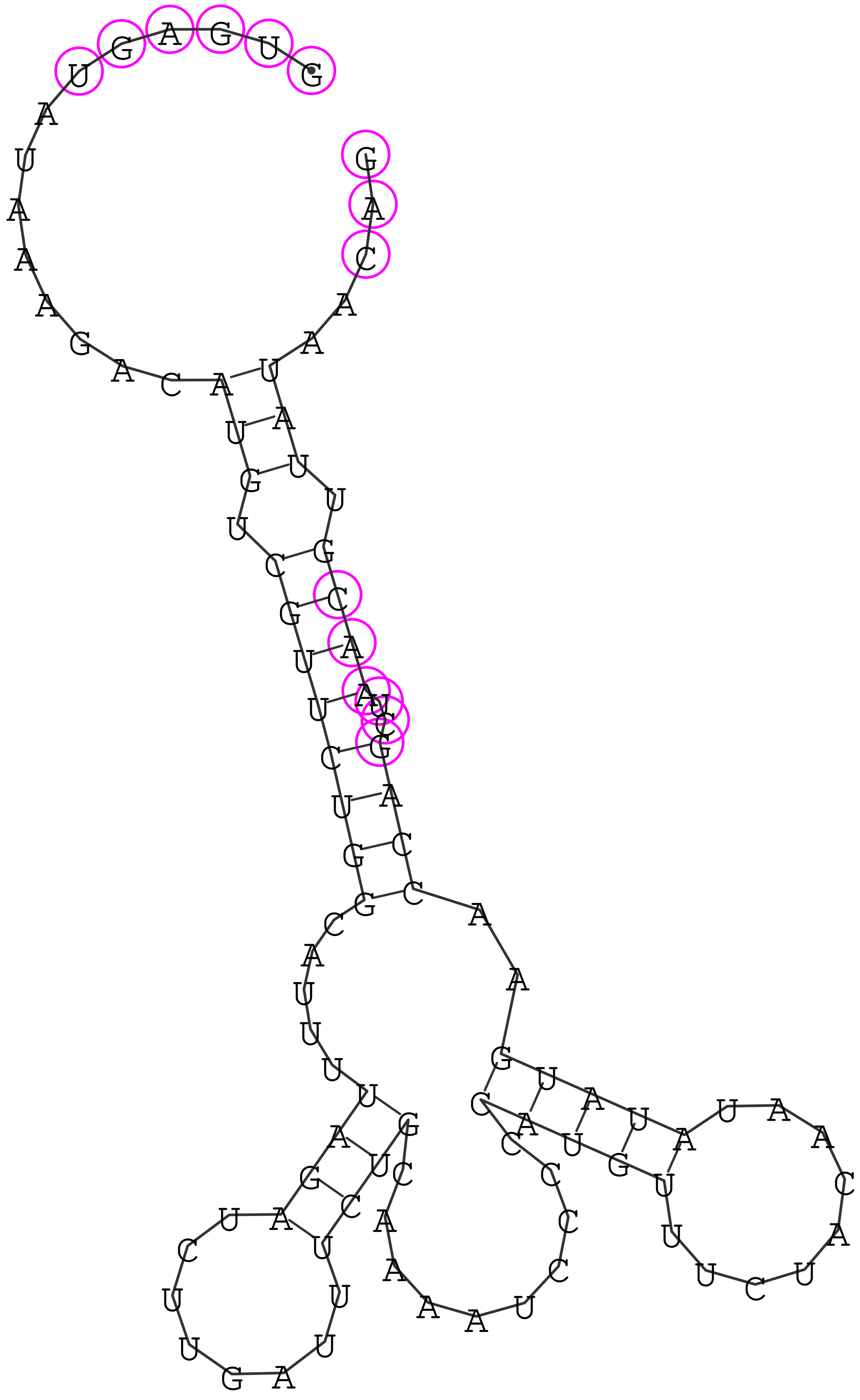

Hruc29A - Internal intron

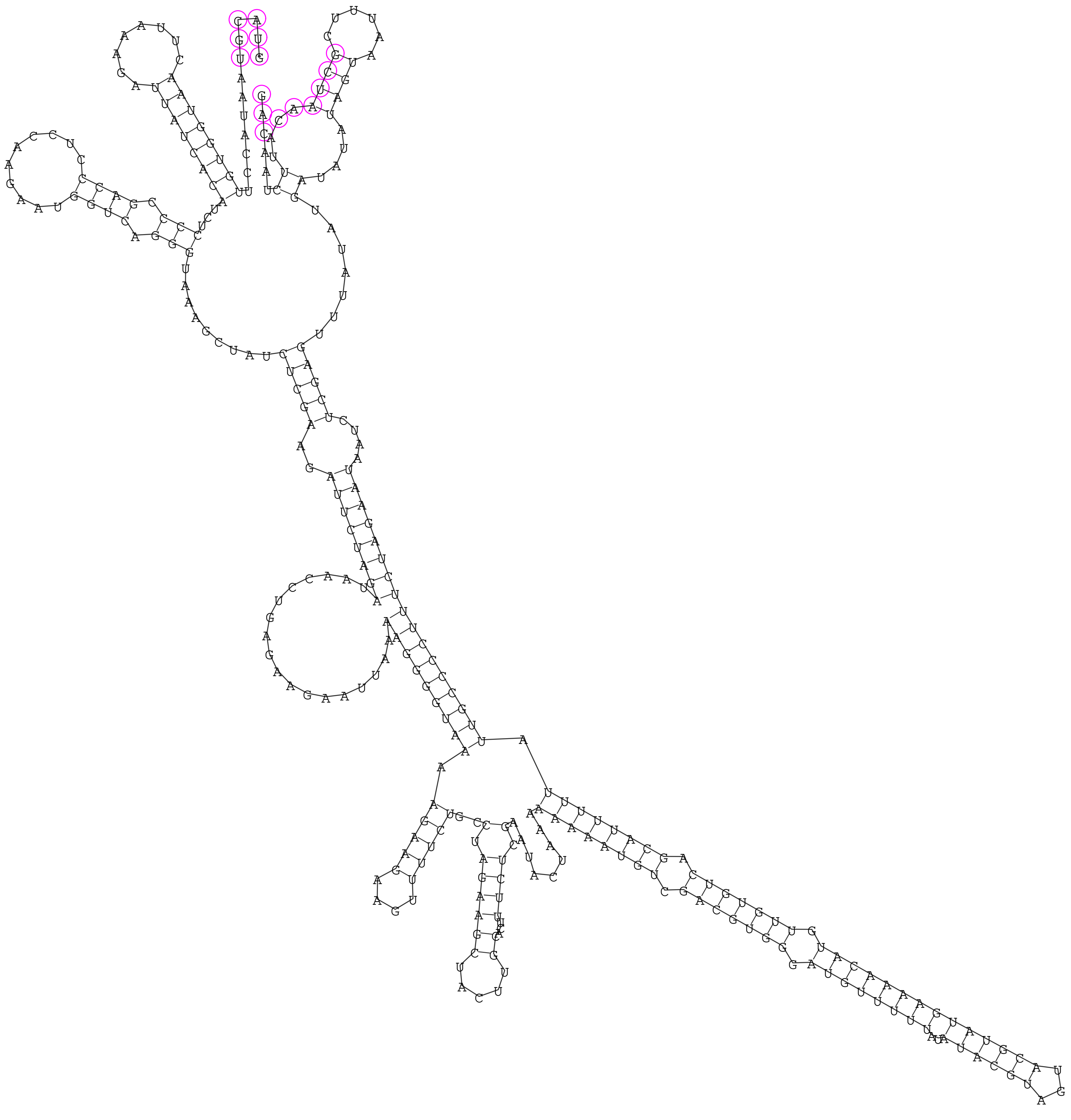

Hruc31A - Internal intron

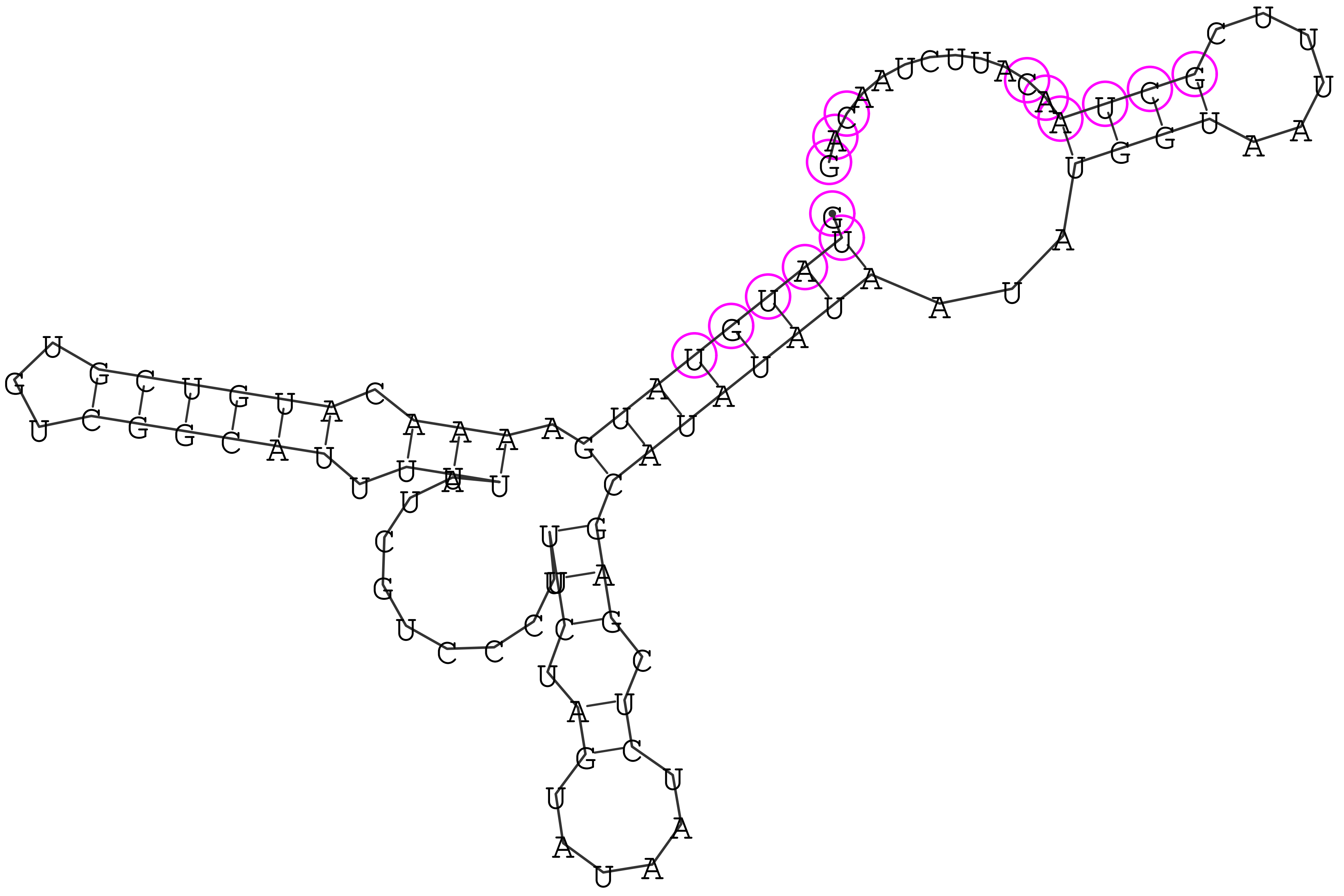

Hruc55A - Internal intron

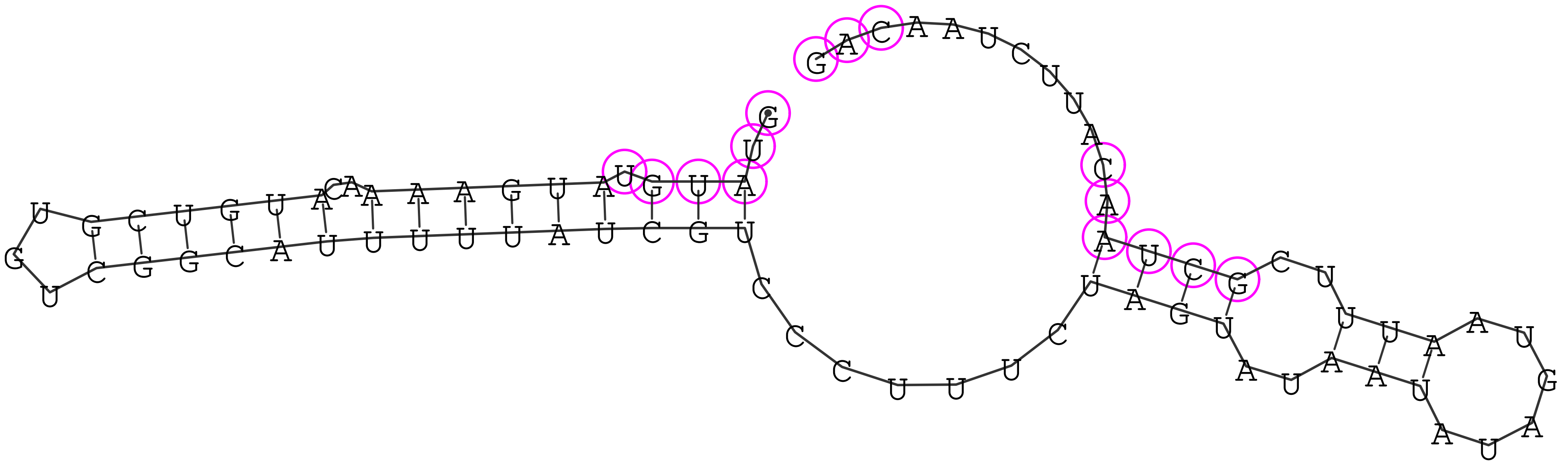

Hruc56A - Internal intron

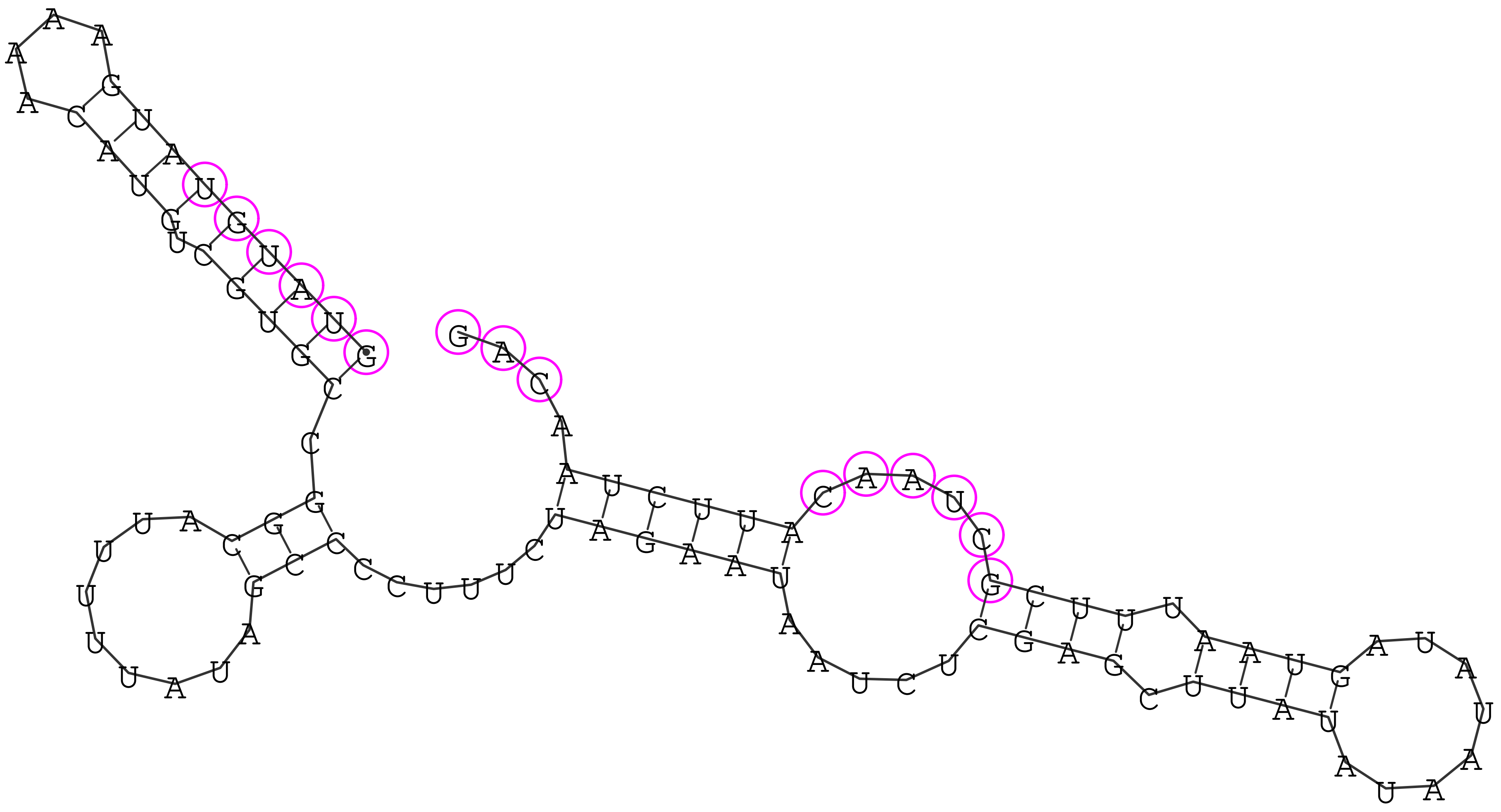

Hruc59A - Internal intron

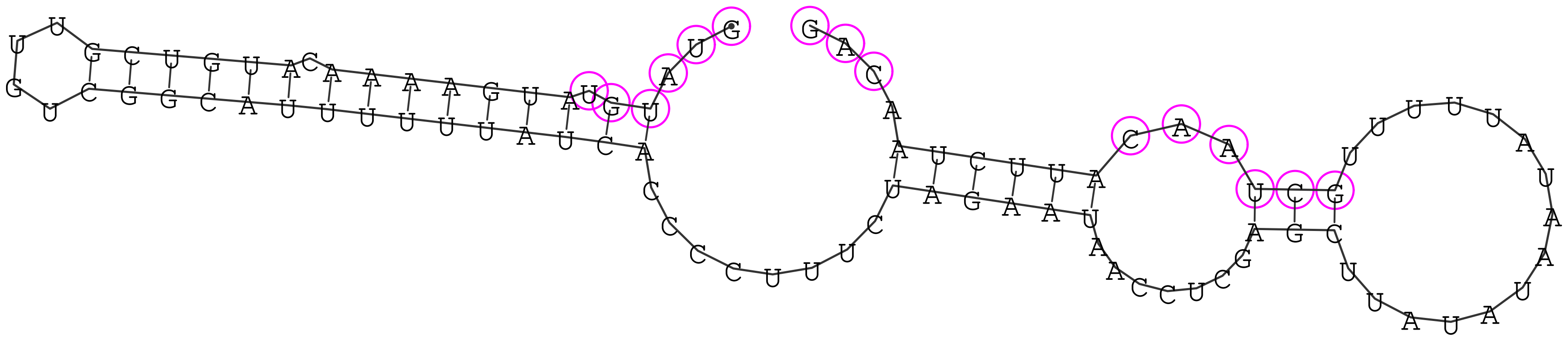

Naboc005A - Internal intron

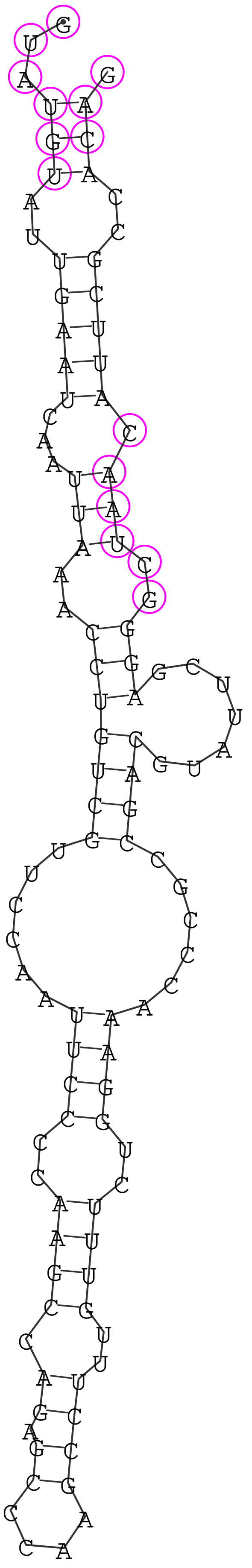

Naboc011A - Internal intron

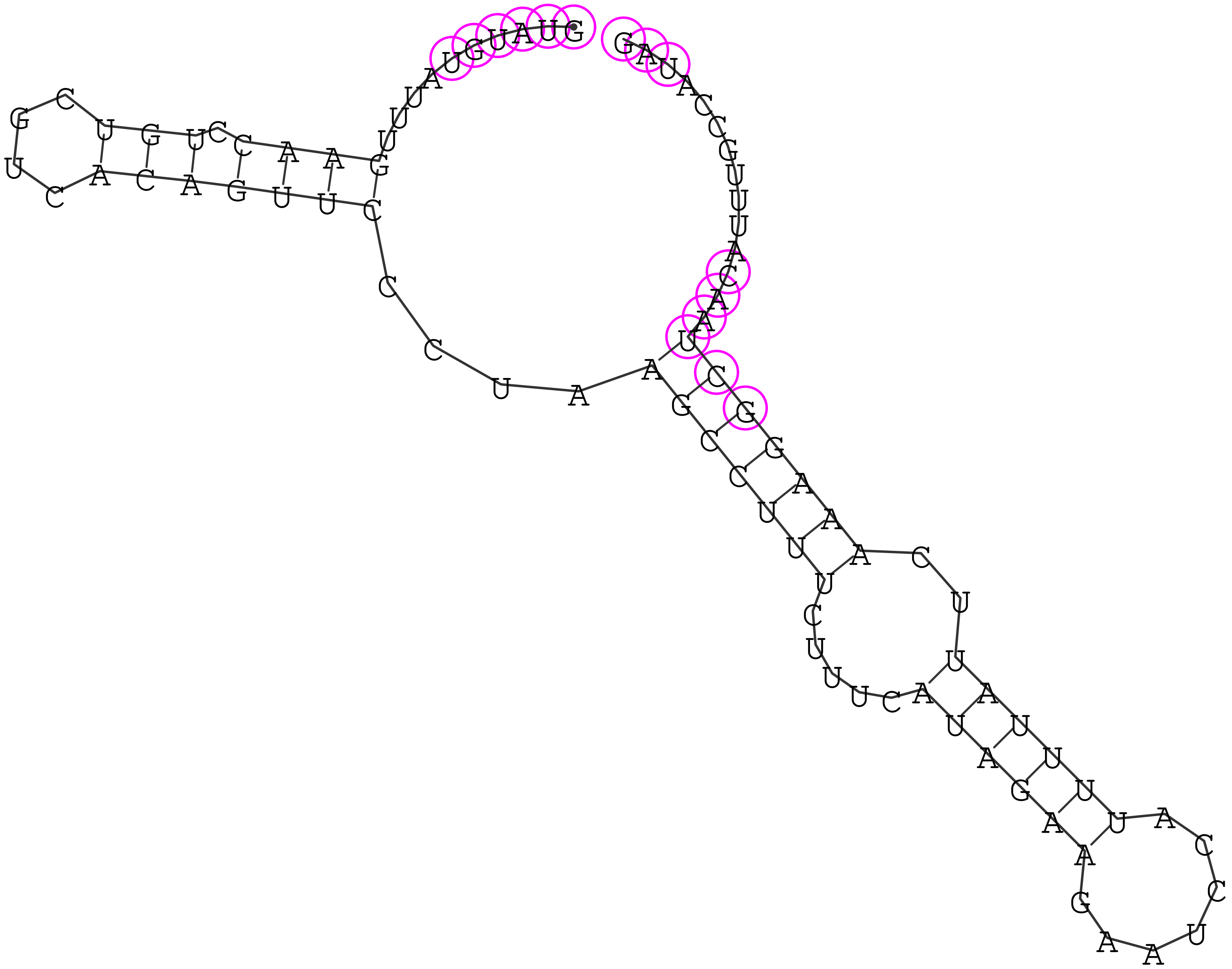

Naboc037A - Internal intron

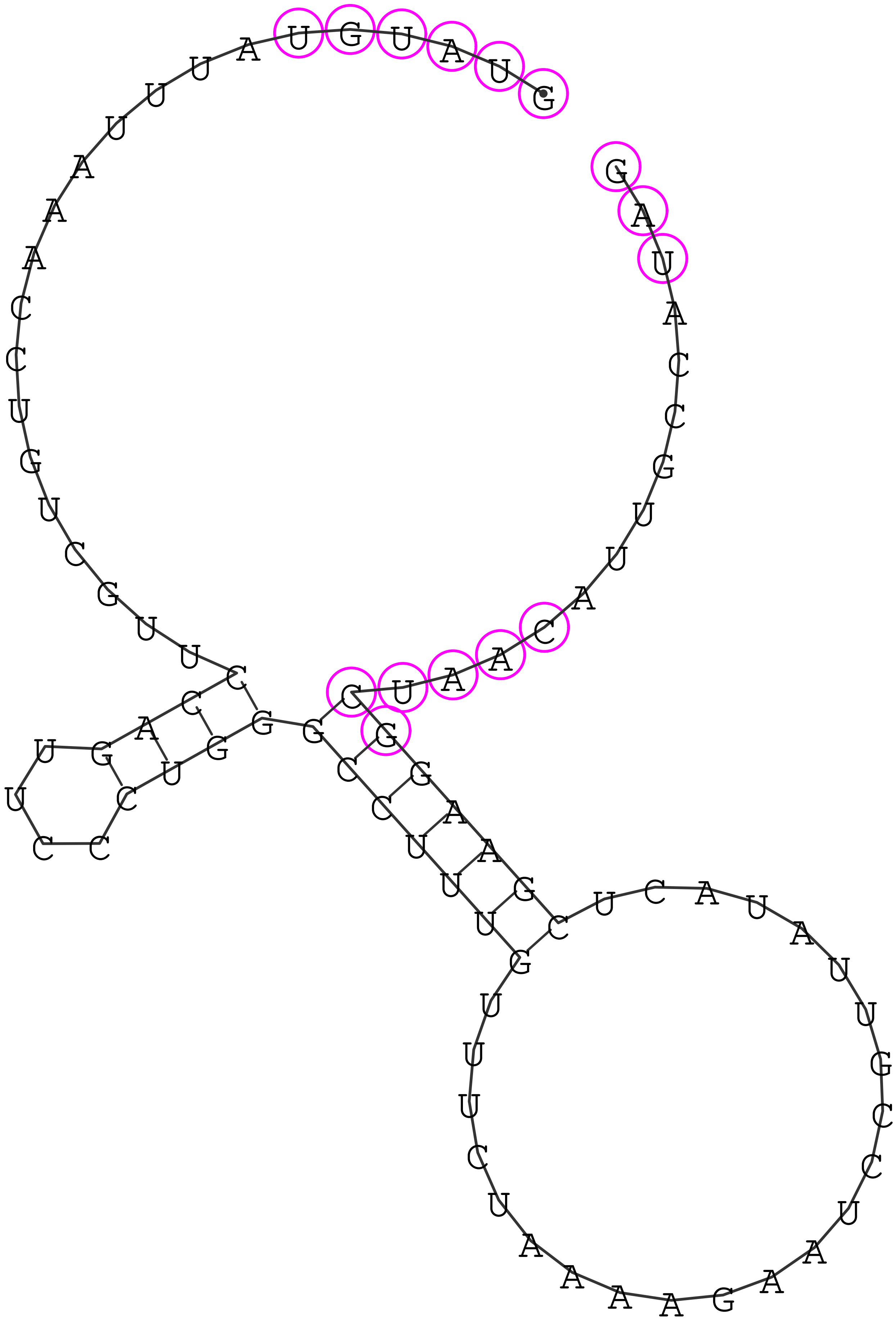

Naboc037B - Internal intron

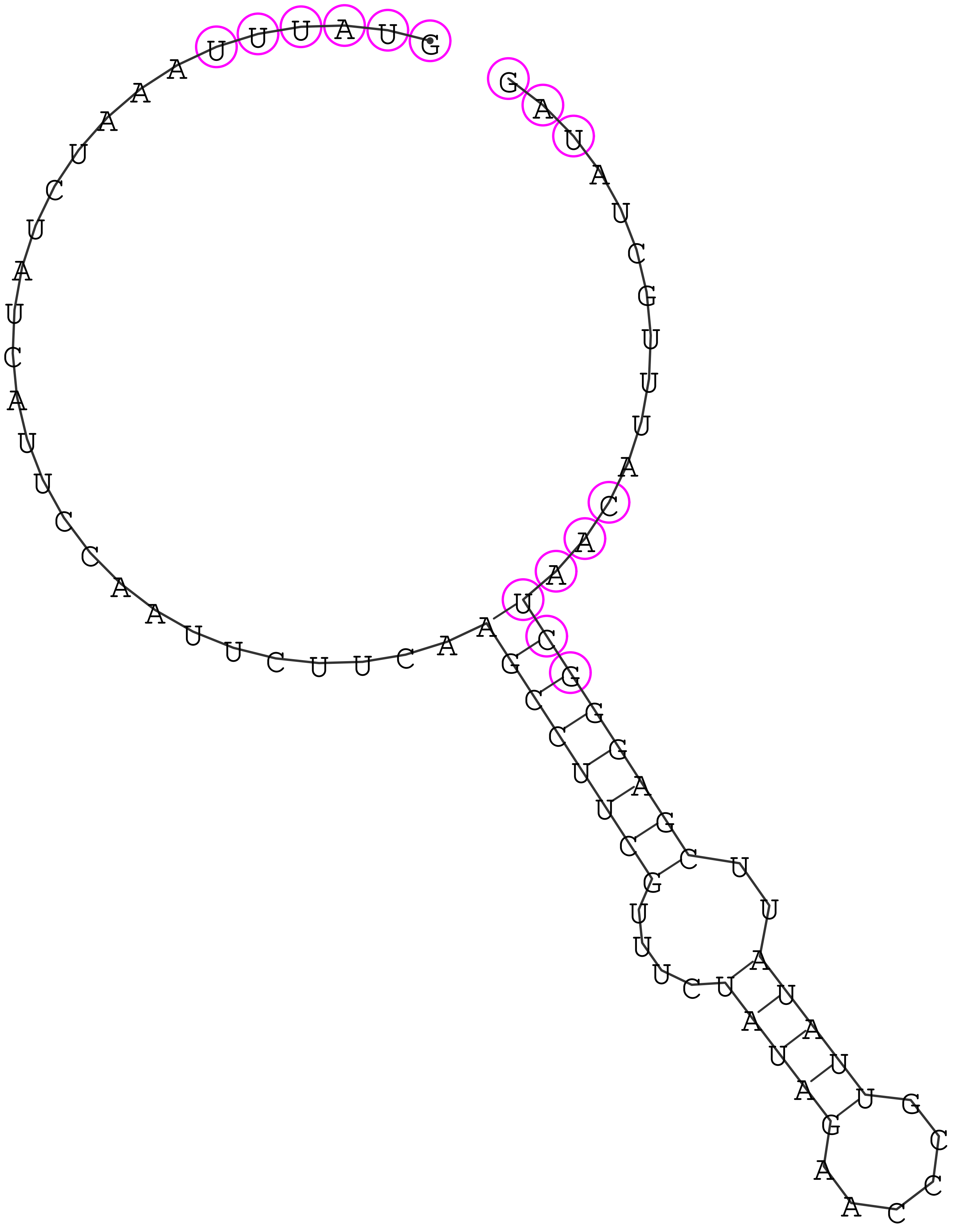

Naboc056A - Internal intron

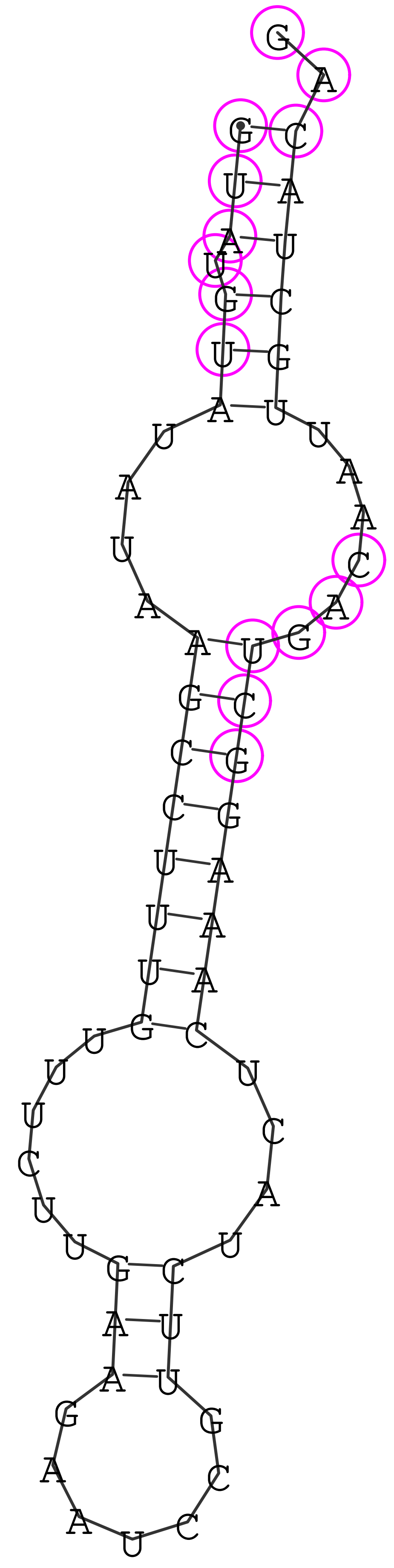

Naboc066A - Internal intron

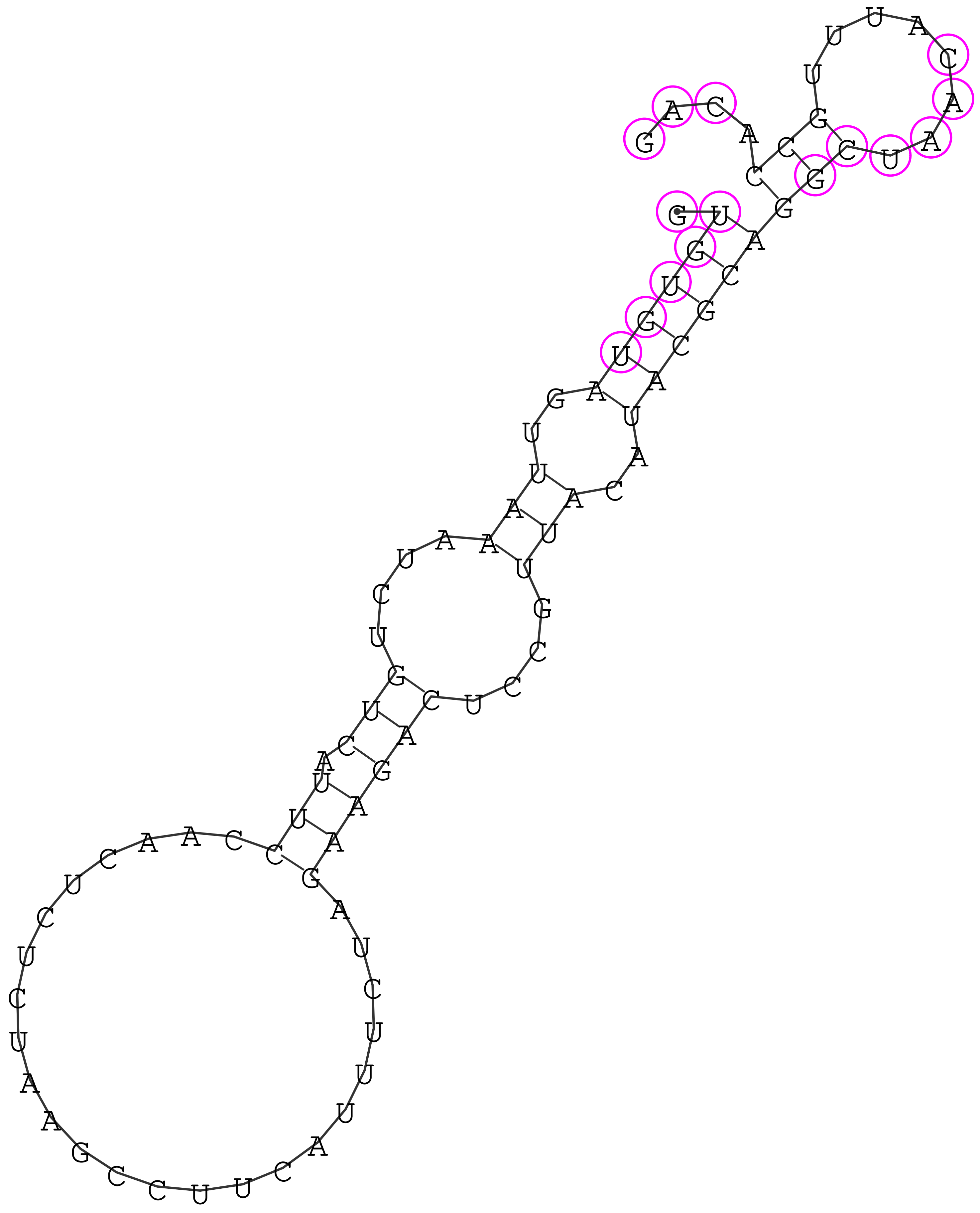

Naboc073A - Internal intron

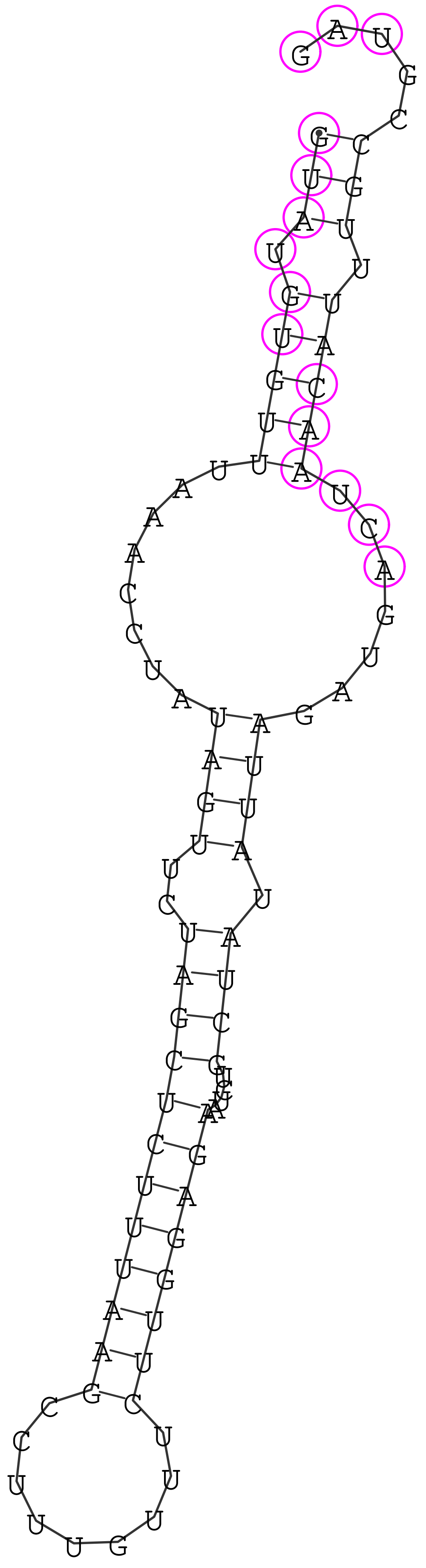

Naboc079A - Internal intron

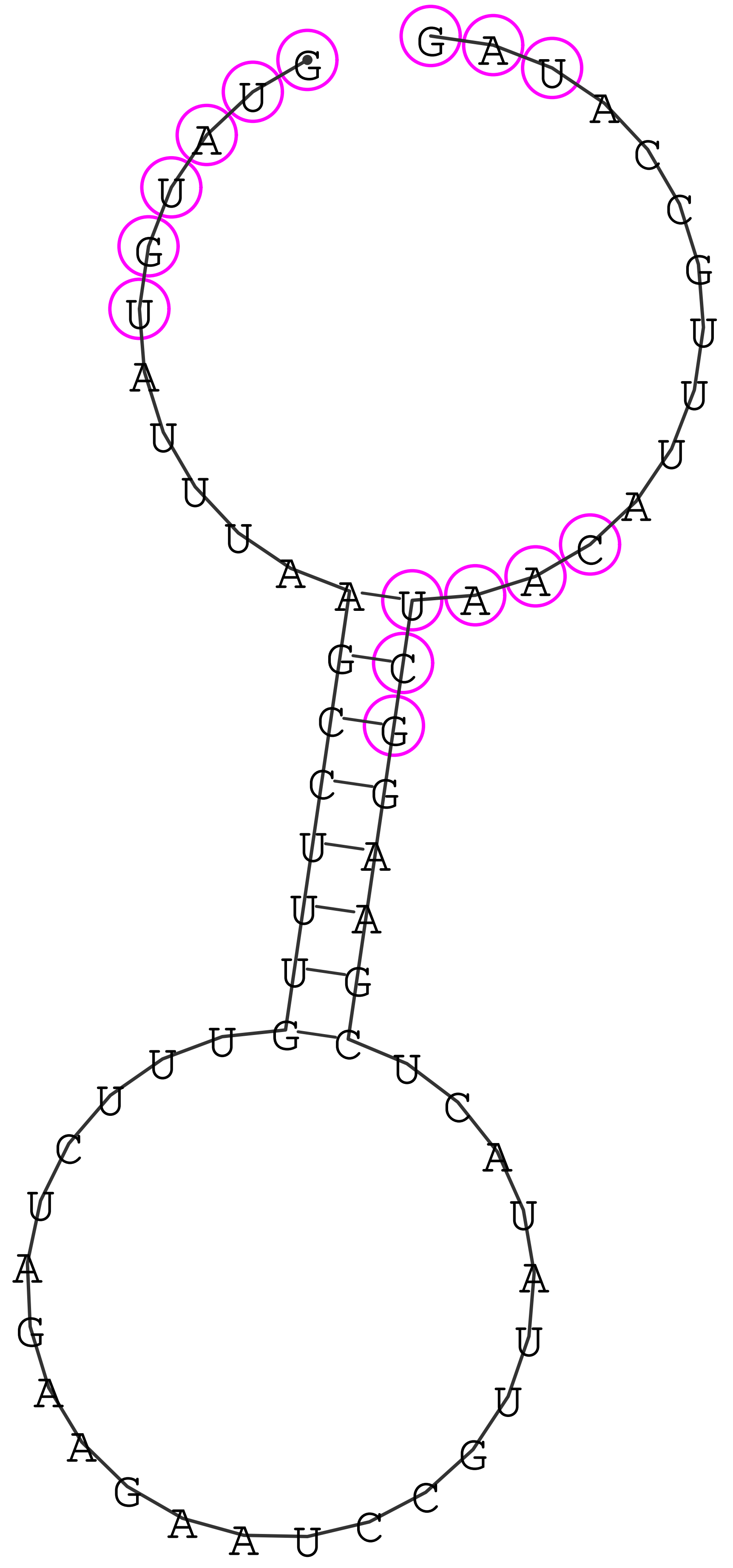

Naboc124A - Internal intron

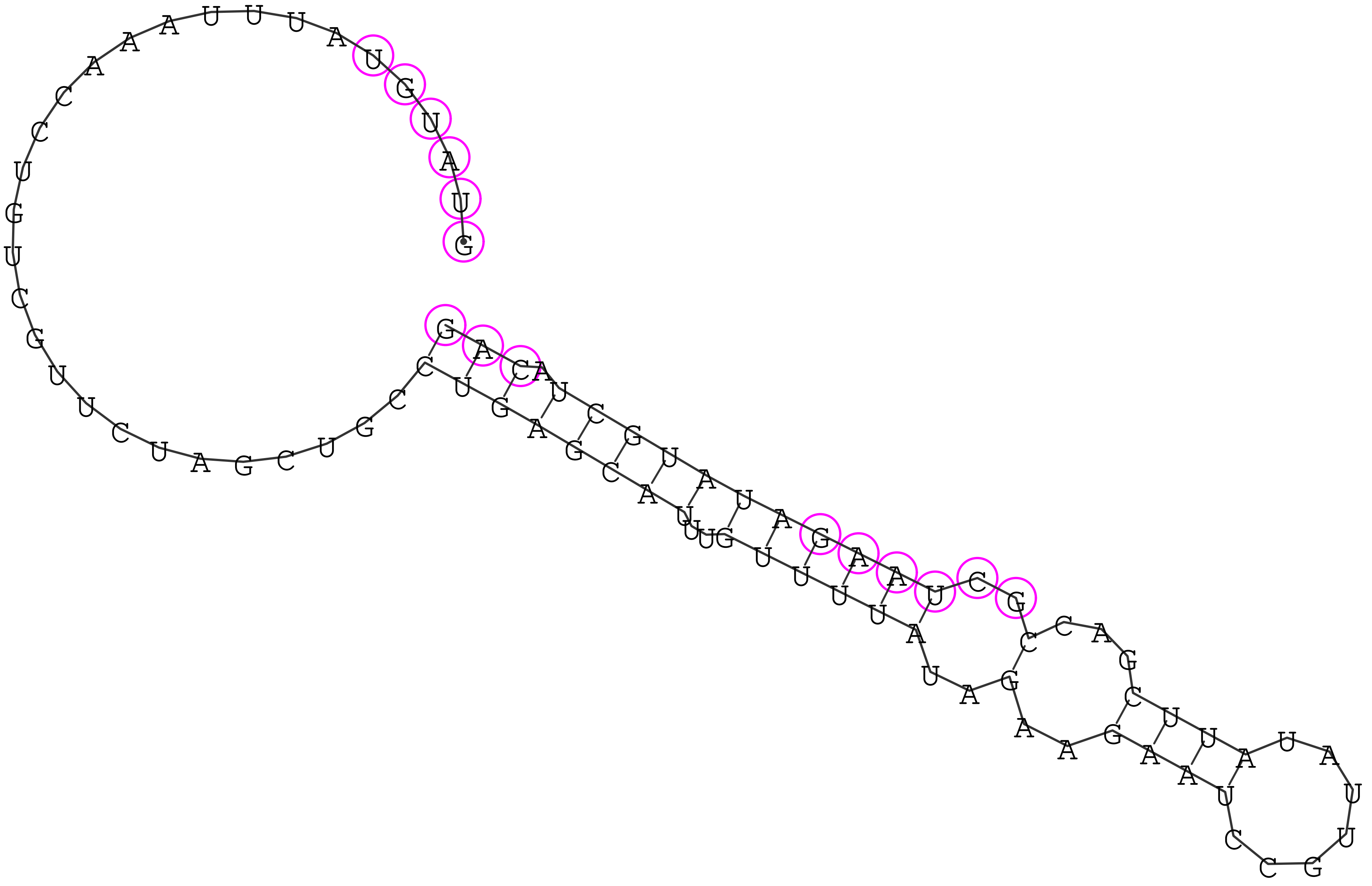

Naboc173A - Internal intron

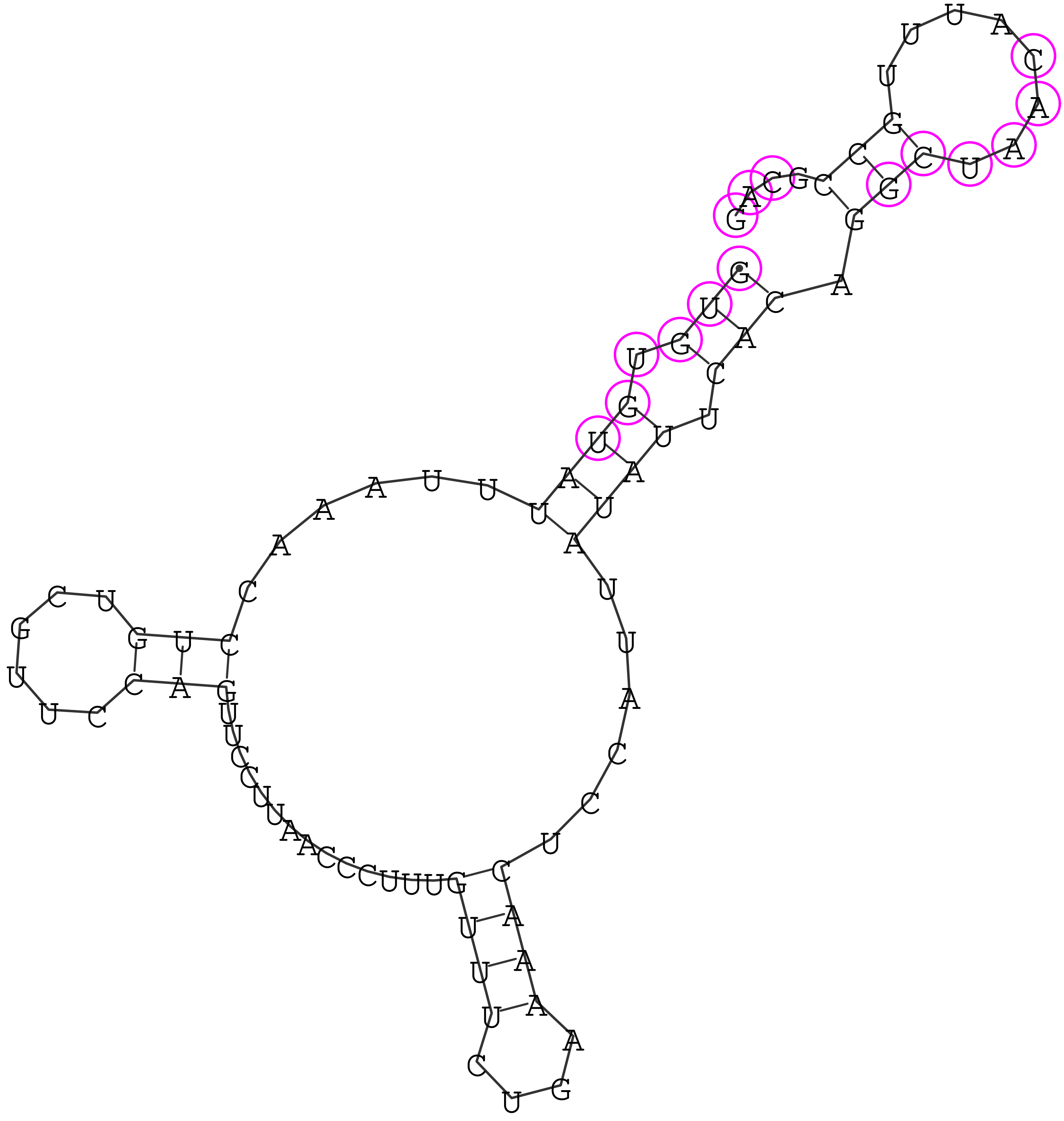

Naboc184A - Internal intron

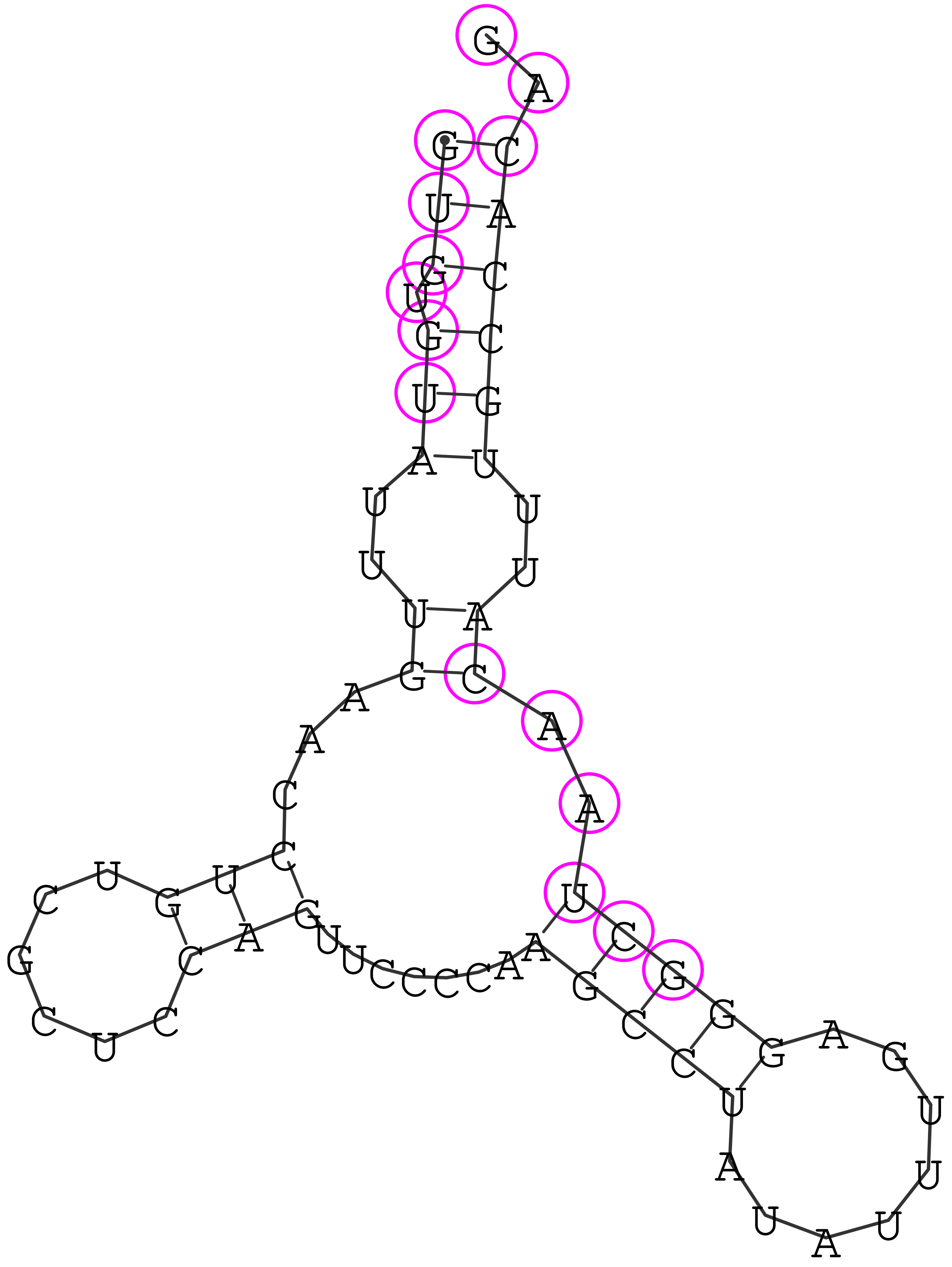

Naboc196A - Internal intron

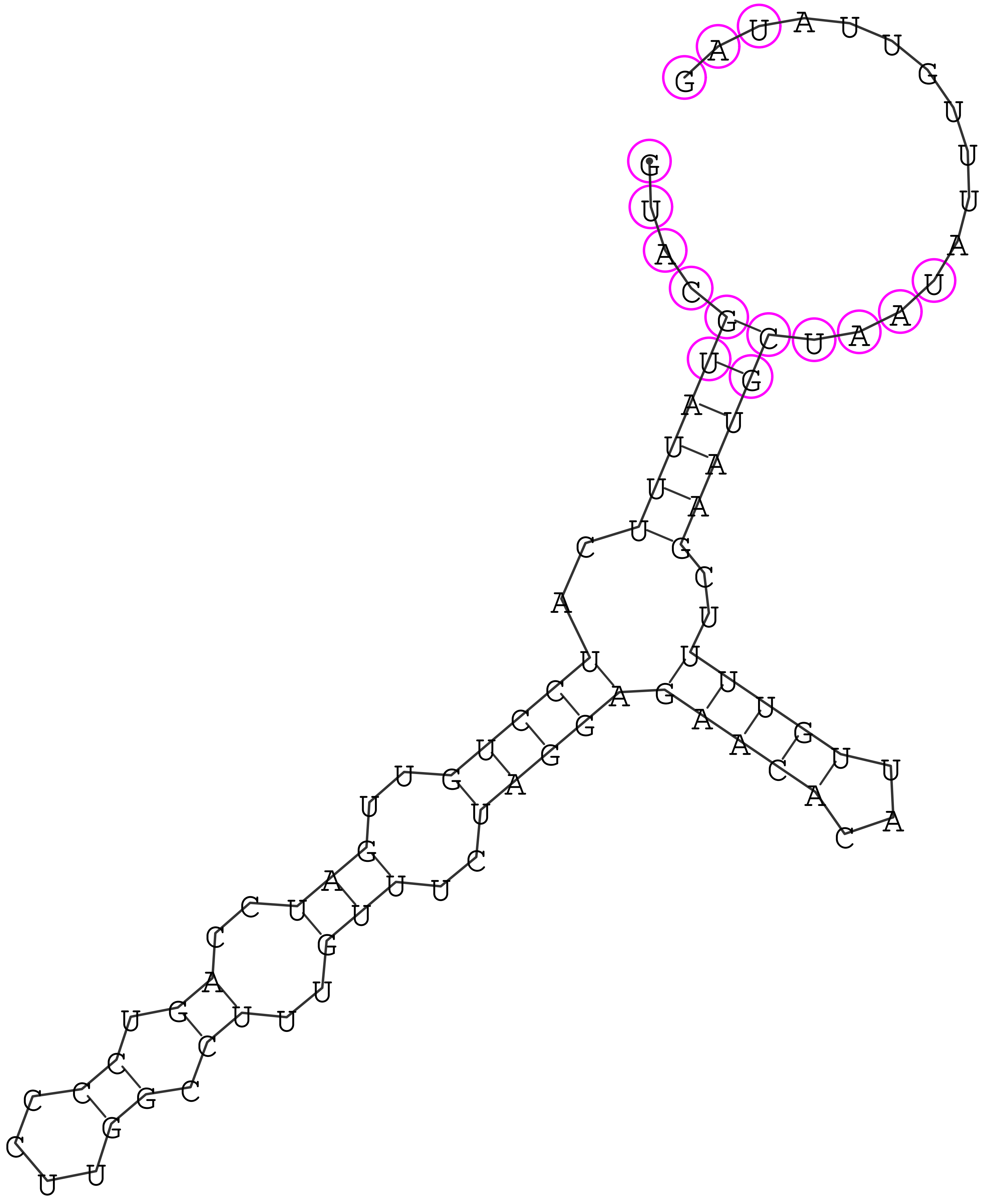

Naboc196B - Internal intron

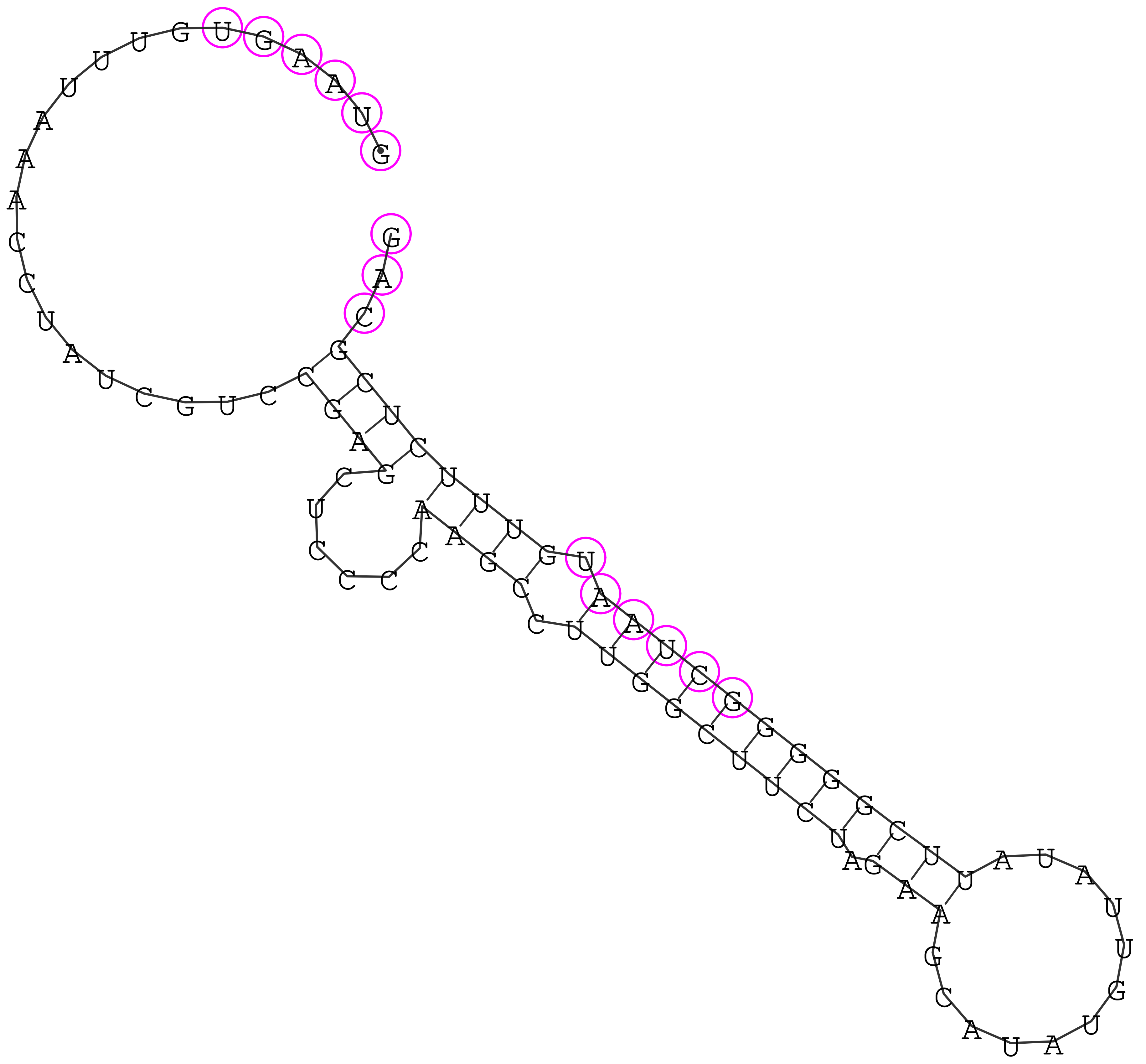

Naboc199A - Internal intron

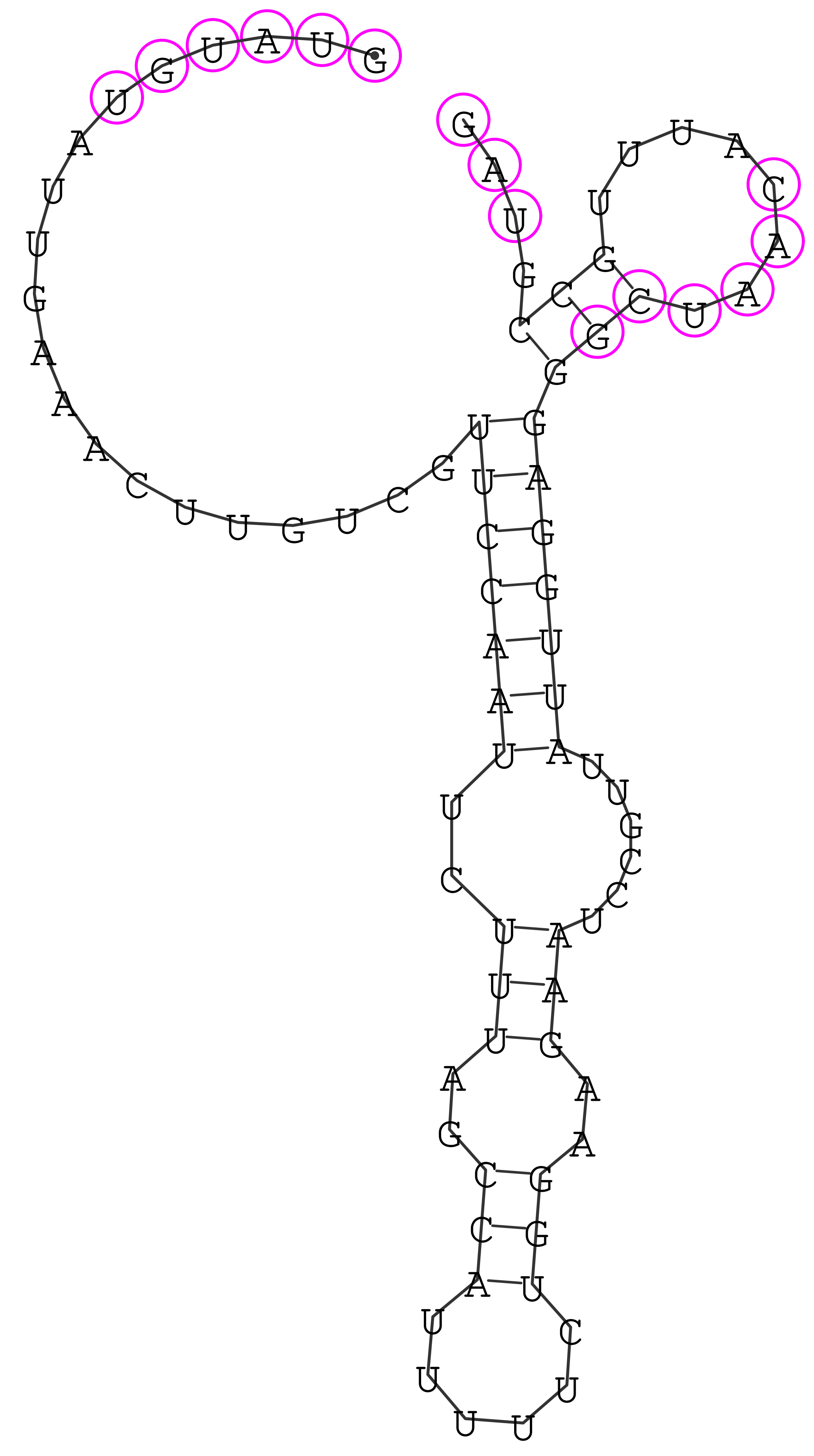

Naboc199B - Internal intron

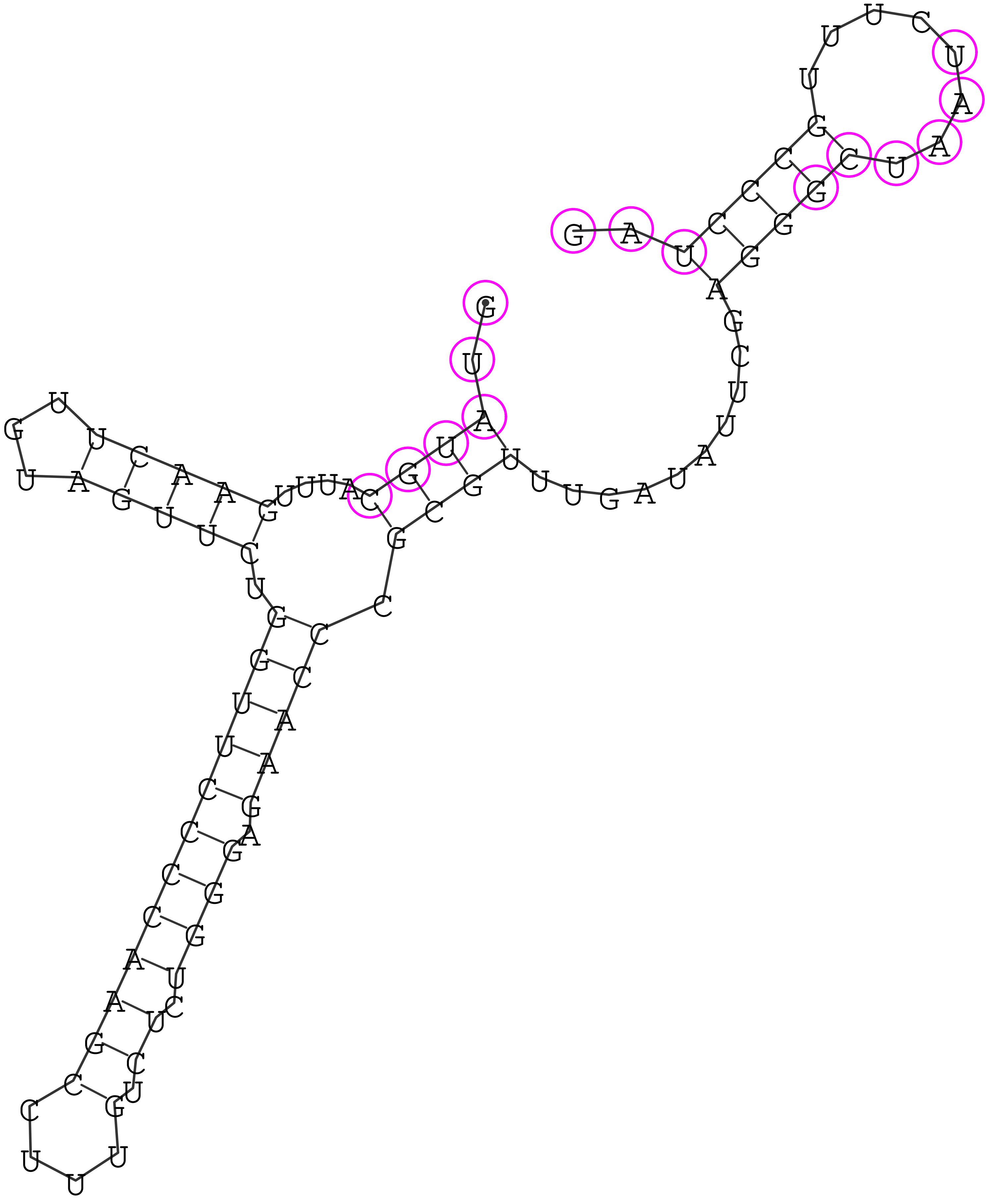

Naboc202A - Internal intron

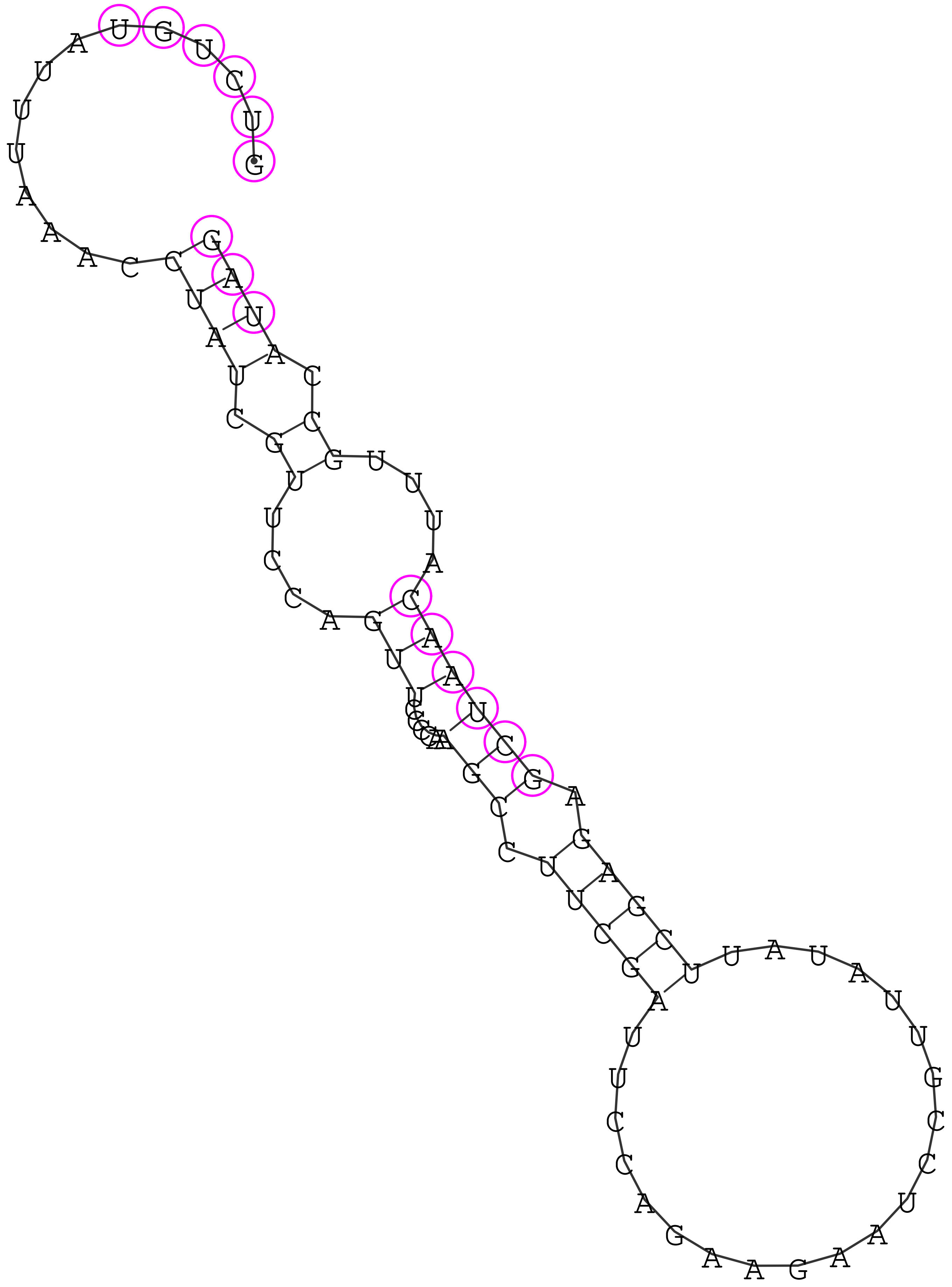

Naboc249A - Internal intron

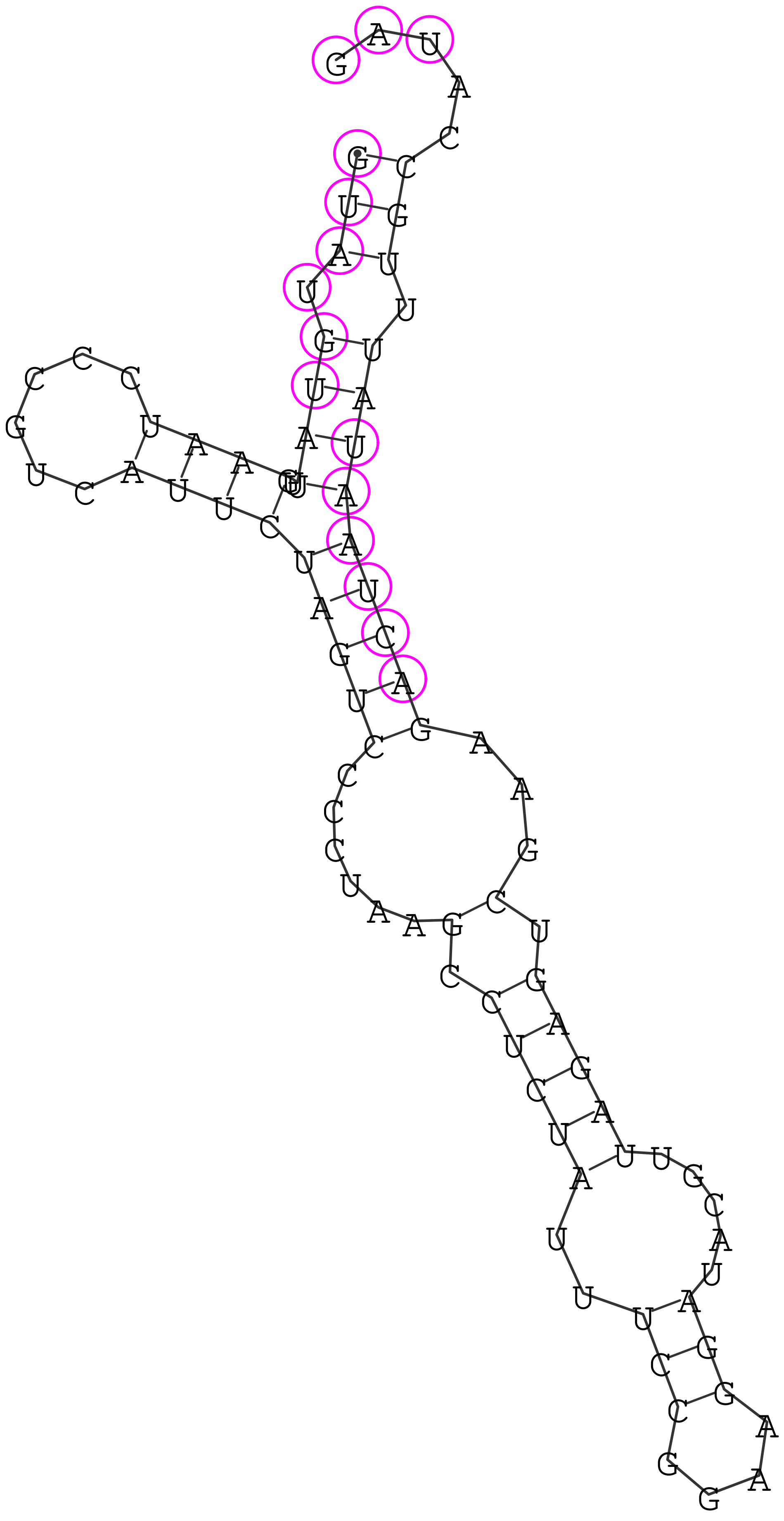

Naboc268A - Internal intron

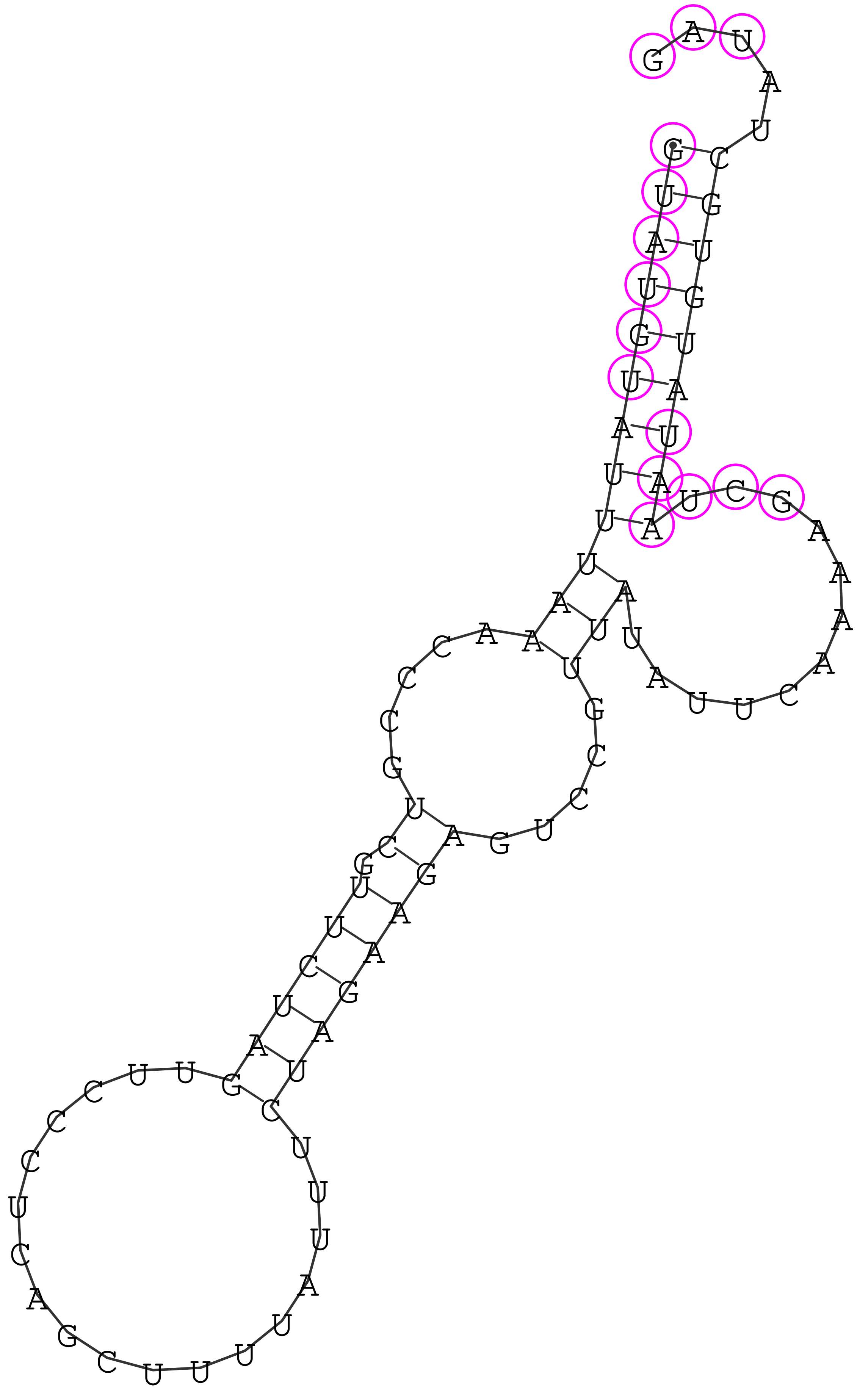

Naboc268B - Internal intron

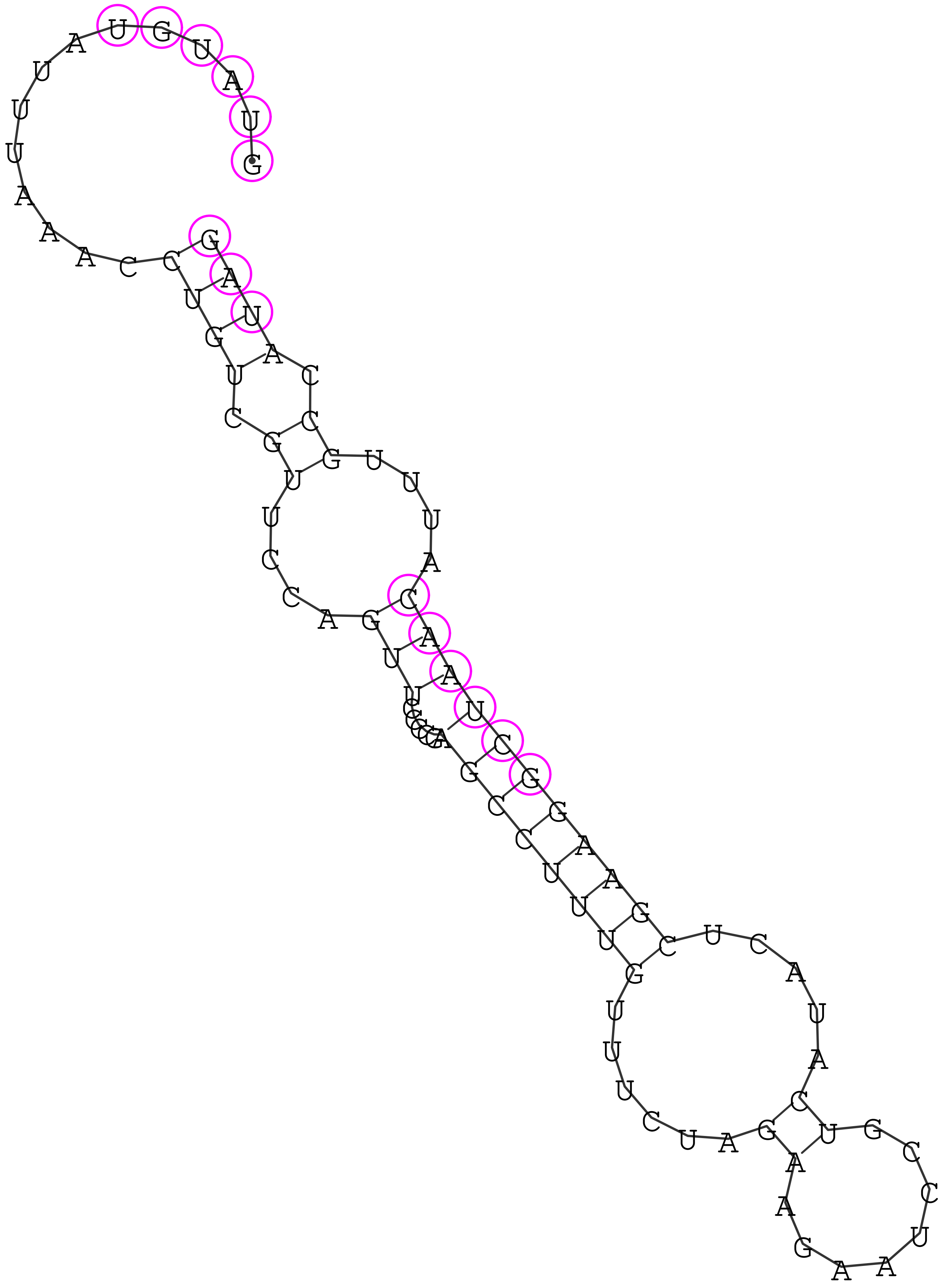

Naboc285A - Internal intron

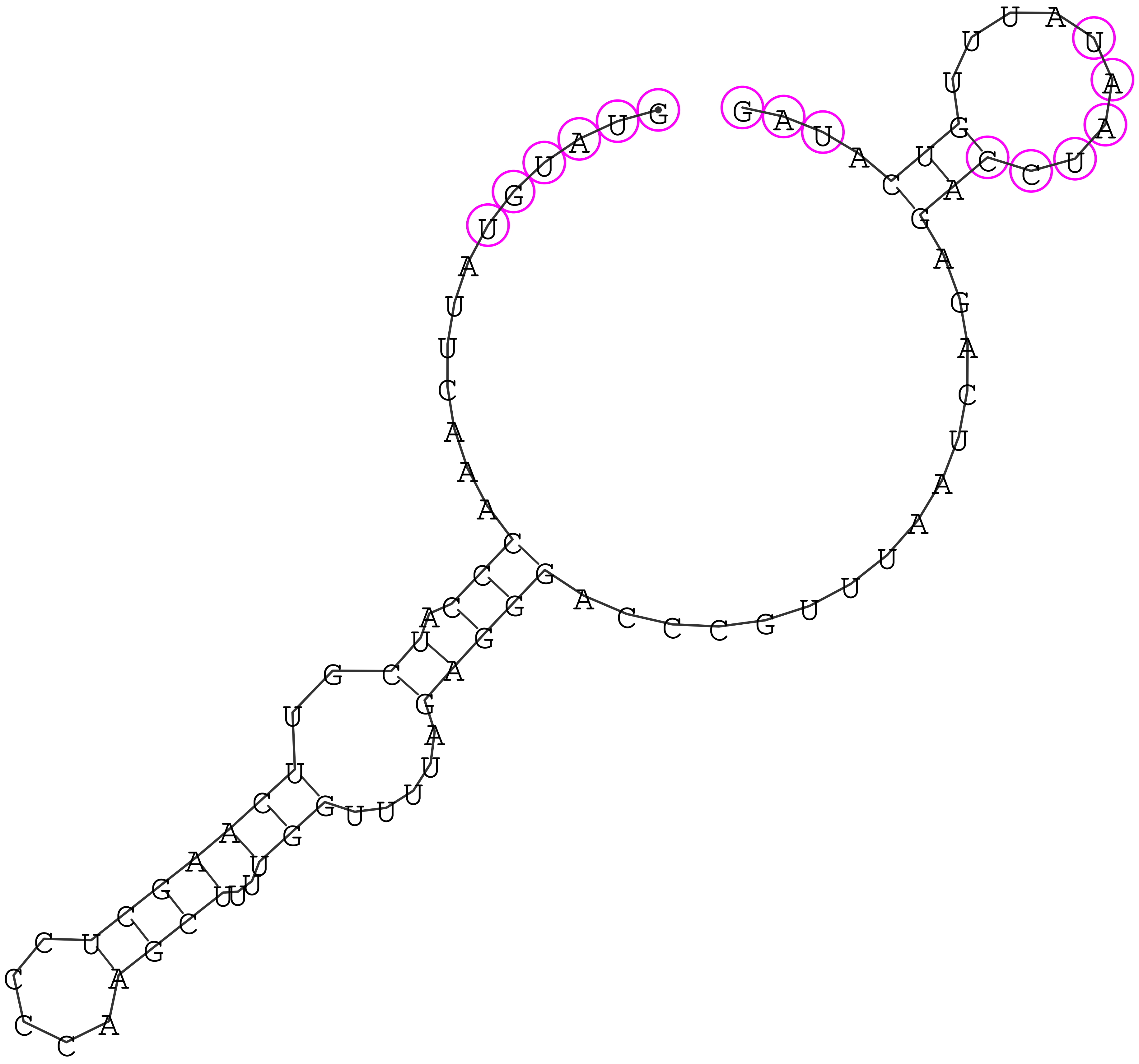

Naboc289A - Internal intron

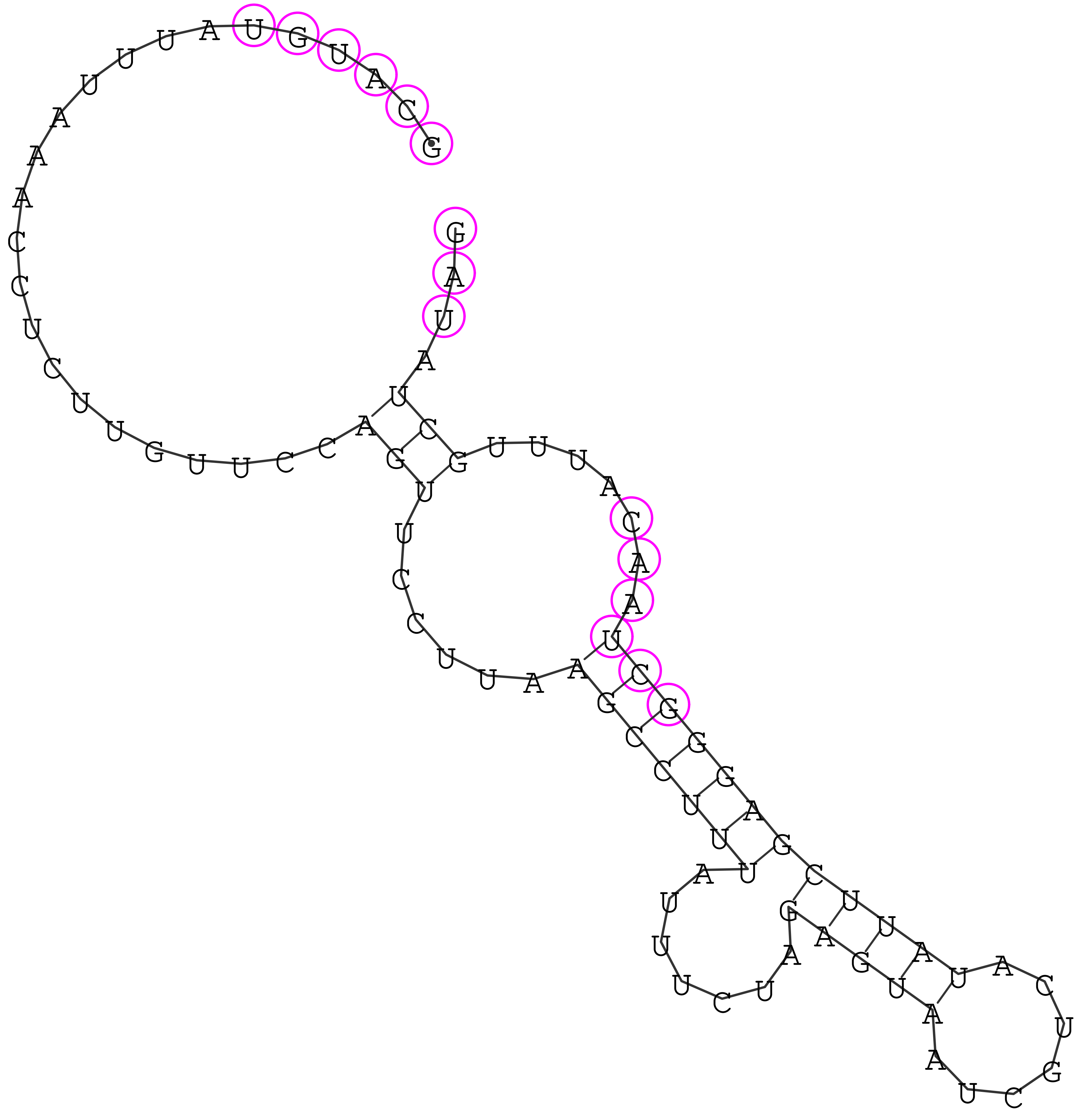

Naboc294A - Internal intron

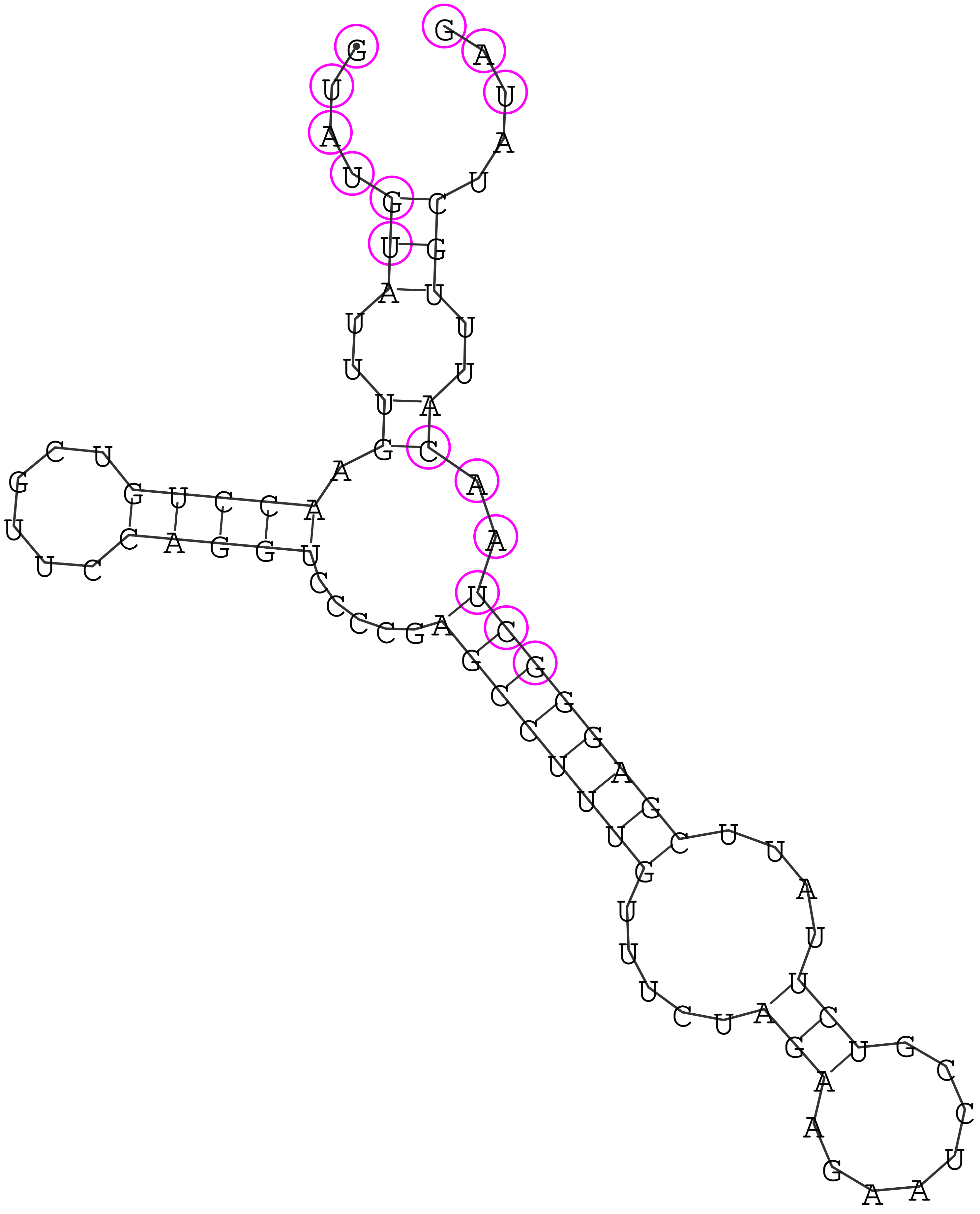

Naboc300A - Internal intron

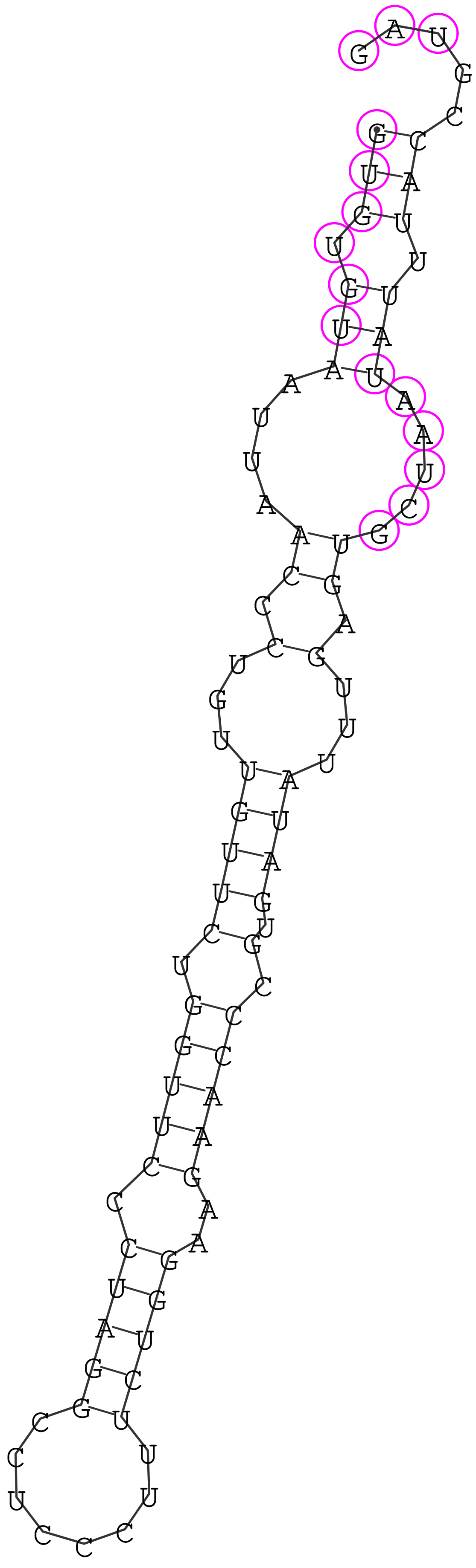

Naboc349A - Internal intron

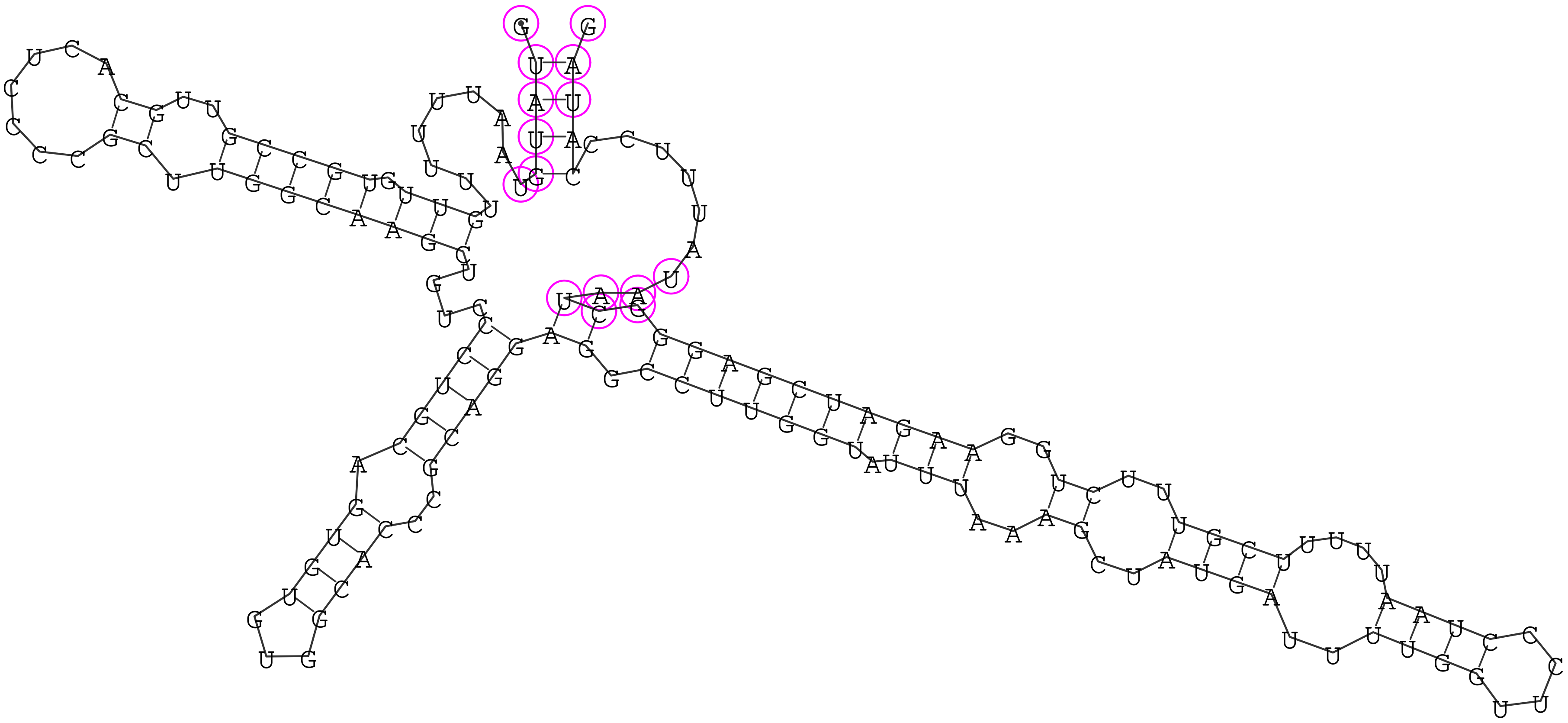

Naboc414A - Internal intron

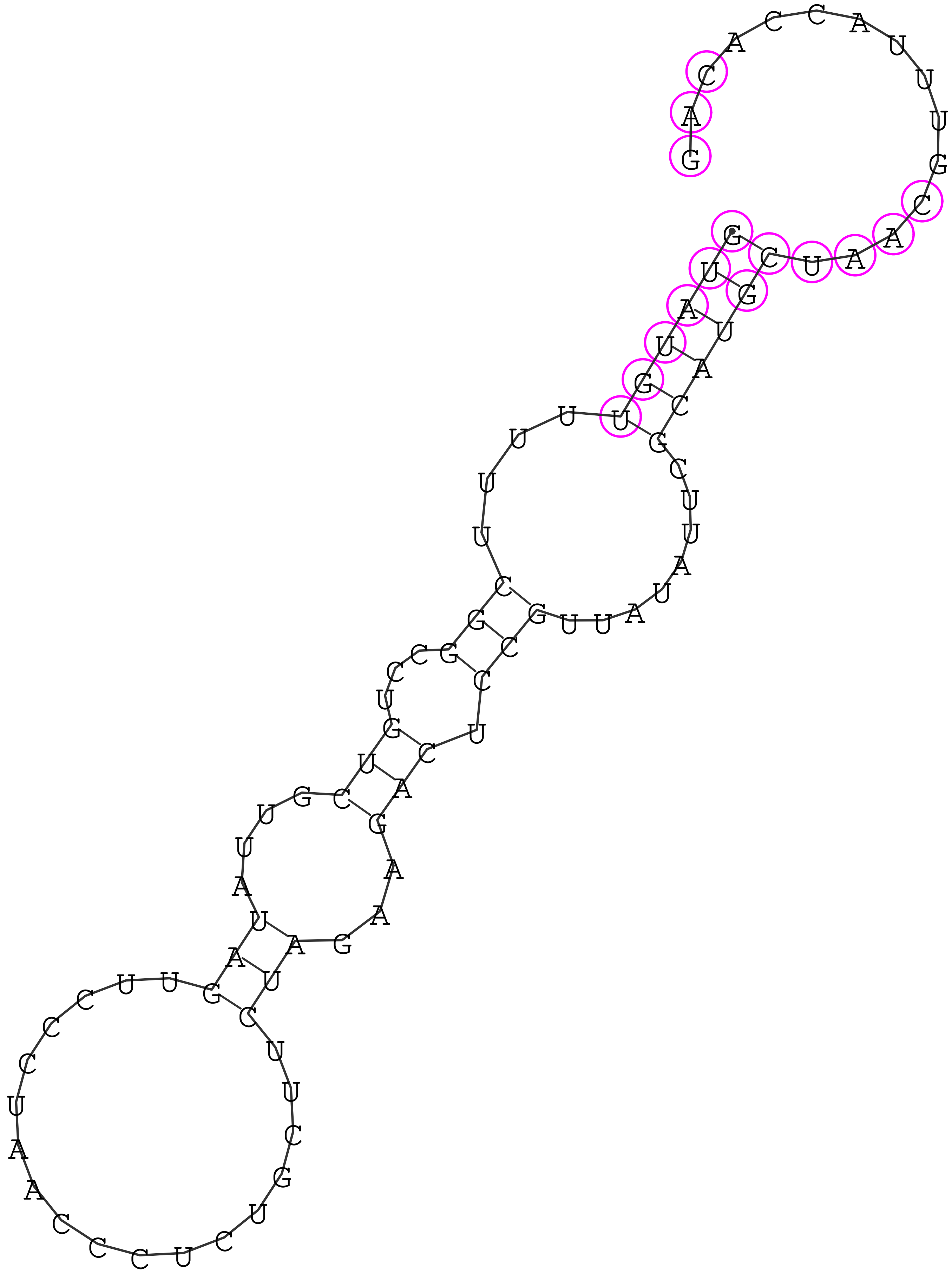

X1651c009A - Internal intron

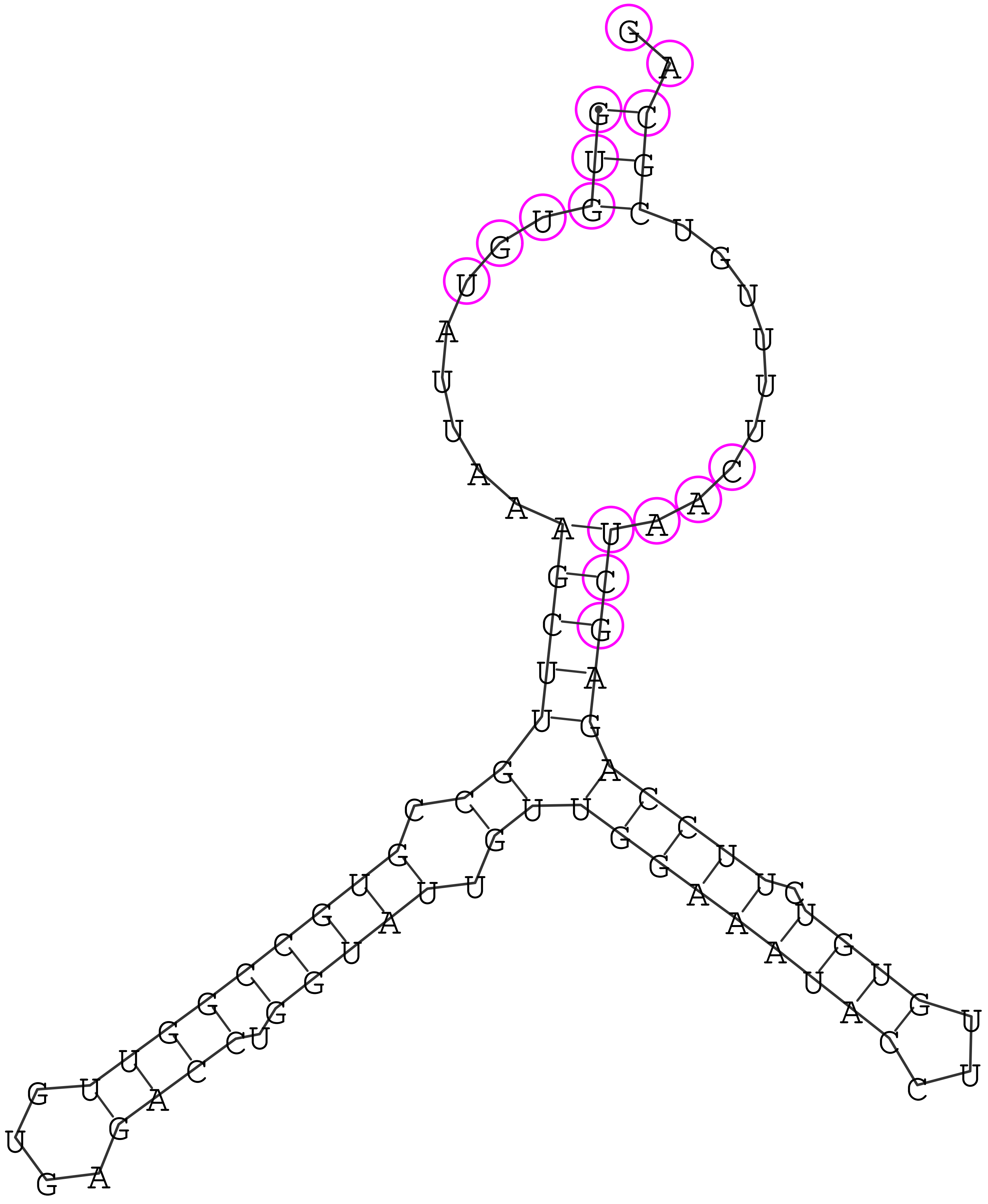

X1651c011A - Internal intron

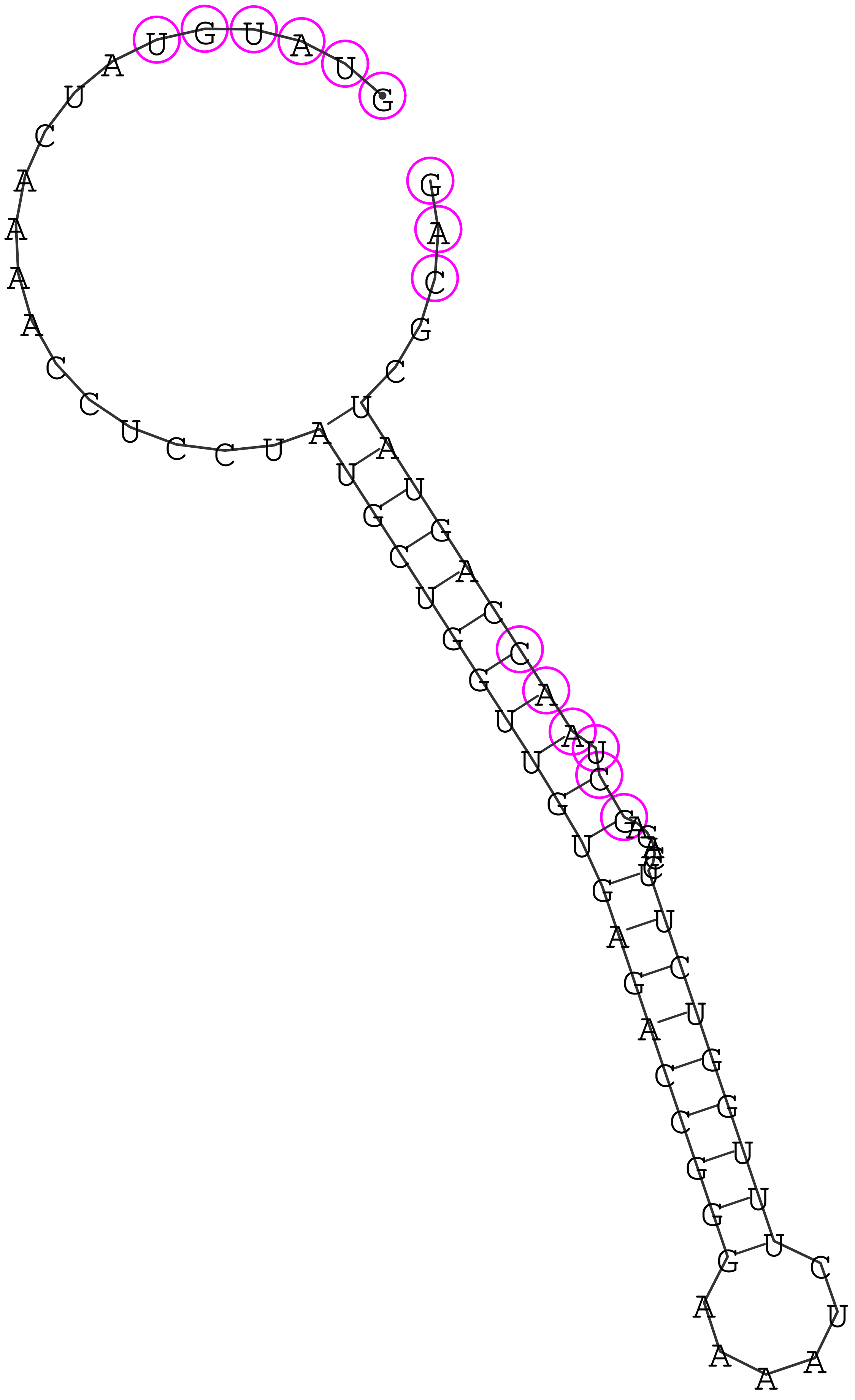

X1651c016A - Internal intron

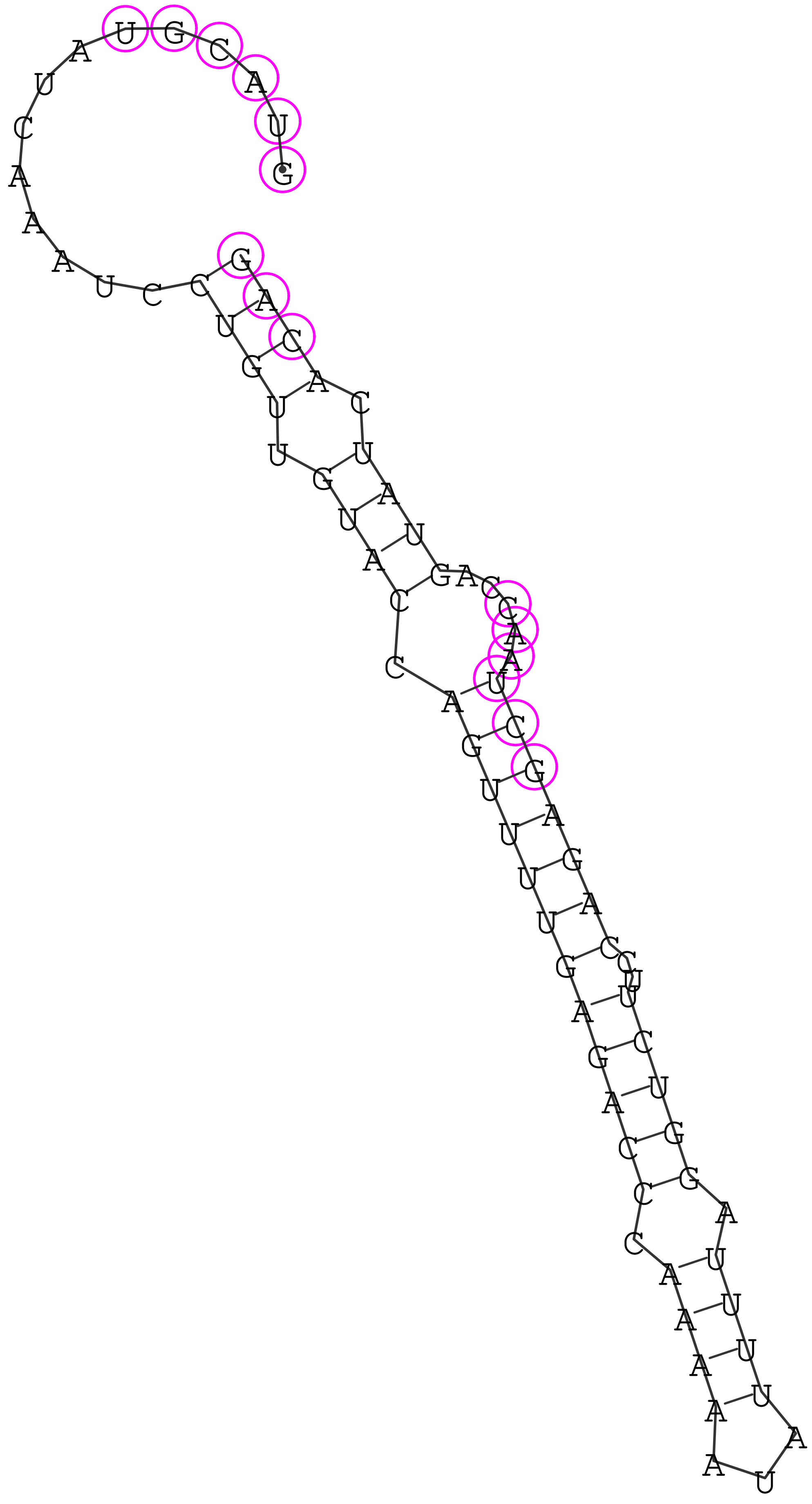

X1651c025A - Internal intron

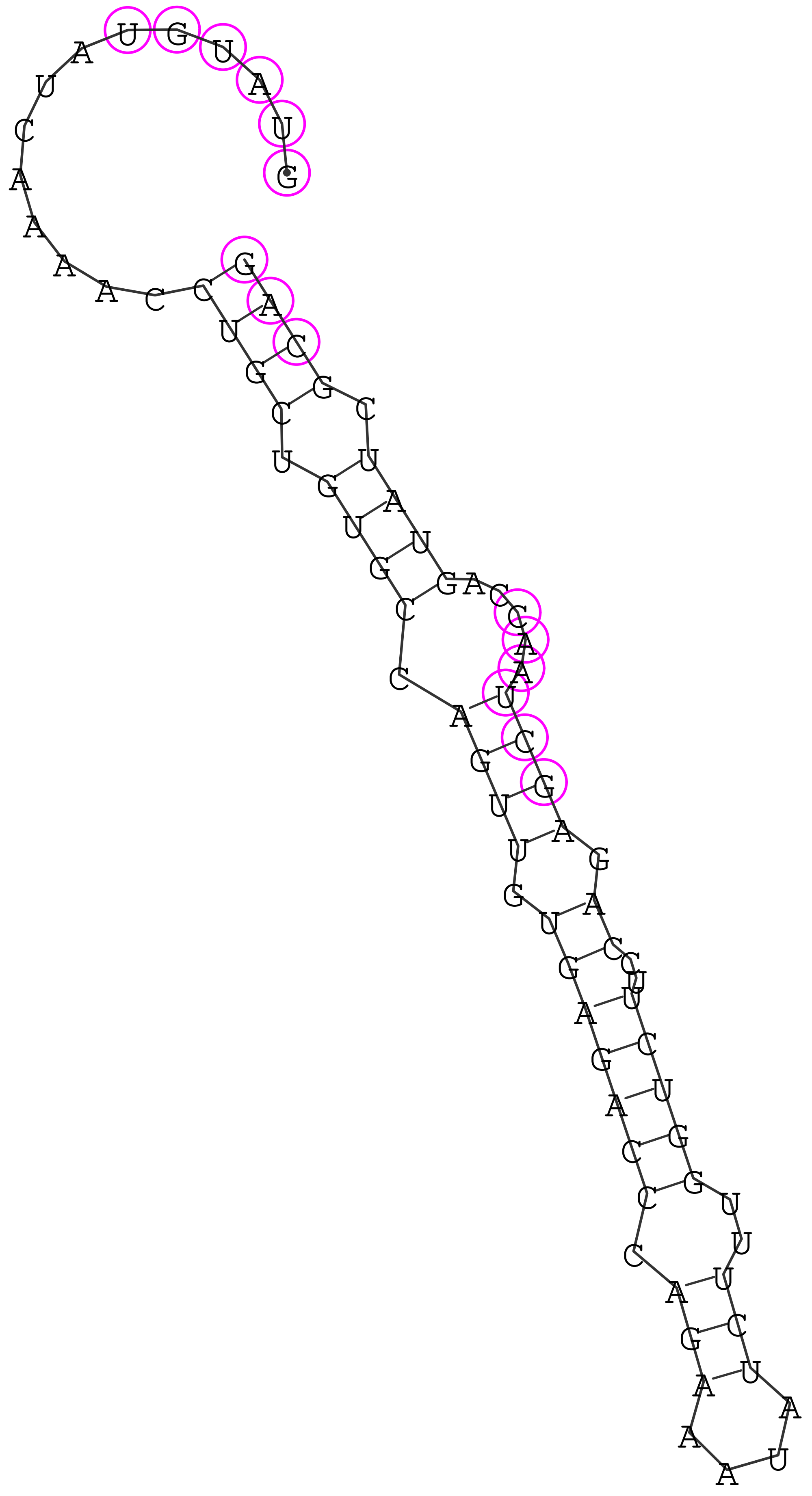

X1651c036A - Internal intron

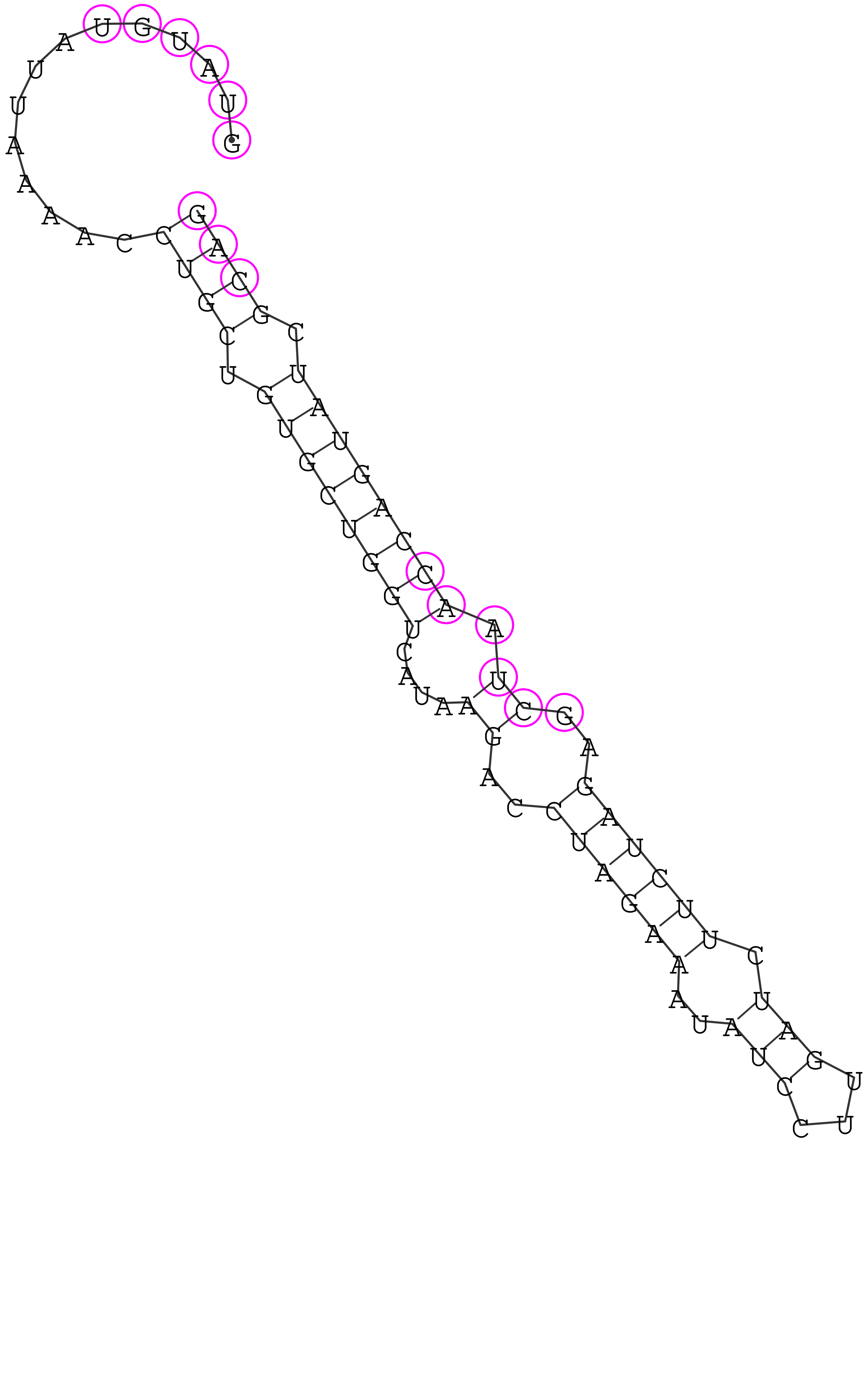

X1651c075A - Internal intron

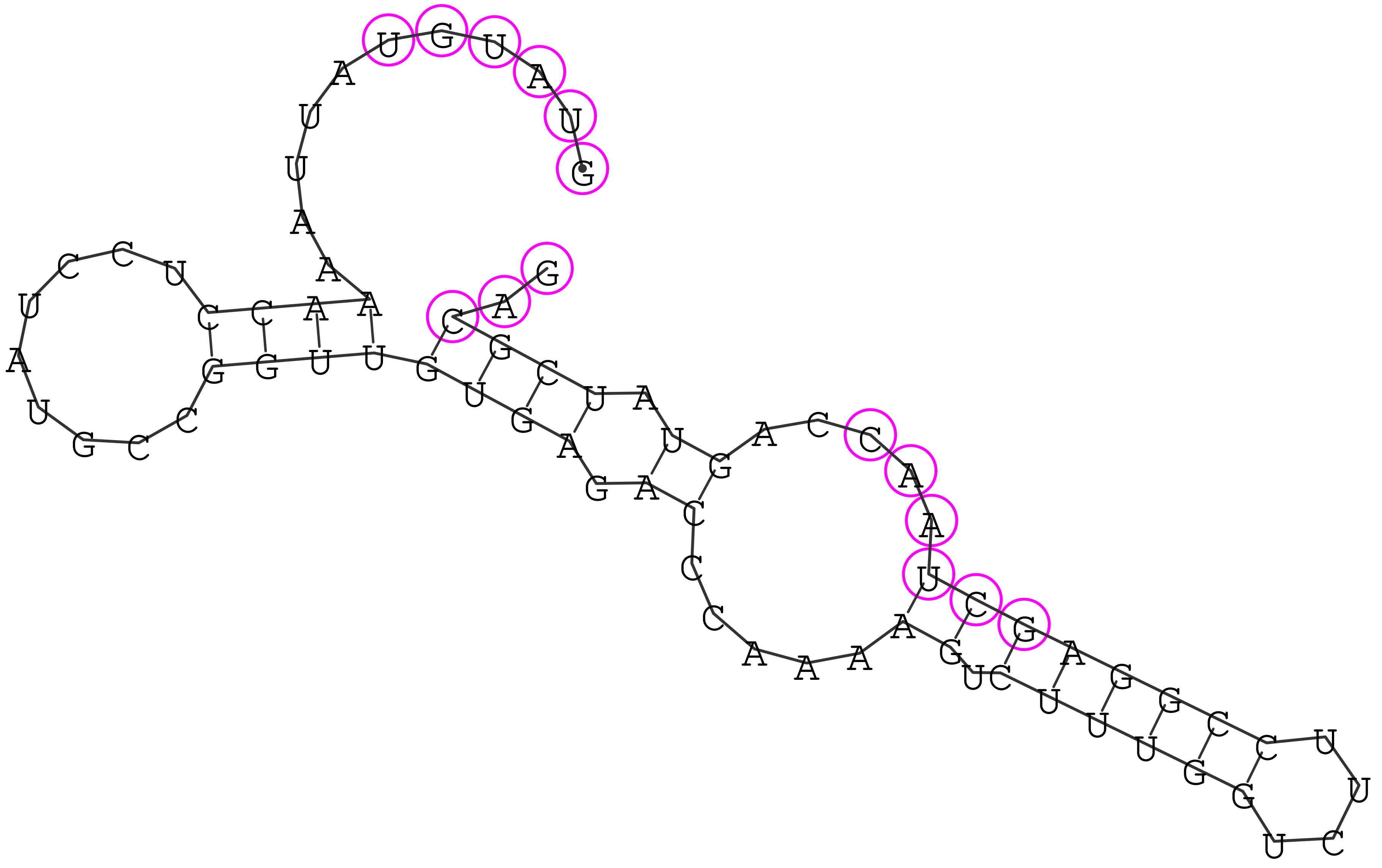

X1651c093A - Internal intron

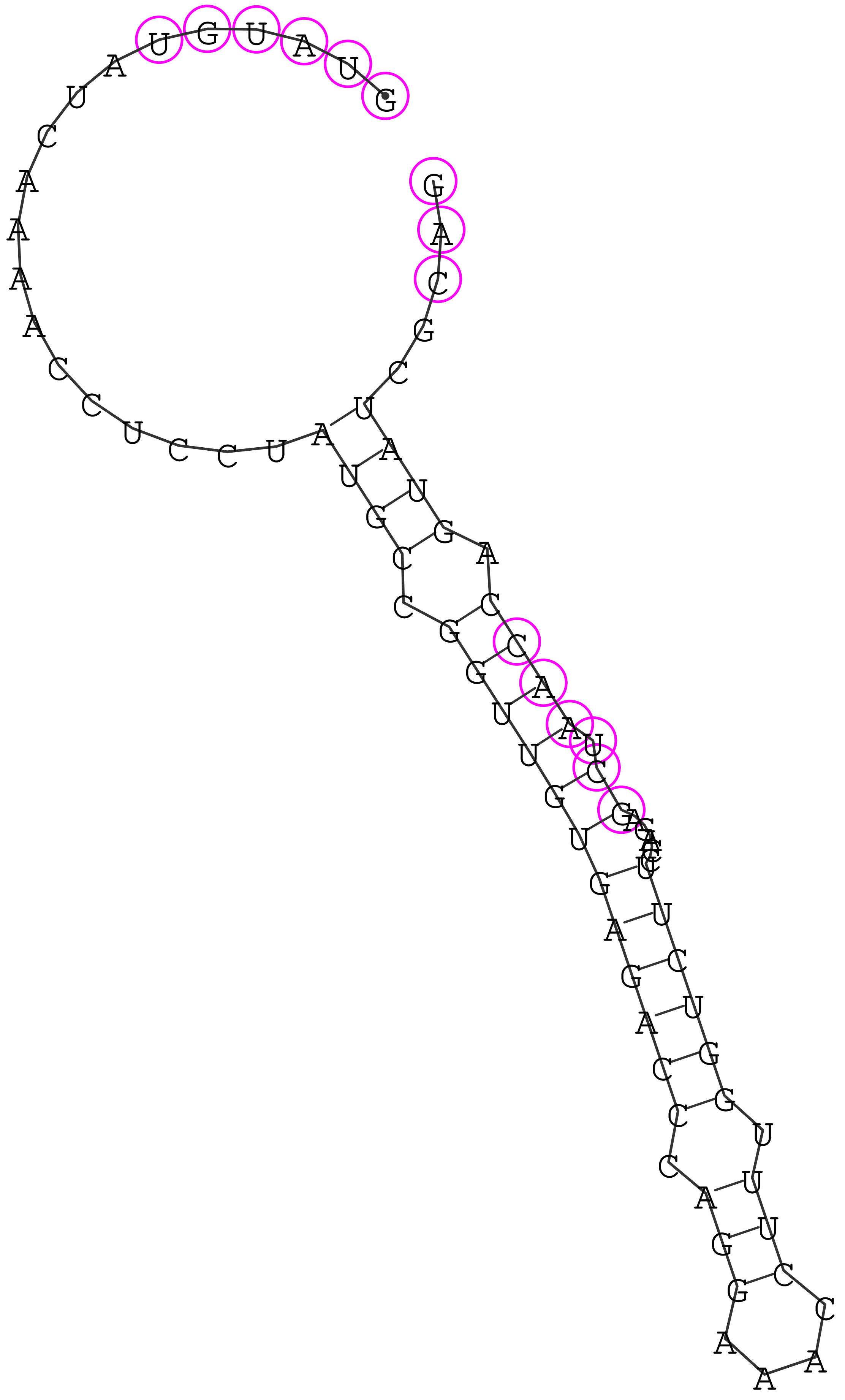

X1651c156A - Internal intron

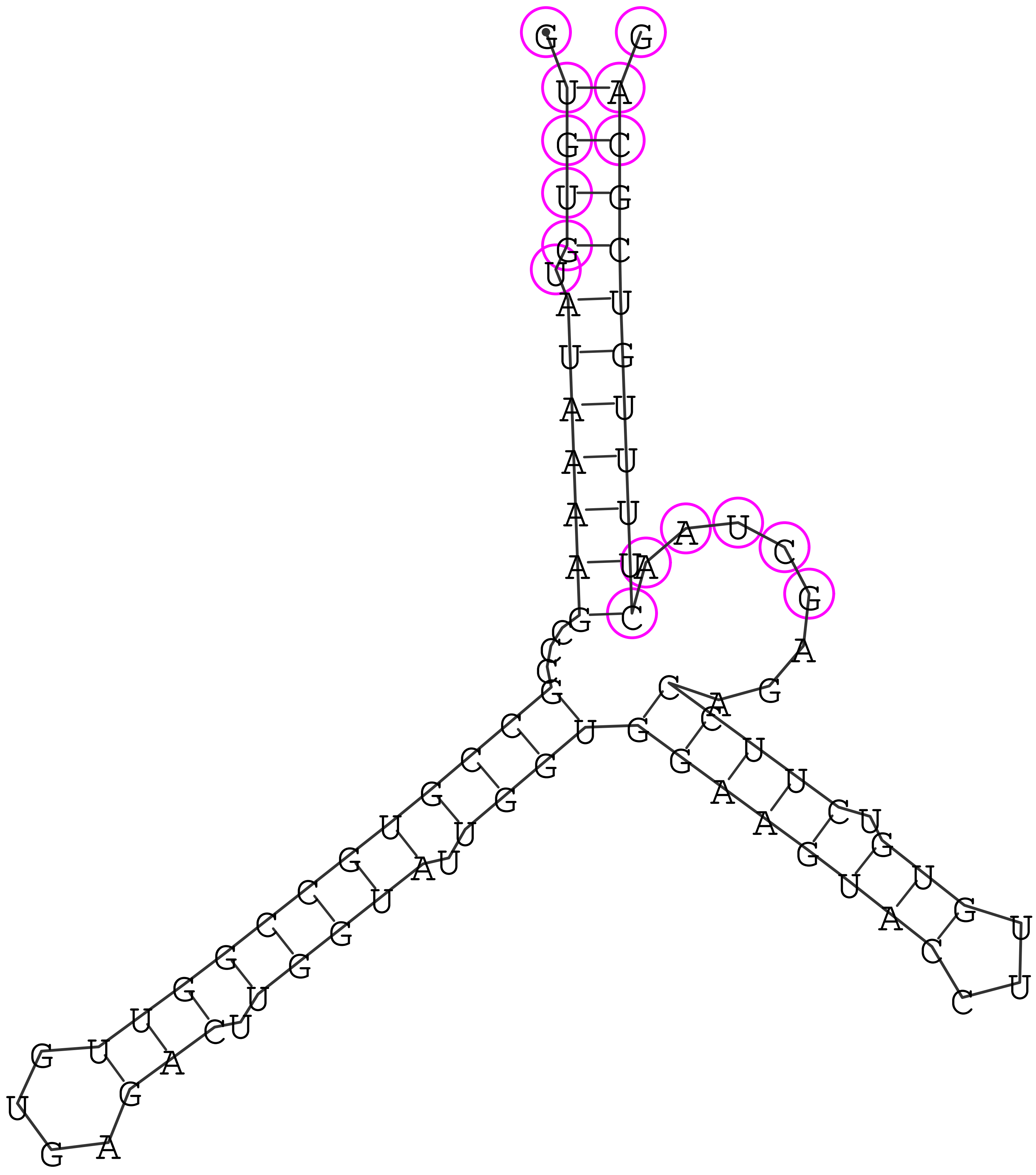

X1651c189A - Internal intron

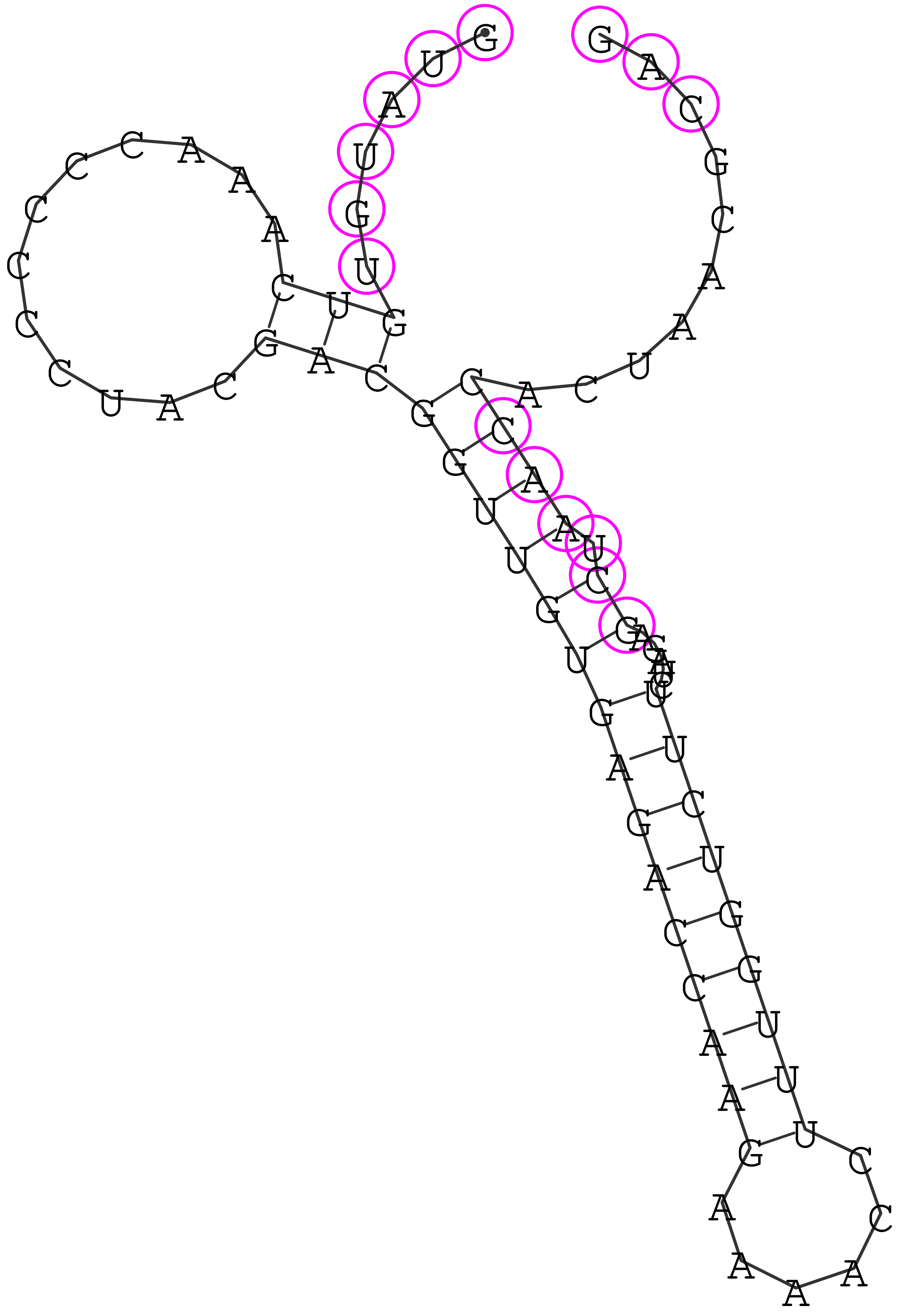

Xarbc0002A - Internal intron

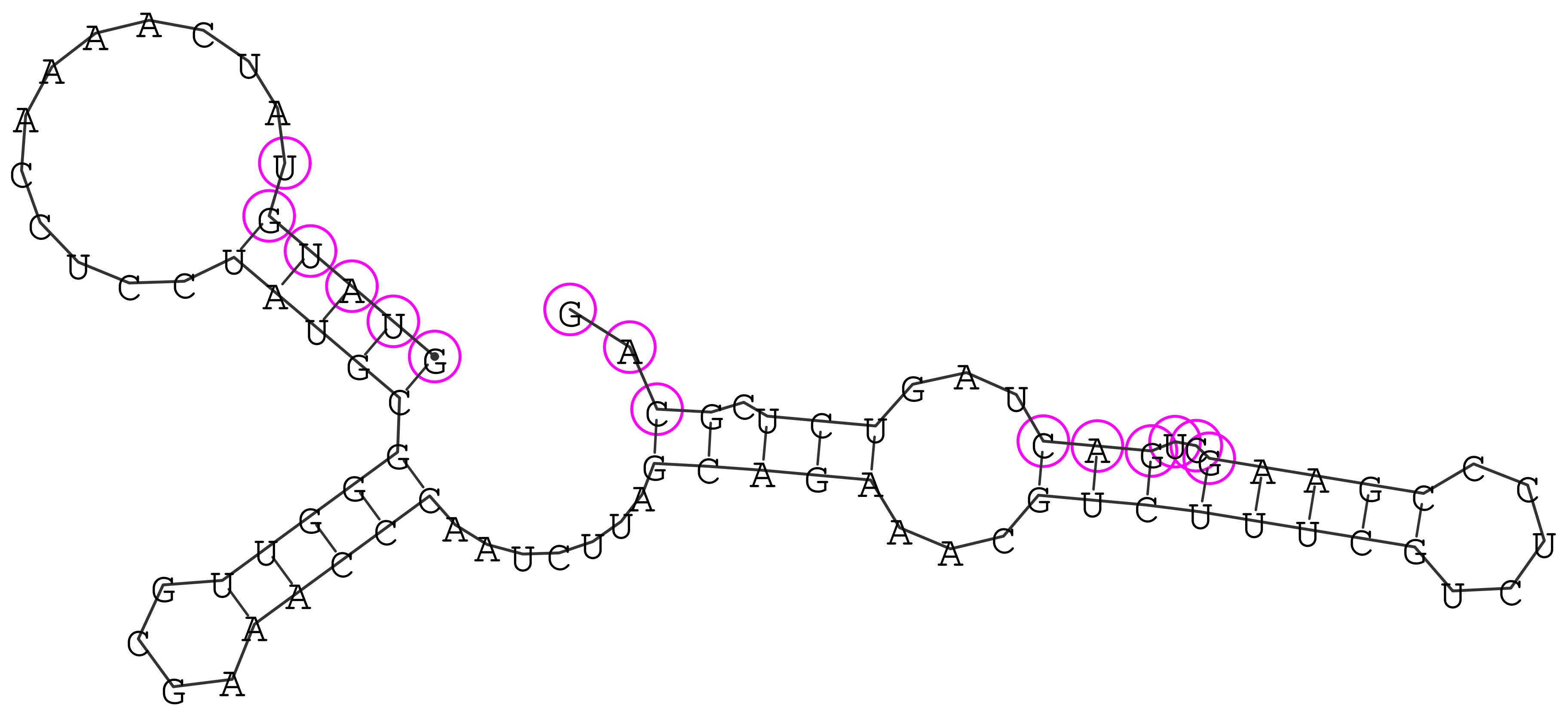

Xarbc0002B - Internal intron

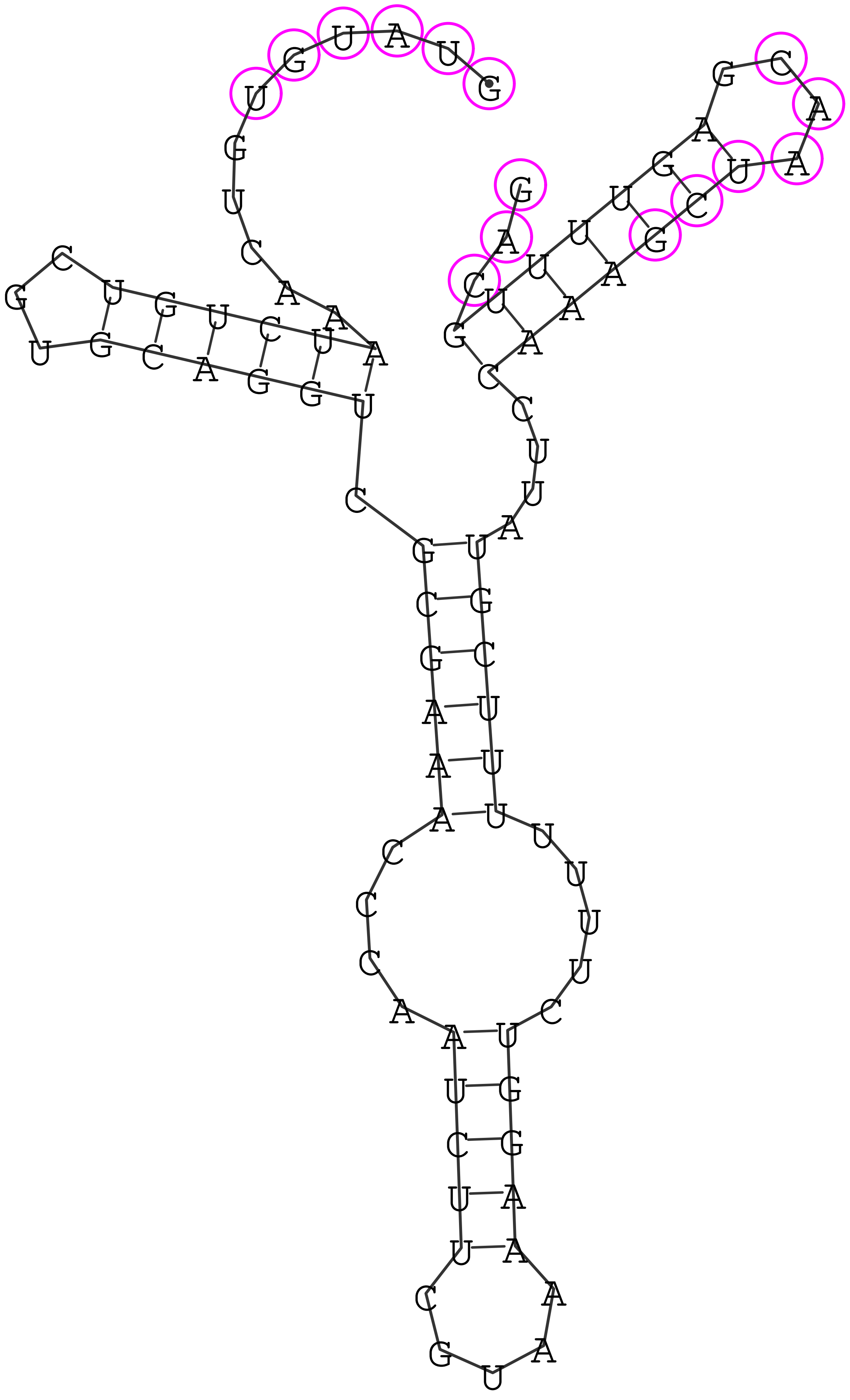

Xarbc0003A - Internal intron

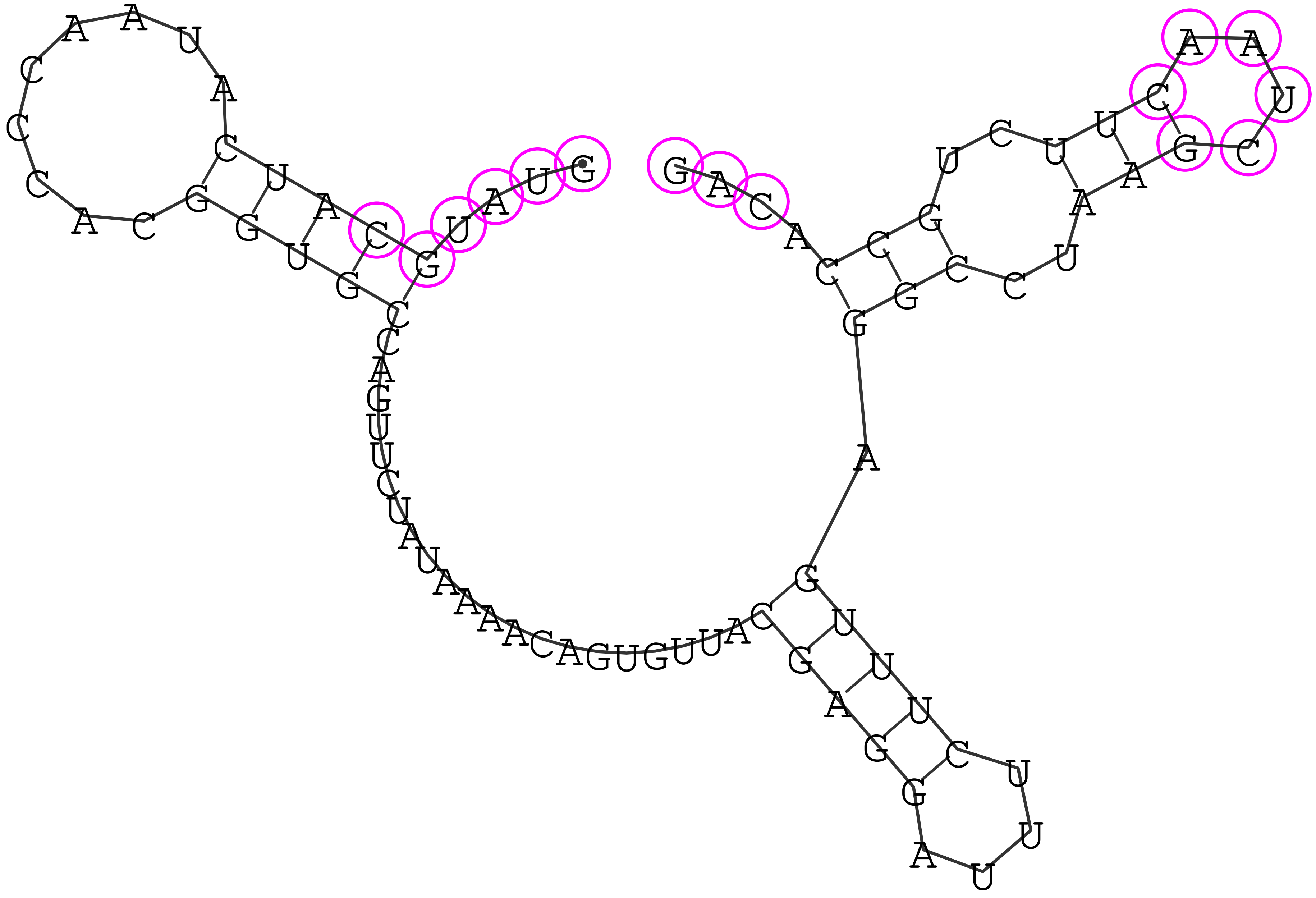

Xarbc0003B - Internal intron

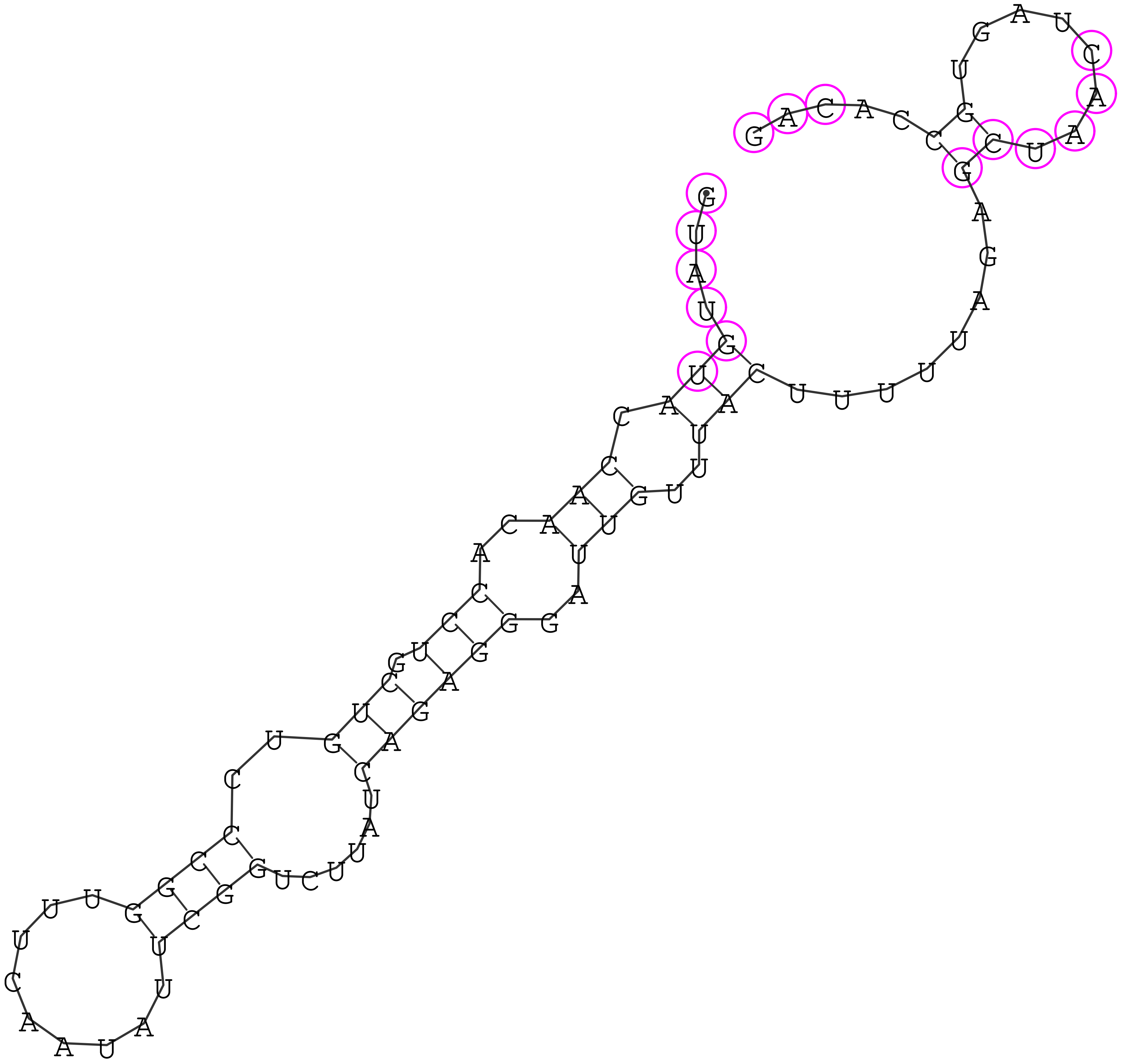

Xarbc0003C - Internal intron

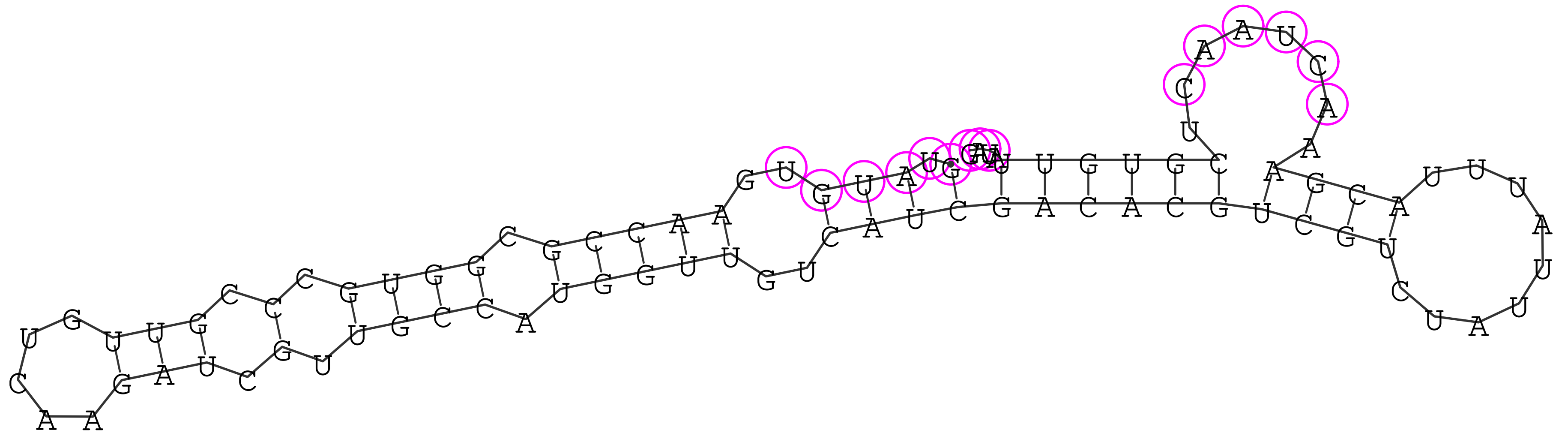

Xarbc0006A - Internal intron

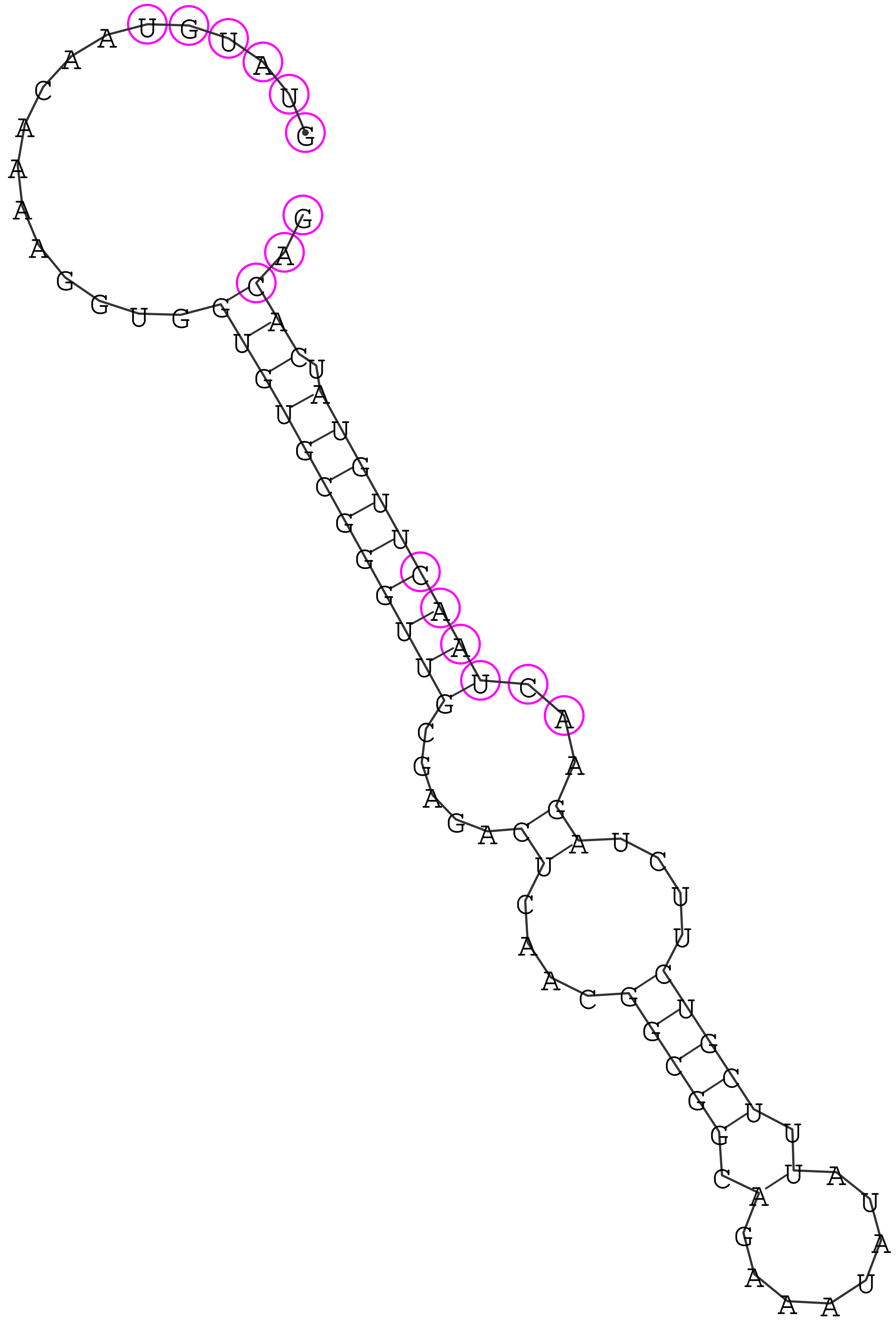

Xarbc0006B - Internal intron

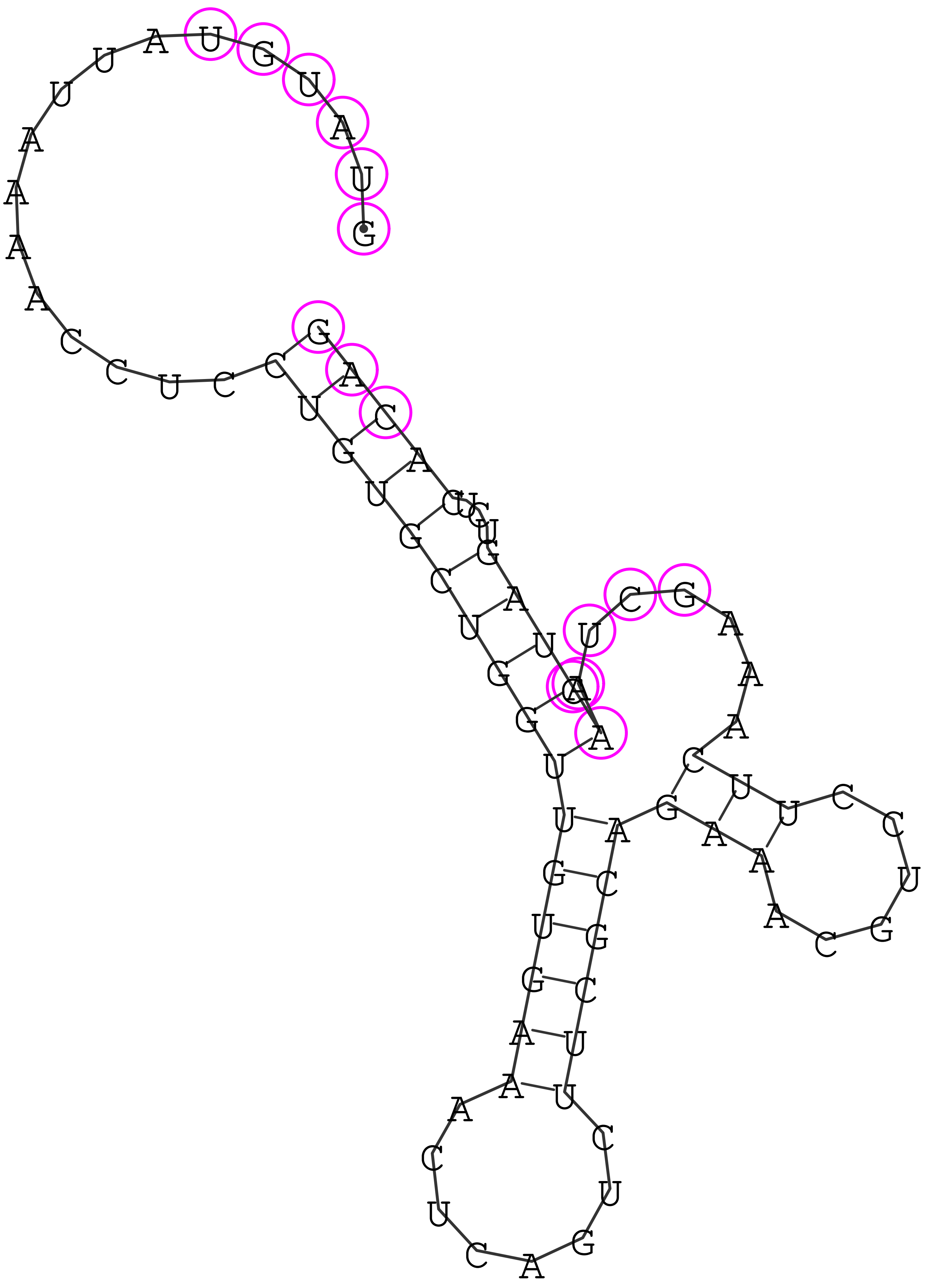

Xarbc0009A - Internal intron

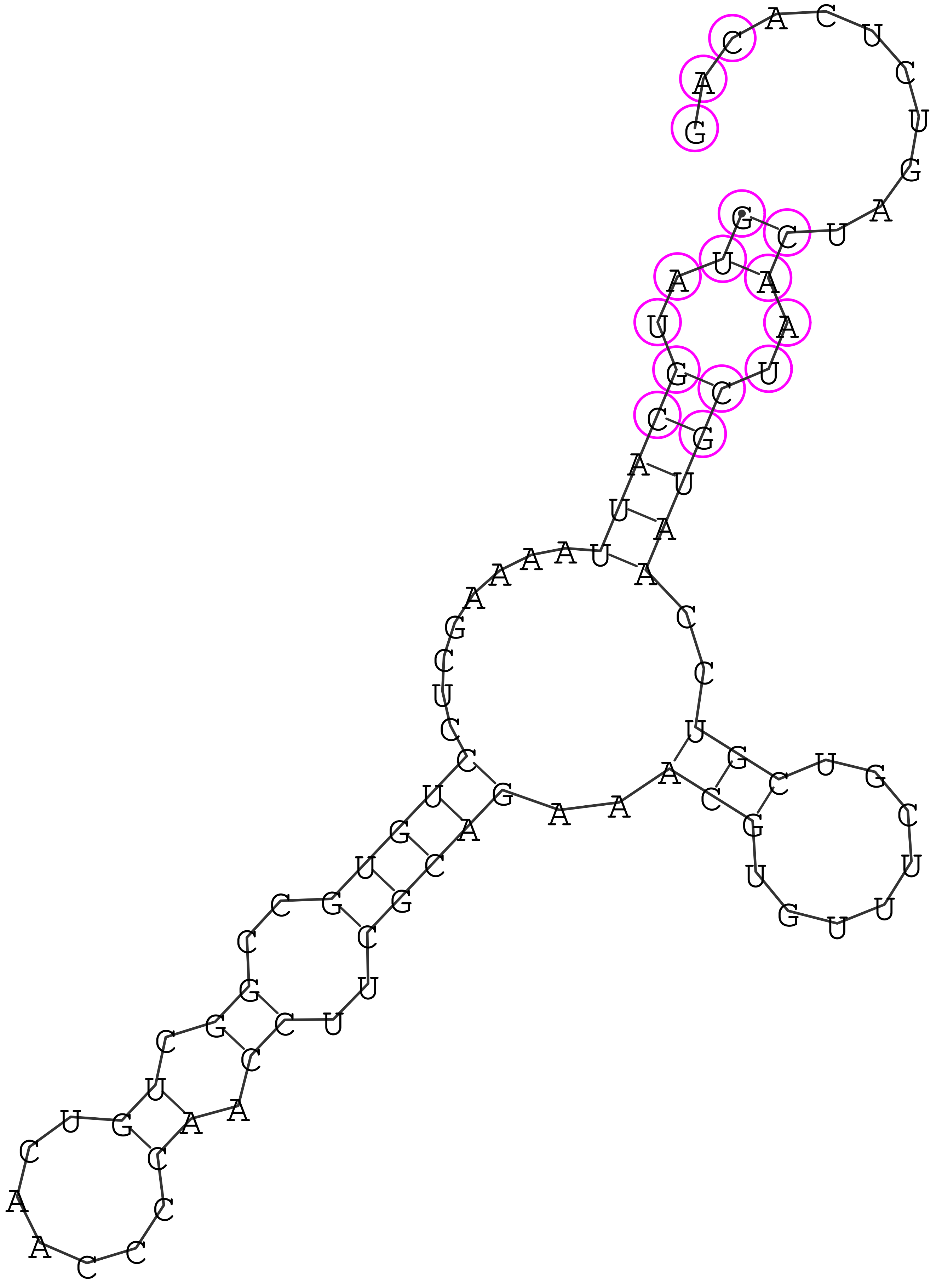

Xarbc0010A - Internal intron

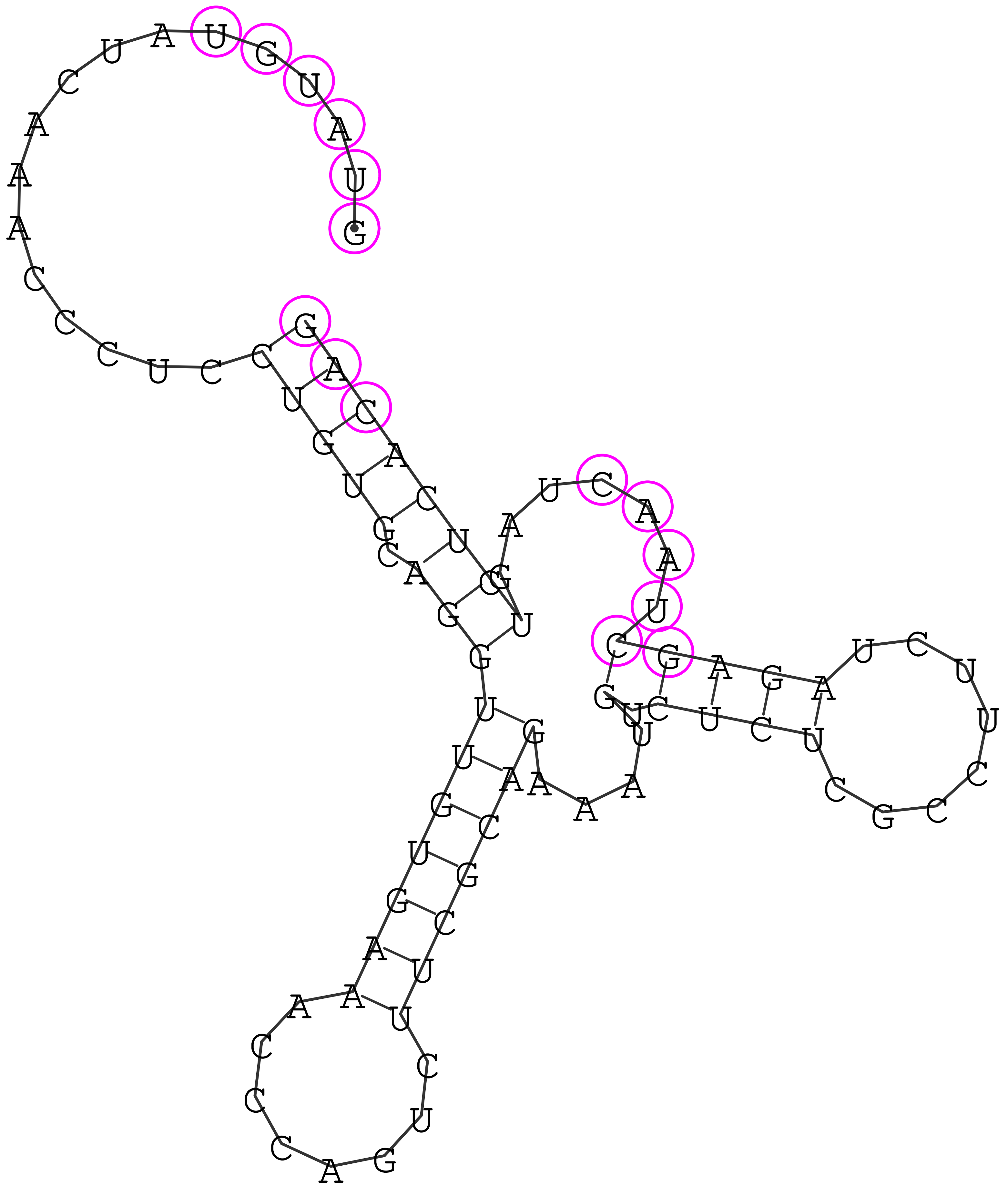

Xarbc0010B - Internal intron

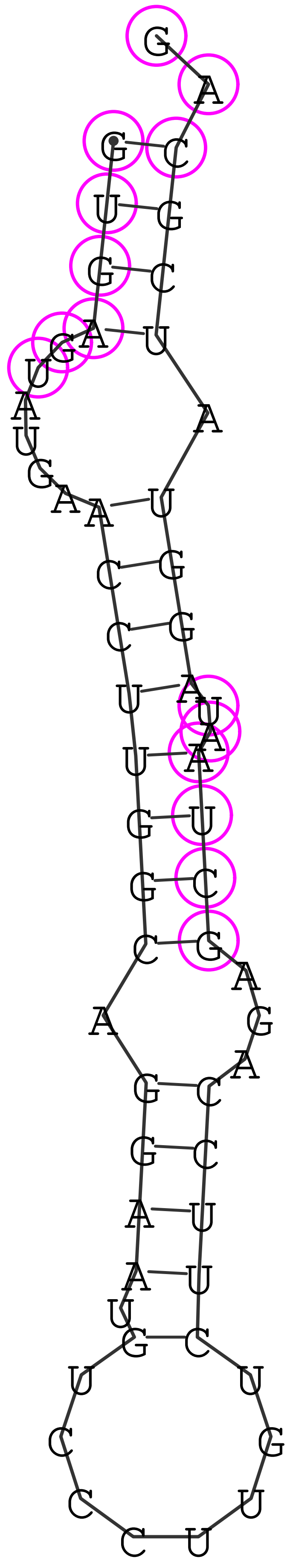

Xarbc0011A - Internal intron

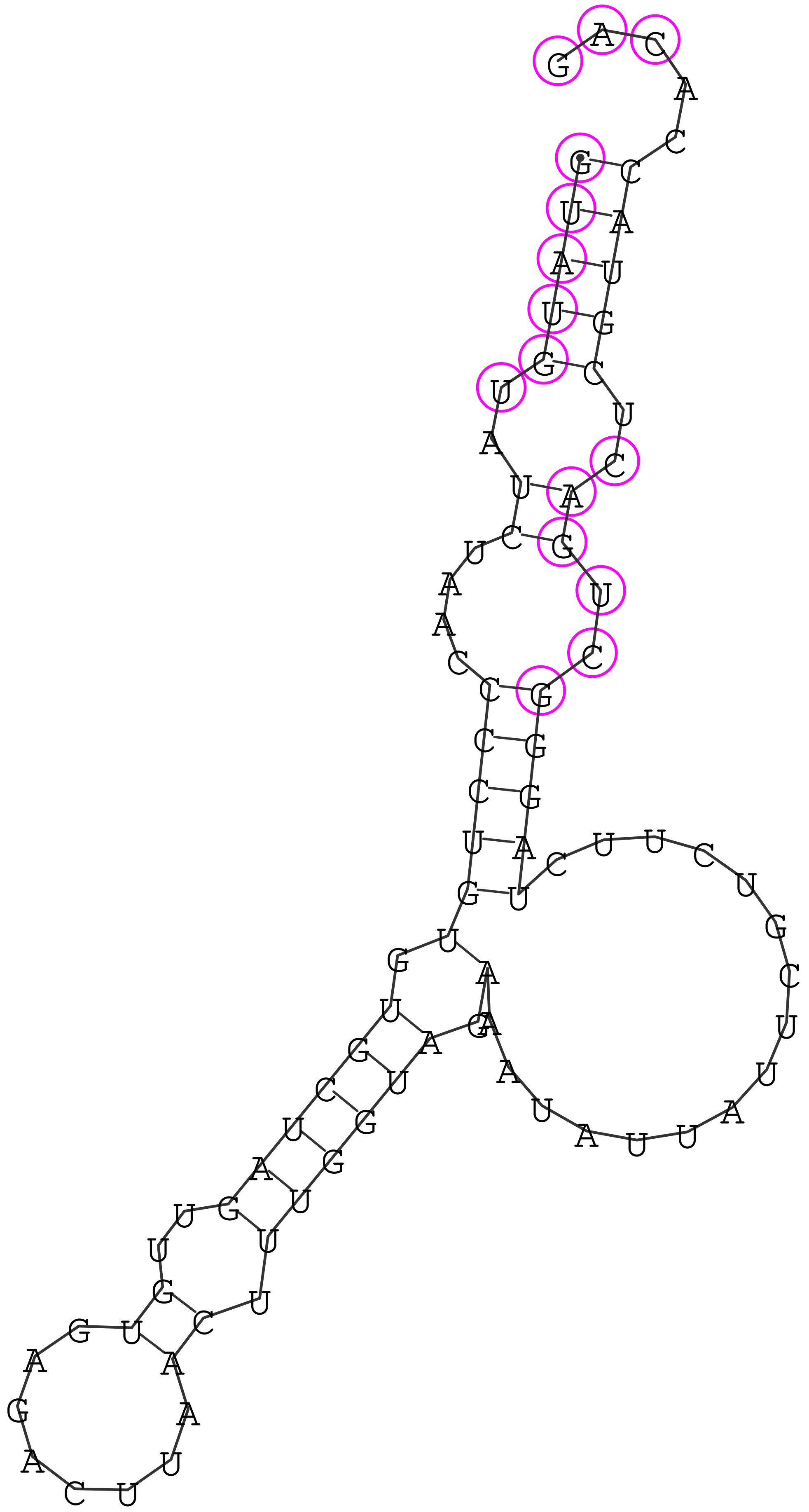

Xarbc0012A - Internal intron

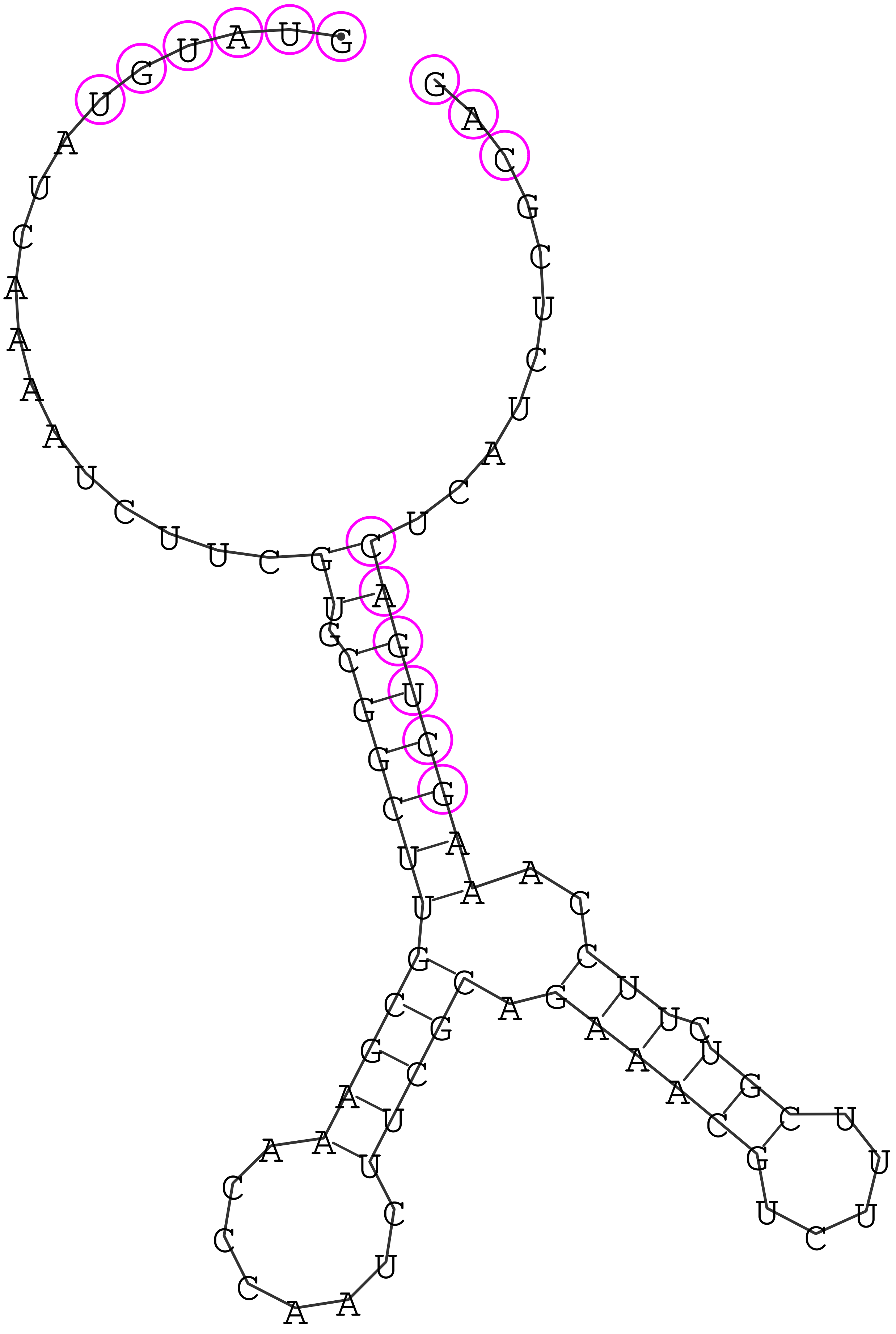

Xarbc0012B - Internal intron

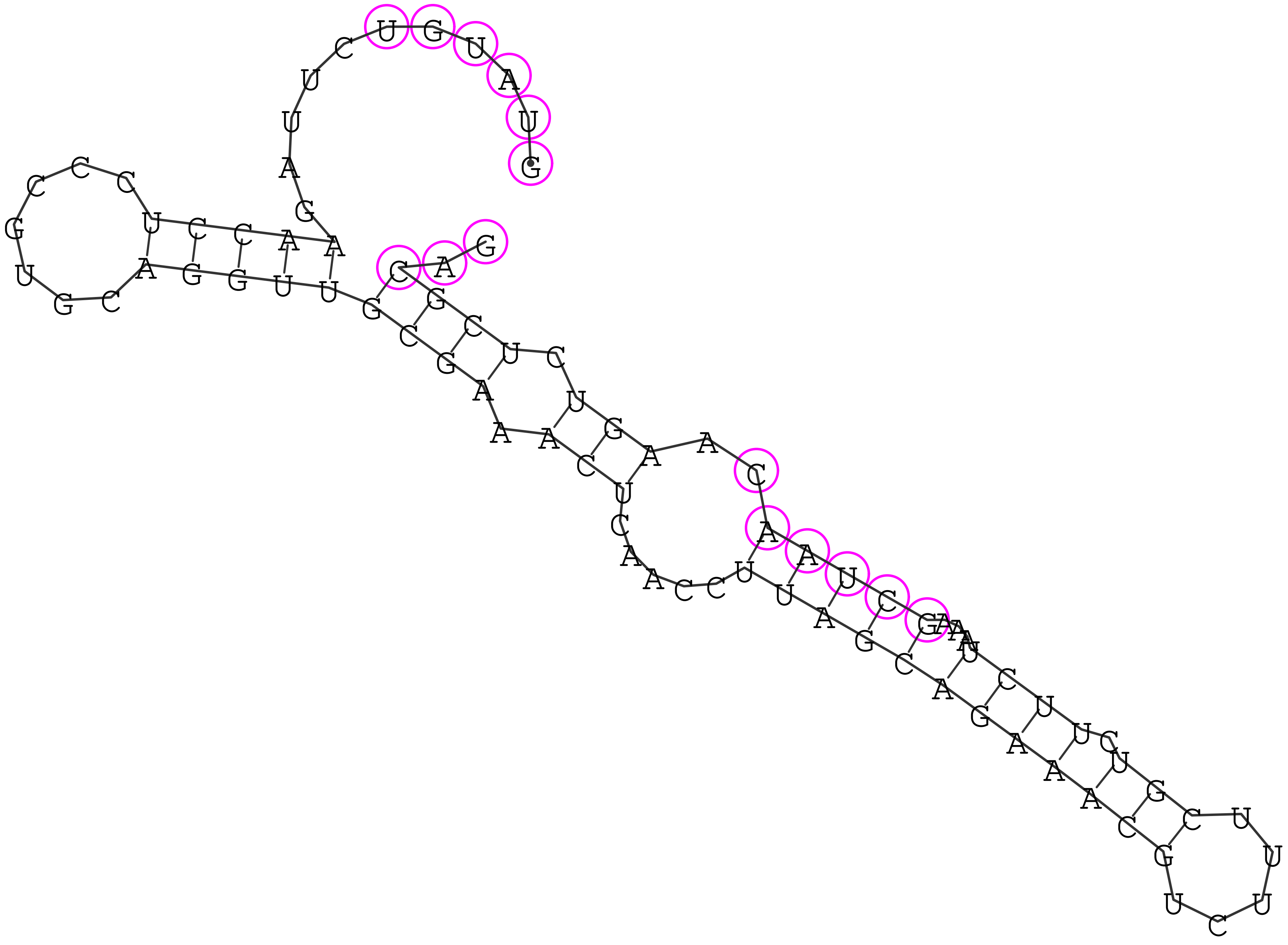

Xarbc0012D - Internal intron

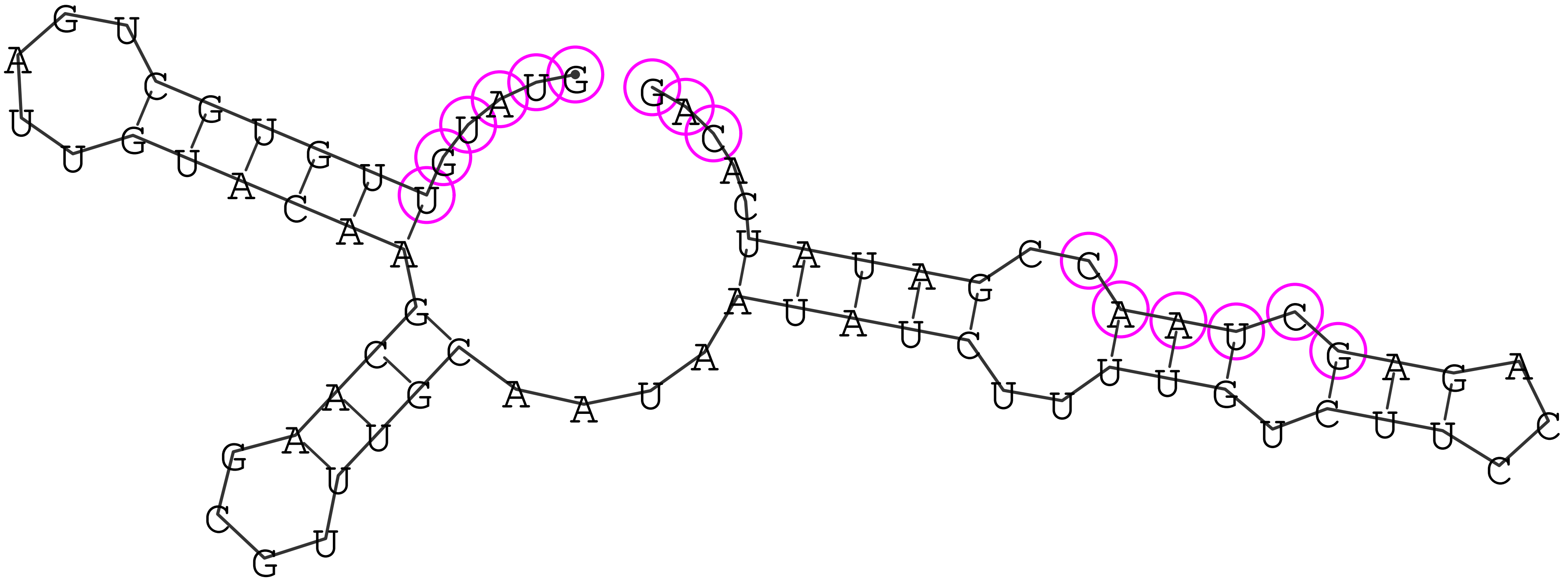

Xarbc0013A - Internal intron

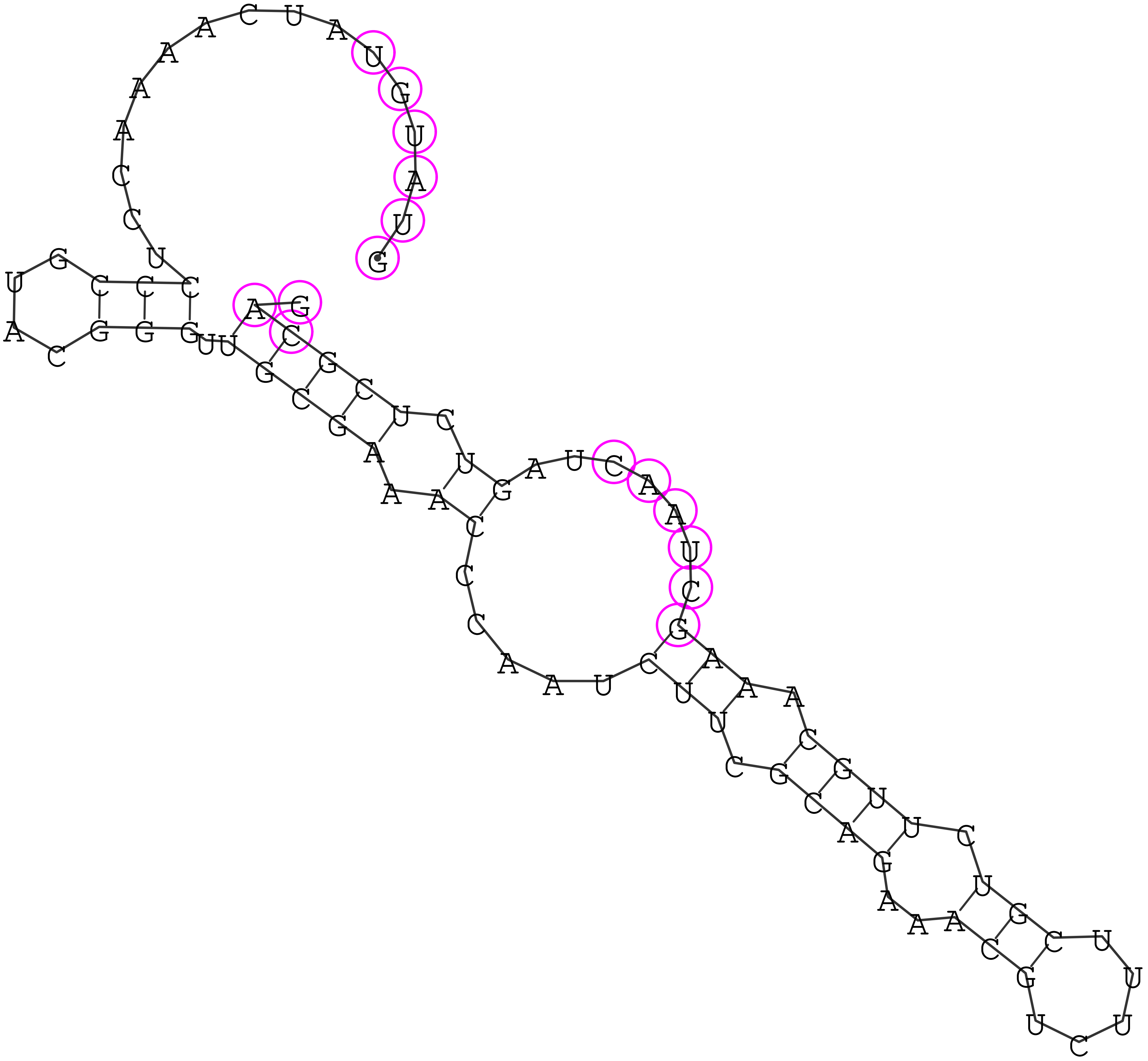

Xarbc0014A - Internal intron

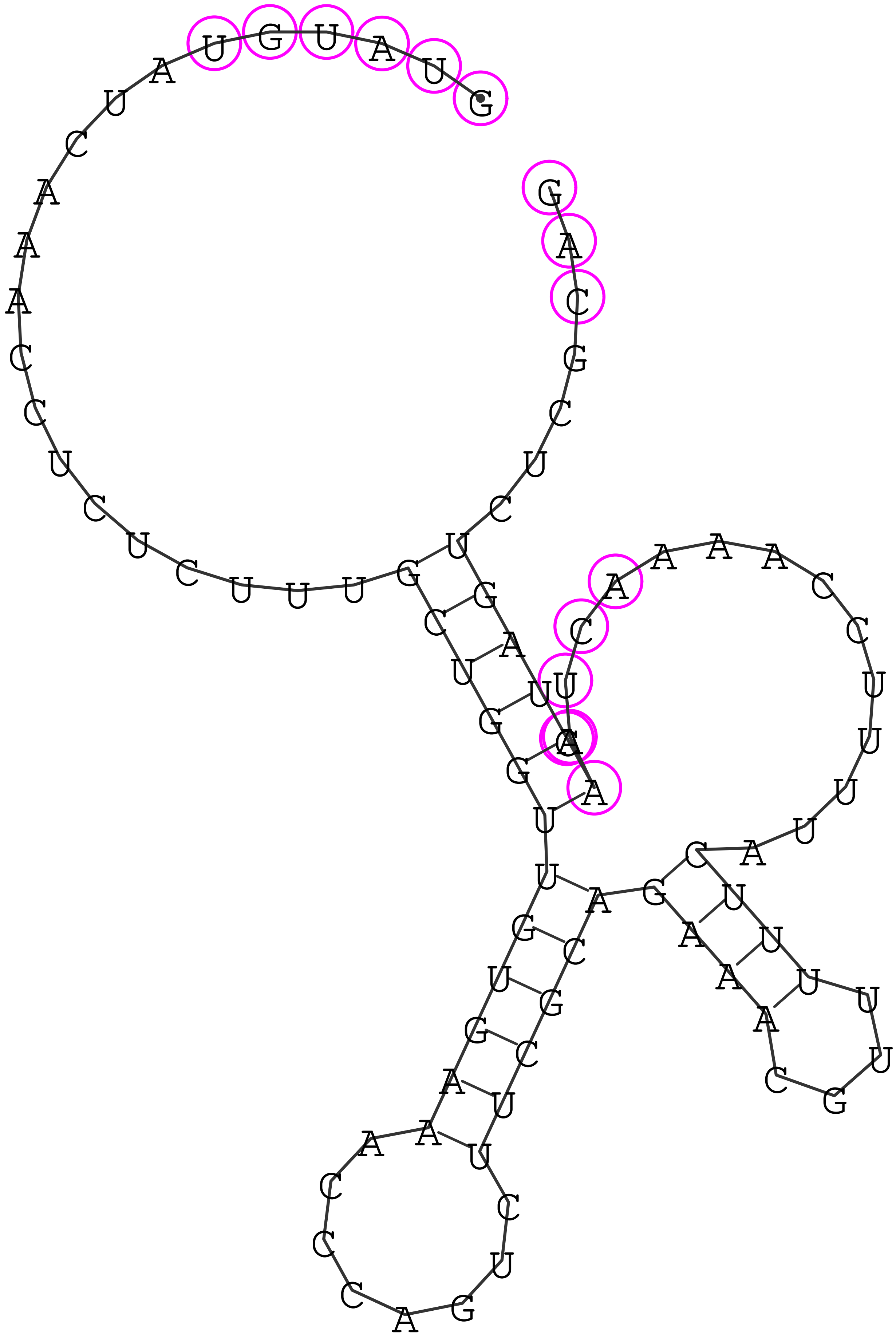

Xarbc0014B - Internal intron

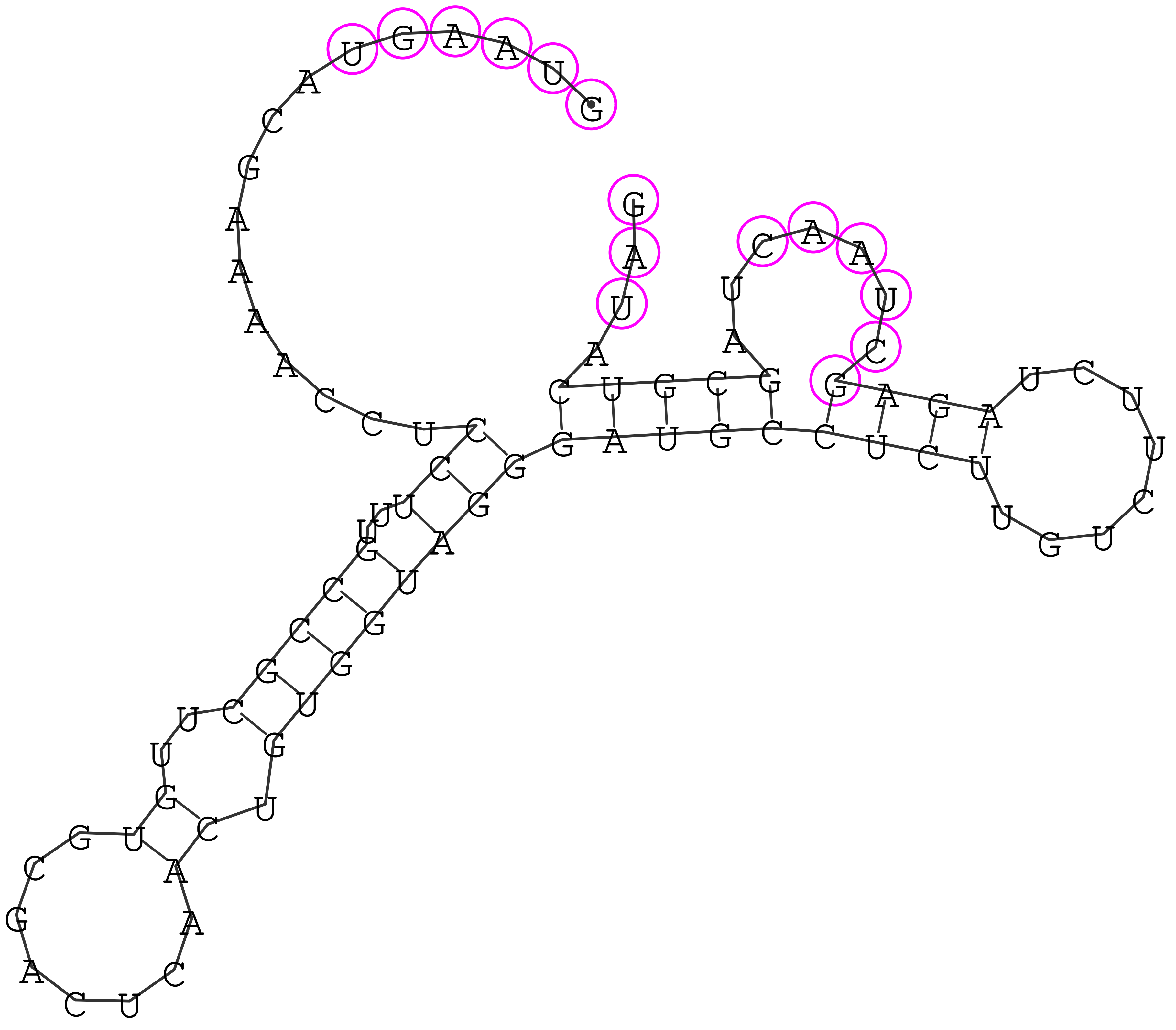

Xarbc0016A - Internal intron

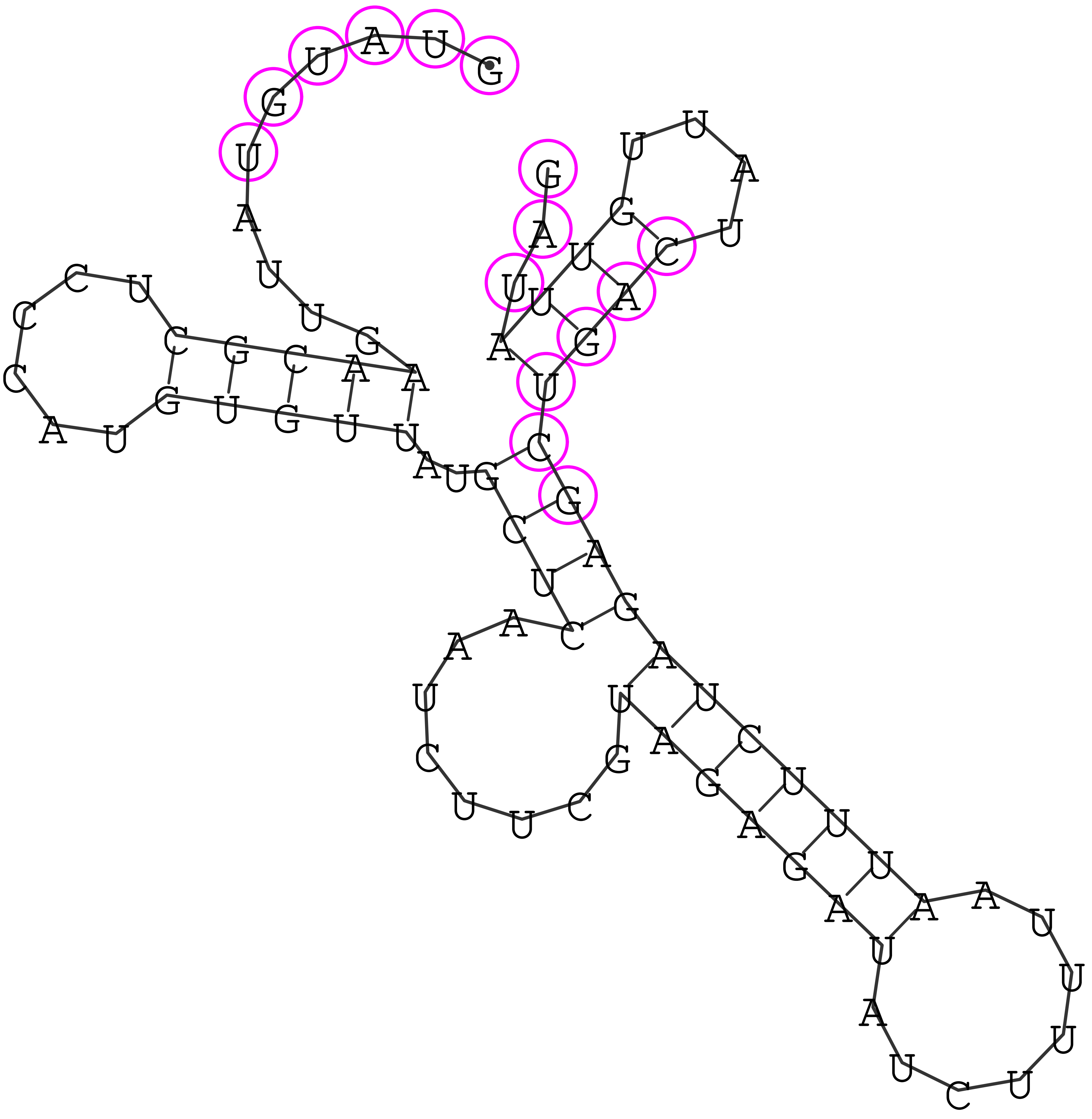

Xarbc0021A - Internal intron

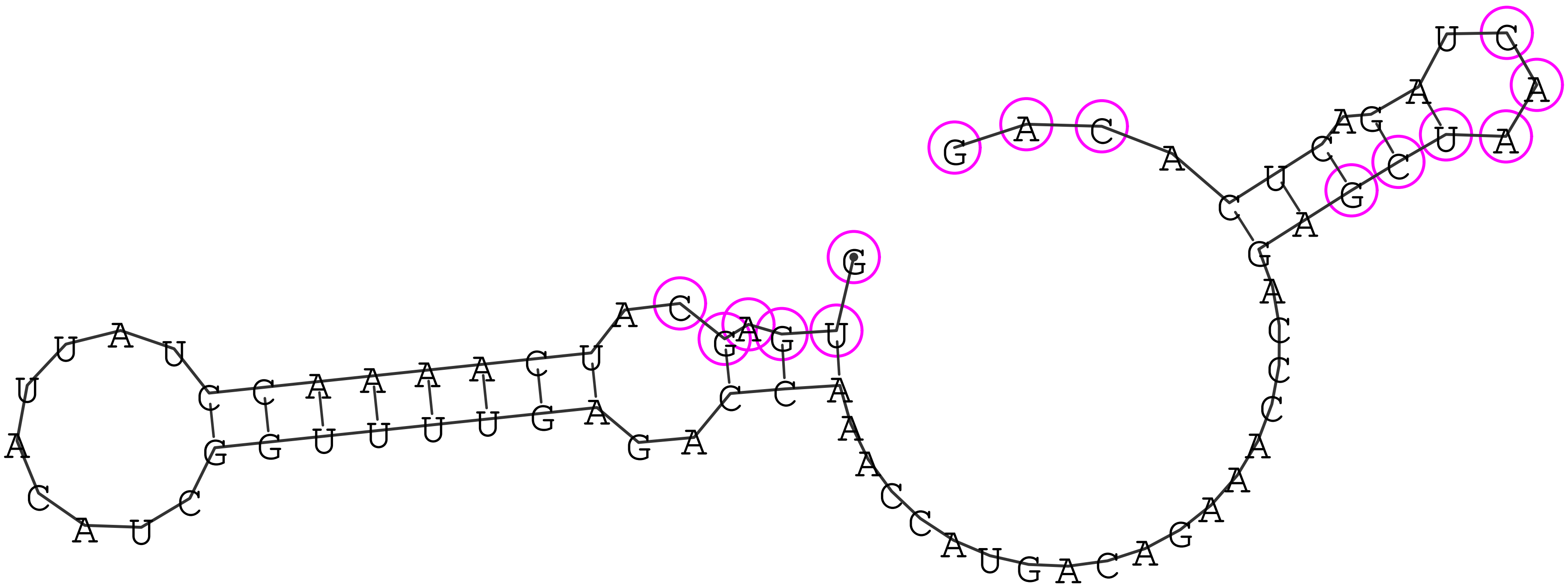

Xarbc0023A - Internal intron

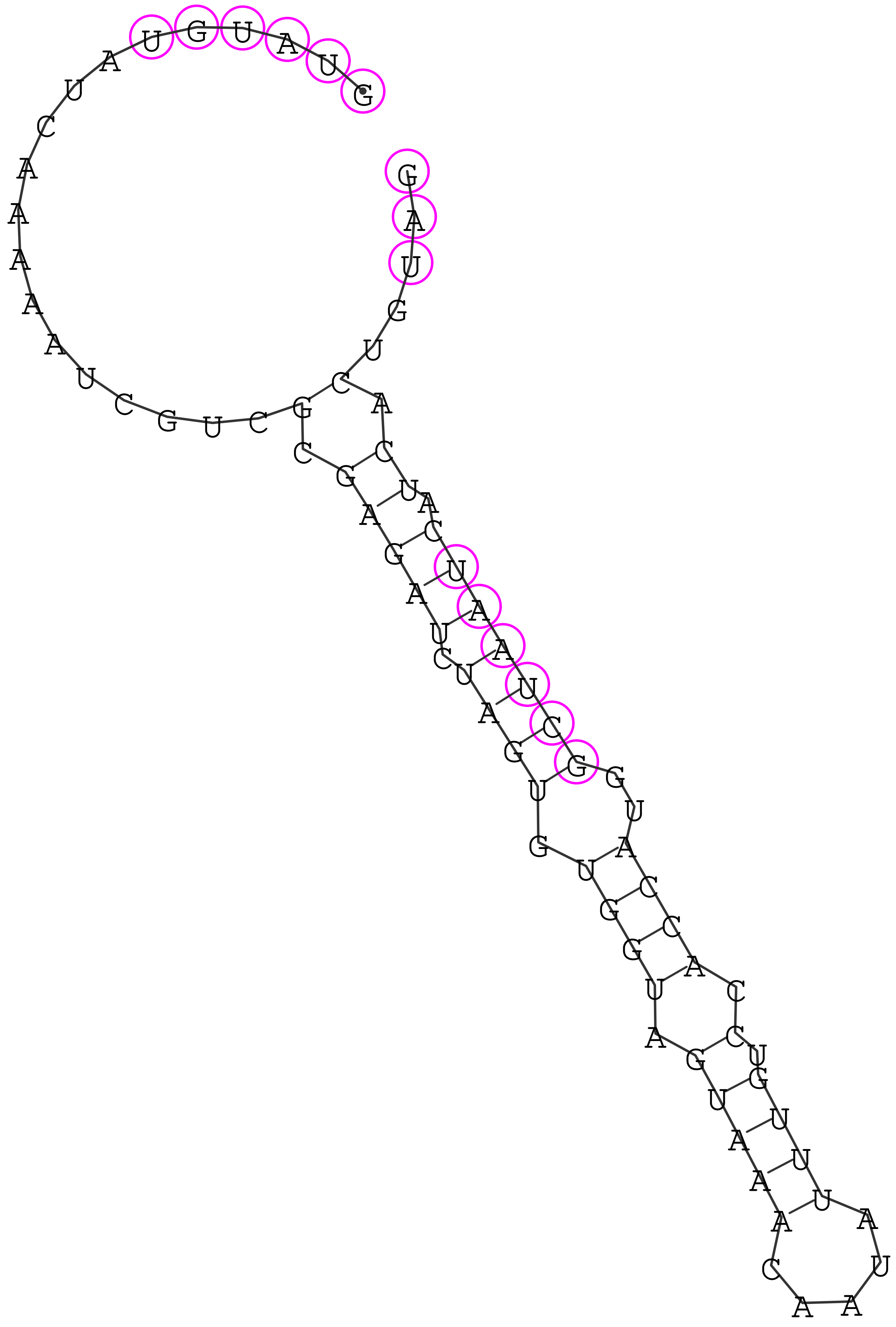

Xarbc0024A - Internal intron

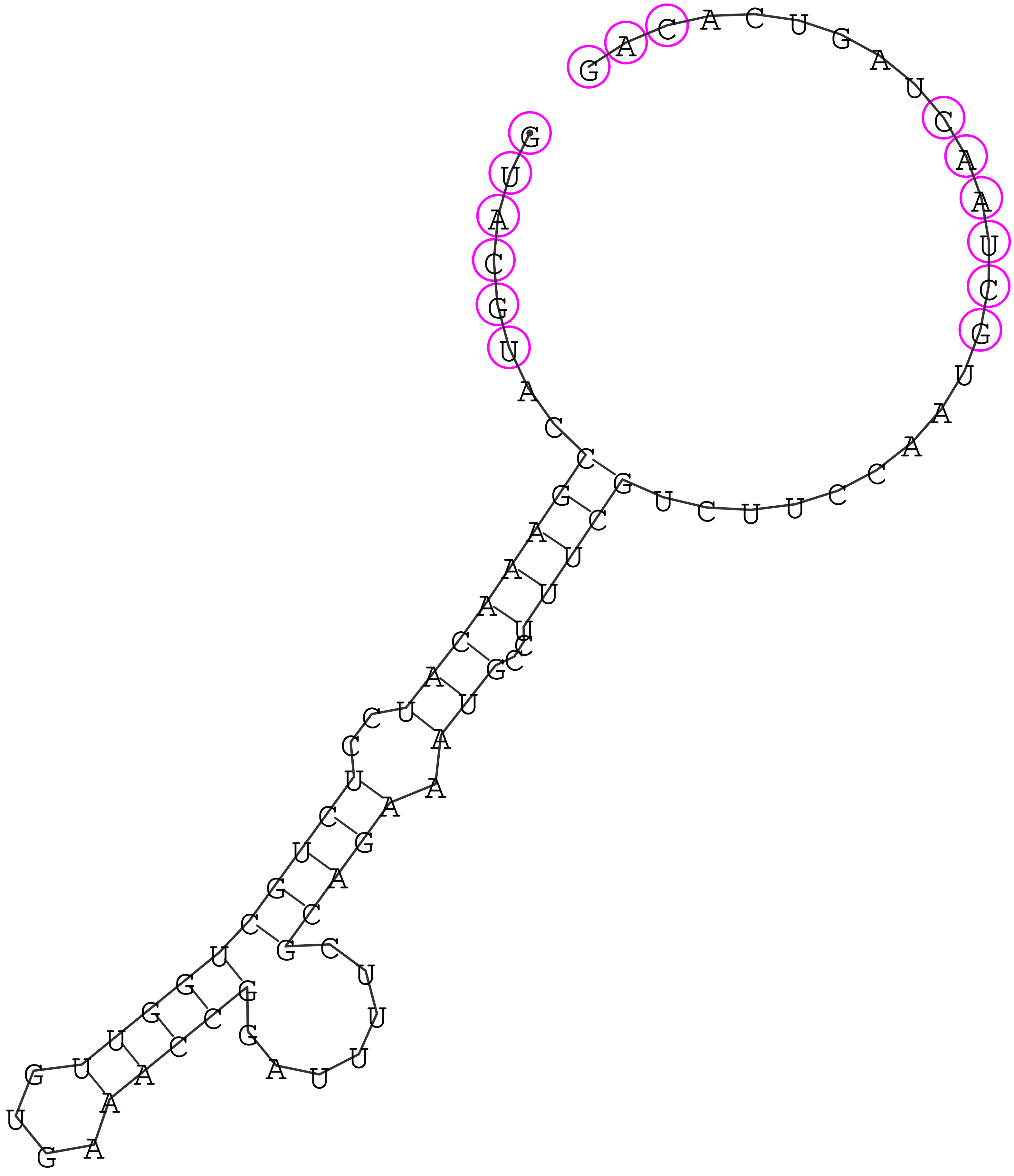

Xarbc0024B - Internal intron

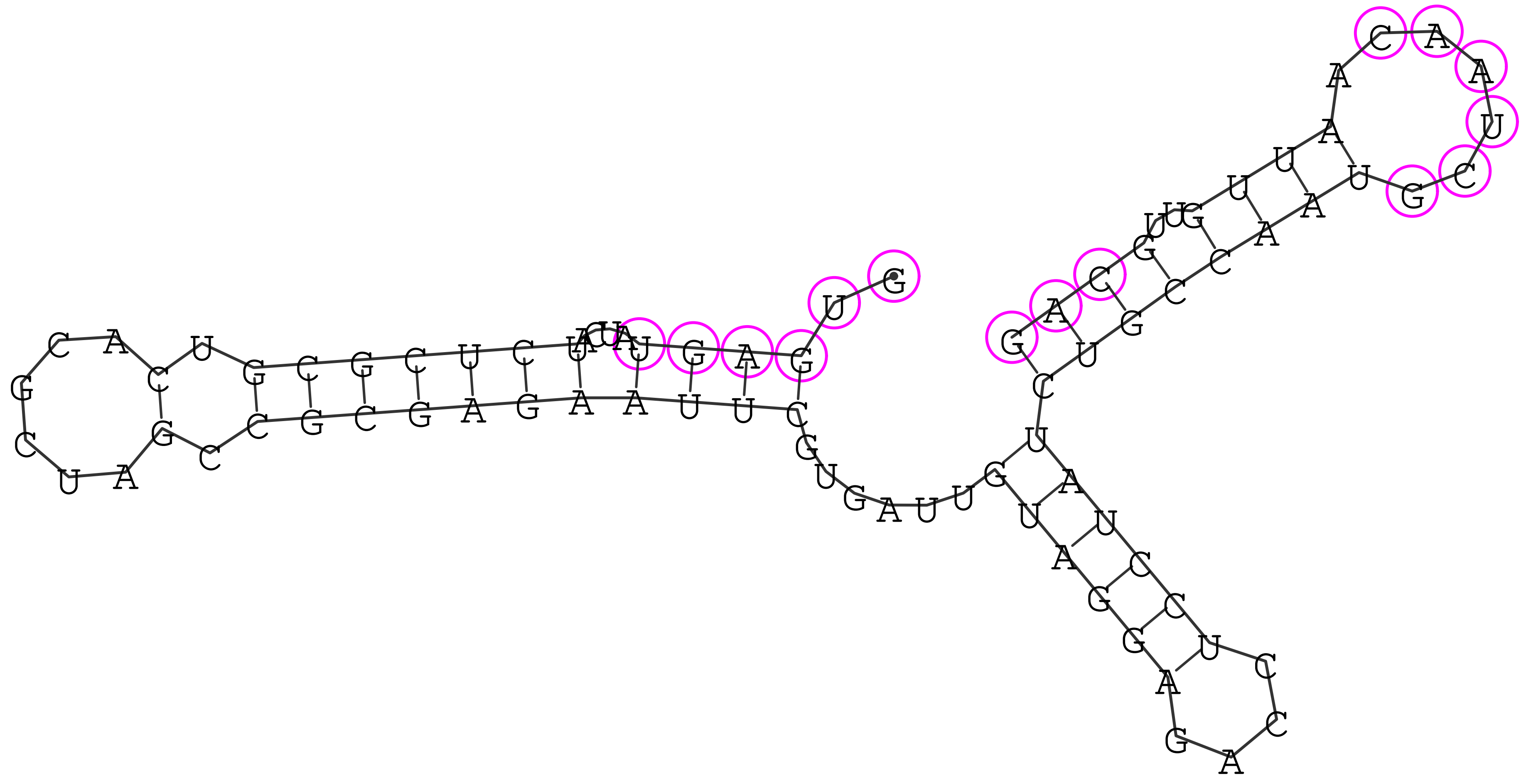

Xarbc0024C - Internal intron

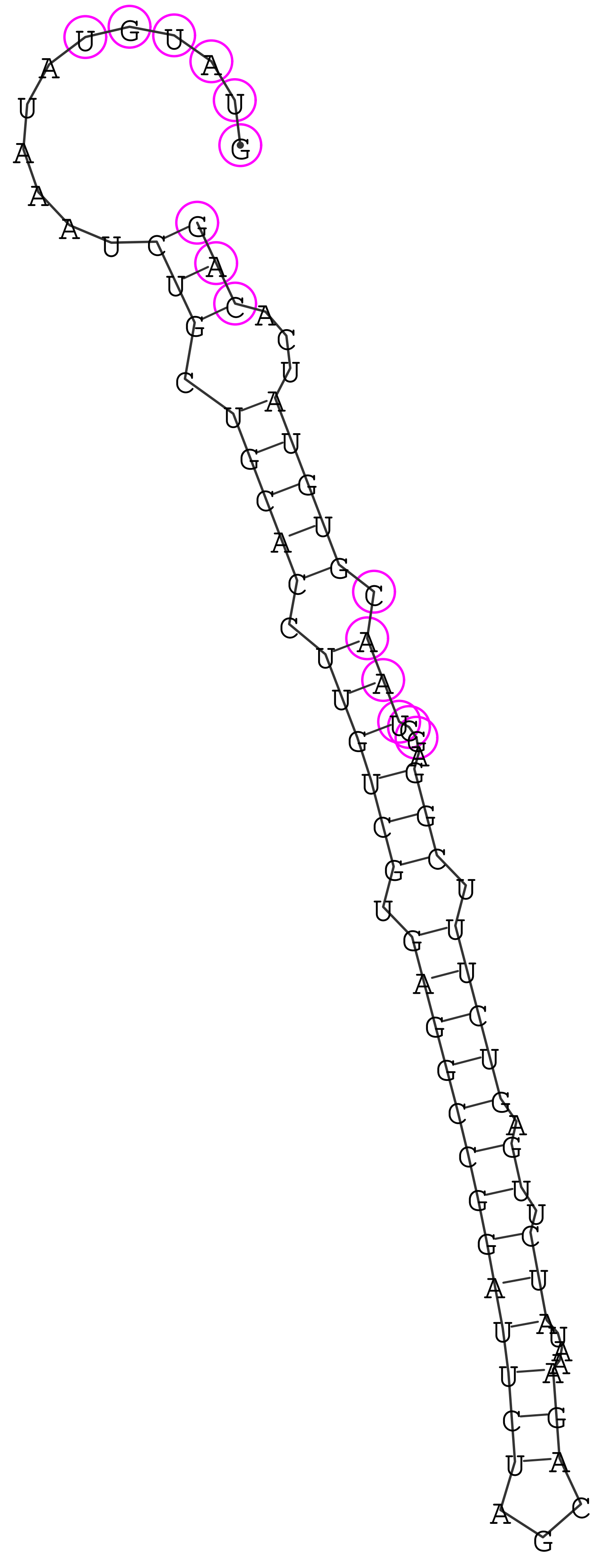

Xarbc0026A - Internal intron

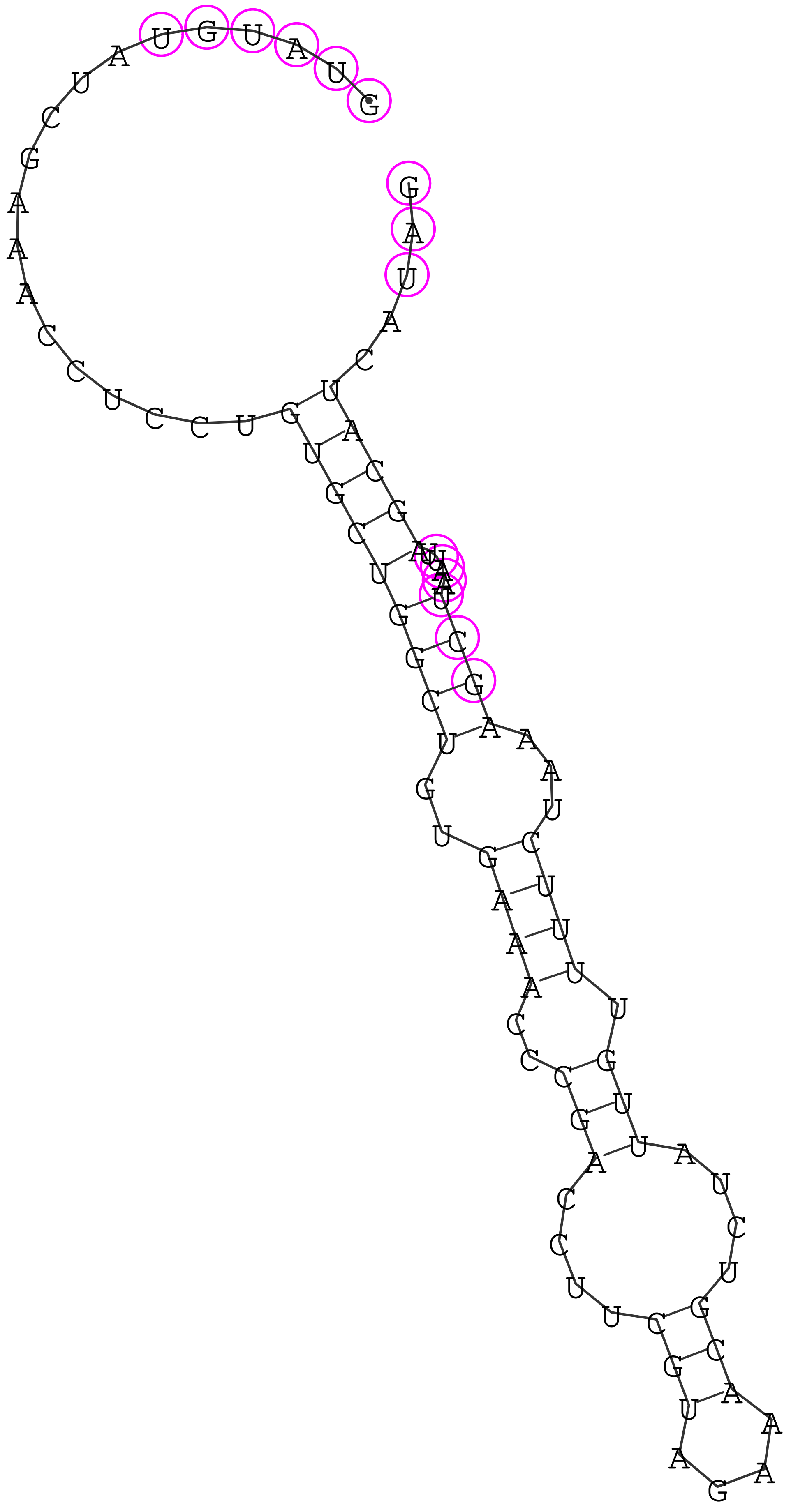

Xarbc0028A - Internal intron

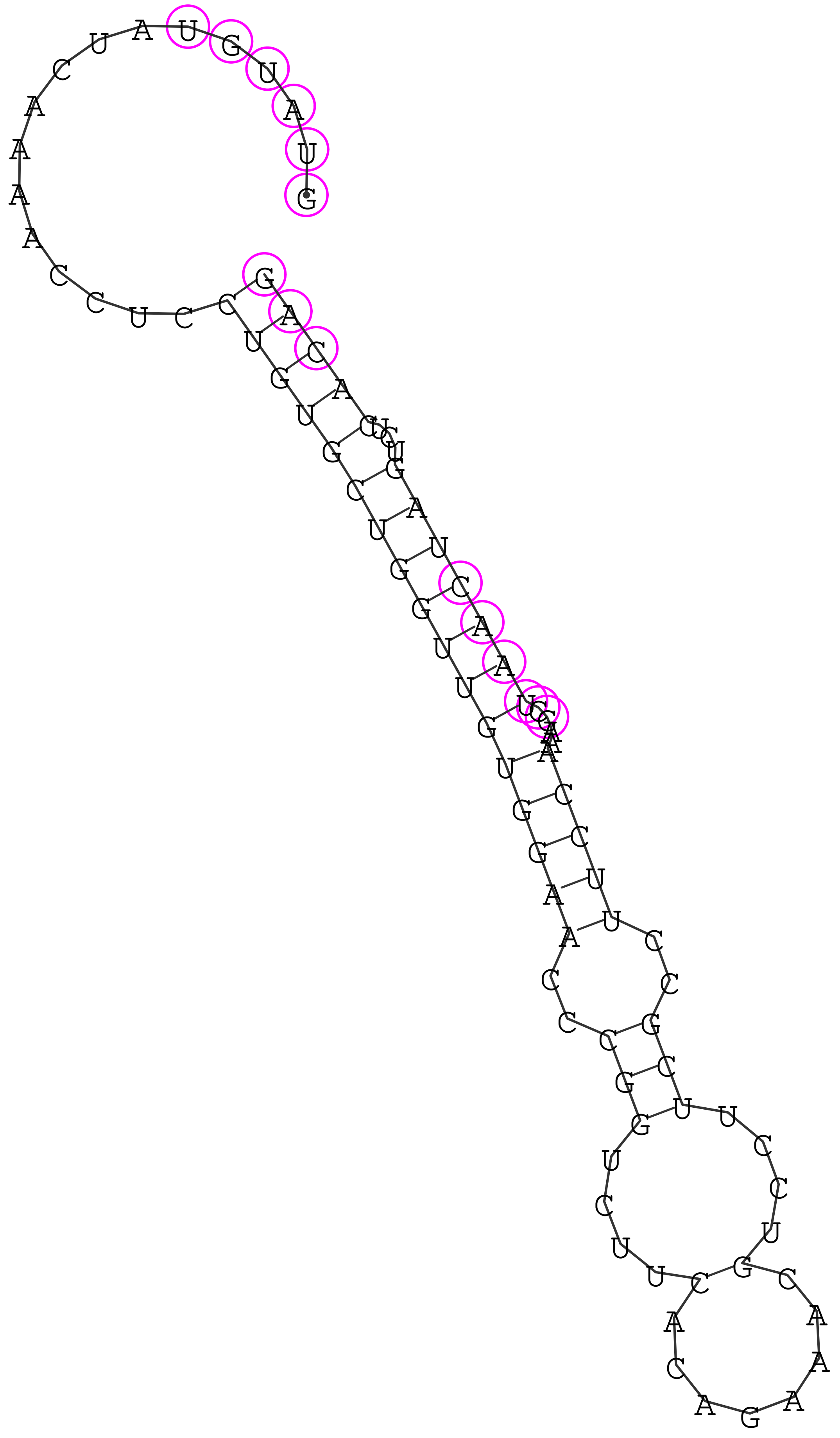

Xarbc0034A - Internal intron

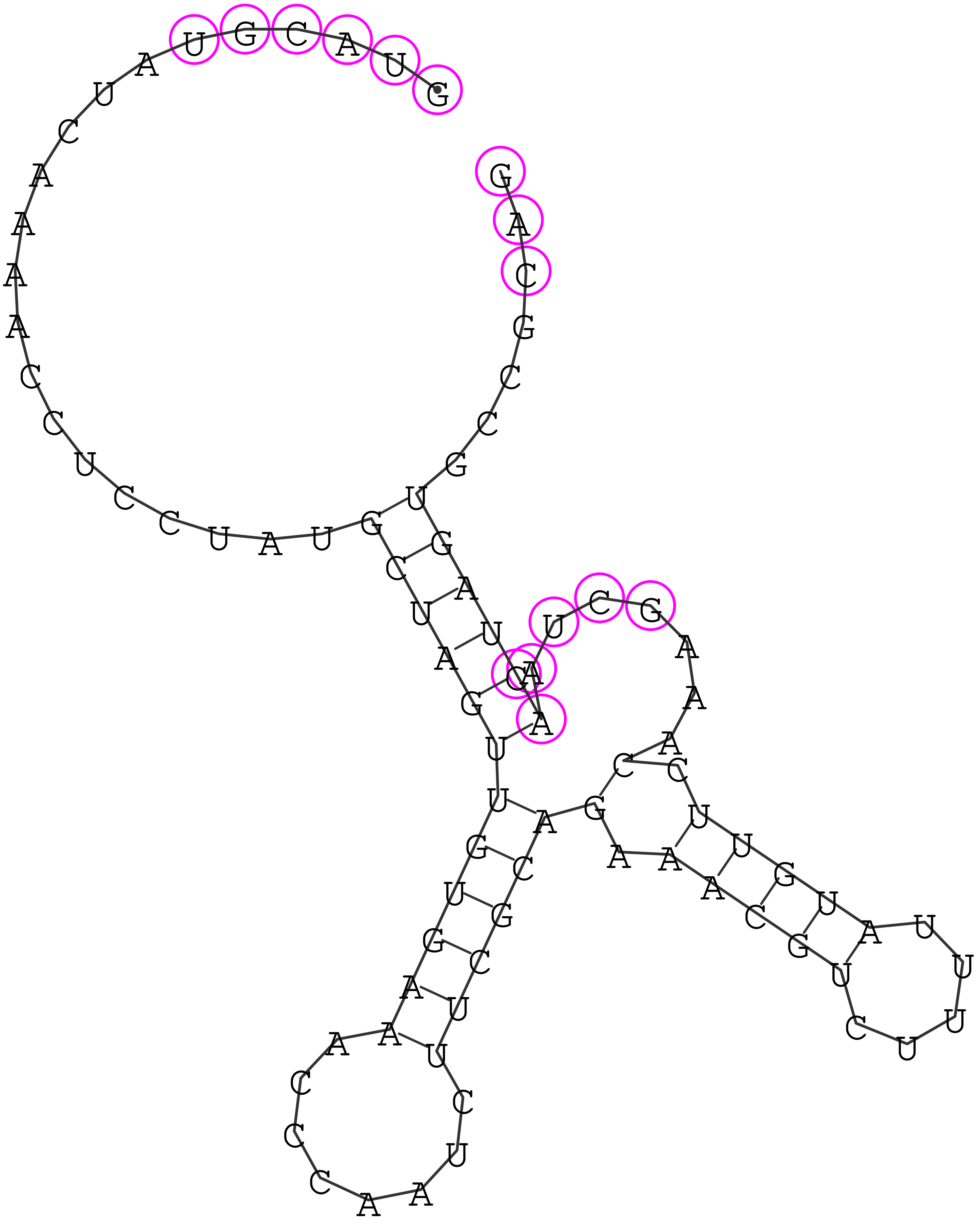

Xarbc0044A - Internal intron

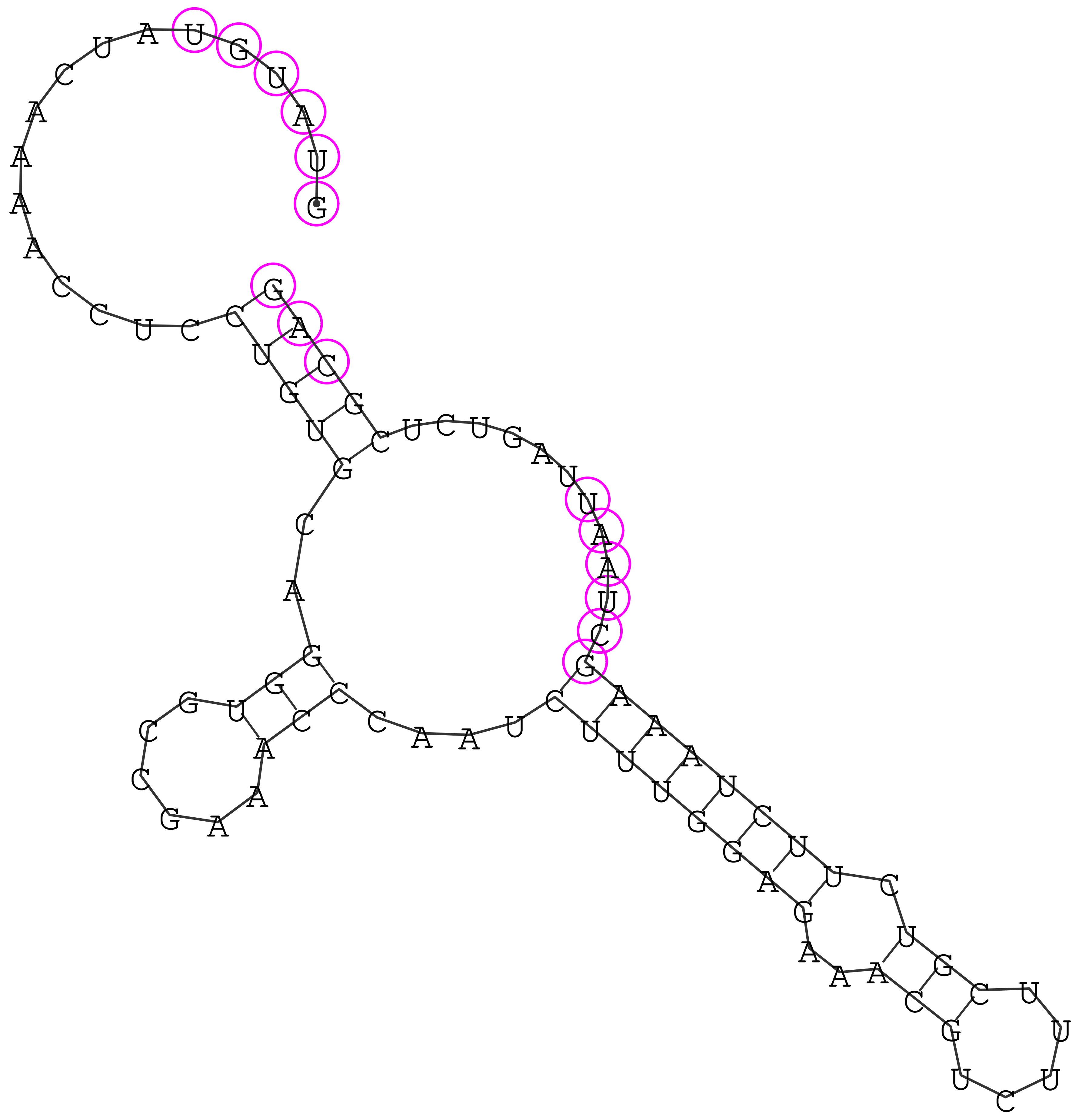

Xarbc0059A - Internal intron

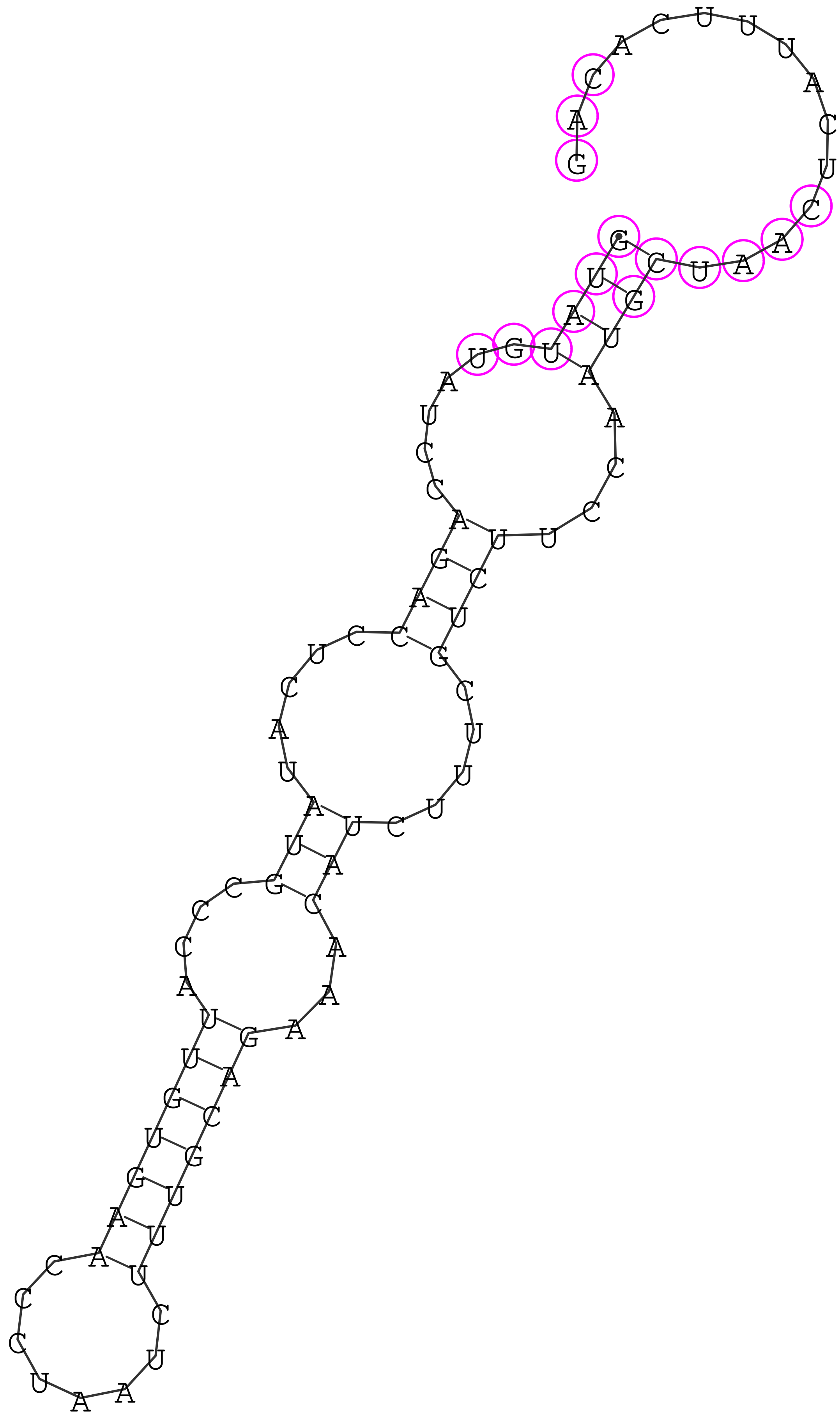

Xarbc0060A - Internal intron

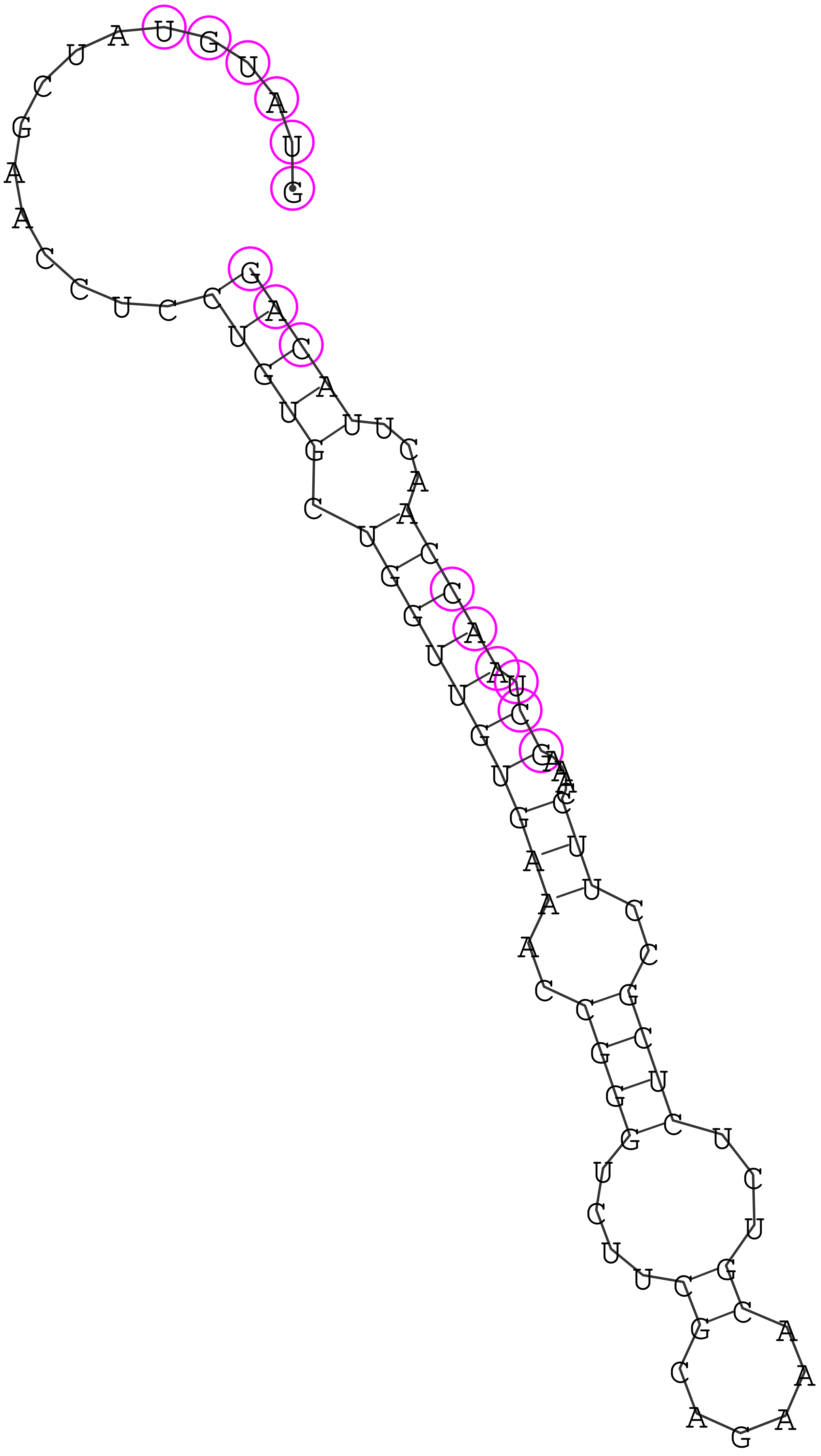

Xarbc0061A - Internal intron

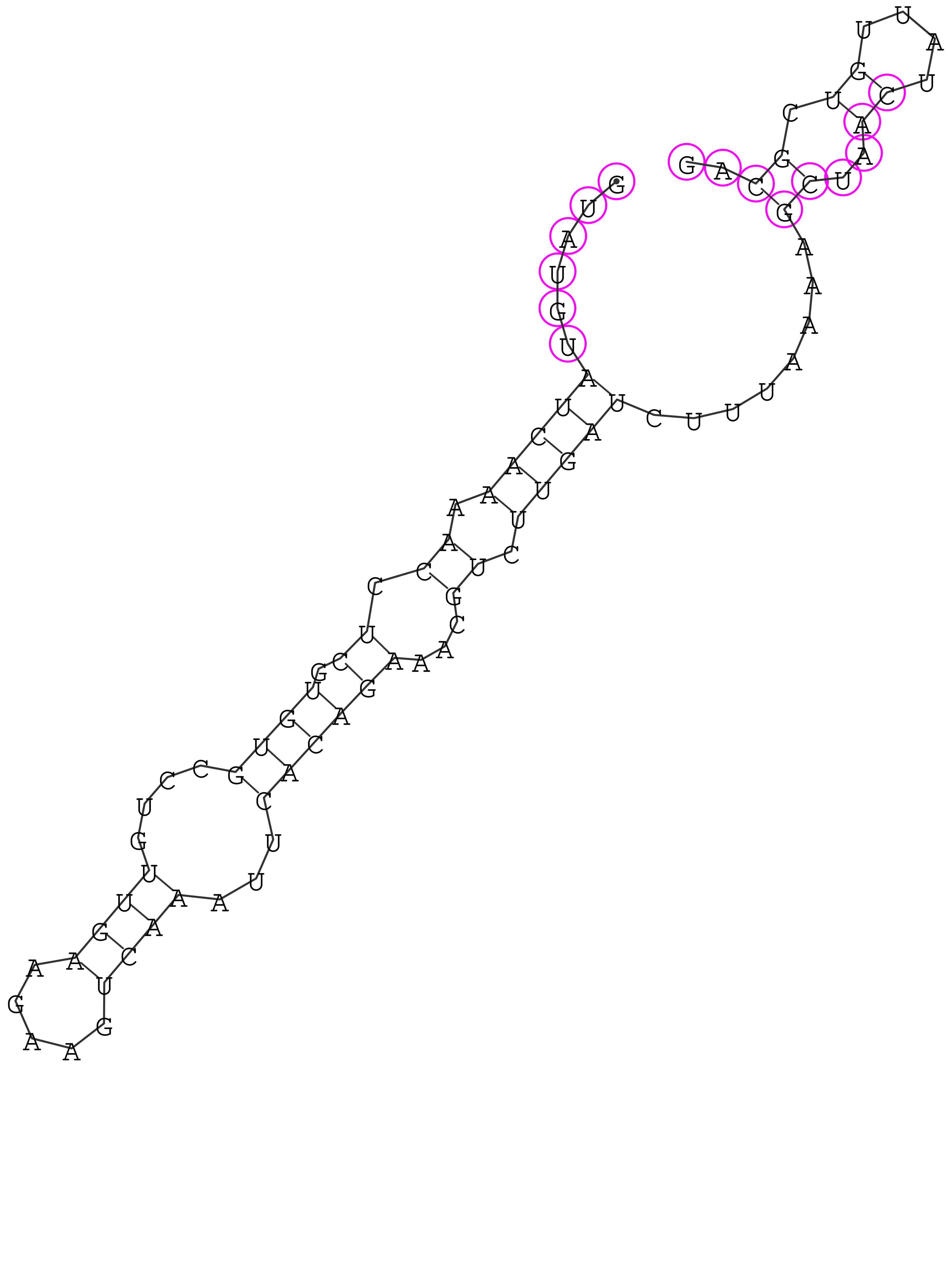

Xarbc0064A - Internal intron

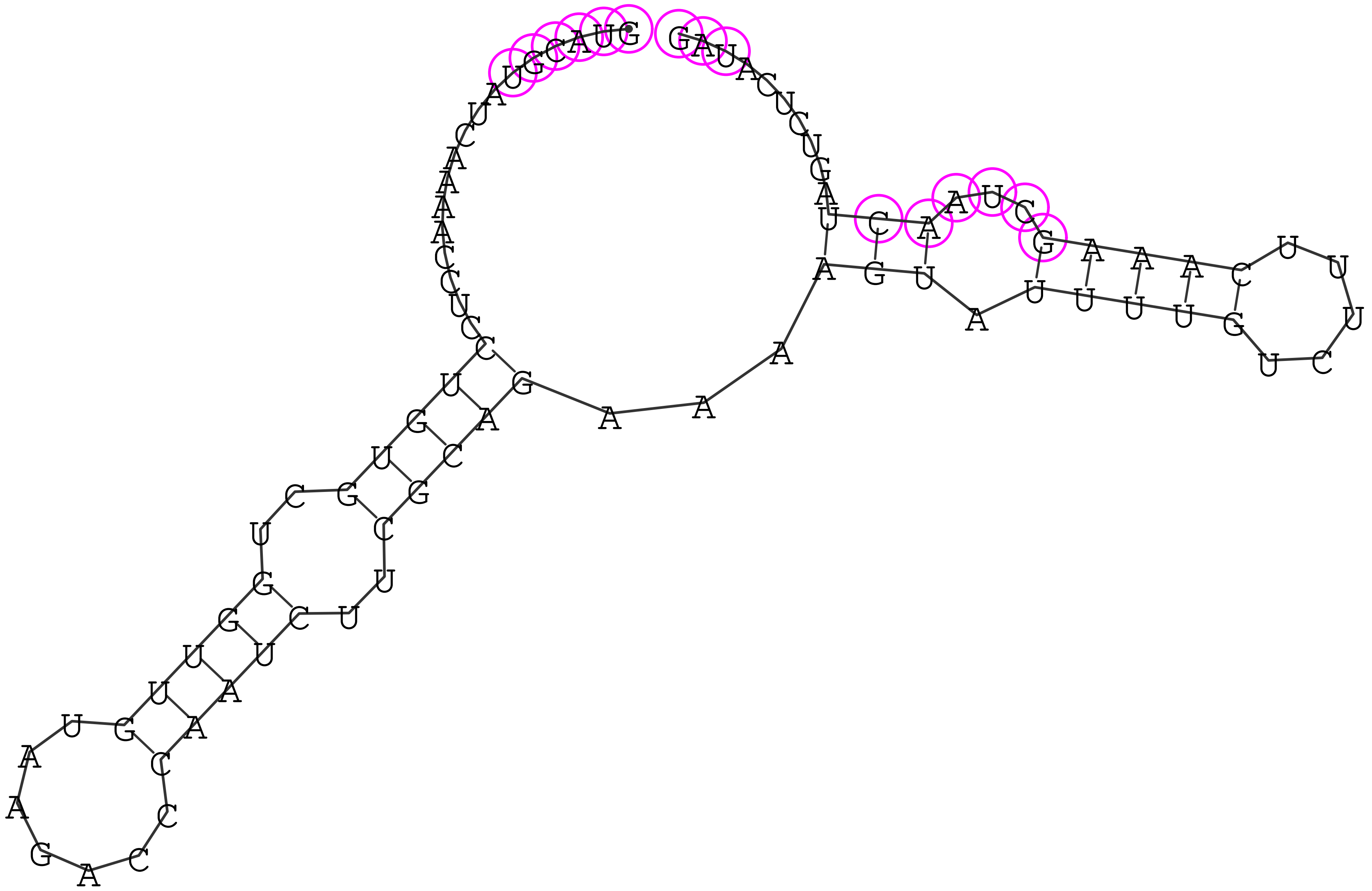

Xarbc0064B - Internal intron

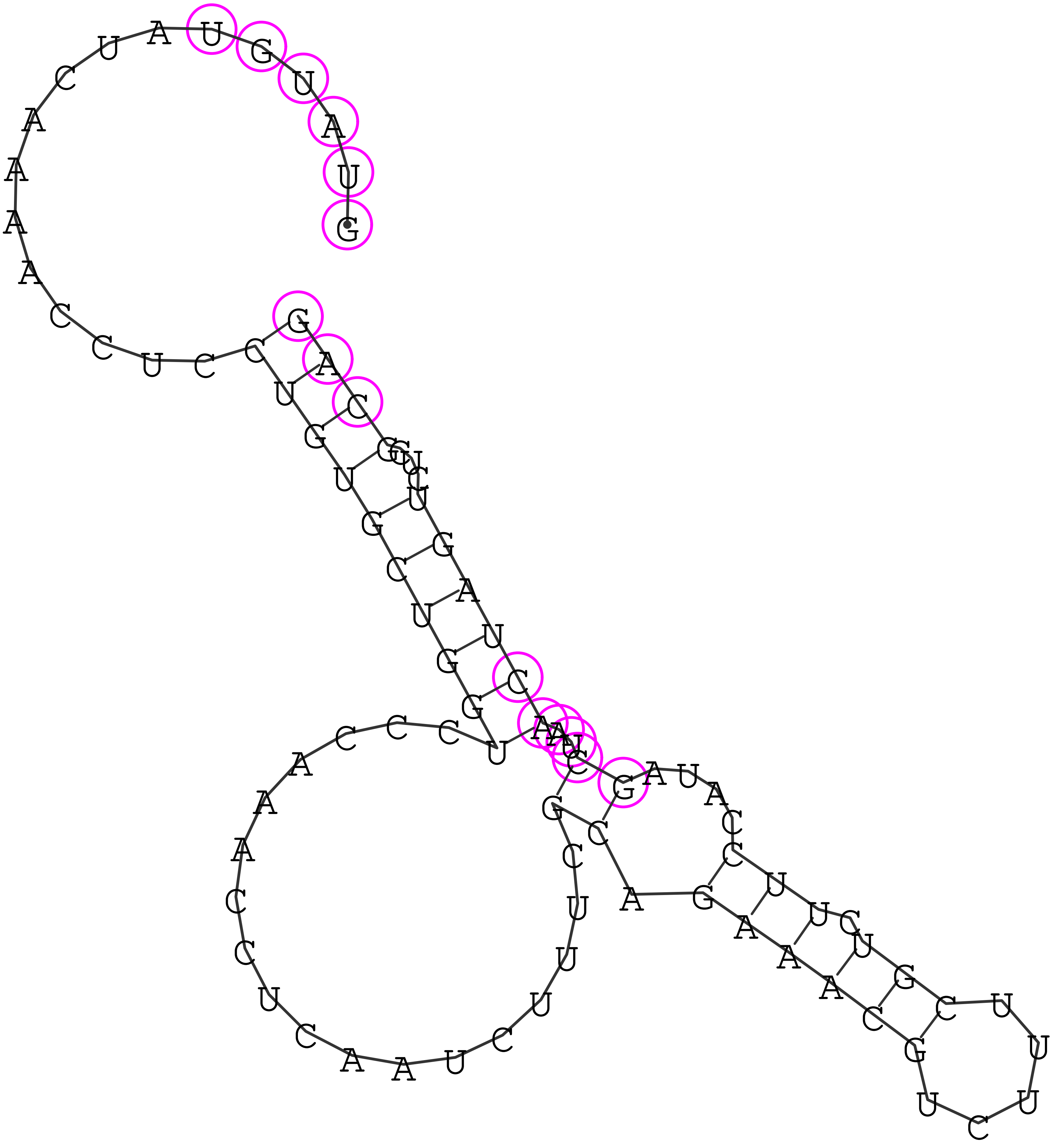

Xarbc0072A - Internal intron

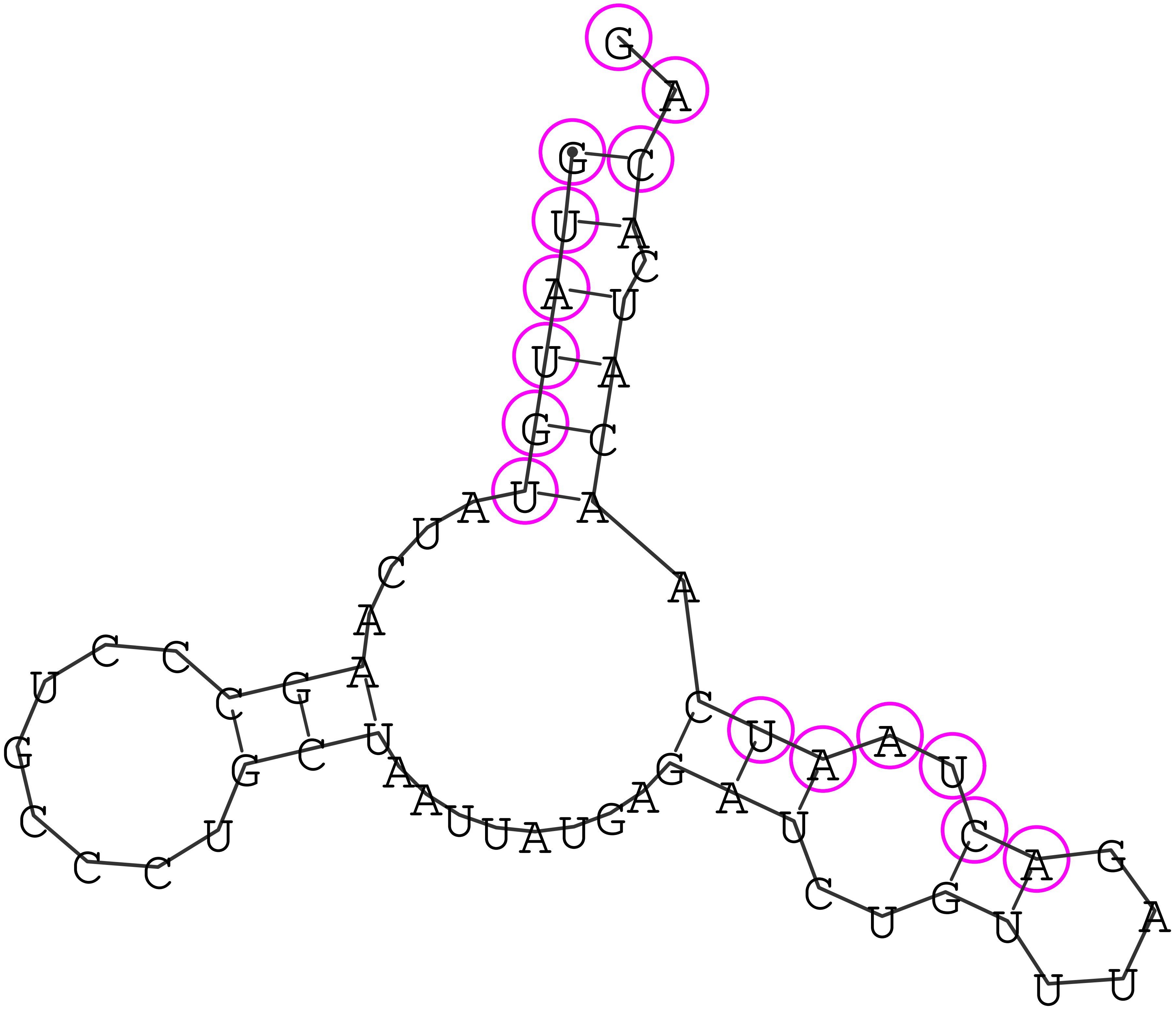

Xarbc0074A - Internal intron

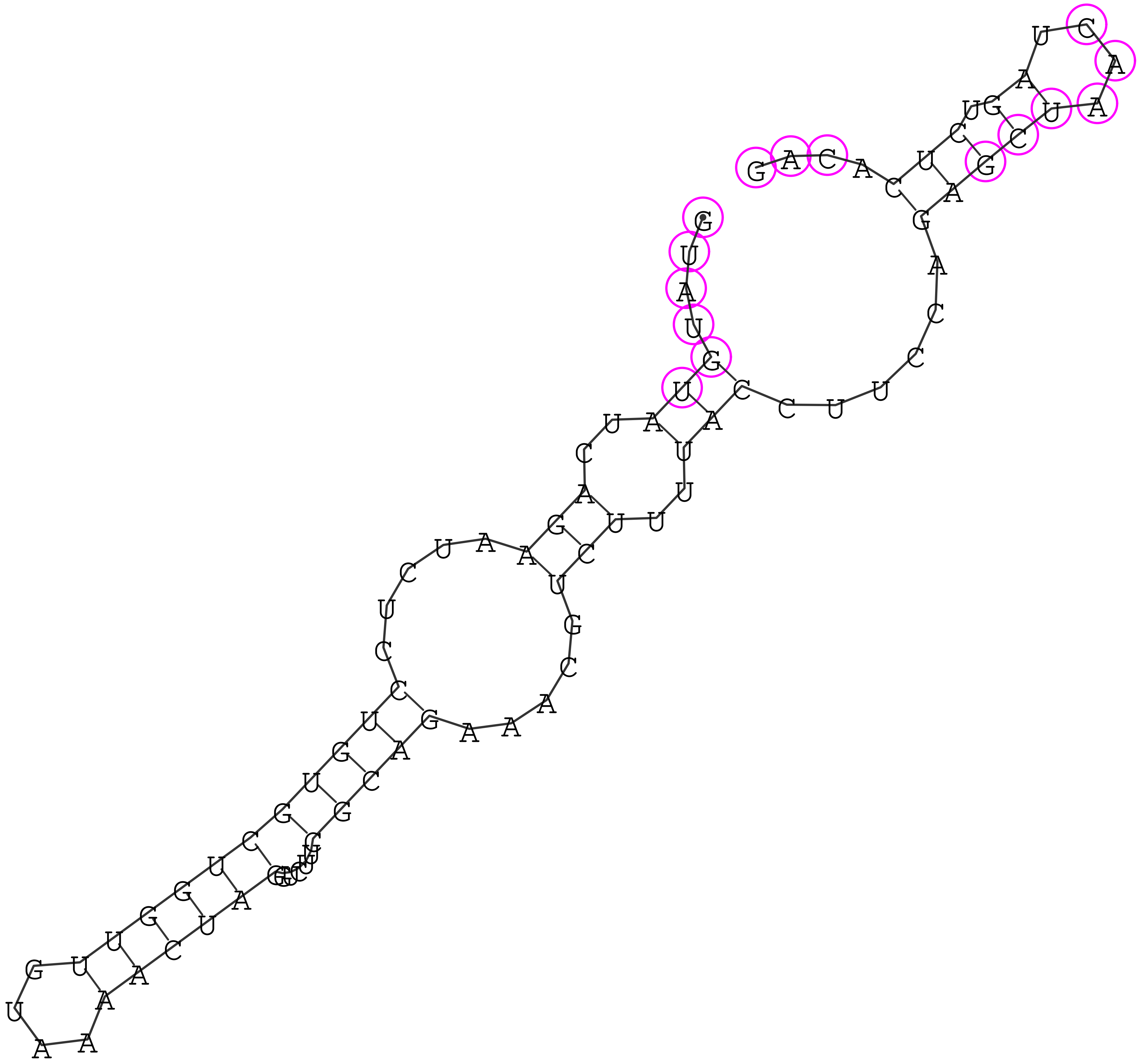

Xarbc0080A - Internal intron

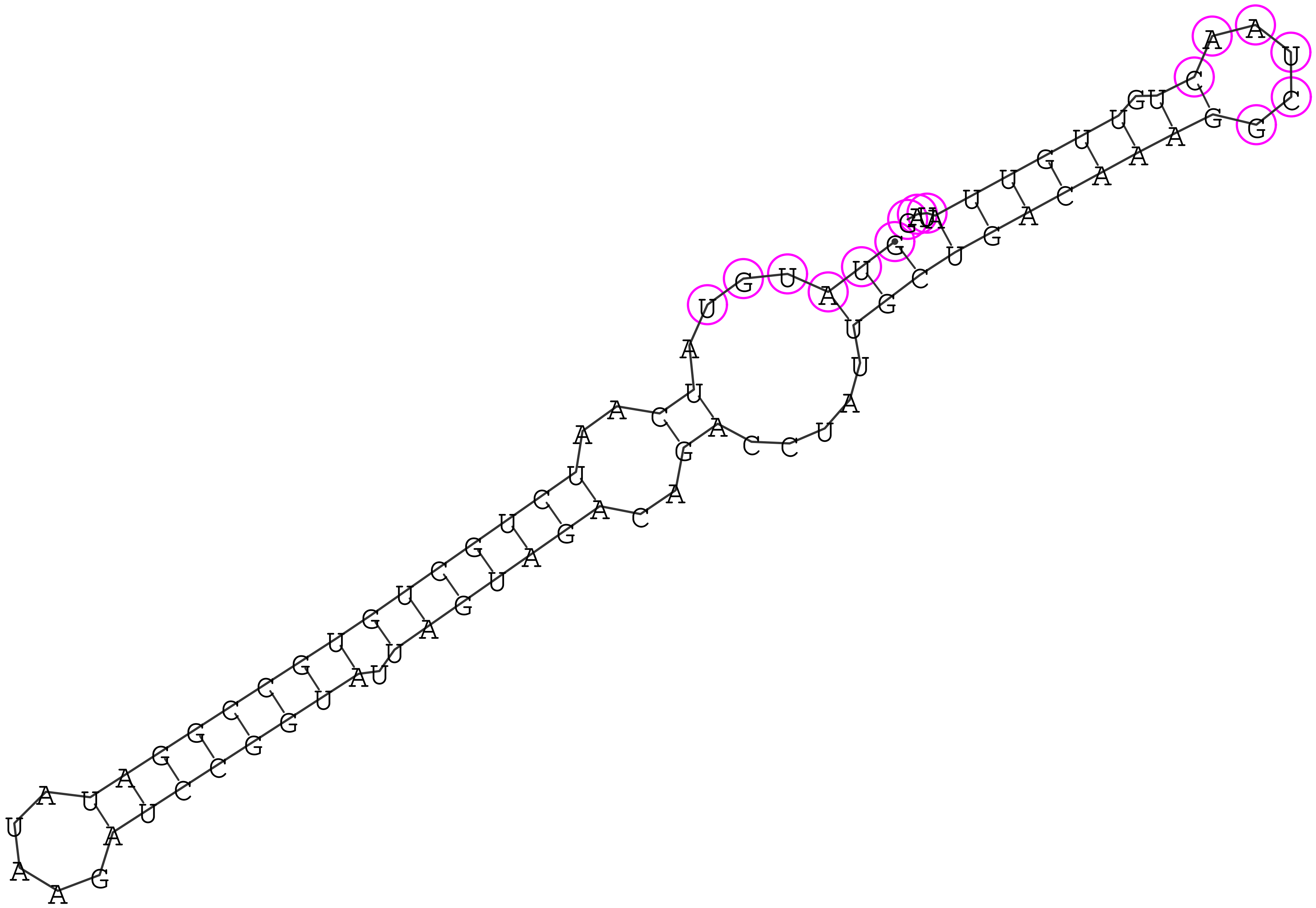

Xarbc0093A - Internal intron

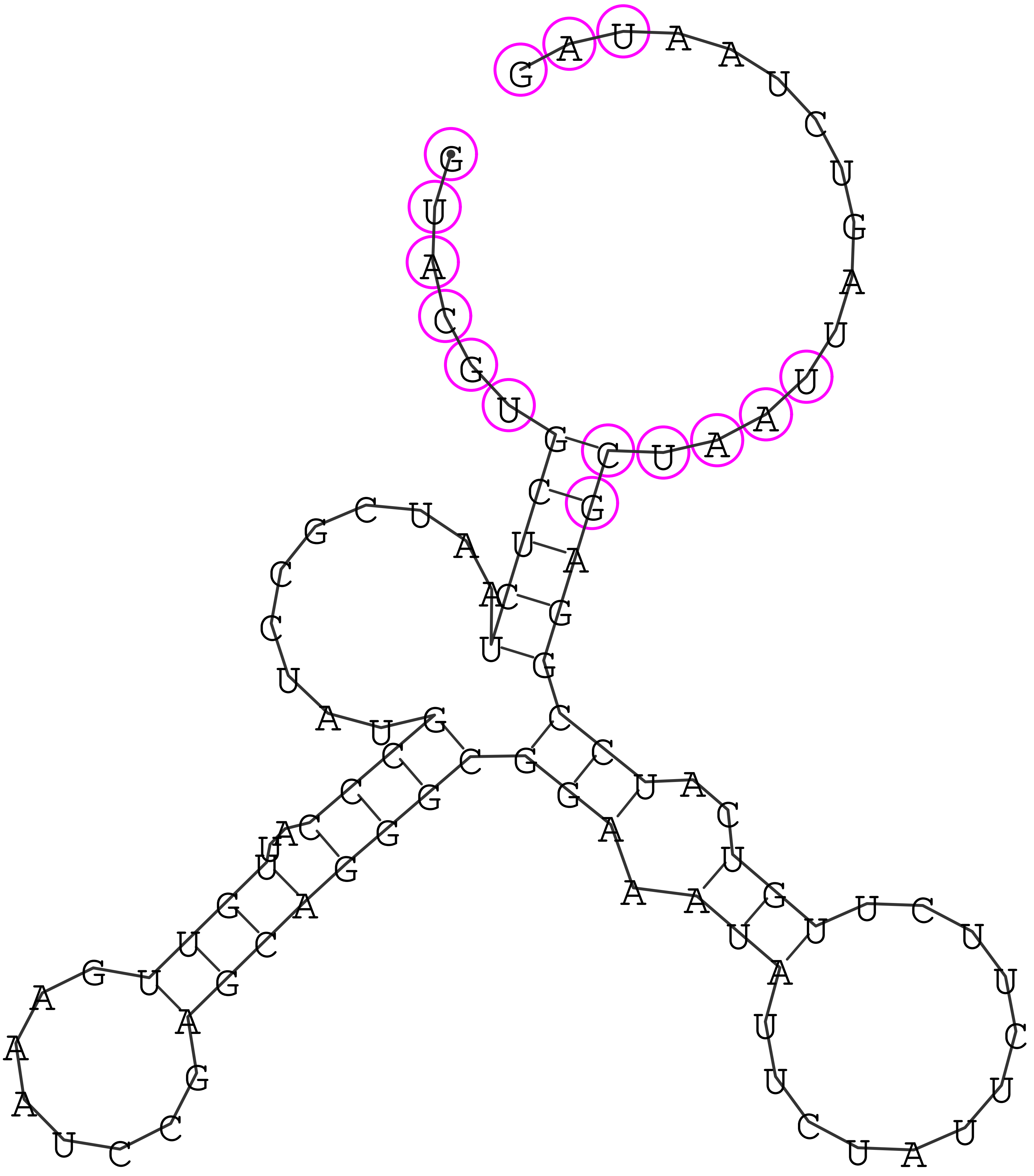

Xarbc0096A - Internal intron

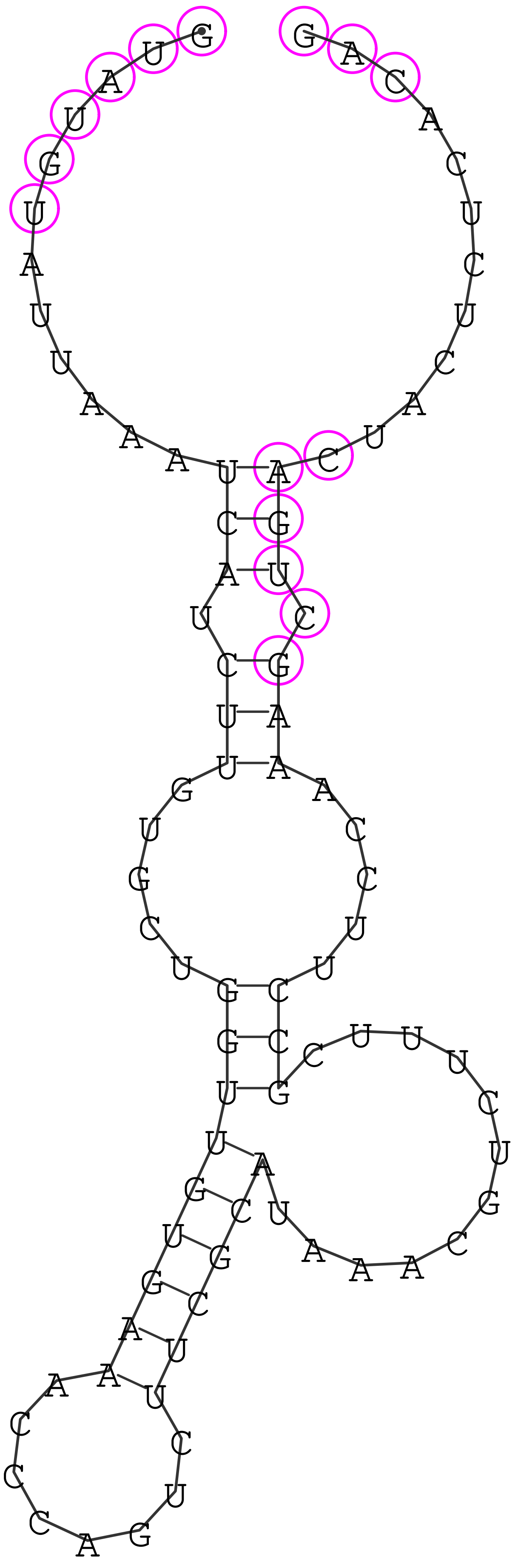

Xarbc0099A - Internal intron

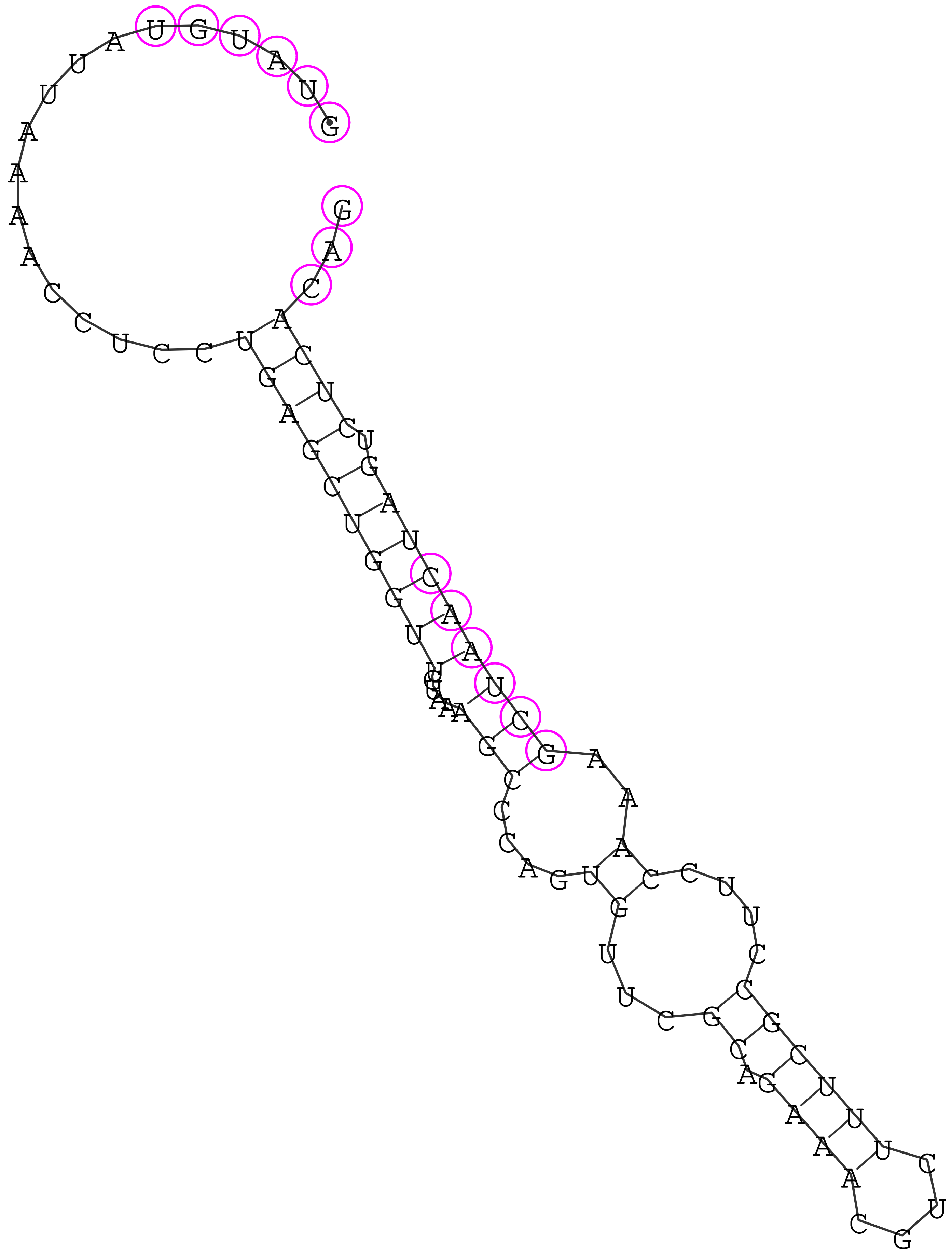

Xarbc0101A - Internal intron

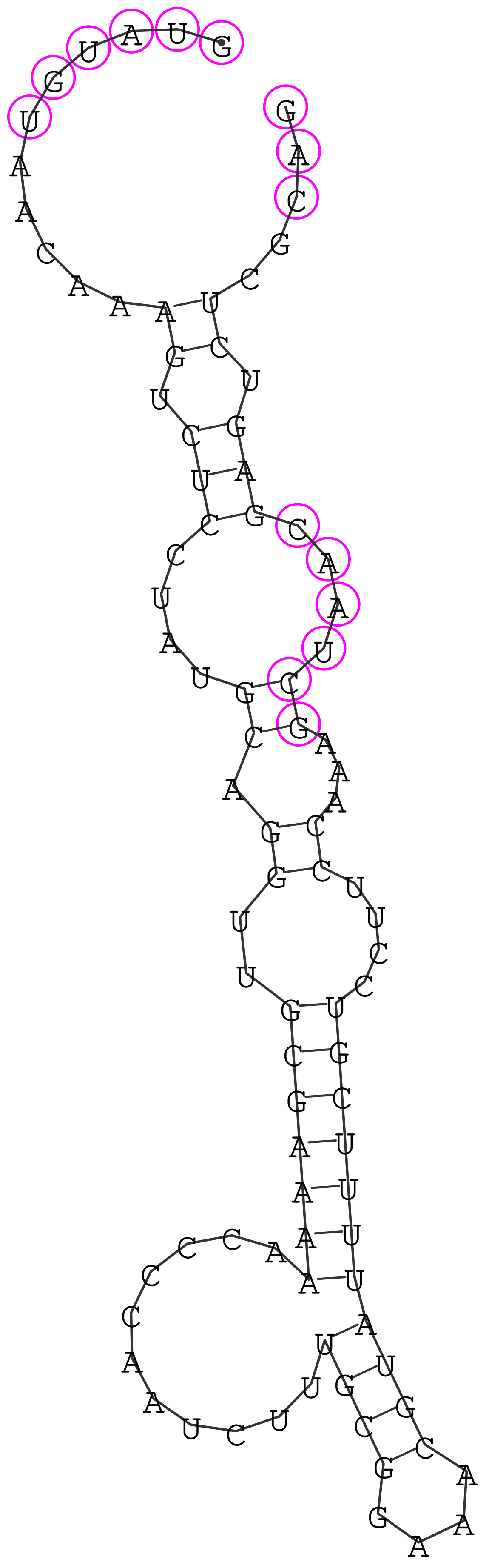

Xarbc0108A - Internal intron

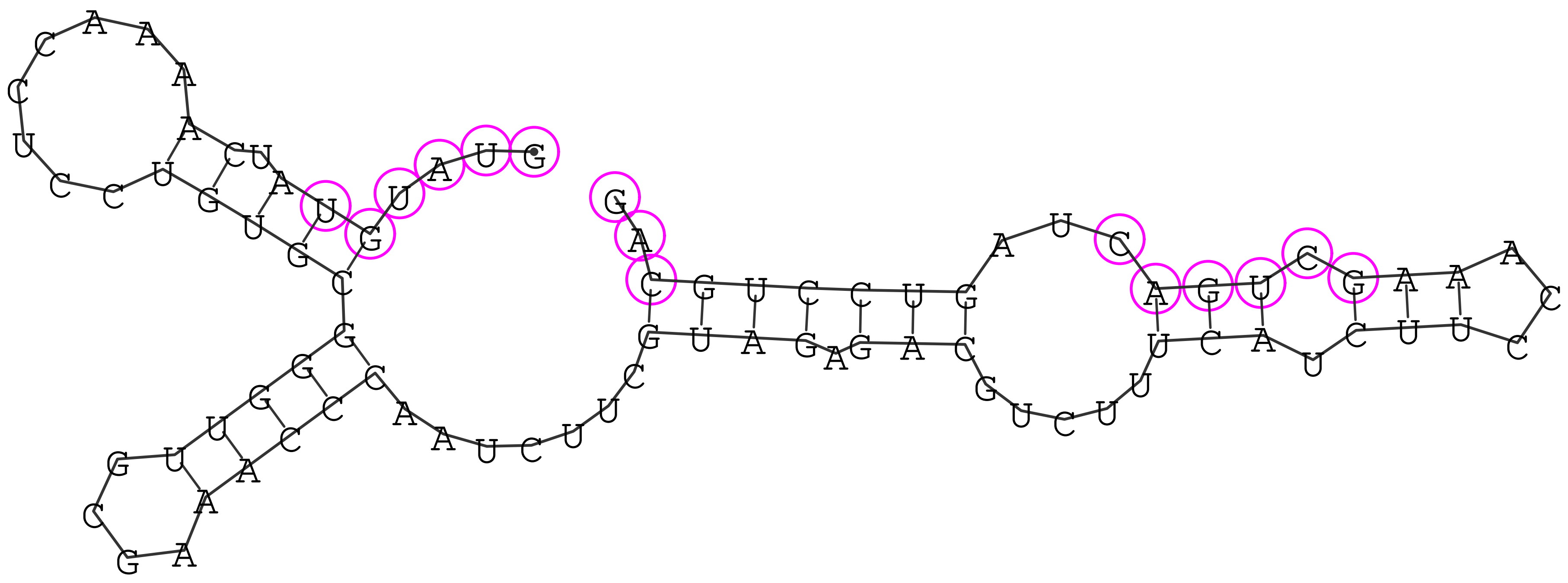

Xarbc0134A - Internal intron

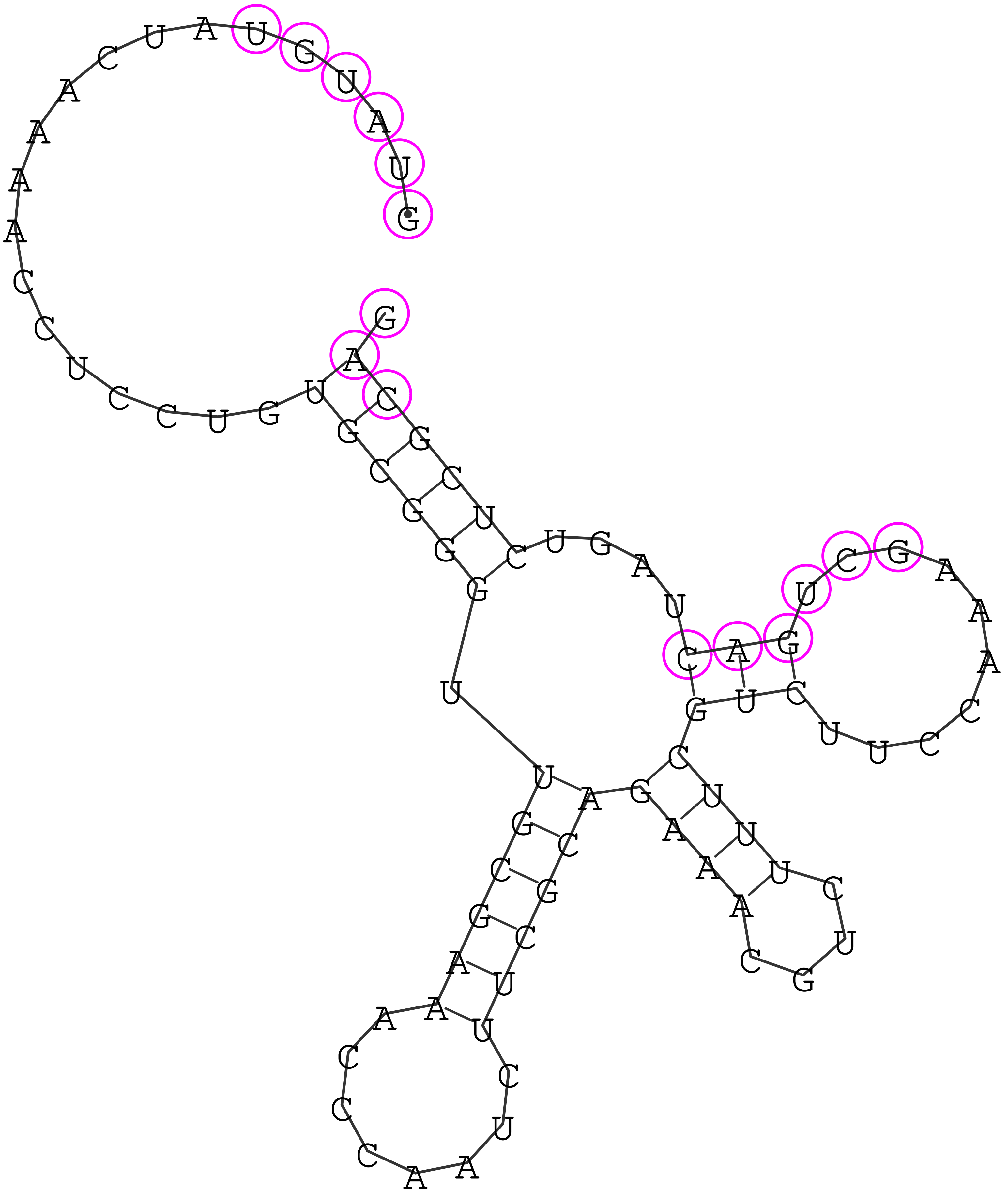

Xarbc0143A - Internal intron

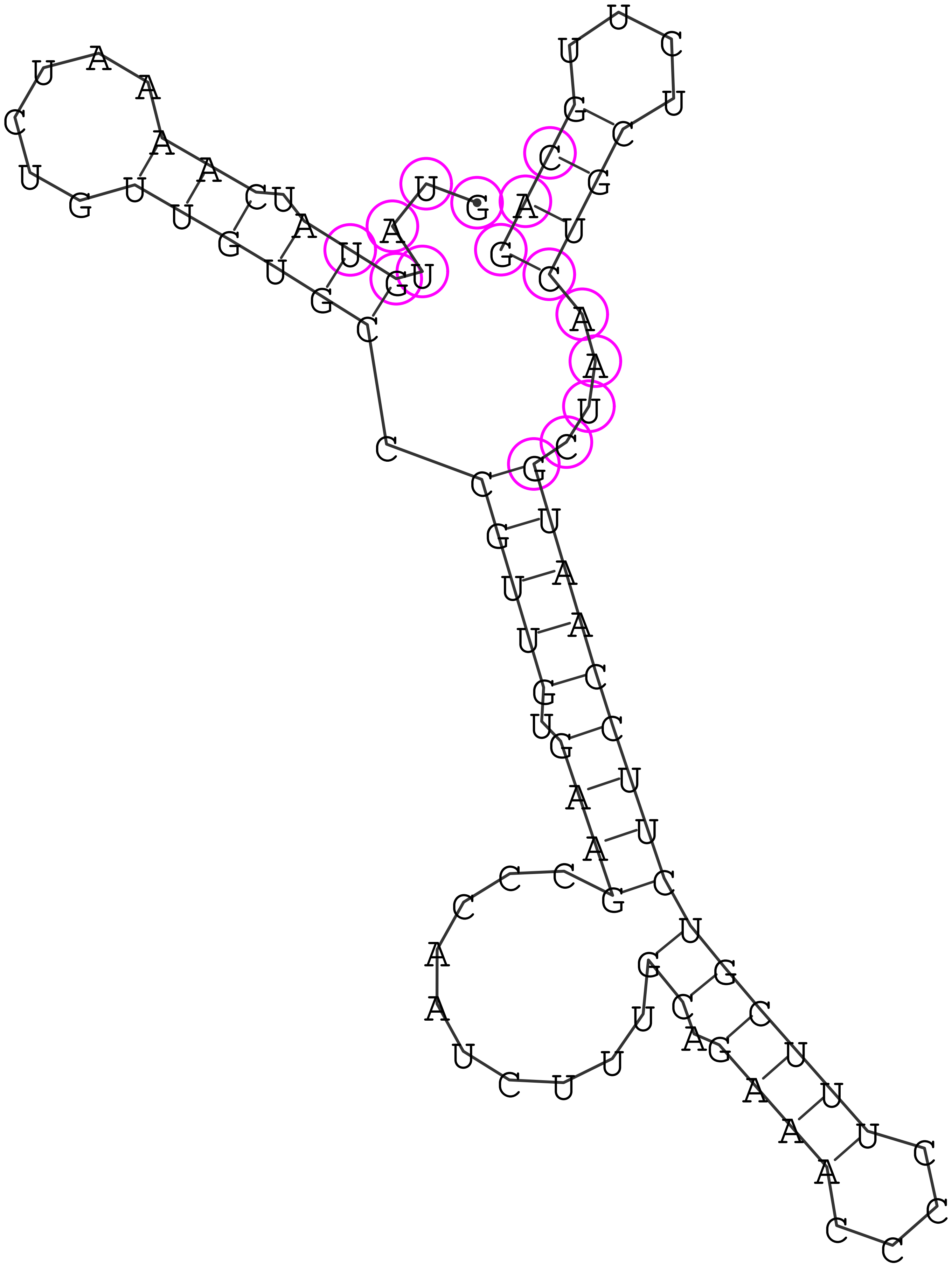

Xarbc0164A - Internal intron

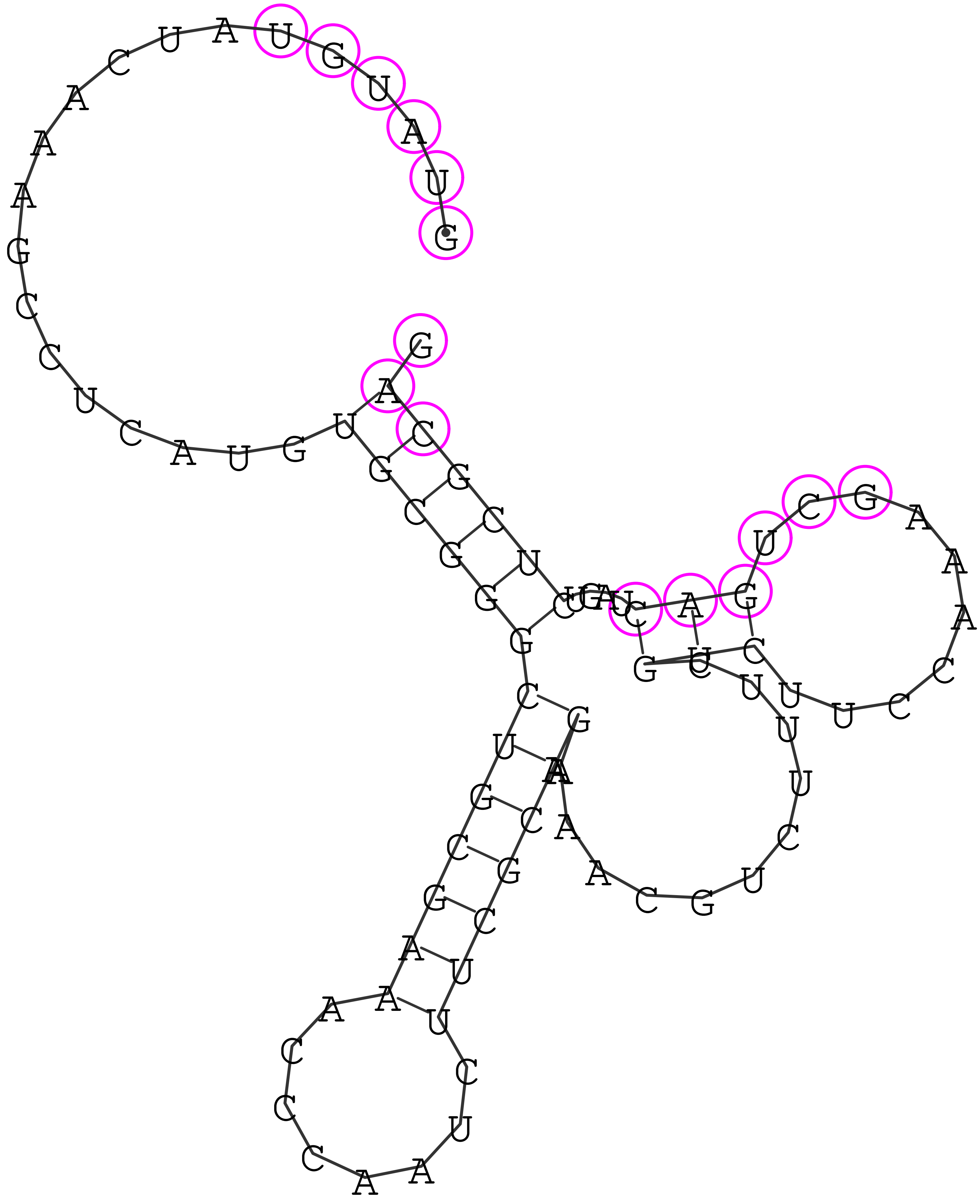

Xarbc0169A - Internal intron

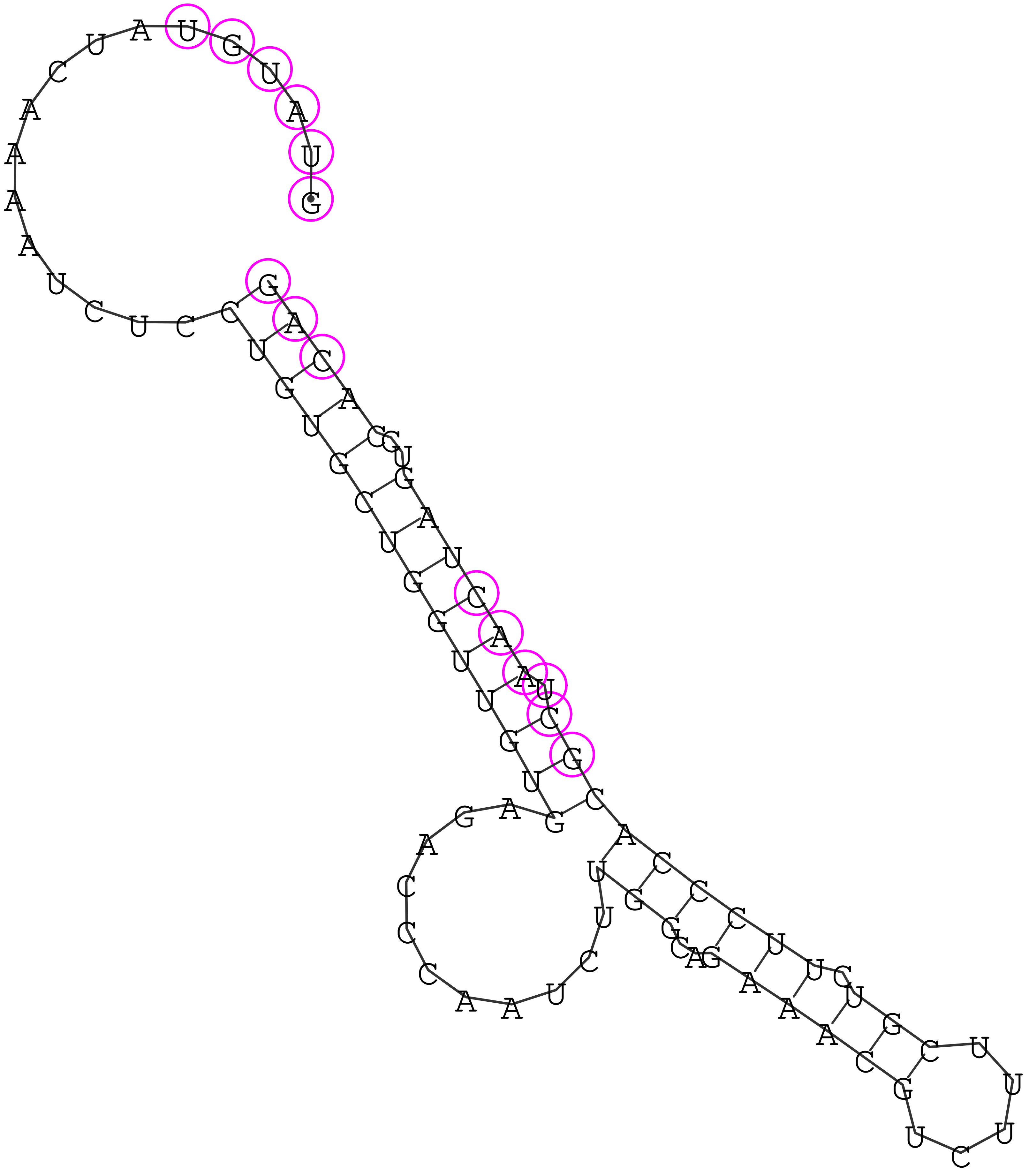

Xarbc0172A - Internal intron

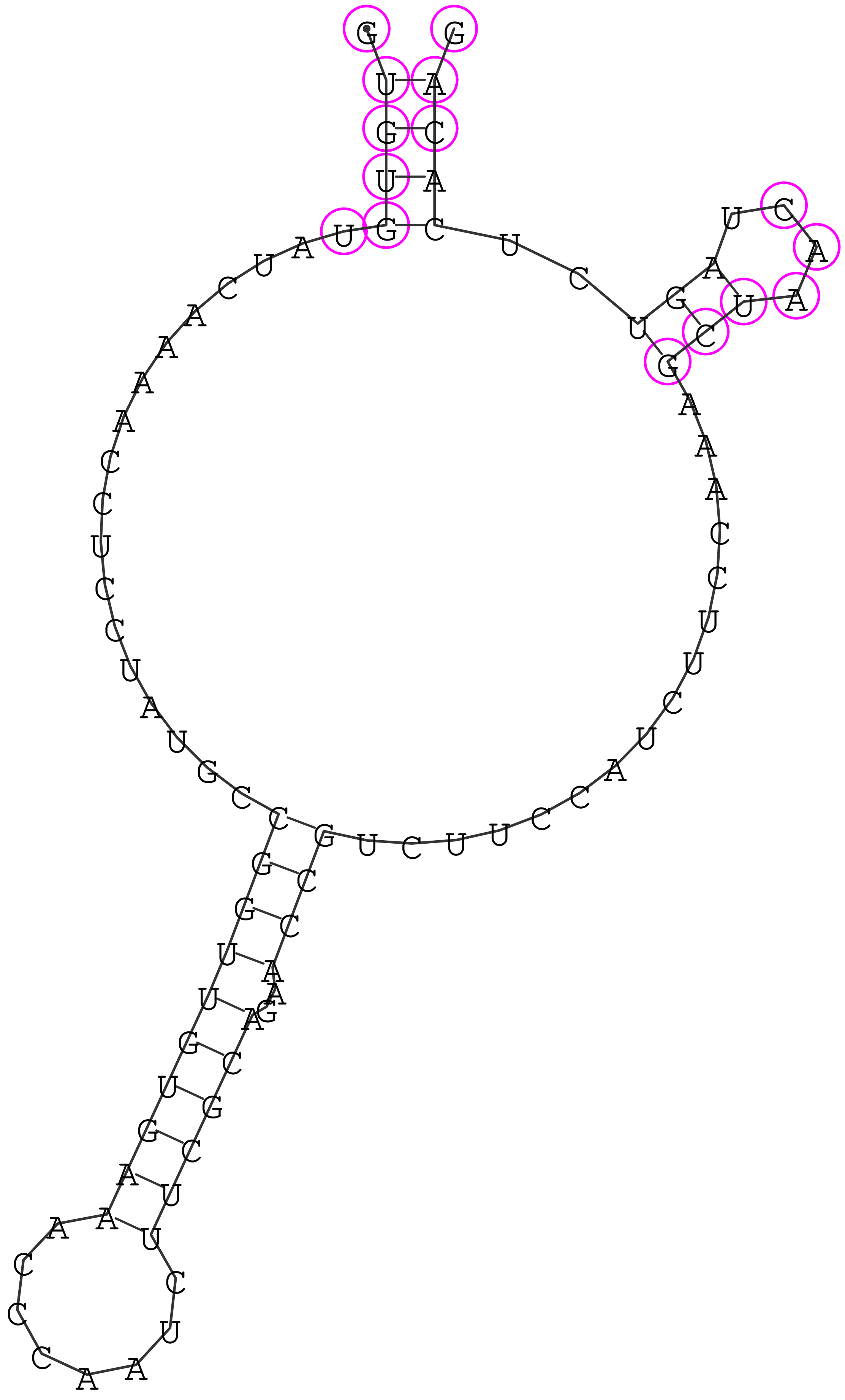

Xarbc0174A - Internal intron

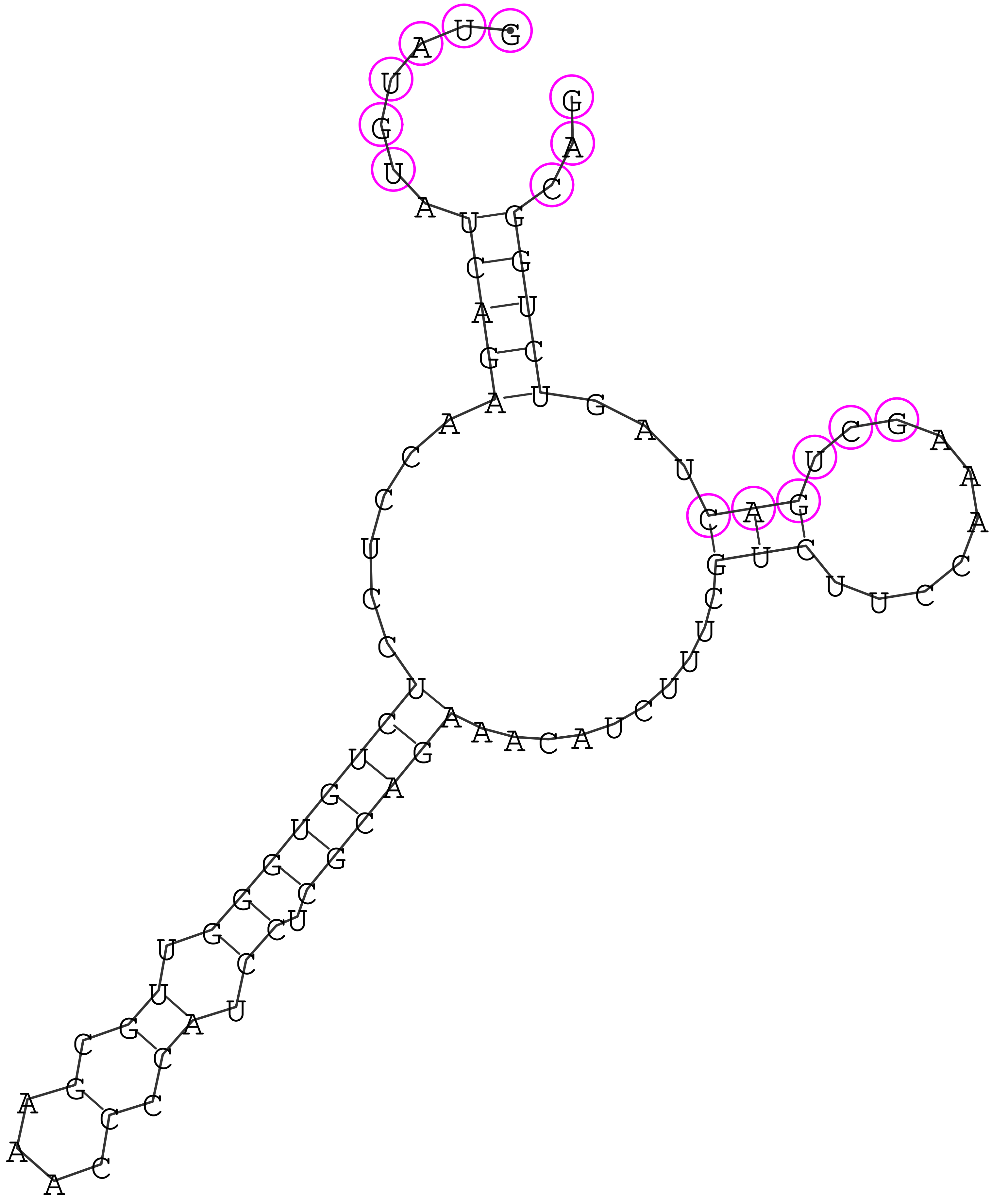

Xarbc0175A - Internal intron

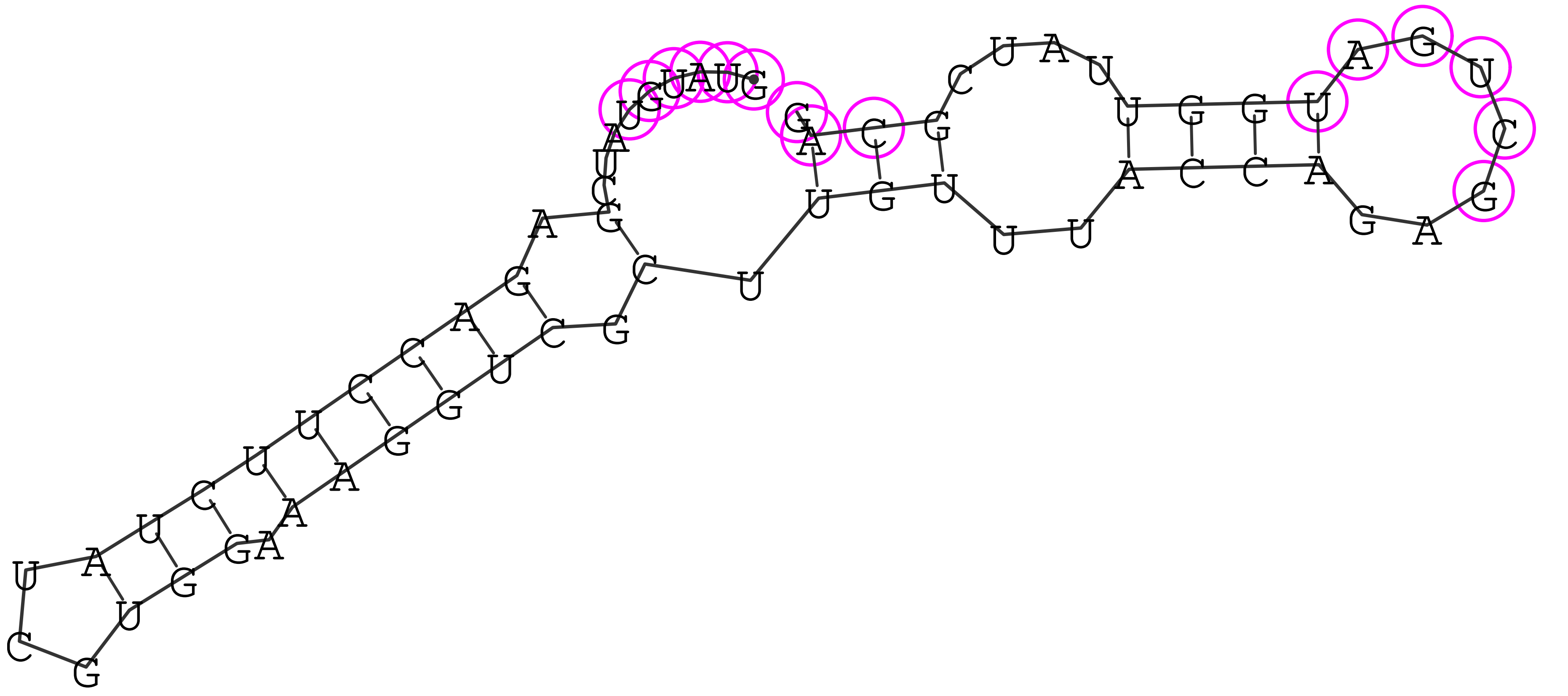

Xarbc0192A - Internal intron

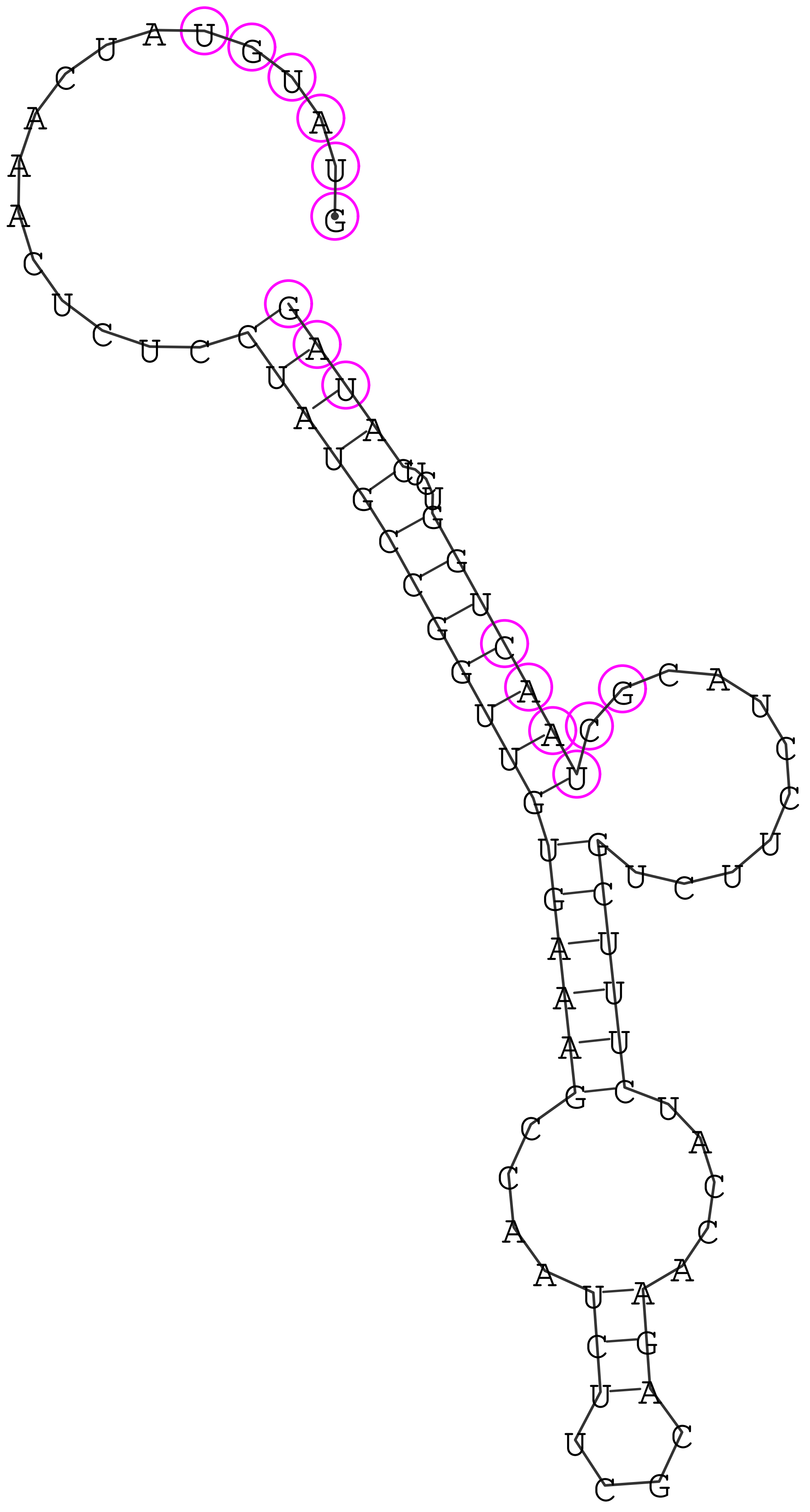

Xarbc0195A - Internal intron

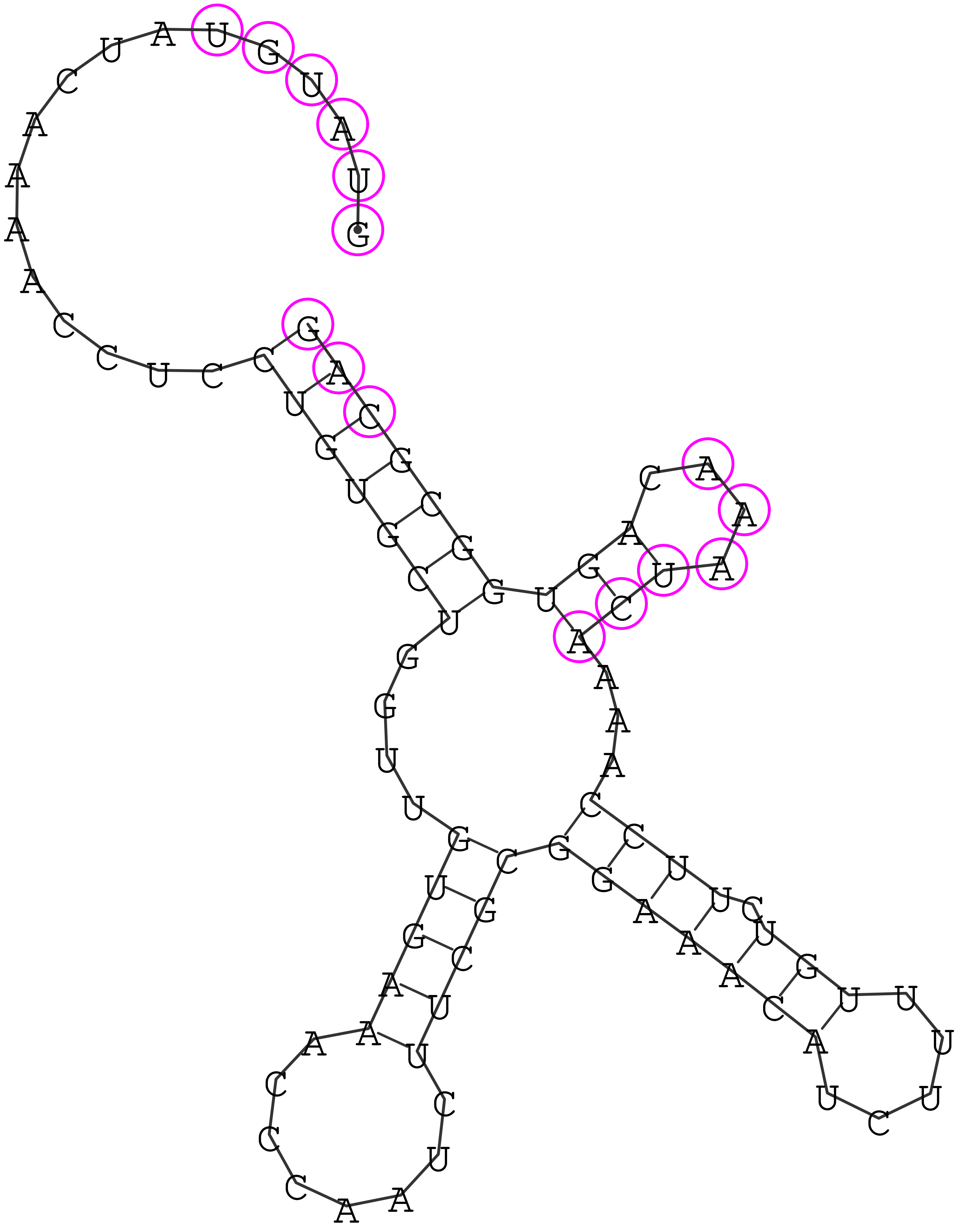

Xarbc0220A - Internal intron

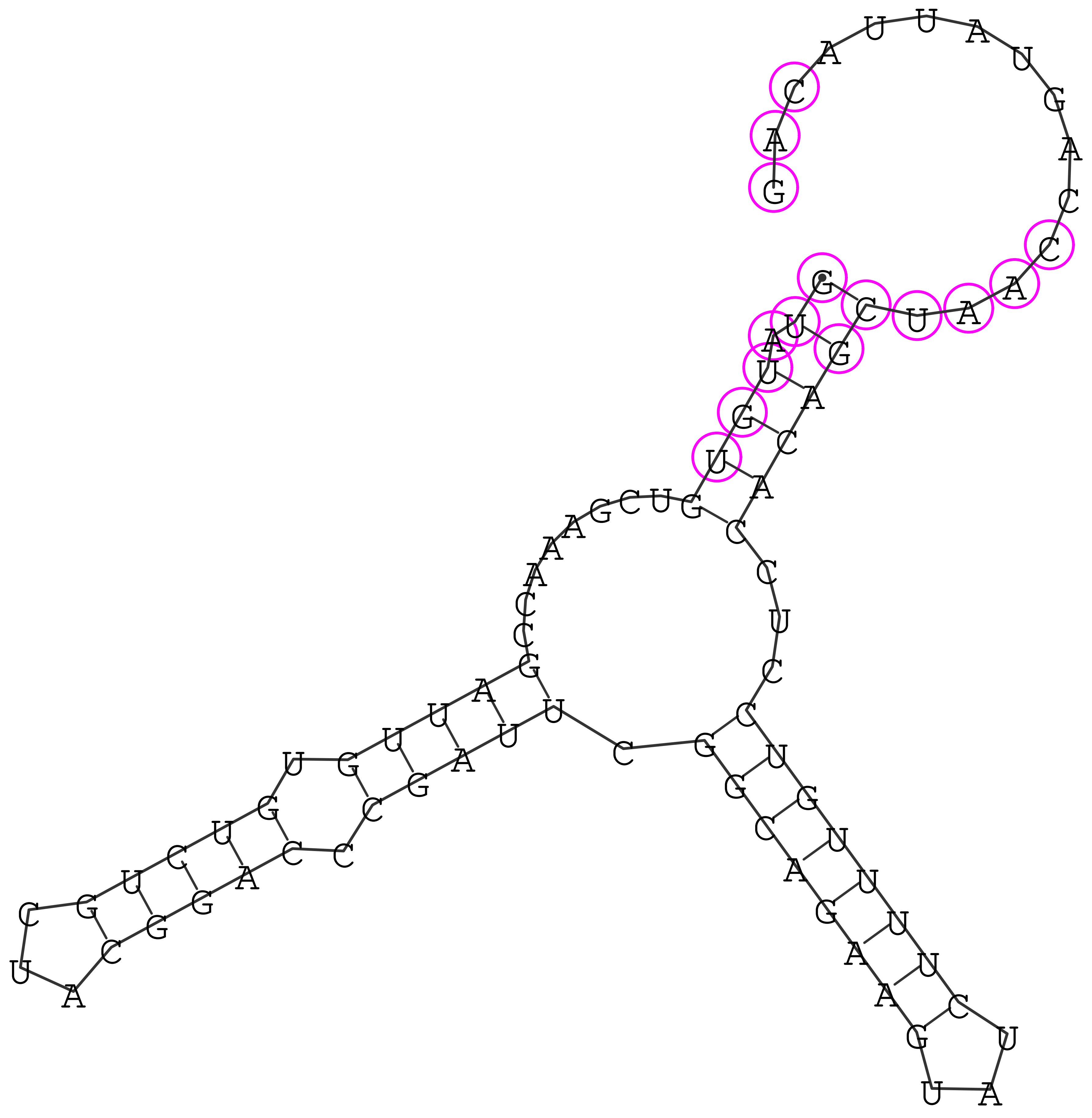

Xarbc0240A - Internal intron

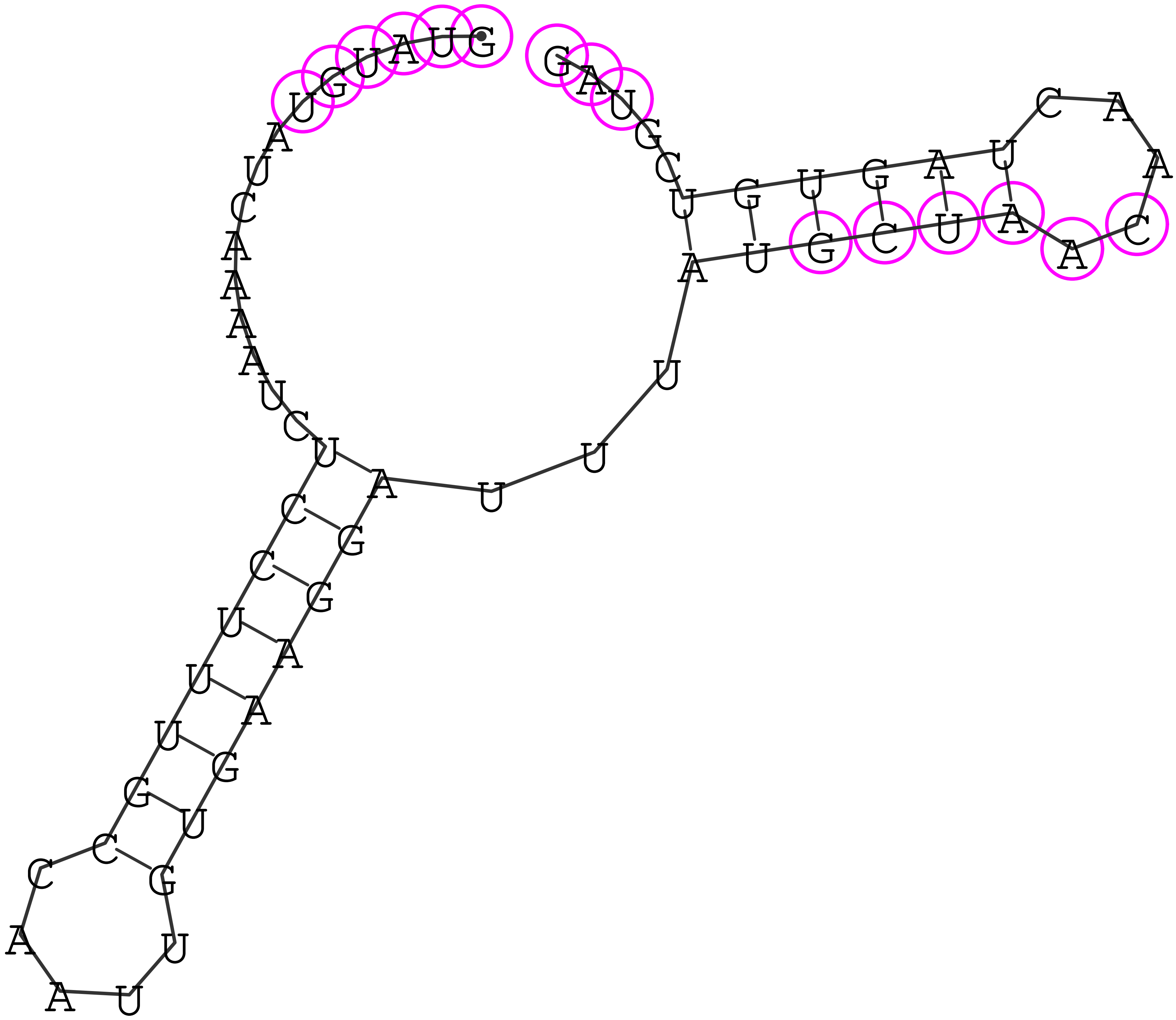

Xarbc0240B - Internal intron

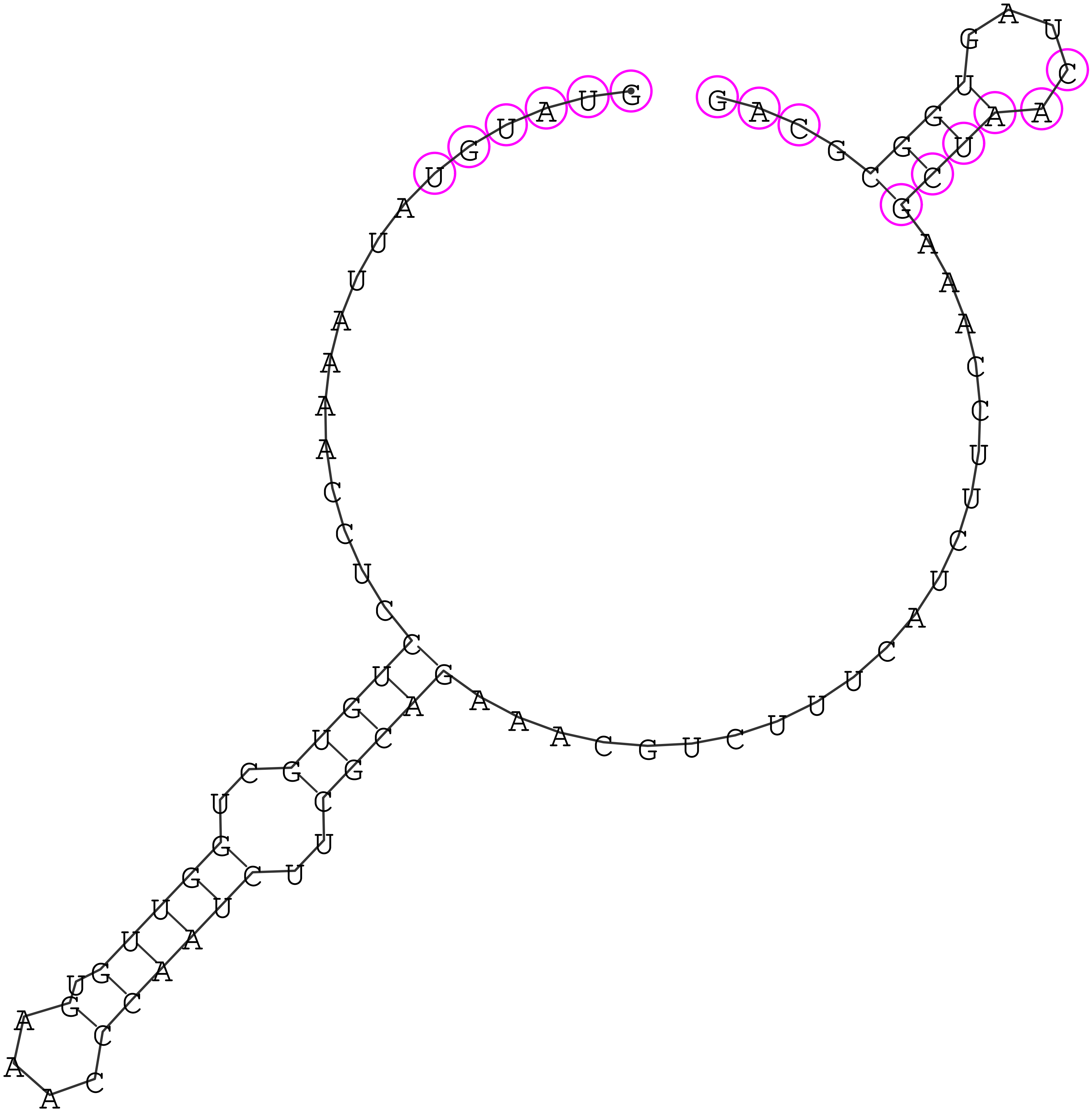

Xarbc0253A - Internal intron

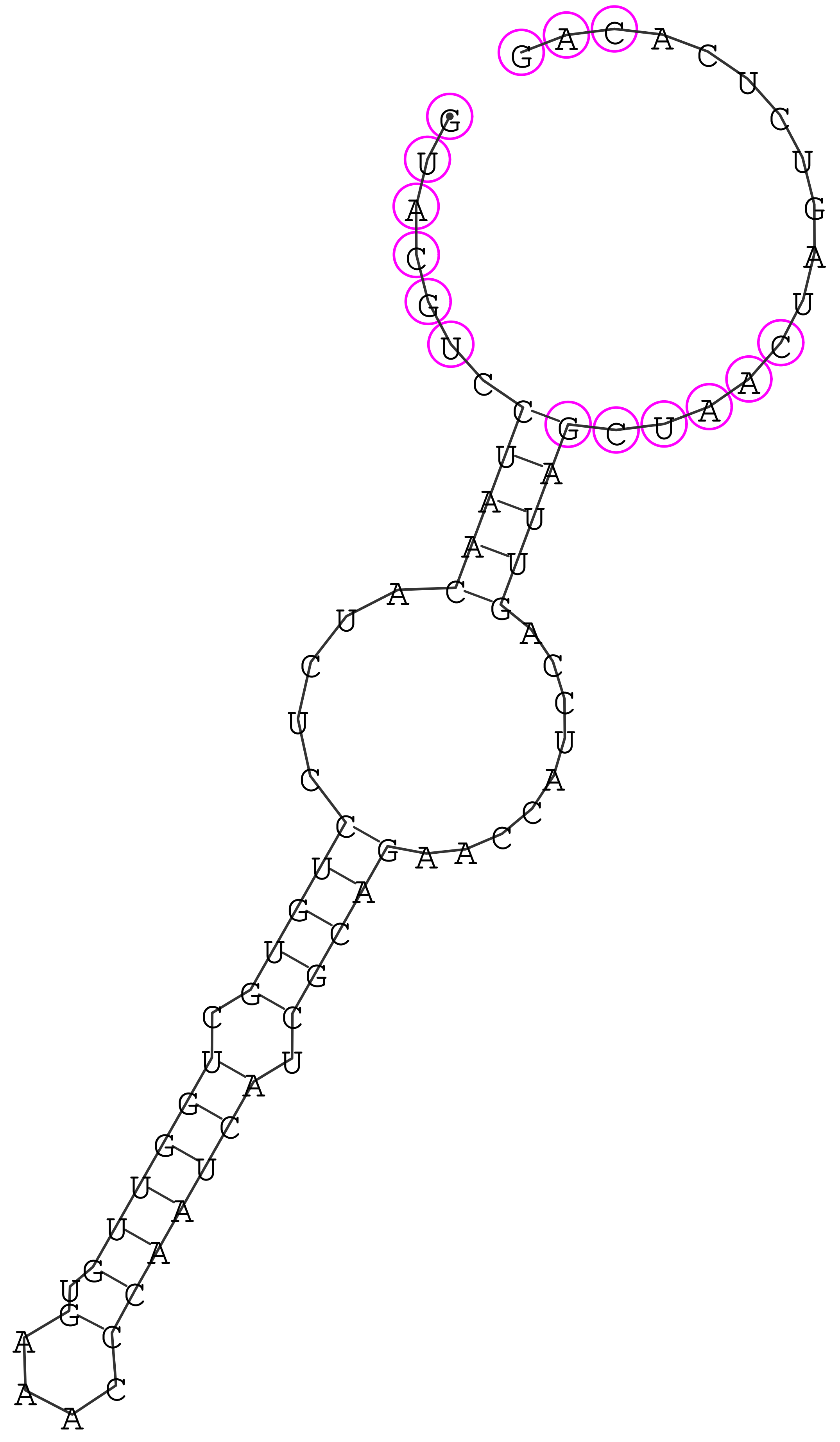

Xarbc0274A - Internal intron

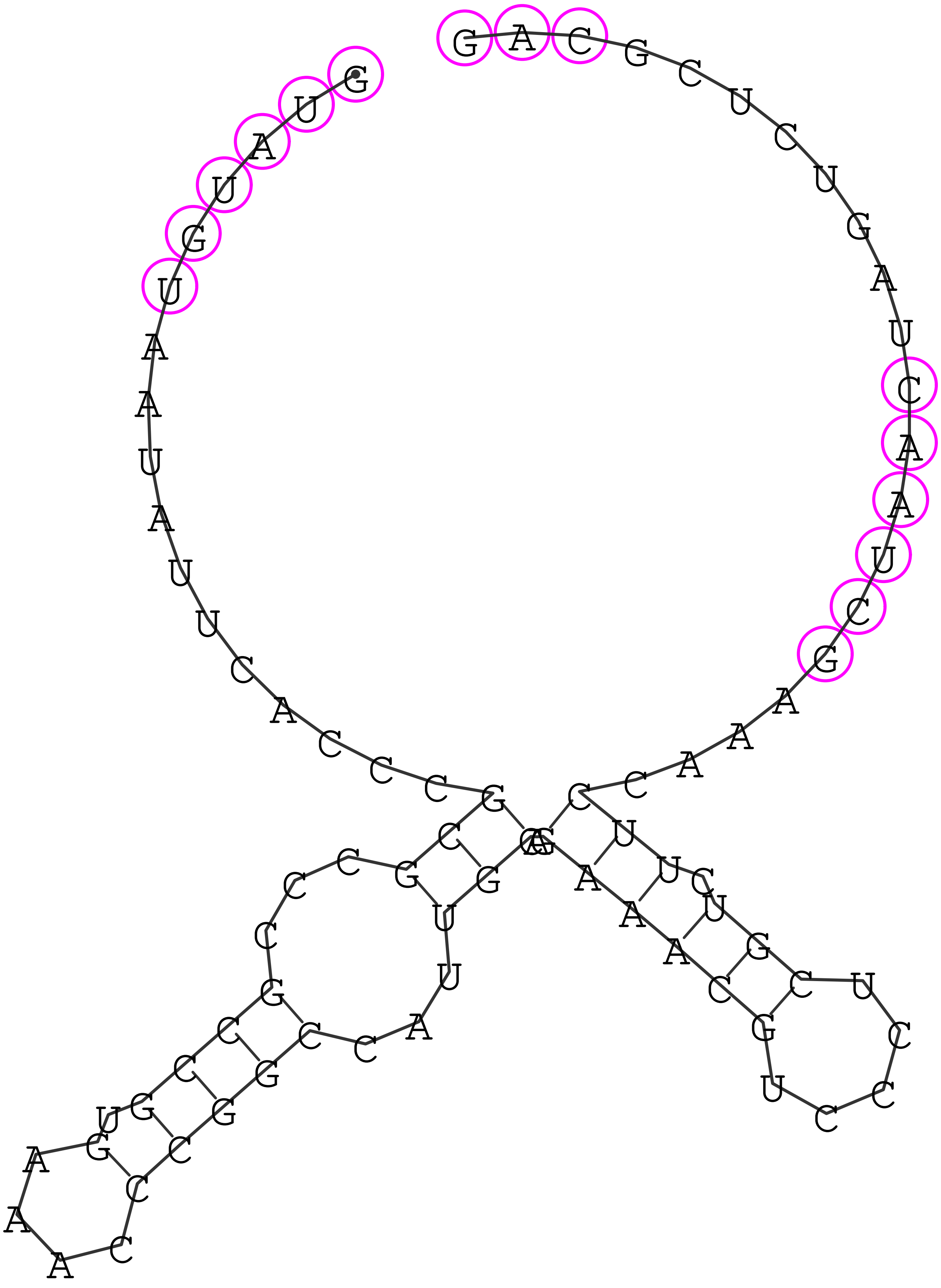

Xarbc0299A - Internal intron

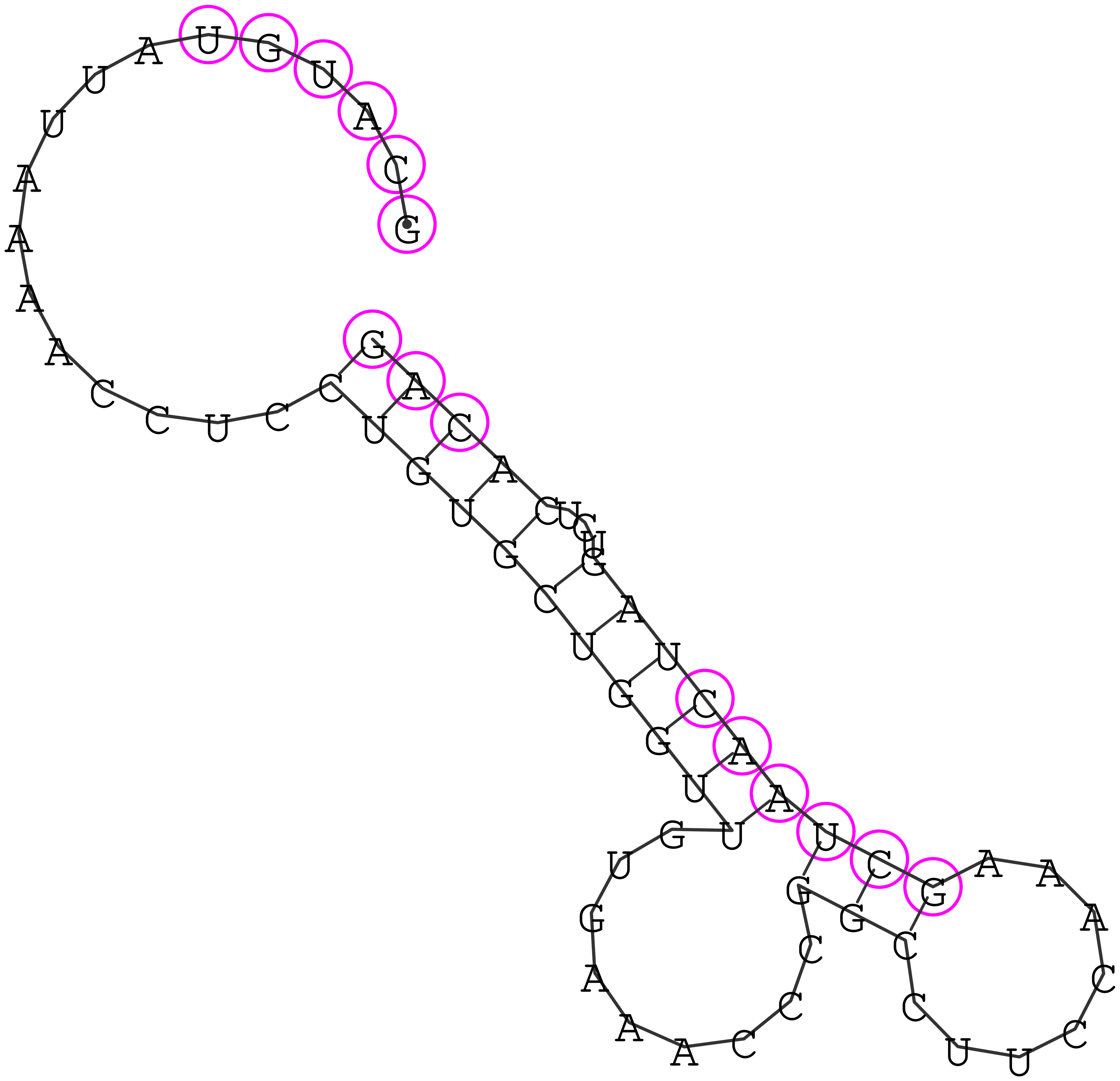

Xarbc0299B - Internal intron

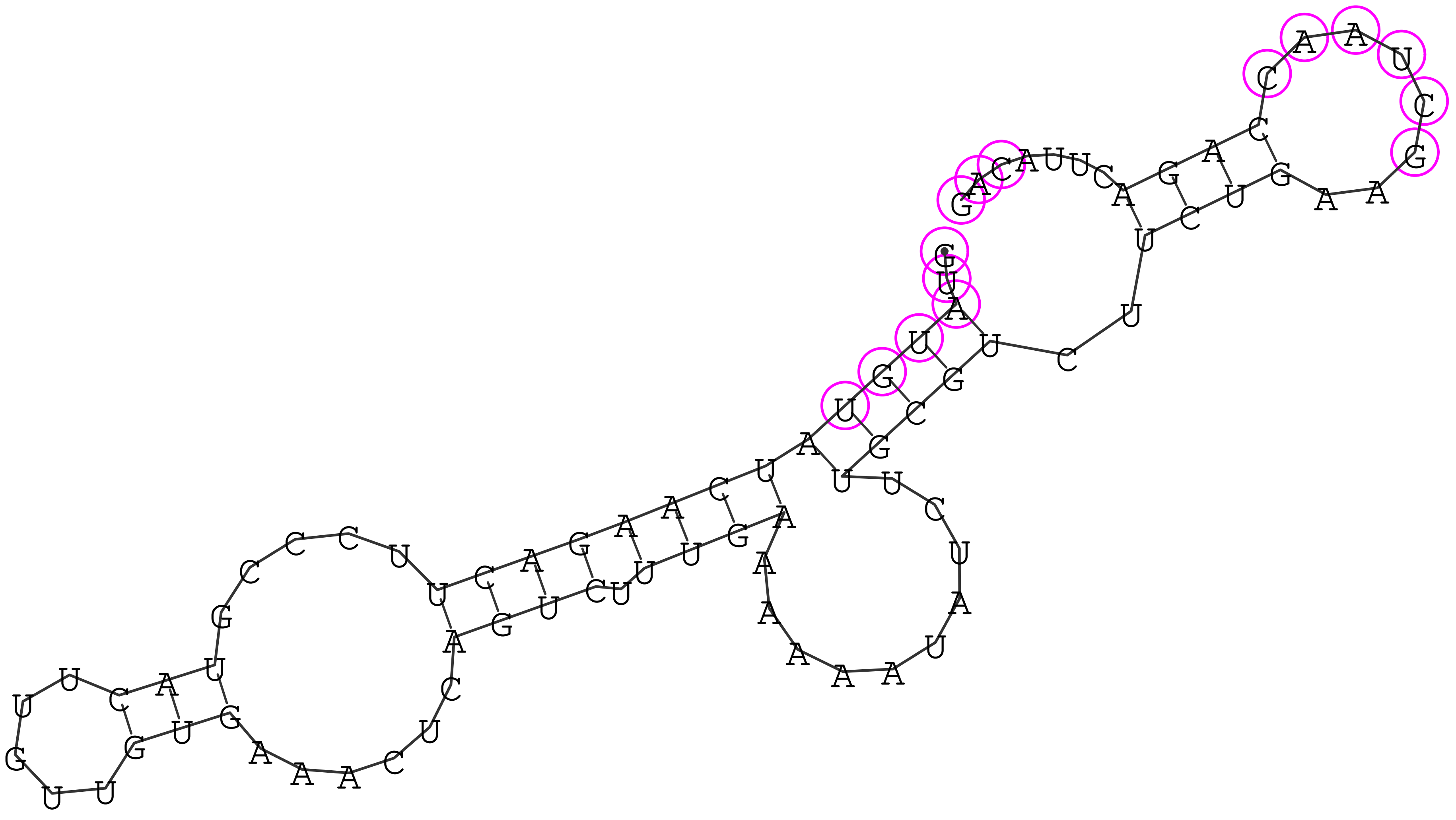

Xarbc0301A - Internal intron

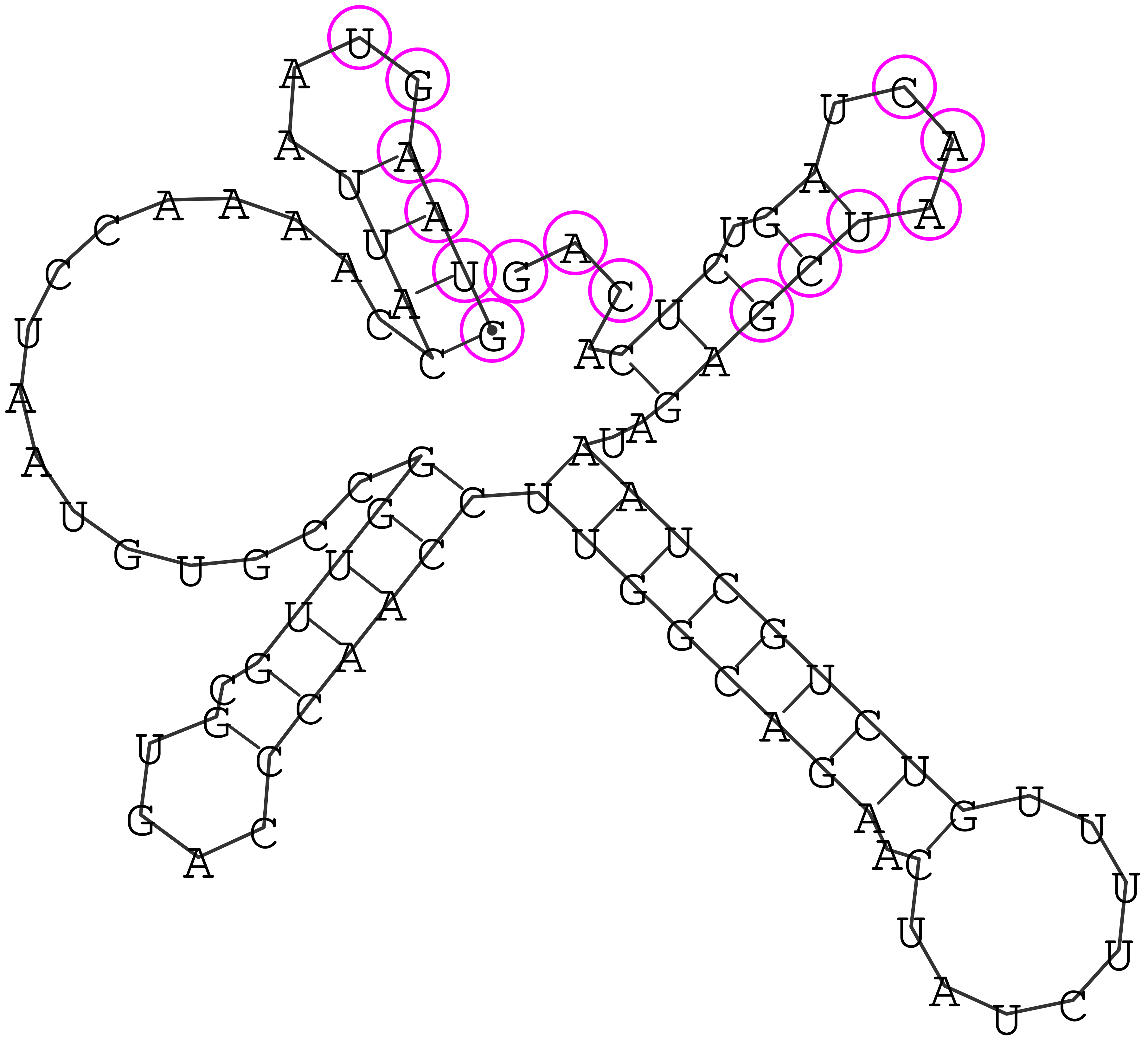

Xarbc0309A - Internal intron

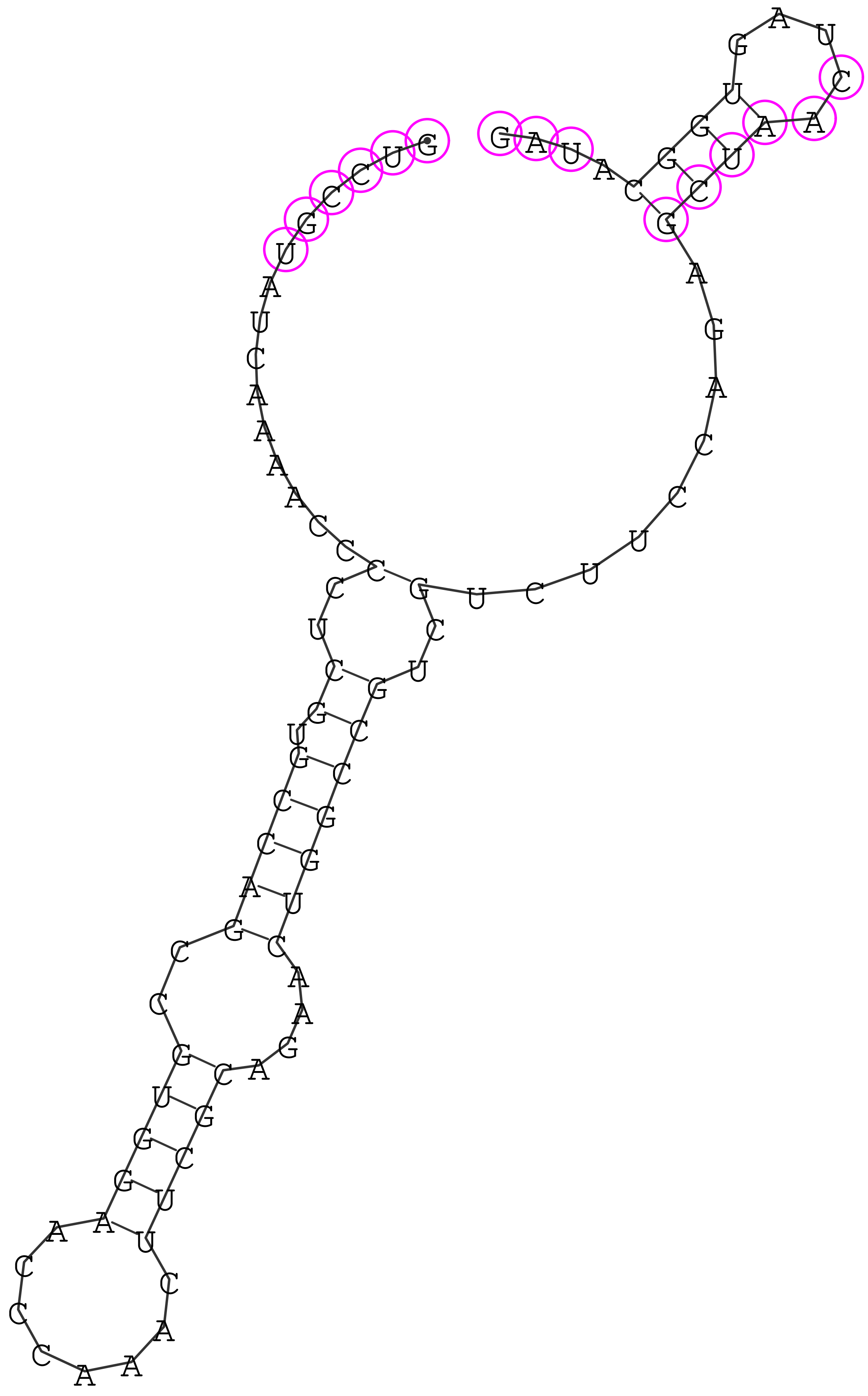

Xarbc0311A - Internal intron

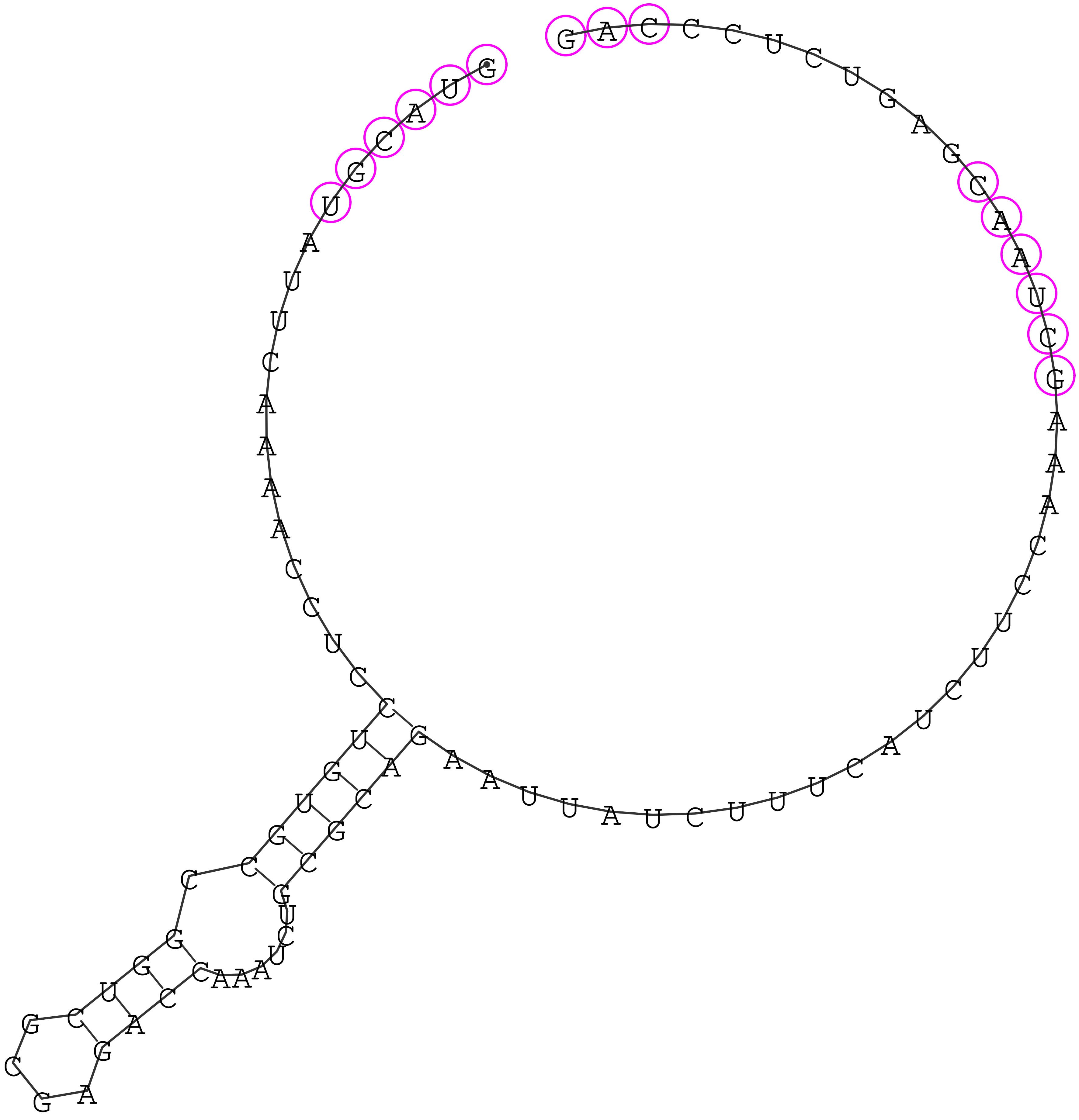

Xarbc0324A - Internal intron

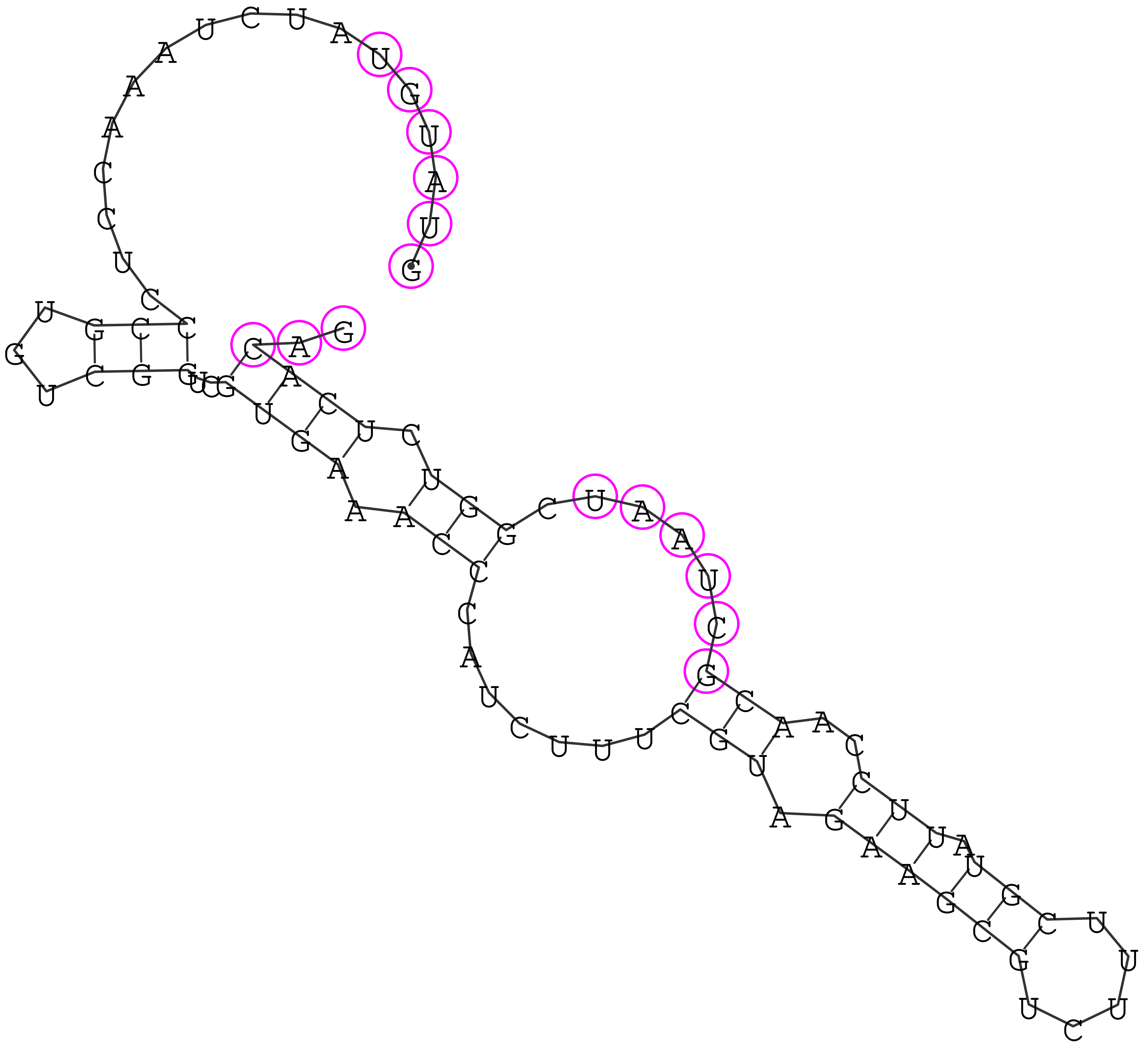

Xarbc0447A - Internal intron

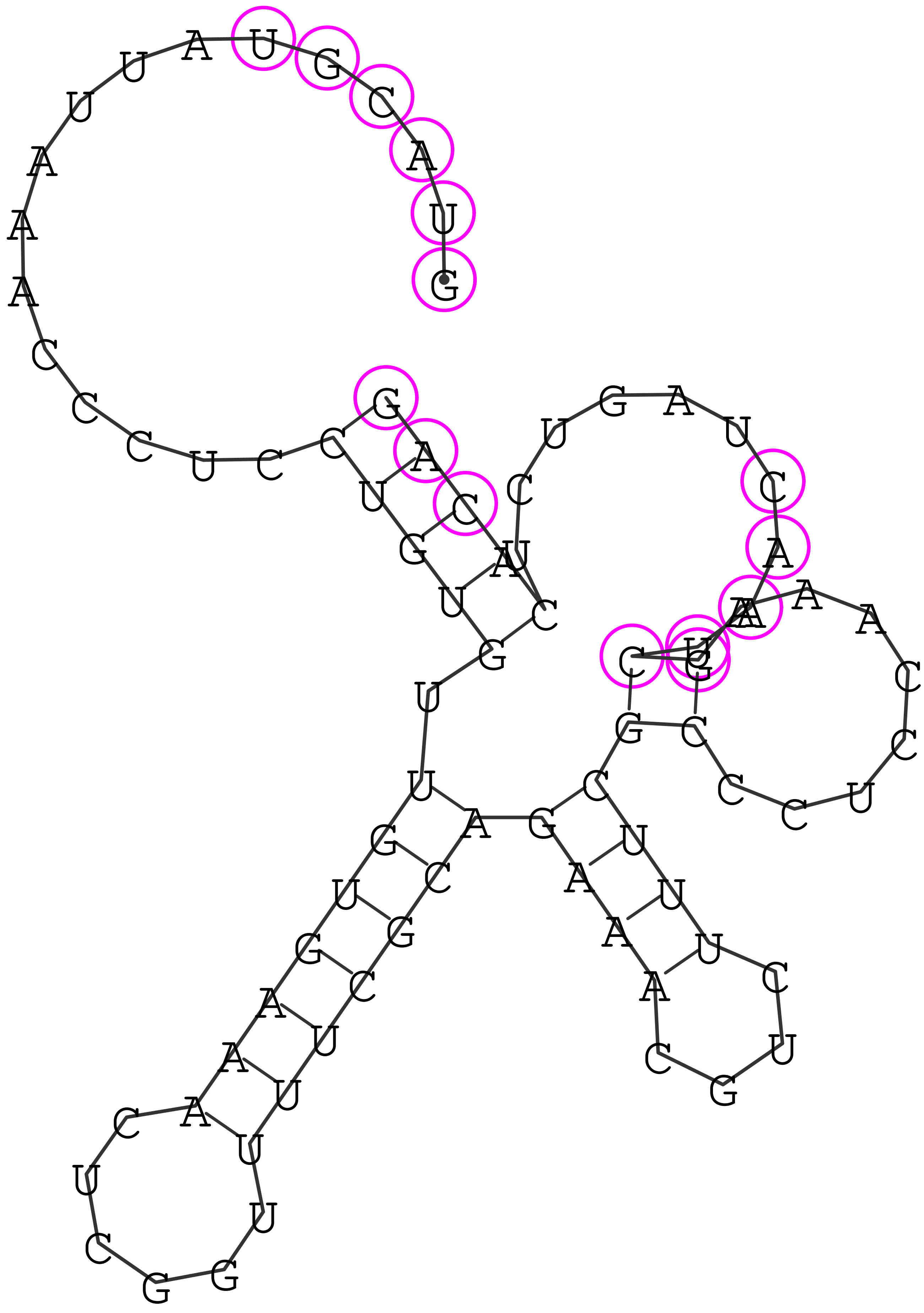

Xbamc009A - Internal intron

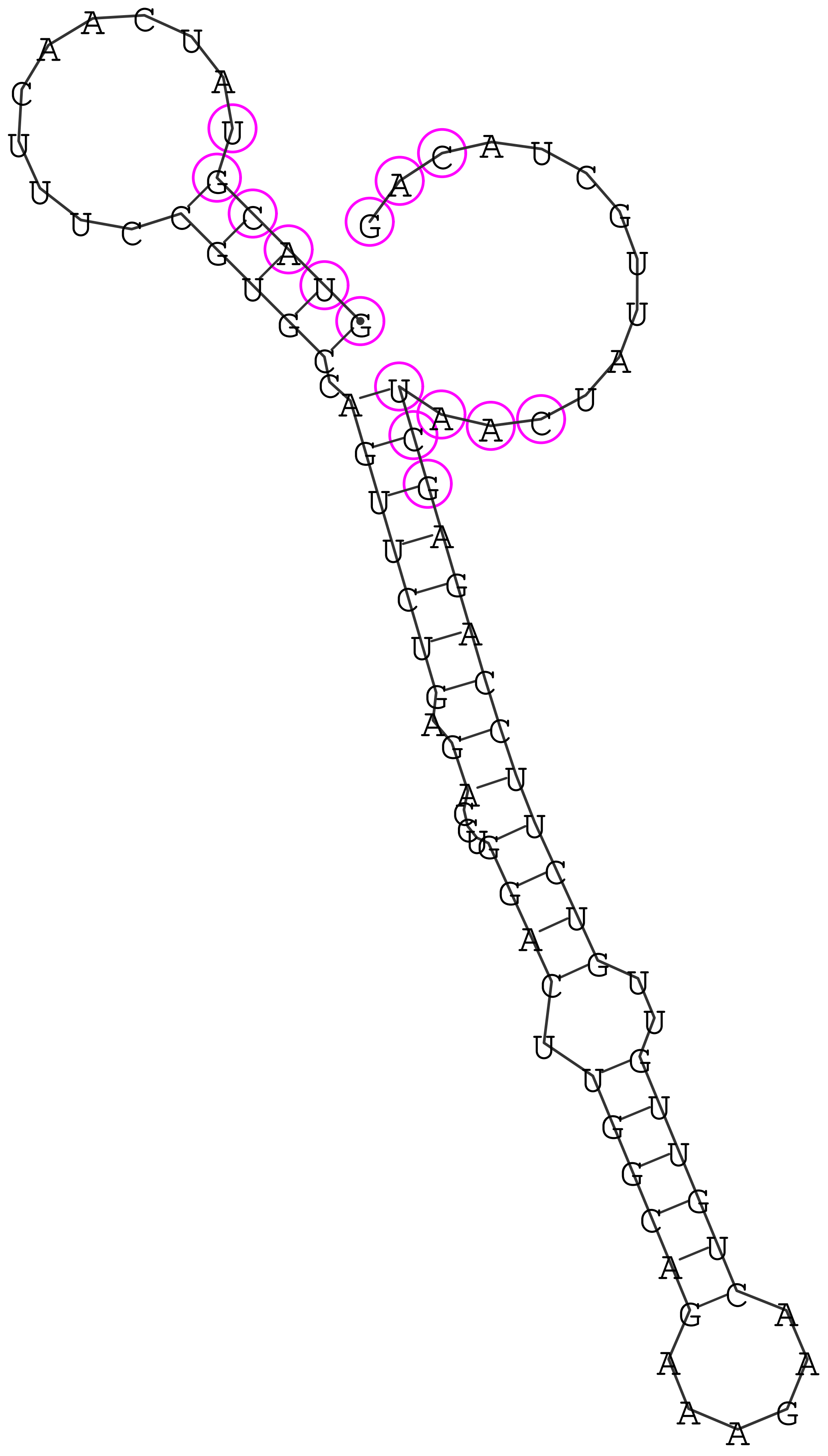

Xbamc015A - Internal intron

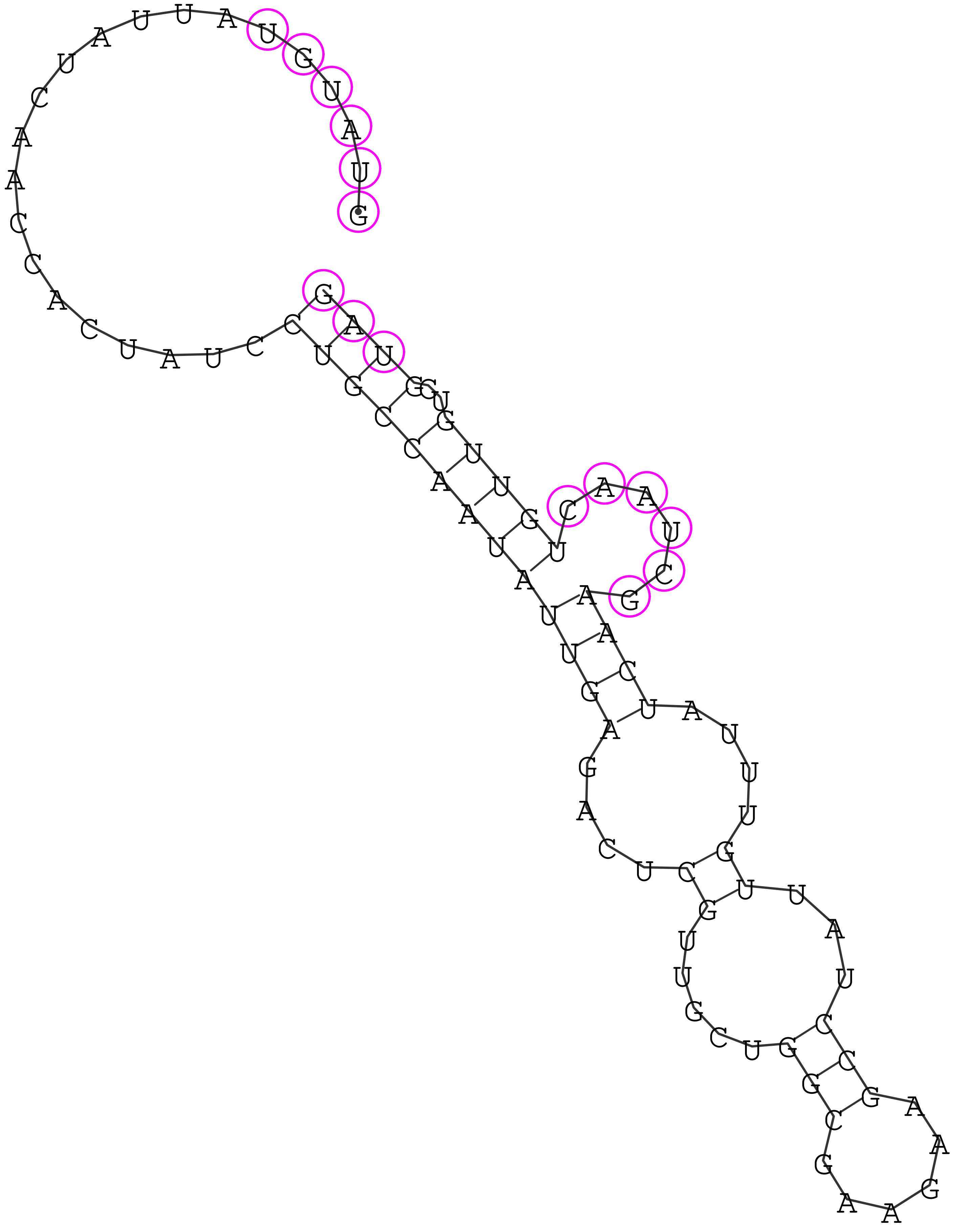

Xbamc019A - Internal intron

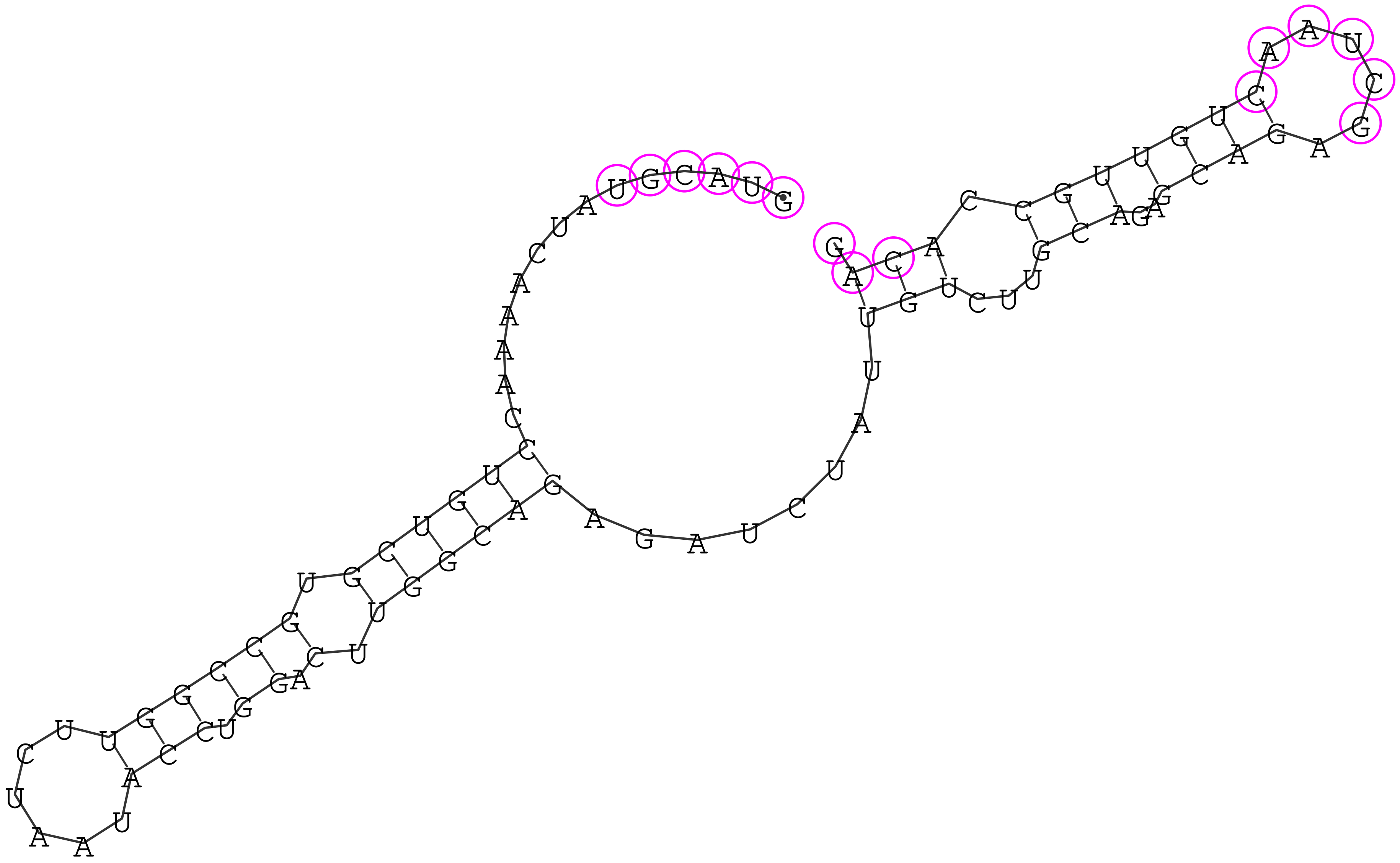

Xbamc020A - Internal intron

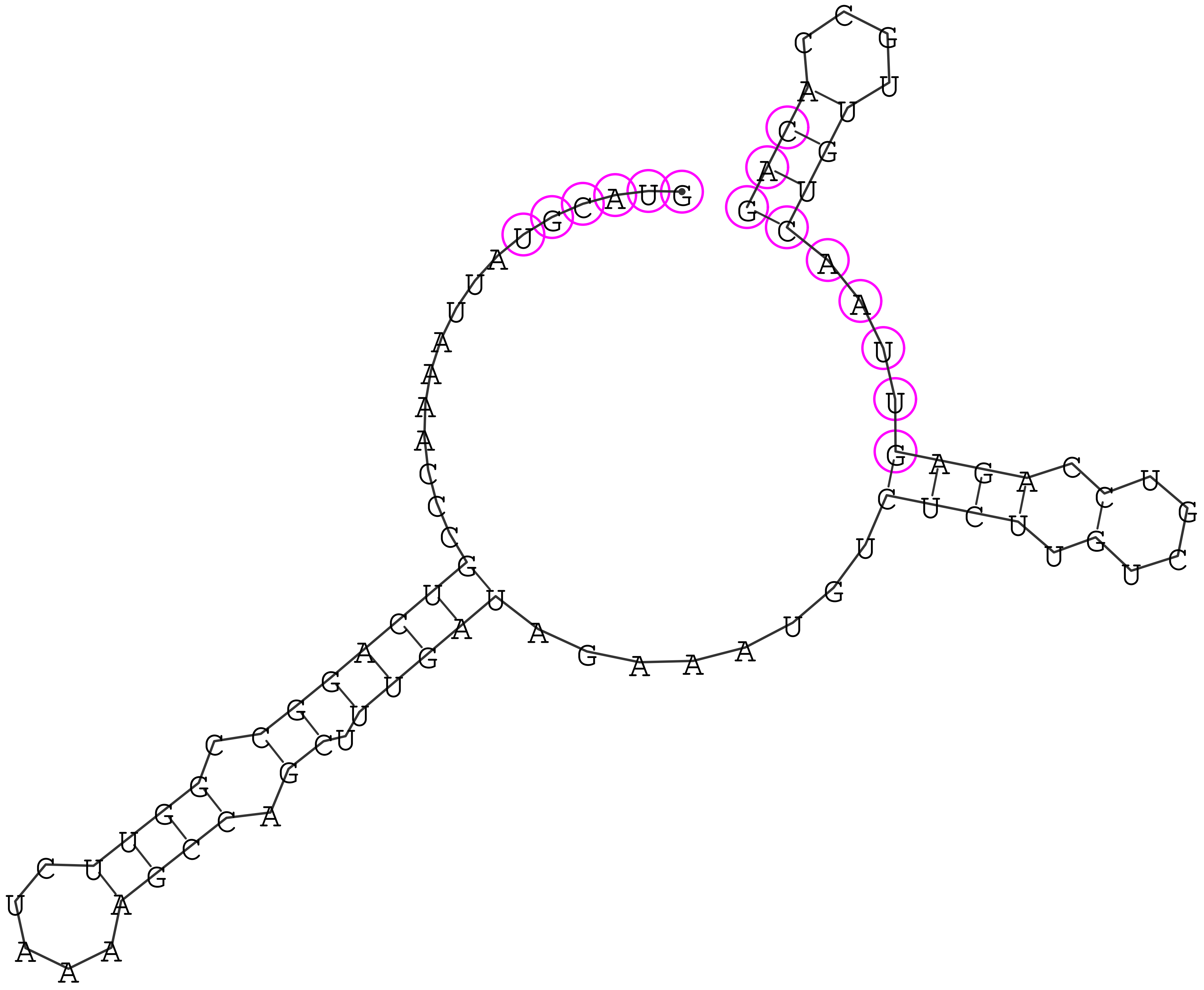

Xbamc022A - Internal intron

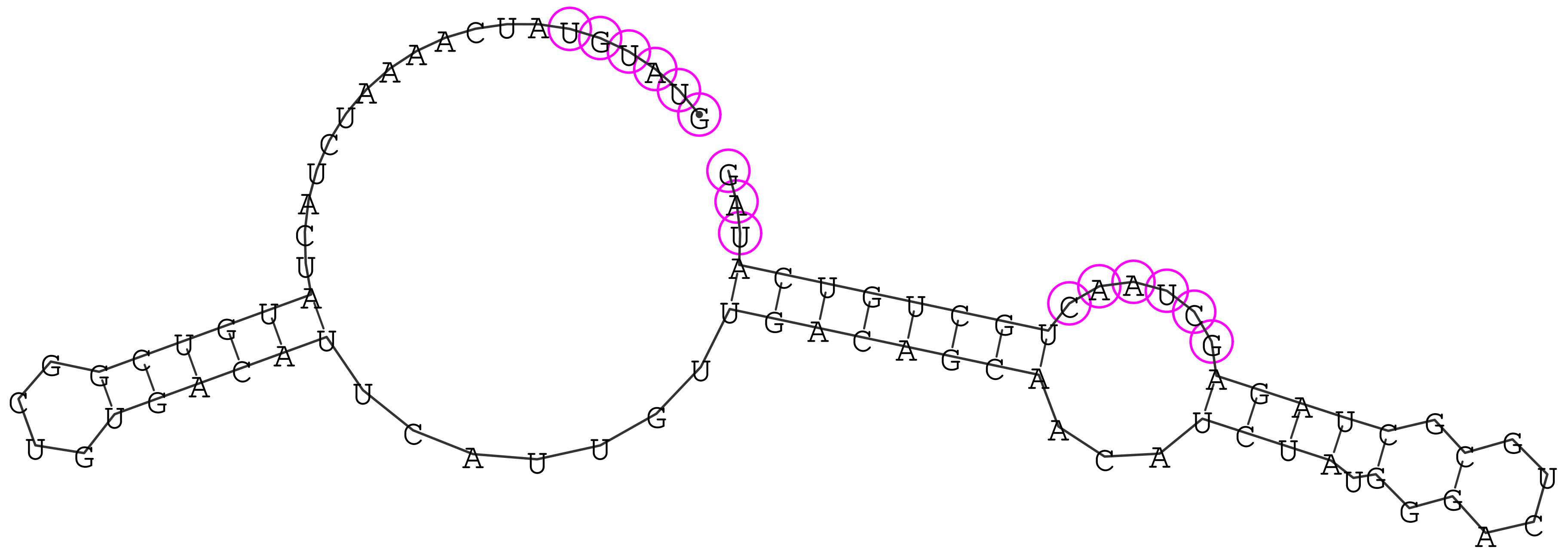

Xbamc024A - Internal intron

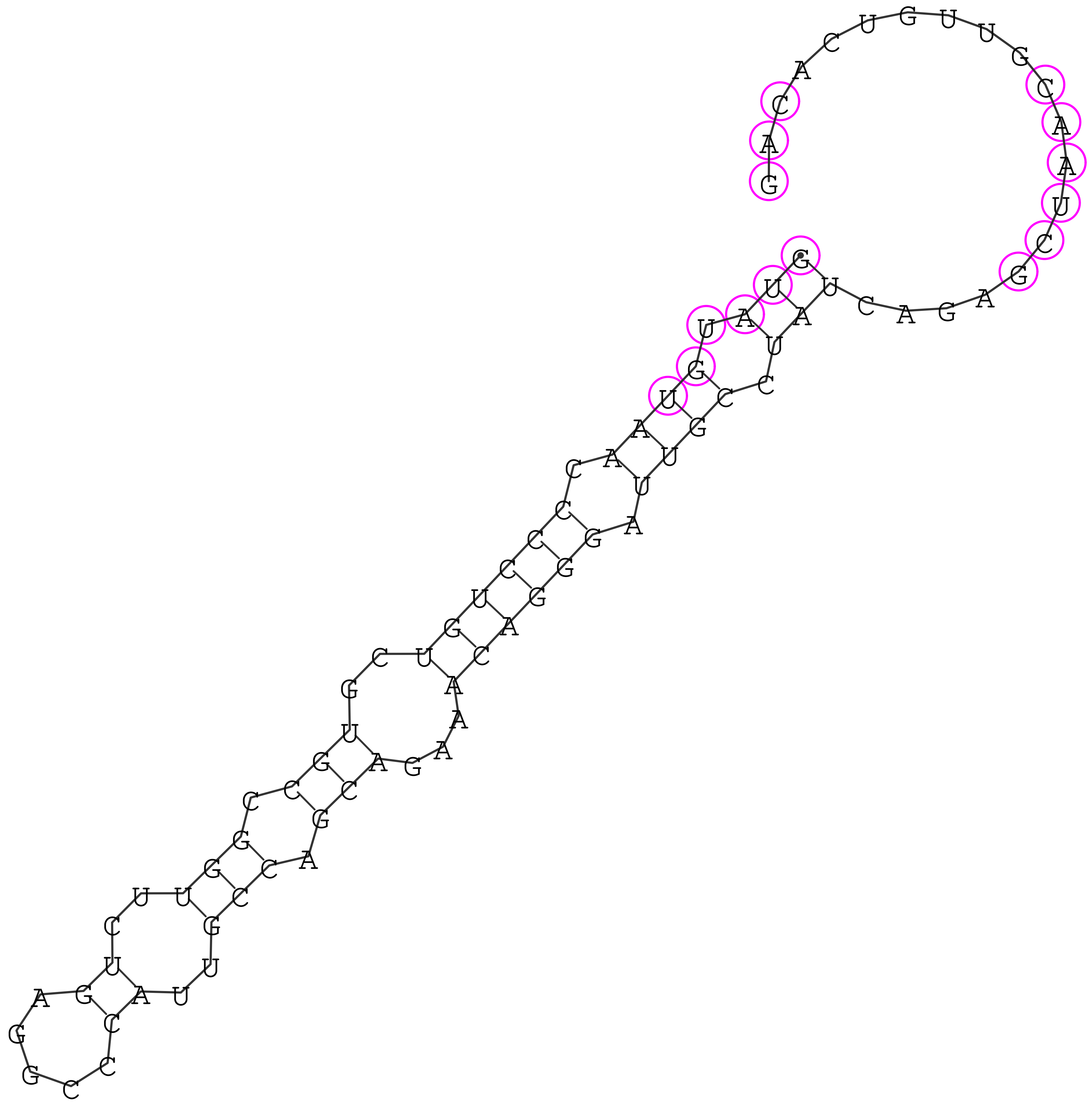

Xbamc025A - Internal intron

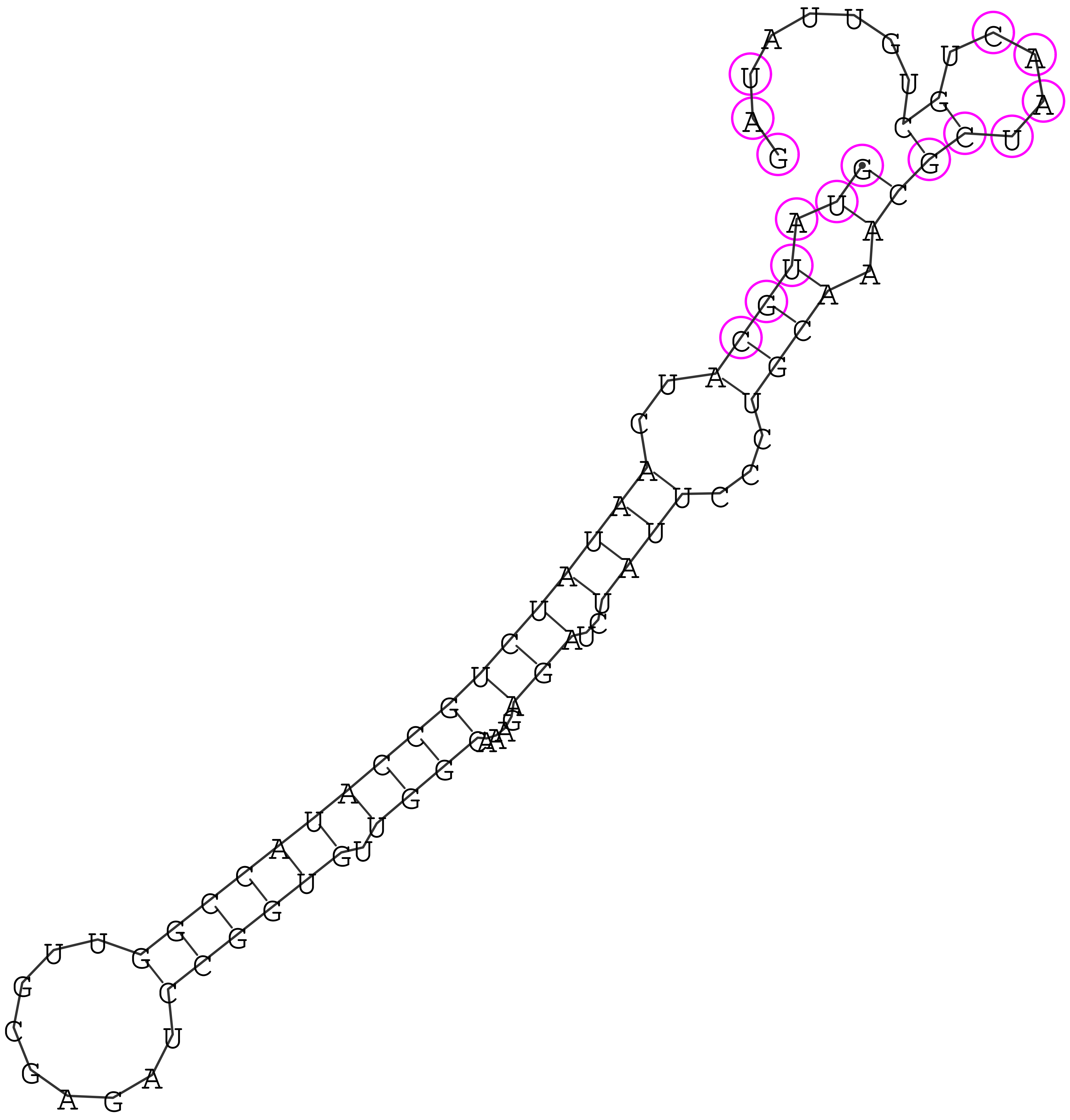

Xbamc027A - Internal intron

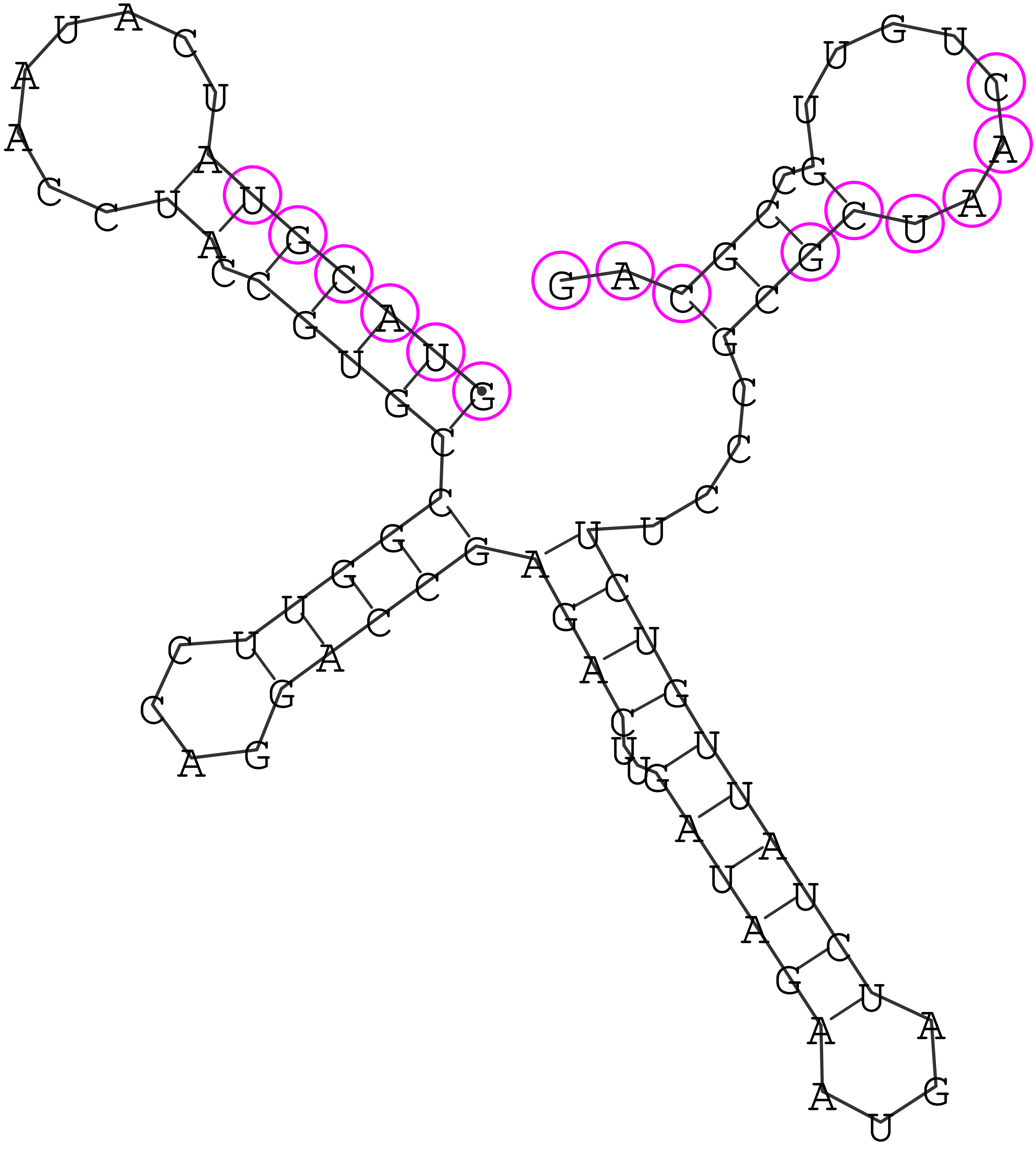

Xbamc027B - Internal intron

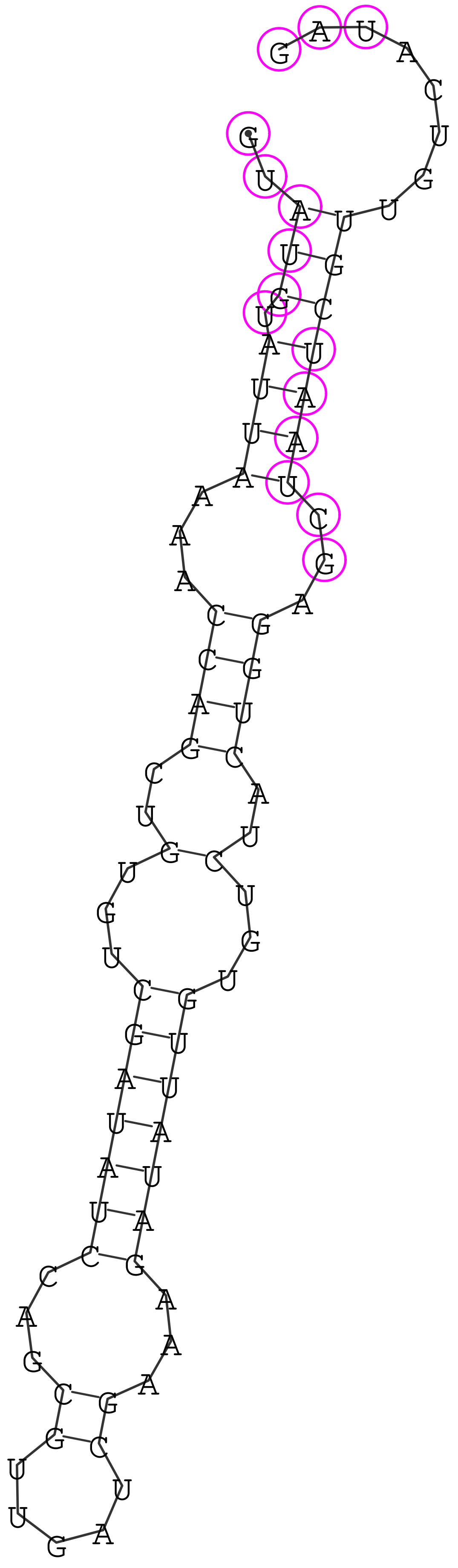

Xbamc027C - Internal intron

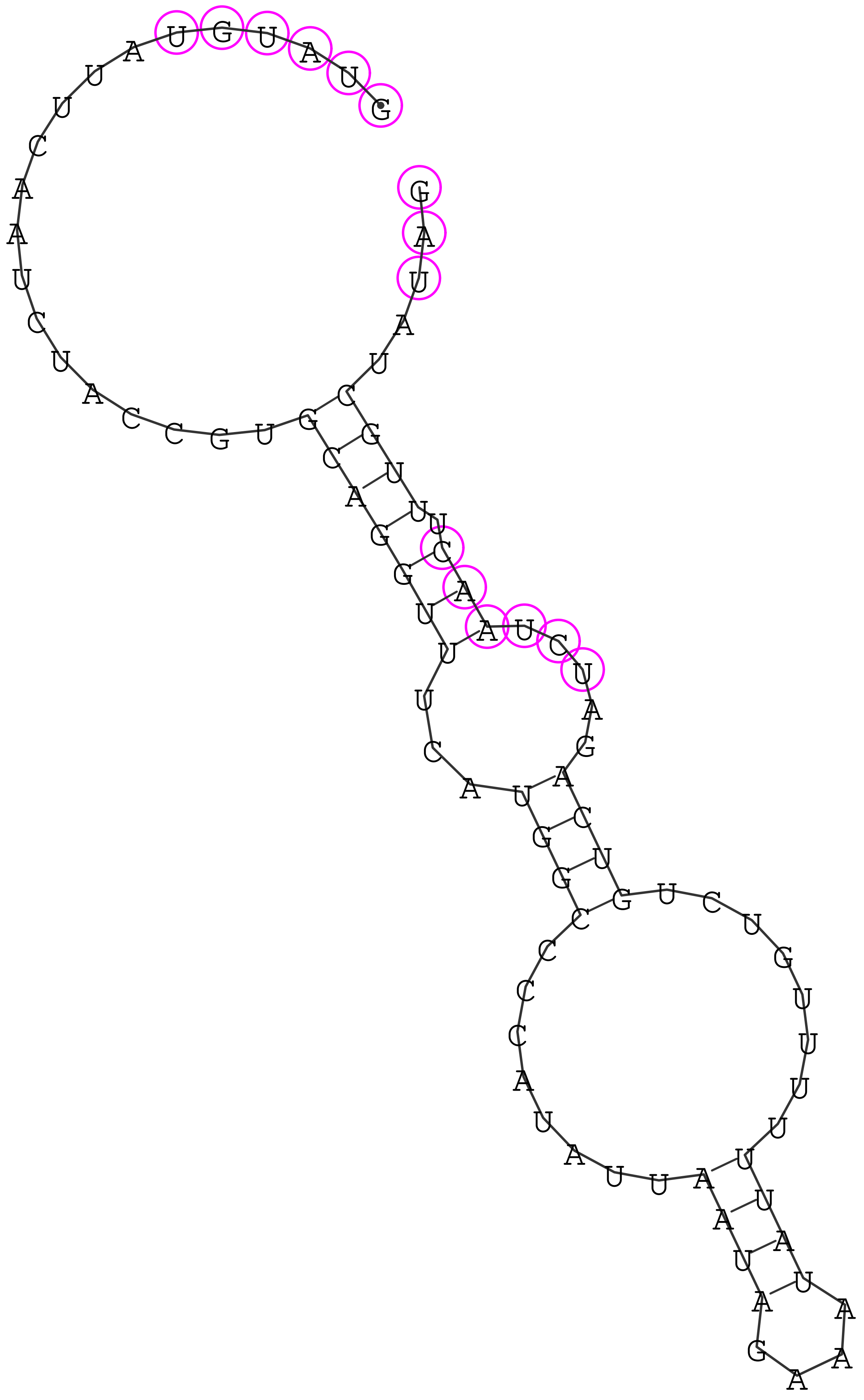

Xbamc040A - Internal intron

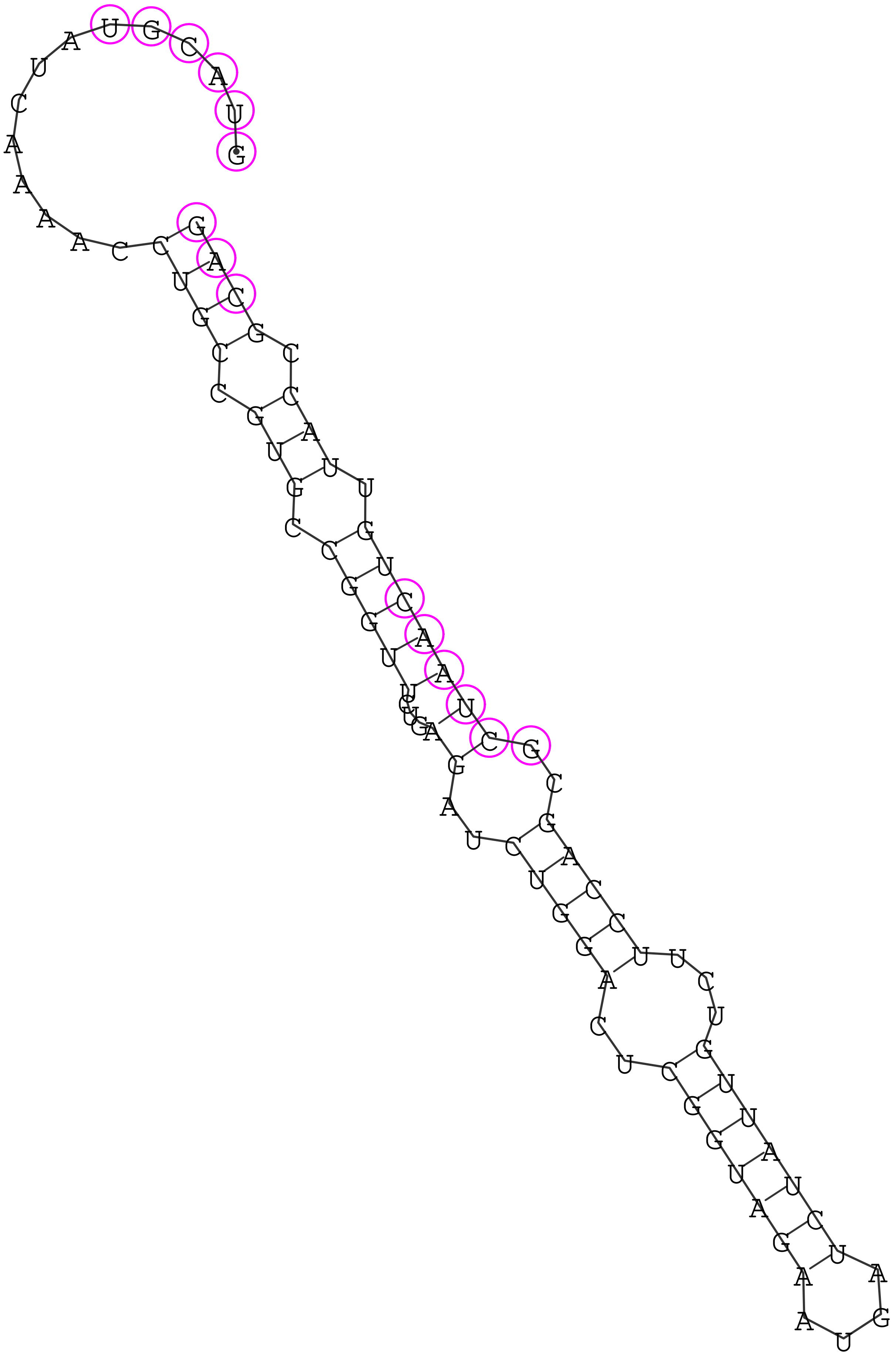

Xbamc041A - Internal intron

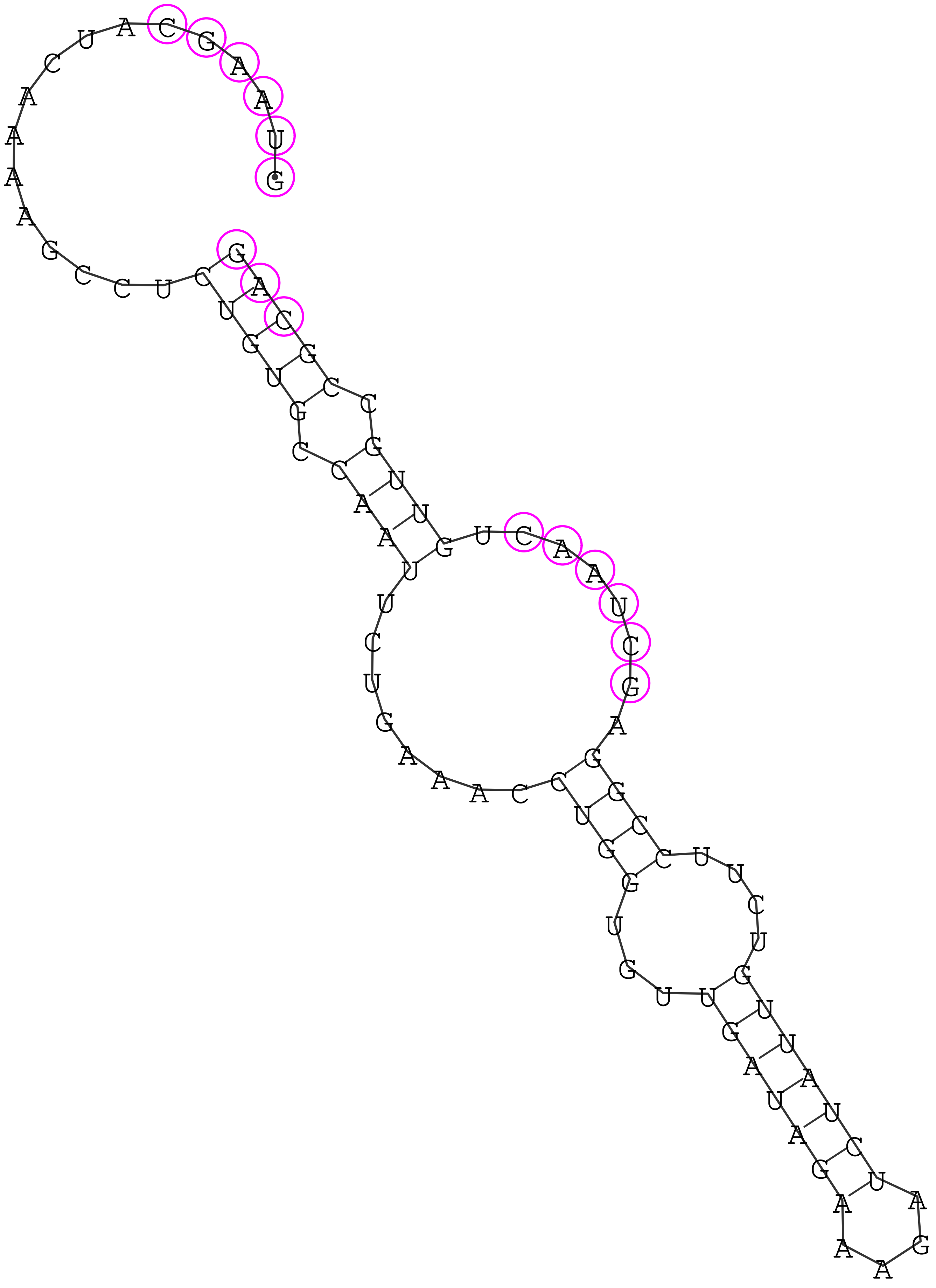

Xbamc051A - Internal intron

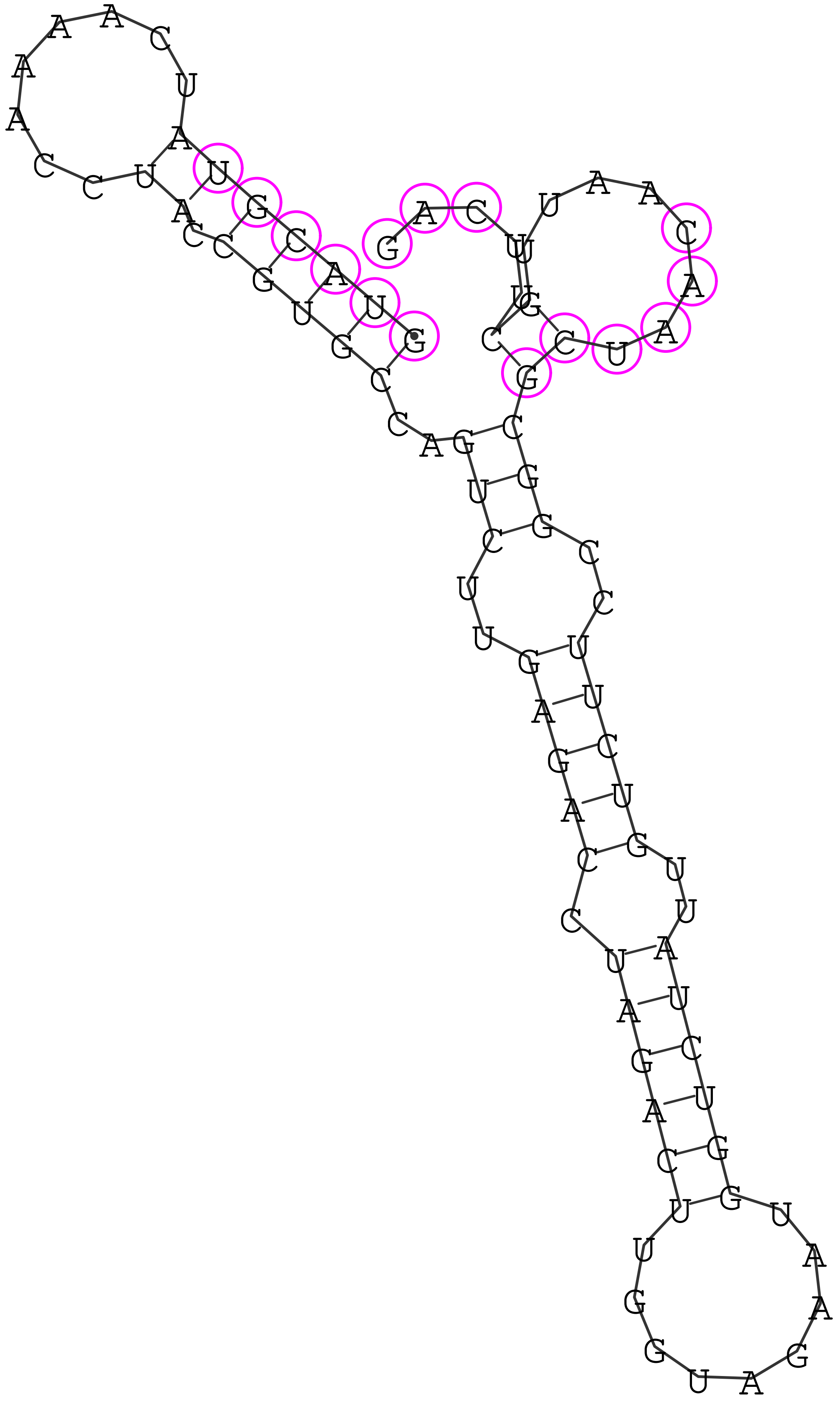

Xbamc053A - Internal intron

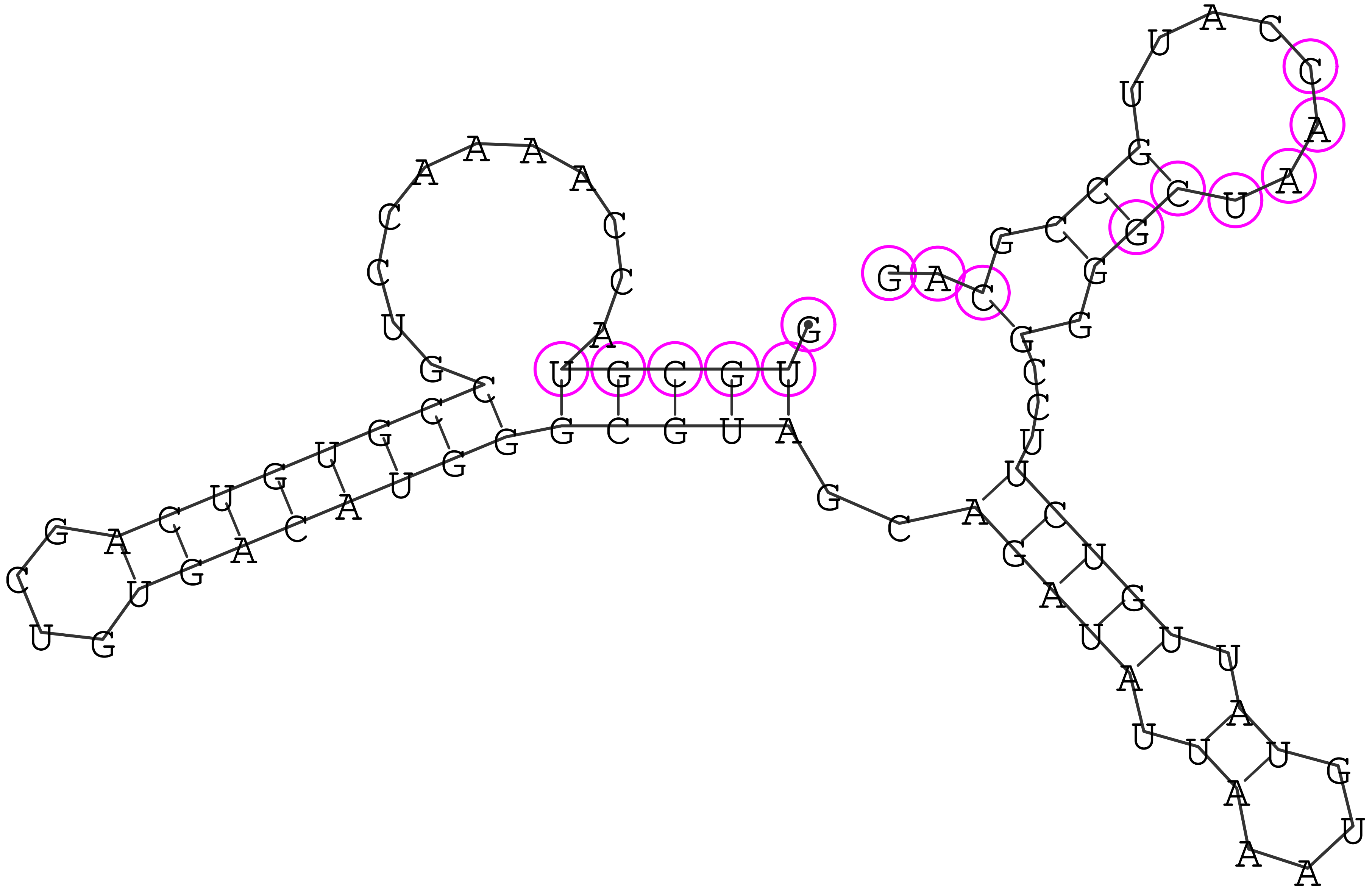

Xbamc053B - Internal intron

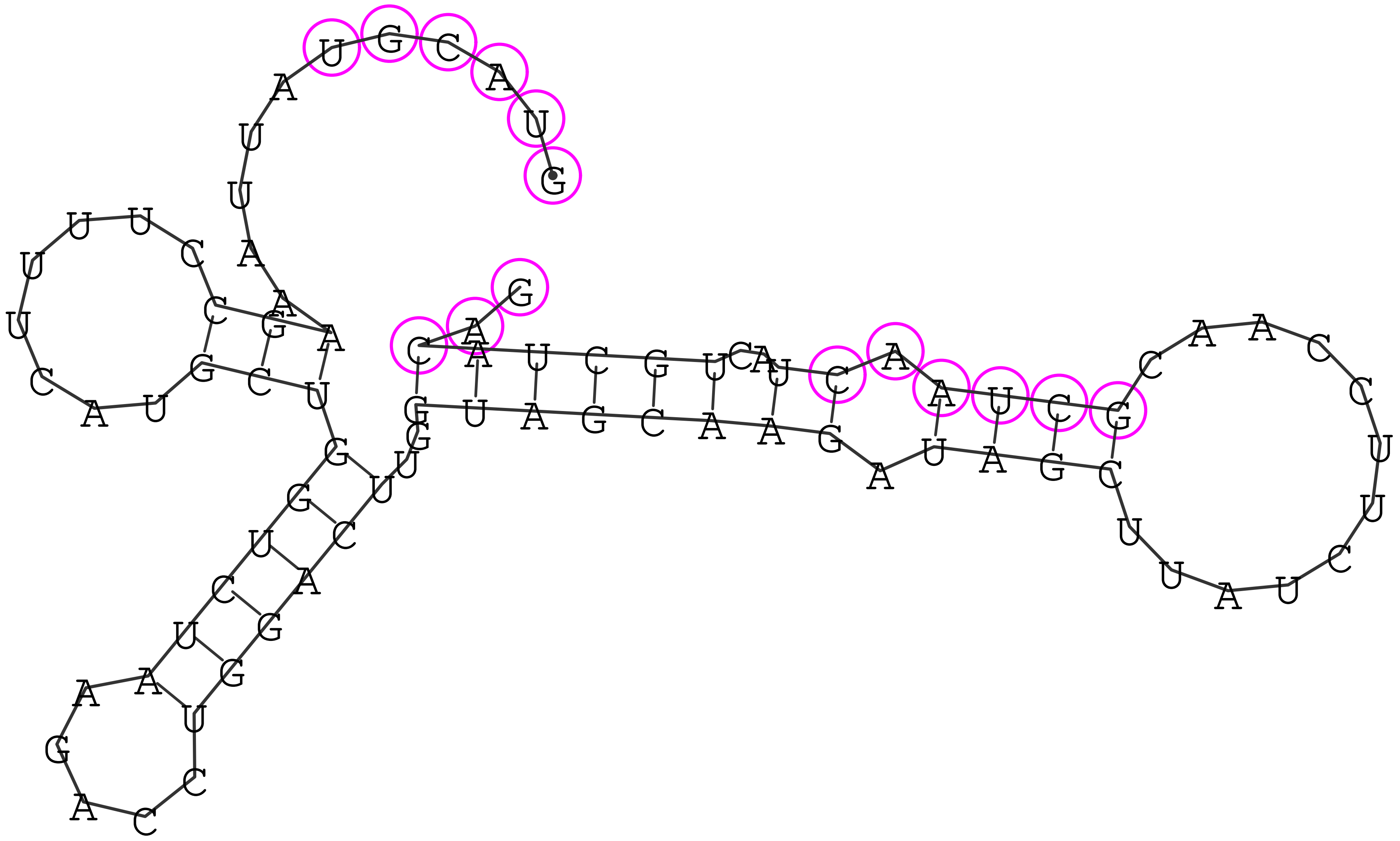

Xbamc053C - Internal intron

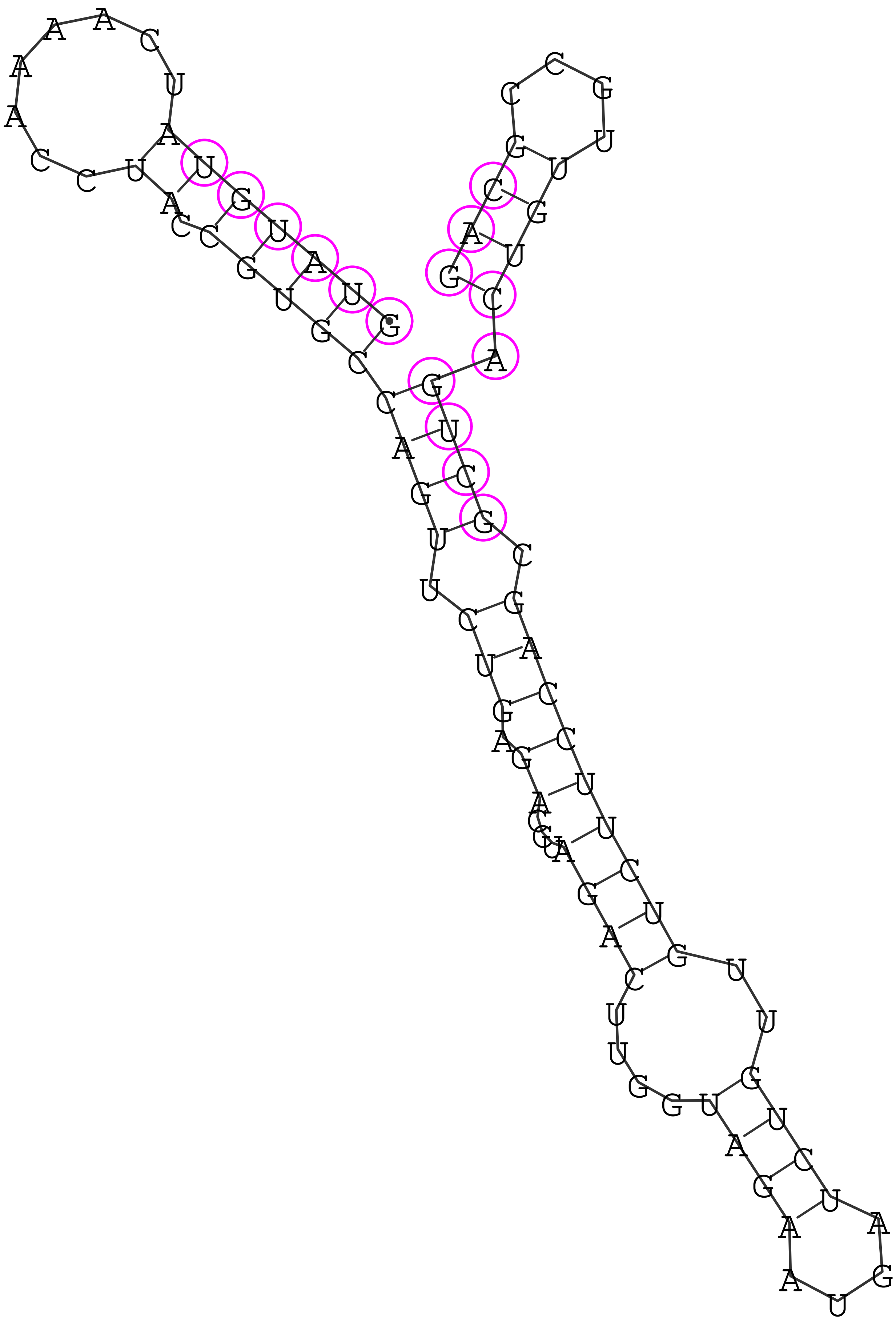

Xbamc067A - Internal intron

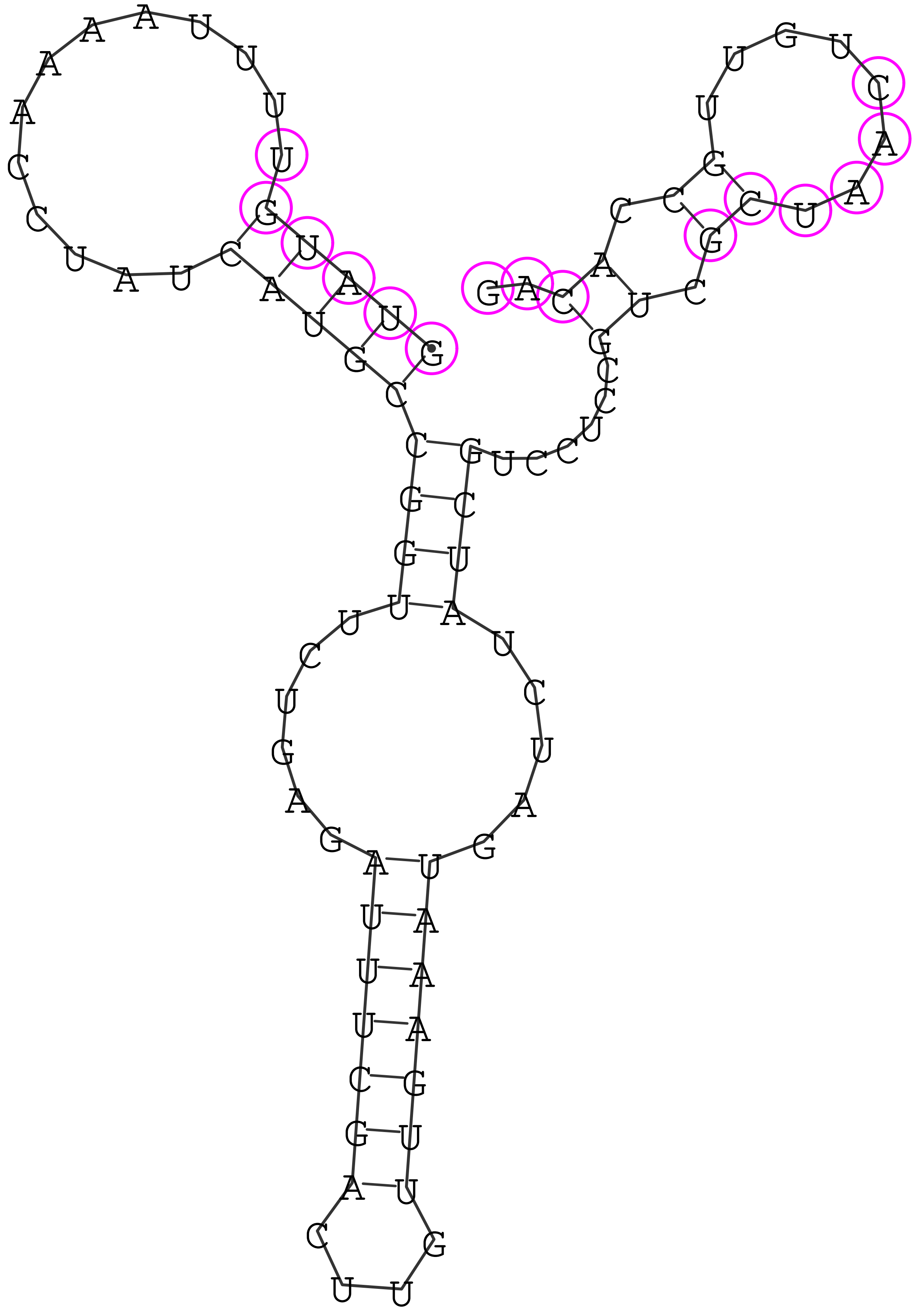

Xbamc067B - Internal intron

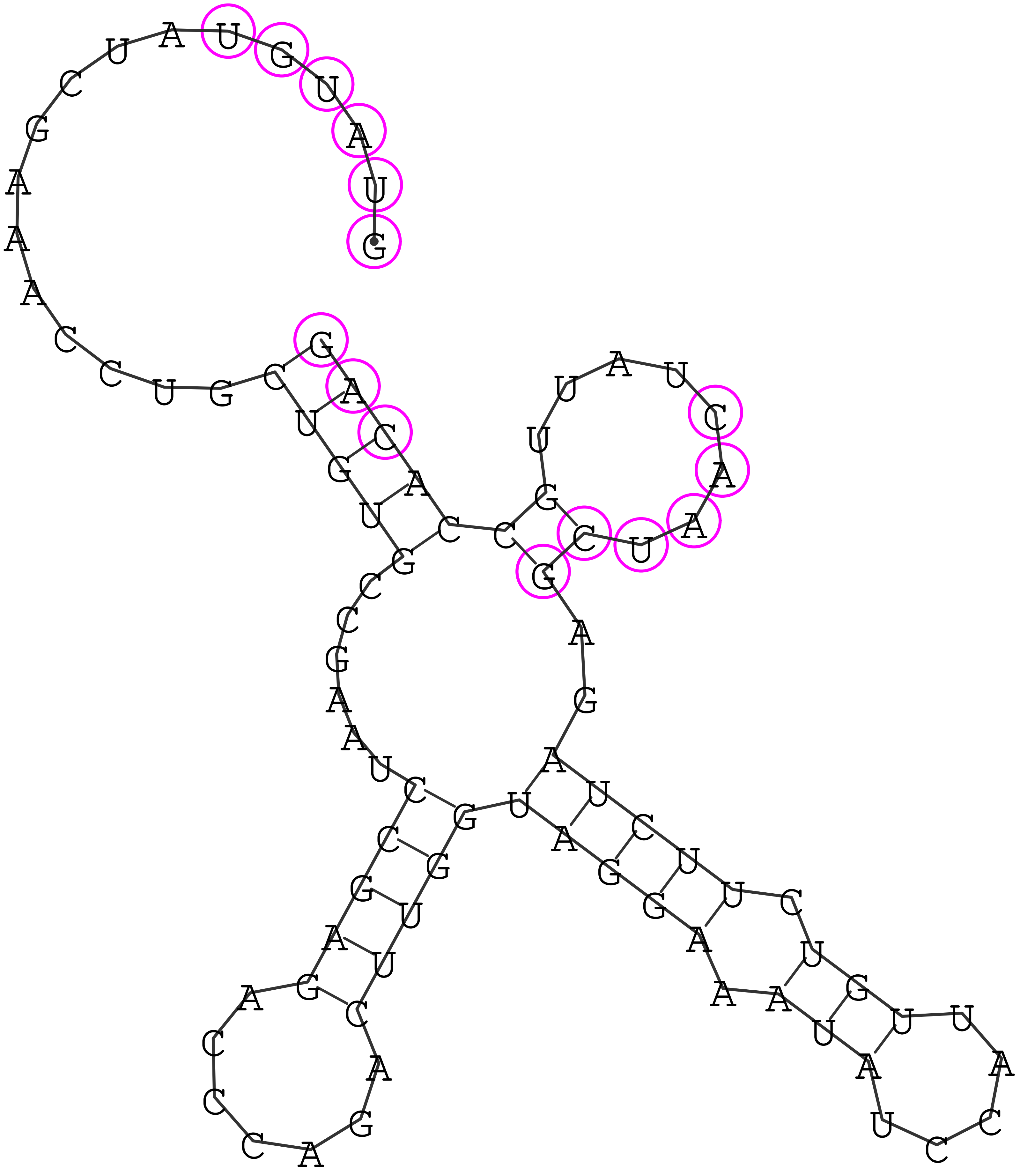

Xbamc080A - Internal intron

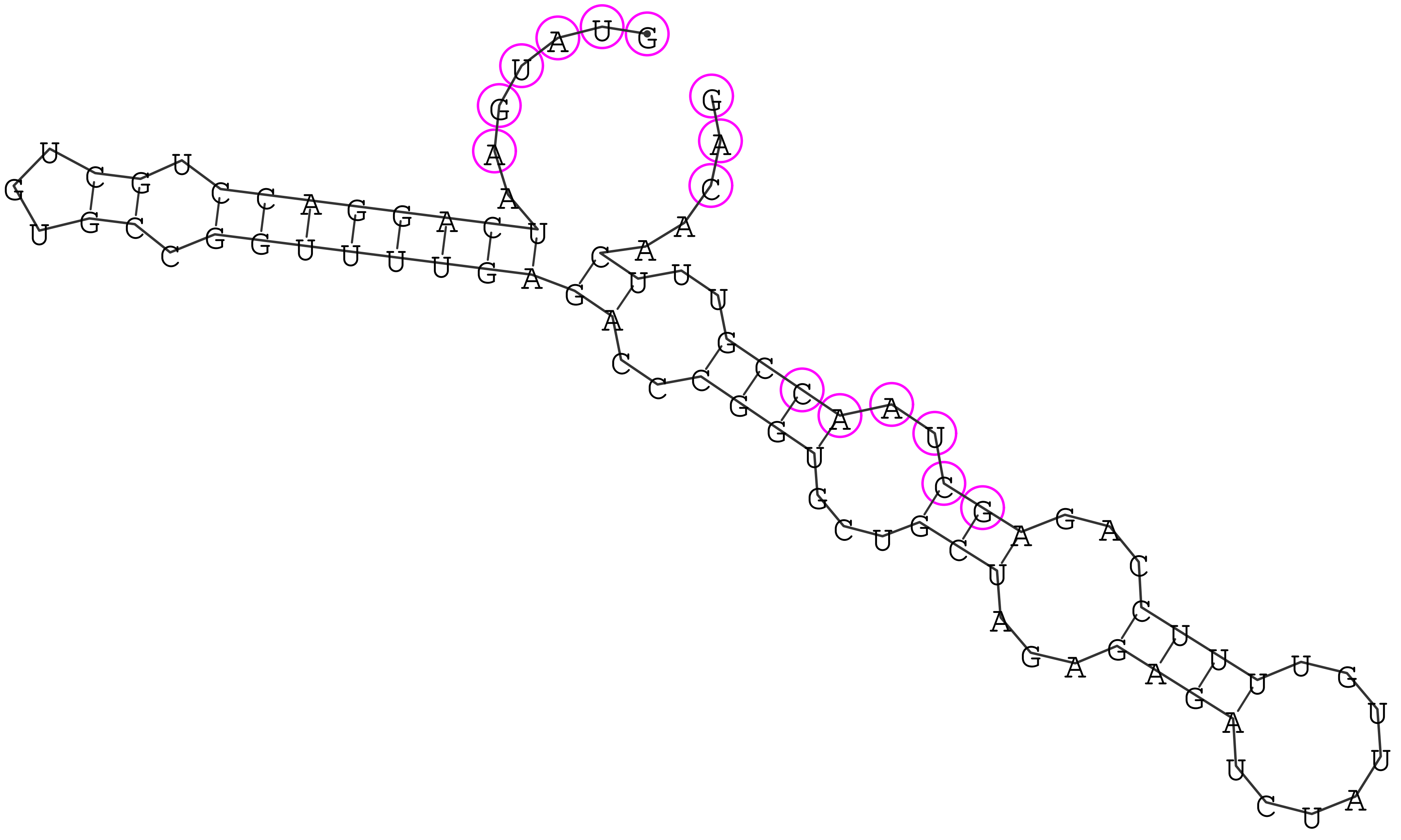

Xbamc083A - Internal intron

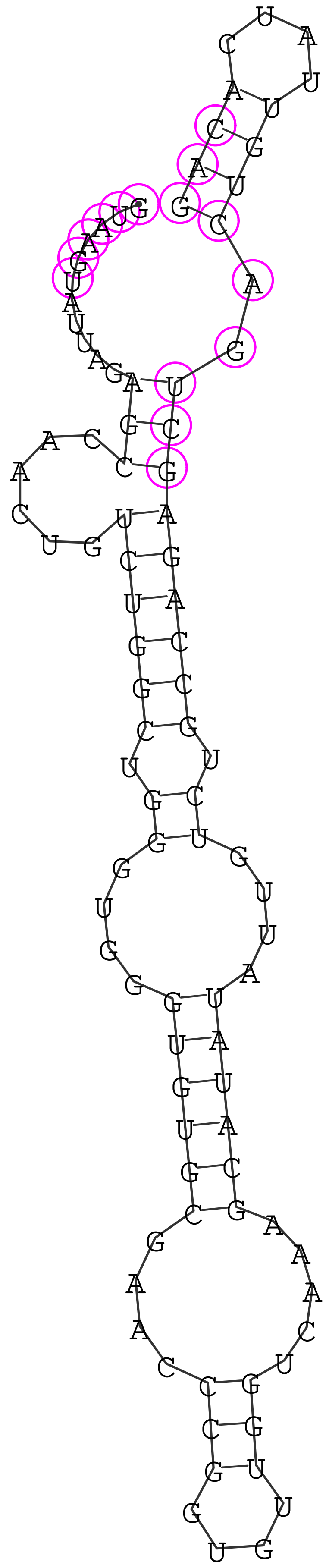

Xbamc083B - Internal intron

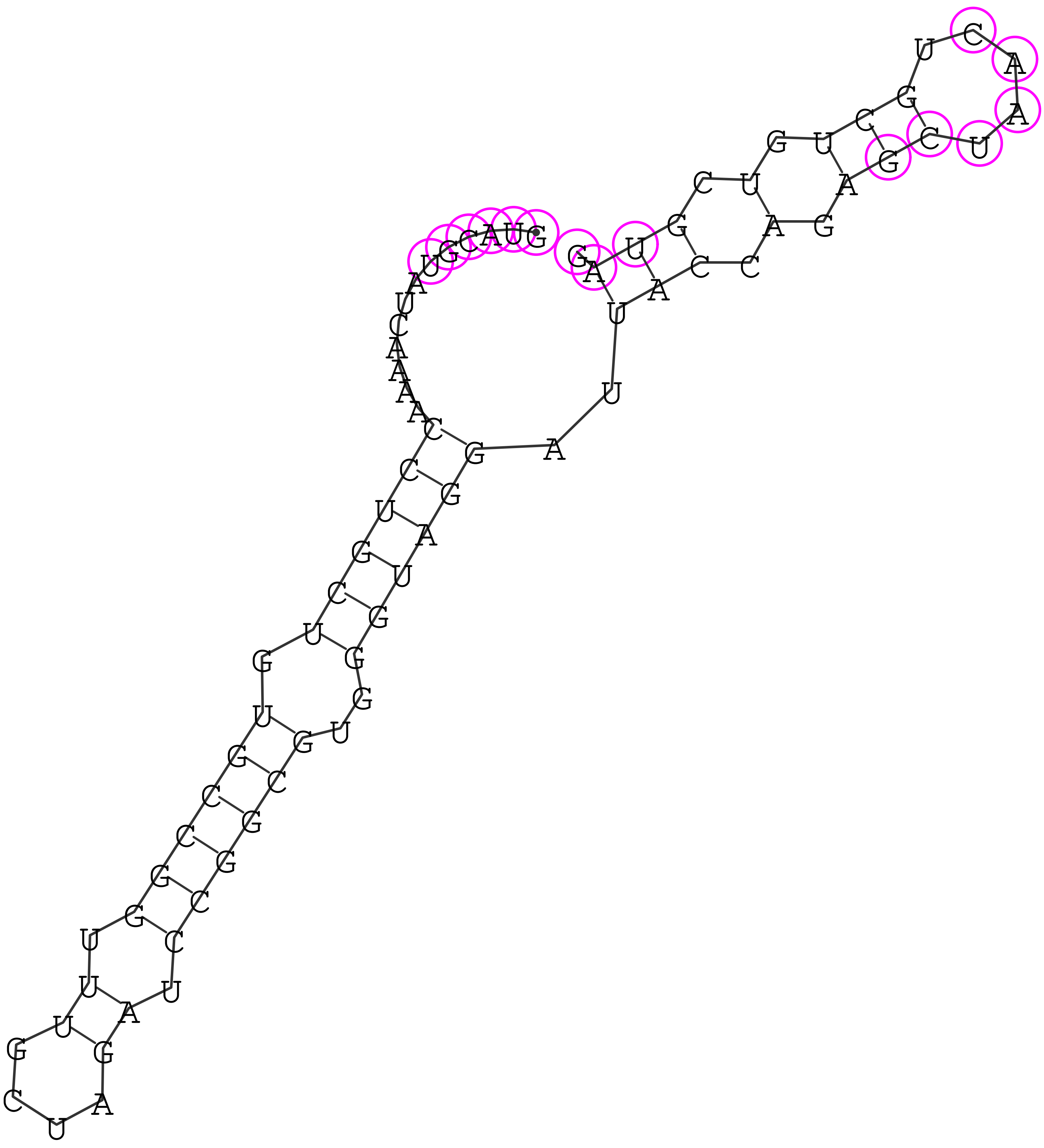

Xbamc084A - Internal intron

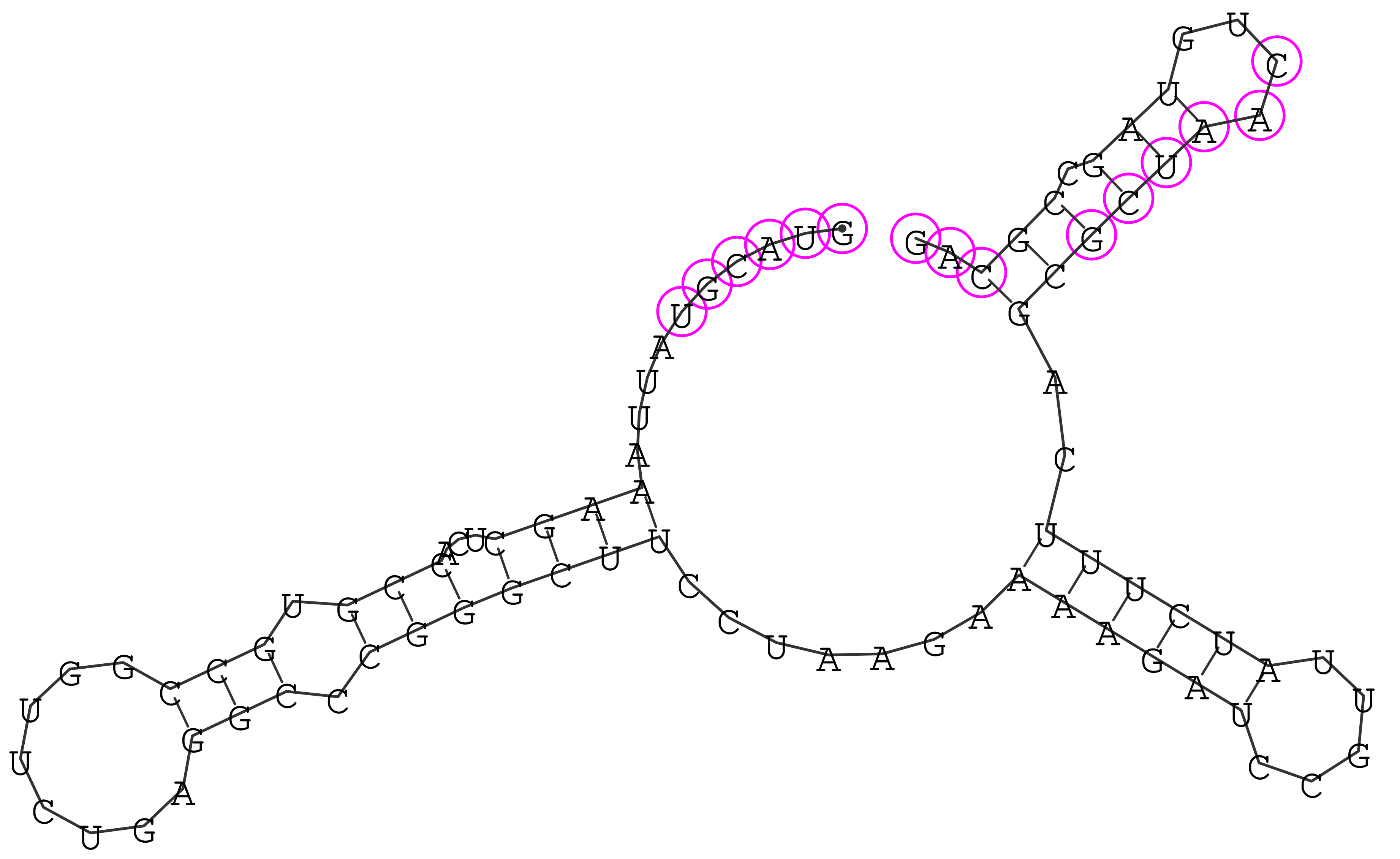

Xbamc086A - Internal intron

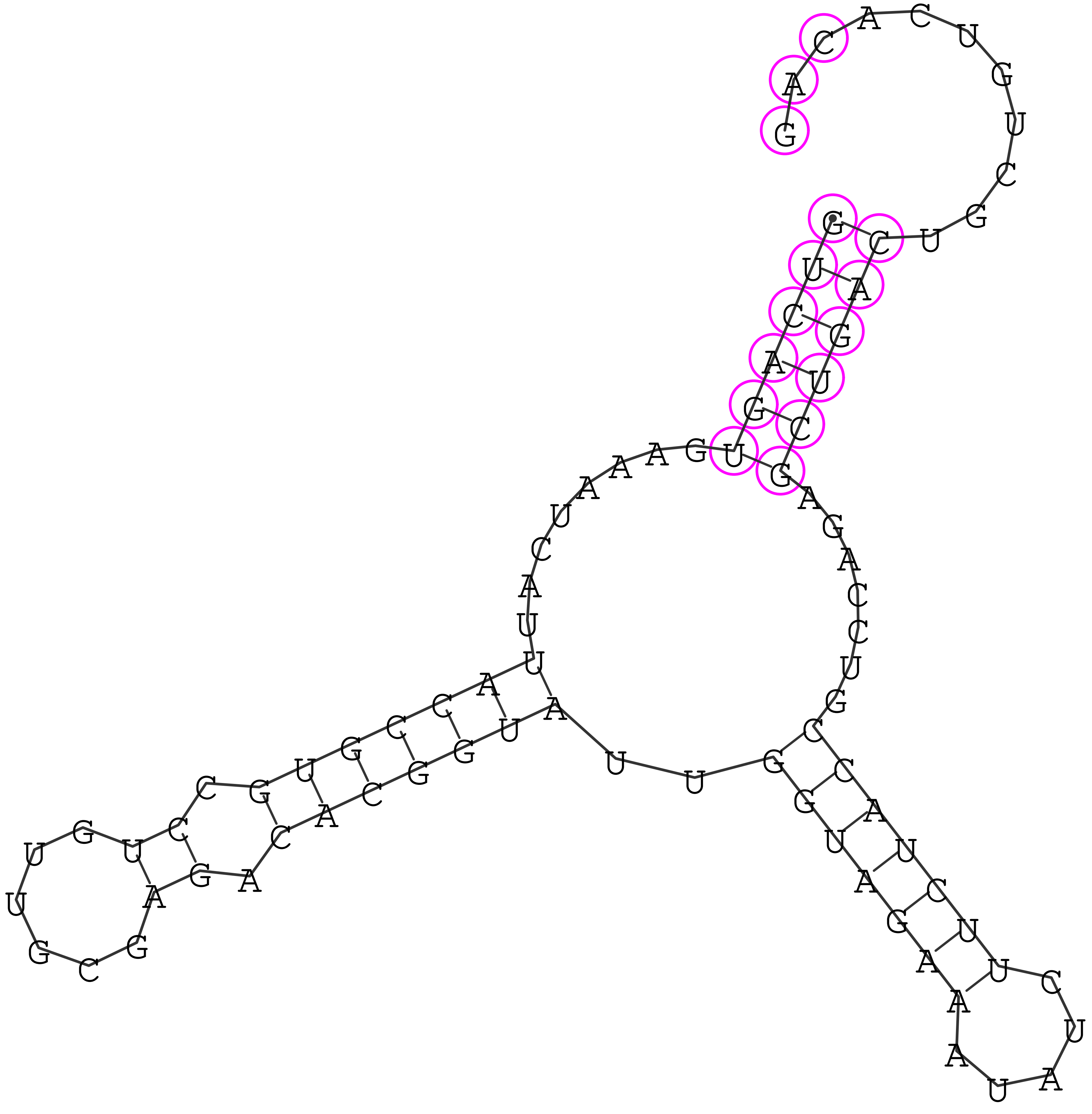

Xbamc089A - Internal intron

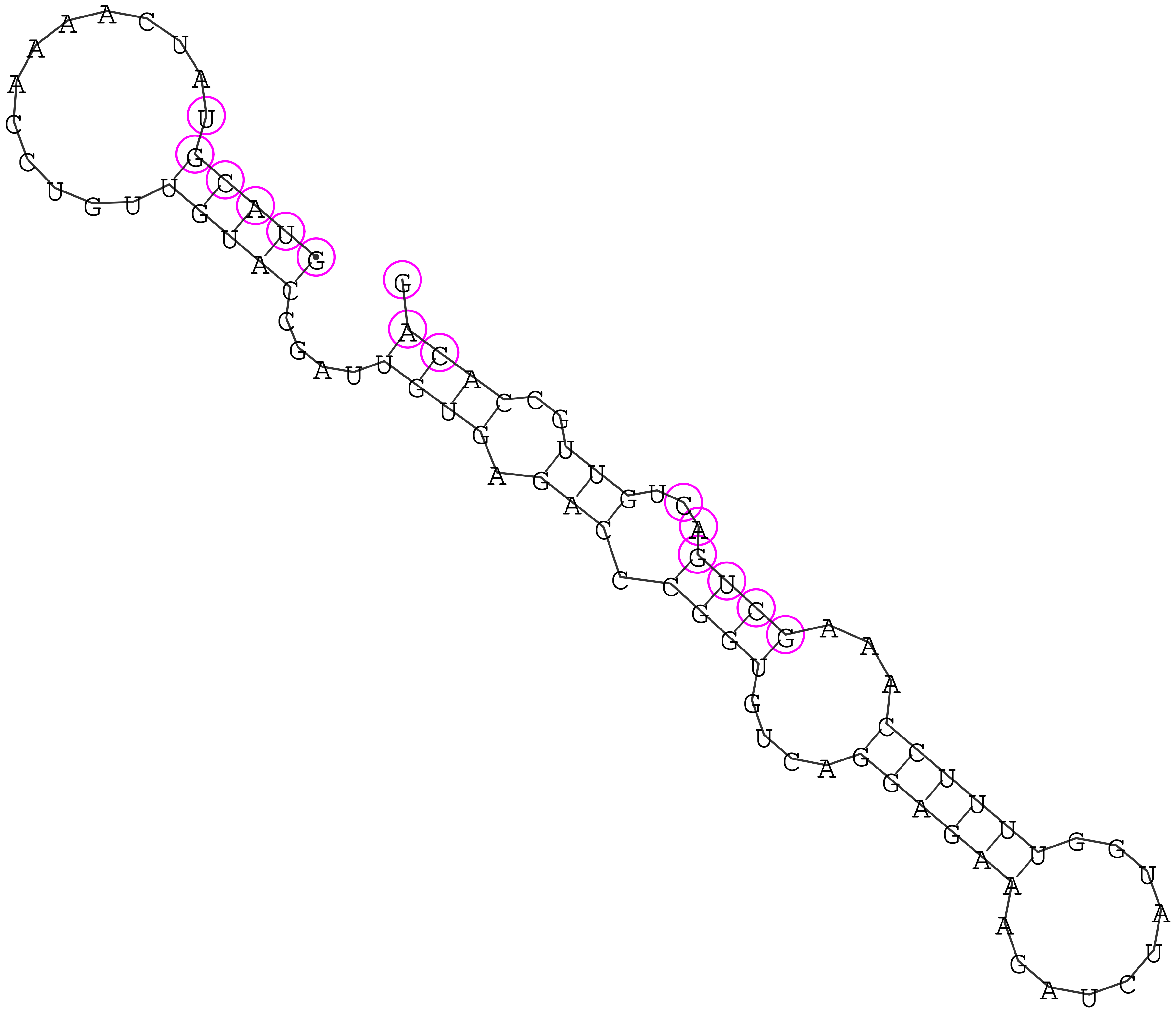

Xbamc096A - Internal intron

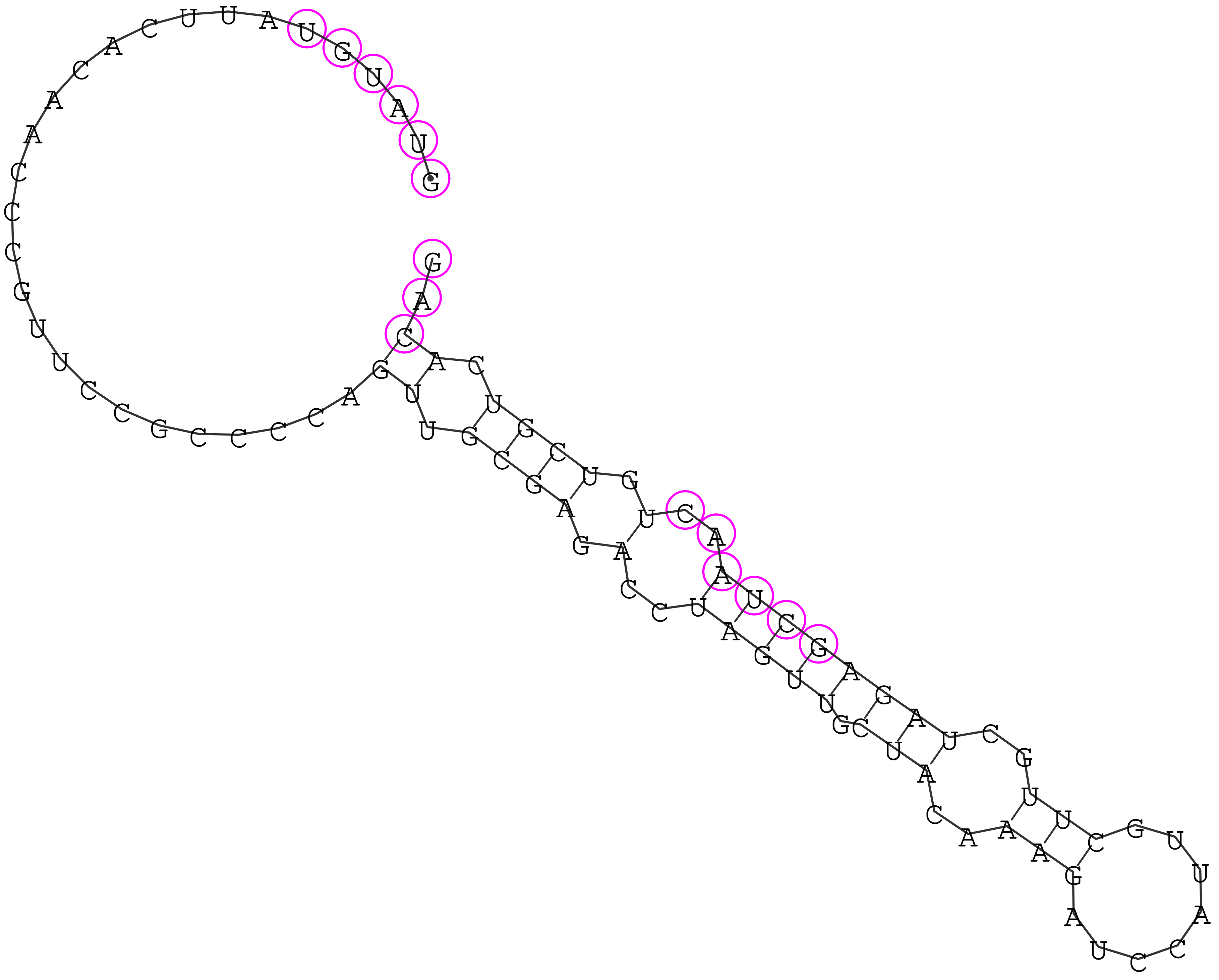

Xbamc102A - Internal intron

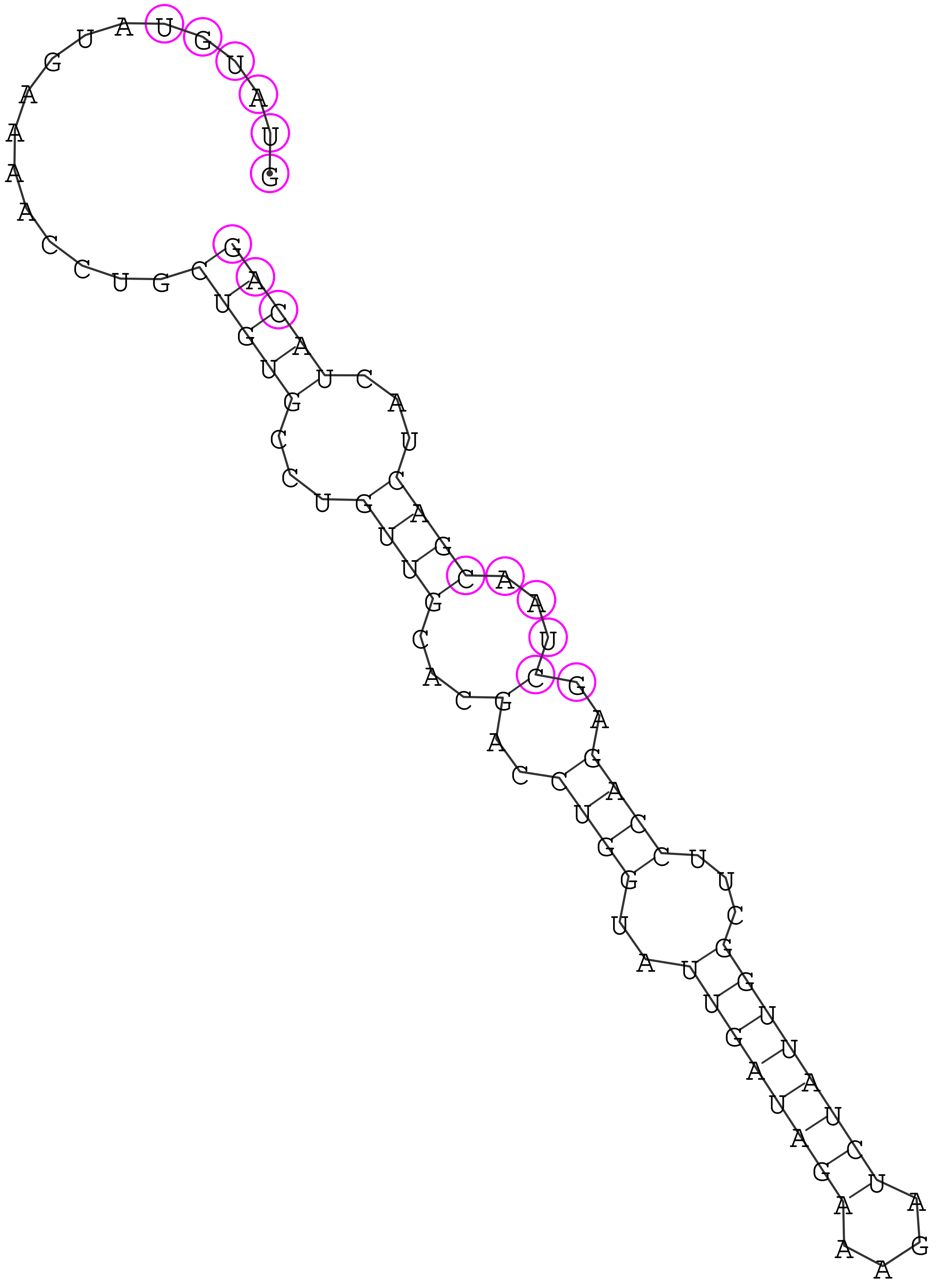

Xbamc106A - Internal intron

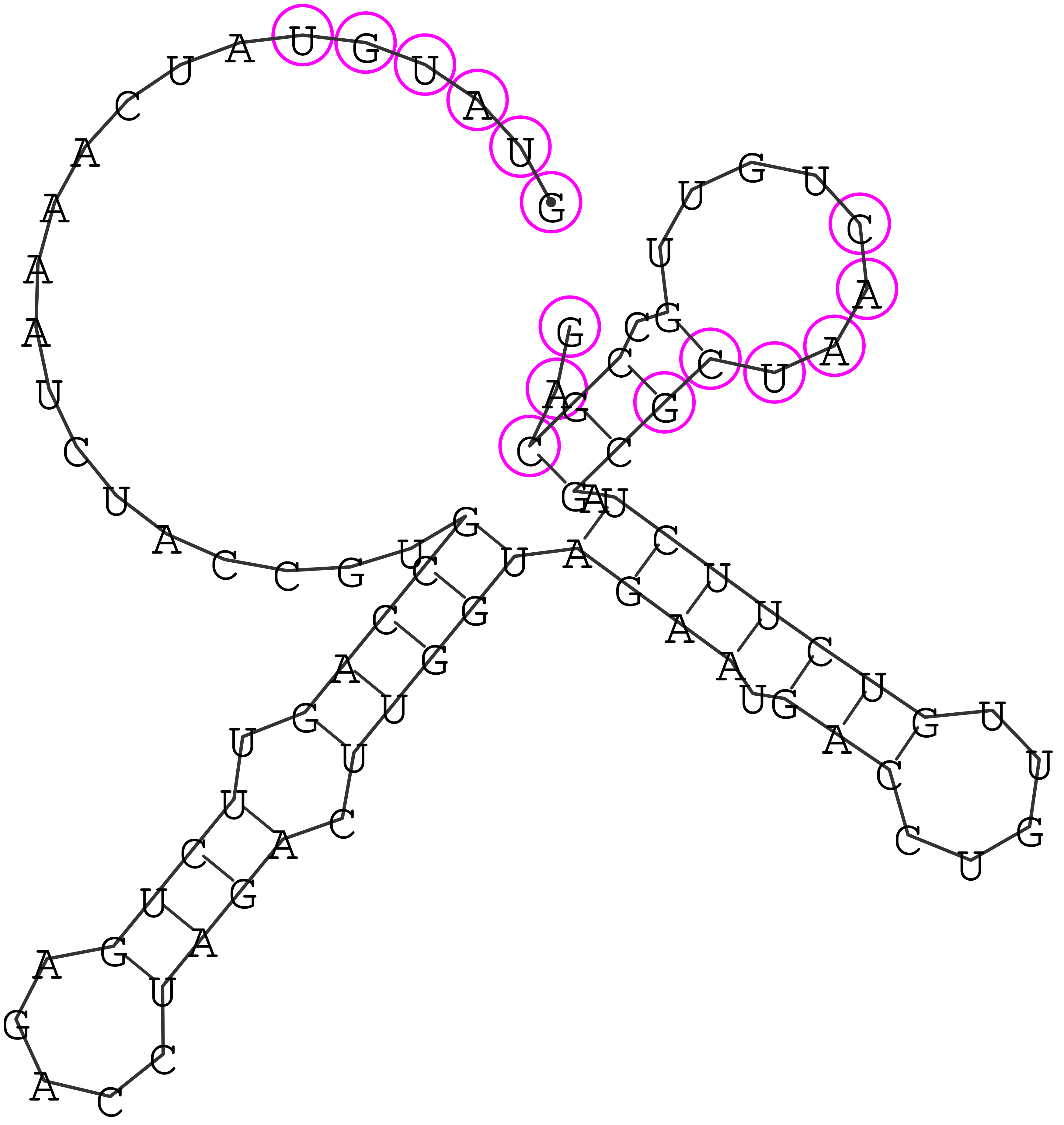

Xbamc106B - Internal intron

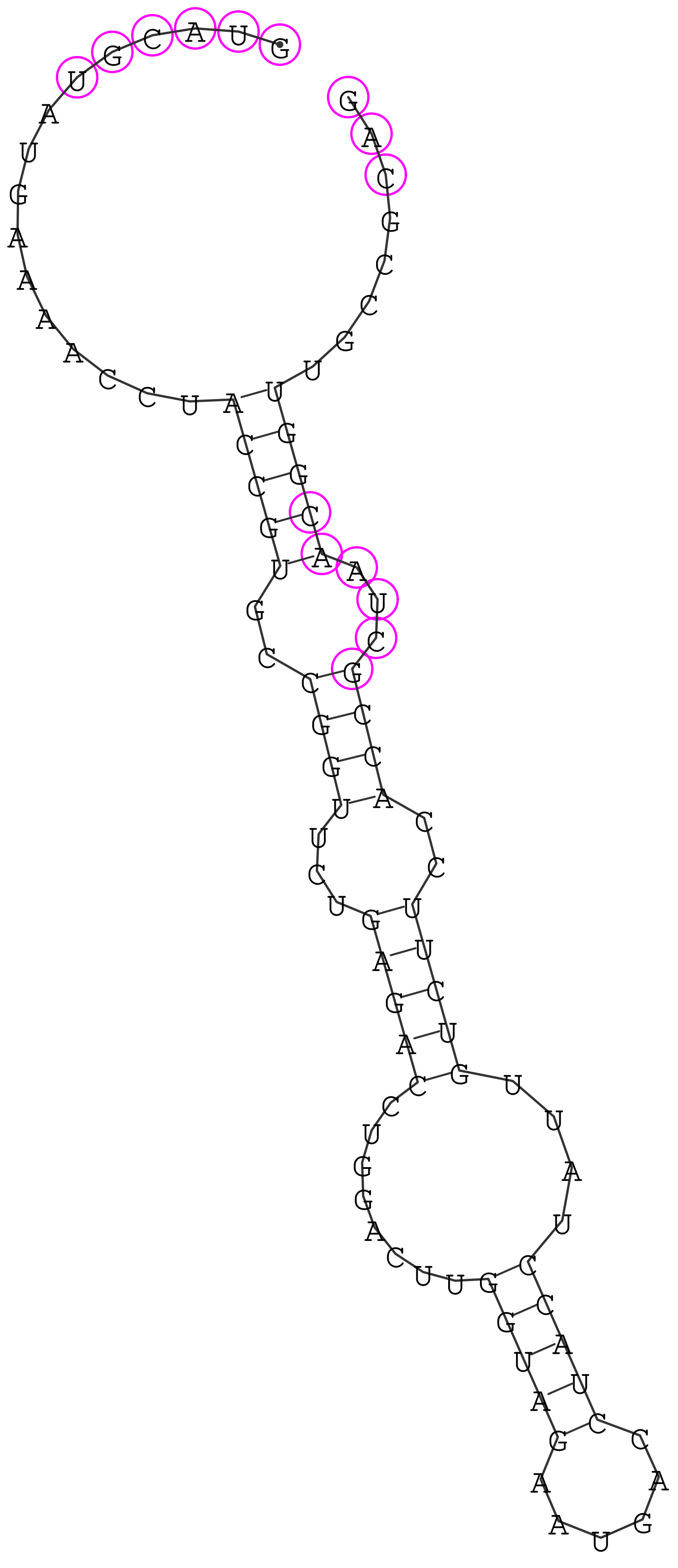

Xbamc107A - Internal intron

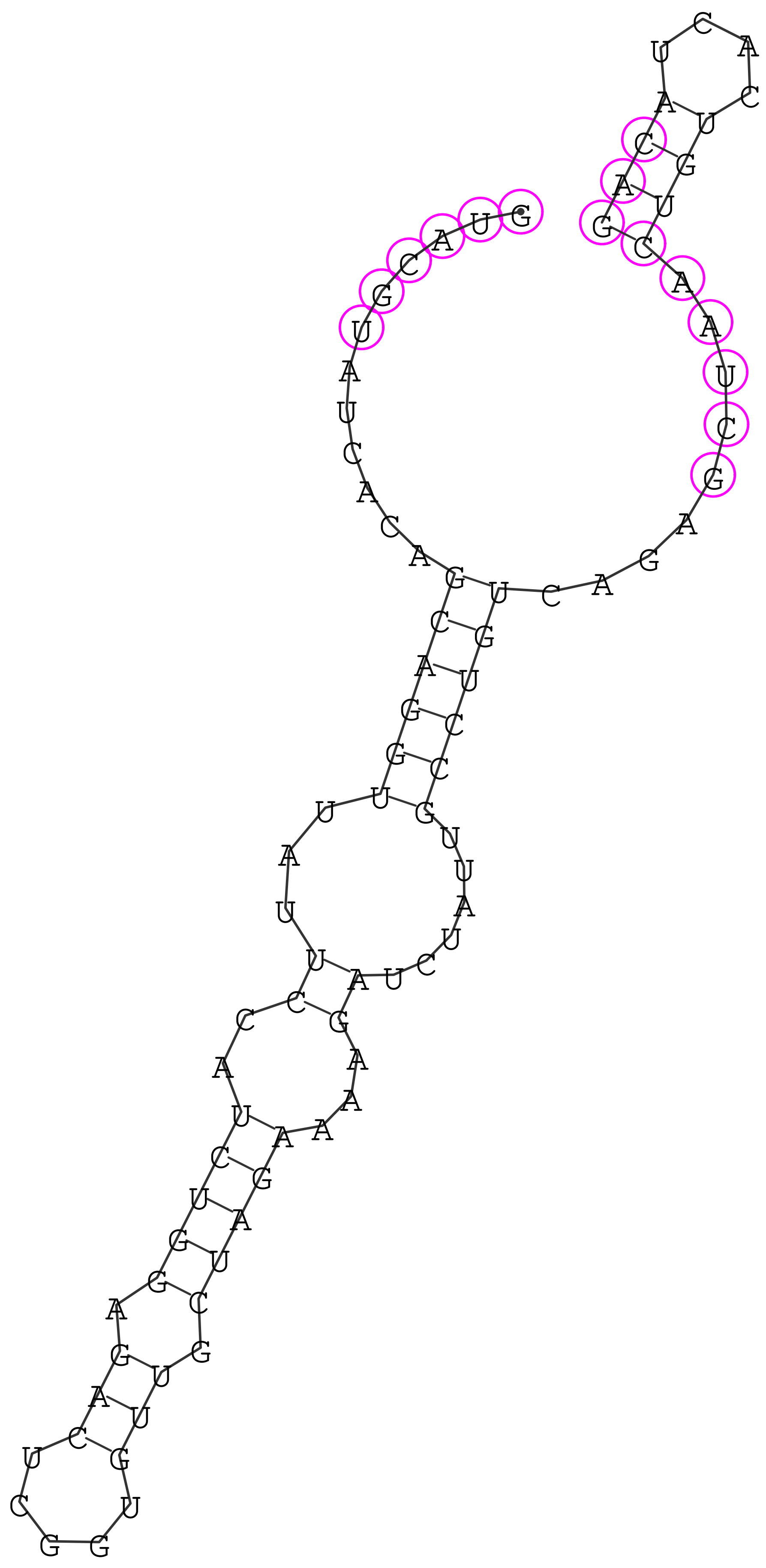

Xbamc109A - Internal intron

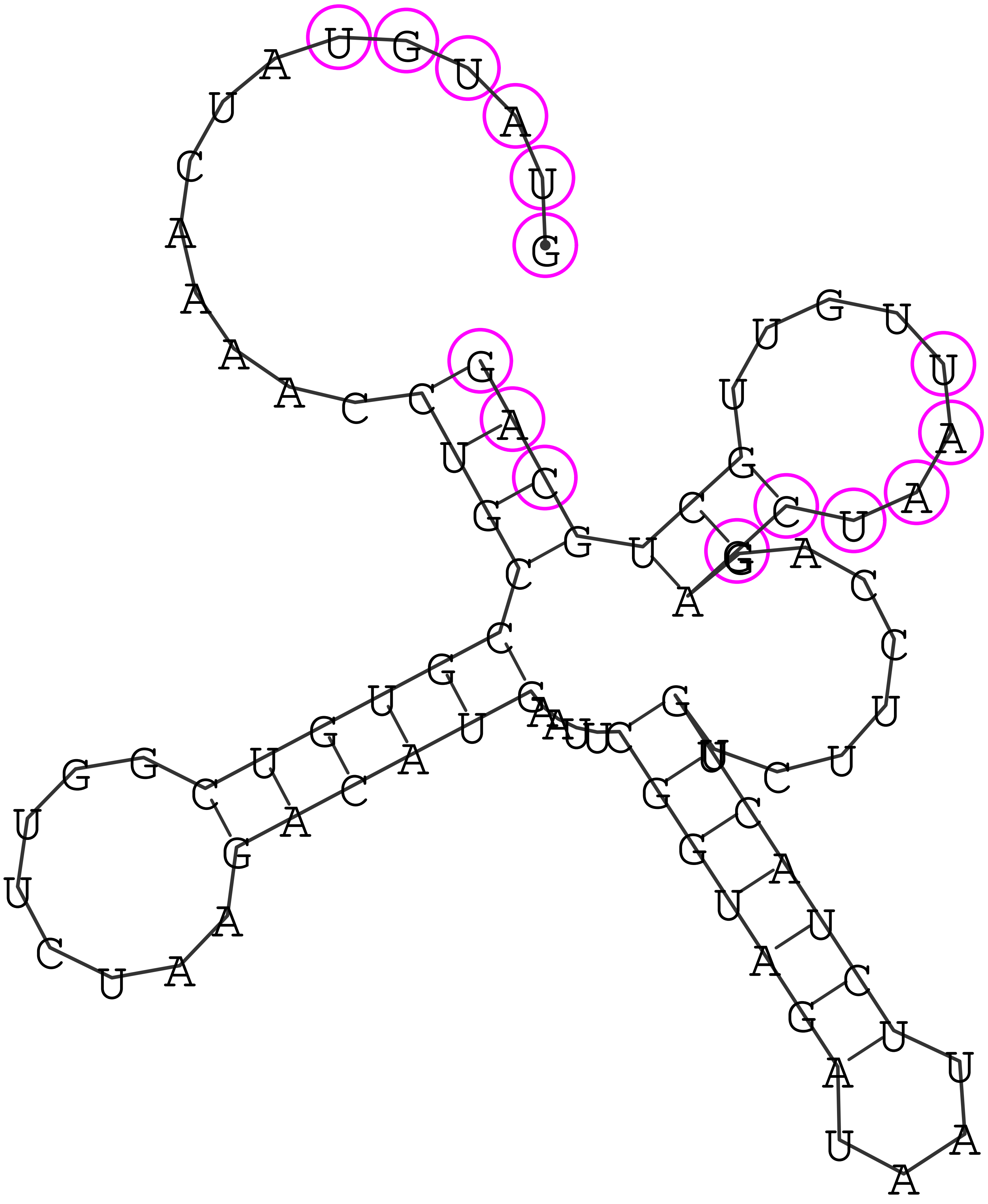

Xbamc110A - Internal intron

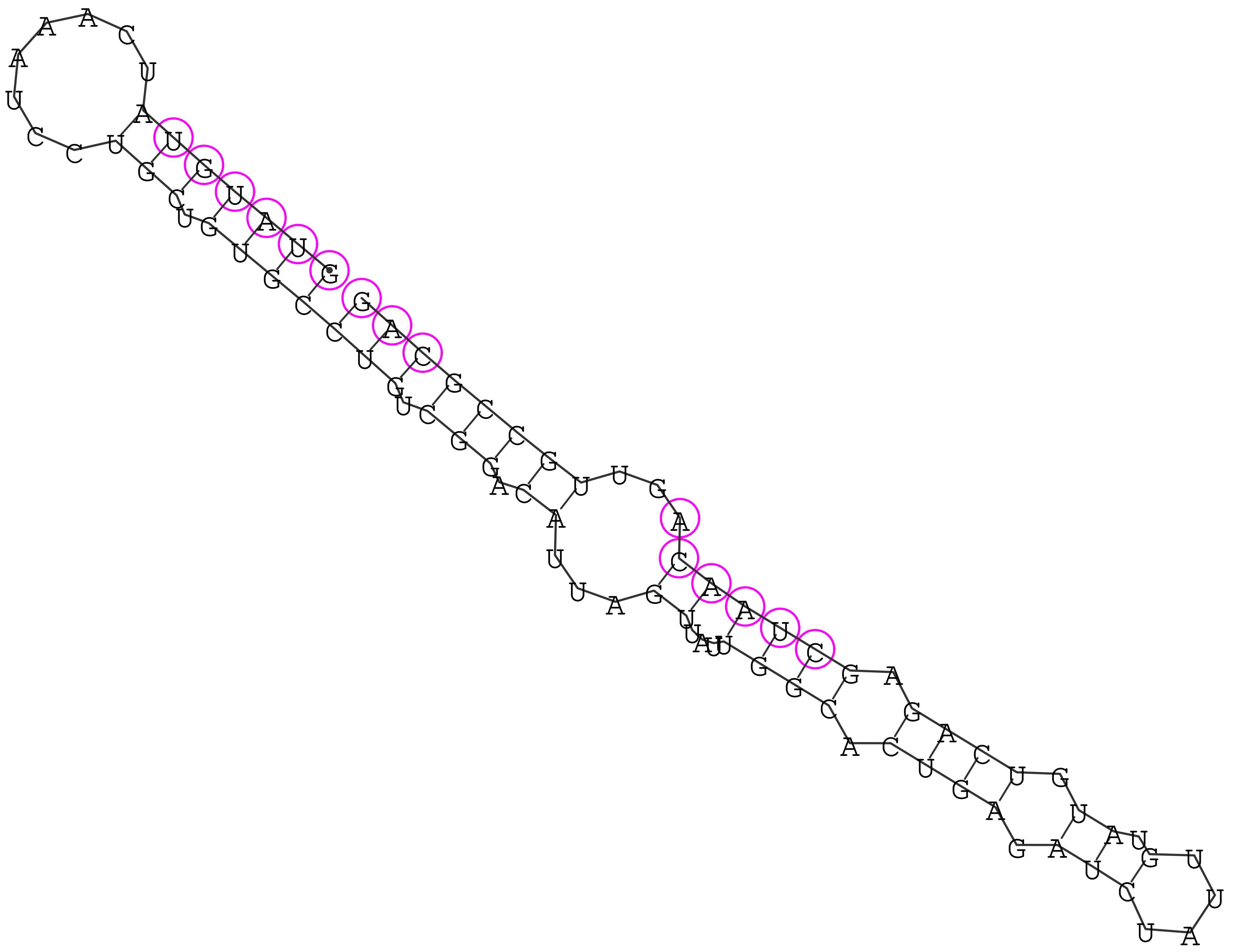

Xbamc124A - Internal intron

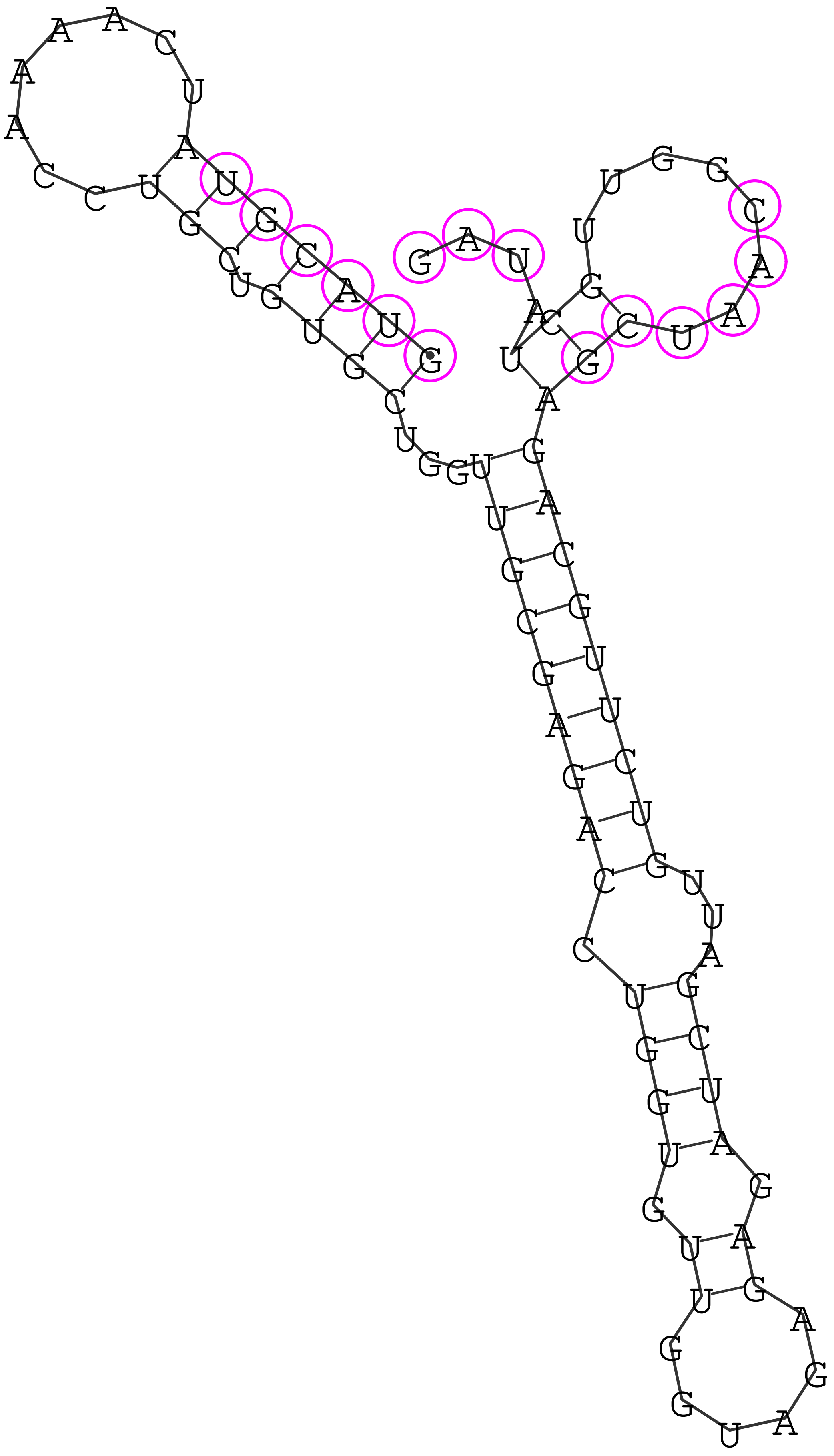

Xbamc132A - Internal intron

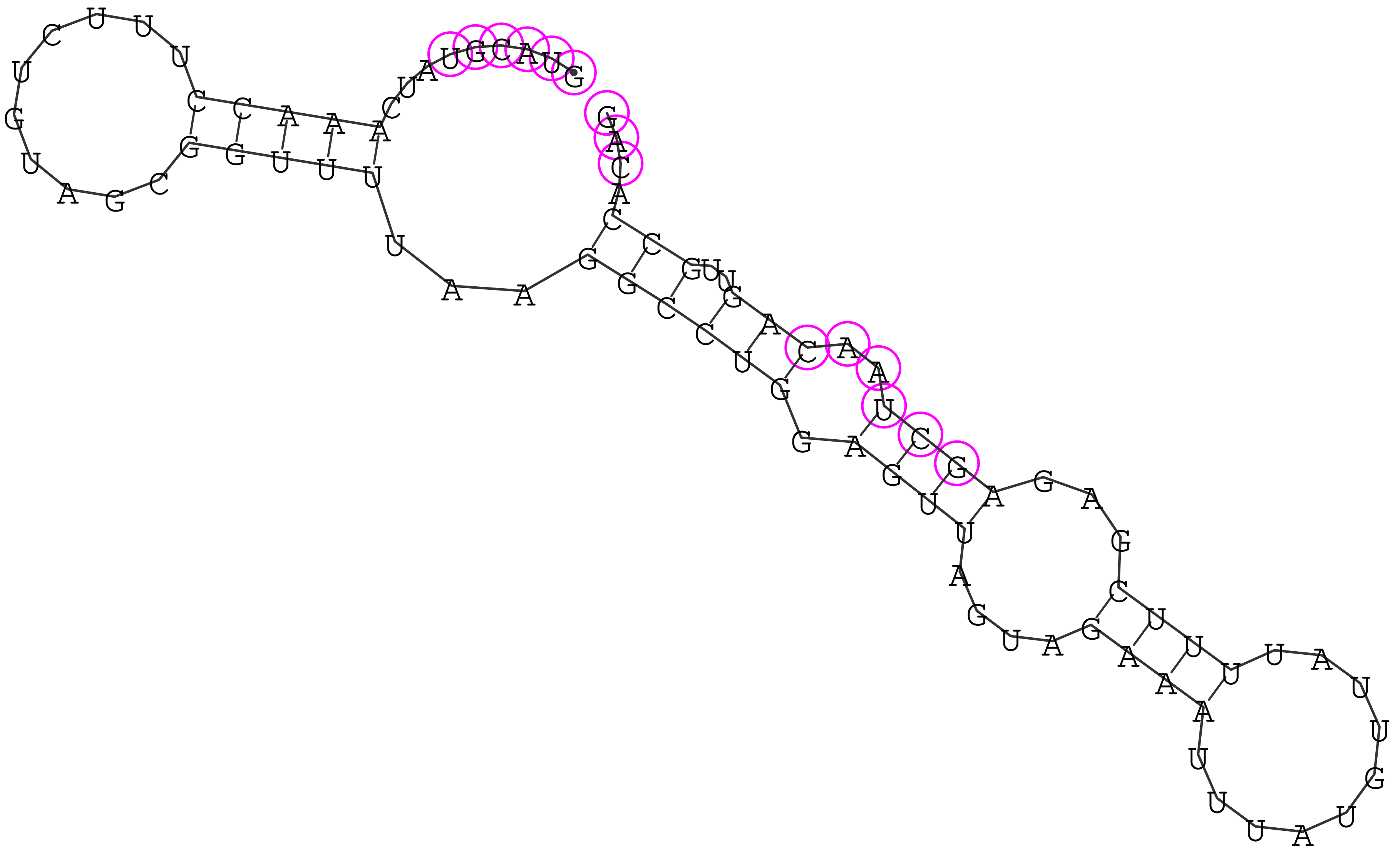

Xbamc132B - Internal intron

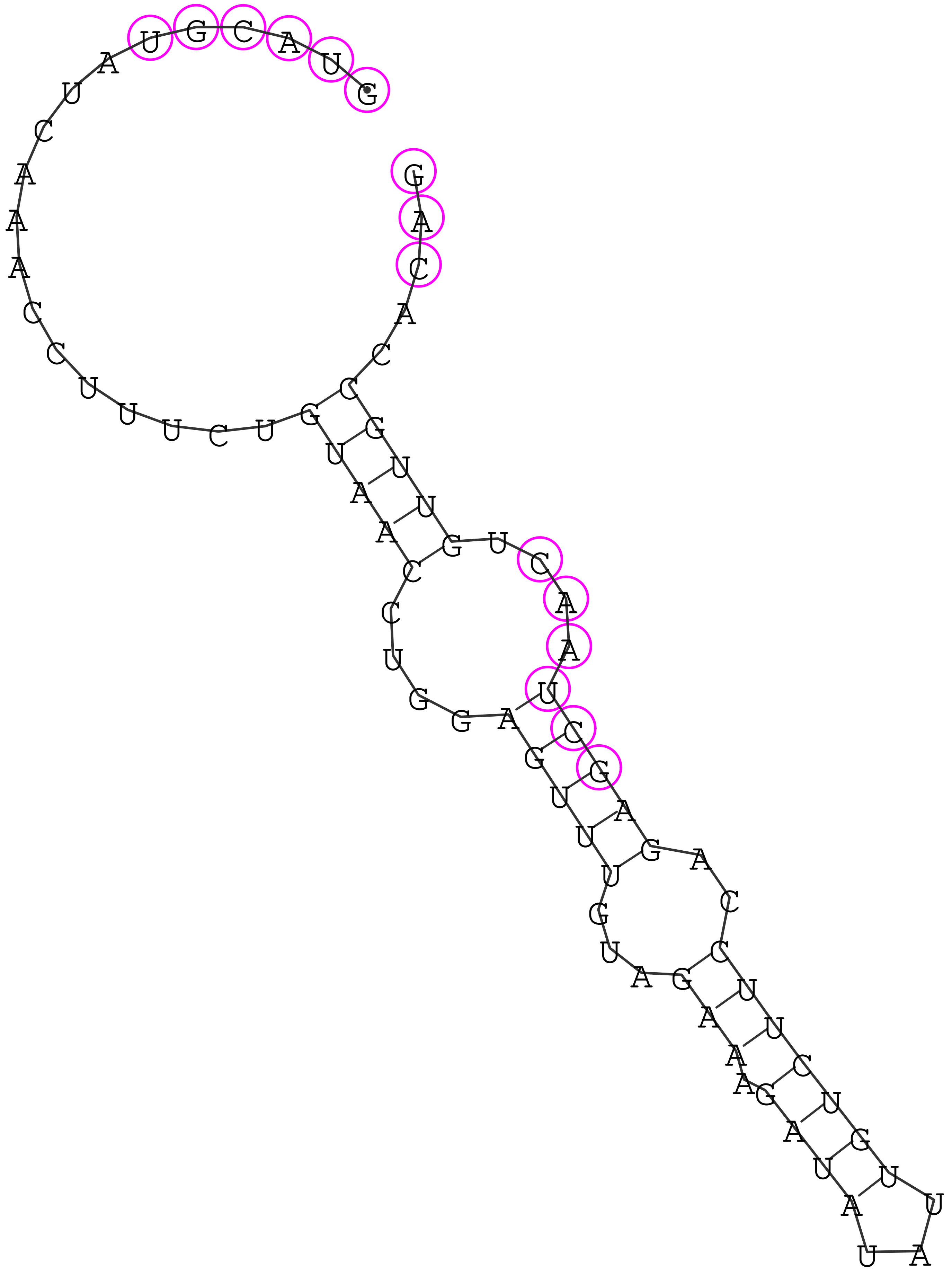

Xbamc152A - Internal intron

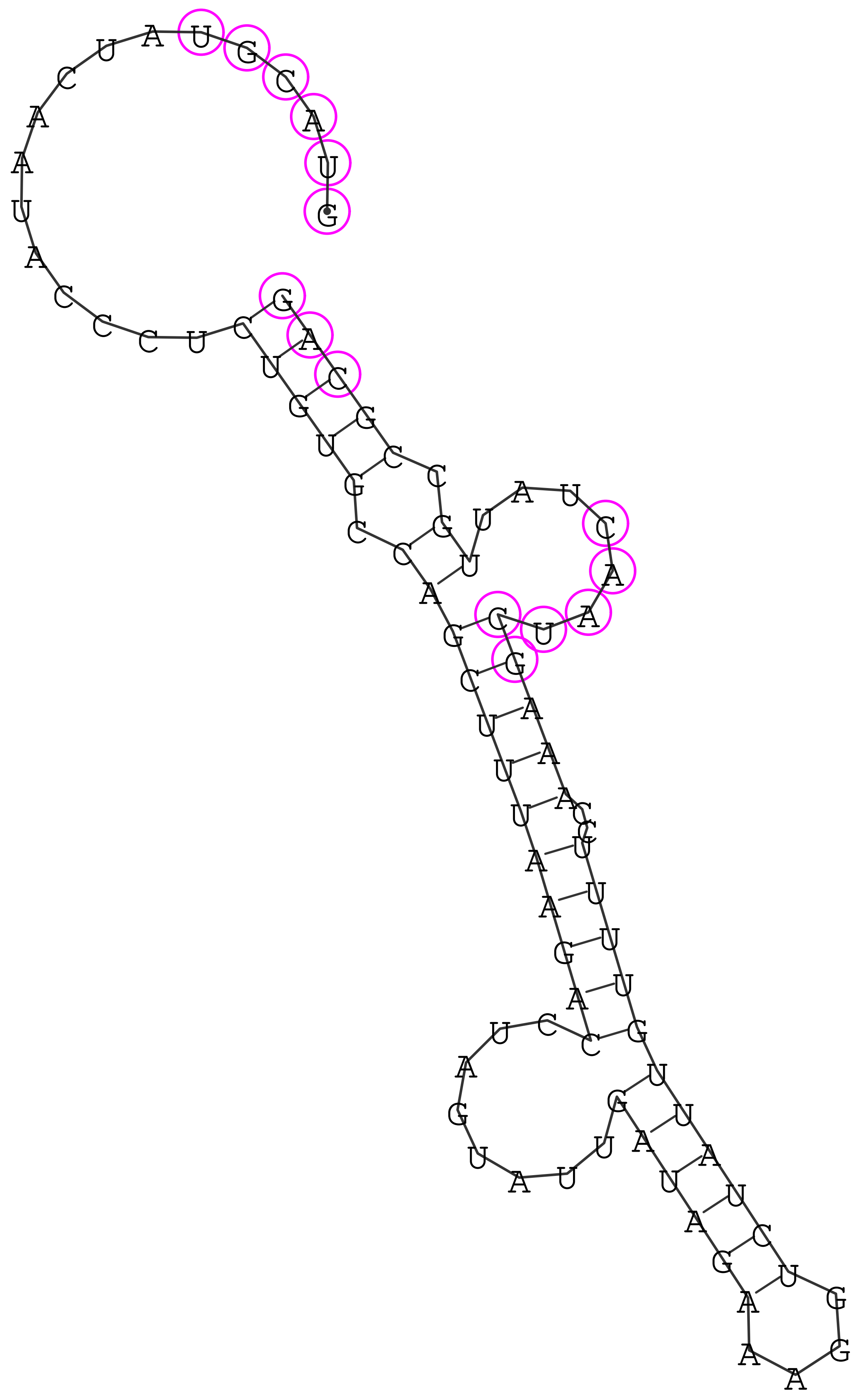

Xbamc152B - Internal intron

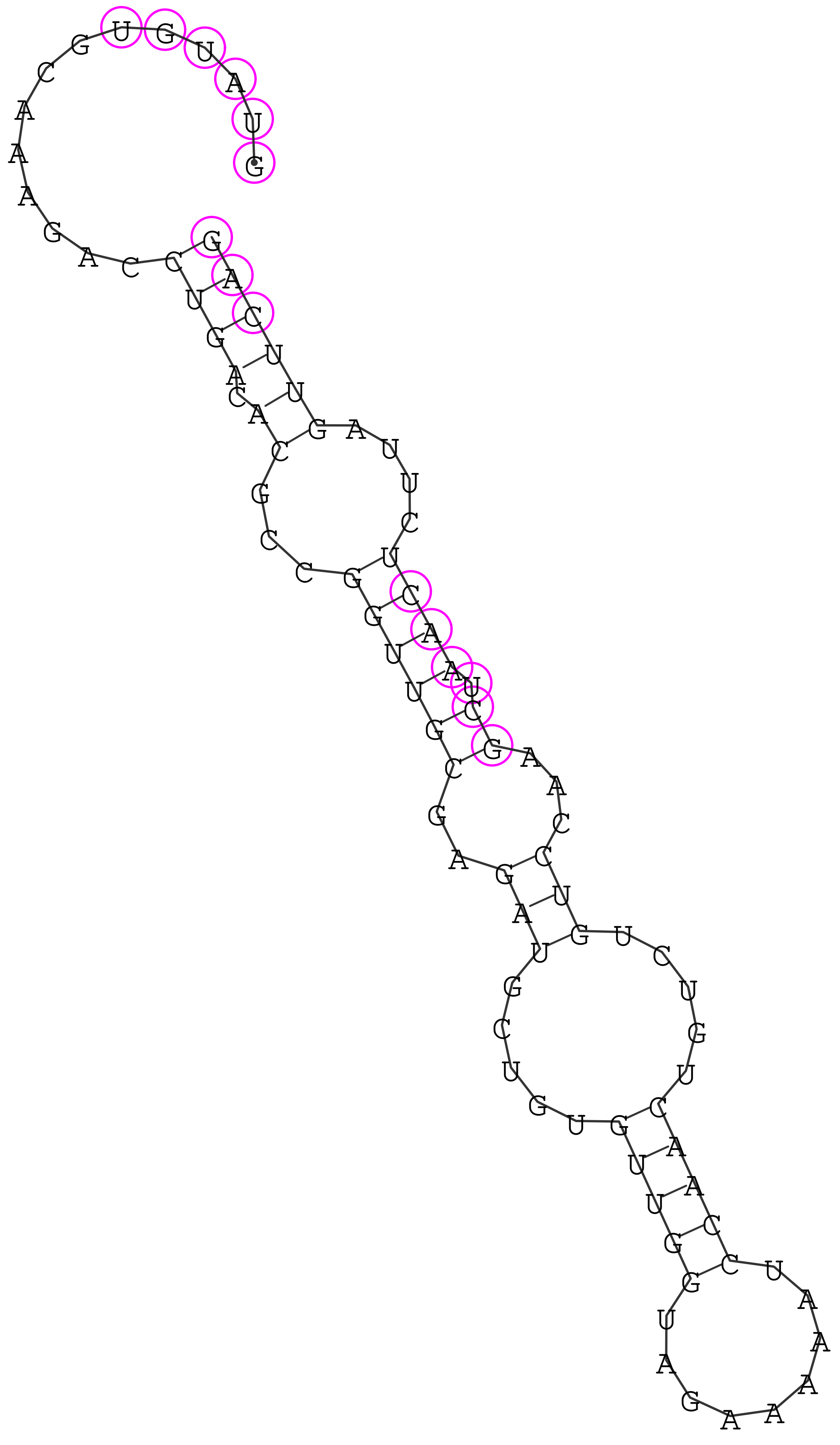

Xbamc152C - Internal intron

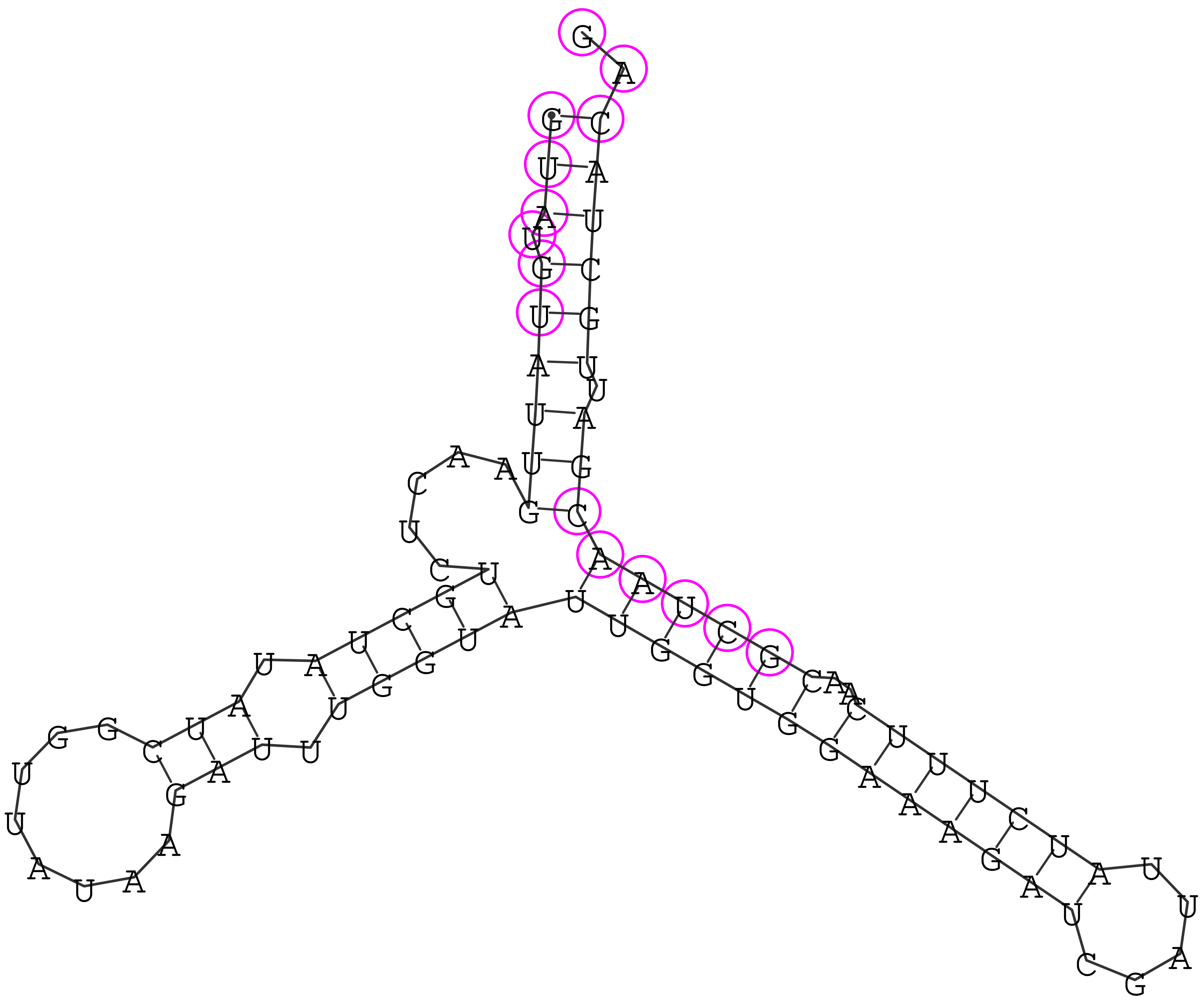

Xbamc153A - Internal intron

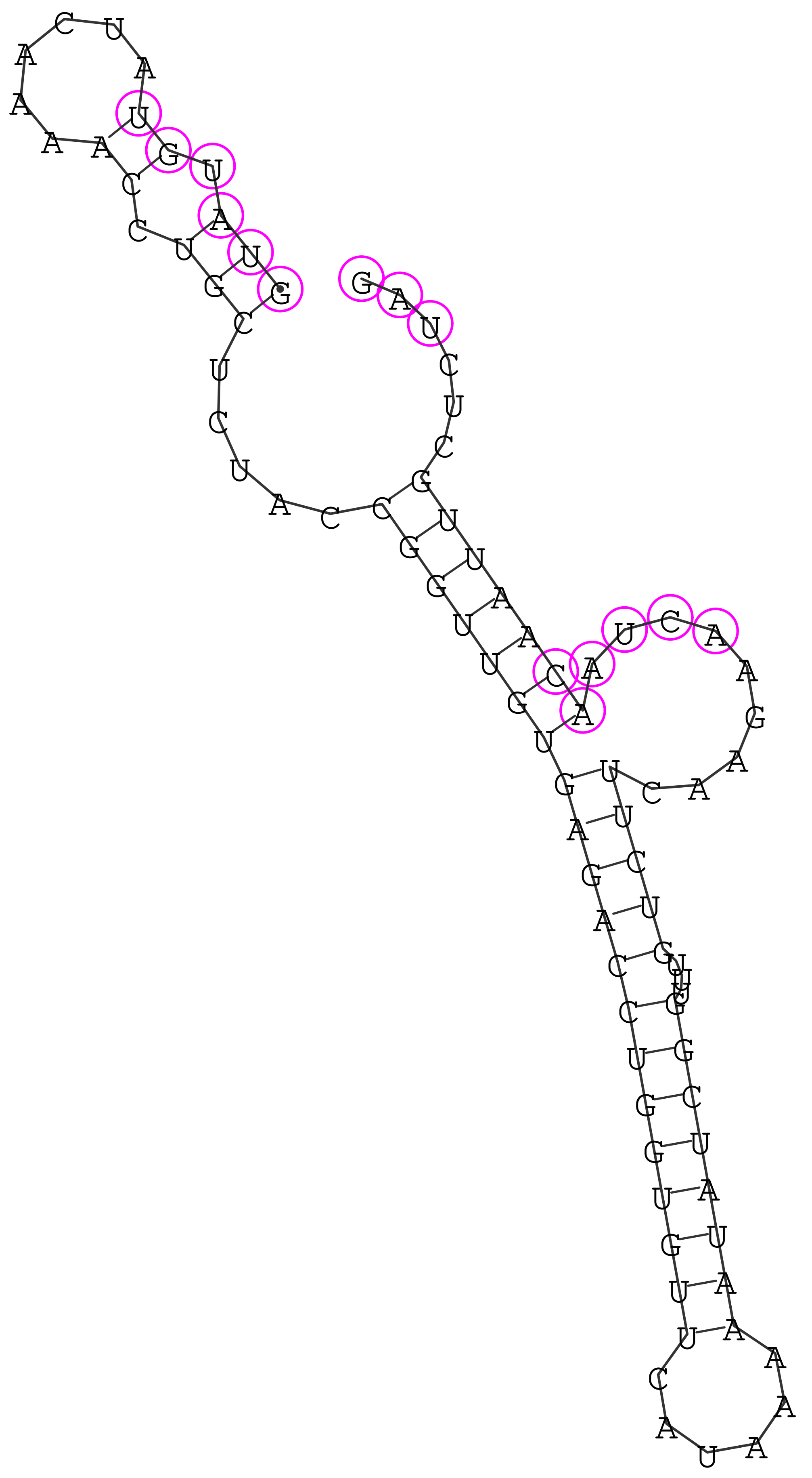

Xbamc155A - Internal intron

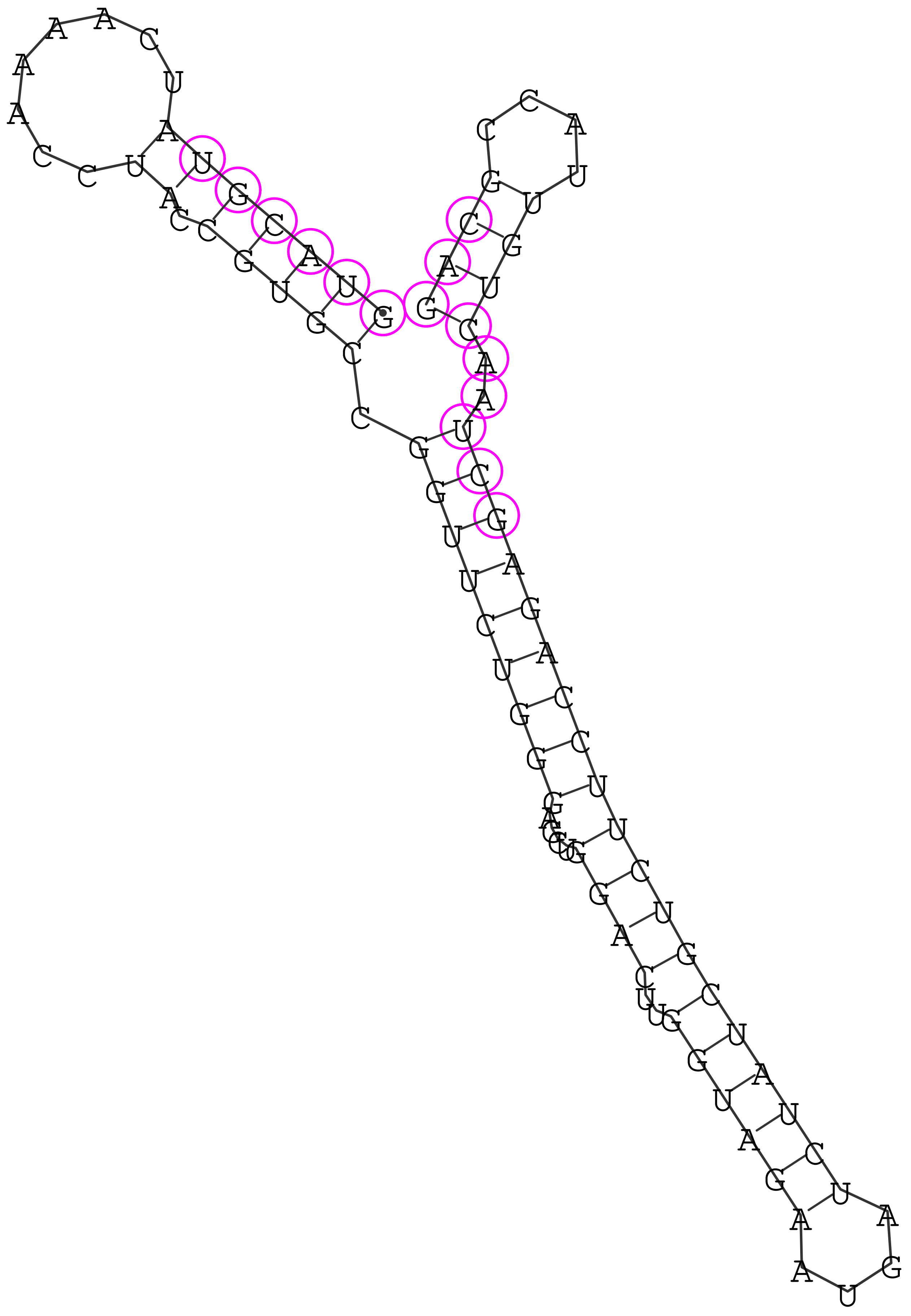

Xbamc156A - Internal intron

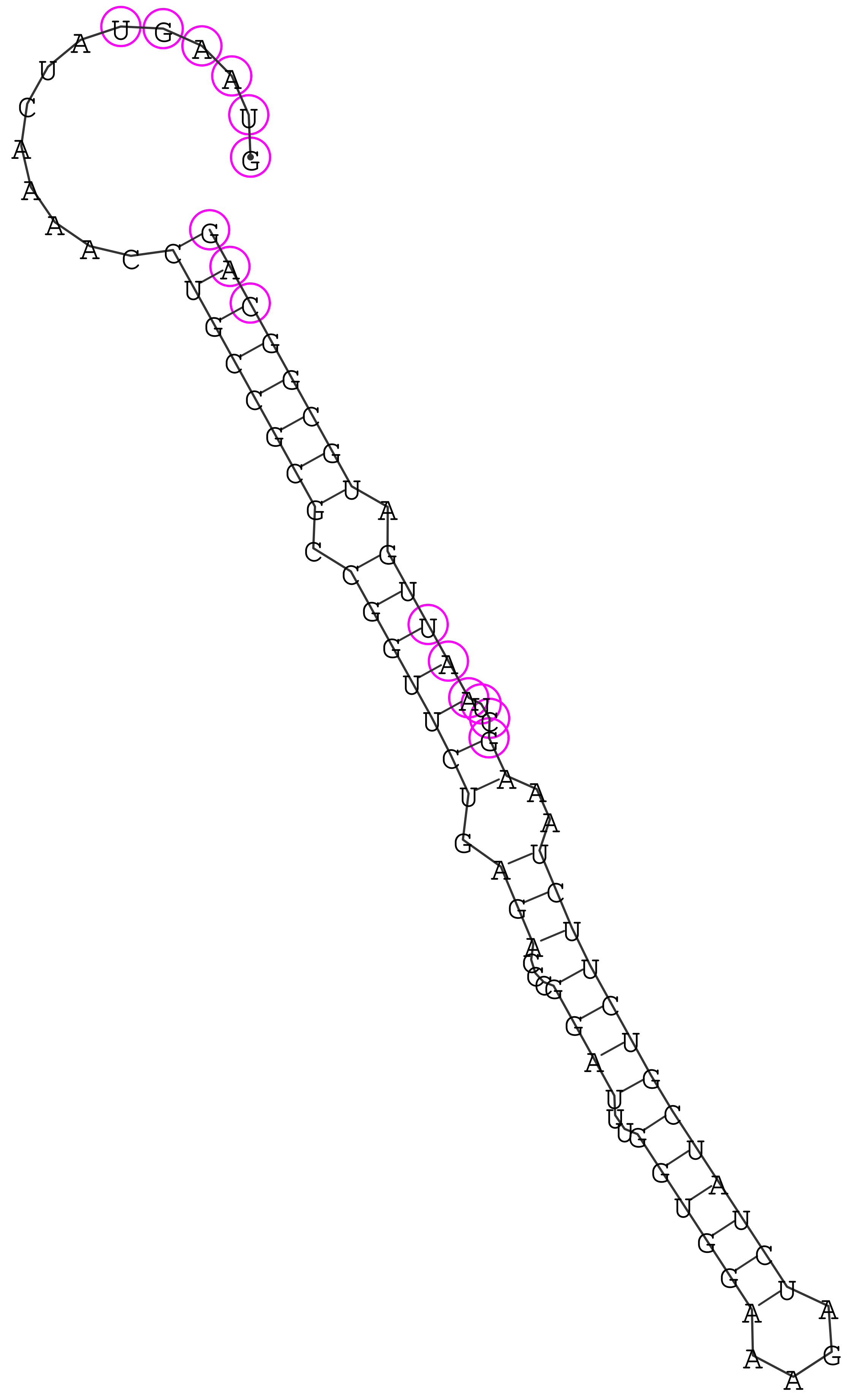

Xbamc159A - Internal intron

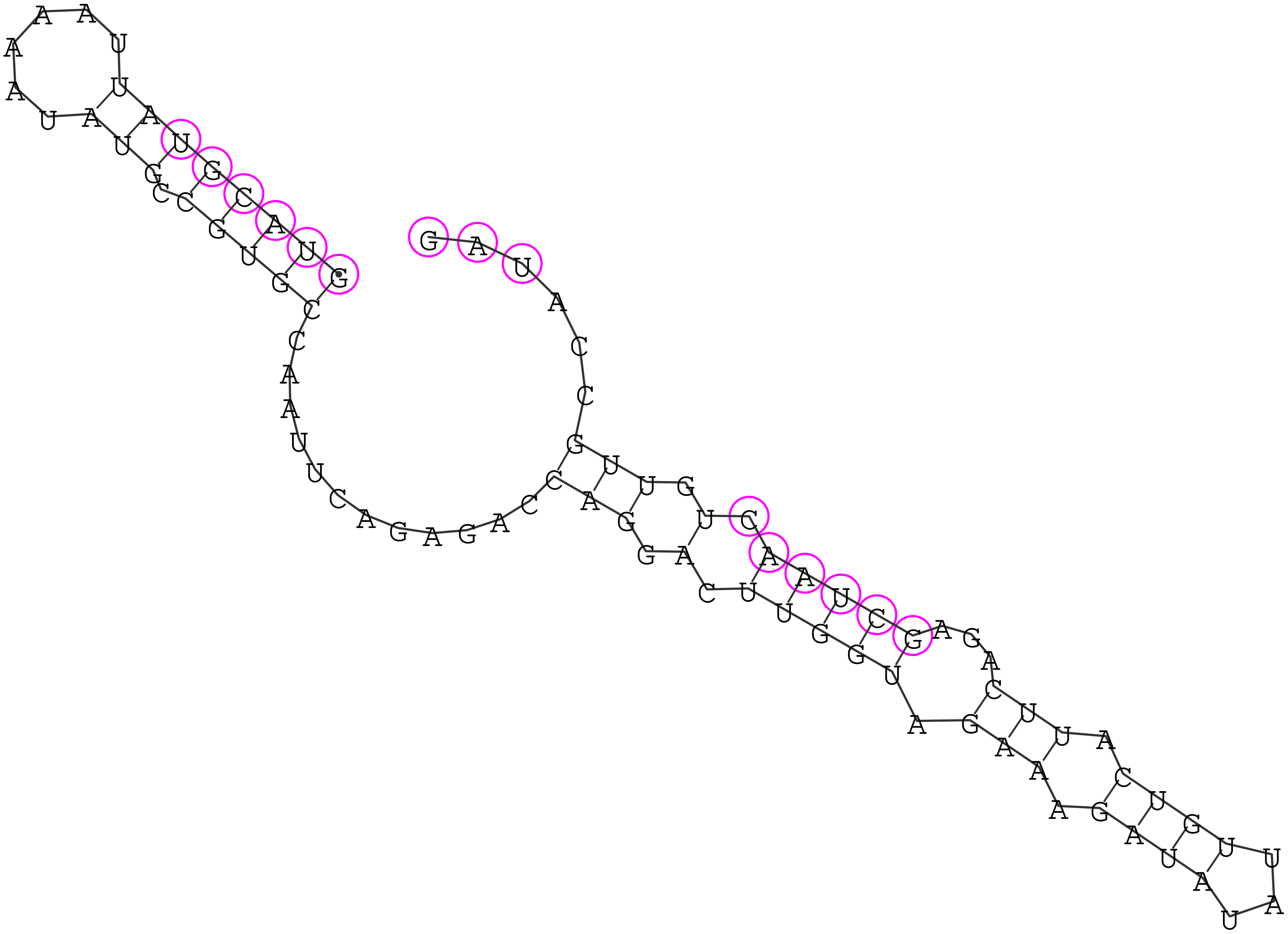

Xbamc164A - Internal intron

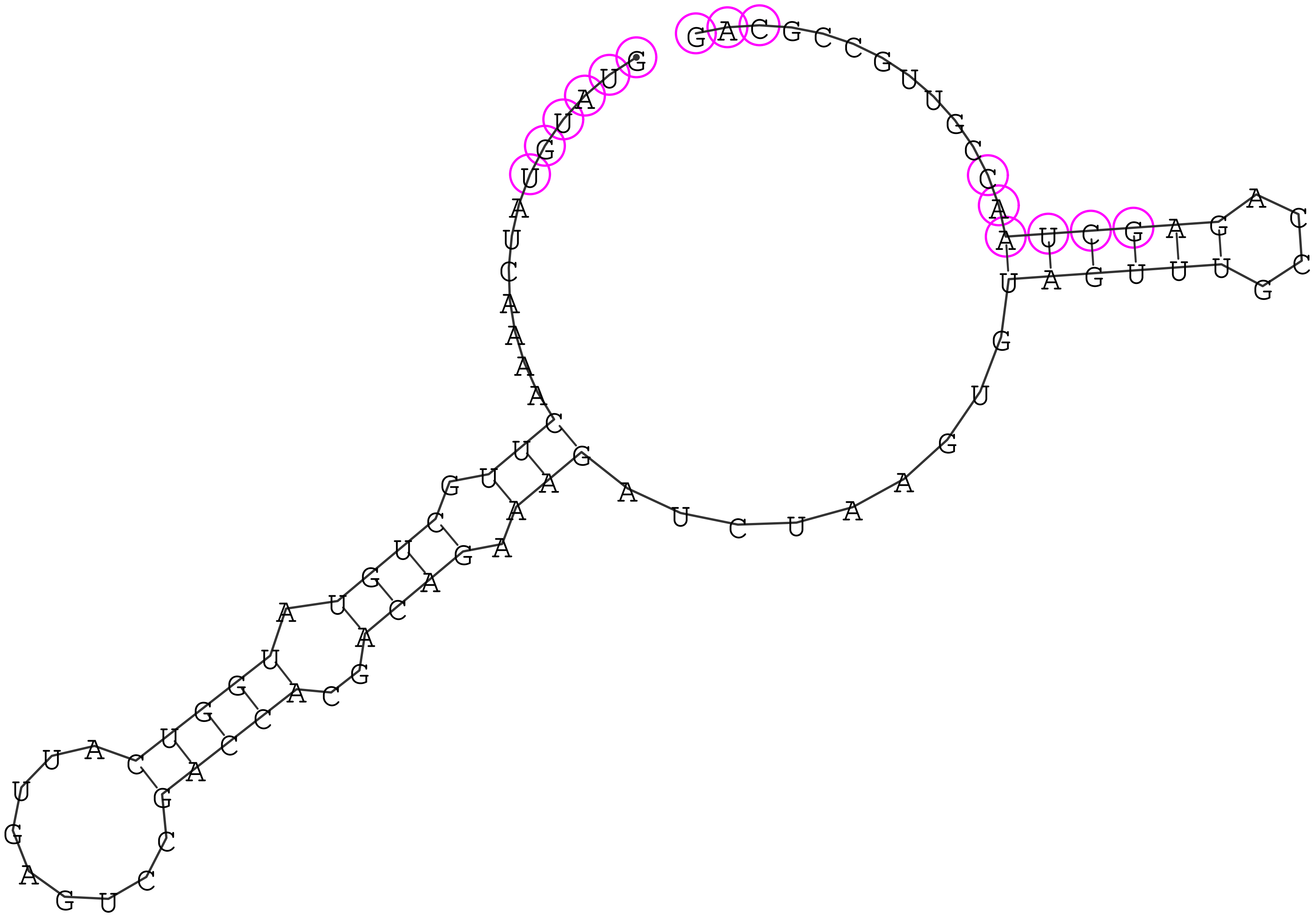

Xbamc176A - Internal intron

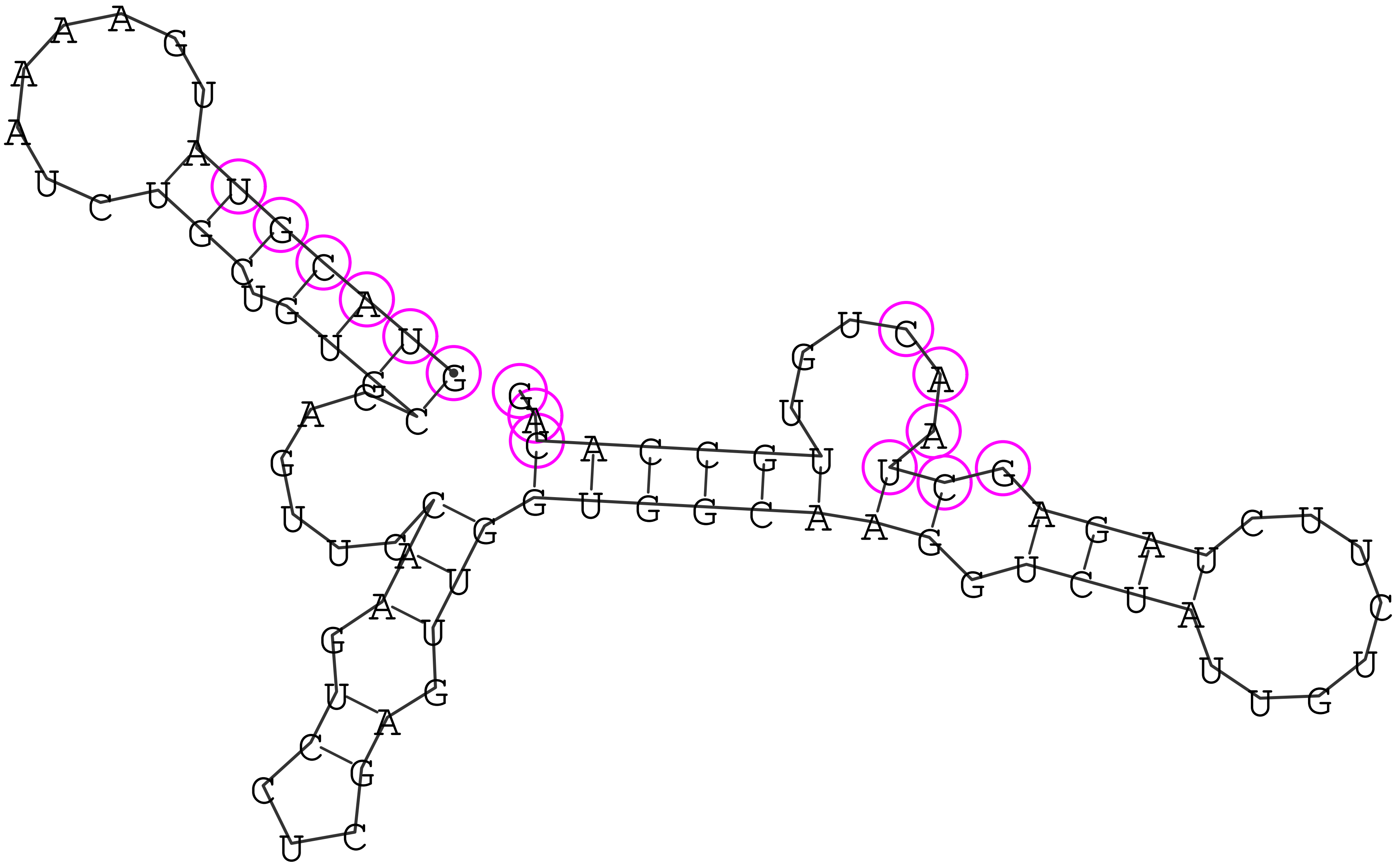

Xbamc177A - Internal intron

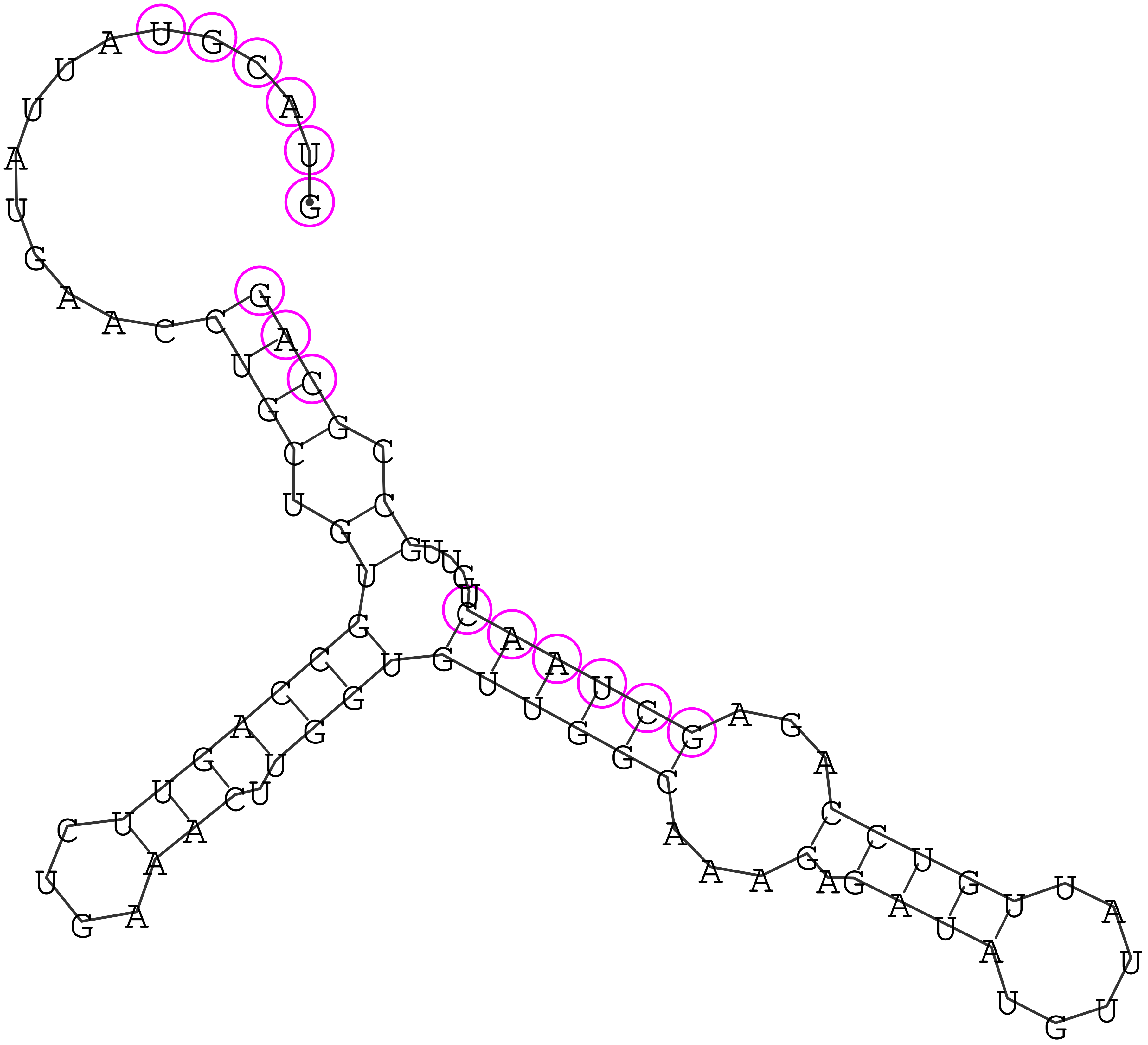

Xbamc191A - Internal intron

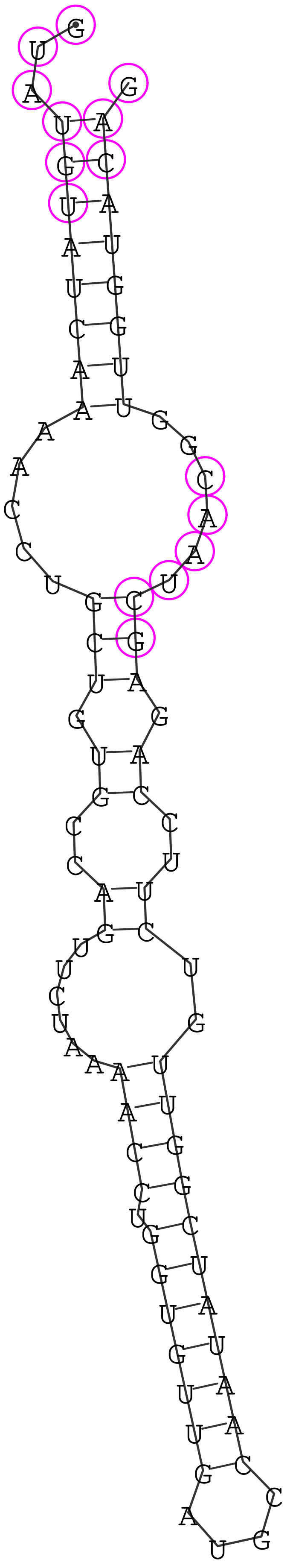

Xbamc198A - Internal intron

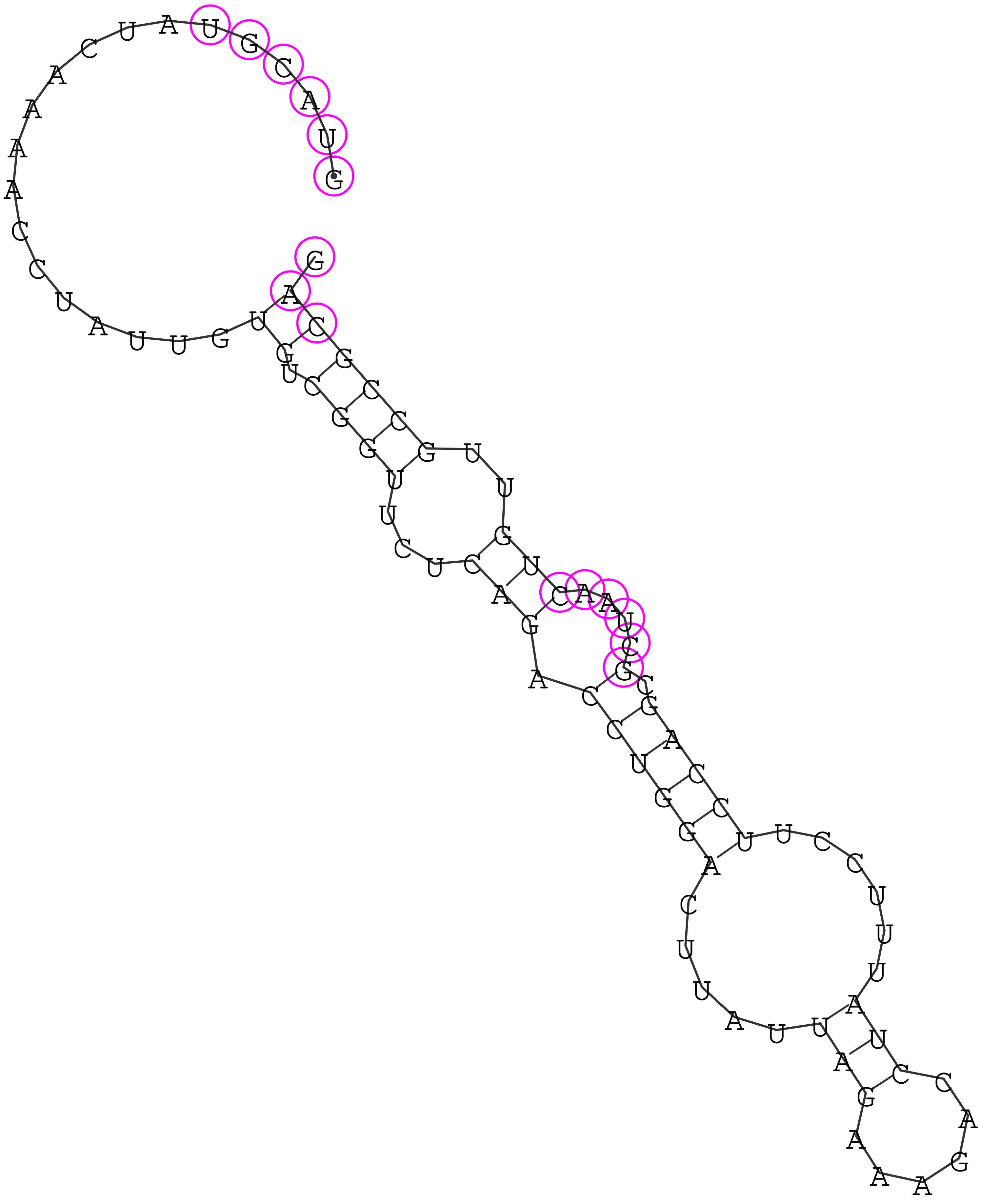

Xbamc199A - Internal intron

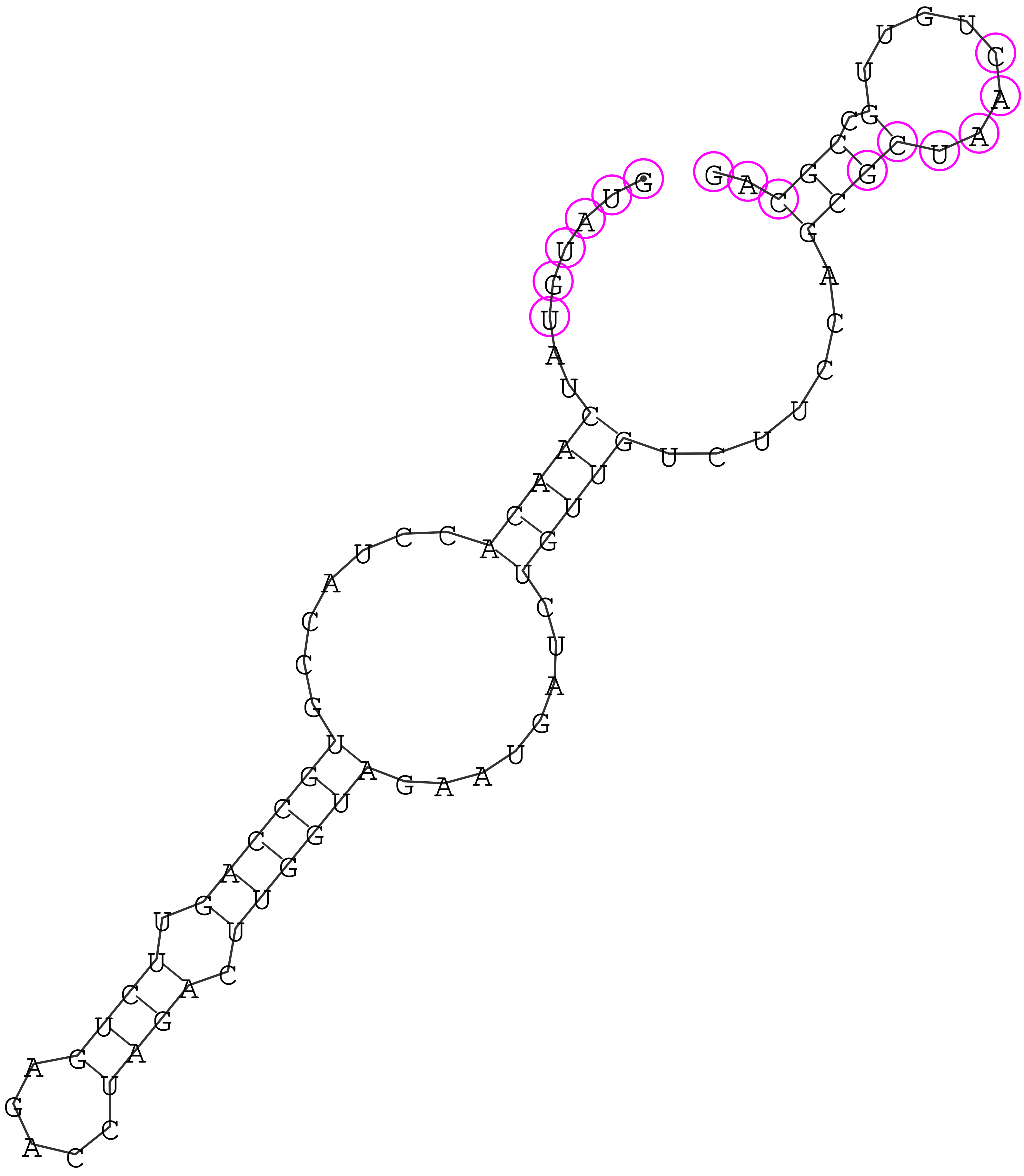

Xbamc236C - Internal intron

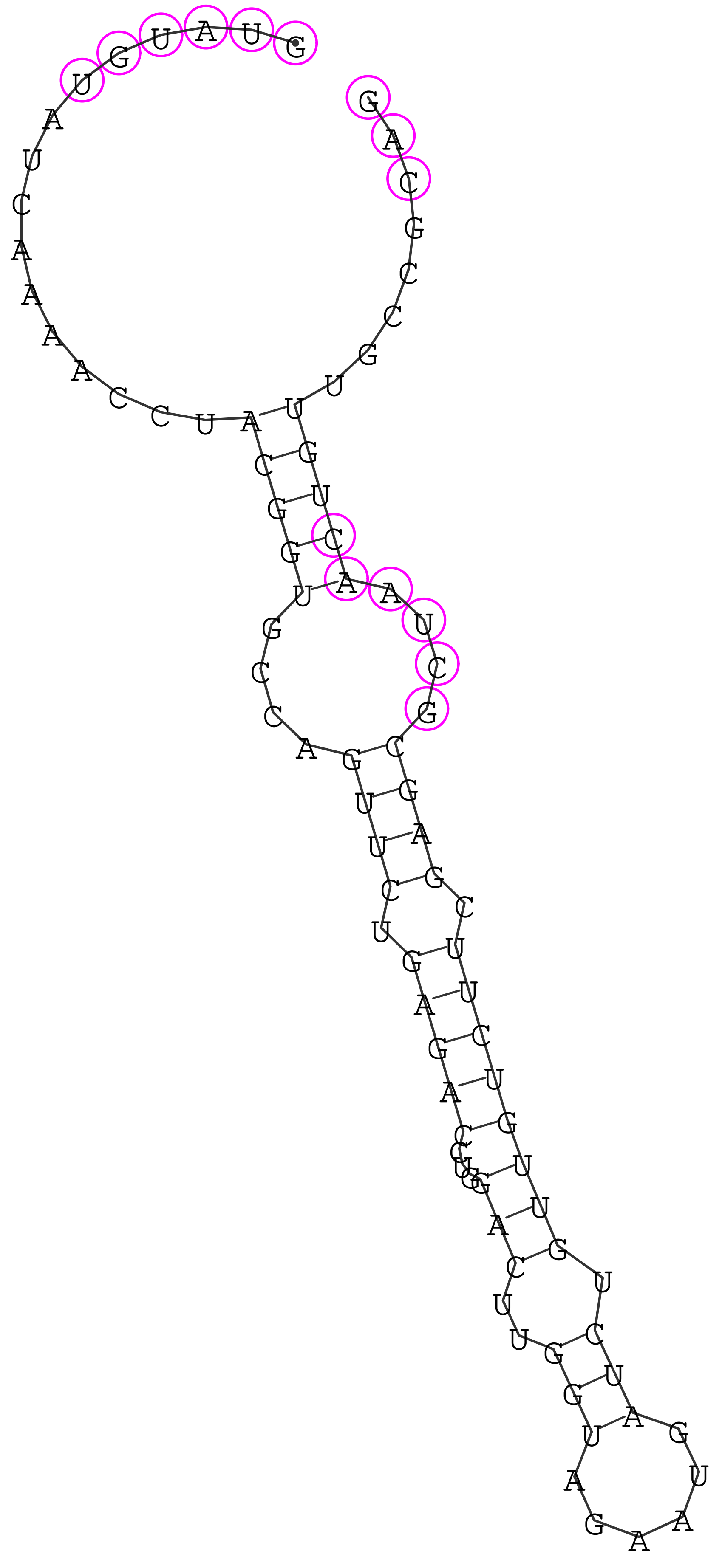

Xbccc01A - Internal intron

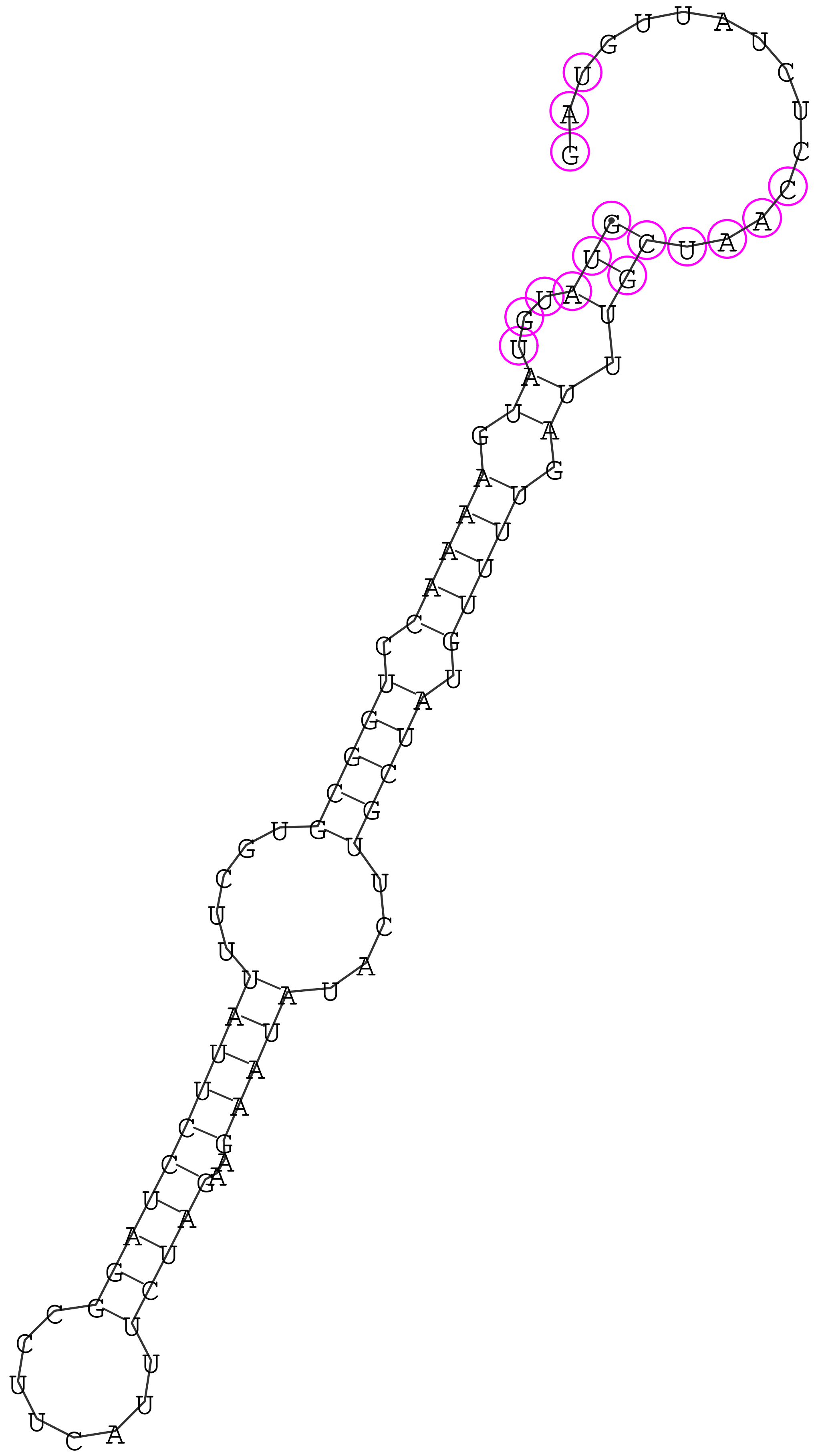

Xbccc01B - Internal intron

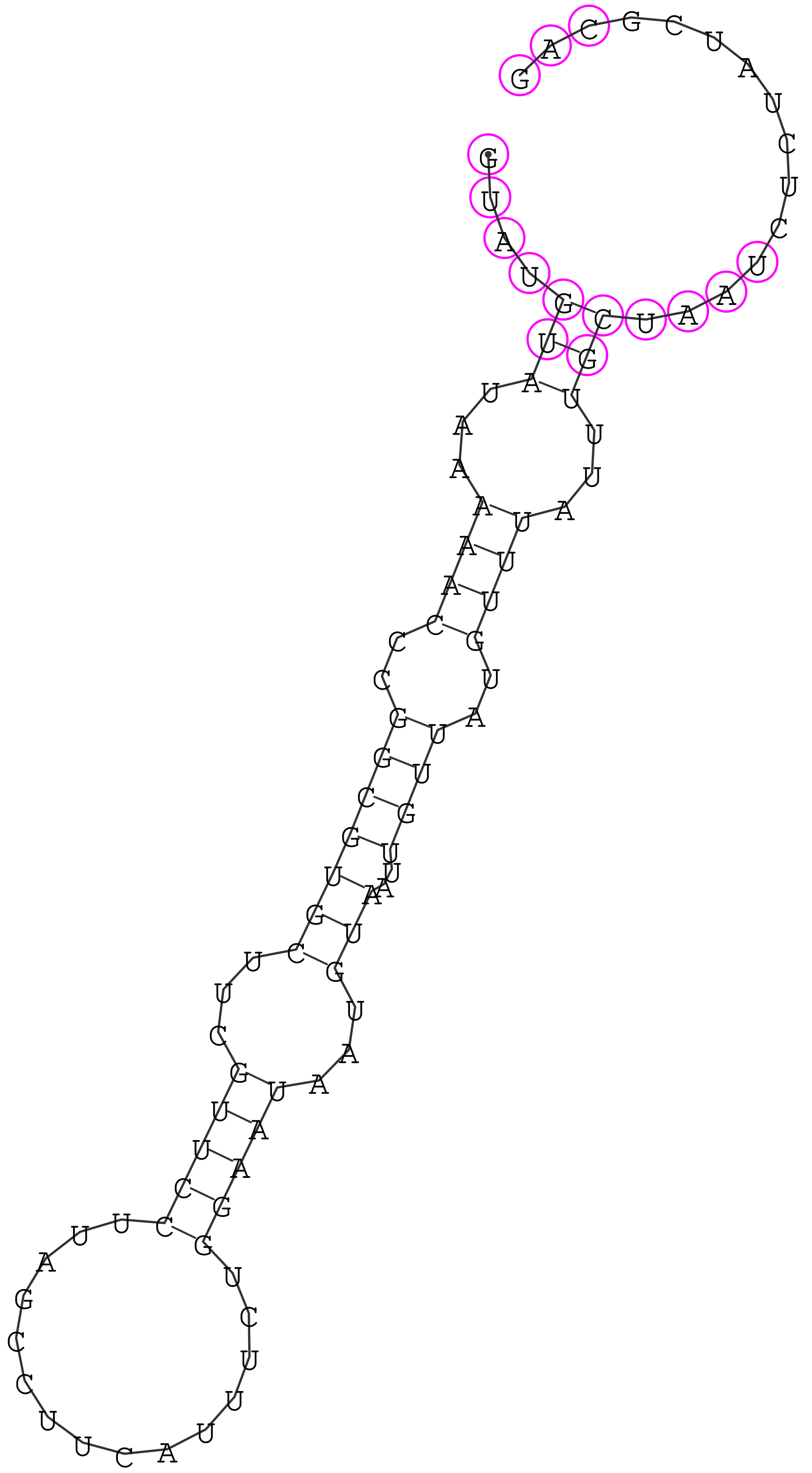

Xbccc05A - Internal intron

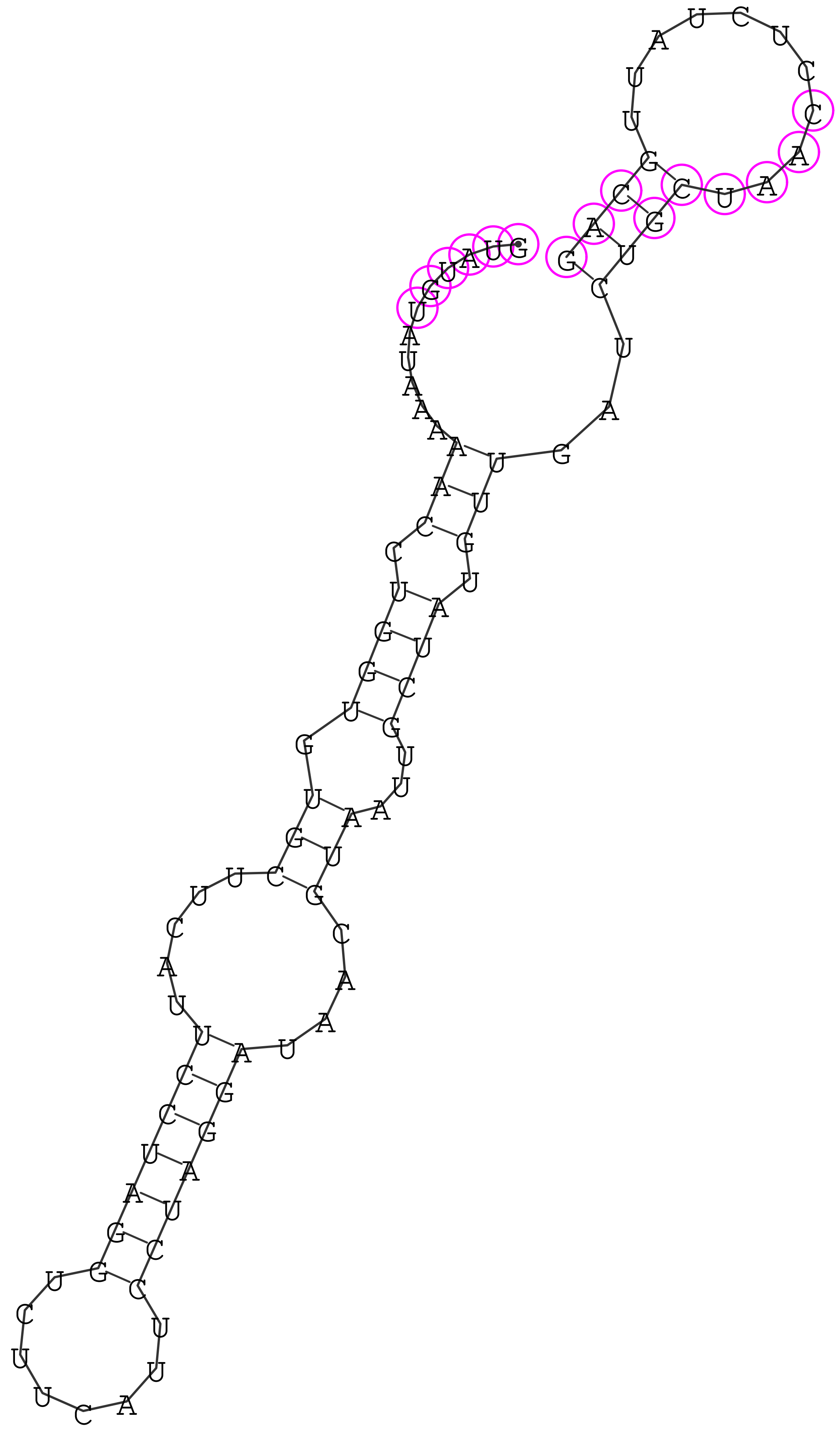

Xbccc05B - Internal intron

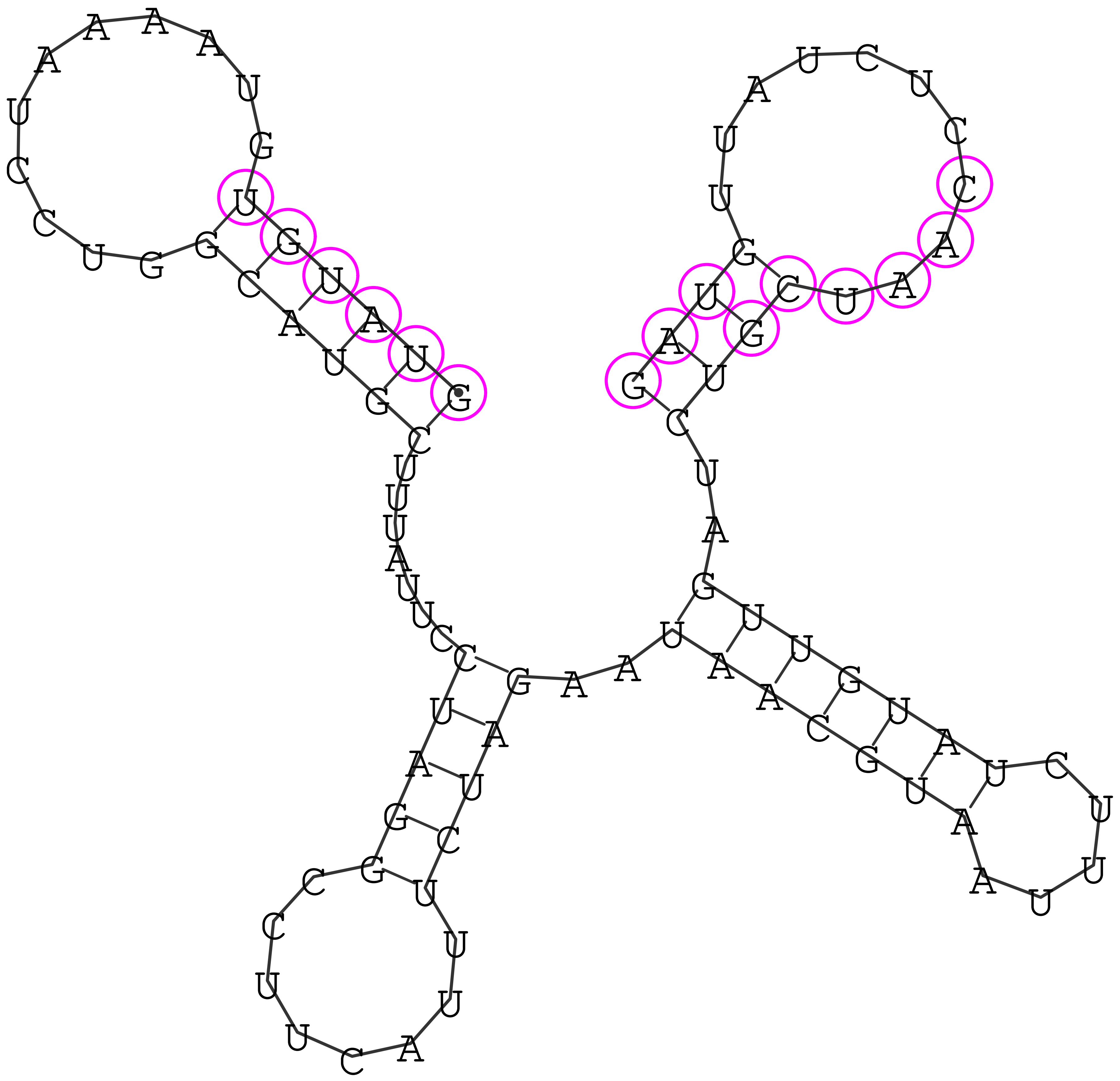

Xbccc05C - Internal intron

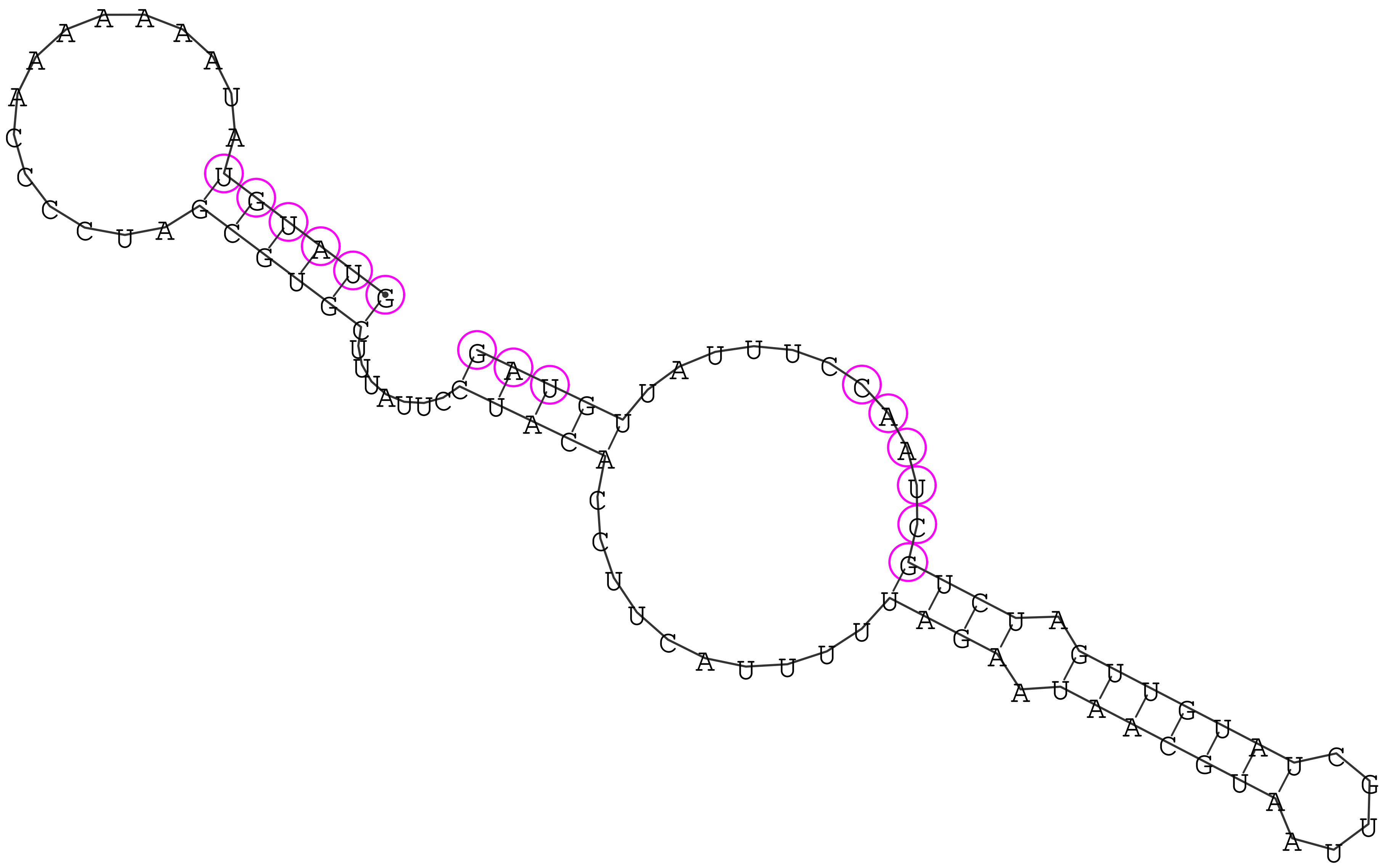

Xbccc06A - Internal intron

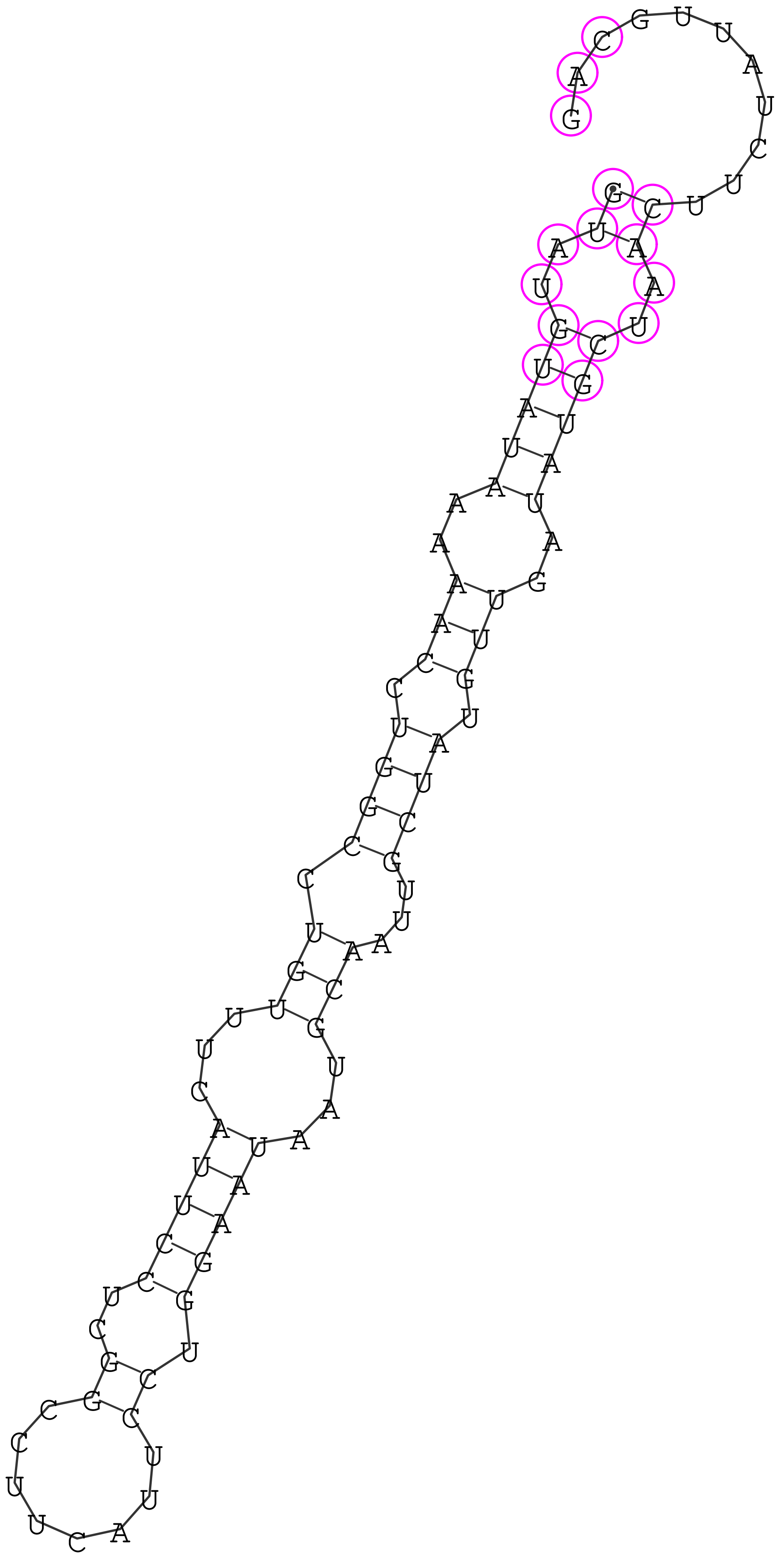

Xbccc07A - Internal intron

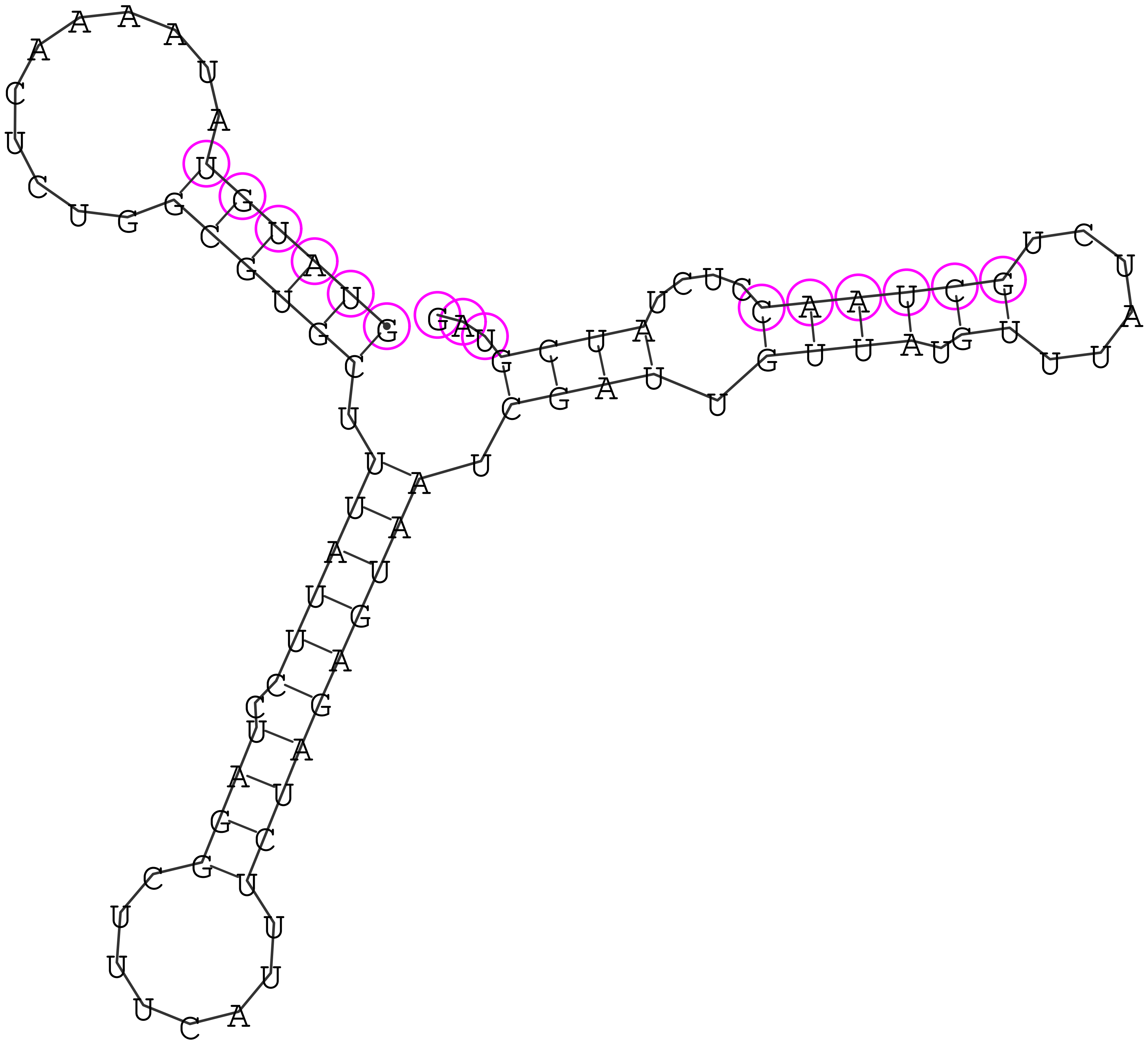

Xbccc09A - Internal intron

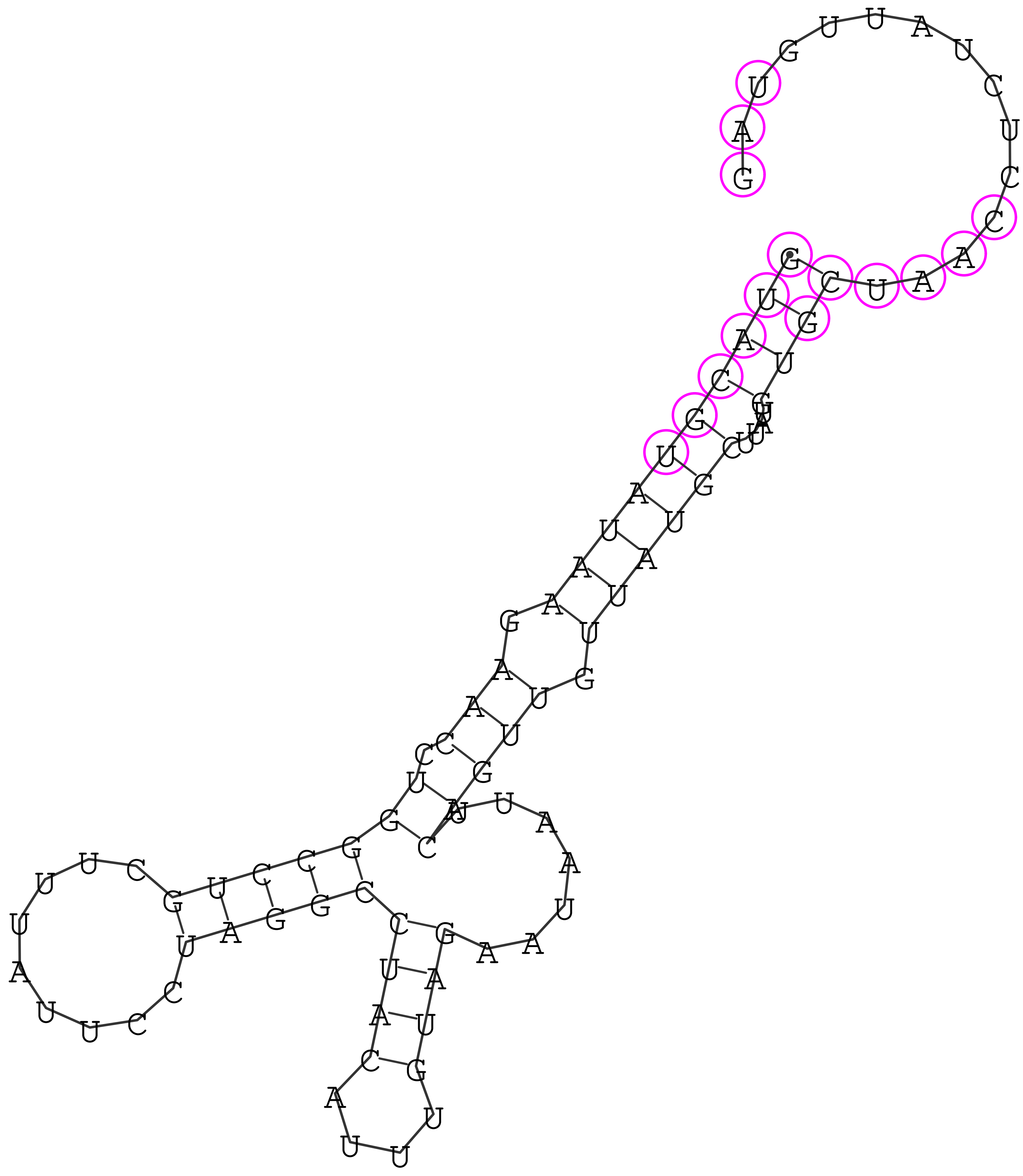

Xlonc0002A - Internal intron

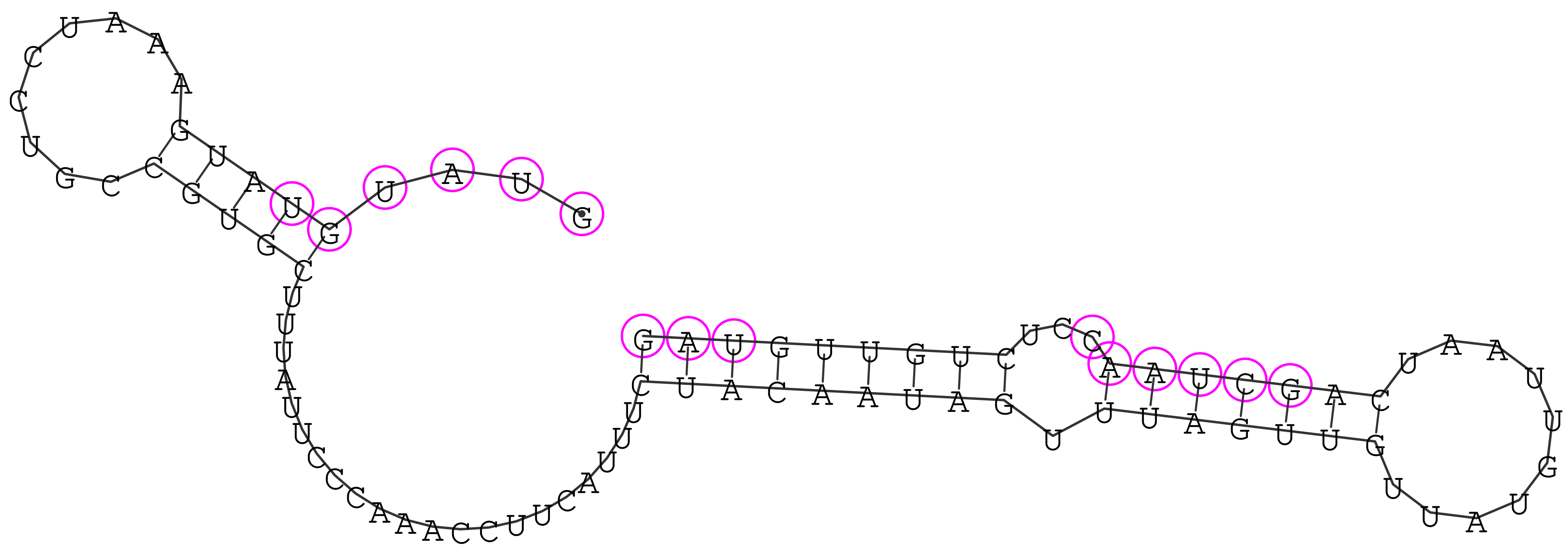

Xlonc0025A - Internal intron

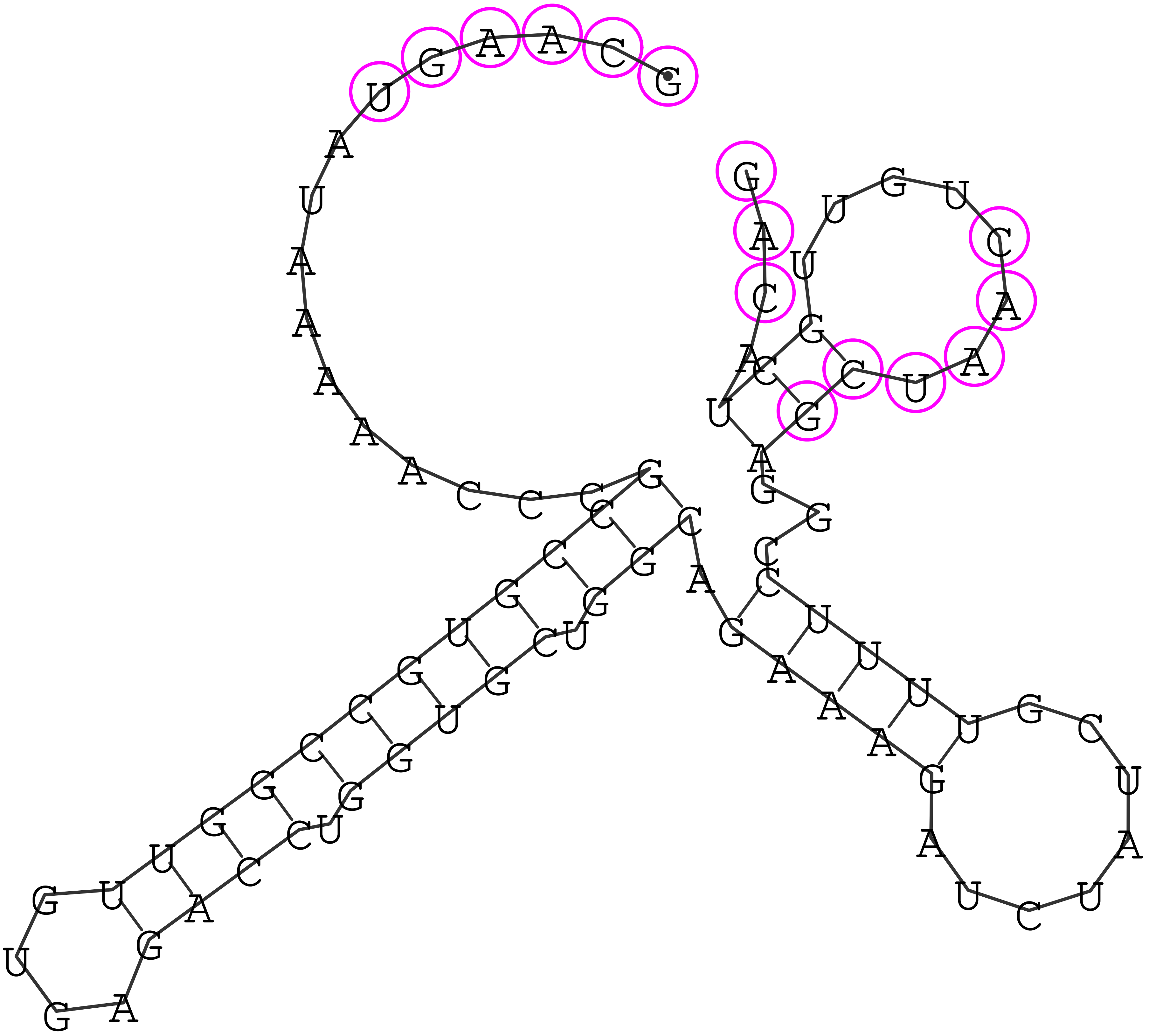

Xlonc0058A - Internal intron

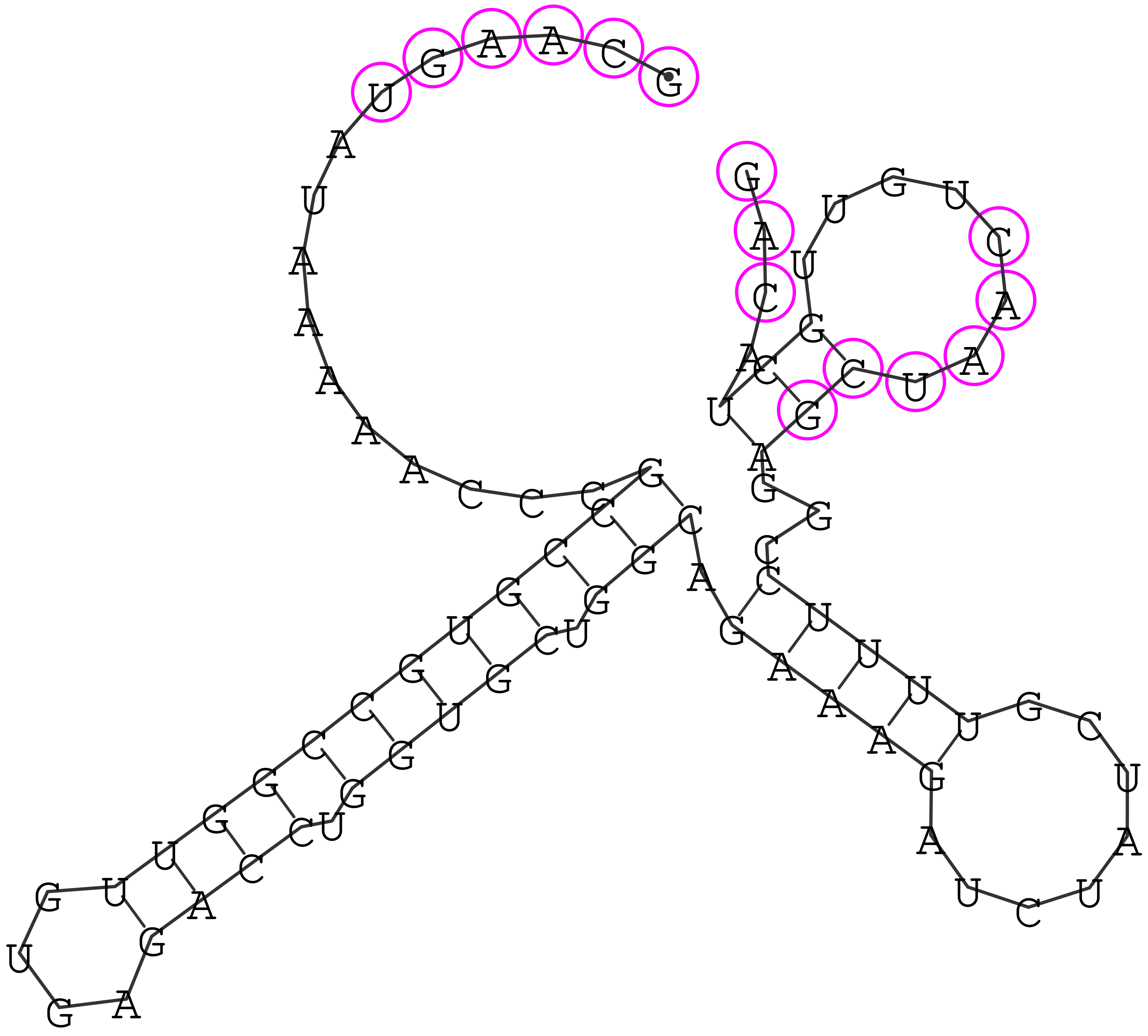

Xlonc0112A - Internal intron

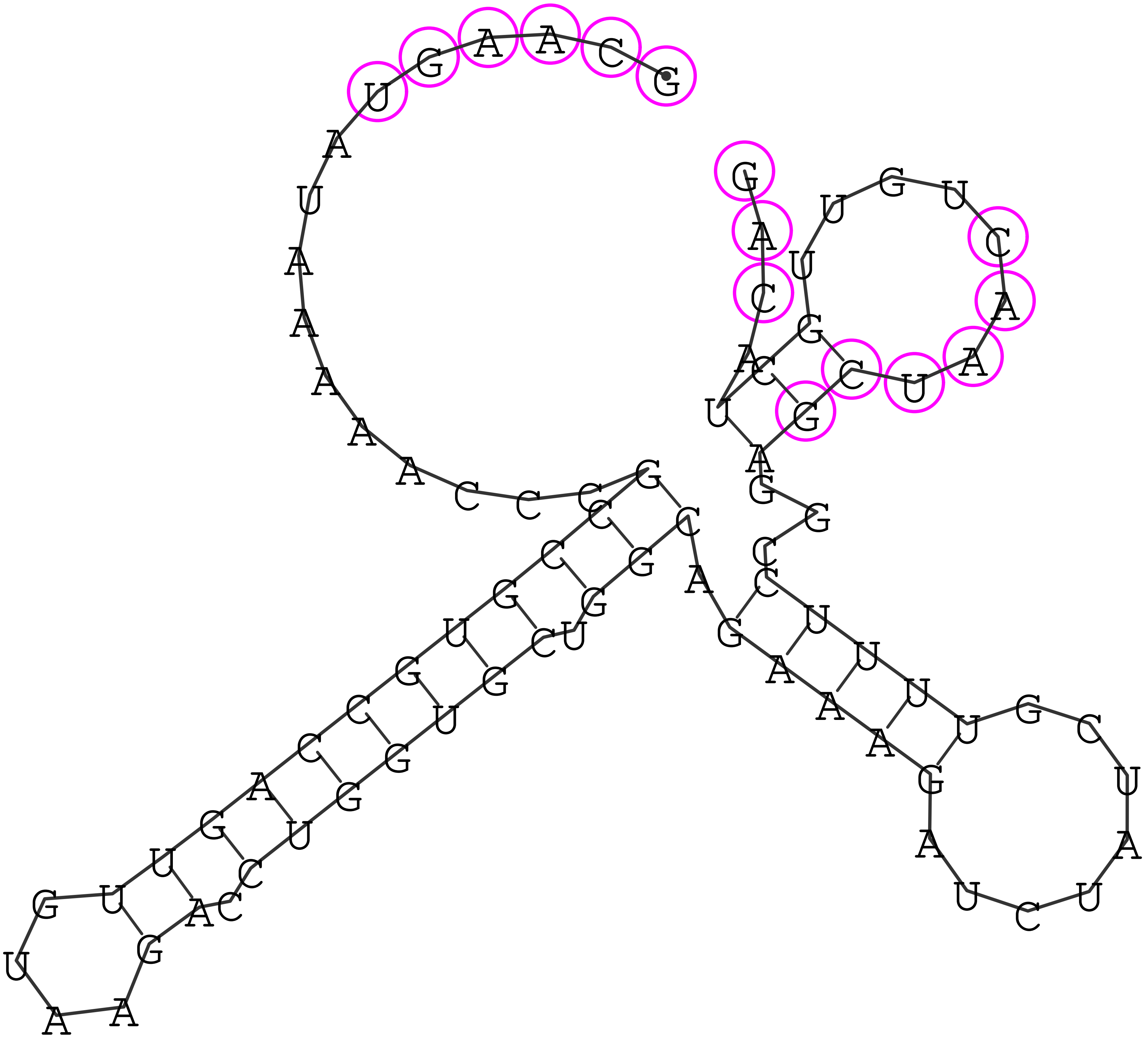

Xmsuc0005A - Internal intron

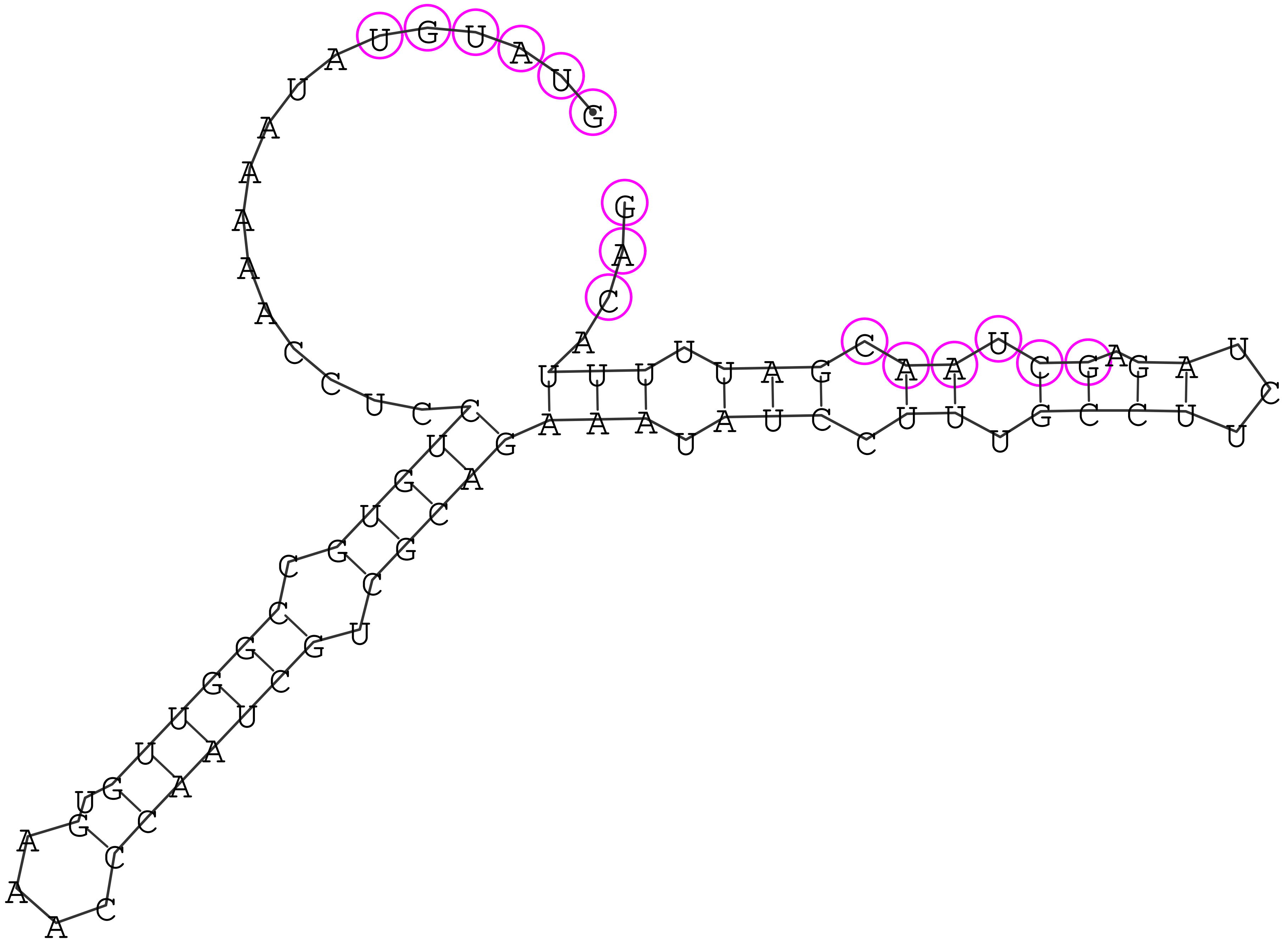

Xmsuc0006A - Internal intron

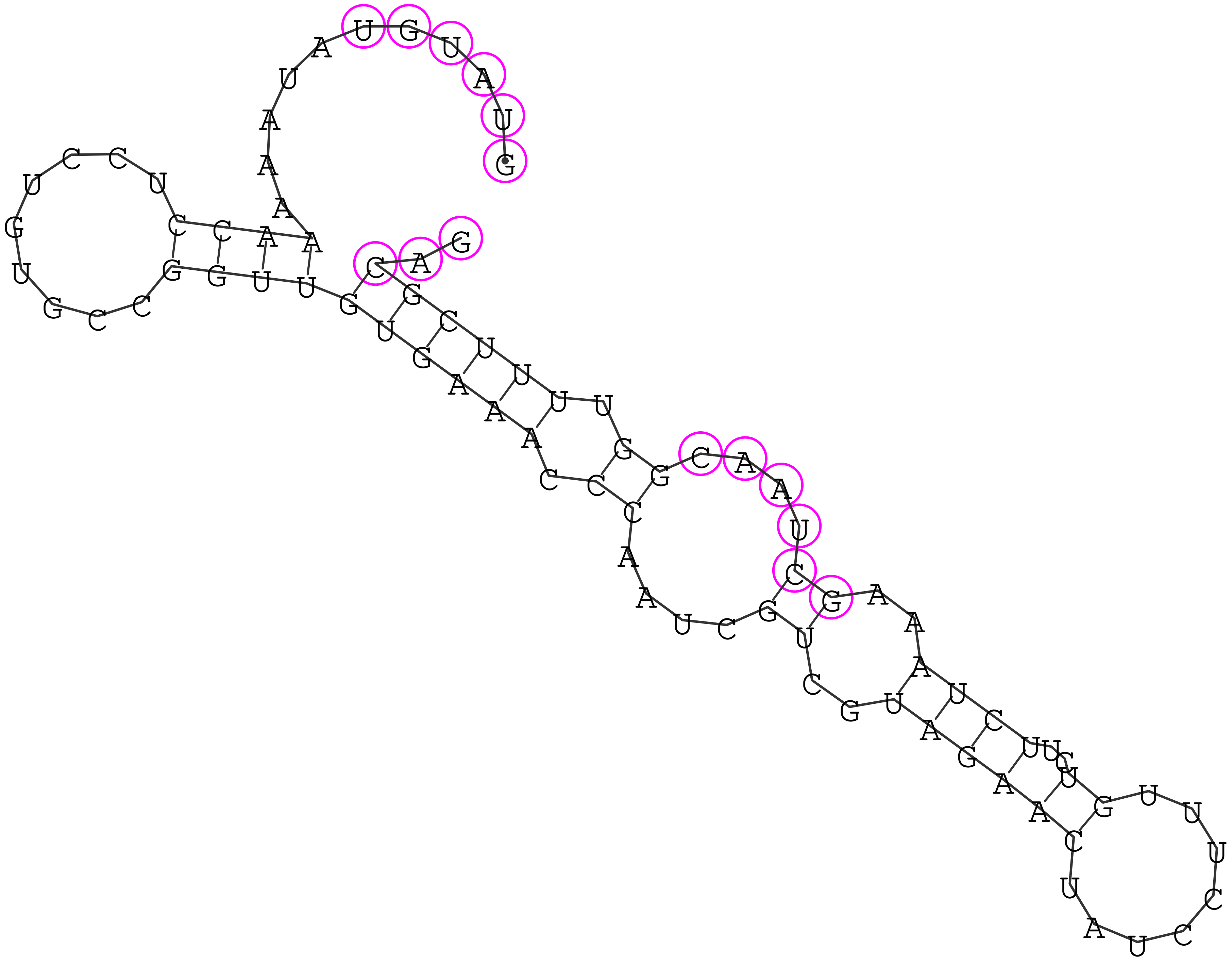

Xmsuc0006B - Internal intron

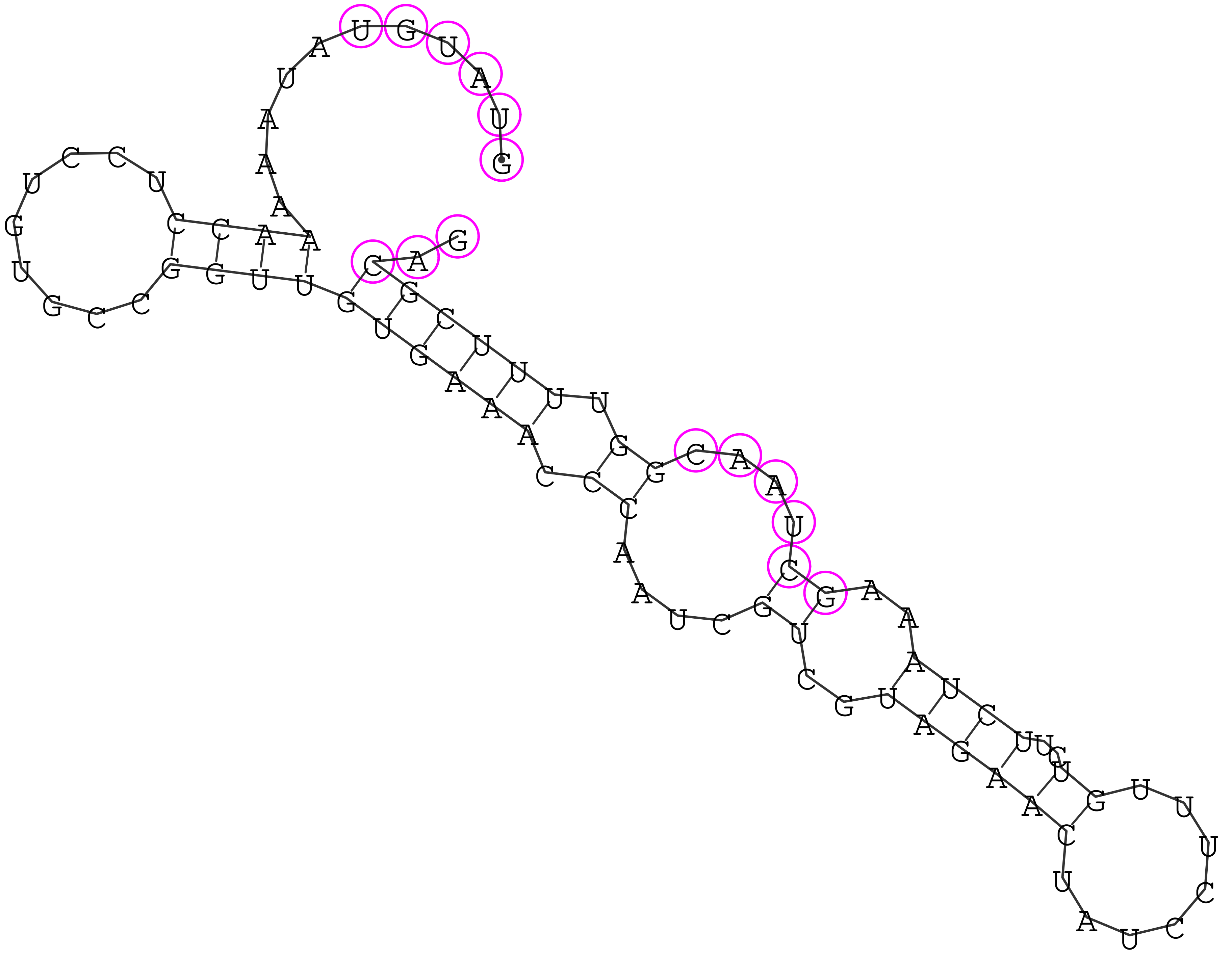

Xmsuc0009A - Internal intron

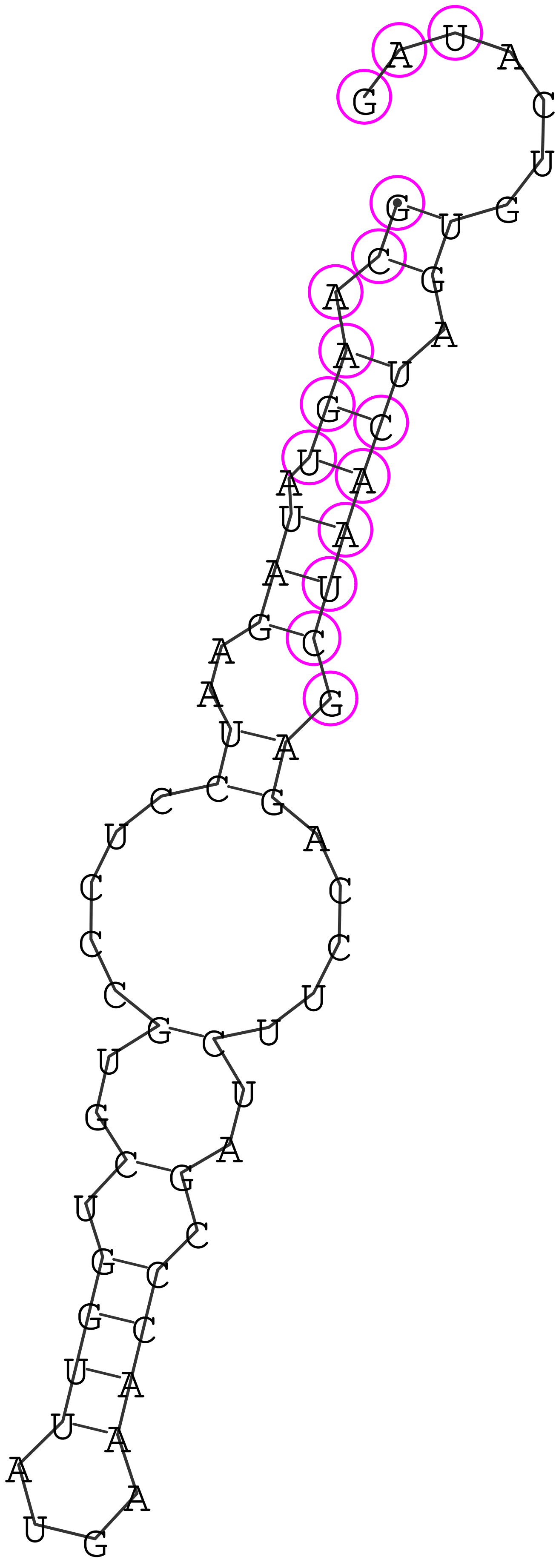

Xmsuc0018A - Internal intron

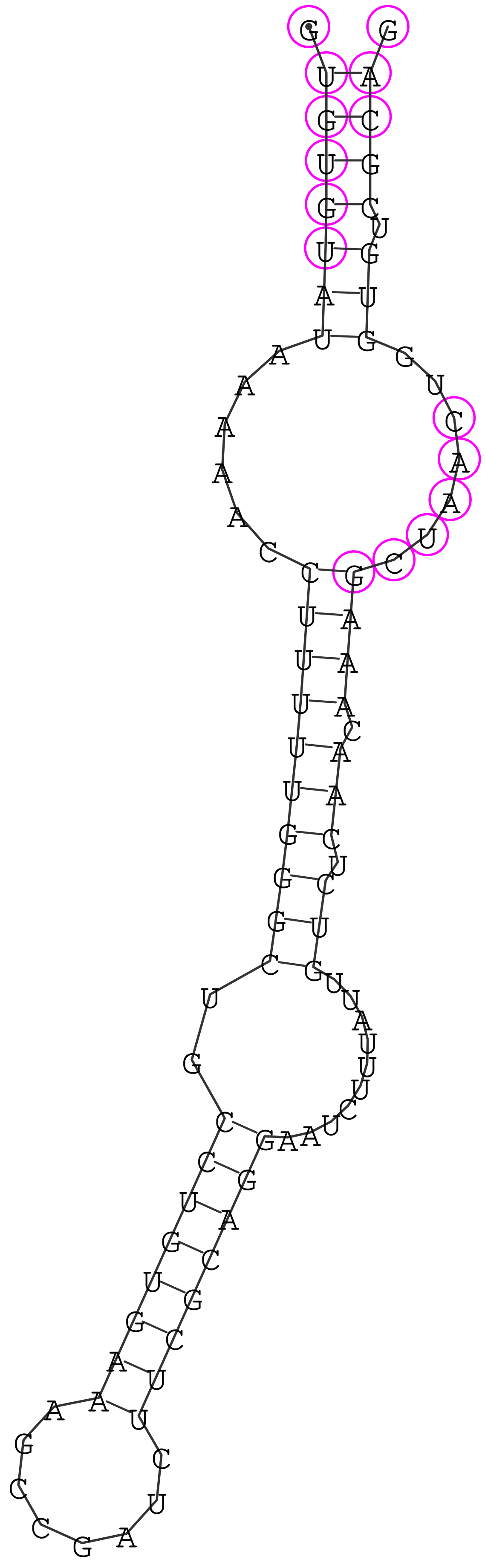

Xmsuc0018B - Internal intron

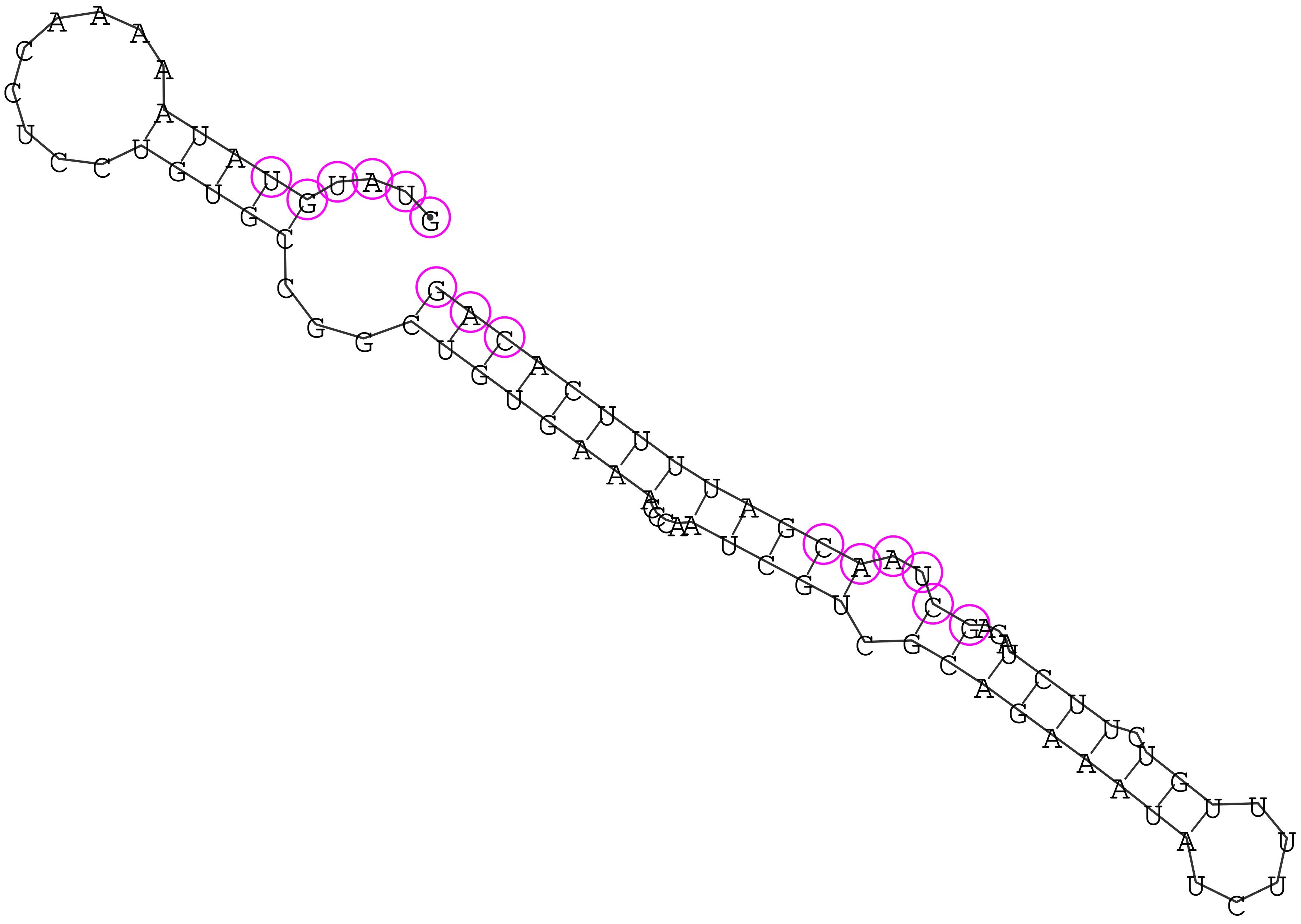

Xmsuc0019A - Internal intron

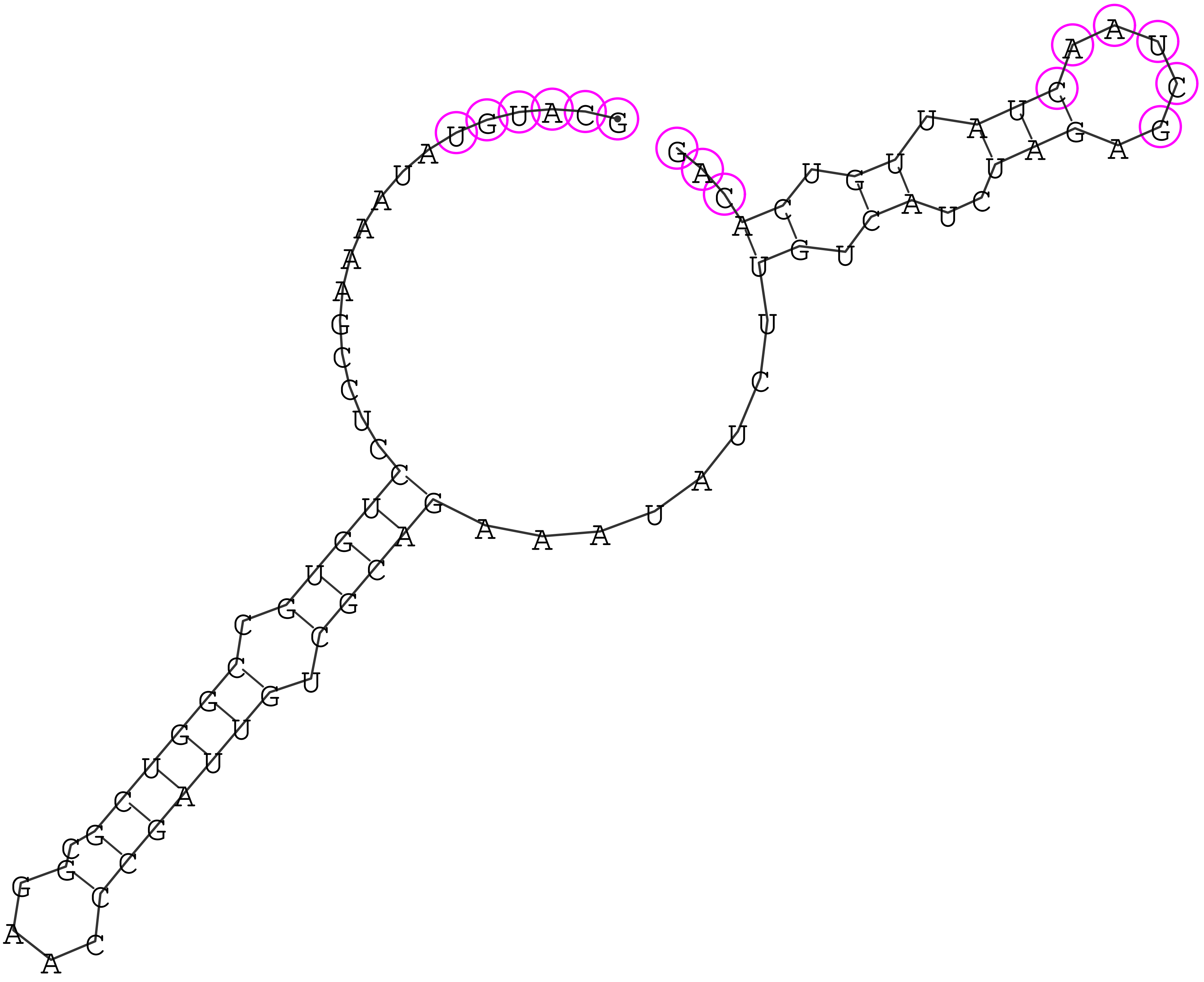

Xmsuc0028A - Internal intron

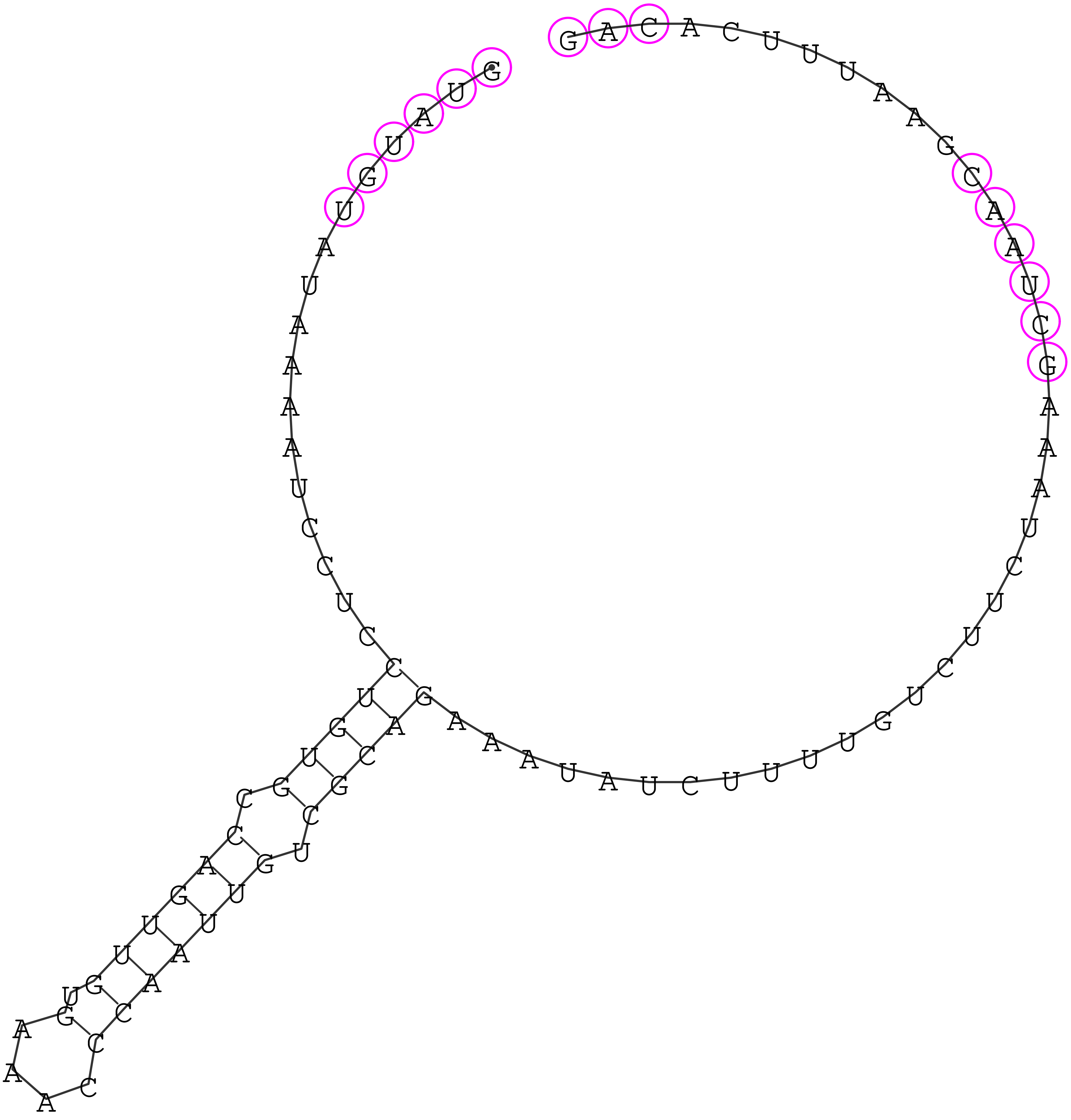

Xmsuc0031A - Internal intron

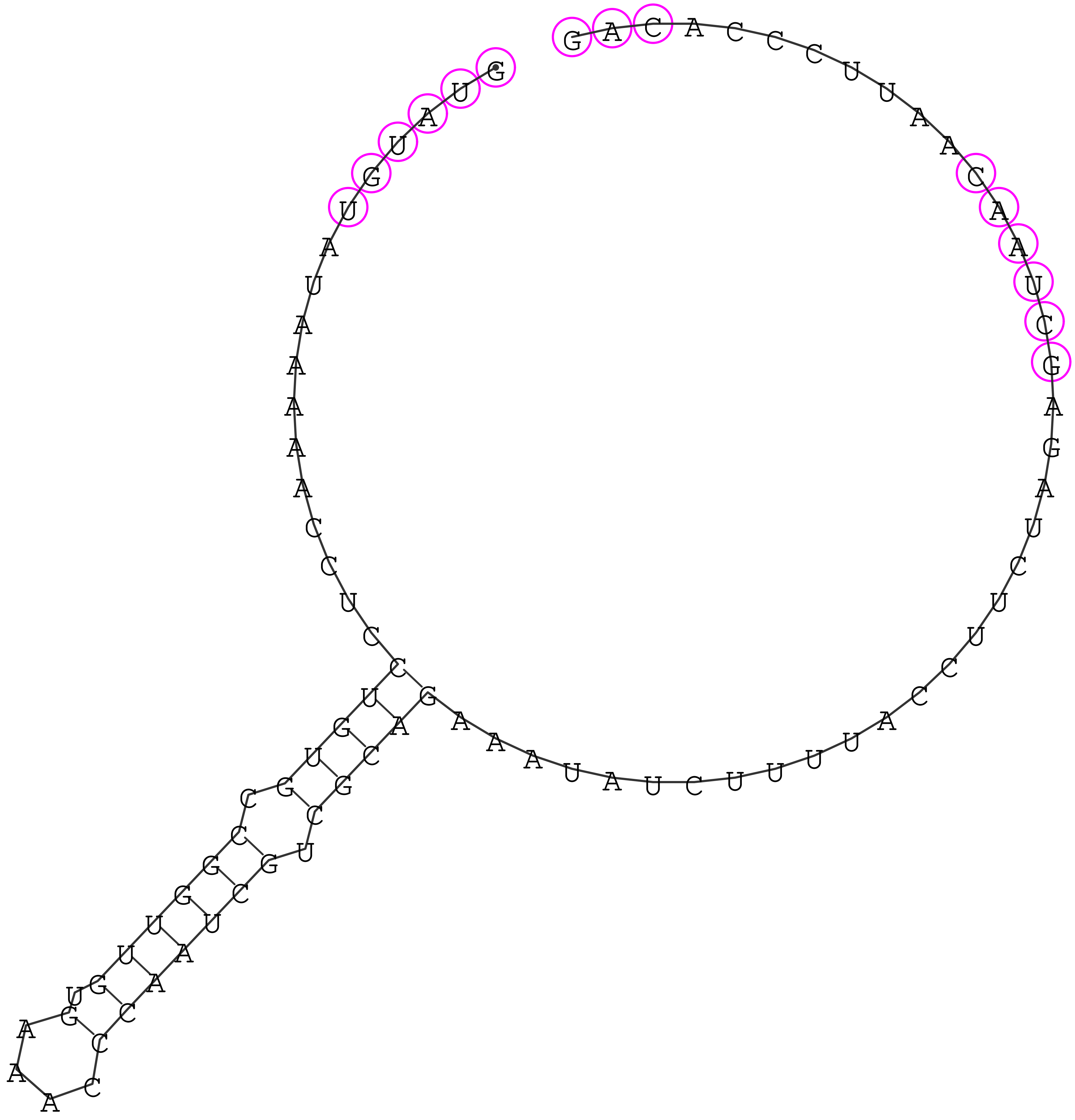

Xmsuc0045A - Internal intron

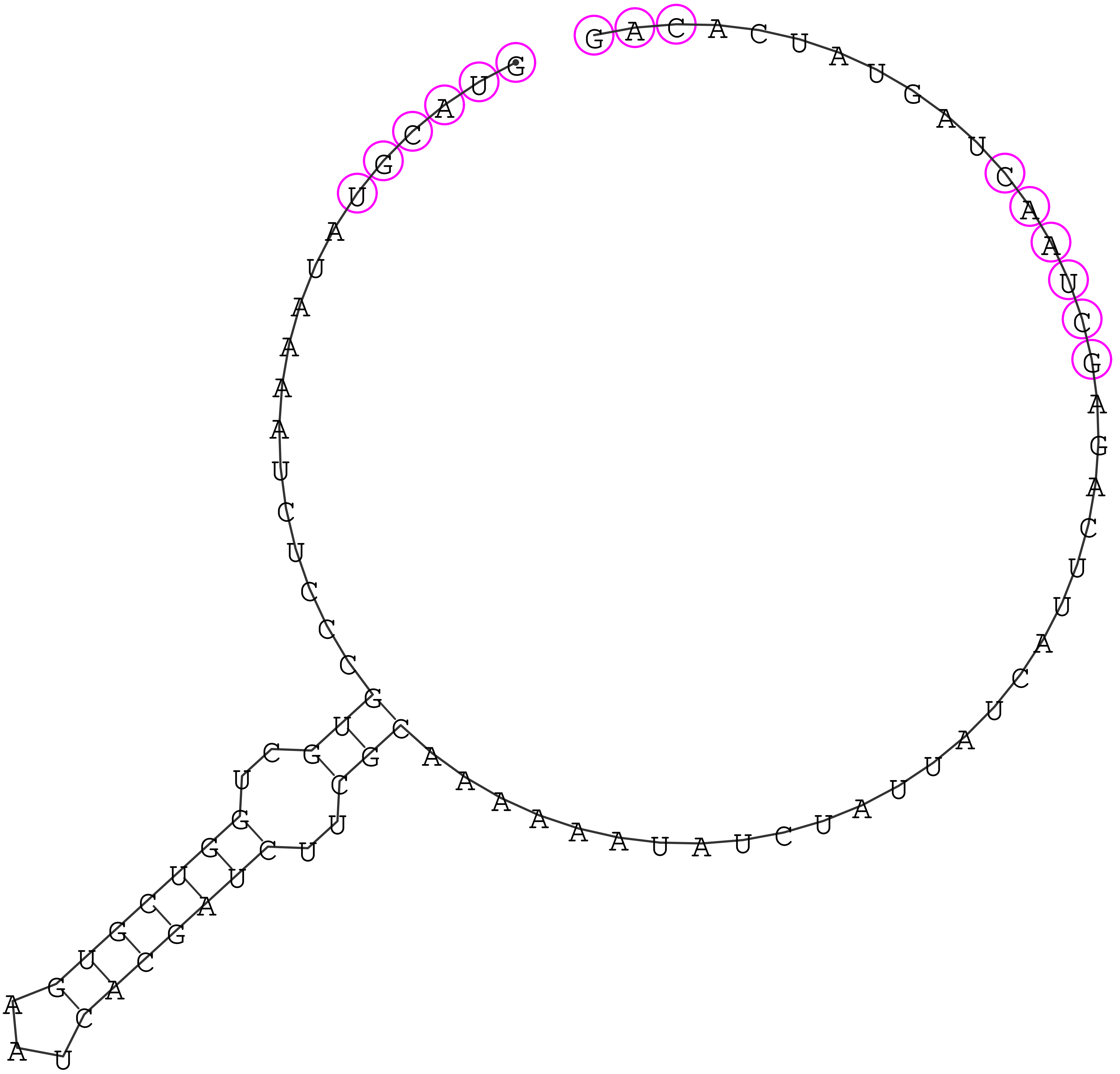

Xmsuc0067A - Internal intron

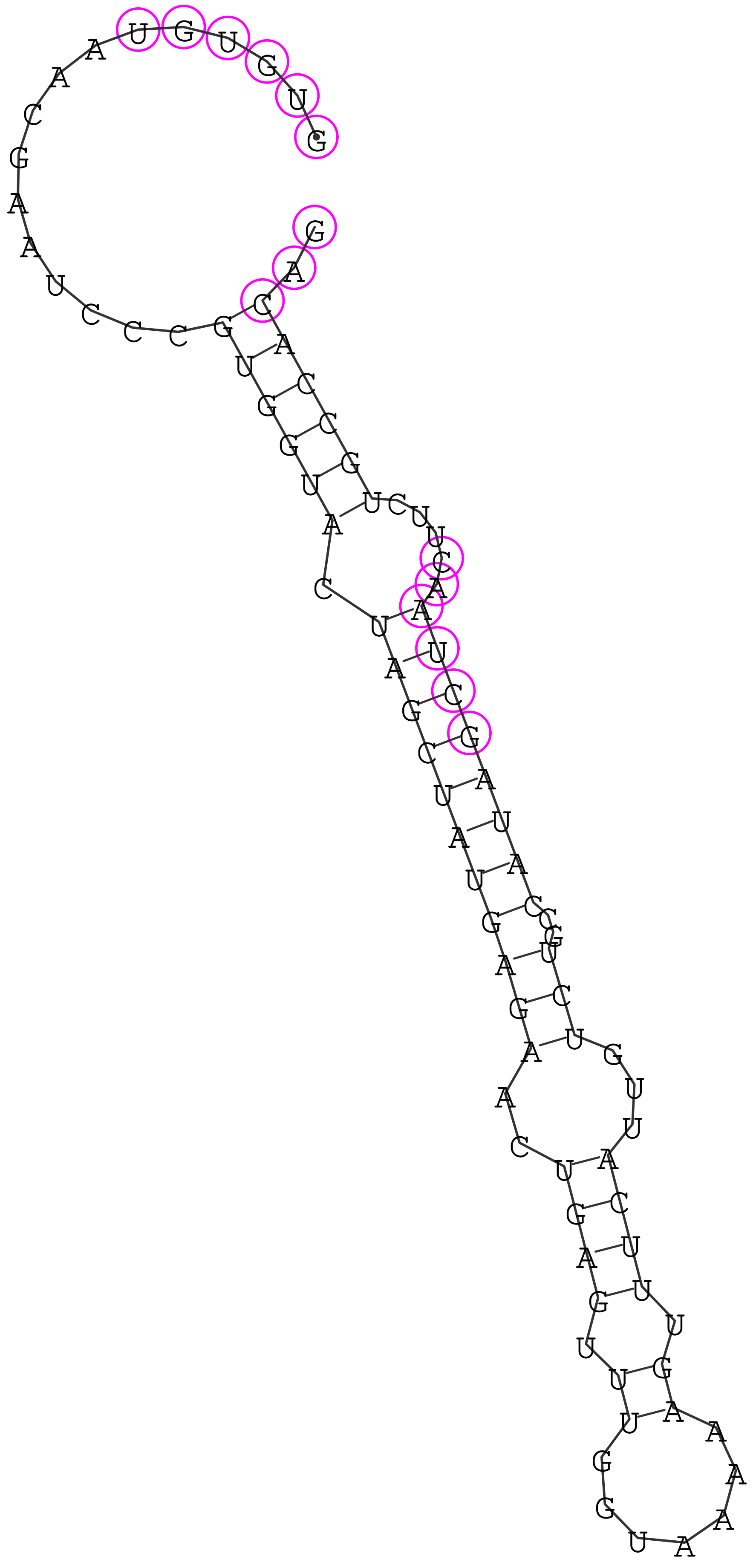

Xmsuc0070A - Internal intron

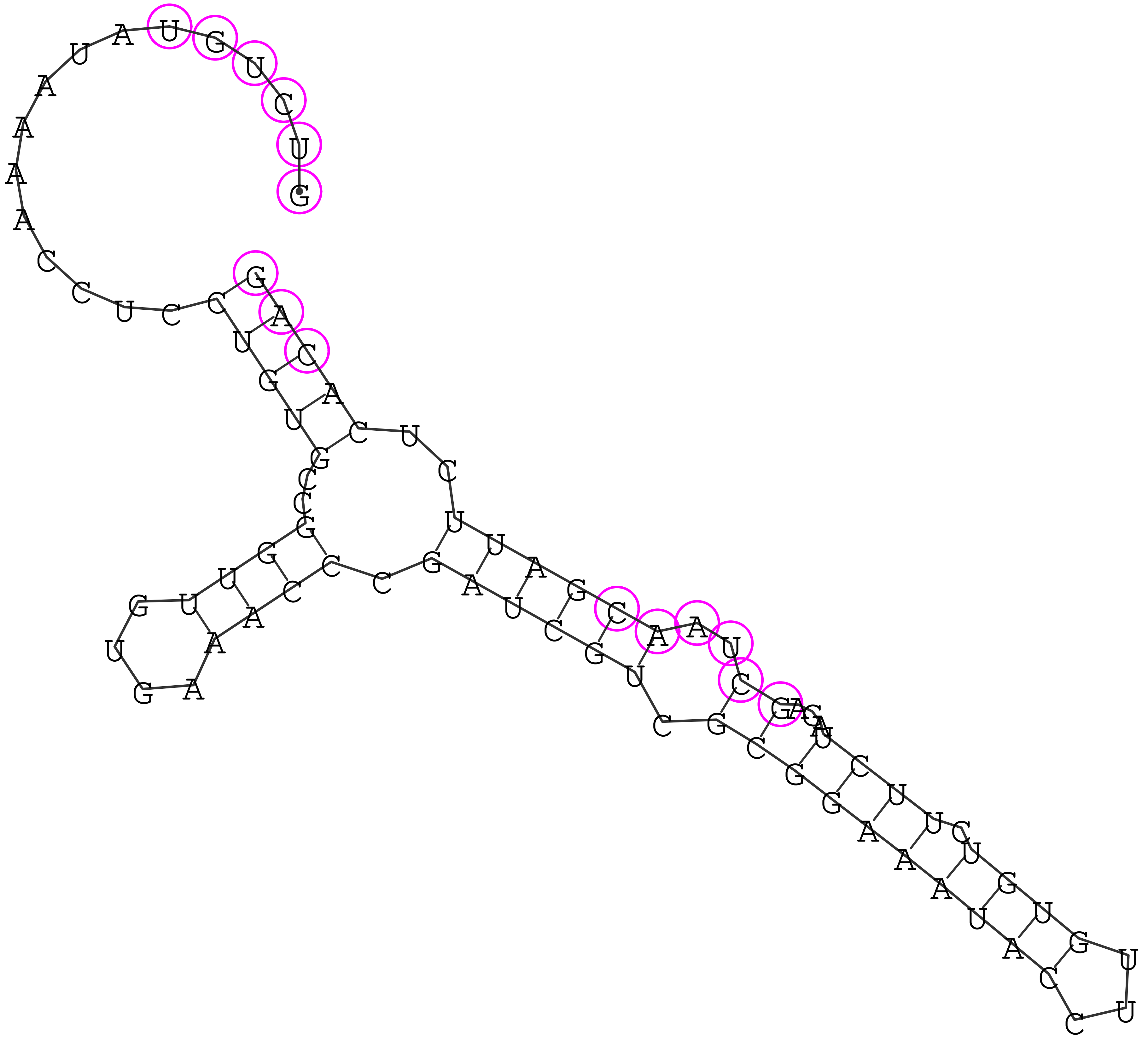

Xmsuc0075A - Internal intron

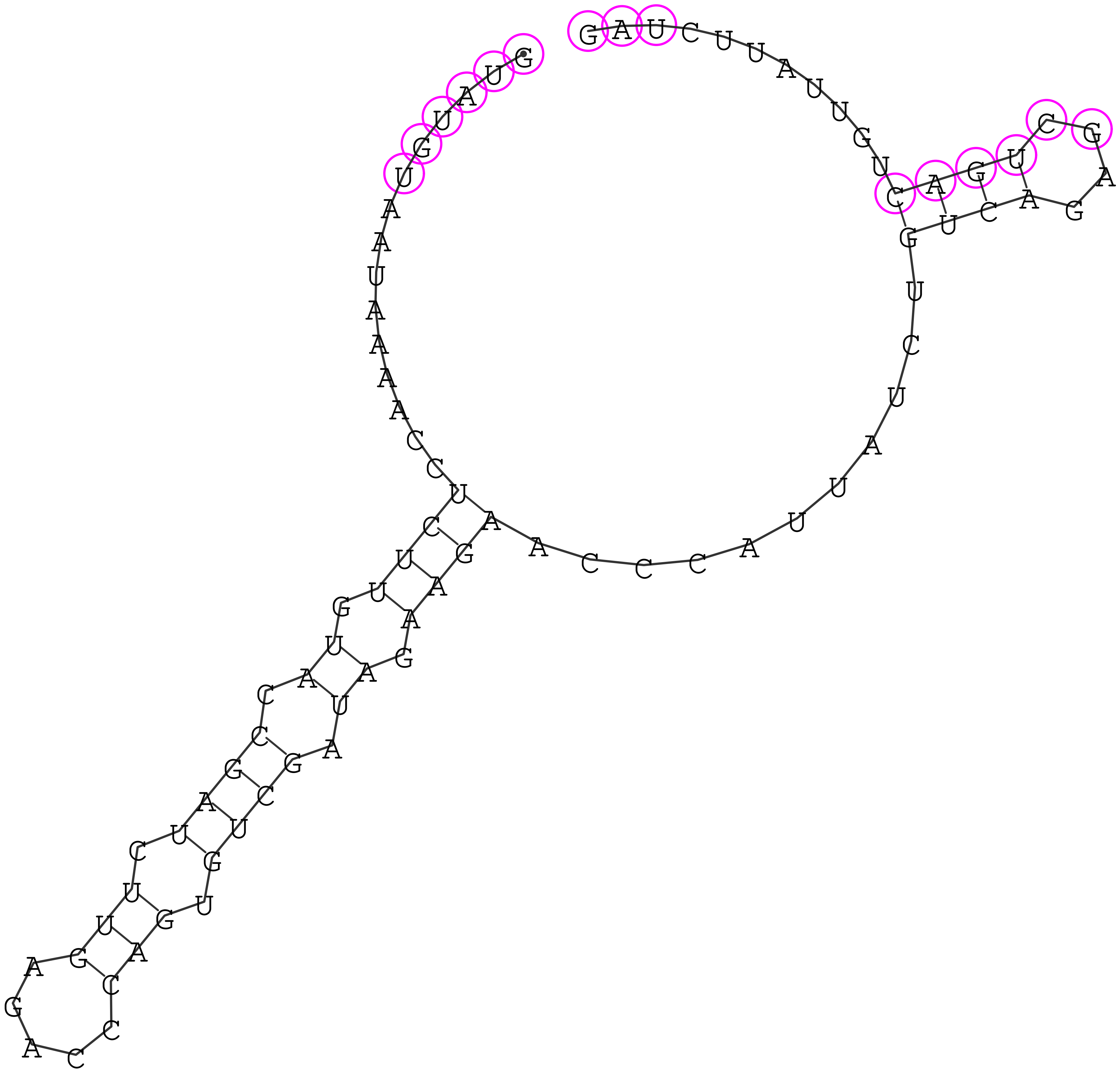

Xmsuc0077A - Internal intron

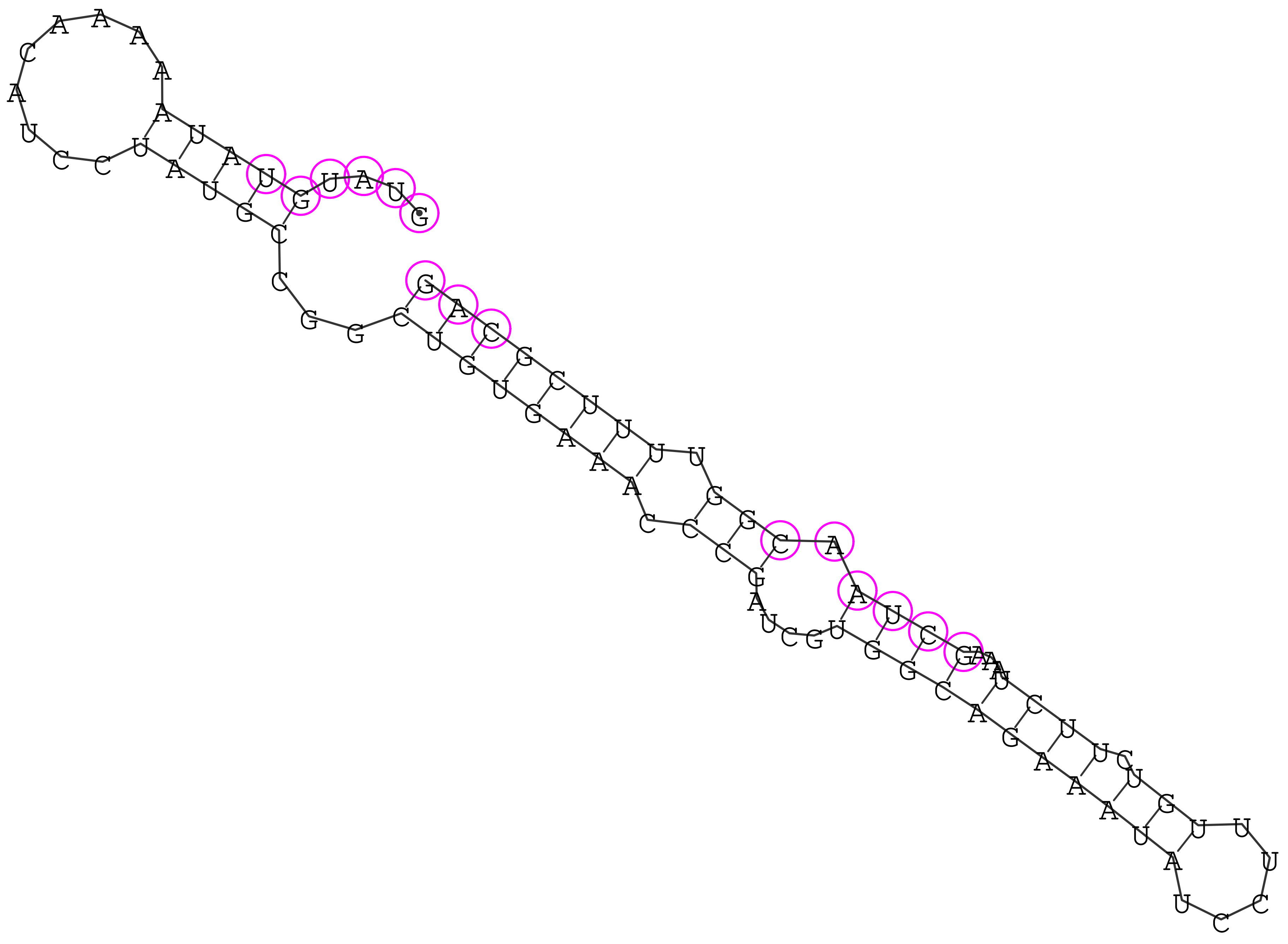

Xmsuc0082A - Internal intron

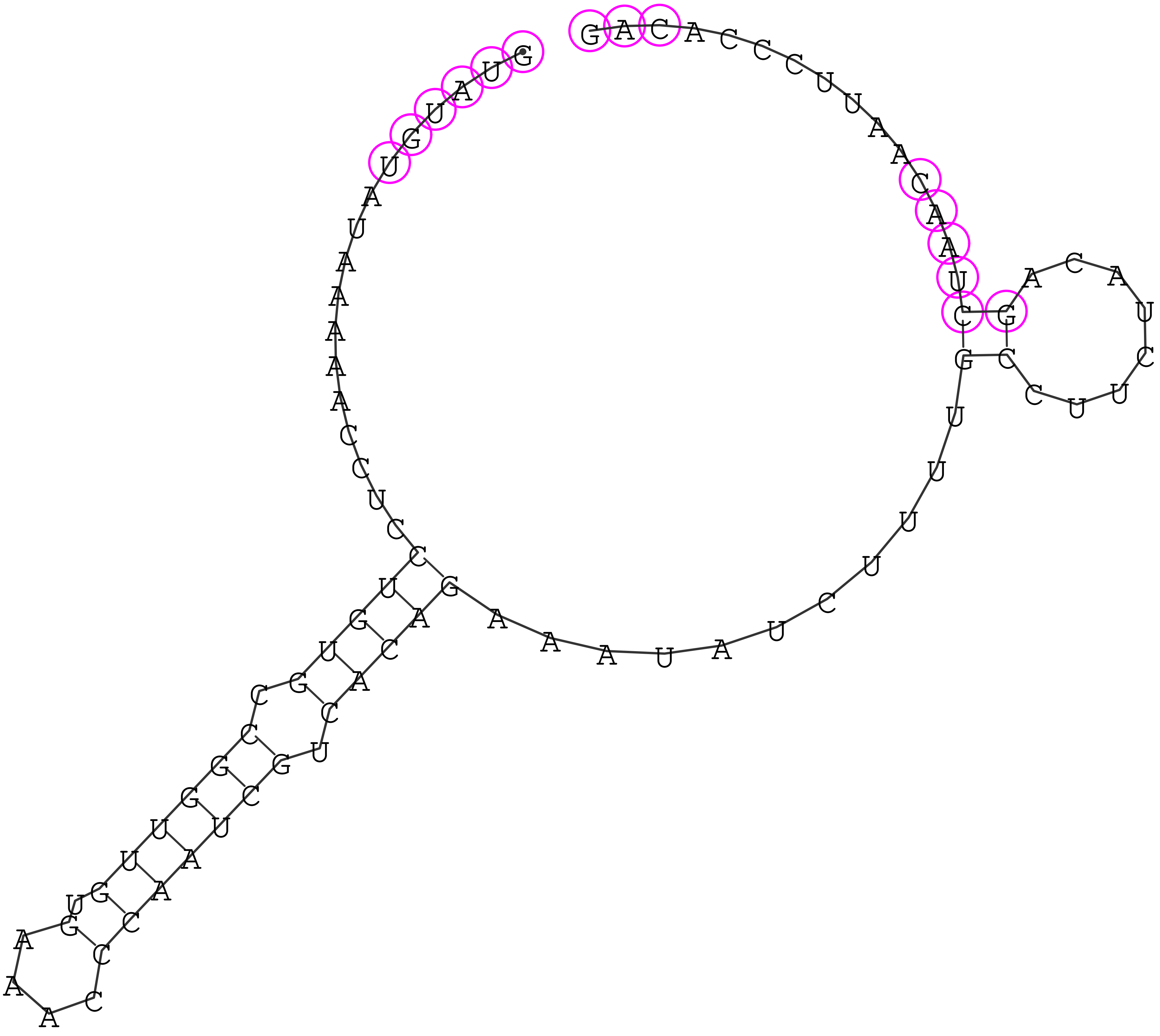

Xmsuc0086A - Internal intron

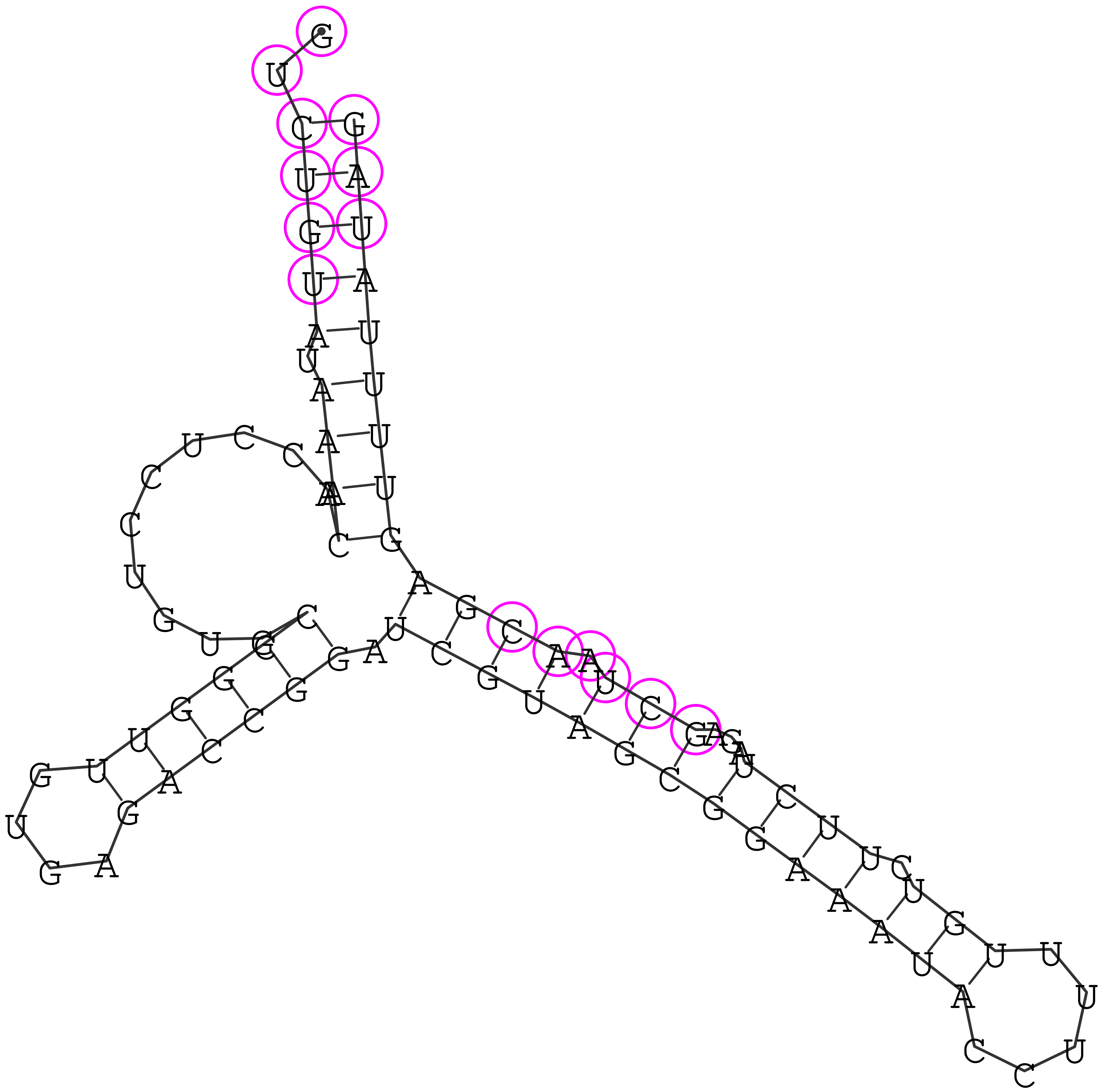

Xmsuc0086B - Internal intron

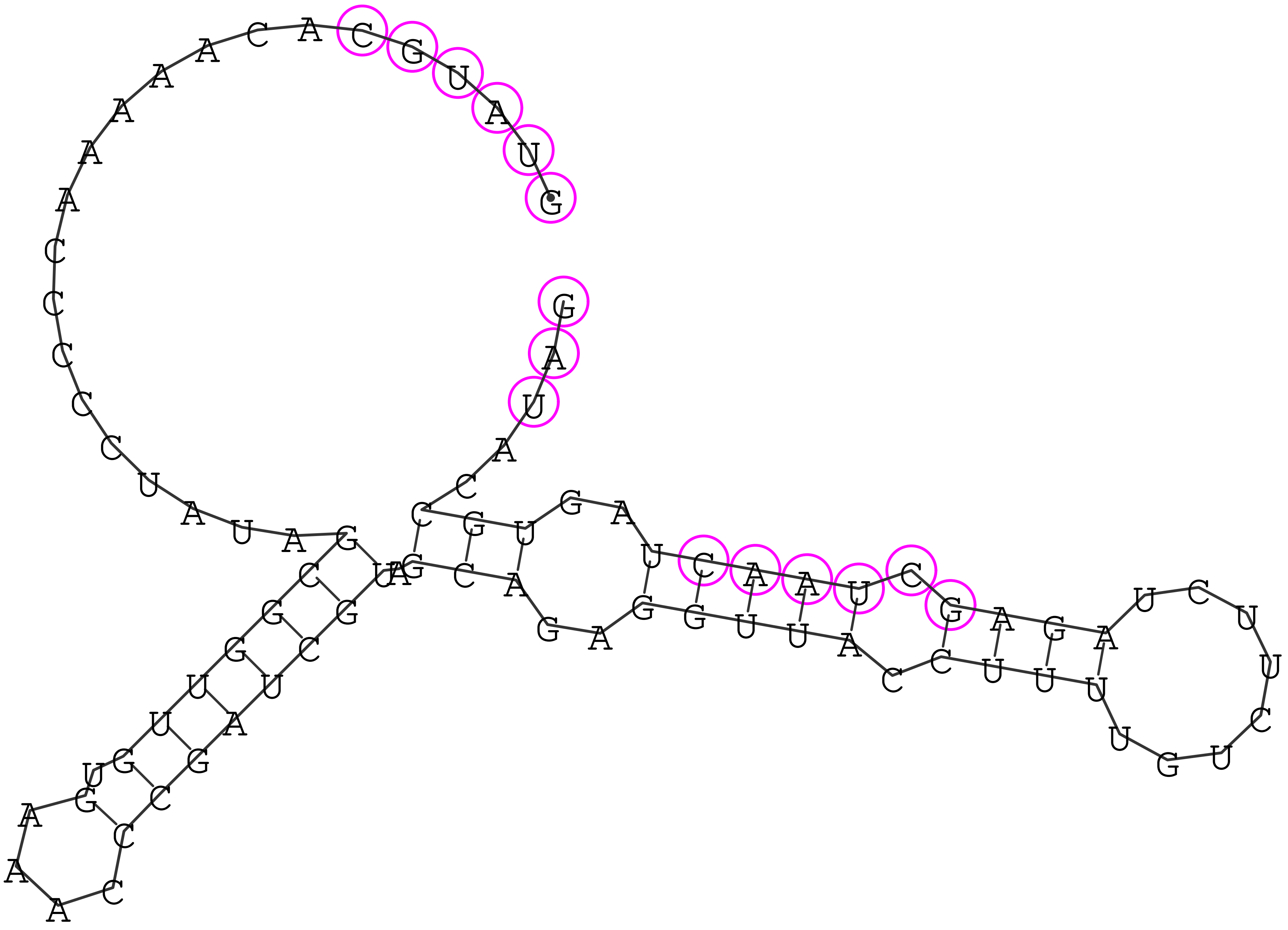

Xmsuc0098A - Internal intron

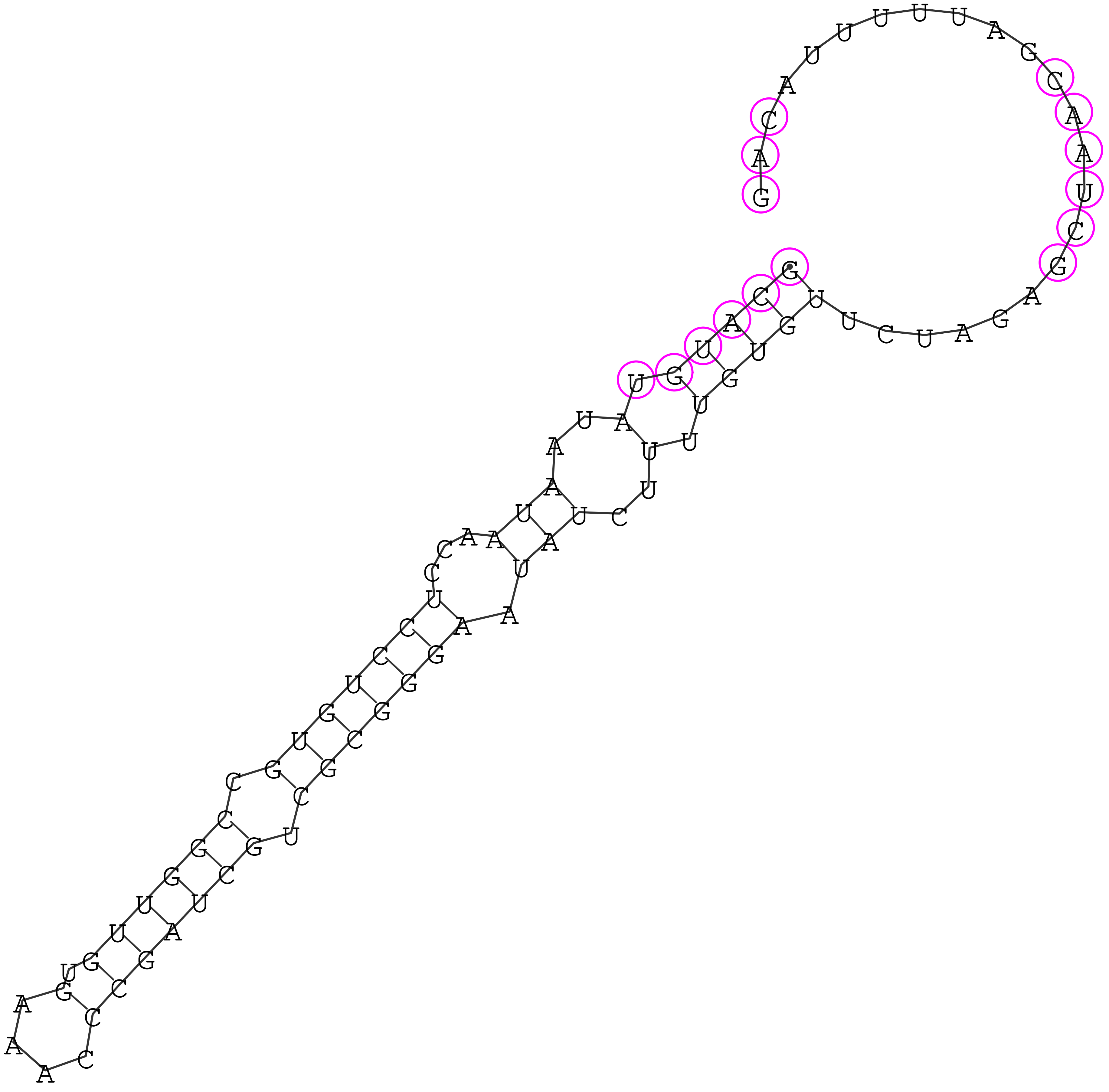

Xmsuc0110A - Internal intron

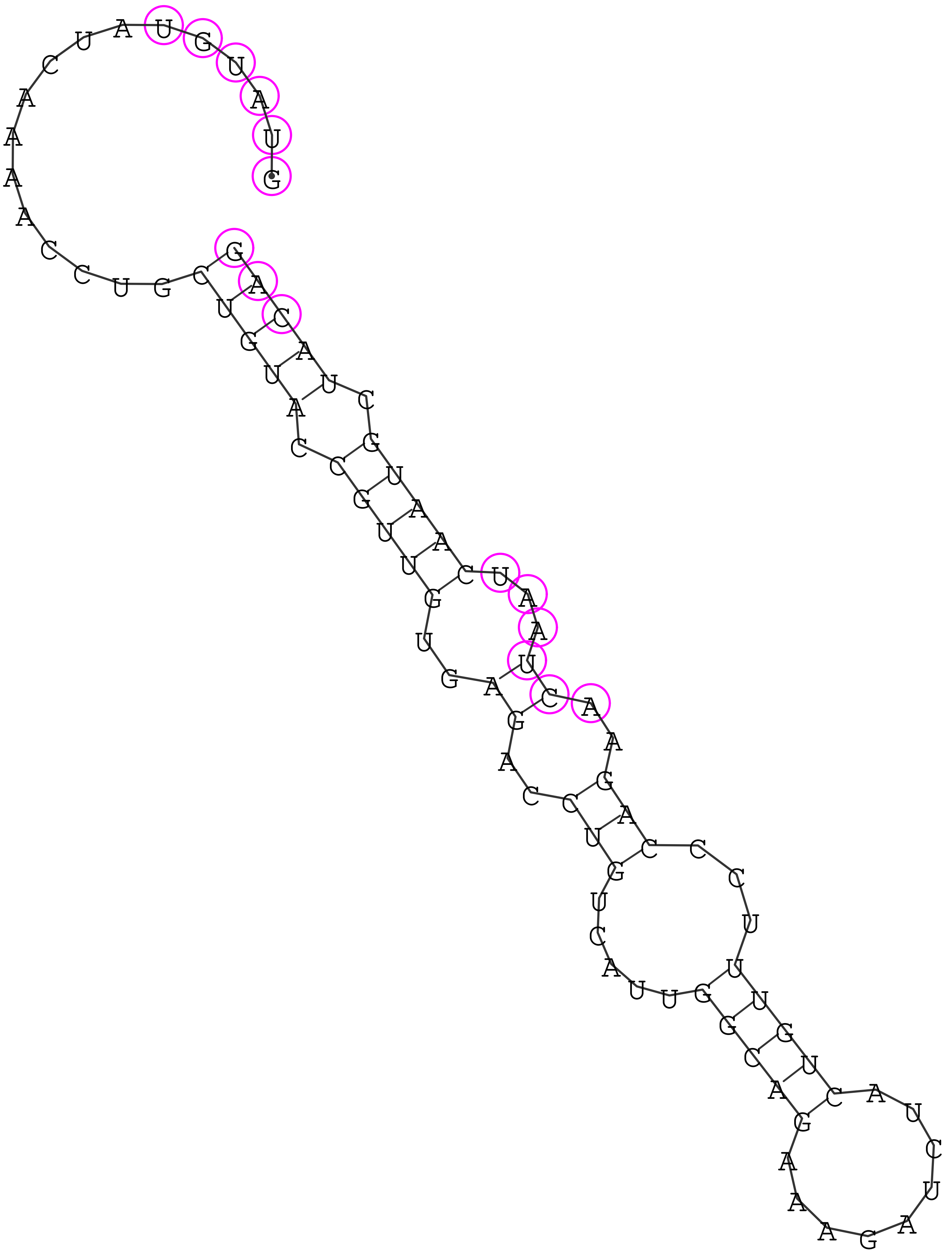

Xmsuc0111A - Internal intron

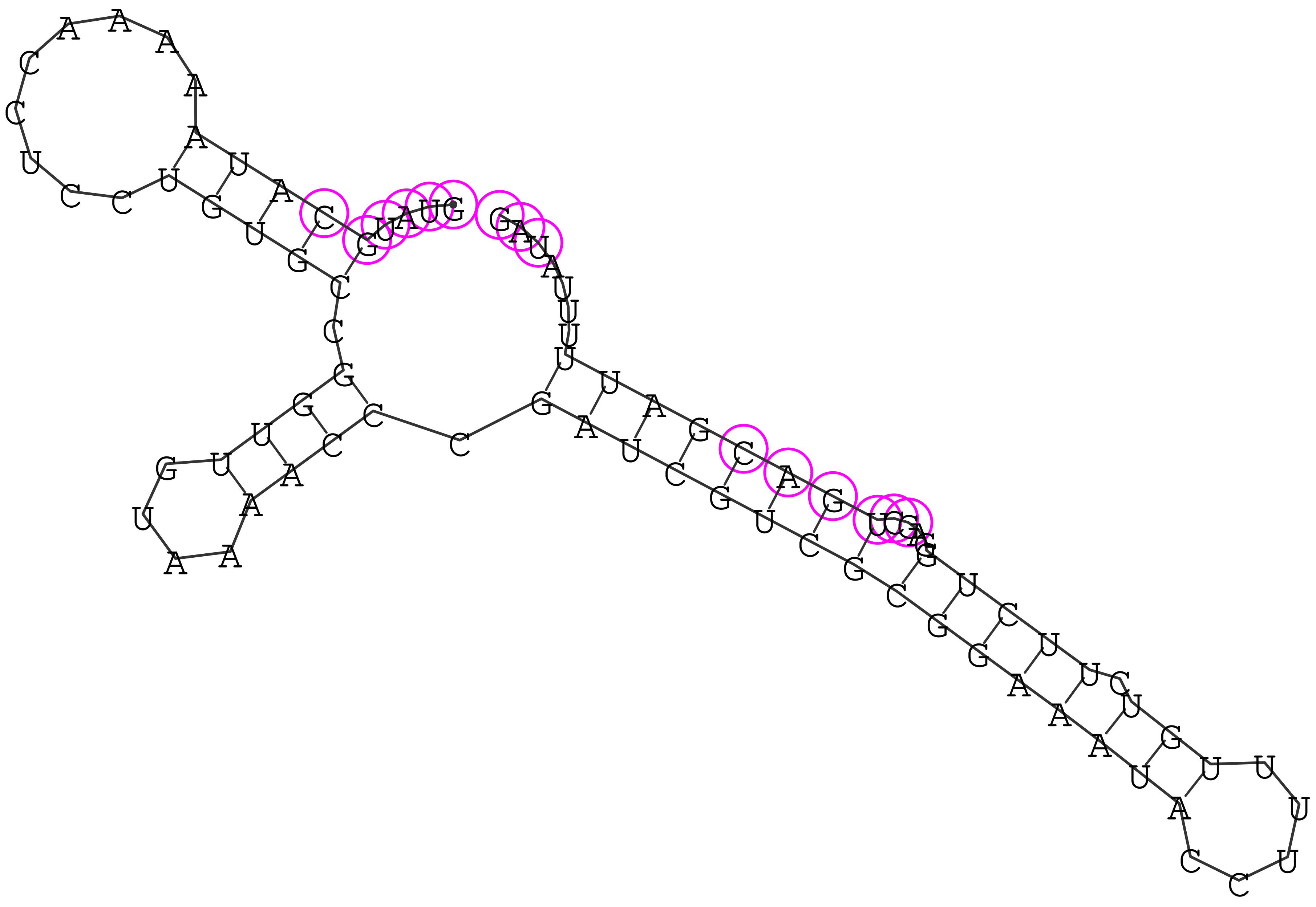

Xmsuc0114A - Internal intron

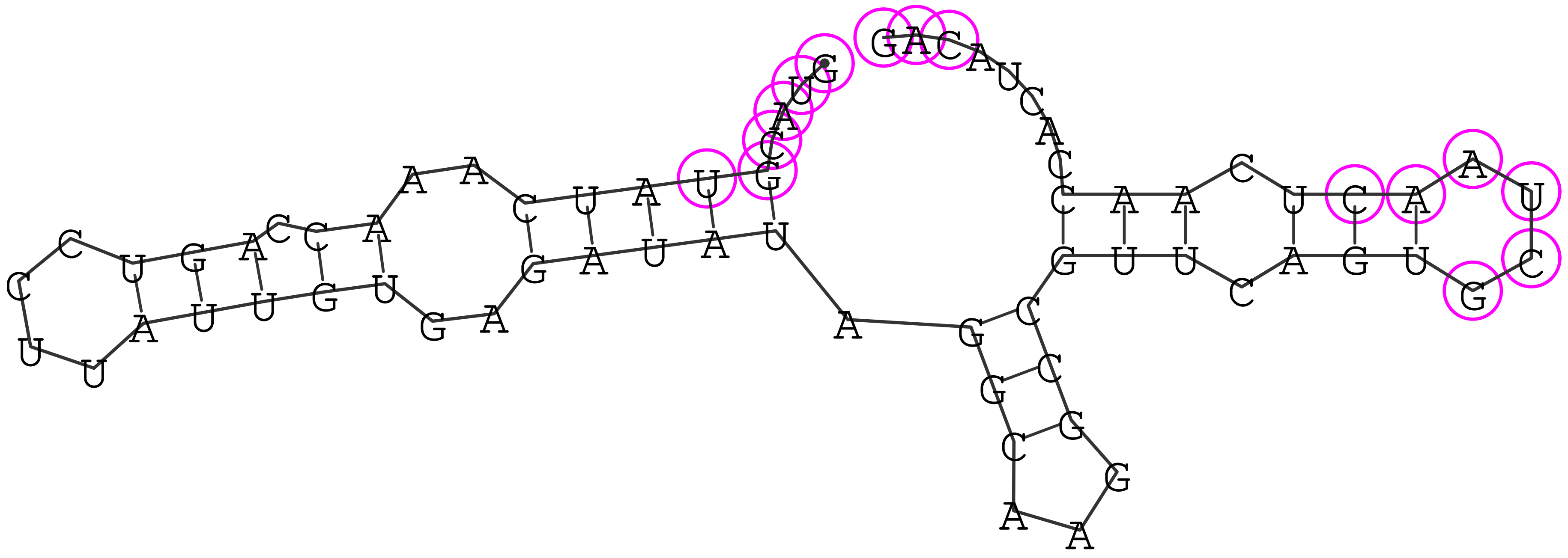

Xmsuc0121A - Internal intron

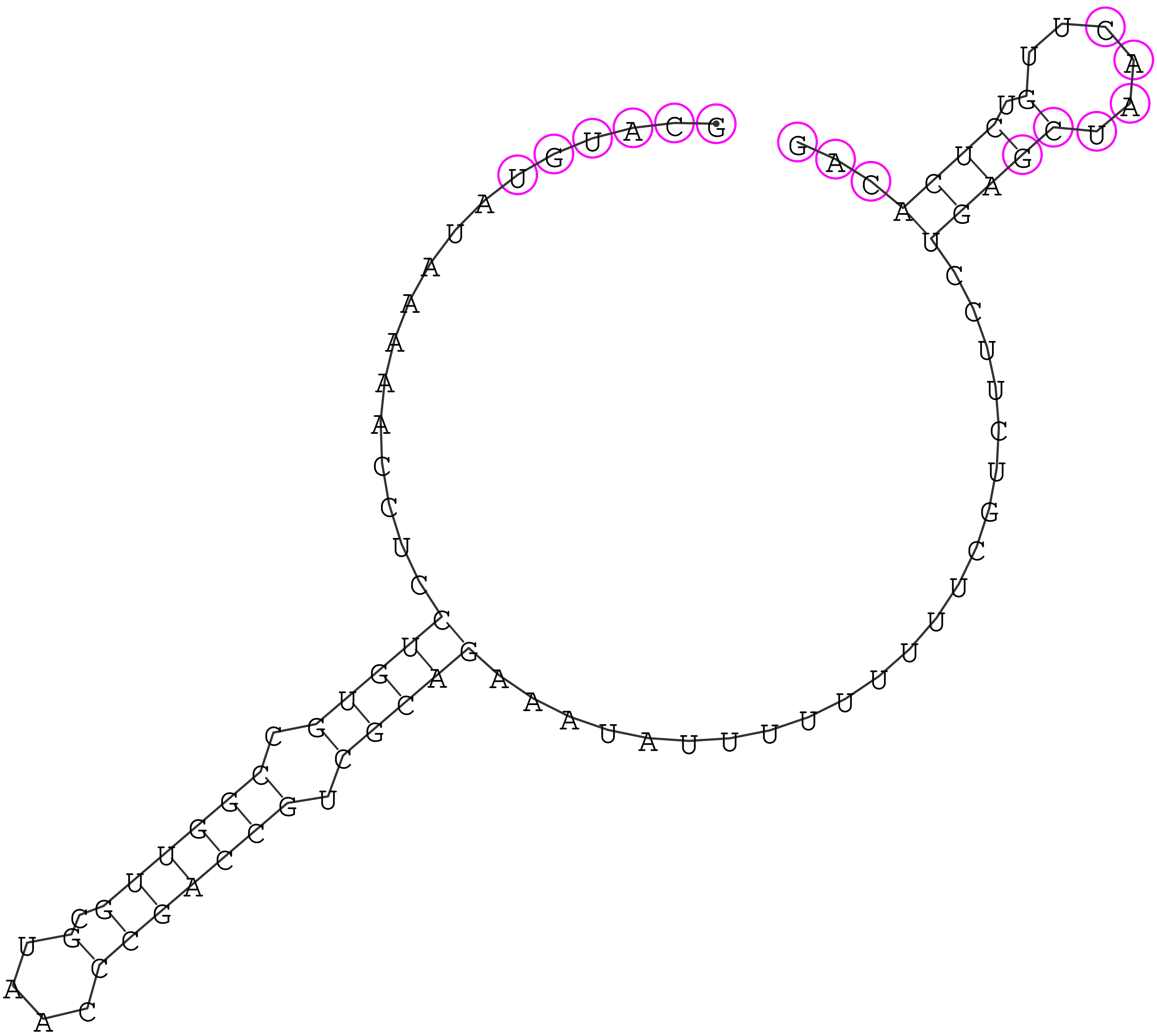

Xmsuc0131A - Internal intron

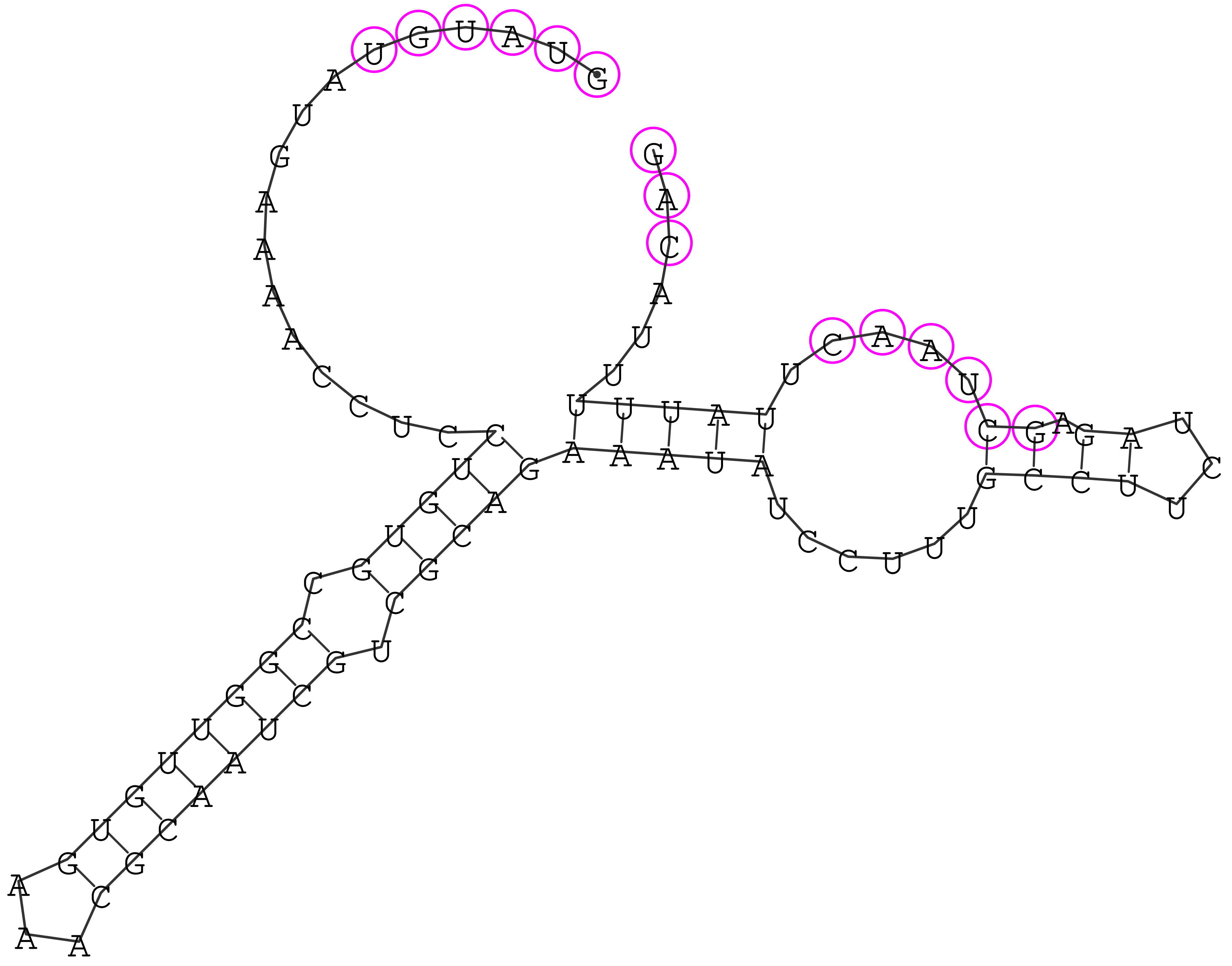

Xmsuc0137A - Internal intron

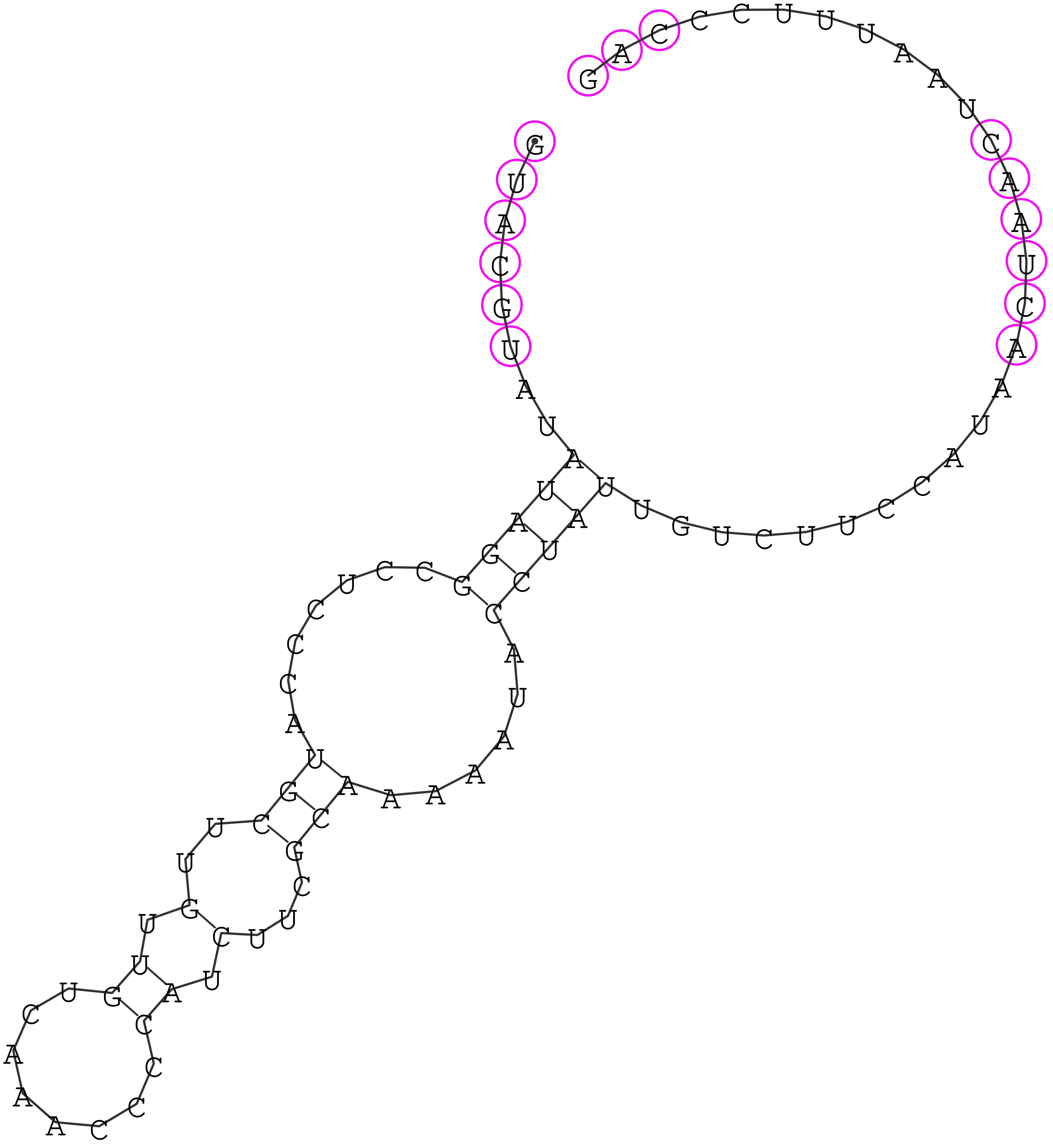

Xmsuc0141A - Internal intron

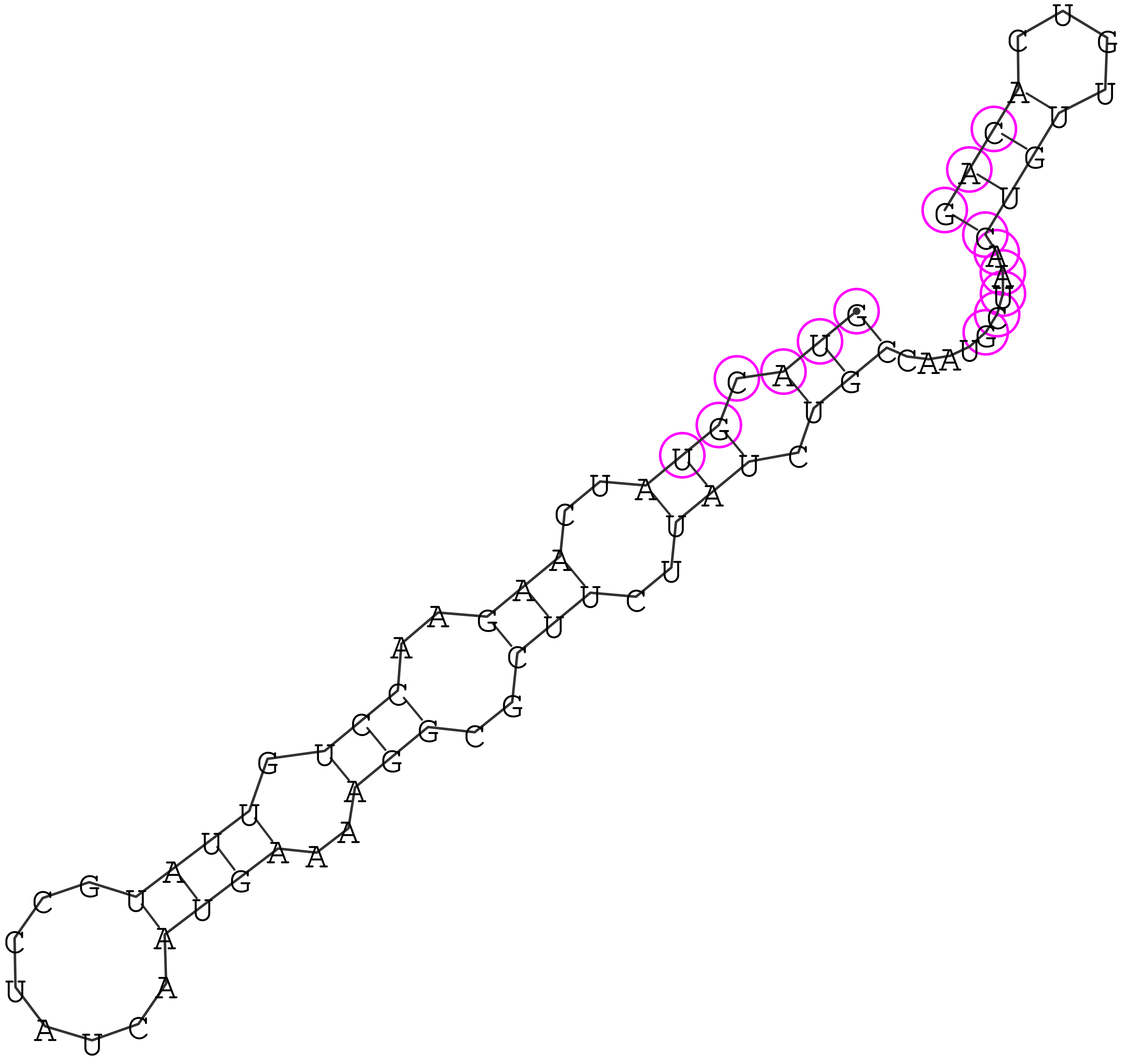

Xmsuc0143A - Internal intron

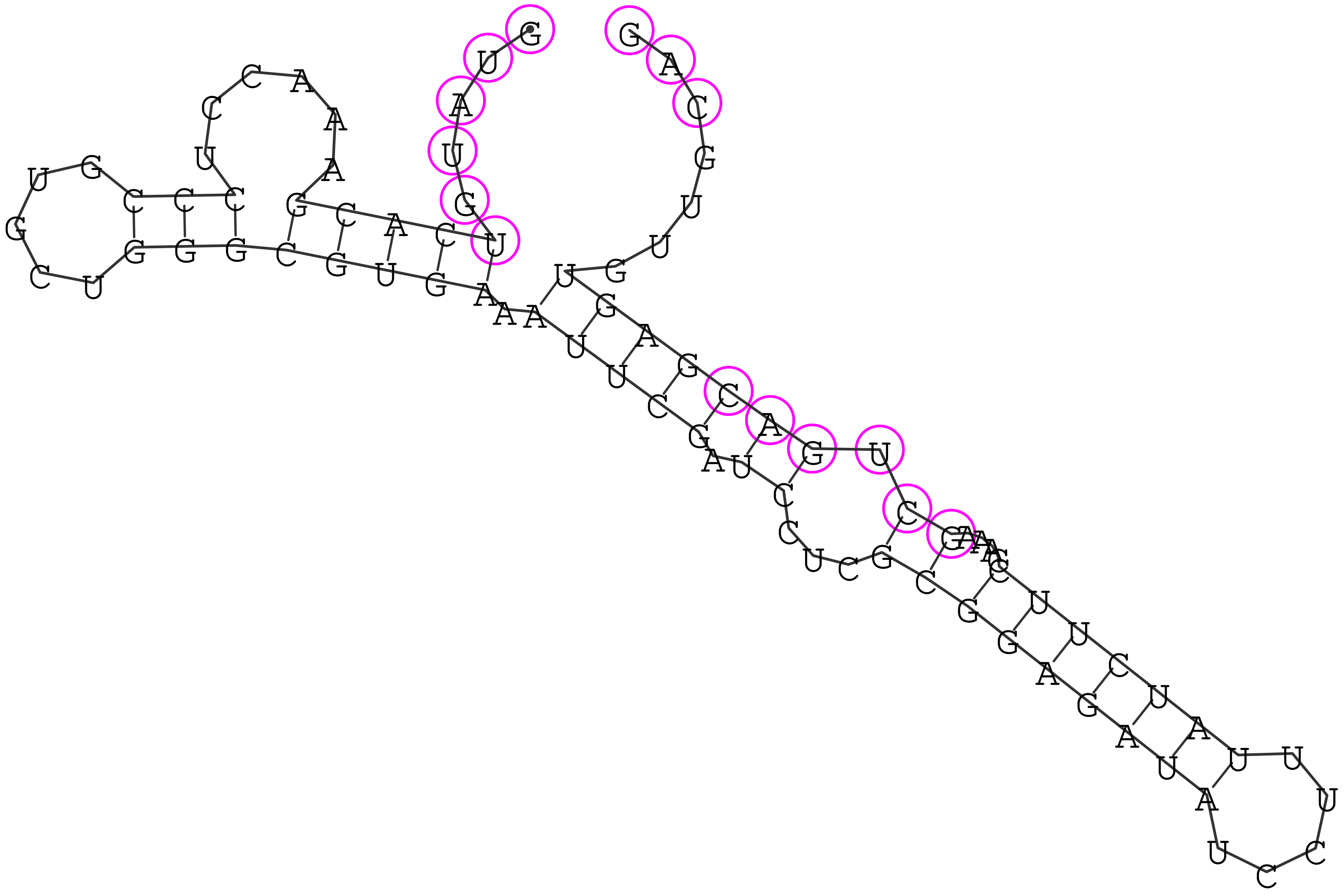

Xmsuc0146A - Internal intron

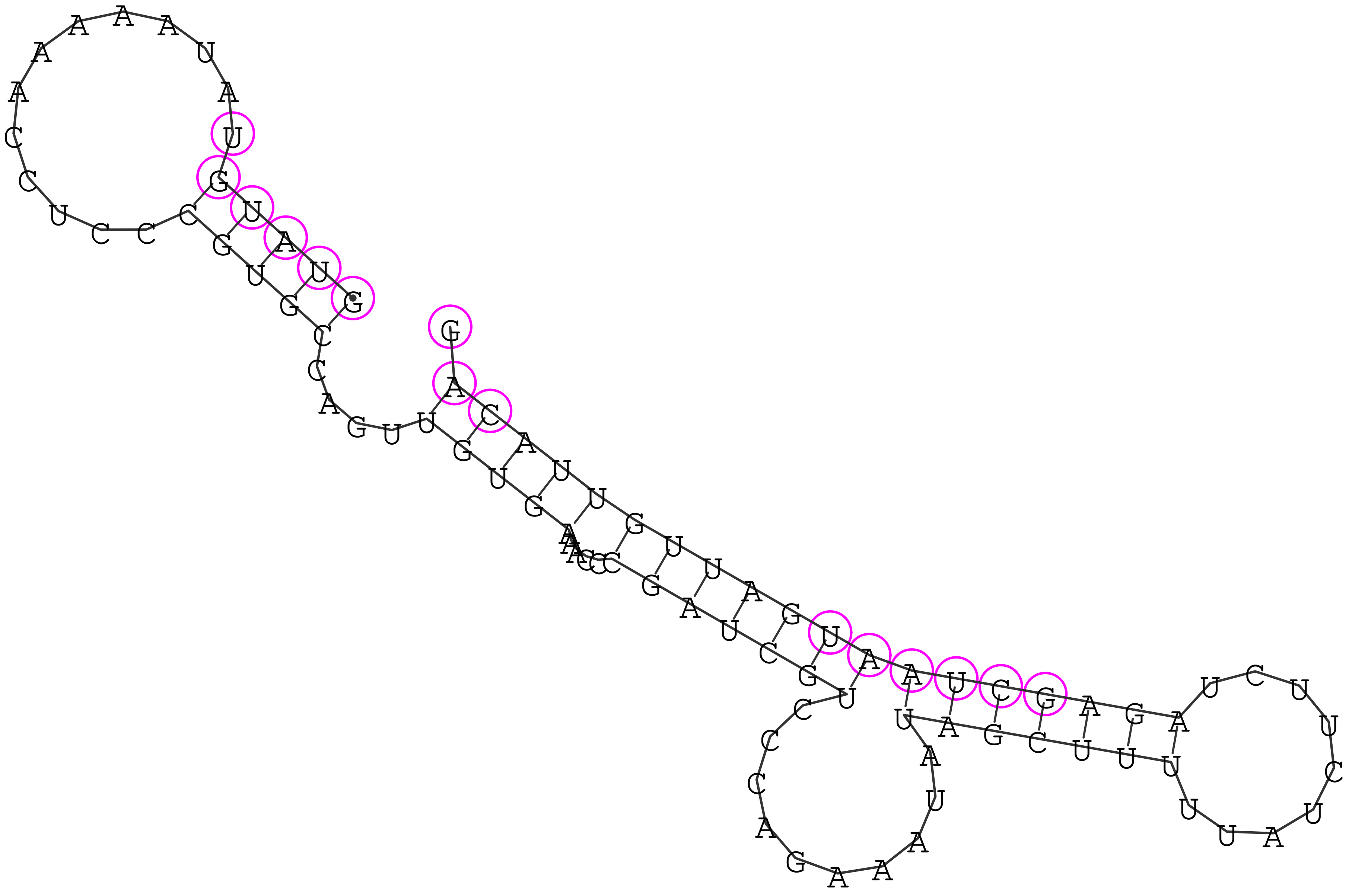

Xmsuc0153A - Internal intron

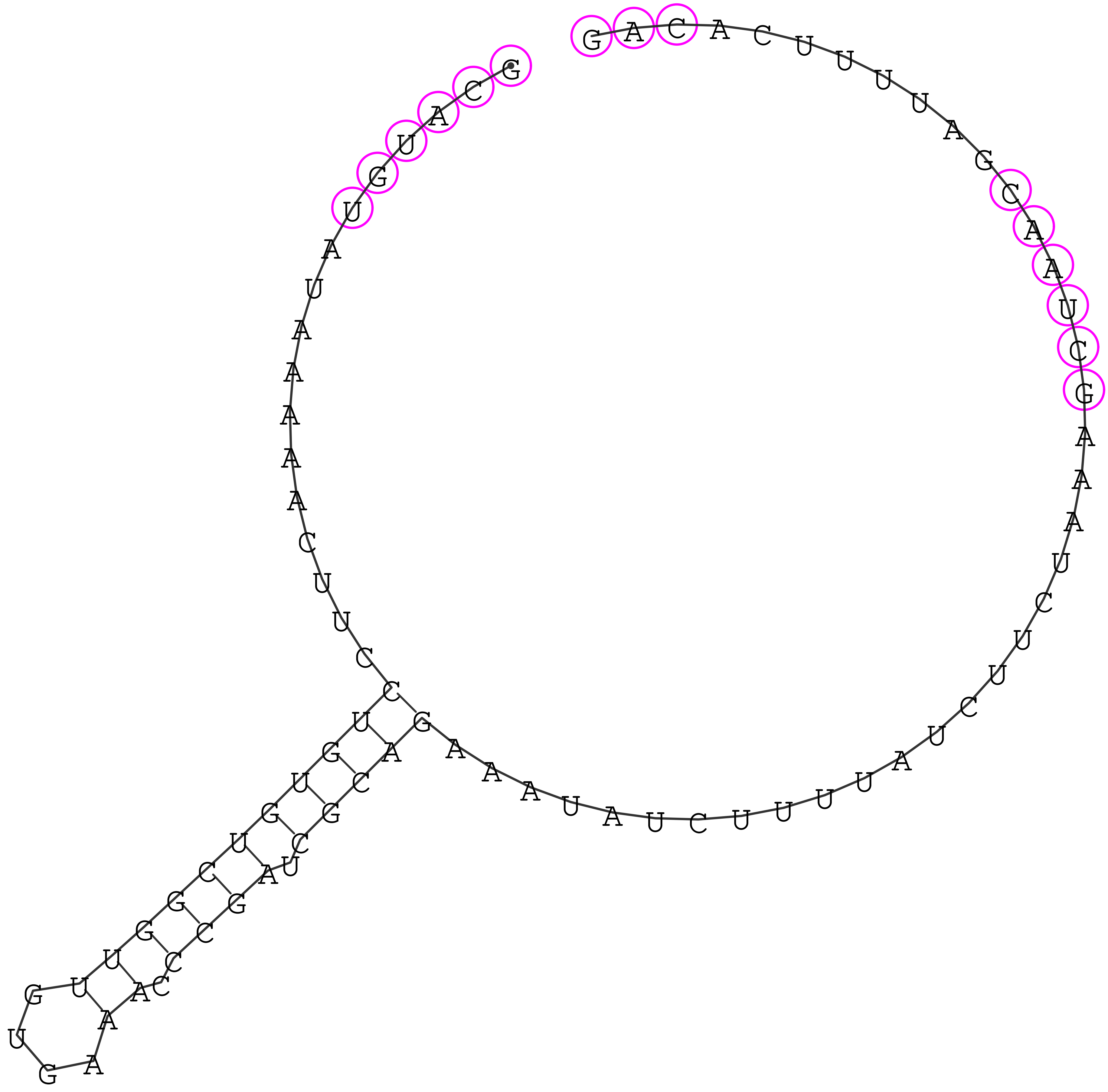

Xmsuc0159A - Internal intron

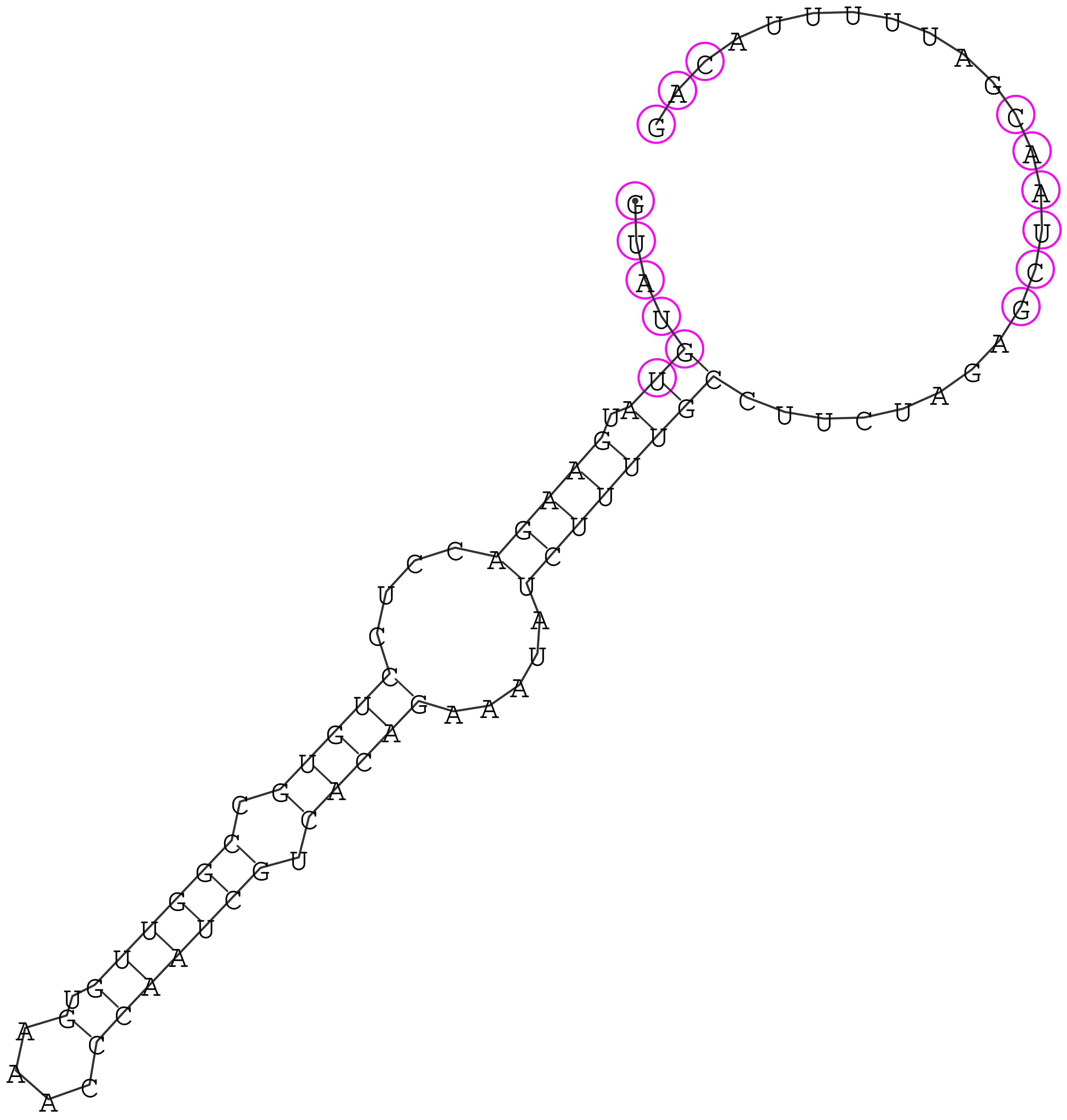

Xmsuc0162A - Internal intron

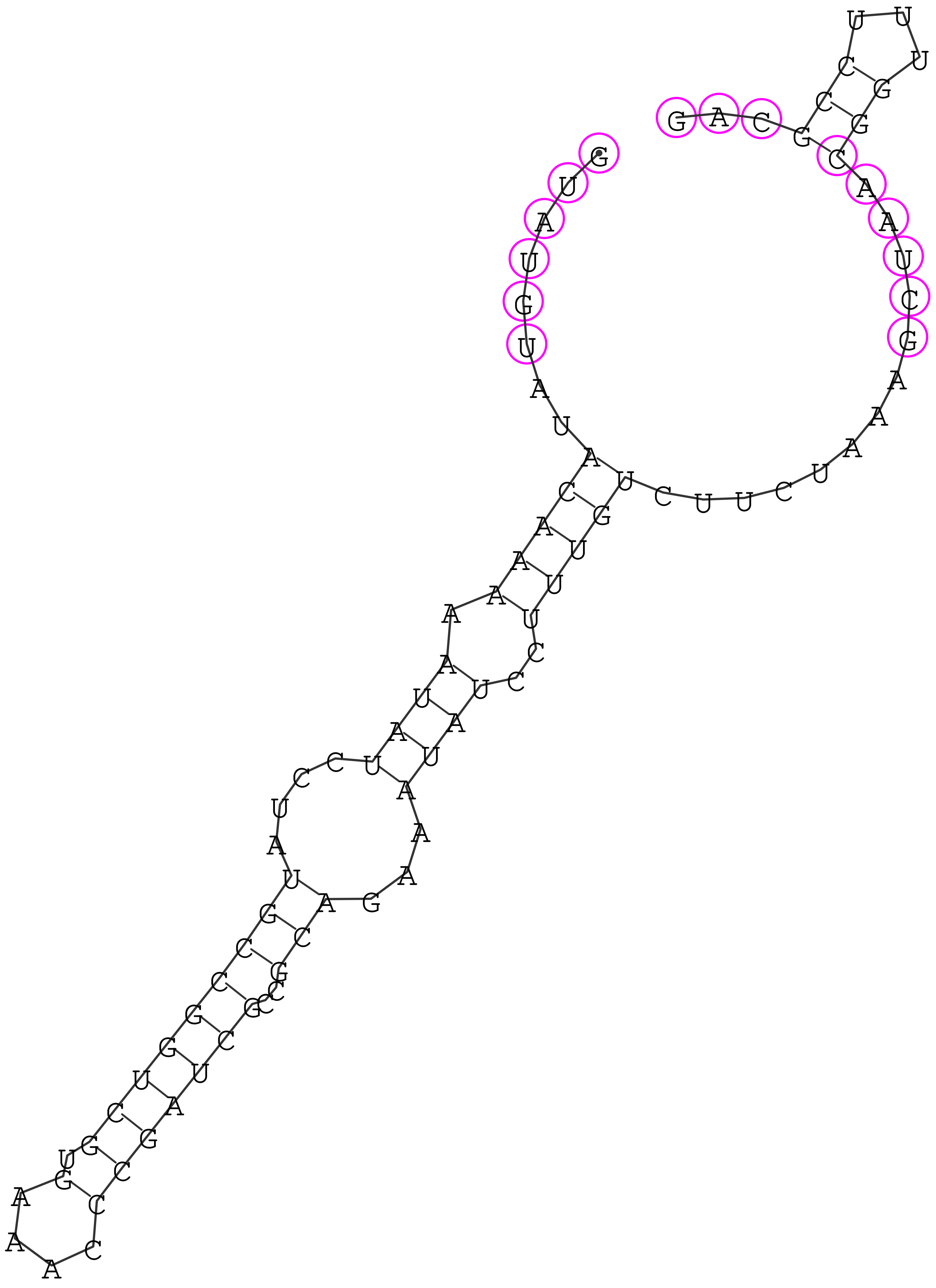

Xmsuc0168A - Internal intron

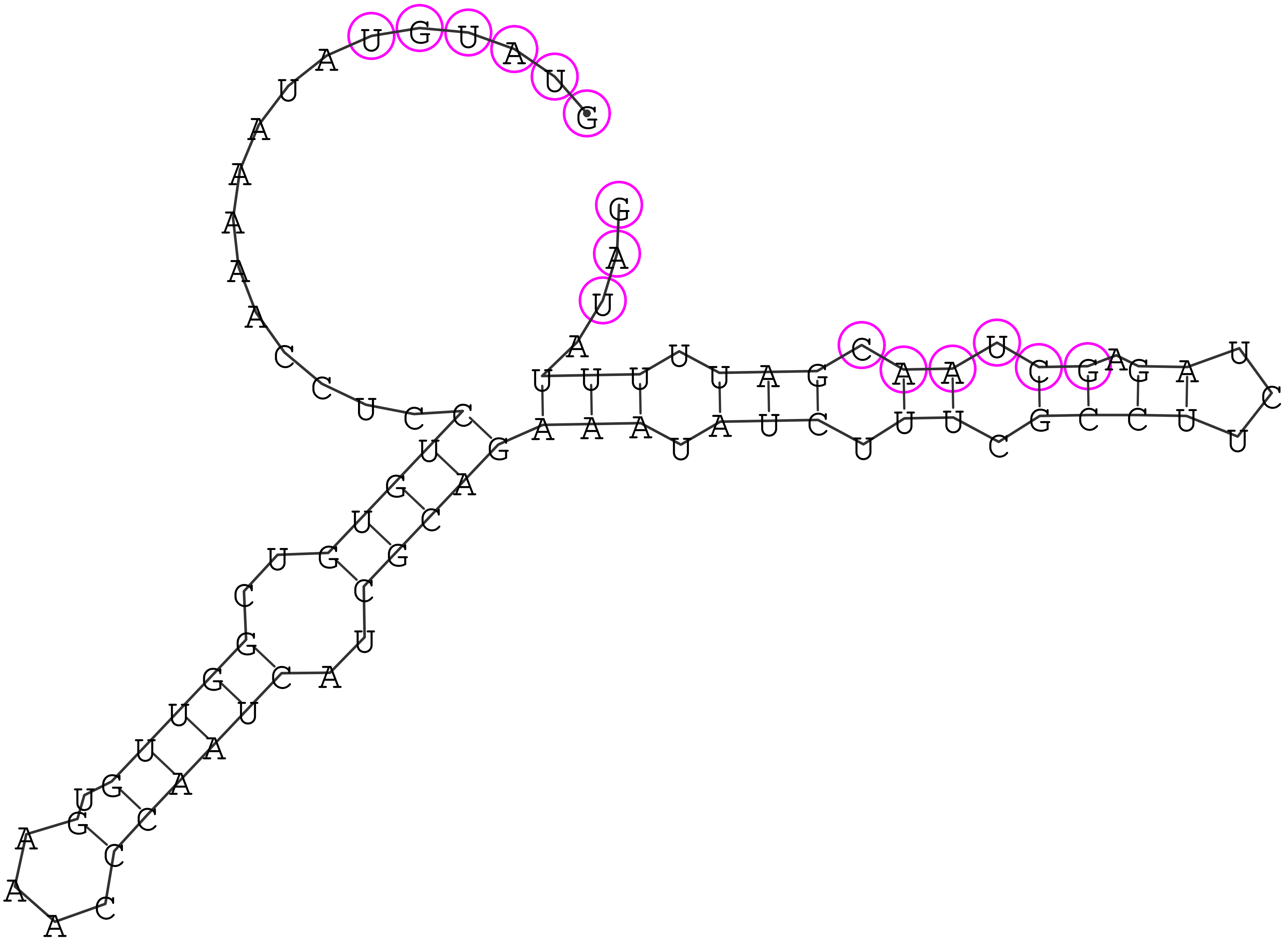

Xmsuc0170A - Internal intron

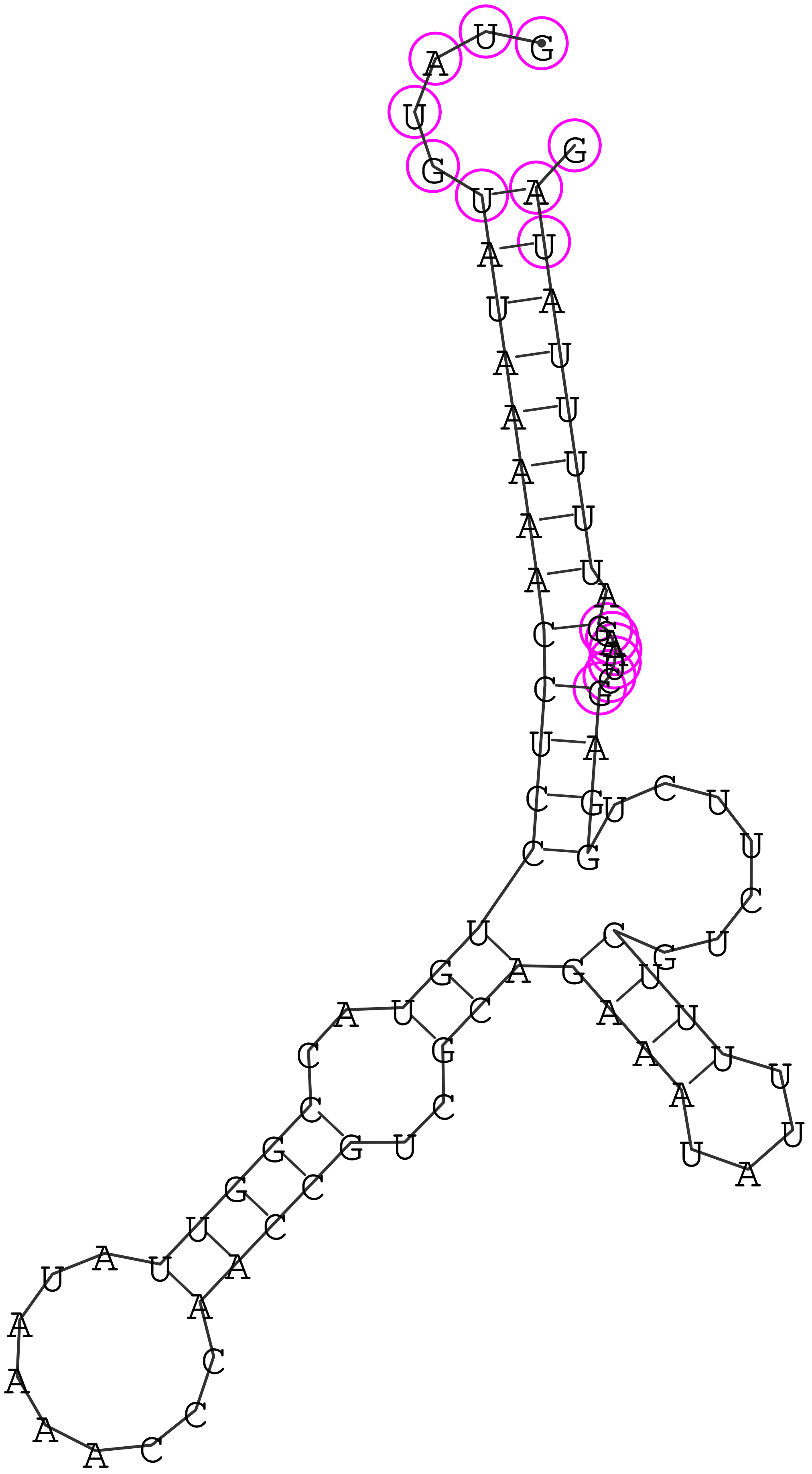

Xmsuc0171A - Internal intron

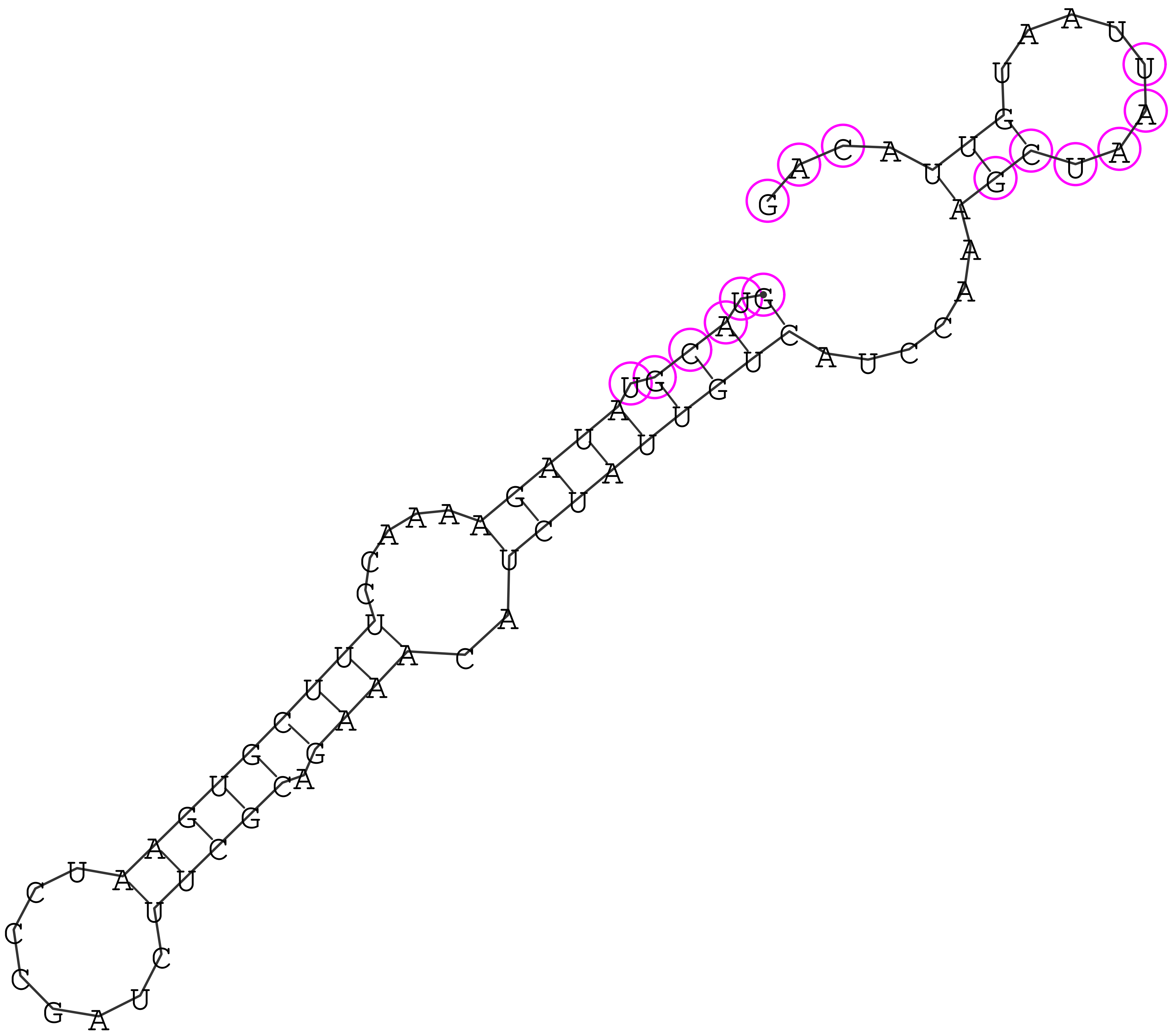

Xmsuc0178A - Internal intron

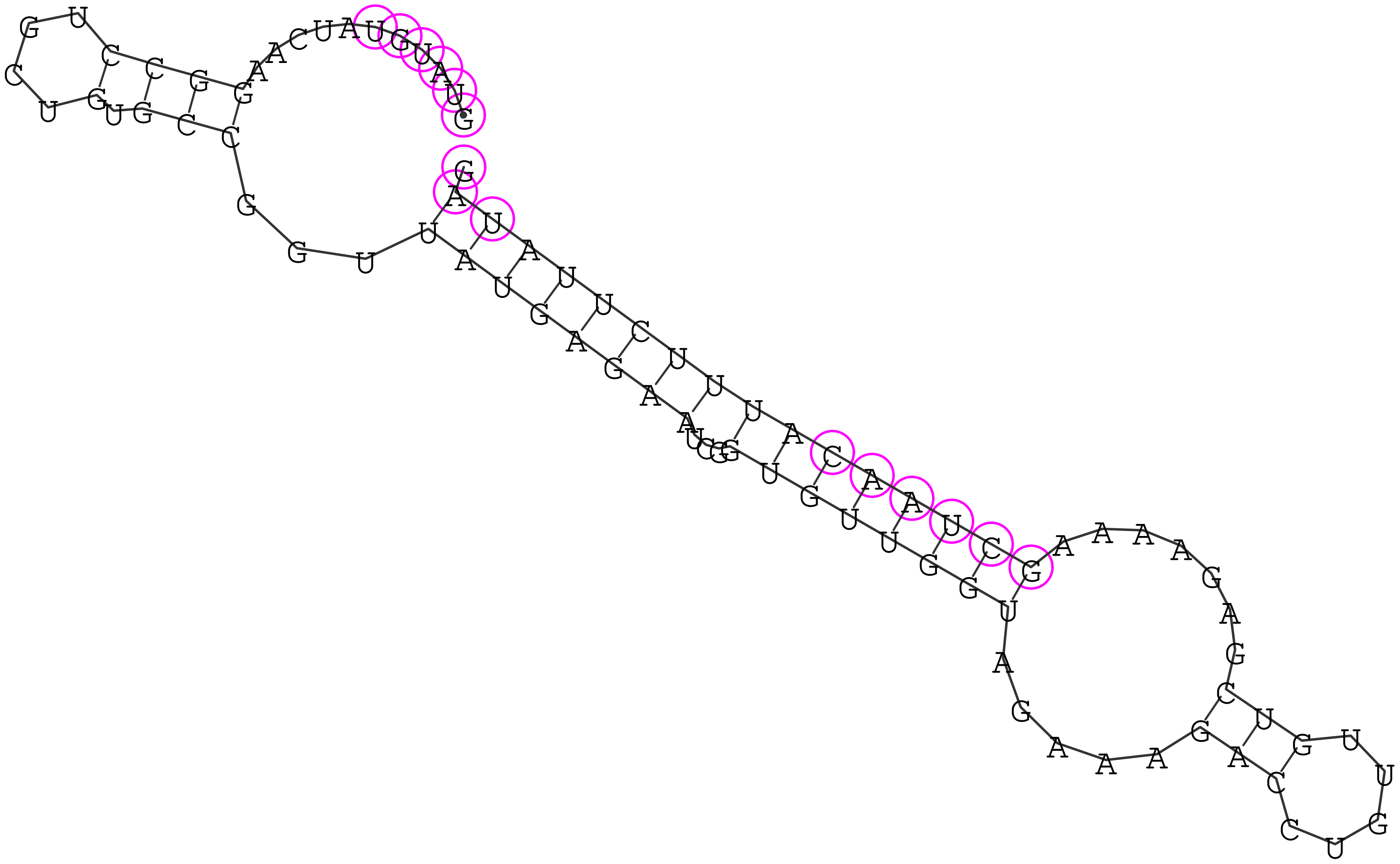

Xmsuc0185A - Internal intron

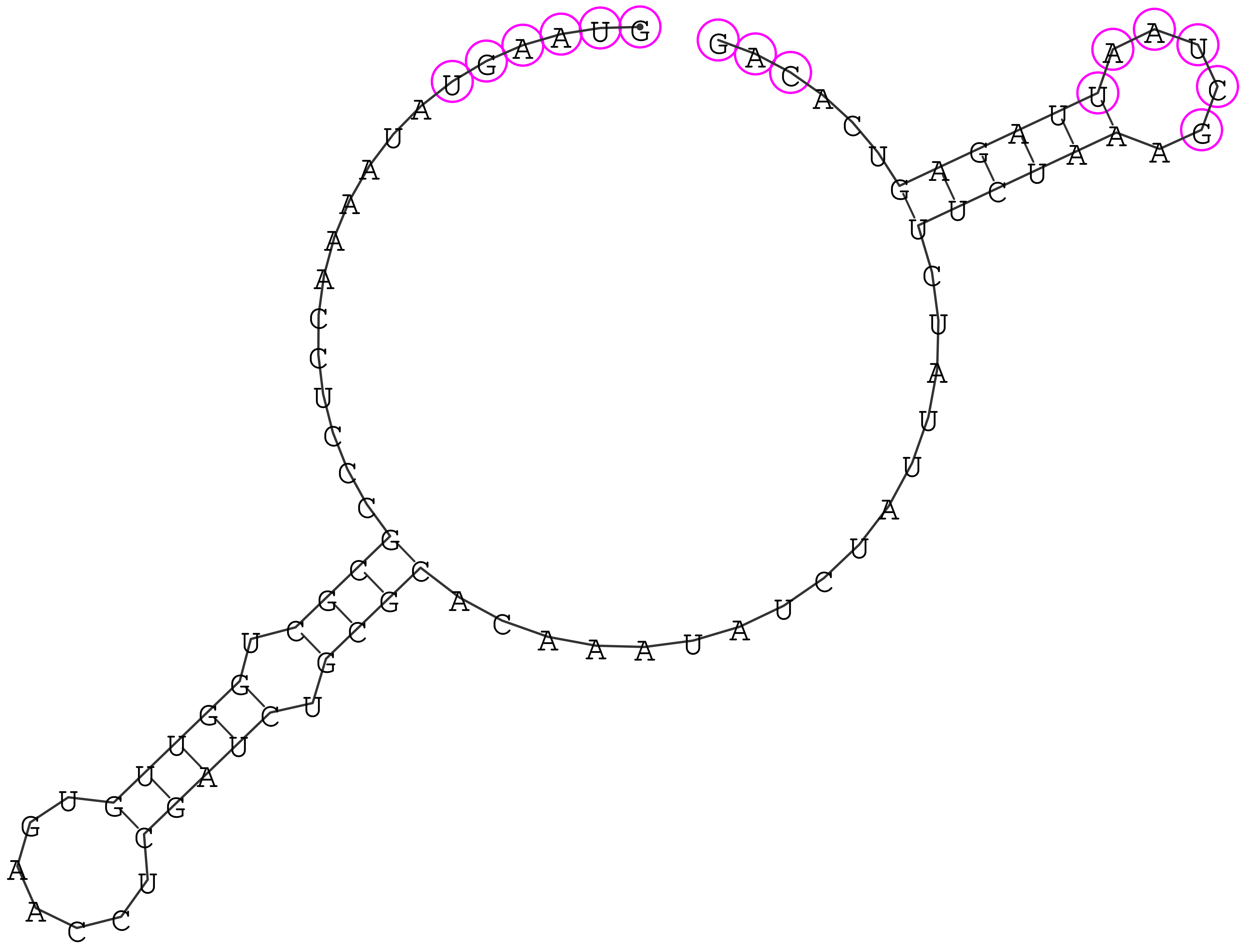

Xmsuc0187A - Internal intron

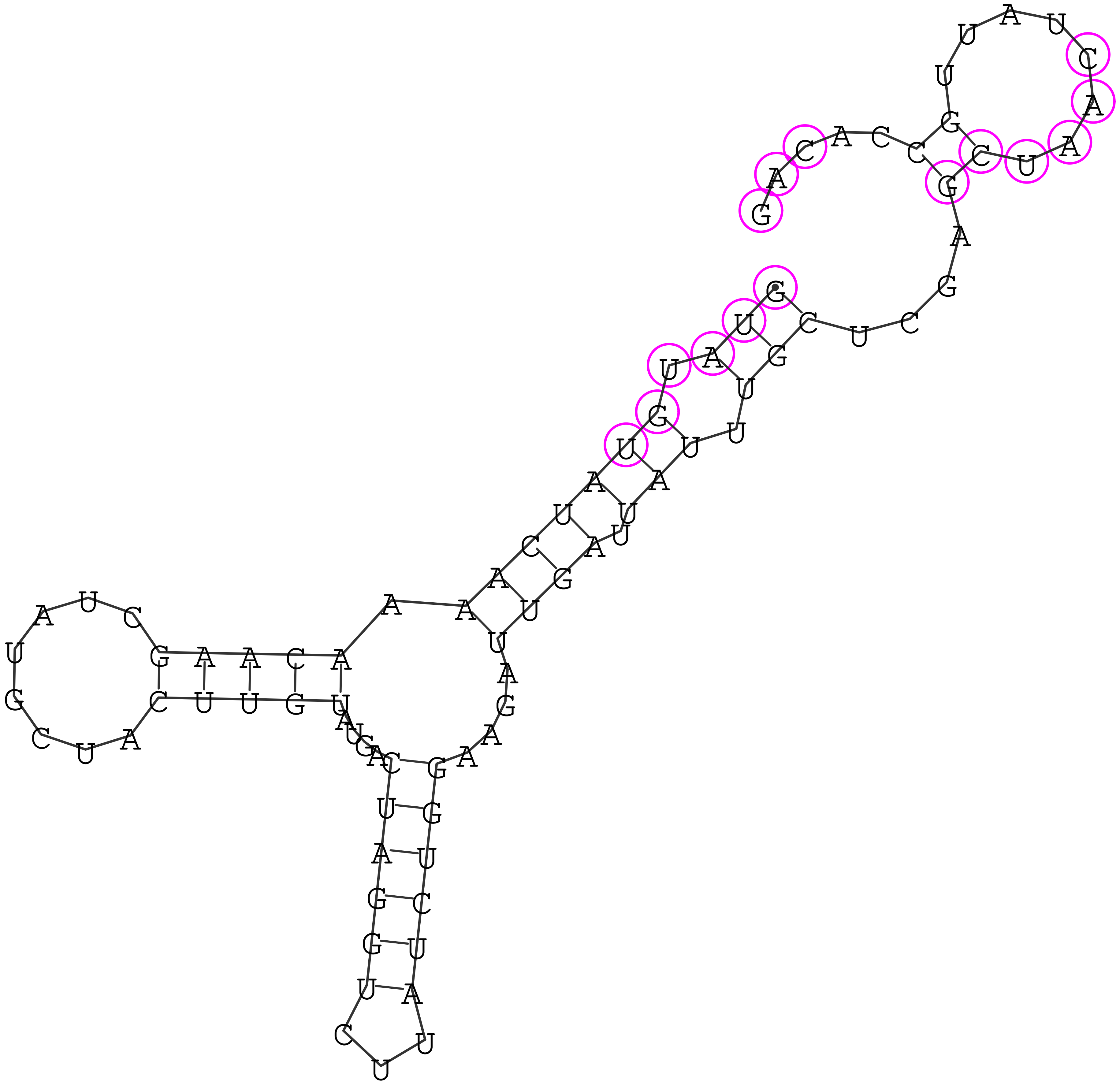

Xmsuc0237A - Internal intron

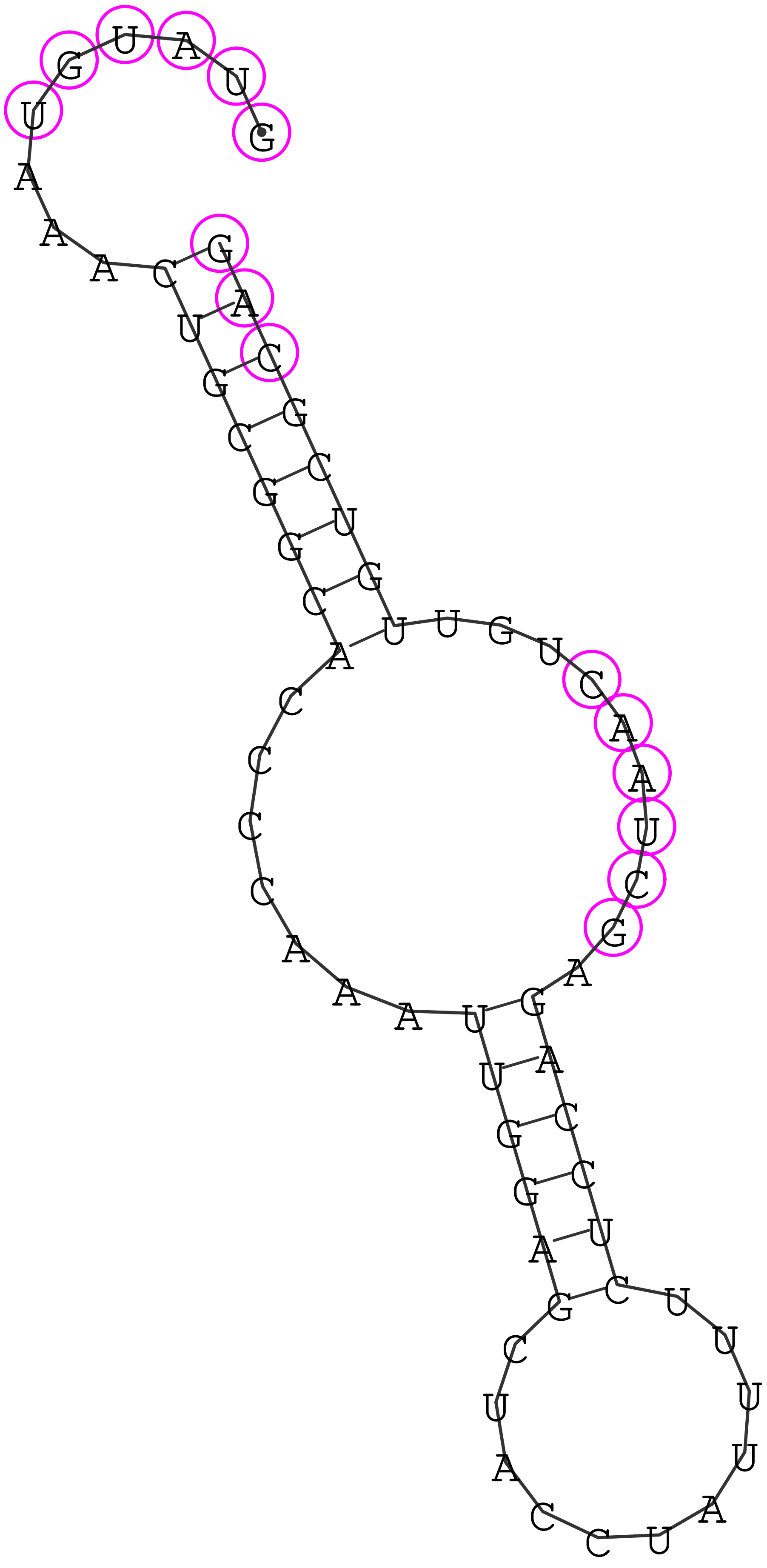

Xmsuc0285A - Internal intron

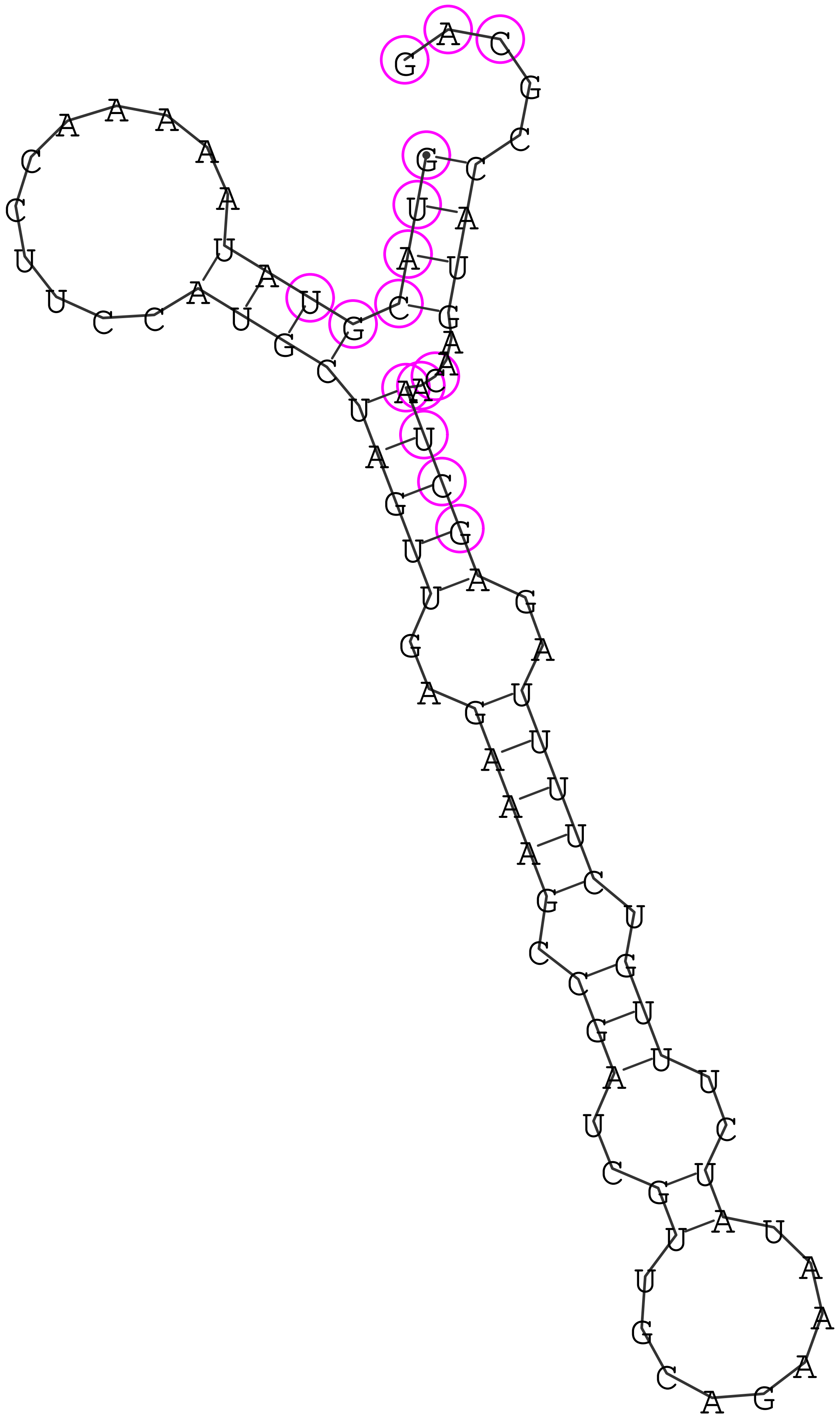

Xmsuc0293A - Internal intron

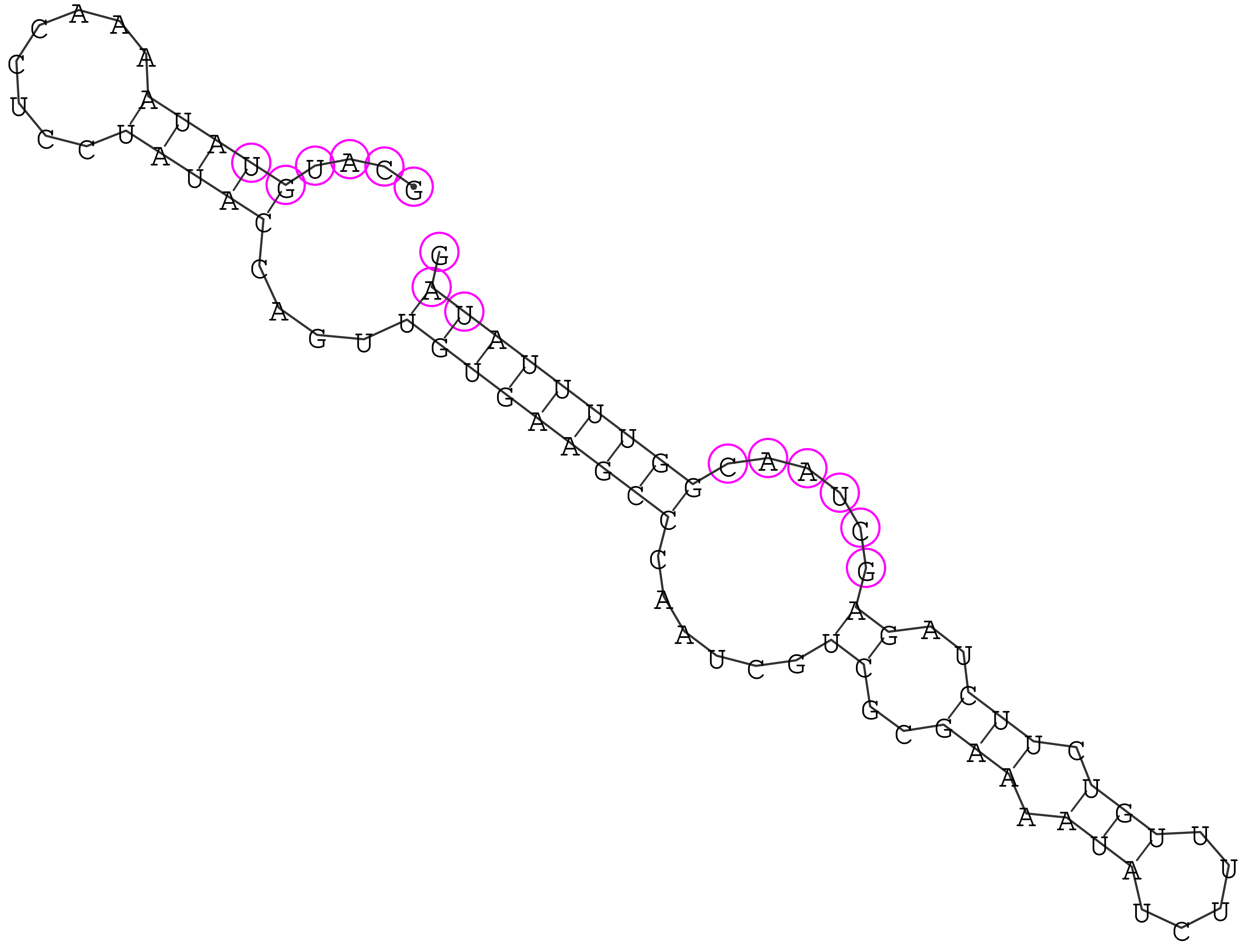

Xmsuc0301A - Internal intron

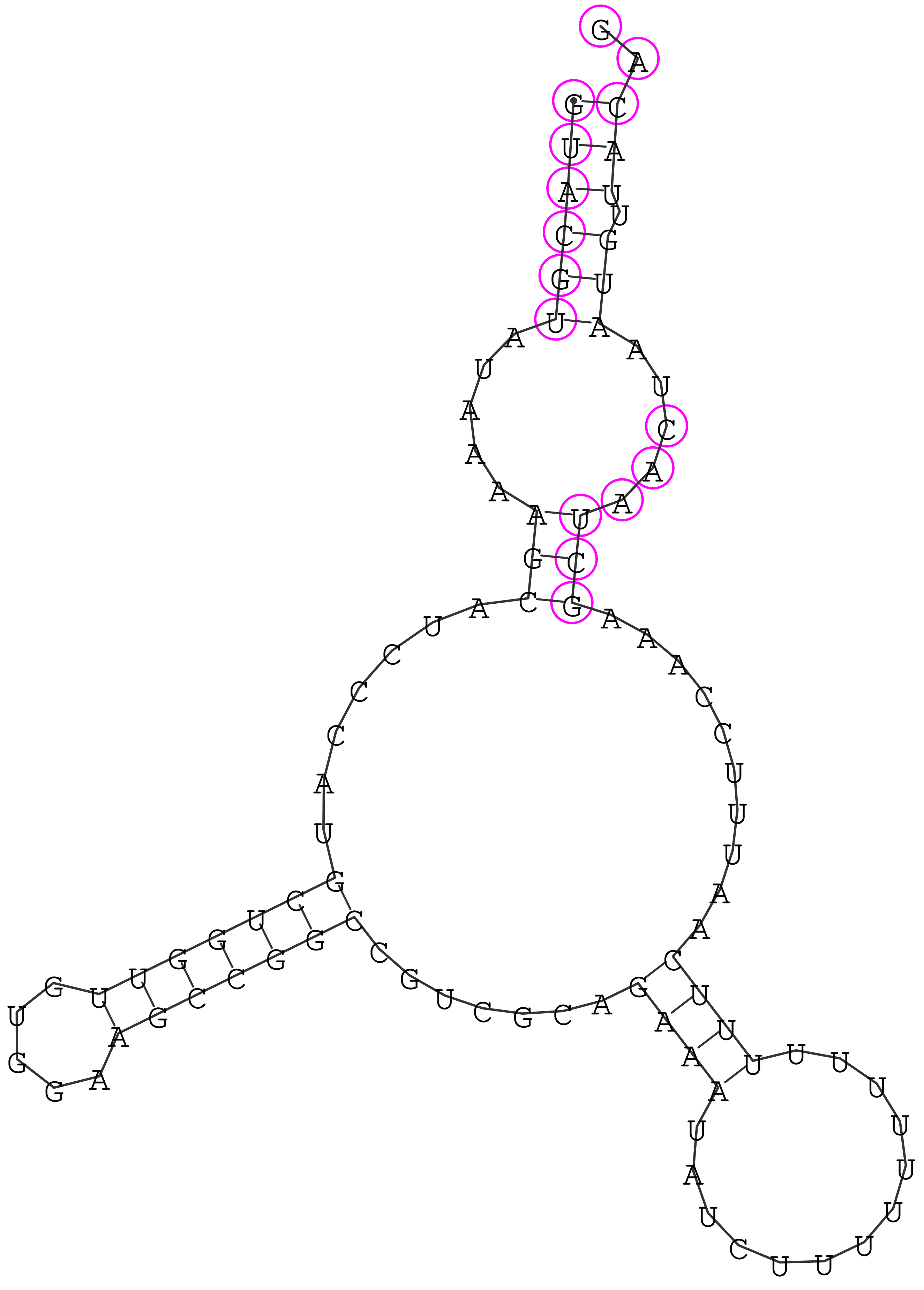

Xmsuc0306A - Internal intron

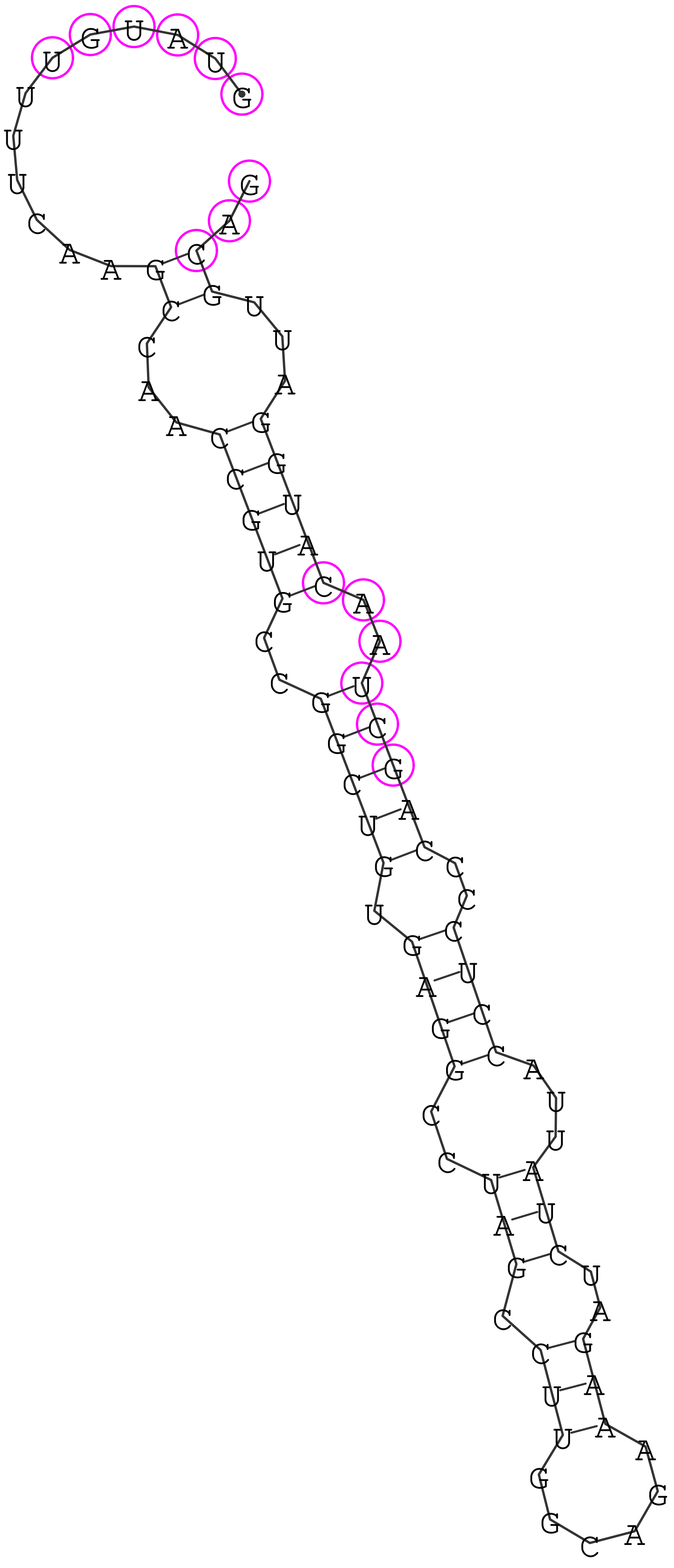

Xmsuc0348A - Internal intron

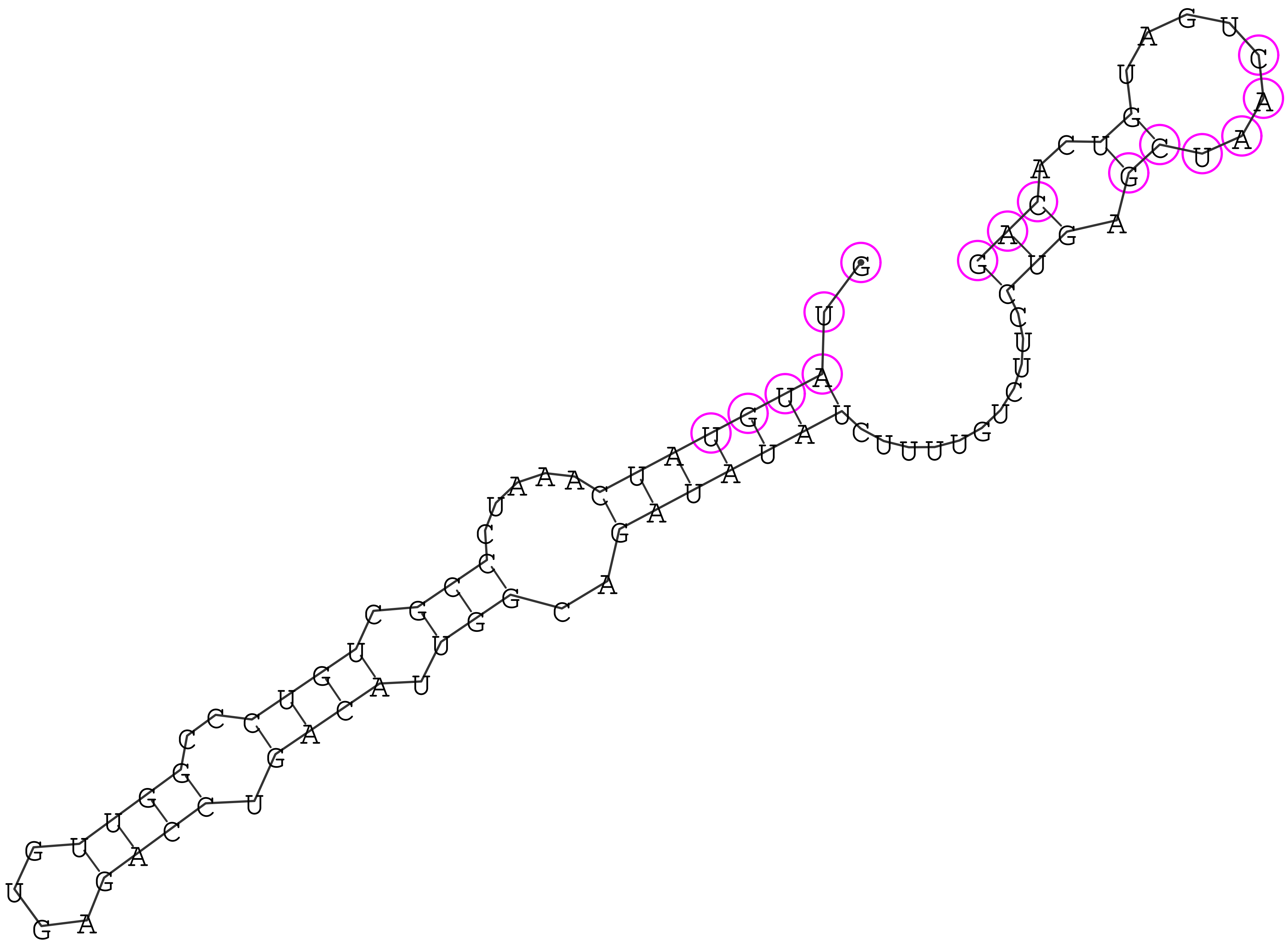

Xmsuc0374A - Internal intron

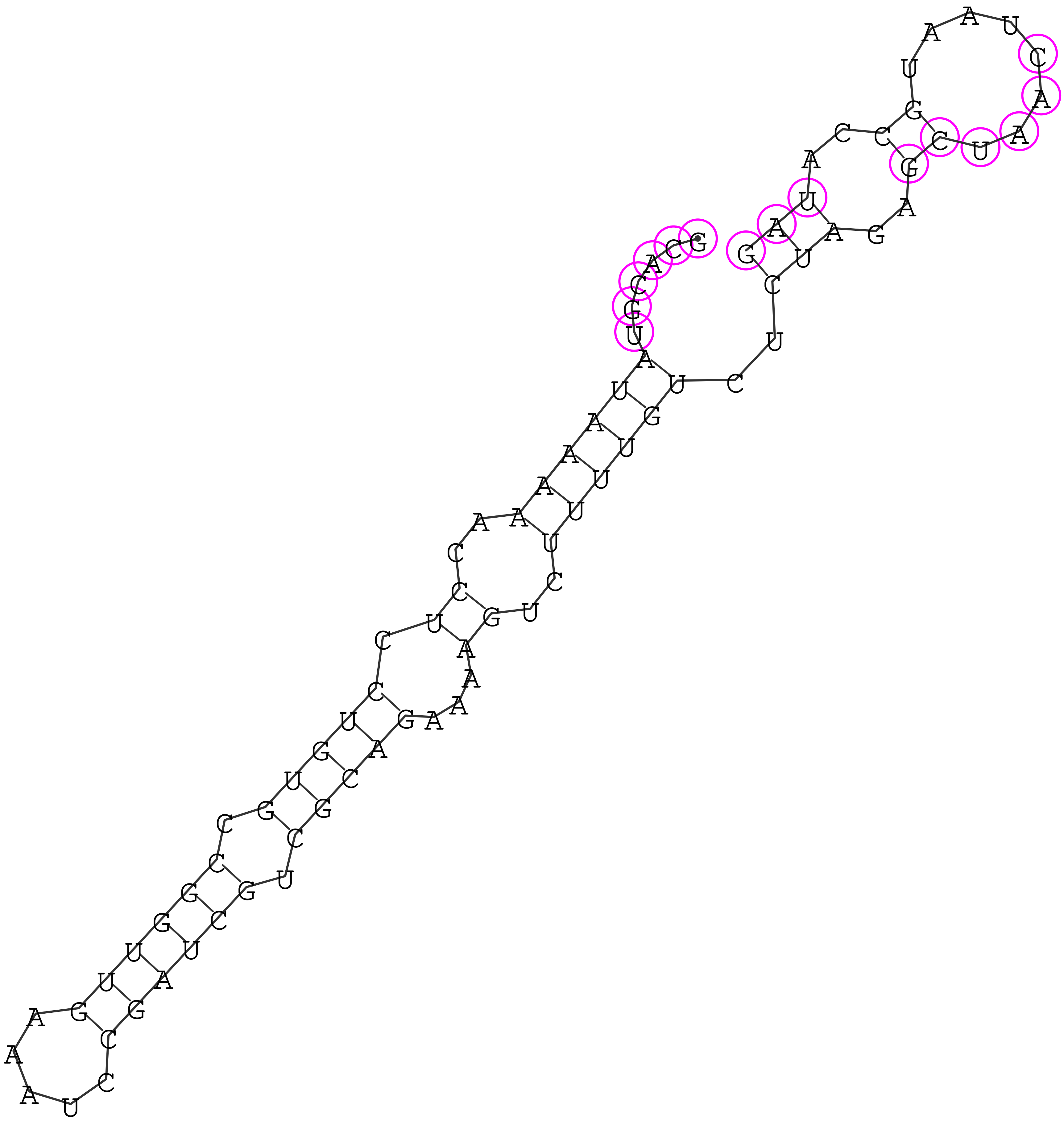

Xmsuc0374B - Internal intron

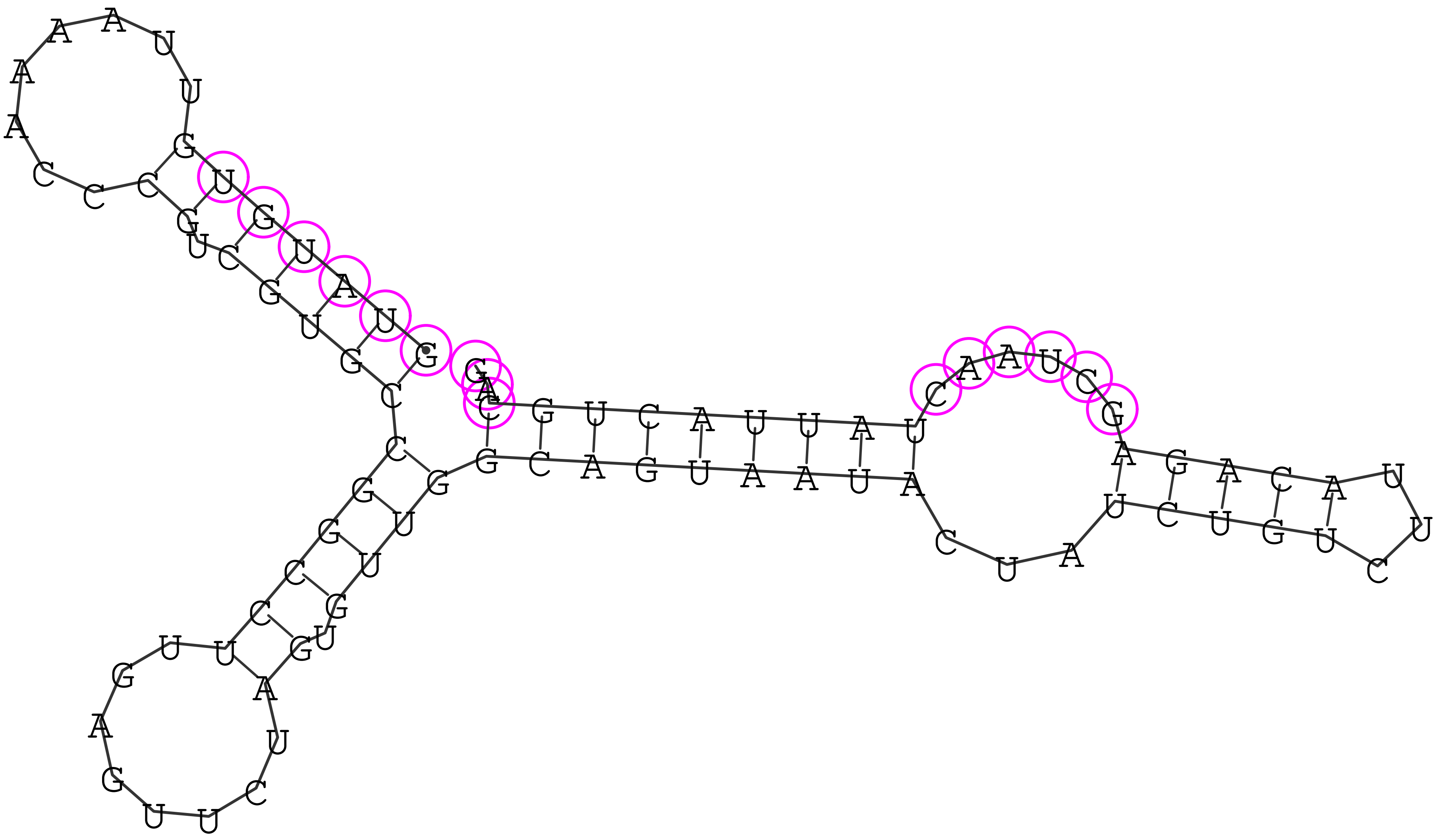

Xmsuc0378A - Internal intron

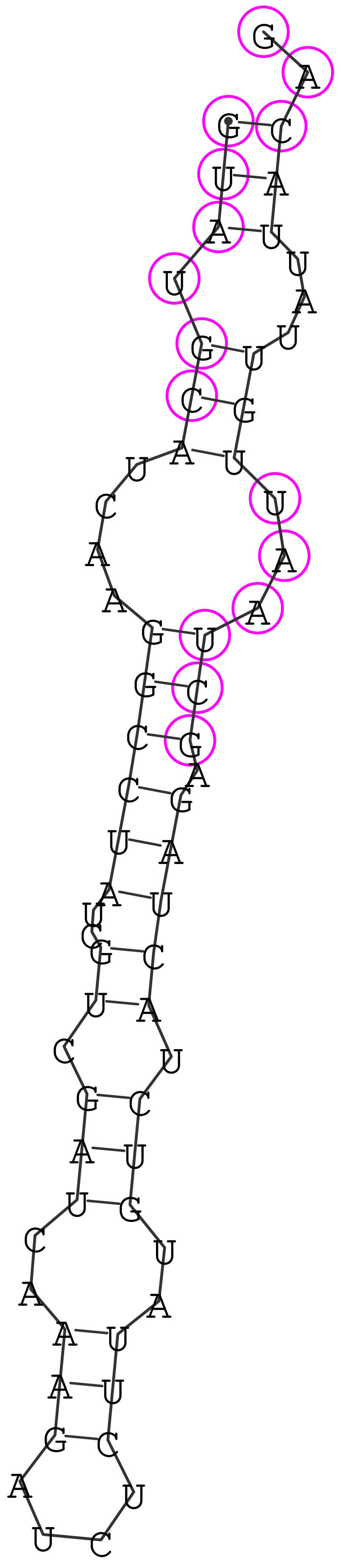

Xmsuc0385A - Internal intron

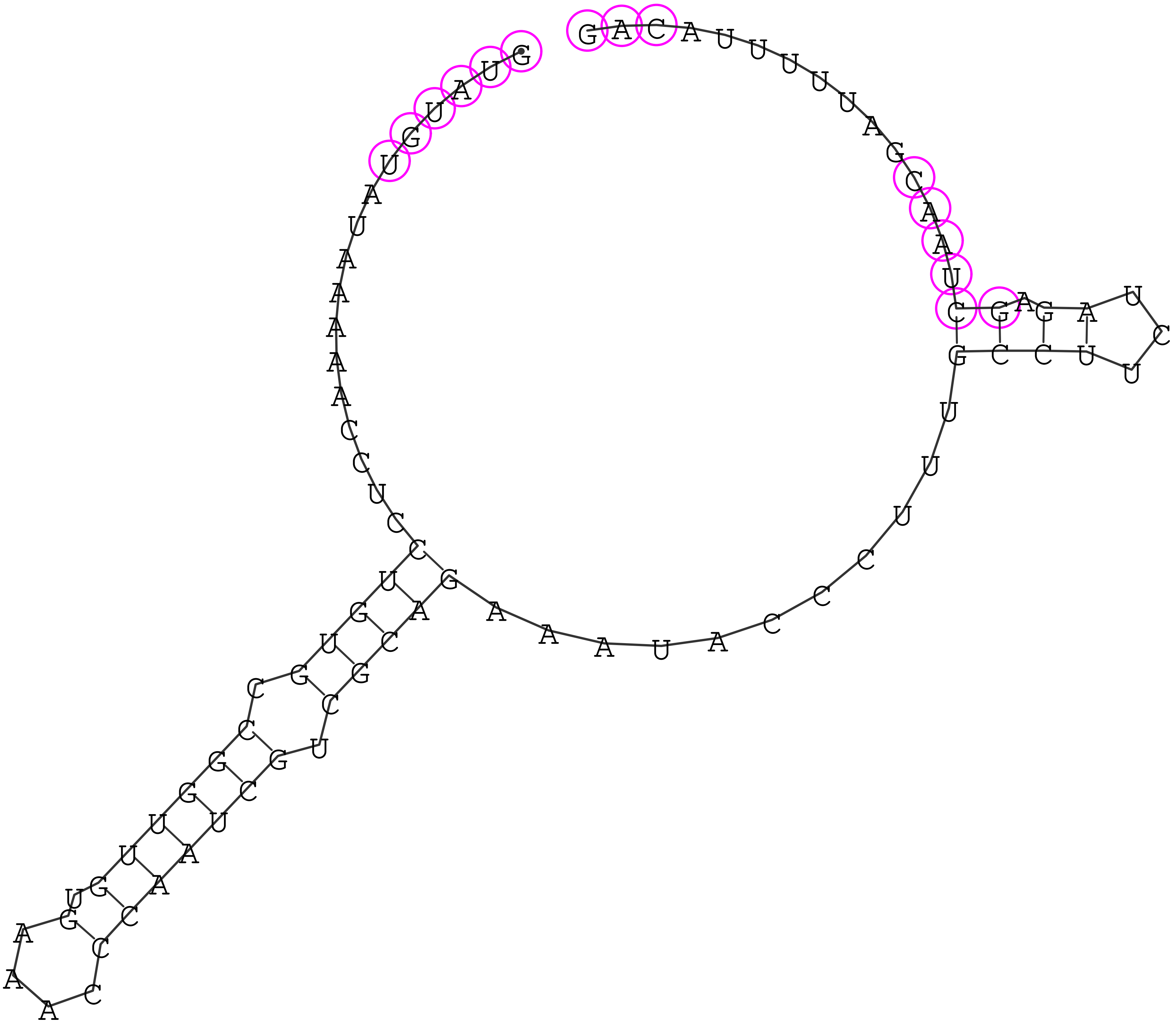

Xmsuc0412A - Internal intron

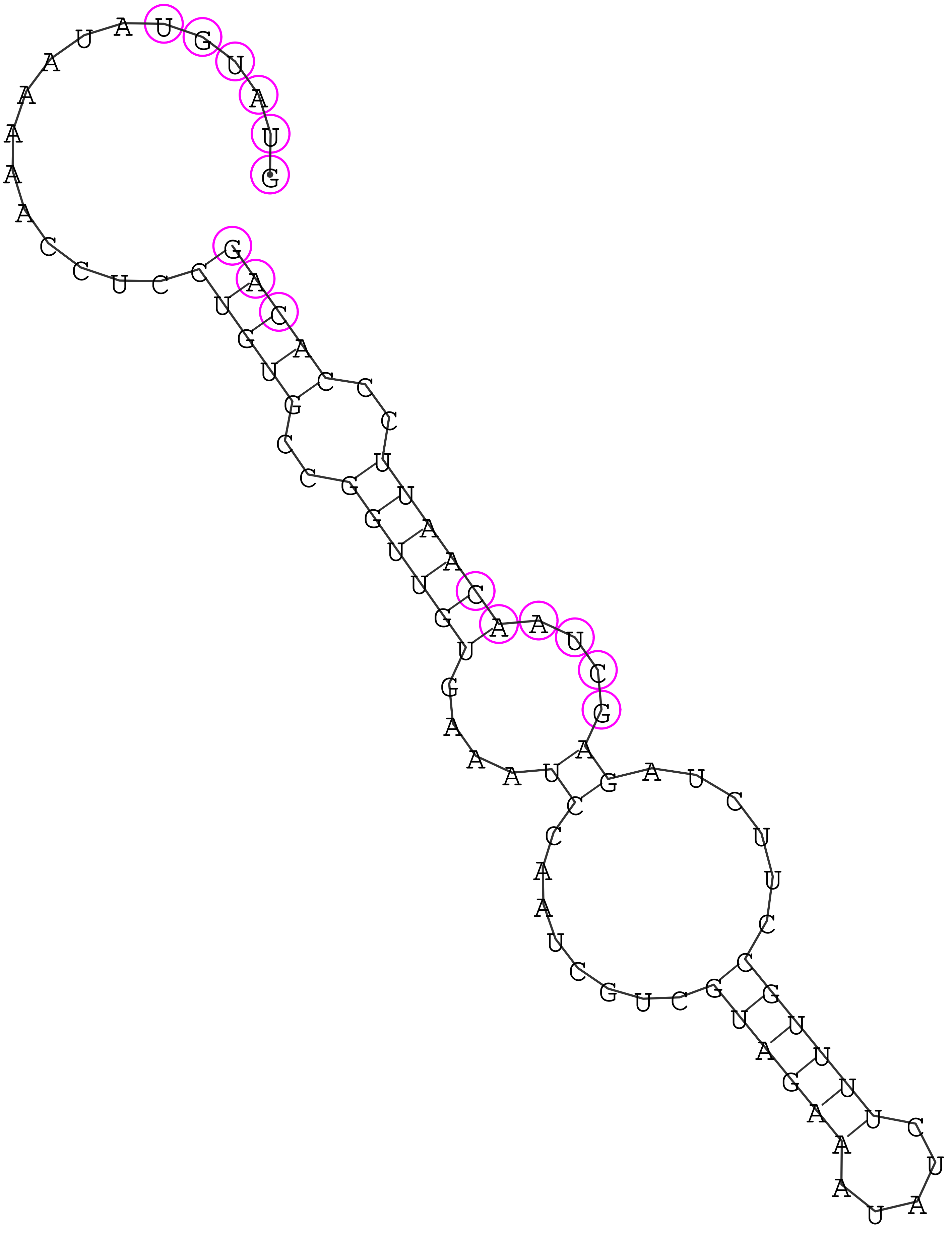

Xmsuc0520A - Internal intron

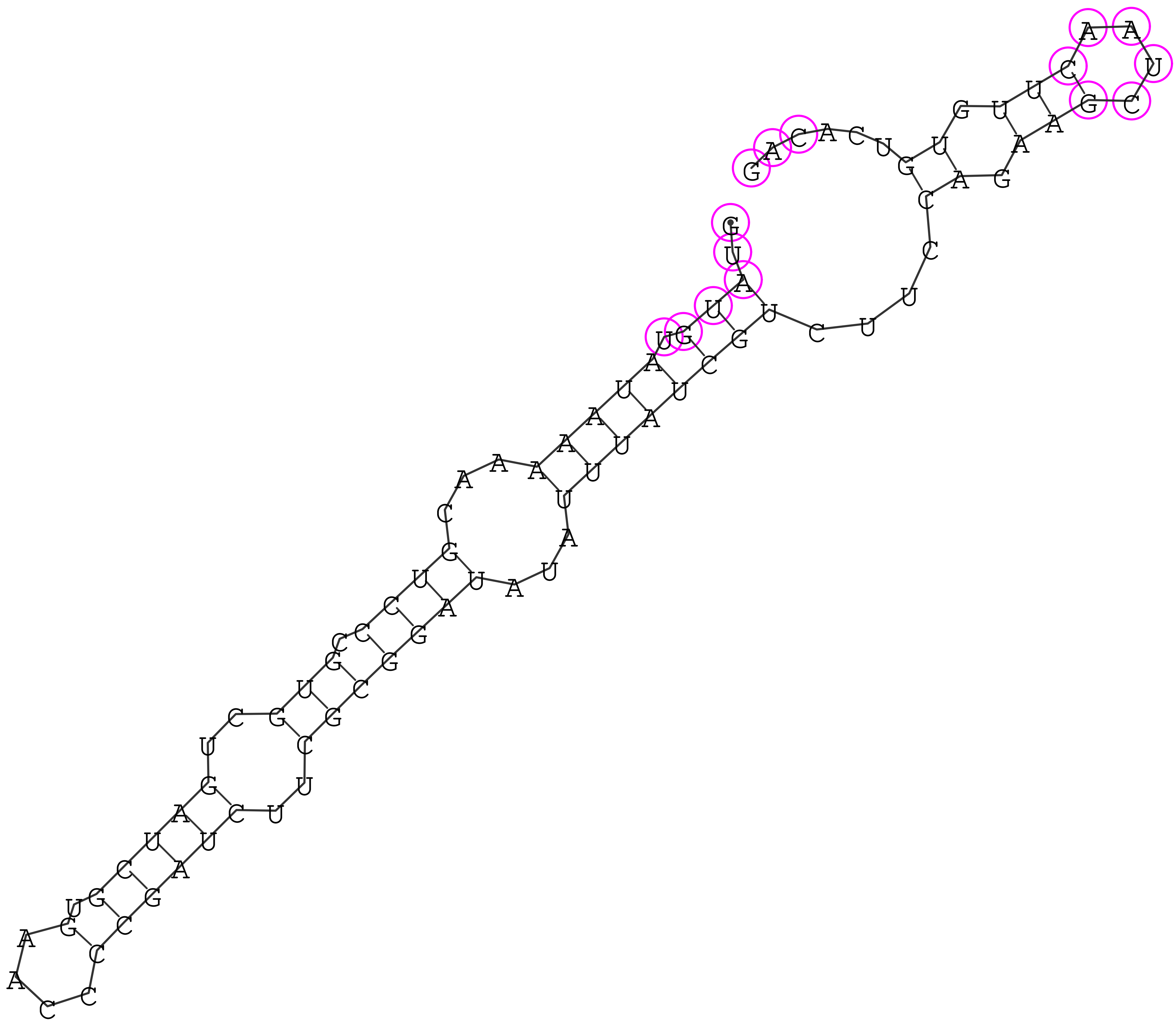

Xmsuc0671A - Internal intron

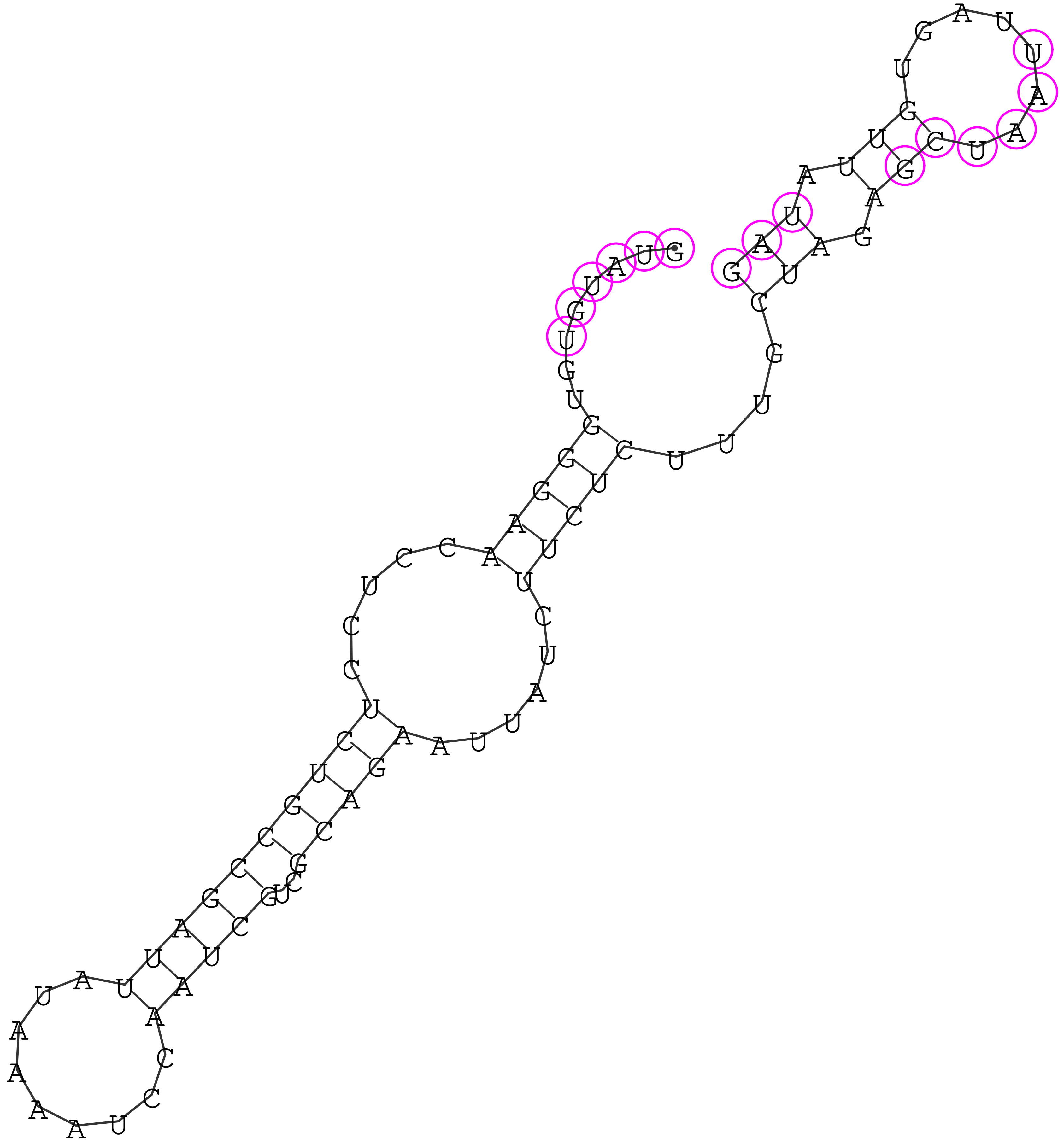

Xmsuc0710A - Internal intron

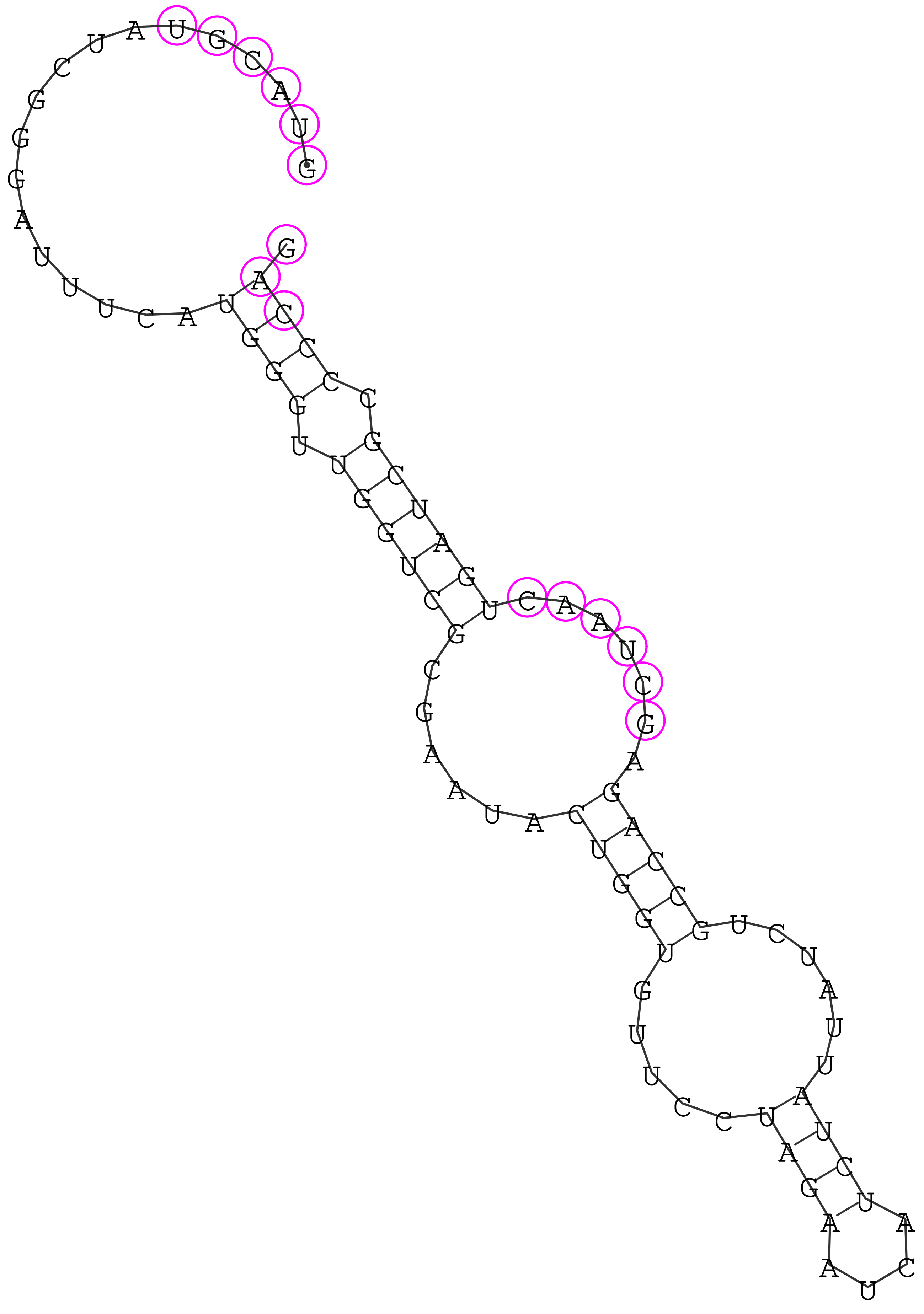

Xmsuc0775A - Internal intron

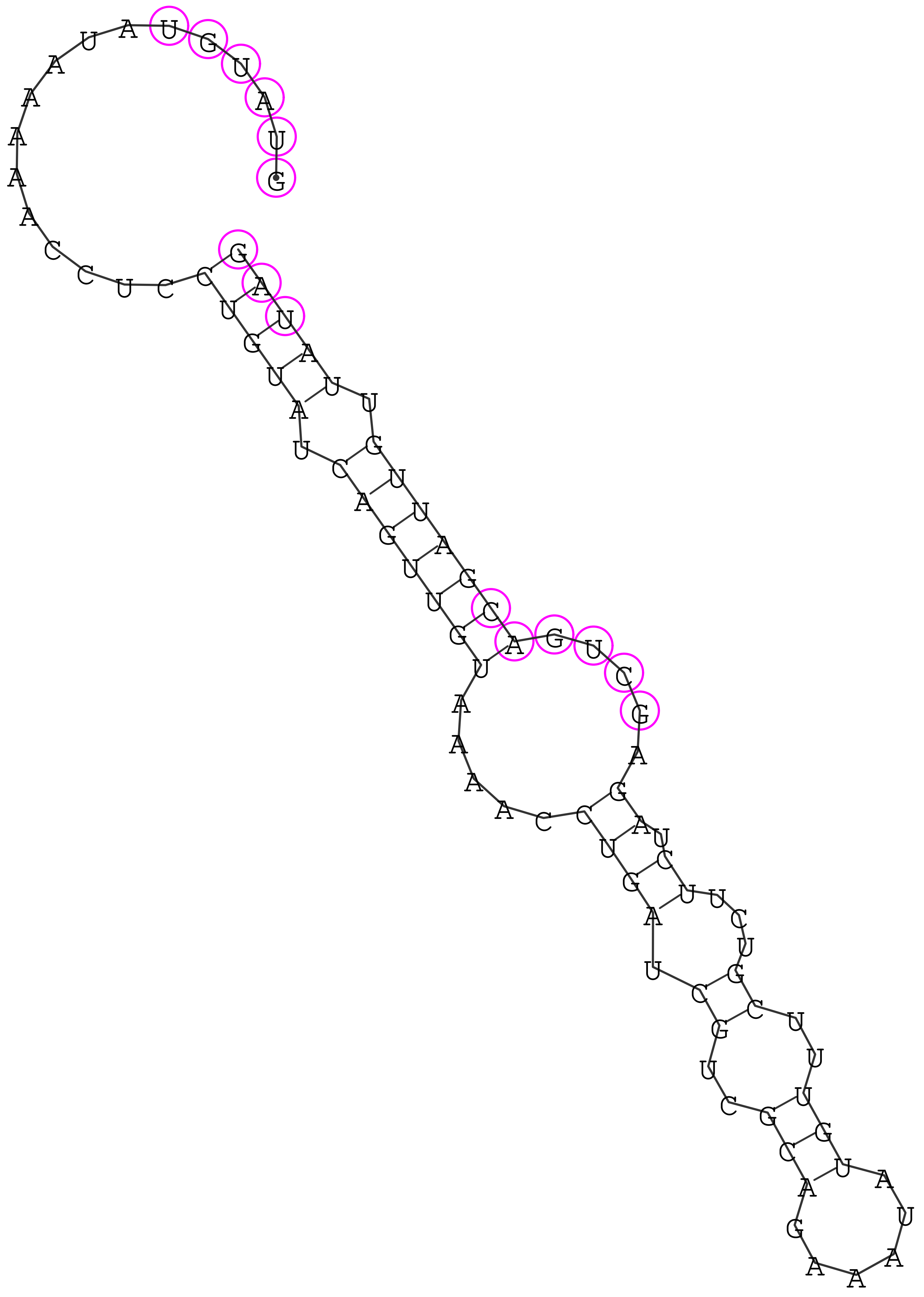

Xmsuc0776A - Internal intron

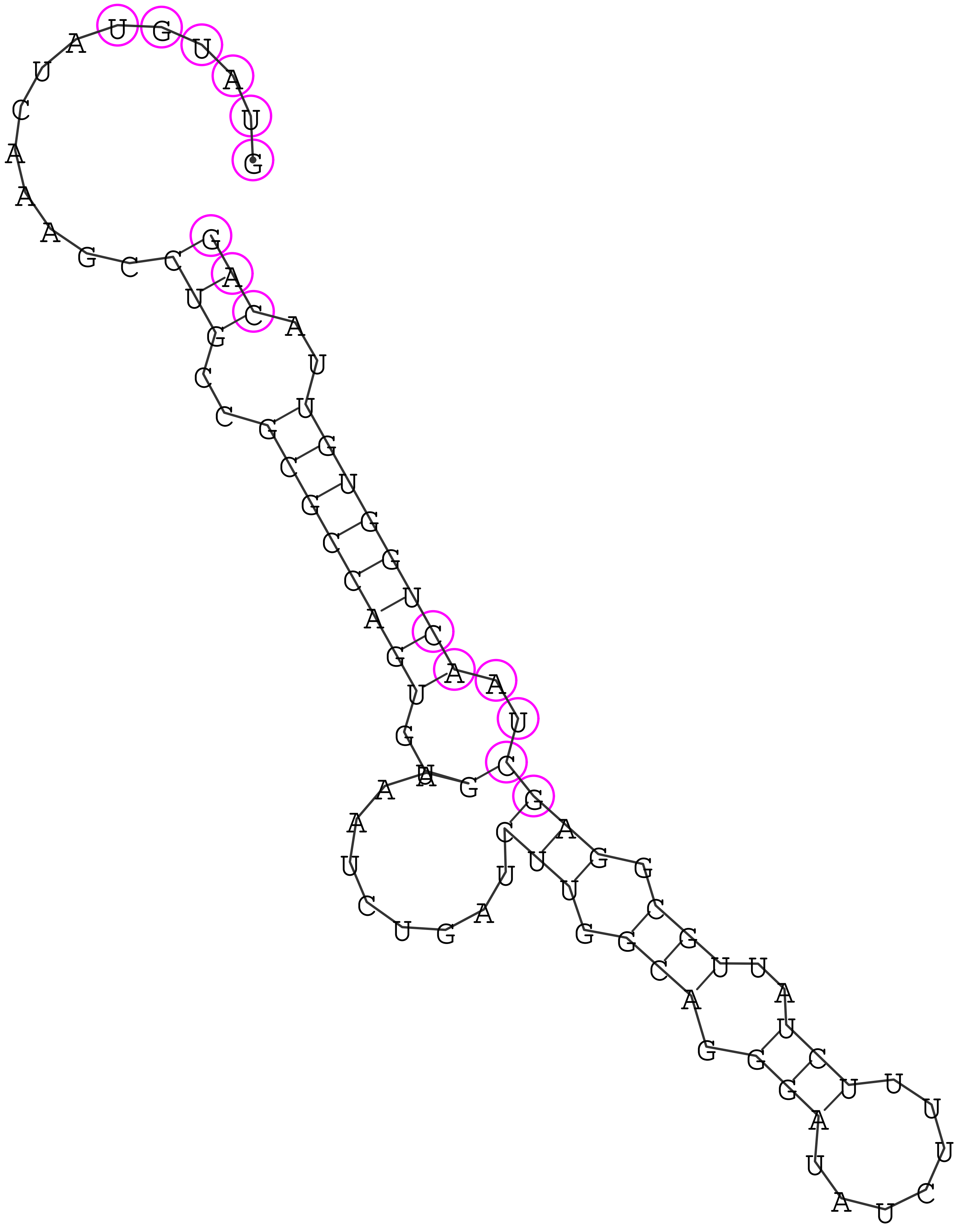

Xmsuc0776B - Internal intron

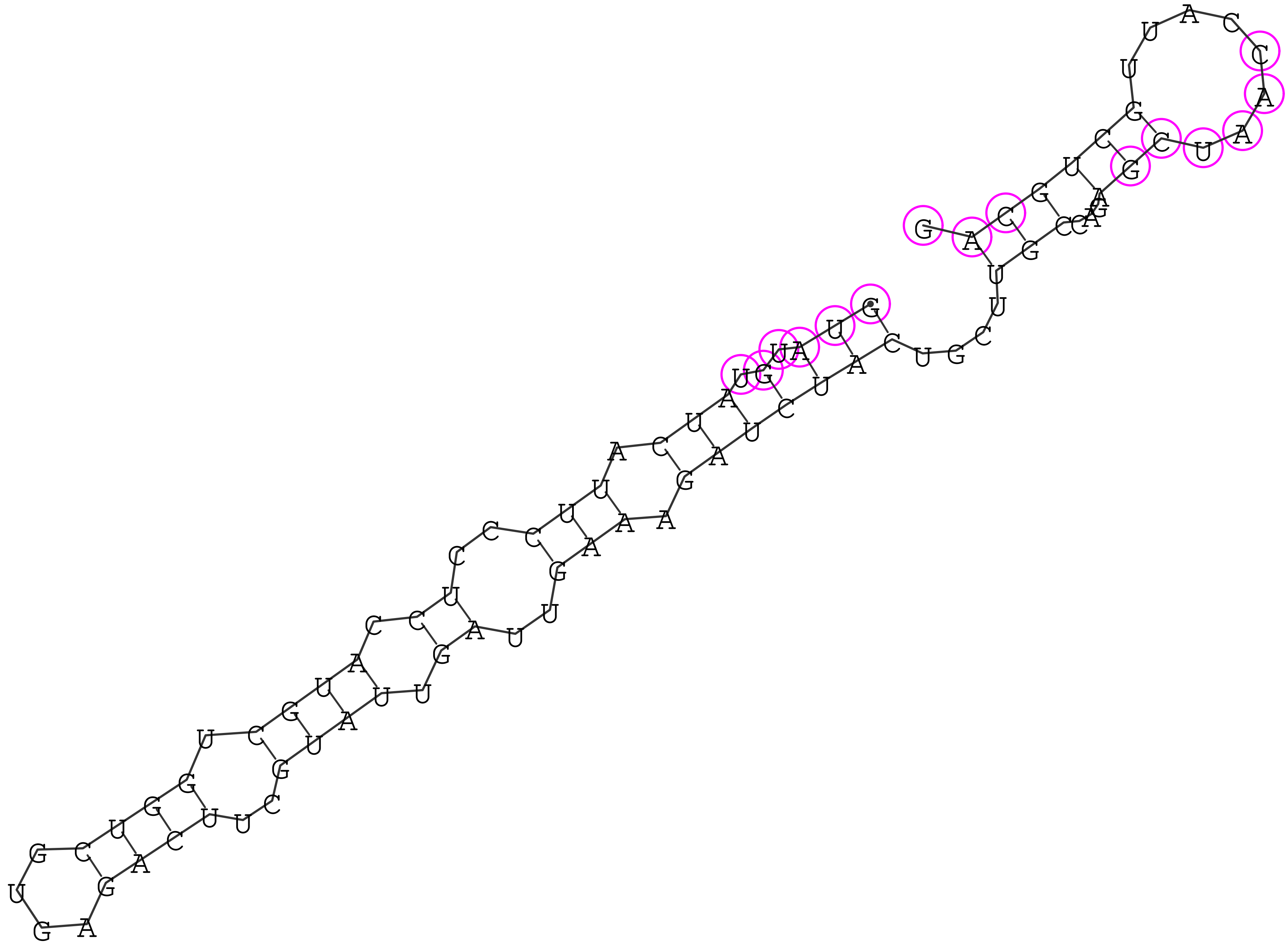

Xmsuc0776C - Internal intron

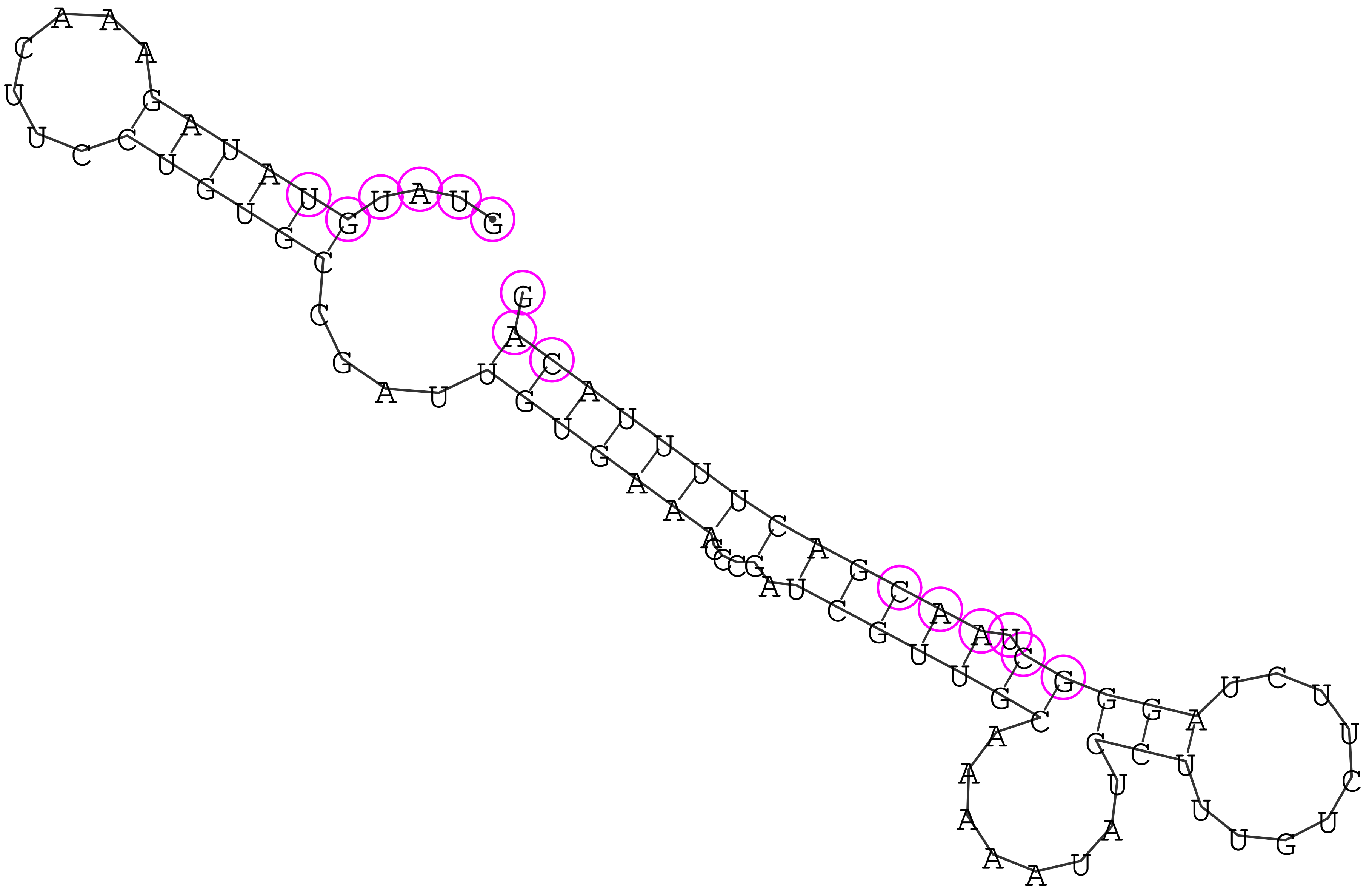

Xmsuc0808A - Internal intron

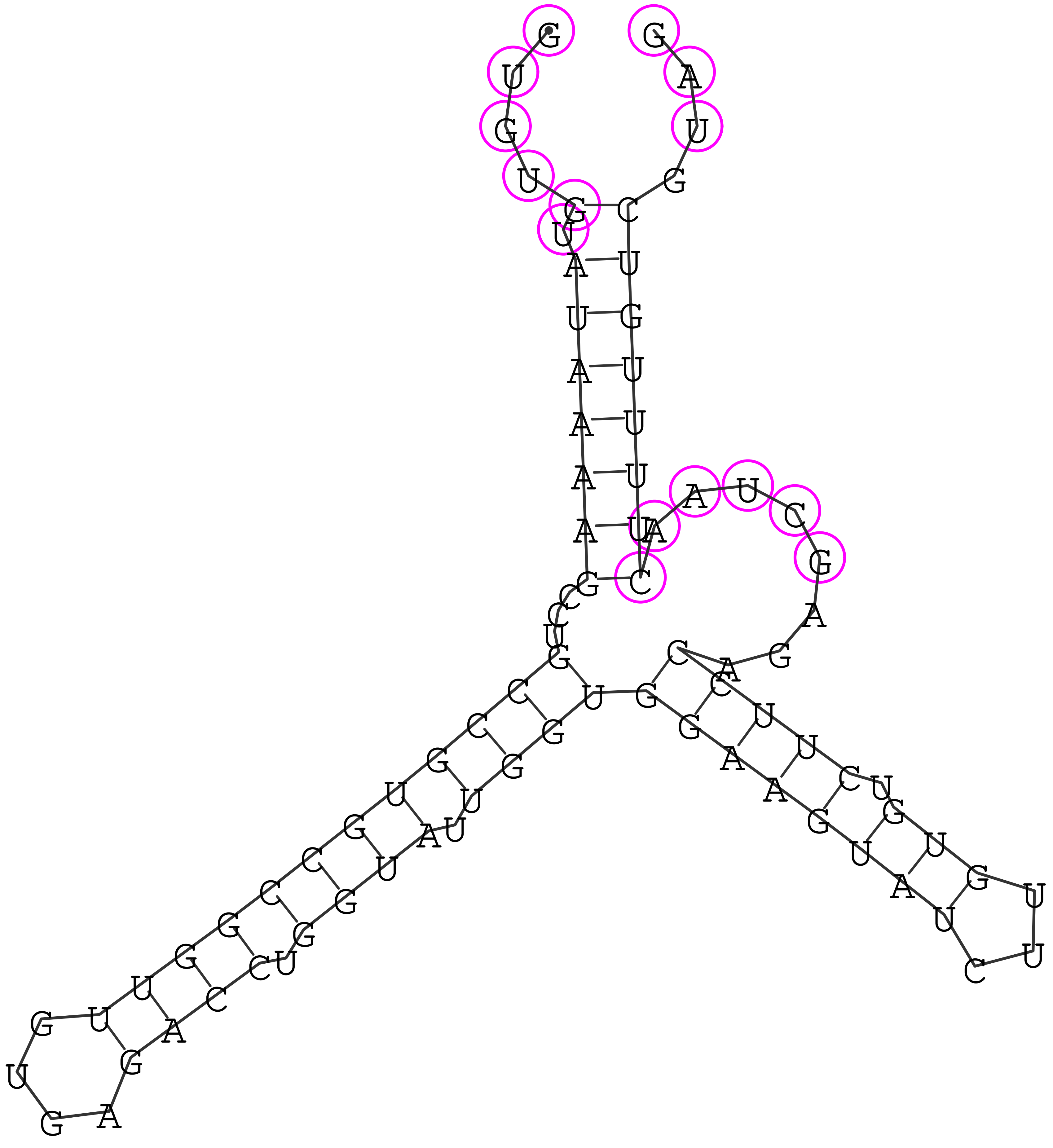

Xmsuc0819A - Internal intron

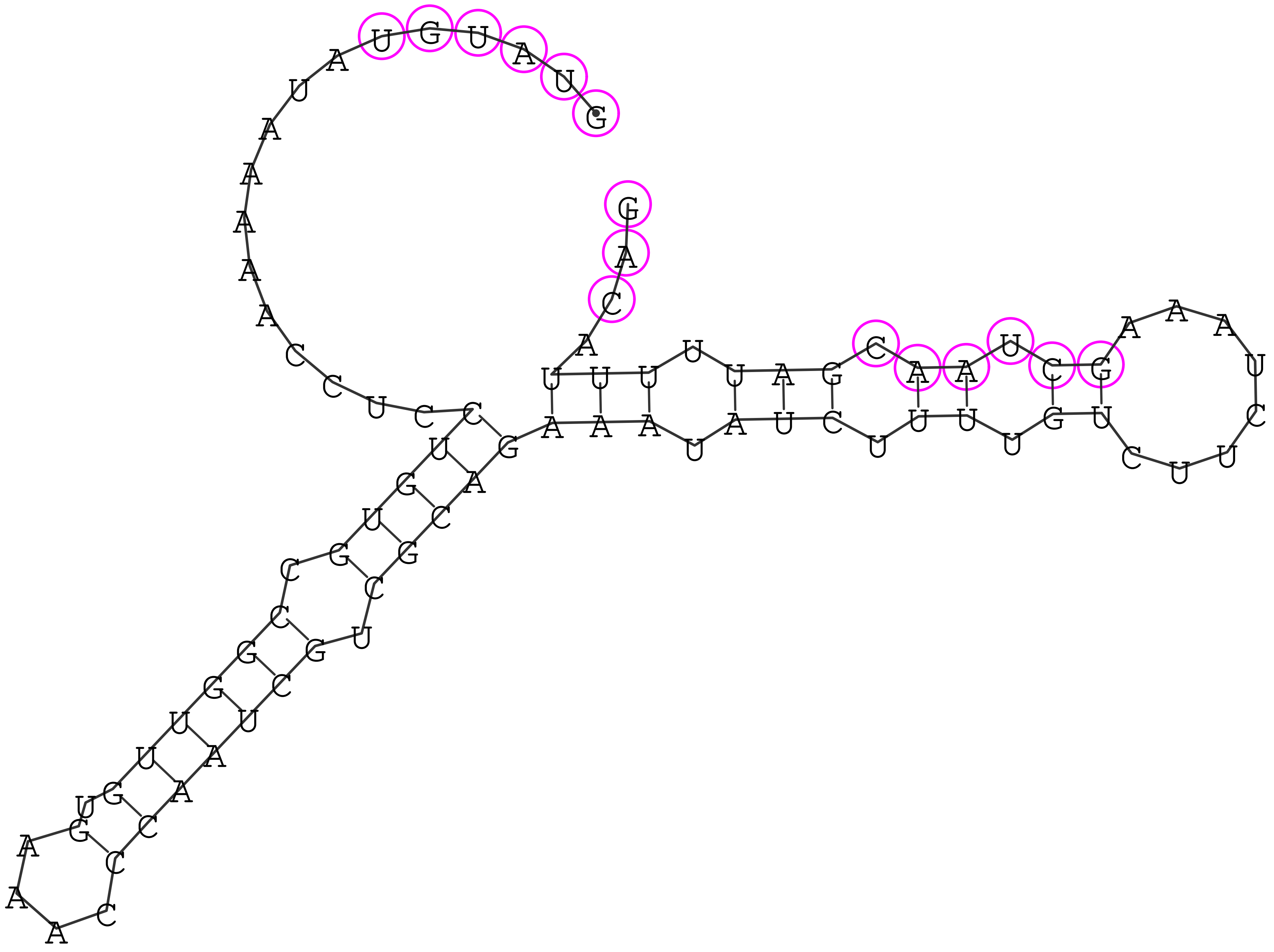

Xmsuc0904A - Internal intron

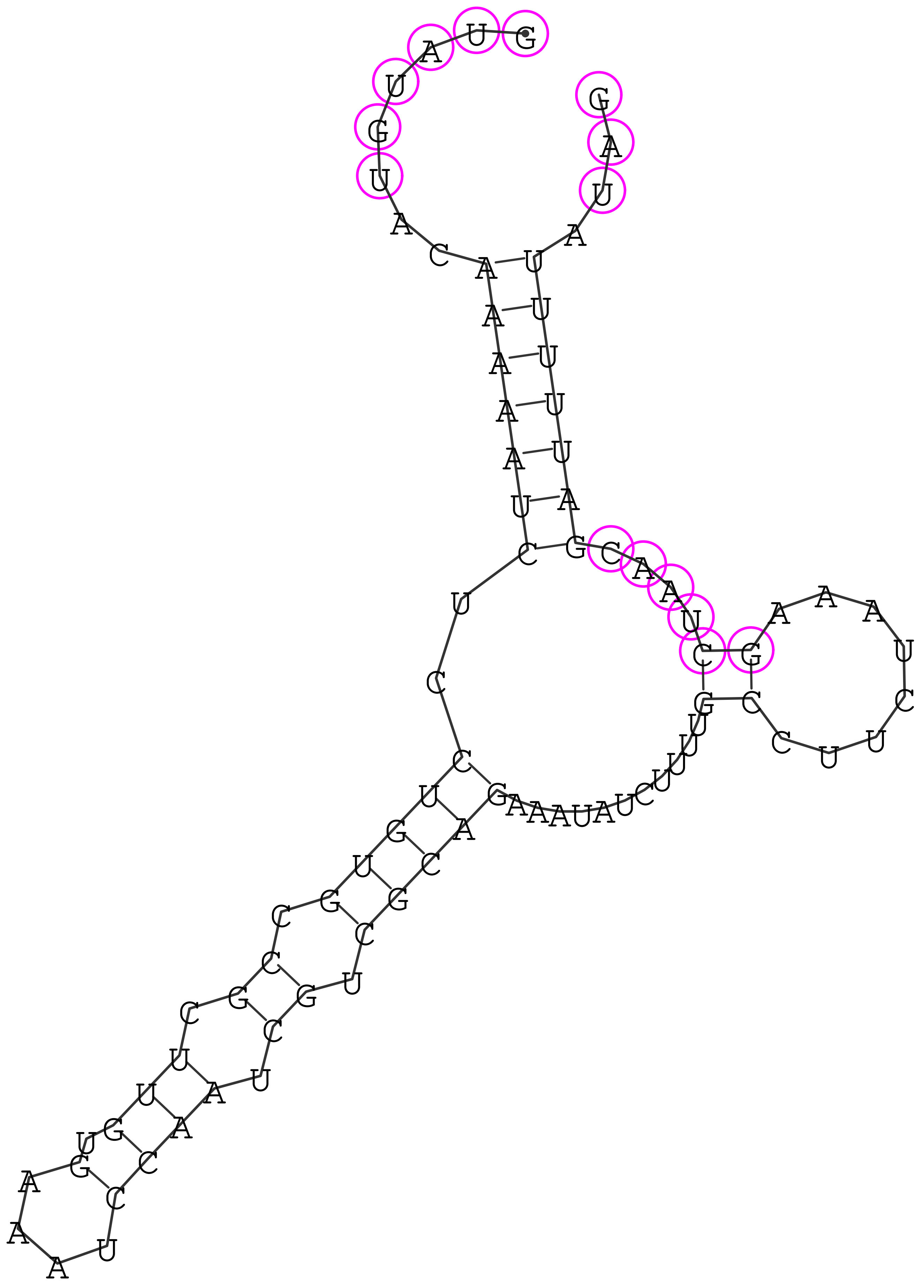

Xmsuc1083A - Internal intron

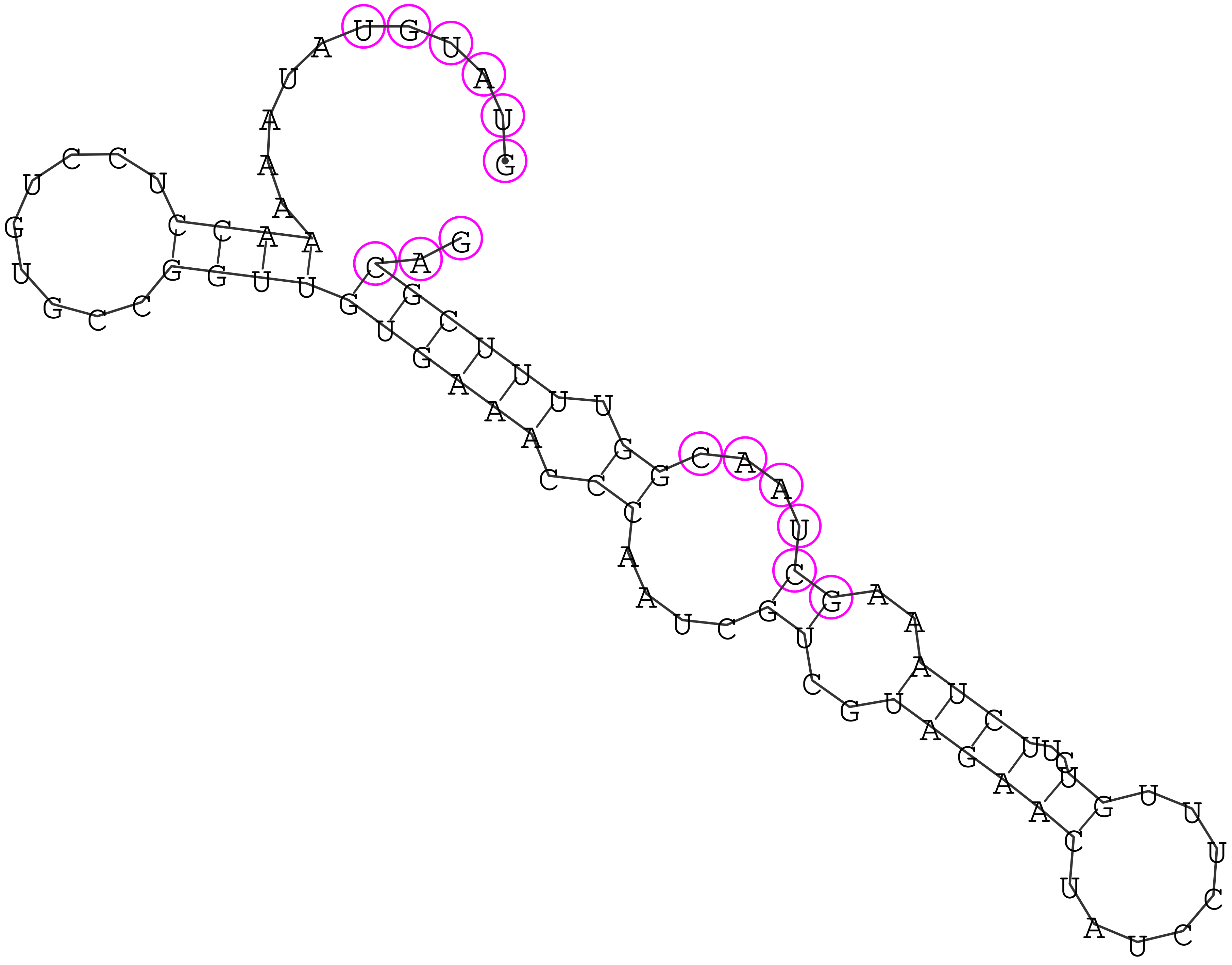

Xmsuc1127A - Internal intron

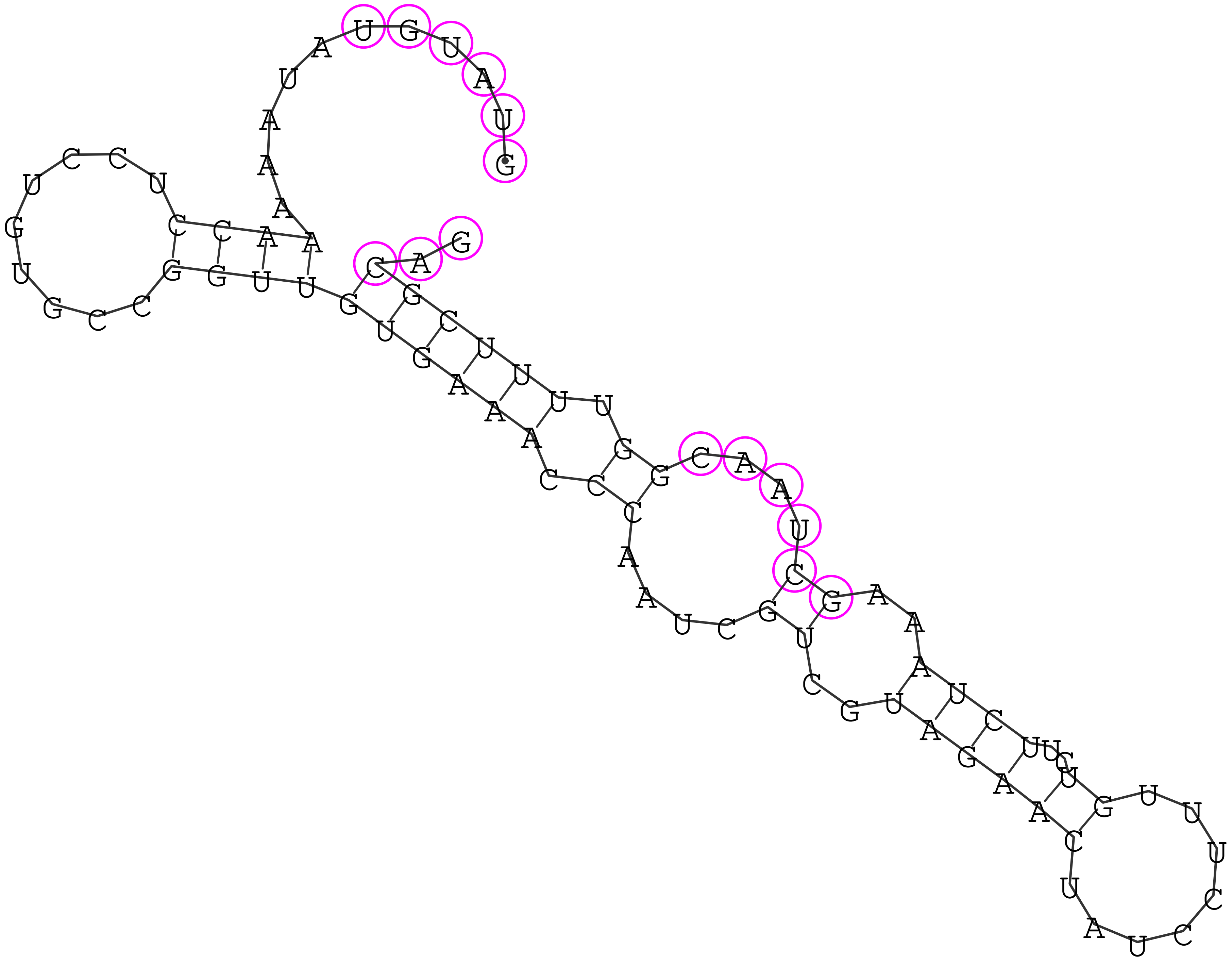

Xmsuc1145A - Internal intron

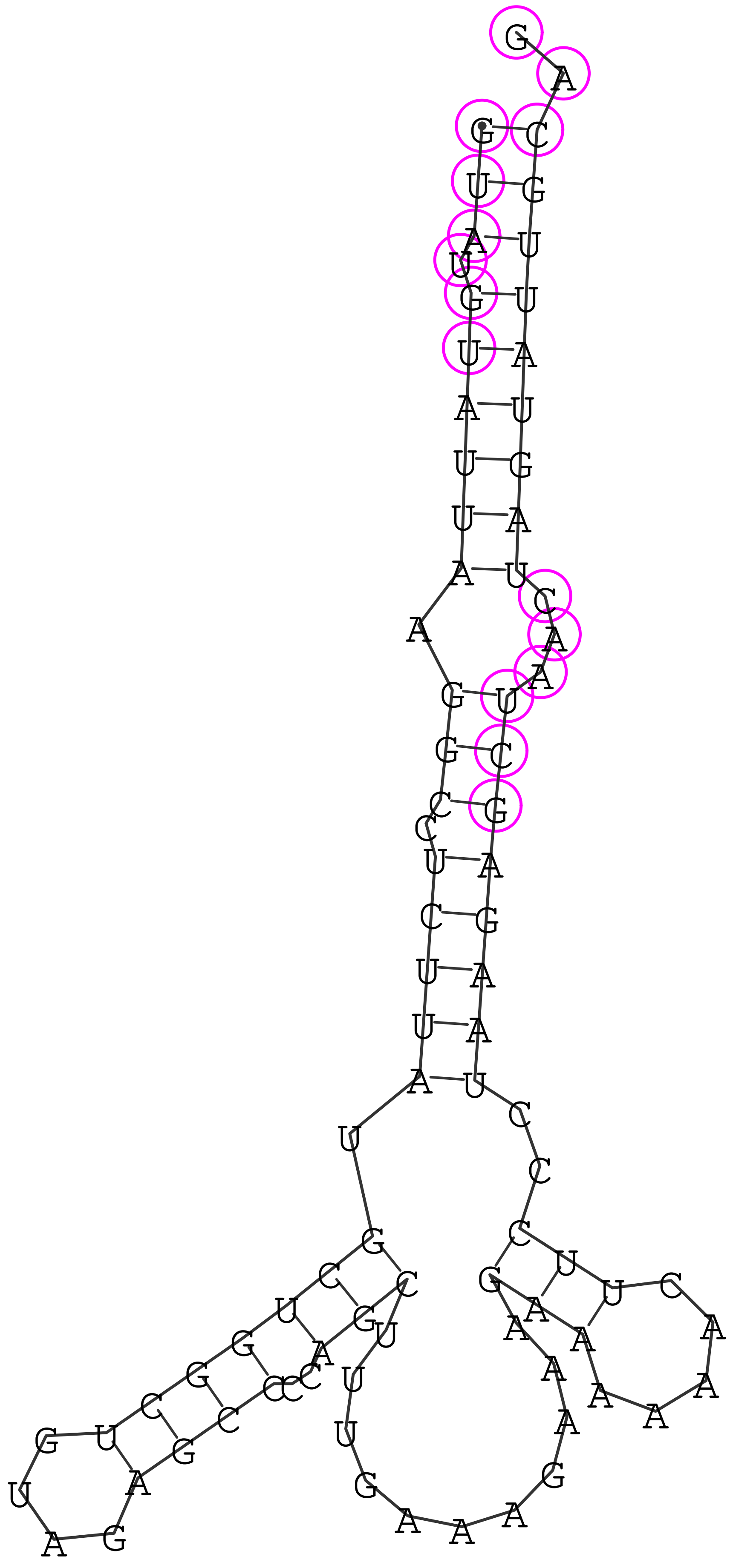

Supplement: Data S2 — Collection of the RNAFold predicted secondary structures of the internal introns of the 288 [D1,2] sister stwintrons. [file spectrum.02926-24-s0002.pdf]
