# Supplementary material for: Propagation of [D1,2]-type spliceosomal twin introns (stwintrons) in Hypoxylaceae and Xylariaceae fungi
Source: Microbiol Spectr. 2025 Aug 8;13(9):e02926-24. doi: 10.1128/spectrum.02926-24 (PMC12403724; doi:10.1128/spectrum.02926-24)

Supplementary datafile S3. Collection of the RNAFold predicted secondary structures of the external introns of the 288 [D1,2] sister stwintrons. The intron sequences are given in Table S1. The 5'-donor of the external intron is interrupted by the internal intron between its first and second nt ([D1,2]). The 5'-donor (6-nt core) [D] and 3'-acceptor (3-nt core) [A] elements at the splice sites are marked with turquoise circles around the nt. The conserved sequence element including the branch point adenosine [L] (6-nt core) near the 3'-splice site is likewise highlighted.

Dchc001A - External intron

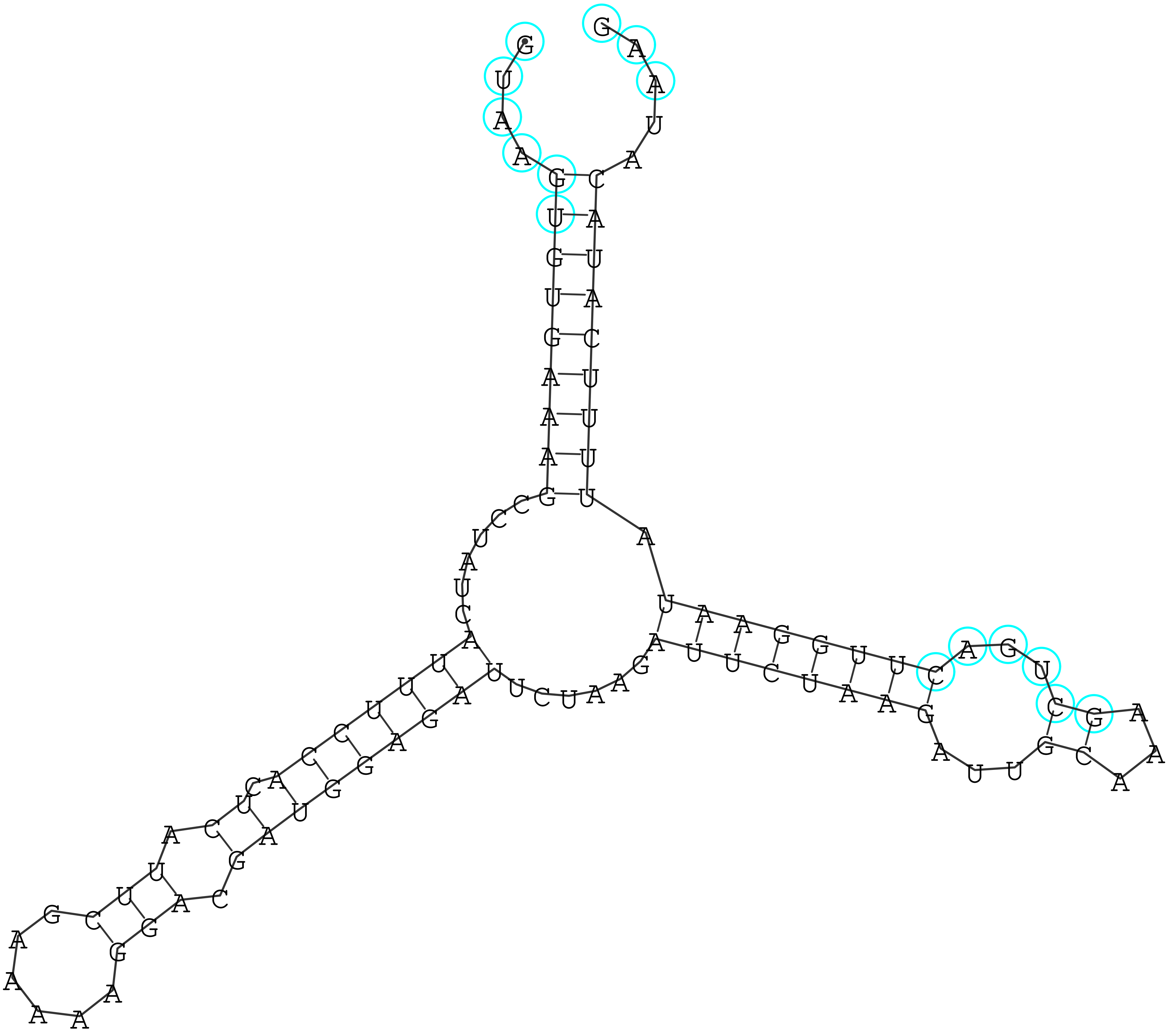

Dchc001B - External intron

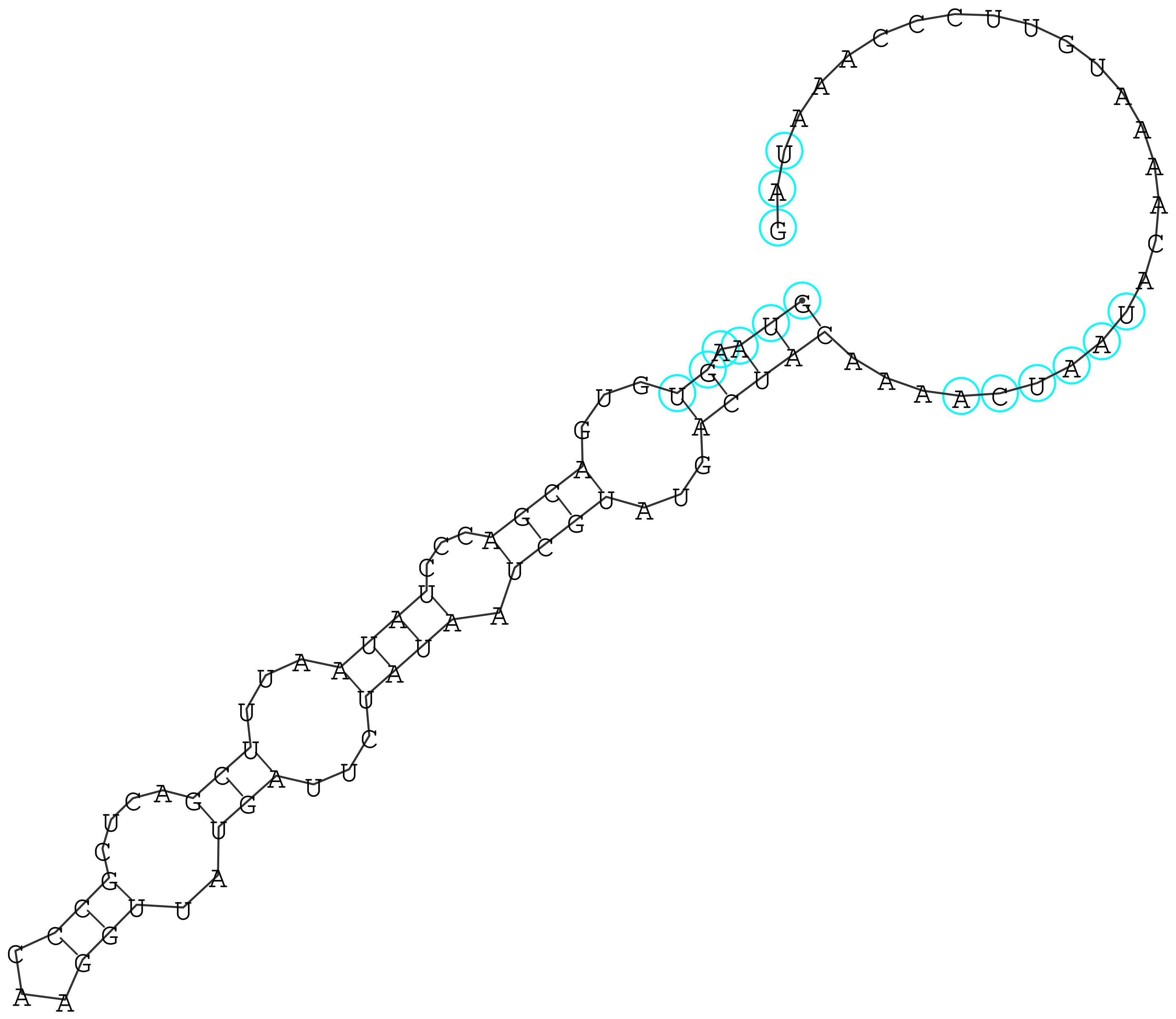

Dchc001C - External intron

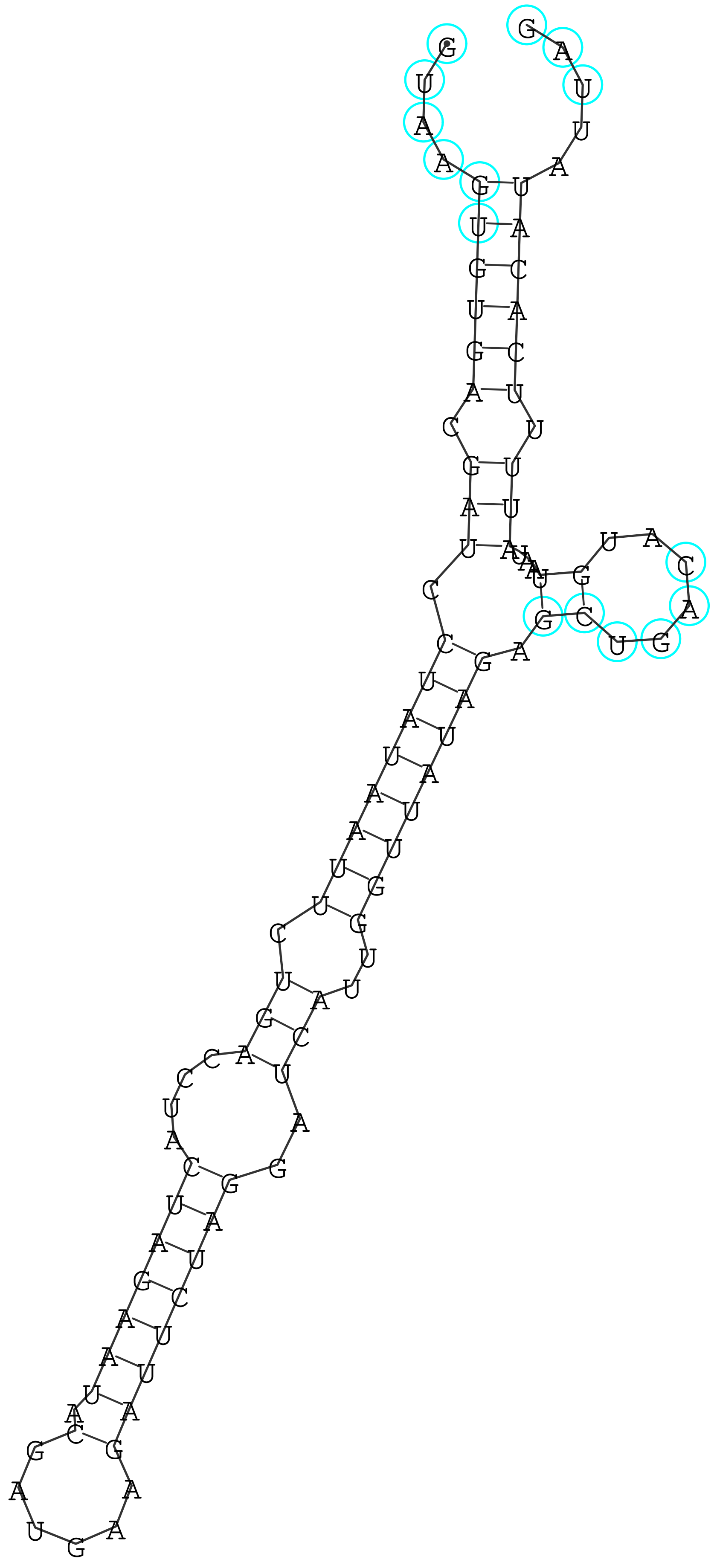

Dchc001D - External intron

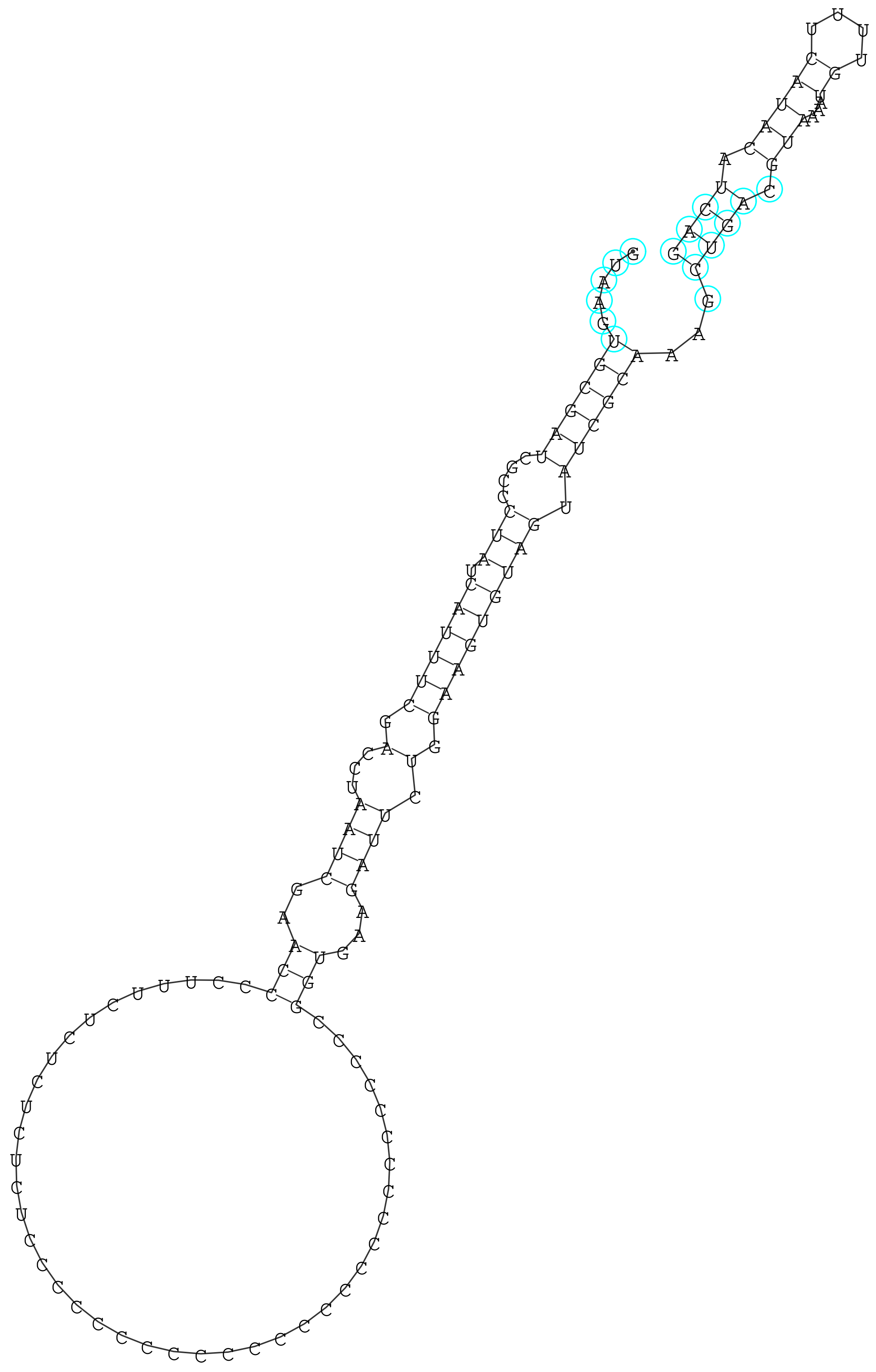

Dchc002A - External intron

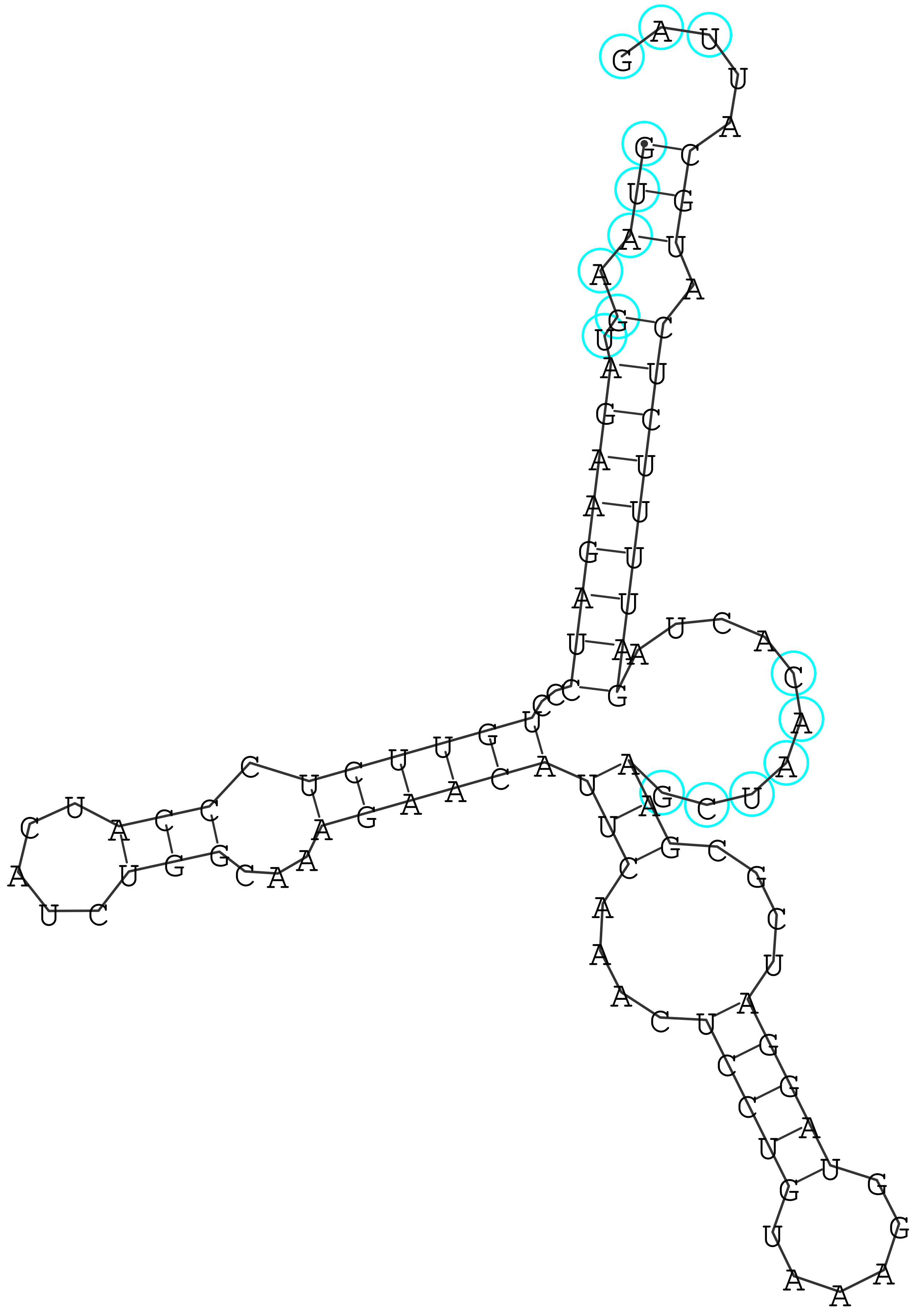

Dchc003A - External intron

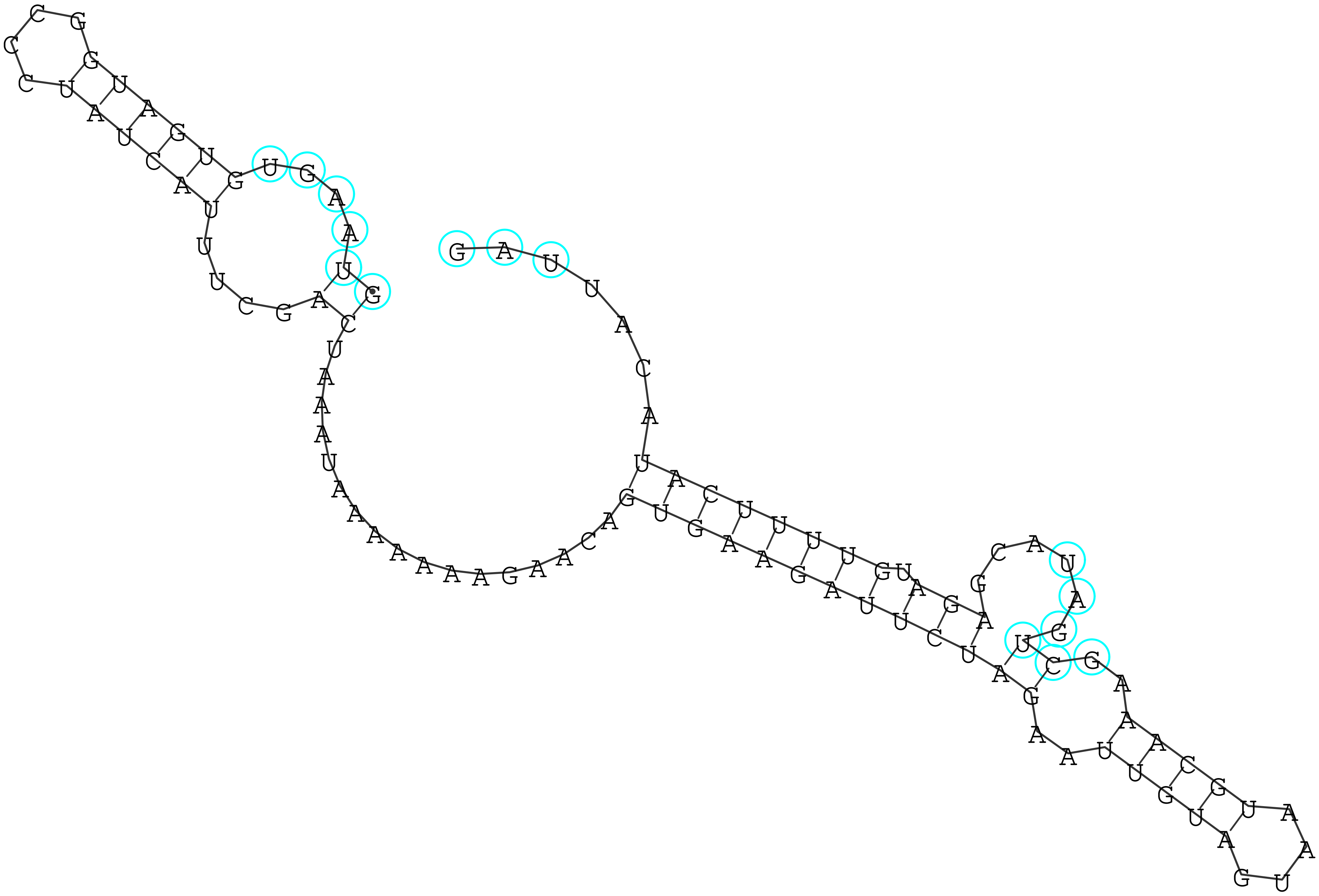

Dchc003B - External intron

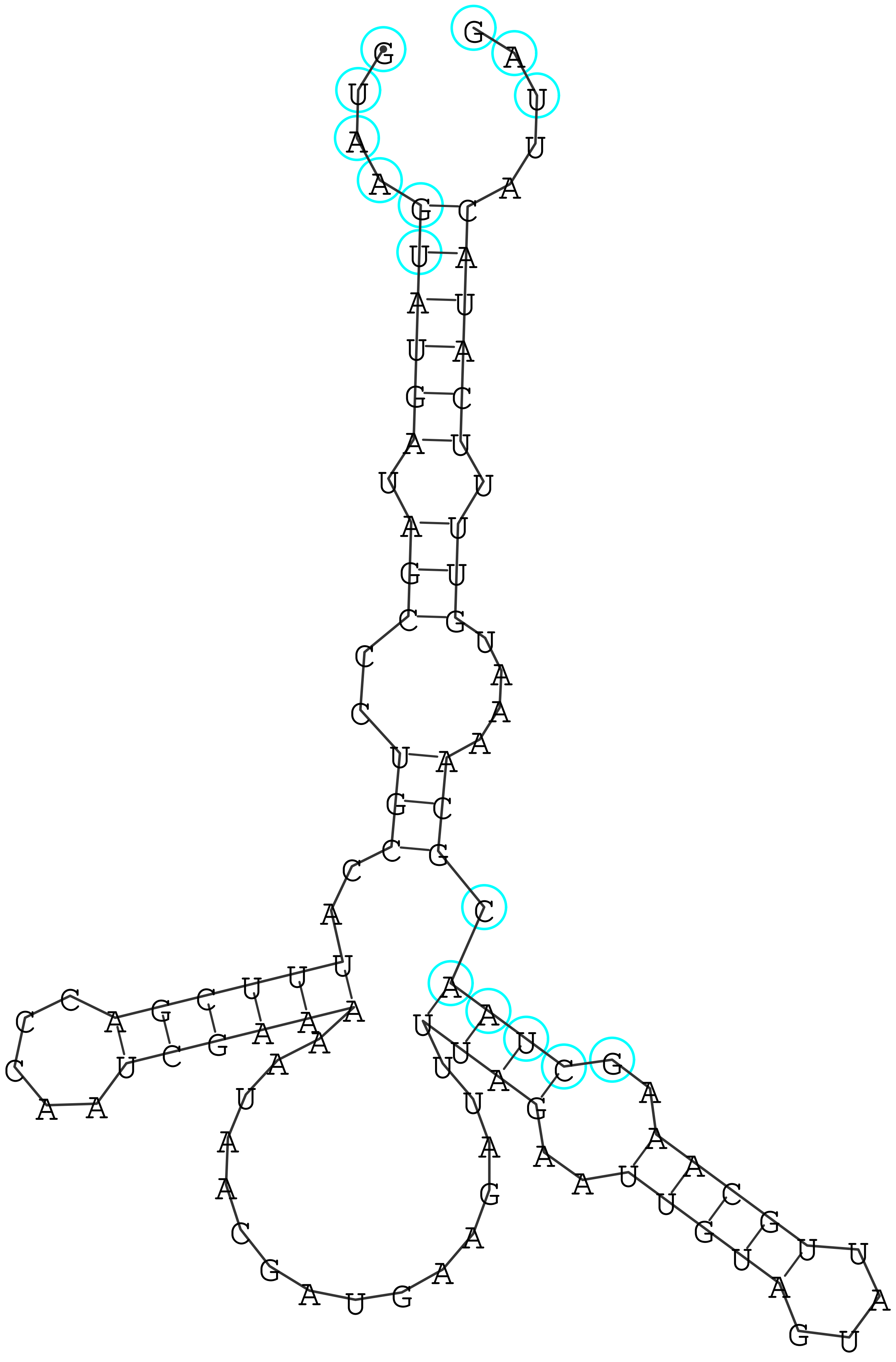

Dchc003C - External intron

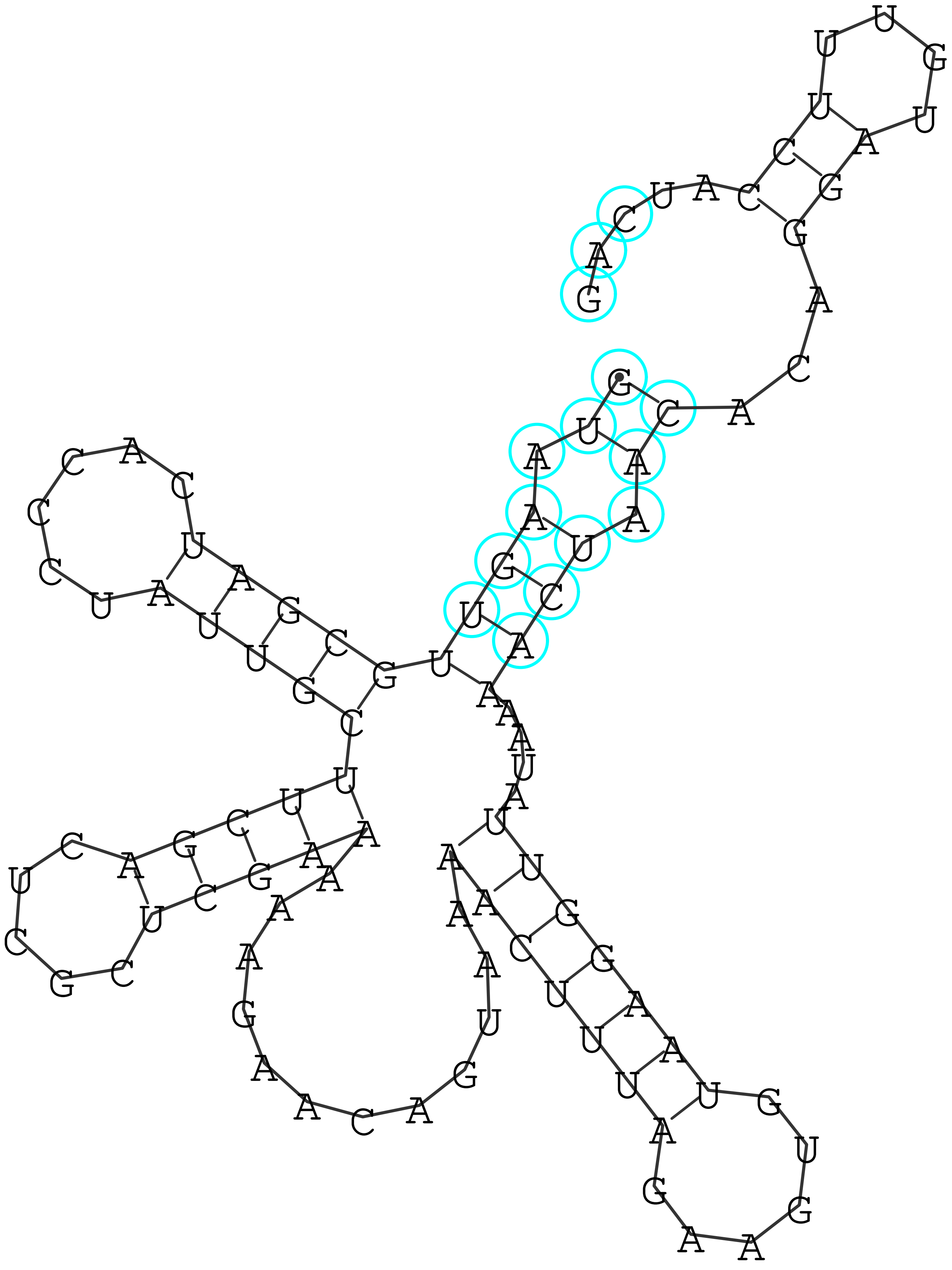

Dchc004A - External intron

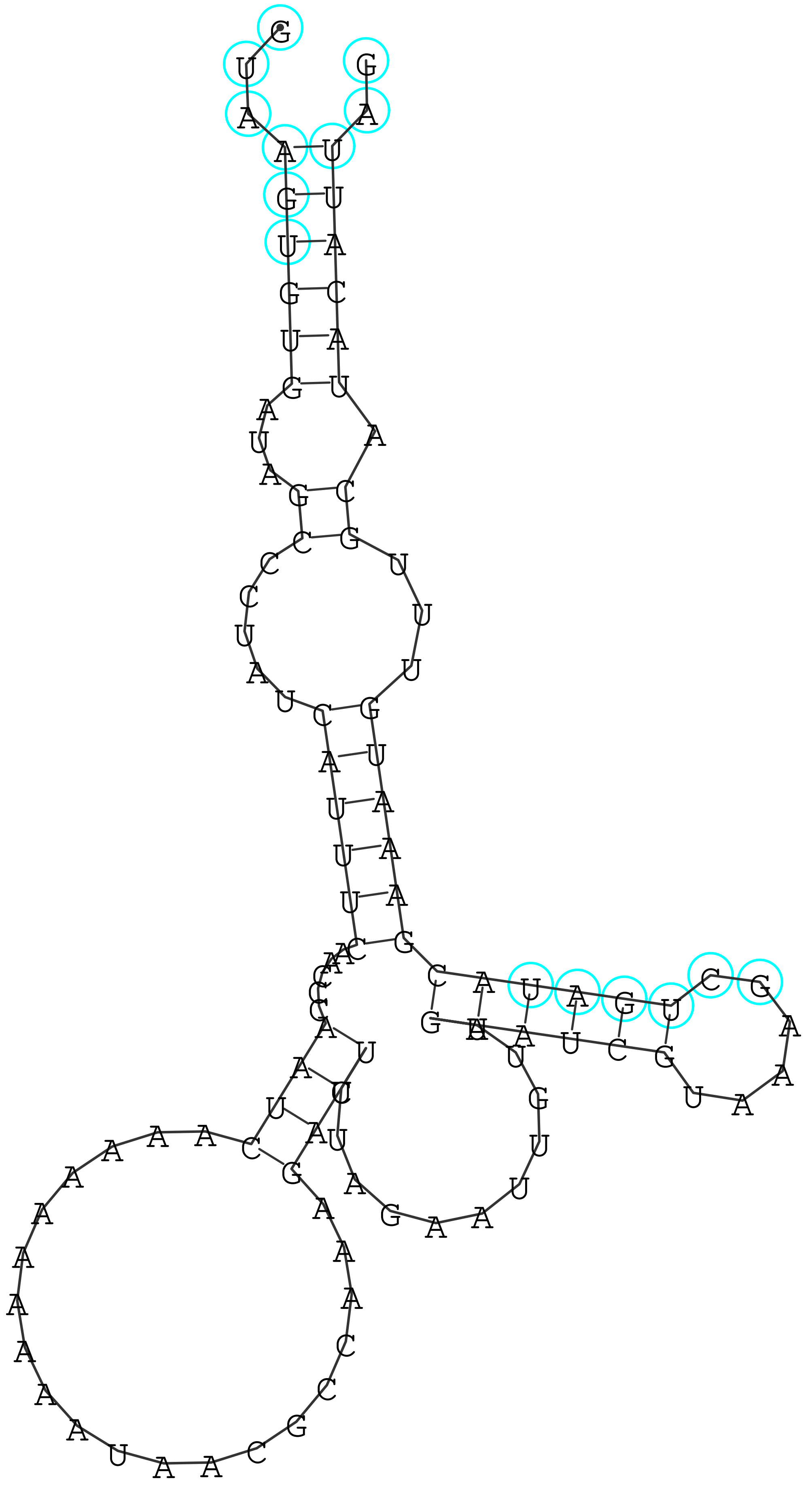

Dchc004B - External intron

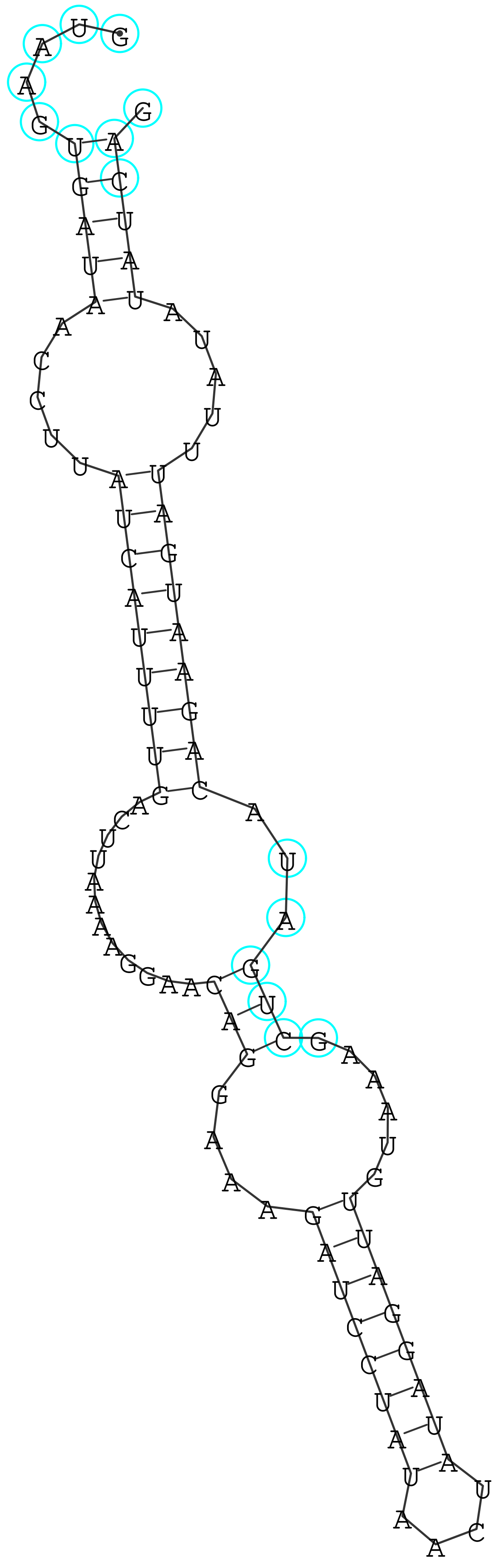

Dchc004C - External intron

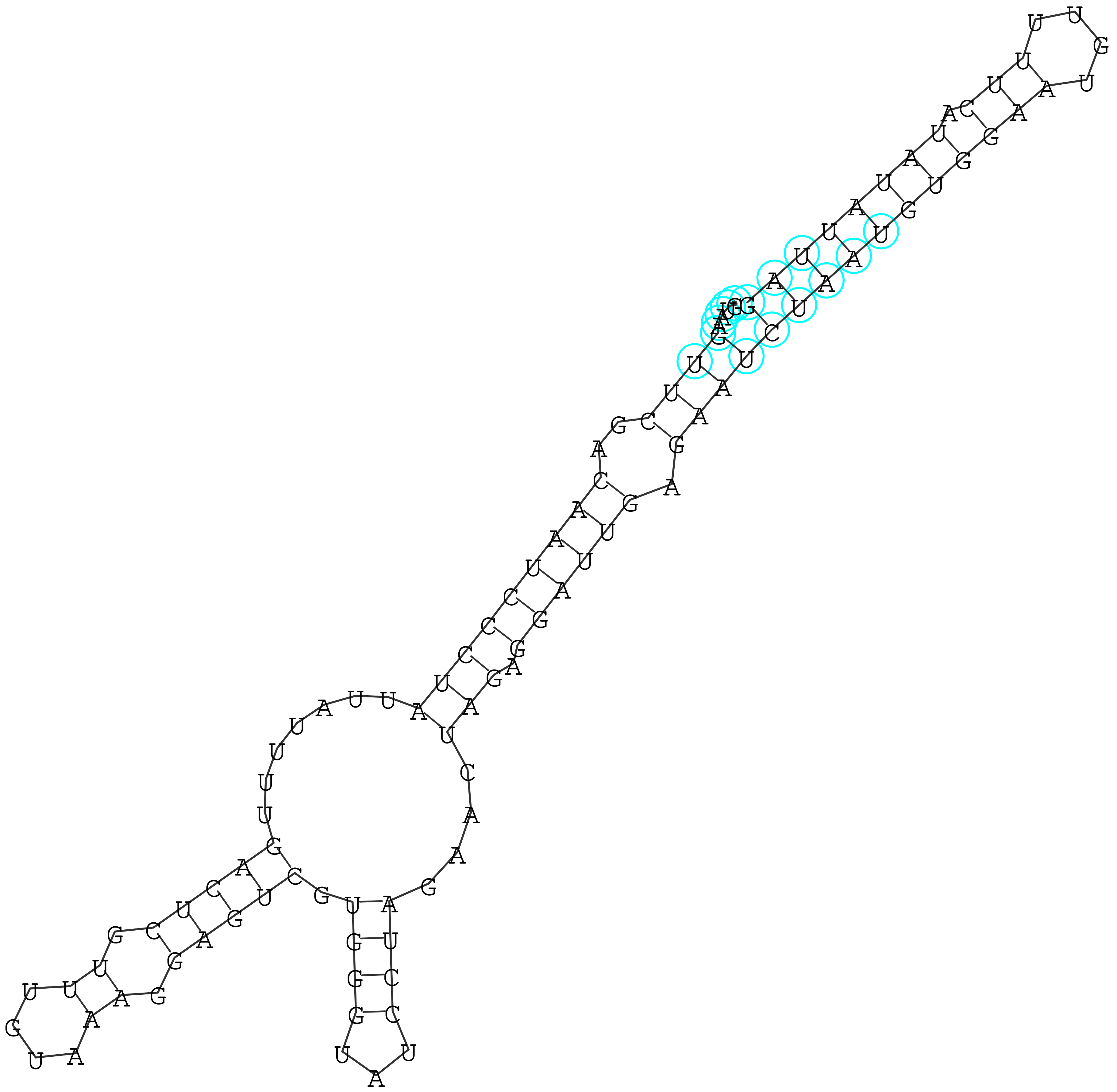

Dchc005A - External intron

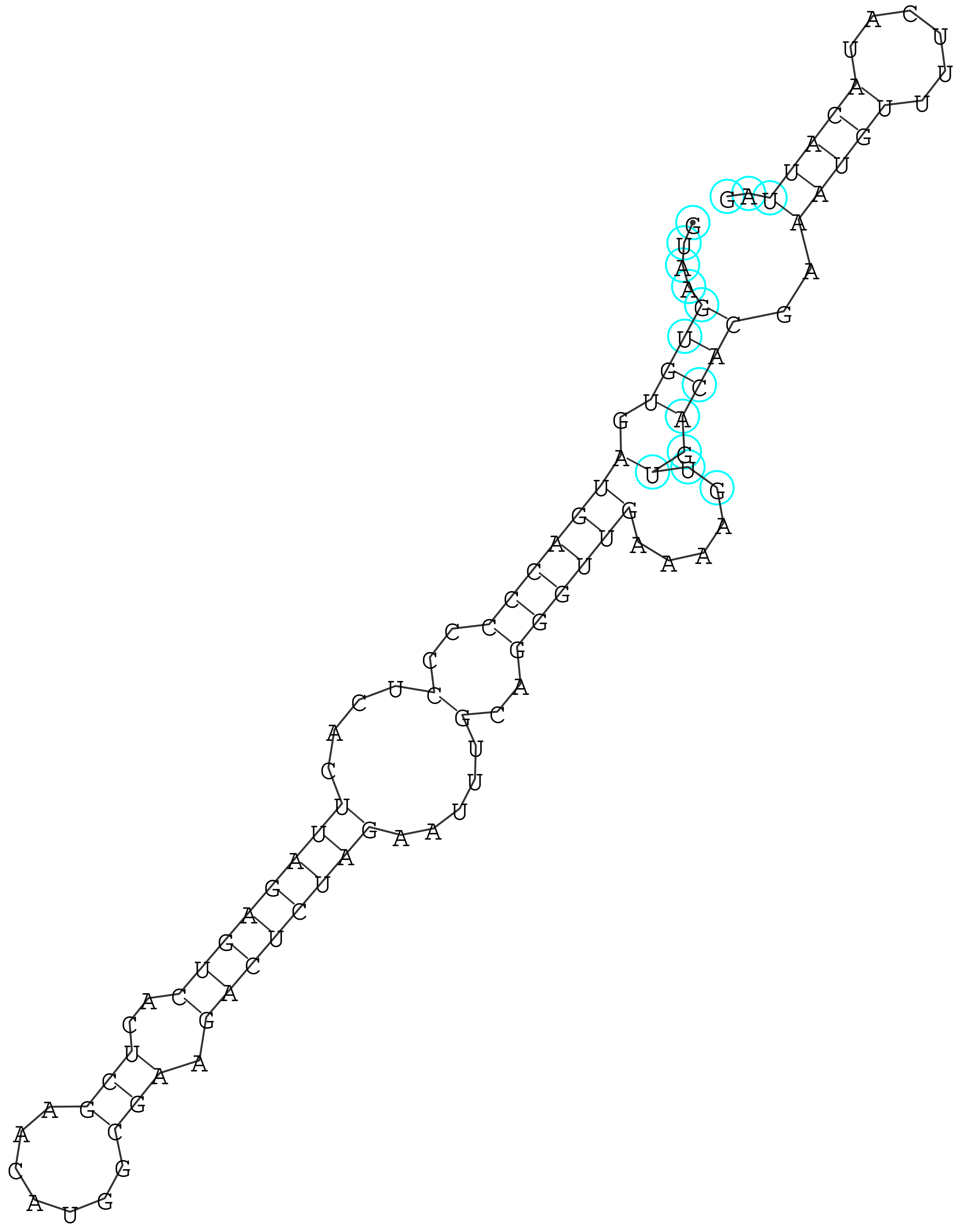

Dchc005B - External intron

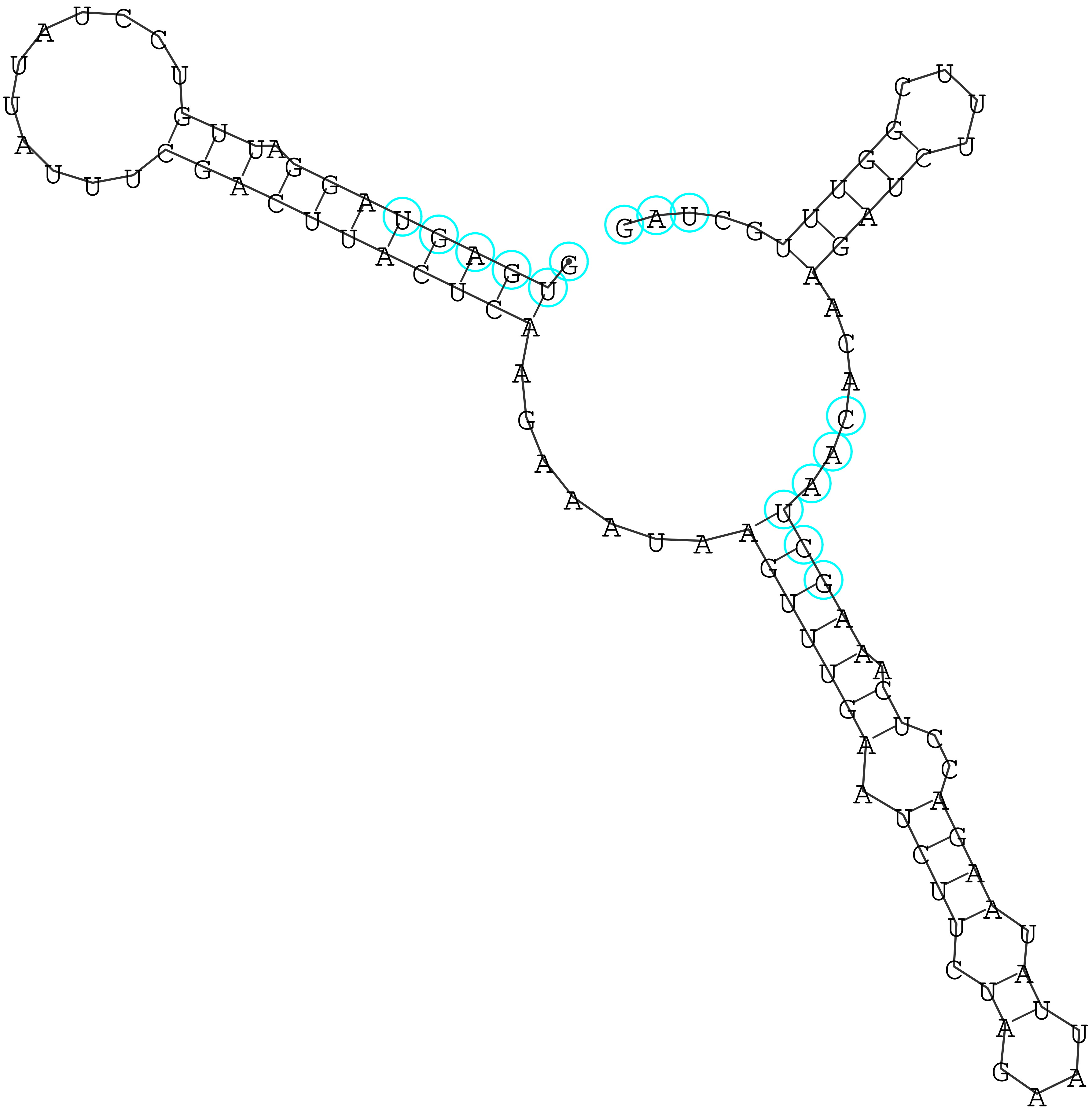

Dchc007A - External intron

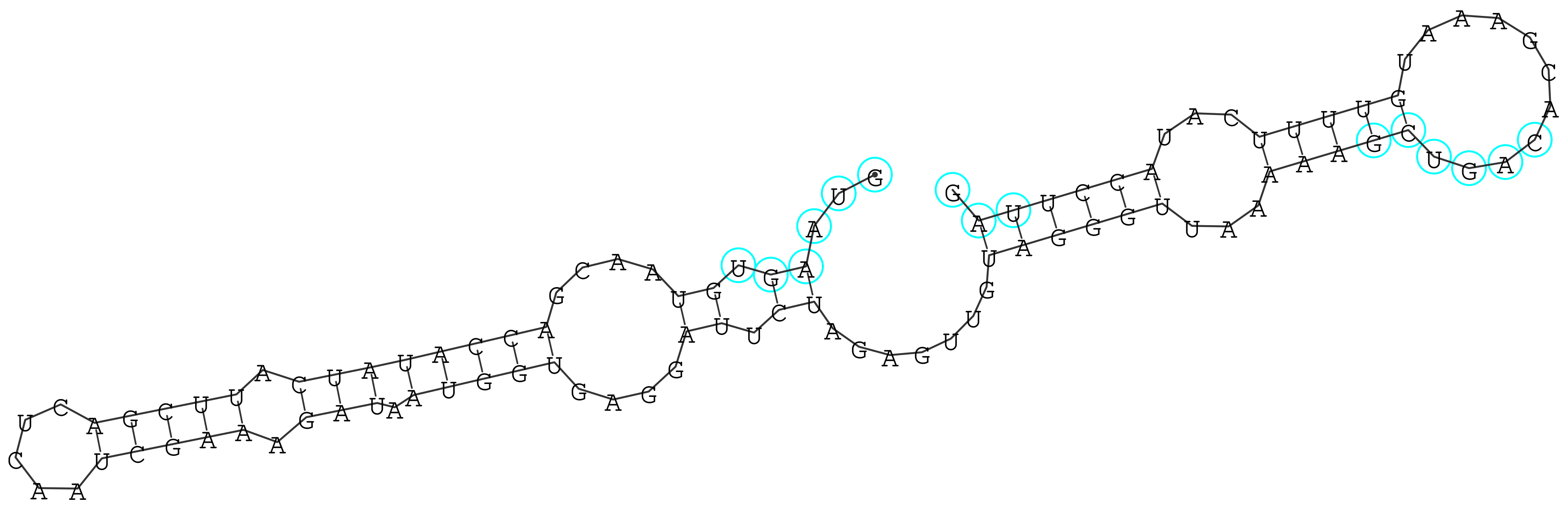

Dchc007B - External intron

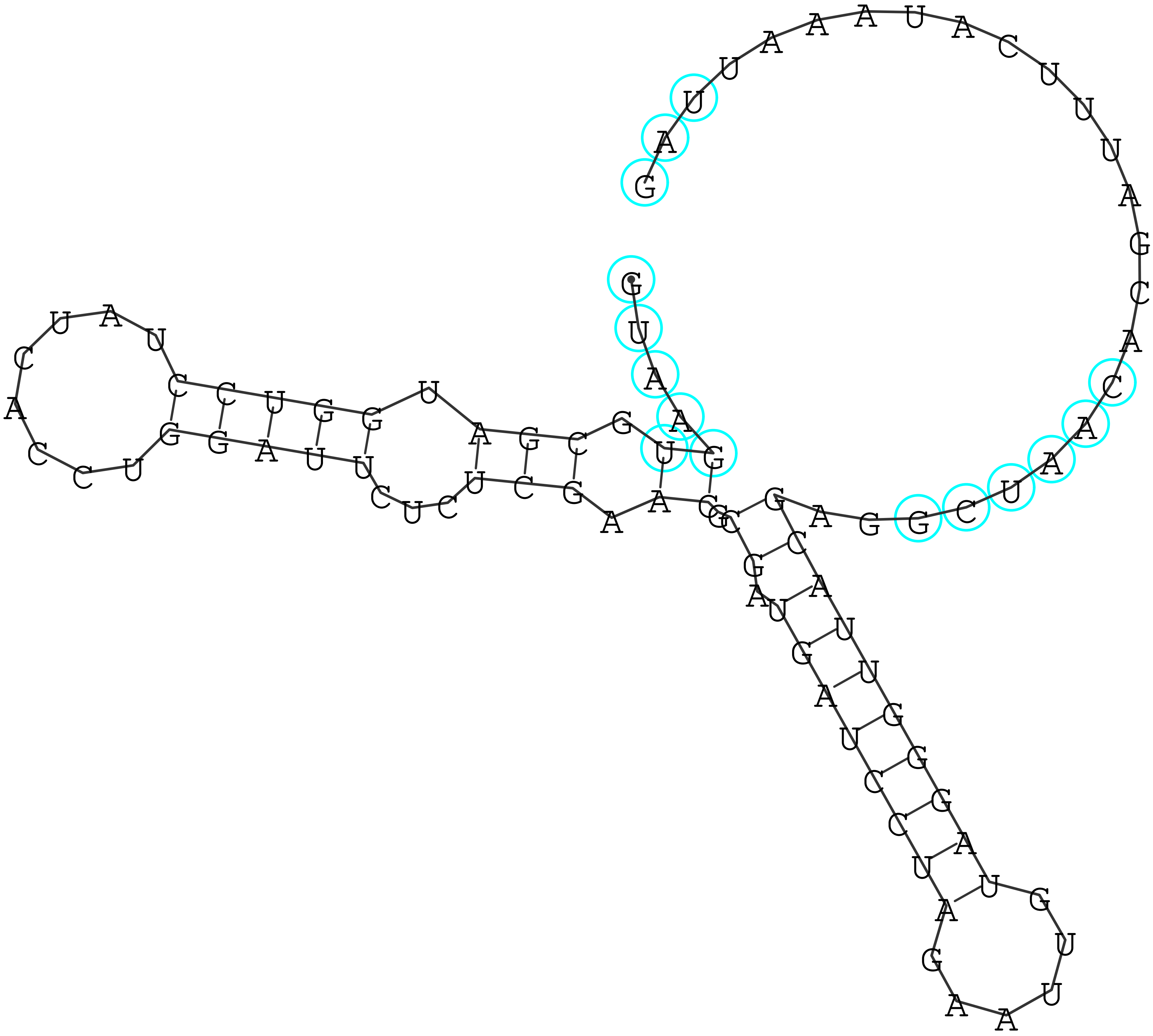

Dchc008A - External intron

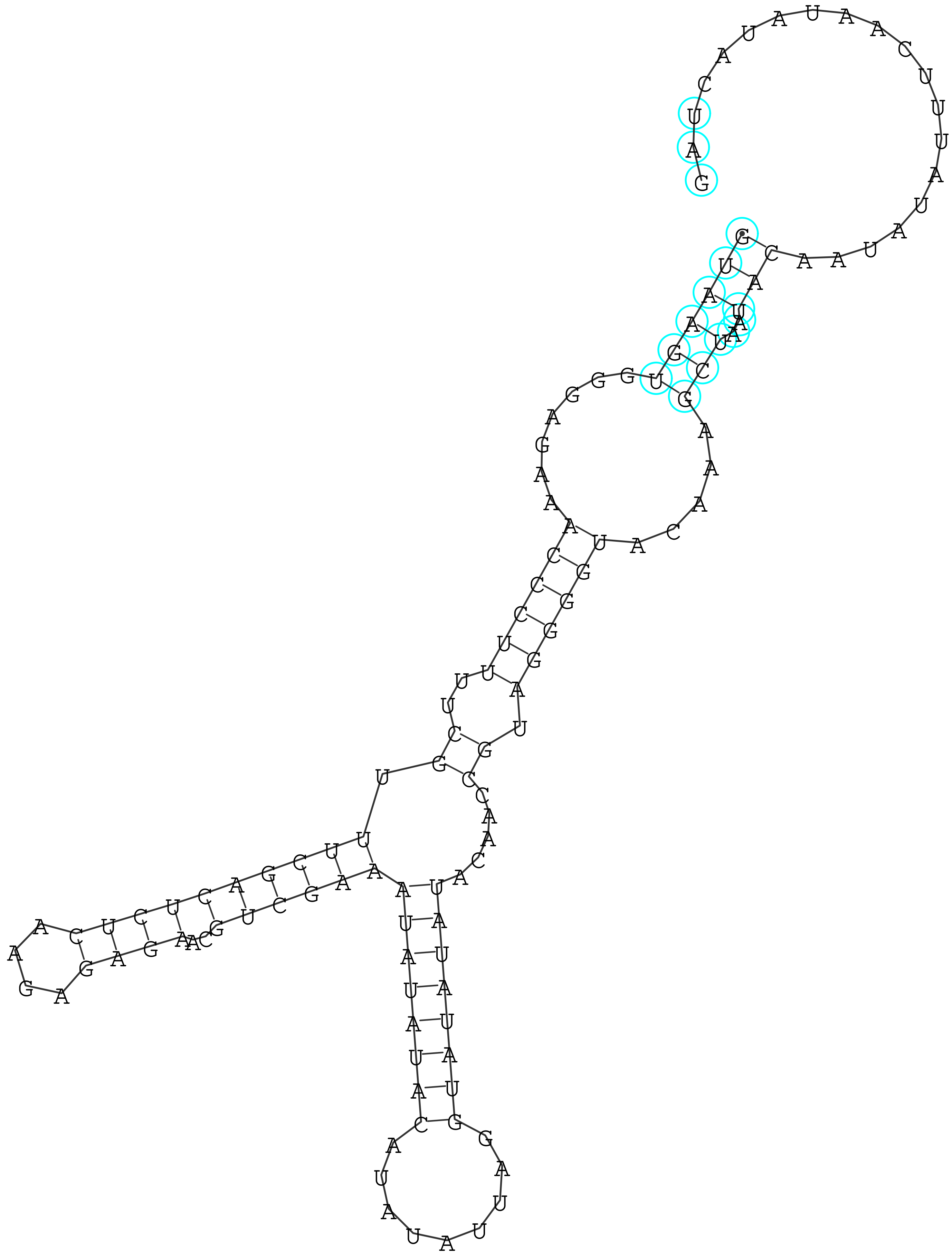

Dchc011A - External intron

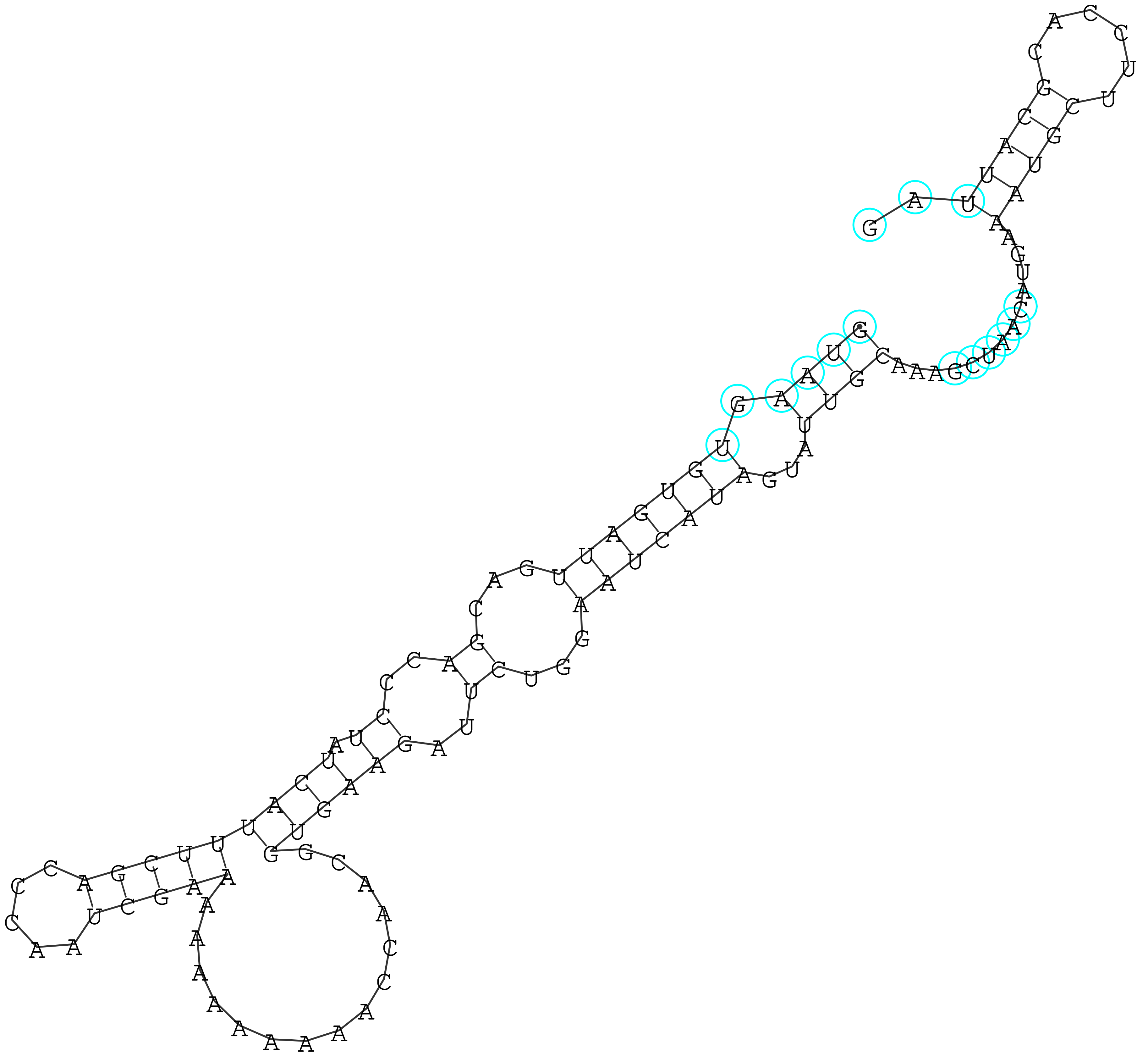

Dchc013A - External intron

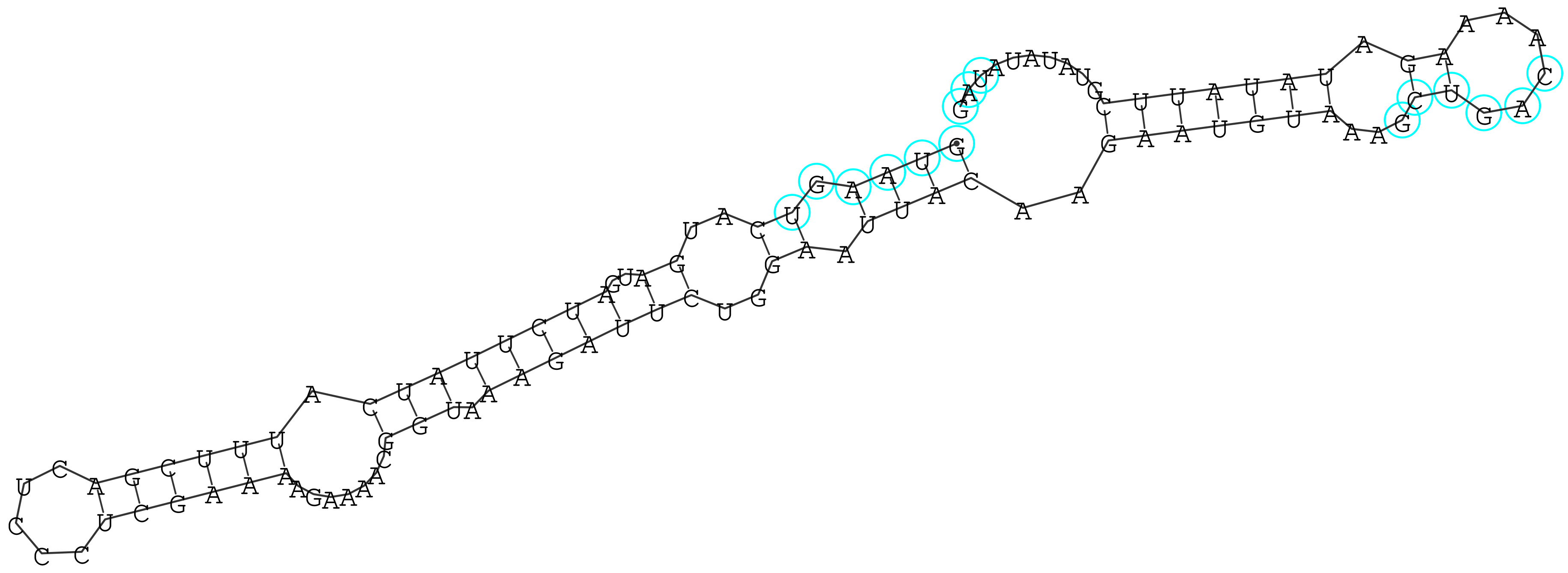

Dchc014A - External intron

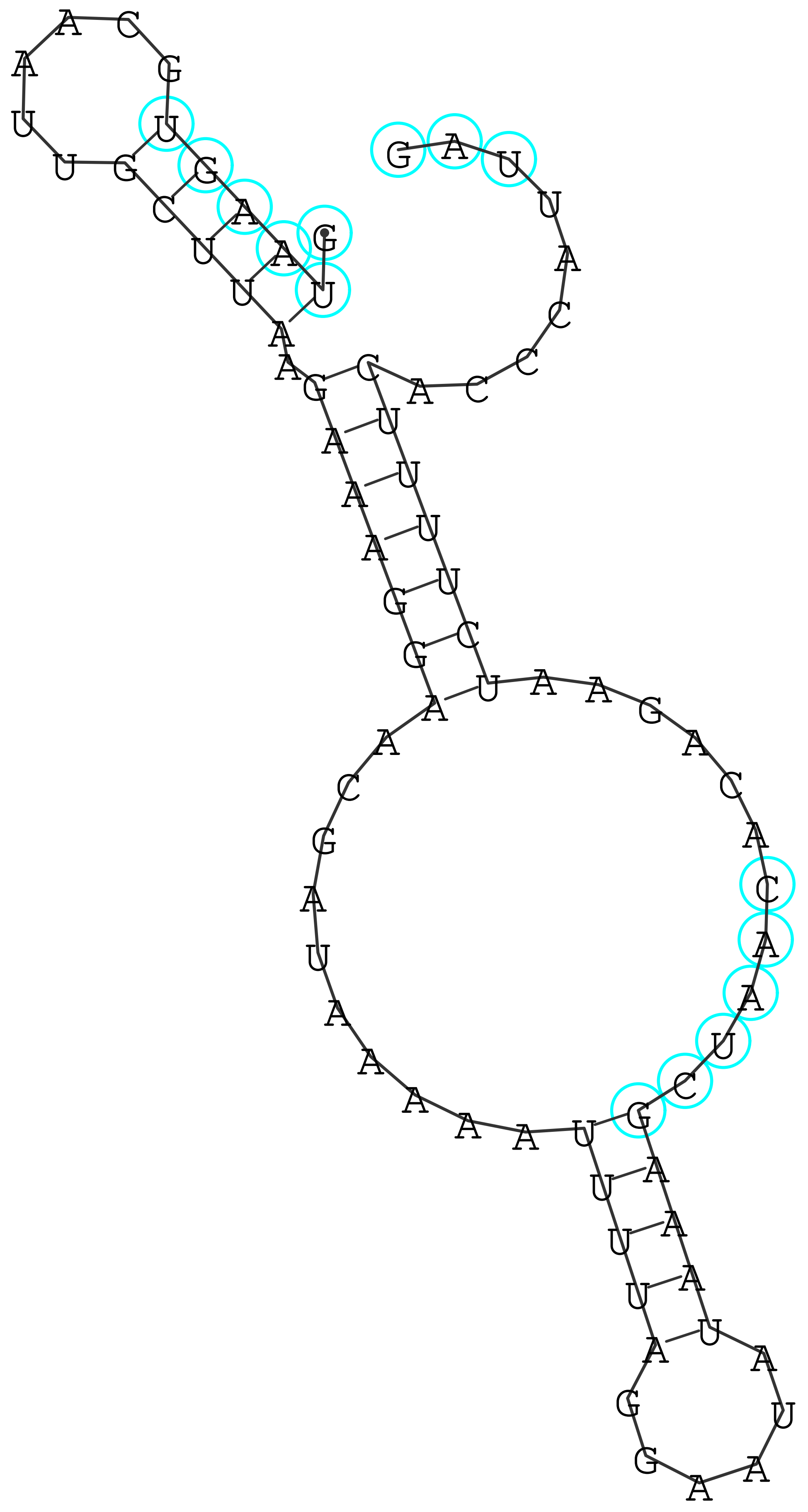

Dcoc02A - External intron

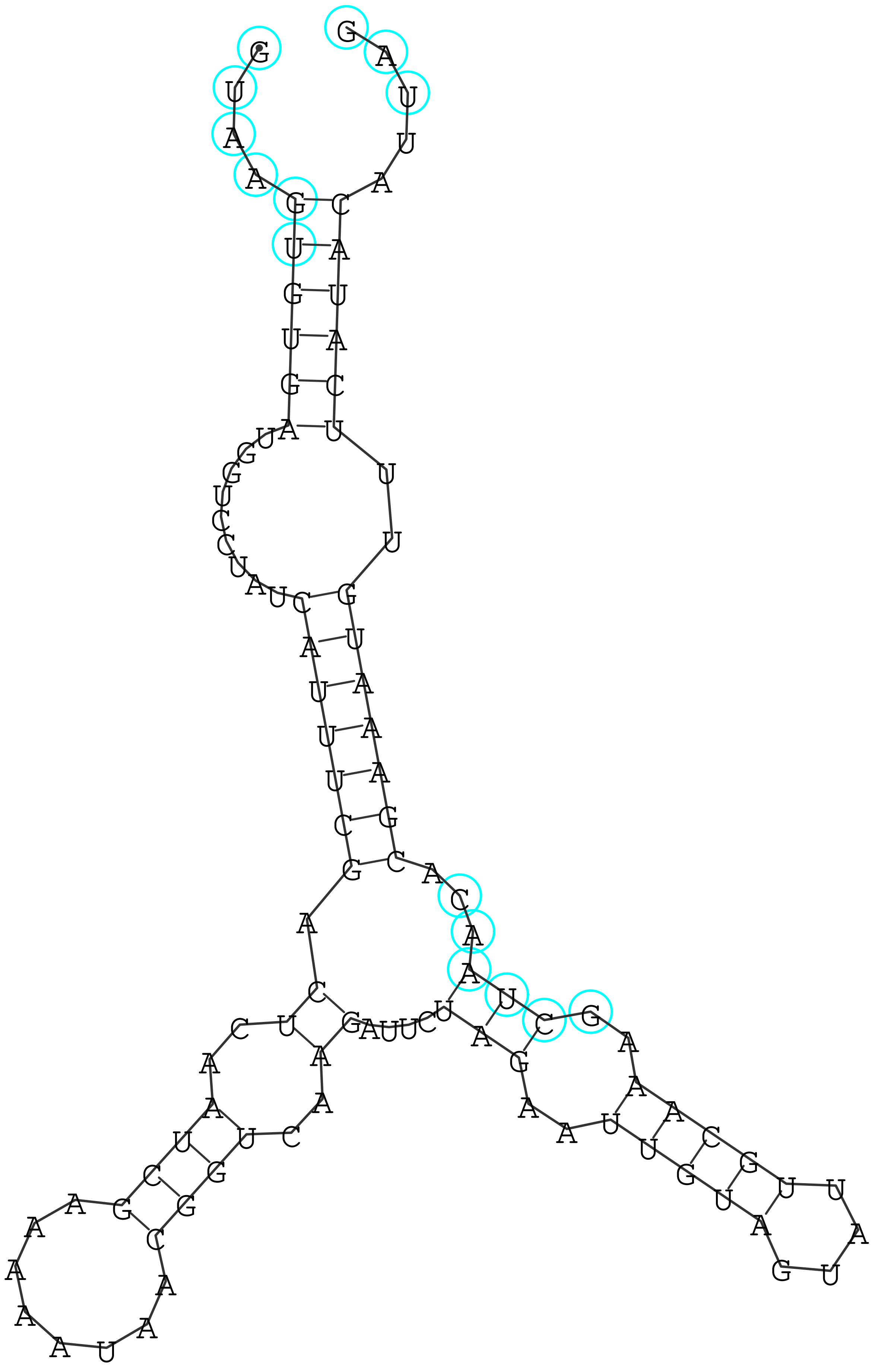

Dcoc02B - External intron

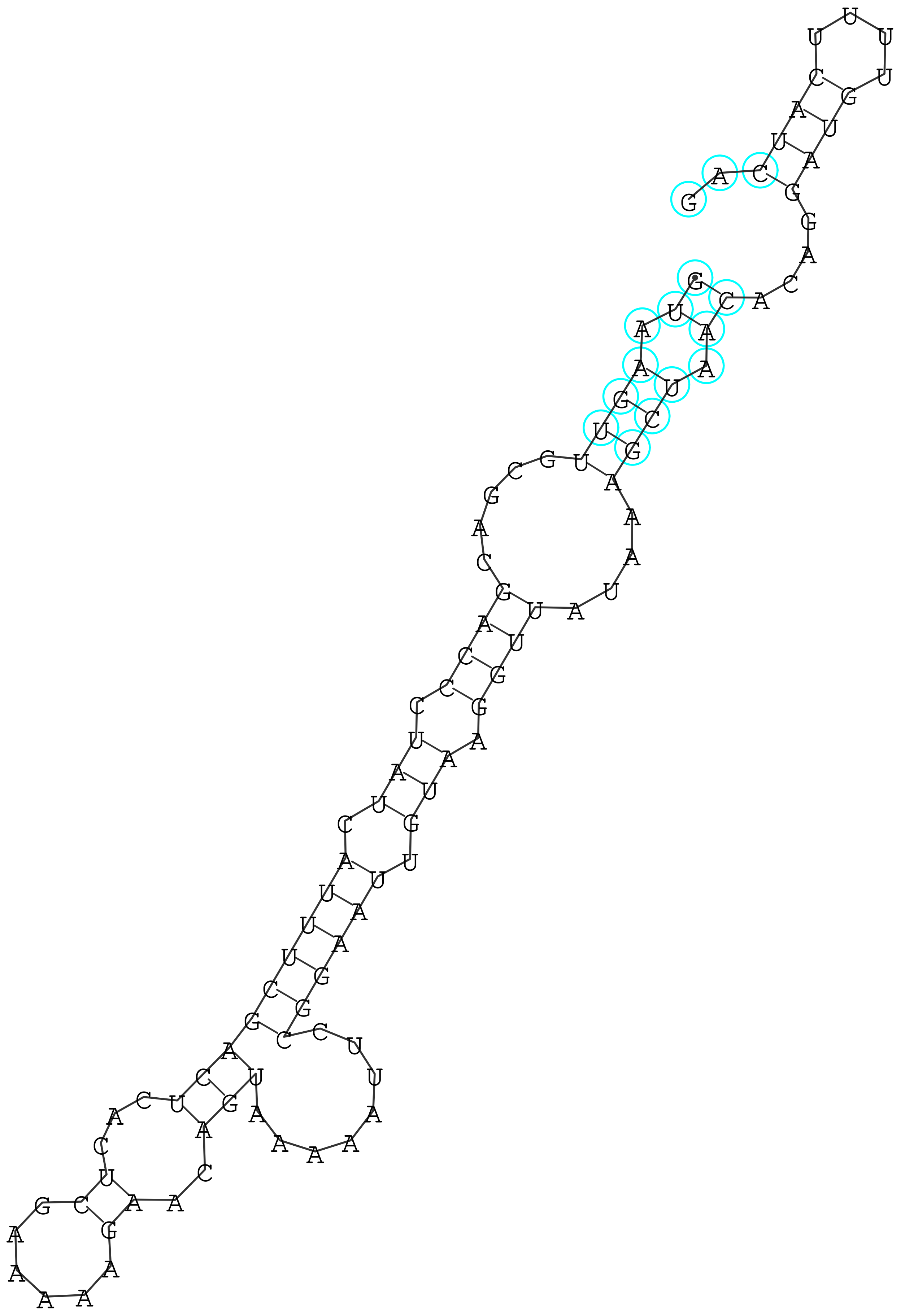

Dcoc03A - External intron

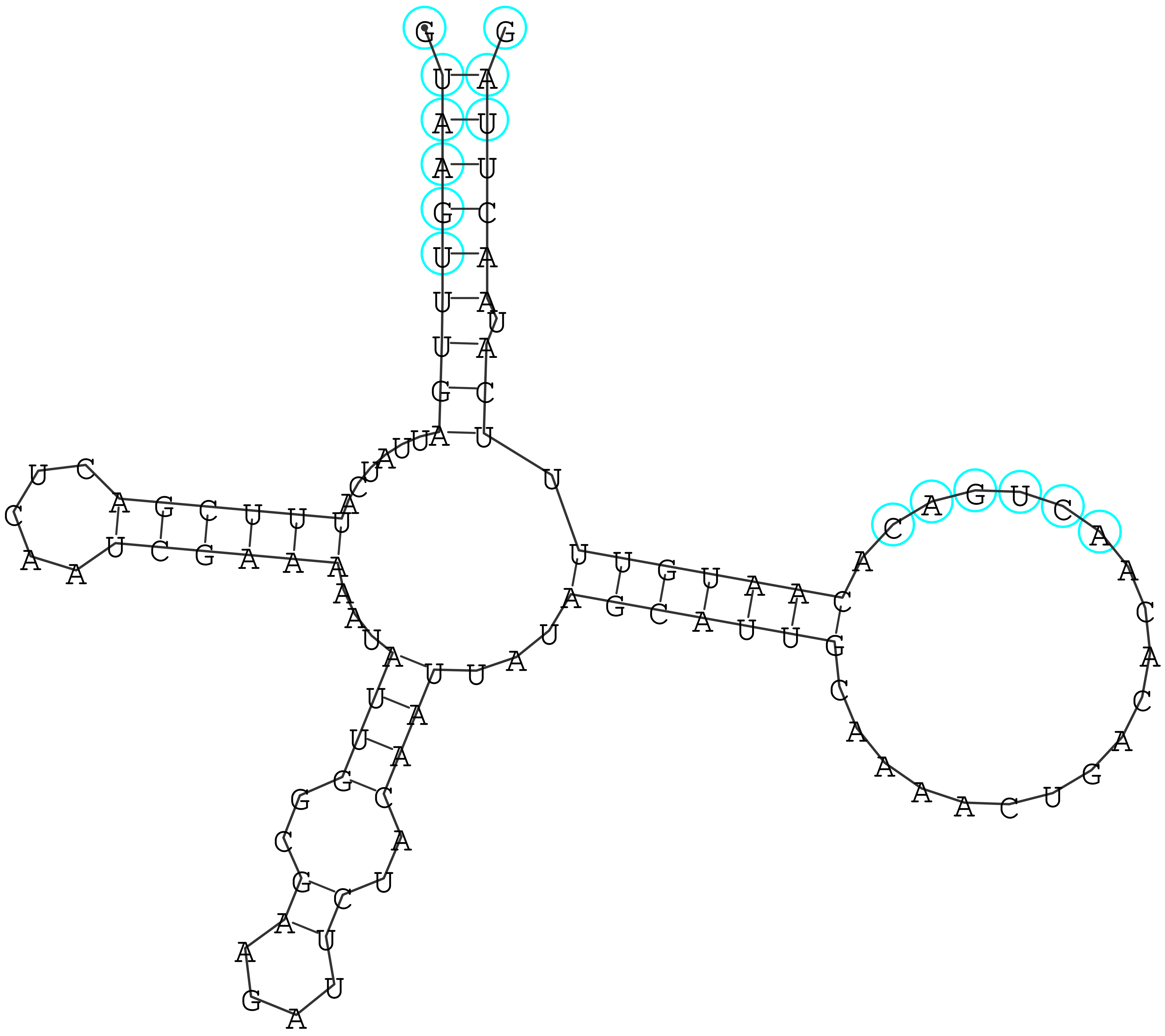

Dcoc05A - External intron

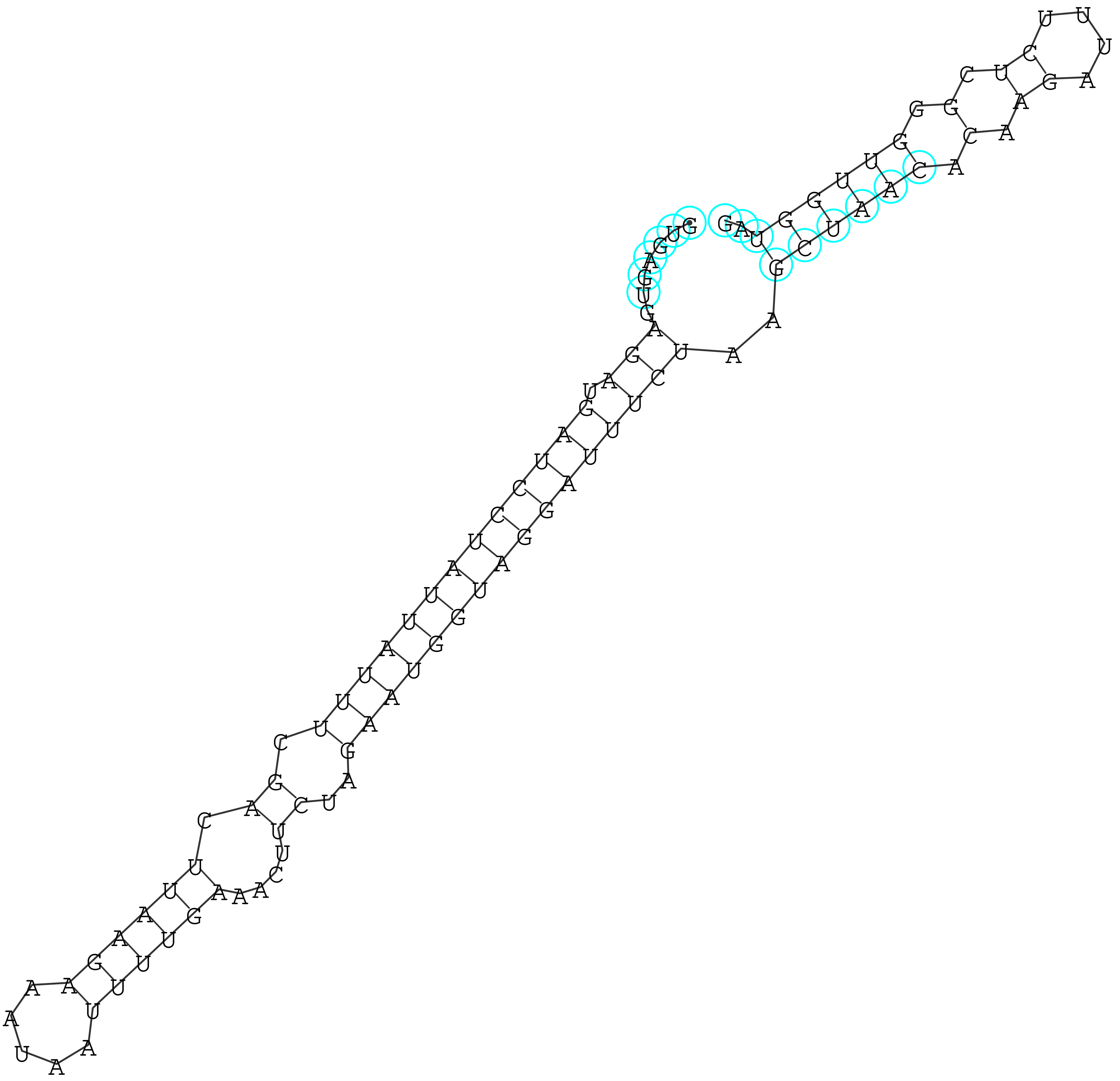

Dcoc06A - External intron

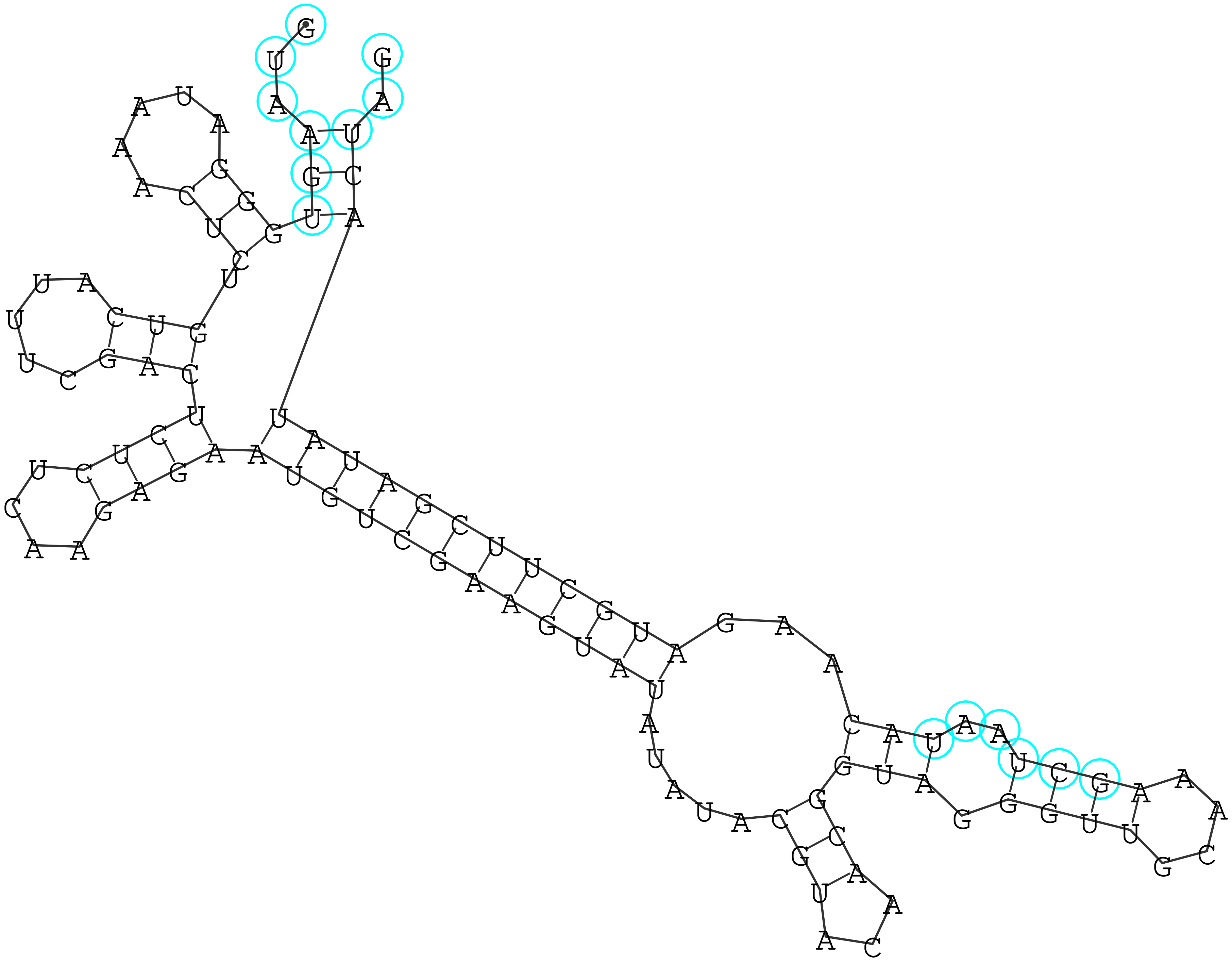

Dcoc11A - External intron

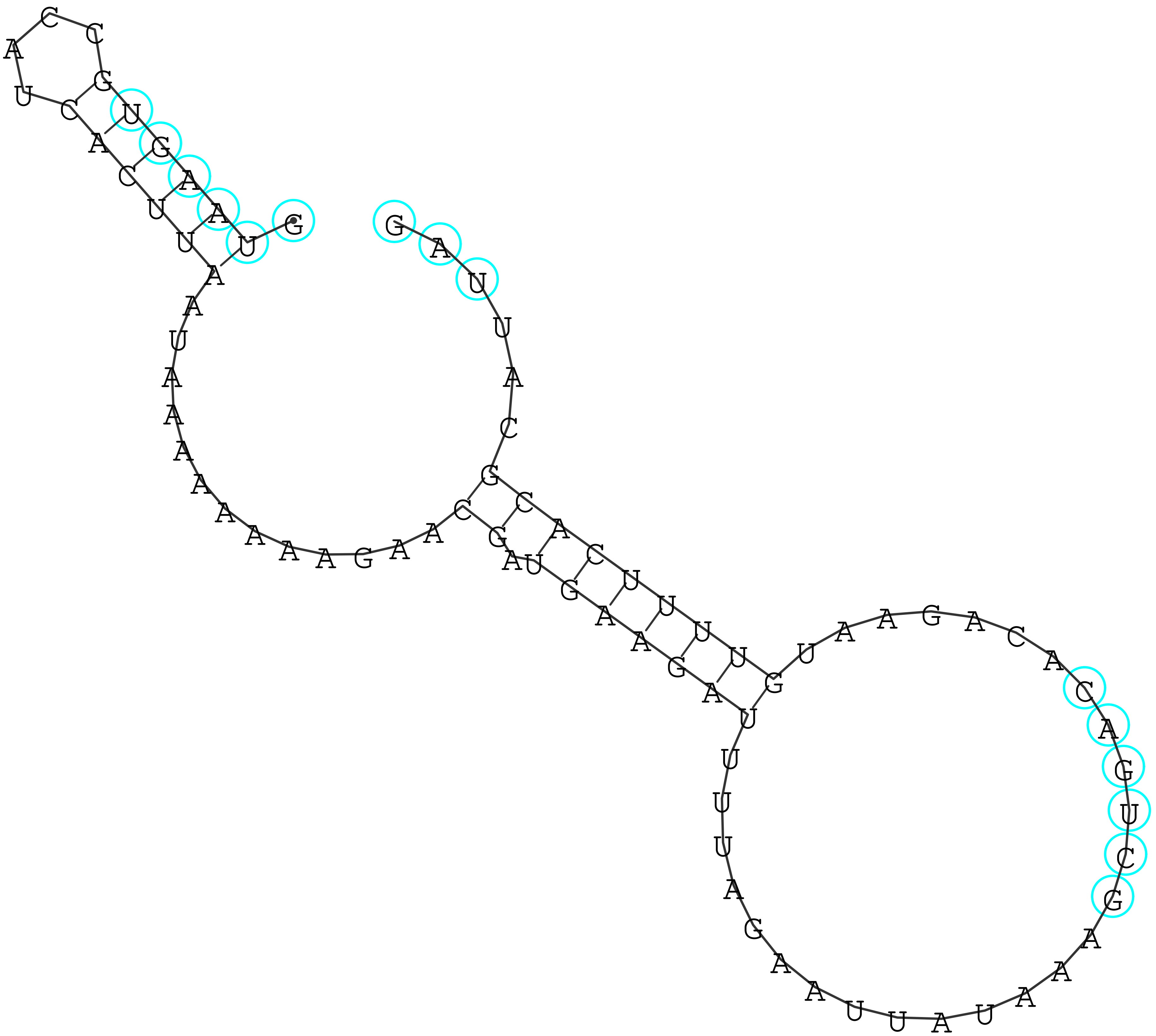

Dcoc20A - External intron

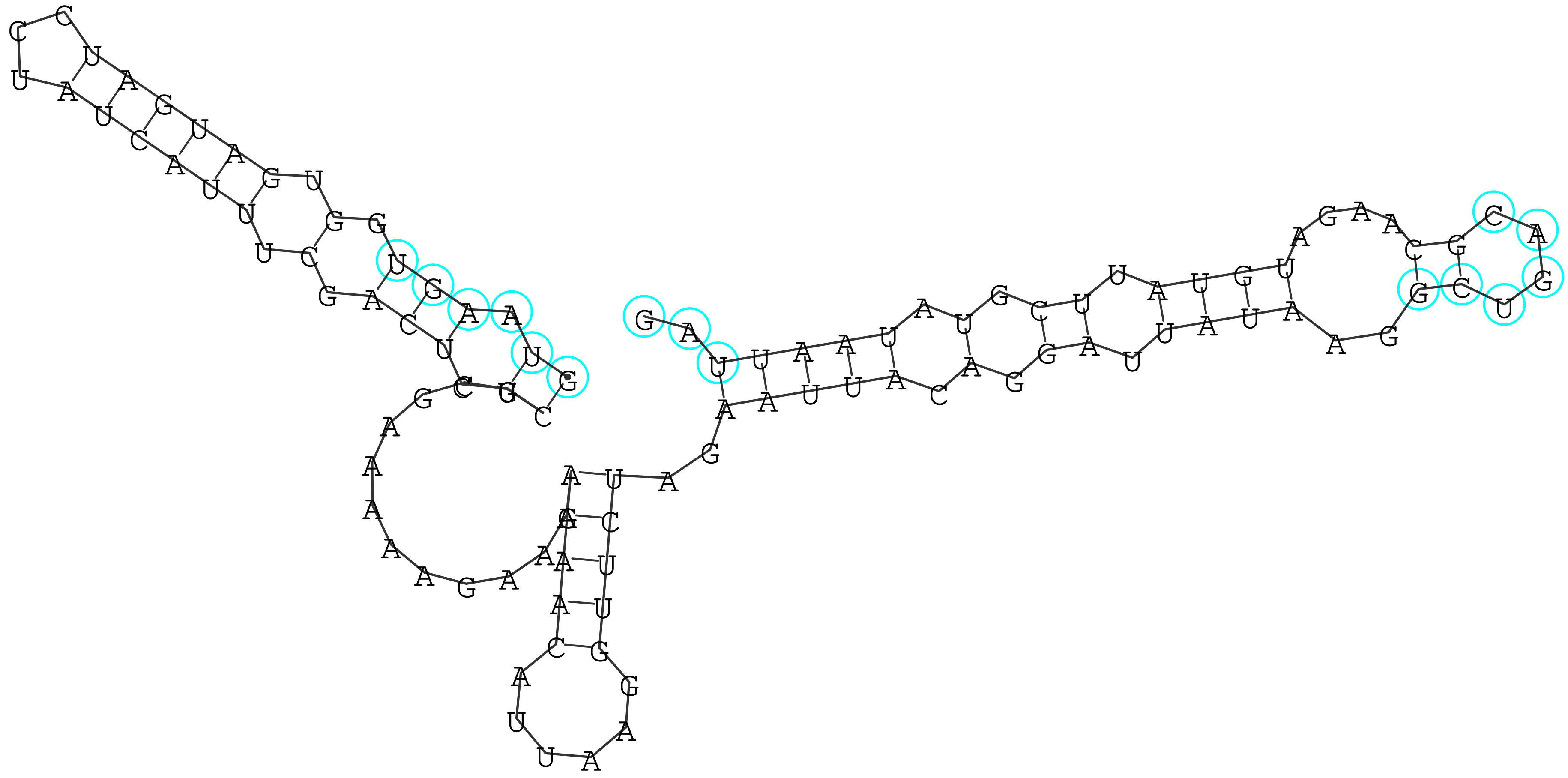

Dcoc38A - External intron

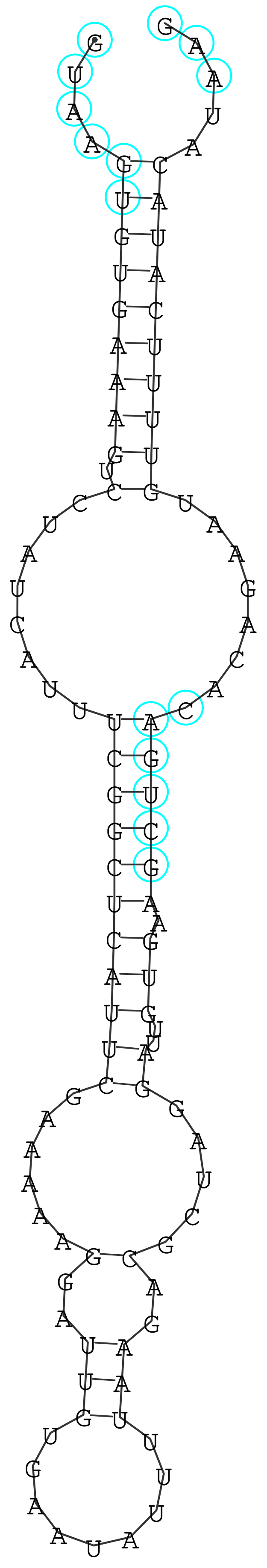

Dcoc41A - External intron

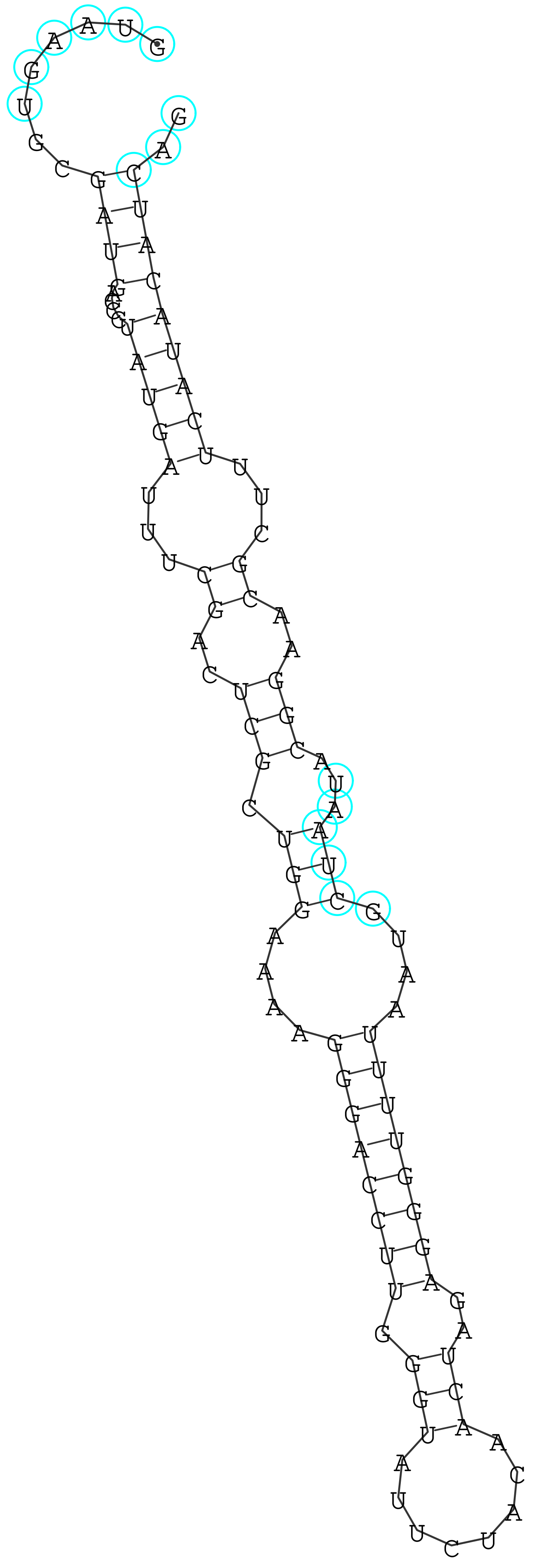

Desc187A - External intron

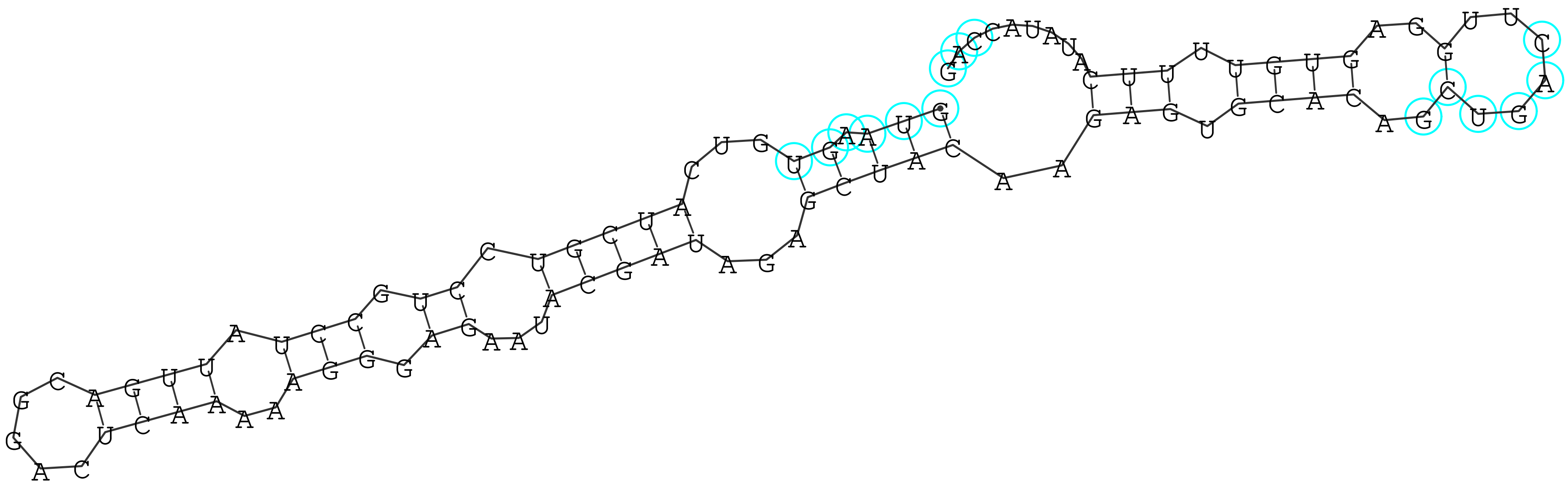

Desc198A - External intron

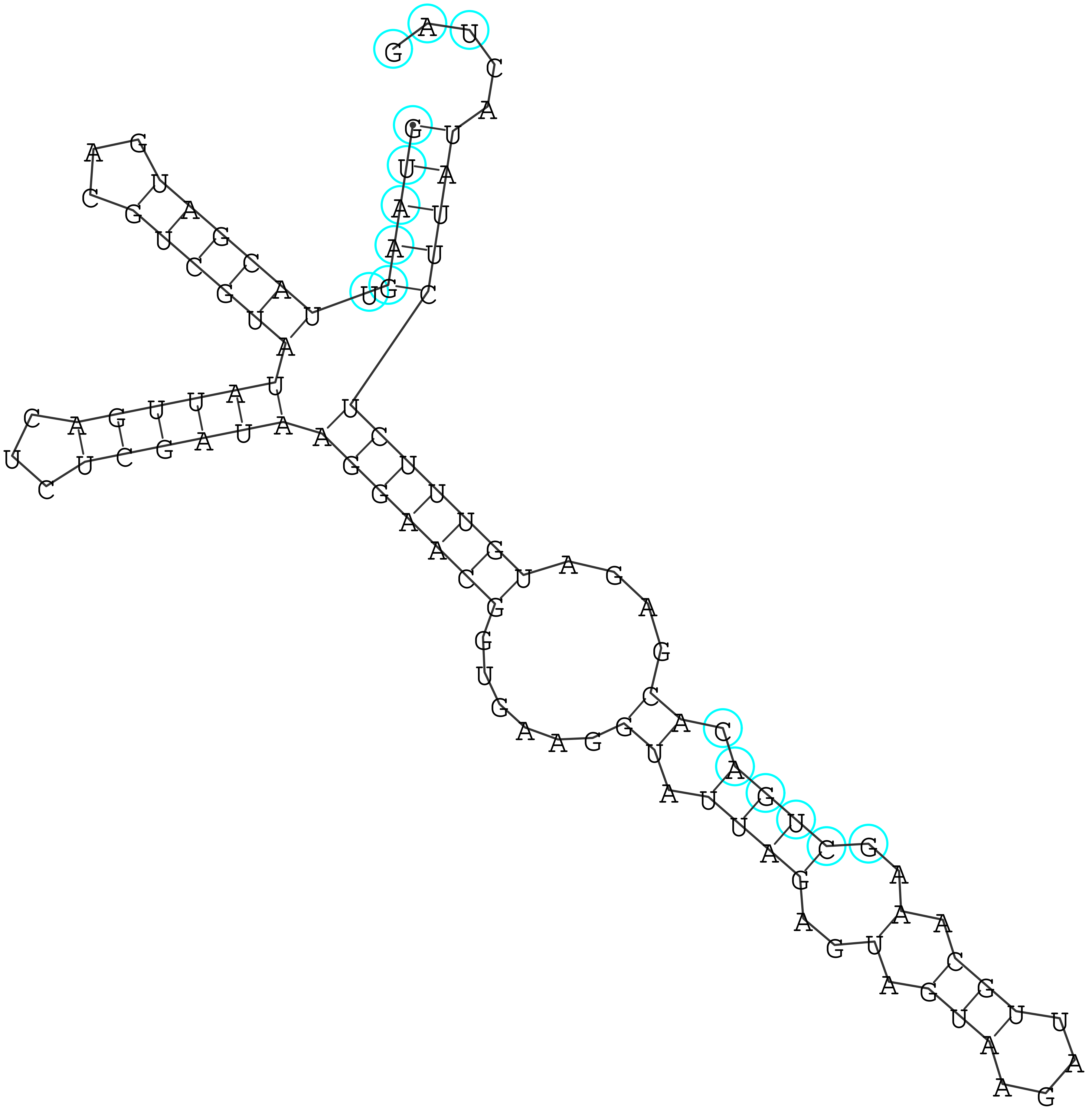

Desc274A - External intron

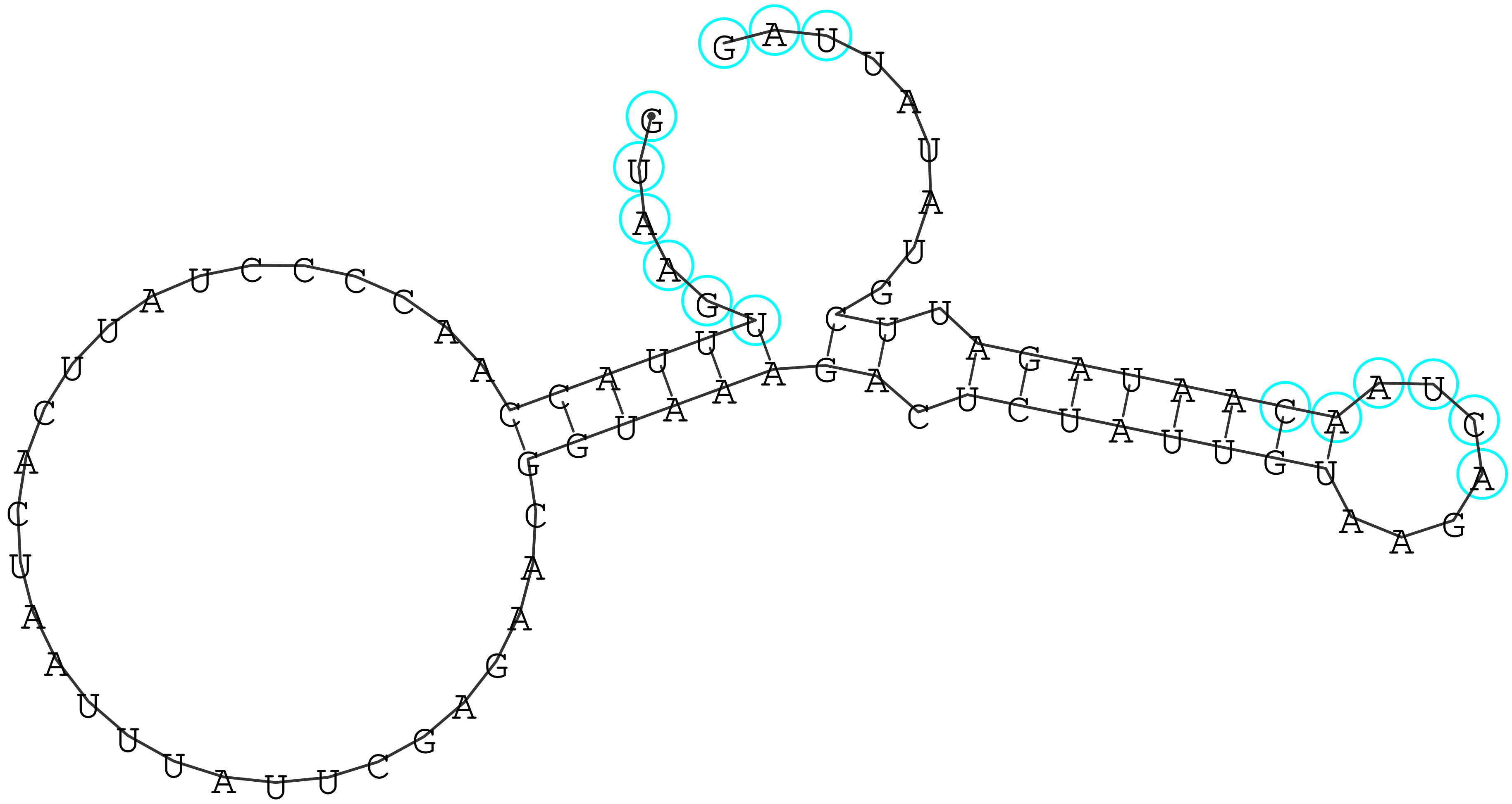

Desc420A - External intron

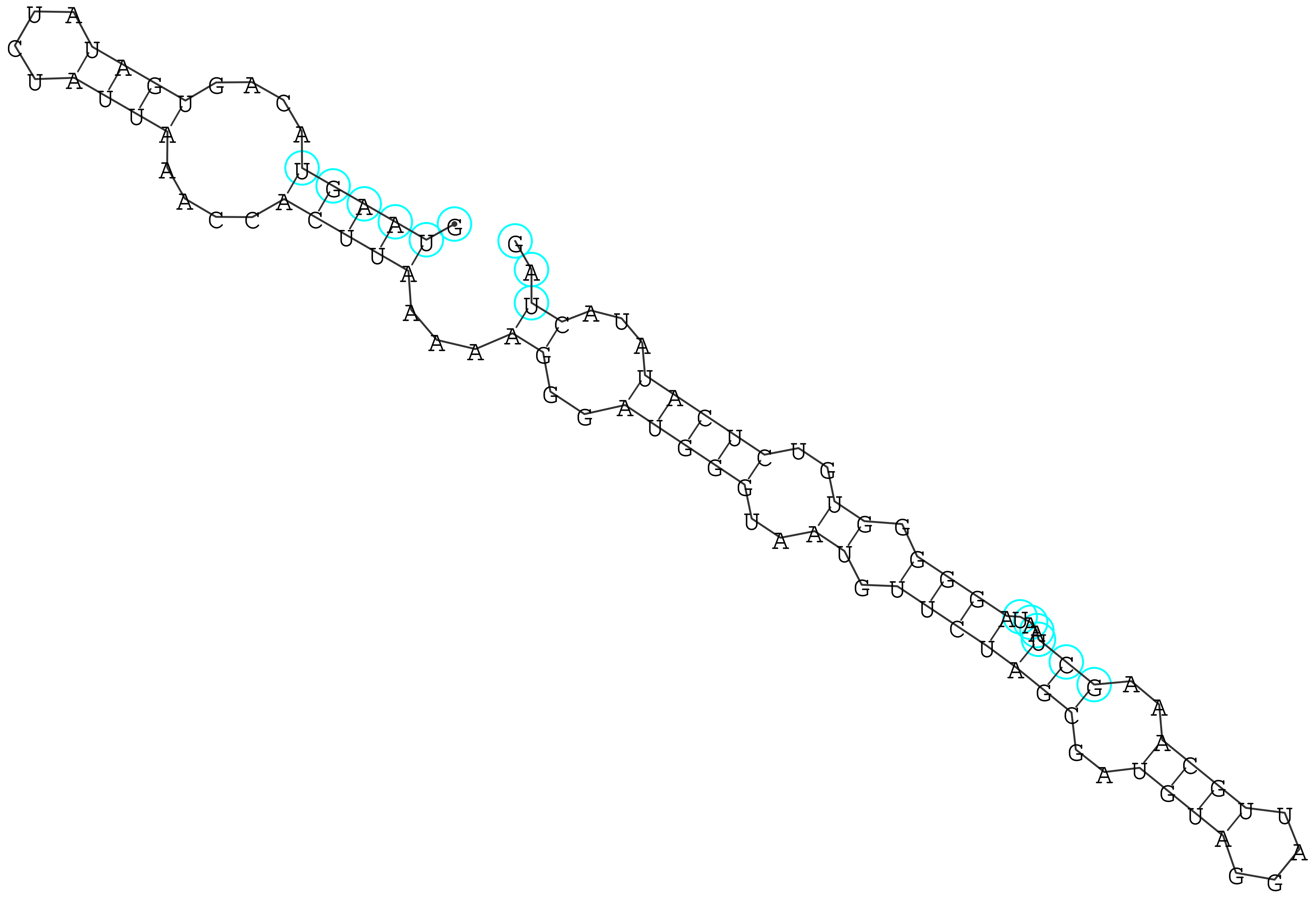

Desc618A - External intron

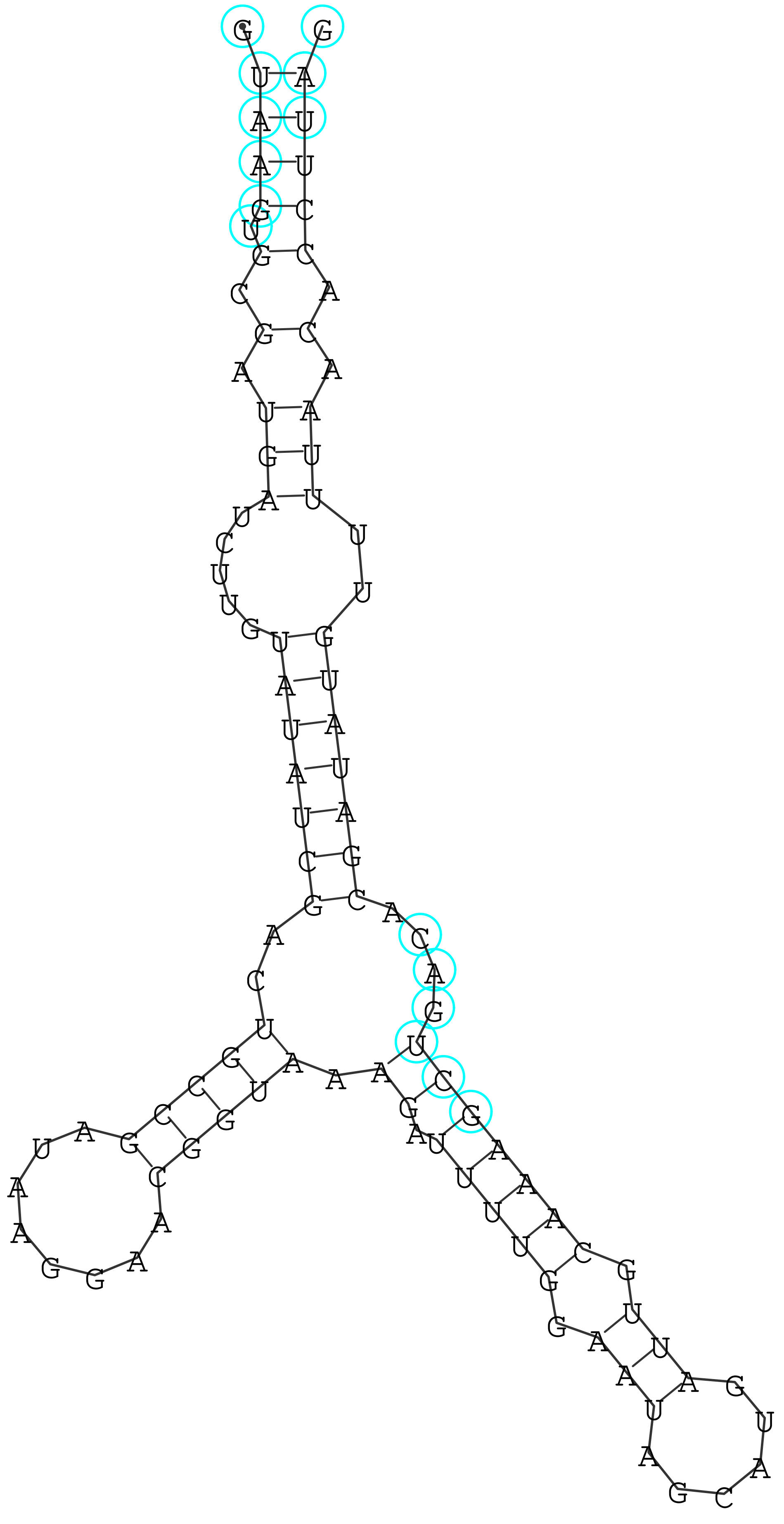

Desc640A - External intron

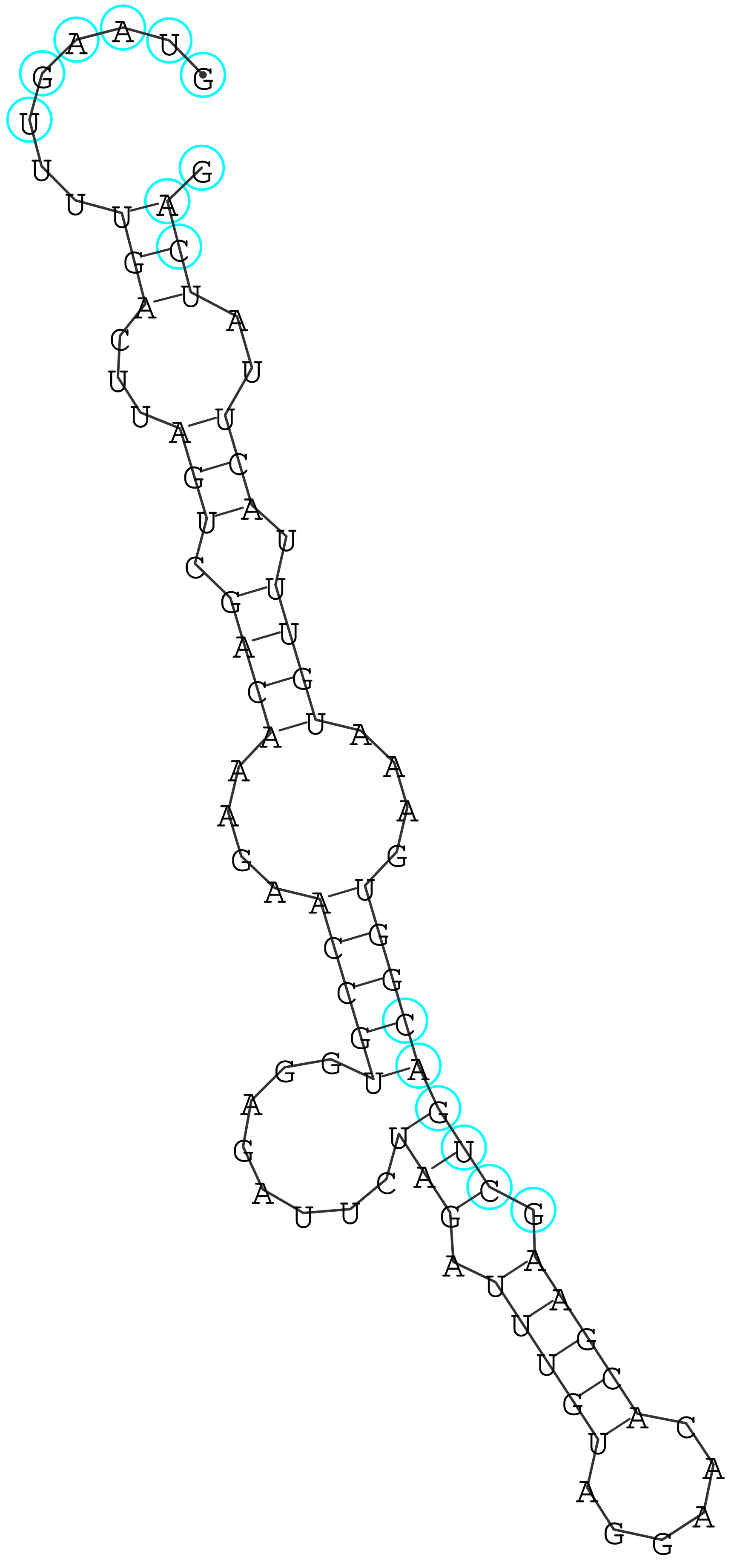

HCOc002A - External intron

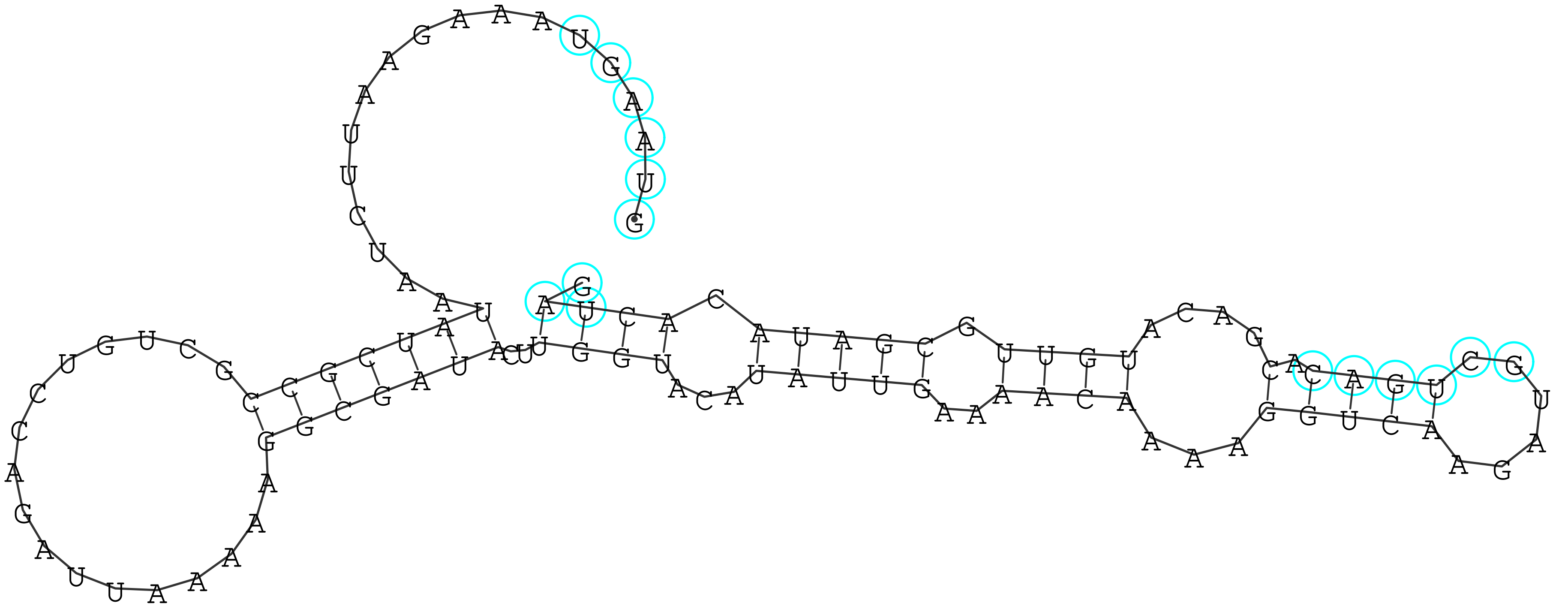

HCOc004A - External intron

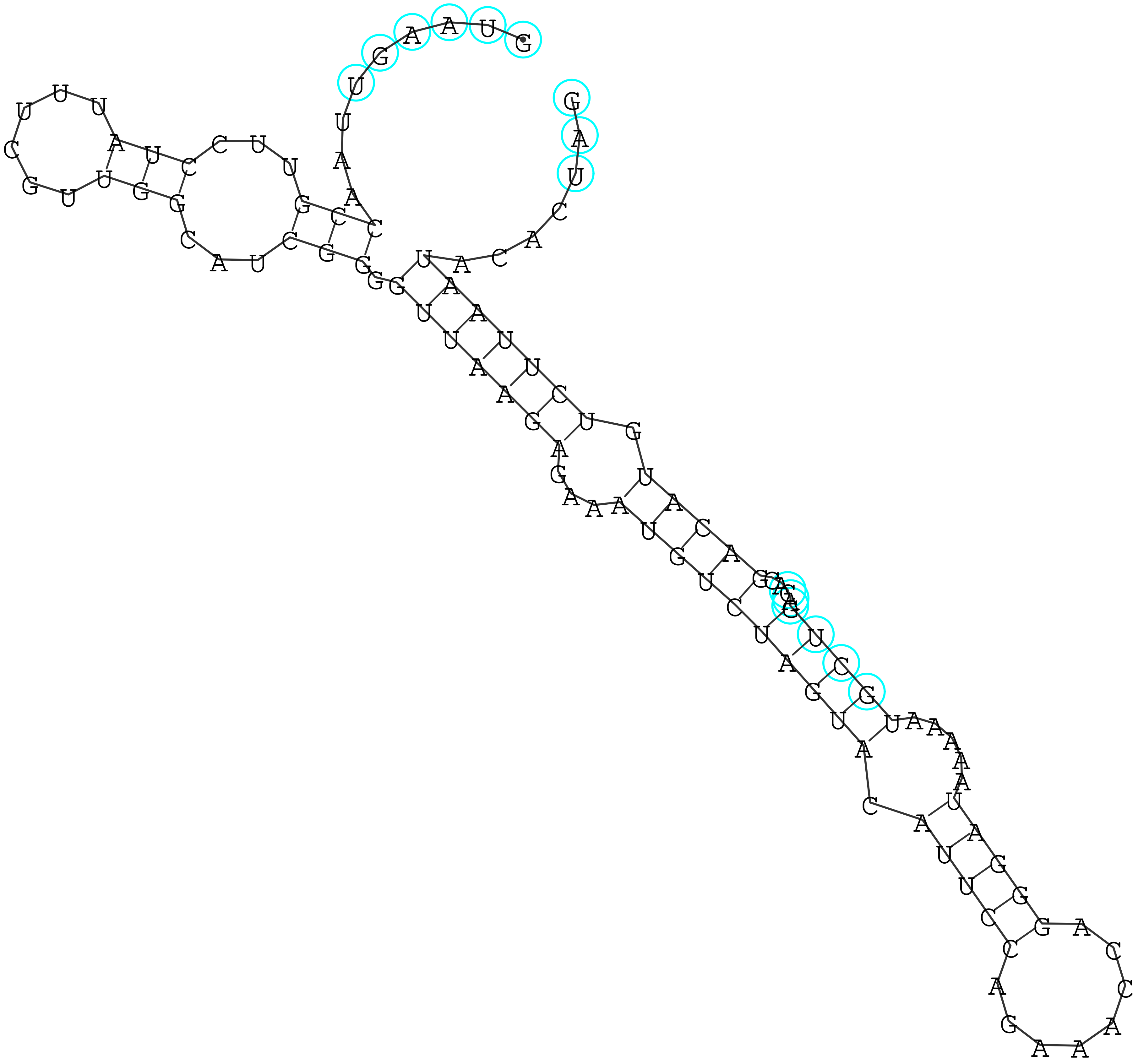

HCOc017A - External intron

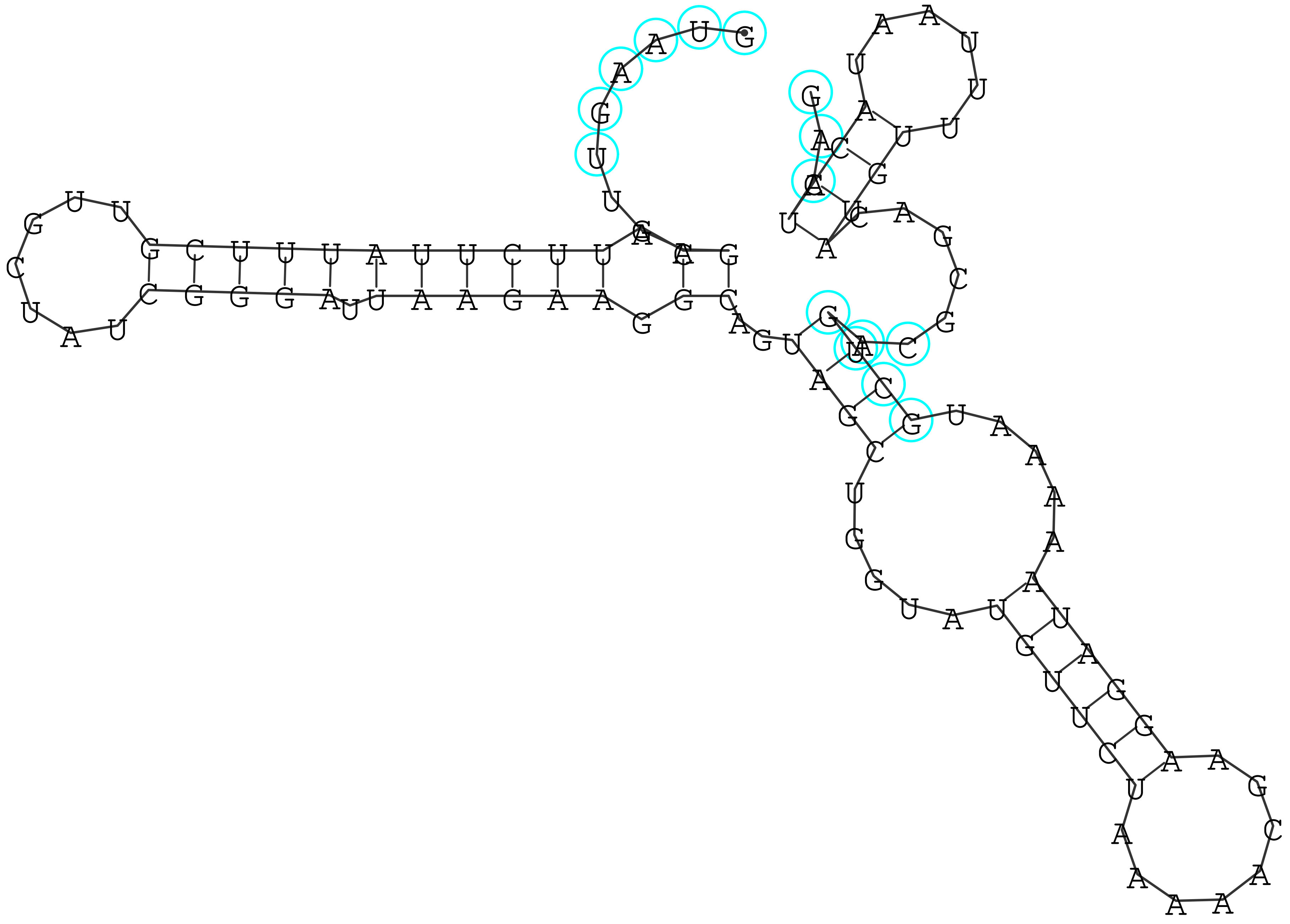

HCOc017B - External intron

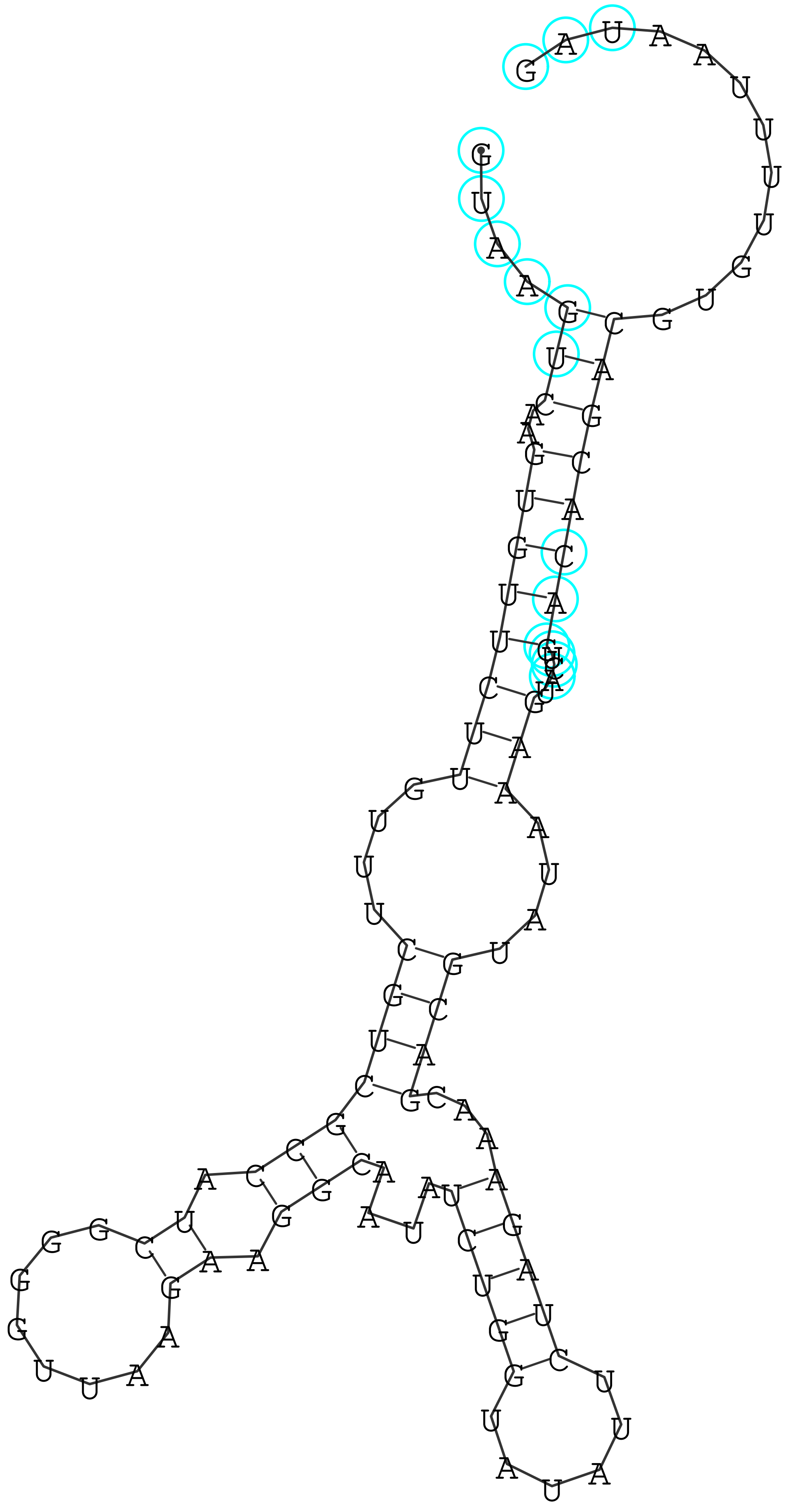

HCOc021A - External intron

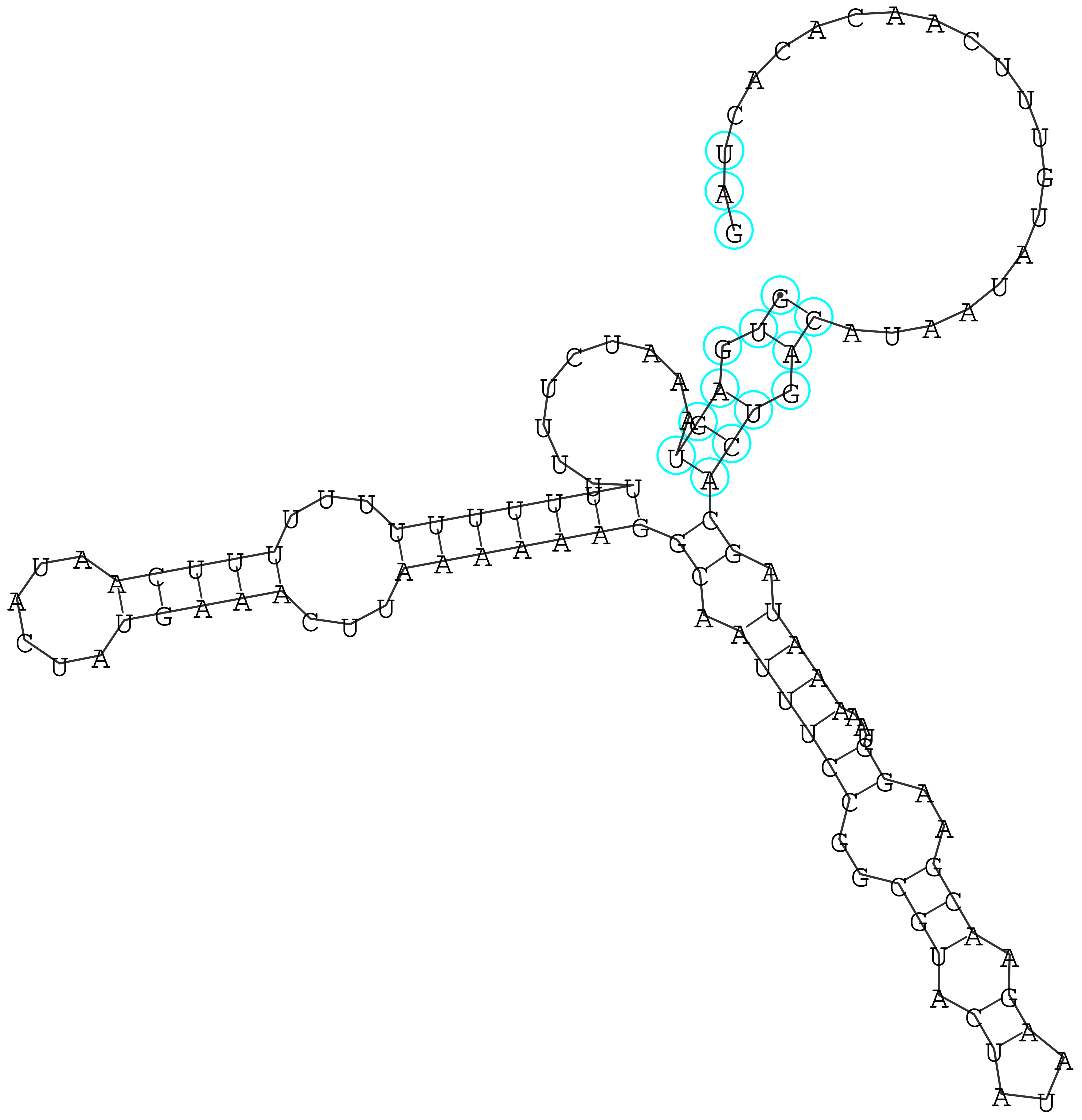

HCOc047A - External intron

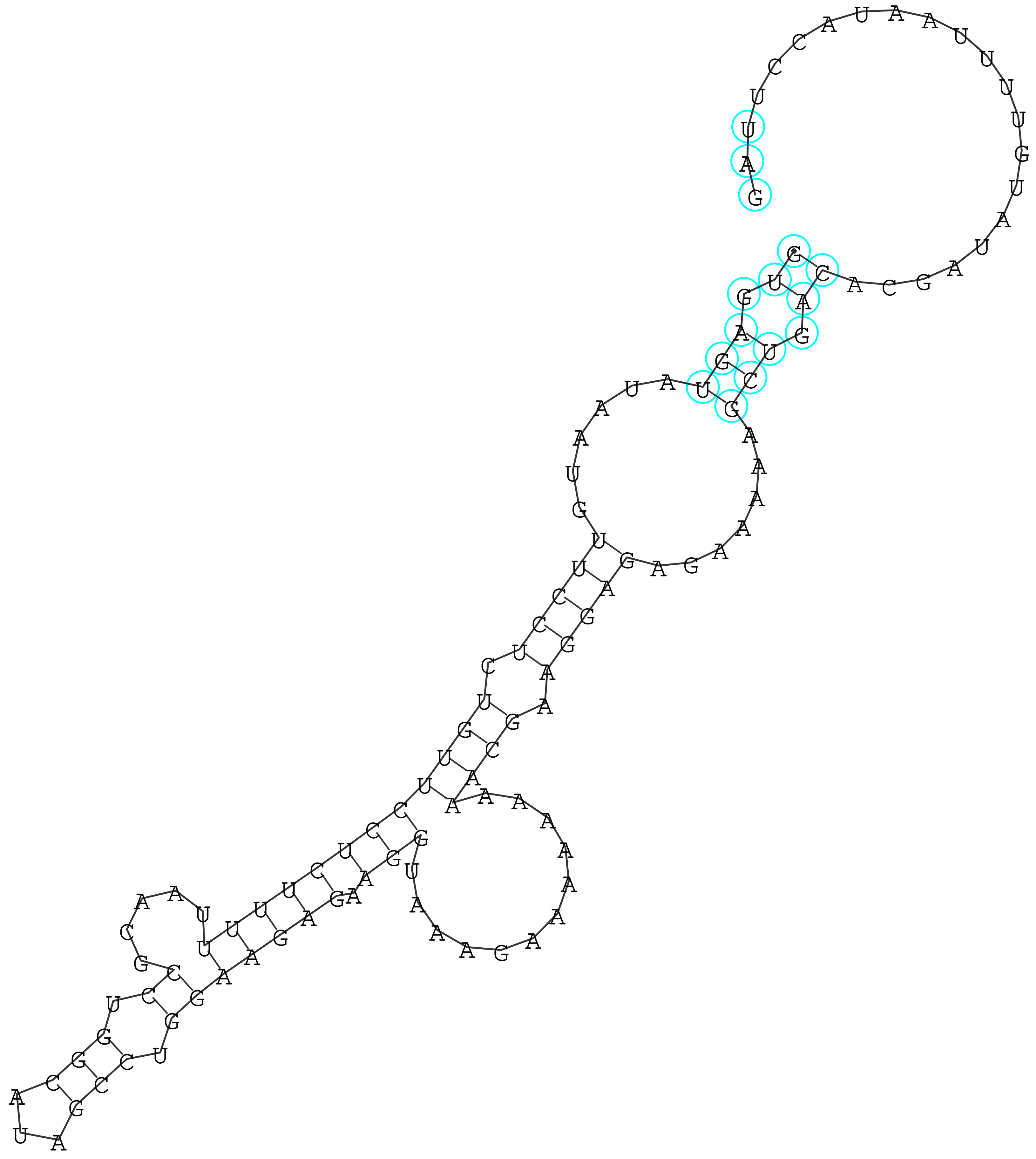

HCOc052A - External intron

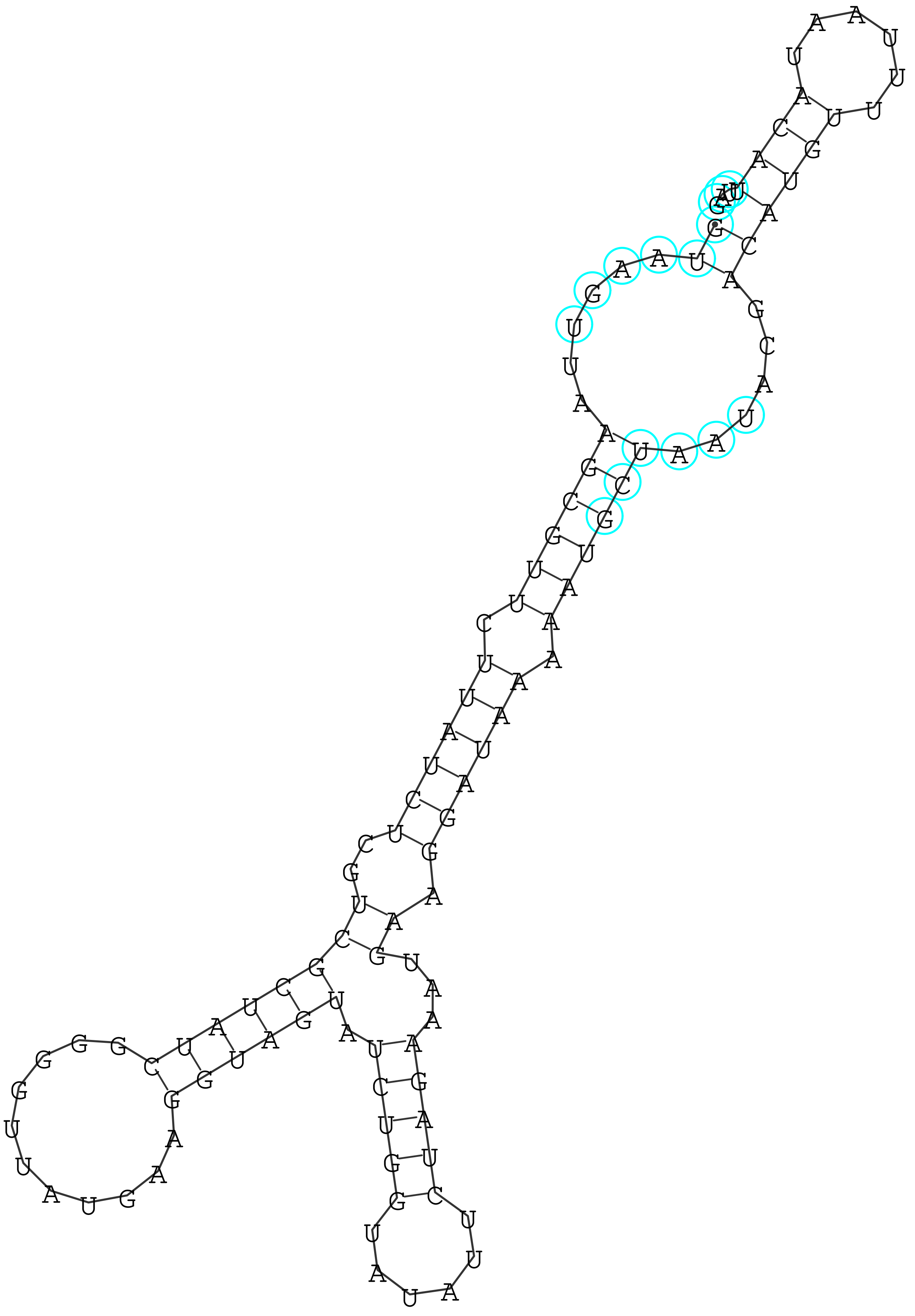

HCOc058A - External intron

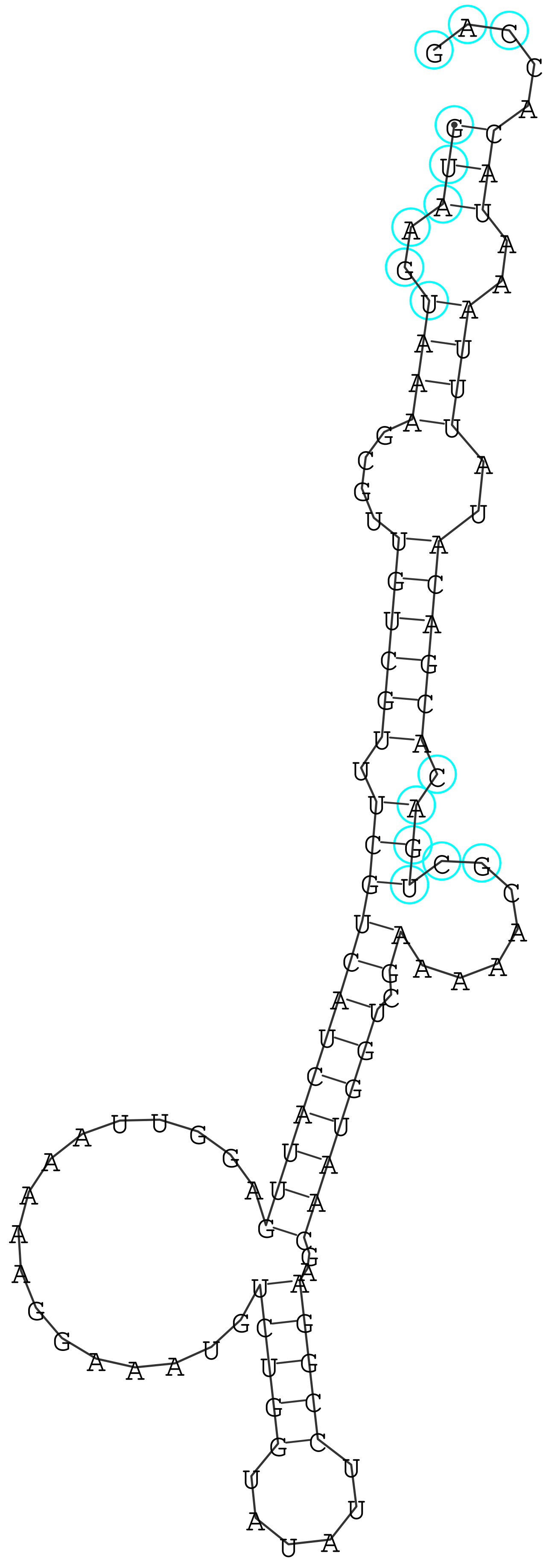

HCOc061A - External intron

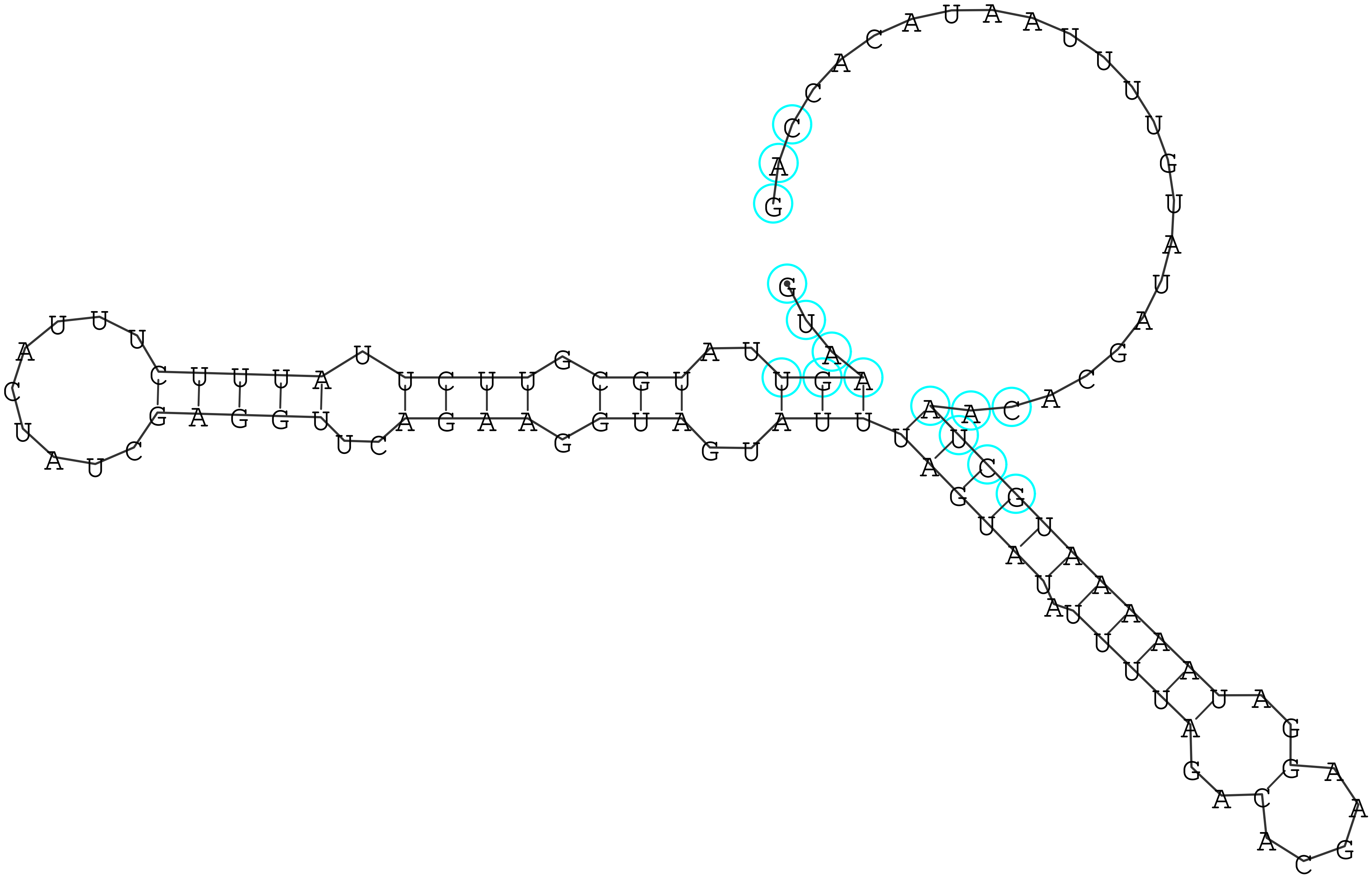

HCOc066A - External intron

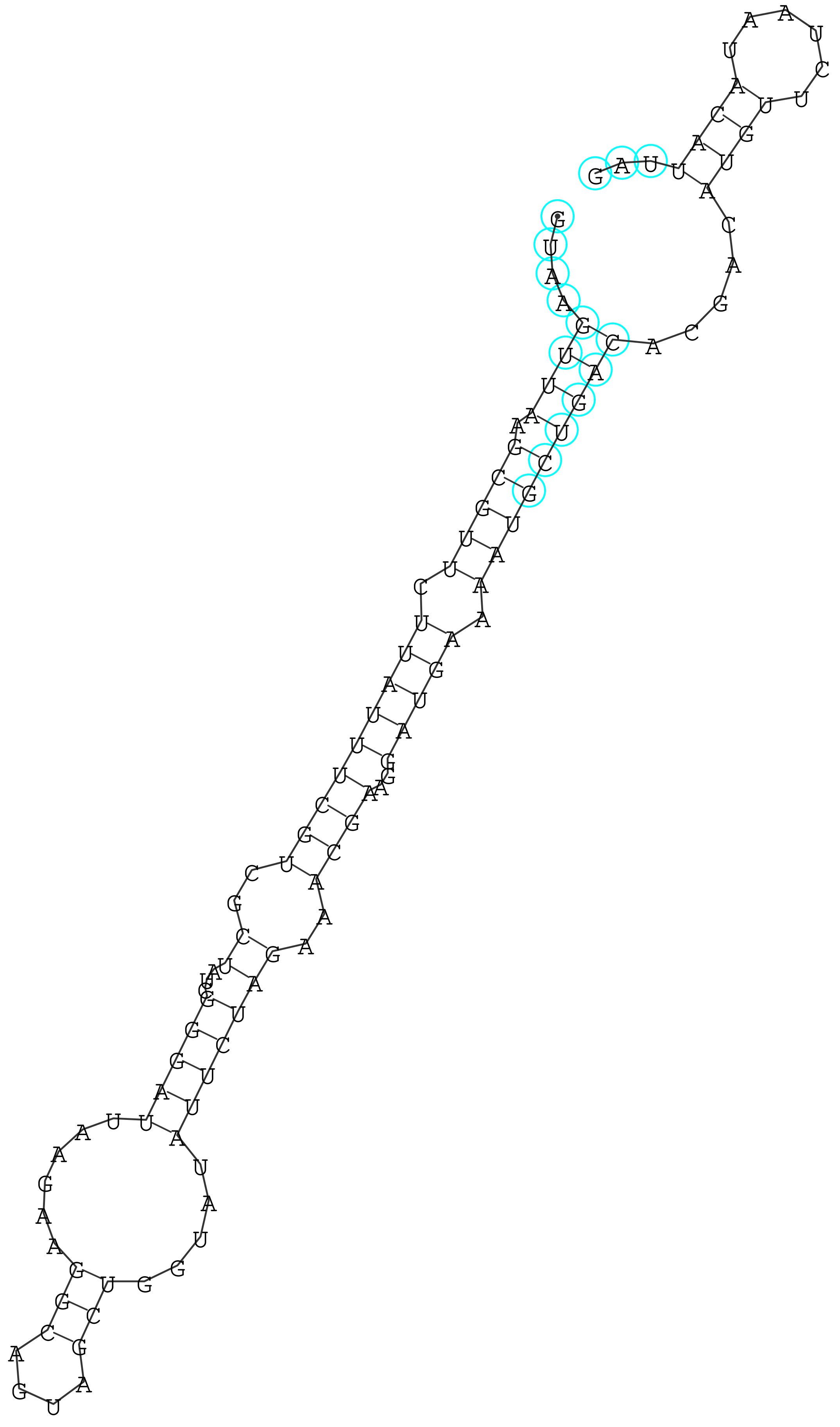

HCOc070A - External intron

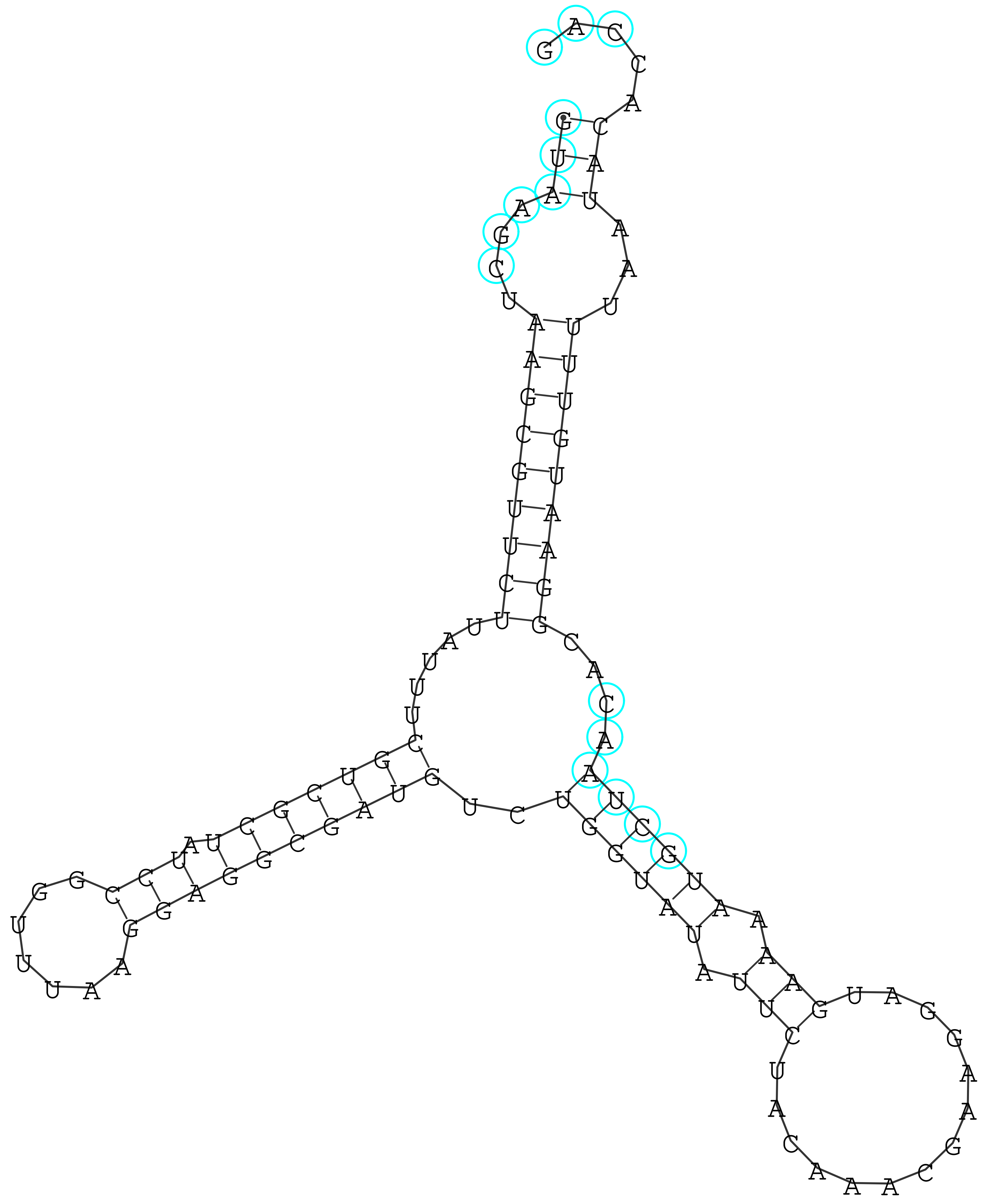

HCOc076A - External intron

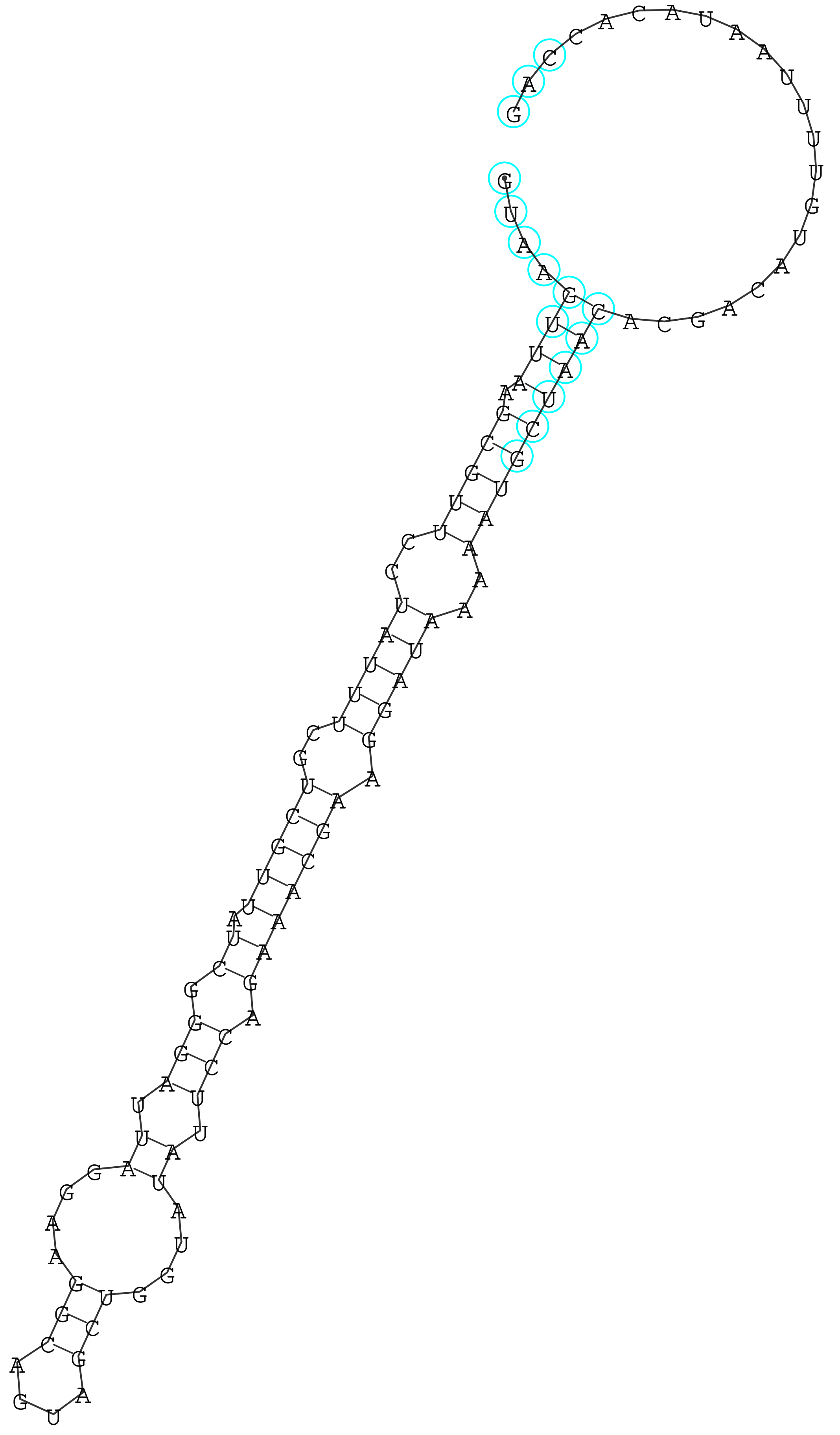

HCOc102A - External intron

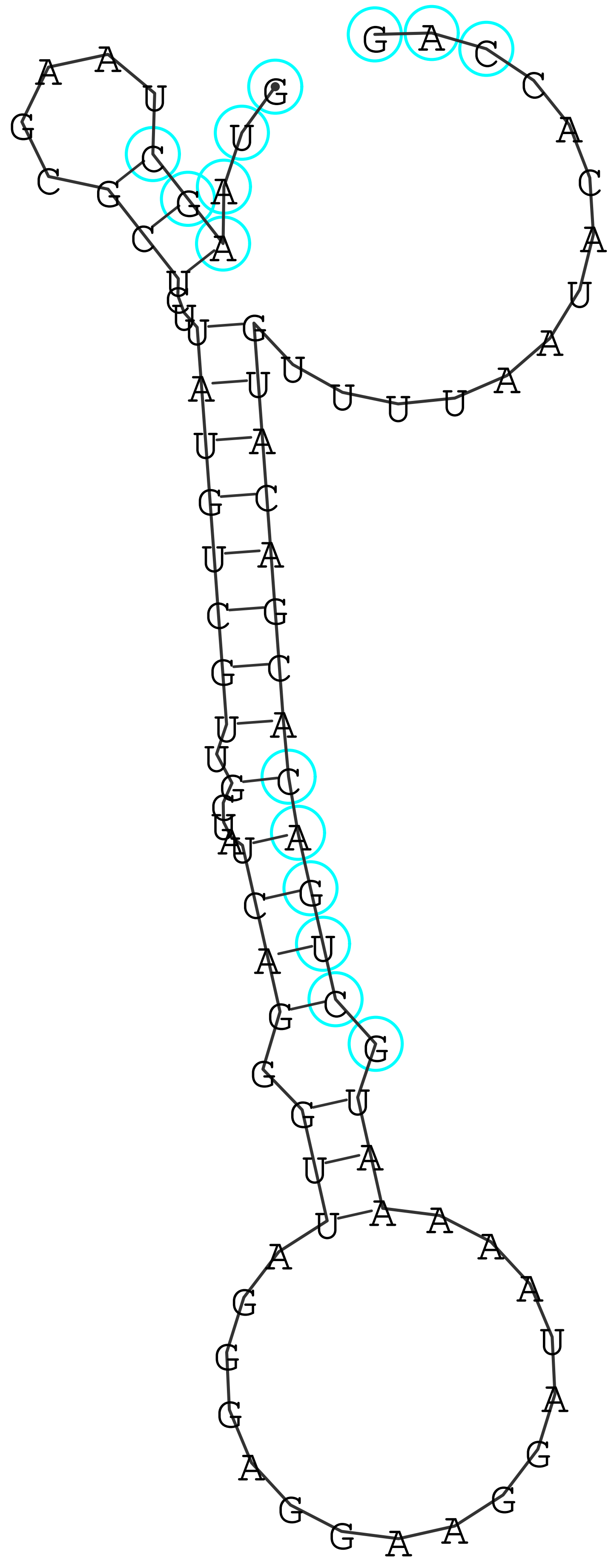

HCOc164A - External intron

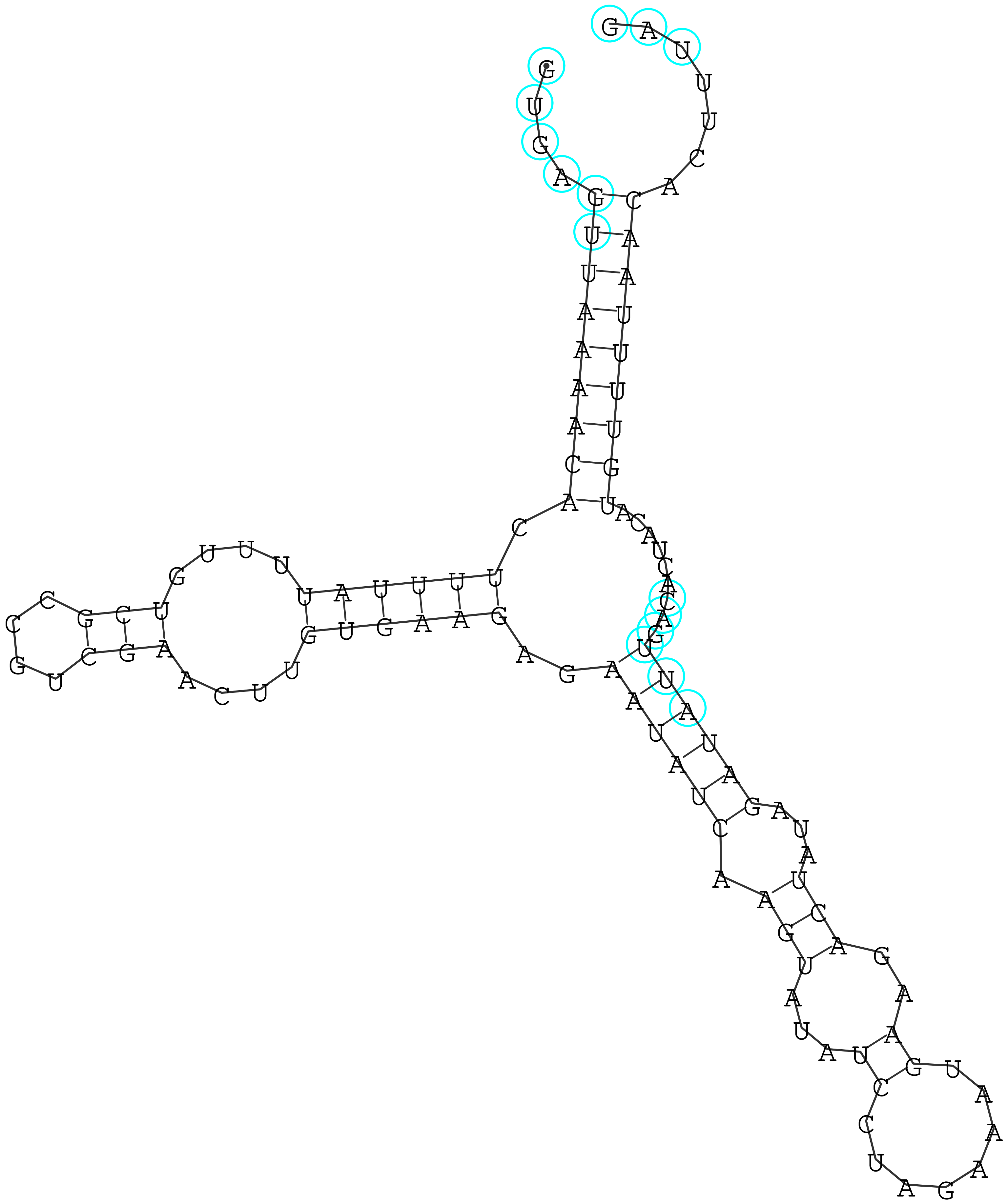

HCOc178A - External intron

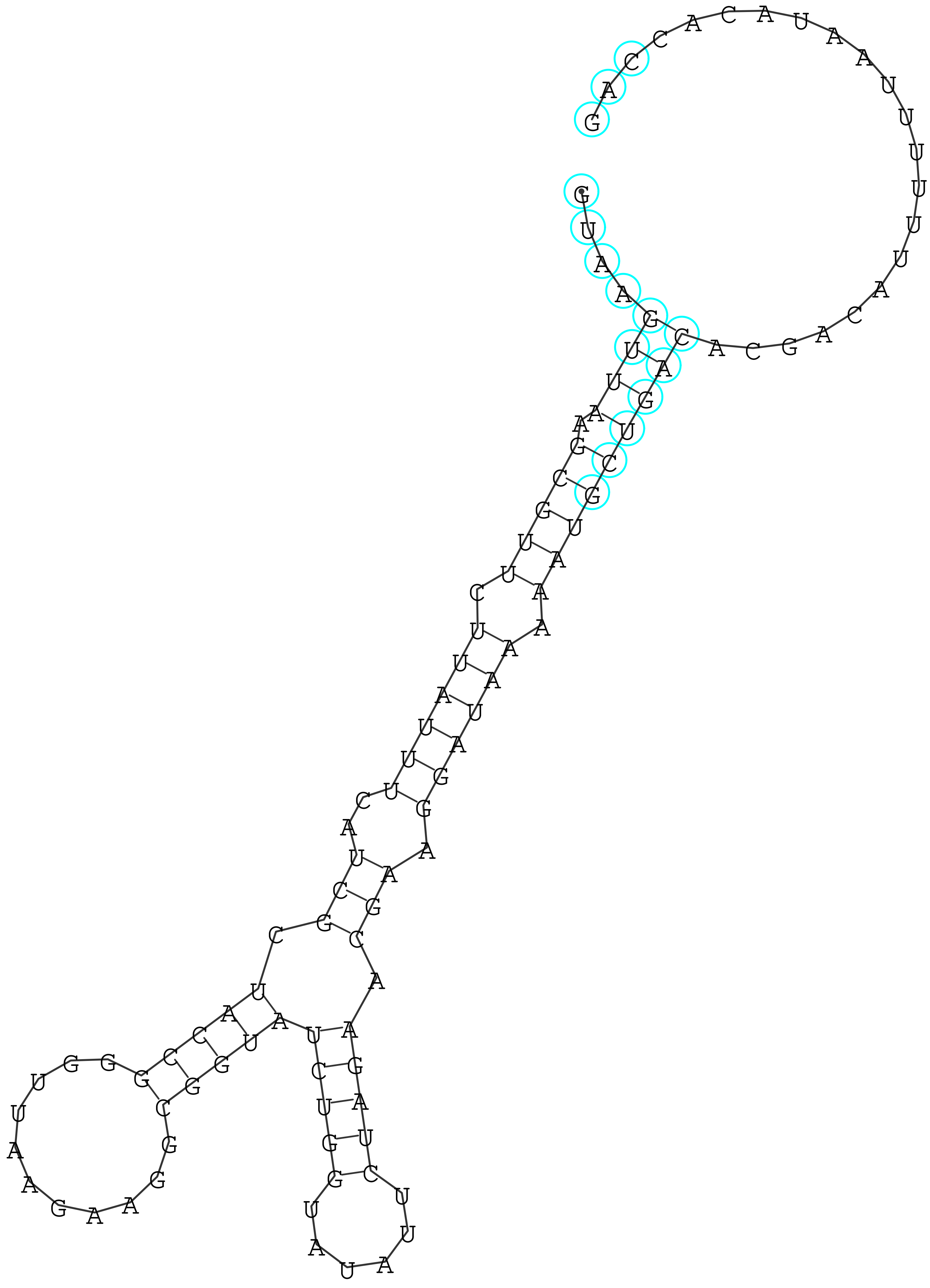

HCOc224-179 - External intron

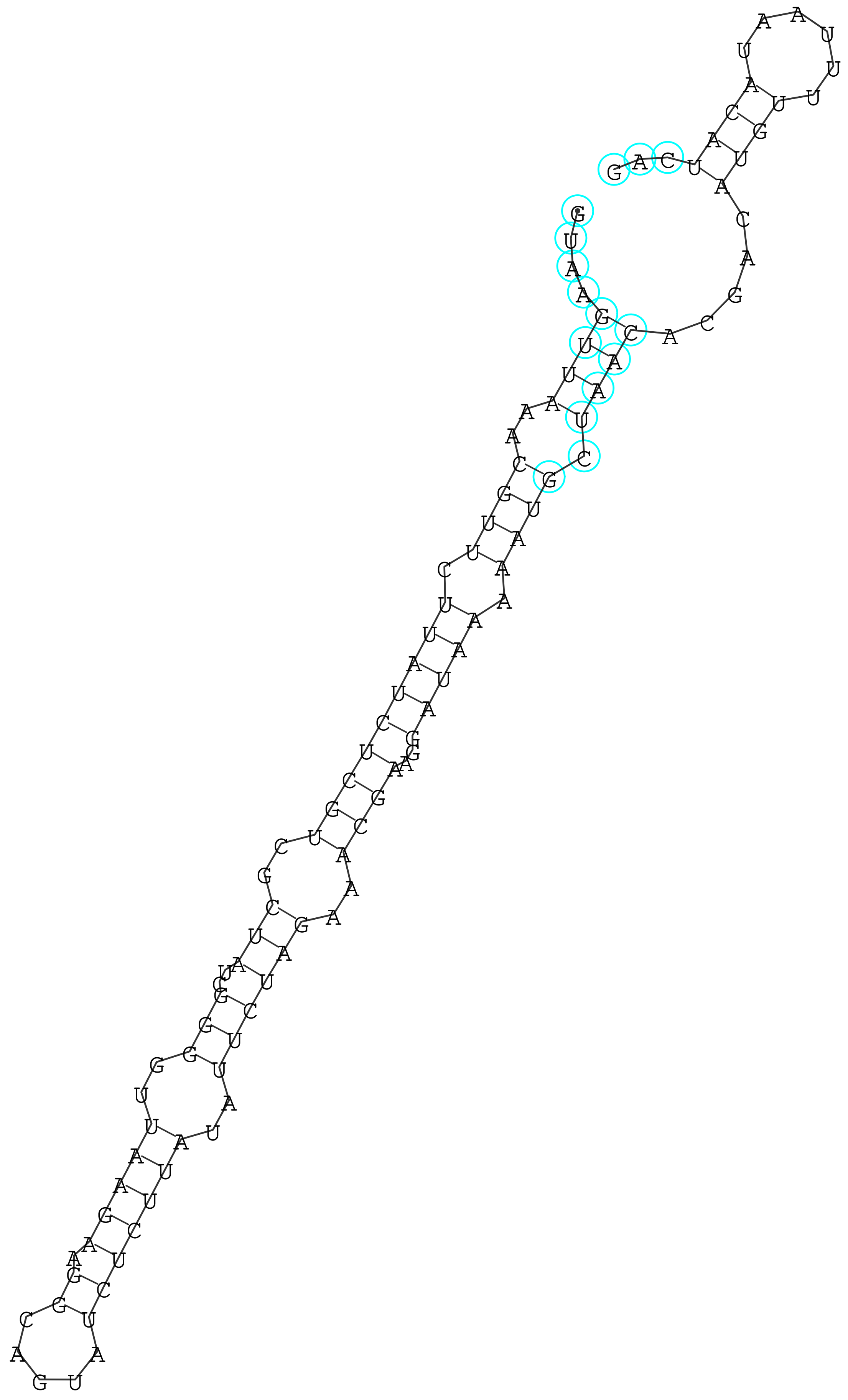

HCOc236A - External intron

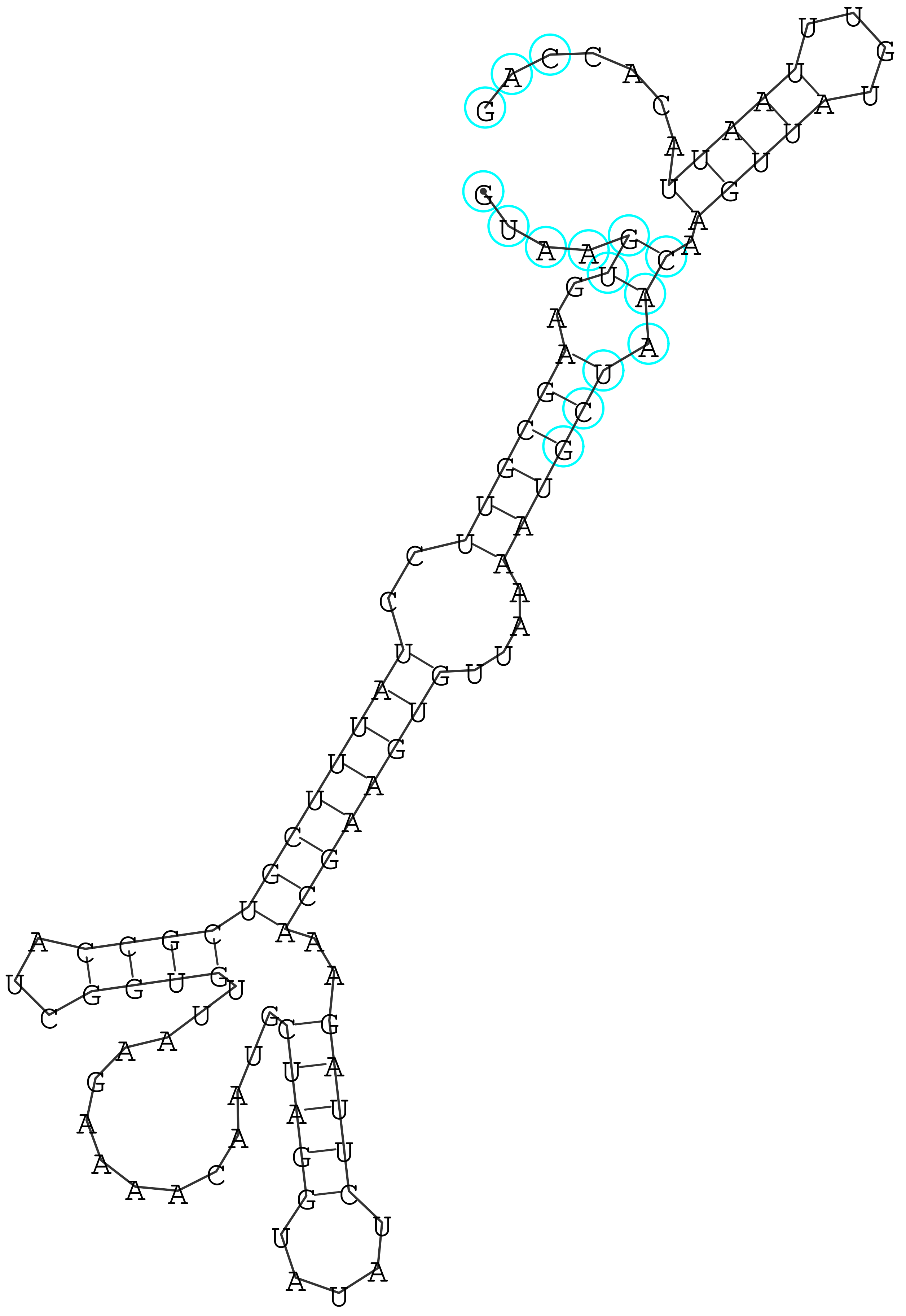

HCOc252A - External intron

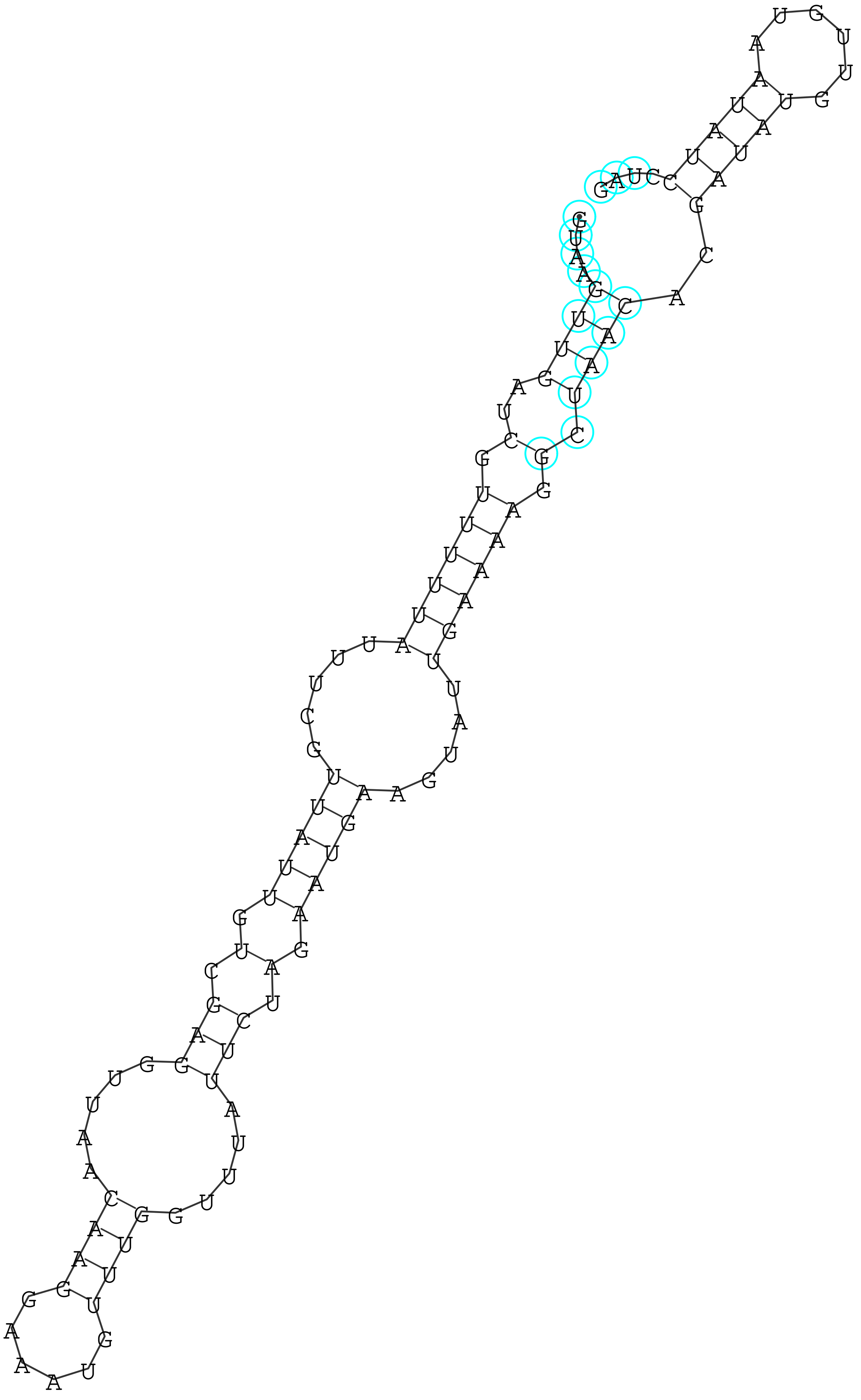

HCOc271A - External intron

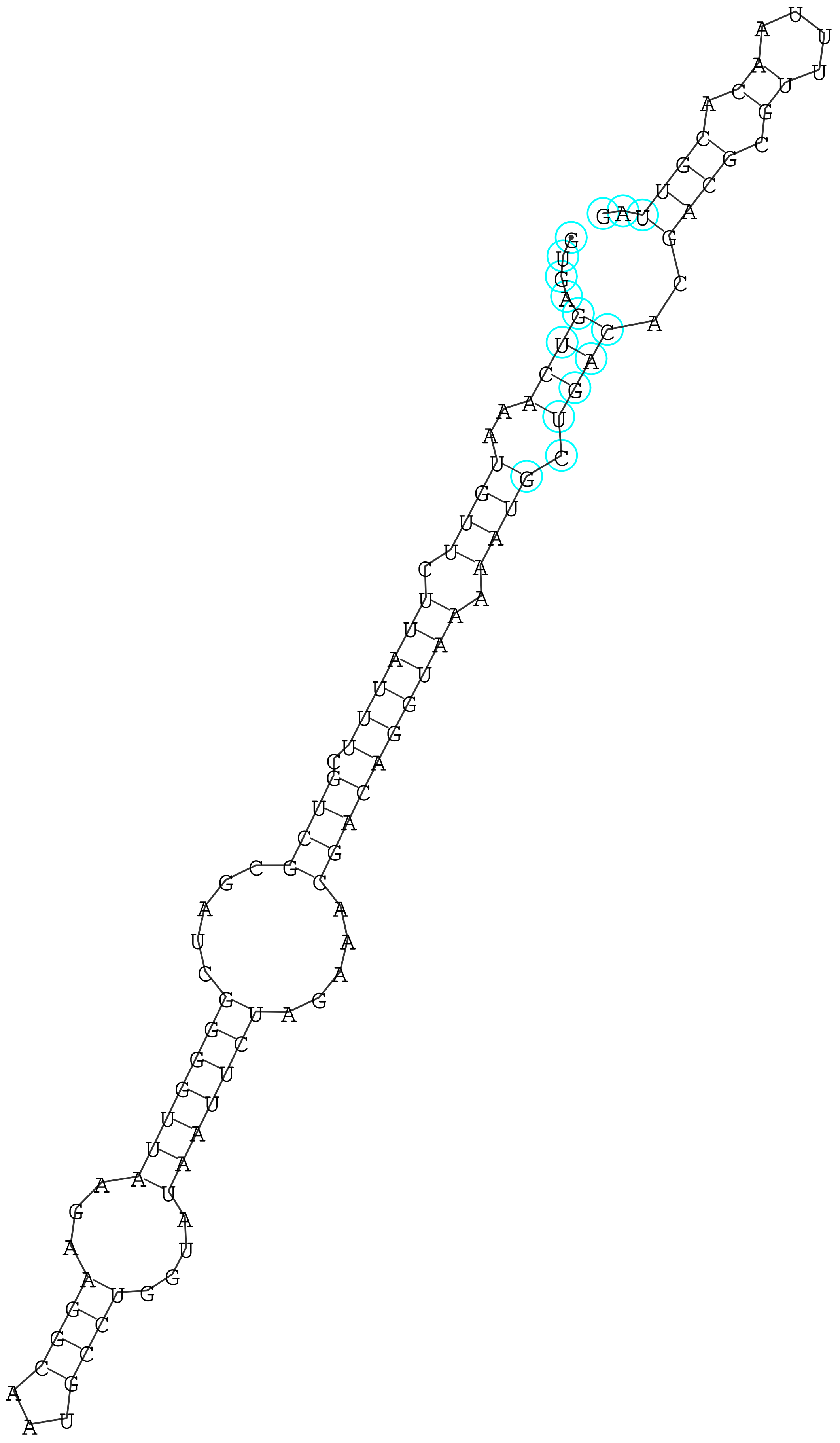

HCOc332A - External intron

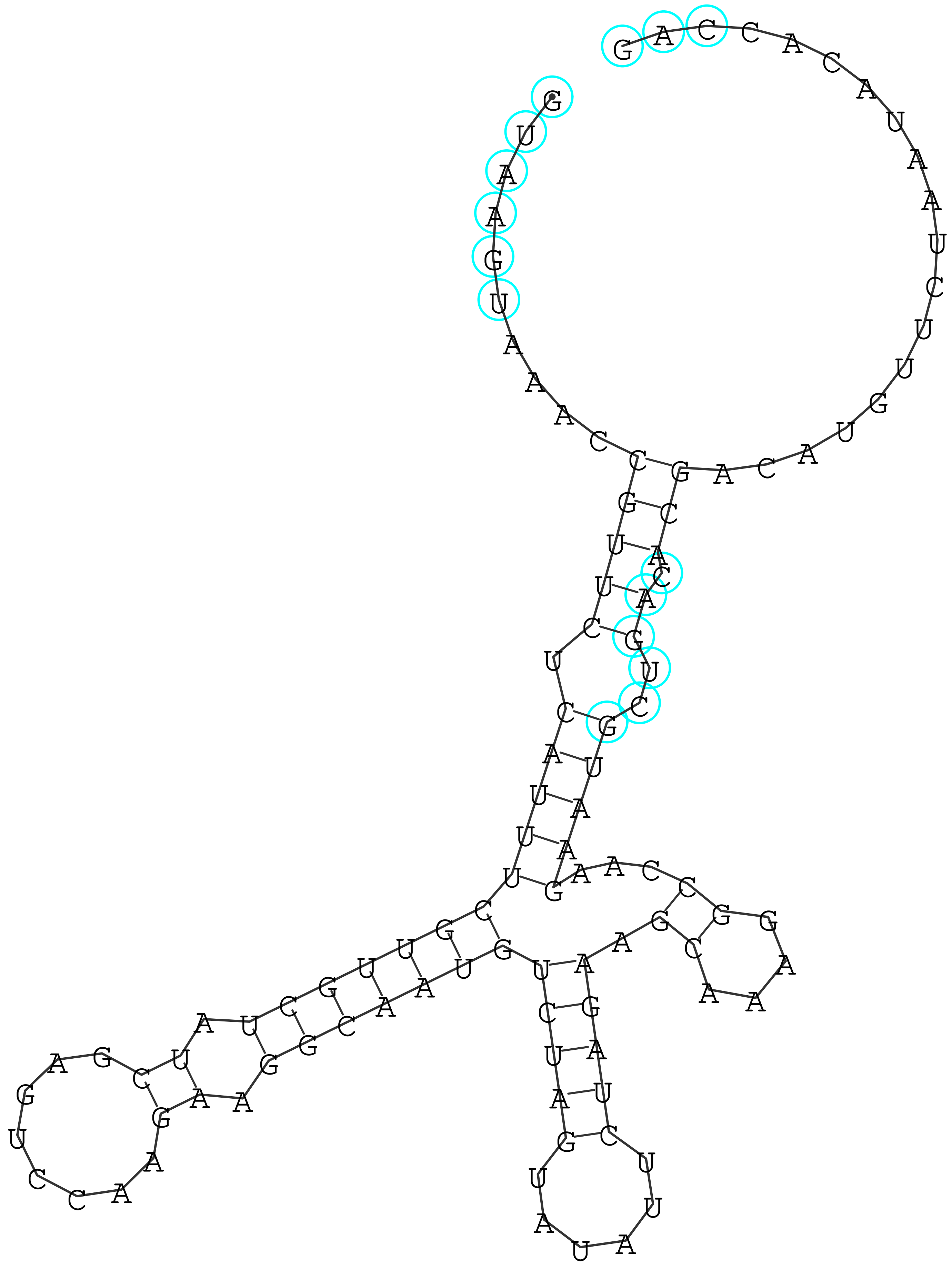

HCOc378A - External intron

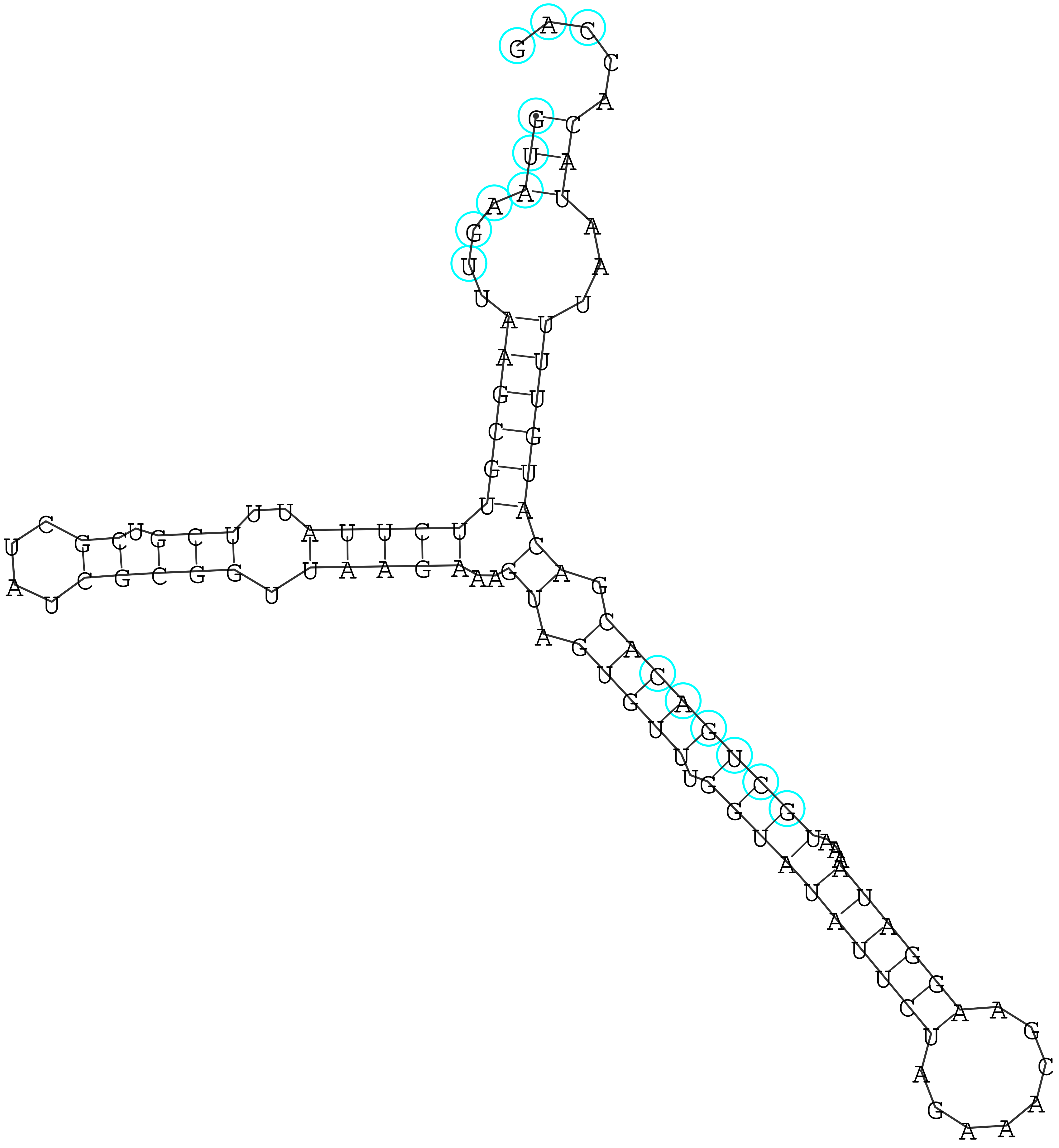

HCOc406A - External intron

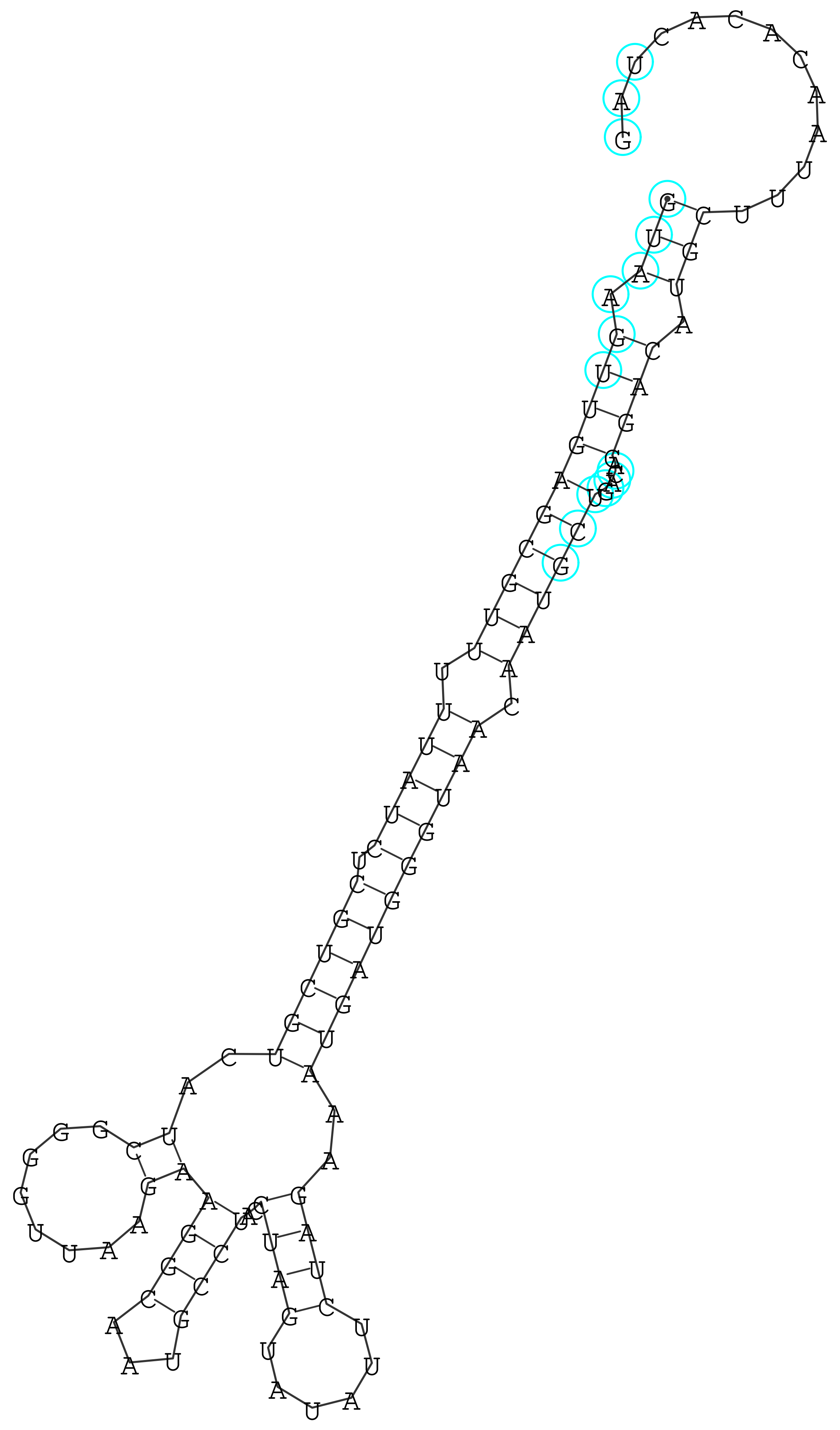

HCOc522A - External intron

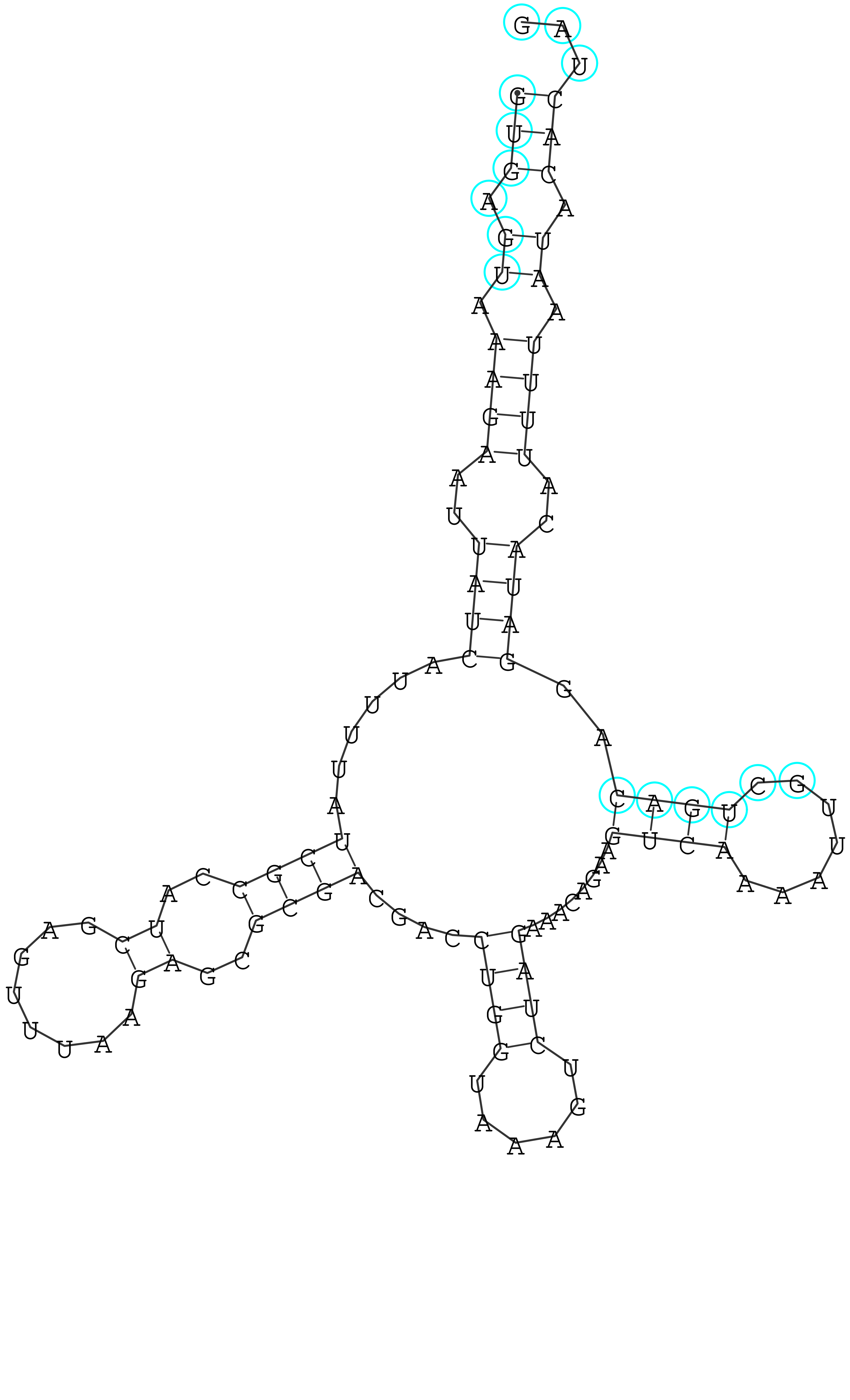

HE7c016A - External intron

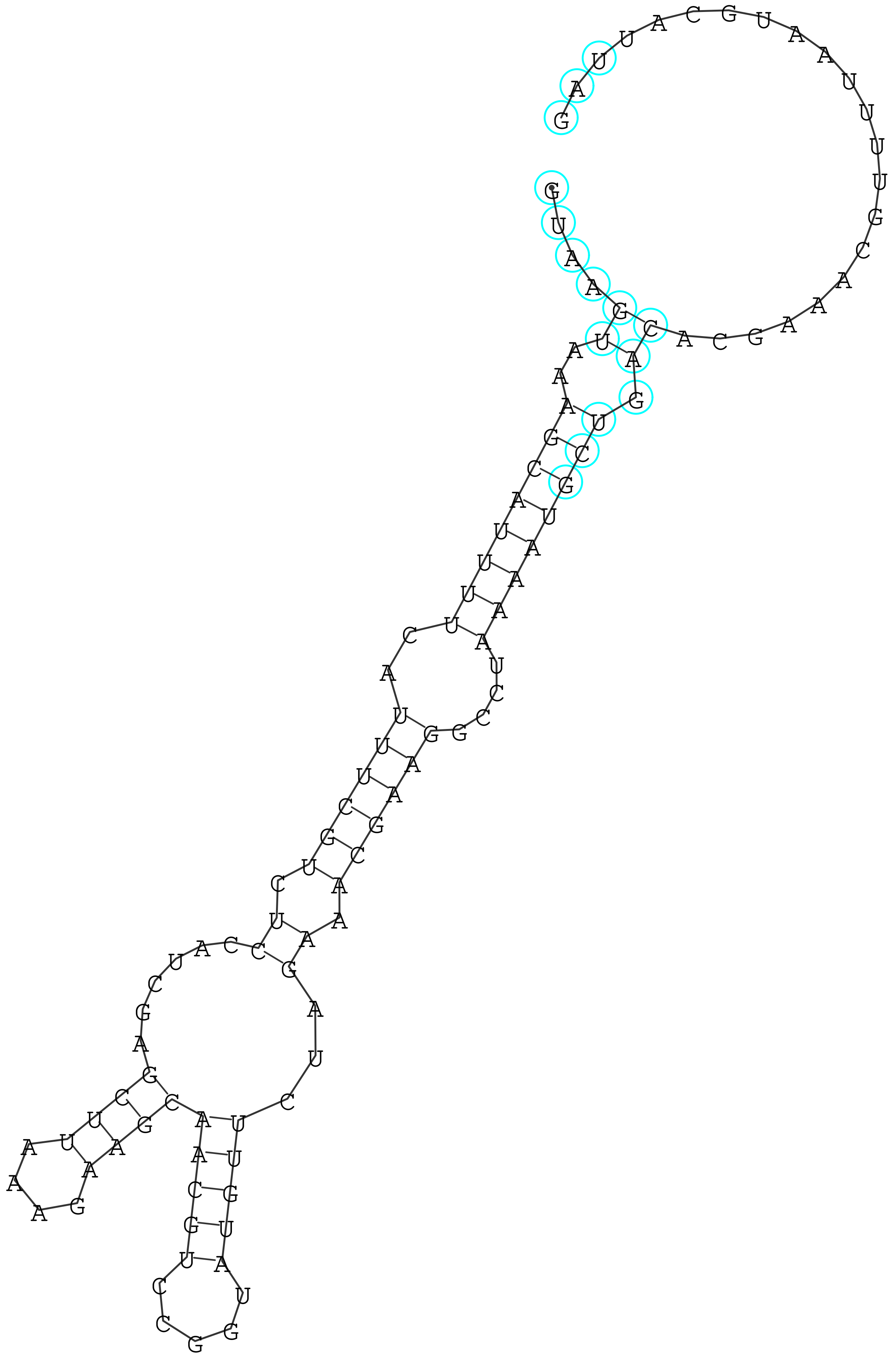

HE7c026A - External intron

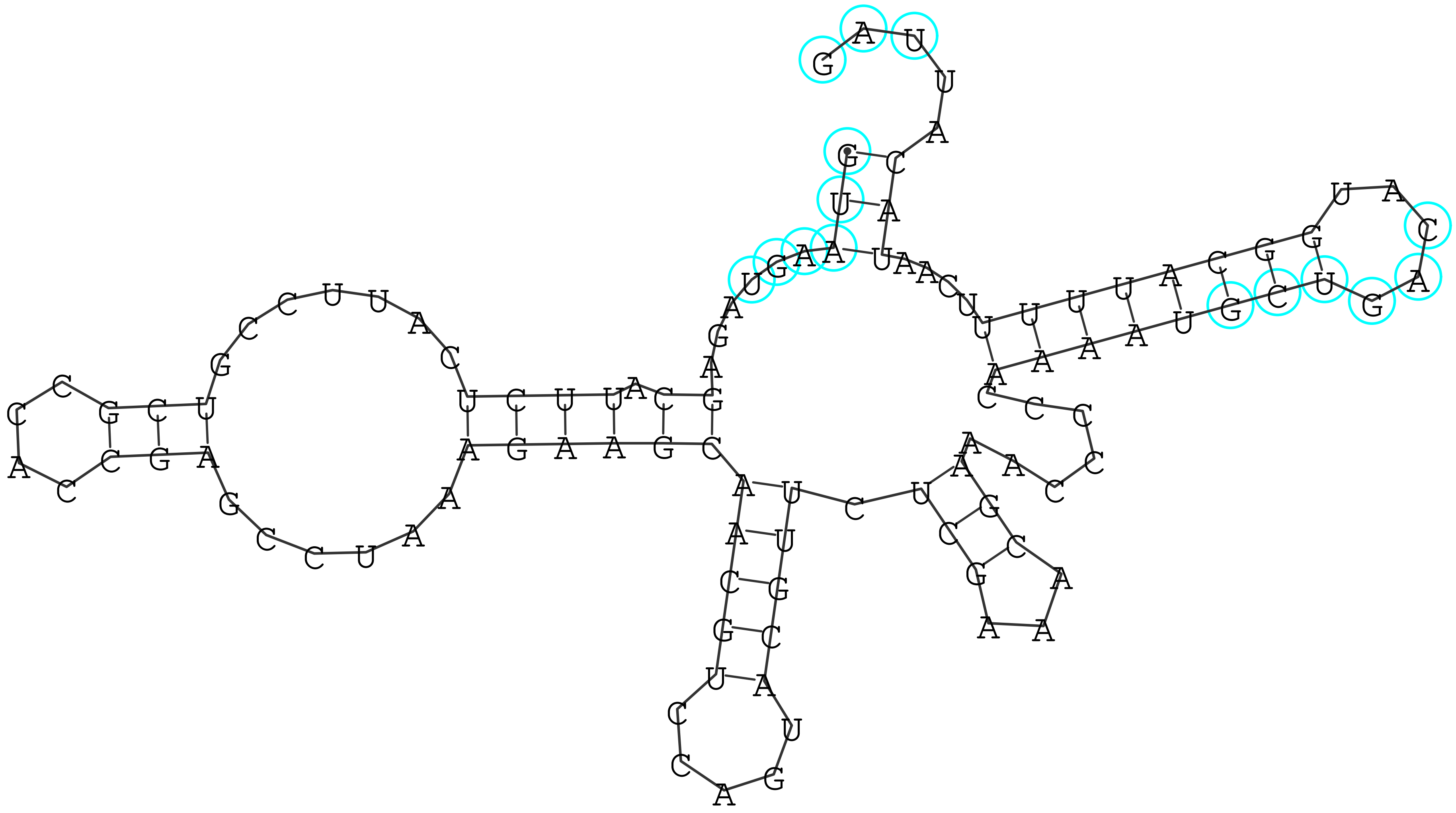

HE7c026B - External intron

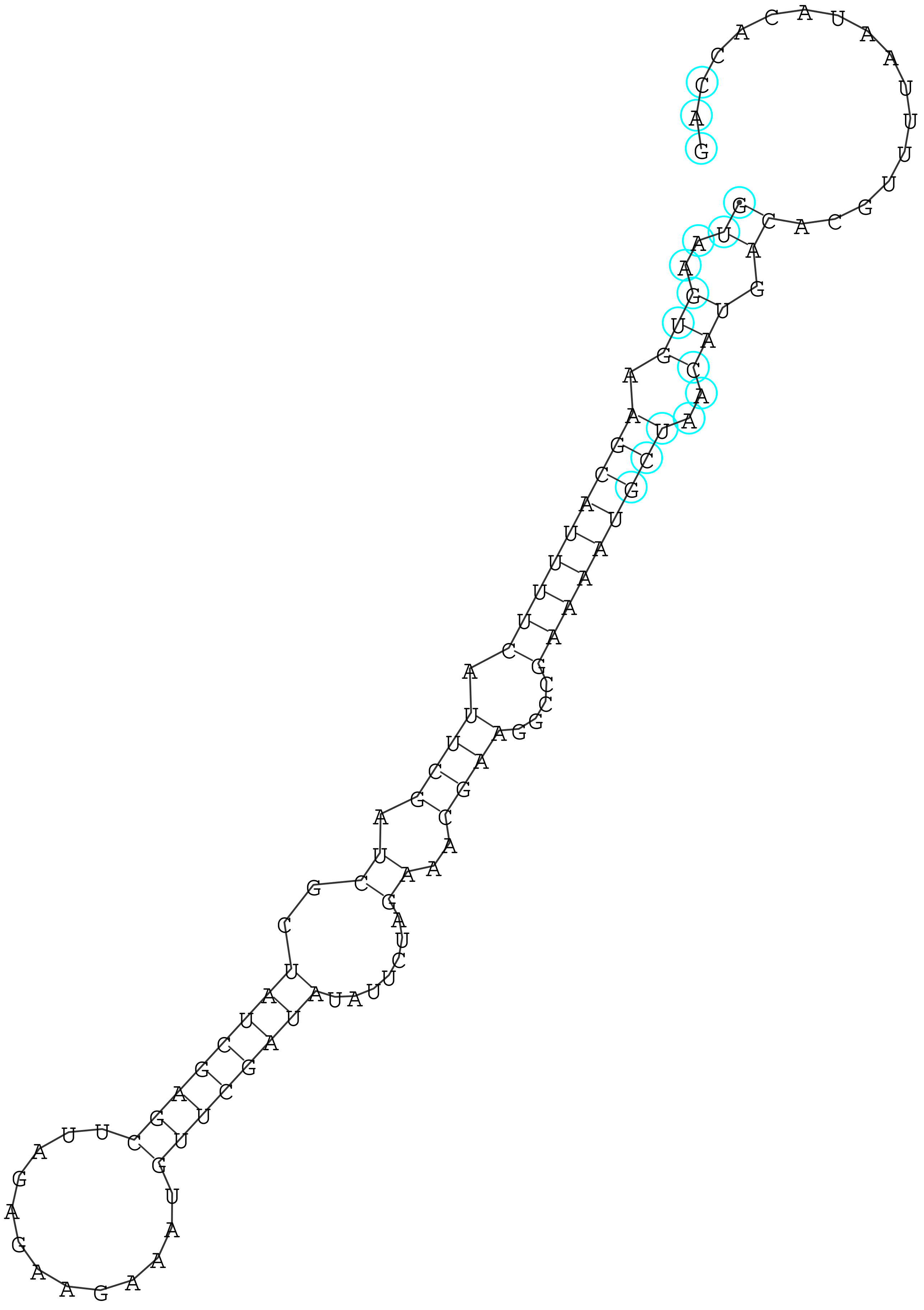

HE7c035A - External intron

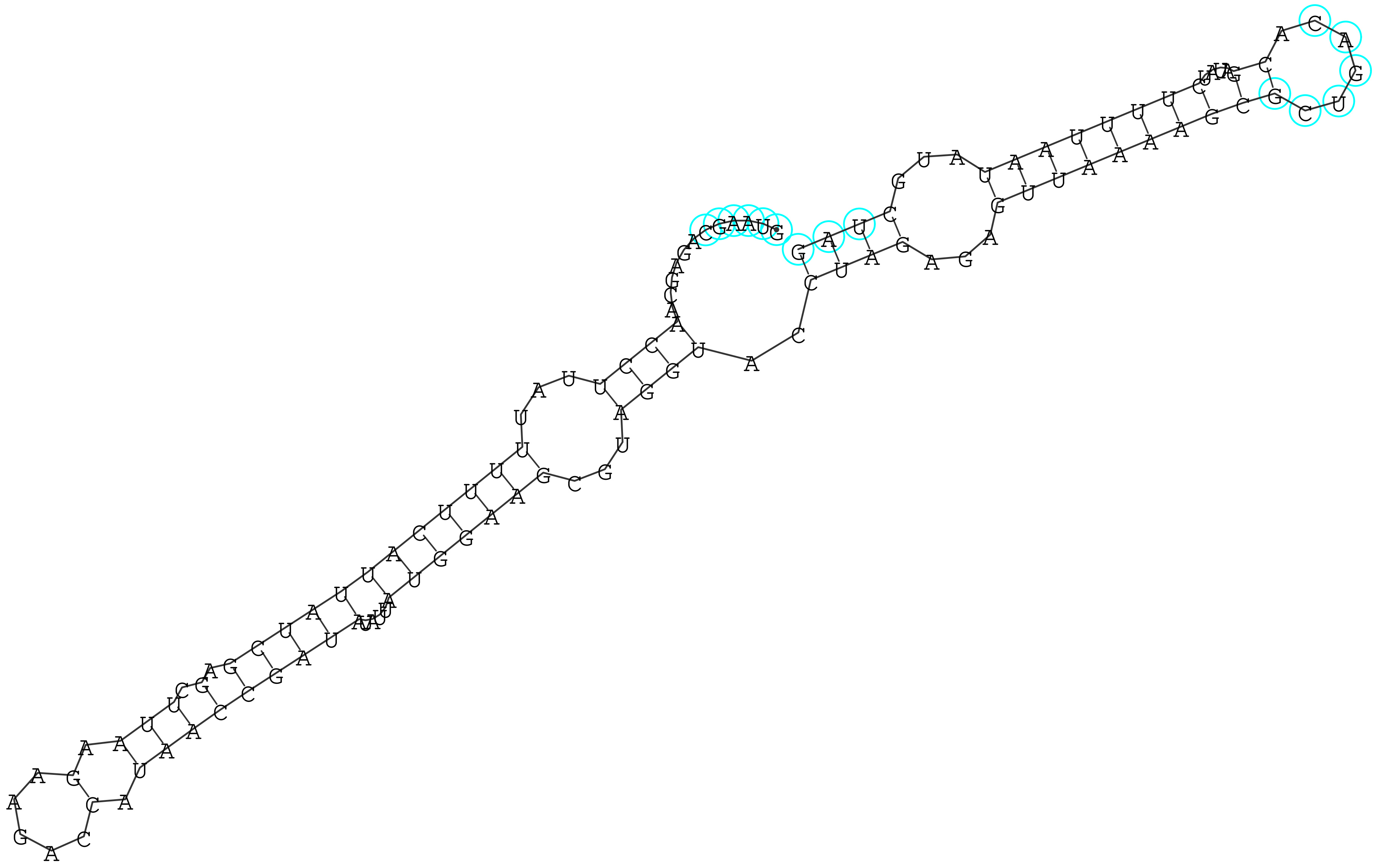

HE7c050A - External intron

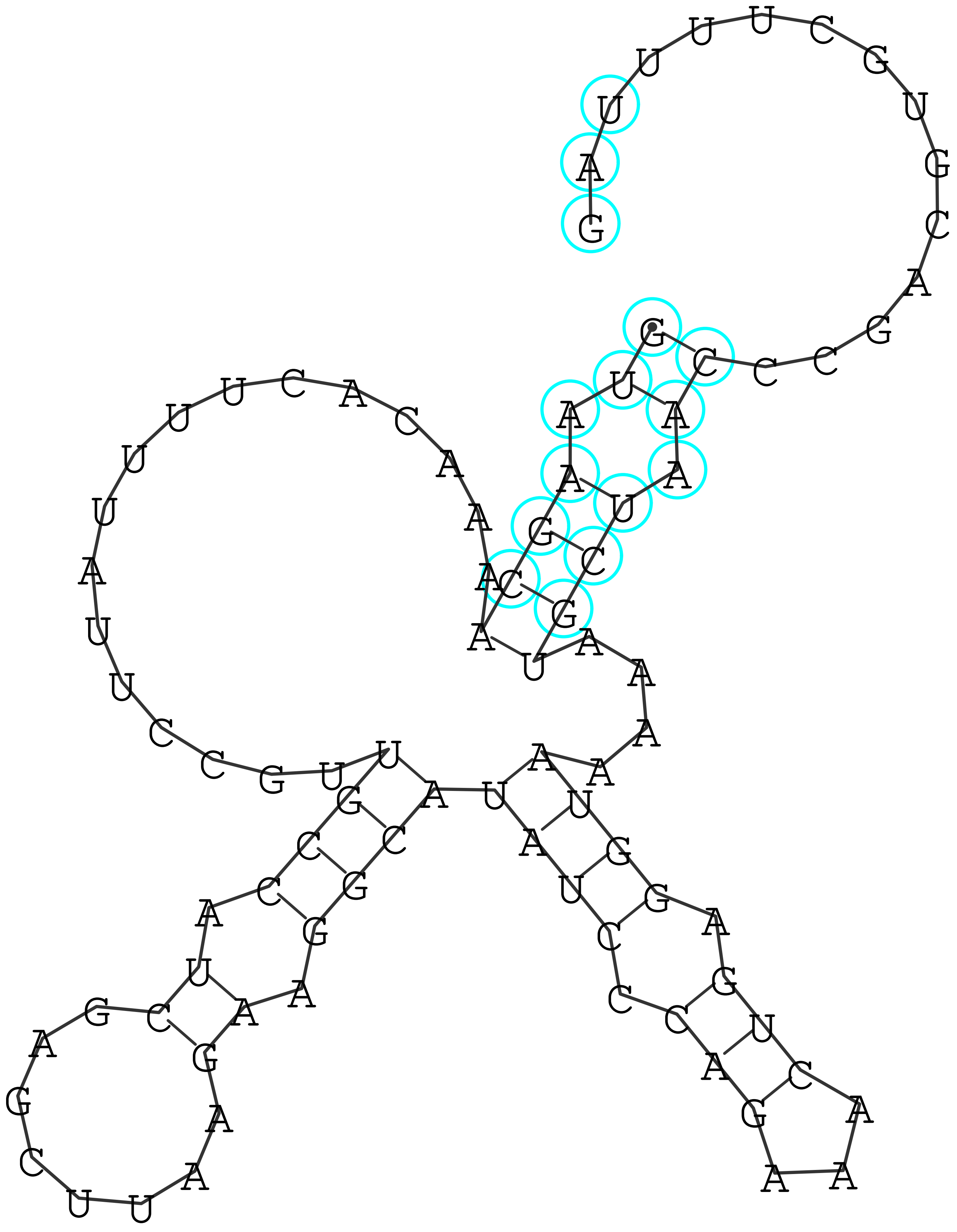

HE7c129A - External intron

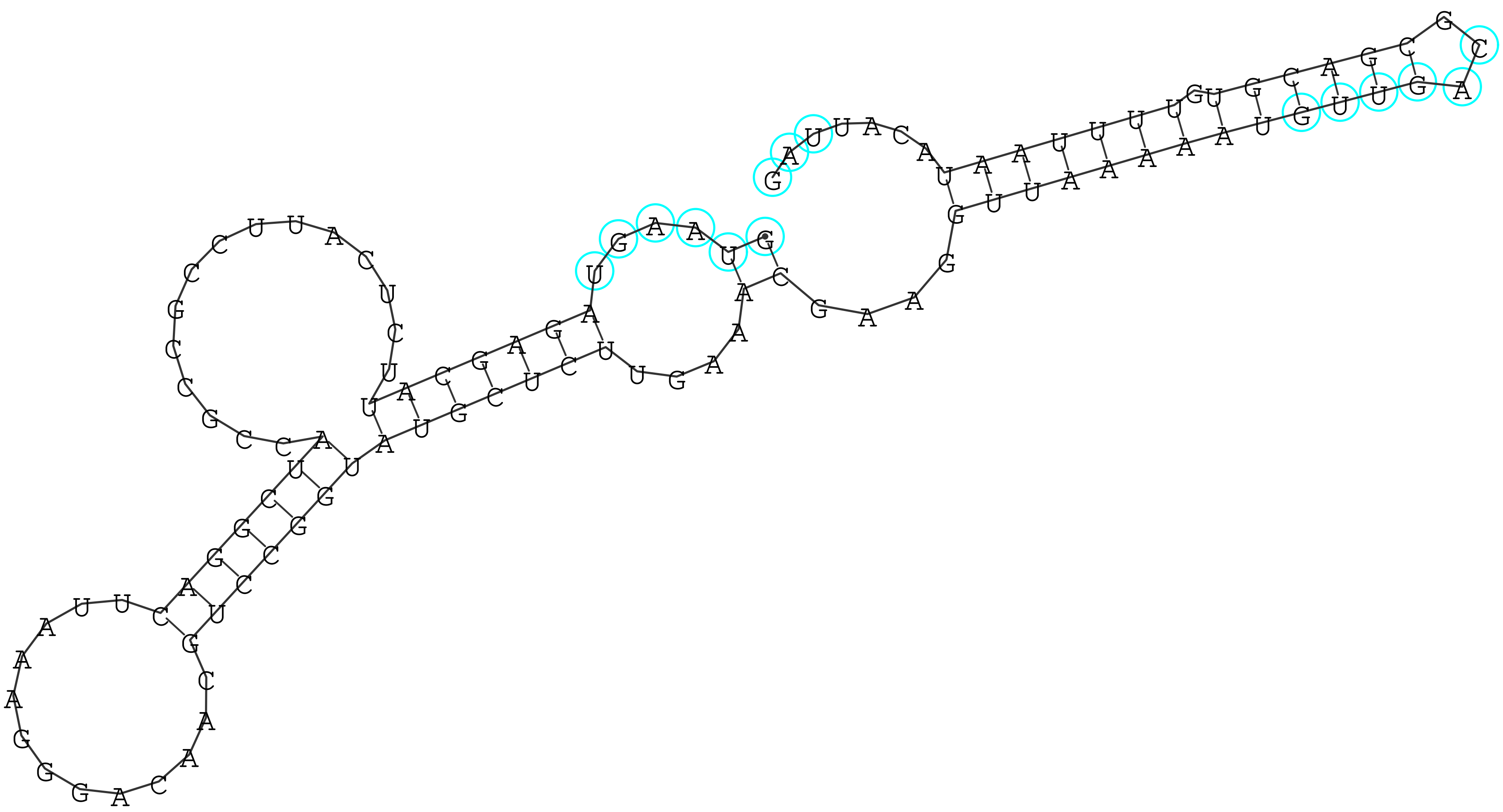

HE7c137A - External intron

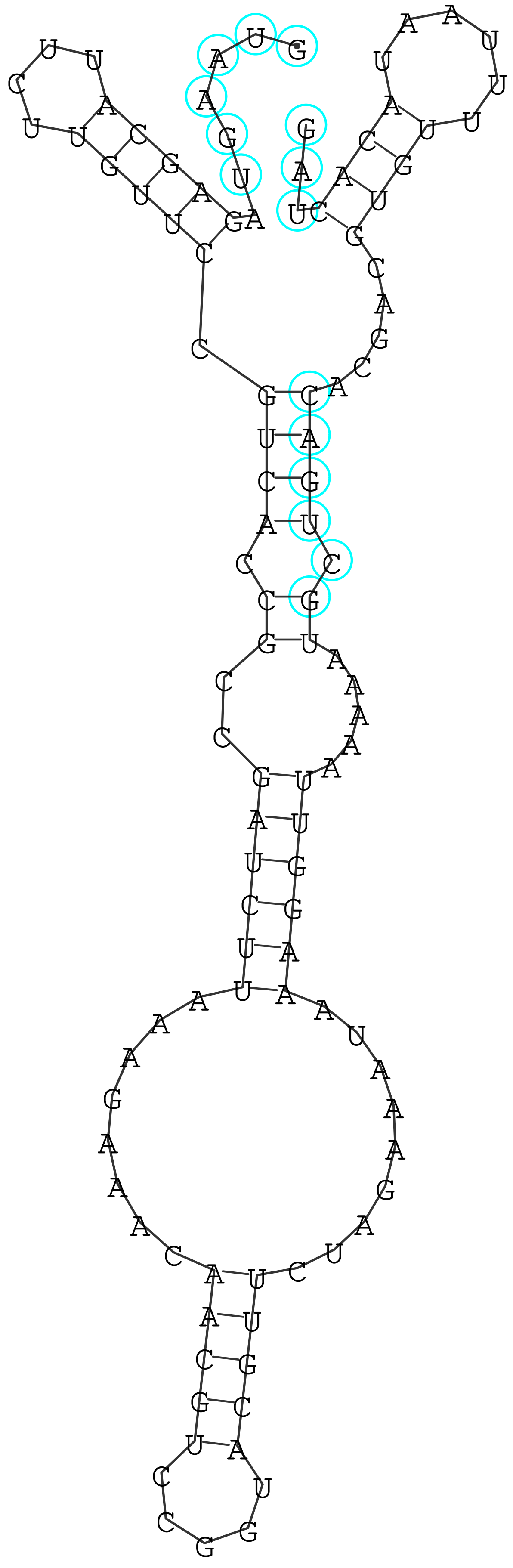

HE7c276A - External intron

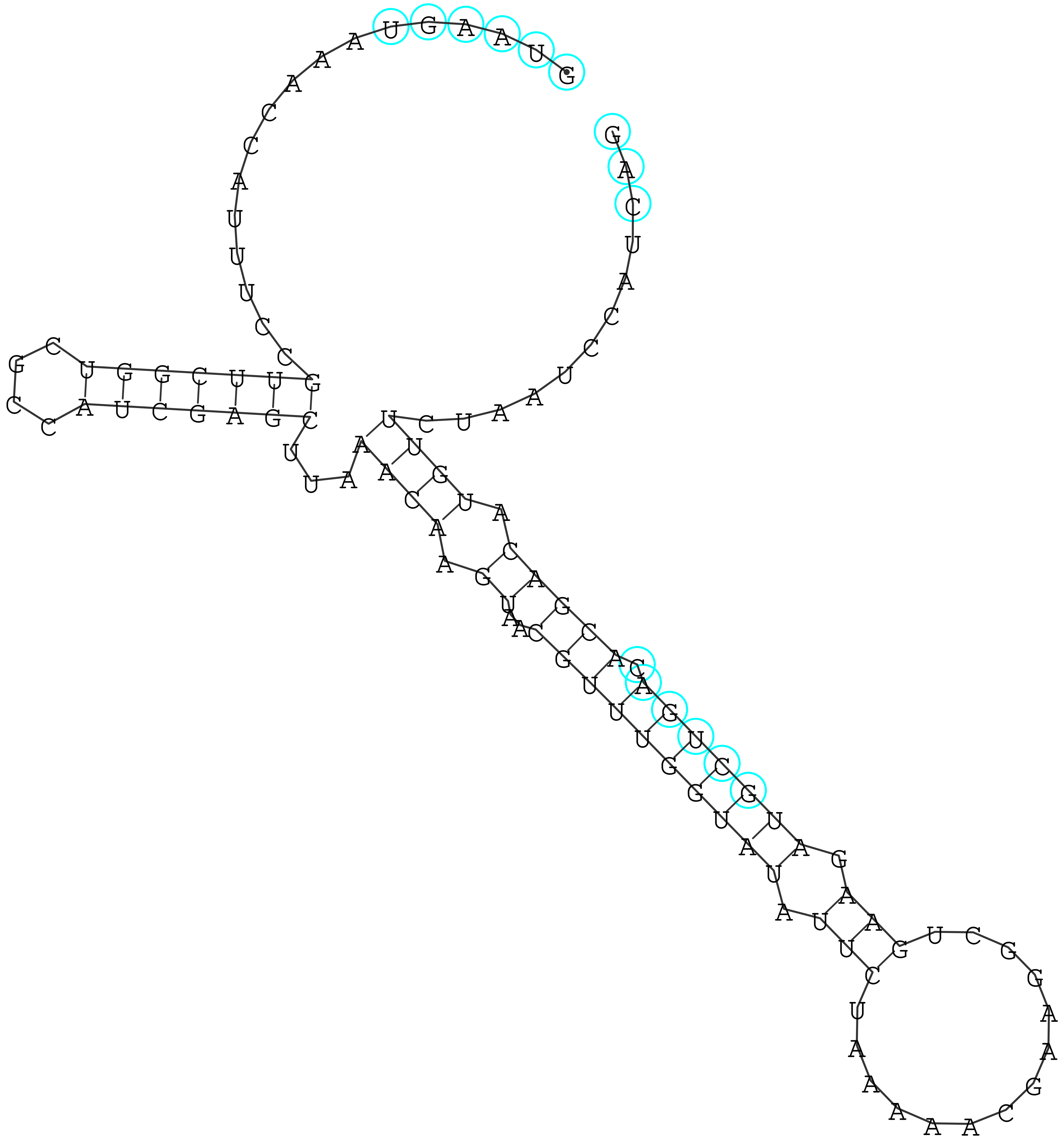

HE7c301A - External intron

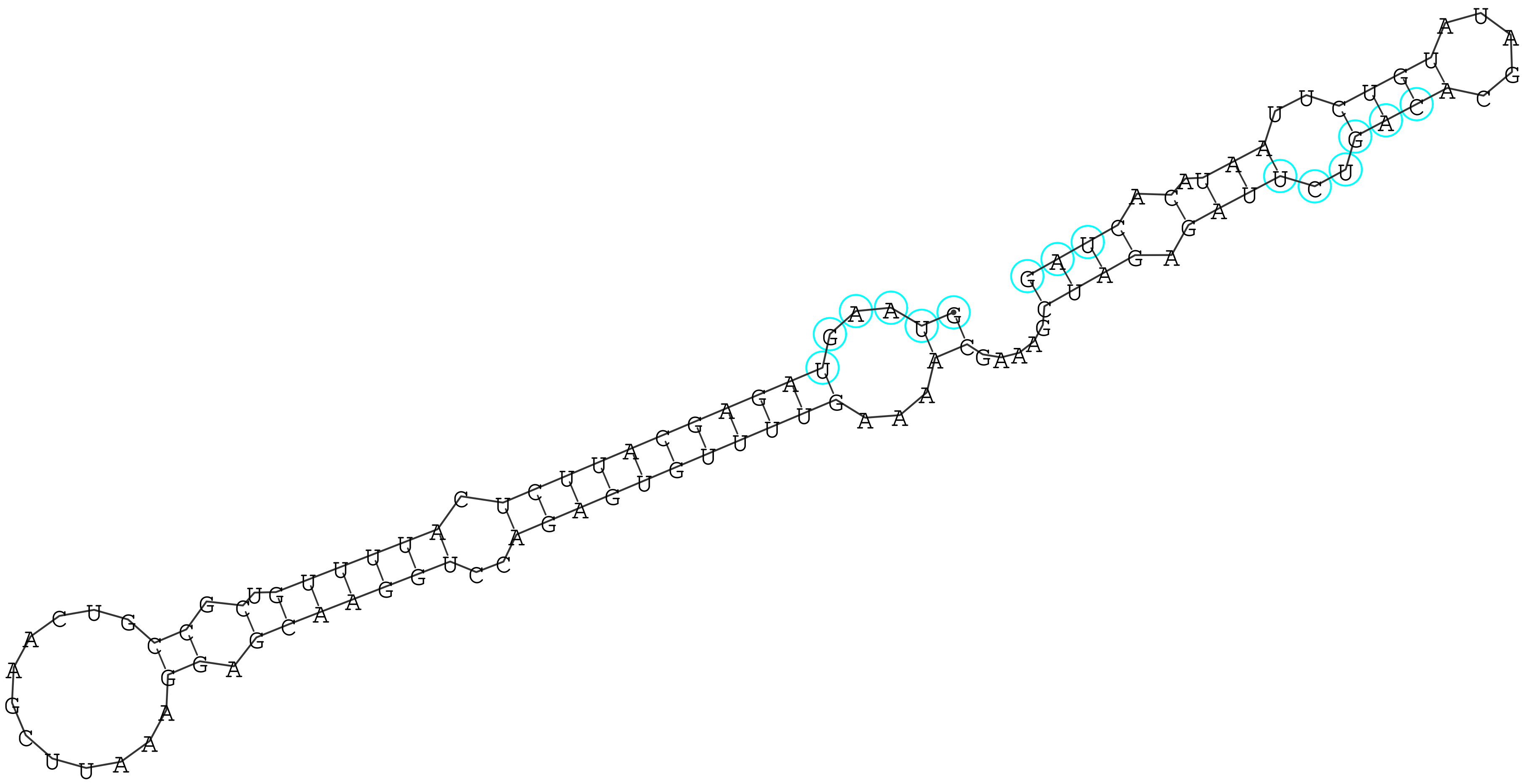

HECc114A - External intron

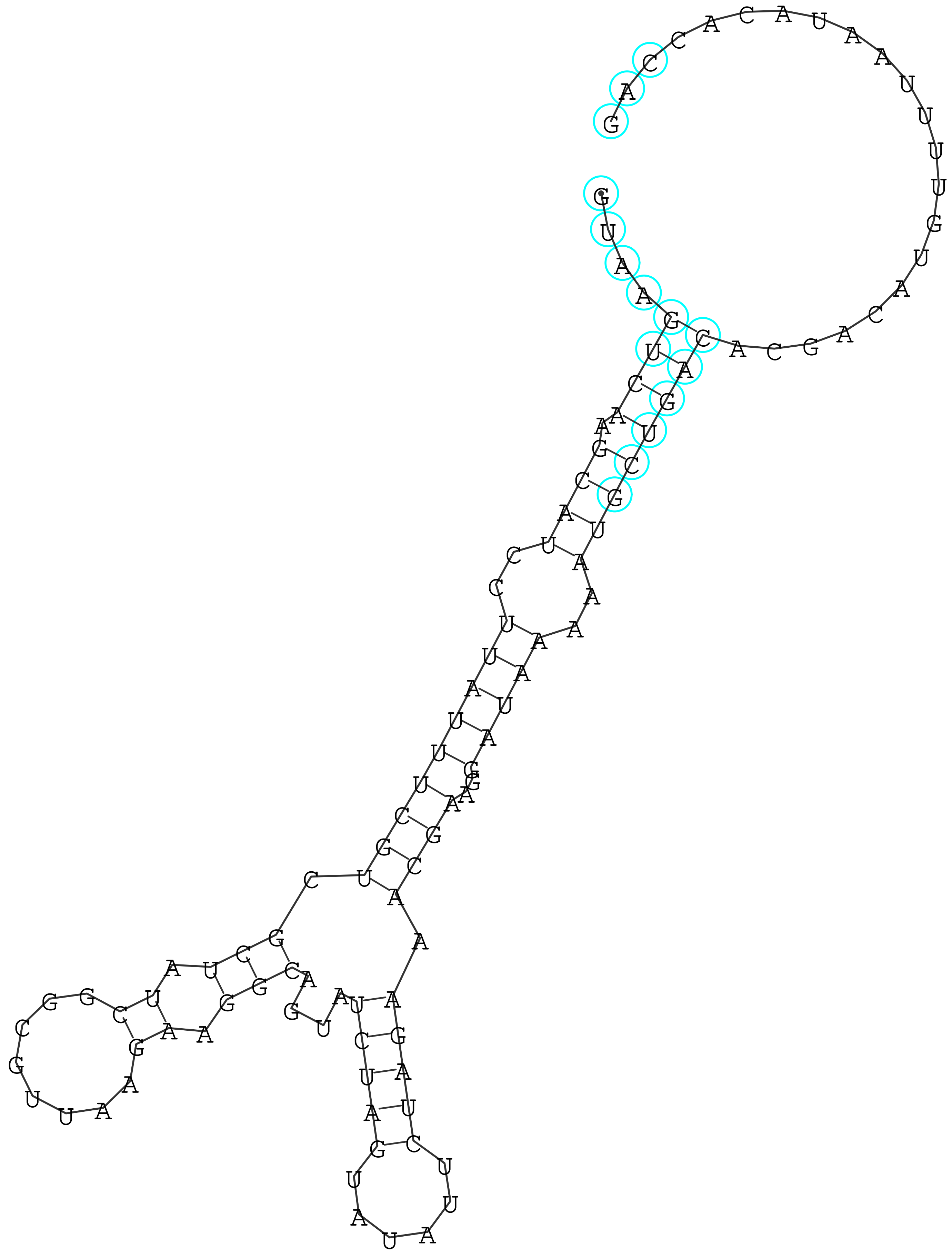

HECc217A - External intron

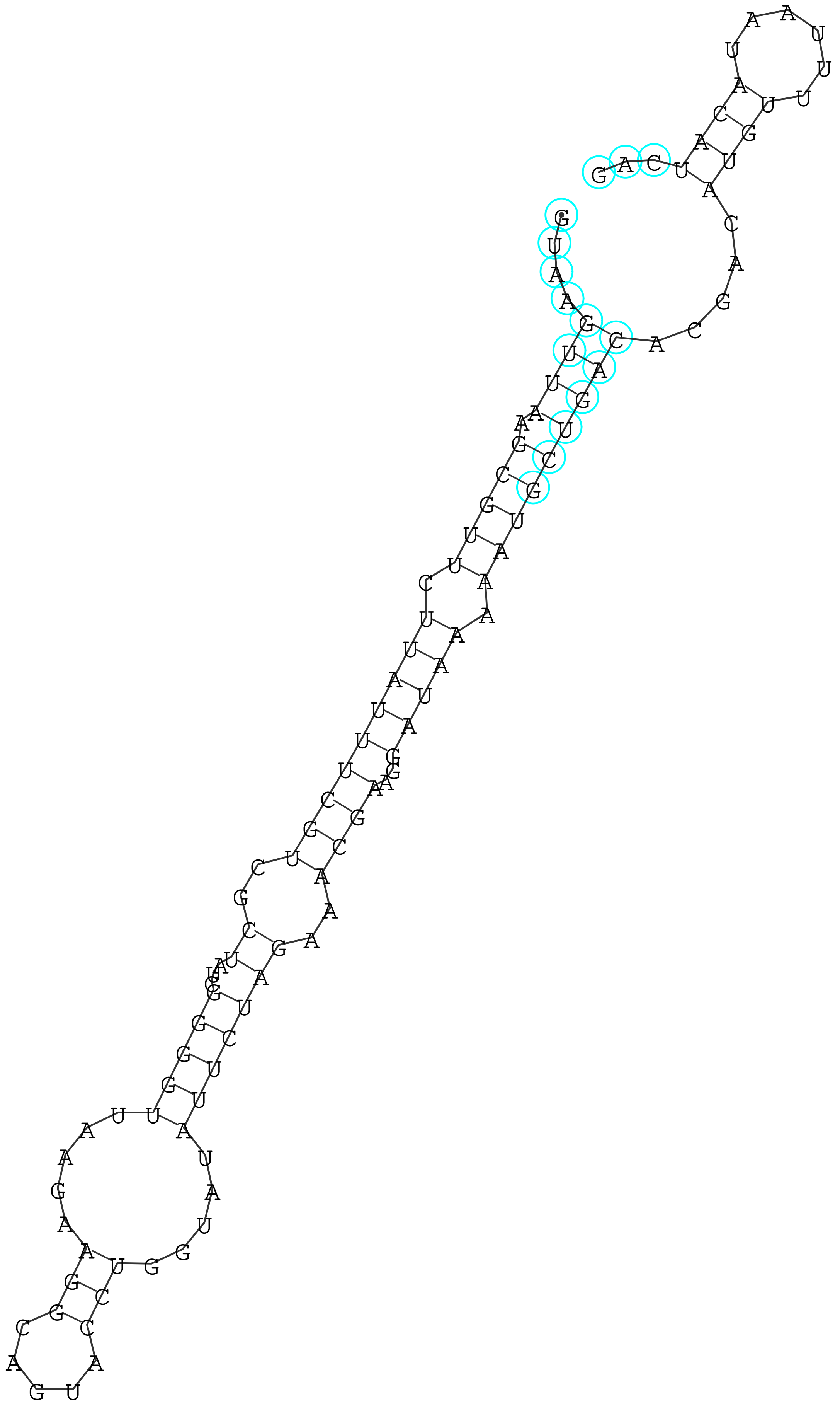

Hruc29A - External intron

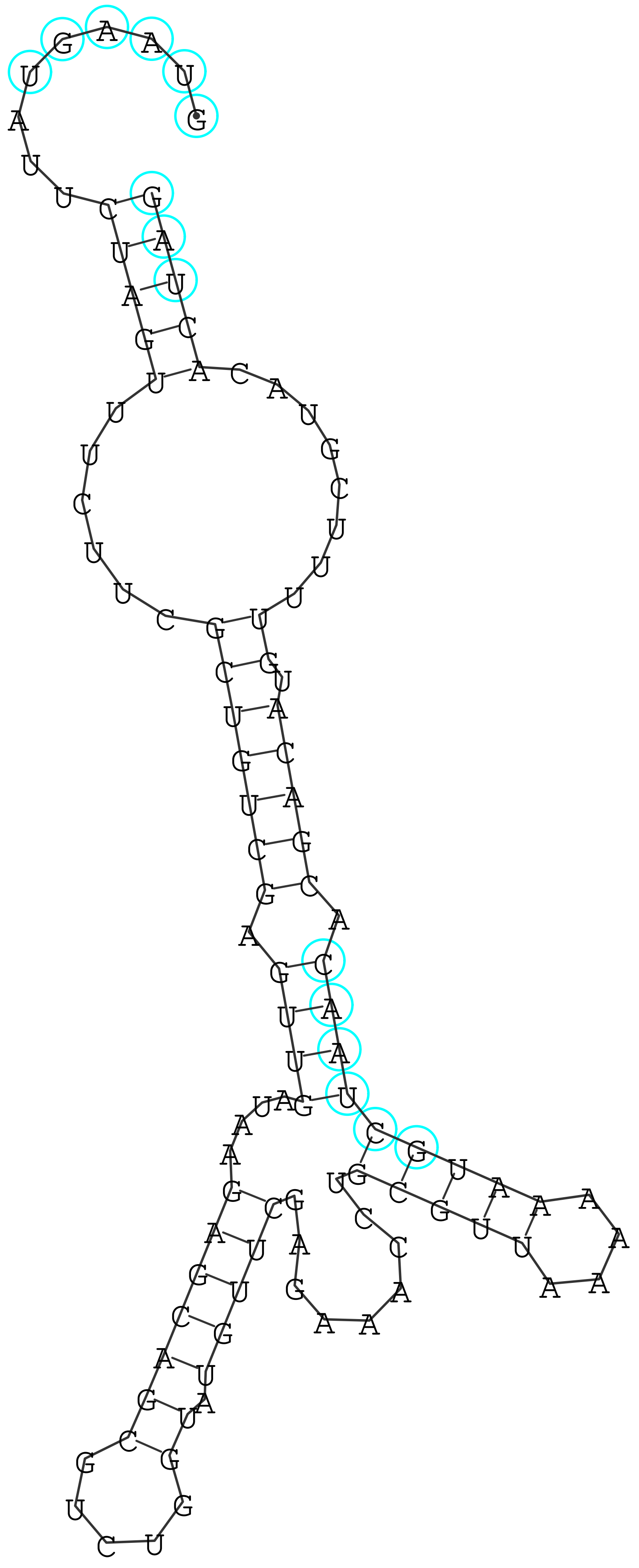

Hruc31A - External intron

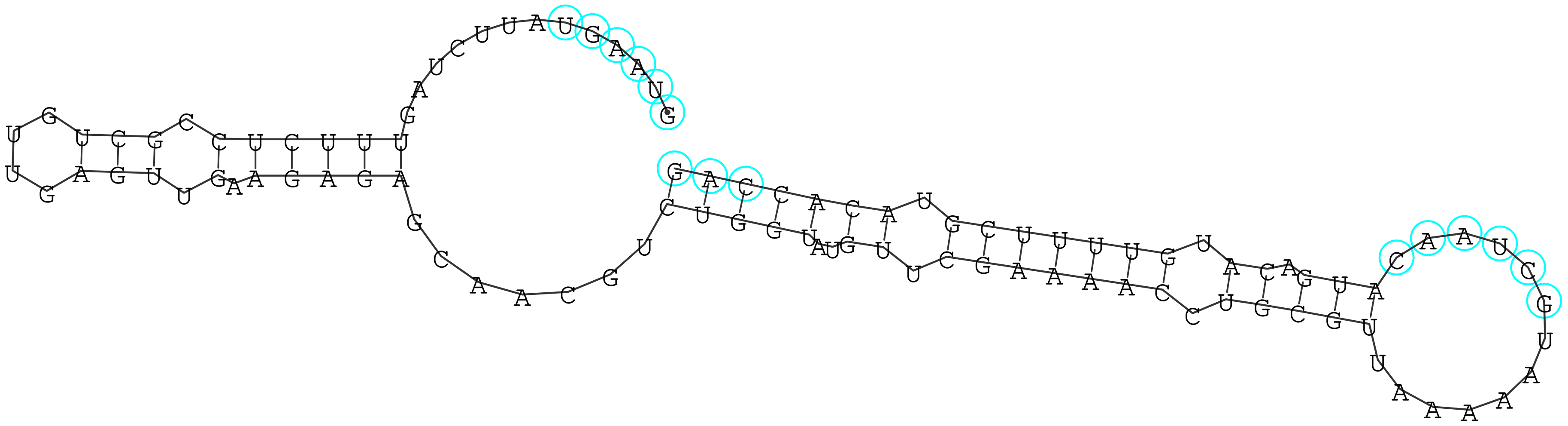

Hruc55A - External intron

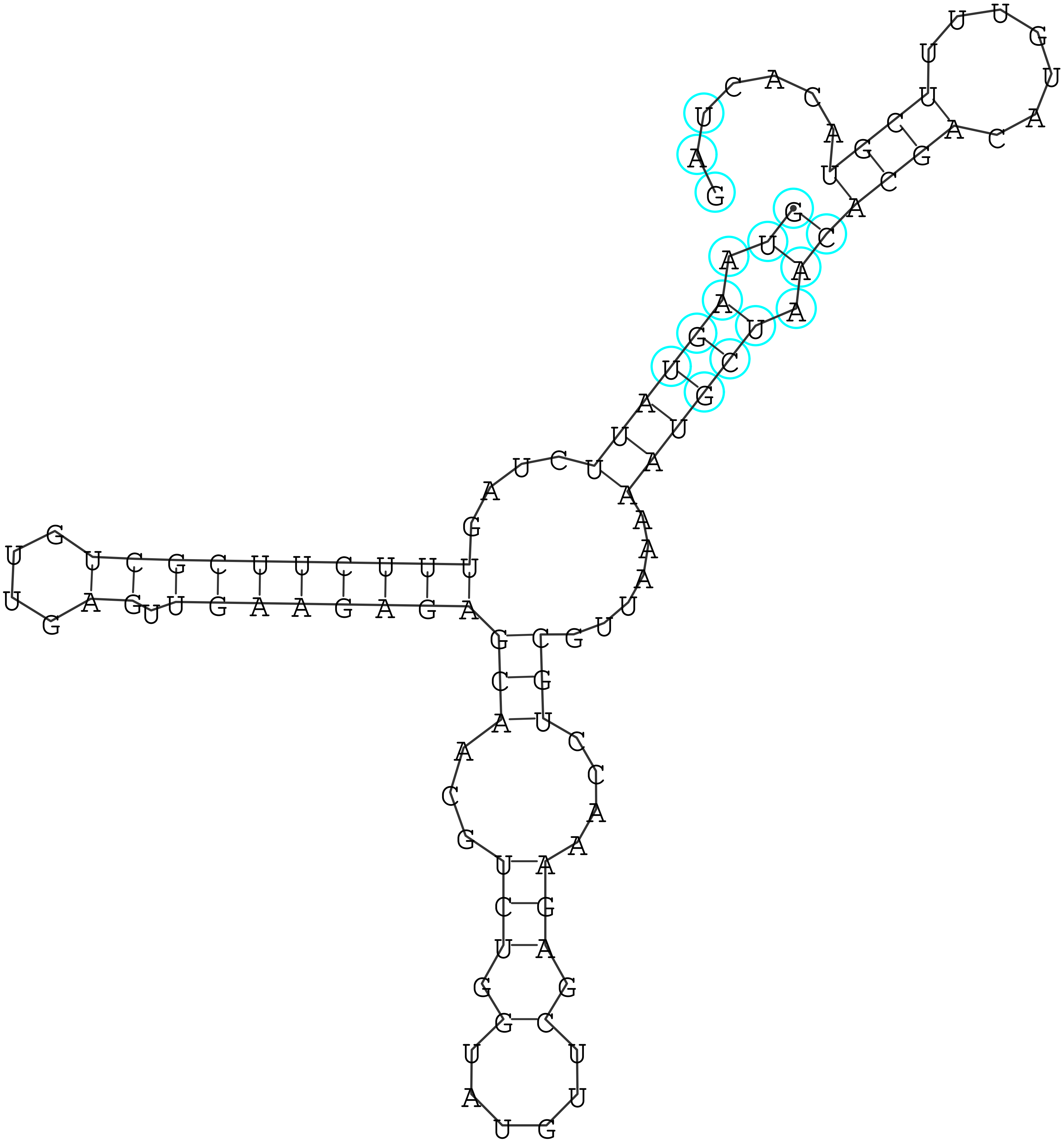

Hruc56A - External intron

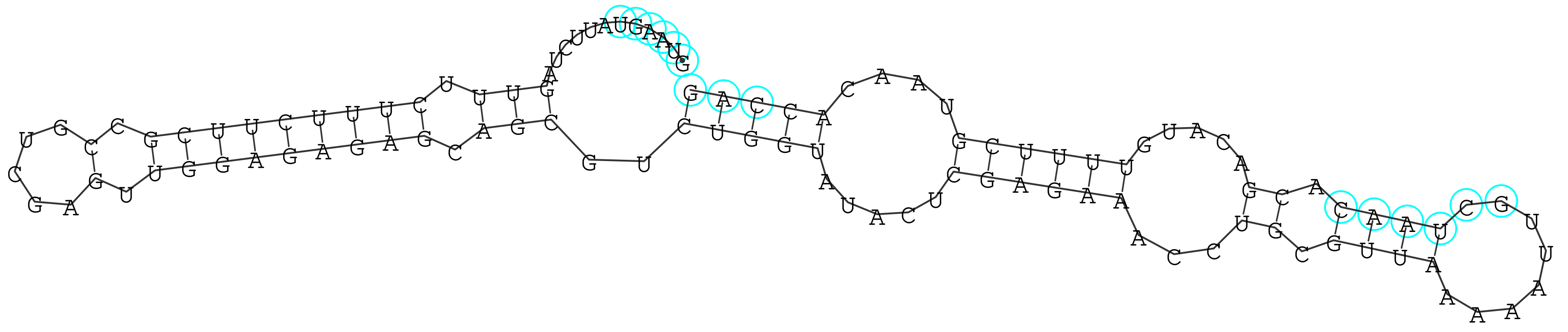

Hruc59A - External intron

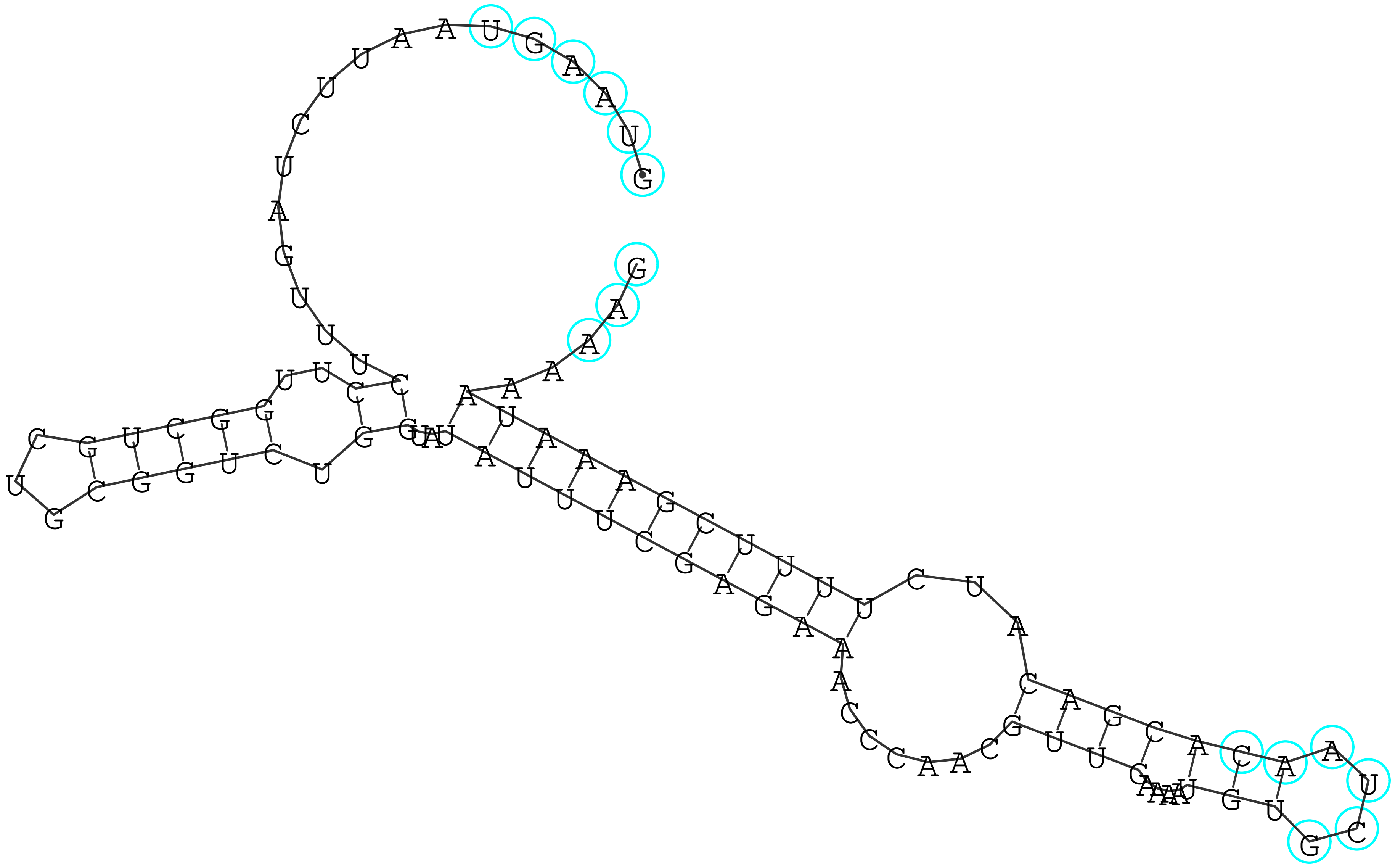

Naboc005A - External intron

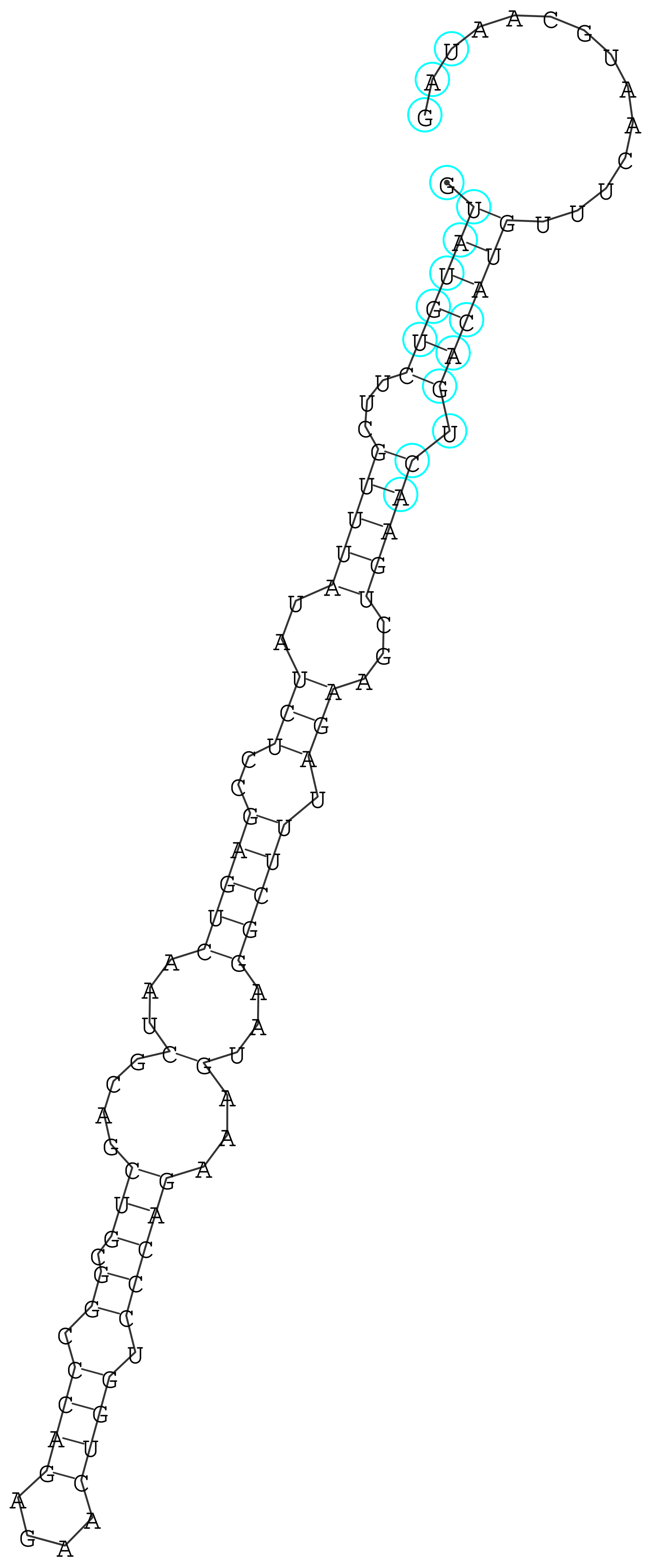

Naboc011A - External intron

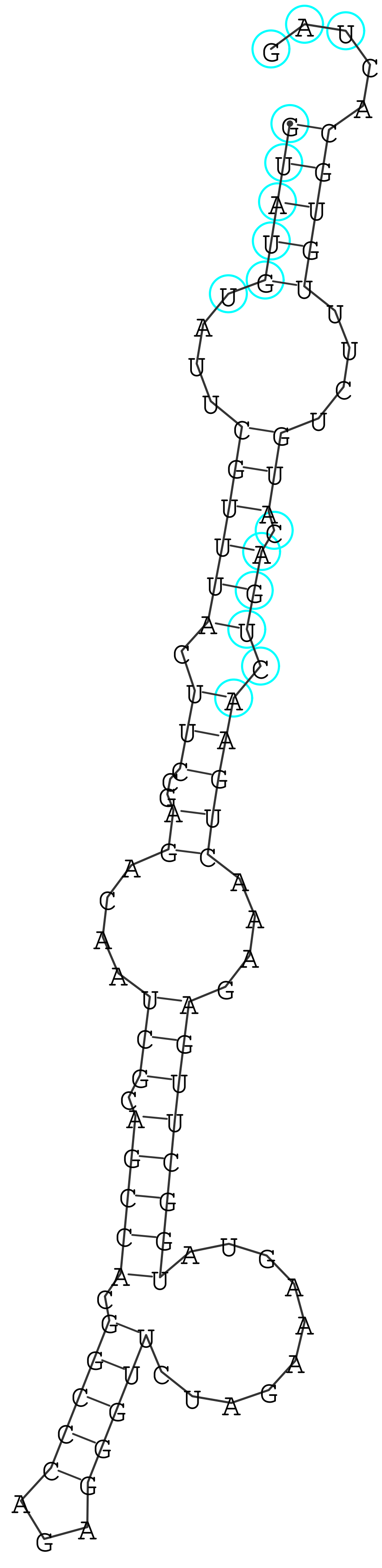

Naboc037A - External intron

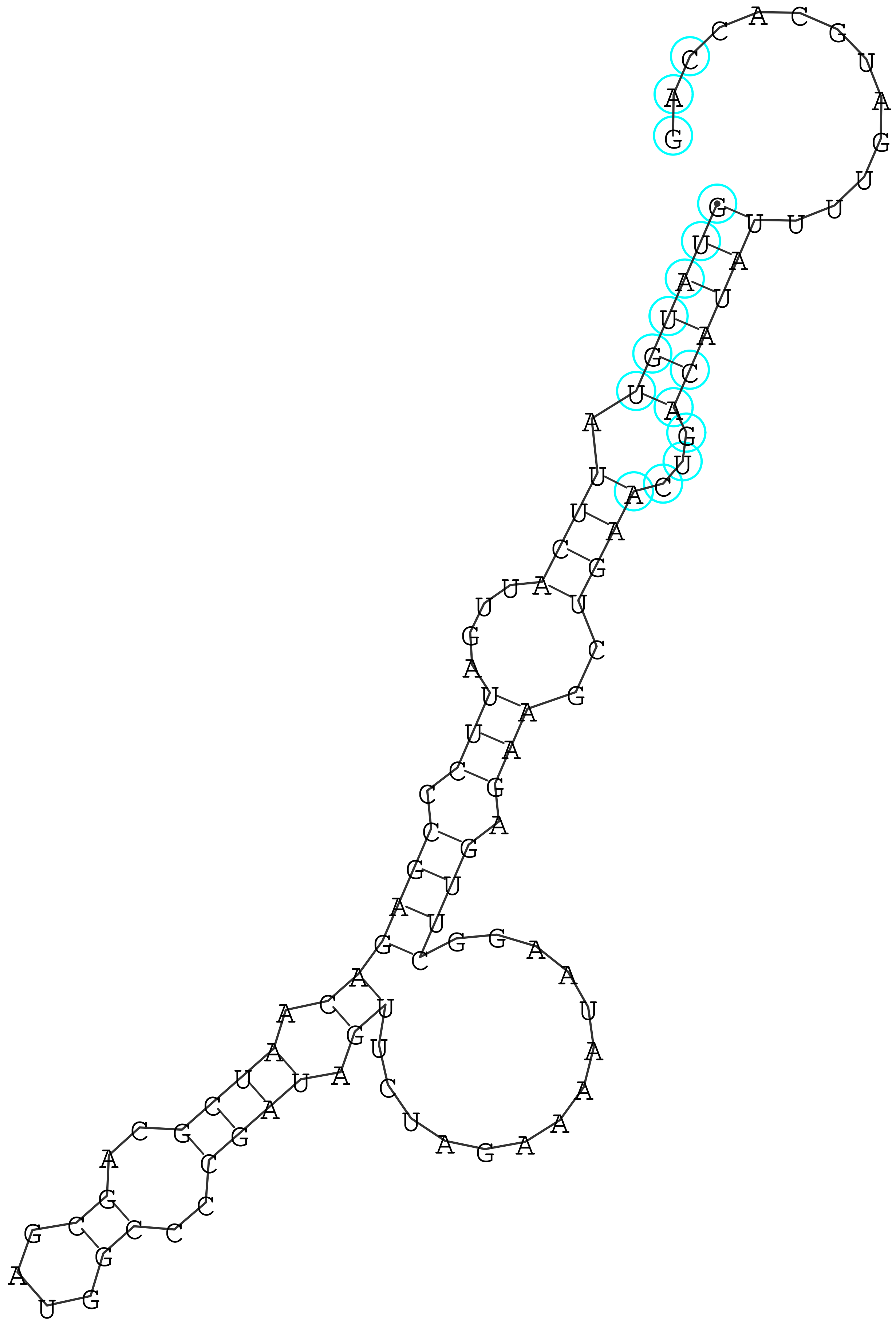

Naboc037B - External intron

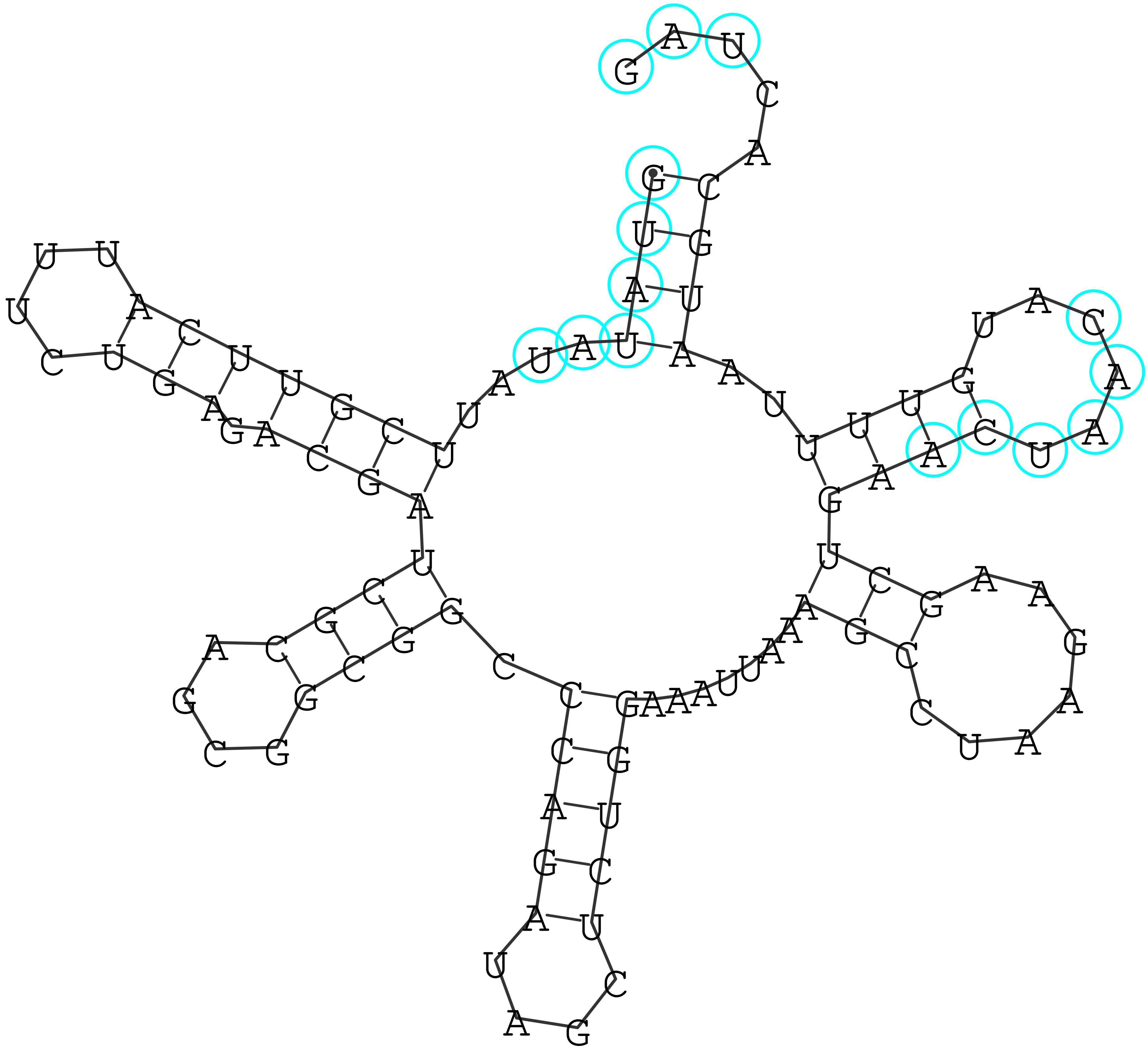

Naboc056A - External intron

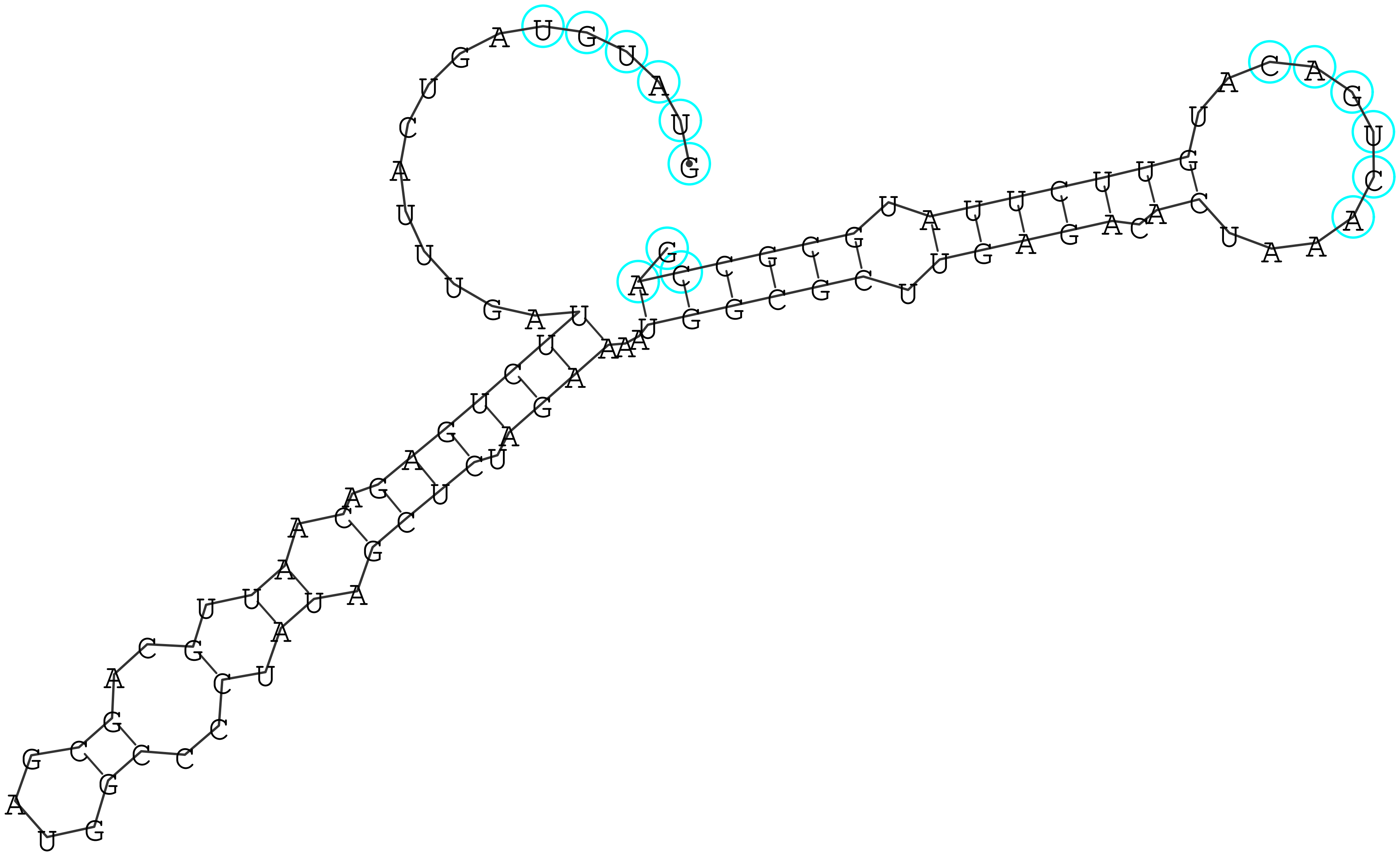

Naboc066A - External intron

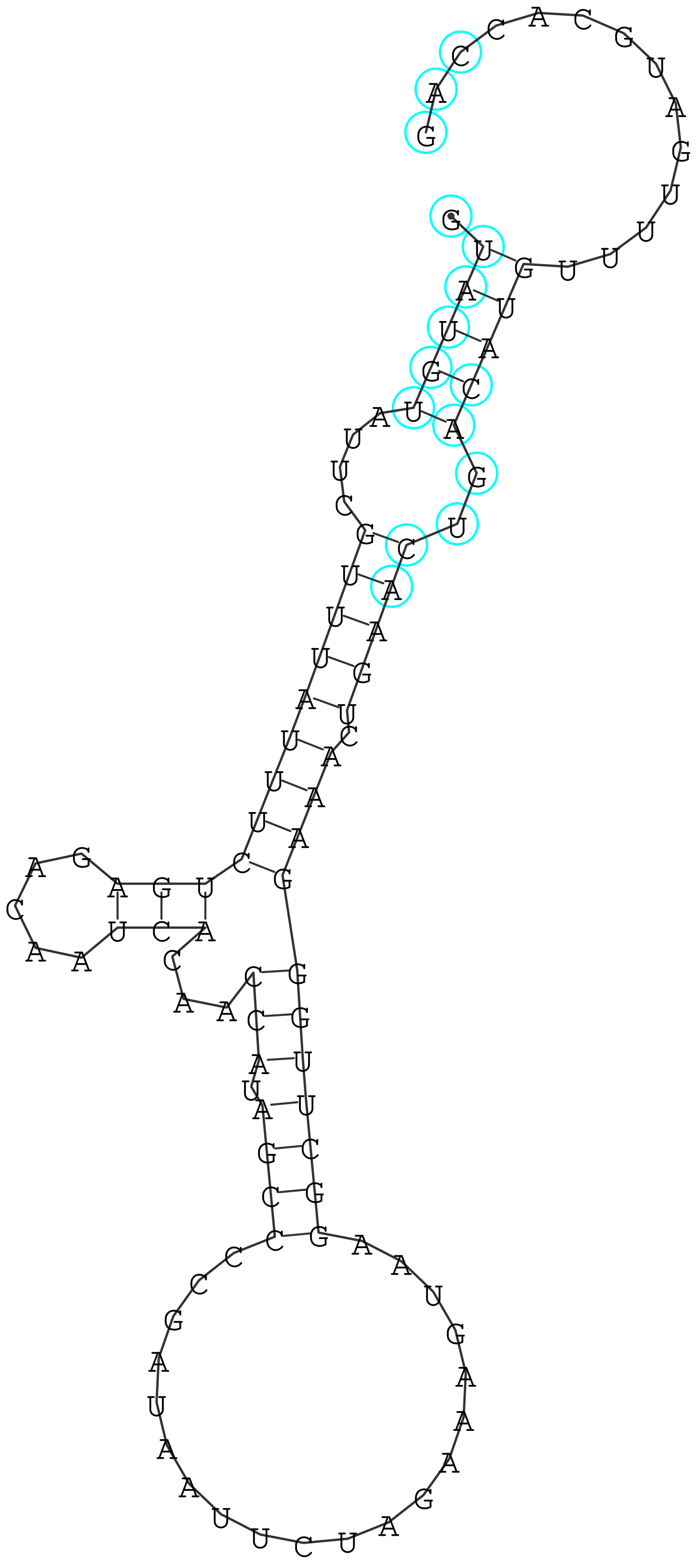

Naboc073A - External intron

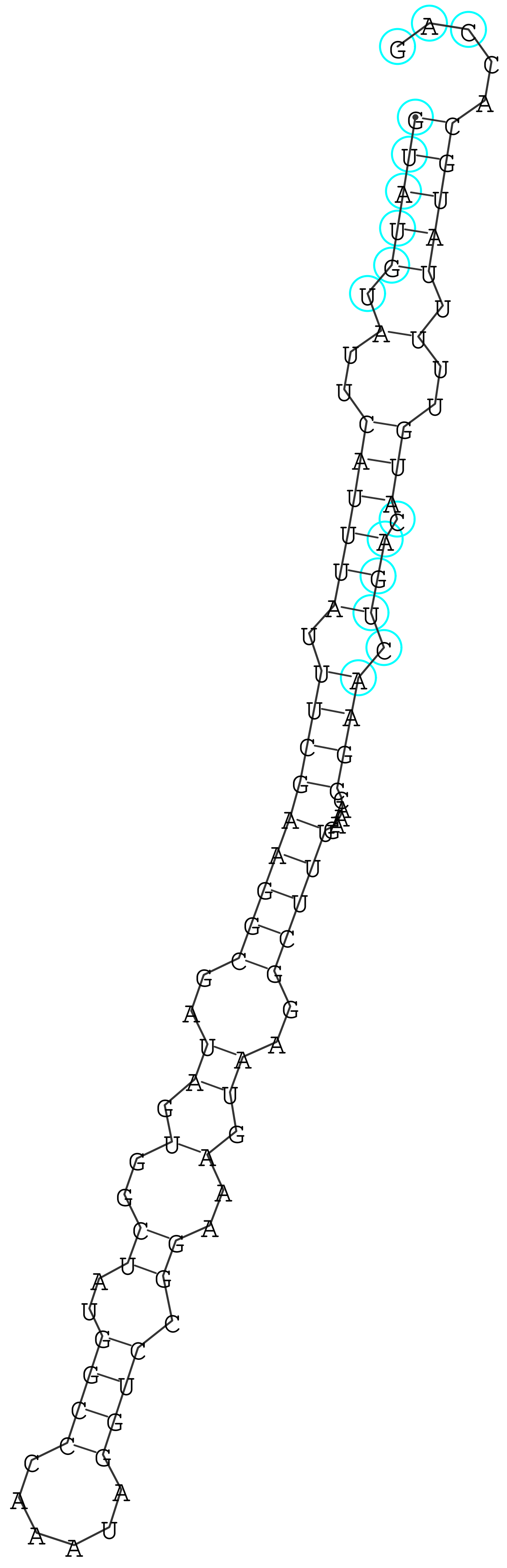

Naboc079A - External intron

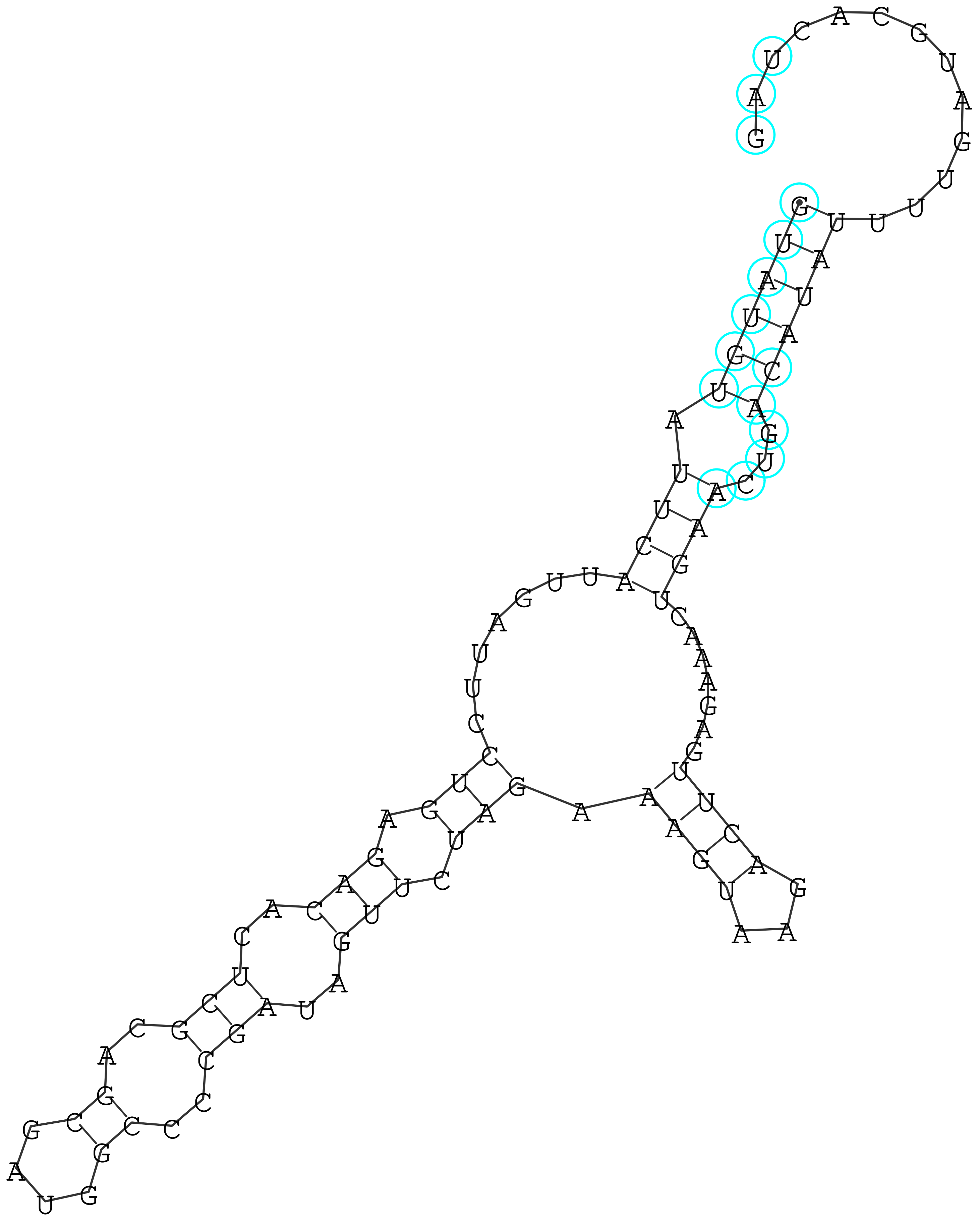

Naboc124A - External intron

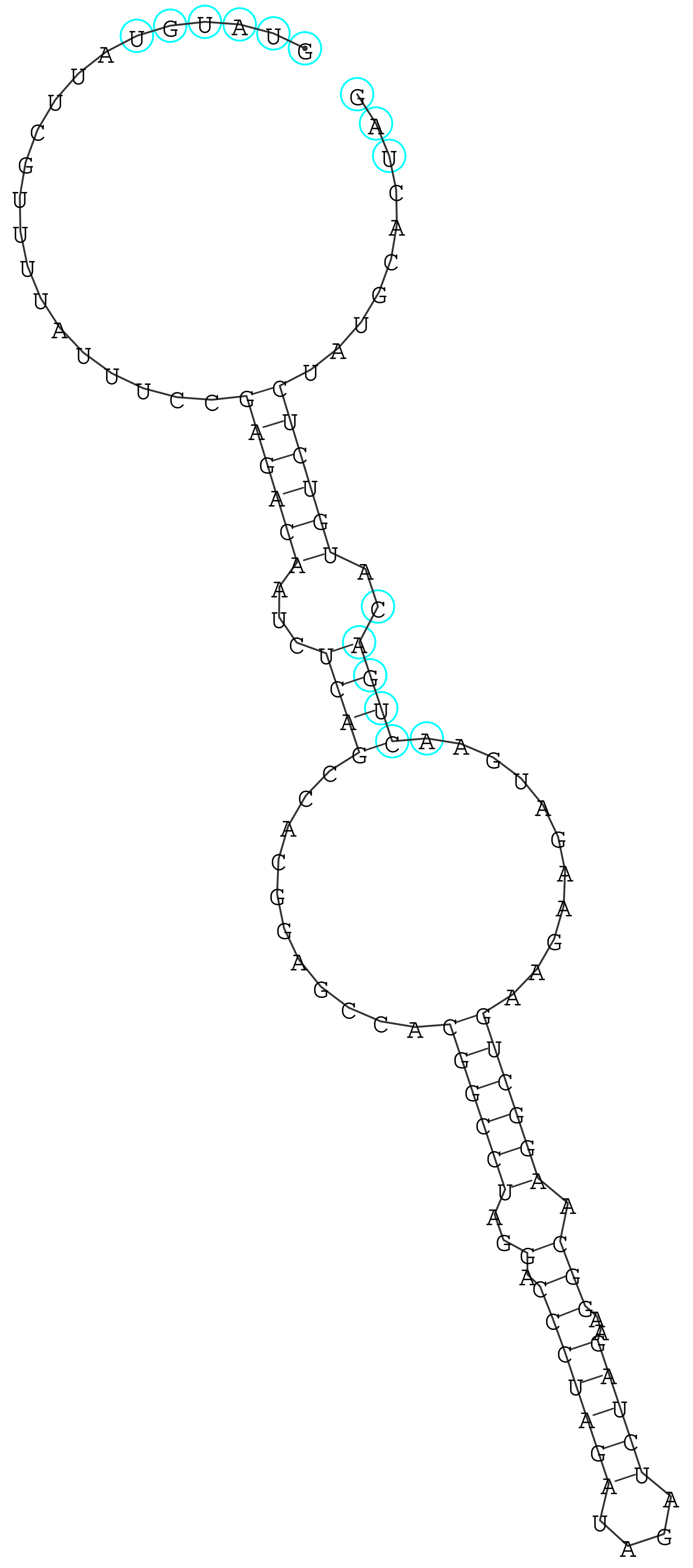

Naboc173A - External intron

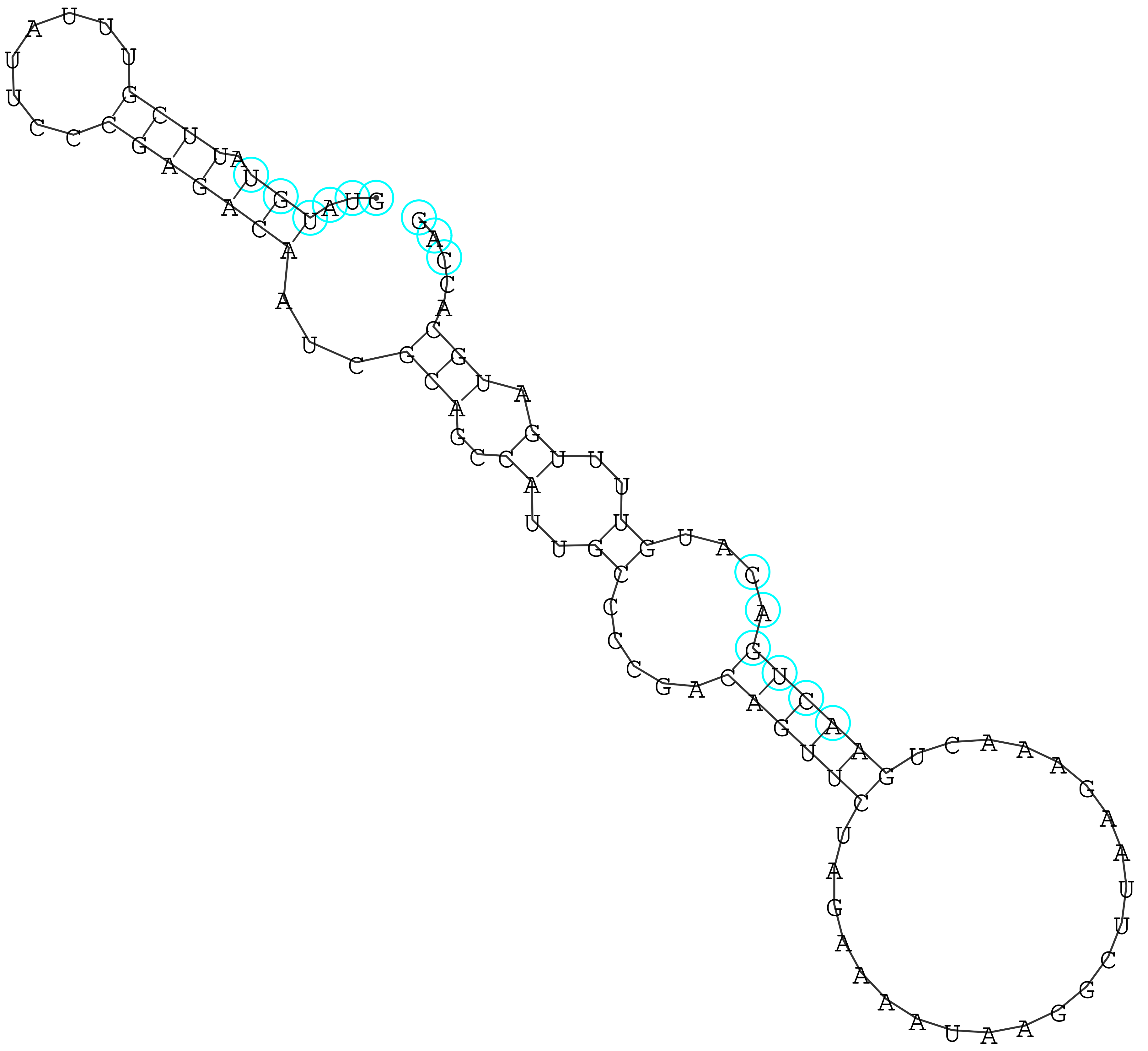

Naboc184A - External intron

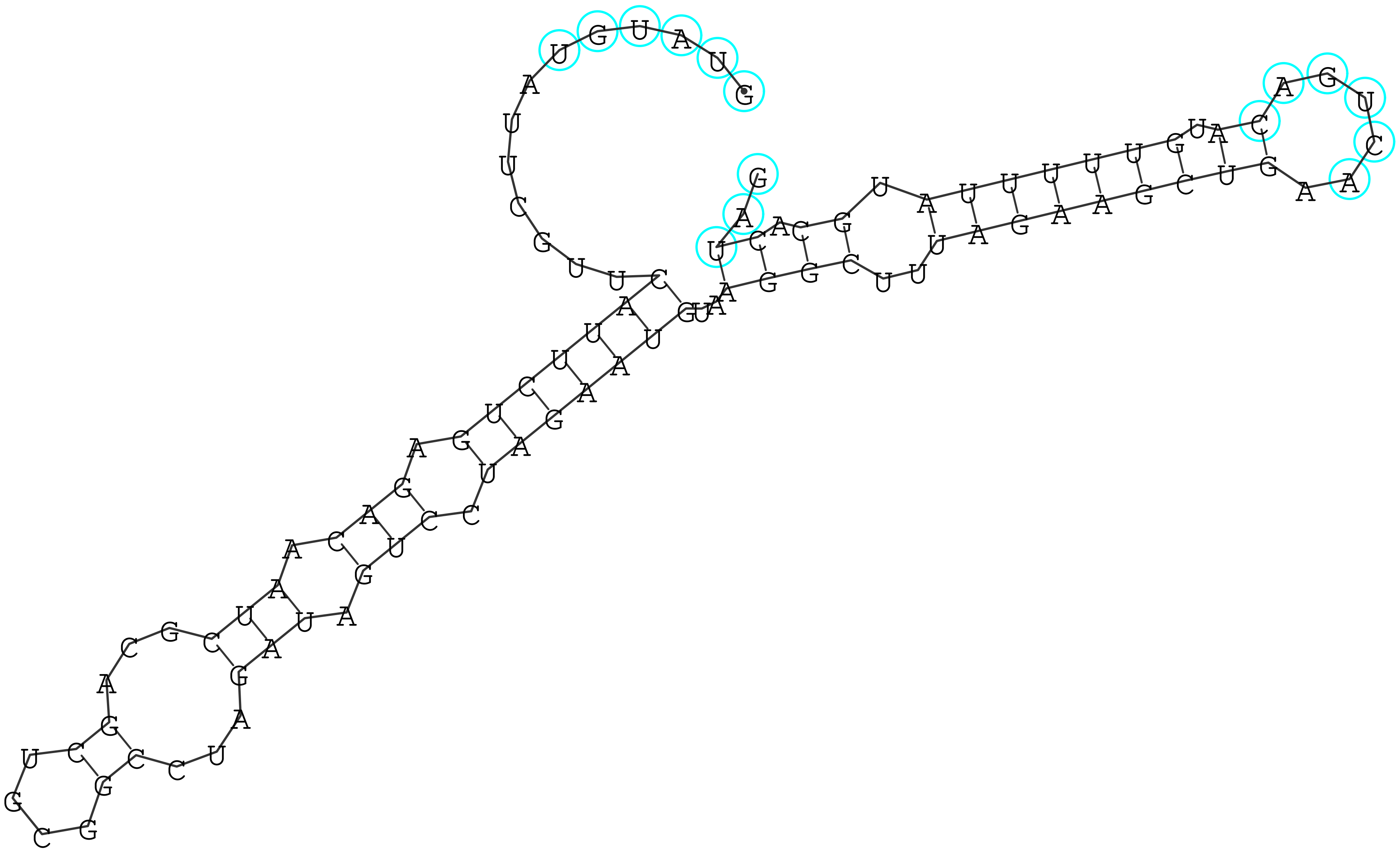

Naboc196A - External intron

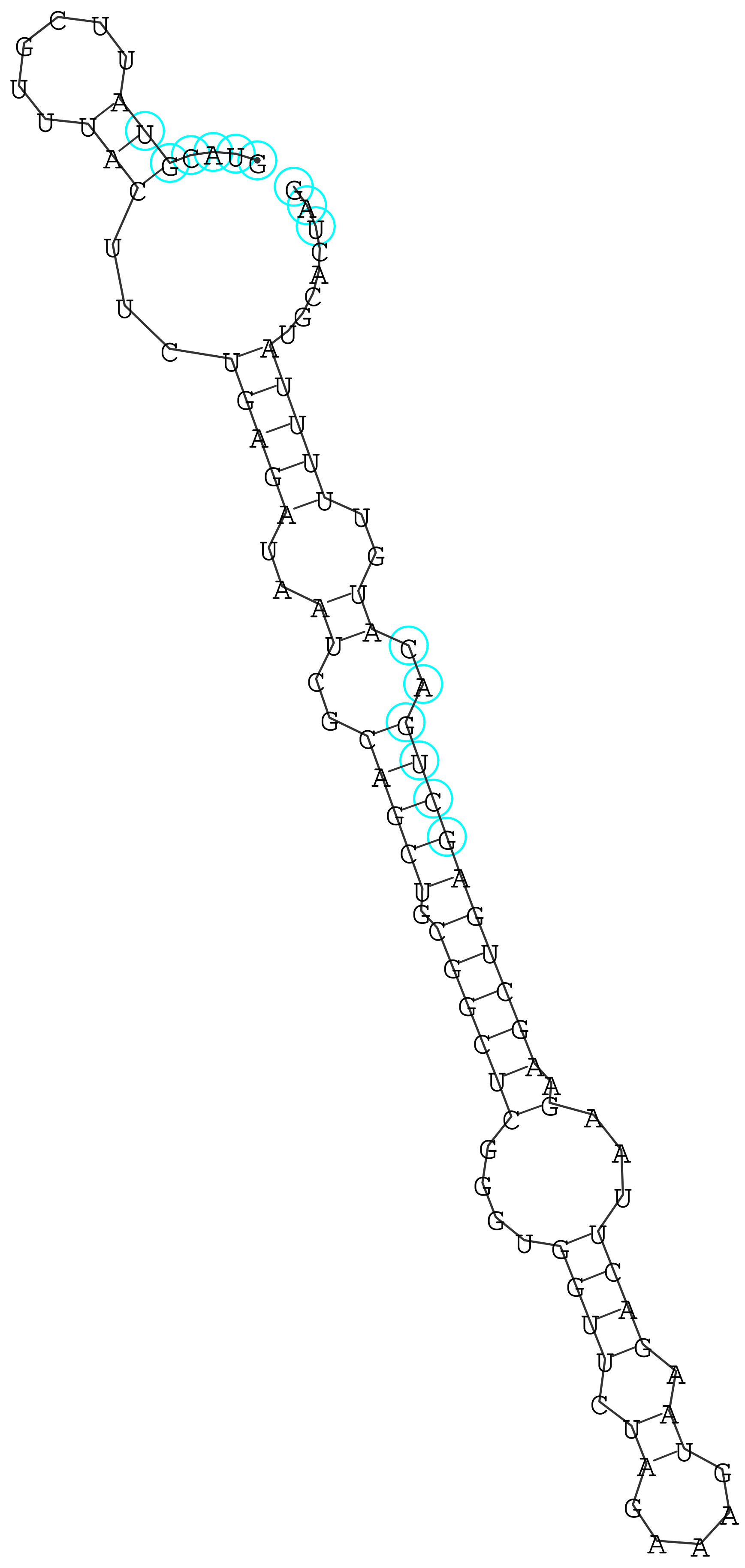

Naboc196B - External intron

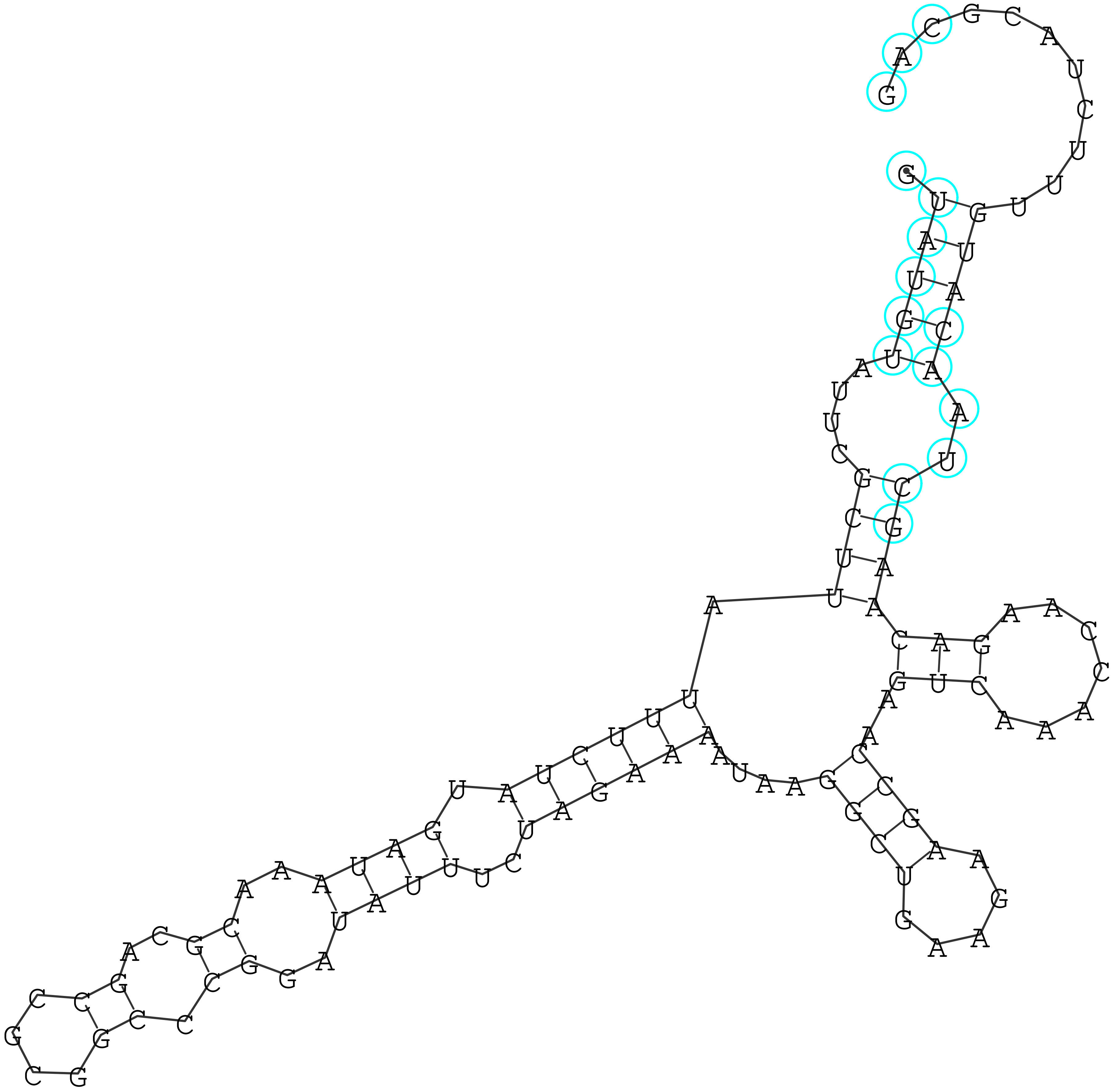

Naboc199A - External intron

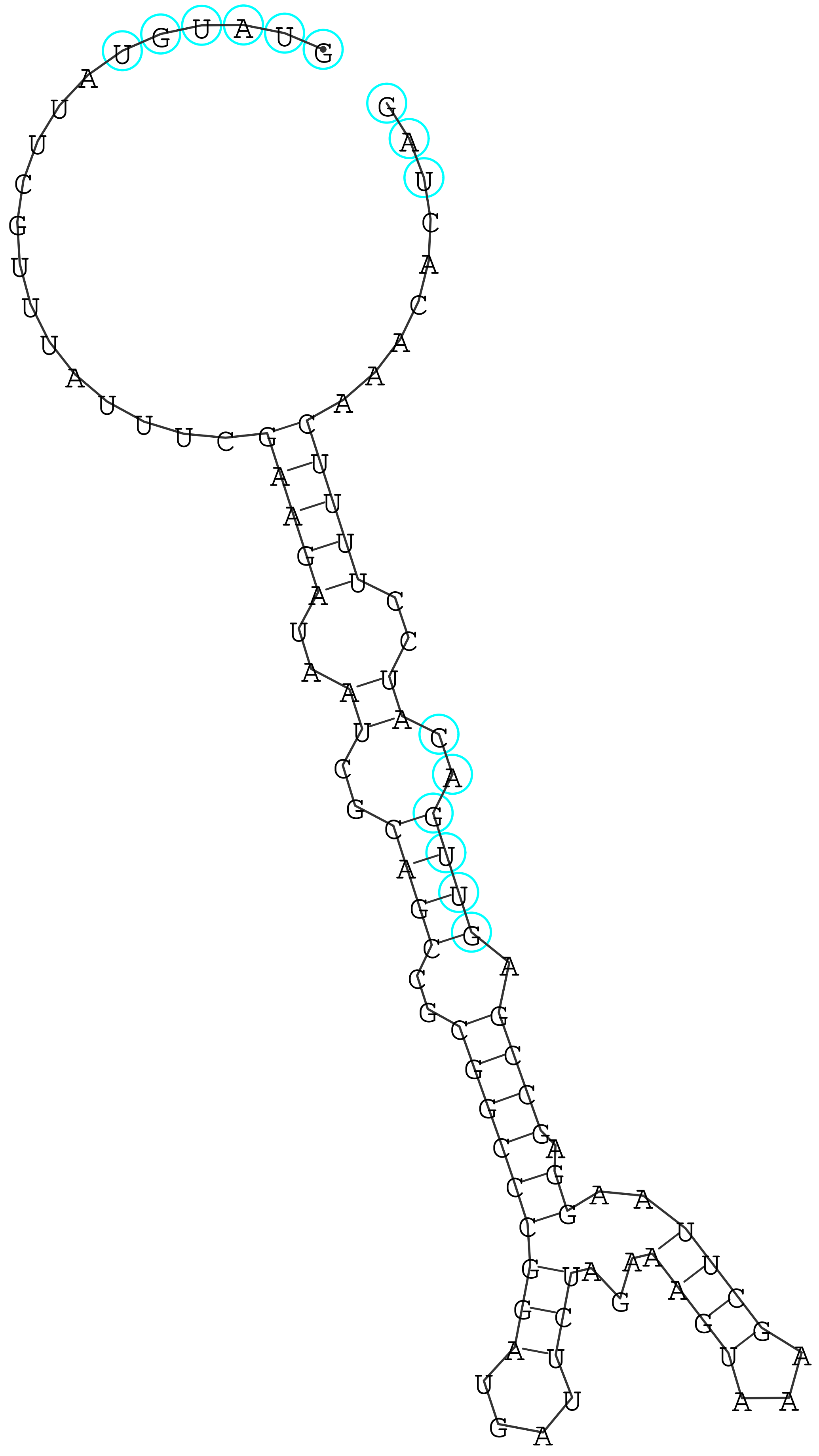

Naboc199B - External intron

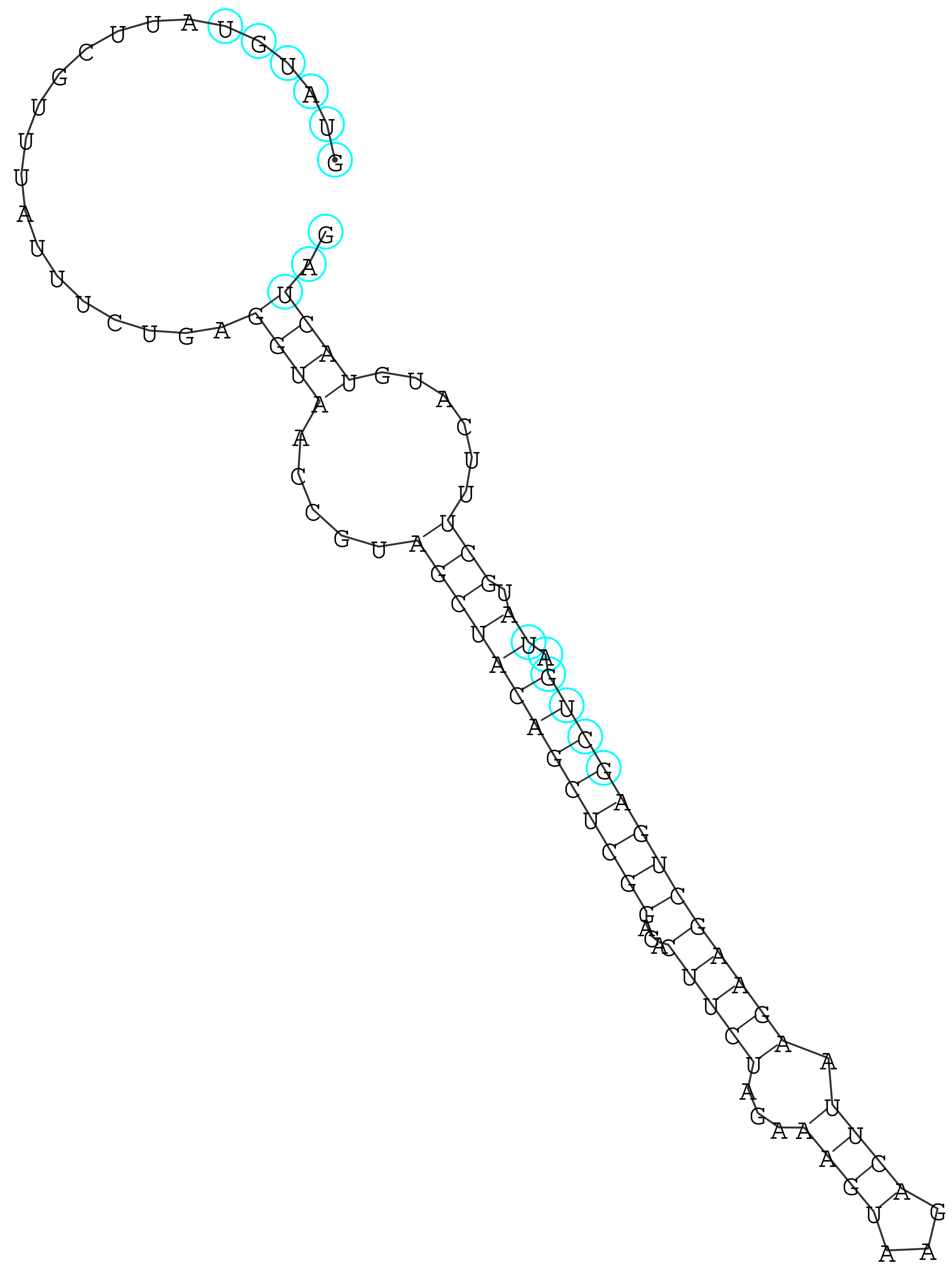

Naboc202A - External intron

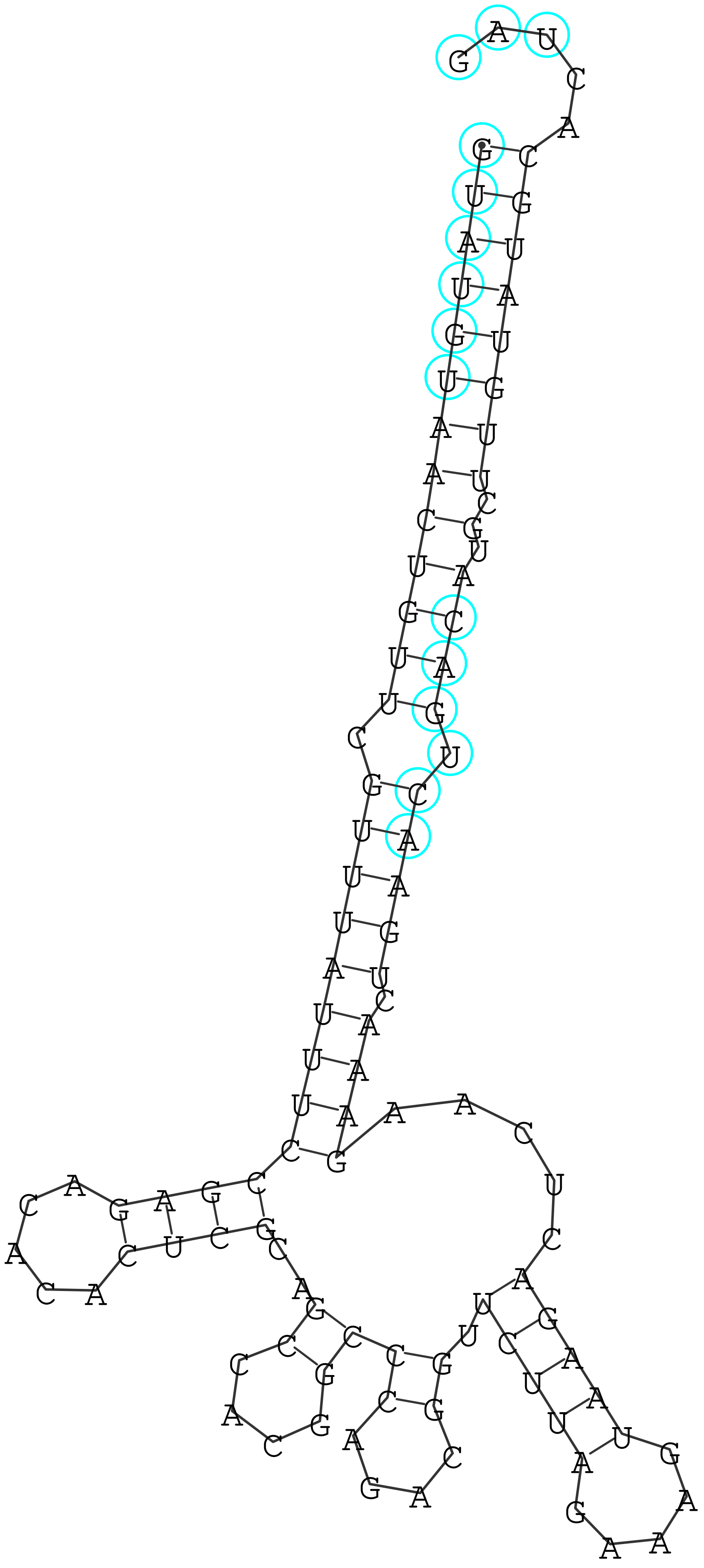

Naboc249A - External intron

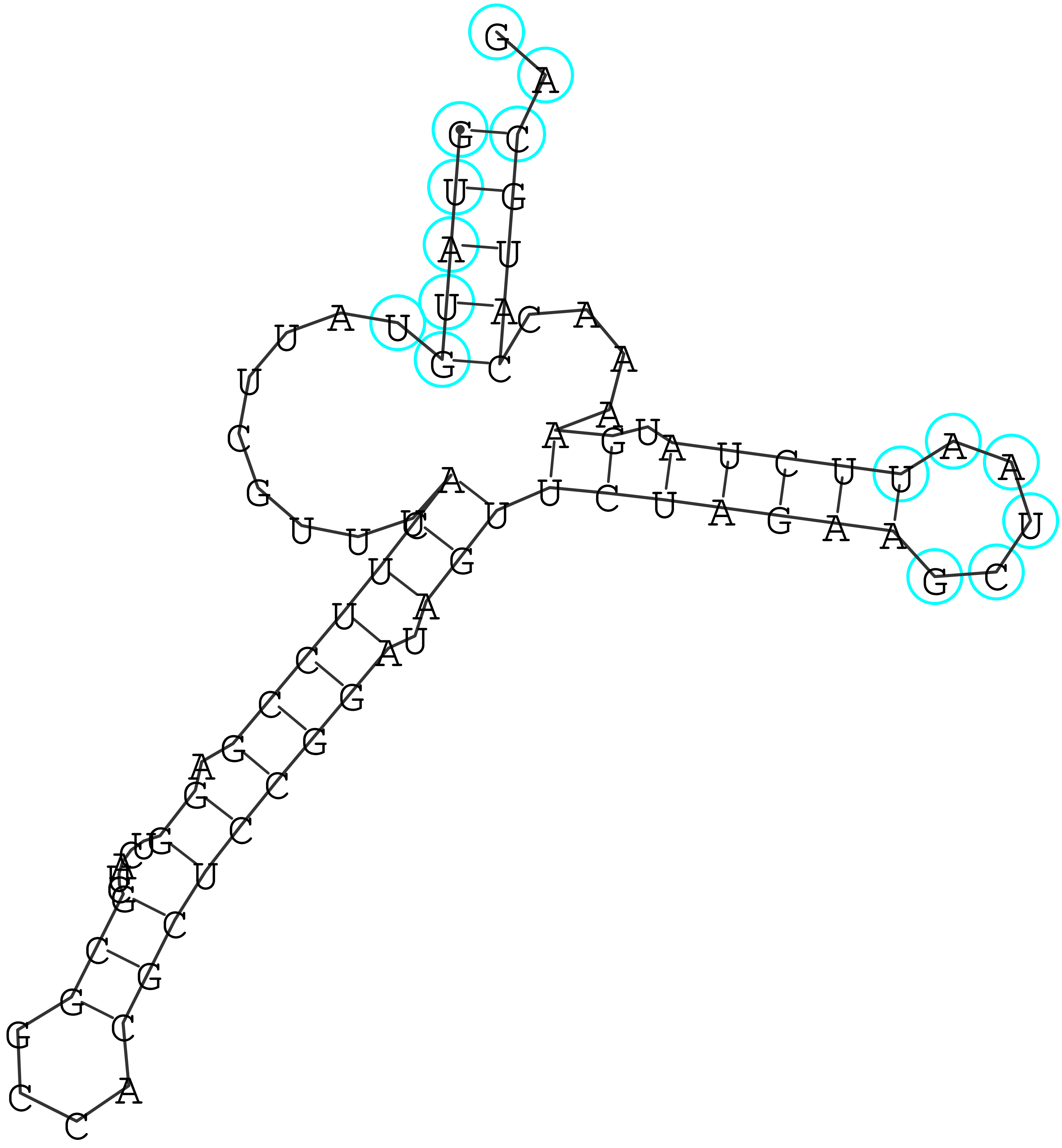

Naboc268A - External intron

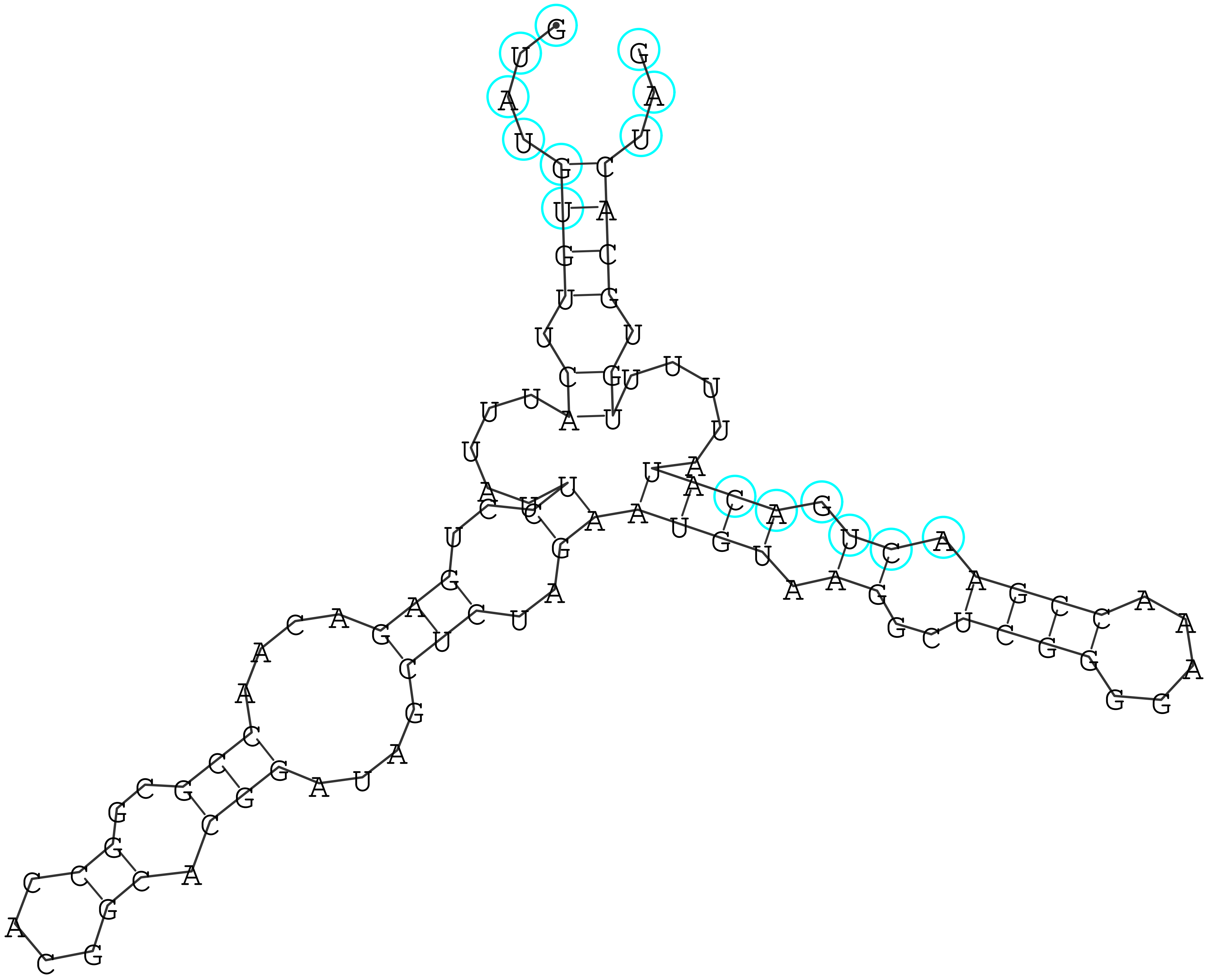

Naboc268B - External intron

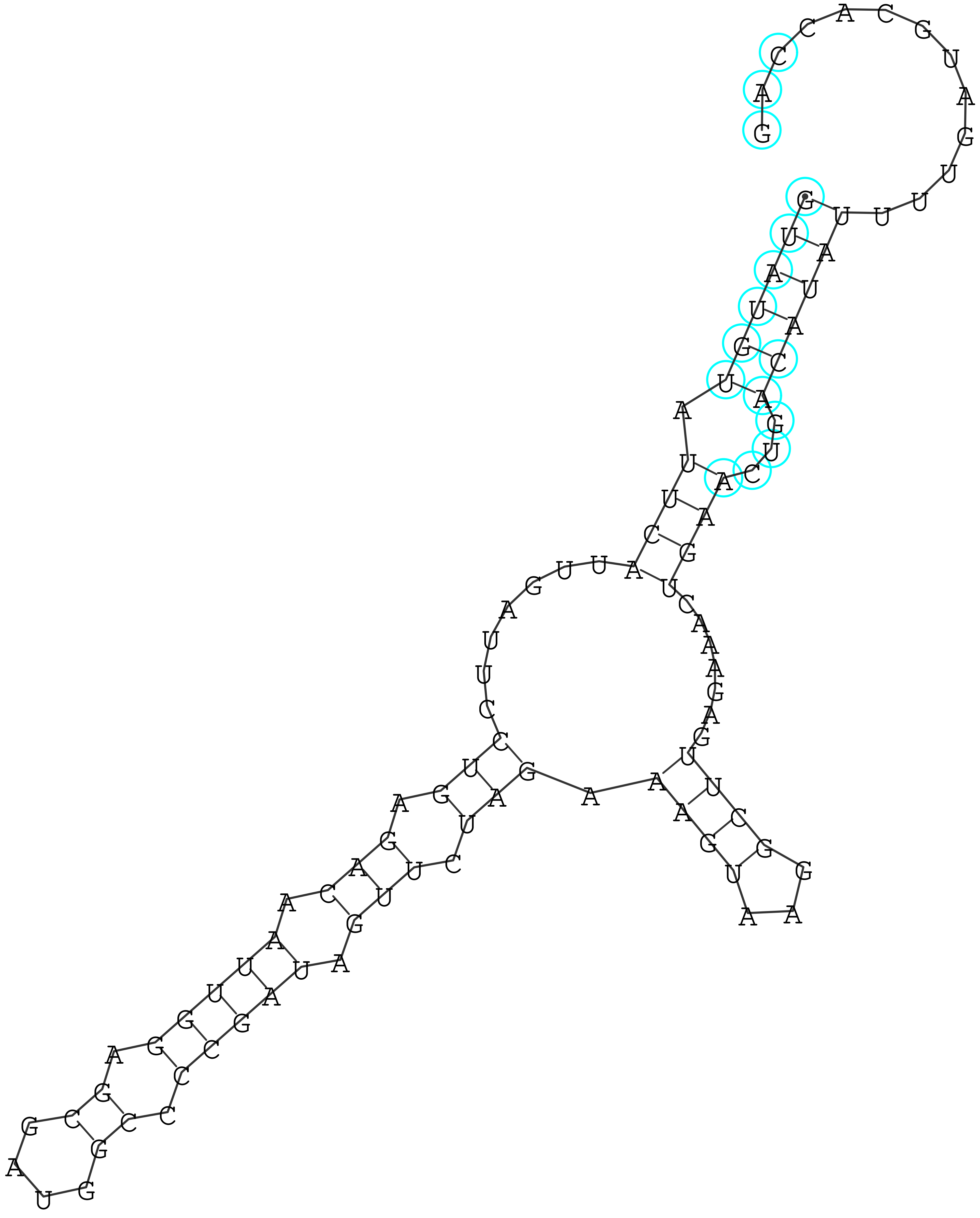

Naboc285A - External intron

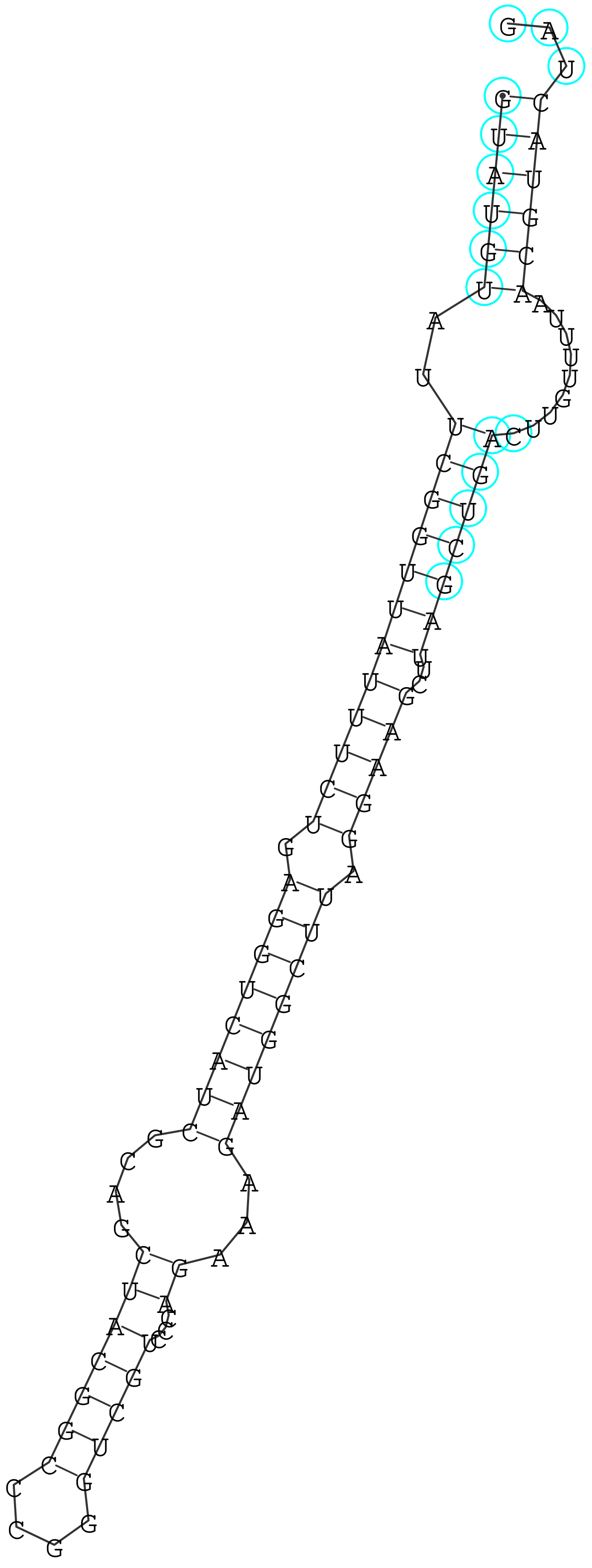

Naboc289A - External intron

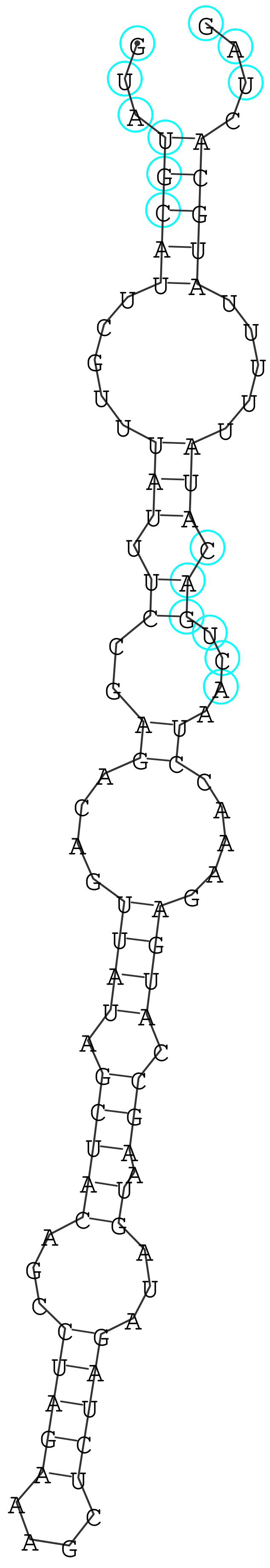

Naboc294A - External intron

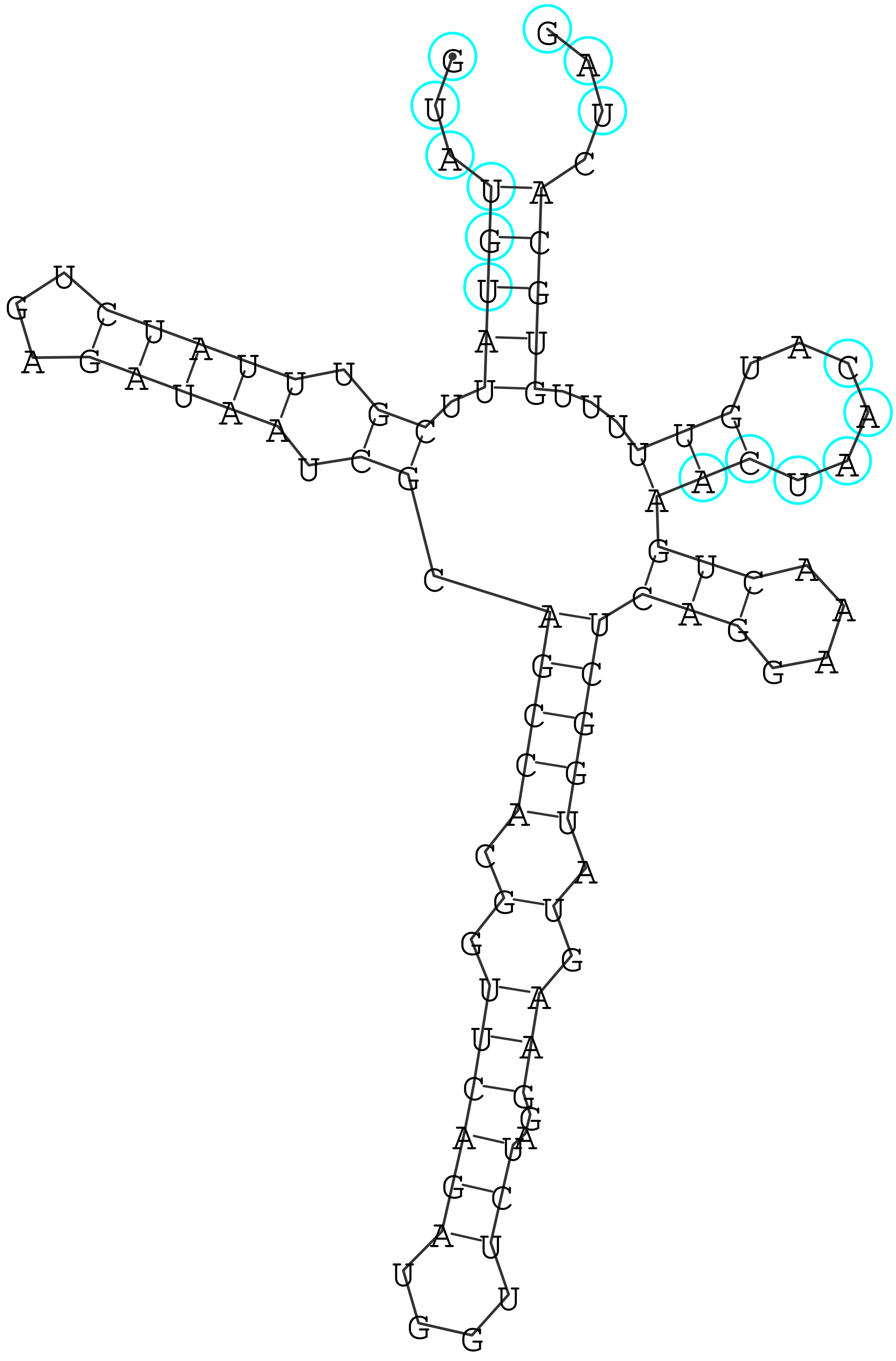

Naboc300A - External intron

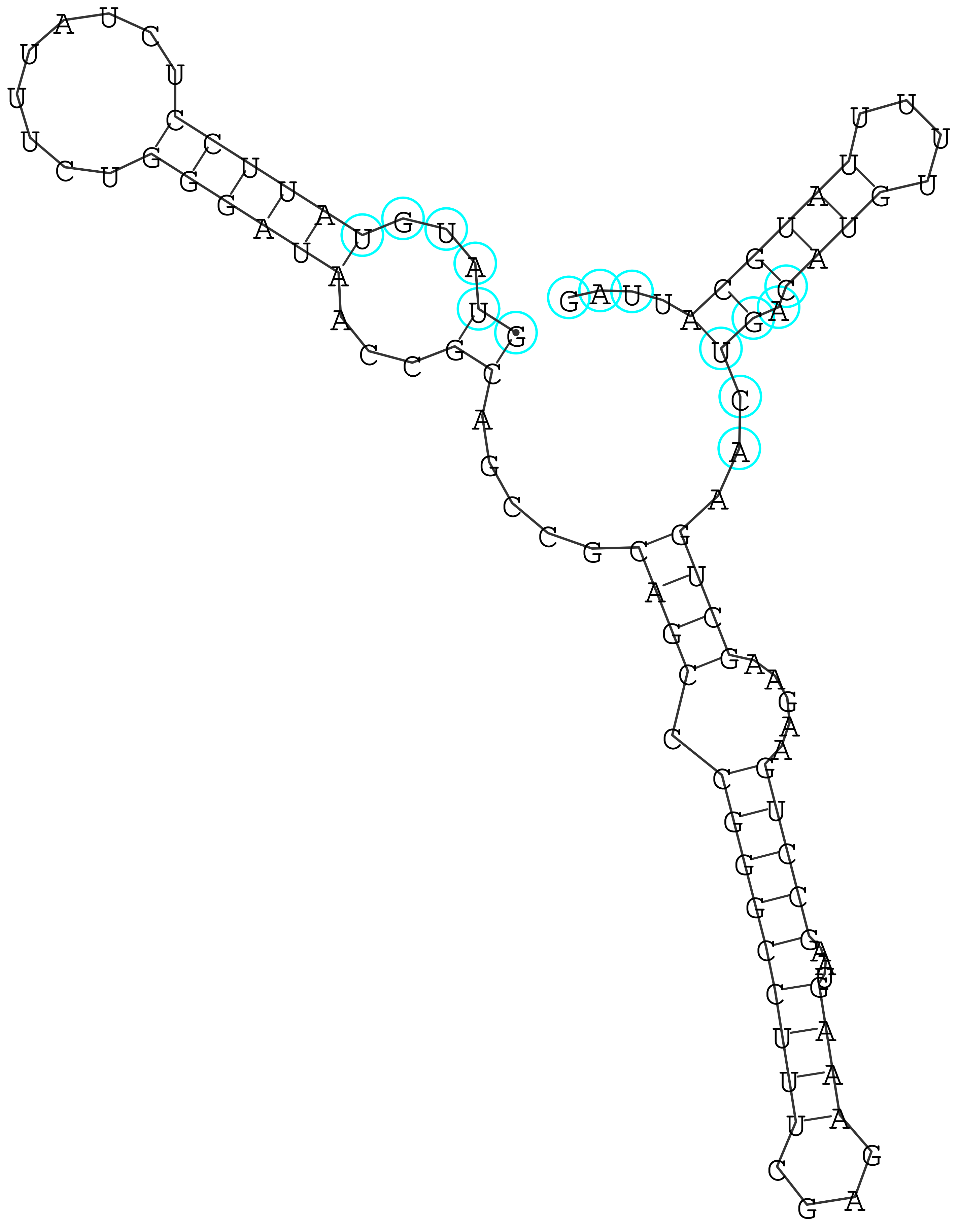

Naboc349A - External intron

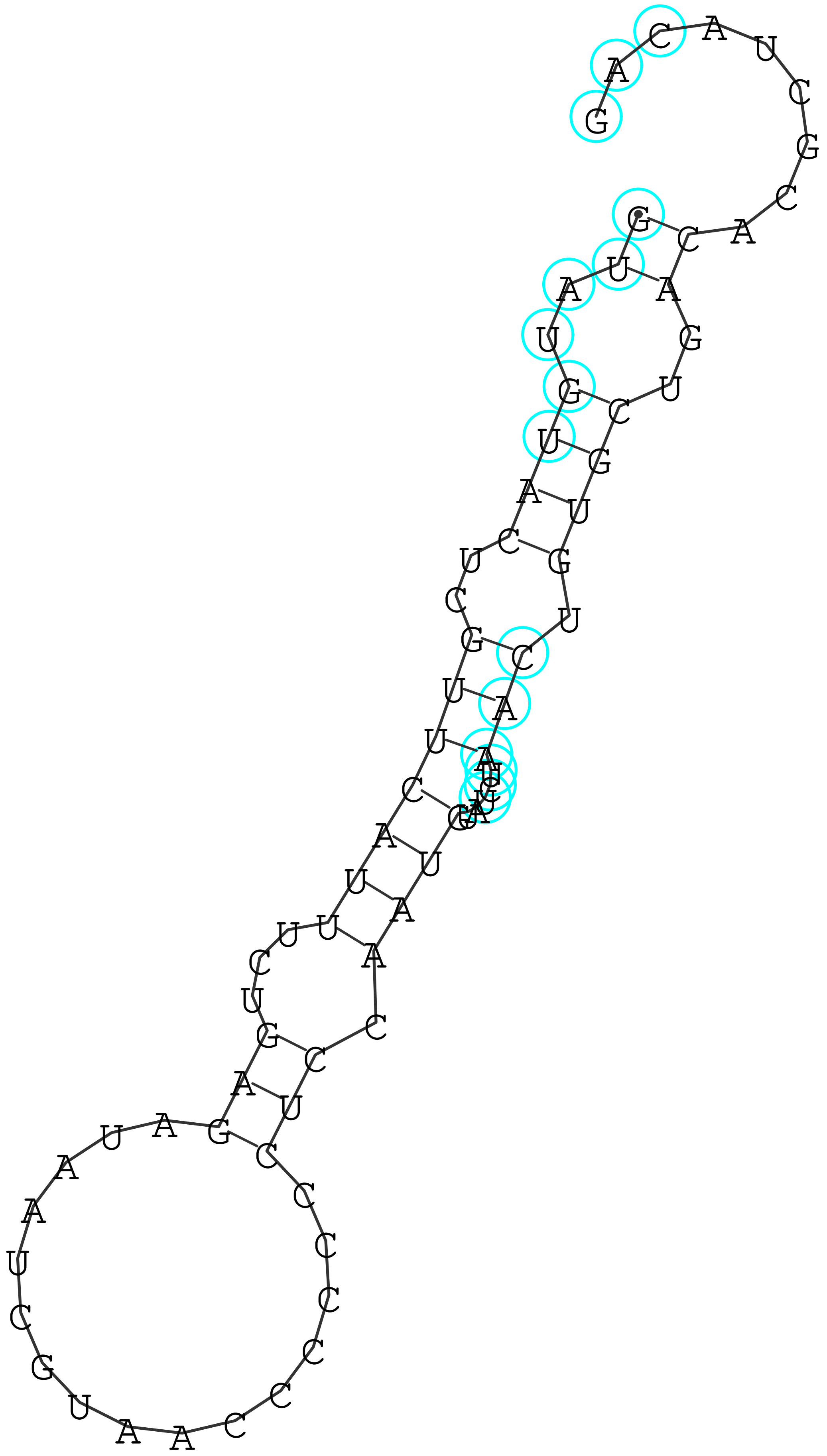

Naboc414A - External intron

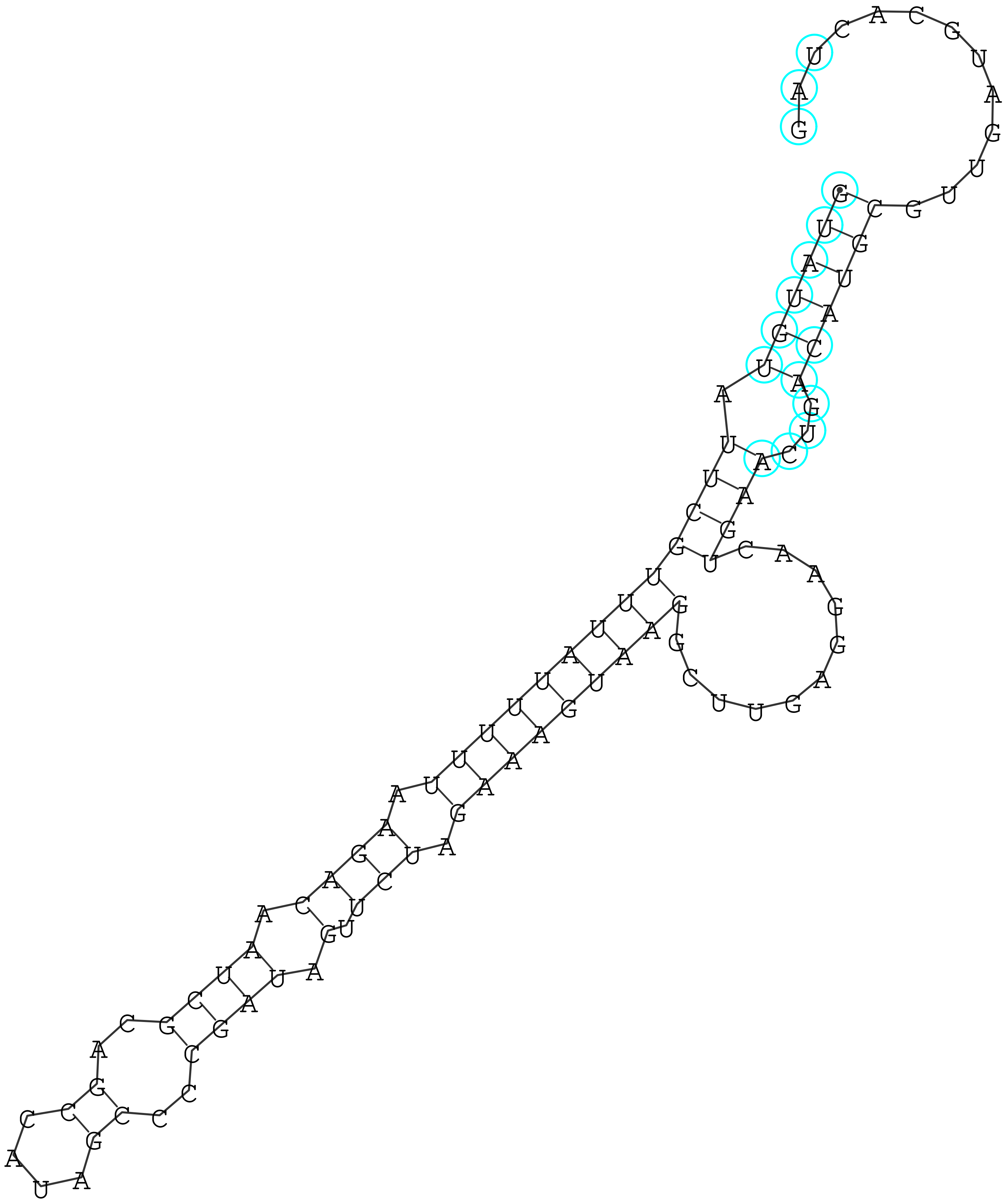

X1651c009A - External intron

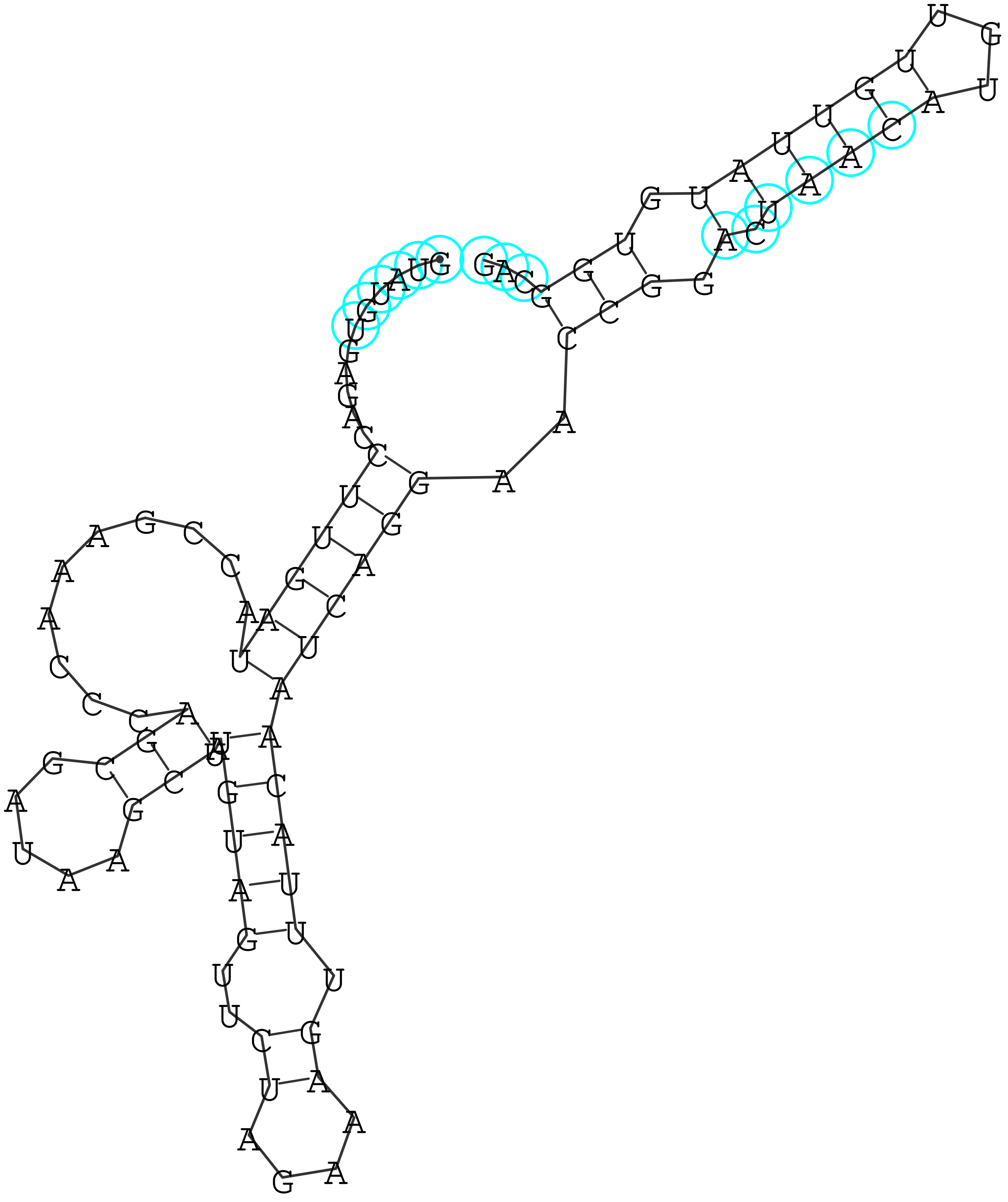

X1651c011A - External intron

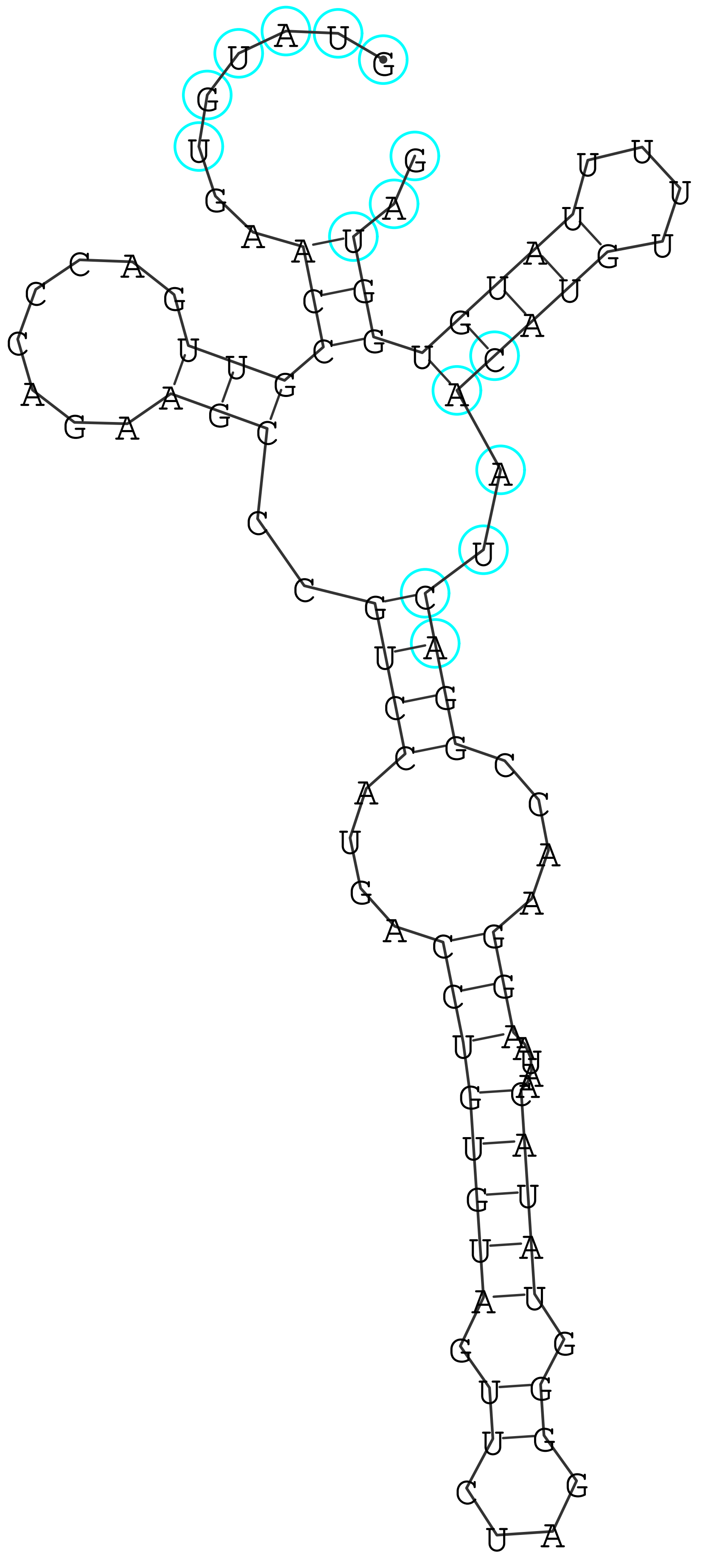

X1651c016A - External intron

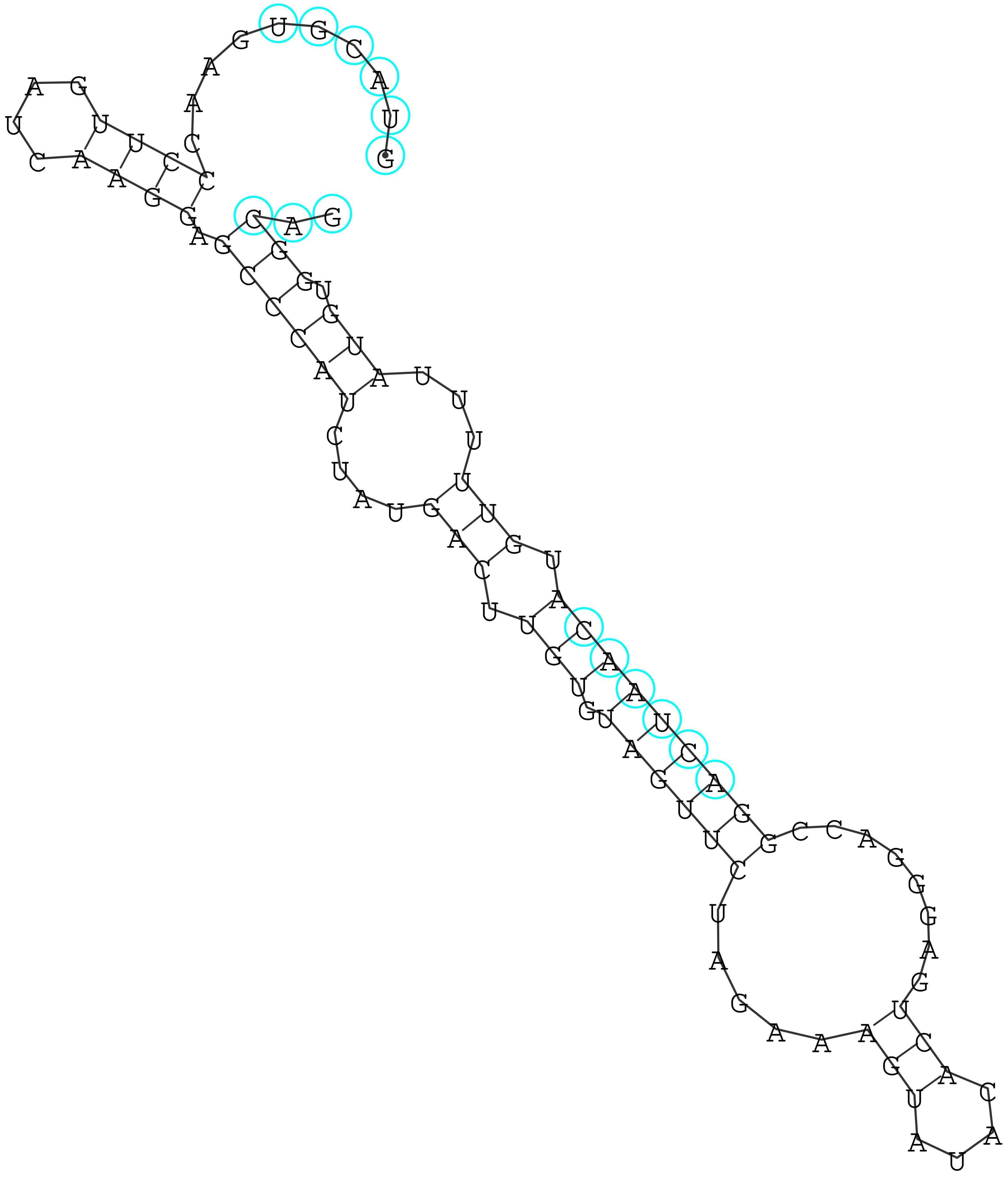

X1651c025A - External intron

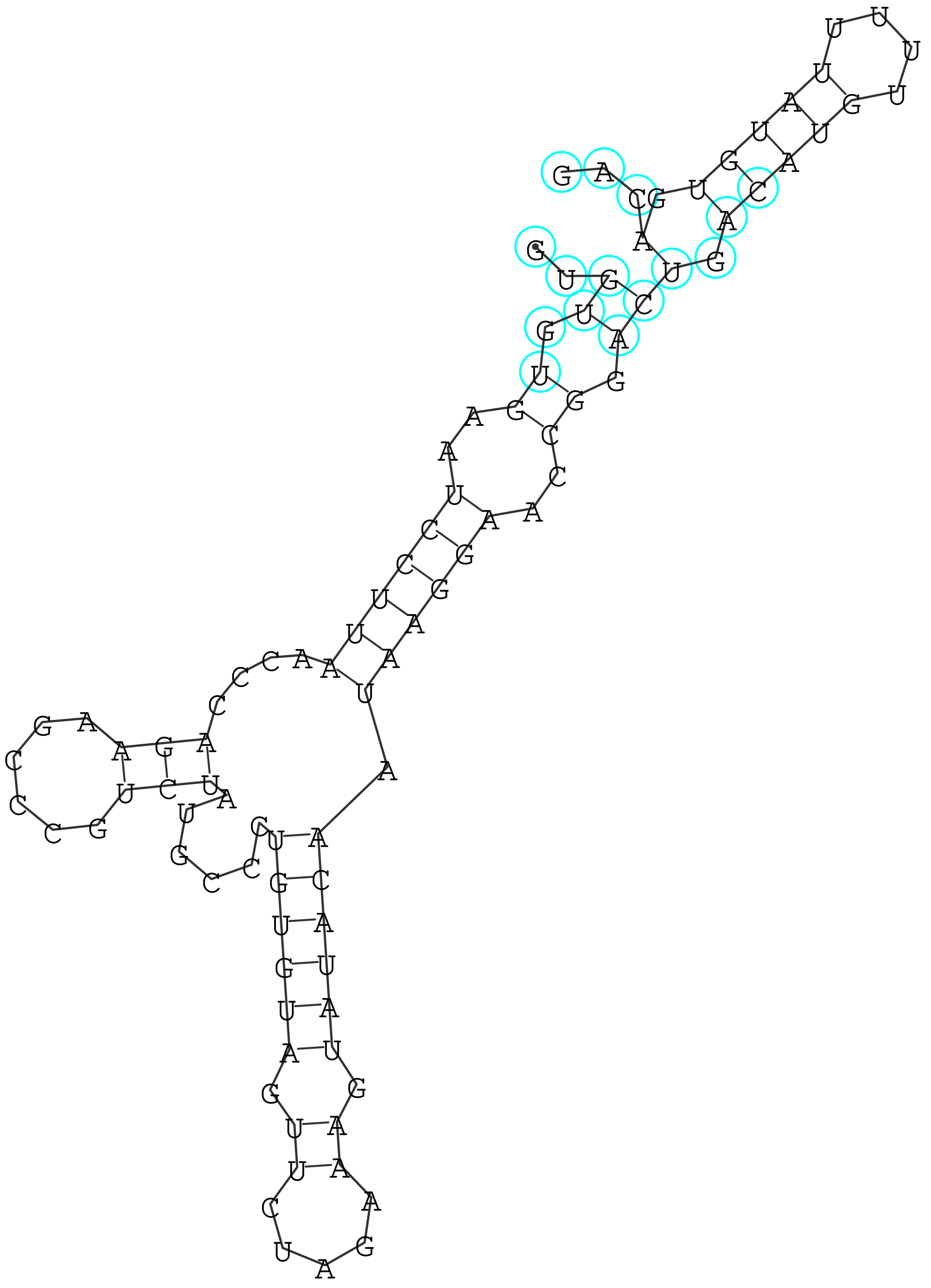

X1651c036A - External intron

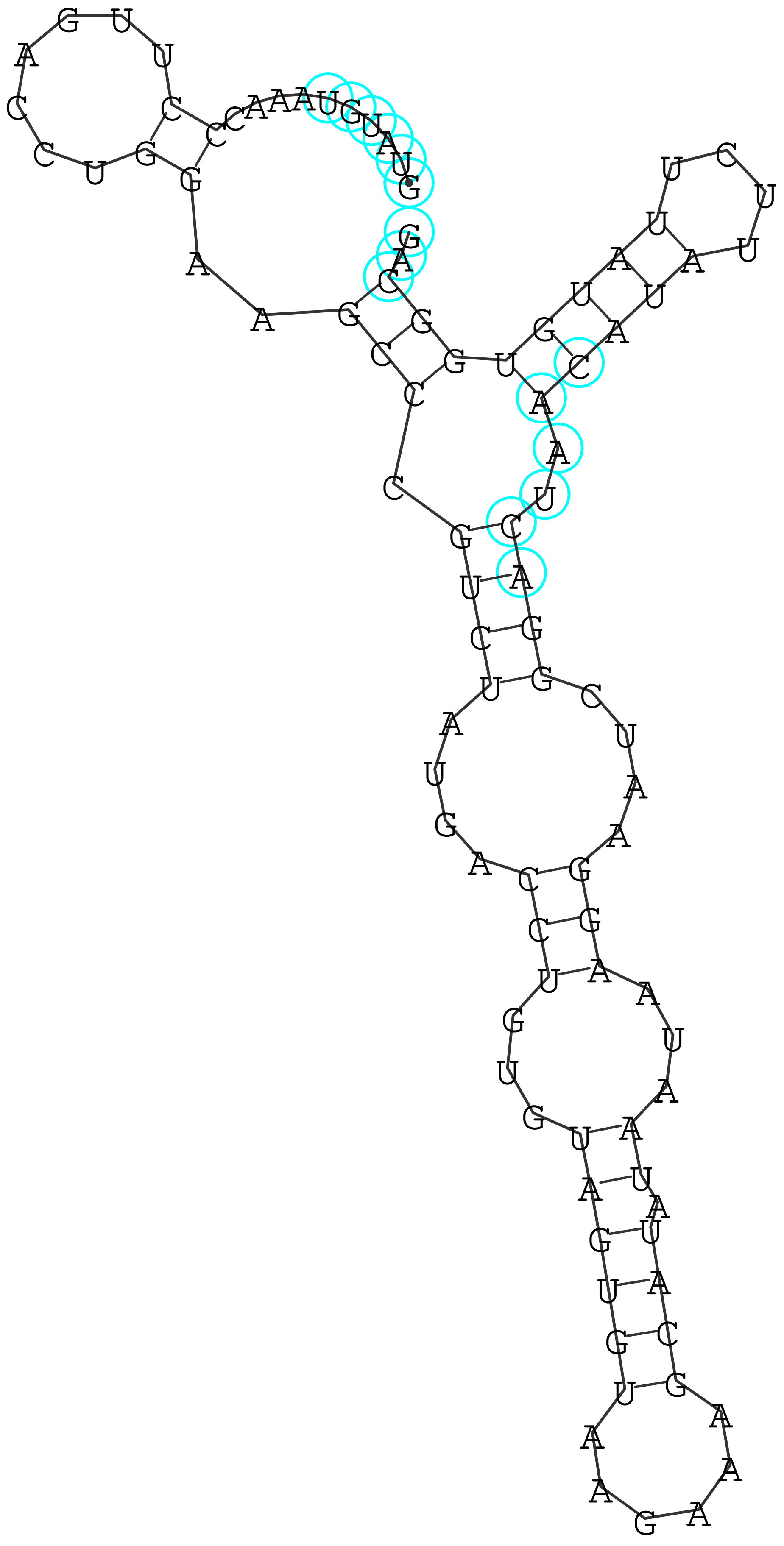

X1651c075A - External intron

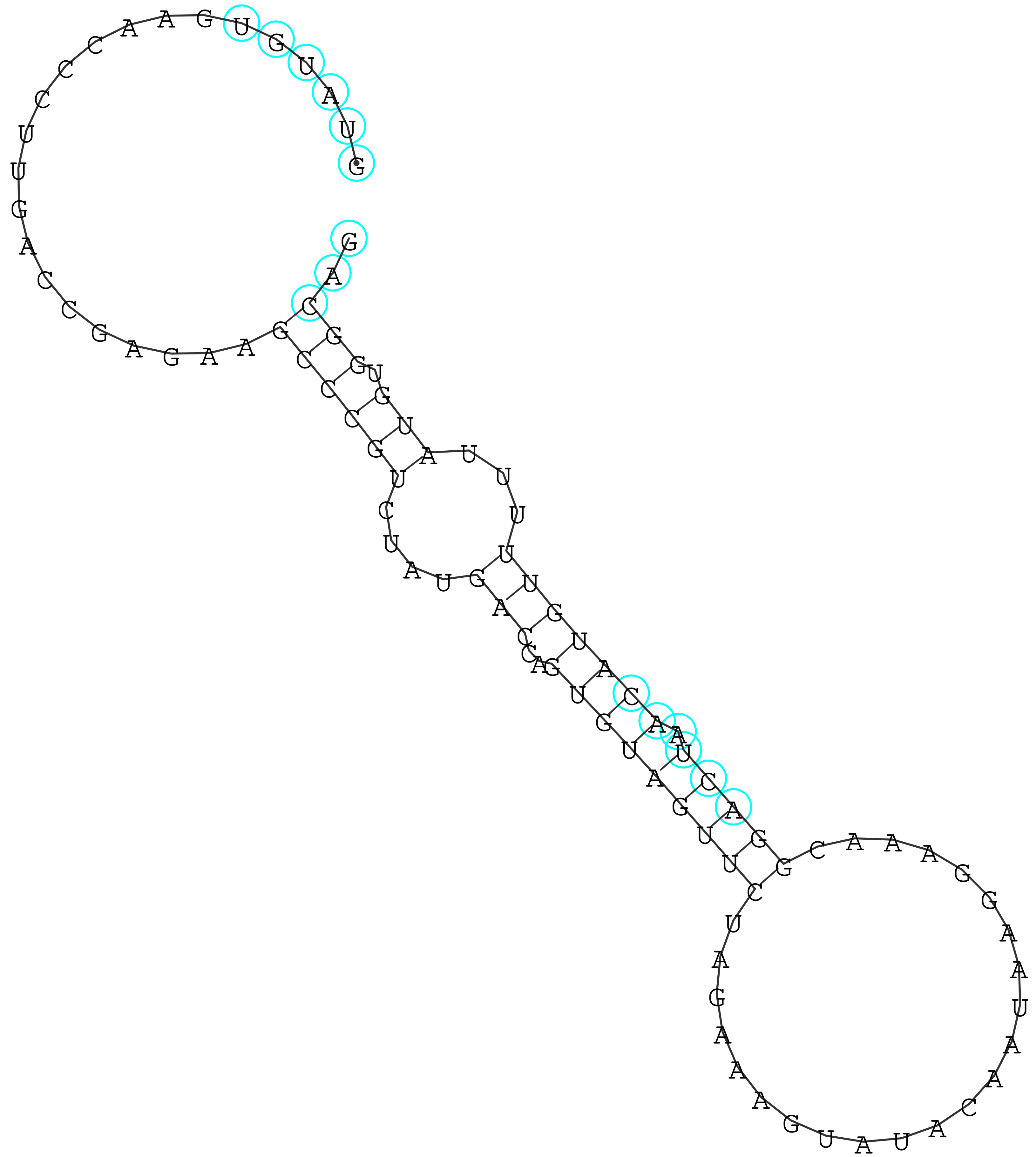

X1651c093A - External intron

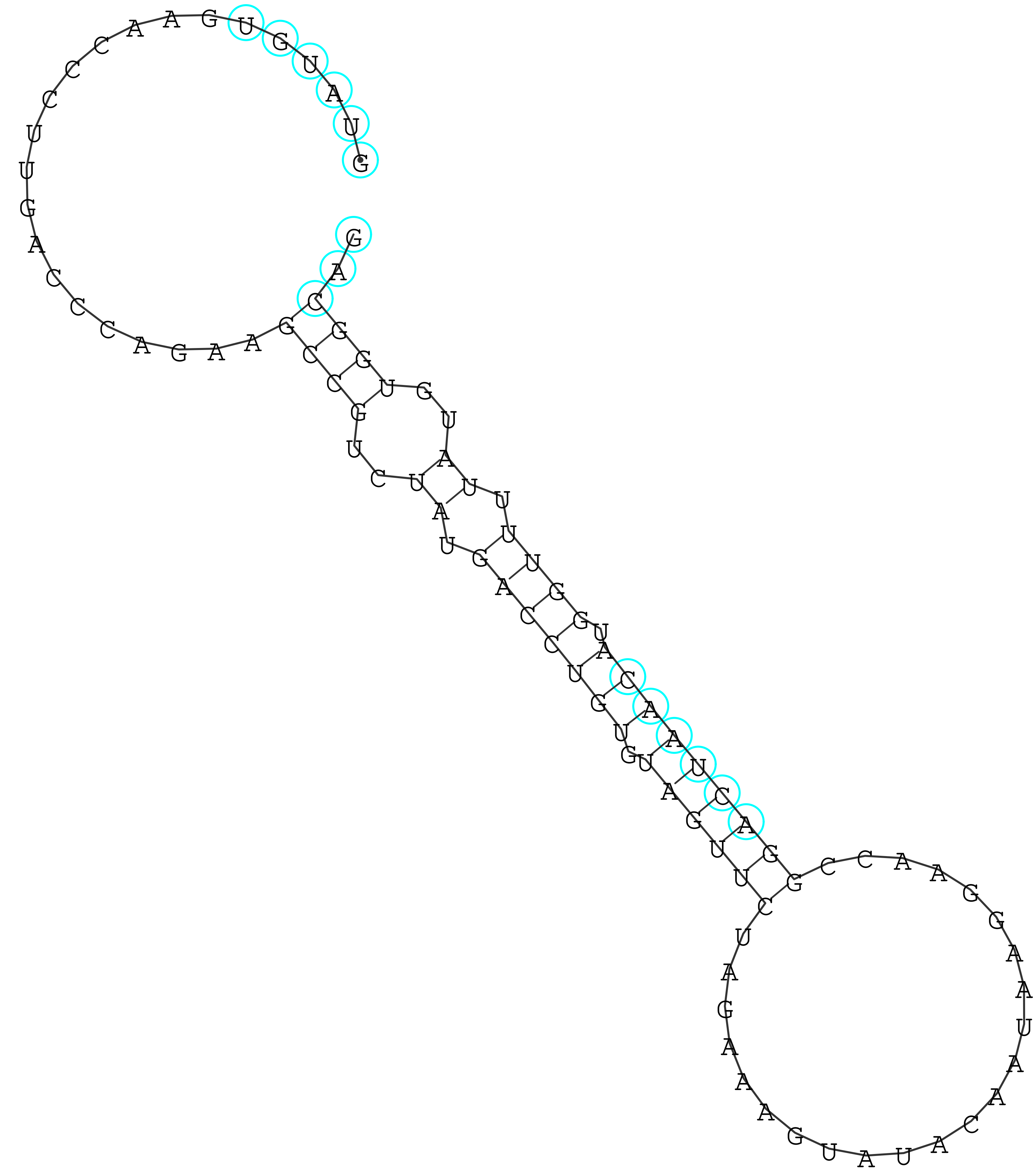

X1651c156A - External intron

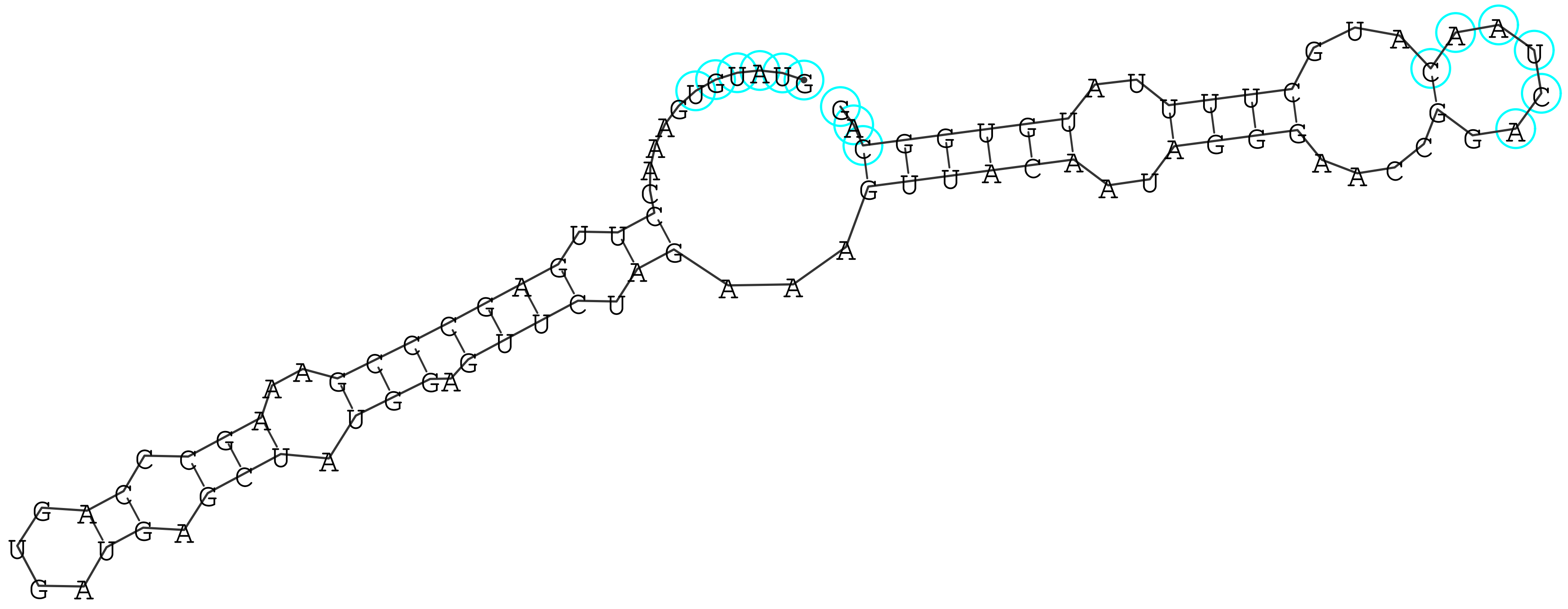

X1651c189A - External intron

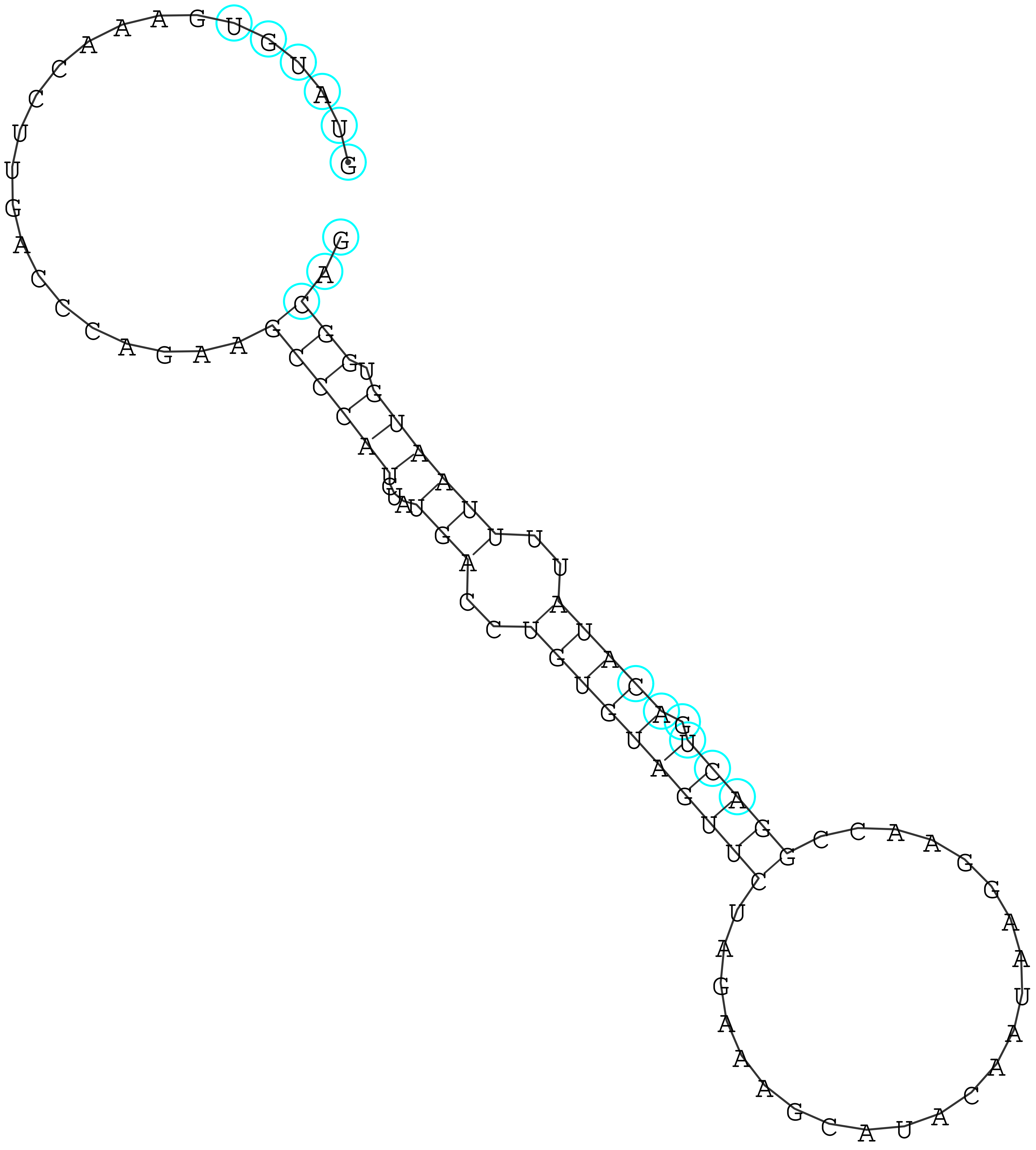

Xarbc0002A - External intron

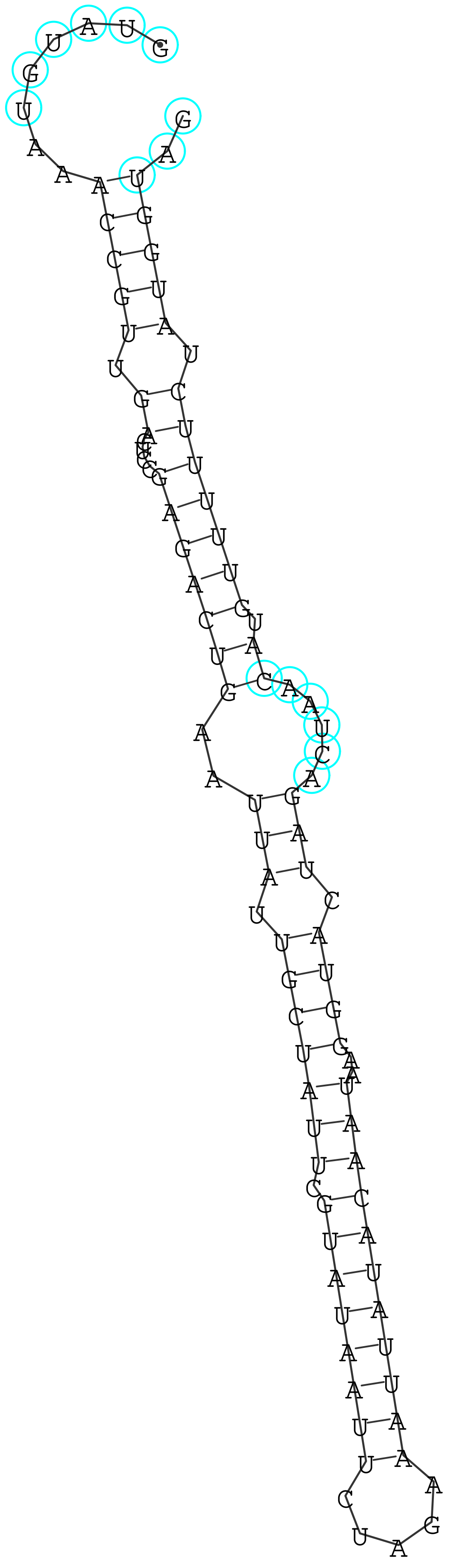

Xarbc0002B - External intron

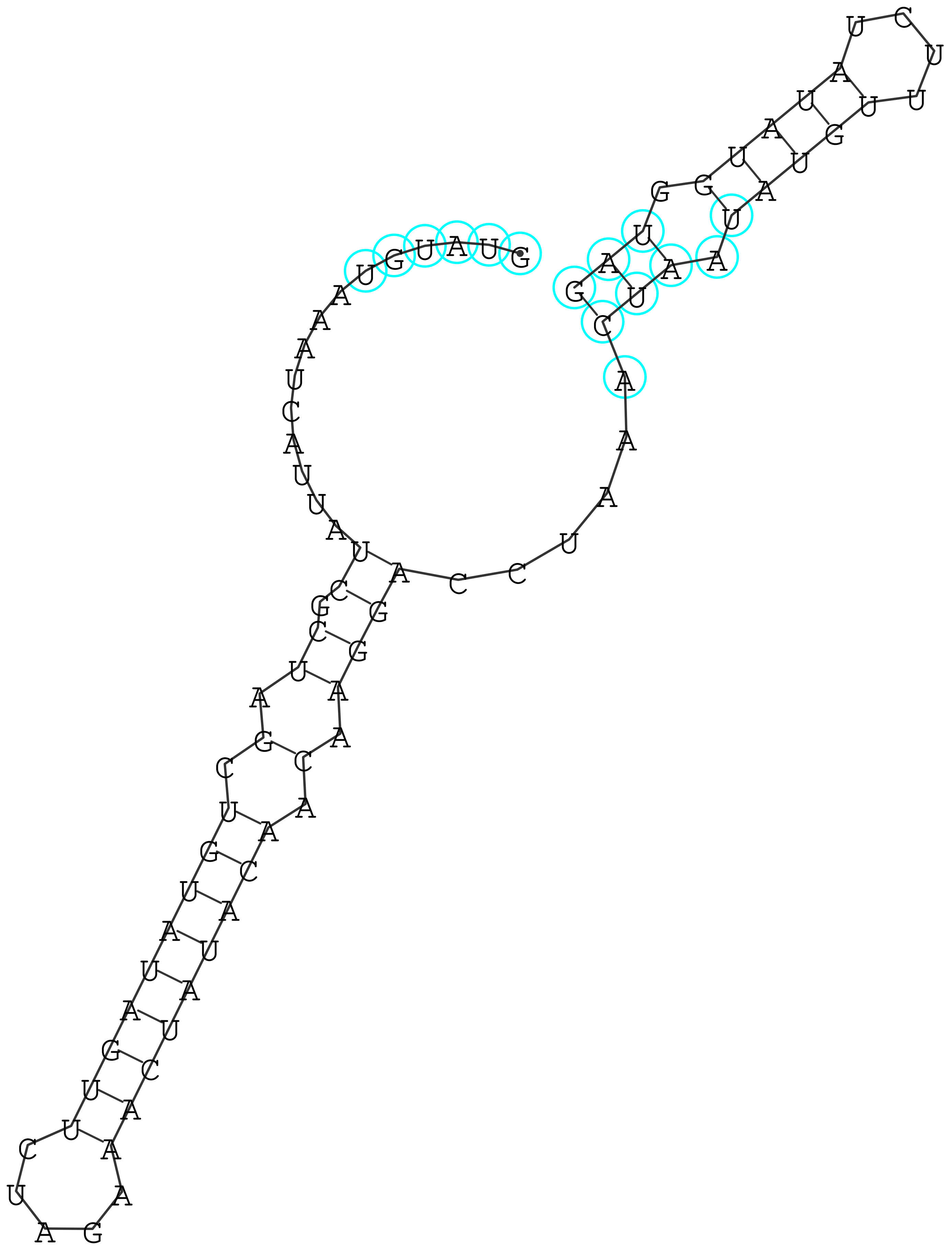

Xarbc0003A - External intron

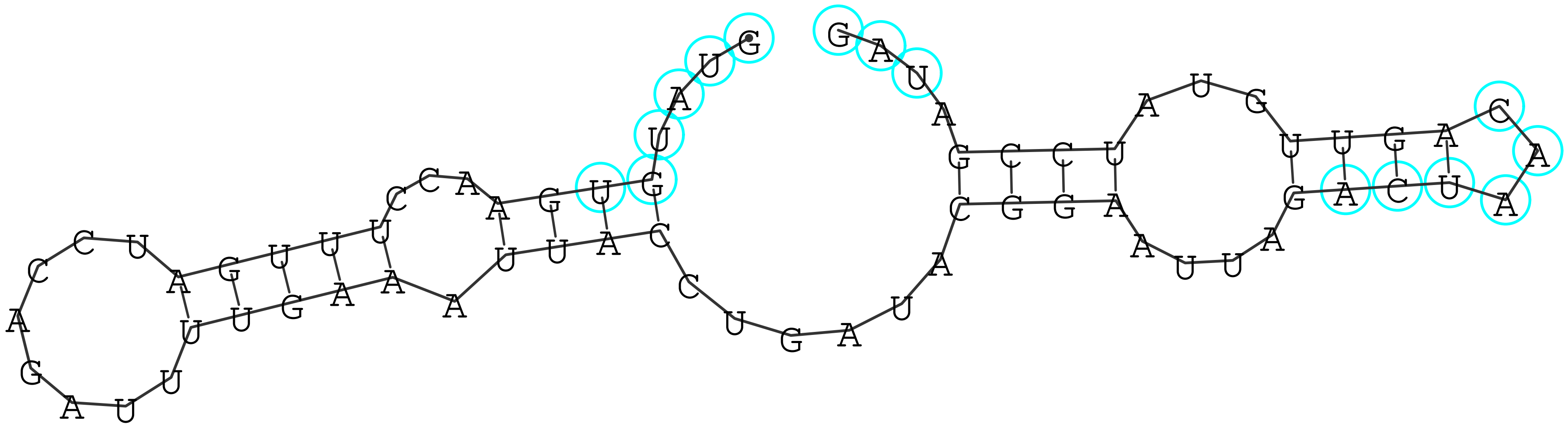

Xarbc0003B - External intron

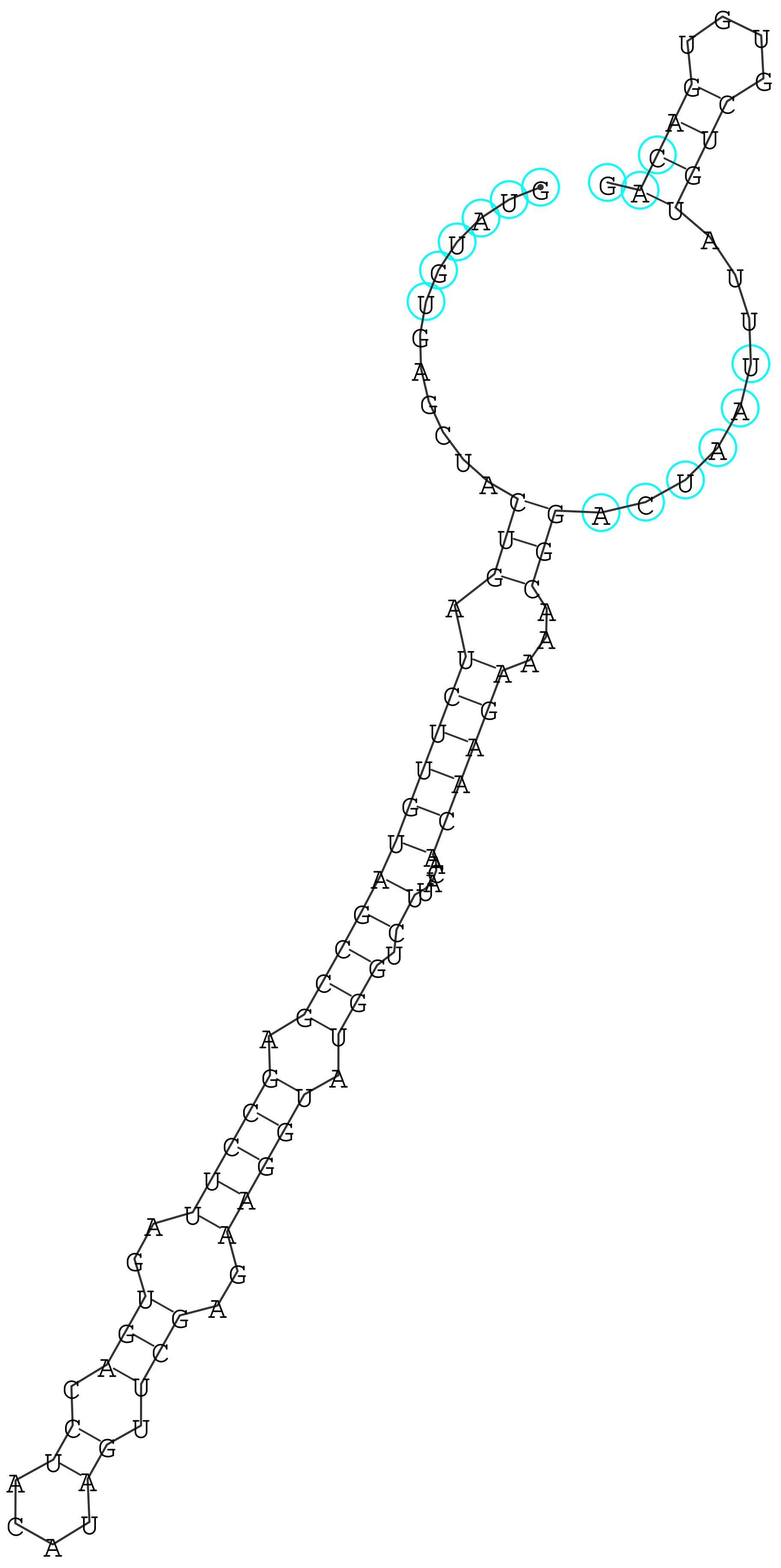

Xarbc0003C - External intron

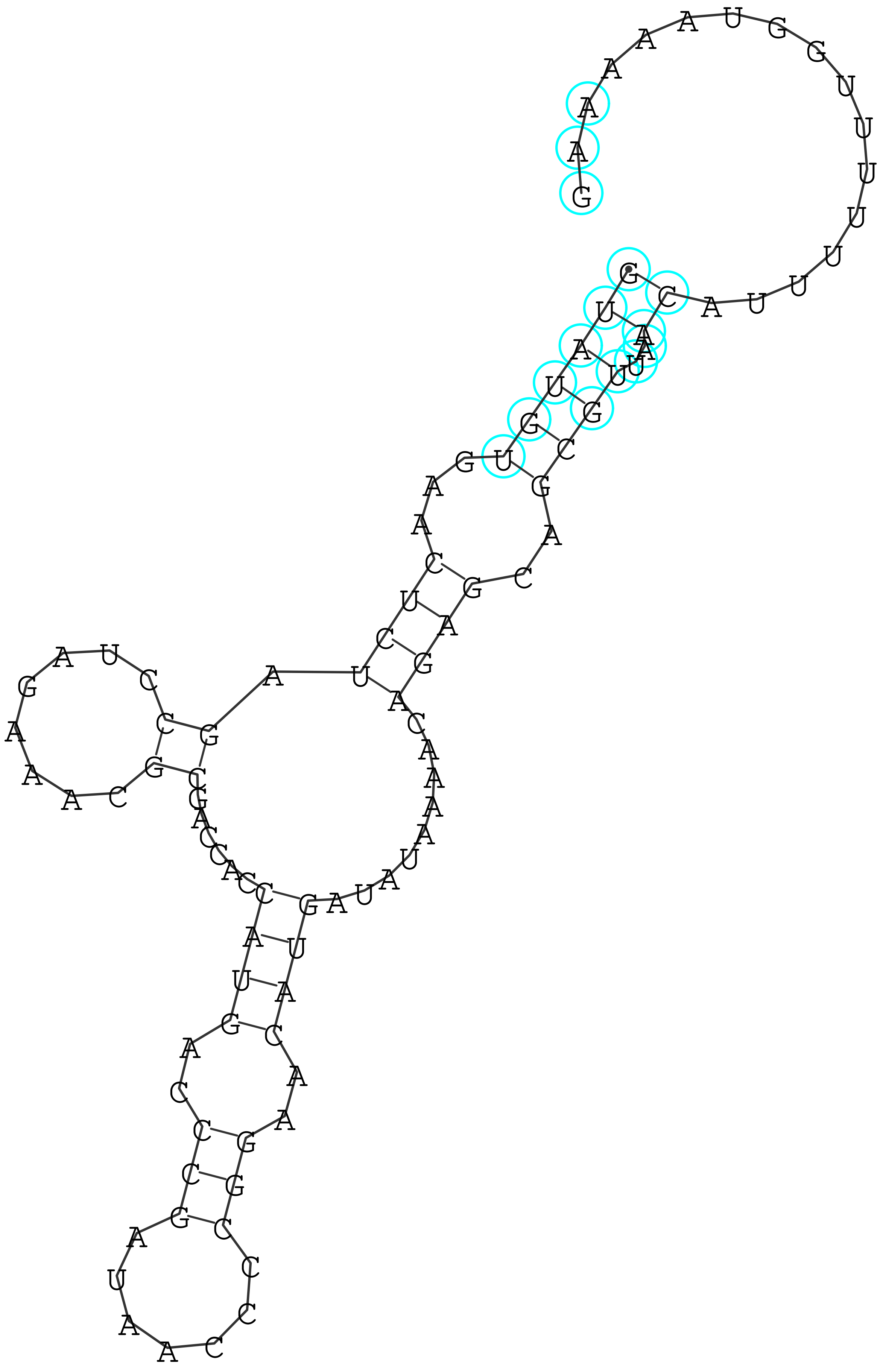

Xarbc0006A - External intron

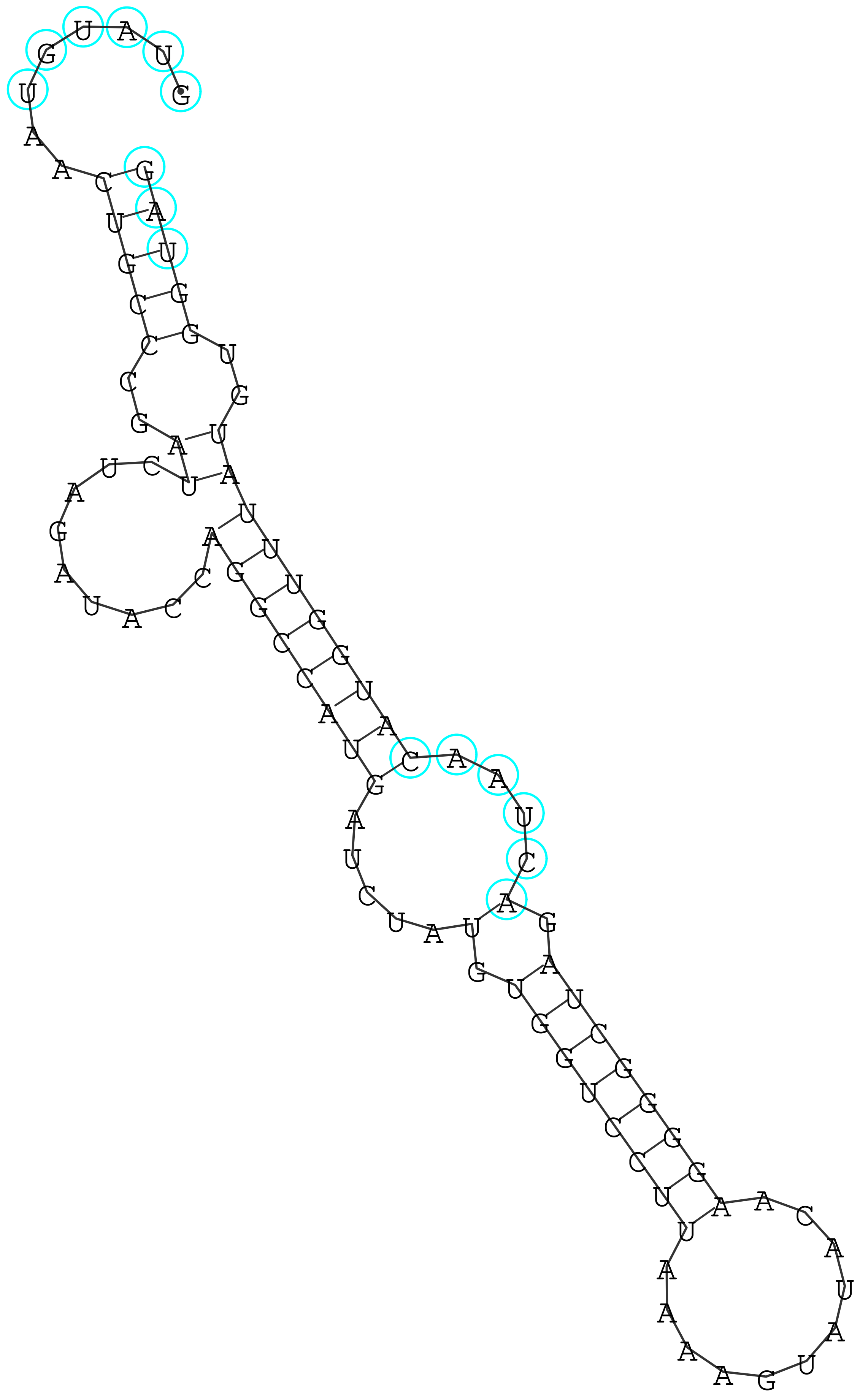

Xarbc0006B - External intron

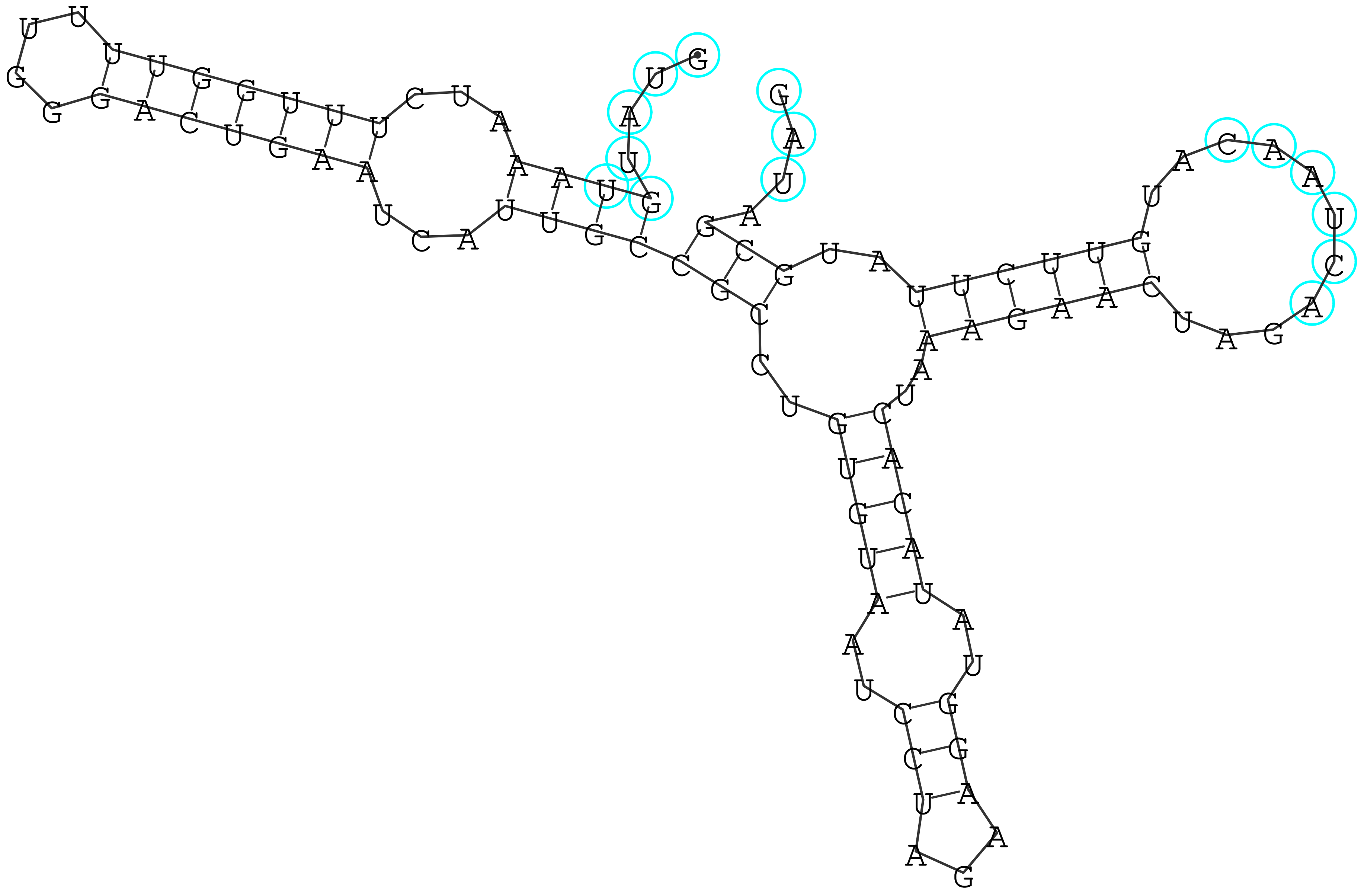

Xarbc0009A - External intron

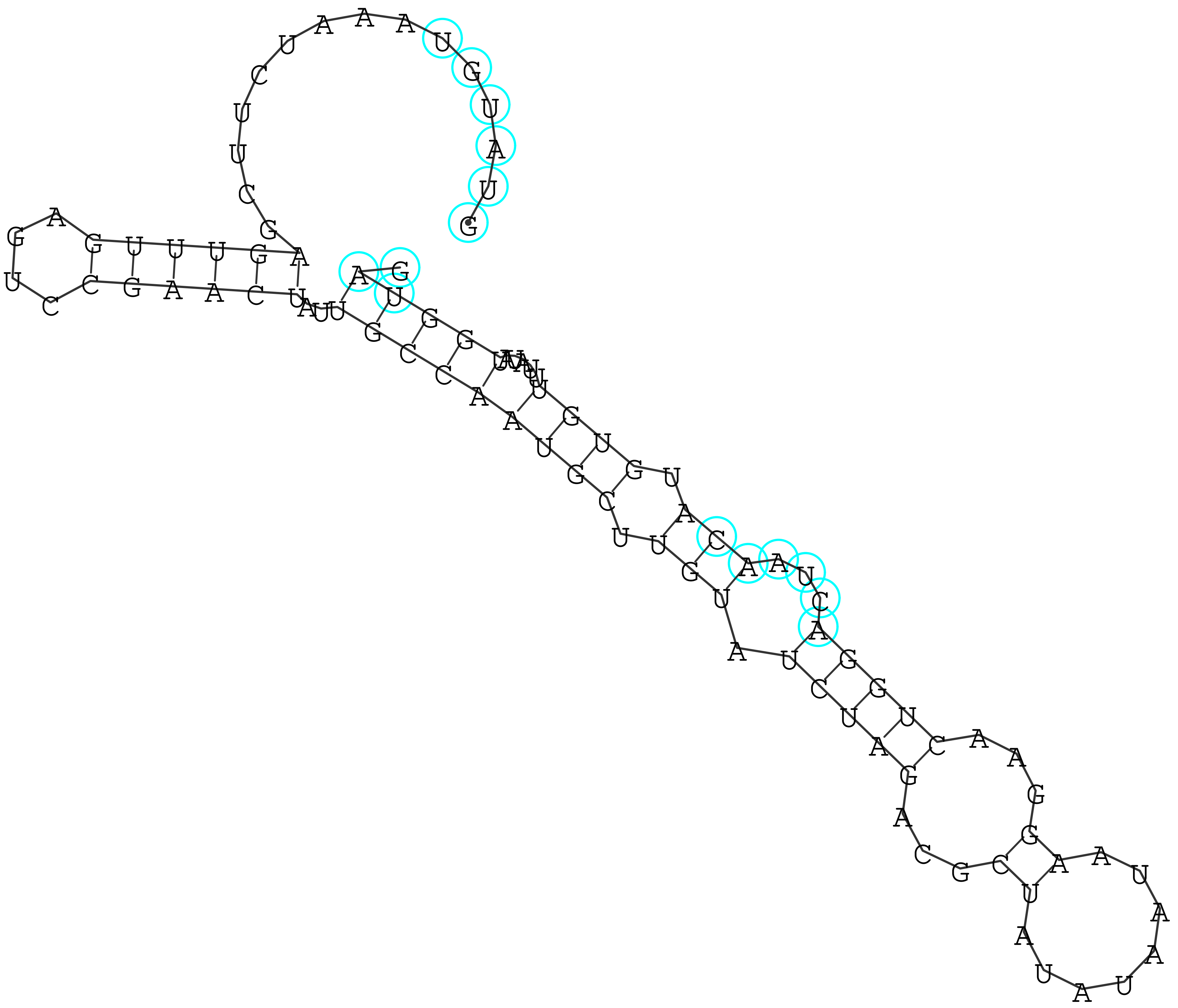

Xarbc0010A - External intron

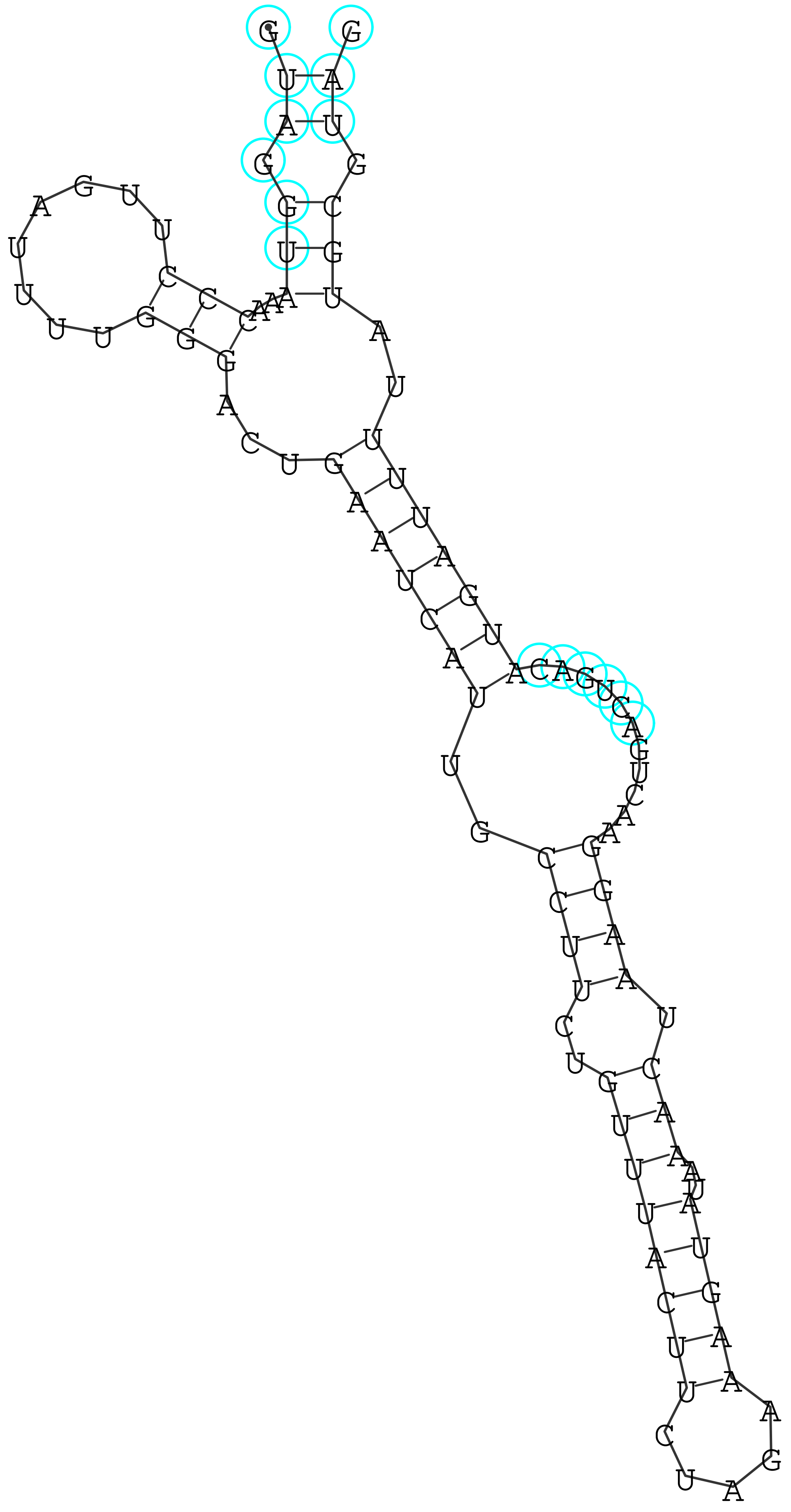

Xarbc0010B - External intron

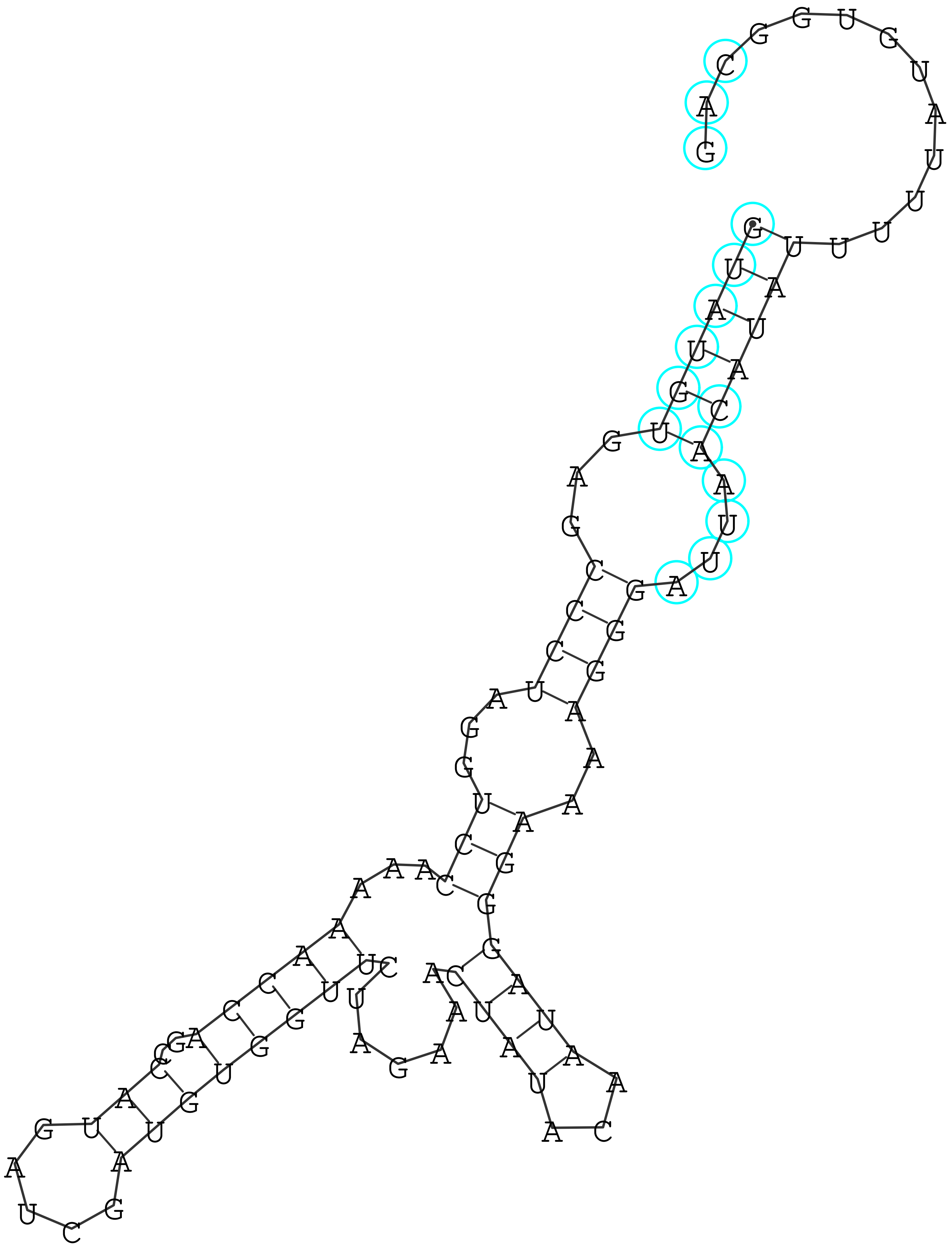

Xarbc0011A - External intron

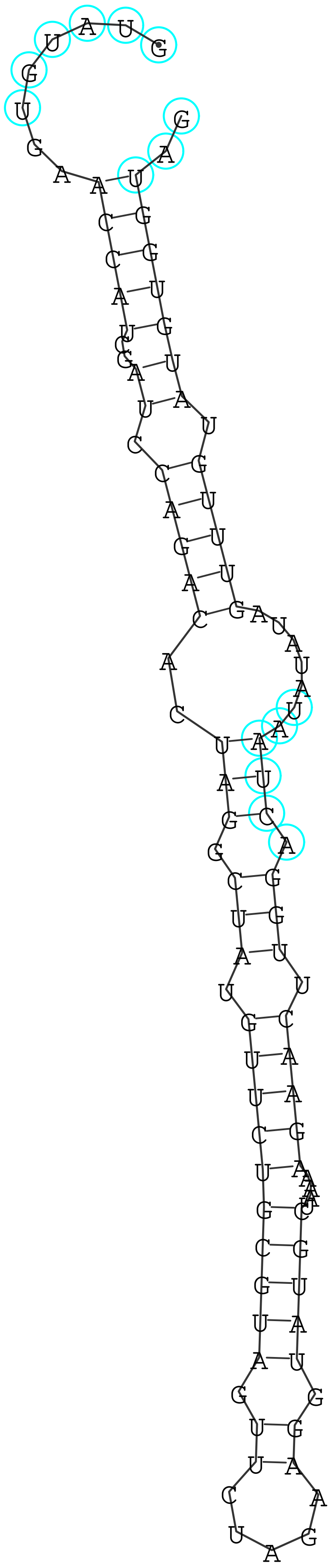

Xarbc0012A - External intron

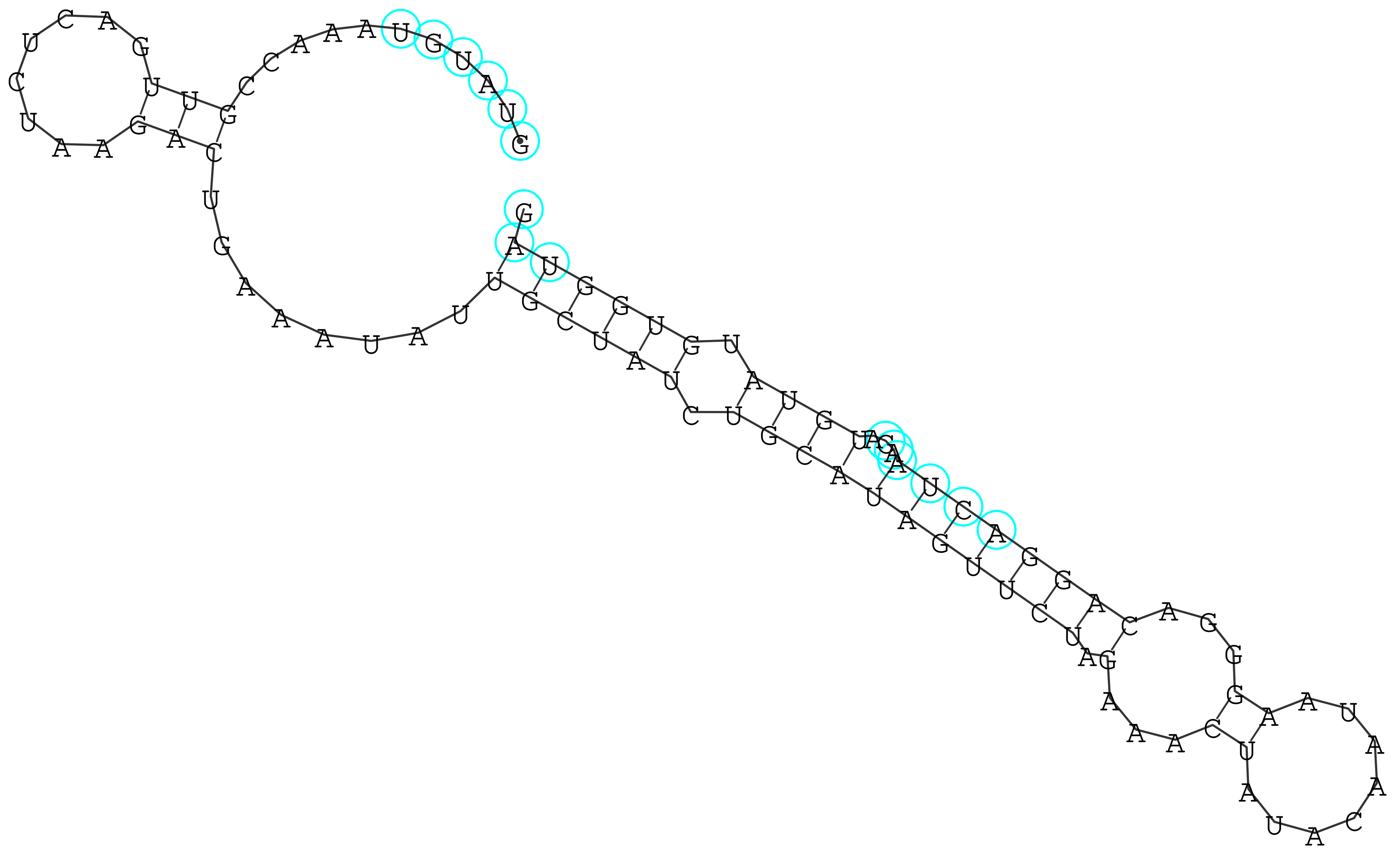

Xarbc0012B - External intron

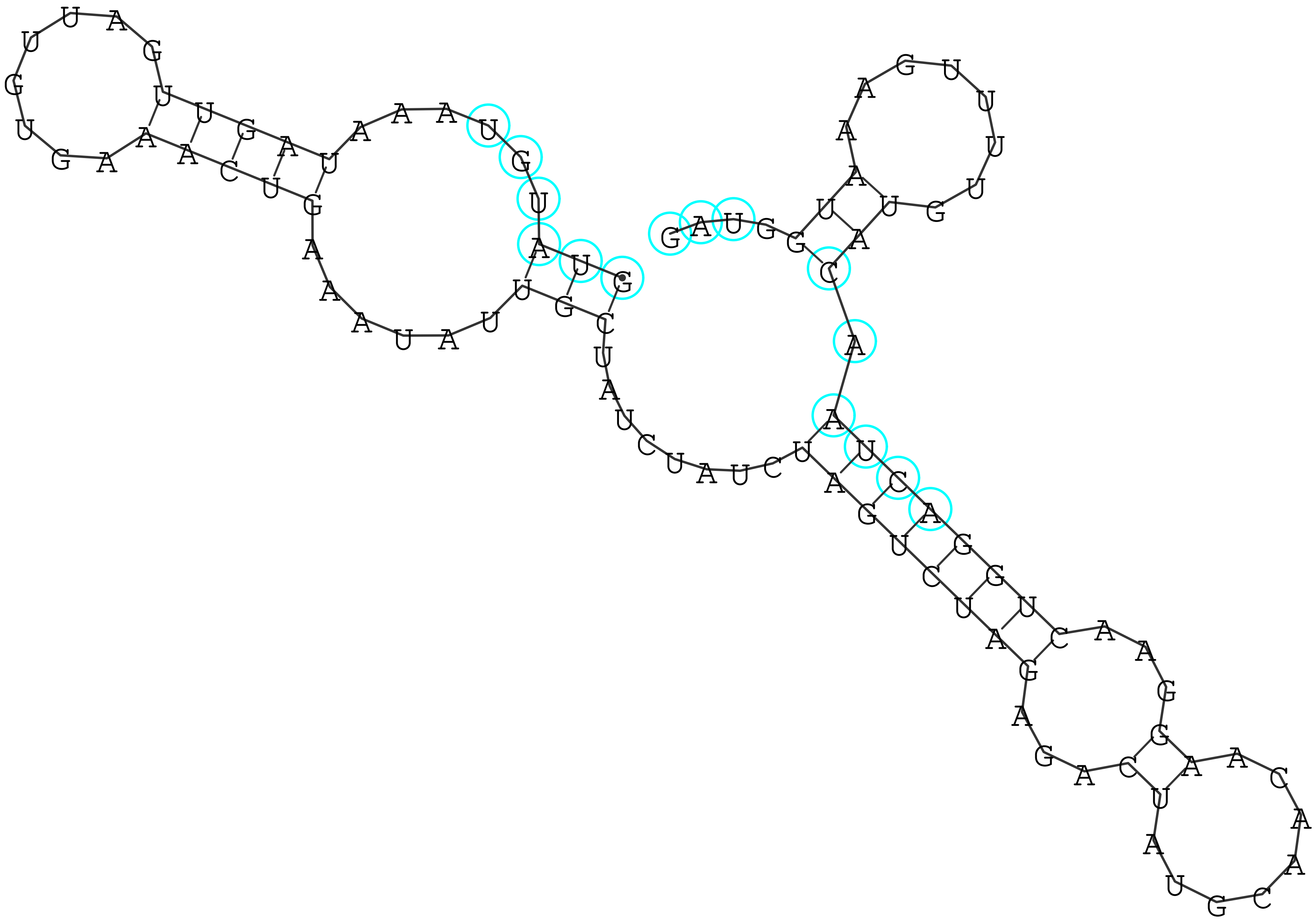

Xarbc0012D - External intron

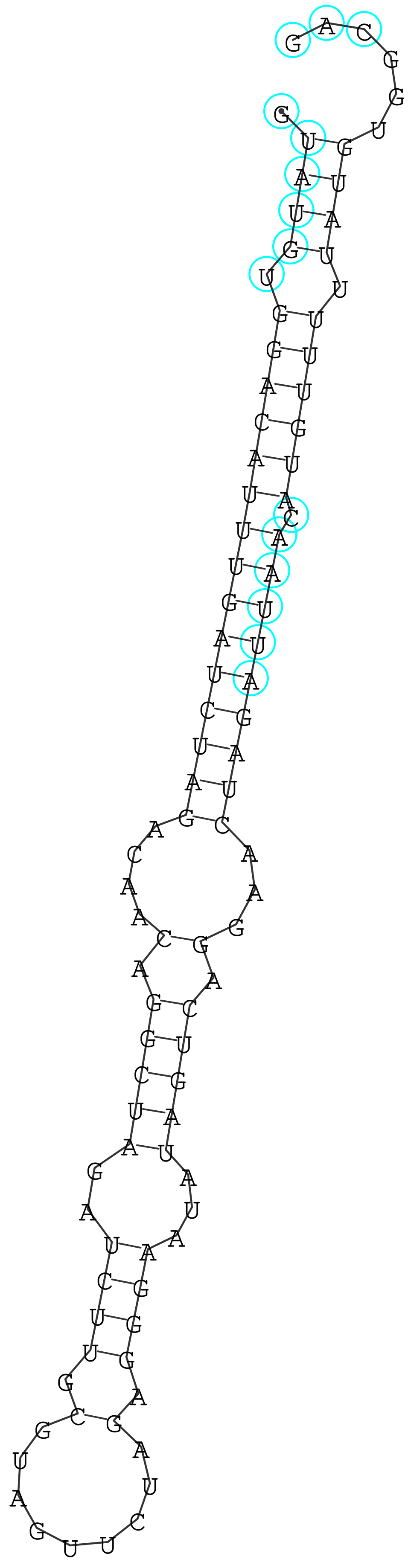

Xarbc0013A - External intron

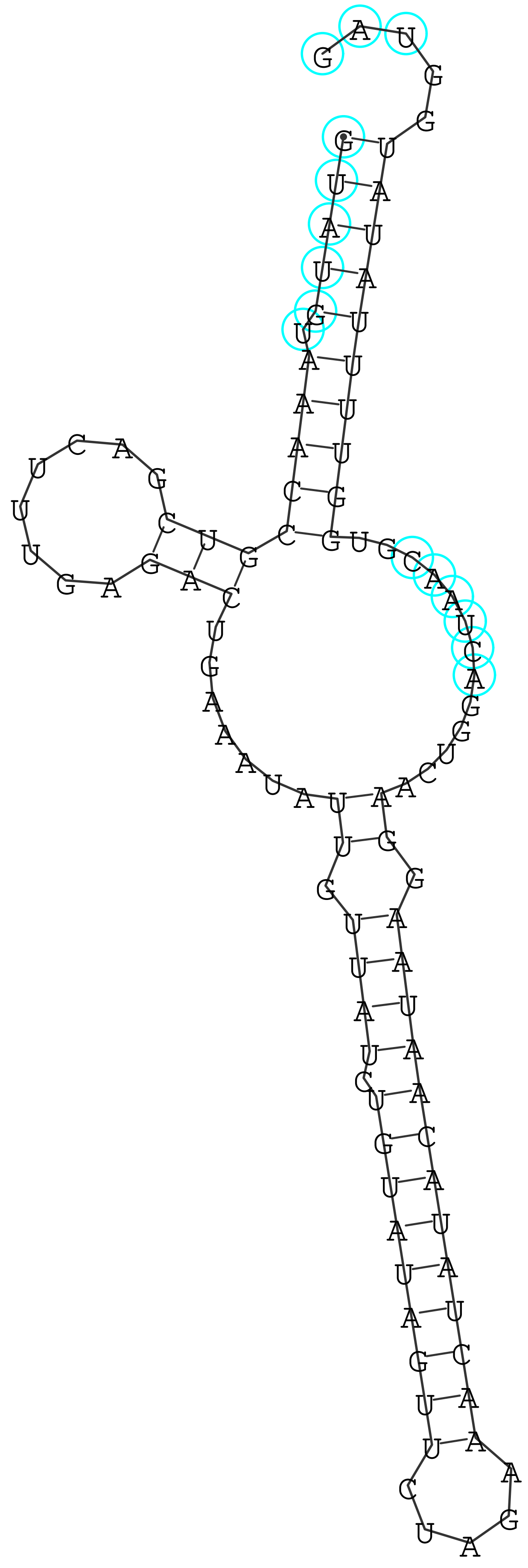

Xarbc0014A - External intron

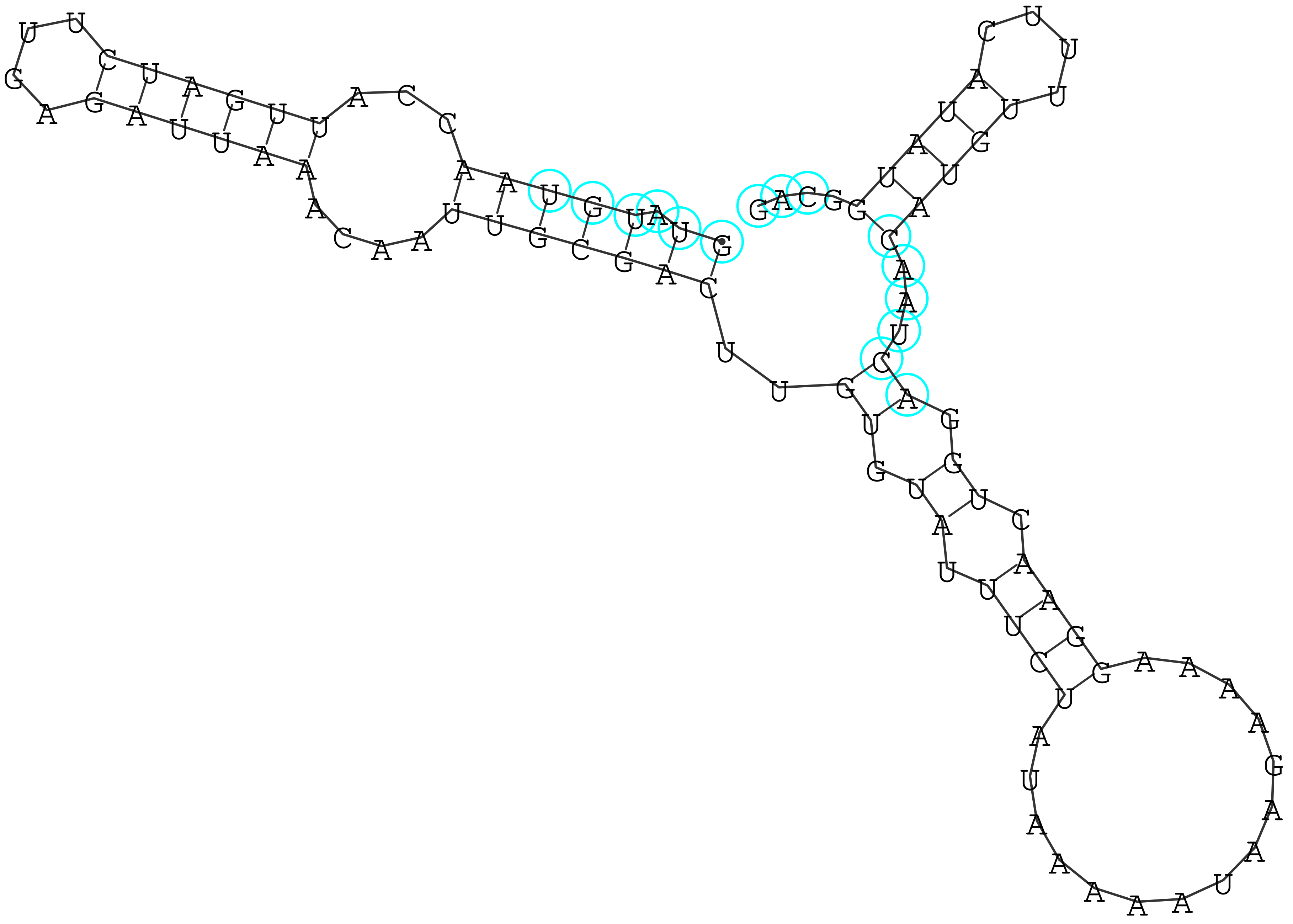

Xarbc0014B - External intron

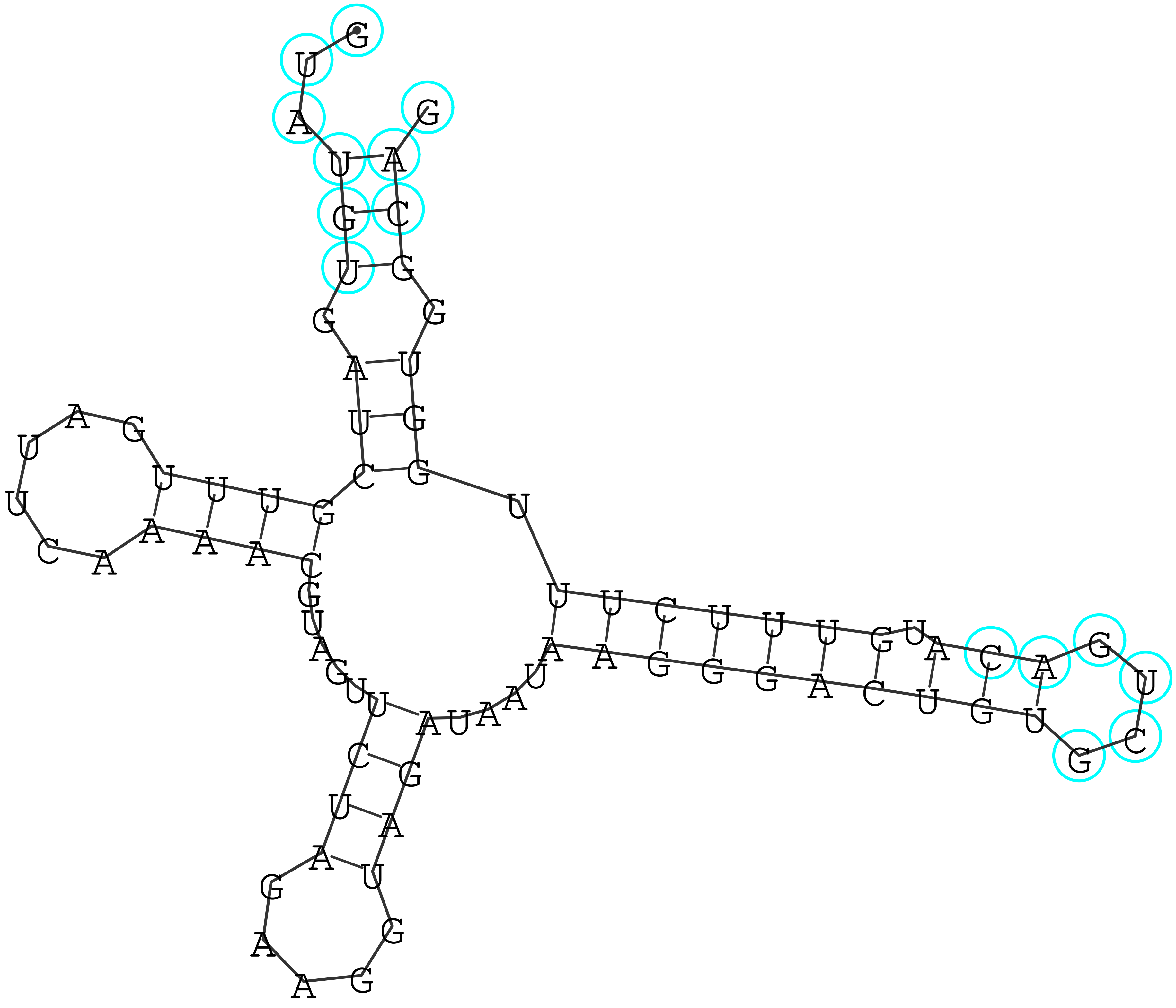

Xarbc0016A - External intron

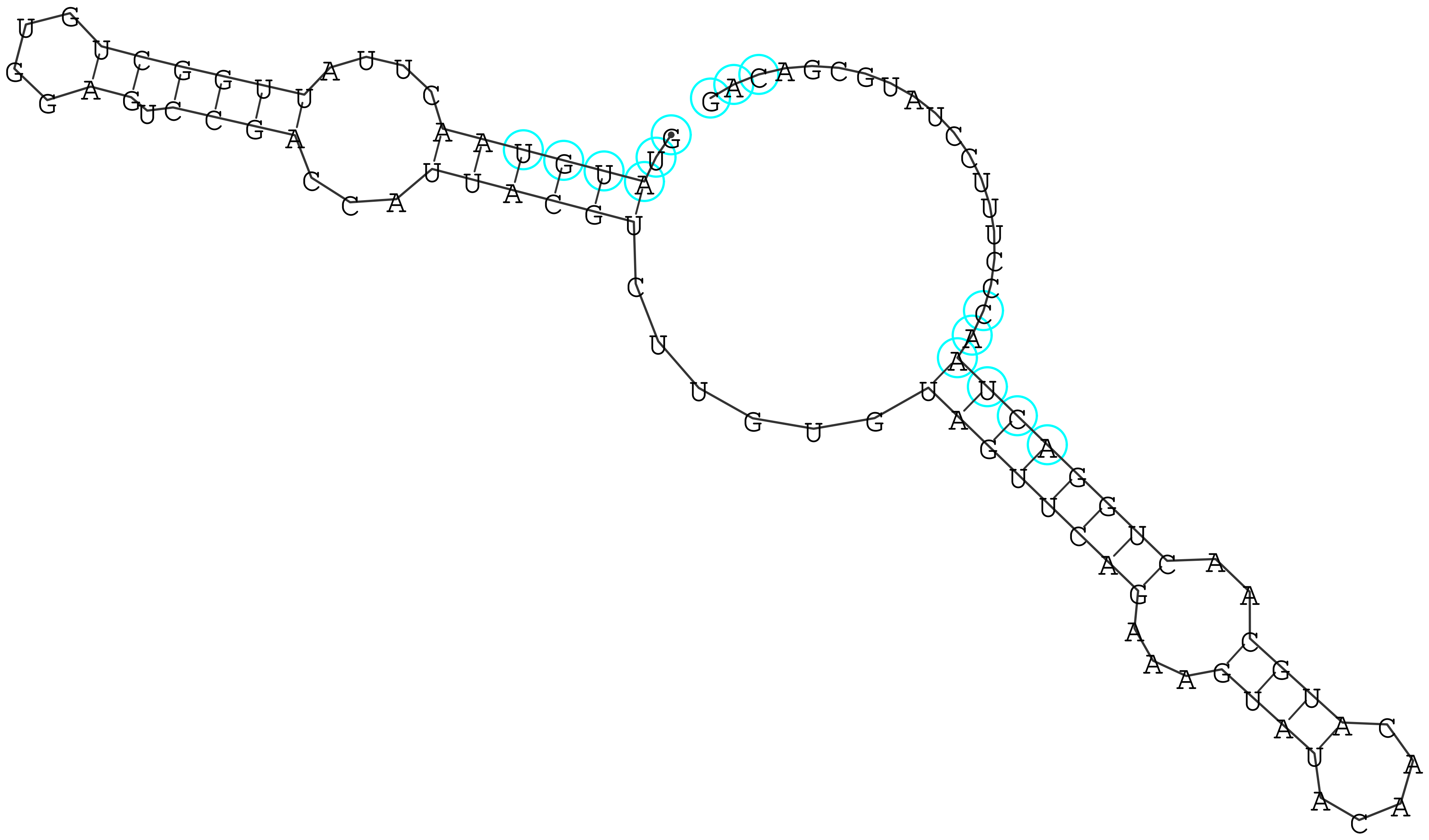

Xarbc0021A - External intron

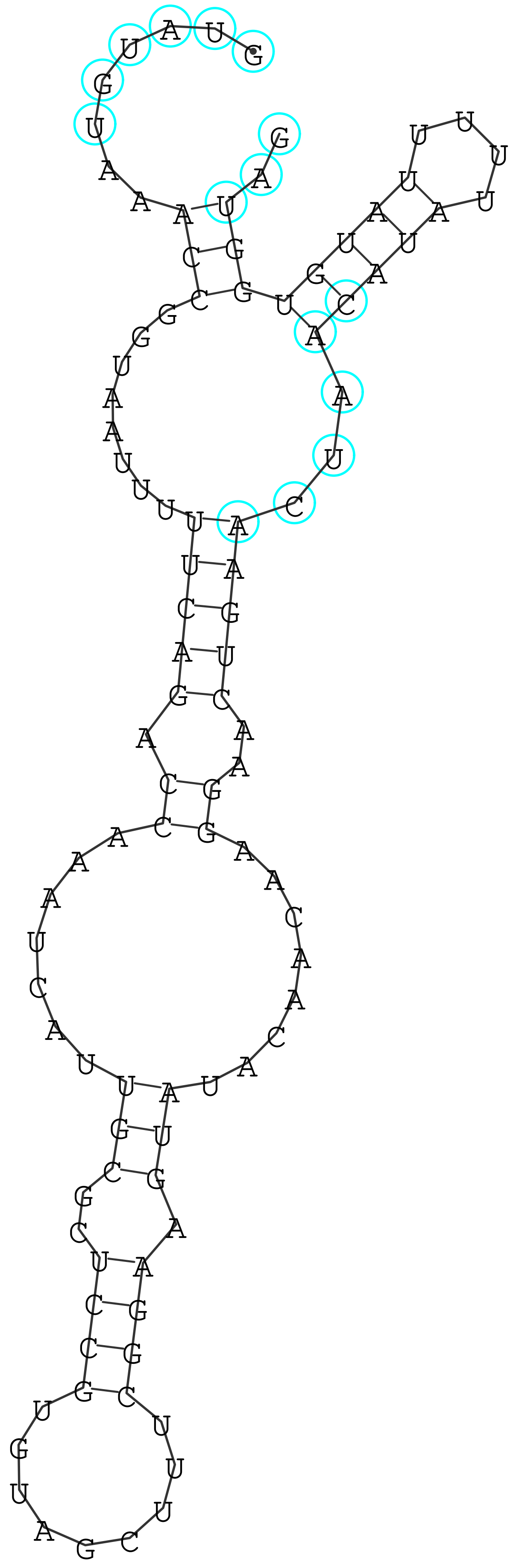

Xarbc0023A - External intron

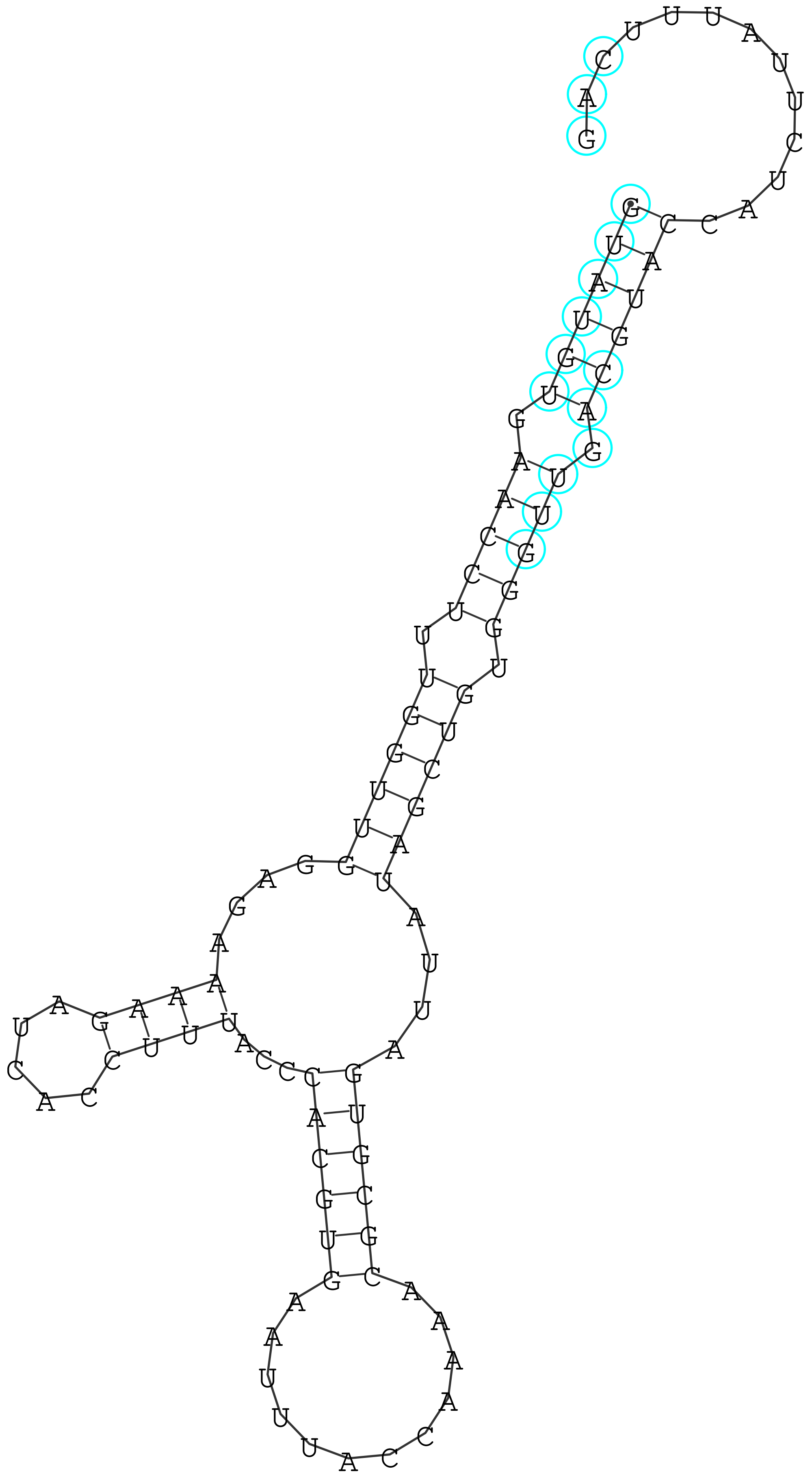

Xarbc0024A - External intron

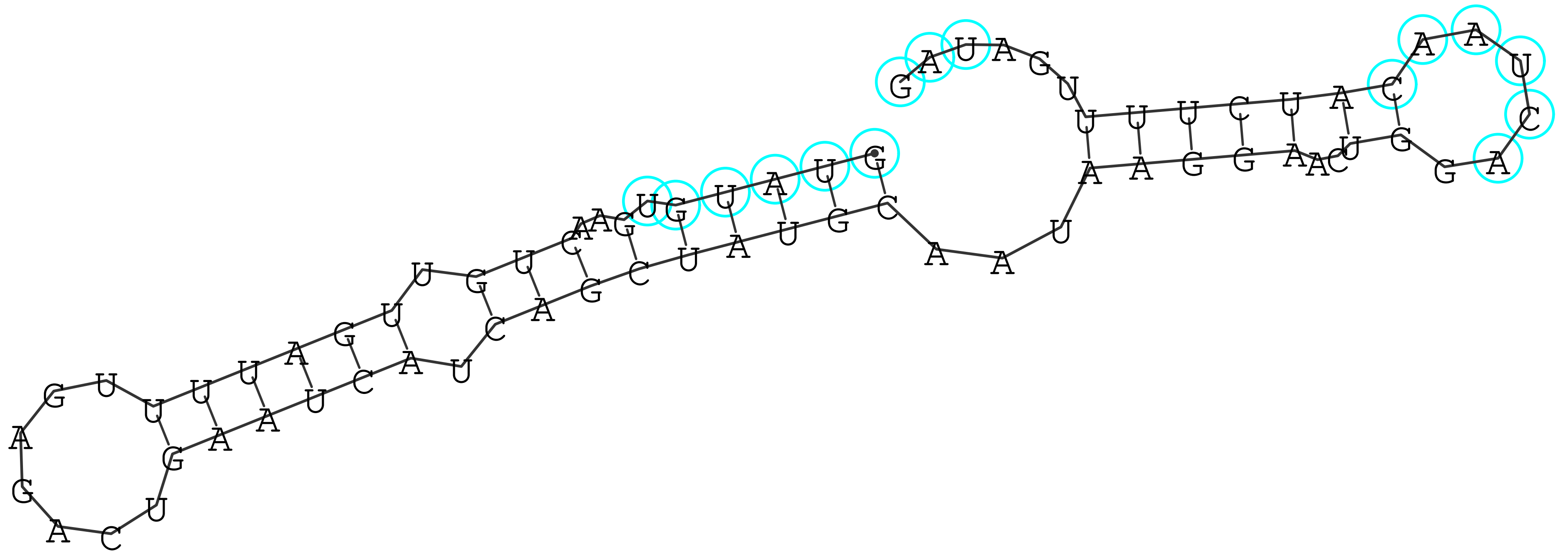

Xarbc0024B - External intron

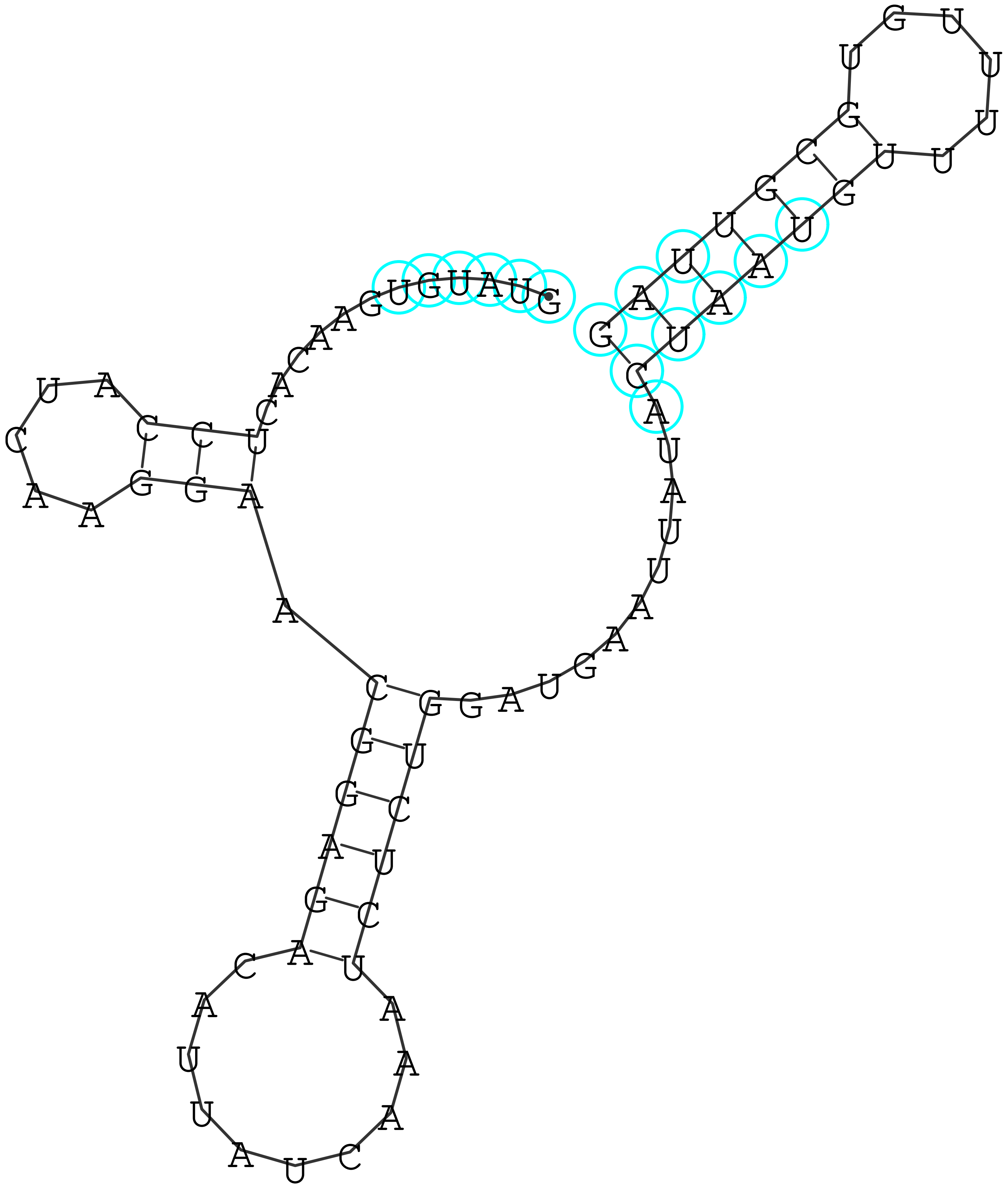

Xarbc0024C - External intron

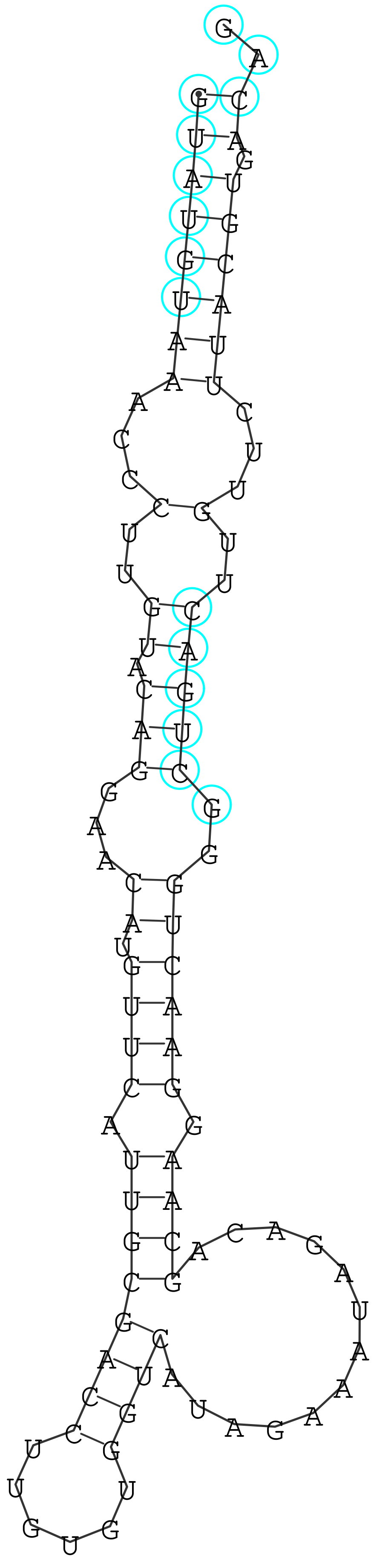

Xarbc0026A - External intron

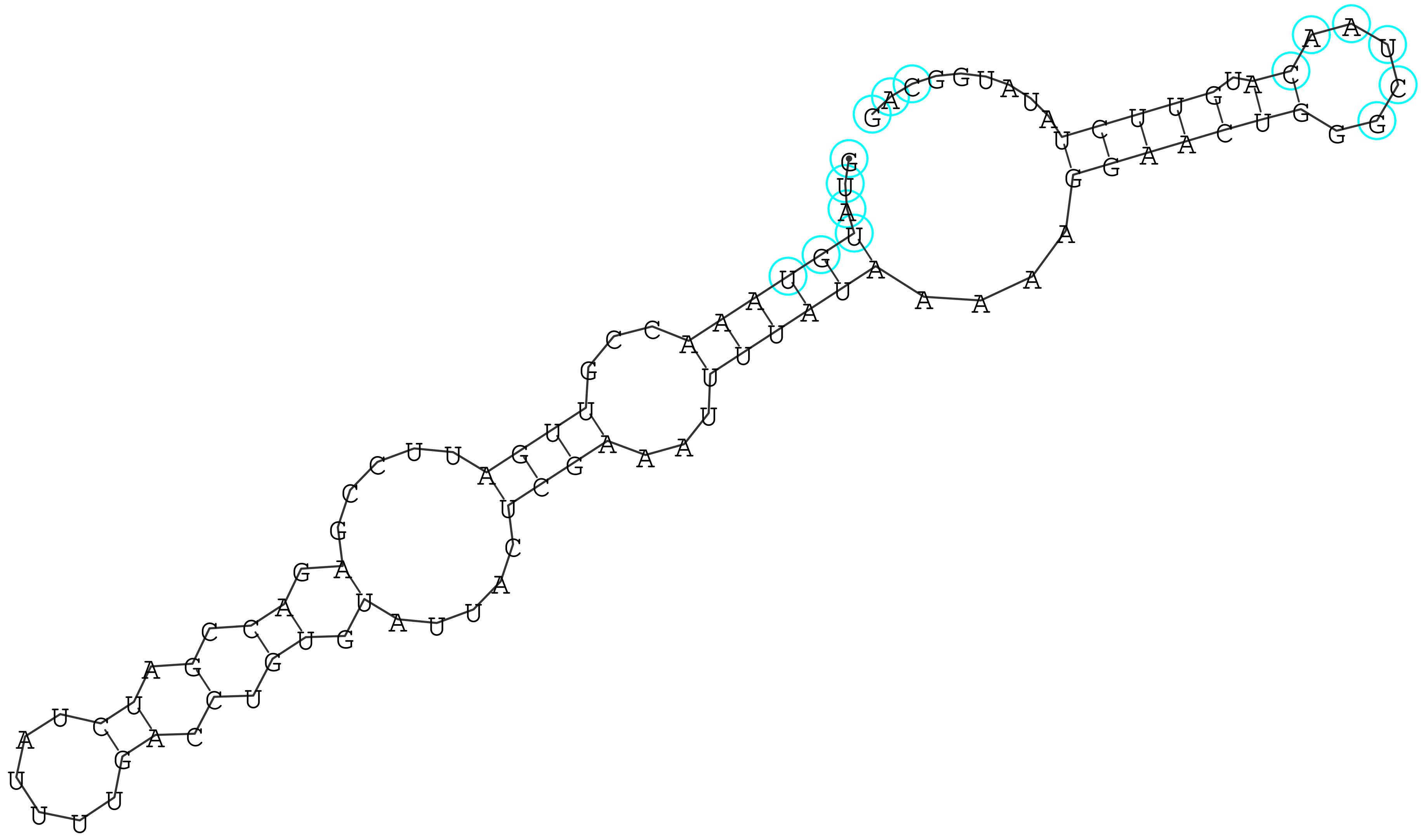

Xarbc0028A - External intron

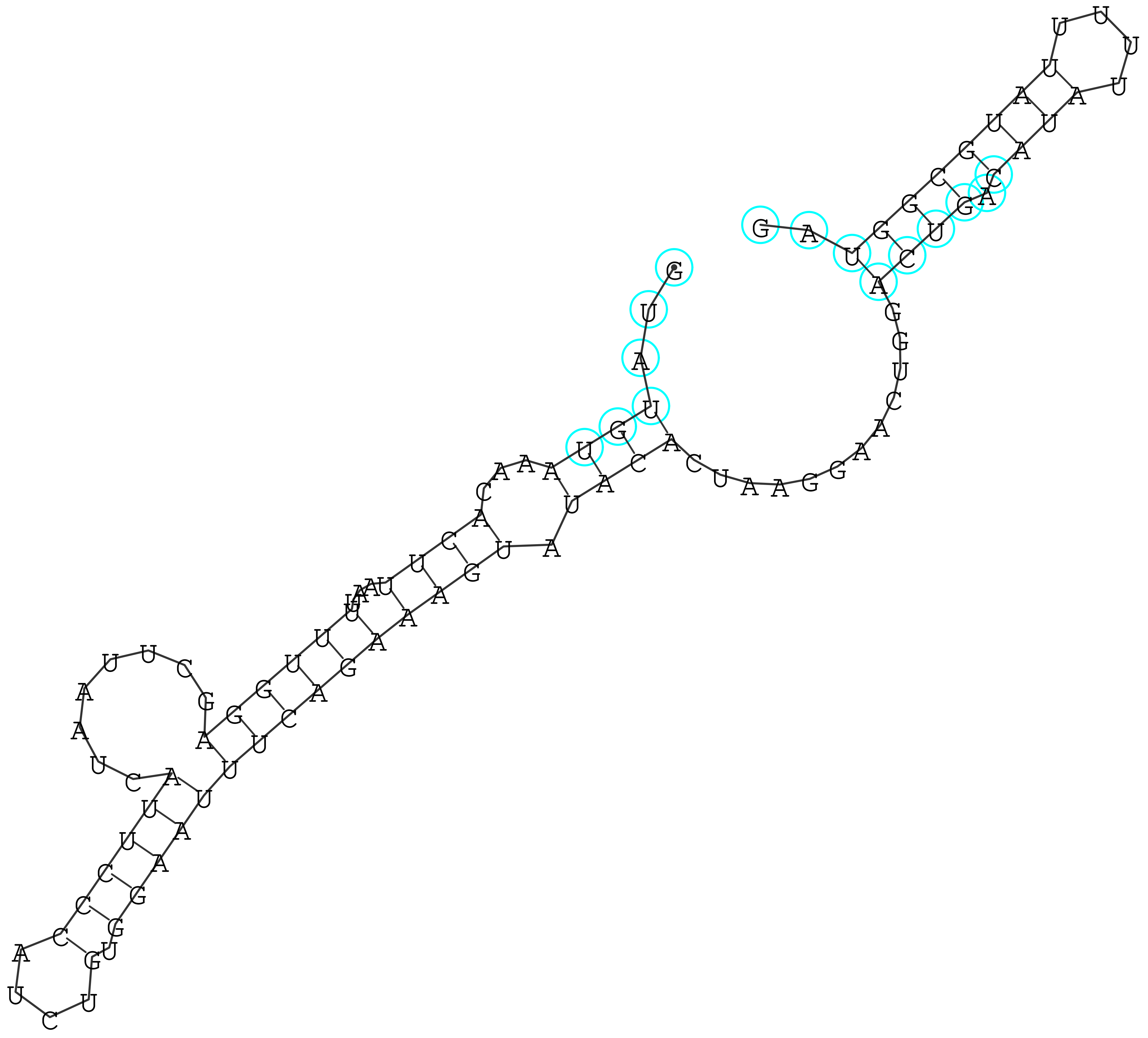

Xarbc0034A - External intron

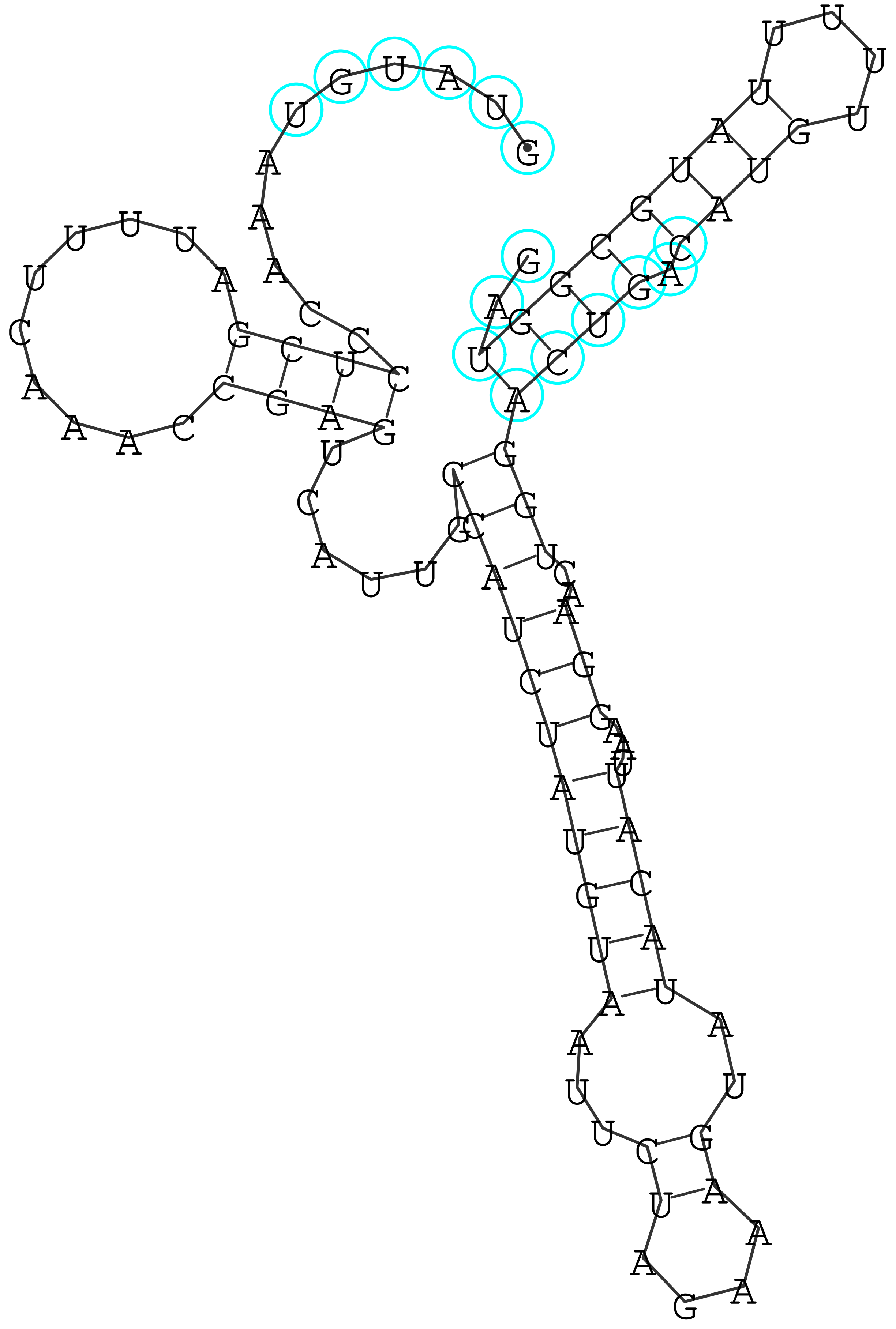

Xarbc0044A - External intron

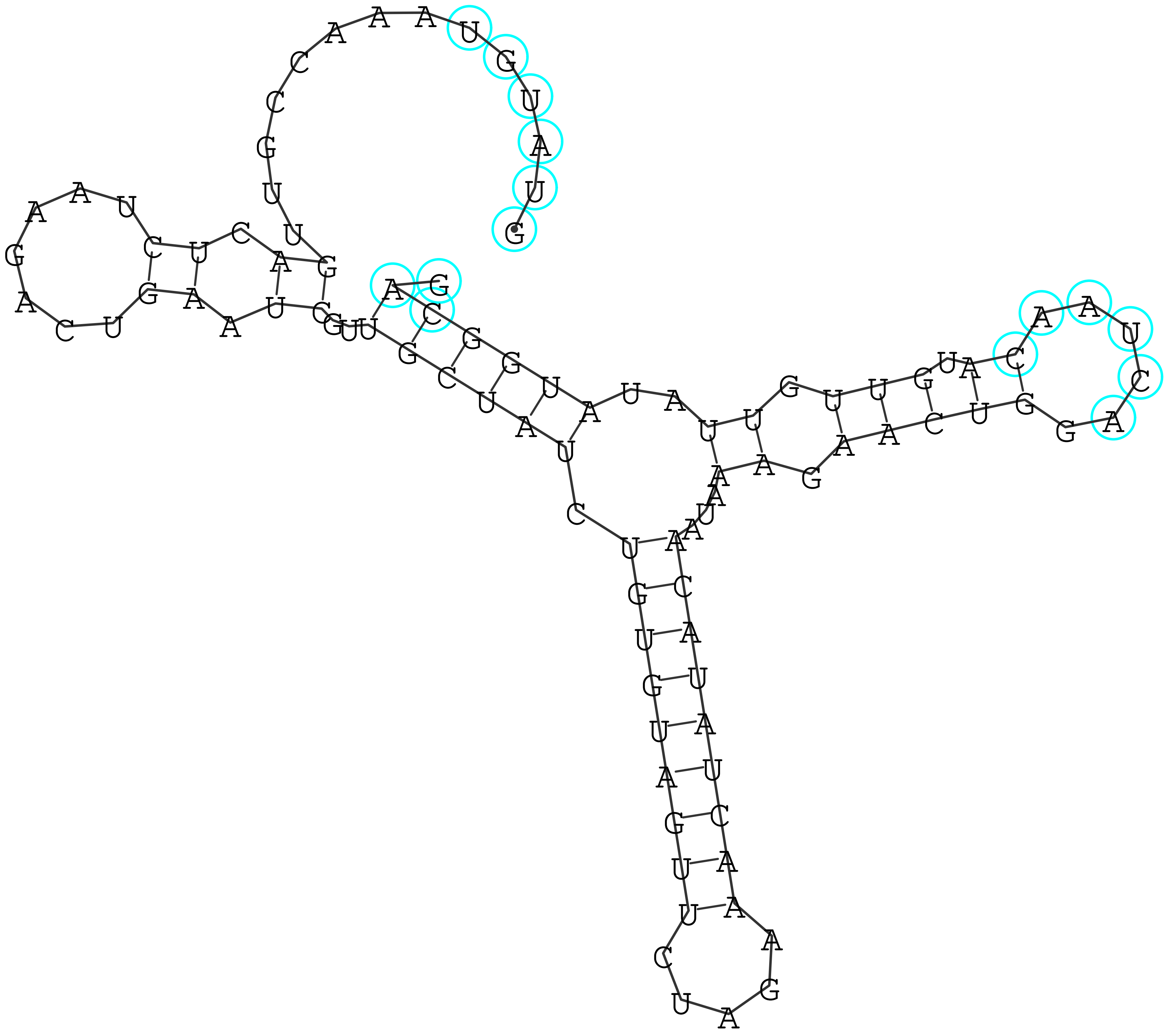

Xarbc0059A - External intron

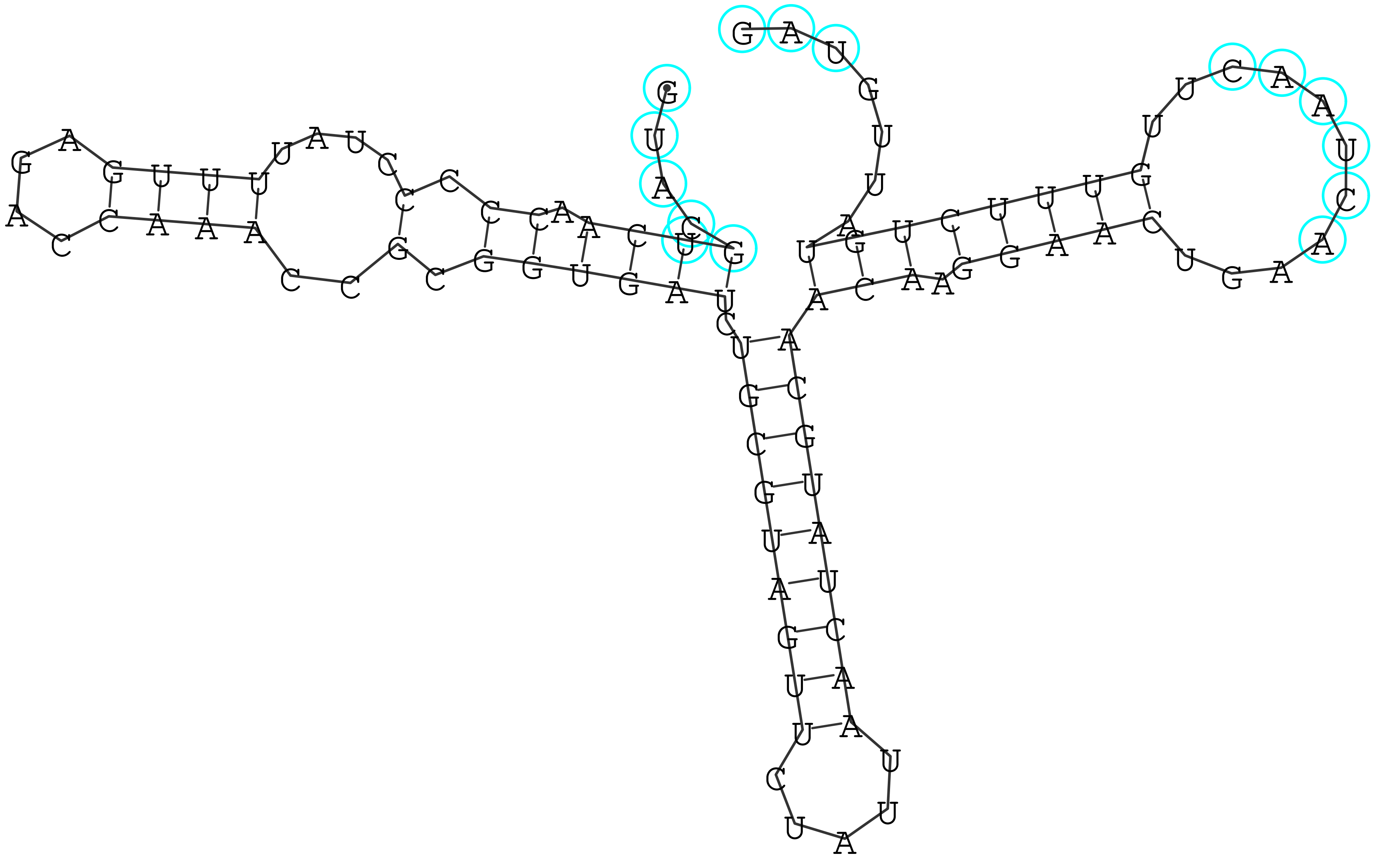

Xarbc0060A - External intron

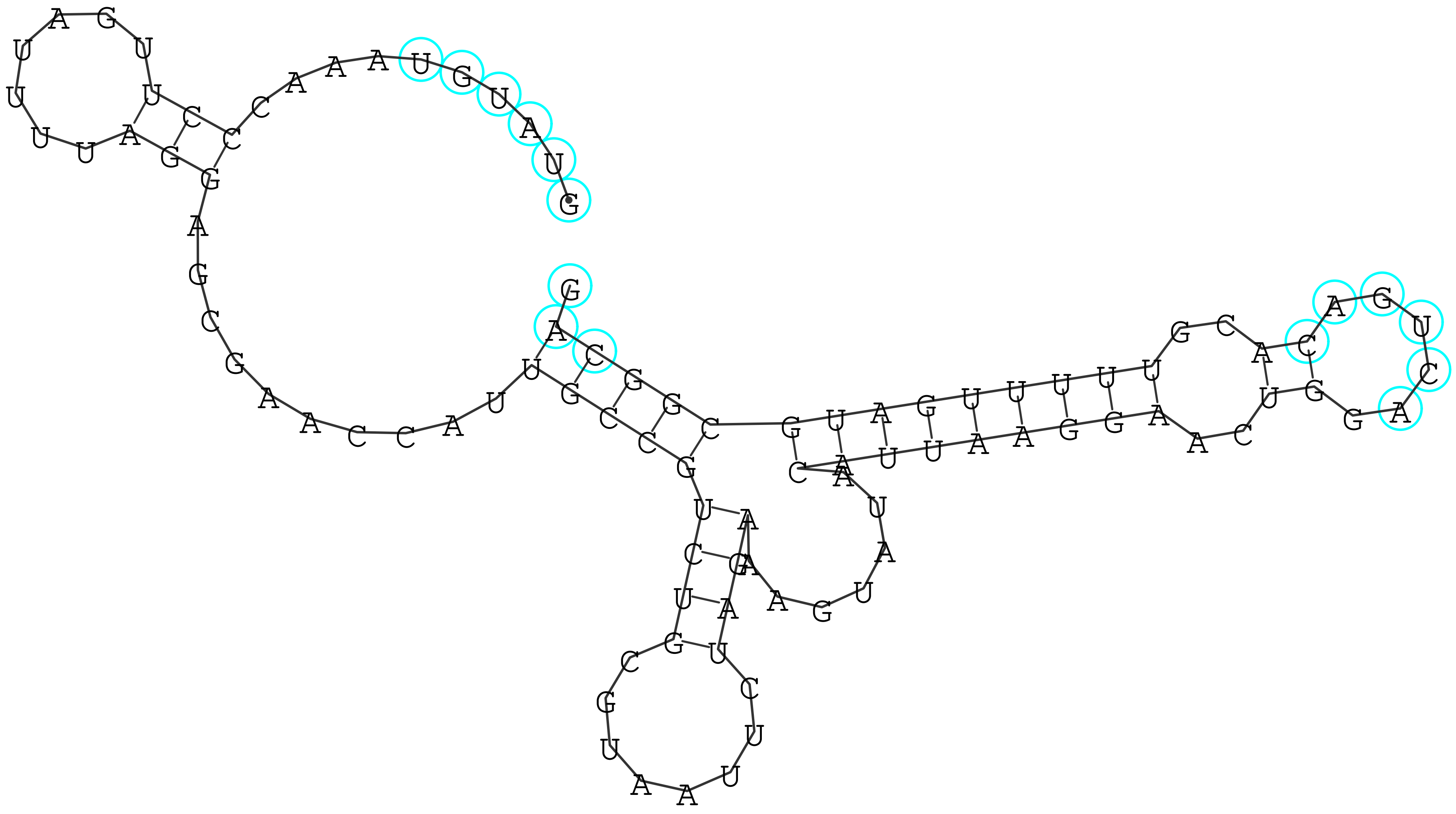

Xarbc0061A - External intron

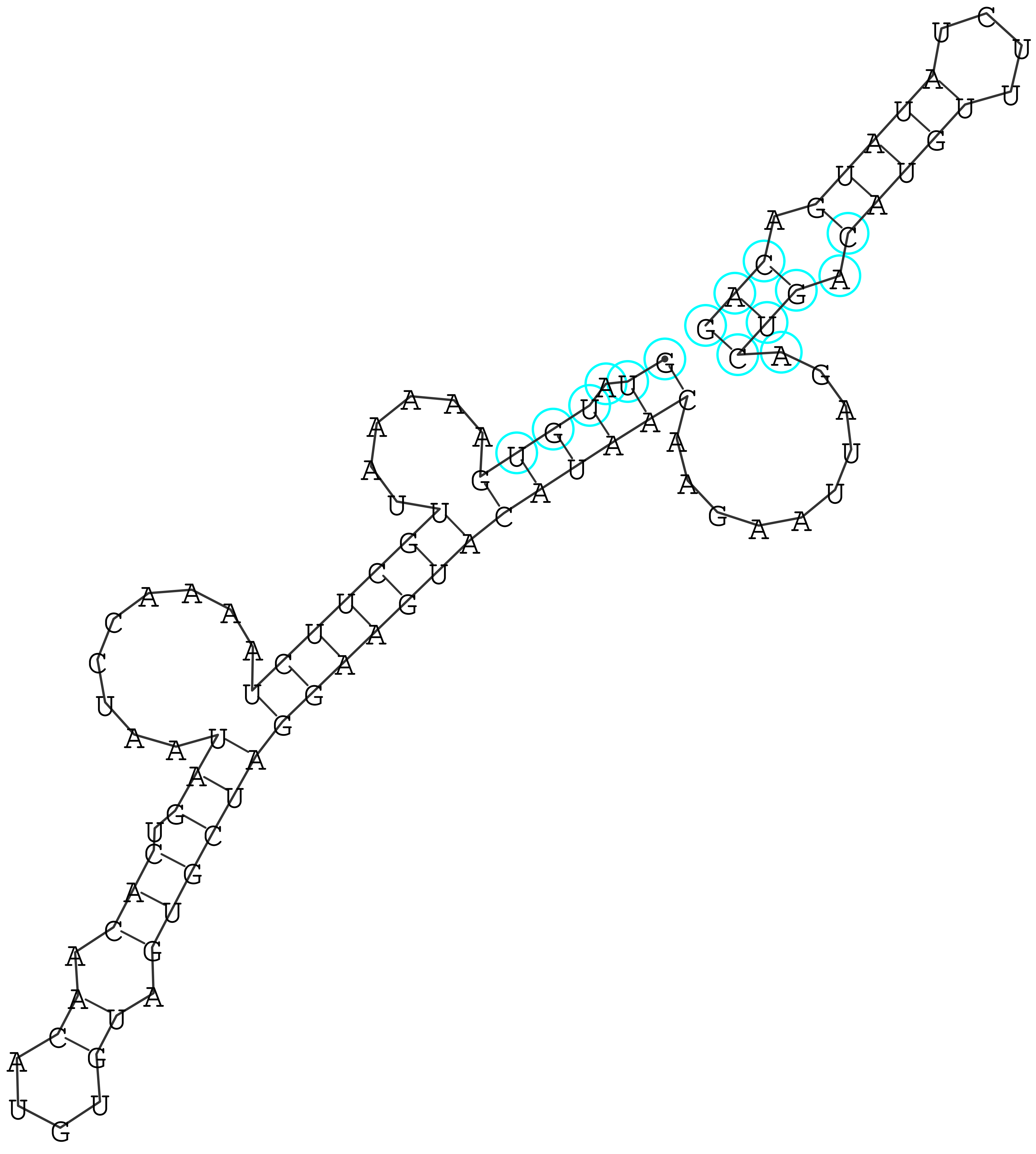

Xarbc0064A - External intron

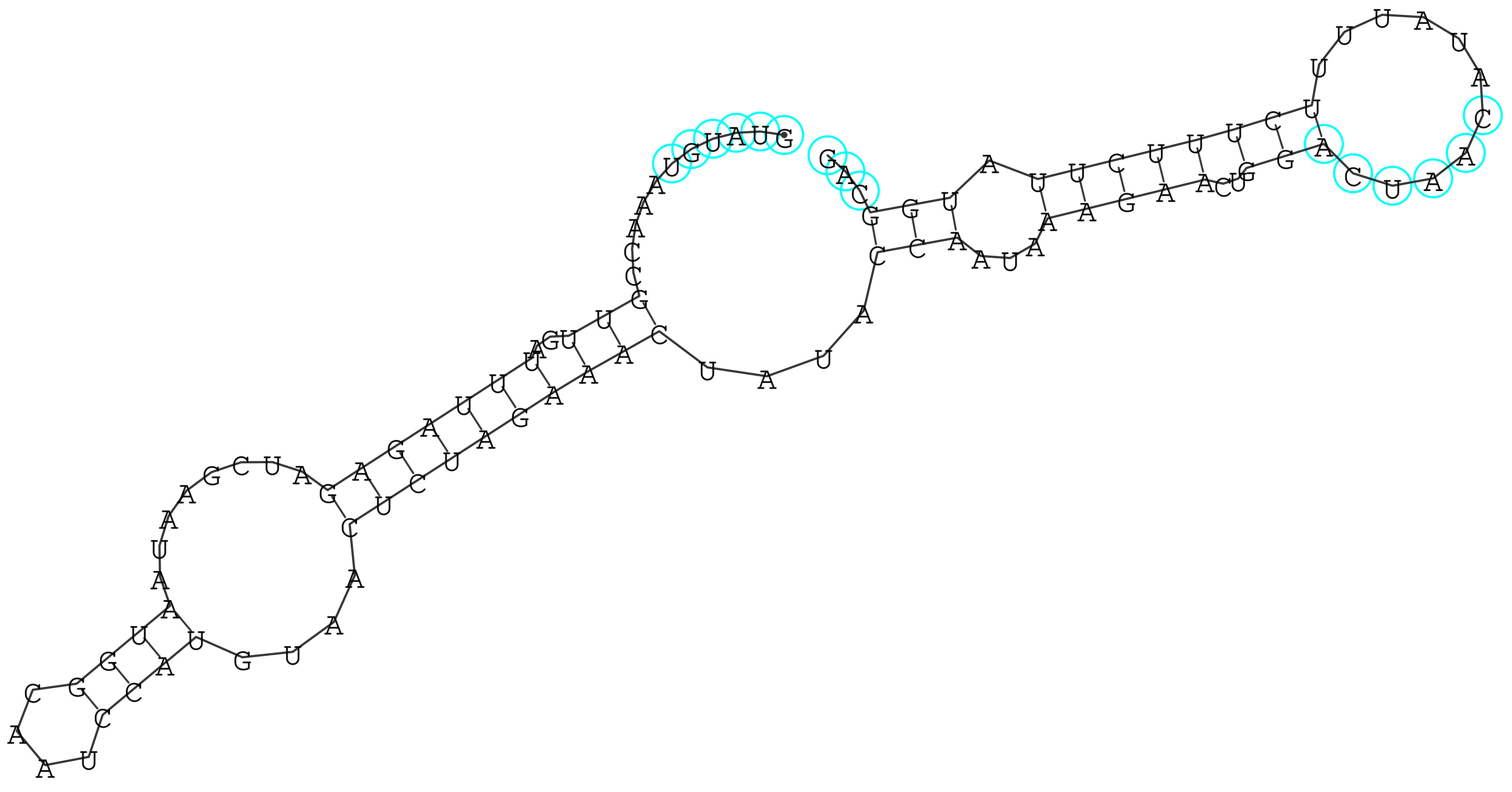

Xarbc0064B - External intron

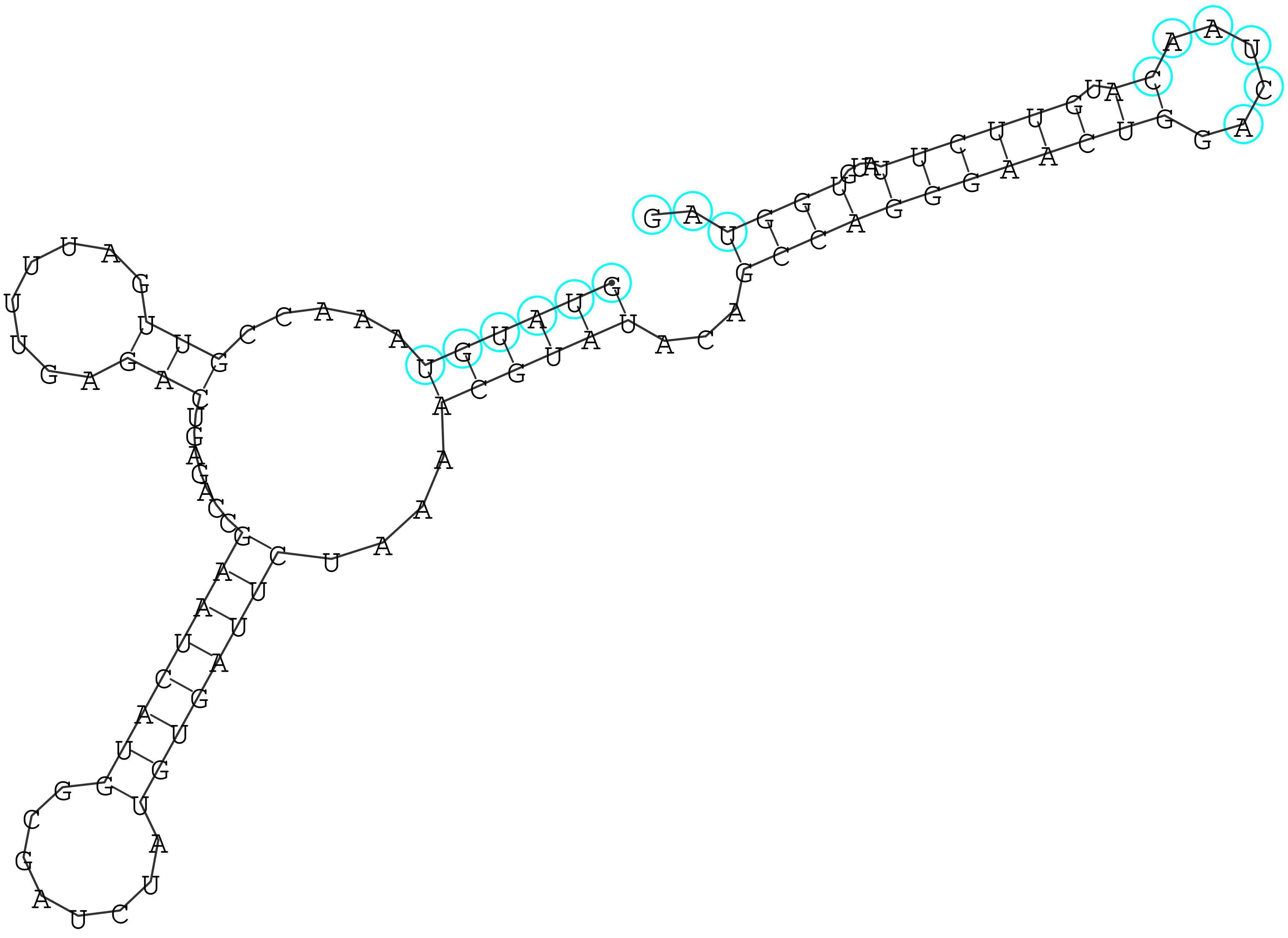

Xarbc0072A - External intron

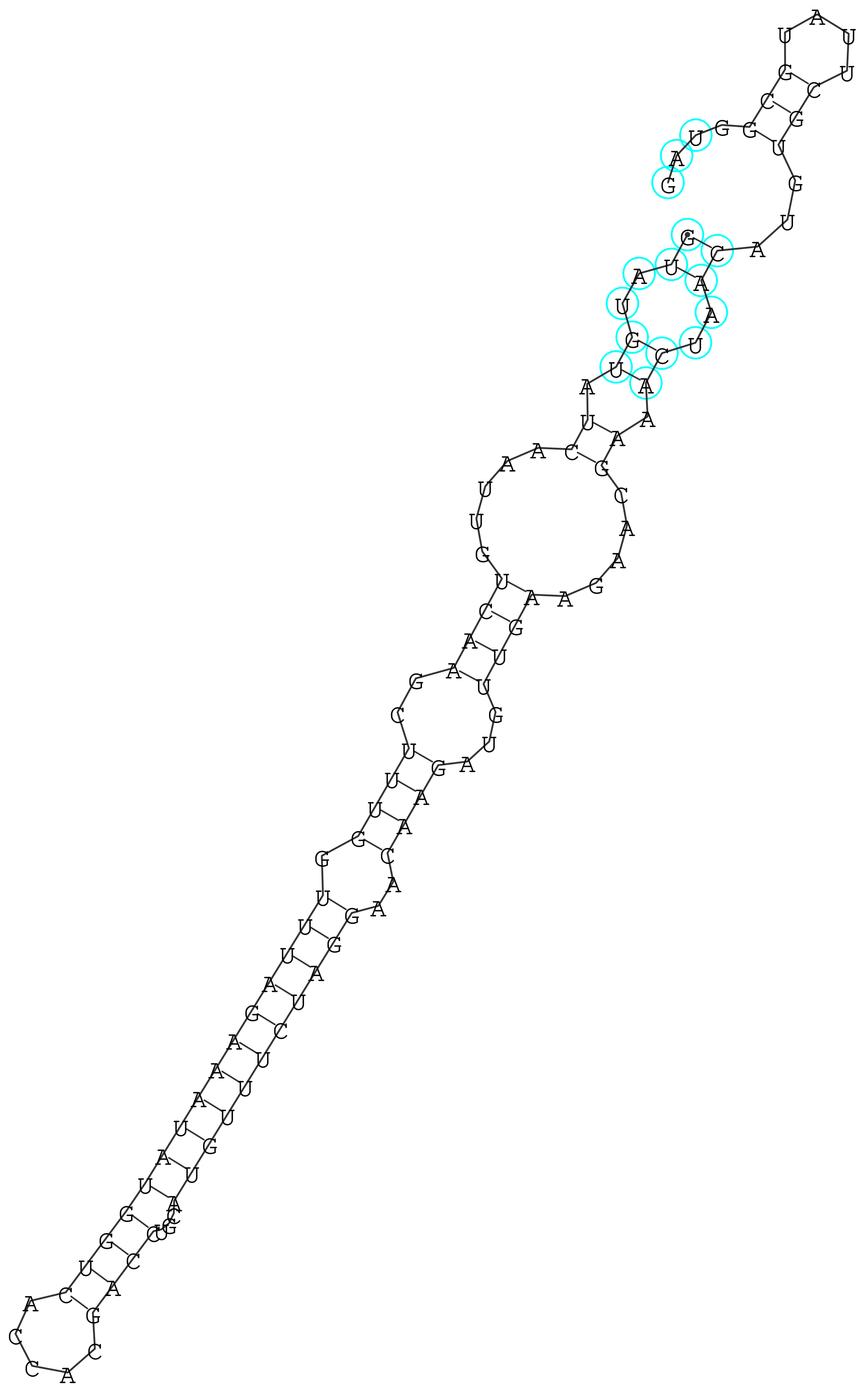

Xarbc0074A - External intron

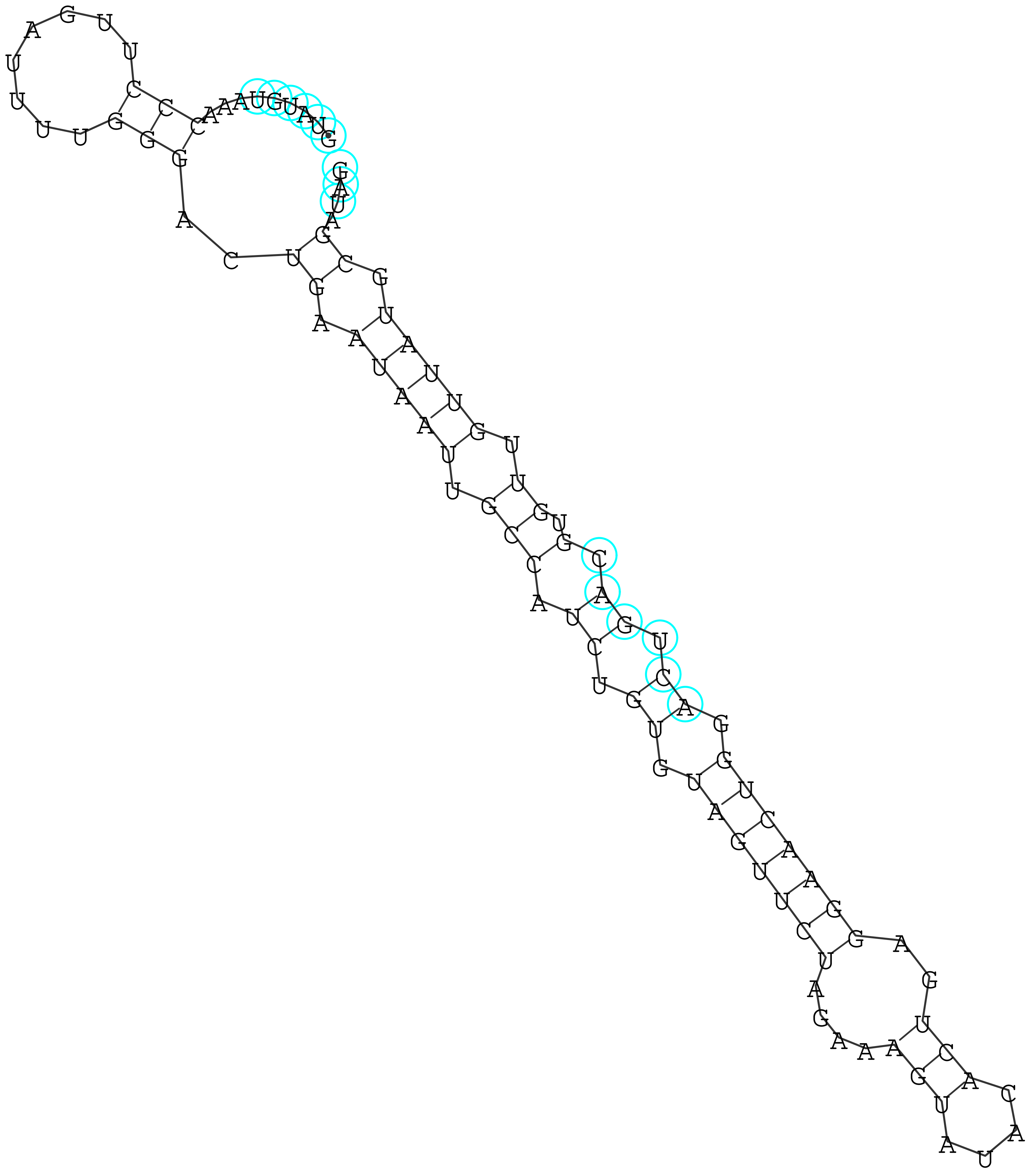

Xarbc0080A - External intron

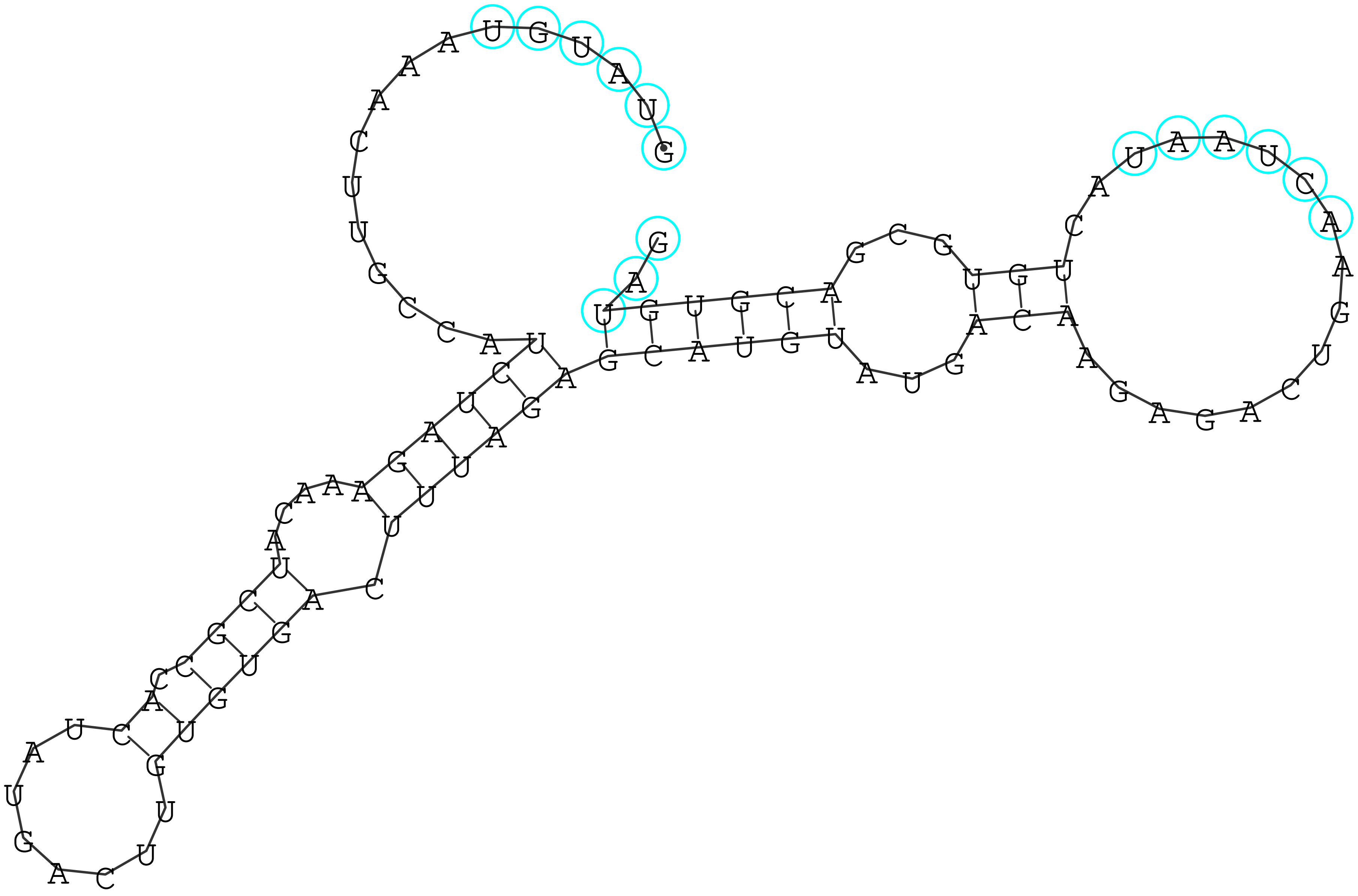

Xarbc0093A - External intron

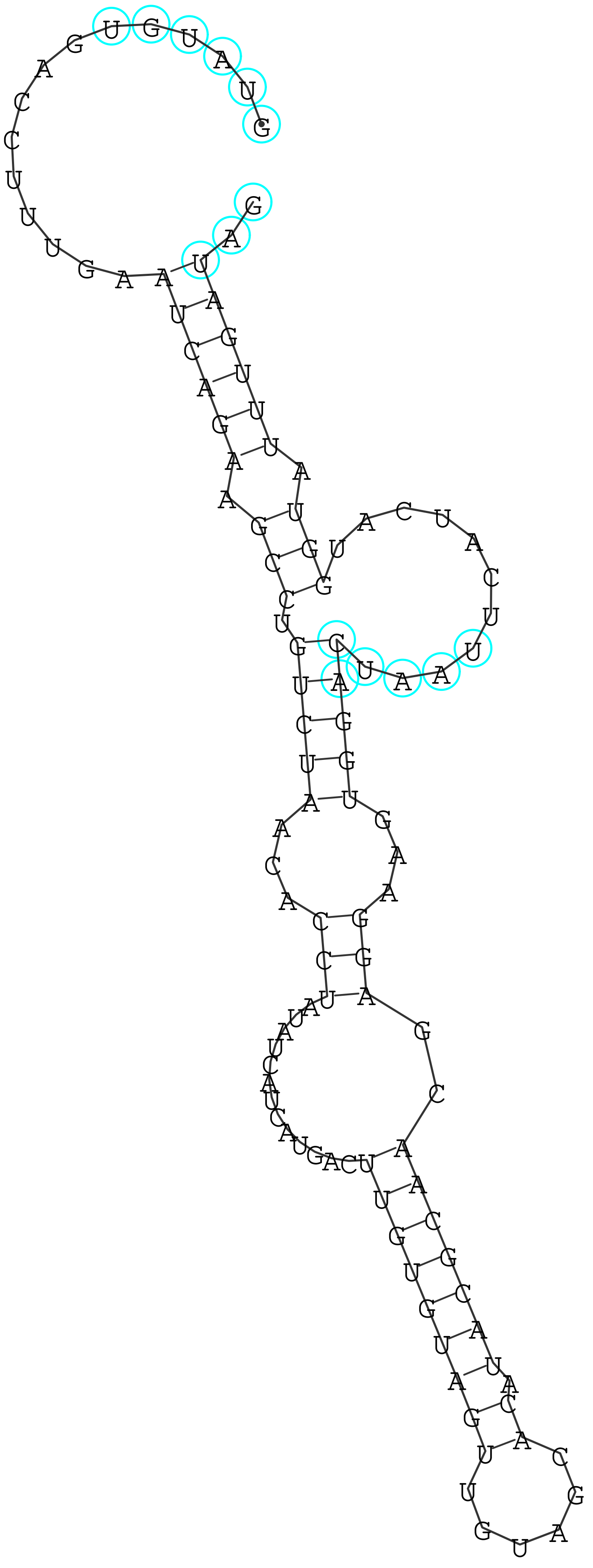

Xarbc0096A - External intron

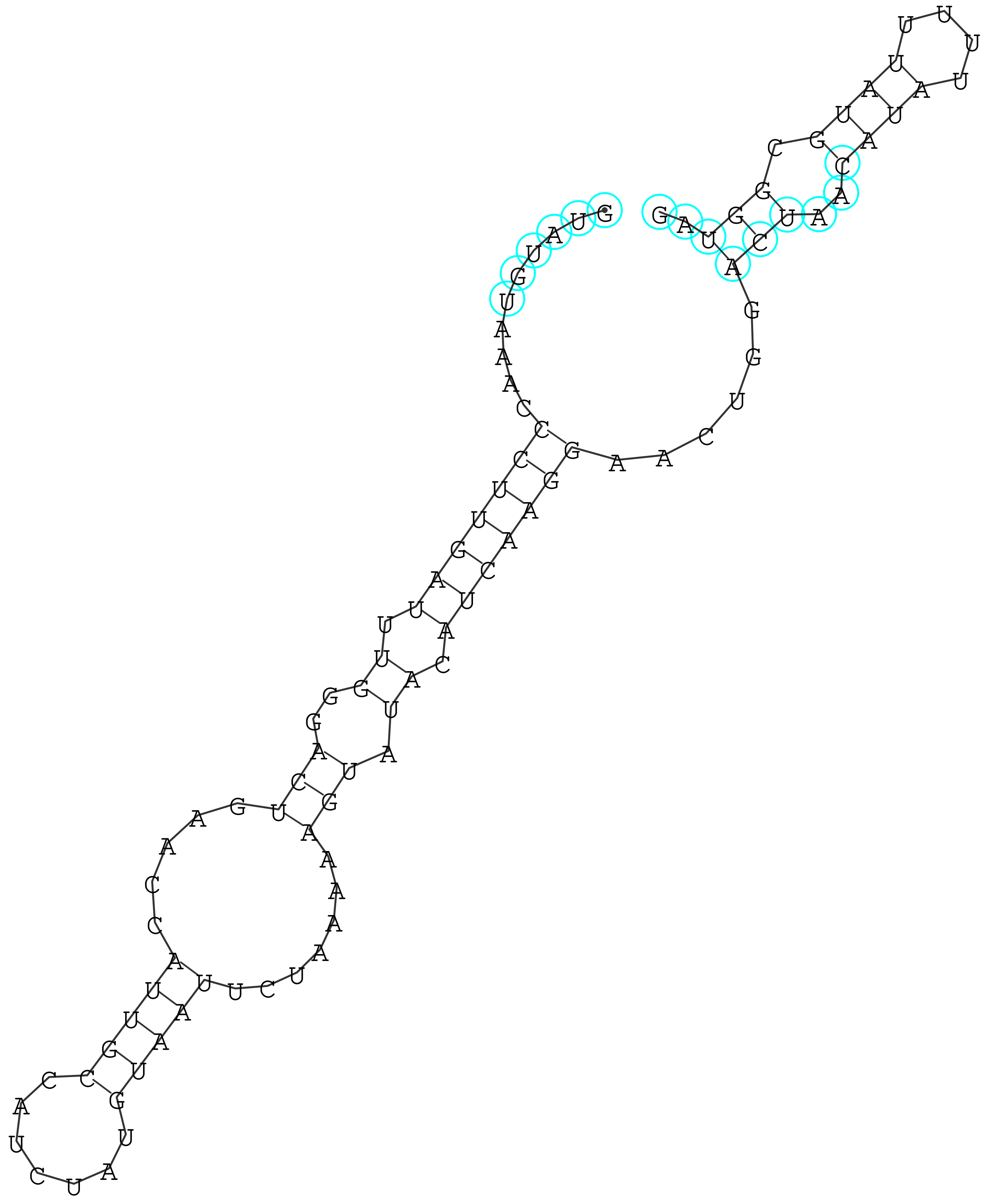

Xarbc0099A - External intron

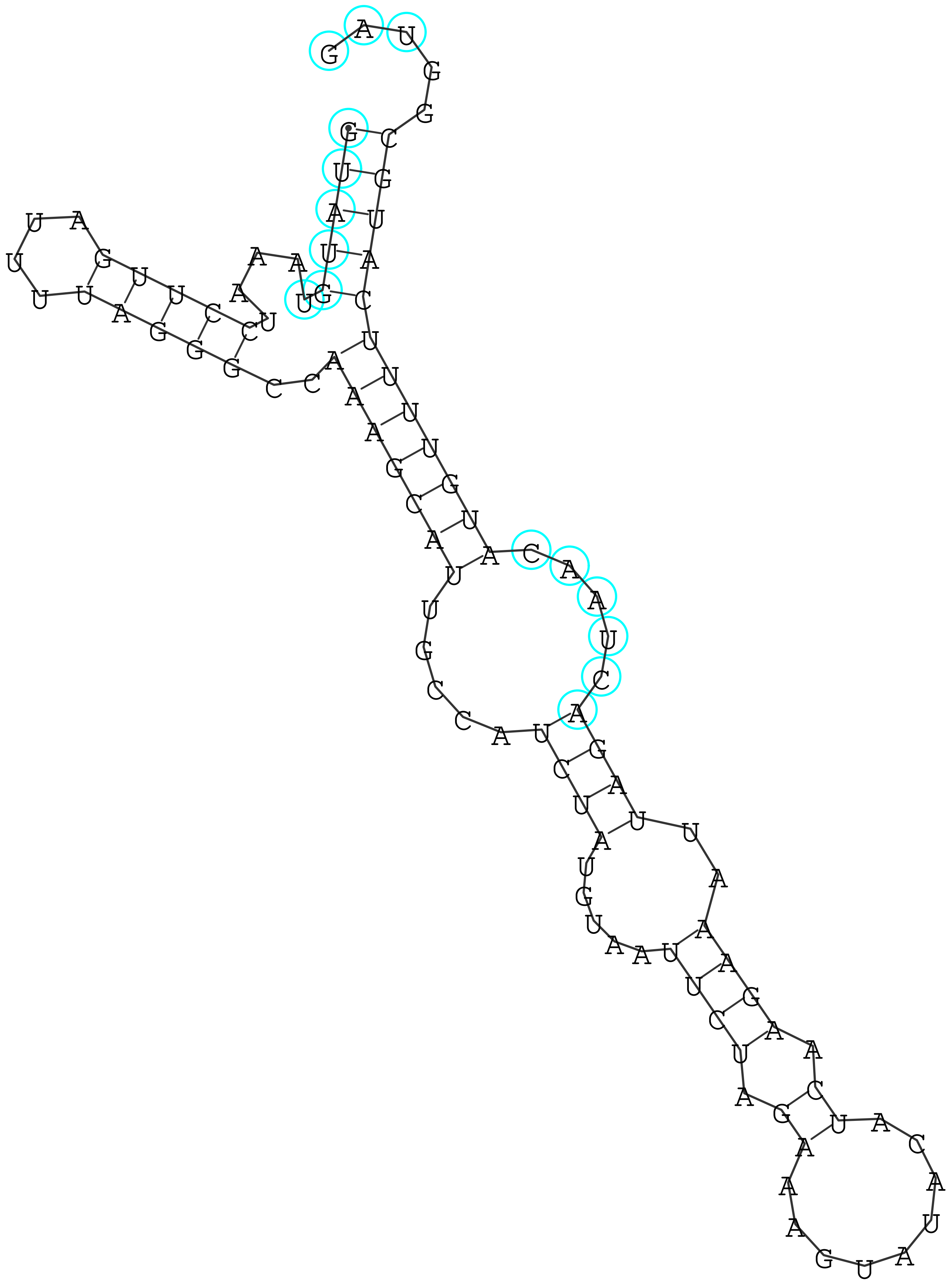

Xarbc0101A - External intron

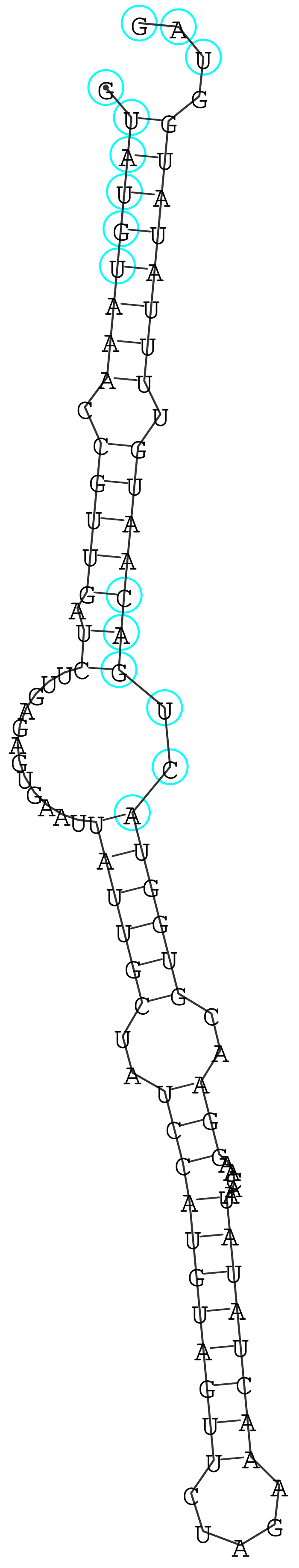

Xarbc0108A - External intron

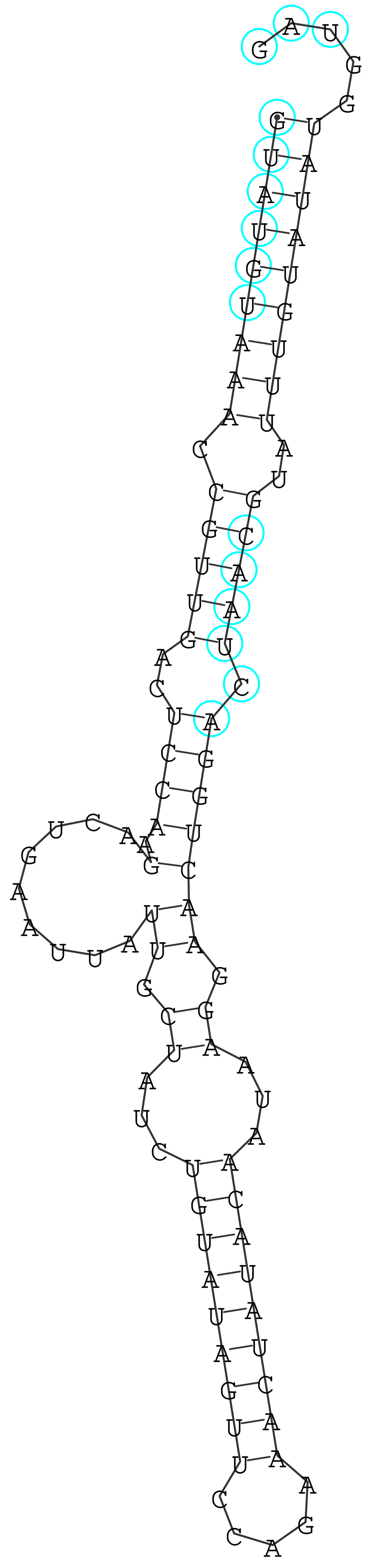

Xarbc0134A - External intron

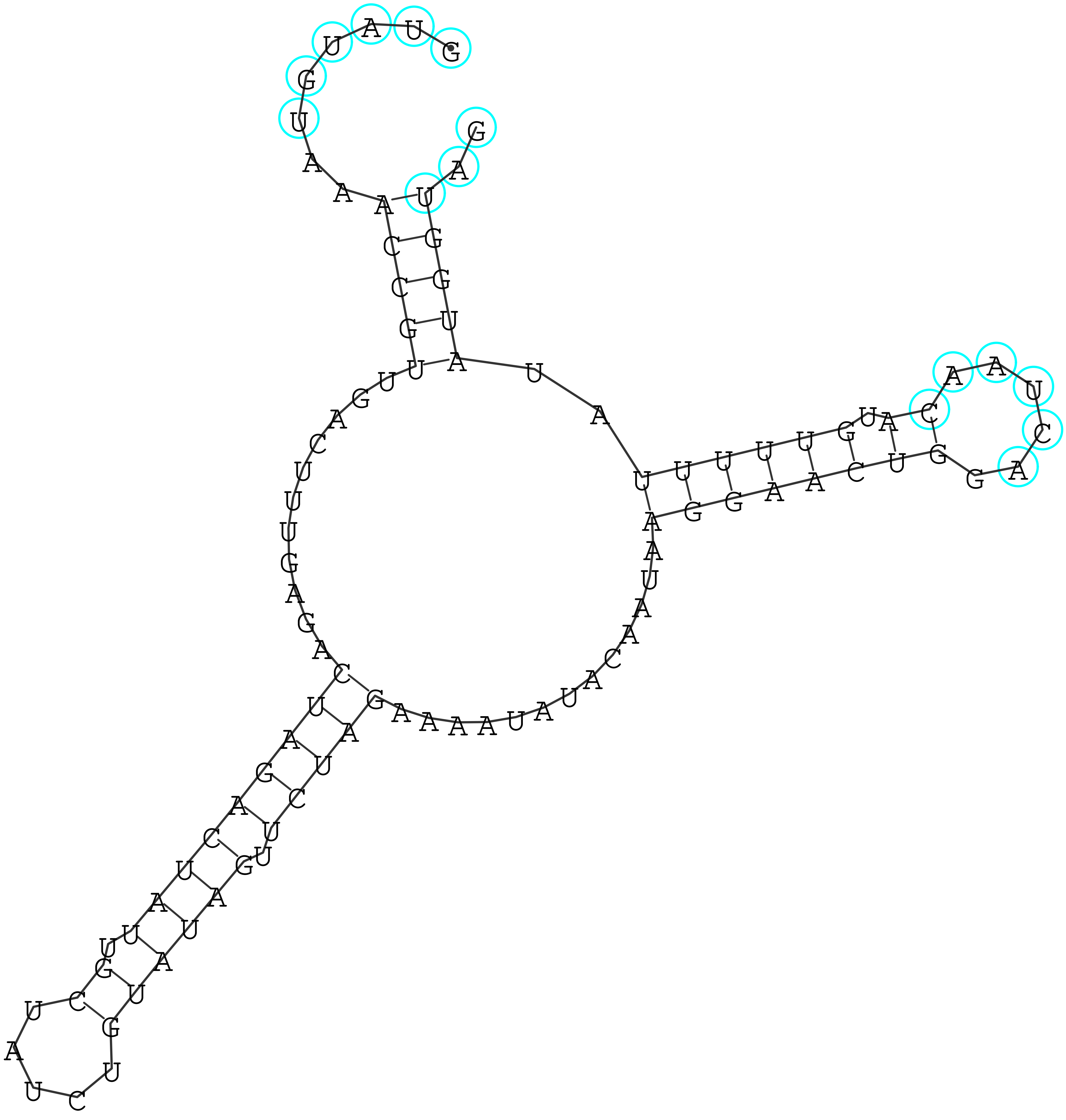

Xarbc0143A - External intron

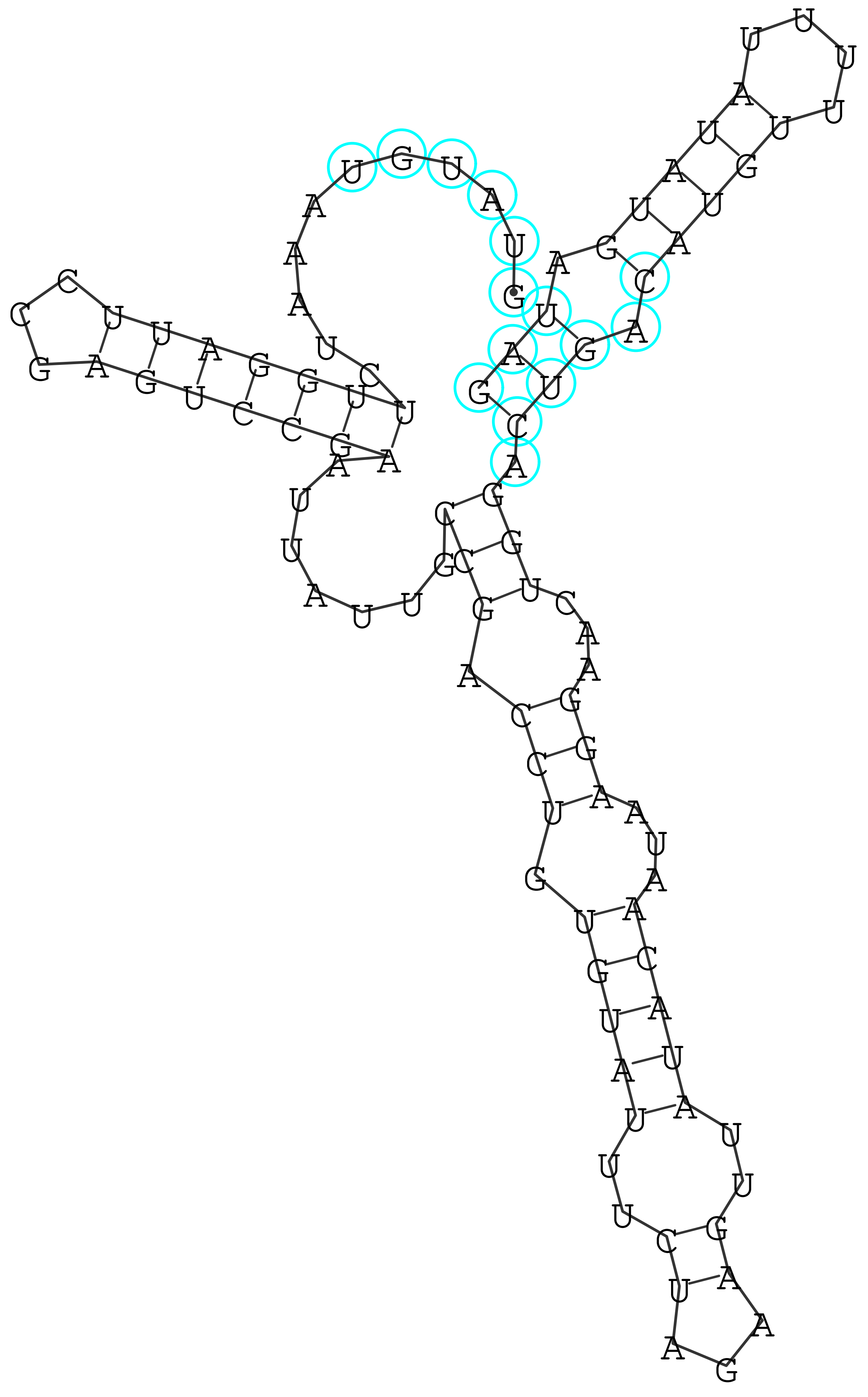

Xarbc0164A - External intron

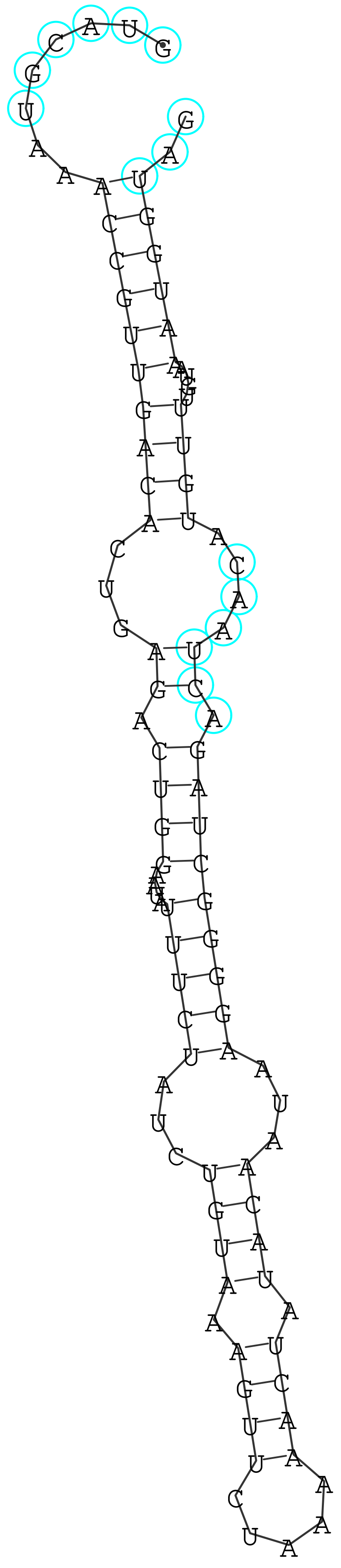

Xarbc0169A - External intron

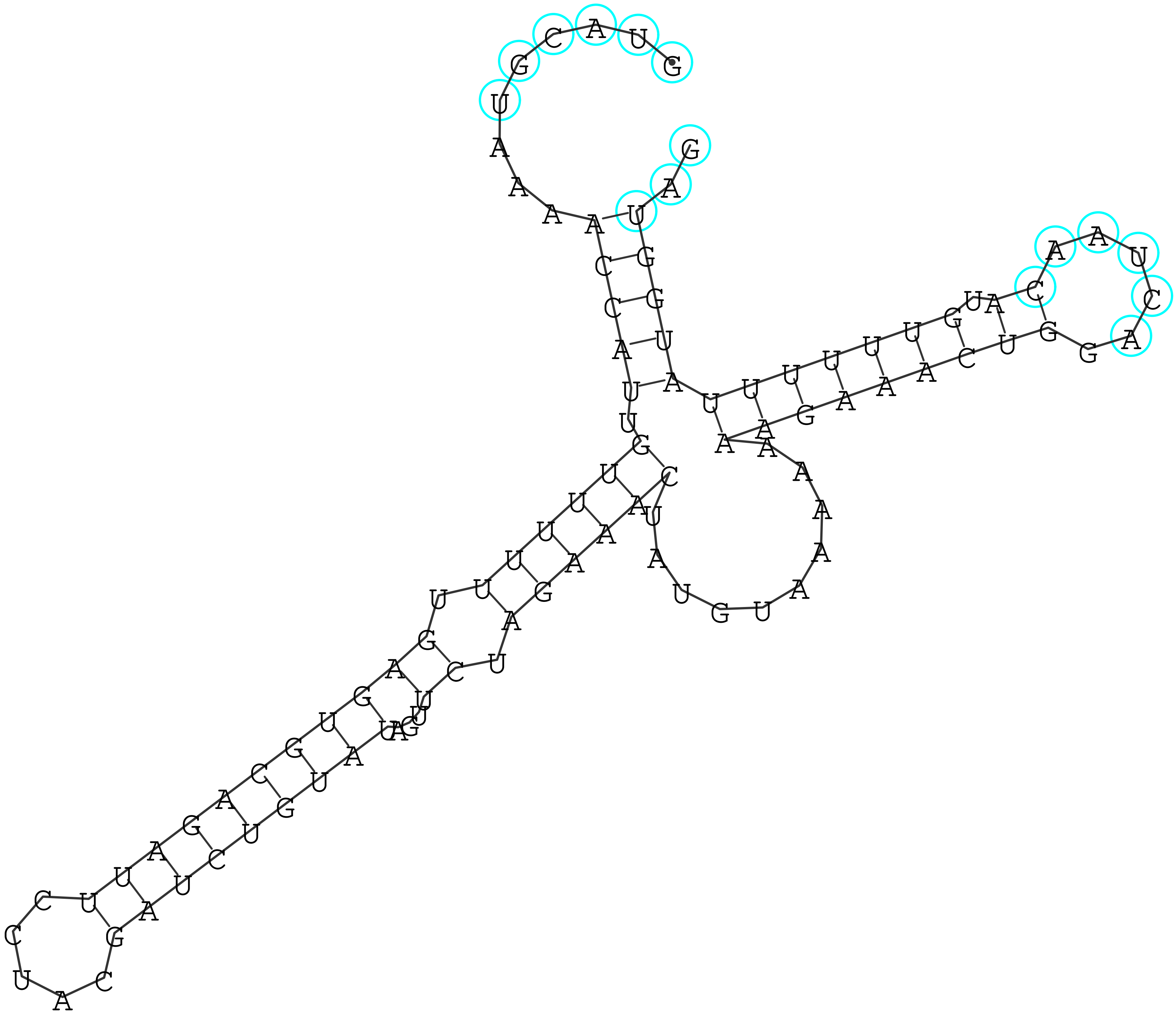

Xarbc0172A - External intron

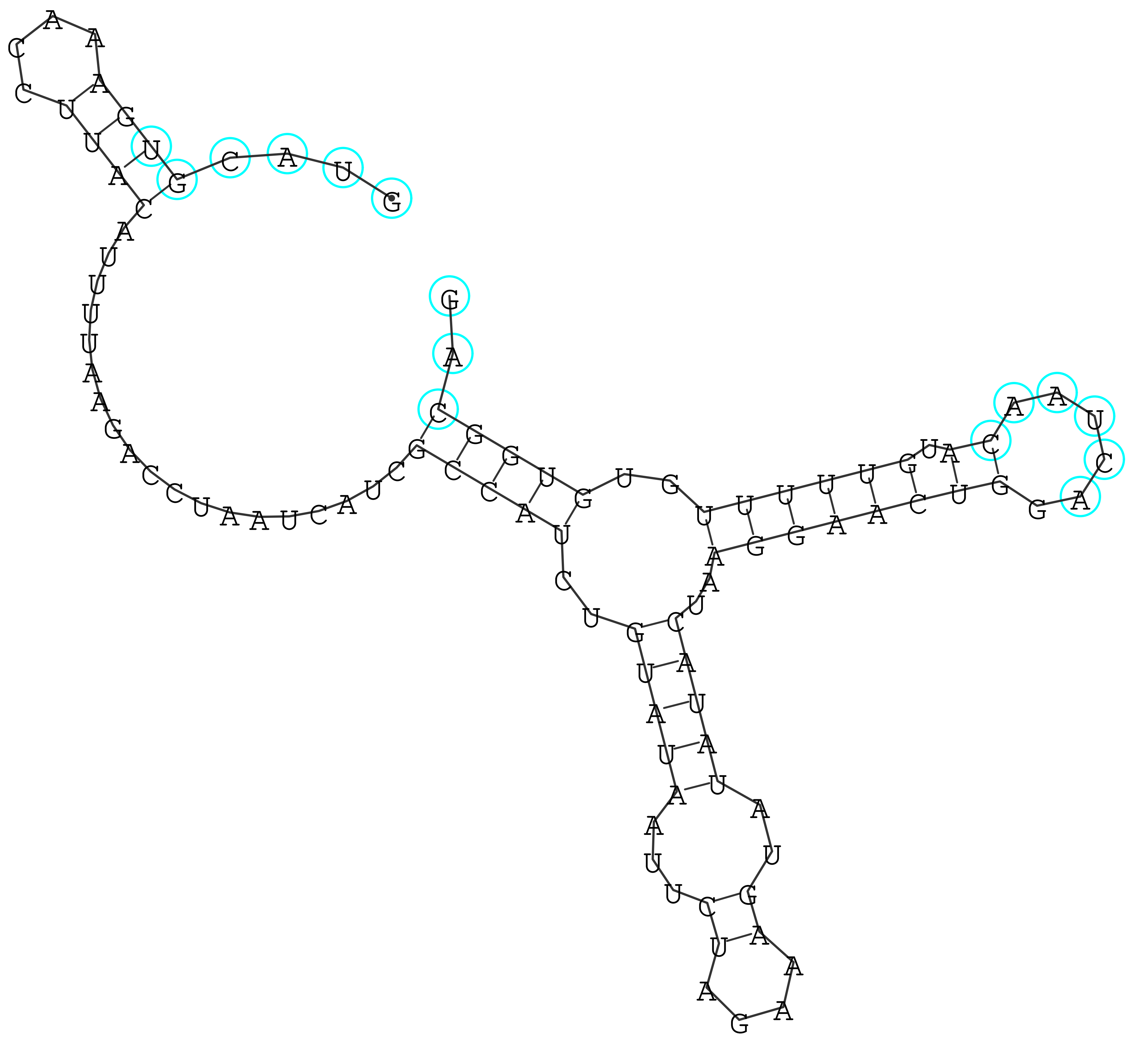

Xarbc0174A - External intron

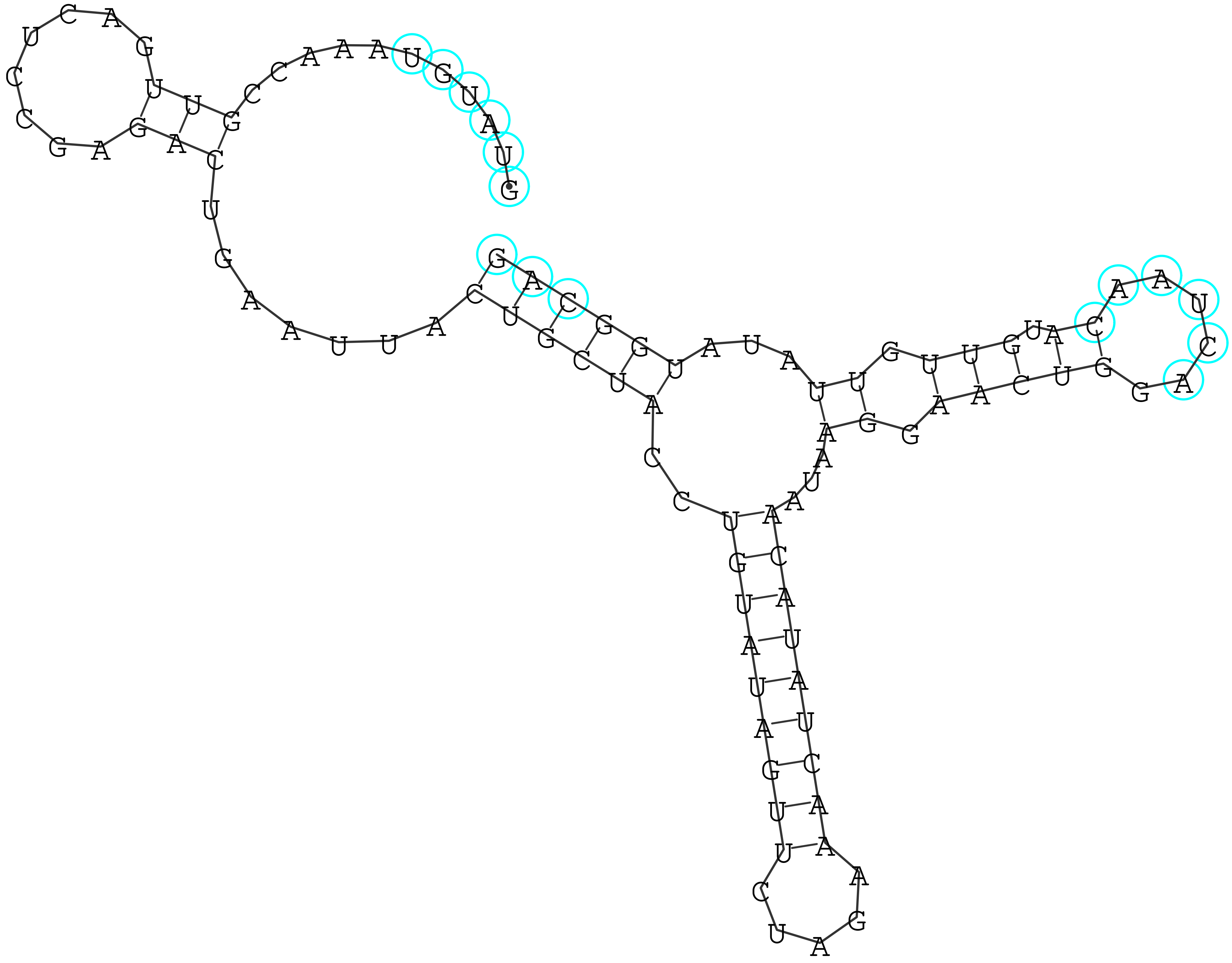

Xarbc0175A - External intron

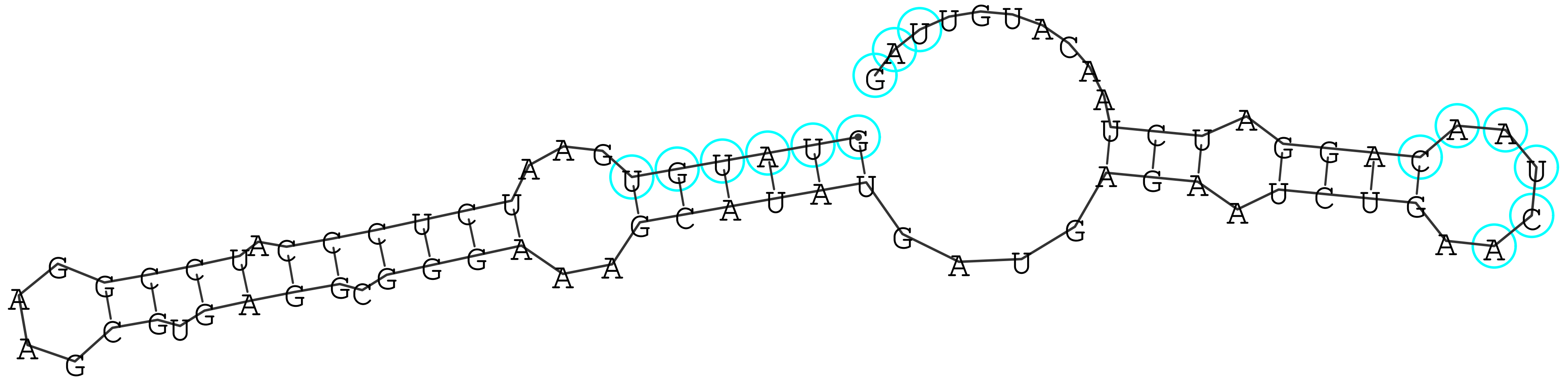

Xarbc0192A - External intron

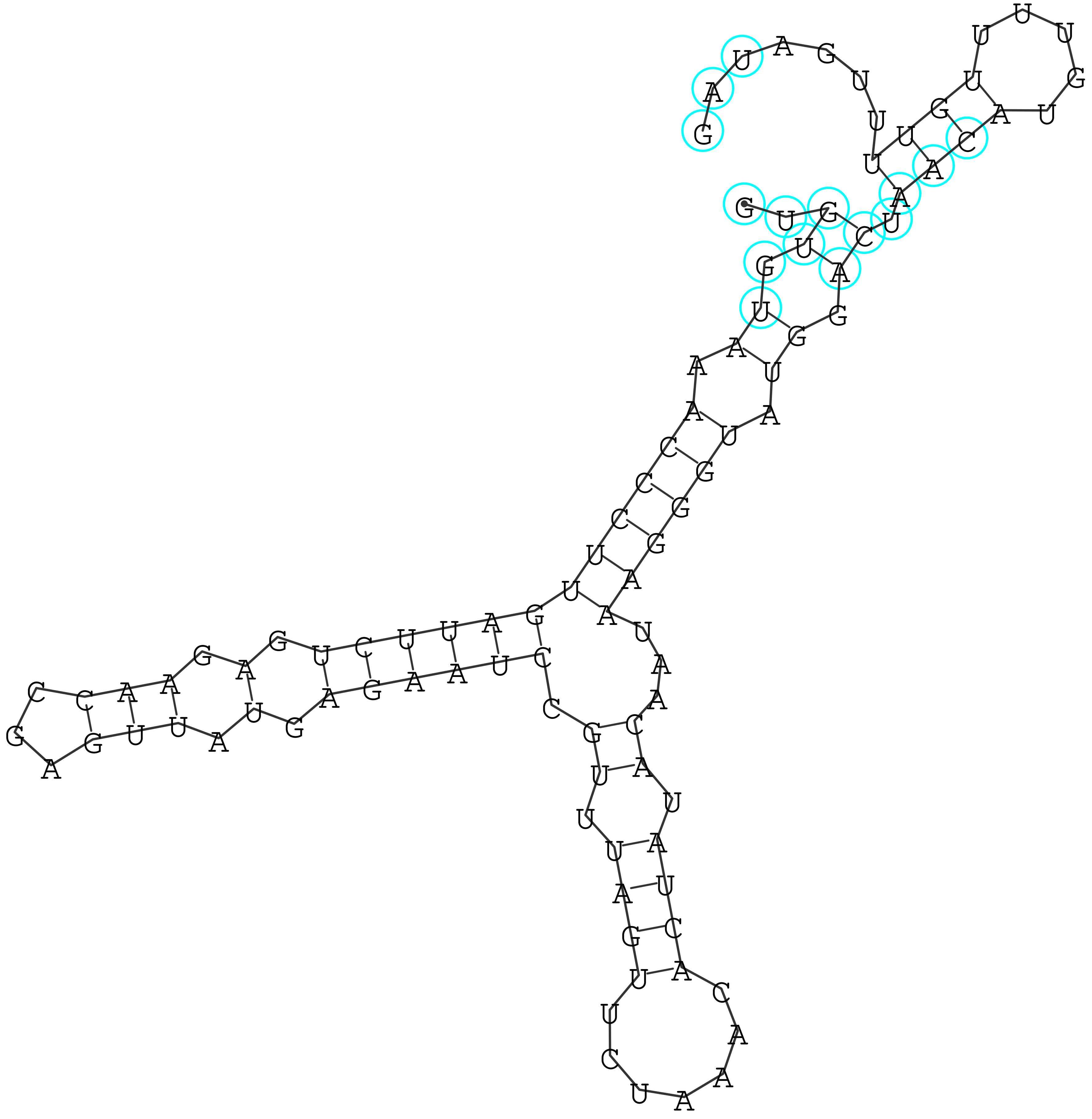

Xarbc0195A - External intron

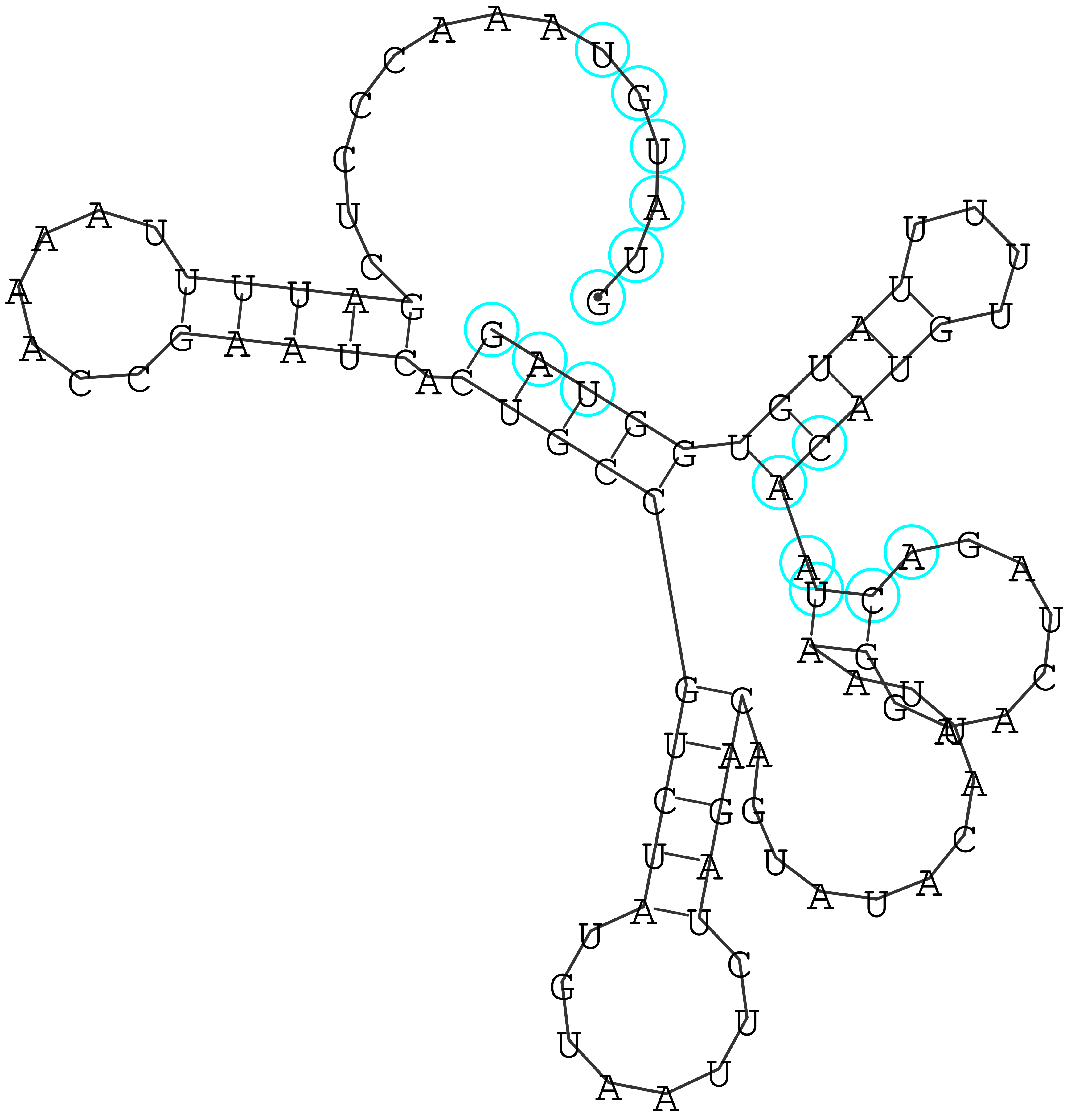

Xarbc0220A - External intron

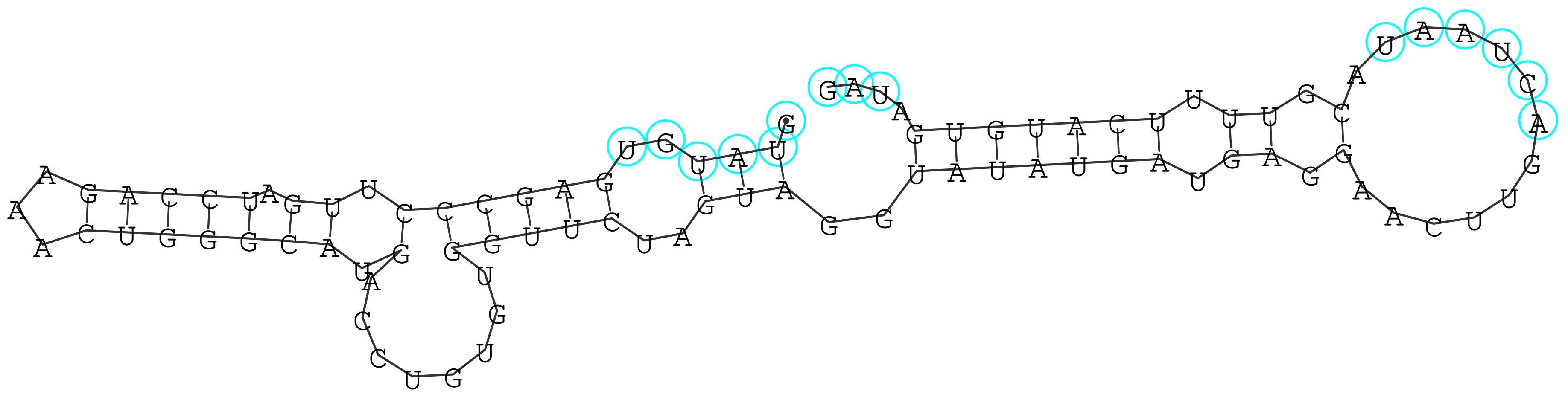

Xarbc0240A - External intron

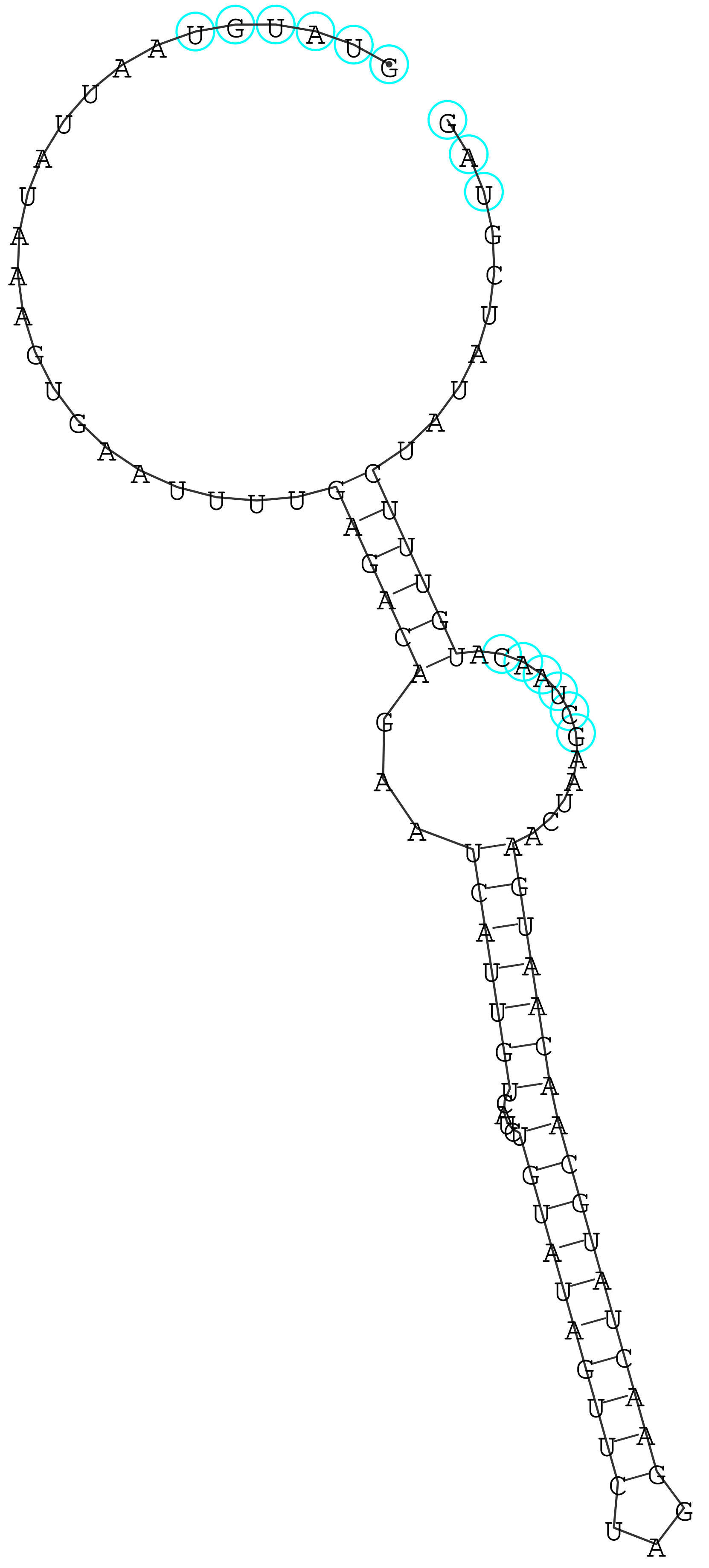

Xarbc0240B - External intron

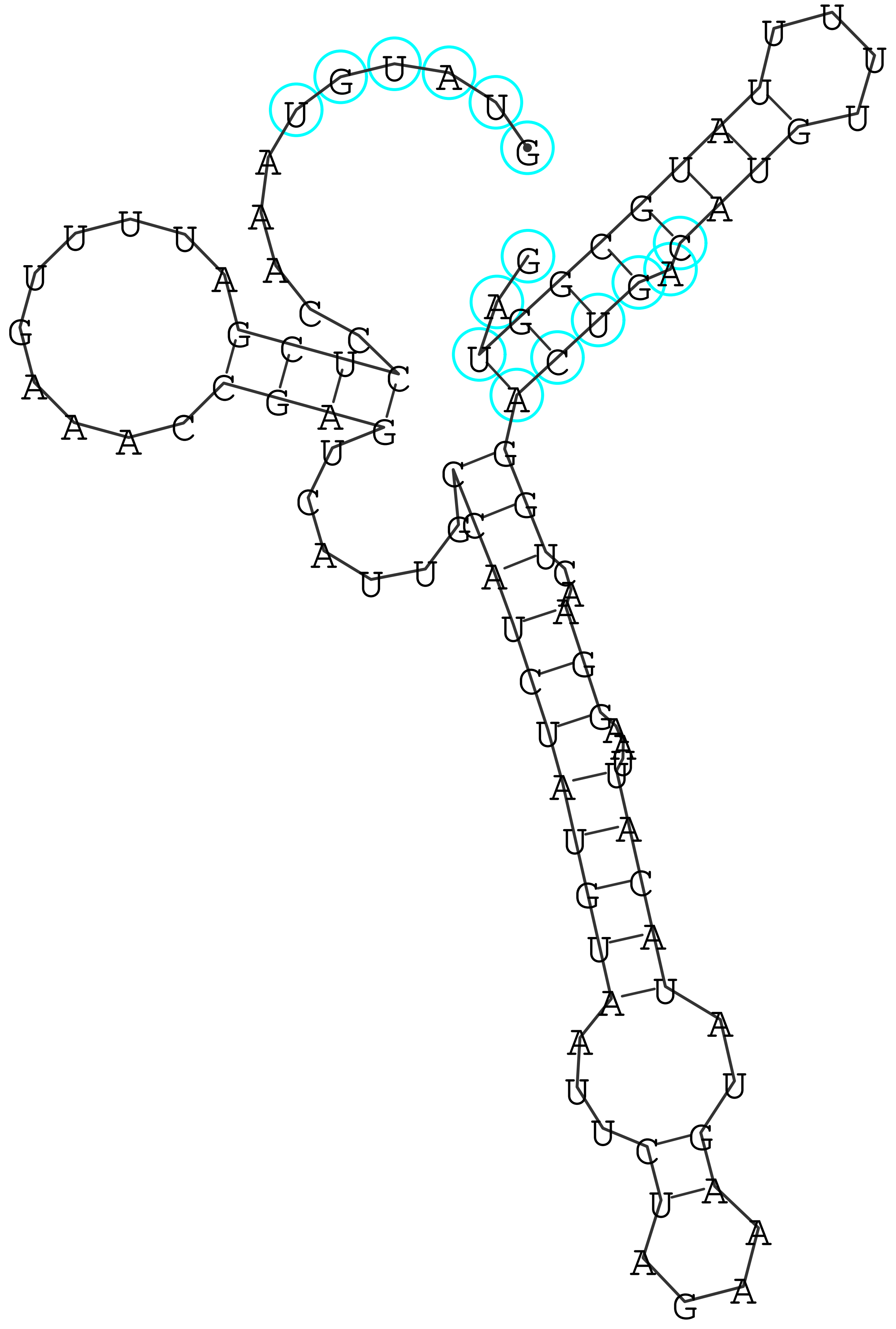

Xarbc0253A - External intron

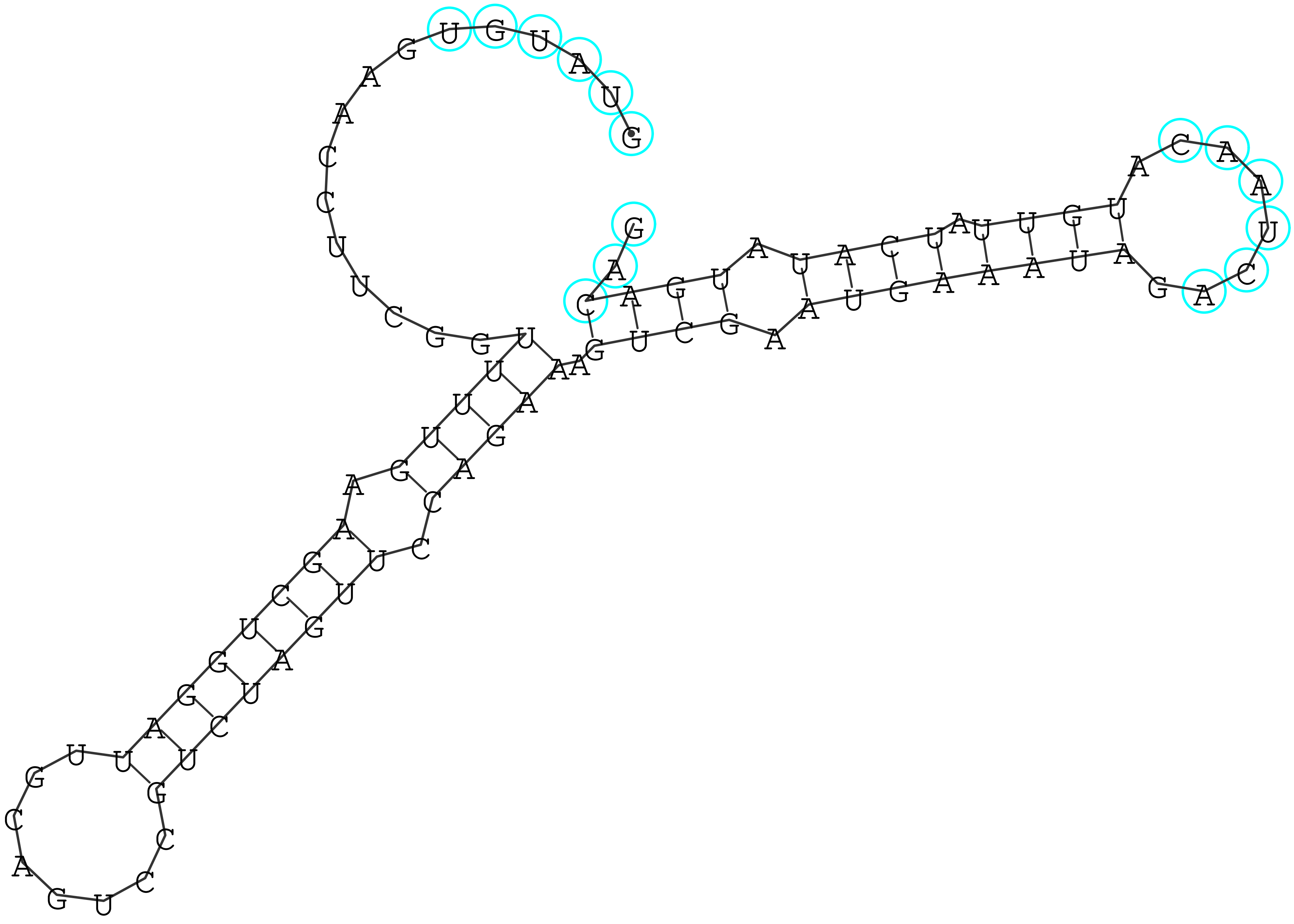

Xarbc0274A - External intron

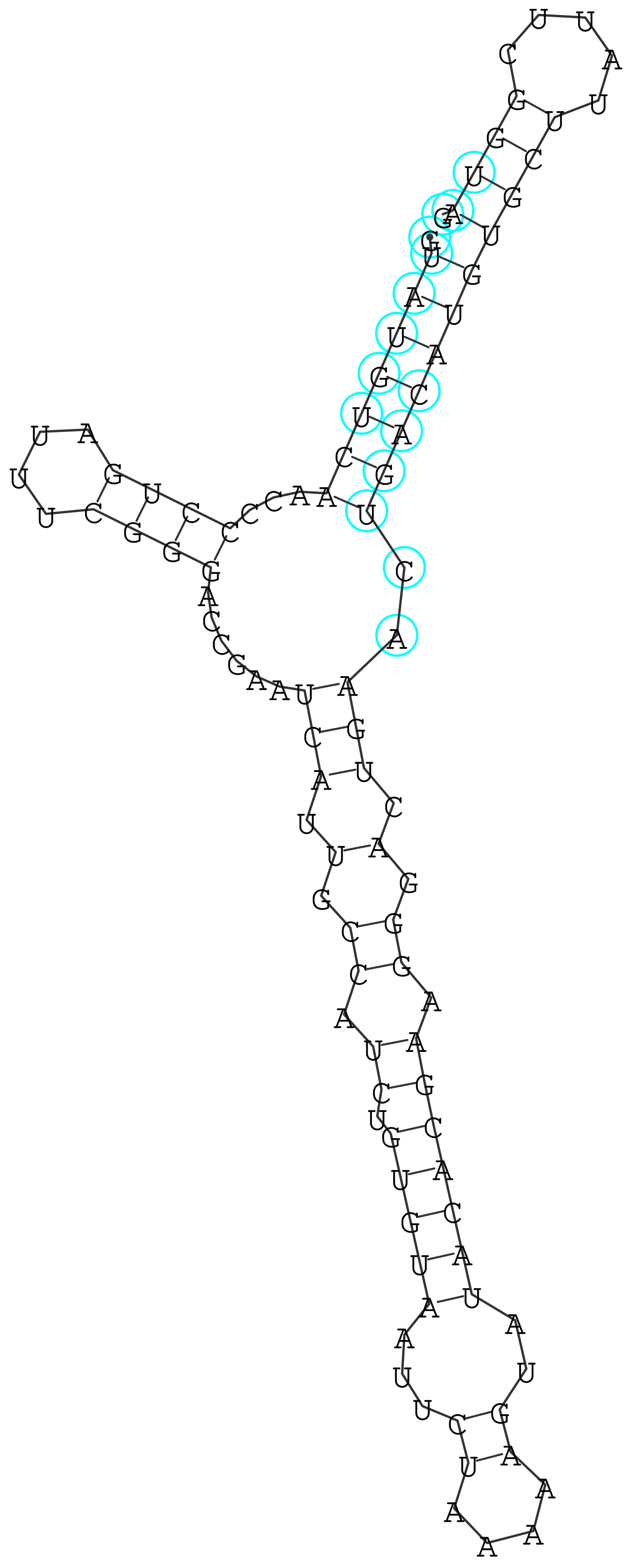

Xarbc0299A - External intron

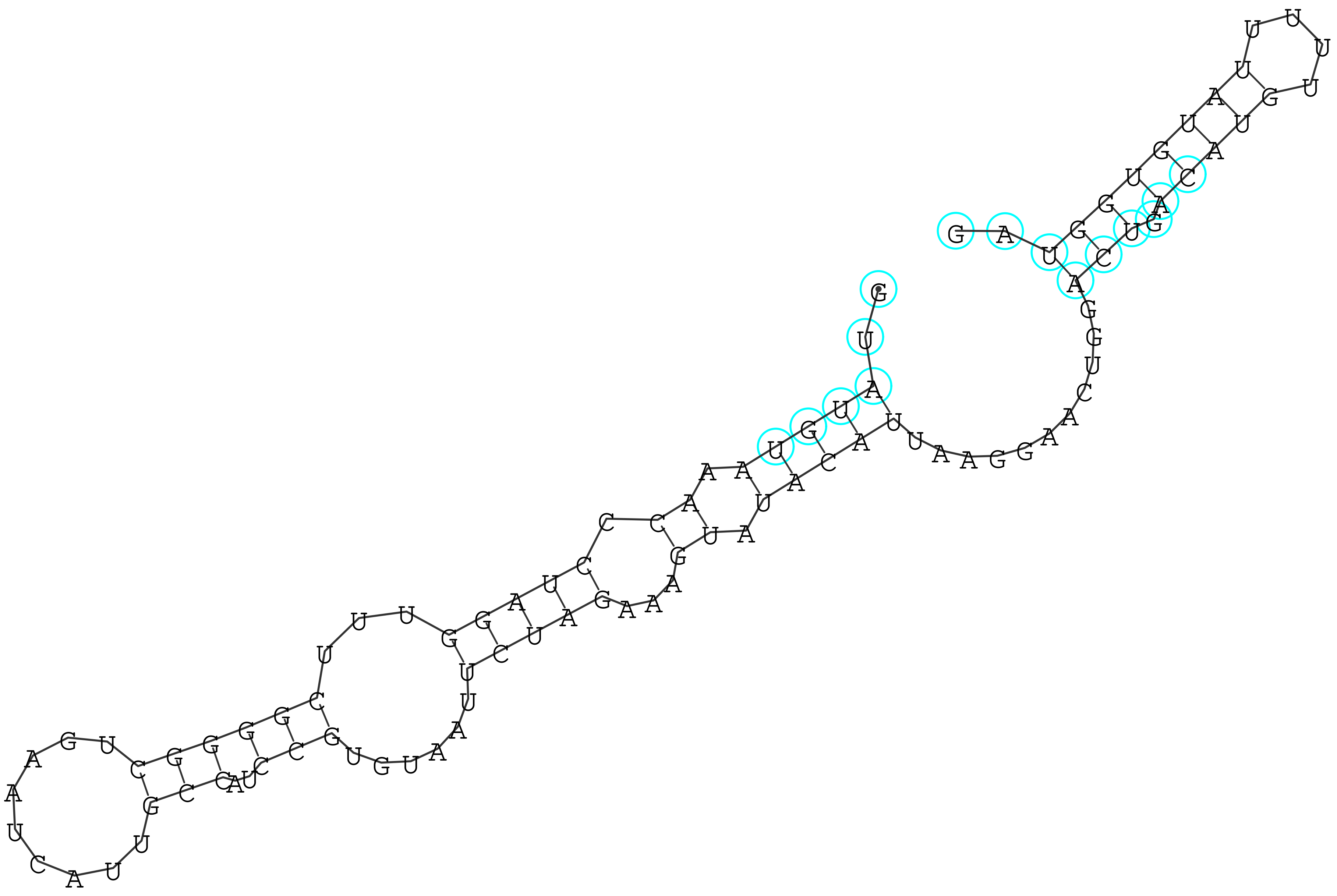

Xarbc0299B - External intron

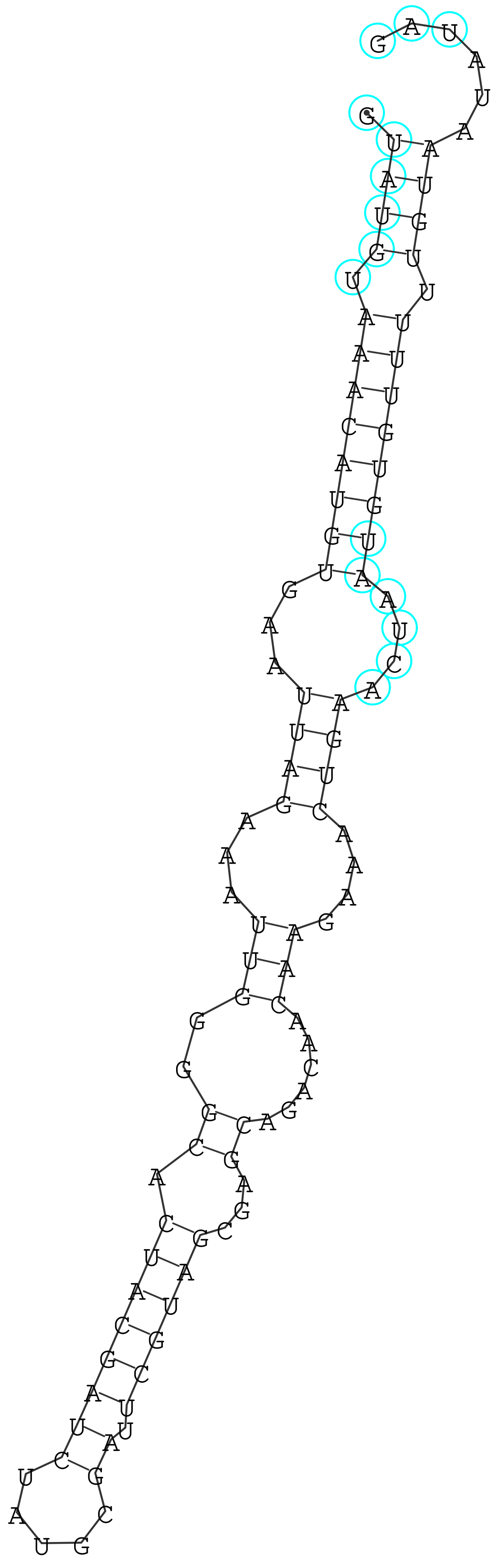

Xarbc0301A - External intron

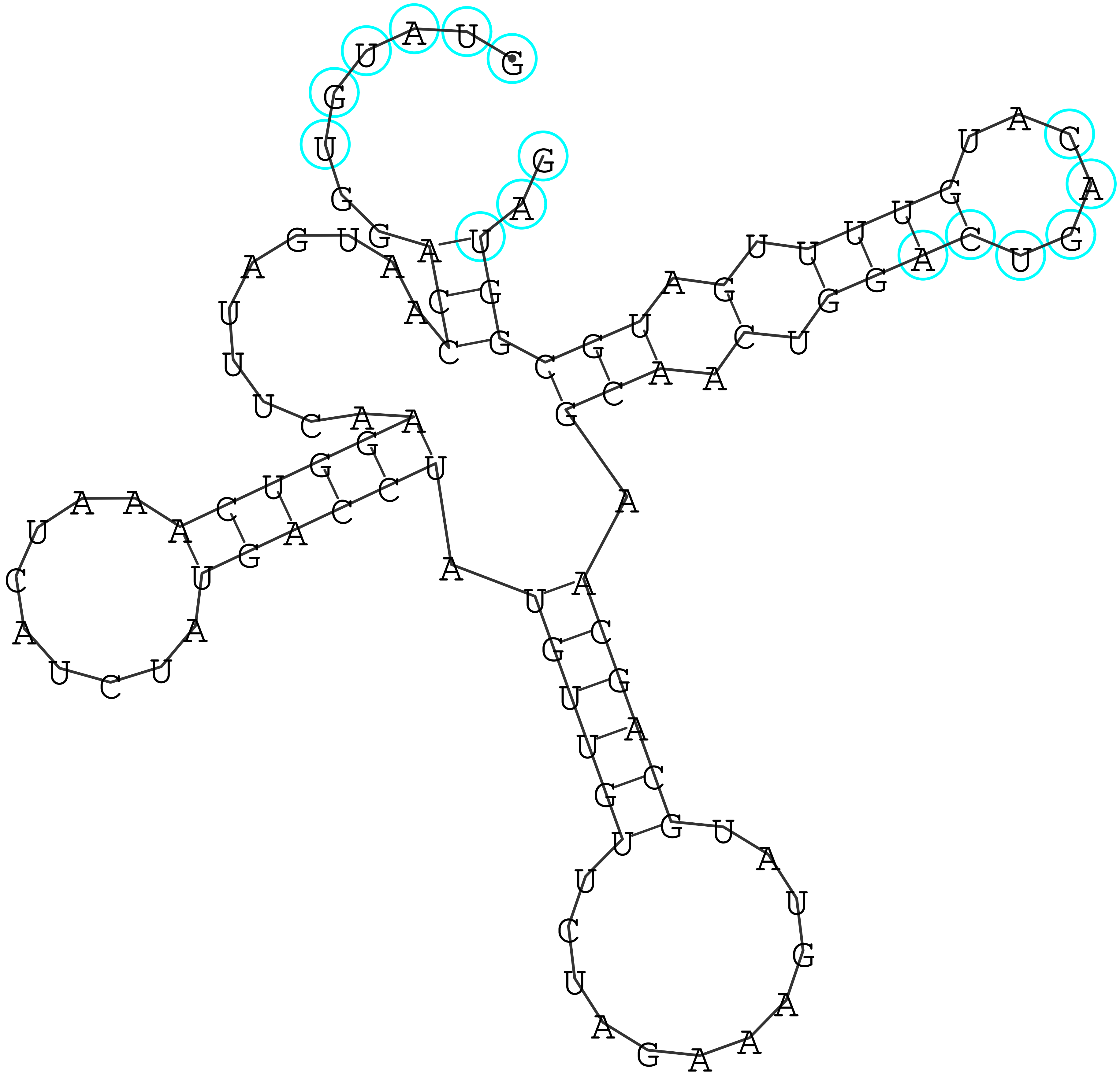

Xarbc0309A - External intron

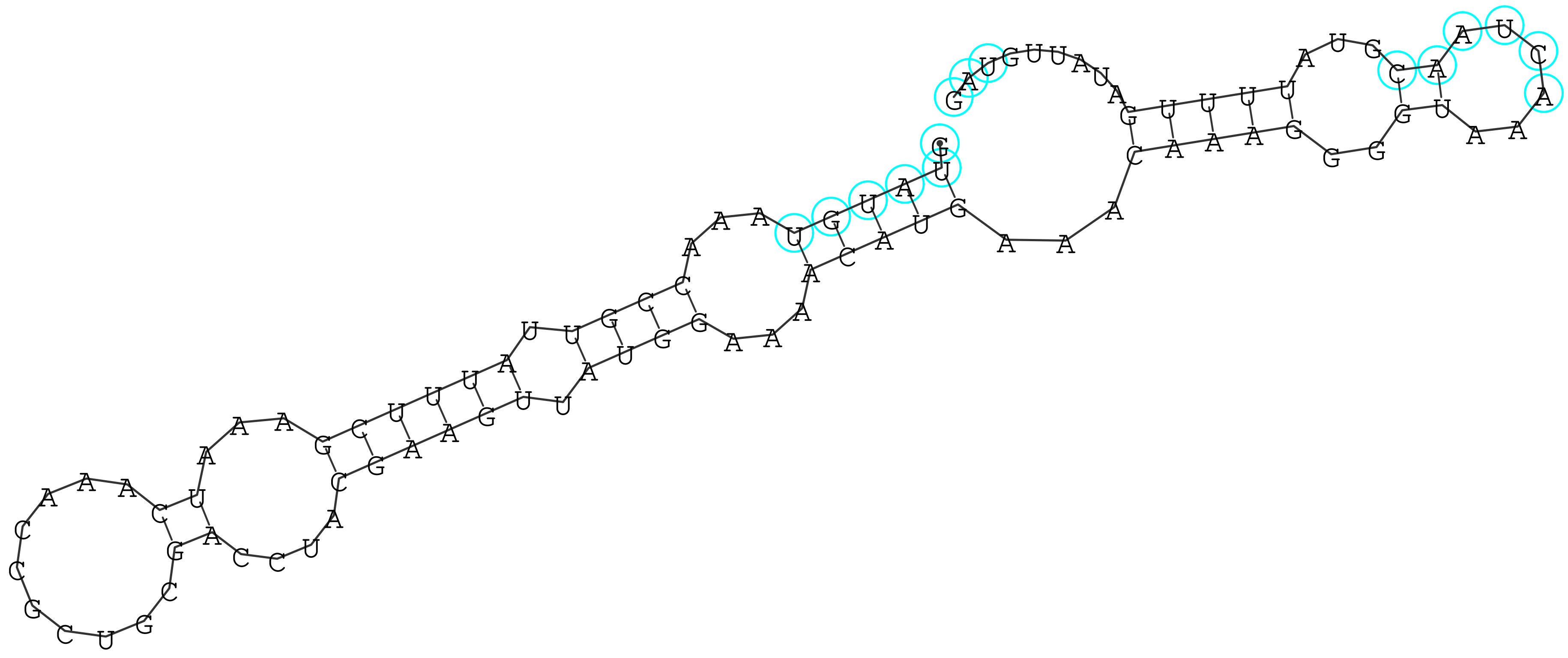

Xarbc0311A - External intron

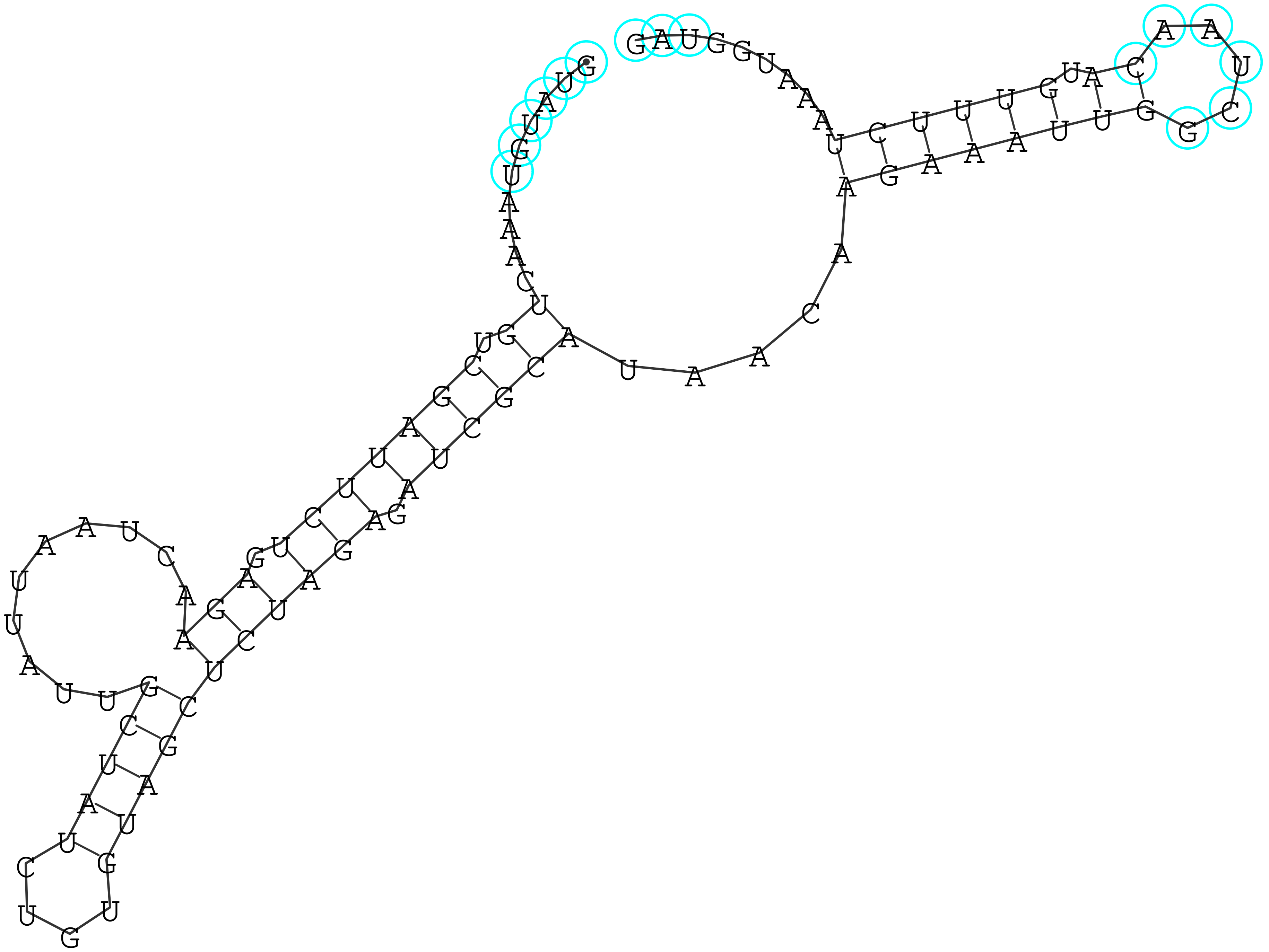

Xarbc0324A - External intron

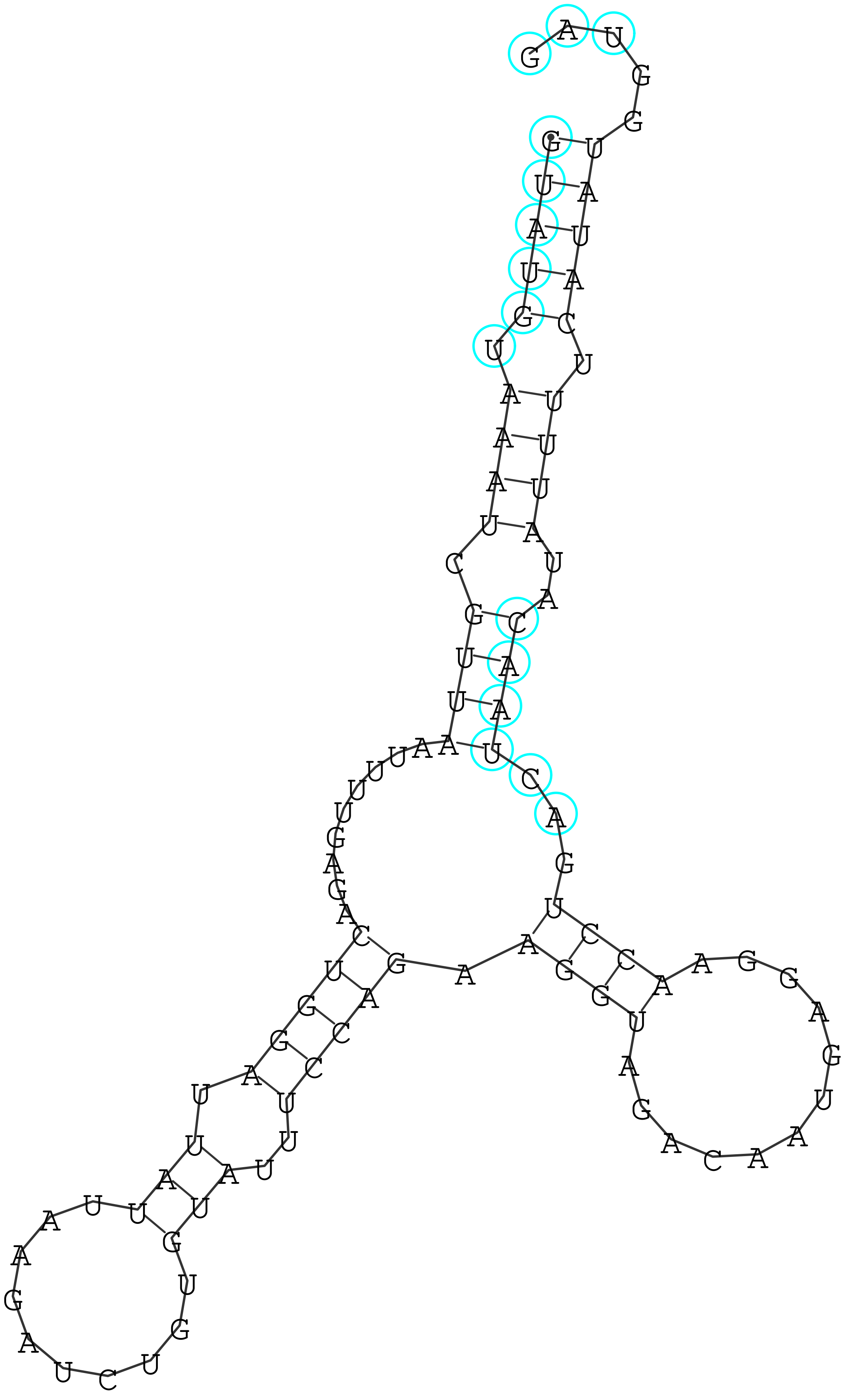

Xarbc0447A - External intron

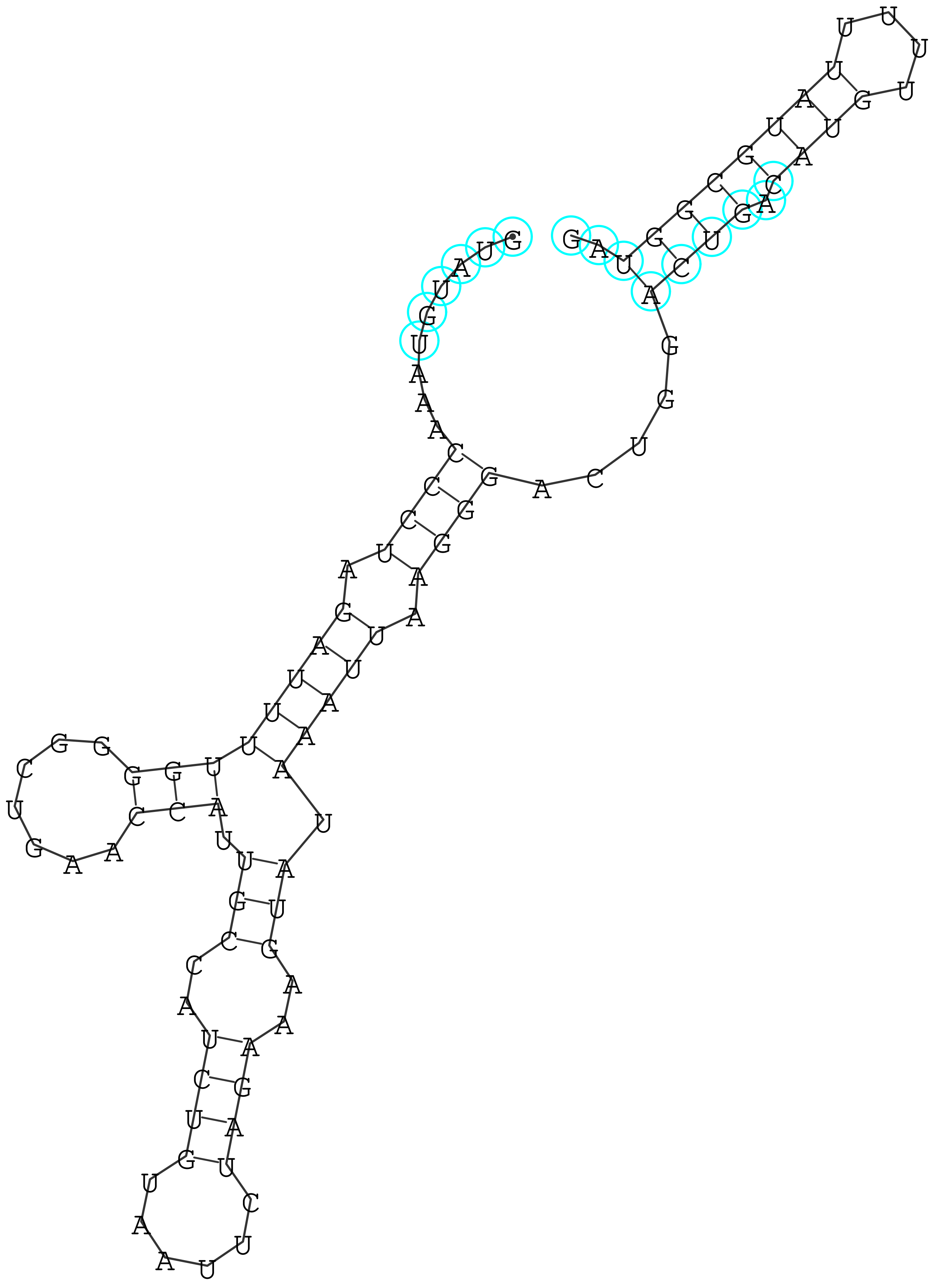

Xbamc009A - External intron

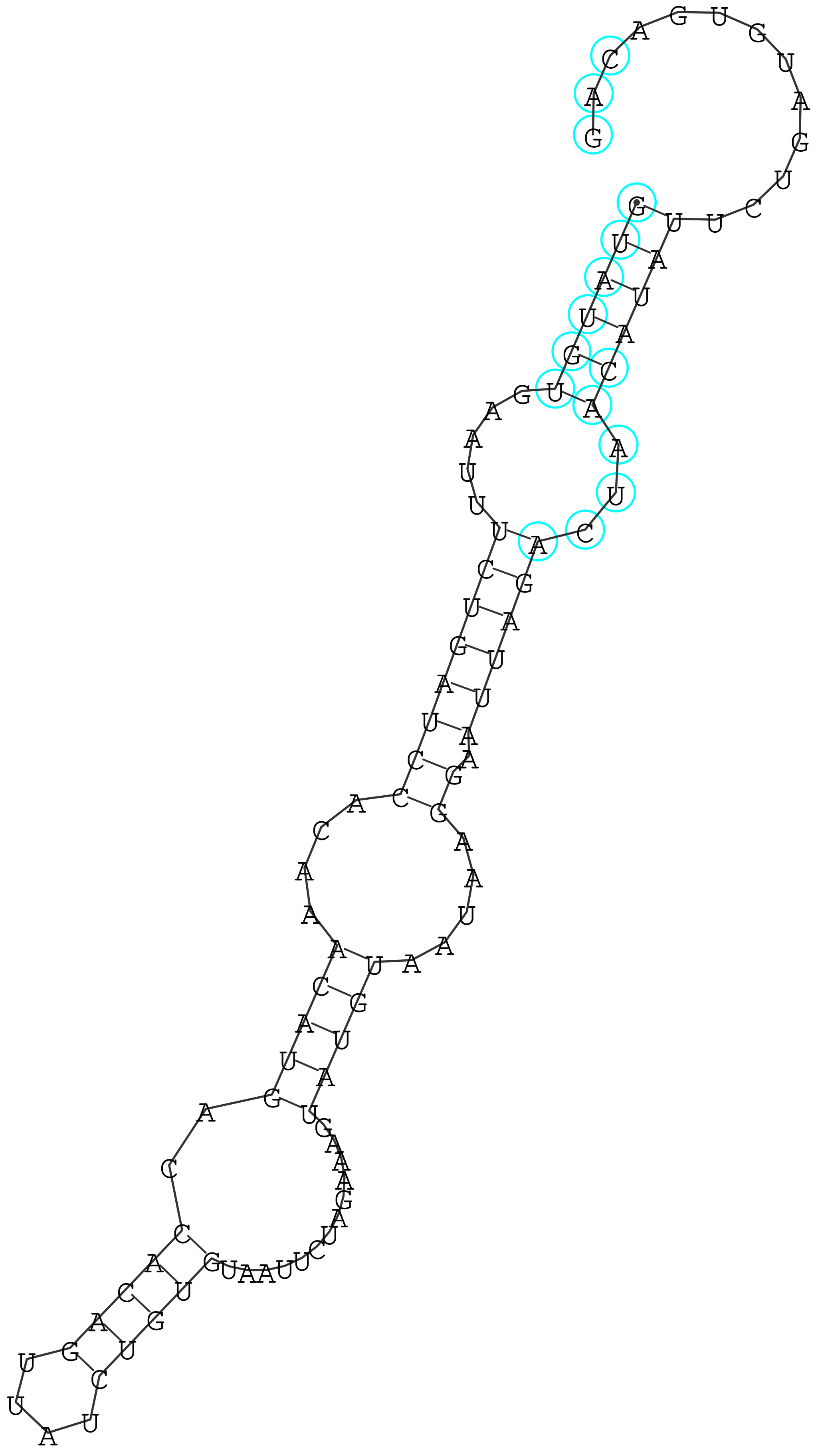

Xbamc015A - External intron

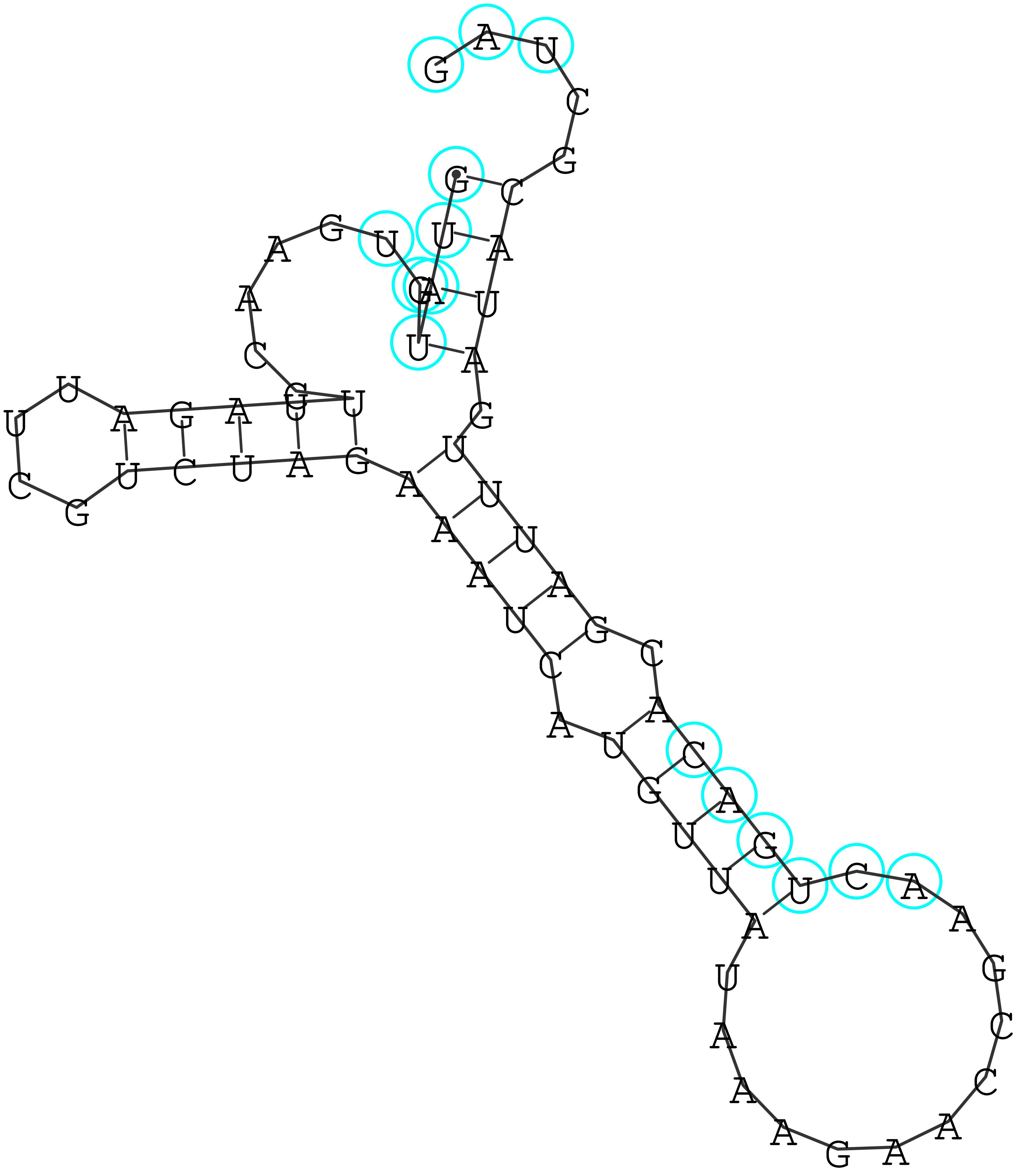

Xbamc019A - External intron

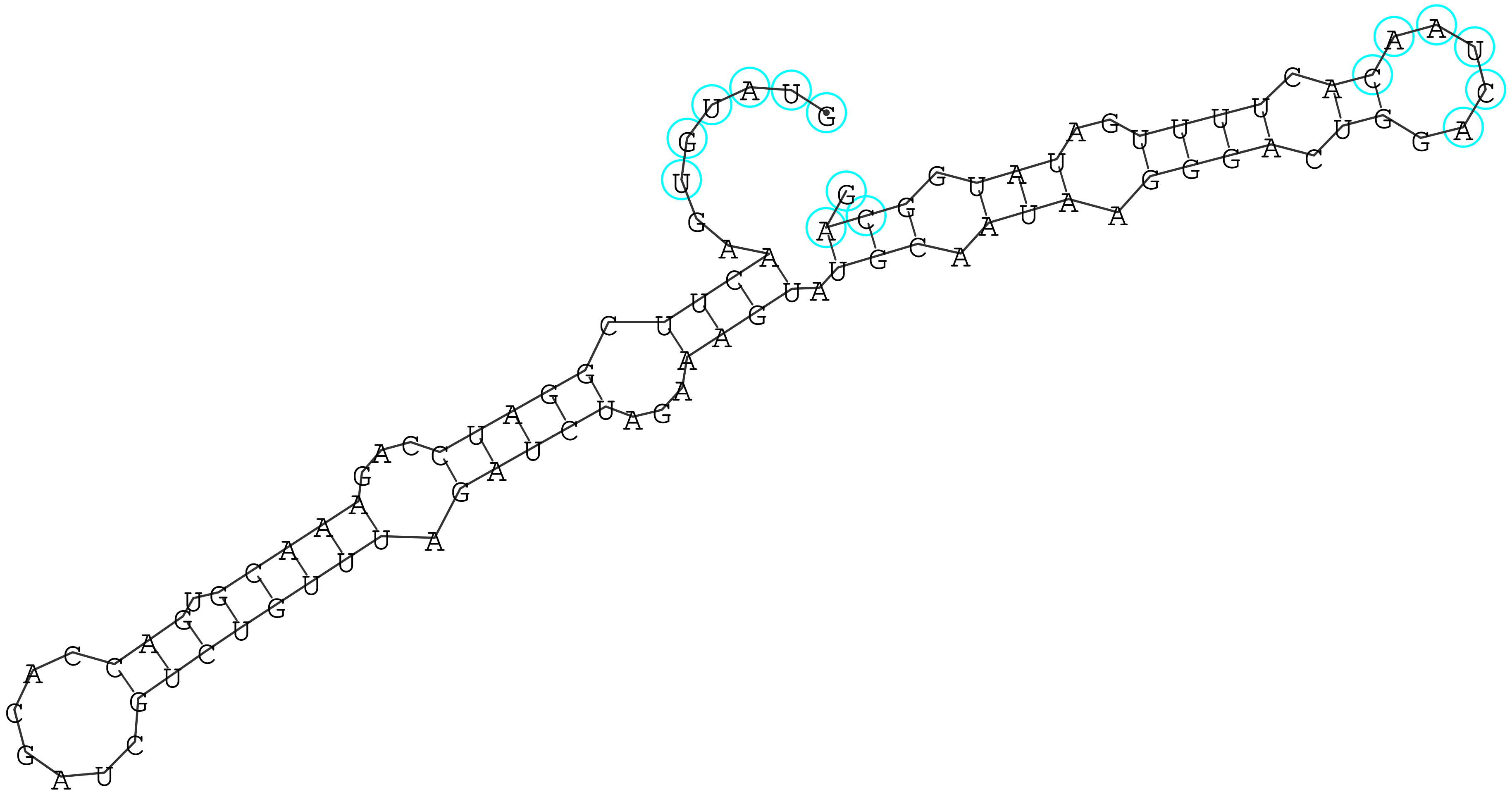

Xbamc020A - External intron

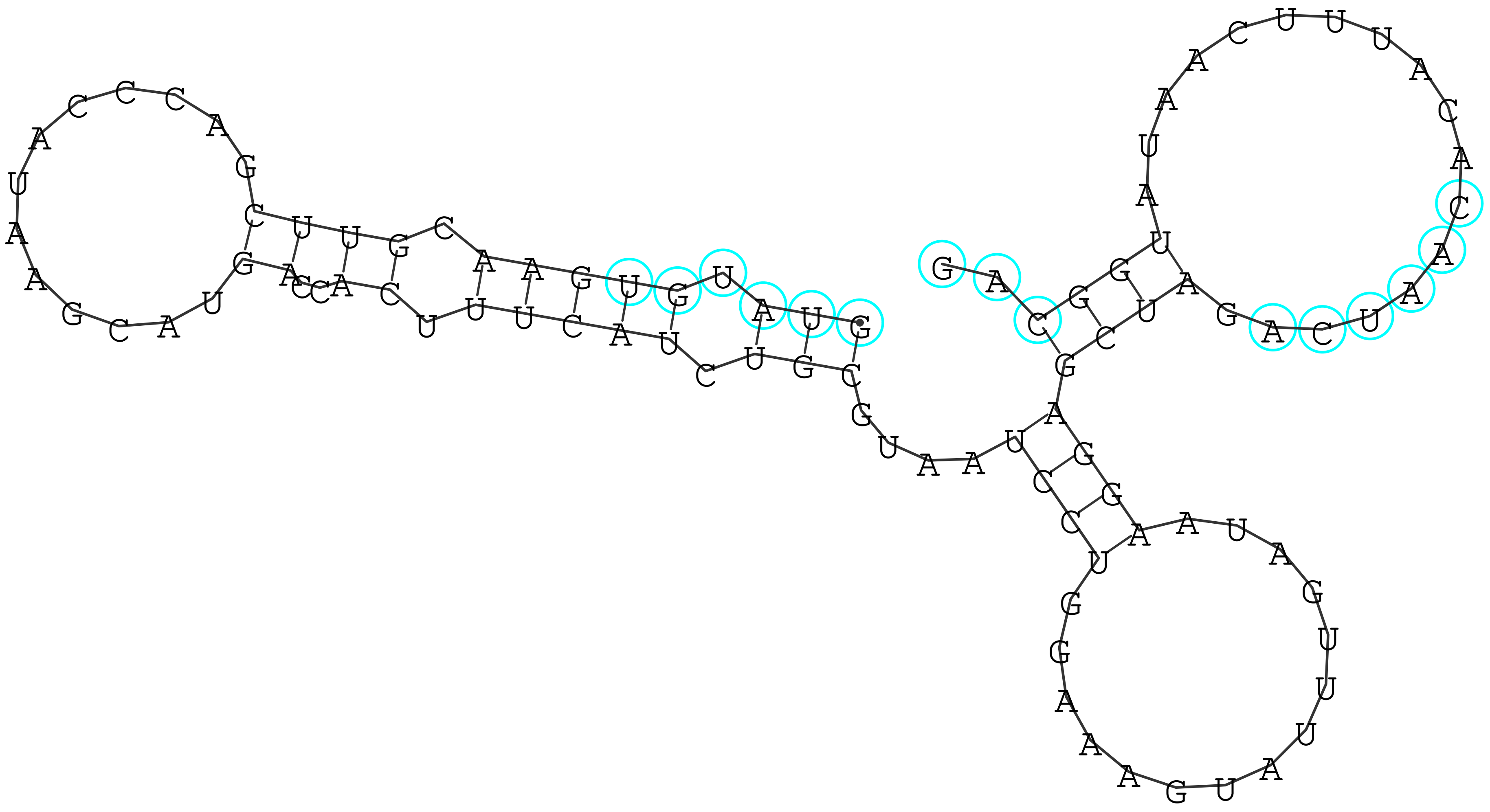

Xbamc022A - External intron

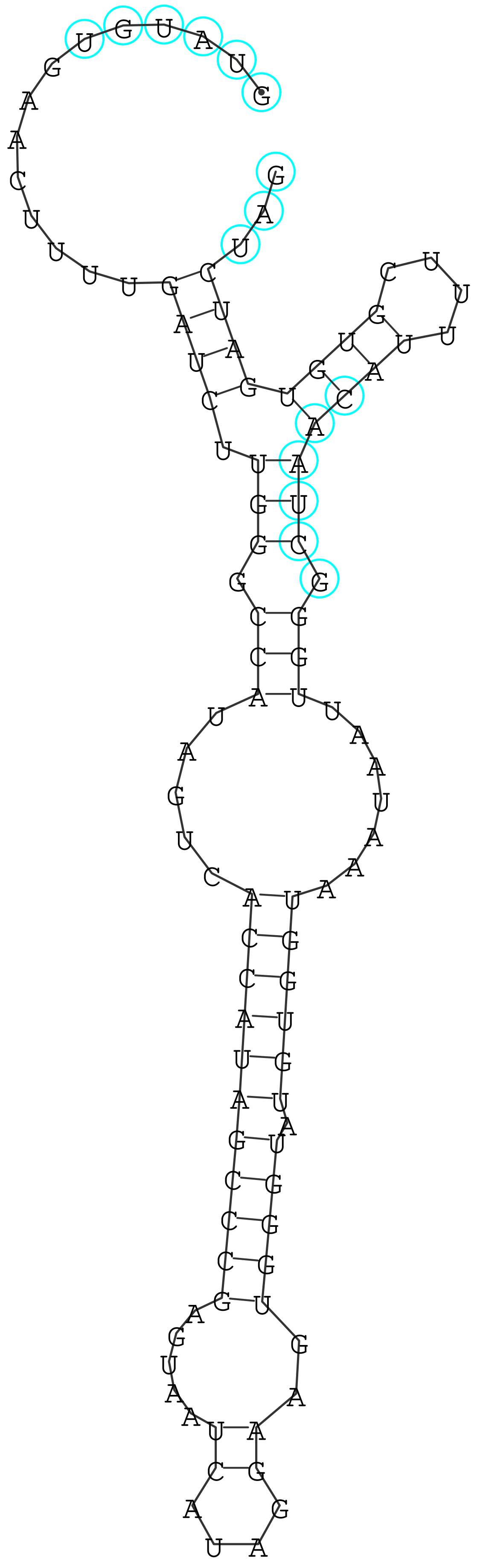

Xbamc024A - External intron

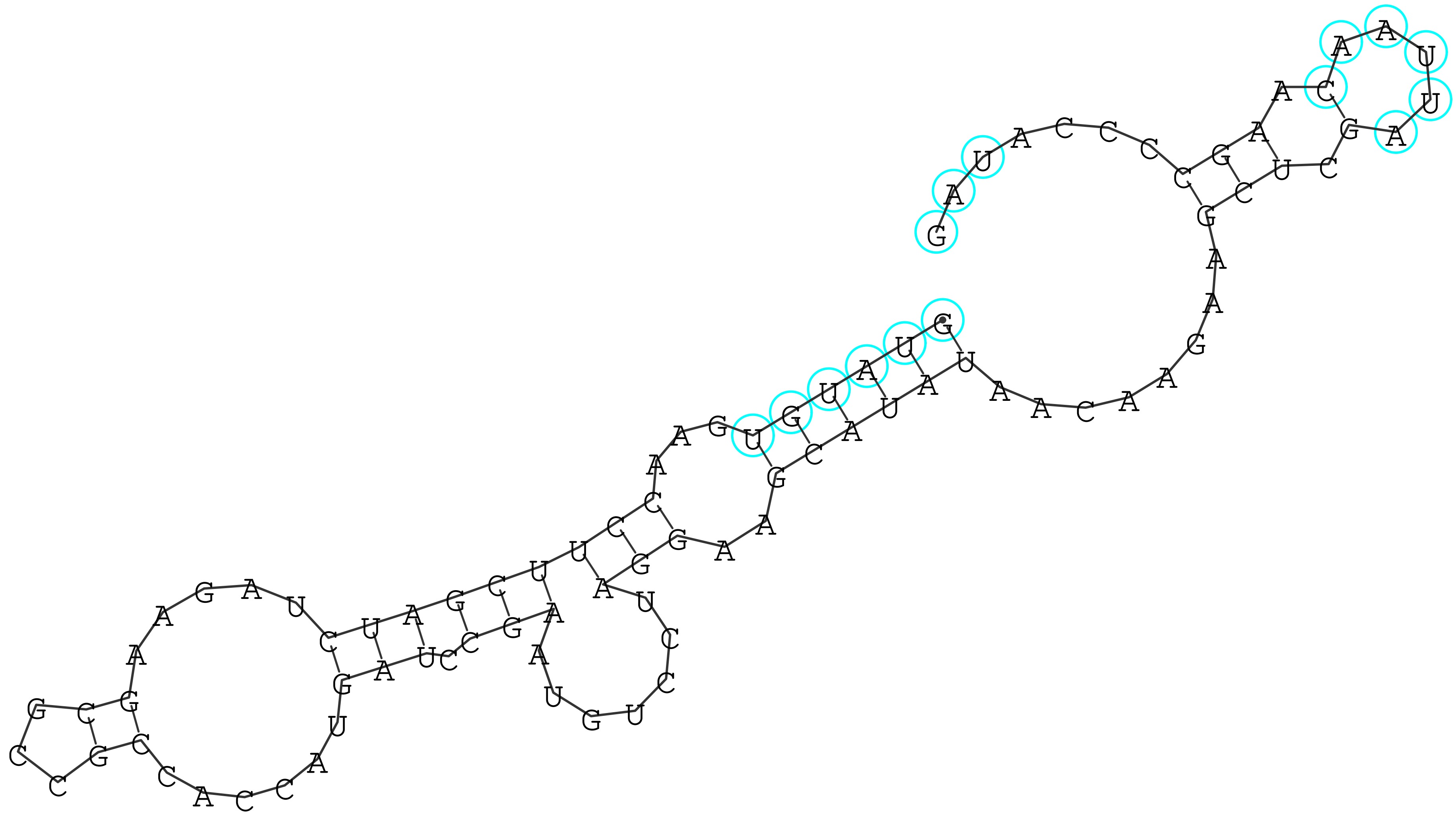

Xbamc025A - External intron

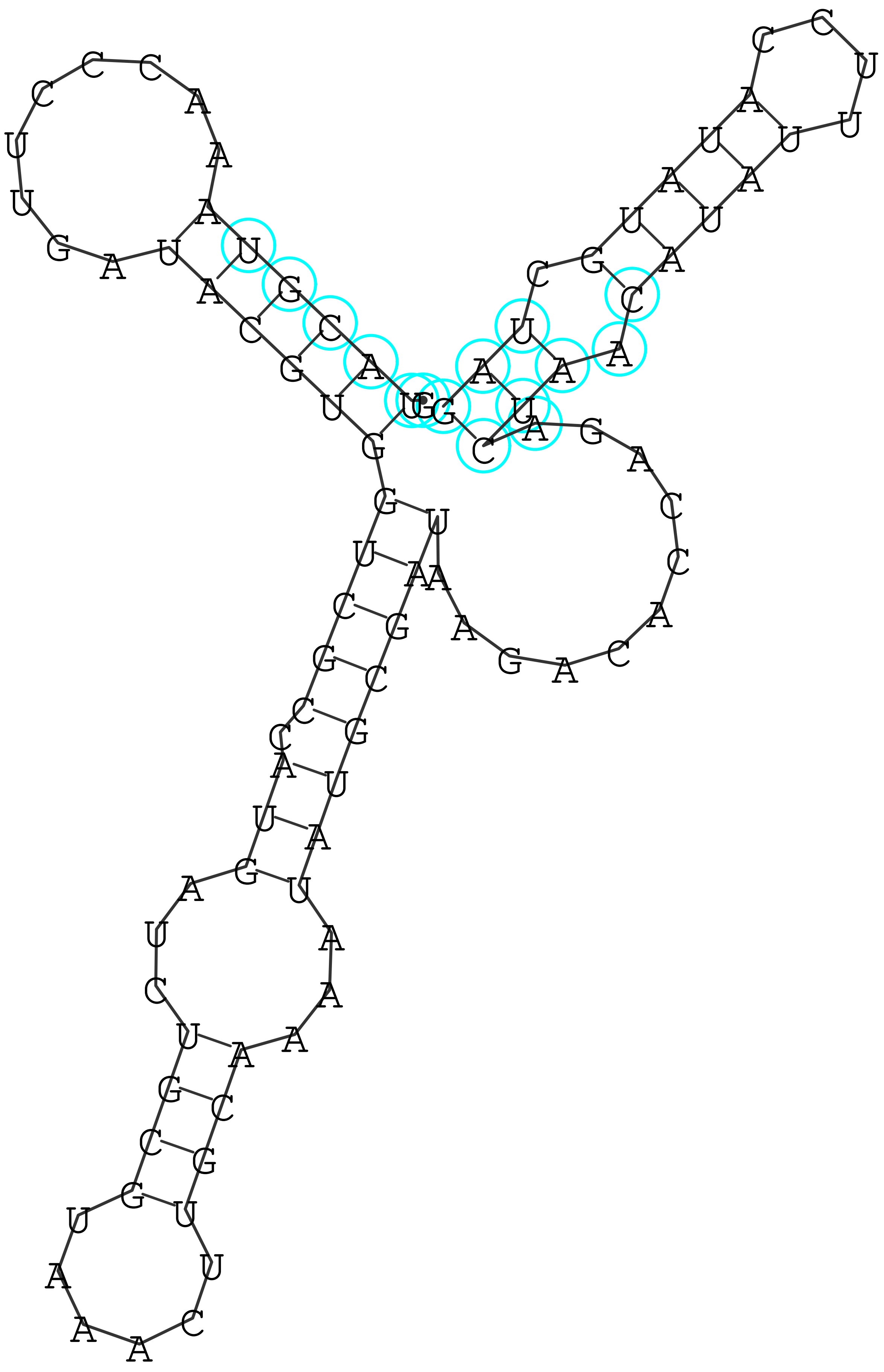

Xbamc027A - External intron

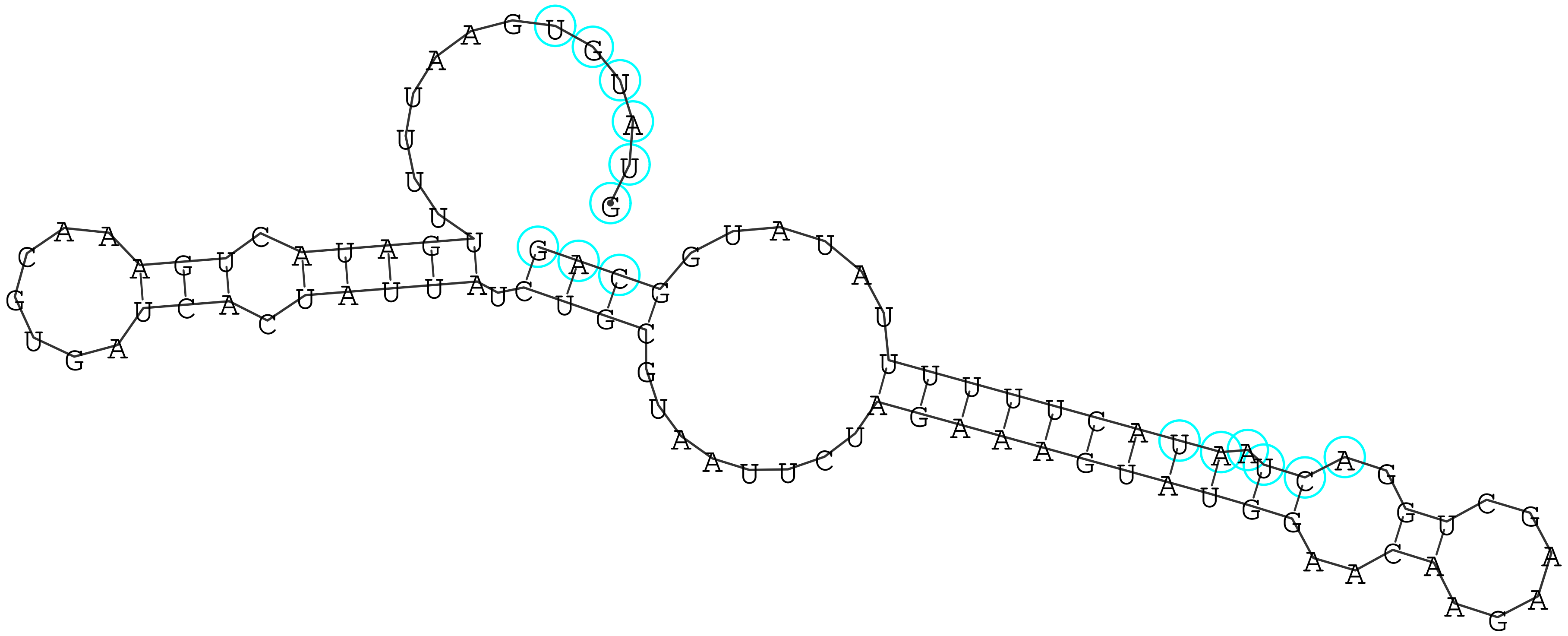

Xbamc027B - External intron

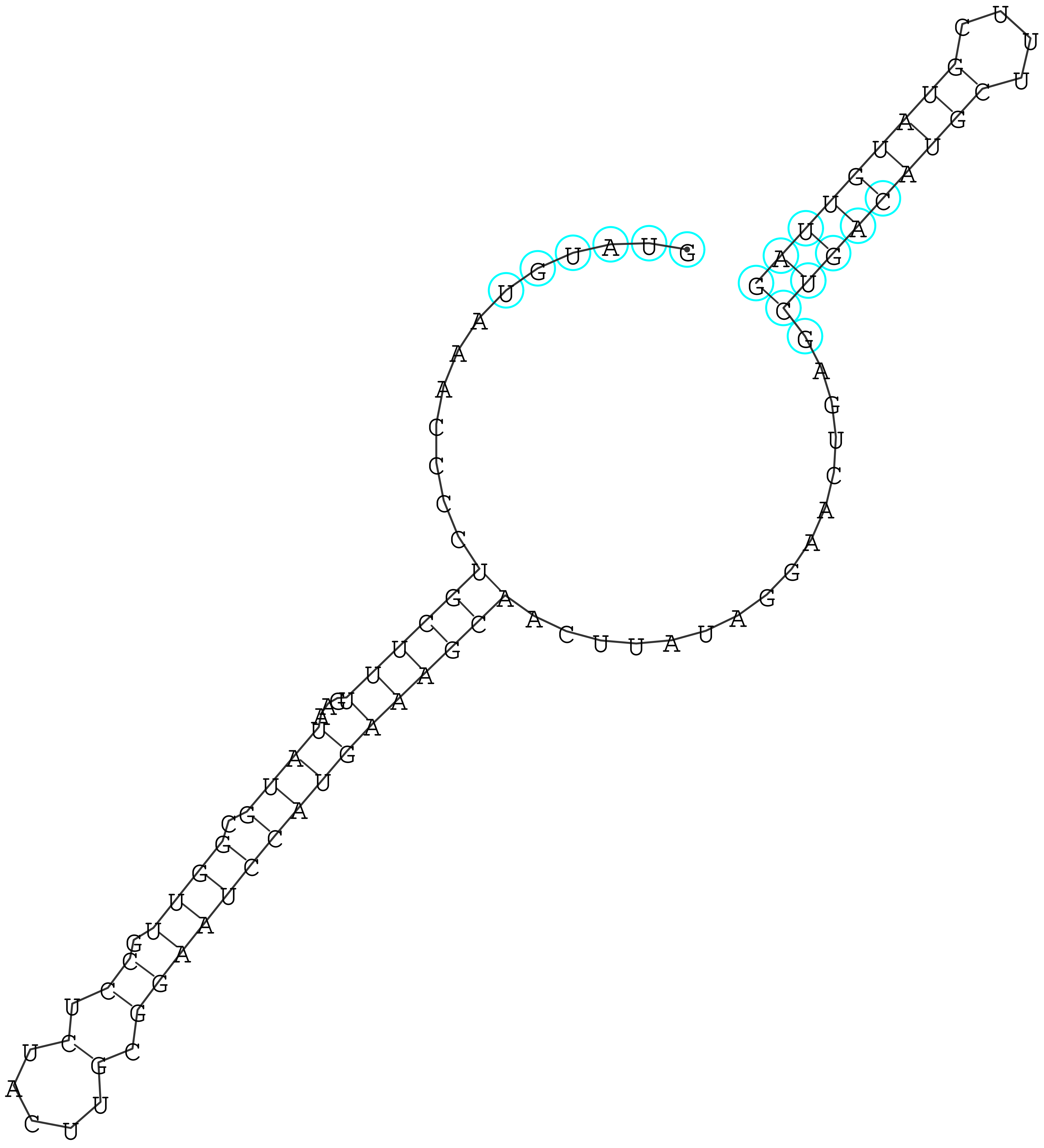

Xbamc027C - External intron

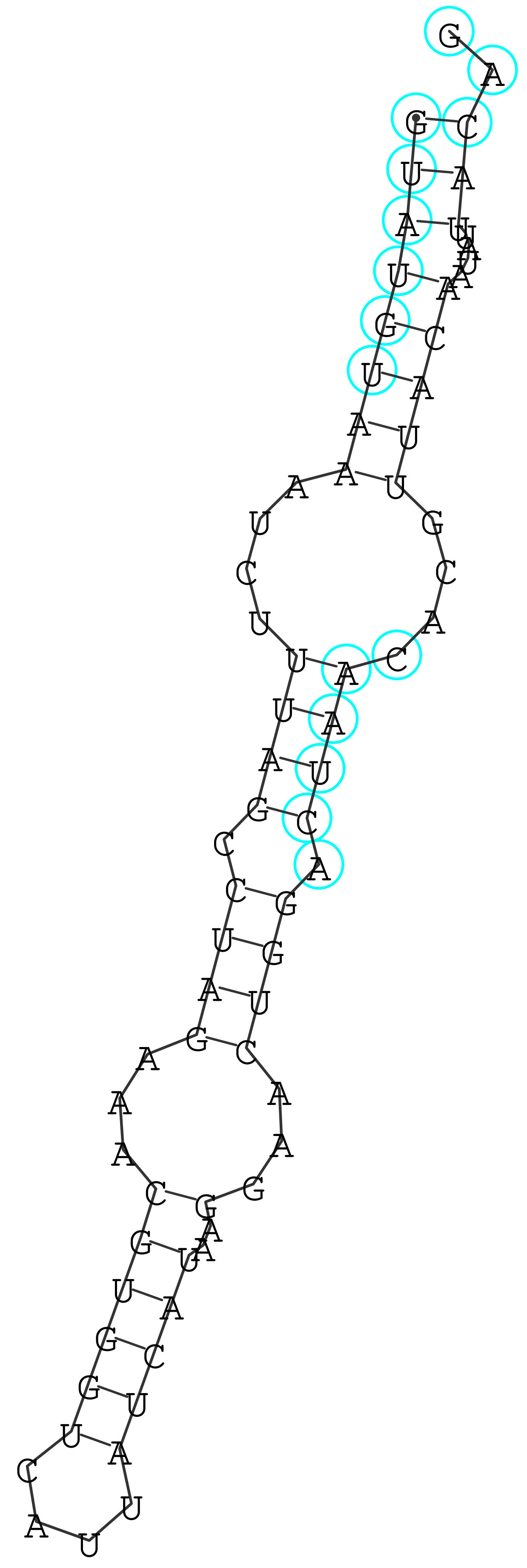

Xbamc040A - External intron

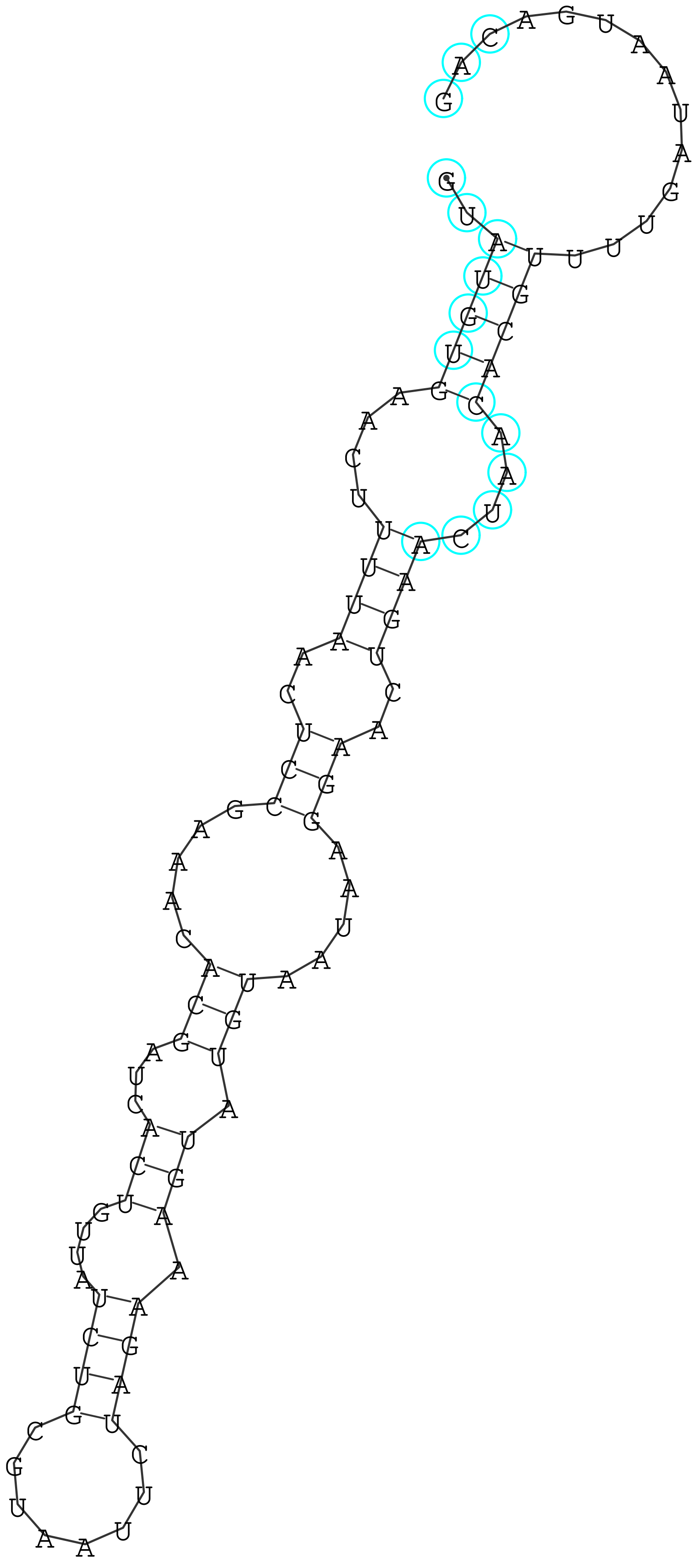

Xbamc041A - External intron

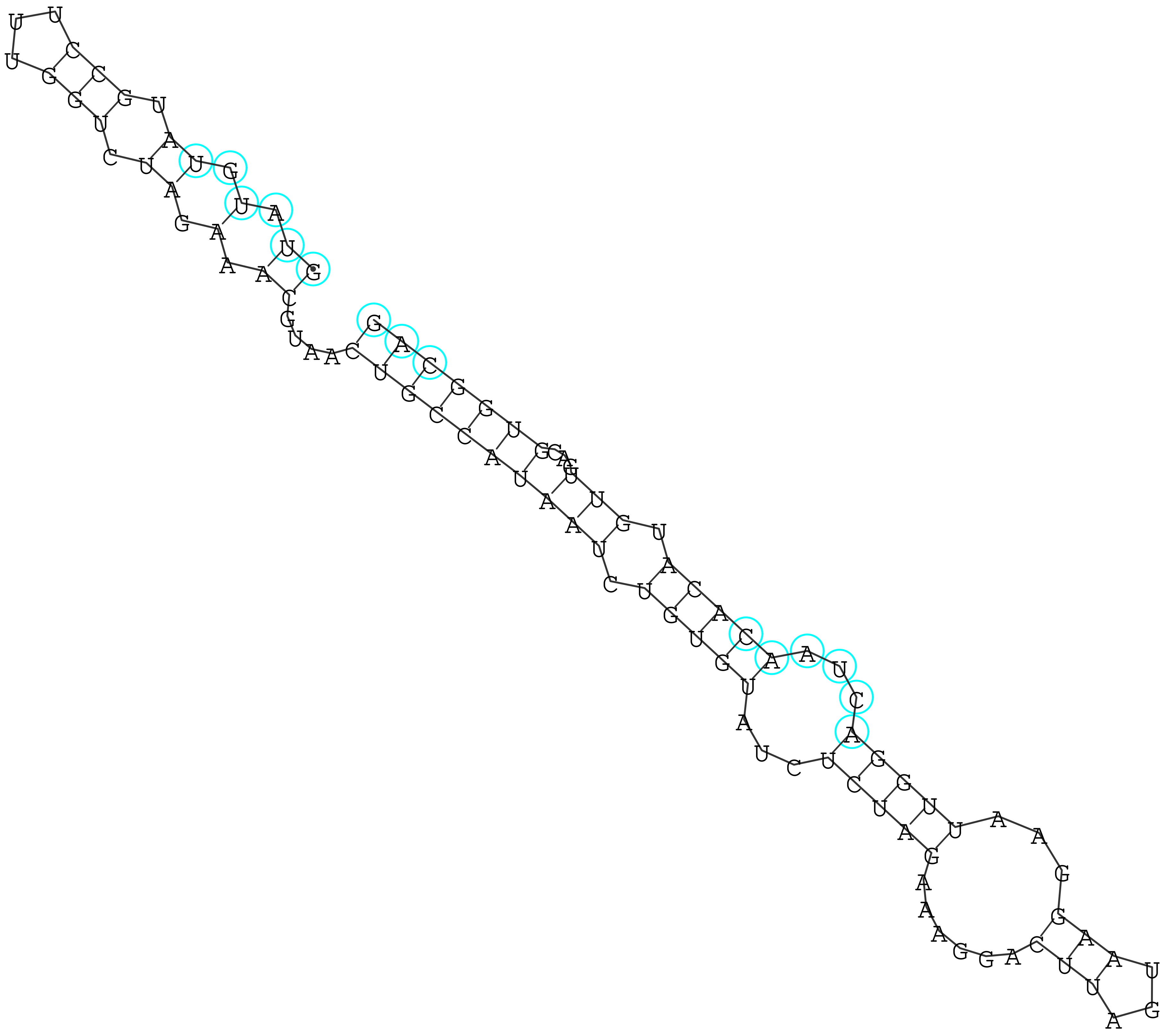

Xbamc051A - External intron

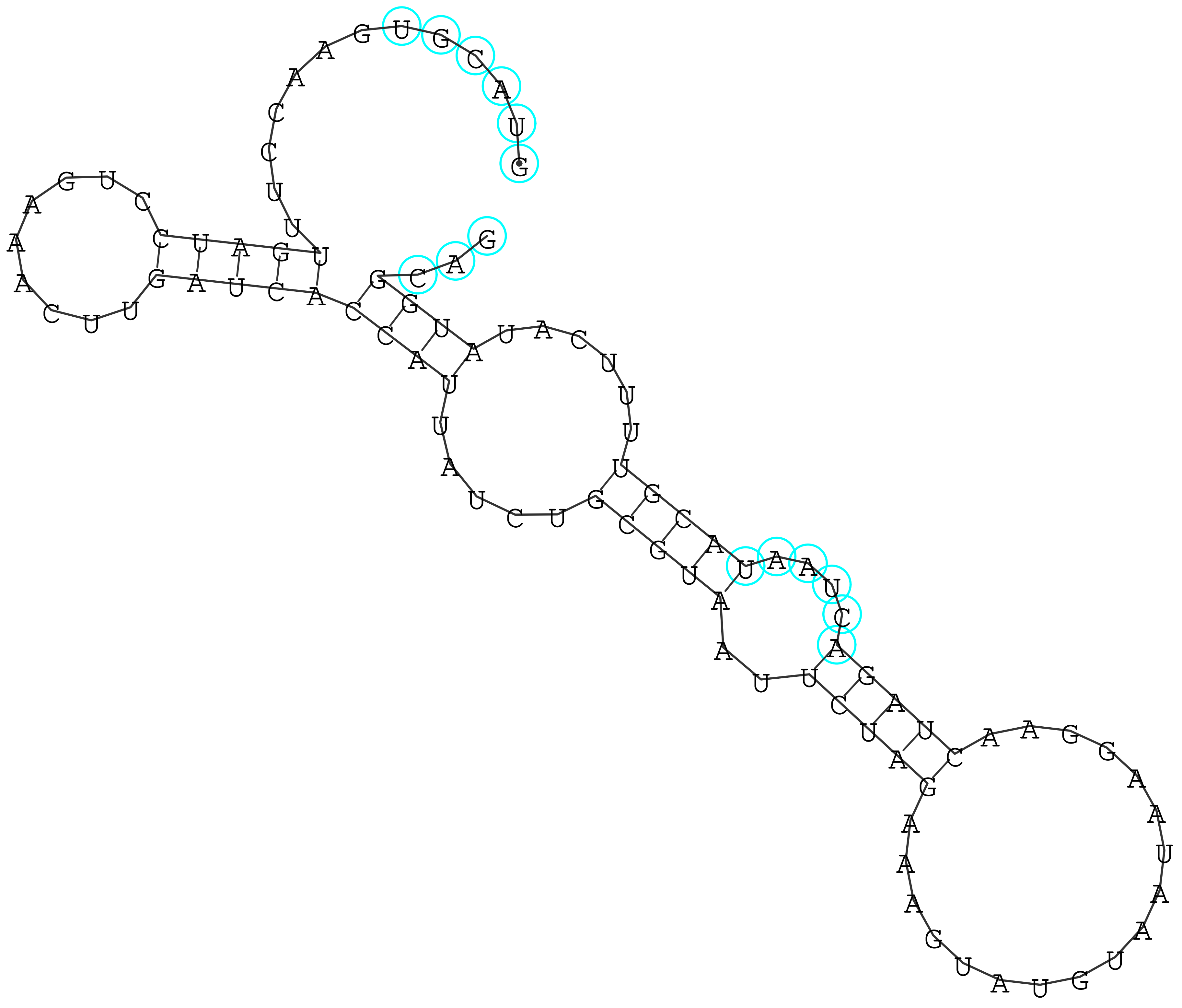

Xbamc053A - External intron

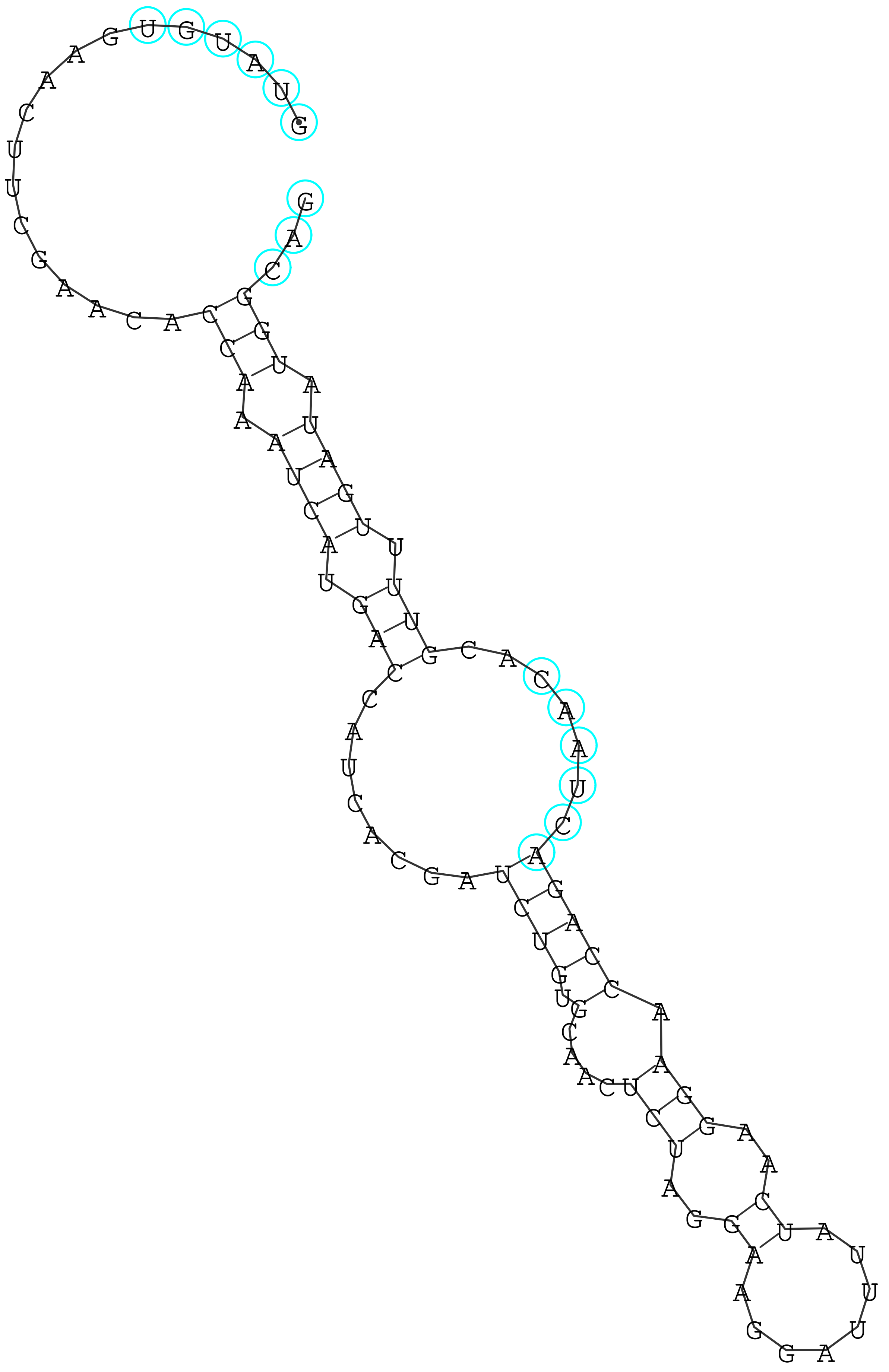

Xbamc053B - External intron

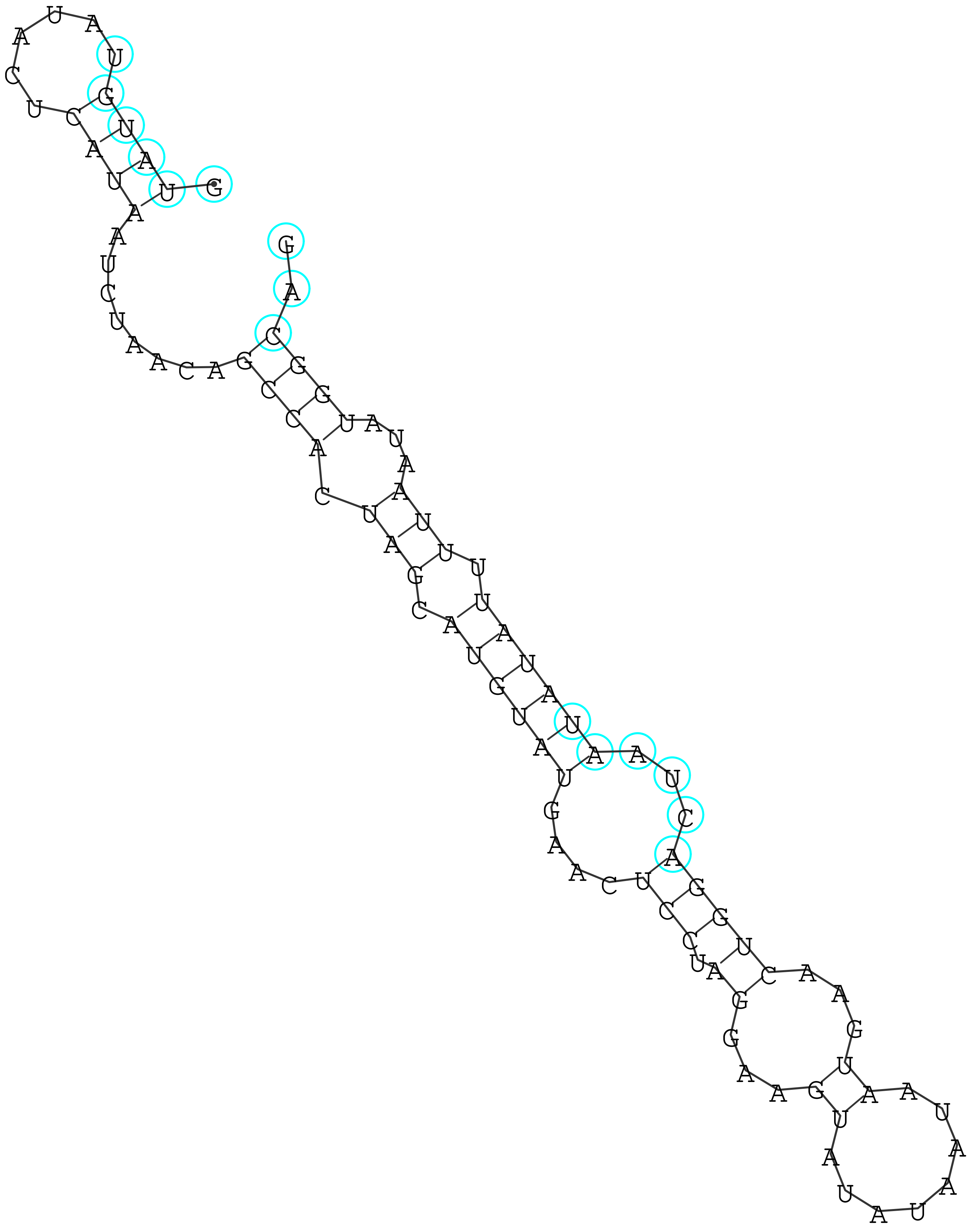

Xbamc053C - External intron

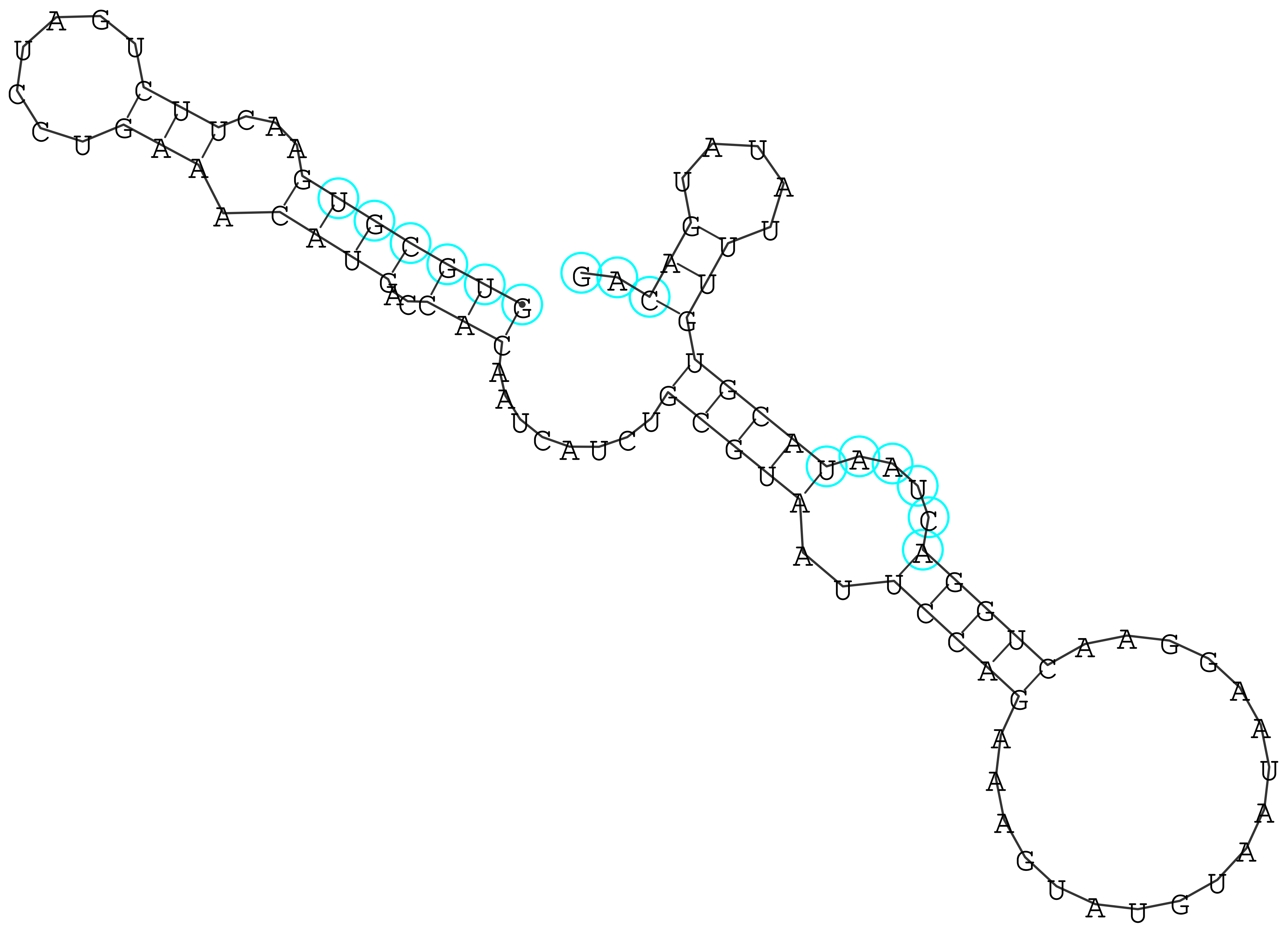

Xbamc067A - External intron

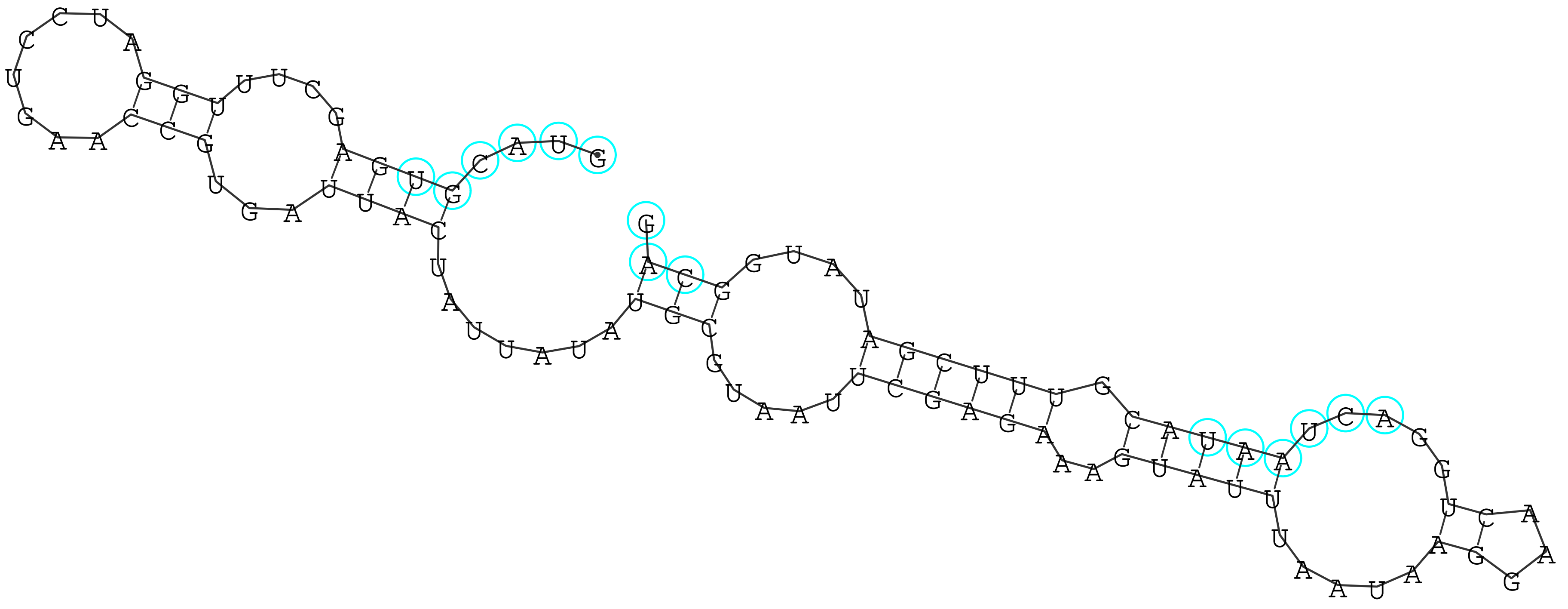

Xbamc067B - External intron

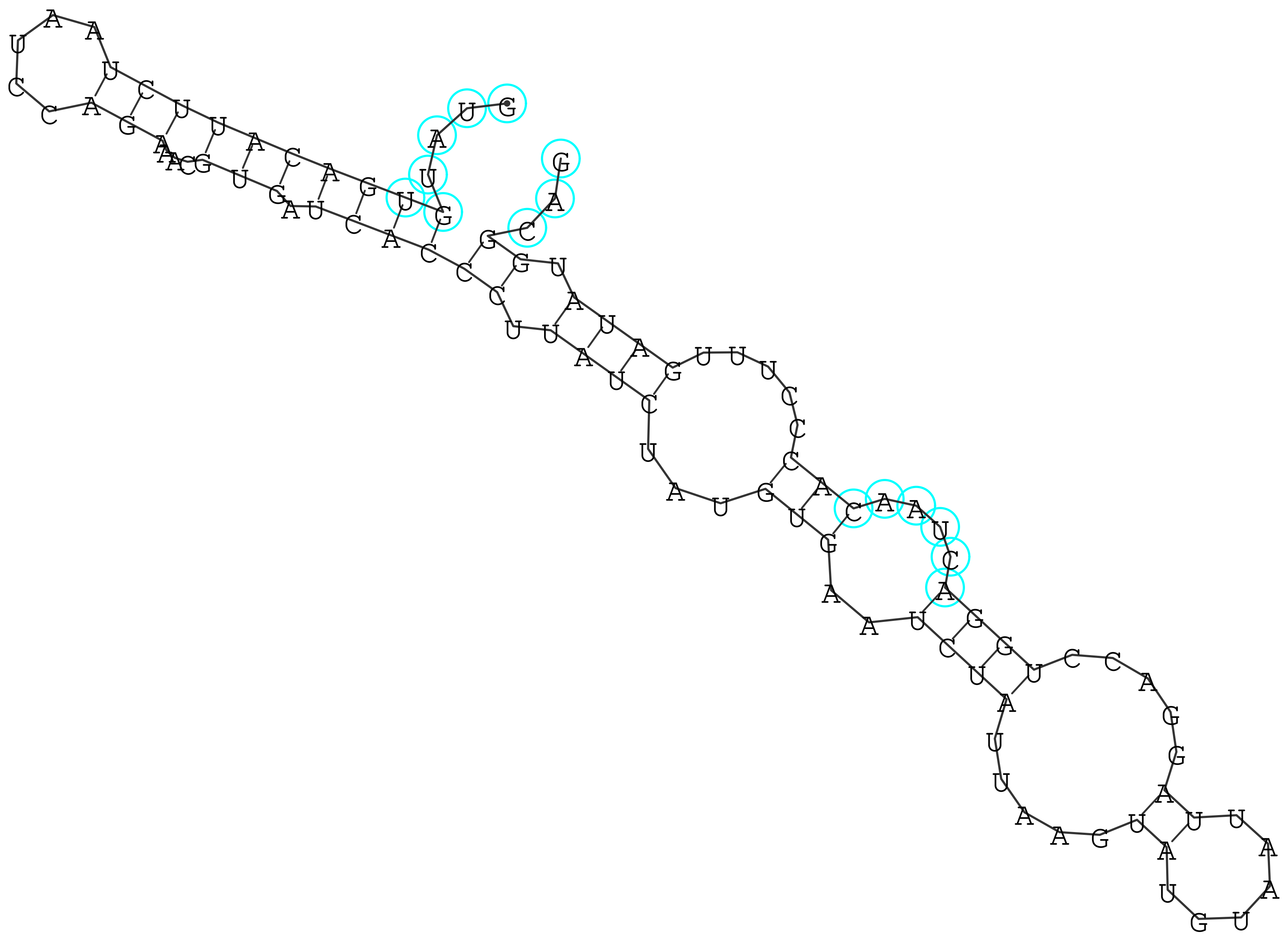

Xbamc080A - External intron

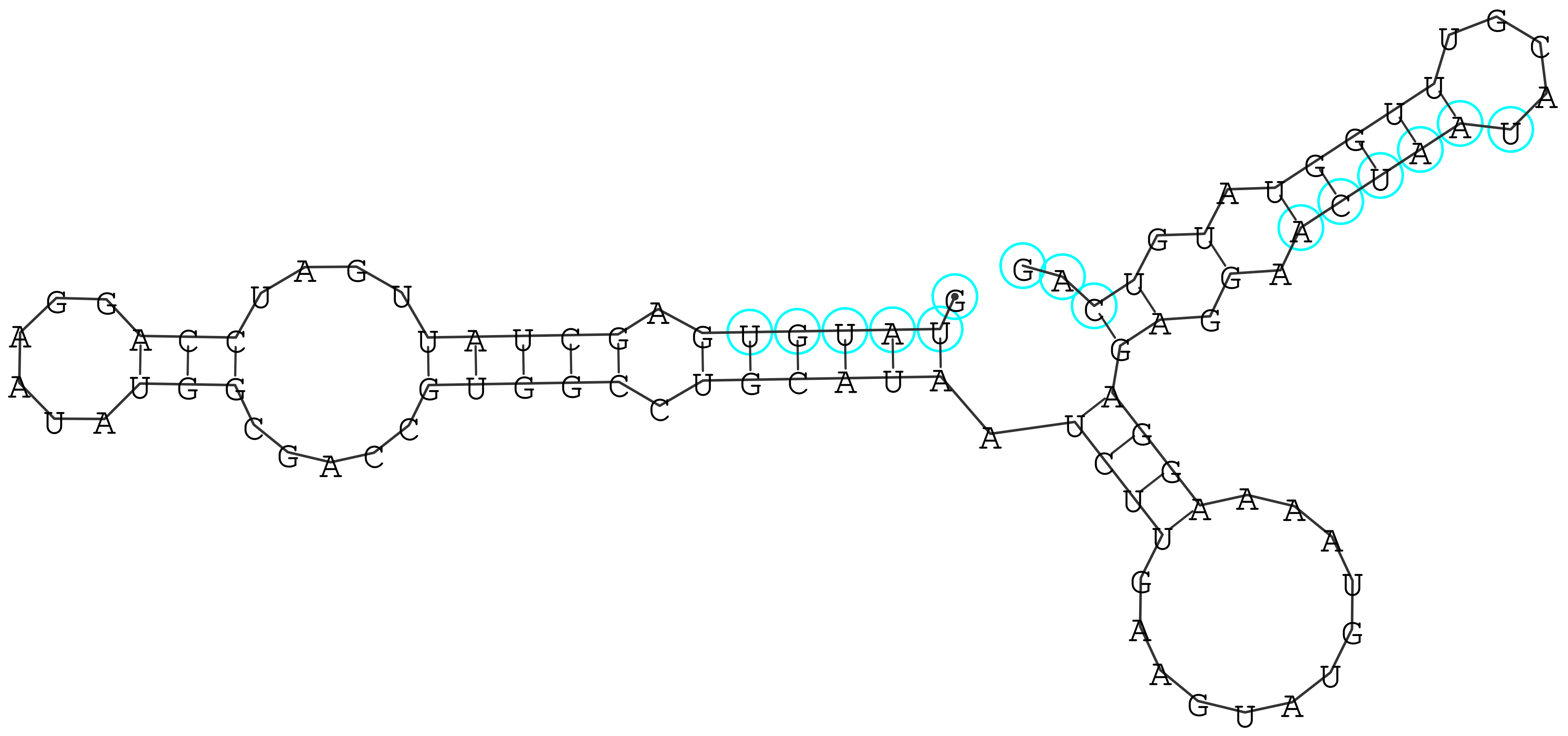

Xbamc083A - External intron

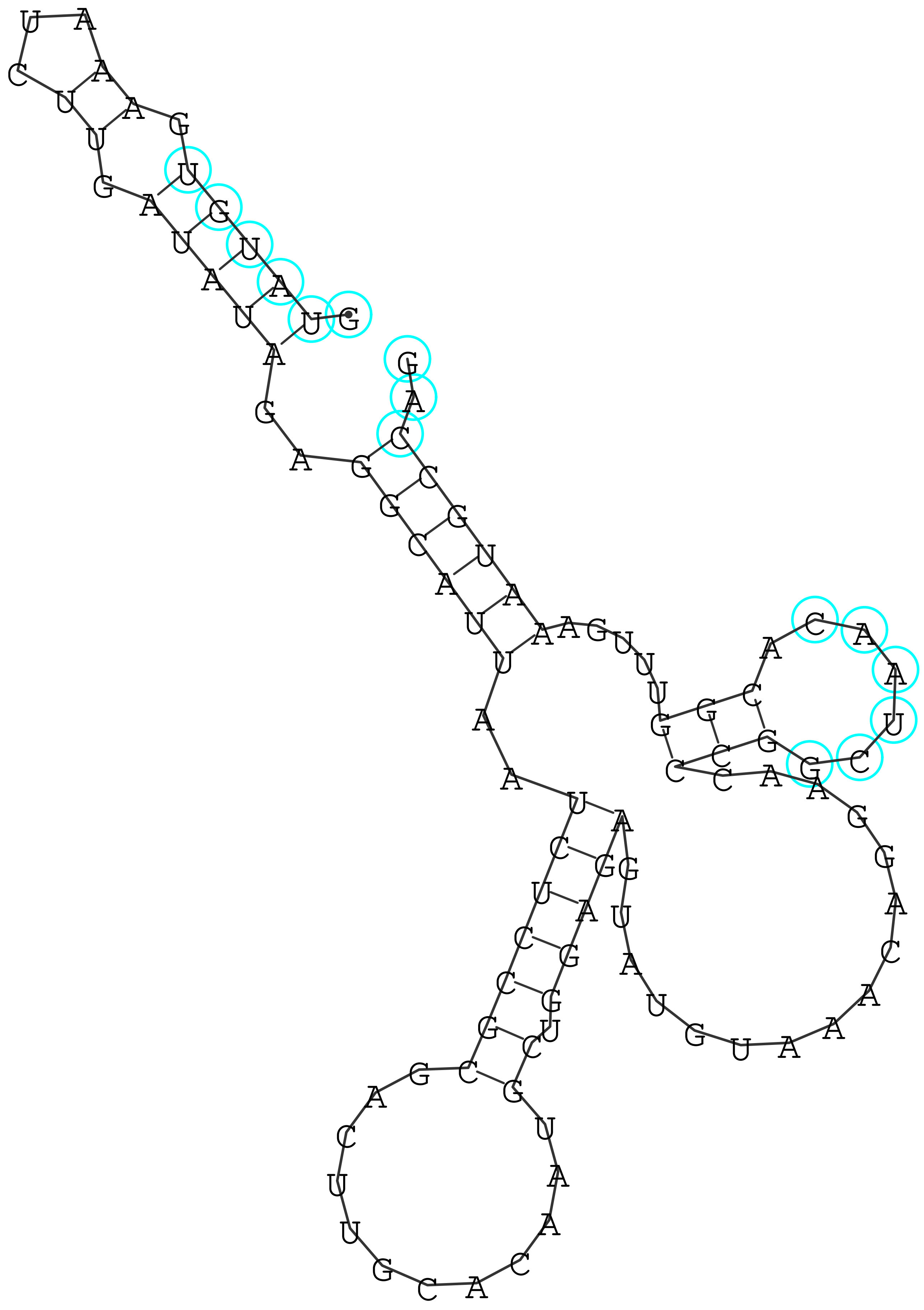

Xbamc083B - External intron

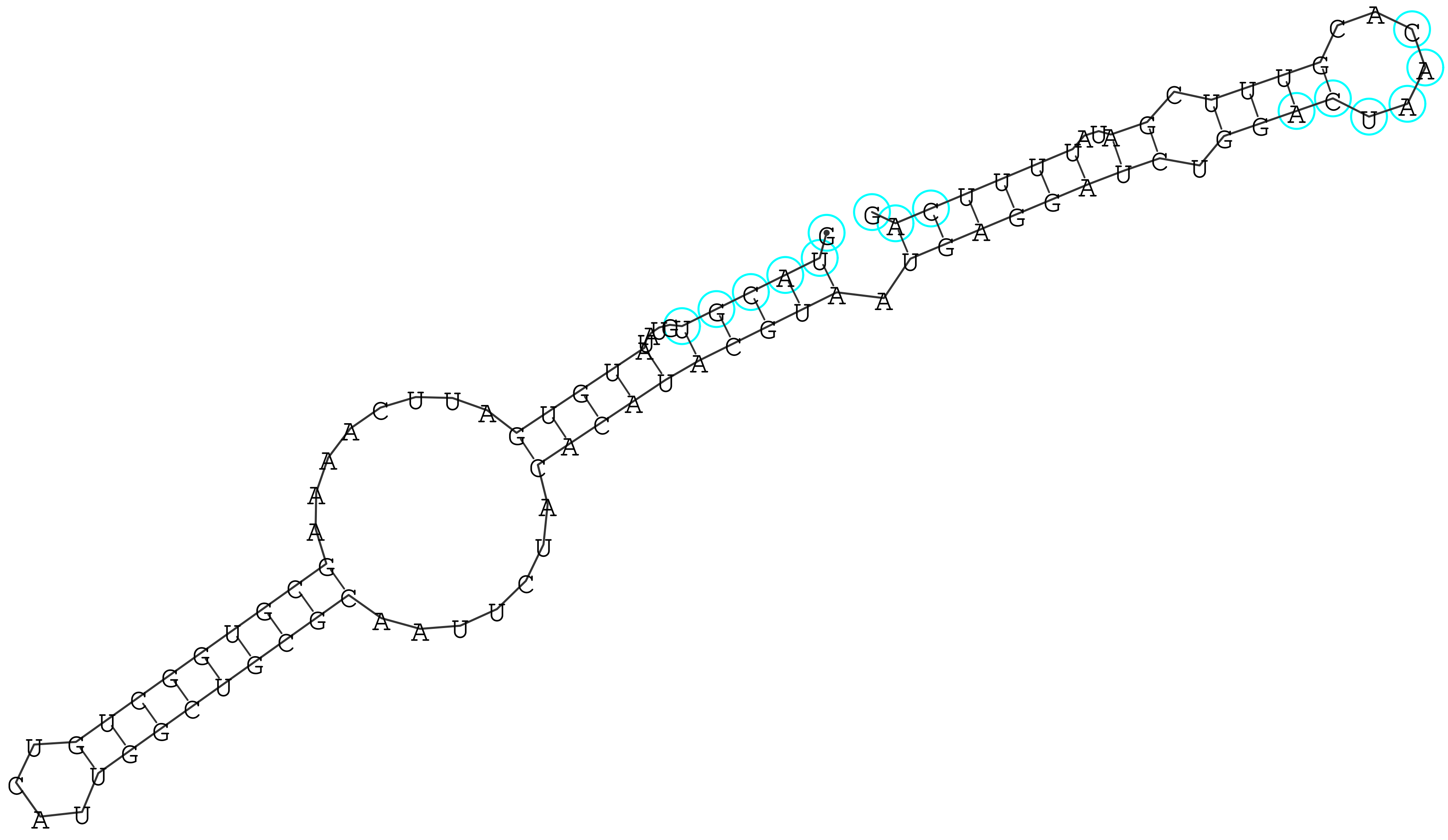

Xbamc084A - External intron

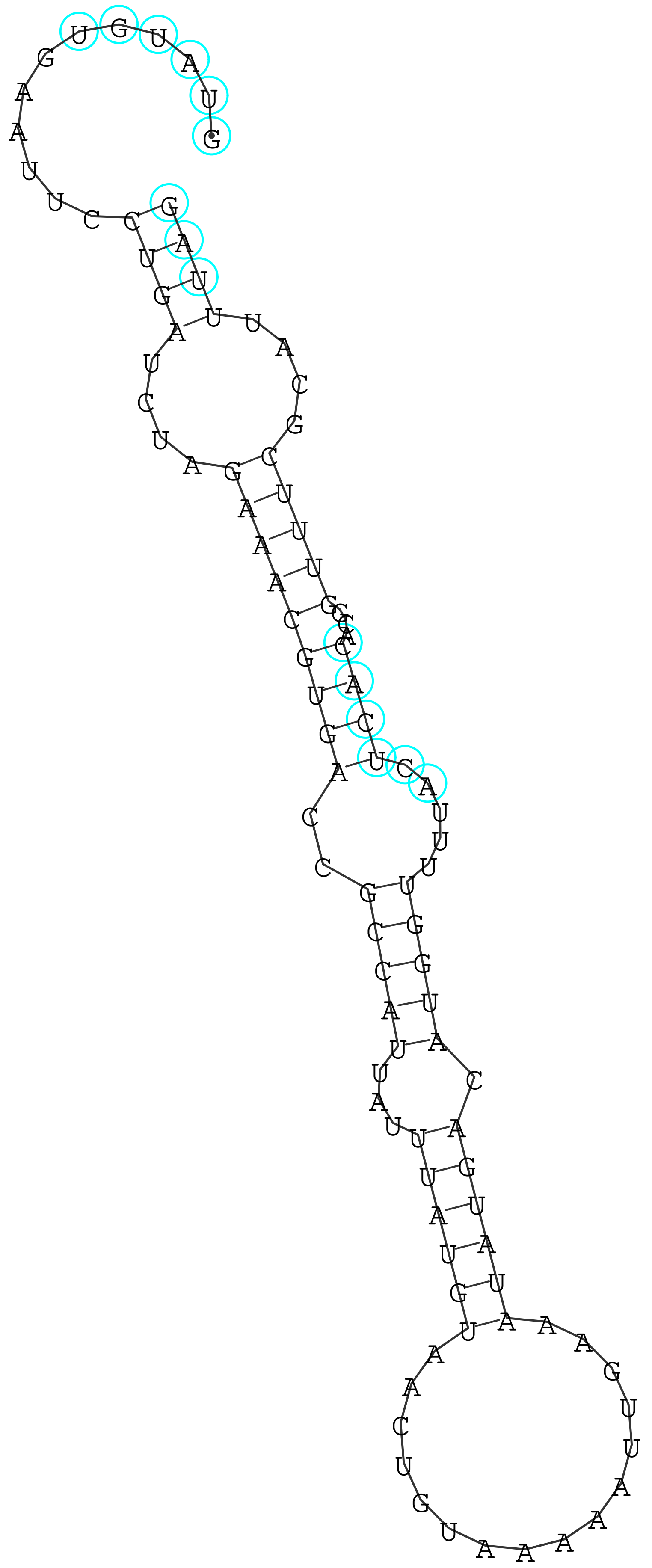

Xbamc086A - External intron

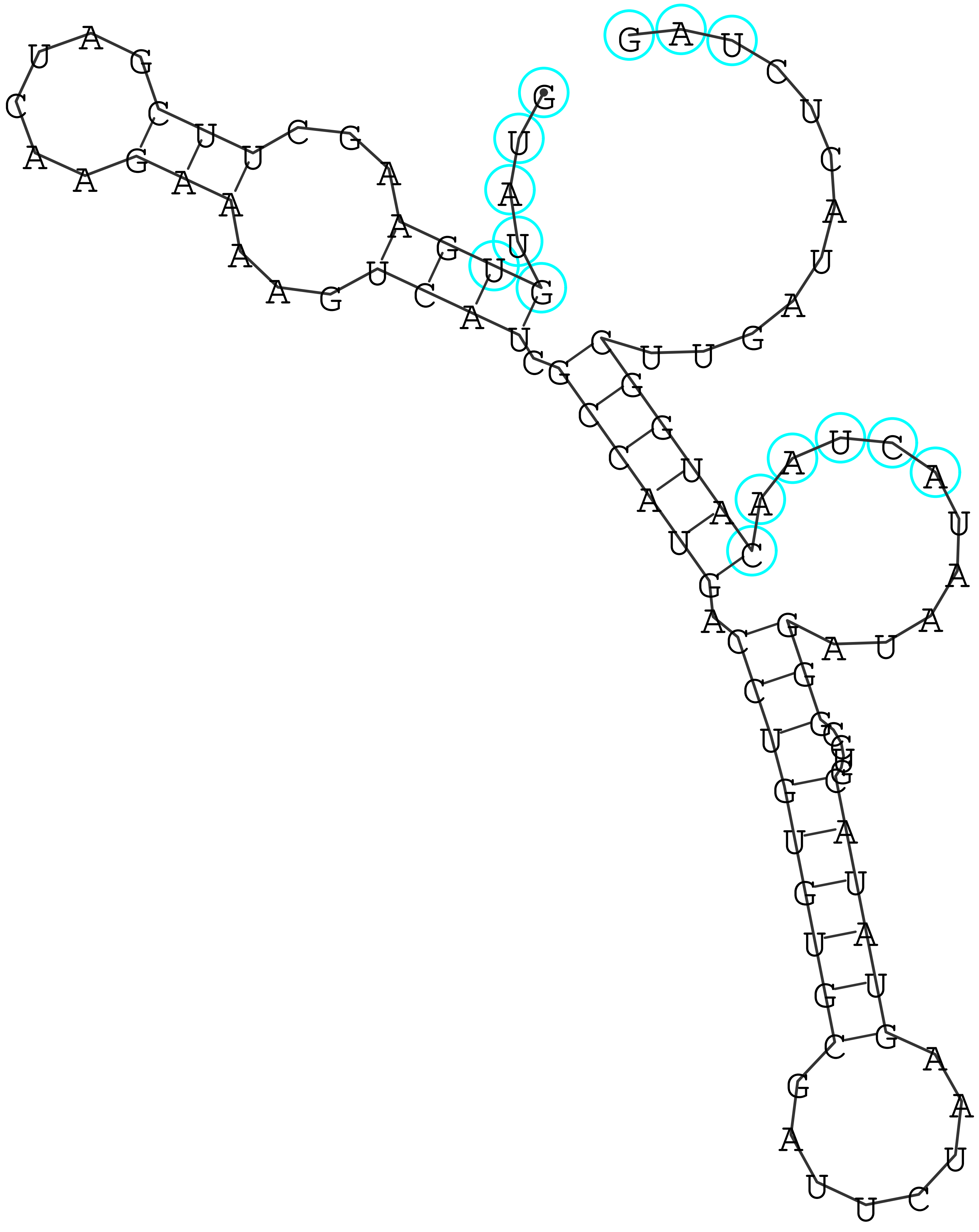

Xbamc089A - External intron

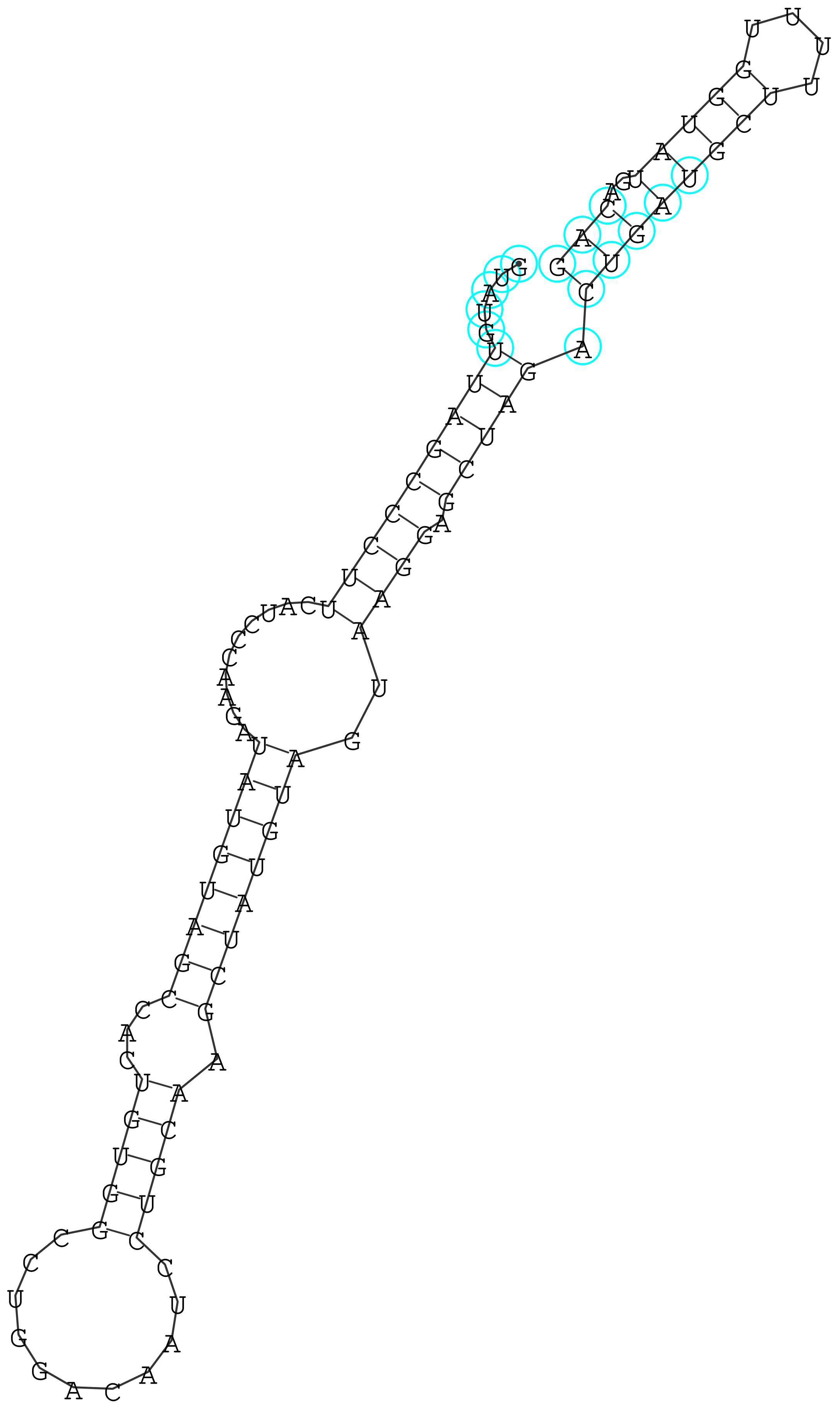

Xbamc096A - External intron

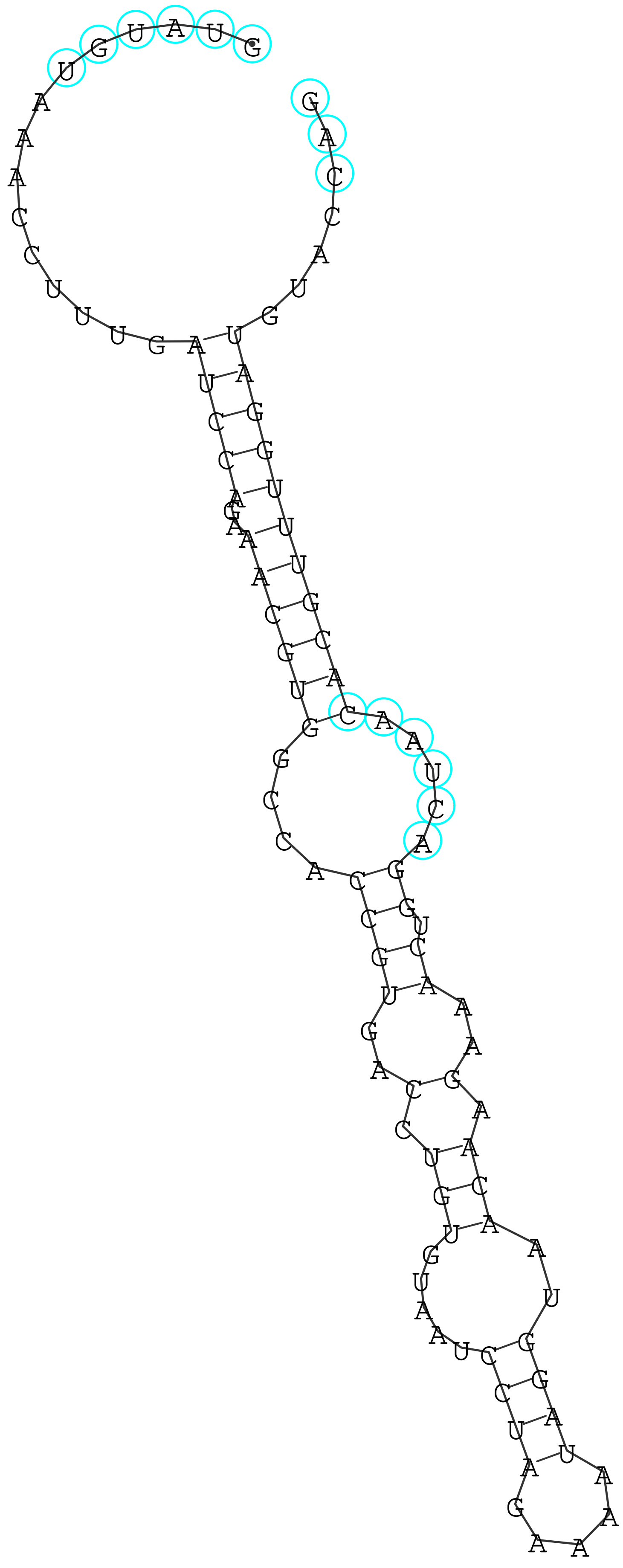

Xbamc102A - External intron

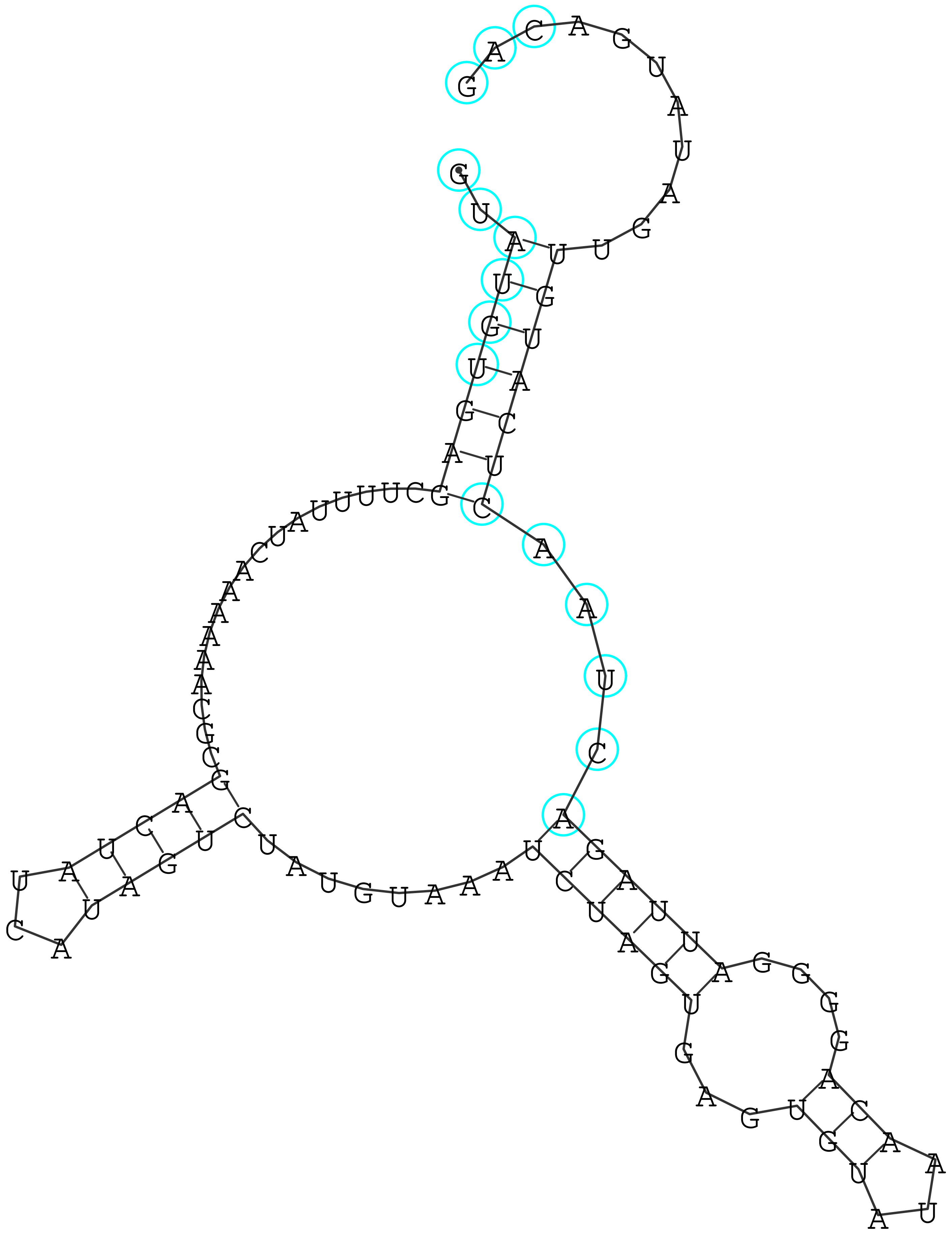

Xbamc106A - External intron

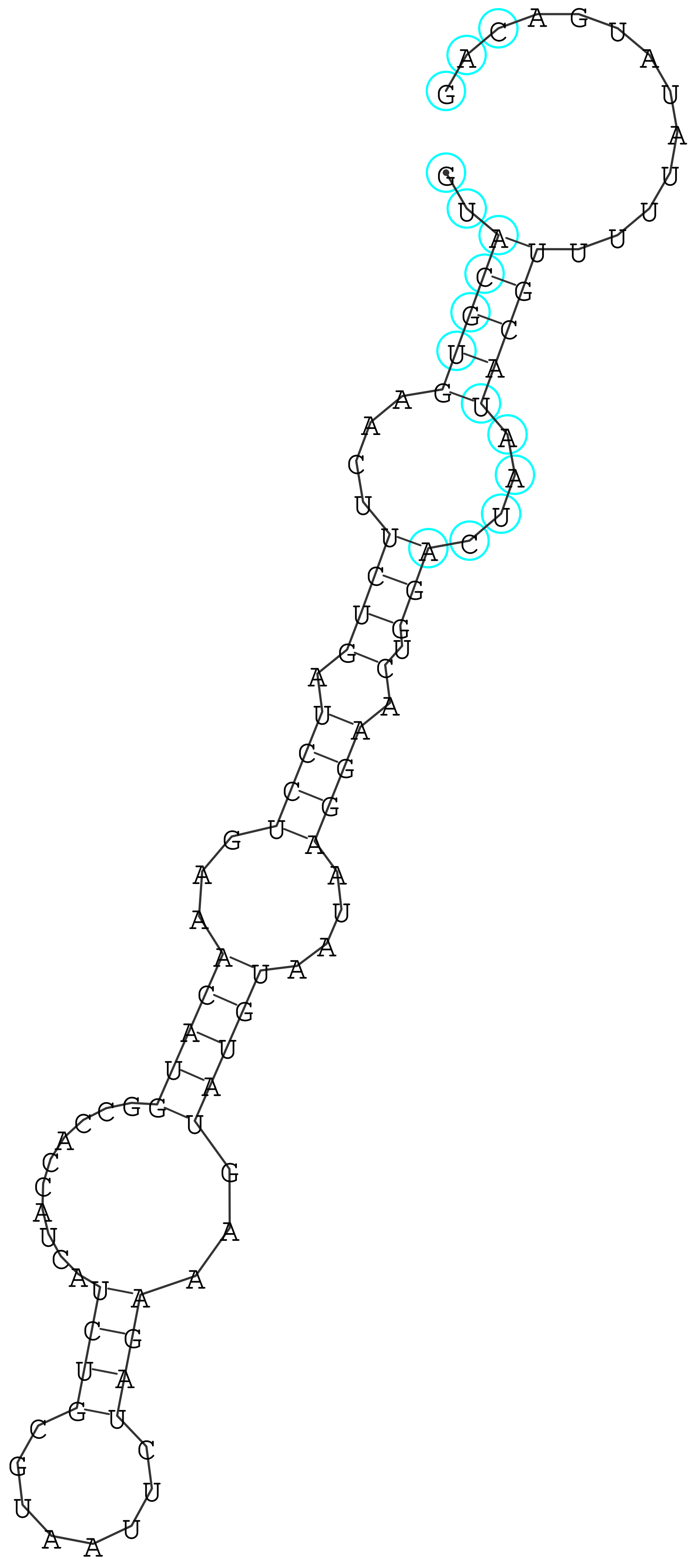

Xbamc106B - External intron

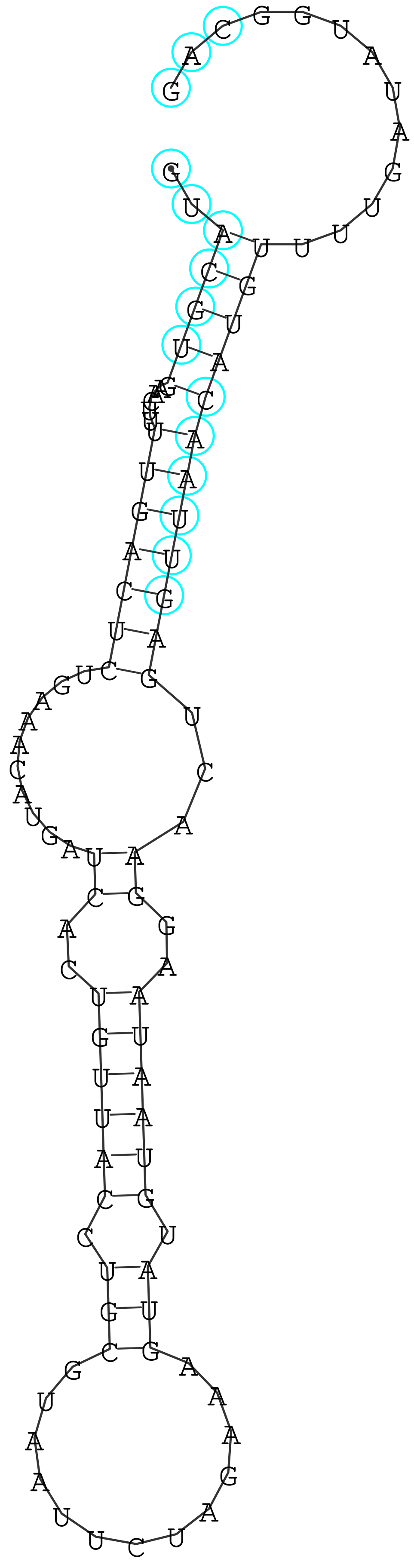

Xbamc107A - External intron

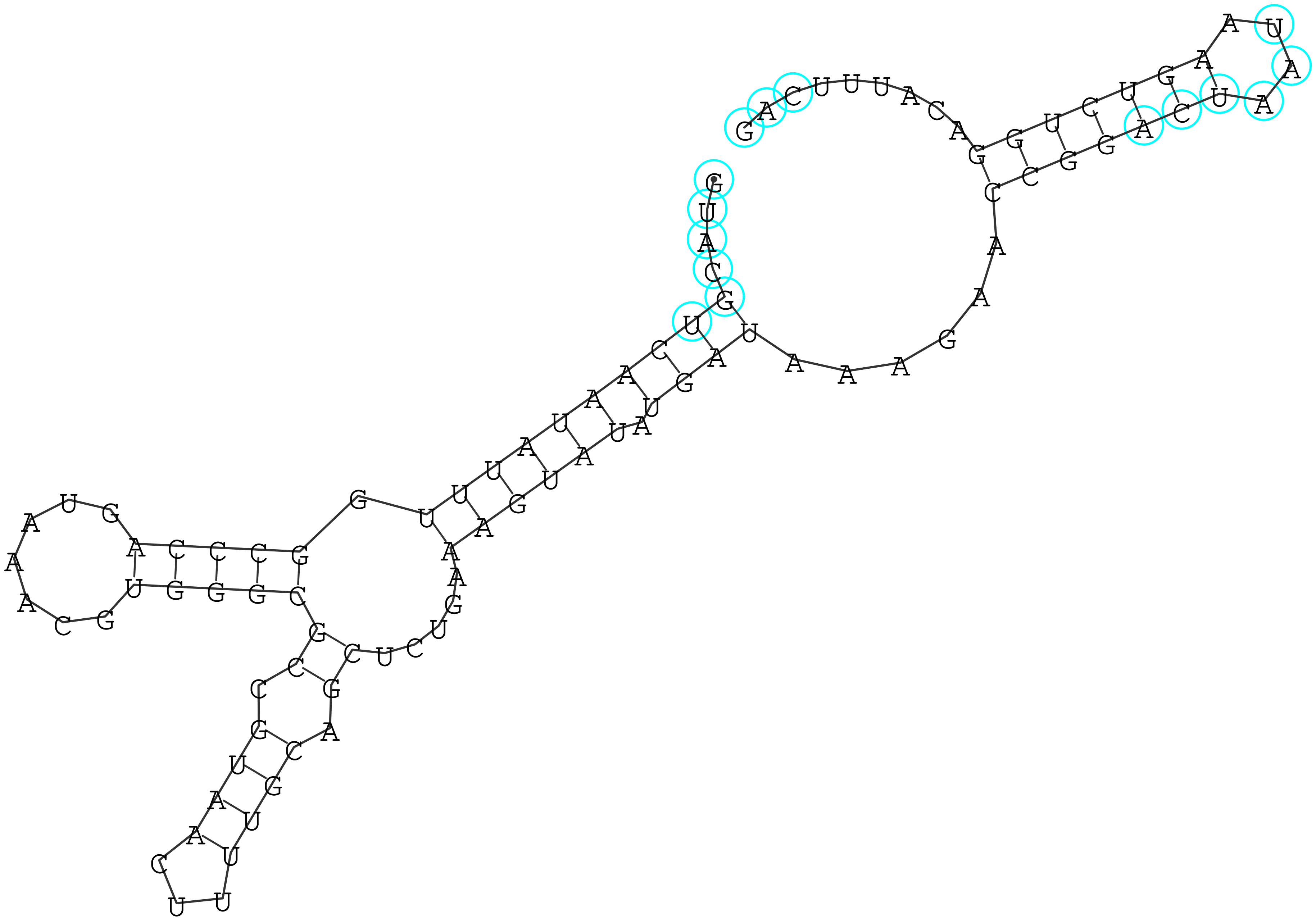

Xbamc109A - External intron

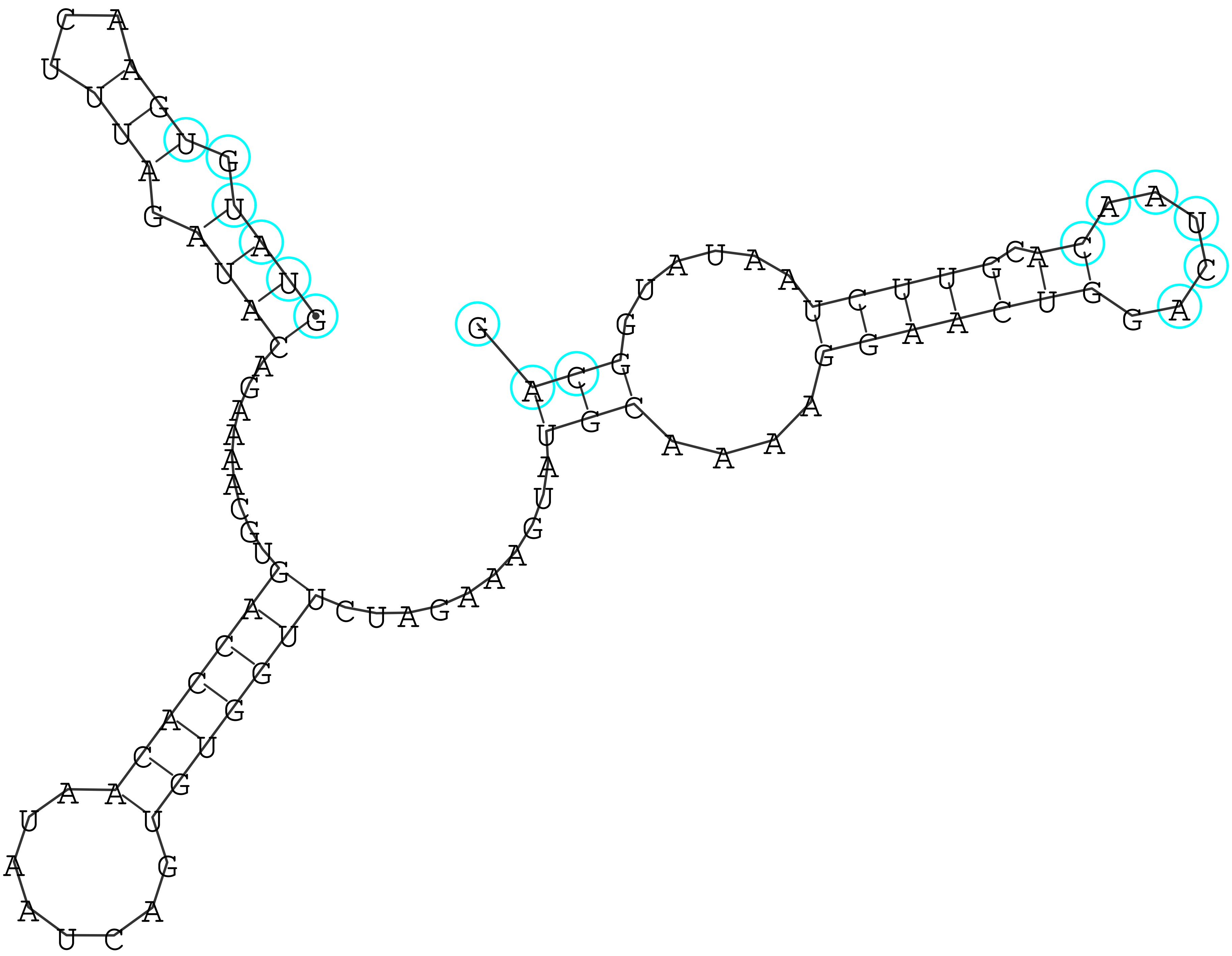

Xbamc110A - External intron

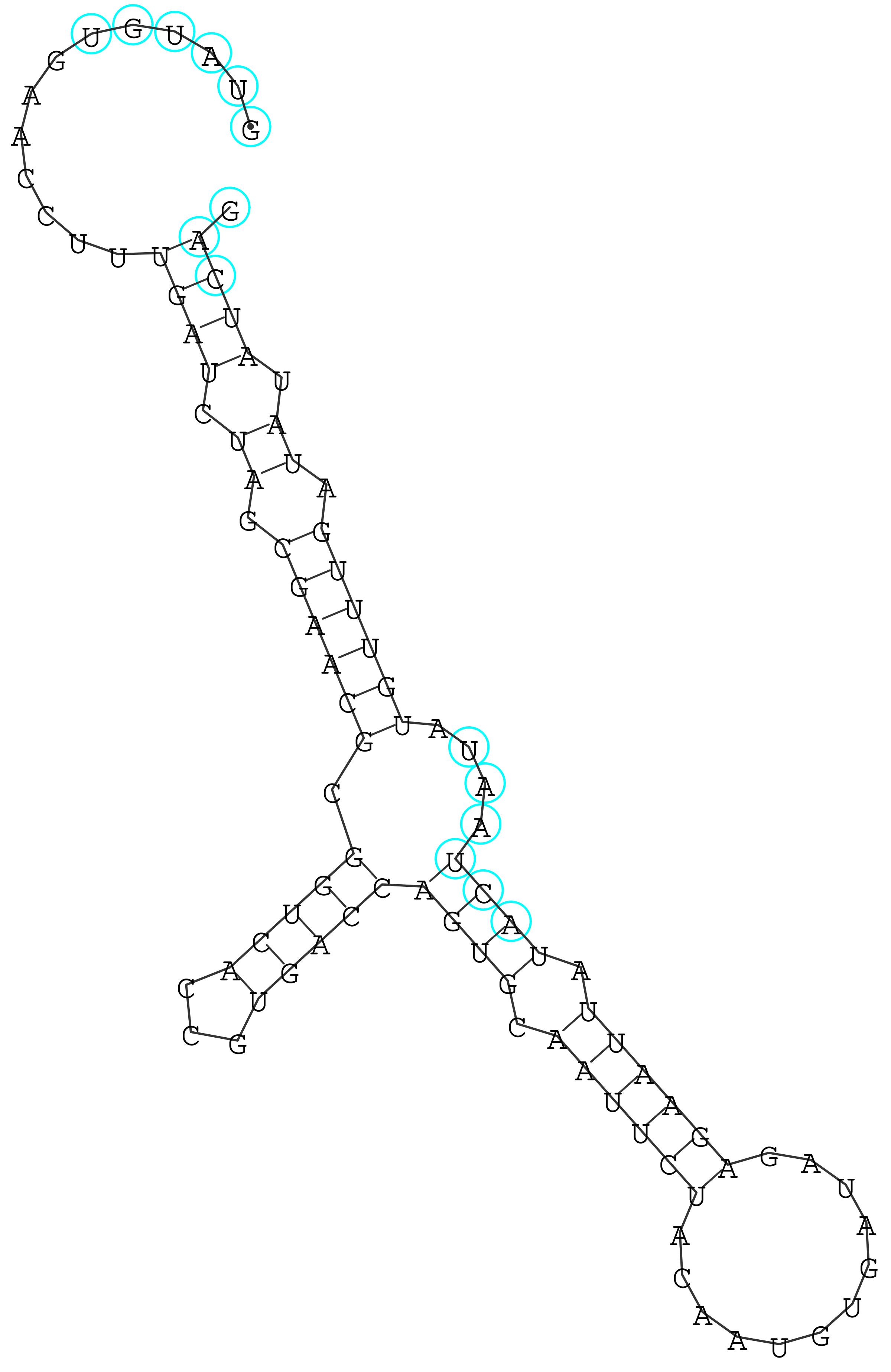

Xbamc124A - External intron

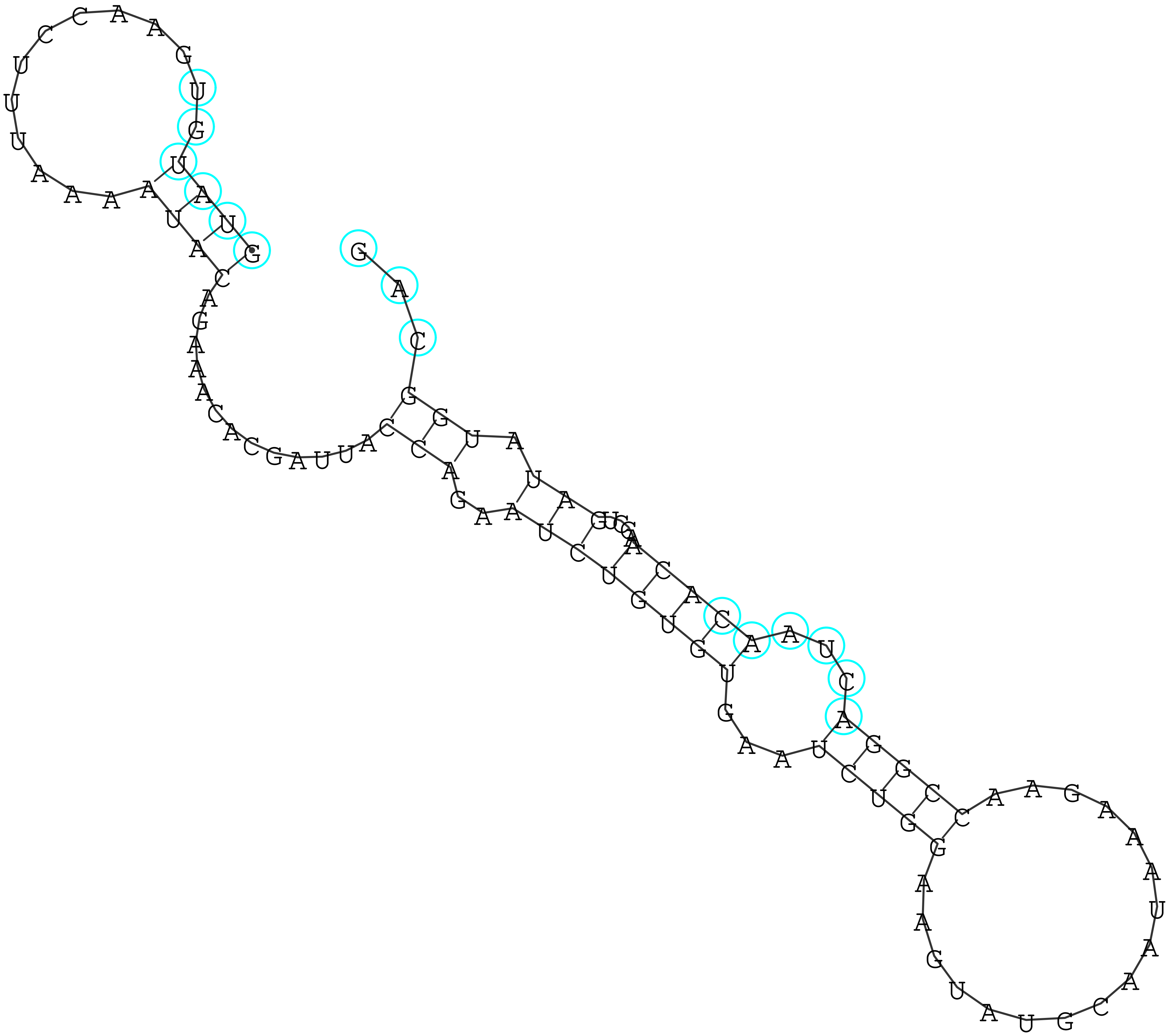

Xbamc132A - External intron

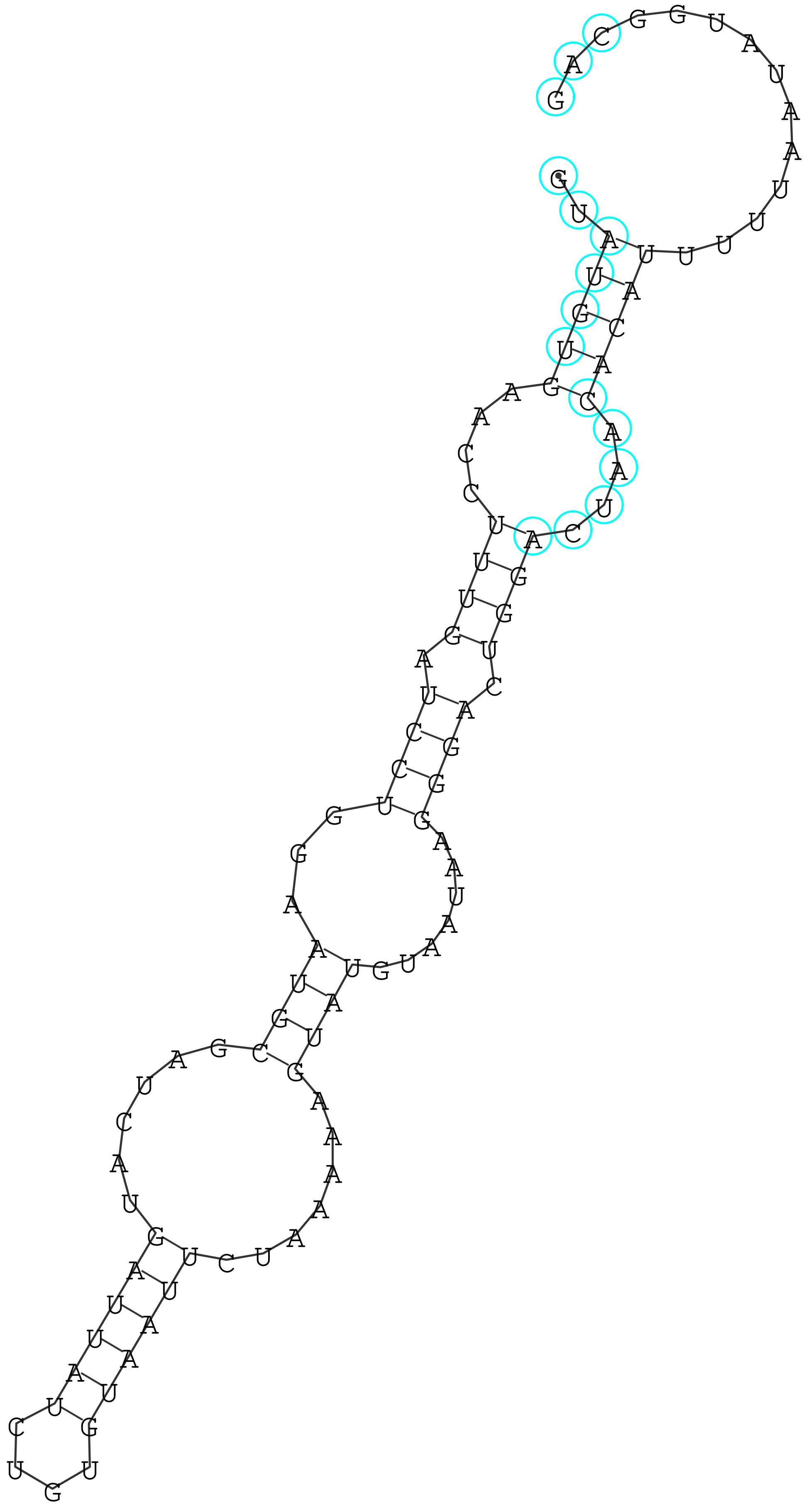

Xbamc132B - External intron

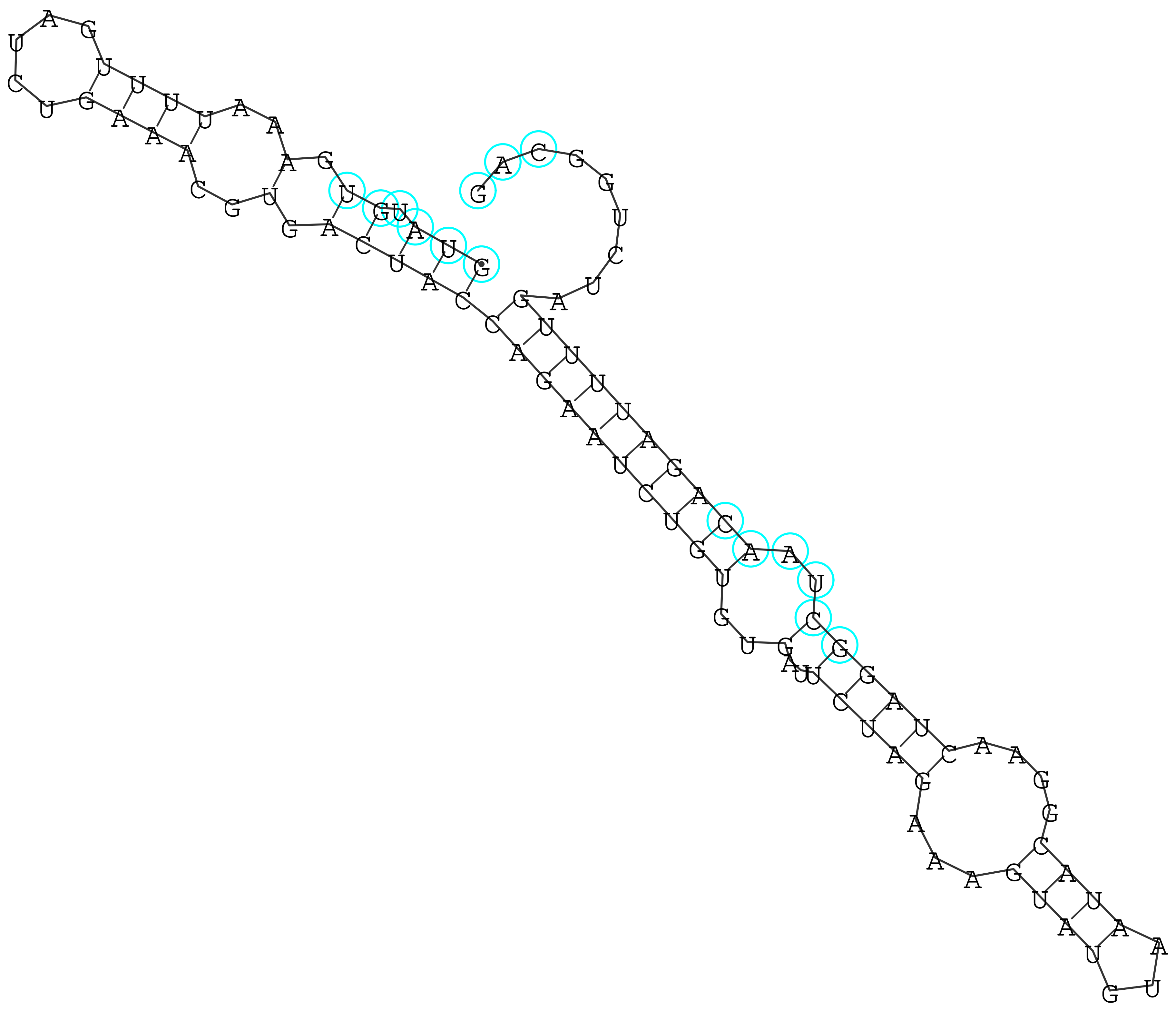

Xbamc152A - External intron

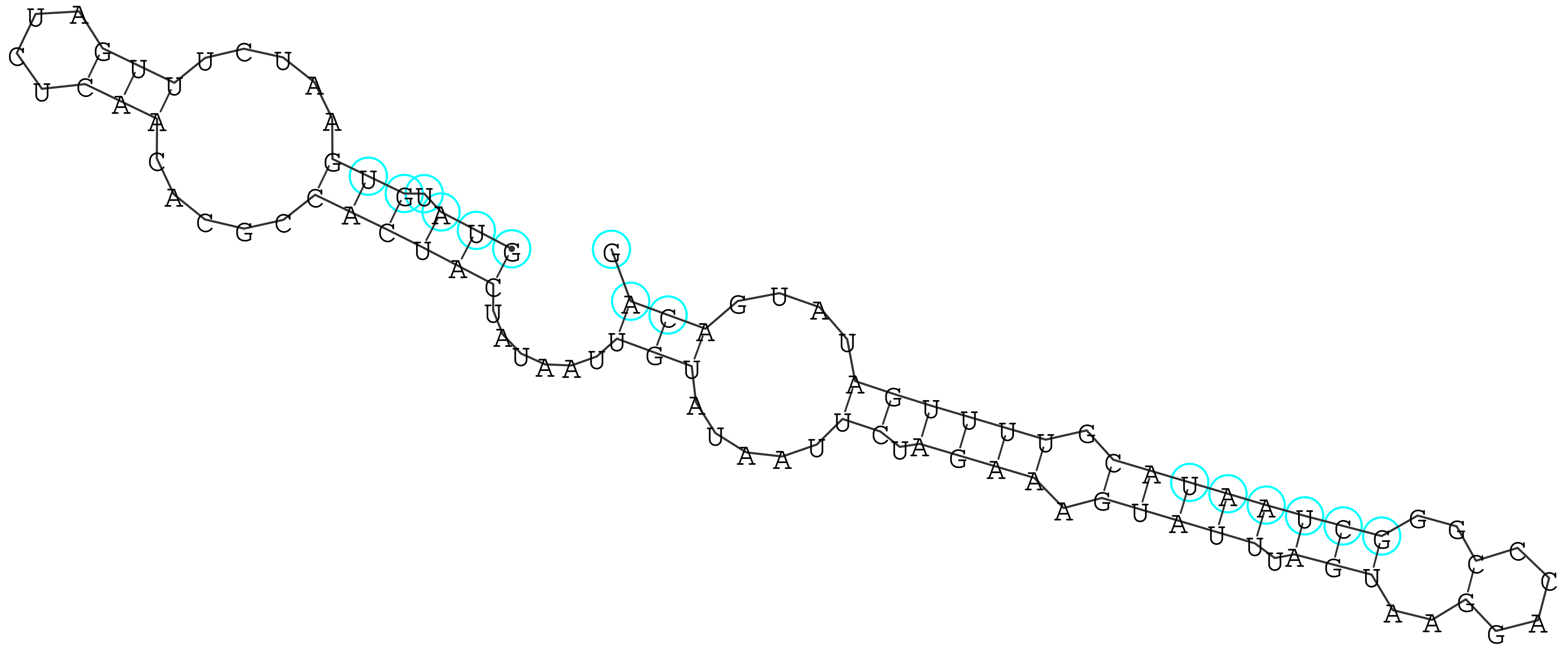

Xbamc152B - External intron

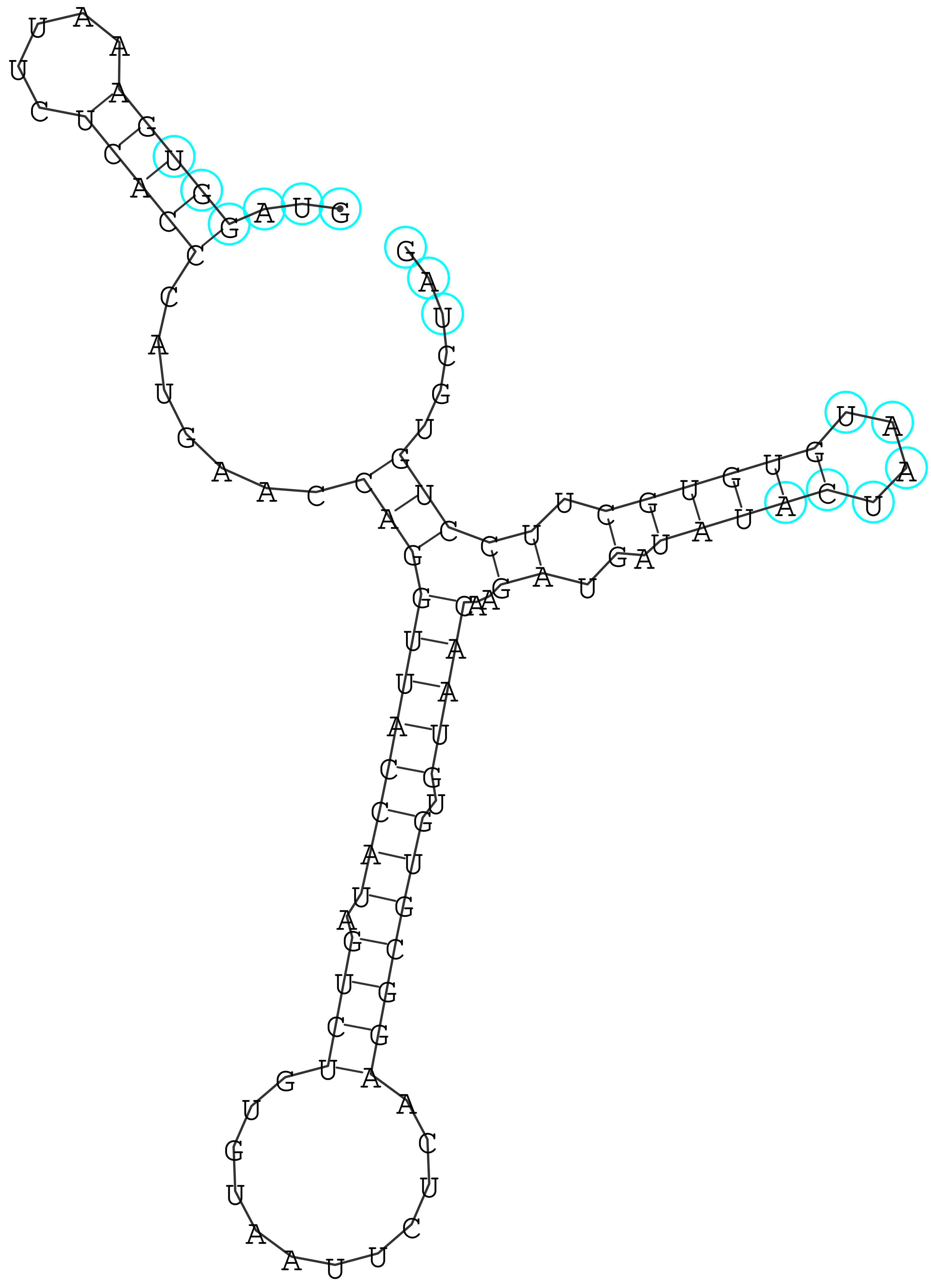

Xbamc152C - External intron

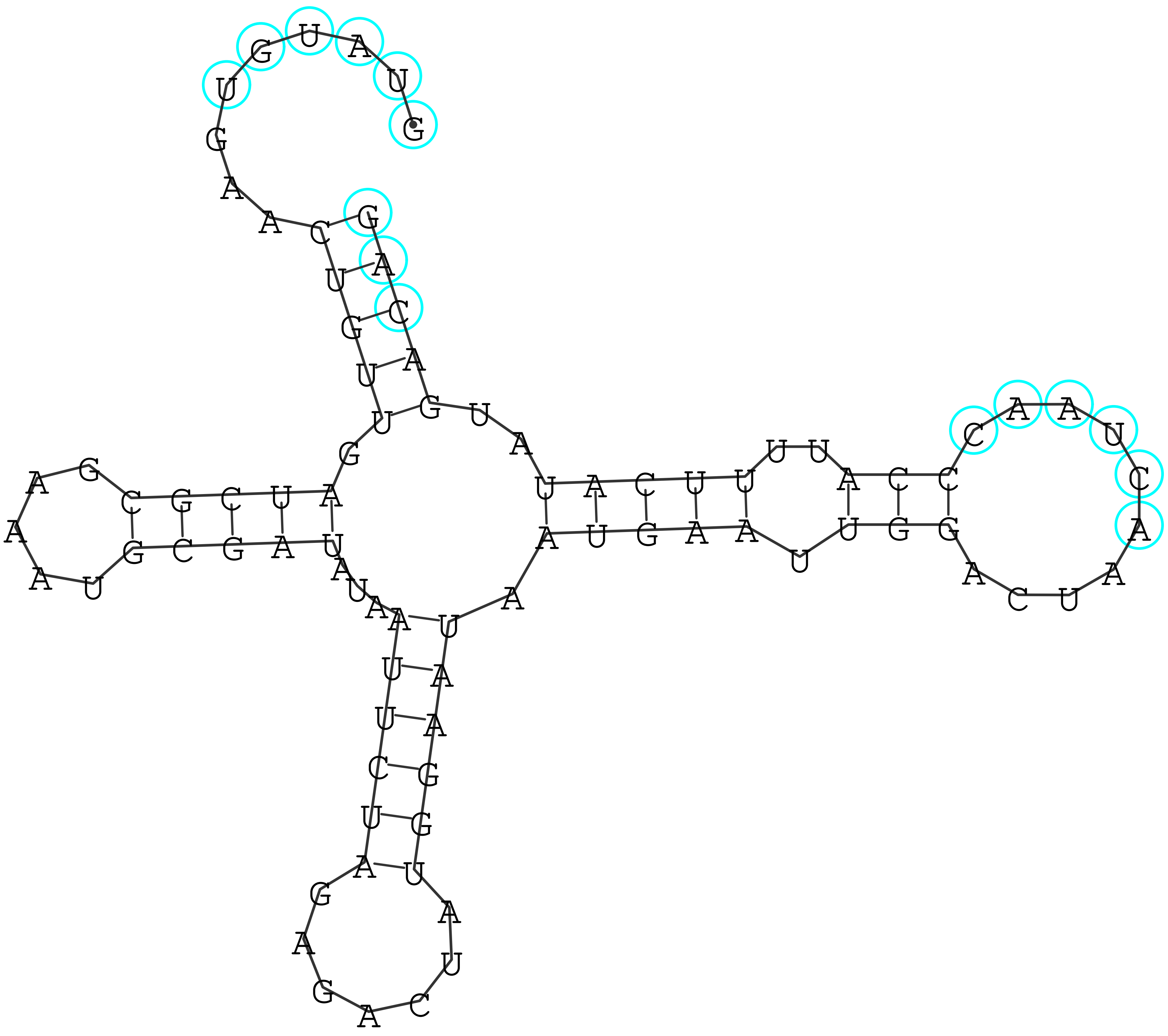

Xbamc153A - External intron

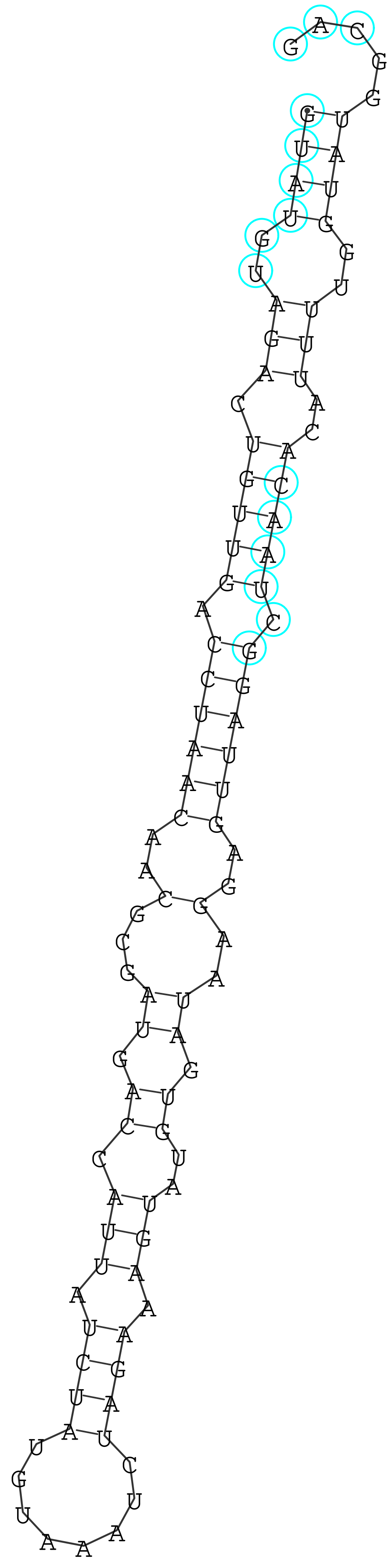

Xbamc155A - External intron

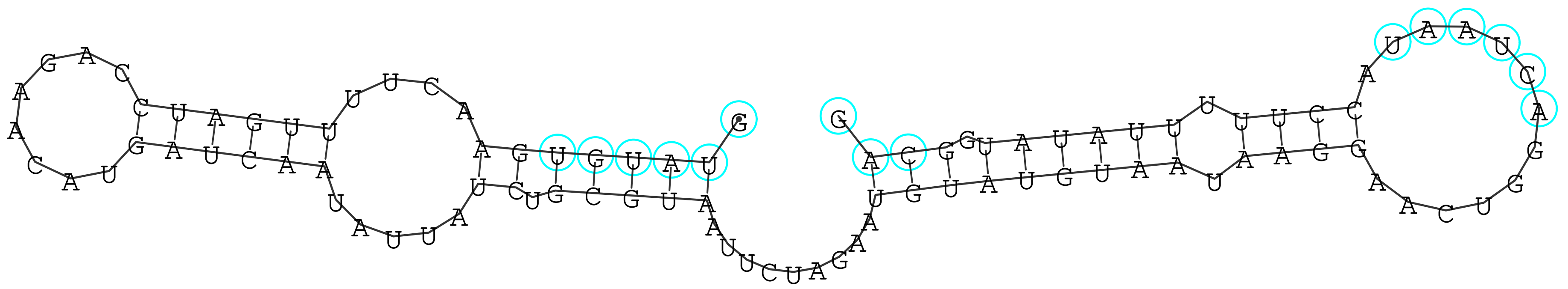

Xbamc156A - External intron

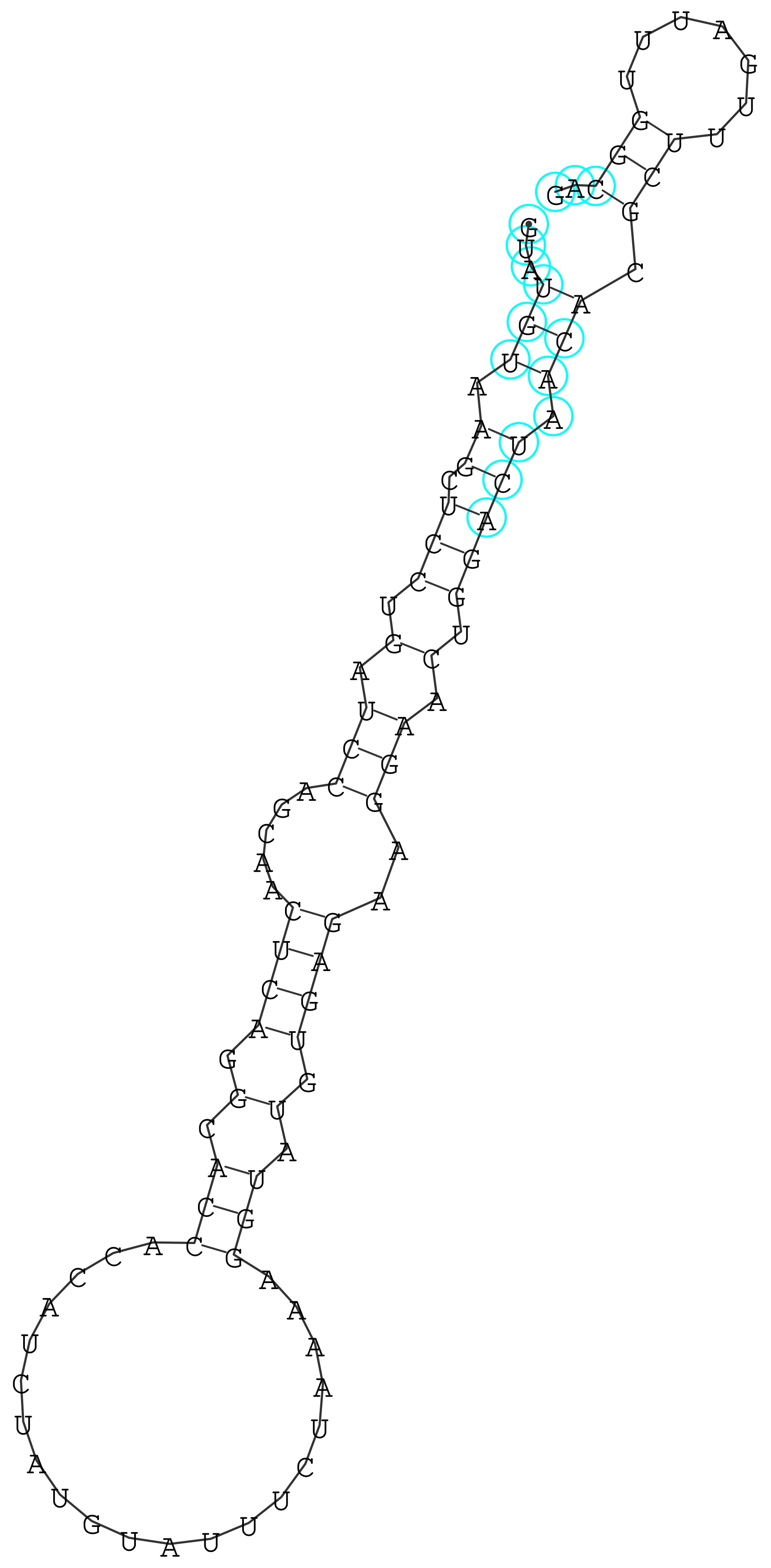

Xbamc159A - External intron

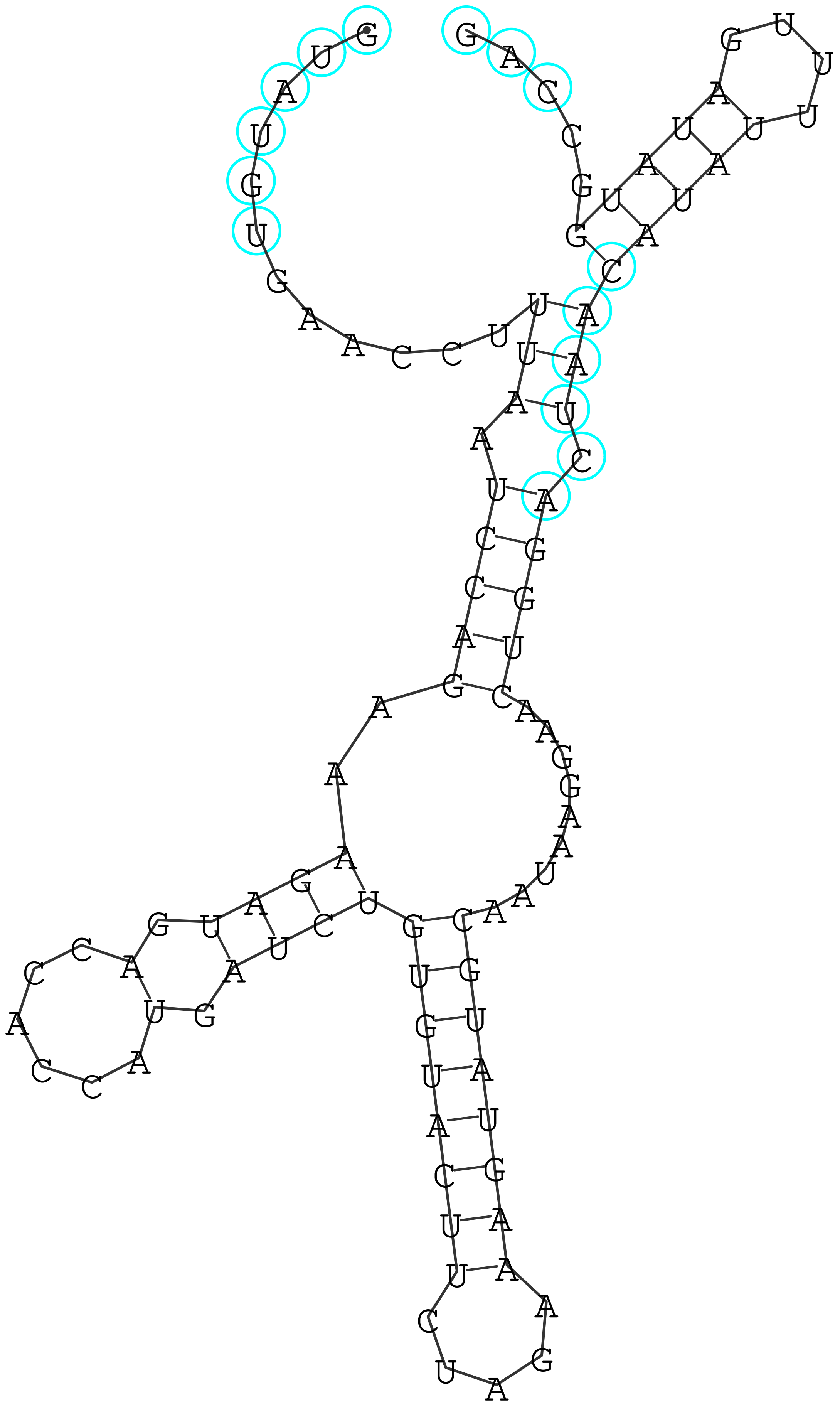

Xbamc164A - External intron

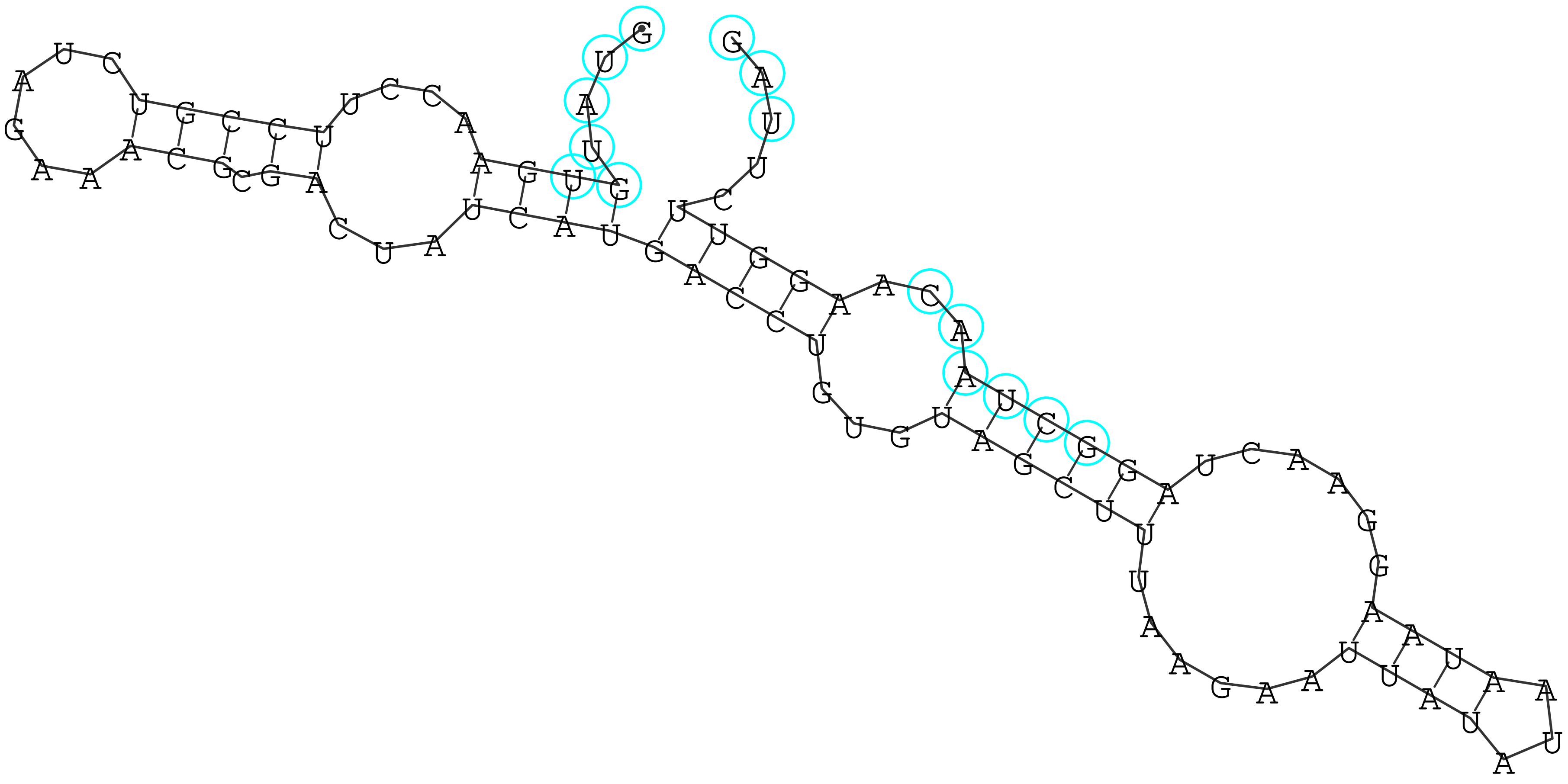

Xbamc176A - External intron

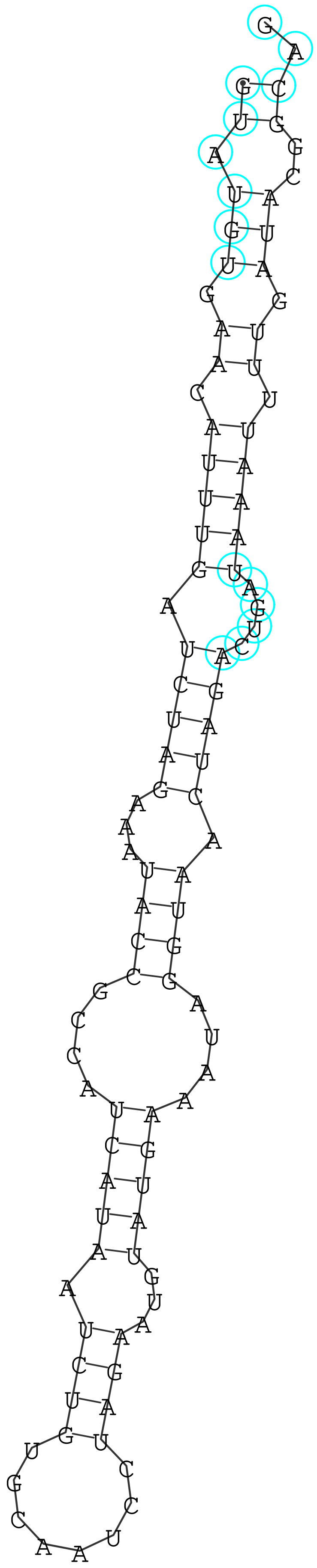

Xbamc177A - External intron

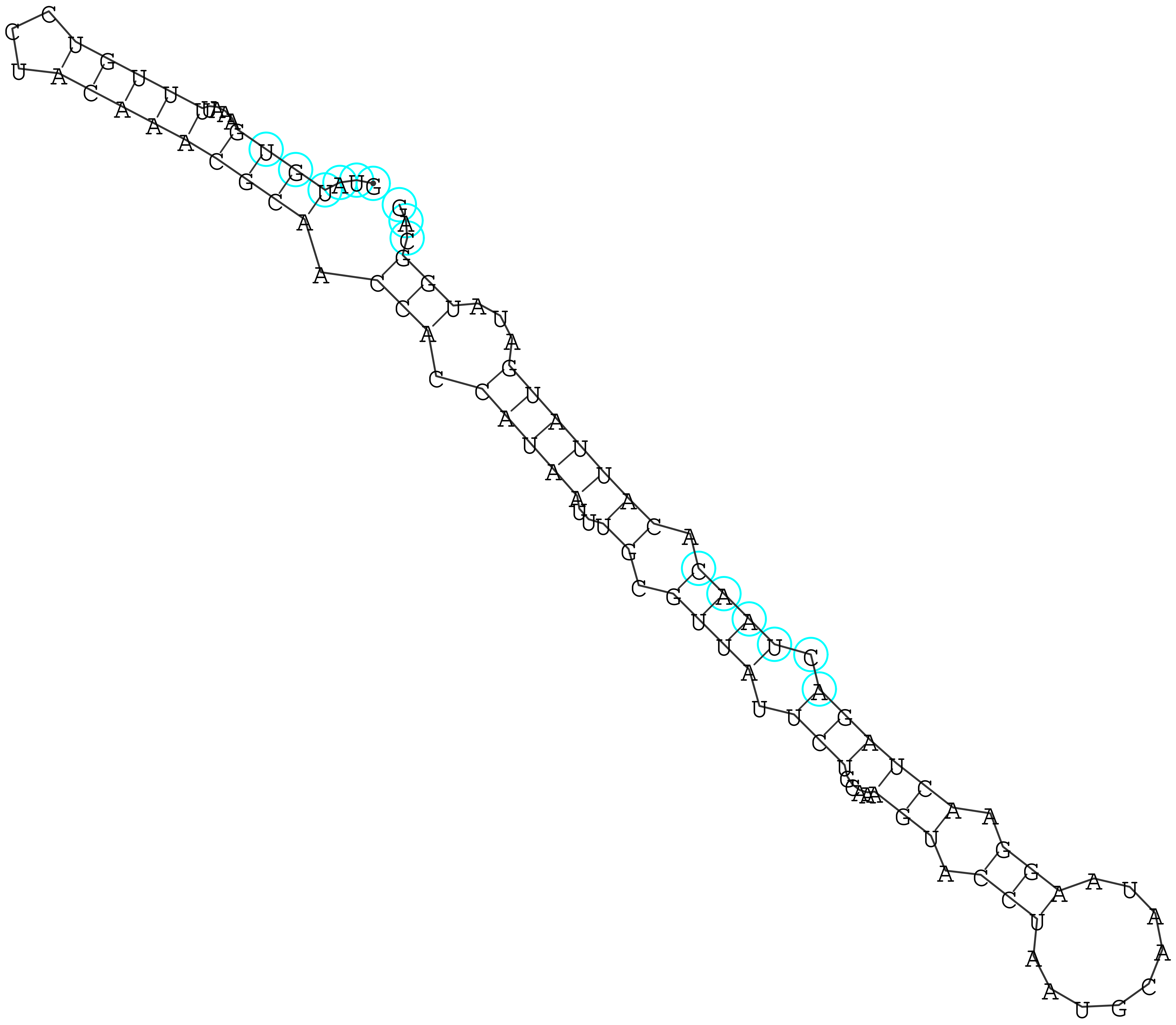

Xbamc191A - External intron

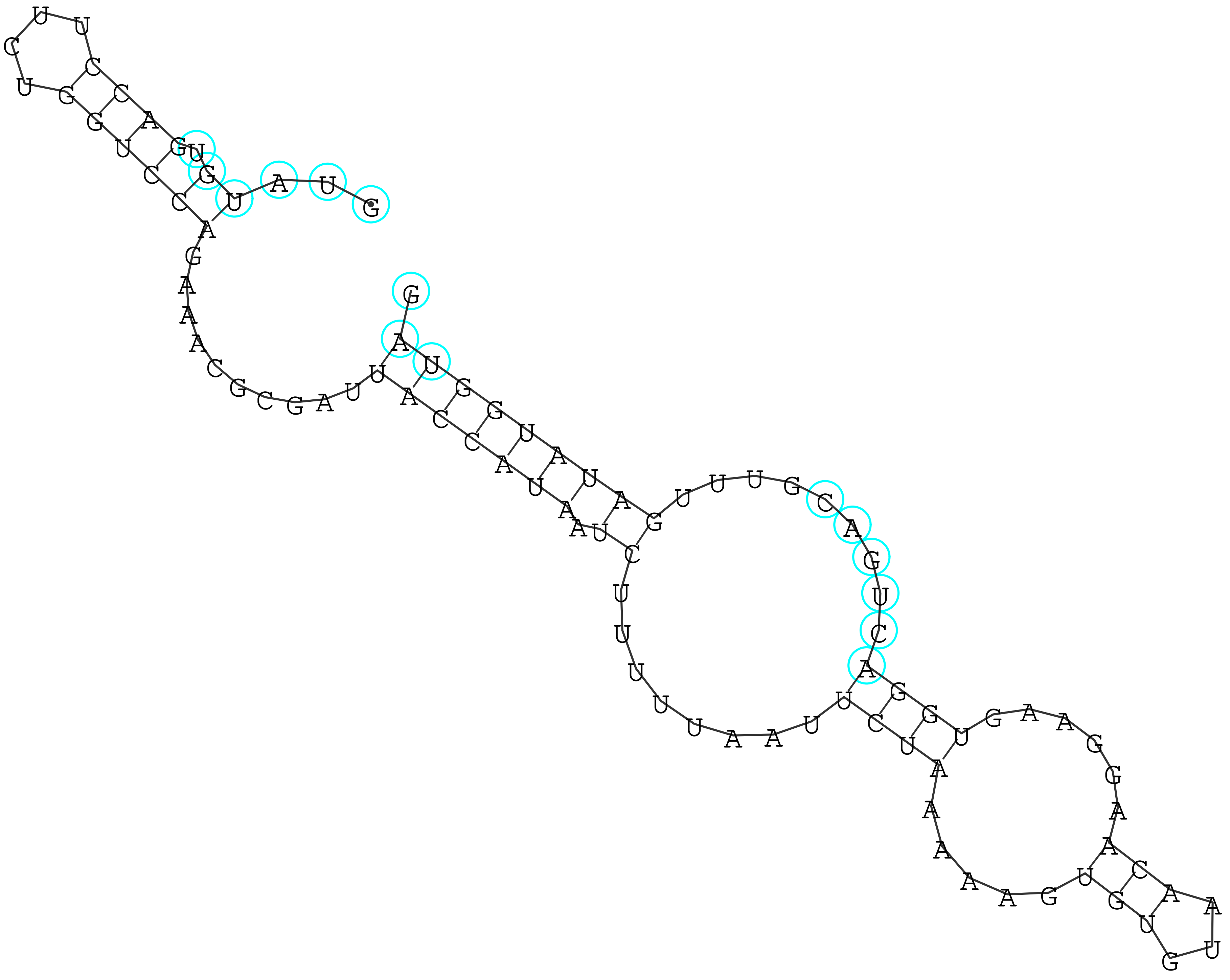

Xbamc198A - External intron

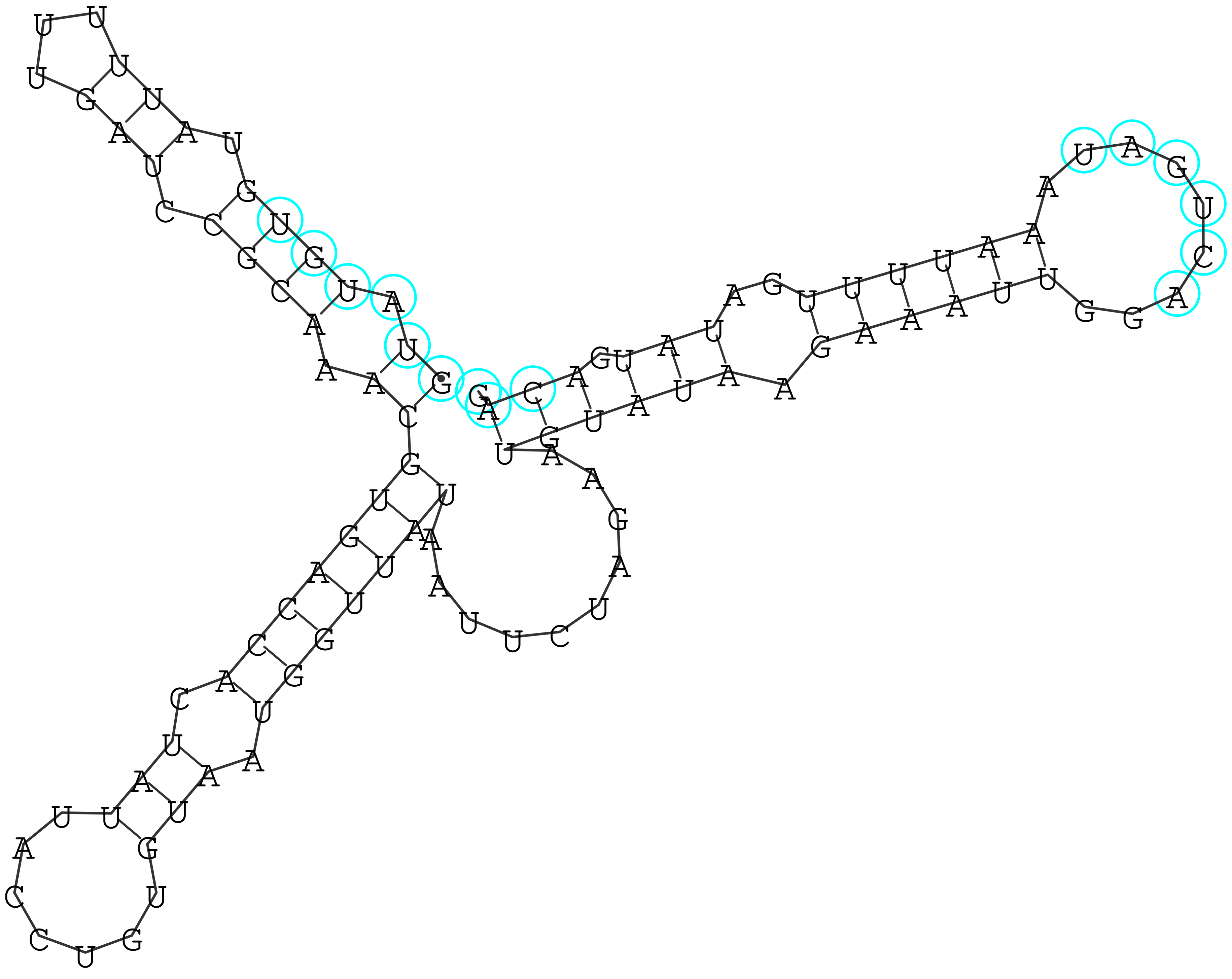

Xbamc199A - External intron

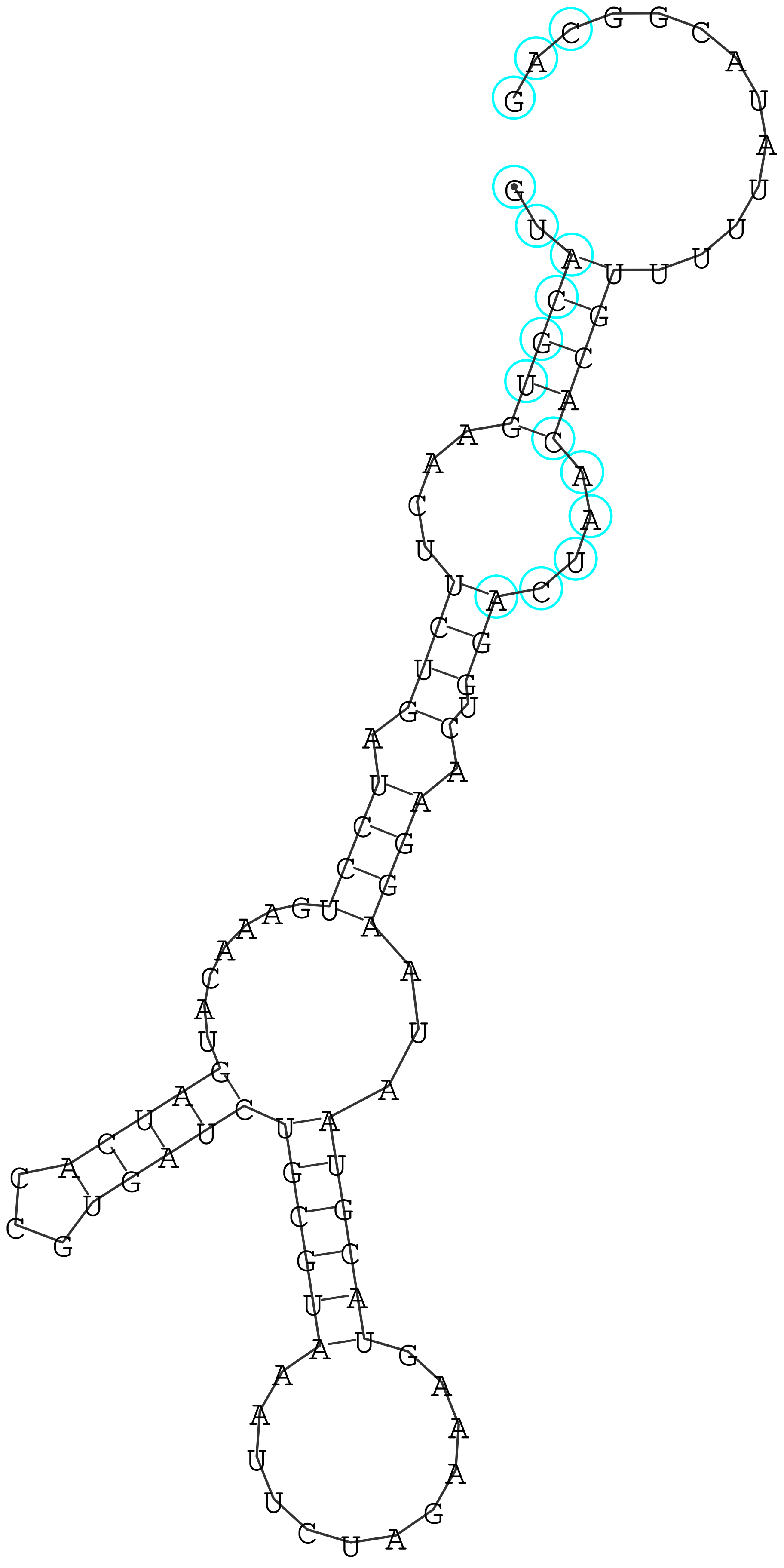

Xbamc236C - External intron

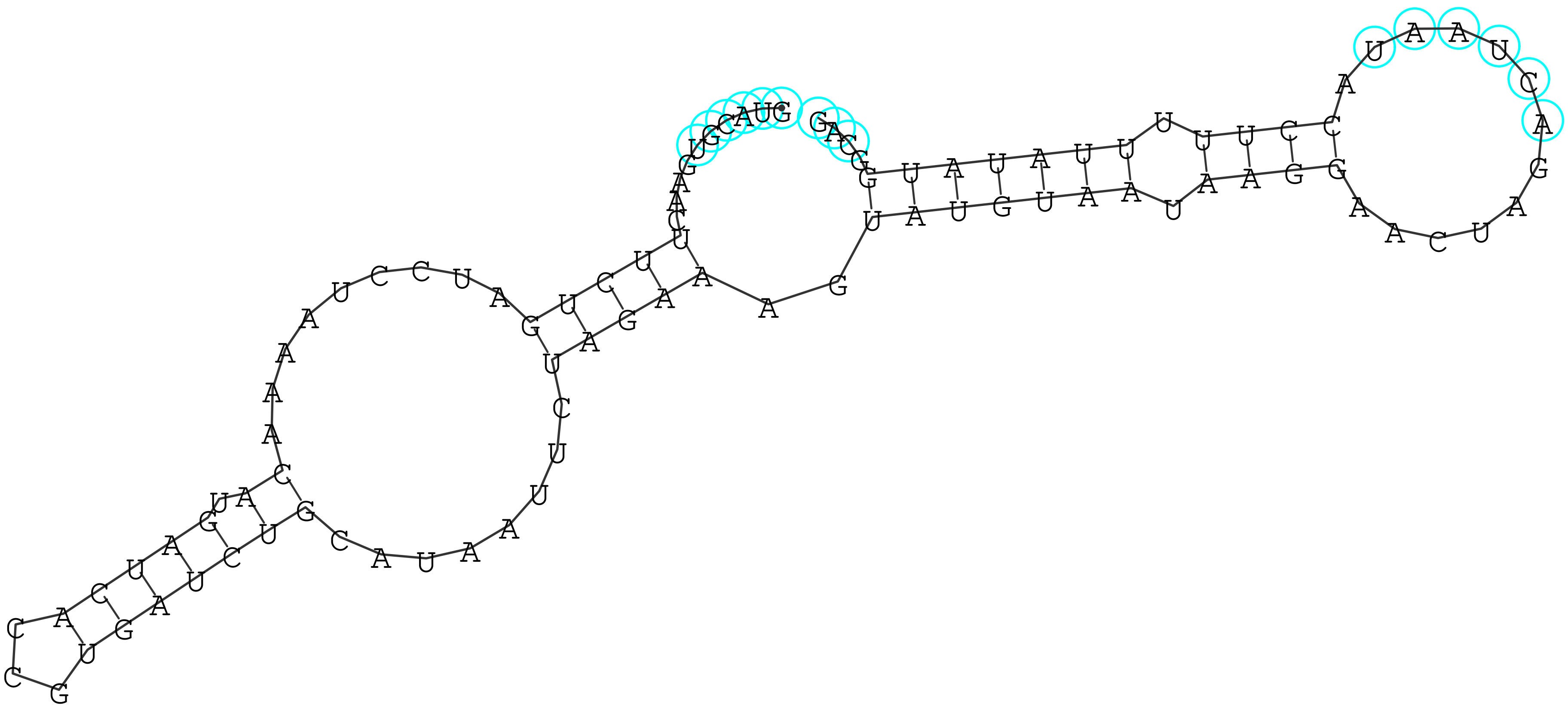

Xbccc01A - External intron

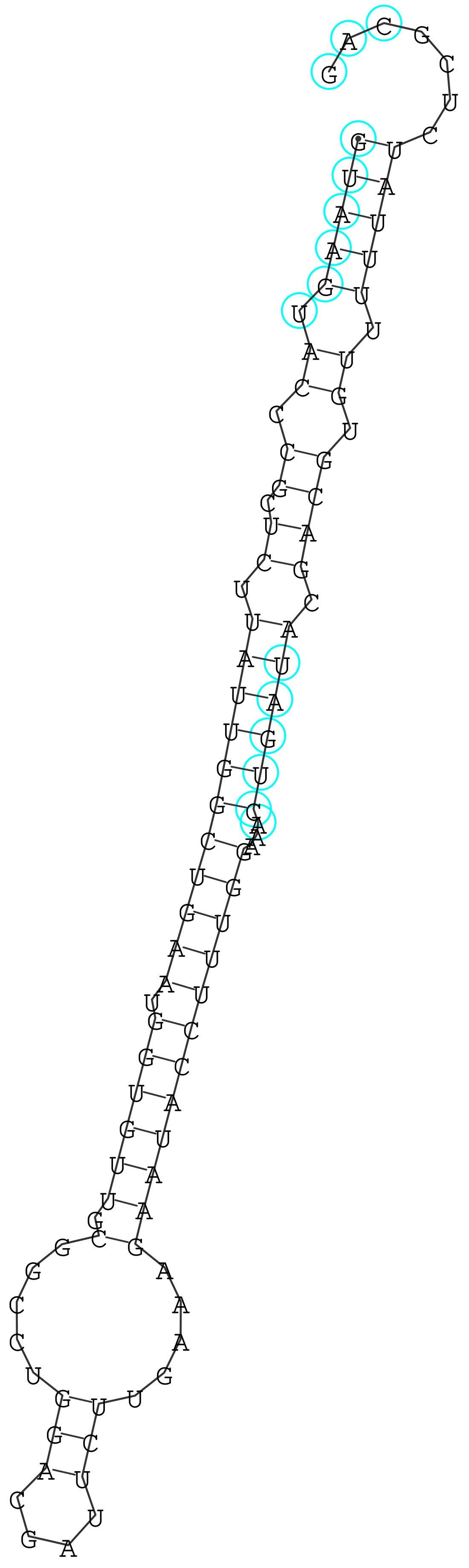

Xbccc01B - External intron

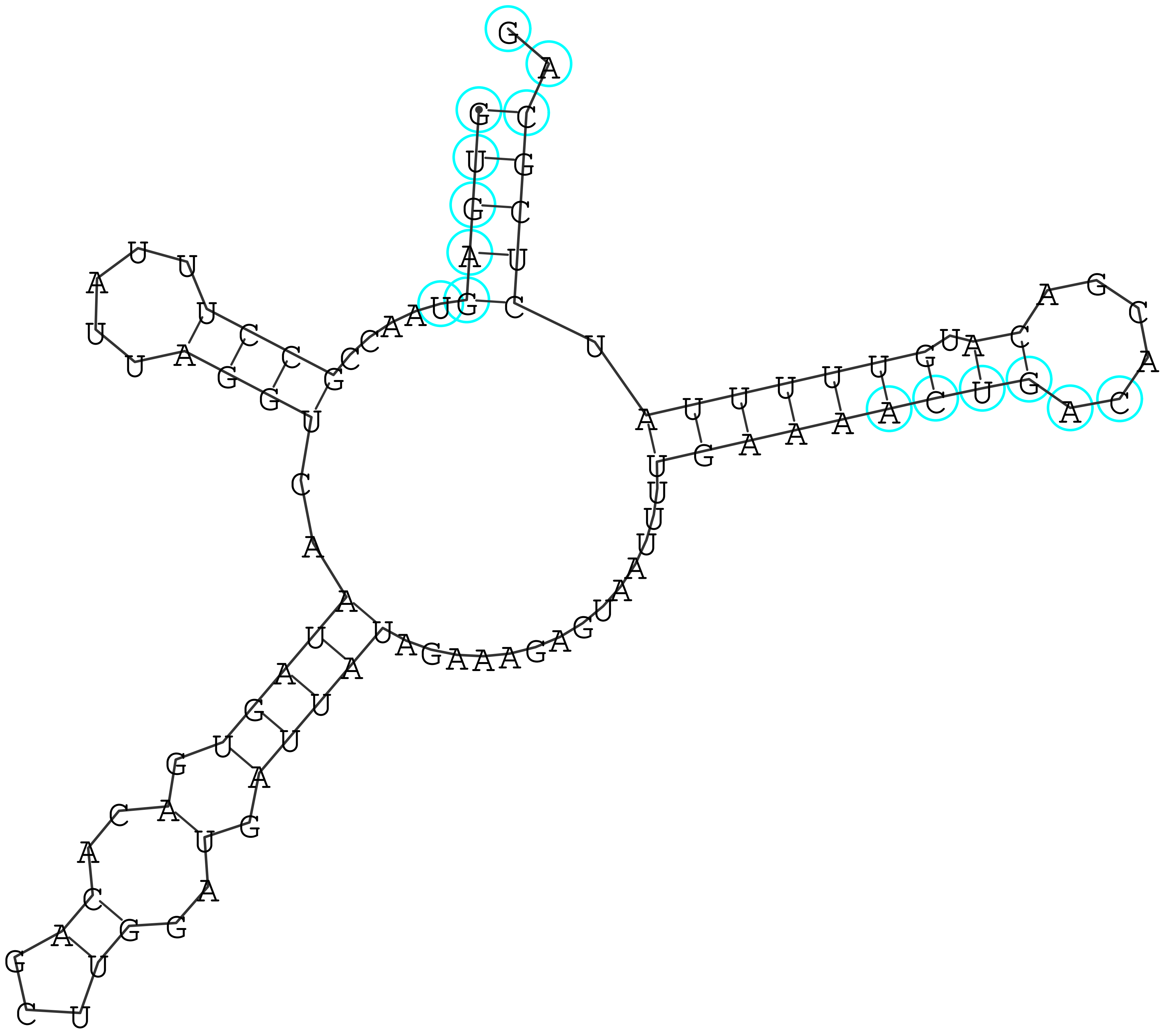

Xbccc05A - External intron

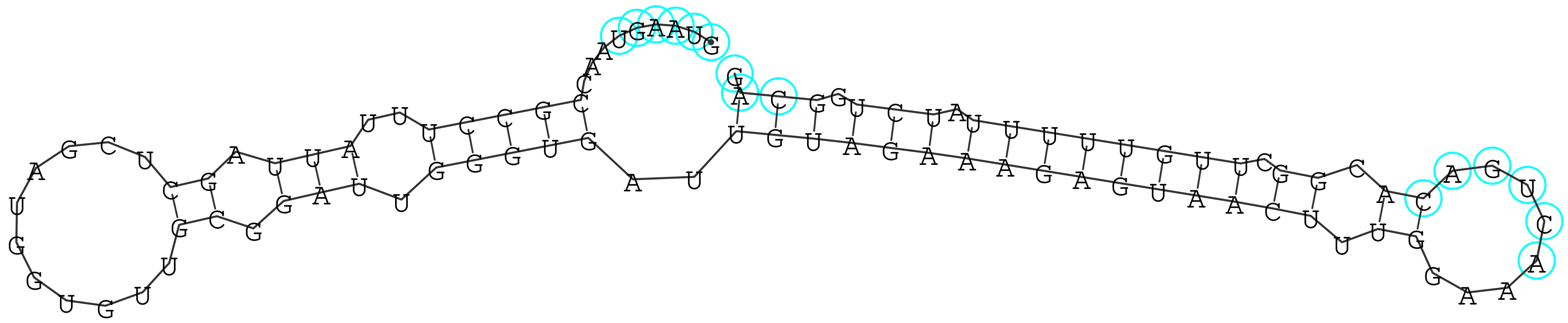

Xbccc05B - External intron

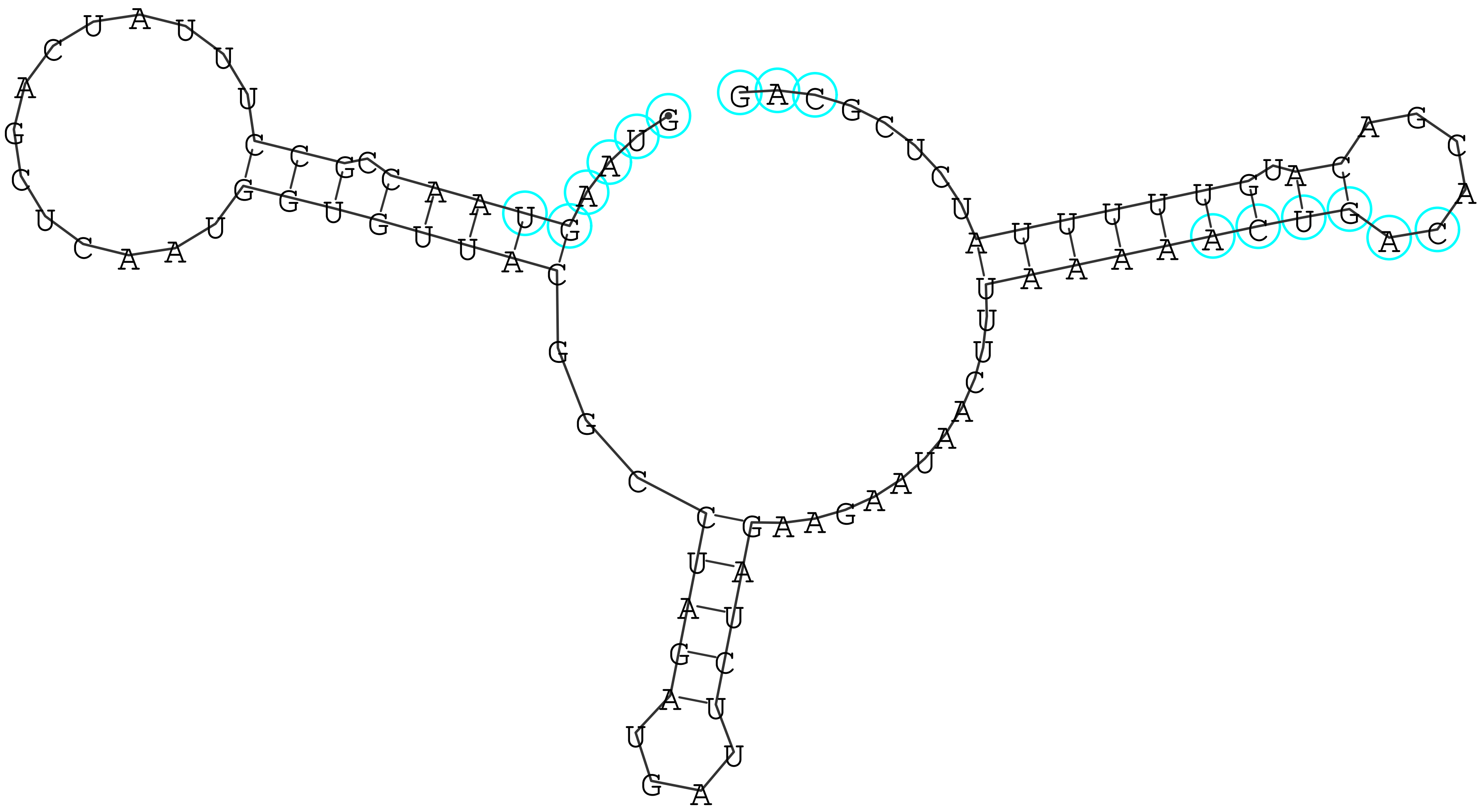

Xbccc05C - External intron

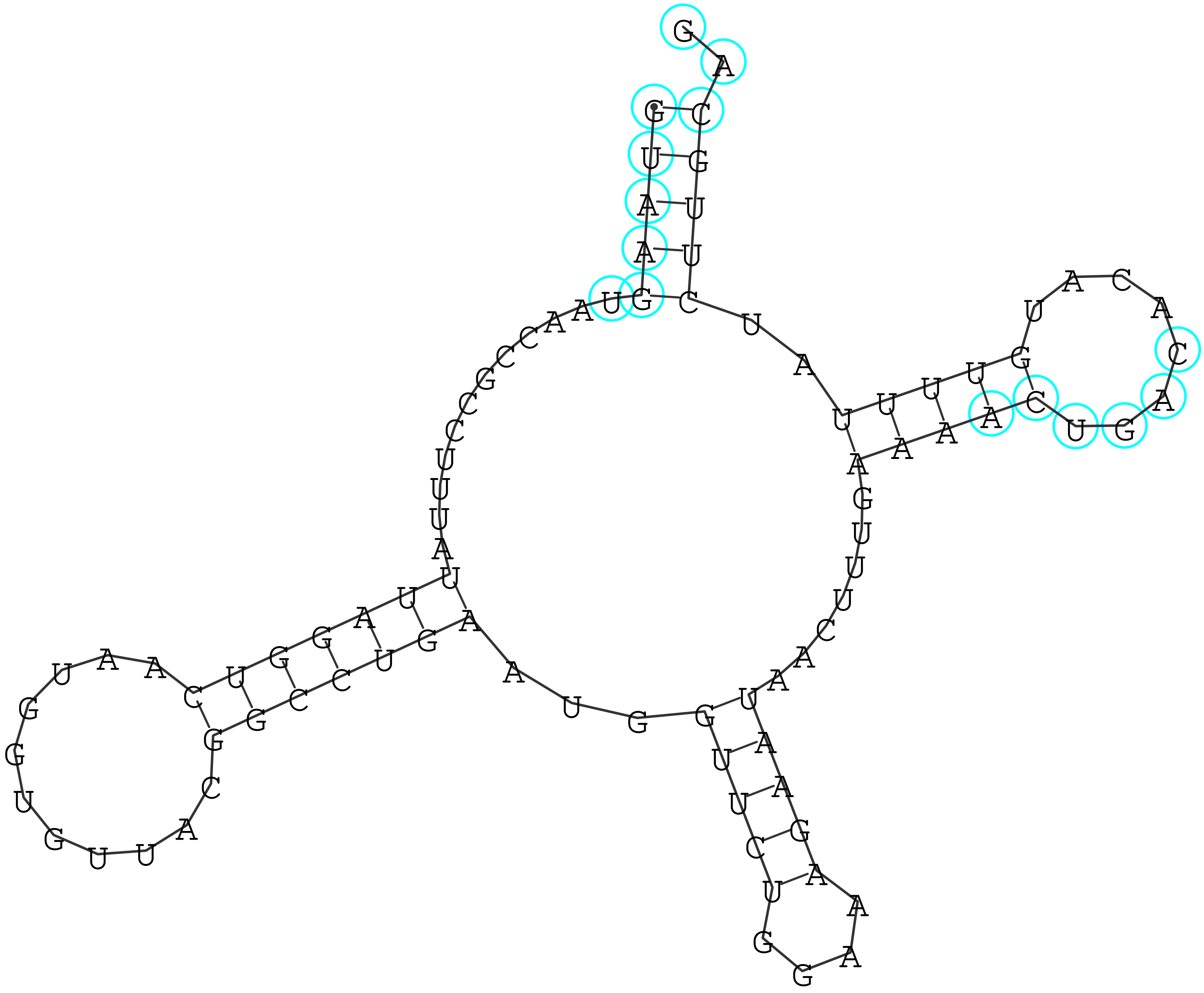

Xbccc06A - External intron

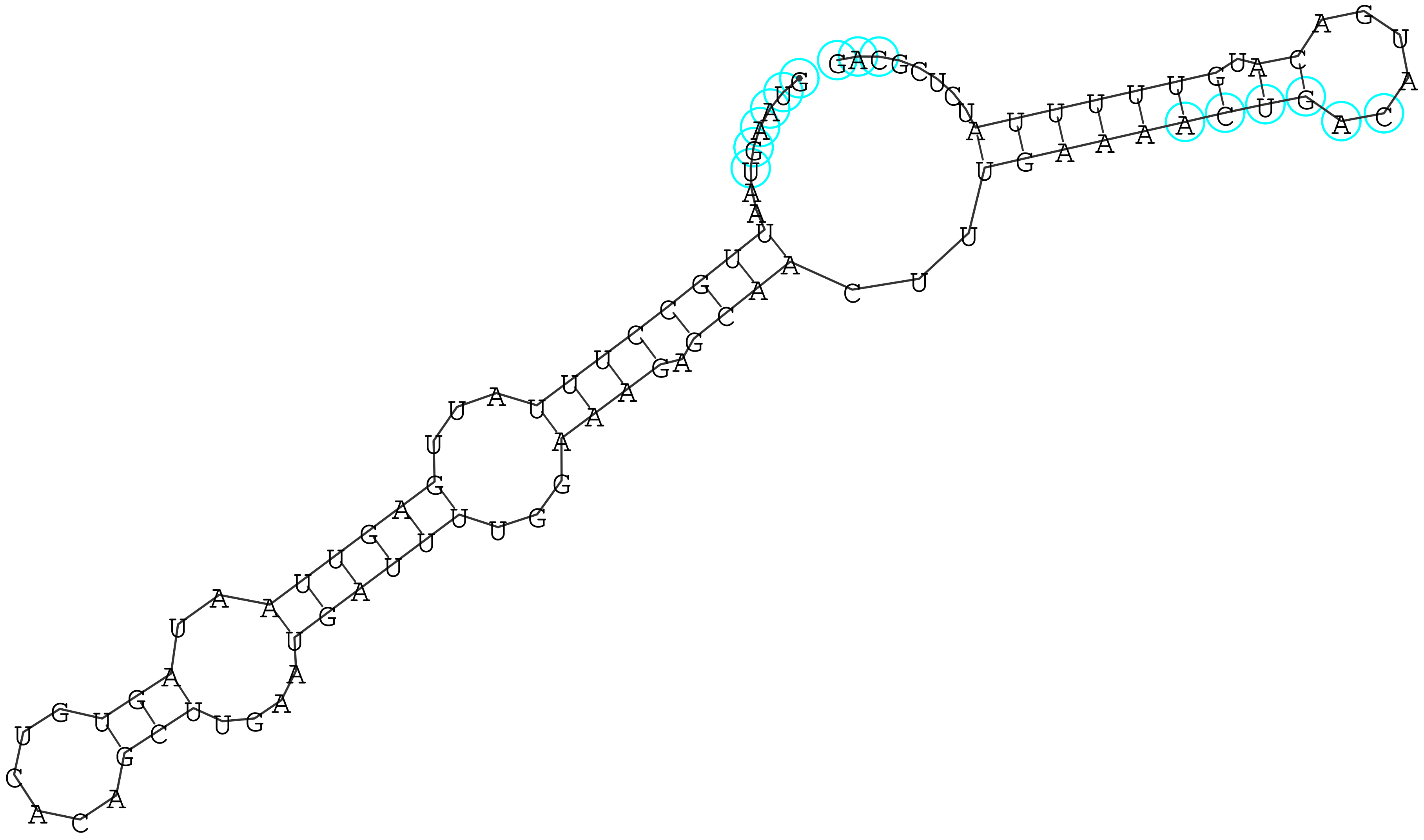

Xbccc07A - External intron

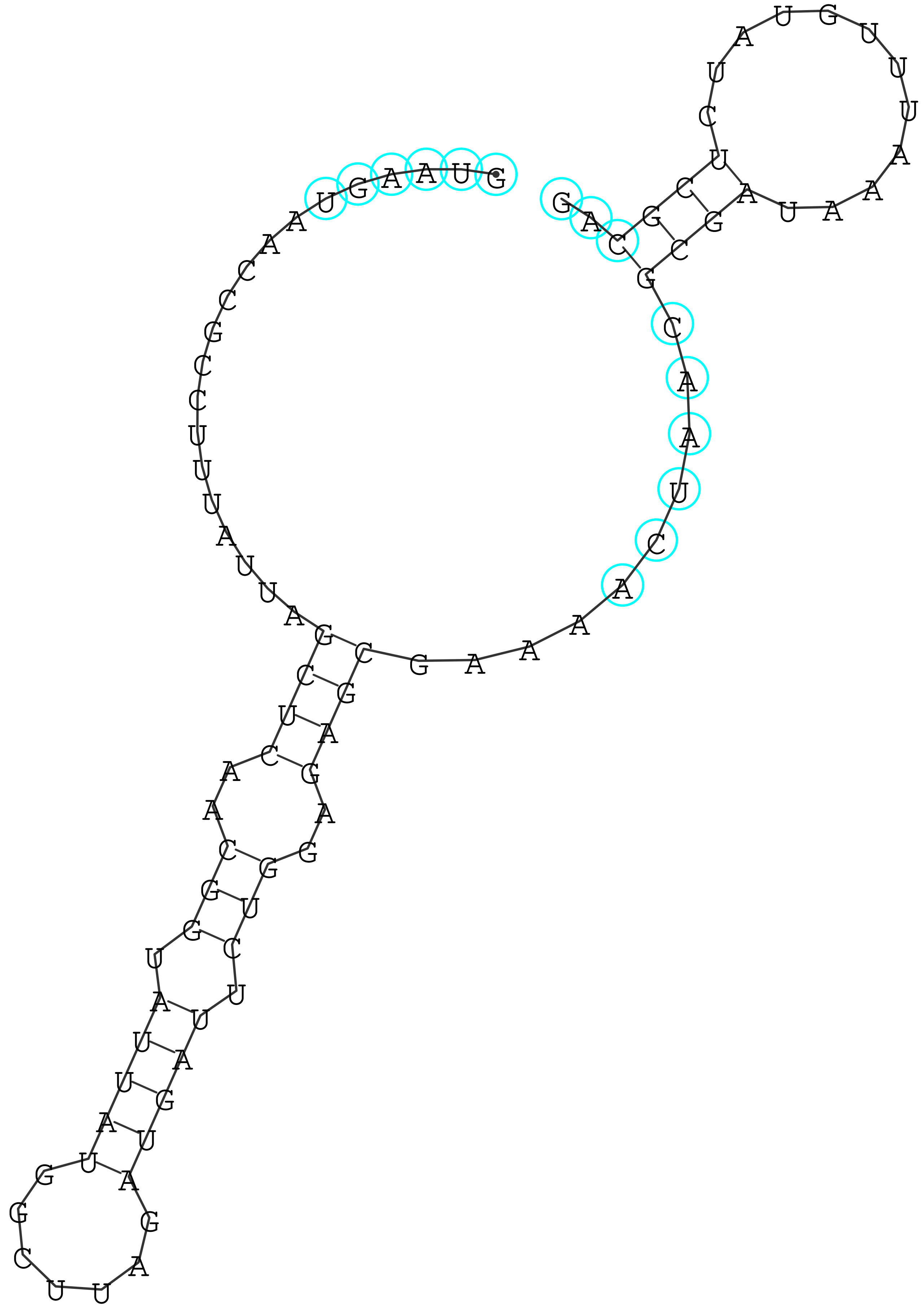

Xbccc09A - External intron

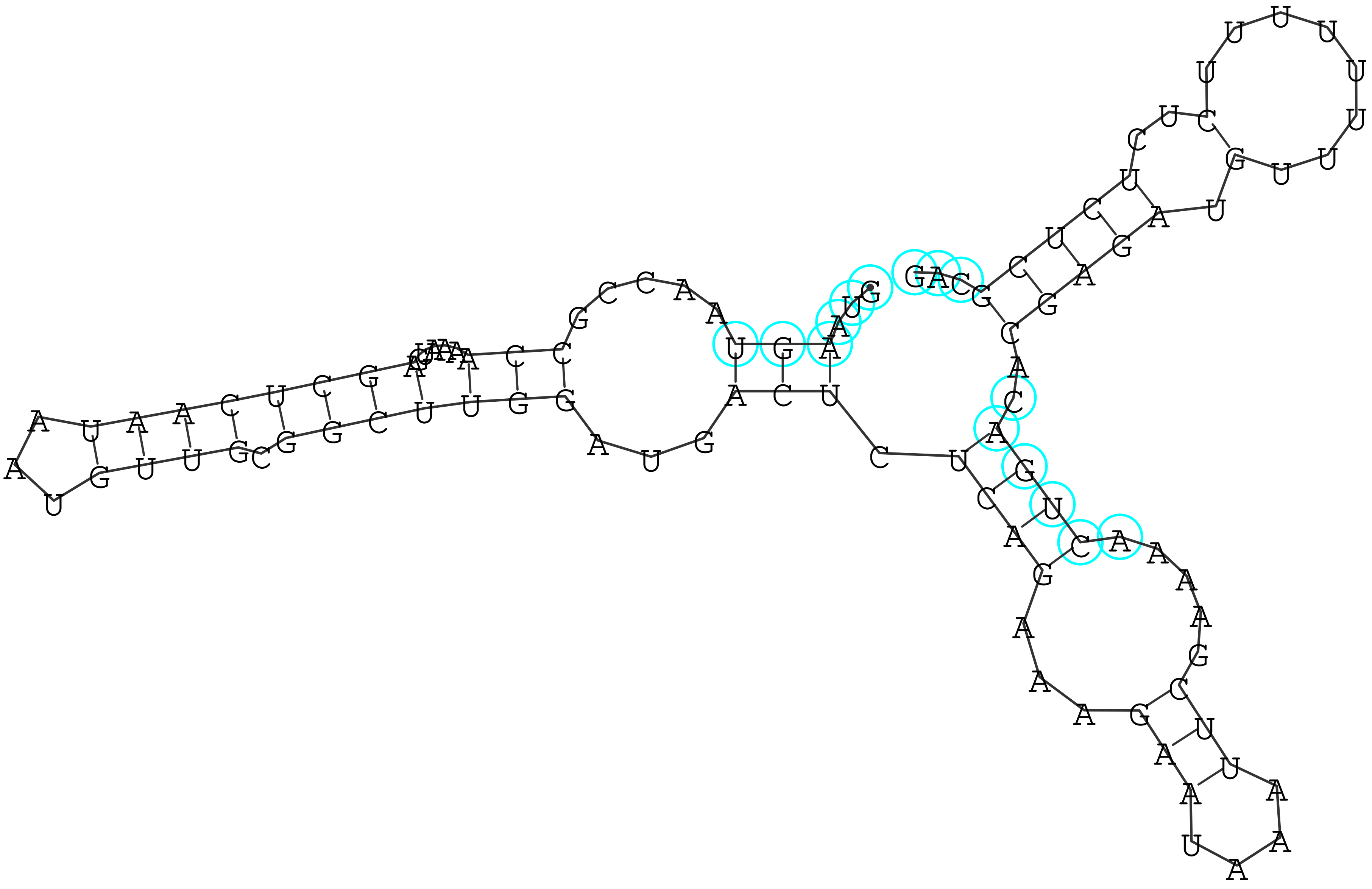

Xlonc0002A - External intron

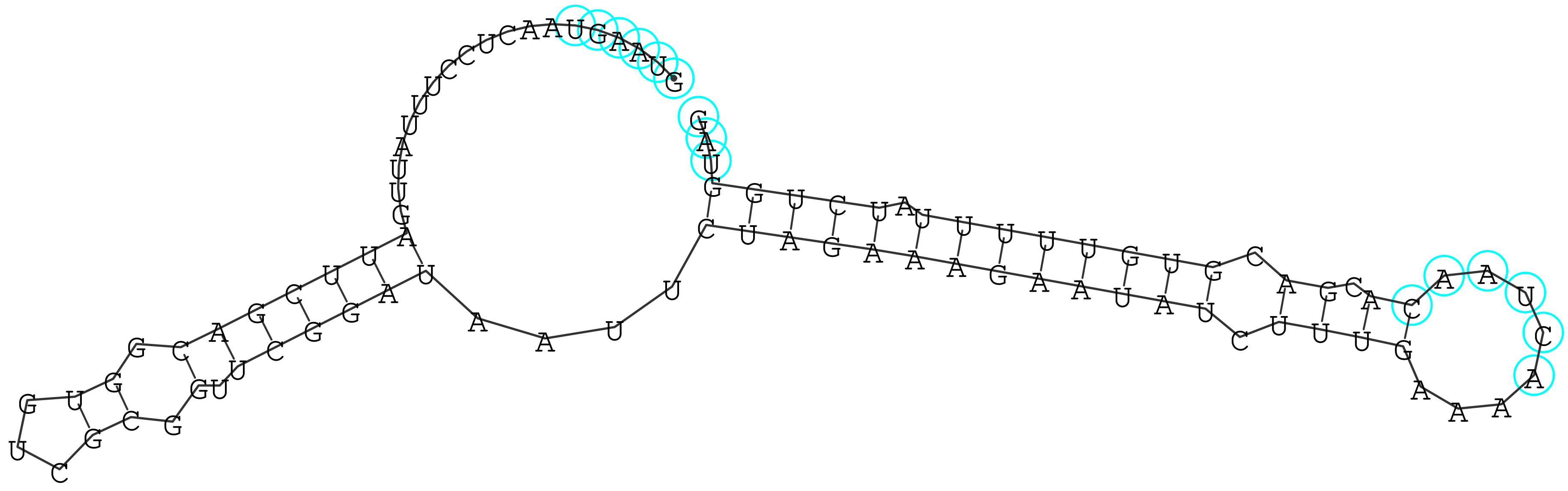

Xlonc0025A - External intron

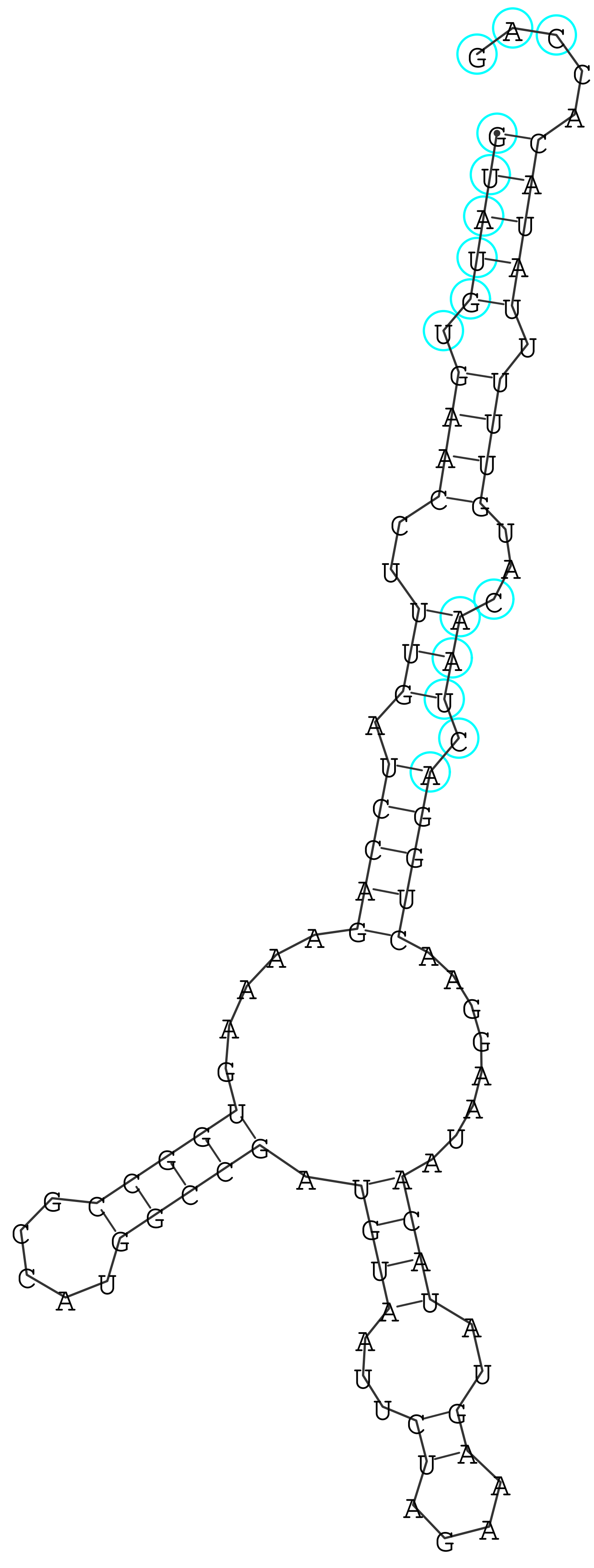

Xlonc0058A - External intron

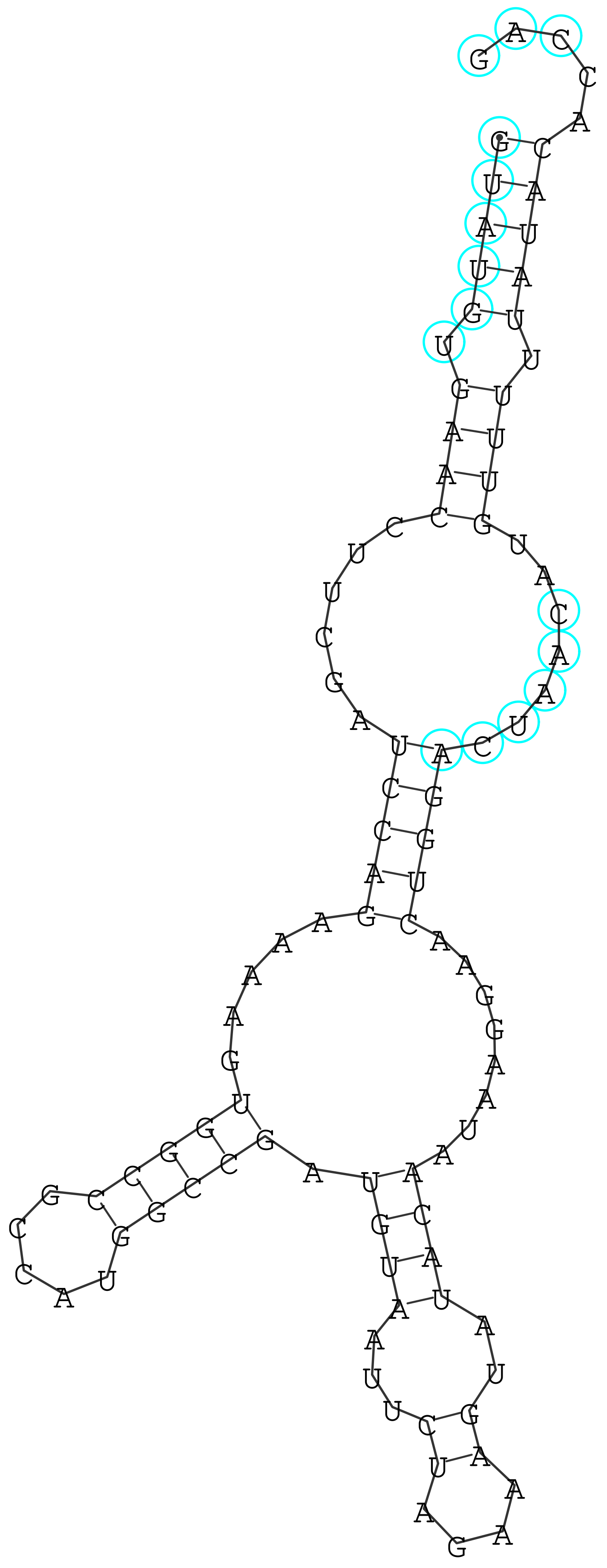

Xlonc0112A - External intron

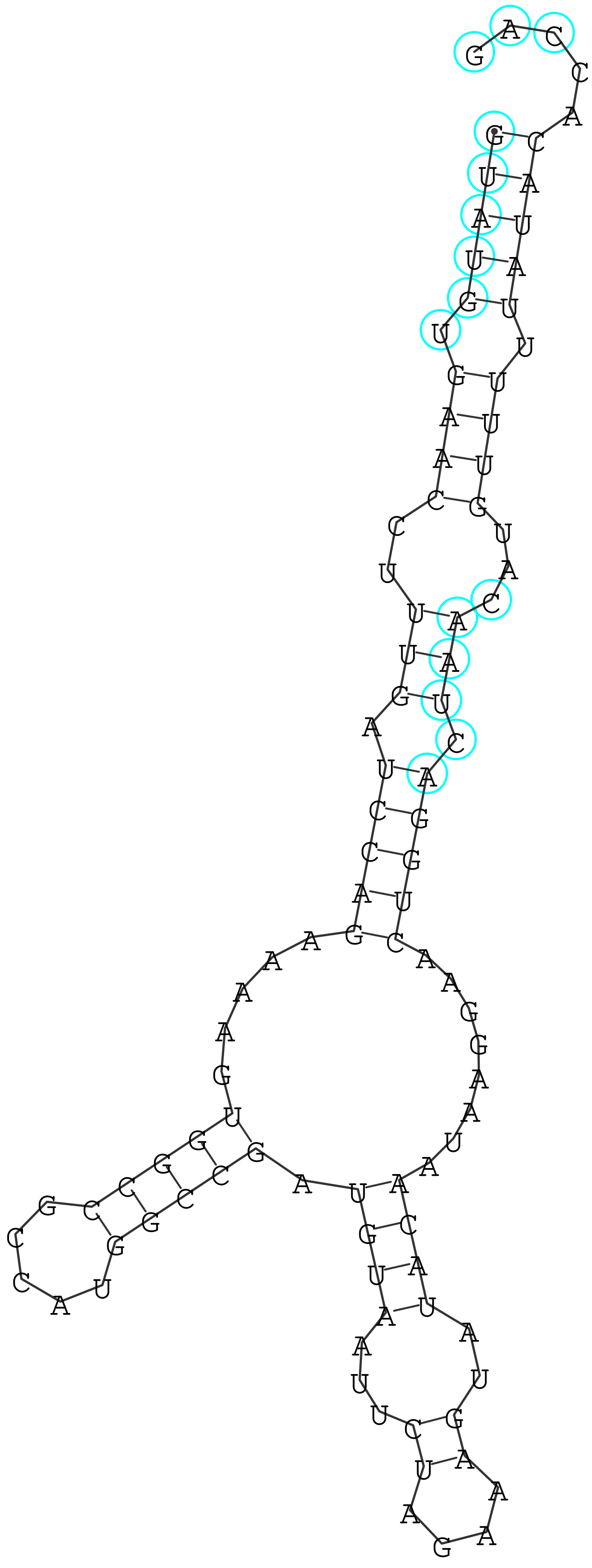

Xmsuc0005A - External intron

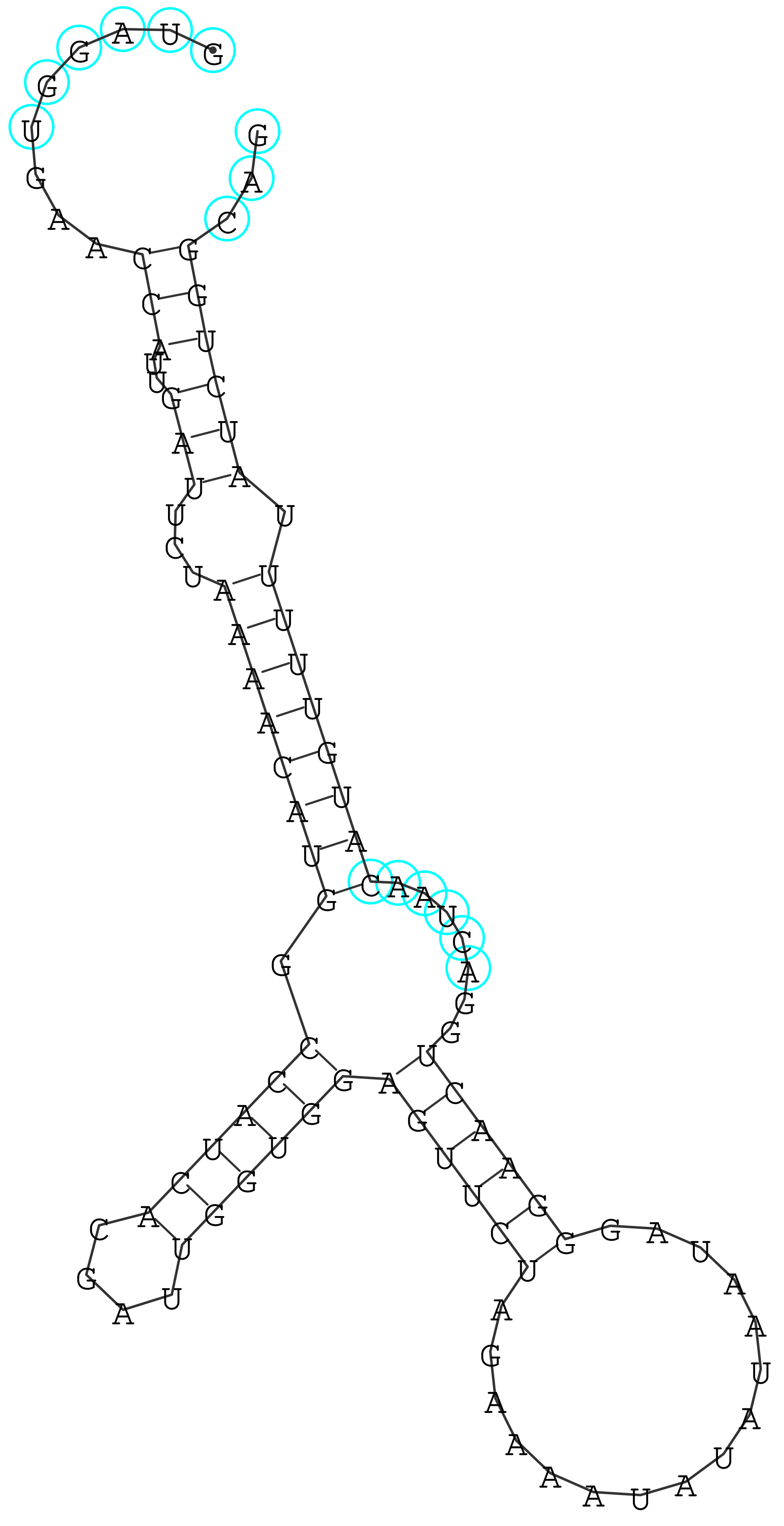

Xmsuc0006A - External intron

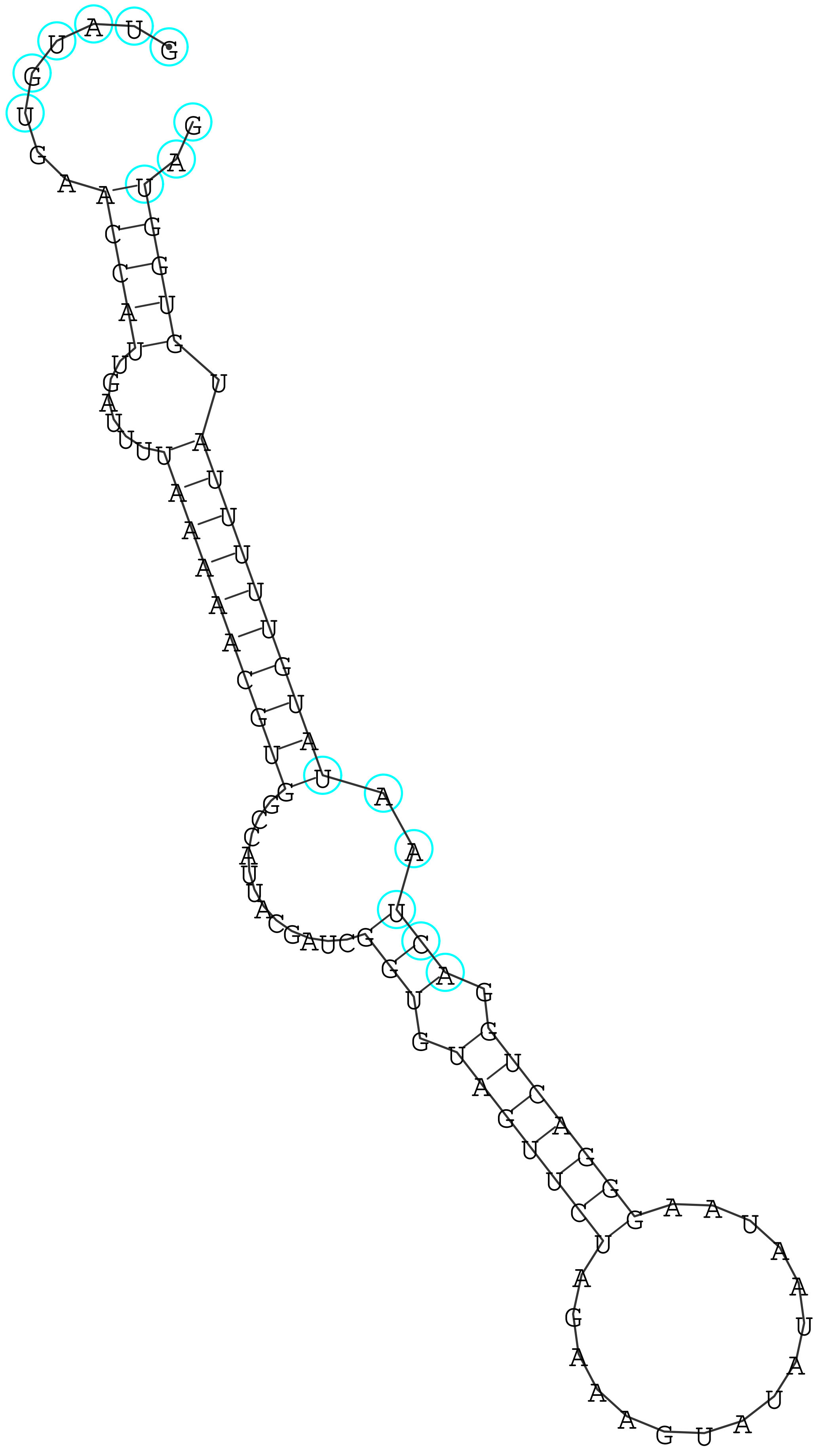

Xmsuc0006B - External intron

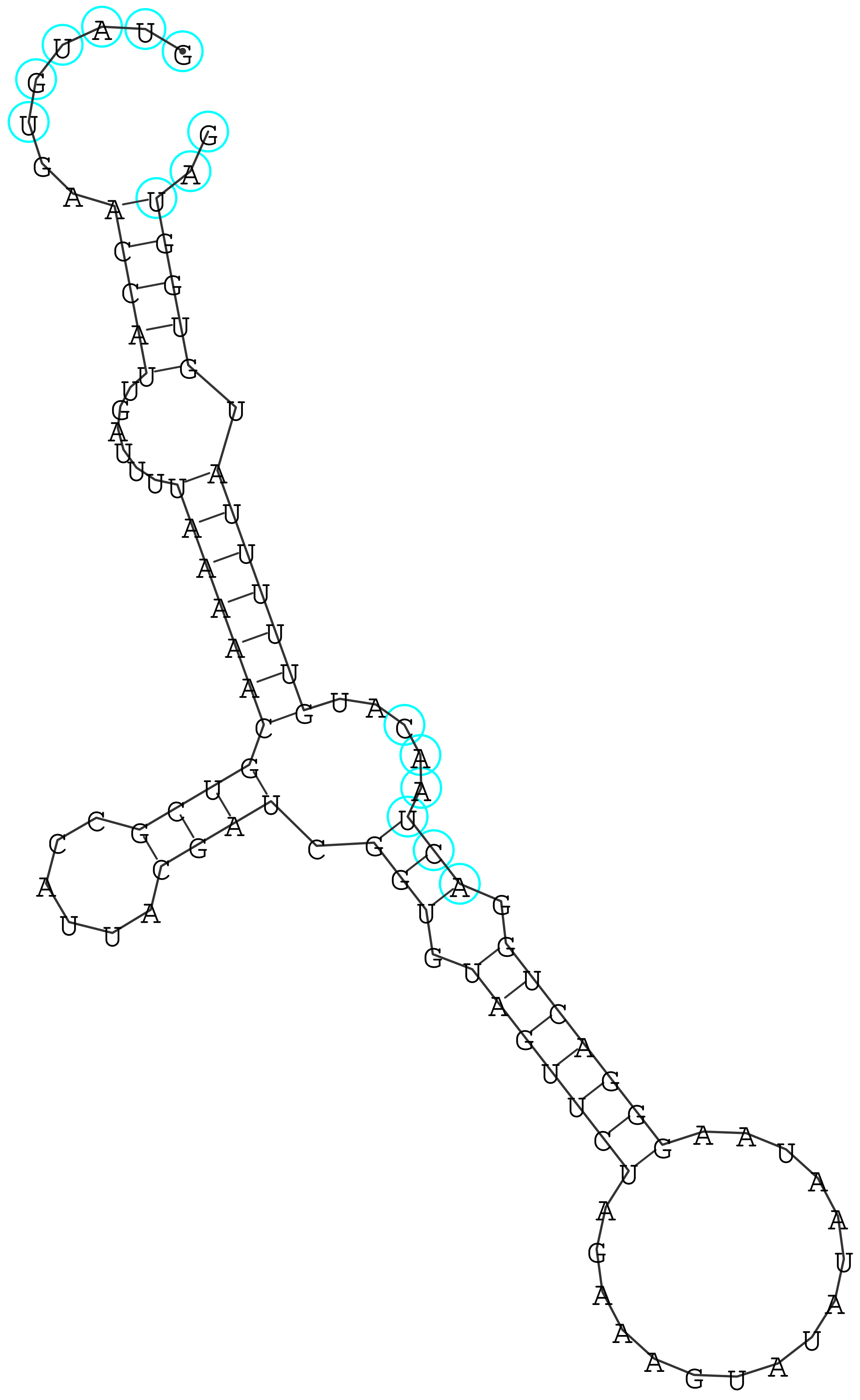

Xmsuc0009A - External intron

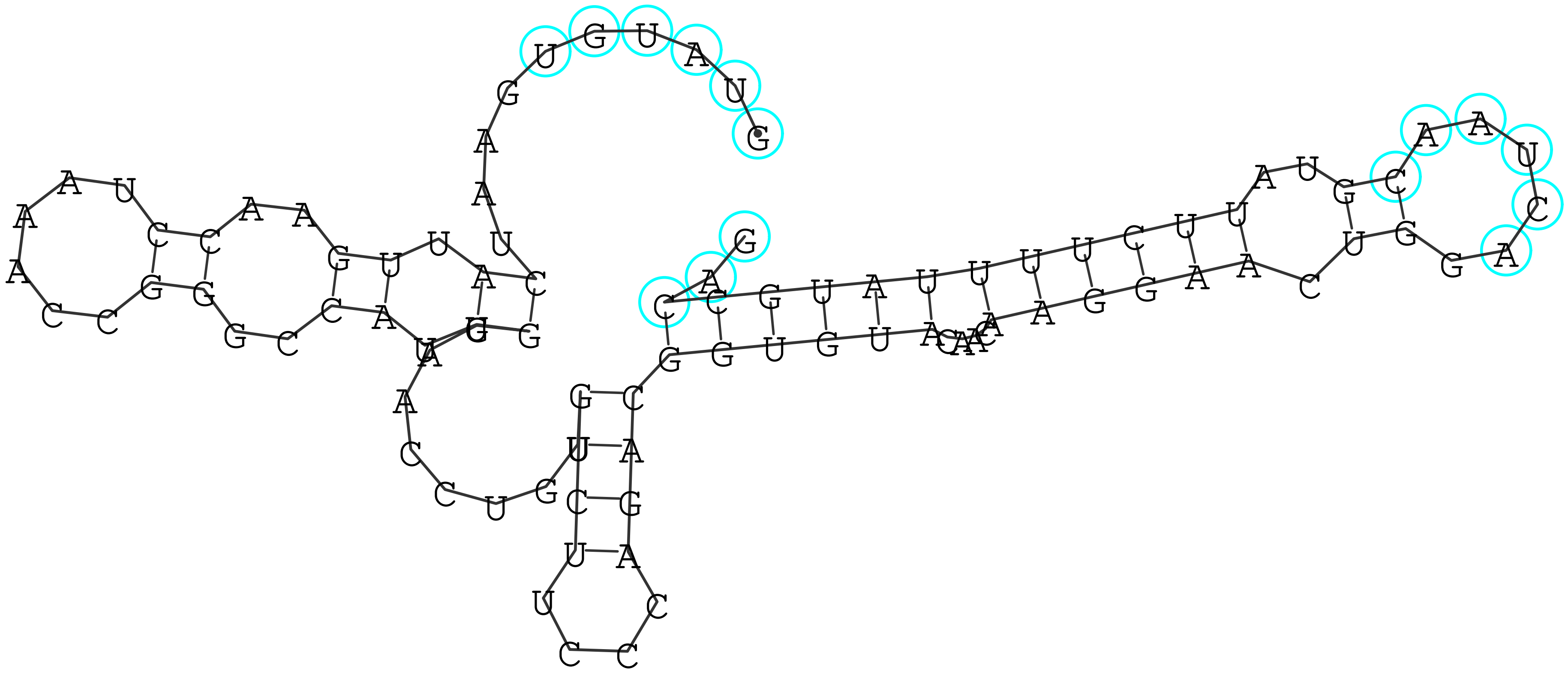

Xmsuc0018A - External intron

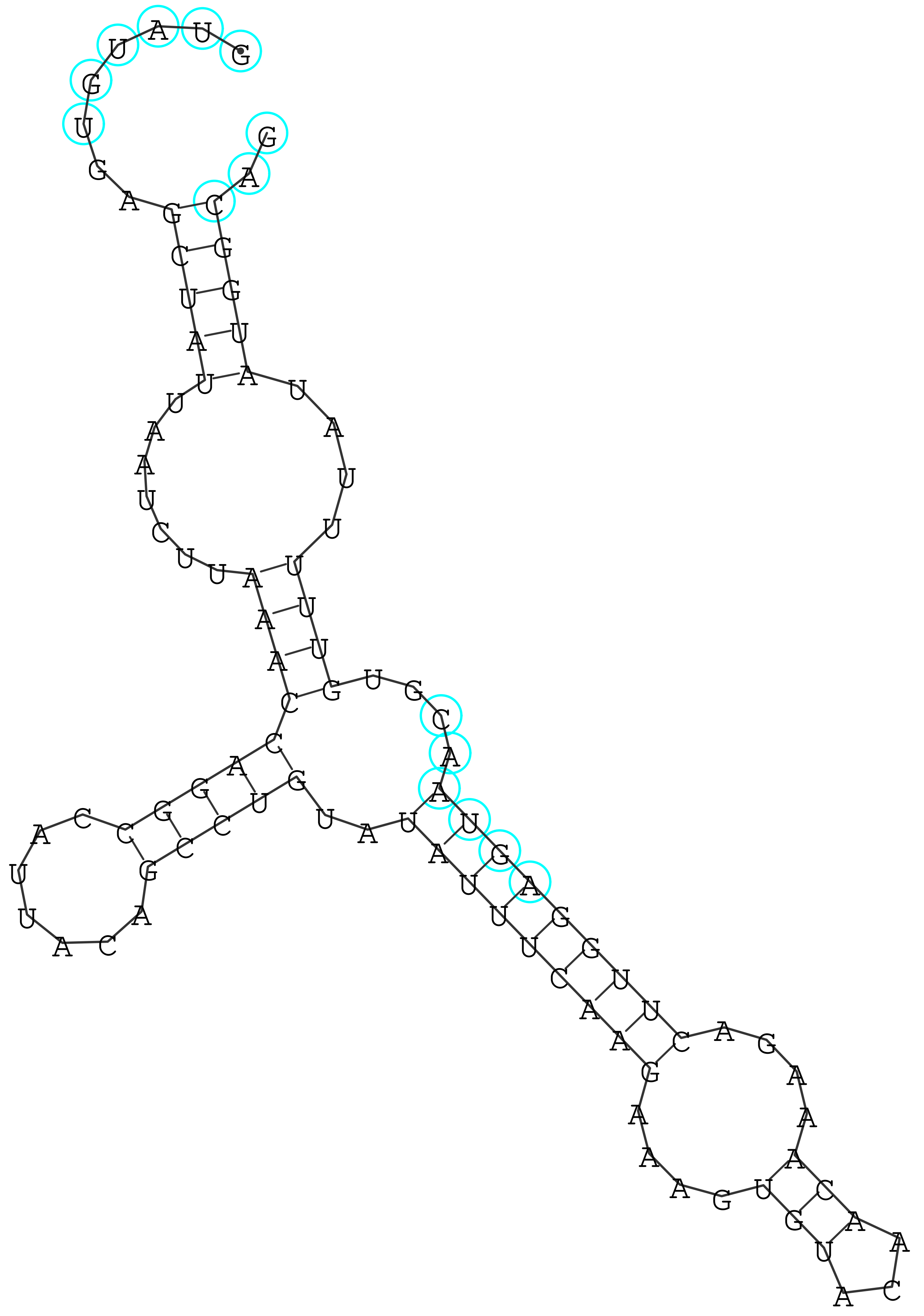

Xmsuc0018B - External intron

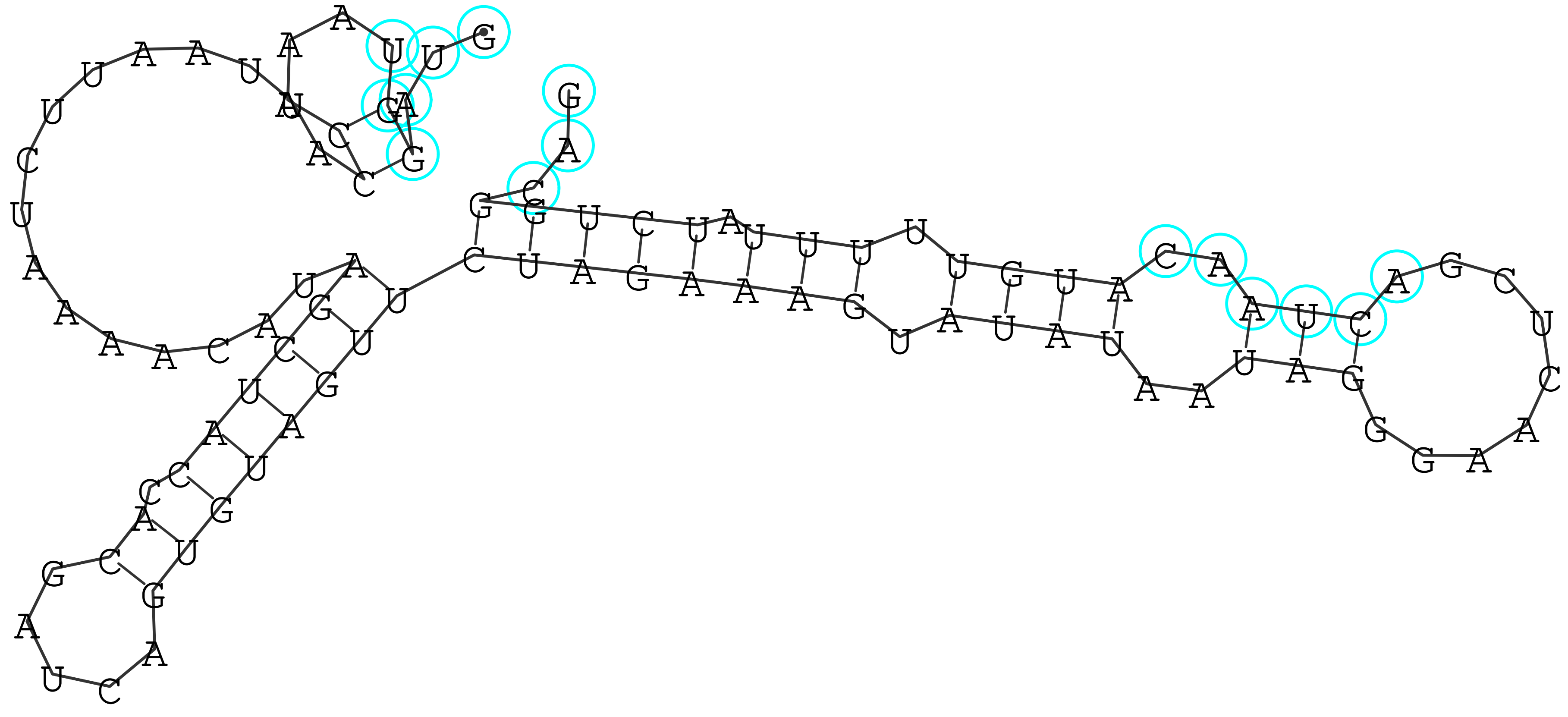

Xmsuc0019A - External intron

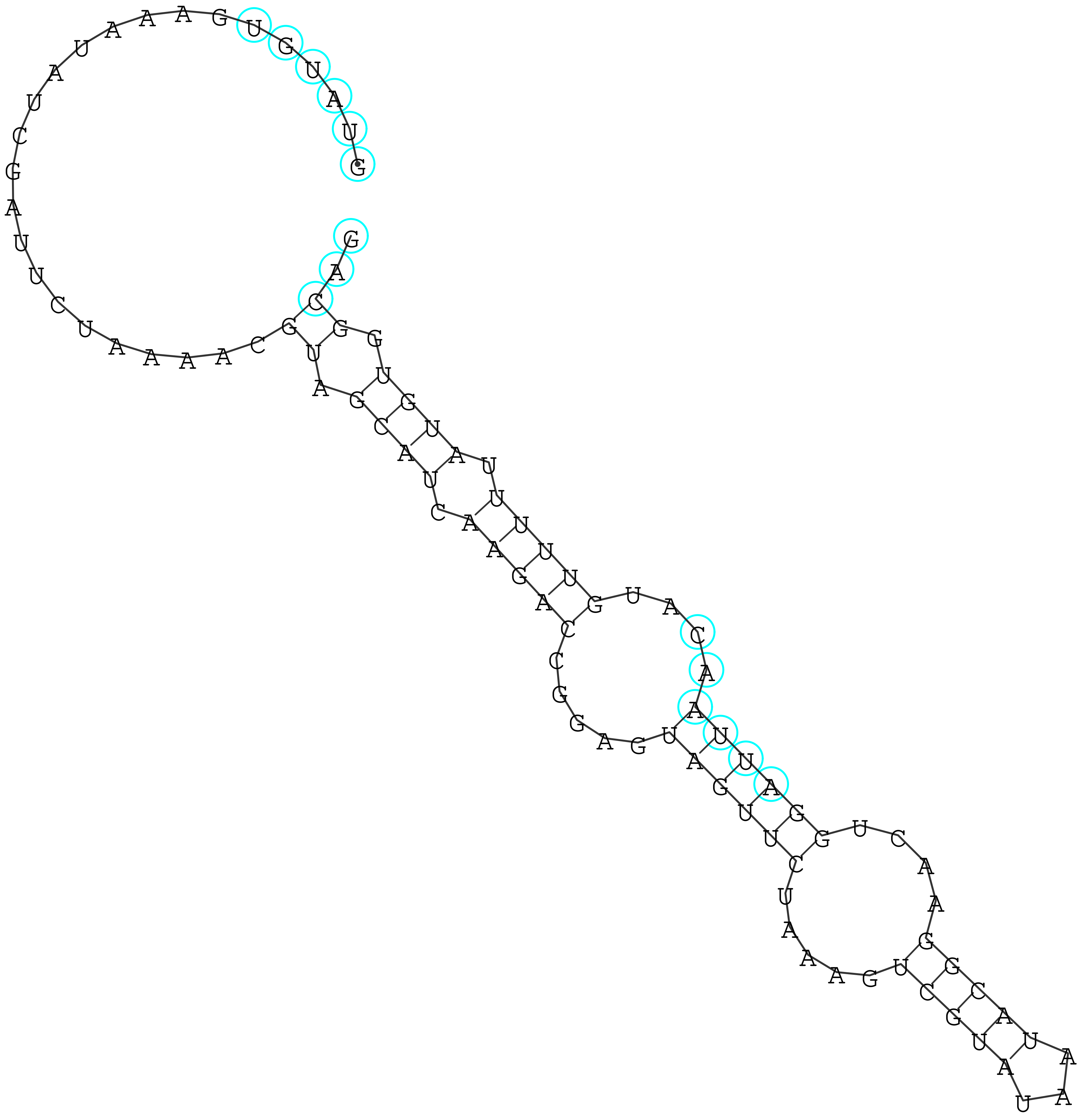

Xmsuc0028A - External intron

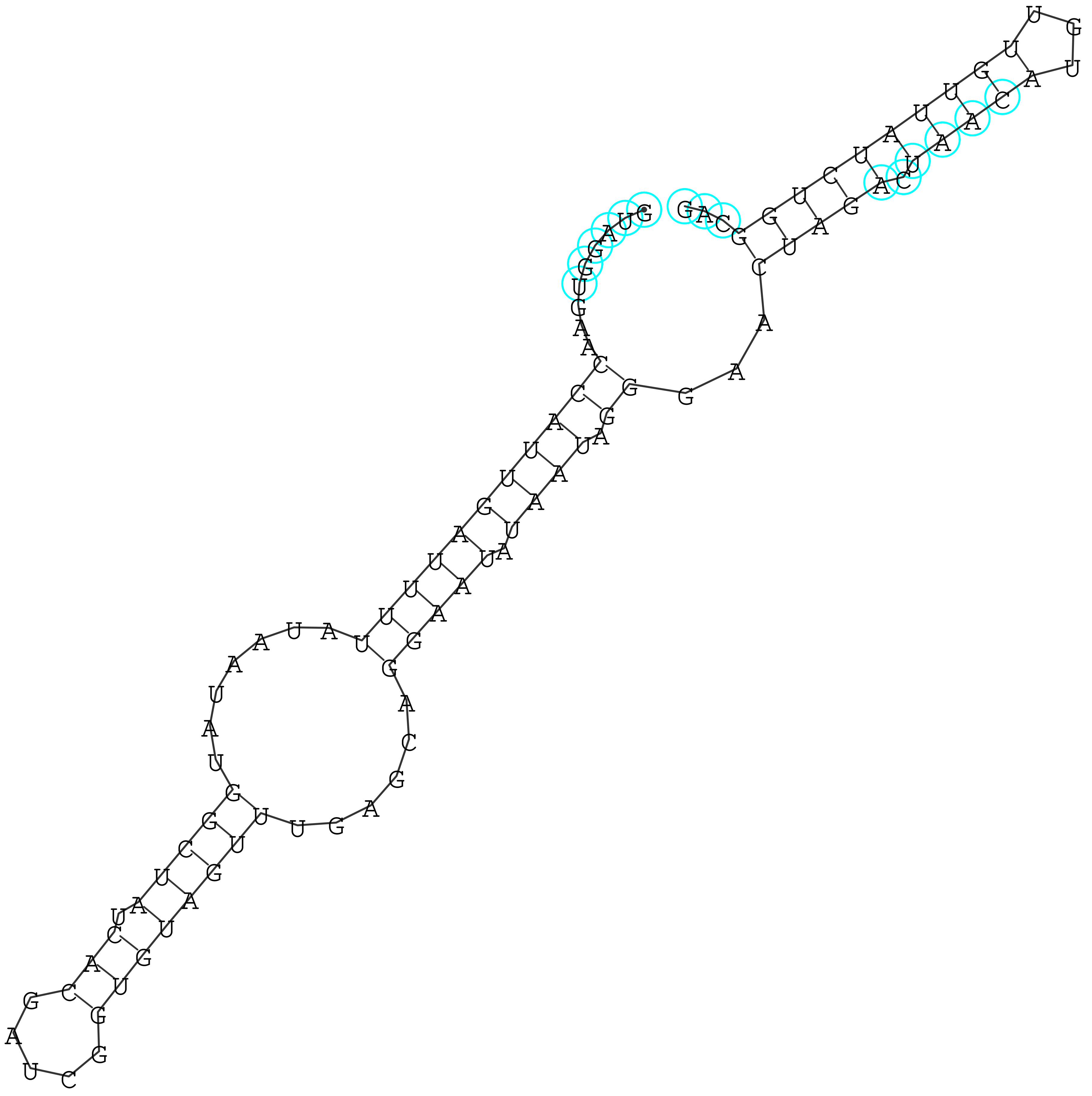

Xmsuc0031A - External intron

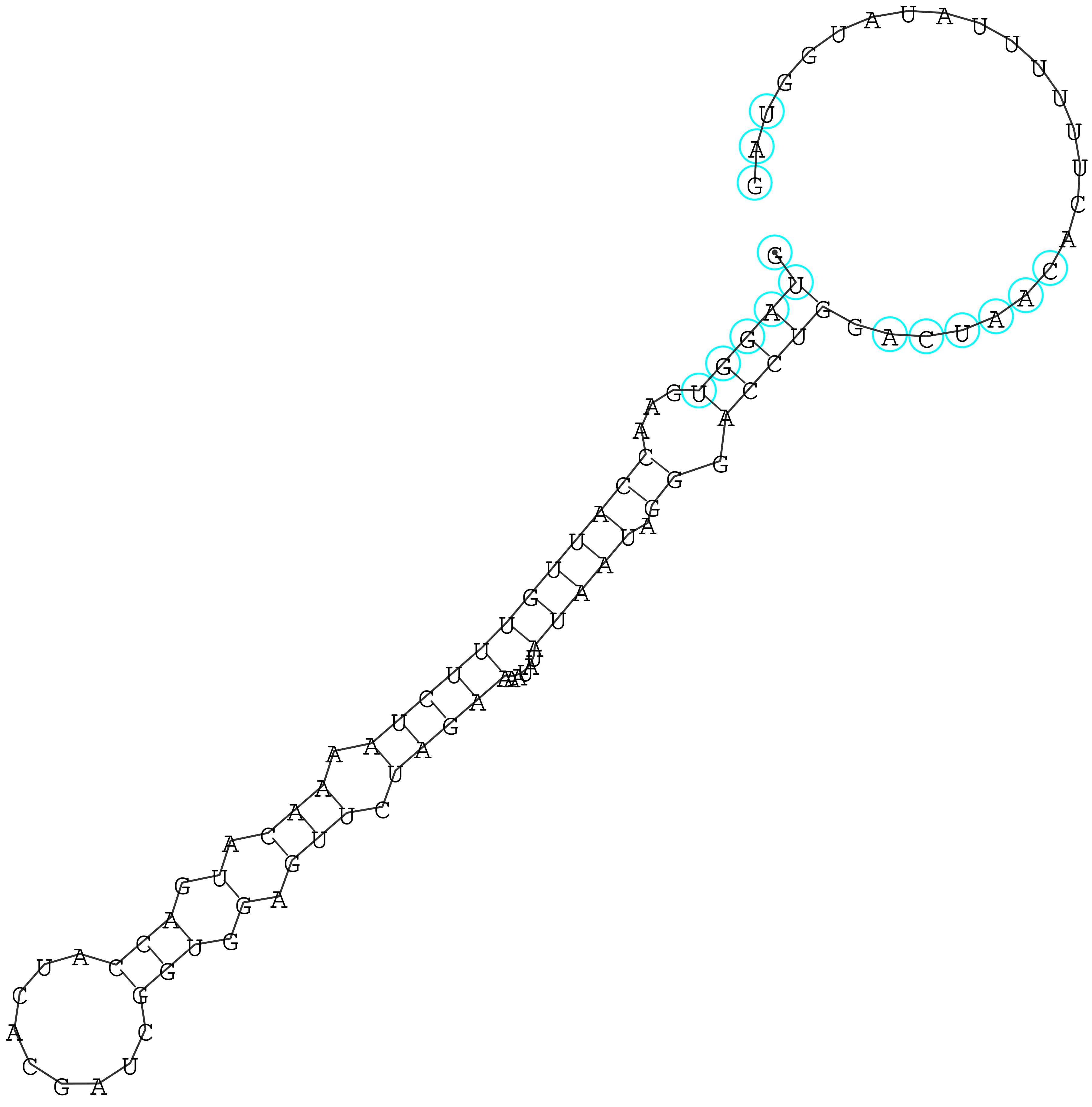

Xmsuc0045A - External intron

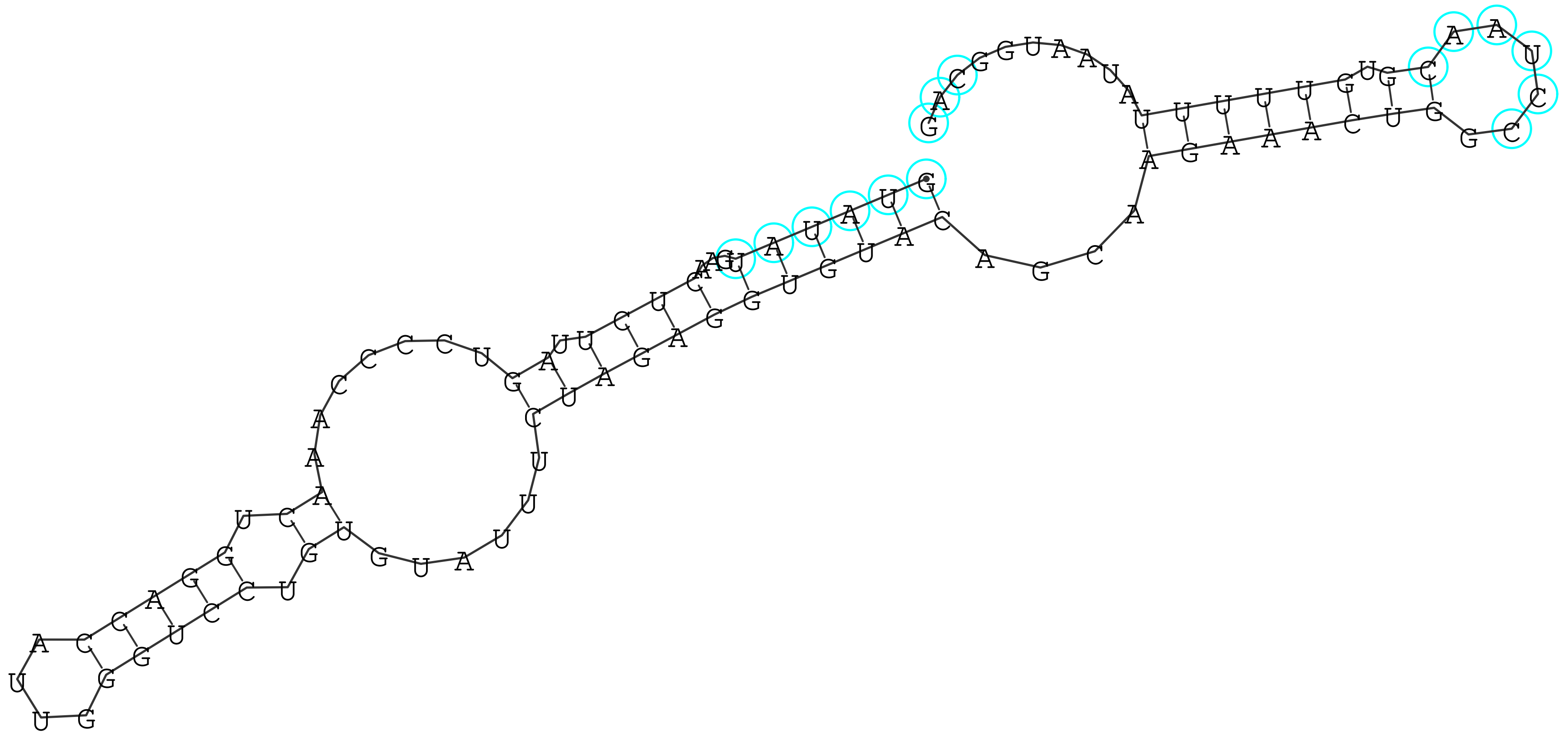

Xmsuc0067A - External intron

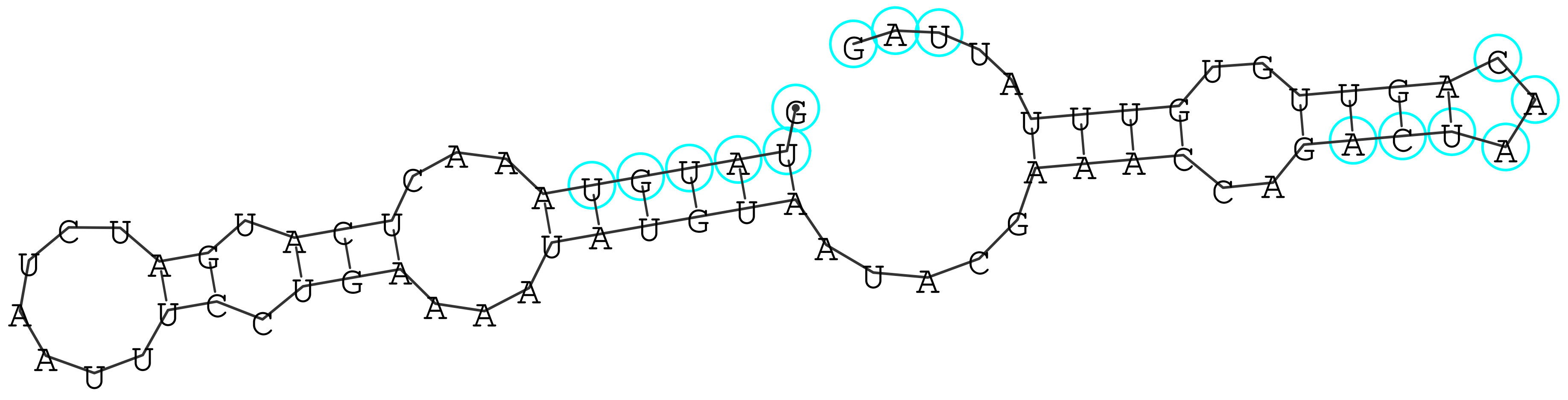

Xmsuc0070A - External intron

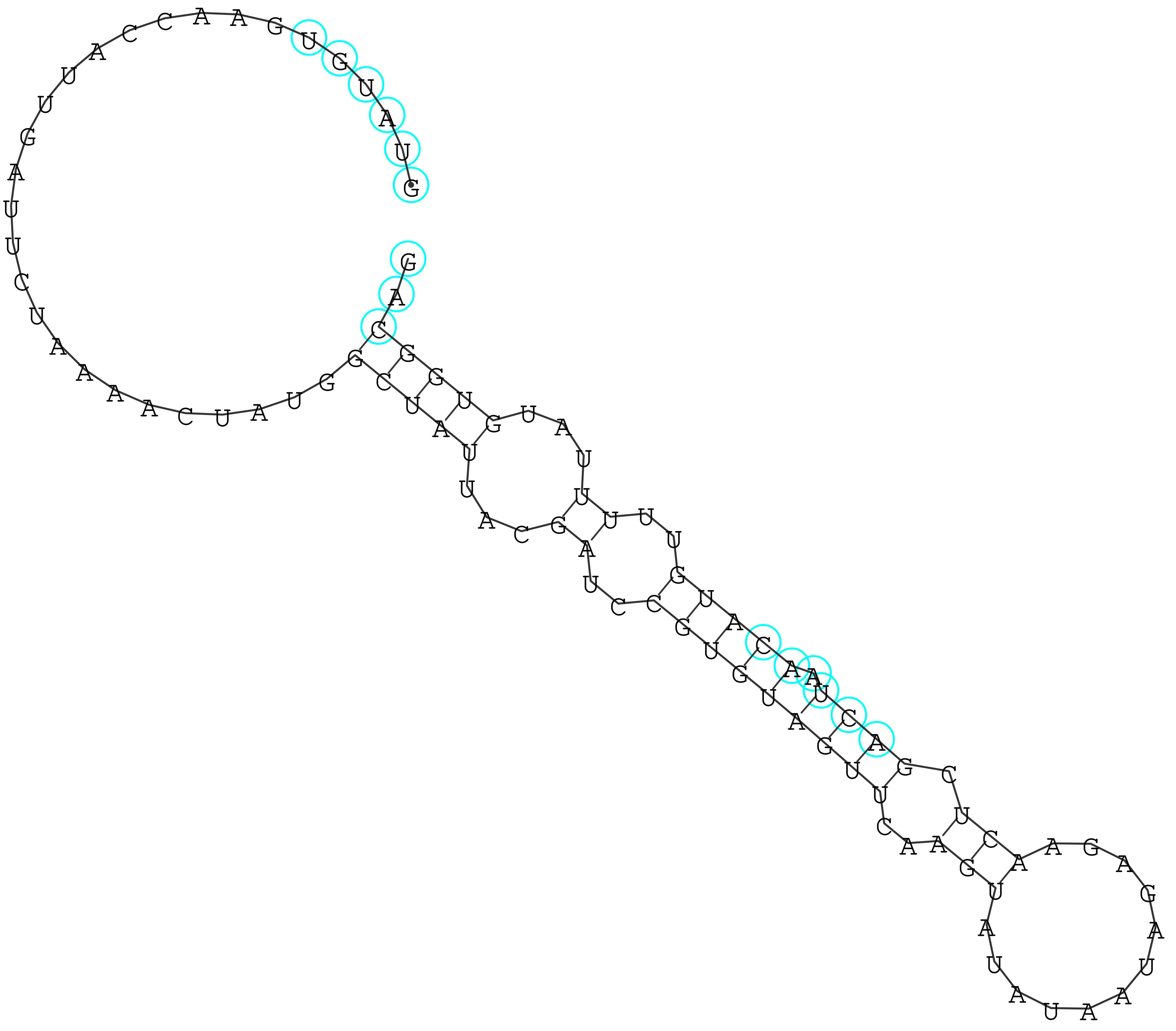

Xmsuc0075A - External intron

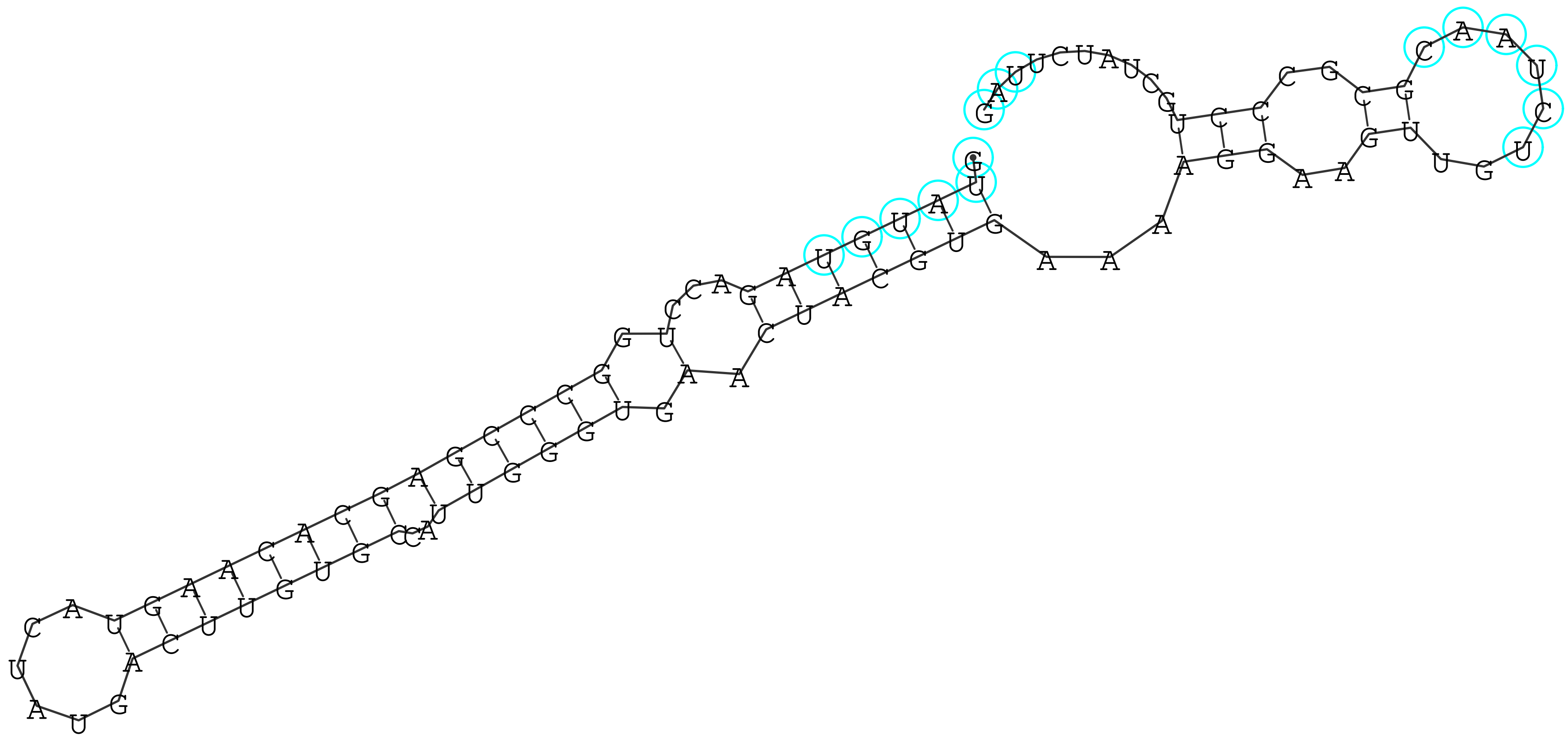

Xmsuc0077A - External intron

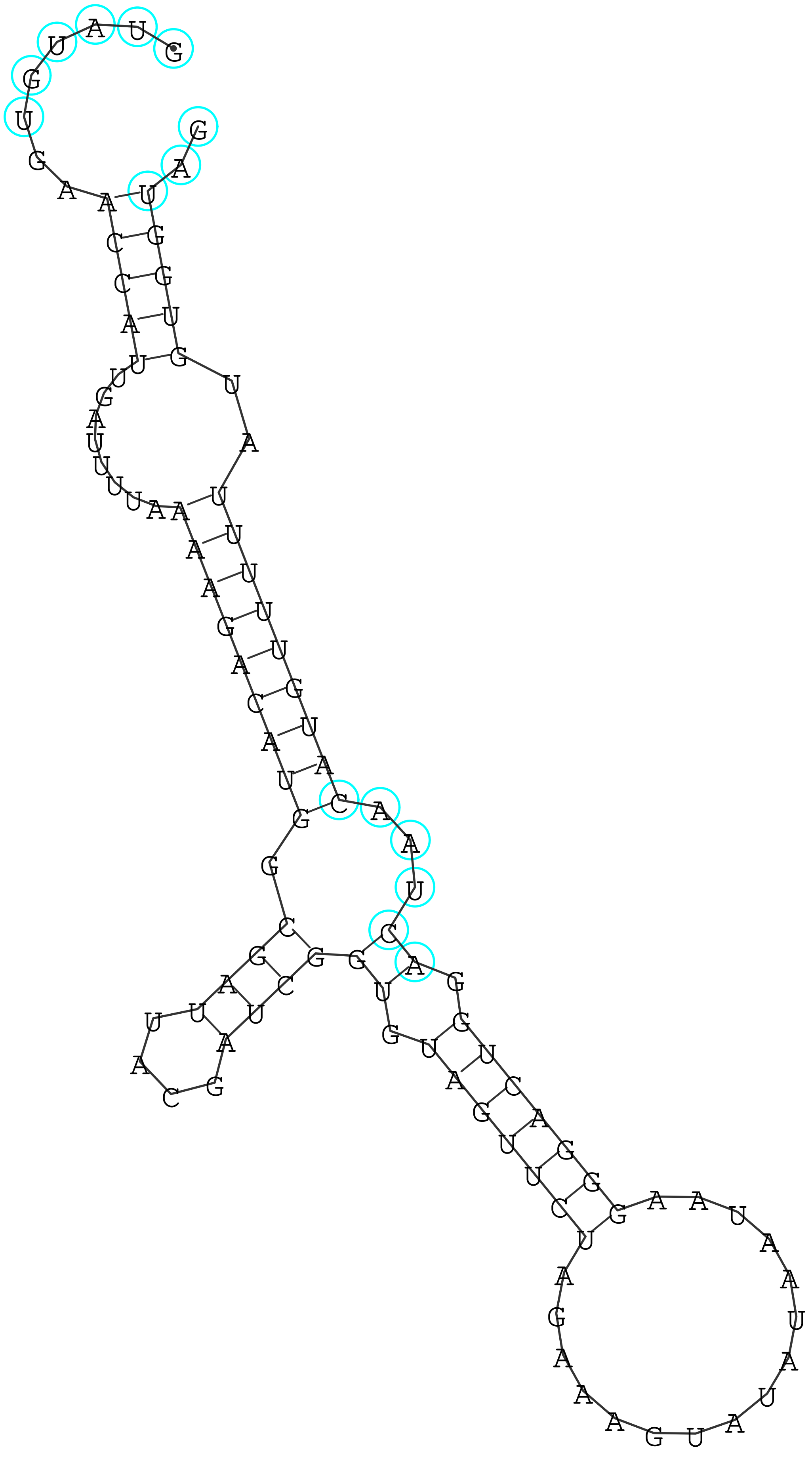

Xmsuc0082A - External intron

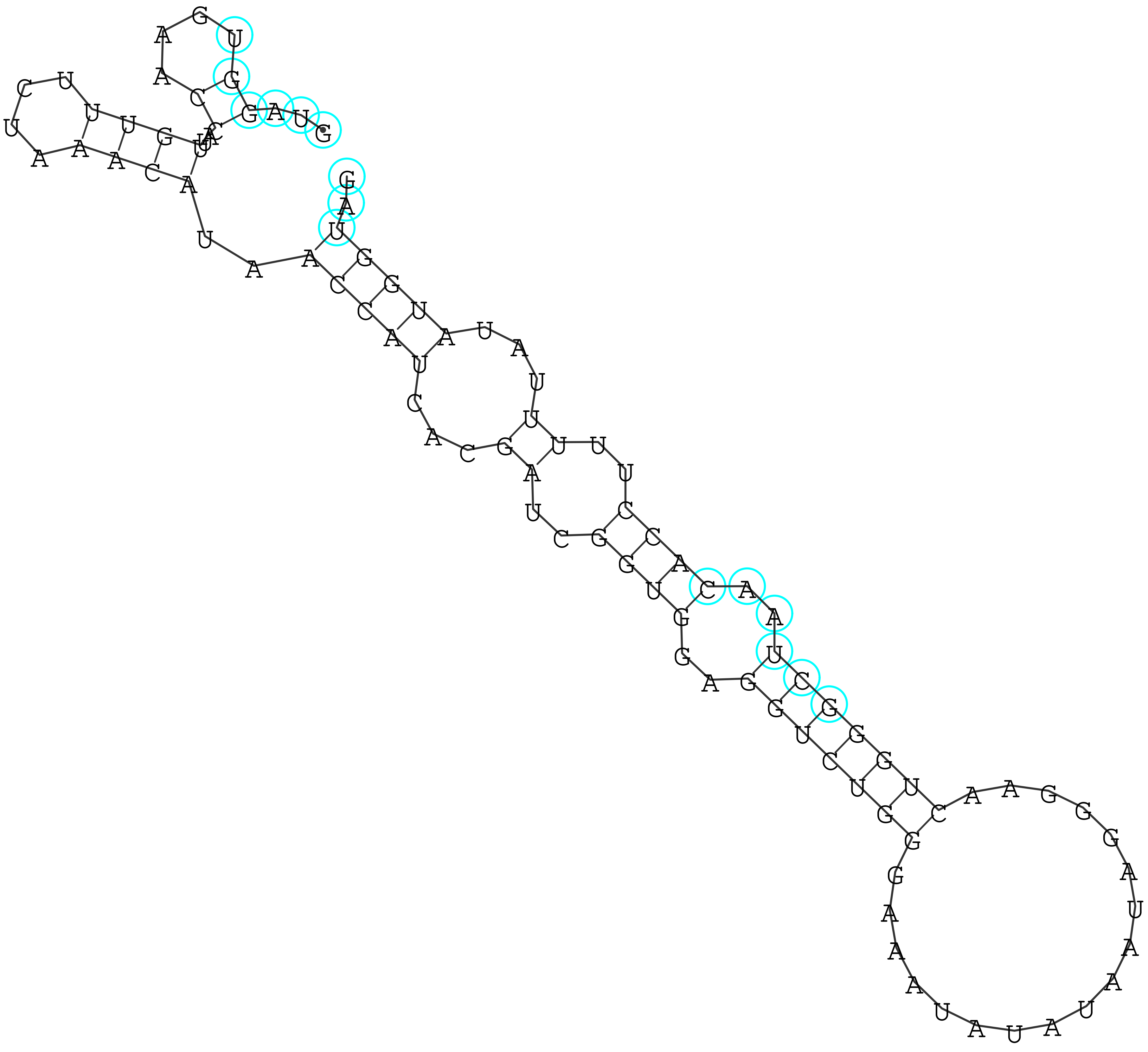

Xmsuc0086A - External intron

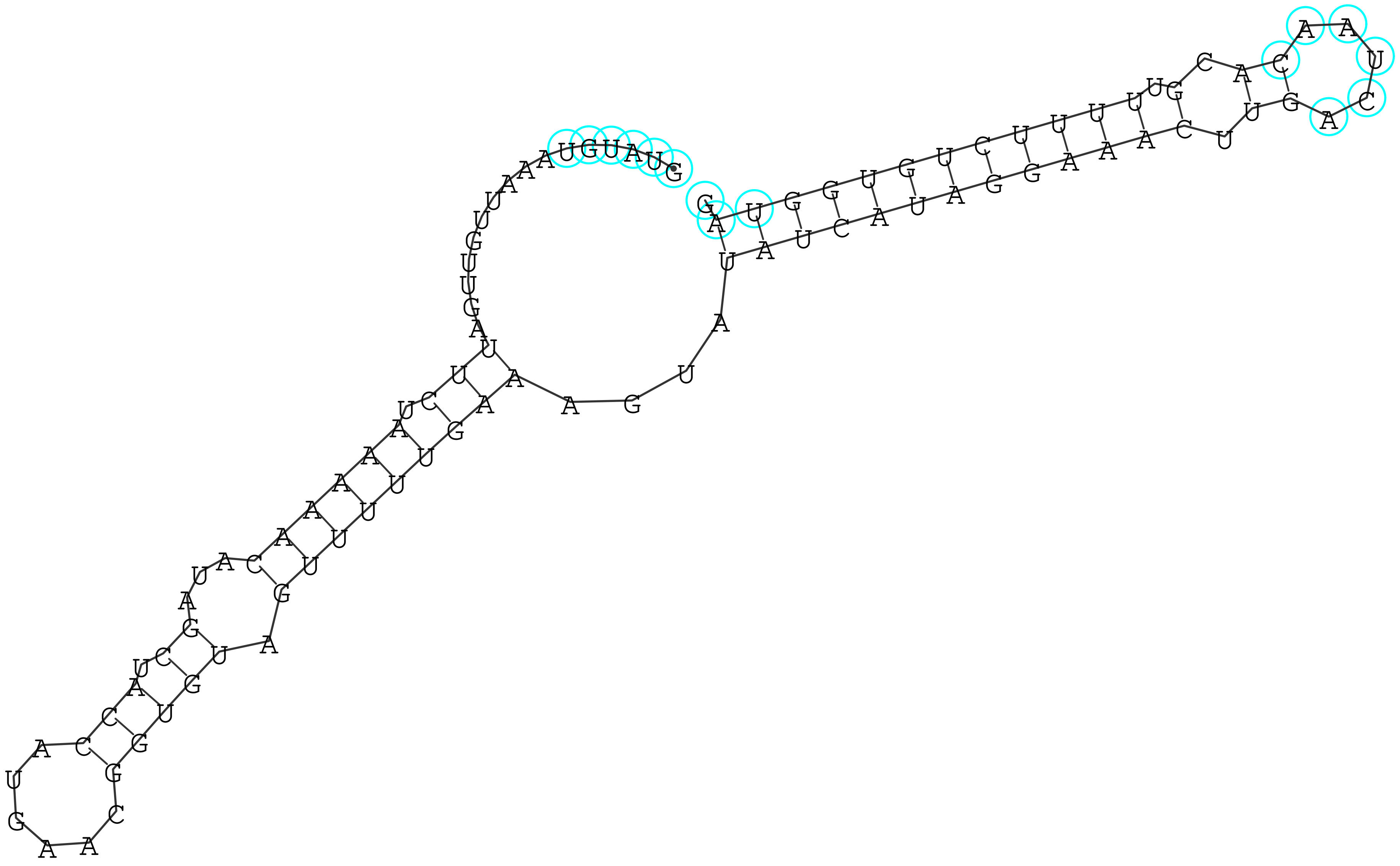

Xmsuc0086B - External intron

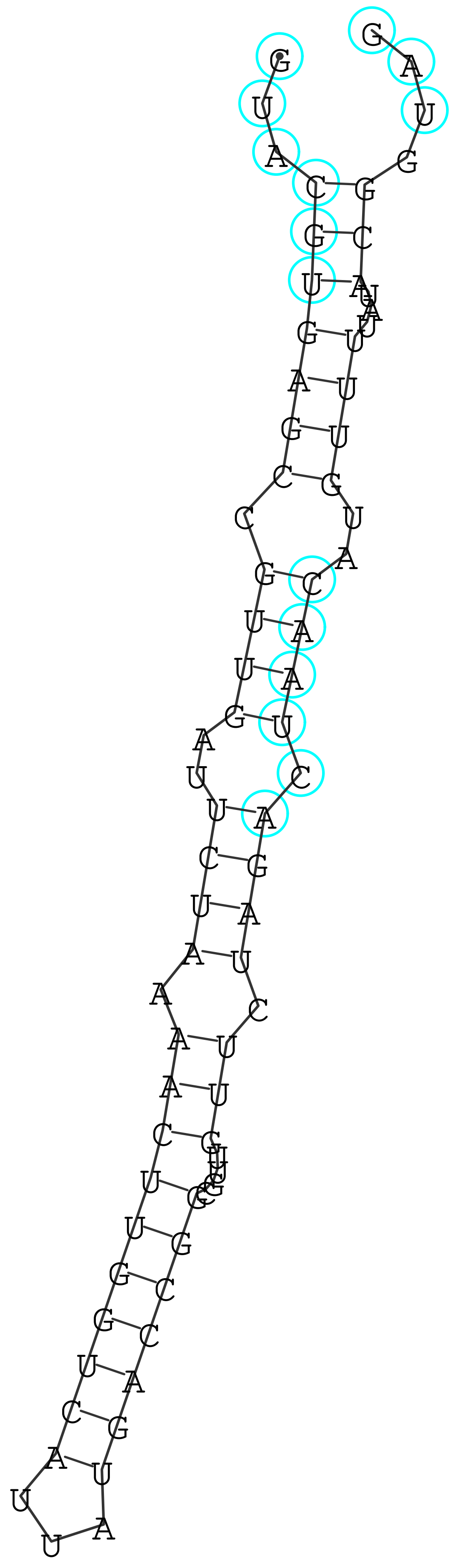

Xmsuc0098A - External intron

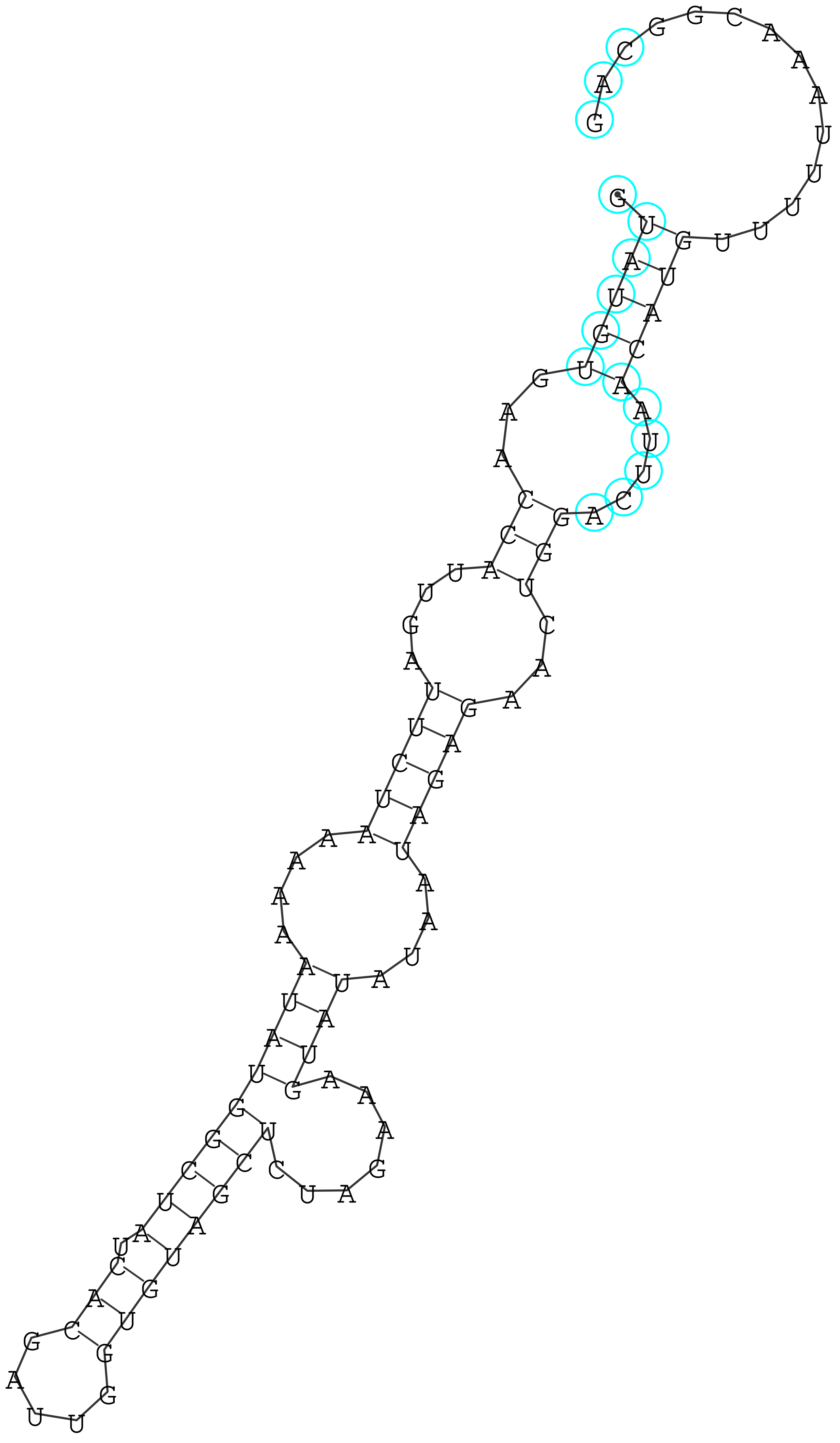

Xmsuc0110A - External intron

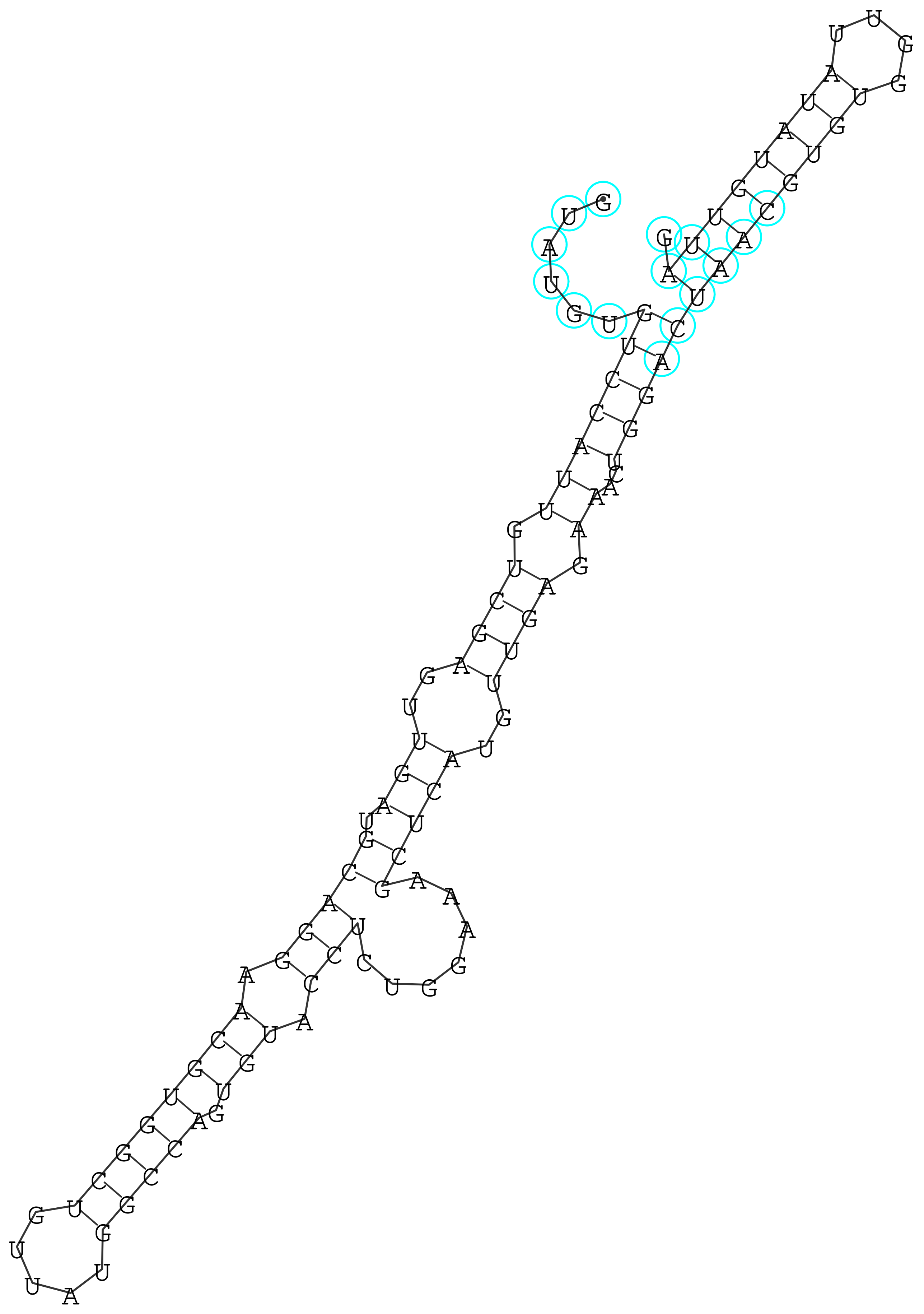

Xmsuc0111A - External intron

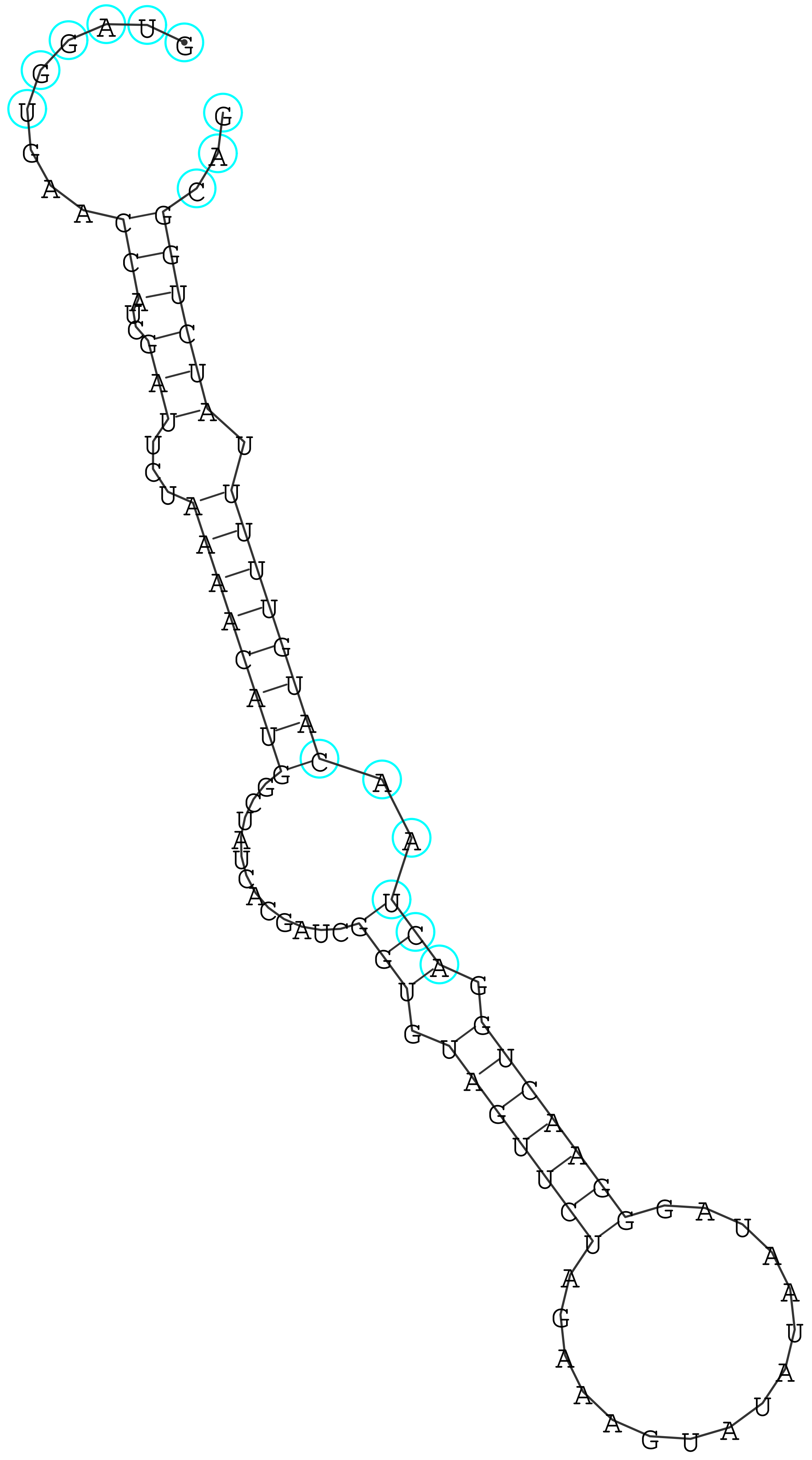

Xmsuc0114A - External intron

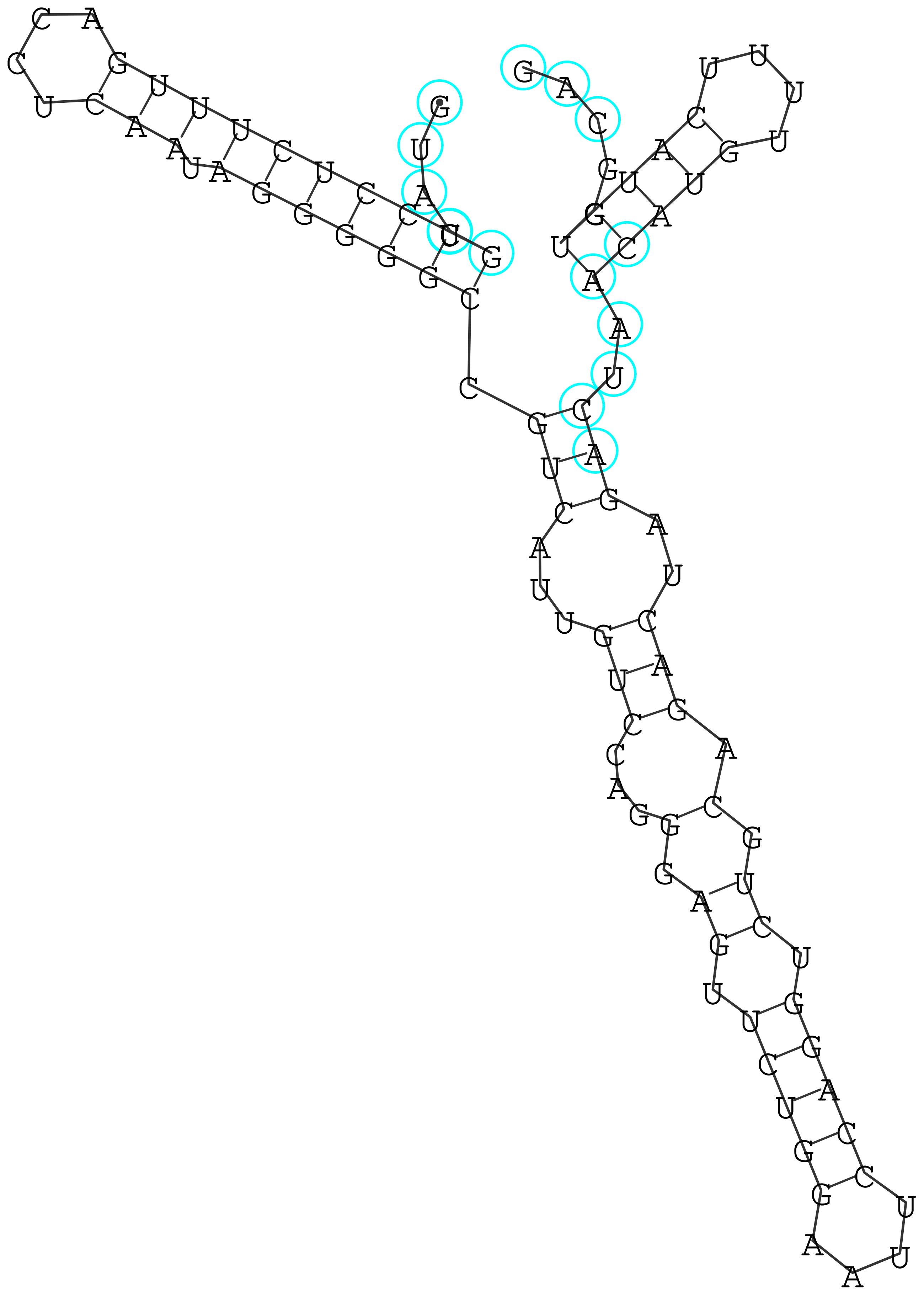

Xmsuc0121A - External intron

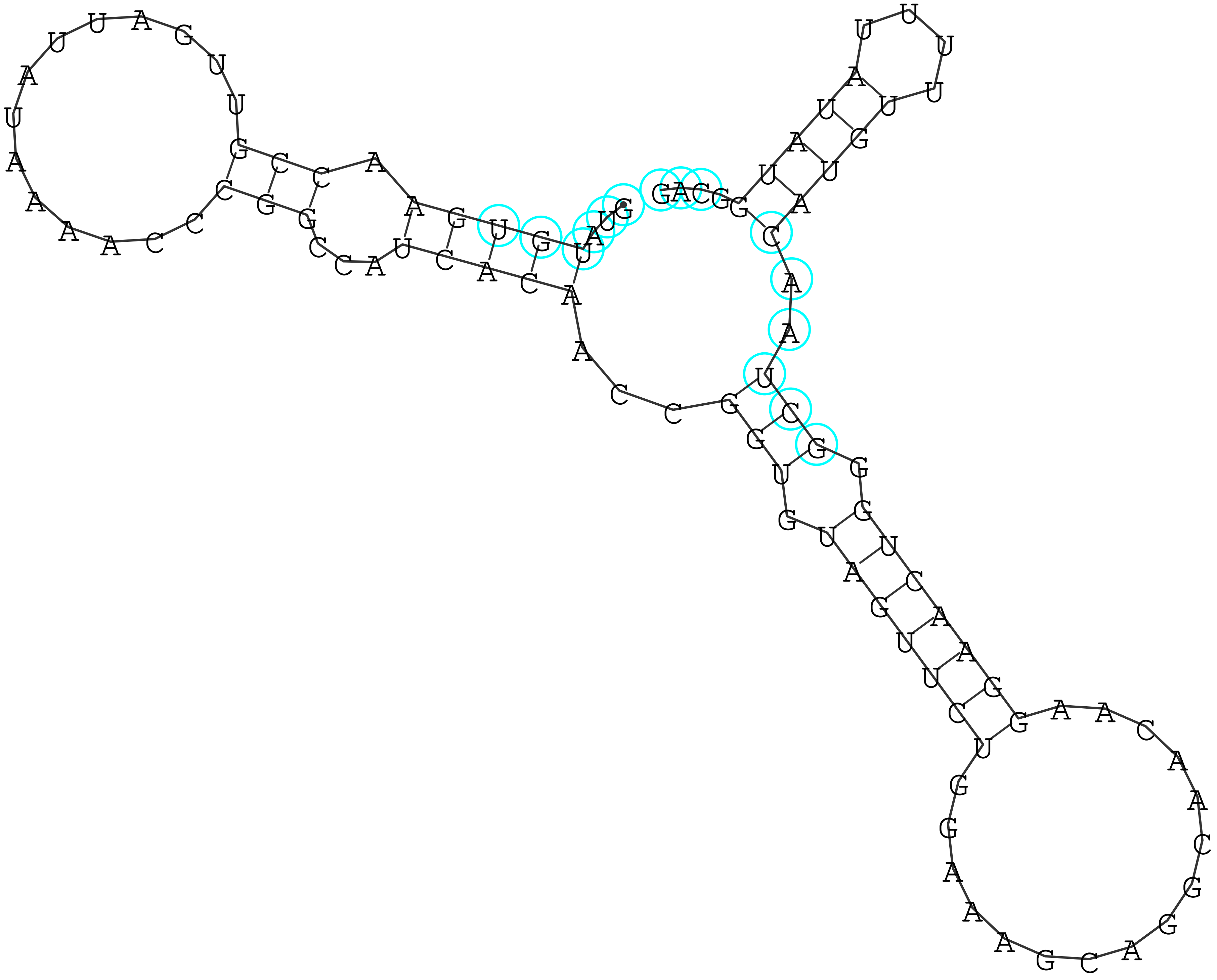

Xmsuc0131A - External intron

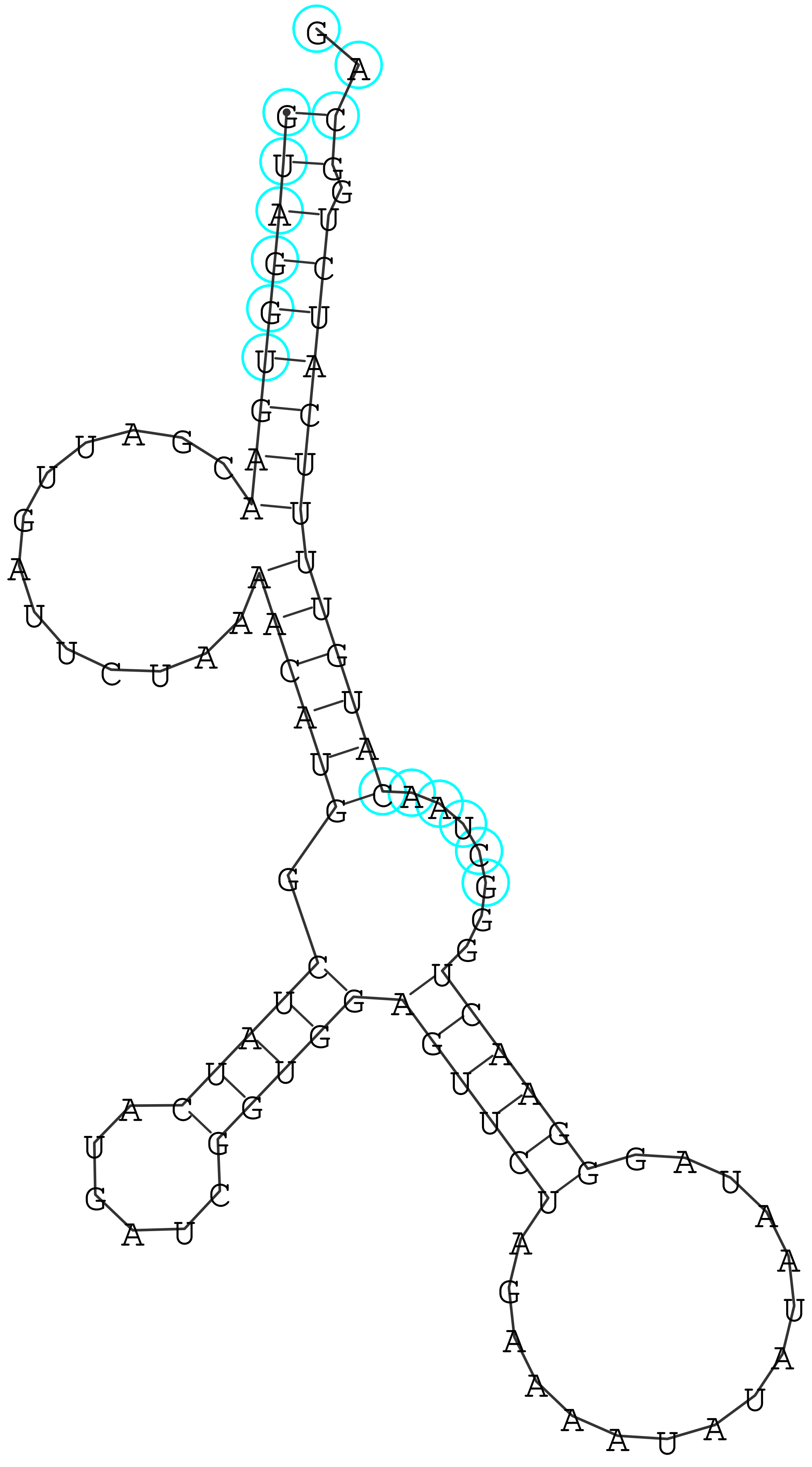

Xmsuc0137A - External intron

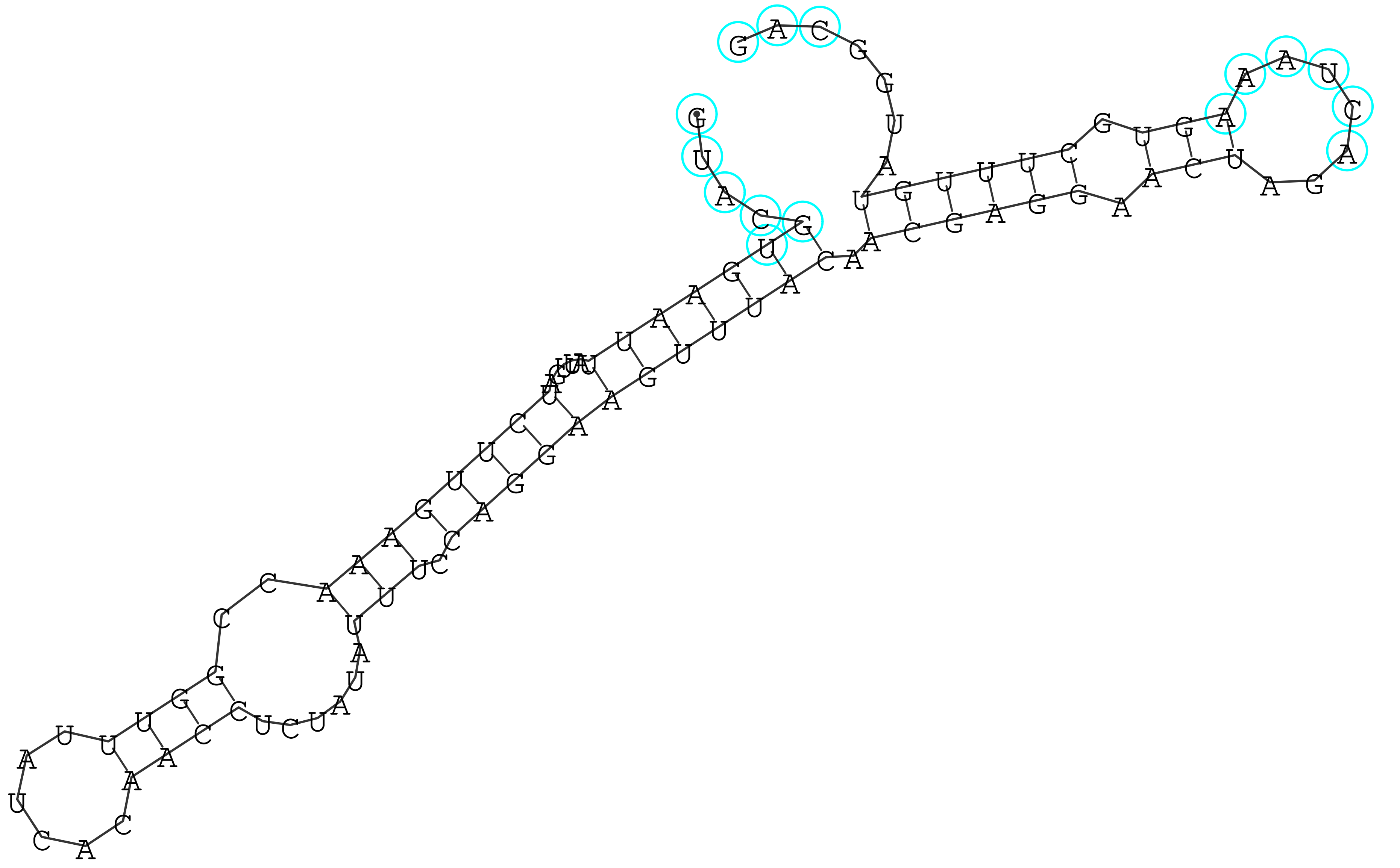

Xmsuc0141A - External intron

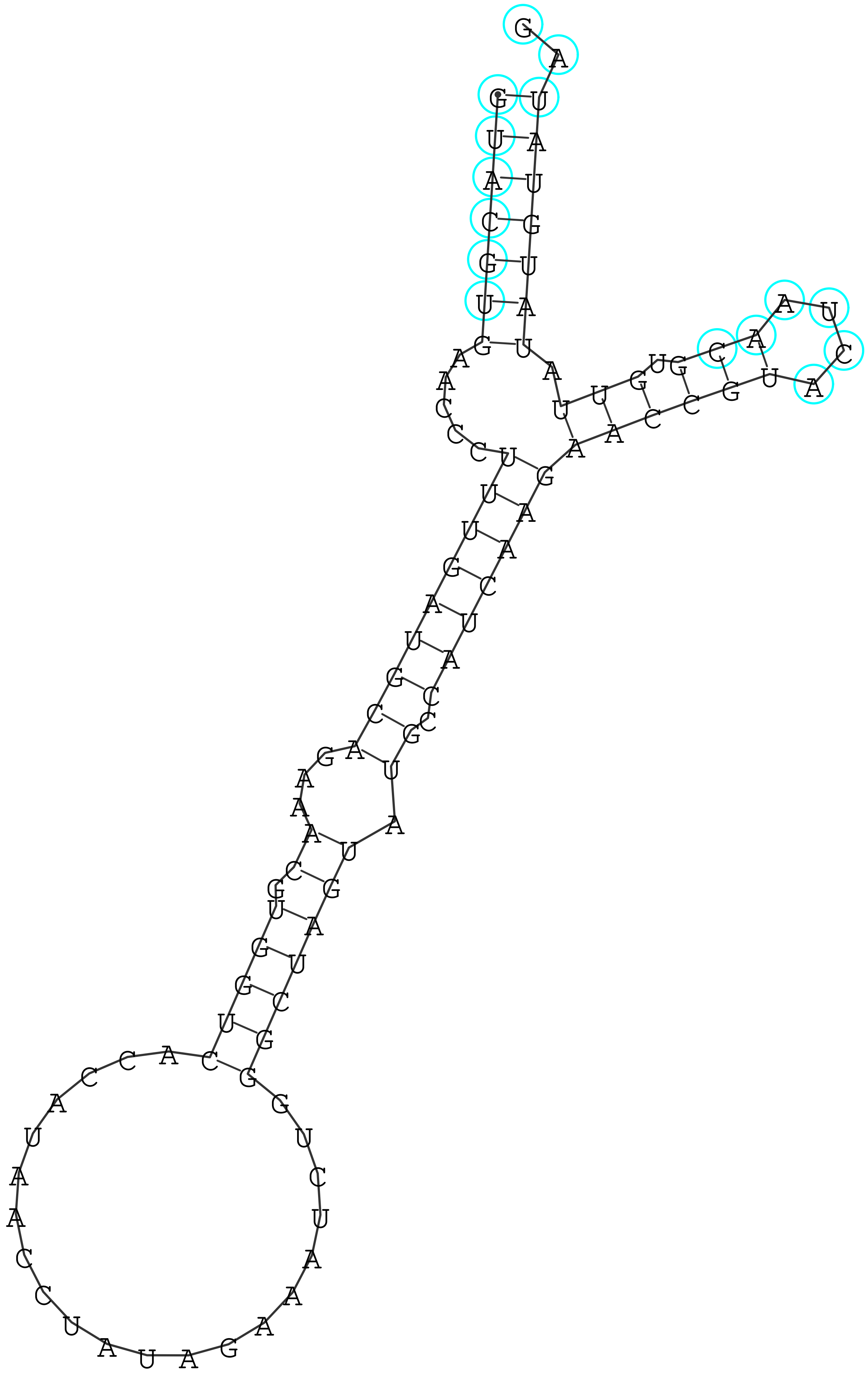

Xmsuc0143A - External intron

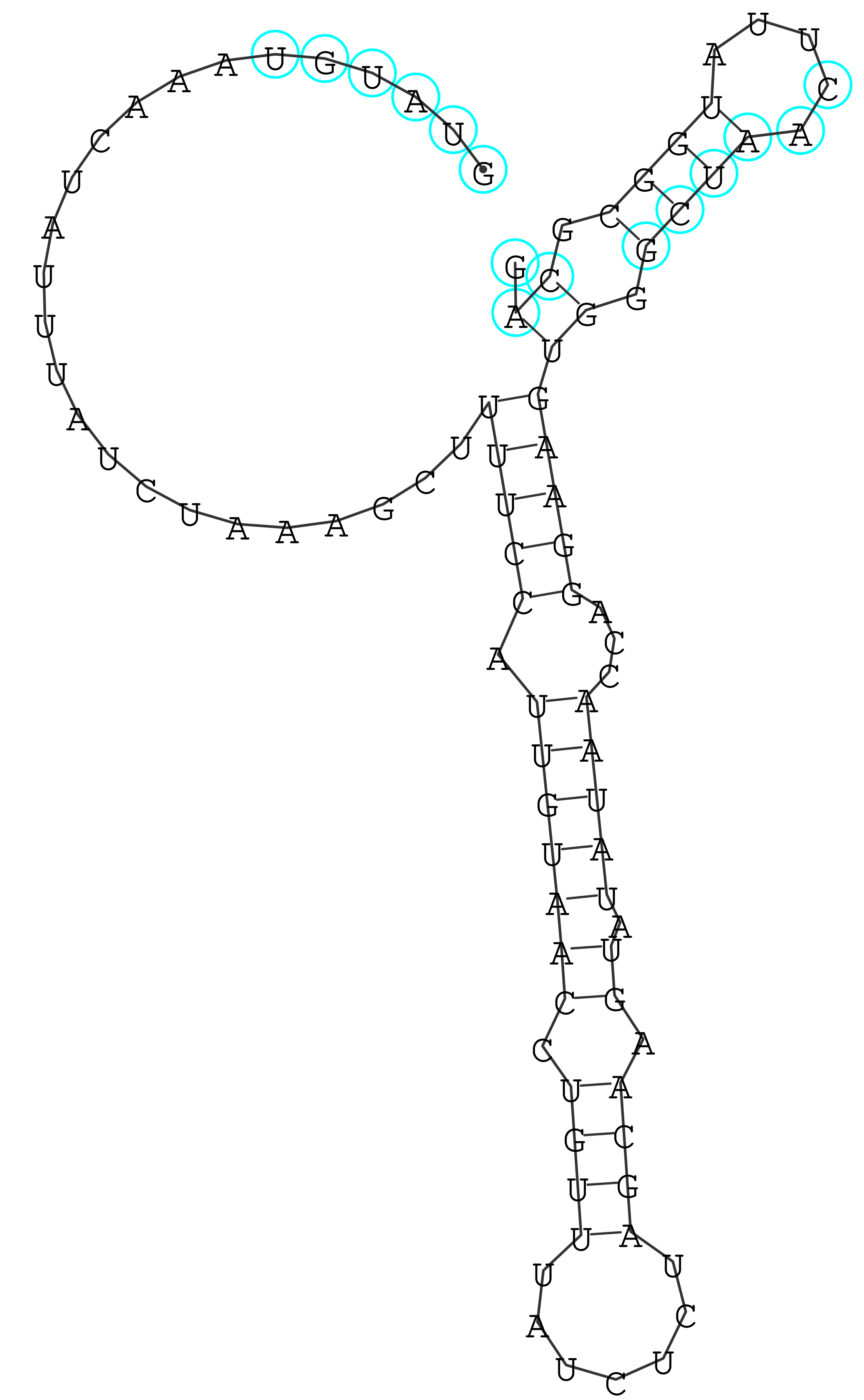

Xmsuc0146A - External intron

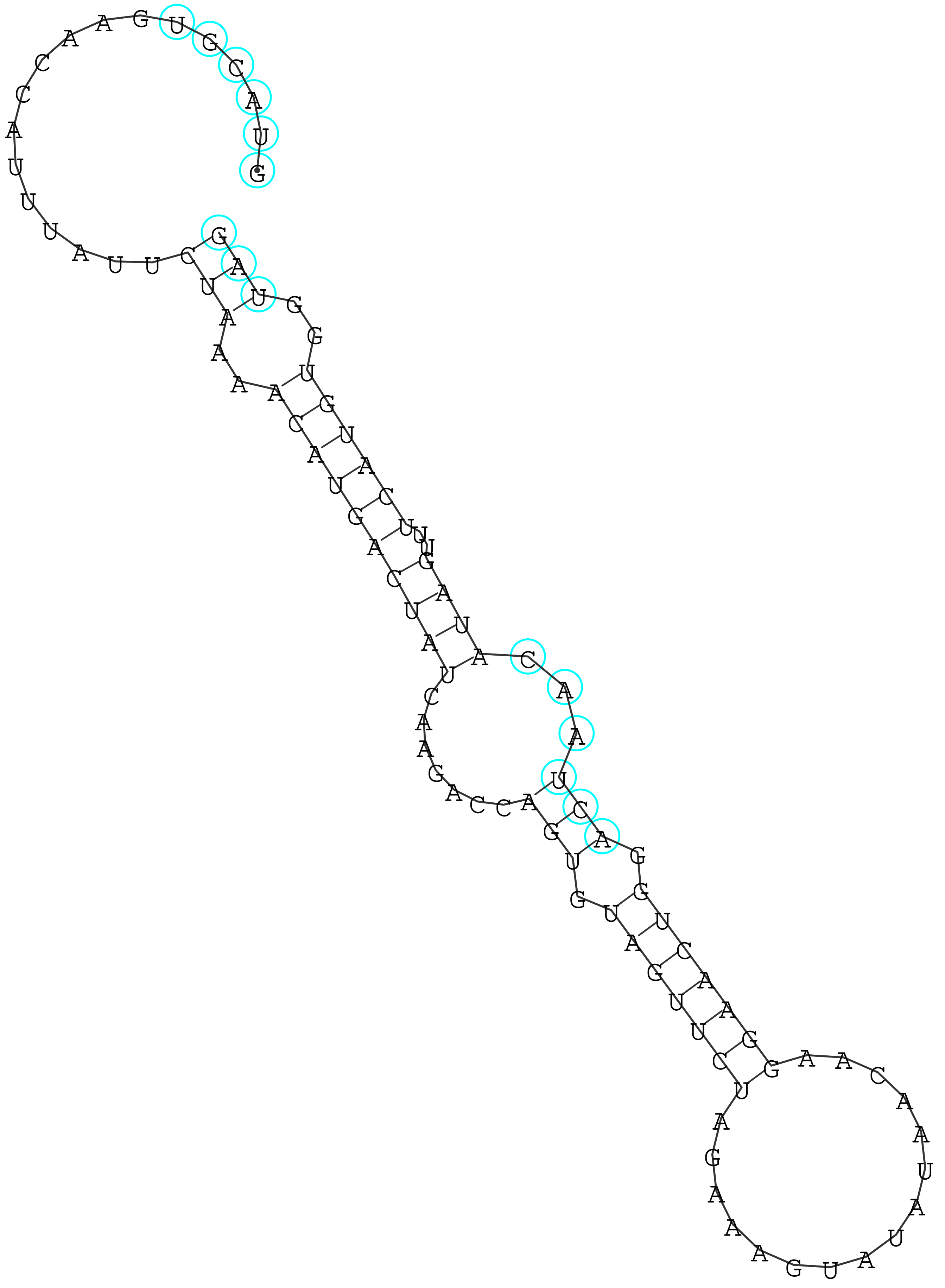

Xmsuc0153A - External intron

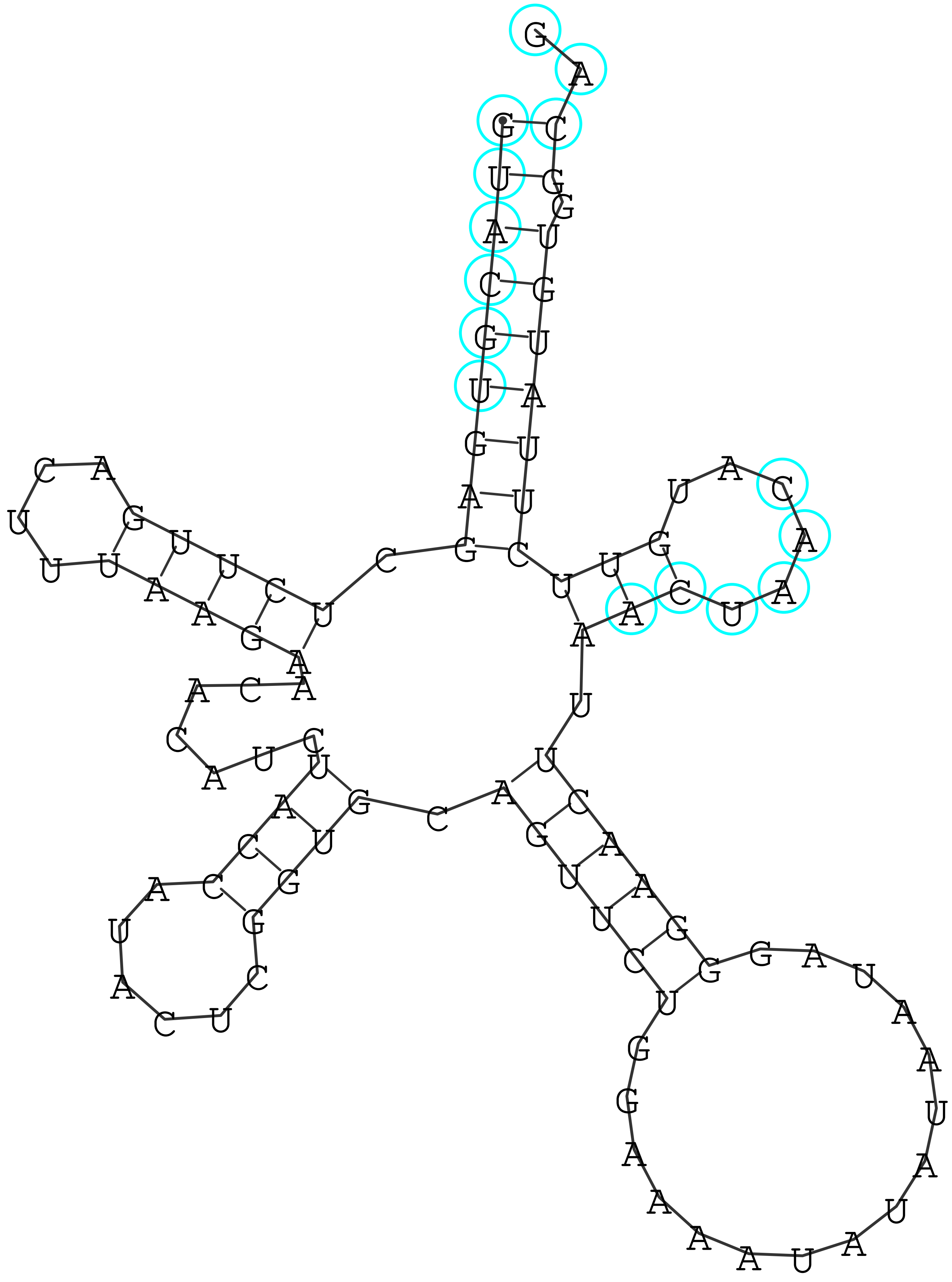

Xmsuc0159A - External intron

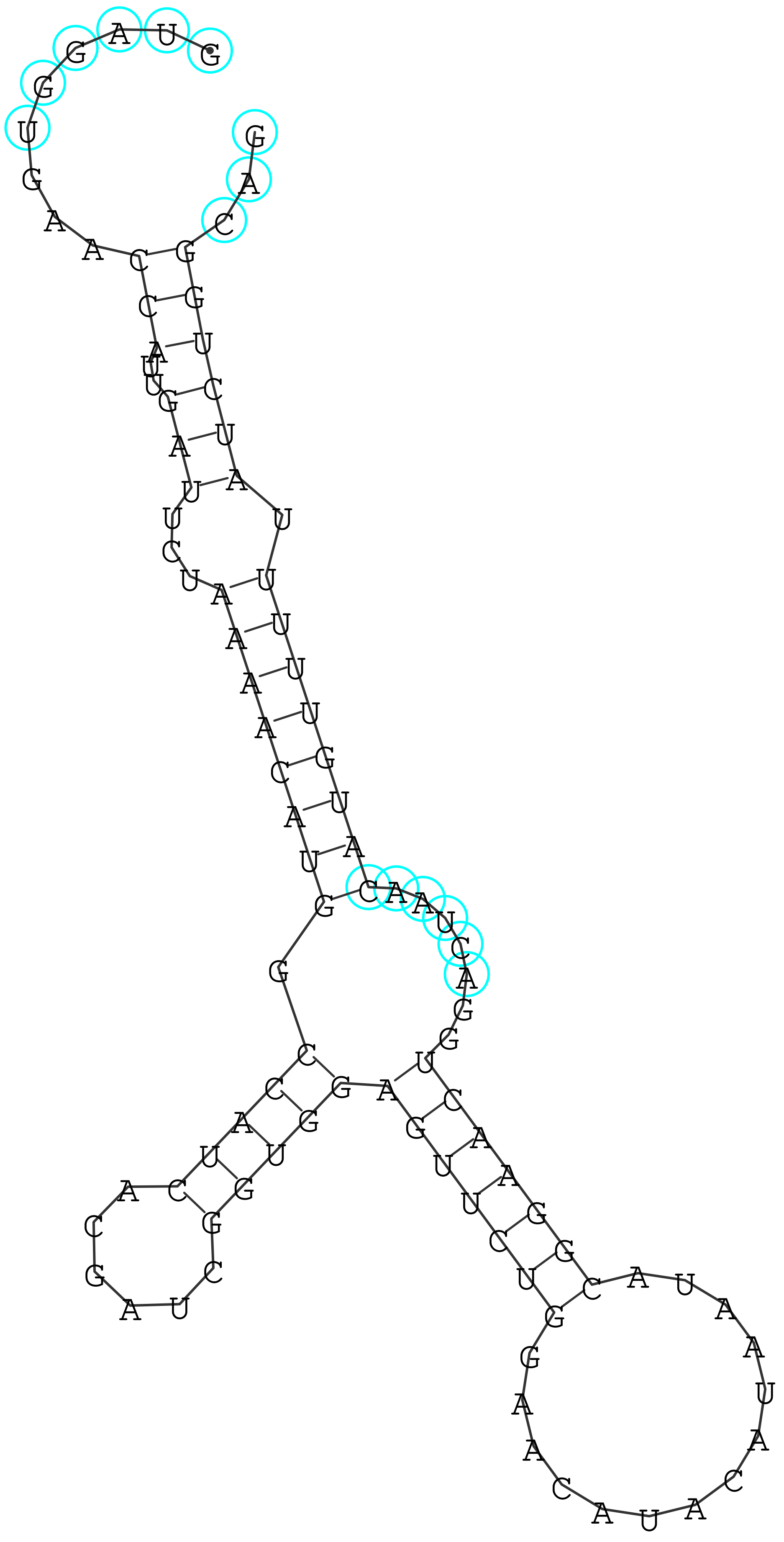

Xmsuc0162A - External intron

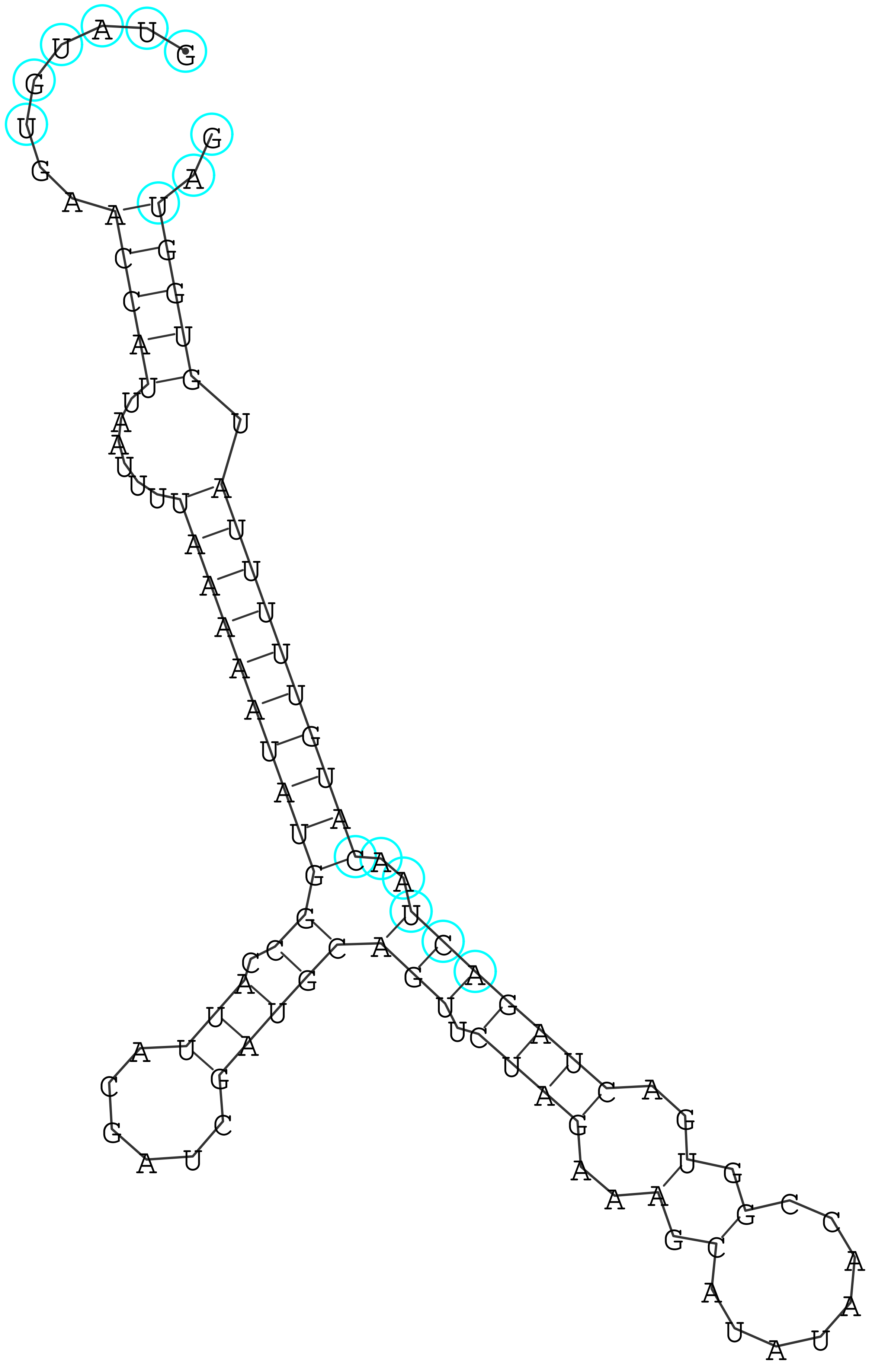

Xmsuc0168A - External intron

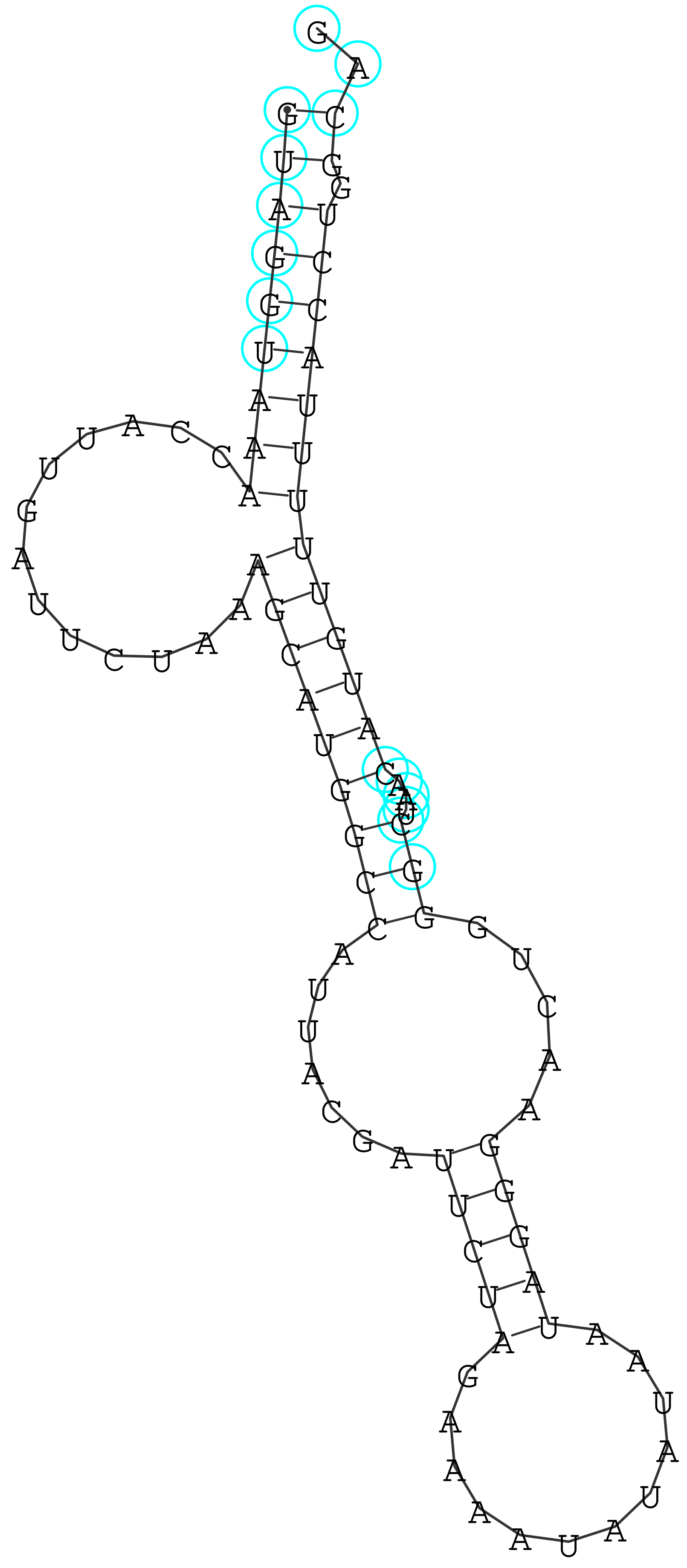

Xmsuc0170A - External intron

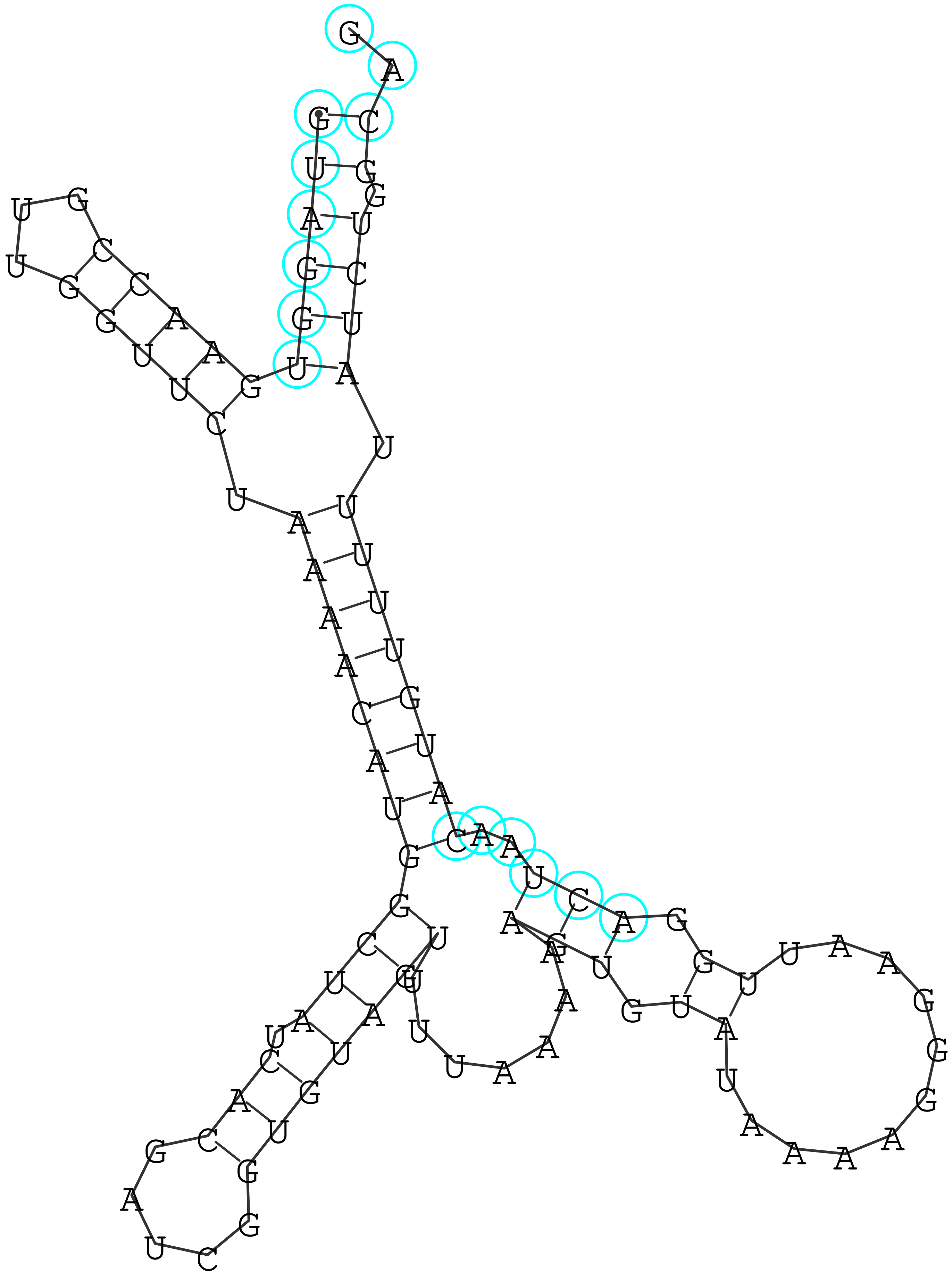

Xmsuc0171A - External intron

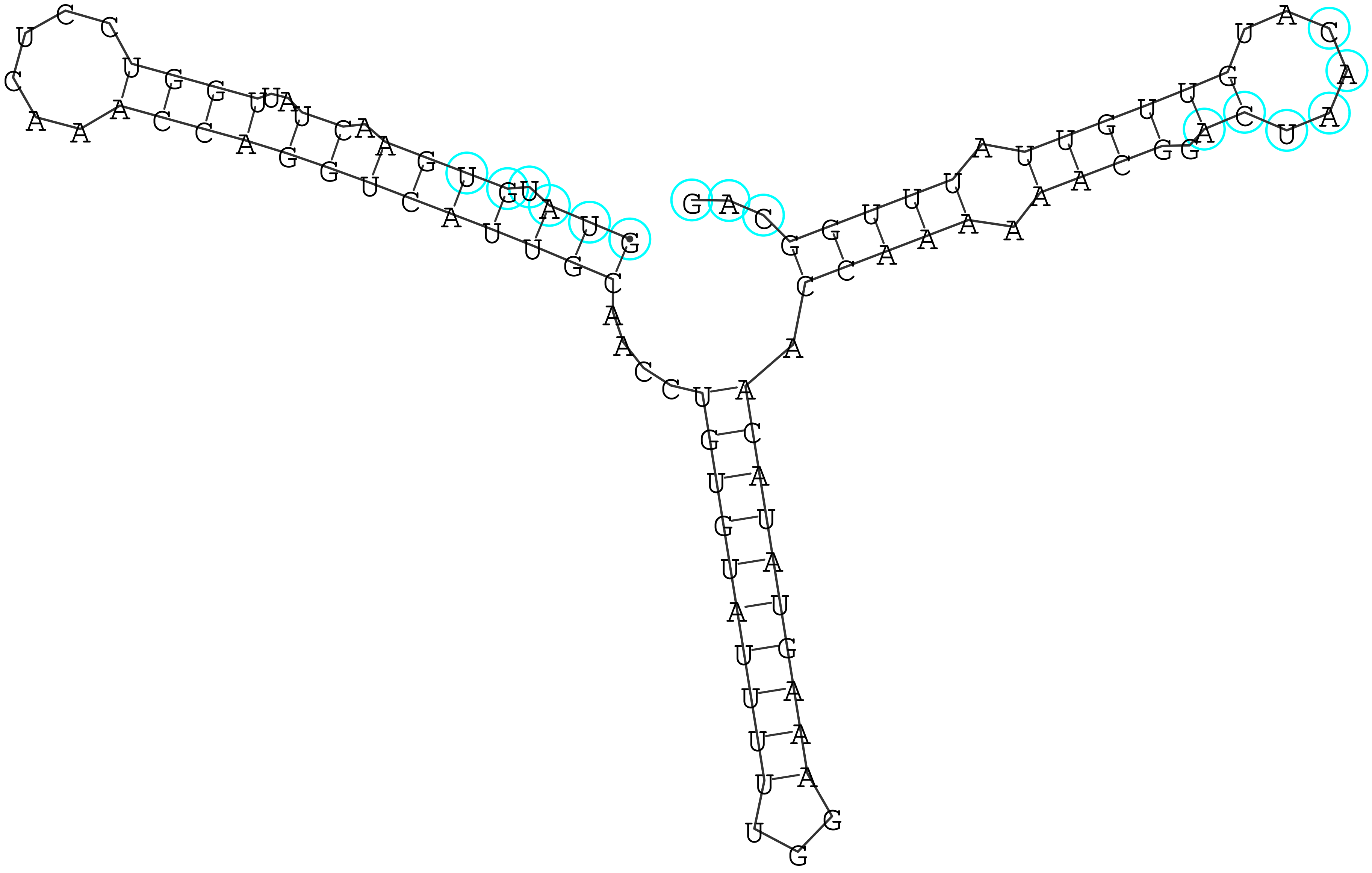

Xmsuc0178A - External intron

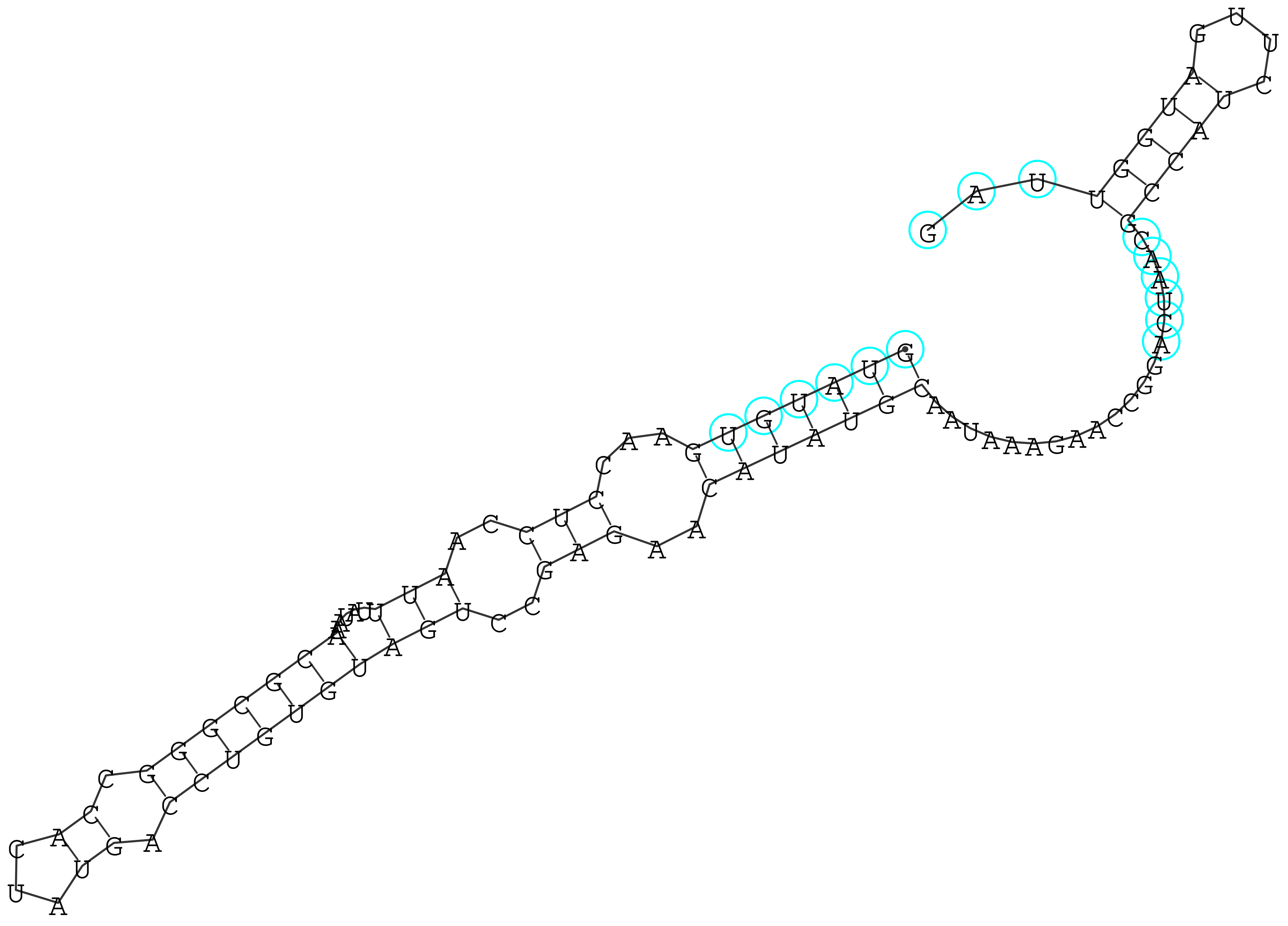

Xmsuc0185A - External intron

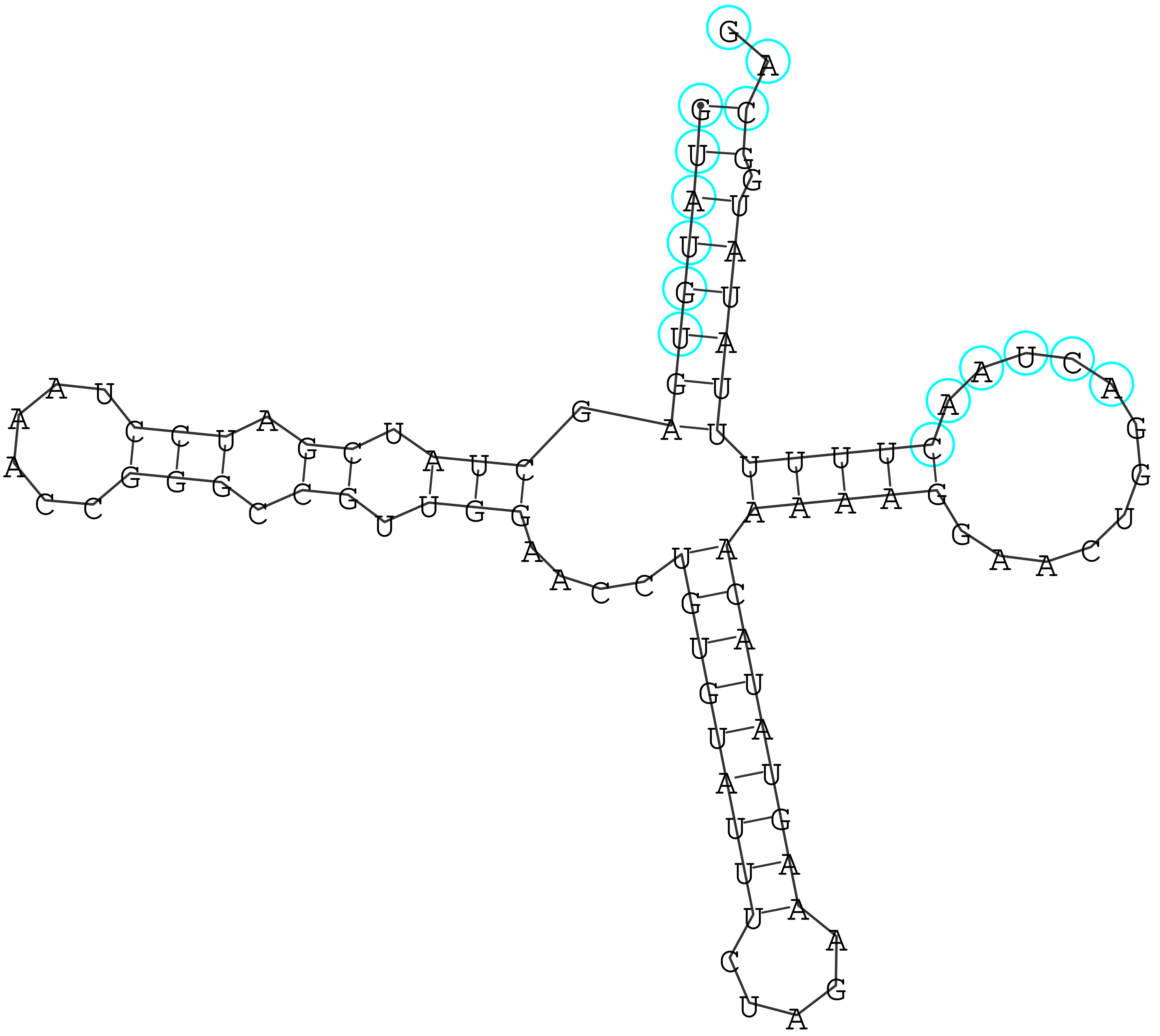

Xmsuc0187A - External intron

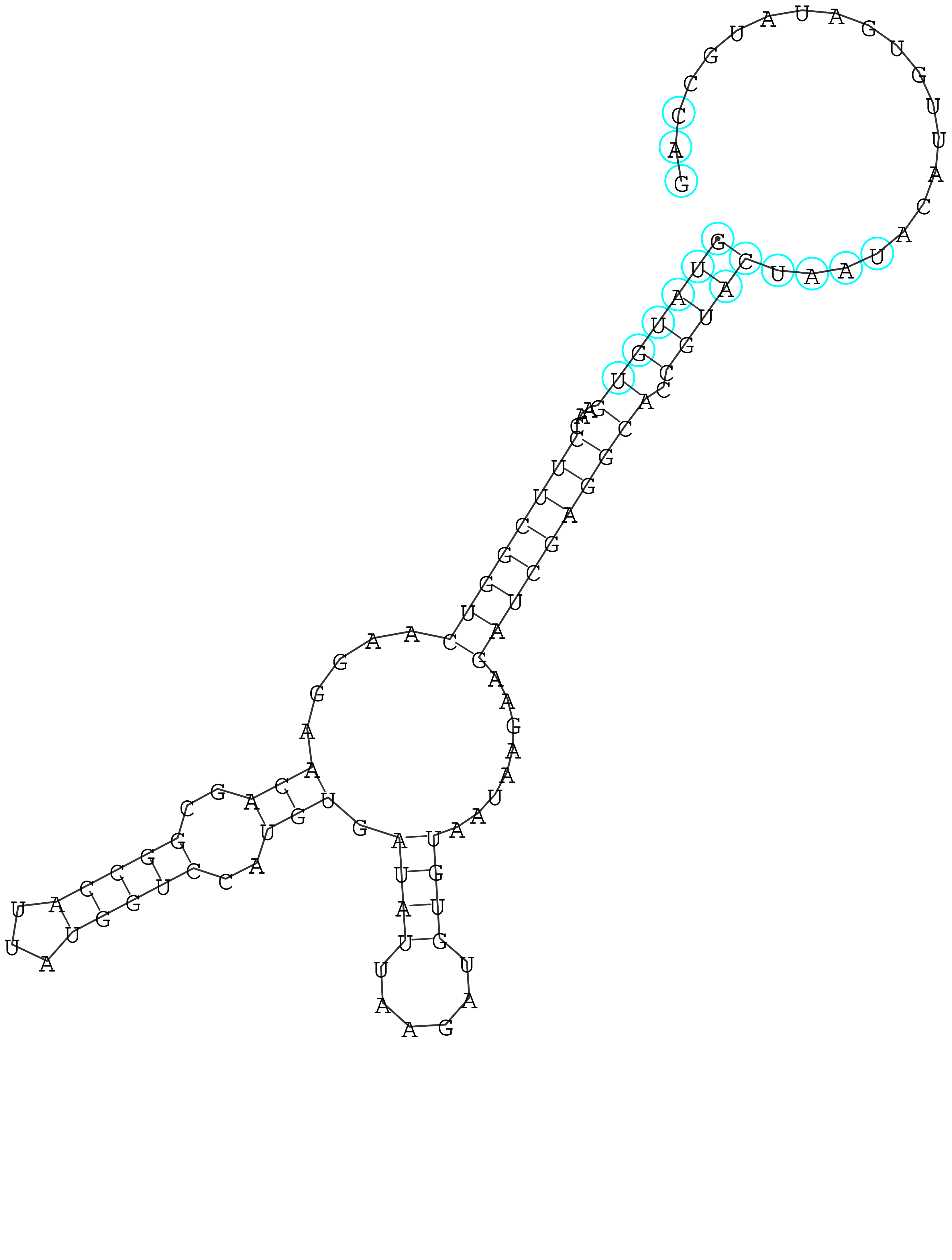

Xmsuc0237A - External intron

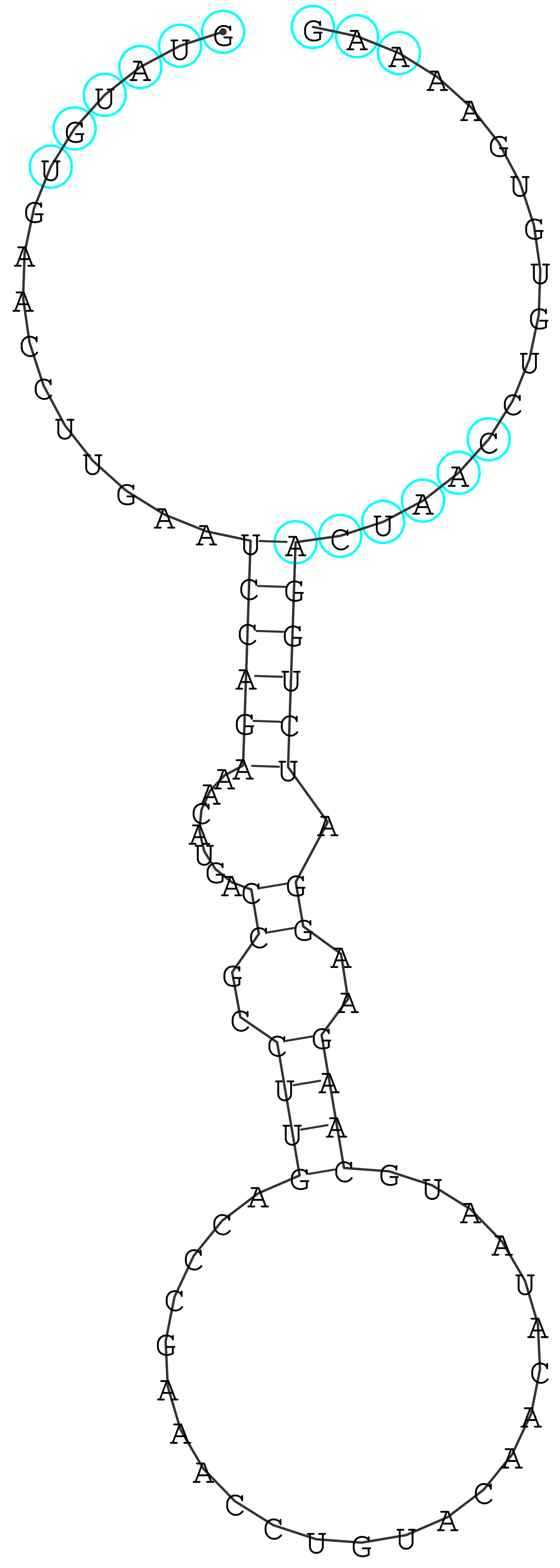

Xmsuc0285A - External intron

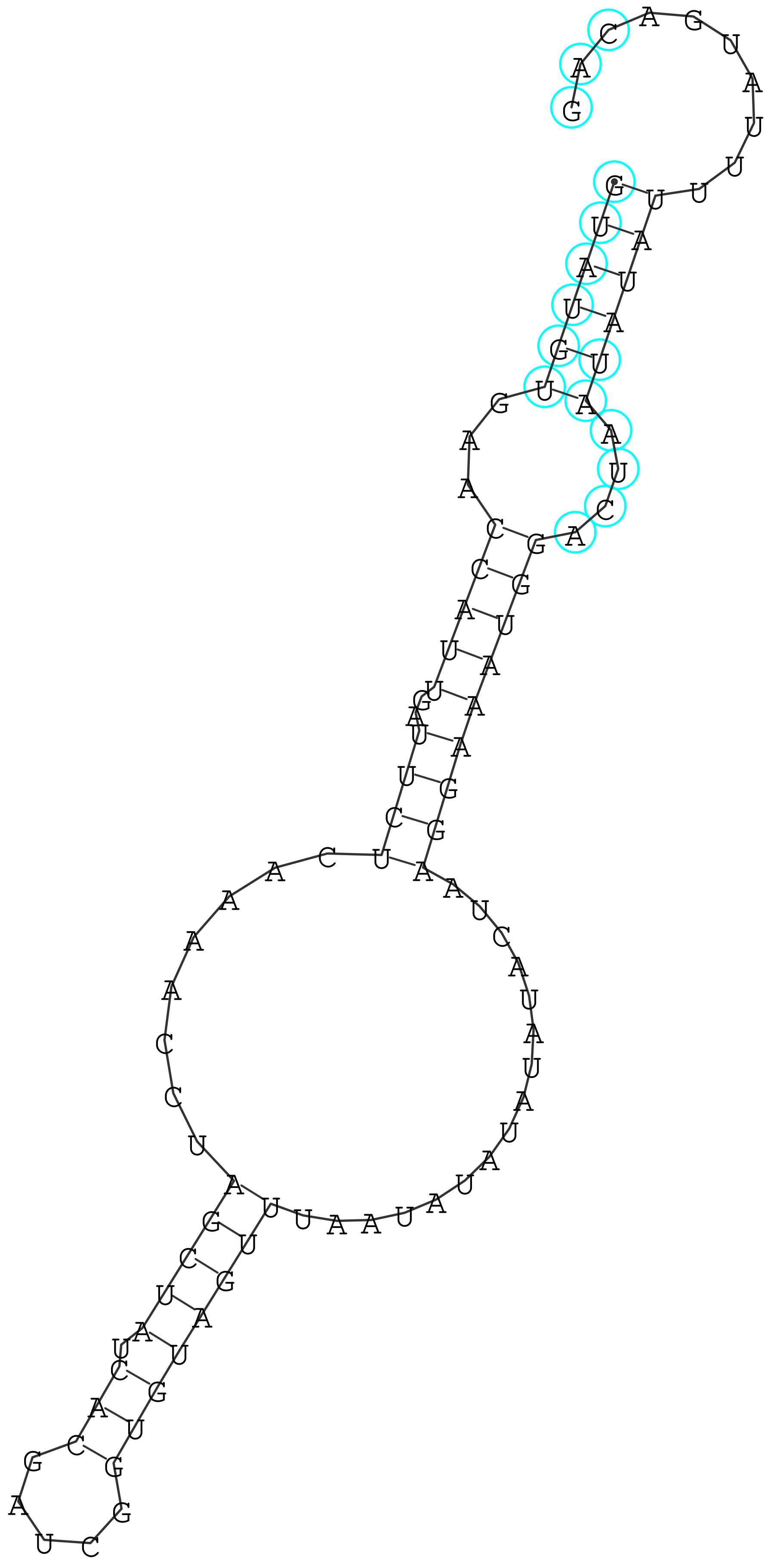

Xmsuc0293A - External intron

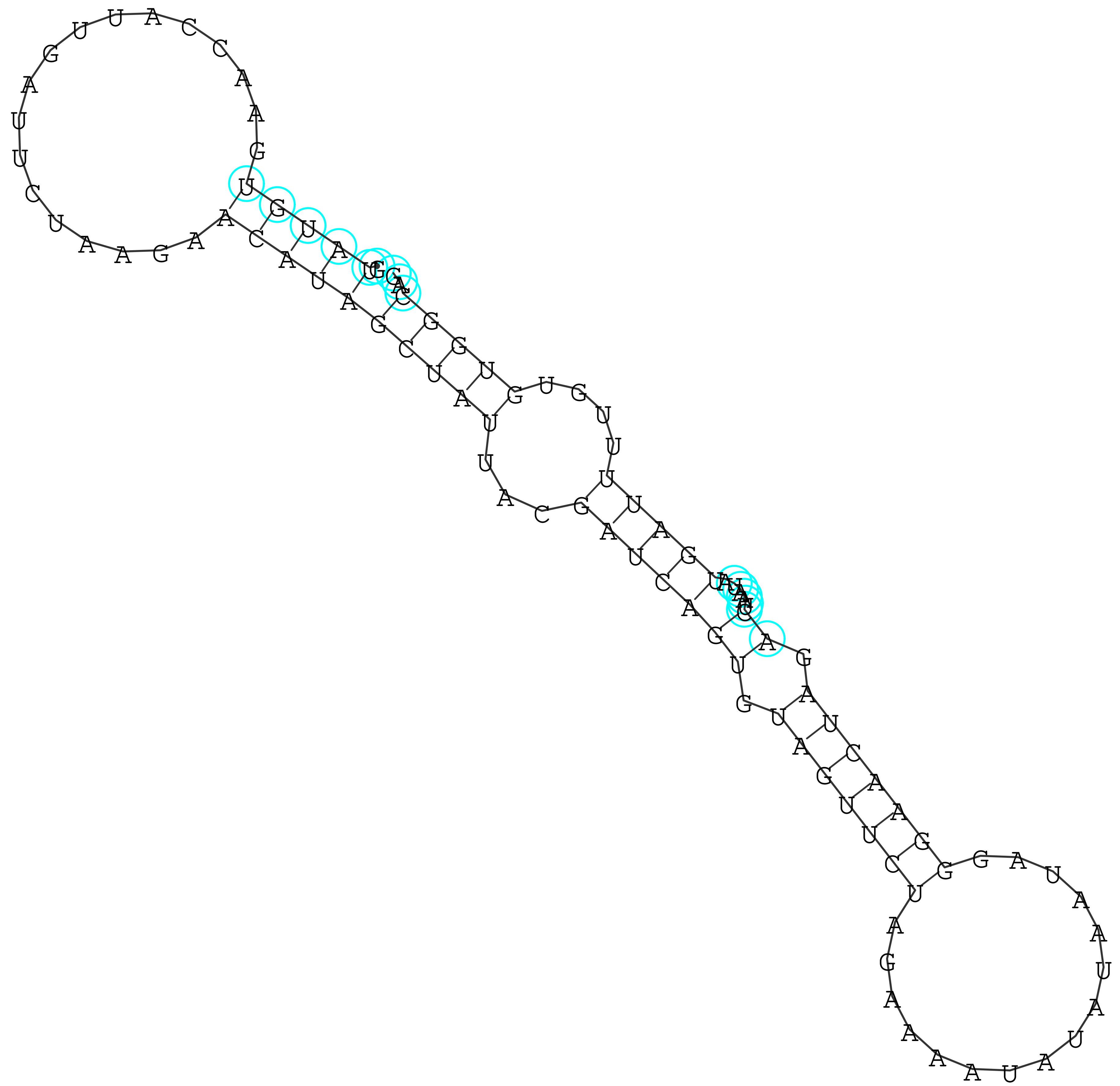

Xmsuc0301A - External intron

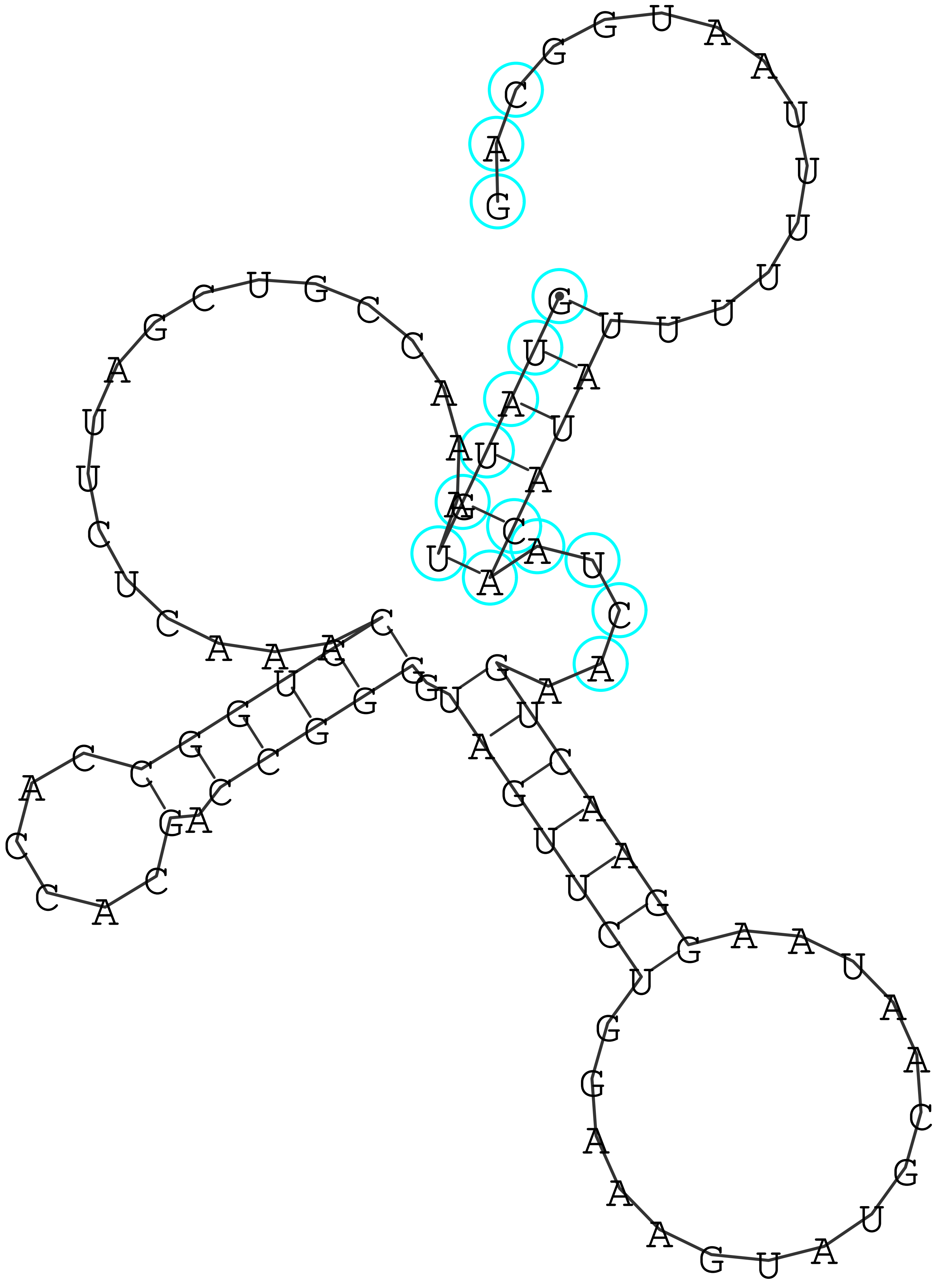

Xmsuc0306A - External intron

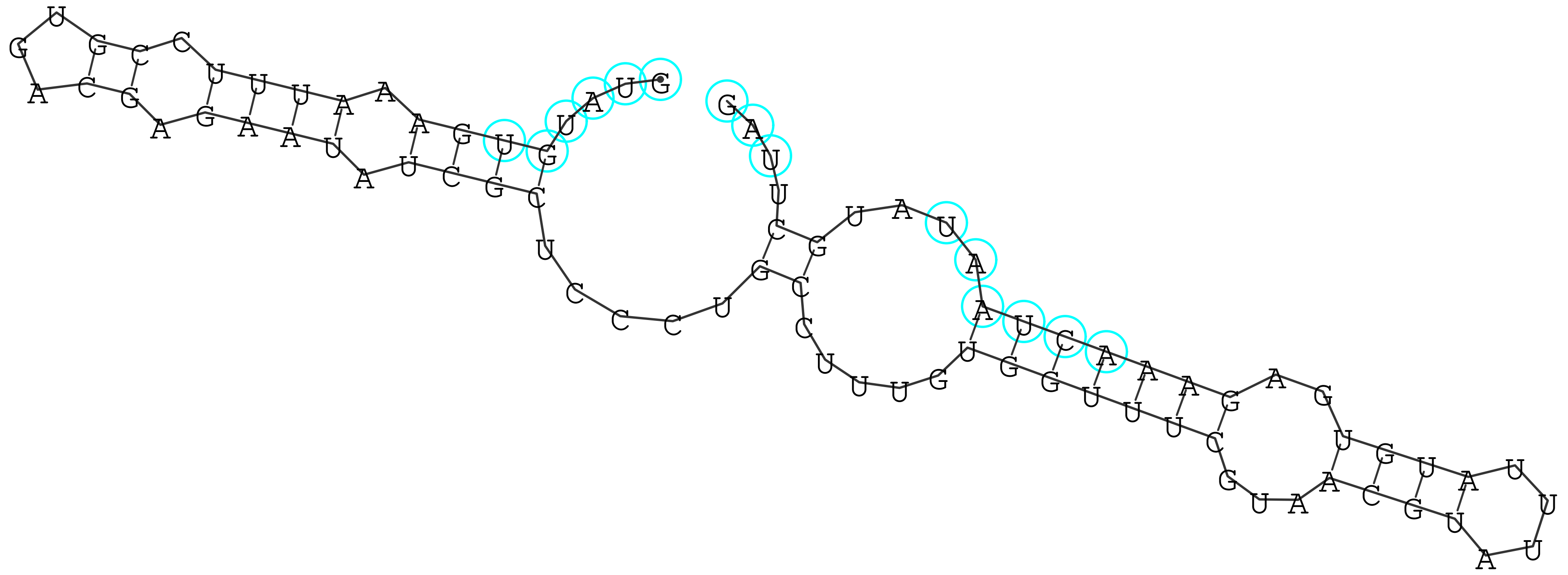

Xmsuc0348A - External intron

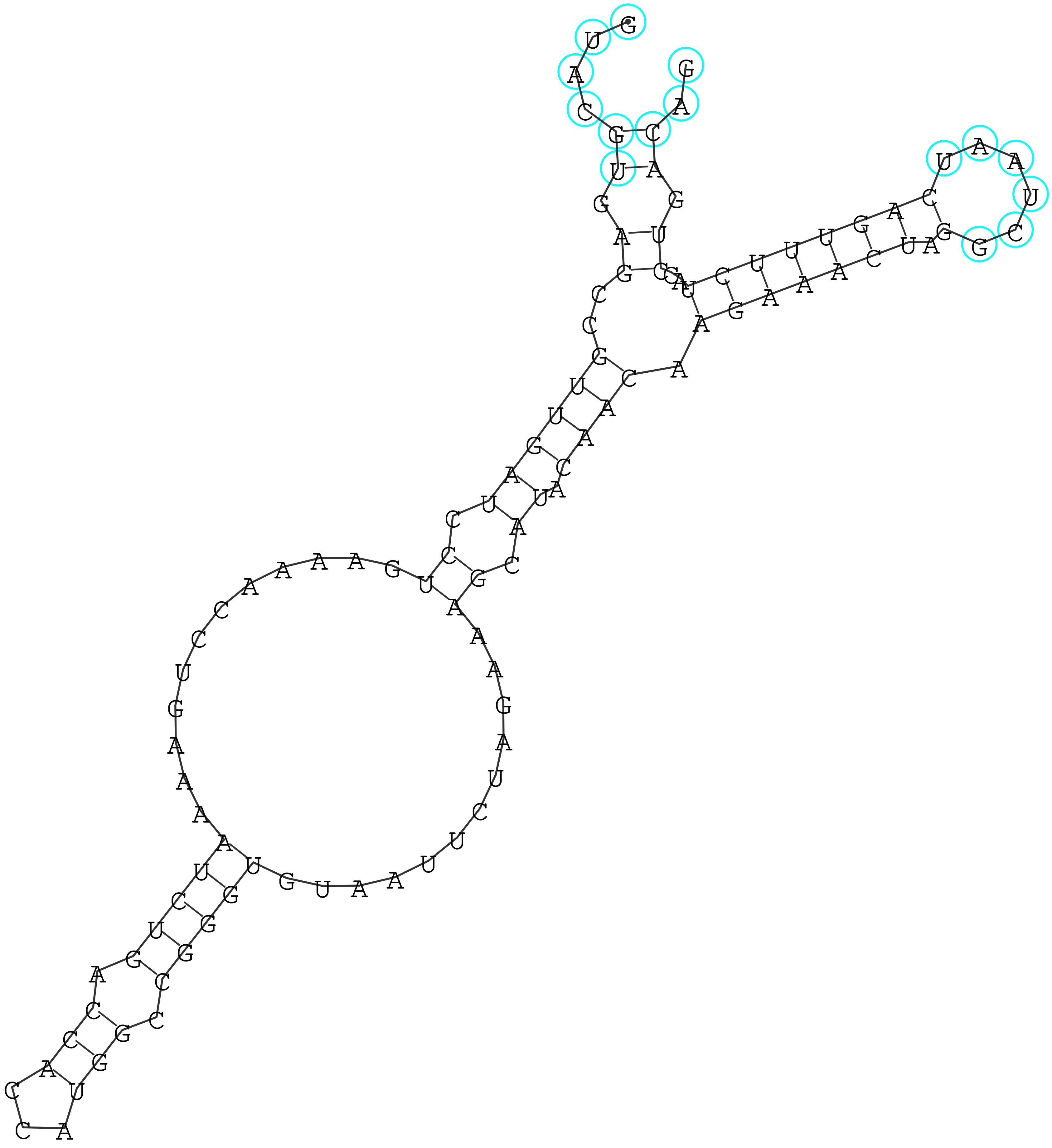

Xmsuc0374A - External intron

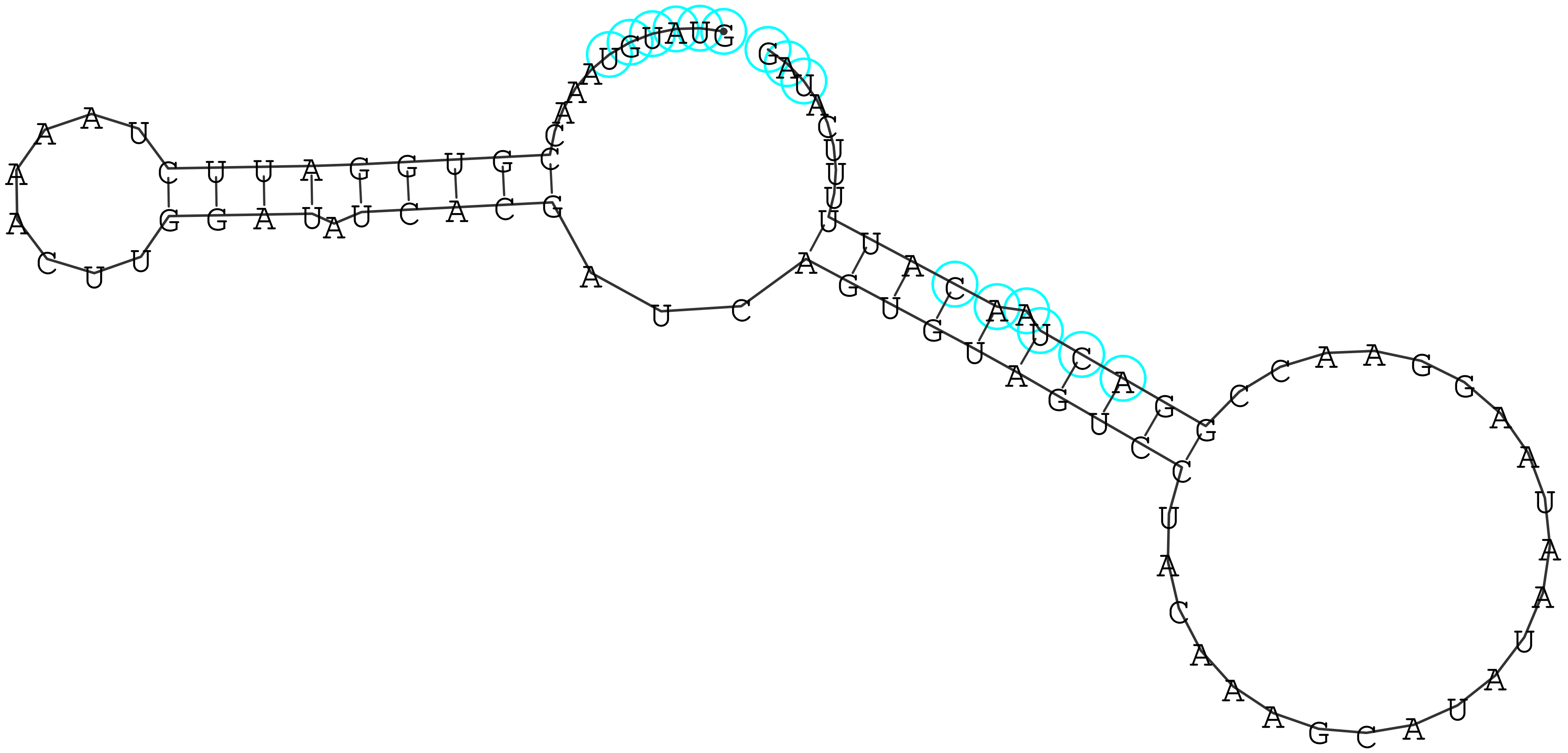

Xmsuc0374B - External intron

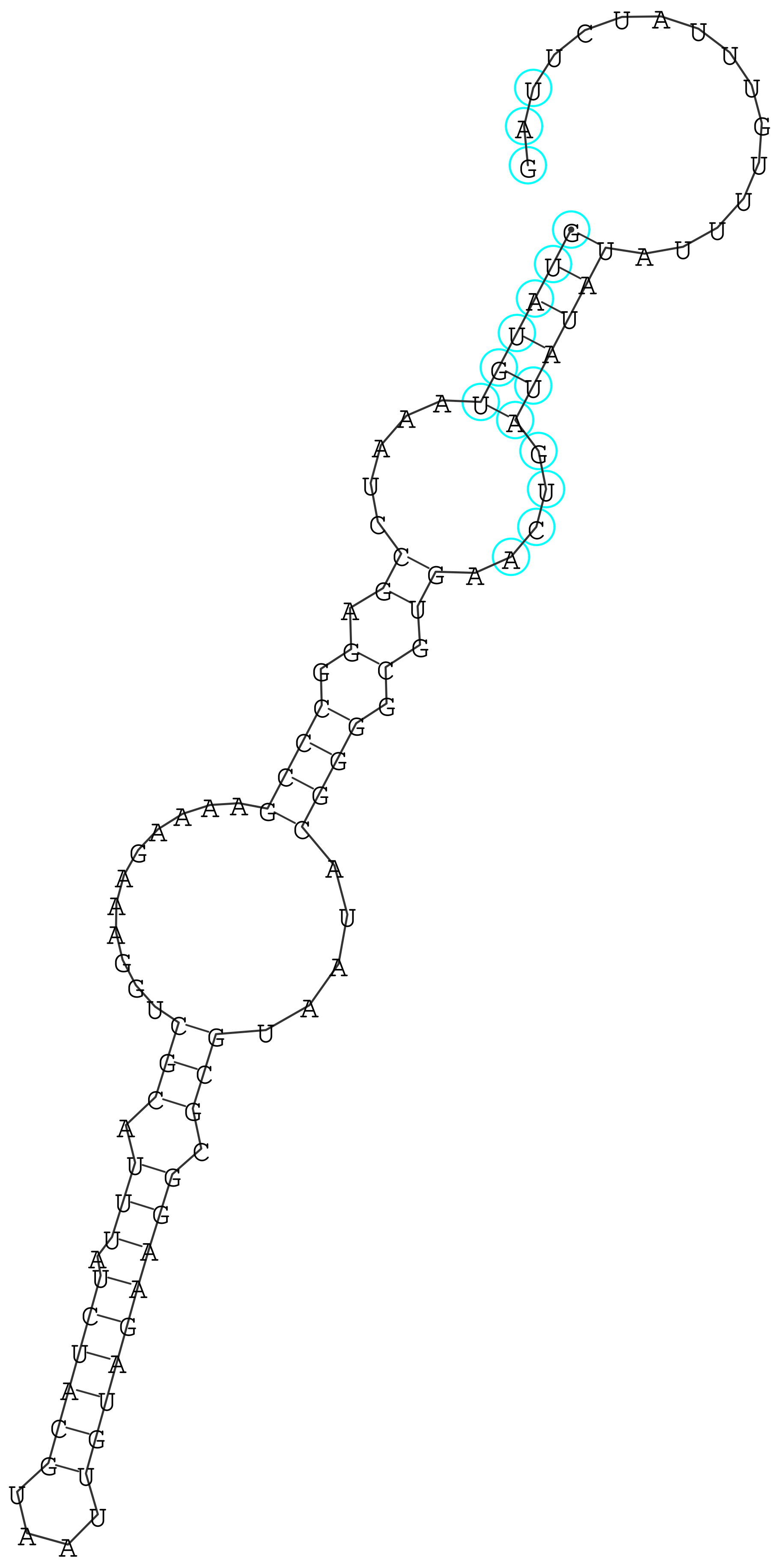

Xmsuc0378A - External intron

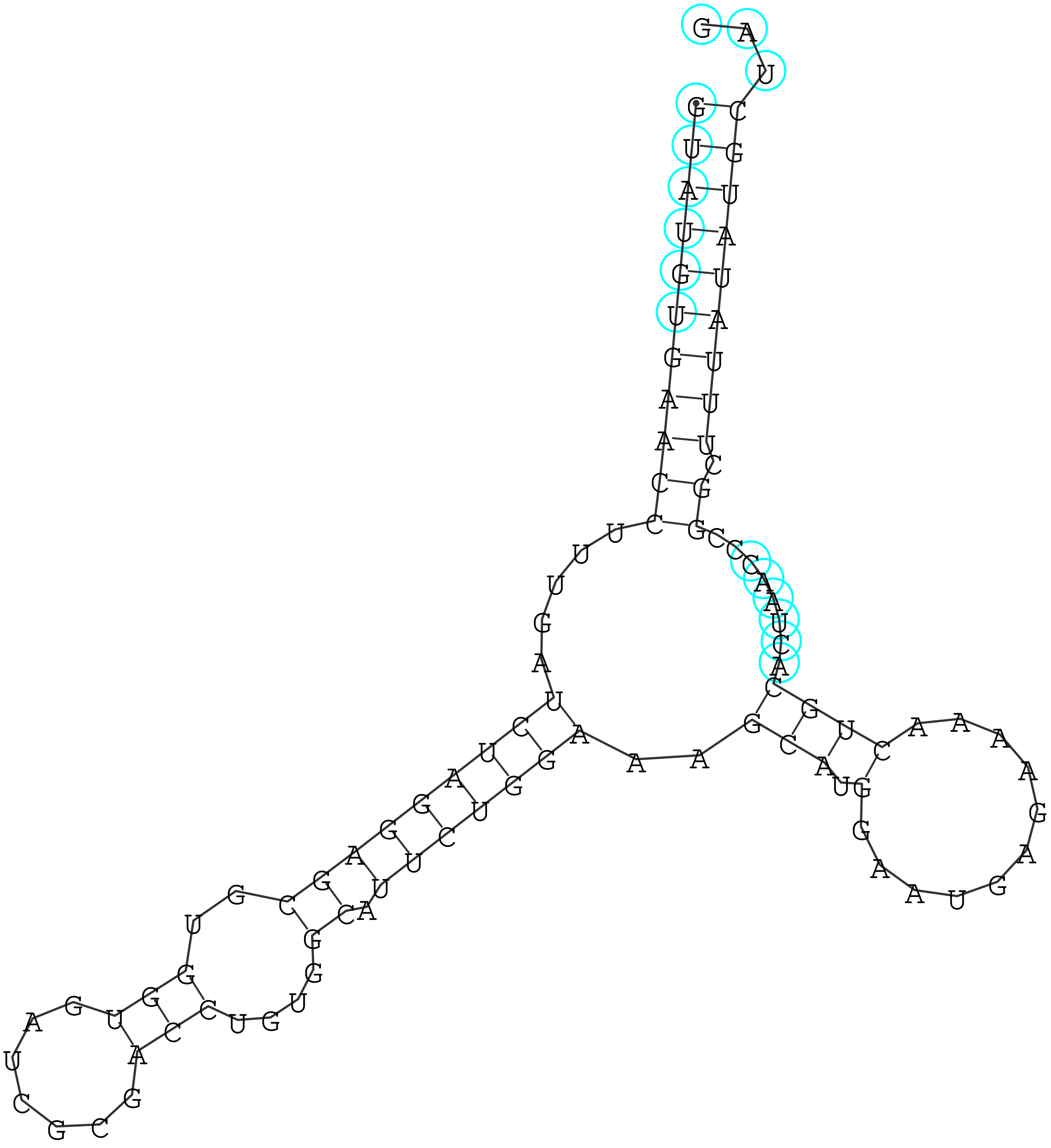

Xmsuc0385A - External intron

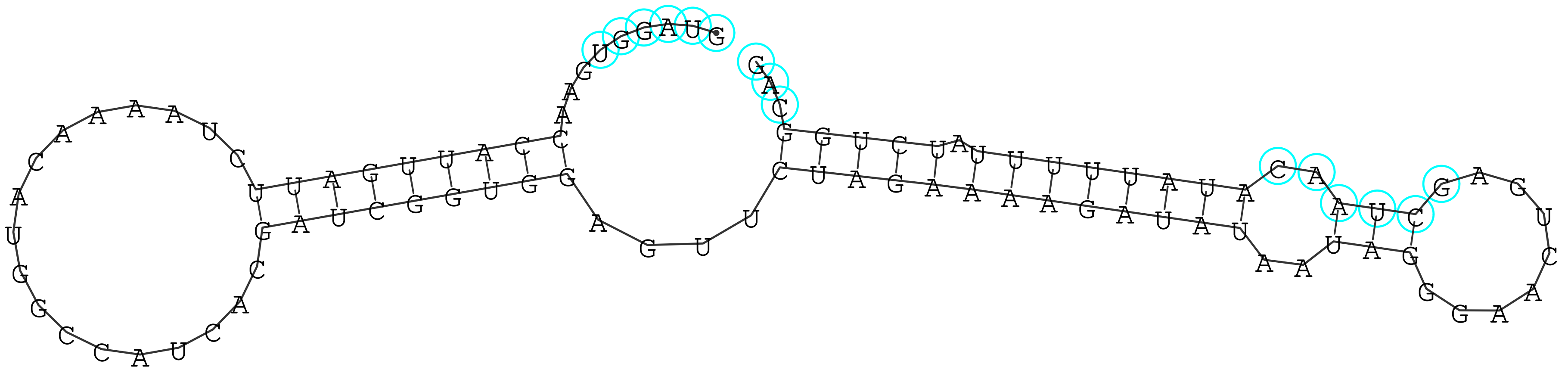

Xmsuc0412A - External intron

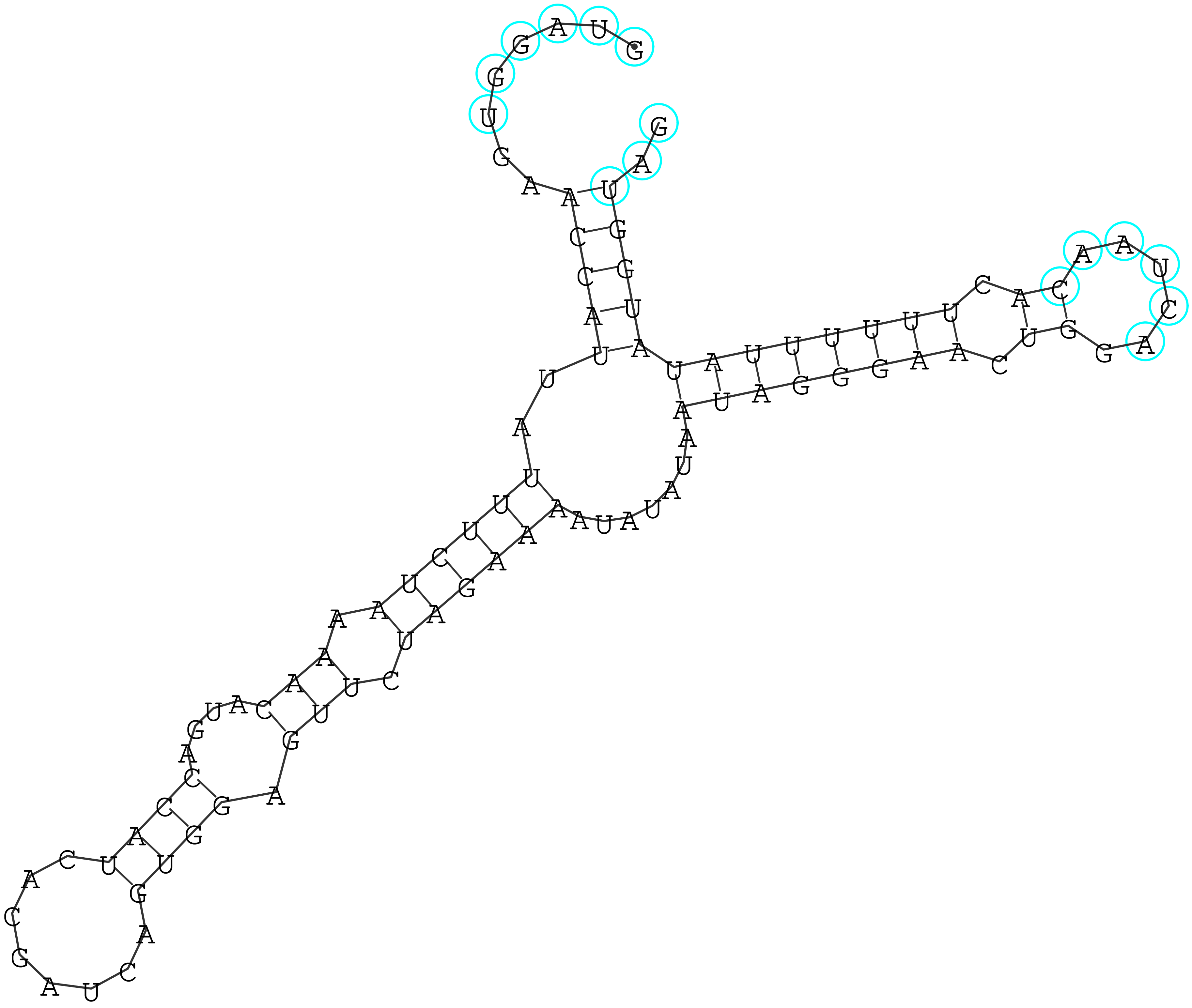

Xmsuc0520A - External intron

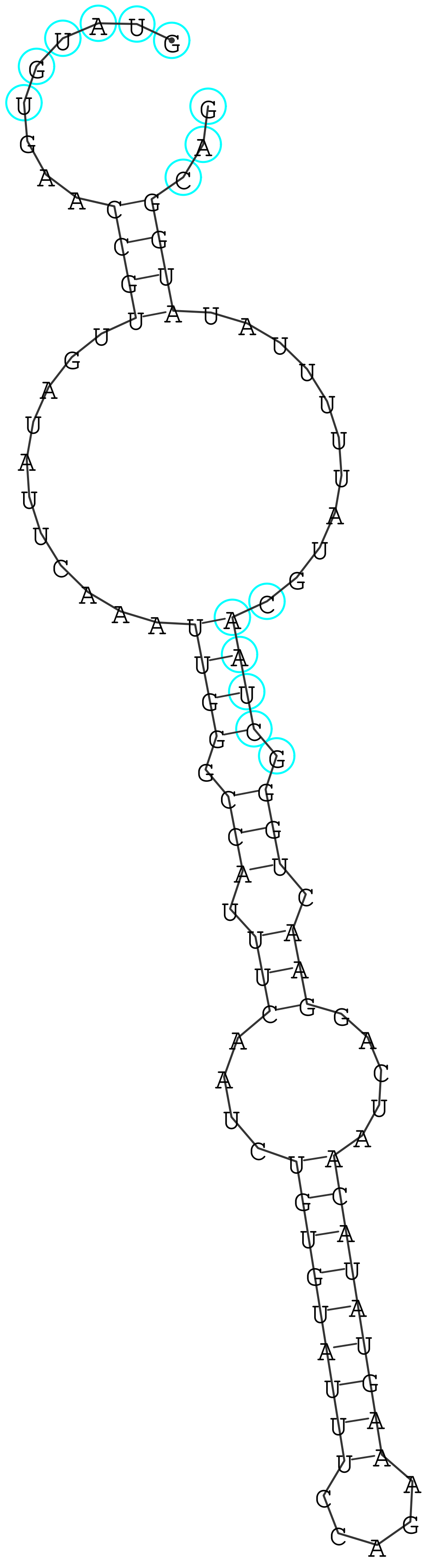

Xmsuc0671A - External intron

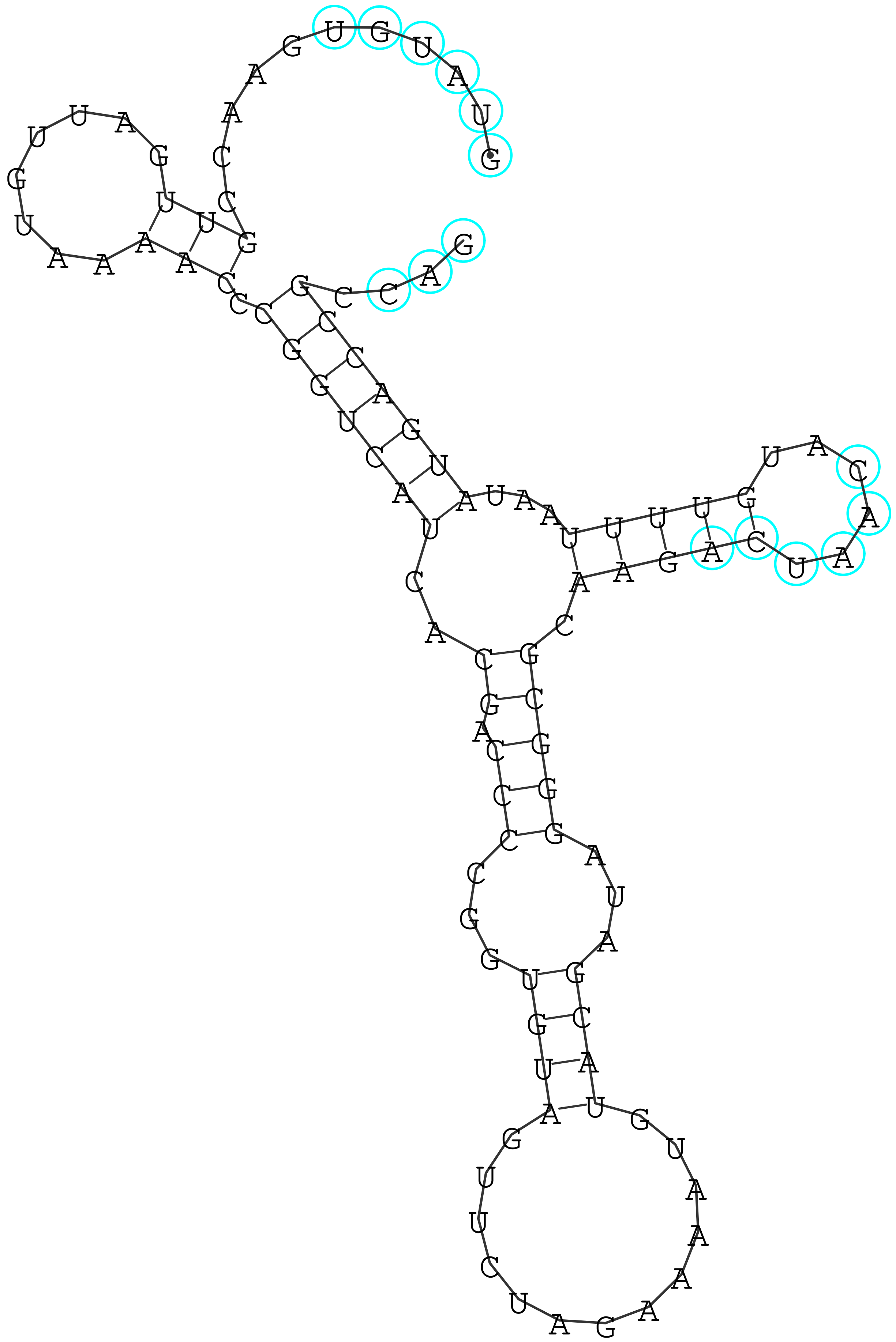

Xmsuc0710A - External intron

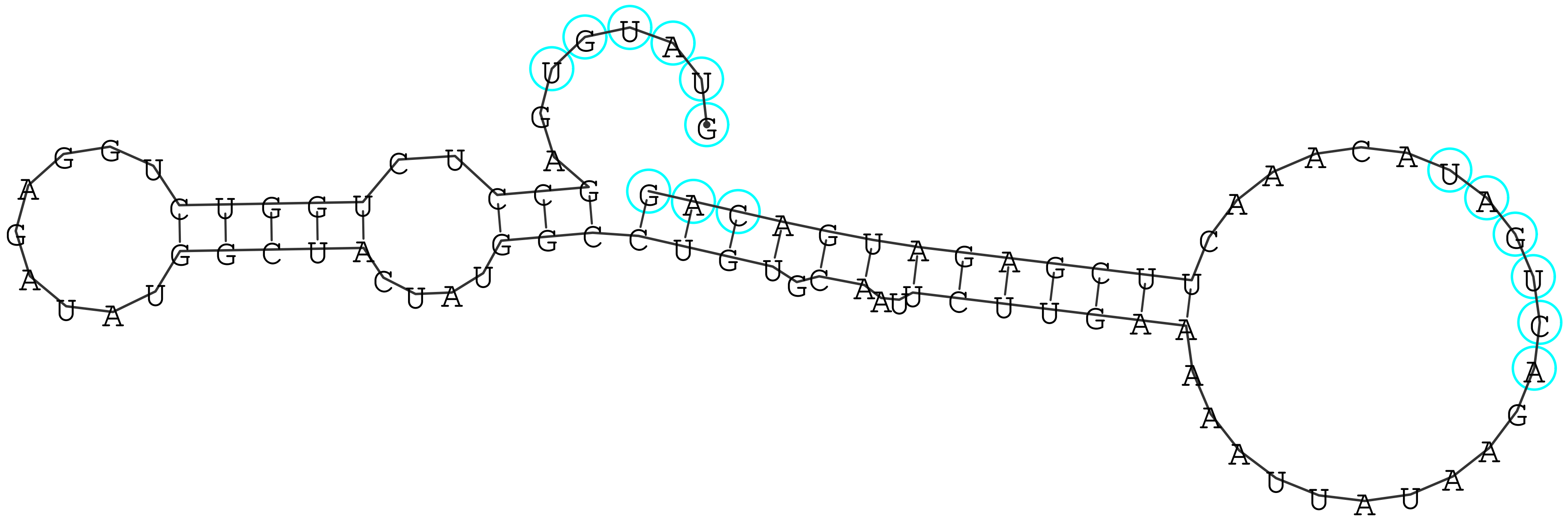

Xmsuc0775A - External intron

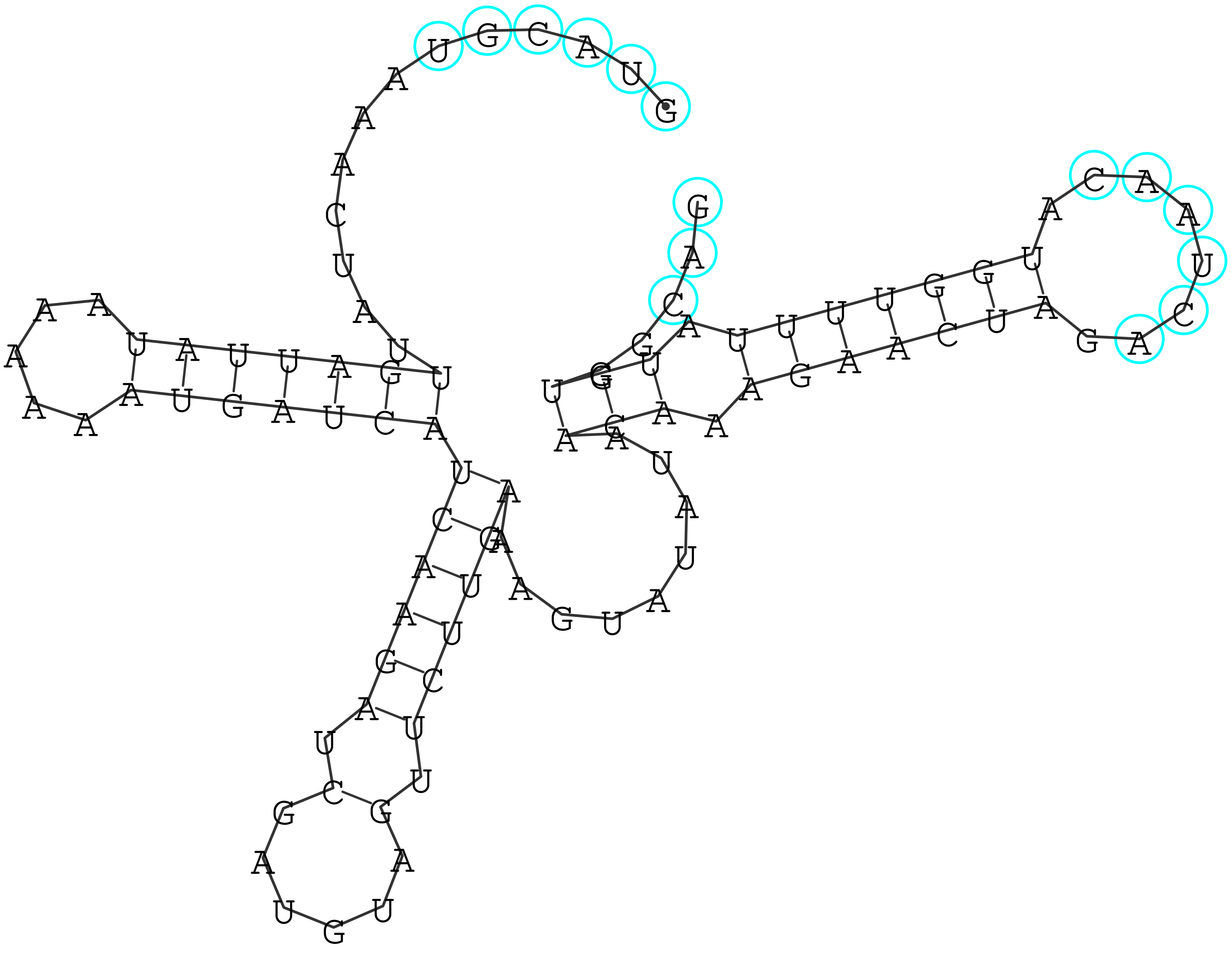

Xmsuc0776A - External intron

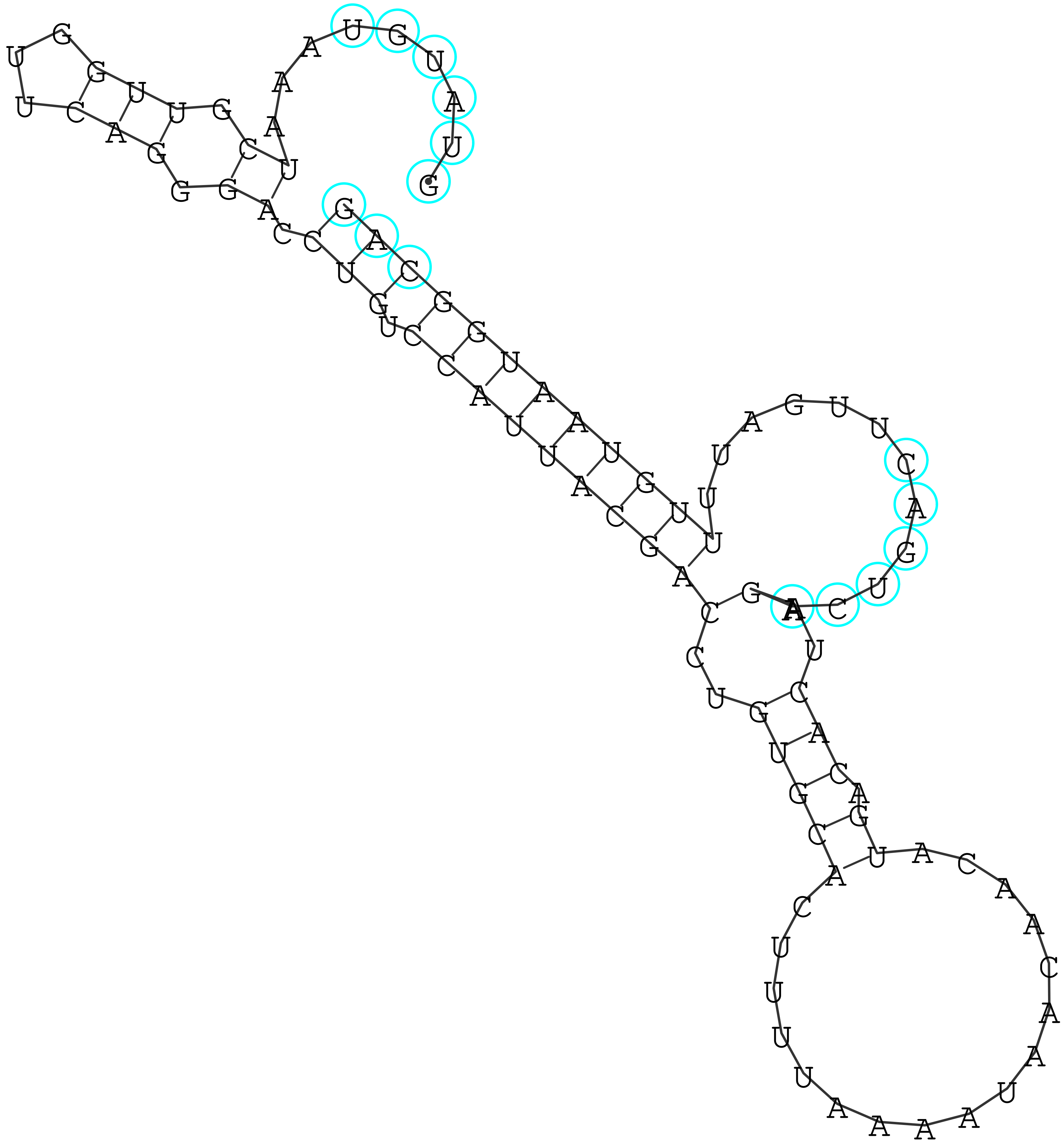

Xmsuc0776B - External intron

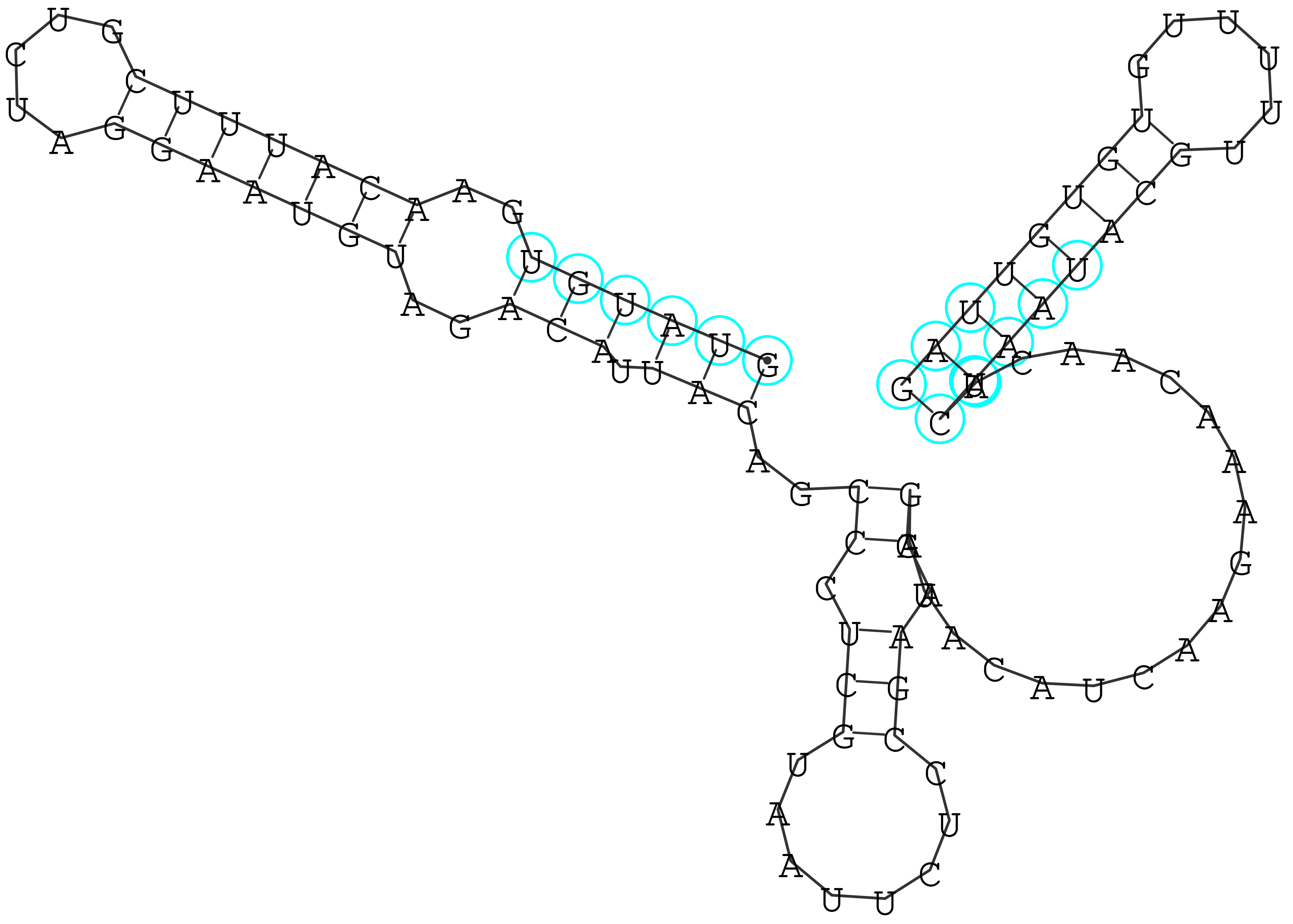

Xmsuc0776C - External intron

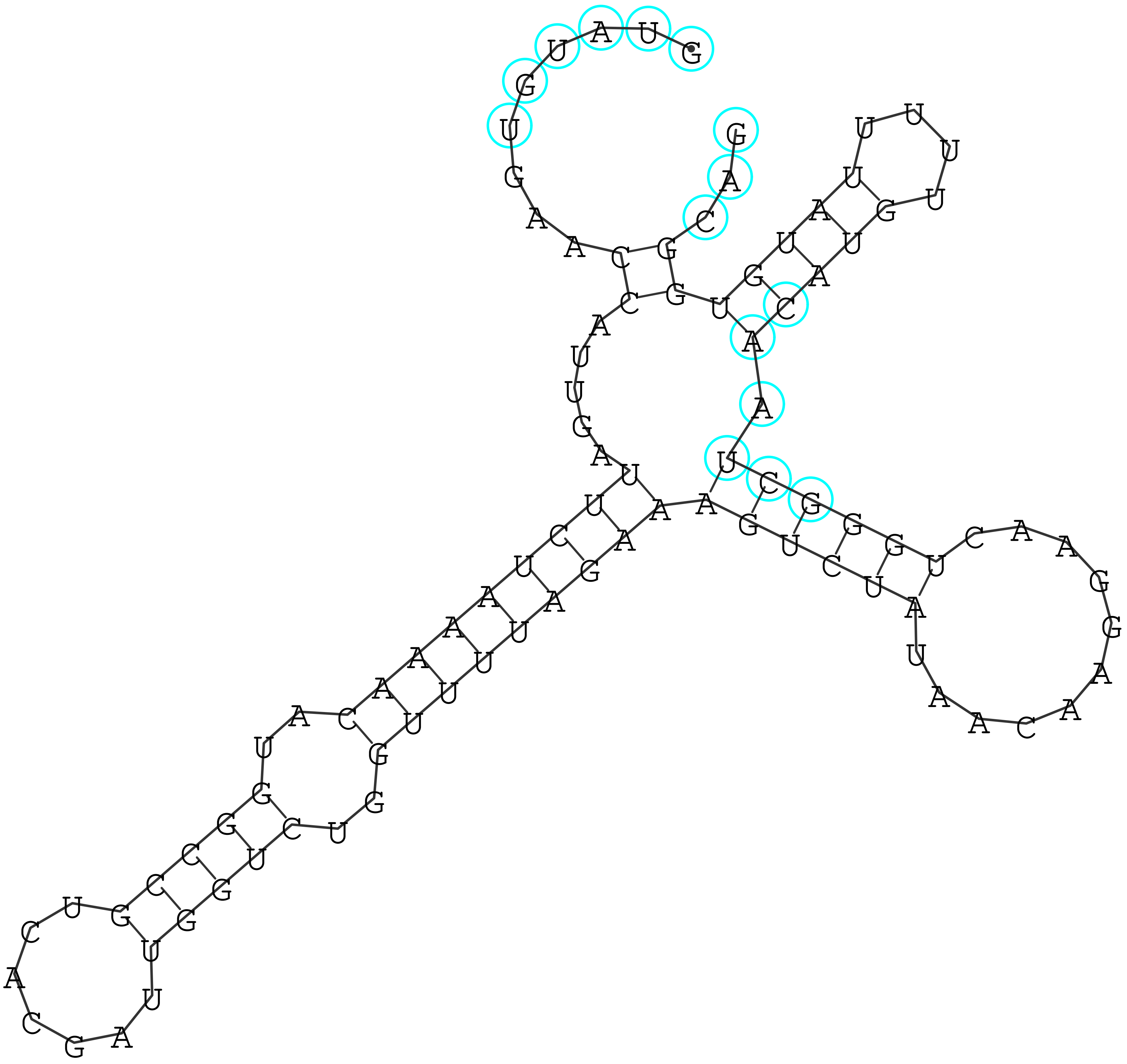

Xmsuc0808A - External intron

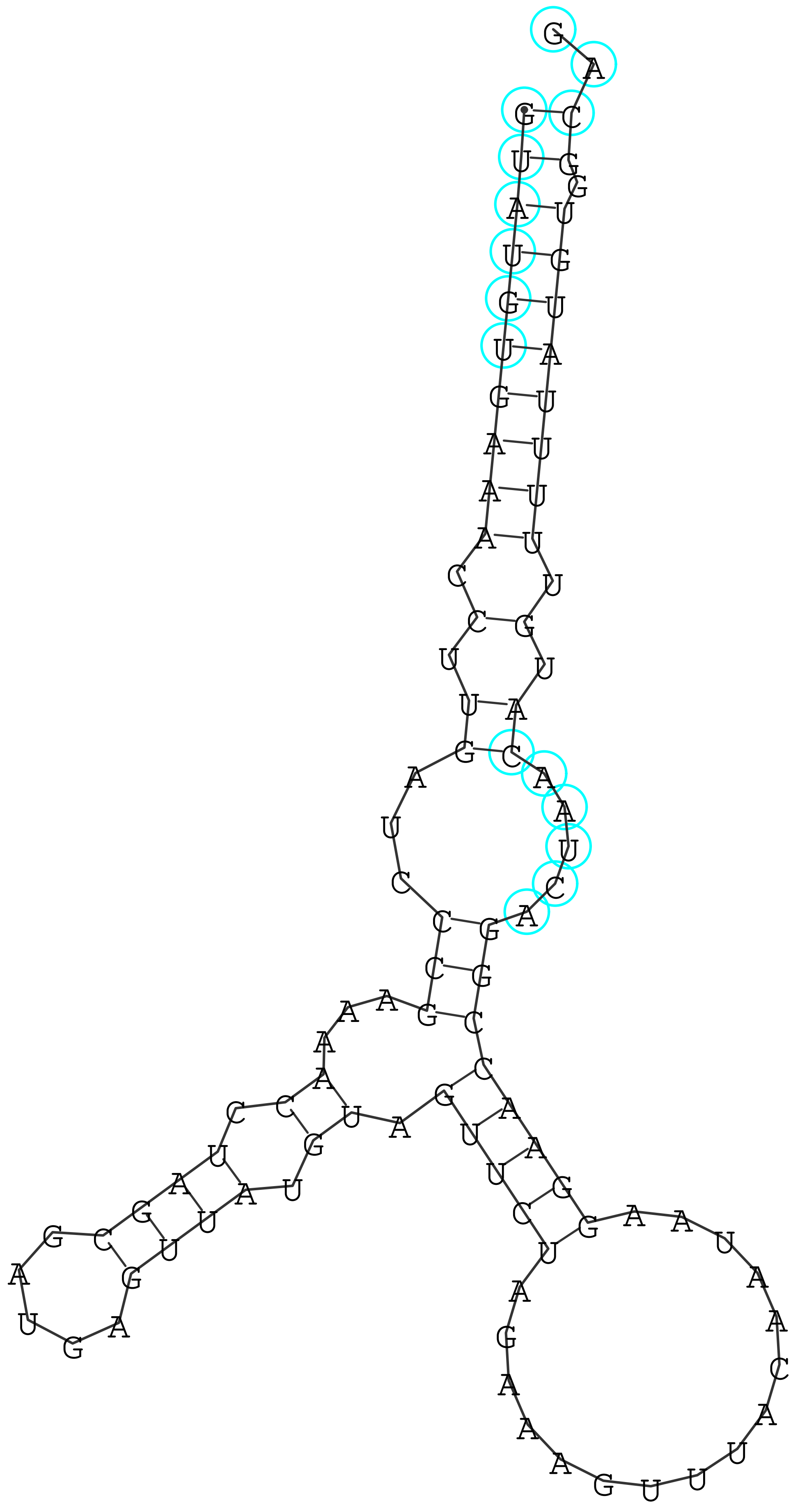

Xmsuc0819A - External intron

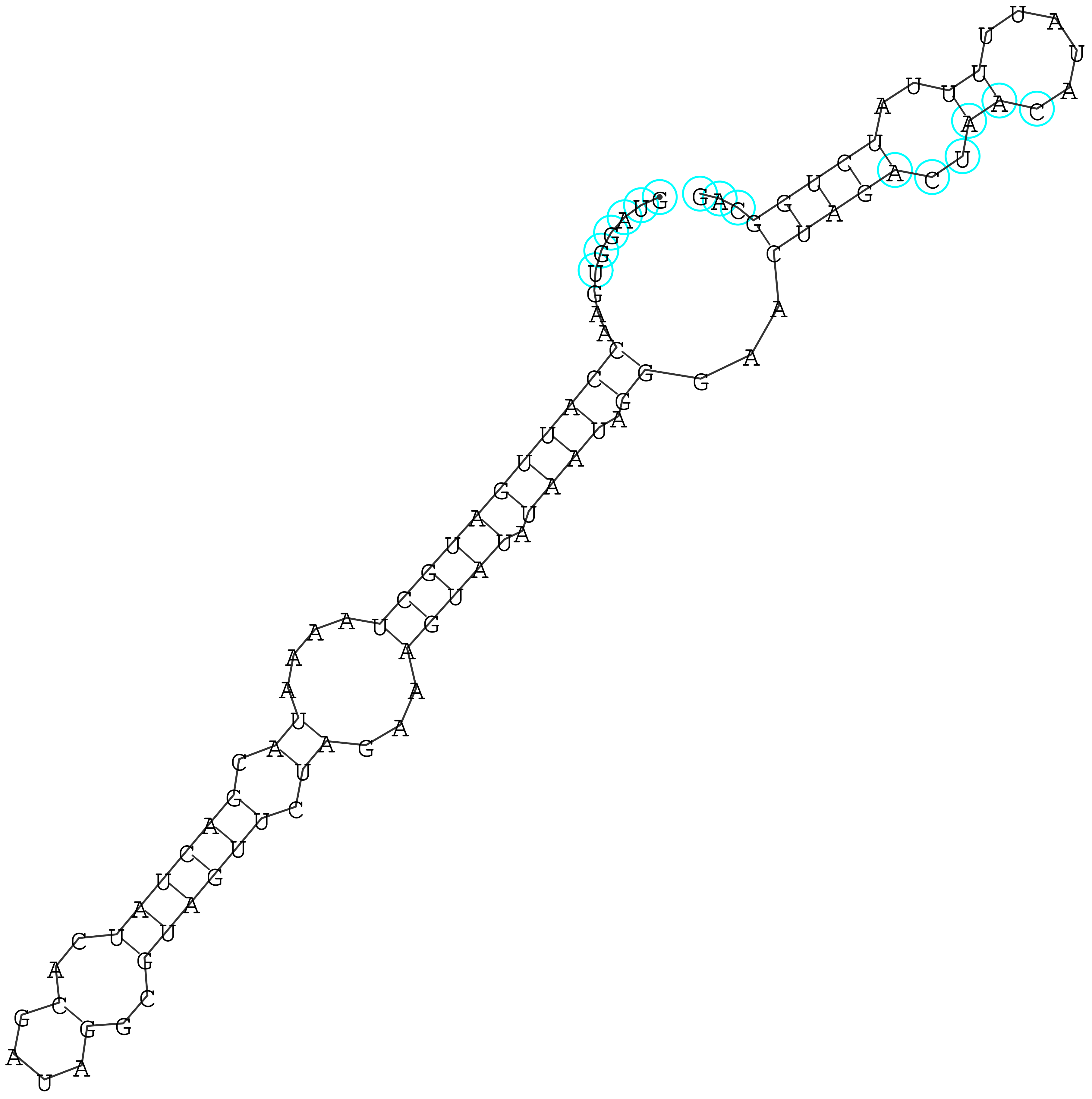

Xmsuc0904A - External intron

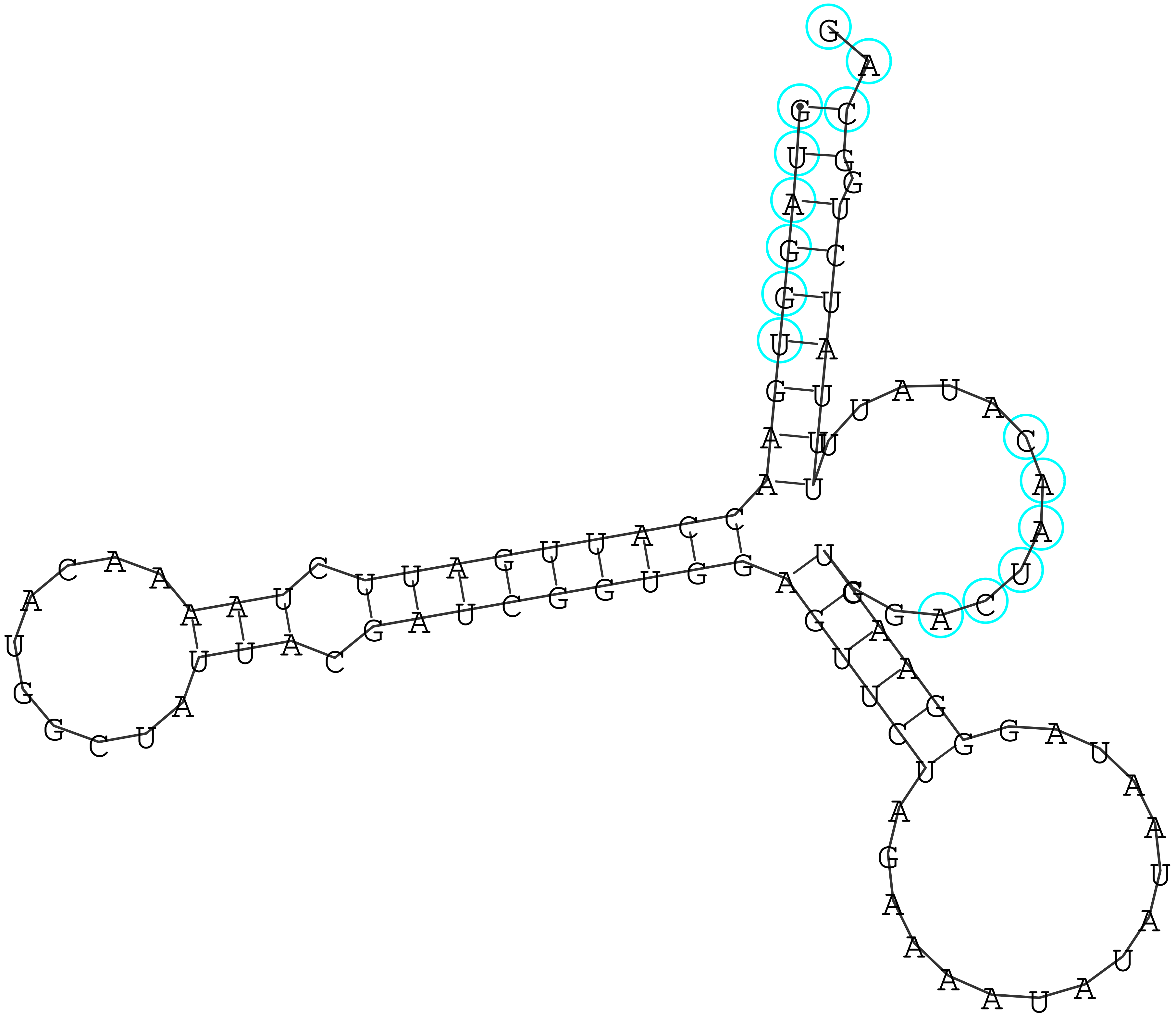

Xmsuc1083A - External intron

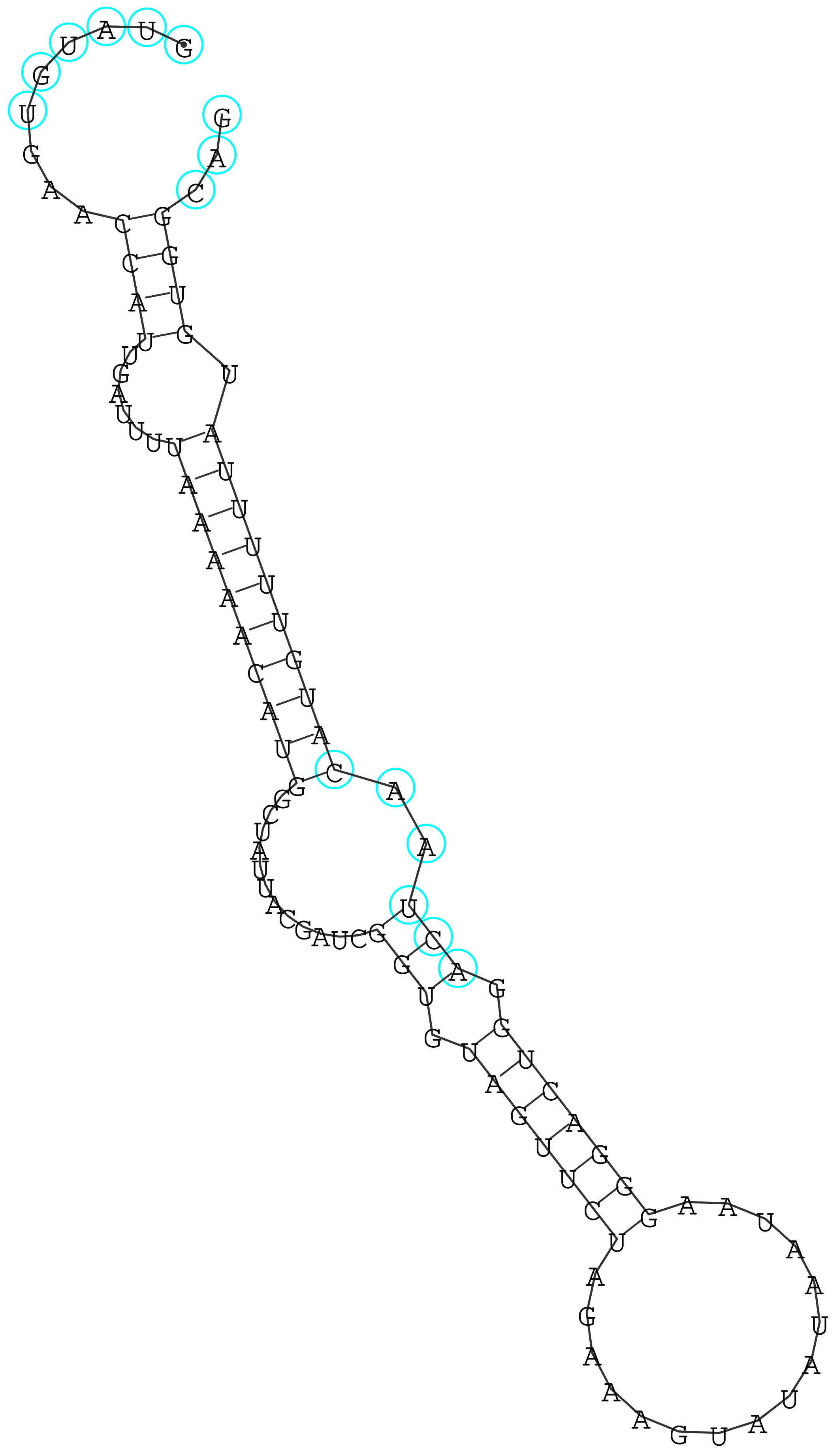

Xmsuc1127A - External intron

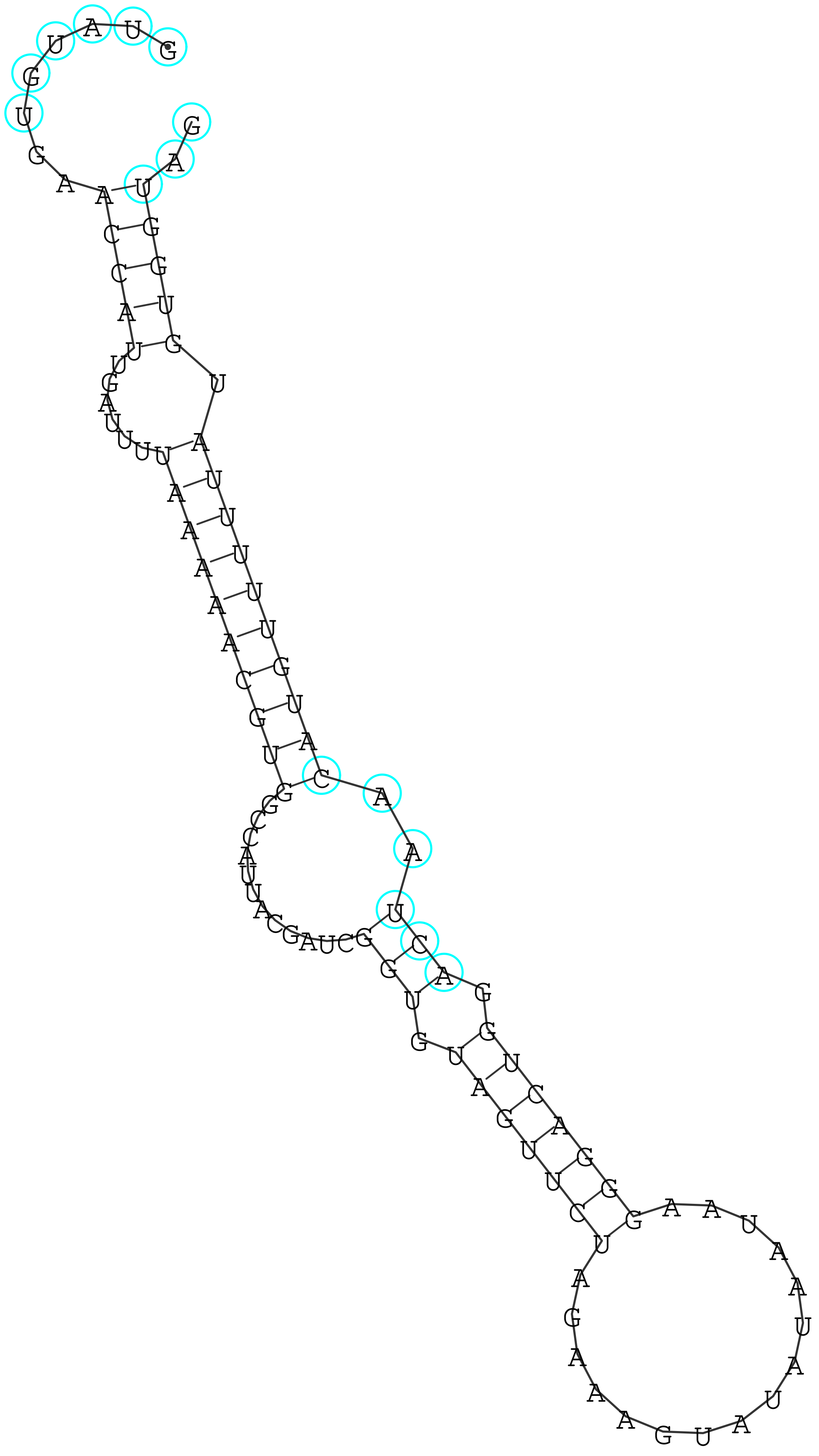

Xmsuc1145A - External intron

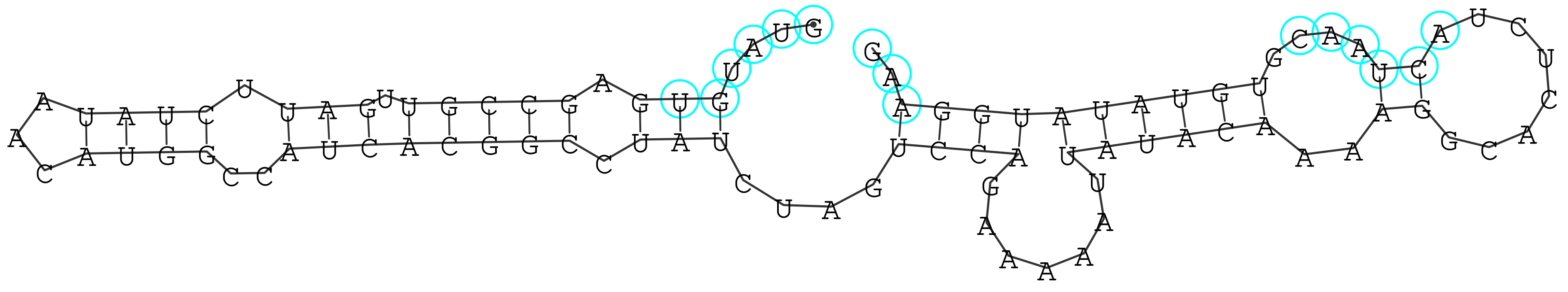

Supplement: Data S3 — Collection of the RNAFold predicted secondary structures of the external introns of the 288 [D1,2] sister stwintrons. [file spectrum.02926-24-s0003.pdf]
