# Supplementary material for: Propagation of [D1,2]-type spliceosomal twin introns (stwintrons) in Hypoxylaceae and Xylariaceae fungi
Source: Microbiol Spectr. 2025 Aug 8;13(9):e02926-24. doi: 10.1128/spectrum.02926-24 (PMC12403724; doi:10.1128/spectrum.02926-24)
Supplement: Data S4 — Collection of the one-on-one sequence alignments of the 288 stwintrons and of their constituent U2 introns, with their respective reverse complement sequences. [file spectrum.02926-24-s0004.pdf]

Supplementary datafile S4. Collection of the one-on-one sequence alignments of the 288 stwintrons and of their constituent U2 introns, with their respective reverse complement sequences. MAFFT alignments are used to detect terminal symmetry (near terminal inverted repeats). For experimental detail, see Material and Methods subsection 2.4. The consensus line underneath the two aligned sequences indicates nucleotide identity with the star symbol and the GU interactions with the number symbol. The centre of the symmetry is indicated by the vertical line symbol | or neighbouring vertical line symbols || in the consensus line. Virtually all constituent introns are symmetrical in their centre.

ALIGNMENT RESULTS - Dchc001A

=== Stwintron Sequence ===

Multiple sequence alignment

```
Dchc001A_fwrd       gguauguaugaua----------uacguuucguaguauu----------auuuccaaccaucccuuucuaaaauag-------------ucucaaaucugccuuuaggagguaacaguagcuaacccucgguaguaagugugaaagccuaucauuuccacucauucgaaaaaggacgauggagauucuaagauucuaagauugcaaagcugacuuggaauauuuucauacauaag
Dchc001A_RevC       cuuauguaugaaaauauuccaagucagcuuugcaaucuuagaaucuuagaaucuccaucguccuuuuucgaaugaguggaaaugauaggcuuucacacuuacuaccgaggguuagcuacuguuaccuccuaaaggcagauuugag-------------acuauuuuagaaagggaugguuggaa----------auaauacuacgaaacgua----------uaucauacauacc
                      ********* *          *  *#** *#*#* **          * *# * * * *** *** # **  **              * *** *# *#*  ## #* * ** * || * ** * *# ##  *#* #* ***               ***  ** # *** *** * * * #*           *** *#*#* **#*  *          * *********
```

=== Internal intron ===

Multiple sequence alignment

```
Dchc001A_fwrd       guauguaugauauacguuucguaguauuauuuccaaccaucccuuucuaaaauagucucaaaucugccuuuaggagguaacaguagcuaacccucg------guag
Dchc001A_RevC       cuac------cgaggguuagcuacuguuaccuccuaaaggcagauuugagacuauuuuagaaagggaugguuggaaauaauacuacgaaacguauaucauacauac
                     **#      #    ***    ** * ***##*** *    *    **   * * || * *  **    *    * ***##*** * **   ***    #      #**
```

=== External intron ===

Multiple sequence alignment

```
Dchc001A_fwrd       -guaagugugaaagccuaucauuuccacucauucgaaaaaggacgauggagauucuaagauucuaagauugcaaagcugacuuggaauauuuucauacauaag
Dchc001A_RevC       cuuauguaugaaaauauuccaagucagcuuugcaaucuuagaaucuuagaaucuccaucguccuuuuucgaaugaguggaaaugauaggcuuucacacuuac-
                      ** **#*****#  * #**  **  **   # #    **#*   *#**#|#**#*   *#**    # #   **  **  **# *  #*****#** **
```

ALIGNMENT RESULTS - Dchc001B

=== Stwintron Sequence ===

Multiple sequence alignment

```
Dchc001B_fwrd       gguauguaccuaaugaaaacauuccguuuugcuguuuucgaaccuucuuuuuagaauugucccc------------------------gaguuauaguugcuaacgaucaauaguaagugugacgacccuauaauuucgacucgcccaagguuaugauucuauaaucguaugacuacaaaacuaauacaaaauguucccaaauag--------
Dchc001B_RevC       --------cuauuugggaacauuuuguauuaguuuuguagucauacgauuauagaaucauaaccuugggcgagucgaaauuauagggucgucacacuuacuauugaucguuagcaacuauaac------------------------ucgggacaauucuaaaaagaagguucgaaaacagcaaaacggaauguuuucauuagguacauacc
                            *    **  ******  ** **# * ** *  *     #  ** ******##*  *                         **#*#* **#***  *||*  ***#** *#*#**                           *  *##****** **  #     * * ** * #** **  ******  **    *
```

=== Internal intron ===

Multiple sequence alignment

```
Dchc001B_fwrd       ---------------------------guauguaccuaaugaaaacauuccguuuugcuguuuucgaaccuucuuuuuagaauugucccgaguuauaguugcuaacgaucaauag
Dchc001B_RevC       cuauugaucguuagcaacuauaacucgggacaauucuaaaaagaagguucgaaaacagcaaaacggaauguuuucauuagguacauac---------------------------
                                               * *##   **** #* **  *** #   ##|##   # ***  ** *# ****   ##* *
```

=== External intron ===

Multiple sequence alignment

```
Dchc001B_fwrd       guaagugugacgacccuauaauuucgacucgcccaagguuaugauucuauaaucguaugacuacaaaacu--------------------aauacaaaauguucccaaauag
Dchc001B_RevC       cuauuugggaacauuuugua--------------------uuaguuuuguagucauacgauuauagaaucauaaccuugggcgagucgaaauuauagggucgucacacuuac
                     **  ** **   *   * **                    *# ** * ** **#||#** ** * ** #                    * ** *   *  ** **  **
```

ALIGNMENT RESULTS - Dchc001C

=== Stwintron Sequence ===

Multiple sequence alignment

```
Dchc001C_fwrd    gguaaguacaaugaacuccuuucuaaaauuguuucgaaauauaguugcuaacagucaguaguaagugugacgauccuauaauucugaccuacuagaauacgaugaagauucuaggaucauugguuauagagcugacauguaauauuuucac---------auauuag----------------------
Dchc001C_RevC    ----------------------cuaauau---------gugaaaauauuacaugucagcucuauaaccaaugauccuagaaucuucaucguauucuaguaggucagaauuauaggaucgucacacuuacuacugacuguuagcaacuauauuucgaaacaauuuuagaaaggaguucauuguacuuacc
                                       **** **          *  *# *# **   *****#  ** #  ##* ******* ***# * * *   * | *   * * * #*** ******* *##  # **  #*****   ** #* #*  *          ** ****
```

=== Internal intron ===

Multiple sequence alignment

```
Dchc001C_fwrd    ---guaaguacaaugaacuccuuucuaaaauuguuucgaaauauaguugcuaacagucaguag
Dchc001C_RevC    cuacugacuguuagcaacuauauuucgaaacaauuuuagaaaggaguucauuguacuuac---
                     * * *   *  ****   ** # ***#|#*** # **   ****  *   * * *
```

=== External intron ===

Multiple sequence alignment

```
Dchc001C_fwrd    guaa-gugugacgauccuauaauucugaccuacuagaauacgaugaagauucuaggaucauug-------guuauagagcugacauguaauauuuucacauauuag
Dchc001C_RevC    cuaauaugugaaaauauuacaugucagcucuauaa-------ccaaugauccuagaaucuucaucguauucuaguaggucagaauuauaggaucgucacac-uuac
                  *** #***** #**  **#*  ** *  ***  *        ##* ***#*||*#*** *##        *  ***  * **  *#**  **# *****# ***
```

ALIGNMENT RESULTS - Dchc001D

=== Stwintron Sequence ===

Multiple sequence alignment

```
Dchc001D_fwrd       gguauguaugaaaacaucccuuuuauagagugguuucgaaauuaucuugauaagauaccuauacaguggcugacgcucagcaguaagugcga-----------ucgcccuaucauuucgaccuaaucgaaccccuuucucucucucucccccccccccccccccccccccccccggugaagauucuggaaguguaguaucgcaaagcugacguaaa----auguuuucauacaucag
Dchc001D_RevC       cugauguaugaaaaca----uuuuacgucagcuuugcgauacuacacuuccagaaucuucaccgggggggggggggggggggggggggagagagagagaaaggguucgauuaggucgaaaugauaggg-----------cgaucgcacuuacugcugagcgucagccacuguauagguaucuuaucaagauaauuucgaaaccacucuauaaaagggauguuuucauacauacc
                       *************    *****#   #   ** *** *#**# #*  #* #**   #*#   * **  *  *    *  *   * * **            *  * ** *  *||*  * ** *            *  ** * *   *  *    *  *  ** *   #*#   **# *#  *# #**#* *** **   #   #****    **************
```

=== Internal intron ===

Multiple sequence alignment

```
Dchc001D_fwrd       guauguaugaaaacaucccuuuuauagagugguuucgaaauuaucuugauaagauaccuauacaguggcugacgcucagcag--
Dchc001D_RevC       --cugcugagcgucagccacuguauagguaucuuaucaagauaauuucgaaaccacucuauaaaagggauguuuucauacauac
                       **#  #    ** ** #* *****     **   **  ||  **   **     ***** *# ** **    #  #**
```

=== External intron ===

Multiple sequence alignment

```
Dchc001D_fwrd       guaagugcgaucgcccuaucauuucgaccuaaucgaaccccuuucucucucucucccccccccccccccccccccccccccggugaagauucuggaaguguaguaucgcaaagcugacguaaaauguuuucauacaucag-----------------------
Dchc001D_RevC       -----------------------cugauguaugaaaacauuuuacgucagcuuugcgauacuacacuuccagaaucuucaccgggggggggggggggggggggggggagagagagagaaaggguucgauuaggucgaaaugauagggcgaucgcacuuac
                                           # **  **   #***   ** *  #  ** * *    *  * *  **     *  * || *  *     **  * *  *    * * **  #  * **   ***#   **  ** #
```

ALIGNMENT RESULTS - Dchc002A

=== Stwintron Sequence ===

Multiple sequence alignment

```
Dchc002A_fwrd       gguauguauaaagacgagacauguuaccccuggcccuucuucaaccuaccucucuguucuuucauguauaguugcuaauaauuaguaguaaguagaagaucccuguucucccaucaucuggcaaagaacauuca--aacuccuguaaagguaggaucgcgaa--------gcuaacacuagauuuucucaugcauuag
Dchc002A_RevC       cuaaugcaugagaaaaucuaguguua--------gcuucgcgauccuaccuuuacaggaguu--ugaauguucuuugccagaugaugggagaacagggaucuucuacuuacuacuaauuauuagcaacuauacaugaaagaacagagagguagguugaagaagggccaggggguaacaugucucgucuuuauacauacc
                       ***#** * #* #     *****         **** # * ******* * ##    **  ** **  *#  *  #*  * #* * *  # #  *||*  #  # * * *# *  *#  *  #*  ** **  **    ## * ******* * # ***        * *****     # *# * **#***
```

=== Internal intron ===

Multiple sequence alignment

```
Dchc002A_fwrd       gua------------------------------------------uguauaaagacgagacauguuaccccuggcccuucuucaaccuaccucucuguucuuucauguauaguugcuaauaauuaguag
Dchc002A_RevC       cuacuaauuauuagcaacuauacaugaaagaacagagagguagguugaagaagggccaggggguaacaugucucgucuuuauaca------------------------------------------uac
                     **                                          ** * ** * * **   *#|#*   ** * * ** * **                                          **
```

=== External intron ===

Multiple sequence alignment

```
Dchc002A_fwrd       gua----aguagaagaucc-------------cuguucucccaucaucuggcaaagaacauucaaacuccuguaaagguaggaucgcgaagcuaacacuagauuuucucaugcauuag
Dchc002A_RevC       cuaaugcaugagaaaaucuaguguuagcuucgcgauccuaccuuuacaggaguuugaauguucuuugccagaugaugggagaacag-------------ggaucuucu----acuuac
                     **    *  ****#***              * #*#** ** * *#  *#    ***||***    #*  #* * ** **#*# *              ***#****      ***
```

ALIGNMENT RESULTS - Dchc003A

=== Stwintron Sequence ===

Multiple sequence alignment

```
Dchc003A_fwrd       gguauguaugaaaacaucccguguuauucuuuugaaacccccuuucuagaacgguuucggaaccaccuagguauacaguagcuaacgcucaguaguaagugugauggcccuaucauuucgac-------------------uaaauaaaaaaagaacagugaagauucuagaauuguaguaaugcaaagcugauacgagauguuuucauacauuag
Dchc003A_RevC       cuaauguaugaaaacaucucguaucagcuuugcauuacuacaauucuagaaucuucacuguucuuuuu-------------------uuauuuagucgaaaugauagggccaucacacuuacuacugagcguuagcuacuguauaccuaggugguuccgaaaccguucuagaaaggggguuucaaaagaauaacacgggauguuuucauacauacc
                       *************** ***#*#* # ** ##  **  *  ********   *# * *  *    *                    *   ****  # #****#*||*#****# #  **                   ** *#  *    *  * * #*   ********  *  **  ## ** # *#*#*** ***************
```

=== Internal intron ===

Multiple sequence alignment

```
Dchc003A_fwrd       -------------------guauguaugaaaacaucccguguuauucuuuugaaacccccuuucuagaacgguuucggaaccaccuagguauacaguagcuaacgcucaguag
Dchc003A_RevC       cuacugagcguuagcuacuguauaccuaggugguuccgaaaccguucuagaaaggggguuucaaaagaauaacacgggauguuuucauacauac-------------------
                                       ****## *#      *** # ### ****   #*   |   *#   **** ### # ***      #* ##****
```

=== External intron ===

Multiple sequence alignment

```
Dchc003A_fwrd       guaagugugauggcccuaucauuucgacuaaauaaaaaaagaacagugaagauucuagaauuguaguaaugcaaagcugauacgagauguuuucauacauuag
Dchc003A_RevC       cuaauguaugaaaacaucucguaucagcuuugcauuacuacaauucuagaaucuucacuguucuuuuuuuauuuagucgaaaugauagggccaucacacuuac
                     ***       ## * * ** * **# **   #*  *  * **   *# *#|#* #*   ** *  *  *#   ** #** * ** * * ##       ***
```

ALIGNMENT RESULTS - Dchc003B

=== Stwintron Sequence ===

Multiple sequence alignment

```
Dchc003B_fwrd      gguauguaugaaaacaucccguguuguuauuuucaagcccccuuucuagaaaggucucgaaacuauccucguaagguauacaguggcuaacgcucaguaguaagugaugauagcccugccauuucgac-------------ccaaucgaaaaauaacgaugaagauuuuagaauu-----------guaguauugcaaagcuaacgcaaaauguuucauacauuag
Dchc003B_RevC      cuaauguaugaaaacauuuugcguuagcuuugcaauacu------------acaauucuaaaaucuucaucguuauuuuucgauugg------------gucgaaauggcagggcuaucauacuuacuacugagcguuagccacuguauaccuuacgaggauaguuucgagaccuuucuagaaagggggcuugaaaauaacaacacgggauguuuucauacauacc
                      **************   *#***# # ** # * #*             * ##* # #*** # ** **** *  * *  * ***             **  # *** #**||**# *** #  **             *** *  * *  * **** ** # ***# # *##           *  *# * # ** # #***#*   *************
```

=== Internal intron ===

Multiple sequence alignment

```
Dchc003B_fwrd      guau---------guaugaaaacaucccguguuguuauuuucaagcccccuuucuagaaaggucucgaaacuauccucguaagguauacaguggcuaacgcucaguag
Dchc003B_RevC      cuacugagcguuagccacuguauaccuuacgaggauaguuucgagaccuuucuagaaagggggcuugaaaauaacaacacgggauguuu---------ucauacauac
                    **#         *#      * *#*  ##*  * ** **** ** **   #* | *#   ** ** **** ** *  *##  *#* *              *  #**
```

=== External intron ===

Multiple sequence alignment

```
Dchc003B_fwrd      guaa-------------guaugauagcccugccauuucgacccaaucgaaaaauaacgaugaagauuuuagaauug---uaguauugcaaagcuaacgcaaaauguuucauacauuag
Dchc003B_RevC      cuaauguaugaaaacauuuugcguuagcuuugcaauacua---caauucuaaaaucuucaucguuauuuuucgauugggucgaaauggcagggcuaucaua-------------cuuac
                    ***               * #* ****  *** ** # #   ****   *****    ||    *****   ****   # # ** ***  **** *# *               ***
```

ALIGNMENT RESULTS - Dchc003C

=== Stwintron Sequence ===

Multiple sequence alignment

```
Dchc003C_fwrd      ------------------gguauguauaaaagcgcccccuucuuaugauuuucaucccccacucccuaaaacagguuucgaaguauacaguaacuaacguucaguaguaaguugcgaucacccuauugcuucgacucgcuc-----gaaaaagaacaguaaaacuuuagaaguguaagguuauaaaacuaacacaggauguuuccaucag
Dchc003C_RevC      cugauggaaacauccuguguuaguuuuauaaccuuacacuucuaaaguuuuacuguucuuuuuc-----gagcgagucgaagcaauagggugaucgcaacuuacuacugaacguuaguuacuguauacuucgaaaccuguuuuagggagugggggaugaaaaucauaagaagggggcgcuuuuauacauacc------------------
                                     * **  * ** ** *   * ***** * * *** *    *    **      *  *# *# #*   ***  ** * #   ##* * || * *##   # * **  ***   *# #* #*  *      **    *    * *** * * ***** *   * ** ** *  ** *
```

=== Internal intron ===

Multiple sequence alignment

```
Dchc003C_fwrd      gua-uguauaaaagcgcccccuucuuaugauuuucaucccccacucccuaaaacagguuucgaaguauacaguaa-------cuaacguucaguag
Dchc003C_RevC      cuacugaacguuag-------uuacuguauacuucgaaaccuguuuuagggagugggggaugaaaaucauaagaagggggcgcuuuuauaca-uac
                    ** ** *#   **       ** #* *#  #***    **   *  ||  *   **    ***#  #* *# **       **   #* ** **
```

=== External intron ===

Multiple sequence alignment

```
Dchc003C_fwrd      ---------------------------------guaaguugcgaucacccuauugcuucgacucgcucgaaaaagaacaguaaaacuuuagaaguguaagguuauaaaacuaacacaggauguuuccaucag
Dchc003C_RevC      cugauggaaacauccuguguuaguuuuauaaccuuacacuucuaaaguuuuacuguucuuuuucgagcgagucgaagcaauagggugaucgcaacuuac---------------------------------
                                                    ** ##* * *      **#** *#    ***||***    #* **#**      * * *## **
```

ALIGNMENT RESULTS - Dchc004A

=== Stwintron Sequence ===

Multiple sequence alignment

```
Dchc004A_fwrd       gguauguaucaaaaauacucuaugcuuguuauuuugaaaccccuuucuaua--auagucucgaaaccgccuucguaagguauacaguggcuaacguuuaguaguaagugugauagcccuaucauuucaacccaaucaaaaaaaaaauaacgccaaagauucuagaauuguaguaucguaaagcugauacgaaaug--------------uuugcauacauuag
Dchc004A_RevC       cuaauguaugcaaa--------------cauuucguaucagcuuuacgauacuacaauucuagaaucuuuggcguuauuuuuuuuuugauuggguugaaaugauagggcuaucacacuuacuacuaaacguuagccacuguauaccuuacgaaggcgguuucgagacuau--uauagaaaggggguuucaaaauaacaagcauagaguauuuuugauacauacc
                       ******  ***              #****#* * *  * ** * ***  *#*#* # # ** *     *** *  * *    **# *    *  *#* #** *  *#|#*  * **# *#*  *    * #**    * *  * ***     * ** # # *#*#*  *** * ** *  * * *#****#              ***  ******
```

=== Internal intron ===

Multiple sequence alignment

```
Dchc004A_fwrd       guauguaucaaaa--------auacucuaugcuuguuauuuugaaaccccuuucuauaauagucucgaaaccgccuucguaagguauacaguggcuaacguuuaguag
Dchc004A_RevC       cuacuaaacguuagccacuguauaccuuacgaaggcgguuucgagacuauuauagaaaggggguuucaaaauaacaagcauagaguau--------uuuugauacauac
                     **#  * *   *        ****# **#*   *#  ***#** **     * | *     ** **#***  #*   *#** #****        *   * *  #**
```

=== External intron ===

Multiple sequence alignment

```
Dchc004A_fwrd       guaagugugauagcccuaucauuucaacccaaucaaaaaaaaaauaacgccaaagauucuagaauuguaguaucguaaagcugauacgaaauguuugcauac----auuag
Dchc004A_RevC       cuaa----uguaugcaaacauuucguaucagcuuuacgauacuacaauucuagaaucuuuggcguuauuuuuuuuuugauuggguugaaaugauagggcuaucacacuuac
                     ***      **  *  *#  **#  * *   *  *  * *  *#**  * * *#|#* * *  **#*  * *  *  *   * *  #**  #*  *  **      ***
```

ALIGNMENT RESULTS - Dchc004B

=== Stwintron Sequence ===

Multiple sequence alignment

```
Dchc004B_fwrd   gguacguguaaaaauacucugucguugcuuu-caaccucccuauuauagaaugacaccuacacuaccuccuuuggguacacagugacugacguuuaauaguaagugauaaccuuaucauuuugacuuaaaag---------------------------gaacaggaaagauccuauaacuauaggauuguaaagcugauacagaaugauuu-auauaucag
Dchc004B_RevC   cugauauau-aaaucauucuguaucagcuuuacaauccuauaguuauaggaucuuuccuguuc---------------------------cuuuuaagucaaaaugauaagguuaucacuuacuauuaaacgucagucacuguguacccaaaggagguaguguaggugucauucuauaauagggagguug-aaagcaacgacagaguauuuuuacacguacc
                   * #*#* *** #* *****  # ***** *** *#     ****** **    ***   *                           * *****    **#******||******#**    ***** *                           *   ***    ** ******     #* *** ***** #  ***** *# *** *#*# *
```

=== Internal intron ===

Multiple sequence alignment

```
Dchc004B_fwrd   guacguguaaaaauacucugucguugcuuucaaccucccuauuauagaaugacaccuacacuaccuccuuuggguacacagugacugacguuuaauag-----------------------------------------
Dchc004B_RevC   -----------------------------------------cuauuaaacgucagucacuguguacccaaaggagguaguguaggugucauucuauaauagggagguugaaagcaacgacagaguauuuuuacacguac
                                                         #*** #**#* **  #**  *   #** | **#   *  **#  ** *#**# ***#
```

=== External intron ===

Multiple sequence alignment

```
Dchc004B_fwrd   guaagugauaaccuuaucauuuugacuuaaaaggaacaggaaagauccuauaacuauaggauu-------guaaagcugauacagaaugauuuauauaucag----
Dchc004B_RevC   ----cugauauauaaaucauucuguaucag-------cuuuacaauccuauaguuauaggaucuuuccuguuccuuuuaagucaaaaugauaagguuaucacuuac
                     *****     ******#**  *#*            * #********||********#        *     *#*  **#******     *****
```

ALIGNMENT RESULTS - Dchc004C

=== Stwintron Sequence ===

Multiple sequence alignment

```
Dchc004C_fwrd       gguauguauuaaaaugucccauauccuuauuuuguuagcccuauuccaaaauauucccaaacugcauucauuaggcauacaaugacuaacgcucaguaguaaguucgacaaucccuauuauuuugacucguuuguaaaggagucguggguauccuagaacuagaggauugagaaucuaauguggaauguuuucauauauuag
Dchc004C_RevC       cuaauauaugaaaacauuccacauuagauucucaauccucuaguucuaggauacccacgacuccuuuacaaacgagucaaaauaauagggauugucgaacuuacuacugagcguuagucauuguaugccuaaugaaugcaguuugggaauauuuuggaauagggcuaacaaaauaaggauaugggacauuuuaauacauacc
                       **#*** ****##* ***#**     *#*## *   *   *** *  ***##* * *  #   * **   *#  #* ***#*     # *    *##||##*    * #     *#*** *#  #*   ** *   #  * * *##***  * ***   *   * ##*#*     **#*** *##**** ***#**
```

=== Internal intron ===

Multiple sequence alignment

```
Dchc004C_fwrd       ---------guauguauuaaaaugucccauauccuuauuuuguuagcccuauuccaaaauauucccaaacugcauucauuaggcauacaaugacuaacgcucaguag
Dchc004C_RevC       cuacugagcguuagucauuguaugccuaaugaaugcaguuugggaauauuuuggaauagggcuaacaaaauaaggauaugggacauuuuaauacauac---------
                             **  **  *   ***#*  **     #* ****  *#   * * | * *   #*  **** *#     **  *#***   *  **  **
```

=== External intron ===

Multiple sequence alignment

```
Dchc004C_fwrd       guaaguucgacaaucccuauuauuuugacucguuuguaaaggagucguggguauccuagaacuagaggauugagaaucuaauguggaauguuuucauauauuag----------------
Dchc004C_RevC       ----------------cuaauauaugaaaacauuccacauuagauucucaauccucuaguucuaggauacccacgacuccuuuacaaacgagucaaaauaauagggauugucgaacuuac
                                    *** *** * #*  *#**#   *  # #*  * ##* # ****||**** # *## *  *# #  *   #**#*  *# * *** ***
```

ALIGNMENT RESULTS - Dchc005A

=== Stwintron Sequence ===

Multiple sequence alignment

```
Dchc005A_fwrd       gguauguaugaaaacauccccuguuguuauuuuuggacucucucucuauaauggucuugaaauauaguagcuaacucucaguaguaagugugaugaccccccucacuuagagucacucgaacauggcgaagacucuagaauuugcaggguugaaaagu--ugacacgaaauguuuucauacauuag
Dchc005A_RevC       cuaauguaugaaaacauuucguguca--acuuuucaacccugcaaauucuagagucuucgccauguucgagugacucuaagugaggggggucaucacacuuacuacugagaguuagcuacuauauuucaagaccauuauagagagagaguccaaaauaacaacaggggauguuuucauacauacc
                       **************  * ***##  *#**** #**#** #   * # * #*****         # # * ***** *** #   * ** || ** *   # *** ***** * # #         *****# * # *   # **#**# ****#*  ##*** *  **************
```

=== Internal intron ===

Multiple sequence alignment

```
Dchc005A_fwrd       guauguaugaaaacauccccuguuguuauuuuuggacucucucucuauaauggucuugaaauauaguagcuaacucucaguag------------------
Dchc005A_RevC       -------------------cuacugagaguuagcuacuauauuucaagaccauuauagagagagaguccaaaauaacaacaggggauguuuucauacauac
                                       **##**  * **    *** * * ** * * || * * ** * * ***   ** *  **##**
```

=== External intron ===

Multiple sequence alignment

```
Dchc005A_fwrd       guaa-gugugaugaccccccucacuuagagucacucgaacauggcgaagacucuagaauuugcaggguugaaaag----uugacacgaaauguuuucauacauuag
Dchc005A_RevC       cuaauguaugaaaacauuucgugucaa----cuuuucaacccugcaaauucuagagucuucgccauguucgagugacucuaagugaggggggucaucacac-uuac
                     *** **#*** #**    *    # *    *  *  *** # **#**  **||**  **#** # ***  *  *    * #    *    **# ***#** ***
```

ALIGNMENT RESULTS - Dchc005B

=== Stwintron Sequence ===

Multiple sequence alignment

```
Dchc005B_fwrd       ---------------gguauguauugacaauuauauauuguaauauccaaaaucuccuuuccuauaucguuucaaacauuuauuucaucuacaucggcuaacgaauaguagugaguaggauuguccuauuauuu-------------cgacuuacucaagaaauaaguuugaauc-----------------uucuagaauuauaagaccucaaagcuaacacaagaucuuucgguuugcuag
Dchc005B_RevC       cuagcaaaccgaaagaucuuguguuagcuuugaggucuuauaauucuagaa-----------------gauucaaacuuauuucuugaguaagucg-------------aaauaauaggacaauccuacucacuacuauucguuagccgauguagaugaaauaaauguuugaaacgauauaggaaaggagauuuuggauauuacaauauauaauugucaauacauacc---------------
                                   # # *** **# *  * *  * **#**** #   **                 * ******* * * *#*    **   ***            *#  *#*****#|#*****#*  #*              ***  **    *#* * * ******* *                 **   # ****#** *  * *  * #** *** # #
```

=== Internal intron ===

Multiple sequence alignment

```
Dchc005B_fwrd       guauguauug------------------acaauuauauauuguaauauccaaaaucuccuuuccuauaucguuucaaacauuuauuucaucuacaucggcuaacgaauaguag
Dchc005B_RevC       cuacuauucguuagccgauguagaugaaauaaauguuugaaacgauauaggaaaggagauuuuggauauuacaauauauaauugu------------------caauacauac
                     **#   *#*                  * ** * * *   ## ****   ***  |  ***   **** ##   * * * ** *                  *#*   #**
```

=== External intron ===

Multiple sequence alignment

```
Dchc005B_fwrd       ----------------------gugaguaggauuguccuauuauuucgacuuacucaagaaauaaguuugaaucuucuagaauuauaagaccucaaagcuaacacaagaucuuucgguuugcuag
Dchc005B_RevC       cuagcaaaccgaaagaucuuguguuagcuuugaggucuuauaauucuagaagauucaaacuuauuucuugaguaagucgaaauaauaggacaauccuacucac----------------------
                                          ** **#      *** *** ***# #    * ****#   |   #**** *    # #*** *** ***      #** **
```

ALIGNMENT RESULTS - Dchc007A

=== Stwintron Sequence ===

Multiple sequence alignment

```
Dchc007A_fwrd      gguacguacgaaaccaucccguguuuauuuugaaacuuccuuucuagaguagucucaaaauugccuucguagaguauacaguggc-------uaacguuuaguaguaaguguaacgaccauaucauucgacucaaucgaaagauaauggugaggauucuagaguuguaggguuaaaaagcugacacgaaauguuuucauaccuuag
Dchc007A_RevC      cuaagguaugaaaacauuucgugucagcuuuuuaacccuacaacucuagaauccucaccauuaucuuucgauugagucgaaugauauggucguuacacuuacuacuaaacguua-------gccacuguauacucuacgaaggcaauuuugagacuacucuagaaaggaaguuucaaaauaaacacgggaugguuucguacguacc
                      * *** **** ***  *****#  #***  ***##  #  **  ** * #****  ***# ***   *  *  *  *#**#        * **##*** || ***##** *        #**#*  *  *  *   *** #***  ****# * **  **  #  ##***  ***#  #*****  *** **** *** *
```

=== Internal intron ===

Multiple sequence alignment

```
Dchc007A_fwrd      gua------------cguacgaaaccaucccguguuuauuuugaaacuuccuuucuagaguagucucaaaauugccuucguagaguauacaguggcuaacguuuaguag
Dchc007A_RevC      cuacuaaacguuagccacuguauacucuacgaaggcaauuuugagacuacucuagaaaggaaguuucaaaauaaacacgggaugguuucguac------------guac
                    **            *##   * **  * * # * # ******* *** * #* | *# * *** ******* # * # * *  ** *   ##            ***
```

=== External intron ===

Multiple sequence alignment

```
Dchc007A_fwrd      guaa-guguaacgaccauaucauucgacucaaucgaaagauaauggugaggauucuagaguuguaggguuaaaaagcugacacgaaauguuuucauaccuuag
Dchc007A_RevC      cuaagguaugaaaacauuucgugucagcuuuuuaacccuacaacucuagaauccucaccauuaucuuucgauugagucgaaugauauggucguuacac-uuac
                    *** **#* * #**  * #   **# **   * #    *#**#  *# ##|## #*  #**#*    # *   ** #**   # *  **# * *#** ***
```

ALIGNMENT RESULTS - Dchc007B

=== Stwintron Sequence ===

Multiple sequence alignment

```
Dchc007B_fwrd      gguacguauuaaagcaucucauguuguuauuuucgacucuccuuuuuagaaugaucucgaauacaauggcuaauguuuaguaguaagugcgaugguccuaucaccuggauucucucgaacgcgaugauccuagaauuguaggguuacgaggcuaacacga-----uuucauaaauuag
Dchc007B_RevC      cuaauuuaugaaa-----ucguguuagccucguaacccuacaauucuaggaucaucgcguucgagagaauccaggugauaggaccaucgcacuuacuacuaaacauuagccauuguauucgagaucauucuaaaaaggagagucgaaaauaacaacaugagaugcuuuaauacguacc
                     *  *** ***     ** ****# # *# * # *#  *  **#*** ** *** **  #   * ## # * **   #  ## * #||# * ##  #    ** * # ## *   #  ** *** ** ***#**  *  #* # * #* # #**** **     *** ***  *
```

=== Internal intron ===

Multiple sequence alignment

```
Dchc007B_fwrd      guac---guauuaaagcaucucauguuguuauuuucgacucuccuuuuuagaaugaucucgaauacaauggc-----------uaauguuuaguag
Dchc007B_RevC      cuacuaaacauua-----------gccauuguauucgagaucauucuaaaaaggagagucgaaaauaacaacaugagaugcuuuaauac---guac
                    ***   ##****           *###** * *****   #  *#*||*#*  #   ***** * **###*           ****##   ***
```

=== External intron ===

Multiple sequence alignment

```
Dchc007B_fwrd      guaagugcgaugguccuaucaccuggauucucucgaacgcgaugauccuagaauuguaggguuacgaggcuaacacgauuucauaaauuag---------------
Dchc007B_RevC      ---------------cuaauuuaugaaaucguguuagccucguaacccuacaauucuaggaucaucgcguucgagagaauccaggugauaggaccaucgcacuuac
                                  ***     **#* **     * *    *#*#**** *||* ****#*#*    * *     ** *#**     ***
```

ALIGNMENT RESULTS - Dchc008A

=== Stwintron Sequence ===

Multiple sequence alignment

```
Dchc008A_fwrd       --gguauguauaaaaugcauuuaucauuuuuacaauguucgacuug---aaguggcuaauacucaguaguaagugggagaaacccuuuucguuucgacucucaagagagaacgucgaaauauauacauauauuagguauauauacaaccguagggguacaaagcuaauacaauauauuucaauauacuag
Dchc008A_RevC       cuaguauauugaaauauauuguauuagcuuuguaccccuacgguuguauauauaccuaauauauguauauauuucgacguucucucuugagagucgaaacgaaaaggguuucucccacuuacuacugaguauuagccac---uucaagucgaacauuguaaaaaugauaaaugcauuuuauacauacc--
                      #****#*  ***     * *** * #***  * # #*    ***   * #*# ******       #**  * *# *    * #**  *  *||*  *  **# *    * #* *  **#       ****** #*#   * ***    *# # *  ***# * *** *     ***  *#****#
```

=== Internal intron ===

Multiple sequence alignment

```
Dchc008A_fwrd       guauguauaaaaugcauuuaucauuuuuacaauguucgacuugaaguggcuaauacucaguag------
Dchc008A_RevC       ------cuacugaguauuagccacuucaagucgaacauuguaaaaaugauaaaugcauuuuauacauac
                           **    * ***  #**#**# *    #|#    * #**#**#  *** *    **
```

=== External intron ===

Multiple sequence alignment

```
Dchc008A_fwrd       guaagugggagaaacccuuuucguuucgacucucaagagagaacgucgaaauauauacauauauuagguauauauacaaccguagggguacaaagcuaauaca---auauauuucaauauacu-------------------------------------ag
Dchc008A_RevC       cu-------------------------------------aguauauugaaauauau---uguauuagcuuuguaccccuacgguuguauauauaccuaauauauguauauauuucgacguucucucuugagagucgaaacgaaaaggguuucucccacuuac
                     *                                     ** * #* *********   * ****** * * **# *   ||   * #** * * ****** *   ********* *# * **                                     *
```

ALIGNMENT RESULTS - Dchc011A

=== Stwintron Sequence ===

Multiple sequence alignment

```
Dchc011A_fwrd       gguaugu augaaaacgccccacguuguuacuuucgagcca-------------------------uucuuuauagaauggccucgaaacuacuucccuaagguauacagugg cuaacgcucaguaguaagugugauugacgacccuaucauuucgacccaaucgaaaaaaaaaaaccaacggugaagauucuggaaucauaguauugcaaagcuaacaugaaaugcuuccacgcauuag
Dchc011A_RevC       cuaaugcguggaagcauuucauguuagcuuugcaauacuaugauuccagaaucuucaccguugguuuuuuuuuuucgauugggucgaaaugauagggucgucaaucacacuuacuacugagcguuagccacuguauaccuuagggaaguaguuucgaggccauucuauaaaga-------------------------auggcucgaaaguaacaacguggggcguuucauacauacc
                       ***# ** ** *#   ** ***# #  * # # #* *                          ** *** *   ** *  ******  *      #   #  #*** * #***  *||*  ***# * ***#  #   #      *  ******  * **   * *** *                          ** *# # # *  # #*** **   #* ** ** #***
```

=== Internal intron ===

Multiple sequence alignment

```
Dchc011A_fwrd       guau---------guaugaaaacgccccacguuguuacuuucga-gccauucuuuauagaauggccucgaaacuacuucccuaagguauacaguggcuaacgcucaguag
Dchc011A_RevC       cuacugagcguuagccacuguauaccuuagggaaguaguuucgaggccauucuauaaagaaugg-cucgaaaguaacaacguggggcguuu---------ucauacauac
                     **#         *#      * #**  * *  # ** ****** ******** || ******* ******* ** #  * *  **# *              *  #**
```

=== External intron ===

Multiple sequence alignment

```
Dchc011A_fwrd       gu-----------------------------------------------aagugugauugacgacccuaucauuucgacccaaucgaaaaaaaaaaaccaacggugaagauucuggaaucauaguauugcaaagcuaacaugaaaugcuuccacgcauuag
Dchc011A_RevC       cuaaugcguggaagcauuucauguuagcuuugcaauacuaugauuccagaaucuucaccguugguuuuuuuuuuucgauugggucgaaaugauagggucgucaaucacacuu-----------------------------------------------ac
                     *                                               ** # * *##*  *    * *  ******  |  ******  * *    *  *##* * # **                                               *
```

ALIGNMENT RESULTS - Dchc013A

=== Stwintron Sequence ===

Multiple sequence alignment

```
Dchc013A_fwrd       -----------gguauaaaauuaauuugaaccccccuuuagaauguaugcaaucgcuaacguucagu-----aguaagucaugaugaucuuaucauuucgacucccucgaaaagaaaacgguaaagauucuggaauuacaagaauguaaagcugacaaaagauauauucguauauauag
Dchc013A_RevC       cuauauauacgaauauaucuuuugucagcuuuacauucuuguaauuccagaaucuuuaccguuuucuuuucgagggagucgaaaugauaagaucaucaugacuuacu-----acugaacguuagcgauugcauacauucuaaagggggguucaaauuaauuuuauacc-----------
                               ##****   **  *# *     *  *#* * *  * ## ****  ** ****   *     **  ****  #***** | *****#  ****  **     *   **** **  **** ## *  * * *#*  *     * #*  **   ****##
```

=== Internal intron ===

Multiple sequence alignment

```
Dchc013A_fwrd       guauaaaauuaauuugaaccccccuuuagaauguaugcaaucgcuaacguucaguag-----------------------
Dchc013A_RevC       -----------------------cuacugaacguuagcgauugcauacauucuaaagggggguucaaauuaauuuuauac
                                           ** # ***#**  ** || **  **#*** # **
```

=== External intron ===

Multiple sequence alignment

```
Dchc013A_fwrd       ------------------------------guaagucaugaugaucuuaucauuucgacucccucgaaaagaaaacgguaaagauucuggaauuacaagaauguaaagcugacaaaagauauauucguauauauag
Dchc013A_RevC       cuauauauacgaauauaucuuuugucagcuuuacauucuuguaauuccagaaucuuuaccguuuucuuuucgagggagucgaaaugauaagaucaucaugacuuac------------------------------
                                                   ** #*  *  *#** ##*  **#*  **#   *   ||   *   #**  *#**  *## **#*  *  *# **
```

ALIGNMENT RESULTS - Dchc014A

=== Stwintron Sequence ===

Multiple sequence alignment

```
Dchc014A_fwrd       ---------------gguauguauuaaacccccuauucuuccauaagguauauagugacuaacguuuaguaguaagugcaauugcuuaagaa----aggaacgauaaaaauuuuaggaauauaaag---cuaacacagaaucuuucacccauuag
Dchc014A_RevC       cuaaugggugaaaagauucuguguuag---cuuuauauuccuaaaauuuuuaucguuccu----uucuuaagcaauugcacuuacuacuaaacguuagucacuauauaccuuauggaagaauaggggguuuaauacauacc--------------
                                   # * *** ***    *  ***  *#* * **  * *** **  **    **#   **#** *||* **#**   #**    **  ** *** *  ** * *#*  ***  *    *** *** * #
```

=== Internal intron ===

Multiple sequence alignment

```
Dchc014A_fwrd       ---guauguauuaaacccccuauucuuccauaagguauauagugacuaacguuuaguag
Dchc014A_RevC       cuacuaaacguuagucacuauauaccuuauggaagaauaggggguuuaauacauac---
                        ** ## ***  * *  *** *#*  |  *#* ***  * *  *** ## **
```

=== External intron ===

Multiple sequence alignment

```
Dchc014A_fwrd       guaagugcaauugcuuaagaaaggaacgauaaaaauuuuaggaauauaaagcuaacacagaaucuuuuc--------acccauuag
Dchc014A_RevC       cuaaugg--------gugaaaagauucuguguuagcuuuauauuccuaaaauuuuuaucguuccuuucuuaagcaauugcacuuac
                     ***  *           #****#  *  *   * #**** #||# ****# *   *  *  #****#           *  ***
```

ALIGNMENT RESULTS - Dcoc02A

=== Stwintron Sequence ===

Multiple sequence alignment

```
Dcoc02A_fwrd        gguacguaugaaaacaucccguguuguuauuuucgaauccccuuucuaga--------------------auggucucgaaaccaccuucuuaagguauacaguggcuaacgcucacuaguaagugugaugguccuaucauuucgacucaaucgaaaaauaacggucaagauucuagaauuguaguauugcaaagcuaacacgaaaug-uuucauacauuag
Dcoc02A_RevC        cuaauguaug-aaacauuucguguuagcuuugcaauacuacaauucuagaaucuugaccguuauuuuucgauugagucgaaaugauaggaccaucacacuuacuagugagcguuagccacuguauaccuuaagaagggugguuucga--------------------gaccauucuagaaaggggauucgaaaauaacaacacgggauguuucauacguacc
                       * ***** ******  ******# # ** # # *#  *  *******                    ** *  ******  *     ##*  ##*#  * *#*  * || *  *#* *  #*##   *##     *  ******                     *  *********  * #* # # ** # #******  *** ******** *
```

=== Internal intron ===

Multiple sequence alignment

```
Dcoc02A_fwrd        guacguauga------------aaacaucccguguuguuauuuucgaauccccuuucuagaauggucucgaaaccaccuucuuaagguauacaguggcuaacgcucac---uag
Dcoc02A_RevC        cua---gugagcguuagccacuguauaccuuaagaagggugguuucgagaccauucuagaaaggggauucgaaaauaacaacacgggaugu-----------uuucauacguac
                     **    ***              * *#*  # *  * *  ******  **  *#*||*#*  **  ******  * *  * #  *#* *              ***    **
```

=== External intron ===

Multiple sequence alignment

```
Dcoc02A_fwrd        guaagugugaugguccuaucauuucgacucaaucgaaaaauaacggucaagauucuagaauuguag----uauugcaaagcuaacacgaaauguuucauacauuag------
Dcoc02A_RevC        ------cuaauguaugaaacauuucguguuagcuuugcaaua----cuacaauucuagaaucuugaccguuauuuuucgauugagucgaaaugauaggaccaucacacuuac
                           *#***     * *******  * * #     ****    # * #****||****# * #    ****     # * *  ******* *     ***#*
```

ALIGNMENT RESULTS - Dcoc02B

=== Stwintron Sequence ===

Multiple sequence alignment

```
Dcoc02B_fwrd        gguaug--------------------uauaaccc-------------------cccuaccuccuagaaugguuuccaaguauacaguaacuaacacucagcaguaaguugcgacgacccuaucauuucgacucacucgaaaaagaacaguaaaaauuccggaauuguaagguuauaaagcuaacacaggauguuuucaucag
Dcoc02B_RevC        cugaugaaaacauccuguguuagcuuuauaaccuuacaauuccggaauuuuuacuguucuuuuucgagugagucgaaaugauagggucgucgcaacuuacugcugaguguuaguuacuguauacuuggaaaccauucuaggagg-------------------uagggggguuaua--------------------cauacc
                       ***                    *******                    *  * * *  * ** **# *#  **  ***  **   #   *** * || * ***   #   **  ***  **  #* #** ** *  * *                   * *   *******                    ***
```

=== Internal intron ===

Multiple sequence alignment

```
Dcoc02B_fwrd        ---guauguauaaccccccuaccuccuagaaugguuuccaaguauacaguaacuaacacucagcag
Dcoc02B_RevC        cugcugaguguuaguuacuguauacuuggaaaccauucuaggagguagggggguuauacauac---
                        *  ** * *    *      * * *** || *** * *      *    * * **  *
```

=== External intron ===

Multiple sequence alignment

```
Dcoc02B_fwrd        ---------guaaguugcgacgacccuaucauuucgacucacucgaaaaagaacaguaaaaauuccggaauugu-----------------------aagguuauaaagcuaacacaggauguuuucaucag
Dcoc02B_RevC        cugaugaaaacauccuguguuagcuuuauaaccuu-----------------------acaauuccggaauuuuuacuguucuuuuucgagugagucgaaaugauagggucgucgcaacuuac---------
                             ##*  #** *  # *  *** *##*                        * *****||***** *                        *##* ***  * #  * **#  *##
```

ALIGNMENT RESULTS - Dcoc03A

=== Stwintron Sequence ===

Multiple sequence alignment

```
Dcoc03A_fwrd        gguauguaugaaaacauc---------------------ccguguuauuauucuagaa-------------------------------------------------------------------auaucuuucgagaauagucucggaaucauccucguaggguauacaguagcuaacgcucaguaguaaguuugauuaucauuucgacucaaucgaaaaauauuggcgaagauucuacaauuauagcauugcaaaacugacacaacugacacaauguuuucauaacuuag
Dcoc03A_RevC        cuaaguuaugaaaacauugugucaguugugucaguuuugcaaugcuauaauuguagaaucuucgccaauauuuuucgauugagucgaaaugauaaucaaacuuacuacugagcguuagcuacuguauacccuacgaggaugauuccgagacuauucucgaaagauau-------------------------------------------------------------------uucuagaauaauaac---------------------acgggauguuuucauacauacc
                       *  ***********                      * #**#*** *** *****                                                                   ***#*#* **** ** #* #||# *# ** **** *#*#***                                                                   ***** *** ***#*                     **   ***********   *
```

=== Internal intron ===

Multiple sequence alignment

```
Dcoc03A_fwrd        guauguaugaaaacaucccguguuauuauucuagaaauaucuuucgagaauagucucggaaucauccucguaggguauacaguagcuaacg---------------cucaguag
Dcoc03A_RevC        cua---------------cugagcguuagcuacuguauacccuacgaggaugauuccgagacuauucucgaaagauauuucuagaauaauaacacgggauguuuucauacauac
                     **               *  # # *** #      ***#*#* **** ** #* #||# *# ** **** *#*#***      # *** #                *  #**
```

=== External intron ===

Multiple sequence alignment

```
Dcoc03A_fwrd        --------------------guaaguuugauuaucauuucgacuc---aaucgaaaaauauuggcgaagauucuacaauuauagcauugcaaaacugacacaacugacacaauguuuucauaacuuag
Dcoc03A_RevC        cuaaguuaugaaaacauugugucaguugugucaguuuugcaaugcuauaauuguagaaucuucgccaauauuuuucgauu---gagucgaaaugauaaucaaacuuac--------------------
                                        ** ****   *#*   ** *#*  *   *** * * *** ** || ** *** * * ***   *  *#* **   *#*   **** **
```

ALIGNMENT RESULTS - Dcoc05A

=== Stwintron Sequence ===

Multiple sequence alignment

```
Dcoc05A_fwrd        gguauguauaaau---aauuauguauugugaccuccaaaacucccuuuaucggauaguuuccuagauuucuuuuauguacaauggcuaaugcauagcagugagugagaugauccuauuauuucg------------------------acuuaagaaauaauuuugaaacuucuagaaugguaggauuucuaagcuaacacaagau--uucucggguugguag
Dcoc05A_RevC        -----cuaccaacccgagaaaucuuguguuagcuuagaaauccuaccauucuagaaguuucaaaauuauuucuuaagu------------------------cgaaauaauaggaucaucucacucacugcuaugcauuagccauuguacauaaaagaaaucuaggaaacuauccgauaaagggaguuuuggaggucacaauacauaauuauuuauacauacc
                          **# **#   *   ** *  *** * **   *** #*  ##  ** #  ******  *# * * *#*** **                         #**#**#*||  **#**#**#                         ** ***#* * * #*  ******  # **  ##  *# ***   ** * ***  * **  ** *  #  ##
```

=== Internal intron ===

Multiple sequence alignment

```
Dcoc05A_fwrd        ---guauguauaaauaauuauguau---ugugaccuccaaaacucccuuuaucggauaguuuccuagauuucuuuuauguacaauggcuaaugcauagcag
Dcoc05A_RevC        cugcuaugcauuagccauuguacauaaaagaaaucuaggaaacuauccgauaaagggaguuuuggaggucac---aauacauaauuauuuauacauac---
                        ****#** * # *** *##**    * #* **   *****  *#  |  #*  *****   ** *# *    **##* *** # * **#****
```

=== External intron ===

Multiple sequence alignment

```
Dcoc05A_fwrd        gugagugagaugauccuauuauuucgacuuaagaaa-------uaauuuugaaacuucuagaaugguaggauuucuaagcuaacacaagauuucucggguugguag--------------------
Dcoc05A_RevC        --------------------cuaccaacccgagaaaucuuguguuagcuuagaaauccuaccauucuagaaguuucaaa-------auuauuucuuaagucgaaauaauaggaucaucucacucac
                                         * #*#**## *****      * * #**# ** *#***  ||  ***#* ** #**#      *  ****** ##**#*# *
```

ALIGNMENT RESULTS - Dcoc06A

=== Stwintron Sequence ===

Multiple sequence alignment

```
Dcoc06A_fwrd        gguauguauaaaaaagcauuucguaugauuguu------------------------------------------------uucgaaauguuugacuugcaguagcuaauaguuaauaguaagugggauaaacucugucauuucgacucucucaagagaaugucgaaguauauauacguacaacgguaggguugcaaagcuaauacaagaugcuucgauauacuag--
Dcoc06A_RevC        --cuaguauaucgaagcaucuuguauuagcuuugcaacccuaccguuguacguauauauacuucgacauucucuugagagagucgaaaugacagaguuuaucccacuuacuauuaacuauuagcuacugcaagucaaacauuucga------------------------------------------------aaacaaucauacgaaaugcuuuuuuauacauacc
                      #  *****   ******#* **** * # **                                                 ******** # ** **    # #** *# #*||*# #* **# #    ** ** # ********                                                 ** # * **** *#******   *****  #
```

=== Internal intron ===

Multiple sequence alignment

```
Dcoc06A_fwrd        guauguauaaaaaagcauuucguaugauuguuuucgaaauguuugacuugcaguagcuaauaguuaauag
Dcoc06A_RevC        cuauuaacuauuagcuacugcaagucaaacauuucgaaaacaaucauacgaaaugcuuuuuuauacauac
                     ***  *# *  *   *#* *#  * *    ***||***    * *  #* *#*   *  * #*  ***
```

=== External intron ===

Multiple sequence alignment

```
Dcoc06A_fwrd        -------------guaagugggauaaacucugu-----------------------cauuucgacucucucaagagaaugucgaaguauauauacguacaacgguaggguugcaaagcuaauacaagaugcuucgauauacuag
Dcoc06A_RevC        cuaguauaucgaagcaucuuguauuagcuuugcaacccuaccguuguacguauauauacuucgacauucucuugagagagucgaaaug-----------------------acagaguuuaucccacuuac-------------
                                 *#*  * * ** * ** **#                        *#******  ****||****  ******#*                        #** ** * ** * *  *#*
```

ALIGNMENT RESULTS - Dcoc11A

=== Stwintron Sequence ===

Multiple sequence alignment

```
Dcoc11A_fwrd        gguauguauaaaaaugacccgu-----uuuauuacuucccaaccccuauuccuucguaagguauacaguugcuaacauuuaguaguaagugccaucacuuaauaaaaaaaaagaacgaugaagauuuuagaauuauaaagcugacacagaauguuuucacgcauuag
Dcoc11A_RevC        cuaaugcgugaaaacauucugugucagcuuuauaauucuaaaaucuucaucguucuuuuuuuuuauuaagugauggcacuuacuacuaaauguuagcaacuguauaccuuacgaaggaauagggguugggaagua-----auaaaacgggucauuuuuauacauacc
                       ***# * ****##  * **     #**  ** ***  **  * *  ** *** *    *    *# ** *  **#*** || ***#**  * ** #*     *    * *** **  * *  **  *** **      *#* ** *  ##**** * #***
```

=== Internal intron ===

Multiple sequence alignment

```
Dcoc11A_fwrd        guauguauaaaaaugacccguuuuauuacuucccaaccccuauuccuucguaagguauacaguugcuaacauuua---guag
Dcoc11A_RevC        cuac---uaaauguuagcaacuguauaccuuacgaaggaauagggguugggaaguaauaaaacgggucauuuuuauacauac
                     **#   ****  * * * ##* ***  *** * **    ||    ** * ***  *** *## * * *  ****   #**
```

=== External intron ===

Multiple sequence alignment

```
Dcoc11A_fwrd        guaagugccaucacuuaauaaaaaaaaagaacgaugaagauuuuagaauuauaaagcugacacagaauguuuucacgc------------auuag
Dcoc11A_RevC        cuaa------------ugcgugaaaacauucugugucagcuuuauaauucuaaaaucuucaucguucuuuuuuuuuauuaagugauggcacuuac
                     ***              #   **** #  *  **  # #**** *||* ****# #  **  *  # ****   #               ***
```

ALIGNMENT RESULTS - Dcoc20A

=== Stwintron Sequence ===

Multiple sequence alignment

```
Dcoc20A_fwrd        gguauacaauuaacccauucuacuuuuaacccccccuuuaggagguuuacaaccgcuaacgcccaau---------aguaaguggugaugauccuaucauuucgacucgcucgaaaaagaaaagaacauuaagguucuagaauuacaggauuauaaggcugacgcaagauguauucguauaauuag
Dcoc20A_RevC        cuaauuauacgaauacaucuugcgucagccuuauaauccuguaauucuagaaccuuaauguucuuuucuuuuucgagcgagucgaaaugauaggaucaucaccacuuacu---------auugggcguuagcgguuguaaaccuccuaaaggggguuaaaaguagaaugguuaauuguauacc
                       **   *# **  ***# * * *#   *      *#  * ##**#    * *  *** *  *  *         **# *** * #***** | *****# * *** #**         *  *  * ***  * *   #**## *  #*      *   #* * * #***  ** #*   **
```

=== Internal intron ===

Multiple sequence alignment

```
Dcoc20A_fwrd        guauacaauuaacccauucuacuuuuaacccccccuuuaggagguuuacaaccgcuaacgcccaauag-----
Dcoc20A_RevC        -----cuauugggcguuagcgguuguaaaccuccuaaaggggguuaaaaguagaaugguuaauuguauac
                         * ***   *  *  #  ** *** ** ***||*** ** *** **  #  *  *   *** *
```

=== External intron ===

Multiple sequence alignment

```
Dcoc20A_fwrd        guaaguggugaugauccuaucauuucgacucgcucgaaaaagaaaagaacauuaagguucuagaauuacaggauuauaagg-----------cugacgcaagauguauucguauaauuag----------
Dcoc20A_RevC        ----------cuaauuauacgaauacaucuugc-----------gucagccuuauaauccuguaauucuagaaccuuaauguucuuuucuuuuucgagcgagucgaaaugauaggaucaucaccacuuac
                               *#**  **# * * *# ** **              * * *** ##*#**  *||*  **#*## *** *            *   ** ** #* * * #**  **#*
```

ALIGNMENT RESULTS - Dcoc38A

=== Stwintron Sequence ===

Multiple sequence alignment

```
Dcoc38A_fwrd        gguacgucugauaugcgucccauguuguuauuuucaaucaucccuuucuaaagugaucucgaauccgccuuuauaagguauauaguggcuaacgcucaguaguaagugugaaaguccuaucauuucggcucauucgaaaaaggauu-gugaauauuuuaagacgcuaggauugugaa-gcugacacagaaugu-uuucauacauaag
Dcoc38A_RevC        cuuauguauga-aaacauucugugucagc-uucacaauccuagcgucuuaaaauauucac-aauccuuuuucgaaugagccgaaaugauaggacuuucacacuuacuacugagcguuagccacuauauaccuuauaaaggcggauucgagaucacuuuagaaagggaugauugaaaauaacaacaugggacgcauaucagacguacc
                      ** ** *** * #*#* *  ***## # **# ***** *  * *# ****#*# ** * *****   **#  * *#  #  *#**#       *  ##* *|* *##  *       #**#*  #  #* *  #**   ***** * ** #*#**** #* *  * ***** #** # ##***  * *#*# * *** ** **
```

=== Internal intron ===

Multiple sequence alignment

```
Dcoc38A_fwrd        guacgucugauaugcgucccauguuguuauuuucaaucaucccuuucuaaagugaucucgaauccgccuuuauaagguau-----------auaguggcuaacgcucaguag------
Dcoc38A_RevC        ------cuacugagcguuagccacuau-----------auaccuuauaaaggcggauucgagaucacuuuagaaagggaugauugaaaauaacaacaugggacgcauaucagacguac
                          **# *  ****    ###*#*           ** ****   ** *#*   *||*   *#* **   **** **           *#*###    ****  * #**
```

=== External intron ===

Multiple sequence alignment

```
Dcoc38A_fwrd        ----------------------guaagugugaaaguccuaucauuucggcucauucgaaaaaggauugugaauauuuuaagacgcuaggauugugaagcugacacagaauguuuucauacauaag
Dcoc38A_RevC        cuuauguaugaaaacauucugugucagcuucacaauccuagcgucuuaaaauauucacaauccuuuuucgaaugagccgaaaugauaggacuuucacacuuac----------------------
                                          ** **# * * *#***** * *#* ##   ****# **  |  ** #****   ## *#* * *****#* * * #** **
```

ALIGNMENT RESULTS - Dcoc41A

=== Stwintron Sequence ===

Multiple sequence alignment

```
Dcoc41A_fwrd      gguacguaugaaaacgucccaugcuguuauuuucaaauccaauucuagaacagucucgaacugcauucauuagguuuacaguagcuaacgcucaguaguaagugcgaugacccuaugauuucgacucgcuggaaaagggaccuuggguauucuacaacuagaggguuu------------------------------uaaugcuaauacggaacgcuuucauacaucag
Dcoc41A_RevC      cugauguaugaaagcguuccguauuagcauu------------------------------aaaacccucuaguuguagaauacccaaggucccuuuuccagcgagucgaaaucauagggucaucgcacuuacuacugagcguuagcuacuguaaaccuaaugaaugcaguucgagacuguucuagaauuggauuugaaaauaacagcaugggacguuuucauacguacc
                     * ******** *** ** *# *# #***                              # *##* #*** * ** *#** *#** * #*  *  # **#* * #**   #||#   **# * *#** #  *  *# * **#* **#* ** * ***# *##* #                              ***# #* #* ** *** ******** *
```

=== Internal intron ===

Multiple sequence alignment

```
Dcoc41A_fwrd      guacguaugaaaacgucccaugcuguuauuuu--caaauccaauucuagaacagucucgaacugcauucauuagguuuacaguagcuaacgcuc-----aguag
Dcoc41A_RevC      cuac-----ugagcguuagcuacuguaaaccuaaugaaugcaguucgagacuguucuagaauuggauuug--aaaauaacagcaugggacguuuucauacguac
                   ***       * ***    *#**** * ##*    *** ** *** *** || *** *** ** ***    *## * ****#*    *** *       ***
```

=== External intron ===

Multiple sequence alignment

```
Dcoc41A_fwrd      ----------------------guaagugcgaugacccuaugauuucgacucgcuggaaaagggaccuuggguauucuacaacuagaggguuuuaaugcuaauacggaacgcuuucauacaucag
Dcoc41A_RevC      cugauguaugaaagcguuccguauuagcauuaaaacccucuaguuguagaauacccaaggucccuuuuccagcgagucgaaaucauagggucaucgcacuuac----------------------
                                        #* **##  * #***** *# **  #    #*# #*    |    *# #*#    #  ** #* *****# *  ##** *#
```

ALIGNMENT RESULTS - Desc187A

=== Stwintron Sequence ===

Multiple sequence alignment

```
Desc187A_fwrd       --gguacguauaaacacgucccgc--auuguuguuuuucguaaccuaauucuagaaugguucuaaagcugcuuuuauauaacuaagcuuuacgauugcugaccuucaguaguaagugucaucguccugccuauugacggacucaaaaagggagaauacgauagagcuacaagagugcacagcugacuuggaguguuuucauauaccag
Desc187A_RevC       cugguauaugaaaacacuccaagucagcugugcacucuuguagcucuaucguauucucccuuuuugaguccgucaauaggcaggacgaugacacuuacuacugaaggucagcaaucguaaagcuuaguuauauaaaagcagcuuuagaaccauucuagaauuagguuacgaaaaacaacaau--gcgggacguguuuauacguacc--
                      **** #*  ****** #*  *    #***   #*#* *** * # **# **   *  #* *   # * * *# ***      *   * **# **#**#   ||   #**#** #** *   *      *** #* * * #   * *#  *   ** #** # * *** *#*#   ***#   *  *# ****** *# ****
```

=== Internal intron ===

Multiple sequence alignment

```
Desc187A_fwrd       guacguauaaacacgucccgcauug-uuguuuuucguaaccuaauucuagaaugguucuaaagcugcuuuuauauaacuaagcuuuacgauugcugaccuucaguag
Desc187A_RevC       cuacugaaggucagcaaucguaaagcuuaguuauauaaaagcagcuuuagaaccauucuagaauuagguuacgaaaaacaa-caaugcgggacguguuuauacguac
                     ***  *    **     ** *  * **# ** *   **  #* #* *****#|#***** *# *#  **   * ** #** *  * **     **    *  ***
```

=== External intron ===

Multiple sequence alignment

```
Desc187A_fwrd       guaagugucaucguccugccuauugacggacucaaaaagggagaauacgauagagcuacaagagugcacagcugacuuggaguguuuucauauacc---------------------------------------ag
Desc187A_RevC       cu---------------------------------------gguauaugaaaacacuccaagucagcugugcacucuuguagcucuaucguauucucccuuuuugaguccgucaauaggcaggacgaugacacuuac
                     *                                        * *** ** *# #** ****   ** | **   **** **# #* ** *** *                                        *
```

ALIGNMENT RESULTS - Desc198A

=== Stwintron Sequence ===

Multiple sequence alignment

```
Desc198A_fwrd       gguacgu-augaaaacauccugcuuuguuauuuucuuaucgccauucu---------auacugcuuuacaaacugccuuuauaagaugu-gcaauagcuaacguuuaauaguaaguuacgaugacgucguauauugacucucgauaaggaacggugaagguauuagaguaguaagauugcaaagcugacacgagauguuucucuuauacuag
Desc198A_RevC       cuaguauaagagaaacaucucgugucagcuuugcaaucuuacuacucuaauaccuucaccguuccuuaucgagagucaauauacgacgucaucguaacuuacuauuaaacguuagcuauugc-acaucuuauaaaggcaguuuguaaagcagua---------uagaauggcgauaagaaaauaacaaagcaggauguuuucau-acguacc
                         #* * # ******* #*  *## # ** #  * * #* *#***         *#  * *#***   *  * *  **** **#** #   **#** **  *||*  ** **#**   # **#** ****  * *  *   ***#* *  #         ****#* *# * *  # ** # ##*  *# ******* # * *#
```

=== Internal intron ===

Multiple sequence alignment

```
Desc198A_fwrd       guacguaugaaaacauccugcuuuguuauuuucuuaucgccauucuauacugcuuuacaaacugccuuuauaagaugugcaauagcuaacguuu-----aauag
Desc198A_RevC       cuau-----uaaacguuagcuauugcacaucuuauaaaggcaguuuguaaagcaguauagaauggcgauaagaaaauaacaaagcaggauguuuucauacguac
                     **       **** *      ***#   *#*  **  * ** * * **  ||  ** * * ** *  **  *#*   #***      * ****       **
```

=== External intron ===

Multiple sequence alignment

```
Desc198A_fwrd       guaaguuacgaugacgucguauauugacucucgauaaggaacggugaagguauuagaguaguaagauugcaaagcugacacgagauguuucucuuau----------------------------acuag
Desc198A_RevC       cuag----------------------------uauaagagaaacaucucgugucagcuuugcaaucuuacuacucuaauaccuucaccguuccuuaucgagagucaauauacgacgucaucguaacuuac
                     **                              *****# * #      ** *#**  * *#**||**#* *  **#* **      # * #*****                              **
```

ALIGNMENT RESULTS - Desc274A

=== Stwintron Sequence ===

Multiple sequence alignment

```
Desc274A_fwrd       gguacguacaaaauuguccuguguuguuauuuucagaauuucauucuagaauggucucaaauagcuaacguuuaguaguaaguuuaccaaccccuauucacuaauuuauucgagaacgguaaagacucuauuguaagacuaacaauaga-------uucguauauuag
Desc274A_RevC       cuaauauacgaa-------ucuauuguuagucuuacaauagagucuuuaccguucucgaauaaauuagugaauagggguugguaaacuuacuacuaaacguuagcuauuugagaccauucuagaaugaaauucugaaaauaacaacacaggacaauuuuguacguacc
                       * #*** **       * *#****** *#* * ***    *# * #     # # **  *# **  *  ***  **  **||**  **  ***  *  ** #*  ** # #     # * #*    *** * *#* ******#* *       ** ***# *
```

=== Internal intron ===

Multiple sequence alignment

```
Desc274A_fwrd       guac------------guacaaaauuguccuguguuguuauuuucagaauuucauucuagaauggucucaaauagcuaacguuuaguag
Desc274A_RevC       cuacuaaacguuagcuauuugagaccauucuagaaugaaauucugaaaauaacaacacaggacaauuuuguac------------guac
                     ***            #*   * *###* **# # **  ***#*|*#***  ** # #** *###* *   *#            ***
```

=== External intron ===

Multiple sequence alignment

```
Desc274A_fwrd       guaaguuuaccaaccccuauucacuaauuuauucgagaacgguaaagacucuauuguaagacuaacaauagauuc--------------guauauuag-----------------------
Desc274A_RevC       -----------------------cuaauauac--------------gaaucuauuguuagucuuacaauagagucuuuaccguucucgaauaaauuagugaauagggguugguaaacuuac
                                           ***** **#              ** ******** **|** ******** **              #** *****
```

ALIGNMENT RESULTS - Desc420A

=== Stwintron Sequence ===

Multiple sequence alignment

```
Desc420A_fwrd       gguacguauggaaacgucucguauuauuauauuuaaaucaucauuccuaaauaaucaagcauuaauagcuaacu--uucaguaguaaguacagugauaucuauuaaaccacuuaaaaagggaugggguaauguucuagcgauguaggauugca-aagcuaauagggggugucuc-auauacuag
Desc420A_RevC       cuaguauau-gagacacccccuauuagc-uuugcaauccuacaucgcuagaacauuacccaucccuuuuuaagugguuuaauagauaucacuguacuuacuacugaa--aguuagcuauuaaugcuugauuauuuaggaaugaugauuuaaauauaauaauacgagacguuuccauacguacc
                         #*** ** **##*#* ***** # * * #** #*  ***# *** *  ** *  ***#  *   *** *  ** *#***  * #**|**# *  ***#* **  * ***   *  #***  * **  * *** #***  *# **# * * # ***** *#*##** ** ***#
```

=== Internal intron ===

Multiple sequence alignment

```
Desc420A_fwrd       guacguauggaaacgucucguauuauuauauuuaaaucaucauuccuaaauaaucaagcauuaauagcuaacuuucaguag----------------------
Desc420A_RevC       ----------------------cuacugaaaguuagcuauuaaugcuugauuauuuaggaaugaugauuuaaauauaauaauacgagacguuuccauacguac
                                          #**#*  *  * * # ** * * **  **|**  ** * * ** # * *  *  *#**#
```

=== External intron ===

Multiple sequence alignment

```
Desc420A_fwrd       guaaguacagugauaucuauuaaaccacuuaaaaagggaugggguaauguucuagcgauguaggauugcaaagcuaa-------------------uagggggugucucauauacuag---------
Desc420A_RevC       ---------cuaguauaugagacacccccua-------------------uuagcuuugcaauccuacaucgcuagaacauuacccaucccuuuuuaagugguuuaauagauaucacuguacuuac
                              *# *** *   * *** *#**                    ****  **#*#||#*#**  ****                    **#* *** *   * *** #*
```

ALIGNMENT RESULTS - Desc618A

=== Stwintron Sequence ===

Multiple sequence alignment

```
Desc618A_fwrd       --gguauguauaaaaaugucauucuagaauacgcuuugaaaaugcccuuacaagauau---acgauagcuaacguuuaguaguaagugcgaugaucuuguauaucgacugccgauaaggaacgguaaagauuuuggaauagcaugauugcaaag----cugacacgauauguuuuaacaccuuag
Desc618A_RevC       cuaagguguuaaaacauaucgugucag----cuuugcaaucaugcuauuccaaaaucuuuaccguuccuuaucggcagucgauauacaagaucaucgcacuuacuacuaaacguuagcuaucg---uauaucuuguaagggcauuuucaaagcguauucuagaaugacauuuuuauacauacc--
                      ##  ***  *** **#** *  #**    *  * ##*  ****  ** ***#** *    ** *   ** ** #   # #** ### ***|*** ### **# #   # ** **   * **    * **#*** **  ****  *## *  *    **#  * **#** ***  ***  ##
```

=== Internal intron ===

Multiple sequence alignment

```
Desc618A_fwrd       -------------------guauguauaaaaaugucauucuagaauacgcuuugaaaaugcccuuacaagauauacgauagcuaacguuuaguag
Desc618A_RevC       cuacuaaacguuagcuaucguauaucuuguaagggcauuuucaaagcguauucuagaaugacauuuuuauacauac--------------------
                                       ****#* *   ** * **** * #**  |  **# * **** * **   * *#****
```

=== External intron ===

Multiple sequence alignment

```
Desc618A_fwrd       -------------------------------guaagugcgaugaucuuguauaucgacugccgauaaggaacgguaaagauuuuggaauagcaugauugcaaagcugacacgauauguuuuaacaccuuag
Desc618A_RevC       cuaagguguuaaaacauaucgugucagcuuugcaaucaugcuauuccaaaaucuuuaccguuccuuaucggcagucgauauacaagaucaucgcacuuac-------------------------------
                                                   *#** ## * *# **# # ** *  **#*    *|*    *#**  * ** # #** #* * ## **#*
```

ALIGNMENT RESULTS - Desc640A

=== Stwintron Sequence ===

Multiple sequence alignment

```
Desc640A_fwrd      gguauguauaaaaaugucuuggauuguuauuuccaugcccccuuuucauaaucaucucgaaaauugucuuu--augauguauacaguggcuaauguucaguaguaaguuuugacuuagucgacaaagaaccguggagauucuagauuuguaggaacacgaagcugacggugaaauguuuacuuaucag----
Desc640A_RevC      ----cugauaaguaaacauuucaccgucagcuucguguuccuacaaaucuagaaucuccacgguucuuugucgacuaagucaaaacuuacuacugaacauuagccacuguauacaucau--aaagacaauuuucgagaugauuaugaaaagggggcauggaaauaacaauccaagacauuuuuauacauacc
                       #  ****  * ## **  *##**#* #* * **  **  #    # *  ***** *   ** * * *  *# * **  * * * #*** **||** ***# * * *  ** * #*  * * * **   * *****  * #    #  **  ** * *# *#**##*  ** ## *  ****  #
```

=== Internal intron ===

Multiple sequence alignment

```
Desc640A_fwrd      guauguauaaaaaugucuuggauuguuauuuccaugcccccuuuucauaaucaucucgaaaauugucuuuaugauguauacaguggcuaauguucag------------uag
Desc640A_RevC      cua------------cugaacauuagccacuguauacaucauaaagacaauuuucgagaugauuaugaaaagggggcauggaaauaacaauccaagacauuuuuauacauac
                    **            #   # ***# #  #*  **#*  * *    *#***  **||**  ***#*    * *  *#**  *#  # #*** #   #            **
```

=== External intron ===

Multiple sequence alignment

```
Desc640A_fwrd      guaaguuuugacuuagucgacaaagaaccguggagauucuagauuuguaggaacacgaagcugacggugaaauguuuacuuaucag----------------
Desc640A_RevC      ----------------cugauaaguaaacauuucaccgucagcuucguguuccuacaaaucuagaaucuccacgguucuuugucgacuaagucaaaacuuac
                                   # ** **  ** *#*   # #  #** **#**  ||  **#** **#  # #   *#* **  ** ** #
```

ALIGNMENT RESULTS - HCOc002A

=== Stwintron Sequence ===

Multiple sequence alignment

```
HCOc002A_fwrd       ------------------------------gguauguucagguacucagugucuagaaa----------------------------gaccucaaaauuucuaaaauauaggaacaagcuaacaucauaacaguaaguaaagaauucuaauaucgccgcuguccagauuaaaaaggcgauacuugguacauauugaaaacaaaaggucaagaugcugacacgacauguugcgauacacuag
HCOc002A_RevC       cuaguguaucgcaacaugucgugucagcaucuugaccuuuuguuuucaauauguaccaaguaucgccuuuuuaaucuggacagcggcgauauuagaauucuuuacuuacuguuaugauguuagcuuguuccuauauuuuagaaauuuugaggu----------------------------cuuucuagacacugaguaccuga------------------------------acauacc
                                                     *   #*   **  ***#*#* **  **                            **  * * ****# * *  **# *  *  *  *|*  *  *  * #**  * * #**** * *  *                            ***  ** *#*#***  **   *                               ***
```

=== Internal intron ===

Multiple sequence alignment

```
HCOc002A_fwrd       gu------auguucagguacucagugucuagaaagaccucaaaauuucuaaaauauaggaacaagcuaacaucauaacag
HCOc002A_RevC       cuguuaugauguuagcuuguuccuauauuuuagaaauuuugaggucuuucuagacacugaguaccugaacau------ac
                     *      *****    *  **      *  * *#*  *||*  *#*  *  *      **  *    *****      *
```

=== External intron ===

Multiple sequence alignment

```
HCOc002A_fwrd       guaaguaaagaauucuaauaucgccgcuguccagauuaaaaaggcgauacuugguacauauugaaaacaaaaggucaagaugcugacacgacauguugcgauac------------acuag
HCOc002A_RevC       cuag------------uguaucgcaacaugucgugucagcaucuugaccuuuuguuuucaauauguaccaaguaucgccuuuuuaaucuggacagcggcgauauuagaauucuuuacuuac
                     **               ****** #*    *   *#*  *    **#  ** **   #*|*#   ** **  #**     *  *#*   *    *# ******               **
```

ALIGNMENT RESULTS - HCOc004A

=== Stwintron Sequence ===

Multiple sequence alignment

```
HCOc004A_fwrd       ggugaguaucaaaacacgucguccuggcauuuuaguccuuaauuucuaaagaaaaaaaaac-----------ccuaaauuucuacaacauaggaa----uaaacuaacauuaugaauaguaaguuaaccguuccuauuucguuggcaucgggguuaagagaaaugucuaguacauuccagaaaccagggauaaaaaugcugacacgacaugucuuaauacacuag
HCOc004A_RevC       cuaguguauuaagacaugucgugucagcauuuuuaucccugguuucuggaauguacuagacauuucucuuaaccccgaugccaacgaaauaggaacgguuaacuuacuauucauaauguuaguuua----uuccuauguuguagaaauuuagg-----------guuuuuuuuucuuuagaaauuaaggacuaaaaugccaggacgacguguuuugauacucacc
                       * **** ** *** *****  ##******* #***#*  *****  *#   *  * **           **#  ** #* ** * *******    ***  **  *** | ***  **  ***    ******* * ** *# **  #**           ** *  *   #*  *****  *#***# *******##  ***** *** ** **** *
```

=== Internal intron ===

Multiple sequence alignment

```
HCOc004A_fwrd       gugaguaucaaaacacgucguccuggcauuuuaguccuuaauuucuaaagaaaaaaaaacccuaaauuucuacaacauaggaauaaacuaacauuauga-------------auag
HCOc004A_RevC       cua-------------uucauaauguuaguuuauuccuauguuguagaaauuuaggguuuuuuuuucuuuagaaauuaaggacuaaaaugccaggacgacguguuuugauacucac
                     *#              **#*  **  * **** ****   **    **#   *   ||   *   #**    **   **** **** *  **  *#**              #*
```

=== External intron ===

Multiple sequence alignment

```
HCOc004A_fwrd       guaa---------------guuaaccguuccuauuucguuggcaucgggguuaagagaaaugucuaguacauuccagaaaccagggauaaaaaugcugacacgacaugucuuaauacacuag
HCOc004A_RevC       cuaguguauuaagacaugucgugucagcauuuuuaucccugguuucuggaauguacuagacauuucucuuaaccccgaugccaacgaaauaggaacgguua---------------acuuac
                     **                  *  * *#   * * ** #***  ** **# *  #  * *||* *  #  * #** **  ***# ** * *   #* *  *                  **
```

ALIGNMENT RESULTS - HCOc017A

=== Stwintron Sequence ===

Multiple sequence alignment

```
HCOc017A_fwrd       ggugaguauaaaaacaugucguucuggcauuuuagauauugauuucuagaaaaaacccccguuucuacaauauaugaaucagcuaacguuauaacaguaaguuaagcguucuuauuucguugcuaucgggau------------uaagaaggcaguagcugguauguucuaaaaacgaaggauaaaaaugcugacgcgacauguuuuaauacaucag
HCOc017A_RevC       cugauguauuaaaacaugucgcgucagcauuuuuauccuucguuuuuagaacauaccagcuacugccuucuua------------aucccgauagcaacgaaauaagaacgcuuaacuuacuguuauaacguuagcugauucauauauuguagaaacggggguuuuuucuagaaaucaauaucuaaaaugccagaacgacauguuuuuauacucacc
                       # **** ***********#  ##******* # # **  *** ***** * ***  * ##* *#    **            * * # *** **## *# **** || **** #* ##** *** # * *            **    #* *## *  *** * ***** ***  ** # # *******##  #*********** **** #
```

=== Internal intron ===

Multiple sequence alignment

```
HCOc017A_fwrd       gugaguauaaaaacaugucguucuggcauuuuagauauuga------uuucuagaaaaaacccccguuucuacaauauaugaaucagcuaacguuaua-acag
HCOc017A_RevC       cug-uuauaacguuagcugauucauauauuguagaaacgggggguuuuuucuagaaa------ucaauaucuaaaaugccagaacgacauguuuuuauacucac
                     **  *****    *  * #***  # *** **** *# *       ****||****       * #* **** *** #  ***# *  *    *****  **
```

=== External intron ===

Multiple sequence alignment

```
HCOc017A_fwrd       gu---------aaguuaagcguucuuauuucguugcuaucgggauuaagaaggcaguagcugguauguucuaaaaacgaaggauaaaaaugcugacgcgacauguuuuaauacaucag
HCOc017A_RevC       cugauguauuaaaacaugucgcgucagcauuuuuauccuucguuuuuagaacauaccagcuacugccuucuuaaucccgauagcaacgaaauaagaacgcuuaacuu---------ac
                     *         **##    **#  #  # *  **# # *  *  ** **** # * #*||*# * # **** **  *  * # #**  * #  #  #**    ##**         *
```

ALIGNMENT RESULTS - HCOc017B

=== Stwintron Sequence ===

Multiple sequence alignment

```
HCOc017B_fwrd      gguaagugaaaaaucugccguuuugguguuuuauacuucaauuucuugaaagauc----accaaauucgcuaacguuaugacaguaagucaaguguucuuguuucgucgccaucggguuaagaaggcaauaucugguauauucuagaaacgacguauaaaguacugacacgacguguuuuaauag----
HCOc017B_RevC      ----cuauuaaaacacgucgugucaguacuuuauacgucguuucuagaauauaccagauauugccuucuuaaccccgauggcgacgaaacaagaacacuugacuuacugucauaacguuagcgaauuugg----ugaucuuucaagaaauugaaguauaaaacaccaaaacggcagauuuuucacuuacc
                            ****# #* *** *##**##******* **  **#   #* * *#*    *     ***   * * # *** * ## *# **** || **** #* ## * *** # * *   ***         **#* * *#   #**  ** *******##**##* *** *# #****
```

=== Internal intron ===

Multiple sequence alignment

```
HCOc017B_fwrd      guaagugaaaaaucugccguuuugguguuuuauacuucaauuucuugaaagaucaccaaauucgcuaacguuaug-acag
HCOc017B_RevC      cu-gucauaacguuagcgaauuuggugaucuuucaagaaauugaaguauaaaacaccaaaacggcagauuuuucacuuac
                    *   ## **  *  ** # ******* *#* *     *||*     * *#* ******* # **  *  ** ##   *
```

=== External intron ===

Multiple sequence alignment

```
HCOc017B_fwrd      ---guaagucaagu-------guucuuguuucgucgccaucggguuaagaaggcaauaucugguauauucuagaaacgacguauaaaguacugacacgacguguuuuaauag
HCOc017B_RevC      cuauuaaaacacgucgugucaguacuuuauacgucguuucuagaauauaccagauauugccuucuuaaccccgauggcgacgaaacaagaac-------acuugacuuac---
                      ***# ** **      ** ***  * *****   # #*##*  *  **# *|* #**  *  *##*# #   ***** *  *** **     ** ** #***
```

ALIGNMENT RESULTS - HCOc021A

=== Stwintron Sequence ===

Multiple sequence alignment

```
HCOc021A_fwrd       gguaccuauguauuaagauauaucauguuag---gauuucggccuuugauuucuagaagaaccccuaugacauagaaacuagcuaacguuauagcagugaguaaaucuuuuuuuuuuuuuuuuuucaauacuaugaaacuuaaaaaaggcaauuuccggcguacuaua-------------------------------------agaacgaagguaaaaauagcacugacauaauauguuucaacacacuag----
HCOc021A_RevC       ----cuaguguguugaaacauauuaugucagugcuauuuuuaccuucguuc-------------------------------------uuauaguacgccggaaauugccuuuuuuaaguuucauaguauugaaaaaaaaaaaaaaaaaagauuuacucacugcuauaacguuagcuaguuucuaugucauagggguucuucuagaaaucaaaggccgaaau---ccuaacaugauauaucuuaauacauagguacc
                        *   *** ** *#*#**** ****#**    ****  #****#* *#                                     ****** *    * ****  ##******   ***#  * || *  #***   ******##  **** *    * *****                                      *#* *#****#  ****    **#**** ****#*#* ** ***   *
```

=== Internal intron ===

Multiple sequence alignment

```
HCOc021A_fwrd       guaccuauguauuaagauauaucauguuaggauuucggccuuugauuucuagaagaaccccuaugacauagaaacuag----cuaacguuaua---------------gcag
HCOc021A_RevC       cugc---------------uauaacguuag----cuaguuucuaugucauagggguucuucuagaaaucaaaggccgaaauccuaacaugauauaucuuaauacauagguac
                     * *               *** *#*****    # #*  *#*#  *# ***  *||*  *** #*  #*#*  *# #    *****#* ***               * *
```

=== External intron ===

Multiple sequence alignment

```
HCOc021A_fwrd       ----------------------gugaguaaaucuuuuuuuuuuuuuuuuuucaauacuaugaaacuuaaaaaaggcaauuuccggcguacuauaagaacgaagguaaaaauagcacugacauaauauguuucaacacacuag
HCOc021A_RevC       cuaguguguugaaacauauuaugucagugcuauuuuuaccuucguucuuauaguacgccggaaauugccuuuuuuaaguuucauaguauugaaaaaaaaaaaaaaaaaagauuuacucac----------------------
                                          ** ***     **** ##**# **#**  * ***  #* ** **     |    ** ** *#  *** *  **#** #**## ****     *** **
```

ALIGNMENT RESULTS - HCOc047A

=== Stwintron Sequence ===

Multiple sequence alignment

```
HCOc047A_fwrd       gguacguacaaaaacuuccuguggagguuuuaaacccuugacgucugaaagaaugucgaauaauaacuaauuuuauaacagugaguauaauguuccucuguuccucuuuuuaacgccuggcauagccuggaagagaagguaaagaaaaaa--------------------------------------------aaacgaaggagagaaaaagcugacacgauauguuuuaauaccuuag
HCOc047A_RevC       cuaagguauuaaaacauaucgugucagcuuuuucucuccuucguuu--------------------------------------------uuuuuucuuuaccuucucuuccaggcuaugccaggcguuaaaaagaggaacagaggaacauuauacucacuguuauaaaauuaguuauuauucgacauucuuucagacgucaaggguuuaaaaccuccacaggaaguuuuuguacguacc
                       * ***  ***** *  #***  #*#***    * ##  *** *                                            * **  # *  #* ***#**# * **   ||   ** * #**#*** *#  * #  ** *                                            * ***  ## *    ***#*#  ***#  * *****  *** *
```

=== Internal intron ===

Multiple sequence alignment

```
HCOc047A_fwrd       guacguacaaaaacuuccuguggagguuuuaaacccuugacgucugaaagaaugucgaauaauaacuaauuuuauaacag--------------
HCOc047A_RevC       --------------cuguuauaaaauuaguuauuauucgacauucuuucagacgucaaggguuuaaaaccuccacaggaaguuuuuguacguac
                                  #*  *#*##*# *  * *    *#***#* # || # *#***#*    * *  * #*##*#*  *#
```

=== External intron ===

Multiple sequence alignment

```
HCOc047A_fwrd       gu------------------gaguauaauguuccucuguuccucuuuuuaacgccuggcauagccuggaagagaagguaaagaaaaaaaaacgaaggagagaaaaagcugacacgauauguuuuaauaccuuag
HCOc047A_RevC       cuaagguauuaaaacauaucgugucagcuuuuucucuccuucguuuuuuuuucuuuaccuucucuuccaggcuaugccaggcguuaaaaagaggaacagaggaacauuauacuc------------------ac
                     *                  * **    * ** **** #* *  *****      *# * *  * *||* *  * * #*      *****  * *# **** ** *    ** *                  *
```

ALIGNMENT RESULTS - HCOc052A

=== Stwintron Sequence ===

Multiple sequence alignment

```
HCOc052A_fwrd       ggugaguauuaaaaacauaucguucuggcauuuuagaccuuugauuucuagaagacuccccauauuuccaguguauaugaauuagcuaacguuaugacaguaaguuaagcguucuuaucucgucgcuaucggggu------------uaugaagguaguaucugguauauucuagaaau-gaaggauaaaaaaugcuaauacgacauguuuu-aauacauuag
HCOc052A_RevC       cuaauguauu-aaaacaugucguauuagcauuuu-uauccuucauuucuagaauauaccagauacuacc------------uucauaaccccgauagcgacgagauaagaacgcuuaacuuacugucauaacguuagcuaauucauauacacuggaaauauggggagucuucuagaaaucaaaggucuaaaaugccagaacgauauguuuuuaauacucacc
                       # ***** ******* ****   *#*******   * *#** ********** *  **   ***#* **            ** #   * * # **# * ## ** **** || **** ** ## * #** # * *            **##  ** *#***   **   * ********** #**** # *******#*   **** ******* ***** #
```

=== Internal intron ===

Multiple sequence alignment

```
HCOc052A_fwrd       ---------------------gugaguauuaaaaacauaucguucuggcauuuuagaccuuugauuucuagaagacu----ccccauauuuccaguguauaugaauuagcuaacguuaugacag
HCOc052A_RevC       cugucauaacguuagcuaauucauauacacuggaaauaugggg----agucuucuagaaaucaaaggucuaaaaugccagaacgauauguuuuuaauacucac---------------------
                                         #*# ##*#*  *** **   *    #*  **#****  *#|#*  ****#**  *#    *   ** ***  *#*## #*#
```

=== External intron ===

Multiple sequence alignment

```
HCOc052A_fwrd       -----------------guaaguuaagcguucuuaucucgucgcuaucggggguuaugaagguaguaucugguauauucuagaaaugaaggauaaaaaaugcuaauacgacauguuuuaauacauuag
HCOc052A_RevC       cuaauguauuaaaacaugucguauuagcauuuuuauccuucauuucuagaauauaccagauacuaccuucauaaccccgauagcgacgagauaagaacgcuuaacuuac-----------------
                                     **    * ***#** *****#  #   * * *##  **# * #  ||  # * #**  ##* * *   #  #***** **#*** *    **
```

ALIGNMENT RESULTS - HCOc058A

=== Stwintron Sequence ===

Multiple sequence alignment

```
HCOc058A_fwrd       gguauguaugaagacauaccgucuuggcguauuagaccuugauuucuagaaagaccccugaguuuucacaauauagggauuagcuaacguuaggacaguaaguaaagcguugucguuucgucaucauugagguuaaaaaggaaaugucugguauauuccggaagcaauggucgaaaaacgcugacacgacauauuuaaauacaccag
HCOc058A_RevC       cugguguauuuaaauaugucgugucagcguuuuucgaccauugcuuccggaauauaccagacauuuccuuuuuaaccucaaugaugacgaaacgacaacgcuuuacuuacuguccuaacguuagcuaaucccuauauugugaaaacucaggggucuuucuagaaaucaaggucuaauacgccaagacgguaugucuucauacauacc
                        *****  *#* **  *** *##**** **    *#    #* # * ** *  ** **  ****    *  *       * * ***  * ****##   *|*   ##**** *  *** * *       *  *    ****  ** **  * ** * # *#    #*    ** ****##* ***  ** *#*  *****
```

=== Internal intron ===

Multiple sequence alignment

```
HCOc058A_fwrd       guauguaugaagacauaccgucuuggcgu--auuagaccuugauuucuagaaagaccccugaguuuucacaauauagggauuagcuaacguuaggacag-----------------
HCOc058A_RevC       -----------------cuguccuaacguuagcuaaucccuauauugugaaaacucaggggucuuucuagaaaucaaggucuaau--acgccaagacgguaugucuucauacauac
                                     * ***#*##***   #**# **#*#  ** * #***  * || *  ***# * **  #*#** #**#   ***##*#*** *
```

=== External intron ===

Multiple sequence alignment

```
HCOc058A_fwrd       gu-----------aaguaaagcguugucguuucgucaucauugagguuaaaaaggaaaugucugguauauuccggaagcaauggucgaaaaacgcugacacgacauauuuaaauacaccag
HCOc058A_RevC       cugguguauuuaaauaugucgugucagcguuuuucgaccauugcuuccggaauauaccagacauuuccuuuuuaaccucaaugaugacgaaacgacaacgcuuuacuu-----------ac
                     *           * #*   * **## *****  # *#*****   ##  ** # *   *|*   * # **  ##   *****#* #  ***** ##** *   *# *           *
```

ALIGNMENT RESULTS - HCOc061A

=== Stwintron Sequence ===

Multiple sequence alignment

```
HCOc061A_fwrd       ggugagcacaaaaacaugucguucugacauuuuauaccuuaauuucuacaa------------accccccuauauuuuuacaacauauaaauuagcuaacguuaugauaguaaguuaugcguucuuauuucuuuacuaucgagguucagaagguaguauuuaguauauuuuagacacgaaggauaaaaaugcuaacacgauauguuuuaauacaccag
HCOc061A_RevC       cugguguauuaaaacauaucguguuagcauuuuuauccuucgugucuaaaauauacuaaauacuaccuucugaaccucgauaguaaagaaauaagaacgcauaacuuacuaucauaacguuagcuaauuuauauguuguaaaaauauaggggg------------guuuguagaaauuaagguauaaaaugucagaacgacauguuuuugugcucacc
                       * * *  *******#****  *# ******   ****  * **** **              **  **  *##*# * *  * * **** **    *#* *#   ||   #* *#*    ** **** * *   * * #*##*  **  **              ** **** *  ****   ****** #*  ****#*******  * * *
```

=== Internal intron ===

Multiple sequence alignment

```
HCOc061A_fwrd       gugagcacaaaaacaugucguucugacauuuuauaccuuaauuucuacaaacccccccuauauuuuuacaacauauaaauuagcuaacguuaugauag-----------------
HCOc061A_RevC       -----------------cuaucauaacguuagcuaauuuauauguuguaaaaauauaggggguuuguagaaauuaagguauaaaaugucagaacgacauguuuuugugcucac
                                     # #*# *#** **   **  ***  *  *  ***     ||     ***  *  *  ***  **   ** **#* #*# #
```

=== External intron ===

Multiple sequence alignment

```
HCOc061A_fwrd       gu---------aaguuaugcguucuuauuucuuuacuaucgagguucagaagguaguauuuaguauauuuuagacacgaaggauaaaaaugcuaacacgauauguuuuaauacaccag
HCOc061A_RevC       cugguguauuaaaacauaucguguuagcauuuuuauccuucgugucuaaaauauacuaaauacuaccuucugaaccucgauaguaaagaaauaagaacg---------cauaacuuac
                     *         **##    ***  *  # * **** # *    **# *#** #** **||** **# **#* #**    * # **** * #  *  ***          ***    *
```

ALIGNMENT RESULTS - HCOc066A

=== Stwintron Sequence ===

Multiple sequence alignment

```
HCOc066A_fwrd       ggugaguauaaaaacaugucguucuggcauuuuaaaccuaaauuuucuagaaaaucucccauguuuuuacaauauaugaauua--------gcuaacauuauaacaguaaguuaagcguucuuauuucgucgcuaucgggauuaagaaggcaguagcugguauauucuagaaacgaagga---------------------ugaaaaugcugacacgacauguucuaauacauuag
HCOc066A_RevC       cuaauguauuagaacaugucgugucagcauuuuca---------------------uccuucguuucuagaauauaccagcuacugccuucuuaaucccgauagcgacgaaauaagaacgcuuaacuuacuguuauaauguu--------agcuaauucauauauuguaaaaacaugggagauuuucuagaaaauuuagguuuaaaaugccagaacgacauguuuuuauacucacc
                       # **** * **********  ##******* *                     ***  #****#** ******# * #**           * * # *** * ## *# **** || **** #* ## * *** # * *           **# * #****** **#****#  ***                     * *******##  ********** * **** #
```

=== Internal intron ===

Multiple sequence alignment

```
HCOc066A_fwrd       gugaguauaaaaacaugucguucuggcauuuuaaaccu-----aaauuuucuagaaaaucucccauguuuuuacaauauaugaauuagcuaacauuaua-acag
HCOc066A_RevC       cug-uuauaauguuagcuaauucauauauuguaaaaacaugggagauuuucuagaaaauu-----uagguuuaaaaugccagaacgacauguuuuuauacucac
                     **   *****    *  * #***  # *** ****  #     * ******||******      *#  **** *** #  ***# *  *    *****  **
```

=== External intron ===

Multiple sequence alignment

```
HCOc066A_fwrd       -----------------guaaguuaagcguucuuauuucgucgcuaucgggauuaagaaggcaguagcugguauauucuagaaacga-aggaugaaaaugcugacacgacauguucuaauacauuag
HCOc066A_RevC       cuaauguauuagaacaugucgugucagcauuuucauccu-ucguuucuagaauauaccagcuacugccuucuuaaucccgauagcgacgaaauaagaacgcuuaacuuac-----------------
                                     **    * ***#** *#**##  *** * # #*#**  *  **  *|*  **  *  **#*# # * ***  ##**#* **#*** *    **
```

ALIGNMENT RESULTS - HCOc070A

=== Stwintron Sequence ===

Multiple sequence alignment

```
HCOc070A_fwrd      gguaaguauaaaaauaugucguuccagcauuuuagaccuugauuucuagaaaccccuauuucuacgauauaugaauuagcuaacauuaugauaguaagcuaagcguucuuauuucgucgcuauccgguuuaagga--ggcgaugucugguauauucuacaaacgaaggaugaaaaugcuaacacggaauguuuuaauacaccag
HCOc070A_RevC      cugguguauuaaaacauuccguguuagcauuuucauccuucguuuguagaauauaccagacaucgcc--uccuuaaaccggauagcgacgaaauaagaacgcuuagcuuacuaucauaauguuagcuaauucauauaucguagaaauaggggguuuucuagaaaucaaggucuaaaaugcuggaacgacauauuuuuauacuuacc
                        **** ****#** #***   ******** # ****   *** ***** *  **  *# **  *  * # *#**## * ** *  #  #***# **  #||#  ** #***#  #  * ** * ##**#* # *  *  ** #*  **  * ***** ***  **** # ********   ***# **#**** ****
```

=== Internal intron ===

Multiple sequence alignment

```
HCOc070A_fwrd      gua-------------------------aguauaaaaauaugucguuccagcauuuuagaccuugauuucuagaaaccccuauuucuacgauauaugaauuagcuaacauuaugauag
HCOc070A_RevC      cuaucauaauguuagcuaauucauauaucguagaaauaggggguuuucuagaaaucaaggucuaaaaugcuggaacgacauauuuuuauac-------------------------uuac
                    **                          *** *** *   *   *** ** * *# **||** #* * ** ***   *   * *** ***                         **
```

=== External intron ===

Multiple sequence alignment

```
HCOc070A_fwrd      gu---------aagcuaagcguucuuauuucgucgcuauccgguuuaaggaggcgaugucugguauauucuacaaacgaaggaugaaaaugcuaacacggaauguuuuaauacaccag
HCOc070A_RevC      cugguguauuaaaacauuccguguuagcauuuucauccuucguuuguagaauauaccagacaucgccuccuuaaaccggauagcgacgaaauaagaacgcuuagcuu---------ac
                    *         **#*    ***  *  # *  **# # * ** **  **#* # # ##||## # # *#**  ** ** * # #**  * #  *  ***    *#**         *
```

ALIGNMENT RESULTS - HCOc076A

=== Stwintron Sequence ===

Multiple sequence alignment

```
HCOc076A_fwrd       ggugaguauaaaaacaugucguucuagcauuuuagaccuugauuucuagagaaaccccuauaucuuuacaauauaugaauuagcuaacguuauaauaguaaguuaagcguuccuauuucgucguuaucgggauuaggaa----ggcaguagcugguauauuccagaaacgaaggauaaaaaugcuaacacgacauguuuuaauacaccag
HCOc076A_RevC       cugguguauuaaaacaugucguguuagcauuuuuauccuucguuucuggaauauaccagcuacugcc----uuccuaaucccgauaacgacgaaauaggaacgcuuaacuuacuauuauaacguuagcuaauucauauauuguaaagauauaggguuucucuagaaaucaaggucuaaaaugcuagaacgacauguuuuuauacucacc
                       * **** ************  ********* # ****  ***** **# *  **   **#  ##    * # *#* ## * ***** #  ***** **  #||#  ** *****  # ***** * ## *#* # *    ##  #**   **  * #** *****  **** # *********  *********** **** *
```

=== Internal intron ===

Multiple sequence alignment

```
HCOc076A_fwrd       gug-----------------------aguauaaaaacau----gucguucuagcauuuuagaccuugauuucuagagaaaccccuauaucuuuacaauauaugaauuagcuaacguuauaauag
HCOc076A_RevC       cuauuauaacguuagcuaauucauauauuguaaagauauaggguuucucuagaaaucaaggucuaaaaugcuagaacgac----auguuuuuauacu-----------------------cac
                     *#                       * * **** * **    **  #***** * *# **||** #* * *****#  **     ** * **** * *                       #*
```

=== External intron ===

Multiple sequence alignment

```
HCOc076A_fwrd       -----------------guaaguuaagcguuccuauuucgucguuaucgggauuaggaaggcaguagcugguauauuccagaaacga-aggauaaaaaugcuaacacgacauguuuuaauacaccag
HCOc076A_RevC       cugguguauuaaaacaugucguguuagcauuuuuauccu-ucguuucuggaauauaccagcuacugccuuccuaaucccgauaacgacgaaauaggaacgcuuaacuuac-----------------
                                     **    * ***#**  ***##  ***** # **#**  #  **  *|*  **  #  **#** # *****  ##***  **#*** *    **
```

ALIGNMENT RESULTS - HCOc102A

=== Stwintron Sequence ===

Multiple sequence alignment

```
HCOc102A_fwrd       ggugaguauaaaaacaugucauucuggcauuuuaggccuuguuucuagaaaaccccucauauuucuacaauauaugaa---uuagcuaacguuaugauaguaagcuaagcgcucuuaugucguugcuaucaggg------uuagggaggaaggauaaaaaugcugacacgacauguuuuaauacaccag
HCOc102A_RevC       cugguguauuaaaacaugucgugucagcauuuuuauccuuccucccuaa------cccugauagcaacgacauaagagcgcuuagcuuacuaucauaacguuagcuaa---uucauauauuguagaaauaugagggguuuucuagaaacaaggccuaaaaugccagaaugacauguuuuuauacucacc
                       * **** ********** *  ##******* # **** #*#*# #*      *#*  **  * ** *#*** **    ****** **  * | *  ** ******    ** ***#* ** *  **  *#*      *# #*#*# **** # *******##  * ********** **** *
```

=== Internal intron ===

Multiple sequence alignment

```
HCOc102A_fwrd       --------gugaguauaaaaacaugucauucuggcauuuuaggccuuguuucuagaaaaccccucauauuucuacaauauaugaauuagcuaacguuaugauag
HCOc102A_RevC       cuaucauaacguuagcuaauucauauauuguagaaauaugagggguuuucuagaaacaaggccuaaaaugccagaaugacauguuuuuauacucac--------
                            ##*    # **  ***#*  *   *# ** * ***  ** *#*||*#* **  *** * ** #*   *  *#***  ** #    *##
```

=== External intron ===

Multiple sequence alignment

```
HCOc102A_fwrd       ---guaagcuaagcgcucuuaugucguugcuaucaggguuagggaggaaggauaaaaaugcugacacgacauguuuuaauacaccag
HCOc102A_RevC       cugguguauuaaaacaugucgugucagcauuuuuauccuuccucccuaacccugauagcaacgacauaagagcgcuuagcuuac---
                       **  # ***#   * *# ****# ## * * *   **   |   **   * * * ## #**** #* *   #*** #  **
```

ALIGNMENT RESULTS - HCOc164A

=== Stwintron Sequence ===

Multiple sequence alignment

```
HCOc164A_fwrd       gguauguauuagguagauauaugguguggugggauuccggcccuugcuuccuagaaaagccuucauuucucuaugaugcaggaaccagcuaauuuuuaugacagugaguuaaaacacuuuuauuuugucgccgucgaacuu-------------gugaagagaauaucaaguauauccuagaaaugaagacuauagauauugacacuacauguuuuaacacuuuag-----
HCOc164A_RevC       -----cuaaaguguuaaaacauguagugucaauaucuauagucuucauuucuaggauauacuugauauucucuuca-------------caaguucgacggcgacaaaauaaaaguguuuuaacucacugucauaaaaauuagcugguuccugcaucauagagaaaugaaggcuuuucuaggaagcaagggccggaaucccaccacaccauauaucuaccuaauacauacc
                          **    ** #* *#***  *** ### **#   #  ***  ** **** * *  *** ** * # # * *             #** **# *#* * ###*# ***** | ***** #*### * *#* #** **             #* * # # * ** ***  * * **** **  ***  #   #** ### ***  ***#* *# **    **
```

=== Internal intron ===

Multiple sequence alignment

```
HCOc164A_fwrd       guauguauuagguagauauaugguguggugggauuccggcccuugcuuccuagaaaagccuucauuucucuaugaugcaggaaccagcuaauuuuuaugacag-------------------
HCOc164A_RevC       -------------------cugucauaaaaaauuagcugguuccugcaucauagagaaaugaaggcuuuucuaggaagcaagggccggaaucccaccacaccauauaucuaccuaauacauac
                                        ** ##*## ##    * **  *#*** ** **** **#  ||  #** **** ** ***#*  ** *    ## ##*## **
```

=== External intron ===

Multiple sequence alignment

```
HCOc164A_fwrd       guga---guuaaaacacuuuuauuuugucgccgucgaacuugugaagagaauaucaaguauauccuagaaaugaagacuauagauauugacacuacauguuuuaacacuuuag
HCOc164A_RevC       cuaaaguguuaaaacauguagugucaauaucuauagucuucauuucuaggauauacuugauauucucuucacaaguucgacggcgacaaaauaaaaguguuuuaac---ucac
                     *#*   *********  *    *# #*  * #* *   *##*    ** ****  |  **** **    *##*   * *# *  *# #*    *  *********   *#*
```

ALIGNMENT RESULTS - HCOc178A

=== Stwintron Sequence ===

Multiple sequence alignment

```
HCOc178A_fwrd       ggugaguauaaaaacaugucauucuggcauuuuaaacauugauuucuagaaaccccccauauuucuaccguauaugaauuagcugacguugugauaguaaguuaagcguucuuauuucaucgcuaccggguuaagaaggcgguaucuggua-------------uauucuagaacgaaggauaaaaaugcugacacgacauuuuuuaauacaccag
HCOc178A_RevC       cuggguguauuaaaaaaugucgugucagcauuuuuauccuucguucuagaa-------------uauaccagauaccgccuucuuaacccgguagcgaugaaauaagaacgcuuaacuuacuaucacaacgucagcuaauucauauacgguagaaauaugggggguuuucuagaaaucaauguuuaaaaugccagaaugacauguuuuuauacucacc
                       * **** **** ***** *  ##******* * * **  **#   #*              * ****# ***#   #*   *#** # **# # #* *# **** || **** #* *# # #** # **#*   *#   #*** #****              * *#   #**  ** * * *******##  * ***** **** **** *
```

=== Internal intron ===

Multiple sequence alignment

```
HCOc178A_fwrd       gugaguauaaaaacaugucauucuggcauuuuaaacau------ugauuucuagaaaccccccauauuucuaccguauaugaauuagcugacguugugau-ag
HCOc178A_RevC       cu-aucacaacgucagcuaauucauauacgguagaaauaugggggguuuucuagaaauc------aauguuuaaaaugccagaaugacauguuuuuauacucac
                     * * #*#**   **  * ****  # *#  ** * **       * ****||**** *       ** * **  #* #  **** *  **   **#*# * *
```

=== External intron ===

Multiple sequence alignment

```
HCOc178A_fwrd       guaaguuaagcguucuuauuucaucgcuaccggguuaagaaggcgguaucugguauauucuagaacgaaggauaaaaaugcugacacgacauuuuuuaauacaccag-------------------------------------------------
HCOc178A_RevC       -------------------------------------------------cuggguguauuaaaaaaugucgugucagcauuuuuauccuucguucuagaauauaccagauaccgccuucuuaacccgguagcgaugaaauaagaacgcuuaacuuac
                                                                     ***** ****  *#** *  *  * *  ||  * *  *  * **#*  **** *****
```

ALIGNMENT RESULTS - HCOc224-179

=== Stwintron Sequence ===

Multiple sequence alignment

```
HCOc224-179_fwr     ggugaguauaaaaacaugucguucuggcauuuuagaccuugauuucuagaagaccccgacccccccccccaacauauuucuacaagauaugaauuagcuaacguu-augguaguaaguuaaacguucuuaucucgucgcuaucgggguuaagaaggcaguaucucuua---------------------------uauucuagaaacgaaggauaaaaaugcuaacacgacauguuuuaauacaucag
HCOc224-179_Rev     cugauguauuaaaacaugucguguuagcauuuuuauccuucguuucuagaa---------------------------uauaagagauacugccuucuuaaccccgauagcgacgagauaagaacguuuaacuuacuaccau-aacguuagcuaauucauaucuuguagaaauauguuggggggggggggucggggucuucuagaaaucaaggucuaaaaugccagaacgacauguuuuuauacucacc
                       # **** ************  *#******* # ****  *********                           * **  *****#   #*   **** ## **#*# ## ** ***  ||  *** ** ## #*#** ## ****   *#   #*****  **                           * *********  **** # *******#*  ************ **** #
```

=== Internal intron ===

Multiple sequence alignment

```
HCOc224-179_fwr     gug-----------------------aguauaaaaacaugucg------------------uucuggcauuuuagaccuugauuucuagaagaccccgacccccccccccaacauauuucuacaagauaugaauuagcuaacguuaugguag
HCOc224-179_Rev     cuaccauaacguuagcuaauucauaucuuguagaaauauguuggggggggggggucggggucuucuagaaaucaaggucuaaaaugccagaa------------------cgacauguuuuuaua-----------------------cucac
                     *#                         * ** *** **** *                  ****#* * *# **||** #* * *#****                  * **** *** ** *                        #*
```

=== External intron ===

Multiple sequence alignment

```
HCOc224-179_fwr     -----------------guaaguuaaacguucuuaucucgucgcuaucgggguuaagaaggcaguaucucuuauauucuagaaacga-aggauaaaaaugcuaacacgacauguuuuaauacaucag
HCOc224-179_Rev     cugauguauuaaaacaugucguguuagcauuuuuauccu-ucguuucuagaauauaagagauacugccuucuuaaccccgauagcgacgagauaagaacguuuaacuuac-----------------
                                     **    * * *#** *****#  *** * # #*##*  *# **# *|* #** #*  *##*# # * ***  #***** **#* * *    **
```

ALIGNMENT RESULTS - HCOc236A

=== Stwintron Sequence ===

Multiple sequence alignment

```
HCOc236A_fwrd      ggugaguauacgaauaugucguuuuugucacuuuagaccuugauuucuagaaagauccucaaaugucuucaauguaggaauuagcuaacgucgugacaguaagugaagcguuccuauuucgucgccaucgguguuaa----------gaaaacaaugcuagguauaucuuagaaacgaaguguuaaaaugcuaacaaguuauguuuaauuacaccag
HCOc236A_RevC      cugguguaauuaaacauaacuuguuagcauuuuaacacuucguuucuaagauauaccuagcauuguuuuc----------uuaacaccgauggcgacgaaauaggaacgcuucacuuacugucacgacguuagcuaauuccuacauugaagacauuugaggaucuuucuagaaaucaaggucuaaagugacaaaaacgacauauucguauacucacc
                      * ***   #**#**# * * ** *#   ** * ** *#* **#  *** * *#*    * *** ***          ***#*    #* *#*** # * # *** | *** # * # ***#* *#    *#***          *** *** *    *#* * ***  #** *#* ** * **   #* ** * * #**#**#   *** *
```

=== Internal intron ===

Multiple sequence alignment

```
HCOc236A_fwrd      gugaguauacgaauaugucguuuuugucacuuuagaccuugauuucuagaaagauccucaaaugucuucaauguaggaauuagcuaacgucgugacag--------------------
HCOc236A_RevC      --------------------cugucacgacguuagcuaauuccuacauugaagacauuugaggaucuuucuagaaaucaaggucuaaagugacaaaaacgacauauucguauacucac
                                       #* *### ** ****    *  #* *    ****#  *||*  #****    * *#  *    **** ** ###* *#
```

=== External intron ===

Multiple sequence alignment

```
HCOc236A_fwrd      --------------------------------------------------guaagugaagcguuccuauuucgucgccaucgguguuaagaaaacaaugcuagguauaucuuagaaacgaaguguuaaaaugcuaacaaguuauguuuaauuacaccag
HCOc236A_RevC      cugguguaauuaaacauaacuuguuagcauuuuaacacuucguuucuaagauauaccuagcauuguuuucuuaacaccgauggcgacgaaauaggaacgcuucacuuac--------------------------------------------------
                                                                   #** ##  ***#**  * *#* # *#** | **#* # *#* *  **#***  ## **#
```

ALIGNMENT RESULTS - HCOc252A

=== Stwintron Sequence ===

Multiple sequence alignment

```
HCOc252A_fwrd       gguacgua-uaaacuaugccauuucggcauuuuagauccuggucccuaccuagagguuuagcuaacguuaagacaguaaguugaucguuuuuauuucguuauugucgagguuaacaaggaaauguuugguuuauu------------------------cuagaaugaaguauugaaaaggcuaacacgauauguuguaauauccuag
HCOc252A_RevC       cuaggauauuacaacauaucguguuagccuuuucaauacuucauucu------------------------agaauaaaccaaacauuuccuuguuaaccucgacaauaacgaaauaaaaacgaucaacuuacugucuuaacguuagcuaaaccucuagguagggaccaggaucuaaaaugccgaaauggcauaguuua-uacguacc
                         #** ** * #**# * * * #** **** #** **    **                         * *#***####* *#***## * * # # *#*|*#* # # * * ##***#* *####***#*                        ***     ** **# **** **# * * * #**# * ** **#
```

=== Internal intron ===

Multiple sequence alignment

```
HCOc252A_fwrd       guacguauaaacuaugccauuucggcauuuuagauccuggucccuaccuagagguuuagcuaacguuaagacag-------------
HCOc252A_RevC       -------------cugucuuaacguuagcuaaaccucuagguagggaccaggaucuaaaaugccgaaauggcauaguuuauacguac
                                  ** * *  **  * #* *# # **#*   |   *#** # #* *# *  **  * * **
```

=== External intron ===

Multiple sequence alignment

```
HCOc252A_fwrd       guaag-----------------uugaucguuuuuauuucguuauugucgagguuaacaaggaaauguuugguuuauucuagaaugaaguauugaaaaggcuaacacgauauguuguaauauccuag
HCOc252A_RevC       cuaggauauuacaacauaucguguuagccuuuucaauacuucauucuagaauaaaccaaacauuuccuuguuaaccucgacaauaacgaaauaaaaacgauca-----------------acuuac
                     ** *                  * * * ****#* * * *#*** * **#   * ***# *||* #*** *   #** * ***#* * * *#**** * * *                  * **
```

ALIGNMENT RESULTS - HCOc271A

=== Stwintron Sequence ===

Multiple sequence alignment

```
HCOc271A_fwrd       ggugaguauaaaacuaugucguuuuggcguuuuagacauuugguuucugg-------------------------------------------aaagaccaucaaaucucaauauaaaaaucagcuaacguuaugacagugagucaaauguucuuauuucgucgcgaucgggguuaagaaggcaaugccugguauaauucuagaaacgacagguaaaaaugcugacacgacgcguuuuaacacguuag
HCOc271A_RevC       cuaacguguuaaaacgcgucgugucagcauuuuuaccugucguuucuagaauuauaccaggcauugccuucuuaaccccgaucgcgacgaaauaagaacauuugacucacugucauaacguuagcugauuuuuauauugagauuugauggucuu-------------------------------------------uccagaaaccaaaugucuaaaacgccaaaacgacauaguuuuauacucacc
                       # ** * *** # #***** *##**#**** # *  *#* **#  *#                                           ** #**  *  * ** *  *   **  * *||* *  **   *  * ** *  *  **# *                                           *#*  #** *#*  * # ****#**##* *****# # *** * ** #
```

=== Internal intron ===

Multiple sequence alignment

```
HCOc271A_fwrd       gugaguauaaaacuaugucguuuuggcguuuuagacauuugguuucuggaaagaccaucaaaucucaauauaaaaaucagcuaacguuaug-acag
HCOc271A_RevC       cug-ucauaacguuagcugauuuuuauauugagauuugauggucuuuccagaaaccaaaugucuaaaacgccaaaacgacauaguuuuauacucac
                     **  #****   **  * #**** # #**   #     ****#* *||* *#****     #   **# # ****# *  **   ****#  **
```

=== External intron ===

Multiple sequence alignment

```
HCOc271A_fwrd       -----------------gugagucaaauguucuuauuucgucgcgaucgggguuaagaaggcaaugccugguauaauucuagaaacgacagguaaaaaugcugacacgacgcguuuuaacacguuag
HCOc271A_RevC       cuaacguguuaaaacgcgucgugucagcauuuuuaccugucguuucuagaauuauaccaggcauugccuucuuaaccccgaucgcgacgaaauaagaacauu-----------------ugacucac
                                     **      * ##** ***##*  #     * *## *  *  *****|*****  *  * ##* *     #  *##*** **## *                     *#*
```

ALIGNMENT RESULTS - HCOc332A

=== Stwintron Sequence ===

Multiple sequence alignment

```
HCOc332A_fwrd      gguauguauaaaaauaugucgu-uuggcaucuuggagacugguuuucauaaaaua-----------uauaugaauuagcuaacacugggacaguaaguaaaccguucucauuucguugcuaucgaguccaagaaggcaaugucuaguauauucuagaagcaaaggccaagaaugcugacacgacauguucuaauacaccag
HCOc332A_RevC      cuggugguauuagaacaugucgugucagcauucuuggccuuugcuucuagaauauacuagacauugccuucuuggacucgauagcaacgaaaugagaacgguuuacuuacugucccaguguuagcuaauucauaua-----------uauuuuaugaaaaccagucuccaagaugccaa-acgacauauuuuuauacauacc
                       ***** * **#******** *##**** #* *   * *#**# * ** ***            * # ** *#* * ** ** #*##*  # **   ||   ** #  *##*# ** ** * *#* ** # *            *** ** * #**#* *    * *# ****##* *******#** * *****
```

=== Internal intron ===

Multiple sequence alignment

```
HCOc332A_fwrd      guauguauaaaaauaugucguuuggcaucuuggagacugguuuucauaaaauauauaugaauuagcuaacacugggacag---------------------
HCOc332A_RevC      ---------------------cugucccaguguuagcuaauucauauauauuuuaugaaaaccagucuccaagaugccaaacgacauauuuuuauacauac
                                        #** * #  **  # **##**#  *** *|* ***  #**##** #  **  # * **#
```

=== External intron ===

Multiple sequence alignment

```
HCOc332A_fwrd      gu---------aaguaaaccguucucauuucguugcuaucgaguccaagaaggcaaugucuaguauauucuagaagcaaaggccaagaaugcugacacgacauguucuaauacaccag
HCOc332A_RevC      cuggugguauuagaacaugucgugucagcauucuuggccuuugcuucuagaauauacuagacauugccuucuuggacucgauagcaacgaaaugagaacgguuuacuu---------ac
                    *          *##*   ***  #  # *  *** # *    * * **** # * *#||#* * # **** * *    * # ***  * #  #  ***   *##*          *
```

ALIGNMENT RESULTS - HCOc378A

=== Stwintron Sequence ===

Multiple sequence alignment

```
HCOc378A_fwrd       ggugaguacaaaagcaugucauucuggcauuuuagaccuugaucucuaagaaaaccacuauauuucuauaauauauuaa--------uuggcuaacguuaugacaguaaguuaagcguucuuauuucgucgcuaucgcgguuaagaaaguaguguuugguauauucuagaaa---------------------cgaaggauaaaaugcugacacgacauguuuuaauacaccag
HCOc378A_RevC       cugguguauuaaaacaugucgugucagcauuuuauccuucg---------------------uuucuagaauauaccaaacacuacuuucuuaaccgcgauagcgacgaaauaagaacgcuuaacuuacugucauaacguuagccaa--------uuaauauauuauagaaauauagugguuuucuuagagaucaaggucuaaaaugccagaaugacaugcuuuuguacucacc
                       * ***  ***#******  *  ##********* * *#*                      ****** ******##**      **    * **# **# * ## *# **** || **** #* ## * #** #** *    **        **##****** ******                     *#* *  ********##  * ******#***  *** *
```

=== Internal intron ===

Multiple sequence alignment

```
HCOc378A_fwrd       gug-----------------------aguacaaaagcaugucauucuggcauuuuagaccuugaucucuaaga----aaaccacuauauuucuauaauauauuaauuggcuaacguuaugacag
HCOc378A_RevC       cugucauaacguuagccaauuaauauauuauagaaauauagugguuu----ucuuagagaucaaggucuaaaaugccagaaugacaugcuuuuguacu-----------------------cac
                     **                       * ** * **# **#    * *    *#*****  *||*  *****#*    * *    #** #** * ** *                       **
```

=== External intron ===

Multiple sequence alignment

```
HCOc378A_fwrd       guaa------------------guuaagcguucuuauuucgucgcuaucgcgguuaagaaaguaguguuugguauauucuagaaacgaaggauaaaaugcugacacgacauguuuuaauacaccag
HCOc378A_RevC       cugguguauuaaaacaugucgugucagcauuuuauccuucguuucuagaauauaccaaacacuacuuucuuaaccgcgauagcgacgaaauaagaacgcuuaac------------------uuac
                     *                    **#*    **  * #*****  ***  # #  # *#* * || * *#* #  # #  ***  *****# *  **    *#**                    *
```

ALIGNMENT RESULTS - HCOc406A

=== Stwintron Sequence ===

Multiple sequence alignment

```
HCOc406A_fwrd       ggugaguauaaaaagacgucauuuuggcauuucauacccagauucccacaauauaggaauucgcuaacacuauagaaguaaguugagcguuuuuaucucgucgucaucgggguuaagaaggcaaugccuaccuag-----------------------uauauucuagaaaugauggguaacaaugcugacacgacaugcuuuaacacacuag
HCOc406A_RevC       cuaguguguuaaagcaugucgugucagcauuguuacccaucauuucuagaauaua-----------------------cuagguaggcauugccuucuuaaccccgaugacgacgagauaaaaacgcucaacuuacuucuauaguguuagcgaauuccuauauugugggaaucuggguaugaaaugccaaaaugacgucuuuuuauacucacc
                       * ** * ***  * *** * *##*****     **   *** * * ******                       # ** *# **#** ## *** # * #* | *# * # *** ## **#** #* ** #                       ****** * * ***   **     *****##* * *** *   *** * ** *
```

=== Internal intron ===

Multiple sequence alignment

```
HCOc406A_fwrd       -------------------------------------gugaguauaaaaagacgucauuuuggcauuucauacccagauucccacaauauaggaauucgcuaacacuauagaag
HCOc406A_RevC       cuucuauaguguuagcgaauuccuauauugugggaaucuggguaugaaaugccaaaaugacgucuuuuuauacucac-------------------------------------
                                                          ** **** *** * *#  ||  #* * *** **** **
```

=== External intron ===

Multiple sequence alignment

```
HCOc406A_fwrd       ---guaaguugagcguuuuuaucucgucgucaucg-----------------ggguuaagaaggcaaugccuaccuaguauauucuagaaaugauggguaacaaugcugacacgacaugcuuuaacacacuag
HCOc406A_RevC       cuaguguguuaaagcaugucgugucagcauuguuacccaucauuucuagaauauacuagguaggcauugccuucuuaaccc-----------------cgaugacgacgagauaaaaacgcucaacuuac---
                       **  ***#*#   * *# * **# *#*  * #                 # ##** * *****|***** * **## #                 # *  *#* #** * #* *   #*#***  **
```

ALIGNMENT RESULTS - HCOc522A

=== Stwintron Sequence ===

Multiple sequence alignment

```
HCOc522A_fwrd       gguaug---------------uauaaaacauguugccuuggauuuccaagcauugaucucuggaaagacucguaaauuuccauaguuuaggaauuagcuaacguugggauagugaguaaagaauuaucauuuuaucgccauc----------------------------gaguuuaagagcgcgacgaccugguaaagucuagaaacagaagucaaaauugcugacaggauacauuuuaauacacuag
HCOc522A_RevC       cuaguguauuaaaauguauccugucagcaauuuugacuucuguuucuagacuuuaccaggucgucgcgcucuuaaacuc----------------------------gauggcgauaaaaugauaauucuuuacucacuaucccaacguuagcuaauuccuaaacuauggaaauuuacgagucuuuccagagaucaaugcuuggaaauccaaggcaacauguuuuaua---------------cauacc
                        **               * * *   ** *** ***   **** * #* **# #   * *     *** ****#*#                            *** *#**  ***  **|**  ***  **#* ***                            #*#**** ***     * *   # #** *# * ****   *** *** **   * * *               **
```

=== Internal intron ===

Multiple sequence alignment

```
HCOc522A_fwrd       guauguauaaaacauguugccuuggauuuccaagcauugaucucuggaaagacucguaaauuuccauaguuuaggaauuagcuaacguuggga--------------uag
HCOc522A_RevC       cua--------------ucccaacguuagcuaauuccuaaacuauggaaauuuacgagucuuuccagagaucaaugcuuggaaauccaaggcaacauguuuuauacauac
                     **              * **   * *  * **   #*#* ** ******    ||    ****** ** *#*#   ** *  * *   ** *              **
```

=== External intron ===

Multiple sequence alignment

```
HCOc522A_fwrd       ---gugaguaaagaauuaucauuuuaucgccaucgaguuuaagagcgcgacgaccugguaaagucuagaaacagaagucaaaauugcugacaggauacauuuuaauacacuag
HCOc522A_RevC       cuaguguauuaaaauguauccugucagcaauuuugacuucuguuucuagacuuuaccaggucgucgcgcucuuaaacucgauggcgauaaaaugauaauucuuuacucac---
                       *** #* **#*  **** * *#* *#   * ** **#     *  ***    #|#    ***  *     #** ** *   #* *#* * ****  *#** *# ***
```

ALIGNMENT RESULTS - HE7c016A

=== Stwintron Sequence ===

Multiple sequence alignment

```
HE7c016A_fwrd       gguacguaugaaaauauucccagauuuagauaauguagacgucagcuaacauuuaagcaguaaguaaagcauuuucauuucgucuccaucgagcuuaaagaagcaacguccgguauguucua---------------gaaacgaaggccuaaaaugcugacac---gaaacguuuuaaugcauuag
HE7c016A_RevC       cuaaugcauuaaaacguuuc---gugucagcauuuuaggccuucguu---------------ucuagaacauaccggacguugcuucuuuaagcucgauggagacgaaaugaaaaugcuuuacuuacugcuuaaauguuagcugacgucuacauuaucuaaaucugggaauauuuucauacguacc
                       * *#** ****# ** *    * * # #* * *** * *  * *                 ** *#*** ##   #    ** * * #*||*# * * **    #   ## ***#* **                 * *  * * *** * *# # * *    * ** #**** **#* *
```

=== Internal intron ===

Multiple sequence alignment

```
HE7c016A_fwrd       guacguaugaaaauauucccagauuuagauaauguagacgucagcuaacauuu----aagcag
HE7c016A_RevC       cugcuu----aaauguuagcugacgucuacauuaucuaaaucugggaauauuuucauacguac
                     * * *    **** **  * **# *  *#*|*#*  * #** *  ** ****    * * *
```

=== External intron ===

Multiple sequence alignment

```
HE7c016A_fwrd       guaaguaa-----------------agcauuuucauuucgucuccaucgagcuuaaagaagcaacguccgguauguucuagaaacgaaggccuaaaaugcugacacgaaacguuuuaaugcauuag
HE7c016A_RevC       cuaaugcauuaaaacguuucgugucagcauuuuaggccuucguuucuagaacauaccggacguugcuucuuuaagcucgauggagacgaaaugaaaaugcu-----------------uuacuuac
                     ***   *                 ********   ##  # *   * **#* **  * *  ||  * *  ** *#** *   * #  ##   ********                 *   ***
```

ALIGNMENT RESULTS - HE7c026A

=== Stwintron Sequence ===

Multiple sequence alignment

```
HE7c026A_fwrd      gguacguauaaaaacgugucgucccggcauuuuagccccugauuucuagagauaccccuauacauaccu----------------------------accucccauguauuggcuaacauuaaacaguaaguagagcauucucauuccgucgccaccgagccuaaagaagcaacguccaguacguucucgaaacgaaacccccaaaaugcugacauggcauuuuucaauacauuag
HE7c026A_RevC      cuaauguauugaaaaaugccaugucagcauuuugggguuucguuucgagaacguacuggacguugcuucuuuaggcucgguggcgacggaaugagaaugcucuacuuacuguuuaauguuagccaauacauggg----------------------------agguagguauguauaggguaucucuagaaaucaggggcuaaaaugccgggacgacacguuuuuauacguacc
                      * ****  *** #**#*#*  *#******* *    *    ** # # #*#   *       * * *                            *  # * *# **#**  ***||***  **#** #* *                            *   * * *      *   #*# # # **   *    * *******#*  *#*#**# ***  **** *
```

=== Internal intron ===

Multiple sequence alignment

```
HE7c026A_fwrd      ----------------------------guacguauaaaaacgugucgucccggcauuuuagccccugauuucuagagauaccccuauacauaccuaccucccauguauuggcuaacauuaaacag
HE7c026A_RevC      cuguuuaauguuagccaauacaugggagguagguauguauaggguaucucuagaaaucaggggcuaaaaugccgggacgacacguuuuuauacguac----------------------------
                                               *** ****  * * * **  # * #* * *#  *||*  #* * *# * #  ** * * *  **** ***
```

=== External intron ===

Multiple sequence alignment

```
HE7c026A_fwrd      guaaguagagcauucucauuccgucgccaccgagccuaaagaagcaacguccaguacguucucgaaacgaaacccccaaaaug---cugacauggcauuuuucaauacauuag--------------------------
HE7c026A_RevC      --------------------------cuaauguauugaaaaaugccauguc---agcauuuuggggguuucguuucgagaacguacuggacguugcuucuuuaggcucgguggcgacggaaugagaaugcucuacuuac
                                             * *  * #   ***#* ** * ***     *#** * *     |     * * **#*     *** * ** *#***   # *  * *
```

ALIGNMENT RESULTS - HE7c026B

=== Stwintron Sequence ===

Multiple sequence alignment

```
HE7c026B_fwrd     ggguacgug-ugaaacaugccgugccgguauuuuagcccuugauuucuagaaauauacccggauuuguauaacguaggcauuaucuaacaucaaaauaguaagugaagcauuuucauucgaucgcuaucgagcuuagagaagaaauguucgauaua--------------uucuagaaacgaaggccgaaaaugcuaacaugacacguuuuaauacaccag
HE7c026B_RevC     cugguguauuaaaacgugucauguuagcauuuucggccuucguuucuagaa--------------uauaucgaacauuucuucucuaagcucgauagcgaucgaaugaaaaugcuucacuuacuauuuugauguuagauaaugccuacguuauacaaauccggguauauuucuagaaaucaagggcuaaaauaccggcacggcauguuuca-cacguacc
                      **# *#**** ** *#**  #*#***** * ****  *********              **** ** * #  *# ***   * *   ****# * #***  * | *  ***# * #****   * *   *** #*  # * ** ****              *********  **** * *****#*#  **#* ** ****#* #**
```

=== Internal intron ===

Multiple sequence alignment

```
HE7c026B_fwrd     guacgugugaaacaugccgugccgguauuuuagcccuugauuucuagaaauauacccggauuuguauaacguaggcauuaucuaacau------------------caaaauag
HE7c026B_RevC     cuauuu------------------ugauguuagauaaugccuacguuauacaaauccggguauauuucuagaaaucaagggcuaaaauaccggcacggcauguuucacacguac
                   **  *                    ** ****    ** #* *   * *#* * *||* * *#* *   * *# **    **** **                   *  **
```

=== External intron ===

Multiple sequence alignment

```
HE7c026B_fwrd     guaaguga-----------------agcauuuucauucgaucgcuaucgagcuuagagaagaaauguucgauauauucuagaaacgaaggccgaaaaugcuaacaugacacguuuuaauacaccag
HE7c026B_RevC     cugguguauuaaaacgugucauguuagcauuuucggccuucguuucuagaauauaucgaacauuucuucucuaagcucgauagcgaucgaaugaaaaugcu-----------------ucacuuac
                   *     *                 *********  #*  #   * * **#  **  *** *||* ***  **  #** * *   #  *#  *********                 *     *
```

ALIGNMENT RESULTS - HE7c035A

=== Stwintron Sequence ===

Multiple sequence alignment

```
HE7c035A_fwrd      gguauguacaacauuaugccguguuggcacuuugguccuuguguuguaguuaagucccuaaauuucuauaauaugagaauua--------gcuaacguuaugacaguaagcagagcaaccuuauuuuucauuaucgagcuuaagaagaccauaaccgauauauuauggaagcguagguaccuag---------agaguuaaaagcgcugacacgauaucuuuuaauaugcuag
HE7c035A_RevC      cuagcauauuaaaagauaucgugucagcgcuuuuaacucu---------cuagguaccuacgcuuccauaauauaucgguuauggucuucuuaagcucgauaaugaaaaauaagguugcucugcuuacugucauaacguuag--------cuaauucucauauuauagaaauuuagggacuuaacuacaacacaaggaccaaagugccaacacggcauaauguuguacauacc
                       ##**  * *  **# *****##** **** # * #*         #** ** ****  #***#*******#    ***           * * # **#*  # **# *# * | * #* #** #  *#** # * *           ***    #*******#***#  **** ** **#         *# * # **** **##***** #**  * *  **##
```

=== Internal intron ===

Multiple sequence alignment

```
HE7c035A_fwrd      guauguacaacauuaugccguguuggcacuuugguccuuguguuguaguuaagucccuaaauuucuauaauaugagaauuagcuaacguuaugacag------------------------------------------
HE7c035A_RevC      ------------------------------------------cugucauaacguuagcuaauucucauauuauagaaauuuagggacuuaacuacaacacaaggaccaaagugccaacacggcauaauguuguacauac
                                                             #*** #* * **   # ****# #***|***# #**** #   ** * *# ***#
```

=== External intron ===

Multiple sequence alignment

```
HE7c035A_fwrd      gu-----------aagcagagcaaccuuauuuuucauuaucgagcuuaagaagaccauaaccgauauauuauggaagcguagguaccuagagaguuaaaagcgcugacacgauaucuuuuaauaugcuag
HE7c035A_RevC      cuagcauauuaaaagauaucgugucagcgcuuuuaacucucuagguaccuacgcuuccauaauauaucgguuauggucuucuuaagcucgauaaugaaaaauaagguugcucugcuu-----------ac
                    *           * # *  *   *  # #**** *#* ** ** *    * *    #*    *||*    *#    * *    * ** ** *#* ****# #  *   *  * # *           *
```

ALIGNMENT RESULTS - HE7c050A

=== Stwintron Sequence ===

Multiple sequence alignment

```
HE7c050A_fwrd       gguauguauaagaucaagucguuccggcauuuagccccugauuuccagguauagacauuagcuaacauugugggcaguaagcaaaacacuuuuauuccguugccaucgagcuuaagaaggcauaucccca--gaaacugagguaaaaaugcuaacccgacgugcuuuuag-------
HE7c050A_RevC       -------cuaaaagcacgucggguuagcauuuuuaccucaguuuc--ugggauaugccuucuuaagcucgauggcaacggaauaaaaguguuuugcuuacugccacaauguuagcuaaugucuauaccauggaaaucaggggcuaaaugccggaacgacuugaucuuauacauacc
                            ***#* ** ****    #******   ** ## ****  *** ***  * **    * * # #*****##  #  *** || ***  #  ##*****# # * *    ** *  *** ***  **** ## **   ******#    **** ** *#***
```

=== Internal intron ===

Multiple sequence alignment

```
HE7c050A_fwrd       guauguauaagaucaagucguuccggcauuuagccccugauuuccagguauagacauuag--------------------cuaacauugug-gcag
HE7c050A_RevC       cugc-cacaauguuag--------------------cuaaugucuauaccauggaaaucaggggcuaaaugccggaacgacuugaucuuauacauac
                     * # #*#**  * *                     **#** ** **#|#** ** **#**                    * *  **#*# # *
```

=== External intron ===

Multiple sequence alignment

```
HE7c050A_fwrd       gu--aagcaaaacacuuuuauuccguugccaucgagcuuaagaaggcauaucccagaaacugagguaaaaaugcuaacccgacgugcuuuuag
HE7c050A_RevC       cuaaaagcacgucggguuagcauuuuuaccucaguuucugggauaugccuucuuaagcucgauggcaacggaauaaaaguguuuugcuu--ac
                     *  *****   *   **  #    **#** # *   #*  ** # | # **  *#   * # **#**    #  **   *   *****  *
```

ALIGNMENT RESULTS - HE7c129A

=== Stwintron Sequence ===

Multiple sequence alignment

```
HE7c129A_fwrd       gguacguauaaagauaugcuguccuggcauuuuaaccccugauuuuagaaguauccc--------uacauuuagauauuauagggauuagcuaacauuaaacaguaaguagagcauucucauuccgccgccaucggacuuaaagggacaacguccgguaugcucuugaaacgaagguuaaaaauguugacgcgacguguuuuaauacauuag
HE7c129A_RevC       cuaauguauuaaaacacgucgcgucaacauuuuuaaccuucguuucaagagcauaccggacguugucccuuuaaguccgauggcggcggaaugagaaugcucuacuuacuguuuaauguuagcuaaucccuauaauaucuaaaugua--------gggauacuucuaaaaucagggguuaaaaugccaggacagcauaucuuuauacguacc
                       * **** **#*#*#* #*#  ###****** * ** *  ***#*# **#** **        * * ****# * # ** * * #  # * * * *   * ##||## *   * * * * #  # * * ** # * #**** * *        ** **#** #*#***  * ** * ******###  #*# *#*#*#** **** *
```

=== Internal intron ===

Multiple sequence alignment

```
HE7c129A_fwrd       guacguauaaagauaugcuguccuggcauuuuaaccccugauuuuagaaguaucccuacauuuagauauuauagggauuagcuaacau-------uaaacag
HE7c129A_RevC       cuguuua-------auguuagcuaaucccuauaauaucuaaauguagggauacuucuaaaaucagggguuaaaaugccaggacagcauaucuuuauacguac
                     *   **       *** *# *  # * #* ***   **#* * ***  #||#  *** * *#**   *** *# * #  * #* ***       **   *
```

=== External intron ===

Multiple sequence alignment

```
HE7c129A_fwrd       gu---------aaguagagcauucucauuccgccgccaucggacuuaaagggacaacguccgguaugcucuugaaacgaagguuaaaaauguugacgcgacguguuuuaauacauuag
HE7c129A_RevC       cuaauguauuaaaacacgucgcgucaacauuuuuaaccuucguuucaagagcauaccggacguugucccuuuaaguccgauggcggcggaaugagaaugcucuacuu---------ac
                     *         **##*   * #  # *#      # * *  *  *#** #* * * **||** * * *# **#*  *  * * #      #* #  # *   *##**         *
```

ALIGNMENT RESULTS - HE7c137A

=== Stwintron Sequence ===

Multiple sequence alignment

```
HE7c137A_fwrd       gguacguauaaaaauguguugccccggcauuuuaaccccugauuucuggaaauaucauuauguuuacguaguguaagcaucggcuaacguuaaacaguaaguagagcauucuuguuccgucaccgccgaucuuaaagaaacaacguccgguacguucuagaaauaaagguuaaaaaugcugacacgacguguuuuaauacacuag
HE7c137A_RevC       cuaguguauuaaaacacgucgugucagcauuuuuaaccuuuauuucuagaacguaccggacguuguuucuuuaagaucggcggugacggaacaagaaugcucuacuuacuguuuaacguuagccgaugcuuacacuacguaaacauaaugauauuuccagaaaucaggggguuaaaaugccggggcaacacauuuuuauacguacc
                         **** ****###**#*   *#******* * ** * ******#***    *   *#***    #  *#  * *  ***  *  *   ** *#*   #|#   *#* **   *  *  ***  * *  #*  #    ***#*   *    ***#****** * ** * *******#*   *#**###**** ****
```

=== Internal intron ===

Multiple sequence alignment

```
HE7c137A_fwrd       guacguauaaaaauguguugccccggcauuuuaaccccugauuucuggaaauaucauuauguuuacguaguguaagcauc---------ggcuaacgu-------uaaacag
HE7c137A_RevC       cuguuua-------acguuagcc---------gaugcuuacacuacguaaacauaaugauauuuccagaaaucaggggguuaaaaugccggggcaacacauuuuuauacguac
                     *   **       ##***# **          *  * *#  #* #* ***#** || **#*** *# *#  #* *  *          ** #***##       **   *
```

=== External intron ===

Multiple sequence alignment

```
HE7c137A_fwrd       gu---------aaguagagcauucuuguuccgucaccgccgaucuuaaagaaacaacguccgguacguucuagaaauaaagguuaaaaaugcugacacgacguguuuuaauacacuag
HE7c137A_RevC       cuaguguauuaaaacacgucgugucagcauuuuuaaccuuuauuucuagaacguaccggacguuguuucuuuaagaucggcggugacggaacaagaaugcucuacuu---------ac
                     *         **##*   * *  # *#    * * *    ** *# * #*   * **||** *   *# * #* **    * * *    #* #  * *   *##**         *
```

ALIGNMENT RESULTS - HE7c276A

=== Stwintron Sequence ===

Multiple sequence alignment

```
HE7c276A_fwrd       gguauguauggaaaauaugucgucccggcauuucagcccuuuauuuuuuuauuuuuauuuuuuagaaauaccccagguuuauuauauaaacguuaacugacauuuaaacaguaaguaaaccauuuccguucggucgccaucgagcuuaaaca-aguaacguuugguauauucu----------------aaaaacgaaggcugaagaugcugacacgacauguucu-aauccaucag
HE7c276A_RevC       cugauggau-uagaacaugucgugucagcaucuucagccuucguuuuu----------------agaauauaccaaacguuacu-uguuuaagcucgauggcgaccgaacggaaaugguuuacuuacuguuuaaaugucaguuaacguuuauauaauaaaccugggguauuucuaaaaaauaaaaauaaaaaaauaaagggcugaaaugccgggacgacauauuuuccauacauacc
                       *** **  * **#*******  *#****#*  # ****# *****                # ****  ** *#  ***#* * *  * *#*   ** *  ## *** * **   ||   ** * *** ##  * **   *#* *  * * *#***  #* **  **** #                ***** #**** #  *#****#*  *******#** *  ** ***
```

=== Internal intron ===

Multiple sequence alignment

```
HE7c276A_fwrd       guauguauggaaaauaugucgucccggcauuucagcccuuuauuuuuuuauuuuuauuuuuuagaaauaccccagguuuauuauauaaacguuaacugacauuuaaacag-----------------------------------
HE7c276A_RevC       -----------------------------------cuguuuaaaugucaguuaacguuuauauaauaaaccugggguauuucuaaaaaauaaaaauaaaaaaauaaagggcugaaaugccgggacgacauauuuuccauacauac
                                                       *  ****  * *#  **  # *** *  #* * *** || *** * *#  * *** #  **  #* *  ****  *
```

=== External intron ===

Multiple sequence alignment

```
HE7c276A_fwrd       gu---------aaguaaaccauuuccguucggucgccaucgagcuuaaacaaguaacguuugguauauucuaaaaacgaaggcugaagaugcugacacgacauguucuaauccaucag
HE7c276A_RevC       cugauggauuagaacaugucgugucagcaucuucagccuucguuuuuagaauauaccaaacguuacuuguuuaagcucgauggcgaccgaacggaaaugguuuacuu---------ac
                     *          *##*   * * ** *#    **# * *     ** *  * #** *#||#* **# *  * **     * * #**    #* ** * *   *##*          *
```

ALIGNMENT RESULTS - HE7c301A

=== Stwintron Sequence ===

Multiple sequence alignment

```
HE7c301A_fwrd       gguacguauggaaaagugccuucccggcauuuuaaccucugauauccagaaguaacccuagauuuauauaguacgggcauuagcuaacauuaaacaguaaguagagcauucucauuuugucgccgucaagcuuaaaggagcaagguccagagug-------------------------------------------------------uuuugaaaacgaaagcuagagauucugacacgauaugucuuaauacacuag
HE7c301A_RevC       cuaguguauuaagacauaucgugucagaaucucuagcuuucguuuucaaaa-------------------------------------------------------cacucuggaccuugcuccuuuaagcuugacggcgacaaaaugagaaugcucuacuuacuguuuaauguuagcuaaugcccguacuauauaaaucuaggguuacuucuggauaucagagguuaaaaugccgggaaggcacuuuuccauacguacc
                         **** #* * #*# * *  *#* **#*# * ** *  * * **#**                                                       **#***   ##*  * ** * **||** * ** *  *##    ***#**                                                       **#** * *  * ** * #*#** *#*  * * #*# * *# ****
```

=== Internal intron ===

Multiple sequence alignment

```
HE7c301A_fwrd       guacguaugg---------------------------aaaagugccuucccggcauuuuaaccucugauauccagaaguaacccuagauuuauauaguacgggcauuagcuaacauuaaacag
HE7c301A_RevC       cuguuuaauguuagcuaaugcccguacuauauaaaucuaggguuacuucuggauaucagagguuaaaaugccgggaaggcacuuu---------------------------uccauacguac
                     *   **  *                            *  **  ****  *# **#  * | *  #** #*  ****  **  *                            *  **   *
```

=== External intron ===

Multiple sequence alignment

```
HE7c301A_fwrd       -----------------------------------------guaaguagagcauucucauuuugucgccgucaagcuuaaaggagcaagguccagaguguuuugaaaacgaaagcuagagauucugacacgauaugucuuaauacacuag
HE7c301A_RevC       cuaguguauuaagacauaucgugucagaaucucuagcuuucguuuucaaaacacucuggaccuugcuccuuuaagcuugacggcgacaaaaugagaaugcucuacuuac-----------------------------------------
                                                             **   #*#*#**#***   ##*  * ** * **||** * ** *  *##   ***#**#*#*#   **
```

ALIGNMENT RESULTS - HECc114A

=== Stwintron Sequence ===

Multiple sequence alignment

```
HECc114A_fwrd       ggugaguauaaaaacaugucguuccgacauuuuagaccuugauuucuagaaaaucccauauuucuacaauauaugaauuaacuaacguu-augacaguaagucaagcauccuuauuucgucgcuaucggcguuaagaaggcaguaucuagu-----------auauucuagaaacgaaggauaaaaaugcugacacgacauguuuuaauacaccag
HECc114A_RevC       cugguguauuaaaacaugucgugucagcauuuuuauccuucguuucuagaa-----------uauacuagauacugccuucuuaacgccgauagcgacgaaauaaggaugcuugacuuacugucau-aacguuaguuaauucauauauuguagaaauaugggauuuucuagaaaucaaggucuaaaaugucggaacgacauguuuuuauacucacc
                       * **** ************  *# ****** # ****  *********           * *** * ***#   #*   *****## **# * ## *#  *** || ***  #* ## * #** ##*****   *#   #*** * **           ** *********  **** # ****** #*  ************ **** *
```

=== Internal intron ===

Multiple sequence alignment

```
HECc114A_fwrd       gugaguauaaaaacaugucguuccgacauuuuagaccuugauuucuagaaaaucccauauuucuacaauauaugaauuaacuaacguuaugacag-------------
HECc114A_RevC       -------------cugucauaacguuaguuaauucauauauuguagaaauaugggauuuucuagaaaucaaggucuaaaaugucggaacgacauguuuuuauacucac
                                   ****#*  **  * ** *  * *  ***    #** ** | ** **#    ***  * *  * ** *  **  *#****
```

=== External intron ===

Multiple sequence alignment

```
HECc114A_fwrd       ---guaagucaagcauccuuauuucgucgcuaucggcguuaagaaggcaguaucuaguauauucuagaaac--------gaaggauaaaaaugcugacacgacauguuuuaauacaccag
HECc114A_RevC       cugguguauuaaaacaugucgugucagcauuuuuauccuu--------cguuucuagaauauacuagauacugccuucuuaacgccgauagcgacgaaauaaggaugcuugacuuac---
                       **  #* **#     *# * **# *# * * # * **         ** ***** *||* ***** **         ** * # * * #* #** * #*     #** *#  **
```

ALIGNMENT RESULTS - HECc217A

=== Stwintron Sequence ===

Multiple sequence alignment

```
HECc217A_fwrd       ggugaguauaaagacaugucguucuggcauuuuagaucuugauuucugcaaaaucccccauguuucuacaauauaugaaccagcuaacguuauaacaguaaguuaagcguucuuauuucgucgcuaucggggu------------uaagaaggcaguaccugguauauucuagaaacgaaggauaaaaaugcugacacgacauguuuuaauacaucag
HECc217A_RevC       cugauguauuaaaacaugucgugucagcauuuuuauccuucguuucuagaauauaccagguacugccuucuua------------accccgauagcgacgaaauaagaacgcuuaacuuacuguuauaacguuagcugguucauauauuguagaaacaugggggauuuugcagaaaucaagaucuaaaaugccagaacgacaugucuuuauacucacc
                       # **** **#*********  ##******* # #***  *****# ** ** **   *##* *#    **            * * # *** * ## *# **** || **** #* ## * *** # * *            **    #* *##*   ** ** ** #*****  ***# # *******##  *********#** **** #
```

=== Internal intron ===

Multiple sequence alignment

```
HECc217A_fwrd       gugaguauaaagacaugucguucuggcauuuuag----aucuugauuucugcaaaaucccccauguuucuacaauauaugaaccagcuaacguuaua-acag
HECc217A_RevC       cug-uuauaacguuagcugguucauauauuguagaaacauggggauuuugcagaaaucaaga----ucuaaaaugccagaacgacaugucuuuauacucac
                     **  ***** *  *  * ****  # *** ***    **   *  ** *||* **  *   *    **** *** #  **** *  *  * *****  **
```

=== External intron ===

Multiple sequence alignment

```
HECc217A_fwrd       -----------------guaaguuaagcguucuuauuucgucgcuaucgggguuaagaaggcaguaccugguauauucuagaaacga-aggauaaaaaugcugacacgacauguuuuaauacaucag
HECc217A_RevC       cugauguauuaaaacaugucgugucagcauuuuuauccu-ucguuucuagaauauaccagguacugccuucuuaaccccgauagcgacgaaauaagaacgcuuaacuuac-----------------
                                     **    * ***#** ****##  *** * # #*##*  *  *** *|* ***  *  *##*# # * ***  ##**** **#*** *    **
```

ALIGNMENT RESULTS - Hruc29A

=== Stwintron Sequence ===

Multiple sequence alignment

```
Hruc29A_fwrd     gguacgu-----aauaccuugugguaacuuaaagauuaucacaucuccccgacccuccaag-aauggucaggguaaagcuaucucgaagauucuagauaaccugagaagaauuaaagggguaaaagaagaaguuuucugccuagaagcuacuugcacuuucuc----gaauacuaaaaaaaaugucgacgugggauguuuuuauauacguaguacguaugaaaacauguugugucagcauuuuuauugccccuuucuagaauaaucucgaguuuauaugauauauaguaauuucgcuaacauucuaacag--------uaaguauucuaguuucuucgcugucgaguugauaagagcagcgucugguauguucgagaaaccugcguuaaaaaaugcuaacacgacauguuuucguacacuag
Hruc29A_RevC     cuaguguacgaaaacaugucguguuagcauuuuuuaacgcagguuucucgaacauaccagacgcugcucuuaucaacucgacagcgaagaaacuagaauacuua--------cuguuagaauguuagcgaaauuacuauauaucauauaaacucgagauuauucuagaaaggggcaauaaaaaugcugacacaacauguuuucauacguacuacguauauaaaaacaucccacgucgacauuuuuuuuaguauuc----gagaaagugcaaguagcuucuaggcagaaaacuucuucuuuuaccccuuuuaauucuucucagguuaucuagaaucuucgagauagcuuuacccugacca-uucuuggagggucggggagaugugauaaucuuuaaguuaccacaagguauu-----acguacc
                     **     **#*  *#*** ** * *         **  * ** * #**   *** #   ** **  # #**  * *#  ******  *****  ** *#        #    #*##*   **  #** * #* *#  * # *   ***#*   **   **    #*    #** *******# ***#### *******#***#  ##|##  #***#******* ####*** #******* **#    *#    **  **   *#***   * # *  #* *# * **#  **   *##*#    #        #* **  *****  ******  #* *  **# #  ** **   # ***   **# * ** *  **         * * ** ***#*  *#**     **
```

=== Internal intron ===

Multiple sequence alignment

```
Hruc29A_fwrd     guacguaauaccuugugguaacuuaaagauuaucacaucuccccgacccuccaagaauggucaggguaaagcuaucucgaagauucuagauaaccugagaagaauuaaaagggguaaaagaagaaguuuucugccuagaagcuacuugcacuuucucgaauacuaaaaaaaaugucgacgugggauguuuuuauauacguaguacguaugaaaacauguugugucagcauuuuuauugcc---------------------------------------------------------ccuuucuagaauaaucucgaguuuauaugau------------------------------------auauaguaauuucgcuaacauucuaa---------cag
Hruc29A_RevC     cug---------uuagaauguuagcgaaauuacuaua-----------------------------------uaucauauaaacucgagauuauucuagaaagg---------------------------------------------------------ggcaauaaaaaugcugacacaacauguuuucauacguacuacguauauaaaaacaucccacgucgacauuuuuuuuaguauucgagaaagugcaaguagcuucuaggcagaaaacuucuucuuuuaccccuuuuaauucuucucagguuaucuagaaucuucgagauagcuuuacccugaccauucuuggagggucggggagaugugauaaucuuuaaguuaccacaagguauuacguac
                  *          **# ##*       *#****# * *                                   ****  # *#*#** **** *  # ****#                                                           #** *******# ***#### *******#***#  ##|##  #***#******* ####*** #******* **#                                                  #**** #  * **** **#*#* #  ***                                   ** * #****#*       *## #**          *
```

=== External intron ===

Multiple sequence alignment

```
Hruc29A_fwrd     guaaguauucuaguuucuucgcugucgaguugauaagagcagcgucugguauguucgagaaaccugcguuaaaaaaugcuaacacgacauguuuucguacacuag---------------------------------------------
Hruc29A_RevC     ---------------------------------------------cuaguguacgaaaacaugucguguuagcauuuuuuaacgcagguuucucgaacauaccagacgcugcucuuaucaacucgacagcgaagaaacuagaauacuuac
                                                              **#** *##  #*# *   #* ****  *||*  **** *#   * #*#  ##* **#**
```

ALIGNMENT RESULTS - Hruc31A

=== Stwintron Sequence ===

Multiple sequence alignment

```
Hruc31A_fwrd        gguauguaugaaaacaugucgugucggcauuuuuaucgucccuuucuaguauaaucucgagcauauauaauaugguaauuucgcuaacauucuaacaguaaguauucuaguuucuccgcuguugaguugaagagagcaacgucugguaugu-------------ucgaaaccugcguuaaaaaugcuaacaugacauguuuucguacaccag
Hruc31A_RevC        cugguguacgaaaacaugucauguuagcauuuuuaacgcagguuuucga-------------acauaccagacguugcucucuucaacucaacagcggagaaacuagaauacuuacuguuagaauguuagcgaaauuaccauauuauauaugcucgagauuauacuagaaagggacgauaaaaaugccgacacgacauguuuucauacauacc
                        ****#***********#*** #********* **#   *** # #             *#***# * *#* *  *#**  #*** #  #* * *  *#  * | *  #*  * * *#  # ***#  **#*  * *#* * #***#*             # # ***   #** *********# ***#***********#****
```

=== Internal intron ===

Multiple sequence alignment

```
Hruc31A_fwrd        --------------------------------guauguaugaaaacaugucgugucggcauuuuuaucgucccuuucuaguauaaucucgagcauauauaauaugguaauuucgcuaacauucuaacag
Hruc31A_RevC        cuguuagaauguuagcgaaauuaccauauuauauaugcucgagauuauacuagaaagggacgauaaaaaugccgacacgacauguuuucauacauac--------------------------------
                                                    #****# #** *  **## # #  ** *#  *|*  #* **  # # ##**  * **# #****#
```

=== External intron ===

Multiple sequence alignment

```
Hruc31A_fwrd        -------------------------------------guaaguauucuaguuucuccgcuguugaguugaagagagcaacgucugguauguucgaaaccugcguuaaaaaugcuaacaugacauguuuucguacaccag
Hruc31A_RevC        cugguguacgaaaacaugucauguuagcauuuuuaacgcagguuuucgaacauaccagacguugcucucuucaacucaacagcggagaaacuagaauacuuac-------------------------------------
                                                         *#* ** *** *## * #* * #****  #* || *#  ****# * *# * ##* *** ** *#*
```

ALIGNMENT RESULTS - Hruc55A

=== Stwintron Sequence ===

Multiple sequence alignment

```
Hruc55A_fwrd        gguauguaugaaaacaugucgugucggcauuuuuaucgucccuuucuaguauaauauaguaauuucgcu-aacauucuaacaguaaguauucuaguuucuucgcuguugaguugaagagagcaacgucugguauguucgagaaaccugcguuaaaaaugcuaacacgacauguuuucguacacuag
Hruc55A_RevC        cuaguguacgaaaacaugucguguuagcauuuuuaacgcagguuucucgaacauaccagacguugcucucuucaacucaacagcgaagaaacuagaauacuuacuguuagaaug-uuagcgaaauuacuauauuauacuagaaagggacgauaaaaaugccgacacgacauguuuucauacauacc
                        ****#*************** #********* **#   ***** * *#*   #**   ** * **   ** # #*****# *# *  *||*  * #* #*****# # **   ** * **   **#   *#* * *****   #** *********# ***************#****
```

=== Internal intron ===

Multiple sequence alignment

```
Hruc55A_fwrd        guauguaugaaaacaugucgugucggcauuuuuaucgucccuuucuaguauaauauaguaauuucgcuaacauucua-----------acag
Hruc55A_RevC        cugu-----------uagaauguuagcgaaauuacuauauuauacuagaaagggacgauaaaaaugccgacacgacauguuuucauacauac
                     * *           *#  #*** #**    ***# #*    * *||* *    *# #***    **# ***#  #*           * *
```

=== External intron ===

Multiple sequence alignment

```
Hruc55A_fwrd        gua--------------aguauucuaguuucuucgcuguugaguugaagagagcaacgucugguauguucgagaaaccugcguuaaaaaugcuaacacgacauguuuucguacacuag
Hruc55A_RevC        cuaguguacgaaaacaugucguguuagcauuuuuaacgcagguuucucgaacauaccagacguugcucucuucaacucaacagcgaagaaacuagaaua--------------cuuac
                     **                 # *  ***# * ** # #*# *  **   **# # * *#||#* * # #**   **  * #*# # ** * #***  * #                **
```

ALIGNMENT RESULTS - Hruc56A

=== Stwintron Sequence ===

Multiple sequence alignment

```
Hruc56A_fwrd        gguaug-uaugaaaacaugucgugccggcauuuuuauagccccuuucuagaauaaucucgagcuuauauaauauaguaauuucgcuaacauucuaacaguaaguauucuaguuucuuucuucgccgucgaguuggagagagcagcgucugguauacu-----------------cgagaaaccugcguua---------------------------------aaaauugcuaacacgacauguuuucguaacaccag
Hruc56A_RevC        cugguguuacgaaaacaugucguguuagcaauuuu---------------------------------uaacgcagguuucucg-----------------aguauaccagacgcugcucucuccaacucgacggcgaagaaagaaacuagaauacuuacuguuagaauguuagcgaaauuacuauauuauauaagcucgagauuauucuagaaaggggcuauaaaaaugccggcacgacauguuuucaua-cauacc
                        ** **#**************  #*** ****                                 ***# #**   *#***                 ***** *#** # ** # #** **# *||* #** **# # ** # **#* *****                 ***#*   **# #***                                 **** ***#  *************#** **
```

=== Internal intron ===

Multiple sequence alignment

```
Hruc56A_fwrd        guauguaugaaaacaugucgugccggcauuuuuauagccccuuucuagaauaaucucgagcuuauauaauauaguaauuucgcuaacauucuaacag-----------------
Hruc56A_RevC        -----------------cuguuagaauguuagcgaaauuacuauauuauauaagcucgagauuauucuagaaaggggcuauaaaaaugccggcacgacauguuuucauacauac
                                     # **   ##  **  #  *#   ** * * # **** **||** **** # * * **   #*  #  **  ##   ** #
```

=== External intron ===

Multiple sequence alignment

```
Hruc56A_fwrd        gu------------------------------------------------aaguauucuaguuucuuucuucgccgucgaguuggagagagcagcgucugguauacucgagaaaccugcguuaaaaauugcuaacacgacauguuuucguaacaccag
Hruc56A_RevC        cugguguuacgaaaacaugucguguuagcaauuuuuaacgcagguuucucgaguauaccagacgcugcucucuccaacucgacggcgaagaaagaaacuagaauacuu------------------------------------------------ac
                     *                                                 ***** *#** # ** # #** **# *||* #** **# # ** # **#* *****                                                 *
```

ALIGNMENT RESULTS - Hruc59A

=== Stwintron Sequence ===

Multiple sequence alignment

```
Hruc59A_fwrd        gguau--guaugaaaacaugucguugucggcauuuuuu----------------------------------------------------aucaccccuuucuagaauaaccucgagcuuauauaauauuuuugcuaacauucuaacaguaaguaauucuaguuuccuuggcugcugcggucugguauauuucgagaaacccaacguugaaaaugugcuaac-acgacaucuuuucgaaauaaaaag
Hruc59A_RevC        cuuuuuauuucgaaaagaugucg-uguuagcacauuuucaacguuggguuucucgaaauauaccagaccgcagcagccaaggaaacuagaauuacuuacuguuagaauguuagcaaaaauauuauauaagcucgagguuauucuagaaaggggugau----------------------------------------------------aaaaaaugccgacaacgacauguuuucaua--cauacc
                      * *   * #***** ****** *** #***# ****                                                    ** **   #*  ******     *#*#  *** | ***  #*#*     ******  *#   ** **                                                    **** #***# ** ******* *****# *   * *
```

=== Internal intron ===

Multiple sequence alignment

```
Hruc59A_fwrd        ------guauguaugaaaacaugucguugucggcauuuuuuaucaccccuuucuagaauaaccucgagcuuau------------------------------auaauauuuuugcuaacauucuaacag
Hruc59A_RevC        cuguuagaauguuagcaaaaauauuau------------------------------auaagcucgagguuauucuagaaaggggugauaaaaaaugccgacaacgacauguuuucauacauac------
                          * ****  * *** **#* #*                              **** **||** ****                              *# *#** *** *  **** *
```

=== External intron ===

Multiple sequence alignment

```
Hruc59A_fwrd        guaaguaauucuaguuuccuuggcugcugcggucugguauauuucgagaaacccaacguugaaaaugugcuaacacgacauc----------uuuucgaaauaaa----------------------------------aag
Hruc59A_RevC        cu----------------------------------uuuuauuucgaaaa----------gaugucguguuagcacauuuucaacguuggguuucucgaaauauaccagaccgcagcagccaaggaaacuagaauuacuuac
                     *                                   * ********#**          **   #*** || ***#   **          **#******** *                                   *
```

ALIGNMENT RESULTS - Naboc005A

=== Stwintron Sequence ===

Multiple sequence alignment

```
Naboc005A_fwrd      gguauguauugaaucaauuaaaccugucguuccaauuccccaagccaga----------------gcccaagccuuuguuucuggaaacccgccgacguauucgagggcuaacauucgccacaguauguсuucguuuauaucuccgagucaaucgcagcugcggcccagagaacuggucccagaaaguaaggcuuuagaagcugaacugacauguuucaaug---caauag-----
Naboc005A_RevC      -----cuauug---cauugaaacaugucaguucagcuucuaaagccuuacuuucugggaccaguucucugggccgcagcugcgauugacucggagauauaaacgaagacauacuguggcgaauguuagcccucgaauacgucggcggguuuccagaaacaaaggcuugg----------------gcucuggcuuggggaauuggaacgacagguuuaauugauucaauacauacc
                          *****   ** * **** ****# * ** #* *  *****  *                  *   *** # *#* * #   ** **  ** #**  ***#*#*  **  * || *  **  *#*#***  **# **  ** **   # * *#* # ***   *                  *  *****  * *# ** * #**** **** * **   *****
```

=== Internal intron ===

Multiple sequence alignment

```
Naboc005A_fwrd      gu---auguauugaaucaauuaaaccugucguuccaauuccccaagccagagcccaagccuuuguuucuggaaacccgccgacguauucgagggcuaacauucgccacag
Naboc005A_RevC      cuguggcgaauguuagcccucgaauacgucggcggguuuccagaaacaaaggcuugggcucuggcuuggggaauuggaacgacagguuuaauugauucaauacau---ac
                     *    #* **   * *  *# **  #**** #    ****  **#* *# ** || ** #* *#**  ****    # ****#  ** #*  * *   ** *#    *
```

=== External intron ===

Multiple sequence alignment

```
Naboc005A_fwrd      gu-----------augucuucguuuauaucuccgaguc-----------------------aaucgcagcugcggcccagagaacuggucccagaaaguaaggcuuuagaagcugaacugacauguuucaaugcaauag
Naboc005A_RevC      cuauugcauugaaacaugucaguucagcuucuaaagccuuacuuucugggaccaguucucugggccgcagcugcgauu-----------------------gacucggagauauaaacgaagacau-----------ac
                     *           *##* *# ***#*  * #  #**#*                          #****||****#                          *#**#  # *  *#*** #* *##*           *
```

ALIGNMENT RESULTS - Naboc011A

=== Stwintron Sequence ===

Multiple sequence alignment

```
Naboc011A_fwrd      gguauguauuugaaccugucgucacaguucccuaagccuuucuuuc-------------------------------------auagaagaauccauuuuauucaaaggcuaacauuugccauaguauguauucguuuacuuccgagacaaucgcagccacggcccagaggguucuagaaaguauggcuugagaaacugaacugacaugucuuugugcacuag
Naboc011A_RevC      cuagugcacaaagacaugucaguucaguuucucaagccauacuuucuagaacccucugggccguggcugcgauugucucggaaguaaacgaauacauacuauggcaaauguuagccuuugaauaaaauggauucuucuau-------------------------------------gaaagaaaggcuuagggaacugugacgacagguucaaauacauacc
                        **#*#  # ** ****#   ***** * #***** * *****                                      **#* **** *** #***   **#  *|*  #**   ***# *** **** *#**                                      ***** * *****# * *****   #**** ** #  #*#**
```

=== Internal intron ===

Multiple sequence alignment

```
Naboc011A_fwrd      guauguauuugaaccugucgucacaguucccuaagccuuucuuucauagaagaauccauuuuauucaaaggcuaacauuugc--cauag
Naboc011A_RevC      cuaug--gcaaauguuagccuuugaauaaaauggauucuucuaugaaagaaaggcuuagggaacugugacgacagguucaaauacauac
                     ****   # #*   *# * *   *#*    *  #  #**** *|* ****#  #  *    *#*   * * #*   *# #   ****
```

=== External intron ===

Multiple sequence alignment

```
Naboc011A_fwrd      gu-----------auguauucguuua----------------cuuccgagacaaucgcagccacggcccagaggguucuagaaaguauggcuugagaaacugaacugacaugucuuugugcacuag
Naboc011A_RevC      cuagugcacaaagacaugucaguucaguuucucaagccauacuuucuagaacccucugggccguggcugcgauugucucggaag----------------uaaacgaauacau-----------ac
                     *           *##* *# ***#*                 *** # #**  **   ***||***   **  **# # ***                 *#*** #* *##*           *
```

ALIGNMENT RESULTS - Naboc037A

=== Stwintron Sequence ===

Multiple sequence alignment

```
Naboc037A_fwrd      gguauguauuuaaaccugucguuccaguucccugggccuuuguuucuaaaagaauc-------------cguuauacucgaaggcuaacauugccauaguauguauucauugauucccgagacaaucgcagcgauggccccgauaguucuagaaaauaaggcuugagaagcugaacugacauauuuugaugcaccag
Naboc037A_RevC      cuggugcaucaaaauaugucaguucagcuucucaagccuuauuuucuagaacuaucggggccaucgcugcgauugucucgggaaucaaugaauacauacuauggcaauguuagccuucgaguauaacg-------------gauucuuuuagaaacaaaggcccagggaacuggaacgacagguuuaaauacauacc
                        **#**# ***  ****# * ***#* * ###*****  ****** **  ***             ** *   ****  ## #**      ****|****      **# ##  ****   * **             ***  ** ******  *****### * *#*** * #****  *** #**#**
```

=== Internal intron ===

Multiple sequence alignment

```
Naboc037A_fwrd      guauguauuuaaaccugucguuccaguucccugggccuuuguuucuaaaagaauccguuauacucgaaggcuaacauugc--------------------cauag
Naboc037A_RevC      cuaug--------------------gcaauguuagccuucgaguauaacggauucuuuuagaaacaaaggcccagggaacuggaacgacagguuuaaauacauac
                     ****                    *#    * #*****#*  * ***  **|**  *** *  *#*****# *    #*                    ****
```

=== External intron ===

Multiple sequence alignment

```
Naboc037A_fwrd      ---------------------------guauguauucauugauucccgagacaaucgcagcgauggccccgauaguucuagaaaauaaggcuugagaagcugaacugacauauuuugaugcaccag
Naboc037A_RevC      cuggugcaucaaaauaugucaguucagcuucucaagccuuauuuucuagaacuaucggggccaucgcugcgauugucucgggaaucaaug--------------------------aauaca-uac
                                                * # #*  * **# ** * # #** ****  ** || **  **** **# # * ** #** *                          #**#**  *
```

ALIGNMENT RESULTS - Naboc037B

=== Stwintron Sequence ===

Multiple sequence alignment

```
Naboc037B_fwrd      ----gguauuuaaaucuaucauuccaauucuucaagccuucguuucuaua-----------------gaacccguuauauucgagggcuaacauuugcuauaguauauauucguucauuucugagacgaucgcagcggcggcccagauagcucuggaaauuaaagccuaagaagcugaacuaacauguuuuaaugcacuag
Naboc037B_RevC      cuagugcauuaaaacauguuaguucagcuucuuaggcuuuaauuuccagagcuaucugggccgccgcugcgaucgucucagaaaugaacgaauauauacuauagcaaauguuagcccucgaauauaacgggu-----------------ucuauagaaacgaaggcuugaagaauuggaaugauagauuuaaauacc----
                         *#*** ***# * * * * ** #* #* * ** ** #****#* *                  *    ***# #*   # *##* ** ** *#**||**#* ** ** *##* #   *# #***                      ** *#****# ** ** * *# *# ** * * * * #*** ***#*
```

=== Internal intron ===

Multiple sequence alignment

```
Naboc037B_fwrd      guauuuaaaucuaucauuccaauucuucaagccuucguuucuauagaacccguuauauucgagggcu--aacauuugcuauag-------------
Naboc037B_RevC      -------------cuauagcaaauguu--agcccucgaauauaacggguucuauagaaacgaaggcuugaagaauuggaaugauagauuuaaauac
                                 # **  *** * **  ****#***  * **  * || *  ** *  ***#****  ** * ***  ** #
```

=== External intron ===

Multiple sequence alignment

```
Naboc037B_fwrd      gu-----------auauauucguuc----------------auuucugagacgaucgcagcggcggcccagauagcucuggaaauuaaagccuaagaagcugaacuaacauguuuuaaugcacuag
Naboc037B_RevC      cuagugcauuaaaacauguuaguucagcuucuuaggcuuuaauuuccagagcuaucugggccgccgcugcgaucgucucagaaa---------------ugaacgaauauau-----------ac
                     *           *#** ** ****                *****## # * ***   ** || **   *** * # ##****               ***** ** **#*           *
```

ALIGNMENT RESULTS - Naboc056A

=== Stwintron Sequence ===

Multiple sequence alignment

```
Naboc056A_fwrd      --gguauguauauaagccuuuguuucuugaagaauccguucuacucaaaggcug--------------acaauugcuacaguauguagucauuugauucugagacaauugcagcgauggccccuauagcucuagaaaauggcgcuugagacacuaaacugacauguucuuaugcgccag
Naboc056A_RevC      cuggcgcauaagaacaugucaguuuagugucucaagcgccauuuucuagagcuauaggggccaucgcugcaauugucucagaaucaaaugacuacauacuguagcaauugu--------------cagccuuugaguagaacggauucuucaagaaacaaaggcuuauauacauacc--
                      **# ##**   * #  *# ****  **    *  **## *  ** * #***#               ****** # *** **  *#*|*#*  ** *** # ******               #***# * **  * ##**  *    **  **** #*  # *   **## #**
```

=== Internal intron ===

Multiple sequence alignment

```
Naboc056A_fwrd      guauguauauaagccuuuguuucuugaagaauccguucuacucaaaggcugacaauugcuacag----
Naboc056A_RevC      ----cuguagcaauugucagccuuugaguagaacggauucuucaagaaacaaaggcuuauauacauac
                         * **  *#   *## ## ****  *   ||   *  **** ## ##*   #*  ** *
```

=== External intron ===

Multiple sequence alignment

```
Naboc056A_fwrd      ---guauguagucauuugauucugagacaauugcagcgauggccccuauagcucuagaaaauggcgcuugagacacuaaacugacauguucuuaugcgccag
Naboc056A_RevC      cuggcgcauaagaacaugucaguuuagugucucaagcgccauuuucuagagcuauaggggccaucgcugcaauugucucagaaucaaaugacuacauac---
                       *# ##**#  *# ** #  *  #    #*  **** ##    *** *||* ***    ## ****  *#    #  *  # ** #*  #**## #*
```

ALIGNMENT RESULTS - Naboc066A

=== Stwintron Sequence ===

Multiple sequence alignment

```
Naboc066A_fwrd      ggugugugaguuaaaucugucauuccaacucucuaagccuucauuucuagaagacuc-----------------cguuacauacgcaggcuaacauuugccacaguaugugauucguuuauuucugagacaaucacaaccauagccccgauaauucuagaaaguaaggcuugggaaacugaacugacauguuuugaugcaccag
Naboc066A_RevC      cuggugcaucaaaacaugucaguucaguuucccaagccuuacuuucuagaauuaucggggcuaugguugugauugucucagaaauaaacgaauacauacuguggcaaauguuagccugcguauguaacg-----------------gagucuucuagaaaugaaggcuuagagaguuggaaugacagauuuaacuacacacc
                       ***#* # ***# ***** * **  * #*#*******  *********   **                  **# ** * # *##* ** *# *#* || *#* #* ** *##* # * ** #**                 **   *********  *******#*# *  ** * ***** #*** # *#***
```

=== Internal intron ===

Multiple sequence alignment

```
Naboc066A_fwrd      guguguaguuaaaucugucauuccaacucucuaagccuucauuucuagaagacuccguuacauacgcaggcuaaca----------------uuug---ccacag
Naboc066A_RevC      cugug--gcaaa-----------------uguuagccugcguauguaacggagucuucuagaaaugaaggcuuagagaguuggaaugacagauuuaacuacacac
                     ****  *# **                 * * ***** * * * **#  **|**  #** * * * ***** * *                ***#    ****
```

=== External intron ===

Multiple sequence alignment

```
Naboc066A_fwrd      gu-----------auguauucguuua----------------uuucugagacaaucacaaccauagccccgauaauucuagaaaguaaggcuugggaaacugaacugacauguuuugaugcaccag
Naboc066A_RevC      cuggugcaucaaaacaugucaguucaguuucccaagccuuacuuucuagaauuaucggggcuaugguugugauugucucagaaa----------------uaaacgaauacau-----------ac
                     *           *##* *# ***#*                *****# #*  ***    * || *    ***  *# #*****                *#*** #* *##*           *
```

ALIGNMENT RESULTS - Naboc073A

=== Stwintron Sequence ===

Multiple sequence alignment

```
Naboc073A_fwrd      gguauguguuuaaaccuauaguucuagcucuuuaagccuuuguuucuuggagaaucug--------------------cuauauuagaugacuaacauuugccguaguauguauucauuuauuucgaaggcgauaguggcuauggcccaaauagguccggaaaguaaggcuuuga-aaccgaacugacauguuuuuaugcaccag
Naboc073A_RevC      cuggugcauaaaaacaugucaguucgg-uuucaaagccuuacuuuccggaccuauuugggccauagccacuaucgccuucgaaauaaaugaauacauacuacggcaaauguuagucaucuaauauag--------------------cagauucuccaagaaacaaaggcuuaaagagcuagaacuauagguuuaaacacauacc
                        **##*  **** * * # * # * * *# *******  ****# *#   ** **                     #  * **#**** **    #*#*|*#*#    ** ****#** *  #                     ** **   #* #****  ******* #* * * # * # * * ****  *##**
```

=== Internal intron ===

Multiple sequence alignment

```
Naboc073A_fwrd      gu-------auguguuuaaaccuauaguucuagcucuuuaagccuuuguuucuuggagaaucugcuauauuagaugacuaacauuugccguag
Naboc073A_RevC      cuacggcaaauguuagucaucuaauauagcagauucuccaagaaacaaaggcuuaaagagcuagaacuauagguuuaaacacau-------ac
                     *       ****   * * *  ***   *  # ***##***   #|#   ***##*** #  *   ***  * * *   ****       *
```

=== External intron ===

Multiple sequence alignment

```
Naboc073A_fwrd      gu-----------auguauucauuuauuucgaaggcgauaguggcuauggcccaaauagguccggaaaguaaggcuuugaaaccgaacugacauguuuuuaugcaccag
Naboc073A_RevC      cuggugcauaaaaacaugucaguucgguuucaaagccuuacuuuccggaccuauuugggccauagccacuaucgccuucgaaauaaaugaauacau-----------ac
                     *           *##* *#  **#  **  **#**  ** *  *#  # *   |   * #  #*  * **  **#**  **  #**  #* *##*           *
```

ALIGNMENT RESULTS - Naboc079A

=== Stwintron Sequence ===

Multiple sequence alignment

```
Naboc079A_fwrd      ----------------------gguauguauuuaagccuuuguuucuagaagaauccg---------------------------------uuauacucgaaggcuaacauuugccauaguauguauucauugauuccugagacacucgcagcgauggccccgauaguucuagaaaguaagacuugagaaacugaacugacauauuuugaugcacuag
Naboc079A_RevC      cuagugcaucaaaauaugucaguucaguuucucaagucuuacuuucuagaacuaucggggccaucgcugcgagugucucaggaaucaaugaauacauacuauggcaaauguuagccuucgaguaua---------------------------------acggauucuucuagaaacaaaggcuuaaaua----------------------cauacc
                                            #*  * #*#*** ***  *********  *** *                                  **#*  * * *** **||** *** * *  *#**                                  * ***  *********  *** ***#*# *                      **
```

=== Internal intron ===

Multiple sequence alignment

```
Naboc079A_fwrd      guaug---uauuuaagccuuuguuucuagaagaauccguuauacucgaaggcuaacauuugccauag
Naboc079A_RevC      cuauggcaaauguuagccuucgaguauaacggauucuucuagaaacaaaggcuuaaaua---cauac
                     ****    ** * ******#*  * **#  **|**  #** *  *#****** * **    ****
```

=== External intron ===

Multiple sequence alignment

```
Naboc079A_fwrd      gua-uguauuca--------------------------uugauuccugagacacucgcagcgauggccccgauaguucuagaaaguaagacuugagaaacugaacugacauauuuugaugcacuag
Naboc079A_RevC      cuagugcaucaaaauaugucaguucaguuucucaagucuuacuuucuagaacuaucggggccaucgcugcgagugucucaggaaucaa--------------------------ugaauaca-uac
                     ** **#**# *                          **# ** **# #**  ***  ** || **  ***  **# #** ** #**                          * #**#** **
```

ALIGNMENT RESULTS - Naboc124A

=== Stwintron Sequence ===

Multiple sequence alignment

```
Naboc124A_fwrd      gguauguauuuaaaccugucguucuagcugccugagcauuuguuuuauagaagaauccguuauauucgaccgcuaagauaugcuacaguauguauucguuuuauuuccgagacaaucucagccacggagccacggccuaggacccuagauagaucuagaaggcaag-gcugaagaagaugaacugacaugucucuaugcacuag
Naboc124A_RevC      cuagugcauagagacaugucaguucaucuucuucagc-cuugccuucuagaucuaucuaggguccuaggccguggcuccguggcugagauugucucggaaauaaaacgaauacauacuguagcauaucuuagcggucgaauauaacggauucuucuauaaaacaaaugcucaggcagcuagaacgacagguuuaaauacauacc
                        **#**  * ** ****# * #* ** * * *** #***##** ****   *** #   * #* * ***      # ** #  **  *** *# *   ||   * #* ***  **  # ** #      *** * *# *   # ***   **** **##***# *** * * ** *# * #**** ** *  **#**
```

=== Internal intron ===

Multiple sequence alignment

```
Naboc124A_fwrd      guauguauuuaaaccugucguucuagcugccugagcauuuguuuuauagaagaauccguuauauucgaccgcuaagauaugcuacag--
Naboc124A_RevC      --cuguagcauaucuuagcggucgaauauaacggauucuucuauaaaacaaaugcucaggcagcuagaacgacagguuuaaauacauac
                       **** #  * * *# ** ** *#     #* #  #** * *|* * **#  # *#     #* ** ** #* * *  # ****
```

=== External intron ===

Multiple sequence alignment

```
Naboc124A_fwrd      guauguauucguuuuauuuccgagacaaucucagccacggagccacggccuaggacccuagauagaucuagaaggcaaggcugaagaagaugaacugacaugucucuaugcacuag------------
Naboc124A_RevC      ------------cuagugcauagagacaugucaguucaucuucuucagccuugccuucuagaucuaucuaggguccuaggccguggcuccguggcugagauugucucggaaauaaaacgaauacauac
                                #*  * #  # #   ** ****        *  *#**** *    ******||******    * ****#*  *        **** **   # #  # *  *#
```

ALIGNMENT RESULTS - Naboc173A

=== Stwintron Sequence ===

Multiple sequence alignment

```
Naboc173A_fwrd      ggugugu auuuaaaccugucguuccaguuccuuaacccuuuguuucugaaaacuccauuauauu------------------cacaggcuaacauuugccgcaguauguauucguuuauucccgagacaaucgcagccauugccccgacaguucuagaaaauaaggcuuaagaaacugaacugacauguuuugaugcaccag
Naboc173A_RevC      cuggugcaucaaaacaugucaguucaguuucuuaagccuuauuuucuagaacugucggggcaauggcugcgauugucucgggaauaaacgaauacauacugcggcaaauguuagccu------------------gugaauauaauggaguuuucagaaacaaaggguuaaggaacuggaacgacagguuuaaauacacacc
                       ***#**# **** ****# * ***** ***** ****  *****# **    *    #* *                  * *##* ** *# *#* || *#* #* ** *###*                  *  * *#    *    ** #*****  **** ***** ***** * #**** **** #**#***
```

=== Internal intron ===

Multiple sequence alignment

```
Naboc173A_fwrd      guguguauuuaaaccugucguuccaguuccuuaacccuuuguuucugaaaacuccauuauauucacaggcuaacauuugc-------------------------cgcag
Naboc173A_RevC      cugcg-------------------------gcaaauguuagccugugaauauaauggaguuuucagaaacaaaggguuaaggaacuggaacgacagguuuaaauacacac
                     **#*                         #**   ** *##* **** *   ||   * **** *##* **   **#                          *#**
```

=== External intron ===

Multiple sequence alignment

```
Naboc173A_fwrd      ---------------------------guauguauucguuuauucccgagacaaucgcagccauugccccgacaguucuagaaaauaaggcuuaagaaacugaacugacauguuuugaugcaccag
Naboc173A_RevC      cuggugcaucaaaacaugucaguucaguuucuuaagccuuauuuucuagaacugucggggcaauggcugcgauugucucgggaauaaacgaauaca---------------------------uac
                                                * # **  * **  ** * # #**  ***  ** || **  ***  **# # * **  ** *  ** #                           *
```

ALIGNMENT RESULTS - Naboc184A

=== Stwintron Sequence ===

Multiple sequence alignment

```
Naboc184A_fwrd      gguguguauuugaaccugucgcuccaguuccccaagccuauauuugagggcuaacauuugccacaguauguauucguucauucugagacaaucgcagcugcggccuagauaguccuagaaugua-------------------------------------aggcuuuagaagcugaacugacauguuuuuaugcacuag
Naboc184A_RevC      cuagugcauaaaaacaugucaguucagcuucuaaagccu-------------------------------------uacauucuaggacuaucuaggccgcagcugcgauugucucagaaugaacgaauacauacuguggcaaauguuagcccucaaauauaggcuuggggaacuggagcgacagguucaaauacacacc
                       ***#**  #*** ****# * ***#* *  ******                                     * ******# *** ***   **#||#**   *** *** #****** *                                     ******  * *#*** * #**** ***#  **#***
```

=== Internal intron ===

Multiple sequence alignment

```
Naboc184A_fwrd      gugug---uauuugaaccugucgcuccaguuccccaagccuauauuugagggcuaacauuugccacag
Naboc184A_RevC      cuguggcaaauguuagcccucaaauauaggcuuggggaacuggagcgacagguucaaaua---cacac
                     ****    ** * * **# # # *  ** #   || # **  * # # #** * * **    ****
```

=== External intron ===

Multiple sequence alignment

```
Naboc184A_fwrd      gua----------------------------uguauucguucauucugagacaaucgcagcugcggccuagauaguccuagaauguaaggcuuuagaagcugaacugacauguuuuuaugcacuag
Naboc184A_RevC      cuagugcauaaaaacaugucaguucagcuucuaaagccuuacauucuaggacuaucuaggccgcagcugcgauugucucagaaugaacgaauaca----------------------------uac
                     **                            *# * #* * ******# *** ***   **#||#**   *** *** #****** * *# * #*                            **
```

ALIGNMENT RESULTS - Naboc196A

=== Stwintron Sequence ===

Multiple sequence alignment

```
Naboc196A_fwrd      gguacguauuucauccuguugauccaguccccuuggccuuuguuucuaggagaacac--------------auuguuuucga---augcuaauauuuguuauaguacguauucguuuacuucugagauaaucgcagcugcggcucgggugguucuagaaaguaagacuuaagaagcugagcugacauguuuuuaugcacuag
Naboc196A_RevC      cuagugcauaaaaacaugucagcucagcuucuuaagucuuacuuucuagaaccacccgagccgcagcugcgauuaucucagaaguaaacgaauacguacuauaacaaauauuagc---auucgaaaacaau--------------guguucuccuagaaacaaaggccaaggggacuggaucaacaggaugaaauacguacc
                         *#**   * * ***## # ***#  * * #* ***  *******#*  ** *              ***#*#*# **   * #* ****# *##*||*##* #**** *#    *** #*#*#***              * **  *#*******  *** *# * *  #*** # ##*** * *   **#*
```

=== Internal intron ===

Multiple sequence alignment

```
Naboc196A_fwrd      guac------------------------guauuucauccuguugauccaguccccuuggccuuuguuucuaggagaacacauuguuuucgaaugcuaauauuuguuauag
Naboc196A_RevC      cuauaacaaauauuagcauucgaaaacaauguguucuccuagaaacaaaggccaaggggacuggaucaacaggaugaaauac------------------------guac
                     **                         #* * *  ****#  #*#  ** ** || ** **  #*#  #****  * * *#                         **
```

=== External intron ===

Multiple sequence alignment

```
Naboc196A_fwrd      gua---------------------------cguauucguuuacuucugagauaaucgcagcugcggcucgggugguucuagaaaguaagacuuaagaagcugagcugacauguuuuuaugcacuag
Naboc196A_RevC      cuagugcauaaaaacaugucagcucagcuucuuaagucuuacuuucuagaaccacccgagccgcagcugcgauuaucucagaaguaaacgaauacg---------------------------uac
                     **                           * **    **   ****# #*# *#*  ***#||#***  *#* #*# #****   **    ** *                           **
```

ALIGNMENT RESULTS - Naboc196B

=== Stwintron Sequence ===

Multiple sequence alignment

```
Naboc196B_fwrd      -----gguaaguguuuaaaccuauc-------guccgagcuccccaagccuuggcuucuagaa-----------------gcauauguuauauucgggggcuaauguuucucgcaguauguauucgcuuauuucuaugauaaacgcagccgcggcccggauauuucuagaaaauaaggcugaagaagccaagucaaaccaagacaagcuaacauguuucuacgcag
Naboc196B_RevC      cugcguagaaacauguuagcuugucuugguuugacuuggcuucuucagccuuauuuucuagaaauauccgggccgcggcugcguuuaucauagaaauaagcgaauacauacugcgagaaacauuagcccccgaauauaacauaugc-----------------uucuagaagccaaggcuuggggagcucgg-------acgauagguuuaaacacuuacc-----
                          # **###* * * * * **       * *   *** *   ******#  ********                 ** * *#*#***   # ##** ***## * # || # * ##*** **## #   ***#*#* * **                 ********  #******   * ***   *       * ** * * * * *###** #
```

=== Internal intron ===

Multiple sequence alignment

```
Naboc196B_fwrd      guaagug----------------uuuaaaccuaucguccgagcuccccaagccuuggcuucuagaagcauauguuauauucgggggcuaauguuucucgcag
Naboc196B_RevC      cugcgagaaacauuagcccccgaauauaacauaugcuucuagaagccaaggcuuggggagcucggacgauagguuuaa----------------acacuuac
                     *  * *                 *  *** ***  * * **   ** * || * **   ** * *  *** ***  *                 * *  *
```

=== External intron ===

Multiple sequence alignment

```
Naboc196B_fwrd      gu--------auguauucgcuuauuucuaugauaaacgcagccgcggcccggauauuucuagaaaauaaggcugaagaagccaagucaaaccaagacaagcuaacauguuucuacgcag
Naboc196B_RevC      cugcguagaaacauguuagcuugucuugguuugacuuggcuucuucagccuuauuuucuagaaauauccgggccgcggcugcguuuaucauagaaauaagcgaauacau--------ac
                     *        *##* ** **** *#*   *   *   *    *   # **  ** **# | #** **  ** #   *    *   *   *   *#* **** ** *##*        *
```

ALIGNMENT RESULTS - Naboc199A

=== Stwintron Sequence ===

Multiple sequence alignment

```
Naboc199A_fwrd      gguauguau-ugaaacuugucguuccaauucuuuagccauuuuucuggaagaauccguuauuggagggcuaacauuugccguaguauguauucguuuauuucgaagauaaucgcagccgcggcccggaugauucuag----------------------aaaguaaagcuuaaggagccgaguugacauccuuuucaaacacuag
Naboc199A_RevC      cuaguguuugaaaaggaugucaacucggcuccuuaagcuuuacuuu---------------------cuagaaucauccgggccgcggcugcgauuaucuucgaaauaaacgaauacauacuacggcaaauguuagcccuccaauaacggauucuuccagaaaaauggcuaaagaauuggaacgacaaguuuc-aauacauacc
                        *** *  #**   ****# # *  #**#***# * ** #* *                      ***  **#  ***   # #*  * ** ****#* | *#**** ** *  *# #   ***  #**  ***                      * *# ** * #***#**#  * # #****   **#  * ***
```

=== Internal intron ===

Multiple sequence alignment

```
Naboc199A_fwrd      guauguauugaaacuugucguuccaauucuuuagccauuuuucuggaagaauccgu----uauuggagggcuaacauuugccguag--
Naboc199A_RevC      --cuacggcaaauguuagcccuccaa----uaacggauucuuccagaaaaauggcuaaagaauuggaacgacaaguuucaauacauac
                       *##  ##**  **# * #*****    * *   ***#***||***#***   *     ******# * #**  **##  ##*
```

=== External intron ===

Multiple sequence alignment

```
Naboc199A_fwrd      gua----------------------------uguauucguuuauuucgaagauaaucgcagccgcggcccggaugauucuagaaaguaaagcuuaaggagccgaguugacauccuuuucaaacacuag
Naboc199A_RevC      cuaguguuugaaaaggaugucaacucggcuccuuaagcuuuacuuucuagaaucauccgggccgcggcugcgauuaucuucgaaauaaacgaauaca----------------------------uac
                     **                            # **  * **  **** * #** ***   ***||***   *** **# * ****  ** *  ** #                            **
```

ALIGNMENT RESULTS - Naboc199B

=== Stwintron Sequence ===

Multiple sequence alignment

```
Naboc199B_fwrd      gguaugcauuugaacuuguaguucugguuccccaagccuuugucucugggagaacccgcguu-----------------------------ugauauucgagggcuaaucuuugcccuaguaugauaucguuuauuucugagguaaccguagcuacagcucggacacuucuagaaaguaagacuuaagaagcugagcugauaugcuuucauguacuag
Naboc199B_RevC      cuaguacaugaaagcauaucagcucagcuucuuaagucuuacuuucuagaaguguccgagcuguagcuacgguuaccucagaaauaaacgaauacauacuagggcaaagauuagcccucgaaua-----------------------------ucaaacgcggguucucccagagacaaaggcuuggggaaccagaacuacaaguucaaaugcauacc
                        *#***  #* * *#* # # ##*#* *  *** ***  * ***#*#**   *** *#*                              *#** * ***** **||** ***** * **#                             *  *#* ***   **#*#*** *  *** ***  * *#*## # # *#* * *#  ***#*
```

=== Internal intron ===

Multiple sequence alignment

```
Naboc199B_fwrd      -----------guaugcauuugaacuuguaguucugguuccccaagccuuugucucugggagaacccgcguuugauauucgagggcuaaucuuugcccuag
Naboc199B_RevC      cuagggcaaagauuagcccucgaauaucaaacgcggguucucccagagacaaaggcuuggggaaccagaacuacaaguucaaaugc-----------auac
                               #*  ** #*#***  *  *## * ***** ** **   #|#   ** ** ***** * ##*  *  ***#*# **            **
```

=== External intron ===

Multiple sequence alignment

```
Naboc199B_fwrd      gua---------------------------uguauucguuuauuucugagguaaccguagcuacagcucggacacuucuagaaaguaagacuuaagaagcugagcugauaugcuuucauguacuag
Naboc199B_RevC      cuaguacaugaaagcauaucagcucagcuucuuaagucuuacuuucuagaaguguccgagcuguagcuacgguuaccucagaaauaaacgaauaca---------------------------uac
                     **                           # **    **  *****# ##    *  ****||****  *    ## #*****  **    ** #                           **
```

ALIGNMENT RESULTS - Naboc202A

=== Stwintron Sequence ===

Multiple sequence alignment

```
Naboc202A_fwrd      ggucuguauuuaaaccuaucguuccaguuccccaagccuucgauuccagaag----------------------------------------aauccguuauauucgagagcuaacauuugccauaguauguaacuguucguuuauuuccgagacacacucgcagccacggcccagacgguucuuagaaaguaagacucaagaaacugaacugacaugcuuguaugcacuag
Naboc202A_RevC      cuagugcauacaagcaugucaguucaguuucuugagucuuacuuucuaagaaccgucugggccguggcugcgagugugucucggaaauaaacgaacaguuacauacuauggcaaauguuagcucucgaauauaacggauu----------------------------------------cuucuggaaucgaaggcuuggggaacuggaacgauagguuuaaauacagacc
                        **#** #** * * **# * ***** *   ** ***   *** *# *#                                         * * ****#** * *  ** **||** **  * * **#**** * *                                         #* #* ***   *** **   * ***** * #** * * **# **#**
```

=== Internal intron ===

Multiple sequence alignment

```
Naboc202A_fwrd      ----------------gucuguauuuaaaccuaucguuccaguuccccaagccuucgauuccagaagaauccguuauauucgagagcuaacauuugccauag
Naboc202A_RevC      cuauggcaaauguuagcucucgaauauaacggauucuucuggaaucgaaggcuuggggaacuggaacgauagguuuaaauacagac----------------
                                     ***  * *  ***  **  ***  *   *  * || *  *   *  ***  **  ***  * *  ***
```

=== External intron ===

Multiple sequence alignment

```
Naboc202A_fwrd      gua-----------uguaacuguucguuuauuuccgagacacacucgcagccacggcccagacgguucuuagaaaguaagacucaagaaacugaacugacaugcuuguaugcacuag
Naboc202A_RevC      cuagugcauacaagcaugucaguucaguuucuugagucuuacuuucuaagaaccgucugggccguggcugcgagugugucucggaaauaaacgaacaguuaca-----------uac
                     **           ##*  * ****# ** #**  *    **  **  **   ** * | * **   **  **  **    *  **# ** #**** *  *##           **
```

ALIGNMENT RESULTS - Naboc249A

=== Stwintron Sequence ===

Multiple sequence alignment

```
Naboc249A_fwrd      gguauguauugaaucccgucauucuaguccccuaagccucuauuuccggaaggauacguuagagucgaagacuaauauuugccauaguauguauucguuuacuuccgaggucaucgcggccacgcuccggauaguucuagaagcuaauucuaugaaaaccaugcag---------------------------------------------
Naboc249A_RevC      ---------------------------------------------cugcaugguuuucauagaauuagcuucuagaacuauccggagcguggccgcgaugaccucggaaguaaacgaauacauacuauggcaaauauuagucuucgacucuaacguauccuuccggaaauagaggcuuaggggacuagaaugacgggauucaauacauacc
                                                             * * * ** *    ****#* #    ***  *#*  **  **# **  # ** * **#**|**#** * ** #  ** #**  **  *#*  ***    # *#****    * ** * * *
```

=== Internal intron ===

Multiple sequence alignment

```
Naboc249A_fwrd      -----guauguauugaaucccgucauucuaguccccuaagccucuauuuccggaaggauacguu-----------agagucgaagacuaauauuugccauag
Naboc249A_RevC      cuauggcaaauauuagucuucgacucu-----------aacguauccuuccggaaauagaggcuuaggggacuagaaugacgggauucaauacauac-----
                         *#* #****#  #  ** * #*           *#* * * #***||***# * * *#*           *# * **  #  #****# *#*
```

=== External intron ===

Multiple sequence alignment

```
Naboc249A_fwrd      guauguauuc--------guuuacuuccgaggucaucgcggccacgcuccggauaguucuagaagcuaauucuaugaaaaccaugcag
Naboc249A_RevC      cugcaugguuuucauagaauuagcuucuagaacuauccggagcguggccgcgaugaccucggaaguaaac--------gaauacauac
                     * ##*  *         #**  **** # ### ***  *# *||* #*  *** ### # ****  **#         *  *## *
```

ALIGNMENT RESULTS - Naboc268A

=== Stwintron Sequence ===

Multiple sequence alignment

```
Naboc268A_fwrd      gguauguauuuaaacccgucguucuaguucccucagcuuuuau-uucuagaagagucc-----------------guuauauucaaaagcuaauaugugcuauaguauguguucauuuauuccugagacaaccgcggccacggcacggauagcucuagaauguaaggcucggggaaaccgaacugacauauuuuugugcacuag
Naboc268A_RevC      cuagugcacaaaaauaugucaguucgguuuccccgagccuuacauucuagagcuauccgugccguggccgcgguugucucaggaauaaaugaacacauacuauagcacauauuagcuuuu-----------------gaauauaacggacucuucuagaa-auaaaagcugagggaacuagaacgacggguuuaaauacauacc
                        **#*#  ***   ***# * # *** **#* # ##***# *******   #***                 **# #*   * **#  **#*##*#**||**#*##*#**  #** *                   * #  *****#   ******* #***## # *#** *** # * #***   ***  #*#**
```

=== Internal intron ===

Multiple sequence alignment

```
Naboc268A_fwrd      guau-guauuuaaacccgucguucuaguucccucagcuuuuauuucuagaagaguccguuauauucaaaagcuaa--------------------uaugugcuauag
Naboc268A_RevC      cuauagcacaua--------------------uuagcuuuugaauauaacggacucuucuagaaauaaaagcugagggaacuagaacgacggguuuaaauac-auac
                     *** *#*# **                     * *******   * **#  **|**  #** *   ******* *                    ** #*#* ***
```

=== External intron ===

Multiple sequence alignment

```
Naboc268A_fwrd      gu-----------auguguucauu------------------uauuccugagacaaccgcggccacggcacggauagcucuagaauguaaggcucggggaaaccgaacugacauauuuuugugcacuag
Naboc268A_RevC      cuagugcacaaaaauaugucaguucgguuuccccgagccuuacauucuagagcuauccgugccguggccgcgguugucucaggaau------------------aaaugaacacau-----------ac
                     *           **#***#  **                  #****  ***  * *** * * | * * *** *  ***  ****                  #**  #***#**           *
```

ALIGNMENT RESULTS - Naboc268B

=== Stwintron Sequence ===

Multiple sequence alignment

```
Naboc268B_fwrd      gguauguauuuaaaccugucguuccaguuccccgagccuuuguuucuagaagaauc-----------------cgucauacucgaaggcuaacauuugccauaguauguauucauugauuccugagacaauuggagcgauggccccgauaguucuagaaaguaaggcuugagaaacugaacugacauauuuugaugcaccag
Naboc268B_RevC      cuggugcaucaaaauaugucaguucaguuucucaagccuuacuuucuagaacuaucggggccaucgcuccaauugucucaggaaucaaugaauacauacuauggcaaauguuagccuucgaguaugacg-----------------gauucuucuagaaacaaaggcucggggaacuggaacgacagguuuaaauacauacc
                        **#**# ***  ****# * ***** * *#******  *********  ***                  *** #*   #  ##  ** *# *#* || *#* #* **  ##  #   *# ***                  ***  *********  ******#* * ***** * #****  *** #**#**
```

=== Internal intron ===

Multiple sequence alignment

```
Naboc268B_fwrd      guauguauuuaaaccugucguuccaguuccccgagccuuuguuucuagaagaauccgucauacucgaaggcuaacauuugc--cauag
Naboc268B_RevC      cuaug--gcaaauguuagccuucgaguaugacggauucuucuagaaacaaaggcucggggaacuggaacgacagguuuaaauacauac
                     ****   # **   *# * *** ***    ** #  #** * || * **#  # **    *** *** * #*   ** #   ****
```

=== External intron ===

Multiple sequence alignment

```
Naboc268B_fwrd      ---------------------------guauguauucauugauuccugagacaauuggagcgauggccccgauaguucuagaaaguaaggcuugagaaacugaacugacauauuuugaugcaccag
Naboc268B_RevC      cuggugcaucaaaauaugucaguucaguuucucaagccuuacuuucuagaacuaucggggccaucgcuccaauugucucaggaaucaaug--------------------------aauaca-uac
                                                * # #*  * **# ** **# #** **#** ** || ** **#** **# #** ** #** *                          #**#**  *
```

ALIGNMENT RESULTS - Naboc285A

=== Stwintron Sequence ===

Multiple sequence alignment

```
Naboc285A_fwrd      gguauguauucaaacccaucguucaagcuccccaagcuuuugguuuuagagggacccg----------------uuuaaucagagaccuaauauuugucauaguauguauucgguuauuucugaggucaucgcagcuacggcccgggucgucccagaaagauggcuuaggaagcuuagcugacuuguuuuaacguacuag
Naboc285A_RevC      cuaguacguuaaaacaagucagcuaagcuuccuaagccaucuuucugggacgacccgggccguagcugcgaugaccucagaaauaaccgaauacauacuaugacaaauauuaggucucugauuaaa----------------cgggucccucuaaaaccaaaagcuuggggagcuugaacgauggguuugaauacauacc
                        *## ** ****   **# # ***** ** ****# *#  *#*  ** *# ** *                ##* *  *# #*** ****# *## || ##* #**** ***# #*  * *##                * ** #* **  *#*  #* #**** ** ***** # #**   **** ** ##*
```

=== Internal intron ===

Multiple sequence alignment

```
Naboc285A_fwrd      ----------------guauguauucaaacccaucguucaagcuccccaagcuuuugguuuuagagggacccguuuaaucagagaccuaauauuugucauag
Naboc285A_RevC      cuaugacaaauauuaggucucugauuaaacgggucccucuaaaaccaaaagcuuggggagcuugaacgauggguuugaauacauac----------------
                                    ** * *  * ****   ** #** *#  **  **||**  **  #* **# **   **** *  * * **
```

=== External intron ===

Multiple sequence alignment

```
Naboc285A_fwrd      ---guaugu-------auucgguua----------------uuucugaggucaucgcagcuacggcccgggucgucccagaaagauggcuuaggaagcuuagcugacuuguuuuaacguacuag
Naboc285A_RevC      cuaguacguuaaaacaagucagcuaagcuuccuaagccaucuuucugggacgacccgggccguagcugcgaugaccucagaaa----------------uaaccgaau-------acauac---
                       ***#**       * **#*#**                ****** *## *#*   **#||#**   *#* ##* ******                **#*#** *       **#***
```

ALIGNMENT RESULTS - Naboc289A

=== Stwintron Sequence ===

Multiple sequence alignment

```
Naboc289A_fwrd      ggcauguauuuaaaccucuuguuccaguuccuuaagccuuuauuucuagag------------------uaaucgucauauucgagggcuaacauuugcuauaguaugcauucguuuauuuccgagacaguuauagcuacagccuagaaagcucuagauaguaagccaugagaaaccuaacugacauauuuuuaugcacuag
Naboc289A_RevC      cuagugcauaaaaauaugucaguuagguuucucauggcuuacuaucuagagcuuucuaggcuguagcuauaacugucucggaaauaaacgaaugcauacuauagcaaauguuagcccucgaauaugacgauua------------------cucuagaaauaaaggcuuaaggaacuggaacaagagguuuaaauacaugcc
                        **#**  ***  * *## *   *** **#* * ***  * *******                  ***# *** #    # ###* **  # *#**||**#* #  ** *### #    # *** #***                  ******* *  *** * *#** ***   * ##* *  ***  **#**
```

=== Internal intron ===

Multiple sequence alignment

```
Naboc289A_fwrd      gcauguauuuaaaccucuuguuccaguuccuuaagccuuuauuucuagaguaaucgucauauucgagggcuaacauuugcuauag--------------------------------------------
Naboc289A_RevC      --------------------------------------------cuauagcaaauguuagcccucgaauaugacgauuacucuagaaauaaaggcuuaaggaacuggaacaagagguuuaaauacaugc
                                                                *** **#**  ** *  ## | ##  * **  **#** ***
```

=== External intron ===

Multiple sequence alignment

```
Naboc289A_fwrd      --------------------------guaugcauucguuuauuuccgagacaguuauagcuacagccuagaaagcucuagauaguaagccaugagaaaccuaacugacauauuuuuaugcacuag
Naboc289A_RevC      cuagugcauaaaaauaugucaguuagguuucucauggcuuacuaucuagagcuuucuaggcuguagcuauaacugucucggaaauaaacgaaugcauac--------------------------
                                              ** *    * *#***#*  * ***   ** *** # | # *** **   *** *  *#***#* *    * **
```

ALIGNMENT RESULTS - Naboc294A

=== Stwintron Sequence ===

Multiple sequence alignment

```
Naboc294A_fwrd      gguauguauuugaaccugucguuccagguccccgagccuuuguuucuagaagaauccgucuuauucgagggcuaacauuugcuauaguauguauucguuuaucugagauaaucgcagccacggu--------------------------ucagaugguucuaggaaguauggcucaggaaacugaacuaacauguuuuugugcacuag
Naboc294A_RevC      cuagugcacaaaaacauguuaguucaguuuccugagccauacuuccuagaaccau--------------------------cugaaccguggcugcgauuaucucagauaaacgaauacauacuauagcaaauguuagcccucgaauaagacggauucuucuagaaacaaaggcucggggaccuggaacgacagguucaaauacauacc
                        **#*#  #*** *** # * *** * ** ***** *  **#******  **                          **  * # **  * ** ******|****** ** *  ** # *                           * ***  ******#**  * ***** ** * *** * # *** ***#  #*#**
```

=== Internal intron ===

Multiple sequence alignment

```
Naboc294A_fwrd      guaug----------------uauuugaaccugucguuccagguccccgagccuuuguuucuagaagaauccgucuuauucgagggcuaacauuugcuauag
Naboc294A_RevC      cuauagcaaauguuagcccucgaauaagacggauucuucuagaaacaaaggcucggggaccuggaacgacagguucaaau----------------acauac
                     ***#                 * * # **  #*  *** **#  *  # || #  *  #** ***  *#  ** # * *                 #***
```

=== External intron ===

Multiple sequence alignment

```
Naboc294A_fwrd      gu-----------auguauucguuua------------------ucugagauaaucgcagccacgguucagaugguucuaggaaguauggcucaggaaacugaacuaacauguuuuugugcacuag
Naboc294A_RevC      cuagugcacaaaaacauguuaguucaguuuccugagccauacuuccuagaaccaucugaaccguggcugcgauuaucucaga------------------uaaacgaauacau-----------ac
                     *           *##* ** ***#*                  #**# #*# ***  *#**||**#*  *** #*# #**#                  *#*** ** *##*           *
```

ALIGNMENT RESULTS - Naboc300A

=== Stwintron Sequence ===

Multiple sequence alignment

```
Naboc300A_fwrd      ggugugugaauuaacccuguuguucugguucccuaggccucccuuucuggaagaacccgugauauuugagugcuaauauuuaccguaguaugauuccucuauuucugggauaaccgcagccgcagcccgggccuuucgagaaaguaagccugaagaagcugaacugacauguuuuuaugcauuag
Naboc300A_RevC      cuaaugcauaaaaacaugucaguucagcuucuucaggcuuacuuucucgaaaggcccgggcugcggcugcgguuaucccagaaauagaggaauacauacuacgguaaauauuagcacucaaauaucacggguucuuccagaaagggaggccuagggaaccagaacaacaggguuaauuacacacc
                       #**#*   ** * ***## * ##*#* * * #* **  ****** ***#  **** * * #    *#* * ** ##    #***   # | #   ***#    ## ** * *#*    # * * ****  #*** ******  ** *# * * *#*## * ##*** * **   *#**#
```

=== Internal intron ===

Multiple sequence alignment

```
Naboc300A_fwrd      guguguaauuaacccuguuguucugguucccuaggccucccuuucuggaagaacccgugauauuugagugcuaauauuuaccguag--
Naboc300A_RevC      --cuacgguaaauauuagcacucaaauaucacggguucuuccagaaagggaggccuagggaaccagaacaacaggguuaauuacacac
                       *##  * **   *# ###** ##*  * # **  #  *# || #*  #  ** # *  *## **### #*   ** *  ##*
```

=== External intron ===

Multiple sequence alignment

```
Naboc300A_fwrd      ---------------guauguauuc------------cucuauuucugggauaaccgcagccgcagcccgggccuuucgagaaaguaagccugaagaagcugaacugacauguuuuuaugcauuag
Naboc300A_RevC      cuaaugcauaaaaacaugucaguucagcuucuucaggcuuacuuucucgaaaggcccgggcugcggcugcgguuaucccagaaauagag------------gaauacauac---------------
                                   #* *   ***            **   ***** *#*   **   ** || **   **   *#* *****   **            ***   * *#
```

ALIGNMENT RESULTS - Naboc349A

=== Stwintron Sequence ===

Multiple sequence alignment

```
Naboc349A_fwrd      gguauguaauuuuuuguugugccguugcacuccccgcuuggcaagcugucccugcaguguguggcacccgcaggaggccuugguauuuaaagcuaugauuuugguucccuaauuuuucguuucuggaagaucgagggcuaauauuucccauaguauguacucguucauuucugagauaaucguaacccccccu----------------------------------------------ccaauguaucuaacugugcugacacgcuacag---------------------------------------------------
Naboc349A_RevC      ---------------------------------------------------cuguagcgugucagcacaguuagauacauug----------------------------------------------gaggggggguuacgauuaucucagaaaugaacgaguacauacuaugggaaauauuagcccucgaucuuccagaaacgaaaaauuagggaaccaaaaucauagcuuuaaauaccaaggccuccugcgggugccacacacugcagggacagcuugccaagcggggagugcaacggcacaacaaaaaauuacauacc
                                                                       *** **#**** #   * *  #** #* ***                                              **  * *** **  ***  *    # *** **||** *** #    *  ***  ** *** *  *                                              **** *# **#  * *   # ****#** ***
```

=== Internal intron ===

Multiple sequence alignment

```
Naboc349A_fwrd      guaug--------------------------------------------------uaauuuuuuguugugccguugcacuccccgcuuggcaagcugucccugcaguguguggcacccgcaggaggccuugguauuuaaagcuaugauuuugguucccuaauuuuucguuucuggaagaucgagggcuaauauuucccauag
Naboc349A_RevC      cuaugggaaauauuagcccucgaucuuccagaaacgaaaaauuagggaaccaaaaucauagcuuuaaauaccaaggccuccugcgggugccacacacugcagggacagcuugccaagcggggagugcaacggcacaacaaaaaauua--------------------------------------------------cauac
                     ****                                                  * **  #**   #*#**#  **  #*  **  ** ** #*  * *||* *  *# ** **  **  *#  **  #**#*#   **#  ** *                                                  ****
```

=== External intron ===

Multiple sequence alignment

```
Naboc349A_fwrd      -------------guauguacucguucauuucugagauaaucguaacccccccuccaauguaucuaacugugcugacacgcuacag
Naboc349A_RevC      cuguagcgugucagcacaguuagauacauuggaggggggguuacgauuaucucagaaaugaacgaguacauac-------------
                                 *#*##     #* ****   * *    * || *    * *   **** *#     ##*#*
```

ALIGNMENT RESULTS - Naboc414A

=== Stwintron Sequence ===

Multiple sequence alignment

```
Naboc414A_fwrd      gguauguuuuucggccugucguuauaguucccuaacccucugcuucuagaagacuccguuauauucgcaugcuaacguuuaccacaguauguauucguuuauuuuuaagacaaucgcagccauagccccgauag--------------------------uucuagaaaguaaggcuugaggaacugaacugacaugcguugaugcacuag
Naboc414A_RevC      cuagugcaucaacgcaugucaguucaguucccucaagccuuacuuucuagaa--------------------------cuaucggggcuauggcugcgauugucuuaaaaauaaacgaauacauacugugguaaacguuagcaugcgaauauaacggagucuucuagaagcagaggguuagggaacuauaacgacaggccgaaaaacauacc
                        **# *#   ** ****# * #****** #** ***    ********                          #*  *     ****  * ** ** *#**|**#* ** ** *  ****     *  *#                          ********    *** **# ******# * #**** **   #* #**
```

=== Internal intron ===

Multiple sequence alignment

```
Naboc414A_fwrd      guauguuuuucggccugucguuauaguucccuaacccucugcuucuagaagacuccguuauauucgcaugcuaacguuuaccacag------------------------------------------
Naboc414A_RevC      ------------------------------------------cugugguaaacguuagcaugcgaauauaacggagucuucuagaagcagaggguuagggaacuauaacgacaggccgaaaaacauac
                                                              #* * * *#**   # #** #||# **# #   **#* * * *#
```

=== External intron ===

Multiple sequence alignment

```
Naboc414A_fwrd      gua---------------------------uguauucguuuauuuuuaagacaaucgcagccauagccccgauaguucuagaaaguaaggcuugaggaacugaacugacaugcguugaugcacuag
Naboc414A_RevC      cuagugcaucaacgcaugucaguucaguucccucaagccuuacuuucuagaacuaucggggcuauggcugcgauugucuuaaaaauaaacg--------------------------aauaca-uac
                     **                           # #*  * **  ***#** #** ****  ** || **  **** **# **#***  ** *                          #**#** **
```

ALIGNMENT RESULTS - X1651c009A

=== Stwintron Sequence ===

Multiple sequence alignment

```
X1651c009A_fwrd     ggugugugauuaaagcuugccgugccgguugugagaccugguauuguuggaaauaccuug--------ugucuuccagagcuaacuuuugucgcaguaugugagaccuugauaccgaaacccagcgauaagcuauguaguucuagaaaguuuacaaucaggaaccggacuaacauguuguuauguggcag
X1651c009A_RevC     cugccacauaacaacauguuaguccgguuccugauuguaaacuuucuagaacuacauagcuuaucgcuggguuucgguaucaaggucucacauacugcgacaaaaguuagcucuggaagaca--------caagguauuuccaacaauaccaggucucacaaccggcacggcaagcuuuaauacacacc
                        ###** * *#* **  #  ****** #  #   *##  ** #*#*** *** * *        **  ** * * # #**  *#*  *# *|* #*  *#*  **# # * * **  **        * * *** ***#*# **  ##*   #  # ******  #  ** *#* * **###
```

=== Internal intron ===

Multiple sequence alignment

```
X1651c009A_fwrd     guguguauuaaagcuugccgugccgguugugagaccugguauuguuggaaauac-----cuugugucuuccagagcuaacuuuugu-cgcag
X1651c009A_RevC     cugcg-acaaaaguuagcucuggaagacacaa-----gguauuuccaacaauaccaggucucacaaccggcacggcaagcuuuaauacacac
                     **#* *# **** * **  **  #* ####*     ****** #||# *****     **#### *#  **  ** * **** #* *#**
```

=== External intron ===

Multiple sequence alignment

```
X1651c009A_fwrd     gu---augugagaccuugaua--ccgaaacccagcgauaagcuauguaguucuagaaaguuuacaaucaggaaccggacuaacauguuguuauguggcag
X1651c009A_RevC     cugccacauaacaacauguuaguccgguuccugauuguaaacuuucuagaacuacauagcuuaucgcuggguuucgg--uaucaaggucucacau---ac
                     *   *##*#* * * ** **  ***   **  #   ***#** * ***||*** * **#***   #  **   ***  ** ** * * *#*##*   *
```

ALIGNMENT RESULTS - X1651c011A

=== Stwintron Sequence ===

Multiple sequence alignment

```
X1651c011A_fwrd     gguauguaucaaaaccuccuaugcugguugugagaccgggaaaaucuuuggucuuccagagcuaaccaguaucgcaguaugugaaccguugacccagaagcccguccaugac----cuguguaguucuagggguauacaa-----------uaaggaaccggacuaacauguuuuuaugugguag
X1651c011A_RevC     cuaccacauaaaaacauguuaguccgguuccuua-----------uuguauaccccuagaacuacacag----gucauggacgggcuucugggucaacgguucacauacugcgauacugguuagcucuggaagaccaaagauuuucccggucucacaaccagcauaggagguuuugauacauacc
                        ###** ***** *  **  *#**** #  #            * *#  *##* ***#***  ***    *  #*  ##*  *  #** | **#  *  *##  *#  *    ***  ***#*** *##*  #* *            #  # ****#*  **  * ***** **###
```

=== Internal intron ===

Multiple sequence alignment

```
X1651c011A_fwrd     guauguaucaaaaccuccuaugcugguugugagaccgggaaaaucuuuggucuuccagagcuaaccaguaucgcag-----------------
X1651c011A_RevC     -----------------cugcgauacugguuagcucuggaagaccaaagauuuucccggucucacaaccagcauaggagguuuugauacauac
                                     ** #* *# * ** **  * **** *#* | *#* **** *  ** ** * #* *# **
```

=== External intron ===

Multiple sequence alignment

```
X1651c011A_fwrd     gu---augugaaccguugacccagaagcccguccaugaccuguguaguucuagggguauacaauaaggaaccggacuaacauguu-----uuuaugugguag
X1651c011A_RevC     cuaccacauaaa-----aacauguuaguccgguuccuuauuguauaccccuagaacuacacaggucauggacgggcuucugggucaacgguucac---auac
                     *   *##*#**     #**     ** ***    #    ***#** ##*||*## **#***    #    *** **     **#     **#*#   #**
```

ALIGNMENT RESULTS - X1651c016A

=== Stwintron Sequence ===

Multiple sequence alignment

```
X1651c016A_fwrd     gguacguaucaaauccuguuguaccaguuuugagacccaaaaauauuuuaggucuuccagagcuaaccaguaucacaguacgugaacccuugaucaaggagcccaucuaugac----uuguguaguucuagaaaguauacacug-----------agggaccggacuaacauguuuuuauguggcag
X1651c016A_RevC     cugccacauaaaaacauguuaguccgguccc-----------ucaguguauacuuucuagaacuacacaa----gucauagaugggcuccuugaucaaggguucacguacugugauacugguuagcucuggaagaccuaaaauauuuuugggucucaaacugguacaacaggauuugauacguacc
                        *##** *** * ****#  ** **###            #* * ** ## *** ***#***  **#       #** #**  * *#*  |  *#* *  **# **#       #**  ***#*** *** ## ** * *#            ###** **  #**** * *** **##*
```

=== Internal intron ===

Multiple sequence alignment

```
X1651c016A_fwrd     guacguaucaaauccuguuguaccaguu-----uugagacccaaaaauauuuuaggucuuccagagcuaaccaguaucacag-------------
X1651c016A_RevC     --------------cugugauacugguuagcucuggaagaccuaaaauauuuuugggucucaa-----aacugguacaacaggauuugauacguac
                                  **** #***  ***     * **#  ** ****||**** **  #** *     ***  ***# ****
```

=== External intron ===

Multiple sequence alignment

```
X1651c016A_fwrd     gu---acgugaacccuugaucaaggagcccaucuaugacuuguguaguucuagaaaguaua-----cacugagggaccggacuaacauguuuuuauguggcag
X1651c016A_RevC     cugccacauaaaaacauguuaguccggucccucag-----uguauacuuucuagaacuacacaagucauagaugggcuccuugaucaaggguucacgu---ac
                     *   **#*#**  * ** *      * ** **       ***#** ** #|# ** **#*      **  ** ** *      * ** *  **#*#**   *
```

ALIGNMENT RESULTS - X1651c025A

=== Stwintron Sequence ===

Multiple sequence alignment

```
X1651c025A_fwrd     gguauguaucaaaaccugcugugccaguugugagacccagaaauaucuuuggucuuccagagcuaaccaguaucgcagugugugaauccuuaacccagaagcccgucuau----gcccuguguaguucuagaaaguauacaaua-----------aggaaccggacugacauguuuuuaugugacag
X1651c025A_RevC     cugucacauaaaaacaugucaguccgguucc-----------uuauuguauacuuucuagaacuacacagggc----auagacgggcuucuggguuaaggauucacacacugcgauacugguuagcucuggaagaccaaagauauuucugggucucacaacuggcacagcagguuuugauacauacc
                        ###** ***** ** ##  ** *** #           ***  *  ## *** ***#***  ***  #    #*# ##*  #  #*  |  *#  #  *## #*#    #  ***  ***#*** *** ##  *  ***           # *** **  ## ** ***** **###
```

=== Internal intron ===

Multiple sequence alignment

```
X1651c025A_fwrd     guauguaucaaaaccugcugugccaguug-----ugagacccagaaauaucuuuggucuuccagagcuaaccaguaucgcag-------------
X1651c025A_RevC     --------------cugcgauacugguuagcucuggaagaccaaagauauuucugggucuc-----acaacuggcacagcagguuuugauacauac
                                  **** #*#*  ***#      **#  ***#* *||* *#***  #**      #***  *#*# ****
```

=== External intron ===

Multiple sequence alignment

```
X1651c025A_fwrd     gugugugaauccuuaacccagaagcccgucuaugcccuguguaguucuagaaaguauacaauaaggaaccggacugacauguuuuuaugugacag--
X1651c025A_RevC     --cugucacauaaaaacaugucaguccgguuccuuauuguauacuuucuagaacuacacagggcauagacgggcuucuggguuaaggauucacacac
                       *** *      ***     ** ***  * #    ***#** ** #|# ** **#***    # *  *** **     ***      * ***
```

ALIGNMENT RESULTS - X1651c036A

=== Stwintron Sequence ===

Multiple sequence alignment

```
X1651c036A_fwrd     gguauguauuaaaaccugcugugcuggucauaagaccuagaaauauccuugaucuucuagagcuaaccaguaucgcaguauguaaacccuugaccuggaagcccgucuaugaccuguguaguguaaga--------------------aagcauauaauaagg------aaucggacuaacauauucuuauguggcag
X1651c036A_RevC     cugccacauaagaauauguuaguccgauu------ccuuauuauaugcuu--------------------ucuuacacuacacaggucauagacgggcuuccaggucaaggguuuacauacugcgauacugguuagcucuagaagaucaaggauauuucuaggucuuaugaccagcacagcagguuuuaauacauacc
                        ###** * **  ** *#  *#*#*       *** #  **** ***                    * * #** **###*   * * ***  * || *  *** * *    *###** **# * *                    *** ****  # ***       *#*#*  #* **  ** * **###
```

=== Internal intron ===

Multiple sequence alignment

```
X1651c036A_fwrd     guauguauuaaaaccugcugugcuggu------cauaagaccuagaaauauccuugaucuucuagagcuaaccaguaucgcag-------------
X1651c036A_RevC     -------------cugcgauacugguuagcucuagaagaucaaggauauuucuaggucuu------augaccagcacagcagguuuuaauacauac
                                  **** #*#*****       * **** * ** * | * ** * ****       * *****#*# ****
```

=== External intron ===

Multiple sequence alignment

```
X1651c036A_fwrd     -----guauguaaacccuugaccuggaagcccgucuaugaccuguguaguguaagaaagcauauaauaaggaaucggacuaacauauucuuauguggcag
X1651c036A_RevC     cugccacauaagaauauguuaguccgauuccuuauuauaugcuuucuuacacuacacaggucauagacgggcuuccaggucaaggguuuacauac-----
                         ##**#  **    * *  # **  **    ***#  ** * * #||# * * **  #***    **  ** #  * *    **  #**##
```

ALIGNMENT RESULTS - X1651c075A

=== Stwintron Sequence ===

Multiple sequence alignment

```
X1651c075A_fwrd     gguauguauuaaaaccuccuaugccgguugugagacccaaaagucuuuggucuuccggagcua---accaguaucgcaguaugugaacccuugaccgagaagcccgucuaugaccaguguaguucuagaaaguauacaauaaggaaacggacuaacauguuuuauguggcag
X1651c075A_RevC     cugccacauaaaaacauguuaguccguuuccuuauuguauacuuucuagaacuacacuggucauagacgggcuucucggucaaggguucacauacugcgauacuggu---uagcuccggaagaccaaagacuuuugggucucacaaccggcauaggagguuuuaauacauacc
                        ###** ***** *  **  *** ** #  #    * *  * #* *# ** *    * #*   **  *# ** * **  # * | * #  ** * ** #*  **   *# *    * ** #* *# *  * *    #  # ** ***  **  * ***** **###
```

=== Internal intron ===

Multiple sequence alignment

```
X1651c075A_fwrd     guauguauuaaaaccuccuaugccgguugugagacccaaaagucuuuggucuuccggagcuaaccaguaucgcag-----------------
X1651c075A_RevC     -----------------cugcgauacugguuagcuccggaagaccaaagacuuuugggucucacaaccggcauaggagguuuuaauacauac
                                     ** #*  # * ** **  **  *** *#||#* ***  **  ** ** * #  *# **
```

=== External intron ===

Multiple sequence alignment

```
X1651c075A_fwrd     gu---augugaacccuugaccgagaagcccgucuaugaccaguguaguucuagaaaguauacaauaaggaaacggacu-----aacauguuuuauguggcag
X1651c075A_RevC     cugccacauaaa-----aacauguuaguccguuuccuuauuguauacuuucuagaacuacacuggucauagacgggcuucucggucaaggguucacau---ac
                     *   *##*#**     #**     ** **** * #     **#** ** #|# ** **#**     # * **** **       ** *  **#*##*   *
```

ALIGNMENT RESULTS - X1651c093A

=== Stwintron Sequence ===

Multiple sequence alignment

```
X1651c093A_fwrd     gguauguaucaaaaccuccuaugccgguugugagacccaggaaaccuuuggucuuccagagcuaaccaguaucgcaguaugugaacccuugacccagaagccgucuaugac----cuguguaguucuagaaaguauacaau----------aaggaaccggacuaacaugguuuuauguggcag
X1651c093A_RevC     cugccacauaaaaccauguuaguccgguucc----------uuauuguauacuuucuagaacuacacag----gucauagacggcuucugggucaaggguucacauacugcgauacugguuagcucuggaagaccaaagguuuccugggucucacaaccggcauaggagguuuugauacauacc
                        ###** *** * *  **  ****** #            *   *  ## *** ***#***  ***   *  #** ##*    ** *||* **    *## **#  *    ***  ***#*** *** ##  *   *           # ******  **  * * *** **###
```

=== Internal intron ===

Multiple sequence alignment

```
X1651c093A_fwrd     guauguaucaaaaccuccuaugccgguugugagacccaggaaaccuuuggucuuccagagcuaaccaguaucgcag----------------
X1651c093A_RevC     ----------------cugcgauacugguuagcucuggaagaccaaagguuuccugggucucacaaccggcauaggagguuuugauacauac
                                     ** #*  # * ** **  *  *#* *** | *** *#*  *  ** ** * #  *# **
```

=== External intron ===

Multiple sequence alignment

```
X1651c093A_fwrd     gu---augugaacccuugacccagaagccgucuaugaccuguguaguucuagaaaguauacaauaaggaaccggacu----aacaugguuuuauguggcag
X1651c093A_RevC     cugccacauaaaaccauguu----aguccgguuccuuauuguauacuuucuagaacuacacaggucauagacggcuucugggucaaggguucacau---ac
                     *   *##*#** ** **      *  ***  * #    ***#** ** #|# ** **#***    # *  ***  *      ** ** **#*##*   *
```

ALIGNMENT RESULTS - X1651c156A

=== Stwintron Sequence ===

Multiple sequence alignment

```
X1651c156A_fwrd     gguguguauaaaagcccgccgugccgguugugagacuugguauugguggaaguaccuugugucuuccagagcuaacuuuugucgcaguaugugaaaccuugagcccgaaagcccagugaugagcuauggaguucuag------------------aaaguuacaauaggg-----------------------------------aaccggacuaacaugcuuuuaugugcag
X1651c156A_RevC     cugccacauaaaagcauguuaguccggu-----------------------------------ucccuauuguaacuuu------------------cuagaacuccauagcucaucacugggcuuucgggcucaagguuucacauacugcgacaaaaguuagcucuggaagacacaagguacuuccaccaauaccaagucucacaaccggcacggcgggcuuuuauacacacc
                        ###********  *  #  *****                                   *#** #   *******                  ** **#* * * *** ** || ** *** * * *#** **                  *******   # **#                                   ******  #  *  ********###
```

=== Internal intron ===

Multiple sequence alignment

```
X1651c156A_fwrd     guguguauaaaagcccgccgugccgguugugagacuugguauugguggaaguaccuugugucuuccagagcuaacuuuugucgcag-----------------------
X1651c156A_RevC     -----------------------cugcgacaaaaguuagcucuggaagacacaagguacuuccaccaauaccaagucucacaaccggcacggcgggcuuuuauacacac
                                           * *# ###*#* **#*# #*** #** ##* | *## **# ***# #*#** *#*### #* *
```

=== External intron ===

Multiple sequence alignment

```
X1651c156A_fwrd     guaugugaaaccuugagcccgaaagcccagugaugagcuauggaguucuagaaaguuacaauagggaaccggacuaacaugcuu---uuauguggcag
X1651c156A_RevC     cugccacaua---aaagcauguuaguccgguucccuauuguaacuuucuagaacuccauagcucaucacugggcuuucgggcucaagguuucacauac
                     * #   * *    #***  *  ** ** **  #  # * *##  ***||***  ##* * #  #  ** ** **  *  ***#    * *   # *
```

ALIGNMENT RESULTS - X1651c189A

=== Stwintron Sequence ===

Multiple sequence alignment

```
X1651c189A_fwrd     -----------------------------------------------------------------------gguaugugucaaaccccc-----cuacgacgguugugagaccaagaaaaccuuuggucuucuagagcuaaccacuaacgcaguaugugaaaccuugacccagaagcccaucuaugaccuguguaguucuagaaagcauacaauaaggaaccggacugacauauuuuaauguggcag
X1651c189A_RevC     cugccacauuaaaauaugucaguccgguuccuuauuguaugcuuucuagaacuacacaggucauagaugggcuucugggucaagguuucacauacugcguuagugguuagcucuagaagaccaaagguuuucuuggucucacaaccgucguag-----ggggguuugacacauacc-----------------------------------------------------------------------
                                                                                             *  ** *****     *     ** **  #** ** **  * **** *** | *** **** *  ** ** **#  ** **     *     ***** ** *
```

=== Internal intron ===

Multiple sequence alignment

```
X1651c189A_fwrd     guaugugucaaaccccccuacgacgguugugagaccaagaaaaccuuuggucuucuagagcuaaccacuaacgcag-----------------
X1651c189A_RevC     -----------------cugcguuagugguuagcucuagaagaccaaagguuuucuuggucucacaaccgucguagggggguuugacacauac
                                     ** **  #** ** **  * **** *** | *** **** *  ** ** **#  ** **
```

=== External intron ===

Multiple sequence alignment

```
X1651c189A_fwrd     guaugugaaaccuugacccagaagcccaucuaugaccuguguaguucuagaaagcauacaauaaggaaccggacugacauauuuuaauguggcag--
X1651c189A_RevC     --cugccacauuaaaauaugucaguccgguuccuuauuguaugcuuucuagaacuacacaggucauagaugggcuucugggucaagguuucacauac
                       **# * *    #*      ** **   * #    ***#*  ** #|# **  *#***    # *   ** **      *#    * * #**
```

ALIGNMENT RESULTS - Xarbc0002A

=== Stwintron Sequence ===

Multiple sequence alignment

```
Xarbc0002A_fwrd     ----------------------------gguauguaucaaaaccuc-----cuaugcggguugcga-aacccaaucuuagcagaaacgucuuucgucucccgaagcugacuagucucgcaguauguaaaccguugacuccgagacugaauuauugcuauucguauaauucuagaaauuauacaauaagguacuagacuaacauguuuuucuaugguag
Xarbc0002A_RevC     cuaccauagaaaaacauguuagucuaguaccuuauuguauaauuucuagaauuauacgaauagcaauaauucagucucggagucaacgguuuacauacugcgagacuagucagcuucgggagacgaaagacguuucugcuaagauuggguu-ucgcaacccgcaua-----ggagguuuugauacauacc----------------------------
                                                # # *#*   * **  **      ***#**##* **#* **  ** ***# *    ****  ** *#* #  *** #**#||#**# ***  # *#* **  ****    * #*** **  ** *#** *##**#***      **  ** *   *#* # #
```

=== Internal intron ===

Multiple sequence alignment

```
Xarbc0002A_fwrd     guauguaucaaaaccuccuaugcggguugcgaaacccaaucuuagcagaaacgucuuucgucucccgaagcuga---cuagucucgcag
Xarbc0002A_RevC     cugcgagacuag---ucagcuucgggagacgaaagacguuucugcuaagauuggguuucgcaacccgcauaggagguuuugauacauac
                     * #*   * *    **   * ****  #*****  *  * #* | *# *  *  *****#  **** *   **    * *   *# *
```

=== External intron ===

Multiple sequence alignment

```
Xarbc0002A_fwrd     -----guauguaaaccguugacuccgagacugaauuauugcuauucguauaauucuagaaauuauac----------aauaagguacuagacuaacauguuuuucuaugguag
Xarbc0002A_RevC     cuaccauagaaaaacauguuagucuaguac----------cuuauuguauaauuucuagaauuauacgaauagcaauaauucagucucggagucaacgguuuacauac-----
                         #** # ****   * * ** #  **          **  * ******** #|# ********          ***  #**  # ** * *   **** # **#
```

ALIGNMENT RESULTS - Xarbc0002B

=== Stwintron Sequence ===

Multiple sequence alignment

```
Xarbc0002B_fwrd     gguaugugucaaa-----------------ucugucgugcaggucgcgaaacccaaucuucguaaaaggucuuuuuuucguauuccaaagcuaacgaguuuugcaguaugaaaucauuaucgcuagcuguau--aguucuagaaacuauacaacaaggaccuaaacuaauauguuucuauaugguag
Xarbc0002B_RevC     cuaccauauagaaacauauuaguuuagguccuuguuguauaguuucuagaacu--auacagcuagcgauaaugauuuacauacugcaaaacucguuagcuuuggaauacgaaaaaaagaccuuuuacgaagauuggguuucgcgaccugcacgacaga-----------------uuugacacauacc
                        ##*#*  **                 # *** **# ** *   # ***   ** #   **   #   *  *** *#**#* ****#** || **#**** *#**#* ***   *   #   **   # **   *** #   * ** #** *** #                 **  *#*##
```

=== Internal intron ===

Multiple sequence alignment

```
Xarbc0002B_fwrd     guaugugucaaaucugucgugcaggucgcgaaacccaaucuucguaaaaggucuuuuuuucguauuccaaagcuaacgaguuuugcag----------------
Xarbc0002B_RevC     ----------------cugcaaaacucguuagcuuuggaauacgaaaaaaagaccuuuuacgaagauuggguuucgcgaccugcacgacagauuugacacauac
                                    # *## *# ***  *         * ** ****##||##**** ** *         *  *** #* ##* #
```

=== External intron ===

Multiple sequence alignment

```
Xarbc0002B_fwrd     gu---auguaaaucauuaucg---------cuagcuguauaguucuagaaacuauacaacaaggaccuaaacuaauauguuucuauaugguag
Xarbc0002B_RevC     cuaccauauagaaacauauuaguuuagguccuuguuguauaguuucuagaacuauacagcuag---------cgauaaugauuuacau---ac
                     *   **#** *    *** #         ** * ********* #|# ********* * **         # ***    * **#**   *
```

ALIGNMENT RESULTS - Xarbc0003A

=== Stwintron Sequence ===

Multiple sequence alignment

```
Xarbc0003A_fwrd     gguaugcaucauaacccacggugccaguucuauaaaacaguguuacgaggauuucuuugaggccuaagcuaacuucugccacaguaugugaaccuuugauccagauuuugaaauuacc----ugauacggaauuagacuaacaguuguauccgauag----------
Xarbc0003A_RevC     ----------cuaucggauacaacuguuagucuaauuccguauca----gguaauuucaaaaucuggaucaaagguucacauacuguggcagaaguuagcuuaggccucaaagaaauccucguaacacuguuuuauagaacuggcaccguggguuaugaugcauacc
                               ** *  * #  #*   *  * ***  * *#*#*    * *   **##*## **  # #**    *  ** *|* **  *    **# #  ** ##*##**   * *    *#*#** *  ***  *  *   *#  # *  * **
```

=== Internal intron ===

Multiple sequence alignment

```
Xarbc0003A_fwrd     guaug--------caucauaacccacggugccaguucuauaaaacaguguuacgaggauuucuuugaggccuaagcuaacuucugccacag
Xarbc0003A_RevC     cuguggcagaaguuagcuuaggccucaaagaaauccucguaacacuguuuuauagaacuggcaccguggguuaugaug--------cauac
                     * **         * * **  ** *## *  * ## # *** **|** *** # ## *  * ##* **  ** * *         ** *
```

=== External intron ===

Multiple sequence alignment

```
Xarbc0003A_fwrd     guaugugaaccuuugauccagauuuugaaauuaccugauacggaauuagacuaacaguuguauccga--------uag
Xarbc0003A_RevC     cua--------ucggauacaacuguuagucuaauuccguaucagguaauuucaaaaucuggaucaaagguucacauac
                     **        *# *** **# * **#   * *  #  ||  #  * *   #** * #** *** #*        **
```

ALIGNMENT RESULTS - Xarbc0003B

=== Stwintron Sequence ===

Multiple sequence alignment

```
Xarbc0003B_fwrd     gguauguaccaacaccugcugucccgguuucaauauucggucuuaucagagggauuguuuacuuuuuagagcuaacuagugccacaguaugugagcuacugaucuuguagccgagccuuagugac-cuacauaguucgagaagguauggucuuacaacaagaaaacggacuaauuuaugucgugugacag---------------
Xarbc0003B_RevC     ---------------cugucacacgacauaaauuaguccguuuucuuguuguaagaccauaccuucucgaacuauguag-gucacuaaggcucggcuacaagaucaguagcucacauacuguggcacuaguuagcucuaaaaaguaaacaaucccucugauaagaccgaauauugaaaccgggacagcagguguugguacauacc
                                   *** ### * #  *  * ** ** ** ** *    * #*   # ***#**#* **#***  *** * *** #    *  ***** # | # *****  *    # *** * ***  ***#** *#**#*** #   *# *    * ** ** ** ** *  *  # * ### ***
```

=== Internal intron ===

Multiple sequence alignment

```
Xarbc0003B_fwrd     guauguaccaacaccugcugucccgguuucaauauucggucuuaucagagggauuguuuacuuuuuagagcuaacuagugccacag--
Xarbc0003B_RevC     --cuguggcacuaguuagcucuaaaaaguaaacaaucccucugauaagaccgaauauugaaaccgggacagcagguguugguacauac
                       ***  **  *  *# # #   ##  * **#* **  *** || ***  ** *#** *  ##   # # #*  *  **  ***
```

=== External intron ===

Multiple sequence alignment

```
Xarbc0003B_fwrd     ------------guaugugagcuacugaucuuguagccgagccuuagugaccuacauaguucgagaagguauggucuuacaacaagaaaacggacuaauuuaugucgugugacag
Xarbc0003B_RevC     cugucacacgacauaaauuaguccguuuucuuguuguaagaccauaccuucucgaacuauguaggucacuaaggcucggcuacaagaucaguagcucacauac------------
                                #** #* ** #  *  ****** *  # #** ** #  * #  *#|#*  # *  # ** **# #  * ******  *  # ** *# **#
```

ALIGNMENT RESULTS - Xarbc0003C

=== Stwintron Sequence ===

Multiple sequence alignment

```
Xarbc0003C_fwrd     gguaugugaaccgcggugcccguugucaagaucguugccaugguugucaucgacacgucgucuauuauuuacgaaacuaacucguguuauaguaugugaacucuagccuagaaacgcgaccaccaugacccga-uaaccccggaacaugauauaaaacagagcagcguuaac-------auuuuuuugguaaaaag------------------
Xarbc0003C_RevC     ------------------cuuuuuaccaaaaaaau-------guuaacgcugcucuguuuuauaucauguuccgggguua-ucgggucaugguggucgcguuucuaggcuagaguucacauacuauaacacgaguuaguuucguaaauaauagacgacgugucgaugacaaccauggcaacgaucuugacaacgggcaccgcgguucacauacc
                                      *   **##***#*  #*       ***# * # *    **  * ***#** * *     * * *** **#** **   #*   * # #||# # *   *#   ** **#** *** * *     * * **#*** *  **    * # * #***       *#  *#***##**   *
```

=== Internal intron ===

Multiple sequence alignment

```
Xarbc0003C_fwrd     gu----------------------------------------augugaaccgcggugcccguugucaagaucguugccaugguugucaucgacacgucgucuauuauuuacgaaacuaacucguguuauag
Xarbc0003C_RevC     cuauaacacgaguuaguuucguaaauaauagacgacgugucgaugacaaccauggcaacgaucuugacaacgggcaccgcgguucacau----------------------------------------ac
                     *                                        ***  ****# **## * #*# *|* #*# * ##** #****  ***                                        *
```

=== External intron ===

Multiple sequence alignment

```
Xarbc0003C_fwrd     guaugugaacucuagccuagaaacgcgacc---------------accaugacccgauaaccccggaacaugauauaaaacagagcagcguuaacauuuuuuugguaaaa-------ag
Xarbc0003C_RevC     cu-------uuuuaccaaaaaaauguuaacgcugcucuguuuuauaucauguuccgggguuaucgggucaug---------------gguggucgcguuucuaggcuagaguucacauac
                     *        * ** *  *#*** *  * *               * ****  ***   |   ***  ****               * * *  * ***#*  * ** *        *
```

ALIGNMENT RESULTS - Xarbc0006A

=== Stwintron Sequence ===

Multiple sequence alignment

```
Xarbc0006A_fwrd     ----gguauguaaca------------------------------------aaagguggugugcggguugcgagacucaacggcggca-----------------------------gaaauauauuucgucuucuagaacuaacuuguaucacaguauguaacugcccgaucuagauaccaggccaugaucuaugugguccuuaaaaguauacaaggggcuagacuaacaugguuuauguggua g
Xarbc0006A_RevC     cuaccacauaaaccauguuagucuagccccuuguauacuuuuaaggaccacauagaucauggccugguaucuagaucgggcaguuacauacugugauacaaguuaguucuagaagacgaaauauauuuc-----------------------------ugccgccguugagucucgcaacccgcacaccaccuuu------------------------------------uguuacauacc----
                         ##**# * **                                    * **#* #**  * ***  * *** #   *#*  #**                             *****||*****                             **#  *#*   # *** *  *** *  **# *#** *                                    ** * #**##
```

=== Internal intron ===

Multiple sequence alignment

```
Xarbc0006A_fwrd     guauguaacaaaagguggugugcggguugcgagacucaacggcggcagaaauauauuucgucuucua------------------gaacuaacuuguaucacag--
Xarbc0006A_RevC     --cugugauacaaguuaguuc------------------uagaagacgaaauauauuucugccgccguugagucucgcaacccgcacaccaccuuuuguuacauac
                       *** * * *** *#** #                   #* #*  *****||*****  *# *#                   # **#* *** * * ***
```

=== External intron ===

Multiple sequence alignment

```
Xarbc0006A_fwrd     guauguaacugcccgaucuagauaccaggccaugaucuaugugguccuuaaaaguauacaaggggcuagacuaacaugguuua-----------------ugugguag
Xarbc0006A_RevC     cuaccaca-----------------uaaaccauguuagucuagccccuuguauacuuuuaaggaccacauagaucauggccugguaucuagaucgggcaguuacauac
                     **#   *                  *##***** *   #  * #****  * || *  ****# *  #   * *****##*                  *   #**
```

ALIGNMENT RESULTS - Xarbc0006B

=== Stwintron Sequence ===

Multiple sequence alignment

```
Xarbc0006B_fwrd     gguauguauuaaaaccuccugugcugguugugaaacucagucuucgcagaaacgucc------------------uucaaagcuaacuagucucacaguauguaaaucuuugguuuugggacugaaucauugccgccuguguaauccuagaagguauacacuaaagaacuagacuaacauguucuuaugcgauag
Xarbc0006B_RevC     cuaucgcauaagaacauguuagucuaguucuuuaguguauaccuucuaggauuacacaggcggcaaugauucagucccaaaaccaaagauuuacauacugugagacuaguuagcuuugaa------------------ggacguuucugcgaagacugaguuucacaaccagcacaggagguuuuaauacauacc
                        #*#** * *** *  *#  **#*** *  *    *  *#*   ** *  ## *                  ##****#*#**  * *  ** *|* **  * *  **#*#****##                  * ##  * **   *#*  *    *  * ***#**  #*  * *** * **#*#
```

=== Internal intron ===

Multiple sequence alignment

```
Xarbc0006B_fwrd     guauguauuaaaaccuccugugcugguugugaaacucagucuucgcagaaacguccuucaaagcuaac----------uagucucacag
Xarbc0006B_RevC     cugugagacua----------guuagcuuugaaggacguuucugcgaagacugaguuucacaaccagcacaggagguuuuaauacauac
                     * **   # *          * *#*#* ****   *  * #* | *# *  *   **** *#*#* *          * #   ** *
```

=== External intron ===

Multiple sequence alignment

```
Xarbc0006B_fwrd     gua-------------------uguaaaucuuugg--------uuuugggacugaaucauugccgccuguguaauccuagaagguauacacuaaagaacuagacuaacauguucuuaugcgauag
Xarbc0006B_RevC     cuaucgcauaagaacauguuagucuaguucuuuaguguauaccuucuaggauuacacaggcggcaaugauucagucccaaa--------accaaagauuuaca-------------------uac
                     **                   * **  *****#*        **#*#*** *# *#   #*|*#   #* #* ***#*#*        **#*****  ** *                   **
```

ALIGNMENT RESULTS - Xarbc0009A

=== Stwintron Sequence ===

Multiple sequence alignment

```
Xarbc0009A_fwrd     gguaugcauuaaaagcuccugugccggcugucaaccccaaccuucgcagaaacguguuucgucguccaaugcuaacuagucucacaguauguaaaucuucgaguuugaguccgaacuauugccaaugcuuguaucuagacgcuauauaauaaggaacuggacuaacauguguuuauaugguag
Xarbc0009A_RevC     cuaccauauaaacacauguuaguccaguuccuuauuauauagcgucuagauacaagcauuggcaauaguucggacucaaacucgaagauuuacauacugugagacuaguuagcauuggacgacgaaacacguuucugcgaagguugggguugacagccggcacaggagcuuuuaaugcauacc
                        ## ** ** *  *  *#  **#* * #  *    *   #    *** **# *# * * *#     *   *  #*# ***  **  * | *  **  *** #*#  *   *     #* * * #* #** ***    #   *    *  # * *#**  #*  *  * ** ** ##
```

=== Internal intron ===

Multiple sequence alignment

```
Xarbc0009A_fwrd     guaug---------------------cauuaaaagcuccugugccggcugucaaccccaaccuucgcagaaacguguuucgucguccaaugcuaacuagucucacag
Xarbc0009A_RevC     cugugagacuaguuagcauuggacgacgaaacacguuucugcgaagguugggguugacagccggcacaggagcuuuuaaug---------------------cauac
                     * **                     *   * * * * ***#*  ** **   |   ** **  *#*** * * * *   *                     ** *
```

=== External intron ===

Multiple sequence alignment

```
Xarbc0009A_fwrd     -----guauguaaaucuucgaguuugaguccgaacuauugccaaugcuuguaucuagacgcua--------uauaauaaggaacuggacuaacauguguuuauaugguag
Xarbc0009A_RevC     cuaccauauaaacacauguuaguccaguuccuuauuau--------auagcgucuagauacaagcauuggcaauaguucggacucaaacucgaagauuuacauac-----
                         #***# * *# *   ***###  ***  * ***         * *# **||** #* *         *** *  ***  ###***   * #* * #***#
```

ALIGNMENT RESULTS - Xarbc0010A

=== Stwintron Sequence ===

Multiple sequence alignment

```
Xarbc0010A_fwrd     gguauguaucaaacccuccugugcagguugugaaacccagucuucgcagaaaugucucucgccuucuagagcuaacuagucucacaguagguaaacccuugauuuugggacugaaucauugccuucugu--------uuacuucuagaaaguauaaa--------------------cuaaggaacugacugacaugauuuuaugcguag-
Xarbc0010A_RevC     -cuacgcauaaaaucaugucagucaguuccu--------------------uaguuuauacuuucuagaaguaa--------acagaaggcaaugauucagucccaaaaucaaggguuuaccuacugugagacuaguuagcucuagaaggcgagagacauuucugcgaagacuggguuucacaaccugcacaggaggguuugauacauacc
                      **#*#** *** * *  ##  *** *# *                    *#  *   #* *******# ***        **** ***#**    *## *##||##* ##*    **#*** ****        *** #******* *#   *                       * * #* ***  ##  * * *** **#*#**
```

=== Internal intron ===

Multiple sequence alignment

```
Xarbc0010A_fwrd     --------------------guauguaucaaacccuccugugcagguugugaaacccagucuucgcagaaaugucucucgccuucuagagcuaacuagucucacag
Xarbc0010A_RevC     cugugagacuaguuagcucuagaaggcgagagacauuucugcgaagacuggguuucacaaccugcacaggaggguuugauacauac--------------------
                                        * * *#   * **  * ***#* **##** * | * **##** *#*** *  ** *   #* * *
```

=== External intron ===

Multiple sequence alignment

```
Xarbc0010A_fwrd     ----guagguaaacccuugauuuugggacugaaucauugccuucuguuuacuucuagaaaguauaaacuaaggaacugacugacaugauuuuaugcguag
Xarbc0010A_RevC     cuacgcauaaaaucaugucagucaguuccuuaguuuauacuuucuagaaguaaacagaaggcaaugauucagucccaaaaucaaggguuuaccuac----
                        *#* # ** *   * * *# *   ** * *   *#* ****#   ||   #**** *#*   * * **   * #* * *   * ** # *#*
```

ALIGNMENT RESULTS - Xarbc0010B

=== Stwintron Sequence ===

Multiple sequence alignment

```
Xarbc0010B_fwrd     ggugaguaugaaccuuggcaggaaugucccuugucuuccagagcuaauagg-----------uaucgcaguaugugagcccuagguccaaaaaccagccaugaucgaugugguucuagaaacuauacaauagggaaaagggauuaacauauuuuuauguggcag
Xarbc0010B_RevC     cugccacauaaaaauauguuaaucccuuuucccuauuguauaguuucuagaaccacaucgaucauggcugguuuuuggaccuagggcucacauacugcgaua-----------ccuauuagcucuggaagacaagggacauuccugccaagguucauacucacc
                         ##**#**  *  *  ##  # *   ## * **  * ** *  ***#           #** ** *  * *  * **||** *  * *  * ** **#           #***  * ** *  ** * ##   * #  ##  *  *  **#**##
```

=== Internal intron ===

Multiple sequence alignment

```
Xarbc0010B_fwrd     -----gugaguaugaaccuuggcaggaaugucccuugucuuccagagcuaauagguaucgcag
Xarbc0010B_RevC     cugcgauaccuauuagcucuggaagacaagggacauuccugccaagguucauacucac-----
                         #*#  *** * * #*** **# * * | * * #** ***# * * ***  #*#
```

=== External intron ===

Multiple sequence alignment

```
Xarbc0010B_fwrd     -------------guaugugagccc---------------uagguccaaaaaccagccaugaucgaugugguucuagaaacuauacaauagggaaaagggauuaacauauuuuuauguggcag
Xarbc0010B_RevC     cugccacauaaaaauauguuaaucccuuuucccuauuguauaguuucuagaaccacaucgaucauggcugguuuuuggaccua---------------gggcucacauac-------------
                                 #***** *# **               *** * * * *****     #|#     ***** * * * ***               ** #* *****#
```

ALIGNMENT RESULTS - Xarbc0011A

=== Stwintron Sequence ===

Multiple sequence alignment

```
Xarbc0011A_fwrd     gguauguaucuaaccccugugugcuaguugugagacuuaacuuugguagaaauauuauucgucuucuagggcu----gacucguaccacaguaugugaaccaucgauccagacacuaggcuauguucugcguaguucuagaaggua--------ugcuaaaagaacuuggacuaauauauaguuuguauguggSuag---------
Xarbc0011A_RevC     ---------cuaccacauacaaacuauauauuaguccaaguucuuuuagca--------uaccuucuagaacuacgcagaacauagccuagugucuggaucgaugguucacauacugugguacg----agucagcccuagaagacgaauaauauuucuaccaaaguuaagucucacaacuagcacacaggguuagauacauacc
                             *** * * *### #***  *#* ** *# *  *#*  *** *         ##*******##**    #   *#** *  *** * ** * * | * * ** * ***  * **#*    #  #**##*******##         * ***  *#*  * #* ** *#*  ***# ###* * * ***
```

=== Internal intron ===

Multiple sequence alignment

```
Xarbc0011A_fwrd     guauguaucuaaccccugugugcuaguugugagacuuaacuuugguagaaauauuauucgucuucuagggcugacucguaccac-----------------------ag
Xarbc0011A_RevC     cu-----------------------gugguacgagucagcccuagaagacgaauaauauuucuaccaaaguuaagucucacaacuagcacacaggguuagauacauac
                     *                       ** **# ** *#* *##*#* ***   **|**   *** *#*##* *#* ** #** **                       *
```

=== External intron ===

Multiple sequence alignment

```
Xarbc0011A_fwrd     guaugugaaccaucgauccagacacuaggcuauguucu----gcguaguucuagaagguaugcuaaaagaacuuggacuaauauauaguuuguaugu---gguag
Xarbc0011A_RevC     cuac---cacauacaaacuauauauuaguccaaguucuuuuagcauaccuucuagaacuacgc----agaacauagccuagugucuggaucgaugguucacauac
                     **#    **   *#* * * * * *** *#* *****    **#** #* #|# *# **#**    ***** *#* *** * * * * *#*   **    #**
```

ALIGNMENT RESULTS - Xarbc0012A

=== Stwintron Sequence ===

Multiple sequence alignment

```
Xarbc0012A_fwrd     ------gguauguaucaa-------aaucuucgugcggcuugcgaaac------------------ccaaucuucgcagaaacgucuuucgucuuccaaagcugacucaucucgcaguauguaaaccguugacucuaagacugaaauauugcuaucugcauaguucuagaaacuauacaauaagggacaggacuaacauguaugugguag
Xarbc0012A_RevC     cuaccacauacauguuaguccugucccuuauuguauaguuucuagaacuaugcagauagcaauauuucagucuuagagucaacgguuuacauacugcgagaugagucagcuuuggaagacgaaagacguuucugcgaagauug------------------gguuucgcaagccgcacgaaga-------uuuugauacauacc------
                           #**##* * *          *  * **# #* **  # ***                   ** **** *    ****  ** *#* #* * * #  #||#  # * * *# *#* **  ****    * **** **                   *** #  ** *# #** *  *          * * *##**#
```

=== Internal intron ===

Multiple sequence alignment

```
Xarbc0012A_fwrd     guauguauc----------------------aaaaucuucgugcggcuugcgaaacccaaucuucgcagaaacgucuuucgucuuccaaagcugacucaucucgcag
Xarbc0012A_RevC     cugcgagaugagucagcuuuggaagacgaaagacguuucugcgaagauuggguuucgcaagccgcacgaagauuuu----------------------gauacauac
                     * #*                           *  * *# *#* #* *** * | * *** *# *#* #* *  *                           *# *
```

=== External intron ===

Multiple sequence alignment

```
Xarbc0012A_fwrd     guauguaaaccguugacucuaagacugaaauauugcuaucugcauaguucuagaaacuauacaauaagggacaggacuaacauguaugugguag---------
Xarbc0012A_RevC     ---------cuaccacauacauguuaguccugucccuuauuguauaguuucuagaacuaugcagauagcaauauuucagucuuagagucaacgguuuacauac
                             * ####  * #* *   *   * *# **   ** ****** #|# ****** **   ** #* *   *   * *# *  #### *
```

ALIGNMENT RESULTS - Xarbc0012B

=== Stwintron Sequence ===

Multiple sequence alignment

```
Xarbc0012B_fwrd     ----------------------------gguaugucuuagaaccucccgugcagguugcga-----aacucaaccuuagcagaaacgucuuucgucuucuaaagcuaacaagucucgcaguauguaaauaguugauugugaaacugaaauauugcuaucuaucuagucuagagacuaugcaacaaggaacuggacuaacauguuuugaaaugguag
Xarbc0012B_RevC     cuaccauuucaaaacauguuaguccaguuccuuguugcauagucucuagacuagauagauagcaauauuucaguuucacaaucaacuauuuacauacugcgagacuuguuagcuuuagaagacgaaagacguuucugcuaagguugaguu-----ucgcaaccugcacgggagguucuaagacauacc----------------------------
                                                  # ***  #* *  ***  *   **#* *  *     *  ***   *#*  *  ***   ** *#* #* # * #** || **# * # *# *#* **   ***  *  *#*   ***  *     *  * *#**   *  ***  * *#  *** #
```

=== Internal intron ===

Multiple sequence alignment

```
Xarbc0012B_fwrd     guaug---------------------ucuuagaaccucccgugcagguugcgaaacucaaccuuagcagaaacgucuuucgucuucuaaagcuaacaagucucgcag
Xarbc0012B_RevC     cugcgagacuuguuagcuuuagaagacgaaagacguuucugcuaagguugaguuucgcaaccugcacgggagguucu---------------------aagacauac
                     * #*                     #   ***   * * *#  ****** * | * ******  #* * *   ***                     #   *# *
```

=== External intron ===

Multiple sequence alignment

```
Xarbc0012B_fwrd     gu---auguaaauaguugauugugaaacugaaauauugcuaucuaucuagucuagagacuaugcaacaaggaa--------cuggacuaacauguuuugaaaugguag
Xarbc0012B_RevC     cuaccauuucaaaacauguuagucca--------guuccuuguugcauagucucuagacuagauagauagcaauauuucaguuucacaaucaacuauuuacau---ac
                     *   ** * ** *  ** * **  *         ** **   * # ******||****** # *   ** **         *  ** * **  * ** * **   *
```

ALIGNMENT RESULTS - Xarbc0012D

=== Stwintron Sequence ===

Multiple sequence alignment

```
Xarbc0012D_fwrd     ------------gguauguugugcugauu------------------------------------------------guacaagcaagcguuugcaauaauaucuuuugucuuccagagcuaaccgauaucacaguauguggacauuugaucuagacaacaggcuagaucuugcguaguucuagagggaauauagucaggaacuagauuaacauguuuuuauguggcag
Xarbc0012D_RevC     cugccacauaaaaacauguuaaucuaguuccugacuauauucccucuagaacuacgcaagaucuagccuguugucuagaucaaauguccacauacugugauaucgguuagcucuggaagacaaaagauauuauugcaaacgcuugcuuguac------------------------------------------------aaucagcacaacauacc------------
                                ###*****#  **# **                                                *  ***#    *## *#*  * *****  **# **# | #** #**  ***** *  *#* ##*    #***  *                                                 ** #**  #*****###
```

=== Internal intron ===

Multiple sequence alignment

```
Xarbc0012D_fwrd     guauguugugcugauuguacaagcaagcguuugcaauaauaucuuuugucuuccagagcuaaccgauaucacag---------------------------------
Xarbc0012D_RevC     ---------------------------------cugugauaucgguuagcucuggaagacaaaagauauuauugcaaacgcuugcuuguacaaucagcacaacauac
                                                     *  * *****  **# **# | #** #**  ***** *  *
```

=== External intron ===

Multiple sequence alignment

```
Xarbc0012D_fwrd     -----guauguggacauuugaucuagacaacaggcuagaucuugcguaguucuagagggaauauagucaggaacuagauuaacauguuuuuauguggcag
Xarbc0012D_RevC     cugccacauaaaaacauguuaaucuaguuccugacuauauucccucuagaacuacgcaagaucuagccuguugucuagaucaaauguccacauac-----
                         ##**# ##**** * *  # #    * *#*** ** ##   ***||***   ## ** ***#* *    # #  * * ****## #**##
```

ALIGNMENT RESULTS - Xarbc0013A

=== Stwintron Sequence ===

Multiple sequence alignment

```
Xarbc0013A_fwrd     ----------------gguauguaucaaaaccucccguacggguugcgaaac------------------ccaaucuucgcagaaacgucuuucgucuugcaaagcuaacuagucucgcaguauguaaaccgucgacuuugagacugaaauauuguuaucuguauaguucuagaaacuauacaauaaggaacuggacuaacgugguuuuauauggaug
Xarbc0013A_RevC     cuaccauauaaaaccacguuaguccaguuccuuauuguauaguuucuagaacuauacagauaacaauauuucagucucaaagucgacgguuuacauacugcgagacuaguuagcuuugcaagacgaaagacguuucugcgaagauug------------------gguuucgcaacccguacgggagguuuugauacauacc----------------
                     **  ** #     * *   *** #* **  # ***                   ** ***# #     ***  ** *#* #*** * #***||***# * ***# *#* **  ***     # #*** **                   *** #  ** *# ***   * *     # **  **
```

=== Internal intron ===

Multiple sequence alignment

```
Xarbc0013A_fwrd     guauguauc---------------------aaaaccucccguacggguugcgaaacccaaucuucgcagaaacgucuuucgucuugcaaagcuaacuagucucgcag
Xarbc0013A_RevC     cugcgagacuaguuagcuuugcaagacgaaagacguuucugcgaagauuggguuucgcaacccguacgggagguuuu---------------------gauacauac
                     * #*   *                     * *   * * *#  #*#*** * | * ***#*#  #* * *   * *                     *   *# *
```

=== External intron ===

Multiple sequence alignment

```
Xarbc0013A_fwrd     guauguaaaccgucgacuuugagacugaaaua------uuguuaucuguauaguucuagaaacuauacaauaaggaacuggacuaacgugguuuuaua-----------ugguag
Xarbc0013A_RevC     cuac-----------cauauaaaaccacguuaguccaguuccuuauuguauaguuucuagaacuauacagauaacaa------uauuucagucucaaagucgacgguuuacauac
                     **#             * *#*#**##   **      ** #*   ********* #|# *********   *# **      **   ##**#*#* *            #**
```

ALIGNMENT RESULTS - Xarbc0014A

=== Stwintron Sequence ===

Multiple sequence alignment

```
Xarbc0014A_fwrd     gguauguaucaaaccucucuuugcugguugugaaacccagucuucgcagaaacguuuuucauuuuccaaaacuaacuagucucgcaguauguaaccauugaucuugagauuaaacaauugcgacuuguguauuucuauaaaaauaagaaaaggaacuggacuaacauguuuucaua--uggcag--------------------
Xarbc0014A_RevC     --------------------cugcca--uaugaaaacauguuaguccaguuccuuuucuuauuuuuauagaaauacacaagucgcaauuguuuaaucucaagaucaaugguuacauacugcgagacuaguuaguuuuggaaaaugaaaaacguuucugcgaagacuggguuucacaaccagcaaagagagguuugauacauacc
                                        #***##  *#***** *  **     ***   * ***#* *****   * *   **  #  *****#*   * *   *##*||*##*   * *   *#*****  #  **   * *   ***** *#*** *   ***     **  * *****#*  ##***#
```

=== Internal intron ===

Multiple sequence alignment

```
Xarbc0014A_fwrd     ---------------------guauguaucaaaccucucuuugcugguugugaaacccagucuucgcagaaacguuuuucauuuuccaaaacuaacuagucucgcag
Xarbc0014A_RevC     cugcgagacuaguuaguuuuggaaaaugaaaaacguuucugcgaagacuggguuucacaaccagcaaagagagguuugauacauac---------------------
                                         * * #*   **** * *** #*  *##** * | * **##*  *# *** * ****   *# * *
```

=== External intron ===

Multiple sequence alignment

```
Xarbc0014A_fwrd     -----guauguaaccauugaucuugagauuaaacaauugcgacuuguguauuucuauaaaaauaagaaaaggaacuggacuaac---------auguuuucauauggcag
Xarbc0014A_RevC     cugccauaugaaaacau---------guuaguccaguuccuuuucuuauuuuuauagaaauacacaagucgcaauuguuuaaucucaagaucaaugguuacauac-----
                         #**** ** ***         * *    ** ** *   *# *#* *** || *** *#* #*   * ** **    * *         *** ** ****#
```

ALIGNMENT RESULTS - Xarbc0014B

=== Stwintron Sequence ===

Multiple sequence alignment

```
Xarbc0014B_fwrd     gguaaguacgaaaaccuccuuugccgcuugugcgacucaacugugguagggaugccucuugucuucua-------------------gagcuaacuagcgucauaguaugugaucguuugauucaaaacguaguucuagaagguagauaauaagggacugugcugacauguuucuuugguggcag--------------------------
Xarbc0014B_RevC     --------------------------cugccaccaaagaaacaugucagcacagucccuuauuaucuaccuucuagaacuacguuuugaaucaaacgaucacauacuaugacgcuaguuagcuc-------------------uagaagacaagaggcaucccuaccacaguugagucgcacaagcggcaaaggagguuuucguacuuacc
                                              **  ##* *   ** ##** #** #  * *#***#*  ****                   **# #** # #   ****|****   # # **# #**                   ****  *#***#* *  # **# **## **   * *##  **
```

=== Internal intron ===

Multiple sequence alignment

```
Xarbc0014B_fwrd     gua-------------------aguacgaaaaccuccuuugccgcuugugcgacucaacugugguagggaugccucuugucuucuagagcuaacuagcgucauag
Xarbc0014B_RevC     cuaugacgcuaguuagcucuagaagacaagaggcaucccuaccacaguugagucgcacaagcggcaaaggagguuuucguacu-------------------uac
                     **                   *# **#* *  *  *##*#**#*   ** *|* **   *#**#*##*  *  * *#** #*                   **
```

=== External intron ===

Multiple sequence alignment

```
Xarbc0014B_fwrd     guaugugaucguuugauucaaaacguaguucuagaagguagauaauaagggacugugcugacauguuucuuugguggc--------------------------------------ag
Xarbc0014B_RevC     cu--------------------------------------gccaccaaagaaacaugucagcacagucccuuauuaucuaccuucuagaacuacguuuugaaucaaacgaucacauac
                     *                                      * #* #**#*#* ##** || **## *#*#**# *# *                                      *
```

ALIGNMENT RESULTS - Xarbc0016A

=== Stwintron Sequence ===

Multiple sequence alignment

```
Xarbc0016A_fwrd     -----------gguauguauugaacgcucccauguguuaugcuc----aaucuucguagagauaucuuuuaauuucuagagcugacuauuguuauaguauguaacuuauuggcuguggaguccgaccauuacgucuuguguaguucagaaaguauacaacaugcaacuggacuaacccuuuccuaugcgacag
Xarbc0016A_RevC     cugucgcauaggaaaggguuaguccaguugcauguuguauacuuucugaacuacacaagacguaauggucggacuccacagccaauaaguuacauacuauaacaauagucagcucuagaaauuaaaagauaucucuacgaagauu----gagcauaacacaugggagcguucaauacauacc-----------
                               *# * *  * *  *# *  *****  ***#**     **#  #   ***  **    *#   #**#* ***##*  * *  #***|***#  * *  *##*** *#**#   #*     **  ***   #  #**     **#***  *****  * #*  * *  * * #*
```

=== Internal intron ===

Multiple sequence alignment

```
Xarbc0016A_fwrd     guau-guauugaacgcucccauguguuaugcucaaucuucguagagauaucuuuuaauuucuagagcugacuauuguuauag
Xarbc0016A_RevC     cuauaacaauagucagcucuagaaauuaaaagauaucucuacgaagauugagcauaacacaugggagcguucaauac-auac
                     *** ##* *#  *# # * * # #*** #    ****# || #****    # ***# # * * # #*  #* *## ***
```

=== External intron ===

Multiple sequence alignment

```
Xarbc0016A_fwrd     guauguaacuuauuggcuguggaguccgaccauuacgucuuguguaguucagaaaguauacaacaugcaa---cuggacuaacccuuuccuaugcgacag--
Xarbc0016A_RevC     --cugucgcauaggaaaggguuagucca---guugcauguuguauacuuucugaacuacacaagacguaauggucggacuccacagccaauaaguuacauac
                       ***  * **  ##  *   *****#    ** *#* ****#** ** || ** **#**** *#* **    #*****   *  ##  ** *  ***
```

ALIGNMENT RESULTS - Xarbc0021A

=== Stwintron Sequence ===

Multiple sequence alignment

```
Xarbc0021A_fwrd     ggugagcaucaaaaccuauuacaucgguuuugagaccaaaccaugacagaaacccagagcuaacuagacucacaguauguaaaccgguaauuuuucagaccaaaucauugcgcuccguguagcuuucggaaguauacaacaa---------ggaacugaacuaacauauuuuuauguggThe
Xarbc0021A_RevC     cuaccacauaaaaauauguuaguucaguucc---------uuguuguauacuuccgaaagcuacacggagcgcaaugauuuggucugaaaaauuaccgguuuacauacugugagucuaguuagcucuggguuucugucaugguuuggucucaaaaccgauguaauagguuuugaugcucacc
                         #*** ****  * ***  **#***##            *   * *   ** #*# # **  *   * **#*   *    * * **||** * *    *   *#** *   *  ** # #*# **   * *   *            ##***#** *** *  **** ***#
```

=== Internal intron ===

Multiple sequence alignment

```
Xarbc0021A_fwrd     gug-----------------------------------agcaucaaaaccuauuacaucgguuuugagaccaaaccaugacagaaacccagagcuaacuagacucacag
Xarbc0021A_RevC     cugugagucuaguuagcucuggguuucugucaugguuuggucucaaaaccgauguaauagguuuugaugcu-----------------------------------cac
                     **                                    *  ******** ** | ** ********  *                                    **
```

=== External intron ===

Multiple sequence alignment

```
Xarbc0021A_fwrd     guauguaaaccgguaauuuuucagaccaaaucauugcgcuccguguagcuuucggaaguauaca------acaaggaacugaacuaacauauuuuuaugugguag--
Xarbc0021A_RevC     --cuaccacauaaaaauauguuaguucaguuccuugu------uguauacuuccgaaagcuacacggagcgcaaugauuuggucugaaaaauuaccgguuuacauac
                       *## *   ## *** * * **  **  ** ***       ****  #***|***#  ****       *** **  **  ** * * *** ##   * ##*
```

ALIGNMENT RESULTS - Xarbc0023A

=== Stwintron Sequence ===

Multiple sequence alignment

```
Xarbc0023A_fwrd     gguauguaucaaaaaucgucgcgagaucuagugugguagua-----------aacaauauuuguccaccauggcuaaucaucacuguaguaugugaaccuuugguuggagaaaagaucaccuuuacccacgugaauuuaccaaaacgcgugauuauagcugugguugacguaccaucuuauuucag-------------------------
Xarbc0023A_RevC     -------------------------cugaaauaagaugguacgucaacccacagcuauaaucacgcguuuugguaaauucacgugggguaaaggugaucuuuucuccaaccaaagguucacauacuacagugaugauuagccaugguggacaaauauuguu-----------uacuaccacacuagaucucgcgacgauuuuugauacauacc
                                              *  *#*# *#* ***           * * *** *### *    ***  ***   *   *  # * **** * ***  #||#  *** * **** * #  *   *   ***  ***    * ###* *** * *           *** *#* #*#*  *
```

=== Internal intron ===

Multiple sequence alignment

```
Xarbc0023A_fwrd     -----guauguaucaaaaaucgucgcgagaucuagugugguaguaaacaauauuuguccaccauggcuaaucaucacuguag
Xarbc0023A_RevC     cuacagugaugauuagccaugguggacaaauauuguuuacuaccacacuagaucucgcgacgauuuuugauacauac-----
                         **    ** *   ** ** *  *#** * ** *# || #* ** * **#*  * ** **   * **    **
```

=== External intron ===

Multiple sequence alignment

```
Xarbc0023A_fwrd     -------------guaugugaaccuuugguuggagaaaagaucaccuuuacccacgugaauuuaccaaaacgcgugauua----------------uagcugugguugacguaccaucuuauuucag
Xarbc0023A_RevC     cugaaauaagaugguacgucaacccacagcua----------------uaaucacgcguuuugguaaauucacgugggguaaaggugaucuuuucuccaaccaaagguucacauac-------------
                                 ***#** ****# ##*#*#                **  ****#*  ** || **  *#****  **                #*#*## #**** **#***
```

ALIGNMENT RESULTS - Xarbc0024A

=== Stwintron Sequence ===

Multiple sequence alignment

```
Xarbc0024A_fwrd     gguacguaccgaaacauccucugcugguugugaaaccggauuuucgcagaaaugccuuucgucuuccaaugcuaacuagucacaguaugugaacuguugauuuugagacugaaucaucagcuaugcaauaa---------ggaacuggacuaacaucuuuugauag-----
Xarbc0024A_RevC     -----cuaucaaaagauguuaguccaguucc---------uuauugcauagcugaugauucagucucaaaaucaacaguucacauacugugacuaguuagcauuggaagacgaaaggcauuucugcgaaaauccgguuucacaaccagcagaggauguuucgguacguacc
                          ** *#*** **  *   *##*** #         ** * *** *  **    *    *# *** # #***   ***** | *****   ***# # *** #*    *    **  * *** * **         # ***##*   *  ** ***#* **
```

=== Internal intron ===

Multiple sequence alignment

```
Xarbc0024A_fwrd     guacguaccgaaacauccucugcugguugugaaaccggauuuucgcagaaaugccuuucgucuuccaaugcuaacuagu------cacag
Xarbc0024A_RevC     cugug------acuaguuagcauuggaagacgaaaggcauuucugcgaaaauccgguuucacaaccagcagaggauguuucgguacguac
                     *  *      *  *     ## ***  *   **  * ****# || #**** *  **   *  *** ##     *  *      *  *
```

=== External intron ===

Multiple sequence alignment

```
Xarbc0024A_fwrd     guau-gugaacuguugauuuugagacugaaucaucagcuaugcaauaaggaacuggacuaacaucuuuugauag
Xarbc0024A_RevC     cuaucaaaagauguuaguccaguuccuuauugcauagcugaugauucagucucaaaaucaacaguucac-auac
                     *** # #*  ****# *## *   ** * *    *||*    * * **   * ##* #****  *# # ***
```

ALIGNMENT RESULTS - Xarbc0024B

=== Stwintron Sequence ===

Multiple sequence alignment

```
Xarbc0024B_fwrd     ------------------ggugaguaucaucucgcgucacgcuagccgcgagaauucgugauuguaggagaccuccuaucugccaaugcuaacaauuguugcaguaugugaacacucc----aucaaggaacggagacauuaucaaaucucuggaugaauuauacuaauguuuuugugcguuag
Xarbc0024B_RevC     cuaacgcacaaaaacauuaguauaauucauccagagauuugauaaugucuccguuccuugau----ggaguguucacauacugcaacaauuguuagcauuggcagauaggaggucuccuacaaucacgaauucucgcggcuagcgugacgcgagaugauacucacc------------------
                                      #**# #  *****# * *    * **#   *     *#* ****    ****   ** #** #  ***## * || * ##***  # **# **   ****    **** *#*     *   #** *    * * #*****  # #**#
```

=== Internal intron ===

Multiple sequence alignment

```
Xarbc0024B_fwrd     gugaguaucaucucgcgucacgcuagccgcgagaauucgugauuguaggagaccuccuaucugccaaugcuaacaauuguugcag--------------------------
Xarbc0024B_RevC     --------------------------cugcaacaauuguuagcauuggcagauaggaggucuccuacaaucacgaauucucgcggcuagcgugacgcgagaugauacucac
                                              * **#* ****  *# #  * * ***   |   *** * *  # #*  **** *#** *
```

=== External intron ===

Multiple sequence alignment

```
Xarbc0024B_fwrd     guaugugaacacuccaucaaggaacggagacauuaucaaaucucuggaugaauuauacuaauguuuuugugcguuag----------------
Xarbc0024B_RevC     ----------------cuaacgcacaaaaacauuaguauaauucauccagagauuugauaaugucuccguuccuugauggaguguucacauac
                                    # ** * **##*#******  * *  **  |  **  * *  ******#*##** * ** #
```

ALIGNMENT RESULTS - Xarbc0024C

=== Stwintron Sequence ===

Multiple sequence alignment

```
Xarbc0024C_fwrd     ------gguauguauaaaucugcu--gcaccuugucgugaggccggauucuagcagaaauaucuugagucuuucggagcuaacguguaucacaguauguaaacccuuguacaggaacauguucauugcgac----------------------------cuuguguggucauagaaauagacagcaaggaacugggcugacuuguucuuacgugacag
Xarbc0024C_RevC     cugucacguaagaacaagucagcccaguuccuugcugucuauuucuaugaccacacaag----------------------------gucgcaaugaacauguuccuguacaaggguuuacauacugugauacacguuagcuccgaaagacucaagauauuucugcuagaauccggccucacgacaaggugc--agcagauuuauacauacc------
                           *** * *#** ** **#  *  *****# **  #     **  # #** **                              ** **#*  ##*    *#**||**#*    *##  *#** **                              ** **# #  **     #  ** #*****  *  #** ** **#* * ***
```

=== Internal intron ===

Multiple sequence alignment

```
Xarbc0024C_fwrd     guaug--------------------------uauaaaucugcugcaccuugucgugaggccggauucuagcagaaauaucuugagucuuucggagcuaacguguaucacag
Xarbc0024C_RevC     cugugauacacguuagcuccgaaagacucaagauauuucugcuagaauccggccucacgacaaggugcagcagauuuau--------------------------acauac
                     * **                           ***  ******# *  ##* * *|* * *##  * #******  ***                           ** *
```

=== External intron ===

Multiple sequence alignment

```
Xarbc0024C_fwrd     guauguaaacccuuguacaggaacauguucauugcgaccuuguguggucauagaaauagacagcaaggaacugggcugacuuguucuuacgugacag---------
Xarbc0024C_RevC     ---------cugucacguaagaacaagucagcccaguuccuugcugucuauuucuaugaccacacaaggucgcaaugaacauguuccuguacaaggguuuacauac
                             *  *###  *#***** **#  ##  *  *#*   ** # ** || ** # **   *#*  *  ##  #** *****#*  ###*  *
```

ALIGNMENT RESULTS - Xarbc0026A

=== Stwintron Sequence ===

Multiple sequence alignment

```
Xarbc0026A_fwrd     gguauguau---------------------------cgaaaccuccugugcu------ggcugugaaacccgaccuucguagaaacgucuauuguuuucuaaagcuaauuagcaucauaguaugugaaaccguugauuccgagaccgaucuauuuugaccuguguauuacucgaaauuuuauaaaaaggaacugggcuaacauguucuauauggcag
Xarbc0026A_RevC     cugccauauagaacauguuagcccaguuccuuuuuauaaaauuucgaguaauacacaggucaaaauagaucggucucggaaucaacgguuuacauacuaugaugcuaauuagcuuuagaaaacaauagacguuucuacgaaggucggguuucacagcc------agcacaggagguuucg---------------------------auacauacc
                        ##***                            #***   **   **# *      * * # # *   **   **# * *   ****  *  ##* #* * * ****||**** * * *# *##  *  ****  * * #**  **   * # # * *      * #**  **  ***#                            ***##
```

=== Internal intron ===

Multiple sequence alignment

```
Xarbc0026A_fwrd     guaug------------uaucgaaaccuccugugcuggcugugaaacccgaccuucguagaaacgucuauuguuuucuaaagcuaauuagcaucauag
Xarbc0026A_RevC     cuaugaugcuaauuagcuuuagaaaacaauagacguuucuacgaaggucggguuucacagccagcacaggagguuucgaua------------cauac
                     ****            * * **** *    *   *  **##***   ||   ***##**  *   *    * **** * *            ****
```

=== External intron ===

Multiple sequence alignment

```
Xarbc0026A_fwrd     -------------------------------guauguaaaccguugauuccgagaccgaucuauuuugaccuguguauuacucgaaauuuuauaaaaaggaacugggcuaacauguucuauauggcag
Xarbc0026A_RevC     cugccauauagaacauguuagcccaguuccuuuuuauaaaauuucgaguaauacacaggucaaaauagaucggucucggaaucaacgguuuacauac-------------------------------
                                                    * *#****   *#** *   * ** * ** *||* ** * ** *   * **#*   ****#* *
```

ALIGNMENT RESULTS - Xarbc0028A

=== Stwintron Sequence ===

Multiple sequence alignment

```
Xarbc0028A_fwrd     gguauguaucaaaaccuccugugcugguuguggaacccggucuucacagaaacgucc-----------uucgccuuccaaagcuaacuagucucacaguauguaaacacuuaauuuuggagcuuaaucauucccaucuguggaauuucagaaaguauacacuaaggaacuggacugacauauuuuuaugcgguag
Xarbc0028A_RevC     cuaccgcauaaaaauaugucaguccaguuccuuaguguauacuuucugaaauuccacagaugggaaugauuaagcuccaaaauuaaguguuuacauacugugagacuaguuagcuuuggaaggcga-----------aggacguuucugugaagaccggguuccacaaccagcacaggagguuuugauacauacc
                        #*#** ****  *  ##  *##*** #  *    #  ***    #**   # *            * #  #******# *** *  *  ** *|* **  *  * *** #******#  # *            * #   **#    ***  #    *  # ***##*  ##  *  **** **#*#
```

=== Internal intron ===

Multiple sequence alignment

```
Xarbc0028A_fwrd     guauguaucaaaaccuccugugcugguuguggaacccggucuucacagaaacguccuucgccuuccaaagcuaacuagucu----cacag
Xarbc0028A_RevC     cugug----agacuaguuagcuuuggaaggcgaaggacguuucugugaagaccggguuccacaaccagcacaggagguuuugauacauac
                     * **    * *       *#  ***  *  ***    ** *# || #* **    ***  *  ***  #*       * *    ** *
```

=== External intron ===

Multiple sequence alignment

```
Xarbc0028A_fwrd     -----guauguaaacacuuaauuuuggagcuuaaucauucccaucuguggaauuucagaaaguauacacuaaggaa----------cuggacugacauauuuuuaugcgguag
Xarbc0028A_RevC     cuaccgcauaaaaauaugucaguccag----------uuccuuaguguauacuuucugaaauuccacagaugggaaugauuaagcuccaaaauuaaguguuuacauac-----
                         *#**# *** *  * * *###*          ****    ***# * ****|**** * #***    ****          *###* * *  * *** #**#*
```

ALIGNMENT RESULTS - Xarbc0034A

=== Stwintron Sequence ===

Multiple sequence alignment

```
Xarbc0034A_fwrd     gguacguaucaaaaccuccuaugcuaguugugaaacccaaucuucgcagaaacgucuuuuauguuccaaagcuaacuagugccgcaguauguaaacccucgauuuucaaaccgagucauu-gccaucuauguaa------------uucuagaaaguauacauuaaggaacuggacugacauguuuuuaugcgguag
Xarbc0034A_RevC     cuaccgcauaaaaacaugucaguccaguuccuuaauguauacuuucuagaa------------uuacauagauggc-aaugacucgguuugaaaaucgaggguuuacauacugcggcacuaguuagcuuuggaacauaaaagacguuucugcgaagauuggguuucacaacuagcauaggagguuuugauacguacc
                        **#** ***** *  #*  *#**** #  **   *  ***   ****            ** ** ** *  * *#** * * ** ** *** * | * *** ** ** * * **#* *  * ** ** **            ****   ***  *   **  # ****#*  *#  * ***** **#**
```

=== Internal intron ===

Multiple sequence alignment

```
Xarbc0034A_fwrd     ---------------------guacguaucaaaaccuccuaugcuaguugugaaacccaaucuucgcagaaacgucuuuuauguuccaaagcuaacuagugccgcag
Xarbc0034A_RevC     cugcggcacuaguuagcuuuggaacauaaaagacguuucugcgaagauuggguuucacaacuagcauaggagguuuugauacguac---------------------
                                         * **#**  * *   * ** #*   #*** * | * ***#   *# ** *   * *  **#** *
```

=== External intron ===

Multiple sequence alignment

```
Xarbc0034A_fwrd     -----guauguaaacccucgauuuucaaaccgagucauugccaucuauguaauucuagaaaguauacauuaaggaacuggacugac-------------------------auguuuuuaugcgguag
Xarbc0034A_RevC     cuaccgcauaaaaac-------------------------augucaguccaguuccuuaauguauacuuucuagaauuacauagauggcaaugacucgguuugaaaaucgaggguuuacauac-----
                         *#**# ****                            **  *  #* ***#  ** **||** **  #*** *# *  **                            **** #**#*
```

ALIGNMENT RESULTS - Xarbc0044A

=== Stwintron Sequence ===

Multiple sequence alignment

```
Xarbc0044A_fwrd     --gguauguaucaaaaccuc-------------------------------cugugcaggugccga-aacccaaucuuuggagaaacgucuuucgucuucuaaagcuaauuagucucgcaguauguaaaccguugacucuaagacugaaucguugcuaucuguguaguucuagaaacuauacaauaaagaacuggacuaacauguuguuauauggcag
Xarbc0044A_RevC     cugccauauaacaacauguuaguccaguucuuuauuguauaguuucuagaacuacacagauagcaacgauucagucuuagagucaacgguuuacauacugcgagacuaauuagcuuuagaagacgaaagacguuucuccaaagauuggguu-ucggcaccugcacag-------------------------------gagguuuugauacauacc--
                      * #**#** *** *  *                                **###***#*# *#*  *  ** **** *#   ****  ** *#* #* # * #***||***# * # *# *#* **  ****   #* **** **  *  *#* #*#***###**                               *  * *** **#**# *
```

=== Internal intron ===

Multiple sequence alignment

```
Xarbc0044A_fwrd     guauguaucaaaaccuccugugcaggugccgaaacccaaucuuuggagaaacgucuuucgucuucuaaa---gcuaauuagucucgcag
Xarbc0044A_RevC     cugcgagacuaa---uuagcuuuagaagacgaaagacguuucuccaaagauuggguuucggcaccugcacaggagguuuugauacauac
                     * #*   * **   *    *  **# * *****  *  * #*#|#*# *  *  ***** * #**  *   *    ** *   *# *
```

=== External intron ===

Multiple sequence alignment

```
Xarbc0044A_fwrd     guauguaaaccguugacucuaagacugaaucguugcuaucuguguaguucuagaaacuauacaauaaagaacuggacuaacauguuguua-----uauggcag
Xarbc0044A_RevC     cugc-----cauauaacaacauguuaguccaguucuuuauuguauaguuucuagaacuacacagauagcaacgauucagucuuagagucaacgguuuacauac
                     * #     *   *#**  #* *   *  # ***  *   ***#***** #|# *****#***   *  *** #  *   * *#  **#*     *   # *
```

ALIGNMENT RESULTS - Xarbc0059A

=== Stwintron Sequence ===

Multiple sequence alignment

```
Xarbc0059A_fwrd     gguauguauccagaccucauaugcccauugugaacccuaaucuuugcagaaacaucuuucgucuuccaaugcuaacucauuucacaguacgucaacccccuauuuugagaccaaaccgcggugaucugcguaguucuauuaacuaugcaacaaggaacugaacuaacuu-----guuucuguauuguag
Xarbc0059A_RevC     cuacaauacagaaac-----aaguuaguucaguuccuuguugcauaguuaaauagaacuacgcagaucaccgcgguuuggucucaaaauaggggguugacguacugugaaaugaguuagcauuggaagacgaaagauguuucugcaaagauuaggguucacaaugggcauaugaggucuggauacauacc
                         #**#  *#**     * *    **  *  ** *  * # *#   **     #* **#    ** #**    *  *#*** *#** *   |   * **#* ***#*  *    **# **    #** *#     **   #* # *  * **  *  **    * *     **#*  #**#
```

=== Internal intron ===

Multiple sequence alignment

```
Xarbc0059A_fwrd     guauguauccagaccucauaugcccauugugaacccuaaucuuugcagaaacaucuuucgucuuccaaugcuaa--------cucauuucacag
Xarbc0059A_RevC     cugugaaaugag--------uuagcauuggaagacgaaagauguuucugcaaagauuaggguucacaaugggcauaugaggucuggauacauac
                     * ** *   **        *   ***** #*  *  **  * *  ||  * *  **  *  *# *****   *        **   * ** *
```

=== External intron ===

Multiple sequence alignment

```
Xarbc0059A_fwrd     guacgucaacccccuauuuugagaccaaaccgcggugaucugcguaguucuauuaacuaugcaacaaggaacugaacuaacuuguuucuguauuguag----------
Xarbc0059A_RevC     ----------cuacaauacagaaacaaguuaguucaguuccuuguugcauaguuaaauagaacuacgcagaucaccgcgguuuggucucaaaauaggggguugacguac
                              *  * ** # **#** *    *    * **#  ** *#    *||*    #* **  #** *    *    * **#** # ** *  *
```

ALIGNMENT RESULTS - Xarbc0060A

=== Stwintron Sequence ===

Multiple sequence alignment

```
Xarbc0060A_fwrd     gguauguaucgaaccuccugugcugguugugaaaccgggucuucgcagaaacgucuc------------------ucgccuuccaaagcuaaccaacuuacaguauguaaacccuugauuuuaggagcgaaccauugccgucugcguaauucuagaaaguauacauuaaggaacuggacugacacguuuuugaugcggcag------------------------------------
Xarbc0060A_RevC     ------------------------------------cugccgcaucaaaaacgugucaguccaguuccuuaauguauacuuucuagaauuacgcagacggcaaugguucgcuccuaaaaucaaggguuuacauacuguaaguugguuagcuuuggaaggcg------------------agagacguuucugcgaagacccgguuucacaaccagcacaggagguucgauacauacc
                                                          *#* #  **#****** **                    #* *** * *# **  **  #  **#*   *    *** #*|*# ***    *   *#**  #  **  ** #* * *** *#                    ** ******#**  # *#*
```

=== Internal intron ===

Multiple sequence alignment

```
Xarbc0060A_fwrd     guauguaucgaaccuccugugcugguugugaaaccgggucuucgcagaaacgucucucgccuuccaaagcuaaccaacuuacag------------------
Xarbc0060A_RevC     ------------------cuguaaguugguuagcuuuggaaggcgagagacguuucugcgaagacccgguuucacaaccagcacaggagguucgauacauac
                                       **  #****   * *   *       *** *||* ***       *   * *   ****#  **
```

=== External intron ===

Multiple sequence alignment

```
Xarbc0060A_fwrd     guauguaaacccuugauuuuaggagcgaaccauugccgucugcguaauucuagaaaguauacauuaaggaacuggacugaca---------cguuuuugaugcggcag----------
Xarbc0060A_RevC     ----------cugccgcaucaaaaacg---------ugucaguccaguuccuuaauguauacuuucuagaauuacgcagacggcaaugguucgcuccuaaaaucaaggguuuacauac
                              *  #  # *#*##*#**          *** *  #* ***#  ** **||** **  #*** *#  * ***          **#*##*#* #  #  *
```

ALIGNMENT RESULTS - Xarbc0061A

=== Stwintron Sequence ===

Multiple sequence alignment

```
Xarbc0061A_fwrd     ----------gguaugugaucaaaccucgugugccuguugaagaagucaaauucacagaaacgucuugauc------uuuaaaagcuaacuauugucgcaguaugugaaaaauugcuucuaaaaccuaauagucacaacauguguagugcuaggaaguacauaacaagaauuaga----cugacauguuucuauaugacag
Xarbc0061A_RevC     cugucauauagaaacaugucag----ucuaauucuuguuauguacuuccuagcacuacacauguugugacuauuagguuuuagaagcaauuuuucacauacugcgacaauaguuagcuuu------uaaagaucaagacguuucugugaauuugacuucuucaacaggcacacgagguuuugauacauacc----------
                              *# *##* ***     **  #* * ****#   * **  * #   * * * **  ***#       *** * *# #** * **  *# *|* #*  ** * **# #* *      ***  #***  ** * * *   # *  **  *   #**** * *#  **     *** *##* #*
```

=== Internal intron ===

Multiple sequence alignment

```
Xarbc0061A_fwrd     guauguaucaaaccucgugugccuguugaagaagucaaauucacagaaacgucuugaucuuuaaaagcuaacuauugucgcag-----------------------
Xarbc0061A_RevC     ----------------------cugcgacaauaguuagcuuuuaaagaucaagacguuucugugaauuugacuucuucaacaggcacacgagguuuugauacauac
                                          ***# # *# *** *  **   *# * *# | #* * #*   **  * *** #* # #***
```

=== External intron ===

Multiple sequence alignment

```
Xarbc0061A_fwrd     gu---augugaaaaauugcuucuaaaaccuaauagucacaacauguguagugcuaggaaguacauaacaagaauuagacu-----gacauguuucuauaugacag
Xarbc0061A_RevC     cugucauauagaaacauguc-----agucuaauucuuguuauguacuuccuagcacuacacauguugugacuauuagguuuuagaagcaauuuuucacau---ac
                     *   **#*# ***  ** #     *  *****  *    *  *## *  *#|#*  * ##*  *    *  *****  *     # **  *** #*#**   *
```

ALIGNMENT RESULTS - Xarbc0064A

=== Stwintron Sequence ===

Multiple sequence alignment

```
Xarbc0064A_fwrd     ----gguacguaucaaaaccuccugugcugguugu-----------------------------------aagacccaaucuucgcagaaaaguauuuugucuuucaaagcuaacuagucucauaguauguaaaccguugauuuagagaucgaauaauggcaauccauguaacucuagaaacuauaccaauaaagaacuggacuaacauauuuucuuucuuauggcag
Xarbc0064A_RevC     cugccauaagaaagaaaauauguuaguccaguucuuuauugguauaguuucuagaguuacauggauugccauuauucgaucucuaaaucaacgguuuacauacuaugagacuaguuagcuuugaaagacaaaauacuuuucugcgaagauugggucuu-----------------------------------acaaccagcacaggagguuuugauacguacc----
                         #** * *  ****  *  *#  *##*** *                                   *  *  * ****# # *  ** *  ** ##* #*   * #***||***# *   *# *## **  * **  * # #**** *  *  *                                   * ***##*  #*  *  ****  * * **#
```

=== Internal intron ===

Multiple sequence alignment

```
Xarbc0064A_fwrd     guacguaucaaaaccuccugugcugguuguaagacccaaucuucgcagaaaaguauuuugucuuucaa--------agcuaacuagucucauag
Xarbc0064A_RevC     cuaugagacuaguuagcu--------uugaaagacaaaauacuuuucugcgaagauugggucuuacaaccagcacaggagguuuugauacguac
                     ** *   * *     *         *** *****  *** #*   ||   *# ***  ***** ***         *     * *   * **
```

=== External intron ===

Multiple sequence alignment

```
Xarbc0064A_fwrd     guauguaaaccguugauuuagagaucgaauaauggcaauccauguaacucuagaaacuauaccaauaaagaacuggacuaacauauuuucuuucuuauggcag------------
Xarbc0064A_RevC     ------------cugccauaagaaagaaaauauguuaguccaguucuuuauugguauaguuucuagaguuacauggauugccauuauucgaucucuaaaucaacgguuuacauac
                                #** # **# #*  #**  ***  * ****  *   * * *  * | *  * * *   *  **** *  ***  **#  *# #** # **#
```

ALIGNMENT RESULTS - Xarbc0064B

=== Stwintron Sequence ===

Multiple sequence alignment

```
Xarbc0064B_fwrd     gguauguaucaaaaccuccugugcuggucccaaaccucaaucuuucgcaga----------------------------------------aacgucuuucgucuuccauagcuaacuagucucgcaguauguaaaccguugauuuugagacugagaccgaaucauggcgaucuaugugauucuaaaacguauacagccagggaacuggacuaacauguucuuaugugguag
Xarbc0064B_RevC     cuaccacauaagaacauguuaguccaguucccuggcuguauacguuuuagaaucacauagaucgccaugauucggucucagucucaaaaucaacgguuuacauacugcgagacuaguuagcuauggaagacgaaagacgu----------------------------------------uucugcgaaagauugagguuugggaccagcacaggagguuuugauacauacc
                        ###** * *** *  *#  *##** **    **  ** # *   ***                                        ****  ** *#* #* *   #***||***#   * *# *#* **  ***                                        ****   * # **  **    ** **##*  #*  * *** * **###
```

=== Internal intron ===

Multiple sequence alignment

```
Xarbc0064B_fwrd     ---------------------guauguaucaaaaccuccugugcuggucccaaaccucaaucuuucgcagaaacgucuuucgucuuccauagcuaacuagucucgcag
Xarbc0064B_RevC     cugcgagacuaguuagcuauggaagacgaaagacguuucugcgaaagauugagguuugggaccagcacaggagguuuugauacauac---------------------
                                         * * ##   * *   * ***#*  #*    * || *    *#  *#*** *   * *   ## * *
```

=== External intron ===

Multiple sequence alignment

```
Xarbc0064B_fwrd     gu----------------------------------------------------auguaaaccguugauuuugagacugagaccgaaucauggcgaucuaugugauucuaaaacguauacagccagggaacuggacuaacauguucuuaugugguag
Xarbc0064B_RevC     cuaccacauaagaacauguuaguccaguucccuggcuguauacguuuuagaaucacauagaucgccaugauucggucucagucucaaaaucaacgguuuacau----------------------------------------------------ac
                     *                                                    *##** * **###   **  * **|** *  **   ###** * **##*                                                    *
```

ALIGNMENT RESULTS - Xarbc0072A

=== Stwintron Sequence ===

Multiple sequence alignment

```
Xarbc0072A_fwrd     gguauguaucaagcccugcccugcuaauuaugagaucuguuuagacuaaucaacaucacaguauguaucaauugucaagcuuugguu--uagaaauauggucaccacgaccugcauguuucuaggaacaagauguugaagaacgaaacuaac----------augugcuuaugcgg----------uag-----------------------
Xarbc0072A_RevC     -----------------------cua----------ccgcauaagcacau----------guuaguuucguucuucaacaucuuguuccuagaaacaugcaggucguggugaccauauuucua--aaccaaagcuugacaauugauacauacugugauguugauuagucuaaacagaucucauaauuagcagggcagggcuugauacauacc
                                           ***          *#*# **# *  **          **  ** **  *# ****  *#* ***  ******#***     *||*     ***#******  *** *#*  **** #*  ** **  **          **  * #** #*#*          ***
```

=== Internal intron ===

Multiple sequence alignment

```
Xarbc0072A_fwrd     guauguaucaagcccugcccugcuaauuaugagaucuguuuagacuaaucaacaucacag--------------
Xarbc0072A_RevC     --------------cugugauguugauuagucuaaacagaucucauaauuagcagggcagggcuugauacauac
                                  ***   ** * ****    *  ||  *    **** * **   ***
```

=== External intron ===

Multiple sequence alignment

```
Xarbc0072A_fwrd     -----------------guauguaucaauugucaagcuuugguu--uagaaauauggucaccacgaccugcauguuucuaggaacaagauguugaagaacgaaacuaacaugugcuuaugcgguag
Xarbc0072A_RevC     cuaccgcauaagcacauguuaguuucguucuucaacaucuuguuccuagaaacaugcaggucguggugaccauauuucua--aaccaaagcuugacaauugauacauac-----------------
                                     **  ** **  *# ****  *#* ***  ******#***     *||*     ***#******  *** *#*  **** #*  ** **  **
```

ALIGNMENT RESULTS - Xarbc0074A

=== Stwintron Sequence ===

Multiple sequence alignment

```
Xarbc0074A_fwrd     gguauguaucagaaucuccugugcugguuguaaaacuaggucuucgcagaaacgucuuuuaccuuccagagcuaacuagucucacaguauguaaacccuugauuuugggacugaauaauugccaucuguguaguucuagaaagua--------uacacugaggaacuggacugacg------------uguuguuaugcgauag
Xarbc0074A_RevC     cuaucgcauaacaac------------acgucaguccaguuccucagug--------uauacuuucuagaacuacacagauggcaauuauucagucccaaaaucaaggguuuacauacugugagacuaguuagcucuggaagguaaaagacguuucugcgaagaccuaguuuuacaaccagcacaggagauucugauacauacc
                        #*#** * **#           #** *  *#** **#**#  *        * *** *** ***#***  #**      * *** #*  ***  #||#  ***  *# *** *      **#  ***#*** *** ***        * *  #**#** **#*  * **#            #** * **#*#
```

=== Internal intron ===

Multiple sequence alignment

```
Xarbc0074A_fwrd     guauguaucagaaucuccugugcugguuguaaaacuaggucuucgcagaaacgucuuuuaccuuccagagcuaacuagucucacag----
Xarbc0074A_RevC     ----cugugagacuaguuagcucuggaagguaaaagacguuucugcgaagaccuaguuuuacaaccagcacaggagauucugauacauac
                         * * *** *     *# ****  *  ***  * ** *# || #* ** *  ***  *  **** #*     * *** * *
```

=== External intron ===

Multiple sequence alignment

```
Xarbc0074A_fwrd     guauguaaacccuugauuuugggacugaauaauugc-caucuguguaguucuagaaaguauacacugaggaacuggacugacguguuguuaugcgau---------------------ag
Xarbc0074A_RevC     cu---------------------aucgcauaacaacacgucaguccaguuccucaguguauacuuucuagaacuacacagaug-gcaauuauucagucccaaaaucaaggguuuacauac
                     *                     * #* ****# #* * ** ** #*****#  *  **||**  *  #*****# ** ** * *# #**** *# *                     *
```

ALIGNMENT RESULTS - Xarbc0080A

=== Stwintron Sequence ===

Multiple sequence alignment

```
Xarbc0080A_fwrd     gguauguaucaaucugcugugccggauauaagauccgguauuag---------uagacagaccuauugcugacaaaggcuaacuguuguuauaguauguaaacuugccaucuagaaacaucgccacuaugacuugugugacuuuagagcauguaugacaagagacugaacuaauacugugcgacguguag-----------------------
Xarbc0080A_RevC     -----------------------cuacacgucgcacaguauuaguucagucucuugucauacaugcucuaaagucacacaagucauaguggcgauguuucuagauggcaaguuuacauacuauaacaacaguuagccuuugucagcaauaggucug---------ucuacuaauaccggaucuuauauccggcacagcagauugauacauacc
                                             *#*#    # *#*******         * * ** ** * #*   #*   * #* *  ##* **  # #* * *  *  **|**  *  * * *# #  ** *##  * *# *   *#   *# * ** **         *  ********#* #    #*#*
```

=== Internal intron ===

Multiple sequence alignment

```
Xarbc0080A_fwrd     gu--------------------------auguaucaaucugcu-gugccggauauaagauccgguauuaguagacagaccuauugcugacaaaggcuaacuguuguuauag
Xarbc0080A_RevC     cuauaacaacaguuagccuuugucagcaauaggucugucuacuaauaccggaucuuauauccggca-cagcagauugauacau--------------------------ac
                     *                          **#  **  ***#** #*#****** *|* ******#* #**#***  **  #**                          *
```

=== External intron ===

Multiple sequence alignment

```
Xarbc0080A_fwrd     guauguaaacuugccaucuagaaacaucgccacuaugacuugugugacuuuagagcauguaugacaagagacugaacuaauacugugcgacg---------uguag
Xarbc0080A_RevC     cuaca---------cgucgcacaguauuaguucagucucuugucauacaugcucuaaagucacacaagucauaguggcgauguuucuagauggcaaguuuacauac
                     **##         * **  # *  ** #   *  *  *****   ** *  ||  * **   *****  *  *   # **  * #  ** *         ##**
```

ALIGNMENT RESULTS - Xarbc0093A

=== Stwintron Sequence ===

Multiple sequence alignment

```
Xarbc0093A_fwrd     ----------------------------------------gguacgugcucuaaucgccuaugcccauuguugaaauccgagcagggcgga----------------aauauucuauucuucuugucauccggagcuaauuagucuaauaguaugugaccuuugaaucagaagccugucuaacaccuauaucauca--ugacuuguguaguuguagcacauacgcaacgaggaagugacuaauucaucaugguauuugauag
Xarbc0093A_RevC     cuaucaaauaccaugaugaauuaguccacuuccucguugcguaugugcuacaacuacacaaguc--augaugauauagguguuagacaggcuucugauucaaaggucacauacuauuagacuaauuagcuccggaugacaagaagaauagaauau----------------uuccgcccugcucggauuucaacaaugggcauaggcgauuagagcacguacc----------------------------------------
                                                         *** ***** #**# #* #* * *   ** *** **  * *  #*#*#*                 *#** *****   ** #* * * || * * *# **   ***** **#*                  *#*#*#  * *  ** *** **   * * *# *# #**# ***** ***
```

=== Internal intron ===

Multiple sequence alignment

```
Xarbc0093A_fwrd     guacgugcucuaaucgccuaugcccauuguugaaauccgagcagggcggaaauauucuauucuucuugucauccggagcuaauua-----------gucuaauag
Xarbc0093A_RevC     cuauuagac-----------uaauuagcuccggaugacaagaagaauagaauauuuccgcccugcucggauuucaacaaugggcauaggcgauuagagcacguac
                     **   * #           *#   * # ##* *   *#** **## #*** | ***# ##** **#*   * *## # *   #*           # *   **
```

=== External intron ===

Multiple sequence alignment

```
Xarbc0093A_fwrd     guaugugaccuuugaaucagaagccugucuaacac--------------cuauaucaucaugacuuguguaguuguagcacauacgcaacgaggaaguggacuaauucaucaugguauuugauag------------------------
Xarbc0093A_RevC     ------------------------cuaucaaauaccaugaugaauuaguccacuuccucguugcguaugugcuacaacuacacaagucaugaugauaua--------------gguguuagacaggcuucugauucaaaggucacauac
                                            **#** ** **              *#*# ** ** *  * *#***  * | *  ***#* *  * ** ** #*#              *** ** **#**
```

ALIGNMENT RESULTS - Xarbc0096A

=== Stwintron Sequence ===

Multiple sequence alignment

```
Xarbc0096A_fwrd     gguauguauuaaaucaucuugugcugguugugaaacccagucu--------ucgcauaaacgucuuucgccu--uccaaagcugacuacucucaguauguaaacccuugauuugggacugaaccauugccaucuauguaauucuaaaaaguauacaucaaggaacuggacuaacauauuuuuaugcgguag
Xarbc0096A_RevC     cuaccgcauaaaaauauguuaguccaguuccuugauguauacuuuuuagaauuacauagauggcaaugguucagucccaaaucaaggguuuacauacugugagaguagucagcuuugga--aggcgaaagacguuuaugc--------gaagacuggguuucacaaccagcacaagaugauuuaauacauacc
                        #*#** ***  ** **#  *##*** #   *   *  **        * #**** * * *  * *  #  *** **# ##*    *  ** *|* **  *    *## #** ***  #  * *  * * * ****#         ***  *   *   # ***##*  #** **  *** **#*#
```

=== Internal intron ===

Multiple sequence alignment

```
Xarbc0096A_fwrd     guauguauuaaaucaucuugugcugguugugaaacccagucuucgcauaaacgucuuucgccuuccaaagcugacuacucu----cacag
Xarbc0096A_RevC     cugug----agaguagucagcuuuggaaggcgaaagacguuuaugcgaagacuggguuucacaaccagcacaagaugauuuaauacauac
                     * **    * *  *  # *#  ***  *   **    ** *  ||  * **    **   *  ***  #* #  *  * *    ** *
```

=== External intron ===

Multiple sequence alignment

```
Xarbc0096A_fwrd     -------------guauguaaacccuugauuugggacugaaccauugccaucuauguaauucuaaaaaguauacaucaaggaacuggacuaacauauuuuuaugcgguag
Xarbc0096A_RevC     cuaccgcauaaaaauauguuaguccaguuccuugauguauacuuuuuagaauuacauagauggcaaugguucagucccaaaucaaggguuuacauac-------------
                                 #***** *  **    ##* *#  *# **  **   *  **||**  *   **  ** #*  #* *##    **  * *****#
```

ALIGNMENT RESULTS - Xarbc0099A

=== Stwintron Sequence ===

Multiple sequence alignment

```
Xarbc0099A_fwrd     gguauguauuaaaaccuccugagcugguuguaaagcccaguguucgcagaaacgucuuucgccuuccaaagcuaacuagucucacaguauguaaauccuugauuuuagggccaaagcauugccaucuauguaa----------------------------uucuagaaaguauacaucaagaaauuagacuaacauguuuucaugcgguag
Xarbc0099A_RevC     cuaccgcaugaaaacauguuagucuaauuucuugauguauacuuucuagaa----------------------------uuacauagauggcaaugcuuuggcccuaaaaucaaggauuuacauacugugagacuaguuagcuuuggaaggcgaaagacguuucugcgaacacugggcuuuacaaccagcucaggagguuuuaauacauacc
                        #*#** ***** *  *#  **##** #   #   *   **   ****                            *  ** **   *#**  * *** ###||### *** *  **#*   ** **  *                            ****   **   *   #   # **##**  #*  * ***** **#*#
```

=== Internal intron ===

Multiple sequence alignment

```
Xarbc0099A_fwrd     -----------------------------------------------guauguauuaaaaccuccugagcugguuguaaagcccaguguucgcagaaacgucuuucgccuuccaaagcuaacuagucucacag
Xarbc0099A_RevC     cugugagacuaguuagcuuuggaaggcgaaagacguuucugcgaacacugggcuuuacaaccagcucaggagguuuuaauacauac-----------------------------------------------
                                                                    *  *# *** ****  **|**  **** *** #*  *
```

=== External intron ===

Multiple sequence alignment

```
Xarbc0099A_fwrd     -----guauguaaauccuugauuuuagggccaaagcauugccaucuauguaauucuagaaaguauacaucaagaaau----------uagacuaacauguuuucaugcgguag
Xarbc0099A_RevC     cuaccgcaugaaaacauguuagucua----------auuucuugauguauacuuucuagaauuacauagauggcaaugcuuuggcccuaaaaucaaggauuuacauac-----
                         *#*** ***#    * * *#**          *** *    * *#** ** #|# ** **#* *    * ***          **#* * *   #*** ***#*
```

ALIGNMENT RESULTS - Xarbc0101A

=== Stwintron Sequence ===

Multiple sequence alignment

```
Xarbc0101A_fwrd     ----------------gguauguaacaaagucuccuaugcagguugcgaaaaaccccaaucuuugcggaaacguauuuucguccuuccaaagcuaacgagucucgcaguauguaaaccguugaucuu--------gagagugaauuauugcuauccauguaguucuagaaacuauauaacaaggaacgugguacugacaauguuuuauaugguag
Xarbc0101A_RevC     cuaccauauaaaacauugucaguaccacguuccuuguuauauaguuucuagaacuacauggauagcaauaauuca--------cucucaagaucaacgguuuacauacugcgagacucguuagcuuuggaaggacgaaaauacguuuccgcaaagauuggggguuuuucgcaaccugcauaggagacuuuguuacauacc----------------
                                     **  *** **   **#    *# * # *    * ***  **    * **## **  #*        **# *** # #****  *  *# *|* #*  *  ****# # **        **#*#*#  ** ##** *    **  *** *    * # * #*    #**   ** ***  **
```

=== Internal intron ===

Multiple sequence alignment

```
Xarbc0101A_fwrd     guauguaacaaagucuccuaugcagguugcgaaaaaccccaaucuuugcggaaacguauuuucguccuuccaaagcuaacgagucucgcag--------------------------
Xarbc0101A_RevC     --------------------------cugcgagacucguuagcuuuggaaggacgaaaauacguuuccgcaaagauuggggguuuuucgcaaccugcauaggagacuuuguuacauac
                                              #***** *  *   * # ** * #* *  # *|* #  * *# * ** # *   *  * *****#
```

=== External intron ===

Multiple sequence alignment

```
Xarbc0101A_fwrd     gu---auguaaaccguugaucuugagagugaauuauugcuauccauguaguucuagaaacuauauaacaaggaacgugguacu------gacaauguuuuauaugguag
Xarbc0101A_RevC     cuaccauauaaaacauuguc------aguaccacguuccuuguuauauaguuucuagaacuacauggauagcaauaauucacucucaagaucaacgguuuacau---ac
                     *   **#**** *#*** #      ***#   # ** **    **#***** #|# *****#**    ** ** #   #***      # ***#* ****#**   *
```

ALIGNMENT RESULTS - Xarbc0108A

=== Stwintron Sequence ===

Multiple sequence alignment

```
Xarbc0108A_fwrd     --gguauguaucaaaaccuc-------------------------------cugugcggguugcga-aacccaaucuucguagagacgucuuucaucuuccaaagcugacuaguccugcaguauguaaaccguugacuccaagacugaauuauugcuaucuguauaguuccagaaacuauacaauaaggaacuggacuaacguauuuguauaugguag
Xarbc0108A_RevC     cuaccauauacaaauacguuaguccaguuccuuauuguauaguuucuggaacuauacagauagcaauaauucagucuuggagucaacgguuuacauacugcaggacuagucagcuuuggaagaugaaagacgucucuacgaagauuggguu-ucgcaacccgcacag-------------------------------gagguuuugauacauacc--
                      # #**#**# ** ** *                                **#*#*#*#* **#* **  ** **** *    #***  ** *** #* **  #**#||#**#  ** *# *** **  ***#    * **** **  ** *#** *#*#*#*#**                               * ** ** #**#**# #
```

=== Internal intron ===

Multiple sequence alignment

```
Xarbc0108A_fwrd     ---------------------guauguaucaaaaccuccugugcggguugcgaaacccaaucuucguagagacgucuuucaucuuccaaagcugacuaguccugcag
Xarbc0108A_RevC     cugcaggacuagucagcuuuggaagaugaaagacgucucuacgaagauuggguuucgcaacccgcacaggagguuuugauacauac---------------------
                                         * * #*   * *   # **##* #*#*** * | * ***#*# *##** #   * *   *# * *
```

=== External intron ===

Multiple sequence alignment

```
Xarbc0108A_fwrd     -----guauguaaaccguugacuccaagacugaauuauugcuaucuguauaguuccagaaacuauacaauaaggaa----------cuggacuaacguauuuguauaugguag
Xarbc0108A_RevC     cuaccauauacaaauacguuagucca----------guuccuuauuguauaguuucuggaacuauacagauagcaauaauucagucuuggagucaacgguuuacauac-----
                         #***##***    * * ****           ** **   ********* *|* *********   ** **           **** * *    ***##***#
```

ALIGNMENT RESULTS - Xarbc0134A

=== Stwintron Sequence ===

Multiple sequence alignment

```
Xarbc0134A_fwrd     gguauguaucaaaaccuccugugcggguugc----------------------------------gaaacccaaucuucgcagaaacgucuuucgucuuccaaagcugacuagucucgcaguauguaaaccguugacuuugagacuagacuauugcuaucuguauaguucuagaaaauauacaauaaggaacuggacuaacauguuuuuauaugguag
Xarbc0134A_RevC     cuaccauauaaaaacauguuaguccaguuccuuauuguauauuuucuagaacuauacagauagcaauagucuagucucaaagucaacgguuuacauacugcgagacuagucagcuuuggaagacgaaagacguuucugcgaagauuggguuuc----------------------------------gcaacccgcacaggagguuuugauacauacc
                        ##*** ***** *  *#  * #*** *                                  # *  * * ***# #    ****  ** *#* #* * * #**#||#**# * * *# *#* **  ****    # #*** * *  * #                                  * ***# *  #*  * ***** ***##
```

=== Internal intron ===

Multiple sequence alignment

```
Xarbc0134A_fwrd     ---------------------guauguaucaaaaccuccugugcggguugcgaaacccaaucuucgcagaaacgucuuucgucuuccaaagcugacuagucucgcag
Xarbc0134A_RevC     cugcgagacuagucagcuuuggaagacgaaagacguuucugcgaagauuggguuucgcaacccgcacaggagguuuugauacauac---------------------
                                         * * ##   * *   * ***#* #*#*** * | * ***#*# *#*** *   * *   ## * *
```

=== External intron ===

Multiple sequence alignment

```
Xarbc0134A_fwrd     -----guauguaaaccguugacuuugagacuagacuauugcuaucuguauaguucuagaaaauauacaauaaggaa----------cuggacuaacauguuuuuauaugguag
Xarbc0134A_RevC     cuaccauauaaaaacauguuagucca----------guuccuuauuguauauuuucuagaacuauacagauagcaauagucuagucucaaagucaacgguuuacauac-----
                         #***# ****   * * *###           ** **   ****** ** #|# ** ******   ** **           ###* *  *   **** #***#
```

ALIGNMENT RESULTS - Xarbc0143A

=== Stwintron Sequence ===

Multiple sequence alignment

```
Xarbc0143A_fwrd     gguauguaucaaaaucuguugugcccguugugaagcccaaucuuugcagaaaccccuuucgucuuccaaugcuaacugcucuugcaguauguaaaucuuggauuccgaguccgaauuauugccgaccugugua--------------------uuucuagaaguuauacaauaaggaacuggacugacauguuuuuauaugauag
Xarbc0143A_RevC     cuaucauauaaaaacaugucaguccaguuccuuauuguauaacuucuagaaa--------------------uacacaggucggcaauaauucggacucggaauccaagauuuacauacugcaagagcaguuagcauuggaagacgaaagggguuucugcaaagauugggcuucacaacgggcacaacagauuuugauacauacc
                        ##*** ****# ***##  ** *** #  *    *   #**  *****                    **  ##   # ***#**  *    **#***|***#**    *  **#*** #   ##  **                    *****  **#   *    *  # *** **  ##*** #**** ***##
```

=== Internal intron ===

Multiple sequence alignment

```
Xarbc0143A_fwrd     guaugua------------ucaaaaucuguugugcccguugugaagcccaaucuuugcagaaaccccuuucgucuuccaaugcuaacugcucuugcag
Xarbc0143A_RevC     cugcaagagcaguuagcauuggaagacgaaagggguuucugcaaagauugggcuucacaacgggcacaacagauuuuga------------uacauac
                     * ##              *  **  * #  * *    #**##***  ||  ***##**#    * *  # *  **  *              ## *
```

=== External intron ===

Multiple sequence alignment

```
Xarbc0143A_fwrd     -----guauguaaaucuuggauuccgaguccgaauuauugccgaccuguguauuucuagaaguuauacaauaaggaacuggacugacauguuuuuauaugauag
Xarbc0143A_RevC     cuaucauauaaaaacaugucaguccaguuccuuauuguauaacuucuagaaauacacaggucggcaauaauucggacucggaauccaagauuuacauac-----
                         #***# ***# *   * ***#  ***  *** *       **# # || # #**       * ***  ***  #*** *   * #*** #***#
```

ALIGNMENT RESULTS - Xarbc0164A

=== Stwintron Sequence ===

Multiple sequence alignment

```
Xarbc0164A_fwrd     ----------------gguauguaucaaagccucaugugcgggcugcgaaac------------------ccaaucuucgcagaaacgucuuucgucuuccaaagcugacuagucucgcaguacguaaaccguugacacugagacuggaauauuucuaucuguaaaguucuaaaaacuauacaauaaggggcuagacuaacauguuuguaaauggguag
Xarbc0164A_RevC     cuaccauuuacaaacauguuagucuagccccuuauuguauaguuuuuagaacuuuacagauagaaauauuccagucucagugucaacgguuuacguacugcgagacuagucagcuuuggaagacgaaagacguuucugcgaagauugg------------------guuucgcagcccgcacaugaggcuuugauacauacc----------------
                                     **  ** *     * *  ***# #*  *  # ***                  *** ***# *    ****  ** *** #* * * #**#||#**# * * *# *** **  ****    * #*** ***                  *** #  *  *# #***  * *       * **  **
```

=== Internal intron ===

Multiple sequence alignment

```
Xarbc0164A_fwrd     guauguaucaaagccucaugugcgggcugcgaaacccaaucuucgcagaaacgucuuucgucuuccaaagcuga---cuagucucgcag
Xarbc0164A_RevC     cugcgagacuag---ucagcuuuggaagacgaaagacguuucugcgaagauuggguuucgcagcccgcacaugaggcuuugauacauac
                     * #*   * *    ***  *  **#  #*****  *  * #* | *# *  *  *****#  #**  *  ***    * *   *# *
```

=== External intron ===

Multiple sequence alignment

```
Xarbc0164A_fwrd     guacguaaaccguugacacugagacuggaaua-uuucuaucuguaaaguucuaaaaacuauacaauaaggggcuagacuaacauguuuguaaauggu------ag
Xarbc0164A_RevC     cu------accauuuacaaacauguuagucuagccccuuauuguauaguuuuuagaacuuuacag-auagaaauauuccagucucagugucaacgguuuacguac
                     *      ***#** ***   *   *#*  ** ###**   **** **** *|* **** ****  * #*## **  *#*   *   *** **#***      *
```

ALIGNMENT RESULTS - Xarbc0169A

=== Stwintron Sequence ===

Multiple sequence alignment

```
Xarbc0169A_fwrd     gguauguaucaaaaucuccugugcugguugugagacccaaucuuggcagaaacgucuuucgucuucccacgcuaacuaguccacaguacguaaaaccauuguuuuuugagugcagauuccuacgaucuguauaguucuagaaacuauguaaa------------------aaaagaaacuggacuaacauguuuu--------------------------------------------------------------------uuaugguag-----
Xarbc0169A_RevC     -----------------------------------------------------------------------cuacc--auaaaaaacauguuaguccaguuucuuuu------------------uuuacauaguuucuagaacuauacagaucguaggaaucugcacucaaaaaacaaugguuuuacguacuguggacuaguuagcgugggaagacgaaagacguuucugccaagauugggucucacaaccagcacaggagauuuugauacauacc
                                                                                           *** *  #*  * *##* ** *  *** * *#****                  * *##****** #|# ******##* *                  ****#* * ***  * ** *##* *                                                                    ** **#**
```

=== Internal intron ===

Multiple sequence alignment

```
Xarbc0169A_fwrd     guaug------------------uaucaaaaucuccugugcugguugugagacccaaucuuggcagaaacgucuuucgucuucccacgcuaacuaguccacag
Xarbc0169A_RevC     cuguggacuaguuagcgugggaagacgaaagacguuucugccaagauugggucucacaaccagcacaggagauuuugau------------------acauac
                     * **                   *# ***  *   * ***###   ** *|* **   ###*** *   *  *** #*                   ** *
```

=== External intron ===

Multiple sequence alignment

```
Xarbc0169A_fwrd     gua-cguaaaaccauuguuuuuugagugcagauuccuacgaucuguauaguucuagaaacuauguaaaaaaagaaacuggacuaacauguuuuuuaugguag
Xarbc0169A_RevC     cuaccauaaaaaacauguuaguccaguuucuuuuuuuacauaguuucuagaacuauacagaucguaggaaucugcacucaaaaaacaaugguuuuacg-uac
                     ** *#*****    ****  *# ***     **  ***#   * * ***||*** * *   #***  **     *** #*  ****    *****#* **
```

ALIGNMENT RESULTS - Xarbc0172A

=== Stwintron Sequence ===

Multiple sequence alignment

```
Xarbc0172A_fwrd     gguguguaucaaaaccuccuaugccgguugugaaacccaaucuucgcagaacc-----------------------------------gucuuccaucuuccaaagcuaacuagucucacaguacgugaaaccuuacauuuuaagaccuaaucaucgccaucuguauaauucuagaaaguauauacuaaggaacuggacuaacauguuuuugugugg cag
Xarbc0172A_RevC     cugccacacaaaaacauguuaguccaguuccuuaguauauacuuucuagaauuauacagauggcgaugauuaggucuuaaaauguaagguuucacguacugugagacuaguuagcuuuggaagauggaagac-----------------------------------gguucugcgaagauuggguuucacaaccggcauaggagguuuugauacacacc
                        ###*# ***** *  **  **#*** #  *    *  ***   ****                                    ** *# * * #*   * #***||***# *   *# * * #* **                                     ****   ***  *    *  # ***#**  **  * ***** #*###
```

=== Internal intron ===

Multiple sequence alignment

```
Xarbc0172A_fwrd     -----------------------------------------------guguguaucaaaaccuccuaugccgguugugaaacccaaucuucgcagaaccgucuuccaucuuccaaagcuaacuagucucacag
Xarbc0172A_RevC     cugugagacuaguuagcuuuggaagauggaagacgguucugcgaagauuggguuucacaaccggcauaggagguuuugauacacac-----------------------------------------------
                                                                    ** ** *** ****  * | *  **** *** ** **
```

=== External intron ===

Multiple sequence alignment

```
Xarbc0172A_fwrd     --------------------------guacgugaaaccuuacauuuuaagaccuaaucaucgccaucuguauaauucuagaaaguauauacuaaggaacuggacuaacauguuuuugugugg cag
Xarbc0172A_RevC     cugccacacaaaaacauguuaguccaguuccuuaguauauacuuucuagaauuauacagauggcgaugauuaggucuuaaaauguaagguuucacguac--------------------------
                                              ** * * *     *** **#** #*    *#    *|*    #*    *# **#** ***     * * * **
```

ALIGNMENT RESULTS - Xarbc0174A

=== Stwintron Sequence ===

Multiple sequence alignment

```
Xarbc0174A_fwrd     --gguauguaucagaaccuc-------------------------------cucuguggguugcgaaaccccauccucgcag-aaacaucuuucgucuuccaaagcugacuagucuggcaguauguaaaccguugacuccgagacugaauuacugcuaccuguauaguucuagaaacuauacaauaaggaacuggacuaacauguuguuauauggcag
Xarbc0174A_RevC     cugccauauaacaacauguuaguccaguuccuuauuguauaguuucuagaacuauacagguagcaguaauucagucucggagucaacgguuuacauacugccagacuagucagcuuuggaagacgaaagauguu-ucugcgaggaugggguuucgcaacccacagag-------------------------------gagguucugauacauacc--
                      * #**#** **# *  *                                ** *###*** **#  *   **  **** **  ***   ** *#* #* * * #**#||#**# * * *# *#* **   ***  ** ****  **   *  #** ***###* **                                *  * #** **#**# *
```

=== Internal intron ===

Multiple sequence alignment

```
Xarbc0174A_fwrd     guauguaucagaaccuccucuguggguugcgaaaccccauccucgcagaaacaucuuucgucuuccaaagcuga---cuagucuggcag
Xarbc0174A_RevC     cugccagacuag---ucagcuuuggaagacgaaagauguuucugcgaggaugggguuucgcaacccacagaggagguucugauacauac
                     * #    * #    **  ** ***#  #*****     * ** | ** *     *****#  #*** **  **    # *    # *
```

=== External intron ===

Multiple sequence alignment

```
Xarbc0174A_fwrd     guauguaaaccguugacuccgagacugaauuacugcuaccuguauaguucuagaaacuauacaauaaggaacuggacuaacauguuguua-----uauggcag
Xarbc0174A_RevC     cugc-----cauauaacaacauguuaguccaguuccuuauuguauaguuucuagaacuauacagguagcaguaauucagucucggagucaacgguuuacauac
                     * #     *   *#**  *# *   *  #   * **   ********* #|# *********   ** *   #  *   * #*  **#*     *   # *
```

ALIGNMENT RESULTS - Xarbc0175A

=== Stwintron Sequence ===

Multiple sequence alignment

```
Xarbc0175A_fwrd     gguauguaucgagaccuucuaucguggaaaggucgcuuguuuaccagagcugaugguuaucgcaguaugugaaucucccauccggaagcgugaggcgggaaagcauaugaugagaa----------------------------ucugaacuaacaggaucuaacauguuag
Xarbc0175A_RevC     cuaacauguuagauccuguuaguucaga----------------------------uucucaucauaugcuuucccgccucacgcuuccggaugggagauucacauacugcgauaaccaucagcucugguaaacaagcgaccuuuccacgauagaaggucucgauacauacc
                       *##* * # # ***  **   ##**                            ** **#  #****#   #*# ** # ** || ** # ** #*#   #****#  #** **                            **##   **  *** # # * *##*
```

=== Internal intron ===

Multiple sequence alignment

```
Xarbc0175A_fwrd     guauguaucgagaccuucuaucguggaaaggucgcuuguuuaccagagcugaugguuau----cgcag
Xarbc0175A_RevC     cugcg----auaaccaucagcucugguaaacaagcgaccuuuccacgauagaaggucucgauacauac
                     * #*    # #*** **  #  *** **#   ||   #** ***  #  ** ***# #    *# *
```

=== External intron ===

Multiple sequence alignment

```
Xarbc0175A_fwrd     guaugugaaucucccauccggaagcgugaggcgggaaagcauaugaugagaaucugaacuaacaggaucuaacauguuag----------------------------------------------------------
Xarbc0175A_RevC     ----------------------------------------------------------cuaacauguuagauccuguuaguucagauucucaucauaugcuuucccgccucacgcuuccggaugggagauucacauac
                                                                              ****** * *||* * ******
```

ALIGNMENT RESULTS - Xarbc0192A

=== Stwintron Sequence ===

Multiple sequence alignment

```
Xarbc0192A_fwrd     gguauguaucaaacucuccuaugccgguugugaaagccaaucuucgcagaaccaucuuucgucuuccuacgcuaacuggucucauaguguguaaaccc---uugauucugagaaccgaguuaugagaauccguuuaguucuaaacacuauacaauaaggguaug-----------------------------------------gacuaacauguuuuguuuugauag-----
Xarbc0192A_RevC     -----cuaucaaaacaaaacauguuaguc----------------------------------------cauacccuuauuguauaguguuuagaacuaaacggauucucauaacucgguucucagaaucaa---ggguuuacacacuaugagaccaguuagcguaggaagacgaaagaugguucugcgaagauuggcuuucacaaccggcauaggagaguuugauacauacc
                          ******* #    #***  #**#                                        *#    ** #*   ******* ** * *    # ****** * *** | *** * ****** #     * * ** *******   *# **    #*                                        #**#  ***#    # *******
```

=== Internal intron ===

Multiple sequence alignment

```
Xarbc0192A_fwrd     guauguaucaaacucuccuaugccgguugugaaagccaaucuucgcagaaccaucuuucgucuuccuacgcuaacuggucu----cauag
Xarbc0192A_RevC     cuaug----agaccaguuagcguaggaagacgaaagaugguucugcgaagauuggcuuucacaaccggcauaggagaguuugauacauac
                     ****    * **#      #*  **  *   **#      *# || #*      #**   *  **  *#      #** *    ****
```

=== External intron ===

Multiple sequence alignment

```
Xarbc0192A_fwrd     gu-------------------guguaaacccuu-------------------------gauucugagaaccgaguuaugagaauccguuuaguucuaaacacuauacaauaaggguauggacuaacauguuuuguuuugauag
Xarbc0192A_RevC     cuaucaaaacaaaacauguuaguccauacccuuauuguauaguguuuagaacuaaacggauucucauaacucgguucucagaauc-------------------------aaggguuuacac-------------------ac
                     *                   ** #* ******                         ****** * *** | *** * ******                         ****** *# **                   *
```

ALIGNMENT RESULTS - Xarbc0195A

=== Stwintron Sequence ===

Multiple sequence alignment

```
Xarbc0195A_fwrd     gguauguaucaaaaccuccugugcugguugugaaacccaaucuucgcggaaacaucuuuugucuuccaaaacuaaacaguggcgcaguauguaaacccucgauuuuaaaaccgaaucacugccgucu-------------auguaauucuagacaguauacauuaaggaacuagacuaacauguuuuuauguggquag
Xarbc0195A_RevC     cuaccacauaaaaacauguuagucuaguuccuuaauguauacugucuagaauuac-------------auagacggcagugauucgguuuuaaaaucgaggguuuacauacugcgccacuguuuaguuuuggaagacaaaagauguuuccgcgaagauuggguuucacaaccagcacaggagguuuugauacauacc
                        ###** ***** *  *#  **#*** #  **   *  **    #***  *#             * *     *****#  * ** *  *** * | * ***  * ** *  #*****     *             * #*  ***#    **  *   **  # ***#**  #*  * ***** **###
```

=== Internal intron ===

Multiple sequence alignment

```
Xarbc0195A_fwrd     ---------------------guauguaucaaaaccuccugugcugguugugaaacccaaucuucgcggaaacaucuuuugucuuccaaaacuaaacaguggcgcag
Xarbc0195A_RevC     cugcgccacuguuuaguuuuggaagacaaaagauguuuccgcgaagauuggguuucacaaccagcacaggagguuuugauacauac---------------------
                                         * * ##*  * *   * *#*#*  *#*** * | * ***#*  *#*#* *   * *  *## * *
```

=== External intron ===

Multiple sequence alignment

```
Xarbc0195A_fwrd     guauguaaacccucgauuuuaaaaccgaaucacugccgucuauguaauucuagacaguauacauuaaggaacuagacuaaca----------uguuuuuaugugguag----------
Xarbc0195A_RevC     ----------cuaccacauaaaaac----------auguuagucuaguuccuuaauguauacugucuagaauuacauagacggcagugauucgguuuuaaaaucgaggguuuacauac
                              *  * *# * *****            **   * ** ***#  *  **||**  *  #*** ** *   **            ***** * #* *  *
```

ALIGNMENT RESULTS - Xarbc0220A

=== Stwintron Sequence ===

Multiple sequence alignment

```
Xarbc0220A_fwrd     gguaugugucgaaaccgauugugucugcuacggacccgauucggcagaaguaucuuuuguccuccacagcuaaccaguauuacaguaugugagcccuugauccagaaacugggcaugaccugugugguucuaguagguauaugaugaggaacuugacuaauac----------------------------guuuucaugugauag
Xarbc0220A_RevC     cuaucacaugaaaac----------------------------guauuagucaaguuccucaucauauaccuacuagaaccacacaggucaugcccaguuucuggaucaagggcucacauacuguaauacugguuagcuguggaggacaaaagauacuucugccgaaucggguccguagcagacacaaucgguuucgacacauacc
                        ####* #****                            * *  ***    **# ** **    #*# ** ** *##***     ## ****    **||**    **** ##     ***##* ** ** #*#    ** ** #**    ***  * *                            ****# *####
```

=== Internal intron ===

Multiple sequence alignment

```
Xarbc0220A_fwrd     ------------guaugugucgaaaccgauugugucugcuacggacccgauucggcagaaguaucuuuuguccuccacagcuaaccaguauuacag
Xarbc0220A_RevC     cuguaauacugguuagcuguggaggacaaaagauacuucugccgaaucggguccguagcagacacaaucgguuucgacacauac------------
                                 **  *** **   *#*  *   ** ** * **  ||  ** * ** **   *  *#*   ** ***  **
```

=== External intron ===

Multiple sequence alignment

```
Xarbc0220A_fwrd     guau---gugagcccuugauccagaaacugggcaugaccugugugguucuaguagguauaugaugaggaacuugacuaauacguuuucaugugauag
Xarbc0220A_RevC     cuaucacaugaaaacguauuagucaaguuccucaucauauaccuacuagaaccacacaggucaugcccaguuucuggaucaagggcucac---auac
                     ***   #***#  * *# *    **  *   *** *  *## *# * | * #* ##*  * ***   *  **    * #* *  #***#   ***
```

ALIGNMENT RESULTS - Xarbc0240A

=== Stwintron Sequence ===

Multiple sequence alignment

```
Xarbc0240A_fwrd     ----gguauguaucaaaaucuccuuugccaauugu----------------------------gaaggauuuaugcuaacaacuagugucguaguauguaauuauaaagugaauuuugagacagaaucauugucaucuguauaguucuaggaacuaugcaacaaugaacuaagcuaacauguuucuauaucguag
Xarbc0240A_RevC     cuacgauauagaaacauguuagcuuaguucauuguugcauaguuccuagaacuauacagaugacaaugauucugucucaaaauucacuuuauaauuacauacuacgacacuaguuguuagcauaaauccuu----------------------------cacaauuggcaaaggagauuuugauacauacc----
                        *#***# *   *  *   *** *   *****                             ** ****#   ** * ** * ## * #**#*  |  *#**# * ## * ** * **   #**** **                             *****   * ***   *  *   * #***#*
```

=== Internal intron ===

Multiple sequence alignment

```
Xarbc0240A_fwrd     gua----------uguaucaaaaucuccuuugccaauugugaaggauuuaugcuaacaacuagugucguag
Xarbc0240A_RevC     cuacgacacuaguuguuagcauaaauccuucacaauuggcaaaggagauuuugauaca----------uac
                     **          ***    * *  *****##* *|* *##*****  * *    ***          **
```

=== External intron ===

Multiple sequence alignment

```
Xarbc0240A_fwrd     guauguaauuauaaagugaauuuugagacagaaucauugucaucuguauaguucuaggaacuaugca----acaaugaacuaagcuaacauguuucuauaucgu-----ag
Xarbc0240A_RevC     cu-----acgauauagaaacauguuagcuuaguucauugu----ugcauaguuccuagaacuauacagaugacaaugauucugucucaaaauucacuuuauaauuacauac
                     *     *# *** ** #*  * * **   #  *******    **#*******#|#*******#**    *******  #   ** * *  *# ** *** #*     *
```

ALIGNMENT RESULTS - Xarbc0240B

=== Stwintron Sequence ===

Multiple sequence alignment

```
Xarbc0240B_fwrd     gguauguauuaaaaccuccugugcugguugugaaacccaaucuucgcagaaacgucuuucaucuuccaaagcuaacuaguggcgcaguauguaaacccucgauuuugaaaccgagucauugccaucuauguaa----------------------------uucuagaaaguauacauuaaggaacuggacugacauguuuuuaugcgguag
Xarbc0240B_RevC     cuaccgcauaaaaacaugucaguccaguuccuuaauguauacuuucuagaa----------------------------uuacauagauggcaaugacucgguuucaaaaucgaggguuuacauacugcgccacuaguuagcuuuggaagaugaaagacguuucugcgaagauuggguuucacaaccagcacaggagguuuuaauacauacc
                        #*#** ***** *  ##  *##*** #  **   *  ***   ****                            * #*# **   *#**   **** ***||*** ****   **#*   ** #*# *                            ****   ***  *   **  # ***##*  ##  * ***** **#*#
```

=== Internal intron ===

Multiple sequence alignment

```
Xarbc0240B_fwrd     ---------------------guauguauuaaaaccuccugugcugguugugaaacccaaucuucgcagaaacgucuuucaucuuccaaagcuaacuaguggcgcag
Xarbc0240B_RevC     cugcgccacuaguuagcuuuggaagaugaaagacguuucugcgaagauuggguuucacaaccagcacaggagguuuuaauacauac---------------------
                                         * * #*   * *   * ***#*  *#*** * | * ***#*  *#*** *   * *   *# * *
```

=== External intron ===

Multiple sequence alignment

```
Xarbc0240B_fwrd     guauguaaacccucgauuuugaaaccgagucauugccaucuauguaauucuagaaaguauacauuaaggaacuggac---------ugacauguuuuua-----------ugcgguag
Xarbc0240B_RevC     cuaccgca-----------uaaaaacauguca---------guccaguuccuuaauguauacuuucuagaauuacauagauggcaaugacucgguuucaaaaucgaggguuuacauac
                     **#   *           *#*** *# ****          * #* ***#  ** **||** **  #*** *# *          **** #* ***#*           *   #**
```

ALIGNMENT RESULTS - Xarbc0253A

=== Stwintron Sequence ===

Multiple sequence alignment

```
Xarbc0253A_fwrd     gguacguccuaacaucuccugugcugguugugaaacccaauca---ucgcagaaccauccaguuagcuaacuagucucacaguaugugaaccuucgguuuugaagcuggauugcaguccgucuaguuccagaaagucgaaugaaauagacuaacauguuaucauaugacag--------
Xarbc0253A_RevC     --------cugucauaugauaacauguuagucuauuucauucgacuuucuggaacuagacggacugcaauccagcuucaaaaccgaagguucacauacugugagacuaguuagcuaacuggaugguucugcga---ugauuggguuucacaaccagcacaggagauguuaggacguacc
                            **  *** *  *#   ** * **  *   ** **    *    **** *  * * # ** * *#**# *** *##  # * | * #  ##* *** #**#* * ** # * *  * ****    *    ** **   *  ** * **   #*  * ***  **
```

=== Internal intron ===

Multiple sequence alignment

```
Xarbc0253A_fwrd     guacguccuaacaucuccugugcugguugugaaacccaaucaucgcagaaccauccaguuagcuaacuagucucacag--------------
Xarbc0253A_RevC     --------------cugugagacuaguuagcuaacuggaugguucugcgaugauuggguuucacaaccagcacaggagauguuaggacguac
                                  **   # #**#***#   ***   **  *  ||  *  **   ***   #***#**# #   **
```

=== External intron ===

Multiple sequence alignment

```
Xarbc0253A_fwrd     guaugugaaccuucgguuuugaagcuggauugcaguccgucuaguuccagaaagucgaaugaaauagacuaacauguuaucauaugacag-------------
Xarbc0253A_RevC     -------------cugucauaugauaacauguuagucuauuucauucgacuuucuggaacuagacggacugcaauccagcuucaaaaccgaagguucacauac
                                 * **# *#  #  # **   **** #* * #*** *  |  * ***# * *# ****   ** #  #  #* #** *
```

ALIGNMENT RESULTS - Xarbc0274A

=== Stwintron Sequence ===

Multiple sequence alignment

```
Xarbc0274A_fwrd     ------gguaugua-auauucacccgcgcccgccgugaa-----------------------------acccggccauugcagaaacgucccucgucuuccaaagcuaacuagucucgcaguaugucaaccccugauuucgggaccgaaucauugccaucuguguaauucuaaaaaguauacacgaagggacugaacugacaugugcuuauucgguag
Xarbc0274A_RevC     cuaccgaauaagcacaugucaguucagucccuucguguauacuuuuuagaauuacacagauggcaaugauucggucccgaaaucaggguugacauacugcgagacuaguuagcuuuggaagacgagggacguuucugcaauggccgggu-----------------------------uucacggcgggcgcgggugaauau-uacauacc------
                          ##** *#* ** *#    *#  ***  **** *                             *  *** * # # *  *  *     *#* #* * * #***||***# * * *# *#*     *  *  * # # * ***  *                             * ****  ***  #*    #* ** *#* **##
```

=== Internal intron ===

Multiple sequence alignment

```
Xarbc0274A_fwrd     guauguaa-uauucacccgcgcccgccgugaaacccggccauugcagaaacgucccucgucuuccaaagcuaacuagucucgcag
Xarbc0274A_RevC     cugcgagacuaguuagcuuuggaagacgagggacguuucugcaauggccggguuucacggcgggcgcgggugaaua-uuacauac
                     * #*  * ** * * *   *   * ** *  **    *  #|#  *    **  * ** *   *   * * * ** *  *# *
```

=== External intron ===

Multiple sequence alignment

```
Xarbc0274A_fwrd     guaugucaaccccugauuucgggaccgaaucauugccaucuguguaauucuaaaaaguauacacgaagggacugaacugacaugugcuua-----uucgguag
Xarbc0274A_RevC     -----cuaccgaauaagcacaugucaguucagucccuucguguauacuuuuuagaauuacacagauggcaaugauucggucccgaaaucaggguugacauac
                         # * *   *#* # *# * * *  #  *# *  # ***#** ** *|* ** **#*** #  * #*  #  * * * #* # *#*     *   #**
```

ALIGNMENT RESULTS - Xarbc0299A

=== Stwintron Sequence ===

Multiple sequence alignment

```
Xarbc0299A_fwrd     ggcauguauuaaaaccuccugugcugguugugaaacccggccuuccaaagcuaacuagucucacaguauguaaacccuagguuucggggcugaaucauugccauccguguaauucuagaaaguauacauuaaggaacuggacugacauguuuuuaugugguag
Xarbc0299A_RevC     cuaccacauaaaaacaugucaguccaguuccuuaauguauacuuucuagaauuacacggauggcaaugauucagccccgaaaccuaggguuuacauacugugagacuaguuagcuuuggaaggccggguuucacaaccagcacaggagguuuuaauacaugcc
                        ###** ***** *  ##  *##*** #  **   #  *** * * # * **  *  *  **#*   * * ***# ##|## #*** * *   *#**  *  *  ** * # * * ***  #   **  # ***##*  ##  * ***** **###
```

=== Internal intron ===

Multiple sequence alignment

```
Xarbc0299A_fwrd     gcauguauuaaaaccuccugugcugguugugaaacccggccuuccaaagcuaac----------uagucucacag
Xarbc0299A_RevC     cugugagacua----------guuagcuuuggaaggccggguuucacaaccagcacaggagguuuuaauacaugc
                       **   # *          * *#*#* ** **  *|*  ** ** *#*#* *          * #   **
```

=== External intron ===

Multiple sequence alignment

```
Xarbc0299A_fwrd     -----guauguaaacccuagguuucggggcugaaucauugccauccguguaauucuagaaaguauacauuaaggaacuggacugaca-------------------------uguuuuuaugugguag
Xarbc0299A_RevC     cuaccacauaaaaac-------------------------augucaguccaguuccuuaauguauacuuucuagaauuacacggauggcaaugauucagccccgaaaccuaggguuuacauac-----
                         ##**# ****                            ** ** #* ***#  ** **||** **  #*** *# ** **                            **** #**##
```

ALIGNMENT RESULTS - Xarbc0299B

=== Stwintron Sequence ===

Multiple sequence alignment

```
Xarbc0299B_fwrd     gguauguau----------caagacuucccguacuuguugu----------------------------gaaacucagucuuugaaaaaauaucuugcgucuucugaagcuaaccagacuuacaguauguaaacaugugaauuagaaauuggggcacuacgaucuaugcgauucguagcgagcagacaacaagaaacugaacuaauguguuuuuguaauauag---
Xarbc0299B_RevC     ---cuauauuacaaaaacacauuaguucaguuucuuguugucugcucgcuacgaaucgcauagaucguagugccccaauuucuaauucacauguuuacauacuguaagucugguuagcuucagaagacgcaagauauuuuuucaaagacugaguuuc----------------------------acaacaaguacgggaagucuug----------auacauacc
                        *#***          **  * ***   * ********                            *   *#**#* *#*#*   *     **#*#* #* *#*  ** || **  *#* *# *#*#**     *   *#*#* *#**#*   *                            ******** *   ***  * **          ***#*
```

=== Internal intron ===

Multiple sequence alignment

```
Xarbc0299B_fwrd     guauguauc-------------aagacuucccguacuuguugugaaacucagucuuugaaaaaauaucuugcgucuucugaagcuaaccagacuuacag
Xarbc0299B_RevC     cuguaagucugguuagcuucagaagacgcaagauauuuuuucaaagacugaguuucacaacaaguacgggaagucuu-------------gauacauac
                     * *#  **             ***** #   #** ** **  #* ***|*** *#  ** ** **#   # *****             **  #* *
```

=== External intron ===

Multiple sequence alignment

```
Xarbc0299B_fwrd     -----guauguaaacaugugaauuaga--------aauuggggcacuacgaucuaugcgauucguagcgagcagacaacaagaaacugaacuaauguguuuuuguaauauag
Xarbc0299B_RevC     cuauauuacaaaaacacauuaguucaguuucuuguugucugcucgcuacgaaucgcauagaucguagugccccaau--------uucuaauucacauguuuacauac-----
                          **## *****##* * ** #           *# *  * ******  # || #  ****** *  * #*           # ** * *##***** ##**
```

ALIGNMENT RESULTS - Xarbc0301A

=== Stwintron Sequence ===

Multiple sequence alignment

```
Xarbc0301A_fwrd     gguaaguaauuaccaaaaccuaaugugccgguugcgugacccaaccuuggcagaac---------uaucuuuugucugcuaauagagcuaacuagucucacaguauguggaccaaugauuucaaggucaaaucaucuaugaccuauguuguucuagaaaguaugcagcaagcaacuggacugacauguuuugaug----cgguag
Xarbc0301A_RevC     cuaccg----caucaaaacaugucaguccaguugcuugcugcauacuuucuagaacaacauaggucauagaugauuugaccuugaaaucauugguccacauacugugagacuaguuagcucuauuagcagacaa---------aagauaguucugccaagguugggucacgcaaccggcacauuagguuuugguaauuacuuacc
                         *    #* ****** *  ##  **#***** **   **  ***   *****         #**   * #* ** #  * #*# #*     #* ** *|* ** *#     *# #*# *  # ** *# *          * #* *****   ***  **   ** *****#**  ##  * ****** *#    *
```

=== Internal intron ===

Multiple sequence alignment

```
Xarbc0301A_fwrd     --------------------guaaguaauuaccaaaaccuaaugugccgguugcgugacccaaccuuggcagaacuaucuuuugucugcuaauagagcuaacuagucucacag
Xarbc0301A_RevC     cugugagacuaguuagcucuauuagcagacaaaagauaguucugccaagguugggucacgcaaccggcacauuagguuuugguaauuacuuac--------------------
                                        #* **#*  #*  * *   *  **#   ***** **|** *****   #**  *   * *  *#  *#** *#
```

=== External intron ===

Multiple sequence alignment

```
Xarbc0301A_fwrd     guauguggaccaaugauuucaaggucaaaucaucuaugaccuauguuguucuagaaaguaugcagcaagcaacuggacugacauguuuugaugcgguag----------------
Xarbc0301A_RevC     ----------------cuaccgcaucaaaacaugucaguccaguugcuugcugcauacuuucuagaacaacauaggucauagaugauuugaccuugaaaucauugguccacauac
                                    #* *   #***** *** *  * **  *  # * **  * *|* *  ** * #  *  ** *  * *** *****#   * *#
```

ALIGNMENT RESULTS - Xarbc0309A

=== Stwintron Sequence ===

Multiple sequence alignment

```
Xarbc0309A_fwrd     gguccguaucaaaaccccucgugccagccguggaaccca----------aacuucgcagaacuggccgucgucuuccagagcuaacuaguggcauaguauguaaaccguuauuucgaaaucaaaccgcugcgaccuacgaaguuauggaaaacaugaaacaaagggguaaacuaacguauuuugauauuguag
Xarbc0309A_RevC     cuacaauaucaaaauacguuaguuuaccccuuuguuucauguuuuccauaacuucguaggucgcagcgguuugauuucgaaauaacgguuuacauacuaugccacuaguuagcucuggaagacgacggccaguucugcgaaguu----------uggguuccacggcuggcacgagggguuuugauacggacc
                       * #********  * * #    * ** *      **          ******* **   *  # **   *  *   **# ****   * #****|****# *   **** #**   *  *   ** #  *  ** *******          **      * ** *     # * *  ********# *
```

=== Internal intron ===

Multiple sequence alignment

```
Xarbc0309A_fwrd     guccguaucaaaaccccucgugccagccguggaacccaaacuucgcagaacuggccgucgucuuccagagcuaacuaguggcau----------ag
Xarbc0309A_RevC     cu----------augccacuaguuagcucuggaagacgacggccaguucugcgaaguuuggguuccacggcuggcacgagggguuuugauacggac
                     *          *  ** *  *  ***  *****  * *   #*#  ||  #*#   * *  *****  ***  *  * **  *          *
```

=== External intron ===

Multiple sequence alignment

```
Xarbc0309A_fwrd     guauguaaaccguuauuucgaaaucaaaccgcugcgaccuacgaaguuauggaaaacaugaaacaaagggguaaacuaacguauuuugauauuguag---------------
Xarbc0309A_RevC     ---------------cuacaauaucaaaauacguuaguuuaccccuuuguuucauguuuuccauaacuucguaggucgcagcgguuugauuucgaaauaacgguuuacauac
                                   #* *#* ******  #*   #   ***    ** *   * || *   * **    ***   #   *#  ****** *#* *#
```

ALIGNMENT RESULTS - Xarbc0311A

=== Stwintron Sequence ===

Multiple sequence alignment

```
Xarbc0311A_fwrd     gguacguauucaaaaccuccugugccggucgcgagaccaaaucugcgcagaauuaucuuucaucuuccaaagcuaacgagucucccaguauguaaacugucgauucugagaacuaauuauugcuaucuguguagcucuagagaucgcauaacaagaaauuggcuaacau----------------------------------------------------guuucuaaaugguag--
Xarbc0311A_RevC     --cuaccauuuagaaac----------------------------------------------------auguuagccaauuucuuguuaugcgaucucuagagcuacacagauagcaauaauuaguucucagaaucgacaguuuacauacugggagacucguuagcuuuggaagaugaaagauaauucugcgcagauuuggucucgcgaccggcacaggagguuuugaauacguacc
                      #   #*** * ** *                                                    * * ** * *#* **    ****# * ** * ** #   * *  ** # || # **  * *   # ** * ** * #****    ** *#* * ** * *                                                     * ** * ***#   #
```

=== Internal intron ===

Multiple sequence alignment

```
Xarbc0311A_fwrd     guacguauucaaaaccuccugugccggucgcgagaccaaaucugcgcagaauuaucuuucaucuuccaaagcuaacgagucucccag------------------
Xarbc0311A_RevC     ------------------cugggagacucguuagcuuuggaagaugaaagauaauucugcgcagauuuggucucgcgaccggcacaggagguuuugaauacguac
                                      *** *  # ***  **         # * *# **|** #* * #         **  *** #  * ***
```

=== External intron ===

Multiple sequence alignment

```
Xarbc0311A_fwrd     ---------------------------------guauguaaacugucgauucugagaacuaauuauugcuaucuguguagcucuagagaucgcauaacaagaaauuggcuaacauguuucuaaaugguag
Xarbc0311A_RevC     cuaccauuuagaaacauguuagccaauuucuuguuaugcgaucucuagagcuacacagauagcaauaauuaguucucagaaucgacaguuuacauac---------------------------------
                                                      ****# * ** * ** #   * *  ** # || # **  * *   # ** * ** * #****
```

ALIGNMENT RESULTS - Xarbc0324A

=== Stwintron Sequence ===

Multiple sequence alignment

```
Xarbc0324A_fwrd     --------------gguaugu aucuaaaccucccgugucggucgu--------------------gaaacccaucuuucguagaagcgucuuucguauuccaacgcuaaucggucucacaguauguaaaucguuaauuuugagacuggauuauuaagaucuguguauuuccagaagguagacaaugaggaaccugacuaacauauuuucauaugguag
Xarbc0324A_RevC     cuaccauaugaaaauauguuagucagguuccucauugucuaccuucuggaaauacacagaucuuaauaauccagucucaaaauuaacgauuuacauacugugagaccgauuagcguuggaauacgaaagacgcuucuacgaaagauggguuuc--------------------acgaccgacacgggagguuuagauacauacc--------------
                                   #* *   **     *# *  **** ##* *                    # ** ***  #*# # *  *#**  ** *#**#*   * #*# || #*# *   *#**#* **  **#*  * # #*#  *** ** #                    * *## ****  * #*     **   * *#
```

=== Internal intron ===

Multiple sequence alignment

```
Xarbc0324A_fwrd     guauguaucuaaaccucccgugucggucgugaaacccaucuuucguagaagcgucuuucguauuccaacgcuaaucggucucacag--------------------------
Xarbc0324A_RevC     --------------------------cugugagaccgauuagcguuggaauacgaaagacgcuucuacgaaagauggguuucacgaccgacacgggagguuuagauacauac
                                              # **** *** **   #  * ***     ||     *** *  #   ** *** **** #
```

=== External intron ===

Multiple sequence alignment

```
Xarbc0324A_fwrd     -----guauguaaaucguuaauuuugagacuggauuauuaagaucuguguauuuccagaagguagacaaugaggaaccugacuaacauauuuucauaugguag
Xarbc0324A_RevC     cuaccauaugaaaauauguuagucagguuccucauugucuaccuucuggaaauacacagaucuuaauaauccagucucaaaauuaacgauuuacauac-----
                         #**** ****   * * *# *   *#  *** *# *  * #  * *|* *  # *  * #* ***  #*   * #* * *   **** ****#
```

ALIGNMENT RESULTS - Xarbc0447A

=== Stwintron Sequence ===

Multiple sequence alignment

```
Xarbc0447A_fwrd     gguacguauuaaacccuccuguguugugaaacucgguuuucgcagaaacgucuuucgcccuccaaagcuaacuagucucacaguauguaaacccuagauuuuggggcugaaccauugccaucuguaauucuagaaaguauaaauuaagggacu------------ggacugacauguuuuuaugcgguag------------------------------------
Xarbc0447A_RevC     ------------------------------------cuaccgcauaaaaacauguca------------guccagucccuuaauuuauacuuucuagaauuacagauggcaagguucagccccaaaaucuaggguuuacauacugugagacuaguuagcuuuggagggcgaaagacguuucugcgaaaaccgaguuucacaacacaggaggguuuaauacguacc
                                                    #* #**** *** ## * **#              *#****#*  *#* *#**    ***** **  #*#  * * || * *  #*#  ** *****    **#* *#*  *#****            #*  #** * ## *** ****# *#
```

=== Internal intron ===

Multiple sequence alignment

```
Xarbc0447A_fwrd     guacg-uauuaaacccuccuguguugugaaacucgguuuucgcagaaacgucuuucgcccuccaaagcuaacuagucucacag
Xarbc0447A_RevC     cugugagacuaguuagcuuuggagggcgaaagacguuucugcgaaaaccgaguuucacaacacaggaggguuuaau-acguac
                     *  *  *#**     #  ** #  *#****  ** **#* | *#** **  ****#*  # **  #     **#*  *  *
```

=== External intron ===

Multiple sequence alignment

```
Xarbc0447A_fwrd     -----guauguaaac-ccuagauuuuggggcugaaccauugccaucuguaauucuagaaaguauaaauuaagggacuggacugacauguuuuuaugcgguag
Xarbc0447A_RevC     cuaccgcauaaaaacaugucaguccagucccuuaauuuauacuuucuagaauuacagauggcaagguucagccccaaaaucua-ggguuuacauac-----
                         *#**# ****   * # *## *   ** **    *#*  ***# *||* #***  *#*    ** **   * ##* # *   **** #**#*
```

ALIGNMENT RESULTS - Xbamc009A

=== Stwintron Sequence ===

Multiple sequence alignment

```
Xbamc009A_fwrd      ----------gguacguaucaacuuuccgugccaguucugagaccuggacuuggcagaaagaacuguuguugucuuccagagcuaacuauugcuacaguaugugaauuucugauccacaaacaugaccacaguuaucuguguaauucuagaaaguauguaauaaggaauuagacuaacauauucugaugugacag
Xbamc009A_RevC      cugucacaucagaauauguuagucuaauuccuuauuacauacuuucuagaauuacacagauaacugugguc auguuuguggaucagaaauucacauacuguagcaauaguuagcucuggaagacaacaacaguucuuucugccaaguccaggucucagaacuggcacggaaaguugauacguacc----------
                              #* * #* * *  #*    #   * * *  *    # #   * #** * * ****** **##* **   * # #*   ***  #* *|* *#  ***   *# # *   ** *##** ****** * * **# *   # #    *  * * *   #    *#  * * *# * *#
```

=== Internal intron ===

Multiple sequence alignment

```
Xbamc009A_fwrd      guac------------------------guaucaacuuuccgugccaguucugagaccuggacuuggcagaaagaacuguuguugucuuccagagcuaacuauugcuacag
Xbamc009A_RevC      cuguagcaauaguuagcucuggaagacaacaacaguucuuucugccaaguccaggucucagaacuggcacggaaaguugauac------------------------guac
                     *                          ##* **  *#*   *****# **## *|* ##** #*****   *#*  ** *##                          *
```

=== External intron ===

Multiple sequence alignment

```
Xbamc009A_fwrd      ----------------------------------guaugugaauuucugauccacaaacaugaccacaguuaucuguguaauucuagaaaguauguaauaaggaauuagacuaacauauucugaugugacag
Xbamc009A_RevC      cugucacaucagaauauguuagucuaauuccuuauuacauacuuucuagaauuacacagauaacuguggucauguuuguggaucagaaauucacauac----------------------------------
                                                       **##*#  **#  **   *** * **#** || **#** * ***   **  #**  #*##**
```

ALIGNMENT RESULTS - Xbamc015A

=== Stwintron Sequence ===

Multiple sequence alignment

```
Xbamc015A_fwrd      gguauguauuaucaaccacuauccugccaauauugagacucguugcuggcgaagaagccuauuguu----uaucaagcuaacuguugucguaguaugugaaccuuagauucgucuagaaaucauguuauaaagaaccgaacugacacgauuugaua---cgcuag
Xbamc015A_RevC      cuagcg---uaucaaaucgugucaguucgguucuuuauaacaugauuucuagacgaaucuaagguucacauacuacgacaacaguuagcuugaua----aacaauaggcuucuucgccagcaacgagucucaauauuggcaggauagugguugauaauacauacc
                        #*   ******    * **    *  * #*  #   *#* # *   # *  *# ***  ***    **# * * #***|***# * * #**    ***  *** #*  * #   * # *#*   #  *# *  *    ** *    ******   *#
```

=== Internal intron ===

Multiple sequence alignment

```
Xbamc015A_fwrd      guauguauuaucaaccacuauccugccaauauugagacucguugcuggcgaagaagccuauuguuuaucaagcuaacuguug-----ucguag
Xbamc015A_RevC      cuacg-----acaacaguuagcuugauaaacaauaggcuucuucgccagcaacgagucucaauauuggcaggauagugguugauaauacauac
                     **#*      ****   ** * **  **     ** **  **  #|#  **  ** **     **  ** * **   ****      *#**
```

=== External intron ===

Multiple sequence alignment

```
Xbamc015A_fwrd      gua-----------ugugaaccuuagauucgucuagaaaucauguuauaaagaaccgaacugacacgauuugauacgcuag
Xbamc015A_RevC      cuagcguaucaaaucgugucaguucgguucuuuauaacaugauuucuagacgaaucuaagguucaca-----------uac
                     **           #***    ** * *** *   #* **|** *#   * *** * **    ***#           **
```

ALIGNMENT RESULTS - Xbamc019A

=== Stwintron Sequence ===

Multiple sequence alignment

```
Xbamc019A_fwrd      gguacguaucaaaaccugucgugccgguucuaauaccuggacuuggcagagaucuauugucuugcagagcagagcuaacuguugccacaguaugugaacuucggauccagaaacgugaccacgaucgucuguuuagaucuagaaaguaugca--------------------------------------------------------auaagggacuggacuaacacuuuugauauggcag
Xbamc019A_RevC      cugccauaucaaaaguguuaguccagucccuuau--------------------------------------------------------ugcauacuuucuagaucuaaacagacgaucguggucacguuucuggauccgaaguucacauacuguggcaacaguuagcucugcucugcaagacaauagaucucugccaaguccagguauuagaaccggcacgacagguuuugauacguacc
                        *#********    * ** * * ##** **                                                        * ##*#   *# #**** *#* * ##** *||* **## * *#* ****# #*   #*## *                                                        ** **## * * ** *    ********#*
```

=== Internal intron ===

Multiple sequence alignment

```
Xbamc019A_fwrd      gu--------------------------------------------------acguaucaaaaccugucgugccgguucuaauaccuggacuuggcagagaucuauugucuugcagagcagagcuaacuguugccacag
Xbamc019A_RevC      cuguggcaacaguuagcucugcucugcaagacaauagaucucugccaaguccagguauuagaaccggcacgacagguuuugauacgu--------------------------------------------------ac
                     *                                                  * **** * **** *# | #* **** * **** *                                                  *
```

=== External intron ===

Multiple sequence alignment

```
Xbamc019A_fwrd      ---------------------------------guaugugaacuucggauccagaaacgugaccacgaucgucuguuuagaucuagaaaguaugcaauaagggacuggacuaacacuuuugauauggcag
Xbamc019A_RevC      cugccauaucaaaaguguuaguccagucccuuauugcauacuuucuagaucuaaacagacgaucguggucacguuucuggauccgaaguucacauac---------------------------------
                                                      * ##*#   *# #**** *#* * ##** *||* **## * *#* ****# #*   #*## *
```

ALIGNMENT RESULTS - Xbamc020A

=== Stwintron Sequence ===

Multiple sequence alignment

```
Xbamc020A_fwrd      gguacguauuaaaacccgucaggccgguuc--------------uaaaagccagcuuugauagaaaugucucuugucguccagaguuaacuguugccacaguaugugaacguucgaccc---auaagcaugaccacuuucaucugcguaauccuggaaaguauuugauaaggagcuagacuaacacauuucaauauggcag
Xbamc020A_RevC      cugccauauugaaauguguuagucuagcuccuuaucaaauacuuuccaggauuacgcagaugaaagugguc augcuuau---gggucgaacguucacauacuguggcaacaguuaacucuggacgacaagagacauuucuaucaaagcuggcuuuua-------------gaaccggccugacggguuuuaauacguacc
                        *#**** ***   ** ** * #*#**              *  * *   #* # *** #** **  # *  * #*   * **# * #***  ** *|* **  ***# * #** *   *# *  * #  ** **# *** # *#   * *  *              **#*# * ** **   *** ****#*
```

=== Internal intron ===

Multiple sequence alignment

```
Xbamc020A_fwrd      guacguauuaaaacccgucaggccgguucuaaaagccagcuuugauagaaaugucucuugucguccagaguuaacuguugccacag----------------------
Xbamc020A_RevC      ----------------------cuguggcaacaguuaacucuggacgacaagagacauuucuaucaaagcuggcuuuuagaaccggccugacggguuuuaauacguac
                                           * **  #** **  *#**#**   ** * * | * * **   **#**#*  ** **#  ** *
```

=== External intron ===

Multiple sequence alignment

```
Xbamc020A_fwrd      guaugugaacguucgacccauaagcaugaccacuuucaucugcguaauccuggaaaguauuugauaaggagcuagacuaacacauuucaauauggc----------------------------------------ag
Xbamc020A_RevC      cu----------------------------------------gccauauugaaauguguuagucuagcuccuuaucaaauacuuuccaggauuacgcagaugaaaguggucaugcuuaugggucgaacguucacauac
                     *                                         *# **  **#** ** ** * # ** | ** # * ** ** **#**  ** #*                                            *
```

ALIGNMENT RESULTS - Xbamc022A

=== Stwintron Sequence ===

Multiple sequence alignment

```
Xbamc022A_fwrd      --------------------------gguaugugaucaaaaucu--------------------acuaugucggcugug------------------acauucauuguugacagcaacaucuaugggacugcgcuagagcuaacugcugucauaguaugugaacuuuugaucuugggccauagucaccauagcccgaguaaucauaggaaguggguaugugguaaauaauugggcuaacauuucgugugaucuag
Xbamc022A_RevC      cuagaucacacgaaaauguuagcccaauuauuuaccacauacccacuuccuaugauuacucgggcuauggugacuauggcccaagaucaaaaguucacauacuaugacagcaguuagcucuagcgcagucccauagauguugcugucaacaaugaaugu------------------cacagccgacauagu--------------------agauuuugauacauacc--------------------------
                              # *** **#** *  *#                     *****  *#**#**                  **** *  ** ## ***  *  ****  * *|* *  ****  *  *** ## **  * ****                  **#**#*  *****                     #*  * **#** *** #
```

=== Internal intron ===

Multiple sequence alignment

```
Xbamc022A_fwrd      guauguaucaaaaucuacuaugucggcugugacauucauuguugacagcaacaucuaugggacugcgcuagagcuaacugcugucauag-----------------
Xbamc022A_RevC      -----------------cuaugacagcaguuagcucuagcgcagucccauagauguugcugucaacaaugaaugucacagccgacauaguagauuuugauacauac
                                     ***** *#** ** *  *# * #*# * *    * || *    * * #*# * #*  * ** **#* *****
```

=== External intron ===

Multiple sequence alignment

```
Xbamc022A_fwrd      ------------------------------------guaugugaacuuuugau-----cuugggccauagucaccauagcccgaguaaucauaggaaguggguaugugguaaauaauugggcuaacauuucgugugaucuag
Xbamc022A_RevC      cuagaucacacgaaaauguuagcccaauuauuuaccacauacccacuuccuaugauuacucgggcuauggugacuauggcccaag-----aucaaaaguucacauac------------------------------------
                                                        ##**##  ****## **     **#**** ** **|** ** ****#**     ** ##****  ##**##
```

ALIGNMENT RESULTS - Xbamc024A

=== Stwintron Sequence ===

Multiple sequence alignment

```
Xbamc024A_fwrd      gguauguaaccccugucgugccgguucugaggcccauugccagcagaaacagggauugccuaucagagcuaacguugucacaguaugugaaccuucgaucuagaagcgccgccaccaugauccgaauguccuaggaagcauauaacaagaagcucgauuaacaagccccauag-----------------------------------------------
Xbamc024A_RevC      ----------------------------------------------cuauggggcuuguuaaucgagcuucuuguuauaugcuuccuaggacauucggaucaugguggcggcgcuucuagaucgaagguucacauacugugacaacguuagcucugauaggcaaucccuguuucugcuggcaaugggccucagaaccggcacgacagggguuacauacc
                                                                    *  *** ***   *** #   *   ***#*     * #  * ** ****   #*     **|**     *#   **** ** *  # *     *#***   *   # ***   *** ***  *
```

=== Internal intron ===

Multiple sequence alignment

```
Xbamc024A_fwrd      --------------------guauguaaccccug-ucgugccgguucugaggcccauugccagcagaaacagggauugccuaucagagcuaacguugucacag
Xbamc024A_RevC      cugugacaacguuagcucugauaggcaaucccuguuucugcuggcaaugggccucagaaccggca-cgacagggguuacauac--------------------
                                        #** *#** ***** *  *** **#  ** *|* **  #** ***   ****** **#* **#
```

=== External intron ===

Multiple sequence alignment

```
Xbamc024A_fwrd      guaugugaaccuucgaucuagaagcgccgccaccaugauccgaauguccuaggaagcauauaacaagaag---------cucgauuaacaagccccauag
Xbamc024A_RevC      cuauggggcuuguuaaucgag---------cuucuuguuauaugcuuccuaggacauucggaucaugguggcggcgcuucuagaucgaagguucacauac
                     **** *     * #*** **         *  * ** *  #  # ***||*** #  #  * ** *  *         ** ***# *     * ****
```

ALIGNMENT RESULTS - Xbamc025A

=== Stwintron Sequence ===

Multiple sequence alignment

```
Xbamc025A_fwrd      gguaugcaucaauaucugccauaccgguugcgagauccgguguuggcaa-----------------------------agagaucuauucccugcaaacgcuaacugcuguuauaguacguaaacccuugauacguggucgccaugaucugcguaaacuugcaaaauaugcgauaagacaccagacuaacauauuuccauaugcuag
Xbamc025A_RevC      cuagcauauggaaauauguuagucuggugucuuaucgcauauuuugcaaguuuacgcagaucauggcgaccacguaucaaggguuuacguacuauaacagcaguuagcguuugcagggaauagaucucu-----------------------------uugccaacaccggaucucgcaaccgguauggcagauauugaugcauacc
                        ## **  * ** **  *  * ***  *  # # *#   ** ****                             *# * * **#   **# **  **  |  **  ** #**   #** * * #*                             **** **   #* # #  *  *** *  *  ** ** *  ** ##
```

=== Internal intron ===

Multiple sequence alignment

```
Xbamc025A_fwrd      guaugcaucaauaucugccauaccgguugcgagauccgguguuggcaaagagaucuauucccugcaaacgcuaacugcuguuauag-----------------------
Xbamc025A_RevC      -----------------------cuauaacagcaguuagcguuugcagggaauagaucucuuugccaacaccggaucucgcaaccgguauggcagauauugaugcauac
                                           * #* #*#  *   #*#*** ***  **#  |  #**  *** ***#*#   *  #*# *# *
```

=== External intron ===

Multiple sequence alignment

```
Xbamc025A_fwrd      guacguaaacccuugauacguggucgccaugaucugcguaaacuugcaaaauaugcgauaagacaccagacuaacauauuuccauaugcu-----------------------------------------------ag
Xbamc025A_RevC      cu-----------------------------------------------agcauauggaaauauguuagucuggugucuuaucgcauauuuugcaaguuuacgcagaucauggcgaccacguaucaaggguuuacguac
                     *                                               * #**# *  ** *    **|**    * **  * #**# *                                               *
```

ALIGNMENT RESULTS - Xbamc027A

=== Stwintron Sequence ===

Multiple sequence alignment

```
Xbamc027A_fwrd      gguacguaucauaaccuaccgugccgguuccaggaccgagacuugauagaaugau-----------cuauugucuucccgcgcuaacuguugccgcaguaugugaauuuuugauacugaaacgugaucacuauuaucugcguaauucuagaaaguauggaacaagaagcuggacuaauacuuuuuuauauggcag
Xbamc027A_RevC      cugccauauaaaaaaguauuaguccagcuucuuguuccauacuuucuagaauuacgcagauaauagugaucacguuucaguaucaaaaauucacauacugcggcaacaguuagcgcgggaagacaa-----------uagaucauucuaucaagucucgguccuggaaccggcacgguagguuaugauacguacc
                        *#*** * **  **  #  **#*#* *  *  * * ****  ****** *#             **### ** * * # #**  #**  *# *|* #*  **#  **# # * * ** ###*           *# #* ******  **** * *  *  * *#*#**  #  **  ** * ***#*
```

=== Internal intron ===

Multiple sequence alignment

```
Xbamc027A_fwrd      gu--------------------------acguaucauaaccuaccgugccgguuccaggaccgagacuugauagaaugaucuauugucuucccgcgcuaacuguugccgcag
Xbamc027A_RevC      cugcggcaacaguuagcgcgggaagacaauagaucauucuaucaagucucgguccuggaaccggcacgguagguuaugauacgu--------------------------ac
                     *                          * # *****    *   **  ****#*||*#****  **   *    ***** # *                          *
```

=== External intron ===

Multiple sequence alignment

```
Xbamc027A_fwrd      guau------gugaauuuuugauacugaaacgugaucacuauuaucugcguaauucuagaaaguauggaacaagaagcuggacuaauacuuuuuuauauggcag
Xbamc027A_RevC      cugccauauaaaaaaguauuaguccagcuucuuguuccauacuuucuagaauuacgcagauaauagugaucacguuucaguaucaaaaauucac------auac
                     * #      # #** * **# * * *   * ** **  **#* ***# # || # #*** *#**  ** ** *   * * * #** * **# #      # *
```

ALIGNMENT RESULTS - Xbamc027B

=== Stwintron Sequence ===

Multiple sequence alignment

```
Xbamc027B_fwrd      gguauguauuaaaaccagcugugucgauauccagcguugaucgaaagauauu----------------gugucuacuggagcuaaucguugucauaguauguaaaccccugcuuugaauaugcgguugccucuacuugcggaauccaugaaagcaacuuauaggaacugagcugacaugcuuucguauguuag
Xbamc027B_RevC      cuaacauacgaaagcaugucagcucaguuccuauaaguugcuuucauggauuccgcaaguagaggcaaccgcauauucaaagcagggguuuacauacuaugacaacgauuagcuccagua----------------gacacaauaucuuucgaucaacgcuggauaucgacacagcugguuuuaauacauacc
                       *##**# *** *  * ##  **# * #* *  # *  #    *   ***                 #*# ** * #*# #*   ***  ****|****  ***   *# #*# * **                #*# ***   *    #  * #  * *# * #**  ## *  * *** #**##*
```

=== Internal intron ===

Multiple sequence alignment

```
Xbamc027B_fwrd      guaug-----uauuaaaaccagcugugucgauauccagcguugaucgaaagauauugugucuacuggagcuaaucguugucauag
Xbamc027B_RevC      cuaugacaacgauuagcuccaguagacacaauaucuuucgaucaacgcuggauaucgacacagcugguuuuaau-----acauac
                     ****      ****   ****  *   *#*****   ** *|* **   *****#*   *  ****   ****      ****
```

=== External intron ===

Multiple sequence alignment

```
Xbamc027B_fwrd      guauguaaaccccugcuuugaauaugcgguugccucuacuugcggaauccaugaaagcaacuuauaggaacugagcugacaugcuuucguauguu------------------ag
Xbamc027B_RevC      cu------------------aacauacgaaagcaugucagcucaguuccuauaaguugcuuucauggauuccgcaaguagaggcaaccgcauauucaaagcaggguuuacauac
                     *                  **#**#**#  ** * *   # *#*  #* **#*   |   *#** *#  *#* #   * * **  #**#**#**                  *
```

ALIGNMENT RESULTS - Xbamc027C

=== Stwintron Sequence ===

Multiple sequence alignment

```
Xbamc027C_fwrd      gguauguauucaaucuaccgugcagguuucauggccccauauuaauagaaauauuuuuugucugucagaucuaacuuugcuauaguauguaaaucuuuagccuagaaacgguggucauuaucauaagg-aacuggacuaacacguuacaau-auuacag---
Xbamc027C_RevC      ---cuguaau-auuguaacguguuaguccagu-uccuuaugauaaugaccacguuucuaggcuaaagauuuacauacuauagcaaaguuagaucugacagacaaaaaauauuucuauuaauauggggccaugaaaccugcacgguagauugaauacauacc
                        **** * * * ** ****  #**##  *  **  **  **** #  *# ***#* * **#   # *   *  #*# | #*#  *   * #   #** * *#*** #*  # ****  **  **  *  ##**#  **** ** * * * ****
```

=== Internal intron ===

Multiple sequence alignment

```
Xbamc027C_fwrd      guau----guauucaaucuaccgugcagguuucauggccccauauuaauagaaauauuuuuugucugucagaucuaacuuugcuauag
Xbamc027C_RevC      cuauagcaaaguuagaucugacagacaaaaaauauuucuauuaauauggggccaugaaaccugcacgguagauugaau----acauac
                     ***    #  **  ****  *# #**##    **  *     ||     *  **    ##**# #*  ****  **      #***
```

=== External intron ===

Multiple sequence alignment

```
Xbamc027C_fwrd      guauguaaaucuuuagccuagaaacgugggucauuaucauaaggaacuggacuaacacguuacaauauuacag--
Xbamc027C_RevC      --cuguaauauuguaacguguuaguccaguuccuuaugauaaugaccacguuucuaggcuaaagauuuacauac
                       *****   * **#* *   *   ##**  #*  ||  *#  **##   *   * *#** *   *****
```

ALIGNMENT RESULTS - Xbamc040A

=== Stwintron Sequence ===

Multiple sequence alignment

```
Xbamc040A_fwrd      -gguacguaucaaaaccugccgugccgguucugagaucuggacucgguagaaugau-----------cuauugucuuccagcgcuaacuguuaccgcaguaugugaacuuuuaacuccgaaacacgaucacuguuaucugcguaauucuagaaaguauguaauaaggaacugaacuaacacguuuugauaaugacag
Xbamc040A_RevC      cugucauuaucaaaacguguuaguucaguuccuuauuacauacuuucuagaauuacgcagauaacagugaucguguuucggaguuaaaaguucacauacugcgguaacaguuagcgcuggaagacaa-----------uagaucauucuaccgaguccagaucucagaaccggcacggcagguuuugauacguacc-
                      **   ********* **  #   *#****#  # * ## ***   ****** *#             **#** ** * * * ***  ***  *# *|* #*  ***  *** * * * ** **#*           *# #* ******   *** ## * #  #****#*   #  ** *********   **
```

=== Internal intron ===

Multiple sequence alignment

```
Xbamc040A_fwrd      guacguaucaaaaccugccgugccgguucugagaucuggacucgguagaaugaucuauugucuuccagcgcu--aacuguuaccgcag--------------
Xbamc040A_RevC      --------------cugcgguaacagu--uagcgcuggaagacaauagaucauucuaccgaguccagaucucagaaccggcacggcagguuuugauacguac
                                  **** **# *#**  *#   #  *#*  *##**** || ****##*  *#*  #   #  ***#* #** ****
```

=== External intron ===

Multiple sequence alignment

```
Xbamc040A_fwrd      ------guaugugaacuuuuaacuccgaaacacgaucacuguuaucugcguaauucuagaaaguauguaauaaggaacugaacuaacacguuuugauaaugacag
Xbamc040A_RevC      cugucauuaucaaaacguguuaguucaguuccuuauuacauacuuucuagaauuacgcagauaacagugaucguguuucggaguuaaaaguucacauac------
                           ***  #*** * * * * *#   *   ** **   # * #  * *|* *  # * #   ** **   *   #* * * * * ***#  ***
```

ALIGNMENT RESULTS - Xbamc041A

=== Stwintron Sequence ===

Multiple sequence alignment

```
Xbamc041A_fwrd      gguaagcaucaaaagccucugugccaauucugaaaccugguguugauagaaagau-----------cuauugucuuccggagcuaacuguugccgcaguaugaugccuuuggucuagaaacguaacugccauaaucuguguaucucuagaaaggacuuaguaaggaauuggacuaacacauguugacguggcag
Xbamc041A_RevC      cugccacgucaacauguguuaguccaauuccuuacuaaguccuuucuagagauacacagauuauggcaguuacguuucuagaccaaaggcauacauacugcggcaacaguuagcuccggaagacaa-----------uagaucuuucuaucaacaccagguuucagaauuggcacagaggcuuuugaugcuuacc
                         #* **** *     *#  *******#  *    * # **  **** * *#           *  **## ** * # #*#**  *#   *# *|* #*   #*  **#*# # * ** ##**           *# #* * ****  ** # *    *  #*******  #*     * **** *#
```

=== Internal intron ===

Multiple sequence alignment

```
Xbamc041A_fwrd      gu-------------------aagcaucaaaagccucugugccaauucugaaaccugguguugauagaaagaucuauugucuuccggagcuaacuguugccgcag
Xbamc041A_RevC      cugcggcaacaguuagcuccggaagacaauagaucuuucuaucaacaccagguuucagaauuggcacagaggcuuuugaugcuu-------------------ac
                     *                    *# *# * * # ** * *# ***# *##  |  ##* #*** #* * ** # * * #* #*                    *
```

=== External intron ===

Multiple sequence alignment

```
Xbamc041A_fwrd      guau------------------------------guaugccuuuggucuagaaacguaacugccauaaucuguguaucucuagaaaggacuuaguaaggaauuggacuaacacauguugacguggcag
Xbamc041A_RevC      cugccacgucaacauguguuaguccaauuccuuacuaaguccuuucuagagauacacagauuauggcaguuacguuucuagaccaaaggcaua------------------------------cauac
                     * #                               ** * *#**  *  *** **##*  *  ||  *  *##** ***  *  **#* * **                               # *
```

ALIGNMENT RESULTS - Xbamc051A

=== Stwintron Sequence ===

Multiple sequence alignment

```
Xbamc051A_fwrd      gguacguaucaaaaccuaccgugccagucuugagaccuagacuugguagaauggu-----------cuauugucuuccggcgcuaacaauugcuucaguacgugaaccuuugauccugaaacuugaucaccauuaucugcguaauucuagaaaguauguaauaaggaacuagacuaauacguuuucauauggcag
Xbamc051A_RevC      cugccauaugaaaacguauuagucuaguuccuuauuacauacuuucuagaauuacgcagauaauggugaucaaguuucaggaucaaagguucacguacugaagcaauuguuagcgccggaagacaa-----------uagaccauucuaccaagucuaggucucaagacuggcacgguagguuuugauacguacc
                        *#*** ***** **  #  * *** ##  #   #* ****  ****** ##             **##  ** *#* # #**   **  #  *|*  #  **   **# # *#* **  ##*           *# ## ******  **** *#   #  ## *** *  #  ** ***** ***#*
```

=== Internal intron ===

Multiple sequence alignment

```
Xbamc051A_fwrd      guacguaucaaaaccuaccgugccagucuugagaccuagacuugguagaauggucuauugucuuccggcgcuaacaauugc----------------uucag
Xbamc051A_RevC      cug----------------aagcaauuguuagcgccggaagacaauagaccauucuaccaagucuaggucucaagacuggcacgguagguuuugauacguac
                     *                 # ** * * **#   **  #*  ###**** || ****###  *#  **   #** * * **                #  *
```

=== External intron ===

Multiple sequence alignment

```
Xbamc051A_fwrd      -----guacgugaaccuuugauccugaaacuugaucaccauuaucugcguaauucuagaaaguauguaauaaggaacuagacuaauacguuuucauauggcag
Xbamc051A_RevC      cugccauaugaaaacguauuagucuaguuccuuauuacauacuuucuagaauuacgcagauaauggugaucaaguuucaggaucaaagguucacguac-----
                         #** * #*** * * *  **#   *#* ** **   # * #  * *|* *  # * #   ** ** *#*   #**  * * * ***# * **#
```

ALIGNMENT RESULTS - Xbamc053A

=== Stwintron Sequence ===

Multiple sequence alignment

```
Xbamc053A_fwrd      ggugcguaccaaaaccugccgugucagcugugacaugggc----------guagcagauauuaaauguauugucuuccggggcuaaccauugccgcaguaugugaacuucgaacaccaaaucaugaccaucacgaucugugcaacucuaggaaggauuuaucaaggaaccagacuaacacguuuugauauggcag
Xbamc053A_RevC      cugccauaucaaaacguguuagucugguuccuugauaaauccuuccuagaguugcacagaucgugauggucaugauuugguguucgaaguucacauacugcggcaaugguuagccccggaagacaauacauuuaauaucugcuac----------gcccaugucacagcugacacggcagguuuugguacgcacc
                        *#** ****** **  #  #  * * #   **###           ** *** * **#       *##*  *  ** * *     **  *# *|* #*  **     * * **  *  *##*       #** * *** **           ###**   # * *  #  #  ** ****** **#*
```

=== Internal intron ===

Multiple sequence alignment

```
Xbamc053A_fwrd      gugcg------------------uaccaaaaccugccgugucagcugugacaugggcguagcagauauuaaauguauugucuuccggggcuaaccauugccgcag
Xbamc053A_RevC      cugcggcaaugguuagccccggaagacaauacauuuaauaucugcuacgcccaugucacagcugacacggcagguuuuggu------------------acgcac
                     ****                     *** ** *   #*#** ***##* * | * *##*** **#*#   * ** ***                     ****
```

=== External intron ===

Multiple sequence alignment

```
Xbamc053A_fwrd      guaugugaacuucgaacaccaaaucaugaccaucacgaucugugcaacucuaggaaggauuuaucaaggaaccagacuaacacguuuugauau----------ggcag
Xbamc053A_RevC      cugc----------cauaucaaaacguguuagucugguuccuugauaaauccuuccuagaguugcacagaucgugauggucaugauuugguguucgaaguucacauac
                     * #           * * **** * **    **  * **# **  *   #  ||  #   *  ** #** *  **    ** * **** * *           # *
```

ALIGNMENT RESULTS - Xbamc053B

=== Stwintron Sequence ===

Multiple sequence alignment

```
Xbamc053B_fwrd      gguacguauuaaagccuuuucaugcuggucuaagaccuggacuugguagcaagauagcuuaucuuccaacgcuaacuacugcuacaguauguauacucauaaucuaacagccacuagcauguaugaa---------cuccuaggaaguauauaauaaugaacuggacuaauauauuuuaauauggcag
Xbamc053B_RevC      cugccauauuaaaauauauuaguccaguucauuauuauauacuuccuagga---------guucauacaugcuaguggcuguuagauuaugaguauacauacuguagcaguaguuagcguuggaagauaagcuaucuugcuaccaaguccaggucuuagaccagcaugaaaggcuuuaaauacguacc
                        *#*******#  * **  * * * **   #   *# ****  *** *          * #    * ****    *** ** * ****  ||  **** * ** ***    **** *    # *          * ***  **** #*   #   ** * * *  ** *  #*******#*
```

=== Internal intron ===

Multiple sequence alignment

```
Xbamc053B_fwrd      guacguauuaaagccuuuucaugcuggucuaagaccuggacuugguagcaagauagcuuaucuuccaacgcuaacuacugcuacag-----------------------
Xbamc053B_RevC      -----------------------cuguagcaguaguuagcguuggaagauaagcuaucuugcuaccaaguccaggucuuagaccagcaugaaaggcuuuaaauacguac
                                           ***   #*  *  *#*  **** **  *# #|# #*  ** ****  *#*  *  *#   ***
```

=== External intron ===

Multiple sequence alignment

```
Xbamc053B_fwrd      guauguauacucauaaucuaacagccacuagcauguaugaacuccuaggaaguauauaauaaugaacuggacuaauauauuuuaauaug------------------------------------------------gcag
Xbamc053B_RevC      cugc------------------------------------------------cauauuaaaauauauuaguccaguucauuauuauauacuuccuaggaguucauacaugcuaguggcuguuagauuaugaguauacauac
                     * #                                                #**** * ***# * *#*|*#* * #*** * ****#                                                # *
```

ALIGNMENT RESULTS - Xbamc053C

=== Stwintron Sequence ===

Multiple sequence alignment

```
Xbamc053C_fwrd      gguaugaucaaaaccuaccgugccaguucugagaccuagacuugguagaaugau-----------cuguugucuuccagcgcugacuguugccgcagugcgugaacuucugauccugaaacaugaccacaaucaucugcguaauuccagaaaguauguaauaaggaacuggacuaauacguguuuauaugacag
Xbamc053C_RevC      cugucauauaaacacguauuaguccaguuccuuauuacauacuuucuggaauuacgcagaugauuguggucauguuucaggaucagaaguucacgcacugcggcaacagucagcgcuggaagacaac-----------agaucauucuaccaagucuaggucucagaacuggcacgguagguuuugauacauacc
                        ##*** ** ** **  #  *******#  #   #* ****  * **** *#             **##* ** *** # ##   ***  ****|****  ***   ## # *** ** *##**           # #* **** *  **** *#   #  #*******  #  ** ** ** ***##
```

=== Internal intron ===

Multiple sequence alignment

```
Xbamc053C_fwrd      guaug-------------------uaucaaaaccuaccgugccaguucugagaccuagacuugguagaaugaucuguugucuuccagcgcugacuguugccgcag
Xbamc053C_RevC      cugcggcaacagucagcgcuggaagacaacagaucauucuaccaagucuaggucucagaacuggcacgguagguuuugau-------------------acauac
                     * #*                    *# * *   #*   *#***# ***# *|* #*** #***#*   *#   * * #*                    *# *
```

=== External intron ===

Multiple sequence alignment

```
Xbamc053C_fwrd      gugcgugaacuucugauccugaaacaugaccacaaucaucugcguaauuccagaaaguauguaauaaggaacuggacuaauacguguuuauaug--------------------acag
Xbamc053C_RevC      cugu--------------------cauauaaacacguauuaguccaguuccuuauuacauacuuucuggaauuacgcagaugauuguggucauguuucaggaucagaaguucacgcac
                     **                     ***#   ***   **  *  #* ****  *  ##||##  *  **** *#  *  **   ***   #***                     **
```

ALIGNMENT RESULTS - Xbamc067A

=== Stwintron Sequence ===

Multiple sequence alignment

```
Xbamc067A_fwrd      gguauguuuuaaaaccuaucaugccgguucugagauuucgacuuguugaaaugaucuaucguccuccgucgcuaacuguugccacaguacgugagcuuuggauccugaaccgugauuacuauuauaugcguaauucgagaaaguauuuaauaa------------ggaacuggacuaauacguuucgauauggcag
Xbamc067A_RevC      cugccauaucgaaacguauuaguccaguu------------ccuuauuaaauacuuucucgaauuacgcauauaauaguaaucacgguucaggauccaaagcucacguacuguggcaacaguuagcgacggaggacgauagaucauuucaacaagucgaaaucucagaaccggcaugauagguuuuaaaacauacc
                        ##* *# **** *** *  **#***            *#*  * ****# * * ***   * **#   ***  ** # *** ** *# ** *#||#* ** #* ** *** # **  ***   #** *   *** * * #**** *  *            #****#**  * *** **** #* *##
```

=== Internal intron ===

Multiple sequence alignment

```
Xbamc067A_fwrd      gu------auguuuuaaaaccuaucaugccgguucugagauuucgacuuguugaaau----gaucuaucguccuccgucgcuaacuguugccacag
Xbamc067A_RevC      cuguggcaacaguuagcgacggaggacgauagauc----auuucaacaagucgaaaucucagaaccggcaugauagguuuuaaaacau------ac
                     *      *## **    **   *  *#*  #* **    *****#**||**#*****    ** *#  *#*  *  **    ** ##*      *
```

=== External intron ===

Multiple sequence alignment

```
Xbamc067A_fwrd      gu-----------acgugagcuuuggauccugaaccgugauuacuauuauaugcguaauucgagaaaguauuuaauaaggaacuggacuaa------------uacguuucgauauggcag
Xbamc067A_RevC      cugccauaucgaaacgua------------uuaguccaguuccuuauuaaauacuuucucgaauuacgcauauaauaguaaucacgguucaggauccaaagcucacgu-----------ac
                     *           ****#            * *  *  * *#  ***** **#* *  *#|#*  * *#** *****  #* *  *  * *            #****           *
```

ALIGNMENT RESULTS - Xbamc067B

=== Stwintron Sequence ===

Multiple sequence alignment

```
Xbamc067B_fwrd      gguauguaucgaaaccugcugugccgaauccgagacccagacuugguaggaaauauccauugucuucuagagcuaacuauugccacaguaugugacauucuaauccagaaacgugaucacccuuaucuaugugaau-----------------------------------------------cuauuaaguauguaauuaggaccuggacuaacacccuuugauauggcag
Xbamc067B_RevC      cugccauaucaaaggguguuaguccagguccuaauuacauacuuaauag-----------------------------------------------auucacauagauaagggugaucacguuucuggauuagaaugucacauacuguggcaauaguuagcucuagaagacaauggauauuuccuaccaagucugggucucggauucggcacagcagguuucgauacauacc
                        ##****#**   ** *#  **#  *** *#   ** ****##***                                               ****  **  * **  ***||***  ** *  **  ****                                               ***##**** **   #* ***  #**  #* **   **#****##
```

=== Internal intron ===

Multiple sequence alignment

```
Xbamc067B_fwrd      guauguaucgaaaccugcugugccgaauccgagacccagacuugguaggaaauauccauugucuucuagagcuaacuauugccacag-----------------
Xbamc067B_RevC      -----------------cuguggcaauaguuagcucuagaagacaauggauauuuccuaccaagucugggucucggauucggcacagcagguuucgauacauac
                                     ***** *#*     **  * ***    #  *** || ***  #    *** *  **     *#* *****
```

=== External intron ===

Multiple sequence alignment

```
Xbamc067B_fwrd      guaugugacauucuaauccagaaacgugaucacccuuaucuaugugaaucuauuaaguauguaauuaggaccuggacuaacacccuuugauaugg------------------------------cag
Xbamc067B_RevC      cug------------------------------ccauaucaaaggguguuaguccagguccuaauuacauacuuaauagauucacauagauaagggugaucacguuucuggauuagaaugucacauac
                     *                               ** **** * * *  *   *# **  # **||** #  ** #*   *  * * * **** **                               *
```

ALIGNMENT RESULTS - Xbamc080A

=== Stwintron Sequence ===

Multiple sequence alignment

```
Xbamc080A_fwrd      gguaugaaucaggaccugcugugccgguuuugagacccggugcugcuagagagaucuauuguuuuccagagcuaaccguuucaacaguaugugagcuauugauccaggaauauggcgaccguggccugcauaaucuugaaguauguaa--aaggagaggaacuaauacguuuggua-------------------------------------ugucag
Xbamc080A_RevC      cugaca-------------------------------------uaccaaacguauuaguuccucucc--uuuuacauacuucaagauuaugcaggccacggucgccauauuccuggaucaauagcucacauacuguugaaacgguuagcucuggaaaacaauagaucucucuagcagcaccgggucucaaaaccggcacagcagguccugauucauacc
                       *##                                     *#*#*#*   **   ** #*#***     **   ##***** * ****## **#*# * # * # *|* # * # * #*#** ##**** * *****##   **     ***#*# **   **   *#*#*#                                      ##*
```

=== Internal intron ===

Multiple sequence alignment

```
Xbamc080A_fwrd      guaugaa----------ucagga---------------ccugcugugccgguuuugagacccggugcugcuagagagaucuauuguuuuccagagcuaaccguuucaacag
Xbamc080A_RevC      cuguugaaacgguuagcucuggaaaacaauagaucucucuagcagcaccgggucucaaaaccggcacagcagg---------------uccuga----------uucauac
                     * *  *          ** ***               *  ** *##**** *#*|*#* ****##* **  *               *** **          *  * *
```

=== External intron ===

Multiple sequence alignment

```
Xbamc080A_fwrd      ------------guaugugagcuau------------ugauccaggaauauggcgaccguggccugcauaaucuugaaguauguaaaaggagaggaacuaauacguuugguaugucag
Xbamc080A_RevC      cugacauaccaaacguauuaguuccucuccuuuuacauacuucaagauuaugcaggccacggucgccauauuccuggauca------------auagcucacauac------------
                                ## *#* ** * #            *# * **#** ****  * **||** *  **** **#** * #*            # * ** *#* ##
```

ALIGNMENT RESULTS - Xbamc083A

=== Stwintron Sequence ===

Multiple sequence alignment

```
Xbamc083A_fwrd      --------gguaaguauuagagccaacugucuggcugggug---------ggugugcgaacccgguguuggucaaagcauauauugucugccagagcugacuguuaucacaguaugugaaaucuugauauagaggcauuaaucuccgc--gacuugcacaaugcuggagaguauguaaacaggaacccggcuaacacgguuugaaaugccag
Xbamc083A_RevC      cuggcauuucaaaccguguuagccggguuccuguuuacauacucuccagcauugugcaaguc--gcggagauuaaugccucuauaucaagauuucacauacugugauaacagucagcucuggcagacaauauaugcuuugaccaacaccggguucgcacacc---------cacccagccagacaguuggcucuaauacuuacc--------
                               ** # *   ****   * #***  *# #*#         # *****#*  *  *#*  *#* ** ** * ***  #  *     #*  ***** || *****  *#     *  #  *** * ** ** *#*  *#*  *  *#***** #         #*# #*  ***# *   ****   * # **
```

=== Internal intron ===

Multiple sequence alignment

```
Xbamc083A_fwrd      guaaguauuagagccaacugucuggcuggguggugugcgaacccgguguuggucaaagcauauauugucugccagagcugacuguuau-cacag
Xbamc083A_RevC      cugug-auaacagucagcucuggcagacaauauaugcuuugaccaacaccggguucgcacacccacccagccagacaguuggcucuaauacuuac
                     *  * ** * ** ** ** *   #   ##*# #**#    *** # | # ***    #**# #*##   #   * ** ** ** * ** *  *
```

=== External intron ===

Multiple sequence alignment

```
Xbamc083A_fwrd      ---guaugugaaaucuugauauagaggcauuaaucuccgcgacuugcacaaugcuggagaguauguaaacaggaacccggcuaacacgguuugaaaugccag
Xbamc083A_RevC      cuggcauuucaaaccguguuagccggguuccuguuuacauacucuccagcauugugcaagucgcggagauuaaugccucuauaucaagauuucacauac---
                       *#** * ***#* ** **    **  ##  * * *# #  #* **  ||  ** *#  # #* * *  ##  **    ** ** *#*** * **#*
```

ALIGNMENT RESULTS - Xbamc083B

=== Stwintron Sequence ===

Multiple sequence alignment

```
Xbamc083B_fwrd      -gguacguaucaaaaccugcugugccgguuugcuagauccggcguggguaggauu-----accagagcuaacugcugucguaguacguguauaugugauucaaaagcguggcugucauuggcugcgcaauucuacacauacguaa--ugaggaucuggacuaacacguuucgauauuuucag
Xbamc083B_RevC      cugaaaauaucgaaacguguuaguccagauccuc--auuacguauguguagaauugcgcagccaaugacagccacgcuuuugaaucacauauacacguacuacgacagcaguuagcucug-----guaauccuacccacgccggaucuagcaaaccggcacagcagguuuugauacguacc-
                      * * #**** **** ** *#  **#* *#  #  **   * #** ****#***      ***# * #* *##*  *  * #  *###*||*###*  # *  *  *##* *# * #**      * ***#**** **# *   *  *#  #* *#**  #* ** **** ****# * *
```

=== Internal intron ===

Multiple sequence alignment

```
Xbamc083B_fwrd      guacguaucaaaaccugcugugccgguuugcuagauccggcguggguaggauuaccagagcuaacugcugucguag-------------
Xbamc083B_RevC      --------------cuacgacagcaguuagcucugguaauccuacccacgccggaucuagcaaaccggcacagcagguuuugauacguac
                                  **#* ### *#*** ***      # * *#||#* * #      *** ***#* ### *#**
```

=== External intron ===

Multiple sequence alignment

```
Xbamc083B_fwrd      -----------------------------------guacguguauaugugauucaaaagcguggcugucauuggcugcgc-----aauucuacacauacguaaugaggaucuggacuaacacguuucgauauuuucag
Xbamc083B_RevC      cugaaaauaucgaaacguguuaguccagauccucauuacguauguguagaauu-----gcgcagccaaugacagccacgcuuuugaaucacauauacacguac-----------------------------------
                                                       *****#* * *# #***     ***##**## || ##**##***     ***# #* * *#*****
```

ALIGNMENT RESULTS - Xbamc084A

=== Stwintron Sequence ===

Multiple sequence alignment

```
Xbamc084A_fwrd      gguacguauuaaagcucaccgugccgguucugaggcccgggcuuccuaagaaaagauccguuaucuuucagcgcuaacuguagccgcaguaugugaauuccugaucuagaaacgugaccgccauuauuuauguaacuguaaaaauugaaauaugacaugguuuuacucacacgguuucgcauuuag-----------------------------
Xbamc084A_RevC      -----------------------------cuaaaugcgaaaccgugugaguaaaaccaugucauauuucaauuuuuacaguuacauaaauaauggcggucacguuucuagaucaggaauucacauacugcggcuacaguuagcgcugaaagauaacggaucuuuucuuaggaagcccgggccucagaaccggcacggugagcuuuaaauacguacc
                                                 **#*#  * ###*#   * ** ***# #  **#** *****#   * ** ** #*   *#**   *   ** #* * #|# * *# **   *   **#*   *# ** ** *   #***** **#**  # #*** ** *   #*### *  #*#**
```

=== Internal intron ===

Multiple sequence alignment

```
Xbamc084A_fwrd      -----------guacguauuaaagcucaccgugccgguucu---gaggcccgggcuuccuaagaaaagauccguuaucuuucagcgcuaacuguagccgcag
Xbamc084A_RevC      cugcggcuacaguuagcgcugaaagauaacggaucuuuucuuaggaagcccgggccuc---agaaccggcacggugagcuuuaaauacguac-----------
                               **  *# #* **#   * ** # *  ****   **#***||***#**   ****  * # ** *   #** *# #*  **
```

=== External intron ===

Multiple sequence alignment

```
Xbamc084A_fwrd      guaugugaauuccugaucuagaaacgugaccgccauuauuuauguaacuguaaaaauugaaauaugacaugguuuuacucacacgguuucgcauuuag-----------------------------------
Xbamc084A_RevC      -----------------------------------cuaaaugcgaaaccgugugaguaaaaccaugucauauuucaauuuuuacaguuacauaaauaauggcggucacguuucuagaucaggaauucacauac
                                                       #**  * #* ***#**   * * #** #***|***# **# * *   **#*** *# *  **#
```

ALIGNMENT RESULTS - Xbamc086A

=== Stwintron Sequence ===

Multiple sequence alignment

```
Xbamc086A_fwrd      ----------------ggucagugaaaucauuaccgugccuguugcgagacacgguauugguagaaauaucuucuaccguccagagcugacugcugucacaguauguga--------agcuucgaucaagaaaagucaucgccaugaccugugugcgauucuaaguauacguccgggauaauacuaacauggcuugauacucuag
Xbamc086A_RevC      cuagaguaucaagccauguuaguauuaucccggacguauacuuagaaucgcacacaggucauggcgaugacuuuucuugaucgaagcu--------ucacauacugugacagcagucagcucuggacgguagaagauauuucuaccaauaccgugucucgcaacaggcacgguaaugauuucacugacc----------------
                                     ** ***#  *** #   ***#  # * * #   ***#    * #* *  **  *** *   *  * #****        ***** | *****        ****# *  *   * ***  **  * *# *    #***   # * * #  #***   # ***  #*** **
```

=== Internal intron ===

Multiple sequence alignment

```
Xbamc086A_fwrd      -------------------------------gucagugaaaucauuaccgugccuguugcgagacacgguauugguagaaauaucuucuaccguccagagcugacugcugucacag
Xbamc086A_RevC      cugugacagcagucagcucuggacgguagaagauauuucuaccaauaccgugucucgcaacaggcacgguaaugauuucacugac-------------------------------
                                                   *  * *   *#** ******* **  ||  ** ******* **#*   * *  *
```

=== External intron ===

Multiple sequence alignment

```
Xbamc086A_fwrd      guaugugaagcuucgaucaagaaaagucaucgccaugaccugugugcgauucuaaguauacguccgggauaauacuaacauggcuugau--------acucuag
Xbamc086A_RevC      cuaga--------guaucaagccauguuaguauuaucccggacguauacuuagaaucgcacacaggucauggcgaugacuuuucuugaucgaagcuucacauac
                     ** #          ******  * ** *  #  **  *  ##**# # **||** # #**##  *  **  #  * ** *  ******          # **
```

ALIGNMENT RESULTS - Xbamc089A

=== Stwintron Sequence ===

Multiple sequence alignment

```
Xbamc089A_fwrd      gguacguaucaaaa----------------ccuguuguaccgauugugagac--------------------ccggugucaggagaaagaucuaugguuuuccaaagcugacuguugccacaguauguuagcccuucaucccaagauauguagccacuguggccuggacaauccugcaagcuauguaguaaggagcuagacugaugcuuuuugguaugacag
Xbamc089A_RevC      cugucauaccaaaaagcaucagucuagcuccuuacuacauagcuugcaggauuguccaggccacaguggcuacauaucuugggaugaagggcuaacauacuguggcaacagucagcuuuggaaaaccauagaucuuucuccugacaccgg--------------------gucucacaaucgguacaacagg----------------uuuugauacguacc
                        *#**#*****                * *##*##*  * ***## **                    *  #* *  ***  ***  ***  #* #*     # ##||## #     *# *#  ***  ***  ***  * *#  *                    ** ##*** *  *##*##* *               *****#**#*
```

=== Internal intron ===

Multiple sequence alignment

```
Xbamc089A_fwrd      guacg-------------------uaucaaaaccuguuguaccgauugugagacccggugucaggagaaagaucuaugguuuuccaaagcugacuguugccacag
Xbamc089A_RevC      cuguggcaacagucagcuuuggaaaaccauagaucuuucuccugacaccgggucucacaaucgguacaacagguuuugau-------------------acguac
                     *  *                    *#** *   # ** * * **#  #* *|* *#  #** * * ** #   * **#*                    *  *
```

=== External intron ===

Multiple sequence alignment

```
Xbamc089A_fwrd      gu------------------auguuagcccuucaucccaagauaugua-----------gccacuguggccuggacaauccugcaagcuauguaguaaggagcuagacugaugcuuuuugguaugacag
Xbamc089A_RevC      cugucauaccaaaaagcaucagucuagcuccuuacuacauagcuugcaggauuguccaggccacagugg-----------cuacauaucuugggaugaagggcuaa------------------cauac
                     *                  *  #**** *#* *#  ** # # **#*           *****|****           **#** # # **  #* *#* ****#                  * *
```

ALIGNMENT RESULTS - Xbamc096A

=== Stwintron Sequence ===

Multiple sequence alignment

```
Xbamc096A_fwrd      gguauguauucacaacccguuccgccccaguugcgagaccuaguugcuacaaagauccauugcuugcuagagcuaacugucgucacaguauguaaaccuuugauccagaaacguggccaccgugac---------------------cuguguaauccuagaaaauagguaacaagaaacuggacuaacacguu-uggauguaccag
Xbamc096A_RevC      cugguacaucca-aacguguuaguccaguuucuuguuaccuauuuucuaggauuacaca---------------------ggucacgguggccacguuucuggaucaaagguuuacauacugugacgacaguuagcucuagcaagcaauggaucuuuguagcaacuaggucucgcaacuggggcggaacggguugugaauacauacc
                        *##**#** ***  ***   **    *#  *  ***** ** ***  *  *# **                      ***** **   #*    *#** | **#*    *#   ** *****                      ** #*  *  *** ** *****  *  #*    **   ***  *** **#**##*
```

=== Internal intron ===

Multiple sequence alignment

```
Xbamc096A_fwrd      guauguauucacaacccguuccgccccaguugcgagaccuaguugcuacaaagauccauugcuugcuagagcuaacugucgucacag--------------------------
Xbamc096A_RevC      --------------------------cugugacgacaguuagcucuagcaagcaauggaucuuuguagcaacuaggucucgcaacuggggcggaacggguugugaauacauac
                                              * ** #*** *  ***#*    ***  *  |  *  ***    *#***  * ***# ** *
```

=== External intron ===

Multiple sequence alignment

```
Xbamc096A_fwrd      guauguaaaccuuugauccagaaacguggccaccgugaccuguguaauccuagaaaauagguaacaagaaacuggacuaacacguuuggaugua--------ccag
Xbamc096A_RevC      cug--------guacauccaaacguguuaguccaguuucuuguuaccuauuuucuaggauuacacaggucacgguggccacguuucuggaucaaagguuuacauac
                     *          *  *****#*   ** #   * **  * ***    *  * || *  *    *** *  ** *   # **   *#*****  *          *
```

ALIGNMENT RESULTS - Xbamc102A

=== Stwintron Sequence ===

Multiple sequence alignment

```
Xbamc102A_fwrd      gguaugu augaaaaccugcugugccuguugcacgaccugguauugauagaaagaucuauug---gcuuccagagcuaacgacuacuacaguaugugagcuuuuaucaaaaaacgcgacuaucauagucuauguaaaucuagugaguguauaacaggggauuagacuaacucauguugauaugacag------------------------
Xbamc102A_RevC      ------------------------cugucauaucaacaugaguuagucuaaucccuguuauacacucacuagauuuacauagacuaugauagucgcguuuuuugauaaaagcucacauacuguaguagucguuagcucuggaagc---caauagaucuuucuaucaauaccaggucgugcaacaggcacagcagguuuucauacauacc
                                            ****## *  * *  *  **# *  **   #** **#   #**# * # # * **#   ****  #**  #* * **** || **** * *#  **#  ****   #** * # # * #**#   #** **#   **  * #**  *  * *  * ##****
```

=== Internal intron ===

Multiple sequence alignment

```
Xbamc102A_fwrd      guauguaugaaaaccugcugugccuguugcacgaccugguauugauagaaagaucuauuggcuuccagagcuaacgacuacuacag-----------------
Xbamc102A_RevC      -----------------cuguaguagucguuagcucuggaagccaauagaucuuucuaucaauaccaggucgugcaacaggcacagcagguuuucauacauac
                                     ****#   **#*   *  **** * # *  # * | * #  * # * ****  *   *#**   #****
```

=== External intron ===

Multiple sequence alignment

```
Xbamc102A_fwrd      gu---augugagcuuuuaucaaaaaacgcgacuaucauagucuauguaaaucuagugaguguauaacaggggauuagacuaacucauguugauaugacag
Xbamc102A_RevC      cugucauaucaacaugaguuagucuaaucccuguuauacacucacuagauuuacauagacuaugauagucgcguuuuuugauaaaagcucacau---ac
                     *   **#* *#* *   * *    *  *  ** * ***  **   ** || **   **  *** * **  *  *    * *   * *#* *#**   *
```

ALIGNMENT RESULTS - Xbamc106A

=== Stwintron Sequence ===

Multiple sequence alignment

```
Xbamc106A_fwrd      gguauguaucaaaaucuaccgugccaguucugagaccuagacuugguagaaugac-----------cuguugucuucuagcgcuaacuguugccgcaguacgugaacuucugauccugaaacauggccaccaucaucugcguaauucuagaaaguauguaauaaggaacuggacuaauacguuuuuauaugacag
Xbamc106A_RevC      cugucauauaaaaacguauuaguccaguuccuuauuacauacuuucuagaauuacgcagaugaugguggccauguuucaggaucagaaguucacguacugcggcaacaguuagcgcuagaagacaac-----------aggucauucuaccaagucuaggucucagaacuggcacgguagauuuugauacauacc
                        ##*** ****# **  #  *******#  #   #* ****  ****** **             *###* ** #** # #*   ***  ** *|* **  ***   *# # **# ** *###*           # ** ******  **** *#   #  #*******  #  ** #**** ***##
```

=== Internal intron ===

Multiple sequence alignment

```
Xbamc106A_fwrd      guaug-------------------uaucaaaaucuaccgugccaguucugagaccuagacuugguagaaugaccuguugucuucuagcgcuaacuguugccgcag
Xbamc106A_RevC      cugcggcaacaguuagcgcuagaagacaacaggucauucuaccaagucuaggucucagaacuggcacgguagauuuugau-------------------acauac
                     * #*                    *# * *   #*   *#***# ***# *|* #*** #***#*   *#   * * #*                   *# *
```

=== External intron ===

Multiple sequence alignment

```
Xbamc106A_fwrd      -----guacgugaacuucugauccugaaacauggccaccaucaucugcguaauucuagaaaguauguaauaaggaacuggacuaauacguuuuuauaugacag
Xbamc106A_RevC      cugucauauaaaaacguauuaguccaguuccuuauuacauacuuucuagaauuacgcagaugaugguggccauguuucaggaucagaaguucacguac-----
                         #** # #*** * * *  *##   * * #  **   * * #  * *|* *  # * *   **  # * *   ##*  * * * ***# # **#
```

ALIGNMENT RESULTS - Xbamc106B

=== Stwintron Sequence ===

Multiple sequence alignment

```
Xbamc106B_fwrd      gguacguaugaaaaccuaccgugccgguucugagaccuggacuugguagaaugaccuaccu-------auugucuuccaccgcuaacgguugccgcaguacgugaacuuuugacucugaaacaugaucacuguuaccugcguaauucuagaaaguauguaauaaggaacugaguuaacauguuuugauauggcag
Xbamc106B_RevC      cugccauaucaaaacauguuaacucaguuccuuauuacauacuuucuagaauuacgcagguaacagugaucauguuucagagucaaaaguucacguacugcggcaaccguuagcgguggaagacaa-------uagguaggucauucuaccaaguccaggucucagaaccggcacgguagguuuucauacguacc
                        *#*** ***** *   #   *#****#  #   ## ****  ****** ** #*  *       **##* ** **  * #** #***  ** *|* **  ***# **# *  ** ** *##*       **  *# ** ******  **** ##   #  #****#*   #   * ***** ***#*
```

=== Internal intron ===

Multiple sequence alignment

```
Xbamc106B_fwrd      ----------------------------guacguaugaaaaccuaccgugccgguucugagaccuggacuugguagaaugaccuaccuauugucuuccaccgcuaacgguugccgcag
Xbamc106B_RevC      cugcggcaaccguuagcgguggaagacaauagguaggucauucuaccaaguccaggucucagaaccggcacgguagguuuucauacguac----------------------------
                                                #** *** *  *  *****# * * #   #||#   # * * #*****  *  * *** **#
```

=== External intron ===

Multiple sequence alignment

```
Xbamc106B_fwrd      -------------guacgugaacuuuugacucugaaacaugaucacuguuaccugcguaauucuagaaaguauguaauaaggaacugaguuaacauguuuugauauggcag
Xbamc106B_RevC      cugccauaucaaaacauguuaacucaguuccuuauuacauacuuucuagaauuacgcagguaacagugaucauguuucagagucaaaaguucacguac-------------
                                 ##* ** ****#    *# *#  ****# *  **#  *    |    *  #**  * #****  #* #*    #**** ** *##
```

ALIGNMENT RESULTS - Xbamc107A

=== Stwintron Sequence ===

Multiple sequence alignment

```
Xbamc107A_fwrd      gguacguaucacagcagguuauuccaucuggagacucgguguu------gcuagaaaagaucuauugccugu-----cagagcuaacugucacuacaguacgucaauauuuggcccaguaaacgugggcgccguaacuuuugcagcucugaaaguauaugauaaagaaccggacuaauaagucuggacauuucag
Xbamc107A_RevC      cugaaauguccagacuuauuaguccgguucuuuaucauauacuuucagagcugcaaaaguuacggcgcccacguuuacugggccaaauauugacguacuguagugacaguuagcucug-----acaggcaauagaucuuuucuagc------aacaccgagucuccagauggaauaaccugcugugauacguacc
                       * #* **   #*  #*** ***   *    * #  #*##*      ***  ***** * #  #***###     * * **#** *#*   #  *|*  #   *#* **#** * *     ###***#  # * *****  ***      *##*#  # *    *   *** ***#  *#   ** *# *
```

=== Internal intron ===

Multiple sequence alignment

```
Xbamc107A_fwrd      guacguaucacagcagguuauuccaucuggagacucgguguugcuagaaaagaucuauugccugucagagcuaacugucacuacag----
Xbamc107A_RevC      ----cuguagugacaguuagcucugacaggcaauagaucuuuucuagcaacaccgagucuccagauggaauaaccugcugugauacguac
                         * *    #*** *  #**   * ** #*   # # ** *||* ** # #   *# ** *   **#  * ***#    * *
```

=== External intron ===

Multiple sequence alignment

```
Xbamc107A_fwrd      gua--------------------------------cgucaauauuuggcccaguaaacgugggcgccguaacuuuugcagcucugaaaguauaugauaaagaaccggacuaauaagucuggacauuucag
Xbamc107A_RevC      cugaaauguccagacuuauuaguccgguucuuuaucauauacuuucagagcugcaaaaguuacggcgcccacguuuacugggccaaauauugacg--------------------------------uac
                     *                                 *#*  *# **##*  * *#*** ** #  ||  # ** ***#* *  *##** #*  *#*                                 *
```

ALIGNMENT RESULTS - Xbamc109A

=== Stwintron Sequence ===

Multiple sequence alignment

```
Xbamc109A_fwrd      gguauguaucaaaaccugccgugucgguucuaagacaugaauucgguagauaauucuacugucuuccagagcua--------auuguugcugcaguaugugaacuuuagauacagaaaacgugaccacaauaaucagugugguucuagaaaguaugcaaa---------------------aggaacuggacuaacacguucuaauauggcag
Xbamc109A_RevC      cugccauauuagaacguguuaguccaguucc---------------------uuuugcauacuuucuagaaccacacugauuauugguggucacguuuucuguaucuaaaguucacauacugcagcaacaau--------uagcucuggaagacaguagaauuaucuaccgaauucaugucuuagaaccgacacggcagguuuugauacauacc
                        ##*** * *** **  #  #*#****#                      **    *## *** ***#*#*        ***** * ##*  * * ** * #*|*# * ** * *  *## * *****        *#*#*** *** ##*    **                      #****#*#  #  ** *** * ***##
```

=== Internal intron ===

Multiple sequence alignment

```
Xbamc109A_fwrd      guauguaucaaaaccugccgugucgguucuaagacaugaauucgguagauaauucuacugucuuccagagcuaauuguugcugcag-----------------------
Xbamc109A_RevC      -----------------------cugcagcaacaauuagcucuggaagacaguagaauuaucuaccgaauucaugucuuagaaccgacacggcagguuuugauacauac
                                           * *#  #** *  *#  *# ** ***#* * | * *#*** ** #*  #*  * **#  #* *
```

=== External intron ===

Multiple sequence alignment

```
Xbamc109A_fwrd      guaugugaacuuuagauacagaaaacgugaccacaauaaucagugugguucuagaaaguaugcaaaaggaacuggacuaacacguucuaauauggc---------------------------ag
Xbamc109A_RevC      cu--------------------------gccauauuagaacguguuaguccaguuccuuuugcauacuuucuagaaccacacugauuauugguggucacguuuucuguaucuaaaguucacauac
                     *                            *** * **    **** # **#**    * * | * *    **#** # ****    ** * ***                            *
```

ALIGNMENT RESULTS - Xbamc110A

=== Stwintron Sequence ===

Multiple sequence alignment

```
Xbamc110A_fwrd      gguauguaucaaauccugcugugccugucggacauuaguuauuggcacugagaucuauuguaugucagagcua--------acaguugccgcaguaugugaaccuuugaucuagcgaacgcggucaccgugaccagugcaauucuacaaugugauagagaauuauacuaaua------------------------uguuugauauaucag
Xbamc110A_RevC      cugauauaucaaac------------------------auauuaguauaauucucuaucacauuguagaauugcacuggucacggugaccgcguucgcuagaucaaagguucacauacugcggcaacugu--------uagcucugacauacaauagaucucagugccaauaacuaauguccgacaggcacagcaggauuugauacauacc
                       **#*******#                         ****#* *  #   *****###**   ***# *         ** ** #****  *   *# * * | * * #*   *  ****# ** **         * #***   **###*****   #  * *#****                         #*******#**
```

=== Internal intron ===

Multiple sequence alignment

```
Xbamc110A_fwrd      guauguaucaaauccugcugugccugucggacauuaguuauuggcacugagaucuauuguaugucagagcuaacaguugc-----cgcag
Xbamc110A_RevC      cug-----cggcaacuguuagcucugacauacaauagaucucagugccaauaacuaauguccgacaggcacagcaggauuugauacauac
                     *      *     *** *#   *** *# *** *** * *##*||*##* * *** *** #* ***   #* ***         *# *
```

=== External intron ===

Multiple sequence alignment

```
Xbamc110A_fwrd      guaugugaaccuuugaucuagcgaacgcggucaccgugaccagugcaauucuacaaugugauagagaauuauacuaau----------auguuugauauaucag------------
Xbamc110A_RevC      ------------cugauauaucaaacau----------auuaguauaauucucuaucacauuguagaauugcacuggucacggugaccgcguucgcuagaucaaagguucacauac
                                #**** ** *#***#           *  ***# ******  * #||# *  ****** #***   *          #***#* ** ****#
```

ALIGNMENT RESULTS - Xbamc124A

=== Stwintron Sequence ===

Multiple sequence alignment

```
Xbamc124A_fwrd      gguacguaucaaaccugcugugcugg-------uugcgagaccugguguugguagagagaucgau-----ugucuugcagagcuaacgguugcuauaguaugugaaccuuuaaaauacagaaacacgauuaccagaaucugugugaaucuggaaguaugcaauaaagaaccggacuaacacaaccugauauggcag
Xbamc124A_RevC      cugccauaucagguuguguuaguccgguucuuuauugcauacuuccagauucacacagauucugguaaucguguuucuguauuuuaaagguucacauacuauagcaaccguuagcuc-----ugcaagacaaucgaucucucuaccaacaccaggucucgc-------aaccagcacagcagguuuugauacguacc
                        *#*****     ** *#  *#**       ****# #   # # #** ##* ***  # * *     *** *#   #   *** ****  #***|***#  **** ***   #       ** **# * * #  *** *## **# # #   # #**       ****#*  #* **     *****#*
```

=== Internal intron ===

Multiple sequence alignment

```
Xbamc124A_fwrd      guacguaucaaaccugcugugcugguugcgagaccugguguugguagagagaucgauugucuugcagagcuaacgguugcuauag---------------------------------------------
Xbamc124A_RevC      --------------------------------------------cuauagcaaccguuagcucugcaagacaaucgaucucucuaccaacaccaggucucgcaaccagcacagcagguuuugauacguac
                                                                 ** ** #*#** * *# #*||*# #* * **#*# ** **
```

=== External intron ===

Multiple sequence alignment

```
Xbamc124A_fwrd      guaugugaaccuuuaaaauacagaaacacgauuaccagaaucugugugaaucuggaaguaugcaauaaagaaccggacuaacacaaccugauauggcag----------------------------
Xbamc124A_RevC      ----------------------------cugccauaucagguuguguuaguccgguucuuuauugcauacuuccagauucacacagauucugguaaucguguuucuguauuuuaaagguucacauac
                                                *  ##*    *   ***** * **#**   * *# | #* *   **#** * *****   *    *##  *
```

ALIGNMENT RESULTS - Xbamc132A

=== Stwintron Sequence ===

Multiple sequence alignment

```
Xbamc132A_fwrd      -----------gguacguaucaaaccuuucuguagcgguuuuaaggccuggaguuaguagaaauuuaug-uuauuuucgagagcuaacaguugccacaguaugugaaccuuugauccuggaaugcgaucaugauuaucuguguaauucuaaaaaguauguaauaagggacuggacuaacacauuuuuaauauggcag
Xbamc132A_RevC      cugccauauuaaaaauguguuaguccagucccuuauuacauacuuuuuagaauuacacagauaaucaugaucgcauuccaggaucaaagguucacauacuguggcaacuguuagcucucgaaaauaa-cauaaauuucuacuaacuccaggccuuaaaaccgcuacagaaagguuugauacguacc-----------
                               ## * ** * *  **  **# * #  ## *        *#* *  ##*** * *#*** *# # *** ** # #**  ***  ** *|* **  ***  **# # ** *** # #* ***#* * ***##  * *#*        * ##  # * #**  **  * * ** * ##
```

=== Internal intron ===

Multiple sequence alignment

```
Xbamc132A_fwrd      guacguaucaaaccuuucuguagcgguu-uuaaggccuggaguuaguagaaauuuauguuauuuucgagagcuaacaguugccacag-----------------
Xbamc132A_RevC      -----------------cuguggcaacuguuagcucucgaaaauaacauaaauuucuacuaacuccaggccuuaa-aaccgcuacagaaagguuugauacguac
                                     **** **###* ***   * #*#*# **##* **||** *##** #*#*# *   *** *###** ****
```

=== External intron ===

Multiple sequence alignment

```
Xbamc132A_fwrd      -----------------------------------guaugugaaccuuugauccuggaaugcgaucaugauuaucuguguaauucuaaaaaguauguaauaagggacuggacuaacacauuuuuaauauggcag
Xbamc132A_RevC      cugccauauuaaaaauguguuaguccagucccuuauuacauacuuuuuagaauuacacagauaaucaugaucgcauuccaggaucaaagguucacauac-----------------------------------
                                                        **##*#    ** **     # * # #***||***# # * #     ** **    #*##**
```

ALIGNMENT RESULTS - Xbamc132B

=== Stwintron Sequence ===

Multiple sequence alignment

```
Xbamc132B_fwrd      gguacguaucaaaccu-----------uucuguaaccuggaguuuguagaaagauauauugucuuccagagcuaacuguugccacaguaugugaaauuuugaucugaaacgugacuaccagaaucugugugauucuagaaaguauguaauacggaacuaggcuaacagauuuugaucuggcag
Xbamc132B_RevC      cugccagaucaaaaucuguuagccuaguuccguauuacauacuuucuagaaucacacagauucugguagucacguuucagaucaaaauuucacauacuguggcaacaguuagcucuggaagacaauauaucuuucuacaaacuccagguuacaga-----------aagguuugauacguacc
                        *# ******  #           ***#***   ## * *** *****  *#*#*   ***   **   #   *   # ** *#* * | * *#* ** #   *   #   **   ***   *#*#*  ***** *** * ##   ***#**           *#  ****** #*
```

=== Internal intron ===

Multiple sequence alignment

```
Xbamc132B_fwrd      ---------------------guacguaucaaaccuuucuguaaccuggaguuuguagaaagauauauugucuuccagagcuaacuguugccacag
Xbamc132B_RevC      cuguggcaacaguuagcucuggaagacaauauaucuuucuacaaacuccagguuacagaaagguuugauacguac---------------------
                                         * * ##*  * * ******##** **||** **##****** * *  *## * *
```

=== External intron ===

Multiple sequence alignment

```
Xbamc132B_fwrd      guaugugaaauuuugaucugaaacgugacuaccagaaucugugugauucuagaaaguauguaauacggaacuaggcuaacagauuuugaucuggcag--
Xbamc132B_RevC      --cugccagaucaaaaucuguuagccuaguuccguauuacauacuuucuagaaucacacagauucugguagucacguuucagaucaaaauuucacauac
                       **# * **#  #*****  *  # * * **  * * ##*##  *# | #*  ##*## * *  ** * * #  *  *****#  #** * #**
```

ALIGNMENT RESULTS - Xbamc152A

=== Stwintron Sequence ===

Multiple sequence alignment

```
Xbamc152A_fwrd      gguacguaucaauacccucugugccagcuuuaagaccuaguauugauagaaaggucuauuguuuuccaaagcu----aacuauugccgcaguaugugaaucuuugaucucaacacgccacuacuauaauuguauaauucuagaaaguauu---------------uaguaaggacccgggcuaauac-----guuuugauaugacag
Xbamc152A_RevC      cugucauaucaaaac-----guauuagcccggguccuuacua---------------aauacuuucuagaauuauacaauuauaguaguggcguguugagaucaaagauucacauacugcggcaauagu----uagcuuuggaaaacaauagaccuuucuaucaauacuaggucuuaaagcuggcacagaggguauugauacguacc
                        *#****** **     **#  ***##     * ** **               * *##**** * *# *    ** *** *  *  *# ***  *  *#|#*  *  *** #*  *  * *** *    ** #* * ****##* *               ** ** *     ##***  #**     ** ******#*
```

=== Internal intron ===

Multiple sequence alignment

```
Xbamc152A_fwrd      guacg-------------------uaucaauacccucugugccagcuuuaagaccuaguauugauagaaaggucuauuguuuuccaaagcuaacuauugccgcag
Xbamc152A_RevC      cugcggcaauaguuagcuuuggaaaacaauagaccuuucuaucaauacuaggucuuaaagcuggcacagagg-guauugau------------------acguac
                     * **                    *# *    *** * *# **#  #** *|* **#  #** #* * ***  ***** *                   ** *
```

=== External intron ===

Multiple sequence alignment

```
Xbamc152A_fwrd      -------------guaugugaaucuuugaucucaacacgccacuacuauaauuguauaauucuagaaaguauuuaguaaggacccgggcuaauacguuuugauaugacag
Xbamc152A_RevC      cugucauaucaaaacguauuagcccggguccuuacuaaauacuuucuagaauuauacaauuauaguaguggcguguugagaucaaagauucacauac-------------
                                 ## *#* * #*#  * #** *  * #    * *** ****#||#**** *** *    # *  * **# *  #*# * *#* ##
```

ALIGNMENT RESULTS - Xbamc152B

=== Stwintron Sequence ===

Multiple sequence alignment

```
Xbamc152B_fwrd      gguaugugcaaagaccugacacgccgguugcgagaugcuguguugguagaaaaauccaacugucuguccaagcua--acucuuaguucaguaggugaaauucucacccaugaaccagguuaccauagucuguguaauucucaaggcguguguaacaagaugauau------acuaaugugugcuuccugugcuag----
Xbamc152B_RevC      ----cuagcacaggaagcacacauuagu------auaucaucuuguuacacacgccuugagaauuacacagacuaugguaaccugguucaugggugagaauuucaccuacugaacuaagagu--uagcuuggacagacaguuggauuuuucuaccaacacagcaucucgcaaccggcgugucaggucuuugcacauacc
                        #  *** **     ****#  #**      **# ##* *** ** * *  #*      # *## ** #***      ## * *  #* ***** *|* ***** *#   * * ##      ***# ** ##*  #     *#  * * ** *** *## #**      **#  #****     ** ***  #
```

=== Internal intron ===

Multiple sequence alignment

```
Xbamc152B_fwrd      guaugugcaaagaccugacacgccgguugcgagaugcuguguugguagaaaaauccaacugucuguccaagcuaacucuuagu------------------------ucag
Xbamc152B_RevC      cug------------------------aacuaagaguuagcuuggacagacaguuggauuuuucuaccaacacagcaucucgcaaccggcgugucaggucuuugcacauac
                     *                          #* *#  * *#  ****  # * * * | * * * #  ****  #* *  #* *#                          *
```

=== External intron ===

Multiple sequence alignment

```
Xbamc152B_fwrd      ---guaggugaaauucucacccaugaaccagguuaccauagucuguguaauucucaaggcguguguaacaagaugauauacuaaugugugcuuccugugcuag
Xbamc152B_RevC      cuagcacaggaagcacacauuaguauaucaucuuguuacacacgccuugagaauuacacagacuaugguaaccugguucaugggugagaauuucaccuac---
                       *#* # *** # * **    *# * **  **   *#*  *  # * * | * * #  *  *#*   **  ** * #*    ** * # *** # *#*
```

ALIGNMENT RESULTS - Xbamc152C

=== Stwintron Sequence ===

Multiple sequence alignment

```
Xbamc152C_fwrd      ----gguauguauugaacucugcuauaucgguuauaagauuugguauugguggaaagaucgauuaucuuucaacgcuaacgauugcuacaguaugugaacuguugaucgcgaaaugcgau--auaauucuagagacuauggaauaaugaauuggacuaacuaacccauuucauaugacag
Xbamc152C_RevC      cugucauaugaaaauggguuaguuaguccaauucauuauuccauagucucuagaauuau--aucgcauuucgcgaucaacaguucacauacuguagcaaucguuagcguugaaagauaaucgaucuuuccaccaauaccaaaucuuauaaccgauauagcagaguucaauacauacc----
                         #**** *      *  * **  #*##**    # *###   *#  *#***  **  **# # ****   # #***# **  #* *|* *#  ** #***# #   **** # #**  **  ***#*  #*   ###* #    **##*#  ** *  *      * ****#
```

=== Internal intron ===

Multiple sequence alignment

```
Xbamc152C_fwrd      guauguauugaacucugcuauaucgguuauaagauuugguauugguggaaagaucgauuaucuuucaacgcuaacgauugcuacag-----------------------
Xbamc152C_RevC      -----------------------cuguagcaaucguuagcguugaaagauaaucgaucuuuccaccaauaccaaaucuuauaaccgauauagcagaguucaauacauac
                                           * **  #**   **#*# ***# #** *# #|# #* **# #*** #*#**   **#  ** *
```

=== External intron ===

Multiple sequence alignment

```
Xbamc152C_fwrd      guaugugaacuguugaucgcgaaaugcgauauaauucuagagacuauggaauaaugaauuggacuaacuaacccauuucauaugacag---------------------------------------------
Xbamc152C_RevC      ---------------------------------------------cugucauaugaaaauggguuaguuaguccaauucauuauuccauagucucuagaauuauaucgcauuucgcgaucaacaguucacauac
                                                                  **   ***  #** ***  **||**  *** **#  ***  **
```

ALIGNMENT RESULTS - Xbamc153A

=== Stwintron Sequence ===

Multiple sequence alignment

```
Xbamc153A_fwrd      gguauguaucaaaaccugcucuaccgguugugagaccugguguucauaaaaauaucgguuugucuucaagaacuaacaauugcucuaguauguagacuguugaccuaacaacgcgaugaccauuaucuauguaaaucuagaaaguaugugauaa-----------------ggaguuaggcuaacacauuuugguauggcag------------------------------------
Xbamc153A_RevC      ------------------------------------cugccauaccaaaauguguuagccuaacu-----------------ccuuaucacauacuuucuagauuuacauagauaauggucaucgcguuguuaggucaacagucuacauacuagagcaauuguuaguucuugaagacaaaccgauauuuuuaugaacaccaggucucacaaccgguagagcagguuuugauacauacc
                                                    *** ##* *  ***  * * #*##*# **                 *# ** #*##**   * * **  **   * # #***||***# # *   **  ** * *   **##*# **                  *#** #*##*# * *  ***  * *## ***
```

=== Internal intron ===

Multiple sequence alignment

```
Xbamc153A_fwrd      guauguaucaaaaccugcucuaccgguugugagaccugguguucauaaaaauaucgguuugucuucaagaacuaacaauugcucuag-----------------
Xbamc153A_RevC      -----------------cuagagcaauuguuaguucuugaagacaaaccgauauuuuuaugaacaccaggucucacaaccgguagagcagguuuugauacauac
                                     **  * *##**** **  ** * #  ** *   *||*   * **  # * **  ** ****##* *  **
```

=== External intron ===

Multiple sequence alignment

```
Xbamc153A_fwrd      gua------uguagacuguugaccuaacaacgcgaugaccauuaucuauguaaaucuagaaaguaugugauaaggaguuaggcuaacacauuuugguauggcag
Xbamc153A_RevC      cugccauaccaaaauguguuagccuaacuccuuaucacauacuuucuagauuuacauagauaauggucaucgcguuguuaggucaacagucuac------auac
                     *       ## *#  ****# ******  *  # ##   *#* **** #*||*# **** *#*   ## #  *  ****** #****  #* #      # *
```

ALIGNMENT RESULTS - Xbamc155A

=== Stwintron Sequence ===

Multiple sequence alignment

```
Xbamc155A_fwrd      gguacguaucaaaaccuaccgugccgguucugggagcuggacuugguagaau-----------gaucuaucgucuuccagagcuaacuguuaccgcaguaugugaacuuuugauccagaacaugaucaauauuaucugcguaauucuagaauguauguaauaaggaacuggacuaauaccuuuuuauauggcag
Xbamc155A_RevC      cugccauauaaaaagguauuaguccaguuccuuauuacauacauucuagaauuacgcagauaauauugaucauguucuggaucaaaaguucacauacugcgguaacaguuagcucuggaagacgauaga-----------ucauucuaccaaguccagcucccagaaccggcacgguagguuuugauacguacc
                        *#*** ****  **  #  **#****#  #   ## ** *  ******            **  ***#* ***  ** * **   *#**   ||   **#*   ** * **  ***  *#***  *           * ******  * ** ##   #  #****#**  #  **  **** ***#*
```

=== Internal intron ===

Multiple sequence alignment

```
Xbamc155A_fwrd      guacguaucaaaaccuaccgugccgguucugggagcuggacuugguagaaugaucuaucgucuuccagagcu----------aacuguuaccgcag--------------
Xbamc155A_RevC      --------------cugcgguaacaguu----------agcucuggaagacgauagaucauucuaccaaguccagcucccagaaccggcacgguagguuuugauacguac
                                  ** * **# *#***          # **# * *# *#***||***#* #* * #** #          ***#* #** * **
```

=== External intron ===

Multiple sequence alignment

```
Xbamc155A_fwrd      guaugugaacuuuugauccagaacaugaucaauauuaucugcguaauucuagaauguauguaauaaggaacuggacuaauaccuuuuuauaug-------------------gcag
Xbamc155A_RevC      cugc-------------------cauauaaaaagguauuaguccaguuccuuauuacauacauucuagaauuacgcagauaauauugaucauguucuggaucaaaaguucacauac
                     * #                   ***#   **   ***  *  #* ***#  * *##||##* *  #*** *#  *  ***   **   #***                   # *
```

ALIGNMENT RESULTS - Xbamc156A

=== Stwintron Sequence ===

Multiple sequence alignment

```
Xbamc156A_fwrd      gguaaguaucaaaaccugccgcgccgguucugagacccggauuugguggaaagaucuaucgucuucuaaagcuaauugaugcggcaguauguaagcuccugauccagcaacucaggcaccaccau---------------------cuauguauuucuaaaagguaugugagaaggaacuggacuaacacgcuuugauuuggcag
Xbamc156A_RevC      cugccaaaucaaagcguguuaguccaguuccuucucacauaccuuuuagaaauacau---------------------agaugguggugccugaguugcuggaucaggagcuuacauacugccgcaucaauuagcuuuagaagacgauagaucuuuccaccaaauccgggucucagaaccggcgcggcagguuuugauacuuacc
                         # ****** * **  #  **#****#    * *# *##*  *#**** *# *                     * # **  ** # * ** * *** | *** * ** * # **  ** #                     ** #* ****#*  *##* #* *    #****#**  #  ** * ****** #
```

=== Internal intron ===

Multiple sequence alignment

```
Xbamc156A_fwrd      guaaguaucaaaaccugccgcgccgguucugagacccggauuugguggaaagaucuaucgucuucuaaagcu----------aauugaugcggcag--------------
Xbamc156A_RevC      --------------cugccgcaucaauu----------agcuuuagaagacgauagaucuuuccaccaaauccgggucucagaaccggcgcggcagguuuugauacuuac
                                  *******# *##**          # #** # ## * ***||*** * ## # **# #          **##* #*******
```

=== External intron ===

Multiple sequence alignment

```
Xbamc156A_fwrd      guauguaagcuccugauccagcaacucaggcaccaccaucuauguauuucuaaaagguaugugagaaggaacuggacuaacacgcuuugauu----------------uggcag
Xbamc156A_RevC      cugc----------------caaaucaaagcguguuaguccaguuccuucucacauaccuuuuagaaauacauagauggugguggccugaguugcuggaucaggagcuuacauac
                     * #                  ** # *#**       **#*  * #**** * * || * * ****# *  *#**       **#* # **                  # *
```

ALIGNMENT RESULTS - Xbamc159A

=== Stwintron Sequence ===

Multiple sequence alignment

```
Xbamc159A_fwrd      -gguacguauuaaaauaugccgugccaauucagagaccaggacuugguagaaagauaua-----------uugucauucagagcuaacuguugccauaguaugugaaccuuuaauccagaaagaugaccaccaugaucuguguacuucuagaaaguaugcaauaaggaacuggacuaacauauuuugauauggccag
Xbamc159A_RevC      cuggccauaucaaaauauguuaguccaguuccuuauugcauacuuucuagaaguacacagaucauggugucaucuuucuggauuaaagguucacauacuauggcaacaguuagcucugaaugacaa-----------uauaucuuucuaccaaguccuggucucugaauuggcacggcauauuuuaauacguacc-
                      *  *#***#********  #  *** ***   #    # ****  *****  *#*#*           *##** *** * # ***  ***  ****|****  ***  *** # * *** **##*           *#*#*  *****  **** #    #   *** ***  #  *******#***#*   *
```

=== Internal intron ===

Multiple sequence alignment

```
Xbamc159A_fwrd      guacguauuaaaauaugccgugccaauucagagaccaggacuugguagaaagauauauugucauucagagcuaacuguugccauag----------------------
Xbamc159A_RevC      ----------------------cuauggcaacaguuagcucugaaugacaauauaucuuucuaccaaguccuggucucugaauuggcacggcauauuuuaauacguac
                                          * *   **# #   **  ** ##* # ** *||* ** # *## **  **   # #**   * *
```

=== External intron ===

Multiple sequence alignment

```
Xbamc159A_fwrd      guaugugaaccuuuaauccagaaagaugaccaccaugauc-----uguguacuucuagaaaguaugcaauaaggaacuggacuaacauauuuugauauggccag---------
Xbamc159A_RevC      ---------cuggccauaucaaaauauguuaguccaguuccuuauugcauacuuucuagaaguacaca-----gaucaugguggucaucuuucuggauuaaagguucacauac
                             *   # **   #*** ***     *  * **     **##***** #|# *****##**     ** *  *     *** ***#   ** #   *
```

ALIGNMENT RESULTS - Xbamc164A

=== Stwintron Sequence ===

Multiple sequence alignment

```
Xbamc164A_fwrd      ----------gguaugugaucaaaacuugcuguauggucauugaguccgaccacgacagaaagaucuaaguguaguuugccagagcuaaccguugccgcaguaugugaaccuuccgucuagaaacgcgacuaucaugaccuguguagcuuuaagaauuauauaauaaggaacuaggcuaacaagguucuuag
Xbamc164A_RevC      cuaagaaccuuguuagccuaguuccuuauuauauaauucuuaaagcuacacaggucaugauagucgcguuucuagacggaagguucacauacugcggcaacgguuagcucuggcaaacuacacuuagaucuuucugucguggucggacucaaugaccauacagcaaguuuugauacauacc----------
                               **  *# *     ***# *#***##*  **#*# * #  ** * **  * # **    * # #  #*  **   #*   ##***|***##   *#   **  *#  # # *    ** # *  ** * **  # * #*#**  *##***#* #***     * #*  **
```

=== Internal intron ===

Multiple sequence alignment

```
Xbamc164A_fwrd      guaug------------uaucaaaacuugcuguauggucauugaguccgaccacgacagaaagaucuaaguguaguuugccagagcuaaccguugccgcag
Xbamc164A_RevC      cugcggcaacgguuagcucuggcaaacuacacuuagaucuuucugucguggucggacucaaugaccauacagcaaguuuugaua------------cauac
                     * #*            * *   ** #*#*  *  *#** **  ***   |   ***  ** **#*  *  *#*# **   * *            *# *
```

=== External intron ===

Multiple sequence alignment

```
Xbamc164A_fwrd      ----------------------------guaugugaaccuuccgucuagaaacgcgacuaucaugaccuguguagcuuuaagaauuauauaauaaggaacuaggcuaacaagguucuuag
Xbamc164A_RevC      cuaagaaccuuguuagccuaguuccuuauuauauaauucuuaaagcuacacaggucaugauagucgcguuucuagacggaagguucacauac----------------------------
                                                 ***#*#*  ***  # *** * * *  *  ||  *  * * * *** #  ***  *#*#***
```

ALIGNMENT RESULTS - Xbamc176A

=== Stwintron Sequence ===

Multiple sequence alignment

```
Xbamc176A_fwrd      gguacguaugaaaaucugcugugccaguuccaaguccucgaguugguggcaaggucuauugucuucuagagcuaacuguugccacaguaugugaacauuugaucuagaaauaccgccaucauaaucugugcaauccuagaauguau---------------gaaauagguaacuagacugauaaauuuugauacggcag
Xbamc176A_RevC      cugccguaucaaaauuuaucagucuaguuaccuauuuc---------------auacauucuaggauugcacagauuaugauggcgguauuucuagaucaaauguucacauacuguggcaacaguuagcucuagaagacaauagaccuugccaccaacucgaggacuuggaacuggcacagcagauuuucauacguacc
                        ***** ***** *# ##  * **** *  #*  #               #* #*** *    * * #*  * *#* #   * **** *  * **#|#** *  * **** *   # *#* *  *# * *    * ***# *#               #  *#  * **** *  ## #* ***** *****
```

=== Internal intron ===

Multiple sequence alignment

```
Xbamc176A_fwrd      guacg-------------------uaugaaaaucugcugugccaguuccaaguccucgaguugguggcaaggucuauugucuucuagagcuaacuguugccacag
Xbamc176A_RevC      cuguggcaacaguuagcucuagaagacaauagaccuugccaccaacucgaggacuuggaacuggcacagcagauuuucau-------------------acguac
                     *  *                    *##* *  *#    ##***##** * *|* * **##***##    #*  * *##*                    *  *
```

=== External intron ===

Multiple sequence alignment

```
Xbamc176A_fwrd      guaugugaacauuugaucuagaaauaccgccaucauaaucugugcaauccuagaauguaugaaauagguaacuagacugauaaauuuugauacggcag-----------------------------------------
Xbamc176A_RevC      -----------------------------------------cugccguaucaaaauuuaucagucuaguuaccuauuucauacauucuaggauugcacagauuaugauggcgguauuucuagaucaaauguucacauac
                                                              ***  *  #*#*** *** *  # #**|**# #  * *** ***#*#  *  ***
```

ALIGNMENT RESULTS - Xbamc177A

=== Stwintron Sequence ===

Multiple sequence alignment

```
Xbamc177A_fwrd      gguacguauuaugaaccugcugugccaguucugaaacuugguguuggcaaagagauauguuauuguccagagcuaacuguugccgcaguaugugaaauuuuguccuacaaacgcaaccaccauaauuugcguuauucugcaaaguaccuaau-----gcaauaaggaacuagacuaacaca------uuaugauauggcag
Xbamc177A_RevC      cugccauaucauaa------uguguuagucuaguuccuuauugc-----auuagguacuuugcagaauaacgcaaauuaugguggguugcguuuguaggacaaaauuucacauacugcggcaacaguuagcucuggacaauaacauaucucuuugccaacaccaaguuucagaacuggcacagcagguucauaauacguacc
                        *#***#**#*      ****  ***#  *   ***# **#     *  ** **# ** # *   *# ** ** *#* *  *  *# * ** *   #|#   * ** * #*  *  *#* ** ** #*   * # ** #** **  *     #** #***   *  #***  ****      *#**#***#*
```

=== Internal intron ===

Multiple sequence alignment

```
Xbamc177A_fwrd      guacg------------------uauuaugaaccugcugugccaguucugaaacuugguguuggcaaagagauauguuauuguccagagcuaacuguugccgcag
Xbamc177A_RevC      cugcggcaacaguuagcucuggacaauaacauaucucuuugccaacaccaaguuucagaacuggcacagcagguucauaaua------------------cguac
                     * **                  #* **  *   # ** *****## *##* | *##* ##***** ** #   *  ** *#                  ** *
```

=== External intron ===

Multiple sequence alignment

```
Xbamc177A_fwrd      guaugugaaauuuuguccuacaaacgcaaccaccauaauuugcguuauucugcaaaguaccuaaugcaauaaggaacuagacuaacacauuaugauauggcag-----------------------------
Xbamc177A_RevC      -----------------------------cugccauaucauaauguguuagucuaguuccuuauugcauuagguacuuugcagaauaacgcaaauuauggugguugcguuuguaggacaaaauuucacauac
                                                 *  ***** # *#   * **   * *  * * ** *||* ** * *  * *   ** *   #* # *****  *
```

ALIGNMENT RESULTS - Xbamc191A

=== Stwintron Sequence ===

Multiple sequence alignment

```
Xbamc191A_fwrd      gguauguaucaaa-------------accugcugugccaguucuaaaaccugguguugaugccaauaucgguugucuuccagagcuaacgguugguacaguaugugaccuucugguccagaaacgcgauuaccauaaucuu-----uuuaauucuaaaaaguguguaacaaggaaguggacugacguuugauaugguag
Xbamc191A_RevC      cuaccauaucaaacgucaguccacuuccuuguuacacacuuuuuagaauuaa-----aaagauuaugguaaucgcguuucuggaccagaaggucacauacuguaccaaccguuagcucuggaagacaaccgauauuggcaucaacaccagguuuuagaacuggcacagcagg-------------uuuugauacauacc
                        ##*******              * ** *###*   ** ** **   #     #* *   **   ##*#*# ** * * #*#*  #* * ##* *|* *## * *#  *#*# * * ** #*#*##   **   * *     ##   ** ** **   *###* ** *              *******##
```

=== Internal intron ===

Multiple sequence alignment

```
Xbamc191A_fwrd      guauguaucaaaaccugcugugccaguucuaaaaccugg-----------uguugaugccaauaucgguugucuuccagagcuaacgguugguacag-----------------
Xbamc191A_RevC      -----------------cuguaccaaccguuagcucuggaagacaaccgauauuggcaucaaca-----------ccagguuuuagaacuggcacagcagguuuugauacauac
                                     ****#***### * *   ****           *#*** || ***#*           ****   * * ###***#****
```

=== External intron ===

Multiple sequence alignment

```
Xbamc191A_fwrd      gu---augugaccuucugguccagaaacgcgauuaccauaaucuuuuuaauucuaaaaaguguguaacaaggaag----uggacugacguuugauaugguag
Xbamc191A_RevC      cuaccauaucaaacgucagucc----acuuccuuguuacacacuuuuuagaauuaaaaagauuaugguaaucgcguuucuggaccagaaggucacau---ac
                     *   **#* *  #  ##****    **    **   *#*  ******* || *******  *#*   **    *    *****##  #  * *#**   *
```

ALIGNMENT RESULTS - Xbamc198A

=== Stwintron Sequence ===

Multiple sequence alignment

```
Xbamc198A_fwrd      gguacguaucaaaaccuauugugucgguucucagaccuggacuuauuagaaagaccuauuuccuuccagcgcuaacuguugccgcaguauguguauuuuugauccgcaaacgugaccacuauuaccuguguaaugguua------------------uaauucuagaauguauaag-----aaauuggacugauaaauuuugauaugacag
Xbamc198A_RevC      cugucauaucaaaauuuaucaguccaauuu-----cuuauacauucuagaauua------------------uaaccauuacacagguaauaguggucacguuugcggaucaaaaauacacauacugcggcaacaguuagcgcuggaaggaaauaggucuuucuaauaaguccaggucugagaaccgacacaauagguuuugauacguacc
                        *#********  ***##  #*##**      * *# ** * #*****  *                  ****##**#*    ***   **  *# #* *  |  * *# #*  **   ***    *#**##****                  *  *****# * ** #* *      **##*#  ##***  ********#*
```

=== Internal intron ===

Multiple sequence alignment

```
Xbamc198A_fwrd      guacguaucaaaaccuauugugucgguucucagaccuggacuuauuagaaagaccuauuuccu---uccagcgcuaacuguugccgcag-----------------
Xbamc198A_RevC      -----------------cugcggcaacaguuagcgcugga---aggaaauaggucuuucuaauaaguccaggucugagaaccgacacaauagguuuugauacguac
                                     #**#* *###  * **  *****   *  *#* **||** *#*  *   *****  ** *  ###* *#**#
```

=== External intron ===

Multiple sequence alignment

```
Xbamc198A_fwrd      gua-----------------------------uguguauuuuugauccgcaaacgugaccacuauuaccuguguaaugguuauaauucuagaauguauaagaaauuggacugauaaauuuugauaugacag
Xbamc198A_RevC      cugucauaucaaaauuuaucaguccaauuucuuauacauucuagaauuauaaccauuacacagguaauaguggucacguuugcggaucaaaaauacaca-----------------------------uac
                     *                              *#*##***#* **   # ** *#* **     *|*     ** *#* ** #   ** *#***##*#*                              *
```

ALIGNMENT RESULTS - Xbamc199A

=== Stwintron Sequence ===

Multiple sequence alignment

```
Xbamc199A_fwrd      gguauguaucaacaccuaccgugccaguucugagaccuagacuugguagaau------------gaucuguugucuuccagcgcuaacuguugccgcaguacgugaacuucugauccugaaacaugaucaccgugaucugcguaaauucuagaaaguacguaauaaggaacuggacuaacacguuuuuauacggcag
Xbamc199A_RevC      cugccguauaaaaacguguuaguccaguuccuuauuacguacuuucuagaauuuacgcagaucacggugaucauguuucaggaucagaaguucacguacugcggcaacaguuagcgcuggaagacaacagau------------cauucuaccaagucuaggucucagaacuggcacgguagguguugauacauacc
                        #**** ** ** *   #  *******#  #   #  ****  ******                 #*##* ** *** # #*   ***  ** *|* **  ***   *# # *** ** *##*#                 ******  ****  #   #  #*******  #   * ** ** ****#
```

=== Internal intron ===

Multiple sequence alignment

```
Xbamc199A_fwrd      guauguaucaacaccuaccg---------------------ugccaguucugagaccuagacuugguagaaugaucuguugucuuccagcgcuaacuguugccgcag--
Xbamc199A_RevC      --cugcggcaacaguuagcgcuggaagacaacagaucauucuaccaagucuaggucucagaacuggca---------------------cgguagguguugauacauac
                       **#  *****  ** **                     *#***# ***# *|* #*** #***#*                     ** **  *****  #**
```

=== External intron ===

Multiple sequence alignment

```
Xbamc199A_fwrd      -----guacgugaacuucugauccugaaacaugaucaccgugaucugcguaaauucuagaaaguac----------guaauaaggaacuggacuaacacguuuuuauacggcag
Xbamc199A_RevC      cugccguauaaaaacguguuaguccaguuccuuau----------uacguacuuucuagaauuuacgcagaucacggugaucauguuucaggaucagaaguucacguac-----
                         *** # #*** * * *  *##   * * **          *#****  ***||***  ***          ** ** * *   ##*  * * * ***# # ***
```

ALIGNMENT RESULTS - Xbamc236C

=== Stwintron Sequence ===

Multiple sequence alignment

```
Xbamc236C_fwrd      gguauguaucaaaaccuacggugccaguucugagaccuggacuugguagaaugau-----------cuguugucuucgagcgcuaacuguugccgcaguacgugaacuucugauccuaaaacaugaucaccgugaucugcauaauucuagaaaguauguaauaaggaacuagacuaauaccuuuuuauauggcag
Xbamc236C_RevC      cugccauauaaaaagguauuagucuaguuccuuauuacauacuuucuagaauuaugcagaucacggugaucauguuuuaggaucagaaguucacguacugcggcaacaguuagcgcucgaagacaa-----------cagaucauucuaccaaguccaggucucagaacuggcaccguagguuuugauacauacc
                        ##*** ****  **  #  * *****#  #   ## ****  ****** **            #*##* **  ** # #*   ***  ** *|* **  ***   *# # **  ** *##*           ## ** ******  **** ##   #  #***** *  #  **  **** ***##
```

=== Internal intron ===

Multiple sequence alignment

```
Xbamc236C_fwrd      guauguaucaaaaccuacggugccaguucugagaccuggacuugguagaaugaucuguugucuucgagcgcu--aacuguugccgcag--------------
Xbamc236C_RevC      --------------cugcggcaacagu--uagcgcucgaagacaacagaucauucuaccaaguccaggucucagaacuggcaccguagguuuugauacauac
                                  ** ***## ****  *#   * #*#*  ####*** || ***####  *#*# *   #  ***** ##*** **
```

=== External intron ===

Multiple sequence alignment

```
Xbamc236C_fwrd      guacgugaacuucugauccuaaaacaugaucaccgugaucugcauaauucuagaaaguauguaauaaggaacuagacuaauaccuuuuuauaug-----------------------------------------------------gcag
Xbamc236C_RevC      cugc-----------------------------------------------------cauauaaaaagguauuagucuaguuccuuauuacauacuuucuagaauuaugcagaucacggugaucauguuuuaggaucagaaguucacguac
                     * *                                                     #**#*** **** * ***|*** * **** ***#**#                                                 * *
```

ALIGNMENT RESULTS - Xbccc01A

=== Stwintron Sequence ===

Multiple sequence alignment

```
Xbccc01A_fwrd       gguauguaugaaaaccuggcgugcuuuauuccuaggccuucauuuc----------------------------------uagaagaauauacuugcuauguuuugauuugcuaaccucuauuguaguaaguacccgcucuuauuggcugaaugguguugcggccuggacgauucuugaaagaauaccuuuggaaacugauacgacguguuuuuaucucgcag
Xbccc01A_RevC       cugcgagauaaaaacacgucguaucaguuuccaaagguauucuuucaagaaucguccaggccgcaacaccauucagccaauaagagcggguacuuacuacaauagagguuagcaaaucaaaacauagcaaguauauucuucua----------------------------------gaaaugaaggccuaggaauaaagcacgccagguuuucauacauacc
                         # **#***** #* ***# #   **** *#*   *  ****                                  **# **    *****#***## *  #  **#|#**  #  * ##***#*****    ** #**                                  ****  *   *#* ****   # #*** *# ****#** #
```

=== Internal intron ===

Multiple sequence alignment

```
Xbccc01A_fwrd       gua-------------uguaugaaaaccuggcgugcuuuauuccuaggccuucauuucuagaagaauauacuugcuauguuuugauuugcuaaccucuauuguag
Xbccc01A_RevC       cuacaauagagguuagcaaaucaaaacauagcaaguauauucuucuagaaaugaaggccuaggaauaaagcacgccagguuuucauaca-------------uac
                     **             ## ** ***** *#**# *  *  *#  # #*   *|*   *# #  #*  *  * #**#* ***** ** ##             **
```

=== External intron ===

Multiple sequence alignment

```
Xbccc01A_fwrd       ----------------------guaaguacccgcucuuauuggcugaaugguguugcggccuggacgauucuugaaagaauaccuuuggaaacugauacgacguguuuuuaucucgcag
Xbccc01A_RevC       cugcgagauaaaaacacgucguaucaguuuccaaagguauucuuucaagaaucguccaggccgcaacaccauucagccaauaagagcggguacuuac----------------------
                                          #* ***  **#    ****   * ** ##*  * *#*|*#* *  *## ** *   ****    #**  *** *#
```

ALIGNMENT RESULTS - Xbccc01B

=== Stwintron Sequence ===

Multiple sequence alignment

```
Xbccc01B_fwrd       gguauguauaaaaacccggcgugcuucguuccuuagccuucauuucuggaauaauguaauuguuauguuuauuugcuaaucucuaucgcagugaguaaccgccuuuauuag------gucaauagugacacagcuuggaugauuauagaaagaguaauuuugaaaacugacacgacauguuuuuaucucgcag
Xbccc01B_RevC       cugcgagauaaaaacaugucgugucaguuuucaaaauuacucuuucuauaaucauccaagcugugucacuauugac------cuaauaaaggcgguuacucacugcgauagagauuagcaaauaaacauaacaauuacauuauuccagaaaugaaggcuaaggaacgaagcacgccggguuuuuauacauacc
                         # ********  * **** #   ** *  *#   #  *****# *** ** #** #  * *  #**** #*      ***  # **   **|**   ** #  ***      *# ****#  * *  # **# ** *** #*****  #   #*  * **   # **** *  ******** #
```

=== Internal intron ===

Multiple sequence alignment

```
Xbccc01B_fwrd       gu-------------auguauaaaaacccggcgugcuucguuccuuagccuucauuucuggaauaauguaauuguuauguuuauuugcuaaucucuaucgcag
Xbccc01B_RevC       cugcgauagagauuagcaaauaaacauaacaauuacauuauuccagaaaugaaggcuaaggaacgaagcacgccggguuuuuauacau-------------ac
                     *              ## ***** *    #  *#* * #****  *#   |   #*  ****# * *#*  #    * ***** ##              *
```

=== External intron ===

Multiple sequence alignment

```
Xbccc01B_fwrd       ----------------------gugaguaaccgccuuuauuaggucaauagugacacagcuuggaugauuauagaaagaguaauuuugaaaacugacacgacauguuuuuaucucgcag
Xbccc01B_RevC       cugcgagauaaaaacaugucgugucaguuuucaaaauuacucuuucuauaaucauccaagcugugucacuauugaccuaauaaaggcgguuacucac----------------------
                                          ** ***   *#   ***#*   ** ***#* *  **#|#**  * *#*** **   *#***   #*   *** **
```

ALIGNMENT RESULTS - Xbccc05A

=== Stwintron Sequence ===

Multiple sequence alignment

```
Xbccc05A_fwrd       gguauguauaaaaaccuggugugcuucauuccuaggucuucauuccuaggauaacguaauu----gcuauguugaucugcuaaccucuauugcaguaaguaaccgccuuuauuagcucgaugguguugcggauuggguGauuguagaaagaguaacuuuggaaacugacacggcuuguuuuuaucuggcag
Xbccc05A_RevC       cugccagauaaaaacaagccgugucaguuuccaaaguuacucuuucuacaaucacccaauccgcaacaccaucgagcuaauaaaggcgguuacuuacugcaauagagguuagcagaucaaca----uagcaauuacguuauccuaggaaugaagaccuaggaaugaagcacaccagguuuuuauacauacc
                        ## ********  * #*** #   **** *#**  #  ** *** #** ** #***#    #*  ##*#** **# ***   *  **#*  |  *#**  *   *** #** **#*##    *#  #***# ** **# *** **  #  **#* ****   # ***# *  ******** ##
```

=== Internal intron ===

Multiple sequence alignment

```
Xbccc05A_fwrd       guauguauaaaaaccuggugugcuucauuccuaggucuucauuccuaggauaacguaauugcuauguugaucugcuaaccucuau-ugcag
Xbccc05A_RevC       cugca-auagagguuagcagaucaacauagcaauuacguuauccuaggaaugaagaccuaggaaugaagcacaccagguuuuuauacauac
                     * ## *** *     *  *  *  ***  * *   * * **#* | *#** * *   * *  ***  *  *  *     * *** ## *
```

=== External intron ===

Multiple sequence alignment

```
Xbccc05A_fwrd       ----------------------guaaguaaccgccuuuauuagcucgaugguguugcggauuggguGauuguagaaagaguaacuuuggaaacugacacggcuuguuuuuaucuggcag
Xbccc05A_RevC       cugccagauaaaaacaagccgugucaguuuccaaaguuacucuuucuacaaucacccaauccgcaacaccaucgagcuaauaaaggcgguuacuuac----------------------
                                          ** ***  **#   ***#*   ** *###*  # *##|##* #  *###* **   *#***   #**  *** **
```

ALIGNMENT RESULTS - Xbccc05B

=== Stwintron Sequence ===

Multiple sequence alignment

```
Xbccc05B_fwrd      gguaugguaaaauccuggcaugcuuuauuccuaggccuucauuucuagaauaacguaauuucua----uguugaucugcuaaccucuauuguaguaaguaaccgccuuuaucagcucaaugguguuacggccuagaugauucuagaagaauaacuuuaaaaacuga-cacgacauguuuuuaucucgcag
Xbccc05B_RevC      cugcgagauaaaaacaugucgug-ucaguuuuuaaaguuauucuucuagaaucaucuaggccguaacaccauugagcugauaaaggcgguuacuuacuacaauagagguuagcagaucaaca----uagaaauuacguuauucuagaaaugaaggccuaggaauaaagcaugccaggauuuuacacauacc
                        # #***** * ** * ** *#  **  **##  *   #********* *  **  ## **    ##**** *** ***   *  **##  |  ##**  *   *** *** ****##    ** ##  **  * *********#   *  ##**  **  #* ** * ** * *****# #
```

=== Internal intron ===

Multiple sequence alignment

```
Xbccc05B_fwrd      guaug--------------------uguaaaauccuggcaugcuuuauuccuaggccuucauuucuagaauaacguaauuucuauguugaucugcuaaccucuauuguag
Xbccc05B_RevC      cuacaauagagguuagcagaucaacauagaaauuacguuauucuagaaaugaaggccuaggaauaaagcaugccaggauuuu--------------------acacauac
                    **##                       ****  #*  ** **  *     **||**     *  ** **  *#  ****                       ##**
```

=== External intron ===

Multiple sequence alignment

```
Xbccc05B_fwrd      guaaguaaccgccuuuaucagcucaaugguguuacggccuagaugauucuagaagaauaacuuuaaaaacugacacgacauguuuuuaucucgcag------------------
Xbccc05B_RevC      ------------------cugcgagauaaaaacaugucgugucaguuuuuaaaguuauucuucuagaaucaucuaggccguaacaccauugagcugauaaaggcgguuacuuac
                                     * **   **## # #* * * *    * ** **#*   ||   *#** ** *    * * * *# # ##**   ** *
```

ALIGNMENT RESULTS - Xbccc05C

=== Stwintron Sequence ===

Multiple sequence alignment

```
Xbccc05C_fwrd       gguauguauaaaaaaaccccuagcgugcuuuauuccuacaccuucauuuuuagaa--------------------uaacguaauugcuauguugaucugcuaaccuuuauuguaguaaguaaccgccuuuauuaggucaaugguguuacggccugaaugguucuggaaagaauaacuuugaaaacugacacau-----guuuuaucuugcag---
Xbccc05C_RevC       ---cugcaagauaaaac-----augugucaguuuucaaaguuauucuuuccagaaccauucaggccguaacaccauugaccuaauaaaggcgguuacuuacuacaauaaagguuagcagaucaacauagcaauuacguua--------------------uucuaaaaaugaagguguaggaauaaagcacgcuagggguuuuuuuauacauacc
                        **#*  * *****     # *** #   ** * *     *  ***##****                     * ** **** #   #* * *# *#***   *||*   ***#* #* * *#   # **** ** *                     ****##***  *     * * **   # *** #     ***** *  *#**
```

=== Internal intron ===

Multiple sequence alignment

```
Xbccc05C_fwrd       guauguauaaaaaaaccccuagcgugcuuuauuccuacaccuucauuuuuagaauaacguaauugcuauguugaucugcuaac----------------------------cuuuauuguag----
Xbccc05C_RevC       ----cuacaauaaag-----------------------------guuagcagaucaacauagcaauuacguuauucuaaaaaugaagguguaggaauaaagcacgcuagggguuuuuuuauacauac
                         **#** ***                               **  #*** #***#** #|# **#***# ***#  **                               *** **#**
```

=== External intron ===

Multiple sequence alignment

```
Xbccc05C_fwrd       --guaaguaaccgccuuuauuag--gucaaugguguuacggccugaaugguucuggaaagaauaacuuugaaaacugacacauguuuuaucuugcag
Xbccc05C_RevC       cugcaagauaaaacaugugucaguuuucaaaguuauucuuuccagaaccauucaggccguaacaccauug--accuaauaaaggcgguuacuuac--
                      *#***  *  #* * * *#**   **** * *#**    ** ***#|#*** **    **#* * ***  * **#* * * *#  *  ***#*
```

ALIGNMENT RESULTS - Xbccc06A

=== Stwintron Sequence ===

Multiple sequence alignment

```
Xbccc06A_fwrd       gguauguauaaaaaccuggccuguuucauuccucggccuucauuccuggaauaaugcaauugcuauguugauaugcuaacuucuauugcaguaaguaauugccuuuauugaguuaauagugucacagc-----uugaaugauuuuggaaagagcaacuuugaaaacugacaugacauguuuuuaucucgcag
Xbccc06A_RevC       cugcgagauaaaaacaugucaugucaguuuucaaaguugcucuuuccaaaaucauucaa-----gcugugacacuauuaacucaauaaaggcaauuacuuacugcaauagaaguuagcauaucaacauagcaauugcauuauuccaggaaugaaggccgaggaaugaaacaggccagguuuuuauacauacc
                         # ******** ** * ***#   ** *  #*   #  ** *###*** ** ***      #  ***#*#  * * #** ** #  *#** || **#*  # ** **# * *  #*#***  #      *** ** ***###* ** #   *#  * **   #*** * ** ******** #
```

=== Internal intron ===

Multiple sequence alignment

```
Xbccc06A_fwrd       gu-------------auguauaaaaaccuggccuguuucauuccucggccuucauuccuggaauaaugcaauugcuauguugauaugcuaacuucuauugcag
Xbccc06A_RevC       cugcaauagaaguuagcauaucaacauagcaauugcauuauuccaggaaugaaggccgaggaaugaaacaggccagguuuuuauacau-------------ac
                     *              ##*** ** *    #  **# * *****  *#   |   #*  ***** * #**  #    * ** ***##              *
```

=== External intron ===

Multiple sequence alignment

```
Xbccc06A_fwrd       -------guaaguaauugccuuuauugaguuaauagugucacagcuugaaugauuuuggaaagagcaacuuugaaaacugacaugacauguuuuuaucucgcag
Xbccc06A_RevC       cugcgagauaaaaacaugucaugucaguuuucaaaguugcucuuuccaaaaucauucaagcugugacacuauuaacucaauaaaggcaauuacuuac-------
                           #***# *  ** * *  # *  ** * ***  * *   ###** || **###   * *  *** * **  * #  * * **  * #***#
```

ALIGNMENT RESULTS - Xbccc07A

=== Stwintron Sequence ===

Multiple sequence alignment

```
Xbccc07A_fwrd       --gguauguauaaaacucuggcgugcuuuauuccuaggcuuucauuucuagaguaauc----------------------gauuguuauguuuaucugcuaaccucuaucguaguaaguaaccgccuuuauuagcucaacgguauuauggcuuagaugauucuggagagcgaaaacua----------acgcgauaaauuuguaucucgcag
Xbccc07A_RevC       cugcgagauacaaauuuaucgcgu----------uaguuuucgcucuccagaaucaucuaagccauaauaccguugagcuaauaaaggcgguuacuuacuacgauagagguuagcagauaaacauaacaauc----------------------gauuacucuagaaaugaaagccuaggaauaaagcacgccagaguuuuauacauacc--
                      *  * #**#***  * * ****          ***  **#  *#**#***#* ***                      #** #   #* ***# *#***   *||*   ***#* #*** *#   # **#                      *** *#***#**#*  #**  ***          **** * *  ***#**# *  *
```

=== Internal intron ===

Multiple sequence alignment

```
Xbccc07A_fwrd       gu-----------------auguauaaaacucuggcgugcuuuauuccuaggcuuucauuucuagaguaaucgauuguuauguuuaucugcuaaccucuaucguag
Xbccc07A_RevC       cuacgauagagguuagcagauaaacauaacaaucgauuacucuagaaaugaaagccuaggaauaaagcacgccagaguuuuauacau-----------------ac
                     *                 **# *#* ***  * *  *#**#**    * ##||## *    **#**#*  * *  *** *#* #**                 *
```

=== External intron ===

Multiple sequence alignment

```
Xbccc07A_fwrd       gu----------aaguaaccgccuu--uauuagcucaacgguauuauggcuuagaugauucuggagagcgaaaacuaacgcgauaaauuuguaucucgcag
Xbccc07A_RevC       cugcgagauacaaauuuaucgcguuaguuuucgcucuccagaaucaucuaagccauaauaccguugagcuaaua--aaggcgguuacuu----------ac
                     *          ** * * *** **  * ** ****  *#* **#**   |   **#** *#*  **** ** *  ** *** * * **          *
```

ALIGNMENT RESULTS - Xbccc09A

=== Stwintron Sequence ===

Multiple sequence alignment

```
Xbccc09A_fwrd       --------------------gguacguauaagaaccuggccugcuuuauuccuaggccuac---auuuguagaauaauucaguuguuaugcuuaugugcuaaccucuauuguaguaaguaaccgccaaaaugagcucaauaauguugcggcuuggaugacucucagaaagaauaaauucgaaaacugacacgagauguuuuuuuucucucucgcag
Xbccc09A_RevC       cugcgagagagaaaaaaaacaucucgugucaguuuucgaauuuauucuuucugagagucauccaagccgcaacauuauugagcucauuuuggcgguuacuuacuacaauagagguuagcacauaagcauaacaacugaauuauucuacaaau---guaggccuaggaauaaagcaggccagguucuuauacguacc--------------------
                                        # # *** * **    #*#  *  **# ***  **#  #*    * ##*#*# ** *** **#*  * *   #   *#** **  * || *  ** **#*   #   * *  *#** *** ** #*#*## *    *#  #**  *** #**  *  #*#    ** * *** # #
```

=== Internal intron ===

Multiple sequence alignment

```
Xbccc09A_fwrd       gua-------------cguauaagaaccuggccugcuuuauucc-------uaggccuacauuuguagaauaauucaguuguuaugcuuaugugcuaaccucuauuguag
Xbccc09A_RevC       cuacaauagagguuagcacauaagcauaacaacugaauuauucuacaaauguaggccua-------ggaauaaagcaggccagguucuuauacg-------------uac
                     **             *##***** *    # ***  ******        ***||***        ******  *** #    * *****##*             **
```

=== External intron ===

Multiple sequence alignment

```
Xbccc09A_fwrd       --guaaguaaccgccaaaaugagcucaauaauguugcggcuuggaugacucucagaaagaauaaauucgaaaacugacacgagauguuuuuuuucucucucgcag
Xbccc09A_RevC       cugcgagagagaaaaaaaacaucucgugucaguuuucgaauuuauucuuucugagagucauccaagccgcaacauuauugagcucauuuuggcgguuacuuac--
                      *# **  *  #  ****##   #   * *  ** **# ** # *   ***|***   * # ** #** **  * *   #   ##****  #  *  ** #*
```

ALIGNMENT RESULTS - Xlonc0002A

=== Stwintron Sequence ===

Multiple sequence alignment

```
Xlonc0002A_fwrd     gguauguaugaaauccugccgugcuuuauucccaaaccuucauuucuacaauaguuuaguuguuauguuaaucagcuaaccucuguuguaguaaguaacuccuuuauugauucgacggugucgcgguucggaua-----------auucuagaaagaauaucuuugaaaacuaacacgacguguuuuuaucugguag
Xlonc0002A_RevC     cuaccagauaaaaacacgucguguuaguuuucaaagauauucuuucuagaa-----------uuauccgaaccgcgacaccgucgaaucaauaaaggaguuacuuacuacaacagagguuagcugauuaacauaacaacuaaacuauuguagaaaugaagguuugggaauaaagcacggcaggauuucauacauacc
                        ## **#*** * #* **** *   ** * **    *  ****** **           **** # **#*     ***  #*   #*#***#  *|*  #***#*#   *#  ***     *#** # ***           *** ******  *    ** * **   * **** *# * ***#** ##
```

=== Internal intron ===

Multiple sequence alignment

```
Xlonc0002A_fwrd     guauguaugaaauccugccgugcuuuauucccaaaccuucauuucuacaauaguuuaguuguuauguuaaucagcuaaccucuguuguag-----------------------------------------
Xlonc0002A_RevC     -----------------------------------------cuacaacagagguuagcugauuaacauaacaacuaaacuauuguagaaaugaagguuugggaauaaagcacggcaggauuucauacauac
                                                             #* * ***   ***   * #*** | ***# *   ***   *** * *#
```

=== External intron ===

Multiple sequence alignment

```
Xlonc0002A_fwrd     gu------------------------------aaguaacuccuuuauugauucgacggugucgcgguucggauaauucuagaaagaauaucuuugaaaacuaacacgacguguuuuuaucugguag
Xlonc0002A_RevC     cuaccagauaaaaacacgucguguuaguuuucaaagauauucuuucuagaauuauccgaaccgcgacaccgucgaaucaauaaaggaguuacuu------------------------------ac
                     *                              **# *  * **** * ** * # * * ##*||*## * * # * ** * **** *  * #**                              *
```

ALIGNMENT RESULTS - Xlonc0025A

=== Stwintron Sequence ===

Multiple sequence alignment

```
Xlonc0025A_fwrd     ggcaaguauaaaaacccgccgugccgguugugagaccuggugcuggcagaa-----------agaucuaucguuuuccggagcuaacuguugcuacaguaugugaaccuuugauccagaaaaguggccgccauggccgauguaauucuagaaaguauacaauaaggaacuggacuaacauguuuuuauacaccag
Xlonc0025A_RevC     cugguguauaaaaacauguuaguccaguuccuuauuguauacuuucuagaauuacaucggccauggcggccacuuuucuggaucaaagguucacauacuguagcaacaguuagcuccggaaaacgau-----------agaucuuucugccagcaccaggucucacaaccggcacggcggguuuuuauacuugcc
                         **********  *  #  **#*** #  #   *#    *   ****           *   *  #*##*** * * # #**  ***  #* *|* *#  ***  **# # * * ***##*#            * #*  ****   *    #*   #  # ***#**  #  *  **********
```

=== Internal intron ===

Multiple sequence alignment

```
Xlonc0025A_fwrd     gc-------------------aaguauaaaaacccgccgugccgguugugagaccuggugcuggcagaaagaucuaucguuuuccggagcuaacuguugcuacag
Xlonc0025A_RevC     cuguagcaacaguuagcuccggaaaacgauagaucuuucugccagcaccaggucucacaaccggcacggcggguuuuuauacuu-------------------gc
                                          *# *# * *   *    ****#*#  ## *|* ##  #*#****    *   * * #* #*
```

=== External intron ===

Multiple sequence alignment

```
Xlonc0025A_fwrd     -----guaugugaaccuuuga-----------------------uccagaaaaguggccgccauggccgauguaauucuagaaaguauacaauaaggaacuggacuaacauguuuuuauacaccag
Xlonc0025A_RevC     cugguguauaaaaacauguuaguccaguuccuuauuguauacuuucuagaauuacaucggccauggcggccacuuuucugga-----------------------ucaaagguucacauac-----
                         ****# #*** * * *                       ** ****  ### * ***||*** * ###  **** **                       * * * ***# #****
```

ALIGNMENT RESULTS - Xlonc0058A

=== Stwintron Sequence ===

Multiple sequence alignment

```
Xlonc0058A_fwrd     ggcaaguauaaaaacccgccgugccgguugugagaccuggugcuggcagaa-----------agaucuaucguuuuccggagcuaacuguugcuacaguaugugaaccuucgauccagaaaaguggccgccauggccgauguaauucuagaaaguauacaauaaggaacuggacuaacauguuuuuauacaccag
Xlonc0058A_RevC     cugguguauaaaaacauguuaguccaguuccuuauuguauacuuucuagaauuacaucggccauggcggccacuuuucuggaucgaagguucacauacuguagcaacaguuagcuccggaaaacgau-----------agaucuuucugccagcaccaggucucacaaccggcacggcggguuuuuauacuugcc
                         **********  *  #  **#*** #  #   *#    *   ****           *   *  #*##*** * * # # *  ***  #* *|* *#  ***  * # # * * ***##*#            * #*  ****   *    #*   #  # ***#**  #  *  **********
```

=== Internal intron ===

Multiple sequence alignment

```
Xlonc0058A_fwrd     gc-------------------aaguauaaaaacccgccgugccgguugugagaccuggugcuggcagaaagaucuaucguuuuccggagcuaacuguugcuacag
Xlonc0058A_RevC     cuguagcaacaguuagcuccggaaaacgauagaucuuucugccagcaccaggucucacaaccggcacggcggguuuuuauacuu-------------------gc
                                          *# *# * *   *    ****#*#  ## *|* ##  #*#****    *   * * #* #*
```

=== External intron ===

Multiple sequence alignment

```
Xlonc0058A_fwrd     -----guaugugaaccuucga-----------------------uccagaaaaguggccgccauggccgauguaauucuagaaaguauacaauaaggaacuggacuaacauguuuuuauacaccag
Xlonc0058A_RevC     cugguguauaaaaacauguuaguccaguuccuuauuguauacuuucuagaauuacaucggccauggcggccacuuuucugga-----------------------ucgaagguucacauac-----
                         ****# #*** *   *                       ** ****  ### * ***||*** * ###  **** **                       *   * ***# #****
```

ALIGNMENT RESULTS - Xlonc0112A

=== Stwintron Sequence ===

Multiple sequence alignment

```
Xlonc0112A_fwrd     ggcaaguauaaaaaacccgccgugccaguuguaagaccuggugcuggcagaa-----------agaucuaucguuuuccggagcuaacuguugcuacaguaugugaaccuuugauccagaaaaguggccgccauggccgauguaauucuagaaaguauacaauaaggaacuggacuaacauguuuuuauacaccag
Xlonc0112A_RevC     cugguguauaaaaacauguuaguccaguuccuuauuguauacuuucuagaauuacaucggccauggcggccacuuuucuggaucaaagguucacauacuguagcaacaguuagcuccggaaaacgau-----------agaucuuucugccagcaccaggucuuacaacuggcacggcggguuuuuauacuugcc
                         **********  *  #  ****** #  #   *#    *   ****           *   *  #*##*** * * # #**  ***  #* *|* *#  ***  **# # * * ***##*#            * #*  ****   *    #*   #  # ******  #  *  **********
```

=== Internal intron ===

Multiple sequence alignment

```
Xlonc0112A_fwrd     gc-------------------aaguauaaaaacccgccgugccaguuguaagaccuggugcuggcagaaagaucuaucguuuuccggagcuaacuguugcuacag
Xlonc0112A_RevC     cuguagcaacaguuagcuccggaaaacgauagaucuuucugccagcaccaggucuuacaacuggcacggcggguuuuuauacuu-------------------gc
                                          *# *# * *   *    ******#  #* *|* *#  #******    *   * * #* #*
```

=== External intron ===

Multiple sequence alignment

```
Xlonc0112A_fwrd     -----guaugugaaccuuuga-----------------------uccagaaaaguggccgccauggccgauguaauucuagaaaguauacaauaaggaacuggacuaacauguuuuuauacaccag
Xlonc0112A_RevC     cugguguauaaaaacauguuaguccaguuccuuauuguauacuuucuagaauuacaucggccauggcggccacuuuucugga-----------------------ucaaagguucacauac-----
                         ****# #*** * * *                       ** ****  ### * ***||*** * ###  **** **                       * * * ***# #****
```

ALIGNMENT RESULTS - Xmsuc0005A

=== Stwintron Sequence ===

Multiple sequence alignment

```
Xmsuc0005A_fwrd     gguauguauaaaaaccuccugugccgguugugaaacccaaucgucgcagaaauauccu-----------uugccuucuagagcuaacgauuuuuacaguaggugaaccauugauucuaaaacauggccaucacgauugguggaguucuagaaaauauauaauagggaacuggacuaacauguuuuuaucuggcag
Xmsuc0005A_RevC     cugccagauaaaaacauguuaguccaguucccuauuauauauuuucuagaacuccaccaaucgugauggccauguuuuagaaucaagguucaccuacuguaaaaaucguuagcucuagaaggcaa-----------aggauauuucugcgacgauuggguuucacaaccggcacaggagguuuuuauacauacc
                        ## ******** *  *#  **#*** #  *    *    *   **** * # *#          ###  ** ****# #** * **# #  *|*  # #** * **# #**** **  ###          #* # * ****   *    *    *  # ***#**  #*  * ******** ##
```

=== Internal intron ===

Multiple sequence alignment

```
Xmsuc0005A_fwrd     ---------------------guauguauaaaaaccuccugugccgguugugaaacccaaucgucgcagaaauauccuuugccuucuagagcuaacgauuuuuacag
Xmsuc0005A_RevC     cuguaaaaaucguuagcucuagaaggcaaaggauauuucugcgacgauuggguuucacaaccggcacaggagguuuuuauacauac---------------------
                                         * * *#* *  *   * ***#* **#*** * | * ***#** *#*** *   *  * *#* * *
```

=== External intron ===

Multiple sequence alignment

```
Xmsuc0005A_fwrd     guaggugaaccauugauucuaaaacauggccaucacgauugguggaguucuagaaaauauauaauagggaacuggacuaa----------cauguuuua----------ucuggcag
Xmsuc0005A_RevC     cugccaga----------uaaaaacaug----------uuaguccaguucccuauuauauauuuucuagaacuccaccaaucgugauggccauguuuuagaaucaagguucaccuac
                     *    **            ********          **#**  *****#  *  **||**  *  #*****  **#**         ********            **    *
```

ALIGNMENT RESULTS - Xmsuc0006A

=== Stwintron Sequence ===

Multiple sequence alignment

```
Xmsuc0006A_fwrd     gguauguauaaaaaaccuccugugccgguugugaaacccaaucgucguagaacuaucc------------uuugucuucuaaagcuaacgguuuucgcaguaugugaaccauugauuuuaaaaacguggccauuacgaucgguguaguucuagaaaguauauaauaagggacuggacuaauauguuuuuauguugguag
Xmsuc0006A_RevC     cuaccacauaaaaacauauuaguccagucccuuauuauauacuuucuagaacuacaccgaucguaauggccacguuuuuaaaaucaaugguucacauacugcgaaaaccguuagcuuuagaag------------acaaaggauaguucuacgacgauugguuucacaaccggcacaggagguuuuuauacauacc
                        ###******** *  *#  **#**# #  *     *  * *  ********# *           ##    ** ****# #** ****# *# *|* #* #**** **# #**** **            #   #* #********  * *  *    *  # #**#**  #*  * ********###
```

=== Internal intron ===

Multiple sequence alignment

```
Xmsuc0006A_fwrd     ---------------------guauguauaaaaaccuccugugccgguugugaaacccaaucgucguagaacuauccuuugucuucuaaagcuaacgguuuucgcag
Xmsuc0006A_RevC     cugcgaaaaccguuagcuuuagaagacaaaggauaguucuacgacgauugguuucacaaccggcacaggagguuuuuauacauac---------------------
                                         * * ##* *  *   * **##* **#*** * | * ***#** *##** *   *  * *## * *
```

=== External intron ===

Multiple sequence alignment

```
Xmsuc0006A_fwrd     guaugugaaccauugauuuuaaaaacguggccauuacgaucgguguaguucuagaaaguauauaauaagggacuggacuaau----------auguuuuua-----------ugugguag
Xmsuc0006A_RevC     cuaccaca-----------uaaaaacau----------auuaguccagucccuuauuauauacuuucuagaacuacaccgaucguaauggccacguuuuuaaaaucaaugguucacauac
                     **#   *           *******#*          ** #** #***#*#  *  #*||*#  *  #*#***# **# **          *#*******           *   #**
```

ALIGNMENT RESULTS - Xmsuc0006B

=== Stwintron Sequence ===

Multiple sequence alignment

```
Xmsuc0006B_fwrd    gguauguauaaaaaccuccugugccgguugugaaacccaaucgucguagaacuauccuuugu------------cuucuaaagcuaacgguuuucgcaguaugugaaccauugauuuuaaaaacgucgccauuacgaucggguguaguucuagaaaguauauaauaagggacuggacuaacauguuuuuauguggguag
Xmsuc0006B_RevC    cuaccacauaaaaacauguuaguccagucccuuauuauauacuuucuagaacuacaccgaucguaauggcgacguuuuuaaaaucaaugguucacauacugcgaaaaccguuagcuuuagaag------------acaaaggauaguucuacgacgauuggguuucacaaccggcacaggagguuuuuauacauacc
                       ###******** *  *#  **#**# #  *    *  * *  ********# *#   #             ** ****# #** ****# *# *|* #* #**** **# #**** **             #   #* #********  * *  *    *  # #**#**  #*  * ********###
```

=== Internal intron ===

Multiple sequence alignment

```
Xmsuc0006B_fwrd    ---------------------guauguauaaaaaccuccugugccgguugugaaacccaaucgucguagaacuauccuuugucuucuaaagcuaacgguuuucgcag
Xmsuc0006B_RevC    cugcgaaaaccguuagcuuuagaagacaaaggauaguucuacgacgauuggguuucacaaccggcacaggagguuuuuauacauac---------------------
                                        * * ##* *  *   * **##* **#*** * | * ***#** *##** *   *  * *## * *
```

=== External intron ===

Multiple sequence alignment

```
Xmsuc0006B_fwrd    guaugugaaccauugauuuuaaaaacgucgccauuacgaucggguguaguucuagaaaguauauaauaagggacuggacuaac----------auguuuuua-----------ugugguag
Xmsuc0006B_RevC    cuaccaca-----------uaaaaacau----------guuaguccagucccuuauuauauacuuucuagaacuacaccgaucguaauggcgacguuuuuaaaaucaaugguucacauac
                    **#   *           *******#*           * #** #***#*#  *  #*||*#  *  #*#***# **# *           *#*******           *   #**
```

ALIGNMENT RESULTS - Xmsuc0009A

=== Stwintron Sequence ===

Multiple sequence alignment

```
Xmsuc0009A_fwrd     ------ggcaaguauagaauccucccgugcugguuaugaaacccgaucu---------------------------uccagagcuaacuagugucauaguaugugaaucauugaaccuaaaccgggccauuggaaccugugucuucccagacgguguacaacaa--ggaacuggacuaacguauucuuuuaugccag
Xmsuc0009A_RevC     cuggcauaaaagaauacguuaguccaguucc--uuguuguacaccgucugggaagacacagguuccaauggcccgguuuagguucaaugauucacauacuaugacacuaguuagcucugga---------------------------agaucggguuucauaaccagcacgggaggauucuauacuugcc------
                           # *** ***   *  *** ** *#  ** *   ** *  ***                           *  **   #**  * *  ****|****  * *  **#   **  *                           ***  * **   * **  #* ** ***  *   *** *** #
```

=== Internal intron ===

Multiple sequence alignment

```
Xmsuc0009A_fwrd     gcaaguauagaauccucccgugcugguuaugaaacccgaucuuccagagcuaacuagugucauag--------------------------
Xmsuc0009A_RevC     --------------------------cuaugacacuaguuagcucuggaagaucggguuucauaaccagcacgggaggauucuauacuugc
                                              #***** **  * *  # *|* #  * *  ** *****#
```

=== External intron ===

Multiple sequence alignment

```
Xmsuc0009A_fwrd     guaugugaaucauugaaccuaaaccgggccauuggaaccugugucuucccagacgguguacaacaaggaacuggacuaacguauucuuuuaugcc----------------------------------------------------ag
Xmsuc0009A_RevC     cu----------------------------------------------------ggcauaaaagaauacguuaguccaguuccuuguuguacaccgucugggaagacacagguuccaauggcccgguuuagguucaaugauucacauac
                     *                                                    **##** ** ** #   *#*|*#*   # ** ** **##**                                                    *
```

ALIGNMENT RESULTS - Xmsuc0018A

=== Stwintron Sequence ===

Multiple sequence alignment

```
Xmsuc0018A_fwrd     gguguguauaaaaaccuuuuugggcugccugugaaagccgaucuucgcaggaaucuuuauugucucaacaaagcuaacuggugucgcaguaugugagcuauuaaucuuaaaccaggccauua--cagccuguauauuucaagaaaguguacaacaaagacuuggagu-------------aacguguuuuuauauggcag
Xmsuc0018A_RevC     cugccauauaaaaa-------------cacguuacuccaagucuuuguuguacacuuucuugaaauauacaggcug--uaauggccugguuuaagauuaauagcucacauacugcgacaccaguuagcuuuguugagacaauaaagauuccugcgaagaucggcuuucacaggcagcccaaaaagguuuuuauacacacc
                        ##********             * #** *   * # **** *  * *  **** ***     *   * ***   *##** *   ** *# **   ||   ** #* **   * **##*   *** *   *    *** ****  * *  * **** # *   *             ** # *********##
```

=== Internal intron ===

Multiple sequence alignment

```
Xmsuc0018A_fwrd     gugug---uauaaaaaccuuuuugggcug---------ccugugaaagccgaucuucgcaggaaucuuuauugucucaacaaagcuaacuggugucgcag
Xmsuc0018A_RevC     cugcgacaccaguuagcuuuguugagacaauaaagauuccugcgaagaucggcuuucacagg---------cagcccaaaaagguuuuuaua---cacac
                     **#*  #     * * ** ***#* ##       ****#*** # || # ***#****       ## *#*** ** * *   #   *#**
```

=== External intron ===

Multiple sequence alignment

```
Xmsuc0018A_fwrd     guaugugagcuauuaaucuuaaaccaggccauuacagccuguauauuucaagaaaguguacaacaaagacuuggaguaacguguuuuuauauggcag
Xmsuc0018A_RevC     cugccauauaaaaacacguuacuccaagucuuuguuguacacuuucuugaaauauacaggcuguaauggccugguuuaagauuaauagcucacauac
                     * #   *   *  *# *** ***#* * **  *  ### * #**|**# * ### *  ** * *#*** *** #*   *  *   # *
```

ALIGNMENT RESULTS - Xmsuc0018B

=== Stwintron Sequence ===

Multiple sequence alignment

```
Xmsuc0018B_fwrd     gguauguauaaaaaccuccugugccggcugugaaacccaaucgucgcagaaauaucuu-----------uugucuucuagagcuaacgauuuucacaguagguaaaccauuaauucuaaaacauagcuaccacgaucaguguaguucuagaaaguauauaauagggaacucgacuaacauguuuuuaucuggcag
Xmsuc0018B_RevC     cugccagauaaaaacauguuagucgaguucccuauuauauacuuucuagaacuacacugaucgugguagcuauguuuuagaauuaaugguuuaccuacugugaaaaucguuagcucuagaagacaa-----------aagauauuucugcgacgauugggguuucacagccggcacaggagguuuuuauacauacc
                        ## ******** *  *#  * #* * #  *    *  * *   **** **# #*          #*#* ** ****# *** * *** *  *|*  * *** * *** #**** ** *#*#          *# #** ****   * *  *    *  # * *# *  #*  * ******** ##
```

=== Internal intron ===

Multiple sequence alignment

```
Xmsuc0018B_fwrd     ---------------------guauguauaaaaaccuccugugccggcugugaaacccaaucgucgcagaaauaucuuuugucuucuagagcuaacgauuuucacag
Xmsuc0018B_RevC     cugugaaaaucguuagcucuagaagacaaaagauauuucugcgacgauugggguuucacagccggcacaggagguuuuuauacauac---------------------
                                         * * ##* ** *   * ***#* **# ** * | * ** #** *#*** *   * ** *## * *
```

=== External intron ===

Multiple sequence alignment

```
Xmsuc0018B_fwrd     -------------guagguaaaccauuaauucuaaaacauagcuaccacgaucaguguaguucuagaaaguauauaauagggaacucgacuaacauguuuuuaucuggcag
Xmsuc0018B_RevC     cugccagauaaaaacauguuagucgaguucccuauuauauacuuucuagaacuacacugaucgugguagcuauguuuuagaauuaaugguuuaccuac-------------
                                 ##* ** *  *     ##***  * ***  * * * #*# * | * #*# * * *  *** *  ***##     *  * ** *##
```

ALIGNMENT RESULTS - Xmsuc0019A

=== Stwintron Sequence ===

Multiple sequence alignment

```
Xmsuc0019A_fwrd     ggcauguauaaaagccuccugugccggucgcgga--acccgauugucgcagaaau---------aucuugucaucuagagcuaacuauugucacaguaugugaaauaucgauucuaaaacguagcaucaagaccggaguaguucuaaagucguauaauacggaacuggauuaacauguuuuuauguggcag
Xmsuc0019A_RevC     cugccacauaaaaacauguuaauccaguuccguauuauacgacuuuagaacuacuccggucuugaugcuacguuuuagaaucgauauuucacauacugugacaauaguuagcucuagaugacaagau---------auuucugcgacaaucgggu--uccgcgaccggcacaggaggcuuuuauacaugcc
                        ###******#* *  *#  **#**  ** *  *  ***#* * * *  * *         ** #*##  * ****# # *   **  ** *|* **  **   * # #**** *  ##*# **         * *  * * * *#***  *  * **  **#**  #*  * *#******###
```

=== Internal intron ===

Multiple sequence alignment

```
Xmsuc0019A_fwrd     gcaug-------------------uauaaaagccuccugugccggucgcggaacccgauugucgcagaaauaucuugucaucuagagcuaacuauugucacag
Xmsuc0019A_RevC     cugugacaauaguuagcucuagaugacaagauauuucugcgacaaucggguuccgcgaccggcacaggaggcuuuuau-------------------acaugc
                       **                    *#** *   * ***#* *##*** * | * ***##* *#*** *   * **#*                    **
```

=== External intron ===

Multiple sequence alignment

```
Xmsuc0019A_fwrd     guaugugaaauaucgauucuaaaacguagcaucaagaccggaguaguucuaaagucguauaauacggaacuggauuaacauguuuuuauguggcag-------------------------
Xmsuc0019A_RevC     -------------------------cugccacauaaaaacauguuaauccaguuccguauuauacgacuuuagaacuacuccggucuugaugcuacguuuuagaaucgauauuucacauac
                                              *  **#  *#*   # ** # **#*   #*****|*****#   *#** # ** #   *#*  #**  *
```

ALIGNMENT RESULTS - Xmsuc0028A

=== Stwintron Sequence ===

Multiple sequence alignment

```
Xmsuc0028A_fwrd     ---------------------------------gguauguauaaaauccuccugugccaguugugaaaccca-auugucgcagaaauaucuuuugucuucuaaagcuaacgaauuucacaguaggugaaccauugauuuuauaauauggcuaucacgaucgguguaguuugagcaggaauauaauagggaacuagacuaacauguuguuaucuggcag
Xmsuc0028A_RevC     cugccagauaacaacauguuagucuaguucccuauuauauuccugcucaaacuacaccgaucgugauagccauauuauaaaaucaaugguucaccuacugugaaauucguuagcuuuagaagacaaaagauauuucugcgacaau-uggguuucacaacuggcacaggaggauuuuauacauacc---------------------------------
                                                     # ***#* #    **   **###** #*#**** * *** ***#* # *  ***   *# # * #* * **# * || * #** * *# * # #*   ***  * # *#*** *** * ****#*# **###**   **    # *#*** #
```

=== Internal intron ===

Multiple sequence alignment

```
Xmsuc0028A_fwrd     ---------------------guauguauaaaauccuccugugccaguugugaaacccaauugucgcagaaauaucuuuugucuucuaaagcuaacgaauuucacag
Xmsuc0028A_RevC     cugugaaauucguuagcuuuagaagacaaaagauauuucugcgacaauuggguuucacaacuggcacaggaggauuuuauacauac---------------------
                                         * * ##* ** **  * ***#* **#*** * | * ***#** *#*** *  ** ** *## * *
```

=== External intron ===

Multiple sequence alignment

```
Xmsuc0028A_fwrd     gua----------------------ggugaaccauugauuuuauaauauggcuaucacgaucgguguaguuugagcaggaauauaauagggaacuagacuaacauguuguuaucuggcag
Xmsuc0028A_RevC     cugccagauaacaacauguuagucuaguucccuauuauauuccugcucaaacuacaccgaucgugauagccauauuauaaaaucaaugguucacc----------------------uac
                     *                       #**   * ***#  **# *  *  ##***#  **||**  #***##  *  * #**  #*** *   **#                       *
```

ALIGNMENT RESULTS - Xmsuc0031A

=== Stwintron Sequence ===

Multiple sequence alignment

```
Xmsuc0031A_fwrd     ----------------gguauguauaaaaaaccuccugugccgguugugaaacccaaucgucgcagaaauaucuuuuaccuucuagagcuaacaauucccacaguaggugaaccauuguuucuaaaacaugaccaucac-----gaucgguggaguucuagaaaauauauaauagggaccuggacuaacacuuuuuuauauggguag
Xmsuc0031A_RevC     cuaccauauaaaaaaguguuaguccaggucccuauuauauauuuucuagaacuccaccg-----aucgugauggucauguuuuagaaacaaugguucaccuacugugggaauuguuagcucuagaagguaaaagauauuucugcgacgauuggguuucacaaccggcacaggagguuuuuauacauacc----------------
                                     **  ** #*    ***  *#*#    ** *# *** * *#**     #    *    *#*  ** ****# #**   ***  * *|*   * ***   **# #**** **  *#*     *         **#* * *** #* **    #*#*  ***    *# **  **
```

=== Internal intron ===

Multiple sequence alignment

```
Xmsuc0031A_fwrd     ---------------------guauguauaaaaaccuccugugccgguugugaaacccaaucgucgcagaaauaucuuuuaccuucuagagcuaacaauucccacag
Xmsuc0031A_RevC     cugugggaauuguuagcucuagaagguaaaagauauuucugcgacgauuggguuucacaaccggcacaggagguuuuuauacauac---------------------
                                         * * *** ** *   * ***#* **#*** * | * ***#** *#*** *   * ** *** * *
```

=== External intron ===

Multiple sequence alignment

```
Xmsuc0031A_fwrd     ---------------------------guaggugaaccauuguuucuaaaacaugaccaucacgaucgguggaguucuagaaaauauauaauagggaccuggacuaacacuuuuuuauaugguag
Xmsuc0031A_RevC     cuaccauauaaaaaaguguuaguccaggucccuauuauauauuuucuagaacuccaccgaucgugauggucauguuuuagaaacaaugguucaccuac--------------------------
                                               **   *#    **  ****** *** # ***    |    *** # *** ******  **    #*   **
```

ALIGNMENT RESULTS - Xmsuc0045A

=== Stwintron Sequence ===

Multiple sequence alignment

```
Xmsuc0045A_fwrd     -gguacguauaaaaucucccgugcuggucgugaaucacgaucuucgcaaaaaauaucuauuaucauucagagcuaacuaguaucacaguauaugaacucuuaguccccaaacuggaccauugg---guccuguguauuucuagagguguacagcaagaaacuggccuaac-----------guguuuuuauaauggcag
Xmsuc0045A_RevC     cugccauuauaaaaac-----------acguuaggccaguuucuugcuguacaccucuagaaauacacaggac---ccaaugguccaguuuggggacuaagaguucauauacugugauacuaguuagcucugaaugauaauagauauuuuuugcgaagaucgugauucacgaccagcacgggagauuuuauacguacc-
                      *#   ******* *            *** *  *  * * #* **   * *# ****  *  *# *** #*   *#*#*    **** *  * *** | *** *  * ****    *#*#*   *# *** #*  *  **** #* *   ** *# * *  *  * **           * * *******   #*
```

=== Internal intron ===

Multiple sequence alignment

```
Xmsuc0045A_fwrd     gu-----------------------acguauaaaaucucccgugcuggucgugaaucacgaucuucgcaaaaaauaucuauuaucauucagagcuaacuaguaucacag
Xmsuc0045A_RevC     cugugauacuaguuagcucugaaugauaauagauauuuuuugcgaagaucgugauucacgaccagcacgggagauuuu-----------------------auacguac
                     *                       * #    * ** *   *#*  *#******|******#*  *#*   * ** *                           *  *
```

=== External intron ===

Multiple sequence alignment

```
Xmsuc0045A_fwrd     ------guauaugaacucuuaguccccaaacuggaccauuggguccuguguauuucuagagguguacagcaagaaacuggcc----------uaacguguuuuuauaauggcag
Xmsuc0045A_RevC     cugccauuauaaaaacacgu----------uaggccaguuucuugcuguacaccucuagaaauacacaggacccaaugguccaguuuggggacuaagaguucauauac------
                           **** #*** * *            ** *  **   * ****##*##**||**##*##**** *   **  * **          # * * ***# ****
```

ALIGNMENT RESULTS - Xmsuc0067A

=== Stwintron Sequence ===

Multiple sequence alignment

```
Xmsuc0067A_fwrd     ------------ggugugu aacgaaucccgugguacuagcuaugagaacugaguuugguaaaaaguuu-cauugucugccauagcuaacuucugccacaguauguaaacucaugaucuaauu---uccugaaaauauguaauacgaaaccagacuaacaguuguguuuauuag
Xmsuc0067A_RevC     cuaauaaacacaacuguuagucugguuucguauuacauauuuucagga---aauuagaucaugaguuuacauacuguggcagaaguuagcuauggcagacaaug-aaacuuuuuaccaaacucaguucucauagcuaguaccacgggauucguuacacacc------------
                                # ***    *   *  ***# ***  # * * ** *   *#** *#* *  ***** ***  * ** ** *# *|* #* ** ** *  *** *****  * *#* **#*   * ** * * #  *** #***  *   *    *** #
```

=== Internal intron ===

Multiple sequence alignment

```
Xmsuc0067A_fwrd     guguguaacgaaucccgugguacuagcuaugagaacugaguuugguaaaaaguuucauugucugccauagcuaacuucugc-----------------------cacag
Xmsuc0067A_RevC     cugug-----------------------gcagaaguuagcuauggcagacaaugaaacuuuuuaccaaacucaguucucauagcuaguaccacgggauucguuacacac
                     ****                        ## #*  *#  * ***#* * *#* | *#* * *#*** *  #*  *# ##                        ****
```

=== External intron ===

Multiple sequence alignment

```
Xmsuc0067A_fwrd     guaugua----aacucaugaucuaauuuccugaaaauauguaauacgaaaccagacuaacaguuguguuuauuag
Xmsuc0067A_RevC     cuaauaaacacaacuguuagucugguuucguauuacauauuuucaggaaauuagaucaugaguu----uacauac
                     **   *    ****  *# ***  **** *#  *  |  *  #* ****  *** #*  ****    *   **
```

ALIGNMENT RESULTS - Xmsuc0070A

=== Stwintron Sequence ===

Multiple sequence alignment

```
Xmsuc0070A_fwrd     ggucuguauaaaaccuccugugccgguu-----------------------gugaaacccgaucgucgcggaaauaccuugugucuucuagagcuaacgauucucacaguaugugaaccauugauucuaaaa--------------cuauggcuauuacgauccguguaguucaaguauauaauagagaacucgacuaacauguuuuuauguggcag
Xmsuc0070A_RevC     cugccacauaaaaacauguuagucgaguucucuauuauauacuugaacuacacggaucguaauagccauag--------------uuuuagaaucaaugguucacauacugugagaaucguuagcucuagaagacacaagguauuuccgcgacgaucggguuucac-----------------------aaccggcacaggagguuuuauacagacc
                       *###****** *   *  * **# *                       ##* * *  #** *#*# #*              ** ****# #** * *** ** *|* ** *** * **# #**** **              *# #*#* **#  * * *##                       * #** *  *   * ******###*
```

=== Internal intron ===

Multiple sequence alignment

```
Xmsuc0070A_fwrd     gucuguauaaaaccuccugugccgguugugaaacccgaucgucgcggaaauaccuugugucuucuagagcuaacgauucucacag-------------------------
Xmsuc0070A_RevC     -------------------------cugugagaaucguuagcucuagaagacacaagguauuuccgcgacgaucggguuucacaaccggcacaggagguuuuauacagac
                                             #***** *  ** * *#   #***    *||*    ***#   #* * **  * *****#
```

=== External intron ===

Multiple sequence alignment

```
Xmsuc0070A_fwrd     guaugugaaccauugauucuaaaacuauggcuauuacgauccguguaguucaaguauauaauagagaacucgacuaacauguuuuua-----------uguggcag
Xmsuc0070A_RevC     cugccaca-----------uaaaaacauguuagucgaguucucuauuauauacuugaacuacacggaucguaauagccauaguuuuagaaucaaugguucacauac
                     * #   *           ***** #***    *#  * **  *#* #*  *||*  *# *#*  ** *  #*    ***# *****           *   # *
```

ALIGNMENT RESULTS - Xmsuc0075A

=== Stwintron Sequence ===

Multiple sequence alignment

```
Xmsuc0075A_fwrd     gguauguaauaaaaccucuuguaccgaucuugagacccagugucgauagaagaacccauuaucugucagagcugacuguuauucuaguauguagaccuggcccgagcacaaguacuaugacuugugccauugggugaacuacgugaaaaggaaguuguc------------uaacgcgcccugcuauc----------------------------------------uuag
Xmsuc0075A_RevC     cuaa----------------------------------------gauagcagggcgcguua------------gacaacuuccuuuucacguaguucacccaauggcacaagucauaguacuugugcucgggccaggucuacauacuagaauaacagucagcucugacagauaauggguucuucuaucgacacugggucucaagaucgguacaagagguuuuauuacauacc
                       *                                        ***** **  * * ***            *** ##* ## *  #*#****  *   *    ********  ||  ********    *   *  ****#*#  * ## *## ***            *** * *  ** *****                                        *
```

=== Internal intron ===

Multiple sequence alignment

```
Xmsuc0075A_fwrd     gu-------------------auguaauaaaaccucuuguaccgaucuugagacccagugucgauagaagaacccauuaucugucagagcugacuguuauucuag
Xmsuc0075A_RevC     cuagaauaacagucagcucugacagauaauggguucuucuaucgacacugggucucaagaucgguacaagagguuuuauuacau-------------------ac
                     *                   *## *  *     **** ** ***# #** *|* **# #*** ** ****     *  * ##*                   *
```

=== External intron ===

Multiple sequence alignment

```
Xmsuc0075A_fwrd     gu----------------------------------auguagaccuggcccgagcacaaguacuaugacuugugccauugggugaacuacgugaaaaggaaguugucuaacgcgcccugcuaucuuag
Xmsuc0075A_RevC     cuaagauagcagggcgcguuagacaacuuccuuuucacguaguucacccaauggcacaagucauaguacuugugcucgggccaggucuacau----------------------------------ac
                     *                                  *#****  *   *    ********  ||  ********    *   *  ****#*                                  *
```

ALIGNMENT RESULTS - Xmsuc0077A

=== Stwintron Sequence ===

Multiple sequence alignment

```
Xmsuc0077A_fwrd     gguauguauaaaaacauccuaugccggcugugaaacccgaucguggcagaaaua-------------uccuuugucuucuaaagcuaacgguuuucgcaguaugugaaccauugauuuuaaaagacauggcgauuacgaucgguguaguucuagaaaguauauaauaagggacuggacuaacauguuuuuaugugguag
Xmsuc0077A_RevC     cuaccacauaaaaacauguuaguccagucccuuauuauauacuuucuagaacuacaccgaucguaaucgccaugucuuuuaaaaucaaugguucacauacugcgaaaaccguuagcuuuagaagaca-------------aaggauauuucugccacgaucggguuucacagccggcauaggauguuuuuauacauacc
                        ###**********  **  **#* # #  *    #  * *   **** **             # *# ****** ****# #** ****# *# *|* #* #**** **# #**** ******              #* #** ****   * *  #    *  # # *#**  **  **********###
```

=== Internal intron ===

Multiple sequence alignment

```
Xmsuc0077A_fwrd     guauguauaaaaacauccuaugccggcugugaaacccgaucguggcagaaauauccuuugucuucuaaagcuaacgguuuucgcag--------------------------
Xmsuc0077A_RevC     --------------------------cugcgaaaaccguuagcuuuagaagacaaaggauauuucugccacgaucggguuucacagccggcauaggauguuuuuauacauac
                                              ***#**** *** * *#   ****     ||     ****   #* * *** ****#***
```

=== External intron ===

Multiple sequence alignment

```
Xmsuc0077A_fwrd     --------------------------guaugugaaccauugauuuuaaaagacauggcgauuacgaucgguguaguucuagaaaguauauaauaagggacuggacuaacauguuuuuaugugguag
Xmsuc0077A_RevC     cuaccacauaaaaacauguuaguccagucccuuauuauauacuuucuagaacuacaccgaucguaaucgccaugucuuuuaaaaucaaugguucacauac--------------------------
                                              ** # * *     *# ***# * *#  *## ****#||#**** ##*  #* * #*** #*     * * # **
```

ALIGNMENT RESULTS - Xmsuc0082A

=== Stwintron Sequence ===

Multiple sequence alignment

```
Xmsuc0082A_fwrd     gguauguauaaaaaccuccugugccgguugugaaac--------------------ccaaucgucacagaaauaucu-----------uuugccuucuacagcuaacaauucccacaguaggugaaccauuguuucuaaacauaaccaucacgaucgguggaggucugggaaauauauaauagggaacugggcuaacaccuuuuuauauagguag
Xmsuc0082A_RevC     cuaccauauaaaaagguguuagcccaguucccuauuauauauuucccagaccuccaccgaucgugaugguuauguuuagaaacaaugguucaccuacugugggaauuguuagcuguagaaggcaaa-----------agauauuucugugacgauugg--------------------guuucacaaccggcacaggagguuuuuauacauacc
                        ##********  *  *#  **#*** #  *                      ** ***** *  *  ** * *          **##*** **   *  * || *  *   ** ***##**          * * **  *  * ***** **                    *  # ***#**  #*  *  ********##
```

=== Internal intron ===

Multiple sequence alignment

```
Xmsuc0082A_fwrd     ---------------------guauguauaaaaaccuccugugccgguugugaaacccaaucgucacagaaauaucuuuugccuucuacagcuaacaauucccacag
Xmsuc0082A_RevC     cugugggaauuguuagcuguagaaggcaaaagauauuucugugacgauuggguuucacaaccggcacaggagguuuuuauacauac---------------------
                                         * * *#* ** *   * ***** **#*** * | * ***#** ***** *   * ** *#* * *
```

=== External intron ===

Multiple sequence alignment

```
Xmsuc0082A_fwrd     guaggugaaccauuguuucuaaacauaaccaucacgaucgguggaggucugggaaauauauaauagggaacugggcuaacaccuuuuuauauggu----------------------------------------------------ag
Xmsuc0082A_RevC     cu----------------------------------------------------accauauaaaaagguguuagcccaguucccuauuauauauuucccagaccuccaccgaucgugaugguuauguuuagaaacaaugguucaccuac
                     *                                                    * #****** *#**   *#*|*#*   **#* ******# *                                                    *
```

ALIGNMENT RESULTS - Xmsuc0086A

=== Stwintron Sequence ===

Multiple sequence alignment

```
Xmsuc0086A_fwrd     ggucuguauaaacaccuccugugccgguu--gugagaccggaucguagcggaaauaccuuuugu----------cuucuagagcuaacgaguuuuauaguauguaaauuguugauucuaaaaacauagcuaccaugaacgguguaguuuuugaaaguauaucauaggaaacuugacuaacacguuuuucugugguag
Xmsuc0086A_RevC     cuaccacagaaaaacguguuagucaaguuuccuaugauauacuuucaaaaacuacaccguucaugguagcuauguuuuuagaaucaacaauuuacauacuauaaaacucguuagcucuagaag----------acaaaagguauuuccgcuacgauccggucuca--caaccggcacaggagguguuuauacagacc
                       *###* *** ** *  *#  * #***   *# **   # *  #*# ##  *#*** **##*           ** ****# #***#* ** #***|***# ** *#***# #**** **           *##** ***#*  ## #*#  * #   ** #*   ***# *  #*  * ** *** *###*
```

=== Internal intron ===

Multiple sequence alignment

```
Xmsuc0086A_fwrd     gucuguauaaacaccuccugugccgguugugagaccggaucguagcggaaauaccuuuugucuucuagagcuaacgaguuuuauag----
Xmsuc0086A_RevC     ----cuauaaaacucguuagcucuagaagacaaaagguauuuccgcuacgauccggucucacaaccggcacaggagguguuuauacagac
                         ******   *    *# * #*  *  *#*  * **  # || #  ** *  *#*  *  *# * #*    *   ******
```

=== External intron ===

Multiple sequence alignment

```
Xmsuc0086A_fwrd     gua---------------------------uguaaauuguugauucuaaaaacauagcuaccaugaacgguguaguuuuugaaaguauaucauaggaaacuugacuaacacguuuuucugugguag
Xmsuc0086A_RevC     cuaccacagaaaaacguguuagucaaguuuccuaugauauacuuucaaaaacuacaccguucaugguagcuauguuuuuagaaucaacaauuua---------------------------cauac
                     **                           # **   *#*   *** ****  *#* *   *||*   * *#*  **** ***   *#*   **                            #**
```

ALIGNMENT RESULTS - Xmsuc0086B

=== Stwintron Sequence ===

Multiple sequence alignment

```
Xmsuc0086B_fwrd     gguaugcacaaaaaccccuauagcgguugugaaacccgaucguagcagagguuaccuuuugucuucuagagcuaacuagugccauaguacgugagccguugauucuaaaacuuggucauuaugaccggcguuguucuagacuaacauguuuuauacgguag-------------
Xmsuc0086B_RevC     -------------cuaccguauaaaacauguuagucuagaacaacgccggucauaaugaccaaguuuuagaaucaacggcucacguacuauggcacuaguuagcucuagaagacaaaagguaaccucugcuacgaucggguuucacaaccgcuauaggggguuuuugugcauacc
                                    ** ****# #  *** *  *  ** *#  ** *    **    ###  ** ****# #***   *  * **|** *  *   ***# #**** **  ###    **    * **  #* **  *  * ***  # #**** **
```

=== Internal intron ===

Multiple sequence alignment

```
Xmsuc0086B_fwrd     guaug----------------------cacaaaaaccccuauagcgguugugaaacccgaucguagcagagguuaccuuuugucuucuagagcuaacuagugccauag
Xmsuc0086B_RevC     cuauggcacuaguuagcucuagaagacaaaagguaaccucugcuacgaucggguuucacaaccgcuauaggggguuuuugu----------------------gcauac
                     ****                       * *   * ** ** # #**#*#* * | * *#*#**# # ** ** *   * *                       ****
```

=== External intron ===

Multiple sequence alignment

```
Xmsuc0086B_fwrd     guacgugagccguugauucuaaaacuuggucauuaugaccggcguuguucuagacuaacauguuuuaua-----------cgguag
Xmsuc0086B_RevC     cuac-----------cguauaaaacauguuagucuagaacaacgccggucauaaugaccaaguuuuagaaucaacggcucacguac
                     ***             * ****** ** *  *#  ** *##||##* **  #*  * ** ****** *             ***
```

ALIGNMENT RESULTS - Xmsuc0098A

=== Stwintron Sequence ===

Multiple sequence alignment

```
Xmsuc0098A_fwrd     -----------------ggcauguauaauaaccuccugugccgguugugaaac----ccgaucgucgcgggaauaucuuuuguguucuagagcuaacgauuuuuacaguaugugaaccauugauucuaaaaaauauggcuaucacgauugguguagcucuagaaaguauauaauagagaacuggacuuaacauguuuuuaaacggcag
Xmsuc0098A_RevC     cugccguuuaaaaacauguuaaguccaguucucuauuauauacuuucuagagcuacaccaaucgugauagccauauuuuuuagaaucaaugguucacauacuguaaaaaaucguuagcucuagaacacaaaagauauucccgcgacgaucgg----guuucacaaccggcacaggagguuauuauacaugcc-----------------
                                     *  * ** #* *   **  *#*#    ** *# * *    **#***** # #*  **** ****# # ** *  * * **#  #* || *#  #** * *  * ** # #**** ****  *# # *****#**    * * #* **    #*#*  **   * *# ** *  *
```

=== Internal intron ===

Multiple sequence alignment

```
Xmsuc0098A_fwrd     ---------------------gcauguauaauaaccuccugugccgguugugaaacccgaucgucgcgggaauaucuuuuguguucuagagcuaacgauuuuuacag
Xmsuc0098A_RevC     cuguaaaaaucguuagcucuagaacacaaaagauauucccgcgacgaucggguuucacaaccggcacaggagguuauuauacaugc---------------------
                                         * *###* ** *   ***#*#* **#*#* * | * *#*#** *#*#***   * ** *###* *
```

=== External intron ===

Multiple sequence alignment

```
Xmsuc0098A_fwrd     guaugugaaccauugauucuaaaaaauauggcuaucacgauugguguagcucuagaaaguauauaauagagaacuggacuuaacaug----------uuuuuaaa-----------cggcag
Xmsuc0098A_RevC     cugc-----------cguuuaaaaa----------cauguuaaguccaguucucuauuauauacuuucuagagcuacaccaaucgugauagccauauuuuuuagaaucaaugguucacauac
                     * #             * ******          ** * * #** #** ***  *  #*||*#  *  *** **# **# * * **          ****** *             # *
```

ALIGNMENT RESULTS - Xmsuc0110A

=== Stwintron Sequence ===

Multiple sequence alignment

```
Xmsuc0110A_fwrd     ggua-----------------------uguaucaaaaccugcuguaccguugugagaccugucauuggcagaaagaucuacuguuucccagaacuaaucaaugcuacaguauguguccauugucgaguuga-------ugcaggaacguggcuguuauggccagug--------uaccucuggaaagcucauguugagaaacuggacuaacgugugguuauauguuag
Xmsuc0110A_RevC     cuaacauauaaccacacguuaguccaguuucucaacaugagcuuuccagaggua--------cacuggccauaacagccacguuccugc-------aucaacucgacaauggacacauacuguagcauugauuaguucugggaaacaguagaucuuucugccaaugacaggucucacaacgguacagcagguuuugauaca-----------------------uacc
                       *                       * * **** *   *** * * *  **#        **#**** # ** * *#**  *##  *       *****# * ***#*  #||#  *#*** * #****       **  ##*  **#* * ** # ****#**        #**  * * * ***   * **** * *                       *
```

=== Internal intron ===

Multiple sequence alignment

```
Xmsuc0110A_fwrd     guau-guaucaaaaccugcuguaccguugugagaccugucauuggcagaaagaucuacuguuucccagaacuaaucaaugcuacag
Xmsuc0110A_RevC     cuguagcauugauuaguucugggaaacaguagaucuuucugccaaugacaggucucacaacgguacagcagguuuugauac-auac
                     * * *#**  *    * ***    ## **# # * * #  #||#  # * * # #** ##    *** *    *  **#* * *
```

=== External intron ===

Multiple sequence alignment

```
Xmsuc0110A_fwrd     ---guauguguccauugucgaguuga------------------ugcaggaacguggcuguuauggccaguguaccucuggaaagcucauguugagaaacuggacuaacgugugguuauauguuag
Xmsuc0110A_RevC     cuaacauauaaccacacguuaguccaguuucucaacaugagcuuuccagagguacacuggccauaacagccacguuccugca------------------ucaacucgacaauggacacauac---
                       ##**#*# ***#     ***# *                  * ***#   ###   *##||##*   ###   #*** *                  * #***     #*** #*#**##
```

ALIGNMENT RESULTS - Xmsuc0111A

=== Stwintron Sequence ===

Multiple sequence alignment

```
Xmsuc0111A_fwrd     gguaugcauaaaaaccuccugugccgguuguaaaacccgaucgucgcggaaauaccuu-----------uugucuucuggagcugacgauuuuuauaguaggugaaccaucgauucuaaaacauggcuaucacgaucgguguaguucuagaaaguauauaauagggaacuggacuaacauguuuuuaucuggcag
Xmsuc0111A_RevC     cugccagauaaaaacauguuaguccaguucccuauuauauacuuucuagaacuacaccgaucgugauagccauguuuuagaaucgaugguucaccuacuauaaaaaucgucagcuccagaagacaa-----------aagguauuuccgcgacgaucggguuuuacaaccggcacaggagguuuuuaugcauacc
                        ## ******** *  *#  **#*** #  *    #  * *   #*** *** ##           ###* ** *#**# #** * **# # **|** # #** * **# #**#* ** *###           ## *** ***#   * *  #    *  # ***#**  #*  * ******** ##
```

=== Internal intron ===

Multiple sequence alignment

```
Xmsuc0111A_fwrd     guaugcauaaaaaccuccugugccgguuguaaaacccgaucgucgcggaaauaccuuuugucuucuggagcugacgauuuuuau----ag
Xmsuc0111A_RevC     cu----auaaaaaucgucagcuccagaagacaaaagguauuuccgcgacgaucggguuuuacaaccggcacaggagguuuuuaugcauac
                     *    ******* *  * *# **#*  *  ***    **  #*||*#  **    ***  *  *#** #* *  * *******    *
```

=== External intron ===

Multiple sequence alignment

```
Xmsuc0111A_fwrd     guaggugaaccaucgauucuaaaacauggcuaucacgaucgguguaguucuagaaaguauauaauagggaacuggacuaa----------cauguuuuua----------ucuggcag
Xmsuc0111A_RevC     cug----------ccagauaaaaacaug----------uuaguccaguucccuauuauauacuuucuagaacuacaccgaucgugauagccauguuuuagaaucgaugguucaccuac
                     *           * *    ********          * #** #*****#  *  #*||*#  *  #*****# **# *          ********            **    *
```

ALIGNMENT RESULTS - Xmsuc0114A

=== Stwintron Sequence ===

Multiple sequence alignment

```
Xmsuc0114A_fwrd     ----------gguacguaucaaaccaguccuuauugugagauauaggcaaggccguucagugcuaacucaaccacuacaguacguccucuuugaccucaauagggggccgucauuguccagggaguucuggaauuccaggucugca------------gacuagacuaacauguuuucauguggcag
Xmsuc0114A_RevC     cugccacaugaaaacauguuagucuaguc------------ugcagaccuggaauuccagaacucccuggacaaugacggcccccuauugaggucaaagaggacguacuguagugguugaguuagcacugaacggccuugccuauaucucacaauaaggacugguuugauacguacc----------
                              ## **#* * *  * ****            * #**#*  **   *#*** #**  **  ** *  ** *# * #  *    *|*    *  # * #* **  * **  **  **# ***#*   **  *#**# *            **** *  * * *#** ##
```

=== Internal intron ===

Multiple sequence alignment

```
Xmsuc0114A_fwrd     guacguaucaaaccaguccuuauugugagauauaggcaaggccguucagugcuaacucaaccacuac--ag
Xmsuc0114A_RevC     cu--guagugguugaguuagcacugaacggccuugccuauaucucacaauaaggacugguuugauacguac
                     *  ***       ***   #*#** # * # * *|* * # * # **#*#   ***       ***  *
```

=== External intron ===

Multiple sequence alignment

```
Xmsuc0114A_fwrd     guacguccucuuugaccucaauagggggccgucauuguccag---ggaguucuggaauuccaggucugcagacuagacuaacauguuuucauguggcag---------------
Xmsuc0114A_RevC     ---------------cugccacaugaaaacauguuagucuagucugcagaccuggaauuccagaacucc---cuggacaaugacggcccccuauugaggucaaagaggacguac
                                   *  * *#* *### *#*  * *** **   * ** #*****||*****# ** *   ** *** *  *#* ###* *#* *  *
```

ALIGNMENT RESULTS - Xmsuc0121A

=== Stwintron Sequence ===

Multiple sequence alignment

```
Xmsuc0121A_fwrd     ggcauguauaaaaaaccuccugugccgguugcguaacccgaccgucgcagaaauauuuuuuuu-------ucgucuuccugagcuaacuugucucacaguaugugaaccguugauuauaaaacccggccaucacaaccgguguaguucuggaaagcaggcaacaaggaacugggcuaacauguuuuuauauggcag
Xmsuc0121A_RevC     cugccauauaaaaacauguuagcccaguuccuuguugccugcuuuccagaacuacaccgguugugauggccggguuuuauaaucaacgguucacauacugugagacaaguuagcucaggaagacga-------aaaaaaaauauuucugcgacggucggguuacgcaaccggcacaggagguuuuuauacaugcc
                        ##********* *  *#  **#*** * *    *   * *  ***** **# ##  **       #**  **    *# #***   ** ** *|* ** **   ***# #*    **  **#       **  ## #** *****  * *   *    * * ***#**  #*  * *********##
```

=== Internal intron ===

Multiple sequence alignment

```
Xmsuc0121A_fwrd     -------------------------gcauguauaaaaaccuccugugccgguugcguaacccgaccgucgcagaaauauuuuuuuucgucuuccugagcuaacuugucucacag
Xmsuc0121A_RevC     cugugagacaaguuagcucaggaagacgaaaaaaaauauuucugcgacggucggguuacgcaaccggcacaggagguuuuuauacaugc-------------------------
                                             #*  # * ****   * ***#* ****#* **|** *#**** *#*** *   **** * #  *#
```

=== External intron ===

Multiple sequence alignment

```
Xmsuc0121A_fwrd     -------------guaugugaaccguugauuauaaaacccggccaucacaaccgguguaguucuggaaagcaggcaacaaggaacugggcuaacauguuuuuauauggcag
Xmsuc0121A_RevC     cugccauauaaaaacauguuagcccaguuccuuguugccugcuuuccagaacuacaccgguugugauggccggguuuuauaaucaacgguucacauac-------------
                                 ##**** * **     ## *    ** *    #** *** # | # *** **#    * **    * ##     ** * ****##
```

ALIGNMENT RESULTS - Xmsuc0131A

=== Stwintron Sequence ===

Multiple sequence alignment

```
Xmsuc0131A_fwrd     gguauguaugaaaaccuccugugccgguugugaaacgcaaucgucgcagaaau------------------auccuuugccuucuagagcuaacuuauuuuuacaguaggugaacgauugauucuaaaacauggcuaucaugaucgguggaguucuagaaaauauauaauagggaacugggcuaacauguuuucaucuggcag
Xmsuc0131A_RevC     cugccagaugaaaacauguuagcccaguucccuauuauauauuuucuagaacuccaccgaucaugauagccauguuuuagaaucaaucguucaccuacuguaaaaauaaguuagcucuagaaggcaaaggau------------------auuucugcgacgauugcguuucacaaccggcacaggagguuuucauacauacc
                        ## ******** *  *#  **#*** #  *  # *    *   **** *                  **  ***#   ** *  * * **#**#* *|* *#**#** * *  * **   #***  **                  * ****   *    * #  *  # ***#**  #*  * ******** ##
```

=== Internal intron ===

Multiple sequence alignment

```
Xmsuc0131A_fwrd     ----------------------guauguaugaaaaccuccugugccgguugugaaacgcaaucgucgcagaaauauccuuugccuucuagagcuaacuuauuuuuacag
Xmsuc0131A_RevC     cuguaaaaauaaguuagcucuagaaggcaaaggauauuucugcgacgauugcguuucacaaccggcacaggagguuuucauacauac----------------------
                                          * * *#* #  *   * ***#* **#***#* | *#***#** *#*** *   *  # *#* * *
```

=== External intron ===

Multiple sequence alignment

```
Xmsuc0131A_fwrd     guaggugaacgauugauucuaaaacauggcuaucaugaucgguggaguucuagaaaauauauaauagggaacugggcuaa----------cauguuuuca----------ucuggcag
Xmsuc0131A_RevC     cugccaga----------ugaaaacaug----------uuagcccaguucccuauuauauauuuucuagaacuccaccgaucaugauagccauguuuuagaaucaaucguucaccuac
                     *    **            ********          * #*#  *****#  *  **||**  *  #*****  #*# *          ********            **    *
```

ALIGNMENT RESULTS - Xmsuc0137A

=== Stwintron Sequence ===

Multiple sequence alignment

```
Xmsuc0137A_fwrd     gguacguauauaggccucccaugcuuguugucaaaccccaucuucgcaaaaauaccuauugucuuccauaacuaacuaauuucccaguacgugaauuauugaucuugaaaccgguuuaucacaa-----ccucuauauuuccaggaaguuuacaacgaggaacuagacuaaagugcuu-uguauggcag
Xmsuc0137A_RevC     cugccauaca-aagcacuuuagucuaguuccucguuguaaacuuccuggaaaua-----uagagguugugauaaaccgguuucaagaucaauaauucacguacugggaaauuaguuaguuauggaagacaauagguauuuuugcgaagaugggguuugacaacaagcaugggaggccuauauacguacc
                        *#**#* *#** #   *  ** *** #        * ****    *****     *#       * *  ***#  ****   #*  #*#*|*#*#  *#   ****  #***  * *          *   *****    **** *        # *** **  *   # **#* *#**#*
```

=== Internal intron ===

Multiple sequence alignment

```
Xmsuc0137A_fwrd     ------------guacguauauaggccucccaugcuuguugucaaaccccaucuucgcaaaaauaccuauugucuuccauaacuaacuaauuucccag
Xmsuc0137A_RevC     cugggaaauuaguuaguuauggaagacaauagguauuuuugcgaagaugggguuugacaacaagcaugggaggccuauauacguac------------
                                 **  ***  *#* *        ** ***# **   ||   ** #*** **        * *#*  ***  **
```

=== External intron ===

Multiple sequence alignment

```
Xmsuc0137A_fwrd     -----guacgugaauuauugaucuugaaaccgguuuaucacaaccucuauauuuccaggaaguuuacaacgaggaacuagacuaaagugcuuuguauggcag
Xmsuc0137A_RevC     cugccauacaaagcacuuuagucuaguuccucguuguaaacuuccuggaaauauagagguugugauaaaccgguuucaagaucaauaauucacguac-----
                         #***# #   # **# *** *   *  ***    **  ***  * || *  ***  **    ***  *   * *** #** #   # #***#
```

ALIGNMENT RESULTS - Xmsuc0141A

=== Stwintron Sequence ===

Multiple sequence alignment

```
Xmsuc0141A_fwrd     -gguacguaucaagaaccugu------------------------------------------------uaugccuaucaaugaaaaggcgcuucuuaucugccaaugcuaacuguugucacaguacgugaacccuuugaugcagaaacguggucaccauaaccuauagaaaucugggcuaguaugccaucaagaaccguacuaacguguuauauguauag
Xmsuc0141A_RevC     cuauacauauaacacguuaguacgguucuugauggcauacuagcccagauuucuauagguuauggugaccacguuucugcaucaaaggguucacguacugugacaacaguuagcauuggcagauaagaagcgccuuuucauugauaggcaua------------------------------------------------acagguucuugauacguacc-
                      #***#*** * #     **                                                #*#*  * *  ** *** **  * # *  * ** ***## *|* ##*** ** *  * # *  ** *** **  * *  *#*#                                                **     # * ***#***#
```

=== Internal intron ===

Multiple sequence alignment

```
Xmsuc0141A_fwrd     guacguaucaagaaccuguuaugccuaucaaugaaaaggcgcuucuuaucugccaaugcuaacuguugu------cacag
Xmsuc0141A_RevC     cugug------acaacaguuagcauuggcagauaagaagcgccuuuucauugauaggcauaacagguucuugauacguac
                     *  *      # * * ****    *  **   ** *#*||*#* **   **  *    **** * * #      *  *
```

=== External intron ===

Multiple sequence alignment

```
Xmsuc0141A_fwrd     guacgugaacccuuugaugcagaaacguggucaccauaaccuauagaaaucugggcuaguaugccaucaagaaccguacuaacguguu-----------auauguauag
Xmsuc0141A_RevC     cuauacauau-----------aacacguuaguacgguucuugauggcauacuagcccagauuucuauagguuauggugaccacguuucugcaucaaaggguucacguac
                     ** ### *            #* **** #  **  *     ** * *  **#*|*#**  * * **     *  **  # **** *#            * ### **
```

ALIGNMENT RESULTS - Xmsuc0143A

=== Stwintron Sequence ===

Multiple sequence alignment

```
Xmsuc0143A_fwrd     ------gguaugucacgaaaccucccgugcugggcgugaaauucgauccucgcggagauauccuuuaucuuccaaagcugacgaguguugcaguaugu aaacuauuuaucuaaagcuuuuccauuguaac-cuguuuaucucuagcaaguauauaac--------------------------------caggaagugggcuaacuuauggcgcag
Xmsuc0143A_RevC     cugcgccauaaguuagcccacuuccug--------------------------------guuauauacuugcuagagauaaacag-guuacaauggaaaagcuuuagauaaauaguuuacauacugcaacacucgucagcuuuggaagauaaaggauaucuccgcgaggaucgaauuucacgcccagcacgggagguuucgugacauacc------
                           #** ** *    ** *** *                                 *  * **# * * * ** *#*  ** ***#**#*  # ** ** *  ||  * ** ** #  *#**#*** **  *#* ** * * * #** *  *                               * *** **    * ** **#
```

=== Internal intron ===

Multiple sequence alignment

```
Xmsuc0143A_fwrd     guaugucacgaaaccucccgugcugggcgugaaauucgauccucgcggagauauccuuuaucuuccaaagcugacga----guguugcag
Xmsuc0143A_RevC     cugcaacac----ucgucagcuuuggaagauaaaggauaucuccgcgaggaucgaauuucacgcccagcacgggagguuucgugacauac
                     * ## ***     *  * *#  ***# *  ***    *** #*||*# ***    ***  * #***  #* *  *     *** ## *
```

=== External intron ===

Multiple sequence alignment

```
Xmsuc0143A_fwrd     guauguaaacuauuuaucuaaagcuuuuccauuguaaccuguuuaucucuagcaaguauauaaccaggaagugggcuaacuua------uggcgcag
Xmsuc0143A_RevC     cugcgcca------uaaguuagcccacuuccugguuauauacuugcuagagauaaacagguuacaauggaaaagcuuuagauaaauaguuuacauac
                     * #*# *      **  * *  *# #* * * ** *  *##** #  |  # **##*  * ** * * *# #*  * *  **      * #*# *
```

ALIGNMENT RESULTS - Xmsuc0146A

=== Stwintron Sequence ===

Multiple sequence alignment

```
Xmsuc0146A_fwrd     gguauguauaaaaaccucccgugccaguugugaaacccgaucgucccagaaauaua--------gcuuuuuaucuucuagagcuaaugauuguuacaguacgugaaccauuuauucuaaaacaugacuaucaagaccaguguaguucuagaaaguauauaacaaggaacuggacuaacauaguuucauggguag
Xmsuc0146A_RevC     cuaccacaugaaacuauguuaguccaguuccuuguuauauacuuucuagaacuacacuggucuugauagucauguuuuagaauaaaugguucacguacuguaacaaucauuagcucuagaagauaaaaagc--------uauauuucugggacgaucggguuucacaacuggcacgggagguuuuuauacauacc
                        ###** ***   *   #  ****** #       #  * * * **** **#*        * *  *#** ** ****#  **** **  #  *|*  #  ** ****  #**** ** **#*  * *        *#** **** * * *  #       # ******  #   *   *** **###
```

=== Internal intron ===

Multiple sequence alignment

```
Xmsuc0146A_fwrd     guauguauaaaaaccucccgugccaguugugaaacccgaucgucccagaaauauagcuuuuuaucuucuagagcuaaugauuguuacag-----------------
Xmsuc0146A_RevC     -----------------cuguaacaaucauuagcucuagaagauaaaaagcuauauuucugggacgaucggguuucacaacuggcacgggagguuuuuauacauac
                                     * **# **#*##* *   * #   *    *#*  *||*  *#*    *   # *   * *##*#** #** *
```

=== External intron ===

Multiple sequence alignment

```
Xmsuc0146A_fwrd     -------------guacgugaaccauuuauucuaaaacaugacuaucaagaccaguguaguucuagaaaguauauaacaaggaacuggacuaacauaguuucaugugguag
Xmsuc0146A_RevC     cuaccacaugaaacuauguuaguccaguuccuuguuauauacuuucuagaacuacacuggucuugauagucauguuuuagaauaaaugguucacguac-------------
                                  ** ** *  *   * ## *   * **#  * # * #** * | * **# * # *  #** *   * ## *   *  * ** **
```

ALIGNMENT RESULTS - Xmsuc0153A

=== Stwintron Sequence ===

Multiple sequence alignment

```
Xmsuc0153A_fwrd     ggcauguauaaaaacuuccugugucgguugugaaacccgaucgcagaaauaucuuuuaucuucuaaagcuaacgauuuucacaguacgugagcucuugacuuuaagaacacaucuaccauacucggug-caguucuggaaaauauauaaua-----------------gggaacuuaacuaacauguucuuauguggcag
Xmsuc0153A_RevC     cugccacauaagaacauguuaguuaaguucc-----------------cuauuauauauuuuccagaacu-gcaccgaguaugguagauguguucuuaaagucaagagcucacguacugugaaaaucguuagcuuuagaagauaaaagauauuucugcgaucggguuucacaaccgacacaggaaguuuuuauacaugcc
                        ###**** *** *  *#  * #*** #                  ***  * *** ***#* *#**  *# #    *  *** #** * ****#*||*#**** * **# ***  *    # #*  **#* *#*** *** *  ***                  # ***# *  #*  * *** ****###
```

=== Internal intron ===

Multiple sequence alignment

```
Xmsuc0153A_fwrd     gcauguauaaaaacuuccugugucgguugugaaacccgaucgcagaaauaucuuuuaucuucuaaagcuaacgauuuucacag-----------------
Xmsuc0153A_RevC     -----------------cugugaaaaucguuagcuuuagaagauaaaagauauuucugcgaucggguuucacaaccgacacaggaaguuuuuauacaugc
                                     *****  ##*#** *     #   *  #*** || ***#  *   #     * **#*##  *****
```

=== External intron ===

Multiple sequence alignment

```
Xmsuc0153A_fwrd     guacgugagcucuugacuuuaagaacacaucuaccauacucggugcaguucuggaaaauauauaauagggaacu----------uaacuaacauguucuua-----------uguggcag
Xmsuc0153A_RevC     cugccaca-----------uaagaacauguuaguua----------aguucccuauuauauauuuuccagaacugcaccgaguaugguagauguguucuuaaagucaagagcucacguac
                     * *   *           ********  *     *          *****#  *  **||**  *  #*****          *     *  ********           *   * *
```

ALIGNMENT RESULTS - Xmsuc0159A

=== Stwintron Sequence ===

Multiple sequence alignment

```
Xmsuc0159A_fwrd     gguauguaugaagaccuccugugccgguugugaaacccaaucgucacagaa-----------auaucuuuugccuucuagagcuaacgauuuuuacaguaggugaaccauugauucuaaaacauggccaucacgaucgguggaguucuggaacauacauaauacggaacuggacuaacauguuuuuaucuggcag
Xmsuc0159A_RevC     cugccagauaaaaacauguuaguccaguuccguauuauguauguuccagaacuccaccgaucgugauggccauguuuuagaaucaagguucaccuacuguaaaaaucguuagcucuagaaggcaa-----------aagauauuucugugacgauugguuucacaaccggcacaggaggucuucauacauacc
                        ## **#**#** *  *#  **#*** #* *       **  *****           *     ###  ** ****# #** * **# #  *|*  # #** * **# #**** **  ###           ## # * *****  **        * *# ***#**  #*  * **#**#** ##
```

=== Internal intron ===

Multiple sequence alignment

```
Xmsuc0159A_fwrd     --------------------guauguaugaagaccuccugugccgguugugaaacccaaucgucacagaaauaucuuuugccuucuagagcuaacgauuuuuacag
Xmsuc0159A_RevC     cuguaaaaaucguuagcucuagaaggcaaaagauauuucugugacgauugguuucacaaccggcacaggaggucuucauacauac--------------------
                                        * * *#* #* #   * ***** **#*** * | * ***#** ***** *   # *# *#* * *
```

=== External intron ===

Multiple sequence alignment

```
Xmsuc0159A_fwrd     guaggugaaccauugauucuaaaacauggccaucacgaucgguggaguucuggaacauacauaauacggaacuggacuaa----------cauguuuua----------ucuggcag
Xmsuc0159A_RevC     cugccaga----------uaaaaacaug----------uuaguccaguuccguauuauguauguuccagaacuccaccgaucgugauggccauguuuuagaaucaagguucaccuac
                     *    **           ********          * #**  *****#* *  **||**  * *#*****  **# *          ********            **    *
```

ALIGNMENT RESULTS - Xmsuc0162A

=== Stwintron Sequence ===

Multiple sequence alignment

```
Xmsuc0162A_fwrd     gguaugu auacaaaaauauccuaugccggucgugaaac--------------------ccgaucgccgcagaaauauccuuugucuucuaaagcuaacgguuuccgcaguaugugaaccauuaauuuuaaaa--------auauggccauuacgaucgaugcaguucuagaaagcauauaaccggugacuagacuaacauguuuuuauguggua g--
Xmsuc0162A_RevC     --cuaccacauaaaaacauguuagucuagucaccgguuauaugcuuucuagaacugcaucgaucguaauggccauau--------uuuuaaaauuaaugguucacauacugcggaaaccguuagcuuuagaagacaaaggauauuucugcggcgaucgg--------------------guuucacgaccggcauaggauauuuuuguauacauacc
                      #   #*#* *****#**  **  * #***##                          ******   #  *  ****       ** ****# *** ****# *# *|* #* #**** *** #**** **        ****  *  #  ******                          ##***# *  **  **#***** *#*#   #
```

=== Internal intron ===

Multiple sequence alignment

```
Xmsuc0162A_fwrd     guaug-------------------uauacaaaaauauccuaugccggucgugaaacccgaucgccgcagaaauauccuuugucuucuaaagcuaacgguuuccgcag
Xmsuc0162A_RevC     cugcggaaaccguuagcuuuagaagacaaaggauauuucugcggcgaucggguuucacgaccggcauaggauauuuuuguau-------------------acauac
                     * #*                    *#* *  *   * ** #* **#*** * | * ***#** *# ** *   *  * *#*                    *# *
```

=== External intron ===

Multiple sequence alignment

```
Xmsuc0162A_fwrd     guaugugaaccauuaauuuuaaaaauauggccauuacgaucgaugcaguucuagaaagcauauaaccggugacuagacuaacauguuuuuaugugguag---------
Xmsuc0162A_RevC     ---------cuaccacauaaaaacauguuagucuagucaccgguuauaugcuuucuagaacugcaucgaucguaauggccauauuuuuaaaauuaaugguucacauac
                             * *##*  *  *** ** * #   *    *#** *   #* ** || ** *#   * **#*    *   # * ** ***  *  *##* *
```

ALIGNMENT RESULTS - Xmsuc0168A

=== Stwintron Sequence ===

Multiple sequence alignment

```
Xmsuc0168A_fwrd     gguauguauaaaaaccuccugugucgguugugaaacccaaucaucgcagaa--auaucuuucgccuucuagagcuaacgauuuuuauaguagguaaaccauugauucuaaagcauggccauuacgauucuagaaaauauauaauagggaacugggcuaacauguuuuuaccuggcag
Xmsuc0168A_RevC     cugccagguaaaaacauguuagcccaguucccuauuauauauuuucuagaaucguaauggccaugcuuuagaaucaaugguuuaccuacuauaaaaaucguuagcucuagaaggcga--aagauauuucugcgaugauuggguuucacaaccgacacaggagguuuuuauacauacc
                        ##  ******* *  *#  #*#*** #  *    *    *   ****   **    #*#  #* ****# #** * *** # **|** # *** * **# #**** *#  #*#  *  * # ****   *    *    *  # ***#*#  #*  * *******  ##
```

=== Internal intron ===

Multiple sequence alignment

```
Xmsuc0168A_fwrd     ---------------------guauguauaaaaaccuccugugucgguugugaaacccaaucaucgcagaaauaucuuucgccuucuagagcuaacgauuuuuauag
Xmsuc0168A_RevC     cuauaaaaaucguuagcucuagaaggcgaaagauauuucugcgaugauuggguuucacaaccgacacaggagguuuuuauacauac---------------------
                                         * * *#  ** *   * ***#*  *#*** * | * ***#*  *#*** *   * **  #* * *
```

=== External intron ===

Multiple sequence alignment

```
Xmsuc0168A_fwrd     gu---agguaa----------------------accauugauucuaaagcauggccauuacgauucuagaaaauauauaauagggaacugggcuaacauguuuuuaccuggcag
Xmsuc0168A_RevC     cugccagguaaaaacauguuagcccaguucccuauuauauauuuucuagaaucguaauggccaugcuuuagaaucaaugg----------------------uuuaccu---ac
                     *   ******                       *  **  *** *  ** ** *  ||  * ** **  * ***  **                        *******   *
```

ALIGNMENT RESULTS - Xmsuc0170A

=== Stwintron Sequence ===

Multiple sequence alignment

```
Xmsuc0170A_fwrd     ----------------gguauguauaaaaaccuccuguaccgguuauaaaacccaaccgucgcagaaauauuuuucgucuucuggagcuaacgauuuuuauaguagguugaaccguugguucuaaaacauggcuaucac-----gaucgguguaguuuuaaaaaguguauaaaagggaauuggacuaacauguuuuuaucuggcag
Xmsuc0170A_RevC     cugccagauaaaaacauguuaguccaauucccuuuuauacacuuuuuaaaacuacaccg-----aucgugauagccauguuuuagaaccaacgguucaccuacuauaaaaaucguuagcuccagaagacgaaaaauauuucugcgacgguuggguuuuauaaccgguacaggagguuuuuauacauacc----------------
                                     **  ** #**   ***  *#***   ** ******   ****     #   *  *  #*#* ** *#**#*#**** **# # **|** # #** ****#*#**#* ** *#*#  *  *         ****   ****** **   ***#*  ***   **# **  **
```

=== Internal intron ===

Multiple sequence alignment

```
Xmsuc0170A_fwrd     guauguauaaaaaccuccuguaccgguuauaaaacccaaccgucgcagaaauauuuuucgucuucuggagcuaacgauuuuuauag-----------------
Xmsuc0170A_RevC     -----------------cuauaaaaaucguuagcuccagaagacgaaaaauauuucugcgacgguuggguuuuauaaccgguacaggagguuuuuauacauac
                                     **#**  ##*# * *   ***   * ** *#** | **#* ** *   ***   * * #*##  **#**
```

=== External intron ===

Multiple sequence alignment

```
Xmsuc0170A_fwrd     guaggugaaccguugguucuaaaacauggcuaucacgaucgguguaguuuuaaaaaguguauaaaagggaauug-----gacuaacauguuuuua----------ucuggcag
Xmsuc0170A_RevC     cugccaga----------uaaaaacauguuaguc-----caauucccuuuuauacacuuuuuaaaacuacaccgaucgugauagccauguuuuagaaccaacgguucaccuac
                     *    **            ********    **     *##* #  ***** * *|* * *****  # *##*     **    ********            **    *
```

ALIGNMENT RESULTS - Xmsuc0171A

=== Stwintron Sequence ===

Multiple sequence alignment

```
Xmsuc0171A_fwrd     gguacguauagaaaaccuuucgugaaucccgaucuucgcagaaacaucuauugucauccaaagcuaauuaauguuacaguaugugaacuauugguccucaaaccaggucauu-gcaaccuguguauuuuuggaaaguauacaaccaaaaaacggacuaacauguuguuauuuggcag----
Xmsuc0171A_RevC     ----cugccaaauaacaacauguuaguccguuuuuugguuguauacuuuccaaaaauacacagguug-caaugaccugguuugaggaccaauaguucacauacuguaacauuaauuagcuuuggaugacaauagauguuucugcgaagaucgggauucacgaaagguuuucuauacguacc
                        *   #*#* ***  #  ** * ***   * ** *  * *   * * # #  ** ** ** *   #**** #   ** ** * **#*|*#** * ** **   # **** #  * ** ** **  # # * *   * *  * ** *   *** * **  #  *** *#*#   *
```

=== Internal intron ===

Multiple sequence alignment

```
Xmsuc0171A_fwrd     --guacguauagaaaaccuuucgugaaucccgaucuucgcagaaacaucuauugucauccaaagcuaauuaauguuacag
Xmsuc0171A_RevC     cuguaacauuaauuagcuuuggaugacaauagauguuucugcgaagaucgggauucacgaaagguuuucuauacguac--
                      ***    **#  * * **  #***     *** **  ||  ** ***     ***#  ** * *  #**    ***
```

=== External intron ===

Multiple sequence alignment

```
Xmsuc0171A_fwrd     guaugugaacuauugguccucaaaccaggucauugcaaccuguguauuuuuggaaaguauacaaccaaaaaacggacuaacauguuguuauuugg----------------cag
Xmsuc0171A_RevC     cug----------------ccaaauaacaacauguuaguccguuuuuugguuguauacuuuccaaaaauacacagguugcaaugaccugguuugaggaccaauaguucacauac
                     *                 #****  * # ***   *  *#** * **  * * * || * * *  ** * **#*  *   *** # *  ****#                 *
```

ALIGNMENT RESULTS - Xmsuc0178A

=== Stwintron Sequence ===

Multiple sequence alignment

```
Xmsuc0178A_fwrd     ---------------------------------gguauguaucaaggccugcugugccgguuaugagaaucgguguugguagaaagaccuguugucgagaaaagcuaacauuucuuauaguaugugaaccu--------------ccaauuuauaaacgcgggccacuaugaccuguguaguccgagaacauaugcaauaaagaaccggacuaacgccaucuugaugguuag
Xmsuc0178A_RevC     cuaaccaucaagauggcguuaguccgguucuuuauugcauauguucucggacuacacaggucauaguggcccgcguuuauaaauug--------------gagguucacauacuauaagaaauguuagcuuuucucgacaacaggucuuucuaccaacaccgauucucauaaccggcacagcaggccuugauacauacc---------------------------------
                                                     # * ##***     *  #**###* ***#**#    #* *#*** #**#*  *                * * * **** #  ||  # **** * * *               *  *#**# ***#* *#    #**#*** *###**#  *     ***## * #
```

=== Internal intron ===

Multiple sequence alignment

```
Xmsuc0178A_fwrd     guauguaucaaggccugcugugccgguuaugagaaucgguguugguagaaagaccuguugucgagaaaagc-uaacauuucuu-----auag
Xmsuc0178A_RevC     cuau-----aagaaauguua-gcuuuucucgacaacaggucuuucuaccaacaccgauucucauaaccggcacagcaggccuugauacauac
                     ***     ***#  ** *# **   *# #** **# *** **  ||  ** *** #** **# #*   ** #* **  #***     ***
```

=== External intron ===

Multiple sequence alignment

```
Xmsuc0178A_fwrd     guaugugaaccuccaauuu---auaaacgcgggccacuaugaccuguguaguccgagaacauaugcaauaaagaaccggacuaacgccaucuugaugguu-----ag
Xmsuc0178A_RevC     cu-----aaccaucaagauggcguuaguccgguucuuuauugcauauguucucggacuacacaggucauaguggcccgcguuua---uaaauuggagguucacauac
                     *     ****  ***  *    * *   ***  *  ***  * *#***  **|**  ***#* *  ***  *  ***   * *    *  ***  ****     *
```

ALIGNMENT RESULTS - Xmsuc0185A

=== Stwintron Sequence ===

Multiple sequence alignment

```
Xmsuc0185A_fwrd     --------------gguaaguauaaaaccucc------------------cgcgcugguugugaaccucgaucugcgcacaaauaucuauuaucuucuaaagcuaauuagagucacaguaugugagcuaucgauccuaaaccgggccguuggaaccuguguauuucuagaaaguauacaaaaaggaacuggacuaacuuuuuuauauggcag
Xmsuc0185A_RevC     cugccauauaaaaaaguuaguccaguuccuuuuuguauacuuucuagaaauacacagguuccaacggcccgguuuaggaucgauagcucacauacugugacucuaauuagcuuuagaagauaauagauauuugugcgcagaucgagguucacaaccagcgc------------------gggagguuuuauacuuacc--------------
                                  #** *** #*   ***                     #*#* **** ##*   #*    *  * *   *** **  #** #* * *  ***||***  * * *# **#  ** ***   * *  *    *#   *## **** *#*#                     ***   *# *** **#
```

=== Internal intron ===

Multiple sequence alignment

```
Xmsuc0185A_fwrd     gu----------------------aaguauaaaaccucccgcgcugguugugaaccucgaucugcgcacaaauaucuauuaucuucuaaagcuaauuagagucacag
Xmsuc0185A_RevC     cugugacucuaauuagcuuuagaagauaauagauauuugugcgcagaucgagguucacaaccagcgcgggagguuuuau----------------------acuuac
                     *                       *  *** *   *   **** *#*#* * | * *#*#* ****   *   * ***                       *  *
```

=== External intron ===

Multiple sequence alignment

```
Xmsuc0185A_fwrd     guaugugagcuaucgauccuaaaccgggccguuggaaccuguguauuucuagaaaguauacaaa----aaggaacuggacuaacuuuuuuauauggcag--
Xmsuc0185A_RevC     --cugccauauaaaaaaguuaguccaguuccuu----uuuguauacuuucuagaaauacacagguuccaacggcccgguuuaggaucgauagcucacauac
                       **# *  **  #*   **  **#*  * **      ***#**#** #|# **#**#***      ** *  *#**  **   *#  **  * #**
```

ALIGNMENT RESULTS - Xmsuc0187A

=== Stwintron Sequence ===

Multiple sequence alignment

```
Xmsuc0187A_fwrd     gguauguaucaaaacaagcuaugcuacuuguaugacuaggucuuaucuggaagauugauuauuugcucgagcuaacuauugccacaguaugugaaccuucggucaaggaacagcggccauuaugguccaugugauauuaagauguguaauaagaagaucgaggcaccguacuaauacauugugauaugccag-----
Xmsuc0187A_RevC     -----cuggcauaucacaauguauuaguacggugccucgaucuucuuauuacacaucuuaauaucacauggaccauaauggccgcuguuccuugaccgaagguucacauacuguggcaauaguuagcucgagcaaauaaucaaucuuccagauaagaccuagucauacaaguagcauagcuuguuuugauacauacc
                          *  ** * ** # * *# ** *    ** ** *#**** *    * #  *  * ** *  #   * # *  ** *** * ** # *  *** | ***  * # ** * *** **  * # *   #  * ** *  *  # *    * ****#* ** **    * ** #* * # ** * **  *
```

=== Internal intron ===

Multiple sequence alignment

```
Xmsuc0187A_fwrd     guaug-------------uaucaaaacaagcuaugcuacuuguaugacuaggucuuaucuggaagauugauuauuugcucgagcuaacuauugccacag
Xmsuc0187A_RevC     cuguggcaauaguuagcucgagcaaauaaucaaucuuccagauaagaccuagucauacaaguagcauagcuuguuuugaua-------------cauac
                     * **             #    *** ** * **  * *  #** ***#|#*** **#  * *  ** * ** ***    #             ** *
```

=== External intron ===

Multiple sequence alignment

```
Xmsuc0187A_fwrd     -----guaugugaaccuucggucaaggaacagcggccauuaugguccaugugauauuaagauguguaauaagaagaucgaggcaccguacuaauacauugugauaugccag
Xmsuc0187A_RevC     cuggcauaucacaauguauuaguacggugccucgaucuucuuauuacacaucuuaauaucacauggaccauaauggccgcuguuccuugaccgaagguucacauac-----
                         #***   **  *   #  * **  *  **# * *# *# * **##*  **|**  *##** * #* #* * #**  *  ** *  #   *  **   ***#
```

ALIGNMENT RESULTS - Xmsuc0237A

=== Stwintron Sequence ===

Multiple sequence alignment

```
Xmsuc0237A_fwrd     gguauguaaacugcggcaccccaaauuggagcuaccuauuuucuccagagcuaacuguugucgcaguaugugaaccuugaauccagaaacaugaccgccuugacccgaaaccuguacaacauaaugcaagaaggaucuggacuaa----------ccugugugaaaag----------------
Xmsuc0237A_RevC     cuuuu--------------------------cacacagguuaguccagauccuucuugcauuauguuguacagguuucgggucaaggcggucauguuucuggauucaagguucacauacugcgacaacaguuagcucuggagaaaauagguagcuccaauuuggggugccgcaguuuacauacc
                      * *                          *   *   **  ****** *#  **  ##* #   * *###    *#*  ** **     ||     ** **  *#*    ###* *   # *##  **  #* ******  **          ** #* **    *
```

=== Internal intron ===

Multiple sequence alignment

```
Xmsuc0237A_fwrd     ------------------guauguaaacugcggcaccccaaauuggagcuaccuauuuucuccagagcuaacuguugucgcag
Xmsuc0237A_RevC     cugcgacaacaguuagcucuggagaaaauagguagcuccaauuuggggugccgcaguuuacauac------------------
                                       *  # *** *#  *   * ****|**** *   * #* *** #  *
```

=== External intron ===

Multiple sequence alignment

```
Xmsuc0237A_fwrd     ---------------------------------------guaugugaaccuugaauccagaaacaugaccgccuugacccgaaaccuguacaacauaaugcaagaaggaucuggacuaaccugugugaaaag
Xmsuc0237A_RevC     cuuuucacacagguuaguccagauccuucuugcauuauguuguacagguuucgggucaaggcggucauguuucuggauucaagguucacauac---------------------------------------
                                                            * *###    *#*  ** **     ||     ** **  *#*    ###* *
```

ALIGNMENT RESULTS - Xmsuc0285A

=== Stwintron Sequence ===

Multiple sequence alignment

```
Xmsuc0285A_fwrd     ------------------------------gguacguauaaaaaccuuccaugcuaguugagaaagccgaucguugcagaaauaucuuugucuuuuagagcuaacaaguaccgcaguaugugaaccauugauucucaaaaccuagcuaucacgaucgguguaguuuaaua--------------uauauauacuaaggaaauggacuaauauauuuuaugacag
Xmsuc0285A_RevC     cugucauaaaauauauuaguccauuuccuuaguauauaua--------------uauuaaacuacaccgaucgugauagcuagguuuugagaaucaaugguucacauacugcgguacuuguuagcucuaaaagacaaagauauuucugcaacgaucggcuuucucaacuagcauggaagguuuuuauacguacc------------------------------
                                                  #*** #****              ** * #*  * #******** # **  *  * ** #   *# *  * * *** ##  ||  ## *** * *  * #*   # ** *  *  ** # *******# *  *# * **              ****# ***#
```

=== Internal intron ===

Multiple sequence alignment

```
Xmsuc0285A_fwrd     guacguauaaaaaccuuccaugcuaguugagaaagccgaucguugcagaaauaucuuugucuuuuagagcuaacaaguaccgcag-----------------
Xmsuc0285A_RevC     -----------------cugcgguacuuguuagcucuaaaagacaaagauauuucugcaacgaucggcuuucucaacuagcauggaagguuuuuauacguac
                                     *  #* ** ***  *   * #*  * ## *** || *** ## *  *# *   *  *** ** *#  *
```

=== External intron ===

Multiple sequence alignment

```
Xmsuc0285A_fwrd     guaugugaaccauugauucucaaaaccuagcuaucacgaucgguguaguuuaauauauauauacuaag-------gaaauggacuaauauauuuuauga--------------cag
Xmsuc0285A_RevC     cug--------------ucauaaaauauauuaguccauuuc-------cuuaguauauauauauuaaacuacaccgaucgugauagcuagguuuugagaaucaaugguucacauac
                     *               **  ****  **    **    **       #*** ****||**** ***#       **    **    **  ****  **              *
```

ALIGNMENT RESULTS - Xmsuc0293A

=== Stwintron Sequence ===

Multiple sequence alignment

```
Xmsuc0293A_fwrd     --ggcauguauaaaaccuccuauaccaguugugaagcccaaucgucgcgaaaauaucuuuugucuucuagagcuaacgguuuuauaguaugugaaccauugauucuaagaacauagcuauuacgaucagug---------------------uaguucuagaaaauauauaaaua-------------------gggaacuagacuaauaugauuu-uguguggcag
Xmsuc0293A_RevC     cugccacaca-aaaucauauuagucuaguucc--------------------cuauuauauauuuucuagaacua---------------------cacugaucguaauagcuauguucuuagaaucaagguucacauacuauaaaaccguuagcucuagaagacaaaagauauuuucgcgacgauugggcuucacaacugguauaggagguuuuauacaugcc--
                      * **###* *** * *  **  * **** #                     ***  * *#* *******#***                     **#****# *** * * | * * *** #****#**                     ***#******* *#* *  ***                     # **** *  **  * * *** *###** *
```

=== Internal intron ===

Multiple sequence alignment

```
Xmsuc0293A_fwrd     gcauguauaaaaccuccuauaccaguugugaagcccaaucgucgcgaaaauaucuuuugucuucuagagcuaacgguuuuauag----
Xmsuc0293A_RevC     ----cuauaaaaccguuagcucuagaagacaaaagauauuuucgcgacgauugggcuucacaacugguauaggagguuuuauacaugc
                         *********     # * **  *  **#    **  **||**  **    #**  *  ** * #     *********
```

=== External intron ===

Multiple sequence alignment

```
Xmsuc0293A_fwrd     guaugugaaccauugauucuaagaacauagcuauuacgaucaguguaguucuagaaaauauaaauagggaacuagacuaauaugauuuugugug---------------------gcag
Xmsuc0293A_RevC     cugc---------------------cacacaaaaucauauuagucuaguucccuauuauauauuuucuagaacuacacugaucguaauagcuauguucuuagaaucaagguucacauac
                     * #                     **#*   * *   ** *** ******#  *  **||**  *  #****** *** **   * *   *#**                     # *
```

ALIGNMENT RESULTS - Xmsuc0301A

=== Stwintron Sequence ===

Multiple sequence alignment

```
Xmsuc0301A_fwrd     gguacguauaaaagcaucccaugcugguuguggaagccggccgucgcagaaauau---------cuuuuuuuuuuuucaauuuccaaagcuaac-------uaauguuacaguauguaaaccgucgauucucaaaccuggccaccacgaccgggguaguucuggaaaguaugcaauaaggaacugaacuaacauauuuuuuuaauggcag-------------------------------------
Xmsuc0301A_RevC     -------------------------------------cugccauuaaaaaaauauguuaguucaguuccuuauugcauacuuuccagaacuaccccggucgugguggccagguuugagaaucgacgguuuacauacuguaacauua-------guuagcuuuggaaauugaaaaaaaaaa---------aagauauuucugcgacggccggcuuccacaaccagcaugggaugcuuuuauacguacc
                                                         * ***#* # *#******          **##** ** #  * ****** *#*** *       *  ** #   ** **  ** **|** **  ** **   # **  *       * ***#* ****** *  # ** **##         ** ******#* # *#*** *
```

=== Internal intron ===

Multiple sequence alignment

```
Xmsuc0301A_fwrd     guacguauaaaagcaucccaugcugguuguggaagccggccgucgcagaaauaucuuuuuuuuuuuucaauuuccaaagcuaacuaauguuac----------ag
Xmsuc0301A_RevC     cu----------guaacauuaguuagcuuuggaaauugaaaaaaaaaaaagauauuucugcgacggccggcuuccacaaccagcaugggaugcuuuuauacguac
                     *          * * *    * *#*#* *****#  *#  #  # *#**  |  **#* #  #  #*  #***** *#*#* *    * * *          *
```

=== External intron ===

Multiple sequence alignment

```
Xmsuc0301A_fwrd     guauguaaaccgucgauucucaaaccuggccaccacgaccgggguaguucuggaaaguaugcaauaaggaacugaacuaacauauuuuuuuaaug---------------------------------------------------------gcag
Xmsuc0301A_RevC     cugc---------------------------------------------------------cauuaaaaaaauauguuaguucaguuccuuauugcauacuuuccagaacuaccccggucgugguggccagguuugagaaucgacgguuuacauac
                     * #                                                         ** ***##** *#   ||   #* **##*** **                                                         # *
```

ALIGNMENT RESULTS - Xmsuc0306A

=== Stwintron Sequence ===

Multiple sequence alignment

```
Xmsuc0306A_fwrd     --------------------gguauguuuucaagccaaccgugccggcugugaggccuagccuuggcagaaagaucuauuaccuccccagcuaacauggauugcaguaugugaaauuuccgugacgagaauaucgcucccugccuuugugguuucguaacguauuuaugugagaaacuaauaugcuuag
Xmsuc0306A_RevC     cuaagcauauuaguuucucacauaaauacguuacgaaaccacaaaggcagggagcgauauucucgucacggaaauuucacauacugcaauccauguuagcuggggagguaauagaucuuucugccaaggcuaggccucacagccggcacgguuggcuugaaaacauacc--------------------
                                         #** #* #   *   ****###  *** * ***   **  **#* **   *#** *  #*  #  * * *#* | *#* * *  #  *#  * **#*   ** *#**  **   *** * ***  ###****   *   # *# **#
```

=== Internal intron ===

Multiple sequence alignment

```
Xmsuc0306A_fwrd     -----------guauguuuucaagccaaccgugccggcugugaggccuagccuuggcagaaagaucuauuaccuccccagcuaacauggauugcag
Xmsuc0306A_RevC     cugcaauccauguuagcuggggagguaauagaucuuucugccaaggcuaggccucacagccggcacgguuggcuugaaaacauac-----------
                               **  *#*    **  **  *  *   ***# *#* *||* *#* #***   *  *  **  **    *#*  **
```

=== External intron ===

Multiple sequence alignment

```
Xmsuc0306A_fwrd     guaugugaaauuuccgugacgagaauaucgcucccugccuuugugguuucguaacguauuuaugugagaaacuaauaugcu------------------uag
Xmsuc0306A_RevC     cua------------------agcauauuaguuucucacauaaauacguuacgaaaccacaaaggcagggagcgauauucucgucacggaaauuucacauac
                     **                  **  **** # *  **  * * #  ## * || * ##  # * *  **  * # **** **                  **
```

ALIGNMENT RESULTS - Xmsuc0348A

=== Stwintron Sequence ===

Multiple sequence alignment

```
Xmsuc0348A_fwrd     gguauguaucaaaucccgcugucccgguu---------------------gugagaccugacauuggcagauauaucuuuugucuuccugagcuaacugaugucacaguacgugagccguugauccugaaaccugaaaaucugaccaccauggccggguguaauucuagaaagcauacaacaagaaacuaggcuaaucaguuucuaccugacag
Xmsuc0348A_RevC     cugucagguagaaacugauuagccuaguuucuuguuguaugcuuucuagaauuacacccggccaugguggucagauuuucagguuuucaggaucaacggcucacguacugugacaucaguuagcucaggaagacaaaagauauaucugccaaugucaggucucac---------------------aaccgggacagcgggauuugauacauacc
                        ##  *  ** *  # *# ** #***                     #* * ***#* *  ***  * #* ** **# *  ** * * # #*** * *  *  *|*  *  * * ***# # * * **  * #** ** *# *  ***  * *#*** * *#                     ***# ** #* #  * **  *  ##
```

=== Internal intron ===

Multiple sequence alignment

```
Xmsuc0348A_fwrd     ----------------------------------guauguaucaaaucccgcugucccgguugugagaccugacauuggcagauauaucuuuugucuuccugagcuaacugaugucacag
Xmsuc0348A_RevC     cugugacaucaguuagcucaggaagacaaaagauauaucugccaaugucaggucucacaaccgggacagcgggauuugauacauac----------------------------------
                                                      #*** * #***   * * * ** *#||#* ** * * *   ***# * ***#
```

=== External intron ===

Multiple sequence alignment

```
Xmsuc0348A_fwrd     guacgugagccguugauccugaaaccugaaaaucugaccaccauggccggguguaauucuagaaagcauacaacaagaaacuaggcuaaucaguuucu--------------------accugacag
Xmsuc0348A_RevC     cugucagg--------------------uagaaacugauuagccuaguuucuuguuguaugcuuucuagaauuacacccggccaugguggucagauuuucagguuuucaggaucaacggcucacguac
                     *    *                      * ** ****  * * *#*     ***  *     ||     *  ***     *#* * *  **** ** *                      *    *
```

ALIGNMENT RESULTS - Xmsuc0374A

=== Stwintron Sequence ===

Multiple sequence alignment

```
Xmsuc0374A_fwrd     ggcacguauaaaaaccuccugugccgguugaaau--------------------ccgaucgucgcagaaaagucuuuugucucuagagcuaacuaaugccauaguaugaaaccguggauucuaaaacuuggauaucacgaucaguguaguccuacaaagcauauaauaaggaaccggacuaacauuuuucauag-----
Xmsuc0374A_RevC     -----cuaugaaaaauguuaguccgguuccuuauuauaugcuuuguaggacuacacugaucgugauauccaaguuuuagaauccacgguuuacauacuauggcauuaguuagcucuagagacaaaagacuuuucugcgacgaucgg--------------------auuucaaccggcacaggagguuuuuauacgugcc
                          *** ****      ** * * *#   **                      * ****** # *   **** **  #  #*  *   **  ** *# | #* **  **   *  *#  #  ** ****   * # ****** *                     **   #* * * **       **** ***
```

=== Internal intron ===

Multiple sequence alignment

```
Xmsuc0374A_fwrd     --------------------gcacguauaaaaaccuccugugccgguugaaauccgaucgucgcagaaaagucuuuugucucuagagcuaacuaaugccauag
Xmsuc0374A_RevC     cuauggcauuaguuagcucuagagacaaaagacuuuucugcgacgaucggauuucaaccggcacaggagguuuuuauacgugc--------------------
                                        # * ##* ** *   * ***#* **#*#* *|* *#*#** *#*** *   * ** *## * #
```

=== External intron ===

Multiple sequence alignment

```
Xmsuc0374A_fwrd     guauguaaaccguggauucuaaaacuuggauaucacgaucaguguaguccuacaaagcauauaau----aaggaaccggacuaacauuuuucauag
Xmsuc0374A_RevC     cuaugaaaaauguuagucc----gguuccuuauuauaugcuuuguaggacuacacugaucgugauauccaaguuuuagaauccacgguuuacauac
                     **** ***  ** # *#*      **   *** * #  *  *****||*****  *  # * **    ***     *#* # **  *** ****
```

ALIGNMENT RESULTS - Xmsuc0374B

=== Stwintron Sequence ===

Multiple sequence alignment

```
Xmsuc0374B_fwrd     -------------gguauguguuaaaacccgucgugccggccuugaguucuaguguug---gcaguaauacuaucugucuuacagagcuaacuauuacugcaguauguaaauccgaggcccgaaaagaaaggucgcauuuaucuacguaauuguagaaggcgcguaauacggggcgugaacugauauauauuuuguuuaucuuag
Xmsuc0374B_RevC     cuaagauaaacaaaauauauaucaguucacgccccguauuacgcgccuucuacaauuacguagauaaaugcgaccuuucuuuucgggccucggauuuacauacugcaguaauaguuagcucuguaagacagauaguauuacugc---caacacuagaacucaaggccggcacgacggguuuuaacacauacc-------------
                                 ##***#*#*#*   * **#* #*     * #*  *****  #**#   # *  *** * *#** ****   * **#    ***  ## *|* ##  ***    #** *   **** **#* * ***  * #   #**#  *****  *# *     *# *#** *   *#*#*#***##
```

=== Internal intron ===

Multiple sequence alignment

```
Xmsuc0374B_fwrd     guauguguuaaaacccgucgugccggccuugaguucuaguguuggcaguaauacuaucugucuuacagagcuaacuauuacugcag---------------------------
Xmsuc0374B_RevC     ---------------------------cugcaguaauaguuagcucuguaagacagauaguauuacugccaacacuagaacucaaggccggcacgacggguuuuaacacauac
                                               **  ***  ****     * **** **  |  ** **** *     ****  ***  **
```

=== External intron ===

Multiple sequence alignment

```
Xmsuc0374B_fwrd     ----------------guauguaaauccgaggcccgaaaagaaaggucgcauuuaucuacguaauuguagaaggcgcguaa------------------uacggggcgugaacugauauauauuuuguuuaucuuag
Xmsuc0374B_RevC     cuaagauaaacaaaauauauaucaguucacgccccgua------------------uuacgcgccuucuacaauuacguagauaaaugcgaccuuucuuuucgggccucggauuuacauac----------------
                                    #***#* * * *# * **** *                   ****#  #* #|# *#  #****                   * **** * #* * * *#***#
```

ALIGNMENT RESULTS - Xmsuc0378A

=== Stwintron Sequence ===

Multiple sequence alignment

```
Xmsuc0378A_fwrd     gguaugcaucaaggccuaucgucgaucaaagaucucuuaugucuacuagagcuaauuguuauuacaguaugugaaccuuugaucuaggagcguggugaucgcgaccuguggcauucuggaaagcauggaaugagaaaacugcacu-----aacccggcuuuauaugcuag---------------------------------------------------------------
Xmsuc0378A_RevC     ----------------------------------------------------------------cuagcauauaaagccggguu-----agugcaguuuucucauuccaugcuuuccagaaugccacaggucgcgaucaccacgcuccuagaucaaagguucacauacuguaauaacaauuagcucuaguagacauaagagaucuuugaucgacgauaggccuugaugcauacc
                                                                                       *#**#*#** *#  * *     ** *##**  ** *#  *##**   *#*|*#*   **##*  #* **  **##* **     * *  #* **#*#**#*
```

=== Internal intron ===

Multiple sequence alignment

```
Xmsuc0378A_fwrd     guau----gcaucaaggccuaucgucgaucaaagaucucuuaugucuacuagagcuaauuguuauuacag
Xmsuc0378A_RevC     cuguaauaacaauuagcucuaguagacauaagagaucuuugaucgacgauaggccuugaugc----auac
                     * *    #**   **  ***  #   ** * **||** * **   #  ***  **   **#    * *
```

=== External intron ===

Multiple sequence alignment

```
Xmsuc0378A_fwrd     ---guaugugaaccuuugaucuaggagcguggugaucgcgaccuguggcauucuggaaagcauggaaugagaaaacugcacu-----aacccggcuuuauaugcuag
Xmsuc0378A_RevC     cuagcauauaaagccggguu-----agugcaguuuucucauuccaugcuuuccagaaugccacaggucgcgaucaccacgcuccuagaucaaagguucacauac---
                       *#**#*#** *#  * *     ** *##**  ** *#  *##**   *#*|*#*   **##*  #* **  **##* **     * *  #* **#*#**#*
```

ALIGNMENT RESULTS - Xmsuc0385A

=== Stwintron Sequence ===

Multiple sequence alignment

```
Xmsuc0385A_fwrd     gguauguauaaaaaccuccugugccgguugugaaacccaaucgucgcagaaauacccu-----------uugccuucuagagcuaacgauuuuuacaguaggugaaccauugauucuaaaacauggccaucacgaucgguggaguucuagaaaagauauaauagggaacugagcuaacauauuuuuaucuggcag
Xmsuc0385A_RevC     cugccagauaaaaauauguuagcucaguucccuauuauaucuuuucuagaacuccaccgaucgugauggccauguuuuagaaucaagguucaccuacuguaaaaaucguuagcucuagaaggcaa-----------aggguauuucugcgacgauuggguuucacaaccggcacaggagguuuuuauacauacc
                        ## *******  *  *#   *#*** #  *    * #  *   **** * * *#           ###  ** ****# #** * **# #  *|*  # #** * **# #**** **  ###            #* * * ****   *  # *    *  # ***#*   #*  *  ******* ##
```

=== Internal intron ===

Multiple sequence alignment

```
Xmsuc0385A_fwrd     guauguauaaaaaccuccugugccgguugugaaacccaaucgucgcagaaauacccuuugccuucuagagcuaacgauuuuuacag----
Xmsuc0385A_RevC     ----cuguaaaaaucguuagcucuagaaggcaaaggguauuucugcgacgauuggguuucacaaccggcacaggagguuuuuauacauac
                         * ****** *    *# * #*  *  ***    **  # || #  **    ***  *  *# * #*    * ****** *
```

=== External intron ===

Multiple sequence alignment

```
Xmsuc0385A_fwrd     -------------guaggugaac--------------cauugauucuaaaacauggccaucacgaucgguggaguucuagaaaagauauaauagggaacugagcuaacauauuuuuaucuggcag
Xmsuc0385A_RevC     cugccagauaaaaauauguuagcucaguucccuauuauaucuuuucuagaacuccaccgaucgugauggccauguuuuagaaucaaug-------------guucaccuac-------------
                                 #** ** * *               **#  ***** *** # #**    |    **# # *** *****  #**                * * ** **#
```

ALIGNMENT RESULTS - Xmsuc0412A

=== Stwintron Sequence ===

Multiple sequence alignment

```
Xmsuc0412A_fwrd     gguauguauaaaaaccuccugugccgguugugaaauccaaucgucguagaaauaucuu-----------uugccuucuagagcuaacaauucccacaguaggugaaccauuauuucuaaaacaugaccaucacgaucaguggaguucuagaaaauauauaauagggaacuggacuaacacuuuuuuauaugguag
Xmsuc0412A_RevC     cuaccauauaaaaaaguguuaguccaguucccuauuauauauuuucuagaacuccacugaucgugauggucauguuuuagaaauaaugguucaccuacugugggaauuguuagcucuagaaggcaa-----------aagauauuucuacgacgauuggauuucacaaccggcacaggagguuuuuauacauacc
                        ##********  *  *#  **#*** #  * *  *    *  ***** * # #*           *##  ** ****# ***   *** *  *|*  * ***   *** #**** **  ##*           *# # * *****  *    *  * *  # ***#**  #*  *  ********##
```

=== Internal intron ===

Multiple sequence alignment

```
Xmsuc0412A_fwrd     guauguauaaaaaccuccugugccgguugugaaauccaaucgucguagaaauaucuuuugccuucuagagcuaacaauucccacag-----------------
Xmsuc0412A_RevC     -----------------cugugggaauuguuagcucuagaaggcaaaagauauuucuacgacgauuggauuucacaaccggcacaggagguuuuuauacauac
                                     *****  ##**** *  ** *   * *# *# * | * #* #* *   * **  * ****##  *****
```

=== External intron ===

Multiple sequence alignment

```
Xmsuc0412A_fwrd     guaggugaaccauuauuucuaaaacaugaccaucacgaucaguggaguucuagaaaauauauaauagggaacuggacuaacacuuuuuuauauggu--------------------ag
Xmsuc0412A_RevC     cu--------------------accauauaaaaaaguguuaguccaguucccuauuauauauuuucuagaacuccacugaucgugauggucauguuuuagaaauaaugguucaccuac
                     *                    * ***#   *  *   * ***  *****#  *  **||**  *  #*****  *** *   *  *   #*** *                    *
```

ALIGNMENT RESULTS - Xmsuc0520A

=== Stwintron Sequence ===

Multiple sequence alignment

```
Xmsuc0520A_fwrd     gguauguauaaaaacgucccgugcugaucgugaaccccgaucuucgcggauauauuuaucgucuuccagaagcuaacuugugucacaguaugugaaccguugauauucaaauugggccauuucaauc--uguguauuuccagaaaguauacaaucaggaacugggcuaacguauuuuuauauggcag
Xmsuc0520A_RevC     cugccauauaaaaauacguuagcccaguuccugauuguauacuuucuggaaaua--cacagauugaaauggcccaauuugaauaucaacgguucacauacugugacacaaguuagcuucuggaagacgauaaauauauccgcgaagaucgggguucacgaucagcacgggacguuuuuauacauacc
                        ##********* ##   #  *## *  #  *    #  ***    *** ***  #*# *  *    *    *#** *** #*  **##   *|*   ##**  *# *** **#*     *    *   *   *# #*** ***    ***  #     *  #   * ##*  #    ## ********##
```

=== Internal intron ===

Multiple sequence alignment

```
Xmsuc0520A_fwrd     ----------------------guauguauaaaaacgucccgugcugaucgugaaccccgaucuucgcggauauauuuaucgucuuccagaagcuaacuugugucacag
Xmsuc0520A_RevC     cugugacacaaguuagcuucuggaagacgauaaauauauccgcgaagaucgggguucacgaucagcacgggacguuuuuauacauac----------------------
                                          * * ##   ***     ***#*  ***** * | * *****  *#***     ***   ## * *
```

=== External intron ===

Multiple sequence alignment

```
Xmsuc0520A_fwrd     guaugugaaccguugauauucaaauugggcca-uuucaaucuguguauuuccagaaaguauacaaucaggaacugggcuaacguauuuuuauauggc------ag
Xmsuc0520A_RevC     cu------gccauauaaaaauacguuagcccaguuccugauuguauacuuucuggaaauacacagauugaaa-uggcccaauuugaauaucaacgguucacauac
                     *       **#*  * *   *  **#* *** **#*    ***#**#** *|* **#**#***    *#** *** *#**  *    * *  *#**       *
```

ALIGNMENT RESULTS - Xmsuc0671A

=== Stwintron Sequence ===

Multiple sequence alignment

```
Xmsuc0671A_fwrd     --------------------gguaugugugggaaccuccucugccgauuauaaaauccaaucgucgcagaauuaucuucucuuugcuagagcuaauuaguguuauaguaugugaaccguugauuguaaaacccggucaucacgaccccgguguaguucuagaaaauguacgauagggcgcaagacuaacauguuuuaauaugaccgccag
Xmsuc0671A_RevC     cuggcggucauauuaaaacauguuagucuugcgcccuaucguacauuuucuagaacuacaccgggggucgugaugaccggguuuuacaaucaacgguucacauacuauaacacuaauuagcucuagcaaagagaagauaauucugcgacgauuggauuuuauaaucggcagaggagguucccacacauacc--------------------
                                         **  ** * *   ***  # *#*   ** ** **#   *#**  *  *   *  *#    ***#* *  # #  ** ###*  ||  *### **  # #  * *#***    #*  *   *  *  **#*   #** ** **   *#* #  ***   * * **  **
```

=== Internal intron ===

Multiple sequence alignment

```
Xmsuc0671A_fwrd     guaugugugggaaccuccucugccgauuauaaaauccaaucgucgcagaauuaucuucucuuugcuagagcuaauuaguguuauag-----------------
Xmsuc0671A_RevC     -----------------cuauaacacuaauuagcucuagcaaagagaagauaauucugcgacgauuggauuuuauaaucggcagaggagguucccacacauac
                                     ** *# *# * ** *  ** * # #  # *# **|** #* #  # # * **  * ** * #* #* **
```

=== External intron ===

Multiple sequence alignment

```
Xmsuc0671A_fwrd     guaugugaaccguugauuguaaaacccggucaucacgaccccgguguaguucuagaaaauguacgauagggcgcaagacuaacauguuuuaauaugaccg------ccag
Xmsuc0671A_RevC     cu------ggcggucauauuaaaacauguuagucuugcgcccuaucguacauuuucuagaacuacaccgggggucgugaugaccggguuuuacaaucaacgguucacauac
                     *        ** * **  ******  * *  **  *  *** #*   ##  * || *  ##   *# ***  *  **  * *  ******  ** * **        *
```

ALIGNMENT RESULTS - Xmsuc0710A

=== Stwintron Sequence ===

Multiple sequence alignment

```
Xmsuc0710A_fwrd     gguacguaucgggauuucaugggguuggucgcgaauacugguguuccuagaaucaucuauuaucugccagagcuaacugaucgccccaguaugugagccucuggucuggagauauggcuacuauggc----------------------cugugcaauucuugaaaaauuauaagacugauacaaacuucgagaugacag--------
Xmsuc0710A_RevC     --------cugucaucucgaaguuuguaucagucuuauaauuuuucaagaauugcaca----------------------ggccauaguagccauaucuccagaccagaggcucacauacuggggcgaucaguuagcucuggcagauaauagaugauucuaggaacaccaguauucgcgaccaacccaugaaaucccgauacguacc
                            # *  **#**  #* ***     *  *  *##* ** * *****  # #*                       ***  ****  ## # ***##*|*##*** # ##  ****  ***                       *# #  ***** * ** *##*  *  *     *** *#  **#**  * #
```

=== Internal intron ===

Multiple sequence alignment

```
Xmsuc0710A_fwrd     guacguaucgggauuucaugggguuggucgcgaauacugguguuccuagaaucaucuauuaucugccagagcuaacugaucgccccag--------------------------------------------
Xmsuc0710A_RevC     --------------------------------------------cuggggcgaucaguuagcucuggcagauaauagaugauucuaggaacaccaguauucgcgaccaacccaugaaaucccgauacguac
                                                                ** *  # ***  *** **  |  ** ***  *** #  * **
```

=== External intron ===

Multiple sequence alignment

```
Xmsuc0710A_fwrd     guaugugagccucuggucuggagauauggcuacuauggccugugcaauucuugaaaaauuauaagacugaua-----caaacuucgagaugacag----------
Xmsuc0710A_RevC     ----------cugucaucucgaaguuug-----uaucagucuuauaauuuuucaagaauugcacaggccauaguagccauaucuccagaccagaggcucacauac
                              ** * #*** **# * **     *** #  # *# **** **|** **** #* #  # ***     ** * #** ***# * **
```

ALIGNMENT RESULTS - Xmsuc0775A

=== Stwintron Sequence ===

Multiple sequence alignment

```
Xmsuc0775A_fwrd     gguauguauaaaaaccuccuguaucaguuguaaaaccugaucgucgcagaaauauguu-----------ucgucuucuagagcugacgauuguuauaguacguaaacuauugauuauaaaaaaugaucaucaagaucgauguaguucuugaaaguauauaacaaagaacuagacuaacaugguuuuauguggcag
Xmsuc0775A_RevC     cugccacauaaaaccauguuagucuaguucuuuguuauauacuuucaagaacuacaucgaucuugaugaucauuuuuuauaaucaauaguuuacguacuauaacaaucgucagcucuagaagacga-----------aacauauuucugcgacgaucagguuuuacaacugauacaggagguuuuuauacauacc
                        ###****** * *  *#  # **** *      *#  * *   **** **##*#           **#* ** ** *# ##* # **  # **|** #  ** # *## #* ** ** *#**           #*##** ****   * *  #*      * **** #  #*  * * ******###
```

=== Internal intron ===

Multiple sequence alignment

```
Xmsuc0775A_fwrd     guauguauaaaaaccuccuguaucaguuguaaaaccugaucgucgcagaaauauguuucgucuucuagagcugacgauuguuauag----
Xmsuc0775A_RevC     ----cuauaacaaucgucagcucuagaagacgaaacauauuucugcgacgaucagguuuuacaacugauacaggagguuuuuauacauac
                         ***** ** *  * *# # **  *   ** *  **  # || #  **  * **   *  ** # #* *  * ** *****
```

=== External intron ===

Multiple sequence alignment

```
Xmsuc0775A_fwrd     guacguaaacuauugauuauaaaaaaugaucaucaagaucgauguaguucuugaaaguauauaacaaagaacuagacuaac-------augguuuuauguggc-------------ag
Xmsuc0775A_RevC     cu-------------gccacauaaaaccau-------guuagucuaguucuuuguuauauacuuucaagaacuacaucgaucuugaugaucauuuuuuauaaucaauaguuuacguac
                     *              ##*#* ****# **        * # * ********    #*||*#    ******** * # *        ** #**** *#*##              *
```

ALIGNMENT RESULTS - Xmsuc0776A

=== Stwintron Sequence ===

Multiple sequence alignment

```
Xmsuc0776A_fwrd     gguauguaucaaagccugccgcgccagugugaaaucugaucuuggcagggauaucuuuucuauugcggagcuaacugguguuacaguauguaaaucguugguucagggaccuguccauuacg--accugugcacuuuuaaaauaacaacaugacacuagacugacuugauuuuguaauggcag--------------------
Xmsuc0776A_RevC     --------------------cugccauuacaaaaucaagucagucuagugucauguuguuauuuuaaaagugcacag--gucguaauggacagguccugaaccaacgauuuacauacuguaacaccaguuagcuccgcaauagaaaagauaucccugccaagaucagauuucacacuggcgcggcaggcuuugauacauacc
                                          **** *###***** # **     ** * #** ** *   **  ##**   ** *  **#  *#*  ##*  ** #**#|#**# **  *##  *#*  #   *** **   **##  **    * ** **# * **     ** # *****###* ****
```

=== Internal intron ===

Multiple sequence alignment

```
Xmsuc0776A_fwrd     guauguaucaaagccugccgcgccagugugaaaucugaucuuggcagggauaucuuuucuauugcggagcuaacugguguuacag--
Xmsuc0776A_RevC     --cuguaacaccaguuagcuccgcaauagaaaagauaucccugccaagaucagauuucacacuggcgcggcaggcuuugauacauac
                       **** **  #  *# * *  **#*# #***  *# #*#**|**#*# #*  ***# #*#**  * * #*  #  ** ****
```

=== External intron ===

Multiple sequence alignment

```
Xmsuc0776A_fwrd     guau-----guaaaucguugguucagggaccuguccauuacgaccugugcacuuuuaaaauaacaacaugacacuagacugacuu----------gauuuuguaauggcag
Xmsuc0776A_RevC     cugccauuacaaaauc----------aagucagucuagugucauguuguuauuuuaaaagugcacaggucguaauggacagguccugaaccaacgauuu-----acauac
                     * #       *****          ##  * *** * *   *  *    * ***|*** *    *  *   * * *** *  ##          *****       # *
```

ALIGNMENT RESULTS - Xmsuc0776B

=== Stwintron Sequence ===

Multiple sequence alignment

```
Xmsuc0776B_fwrd     gguaugaucauucccuccaugcuggucgugagacuu--cguauugauugaaagaucuacugcuugccagagcuaaccauugcugcaguaugugaacauuucgucuaggaauguagacauuacagcccucgu------------------aauucuccgaaggauacaucaagaaacaacacuaauacg----uuuuuguguguuag
Xmsuc0776B_RevC     cuaacacacaa----aaacguauuaguguuguuucuugauguauccuucggagaauu------------------acgagggcuguaaugucuacauuccuagacgaaauguucacauacugcagcaagguuagcucuggcaagcaguagaucuuucaaucaauacg--aagucucacgaccagcauggagggaaugauacauacc
                       *###*# *       * *# *#**  **   ***   ****#  *#* * #**                   ** *  **** *#* * *# *  ##* *|* *##  * #* * *#* ****  * **                   **# * *#*  #****   ***   **  **#* #* *       * #*###*
```

=== Internal intron ===

Multiple sequence alignment

```
Xmsuc0776B_fwrd     guau-guaucauucccuccaugcuggucgugagacuucguauugauugaaagaucuacugcuugccagagcuaaccauugcugcag
Xmsuc0776B_RevC     cugcagcaaugguuagcucuggcaagcaguagaucuuucaaucaauacgaagucucacgaccagcauggagggaaugauac-auac
                     * # *#*    *   # *  ** #*# **# # ***   **||**   *** # #** #*# **  * #   *    *#* # *
```

=== External intron ===

Multiple sequence alignment

```
Xmsuc0776B_fwrd     gu-----------augugaacauuucgucuaggaauguagacauuacagcccucguaauucuccgaaggauacaucaagaaacaacacuaauacguuuuuguguguuag
Xmsuc0776B_RevC     cuaacacacaaaaacguauuaguguuguuucuugauguauccuucggagaauuacgagggcuguaaugucuacauuccuagacgaaauguucacau-----------ac
                     *           *#**#    * * ** *    *****  * *#  **   * | *   **  #* *  *****    * ** * *    #**#*           *
```

ALIGNMENT RESULTS - Xmsuc0776C

=== Stwintron Sequence ===

Multiple sequence alignment

```
Xmsuc0776C_fwrd     gguauguauagaaacuuccugugccgauugugaaacccgaucguugcaaaa-----------auauccuuugucuucuagggcuaacgacuuuuacaguaugugaaccauugauucuaaaacauggccgucacgauuggucugguuuuagaaagucuauaacaaggaacugggcuaacauguuuuuauguggcag
Xmsuc0776C_RevC     cugccacauaaaaacauguuagcccaguuccuuguuauagacuuucuaaaaccagaccaaucgugacggccauguuuuagaaucaaugguucacauacuguaaaagucguuagcccuagaagacaa-----------aggauauuuuugcaacgaucggguuucacaaucggcacaggaaguuucuauacauacc
                        ###***#**** *  *#  **# ** #       #  * **  ****            *  *  ###* ** ***## #** *  *# #* *|* *# #*  * **# ##*** ** *###           #*  *# ****  ** *  #       # ** #**  #*  * ****#***###
```

=== Internal intron ===

Multiple sequence alignment

```
Xmsuc0776C_fwrd     ---------------------guauguauagaaacuuccugugccgauugugaaacccgaucguugcaaaaauauccuuugucuucuagggcuaacgacuuuuacag
Xmsuc0776C_RevC     cuguaaaagucguuagcccuagaagacaaaggauauuuuugcaacgaucggguuucacaaucggcacaggaaguuucuauacauac---------------------
                                         * * ##* ** *  **  **## ****#* * | * *#**** ##**  **  * ** *## * *
```

=== External intron ===

Multiple sequence alignment

```
Xmsuc0776C_fwrd     guaugugaaccauugauucuaaaacauggccgucacgauuggucugguuuuagaaagucuauaacaag---gaacugggcuaacauguuuuuaugu----------ggcag
Xmsuc0776C_RevC     cug----------ccacauaaaaacauguuagcccaguu---ccuuguuauagacuuucuaaaaccagaccaaucgugacggccauguuuuagaaucaaugguucacauac
                     *           # *#   ********   *#*  * *   #** *** **** | **** *** **   #* *  *#*   ********   #*           # *
```

ALIGNMENT RESULTS - Xmsuc0808A

=== Stwintron Sequence ===

Multiple sequence alignment

```
Xmsuc0808A_fwrd     ggugugu auaaaagccugccgugccgguugugagaccugguauugguggaaguaucuugu---------gucuuccagagcuaacuuuugucguaguaugugaaaccuugaucccgaaaaccuagcgaugaguuauguaguucuagaaaguuuacaauaaggaaccggacuaacauguuuuuauguggcag
Xmsuc0808A_RevC     cugccacauaaaaacauguuaguccgguuccuuauuguaaacuuucuagaacuacauaacucaucgcuagguuuucgggaucaagguuucacauacuacgacaaaaguuagcucuggaagac---------acaagauacuuccaccaauaccaggucucacaaccggcacggcaggcuuuuauacacacc
                        ###******#* **  #  ****** #  #   *##  **  *#*** **# * ##         *  ** * * # #**  ***  *#**|**#*  ***  **# # * * **  *         ## * #** ***#*  **  ##*   #  # ******  #  ** *#******###
```

=== Internal intron ===

Multiple sequence alignment

```
Xmsuc0808A_fwrd     guguguauaaaagccugccgugccgguugugagaccugguauugguggaaguaucuugugucuuccagagcuaacuuuugu-cguag
Xmsuc0808A_RevC     cuacg-acaaaaguuagcucuggaagacacaagauacuuccaccaauaccaggucucacaaccggcacggcaggcuuuuauacacac
                     *##* *#*****   **  **  #* ####***  #  #  #|#  #  #  ***#### *#  **  **   *****#* *##*
```

=== External intron ===

Multiple sequence alignment

```
Xmsuc0808A_fwrd     guaugugaaaccuugaucccgaaaaccuagcgaugaguuauguaguucuagaaaguuuacaauaaggaaccggacuaacauguuuuuaug-----------uggcag
Xmsuc0808A_RevC     cugc-----------cacauaaaaacauguuaguccgguuccuuauuguaaacuuucuagaacuacauaacucaucgcuagguuuucgggaucaagguuucacauac
                     * #             *  #***** *   # *  * * # * #** **#* | *#** **# * # * *  * #   * *****#  *             # *
```

ALIGNMENT RESULTS - Xmsuc0819A

=== Stwintron Sequence ===

Multiple sequence alignment

```
Xmsuc0819A_fwrd     gguauguauaaaaaccuccugugccgguugugaaacccaaucgucgcagaaauaucuu-----------uugucuucuaaagcuaacgauuuuuacaguaggugaaccauugaugcuaaaauacgacuaucacgauaggcguaguucuagaaaguauauaauagggaacuagacuaacauauuuuuaucuggcag
Xmsuc0819A_RevC     cugccagauaaaaauauguuagucuaguucccuauuauauacuuucuagaacuacgccuaucgugauagucguauuuuagcaucaaugguucaccuacuguaaaaaucguuagcuuuagaagaca-----------aaagauauuucugcgacgauuggguuucacaaccggcacaggagguuuuuauacauacc
                        ## *******  *  *#  * #*** #  *    *  * *   **** **# ##           *#** ** **  # #** * **# #  *|*  # #** * **# #  ** ** **#           *## #** ****   * *  *    *  # ***# *  #*  *  ******* ##
```

=== Internal intron ===

Multiple sequence alignment

```
Xmsuc0819A_fwrd     ---------------------guauguauaaaaaccuccugugccgguugugaaacccaaucgucgcagaaauaucuuuugucuucuaaagcuaacgauuuuuacag
Xmsuc0819A_RevC     cuguaaaaaucguuagcuuuagaagacaaaagauauuucugcgacgauuggguuucacaaccggcacaggagguuuuuauacauac---------------------
                                         * * ##* ** *   * ***#* **#*** * | * ***#** *#*** *   * ** *## * *
```

=== External intron ===

Multiple sequence alignment

```
Xmsuc0819A_fwrd     gu---aggugaaccauugaugcu-------------------------aaaauacgacuaucacgauaggcguaguucuagaaaguauauaauagggaacuagacuaacauauuuuuaucuggcag
Xmsuc0819A_RevC     cugccagauaaaaauauguuagucuaguucccuauuauauacuuucuagaacuacgccuaucgugauagucguauuuu-------------------------agcaucaaugguucaccu---ac
                     *   **#*#**    ** *# *                          ** **** *****||***** **** **                          * #* **    **#*#**   *
```

ALIGNMENT RESULTS - Xmsuc0904A

=== Stwintron Sequence ===

Multiple sequence alignment

```
Xmsuc0904A_fwrd     gguauguacaaaaaucuccugugccgcuugu-----------------gaaauccaaucgucgcagaaauaucuuuugccuucuaaagcuaacgauuuuuauaguaggugaaccauugauucuaaaacaug------gcuauuacgaucgguggaguucuagaaaauauauaauagggaacuggacuaacauauuuuuaucuggcag
Xmsuc0904A_RevC     cugccagauaaaaaauauguuaguccaguucccuauuauauauuuucuagaacuccaccgaucguaauag------ccauguuuuagaaucaaugguucaccuacuauaaaaaucguuagcuuuagaaggcaaaagauauuucugcgacgauuggauuuc-----------------acaagcggcacaggagauuuuuguacauacc
                        ## * ****** *  *#  **# ** #                 *** **** # #*** *# *       ###  ** ** *# #** * **# # **|** # #** * **# #* ** **  ##      # * #* ***# # **** ***                 # ** #**  #*  * ****** * ##
```

=== Internal intron ===

Multiple sequence alignment

```
Xmsuc0904A_fwrd     ---------------------guauguacaaaaaucuccugugccgcuugugaaauccaaucgucgcagaaauaucuuuugccuucuaaagcuaacgauuuuuauag
Xmsuc0904A_RevC     cuauaaaaaucguuagcuuuagaaggcaaaagauauuucugcgacgauuggauuucacaagcggcacaggagauuuuuguacauac---------------------
                                         * * *#* ** *   * ***#* ** *** # | # *** ** *#*** *   * ** *#* * *
```

=== External intron ===

Multiple sequence alignment

```
Xmsuc0904A_fwrd     guaggugaaccauugauucuaaaacauggcuauuacgaucgguggaguucuagaaaauauauaauagggaacuggacuaa----------cauauuuuua----------ucuggcag
Xmsuc0904A_RevC     cugccaga----------uaaaaauaug----------uuaguccaguucccuauuauauauuuucuagaacuccaccgaucguaauagccauguuuuagaaucaaugguucaccuac
                     *    **           **** ***          * #**  *****#  *  **||**  *  #*****  **# *          *** ****            **    *
```

ALIGNMENT RESULTS - Xmsuc1083A

=== Stwintron Sequence ===

Multiple sequence alignment

```
Xmsuc1083A_fwrd     gguauguauaaaaaaccuccugugccgguugugaaacccaaucgucguagaacuaucc------------uuugucuucuaaagcuaacgguuuucgcaguaugugaaccauugauuuuaaaaacauggcuauuacgaucgguguaguucuagaaaguauauaauaagggacuggacuaacauguuuuuauguggcag
Xmsuc1083A_RevC     cugccacauaaaaaacauguuaguccagucccuuauuauauacuuucuagaacuacaccgaucguaauagccauguuuuuaaaaucaaugguucacauacugcgaaaaccguuagcuuuagaag------------acaaaggauaguucuacgacgauuggguuucacaaccggcacaggagguuuuuauacauacc
                        ###******** *  *#  **#**# #  *    *   * *  ********# *            ##    ** ****# #** ****# *# *|* #* #**** **# #**** **             #   #* #********  * *  *    *  # #**#**  #*  * ********###
```

=== Internal intron ===

Multiple sequence alignment

```
Xmsuc1083A_fwrd     ---------------------guauguauaaaaaaccuccugugccgguugugaaacccaaucgucguagaacuauccuuugucuucuaaagcuaacgguuuucgcag
Xmsuc1083A_RevC     cugcgaaaaccguuagcuuuagaagacaaaggauaguucuacgacgauuggguuucacaaccggcacaggagguuuuuauacauac---------------------
                                         * * ##* *  *    * **##* **#*** * | * ***#** *##** *   *  * *## * *
```

=== External intron ===

Multiple sequence alignment

```
Xmsuc1083A_fwrd     guaugugaaccauugauuuuaaaaacauggcuauuacgaucgguguaguucuagaaaguauauaauaagggacuggacuaa----------cauguuuua-----------uguggcag
Xmsuc1083A_RevC     cugccaca-----------uaaaaacaug----------uuaguccagucccuuauuauauacuuucuagaacuacaccgaucguaauagccauguuuuuaaaaucaaugguucacauac
                     * #   *           **********          * #** #***#*#  *  #*||*#   *  #*#***# **# *          **********            *   # *
```

ALIGNMENT RESULTS - Xmsuc1127A

=== Stwintron Sequence ===

Multiple sequence alignment

```
Xmsuc1127A_fwrd     gguauguauaaaaaccuccugugccgguugugaaacccaaucgucguagaacuaucc------------uuugucuucuaaagcuaacgguuuucgcaguaugugaaccauugauuuuaaaaacguggccauuacgaucgguguaguucuagaaaguauauaauaagggacuggacuaacauguuuuuaugugguag
Xmsuc1127A_RevC     cuaccacauaaaaacauguuaguccagucccuuauuauauacuuucuagaacuacaccgaucguaauggccacguuuuuaaaaucaaugguucacauacugcgaaaaccguuagcuuuagaag------------acaaaggauaguucuacgacgauuggguuucacaaccggcacaggagguuuuuauacauacc
                        ###******** *  *#  **#**# #  *    *  * *  ********# *            ##    ** ****# #** ****# *# *|* #* #**** **# #**** **             #   #* #********  * *  *    *  # #**#**  #*  * ********###
```

=== Internal intron ===

Multiple sequence alignment

```
Xmsuc1127A_fwrd     ---------------------guauguauaaaaaccuccugugccgguugugaaacccaaucgucguagaacuauccuuugucuucuaaagcuaacgguuuucgcag
Xmsuc1127A_RevC     cugcgaaaaccguuagcuuuagaagacaaaggauaguucuacgacgauuggguuucacaaccggcacaggagguuuuuauacauac---------------------
                                         * * ##* *  *   * **##* **#*** * | * ***#** *##** *   *  * *## * *
```

=== External intron ===

Multiple sequence alignment

```
Xmsuc1127A_fwrd     guaugugaaccauugauuuuaaaaacguggccauuacgaucgguguaguucuagaaaguauauaauaagggacuggacuaa----------cauguuuuua-----------ugugguag
Xmsuc1127A_RevC     cuaccaca-----------uaaaaacaug----------uuaguccagucccuuauuauauacuuucuagaacuacaccgaucguaauggccacguuuuuaaaaucaaugguucacauac
                     **#   *           *******#**          * #** #***#*#  *  #*||*#  *  #*#***# **# *          **#*******           *   #**
```

ALIGNMENT RESULTS - Xmsuc1145A

=== Stwintron Sequence ===

Multiple sequence alignment

```
Xmsuc1145A_fwrd     gguauguau---------uaaggccucuuaug--------------------cuggcuguagagccccagcuuugaaagaaagaaaaaacuucccuaagagcuaacuaguauugcaguaugugagccguugauucuauaacauggccaucacggccuaucuaguccagaaaaauuauacaaaaggcacucuacuaacguguauauggaag
Xmsuc1145A_RevC     cuuccauauacacguuaguagagugccuuuuguauaauuuuucuggacuagauaggccgugauggccauguuauagaaucaacggcucacauacugcaauacuaguuagcucuuagggaaguuuuuucuuucuuucaaagcuggggcucuacagcc--------------------agcauaagaggccuua---------auacauacc
                      * ##***         ** #*  #*** **                      ***#** # * **  * * *# **  **      ** * *   *# #***||***# #*   * * **      **  ** #* * *  ** * # **#***                    ** ***#  *# **         ***## *
```

=== Internal intron ===

Multiple sequence alignment

```
Xmsuc1145A_fwrd     ------------guauguauuaaggccucuuaugcuggcuguagagccccagcuuugaaagaaagaaaaaacuucccuaagagcuaacuaguauugcag
Xmsuc1145A_RevC     cugcaauacuaguuagcucuuagggaaguuuuuucuuucuuucaaagcuggggcucuacagccagcauaagaggccuuaauacauac------------
                                 **  * *** **    ** * **  ** * #*# * | * #*# * **  ** * **    ** *** *  **
```

=== External intron ===

Multiple sequence alignment

```
Xmsuc1145A_fwrd     -------------guaugugagccguugauucuauaacauggccaucacggccuaucuaguccagaaaaauuauacaaaaggcacucuacuaacguguauauggaag
Xmsuc1145A_RevC     cuuccauauacacguuaguagagugccuuuuguauaauuuuucuggacuagauaggccgugauggccauguuauagaaucaacggcucacauac-------------
                                 **  **# #  *##  ** *****  *  *      #*  |  *#      *  *  ***** **  ##*  # #**  **
```
