# Supplementary material for: Propagation of [D1,2]-type spliceosomal twin introns (stwintrons) in Hypoxylaceae and Xylariaceae fungi
Source: Microbiol Spectr. 2025 Aug 8;13(9):e02926-24. doi: 10.1128/spectrum.02926-24 (PMC12403724; doi:10.1128/spectrum.02926-24)
Supplement: Supplemental material — Fig. S1 to S3; Tables S2 and S3. [file spectrum.02926-24-s0005.pdf]

Supplementary Material (online)

associated with

Propagation of [D1,2]-type spliceosomal twin introns (stwintrons) in *Hypoxylaceae* and *Xylariaceae* fungi

Erzsébet Fekete [1], Norbert Ág [1], Viktória Ág-Rácz [1,2], Alexandra Márton [1], Erzsébet Sándor [3], Claudio Scazzocchio [4,5], Michel Flipphi [1], Levente Karaffa [1]

[1] Department of Biochemical Engineering, Faculty of Science and Technology, University of Debrecen, H-4032 Debrecen, Hungary;
[2] Juhász-Nagy Pál Doctoral School of Biology and Environmental Sciences, University of Debrecen, Debrecen H-4032, Hungary;
[3] Institute of Food Science, Faculty of Agricultural and Food Science and Environmental Management, University of Debrecen, H-4032 Debrecen, Hungary;
[4] Department of Life Sciences, Imperial College London, London SW7 2AZ, UK;
[5] Institute for Integrative Biology of the Cell (I2BC), Université Paris-Saclay, CEA and CNRS (Unité mixte de Recherche UMR 9198), 91190 Gif-sur-Yvette, France

Supplementary Figure S1
Supplementary Figure S2
Supplementary Figure S3

Supplementary Table S1 is provided separately as an Excel file (Supplementary_Table_S1.xlsx)
Identifiers, localisation, statistics and other information concerning 288 sequence-similar sister stwintrons in seven whole genome sequences of *Hypoxylaceae* taxa and seven whole genome sequences of *Xylariaceae* taxa, including SRA evidence of their existence (where applicable).
Supplementary Table S2
Supplementary Table S3

Supplementary Datafile S1
Supplementary Datafile S2
Supplementary Datafile S3
Supplementary Datafile S4

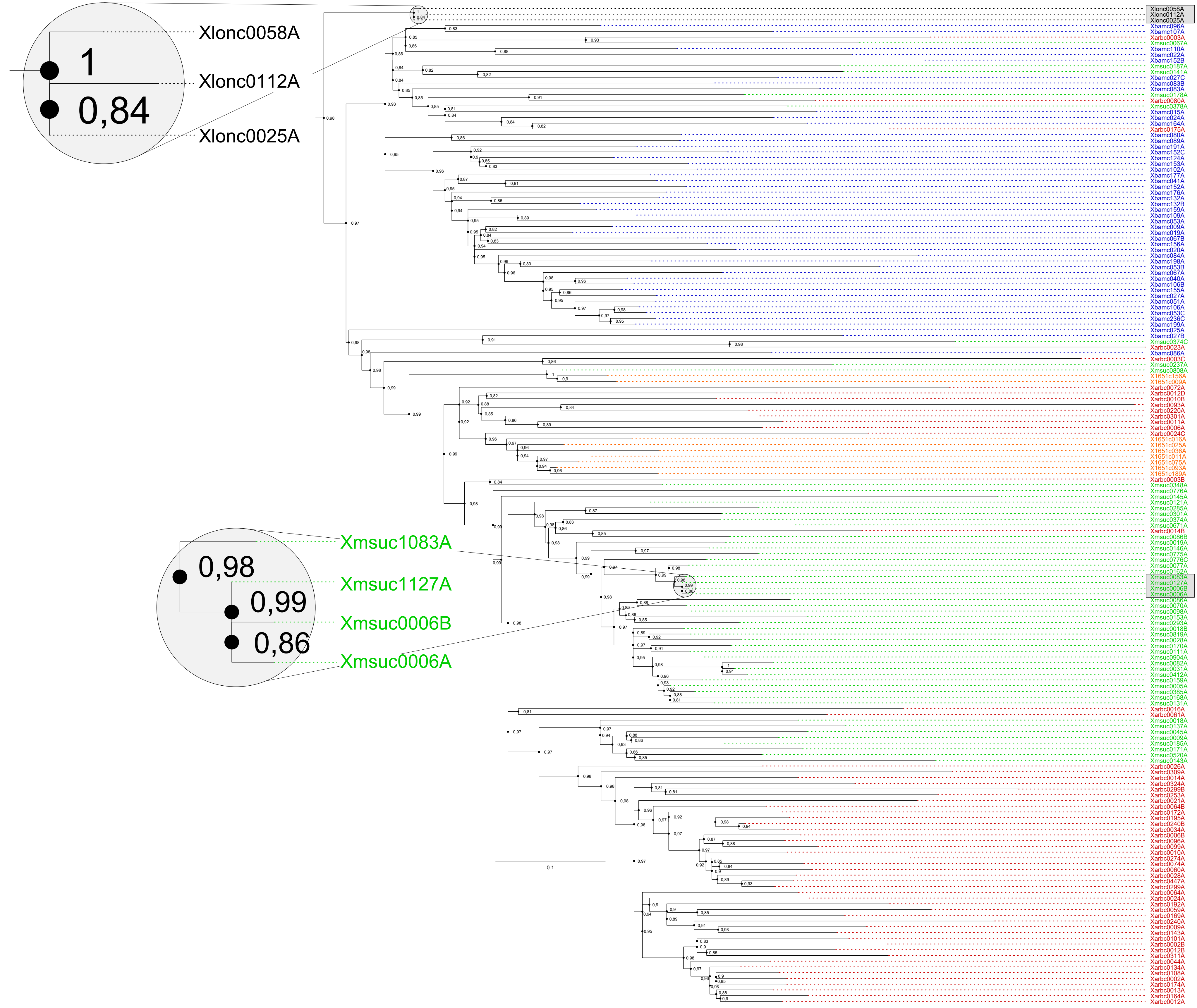

Figure S1. Two small clades of species-specific, near identical stwintrons in a maximum likelihood analysis of sequence-similar sister stwintrons from five species of *Xylaria* (*Xylariaceae*) examplying the general trend of the evolution of species-specific clades of stwintrons. Color code: *X. bambusicola*, blue; *X. arbuscula* FL1030, red; *Xylaria* sp. MSU SB201401, green; *Xylariaceae* sp. FL1651, orange; *X. longipes*, black. The two clades of near identical [D1,2] stwintrons are highlighted with the enlarged inlets. The ML tree appears rooted in a clade of three extremely similar *Xylaria longipes* stwintrons. This is the first of the clades of near identical stwintrons (>99 % identical). The clade of four near-identical [D1,2] stwintrons in MSU SB201401 (>97 % identical) is phylogenetically separated from the *X. longipes* clade. The ML tree at the basis of the Figure was inferred as described in Material and Methods Section 2.5. The number of informative nt in the manually trimmed multiple sequence alignment was 220. Node statistics (from 0 to 1) were calculated with 10,000 iterations of transfer bootstrapping. The scale bar represents 0.1 substitutions per site.

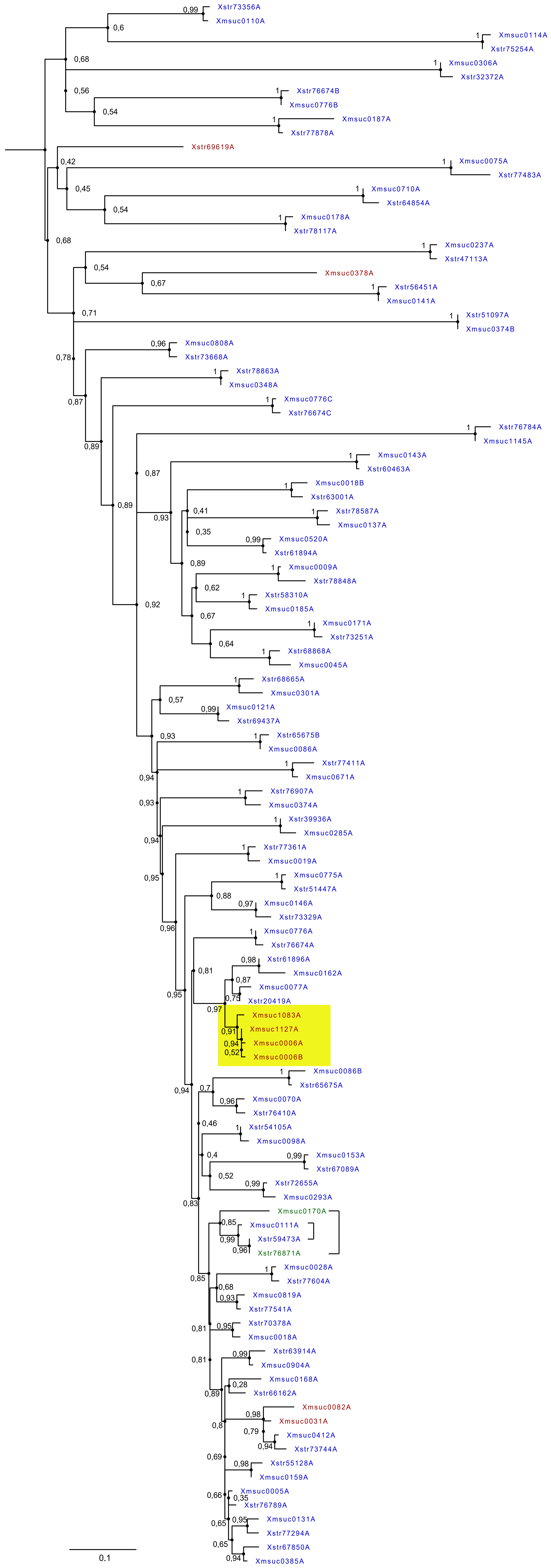

Figure S2. Phylogenetic relations between sister stwintrons in the closely related fungi *Xylaria* sp. MSU SB201401 (Xmsuc) and *Xylaria striata* RK1-1 (Xstr). A ML tree was inferred for 112 [D1,2] stwintrons cross-identified with Blastn screens in the genome sequences of *Xylaria* sp. MSU SB201401 (59 sister stwintrons) and *Xylaria striata* (53 sister stwintrons). These genome-sequenced fungi (see Table 1 main text) were isolated on different continents (North America and Asia, respectively) from the roots of very different host plants (a dicotyl and a monocotyl, respectively) growing in completely different context (monoculture and a natural environment, respectively). Yet, limited comparison focussed on sister stwintrons originally defined in MSU SB201401 and on the genes that carry them, strongly suggested that it concerns strains or variants of the same species, or cryptic species within a species complex. Their sister stwintron content is very similar and the orthologous stwintron sequences are highly similar or even near identical, leading to the many orthologous "duplets" in the joined ML phylogeny on very short end-branches, suggesting very recent divergence. In two cases, orthologous stwintrons did not directly neighbor in the tree; the orthologous couples involved were connected with the hooks to the right of their names, the names of the one "separated" couple written in green. A few stwintrons were unique to one fungus and their names are written in red. Four of the stwintrons unique to MSU SB201401, Xmsuc1083A–Xmsuc1127A–Xmsuc0006A–Xmsuc0006B, are tightly clustered, equal in length and differing in sequence at one to four SNPs; This clade is highlighted in yellow. The four orthologous genes are present in *X. striata* but the stwintrons are absent. This lineage was arguably generated after the geographical separation of the strains as it involves three consecutive, strain-specific sister stwintron duplication events. The ML tree was inferred through multiple sequence alignment of the 112 sister stwintrons, curation and trimming of this alignment, and subsequent maximum likelihood estimation, as described in the Materials and Methods section. The number of informative nt was 185. The inferred phylogram was drawn by FigTree version 1.4.3. from Newick files produced by PhyML with decreasing node order and rooted at midpoint (NB. Settings of the drawing program). Node statistics ranging from 0 to 1 were calculated with 10,000 iterations of transfer bootstrapping. The scale bar represents 0.1 substitutions per site.

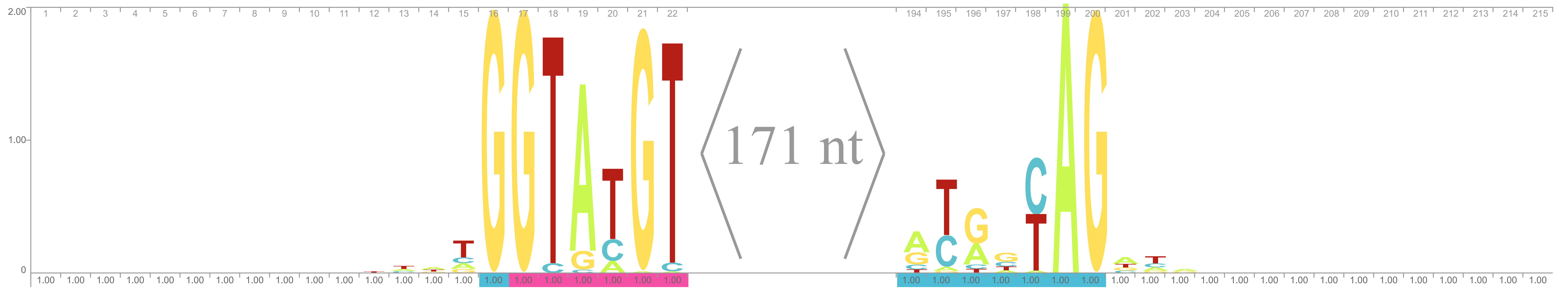

Figure S3. Sequence bias is absent in the sister stwintron-flanking exonic sequences and evidence for splice site co-option at the exon-stwintron interfaces is absent. The sequence logo of the direct environment of the stwintron/exon junctions in pre-mRNAs containing sister stwintrons (all 285) was created by Skylign using default settings (see Materials and Methods section). The mean length of the internal stwintron sequence (not shown) between the depicted terminal sequences is 171 nt. The logo visualises the extent of nucleotide conservation in a multiple sequence alignment and highlights conservation patterns adjacent to the (stw)intron–exon junctions of sister (stw)introns. The relative sizes of the four nts refect their frequency at each individual position. The height of the nt stack equals the information content at each position. The information content is low in the exonic regions testament to seamless, random integration of *Xylariales* [D1,2] sister stwintrons at new gene positions.

Table S2: SRA reads that confirm occasional missplicing of sister [D1,2] stwintrons in one canonical U2 reaction, ignoring the internal splice sites of the stwintrons. Analysis was done in four species of *Xylaria*, *Xylariaceae* sp. FL1651 and in *Hypoxylon rubiginosum*. Results for *Hypoxylon* sp. CO27-5/EC38 were reported in the Supplementary Material of [7]. The use of alternative distal donor- and/or acceptor sites was also confirmed by SRA reads.

	**missplice [D1,2]**	**alternative splice sites**	**database (run)**
X. bambusicola			
**Xbamc155A**	67268001.2<br>62099009.2<br>59009032.1		SRR10915175
**Xbamc027A**		29090613.2<br>[acceptor]	SRR10915175
**Xbamc027C**	84433676.2		SRR10915175
**Xbamc067A**	71807868.1		SRR10915175
**Xbamc109A**		80749179.1<br>[acceptor]	SRR10915175
**Xbamc191A**	28533010.2	46434346.2<br>[acceptor]	SRR10915175
**Xbamc041A**	29036780.2		SRR10915175
**Xbamc177A**		19761803.2<br>[donor]	SRR10915175
	**missplice [D1,2]**	**alternative splice sites**	**database (run)**
N.arbortiva			
**Naboc037A**	24953200.1		SRR10843706
**Naboc037B**	1677634.1	1677634.1<br>[donor]	SRR10843706<br>SRR10843705
**Naboc202A**		27591478.2<br>[acceptor]<br>15417580.1<br>[acceptor]<br>3393253.2<br>[acceptor]	SRR10843706<br><br>SRR10843706<br><br>SRR10843705
**Naboc199B**	11428917.1		SRR10843706
**Naboc184A**		8583171.2<br>30280005.2<br>30280005.1<br>[all acceptor]	SRR10843706<br>SRR10843706<br>SRR10843706<br>SRR10843705
	**missplice [D1,2]**	**alternative splice sites**	**database (run)**
X.arbuscula			
**Xarbc0240A**		24407470.2<br>15711219.2<br>5050876.2<br>[all acceptor]	SRR10843694<br>SRR10843694<br>SRR10843694
**Xarbc0044A**		996477.2<br>microexon 7 nt	SRR10843694

**Xarbc0002A**		9774544.2<br>[acceptor]	SRR10843694
**Xarbc0064A**		32601223.1<br>[acceptor]	SRR10843694
**Xarbc0099A**		2204724.2<br>19183919.2<br>8492814.1<br>[all acceptor]	SRR10843693<br>SRR10843694<br>SRR10843694
**Xarbc0143A**	2302422.1	8900364.2<br>microexon 3 nt	SRR10843694<br>SRR10843694
**Xarbc0026A**	28280668.1	28280668.1<br>[acceptor]	SRR10843694
**Xarbc0169A**		3174136.2<br>17644819.2<br>[all acceptor]	SRR10843694<br>SRR10843693
**Xarbc0010A**		12939707.2<br>18002142.2<br>both splice sites	SRR10843693<br>SRR10843694<br>SRR10843693
**Xarbc0014A**	12920728.2		SRR10843694
**Xarbc0072A**	5042374.2<br>5042374.1	5042374.2<br>5042374.1<br>[all acceptor]	SRR10843694<br>SRR10843694
**Xarbc0024A**		10621715.2<br>[normal D1,2]<br>11138274.1<br>11138005.2<br>microexon 3 nt<br>12172816.1<br>[acceptor]	SRR10843693<br><br>SRR10843694<br>SRR10843694<br><br>SRR10843693
	**missplice [D1,2]**	**alternative splice sites**	**database (run)**
X. longipes			
**Xlonc0002A**	31098352.2		SRR10915188
**Xlonc0112A**	18929947.2		SRR10915188
	**missplice [D1,2]**	**alternative splice sites**	**database (run)**
H.rubiginosum			
**Hruc55A**	59964722.2<br>4189749.2<br>9432374.2<br>[two more]		SRR10914997
	**missplice [D1,2]**	**alternative splice sites**	**database**
**X.sp.FL1651**			
**X1651c016A**	10533179.1		SRR10843721

Table S3. Ribonucleotide sequences of near terminal inverted repeat elements (NTIRE) of which the 5'- and 3'-elements are fully complementary. 25 (stw)introns have fully complementary NTIREs of ten ribonucleotides, NTIRE-10, while an additional 17 (stw)introns have nine basepairing ribonucleotides, NTIRE-9. For clarity, both sequences are located on the same pre-mRNA and on the misspliced [D1,2] intron RNA (Figure 3b). In *Hypoxylon* sp. CO27-5 and EC38, all but one of the complementary 5'- and 3'-NTIREs are found in type-2 cropped sister introns which are marked with the star (*). These canonical U2 introns have been formed from a parent sister stwintron by internal deletion removing the internal splice sites from the latter [7]. The exception is HCOc052A, a sister stwintron. For the 17 NTIRE-9, the position of the basepairing ribonucleotide lacking with respect to the NTIRE-10, is indicated with the number sign (#).

taxon	[D1.2] stwintron	5'-NTIRE-10	3'-NTIRE-10
		(5'-3')	(5'-3')
**H. rubiginosum**	Hruc31A	guaugaaaac	guuuucguac
**H. rubiginosum**	Hruc55A	guaugaaaac	guuuucguac
**D. childiae**	Dchc001D	guaugaaaac	guuuucauac
**D. childiae**	Dchc003A	guaugaaaac	guuuucauac
**D. childiae**	Dchc003B	guaugaaaac	guuuucauac
**D. childiae**	Dchc005A	guaugaaaac	guuuucauac
**Hypoxylon sp. CO27-5**	HCOc105A *	guauaaaaac	guuuuuauac
**Hypoxylon sp. CO27-5**	HCOc153A *	guauaaaaac	guuuuuauac
**Hypoxylon sp. CO27-5**	HCOc171A *	guauaaaaac	guuuuuauac
**Hypoxylon sp. EC38**	HECc321B *	guauaaaaac	guuuuuauac
**X. longipes**	Xlonc0025A	guauaaaaac	guuuuuauac
**X. longipes**	Xlonc0058A	guauaaaaac	guuuuuauac
**X. longipes**	Xlonc0112A	guauaaaaac	guuuuuauac
**Xylaria sp. MSU SB201401**	Xmsuc0006A	guauaaaaac	guuuuuaugu
**Xylaria sp. MSU SB201401**	Xmsuc0006B	guauaaaaac	guuuuuaugu
**Xylaria sp. MSU SB201401**	Xmsuc0077A	guauaaaaac	guuuuuaugu
**Xylaria sp. MSU SB201401**	Xmsuc1083A	guauaaaaac	guuuuuaugu
**Xylaria sp. MSU SB201401**	Xmsuc1127A	guauaaaaac	guuuuuaugu
**Xylaria sp. MSU SB201401**	Xmsuc0018A	guauaaaaac	guuuuuauau
**Xylaria sp. MSU SB201401**	Xmsuc0121A	guauaaaaac	guuuuuauau

**Xylaria sp. MSU SB201401**	Xmsuc0776C	guauagaaac	guuuuuaugu
**Xylaria sp. MSU SB201401**	Xmsuc0019A	guauaaaagc	guuuuuaugu
**Xylaria sp. MSU SB201401**	Xmsuc0808A	guauaaaagc	guuuuuaugu
**Xylariaceae FL1651**	X1651c156A	guauaaaagc	gcuuuuaugu
**X. bambusicola**	Xbamc159A	guauuaaaau	auuuugauau

**taxon**	**[D1.2] stwintron**	**5'-NTIRE-9**	**3'-NTIRE-9**
		(5'-3')	(5'-3')
**H. rubiginosum**	Hruc56A	#uaugaaaac	guuuucgua#
**D. concentrica**	Dcoc03A	#uaugaaaac	guuuucaua#
**Hypoxylon sp. CO27-5**	HCOc052A	guauuaaaa#	#uuuuaauac
**Hypoxylon sp. CO27-5**	HCOc147A *	guauaaaaa#	#uuuuuauac
**Xylaria sp. MSU SB201401**	Xmsuc0031A	guauaaaaa#	#uuuuuauau
**Xylaria sp. MSU SB201401**	Xmsuc0082A	guauaaaaa#	#uuuuuauau
**Xylaria sp. MSU SB201401**	Xmsuc0412A	guauaaaaa#	#uuuuuauau
**Xylaria sp. MSU SB201401**	Xmsuc0520A	guauaaaaa#	#uuuuuauau
**X. arbuscula FL1030**	Xarbc0309A	guaucaaaa#	#uuuugauau
**X. bambusicola**	Xbamc019A	guaucaaaa#	#uuuugauau
**X. bambusicola**	Xbamc040A	#uaucaaaac	guuuugaua#
**X. bambusicola**	Xbamc053B	guauuaaag#	#uuuuaauau
**X. bambusicola**	Xbamc089A	guaucaaaa#	#uuuugguau
**X. bambusicola**	Xbamc110A	guaucaaau#	#guuugauau
**X. bambusicola**	Xbamc153A	guaucaaaa#	#uuuugguau
**X. bambusicola**	Xbamc177A	guauuauga#	#uuaugauau
**X. bambusicola**	Xbamc198A	guaucaaaa#	#uuuugauau
